# Supplementary material for: Efficient inter-species conjugative transfer of a CRISPR nuclease for targeted bacterial killing
Source: Nat Commun. 2019 Oct 4;10:4544. doi: 10.1038/s41467-019-12448-3 (PMC6778077; doi:10.1038/s41467-019-12448-3)
Supplement: Supplementary file 1 — Supplementary Information [file 41467_2019_12448_MOESM1_ESM.pdf]

SUPPLEMENTARY INFORMATION: EFFICIENT INTER-SPECIES CONJUGATIVE TRANSFER OF A CRISPR NUCLEASE FOR TARGETED BACTERIAL KILLING

THOMAS A. HAMILTON, GREGORY M. PELLEGRINO, JASMINE A. THERRIEN, DALTON T. HAM, PETER C. BARTLETT, BOGUMIL J. KARAS, GREGORY B. GLOOR, DAVID R. EDGELL

pNuc_cis (61203 bp)

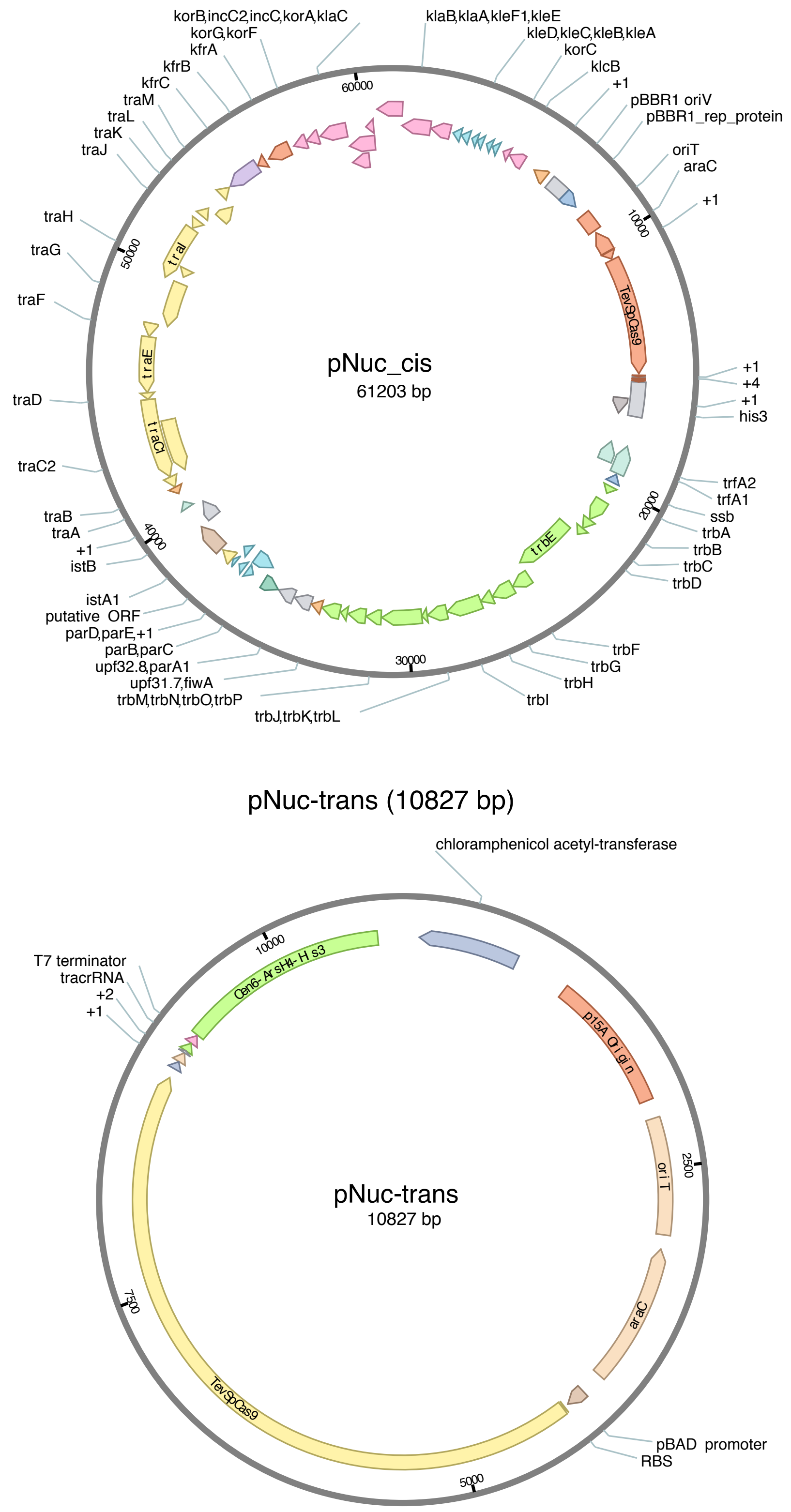

SUPPLEMENTARY FIGURE 1. Plasmid maps of pNuc-*cis* and pNuc-*trans*. The corresponding GenBank formatted files of the nucleotide sequence of each plasmid are provided as Supplementary Data 5-7.

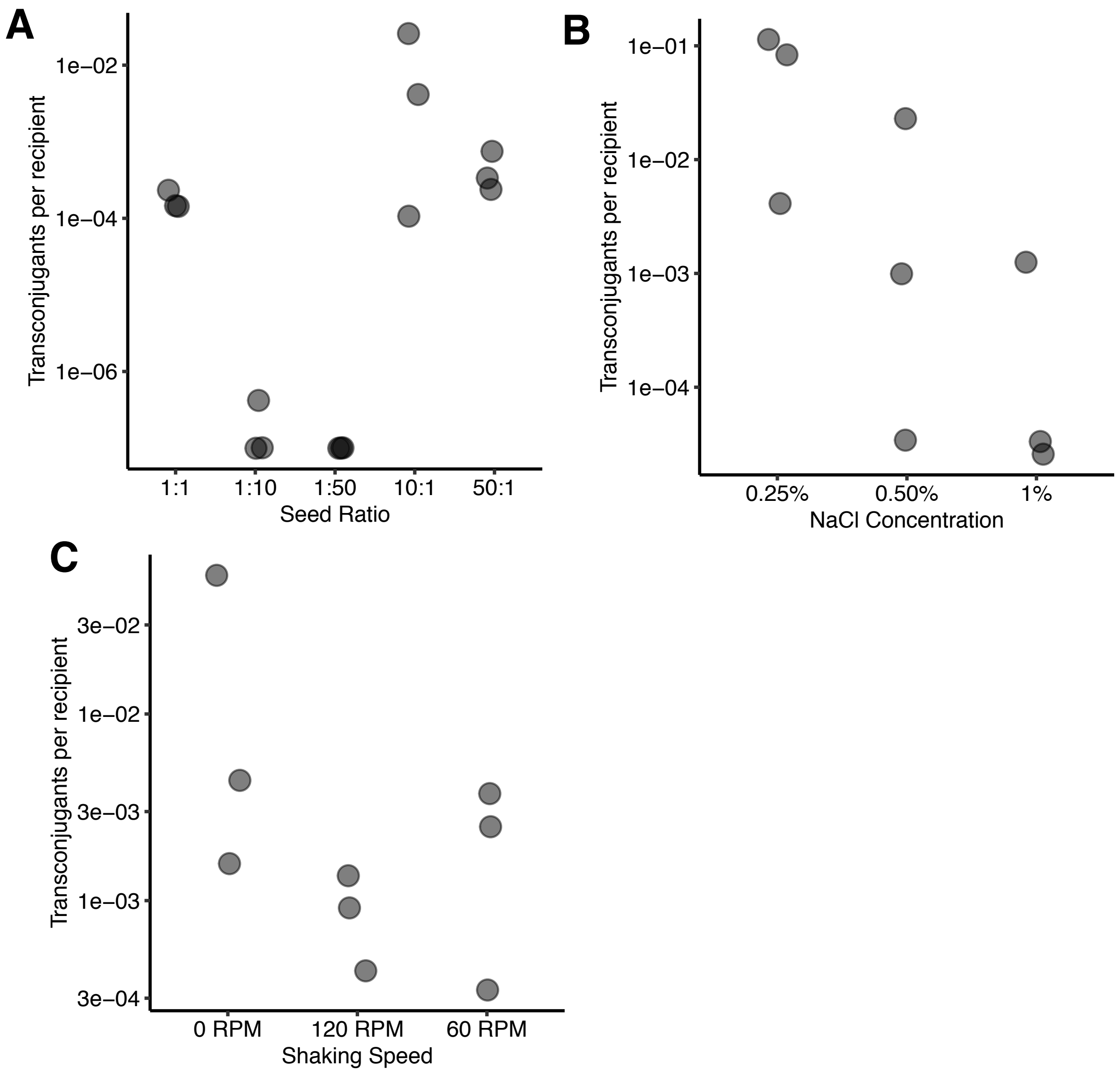

SUPPLEMENTARY FIGURE 2. Optimizing liquid culture conditions for *E. coli* to *S. enterica* conjugation. A: Conjugation frequency for different sodium chloride (NaCl) media conditions. B: Conjugation frequency measured with different *E. coli* donor to*S. enterica* recipient ratios at the start of conjugation. C: Effect of culture agitation on conjugation frequency (RPM - revolutions per minute). For each plot, points indicate conjugation frequency for independent biological replicates.

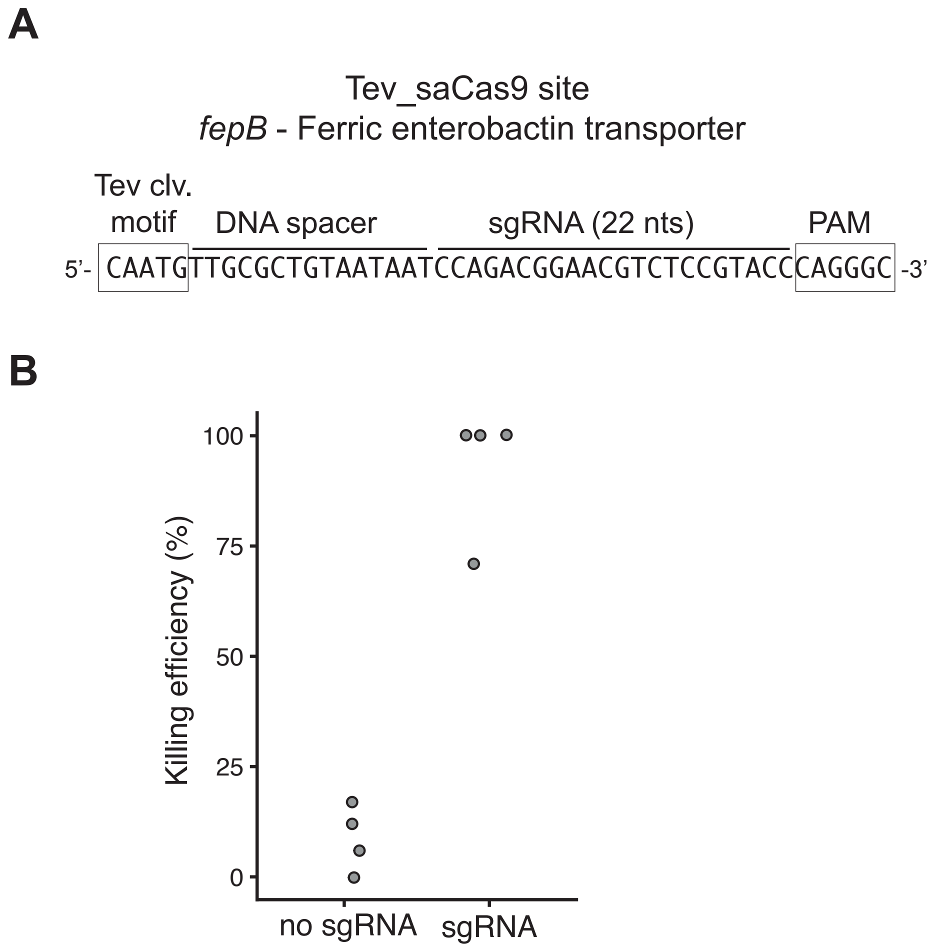

SUPPLEMENTARY FIGURE 3. Killing of *S. enterica* by conjugative delivery of TevSaCas9. A: Schematic of TevSaCas9 target site in the *fepB* gene of *S. enterica*, with I-TevI cleavage motif, DNA spacer, sgRNA binding site and PAM motif indicated. B: Plot of *S. enterica* killing efficiency with no sgRNA cloned in pNuc, or the *fepB* sgRNA cloned in pNuc. Points are independent biological replicates.

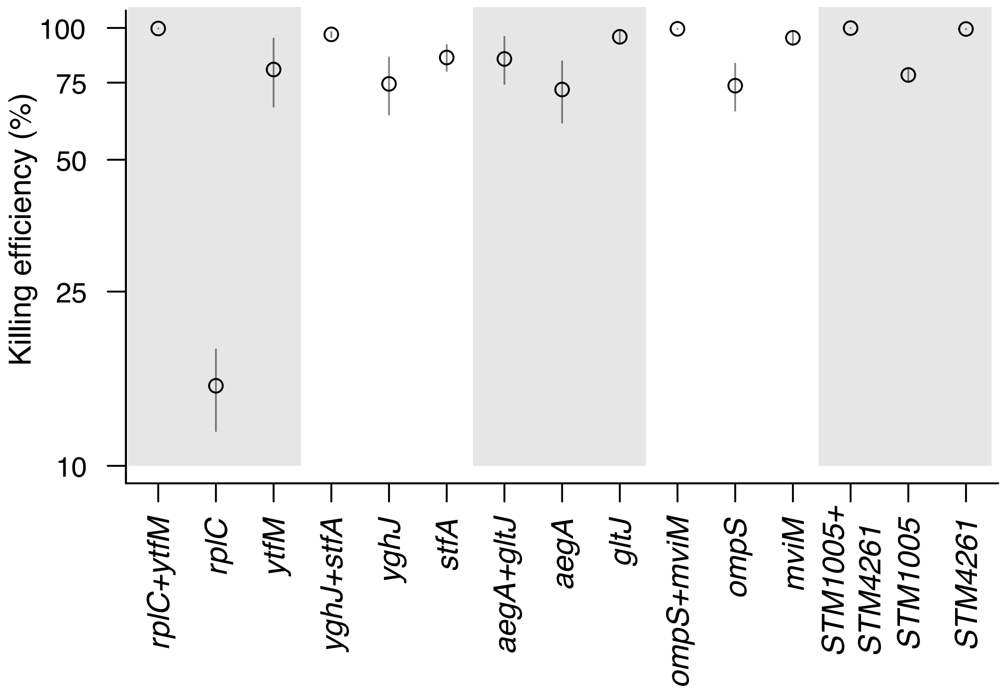

SUPPLEMENTARY FIGURE 4. Killing efficiency of multiplexed pairs of sgRNAs, with single sgRNAs plotted for comparison. Data are plotted on log10 scale as the mean of at least three independent biological replicates, with vertical lines representing the standard error of the mean. A Mann-Whitney Wilcox test comparing if multiplexed sgRNAs had a significantly higher killing efficiency as a group than their single sgRNA constituents yielded a p-value=0.003.

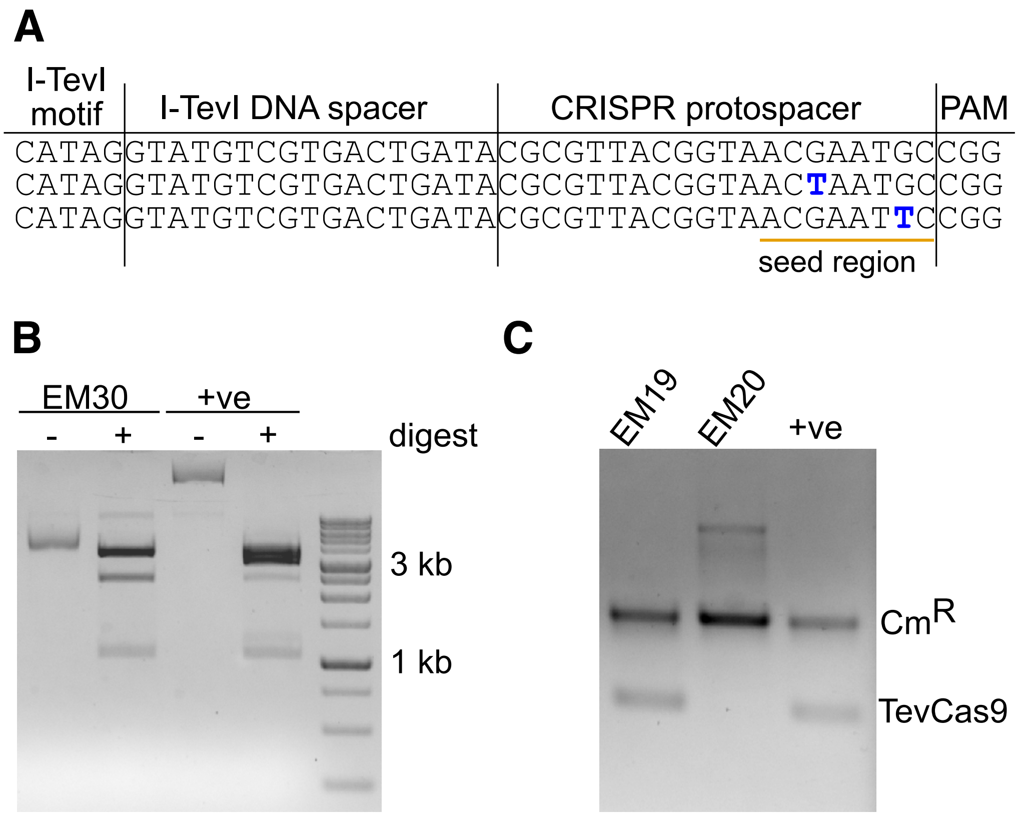

SUPPLEMENTARY FIGURE 5. Examples of *S. enterica* escape mutants. a) Nucleotide sequence of the TevSpCas9 target site for STM sgRNA in the Gifsy prophage. Nucleotide substitutions in the seed region of the sgRNA are indicated and underlined. b) Example of an agarose gel of pNuc DNA isolated from EM30 or from wild-type pNuc (+ve) incubated with (+) or without (-) a mixture of FspI and MslI restriction enzymes. Size standards in kilobase pairs (kb) are indicated to the right of the gel image.c) Example of multiplex PCR with pNuc DNA isolated from EM19, EM20 or wild-type pNuc (+ve) with primers specific for the Cm^R and TevSpCas9 coding regions.

	Model 1
(Intercept)	92.63 ***
	(16.05)
gene.functionN	-19.83
	(17.06)
gene.functionY	-35.58 *
	(16.23)
trans_strand	-21.61
	(14.28)
sgRNA.score	9.23 *
	(3.70)
replication_strand	8.98
	(14.41)
rel.dist.in.gene	2.59
	(3.77)
N	65
AIC	631.54
BIC	648.93
Pseudo R2	0.25

*** p < 0.001; ** p < 0.01; * p < 0.05.

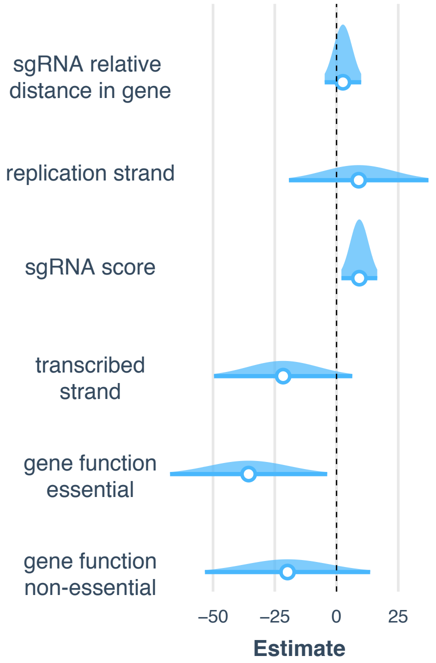

SUPPLEMENTARY FIGURE 6. Summary of generalized linear model of sgRNA parameters that are indicative of killing efficiency with P-values indicated (left), and a graphical representation of the confidence intervals associated with each parameter. Note that parameters with confidence intervals that pass over the 0 line are not considered significant.

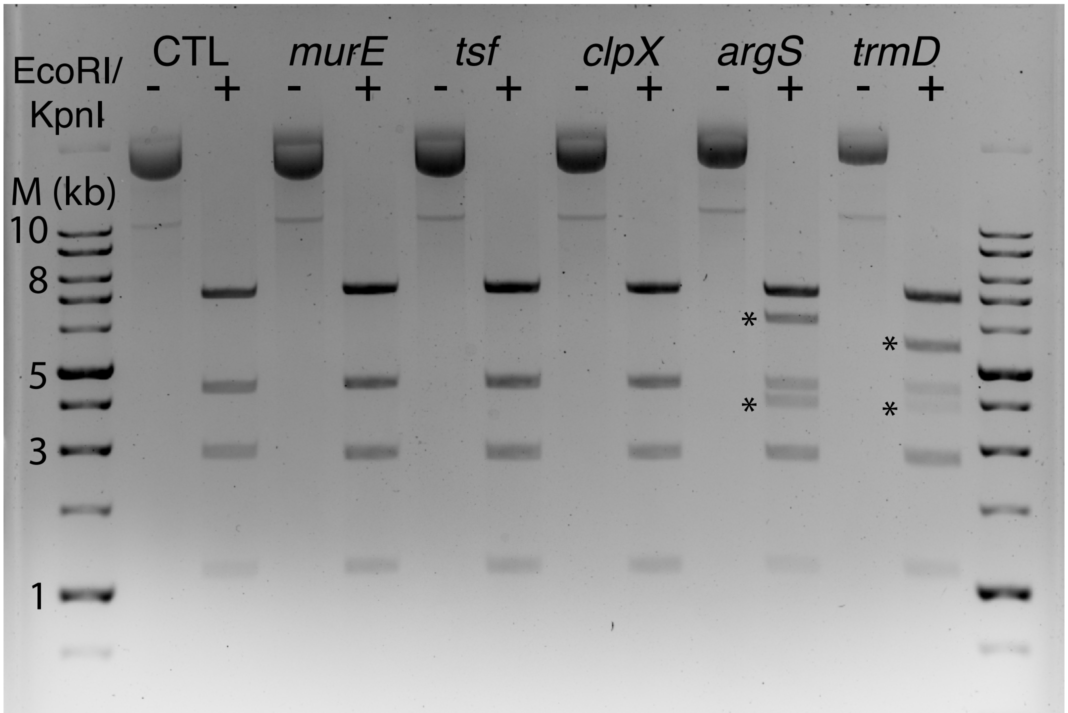

SUPPLEMENTARY FIGURE 7. Example of agarose gel of diagnostic restriction digest of different guideRNAs cloned into pNuc-*trans*. Each plasmid was digested with EcoRI and KpnI and compared to the pNuc-*trans* backbone (CTL). Asterisks indicate unexpected digestion patterns. The size of the ladder is indicated in kilobase pairs (kb) to the left of the gel image. The image was cropped for publication.

sgRNA	off_target	OT_pos	num_mm	pam_mm	mm_score	mm_map	pam_map
STM1005	ACGCTTACTGTAACGAATGCGGC	3018862	5	1	6.5	****....*...........	.*
STM1005	AGCGTTAAGGCAACGAATACCCA	4497514	4	2	16.25	*......*..*.......*.	**
STM1005	AGCGTTACGGGTACAAATGTAGT	200113	5	1	29	*.........**..*....*	.*
STM1005	AGCGTTACGTTAACGATAGCGTC	1353655	4	2	23	*........*......**..	**
STM1005	AGCGTTATGGTAACGCCTGCGAT	465571	4	2	18.75	*......*.......**...	**
STM1005	AGCGTTGCGGTAACCAGCGCCTG	3310101	5	1	26	*.....*.......*.**..	*.
STM1005	AGCGTTTCCGTAACGAATTTATC	3260062	5	2	25	*.....*.*.........**	**
STM1005	AGCGTTTCGGTGGCGAATCCGTT	1308603	5	2	23	*.....*....**.....*.	**

SUPPLEMENTARY FIGURE 8. Example of off-target site predictions in the *E. coli* genome. The sgRNA.off.target.finder.pl inputs a fasta file of sgRNA sequences, searches the sgRNA against a provided reference genome, and outputs (from left to right): the sgRNA on-target site, the predicted off-target site (off_target), the position of the off-target site in the reference genome (OT_pos), the number of nucleotide mismatches relative to the on-target site (num_mm), the number of mismatches to positions 2 and 3 of the NGG PAM (pam_mm) , the mismatch score (mm_score) calculated as described in the Methods, a map of nucleotide mismatches where asterisks (*) indicate mismatches to the on-target site and dots (.) are nucleotide identities, and a mismatch map for positions 2 and 3 of the PAM sequence (pam_map) where asterisks (*) are mismatches and dots (.) are identities.
