# Supplementary material for: Efficient inter-species conjugative transfer of a CRISPR nuclease for targeted bacterial killing
Source: Nat Commun. 2019 Oct 4;10:4544. doi: 10.1038/s41467-019-12448-3 (PMC6778077; doi:10.1038/s41467-019-12448-3)
Supplement: Supplementary file 3 — Description of Additional Supplementary Files [file 41467_2019_12448_MOESM3_ESM.pdf]

Description of Additional Supplementary Files

File Name: Supplementary Data 1

Description: Summary information for sgRNAs targeting genes in the S. enterica genome.

File Name: Supplementary Data 2

Description: Summary of sgRNA cloning attempts for sgRNAs. Note that NS refers to ’not sequenced’.

File Name: Supplementary Data 3

Description: Predicted sgRNA off-target sites in E. coli genome for all 65 sgRNAs used in this study.

File Name: Supplementary Data 4

Description: List of PCR primers used in this study.

File Name: Supplementary Data 5

Description: GenBank formatted file of the pNuc-cis sequence.

File Name: Supplementary Data 6

Description: GenBank formatted file of the pNuc-trans sequence.

File Name: Supplementary Data 7

Description: GenBank formatted file of the pTA-Mob plasmid.

File Name: Supplementary Data 8

Description: Fasta file of sgRNA sequences targeting S.enterica genes for use with sgRNA.off.target.finder.pl.

File Name: Supplementary Data 9

Description: Fasta file of E. coli MG16552 to be used as a reference genome with sgRNA.off.target.finder.pl.

File Name: Supplementary Software 1

Description: Perl script (sgRNA.off.target.finder.pl) to find off-target sites in reference genome. Instructions are provided in the header of the program. Use with Supplementary Data 8 and 9.
