# Supplementary material for: Efficient inter-species conjugative transfer of a CRISPR nuclease for targeted bacterial killing
Source: Nat Commun. 2019 Oct 4;10:4544. doi: 10.1038/s41467-019-12448-3 (PMC6778077; doi:10.1038/s41467-019-12448-3)
Supplement: Supplementary file 8 — Supplementary Data 5 [file 41467_2019_12448_MOESM8_ESM.pdf]

```
LOCUS       pNuc_cis_no_guide      61203 bp ds-DNA     circular     02-AUG-2019
DEFINITION  .
FEATURES             Location/Qualifiers
     CDS             28445..29221
                     /label="trbJ"
     CDS             31674..32378
                     /label="trbN"
     CDS             complement(18304..19452)
                     /label="trfA1"
     CDS             34625..35287
                     /label="upf32.8"
     misc_RNA        15571..15586
                     /label="crRNA"
     CDS             complement(54188..55534)
                     /label="kfrC"
     CDS             33460..33879
                     /label="upf31.7"
     CDS             complement(4533..4790)
                     /label="korC"
     CDS             complement(2416..2730)
                     /label="kleF1 "
     CDS             27037..28428
                     /label="trbI"
     CDS             complement(55563..55910)
                     /label="kfrB"
     CDS             53698..54135
                     /label="traM"
     CDS             52572..52976
                     /label="traK"
     CDS             complement(58291..59367)
                     /label="korB"
     CDS             complement(45068..47281)
                     /label="traE"
     CDS             complement(60140..60445)
                     /label="korA"
     terminator      15648..15709
                     /label="T7 terminator"
     CDS             complement(3193..3411)
                     /label="kleD"
     misc_RNA        15587..15646
                     /label="tracrRNA"
     CDS             complement(4813..5439)
                     /label="klcB"
     CDS             complement(3427..3657)
                     /label="kleC"
     CDS             29233..29442
                     /label="trbK"
     CDS             complement(51963..52334)
                     /label="traJ"
     promoter        15447..15500
                     /label="pTet promoter"
     CDS             39265..40062
                     /label="istB"
```

CDS complement(49730..51928)
/label="traI"
promoter 10370..10672
/label="pBAD promoter"
CDS 33894..34589
/label="fiwA"
CDS 38093..39265
/label="istA1"
CDS complement(40853..41143)
/label="traA"
CDS complement(join(60619..61203,1..369))
/label="klaC"
CDS complement(36461..36754)
/label="parC"
CDS 20671..21567
/label="trbB"
CDS complement(366..1502)
/label="klaB"
CDS complement(2758..3081)
/label="kleE"
CDS complement(50029..50388)
/label="traH"
CDS 37503..38006
/label="putative ORF"
CDS 32709..33443
/label="trbP"
CDS complement(44799..45062)
/label="traD"
CDS complement(41607..43847)
/label="traC2"
CDS complement(59364..60143)
/label="incC2"
CDS complement(41607..44792)
/label="traC1"
CDS complement(35931..36776)
/label="parB"
CDS 21580..22017
/label="trbC"
CDS complement(57180..57707)
/label="korG"
misc_feature 15715..17093
/label="Cen6-ArsH4-His3"
CDS 16407..17069
/label="his3"
CDS complement(56068..56994)
/label="kfrA"
CDS 52976..53701
/label="traL"
CDS complement(9463..10341)
/label="araC"
CDS complement(4074..4307)
/label="kleA"
CDS 20026..20337

/label="trbA"
CDS 22020..22331
/label="trbD"
CDS complement(19501..19851)
/label="ssb"
CDS 26550..27032
/label="trbH"
CDS 7501..8163
/label="pBBR1_rep_protein"
CDS complement(57717..58220)
/label="korF"
CDS complement(5962..6495)
/label="Gentamicin-3-N-acetyltransferase"
CDS 10714..15381
/label="TevSpCas9"
CDS 22328..24886
/label="trbE"
CDS complement(1520..2293)
/label="klaA"
CDS 31059..31658
/label="trbM"
misc_feature 15503..15570
/label="246 bp stuffer"
CDS 32409..32672
/label="trbO"
oriT 8597..9367
/label="oriT"
CDS complement(3810..4025)
/label="kleB"
CDS 37312..37464
/label="parE partial"
CDS 29449..31035
/label="trbL"
CDS 37153..37464
/label="parE"
CDS complement(41151..41591)
/label="traB"
CDS complement(59364..60458)
/label="incC"
CDS 24883..25641
/label="trbF"
CDS 36905..37156
/label="parD"
rep_origin 6730..7500
/label="pBBR1 oriV"
CDS complement(47296..47829)
/label="traF"
CDS 40193..40486
/label="putative_ORF"
CDS 25653..26546
/label="trbG"
CDS complement(35311..35970)
/label="parA1"

CDS complement(18304..19161)
/label="trfA2"
CDS complement(47826..49733)
/label="traG"
ORIGIN
1 ttcaccccg aacacgagca cggcacccgc gaccactatg ccaagaatgc ccAAGGTAAA
61 AATTGCCGGC CCCGCCATGA AGTCCGTGAA TGCCCCGACG GCCGAAGTGA AGGGCAGGCC
121 GCCACCCAGG CCGCCGCCCT CACTGCCCGG CACCTGGTCG CTGAATGTCG ATGCCAGCAC
181 CTGCGGCACG TCAATGCTTC CGGGCGTCGC GCTCGGGCTG ATCGCCCATC CCGTTACTGC
241 CCCGATCCCG GCAATGGCAA GGACTGCCAG CGCCGCGATG AGGAAGCGGG TGCCCCGCTT
301 CTTCATCTTC GCGCCTCGGG CCTCGAGGCC GCCTACCTGG GCGAAAACAT CGGTGTTTGT
361 GGCATTCATA CGGACTCCTG TTGGGCCAGC TCGCGCACGG GCTGGCGGGT CAGCTTGGCT
421 TGAAGATCGC CACGCATTGC GGCGATCTGC TTCTCGGCAT CCTTGCGCTT CTGCACGCCT
481 TCCTGCTGGA TGCGAATAAC GTCCTCGACG GTCTTGATGA GCGTCGTCTG AACCTGCTTG
541 AGCGTGTCCA CGTCGATCAC CAGGCGTTGG TTCTCCTTCG CCGTCTCGAC GGACGTGCGA
601 TGCAGCAGGG CCGCATTGCG CTTCATCAGG TCGTTGGTGG TGTCGTCGAT GGCCGTGGCC
661 AGTTCGACGG CGTTCTTCTG CTCGTTGAGG CTCAAGGCCA GCATGAATTG CCGCTTCCAC
721 GCCGGCACGG TGATTTCGCG GATGGTGTGG AATTTATCGA CCAGCATCTG GTTGTTGGCC
781 TGGATCATGC GGATGGTCGG CAGGCTCTGC ATGGCCGAAT GTTGCAAGGC GATCAGGTCG
841 CCGATGCGCT TGTCCAGGTT GGCAACCATC GCATCGAGGT CGGCCAGCTC CTGCACGCGG
901 CCCGGGTCGT TCCCGACATT GCCGCGCAGA CCCTCGGCCT GCTCGCGCAG CTCGGCAAGG
961 CGGACCTTGC CGGCCGCGAT GTGGACGCCA AGAAGGCGGT GTTCCTCGCG CACGGCTGCG
1021 AACATTTCGT CGAGCGAGGC ATTGCGCTGC GCGATGCCTT GCTGGGTGGT CTGCACTTCG
1081 CTGACCAGGT GTTCGATCTG CTCGCGGGTC GTGTCGAAGC GCGCCATGAA GCCCGTCGAA
1141 CGGACGCGGA AGCGGTCGAT CAGCGGGCCA ATCAGGGGCA GGCGGGAACG GTTGTCGGAC
1201 AAAGGGCCGA CGTTCAGGGA ACGGGCCTTG GCGACAACCT GGGTCAGTTT CTCGCCTGCT
1261 TCGTCCAGGT CGCTGTTGCG CACCTGGTCC AGCAGGCTAT CGGCGTAGCG GGACGTGTGC
1321 TCGGCCACGT CGCGGCCGAA CTCGGCAACG GTCTGCGGAC TGCCGACCTC GATCCGCTGC
1381 GCGACCGCAT GGACTTCCGG CACGTCGCTT TCCTGCAAGC CCAGCTCGCG CAGGGTTGCC
1441 GGGGTCATGT CGAAGGCGAC GATAGGGGCC TTGGCGTCGT GCGTCGTTTT CAGTGCGTTC
1501 ATAGGGTTCT CCCGCCGTGT TATTGGTTGA TGCCTTCCAG GCTCTGCGAA AGGCTCCGCA
1561 TGAGCGCCTG GTGAGCTTTG GCCGCCTCGG CGACCATTGC CGGATTCATG TTCTTGGTGG
1621 TGATGAGCGC GAGGGTGTGC TGACGCCAGA CGGGCACCAG GACGGATGCC GTTTCAGAGA
1681 AGCGGTCCAG CATGTCCACG GCCTGCGCCC GCGTGAGCTT CATCTGAGTG ACGCTCATTT
1741 CATGGGACGC CATGAGGGTT GCCAGGTTGG CGAGCTTGCG CGCGAAGCGT TCGCGCGGCT
1801 TGTCGAACTC GATCACGCCG GCCTTGGCCG CGCCGGCCTC GGGGTTCTCG TCCAGGAACT
1861 CGCGCCCGGC TTGAATGTAG GCTCTGAGCC GGTCTACCTC GGCCTCATGC GTATTGAGCA
1921 TGTCATCCAA GGCGCGCAAC GTGTCCCGCA CGCGCTGCGC TACGCCCTCG GCTTCGTCCA
1981 GCAACTGGTC GAGCGTCTTG CGGGCGACCT GATACCTCAC CTGGCGTTCA ACCTCACGGC
2041 CAAGCATCTT CTCGAACCAG GTAGGCTTTT CCGCGATCTT GCGGGGGTCC GCGTCGGCCA
2101 GCTTCGCCAC GATCTGGCTG ATTTTGTCGG CCAGCGCGGC AACTGCGCCG TGCTCCATCA
2161 GATTCGACAG CTCGTTGAGG GAATCCGCCC CGTCGATGCC GGCCCCGTAC TCGCCAATCG
2221 TCGCCGGCGA CGCGAAGAGG GCGGGCAAAA CCTCCCCCTT CAATCGCGCC ATGTTCACGC
2281 TTTGTTCTTC CATTCGATAC ACCCTCGCGG TGGGTTAATT GCTTTTCGAT GGAAGAAGTT
2341 TAGCTAAACT TTCTATCCCT CGTCAACACC TTTAGCCGCT AAAATTTGGG GACAGGTCAT
2401 TTACAGAAAG CCAGCTCACT CCTGGCGTTG CCCCTTGAGC GCCGCTAGGC GCGCAGCATC
2461 CTTCGCGCTG AGAAAGAACG TCATCAGCGG CCCGACCGTC TTGCTTGAAC CGTCGGCAAA
2521 GCAAACATCC ATCGAACAGC CTTGCGTGTG GGGGTCCACG CCTTCGACCA GTTTCCAAGG
2581 GTCCATGCCC CAGCCCTCGG CCTCCGGATT GAACCAGTAC GCCGATGCGT CGCCGTTTAG
2641 GTCGCTGTCG GCGTAGTCCT TGACCAGCAC GGCCACCCGC CCGGTCGTCG GGCACACGTA
2701 GCCCGGCTGC TTAGGTTCCT GTCTTGGCAT TGCTCAAAGC TCCTTGAAGG GGCCGCTCTA
2761 CAGCCCCTTG GGCTTGTAGA GCGACACGAA ATAGGTGAGT GCGGTCAGTA CCGCGAAATG
2821 CACCAGGAAC GTCCAGCCGG CATGAACGCC AAGGGTGTTC CAGTGGTACA GCATCCGCAG

2881 GAACTGAAAG AAAACGTCGA TAGAGATGAT CCACTTCGCC ACCGGCCACA CCAGGACAGT
2941 AACGACCCAT ACAAAGCGGA CCAGGGCCTG GACACCCTTG GCAAAAGTGA ACCGGGGCGG
3001 GGCCTTGCTC GGGGCCTCAA CGCGCGGGGC AGGGGCCTCG GCCTCCACTT CCACGCCTGG
3061 GAACTTGATA ATCTTCGACA TTGCTTGACC CTCCACGGCG ATGCGTGTTC AATTCGTCCA
3121 GCGCTCGCGC GCCTAGACCG TGATGTGACA GCATCGAGGT CAAGCGCCCC GGAGAAATCC
3181 GGGGCGTCAT CCCTATGCCC CGTCCAACTC GGGAACCGGC TTTTCCCTGG TGCTCAACCT
3241 GGCCGGCTCG ACCCACTTGG TGACTTGCTG CCACTCGTTA CCACTGCGAA CGGCTACCCG
3301 AATCTGCACA CGTTTAGCCG CGATCTTCGT CACTATGCCA GCAACACAAA CGGAATACCC
3361 GTACCCGCCG CGCGGGGTGT GCTGCCAGTT CACCCTATCT CCTACTTGCA TCATCATCCC
3421 CTGGCGTCAG TGACCGGCCC GGAATTTCGC CAAGTCGATT TCGTTGAAGG TCCAGCGCTG
3481 TTTCGCCCGT TCCTCCAACC TCGACGACTC CCGCATGACC TCGATGCGCA GGCGCTCGAC
3541 CTGGTGCATC AGCTCATCAG CGCGCCGCTG CTTCTCGGCA ATAGCATTCC GCTGCGCGAC
3601 CAGCTCCTGG TCTACGTTCG GCAGCTCGTC GATCCACGGC ATGAACTTAT CGGTCATCGG
3661 ATTGGCCTCC GGTAATTGAC CTGGGAATCT ACCCGGCCTC AAAACAAGAA TAGGGCATAA
3721 TGCCCTAACT TGTCAAGCAA TTTTAGCTAA ACAATTGAGG GGATTCAGCG AGGCGTCATG
3781 CTTGAAAACA CCTTTCCCCT GGCGTGCAAT CAGCTTGTCC CGGCGGCAGC GCACTGCCGC
3841 AGCGCGGCCA GCAAGGTGCC TTGCTCGATC CGCTGTCGTT CCTCCGGCGT AATGCAATCG
3901 CCGCAGATAC CGCCCAGCTC TTCGCGCAGG GCGTCCGCCC CGATCAGGCT GCGGCTAAGG
3961 GTCGAAATGG ACTTACCGCA GCGGCGGCAA TTGGTGGTGA CAATCTCGAT CTTGCGGTTG
4021 GGCATGGCCC TATCTCCTTG AGAGAGGCCC GACCGTAGCC GGGCCTCGTT CCGTTACCAG
4081 CCTGCGCGAG CCTTCGCGCG TTCGACTTCC TGGGTGGACC AGTGGCCCTT TGCTTCACCC
4141 TCCAGGGTGC AACCTTGCAG GTACGCCTTG TGCCGCAGCT CTTCGGCCTG GCGGGTAAGC
4201 TCTGCCGCCT GCTCCATCAG CGCCGCGATC TGGTCACGAC GGGCCAGAAA ATCCGTGGCG
4261 GCCGCGCCGG GCAGCTCATC GAGCCAAGAC ATGATCTTGC TTTTCATCGG GGTTGATCCT
4321 CCGGTTGCTG ACCTGGGCGA AGTGCCCGGC CTTGGATTGC TATATTAGGG CATTATGCCC
4381 TAGATAGTCA AGGAAATTTA GCTAAACAAT TTGCGGCGGG CGCACGAAAA AACCCGGCTT
4441 GCGGGCCGGG CTGCTGGCAG CATATCGCAA CGATCAGGGT TGGCGGTTTT AGCCGCTAAA
4501 GTCCTCTCCC TTGGCGTAAA GTCCTGCGGG CGTCAGCCCT GGCCTTTCCA GATCGCCCCA
4561 ATCCCCGCTA GATCGCAAAG GATCGCCCAG GCGGCATAGG GGATCGGCGA ATCCTCGCCA
4621 ACCCAACGCC GCACCGTGCG GTCGCCCTTG GCACCCAAGC CCAAGATGCG CGCAGCCTGT
4681 CCGCCGGTGA GGCCGGCCAA GTGCAAGACT TCCCGGATTT CTGCGCCGGT CGGCTGCACC
4741 CAGCGTTCCG CCGGGCGCAG GCACTCAAGC CGGATATTCA CGTCGCTCAT GCTGCTTTTC
4801 TCCTAATCGT TATCAAATGG CGGCCCGCAA TTGGTCGTAG CCGTAGCACG ACTCGATGCA
4861 ACGCGGGTCA TATTCACAAA CCTTTCTACC GTTGCGATTG ATGCGATAGC GCGGCGGCTT
4921 GTCGTCGTGC GCCCACACCT GTCGCGTTTC GGGACTGATC TTCGTAATGA TGACGTGCTT
4981 CCCGATTTCC TTGGCAAAAA CCGGGTGATG CACGCTCACA ATCTCGCACC GCAGGCCAGG
5041 AAAGAACGTC GCCGGCCACG CCGGCGCGTC CTCGTCCTTG GCCGGCTCCG GCTCCGGCTT
5101 GGCCGGTGTA ACCGGCTCCC TGCGCTGCCC GGCCGGCTCC TGAGCTTTCG CACGCGCGGC
5161 CGCGAGCTTC GCCTTGGCGC TGGCCGCCCA TTTGCGGGCG ATGAATGCCT GGTGCGCCGG
5221 CAGCACGGCA TCAACCCAGG CGAAGCCCGG CGGCAGTTCG TCCGGCTTCC GCCAGTCCTC
5281 GACCACGGCC CGCACGCGGC GCGGCGGTGG CGTGATACCC GCGAGCCACG CCGGCAGTGG
5341 CTCGTCGGCA AGGCGGTCCT GGTCGCGGGC GTCCAGATAC CGGCGATGCG AGCGCAACAG
5401 GGCGCGCAAA ACCCCGTCCG CTACCTGGTC CACCGTCATG CCGCCGCGCG CATGGTCGTA
5461 ATGGGACCGA TAGCCCGTTT CGGAAATAAA AGGGGTCTGA CGCTCAGTGG AACGAAAACT
5521 CACGTTAAGG GATTTTGGTC ATGAGATTAT CAAAAAGGAT CTTCACCTAG ATCCTTTTAA
5581 ATTAAAAATG AAGTTTTAAA TCAATCTAAA GTATATATGA GTAAACTTGG TCTGACAGTT
5641 ACCAATGCTT AATCAGTGAG GCACCTATCT CAGCGATCTG TCTATTTCGT TCATCCATAG
5701 TTGCCTGACT CCCCGTCGTG TAGATAACTA CGATACGGGA GGGCTTACCA TCTGGCCCCA
5761 GTGCTGCAAT GATACCGCGA GATCCACGCT CACCGGCTCC AGATTTATCA GCAATAAACC
5821 AGCCAGCCGG AAGGGCCGAG CGCAGAAGTG GTCCTGCAAC TTTATCCGCC TCCATCCAGT
5881 CTATTAATCC ACCTGGCGGC GTTGTGACAA TTTACCGAAC AACTCCGCGG CCGGGAAGCC
5941 GATCTCGGCT TGAACGAATT GTTAGGTGGC GGTACTTGGG TCGATATCAA AGTGCATCAC
6001 TTCTTCCCGT ATGCCCAACT TTGTATAGAG AGCCACTGCG GGATCGTCAC CGTAATCTGC

6061 TTGCACGTAG ATCACATAAG CACCAAGCGC GTTGGCCTCA TGCTTGAGGA GATTGATGAG
6121 CGCGGTGGCA ATGCCCTGCC TCCGGTGCTC TCCGGAGACT GCGAGATCAT AGATATAGAT
6181 CTCACTACGC GGCTGCTCAA ACTTGGGCAG AACGTAAGCC GCGAGAGCGC CAACAACCGC
6241 TTCTTGGTCG AAGGCAGCAA GCGCGATGAA TGTCTTACTA CGGAGCAAGT TCCCGAGGTA
6301 ATCGGAGTCC GGCTGATGTT GGGAGTAGGT GGCTACGTCT CCGAACTCAC GACCGAAAAG
6361 ATCAAGAGCA GCCCGCATGG ATTTGACTTG GTCAGGGCCG AGCCTACATG TGCGAATGAT
6421 GCCCATACTT GAGCCACCTA ACTTTGTTTT AGGGCGACTG CCCTGCTGCG TAACATCGTT
6481 GCTGCTGCGT AACATCGTTG CTGCTCCATA ACATCAAACA TCGACCCACG GCGTAACGCG
6541 CTTGCTGCTT GGATGCCCGA GGCATAGACT GTACAAAAAA ACAGTCATAA CAAGCCATGA
6601 AAACCGCCAC TGCGCCGTTA CCACCGCTGC GTTCGGTCAA GGTTCTGGAC CAGTTGCGTG
6661 AGCGCATACG CTACTTGCAT TACAGTTTAC GAACCGAACA GGCTTATGTC AACTGGGTTC
6721 GTGCCTTCAT CCGTTTCCAC GGTGTGCGTC CATGGGCAAA TATTATACGC AAGGCGACAA
6781 GGTGCTGATG CCGCTGGCGA TTCAGGTTCA TCATGCCGTT TGTGATGGCT TCCATGTCGG
6841 CAGGAATTCG AATTCATACC CACCGGCTCC AACTGCGCGG CCTGCGGCCT TGCCCCATCA
6901 ATTTTTTTAA TTTTCTCTGG GGAAAAGCCT CCGGCCTGCG GCCTGCGCGC TTCGCTTGCC
6961 GGTTGGACAC CAAGTGGAAG GCGGGTCAAG GCTCGCGCAG CGACCGCGCA GCGGCTTGGC
7021 CTTGACGCGC CTGGAACGAC CCAAGCCTAT GCGAGTGGGG GCAGTCGAAG GCGAAGCCC
7081 GCCCGCCTGC CCCCCGAGCC TCACGGCGGC GAGTGCGGGG GTTCCAAGGG GGCAGCGCCA
7141 CCTTGGGCAA GGCCGAAGGC CGCGCAGTCG ATCAACAAGC CCCGGAGGGG CCACTTTTTG
7201 CCGGAGGGGG AGCCGCGCCG AAGGCGTGGG GGAACCCCGC AGGGGTGCCC TTCTTTGGGC
7261 ACCAAAGAAC TAGATATAGG GCGAAATGCG AAAGACTTAA AAATCAACAA CTTAAAAAAG
7321 GGGGGTACGC AACAGCTCAT TGCGGCACCC CCCGCAATAG CTCATTGCGT AGGTTAAAGA
7381 AAATCTGTAA TTGACTGCCA CTTTTACGCA ACGCATAATT GTTGTCGCGC TGCCGAAAAG
7441 TTGCAGCTGA TTGCGCATGG TGCCGCAACC GTGCGGCACC CCTACCGCAT GGAGATAAGC
7501 ATGGCCACGC AGTCCAGAGA AATCGGCATT CAAGCCAAGA ACAAGCCCGG TCACTGGGTG
7561 CAAACGGAAC GCAAAGCGCA TGAGGCGTGG GCCGGGCTTA TTGCGAGGAA ACCCACGGCG
7621 GCAATGCTGC TGCATCACCT CGTGGCGCAG ATGGGCCACC AGAACGCCGT GGTGGTCAGC
7681 CAGAAGACAC TTTCCAAGCT CATCGGACGT TCTTTGCGGA CGGTCCAATA CGCAGTCAAG
7741 GACTTGGTGG CCGAGCGCTG GATCTCCGTC GTGAAGCTCA ACGGCCCCGG CACCGTGTCG
7801 GCCTACGTGG TCAATGACCG CGTGGCGTGG GGCCAGCCCC GCGACCAGTT GCGCCTGTCG
7861 GTGTTCAGTG CCGCCGTGGT GGTTGATCAC GACGACCAGG ACGAATCGCT GTTGGGGCAT
7921 GGCGACCTGC GCCGCATCCC GACCCTGTAT CCGGGCGAGC AGCAACTACC GACCGGCCCC
7981 GGCGAGGAGC CGCCCAGCCA GCCCGGCATT CCGGGCATGG AACCAGACCT GCCAGCCTTG
8041 ACCGAAACGG AGGAATGGGA ACGGCGCGGG CAGCAGCGCC TGCCGATGCC CGATGAGCCG
8101 TGTTTTCTGG ATGATGGCGA GCCGTTGGAG CCGCCGACAC GGGTCACGCT GCCGCGCCGG
8161 TAGCACTTGG GTTGCGCAGC AACCCGTAAG TGCGCTGTTC CAGACTATCG GCTGTAGCCG
8221 CCTCGCCGCC CTATACCTTG TCTGCCTCCC CGCGTTGCGT CGCGGTGCAT GGAGCCGGGC
8281 CACCTCGACC TGAATGGAAG CCGGCGGCAC CTCGCTAACG GATTCACCGT TTTTATCAGG
8341 CTCTGGGAGG CAGAATAAAT GATCATATCG TCAATTATTA CCTCCACGGG GAGAGCCTGA
8401 GCAAACTGGC CTCAGGCATT TGAGAAGCAC ACGGTCACAC TGCTTCCGGT AGTCAATAAA
8461 CCGGTAAACC AGCAATAGAC ATAAGCGGCT ATTTAACGAC CCTGCCCTGA ACCGACGACC
8521 GGGTCGAATT TGCTTTCGAA TTTCTGCCAT TCATCCGCTT ATTATCACTT ATTCAGGCGT
8581 AGCACCAGGC GTTTAACGAT CGTCTTCCTT GCTCGTCGGT GATGTACTTA CAGCTCGAAG
8641 TGCCTCTTCT TGATGGAGCG CATGGGGACG TGCTTGGCAA TCACGCGCAC CCCCCGGCCG
8701 TTTTAGCGGC TAAAAAAGTC ATGGCTCTGC CCTCGGGCGG ACCACGCCCA TCATGACCTT
8761 GCCAAGCTCG TCCTGCTTCT CTTCGATCTT CGCCAGCAGG GCGAGGATCG TGGCATCACC
8821 GAACCGCGCC GTGCGCGGGT CGTCGGTGAG CCAGAGTTTC AGCAGGCCGC CCAGGCGGCC
8881 CAGGTCGCCA TTGATGCGGG CCAGCTCGCG GACGTGCTCA TAGTCCACGA CGCCCGTGAT
8941 TTTGTAGCCC TGGCCGACGG CCAGCAGGTA GGCCGACAGG CTCATGCCGG CCGCCGCCGC
9001 CTTTTCCTCA ATCGCTCTTC GTTCGTCTGG AAGGCAGTAC ACCTTGATAG GTGGGCTGCC
9061 CTTCCTGGTT GGCTTGGTTT CATCAGCCAT CCGCTTGCCC TCATCTGTTA CGCCGGCGGT
9121 AGCCGGCCAG CCTCGCAGAG CAGGATTCCC GTTGAGCACC GCCAGGTGCG AATAAGGGAC
9181 AGTGAAGAAG GAACACCCGC TCGCGGGTGG GCCTACTTCA CCTATCCTGC CCGGCTGACG

9241 CCGTTGGATA CACCAAGGAA AGTCTACACG AACCCTTTGG CAAAATCCTG TATATCGTGC
9301 GAAAAAGGAT GGATATACCG AAAAAATCGC TATAATGACC CCGAAGCAGG GTTATGCAGC
9361 GGAAGATATC GATGCATAAT GTGCCTGTCA AATGGACGAA GCAGGGATTC TGCAAACCCT
9421 ATGCTACTCC GTCAAGCCGT CAATTGTCTG ATTCGTTACC AATTATGACA ACTTGACGGC
9481 TACATCATTC ACTTTTTCTT CACAACCGGC ACGGAACTCG CTCGGGCTGG CCCCGGTGCA
9541 TTTTTTAAAT ACCCGCGAGA AATAGAGTTG ATCGTCAAAA CCAACATTGC GACCGACGGT
9601 GGCGATAGGC ATCCGGGTGG TGCTCAAAAG CAGCTTCGCC TGGCTGATAC GTTGGTCCTC
9661 GCGCCAGCTT AAGACGCTAA TCCCTAACTG CTGGCGGAAA AGATGTGACA GACGCGACGG
9721 CGACAAGCAA ACATGCTGTG CGACGCTGGC GATATCAAAA TTGCTGTCTG CCAGGTGATC
9781 GCTGATGTAC TGACAAGCCT CGCGTACCCG ATTATCCATC GGTGGATGGA GCGACTCGTT
9841 AATCGCTTCC ATGCGCCGCA GTAACAATTG CTCAAGCAGA TTTATCGCCA GCAGCTCCGA
9901 ATAGCGCCCT TCCCCTTGCC CGGCGTTAAT GATTTGCCCA AACAGGTCGC TGAAATGCGG
9961 CTGGTGCGCT TCATCCGGGC GAAAGAACCC CGTATTGGCA AATATTGACG GCCAGTTAAG
10021 CCATTCATGC CAGTAGGCGC GCGGACGAAA GTAAACCCAC TGGTGATACC ATTCGCGAGC
10081 CTCCGGATGA CGACCGTAGT GATGAATCTC TCCTGGCGGG AACAGCAAAA TATCACCCGG
10141 TCGGCAAACA AATTCTCGTC CCTGATTTTT CACCACCCCC TGACCGCGAA TGGTGAGATT
10201 GAGAATATAA CCTTTCATTC CCAGCGGTCG GTCGATAAAA AAATCGAGAT AACCGTTGGC
10261 CTCAATCGGC GTTAAACCCG CCACCAGATG GGCATTAAAC GAGTATCCCG GCAGCAGGGG
10321 ATCATTTTGC GCTTCAGCCA TACTTTTCAT ACTCCCGCCA TTCAGAGAAG AAACCAATTG
10381 TCCATATTGC ATCAGACATT GCCGTCACTG CGTCTTTTAC TGGCTCTTCT CGCTAACCAA
10441 ACCGGTAACC CCGCTTATTA AAAGCATTCT GTAACAAAGC GGGACCAAAG CCATGACAAA
10501 AACGCGTAAC AAAAGTGTCT ATAATCACGG CAGAAAAGTC CACATTGATT ATTTGCACGG
10561 CGTCACACTT TGCTATGCCA TAGCATTTTT ATCCATAAGA TTAGCGGATC CTACCTGACG
10621 CTTTTTATCG CAACTCTCTA CTGTTTCTCC ATACCCGTTT TTTTGGGCTA GCCCTGTAGA
10681 AATAATTGTT TAACTTTAAT AAGGAGATAT ACCATGGGTA AAAGCGGAAT TTATCAGATT
10741 AAAAATACTT TAAACAATAA AGTATATGTA GGAAGTGCTA AAGATTTTGA AAAGAGATGG
10801 AAGAGGCATT TTAAAGATTT AGAAAAGGGA TGCCATTCTT CTATAAAACT TCAGAGGTCT
10861 TTTAACAAAC ATGGTAATGT GTTTGAATGT TCTATTTTGG AAGAAATTCC ATATGAGAAA
10921 GATTTGATTA TTGAACGAGA AAATTTTTGG ATTAAAGAGC TTAATTCTAA AATTAATGGA
10981 TACAATATTG CTGATGCAAC GTTTGGTGAT ACGTGTTCTA CGCATCCATT AAAAGAAGAA
11041 ATTATTAAGA AACGTTCTGA AACTTTTAAA GCTAAGATGC TTAAACTTGG ACCTGATGGT
11101 CGGAAAGCTC TTTACAGTAA ACCCGGAAGT AAAAACGGGC GTTGGAATCC AGAAACCCAT
11161 AAGTTTTGTA AGTGCGGTGT TCGCATACAA ACTTCTGCTT ATACTTGTAG TAAATGCAGA
11221 AATGGTGGTT CTGGTGGTAC CGGAGGTAGC ATGGATAAAA AGTATTCTAT TGGTTTAGAC
11281 ATCGGCACTA ATTCCGTTGG ATGGGCTGTC ATAACCGATG AATACAAAGT ACCTTCAAAG
11341 AAATTTAAGG TGTTGGGGAA CACAGACCGT CATTCGATTA AAAAGAATCT TATCGGTGCC
11401 CTCCTATTCG ATAGTGGCGA AACGGCAGAG GCGACTCGCC TGAAACGAAC CGCTCGGAGA
11461 AGGTATACAC GTCGCAAGAA CCGAATATGT TACTTACAAG AAATTTTTAG CAATGAGATG
11521 GCCAAAGTTG ACGATTCTTT CTTTCACCGT TTGGAAGAGT CCTTCCTTGT CGAAGAGGAC
11581 AAGAAACATG AACGGCACCC CATCTTTGGA AACATAGTAG ATGAGGTGGC ATATCATGAA
11641 AAGTACCCAA CGATTTATCA CCTCAGAAAA AAGCTAGTTG ACTCAACTGA TAAAGCGGAC
11701 CTGAGGTTAA TCTACTTGGC TCTTGCCCAT ATGATAAAGT TCCGTGGGCA CTTTCTCATT
11761 GAGGGTGATC TAAATCCGGA CAACTCGGAT GTCGACAAAC TGTTCATCCA GTTAGTACAA
11821 ACCTATAATC AGTTGTTTGA AGAGAACCCT ATAAATGCAA GTGGCGTGGA TGCGAAGGCT
11881 ATTCTTAGCG CCCGCCTCTC TAAATCCCGA CGGCTAGAAA ACCTGATCGC ACAATTACCC
11941 GGAGAGAAGA AAAATGGGTT GTTCGGTAAC CTTATAGCGC TCTCACTAGG CCTGACACCA
12001 AATTTTAAGT CGAACTTCGA CTTAGCTGAA GATGCCAAAT TGCAGCTTAG TAAGGACACG
12061 TACGATGACG ATCTCGACAA TCTACTGGCA CAAATTGGAG ATCAGTATGC GGACTTATTT
12121 TTGGCTGCCA AAAACCTTAG CGATGCAATC CTCCTATCTG ACATACTGAG AGTTAATACT
12181 GAGATTACCA AGGCGCCGTT ATCCGCTTCA ATGATCAAAA GGTACGATGA ACATCACCAA
12241 GACTTGACAC TTCTCAAGGC CCTAGTCCGT CAGCAACTGC CTGAGAAATA TAAGGAAATA
12301 TTCTTTGATC AGTCGAAAAA CGGGTACGCA GGTTATATTG ACGGCGGAGC GAGTCAAGAG
12361 GAATTCTACA AGTTTATCAA ACCCATATTA GAGAAGATGG ATGGGACGGA AGAGTTGCTT

12421 GTAAAACTCA ATCGCGAAGA TCTACTGCGA AAGCAGCGGA CTTTCGACAA CGGTAGCATT
12481 CCACATCAAA TCCACTTAGG CGAATTGCAT GCTATACTTA GAAGGCAGGA GGATTTTTAT
12541 CCGTTCCTCA AAGACAATCG TGAAAAGATT GAGAAAATCC TAACCTTTCG CATACCTTAC
12601 TATGTGGGAC CCCTGGCCCG AGGGAACTCT CGGTTCGCAT GGATGACAAG AAAGTCCGAA
12661 GAAACGATTA CTCCCTGGAA TTTTGAGGAA GTTGTCGATA AAGGTGCGTC AGCTCAATCG
12721 TTCATCGAGA GGATGACCAA CTTTGACAAG AATTTACCGA ACGAAAAAGT ATTGCCTAAG
12781 CACAGTTTAC TTTACGAGTA TTTCACAGTG TACAATGAAC TCACGAAAGT TAAGTATGTC
12841 ACTGAGGGCA TGCGTAAACC CGCCTTTCTA AGCGGAGAAC AGAAGAAAGC AATAGTAGAT
12901 CTGTTATTCA AGACCAACCG CAAAGTGACA GTTAAGCAAT TGAAAGAGGA CTACTTTAAG
12961 AAAATTGAAT GCTTCGATTC TGTCGAGATC TCCGGGGTAG AAGATCGATT TAATGCGTCA
13021 CTTGGTACGT ATCATGACCT CCTAAAGATA ATTAAAGATA AGGACTTCCT GGATAACGAA
13081 GAGAATGAAG ATATCTTAGA AGATATAGTG TTGACTCTTA CCCTCTTTGA AGATCGGGAA
13141 ATGATTGAGG AAAGACTAAA AACATACGCT CACCTGTTCG ACGATAAGGT TATGAAACAG
13201 TTAAAGAGGC GTCGCTATAC GGGCTGGGGA CGATTGTCGC GGAAACTTAT CAACGGGATA
13261 AGAGACAAGC AAAGTGGTAA AACTATTCTC GATTTTCTAA AGAGCGACGG CTTCGCCAAT
13321 AGGAACTTTA TGCAGCTGAT CCATGATGAC TCTTTAACCT TCAAAGAGGA TATACAAAAG
13381 GCACAGGTTT CCGGACAAGG GGACTCATTG CACGAACATA TTGCGAATCT TGCTGGTTCG
13441 CCAGCCATCA AAAAGGGCAT ACTCCAGACA GTCAAAGTAG TGGATGAGCT AGTTAAGGTC
13501 ATGGGACGTC ACAAACCGGA AAACATTGTA ATCGAGATGG CACGCGAAAA TCAAACGACT
13561 CAGAAGGGGC AAAAAAACAG TCGAGAGCGG ATGAAGAGAA TAGAAGAGGG TATTAAAGAA
13621 CTGGGCAGCC AGATCTTAAA GGAGCATCCT GTGGAAAATA CCCAATTGCA GAACGAGAAA
13681 CTTTACCTCT ATTACCTACA AAATGGAAGG GACATGTATG TTGATCAGGA ACTGGACATA
13741 AACCGTTTAT CTGATTACGA CGTCGATCAC ATTGTACCCC AATCCTTTTT GAAGGACGAT
13801 TCAATCGACA ATAAAGTGCT TACACGCTCG GATAAGAACC GAGGGAAAAG TGACAATGTT
13861 CCAAGCGAGG AAGTCGTAAA GAAAATGAAG AACTATTGGC GGCAGCTCCT AAATGCGAAA
13921 CTGATAACGC AAAGAAAGTT CGATAACTTA ACTAAAGCTG AGAGGGGTGG CTTGTCTGAA
13981 CTTGACAAGG CCGGATTTAT TAAACGTCAG CTCGTGGAAA CCCGCCAAAT CACAAAGCAT
14041 GTTGCACAGA TACTAGATTC CCGAATGAAT ACGAAATACG ACGAGAACGA TAAGCTGATT
14101 CGGGAAGTCA AAGTAATCAC TTTAAAGTCA AAATTGGTGT CGGACTTCAG AAAGGATTTT
14161 CAATTCTATA AAGTTAGGGA GATAAATAAC TACCACCATG CGCACGACGC TTATCTTAAT
14221 GCCGTCGTAG GGACCGCACT CATTAAGAAA TACCCGAAGC TAGAAAGTGA GTTTGTGTAT
14281 GGTGATTACA AAGTTTATGA CGTCCGTAAG ATGATCGCGA AAAGCGAACA GGAGATAGGC
14341 AAGGCTACAG CCAAATACTT CTTTTATTCT AACATTATGA ATTTCTTTAA GACGGAAATC
14401 ACTCTGGCAA ACGGAGAGAT ACGCAAACGA CCTTTAATTG AAACCAATGG GGAGACAGGT
14461 GAAATCGTAT GGGATAAGGG CCGGGACTTC GCGACGGTGA GAAAAGTTTT GTCCATGCCC
14521 CAAGTCAACA TAGTAAAGAA AACTGAGGTG CAGACCGGAG GGTTTTCAAA GGAATCGATT
14581 CTTCCAAAAA GGAATAGTGA TAAGCTCATC GCTCGTAAAA AGGACTGGGA CCCGAAAAAG
14641 TACGGTGGCT TCGATAGCCC TACAGTTGCC TATTCTGTCC TAGTAGTGGC AAAAGTTGAG
14701 AAGGGAAAAT CCAAGAAACT GAAGTCAGTC AAAGAATTAT TGGGGATAAC GATTATGGAG
14761 CGCTCGTCTT TTGAAAAGAA CCCCATCGAC TTCCTTGAGG CGAAAGGTTA CAAGGAAGTA
14821 AAAAAGGATC TCATAATTAA ACTACCAAAG TATAGTCTGT TTGAGTTAGA AAATGGCCGA
14881 AAACGGATGT TGGCTAGCGC CGGAGAGCTT CAAAAGGGGA ACGAACTCGC ACTACCGTCT
14941 AAATACGTGA ATTTCCTGTA TTTAGCGTCC CATTACGAGA AGTTGAAAGG TTCACCTGAA
15001 GATAACGAAC AGAAGCAACT TTTTGTTGAG CAGCACAAAC ATTATCTCGA CGAAATCATA
15061 GAGCAAATTT CGGAATTCAG TAAGAGAGTC ATCCTAGCTG ATGCCAATCT GGACAAAGTA
15121 TTAAGCGCAT ACAACAAGCA CAGGGATAAA CCCATACGTG AGCAGGCGGA AAATATTATC
15181 CATTTGTTTA CTCTTACCAA CCTCGGCGCT CCAGCCGCAT TCAAGTATTT TGACACAACG
15241 ATAGATCGCA AACGATACAC TTCTACCAAG GAGGTGCTAG ACGCGACACT GATTCACCAA
15301 TCCATCACGG GATTATATGA AACTCGGATA GATTTGTCAC AGCTTGGGGG TGACGGATCC
15361 CATCATCACC ACCACCATTG AGCGGCCGCA TAATGCTTAA GTCGAACAGA AAGTAATCGT
15421 ATTGTACACG GCCGCATAAT CGAAATTCCC TATCAGTGAT AGAGATTGAC ATCCCTATCA
15481 GTGATAGAGA TACTGAGCAC GGGAGACCCA TGCCATAGCG TTGTTCGGAA TATGAATTTT
15541 TGAACAGATT CACCAACACC TAGTGGTCTC GTTTTAGAGC TAGAAATAGC AAGTTAAAAT

15601 AAGGCTAGTC CGTTATCAAC TTGAAAAAGT GGCACCGAGT CGGTGCTCCG CTGAGCAATA
15661 ACTAGCATAA CCCCTTGGGG CCTCTAAACG GGTCTTGAGG GGTTTTTTGG CGAGCATCAC
15721 GTGCTATAAA AATAATTATA ATTTAAATTT TTTAATATAA ATATATAAAT TAAAAATAGA
15781 AAGTAAAAAA AGAAATTAAA GAAAAAATAG TTTTTGTTTT CCGAAGATGT AAAAGACTCT
15841 AGGGGGATCG CCAACAAATA CTACCTTTTA CCTTGCTCTT CCTGCTCTCA GGTATTAATG
15901 CCGAATTGTT TCATCTTGTC TGTGTAGAAG ACCACACACG AAAATCCTGT GATTTTACAT
15961 TTTACTTATC GTTAATCGAA TGTATATCTA TTTAATCTGC TTTTCTTGTC TAATAAATAT
16021 ATATGTAAAG TACGCTTTTT GTTGAAATTT TTTAAACCTT TGTTTATTTT TTTTTCTTCA
16081 TTCCGTAACT CTTCTACCTT CTTTATTTAC TTTCTAAAAT CCAAATACAA AACATAAAAA
16141 TAAATAAACA CAGAGTAAAT TCCCAAATTA TTCCATCATT AAAAGATACG AGGCGCGTGT
16201 AAGTTACAGG CAAGCGATCC TAGTACACTC TATATTTTTT TATGCCTCGG TAATGATTTT
16261 CATTTTTTTT TTCCACCTAG CGGATGACTC TTTTTTTTTC TTAGCGATTG GCATTATCAC
16321 ATAATGAATT ATACATTATA TAAAGTAATG TGATTTCTTC GAAGAATATA CTAAAAAATG
16381 AGCAGGCAAG ATAAACGAAG GCAAAGATGA CAGAGCAGAA AGCCCTAGTA AAGCGTATTA
16441 CAAATGAAAC CAAGATTCAG ATTGCGATCT CTTTAAAGGG TGGTCCCCTA GCGATAGAGC
16501 ACTCGATCTT CCCAGAAAAA GAGGCAGAAG CAGTAGCAGA ACAGGCCACA CAATCGCAAG
16561 TGATTAACGT CCACACAGGT ATAGGGTTTC TGGACCATAT GATACATGCT CTGGCCAAGC
16621 ATTCCGGCTG GTCGCTAATC GTTGAGTGCA TTGGTGACTT ACACATAGAC GACCATCACA
16681 CCACTGAAGA CTGCGGGATT GCTCTCGGTC AAGCTTTTAA AGAGGCCCTA GGGGCCGTGC
16741 GTGGAGTAAA AAGGTTTGGA TCAGGATTTG CGCCTTTGGA TGAGGCACTT TCCAGAGCGG
16801 TGGTAGATCT TTCGAACAGG CCGTACGCAG TTGTCGAACT TGGTTTGCAA AGGGAGAAAG
16861 TAGGAGATCT CTCTTGCGAG ATGATCCCGC ATTTTCTTGA AAGCTTTGCA GAGGCTAGCA
16921 GAATTACCCT CCACGTTGAT TGTCTGCGAG GCAAGAATGA TCATCACCGT AGTGAGAGTG
16981 CGTTCAAGGC TCTTGCGGTT GCCATAAGAG AAGCCACCTC GCCCAATGGT ACCAACGATG
17041 TTCCCTCCAC CAAAGGTGTT CTTATGTAGT TTTACACAGG AGTCTGGACT TGACTAGGAG
17101 TGCGGTTGGA ACGTTGGCCC AGCCAGATAC TCCCGATCAC GAGCAGGACG CCGATGATTT
17161 GAAGCGCACT CAGCGTCTGA TCCAAGAACA ACCATCCTAG CAACACGGCG GTCCCCGGGC
17221 TGAGAAAGCC CAGTAAGGAA ACAACTGTAG GTTCGAGTCG CGAGATCCCC CGGAACCAAA
17281 GGAAGTAGGT TAAACCCGCT CCGATCAGGC CGAGCCACGC CAGGCCGAGA ACATTGGTTC
17341 CTGTAGGCAT CGGGATTGGC GGATCAAACA CTAAAGCTAC TGGAACGAGC AGAAGTCCTC
17401 CGGCCGCCAG TTGCCAGGCG GTAAAGGTGA GCAGAGGCAC GGGAGGTTGC CACTTGCGGG
17461 TCAGCACGGT TCCGAACGCC ATGGAAACCG CCCCCGCCAG GCCCGCTGCG ACGCCGACAG
17521 GATCTAGCGC TGCGTTTGGT GTCAACACCA ACAGCGCCAC GCCCGCAGTT CCGCAAATAG
17581 CCCCCAGGAC CGCCATCAAT CGTATCGGGC TACCTAGCAG AGCGGCAGAG ATGAACACGA
17641 CCATCAGCGG CTGCACAGCG CCTACCGTCG CCGCGACCCC GCCCGGCAGG CGGTAGACCG
17701 AAATAAACAA CAAGCTCCAG AATAGCGAAA TATTAAGTGC GCCGAGGATG AAGATGCGCA
17761 TCCACCAGAT TCCCGTTGGA ATCTGTCGGA CGATCATCAC GAGCAATAAA CCCGCCGGCA
17821 ACGCCCGCAG CAGCATACCG GCGACCCCTC GGCCTCGCTG TTCGGGCTCC ACGAAAACGC
17881 CGGACAGATG CGCCTTGTGA GCGTCCTTGG GGCCGTCCTC CTGTTTGAAG ACCGACAGCC
17941 CAATGATCTC GCCGTCGATG TAGGCGCCGA ATGCCACGGC ATCTCGCAAC CGTTCAGCGA
18001 ACGCCTCCAT GGGCTTTTTC TCCTCGTGCT CGTAAACGGA CCCGAACATC TCTGGAGCTT
18061 TCTTCAGGGC CGACAATCGG ATCTCGCGGA AATCCTGCAC GTCGGCCGCT CCAAGCCGTC
18121 GAATCTGAGC CTTAATCACA ATTGTCAATT TTAATCCTCT GTTTATCGGC AGTTCGTAGA
18181 GCGCGCCGTG CGTCCCGAGC GATACTGAGC GAAGCAAGTG CGTCGAGCAG TGCCCGCTTG
18241 TTCCTGAAAT GCCAGTAAAG CGCTGGCTGC TGAACCCCCA GCCGGAACTG ACCCCACAAG
18301 GCCCTAGCGT TTGCAATGCA CCAGGTCATC ATTGACCCAG GCGTGTTCCA CCAGGCCGCT
18361 GCCTCGCAAC TCTTCGCAGG CTTCGCCGAC CTGCTCGCGC CACTTCTTCA CGCGGGTGGA
18421 ATCCGATCCG CACATGAGGC GGAAGGTTTC CAGCTTGAGC GGGTACGGCT CCCGGTGCGA
18481 GCTGAAATAG TCGAACATCC GTCGGGCCGT CGGCGACAGC TTGCGGTACT TCTCCCATAT
18541 GAATTTCGTG TAGTGGTCGC CAGCAAACAG CACGACGATT TCCTCGTCGA TCAGGACCTG
18601 GCAACGGGAC GTTTTCTTGC CACGGTCCAG GACGCGGAAG CGGTGCAGCA GCGACACCGA
18661 TTCCAGGTGC CCAACGCGGT CGGACGTGAA GCCCATCGCC GTCGCCTGTA GGCGCGACAG
18721 GCATTCCTCG GCCTTCGTGT AATACCGGCC ATTGATCGAC CAGCCCAGGT CCTGGCAAAG

18781 CTCGTAGAAC GTGAAGGTGA TCGGCTCGCC GATAGGGGTG CGCTTCGCGT ACTCCAACAC
18841 CTGCTGCCAC ACCAGTTCGT CATCGTCGGC CCGCAGCTCG ACGCCGGTGT AGGTGATCTT
18901 CACGTCCTTG TTGACGTGGA AAATGACCTT GTTTTGCAGC GCCTCGCGCG GGATTTTCTT
18961 GTTGCGCGTG GTGAACAGGG CAGAGCGGGC CGTGTCGTTT GGCATCGCTC GCATCGTGTC
19021 CGGCCACGGC GCAATATCGA ACAAGGAAAG CTGCATTTCC TTGATCTGCT GCTTCGTGTG
19081 TTTCAGCAAC GCGGCCTGCT TGGCCTCGCT GACCTGTTTT GCCAGGTCCT CGCCGGCGGT
19141 TTTTCGCTTC TTGGTCGTCA TAGTTCCTCG CGTGTCGATG GTCATCGACT TCGCCAAACC
19201 TGCCGCCTCC TGTTCGAGAC GACGCGAACG CTCCACGGCG GCCGATGGCG CGGGCAGGGC
19261 AGGGGGAGCC AGTTGCACGC TGTCGCGCTC GATCTTGGCC GTAGCTTGCT GGACCATCGA
19321 GCCGACGGAC TGGAAGGTTT CGCGGGGCGC ACGCATGACG GTGCGGCTTG CGATGGTTTC
19381 GGCATCCTCG GCGGAAAACC CCGCGTCGAT CAGTTCTTGC CTGTATGCCT TCCGGTCAAA
19441 CGTCCGATTC ATTCACCCTC CTTGCGGGAT TGCCCCGACT CACGCCGGGG CAATGTGCCC
19501 TTATTCCTGA TTTGACCCGC CTGGTGCCTT GGTGTCCAGA TAATCCACCT TATCGGCAAT
19561 GAAGTCGGTC CCGTAGACCG TCTGGCCGTC CTTCTCGTAC TTGGTATTCC GAATCTTGCC
19621 CTGCACGAAT ACCAGCGACC CCTTGCCCAA ATACTTGCCG TGGGCCTCGG CCTGAGAGCC
19681 AAAACACTTG ATGCGGAAGA AGTCGGTGCG CTCCTGCTTG TCGCCGGCAT CGTTGCGCCA
19741 CTCTTCATTA ACCGCTATAT CGAAAATTGC TTGCGGCTTG TTAGAATTGC CATGACGTAC
19801 CTCGGTGTCA CGGGTAAGAT TACCGATAAA CTGGAACTGA TTATGGCTCA TATCGAAAGT
19861 CTCCTTGAGA AAGGAGACTC TAGTTTAGCT AAACATTGGT TCCGCTGTCA AGAACTTTAG
19921 CGGCTAAAAT TTTGCGGGCC GCGACCAAAG GTGCGAGGGG CGGCTTCCGC TGTGTACAAC
19981 CAGATATTTT TCACCAACAT CCTTCGTCTG CTCGATGAGC GGGGCATGAC GAAACATGAG
20041 CTGTCGGAGA GGGCAGGGGT TTCAATTTCG TTTTTATCAG ACTTAACCAA CGGTAAGGCC
20101 AACCCCTCGT TGAAGGTGAT GGAGGCCATT GCCGACGCCC TGGAAACTCC CCTACCTCTT
20161 CTCCTGGAGT CCACCGACCT TGACCGCGAG GCACTCGCGG AGATTGCGGG TCATCCTTTC
20221 AAGAGCAGCG TGCCGCCCGG ATACGAACGC ATCAGTGTGG TTTTGCCGTC ACATAAGGCG
20281 TTTATCGTAA AGAAATGGGG CGACGACACC CGAAAAAAGC TGCGTGGAAG GCTCTGACGC
20341 CAAGGGTTAG GGCTTGCACT TCCTTCTTTA GCCGCTAAAA CGGCCCCTTC TCTGCGGGCC
20401 GTCGGCTCGC GCATCATATC GACATCCTCA ACGGAAGCCG TGCCGCGAAT GGCATCGGGC
20461 GGGTGCGCTT TGACAGTTGT TTTCTATCAG AACCCCTACG TCGTGCGGTT CGATTAGCTG
20521 TTTGTCTTGC AGGCTAAACA CTTTCGGTAT ATCGTTTGCC TGTGCGATAA TGTTGCTAAT
20581 GATTTGTTGC GTAGGGGTTA CTGAAAAGTG AGCGGGAAAG AAGAGTTTCA GACCATCAAG
20641 GAGCGGGCCA AGCGCAAGCT GGAACGCGAC ATGGGTGCGG ACCTGTTGGC CGCGCTCAAC
20701 GACCCGAAAA CCGTTGAAGT CATGCTCAAC GCGGACGGCA AGGTGTGGCA CGAACGCCTT
20761 GGCGAGCCGA TGCGGTACAT CTGCGACATG CGGCCCAGCC AGTCGCAGGC GATTATAGAA
20821 ACGGTGGCCG GATTCCACGG CAAAGAGGTC ACGCGGCATT CGCCCATCCT GGAAGGCGAG
20881 TTCCCCTTGG ATGGCAGCCG CTTTGCCGGC CAATTGCCGC CGGTCGTGGC CGCGCCAACC
20941 TTTGCGATCC GCAAGCGCGC GGTCGCCATC TTCACGCTGG AACAGTACGT CGAGGCGGGC
21001 ATCATGACCC GCGAGCAATA CGAGGTCATT AAAAGCGCCG TCGCGGCGCA TCGAAACATC
21061 CTCGTCATTG GCGGTACTGG CTCGGGCAAG ACCACGCTCG TCAACGCGAT CATCAATGAA
21121 ATGGTCGCCT TCAACCCGTC TGAGCGCGTC GTCATCATCG AGGACACCGG CGAAATCCAG
21181 TGCGCCGCAG AGAACGCCGT CCAATACCAC ACCAGCATCG ACGTCTCGAT GACGCTGCTG
21241 CTCAAGACAA CGCTGCGTAT GCGCCCCGAC CGCATCCTGG TCGGTGAGGT ACGTGGCCCC
21301 GAAGCCCTTG ATCTGTTGAT GGCCTGGAAC ACCGGGCATG AAGGAGGTGC CGCCACCCTG
21361 CACGCAAACA ACCCCAAAGC GGGCCTGAGC CGGCTCGCCA TGCTTATCAG CATGCACCCG
21421 GATTCACCGA AACCCATTGA GCCGCTGATT GGCGAGGCGG TTCATGTGGT CGTCCATATC
21481 GCCAGGACCC CTAGCGGCCG TCGAGTGCAA GAAATTCTCG AAGTTCTTGG TTACGAGAAC
21541 GGCCAGTACA TCACCAAAAC CCTGTAAGGA GTATTTCCAA TGACAACGGC TGTTCCGTTC
21601 CGTCTGACCA TGAATCGCGG CATTTTGTTC TACCTTGCCG TGTTCTTCGT TCTCGCTCTC
21661 GCGTTATCCG CGCATCCGGC GATGGCCTCG GAAGGCACCG GCGGCAGCTT GCCATATGAG
21721 AGCTGGCTGA CGAACCTGCG CAACTCCGTA ACCGGCCCGG TGGCCTTCGC GCTGTCCATC
21781 ATCGGCATCG TCGTCGCCGG CGGCGTGCTG ATCTTCGGCG GCGAACTCAA CGCCTTCTTC
21841 CGAACCCTGA TCTTCCTGGT TCTGGTGATG GCGCTGCTGG TCGGCGCGCA GAACGTGATG
21901 AGCACCTTCT TCGGTCGTGG TGCCGAAATC GCGGCCCTCG GCAACGGGGC GCTGCACCAG

21961 GTGCAAGTCG CGGCGGCGGA TGCCGTGCGT GCGGTAGCGG CTGGACGGCT CGCCTAATCA
22021 TGGCTCTGCG CACGATCCCC ATCCGTCGCG CAGGCAACCG AGAAAACCTG TTCATGGGTG
22081 GTGATCGTGA ACTGGTGATG TTCTCGGGCC TGATGGCGTT TGCGCTGATT TTCAGCGCCC
22141 AAGAGCTGCG GGCCACCGTG GTCGGTCTGA TCCTGTGGTT CGGGGCGCTC TATGCGTTCC
22201 GAATCATGGC GAAGGCCGAT CCGAAGATGC GGTTCGTGTA CCTGCGTCAC CGCCGGTACA
22261 AGCCGTATTA CCCGGCCCGC TCGACCCCGT TCCGCGAGAA CACCAATAGC CAAGGGAAGC
22321 AATACCGATG ATCCAAGCAA TTGCGATTGC AATCGCGGGC CTCGGCGCGC TTCTGTTGTT
22381 CATCCTCTTT GCCCGCATCC GCGCGGTCGA TGCCGAACTG AAACTGAAAA AGCATCGTTC
22441 CAAGGACGCC GGCCTGGCCG ATCTGCTCAA CTACGCCGCT GTCGTCGATG ACGGCGTAAT
22501 CGTGGGCAAG AACGGCAGCT TTATGGCTGC CTGGCTGTAC AAGGGCGATG ACAACGCAAG
22561 CAGCACCGAC CAGCAGCGCG AAGTAGTGTC CGCCCGCATC AACCAGGCCC TCGCGGGCCT
22621 GGGAAGTGGG TGGATGATCC ATGTGGACGC CGTGCGGCGT CCTGCTCCGA ACTACGCGGA
22681 GCGGGGCCTG TCGGCGTTCC CTGACCGTCT GACGGCAGCG ATTGAAGAAG AGCGCCGGCG
22741 GCATTTCGAG AGCCTGGGAA CGATGTACGA GGGCTATTTC GTCCTCACCT TGACCTGGTT
22801 CCCGCCGCTG CTCGCCCAGC GCAAGTTCGT CGAGCTGATG TTTGACGACG ACGCGACCGC
22861 ACCGGATCGC AAGGCGCGCA CGCGGGGCCT CATCGACCAA TTCAAGCGTG ACGTGCGCAG
22921 CATCGAGTCG CGCCTGTCGT CGGCCGTGTC GCTCACTCGC TTGAAGGGGC ACAAGATCGT
22981 CAACGAGGAC GGCACGACCG TCACGCATGA CGACTTCCTG CGCTGGCTGC AATTCTGCGT
23041 GACGGGCCTG CACCATCCGG TGCAGCTCCC CAGCAACCCG ATGTACCTGG ACGCCCTGGT
23101 CGGCGGACAG GAAATGTGGG GCGGGGTAGT GCCCAAGGTC GGCCGCAAGT TCGTCCAGGT
23161 GGTCGCTCTC GAAGGCTTCC CCTTGGAGTC CTATCCCGGC ATCCTGACGG CGCTCGGCGA
23221 GCTGCCCTGC GAGTATCGGT GGTCGAGCCG GTTCATCTTC ATGGACCAGC ACGAAGCCGT
23281 GAAGCACCTC GACAAGTTCC GCAAGAAGTG GCGGCAGAAG ATTCGCGGCT TCTTCGACCA
23341 GGTGTTCAAC ACGAACACCG GCCCGGTCGA TCAGGACGCG CTTTCGATGG TGGCCGATGC
23401 TGAGGCGGCC ATTGCCGAAG TCAACAGCGG CATCGTGGCC GTGGGCTACT ACACCAGCGT
23461 CGTCGTGCTG ATGGATGAGG ACCGCACGCG CCTGGAAGCT GCGGCCCGCG ATGTTGAAAA
23521 GGCCGTCAAC CGGTTGGGCT TTGCCGCGCG CATCGAGTCC ATCAACACCC TGGACGCCTT
23581 CCTTGGTAGT TTGCCGGGCC ACGGCGTGGA AAACGTCCGC CGGCCGCTCA TCAACACGAT
23641 GAACCTGGCC GACCTGCTGC CGACCAGCAC CATCTGGACC GGCAACGCGA ACGCGCCATG
23701 CCCGATGTAC CCGCCGCTGT CGCCGGCGCT CATGCACTGC GTCACGCAAG GATCAACGCC
23761 GTTCCGGCTG AACCTGCACG TGCGCGACCT CGGCCACACC TTTATGTTCG GGCCGACCGG
23821 CGCAGGTAAA TCGACGCACC TGGCGATCCT CGCCGCGCAG CTCCGTCGCT ATGCCGGCAT
23881 GTCGATCTTC GCCTTTGACA AGGGCATGTC GATGTACCCG CTGGCCGCCG GCATCCGTGC
23941 GGCCACGAAG GGCACCAGCG GCCTGCACTT CACCGTGGCG GCCGACGACG AACGCCTGGC
24001 GTTCTGCCCG TTGCAGTTCC TGAGCACCAA GGGCGACCGT GCTTGGGCGA TGGAGTGGAT
24061 CGACACCATC CTGGCGTTGA ACGGCGTCGA AACGACCCCG GCCCAGCGCA ACGAAATCGG
24121 CAACGCGATC ATGAGCATGC ACGCCAGCGG CGCGCGCACG CTCTCCGAGT TCAGCGTGAC
24181 GATTCAGGAT GAGGCGATCC GCGAGGCGAT CCGCCAGTAC ACCGTCGATG GCGCAATGGG
24241 CCATCTGCTC GACGCCGAAG AGGACGGCTT GGCGCTGTCC GACTTTACAG TGTTCGAGAT
24301 CGAAGAGCTG ATGAACCTCG GCGAGAAATT CGCCCTGCCT GTGTTGCTCT ACCTGTTCCG
24361 CCGTATCGAG CGCGCCCTGA CGGGCCAGCC GGCCGTCATC ATCCTGGACG AAGCCTGGTT
24421 GATGCTCGGC CACCCGGCAT TCCGCGCGAA GATCAGGGAA TGGCTCAAGG TGCTGCGTAA
24481 GGCCAACTGC CTTGTGCTGA TGGCAACGCA GAGCCTGTCC GACGCCGCCA ACAGCGGCAT
24541 CCTGGACGTG ATCGTGGAAT CGACCGCGAC CAAGATTTTC CTGCCGAATA TTTACGCCAG
24601 GGATGAGGAC ACGGCGGCCC TGTACCGCCG CATGGGCCTG AACGCTCGCC AGATCGAGAT
24661 TCTGGCCCAG GCCGTTCCCA AGCGTCAGTA CTACTACGTG TCGGAAAACG GCCGCCGTCT
24721 CTACGACCTG GCACTTGGCC CGCTCGCGCT CGCGTTCGTC GGCGCATCCG ACAAGGAATC
24781 CGTCGCCATC ATCAAGAACC TGGAAGCCAA GTTCGGCGAC CAGTGGGTGG ATGAATGGCT
24841 GCGTGGCCGG GGCCTCGCCC TTGATGAATA CCTGGAGGCA GCATGAGTTT TGCAGACACG
24901 ATCAAGGGCT TGATCTTCAA GAAGAAGCCC GCAACGGCCG CAGCAGCGGC GACGCCGGCC
24961 GCGACCGGCC CGCAAACCGA CAACCCGTAC CTGACGGCGC GGCGCACCTG GAACGACCAC
25021 GTTGGTTCCG TTGTGTCGCA AAAGCAGACC TGGCAGGTTG TCGGCATCCT TTCGCTGATG
25081 ATCGTCCTCG CGGCGGTCGG CGGCATCATC CACATCGGCA GCCAGTCGAA GTTCGTGCCC

25141 TATGTCTACG AGGTAGACAA GCTCGGGCAG ACGGCCGCCG TGGGGCCGAT GACCAGGGCG
25201 TCGAAAGCCG ATCCGCGTGT CATTCACGCC TCGGTGGCTG AGTTCGTCGG CGATGCTCGC
25261 CTGGTGACGC CGGACGTAGC TTTGCAGCGC AAGGCCGTCT ACCGCCTCTA TGCCAAGCTC
25321 GGGCCGAATG ACCCGGCCAC CGCCAAGATG AACGAATGGC TCAACGGCAC CGCCGACGCC
25381 AGCCCGTTCG CTCGCGCGGC CGTCGAAACG GTCAGCACCG AAATCACTTC CGTAATCCCG
25441 CAGACGCCCG ACACCTGGCA GGTCGATTGG GTCGAGACGA CGCGCGACAG GCAAGGCGTG
25501 GTGAAAGGCC AGCCCGTGCG CATGCGGGCC TTGGTGACGG TCTACGTCGT CGAGCCGACG
25561 GCGGACACCA AGGAAGAACA ACTGCGAAAC AACCCGGCCG GGATCTACGT CCGGGACTTC
25621 TCCTGGTCGA GACTTCTGTG AGGCACTGAA TTATGAAAAA GGAACTGTTT GCTTTGGTCC
25681 TGGCCGCGTC CGTTAGCGTG CCTGCATTTG CCGCCGATCC CGGCGCGGAC CTGACTGACC
25741 TCTATTTTTC CGGCAAGAAC CCGGAGCTGA CCGCGCAAGA GCGGGCGGCC ATCGCCATCG
25801 CCAAGAAGTG GGAGGCGGGT ACCGCCGGCA TGCGGCCGGT GGCCGGCCCC GGTGGTTCGG
25861 TGCGCTTCCT GTTCGGCGCG CAGCAGCCGA GCATCGTATG CGCCGTGCTG CAAGTGTGCG
25921 ACGTGGCCCT GCAACCCGGC GAGCAAGTCA ACTCGATCAA CCTGGGCGAC ACCGCCCGTT
25981 GGACGGTCGA GCCGGCCATT ACCGGCAGCG GCGCGAACGA AACCCAGCAC CTCATCATCA
26041 AGCCGATGGA TGTGGGCCTG GAAACCAGCC TGGTCGTGAC CACGGACCGC CGCAGCTACC
26101 ACATGCGCCT GCGCTCGCAT CGCACGCAGT ACATGCCGCA GGTGTCGTTC ACCTACCCGG
26161 AAGATGCCCT TGCGAAGTGG GACGCCATCA AGAACCGCGA ACAGCGGGAT CGCGTCGAGA
26221 AAACCATTCC GCAGACCGGC GAGTACCTGG GCAACCTGAG CTTCAACTAC TCCGTCAGCG
26281 GGTCCACGTC GTGGAAGCCG GTGCGCGTCT ACAACGACGG CAAGAAAACC ATCATCCAGA
26341 TGCCGCACTC GATGGAACAG ACCGAAGCGC CGACGCTCCT GGTCGTTCGC AGGGAGGGCG
26401 GCCTGTTCTC CGACGATGAA ACGGTGATGG TCAACTACCG GGTCCAGGGC GACCGCTACA
26461 TCGTCGATAC GATTTTCGAC AAGGCCATCC TCATCGCGGG CGTGGGCAGC AGCCAGGACC
26521 GCGTGACCAT TTCAAGGGGG AACTAAACCA TGCGTAAGAT TCTGACCGTC ATCGCACTCG
26581 CGGCCACGTT GGCCGGCTGC GCGACCTCCA AGTACGGCAG CTTCGTCCAG GACGCGCCGG
26641 CCGCCTACAA CCAGACCATT GCGACCGACG CGGTGAAGCA GCTCGTCAAG CTCTACCCGC
26701 CGGCGCAAAC CAAGCTGGAA TTGCAGCAGG CTACGCCCGA TCCGTTCGGC ATTGCCCTGG
26761 TCACTGACCT TCGCGCCCAG GGCTATGCTG TCATGGAGTA CAAGCCCGAC GGCAACGCGG
26821 CCGCAGCTCC GGCTGCTGCG TCCTCGGCCG CTGCGAAGCC GGCAACGCCG CAAGCCCAGG
26881 GCGGCTATCC GCTGCGCTAC GTGCTGGACC AATTCAGCGA CAGCAACCTG TATCGCCTGA
26941 CCGTCATGGT CGGCTCTCAA TCGCTCACGC GCGCCTACCT CGCCCAAAAC AACACGATGG
27001 TCCCGGCCGG CGCATGGGTT CGGAAGGAGT AAGCCAATGA GCGAAGATCA AATGGCACCG
27061 GACGCATCGC CAGATGCGGT CAAGCCGAAA AGCGGGGTTC GCCGCGTCAA CAACATGCCG
27121 ATGTACCTCA TCGGCGGTGT GCTCGGCATC TTCCTGCTGG TGATGGCCCT GGTTGCTGCG
27181 GATCGCGCTG CGCAGCAGAA CCAGCCGGGA GCTGCGAAGG CTGAGAAGGC CGGCAGCACC
27241 AGCATGTTTG CCGACGAAAT TGCCGGCAAA CAGCAGGACG GCATCATCAA GGCCAAGCCG
27301 CTGGAGATTC CGCCGGAACA AACCGCCCAG CAACCGACGA CGGAGCTGAC GCCAGCCCCG
27361 GCGCAGGGAA CGACTATCAC GGTCGCACGG CCCGAGAACC TGGACCAGCC CCCGACGCCG
27421 CCGCAGGGTG CGCGCAACGA GGACCTGGAC CGCATCCGCA TGGCGAAGTT GCAGATGCTG
27481 GAAGAGGCGA TCAAGGCCAA GACGACGGTG CGCATCGACG CGCCGCGCAG CCAGGGCAGC
27541 GCCGGCGGCG GTGCTCCGCA GGGCCGCGAG GAAACCCTTG CGCGCATCCA GGAGCTGCGT
27601 CGGCAGGCTG AGAACGCCCG CGCCACCGAT CCGACCGCCG CCTATCAGGC CGCGCTTGCG
27661 CAGGCTCGCA CGATGGGCGG CGCGGCAGGG GGTGGCGGTA TGGGCGGCTC GGGTGCGCCG
27721 ACCCTCGTGC AGACCTCGAA CCGCAGTGGT GGCGGCGCTG GCTATGGGTC GTTCGACAAC
27781 CGCAGCGAGG GCGACCGTTG GCGGCTCGAC TCCCAGCCGG AAGCACCTGC AACGCCCTAT
27841 GTGCTGCGCG CTGGCTTCGT CGTTCCGGCT ACGCTTATCT CGGGCATCAA CTCCGATCTG
27901 CCAGGCCAAA TCATGGCCCA GGTATCGCAG TCGGTGTACG ACACGGCGAC CGGCAAGCAC
27961 ATGCTCATCC CCCAAGGCTC GCGCCTGGTG GGCAGCTACT CGAACGATGT GGCCTACGGG
28021 CAGAAGCGCG TTCTGGTGGC ATGGCAGCGC ATCATCTTCC CCGACGGCAA GGCAATGGAC
28081 ATTGGGGGCCA TGCCGGGCGG CGATAGCGCT GGGTATGCAG GCTTCAACGA CAAGGTCAAC
28141 AACCACTACT TCCGCACCTT CGCATCGGCA TTCCTCATGT CGGGCGTCGT TGCGGGCATC
28201 AGCTTGAGTC AGGACCGTGG CAACAGCAAC AGCGGTTACG GACGACAAGA CGCGGGTTCC
28261 GCGATGAGTG AAGCGTTGGG TCAACAGCTC GGCCAAGTAA CGGCGCAGAT GATCGCCAAA

28321 AACTTGAATA TCGCGCCGAC GCTGGAAATC CGTCCGGGCT ATCGCTTCAA CGTCATTGTC
28381 ACGAAAGACA TGACGTTTTC TAAGCCCTAC CAGGCGTTTG ACTATTAACT CCAAGGAGTA
28441 ACTTATGAAG AAGCTCGCTA AGAATGTTTT AGCCGCTAAA GTAGCTCTGG TGCTGGCCCT
28501 CTCGGTCGGC ACCTTGGCGG TCACGCCTGC GCAAGCGGGC ATTCCGGTCA TCGACGGCAC
28561 CAACCTGTCA CAAACCACTG TCACCGCGAT TCAGCAGGTT GCGCAGGTCC AGAAGCAAAT
28621 CGAGGAATAC CGGACGCAGT TGCAGCAGTA CGAAAACATG CTGCAAAACA CGGTGGCCCC
28681 GGCCGCCTAC GTGTGGGACC AGGCGCAGTC CACCATCAAC GGCCTGATGA GCGCCGTTGA
28741 TACCCTGAAC TACTACAAGA ACCAGGCGGG CAGCATCGAC GCTTACCTGG GCAAGTTCAA
28801 GGACGTGTCC TACTACAAGG GGTCGCCGTG CTTCTCCCTG TCGGGCTGCT CGGAAAGCGA
28861 GCGCAAGGCG ATGGAAGAGA ACCGCCGCCT GGCGTCCGAA TCGCAGAAAA AGGCCAACGA
28921 TGCGCTGTTC CGTGGCCTCG ATCAGCAGCA GAGCAACCTC AAGTCCGACG CCGCCACGCT
28981 GGAGCAATTG AAGGGCAAGG CGACGACGGC GCAGGGCCAG TTGGAAGCCC TCGGCTACGC
29041 CAACCAGTTC GCCAGCCAGC AGGCCAACCA GCTCATGCAA ATCCGTGGCC TTCTGCTTGC
29101 GCAGCAGAAC GCCATCGCCA CGCAGATGCA GGCCCAGCAG GACCGGCAGG CCCAGCAGGA
29161 CGCTGCGGGC GCGAAGCTGC GCGAGGGTTC GTACCGCGCA AGCCCGTCTA AGACCTGGTG
29221 AGGGGAGGCG CGATGAAGAA ATCCAACTTC ATCGCAGTTG CCGCGCTGGC CGCCGTCATG
29281 GCGGCCAGCC TGGCAGGCTG CGACAACAAG CCCGACACCG ACAAGCTGAC CTGCGCCGAT
29341 CTGCCGAAGG TCACGGATGC CGCTCAACGC GCGGAGCTGT TGAAGAAGTG CCCGCGCGGA
29401 GAACCGGGAG GCTTCAAGCC CAGCGAAAAG AAAGAGTGGT GATGACGTAT GAAAATCCAG
29461 ACTAGAGCTG CCGCGCTCGC GGTCCTGATG CTGGCCTTGA TGCCGGTAGC GGCATACGCC
29521 CAAATCGACA ATTCGGGCAT CCTCGACAAC GTATTGCAGC GCTACCAGAA CGCCGCGAGC
29581 GGCTGGGCCA CTGTCGTCCA GAACGCCGCA ACCTGGCTGT TCTGGACCTT GACCGTGATT
29641 AGCATGGTCT GGACCTTCGG CATGATGGCA CTGCGCAAGG CCGACATTGG CGAGTTCTTC
29701 GCCGAGTTCG TGCGGTTCAC CATCTTCACC GGCTTCTTCT GGTGGCTGCT GACCAACGGC
29761 CCGAATTTCG CGTCGTCCAT CTATGCGTCC CTGCGGCAGA TTGCAGGCCA GGCAACGGGG
29821 TTGGGGCAGG GGCTTTCGCC GTCCGGCATC GTCGATGTTG GCTTCGAGAT TTTCTTCAAG
29881 GTGATGGACG AAACCTCGTA CTGGTCGCCG GTCGATAGCT TCGTCGGTGC CTCGTTGGCG
29941 GCCGCCATCC TCTGCATCCT GGCCCTGGTC GGCGTGAATA TGCTTCTGCT CCTGGCGTCC
30001 GGATGGATTC TTGCCTACGG CGGTGTGTTC TTCCTGGGCT TCGGCGGCTC GCGCTGGACC
30061 TCGGACATGG CGATCAACTA CTACAAGACC GTCCTCGGGG TCGCCGCGCA GCTCTTCGCA
30121 ATGGTGCTGC TCGTAGGCAT CGGCAAGACC TTCCTCGATG ACTACTACAG CCGCATGAGC
30181 GAAGGCATCA ACTTCAAGGA ACTTGGAGTG ATGCTGATCG TCGGCCTGAT CCTGCTCGTT
30241 CTGGTCAACA AGGTGCCGCA GCTCATCGCC GGCATCATCA CCGGCGCGAG CGTCGGCGGT
30301 GCTGGTATCG GCCAGTTCGG CGCTGGCACG CTCGTCGGTG CGGCCGCGAC GGCCGGCGCG
30361 GCAATCGCAA CTGGCGGCGC ATCTATCGCG GCCGGCGCTG CGGCGGCGGC CGGTGGCGCG
30421 CAGGCCATCA TGGCGGCCGC GTCGAAGGCC AGCGATAACG TCTCTGCCGG CACTGACATT
30481 CTGTCGAGCA TGATGGGCGG CGGCGGTGGC GGCGGCGGTG GTAGCGCCGG CACCAGCGGC
30541 GGCGACGGCG GCGGCTCGGG TGGCGGCGGT GGCTCGGGCG GCGGTGAAAC CCCGATGGCC
30601 TCGGCCGCCG GCGACAACAG CAGCGGCGCA CGCGGCGGCA GTTCGGGCGG CGGCTCGGGT
30661 GGTGGCCGTT CGTCTGGCGG TATCGGTGCC ACGGCGGCCA AGGGCGGCCG GATCGCGGCC
30721 GATACCGTCG CCAACCTGGC GAAAGGTGCC GGCTCGATTG CCAAGGCCAA GGCCGGCGAA
30781 ATGCGCGCAT CGGCCCAGGA ACGCATCGGC GATACCGTAG GCGGCAAGAT CGCGCAGGCA
30841 ATTCGCGGCG CGGGTGCGGC GGCGCAGACC GCTGCAACCG TCGCCGATAG CAACAGCCAG
30901 GCGCAGGAAC AACCTGCACC GGCACCCGCA CCGTCGTTCG ACGACAACAG CCTTTCCGCA
30961 AGCAACAACA GGGAAGCGGC CGCCGACGCG GATTCCGAAG TGGCGAGCTT CGTCAACAAG
31021 CCCGCCCAAT CCTGAAACGA CTCTTAGGAG CTACGACCAT GCAACTGAAA AAAGCGTTCT
31081 CGTCGGCCGC CCTGGTGGTG GCCTTGGGCC TCGGCGCAAC TGGCTCGGCC AGCGCGCAAG
31141 ACGTGCTGAC GGGCGATACC CGCCTGGCCT GCGAGGCCAT TCTGTGCCTG TCCACGGGCA
31201 GCCGGCCCAG CGAGTGCAGC CCGTCGCTCT CGCGGTACTT CGGCATCCAC AAGCGCAAGC
31261 TGTCGGACAC GCTCAAGGCG CGGCTGAACT TCCTCAACCT CTGCCCGGTA TCGAACCAGA
31321 CGCCGGAAAT GCAGACGCTC GTTTCCTCGA TTTCGCGCGG GGCCGGGCGC TGCGATGCGT
31381 CCTCGCTGAA CTCCGTGCTG CGTGAGTGGC GGAGCTGGGA CGACCAGTTC TACATCGGCA
31441 ACCGCCTGCC GGACTACTGC GCGGCCTACA CCGGCCATGC CTATACCGAC TTCAACACGA

31501 CCGCGCCGCG CTACGTCGGC ACGCCGGAAG AGGGCGGCTA TTGGATCGAG GCGGCCGACT
31561 ACGACCGCGC GCTCAAGGAG TACGAGGCGA AGCTGAAAGA GCGGCAGCAG CAGTACGGTC
31621 GCTATGGCAG CGACGCCTAC CGTCGGTTCG AGCGGTAAGG GGAGGGGATA GCGATGCCGT
31681 TTGCCAAGCT GCTGGCACGG AACGCTCTGC CGGTGGTCGC CCTGGTGGCG GCCACTGGCT
31741 TCGGTGCGGC GGATGCGACC GCCGCACGGC TCTTCCCCGA TCTGTCGGAA CAGATGGAAG
31801 AGCGCGTTGT GTGCTCGGTG TCTGCGGCCG CGAAGTACGA GATTCCGGCC AACATTCTTC
31861 TCGCCATTCG GGAAAAGGAG GGCGGCAAGC CGGGCCAGTG GGTCAAGAAC ACCAATGGCA
31921 CCTATGACGT GGGCGAGCTG CAATTCAACA CCGCCTACCT GGGCGACCTG GCGAAGTATG
31981 GGATCACGGC CCAGGACGTT GCTGCGGCAG GCTGCTATCC CTATGACCTG GCGGCCTGGC
32041 GGTTGCGCGG GCACATTCGC AACGACAGGG GCGATCTGTG GACACGCGCC GCTAACTATC
32101 ACTCGCGCAC GCCGTCGAAG AACGCGATCT ATCGCGCCGA TCTGATGGTG AAGGCCGACA
32161 AGTGGGCGAA GTGGCTGGAT GCGCGTTTCG TCACCGTCAA CTATGGCCCC AGCTCGCCGG
32221 CGCAGCCGGC AGGGAAGGGG ACCACACTTG CGGCCGCTGA TACGTCGGCA GCAGCGCCGG
32281 CCGAAGCGCA GCCGATGAAG CAAGGCCGGA TCACCCGCAC CAGCCTCCGC AGCTCGGGTT
32341 ACGTACCCCG GCAGCTCATC ATCAACAACA CGCCATAAGG AGGAACGGCC GTTTAGCGGC
32401 TAAAGCCTAT GGGCATTCGC AACCTGACGC AGCGATACAT GAACGGGGCC AGGGCCTACG
32461 CGGCCTGGGC GGCATCGCAG GCGAAAGCGC CGTTTGATCT TCTGGTACTG GGCATCGGGC
32521 CTGTCATCGT CTTTGGCCTG GTCGCGCATA CGCTGCTCGC GTTCCTGCCC ACATGGGCCA
32581 TGTACGCCGC CGGCGCTCTG CTGGTCCTCG CGGCCCTGCC TTTGGCGCTG CACGTCCTCC
32641 GGGAATACGC GCTGCGCTAT GGGCGCAAAT AGCGCCCTGC AGGGCGTTCT TACTCCAAGG
32701 GGGAGGGCAT GAATACACGC GCCATGAACG ACGCCAGCGG CCGGGCCTCG CTGCCTGCCA
32761 TGGTGATCGC CGACGGCACC ATTGAAGCCT TGAAGTGGCT CGCCTTGCTT GCCATGACCG
32821 GGGATCACGT CAACAAGTAC CTGTTCAACG GTACGCTGCC ATATCTGTTC GAGGCGGGGC
32881 GCTTGGCCCT GCCTCTTTTC GTTTTCGTCC TGGCGTACAA CCTCGCCCGC CCGGGCGCGC
32941 TCGAGCGCGG TTTGTACGGG CGAGCGATGA AACGCCTGTT GGCCTTCGGC CTGGTCGCCT
33001 CGGTCCCGTT CATTGCGTTG GGTGGAGTGG TGGGCGGATG GTGGCCGCTG AACGTCATGT
33061 TCACGCTGTT GGCCGCAACC GCGATGCTCT ACCTGGTCGA GCGCGGCCGC TCGGTCGCTC
33121 CTATAGCGCT GTTCGTCGTG GCCGGCGGCC TGGTCGAGTT CTGTTGGCCG GCGCTGCTGC
33181 TGGCCGCGTC TGTCTGGTTG TACCTCAAGC GCCCGACGTG GGCGGCCGCG TTGATGGCGC
33241 TGCTGTCTTG CGCGTCCCTG TGGTACATCA ATGGCAACCT TTGGGCGCTT GCTGTTGTGC
33301 CCCTGGTGAT CGTCGCCGCC GGCGTCGATC TTCGTGTCCC GCGCCTGCGC TGGGCCTTTT
33361 ACACGTACTA CCCGCTGCAT CTTGCCGCTC TTTGGCTGAT CCGCATTCCG ATGCGCGAGG
33421 CGGGCTACTT GTTTTTCACC TGACCTTTGA GATTCCAATA TGCAATTGCT CAAGAAATGC
33481 ACCATCGCGG CCCTGCCGCT GCTCGCCCTG TCCGGCTGCG CACTGCTGAA CATCCCCATG
33541 CCGACGCCGC CCGGTTCGAC CCCGCCGGAA ATGCTGACCG TGCCAGTGGC GCAAATCTGC
33601 CGCGACGCTG ACAAGAACCC TGTTCGGGCA ACGGAGCTGT ACGGCAAGAA AGGGTTGTCG
33661 GCCACCGGCA AGGTGCAGGT GATTTCCGAA GGCTTCAAGC CTCGCTATCG GGTGCTGCTG
33721 CGCGCTGGCA GCGCCTCGGT CCATGCTGGG ACCGATAACC AGCTCGCCAT CAAGTCGGTT
33781 TCCACCGGCC AGACCACGCG CGTCACTGGC ACCGTGAAGG ACGTGTCCTA CGACCATAAC
33841 GGCTGCTCGA TCTCGCTTGA CGATGCGAAG TTCTACTGAG GGAGGGCGG CGGATGCTGA
33901 CACGGTTGAA GGGCTTCCTT GCTCGTCGCC GCGAGTTGAA GGAACTGGAT GTGTCCGTGG
33961 TGAGCCGGCC CCGGCCGGCT CCGGCGGAAT TGGTCCAGGT TGATGCACGC GAGGCCGTTT
34021 GGCGCGTGCC GGTGCCCGGC CAGGCCGACC GCTTCATGTC GGCCAAGCCT GGCGCGATCA
34081 ACGATGAAAT GTTCGTGGTT CGGGTGGACA CCGAAGCGTT CTATCGGGCT TGGCTGCGCA
34141 GCAGCTCGAC GGGCCGCGAA ACGCGGTCGG ACAACTGCCC GCTGCGCTCG GAAATGCCGC
34201 AGGACTACAA GTTCAAGCAC GCCGTCCAGG GCTTCGCGCA CGGCAGGGAA AATCCTGTGC
34261 CGCTGGCCTT CGCCGGCGCG CACCAGGAGC GCCACCGGGT GGACATTGGT TTCAGCAACG
34321 GGGTCACGCG CTCGTTCTGG CTGATTGCCA ACAAGGCTCC GTCGTTCCCG ATCCAGGTCC
34381 ACGGCCGGGA GTCGGCCGAG CTGCTGAACA AGGTTTGCGG CCTCGATCCT GCGCCGCTGT
34441 CGTTCACGGA ACTGTTCGCG CAGGCCCAAC GCCAGGCTCC GCAGGTCGCC ACACCGGCCC
34501 GGCCTGCGCC GGCAGCGGCC ACCCGGCCAG CTCCCAAGGT GCAGCCACGC CCCGGCCGAA
34561 GCGGCCCGCG CAAAGGCCGC GGACTCTGAC TACAACCGTG CGCAAGGCGC ATTAGGGAGG
34621 ATGTATGTAT GTAATCGCCT GCGGCATCGT TGCCGGCTTG GCGGCTGCGG TGGCCCTGTT

34681 GGGCTTCACG CCGATGATGG AGGCGCTTGC CGCCGGCGAA CGCCGCAAGG CACTCGCGCA
34741 ATGGACGCGG ACGATGTTCC TGGTGCTGCT GCCTGTCGTG CTGATGTGCG CGCCCATCGG
34801 GTCCAGCATT TACGACGCCG TGCAAGCGGA CGCTGGCAAG CCCATCGCTT TCCACAACGG
34861 CCGGATCACG GTCGTCATGG CCCTGGTCGG CAGCTTGGCC GTTGTCCTGG TCGCGGCTGC
34921 GCGTGCGGTG GTCAACCGCA AGCATGCCAG CTTCTGGTTC GTCGGCTGGG TGATGGCGTC
34981 GGTTTTGGCC GGCGGCGTCG GCGCGATCGC CAGCGCGAAG CAACTGGCGT TCCTCGGCGA
35041 ACATAGCGGC ATGGTGGCCT TCGGCTTCTT CCGCGACCAG GTGAAGGACA TGCACTGCGA
35101 TGCGGACGTG ATCCTGGCCC GGTGGGATGA AAAGGCGAAC TCGCCGGTGG TCTACCGCTG
35161 CCCGAAGGCG TACCTGCTCA ACAGGTTCGC ATCCGCGCCC TTCGTGCCCT GGCCGGACTA
35221 CACCGAGGGG GAAAGCGAGG ATCTAGGTAG GGCGCTCGCA GCGGCCCTGC GGGACGCGAA
35281 AAGGTGAGAA AAGCCGGGCA CTGCCCGGCT TTATTTTTGC TGCTGCGCGT TCCAGGCCGC
35341 CCACACTCGT TTGACCTGGC TCGGGCTGCA TCCGACCAGC TTGGCCGTCT TGGCAATGCT
35401 CGATCCGCCG GAGCGAAGCG TGATGATGCG GTCGTGCATG CCGGCGTCAC GTTTGCGGCC
35461 GGTGTAGCGG CCGGCGGCCT TCGCCAACTG GACACCCTGA CGTTGACGCT CGCGCCGATC
35521 CTCGTAGTCG TCGCGGGCCA TCTGCAAGGC GAGCTTCAAA AGCATGTCCT GGACGGATTC
35581 CAGAACGATT TTCGCCACTC CGTTCGCCTC GGCGGCCAGC TCCGACAGGT CCACCACGCC
35641 AGGCACGGCC AGCTTGGCCC CTTTGGCCCG GATCGACGCA ACCAGGCGCT CGGCCTCGGC
35701 CAACGGCAAG CGGCTGATGC GGTCGATCTT CTCCGCAACG ACGACTTCAC CAGGTTGCAG
35761 GTCCGCGATC ATGCGCAGCA GCTCGGGCCG GTCGGCGCGT GCGCCGGACG CCTTCTCGCG
35821 GTAGATGCCG GCGACGTAGT ACCCGGCGGC CCGCGTGGCC GCTACAAGGC TCTCCTGGCG
35881 TTCAAGATTC TGCTCGTCCG TACTGGCGCG CAGGTAGATG CGGGCGACCT TCAACCTTCG
35941 TCCCTCCGGT TGTTGCTCTC GCGTCGCCAT TTCCACGGCT CGACGGCGTG CGGATCGGAC
36001 CAGAGGCCGA CGCGCTTGCC TCGCGCCTCC TGTTCGAGCC GCAGCATTTC AGGGTCGGCC
36061 GCGCGGCCGT GGAAGCGATA GGCCCACGCC ATGCCCTGGT GAACCATCGC GGCGTTGACG
36121 TTGCGCGGCT GCGGCGGCCG GCTGGCCAGC TCCATGTTGA CCCACACGGT GCCCAGCGTG
36181 CGGCCGTAAC GGTCGGTGTC CTTCTCGTCG ACCAGGACGT GCCGGCGGAA CACCATGCCG
36241 GCCAGCGCCT GGCGCGCACG TTCGCCGAAG GCTTGCCGCT TTTCCGGCGC GTCAATGTCC
36301 ACCAGGCGCA CGCGCACCGG CTGCTTGTCT ACCAGCACGT CGATGGTGTC GCCGTCGATG
36361 ATGCGCACGA CCTCGCCGCG CAGCTCGGCC CATGCCGGCG AGGCAACGAC CAGGACGGCC
36421 AGCGCGGCAG CGGCGCGCAG CATGGCGTAG CTTCGGCGCT TCATGCGTGG CCCCATTGCT
36481 GATGATCGGG GTACGCCAGG TGCAGCACTG CATCGAAATT GGCCTTGCAG TAGCCGTCCA
36541 GCGCCACCCG CGAGCCGAAC GCCGGCGAAA GGTACTCGAC CAGGCCGGGC CGGTCGCGGA
36601 CCTCGCGCCC CAGGACGTGG ATGCGCCGGC CGCGTGTGCC GTCGGGTCCA GGCACGAAGG
36661 CCAGCGCCTC GATGTTGAAG TCGATGGATA GAAGTTGTCG GTAGTGCTTG GCCGCCCTCA
36721 TCGCGTCCCC CTTGGTCAAA TTGGGTATAC CCATTTGGGC CTAGTCTAGC CGGCATGGCG
36781 CATTACAGCA ATACGCAATT TAAATGCGCC TAGCGCATTT TCCCGACCTT AATGCGCCTC
36841 GCGCTGTAGC CTCACGCCCA CATATGTGCT AATGTGGTTA CGTGTATTTT ATGGAGGTTA
36901 TCCAATGAGC CGCCTGACAA TCGACATGAC GGACCAGCAG CACCAGAGCC TGAAAGCCCT
36961 GGCCGCCTTG CAGGGCAAGA CCATTAAGCA ATACGCCCTC GAACGTCTGT TCCCCGGTGA
37021 CGCTGATGCC GATCAGGCAT GGCAGGAACT GAAAACCATG CTGGGGAACC GCATCAACGA
37081 TGGGCTTGCC GGCAAGGTGT CCACCAAGAG CGTCGGCGAA ATTCTTGATG AAGAACTCAG
37141 CGGGGATCGC GCTTGACGGC CTACATCCTC ACGGCTGAGG CCGAAGCCGA TCTACGCGGC
37201 ATCATCCGCT ACACGCGCCG GGAGTGGGGC GCGGCGCAGG TGCGCCGCTA TATCGCTAAG
37261 CTGGAACAGG GCATAGCCAG GCTTGCCGCC GGCGAAGGCC CGTTTAAGGA CATGAGCGAA
37321 CTCTTTCCCG CGCTGCGGAT GGCCCGCTGC GAACACCACT ACGTTTTTTG CCTGCCGCGT
37381 GCGGGCGAAC CCGCGTTGGT CGTGGCGATC CTGCATGAGC GCATGGACCT CATGACGCGA
37441 CTTGCCGACA GGCTCAAGGG CTGATTTCAG CCGCTAAAAA TCGCGCCACT CACAACGTCC
37501 TGATGGCGTA CTTACCCAAA GAACAGCTAG GAGAATCATT TATGCTCAGC ACACTTCCAC
37561 AAGCTCATGC AACTTTCTTG AACCGCATCC GCGATGCGGT CGCTTCCGAT GTTCGCTTCC
37621 GCGCTCTTCT GATCGGCGGC TCTTACGTTC ACGGAGGACT CGATGAGCAC TCCGATTTGG
37681 ATTTCGACAT CGTTGTTGAG GACAACTGCT ACGCAGATGT CTTGTCTACA CGCAAGGATT
37741 TTGCCGAGGC ACTGCCCGGC TTCCTCAACG CGTTCACCGG CGAACATGTA GGAGAACCGC
37801 GCCTTCTGAT CTGCCTATAT GGTCCGCCAC TGCTACACAT CGATTTGAAG TTTTCTCTTG

37861 CTTCCGATCT CGACCAGCAA ATCGAGCGGC GGGCGGTTCT GTTTGCTCGT GATCCGGCAG
37921 AGATCGAGAA GCGCATTGAG GCGGCAGCGG TGGCATGGCC AAACCGTCCC TCCGAGTGGT
37981 TCGAAGCACG TTGTCAGCGC CAGTGATATA AGACGGTAAT TCACCATTTG GATTGTCCGC
38041 TCCACCCAAC ATGTTGTTTC CTTAAGGTTC TCACACCAGA AAGGACATCA ACATGCTGAG
38101 CAGAGAGGAC TTTTACATGA TAAAGCAAAT GCGCCAGCAG GGCGCGTACA TTGTCGATAT
38161 TGCGACTCAG ATTGGTTGCT CTGAACGGAC GGTCAGACGC TACCTCAAAT ACCCTGAACC
38221 GCCAGCCAGA AAGACCCGCC ACAAAATGGT TAAGCTGAAA CCGTTTATGG ATTACATCGA
38281 CATGCGCCTG GCAGAGAATG TCTGGAATAG TGAGGTTATC TTTGCGGAGA TTAAGGCAAT
38341 GGGTTATACG GGCGGACGTT CCATGCTGCG TTACTACATC CAGCCCAAAC GTAAAATGCG
38401 TCCGTCAAAA AGAACAGTTC GCTTCGAAAC TCAGCCTGGA TACCAGCTCC AGCACGACTG
38461 GGGCGAAGTT GAGGTGGAGG TTGCCGGGCA ACGGTGCAAA GTTAACTTTG CGGTTAATAC
38521 GCTGGGGTTC TCCCGCCGCT TCCATGTCTT CGCCGCACCA AAACAGGATG CTGAGCATAC
38581 CTACGAATCA CTGGTTCGCG CCTTCCGCTA CTTCGGTGGT TGTGTGAAAA CGGTGCTGGT
38641 TGATAACCAG AAGGCTGCGG TGCTGAAGAA TAACAACGGG AAAGTCGTGT TCAACTCCGG
38701 ATTCCTGTTG CTGGCCGACC ACTATAACTT CCTGCCACGG GCATGCCGTC CACGCAGGGC
38761 CAGAACAAAA GGTAAGGTTG AGCGGATGGT GAAATACCTC AAGGAGAACT TCTTCGTTCG
38821 GTACCGCAGG TTCGACAGCT TCACTCATGT TAATCAACAA CTGGAGCAAT GGATAGCCGA
38881 TGTGGCTGAC AAACGGGAAC TTCGCCAGTT CAAAGAAACG CCGGAACAGC GCTTCGCGCT
38941 GGAGCAGGAA CATCTGCAGC CGTTACCGGA TACGGACTTC GATACCAGTT ACTTCGACAT
39001 CCGCCATGTG TCCTGGGACA GCTATATCGA GGTTGGTGGT AATCGTTACA GCGTTCCCGA
39061 AGCGCTGTGT GGTCAGCCGG TATCGATACG AATATCGCTG GATGACGAGT TGCGGATCTA
39121 CAGTAATGAG AAACTGGTGG CCTCACATCG CCTCTGTTCG GCATCGTCTG GCTGGCAGAC
39181 AGTGCCGGAG CATCACGCCC CGCTCTGGCA GCAGGTCAGT CAGGTGGAAC ATCGACCACT
39241 GAGTGCCTAT GAGGAGCTGT TGTGATGCAT GAGCTGGAAG TCCTGCTGAG TCGCCTGAAA
39301 ATGGAGCATC TGAGTTATCA CGTTGAAAGC CTGCTGGAAC AGGCAGCTAA AAAAGAGCTG
39361 AACTACCGGG AGTTCCTGTG CATGGCGCTA CAGCAGGAAT GGAACGGCAG GCATCAGCGC
39421 GGTATGGAGT CCAGGCTGAA GCAGGCTCGT CTGCCGTGGG TCAAAACGCT GGAGCAGTTC
39481 GACTTTACCT TCCAGCCGGG CATCGACCGT AAGGTTGTCC GGGAACTGGC TGGTCTGGCG
39541 TTCGTGGAGC GCAGCGAAAA CGTGATCCTG CTGGGACCTC CTGGTGTCGG AAAAACTCAT
39601 CTGGCCATAG CTCTTGGCGT GAAAGCGGTG GATGCGGGAC ATCGGGTACT GTTTATGCCA
39661 CTGGACAGAC TGATCGCGAC ACTGATGAAA GCGAAACAGG AAAACCGGCT GGAGCGTCAG
39721 CTGCAGCAAC TGAGTTATGC CCGGGTGTTG ATCCTGGATG AAATAGGCTA TCTGCCGATG
39781 AACAGAGAGG AAGCCAGCCT GTTCTTCCGG CTACTGAACC GTCGATATGA AAAAGCGAGC
39841 ATCATACTGA CGTCAAACAA AGGGTTCGCA GACTGGGGAG AAATGTTCGG AGATCACGTG
39901 CTGGCAACAG CGATACTGGA TCGGTTGCTA CATCACTCAA CCACGCTGAA TATCAAAGGA
39961 GAGAGTTACC GGTTAAAAGA GAAACGTAAA GCTGGAGTGC TGACCAAAAA CACAACGCCA
40021 ATCAGTGATG ATGAAATGGT GAAAAGCGGA CAGCATCAGT AACGAAAGTA TCTTAGCGGG
40081 CATGAAAATG GCAAATAACG GTCAAACATC GTGGCGTTGA CAACGTGCCT GGATCTGGCT
40141 ACACTATGCG GCCACCAAGC TCGCCCGTGG CGAGCTTTAC GAAGCGATCG GCATGCTCGG
40201 TTTCTTCCGT GAGCAAGTGT TAGGACCTTT GCTCTACCGT CGCGCTGGAA AGGACCAGCG
40261 CGGAGTGAGG CGATTGGAAA CCCTTCGACT GGATGAAGAG CGCAGACTAG CCACCACCAT
40321 TGCGCTGCAC GATGCGTTGT CTGTCAGGGA TGCCATCAAA GCATCTGCCT CCATCTATCT
40381 CGACCTCCGA GCCGCCGATC CGTCGTTGGA ACCGACAACG CATATGCCAG GTCTTCTGTA
40441 CGACTTAATA GAACGTGCGG TACCAGGCAC GCCTAACCGT CAGTGAGATT GGATGAGTGA
40501 ACGATATTGA TCGAGAAGAG CCCTGCGCAG CCGCTGCCGT GCCCGAGAGC ATGGCGGCTC
40561 ACGTGATGGG ATACAAATGG GCGCGTGATA AGGTTGGTCA GTCCGGCTGC GCGGTCTATC
40621 GGCTGCATAG CAAGAATTCT GCCGTGTTAT GGAACTGTCT TGAGGAGTTC GAACCTTCGC
40681 TTCAGGAGAG GCTTGTTGCG CAATATGGCA TTGCCGATCC GGATAGGCGC AAGCTGCAAT
40741 TTCATCTCCT GCTGGACGAA CTTTTCTAAG GCGATGCCCC CTCGACCTCG ATCAGGGAGG
40801 CGTTCAGGAC GACTCACAAA GAAAGCCGGG CAATGCCCGG CTTTTTCTGC TGCTACCTCC
40861 GTAGTCGTAA GGTCGTTGCA GGTGCTCGGG TGCGGTACAA CTCGCCGGTC GCCAGCTCAA
40921 GCGCGATCAC GTCGTTGCCG TCGTAGTTGA CGATGATGCT GTTGGGCCGA CTGTCCTCAC
40981 GCTTCGCAGG GAGAGGCCAG CCTTCAATCG AAGCCGGCGC AAGCTCGTAG TGCTTCCCGG

41041 TTTCGACGCT GCGCAGCGTC CAGGTCCTGC AACCGGCCAC GCCGGTCGCA GAAACCACGG
41101 CGAGCGAGCC GCGAAAATCG TGCGGGTACG CCTCGATGTT CATACGCCTC CTAGATCGAG
41161 CGCGAGCGTT TCTGCTCGGC CTTGGCCGCC TGTTCCTGGG ACACCTCGCC GATGACCTTG
41221 CCCTGGCCCC GGCTGTAGGC GATTTCGTAG TTCTTGCCGA CAACCGGCGG CTTCTCAAAG
41281 ATGCCCCGGC TGTGTTTCAC GATCCCGCCT TCGCTGAACT GGTAGACGTT GCGCCCATCG
41341 TCGTGCAGCA CCTGGCCGAC GTGCTTGTGC GGGTGGACGT TTTTGCTTGC GTCCTTCGCA
41401 TCGCTCAACT GGTGAATGCC TTTCGGTAGC CCCGCCTCGG GCAACACCTT CATGGTCAGC
41461 CATTCGCCGT TCACTACCTG GTCCACCTGG CGGCTGCCGT TCATGACGGC GATCTTGACG
41521 CTGCCCTCGG GTTTCATGAT GACGCCAGGG CTTGCCGATG TGCGTGGTGC CCCGATCTGT
41581 ACTTTGTTCA TACGCTCTAG TTCTCCTTAG TAGGTTCTCG CGCGGCGTTG CCGCTGTTCT
41641 TGCTGCTCGA TGTCTTGCTG CTTGAGCTGC TGCACCTTCT GCCGCTGGCC CTCGTCGAGA
41701 AGCACCTTGC CGACAGCACT TCTCACCTGG CGTTCAACCC CGTCCTTGCC CAGGCTGCTG
41761 CGCTCGGACA GGTCGTTGAA ATCGGTGTGC TTCTTCATGT TCGACAGGGC GGCGAGCTGG
41821 CCATCGTTCA ACAGCGATTC CTTGAGCTTG GCCGTGTCGG CCTCGCTCAA CTGGACCTTG
41881 CCGGCCGCCG CGTCCGCGAG GCGCTTTTCA GCGTGCAAAT GGTTGCGGTA GTTCTCCGGG
41941 GTGATCGGCG GCAGCTCCTT CGGGTAGGCG TTCTCGCCCG GCGCGAAGAT TGGGAAGATG
42001 GCCTTGCCGC CGACCGCCTT GGCGGCCTCC TGTGCCTTCG TCCTGCCGGG ATTCACGCCC
42061 TGGGTGATCT GCACCTGGCG GTCGTCGTCG CCGGCGATCA CAACGGGCTT GTCCGGGAAT
42121 TTCGCGTGCA GGGCCTCGGC AACAGCCTGT AGGTTGCCGG AATCGAACGC GGCGACAGTC
42181 GCGTGCCCCA GCGCTTCGGC CACTGTGGCG GCGGTGGCAT AGCCTTCGCC GATCACCAGC
42241 GCCGGCGCGG CCGCGAGCGC ATCCATGCCA CCGACGACAT GGAAGCATCC TTCCTTGCGG
42301 CTGTCCTTGG CGAAGCGCTT GGTGCCGTCC TCCTGGATGT ACTGCATGGT CCATTGCTTG
42361 CCGTCGGCGT CGTAGGCCGG GATGTAGGTT TTCTGGCCCT CCTGGTCGGT AAGGACGCCG
42421 GCGTGCACCT GTAGACCCTT GTCGCGCAGG TACGGCGTCG GTTCCGTGAT GGGAACCAGG
42481 CTTTGCGCCT GGCGGCCGAT GCGCTGCGCC GTGGCTTCGT GCTGGCGTTC TTGTTCCTCG
42541 GCACGCGCGG CCAGCTTGGC CGCCGCCTCG GCCTGCATCT TGGCCTTCTC GGCGGGGTCC
42601 AGGGCGTAGC CCTTGGCCTT CCACTTCATT TCGACGCCGG TGCGGTTGTT TTTGATGTAA
42661 CCGGCCGGGT GGCCGTCGAG GTGGCCGACG TAGAAGCCCG ACTTCTCGCC CTTCTTGTCG
42721 CCCTCGGTCT CGATGCGGTG CTTCTTGCCG TCCATGATGG GGTGCTCGCC GCCTGGGGTG
42781 ACGACGCAGC CCATGCTTTT CAGGGCCTCC GCGAACTCAT CTTCGGGGGT GACGGCCGGG
42841 GATTGCTGGG TGGGCACGTT GTCCGGCAGC CAGCGTTGCA GCTTGCCCAT GTCGGCGTTC
42901 GGTCCGGCGT ACCAGGACTT GGCCACCTTG TCCCACTGCG CGCCGGCCGC CTTGGCAACC
42961 TGGCGCTCGC CGTAGGGCAC GGCCAGGTAG ACGCGCTCCT GGGCCGCGTT GGGGCGCTCG
43021 GCCGTGGGTT GGGTAGGCTG GGCCTCGGCG CGGGCCTCTA CGGCCGCTGT AGCGCCCTCG
43081 CGCGCCCATT TGGCGAACGG GGCAGGGTCA ACCCCTGCCG GAACGTACCA GGCGCGTTCC
43141 TGGCGGTCCC AGCGCGCTCC AAGGGCCTTC ACCTCGTCTT TCTCCTTGAA CGGCACGTTC
43201 AAGTAGGCGC GCTCGGGCTT GGCGGGGGCT TGAGCGGCCG CCGGCTGCTC GGCGGCGTTC
43261 ATGGCCTGGG CCATTTCCTG CTGCTCGCGC TCGTAGTCGG CGATCCGGCG CTGTAGGTCC
43321 TCGTCGTGCA GCATGGCCGT GCCCTCGGCG GCCTTGCGCG CCTCCTTGGC GGCAACGCGG
43381 TCCTCGTCGG TGCTGTTGGG ATCGCGGCGA ACTCGCTCTT CATGGATGCG GGCGAACTTC
43441 GCGGCCTGCT CGTACTCGTT GGCCGTCGCG TAAGCGTCGA TCACGGCCAG GCGGTCGGCG
43501 AGCGCTTCGG CATTGGTCTG CGCGTCGGGG CCGGCGAAGT CGGCAAGCCA TTGGTGGCCG
43561 CCCCACGCAT GGTTCGCATA GACGCCCCAA AACTCCGGCT CTCGGTCGCC GGCCGGCACC
43621 ACGGACCGTT CGCCGTCGTG CTCGACCTCG ACGTTGGCCT GGACCTGGAC GCGGCCGGTC
43681 CAATCGGCAG GCAGCTCAAA GCCCAGCGTG GTTTCGGTCA GCGCGGCCAG CGATTGGTTG
43741 CCTTCCGCCG GCTCCGCGCC GGCGCGGTAC ATGCGCAGGG TCTGCGCGAT CAGCTCGTCG
43801 GCCGGCGCAA TGGCCGGTCG TGCCACCTGG TCTTGCTGTT GCTCCATAGT TGCCCCCTGC
43861 GCAGGCTCGA TGGCCTGCTG GGTCGTTTGT TCTTGAATTT GCTTCTGCTC GAACGCCAGG
43921 ACGAAATCCT GGATCTTCTC CGCGTCGGCG GCCGCGCGGA AAATCTCTAG CGGGTCCTCT
43981 TGTAGCGCCT TGATCCACGA TCCGACATAG GCCGCGTGCT GGCCGGGGTC GTGGCCGATG
44041 CCCAGCTCGT CGCCCAGGAT CATGCTGGCA ATCTCGGCCC GCAGCTCTTC CTTGGCGTAC
44101 CCCTCGCTCC CGAAGGGATG CGCCAGGTCG CGGTCCAGCC GCGACGGGTG GCCGGTCCAG
44161 TGCCCCAGCT CATGGAGCGC GGTTGCGTAG TAGTTGTCGG CGCTCGGGAA CTGGCCTTTG

44221 TCGGGCAGAT GGATGCTGTC CGTGGACGGC CGATAAAACG CGCGGTCGTG CTCGCCGTGG
44281 CGGATGGTGG CACCTGACGC CGCAAGGATG TGCTCGGCCC GCTCGACGGC GCTCCAAGTC
44341 TGTTCCTTGC GTTCCAACGG CGGCAGGCCG TCGATCTGCT CCGCATTGAA CACGGTGGCG
44401 AAGAACACGC GCGGGCGTTC GAGCTGCACC GTCACCTTGA CCGGATCGCC GTTGGCATCG
44461 AGGACCGGCT TGCCGGTCTG CTCGTCGGTC TTGGTCTGCT CTTCGCTGAA CTTCCAATAC
44521 TGGATCGGCG TGCCTTTCTC GCCGCGACGC ACCTGTGCGC CGGCGGCAGC GGCCTGCTTG
44581 TAGGTCATCC AGCGCGGGTC CGCATGGCCC TGGGCCATGA GCTGAATCGC GTTGATGCCC
44641 TTGTAACGCT TCCCGGTAGT CGGGTTGAGC GGGATGAAGG AGCCGGGCAT GCCCGGTTCC
44701 CACGGTTTTT GCCACGGCGC AGTGCCGGCT TTCAGTTGCT CAATGAGGCG TTCGGCAACC
44761 TGCTCGTGGA ACGGCTTTTT GACCTCTGCC ATAGCCAATT ACCTCCCGTC ATTGGCGGCC
44821 GCGGTCGTCG TGTCCTCGGG CACGGTCGCG TCCACCAGGT CAATGTCGCT CTCGGCGGCG
44881 TCCTGCTCGC TCAAGGCGTC CTCGGGGAAG GCCCCGGCCT TCTCCGCTTC TTCCGGGTCG
44941 AACTCGACCT GGAAGCCGGG CGTCATCGCA TCGGCGAGCT TTTCACGCAG GGCGGCGGCG
45001 GCGTTCGCGG TATCGTCGAA CGGCGCAAAG TCGTCCTGCT GCACGGTCTT GGGATCATTC
45061 ATCGCTTTCA CTCCTGGTTG GTGCCGTTAC GGCCTTTGCT GTAGTCCGGC CTGCCTTTCA
45121 GGTCGGGGTA TGTCTGCTTG CACGTCGGGA AGTTGCTGCA CCCCCACCAG AACATGCCGC
45181 GCTTCTTGCC AGGCCGACGG GAAAGGCCGT GGCCGCAGGC CATGCACTTG TGCAGCTCGG
45241 AGACTTTCGG GGCCTCGCGC GGGACGGGCT TGCCGCCCTT GTCGTCGCAC GCGAACTTGC
45301 AGCCGTCGGC AAAGCCGGTG CAGCCCCAAA AGTATTCGTT CTTGTCCTTC TTCTTGAGGC
45361 GTCGCAGCGG CTTGCCGCAG GACGGGCAAG GGTGCGTGTC GATCTTCATG TTGAGGCCGT
45421 TGTCTTTGAT GTTGGCGACC TCGGCGCCGA TGTATTCCAT CAGCTCGTTG ACGAACGACA
45481 GCGTGTCGCG CTCGCCGGCC TGGATGGCCT TCTGCTGCTC ATGCCAGAGC GCGGTCATGT
45541 CGGGGAATCT GGCCGTGTCG GGCAGTGCGT CGTACAGCTC TTCGCCGGTC GGCGTGGACA
45601 CGATGTGCTT GCCCTTCTCC ACCAGGTAGC CGCGCTCGAA AAGCGTGGCG ATGATGGAGT
45661 CTCGCGTTGC CGGCGTGCCG ATCCCGCCGT GCTCGCCTTG CTTGCCCTTG TCCTTTTCGA
45721 TCAAGATTTT CCGCAGGCGG TCATCGCGGA TGTATTTCGC AACGCGGGTA AGGTCCGACA
45781 GCAGAGATTC CATCGTGTAC AGCGGCTGCG GTTTCGTCTC CTGCTGCTCG GCCTTCGCAT
45841 CGGTGCAGGT GCCGGCCTGG CCGTCACGCA GCTTGCGCAG GTCCTGTTCA ATGTCGTCGG
45901 CATTGCCTTC CAGGTCCTCG TTGCCGGCGT CGTTCTTGTA GAGAATCTTC CAGCCCGGCG
45961 ACGTGGTGAC GTTCGAGCGC ACGCCGAAAC GATGATCGCC GACCTGGGCA AGCACGTCGG
46021 TCTGGTCATA CAGATGCTTC GGCCAGAACT GCGCGACGTA GGCGCGCGCG ATCAGCAGGT
46081 AAATCTTCTG CTCGGCATCG GTGAGCTTCG ACAGGTCGGC CGTGCTTTCG GTCGGGATGA
46141 TCGCGTGGTG CGCGGAAACC TTGGACGAGT TGAAGGCGCG GCTCTTGATC GTCGGATTGG
46201 CGCGCTGCGC AGCAGCGGCC AGCATGGGGG CCGTCTGTGC GATGGCCGCC AGCACGCCCG
46261 GCGCATCGCC GTGCTGTTCC TCGCTCAAGT ATTCGCAGTC GGAACGGTTG TAGGTGATGA
46321 GCTTGTGCTT CTCGCGCAGG GCCTGCGTAA TGTCCTTCAC CTGGTCCGGC TTGAAGCCGA
46381 ACTTGCGCGA GGCGTCCATT TGCAGTTTCA GCAGGTTGTA GGGCAGCGGC GCAGCCGCTT
46441 CCTTCGCCTT GGTGGTCACG GACACGATGC GGGCGGGTTG GCCGCTCACG GCGGCCGCGA
46501 TGCCCTCGGC GTGCTCCTTG TTGCTGAGGC GGCCTTTCTC GTCCACCGGA TCGCCGTCGG
46561 CGACCTGGTA ACGGGCCGGG AACTGAATGC CCTCGACCTC GAACTGGCCG TTCACCAGGT
46621 AGTAGTAGGT TTTCTGGTGG GCCGCGTTCT CGCGGCAACG GCGCACGACA AGGCCCAGGA
46681 TCGGAGTCTG CACGCGCCCC ACGCTCAACA GCCCCTGATA GCCCTTCGCG CGTGCCGCAA
46741 GCGTGTACAG GCGCGTGATG TTGAAGCCGT ATAGCTGGTC GCCGACGCTG CGGGCCTCGG
46801 CCGCAGCGGA CAGGCCGGCG AACTCGCGGT TGTCGCGCAT CGCGGCGAGC TGCCGGCGCA
46861 CGATCTTCAC GTTGTTGTCG TTGATAAGCA GCCGCTGCAC CGGCAGACGG CAGTTGGCGT
46921 ATTCCAGGAT TTCATCGACC AGAAGCTGGC CTTCGTCGTC CGGGTCGCCG GCGTGAACCA
46981 CGCTTTTCGC CTGCTTCAAC AGGCTGAGGA TGGTCTTGAA CTGAGCTTTC GCACCGGCAT
47041 CGCCGGACGG TTTCTTGCGC CAGGGAATAT GGACGATGGG CAGGTCGGCC ATGTTCCAGT
47101 TGGCGTAGCG CTCGTCGTAG TCCTCCGGGT CTAGCAAGGC CAGCATGTGA CCGTAGCACC
47161 AGGTCACGCG GTCGGAGCCG CATTCGTAAT AGCCGTCCTT GCGGCTGCCG CCGCCCAGGC
47221 CCTCGACGAT GGCTTTTGCC AGCTCCGGTT TTTCAGCGAT TACAAGGCGT TCAAATTGCA
47281 TATATCCCCC TACCCTCACC AGGTCAGAAC CGGCCTGATG ACGGTGATGA TTTGCGAACG
47341 ATTGACAGGC CCGAAGTAGC GGCCGTCGAA AGACGTGTCG CTTACGTCGG ACATAAGCAG

47401 AACCTCGGCG GTCCCCAGGG TGTAGCTGTC GGACTGATAA CGAGGCAGCG GCCGTCCTGA
47461 TGGATCGGCC TTGATGAGCG CGCTGTGAGG CAGCAGCCCG CCATTCACGC GCACGCCGGC
47521 GTCGGTGATG GCAACCTCGT CGCCTTTAGC GGCTAAAACT CGCTTCATCA TGTAGCCGTA
47581 GTCGCCGGGG CAGAAACCGC CGGCGATGTA GCCCCGCTCC TTGGCGTCCG AAAACACGCC
47641 GACTTGCGGC GGGCAGAACA TGACGTAAGC CCCCTTCTCC ACCGGCGCAT TCGATTTCCA
47701 GTACAGGCCG ACCGGAATGC TTTTGGTGGT GTTGACCTTC GCGCCGGCGA GATAGGCCGC
47761 GCCGGCGAGC AACAAGGCCG CGCCGCCTCC GATGGCGACG TACTTGGTGA GGCGCTGGAA
47821 GCGGCTCATA TCGTGATCCC CTCCCCTTCC TCGACGGTGG CCGTCTGGAT CAGCTTGTCG
47881 CTGACCTTCG GAGCCGGTAC GGCCGCGCGG GCCTGGAATA TCGGGTCTTT GAAGTAGAGC
47941 GGCTGCTTGC CGTAGATCGC GGGATAGCCG GCGACGTACA CAACCATGTC GCCCGCCTCT
48001 TCAATGCTGC CGTCGGCGCT CTTCTTCGGC CCCGGCATGC GCAGGCATTC ATCGGGGGTC
48061 AGCAATGGCC GCTGCACTTC CTGGAAGGTC CGCGAGACGT TGCCCAACAG CGCCGACGTG
48121 CGGCGGCCGC TCGTCGTGAT CTGCTCCTTC ACGATGGTCG TGGTGCCTGT CAGTTTTGAC
48181 AGGTGCTCGG CCGTCTCCAC GCGGTTCGGC GGGTAGGCGT TCTGCACGTG GCAGTTCGAC
48241 GTGATGCTTT CGTCGTGGCC GTAGCCGGTT TCGCGGCTCT TGAGCTGGTT AATGTCCTGG
48301 CAGATGAGGT AGCACTTGAT GCCGTAGCCG GCGACGAAGG CAAGGGACTC TTGCAGGATT
48361 TCGAGCTTGC CCAGGCTGGG GAACTCGTCG AGCATCATCA GCAGACGATG CTTGTAGTGC
48421 GCGACAGGAC GGCCGTTCTC GAAGTCCATC TTGTCGGCCA GCAGCCGGAC GATCATGTTG
48481 ACCATGACGC GCACCAGAGG CCGCAGACGG GCCTTGTCGT TGGGCTGCGT CACGATGAAC
48541 AGGCTTACCG GGTCGTCGTG GTGCATCAGT TGCTTGATGC GGAAGTCGGA CTTGCTGACG
48601 TTGCGGGCCA CAACCGGGTC GCGGTACAGG GCCAGGTAGG ACTTGGCGGT GGACAGCACG
48661 GAACCGGATT CTTCCTCCGG GCGGTCCATC ATGTCGCGGG CCGCAGAGCC GACCGCAGGG
48721 TGGTTCTGCC CGTCAACGTG GCCGTAGGTG GTCATTTCCA TCCAAAGCTC GCCCACGTCG
48781 CGGTTCGGGT CGGCAAGCAT GCCGTCCACC GACGGCAGGG TGGCCGGCGT ACCCTCGTTC
48841 TTAGCCTTGT AGAGCGCGTG CAGGATGACG CCGACAAGCA GCGCCTGGCT GGTTTTCTGC
48901 CAGTGCGATT CCAGGCCCTT GCCGTCCGGA TCGACGATCA GGGTGGCAAG GTTCTGCACG
48961 TCGCCAACCT CGTACTCGGT CCCCAAGCGG ATTTCATCGA GCGGGTTCCA GCACGCGCTA
49021 CCCTGCGCGG ATGCCGGCTC AAAGCGCACG ACCTTGTTGC GGGCATGCTT CTTCCGCCAG
49081 CCGGCGGTCA GCGCCCACAA CTCGCCTTTC AGGTCGGTGA TGACGGCGCT GTGCGCCCAG
49141 GAAAGCAGCG TCGGAACGAC CAGGCCGACG CCCTTGCCGG AGCGCGTCGG CGCGTAGGTC
49201 AAGACGTGCT CGGGGCCGTT GTGCCGCAGG TAGTGGAACT TGCCGTCCTT GTCCTGCCAG
49261 CCGCCCACAT AGACGCCGCT GGAAGTGGGC GGGTGTTTGC CTGACACCAG CTCGACGACG
49321 GTGCGCGGCC GGGGCAGCAG GCCGGCGGCC TGTATGTCCT TCTTGTCGGC CCAGCGGGCC
49381 GAACCGTGCA GATAGTCGTT CGCCTTGCCG GTGTTCGCCT TGACCATCTG CGTGACGGCC
49441 GTGCCCAGCA GGCCCACGGT CGAAACGACC ATACCCATGC TGGCCGCGCG CATGAAATCG
49501 TCGGGATATT GGCCGTACCA CTTGCCGGCC CATTGAAGGA TCGACCAGGG CGTGTAGACG
49561 TGGTTGATAT TCCAGCCAAG TCCGGCCTGA TACTGGAAGG AATGGGCGAA ATATTGCGTC
49621 GCGGTCTGCA AGCCTGCCCC AAGGGACAGG CCGGCGAGGA TGGGAACGGT CTTGCTGGCC
49681 TTCGGTTTTT TCGCCCGTAT CTGTGGCCCC ACGGCGTTGT TTCGGTTCTT CATCTACTCC
49741 TACCTCGGGT AGTTTTAAGG GAGCCTCGCG GGGTCACGGT GACGGGATCA CCGATGGCGA
49801 GGCGCTTCAT GCGTTGCACC GTGGCCTTAT CGACGGGCAG CACCAGAATC TCGTCGTTTT
49861 CTTTCCTCAA CAGGGCCAGC GCCTGGTCCT CGACGTTCCG GGTGCCTGCA TAGGACAGCG
49921 CACCAACATA ATCAGTATAT CGTGCATGCT TCGGTATATC GAAGCCGTTT AGCCGCTTTT
49981 GCTCGCGCTC GGCAACATAT TTCTCGGCCG CCGCGATCTG TTCGGGCTTT AGCCCTCTTC
50041 CTGGCCCAGA AACTCCCCGT CGCAGTGCGT GAGCTGGTTC GGCTCCTTGC TGCTCCACGT
50101 GACCAGGAAC ATCACGCGGC AATAGCATTT CAGCTCCGCC GGCGATGCGA ACCACACCGA
50161 GTTGGGACAG CGCTCGCAAA CGGTTTTGGC TTTGGGGCGG CGGCTTTCGT CCAATGCGTC
50221 CAACGTTGGG CTTGCGGAGT GCGACGGTTC CGCCGGCGCT GACGGCGCGA GCGTCCCGTC
50281 GGTCGCCGTC GCCGCCTGTG GCGTTGAGGG TGGTTCTGGC TGCGGCAGGT CGAATGCCTC
50341 CATCGCCGCC GCGATCTCTT CGTCCGTCAT TTCGTTCGGG TTGCTCATGT GCTTGCTCCT
50401 TCGTCAGTAG TTCTTGACGG CGGCGCTCAA GGGCGGCGTC GTCAAAGGTG ATTGCCAGAC
50461 GGCCAGCGGC GGCCGCCTGC GCGATCCGCT CCTTGAACTC TGCTGTGCCG TTGACGGTGA
50521 TCCGGTCGCC GAAGCGCTCC ATTGCCAGGC GCAGGGCGGC GTCCAGGCCG TCCGTGGTGG

50581 CCTCGCGCGA GACTTGCAGG CGGTCGCCGT CGTCGCGGAC GGCGCTGCTG CCGACGCGAT
50641 AGATGATGGT TCCCTTCTTC GTGATGTTGT CCGTCACGGC CGCATGGCCC GGCTTGGCCT
50701 CGCCGCTGCC CTGGATGGTG TTGCCCTTGA GGTCGCTGCG GCCCTCGCGT GCGCGCAGCG
50761 CGGCCAGGGC CTTGTCGTCG CCCTTCATCG CCTCGGCCTT GAGCCAGTCG GCCCACGCGC
50821 GGCGCTGCGT GCGCTCCTGG ACCGCCTGAC GGCCCTGCCG GTACTCGCGG TTGATCTTGT
50881 CCAGGTCGGC GCGCAGAGCC TTGTGCGCCT GCGCGTACAT CAGTCGCTTT GCAATGCGCC
50941 CCTCGCCCAG CAGCTTGATA GCGGCGCGGC GCAGCCGGTT GCTGCGCATC GCGGCTTCAA
51001 TCAGGCGGTC ACGACGCCGG CGCAGCGTGT CCAGCTCGCC CTTGCGCACG GCCCCCATTT
51061 CCTGGCGTTC AGACTGATAC CGGGCGTATA GCTCGGTGGT GTCGATGCGG GTCTTGAGCG
51121 GCTTCGCTCG ATACTCCCGC CGCCGGGGGG CTTCGCCGCC CTCGGCTGGC GTGAATGCCC
51181 CGAATCGGGC TTCGAGCTTC GGCTTGGACA GGTCGCGCGA AACGGTGCTG GCCTTGACCG
51241 TCGTGCCGTC GCCGGCCTCG AAGATGAAGC CGTTTCCGCG CTCGCGCAGC TTAAGCCCGT
51301 TTTCCCGCAG GACGCGGTGC AGGTCCTCCC AGGATTGCGC CGCTTGCAGC TCCGGCAGGC
51361 ATTCGCGCTT GATCCAGCCG ACCAGGCTTT CCACGCCCGC GTGCCGCTCC ATGTCGTTCG
51421 CGCGGTTCTC GGAAACGCGC TGCCGCGTTT CGTGATTGTC ACGCTCAAGC CCGTAGTCCC
51481 GTTCGAGCGT CGCGCAGAGG TCAGCGAGGG CGCGGTAGGC CCGATACGGC TCATGGATGG
51541 TGTTTCGGGT CGGGTGAATC TTGTTGATGG CGATATGGAT GTGCAGGTTG TCGGTGTCGT
51601 GATGCACGGC ACTGACGCGC TGATGCTCGG CGAAGCCAAG CCCAGCGCAG ATGCGGTCCT
51661 CAATCGCGCG CAACGTCTCC GCGTCGGGCT TCTCTCCCGC GCGGAAGCTA ACCAGCAGGT
51721 GATAGGTCTT GTCGGCCTCG GAACGGGTGT TGCCGTGCTG GGTCGCCATC ACCTCGGCCA
51781 TGACAGCGGG CAGGGTGTTT GCCTCGCAGT TCGTGACGCG CACGTGACCC AGGCGCTCGG
51841 TCTTGCCTTG CTCGTCGGTG ATGTACTTCA CCAGCTCCGC GAAGTCGCTC TTCTTGATGG
51901 AGCGCATGGG GACGTGCTTG GCAATCACGC GCACCCCCCG GCCGTTTTAG CGGCTAAAAA
51961 AGTCATGGCT CTGCCCTCGG GCGGACCACG CCCATCATGA CCTTGCCAAG CTCGTCCTGC
52021 TTCTCTTCGA TCTTCGCCAG CAGGGCGAGG ATCGTGGCAT CACCGAACCG CGCCGTGCGC
52081 GGGTCGTCGG TGAGCCAGAG TTTCAGCAGG CCGCCCAGGC GGCCCAGGTC GCCATTGATG
52141 CGGGCCAGCT CGCGGACGTG CTCATAGTCC ACGACGCCCG TGATTTTGTA GCCCTGGCCG
52201 ACGGCCAGCA GGTAGGCCGA CAGGCTCATG CCGGCCGCCG CCGCCTTTTC CTCAATCTCT
52261 CTTCGTTCGT CTGGAAGGCA GTACACCTTG ATAGGTGGGC TGCCCTTCCT GGTTGGCTTG
52321 GTTTCATCAG CCATCCGCTT GCCGAATTCT GACGCCGTTG GATACACCAA GGAAAGTCTA
52381 CACGAACCCT TTGGCAAAAT CCTGTATATC GTGCGAAAAA GGATGGATAT ACCGAAAAAA
52441 TCGCTATAAT GACCCCGAAG CAGGGTTATG CAGCGGAAAA GCGCTGCTTC CCTGCTGTTT
52501 TGTGGAATAT CTACCGACTG GAAACAGGCA AATGCAGGAA ATTACTGAAC TGAGGGGACA
52561 GGCGAGAGAC GATGCCAAAG AGCTACACCG ACGAGCTGGC CGAGTGGGTT GAATCCCGCG
52621 CGGCCAAGAA GCGCCGGCGT GATGAGGCTG CGGTTGCGTT CCTGGCGGTG AGGGCGGATG
52681 TCGAGGCGGC GTTAGCGTCC GGCTATGCGC TCGTCACCAT TTGGGAGCAC ATGCGGGAAA
52741 CGGGGAAGGT CAAGTTCTCC TACGAGACGT TCCGCTCGCA CGCCAGGCGG CACATCAAGG
52801 CCAAGCCCGC CGATGTGCCC GCACCGCAGG CCAAGGCTGC GGAACCCGCG CCGGCACCCA
52861 AGACGCCGGA GCCACGGCGG CCGAAGCAGG GGGGCAAGGC TGAAAAGCCG GCCCCCGCTG
52921 CGGCCCCGAC CGGCTTCACC TTCAACCCAA CACCGGACAA AAAGGATCTA CTGTAATGGC
52981 GAAAATTCAC ATGGTTTTGC AGGGCAAGGG CGGGGTCGGC AAGTCGGCCA TCGCCGCGAT
53041 CATTGCGCAG TACAAGATGG ACAAGGGGCA GACACCCTTG TGCATCGACA CCGACCCGGT
53101 GAACGCGACG TTCGAGGGCT ACAAGGCCCT GAACGTCCGC CGGCTGAACA TCATGGCCGG
53161 CGACGAAATT AACTCGCGCA ACTTCGACAC CCTGGTCGAG CTGATTGCGC CGACCAAGGA
53221 TGACGTGGTG ATCGACAACG GTGCCAGCTC GTTCGTGCCT CTGTCGCATT ACCTCATCAG
53281 CAACCAGGTG CCGGCTCTGC TGCAAGAAAT GGGGCATGAG CTGGTCATCC ATACCGTCGT
53341 CACCGGCGGC CAGGCTCTCC TGGACACGGT GAGCGGCTTC GCCCAGCTCG CCAGCCAGTT
53401 CCCGGCCGAA GCGCTTTTCG TGGTCTGGCT GAACCCGTAT TGGGGGCCTA TCGAGCATGA
53461 GGGCAAGAGC TTTGAGCAGA TGAAGGCGTA CACGGCCAAC AAGGCCCGCG TGTCGTCCAT
53521 CATCCAGATT CCGGCCCTCA AGGAAGAAAC CTACGGCCGC GATTTCAGCG ACATGCTGCA
53581 AGAGCGGCTG ACGTTCGACC AGGCGCTGGC CGATGAATCG CTCACGATCA TGACGCGGCA
53641 ACGCCTCAAG ATCGTGCGGC GCGGCCTGTT TGAACAGCTC GACGCGGCGG CCGTGCTATG
53701 AGCGACCAGA TTGAAGAGCT GATCCGGGAG ATTGCGGCCA AGCACGGCAT CGCCGTCGGC

53761 CGCGACGACC CGGTGCTGAT CCTGCATACC ATCAACGCCC GGCTCATGGC CGACAGTGCG
53821 GCCAAGCAAG AGGAAATCCT TGCCGCGTTC AAGGAAGAGC TGGAAGGGAT CGCCCATCGT
53881 TGGGGCGAGG ACGCCAAGGC CAAAGCGGAG CGGATGCTGA ACGCGGCCCT GGCGGCCAGC
53941 AAGGACGCAA TGGCGAAGGT AATGAAGGAC AGCGCCGCGC AGGCGGCCGA AGCGATCCGC
54001 AGGGAAATCG ACGACGGCCT TGGCCGCCAG CTCGCGGCCA AGGTCGCGGA CGCGCGGCGC
54061 GTGGCGATGA TGAACATGAT CGCCGGCGGC ATGGTGTTGT TCGCGGCCGC CCTGGTGGTG
54121 TGGGCCTCGT TATGAATCGC AGAGGCGCAG ATGAAAAAGC CCGGCGTTGC CGGGCTTTGT
54181 TTTTGCGTTA GCTGGGCTTG TTTGACAGGC CCAAGCTCTG ACTGCGCCCG CGCTCGCGCT
54241 CCTGGGCCTG TTTCTTCTCC TGCTCCTGCT TGCGCATCAG GGCCTGGTGC CGTCGGGCTG
54301 CTTCACGCAT CGAATCCCAG TCGCCGGCCA GCTCGGGATG CTCCGCGCGC ATCTTGCGCG
54361 TCGCCAGTTC CTCGATCTTG GGCGCGTGAA TGCCCATGCC TTCCTTGATT TCGCGCACCA
54421 TGTCCAGCCG CGTGTGCAGG GTCTGCAAGC GGGCTTGCTG TTGGGCCTGC TGCTGCTGCC
54481 AGGCGGCCTT TGTACGCGGC AGGGACAGCA AGCCGGGGGC ATTGGACTGT AGCTGCTGCA
54541 AACGCGCCTG CTGACGGTCT ACGAGCTGTT CTAGGCGGTC CTCGATGCGC TCCACCTGGT
54601 CATGCTTTGC CTGCACGTAG AGCGCAAGGG TCTGCTGGTA GGTCTGCTCG ATGGGCGCGG
54661 ATTCTAAGAG GGCCTGCTGT TCCGTCTCGG CCTCCTGGGC CGCCTGTAGC AAATCCTCGC
54721 CGCTGTTGCC GCTGGACTGC TTTACTGCCG GGGACTGCTG TTGCCCTGCT CGCGCCGTCG
54781 TCGCAGTTCG GCTTGCCCCC ACTCGATTGA CTGCTTCATT TCGAGCCGCA GCGATGCGAT
54841 CTCGGATTGC GTCAACGGAC GGGGCAGCGC GGAGGTGTCC GGCTTCTCCT TGGGTGAGTC
54901 GGTCGATGCC ATAGCCAAAG GTTTCCTTCC AAAATGCGTC CATTGCTGGA CCGTGTTTCT
54961 CATTGATGCC CGCAAGCATC TTCGGCTTGA CCGCCAGGTC AAGCGCGCCT TCATGGGCGG
55021 TCATGACGGA CGCCGCCATG ACCTTGCCGC CGTTGTTCTC GATGTAGCCG CGTAATGAGG
55081 CAATGGTGCC GCCCATCGTC AGCGTGTCAT CGACAACGAT GTACTTCTGG CCGGGGATCA
55141 CCTCCCCCTC GAAAGTCGGG TTGAACGCCA GGCGATGATC TGAACCGGCT CCGGTTCGGG
55201 CGACCTTCTC CCGCTGCACA ATGTCCGTTT CGACCTCAAG GCCAAGGCGG TCGGCCAGAA
55261 CGACCGCCAT CATGGCCGGA ATCTTGTTGT TCCCCGCCGC CTCGACGGCG AGGACTGGAA
55321 CGATGCGGGG CTTGTCGTCG CCGATCAGCG TCTTGAGCTG GGCAACAGTG TCGTCCGAAA
55381 TCAGGCGCTC GACCAAATTA AGCGCCGCTT CCGCGTCGCC CTGCTTCGCA GCCTGGTATT
55441 CAGGCTCGTT GGTCAAAGAA CCAAGGTCGC CGTTGCGAAC CACCTTCGGG AAGTCTCCCC
55501 ACGGTGCGCG CTCGGCTCTG CTGTAGCTGC TCAAGACGCC TCCCTTTTTA GCCGCTAAAA
55561 CTCTAACGAG TGCGCCCGCG ACTCAACTTG ACGCTTTCGG CACTTACCTG TGCCTTGCCA
55621 CTTGCGTCAT AGGTGATGCT TTTCGCACTC CCGATTTCAG GTACTTTATC GAAATCTGAC
55681 CGGGCGTGCA TTACAAAGTT CTTCCCCACC TGTTGGTAAA TGCTGCCGCT ATCTGCGTGG
55741 ACGATGCTGC CGTCGTGGCG CTGCGACTTA TCGGCCTTTT GGGCCATATA GATGTTGTAA
55801 ATGCCAGGTT TCAGGGCCCC GGCTTTATCT ACCTTCTGGT TCGTCCATGC GCCTTGGTTC
55861 TCGGTCTGGA CAATTCTTTG CCCATTCATG ACCAGGAGGC GGTGTTTCAT TGGGTGACTC
55921 CTGACGGTTG CCTCTGGTGT TAAACGTGTC CTGGTCGCTT GCCGGCTAAA AAAAAGCCGA
55981 CCTCGGCAGT TCGAGGCCGG CTTTCCCTAG AGCCGGGCGC GTCAAGGTTG TTCCATCTAT
56041 TTTAGTGAAC TGCGTTCGAT TTATCAGTTA CTTTCCTCCC GCTTTGTGTT TCCTCCCACT
56101 CGTTTCCGCG TCTAGCCGAC CCCTCAACAT AGCGGCCTCT TCTTGGGCTG CCTTTGCCTC
56161 TTGCCGCGCT TCGTCACGCT CGGCTTGCAC CGTCGTAAAG CGCTCGGCCT GCCTGGCCGC
56221 CTCTTGCGCC GCCAACTTCC TTTGCTCCTG GTGGGCCTCG GCGTCGGCCT GCGCCTTCGC
56281 TTTCACCGCT GCCAACTCCG TGCGCAAACT CTCCGCTTCG CGCCTGGTGG CGTCGCGCTC
56341 GCCGCGAAGC GCCTGCATTT CCTGGTTGGC CGCGTCCAGG GTCTTGCGGC TCTCTTCTTT
56401 GAATGCGCGG GCGTCCTGGT GAGCGTAGTC CAGCTCGGCG CGCAGCTCCT GCGCTCGACG
56461 CTCCACCTCG TCGGCCCGCT GCGTCGCCAG CGCGGCCCGC TGCTCGGCTC CTGCCAGGGC
56521 GGTGCGTGCT TCGGCCAGGG CTTGCCGCTG GCGTGCGGCC AGCTCGGCCG CCTCGGCGGC
56581 CTGCTGCTCT AGCAATGTAA CGCGCGCCTG GGCTTCTTCC AGCTCGCGGG CCTGCGCCTC
56641 GAAGGCGTCG GCCAGCTCCC CGCGCACGGC TTCCAACTCG TTGCGCTCAC GATCCCAGCC
56701 GGCTTGCGCT GCCTGCAACG ATTCATTGGC AAGGGCCTGG GCGGCTTGCC AGAGGGCGGC
56761 CACGGCCTGG TTGCCGGCCT GCTGCACCGC GTCCGGCACC TGGACTGCCA GCGGGGCGGC
56821 CTGCGCCGTG CGCTGGCGTC GCCATTCGCG CATGCCGGCG CTGGCGTCGT TCATGTTGAC
56881 GCGGGCGGCC TTACGCACTG CATCCACGGT CGGGAAGTTC TCCCGGTCGC CTTGCTCGAA

56941 CAGCTCGTCC GCAGCCGCAA AAATGCGGTC GCGCGTCTCT TTGTTCAGTT CCATGTTGGC
57001 TCCGGTAATT GGTAAGAATA ATAATACTCT TACCTACCTT ATCAGCGCAA GAGTTTAGCT
57061 GAACAGTTCT CGACTTAACG GCAGGTTTTT TAGCGGCTGA AGGGCAGGCA AAAAAAGCCC
57121 CGCACGGTCG GCGGGGGCAA AGGGTCAGCG GGAAGGGGAT TAGCGGGCGT CGGGCTTCTT
57181 CATGCGTCGG GGCCGCGCTT CTTGGGATGG AGCACGACGA AGCGCGCACG CGCATCGTCC
57241 TCGGCCCTAT CGGCCCGCGT CGCGGTCAGG AACTTGTCGC GCGCTAGGTC CTCCCTGGTG
57301 GGCACCAGGG GCATGAACTC GGCCTGCTCG ATGTAGGTCC ACTCCATGAC CGCATCGCAG
57361 TCGAGGCCGC GTTCCTTCAC CGTCTCTTGC AGGTCGCGGT ACGCCCGCTC GTTGAGCGGC
57421 TGGTAACGGG CCAATTGGTC GTAAATGGCT GTCGGCCATG AGCGGCCTTT CCTGTTGAGC
57481 CAGCAGCCGA CGACGAAGCC GGCAATGCAG GCCCCTGGCA CAACCAGGCC GACGCCGGGG
57541 GCAGGGGATG GCAGCAGCTC GCCAACCAGG AACCCCGCCG CGATGATGCC GATGCCGGTC
57601 AACCAGCCCT TGAAACTATC CGGCCCCGAA ACACCCCTGC GCATTGCCTG GATGCTGCGC
57661 CGGATAGCTT GCAACATCAG GAGCCGTTTC TTTTGTTCGT CAGTCATGGT CCGCCCTCAC
57721 CAGTTGTTCG TATCGGTGTC GGACGAACTG AAATCGCAAG AGCTGCCGGT ATCGGTCCAG
57781 CCGCTGTCCG TGTCGCTGCT GCCGAAGCAC GGCGAGGGGT CCGCGAACGC CGCAGACGGC
57841 GTATCCGGCC GCAGCGCATC GCCCAGCATG GCCCCGGTCA GCGAGCCGCC GGCCAGGTAG
57901 CCCAGCATGG TGCTGTTGGT CGCCCCGGCC ACCAGGGCCG ACGTGACGAA ATCGCCGTCA
57961 TTCCCTCTGG ATTGTTCGCT GCTCGGCGGG GCAGTGCGCC GCGCCGGCGG CGTCGTGGAT
58021 GGCTCGGGTT GGCTGGCCTG CGACGGCCGG CGAAAGGTGC GCAGCAGCTC GTTATCGACC
58081 GGCTGCGGCG TCGGGGCCGC CGCCTTGCGC TGCGGTCGGT GTTCCTTCTT CGGCTCGCGC
58141 AGCTTGAACA GCATGATCGC GGAAACCAGC AGCAACGCCG CGCCTACGCC TCCCGCGATG
58201 TAGAACAGCA TCGGATTCAT TCTTCGGTCC TCCTTGTAGC GGAACCGTTG TCTGTGCGGC
58261 GCGGGTGGCC CGCGCCGCTG TCTTTGGGGA TCAGCCCTCG ATGAGCGCGA CCAGTTTCAC
58321 GTCGGCAAGG TTCGCCTCGA ACTCCTGGCC GTCGTCCTCG TACTTCAACC AGGCATAGCC
58381 TTCCGCCGGC GGCCGACGGT TGAGGATAAG GCGGGCAGGG CGCTCGTCGT GCTCGACCTG
58441 GACGATGGCC TTTTTCAGCT TGTCCGGGTC CGGCTCCTTC GCGCCCTTTT CCTTGGCGTC
58501 CTTACCGTCC TGGTCGCCGT CCTCGCCGTC CTGGCCGTCG CCGGCCTCCG CGTCACGCTC
58561 GGCATCAGTC TGGCCGTTGA AGGCATCGAC GGTGTTGGGA TCGCGGCCCT TCTCGTCCAG
58621 GAACTCGCGC AGCAGCTTGA CCGTGCCGCG CGTGATTTCC TGGGTGTCGT CGTCAAGCCA
58681 CGCCTCGACT TCCTCCGGGC GCTTCTTGAA GGCCGTCACC AGCTCGTTCA CCACGGTCAC
58741 GTCGCGCACG CGGCCGGTGT TGAACGCATC GGCGATCTTC TCCGGCAGGT CCAGCAGCGT
58801 GACGTGCTGG GTGATGAACG CCGGCGACTT GCCGATTTCC TTGGCGATAT CGCCTTTCTT
58861 CTTGCCCTTC GCCAGCTCGC GGCCAATGAA GTCGGCAATT TCGCGCGGGG TCAGCTCGTT
58921 GCGTTGCAGG TTCTCGATAA CCTGGTCGGC TTCGTTGTAG TCGTTGTCGA TGAACGCCGG
58981 GATGGACTTC TTGCCGGCCC ACTTCGAGCC ACGGTAGCGG CGGGCGCCGT GATTGATGAT
59041 ATAGCGGCCC GGCTGCTCCT GGTTCTCGCG CACCGAAATG GGTGACTTCA CCCCGCGCTC
59101 TTTGATCGTG GCACCGATTT CCGCGATGCT CTCCGGGGAA AAGCCGGGGT TGTCGGCCGT
59161 CCGCGGCTGA TGCGGATCTT CGTCGATCAG GTCCAGGTCC AGCTCGATAG GGCCGGAACC
59221 GCCCTGAGAC GCCGCAGGAG CGTCCAGGAG GCTCGACAGG TCGCCGATGC TATCCAACCC
59281 CAGGCCGGAC GGCTGCGCCG CGCCTGCGGC TTCCTGAGCG GCCGCAGCGG TGTTTTTCTT
59341 GGTGGTCTTG GCTTGAGCCG CAGTCATTGG GAAATCTCCA TCTTCGTGAA CACGTAATCA
59401 GCCAGGGCGC GAACCTCTTT CGATGCCTTG CGCGCGGCCG TTTTCTTGAT CTTCCAGACC
59461 GGCACACCGG ATGCGAGGGC ATCGGCGATG CTGCTGCGCA GGCCAACGGT GGCCGGAATC
59521 ATCATCTTGG GGTACGCGGC CAGCAGCTCG GCTTGGTGGC GCGCGTGGCG CGGATTCCGC
59581 GCATCGACCT TGCTGGGCAC CATGCCAAGG AATTGCAGCT TGGCGTTCTT CTGGCGCACG
59641 TTCGCAATGG TCGTGACCAT CTTCTTGATG CCCTGGATGC TGTACGCCTC AAGCTCGATG
59701 GGGGACAGCA CATAGTCGGC CGCGAAGAGG GCGGCCGCCA GGCCGACGCC AAGGGTCGGG
59761 GCCGTGTCGA TCAGGCACAC GTCGAAGCCT TGGTTCGCCA GGGCCTTGAT GTTCGCCCCG
59821 AACAGCTCGC GGGCGTCGTC CAGCGACAGC CGTTCGGCGT TCGCCAGTAC CGGGTTGGAC
59881 TCGATGAGGG CGAGGCGCGC GGCCTGGCCG TCGCCGGCTG CGGGTGCGGT TTCGGTCCAG
59941 CCGCCGGCAG GGACAGCGCC GAACAGCTTG CTTGCATGCA GGCCGGTAGC AAAGTCCTTG
60001 AGCGTGTAGG ACGCATTGCC CTGGGGGTCC AGGTCGATCA CGGCAACCCG CAAGCCGCGC
60061 TCGAAAAAGT CGAAGGCAAG ATGCACAAGG GTCGAAGTCT TGCCGACGCC GCCTTTCTGG

```
60121 TTGGCCGTGA CCAAAGTTTT CATCGTTTGG TTTCCTGTTT TTTCTTGGCG TCCGCTTCCC
60181 ACTTCCGGAC GATGTACGCC TGATGTTCCG GCAGAACCGC CGTTACCCGC GCGTACCCCT
60241 CGGGCAAGTT CTTGTCCTCG AACGCGGCCC ACACGCGATG CACCGCTTGC GACACTGCGC
60301 CCCTGGTCAG TCCCAGCGAC GTTGCGAACG TCGCCTGTGG CTTCCCATCG ACTAAGACGC
60361 CCCGCGCTAT CTCGATGGTC TGCTGCCCCA CTTCCAGCCC CTGGATCGCC TCCTGGAACT
60421 GGCTTTCGGT AAGCCGTTTC TTCATGGATA ACACCCATAA TTTGCTCCGC GCCTTGGTTG
60481 AACATAGCGG TGACAGCCGC CAGCACATGA GAGAAGTTTA GCTAAACATT TCTCGCACGT
60541 CAACACCTTT AGCCGCTAAA ACTCGTCCTT GGCGTAACAA AACAAAAGCC CGGAAACCGG
60601 GCTTTCGTCT CTTGCCGCTT ATGGCTCTGC ACCCGGCTCC ATCACCAACA GGTCGCGCAC
60661 GCGCTTCACT CGGTTGCGGA TCGACACTGC CAGCCCAACA AAGCCGGTTG CCGCCGCCGC
60721 CAGGATCGCG CCGATGATGC CGGCCACACC GGCCATCGCC CACCAGGTCG CCGCCTTCCG
60781 GTTCCATTCC TGCTGGTACT GCTTCGCAAT GCTGGACCTC GGCTCACCAT AGGCTGACCG
60841 CTCGATGGCG TATGCCGCTT CTCCCCTTGG CGTAAAACCC AGCGCCGCAG GCGGCATTGC
60901 CATGCTGCCC GCCGCTTTCC CGACCACGAC GCGCGCACCA GGCTTGCGGT CCAGACCTTC
60961 GGCCACGGCG AGCTGCGCAA GGACATAATC AGCCGCCGAC TTGGCTCCAC GCGCCTCGAT
61021 CAGCTCTTGC ACTCGCGCGA AATCCTTGGC CTCCACGGCC GCCATGAATC GCGCACGCGG
61081 CGAAGGCTCC GCAGGGCCGG CGTCGTGATC GCCGCCGAGA ATGCCCTTCA CCAAGTTCGA
61141 CGACACGAAA ATCATGCTGA CGGCTATCAC CATCATGCAG ACGGATCGCA CGAACCCGCT
61201 GAA
//
```
