# Supplementary material for: Efficient inter-species conjugative transfer of a CRISPR nuclease for targeted bacterial killing
Source: Nat Commun. 2019 Oct 4;10:4544. doi: 10.1038/s41467-019-12448-3 (PMC6778077; doi:10.1038/s41467-019-12448-3)
Supplement: Supplementary file 9 — Supplementary Data 6 [file 41467_2019_12448_MOESM9_ESM.pdf]

```
LOCUS       pNuc-trans_TevSpCas9   10827 bp ds-DNA     circular     02-JUL-2019
DEFINITION  .
FEATURES             Location/Qualifiers
     terminator      9219..9280
                     /label="T7 terminator"
     misc_RNA        9142..9157
                     /label="crRNA"
     promoter        9018..9071
                     /label="pTet promoter"
     misc_RNA        9158..9217
                     /label="tracrRNA"
     CDS             4285..8952
                     /label="TevSpCas9"
     CDS             complement(3034..3963)
                     /label="araC"
     CDS             complement(110..769)
                     /label="chloramphenicol acetyl-transferase"
     rep_origin      1131..2044
                     /label="p15A Origin"
     promoter        4113..4243
                     /label="pBAD promoter"
     misc_feature    9286..10664
                     /label="Cen6-ArsH4-His3"
     oriT            2168..2938
                     /label="oriT"
     misc_feature    9074..9141
                     /label="246 bp stuffer"
     misc_feature    4272..4278
                     /label="RBS"
ORIGIN
        1 acgaccgggt cgaatttgct ttcgaatttc tgccattcat ccgcttatta tcACTTATTC
       61 AGGCGTAGCA ACCAGGCGTT TAAGGGCACC AATAACTGCC TTAAAAAAAT TACGCCCCGC
      121 CCTGCCACTC ATCGCAGTAC TGTTGTAATT CATTAAGCAT TCTGCCGACA TGGAAGCCAT
      181 CACAAACGGC ATGATGAACC TGAATCGCCA GCGGCATCAG CACCTTGTCG CCTTGCGTAT
      241 AATATTTGCC CATAGTGAAA ACGGGGGCGA AGAAGTTGTC CATATTGGCC ACGTTTAAAT
      301 CAAAACTGGT GAAACTCACC CAGGGATTGG CTGAGACGAA AAACATATTC TCAATAAACC
      361 CTTTAGGGAA ATAGGCCAGG TTTTCACCGT AACACGCCAC ATCTTGCGAA TATATGTGTA
      421 GAAACTGCCG GAAATCGTCG TGGTATTCAC TCCAGAGCGA TGAAAACGTT TCAGTTTGCT
      481 CATGGAAAAC GGTGTAACAA GGGTGAACAC TATCCCATAT CACCAGCTCA CCGTCTTTCA
      541 TTGCCATACG GAACTCCGGA TGAGCATTCA TCAGGCGGGC AAGAATGTGA ATAAAGGCCG
      601 GATAAAACTT GTGCTTATTT TTCTTTACGG TCTTTAAAAA GGCCGTAATA TCCAGCTGAA
      661 CGGTCTGGTT ATAGGTACAT TGAGCAACTG ACTGAAATGC CTCAAAATGT TCTTTACGAT
      721 GCCATTGGGA TATATCAACG GTGGTATATC CAGTGATTTT TTTCTCCATT TTAGCTTCCT
      781 TAGCTCCTGA AAATCTCGAT AACTCAAAAA ATACGCCCGG TAGTGATCTT ATTTCATTAT
      841 GGTGAAAGTT GGAACCTCTT ACGTGCCGAT CAACGTCTCA TTTTCGCCAA AAGTTGGCCC
      901 AGGGCTTCCC GGTATCAACA GGGACACCAG GATTTATTTA TTCTGCGAAG TGATCTTCCG
      961 TCACAGGTAT TTATTCGGCG CAAAGTGCGT CGGGTGATGC TGCCAACTTA CTGATTTAGT
     1021 GTATGATGGT GTTTTTGAGG TGCTCCAGTG GCTTCTGTTT CTATCAGCTG TCCCTCCTGT
     1081 TCAGCTACTG ACGGGGTGGT GCGTAACGGC AAAAGCACCG CCGGACATCA GCGCTAGCGG
     1141 AGTGTATACT GGCTTACTAT GTTGGCACTG ATGAGGGTGT CAGTGAAGTG CTTCATGTGG
     1201 CAGGAGAAAA AAGGCTGCAC CGGTGCGTCA GCAGAATATG TGATACAGGA TATATTCCGC
     1261 TTCCTCGCTC ACTGACTCGC TACGCTCGGT CGTTCGACTG CGGCGAGCGG AAATGGCTTA
     1321 CGAACGGGGC GGAGATTTCC TGGAAGATGC CAGGAAGATA CTTAACAGGG AAGTGAGAGG
```

1381 GCCGCGGCAA AGCCGTTTTT CCATAGGCTC CGCCCCCCTG ACAAGCATCA CGAAATCTGA
1441 CGCTCAAATC AGTGGTGGCG AAACCCGACA GGACTATAAA GATACCAGGC GTTTCCCCCT
1501 GGCGGCTCCC TCGTGCGCTC TCCTGTTCCT GCCTTTCGGT TTACCGGTGT CATTCCGCTG
1561 TTATGGCCGC GTTTGTCTCA TTCCACGCCT GACACTCAGT TCCGGGTAGG CAGTTCGCTC
1621 CAAGCTGGAC TGTATGCACG AACCCCCCGT TCAGTCCGAC CGCTGCGCCT TATCCGGTAA
1681 CTATCGTCTT GAGTCCAACC CGGAAAGACA TGCAAAAGCA CCACTGGCAG CAGCCACTGG
1741 TAATTGATTT AGAGGAGTTA GTCTTGAAGT CATGCGCCGG TTAAGGCTAA ACTGAAAGGA
1801 CAAGTTTTGG TGACTGCGCT CCTCCAAGCC AGTTACCTCG GTTCAAAGAG TTGGTAGCTC
1861 AGAGAACCTT CGAAAAACCG CCCTGCAAGG CGGTTTTTTC GTTTTCAGAG CAAGAGATTA
1921 CGCGCAGACC AAAACGATCT CAAGAAGATC ATCTTATTAA TCAGATAAAA TATTTCTAGA
1981 TTTCAGTGCA ATTTATCTCT TCAAATGTAG CACCTGAAGT CAGCCCCATA CGATATAAGT
2041 TGTAATTCTC ATGTTAGTCA TGCCCCGCGC CCACCGGAAG GAGCTGACTG GGTTGAAGGC
2101 TCTCAAGGGC ATCGGTCGAG ATCCCGGTGC CTAATGAGTG AGCTAACTTA CATTAATTGC
2161 GTTGCGCGAT CGTCTTGCCT TGCTCGTCGG TGATGTACTT ACAGCTCGAA GTGCCTCTTC
2221 TTGATGGAGC GCATGGGGAC GTGCTTGGCA ATCACGCGCA CCCCCCGGCC GTTTTAGCGG
2281 CTAAAAAAGT CATGGCTCTG CCCTCGGGCG GACCACGCCC ATCATGACCT TGCCAAGCTC
2341 GTCCTGCTTC TCTTCGATCT TCGCCAGCAG GGCGAGGATC GTGGCATCAC CGAACCGCGC
2401 CGTGCGCGGG TCGTCGGTGA GCCAGAGTTT CAGCAGGCCG CCCAGGCGGC CCAGGTCGCC
2461 ATTGATGCGG GCCAGCTCGC GGACGTGCTC ATAGTCCACG ACGCCCGTGA TTTTGTAGCC
2521 CTGGCCGACG GCCAGCAGGT AGGCCGACAG GCTCATGCCG GCCGCCGCCG CCTTTTCCTC
2581 AATCGCTCTT CGTTCGTCTG GAAGGCAGTA CACCTTGATA GGTGGGCTGC CCTTCCTGGT
2641 TGGCTTGGTT TCATCAGCCA TCCGCTTGCC CTCATCTGTT ACGCCGGCGG TAGCCGGCCA
2701 GCCTCGCAGA GCAGGATTCC CGTTGAGCAC CGCCAGGTGC GAATAAGGGA CAGTGAAGAA
2761 GGAACACCCG CTCGCGGGTG GGCCTACTTC ACCTATCCTG CCCGGCTGAC GCCGTTGGAT
2821 ACACCAAGGA AAGTCTACAC GAACCCTTTG GCAAAATCCT GTATATCGTG CGAAAAAGGA
2881 TGGATATACC GAAAAAATCG CTATAATGAC CCCGAAGCAG GGTTATGCAG CGGAAGATAT
2941 CGATGCATAA TGTGCCTGTC AAATGGACGA AGCAGGGATT CTGCAAACCC TATGCTACTC
3001 CGTCAAGCCG TCAATTGTCT GATTCGTTAC CAATTATGAC AACTTGACGG CTACATCATT
3061 CACTTTTTCT TCACAACCGG CACGGAACTC GCTCGGGCTG GCCCCGGTGC ATTTTTTAAA
3121 TACCCGCGAG AAATAGAGTT GATCGTCAAA ACCAACATTG CGACCGACGG TGGCGATAGG
3181 CATCCGGGTG GTGCTCAAAA GCAGCTTCGC CTGGCTGATA CGTTGGTCCT CGCGCCAGCT
3241 TAAGACGCTA ATCCCTAACT GCTGGCGGAA AAGATGTGAC AGACGCGACG GCGACAAGCA
3301 AACATGCTGT GCGACGCTGG CGATATCAAA ATTGCTGTCT GCCAGGTGAT CGCTGATGTA
3361 CTGACAAGCC TCGCGTACCC GATTATCCAT CGGTGGATGG AGCGACTCGT TAATCGCTTC
3421 CATGCGCCGC AGTAACAATT GCTCAAGCAG ATTTATCGCC AGCAGCTCCG AATAGCGCCC
3481 TTCCCCTTGC CCGGCGTTAA TGATTTGCCC AAACAGGTCG CTGAAATGCG GCTGGTGCGC
3541 TTCATCCGGG CGAAAGAACC CCGTATTGGC AAATATTGAC GGCCAGTTAA GCCATTCATG
3601 CCAGTAGGCG CGCGGACGAA AGTAAACCCA CTGGTGATAC CATTCGCGAG CCTCCGGATG
3661 ACGACCGTAG TGATGAATCT CTCCTGGCGG GAACAGCAAA ATATCACCCG GTCGGCAAAC
3721 AAATTCTCGT CCCTGATTTT TCACCACCCC CTGACCGCGA ATGGTGAGAT TGAGAATATA
3781 ACCTTTCATT CCCAGCGGTC GGTCGATAAA AAAATCGAGA TAACCGTTGG CCTCAATCGG
3841 CGTTAAACCC GCCACCAGAT GGGCATTAAA CGAGTATCCC GGCAGCAGGG GATCATTTTG
3901 CGCTTCAGCC ATACTTTTCA TACTCCCGCC ATTCAGAGAA GAAACCAATT GTCCATATTG
3961 CATCAGACAT TGCCGTCACT GCGTCTTTTA CTGGCTCTTC TCGCTAACCA AACCGGTAAC
4021 CCCGCTTATT AAAAGCATTC TGTAACAAAG CGGGACCAAA GCCATGACAA AAACGCGTAA
4081 CAAAAGTGTC TATAATCACG GCAGAAAAGT CCACATTGAT TATTTGCACG GCGTCACACT
4141 TTGCTATGCC ATAGCATTTT TATCCATAAG ATTAGCGGAT CCTACCTGAC GCTTTTTATC
4201 GCAACTCTCT ACTGTTTCTC CATACCCGTT TTTTTGGGCT AGCCCTGTAG AAATAATTGT
4261 TTAACTTTAA TAAGGAGATA TACCATGGGT AAAAGCGGAA TTTATCAGAT TAAAAATACT
4321 TTAAACAATA AAGTATATGT AGGAAGTGCT AAAGATTTTG AAAAGAGATG GAAGAGGCAT
4381 TTTAAAGATT TAGAAAAAGG ATGCCATTCT TCTATAAAAC TTCAGAGGTC TTTTAACAAA
4441 CATGGTAATG TGTTTGAATG TTCTATTTTG GAAGAAATTC CATATGAGAA AGATTTGATT
4501 ATTGAACGAG AAAATTTTTG GATTAAAGAG CTTAATTCTA AAATTAATGG ATACAATATT

4561 GCTGATGCAA CGTTTGGTGA TACGTGTTCT ACGCATCCAT TAAAAGAAGA AATTATTAAG
4621 AAACGTTCTG AAACTTTTAA AGCTAAGATG CTTAAACTTG GACCTGATGG TCGGAAAGCT
4681 CTTTACAGTA AACCCGGAAG TAAAAACGGG CGTTGGAATC CAGAAACCCA TAAGTTTTGT
4741 AAGTGCGGTG TTCGCATACA AACTTCTGCT TATACTTGTA GTAAATGCAG AAATGGTGGT
4801 TCTGGTGGTA CCGGAGGTAG CATGGATAAA AAGTATTCTA TTGGTTTAGA CATCGGCACT
4861 AATTCCGTTG GATGGGCTGT CATAACCGAT GAATACAAAG TACCTTCAAA GAAATTTAAG
4921 GTGTTGGGGA ACACAGACCG TCATTCGATT AAAAAGAATC TTATCGGTGC CCTCCTATTC
4981 GATAGTGGCG AAACGGCAGA GGCGACTCGC CTGAAACGAA CCGCTCGGAG AAGGTATACA
5041 CGTCGCAAGA ACCGAATATG TTACTTACAA GAAATTTTTA GCAATGAGAT GGCCAAAGTT
5101 GACGATTCTT TCTTTCACCG TTTGGAAGAG TCCTTCCTTG TCGAAGAGGA CAAGAAACAT
5161 GAACGGCACC CCATCTTTGG AAACATAGTA GATGAGGTGG CATATCATGA AAAGTACCCA
5221 ACGATTTATC ACCTCAGAAA AAAGCTAGTT GACTCAACTG ATAAAGCGGA CCTGAGGTTA
5281 ATCTACTTGG CTCTTGCCCA TATGATAAAG TTCCGTGGGC ACTTTCTCAT TGAGGGTGAT
5341 CTAAATCCGG ACAACTCGGA TGTCGACAAA CTGTTCATCC AGTTAGTACA AACCTATAAT
5401 CAGTTGTTTG AAGAGAACCC TATAAATGCA AGTGGCGTGG ATGCGAAGGC TATTCTTAGC
5461 GCCCGCCTCT CTAAATCCCG ACGGCTAGAA AACCTGATCG CACAATTACC CGGAGAGAAG
5521 AAAAATGGGT TGTTCGGTAA CCTTATAGCG CTCTCACTAG GCCTGACACC AAATTTTAAG
5581 TCGAACTTCG ACTTAGCTGA AGATGCCAAA TTGCAGCTTA GTAAGGACAC GTACGATGAC
5641 GATCTCGACA ATCTACTGGC ACAAATTGGA GATCAGTATG CGGACTTATT TTTGGCTGCC
5701 AAAAACCTTA GCGATGCAAT CCTCCTATCT GACATACTGA GAGTTAATAC TGAGATTACC
5761 AAGGCGCCGT TATCCGCTTC AATGATCAAA AGGTACGATG AACATCACCA AGACTTGACA
5821 CTTCTCAAGG CCCTAGTCCG TCAGCAACTG CCTGAGAAAT ATAAGGAAAT ATTCTTTGAT
5881 CAGTCGAAAA ACGGGTACGC AGGTTATATT GACGGCGGAG CGAGTCAAGA GGAATTCTAC
5941 AAGTTTATCA AACCCATATT AGAGAAGATG GATGGGACGG AAGAGTTGCT TGTAAAACTC
6001 AATCGCGAAG ATCTACTGCG AAAGCAGCGG ACTTTCGACA ACGGTAGCAT TCCACATCAA
6061 ATCCACTTAG GCGAATTGCA TGCTATACTT AGAAGGCAGG AGGATTTTTA TCCGTTCCTC
6121 AAAGACAATC GTGAAAAGAT TGAGAAAATC CTAACCTTTC GCATACCTTA CTATGTGGGA
6181 CCCCTGGCCC GAGGGAACTC TCGGTTCGCA TGGATGACAA GAAAGTCCGA AGAAACGATT
6241 ACTCCCTGGA ATTTTGAGGA AGTTGTCGAT AAAGGTGCGT CAGCTCAATC GTTCATCGAG
6301 AGGATGACCA ACTTTGACAA GAATTTACCG AACGAAAAAG TATTGCCTAA GCACAGTTTA
6361 CTTTACGAGT ATTTCACAGT GTACAATGAA CTCACGAAAG TTAAGTATGT CACTGAGGGC
6421 ATGCGTAAAC CCGCCTTTCT AAGCGGAGAA CAGAAGAAAG CAATAGTAGA TCTGTTATTC
6481 AAGACCAACC GCAAAGTGAC AGTTAAGCAA TTGAAAGAGG ACTACTTTAA GAAAATTGAA
6541 TGCTTCGATT CTGTCGAGAT CTCCGGGGTA GAAGATCGAT TTAATGCGTC ACTTGGTACG
6601 TATCATGACC TCCTAAAGAT AATTAAAGAT AAGGACTTCC TGGATAACGA AGAGAATGAA
6661 GATATCTTAG AAGATATAGT GTTGACTCTT ACCCTCTTTG AAGATCGGGA AATGATTGAG
6721 GAAAGACTAA AACATACGC TCACCTGTTC GACGATAAGG TTATGAAACA GTTAAAGAGG
6781 CGTCGCTATA CGGGCTGGGG ACGATTGTCG CGGAAACTTA TCAACGGGAT AAGAGACAAG
6841 CAAAGTGGTA AAACTATTCT CGATTTTCTA AAGAGCGACG GCTTCGCCAA TAGGAACTTT
6901 ATGCAGCTGA TCCATGATGA CTCTTTAACC TTCAAAGAGG ATATACAAAA GGCACAGGTT
6961 TCCGGACAAG GGGACTCATT GCACGAACAT ATTGCGAATC TTGCTGGTTC GCCAGCCATC
7021 AAAAAGGGCA TACTCCAGAC AGTCAAAGTA GTGGATGAGC TAGTTAAGGT CATGGGACGT
7081 CACAAACCGG AAAACATTGT AATCGAGATG GCACGCGAAA ATCAAACGAC TCAGAAGGGG
7141 CAAAAAAACA GTCGAGAGCG GATGAAGAGA ATAGAAGAGG GTATTAAAGA ACTGGGCAGC
7201 CAGATCTTAA AGGAGCATCC TGTGGAAAAT ACCCAATTGC AGAACGAGAA ACTTTACCTC
7261 TATTACCTAC AAAATGGAAG GGACATGTAT GTTGATCAGG AACTGGACAT AAACCGTTTA
7321 TCTGATTACG ACGTCGATCA CATTGTACCC CAATCCTTTT TGAAGGACGA TTCAATCGAC
7381 AATAAAGTGC TTACACGCTC GGATAAGAAC CGAGGGAAAA GTGACAATGT TCCAAGCGAG
7441 GAAGTCGTAA AGAAAATGAA GAACTATTGG CGGCAGCTCC TAAATGCGAA ACTGATAACG
7501 CAAAGAAAGT TCGATAACTT AACTAAAGCT GAGAGGGGTG GCTTGTCTGA ACTTGACAAG
7561 GCCGGATTTA TTAAACGTCA GCTCGTGGAA ACCCGCCAAA TCACAAAGCA TGTTGCACAG
7621 ATACTAGATT CCCGAATGAA TACGAAATAC GACGAGAACG ATAAGCTGAT TCGGGAAGTC
7681 AAAGTAATCA CTTTAAAGTC AAAATTGGTG TCGGACTTCA GAAAGGATTT TCAATTCTAT

7741 AAAGTTAGGG AGATAAATAA CTACCACCAT GCGCACGACG CTTATCTTAA TGCCGTCGTA
7801 GGGACCGCAC TCATTAAGAA ATACCCGAAG CTAGAAAGTG AGTTTGTGTA TGGTGATTAC
7861 AAAGTTTATG ACGTCCGTAA GATGATCGCG AAAAGCGAAC AGGAGATAGG CAAGGCTACA
7921 GCCAAATACT TCTTTTATTC TAACATTATG AATTTCTTTA AGACGGAAAT CACTCTGGCA
7981 AACGGAGAGA TACGCAAACG ACCTTTAATT GAAACCAATG GGGAGACAGG TGAAATCGTA
8041 TGGGATAAGG GCCGGGACTT CGCGACGGTG AGAAAAGTTT TGTCCATGCC CCAAGTCAAC
8101 ATAGTAAAGA AAACTGAGGT GCAGACCGGA GGGTTTTCAA AGGAATCGAT TCTTCCAAAA
8161 AGGAATAGTG ATAAGCTCAT CGCTCGTAAA AAGGACTGGG ACCCGAAAAA GTACGGTGGC
8221 TTCGATAGCC CTACAGTTGC CTATTCTGTC CTAGTAGTGG CAAAAGTTGA GAAGGGAAAA
8281 TCCAAGAAAC TGAAGTCAGT CAAAGAATTA TTGGGGATAA CGATTATGGA GCGCTCGTCT
8341 TTTGAAAAGA ACCCCATCGA CTTCCTTGAG GCGAAAGGTT ACAAGGAAGT AAAAAAGGAT
8401 CTCATAATTA AACTACCAAA GTATAGTCTG TTTGAGTTAG AAAATGGCCG AAAACGGATG
8461 TTGGCTAGCG CCGGAGAGCT TCAAAAGGGG AACGAACTCG CACTACCGTC TAAATACGTG
8521 AATTTCCTGT ATTTAGCGTC CCATTACGAG AAGTTGAAAG GTTCACCTGA AGATAACGAA
8581 CAGAAGCAAC TTTTTGTTGA GCAGCACAAA CATTATCTCG ACGAAATCAT AGAGCAAATT
8641 TCGGAATTCA GTAAGAGAGT CATCCTAGCT GATGCCAATC TGGACAAAGT ATTAAGCGCA
8701 TACAACAAGC ACAGGGATAA ACCCATACGT GAGCAGGCGG AAAATATTAT CCATTTGTTT
8761 ACTCTTACCA ACCTCGGCGC TCCAGCCGCA TTCAAGTATT TTGACACAAC GATAGATCGC
8821 AAACGATACA CTTCTACCAA GGAGGTGCTA GACGCGACAC TGATTCACCA ATCCATCACG
8881 GGATTATATG AAACTCGGAT AGATTTGTCA CAGCTTGGGG GTGACGGATC CCATCATCAC
8941 CACCACCATT GAGCGGCCGC ATAATGCTTA AGTCGAACAG AAAGTAATCG TATTGTACAC
9001 GGCCGCATAA TCGAAATTCC CTATCAGTGA TAGAGATTGA CATCCCTATC AGTGATAGAG
9061 ATACTGAGCA CGGGAGACCC ATGCCATAGC GTTGTTCGGA ATATGAATTT TTGAACAGAT
9121 TCACCAACAC CTAGTGGTCT CGTTTTAGAG CTAGAAATAG CAAGTTAAAA TAAGGCTAGT
9181 CCGTTATCAA CTTGAAAAAG TGGCACCGAG TCGGTGCTCC GCTGAGCAAT AACTAGCATA
9241 ACCCCTTGGG GCCTCTAAAC GGGTCTTGAG GGGTTTTTTG GCGAGCATCA CGTGCTATAA
9301 AAATAATTAT AATTTAAATT TTTTAATATA AATATATAAA TTAAAAATAG AAAGTAAAAA
9361 AAGAAATTAA AGAAAAAATA GTTTTTGTTT TCCGAAGATG TAAAAGACTC TAGGGGGATC
9421 GCCAACAAAT ACTACCTTTT ACCTTGCTCT TCCTGCTCTC AGGTATTAAT GCCGAATTGT
9481 TTCATCTTGT CTGTGTAGAA GACCACACAC GAAAATCCTG TGATTTTACA TTTTACTTAT
9541 CGTTAATCGA ATGTATATCT ATTTAATCTG CTTTTCTTGT CTAATAAATA TATATGTAAA
9601 GTACGCTTTT TGTTGAAATT TTTTAAACCT TTGTTTATTT TTTTTTCTTC ATTCCGTAAC
9661 TCTTCTACCT TCTTTATTTA CTTTCTAAAA TCCAAATACA AAACATAAAA ATAAATAAAC
9721 ACAGAGTAAA TTCCCAAATT ATTCCATCAT TAAAAGATAC GAGGCGCGTG TAAGTTACAG
9781 GCAAGCGATC CTAGTACACT CTATATTTTT TTATGCCTCG GTAATGATTT TCATTTTTTT
9841 TTTCCACCTA GCGGATGACT CTTTTTTTTT CTTAGCGATT GGCATTATCA CATAATGAAT
9901 TATACATTAT ATAAAGTAAT GTGATTTCTT CGAAGAATAT ACTAAAAAAT GAGCAGGCAA
9961 GATAAACGAA GGCAAAGATG ACAGAGCAGA AAGCCCTAGT AAAGCGTATT ACAAATGAAA
10021 CCAAGATTCA GATTGCGATC TCTTTAAAGG GTGGTCCCCT AGCGATAGAG CACTCGATCT
10081 TCCCAGAAAA AGAGGCAGAA GCAGTAGCAG AACAGGCCAC ACAATCGCAA GTGATTAACG
10141 TCCACACAGG TATAGGGTTT CTGGACCATA TGATACATGC TCTGGCCAAG CATTCCGGCT
10201 GGTCGCTAAT CGTTGAGTGC ATTGGTGACT TACACATAGA CGACCATCAC ACCACTGAAG
10261 ACTGCGGGAT TGCTCTCGGT CAAGCTTTTA AAGAGGCCCT AGGGGCCGTG CGTGGAGTAA
10321 AAAGGTTTGG ATCAGGATTT GCGCCTTTGG ATGAGGCACT TTCCAGAGCG GTGGTAGATC
10381 TTTCGAACAG GCCGTACGCA GTTGTCGAAC TTGGTTTGCA AAGGGAGAAA GTAGGAGATC
10441 TCTCTTGCGA GATGATCCCG CATTTTCTTG AAAGCTTTGC AGAGGCTAGC AGAATTACCC
10501 TCCACGTTGA TTGTCTGCGA GGCAAGAATG ATCATCACCG TAGTGAGAGT GCGTTCAAGG
10561 CTCTTGCGGT TGCCATAAGA GAAGCCACCT CGCCCAATGG TACCAACGAT GTTCCCTCCA
10621 CCAAAGGTGT TCTTATGTAG TTTTACACAG GAGTCTGGAC TTGACTGAAA CCTCAGGCAT
10681 TTGAGAAGCA CACGGTCACA CTGCTTCCGG TAGTCAATAA ACCGGTAAAC CAGCAATAGA
10741 CATAAGCGGC TATTTAACGA CCCTGCCCTG AACCGacgac cgggtcgaat ttgctttcga
10801 atttctgcca ttcatccgct tattatc
//
