# Supplementary material for: Efficient inter-species conjugative transfer of a CRISPR nuclease for targeted bacterial killing
Source: Nat Commun. 2019 Oct 4;10:4544. doi: 10.1038/s41467-019-12448-3 (PMC6778077; doi:10.1038/s41467-019-12448-3)
Supplement: Supplementary file 10 — Supplementary Data 7 [file 41467_2019_12448_MOESM10_ESM.pdf]

```
LOCUS       pTA-Mob_-_sequence_ver 52707 bp ds-DNA     circular     02-AUG-2019
DEFINITION  .
FEATURES             Location/Qualifiers
     CDS             complement(38443..38652)
                     /label="trbK"
     CDS             complement(30729..30980)
                     /label="parD"
     CDS             26294..26734
                     /label="traB"
     CDS             complement(29879..30382)
                     /label="putative ORF"
     CDS             3271..3489
                     /label="kleD"
     CDS             3952..4266
                     /label="kleF1 "
     CDS             2657..2872
                     /label="kleB"
     CDS             4389..5162
                     /label="klaA"
     CDS             7427..8521
                     /label="incC"
     CDS             complement(42999..45557)
                     /label="trbE"
     CDS             complement(40853..41335)
                     /label="trbH"
     CDS             5180..6316
                     /label="klaB"
     CDS             complement(38664..39440)
                     /label="trbJ"
     CDS             9665..10168
                     /label="korF"
     CDS             complement(33296..33991)
                     /label="fiwA"
     CDS             20604..22817
                     /label="traE"
     CDS             22823..23086
                     /label="traD"
     CDS             complement(34006..34425)
                     /label="upf31.7"
     CDS             7440..7745
                     /label="korA"
     CDS             complement(34442..35176)
                     /label="trbP"
     CDS             10891..11817
                     /label="kfrA"
     CDS             11975..12322
                     /label="kfrB"
     CDS             complement(14909..15313)
                     /label="traK"
     CDS             complement(42244..43002)
                     /label="trbF"
     CDS             complement(28620..29792)
                     /label="istA1"
```

```
CDS             8518..9594
              /label="korB"
CDS             complement(45868..46305)
              /label="trbC"
CDS             6313..7266
              /label="klaC"
CDS             complement(35507..36211)
              /label="trbN"
CDS             complement(36850..38436)
              /label="trbL"
CDS             complement(13750..14187)
              /label="traM"
CDS             48724..49581
              /label="trfA2"
CDS             20056..20589
              /label="traF"
CDS             2375..2608
              /label="kleA"
CDS             7742..8521
              /label="incC2"
CDS             complement(30421..30732)
              /label="parE"
CDS             3601..3924
              /label="kleE"
CDS             31131..31424
              /label="parC"
CDS             48433..49581
              /label="trfA1"
CDS             complement(14184..14909)
              /label="traL"
CDS             complement(46318..47214)
              /label="trbB"
CDS             complement(41339..42232)
              /label="trbG"
CDS             18152..20059
              /label="traG"
CDS             complement(47548..47859)
              /label="trbA"
CDS             31915..32574
              /label="parA1"
CDS             complement(27399..27692)
              /label="putative_ORF"
CDS             26742..27032
              /label="traA"
CDS             48034..48384
              /label="ssb"
CDS             3025..3255
              /label="kleC"
CDS             10178..10705
              /label="korG"
CDS             complement(27823..28620)
              /label="istB"
CDS             17497..17856
```

```
                     /label="traH"
     CDS             complement(45554..45865)
                     /label="trbD"
     CDS             187..720
                     /label="Gentamicin-3-N-acetyltransferase"
     rep_origin      51889..52659
                     /label="pBBR1 oriV"
     CDS             complement(36227..36826)
                     /label="trbM"
     CDS             complement(39457..40848)
                     /label="trbI"
     CDS             12351..13697
                     /label="kfrC"
     CDS             1243..1869
                     /label="klcB"
     CDS             15551..15922
                     /label="traJ"
     CDS             24038..26278
                     /label="traC2"
     CDS             complement(32598..33260)
                     /label="upf32.8"
     CDS             23093..26278
                     /label="traC1"
     CDS             1892..2149
                     /label="korC"
     CDS             complement(35213..35476)
                     /label="trbO"
     CDS             31109..31954
                     /label="parB"
     CDS             15957..18155
                     /label="traI"
ORIGIN
        1 aatgcaagta gcgtatgcgc tcacgcaact ggtccagaac cttgaccgaa cgCAGCGGTG
       61 GTAACGGCGC AGTGGCGGTT TTCATGGCTT GTTATGACTG TTTTTTTGTA CAGTCTATGC
      121 CTCGGGCATC CAAGCAGCAA GCGCGTTACG CCGTGGGTCG ATGTTTGATG TTATGGAGCA
      181 GCAACGATGT TACGCAGCAG CAACGATGTT ACGCAGCAGG GCAGTCGCCC TAAAACAAAG
      241 TTAGGTGGCT CAAGTATGGG CATCATTCGC ACATGTAGGC TCGGCCCTGA CCAAGTCAAA
      301 TCCATGCGGG CTGCTCTTGA TCTTTTCGGT CGTGAGTTCG GAGACGTAGC CACCTACTCC
      361 CAACATCAGC CGGACTCCGA TTACCTCGGG AACTTGCTCC GTAGTAAGAC ATTCATCGCG
      421 CTTGCTGCCT TCGACCAAGA AGCGGTTGTT GGCGCTCTCG CGGCTTACGT TCTGCCCAAG
      481 TTTGAGCAGC CGCGTAGTGA GATCTATATC TATGATCTCG CAGTCTCCGG AGAGCACCGG
      541 AGGCAGGGCA TTGCCACCGC GCTCATCAAT CTCCTCAAGC ATGAGGCCAA CGCGCTTGGT
      601 GCTTATGTGA TCTACGTGCA AGCAGATTAC GGTGACGATC CCGCAGTGGC TCTCTATACA
      661 AAGTTGGGCA TACGGGAAGA AGTGATGCAC TTTGATATCG ACCCAAGTAC CGCCACCTAA
      721 CAATTCGTTC AAGCCGAGAT CGGCTTCCCG GCCGCGGAGT TGTTCGGTAA ATTGTCACAA
      781 CGCCGCCAGG TGGATTAATA GACTGGATGG AGGCGGATAA AGTTGCAGGA CCACTTCTGC
      841 GCTCGGCCCT TCCGGCTGGC TGGTTTATTG CTGATAAATC TGGAGCCGGT GAGCGTGGAT
      901 CTCGCGGTAT CATTGCAGCA CTGGGGCCAG ATGGTAAGCC CTCCCGTATC GTAGTTATCT
      961 ACACGACGGG GAGTCAGGCA ACTATGGATG AACGAAATAG ACAGATCGCT GAGATAGGTG
     1021 CCTCACTGAT TAAGCATTGG TAACTGTCAG ACCAAGTTTA CTCATATATA CTTTAGATTG
     1081 ATTTAAAACT TCATTTTTAA TTTAAAAGGA TCTAGGTGAA GATCCTTTTT GATAATCTCA
     1141 TGACCAAAAT CCCTTAACGT GAGTTTTCGT TCCACTGAGC GTCAGACCCC TTTTATTTCC
     1201 GAAACGGGCT ATCGGTCCCA TTACGACCAT GCGCGCGGCG GCATGACGGT GGACCAGGTA
```

1261 GCGGACGGGG TTTTGCGCGC CCTGTTGCGC TCGCATCGCC GGTATCTGGA CGCCCGCGAC
1321 CAGGACCGCC TTGCCGACGA GCCACTGCCG GCGTGGCTCG CGGGTATCAC GCCACCGCCG
1381 CGCCGCGTGC GGGCCGTGGT CGAGGACTGG CGGAAGCCGG ACGAACTGCC GCCGGGCTTC
1441 GCCTGGGTTG ATGCCGTGCT GCCGGCGCAC CAGGCATTCA TCGCCCGCAA ATGGGCGGCC
1501 AGCGCCAAGG CGAAGCTCGC GGCCGCGCGT GCGAAAGCTC AGGAGCCGGC CGGGCAGCGC
1561 AGGGAGCCGG TTACACCGGC CAAGCCGGAG CCGGAGCCGG CCAAGGACGA GGACGCGCCG
1621 GCGTGGCCGG CGACGTTCTT TCCTGGCCTG CGGTGCGAGA TTGTGAGCGT GCATCACCCG
1681 GTTTTTGCCA AGGAAATCGG GAAGCACGTC ATCATTACGA AGATCAGTCC CGAAACGCGA
1741 CAGGTGTGGG CGCACGACGA CAAGCCGCCG CGCTATCGCA TCAATCGCAA CGGTAGAAAG
1801 GTTTGTGAAT ATGACCCGCG TTGCATCGAG TCGTGCTACG GCTACGACCA ATTGCGGGCC
1861 GCCATTTGAT AACGATTAGG AGAAAAGCAG CATGAGCGAC GTGAATATCC GGCTTGAGTG
1921 CCTGCGCCCG GCGGAACGCT GGGTGCAGCC GACCGGCGCA GAAATCCGGG AAGTCTTGCA
1981 CTTGGCCGGC CTCACCGGCG GACAGGCTGC GCGCATCTTG GGCTTGGGTG CCAAGGGCGA
2041 CCGCACGGTG CGGCGTTGGG TTGGCGAGGA TTCGCCGATC CCCTATGCCG CCTGGGCGAT
2101 CCTTTGCGAT CTAGCGGGGA TTGGGGCGAT CTGGAAAGGC CAGGGCTGAC GCCCGCAGGA
2161 CTTTACGCCA AGGGAGAGGA CTTTAGCGGC TAAAACCGCC AACCCTGATC GTTGCGATAT
2221 GCTGCCAGCA GCCCGGCCCG CAAGCCGGGT TTTTTCGTGC GCCCGCCGCA AATTGTTTAG
2281 CTAAATTTCC TTGACTATCT AGGGCATAAT GCCCTAATAT AGCAATCCAA GGCCGGGCAC
2341 TTCGCCCAGG TCAGCAACCG GAGGATCAAC CCCGATGAAA AGCAAGATCA TGTCTTGGCT
2401 CGATGAGCTG CCCGGCGCGG CCGCCACGGA TTTTCTGGCC CGTCGTGACC AGATCGCGGC
2461 GCTGATGGAG CAGGCGGCAG AGCTTACCCG CCAGGCCGAA GAGCTGCGGC ACAAGGCGTA
2521 CCTGCAAGGT TGCACCCTGG AGGGTGAAGC AAAGGGCCAC TGGTCCACCC AGGAAGTCGA
2581 ACGCGCGAAG GCTCGCGCAG GCTGGTAACG GAACGAGGCC CGGCTACGGT CGGGCCTCTC
2641 TCAAGGAGAT AGGGCCATGC CCAACCGCAA GATCGAGATT GTCACCACCA ATTGCCGCCG
2701 CTGCGGTAAG TCCATTTCGA CCCTTAGCCG CAGCCTGATC GGGGCGGACG CCCTGCGCGA
2761 AGAGCTGGGC GGTATCTGCG GCGATTGCAT TACGCCGGAG GAACGACAGC GGATCGAGCA
2821 AGGCACCTTG CTGGCCGCGC TGCGGCAGTG CGCTGCCGCC GGGACAAGCT GATTGCACGC
2881 CAGGGGAAAG GTGTTTTCAA GCATGACGCC TCGCTGAATC CCCTCAATTG TTTAGCTAAA
2941 ATTGCTTGAC AAGTTAGGGC ATTATGCCCT ATTCTTGTTT TGAGGCCGGG TAGATTCCCA
3001 GGTCAATTAC CGGAGGCCAA TCCGATGACC GATAAGTTCA TGCCGTGGAT CGACGAGCTG
3061 CCGAACGTAG ACCAGGAGCT GGTCGCGCAG CGGAATGCTA TTGCCGAGAA GCAGCGGCGC
3121 GCTGATGAGC TGATGCACCA GGTCGAGCGC CTGCGCATCG AGGTCATGCG GGAGTCGTCG
3181 AGGTTGGAGG AACGGGCGAA ACAGCGCTGG ACCTTCAACG AAATCGACTT GGCGAAATTC
3241 CGGGCCGGTC ACTGACGCCA GGGGATGATG ATGCAAGTAG GAGATAGGGT GAACTGGCAG
3301 CACACCCCGC GCGGCGGGTA CGGGTATTCC GTTTGTGTTG CTGGCATAGT GACGAAGATC
3361 GCGGCTAAAC GTGTGCAGAT TCGGGTAGCC GTTCGCAGTG GTAACGAGTG GCAGCAAGTC
3421 ACCAAGTGGG TCGAGCCGGC CAGGTTGAGC ACCAGGGAAA AGCCGGTTCC CGAGTTGGAC
3481 GGGGCATAGG GATGACGCCC CGGATTTCTC CGGGGCGCTT GACCTCGATG CTGTCACATC
3541 ACGGTCTAGG CGCGCGAGCG CTGGACGAAT TGAACACGCA TCGCCGTGGA GGGTCAAGCA
3601 ATGTCGAAGA TTATCAAGTT CCCAGGCGTG GAAGTGGAGG CCGAGGCCCC TGCCCCGCGC
3661 GTTGAGGCCC CGAGCAAGGC CCCGCCCCGG TTCACTTTTG CCAAGGGTGT CCAGGCCCTG
3721 GTCCGCTTTG TATGGGTCGT TACTGTCCTG GTGTGGCCGG TGGCGAAGTG GATCATCTCT
3781 ATCGACGTTT TCTTTCAGTT CCTGCGGATG CTGTACCACT GGAACACCCT TGGCGTTCAT
3841 GCCGGCTGGA CGTTCCTGGT GCATTTCGCG GTACTGACCG CACTCACCTA TTTCGTGTCG
3901 CTCTACAAGC CCAAGGGGCT GTAGAGCGGC CCCTTCAAGG AGCTTTGAGC AATGCCAAGA
3961 CAGGAACCTA AGCAGCCGGG CTACGTGTGC CCGACGACCG GGCGGGTGGC CGTGCTGGTC
4021 AAGGACTACG CCGACAGCGA CCTAAACGGC GACGCATCGG CGTACTGGTT CAATCCGGAG
4081 GCCGAGGGCT GGGGCATGGA CCCTTGGAAA CTGGTCGAAG GCGTGGACCC CCACACGCAA
4141 GGCTGTTCGA TGGATGTTTG CTTTGCCGAC GGTTCAAGCA AGACGGTCGG GCCGCTGATG
4201 ACGTTCTTTC TCAGCGCGAA GGATGCTGCG CGCCTAGCGG CGCTCAAGGG GCAACGCCAG
4261 GAGTGAGCTG GCTTTCTGTA AATGACCTGT CCCCAAATTT TAGCGGCTAA AGGTGTTGAC
4321 GAGGGATAGA AAGTTTAGCT AAACTTCTTC CATCGAAAAG CAATTAACCC ACCGCGAGGG
4381 TGTATCGAAT GGAAGAACAA AGCGTGAACA TGGCGCGATT GAAGGGGGAG GTTTTGCCCG

4441 CCCTCTTCGC GTCGCCGGCG ACGATTGGCG AGTACGGGGC CGGCATCGAC GGGGCGGATT
4501 CCCTCAACGA GCTGTCGAAT CTGATGGAGC ACGGCGCAGT TGCCGCGCTG GCCGACAAAA
4561 TCAGCCAGAT CGTGGCGAAG CTGGCCGACG CGGACCCCCG CAAGATCGCG GAAAAGCCTA
4621 CCTGGTTCGA GAAGATGCTT GGCCGTGAGG TTGAACGCCA GGTGAGGTAT CAGGTCGCCC
4681 GCAAGACGCT CGACCAGTTG CTGGACGAAG CCGAGGGCGT AGCGCAGCGC GTGCGGGACA
4741 CGTTGCGCGC CTTGGATGAC ATGCTCAATA CGCATGAGGC CGAGGTAGAC CGGCTCAGAG
4801 CCTACATTCA AGCCGGGCGC GAGTTCCTGG ACGAGAACCC CGAGGCCGGC GCGGCCAAGG
4861 CCGGCGTGAT CGAGTTCGAC AAGCCGCGCG AACGCTTCGC GCGCAAGCTC GCCAACCTGG
4921 CAACCCTCAT GGCGTCCCAT GAAATGAGCG TCACTCAGAT GAAGCTCACG CGGGCGCAGG
4981 CCGTGGACAT GCTGGACCGC TTCTCTGAAA CGGCATCCGT CCTGGTGCCC GTCTGGCGTC
5041 AGCACACCCT CGCGCTCATC ACCACCAAGA ACATGAATCC GGCAATGGTC GCCGAGGCGG
5101 CCAAAGCTCA CCAGGCGCTC ATGCGGAGCC TTTCGCAGAG CCTGGAAGGC ATCAACCAAT
5161 AACACGGCGG GAGAACCCTA TGAACGCACT GAAAACGACG CACGACGCCA AGGCCCCTAT
5221 CGTCGCCTTC GACATGACCC CGGCAACCCT GCGCGAGCTG GGCTTGCAGG AAAGCGACGT
5281 GCCGGAAGTC CATGCGGTCG CGCAGCGGAT CGAGGTCGGC AGTCCGCAGA CCGTTGCCGA
5341 GTTCGGCCGC GACGTGGCCG AGCACACGTC CCGCTACGCC GATAGCCTGC TGGACCAGGT
5401 GCGCAACAGC GACCTGGACG AAGCAGGCGA GAAACTGACC CAGGTTGTCG CCAAGGCCCG
5461 TTCCCTGAAC GTCGGCCCTT TGTCCGACAA CCGTTCCCGC CTGCCCCTGA TTGGCCCGCT
5521 GATCGACCGC TTCCGCGTCC GTTCGACGGG CTTCATGGCG CGCTTCGACA CGACCCGCGA
5581 GCAGATCGAA CACCTGGTCA GCGAAGTGCA GACCACCCAG CAAGGCATCG CGCAGCGCAA
5641 TGCCTCGCTC GACGAAATGT TCGCAGCCGT GCGCGAGGAA CACCGCCTTC TTGGCGTCCA
5701 CATCGCGGCC GGCAAGGTCC GCCTTGCCGA GCTGCGCGAG CAGGCCGAGG GTCTGCGCGG
5761 CAATGTCGGG AACGACCCGG GCCGCGTGCA GGAGCTGGCC GACCTCGATG CGATGGTTGC
5821 CAACCTGGAC AAGCGCATCG GCGACCTGAT CGCCTTGCAA CATTCGGCCA TGCAGAGCCT
5881 GCCGACCATC CGCATGATCC AGGCCAACAA CCAGATGCTG GTCGATAAAT TCCACACCAT
5941 CCGCGAAATC ACCGTGCCGG CGTGGAAGCG GCAATTCATG CTGGCCTTGA GCCTCAACGA
6001 GCAGAAGAAC GCCGTCGAAC TGGCCACGGC CATCGACGAC ACCACCAACG ACCTGATGAA
6061 GCGCAATGCG GCCCTGCTGC ATCGCACGTC CGTCGAGACG GCGAAGGAGA ACCAACGCCT
6121 GGTGATCGAC GTGGACACGC TCAAGCAGGT TCAGACGACG CTCATCAAGA CCGTCGAGGA
6181 CGTTATTCGC ATCCAGCAGG AAGGCGTGCA GAAGCGCAAG GATGCCGAGA AGCAGATCGC
6241 CGCAATGCGT GGCGATCTTC AAGCCAAGCT GACCCGCCAG CCCGTGCGCG AGCTGGCCCA
6301 ACAGGAGTCC GTATGAATGC CACAAACACC GATGTTTTCG CCCAGGTAGG CGGCCTCGAG
6361 GCCCGAGGCG CGAAGATGAA GAAGCGGGGC ACCCGCTTCC TCATCGCGGC GCTGGCAGTC
6421 CTTGCCATTG CCGGGATCGG GGCAGTAACG GGATGGGCGA TCAGCCCGAG CGCGACGCCC
6481 GGAAGCATTG ACGTGCCGCA GGTGCTGGCA TCGACATTCA GCGACCAGGT GCCGGGCAGT
6541 GAGGGCGGCG GCCTGGGTGG CGGCCTGCCC TTCACTTCGG CCGTCGGGGC ATTCACGGAC
6601 TTCATGGCGG GGCCGGCAAT TTTTACCTTG GGCATTCTTG GCATAGTGGT CGCGGGTGCC
6661 GTGCTCGTGT TCGGGGGTGA ATTCAGCGGG TTCGTGCGAT CCGTCTGCAT GATGGTGATA
6721 GCCGTCAGCA TGATTTTCGT GTCGTCGAAC TTGGTGAAGG GCATTCTCGG CGGCGATCAC
6781 GACGCCGGCC CTGCGGAGCC TTCGCCGCGT GCGCGATTCA TGGCGGCCGT GGAGGCCAAG
6841 GATTTCGCGC GAGTGCAAGA GCTGATCGAG GCGCGTGGAG CCAAGTCGGC GGCTGATTAT
6901 GTCCTTGCGC AGCTCGCCGT GGCCGAAGGT CTGGACCGCA AGCCTGGTGC GCGCGTCGTG
6961 GTCGGGAAAG CGGCGGGCAG CATGGCAATG CCGCCTGCGG CGCTGGGTTT TACGCCAAGG
7021 GGAGAAGCGG CATACGCCAT CGAGCGGTCA GCCTATGGTG AGCCGAGGTC CAGCATTGCG
7081 AAGCAGTACC AGCAGGAATG GAACCGGAAG GCGGCGACCT GGTGGGCGAT GGCCGGTGTG
7141 GCCGGCATCA TCGGCGCGAT CCTGGCGGCG GCGGCAACCG GCTTTGTTGG GCTGGCAGTG
7201 TCGATCCGCA ACCGAGTGAA GCGCGTGCGC GACCTGTTGG TGATGGAGCC GGGTGCAGAG
7261 CCATAAGCGG CAAGAGACGA AAGCCCGGTT TCCGGGCTTT TGTTTTGTTA CGCCAAGGAC
7321 GAGTTTTAGC GGCTAAAGGT GTTGACGTGC GAGAAATGTT TAGCTAAACT TCTCTCATGT
7381 GCTGGCGGCT GTCACCGCTA TGTTCAACCA AGGCGCGGAG CAAATTATGG GTGTTATCCA
7441 TGAAGAAACG GCTTACCGAA AGCCAGTTCC AGGAGGCGAT CCAGGGGCTG GAAGTGGGGC
7501 AGCAGACCAT CGAGATAGCG CGGGGCGTCT TAGTCGATGG GAAGCCACAG GCGACGTTCG
7561 CAACGTCGCT GGGACTGACC AGGGGCGCAG TGTCGCAAGC GGTGCATCGC GTGTGGGCCG

7621 CGTTCGAGGA CAAGAACTTG CCCGAGGGGT ACGCGCGGGT AACGGCGGTT CTGCCGGAAC
7681 ATCAGGCGTA CATCGTCCGG AAGTGGGAAG CGGACGCCAA GAAAAAACAG GAAACCAAAC
7741 GATGAAAACT TTGGTCACGG CCAACCAGAA AGGCGGCGTC GGCAAGACTT CGACCCTTGT
7801 GCATCTTGCC TTCGACTTTT TCGAGCGCGG CTTGCGGGTT GCCGTGATCG ACCTGGACCC
7861 CCAGGGCAAT GCGTCCTACA CGCTCAAGGA CTTTGCTACC GGCCTGCATG CAAGCAAGCT
7921 GTTCGGCGCT GTCCCTGCCG GCGGCTGGAC CGAAACCGCA CCCGCAGCCG GCGACGGCCA
7981 GGCCGCGCGC CTCGCCCTCA TCGAGTCCAA CCCGGTACTG GCGAACGCCG AACGGCTGTC
8041 GCTGGACGAC GCCCGCGAGC TGTTCGGGGC GAACATCAAG GCCCTGGCGA ACCAAGGCTT
8101 CGACGTGTGC CTGATCGACA CGGCCCCGAC CCTTGGCGTC GGCCTGGCGG CCGCCCTCTT
8161 CGCGGCCGAC TATGTGCTGT CCCCCATCGA GCTTGAGGCG TACAGCATCC AGGGCATCAA
8221 GAAGATGGTC ACGACCATTG CGAACGTGCG CCAGAAGAAC GCCAAGCTGC AATTCCTTGG
8281 CATGGTGCCC AGCAAGGTCG ATGCGCGGAA TCCGCGCCAC GCGCGCCACC AAGCCGAGCT
8341 GCTGGCCGCG TACCCCAAGA TGATGATTCC GGCCACCGTT GGCCTGCGCA GCAGCATCGC
8401 CGATGCCCTC GCATCCGGTG TGCCGGTCTG GAAGATCAAG AAAACGGCCG CGCGCAAGGC
8461 ATCGAAAGAG GTTCGCGCCC TGGCTGATTA CGTGTTCACG AAGATGGAGA TTTCCCAATG
8521 ACTGCGGCTC AAGCCAAGAC CACCAAGAAA AACACCGCTG CGGCCGCTCA GGAAGCCGCA
8581 GGCGCGGCGC AGCCGTCCGG CCTGGGGTTG GATAGCATCG GCGACCTGTC GAGCCTCCTG
8641 GACGCTCCTG CGGCGTCTCA GGGCGGTTCC GGCCCTATCG AGCTGGACCT GGACCTGATC
8701 GACGAAGATC CGCATCAGCC GCGGACGGCC GACAACCCCG GCTTTTCCCC GGAGAGCATC
8761 GCGGAAATCG GTGCCACGAT CAAAGAGCGC GGGGTGAAGT CACCCATTTC GGTGCGCGAG
8821 AACCAGGAGC AGCCGGGCCG CTATATCATC AATCACGGCG CCCGCCGCTA CCGTGGCTCG
8881 AAGTGGGCCG GCAAGAAGTC CATCCCGGCG TTCATCGACA ACGACTACAA CGAAGCCGAC
8941 CAGGTTATCG AGAACCTGCA ACGCAACGAG CTGACCCCGC GCGAAATTGC CGACTTCATT
9001 GGCCGCGAGC TGGCGAAGGG CAAGAAGAAA GGCGATATCG CCAAGGAAAT CGGCAAGTCG
9061 CCGGCGTTCA TCACCCAGCA CGTCACGCTG CTGGACCTGC CGGAGAAGAT CGCCGATGCG
9121 TTCAACACCG GCCGCGTGCG CGACGTGACC GTGGTGAACG AGCTGGTGAC GGCCTTCAAG
9181 AAGCGCCCGG AGGAAGTCGA GGCGTGGCTT GACGACGACA CCCAGGAAAT CACGCGCGGC
9241 ACGGTCAAGC TGCTGCGCGA GTTCCTGGAC GAGAAGGGCC GCGATCCCAA CACCGTCGAT
9301 GCCTTCAACG GCCAGACTGA TGCCGAGCGT GACGCGGAGG CCGGCGACGG CCAGGACGGC
9361 GAGGACGGCG ACCAGGACGG TAAGGACGCC AAGGAAAAGG GCGCGAAGGA GCCGGACCCG
9421 GACAAGCTGA AAAAGGCCAT CGTCCAGGTC GAGCACGACG AGCGCCCTGC CCGCCTTATC
9481 CTCAACCGTC GGCCGCCGGC GGAAGGCTAT GCCTGGTTGA AGTACGAGGA CGACGGCCAG
9541 GAGTTCGAGG CGAACCTTGC CGACGTGAAA CTGGTCGCGC TCATCGAGGG CTGATCCCCA
9601 AAGACAGCGG CGCGGGCCAC CCGCGCCGCA CAGACAACGG TTCCGCTACA AGGAGGACCG
9661 AAGAATGAAT CCGATGCTGT TCTACATCGC GGGAGGCGTA GGCGCGGCGT TGCTGCTGGT
9721 TTCCGCGATC ATGCTGTTCA AGCTGCGCGA GCCGAAGAAG GAACACCGAC CGCAGCGCAA
9781 GGCGGCGGCC CCGACGCCGC AGCCGGTCGA TAACGAGCTG CTGCGCACCT TTCGCCGGCC
9841 GTCGCAGGCC AGCCAACCCG AGCCATCCAC GACGCCGCCG GCGCGGCGCA CTGCCCCGCC
9901 GAGCAGCGAA CAATCCAGAG GGAATGACGG CGATTTCGTC ACGTCGGCCC TGGTGGCCGG
9961 GGCGACCAAC AGCACCATGC TGGGCTACCT GGCCGGCGGC TCGCTGACCG GGGCCATGCT
10021 GGGCGATGCG CTGCGGCCGG ATACGCCGTC TGCGGCGTTC GCGGACCCCT CGCCGTGCTT
10081 CGGCAGCAGC GACACGGACA GCGGCTGGAC CGATACCGGC AGCTCTTGCG ATTTCAGTTC
10141 GTCCGACACC GATACGAACA ACTGGTGAGG GCGGACCATG ACTGACGAAC AAAAGAAACG
10201 GCTCCTGATG TTGCAAGCTA TCCGGCGCAG CATCCAGGCA ATGCGCAGGG GTGTTTCGGG
10261 GCCGGATAGT TTCAAGGGCT GGTTGACCGG CATCGGCATC ATCGCGGCGG GGTTCCTGGT
10321 TGGCGAGCTG CTGCCATCCC CTGCCCCCGG CGTCGGCCTG GTTGTGCCAG GGGCCTGCAT
10381 TGCCGGCTTC GTCGTCGGCT GCTGGCTCAA CAGGAAAGGC CGCTCATGGC CGACAGCCAT
10441 TTACGACCAA TTGGCCCGTT ACCAGCCGCT CAACGAGCGG GCGTACCGCG ACCTGCAAGA
10501 GACGGTGAAG GAACGCGGCC TCGACTGCGA TGCGGTCATG GAGTGGACCT ACATCGAGCA
10561 GGCCGAGTTC ATGCCCCTGG TGCCCACCAG GGAGGACCTA GCGCGCGACA AGTTCCTGAC
10621 CGCGACGCGG GCCGATAGGG CCGAGGACGA TGCGCGTGCG CGCTTCGTCG TGCTCCATCC
10681 CAAGAAGCGC GGCCCCGACG CATGAAGAAG CCCGACGCCC GCTAATCCCC TTCCCGCTGA
10741 CCCTTTGCCC CCGCCGACCG TGCGGGGCTT TTTTTGCCTG CCCTTCAGCC GCTAAAAAAC

10801 CTGCCGTTAA GTCGAGAACT GTTCAGCTAA ACTCTTGCGC TGATAAGGTA GGTAAGAGTA
10861 TTATTATTCT TACCAATTAC CGGAGCCAAC ATGGAACTGA ACAAAGAGAC GCGCGACCGC
10921 ATTTTTGCGG CTGCGGACGA GCTGTTCGAG CAAGGCGACC GGGAGAACTT CCCGACCGTG
10981 GATGCAGTGC GTAAGGCCGC CCGCGTCAAC ATGAACGACG CCAGCGCCGG CATGCGCGAA
11041 TGGCGACGCC AGCGCACGGC GCAGGCCGCC CCGCTGGCAG TCCAGGTGCC GGACGCGGTG
11101 CAGCAGGCCG GCAACCAGGC CGTGGCCGCC CTCTGGCAAG CCGCCCAGGC CCTTGCCAAT
11161 GAATCGTTGC AGGCAGCGCA AGCCGGCTGG GATCGTGAGC GCAACGAGTT GGAAGCCGTG
11221 CGCGGGGAGC TGGCCGACGC CTTCGAGGCG CAGGCCCGCG AGCTGGAAGA AGCCCAGGCG
11281 CGCGTTACAT TGCTAGAGCA GCAGGCCGCC GAGGCGGCCG AGCTGGCCGC ACGCCAGCGG
11341 CAAGCCCTGG CCGAAGCACG CACCGCCCTG GCAGGAGCCG AGCAGCGGGC CGCGCTGGCG
11401 ACGCAGCGGG CCGACGAGGT GGAGCGTCGA GCGCAGGAGC TGCGCGCCGA GCTGGACTAC
11461 GCTCACCAGG ACGCCCGCGC ATTCAAAGAA GAGAGCCGCA AGACCCTGGA CGCGGCCAAC
11521 CAGGAAATGC AGGCGCTTCG CGGCGAGCGC GACGCCACCA GGCGCGAAGC GGAGAGTTTG
11581 CGCACGGAGT TGGCAGCGGT GAAAGCGAAG GCGCAGGCCG ACGCCGAGGC CCACCAGGAG
11641 CAAAGGAAGT TGGCGGCGCA AGAGGCGGCC AGGCAGGCCG AGCGCTTTAC GACGGTGCAA
11701 GCCGAGCGTG ACGAAGCGCG GCAAGAGGCA AAGGCAGCCC AAGAAGAGGC CGCTATGTTG
11761 AGGGGTCGGC TAGACGCGGA AACGAGTGGG AGGAAACACA AAGCGGGAGG AAAGTAACTG
11821 ATAAATCGAA CGCAGTTCAC TAAAATAGAT GGAACAACCT TGACGCGCCC GGCTCTAGGG
11881 AAAGCCGGCC TCGAACTGCC GAGGTCGGCT TTTTTTAGC CGGCAAGCGA CCAGGACACG
11941 TTTAACACCA GAGGCAACCG TCAGGAGTCA CCCAATGAAA CACCGCCTCC TGGTCATGAA
12001 TGGGCAAAGA ATTGTCCAGA CCGAGAACCA AGGCGCATGG ACGAACCAGA AGGTAGATAA
12061 AGCCGGGGCC CTGAAACCTG GCATTTACAA CATCTATATG GCCCAAAAGG CCGATAAGTC
12121 GCAGCGCCAC GACGGCAGCA TCGTCCACGC AGATAGCGGC AGCATTTACC AACAGGTGGG
12181 GAAGAACTTT GTAATGCACG CCCGGTCAGA TTTCGATAAA GTACCTGAAA TCGGGAGTGC
12241 GAAAAGCATC ACCTATGACG CAAGTGGCAA GGCACAGGTA AGTGCCGAAA GCGTCAAGTT
12301 GAGTCGCGGG CGCACTCGTT AGAGTTTTAG CGGCTAAAAA GGGAGGCGTC TTGAGCAGCT
12361 ACAGCAGAGC CGAGCGCGCA CCGTGGGGAG ACTTCCCGAA GGTGGTTCGC AACGGCGACC
12421 TTGGTTCTTT GACCAACGAG CCTGAATACC AGGCTGCGAA GCAGGGCGAC GCGGAAGCGG
12481 CGCTTAATTT GGTCGAGCGC CTGATTTCGG ACGACACTGT TGCCCAGCTC AAGACGCTGA
12541 TCGGCGACGA CAAGCCCCGC ATCGTTCCAG TCCTCGCCGT CGAGGCGGCG GGGAACAACA
12601 AGATTCCGGC CATGATGGCG GTCGTTCTGG CCGACCGCCT TGGCCTTGAG GTCGAAACGG
12661 ACATTGTGCA GCGGGAGAAG GTCGCCCGAA CCGGAGCCGG TTCAGATCAT CGCCTGGCGT
12721 TCAACCCGAC TTTCGAGGGG GAGGTGATCC CCGGCCAGAA GTACATCGTT GTCGATGACA
12781 CGCTGACGAT GGGCGGCACC ATTGCCTCAT TACGCGGCTA CATCGAGAAC AACGGCGGCA
12841 AGGTCATGGC GGCGTCCGTC ATGACCGCCC ATGAAGGCGC GCTTGACCTG GCGGTCAAGC
12901 CGAAGATGCT TGCGGGCATC AATGAGAAAC ACGGTCCAGC AATGGACGCA TTTTGGAAGG
12961 AAACCTTTGG CTATGGCATC GACCGACTCA CCCAAGGAGA AGCCGGACAC CTCCGCGCTG
13021 CCCCGTCCGT TGACGCAATC CGAGATCGCA TCGCTGCGGC TCGAAATGAA GCAGTCAATC
13081 GAGTGGGGGC AAGCCGAACT GCGACGACGG CGCGAGCAGG GCAACAGCAG TCCCCGGCAG
13141 TAAAGCAGTC CAGCGGCAAC AGCGGCGAGG ATTTGCTACA GGCGGCCCAG GAGGCCGAGA
13201 CGGAACAGCA GGCCCTCTTA GAATCCGCGC CCATCGAGCA GACCTACCAG CAGACCCTTG
13261 CGCTCTACGT GCAGGCAAAG CATGACCAGG TGGAGCGCAT CGAGGACCGC CTAGAACAGC
13321 TCGTAGACCG TCAGCAGGCG CGTTTGCAGC AGCTACAGTC CAATGCCCCC GGCTTGCTGT
13381 CCCTGCCGCG TACAAAGGCC GCCTGGCAGC AGCAGCAGGC CCAACAGCAA GCCCGCTTGC
13441 AGACCCTGCA CACGCGGCTG GACATGGTGC GCGAAATCAA GGAAGGCATG GGCATTCACG
13501 CGCCCAAGAT CGAGGAACTG GCGACGCGCA AGATGCGCGC GGAGCATCCC GAGCTGGCCG
13561 GCGACTGGGA TTCGATGCGT GAAGCAGCCC GACGGCACCA GGCCCTGATG CGCAAGCAGG
13621 AGCAGGAGAA GAAACAGGCC CAGGAGCGCG AGCGCGGGCG CAGTCAGAGC TTGGGCCTGT
13681 CAAACAAGCC CAGCTAACGC AAAAACAAAG CCCGGCAACG CCGGGCTTTT TCATCTGCGC
13741 CTCTGCGATT CATAACGAGG CCCACACCAC CAGGGCGGCC GCGAACAACA CCATGCCGCC
13801 GGCGATCATG TTCATCATCG CCACGCGCCG CGCGTCCGCG ACCTTGGCCG CGAGCTGGCG
13861 GCCAAGGCCG TCGTCGATTT CCCTGCGGAT CGCTTCGGCC GCCTGCGCGG CGCTGTCCTT
13921 CATTACCTTC GCCATTGCGT CCTTGCTGGC CGCCAGGGCC GCGTTCAGCA TCCGCTCCGC

13981 TTTGGCCTTG GCGTCCTCGC CCCAACGATG GGCGATCCCT TCCAGCTCTT CCTTGAACGC
14041 GGCAAGGATT TCCTCTTGCT TGGCCGCACT GTCGGCCATG AGCCGGGCGT TGATGGTATG
14101 CAGGATCAGC ACCGGGTCGT CGCGGCCGAC GGCGATGCCG TGCTTGGCCG CAATCTCCCG
14161 GATCAGCTCT TCAATCTGGT CGCTCATAGC ACGGCCGCCG CGTCGAGCTG TTCAAACAGG
14221 CCGCGCCGCA CGATCTTGAG GCGTTGCCGC GTCATGATCG TGAGCGATTC ATCGGCCAGC
14281 GCCTGGTCGA ACGTCAGCCG CTCTTGCAGC ATGTCGCTGA AATCGCGGCC GTAGGTTTCT
14341 TCCTTGAGGG CCGGAATCTG GATGATGGAC GACACGCGGG CCTTGTTGGC CGTGTACGCC
14401 TTCATCTGCT CAAAGCTCTT GCCCTCATGC TCGATAGGCC CCCAATACGG GTTCAGCCAG
14461 ACCACGAAAA GCGCTTCGGC CGGGAACTGG CTGGCGAGCT GGGCGAAGCC GCTCACCGTG
14521 TCCAGGAGAG CCTGGCCGCC GGTGACGACG GTATGGATGA CCAGCTCATG CCCCATTTCT
14581 TGCAGCAGAG CCGGCACCTG GTTGCTGATG AGGTAATGCG ACAGAGGCAC GAACGAGCTG
14641 GCACCGTTGT CGATCACCAC GTCATCCTTG GTCGGCGCAA TCAGCTCGAC CAGGGTGTCG
14701 AAGTTGCGCG AGTTAATTTC GTCGCCGGCC ATGATGTTCA GCCGGCGGAC GTTCAGGGCC
14761 TTGTAGCCCT CGAACGTCGC GTTCACCGGG TCGGTGTCGA TGCACAAGGG TGTCTGCCCC
14821 TTGTCCATCT TGTACTGCGC AATGATCGCG GCGATGGCCG ACTTGCCGAC CCCGCCCTTG
14881 CCCTGCAAAA CCATGTGAAT TTTCGCCATT ACAGTAGATC CTTTTTGTCC GGTGTTGGGT
14941 TGAAGGTGAA GCCGGTCGGG GCCGCAGCGG GGGCCGGCTT TTCAGCCTTG CCCCCCTGCT
15001 TCGGCCGCCG TGGCTCCGGC GTCTTGGGTG CCGGCGCGGG TTCCGCAGCC TTGGCCTGCG
15061 GTGCGGGCAC ATCGGCGGGC TTGGCCTTGA TGTGCCGCCT GGCGTGCGAG CGGAACGTCT
15121 CGTAGGAGAA CTTGACCTTC CCCGTTTCCC GCATGTGCTC CCAAATGGTG ACGAGCGCAT
15181 AGCCGGACGC TAACGCCGCC TCGACATCCG CCCTCACCGC CAGGAACGCA ACCGCAGCCT
15241 CATCACGCCG GCGCTTCTTG GCCGCGCGGG ATTCAACCCA CTCGGCCAGC TCGTCGGTGT
15301 AGCTCTTTGG CATCGTCTCT CGCCTGTCCC CTCAGTTCAG TAATTTCCTG CATTTGCCTG
15361 TTTCCAGTCG GTAGATATTC CACAAAACAG CAGGGAAGCA GCGCTTTTCC GCTGCATAAC
15421 CCTGCTTCGG GGTCATTATA GCGATTTTTT CGGTATATCC ATCCTTTTTC GCACGATATA
15481 CAGGATTTTG CCAAAGGGTT CGTGTAGACT TTCCTTGGTG TATCCAACGG CGTCAGAATT
15541 CGGCAAGCGG ATGGCTGATG AAACCAAGCC AACCAGGAAG GGCAGCCCAC CTATCAAGGT
15601 GTACTGCCTT CCAGACGAAC GAAGAGAGAT TGAGGAAAAG GCGGCGGCGG CCGGCATGAG
15661 CCTGTCGGCC TACCTGCTGG CCGTCGGCCA GGGCTACAAA ATCACGGGCG TCGTGGACTA
15721 TGAGCACGTC CGCGAGCTGG CCCGCATCAA TGGCGACCTG GGCCGCCTGG GCGGCCTGCT
15781 GAAACTCTGG CTCACCGACG ACCCGCGCAC GGCGCGGTTC GGTGATGCCA CGATCCTCGC
15841 CCTGCTGGCG AAGATCGAAG AGAAGCAGGA CGAGCTTGGC AAGGTCATGA TGGGCGTGGT
15901 CCGCCCGAGG GCAGAGCCAT GACTTTTTTA GCCGCTAAAA CGGCCGGGGG GTGCGCGTGA
15961 TTGCCAAGCA CGTCCCCATG CGCTCCATCA AGAAGAGCGA CTTCGCGGAG CTGGTGAAGT
16021 ACATCACCGA CGAGCAAGGC AAGACCGAGC GCCTGGGTCA CGTGCGCGTC ACGAACTGCG
16081 AGGCAAACAC CCTGCCCGCT GTCATGGCCG AGGTGATGGC GACCCAGCAC GGCAACACCC
16141 GTTCCGAGGC CGACAAGACC TATCACCTGC TGGTTAGCTT CCGCGCGGGA GAGAAGCCCG
16201 ACGCGGAGAC GTTGCGCGCG ATTGAGGACC GCATCTGCGC TGGGCTTGGC TTCGCCGAGC
16261 ATCAGCGCGT CAGTGCCGTG CATCACGACA CCGACAACCT GCACATCCAT ATCGCCATCA
16321 ACAAGATTCA CCCGACCCGA AACACCATCC ATGAGCCGTA TCGGGCCTAC CGCGCCCTCG
16381 CTGACCTCTG CGCGACGCTC GAACGGGACT ACGGGCTTGA GCGTGACAAT CACGAAACGC
16441 GGCAGCGCGT TTCCGAGAAC CGCGCGAACG ACATGGAGCG GCACGCGGGC GTGGAAAGCC
16501 TGGTCGGCTG GATCAAGCGC GAATGCCTGC CGGAGCTGCA AGCGGCGCAA TCCTGGGAGG
16561 ACCTGCACCG CGTCCTGCGG GAAAACGGGC TTAAGCTGCG CGAGCGCGGA AACGGCTTCA
16621 TCTTCGAGGC CGGCGACGGC ACGACGGTCA AGGCCAGCAC CGTTTCGCGC GACCTGTCCA
16681 AGCCGAAGCT CGAAGCCCGA TTCGGGGCAT TCACGCCAGC CGAGGGCGGC GAAGCCCCCC
16741 GGCGGCGGGA GTATCGAGCG AAGCCGCTCA AGACCCGCAT CGACACCACC GAGCTATACG
16801 CCCGGTATCA GTCTGAACGC CAGGAAATGG GGGCCGTGCG CAAGGGCGAG CTGGACACGC
16861 TGCGCCGGCG TCGTGACCGC CTGATTGAAG CCGCGATGCG CAGCAACCGG CTGCGCCGCG
16921 CCGCTATCAA GCTGCTGGGC GAGGGGCGCA TTGCAAAGCG ACTGATGTAC GCGCAGGCGC
16981 ACAAGGCTCT GCGCGCCGAC CTGGACAAGA TCAACCGCGA GTACCGGCAG GGCCGTCAGG
17041 CGGTCCAGGA GCGCACGCAG CGCCGCGCGT GGGCCGACTG GCTCAAGGCC GAGGCGATGA
17101 AGGGCGACGA CAAGGCCCTG GCCGCGCTGC GCGCACGCGA GGGCCGCAGC GACCTCAAGG

17161 GCAACACCAT CCAGGGCAGC GGCGAGGCCA AGCCGGGCCA TGCGGCCGTG ACGGACAACA
17221 TCACGAAGAA GGGAACCATC ATCTATCGCG TCGGCAGCAG CGCCGTCCGC GACGACGGCG
17281 ACCGCCTGCA AGTCTCGCGC GAGGCCACCA CGGACGGCCT GGACGCCGCC CTGCGCCTGG
17341 CAATGGAGCG CTTCGGCGAC CGGATCACCG TCAACGGCAC AGCAGAGTTC AAGGAGCGGA
17401 TCGCGCAGGC GGCCGCCGCT GGCCGTCTGG CAATCACCTT TGACGACGCC GCCCTTGAGC
17461 GCCGCCGTCA AGAACTACTG ACGAAGGAGC AAGCACATGA GCAACCCGAA CGAAATGACG
17521 GACGAAGAGA TCGCGGCGGC GATGGAGGCA TTCGACCTGC CGCAGCCAGA ACCACCCTCA
17581 ACGCCACAGG CGGCGACGGC GACCGACGGG ACGCTCGCGC CGTCAGCGCC GGCGGAACCG
17641 TCGCACTCCG CAAGCCCAAC GTTGGACGCA TTGGACGAAA GCCGCCGCCC CAAAGCCAAA
17701 ACCGTTTGCG AGCGCTGTCC CAACTCGGTG TGGTTCGCAT CGCCGGCGGA GCTGAAATGC
17761 TATTGCCGCG TGATGTTCCT GGTCACGTGG AGCAGCAAGG AGCCGAACCA GCTCACGCAC
17821 TGCGACGGGG AGTTTCTGGG CCAGGAAGAG GGCTAAAGCC CGAACAGATC GCGGCGGCCG
17881 AGAAATATGT TGCCGAGCGC GAGCAAAAGC GGCTAAACGG CTTCGATATA CCGAAGCATG
17941 CACGATATAC TGATTATGTT GGTGCGCTGT CCTATGCAGG CACCCGGAAC GTCGAGGACC
18001 AGGCGCTGGC CCTGTTGAGG AAAGAAAACG ACGAGATTCT GGTGCTGCCC GTCGATAAGG
18061 CCACGGTGCA ACGCATGAAG CGCCTCGCCA TCGGTGATCC CGTCACCGTG ACCCCGCGAG
18121 GCTCCCTTAA AACTACCCGA GGTAGGAGTA GATGAAGAAC CGAAACAACG CCGTGGGGCC
18181 ACAGATACGG GCGAAAAAAC CGAAGGCCAG CAAGACCGTT CCCATCCTCG CCGGCCTGTC
18241 CCTTGGGGCA GGCTTGCAGA CCGCGACGCA ATATTTCGCC CATTCCTTCC AGTATCAGGC
18301 CGGACTTGGC TGGAATATCA ACCACGTCTA CACGCCCTGG TCGATCCTTC AATGGGCCGG
18361 CAAGTGGTAC GGCCAATATC CCGACGATTT CATGCGCGCG GCCAGCATGG GTATGGTCGT
18421 TTCGACCGTG GGCCTGCTGG GCACGGCCGT CACGCAGATG GTCAAGGCGA ACACCGGCAA
18481 GGCGAACGAC TATCTGCACG GTTCGGCCCG CTGGGCCGAC AAGAAGGACA TACAGGCCGC
18541 CGGCCTGCTG CCCCGGCCGC GCACCGTCGT CGAGCTGGTG TCAGGCAAAC ACCCGCCCAC
18601 TTCCAGCGGC GTCTATGTGG GCGGCTGGCA GGACAAGGAC GGCAAGTTCC ACTACCTGCG
18661 GCACAACGGC CCCGAGCACG TCTTGACCTA CGCGCCGACG CGCTCCGGCA AGGGCGTCGG
18721 CCTGGTCGTT CCGACGCTGC TTTCCTGGGC GCACAGCGCC GTCATCACCG ACCTGAAAGG
18781 CGAGTTGTGG GCGCTGACCG CCGGCTGGCG GAAGAAGCAT GCCCGCAACA AGGTCGTGCG
18841 CTTTGAGCCG GCATCCGCGC AGGGTAGCGC GTGCTGGAAC CCGCTCGATG AAATCCGCTT
18901 GGGGACCGAG TACGAGGTTG GCGACGTGCA GAACCTTGCC ACCCTGATCG TCGATCCGGA
18961 CGGCAAGGGC CTGGAATCGC ACTGGCAGAA AACCAGCCAG GCGCTGCTTG TCGGCGTCAT
19021 CCTGCACGCG CTCTACAAGG CTAAGAACGA GGGTACGCCG GCCACCCTGC CGTCGGTGGA
19081 CGGCATGCTT GCCGACCCGA ACCGCGACGT GGGCGAGCTT TGGATGGAAA TGACCACCTA
19141 CGGCCACGTT GACGGGCAGA ACCACCCTGC GGTCGGCTCT GCGGCCCGCG ACATGATGGA
19201 CCGCCCGGAG GAAGAATCCG GTTCCGTGCT GTCCACCGCC AAGTCCTACC TGGCCCTGTA
19261 CCGCGACCCG GTTGTGGCCC GCAACGTCAG CAAGTCCGAC TTCCGCATCA AGCAACTGAT
19321 GCACCACGAC GACCCGGTAA GCCTGTTCAT CGTGACGCAG CCCAACGACA AGGCCCGTCT
19381 GCGGCCTCTG GTGCGCGTCA TGGTCAACAT GATCGTCCGG CTGCTGGCCG ACAAGATGGA
19441 CTTCGAGAAC GGCCGTCCTG TCGCGCACTA CAAGCATCGT CTGCTGATGA TGCTCGACGA
19501 GTTCCCCAGC CTGGGCAAGC TCGAAATCCT GCAAGAGTCC CTTGCCTTCG TCGCCGGCTA
19561 CGGCATCAAG TGCTACCTCA TCTGCCAGGA CATTAACCAG CTCAAGAGCC GCGAAACCGG
19621 CTACGGCCAC GACGAAAGCA TCACGTCGAA CTGCCACGTG CAGAACGCCT ACCCGCCGAA
19681 CCGCGTGGAG ACGGCCGAGC ACCTGTCAAA ACTGACAGGC ACCACGACCA TCGTGAAGGA
19741 GCAGATCACG ACGAGCGGCC GCCGCACGTC GGCGCTGTTG GGCAACGTCT CGCGGACCTT
19801 CCAGGAAGTG CAGCGGCCAT TGCTGACCCC CGATGAATGC CTGCGCATGC CGGGGCCGAA
19861 GAAGAGCGCC GACGGCAGCA TTGAAGAGGC GGGCGACATG GTTGTGTACG TCGCCGGCTA
19921 TCCCGCGATC TACGGCAAGC AGCCGCTCTA CTTCAAAGAC CCGATATTCC AGGCCCGCGC
19981 GGCCGTACCG GCTCCGAAGG TCAGCGACAA GCTGATCCAG ACGGCCACCG TCGAGGAAGG
20041 GGAGGGGATC ACGATATGAG CCGCTTCCAG CGCCTCACCA AGTACGTCGC CATCGGAGGC
20101 GGCGCGGCCT TGTTGCTCGC CGGCGCGGCC TATCTCGCCG GCGCGAAGGT CAACACCACC
20161 AAAAGCATTC CGGTCGGCCT GTACTGGAAA TCGAATGCGC CGGTGGAGAA GGGGGCTTAC
20221 GTCATGTTCT GCCCGCCGCA AGTCGGCGTG TTTTCGGACG CCAAGGAGCG GGGCTACATC
20281 GCCGGCGGTT TCTGCCCCGG CGACTACGGC TACATGATGA AGCGAGTTTT AGCCGCTAAA

20341 GGCGACGAGG TTGCCATCAC CGACGCCGGC GTGCGCGTGA ATGGCGGGCT GCTGCCTCAC
20401 AGCGCGCTCA TCAAGGCCGA TCCATCAGGA CGGCCGCTGC CTCGTTATCA GTCCGACAGC
20461 TACACCCTGG GGACCGCCGA GGTTCTGCTT ATGTCCGACG TAAGCGACAC GTCTTTCGAC
20521 GGCCGCTACT TCGGGCCTGT CAATCGTTCG CAAATCATCA CCGTCATCAG GCCGGTTCTG
20581 ACCTGGTGAG GGTAGGGGGA TATATGCAAT TTGAACGCCT TGTAATCGCT GAAAAACCGG
20641 AGCTGGCAAA AGCCATCGTC GAGGGCCTGG GCGGCGGCAG CCGCAAGGAC GGCTATTACG
20701 AATGCGGCTC CGACCGCGTG ACCTGGTGCT ACGGTCACAT GCTGGCCTTG CTAGACCCGG
20761 AGGACTACGA CGAGCGCTAC GCCAACTGGA ACATGGCCGA CCTGCCCATC GTCCATATTC
20821 CCTGGCGCAA GAAACCGTCC GGCGATGCCG GTGCGAAAGC TCAGTTCAAG ACCATCCTCA
20881 GCCTGTTGAA GCAGGCGAAA AGCGTGGTTC ACGCCGGCGA CCCGGACGAC GAAGGCCAGC
20941 TTCTGGTCGA TGAAATCCTG GAATACGCCA ACTGCCGTCT GCCGGTGCAG CGGCTGCTTA
21001 TCAACGACAA CAACGTGAAG ATCGTGCGCC GGCAGCTCGC CGCGATGCGC GACAACCGCG
21061 AGTTCGCCGG CCTGTCCGCT GCGGCCGAGG CCCGCAGCGT CGGCGACCAG CTATACGGCT
21121 TCAACATCAC GCGCCTGTAC ACGCTTGCGG CACGCGCGAA GGGCTATCAG GGGCTGTTGA
21181 GCGTGGGGCG CGTGCAGACT CCGATCCTGG GCCTTGTCGT GCGCCGTTGC CGCGAGAACG
21241 CGGCCCACCA GAAAACCTAC TACTACCTGG TGAACGGCCA GTTCGAGGTC GAGGGCATTC
21301 AGTTCCCGGC CCGTTACCAG GTCGCCGACG GCGATCCGGT GGACGAGAAA GGCCGCCTCA
21361 GCAACAAGGA GCACGCCGAG GGCATCGCGG CCGCCGTGAG CGGCCAACCC GCCCGCATCG
21421 TGTCCGTGAC CACCAAGGCG AAGGAAGCGG CTGCGCCGCT GCCCTACAAC CTGCTGAAAC
21481 TGCAAATGGA CGCCTCGCGC AAGTTCGGCT TCAAGCCGGA CCAGGTGAAG GACATTACGC
21541 AGGCCCTGCG CGAGAAGCAC AAGCTCATCA CCTACAACCG TTCCGACTGC GAATACTTGA
21601 GCGAGGAACA GCACGGCGAT GCGCCGGGCG TGCTGGCGGC CATCGCACAG ACGGCCCCCA
21661 TGCTGGCCGC TGCTGCGCAG CGCGCCAATC CGACGATCAA GAGCCGCGCC TTCAACTCGT
21721 CCAAGGTTTC CGCGCACCAC GCGATCATCC CGACCGAAAG CACGGCCGAC CTGTCGAAGC
21781 TCACCGATGC CGAGCAGAAG ATTTACCTGC TGATCGCGCG CGCCTACGTC GCGCAGTTCT
21841 GGCCGAAGCA TCTGTATGAC CAGACCGACG TGCTTGCCCA GGTCGGCGAT CATCGTTTCG
21901 GCGTGCGCTC GAACGTCACC ACGTCGCCGG GCTGGAAGAT TCTCTACAAG AACGACGCCG
21961 GCAACGAGGA CCTGGAAGGC AATGCCGACG ACATTGAACA GGACCTGCGC AAGCTGCGTG
22021 ACGGCCAGGC CGGCACCTGC ACCGATGCGA AGGCCGAGCA GCAGGAGACG AAACCGCAGC
22081 CGCTGTACAC GATGGAATCT CTGCTGTCGG ACCTTACCCG CGTTGCGAAA TACATCCGCG
22141 ATGACCGCCT GCGGAAAATC TTGATCGAAA AGGACAAGGG CAAGCAAGGC GAGCACGGCG
22201 GGATCGGCAC GCCGGCAACG CGAGACTCCA TCATCGCCAC GCTTTTCGAG CGCGGCTACC
22261 TGGTGGAGAA GGGCAAGCAC ATCGTGTCCA CGCCGACCGG CGAAGAGCTG TACGACGCAC
22321 TGCCCGACAC GGCCAGATTC CCCGACATGA CCGCGCTCTG GCATGAGCAG CAGAAGGCCA
22381 TCCAGGCCGG CGAGCGCGAC ACGCTGTCGT TCGTCAACGA GCTGATGGAA TACATCGGCG
22441 CCGAGGTCGC CAACATCAAA GACAACGGCC TCAACATGAA GATCGACACG CACCCTTGCC
22501 CGTCCTGCGG CAAGCCGCTG CGACGCCTCA AGAAGAAGGA CAAGAACGAA TACTTTTGGG
22561 GCTGCACCGG CTTTGCCGAC GGCTGCAAGT TCGCGTGCGA CGACAAGGGC GGCAAGCCCG
22621 TCCCGCGCGA GGCCCCGAAA GTCTCCGAGC TGCACAAGTG CATGGCCTGC GGCCACGGCC
22681 TTTCCCGTCG GCCTGGCAAG AAGCGCGGCA TGTTCTGGTG GGGGTGCAGC AACTTCCCGA
22741 CGTGCAAGCA GACATACCCC GACCTGAAAG GCAGGCCGGA CTACAGCAAA GGCCGTAACG
22801 GCACCAACCA GGAGTGAAAG CGATGAATGA TCCCAAGACC GTGCAGCAGG ACGACTTTGC
22861 GCCGTTCGAC GATACCGCGA ACGCCGCCGC CGCCCTGCGT GAAAAGCTCG CCGATGCGAT
22921 GACGCCCGGC TTCCAGGTCG AGTTCGACCC GGAAGAAGCG GAGAAGGCCG GGGCCTTCCC
22981 CGAGGACGCC TTGAGCGAGC AGGACGCCGC CGAGAGCGAC ATTGACCTGG TGGACGCGAC
23041 CGTGCCCGAG GACACGACGA CCGCGGCCGC CAATGACGGG AGGTAATTGG CTATGGCAGA
23101 GGTCAAAAAG CCGTTCCACG AGCAGGTTGC CGAACGCCTC ATTGAGCAAC TGAAAGCCGG
23161 CACTGCGCCG TGGCAAAAAC CGTGGGAACC GGGCATGCCC GGCTCCTTCA TCCCGCTCAA
23221 CCCGACTACC GGGAAGCGTT ACAAGGGCAT CAACGCGATT CAGCTCATGG CCCAGGGCCA
23281 TGCGGACCCG CGCTGGATGA CCTACAAGCA GGCCGCTGCC GCCGGCGCAC AGGTGCGTCG
23341 CGGCGAGAAA GGCACGCCGA TCCAGTATTG GAAGTTCAGC GAAGAGCAGA CCAAGACCGA
23401 CGAGCAGACC GGCAAGCCGG TCCTCGATGC CAACGGCGAT CCGGTCAAGG TGACGGTGCA
23461 GCTCGAACGC CCGCGCGTGT TCTTCGCCAC CGTGTTCAAT GCGGAGCAGA TCGACGGCCT

23521 GCCGCCGTTG GAACGCAAGG AACAGACTTG GAGCGCCGTC GAGCGGGCCG AGCACATCCT
23581 TGCGGCGTCA GGTGCCACCA TCCGCCACGG CGAGCACGAC CGCGCGTTTT ATCGGCCGTC
23641 CACGGACAGC ATCCATCTGC CCGACAAAGG CCAGTTCCCG AGCGCCGACA ACTACTACGC
23701 AACCGCGCTC CATGAGCTGG GGCACTGGAC CGGCCACCCG TCGCGGCTGG ACCGCGACCT
23761 GGCGCATCCC TTCGGGAGCG AGGGGTACGC CAAGGAAGAG CTGCGGGCCG AGATTGCCAG
23821 CATGATCCTG GGCGACGAGC TGGGCATCGG CCACGACCCC GGCCAGCACG CGGCCTATGT
23881 CGGATCGTGG ATCAAGGCGC TACAAGAGGA CCCGCTAGAG ATTTTCCGCG CGGCCGCCGA
23941 CGCGGAGAAG ATCCAGGATT TCGTCCTGGC GTTCGAGCAG AAGCAAATTC AAGAACAAAC
24001 GACCCAGCAG GCCATCGAGC CTGCGCAGGG GGCAACTATG GAGCAACAGC AAGACCAGGT
24061 GGCACGACCG GCCATTGCGC CGGCCGACGA GCTGATCGCG CAGACCCTGC GCATGTACCG
24121 CGCCGGCGCG GAGCCGGCGG AAGGCAACCA ATCGCTGGCC GCGCTGACCG AAACCACGCT
24181 GGGCTTTGAG CTGCCTGCCG ATTGGACCGG CCGCGTCCAG GTCCAGGCCA ACGTCGAGGT
24241 CGAGCACGAC GGCGAACGGT CCGTGGTGCC GGCCGGCGAC CGAGAGCCGG AGTTTTGGGG
24301 CGTCTATGCG AACCATGCGT GGGGCGGCCA CCAATGGCTT GCCGACTTCG CCGGCCCCGA
24361 CGCGCAGACC AATGCCGAAG CGCTCGCCGA CCGCCTGGCC GTGATCGACG CTTACGCGAC
24421 GGCCAACGAG TACGAGCAGG CCGCGAAGTT CGCCCGCATC CATGAAGAGC GAGTTCGCCG
24481 CGATCCCAAC AGCACCGACG AGGACCGCGT TGCCGCCAAG GAGGCGCGCA AGGCCGCCGA
24541 GGGCACGGCC ATGCTGCACG ACGAGGACCT ACAGCGCCGG ATCGCCGACT ACGAGCGCGA
24601 GCAGCAGGAA ATGGCCCAGG CCATGAACGC CGCCGAGCAG CCGGCGGCCG CTCAAGCCCC
24661 CGCCAAGCCC GAGCGCGCCT ACTTGAACGT GCCGTTCAAG GAGAAAGACG AGGTGAAGGC
24721 CCTTGGAGCG CGCTGGGACC GCCAGGAACG CGCCTGGTAC GTTCCGGCAG GGGTTGACCC
24781 TGCCCCGTTC GCCAAATGGG CGCGCGAGGG CGCTACAGCG GCCGTAGAGG CCCGCGCCGA
24841 GGCCCAGCCT ACCCAACCCA CGGCCGAGCG CCCCAACGCG GCCCAGGAGC GCGTCTACCT
24901 GGCCGTGCCC TACGGCGAGC GCCAGGTTGC CAAGGCGGCC GGCGCGCAGT GGGACAAGGT
24961 GGCCAAGTCC TGGTACGCCG GACCGAACGC CGACATGGGC AAGCTGCAAC GCTGGCTGCC
25021 GGACAACGTG CCCACCCAGC AATCCCCGGC CGTCACCCCC GAAGATGAGT TCGCGGAGGC
25081 CCTGAAAAGC ATGGGCTGCG TCGTCACCCC AGGCGGCGAG CACCCCATCA TGGACGGCAA
25141 GAAGCACCGC ATCGAGACCG AGGGCGACAA GAAGGGCGAG AAGTCGGGCT TCTACGTCGG
25201 CCACCTCGAC GGCCACCCGG CCGGTTACAT CAAAAACAAC CGCACCGGCG TCGAAATGAA
25261 GTGGAAGGCC AAGGGCTACG CCCTGGACCC CGCCGAGAAG GCCAAGATGC AGGCCGAGGC
25321 GGCGGCCAAG CTGGCCGCGC GTGCCGAGGA ACAAGAACGC CAGCACGAAG CCACGGCGCA
25381 GCGCATCGGC CGCCAGGCGC AAAGCCTGGT TCCCATCACG GAACCGACGC CGTACCTGCG
25441 CGACAAGGGT CTACAGGTGC ACGCCGGCGT CCTTACCGAC CAGGAGGGCC AGAAAACCTA
25501 CATCCCGGCC TACGACGCCG ACGGCAAGCA ATGGACCATG CAGTACATCC AGGAGGACGG
25561 CACCAAGCGC TTCGCCAAGG ACAGCCGCAA GGAAGGATGC TTCCATGTCG TCGGTGGCAT
25621 GGATGCGCTC GCGGCCGCGC CGGCGCTGGT GATCGGCGAA GGCTATGCCA CCGCCGCCAC
25681 AGTGGCCGAA GCGCTGGGGC ACGCGACTGT CGCCGCGTTC GATTCCGGCA ACCTACAGGC
25741 TGTTGCCGAG GCCCTGCACG CGAAATTCCC GGACAAGCCC GTTGTGATCG CCGGCGACGA
25801 CGACCGCCAG GTGCAGATCA CCCAGGGCGT GAATCCCGGC AGGACGAAGG CACAGGAGGC
25861 CGCCAAGGCG GTCGGCGGCA AGGCCATCTT CCCAATCTTC GCGCCGGGCG AGAACGCCTA
25921 CCCGAAGGAG CTGCCGCCGA TCACCCCGGA GAACTACCGC AACCATTTGC ACGCTGAAAA
25981 GCGCCTCGCG GACGCGGCGG CCGGCAAGGT CCAGTTGAGC GAGGCCGACA CGGCCAAGCT
26041 CAAGGAATCG CTGTTGAACG ATGGCCAGCT CGCCGCCCTG TCGAACATGA AGAAGCACAC
26101 CGATTTCAAC GACCTGTCCG AGCGCAGCAG CCTGGGCAAG GACGGGGTTG AACGCCAGGT
26161 GAGAAGTGCT GTCGGCAAGG TGCTTCTCGA CGAGGGCCAG CGGCAGAAGG TGCAGCAGCT
26221 CAAGCAGCAA GACATCGAGC AGCAAGAACA GCGGCAACGC CGCGCGAGAA CCTACTAAGG
26281 AGAACTAGAG CGTATGAACA AAGTACAGAT CGGGGCACCA CGCACATCGG CAAGCCCTGG
26341 CGTCATCATG AAACCCGAGG GCAGCGTCAA GATCGCCGTC ATGAACGGCA GCCGCCAGGT
26401 GGACCAGGTA GTGAACGGCG AATGGCTGAC CATGAAGGTG TTGCCCGAGG CGGGGCTACC
26461 GAAAGGCATT CACCAGTTGA GCGATGCGAA GGACGCAAGC AAAAACGTCC ACCCGCACAA
26521 GCACGTCGGC CAGGTGCTGC ACGACGATGG GCGCAACGTC TACCAGTTCA GCGAAGGCGG
26581 GATCGTGAAA CACAGCCGGG GCATCTTTGA GAAGCCGCCG GTTGTCGGCA AGAACTACGA
26641 AATCGCCTAC AGCCGGGGCC AGGGCAAGGT CATCGGCGAG GTGTCCCAGG AACAGGCGGC

26701 CAAGGCCGAG CAGAAACGCT CGCGCTCGAT CTAGGAGGCG TATGAACATC GAGGCGTACC
26761 CGCACGATTT TCGCGGCTCG CTCGCCGTGG TTTCTGCGAC CGGCGTGGCC GGTTGCAGGA
26821 CCTGGACGCT GCGCAGCGTC GAAACCGGGA AGCACTACGA GCTTGCGCCG GCTTCGATTG
26881 AAGGCTGGCC TCTCCCTGCG AAGCGTGAGG ACAGTCGGCC CAACAGCATC ATCGTCAACT
26941 ACGACGGCAA CGACGTGATC GCGCTTGAGC TGGCGACCGG CGAGTTGTAC CGCACCCGAG
27001 CACCTGCAAC GACCTTACGA CTACGGAGGT AGCAGCAGAA AAAGCCGGGC ATTGCCCGGC
27061 TTTCTTTGTG AGTCGTCCTG AACGCCTCCC TGATCGAGGT CGAGGGGGCA TCGCCTTAGA
27121 AAAGTTCGTC CAGCAGGAGA TGAAATTGCA GCTTGCGCCT ATCCGGATCG GCAATGCCAT
27181 ATTGCGCAAC AAGCCTCTCC TGAAGCGAAG GTTCGAACTC CTCAAGACAG TTCCATAACA
27241 CGGCAGAATT CTTGCTATGC AGCCGATAGA CCGCGCAGCC GGACTGACCA ACCTTATCAC
27301 GCGCCCATTT GTATCCCATC ACGTGAGCCG CCATGCTCTC GGGCACGGCA GCGGCTGCGC
27361 AGGGCTCTTC TCGATCAATA TCGTTCACTC ATCCAATCTC ACTGACGGTT AGGCGTGCCT
27421 GGTACCGCAC GTTCTATTAA GTCGTACAGA AGACCTGGCA TATGCGTTGT CGGTTCCAAC
27481 GACGGATCGG CGGCTCGGAG GTCGAGATAG ATGGAGGCAG ATGCTTTGAT GGCATCCCTG
27541 ACAGACAACG CATCGTGCAG CGCAATGGTG GTGGCTAGTC TGCGCTCTTC ATCCAGTCGA
27601 AGGGTTTCCA ATCGCCTCAC TCCGCGCTGG TCCTTTCCAG CGCGACGGTA GAGCAAAGGT
27661 CCTAACACTT GCTCACGGAA GAAACCGAGC ATGCCGATCG CTTCGTAAAG CTCGCCACGG
27721 GCGAGCTTGG TGGCCGCATA GTGTAGCCAG ATCCAGGCAC GTTGTCAACG CCACGATGTT
27781 TGACCGTTAT TTGCCATTTT CATGCCCGCT AAGATACTTT CGTTACTGAT GCTGTCCGCT
27841 TTTCACCATT TCATCATCAC TGATTGGCGT TGTGTTTTTG GTCAGCACTC CAGCTTTACG
27901 TTTCTCTTTT AACCGGTAAC TCTCTCCTTT GATATTCAGC GTGGTTGAGT GATGTAGCAA
27961 CCGATCCAGT ATCGCTGTTG CCAGCACGTG ATCTCCGAAC ATTTCTCCCC AGTCTGCGAA
28021 CCCTTTGTTT GACGTCAGTA TGATGCTCGC TTTTTCATAT CGACGGTTCA GTAGCCGGAA
28081 GAACAGGCTG GCTTCCTCTC TGTTCATCGG CAGATAGCCT ATTTCATCCA GGATCAACAC
28141 CCGGGCATAA CTCAGTTGCT GCAGCTGACG CTCCAGCCGG TTTTCCTGTT TCGCTTTCAT
28201 CAGTGTCGCG ATCAGTCTGT CCAGTGGCAT AAACAGTACC CGATGTCCCG CATCCACCGC
28261 TTTCACGCCA AGAGCTATGG CCAGATGAGT TTTTCCGACA CCAGGAGGTC CCAGCAGGAT
28321 CACGTTTTCG CTGCGCTCCA CGAACGCCAG ACCAGCCAGT TCCCGGACAA CCTTACGGTC
28381 GATGCCCGGC TGGAAGGTAA AGTCGAACTG CTCCAGCGTT TTGACCCACG GCAGACGAGC
28441 CTGCTTCAGC CTGGACTCCA TACCGCGCTG ATGCCTGCCG TTCCATTCCT GCTGTAGCGC
28501 CATGCACAGG AACTCCCGGT AGTTCAGCTC TTTTTTAGCT GCCTGTTCCA GCAGGCTTTC
28561 AACGTGATAA CTCAGATGCT CCATTTTCAG GCGACTCAGC AGGACTTCCA GCTCATGCAT
28621 CACAACAGCT CCTCATAGGC ACTCAGTGGT CGATGTTCCA CCTGACTGAC CTGCTGCCAG
28681 AGCGGGGCGT GATGCTCCGG CACTGTCTGC CAGCCAGACG ATGCCGAACA GAGGCGATGT
28741 GAGGCCACCA GTTTCTCATT ACTGTAGATC CGCAACTCGT CATCCAGCGA TATTCGTATC
28801 GATACCGGCT GACCACACAG CGCTTCGGGA ACGCTGTAAC GATTACCACC AACCTCGATA
28861 TAGCTGTCCC AGGACACATG GCGGATGTCG AAGTAACTGG TATCGAAGTC CGTATCCGGT
28921 AACGGCTGCA GATGTTCCTG CTCCAGCGCG AAGCGCTGTT CCGGCGTTTC TTTGAACTGG
28981 CGAAGTTCCC GTTTGTCAGC CACATCGGCT ATCCATTGCT CCAGTTGTTG ATTAACATGA
29041 GTGAAGCTGT CGAACCTGCG GTACCGAACG AAGAAGTTCT CCTTGAGGTA TTTCACCATC
29101 CGCTCAACCT TACCTTTTGT TCTGGCCCTG CGTGGACGGC ATGCCCGTGG CAGGAAGTTA
29161 TAGTGGTCGG CCAGCAACAG GAATCCGGAG TTGAACACGA CTTTCCCGTT GTTATTCTTC
29221 AGCACCGCAG CCTTCTGGTT ATCAACCAGC ACCGTTTTCA CACAACCACC GAAGTAGCGG
29281 AAGGCGCGAA CCAGTGATTC GTAGGTATGC TCAGCATCCT GTTTTGGTGC GGCGAAGACA
29341 TGGAAGCGGC GGGAGAACCC CAGCGTATTA ACCGCAAAGT TAACTTTGCA CCGTTGCCCG
29401 GCAACCTCCA CCTCAACTTC GCCCCAGTCG TGCTGGAGCT GGTATCCAGG CTGAGTTTCG
29461 AAGCGAACTG TTCTTTTTGA CGGACGCATT TTACGTTTGG GCTGGATGTA GTAACGCAGC
29521 ATGGAACGTC CGCCCGTATA ACCCATTGCC TTAATCTCCG CAAAGATAAC CTCACTATTC
29581 CAGACATTCT CTGCCAGGCG CATGTCGATG TAATCCATAA ACGGTTTCAG CTTAACCATT
29641 TTGTGGCGGG TCTTTCTGGC TGGCGGTTCA GGGTATTTGA GGTAGCGTCT GACCGTCCGT
29701 TCAGAGCAAC CAATCTGAGT CGCAATATCG ACAATGTACG CGCCCTGCTG GCGCATTTGC
29761 TTTATCATGT AAAAGTCCTC TCTGCTCAGC ATGTTGATGT CCTTTCTGGT GTGAGAACCT
29821 TAAGGAAACA ACATGTTGGG TGGAGCGGAC AATCCAAATG GTGAATTACC GTCTTATATC

29881 ACTGGCGCTG ACAACGTGCT TCGAACCACT CGGAGGGACG GTTTGGCCAT GCCACCGCTG
29941 CCGCCTCAAT GCGCTTCTCG ATCTCTGCCG GATCACGAGC AAACAGAACC GCCCGCCGCT
30001 CGATTTGCTG GTCGAGATCG GAAGCAAGAG AAAACTTCAA ATCGATGTGT AGCAGTGGCG
30061 GACCATATAG GCAGATCAGA AGGCGCGGTT CTCCTACATG TTCGCCGGTG AACGCGTTGA
30121 GGAAGCCGGG CAGTGCCTCG GCAAAATCCT TGCGTGTAGA CAAGACATCT GCGTAGCAGT
30181 TGTCCTCAAC AACGATGTCG AAATCCAAAT CGGAGTGCTC ATCGAGTCCT CCGTGAACGT
30241 AAGAGCCGCC GATCAGAAGA GCGCGGAAGC GAACATCGGA AGCGACCGCA TCGCGGATGC
30301 GGTTCAAGAA AGTTGCATGA GCTTGTGGAA GTGTGCTGAG CATAAATGAT TCTCCTAGCT
30361 GTTCTTTGGG TAAGTACGCC ATCAGGACGT TGTGAGTGGC GCGATTTTTA GCGGCTGAAA
30421 TCAGCCCTTG AGCCTGTCGG CAAGTCGCGT CATGAGGTCC ATGCGCTCAT GCAGGATCGC
30481 CACGACCAAC GCGGGTTCGC CCGCACGCGG CAGGCAAAAA ACGTAGTGGT GTTCGCAGCG
30541 GGCCATCCGC AGCGCGGGAA AGAGTTCGCT CATGTCCTTA AACGGGCCTT CGCCGGCGGC
30601 AAGCCTGGCT ATGCCCTGTT CCAGCTTAGC GATATAGCGG CGCACCTGCG CCGCGCCCCA
30661 CTCCCGGCGC GTGTAGCGGA TGATGCCGCG TAGATCGGCT TCGGCCTCAG CCGTGAGGAT
30721 GTAGGCCGTC AAGCGCGATC CCCGCTGAGT TCTTCATCAA GAATTTCGCC GACGCTCTTG
30781 GTGGACACCT TGCCGGCAAG CCCATCGTTG ATGCGGTTCC CCAGCATGGT TTTCAGTTCC
30841 TGCCATGCCT GATCGGCATC AGCGTCACCG GGGAACAGAC GTTCGAGGGC GTATTGCTTA
30901 ATGGTCTTGC CCTGCAAGGC GGCCAGGGCT TTCAGGCTCT GGTGCTGCTG GTCCGTCATG
30961 TCGATTGTCA GGCGGCTCAT TGGATAACCT CCATAAAATA CACGTAACCA CATTAGCACA
31021 TATGTGGGCG TGAGGCTACA GCGCGAGGCG CATTAAGGTC GGGAAAATGC GCTAGGCGCA
31081 TTTAAATTGC GTATTGCTGT AATGCGCCAT GCCGGCTAGA CTAGGCCCAA ATGGGTATAC
31141 CCAATTTGAC CAAGGGGGAC GCGATGAGGG CGGCCAAGCA CTACCGACAA CTTCTATCCA
31201 TCGACTTCAA CATCGAGGCG CTGGCCTTCG TGCCTGGACC CGACGGCACA CGCGGCCGGC
31261 GCATCCACGT CCTGGGGCGC GAGGTCCGCG ACCGGCCCGG CCTGGTCGAG TACCTTTCGC
31321 CGGCGTTCGG CTCGCGGGTG GCGCTGGACG GCTACTGCAA GGCCAATTTC GATGCAGTGC
31381 TGCACCTGGC GTACCCCGAT CATCAGCAAT GGGGCCACGC ATGAAGCGCC GAAGCTACGC
31441 CATGCTGCGC GCCGCTGCCG CGCTGGCCGT CCTGGTCGTT GCCTCGCCGG CATGGGCCGA
31501 GCTGCGCGGC GAGGTCGTGC GCATCATCGA CGGCGACACC ATCGACGTGC TGGTAGACAA
31561 GCAGCCGGTG CGCGTGCGCC TGGTGGACAT TGACGCGCCG GAAAAGCGGC AAGCCTTCGG
31621 CGAACGTGCG CGCCAGGCGC TGGCCGGCAT GGTGTTCCGC CGGCACGTCC TGGTCGACGA
31681 GAAGGACACC GACCGTTACG GCCGCACGCT GGGCACCGTG TGGGTCAACA TGGAGCTGGC
31741 CAGCCGGCCG CCGCAGCCGC GCAACGTCAA CGCCGCGATG GTTCACCAGG GCATGGCGTG
31801 GGCCTATCGC TTCCACGGCC GCGCGGCCGA CCCTGAAATG CTGCGGCTCG AACAGGAGGC
31861 GCGAGGCAAG CGCGTCGGCC TCTGGTCCGA TCCGCACGCC GTCGAGCCGT GGAAATGGCG
31921 ACGCGAGAGC AACAACCGGA GGGACGAAGG TTGAAGGTCG CCCGCATCTA CCTGCGCGCC
31981 AGTACGGACG AGCAGAATCT TGAACGCCAG GAGAGCCTTG TAGCGGCCAC GCGGGCCGCC
32041 GGGTACTACG TCGCCGGCAT CTACCGCGAG AAGGCGTCCG GCGCACGCGC CGACCGGCCC
32101 GAGCTGCTGC GCATGATCGC GGACCTGCAA CCTGGTGAAG TCGTCGTTGC GGAGAAGATC
32161 GACCGCATCA GCCGCTTGCC GTTGGCCGAG GCCGAGCGCC TGGTTGCGTC GATCCGGGCC
32221 AAAGGGGCCA AGCTGGCCGT GCCTGGCGTG GTGGACCTGT CGGAGCTGGC CGCCGAGGCG
32281 AACGGAGTGG CGAAAATCGT TCTGGAATCC GTCCAGGACA TGCTTTTGAA GCTCGCCTTG
32341 CAGATGGCCC GCGACGACTA CGAGGATCGG CGCGAGCGTC AACGTCAGGG TGTCCAGTTG
32401 GCGAAGGCCG CCGGCCGCTA CACCGGCCGC AAACGTGACG CCGGCATGCA CGACCGCATC
32461 ATCACGCTTC GCTCCGGCGG ATCGAGCATT GCCAAGACGG CCAAGCTGGT CGGATGCAGC
32521 CCGAGCCAGG TCAAACGAGT GTGGGCGGCC TGGAACGCGC AGCAGCAAAA ATAAAGCCGG
32581 GCAGTGCCCG GCTTTTCTCA CCTTTTCGCG TCCCGCAGGG CCGCTGCGAG CGCCCTACCT
32641 AGATCCTCGC TTTCCCCCTC GGTGTAGTCC GGCCAGGGCA CGAAGGGCGC GGATGCGAAC
32701 CTGTTGAGCA GGTACGCCTT CGGGCAGCGG TAGACCACCG GCGAGTTCGC CTTTTCATCC
32761 CACCGGGCCA GGATCACGTC CGCATCGCAG TGCATGTCCT TCACCTGGTC GCGGAAGAAG
32821 CCGAAGGCCA CCATGCCGCT ATGTTCGCCG AGGAACGCCA GTTGCTTCGC GCTGGCGATC
32881 GCGCCGACGC CGCCGGCCAA AACCGACGCC ATCACCCAGC CGACGAACCA GAAGCTGGCA
32941 TGCTTGCGGT TGACCACCGC ACGCGCAGCC GCGACCAGGA CAACGGCCAA GCTGCCGACC
33001 AGGGCCATGA CGACCGTGAT CCGGCCGTTG TGGAAAGCGA TGGGCTTGCC AGCGTCCGCT

33061 TGCACGGCGT CGTAAATGCT GGACCCGATG GGCGCGCACA TCAGCACGAC AGGCAGCAGC
33121 ACCAGGAACA TCGTCCGCGT CCATTGCGCG AGTGCCTTGC GGCGTTCGCC GGCGGCAAGC
33181 GCCTCCATCA TCGGCGTGAA GCCCAACAGG GCCACCGCAG CCGCCAAGCC GGCAACGATG
33241 CCGCAGGCGA TTACATACAT ACATCCTCCC TAATGCGCCT TGCGCACGGT TGTAGTCAGA
33301 GTCCGCGGCC TTTGCGCGGG CCGCTTCGGC CGGGGCGTGG CTGCACCTTG GGAGCTGGCC
33361 GGGTGGCCGC TGCCGGCGCA GGCCGGGCCG GTGTGGCGAC CTGCGGAGCC TGGCGTTGGG
33421 CCTGCGCGAA CAGTTCCGTG AACGACAGCG GCGCAGGATC GAGGCCGCAA ACCTTGTTCA
33481 GCAGCTCGGC CGACTCCCGG CCGTGGACCT GGATCGGGAA CGACGGAGCC TTGTTGGCAA
33541 TCAGCCAGAA CGAGCGCGTG ACCCCGTTGC TGAAACCAAT GTCCACCCGG TGGCGCTCCT
33601 GGTGCGCGCC GGCGAAGGCC AGCGGCACAG GATTTTCCCT GCCGTGCGCG AAGCCCTGGA
33661 CGGCGTGCTT GAACTTGTAG TCCTGCGGCA TTTCCGAGCG CAGCGGGCAG TTGTCCGACC
33721 GCGTTTCGCG GCCCGTCGAG CTGCTGCGCA GCCAAGCCCG ATAGAACGCT TCGGTGTCCA
33781 CCCGAACCAC GAACATTTCA TCGTTGATCG CGCCAGGCTT GGCCGACATG AAGCGGTCGG
33841 CCTGGCCGGG CACCGGCACG CGCCAAACGG CCTCGCGTGC ATCAACCTGG ACCAATTCCG
33901 CCGGAGCCGG CCGGGGCCGG CTCACCACGG ACACATCCAG TTCCTTCAAC TCGCGGCGAC
33961 GAGCAAGGAA GCCCTTCAAC CGTGTCAGCA TCCGCCGCCC TCCCCTCAGT AGAACTTCGC
34021 ATCGTCAAGC GAGATCGAGC AGCCGTTATG GTCGTAGGAC ACGTCCTTCA CGGTGCCAGT
34081 GACGCGCGTG GTCTGGCCGG TGGAAACCGA CTTGATGGCG AGCTGGTTAT CGGTCCCAGC
34141 ATGGACCGAG GCGCTGCCAG CGCGCAGCAG CACCCGATAG CGAGGCTTGA AGCCTTCGGA
34201 AATCACCTGC ACCTTGCCGG TGGCCGACAA CCCTTTCTTG CCGTACAGCT CCGTTGCCCG
34261 AACAGGGTTC TTGTCAGCGT CGCGGCAGAT TTGCGCCACT GGCACGGTCA GCATTTCCGG
34321 CGGGGTCGAA CCGGGCGGCG TCGGCATGGG GATGTTCAGC AGTGCGCAGC CGGACAGGGC
34381 GAGCAGCGGC AGGGCCGCGA TGGTGCATTT CTTGAGCAAT TGCATATTGG AATCTCAAAG
34441 GTCAGGTGAA AAACAAGTAG CCCGCCTCGC GCATCGGAAT GCGGATCAGC CAAAGAGCGG
34501 CAAGATGCAG CGGGTAGTAC GTGTAAAAGG CCCAGCGCAG GCGCGGGACA CGAAGATCGA
34561 CGCCGGCGGC GACGATCACC AGGGGCACAA CAGCAAGCGC CCAAAGGTTG CCATTGATGT
34621 ACCACAGGGA CGCGCAAGAC AGCAGCGCCA TCAACGCGGC CGCCCACGTC GGGCGCTTGA
34681 GGTACAACCA GACAGACGCG GCCAGCAGCA GCGCCGGCCA ACAGAACTCG ACCAGGCCGC
34741 CGGCCACGAC GAACAGCGCT ATAGGAGCGA CCGAGCGGCC GCGCTCGACC AGGTAGAGCA
34801 TCGCGGTTGC GGCCAACAGC GTGAACATGA CGTTCAGCGG CCACCATCCG CCCACCACTC
34861 CACCCAACGC AATGAACGGG ACCGAGGCGA CCAGGCCGAA GGCCAACAGG CGTTTCATCG
34921 CTCGCCCGTA CAAACCGCGC TCGAGCGCGC CCGGGCGGGC GAGGTTGTAC GCCAGGACGA
34981 AAACGAAAAG AGGCAGGGCC AAGCGCCCCG CCTCGAACAG ATATGGCAGC GTACCGTTGA
35041 ACAGGTACTT GTTGACGTGA TCCCCGGTCA TGGCAAGCAA GGCGAGCCAC TTCAAGGCTT
35101 CAATGGTGCC GTCGGCGATC ACCATGGCAG GCAGCGAGGC CCGGCCGCTG GCGTCGTTCA
35161 TGGCGCGTGT ATTCATGCCC TCCCCCTTGG AGTAAGAACG CCCTGCAGGG CGCTATTTGC
35221 GCCCATAGCG CAGCGCGTAT TCCCGGAGGA CGTGCAGCGC CAAAGGCAGG GCCGCGAGGA
35281 CCAGCAGAGC GCCGGCGGCG TACATGGCCC ATGTGGGCAG GAACGCGAGC AGCGTATGCG
35341 CGACCAGGCC AAAGACGATG ACAGGCCCGA TGCCCAGTAC CAGAAGATCA AACGGCGCTT
35401 TCGCCTGCGA TGCCGCCCAG GCCGCGTAGG CCCTGGCCCC GTTCATGTAT CGCTGCGTCA
35461 GGTTGCGAAT GCCCATAGGC TTTAGCCGCT AAACGGCCGT TCCTCCTTAT GGCGTGTTGT
35521 TGATGATGAG CTGCCGGGGT ACGTAACCCG AGCTGCGGAG GCTGGTGCGG GTGATCCGGC
35581 CTTGCTTCAT CGGCTGCGCT TCGGCCGGCG CTGCTGCCGA CGTATCAGCG GCCGCAAGTG
35641 TGGTCCCCTT CCCTGCCGGC TGCGCCGGCG AGCTGGGGCC ATAGTTGACG GTGACGAAAC
35701 GCGCATCCAG CCACTTCGCC CACTTGTCGG CCTTCACCAT CAGATCGGCG CGATAGATCG
35761 CGTTCTTCGA CGGCGTGCGC GAGTGATAGT TAGCGGCGCG TGTCCACAGA TCGCCCCTGT
35821 CGTTGCGAAT GTGCCCGCGC AACCGCCAGG CCGCCAGGTC ATAGGGATAG CAGCCTGCCG
35881 CAGCAACGTC CTGGGCCGTG ATCCCATACT TCGCCAGGTC GCCCAGGTAG GCGGTGTTGA
35941 ATTGCAGCTC GCCCACGTCA TAGGTGCCAT TGGTGTTCTT GACCCACTGG CCCGGCTTGC
36001 CGCCCTCCTT TTCCCGAATG GCGAGAAGAA TGTTGGCCGG AATCTCGTAC TTCGCGGCCG
36061 CAGACACCGA GCACACAACG CGCTCTTCCA TCTGTTCCGA CAGATCGGGG AAGAGCCGTG
36121 CGGCGGTCGC ATCCGCCGCA CCGAAGCCAG TGGCCGCCAC CAGGGCGACC ACCGGCAGAG
36181 CGTTCCGTGC CAGCAGCTTG GCAAACGGCA TCGCTATCCC CTCCCCTTAC CGCTCGAACC

36241 GACGGTAGGC GTCGCTGCCA TAGCGACCGT ACTGCTGCTG CCGCTCTTTC AGCTTCGCCT
36301 CGTACTCCTT GAGCGCGCGG TCGTAGTCGG CCGCCTCGAT CCAATAGCCG CCCTCTTCCG
36361 GCGTGCCGAC GTAGCGCGGC GCGGTCGTGT TGAAGTCGGT ATAGGCATGG CCGGTGTAGG
36421 CCGCGCAGTA GTCCGGCAGG CGGTTGCCGA TGTAGAACTG GTCGTCCCAG CTCCGCCACT
36481 CACGCAGCAC GGAGTTCAGC GAGGACGCAT CGCAGCGCCC GGCCCCGCGC GAAATCGAGG
36541 AAACGAGCGT CTGCATTTCC GGCGTCTGGT TCGATACCGG GCAGAGGTTG AGGAAGTTCA
36601 GCCGCGCCTT GAGCGTGTCC GACAGCTTGC GCTTGTGGAT GCCGAAGTAC CGCGAGAGCG
36661 ACGGGCTGCA CTCGCTGGGC CGGCTGCCCG TGGACAGGCA CAGAATGGCC TCGCAGGCCA
36721 GGCGGGTATC GCCCGTCAGC ACGTCTTGCG CGCTGGCCGA GCCAGTTGCG CCGAGGCCCA
36781 AGGCCACCAC CAGGGCGGCC GACGAGAACG CTTTTTTCAG TTGCATGGTC GTAGCTCCTA
36841 AGAGTCGTTT CAGGATTGGG CGGGCTTGTT GACGAAGCTC GCCACTTCGG AATCCGCGTC
36901 GGCGGCCGCT TCCCTGTTGT TGCTTGCGGA AAGGCTGTTG TCGTCGAACG ACGGTGCGGG
36961 TGCCGGTGCA GGTTGTTCCT GCGCCTGGCT GTTGCTATCG GCGACGGTTG CAGCGGTCTG
37021 CGCCGCCGCA CCCGCGCCGC GAATTGCCTG CGCGATCTTG CCGCCTACGG TATCGCCGAT
37081 GCGTTCCTGG GCCGATGCGC GCATTTCGCC GGCCTTGGCC TTGGCAATCG AGCCGGCACC
37141 TTTCGCCAGG TTGGCGACGG TATCGGCCGC GATCCGGCCG CCCTTGGCCG CCGTGGCACC
37201 GATACCGCCA GACGAACGGC CACCACCCGA GCCGCCGCCC GAACTGCCGC CGCGTGCGCC
37261 GCTGCTGTTG TCGCCGGCGG CCGAGGCCAT CGGGGTTTCA CCGCCGCCCG AGCCACCGCC
37321 GCCACCCGAG CCGCCGCCGT CGCCGCCGCT GGTGCCGGCG CTACCACCGC CGCCGCCACC
37381 GCCGCCGCCC ATCATGCTCG ACAGAATGTC AGTGCCGGCA GAGACGTTAT CGCTGGCCTT
37441 CGACGCGGCC GCCATGATGG CCTGCGCGCC ACCGGCCGCC GCCGCAGCGC CGGCCGCGAT
37501 AGATGCGCCG CCAGTTGCGA TTGCCGCGCC GGCCGTCGCG GCCGCACCGA CGAGCGTGCC
37561 AGCGCCGAAC TGGCCGATAC CAGCACCGCC GACGCTCGCG CCGGTGATGA TGCCGGCGAT
37621 GAGCTGCGGC ACCTTGTTGA CCAGAACGAG CAGGATCAGG CCGACGATCA GCATCACTCC
37681 AAGTTCCTTG AAGTTGATGC CTTCGCTCAT GCGGCTGTAG TAGTCATCGA GGAAGGTCTT
37741 GCCGATGCCT ACGAGCAGCA CCATTGCGAA GAGCTGCGCG GCGACCCCGA GGACGGTCTT
37801 GTAGTAGTTG ATCGCCATGT CCGAGGTCCA GCGCGAGCCG CCGAAGCCCA GGAAGAACAC
37861 ACCGCCGTAG GCAAGAATCC ATCCGGACGC CAGGAGCAGA AGCATATTCA CGCCGACCAG
37921 GGCCAGGATG CAGAGGATGG CGGCCGCCAA CGAGGCACCG ACGAAGCTAT CGACCGGCGA
37981 CCAGTACGAG GTTTCGTCCA TCACCTTGAA GAAAATCTCG AAGCCAACAT CGACGATGCC
38041 GGACGGCGAA AGCCCCTGCC CCAACCCCGT TGCCTGGCCT GCAATCTGCC GCAGGGACGC
38101 ATAGATGGAC GACGCGAAAT TCGGGCCGTT GGTCAGCAGC CACCAGAAGA AGCCGGTGAA
38161 GATGGTGAAC CGCACGAACT CGGCGAAGAA CTCGCCAATG TCGGCCTTGC GCAGTGCCAT
38221 CATGCCGAAG GTCCAGACCA TGCTAATCAC GGTCAAGGTC CAGAACAGCC AGGTTGCGGC
38281 GTTCTGGACG ACAGTGGCCC AGCCGCTCGC GGCGTTCTGG TAGCGCTGCA ATACGTTGTC
38341 GAGGATGCCC GAATTGTCGA TTTGGGCGTA TGCCGCTACC GGCATCAAGG CCAGCATCAG
38401 GACCGCGAGC GCGGCAGCTC TAGTCTGGAT TTTCATACGT CATCACCACT CTTTCTTTTC
38461 GCTGGGCTTG AAGCCTCCCG GTTCTCCGCG CGGGCACTTC TTCAACAGCT CCGCGCGTTG
38521 AGCGGCATCC GTGACCTTCG GCAGATCGGC GCAGGTCAGC TTGTCGGTGT CGGGCTTGTT
38581 GTCGCAGCCT GCCAGGCTGG CCGCCATGAC GGCGGCCAGC GCGGCAACTG CGATGAAGTT
38641 GGATTTCTTC ATCGCGCCTC CCCTCACCAG GTCTTAGACG GGCTTGCGCG GTACGAACCC
38701 TCGCGCAGCT TCGCGCCCGC AGCGTCCTGC TGGGCCTGCC GGTCCTGCTG GGCCTGCATC
38761 TGCGTGGCGA TGGCGTTCTG CTGCGCAAGC AGAAGGCCAC GGATTTGCAT GAGCTGGTTG
38821 GCCTGCTGGC TGGCGAACTG GTTGGCGTAG CCGAGGGCTT CCAACTGGCC CTGCGCCGTC
38881 GTCGCCTTGC CCTTCAATTG CTCCAGCGTG GCGGCGTCGG ACTTGAGGTT GCTCTGCTGC
38941 TGATCGAGGC CACGGAACAG CGCATCGTTG GCCTTTTTCT GCGATTCGGA CGCCAGGCGG
39001 CGGTTCTCTT CCATCGCCTT GCGCTCGCTT TCCGAGCAGC CCGACAGGGA GAAGCACGGC
39061 GACCCCTTGT AGTAGGACAC GTCCTTGAAC TTGCCCAGGT AAGCGTCGAT GCTGCCCGCC
39121 TGGTTCTTGT AGTAGTTCAG GGTATCAACG GCGCTCATCA GGCCGTTGAT GGTGGACTGC
39181 GCCTGGTCCC ACACGTAGGC GGCCGGGGCC ACCGTGTTTT GCAGCATGTT TTCGTACTGC
39241 TGCAACTGCG TCCGGTATTC CTCGATTTGC TTCTGGACCT GCGCAACCTG CTGAATCGCG
39301 GTGACAGTGG TTTGTGACAG GTTGGTGCCG TCGATGACCG GAATGCCCGC TTGCGCAGGC
39361 GTGACCGCCA AGGTGCCGAC CGAGAGGGCC AGCACCAGAG CTACTTTAGC GGCTAAAACA

39421 TTCTTAGCGA GCTTCTTCAT AAGTTACTCC TTGGAGTTAA TAGTCAAACG CCTGGTAGGG
39481 CTTAGAAAAC GTCATGTCTT TCGTGACAAT GACGTTGAAG CGATAGCCCG GACGGATTTC
39541 CAGCGTCGGC GCGATATTCA AGTTTTTGGC GATCATCTGC GCCGTTACTT GGCCGAGCTG
39601 TTGACCCAAC GCTTCACTCA TCGCGGAACC CGCGTCTTGT CGTCCGTAAC CGCTGTTGCT
39661 GTTGCCACGG TCCTGACTCA AGCTGATGCC CGCAACGACG CCCGACATGA GGAATGCCGA
39721 TGCGAAGGTG CGGAAGTAGT GGTTGTTGAC CTTGTCGTTG AAGCCTGCAT ACCCAGCGCT
39781 ATCGCCGCCC GGCATGGCCC CAATGTCCAT TGCCTTGCCG TCGGGGAAGA TGATGCGCTG
39841 CCATGCCACC AGAACGCGCT TCTGCCCGTA GGCCACATCG TTCGAGTAGC TGCCCACCAG
39901 GCGCGAGCCT TGGGGGATGA GCATGTGCTT GCCGGTCGCC GTGTCGTACA CCGACTGCGA
39961 TACCTGGGCC ATGATTTGGC CTGGCAGATC GGAGTTGATG CCCGAGATAA GCGTAGCCGG
40021 AACGACGAAG CCAGCGCGCA GCACATAGGG CGTTGCAGGT GCTTCCGGCT GGGAGTCGAG
40081 CCGCCAACGG TCGCCCTCGC TGCGGTTGTC GAACGACCCA TAGCCAGCGC CGCCACCACT
40141 GCGGTTCGAG GTCTGCACGA GGGTCGGCGC ACCCGAGCCG CCCATACCGC CACCCCCTGC
40201 CGCGCCGCCC ATCGTGCGAG CCTGCGCAAG CGCGGCCTGA TAGGCGGCGG TCGGATCGGT
40261 GGCGCGGGCG TTCTCAGCCT GCCGACGCAG CTCCTGGATG CGCGCAAGGG TTTCCTCGCG
40321 GCCCTGCGGA GCACCGCCGC CGGCGCTGCC CTGGCTGCGC GGCGCGTCGA TGCGCACCGT
40381 CGTCTTGGCC TTGATCGCCT CTTCCAGCAT CTGCAACTTC GCCATGCGGA TGCGGTCCAG
40441 GTCCTCGTTG CGCGCACCCT GCGGCGGCGT CGGGGGCTGG TCCAGGTTCT CGGGCCGTGC
40501 GACCGTGATA GTCGTTCCCT GCGCCGGGGC TGGCGTCAGC TCCGTCGTCG GTTGCTGGGC
40561 GGTTTGTTCC GGCGGAATCT CCAGCGGCTT GGCCTTGATG ATGCCGTCCT GCTGTTTGCC
40621 GGCAATTTCG TCGGCAAACA TGCTGGTGCT GCCGGCCTTC TCAGCCTTCG CAGCTCCCGG
40681 CTGGTTCTGC TGCGCAGCGC GATCCGCAGC AACCAGGGCC ATCACCAGCA GGAAGATGCC
40741 GAGCACACCG CCGATGAGGT ACATCGGCAT GTTGTTGACG CGGCGAACCC CGCTTTTCGG
40801 CTTGACCGCA TCTGGCGATG CGTCCGGTGC CATTTGATCT TCGCTCATTG GCTTACTCCT
40861 TCCGAACCCA TGCGCCGGCC GGGACCATCG TGTTGTTTTG GGCGAGGTAG GCGCGCGTGA
40921 GCGATTGAGA GCCGACCATG ACGGTCAGGC GATACAGGTT GCTGTCGCTG AATTGGTCCA
40981 GCACGTAGCG CAGCGGATAG CCGCCCTGGG CTTGCGGCGT TGCCGGCTTC GCAGCGGCCG
41041 AGGACGCAGC AGCCGGAGCT GCGGCCGCGT TGCCGTCGGG CTTGTACTCC ATGACAGCAT
41101 AGCCCTGGGC GCGAAGGTCA GTGACCAGGG CAATGCCGAA CGGATCGGGC GTAGCCTGCT
41161 GCAATTCCAG CTTGGTTTGC GCCGGCGGGT AGAGCTTGAC GAGCTGCTTC ACCGCGTCGG
41221 TCGCAATGGT CTGGTTGTAG GCGGCCGGCG CGTCCTGGAC GAAGCTGCCG TACTTGGAGG
41281 TCGCGCAGCC GGCCAACGTG GCCGCGAGTG CGATGACGGT CAGAATCTTA CGCATGGTTT
41341 AGTTCCCCCT TGAAATGGTC ACGCGGTCCT GGCTGCTGCC CACGCCCGCG ATGAGGATGG
41401 CCTTGTCGAA AATCGTATCG ACGATGTAGC GGTCGCCCTG GACCCGGTAG TTGACCATCA
41461 CCGTTTCATC GTCGGAGAAC AGGCCGCCCT CCCTGCGAAC GACCAGGAGC GTCGGCGCTT
41521 CGGTCTGTTC CATCGAGTGC GGCATCTGGA TGATGGTTTT CTTGCCGTCG TTGTAGACGC
41581 GCACCGGCTT CCACGACGTG GACCCGCTGA CGGAGTAGTT GAAGCTCAGG TTGCCCAGGT
41641 ACTCGCCGGT CTGCGGAATG GTTTTCTCGA CGCGATCCCG CTGTTCGCGG TTCTTGATGG
41701 CGTCCCACTT CGCAAGGGCA TCTTCCGGGT AGGTGAACGA CACCTGCGGC ATGTACTGCG
41761 TGCGATGCGA GCGCAGGCGC ATGTGGTAGC TGCGGCGGTC CGTGGTCACG ACCAGGCTGG
41821 TTTCCAGGCC CACATCCATC GGCTTGATGA TGAGGTGCTG GGTTTCGTTC GCGCCGCTGC
41881 CGGTAATGGC CGGCTCGACC GTCCAACGGG CGGTGTCGCC CAGGTTGATC GAGTTGACTT
41941 GCTCGCCGGG TTGCAGGGCC ACGTCGCACA CTTGCAGCAC GGCGCATACG ATGCTCGGCT
42001 GCTGCGCGCC GAACAGGAAG CGCACCGAAC CACCGGGGCC GGCCACCGGC CGCATGCCGG
42061 CGGTACCCGC CTCCCACTTC TTGGCGATGG CGATGGCCGC CCGCTCTTGC GCGGTCAGCT
42121 CCGGGTTCTT GCCGGAAAAA TAGAGGTCAG TCAGGTCCGC GCCGGGATCG GCGGCAAATG
42181 CAGGCACGCT AACGGACGCG GCCAGGACCA AAGCAAACAG TTCCTTTTTC ATAATTCAGT
42241 GCCTCACAGA AGTCTCGACC AGGAGAAGTC CCGGACGTAG ATCCCGGCCG GGTTGTTTCG
42301 CAGTTGTTCT TCCTTGGTGT CCGCCGTCGG CTCGACGACG TAGACCGTCA CCAAGGCCCG
42361 CATGCGCACG GGCTGGCCTT TCACCACGCC TTGCCTGTCG CGCGTCGTCT CGACCCAATC
42421 GACCTGCCAG GTGTCGGGCG TCTGCGGGAT TACGGAAGTG ATTTCGGTGC TGACCGTTTC
42481 GACGGCCGCG CGAGCGAACG GGCTGGCGTC GGCGGTGCCG TTGAGCCATT CGTTCATCTT
42541 GGCGGTGGCC GGGTCATTCG GCCCGAGCTT GGCATAGAGG CGGTAGACGG CCTTGCGCTG

42601 CAAAGCTACG TCCGGCGTCA CCAGGCGAGC ATCGCCGACG AACTCAGCCA CCGAGGCGTG
42661 AATGACACGC GGATCGGCTT TCGACGCCCT GGTCATCGGC CCCACGGCGG CCGTCTGCCC
42721 GAGCTTGTCT ACCTCGTAGA CATAGGGCAC GAACTTCGAC TGGCTGCCGA TGTGGATGAT
42781 GCCGCCGACC GCCGCGAGGA CGATCATCAG CGAAAGGATG CCGACAACCT GCCAGGTCTG
42841 CTTTTGCGAC ACAACGGAAC CAACGTGGTC GTTCCAGGTG CGCCGCGCCG TCAGGTACGG
42901 GTTGTCGGTT TGCGGGCCGG TCGCGGCCGG CGTCGCCGCT GCTGCGGCCG TTGCGGGCTT
42961 CTTCTTGAAG ATCAAGCCCT TGATCGTGTC TGCAAAACTC ATGCTGCCTC CAGGTATTCA
43021 TCAAGGGCGA GGCCCCGGCC ACGCAGCCAT TCATCCACCC ACTGGTCGCC GAACTTGGCT
43081 TCCAGGTTCT TGATGATGGC GACGGATTCC TTGTCGGATG CGCCGACGAA CGCGAGCGCG
43141 AGCGGGCCAA GTGCCAGGTC GTAGAGACGG CGGCCGTTTT CCGACACGTA GTAGTACTGA
43201 CGCTTGGGAA CGGCCTGGGC CAGAATCTCG ATCTGGCGAG CGTTCAGGCC CATGCGGCGG
43261 TACAGGGCCG CCGTGTCCTC ATCCCTGGCG TAAATATTCG GCAGGAAAAT CTTGGTCGCG
43321 GTCGATTCCA CGATCACGTC CAGGATGCCG CTGTTGGCGG CGTCGGACAG GCTCTGCGTT
43381 GCCATCAGCA CAAGGCAGTT GGCCTTACGC AGCACCTTGA GCCATTCCCT GATCTTCGCG
43441 CGGAATGCCG GGTGGCCGAG CATCAACCAG GCTTCGTCCA GGATGATGAC GGCCGGCTGG
43501 CCCGTCAGGG CGCGCTCGAT ACGGCGGAAC AGGTAGAGCA ACACAGGCAG GGCGAATTTC
43561 TCGCCGAGGT TCATCAGCTC TTCGATCTCG AACACTGTAA AGTCGGACAG CGCCAAGCCG
43621 TCCTCTTCGG CGTCGAGCAG ATGGCCCATT GCGCCATCGA CGGTGTACTG GCGGATCGCC
43681 TCGCGGATCG CCTCATCCTG AATCGTCACG CTGAACTCGG AGAGCGTGCG CGCGCCGCTG
43741 GCGTGCATGC TCATGATCGC GTTGCCGATT TCGTTGCGCT GGGCCGGGGT CGTTTCGACG
43801 CCGTTCAACG CCAGGATGGT GTCGATCCAC TCCATCGCCC AAGCACGGTC GCCCTTGGTG
43861 CTCAGGAACT GCAACGGGCA GAACGCCAGG CGTTCGTCGT CGGCCGCCAC GGTGAAGTGC
43921 AGGCCGCTGG TGCCCTTCGT GGCCGCACGG ATGCCGGCGG CCAGCGGGTA CATCGACATG
43981 CCCTTGTCAA AGGCGAAGAT CGACATGCCG GCATAGCGAC GGAGCTGCGC GGCGAGGATC
44041 GCCAGGTGCG TCGATTTACC TGCGCCGGTC GGCCCGAACA TAAAGGTGTG GCCGAGGTCG
44101 CGCACGTGCA GGTTCAGCCG GAACGGCGTT GATCCTTGCG TGACGCAGTG CATGAGCGCC
44161 GGCGACAGCG GCGGGTACAT CGGGCATGGC GCGTTCGCGT TGCCGGTCCA GATGGTGCTG
44221 GTCGGCAGCA GGTCGGCCAG GTTCATCGTG TTGATGAGCG GCCGGCGGAC GTTTTCCACG
44281 CCGTGGCCCG GCAAACTACC AAGGAAGGCG TCCAGGGTGT TGATGGACTC GATGCGCGCG
44341 GCAAAGCCCA ACCGGTTGAC GGCCTTTTCA ACATCGCGGG CCGCAGCTTC CAGGCGCGTG
44401 CGGTCCTCAT CCATCAGCAC GACGACGCTG GTGTAGTAGC CCACGGCCAC GATGCCGCTG
44461 TTGACTTCGG CAATGGCCGC CTCAGCATCG GCCACCATCG AAAGCGCGTC CTGATCGACC
44521 GGGCCGGTGT TCGTGTTGAA CACCTGGTCG AAGAAGCCGC GAATCTTCTG CCGCCACTTC
44581 TTGCGGAACT TGTCGAGGTG CTTCACGGCT TCGTGCTGGT CCATGAAGAT GAACCGGCTC
44641 GACCACCGAT ACTCGCAGGG CAGCTCGCCG AGCGCCGTCA GGATGCCGGG ATAGGACTCC
44701 AAGGGGAAGC CTTCGAGAGC GACCACCTGG ACGAACTTGC GGCCGACCTT GGGCACTACC
44761 CCGCCCCACA TTTCCTGTCC GCCGACCAGG GCGTCCAGGT ACATCGGGTT GCTGGGGAGC
44821 TGCACCGGAT GGTGCAGGCC CGTCACGCAG AATTGCAGCC AGCGCAGGAA GTCGTCATGC
44881 GTGACGGTCG TGCCGTCCTC GTTGACGATC TTGTGCCCCT TCAAGCGAGT GAGCGACACG
44941 GCCGACGACA GGCGCGACTC GATGCTGCGC ACGTCACGCT TGAATTGGTC GATGAGGCCC
45001 CGCGTGCGCG CCTTGCGATC CGGTGCGGTC GCGTCGTCGT CAAACATCAG CTCGACGAAC
45061 TTGCGCTGGG CGAGCAGCGG CGGGAACCAG GTCAAGGTGA GGACGAAATA GCCCTCGTAC
45121 ATCGTTCCCA GGCTCTCGAA ATGCCGCCGG CGCTCTTCTT CAATCGCTGC CGTCAGACGG
45181 TCAGGGAACG CCGACAGGCC CCGCTCCGCG TAGTTCGGAG CAGGACGCCG CACGGCGTCC
45241 ACATGGATCA TCCACCCACT TCCCAGGCCC GCGAGGGCCT GGTTGATGCG GGCGGACACT
45301 ACTTCGCGCT GCTGGTCGGT GCTGCTTGCG TTGTCATCGC CCTTGTACAG CCAGGCAGCC
45361 ATAAAGCTGC CGTTCTTGCC CACGATTACG CCGTCATCGA CGACAGCGGC GTAGTTGAGC
45421 AGATCGGCCA GGCCGGCGTC CTTGGAACGA TGCTTTTTCA GTTTCAGTTC GGCATCGACC
45481 GCGCGGATGC GGGCAAAGAG GATGAACAAC AGAAGCGCGC CGAGGCCCGC GATTGCAATC
45541 GCAATTGCTT GGATCATCGG TATTGCTTCC CTTGGCTATT GGTGTTCTCG CGGAACGGGG
45601 TCGAGCGGGC CGGGTAATAC GGCTTGTACC GGCGGTGACG CAGGTACACG AACCGCATCT
45661 TCGGATCGGC CTTCGCCATG ATTCGGAACG CATAGAGCGC CCCGAACCAC AGGATCAGAC
45721 CGACCACGGT GGCCCGCAGC TCTTGGGCGC TGAAAATCAG CGCAAACGCC ATCAGGCCCG

45781 AGAACATCAC CAGTTCACGA TCACCACCCA TGAACAGGTT TTCTCGGTTG CCTGCGCGAC
45841 GGATGGGGAT CGTGCGCAGA GCCATGATTA GGCGAGCCGT CCAGCCGCTA CCGCACGCAC
45901 GGCATCCGCC GCCGCGACTT GCACCTGGTG CAGCGCCCCG TTGCCGAGGG CCGCGATTTC
45961 GGCACCACGA CCGAAGAAGG TGCTCATCAC GTTCTGCGCG CCGACCAGCA GCGCCATCAC
46021 CAGAACCAGG AAGATCAGGG TTCGGAAGAA GGCGTTGAGT TCGCCGCCGA AGATCAGCAC
46081 GCCGCCGGCG ACGACGATGC CGATGATGGA CAGCGCGAAG GCCACCGGGC CGGTTACGGA
46141 GTTGCGCAGG TTCGTCAGCC AGCTCTCATA TGGCAAGCTG CCGCCGGTGC CTTCCGAGGC
46201 CATCGCCGGA TGCGCGGATA ACGCGAGAGC GAGAACGAAG AACACGGCAA GGTAGAACAA
46261 AATGCCGCGA TTCATGGTCA GACGGAACGG AACAGCCGTT GTCATTGGAA ATACTCCTTA
46321 CAGGGTTTTG GTGATGTACT GGCCGTTCTC GTAACCAAGA ACTTCGAGAA TTTCTTGCAC
46381 TCGACGGCCG CTAGGGGTCC TGGCGATATG GACGACCACA TGAACCGCCT CGCCAATCAG
46441 CGGCTCAATG GGTTTCGGTG AATCCGGGTG CATGCTGATA AGCATGGCGA GCCGGCTCAG
46501 GCCCGCTTTG GGGTTGTTTG CGTGCAGGGT GGCGGCACCT CCTTCATGCC CGGTGTTCCA
46561 GGCCATCAAC AGATCAAGGG CTTCGGGGCC ACGTACCTCA CCGACCAGGA TGCGGTCGGG
46621 GCGCATACGC AGCGTTGTCT TGAGCAGCAG CGTCATCGAG ACGTCGATGC TGGTGTGGTA
46681 TTGGACGGCG TTCTCTGCGG CGCACTGGAT TTCGCCGGTG TCCTCGATGA TGACGACGCG
46741 CTCAGACGGG TTGAAGGCGA CCATTTCATT GATGATCGCG TTGACGAGCG TGGTCTTGCC
46801 CGAGCCAGTA CCGCCAATGA CGAGGATGTT TCGATGCGCC GCGACGGCGC TTTTAATGAC
46861 CTCGTATTGC TCGCGGGTCA TGATGCCCGC CTCGACGTAC TGTTCCAGCG TGAAGATGGC
46921 GACCGCGCGC TTGCGGATCG CAAAGGTTGG CGCGGCCACG ACCGGCGGCA ATTGGCCGGC
46981 AAAGCGGCTG CCATCCAAGG GGAACTCGCC TTCCAGGATG GGCGAATGCC GCGTGACCTC
47041 TTTGCCGTGG AATCCGGCCA CCGTTTCTAT AATCGCCTGC GACTGGCTGG GCCGCATGTC
47101 GCAGATGTAC CGCATCGGCT CGCCAAGGCG TTCGTGCCAC ACCTTGCCGT CCGCGTTGAG
47161 CATGACTTCA ACGGTTTTCG GGTCGTTGAG CGCGGCCAAC AGGTCCGCAC CCATGTCGCG
47221 TTCCAGCTTG CGCTTGGCCC GCTCCTTGAT GGTCTGAAAC TCTTCTTTCC CGCTCACTTT
47281 TCAGTAACCC CTACGCAACA AATCATTAGC AACATTATCG CACAGGCAAA CGATATACCG
47341 AAAGTGTTTA GCCTGCAAGA CAAACAGCTA ATCGAACCGC ACGACGTAGG GGTTCTGATA
47401 GAAAACAACT GTCAAAGCGC ACCCGCCCGA TGCCATTCGC GGCACGGCTT CCGTTGAGGA
47461 TGTCGATATG ATGCGCGAGC CGACGGCCCG CAGAGAAGGG GCCGTTTTAG CGGCTAAAGA
47521 AGGAAGTGCA AGCCCTAACC CTTGGCGTCA GAGCCTTCCA CGCAGCTTTT TTCGGGTGTC
47581 GTCGCCCCAT TTCTTTACGA TAAACGCCTT ATGTGACGGC AAAACCACAC TGATGCGTTC
47641 GTATCCGGGC GGCACGCTGC TCTTGAAAGG ATGACCCGCA ATCTCCGCGA GTGCCTCGCG
47701 GTCAAGGTCG GTGGACTCCA GGAGAAGAGG TAGGGGAGTT TCCAGGGCGT CGGCAATGGC
47761 CTCCATCACC TTCAACGAGG GGTTGGCCTT ACCGTTGGTT AAGTCTGATA AAAACGAAAT
47821 TGAAACCCCT GCCCTCTCCG ACAGCTCATG TTTCGTCATG CCCCGCTCAT CGAGCAGACG
47881 AAGGATGTTG GTGAAAAATA TCTGGTTGTA CACAGCGGAA GCCGCCCCTC GCACCTTTGG
47941 TCGCGGCCCG CAAAATTTTA GCCGCTAAAG TTCTTGACAG CGGAACCAAT GTTTAGCTAA
48001 ACTAGAGTCT CCTTTCTCAA GGAGACTTTC GATATGAGCC ATAATCAGTT CCAGTTTATC
48061 GGTAATCTTA CCCGTGACAC CGAGGTACGT CATGGCAATT CTAACAAGCC GCAAGCAATT
48121 TTCGATATAG CGGTTAATGA AGAGTGGCGC AACGATGCCG GCGACAAGCA GGAGCGCACC
48181 GACTTCTTCC GCATCAAGTG TTTTGGCTCT CAGGCCGAGG CCCACGGCAA GTATTTGGGC
48241 AAGGGGTCGC TGGTATTCGT GCAGGGCAAG ATTCGGAATA CCAAGTACGA GAAGGACGGC
48301 CAGACGGTCT ACGGGACCGA CTTCATTGCC GATAAGGTGG ATTATCTGGA CACCAAGGCA
48361 CCAGGCGGGT CAAATCAGGA ATAAGGGCAC ATTGCCCCGG CGTGAGTCGG GGCAATCCCG
48421 CAAGGAGGGT GAATGAATCG GACGTTTGAC CGGAAGGCAT ACAGGCAAGA ACTGATCGAC
48481 GCGGGGTTTT CCGCCGAGGA TGCCGAAACC ATCGCAAGCC GCACCGTCAT GCGTGCGCCC
48541 CGCGAAACCT TCCAGTCCGT CGGCTCGATG GTCCAGCAAG CTACGGCCAA GATCGAGCGC
48601 GACAGCGTGC AACTGGCTCC CCCTGCCCTG CCCGCGCCAT CGGCCGCCGT GGAGCGTTCG
48661 CGTCGTCTCG AACAGGAGGC GGCAGGTTTG GCGAAGTCGA TGACCATCGA CACGCGAGGA
48721 ACTATGACGA CCAAGAAGCG AAAAACCGCC GGCGAGGACC TGGCAAAACA GGTCAGCGAG
48781 GCCAAGCAGG CCGCGTTGCT GAAACACACG AAGCAGCAGA TCAAGGAAAT GCAGCTTTCC
48841 TTGTTCGATA TTGCGCCGTG GCCGGACACG ATGCGAGCGA TGCCAAACGA CACGGCCCGC
48901 TCTGCCCTGT TCACCACGCG CAACAAGAAA ATCCCGCGCG AGGCGCTGCA AAACAAGGTC

48961 ATTTTCCACG TCAACAAGGA CGTGAAGATC ACCTACACCG GCGTCGAGCT GCGGGCCGAC
49021 GATGACGAAC TGGTGTGGCA GCAGGTGTTG GAGTACGCGA AGCGCACCCC TATCGGCGAG
49081 CCGATCACCT TCACGTTCTA CGAGCTTTGC CAGGACCTGG GCTGGTCGAT CAATGGCCGG
49141 TATTACACGA AGGCCGAGGA ATGCCTGTCG CGCCTACAGG CGACGGCGAT GGGCTTCACG
49201 TCCGACCGCG TTGGGCACCT GGAATCGGTG TCGCTGCTGC ACCGCTTCCG CGTCCTGGAC
49261 CGTGGCAAGA AAACGTCCCG TTGCCAGGTC CTGATCGACG AGGAAATCGT CGTGCTGTTT
49321 GCTGGCGACC ACTACACGAA ATTCATATGG GAGAAGTACC GCAAGCTGTC GCCGACGGCC
49381 CGACGGATGT TCGACTATTT CAGCTCGCAC CGGGAGCCGT ACCCGCTCAA GCTGGAAACC
49441 TTCCGCCTCA TGTGCGGATC GGATTCCACC CGCGTGAAGA AGTGGCGCGA GCAGGTCGGC
49501 GAAGCCTGCG AAGAGTTGCG AGGCAGCGGC CTGGTGGAAC ACGCCTGGGT CAATGATGAC
49561 CTGGTGCATT GCAAACGCTA GGGCCTTGTG GGGTCAGTTC CGGCTGGGGG TTCAGCAGCC
49621 AGCGCTTTAC TGGCATTTCA GGAACAAGCG GGCACTGCTC GACGCACTTG CTTCGCTCAG
49681 TATCGCTCGG GACGCACGGC GCGCTCTACG AACTGCCGAT AAACAGAGGA TTAAAATTGA
49741 CAATTGTGAT TAAGGCTCAG ATTCGACGGC TTGGAGCGGC CGACGTGCAG GATTTCCGCG
49801 AGATCCGATT GTCGGCCCTG AAGAAAGCTC CAGAGATGTT CGGGTCCGTT TACGAGCACG
49861 AGGAGAAAAA GCCCATGGAG GCGTTCGCTG AACGGTTGCG AGATGCCGTG GCATTCGGCG
49921 CCTACATCGA CGGCGAGATC ATTGGGCTGT CGGTCTTCAA ACAGGAGGAC GGCCCCAAGG
49981 ACGCTCACAA GGCGCATCTG TCCGGCGTTT TCGTGGAGCC CGAACAGCGA GGCCGAGGGG
50041 TCGCCGGTAT GCTGCTGCGG GCGTTGCCGG CGGGTTTATT GCTCGTGATG ATCGTCCGAC
50101 AGATTCCAAC GGGAATCTGG TGGATGCGCA TCTTCATCCT CGGCGCACTT AATATTTCGC
50161 TATTCTGGAG CTTGTTGTTT ATTTCGGTCT ACCGCCTGCC GGGCGGGGTC GCGGCGACGG
50221 TAGGCGCTGT GCAGCCGCTG ATGGTCGTGT TCATCTCTGC CGCTCTGCTA GGTAGCCCGA
50281 TACGATTGAT GGCGGTCCTG GGGGCTATTT GCGGAACTGC GGGCGTGGCG CTGTTGGTGT
50341 TGACACCAAA CGCAGCGCTA GATCCTGTCG GCGTCGCAGC GGGCCTGGCG GGGGCGGTTT
50401 CCATGGCGTT CGGAACCGTG CTGACCCGCA AGTGGCAACC TCCCGTGCCT CTGCTCACCT
50461 TTACCGCCTG GCAACTGGCG GCCGGAGGAC TTCTGCTCGT TCCAGTAGCT TTAGTGTTTG
50521 ATCCGCCAAT CCCGATGCCT ACAGGAACCA ATGTTCTCGG CCTGGCGTGG CTCGGCCTGA
50581 TCGGAGCGGG TTTAACCTAC TTCCTTTGGT TCCGGGGGAT CTCGCGACTC GAACCTACAG
50641 TTGTTTCCTT ACTGGGCTTT CTCAGCCCGG GGACCGCCGT GTTGCTAGGA TGGTTGTTCT
50701 TGGATCAGAC GCTGAGTGCG CTTCAAATCA TCGGCGTCCT GCTCGTGATC GGGAGTATCT
50761 GGCTGGGCCA ACGTTCCAAC CGCACTCCTA GGTTAAACGC CTGGTGCTAC GCCTGAATAA
50821 GTGATAATAA GCGGATGAAT GGCAGAAATT CGAAAGCAAA TTCGACCCGG TCGTCGGTTC
50881 AGGGCAGGGT CGTTAAATAG CCGCTTATGT CTATTGCTGG TTTACCGGTT TATTGACTAC
50941 CGGAAGCAGT GTGACCGTGT GCTTCTCAAA TGCCTGAGGC CAGTTTGCTC AGGCTCTCCC
51001 CGTGGAGGTA ATAATTGACG ATATGATCAT TTATTCTGCC TCCCAGAGCC TGATAAAAAC
51061 GGTGAATCCG TTAGCGAGGT GCCGCCGGCT TCCATTCAGG TCGAGGTGGC CCGGCTCCAT
51121 GCACCGCGAC GCAACGCGGG GAGGCAGACA AGGTATAGGG CGGCGAGGCG GCTACAGCCG
51181 ATAGTCTGGA ACAGCGCACT TACGGGTTGC TGCGCAACCC AAGTGCTACC GGCGCGGCAG
51241 CGTGACCCGT GTCGGCGGCT CCAACGGCTC GCCATCGTCC AGAAAACACG GCTCATCGGG
51301 CATCGGCAGG CGCTGCTGCC CGCGCCGTTC CCATTCCTCC GTTTCGGTCA AGGCTGGCAG
51361 GTCTGGTTCC ATGCCCGGAA TGCCGGGCTG GCTGGGCGGC TCCTCGCCGG GGCCGGTCGG
51421 TAGTTGCTGC TCGCCCGGAT ACAGGGTCGG GATGCGGCGC AGGTCGCCAT GCCCCAACAG
51481 CGATTCGTCC TGGTCGTCGT GATCAACCAC CACGGCGGCA CTGAACACCG ACAGGCGCAA
51541 CTGGTCGCGG GGCTGGCCCC ACGCCACGCG GTCATTGACC ACGTAGGCCG ACACGGTGCC
51601 GGGGCCGTTG AGCTTCACGA CGGAGATCCA GCGCTCGGCC ACCAAGTCCT TGACTGCGTA
51661 TTGGACCGTC CGCAAAGAAC GTCCGATGAG CTTGGAAAGT GTCTTCTGGC TGACCACCAC
51721 GGCGTTCTGG TGGCCCATCT GCGCCACGAG GTGATGCAGC AGCATTGCCG CCGTGGGTTT
51781 CCTCGCAATA AGCCCGGCCC ACGCCTCATG CGCTTTGCGT TCCGTTTGCA CCCAGTGACC
51841 GGGCTTGTTC TTGGCTTGAA TGCCGATTTC TCTGGACTGC GTGGCCATGC TTATCTCCAT
51901 GCGGTAGGGG TGCCGCACGG TTGCGGCACC ATGCGCAATC AGCTGCAACT TTTCGGCAGC
51961 GCGACAACAA TTATGCGTTG CGTAAAAGTG GCAGTCAATT ACAGATTTTC TTTAACCTAC
52021 GCAATGAGCT ATTGCGGGGG GTGCCGCAAT GAGCTGTTGC GTACCCCCCT TTTTTAAGTT
52081 GTTGATTTTT AAGTCTTTCG CATTTCGCCC TATATCTAGT TCTTTGGTGC CCAAAGAAGG

52141 GCACCCCTGC GGGGTTCCCC CACGCCTTCG GCGCGGCTCC CCCTCCGGCA AAAAGTGGCC
52201 CCTCCGGGGC TTGTTGATCG ACTGCGCGGC CTTCGGCCTT GCCCAAGGTG GCGCTGCCCC
52261 CTTGGAACCC CCGCACTCGC CGCCGTGAGG CTCGGGGGGC AGGCGGGCGG GCTTCGCCCT
52321 TCGACTGCCC CCACTCGCAT AGGCTTGGGT CGTTCCAGGC GCGTCAAGGC CAAGCCGCTG
52381 CGCGGTCGCT GCGCGAGCCT TGACCCGCCT TCCACTTGGT GTCCAACCGG CAAGCGAAGC
52441 GCGCAGGCCG CAGGCCGGAG GCTTTTCCCC AGAGAAAATT AAAAAAATTG ATGGGGCAAG
52501 GCCGCAGGCC GCGCAGTTGG AGCCGGTGGG TATGAATTCG AATTCCTGCC GACATGGAAG
52561 CCATCACAAA CGGCATGATG AACCTGAATC GCCAGCGGCA TCAGCACCTT GTCGCCTTGC
52621 GTATAATATT TGCCCATGGA CGCACACCGT GGAAACGGAT GAAGGCACGA ACCCAGTTGA
52681 CATAAGCCTG TTCGGTTCGT AAACTGT
//
