# Supplementary material for: Efficient inter-species conjugative transfer of a CRISPR nuclease for targeted bacterial killing
Source: Nat Commun. 2019 Oct 4;10:4544. doi: 10.1038/s41467-019-12448-3 (PMC6778077; doi:10.1038/s41467-019-12448-3)
Supplement: Supplementary file 11 — Supplementary Data 8 [file 41467_2019_12448_MOESM11_ESM.pdf]

```
>rplC
GTTAAAAAAGTTGACGTAACCGG
>ytfM
CTGAATATCGAGTCATTTCGCGG
>yghJ
GTTGATCGGTTCATAAAACGCGG
>stfA
ACGCCAGTATGATCTTTCGCTGG
>aegA
ACGCGGCTTGGCGAACCGGATGG
>gltJ
CCATAGCCAGCCGAGATAGGTGG
>ompS
ATTAAGGTAAACACCACCGAAGG
>mviM
TGCCGGCGTCCATGTCTGCGTGG
```
