# Supplementary material for: Efficient inter-species conjugative transfer of a CRISPR nuclease for targeted bacterial killing
Source: Nat Commun. 2019 Oct 4;10:4544. doi: 10.1038/s41467-019-12448-3 (PMC6778077; doi:10.1038/s41467-019-12448-3)
Supplement: Supplementary file 12 — Supplementary Data 9 [file 41467_2019_12448_MOESM12_ESM.pdf]

>MG16552
AGCTTTTCATTCTGACTGCAACGGGCAATATGTCTCTGTGTGGATTAAAAAAAGAGTGTCTGATAGCAGCTTCTGAACTG
GTTACCTGCCGTGAGTAAATTAAAATTTTATTGACTTAGGTCACTAAATACTTTAACCAATATAGGCATAGCGCACAGAC
AGATAAAAATTACAGAGTACACAACATCCATGAAACGCATTAGCACCACCATTACCACCACCATCACCATTACCACAGGT
AACGGTGCGGGCTGACGCGTACAGGAAACACAGAAAAAAGCCCGCACCTGACAGTGCGGGCTTTTTTTTTCGACCAAAGG
TAACGAGGTAACAACCATGCGAGTGTTGAAGTTCGGCGGTACATCAGTGGCAAATGCAGAACGTTTTCTGCGTGTTGCCG
ATATTCTGGAAAGCAATGCCAGGCAGGGGCAGGTGGCCACCGTCCTCTCTGCCCCCGCCAAAATCACCAACCACCTGGTG
GCGATGATTGAAAAAACCATTAGCGGCCAGGATGCTTTACCCAATATCAGCGATGCCGAACGTATTTTTGCCGAACTTTT
GACGGGACTCGCCGCCGCCCAGCCGGGGTTCCCGCTGGCGCAATTGAAAACTTTCGTCGATCAGGAATTTGCCCAAATAA
AACATGTCCTGCATGGCATTAGTTTGTTGGGGCAGTGCCCGGATAGCATCAACGCTGCGCTGATTTGCCGTGGCGAGAAA
ATGTCGATCGCCATTATGGCCGGCGTATTAGAAGCGCGCGGTCACAACGTTACTGTTATCGATCCGGTCGAAAAACTGCT
GGCAGTGGGGCATTACCTCGAATCTACCGTCGATATTGCTGAGTCCACCCGCCGTATTGCGGCAAGCCGCATTCCGGCTG
ATCACATGGTGCTGATGGCAGGTTTCACCGCCGGTAATGAAAAAGGCGAACTGGTGGTGCTTGGACGCAACGGTTCCGAC
TACTCTGCTGCGGTGCTGGCTGCCTGTTTACGCGCCGATTGTTGCGAGATTTGGACGGACGTTGACGGGGTCTATACCTG
CGACCCGCGTCAGGTGCCCGATGCGAGGTTGTTGAAGTCGATGTCCTACCAGGAAGCGATGGAGCTTTCCTACTTCGGCG
CTAAAGTTCTTCACCCCCGCACCATTACCCCCATCGCCCAGTTCCAGATCCCTTGCCTGATTAAAAATACCGGAAATCCT
CAAGCACCAGGTACGCTCATTGGTGCCAGCCGTGATGAAGACGAATTACCGGTCAAGGGCATTTCCAATCTGAATAACAT
GGCAATGTTCAGCGTTTCTGGTCCGGGGATGAAAGGGATGGTCGGCATGGCGGCGCGCGTCTTTGCAGCGATGTCACGCG
CCCGTATTTCCGTGGTGCTGATTACGCAATCATCTTCCGAATACAGCATCAGTTTCTGCGTTCCACAAAGCGACTGTGTG
CGAGCTGAACGGGCAATGCAGGAAGAGTTCTACCTGGAACTGAAAGAAGGCTTACTGGAGCCGCTGGCAGTGACGGAACG
GCTGGCCATTATCTCGGTGGTAGGTGATGGTATGCGCACCTTGCGTGGGATCTCGGCGAAATTCTTTGCCGCACTGGCCC
GCGCCAATATCAACATTGTCGCCATTGCTCAGGGATCTTCTGAACGCTCAATCTCTGTCGTGGTAAATAACGATGATGCG
ACCACTGGCGTGCGCGTTACTCATCAGATGCTGTTCAATACCGATCAGGTTATCGAAGTGTTTGTGATTGGCGTCGGTGG
CGTTGGCGGTGCGCTGCTGGAGCAACTGAAGCGTCAGCAAAGCTGGCTGAAGAATAAACATATCGACTTACGTGTCTGCG
GTGTTGCCAACTCGAAGGCTCTGCTCACCAATGTACATGGCCTTAATCTGGAAAACTGGCAGGAAGAACTGGCGCAAGCC
AAAGAGCCGTTTAATCTCGGGCGCTTAATTCGCCTCGTGAAAGAATATCATCTGCTGAACCCGGTCATTGTTGACTGCAC
TTCCAGCCAGGCAGTGGCGGATCAATATGCCGACTTCCTGCGCGAAGGTTTCCACGTTGTCACGCCGAACAAAAAGGCCA
ACACCTCGTCGATGGATTACTACCATCAGTTGCGTTATGCGGCGGAAAAATCGCGGCGTAAATTCCTCTATGACACCAAC
GTTGGGGCTGGATTACCGGTTATTGAGAACCTGCAAAATCTGCTCAATGCAGGTGATGAATTGATGAAGTTCTCCGGCAT
TCTTTCTGGTTCGCTTTCTTATATCTTCGGCAAGTTAGACGAAGGCATGAGTTTCTCCGAGGCGACCACGCTGGCGCGGG
AAATGGGTTATACCGAACCGGACCCGCGAGATGATCTTTCTGGTATGGATGTGGCGCGTAAACTATTGATTCTCGCTCGT
GAAACGGGACGTGAACTGGAGCTGGCGGATATTGAAATTGAACCTGTGCTGCCCGCAGAGTTTAACGCCGAGGGTGATGT
TGCCGCTTTTATGGCGAATCTGTCACAACTCGACGATCTCTTTGCCGCGCGCGTGGCGAAGGCCCGTGATGAAGGAAAAG
TTTTGCGCTATGTTGGCAATATTGATGAAGATGGCGTCTGCCGCGTGAAGATTGCCGAAGTGGATGGTAATGATCCGCTG
TTCAAAGTGAAAAATGGCGAAAACGCCCTGGCCTTCTATAGCCACTATTATCAGCCGCTGCCGTTGGTACTGCGCGGATA
TGGTGCGGGCAATGACGTTACAGCTGCCGGTGTCTTTGCTGATCTGCTACGTACCCTCTCATGGAAGTTAGGAGTCTGAC
ATGGTTAAAGTTTATGCCCCGGCTTCCAGTGCCAATATGAGCGTCGGGTTTGATGTGCTCGGGGCGGCGGTGACACCTGT
TGATGGTGCATTGCTCGGAGATGTAGTCACGGTTGAGGCGGCAGAGACATTCAGTCTCAACAACCTCGGACGCTTTGCCG
ATAAGCTGCCGTCAGAACCACGGGAAAATATCGTTTATCAGTGCTGGGAGCGTTTTTGCCAGGAACTGGGTAAGCAAATT
CCAGTGGCGATGACCCTGGAAAAGAATATGCCGATCGGTTCGGGCTTAGGCTCCAGTGCCTGTTCGGTGGTCGCGGCGCT
GATGGCGATGAATGAACACTGCGGCAAGCCGCTTAATGACACTCGTTTGCTGGCTTTGATGGGCGAGCTGGAAGGCCGTA
TCTCCGGCAGCATTCATTACGACAACGTGGCACCGTGTTTTCTCGGTGGTATGCAGTTGATGATCGAAGAAAACGACATC
ATCAGCCAGCAAGTGCCAGGGTTTGATGAGTGGCTGTGGGTGCTGGCGTATCCGGGGATTAAAGTCTCGACGGCAGAAGC
CAGGGCTATTTTACCGGCGCAGTATCGCCGCCAGGATTGCATTGCGCACGGGCGACATCTGGCAGGCTTCATTCACGCCT
GCTATTCCCGTCAGCCTGAGCTTGCCGCGAAGCTGATGAAAGATGTTATCGCTGAACCCTACCGTGAACGGTTACTGCCA
GGCTTCCGGCAGGCGCGGCAGGCGGTCGCGGAAATCGGCGCGGTAGCGAGCGGTATCTCCGGCTCCGGCCCGACCTTGTT
CGCTCTGTGTGACAAGCCGGAAACCGCCCAGCGCGTTGCCGACTGGTTGGGTAAGAACTACCTGCAAAATCAGGAAGGTT
TTGTTCATATTTGCCGGCTGGATACGGCGGGCGCACGAGTACTGGAAAACTAAATGAAACTCTACAATCTGAAAGATCAC
AACGAGCAGGTCAGCTTTGCGCAAGCCGTAACCCAGGGGTTGGGCAAAAATCAGGGGCTGTTTTTTCCGCACGACCTGCC
GGAATTCAGCCTGACTGAAATTGATGAGATGCTGAAGCTGGATTTTGTCACCCGCAGTGCGAAGATCCTCTCGGCGTTTA
TTGGTGATGAAATCCCACAGGAAATCCTGGAAGAGCGCGTGCGCGCGGCGTTTGCCTTCCCGGCTCCGGTCGCCAATGTT
GAAAGCGATGTCGGTTGTCTGGAATTGTTCCACGGGCCAACGCTGGCATTTAAAGATTTCGGCGGTCGCTTTATGGCACA
AATGCTGACCCATATTGCGGGTGATAAGCCAGTGACCATTCTGACCGCGACCTCCGGTGATACCGGAGCGGCAGTGGCTC
ATGCTTTCTACGGTTTACCGAATGTGAAAGTGGTTATCCTCTATCCACGAGGCAAAATCAGTCCACTGCAAGAAAAACTG
TTCTGTACATTGGGCGGCAATATCGAAACTGTTGCCATCGACGGCGATTTCGATGCCTGTCAGGCGCTGGTGAAGCAGGC
GTTTGATGATGAAGAACTGAAAGTGGCGCTAGGGTTAAACTCGGCTAACTCGATTAACATCAGCCGTTTGCTGGCGCAGA

TTTGCTACTACTTTGAAGCTGTTGCGCAGCTGCCGCAGGAGACGCGCAACCAGCTGGTTGTCTCGGTGCCAAGCGGAAAC
TTCGGCGATTTGACGGCGGGTCTGCTGGCGAAGTCACTCGGTCTGCCGGTGAAACGTTTTATTGCTGCGACCAACGTGAA
CGATACCGTGCCACGTTTCCTGCACGACGGTCAGTGGTCACCCAAAGCGACTCAGGCGACGTTATCCAACGCGATGGACG
TGAGTCAGCCGAACAACTGGCCGCGTGTGGAAGAGTTGTTCCGCCGCAAAATCTGGCAACTGAAAGAGCTGGGTTATGCA
GCCGTGGATGATGAAACCACGCAACAGACAATGCGTGAGTTAAAAGAACTGGGCTACACTTCGGAGCCGCACGCTGCCGT
AGCTTATCGTGCGCTGCGTGATCAGTTGAATCCAGGCGAATATGGCTTGTTCCTCGGCACCGCGCATCCGGCGAAATTTA
AAGAGAGCGTGGAAGCGATTCTCGGTGAAACGTTGGATCTGCCAAAAGAGCTGGCAGAACGTGCTGATTTACCCTTGCTT
TCACATAATCTGCCCGCCGATTTTGCTGCGTTGCGTAAATTGATGATGAATCATCAGTAAAATCTATTCATTATCTCAAT
CAGGCCGGGTTTGCTTTTATGCAGCCCGGCTTTTTTATGAAGAAATTATGGAGAAAAATGACAGGGAAAAAGGAGAAATT
CTCAATAAATGCGGTAACTTAGAGATTAGGATTGCGGAGAATAACAACCGCCGTTCTCATCGAGTAATCTCCGGATATCG
ACCCATAACGGGCAATGATAAAAGGAGTAACCTGTGAAAAAGATGCAATCTATCGTACTCGCACTTTCCCTGGTTCTGGT
CGCTCCCATGGCAGCACAGGCTGCGGAAATTACGTTAGTCCCGTCAGTAAAATTACAGATAGGCGATCGTGATAATCGTG
GCTATTACTGGGATGGAGGTCACTGGCGCGACCACGGCTGGTGGAAACAACATTATGAATGGCGAGGCAATCGCTGGCAC
CTACACGGACCGCCGCCACCGCCGCGCCACCATAAGAAAGCTCCTCATGATCATCACGGCGGTCATGGTCCAGGCAAACA
TCACCGCTAAATGACAAATGCCGGGTAACAATCCGGCATTCAGCGCCTGATGCGACGCTGGCGCGTCTTATCAGGCCTAC
GTTAATTCTGCAATATATTGAATCTGCATGCTTTTGTAGGCAGGATAAGGCGTTCACGCCGCATCCGGCATTGACTGCAA
ACTTAACGCTGCTCGTAGCGTTTAAACACCAGTTCGCCATTGCTGGAGGAATCTTCATCAAAGAAGTAACCTTCGCTATT
AAAACCAGTCAGTTGCTCTGGTTTGGTCAGCCGATTTTCAATAATGAAACGACTCATCAGACCGCGTGCTTTCTTAGCGT
AGAAGCTGATGATCTTAAATTTGCCGTTCTTCTCATCGAGGAACACCGGCTTGATAATCTCGGCATTCAATTTCTTCGGC
TTCACCGATTTAAAATACTCATCTGACGCCAGATTAATCACCACATTATCGCCTTGTGCTGCGAGCGCCTCGTTCAGCTT
GTTGGTGATGATATCTCCCCAGAATTGATACAGATCTTTCCCTCGGGCATTCTCAAGACGGATCCCCATTTCCAGACGAT
AAGGCTGCATTAAATCGAGCGGGCGGAGTACGCCATACAAGCCGGAAAGCATTCGCAAATGCTGTTGGGCAAAATCGAAA
TCGTCTTCGCTGAAGGTTTCGGCCTGCAAGCCGGTGTAGACATCACCTTTAAACGCCAGAATCGCCTGGCGGGCATTCGC
CGGCGTGAAATCTGGCTGCCAGTCATGAAAGCGAGCGGCGTTGATACCCGCCAGTTTGTCGCTGATGCGCATCAGCGTGC
TAATCTGCGGAGGCGTCAGTTTCCGCGCCTCATGGATCAACTGCTGGGAATTGTCTAACAGCTCCGGCAGCGTATAGCGC
GTGGTGGTCAACGGGCTTTGGTAATCAAGCGTTTTCGCAGGTGAAATAAGAATCAGCATATCCAGTCCTTGCAGGAAATT
TATGCCGACTTTAGCAAAAAATGAGAATGAGTTGATCGATAGTTGTGATTACTCCTGCGAAACATCATCCCACGCGTCCG
GAGAAAGCTGGCGACCGATATCCGGATAACGCAATGGATCAAACACCGGGCGCACGCCGAGTTTACGCTGGCGTAGATAA
TCACTGGCAATGGTATGAACCACAGGCGAGAGCAGTAAAATGGCGGTCAAATTGGTAATAGCCATGCAGGCCATTATGAT
ATCTGCCAGTTGCCACATCAGCGGAAGGCTTAGCAAGGTGCCGCCGATGACCGTTGCGAAGGTGCAGATCCGCAAACACC
AGATCGCTTTAGGGTTGTTCAGGCGTAAAAAGAAGAGATTGTTTTCGGCATAAATGTAGTTGGCAACGATGGAGCTGAAG
GCAAACAGAATAACCACAAGGGTAACAAACTCAGCACCCCAGGAACCCATTAGCACCCGCATCGCCTTCTGGATAAGCTG
AATACCTTCCAGCGGCATGTAGGTTGTGCCGTTACCCGCCAGTAATATCAGCATGGCGCTTGCCGTACAGATGACCAGGG
TGTCGATAAAAATGCCAATCATCTGGACAATCCCTTGCGCTGCCGGATGCGGAGGCCAGGACGCCGCTGCCGCTGCCGCG
TTTGGCGTCGAACCCATTCCCGCCTCATTGGAAAACATACTGCGCTGAAAACCGTTAGTAATCGCCTGGCTTAAGGTATA
TCCCGCCGCGCCGCCTGCCGCTTCCTGCCAGCCAAAAGCACTCTCAAAAATAGACCAAATGACGTGGGGAAGTTGCCCGA
TATTCATTACGCAAATTACCAGGCTGGTCAGTACCCAGATTATCGCCATCAACGGGACAAAGCCCTGCATGAGCCGGGCG
ACGCCATGAAGACCGCGAGTGATTGCCAGCAGAGTAAAGACAGCGAGAATAATGCCTGTCACCAGCGGGGGAAAATCAAA
AGAAAAACTCAGGGCGCGGGCAACGGCGTTCGCTTGAACTCCGCTGAAAATTATGCCATAGGCGATGAGCAAAAAGACGG
CGAACAGAACGCCCATCCAGCGCATCCCCAGCCCGCGCGCCATATACCATGCCGGTCCGCCACGAAACTGCCCATTGACG
TCACGTTCTTTATAAAGTTGTGCCAGAGAACATTCGGCAAACGAGGTCGCCATGCCGATAAACGCGGCAACCCCACATCCA
AAAGACGGCTCCAGGTCCACCGGCGGTAATAGCCAGCGCAACGCCGGCCAGGTTGCCGCTACCCACGCGCGCCGCAAGAC
TGGTACACAATGACTGAAATGAGGTTAAACCGCCTGGCTGTGGATGAATGCTATTTTTAAGACTTTTGCCAAACTGGCGG
ATGTAGCGAAACTGCACAAATCCGGTGCGAAAAGTGAACCAACAACCTGCGCCGAAGAGCAGGTAAATCATTACCGATCC
CCAAAGGACGCTGTTAATGAAGGAGAAAAAATCTGGCATGCATATCCCTCTTATTGCCGGTCGCGATGACTTTCCTGTGT
AAACGTTACCAATTGTTTAAGAAGTATATACGCTACGAGGTACTTGATAACTTCTGCGTAGCATACATGAGGTTTTGTAT
AAAAATGGCGGGCGATATCAACGCAGTGTCAGAAATCCGAAACAGTCTCGCCTGGCGATAACCGTCTTGTCGGCGGTTGC
GCTGACGTTGCGTCGTGATATCATCAGGGCAGACCGGTTACATCCCCCTAACAAGCTGTTTAAAGAGAAATACTATCATG
ACGGACAAATTGACCTCCCTTCGTCAGTACACCACCGTAGTGGCCGACACTGGGGACATCGCGGCAATGAAGCTGTATCA
ACCGCAGGATGCCACAACCAACCCTTCTCTCATTCTTAACGCAGCGCAGATTCCGGAATACCGTAAGTTGATTGATGATG
CTGTCGCCTGGGCGAAACAGCAGAGCAACGATCGCGCGCAGCAGATCGTGGACGCGACCGACAAACTGGCAGTAAATATT
GGTCTGGAAATCCTGAAACTGGTTCCGGGCCGTATCTCAACTGAAGTTGATGCGCGTCTTTCCTATGACACCGAAGCGTC
AATTGCGAAAGCAAAACGCCTGATCAAACTCTACAACGATGCTGGTATTAGCAACGATCGTATTCTGATCAAACTGGCTT
CTACCTGGCAGGGTATCCGTGCTGCAGAACAGCTGGAAAAAGAAGGCATCAACTGTAACCTGACCCTGCTGTTCTCCTTC
GCTCAGGCTCGTGCTTGTGCGGAAGCGGGCGTGTTCCTGATCTCGCCGTTTGTTGGCCGTATTCTTGACTGGTACAAAGC
GAATACCGATAAGAAAGAGTACGCTCCGGCAGAAGATCCGGGCGTGGTTTCTGTATCTGAAATCTACCAGTACTACAAAG

AGCACGGTTATGAAACCGTGGTTATGGGCGCAAGCTTCCGTAACATCGGCGAAATTCTGGAACTGGCAGGCTGCGACCGT
CTGACCATCGCACCGGCACTGCTGAAAGAGCTGGCGGAGAGCGAAGGGGCTATCGAACGTAAACTGTCTTACACCGGCGA
AGTGAAAGCGCGTCCGGCGCGTATCACTGAGTCCGAGTTCCTGTGGCAGCACAACCAGGATCCAATGGCAGTAGATAAAC
TGGCGGAAGGTATCCGTAAGTTTGCTATTGACCAGGAAAAACTGGAAAAAATGATCGGCGATCTGCTGTAATCATTCTTA
GCGTGACCGGGAAGTCGGTCACGCTACCTCTTCTGAAGCCTGTCTGTCACTCCCTTCGCAGTGTATCATTCTGTTTAACG
AGACTGTTTAAACGGAAAAATCTTGATGAATACTTTACGTATTGGCTTAGTTTCCATCTCTGATCGCGCATCCAGCGGCG
TTTATCAGGATAAAGGCATCCCTGCGCTGGAAGAATGGCTGACATCGGCGCTAACCACGCCGTTTGAACTGGAAACCCGC
TTAATCCCCGATGAGCAGGCGATCATCGAGCAAACGTTGTGTGAGCTGGTGGATGAAATGAGTTGCCATCTGGTGCTCAC
CACGGGCGGAACTGGCCCGGCGCGTCGTGACGTAACGCCCGATGCGACGCTGGCAGTAGCGGACCGCGAGATGCCTGGCT
TTGGTGAACAGATGCGCCAGATCAGCCTGCATTTTGTACCAACTGCGATCCTTTCGCGTCAGGTGGGCGTGATTCGCAAA
CAGGCGCTGATCCTTAACTTACCCGGTCAGCCGAAGTCTATTAAAGAGACGCTGGAAGGTGTGAAGGACGCTGAGGGTAA
CGTTGTGGTACACGGTATTTTTGCCAGCGTACCGTACTGCATTCAGTTGCTGGAAGGGCCATACGTTGAAACGGCACCGG
AAGTGGTTGCAGCATTCAGACCGAAGAGTGCAAGACGCGACGTTAGCGAATAAAAAAATCCCCCCGAGCGGGGGGATCTC
AAAACAATTAGTGGGATTCACCAATCGGCAGAACGGTGCGACCAAACTGCTCGTTCAGTACTTCACCCATCGCCAGATAG
ATTGCGCTGGCACCGCAGATCAGCCCAATCCAGCCGGCAAAGTGGATGATTGCGGCGTTACCGGCAATGTTACCGATCGC
CAGCAGGGCAAACAGCACGGTCAGGCTAAAGAAAACGAATTGCAGAACGCGTGCGCCTTTCAGCGTGCCGAAGAACATAA
ACAGCGTAAATACGCCCCACAGACCCAGGTAGACACCAAGGAACTGTGCATTTGGCGCATCGGTCAGACCCAGTTTCGGC
ATCAGCAGAATCGCAACCAGCGTCAGCCAGAAAGAACCGTAAGAGGTGAATGCGGTTAAACCGAAAGTGTTGCCTTTTTT
GTACTCCAGCAGACCAGCAAAAATTTGCGCGATGCCGCCGTAGAAAATGCCCATGGCAAGAATAATACCGTCCAGAGCGA
AATAACCCACGTTGTGCAGGTTAAGCAGAATGGTGGTCATGCCGAAGCCCATCAGGCCCAGCGGTGCCGGATTAGCCAAC
TTAGTGTTGCCCATAATTCCTCAAAAATCATCATCGAATGAATGGTGAAATAATTTCCCTGAATAACTGTAGTGTTTTCA
GGGCGCGGCATAATAATCAGCCAGTGGGGCAGTGTCTACGATCTTTTGAGGGGAAAATGAAAATTTTCCCCGGTTTCCGG
TATCAGACCTGAGTGGCGCTAACCATCCGGCGCAGGCAGGCGATTTGCAGTACGGCTGGAATCGTCACGCGATAGGCGCT
GCCGCTGACCGCTTTAACCCCATTTAGTGCCGCACCTACAGGGCCTCCCAGCCCCGCGCCGCGCAGCAAACCATGCCCAA
GTACGCTCATTGCTGCGTGGGTGCGTAAAATGCGGGTCAGTTGGCTGGAAAGCAAATGCGACACACCTTTTGCCAATAAT
TTGTCTTTCATCAGCAGCGGCAGCAGCTCTTCCAGCTCATTCACCCTGGCATCGACCGCGTGCAGAAACTCCTGCTTATG
TTCCTCGTCCATTTTCTTCCAGGTATTACGCAGAAATTGTTCCAGTAACTGTTGCTCAATTTCAAACGTAGACATCTCTT
TGTCGGCTTTCAGCTTCAATCGCTTTGAAACATCGAGCAAAATGGCCCGATACAATTTACCGTGTCCGCGCAGTTTGTTG
GCGATACTATCGCCACCAAAATGCTGTAATTCTCCGGCAATCAGCTGCCAGTTGCGGCGATGTTGCTCGGGATGCCCTTC
CATCGATTTAAACAGTTCGTTGCGCATCAGTACGCTGGAGAGGCGAGTTTTGCCTTTTTCATTATGGGTGAGCAATCGGG
CGAAATTTGCCAACTGTTCCTCACTACAATGCTGAAGAAAATCCAGATCTGAATCATTCAGGTAATTAACATTCATTTTT
TGTGGCTTCTATATTCTGGCGTTAGTCGTCGCCGATAATTTTCAGCGTGGCCATATCCGATGAGTTCACCGTATGACCCG
AAAAGGTGATTTTTGAGACGCAGCGTTTATTGTCGTTATCGCTGTTAATGTTGATCCAGTCAGTGGTTTGCCCTTCTTTT
ATTTCTGAAGGAATATTCAGGCTCTGACTGGCGCTACGGGCGGCTTTGAAATAAACCGATGCACCGCTTAACTGTAAATC
GCCATGGTCGGCAGAGAGTTGTATGCGTTTCACAATGCGACAAACAGGAAGTTTCAGCGCCAGATCGTTGGTTTCGTTAC
GCGGCATTGCAATGGCGCCGAGGAGTTTATGGTCGTTTGCCTGCGCCGTGCAGCACAGCATCAGGCTAATCGCCAGGCTG
GCGGAAATCGTAAAAACGGATTTCATAAGGATTCTCTTAGTGGGAAGAGGTAGGGGGATGAATACCCACTAGTTTACTGC
TGATAAAGAGAAGATTCAGGCACGTAATCTTTTCTTTTTATTACAATTTTTTGATGAATGCCTTGGCTGCGATTCATTCT
TTATATGAATAAAATTGCTGTCAATTTTACGTCTTGTCCTGCCATATCGCGAAATTTCTGCGCAAAAGCACAAAAAATTT
TTGCATCTCCCCCTTGATGACGTGGTTTACGACCCCATTTAGTAGTCAACCGCAGTGAGTGAGTCTGCAAAAAAATGAAA
TTGGGCAGTTGAAACCAGACGTTTCGCCCCTATTACAGACTCACAACCACATGATGACCGAATATATAGTGGAGACGTTT
AGATGGGTAAAATAATTGGTATCGACCTGGGTACTACCAACTCTTGTGTAGCGATTATGGATGGCACCACTCCTCGCGTG
CTGGAGAACGCCGAAGGCGATCGCACCACGCCTTCTATCATTGCCTATACCCAGGATGGTGAAACTCTAGTTGGTCAGCC
GGCTAAACGTCAGGCAGTGACGAACCCGCAAAACACTCTGTTTGCGATTAAACGCCTGATTGGTCGCCGCTTCCAGGACG
AAGAAGTACAGCGTGATGTTTCCATCATGCCGTTCAAAATTATTGCTGCTGATAACGGCGACGCATGGGTCGAAGTTAAA
GGCCAGAAAATGGCACCGCCGCAGATTTCTGCTGAAGTGCTGAAAAAAATGAAGAAAACCGCTGAAGATTACCTGGGTGA
ACCGGTAACTGAAGCTGTTATCACCGTACCGGCATACTTTAACGATGCTCAGCGTCAGGCAACCAAAGACGCAGGCCGTA
TCGCTGGTCTGGAAGTAAAACGTATCATCAACGAACCGACCGCAGCTGCGCTGGCTTACGGTCTGGACAAAGGCACTGGC
AACCGTACTATCGCGGTTTATGACCTGGGTGGTGGTACTTTCGATATTTCTATTATCGAAATCGACGAAGTTGACGGCGA
AAAAACCTTCGAAGTTCTGGCAACCAACGGTGATACCCACCTGGGGGGTGAAGACTTCGACAGCCGTCTGATCAACTATC
TGGTTGAAGAATTCAAGAAAGATCAGGGCATTGACCTGCGCAACGATCCGCTGGCAATGCAGCGCCTGAAAGAAGCGGCA
GAAAAAGCGAAAATCGAACTGTCTTCCGCTCAGCAGACCGACGTTAACCTGCCATACATCACTGCAGACGCGACCGGTCC
GAAACACATGAACATCAAAGTGACTCGTGCGAAACTGGAAAGCCTGGTTGAAGATCTGGTAAACCGTTCCATTGAGCCGC
TGAAAGTTGCACTGCAGGACGCTGGCCTGTCCGTATCTGATATCGACGACGTTATCCTCGTTGGTGGTCAGACTCGTATG
CCAATGGTTCAGAAGAAAGTTGCTGAGTTCTTTGGTAAAGAGCCGCGTAAAGACGTTAACCCGGACGAAGCTGTAGCAAT
CGGTGCTGCTGTTCAGGGTGGTGTTCTGACTGGTGACGTAAAAGACGTACTGCTGCTGGACGTTACCCCGCTGTCTCTGG

GTATCGAAACCATGGGCGGTGTGATGACGACGCTGATCGCGAAAAACACCACTATCCCGACCAAGCACAGCCAGGTGTTC
TCTACCGCTGAAGACAACCAGTCTGCGGTAACCATCCATGTGCTGCAGGGTGAACGTAAACGTGCGGCTGATAACAAATC
TCTGGGTCAGTTCAACCTAGATGGTATCAACCCGGCACCGCGCGGCATGCCGCAGATCGAAGTTACCTTCGATATCGATG
CTGACGGTATCCTGCACGTTTCCGCGAAAGATAAAAACAGCGGTAAAGAGCAGAAGATCACCATCAAGGCTTCTTCTGGT
CTGAACGAAGATGAAATCCAGAAAATGGTACGCGACGCAGAAGCTAACGCCGAAGCTGACCGTAAGTTTGAAGAGCTGGT
ACAGACTCGCAACCAGGGCGACCATCTGCTGCACAGCACCCGTAAGCAGGTTGAAGAAGCAGGCGACAAACTGCCGGCTG
ACGACAAAACTGCTATCGAGTCTGCGCTGACTGCACTGGAAACTGCTCTGAAAGGTGAAGACAAAGCCGCTATCGAAGCG
AAAATGCAGGAACTGGCACAGGTTTCCCAGAAACTGATGGAAATCGCCCAGCAGCAACATGCCCAGCAGCAGACTGCCGG
TGCTGATGCTTCTGCAAACAACGCGAAAGATGACGATGTTGTCGACGCTGAATTTGAAGAAGTCAAAGACAAAAAATAAT
CGCCCTATAAACGGGTAATTATACTGACACGGGCGAAGGGGAATTTCCTCTCCGCCCGTGCATTCATCTAGGGGCAATTT
AAAAAAGATGGCTAAGCAAGATTATTACGAGATTTTAGGCGTTTCCAAAACAGCGGAAGAGCGTGAAATCAGAAAGGCCT
ACAAACGCCTGGCCATGAAATACCACCCGGACCGTAACCAGGGTGACAAAGAGGCCGAGGCGAAATTTAAAGAGATCAAG
GAAGCTTATGAAGTTCTGACCGACTCGCAAAAACGTGCGGCATACGATCAGTATGGTCATGCTGCGTTTGAGCAAGGTGG
CATGGGCGGCGGCGGTTTTGGCGGCGGCGCAGACTTCAGCGATATTTTTGGTGACGTTTTCGGCGATATTTTTGGCGGCG
GACGTGGTCGTCAACGTGCGGCGCGCGGTGCTGATTTACGCTATAACATGGAGCTCACCCTCGAAGAAGCTGTACGTGGC
GTGACCAAAGAGATCCGCATTCCGACTCTGGAAGAGTGTGACGTTTGCCACGGTAGCGGTGCAAAACCAGGTACACAGCC
GCAGACTTGTCCGACCTGTCATGGTTCTGGTCAGGTGCAGATGCGCCAGGGATTCTTCGCTGTACAGCAGACCTGTCCAC
ACTGTCAGGGCCGCGGTACGCTGATCAAAGATCCGTGCAACAAATGTCATGGTCATGGTCGTGTTGAGCGCAGCAAAACG
CTGTCCGTTAAAATCCCGGCAGGGGTGGACACTGGAGACCGCATCCGTCTTGCGGGCGAAGGTGAAGCGGGCGAGCATGG
CGCACCGGCAGGCGATCTGTACGTTCAGGTTCAGGTTAAACAGCACCCGATTTTCGAGCGTGAAGGCAACAACCTGTATT
GCGAAGTCCCGATCAACTTCGCTATGGCGGCGCTGGGTGGCGAAATCGAAGTACCGACCCTTGATGGTCGCGTCAAACTG
AAAGTGCCTGGCGAAACCCAGACCGGTAAGCTATTCCGTATGCGCGGTAAAGGCGTCAAGTCTGTCCGCGGTGGCGCACA
GGGTGATTTGCTGTGCCGCGTTGTCGTCGAAACACCGGTAGGCCTGAACGAAAGGCAGAAACAGCTGCTGCAAGAGCTGC
AAGAAAGCTTCGGTGGCCCAACCGGCGAGCACAACAGCCCGCGCTCAAAGAGCTTCTTTGATGGTGTGAAGAAGTTTTTT
GACGACCTGACCCGCTAACCTCCCCAAAAGCCTGCCCGTGGGCAGGCCTGGGTAAAAATAGGGTGCGTTGAAGATATGCG
AGCACCTGTAAAGTGGCGGGGATCACTCCCATAAGCGCTAACTTAAGGGTTGTGGTATTACGCCTGATATGATTTAACGT
GCCGATGAATTACTCTCACGATAACTGGTCAGCAATTCTGGCCCATATTGGTAAGCCCGAAGAACTGGATACTTCGGCAC
GTAATGCCGGGGCTCTAACCCGCCGCCGCGAAATTCGTGATGCTGCAACTCTGCTACGTCTGGGGCTGGCTTACGGCCCC
GGGGGGATGTCATTACGTGAAGTCACTGCATGGGCTCAGCTCCATGACGTTGCAACATTATCTGACGTGGCTCTCCTGAA
GCGGCTGCGGAATGCCGCCGACTGGTTTGGCATACTTGCCGCACAAACACTTGCTGTACGCGCCGCAGTTACGGGTTGTA
CAAGCGGAAAGAGATTGCGTCTTGTCGATGGAACAGCAATCAGTGCGCCCGGGGGCGGCAGCGCTGAATGGCGACTACAT
ATGGGATATGATCCTCATACCTGTCAGTTCACTGATTTTGAGCTAACCGACAGCAGAGACGCTGAACGGCTGGACCGATT
TGCGCAAACGGCAGACGAGATACGCATTGCTGACCGGGGATTCGGTTCGCGTCCCGAATGTATCCGCTCACTTGCTTTTG
GAGAAGCTGATTATATCGTCCGGGTTCACTGGCGAGGATTGCGCTGGTTAACTGCAGAAGGAATGCGCTTTGACATGATG
GGTTTTCTGCGCGGGCTGGATTGCGGTAAGAACGGTGAAACCACTGTAATGATAGGCAATTCAGGTAATAAAAAAGCCGG
AGCTCCCTTTCCGGCACGTCTCATTGCCGTATCACTTCCTCCCGAAAAAGCATTAATCAGTAAAACCCGACTGCTCAGCG
AGAATCGTCGAAAAGGACGAGTAGTTCAGGCGGAAACGCTGGAAGCAGCGGGCCATGTGCTATTGCTAACATCATTACCG
GAAGATGAATATTCAGCAGAGCAAGTGGCTGATTGTTACCGTCTGCGATGGCAAATTGAACTGGCTTTTAAGCGGCTCAA
AAGTTTGCTGCACCTGGATGCTTTGCGTGCAAAGGAACCTGAACTCGCGAAAGCGTGGATATTTGCTAATCTACTCGCCG
CATTTTTAATTGACGACATAATCCAGCCATCGCTGGATTTCCCCCCCAGAAGTGCCGGATCCGAAAAGAAGAACTAACTC
GTTGTGGAGAATAACAAAAATGGTCATCTGGAGCTTACAGGTGGCCATTCGTGGGACAGTATCCCTGACAGCCTACAAAA
CGCAATTGAAGAACGCGAGGCATCGTCTTAACGAGGCACCGAGGCGTCGCATTCTTCAGATGGTTCAACCCTTAAGTTAG
CGCTTATGGGATCACTCCCCGCCGTTGCTCTTACTCGGATTCGTAAGCCGTGAAAACAGCAACCTCCGTCTGGCCAGTTC
GGATGTGAACCTCACAGAGGTCTTTTCTCGTTACCAGCGCCGCCACTACGGCGGTGATACAGATGACGATCAGGGCGACA
ATCATCGCCTTATGCTGCTTCATTGCTCTCTTCTCCTTGACCTTACGGTCAGTAAGAGGCACTCTACATGTGTTCAGCAT
ATAGGAGGCCTCGGGTTGATGGTAAAATATCACTCGGGGCTTTTCTCTATCTGCCGTTCAGCTAATGCCTGAGACAGACA
GCCTCAAGCACCCGCCGCTATTATATCGCTCTCTTTAACCCATTTTGTTTTATCGATTCTAATCCTGAAGACGCCTCGCA
TTTTTGTGGCGTAATTTTTTAATGATTTAATTATTTAACTTTAATTTATCTCTTCATCGCAATTATTGACGACAAGCTGG
ATTATTTTTGAAATATTGGCCTAACAAGCATCGCCGACTGACAACAAATTAATTATTACTTTTCCTAATTAATCCCTCAG
GAATCCTCACCTTAAGCTATGATTATCTAGGCTTAGGGTCACTCGTGAGCGCTTACAGCCGTCAAAAACGCATCTCACCG
CTGATGGCGCAAATTCTTCAATAGCTCGTAAAAAACGAATTATTCCTACACTATAATCTGATTTTAACGATGATTCGTGC
GGGGTAAAATAGTAAAAACGATCTATTCACCTGAAAGAGAAATAAAAAGTGAAACATCTGCATCGATTCTTTAGCAGTGA
TGCCTCGGGAGGCATTATTCTTATCATTGCCGCTATCCTGGCGATGATTATGGCCAACAGCGGCGCAACCAGTGGATGGT
ATCACGACTTTCTGGAGACGCCGGTTCAGCTCCGGGTTGGTTCACTCGAAATCAACAAAAACATGCTGTTATGGATAAAT
GACGCGCTGATGGCGGTATTTTTCCTGTTAGTCGGTCTGGAAGTTAAACGTGAACTGATGCAAGGATCGCTAGCCAGCTT
ACGCCAGGCCGCATTTCCAGTTATCGCCGCTATTGGTGGGATGATTGTGCCGGCATTACTCTATCTGGCTTTTAACTATG

CCGATCCGATTACCCGCGAAGGGTGGGCGATCCCGGCGGCTACTGACATTGCTTTTGCACTTGGTGTACTGGCGCTGTTG
GGAAGTCGTGTTCCGTTAGCGCTGAAGATCTTTTTGATGGCTCTGGCTATTATCGACGATCTTGGGGCCATCATTATCAT
CGCATTGTTCTACACTAATGACTTATCGATGGCCTCTCTTGGCGTCGCGGCTGTAGCAATTGCGGTACTCGCGGTATTGA
ATCTGTGTGGTGCACGCCGCACGGGCGTCTATATTCTTGTTGGCGTGGTGTTGTGGACTGCGGTGTTGAAATCGGGGGTT
CACGCAACTCTGGCGGGGGTAATTGTCGGCTTCTTTATTCCTTTGAAAGAGAAGCATGGGCGTTCTCCAGCGAAGCGACT
GGAGCATGTGTTGCACCCGTGGGTGGCGTATCTGATTTTGCCGCTGTTTGCATTTGCTAATGCTGGCGTTTCACTGCAAG
GCGTCACGCTGGATGGCTTGACCTCCATTCTGCCATTGGGGATCATCGCTGGCTTGCTGATTGGCAAACCGCTGGGGATT
AGTCTGTTCTGCTGGTTGGCGCTGCGTTTGAAACTGGCGCATCTGCCTGAGGGAACGACTTATCAGCAAATTATGGTGGT
GGGGATCCTGTGCGGTATCGGTTTTACTATGTCTATCTTTATTGCCAGCCTGGCCTTTGGTAGCGTAGATCCAGAACTGA
TTAACTGGGCGAAACTCGGTATCCTGGTCGGTTCTATCTCTTCGGCGGTAATTGGATACAGCTGGTTACGCGTTCGTTTG
CGTCCATCAGTTTGACAGGACGGTTTACCGGGGAGCCATAAACGGCTCCCTTTTCATTGTTATCAGGGAGAGAAATGAGC
ATGTCTCATATCAATTACAACCACTTGTATTACTTCTGGCATGTCTATAAAGAAGGTTCCGTGGTTGGCGCAGCGGAGGC
GCTTTATTTAACTCCACAAACCATTACCGGACAGATTCGAGCGCTGGAAGAGCGCCTGCAAGGCAAATTATTTAAACGCA
AGGGACGTGGTCTCGAACCCAGCGAGCTGGGAGAACTGGTCTATCGCTATGCCGATAAAATGTTCACCTTAAGCCAGGAA
ATGCTGGATATTGTGAACTATCGCAAAGAATCCAATTTATTGTTTGACGTTGGCGTGGCTGATGCACTTTCCAAACGCCT
GGTCAGTAGCGTACTTAACGCCGCAGTGGTAGAAGGCGAGCCCATTCATCTTCGCTGCTTCGAATCCACCCACGAAATGC
TGCTGGAGCAATTAAGTCAGCATAAACTGGATATGATCATTTCTGACTGTCCGATAGACTCTACGCAGCAGGAAGGCCTG
TTCTCCGTGAGAATTGGCGAATGTGGCGTGAGTTTCTGGTGTACAAATCCACCACCAGAAAAACCGTTCCCGGCTTGTCT
GGAAGAACGGCGACTTTTGATTCCTGGGCGACGTTCAATGTTAGGGCGCAAATTGCTTAACTGGTTTAACTCCCAGGGAT
TAAACGTAGAAATCCTCGGCGAGTTTGATGATGCCGCTTTGATGAAAGCTTTTGGTGCGATGCACAATGCAATCTTCGTT
GCCCCAACGCTTTATGCATATGACTTTTATGCCGATAAAACTGTCGTAGAAATTGGTCGCGTCGAGAATGTGATGGAAGA
GTACCATGCTATTTTTGCTGAGCGGATGATTCAGCACCCGGCGGTACAGCGAATCTGCAATACGGATTATTCTGCGCTTT
TTAGTCCAGCGGTGCGTTAATCGGCAGCTCCCCCAAAGTTAAGGTGGGGGAGATAGATTAGTTGTACATTACCACGATTT
TGACTCGGCTCATTATTTGCCCGCTTGAGACATTGTTTCCATATGTACGCGGGCGAATAAATAGAGGAATCTGATTACTT
CCTTCATGGGGATGCTGAAAAGAGTAGTAATTGCTGGTAATGACTCCAACTTATTGATAGTGTTTTATGTTCAGATAATG
CCCGATGACTTTGTCATGCAGCTCCACCGATTTTGAGAACGACAGCGACTTCCGTCCCAGCCGTGCCAGGTGCTGCCTCA
GATTCAGGTTATGCCGCTCAATTCGCTGCGTATATCGCTTGCTGATTACGTGCAGCTTTCCCTTCAGGCGGGATTCATAC
AGCGGCCAGCCATCCGTCATCCATATCACCACGTCAAAGGGTGACAGCAGGCTCATAAGACGCCCCAGCGTCGCCATAGT
GCGTTCACCGAATACGTGCGCAACAACCGTCTTCCGGAGACTGTCATACGCGTAAAACAGCCAGCGCTGGCGCGATTTAG
CCCCGACATAGCCCCACTGTTCGTCCATTTCCGCGCAGACGATGACGTCACTGCCCGGCTGTATGCGCGAGGTTACCGAC
TGCGGCCTGAGTTTTTTAAGTGACGTAAAATCGTGTTGAGGCCAACGCCCATAATGCGGGCTGTTGCCCGGCATCCAACG
CCATTCATGGCCATATCAATGATTTTCTGGTGCGTACCGGGTTGAGAAGCGGTGTAAGTGAACTGCAGTTGCCATGTTTT
ACGGCAGTGAGAGCAGAGATAGCGCTGATGTCCGGCGGTGCTTTTGCCGTTACGCACCACCCCGTCAGTAGCTGAACAGG
AGGGACAGCTGATAGAAACAGAAGCCACTGGAGCACCTCAAAAACACCATCATACACTAAATCAGTAAGTTGGCAGCATC
ACCTACCTCAATGTGTATCACAATATCCATATTCTTTGTGGGGGAGTCTGGAGATTGAGTAGATATTCTTGTTCAGAATG
TATCAGCCGATGGTTCTACGATTCTTAAGCCACGAAGAGTTCAGATAGTACAACGGCATGTCTCTTTTGACTATCTGGCA
ACCGGCAGTGTGTTCTCTCACGCATCACAAAAGCAGCAGGCATAAAAAAACCCGCTTGCGCGGGCTTTTTCACAAAGCTT
CAGCAAATTGGCGATTAAGCCAGTTTGTTGATCTGTGCAGTCAGGTTAGCCTTATGACGTGCAGCTTTGTTTTTGTGGAT
CAGACCTTTAGCAGCCTGACGGTCCACGATCGGTTGCATTTCGTTAAATGCTTTCTGTGCAGCAGCTTTGTCGCCAGCTT
CGATAGCTGCGTATACTTTCTTGATGAAAGTACGCATCATAGAGCGACGGCTTGCGTTGTGCTTACGAGCCTTTTCAGAC
TGAATGGCGCGCTTCTTAGCTGATTTGATATTAGCCAAGGTCCAACTCCCAAATGTGTTCTATATGGACAATTCAAAGGC
CGAGGAATATGCCCTTTTAGCCTTCTTTTGTCAATGGATTTGTGCAAATAAGCGCCGTTAATGTGCCGGCACTCGTTACG
TAGTGATGGCGCAGGATTCTACCAGCTTGCGGGGTGTGAATACAGCTTTTCCGCGATAAAAATTGCAGCAGGCGGTCAGT
TTCTTCCCGTGATTTGCGCCATGGCAATGAAAAGCCACTTCTTTCTGATTTCGGTACTCAATCGCCGGTTAACCTTGACC
GCTGTACAAGGTATACTCGGACGATTTTCACTGTTTTGAGCCAGACATGAAGCTGATACGCGGCATACATAATCTCAGCC
AGGCCCCGCAAGAAGGGTGTGTGCTGACTATTGGTAATTTCGACGGCGTGCATCGCGGTCATCGCGCGCTGTTACAGGGC
TTGCAGGAAGAAGGGCGCAAGCGCAACTTACCGGTGATGGTGATGCTTTTTGAACCTCAACCACTGGAACTGTTTGCTAC
CGATAAAGCCCCGGCAAGACTGACCCGGCTGCGGGAAAAACTGCGTTACCTTGCAGAGTGTGGCGTTGATTACGTGCTGT
GCGTGCGTTTCGACAGGCGTTTCGCGGCGTTAACCGCGCAAAATTTCATCAGCGATCTTCTGGTGAAGCATTTGCGCGTA
AAATTTCTTGCCGTAGGTGATGATTTCCGCTTTGGCGCTGGTCGTGAAGGCGATTTCTTGTTATTACAGAAAGCTGGCAT
GGAATACGGCTTCGATATCACCAGTACGCAAACTTTTTGCGAAGGTGGCGTGCGCATCAGCAGCACCGCCGTGCGTCAGG
CCCTTGCGGATGACAATCTGGCTCTGGCAGAGAGTTTACTGGGGCACCCGTTTGCCATCTCCGGGCGTGTAGTCCACGGT
GATGAATTAGGGCGCACTATAGGTTTCCCGACGGCGAATGTACCGCTGCGCCGTCAGGTTTCCCCGGTGAAAGGGGTTTA
TGCGGTAGAAGTGCTGGGCCTCGGTGAAAAGCCGTTACCCGGCGTGGCAAACATCGGAACACGCCCAACGGTTGCCGGTA
TTCGCCAGCAGCTGGAAGTGCATTTGTTAGATGTTGCAATGGACCTTTACGGTCGCCATATACAAGTAGTGCTGCGTAAA
AAATACGCAATGAGCAGCGATTTGCGTCGCTGGACGAACTGAAAGCGCAGATTGCGCGTGATGAATTAACCGCCCGCGA

ATTTTTTGGGCTAACAAAACCGGCTTAAGCCTGTTATGTAATCAAACCGAAATACGGAACCGAGAATCTGATGAGTGACT
ATAAATCAACCCTGAATTTGCCGGAAACAGGGTTCCCGATGCGTGGCGATCTCGCCAAGCGCGAACCCGGAATGCTGGCG
CGTTGGACTGATGATGATCTGTACGGCATCATCCGTGCGGCTAAAAAAGGCAAAAAAACCTTCATTCTGCATGATGGCCC
TCCTTATGCGAATGGCAGCATTCATATTGGTCACTCGGTTAACAAGATTCTGAAAGACATTATCGTGAAGTCCAAAGGGC
TTTCCGGTTATGACTCGCCGTATGTGCCTGGCTGGGACTGCCACGGTCTGCCGATCGAGCTGAAAGTCGAGCAAGAATAC
GGTAAGCCGGGTGAGAAATTCACCGCCGCCGAGTTCCGCGCCAAGTGCCGCGAATACGCGGCGACCCAGGTTGACGGTCA
ACGCAAAGACTTTATCCGTCTGGGCGTGCTGGGCGACTGGTCGCACCCGTACCTGACCATGGACTTCAAAACTGAAGCCA
ACATCATCCGCGCGCTGGGCAAAATCATCGGCAACGGTCACCTGCACAAAGGCGCGAAGCCAGTTCACTGGTGCGTTGAC
TGCCGTTCTGCGCTGGCGGAAGCGGAAGTTGAGTATTACGACAAAACTTCTCCGTCCATCGACGTTGCTTTCCAGGCAGT
CGATCAGGATGCACTGAAAGCAAAATTTGCCGTAAGCAACGTTAACGGCCCAATCTCGCTGGTAATCTGGACCACCACGC
CGTGGACTCTGCCTGCCAACCGCGCAATCTCTATTGCACCAGATTTCGACTATGCGCTGGTGCAGATCGACGGTCAGGCC
GTGATTCTGGCGAAAGATCTGGTTGAAAGCGTAATGCAGCGTATCGGCGTGACCGATTACACCATTCTCGGCACGGTAAA
AGGTGCGGAGCTTGAGCTGCTGCGCTTTACCCATCCGTTTATGGGCTTCGACGTTCCGGCAATCCTCGGCGATCACGTTA
CCCTGGATGCCGGTACCGGTGCCGTTCACACCGCGCCTGGCCACGGCCCGGACGACTATGTGATCGGTCAGAAATACGGC
CTGGAAACCGCTAACCCGGTTGGCCCGGACGGCACTTATCTGCCGGGCACTTATCCGACGCTGGATGGCGTGAACGTCTT
CAAAGCGAACGACATCGTCGTTGCGCTGCTGCAGGAAAAAGGCGCGCTGCTGCACGTTGAGAAAATGCAGCACAGCTATC
CGTGCTGCTGGCGTCACAAAACGCCGATCATCTTCCGCGCGACGCCGCAGTGGTTCGTCAGCATGGATCAGAAAGGTCTG
CGTGCGCAGTCACTGAAAGAGATCAAAGGCGTGCAGTGGATCCCGGACTGGGGCCAGGCGCGTATCGAGTCGATGGTTGC
TAACCGTCCTGACTGGTGTATCTCCCGTCAGCGCACCTGGGGTGTACCGATGTCACTGTTCGTGCACAAAGACACGGAAG
AGCTGCATCCGCGTACCCTTGAACTGATGGAAGAAGTGGCAAAACGCGTTGAAGTCGATGGCATCCAGGCGTGGTGGGAT
CTCGATGCGAAAGAGATCCTCGGCGACGAAGCTGATCAGTACGTGAAAGTGCCGGACACATTGGATGTATGGTTTGACTC
CGGATCTACCCACTCTTCTGTTGTTGACGTGCGTCCGGAATTTGCCGGTCACGCAGCGGACATGTATCTGGAAGGTTCTG
ACCAACACCGCGGCTGGTTCATGTCTTCCCTAATGATCTCCACCGCGATGAAGGGTAAAGCGCCGTATCGTCAGGTACTG
ACCCACGGCTTTACCGTGGATGGTCAGGGCCGCAAGATGTCTAAATCCATCGGCAATACCGTTTCGCCGCAGGATGTGAT
GAACAAACTGGGCGCGGATATTCTGCGTCTGTGGGTGGCATCAACCGACTACACCGGTGAAATGGCCGTTTCTGACGAGA
TCCTGAAACGTGCTGCCGATAGCTATCGTCGTATCCGTAACACCGCGCGCTTCCTGCTGGCAAACCTGAACGGTTTTGAT
CCAGCAAAAGATATGGTGAAACCGGAAGAGATGGTGGTACTGGATCGCTGGGCCGTAGGTTGTGCGAAAGCGGCACAGGA
AGACATCCTCAAGGCGTACGAAGCATACGATTTCCACGAAGTGGTACAGCGTCTGATGCGCTTCTGCTCCGTTGAGATGG
GTTCCTTCTACCTCGACATCATCAAAGACCGTCAGTACACCGCCAAAGCGGACAGTGTGGCGCGTCGTAGCTGCCAGACT
GCGCTATATCACATCGCAGAAGCGCTGGTGCGCTGGATGGCACCAATCCTCTCCTTCACCGCTGATGAAGTGTGGGGCTA
CCTGCCGGGCGAACGTGAAAAATACGTCTTCACCGGTGAGTGGTACGAAGGCCTGTTTGGCCTGGCAGACAGTGAAGCGA
TGAACGATGCGTTCTGGGACGAGCTGTTGAAAGTGCGTGGCGAAGTGAACAAAGTCATTGAGCAAGCGCGTGCCGACAAG
AAAGTGGGTGGCTCGCTGGAAGCGGCAGTAACCTTGTATGCAGAACCGGAACTGTCGGCGAAACTGACCGCGCTGGGCGA
TGAATTACGATTTGTCCTGTTGACCTCCGGCGCTACCGTTGCAGACTATAACGACGCACCTGCTGATGCTCAGCAGAGCG
AAGTACTCAAAGGGCTGAAAGTCGCGTTGAGTAAAGCCGAAGGTGAGAAGTGCCCACGCTGCTGGCACTACACCCAGGAT
GTCGGCAAGGTGGCGGAACACGCAGAAATCTGCGGCCGCTGTGTCAGCAACGTCGCCGGTGACGGTGAAAAACGTAAGTT
TGCCTGATGAGTCAATCGATCTGTTCAACAGGGCTACGCTGGCTGTGGCTGGTGGTAGTCGTGCTGATTATCGATCTGGG
CAGCAAATACCTGATCCTCCAGAACTTTGCTCTGGGGGATACGGTCCCGCTGTTCCCGTCGCTTAATCTGCATTATGCGC
GTAACTATGGCGCGGCGTTTAGTTTCCTTGCCGATAGCGGCGGCTGGCAGCGTTGGTTCTTTGCCGGTATTGCGATTGGT
ATTAGCGTGATCCTGGCAGTGATGATGTATCGCTCGAAGGCCACGCAGAAGCTAAACAATATCGCTTACGCGCTGATTAT
TGGCGGCGCGCTGGGCAACCTGTTCGACCGCCTGTGGCACGGCTTCGTTGTCGATATGATCGACTTCTACGTCGGCGACT
GGCACTTCGCCACCTTCAACCTTGCCGATACTGCCATCTGTGTCGGTGCGGCACTGATTGTGCTGGAAGGTTTTTTGCCT
TCTAGAGCGAAAAAACAATAATAAACCCTGCCGGATGCGATGCTGACGCATCTTATCCGGCCTACAGATTGCTGCGAAAT
CGTAGGCCGGATAAGGCGTTTACGCCGCATCCGGCAAAAATCCTTAAATATAAGAGCAAACCTGCATGTCTGAATCTGTA
CAGAGCAATAGCGCCGTCCTGGTGCACTTCACGCTAAAACTCGACGATGGCACCACCGCCGAGTCTACCCGCAACAACGG
TAAACCGGCGCTGTTCCGCCTGGGTGATGCTTCTCTTTCTGAAGGGCTGGAGCAACACCTGTTGGGGCTGAAAGTGGGCG
ATAAAACCACCTTCTCGTTGGAGCCAGATGCGGCGTTTGGCGTGCCGTCACCGGACCTGATTCAGTACTTCTCCCGCCGT
GAATTTATGGATGCAGGCGAGCCAGAAATTGGCGCAATCATGCTTTTTACCGCAATGGATGGCAGTGAGATGCCTGGCGT
GATCCGCGAAATTAACGGCGACTCCATTACCGTTGATTTCAACCATCCGCTGGCCGGGCAGACCGTTCATTTTGATATTG
AAGTGCTGGAAATCGATCCGGCACTGGAGGCGTAACATGCAGATCCTGTTGGCCAACCCGCGTGGTTTTTGTGCCGGGGT
AGACCGCGCTATCAGCATTGTTGAAAACGCGCTGGCCATTTACGGCGCACCGATATATGTCCGTCACGAAGTGGTACATA
ACCGCTATGTGGTCGATAGCTTGCGTGAGCGTGGGGCTATCTTTATTGAGCAGATTAGCGAAGTACCGGACGGCGCGATC
CTGATTTTCTCCGCACACGGTGTTTCTCAGGCGGTACGTAACGAAGCAAAAAGTCGCGATTTGACGGTGTTTGATGCCAC
CTGTCCGCTGGTGACCAAAGTGCATATGGAAGTCGCCCGCGCCAGTCGCCGTGGCGAAGAATCTATTCTCATCGGTCACG
CCGGGCACCCGGAAGTGGAAGGGACAATGGGCCAGTACAGTAACCCGGAAGGGGGAATGTATCTGGTCGAATCGCCGGAC
GATGTGTGGAAACTGACGGTCAAAAACGAAGAGAAGCTCTCCTTTATGACCCAGACCACGCTGTCGGTGGATGACACGTC

TGATGTGATCGACGCGCTGCGTAAACGCTTCCCGAAAATTGTCGGTCCGCGCAAAGATGACATCTGCTACGCCACGACTA
ACCGTCAGGAAGCGGTACGCGCCCTGGCAGAACAGGCGGAAGTTGTGTTGGTGGTCGGTTCGAAAAACTCCTCCAACTCC
AACCGTCTGGCGGAGCTGGCCCAGCGTATGGGCAAACGCGCGTTTTTGATTGACGATGCGAAAGACATCCAGGAAGAGTG
GGTGAAAGAGGTTAAATGCGTCGGCGTGACTGCGGGCGCATCGGCTCCGGATATTCTGGTGCAGAATGTGGTGGCACGTT
TGCAGCAGCTGGGCGGTGGTGAAGCCATTCCGCTGGAAGGCCGTGAAGAAAACATTGTTTTCGAAGTGCCGAAAGAGCTG
CGTGTCGATATTCGTGAAGTCGATTAAGTCATTAGCAGCCTAAGTTATGCGAAAATGCCGGTCTTGTTACCGGCATTTTT
TATGGAGAAAACATGCGTTTACCTATCTTCCTCGATACTGACCCCGGCATTGACGATGCCGTCGCCATTGCCGCCGCGAT
TTTTGCACCCGAACTCGACCTGCAACTGATGACCACCGTCGCGGGTAATGTCTCGGTTGAGAAAACTACCCGCAATGCCC
TGCAACTGCTGCATTTCTGGAATGCGGAGATTCCGCTCGCCCAAGGGGCCGCTGTGCCACTGGTACGCGCACCGCGTGAT
GCGGCATCTGTGCACGGCGAATCGGGAATGGCTGGCTACGACTTTGTTGAGCACAACCGAAAGCCGCTCGGGATACCGGC
GTTTCTGGCGATTCGGGATGCCCTGATGCGTGCACCAGAGCCTGTTACCCTGGTGGCCATCGGCCCGTTAACCAATATTG
CGCTGTTACTTTCACAATGCCCGGAATGCAAGCCGTATATTCGCCGTCTGGTGATCATGGGTGGTTCTGCCGGACGCGGC
AACTGTACGCCAAACGCCGAGTTTAATATTGCTGCCGATCCAGAAGCTGCTGCCTGTGTCTTCCGCAGTGGTATTGAAAT
CGTCATGTGCGGTTTGGATGTCACCAATCAGGCAATATTAACTCCTGACTATCTCTCTACACTGCCGCAGTTAAACCGTA
CCGGGAAAATGCTTCACGCCCTGTTTAGCCACTACCGTAGCGGCAGTATGCAAAGCGGCTTGCGAATGCACGATCTCTGC
GCCATCGCCTGGCTGGTGCGCCCGGACCTGTTCACTCTCAAACCCTGTTTTGTGGCAGTGGAAACTCAGGGCGAATTTAC
CTCAGGCACGACGGTGGTTGATATCGACGGTTGCCTGGGCAAGCCAGCCAATGTACAGGTGGCATTGGATCTGGATGTGA
AAGGCTTCCAGCAGTGGGTGGCTGAGGTGCTGGCTCTGGCGTCGTAACCTGTCACATGTTATTGGCATGCAGTCATTCAT
CGACTCATGCCTTTCACTGATATCCCTCCCTGTTTATCATTAATTTCTAATTATCAGCGTTTTTGGCTGGCGGCGTAGCG
ATGCGCTGGTTACTCTGAAAACGGTCTATGCAAATTAACAAAAGAGAATAGCTATGCATGATGCAAACATCCGCGTTGCC
ATCGCGGGAGCCGGGGGGCGTATGGGCCGCCAGTTGATTCAGGCGGCGCTGGCATTAGAGGGCGTGCAGTTGGGCGCTGC
GCTGGAGCGTGAAGGATCTTCTTTACTGGGCAGCGACGCCGGTGAGCTGGCCGGAGCCGGGAAAACAGGCGTTACCGTGC
AAAGCAGCCTCGATGCGGTAAAAGATGATTTTGATGTGTTTATCGATTTTACCCGTCCGGAAGGTACGCTGAACCATCTC
GCTTTTTGTCGCCAGCATGGCAAAGGGATGGTGATCGGCACTACGGGGTTTGACGAAGCCGGTAAACAAGCAATTCGTGA
CGCCGCTGCCGATATTGCGATTGTCTTTGCTGCCAATTTTAGCGTTGGCGTTAACGTCATGCTTAAGCTGCTGGAGAAAG
CAGCCAAAGTGATGGGTGACTACACCGATATCGAAATTATTGAAGCACATCATAGACATAAAGTTGATGCGCCGTCAGGC
ACCGCACTGGCAATGGGAGAGGCGATCGCCCACGCCCTTGATAAAGATCTGAAAGATTGCGCGGTCTACAGTCGTGAAGG
CCACACCGGTGAACGTGTGCCTGGCACCATTGGTTTTGCCACCGTGCGTGCAGGTGACATCGTTGGTGAACATACCGCGA
TGTTTGCCGATATTGGCGAGCGTCTGGAGATCACCCATAAGGCGTCCAGCCGTATGACATTTGCTAACGGCGCGGTAAGA
TCGGCTTTGTGGTTGAGTGGTAAGGAAAGCGGTCTTTTTGATATGCGAGATGTACTTGATCTCAATAATTTGTAACCACA
AAATATTTGTTATGGTGCAAAAATAACACATTTAATTTATTGATTATAAAGGGCTTTAATTTTTGGCCCTTTTATTTTTG
GTGTTATGTTTTTAAATTGTCTATAAGTGCCAAAAATTACATGTTTTGTCTTCTGTTTTTGTTGTTTTAATGTAAATTTT
GACCATTTGGTCCACTTTTTTCTGCTCGTTTTTATTTCATGCAATCTTCTTGCTGCGCAAGCGTTTTCCAGAACAGGTTA
GATGATCTTTTTGTCGCTTAATGCCTGTAAAACATGCATGAGCCACAAAATAATATAAAAAATCCCGCCATTAAGTTGAC
TTTTAGCGCCCATATCTCCAGAATGCCGCCGTTTGCCAGAAATTCGTCGGTAAGCAGATTTGCATTGATTTACGTCATCA
TTGTGAATTAATATGCAAATAAAGTGAGTGAATATTCTCTGGAGGGTGTTTTGATTAAGTCAGCGCTATTGGTTCTGGAA
GACGGAACCCAGTTTCACGGTCGGGCCATAGGGGCAACAGGTTCGGCGGTTGGGGAAGTCGTTTTCAATACTTCAATGAC
CGGTTATCAAGAAATCCTCACTGATCCTTCCTATTCTCGTCAAATCGTTACTCTTACTTATCCCCATATTGGCAATGTCG
GCACCAATGACGCCGATGAAGAATCTTCTCAGGTACATGCACAAGGTCTGGTGATTCGCGACCTGCCGCTGATTGCCAGC
AACTTCCGTAATACCGAAGACCTCTCTTCTTACCTGAAACGCCATAACATCGTGGCGATTGCCGATATCGATACCCGTAA
GCTGACGCGTTTACTGCGCGAGAAAGGCGCACAGAATGGCTGCATTATCGCGGGCGATAACCCGGATGCGGCGCTGGCGT
TAGAAAAAGCCCGCGCGTTCCCAGGTCTGAATGGCATGGATCTGGCAAAAGAAGTGACCACCGCAGAAGCCTATAGCTGG
ACACAAGGGAGCTGGACGTTGACCGGTGGCCTGCCAGAAGCGAAAAAAGAAGACGAGCTGCCGTTCCACGTCGTGGCTTA
TGATTTTGGTGCCAAGCGCAACATCCTGCGGATGCTGGTGGATAGAGGCTGTCGCCTGACCATCGTTCCGGCGCAAACTT
CTGCGGAAGATGTGCTGAAAATGAATCCAGACGGCATCTTCCTCTCCAACGGTCCTGGCGACCCGGCCCCGTGCGATTAC
GCCATTACCGCCATCCAGAAATTCCTCGAAACCGATATTCCGGTATTCGGCATCTGTCTCGGTCATCAGCTGCTGGCGCT
GGCGAGCGGTGCGAAGACTGTCAAAATGAAATTTGGTCACCACGGCGGCAACCATCCGGTTAAAGATGTGGAGAAAAACG
TGGTAATGATCACCGCCCAGAACCACGGTTTTGCGGTGGACGAAGCAACATTACCTGCAAACCTGCGTGTCACGCATAAA
TCCCTGTTCGACGGTACGTTACAGGGCATTCATCGCACCGATAAACCGGCATTCAGCTTCCAGGGGCACCCTGAAGCCAG
CCCTGGTCCACACGACGCCGCGCCGTTGTTCGACCACTTTATCGAGTTAATTGAGCAGTACCGTAAAACCGCTAAGTAAT
CAGGAGTAAAAGAGCCATGCCAAAACGTACAGATATAAAAAGTATCCTGATTCTGGGTGCGGGCCCGATTGTTATCGGTC
AGGCGTGTGAGTTTGACTACTCTGGCGCGCAAGCGTGTAAAGCCCTGCGTGAAGAGGGTTACCGCGTCATTCTGGTGAAC
TCCAACCCGGCGACCATCATGACCGACCCGGAAATGGCTGATGCAACCTACATCGAGCCGATTCACTGGGAAGTTGTACG
CAAGATTATTGAAAAAGAGCGCCCGGACGCGGTGCTGCCAACGATGGGCGGTCAGACGGCGCTGAACTGCGCGCTGGAGC
TGGAACGTCAGGGCGTGTTGGAAGAGTTCGGTGTCACCATGATTGGTGCCACTGCCGATGCGATTGATAAAGCAGAAGAC
CGCCGTCGTTTCGACGTAGCGATGAAGAAAATTGGTCTGGAAACCGCGCGTTCCGGTATCGCACACACGATGGAAGAAGC

GCTGGCGGTTGCCGCTGACGTGGGCTTCCCGTGCATTATTCGCCCATCCTTTACCATGGGCGGTAGCGGCGGCGGTATCG
CTTATAACCGTGAAGAGTTTGAAGAAATTTGCGCCCGCGGTCTGGATCTCTCTCCGACCAAAGAGTTGCTGATTGATGAG
TCGCTGATCGGCTGGAAAGAGTACGAGATGGAAGTGGTGCGTGATAAAAACGACAACTGCATCATCGTCTGCTCTATCGA
AAACTTCGATGCGATGGGCATCCACACCGGTGACTCCATCACTGTCGCGCCAGCCCAAACGCTGACCGACAAAGAATATC
AAATCATGCGTAACGCCTCGATGGCGGTGCTGCGTGAAATCGGCGTTGAAACCGGTGGTTCCAACGTTCAGTTTGCGGTG
AACCCGAAAAACGGTCGTCTGATTGTTATCGAAATGAACCCACGCGTGTCCCGTTCTTCGGCGCTGGCGTCGAAAGCGAC
CGGTTTCCCGATTGCTAAAGTGGCGGCGAAACTGGCGGTGGGTTACACCCTCGACGAACTGATGAACGACATCACTGGCG
GACGTACTCCGGCCTCCTTCGAGCCGTCCATCGACTATGTGGTTACTAAAATTCCTCGCTTCAACTTCGAAAAATTCGCC
GGTGCTAACGACCGTCTGACCACTCAGATGAAATCGGTTGGCGAAGTGATGGCGATTGGTCGCACGCAGCAGGAATCCCT
GCAAAAAGCGCTGCGCGGCCTGGAAGTCGGTGCGACTGGATTCGACCCGAAAGTGAGCCTGGATGACCCGGAAGCGTTAA
CCAAAATCCGTCGCGAACTGAAAGACGCAGGCGCAGATCGTATCTGGTACATCGCCGATGCGTTCCGTGCGGGCCTGTCT
GTGGACGGCGTCTTCAACCTGACCAACATTGACCGCTGGTTCCTGGTACAGATTGAAGAGCTGGTGCGTCTGGAAGAGAA
AGTGGCGGAAGTGGGCATCACTGGCCTGAACGCTGACTTCCTGCGCCAGCTGAAACGCAAAGGCTTTGCCGATGCGCGCT
TGGCAAAACTGGCGGGCGTACGCGAAGCGGAAATCCGTAAGCTGCGTGACCAGTATGACCTGCACCCGGTTTATAAGCGC
GTGGATACCTGTGCGGCAGAGTTCGCCACCGACACCGCTTACATGTACTCCACTTATGAAGAAGAGTGCGAAGCGAATCC
GTCTACCGACCGTGAAAAAATCATGGTGCTTGGCGGCGGCCCGAACCGTATCGGTCAGGGTATCGAATTCGACTACTGTT
GCGTACACGCCTCGCTGGCGCTGCGCGAAGACGGTTACGAAACCATTATGGTTAACTGTAACCCGGAAACCGTCTCCACC
GACTACGACACTTCCGACCGCCTCTACTTCGAGCCGGTAACTCTGGAAGATGTGCTGGAAATCGTGCGTATCGAGAAGCC
GAAAGGCGTTATCGTCCAGTACGGCGGTCAGACCCCGCTGAAACTGGCGCGCGCGCTGGAAGCTGCTGGCGTACCGGTTA
TCGGCACCAGCCCGGATGCTATCGACCGTGCAGAAGACCGTGAACGCTTCCAGCATGCGGTTGAGCGTCTGAAACTGAAA
CAACCGGCGAACGCCACCGTTACCGCTATTGAAATGGCGGTAGAGAAGGCGAAAGAGATTGGCTACCCGCTGGTGGTACG
TCCGTCTTACGTTCTCGGCGGTCGGGCGATGGAAATCGTCTATGACGAAGCTGACCTGCGTCGCTACTTCCAGACGGCGG
TCAGCGTGTCTAACGATGCGCCAGTGTTGCTGGACCACTTCCTCGATGACGCGGTAGAAGTTGACGTGGATGCCATCTGC
GACGGCGAAATGGTGCTGATTGGCGGCATCATGGAGCATATTGAGCAGGCGGGCGTGCACTCCGGTGACTCCGCATGTTC
TCTGCCAGCCTACACCTTAAGTCAGGAAATTCAGGATGTGATGCGCCAGCAGGTGCAGAAACTGGCCTTCGAATTGCAGG
TGCGCGGCCTGATGAACGTGCAGTTTGCGGTGAAAAACAACGAAGTCTACCTGATTGAAGTTAACCCGCGTGCGGCGCGT
ACCGTTCCGTTCGTCTCCAAAGCCACCGGCGTACCGCTGGCAAAAGTGGCGGCGCGCGTGATGGCTGGCAAATCGCTGGC
TGAGCAGGGCGTAACCAAAGAAGTTATCCCGCCGTACTACTCGGTGAAAGAAGTGGTGCTGCCGTTCAATAAATTCCCGG
GCGTTGACCCGCTGTTAGGGCCAGAAATGCGCTCTACCGGGGAAGTCATGGGCGTGGGCCGCACCTTCGCTGAAGCGTTT
GCCAAAGCGCAGCTGGGCAGCAACTCCACCATGAAGAAACACGGTCGTGCGCTGCTTTCCGTGCGCGAAGGCGATAAAGA
ACGCGTGGTGGACCTGGCGGCAAAACTGCTGAAACAGGGCTTCGAGCTGGATGCGACCCACGGCACGGCGATTGTGCTGG
GCGAAGCAGGTATCAACCCGCGTCTGGTAAACAAGGTGCATGAAGGCCGTCCGCACATTCAGGACCGTATCAAGAATGGC
GAATATACCTACATCATCAACACCACCTCAGGCCGTCGTGCGATTGAAGACTCCCGCGTGATTCGTCGCAGTGCGCTGCA
ATATAAAGTGCATTACGACACCACCCTGAACGGCGGCTTTGCCACCGCGATGGCGCTGAATGCCGATGCGACTGAAAAAG
TAATTTCGGTGCAGGAAATGCACGCACAGATCAAATAATAGCGTGTCATGGCAGATATTTTTCATCCGCTAATTTGATCG
AATAACTAATACGGTTCTCTGATGAGGACCGTTTTTTTTTGCCCATTAAGTAAATCTTTTGGGGAATCGATATTTTTGAT
GACATAAGCAGGATTTAGCTCACACTTATCGACGGTGAAGTTGCATACTATCGATATATCCACAATTTTAATATGGCCTT
GTTTAATTGCTTCAAAACGAGTCATAGCCAGACTTTTAATTTGTGAAACTGGAGTTCGTATGTGTGAAGGATATGTTGAA
AAACCACTCTACTTGTTAATCGCCGAATGGATGATGGCTGAAAATCGGTGGGTGATAGCAAGAGAGATCTCTATTCATTT
CGATATTGAACACAGCAAGGCGGTTAATACCCTGACTTATATTCTGTCGGAAGTCACAGAAATAAGCTGCGAAGTTAAGA
TGATCCCTAATAAGCTGGAAGGGCGGGGATGCCAGTGTCAGCGACTGGTTAAAGTGGTCGATATCGATGAGCAAATTTAC
GCGCGCCTGCGCAATAACAGTCGGGAAAAATTAGTCGGTGTAAGAAAGACGCCGCGTATTCCTGCCGTTCCGCTCACGGA
ACTTAACCGCGAGCAGAAGTGGCAGATGATGTTGTCAAAGAGTATGCGTCGTTAATTTTATCTCGTTGATACCGGGCGTC
CTGCTTGCCAGATGCGATGTTGTAGCATCTTATCCAGCAACCAGGTCGCATCCGGCAAGATCACCGTTTAGGCGTCACAT
CCGTCGTCCCCTGCAAACGGGGGCGATTTTCCTCCATTTGCCTCAGTGGCTGCGTTTCATGTAACGATACATGACAGCGC
CCGACAAGATCCTGATACTCTTTGGTATTCAACCGTTTCCAGTGTAACTCGTCGTCACTAACATTGCGTACAGCGCGGGC
TGGCGTACCCATCAACAACTGGCGTTTCTCGCCGCGAAAGCCCGCTTTGACAAAGCTCATGGCGGCAACAATGCTCTCTT
CGCCAATGACCGCGCCATCCATAATCACGCTGTTCATCCCGACCAATGCATCGCGACCAATCAAACAACCATGCAGGATC
GCTCCGTGCCCGATATGGCCGTTTTCCCCAACGATAGTGTCAGTGTCGCAGTAGCCATGCATAATGCAGCCATCCTGAAT
ATTGGCTCCCGCTTGCACGATCAACCGCCCGTAGTCACCACGCAGTGAGGCGAGTGGGCCGATGTAGACACCGGCTCCCA
CAATCACATCGCCAATCAAGACGGCACTGGGATGGACAAACGCCGTCGGGTGAACCACCGGAATTAACCCCTCAAAGGCG
TAATAGCTCACGGTTGTTAACGTCCTTTCCACACCGGATCGCGCTTCTCGGCAAACGCCAGCGGCCCTTCAATGGCATCT
TCCGAATGCAGAACCGATGGATAGTGTTTCAACACGCCGCTGCGAATATAGCGATACGCTTCTTCTACCGGCATTTCGCT
GGTGGTGCGGTAGATCTCTTTCAGCGCCGCAATCGCCAGCGGGGCGCTGTTAACCAGCTGCTGAGCCAGTTCGCGGGCGT
TATCCATCAGTTCCGCCTGGCTAACCACGCGGTTGACTATCCCCCAACGCAGCGCCTCTTCTGCGCCCATTCGTCTGCCG
GTCATCACCATTTCATTGACGATGGCAGGCGGCAGGATCTTCGGCAGACGCAGCACACCGCCGCTGTCAGGAACGATGCC

CAGTTTGGCTTCCGGCAGGGCGAAGCTGGCGTTATCGGCACAAACAATAAAATCTGCCGCCAGCGCCAGTTCAAAGCCGC
CGCCAAAGGCATAGCCGTTCACAGCTGCGATAACCGGTTTGTCGAGATTGAAAATTTCGGTTAATCCCGCAAAACCACCC
GGACCAAAGTCAGCATCCGGTGCTTCGCCTTCTGCTGCCGCTTTTAAATCCCAGCCCGCGGAAAAGAACTTCTCTCCGGC
ACCGGTAATAATGGCGACACGTAATTGCGGATCGTCACGGAAATTTAGAAATACTTCGCCCATTTCAAAGCTGGTTTTTG
CATCAATAGCATTCGCTTTTGGACGATCAAGGGTAATTTCCAGAATTGATCCATTGCGGGTCAGATGTAATGATTCACTC
ATTCCTTTTCTCCATTTTTGCTTTTTCAGGGACGACAACATCCCTGCAAAAAATGCATATTGTTTTAGAGTGTGATTATT
AGCTGGCAGGGTAGTTCCCTGCTGTTTCATTTATTTCAGATTCTTTCTAATTATTTTCCCCGAGCAATTACGTGGCAGAT
CTTTTCTGATCTCCAGATAAGAGGGCACTTTAAATTTCGCCATATTTTGTTCGCAGAAGCGGAAAAATTCCTCTTCGCTC
AATGTTTCACCTTCATTCAGCACCACAAATGCTTTGATGGCTTCATCGCGAATCGAATCTTTAATACCCACAACCACGAT
GTCCTGAATTTTCGGGTGCGCGGCGATAATATTTTCCAGCTCCACGCAGGAGACATTCTCGCCGCCACGTTTAATCATAT
TGCAGCGGCGATCGACGAAATAAAAAAAGTCCTCTTCGTCGCGGTATCCGGTATCGCCGGTATGCAGCCAGCCATCGGCT
TCCAGCACTTTCGCAGTGGCTTGTGGGTTGAGAAAGTACTCTTTGAAGATGGTTTTCCCAGGTATGCCTTTAATGCAGAT
TTCACCGATCTCACCAGCCGGGAGCGGGCGATTGTGATCGTCGCGGATCTCCGCTTCGTAGCAAAACCCCACCCGACCAA
TCGACGGCCAGCGTCGTTTATCGCCAGGACGATCGCCGATAATGCCCACAATGGTTTCCGTCATCCCATAAGACGTCAGC
AAGCGAACGCCGAAGCGTTCACAAAACGCATCTTTTTCCTGCTCCGACAAGTTGAGATAAAACATCACTTCCCGCAGGCG
GTGTTGCTGATCGTTCGCTGAAGGCGGCTGTACCATCAACGTACGGATCATCATCGGAATACATTCGGTAACGGTGGCGC
GGTACTTCTGTACCTGTCCCCAGAAGGCGCGGGCGCTGTATTTCTCGACCAGCACAAAGGTGGCCCCGGCAGAAAACGCC
GCCATCGCCGCAGTACACTGGCAATCGATATGAAACGCAGGCATTACCGTCAGGTAGACGTCATCGTCACGCAGTGCACA
CTGCCAGGCGGAGTAATATCCAGCGAAGCGCAGGTTGTAATGGGTAATCACCACACCTTTCGGTCGGGAGGTGGTGCCGG
AGGTGAAGAGAATTTCCGCCGTATCGTCAGTCGATAGCGGCGGTGCATAGCACAAGGTGGCAGGTTGTTGATTTTTCAGT
TGAGTAAACGAACTCACGCCATCATCAGCGGGAAGTGCCACATCTGTCAGGCAAATGTGCCGCAATTGAGTGGCATCTTC
CTGCTGAATCTGTTGATACATAGGATAGAATTGCGCACTGGTCACCAGCAGGCACGCCTGGCTATTTTGCAGGATCCACG
CGCTTTCCTCGCACAACAGGCGGGCGTTAATCGGCACCATAATCGCGCCAATTTTTGCCAGCCCGAACCAGCAAAAGATA
AATTCCGGGCAGTTGTCGAGATGTAGTGCAACCTTGTCGCCTTTGCGAATCCCCAGCGTATAAAACAGGTTTGCCGTGCG
GTTAATCTCCTGATTTAACTCAAGATAACTATACCGGTTAACGACTCCGCCGCTGGATTCACAAATCAGCGCCGTTTTAT
GACCGTAAACGTCCGCAAGATCGTCCCACATTTGACGTAGATGTTGTCCGCCAATGATATCCATTGCACCTCTATCCATT
TTTGTTCGTTTGTTATTGGGCGGGCGCTAGTCAGGCAAGCCGACTGACGCCACGCGTTTAGTCCTCAACTTTGGCCAGAC
CTTTGCTGACCAACTCCTGAATGTCGTTTTCGCTGTAGCCGATATTTTTCAAAATGGCAGCCGTGTCCATGCCATGTGAG
GGCATTCCGCGCCAGATTTGTCCGGGGTTATTTTTGAATTTCGGCATGATGTTCGGCCCTTTGCAGGTGCGACCATCCAT
CGTTTGCCACTGAGTGATTGATTCGCGAGCCACATACTGTGGATTGCTTTCCAGTTCCGGTACGGTCAGCACTTTGGCGC
AGGCGATATTCAGTTCAGCAAAGCGTTCTTTTACTTCCGCGATGGTATGTGTCGCCAGCCAGGCATCGAGTTTCTCTTCA
ACCAGTGGGCCGTAAGGGCATTCGATACGGTGGATAAGCTGAGTGCCTTCCGGGATTTCTGGCGTGCCAAGCAGATGTGC
GAGGCCAATATCTTTAAAGCACTCTTCAATTTGGGTAATGCCCACCAGTTCCATCACGATGTAGCCGTCGGCACATTTAT
ACAGACCGCAACCGGCGTAGTAGGGATCTTTACCTTTGCTCATGCGCGGGCACATTTCGCCGCCGTTGAAGTAATCCATC
ATGAAGTACTGGCCCATACGCAGCATCACTTCATACATGGCGATGTCGATACTTTCGCCTTTACCGGTTTCACGCACTTT
ATGCAGTGCTGCCAGCGCCGCCGTGGTGGCGGTCAGGCCAGAAAAGTAATCGGCGGTATACGGGAAGGCAGGCATTGGCT
GGTCAACATCACCGTTCTGAATCAGGTAACCACTAAAGGCCTGGGCGATAGTGTTATAGGCCGGAAGATTGGTGTACTCC
TCGGTGCCGTACTGACCAAAACCGGACAGGTGAGCGATAACCAGTTTCGGGTTGTGCTGCCACAGTACTTCATCGGTAAT
GCCACGACGGGCAAAGGCCGGACCTTTACTGGCTTCGATGAAGATATCGGTGGTTTCCATTAATTTCAGAAACGCTTCGC
GGCCTTCATCTTTGAAAATATTTAACGACAGCGCGTGCAAATTGCGGCGGGAGAGTTGCGGGTAGTTCGGTTGAACGCGA
ATGGTGTCGGCCCAGGCGACGTTCTCGATCCAGATAACTTCCGCGCCCCATTCTGCGAACATTTGCCCGGCAAACGGTCC
GGCGATTTCGATACCGGAGAAGACAACGCGCAATCCGGCCAACGGCCCGAATTTCGGCATGGGTAGATGATCCATTATTT
GCTCCTGAAAAATTTATGTAGCGCATGACTGCCGGATGCGGCGTAAACGCTTTATCCGGCCTACATTCGTGCTCCCGTAG
GCCTGATAAGACGCATCAGCGTCGCATCAGGCAGCGCACGGACTTAGCGGTATTGCTTCAGCACCGCACGACCCAGCGTC
AGGATCTGCATTTCGTCAGATCCCCCGGAGACGCGGTCTACACGCAGATCACGCCAGAAGCGGCTGATGCGGTGGTTGCC
CGCAATCCCGACACCGCCCAGCACCTGCATTGCGCTATCCACAACTTCAAATGCCGCATTGGCGCAGAAGTATTTGCACA
TCGCTGCATCGCCAGAGGTGATGGTGCCGTTGTCTGCTTTCCACGCTGCTTCATACAGCATGTTTTTCATGGAGTTTAAT
TTGATCGCCATGTGGGCGAATTTTTCCTGAATCAACTGGAAACGACCAATAGCCTCGCCAAACTGCACGCGCTGATTGGC
GTAGCGCGCCGCATCTTCAAAGGCGCACATCGCCGTACCGTAGTTGGTGAGGGCTACCAGGAAACGTTCATGGTCGAACT
CTTCTTTGACGCGGTTAAAGCCGTTACCTTCCCGACCGAACATGTCTTTCTCGTCCAGTTCCACGTCGTCAAAGGTGATT
TCACAGCAGCTATCCATACGCAGACCGAGCTTTTCAAGTTTGGTCACTTTGATGCCCGGTTTGCTCATATCAACAAACCA
TTCGGTGTAGACAGGTTTGTCCGGAGAAGCCCCGTCGCGCGCCATCACCACGATGTACGGGGTGTAGGCGCTGCTGGTAA
TAAAACACTTACTACCATTAAGATAAATCTTACCATTTCTACGGGTATAAGTCGTTTTCAGGCTACCCACGTCGGAGCCC
GCGCCCGGTTCGGTAATCGCTGAGTTCCACATCTGCTTACCGGTGCCGCGGAAAGCCATAATTTTGTCGATCTGCTCTTG
TGTGCCTTCGCGCAGGAAGGTGTTGAACCCGCCCGGCAACTGGTACAGCACATAGGTTGGTGCCCCCAGACGTCCCAGCT
CCATCCACACGGCGGCGAGAGTAACAAACCCCGCGTCCAGACCACCGTGCTCTTCAGGGATCAGCAGACTGTCGATACCC

ATATCCGCCAGTGCTTTGACAAAACGTTCCGGGTAGACGCTGTCACGGTCGCACTCGGCAAAATAGGCCTCCCAGTTTTC
GCTGGCCATCAGTTCGCGGATACCGGCGACAAACAGTTCCTGCTCATCATTTAAATTAAAATCCATCTTTCAACCTCTTG
ATATTTTGGGGGTTAATTAATCTTTCCAGTTCTGTTTCGCGTCTTTAATAAAGGAGAGCGTCACCATAATGTTGACGAAG
AACAGCGGGCATCCTCCGGCGATAATGGCGGTTTGAATCGGTTTCAGGCCGCCGAGCGCCAGCAGAACAATACCGATAAT
GCCAACCAGAATTGACCAACCGATACGCACCAGCAGAGGTGGTTCTTCACCATCGCGTACTTCGCGGCAAGTGGACATCG
CCAGGGTATAAGAGCAGGCGTTAACCAGCGTAACGGTGGCAATAAAGCAGAGGATGAAGAAGCCCCACATGGTGGCGGTG
CTGAGTGGCAGAGCGGCCCAGGTTTCAATGATGGCGCGCGCCACACCGTACTGTTCGATCAGATTTGGAATGTTGATGAT
GTTTTTATCTATCAACAGCAGAGTGTTACTACCGAGTACAGTCCACAGGATCCAGGTTGACGCTGTCAGCCCCAGCACCA
TGCCGAAGCACAGTTCACGCACAGTACGACCACGGGAGATGCGGGCGAGGAAGATACTCATCTGGATAGCATAAATCACC
CACCATGCCCAGTAGAACACGGTCCAGCCCTGCGGGAAGCCGCCTTTAGCGATGGGATCGGTATAGAACAACATGCGCGG
CAGATACATCAGCAACATCCCCACCGAATCGGTGAAGTAGTTCATGATGAAGCTGGCACCGCTGACAATGAACACCCAAC
CCAGCATCAGGAAGCTCAGGTAACTACGCACGTCACTGGCGATACGTACCCCTTTTTGCAGACCGCAAGCGACGCAAATG
GCGTTGAGGATAATCCAGCAGGTAATGATGATAGCGTCCAGTTGCAGGGTATGCGGAATGCCAAACAACCATTGCATACA
CTCGGTCACCAGCGGCGTGGCAAGGCCCAGACTGGTACCCATCGCGAAGATCAAGGCGACGAGATAGAAGTTGTCGACGA
TAGTGCCGAACAACCCTTTGGCGTGTTTTTCACCTACCAGCGGCACCAGTGTCGAGCTGGGGCGAATCACTTCCATTTTG
CGGACAAAGAAGAAGTAAGCGAAGGCGACTGAAAGGAAGCTGTAAGTGGCCCACGGCAGAGGTCCCCAGTGGAACAAGCT
GTAAGCCAGCCCCAACTCTTTCGCCCCTGTCGAGTTCGGTTCTAAGCCAAACGGCGGGGTGGAGATGTAGTAGTAGATCT
CAATCGATCCCCAGAACAGTACGGCAGCAGACGTACAGGAGGCGAACATCATAAAGATCCAACTGGCGGTGCTAAATTCT
GGCGGTTCGTTACCTAAACGCTTTTTGGCATACGGGCCAAACACCAGCCAGAACCAACCGAAAAGCATCACCACCATATA
CCATTCAAATGCCCATCCCCATACATTGGTGACGTAACTGAATACAGCATTAATAACGACATTCGCTGCATCCAGATCTC
TGACTGTAAGCCAACAAAGTATGCCGACGATTATTAACGGCGGAAAGAAAACCTTCGGTTCTATTCCCGTTTTTCTCTTT
TCATTCTTCATGAGTTAATTCCACTGTGAAAACGAATATTTATTTTGCGTTCCCGTTTGTTTTATTTTTGTTAACATTTA
ATATAATTATTATTAACCTCGTGGACGCGTTAATGGCTAACTCATAATGGGTATTCAATAAGCTGTATTCTGTGATTGGT
ATCACATTTTTGTTTCGGGTGAATAGAGGGCGTTTTTTCGTTAATTTTGATTAATAATCAGTTTGTTATGCTCTGTTGTG
AGTAAAAAATAACATCTGACTTTCAATATTGGTGATCCATAAAACAATATTGAAAATTTCTTTTTGCTACGCCGTGTTTT
CAATATTGGTGAGGAACTTAACAATATTGAAAGTTGGATTTATCTGCGTGTGACATTTTCAATATTGGTGATTAAAGTTT
TATTTCAAAATTAAAGGGCGTGATATCTGTAATTAACACCACCGATATGAACGACGTTTCCTTCATGATTTCTGGAGATG
CAATGAAGATTATTACTTGCTATAAGTGCGTGCCTGATGAACAGGATATTGCGGTCAATAATGCTGATGGTTCATTAGAC
TTCAGCAAAGCCGATGCCAAAATAAGCCAATACGATCTCAACGCTATTGAAGCGGCTTGCCAGCTAAAGCAACAGGCAGC
AGAGGCGCAGGTGACAGCCTTAAGTGTGGGCGGTAAAGCCCTGACCAACGCCAAAGGGCGTAAAGATGTGCTATCGCGCG
GCCCGGATGAACTGATTGTGGTGATTGATGACCAGTTCGAGCAGGCACTGCCGCAACAAACGGCGAGCGCACTGGCTGCA
GCCGCCCAGAAAGCAGGCTTTGATCTGATCCTCTGTGGCGATGGTTCTTCCGACCTTTATGCCCAGCAGGTTGGTCTGCT
GGTGGGCGAAATCCTCAATATTCCGGCAGTTAACGGCGTCAGCAAAATTATCTCCCTGACGGCAGATACCCTCACCGTTG
AGCGCGAACTGGAAGATGAAACCGAAACCTTAAGCATTCCGCTGCCTGCGGTTGTTGCTGTTTCCACTGATATCAACTCC
CCACAAATTCCTTCGATGAAAGCCATTCTCGGCGCGGCGAAAAAGCCCGTCCAGGTATGGTCGGCGGCGGATATTGGTTT
TAACGCAGAGGCAGCCTGGTCAGAACAACAGGTTGCCGCGCCGAAACAGCGCGAACGTCAGCGCATCGTGATTGAAGGCG
ACGGCGAAGAACAGATCGCCGCATTTGCTGAAAATCTTCGCAAAGTCATTTAATTACAGGGGATGCTATGAACACGTTTT
CTCAAGTCTGGGTATTCAGCGATACCCCTTCTCGTCTGCCGGAACTGATGAACGGTGCGCAGGCTTTAGCTAATCAAATC
AACACCTTTGTCCTCAATGATGCCGACGGCGCACAGGCAATCCAGCTCGGCGCTAATCATGTCTGGAAATTAAACGGCAA
ACCGGACGATCGGATGATCGAAGATTACGCCGGTGTCATGGCTGACACTATTCGCCAGCACGGCGCAGACGGCCTGGTGC
TGCTGCCAAACACCCGTCGCGGCAAATTACTGGCGGCAAAACTGGGTTATCGCCTTAAAGCGGCGGTGTCTAACGATGCC
AGCACCGTCAGCGTACAGGACGGTAAAGCGACAGTGAAACACATGGTTTACGGTGGTCTGGCGATTGGCGAAGAACGCAT
TGCCACGCCGTATGCGGTACTGACCATCAGCAGCGGCACGTTCGATGCGGCTCAGCCAGACGCGTCACGCACTGGCGAAA
CGCACACCGTGGAGTGGCAGGCTCCGGCTGTGGCGATTACCCGCACGGCAACCCAGGCGCGCCAGAGCAACAGCGTCGAT
CTCGACAAAGCCCGTCTGGTGGTCAGCGTCGGTCGCGGTATTGGCAGCAAAGAGAACATTGCGCTGGCAGAACAGCTTTG
CAAGGCGATAGGTGCGGAGTTGGCCTGTTCTCGTCCGGTGGCGGAAAACGAAAAATGGATGGAGCACGAACGCTATGTCG
GTATCTCCAACCTGATGCTGAAACCTGAACTGTACCTGGCGGTGGGGATCTCCGGGCAGATCCAGCACATGGTTGGCGCT
AACGCGTCGCAAACCATTTTCGCCATCAATAAAGATAAAAATGCGCCGATCTTCCAGTACGCGGATTACGGCATTGTTGG
CGACGCCGTGAAGATCCTTCCGGCGCTGACCGCAGCTTTAGCGCGTTGATCCACTCTGGCAGGGCTGCATTTTGGCCCTG
CCGCTGACAGGGAGCTCTTATGTCCGAAGATATCTTTGACGCCATCATCGTCGGTGCAGGGCTTGCCGGTTCGGTTGCCG
CACTGGTGCTCGCCCGCGAAGGTGCGCAAGTGTTAGTTATCGAGCGTGGCAATTCCGCAGGTGCCAAGAACGTCACCGGC
GGGCGTCTCTATGCCCACAGTCTGGAACACATTATTCCTGGTTTCGCCGACTCCGCCCCCGTAGAACGCCTGATCACCCA
TGAAAAACTCGCGTTTATGACGGAAAAGTCAGCGATGACTATGGACTACTGCAATGGTGACGAAACCTCGCCATCCCAGC
GTTCTTACTCCGTTTTGCGCAGTAAATTTGATGCCTGGCTGATGGAGCAGGCCGAAGAAGCGGGCGCGCAGTTAATTACC
GGGATCCGCGTCGATAACCTCGTACAGCGCGATGGCAAAGTCGTCGGTGTAGAAGCCGATGGCGATGTGATTGAAGCGAA
AACGGTGATCCTTGCTGATGGGGTGAACTCCATCCTTGCCGAAAAAATTGGGGATGGCAAAACGCGTCAAACCGACGGATG

TGGCGGTTGGCGTGAAGGAACTGATCGAGTTACCGAAGTCGGTTATTGAAGACCGTTTTCAGTTGCAGGGTAATCAGGGG
GCGGCTTGCCTGTTTGCGGGATCACCCACCGATGGCCTGATGGGCGGCGGCTTCCTTTATACCAATGAAAACACCCTGTC
GCTGGGGCTGGTTTGTGGTTTGCATCATCTGCATGACGCGAAAAAATCGGTGCCGCAAATGCTGGAAGATTTCAAACAGC
ATCCGGCCGTTGCACCGCTGATCGCGGGCGGCAAGCTGGTGGAATATTCCGCTCACGTAGTGCCGGAAGCAGGCATCAAC
ATGCTGCCGGAGTTGGTTGGTGACGGCGTATTGATTGCCGGTGATGCCGCCGGAATGTGTATGAACCTCGGTTTTACCAT
TCGCGGTATGGATCTGGCGATTGCCGCCGGGGAAGCCGCAGCAAAAACCGTGCTTTCAGCGATGAAAAGCGACGATTTCA
GTAAGCAAAAACTGGCGGAATATCGTCAGCATCTTGAGAGTGGTCCGCTGCGCGATATGCGTATGTACCAGAAACTACCG
GCGTTCCTTGATAACCCACGCATGTTTAGCGGCTACCCGGAGCTGGCGGTGGGTGTGGCGCGTGACCTGTTCACCATTGA
TGGCAGCGCGCCGGAACTGATGCGCAAGAAAATCCTCCGCCACGGCAAGAAAGTGGGCTTCATCAATCTAATCAAGGATG
GCATGAAAGGAGTGACCGTTTTATGACTTCTCCCGTCAATGTGGACGTCAAACTGGGCGTCAATAAATTCAATGTCGATG
AAGAGCATCCGCACATTGTTGTGAAGGCCGATGCTGATAAACAGGCGCTGGAGCTGCTGGTGAAAGCGTGCCCCGCAGGT
CTGTACAAGAAGCAGGATGACGGCAGTGTGCGCTTCGATTACGCCGGATGTCTGGAGTGCGGCACCTGTCGCATTCTGGG
GCTGGGGTCGGCGCTGGAACAGTGGGAATACCCGCGCGGCACCTTTGGTGTGGAGTTCCGTTACGGCTGATGTTGGTTTG
ATACGTAACGCCGCACTGACTCTCATTGCAAAAAACAGGAATAACCATGCAACCGTCCAGAAACTTTGACGATCTCAAAT
TCTCCTCTATTCACCGCCGCATTTTGCTGTGGGGAAGCGGTGGTCCGTTTCTGGATGGTTATGTACTGGTAATGATTGGC
GTGGCGCTGGAGCAACTGACGCCGGCGCTGAAACTGGACGCTGACTGGATTGGCTTGCTGGGCGCGGGAACGCTCGCCGG
GCTGTTCGTTGGCACATCGCTGTTTGGTTATATTTCCGATAAAGTCGGACGGCGCAAAATGTTCCTCATTGATATCATCG
CCATCGGCGTGATATCGGTGGCGACGATGTTTGTTTCATCCCCCGTCGAACTGTTGGTGATGCGGGTACTTATCGGCATT
GTCATCGGTGCAGATTATCCCATCGCCACCTCAATGATCACCGAGTTCTCCAGTACCCGTCAGCGGGCGTTTTCCATCAG
CTTTATTGCCGCGATGTGGTATGTCGGCGCGACCTGTGCCGATCTGGTCGGCTACTGGCTTTATGATGTGGAAGGCGGCT
GGCGCTGGATGCTGGGTAGCGCGGCGATCCCCTGTTTGTTGATTTTGATTGGTCGATTCGAACTGCCTGAATCTCCCCGC
TGGTTATTACGCAAAGGGCGAGTAAAAGAGTGCGAAGAGATGATGATCAAACTGTTTGGCGAACCGGTGGCTTTCGATGA
AGAGCAGCCGCAGCAAACCCGTTTTCGCGATCTGTTTAATCGCCGCCATTTTCCTTTTGTTCTGTTTGTTGCCGCCATCT
GGACCTGCCAGGTGATCCCAATGTTCGCCATTTACACCTTTGGCCCGCAAATCGTTGGTTTGTTGGGATTGGGGGTTGGC
AAAAACGCGGCACTAGGGAATGTGGTGATTAGCCTGTTCTTTATGCTCGGCTGTATTCCGCCGATGCTGTGGTTAAACAC
TGCCGGACGGCGTCCATTGTTGATTGGCAGCTTTGCCATGATGACGCTGGCGCTGGCGGTTTTGGGGCTAATCCCGGATA
TGGGGATCTGGCTGGTAGTGATGGCCTTTGCGGTGTATGCCTTTTTCTCTGGCGGGCCGGGTAATTTGCAGTGGCTCTAT
CCTAATGAACTCTTCCCGACAGATATCCGCGCCTCTGCCGTGGGCGTGATTATGTCCTTAAGTCGTATTGGCACCATTGT
TTCGACCTGGGCACTACCGATCTTTATCAATAATTACGGTATCAGTAACACGATGCTAATGGGGGCGGGTATCTCGCTGT
TTGGCTTGTTGATTTCCGTAGCGTTTGCCCCGGAGACTCGAGGGATGTCACTGGCGCAGACCAGCAATATGACGATCCGC
GGGCAGAGAATGGGGTAAATTGTTCAGATTTCTCTCTTTTCTGAATCAATATTATTGACTATAAGCCGCGTGAATATATG
ACTACACTTTGTGGGAAAACAAAGGCGTAATCACGCGGGCTACCTATGATTCTTATAATTTATGCGCATCCGTATCCGCA
TCATTCCCATGCGAATAAACGGATGCTTGAACAGGCAAGGACGCTGGAAGGCGTCGAAATTCGCTCTCTTTATCAACTCT
ATCCTGACTTCAATATCGATATTGCCGCCGAGCAGGAGGCGCTGTCTCGCGCCGATCTGATCGTCTGGCAGCATCCGATG
CAGTGGTACAGCATTCCTCCGCTCCTCAAACTTTGGATCGATAAAGTTTTCTCCCACGGCTGGGCTTACGGTCATGGCGG
CACGGCGCTGCATGGCAAACATTTGCTGTGGGCGGTGACGACCGGCGGCGGGGAAAGCCATTTTGAAATTGGTGCGCATC
CGGGCTTTGATGTGCTGTCGCAGCCGCTACAGGCGACGGCAATCTACTGCGGGCTGAACTGGCTGCCACCGTTTGCCATG
CACTGCACCTTTATTTGTGACGACGAAACCCTCGAAGGGCAGGCGCGTCACTATAAGCAACGTCTGCTGGAATGGCAGGA
GGCCCATCATGGATAGCCATACGCTGATTCAGGCGCTGATTTATCTCGGTTCGGCAGCGCTGATTGTACCCATTGCGGTA
CGTCTTGGTCTGGGATCGGTACTTGGCTACCTGATCGCCGGCTGCATTATTGGCCCGTGGGGGCTGCGACTGGTGACCGA
TGCCGAATCTATTCTGCACTTTGCCGAGATTGGGGTGGTGCTGATGCTGTTTATTATCGGCCTCGAACTCGATCCACAAA
GGCTGTGGAAGCTGCGTGCGGCAGTGTTCGGCTGTGGCGCATTGCAGATGGTGATTTGCGGCGGCCTGCTGGGGCTGTTC
TGCATGTTACTTGGGCTGCGCTGGCAGGTCGCGGAATTGATCGGCATGACGCTGGCGCTCTCCTCTACGGCGATTGCCAT
GCAGGCGATGAATGAACGCAATCTGATGGTGACGCAAATGGGTCGCAGTGCCTTTGCGGTGCTGCTGTTCCAGGATATCG
CGGCGATCCCGCTGGTGGCGATGATTCCGCTACTGGCAACGAGCAGTGCCTCGACGACGATGGGCGCATTTGCTCTCTCG
GCGTTAAAAGTGGCGGGTGCGCTGGTGCTGGTGGTATTGCTGGGGCGCTATGTCACGCGTCCGGCGCTGCGTTTTGTAGC
CCGCTCTGGCTTGCGGGAAGTGTTTAGTGCCGTGGCGTTATTCCTCGTGTTTGGCTTTGGTTTGCTGCTGGAAGAGGTCG
GCTTGTCGATGGCGATGGGCGCGTTTCTGGCGGGCGTACTGCTGGCAAGCTCGGAATACCGTCATGCGCTGGAGAGCGAT
ATCGAACCATTTAAAGGTTTGCTGTTGGGGCTGTTTTTCATCGGTGTTGGCATGTCGATAGACTTTGGCACGCTGCTTGA
AAACCCATTGCGCATTGTCATTTTGCTGCTCGGTTTCCTCATCATCAAAATCGCCATGCTGTGGCTGATTGCCCGACCGT
TGCAAGTGCCAAATAAACAGCGTCGTTGGTTTGCGGTGTTGTTAGGGCAGGGCAGTGAGTTTGCCTTTGTGGTATTTGGC
GCGGCGCAGATGGCGAATGTGCTGGAGCCGGAGTGGGCGAAATCGCTGACCCTGGCGGTGGCGCTGTCGATGGCAGCAAC
GCCGATTCTGCTGGTGATCCTCAATCGCCTTGAGCAATCTTCTACTGAGGAAGCGCGTGAAGCCGATGAGATCGACGAAG
AACAGCCGCGCGTGATTATCGCCGGATTCGGTCGTTTTGGGCAGATTACCGGACGTTTACTGCTCTCCAGCGGGGTGAAA
ATGGTGGTACTCGATCACGATCCGGACCATATCGAAACCTTGCGTAAATTTGGTATGAAAGTGTTTTATGGCGATGCCAC
GCGGATGGATTTACTGGAATCTGCCGGAGCGGCGAAAGCGGAAGTGCTGATTAACGCCATCGACGATCCGCAAACCAACC

TGCAACTGACAGAGATGGTGAAAGAACATTTCCCGCATTTGCAGATTATTGCCCGCGCCCGCGATGTCGACCACTACATT
CGTTTGCGTCAGGCAGGCGTTGAAAAGCCGGAGCGTGAAACCTTCGAAGGTGCGCTGAAAACCGGGCGTCTGGCACTGGA
AAGTTTAGGTCTGGGGCCGTATGAAGCGCGAGAACGTGCCGATGTGTTCCGCCGCTTTAATATTCAGATGGTGGAAGAGA
TGGCAATGGTTGAGAACGACACCAAAGCCCGCGCGGCGGTCTATAAACGCACCAGCGCGATGTTAAGTGAGATCATTACC
GAGGACCGCGAACATCTGTCATTAATTCAACGACATGGCTGGCAGGGAACCGAAGAAGGTAAACATACCGGCAACATGGC
GGATGAACCGGAAACGAAACCCTCATCCTAATAAAGAGTGACGTAAATCACACTTTACAGCTAACTGTTTGTTTTTGTTT
CATTGTAATGCGGCGAGTCCAGGGAGAGAGCGTGGACTCGCCAGCAGAATATAAAATTTTCCTCAACATCATCCTCGCAC
CAGTCGACGACGGTTTACGCTTTACGTATAGTGGCGACAATTTTTTTTATCGGGAAATCTCAATGATCAGTCTGATTGCG
GCGTTAGCGGTAGATCGCGTTATCGGCATGGAAAACGCCATGCCGTGGAACCTGCCTGCCGATCTCGCCTGGTTTAAACG
CAACACCTTAAATAAACCCGTGATTATGGGCCGCCATACCTGGGAATCAATCGGTCGTCCGTTGCCAGGACGCAAAAATA
TTATCCTCAGCAGTCAACCGGGTACGGACGATCGCGTAACGTGGGTGAAGTCGGTGGATGAAGCCATCGCGGCGTGTGGT
GACGTACCAGAAATCATGGTGATTGGCGGCGGTCGCGTTTATGAACAGTTCTTGCCAAAAGCGCAAAAACTGTATCTGAC
GCATATCGACGCAGAAGTGGAAGGCGACACCCATTTCCCGGATTACGAGCCGGATGACTGGGAATCGGTATTCAGCGAAT
TCCACGATGCTGATGCGCAGAACTCTCACAGCTATTGCTTTGAGATTCTGGAGCGGCGGTAATTTTGTATAGAATTTACG
GCTAGCGCCGGATGCGACGCCGGTCGCGTCTTATCCGGCCTTCCTATATCAGGCTGTGTTTAAGACGCCGCCGCTTCGCC
CAAATCCTTATGCCGGTTCGACGGCTGGACAAAATACTGTTTATCTTCCCAGCGCAGGCAGGTTAATGTACCACCCCAGC
AGCAGCCGGTATCCAGCGCGTATATACCTTCCGGCGTACCTTTGCCCTCCAGCGATGCCCAGTGACCAAAGGCGATGCTG
TATTCTTCAGCGACAGGGCCAGGAATCGCAAACCACGGTTTCAGTGGGGCAGGGGCCTCTTCCGGCGATTCTTTGCTGTA
CATATCCAGTTGACCGTTCGGGAAGCAAAAACGCATACGGGTAAAAGCGTTGGTGATAAAACGCAGTCTTCCCAGCCCCC
GCAATTCCGGTGACCAGTTATTTGGCATATCGCCGTACATGGCATCAAGAAAGAAGGGATAGGAGTCACTCGATAGCACC
GCTTCTACATCGCGTGCGCACTCTTTGGCGGTCTGCAGATCCCACTGCGGCGTGATCCCTGCGTGGGCCATCACCAGCTT
TTTCTCTTCGTCGATTTGCAGCAGAGGCTGGCGCCGCAGCCAGTTAAGCAGCTCGTCGGCATCCGGCGCTTCCAGCAGCG
GTGTCAGGCGATCTTTCGGTTTATTGCGGCTGATCCCGGCAAATACCGCCAGCAGATGCAGATCGTGATTGCCCAGCACC
AGACGTACGCTGTCGCCTAAGGATTTCACATAGCGCAGAACATCCAGCGAACCCGGCCCGCGCGCGACCAGATCGCCCGT
CAGCCAGAGGGTATCTTTCCCAGGGGTAAATTCTACTTTATGCAGCAATGCGATCAGTTCATCGTAACAACCATGAACGT
CGCCAATAAGGTATGTCGCCATATTCTTTTAATGAATGAGTGTGGGAACGGCGAGTCGGAATACGGGAATGTCGATGCTG
AAAGGGACGCCATTTTCATCGATCATTTCGTAGTGACCCTGCATGGTGCCCAGCGGGGTTTCAATGATTGCACCGCTGGT
GTACTGGTACTCTTCGCCAGGCGCGATAAGTGGCTGGACGCCAACCACTCCTTCGCCCTGGACTTCGGTTTCACGGCCAT
TGCCATTGGTGATCAGCCAGTAACGCCCCAACAACTGCACTGGCGCTCGCCCCAGATTGCGTATGGTTACGGTATAAGCA
AAAACGTAACGTTCATTATCAGGTGAAGATTGAGCCTCAATGTAGACGCTTTGAACCTGAATACACACTCGGGGCGAATT
GATCATCGTTAACTCTCCTGCAAAGGCGCGTTCTCCGCCAGATAGTTCGCCATCTGGCAATATTGCGCGACAGAGATATT
TTCCGCTCGCATCGCCGGGTCGATCCCCATTCCCGTTAACACCTCGACGCTAAACAGGTTGCCGAGGCTGTTACGAATGG
TTTTACGACGCTGGTTAAAGGCTTCGGTGGTGATGCGGCTCAACACACGAACATCTTTAACCGGGTGAGGCATCGTTGCA
TGAGGAACCAGGCGCACGACGGCGGAATCCACTTTGGGTGGTGGTGTAAAGGCTGACGGCGGTACTTCCAGTACCGGGAT
CACATTGCAATAGTATTGCGCCATGACGCTTAATCGACCATACGCTTTGCTGTTCGGTCCTGCAACCAGACGATTCACCA
CCTCTTTTTGCAACATAAAGTGCATGTCGGCAATGGCATCAGTATAGCTAAACAGATGGAACATCAACGGCGTGGAGATG
TTATAAGGCAGGTTGCCGAAAACACGCAGCGGCTGACCCATTTTCTCGGCCAGTTCACCAAAGTTAAAGGTCATCGCATC
CTGCTGATAAATCGTCAGTTTCGGGCCTAAGAATGGATGCGTTTGCAGACGTGCCGCCAGATCGCGGTCAAGTTCGATGA
CCGTCAGCTGGTCCAGACGTTCGCCGACCGGTTCGGTCAATGCCGCCAGACCGGGGCCGATTTCGACCATCGCCTGGCCC
TTTTGCGGGTTAATGGCAGACACAATACTGTCGATCACGAACTGATCGTTGAGAAAGTTTTGCCCGAAGCGTTTACGGGC
TAAGTGGCCCTGGTGGACTCGATTATTCATTGGGTGTTAACAATCATTTTGATGGCGAGATTAAGCGCCGTAATAAAACT
GCCGACATCGGCTTTGCCACGTCCCGCCAGTTCAAGCGCGGTGCCGTGGTCCACTGATGTGCGAATAAAGGGCAGGCCCA
GCGTAATGTTCACACCGCGCCCGAAGCCCTGGTATTTTAGCACGGGAAGACCCTGATCGTGGTACATCGCCAGCACGGCG
TCGGCGTTATCAAGATATTTCGGCTGAAACAGGGTATCGGCAGGCAGCGGCCCGTTGAGTTTCATCCCCTGCGCCCGCAG
CTCATTGAGCACCGGAATAATGGTGTCTATCTCTTCCGTACCCATATGACCGCCTTCGCCCGCGTGCGGATTCAGCCCGC
AGACCAGAATGCGCGGTTCGGCAATACCAAATTTGGTCCGCAAATCGTGATGCAAAATAGCAATCACTTCGTGCAAAAGT
GCAGGGGTGATAGCGTCTGCGATATCGCGCAGCGGTAAATGCGTCGTTGCCAGCGCCACGCGAAGTTCTTCGGTCGCCAG
CATCATCACCACCTTTTTCGCCTGCGAACGCTCTTCGAAAAACTCGGTATGACCGGTAAAAGGAATGCCAGCGTCGTTAA
TAACGCCTTTATGCACCGGACCTGTGATCAGCGCGGCAAATTCGCCGTTCAGACAACCATCGCACGCTCGCGCCAGCGTT
TCCACCACATAATGCCCATTTTCAACCGCTAACTGCCCCGCAGTGACAGGTGCACGTAGCGCGACAGGAAGTAGCGTTAA
TGTGCCCGCAGTTTGCGGTTGTGCAGGGGAGTTGGGGGAATAAGGGCGGAGGGTGAGCGGCAAACCGAGCATCGCTGCCC
GGTTGGTAAGGAGAGTGGCATCGGCACAAACAACCAGTTCGACCGGCCACTCACGCTGTGCAAGCTGGACAACTAAGTCC
GGGCCAATCCCGGCGGGCTCGCCGGGAGTGATCACAACACGTTGGGTTTTAACCATTAGTTGCTCAGGATTTTAACGTAG
GCGCTGGCACGTTGTTCCTGCATCCAGCTTGCTGCTTCTTCCGAGAACTTACGGTTCATCAGCATGCGGTATGCACGATC
TTTCTGCGCAGCGTCGGTTTTATCGACATTACGGGTATCCAGCAGTTCGATTAAATGCCAGCCGAATGAAGAGTGAACCG
GTGCACTCATTTGACCTTTGTTCAGGCGAGTCAGGGCGTCACGGAAGGCCGGATCGAAAATATCTGGTGTAGCCCAGCCG

AGATCGCCGCCCTGGTTAGCAGAGCCTGGATCCTGAGAGAACTCTTTCGCTGCGGCAGCAAAAGTCGTTTTACCACTCTT
GATATCAGCAGCAATCTGTTCCAGTTTCACACGGGCCTGTTCGTCAGTCATGATCGGCGACGGTTTCAGCAGAATATGGC
GAGCATGAACTTCGGTCACCGAGATATTTTTGCTTTCGCCGCGCAGGTCGTTAACTTTCAGAATATGGAAGCCAACGCCG
GAACGAATCGGGCCAACAATGTCGCCTTTCTTCGCGGTGCTTAATGCCTGGGCGAAGATCCCGGGCAACTCCTGAATACG
GCCCCAGCCCATCTGGCCGCCGTTCAGCGCCTGCTGGTCGGCAGAATGAGCAATCGCCAGCTTACCGAAATCAGCGCCGT
TACGCGCCTGATCGACAATGGCGCGCGCCTGGCTTTCCGCTTCGTTCACCTGATCAGAGGTCGGGTTTTCCGGCAGCGGG
ATCAGGATGTGGCTCAGGTTCAGCTCAGTGCTGGCGTCGTTTTGGTTACCCACCTGCTGCGCCAGGGATTCGACTTCCTG
CGGCAGGATGGTGATGCGACGACGCACCTCGTTGTTACGCACTTCAGAGATAATCATCTCTTTGCGGATCTGGTTACGAT
AGGTGTTGTAGTTCAGTCCATCGTAAGCCAGACGGCTGCGCATCTGATCCAGCGTCATGTTGTTCTGTTTCGCGATGTTA
GCAATCGCCTGATCCAGCTGCTCATCGGAGATTTTCACTCCCATTTTCTGCCCCATCTGCAGGATGATTTGATCCATGAT
CAAACGTTCCATGATTTGGTGGCGCAGCGTCGCGTCATCAGGAAGTTGCTGCCTTGCCTGAGCAGCGTTCAGTTTTACCG
ACTGCATTAATCCATCAACGTCGCTTTCCAGCACGACGCCGTTATTGACGACGGCTGCGACTTTATCGACTACCTGGGGG
GCAGCGAAACTGGTATTCGCGATCATGGCGATACCGAGAAGCAGCGTTTTCCAGTTCTTCATACTTTTTCCATTTCAATT
AACCGCACTGCGGATTACGTGGTAAATCAACAAATCACAAAGTGTTTTGATACGGCAGAATGTTCGAACGCAGCATCTCT
TGCGTACCCAGACCGTAGTTGGAGCTCAGGCCGCGAAGTTCGATGTTAAAGCCGATTGCGTTGTCATATACCGCATGTTG
TTTATCGTTATCCCAACCGTTCAGCTTCCGCTCGTAACCGACGCGAATTGCATAGCAGCAGGAGCTGTATTGCACACCTA
ACATAGAGTCGGCTTGCTTGTTAGCATTGGTGTCGTAGTAGTAGGCCCCAACAATGGACCAACGATCGGCAATTGGCCAG
CTGGCGACAGCACCTACCTGCGAAATACCATTCTTATATTGCTCAGCAGTGGAATAGTACTTAGGCAGCGTAGCCTGAAT
ATATTCCGGGCTGGCGTAACGGTAATTCAGCTGTACCAGACGGTCTTCATCCCGACGGTATTCAATGCTGGAGTTACTGG
TCGCTACGTTATCCAGACGTGTATCGTACTGAATCCCGCCACGCAATCCCCAACGCTCGGAGATACGCCAGTAAGTATCG
CCTGCCCACACCAGTGAACCCGTTTTGTCGTCATTCTCCCATGTTATGTTGTCATCGCCAGTGCGAGACTCCGTGAAATA
GTAGATTTGACCAACGGAAATATTAAAACGTTCAACGGCAGCATCATCATATATGCGAGATGTGACACCGGTCGTCACCT
GGTTAGCGGAGGCAATACGGTCAAGACCGCCGTAAGTCCGGTCCCGGAACAGGCCAGAGTAGTCAGATTGCAGCAGAGAC
GAGTCGTAGTTATAGATGTCGCTCTGATCGCGATACGGCACGTACAAATACTGCGCGCGCGGTTCCAGCGTTTGGGTATA
ACCCGGAGCCAGCATTTCCATATCGCGTTCAAAGACCATTTTGCCGTCAACTTTGAATTGCGGCATTACGCGGTTAACGG
ATTCGTCCAGCTTGGTCGTGTTTCTGGAGTTATACCAGTCAAGATTGGTTTGCTGATAATGGGTTGCCAGCAACTTCGCT
TCGGTATTGATGCTGCCCCAGTTATTAGAGAGCGGCAAATTGATGGTCGGTTCCAGGTGAACACGGGTTGCTTCAGGCAT
GTCGTCTCTGGTGTTAACAAAGTGCACTGCCTGGCCGTAAATACGCGTATCAAACGGACCAACATCATTCTGGTAGTAAT
TAACGTCTAACTGCGGCTCTGCCGAGTAGCTACTGGTGTTCTGTTCGCTGAAAACCTGGAACTGCTTGGTTGAAACGGTG
GCATTGAAGTTTTGCACCGCATAGCCAACGCTGAATTTTTGCGTTGCGTAGCCGTCAGTACTGGAACCGTACTTGTTATC
GAAATCATTGAAGTAGCTAGGATCGCTGACCTTGGTGTAGTCGACGTTGAAACGCCACACCTGATCCATGACCCCGGAGT
GGTTCCAGTAGAATAACCAACGACGTGAACTGTCATCGTTCGGGTGTTCATCTTCATAGACTTTATCTGAAGGCAGATAG
TCCAGTTCCATCAAGCCAGCGCCCGCCTGGGAGAGGTAGCGGAATTCGTTCTCCCACATGATGTTGCCACGACGATGCAT
ATAATGCGGCGTGATGGTGGCATCCATATTTGGCGCGATGTTCCAGTAATATGGCAGGTAGAACTCAAAGTAGTTGGTGG
TGGTGTACTTGGCGTTCGGGATCAAGAAACCAGAGCGACGTTTGTCACCCACCGGCAACTGCAAATAGGGGCTATAAAAG
ATCGGTACCGGACCCACCTTAAAGCGGGCGTTCCAGATCTCCGCAACTTGTTCTTCGCGGTCATGAATAATTTCGCTACC
TACCACGCTCCAGGTGTCAGAACCCGGCAGACAGGAGGTAAAGCTACCGTTATCCAGAATGGTATAGCGGTTTTCGCCAC
GTTGTTTCATCAGGTCCGCTTTACCGCGACCCTGGCGACCCACCATCTGGTAATCACCTTCCCAGACGTTGGTATCTTTG
GTGTTCAGATTCGCCCAGCCTTTCGGCCCTTTGAGGATCACCTGGTTATCGTCGTAATGGACATTACCGAGCGCATCAAC
GGTACGTACCGGCTCCGGTTGTCCTGGTGCCTCTTTTTGATGGAGCTGCACTTCGTCGGCCTGCAGACGGCTGTTACCCT
GCATGATATCCACGCTGCCAGTAAACACGGCGTCATCCGGGTAGTCCCCTTTCGCGTGGTCAGCATTGATAGTCACGGGT
AAGTCATTGGTATCGCCCTGTACCAGAGGACGGTCATAGCTTGGCACGCCCAACATGCACTGTGAGGCGAGGTCGGCTGC
CAGTCCCTGTTGACTATAAAGGGCGGTGGCAATCATGGTGGCCAGGAGAGTGGGGATACGTTTTTTCATACGTTGATTTT
ATTGTTCCATCATCGGTAACGTTGCGCGTGACAAACGGTCAGAGACTAACGTACTCGTCATCTCTACGCTAGTGTTAATC
CTGTCCGAATAGCGTCAGTGGTGTTAGGCACGGCATTGAATGACAGGTATGATAATGCAAATTATAGGCGATGTCCCACA
ATTGACCGCAGCCGGAAAACGGTAAAAGCACCTTTATATTGTGGGAGATAGCCCTGATATCCGTGTGTCGATTTGGGGAA
TATATGCAGTATTGGGGAAAAATCATTGGCGTGGCCGTGGCCTTACTGATGGGCGGCGGCTTTTGGGGCGTAGTGTTAGG
CCTGTTAATTGGCCATATGTTTGATAAAGCCCGTAGCCGTAAAATGGCGTGGTTCGCCAACCAGCGTGAGCGTCAGGCGC
TGTTTTTTGCCACCACTTTTGAAGTGATGGGGCATTTAACCAAATCCAAAGGTCGCGTCACGGAGGCTGATATTCATATC
GCCAGCCAGTTGATGGACCGAATGAATCTTCATGGCGCTTCCCGTACTGCGGCGCAAAATGCGTTCCGGGTGGGAAAATC
AGACAATTACCCGCTGCGCGAAAAGATGCGCCAGTTTCGCAGTGTCTGCTTTGGTCGTTTTGACTTAATTCGTATGTTTC
TGGAGATCCAGATTCAGGCGGCGTTTGCTGATGGTTCACTGCACCCGAATGAACGGGCGGTGCTGTATGTCATTGCAGAA
GAATTAGGGATCTCCCGCGCTCAGTTTGACCAGTTTTTGCGCATGATGCAGGGCGGTGCACAGTTTGGCGGCGGTTATCA
GCAGCAAACTGGCGGTGGTAACTGGCAGCAAGCGCAGCGTGGCCCAACGCTGGAAGATGCCTGTAATGTGCTGGGCGTGA
AGCCGACGGATGATGCGACCACCATCAAACGTGCCTACCGTAAGCTGATGAGTGAACACCATCCCGATAAGCTGGTGGCG
AAAGGTTTGCCGCCTGAGATGATGGAGATGGCGAAGCAGAAAGCGCAGGAAATTCAGCAGGCATATGAGCTGATAAAGCA

GCAGAAAGGGTTTAAATGACCCTGTAAATGATGCTGAGTAACTGCCCACGATTAAAGGTGGCCGCCCTGGCGGTCACTTC
TTTGAGAAAAGGCGTTTACTCAGAATGGTGGACAGGCTCAATGCACGGTTTACGGGAGGGGTTCTGTAGGTTTTATCGCG
TTGACCCTGCTTAAGGTTGAGAGCTTTACGACGAGCGGAATTATATTTTTACGTCTTAAAAATAAAAAACACATACCTGA
ATGAGCGATTTTTGAAAGTATATTTATTCAGAACGCGCATCATGAGTTTTTAACTCAATGCGAGGCTATTACCATGAAAG
TATCAGTTCCAGGCATGCCGGTTACACTTTTAAATATGAGCAAGAACGATATTTATAAGATGGTGAGCGGGGACAAGATG
GACGTGAAGATGAATATCTTTCAACGCTTGTGGGAGACGTTACGCCATCTGTTCTGGTCAGATAAACAGACTGAGGCTTA
TAAACTTCTGTTCAATTTCGTGAATAACCAGACTGGCAACATCAACGCCTCAGAATACTTTACTGGGGCTATCAACGAGA
ATGAGAGAGAAAAGTTTATCAATAGCCTGGAATTATTCAATAAACTTAAAACATGCGCAAAAAATCCGGATGAGTTGGTC
GCAAAGGGCAATATGCGCTGGGTCGCCCAGACCTTCGGGGATATCGAGTTAAGTGTCACTTTTTTCATTGAAAAGAATAA
GATATGTACTCAGACGTTGCAGCTGCATAAGGGCCAAGGTAACTTGGGCGTTGATCTTAGAAAGGCTTACCTTCCCGGCG
TTGACATGAGGGATTGTTACCTTGGTAAAAAAACAATGAAAGGTAGCAATGATATCCTTTATGAGAGACCTGGGTGGAAT
GCTAACCTGGGCGTGCTACCCCGGACGGTGCTACCCCGGACGGTGCTAACCCGGACGGTGCTAACCTGGACGGTGCTACC
GTGAACGGTGCTACCTCCTTATATGATGAGGTAATTATTATTAATAAAATCCCCCCCAAAAAAATTGATACTAAAGGAGT
TGCTACTGAAGAAGTTGCTACTAAAAAAGTACTGCTGAACAAATTACTGACAACGCAATTATTGAATGAGCCAGAATAAG
CTAAGGTTGAAGGGGCTGGAACGCCCCTTCAACCTTAGCAGTAGCGTGGGATGATTTCACAATTAGAAAGACCTGCATGA
TGAGCTAGAGAAGAGGCTAGTGACGCAAGGCGTCGTGCAGGACACGGATCACCGAGATGGGCATCGCCAACCAGACTGCT
AATTAGCCCATGAATAACAATCAGAAAGGACCATAACAGACCCGTTAAAATGAAATATAAGAGACGGTCAACGGGTGAAG
AAAAAGTTCAAAAATTCGCTGTGGAGCAGGAAGGGAATTACCGAATGGAAAGCGTAGCCACACGCAACAACTGAAAGCAG
TTTGGCAGAAACAAAAAATCCCCGGACTCGGGGATTTATGTACAAGAGGCAGCCCTTAGGATGAGGGTATAAACGTACAG
GAAAGGTTAAAAATCCGCTGGCGCTTTAAACGTCATACTATTGCCATACGCCGGATGGGTAATCGTCAACATCTCTGCAT
GTAGCAACAAACGTGGTGCCATCGCTCTCGCTTCTGGTGATGCATAAAAACGATCGCCGAGAATCGGATGACCCAGCGCC
AGCATATGCACACGCAATTGATGCGAACGCCCGGTAATCGGTTTTAACACCACTCTTGCCGTGTTATCCGCCGCATACTC
CACCACTTCATATTCCGTCTGCGCAGGTTTACCCGTTTCGTAACAGACTTTCTGTTTCGGGCGGTTTGGCCAGTCGCAAA
TCAGCGGCAGATCCACCAGACCTTCTGCGGGGGATGGATGCCCCCAGACGCGGGCCACATACTGCTTTTTCGGCTCGCGC
TCGCGGAACTGGCGTTTTAACTCCCGCTCCGCGGCTTTGGTCAGCGCCACTACAATCACGCCGCTGGTAGCCATATCCAG
ACGATGCACCGATTCTGCCTGCGGATAATCACGCTGAATGCGCGTCATCACGCTGTCTTTGTGCTCTTCCAGACGACCCG
GCACTGACAACAAACCGCTCGGCTTGTTGACCACCATAATATGGTCATCCTGATACAGGATAACCAACCAGGGTTCCTGC
GGTGGATTGTAGTTTTCCATCCCCATTTTCGGCTCCGTTACTGATGCGTTACAACGATCAAACGCAGGGCATCCAGACGC
CAACCTGCCTGATCCAGGCTTTCCATTACCTGCTGACGGTTGCTCTCAATGGCGGTCAGTTCGTCGTCACGAATGTTCGG
GTTCACTGCACGCAGAGCTTCCAGACGAGACAGCTCGGCAGACAGTTTTTCGTCGGCTTCGTTACGCGCTGCATCAATCA
ATGCACGGGCAGATTTCTCGATCTGCGCTTCACCCAGTTGAAGGATAGCGTGAACATCCTGCTGCACGGCGTTAACCAGT
TTGCTGCCGGTGTGACGGTTAACCGCGTTAAGCTGGCGGTTAAAGGTTTCAAACTCTACCTGCGCCGCCAGGTTGTTGCC
GTTTTTATCCAGCAGCATACGTACCGGCGTCGGTGGCAGGAAGCGGTTGAGCTGCAACTGCTTCGGAGCCTGGGCTTCAA
CCACATAAATCAGTTCCACCAACAGCGTACCTACCGGCAACGCTTTGTTTTTTAACAGTGAAATCGTGCTGCTACCGGTA
TCGCCAGAAAGGATCAGATCCAGACCGTTGCGGATCAGCGGATGCTCCCAGGTAATAAACTGTGCATCTTCACGCGCCAG
CGCCACTTCACGATCAAAGGTGATGGTGATGCCATCTTCCGACAGGCCAGGGAAGTCCGGCACCAGCATATGATCGGACG
GCGTCAGCACGATCATGTTGTCGCCGCGATCGTCCTGATTGATACCGATAATATCGAACAGGTTCATGGCGAAGGCGATC
AGGTTGGTATCGTCATCCTGCTCTTCAATGCTTTCTGCCAGTGCCTGGGCTTTTTCGCCACCGTTGGAGTGGATTTCCAG
CAGGCGGTCACGACCCTGTTCCAGCTGTGCTTTCAGCGCTTCATGTTGCTCGCGGCAGTTTTTGATCAGATCGTCAAAGC
CTTCGGTTTGATCCGGTGAAGCCAGATAGTTAATCAGATCGTTGTATACGCTATCGTAAATAGTGCGTCCGGTCGGGCAG
GTGTGCTCAAATGCATCCAGACCTTCGTGATACCAGCGCACCAGCACCGACTGAGCGGTTTTCTCCAGATAAGGCACATG
GATCTGAATATCGTGCGCCTGGCCGATACGATCCAGACGACCAATACGCTGCTCCAGTAGATCCGGGTTGAATGGCAGGT
CAAACATCACCATGTGGCTGGCGAACTGGAAGTTACGTCCTTCAGAACCGATTTCTGAGCACAGCAGTACCTGTGCGCCG
GTGTCTTCTTCGGCAAACCAGGCGGCAGCGCGGTCACGTTCGATAATCGACATACCTTCGTGGAACACCGCAGCGCGAAT
ACCTTCACGTTCGCGCAGTACCTGCTCCAGTTGCAGCGCAGTGGCAGCTTTGGCGCAGATCACCAGCACTTTCTGAGAGC
GATGGCTGGTCAGGTAGCCCATCAGCCACTCAACGCGCGGATCGAAGTTCCACCAGGTGGCGTTATCACCTTCAAATTCC
TGATAAATACGCTCCGGGTAGAGCATATCGCGAGCACGATCTTCCGCACTTTTACGTGCGCCCATAATGCCGGAGACTTT
AATAGCCGTCTGATACTGCGTCGGTAGCGGCAGCTTAATGGTGTGCAGCTCGCGTTTCGGGAATCCTTTCACACCGTTAC
GCGTGTTACGGAACAGCACGCGGCTGGTGCCGTGGCGATCCATCAGCATCGAAACCAGCTCCTGACGGGCGCTCTGGGCA
TCTTCGCTGTCGCTGTTTGCTGCCTGCAACAGCGGCTCGATATCCTGCTCGCCGATCATCTCGCCGAGCATGTTCAGTTC
GTCATTGCTCAGTTTGTTACCTGCCAGCAGCATGGCAACGGCGTCCGCAACCGGACGATAATTTTTCTGCTCTTCAACGA
ACTGCGCAAAATCGTGGAAACGGTTCGGGTCCAGCAGACGCAGACGGGCGAAGTGGCTTTCCATCCCCAGCTGTTCCGGG
GTCGCGGTCAGCAGCAGAACGCCCGGCACGTGCTCTGCCAGTTGTTCAATGGCCTGATATTCACGGCTTGGCGCATCTTC
GCTCCACACCAGGTGATGCGCTTCATCGACCACCAGCAGGTCCCATTCGGCTTCACAGAGATGTTCCAGGCGCTGTTTGC
TACGACGGGCAAAATCCAGCGAGCAAATCACCAGCTGTTCGGTGTCAAACGGGTTGTAAGCATCGTGCTGAGCTTCGGCA
TAACGCTCATCATCAAATAGCGCAAAGCGCAGGTTGAAACGGCGCAGCATTTCTACCAGCCACTGATGCTGTAAGGTTTC

CGGGACGATAATTAGCACACGTTCAGCAGCGCCAGAGAGCAGTTGCTGATGCAGGATCATCCCGGCTTCAATGGTTTTCC
CTAAACCCACTTCGTCAGCCAGCAGGACGCGCGGCGCGTGGCGGCGACCAACATCATGAGCGATGTTGAGCTGATGCGGG
ATCAGGCTGGTACGCTGACCGCGCAGGCCGCTGTACGGCATACGGAACTGTTCGCTGGAATATTTACGCGCGCGATAACG
CAGCGCAAAGCGGTCCATACGGTCAATCTGCCCGGCAAACAGACGGTCCTGCGGTTTGCTGAACACCAGTTTGCTATCAA
GGAAAACTTCACGCAGGGCTACGCCGGACTCTTCAGTATCCAGGCGAGTACCGATATAGGTCAGCAAGCCATTTTCTTCT
TTTACTTCTTCGACTTGCATCTGCCAGCCGTCATGGCTGGTAATGGTATCACCAGGGTTGAACATCACGCGGGTCACGGG
GGAATCACTGCGTGCGTACAGACGGTTTTCACCAGTAGATGGGAAAAGTAAAGTGACAGTTCGCGCATCCACCGCGACAA
CGGTTCCAAGTCCCAATTCGCTTTCTGTATCGCTGATCCAGCGTTGACCAAGTGTAAAAGGCATATGTGTTCGGCTCTAT
ATCTTTAATTGCAGGCAATAACCACCCGCTACCGTGCTTATGAGGTAGTGGTGTTATTCAGGTCCAGGAATGGAAAGGGC
GCTATGGTACTGGATGGCAAAGCATTCGTCACGCATCAAAATGGTATCTGGCGAACTCTTTTTTTTGCTCAAAATAGCCC
AAGTTGCCCGGTCATAAGTGTAGCAAAATTATCCTCAATAAAAGGGAGTATTCCCTCCGCCACGGGTTGTAGCTGGCGGG
TCAGATAGTGTTCGTAATCCAGTGGTGAACGTTGGTAGTCCAGCGGCTCCGGGCCGTTGGTGGTCCATACGTACTTAATG
GTGCCGCGATTCTGATATTGCAAGGGGCGACCACGCTTTTGGTTTTCTTCATCGGCAAGGCGAGCGGCGCGTACATGAGG
CGGCACATTACGCTGATACTCGCTCAGCGGACGGCGAAGGCGTTTACGGTAAACCAGTCGCGCATCCAGTTCACCCGCCA
TCAGTTTGTCGATGGTTTCGCGTACATATTCCTGATATGGCTCGTTGCGGAAGATGCGCAGGTATAGCTCCTGCTGAAAC
TGCTGGGCCAGCGGCGTCCAGTCGGTGCGCACGGTTTCCAGCCCTTTAAACACCATCCGCTGCTTGTCGCCCTCCTGAAT
CAGTCCGGCATAACGCTTTTTACTGCCGGTATCGGCTCCGCGAATGGTTGGCATCAGAAAACGGCAGAAATGGGTTTCAT
ACTCCAGTTCTAATGCGCTGGTCAGCCGTTGTTTTTGCAGCGTTTCCGCCCACCAGGCGTTAACGTGCTGCACCAGTGCA
CGACCGATTTTCGCCGCTTCTTCTTCCGAATGTGCGCCTTTCAGCCAGACAAACGTTGAGTCGGTATCGCCGTAGATAAC
GTCGTAGCCCTGTGCTTCAATCAACGCTTTGGTTTGCCGCATGATCTGATGACCACGCATGGTGATCGACGATGCCAGCC
GCGGATCGAAGAAGCGGCAGGCGGTGGTGCCGAGCACGCCATAAAAGGCATTCATGATGATTTTCAGCGCCTGCGACAGC
GGTTTGTTACCCTGGCGTTTGGCTTCATCGCGCCCGTGCCAGATGTTAGTCACAATCTCCGGCAGGCAATGTTTTTCTCG
CGAGAACCAGGCATCGAGAAAACCTTCGGTACTGTGCTCTGGATCAGGCTGCGCCATGCCTTCCACCAGCCCGACGGGAT
CAATCAGAAAGGTGCGGATGATCGACGGGTACAGGCTTTTATAGTCCAGCACCAGCACTGAATCATAAAGCCCTGGCCGT
GAATCCATCACGTAGCCGCCAGGGCTGGCGTGCGGCGGCACTTCGCCGAGATTAGGCGCGACATAACCAGCGCGATGCAT
TCGCGGAAAATAGAGATGACCAAATGCCGCCACCGAACCGCCGTGTCGGTCCACCGGCAGGCCGTTCACCGTTGCCCGTT
CGAGTAAAAATGGCATGATTTCAGTTTTGTGGAAGATCTGCGTCACCAGCTCGCAATCTTTCAGGTTATAAGTTGCCAGC
GCAGGTTTATCTTCGGCGAAACGGCGGTCAATTTCGTCCATTCGATCCCACGGGTTATCGATAGATTTTCCTTCGCCTAA
TAGCTCCTGAGCGACAGTTTCCAGCGAGAATGAAGAGAAATTCCAGAACGCGGATTTCAGCGCCTCGATACCGTCGATAA
TTAGCCGACCTTTAGCCTGGGCAAAAAAGACGCCGTTTTTAAAGCCGTGCTCGCGCCACTCCAGCTCGCTATTATCGCGC
CCAAGACGCAGCGGAAGACGGTAACGCTCGGCATGTTTTTGCAGCATTCGCAGATCGAACTGCACCACGTTCCAACCGAT
GATCACATCAGGATCGTAGTTGGCAAACCAGGCGTTGAGTTTTTCCAGCAACTGCGGGCGGCTGGCGACGTATTCCAGTT
CGAAATCAAGCGAGGAGGCGTCGCCATTCTCCGGCCCCAGCATATAAACGATGCGCTGCCCGCAGCCTTCCAGGCCGATG
CAGTACAGCTCACCGTGGCGGGTGGTTTCAATATCTATAGAAACCCACTTGAGCGGCGGACGATAGTCGGGATGCGGTTT
CAGACGGGCATTAACGATAGTGCCATTGTGCATATCACCCTCGACCCACACCGGTGAGGTGATAAACCGCTCCATCAGAT
AGCGTTCTGGCGGACGCACATCGGCCTCGTAGACGGTAACGCCACCTTCACGCAGGCGCTTTTCGTAATTCATCAATTGG
CGATGGGCGCGACAGTAAAGGCCATACACCGGCTGGCGGTGAAAATCCTTTAACGCCAGCGGTGTCAGGCGAAAGCCTTG
TTCACCCTGCAAAATATGCTGAGCGCGGGGAACCTGATCGGCGGGAATAAACGCCACGGACTCTTGCGGTGCAAGCGTAA
CCTGCAACGGCCCGTTGTCCGTCGCCAGCCAGAAGGAGACTTCTGTCCCTTGCGGGGTGTCCCGCCAGTGTCGGGTTAAG
ATAAAACCTGCCTGCGCCACGCTGAAAATCCATCAAAAAACCAGGCTTGAGTATAGCCTGGTTTCGTTTGATTGGCTGTG
GTTTTATACAGTCATTACTGCCCGTAATATGCCTTCGCGCCATGCTTACGCAGATAGTGTTTATCCAGCAGCGTTTGCTG
CATATCCGGTAACTGCGGCGCTAACTGACGGCAGAATATCCCCATATAAGCGACCTCTTCCAGCACGATGGCGTTATGCA
CCGCATCTTCGGCATTTTTGCCCCATGCAAACGGGCCGTGGGAATGGACCAGAACGCCGGGCATTTGCGCTGCATCGATA
CCCTGTTTTTCAAAGGTTTCTACGATGACGTTACCGGTTTCCCACTCATATTCGCCGTTGATTTCTGCGTCGGTCATTTT
GCGGGTGCAGGGAATGGTGCCGTAGAAATAGTCGGCGTGGGTGGTGCCGGTTGCTGGAATCGACTGACCCGCCTGCGCCC
AGATGGTGGCGTGGCGCGAGTGCGTATGCACAATGCCGCCAATGGAGGGGAATGCCTGATAGAGCAGCCGGTGAGTTGGC
GTGTCGGAGGAGGGCTTTTTCGTACCTTCAACCACTTCACCGGTTTCGATGCTAACCACGACCATATCGTCAGCGGTCAT
GACGCTGTAATCGACGCCGGAAGGTTTGATCACAAAGACGCCGCGCTCGCGATCAACGGCGCTGACGTTGCCCCATGTGA
GCGTGACCAGGTTGTGTTTTGGCAGCGCCAGGTTGGCTTCTAATACCTGGCGTTTGAGATCTTCTAACATGTTGACTCCT
TCGTGCCGGATGCGCTTTGCTTATCCGGCCTACAAAATCGCAGCGTGTAGGCCTGATAAGACGCGCCAGCGTCGCATCAG
GCGTTGAATGCCGGATGCGCTTTGCTTATCCGGCCTACAAAATCGCAGCGCGTAGGCCTGATAAGACGCGCCAGCGTCGC
ATCAGGCGTTGAATGCCGGATGCGCTTTGCTTATCCGGCCTACAAAATCGCAGCGTGTAGGCCAGATAAGACGCGTCAGC
GTCGCATCAGGCGTTACATACCGGATGCGGCTACTTAGCGACGAAACCCGTAATACACTTCGTTCCAGCGCAGCGCGTCT
TTAAACGCTGGCAGGCGTGTGTCGTTATCAATCACCGTGATTTCAATGTCGTGCATCTCGGCGAATTGGCGCATATCGTT
GAGGTTCAGTGCATGGCTGAAGACGGTATGGTGCGCGCCACCAGCGAGGATCCACGCTTCGGAAGCAGTTGGCAGATCCG
GTTGCGCTTTCCACAGCGCATTCGCCACCGGCAGTTTCGGCAGGGAGTGCGGTGTTTTCACCGTGTCGATGCAGTTAACC

AGTAGACGGTAACGATCGCCGAGATCAATCAAGCTGGCGACAATCGCTGGGCCGGTTTGGGTATTGAAGATCAGGCGGGC
AGGATCGTCCTTACCACCAATACCGAGATGCTGAACGTCGAGGATCGGTTTCTCTTCTGCGGCGATCGACGGGCAGACTT
CCAGCATATGGGAGCCGAGCACCAGGTCATTACCTTTCTCGAAGTGATAGGTGTAGTCCTCCATAAAGGAGGTGCCGCCC
TGCAGACCGGTTGACATCACCTTCATGATGCGAAGCAGGGCGGCAGTTTTCCAGTCGCCTTCGCCCGCAAAGCCGTAACC
CTGCTGCATCAGACGCTGTACGGCCAGACCAGGAAGCTGTTTCAGACCGTGCAAATCTTCAAAGGTGGTGGTGAACGCGT
GGAAGCCACCTTGTTCCAGGAAACGCTTCATCCCCAGCTCAATACGCGCCGCTTCCAGCACGTTCTGTCGTTTTTTGCCG
TGGATTTGTGTGGCAGGCGTCATGGTGTAGCAGCTTTCGTACTCATCGACCAGCGCGTTAACATCGCCGTCGCTGATGGA
GTTCACCACCTGCACCAGATCGCCAACCGCCCAGGTATTGACGGAGAAACCGAACTTGATCTGTGCGGCAACTTTATCGC
CATCGGTGACCGCCACTTCACGCATGTTATCGCCAAATCGGCAGACTTTCAGATGACGGGTATCCTGTTTAGAGACCGCC
TGACGCATCCAGGAGCCGATACGCTCATGGGCTTGTTTATCCTGCCAGTGACCGGTAACCACGGCATGTTGCTGACGCAT
ACGCGCGCCAATGAAGCCGAACTCGCGACCGCCATGTGCAGTCTGGTTCAGGTTCATAAAGTCCATATCGATACTGTCCC
ACGGCAGCGCCGCGTTGAACTGGGTGTGGAATTGCAGCAACGGTTTGTTGAGCATGGTCAGGCCGTTGATCCACATTTTG
GCCGGGGAGAAGGTGTGCAGCCACACCACCAGACCAGCGCAACGATCGTCGTAATTCGCGTCGCGGCAAATAGCGGTGAT
TTCATCCGGCGTGGTGCCCAGCGGTTTCAACACCAGTTTGCAGGGCAGTTTCGCTTCCGTATTCAGCGCATTAACGACGT
GCTCGGCATGTTGGGTGACCTGACGCAGGGTTTCCGGGCCATACAGATGCTGGCTGCCAATGACAAACCACACTTCATAA
TTATCAAAAATCGTCATTATCGTGTCCTTATAGAGTCGCAACGGCCTGGGCAGCCTGTGCCGGGGCGGAAGTTGGAAGAT
AGTGTTGTTCGGCGCTCATCGCCCATTGCTGATAGCGGCGATAAAGCTGTTCAAAGCGTTGTGCCTGCTCGCTGCACGGT
TGCAGGGTTTTCTCTACCGCACTGGCCATTTTTTGCTGAGCTGATGGGATGTCTGCGTGCACTTTCGCGGCGACGGCAGC
AAAAATCGCCGCACCGAGCGCACAGCACTGGTCAGAGGCAACAATTTGCAGCGGGCGATTCAGCACGTCGCAGCAGGCCT
GCATAATGACCTGGTTTTTCCGCGCGATGCCGCCCAGTGCCATCACGTTATTAACGGCGATCCCCTGATCGGTAAAGCAC
TCCATGATTGCGCGTGCGCCAAAGGCGGTGGCAGCAATCAAACCGCCGAACAGCAGCGGAGCGTCGGTAGCGAGGTTAAG
ATCGGTAATCACCCCTTTCAGGCGTTGGTTAGCGTTCGGTGTGCGGCGGCCGTTAAACCAGTCGAGCACCACCGGCAGGT
GATCCAGAGACGGATTTTTGGCCCATGCTTCGGTCAGCGCCGGAAGCAGTTGTTTCTGGCTGGCGTTGATTTGCGTTTTC
AGTTCCGGATGCTGGGCGGCAAGCTGTTCCAGCGGCCAGCCGAGTACGCGACCAAACCAGGCGTAGATATCACCAAACGC
CGATTGGCCTGCTTCCAGACCGATAAATCCAGGCACCACGCTGCCATCAACCTGACCGCAAATACCTTTAACTGCCCGCT
CGCCAACGCTCTGTTTGTCGGCAATCAGAATGTCGCAGGTGGAAGTACCGATAACTTTTACCAGTGCGTTAGGCTGTGCG
CCTGCGCCAACTGCGCCCATATGGCAGTCAAACGCGCCGCCGGAAATCACCACGCTTTCAGGCAGGCCGAGACGCTGCGC
CCATTCCGGGCATAAGGTGCCCACCGGAATATCGGCAGTCCAAGTGTCAGTGAACAGCGGGGAAGGCAAATGGCGATTGA
GGATCGGGTCCAGCTCATCAAAGAAACTGGCTGGCGGCAGGCCGCCCCAGCTTTCGTGCCACAGAGATTTATGCCCGGCG
CTGCAACGTCCGCGACGAATATCCTGCGGGCGGGTGGTACCGGAAAGCAGAGCTGGCACCCAGTCGCACAGCTCAATCCA
CGATGCGGCAGATTGCGCCACGGCGCTGTCCTGGCGAGTCACATGCAGGATTTTTGCCCAGAACCATTCGCTGGAATAAA
TACCACCAATGTAGCGGGAGTAGTCAACGTTGCCCGGCGCGTGGCACAAACGGGTAATCTCTTCCGCTTCTTCAACCGCA
GTGTGGTCTTTCCACAATACGAACATCGCGTTCGGGTTTTCGGCAAACTCCGGGCGCAGCGCCAGCACGTTTCCGTCGGC
ATCAATCGGTGCGGGCGTCGAGCCGGTACTGTCAACGCCAATCCCGACCACAGCTGCGCGCTGTTCGACGCTAAGCTCTG
CAAGCACGGTTTTCAGTGCCGCTTCCATTGACTCAATGTAGTCACGCGGATGATGACGGAACTGGTTATTCGGGGCATCA
CAAAATTGCCCTTTCTGCCAACGGGGATACCACTCTACGCTGGTGGCGATCTCTTCACCGGTAGCGCAGTCCACCGCCAA
AGCTCGCACAGAATCACTGCCAAAATCGAGGCCAATTGCAATCGCCATCGTTTCACTCCATCCAAAAAAACGGGTATGGA
GAAACAGTAGAGAGTTGCGATAAAAAGCGTCAGGTAGGATCCGCTAATCTTATGGATAAAAATGCTATGGCATAGCAAAG
TGTGACGCCGTGCAAATAATCAATGTGGACTTTTCTGCCGTGATTATAGACACTTTTGTTACGCGTTTTTGTCATGGCTT
TGGTCCCGCTTTGTTACAGAATGCTTTTAATAAGCGGGGTTACCGGTTGGGTTAGCGAGAAGAGCCAGTAAAAGACGCAG
TGACGGCAATGTCTGATGCAATATGGACAATTGGTTTCTTCTCTGAATGGTGGGAGTATGAAAAGTATGGCTGAAGCGCA
AAATGATCCCCTGCTGCCGGGATACTCGTTTAACGCCCATCTGGTGGCGGGTTTAACGCCGATTGAGGCCAACGGTTATC
TCGATTTTTTTATCGACCGACCGCTGGGAATGAAAGGTTATATTCTCAATCTCACCATTCGCGGTCAGGGGGTGGTGAAA
AATCAGGGACGAGAATTTGTCTGCCGACCGGGTGATATTTTGCTGTTCCCGCCAGGAGAGATTCATCACTACGGTCGTCA
TCCGGAGGCTCGCGAATGGTATCACCAGTGGGTTTACTTTCGTCCGCGCGCCTACTGGCATGAATGGCTTAACTGGCCGT
CAATATTTGCCAATACGGGTTTCTTTCGCCCGGATGAAGCGCACCAGCCGCATTTCAGCGACCTGTTTGGGCAAATCATT
AACGCCGGGCAAGGGGAAGGGCGCTATTCGGAGCTGCTGGCGATAAATCTGCTTGAGCAATTGTTACTGCGGCGCATGGA
AGCGATTAACGAGTCGCTCCATCCACCGATGGATAATCGGGTACGCGAGGCTTGTCAGTACATCAGCGATCACCTGGCAG
ACAGCAATTTTGATATCGCCAGCGTCGCACAGCATGTTTGCTTGTCGCCGTCGCGTCTGTCACATCTTTTCCGCCAGCAG
TTAGGGATTAGCGTCTTAAGCTGGCGCGAGGACCAACGCATTAGTCAGGCGAAGCTGCTTTTGAGCACTACCCGGATGCC
TATCGCCACCGTCGGTCGCAATGTTGGTTTTGACGATCAACTCTATTTCTCGCGAGTATTTAAAAAATGCACCGGGGCCA
GCCCGAGCGAGTTTCGTGCCGGTTGTGAAGAAAAAGTGAATGATGTAGCCGTCAAGTTGTCATAATTGGTAACGAATCAG
ACAATTGACGGCTTGACGGAGTAGCATAGGGTTTGCAGAATCCCTGCTTCGTCCATTTGACAGGCACATTATGCAAGCAT
TGCTGGAACACTTTATTACCCAATCCACCGTGTATTCATTGATGGCGGTGGTGTTGGTGGCCTTTCTGGAGTCGCTGGCG
CTGGTCGGTTTGATTCTACCCGGTACGGTGCTGATGGCGGGGCTGGGAGCGCTGATTGGCAGCGGCGAGTTAAGTTTCTG
GCACGCCTGGCTGGCAGGGATTATTGGCTGCTTGATGGGCGACTGGATTTCTTTCTGGCTGGGTTGGCGTTTTAAAAAGC

CGTTGCATCGCTGGTCATTTCTGAAGAAAAACAAAGCACTACTTGATAAAACTGAACATGCGTTGCATCAACACAGCATG
TTCACCATTCTGGTCGGTCGTTTTGTTGGCCCGACGCGTCCGCTGGTGCCAATGGTGGCGGGAATGCTGGATCTGCCGGT
GGCTAAATTTATTACGCCGAATATTATCGGCTGCCTGCTGTGGCCGCCGTTTTACTTCCTGCCAGGGATTCTGGCGGGCG
CGGCGATCGATATTCCTGCCGGAATGCAGAGCGGTGAGTTTAAATGGTTGCTGCTGGCAACAGCGGTGTTTTTGTGGGTT
GGTGGCTGGCTGTGCTGGCGGTTATGGCGCAGCGGTAAAGCGACTGACCGTTTGAGTCATTATTTGTCCCGCGGTCGTTT
GTTGTGGCTGACGCCGTTGATTTCTGCCATCGGCGTGGTGGCGCTGGTGGTGTTAATTCGCCACCCGTTGATGCCGGTGT
ATATCGATATTTTGCGTAAAGTGGTTGGGGTTTAGGAGATAGTCTTGTGCGGGTTGCCTGATGCGACGCTTGCCGCGTCT
TATCAGGCCTACAAAACGCACTACCCGTAGGTCGGATAAGGCGTTCACGCCGCATCCGACAGTGCATACTAACCCGTAAT
CCCCAATAGTGCCGAAGCACTCGCCTTACCGCTCAACAACTCATTGGTCATACCCTGCCAGGCGATGCGCCCGTCGGCGA
CTACTACCGAGCGCGTGGCGATCCGCGCCGCATCTTCCACGCTGTGCGACACCATCAATAGCGTCATTTTTTGCTGCTGG
CAGCTCGTGCTCACCAGCGTCAACATCTCCTGACGTAACGCCGGATCGAGCGCAGAGAACGGTTCATCGAGCAATAAAAT
CGGCTGTTCGCGTACCAGACAACGCGCTAACGCCACTCGCTGTCGCTGACCGCCGGAAAGCTCGCCCGGTAACCGCGCCA
TTAAATTATCAATCCCCATCTGGCGGGCGATAGCGTGCATTTTCCCCTGCTGTACCGCGTTCAGTTTCAATCCCGGATTT
AGCCCCAGCCCGATGTTCTGTGCGACCGTCAGGTGGCTGAACAGGTTGTTCTCCTGAAACAGCATCGACACCGGACGGCG
TGACGGCGGCATAGTTGTGTGATCTACGCCATCGATAGTCAGCGAACCGCTGGCTGGCGTCAGAAAACCGGCGATCAAAT
TCAGCAGGGTACTTTTACCCGCGCCGCTTGGCCCGAGGATCGCCACCTGCTCGCCGCGTTCCACCGTTAAGCTAAAACGC
ATCGGCAAATGGTGGTAAAGCCAGGTGATATCAGTCAGTTTTAACATTTCGCCCCGGTAGTTTTTCAATCACGGTAAACA
GCAGAAAACAGAGCAGCAGCAGAATTAACGCGGTGACCGCACCGTCCTGGCTGCGATAGGAGCCAATTTGCTGGTAGAGA
TAAAACGGCAGGGTGCGGAAATCATCGTTACCGAACAACGCCACCACGCCAAAATCACCAATCGACAGCACGCATGCAAA
GGCCAGCGCCTGCGCCAGTGGACGTTTCAGGGCGCGCAGCTCCACCACTTTTAAGCGTGACCAGCCTTCAATCCCCAGCG
ACTGACATAACATGCTGTAGCGGGCGGTGATATCGCGCATCGGGTTTTCCAGCACTTTCAGCGCATAAGGGATCGCCATT
AACGCATTGGTGAAAATCACAATGCCGTCAGCAGATTGTGGCAGGCCGATAGTGTTGTTGAGCAGTAAAAAGAAGCCGGT
AGCCAGCACAATCCCCGGCATGGCGAGGATCAACATGCCGCTCATCTCCAGCACCTGACCCGCCAGCATTTTCTGCCGCG
CCCGCAGTTCGCGACTGCTCCATAGCAGCATCATGGTCAGCACTACGCACAATACACCTGCCGCCAGCGCAATACGCAAC
GAGGTCCACAGCGCCTGCCACAGCACCGGTTGTGCCAGCACTTCCGGCAACTGGCGATTTACCCCATCGACGATCACCGC
CAGTAACGGTGGCAGCAACAGCAGCAGCGCCAGCACAATTAACACCGTGTCGCAAATGCGGCTATGCAGACGATCGTCCG
GGTCGCGCCAGCCTTGCAGCAGCGTGGTGCCGGGCGCAATGGCCTTACTCAATCGCTGACTCAACAGCACCAGCCCGAGG
CAGCACACCATCTGGAGCAGCGCCAGCATTGCCGCGCGGGCAGGATCGTAGTCGTAACTCAGCGCCTGATAGATTGCCAG
CTCGATAGTGGTCGCCTGCGGACCGCCCCCCAGCGATAGCACGGTGGCGAAGCTGGCGAAACAGAGCATAAAGATAAGCG
CAGCAACCGGCGGGATTTGTCGCCGTAACCACGGCCATTCGACGAAGCGGAAAAAAATGCCAGCTACGCATCCCAAGCTGG
GCGGCAAGTTGACGCTGTTCGCCGGGGATGTTTTCCAGTGCCTGGAGTAATAAGCGGCTCGCCATCGGCAGATTAAAAAA
CACATGGGCCAGCAAAATACCTTGCAGGCCGTAGGGCGAAAAGGTCCACTCCAGACCGAGCGATTGGCAGAGTGTTGCCA
GCCAGCCCTGGCGACCATAGACGCTAAGAATGCCGAAAACAGCGACCAACACCGGGAGGATCAAGGTCATTGCACACAGA
CGCAACAGCGCCAGCCGACCCGGAAAGCGCCTGCGATAGAGCGCGCGGGCGAGGAATATCGCGGGTATGACAGAGAGCAG
TGCCGAGAGAAACGCCTGCCAGAAGGAGAAGCGCACCACATGCCACAGATAGCTGTCCTGCCAGACTGCCACCCAGTCAT
CCTGCGGCGCGTTCCACCACAGGGCGAGAAACGCCGCCAGCGCAACCGCTACCACCAGCGTGGTGGCGCTTACACCTGGA
ATTAACCAGCCGGGAATTAACGGCTGACGGCGCGTTGCCATTCGCTAATCCATGCCTGACGTTGTGCCGCCACTTCGGCT
GGCGTGAACTCCAACGTGGTTGCGGGTTTGGTCAATTTTTCAAAACCGGCAGGCAGCGTGACGTTTGCCACCGGATACAT
CCAGTTGCCGGTTGGGATCGCATTCTGGAAAGCCGGAGAAACCATAAACTGGAGGAATTTTTGCGCCAGCTCCGGCTGCT
TGCTGGCAGCGGTGCGGGCGGCGACTTCCACTTGCAGATAGTGACCTTCGCTGAAGTTCGCGGCGGCGTAGTTATCTTTC
TTCTCTTCGAGAATGTGATAAGCCGGAGAGGTGGTGTAACTCAGTACCAGATCGCTTTCACCTTTTAAAAACAGGCCGTA
GGCTTCGCTCCAGCCTTTGGTGACCGTGACCGTTTTCTTCGCCAGTTTCTGCCAGGCTTGTGGGGCGTCATCGCCATAGA
CTTTTTGCATCCATAGCAACAGACCCAGCCCCGGTGTACTGGTGCGCGGATCCTGATAAATCACCCGCCAGTTTTGATCG
CTCTCAACCAGTTCTTTCAGGCTTTGTGGCGGGTTTTTCAGTTTGTTCTTGTCATAAACGAAGGCGAAGTAGCCATAATC
AAACGGTACGAAAGTGTCATTATTCCAGCCGCCGGGAACGTTAACGGCATCCGCTGCCACACCGCTTTTGGCAAACAGTC
CGGTTTTACTGGCGGCGTCTAACAGGTTGTTATCCAGCCCCAGCACCACATCGGCTTTACTGTTTTTGCCTTCCATCCGT
AGACGGTTGAGAAGCGAAACGCCATCTTCCAGCGCCACCAGTTTCAGTTCGCAATTACAGTCGGCTTCAAAGGCTTTTTT
AACCACCGGACCAGGCCCCCAGTCGGCGGCGAAGGAATCGTAGGTATAAACAGTCAGAACGGGTTTAGCGAAAACGGGCG
CTGTGCACAGCAACAGCAGGGGCAGACATTTTTTTAACACTTTGCACCTCAAAAAAGAGTGGCAAAGGACTTGAGAAGGA
GCCTCAAATCCCTTCGCCGGCGTTATCCGGATCAGGTTCGACGGGTATTTTCTCAGCGCACGCGTACGCGTGGCACCCCG
TTGAGAACGGCGTTAGTGTAGTGATTTTGTTATCAACCAGCAATCATGGATCCGGTGGCGCAAACCACGCTGATTTAAAA
TCGAACCAGCCGAGGGTATTCATGCGCAGGCCGCGCATACTGCGTTGCCCCTGAATGATCAGCCAGTGGTGCAATAATGG
CACCATCGCTTTGCTGGCGACCAGTTGCTGGCACCAGTTCGCCAGATTCATCTCGCCATTGCGCCAGCGAGCAGCGTCGG
CTTGCCAGTCAATGGGAATGCAATGTTGTAGCAGTGGCACTTCGCATAAATGTGCGAAAACAGAGAAGTCCAGCGGCAGG
GTAAAGTTGGCGCTGTTTAGCCAGATATCACTTTCGATCTCTCCTGTATGCCACTGATCGTAGTCGATCTCTTTGATTTT
CAGCGTGACCTGGTGACTTGCCAGAATCTGCTGCATGATCCCGGCAATCACCCGATGCTCACTGTGATCCTGATAAAAGG

TTAGGGTGAGGCTTTCCAGGCCAGCCGGTTTTTCGCTCTTTATGGTGCGGGCATGGTGCCAACGGGGGAGCAGTCCATAA
GCCGGGAACCACAGTTGCTGGTACTGTTCCTCAGCGAAATAGACCAGATTAGTTGGAGAAAGCACATAGCTTACCCAGTC
CCTGACTTGCTGATTCGCCCCGCGATGGGTGCGGCTGTCGAACAGTAAATAGTAGCAACCTTCCTCCAGGCGGCTTTCAA
TCTCTTTTTCCTCGCCCTGTGGACCTTTTAGCATCAGCCCTCCGGCTGGCTCGTCGGCAATTTCCGGCAGAACCCAGACG
TTAACTTCGTCGATTAATGCCCGGTAACCGAAGAAGTCATCGAATGCCTGAATTTTCAGTTGATTGGTGCTGTTGCGAAT
CACCGCATACGGACCGGTGCCGATGGGATGGCTGGCAAAGTTACTGAGGGTTTCCCATTCGCGCGGCAGGATCATCGCCG
GAACTTGCCCCAGCAGTAACGGTAACCAGCGGTCCGGTTGCGTGAGATGGATATCCAGCGTCCAGGGCGTCGGCGACACA
ATGTCAGCAATATGCGAATAGAGCGGCAGCGTATTGATTCGTTTTAAAGAGGCGATCACATCGTCCATTTCCAGTTCACG
ACCATGGTGAAAATGGACTCCTGGACGCAAAAAGAAACGCCAGTGAAGCGGTGAAATTTGCTGCCAGTGGTGGGCGATGT
CTGCTTCCAGTTCCCCATTTTCCTCATTTATGCGCGTTAGCGAACTGAAGATTTGCCGGGCGATATGGGTTTCGGAACGG
CGCAATGCGCTGCCAGGTAGCAGATTACGCAACGGACGATAGTAGAGCACGCGCAGGATGTGCCGCCCCTGGCGGAAGCT
GCGGCCCAGATGAGAAACCAGCATTTGCCGCACAGTCGCTTTGTCGCCAACCAACTGCACCAGTTGATCGATACGATCCT
GCTCCAGCAGGTCTTCCGCCCGCTGTTGCTGAAGCGCCAGCCCGGTATAGAGGAATGTCAGACGCGAGCGTTTACCGCGC
CCGACTTCCGCTTCCCACGTCAGCCAGCCGCGATCCTGCATGGTGTTGAGCAGGGTGCGCATATGACGACGCGAGCAGCT
CAATAACGCTGCCAGTTCGTTGAGCGTTGTGTCCTGCGATTTACCCTCGCAGCATTGCCACAGGCGGATGAACTGTTGTT
GCAGACGAGCAGATGGCATAAAAGGGGAACTCCTGTGCAAAAGACAGCAATTTTATTTTCCCTATATTAAGTCAATAATT
CCTAACGATGAAGCAAGGGGGTGCCCCATGCGTCAGTTTTATCAGCACTATTTTACCGCGACAGCGAAGTTGTGCTGGTT
GCGTTGGTTAAGCGTCCCACAACGATTAACCATGCTTGAAGGACTGATGCAGTGGGATGACCGCAATTCTGAAAGTTGAC
TTGCCTGCATCATGTGTGACTGAGTATTGGTGTAAAATCACCCGCCAGCAGATTATACCTGCTGGTTTTTTTTATTCTCG
CCGCGCTAAAAAGGGAACGTATGATCTGGATAATGACGATGGCTCGCCGTATGAACGGTGTTTACGCGGCATTTATGCTG
GTCGCTTTTATGATGGGGGTGGCCGGGGCGCTACAGGCTCCTACATTGAGCTTATTTCTGAGTCGTGAGGTTGGCGCGCA
ACCTTTCTGGATCGGCCTCTTTTATACGGTGAATGCTATTGCTGGGATCGGCGTAAGCCTCTGGTTGGCAAAACGTTCTG
ACAGTCAGGGCGATCGGCGAAAACTGATTATATTTTGCTGTTTGATGGCTATCGGCAATGCGCTATTGTTTGCATTTAAT
CGTCATTATCTGACGCTTATCACCTGTGGTGTGCTTCTGGCATCTCTGGCCAATACGGCAATGCCACAGTTATTTGCTCT
GGCGCGGGAATATGCGGATAACTCGGCGCGAGAAGTGGTGATGTTTAGCTCGGTGATGCGTGCGCAGCTTTCTCTGGCAT
GGGTTATCGGTCCACCGTTGGCCTTTATGCTGGCGTTGAATTACGGCTTTACGGTGATGTTTTCGATTGCCGCCGGGATA
TTCACACTCAGTCTGGTATTGATTGCATTTATGCTTCCGTCTGTGGCGCGGGTAGAACTGCCGTCGGAAAATGCTTTATC
AATGCAAGGTGGCTGGCAGGATAGTAACGTACGGATGTTATTTGTCGCCTCGACGTTAATGTGGACCTGCAACACCATGT
ACATTATTGATATGCCGTTGTGGATCAGTAGCGAGTTAGGATTGCCAGACAAACTGGCGGGTTTCCTGATGGGGACGGCA
GCTGGACTGGAAATACCAGCAATGATTCTGGCTGGCTACTATGTCAAACGTTATGGTAAGCGGCGAATGATGGTCATAGC
AGTGGCGGCAGGAGTACTGTTTTACACCGGATTGATTTTCTTTAATAGCCGTATGGCGTTGATGACGCTGCAACTTTTTA
ACGCTGTATTTATCGGCATTGTTGCGGGTATTGGGATGCTATGGTTTCAGGATTTAATGCCTGGAAGAGCGGGGGCAGCT
ACCACCTTATTTACTAACAGTATTTCTACCGGGGTAATTCTGGCTGGCGTTATTCAGGGAGCAATTGCACAAAGTTGGGG
GCACTTTGCTGTCTACTGGGTAATTGCGGTTATTTCTGTTGTCGCATTATTTTTAACCGCAAAGGTTAAAGACGTTTGAT
GACGTGGACGATAGCGGAAAGCCCGGTCATTTGACCGGGCAAGGGGATTAATTCATAAACGCAGGTTGTTTTGCTTCATA
AGCGGCAATGGCGTCGTCGTGCTGCAAGGTAAGCCCAATACTGTCCAGACCGTTCATCATGCAGTGGCGGCGGAAGGCAT
CGATGGTAAAGCGATAGGTTTTCTCTCCCGCTTTCACCTCTTGCGCTTCCAGATCCACGTCGAAATGGATCCCCGGATTA
GCTTTCACCAGCGCAAACAGTTCGTCCACTTCTGCATCGCTTAATTTCACCGGCAGCAGCTGGTTGTTAAAGCTATTGCC
GTAGAAGATGTCAGCAAAACTCGGCGCAATCACCACTTTAAAACCGTAGTCGGTCAATGCCCAGGGCGCGTGCTCACGCG
AAGAGCCACAGCCGAAGTTTTCTCGTGCCAGCAAAATGGAAGCGCCCTGATACTGCGGGAAGTTCAGCACGAAGTCCGGG
TTTGGCTGTTGGCCTTTTTCATCCAGAAAACGCCAGTCGTTAAACAGATGCGCGCCAAAACCCGTACGGGTCACTTTCTG
CAAAAACTGTTTCGGGATGATTGCATCGGTATCGACATTGGCGGCATCCAGCGGAACCACCAGGCCTGTGTGTTTGATAA
ATTTCTCTGCCATGGTGTGCTCCTTATTTAATGTTGCGAATGTCGGCGAAATGTCCGGTCACAGCAGCAGCGGCAGCCAT
TGCCGGGCTGACCAGATGCGTGCGCCCGCCGCGCCCCTGGCGGCCTTCAAAGTTACGGTTGCTGGTGGAGGCACAACGTT
CGCCCGGATTCAGACGGTCGTTGTTCATCGCCAGACACATTGAGCAGCCAGGCAAGCGCCATTCAAAACCGGCTTCAATA
AAGATTTTATCCAGACCTTCCGCTTCCGCCTGGGCTTTTACCGGGCCAGAGCCGGGAACCACCAGTGCCTGCACGCCTGG
CGCGACTTTTCGCCCTTTGGCGATCTCCGCTGCCGCGCGTAAATCTTCAATGCGCGAGTTGGTACAGGAACCGATAAACA
CTTTGTCGATAGCCACTTCGGTCAGCGGAATACCCGGTTTCAGCCCCATATAGGCCAGCGCTTTTTCTGCCGACGCGCGT
TCAACCGGATCGGCAAACGAAGCCGGATCGGGAATATTGTCGTTCACGGAAATCACCTGGCCGGGATTGGTGCCCCAGGT
GACCTGCGGTGAAATTTCTTCTGCTTGCAGAGTGACAACGGTATCGAAAGTTGCGCCTTCGTCGGTTTGCAGGGTTTTCC
AGTAGGCAACGGCGTCGTCGAAATCTTTGCCTTTCGGCGCATGCAGACGGCCTTTGACATAGTTAAAGGTGGTTTCGTCC
GGTGCAACCAGACCGGCTTTTGCGCCCATTTCGATTGCCATATTGCACAGGGTCATACGACCTTCCATGCTTAAATCACG
GATTGCTTCGCCGCAAAACTCCACCACATGCCCGGTGCCGCCTGCGCTACCGGTTTTACCGATAATTGCCAGCACGATAT
CTTTTGCGGTAATGCCCGGCGCGGCTTTGCCCTGGACTTCAATTTTCATGGTTTTTGCGCGGCCCTGTTTCAGGGTTTGC
GTTGCCAGTACGTGTTCAACTTCGGAAGTGCCGATACCAAAGGCCAGTGCGCCAAACGCGCCGTGGGTGGCGGTATGCGA
GTCGCCGCAGACAATGGTCATCCCCGGCAAGGTGACGCCCTGTTCCGGCCCCATTACGTGGACGATCCCCTGATACGGGT

GATTCAGGTCATACAGTTCGACGCCAAATTCTTTGCAGTTTTTGATCAGTTCCTGCATCTGGATACGCGCCATTTCACCG
CAGGCATTAATGTCTTTGGTCTGGGTAGAGACGTTGTGATCCATGGTAGCGAAGGTTTTGCCCGGCTGACGTACCGGGCG
ACCGTGGGCGCGCAGACCATCGAACGCCTGCGGTGAGGTCACTTCATGCACCAGGTGGCGGTCGATATATAACAGTGGGG
TTTCGTTTTCGGCTTCGTACACAACGTGAGCGTCGAACAATTTTTCGTATAACGTCTTAGCCATGATTACACCCCTTCTG
CTACATAGCGGGCAATGATATCGCCCATTTCATCGGTACTAACGGCGGCAGCGCCACGGGCTAAATCCCCGGTGCGAATG
CCTTCTTCTAATGCGCGGTTAATGGCGCGTTCAATGGCGCAAGCCGCATCATCGGCATCCAGGCTGTAACGCAGCAGCAG
TGCCAGCGAAAGGATTTGTGCAATCGGGTTGGCGATGTTTTTGCCTGCGATATCTGGTGCCGAGCCGCCCGCCGGTTCAT
ACAGTCCAAAACCTTGCTCGTTCAGGCTGGCGGAAGGCAACATCCCCATCGAGCCAGTGATCATTGCGCACTCGTCAGAC
AGAATGTCGCCAAACAGGTTGGAGCACAGCAGAACGTCAAACTGTGATGGATCTTTAATCAGCTGCATGGTGGCGTTGTC
GATGTACATATGCGCCAGTTCGACATCCGGGTATTCCGTGGCGATCTCGTTAACGATCTCCCGCCATAAAATAGAGGATT
GCAGCACGTTGGCTTTATCGATCGACGTCACTTTGTGGCGACGCTTGCGAGCAGATTCAAACGCGATGCGGGCGATACGT
TCGATCTCAAAACGGTGATACACCTCGGTATCAAAGGCTTTTTCATATTGTCCGCTACCTTCGCGGCCTTTTGGCTGACC
GAAATAGATGCCGCCGGTCAGTTCGCGCACACACAGGATGTCGAAGCCGTTTGCGGCAATGTCTGCACGCAGCGGACAGA
ATGCTTCCAGCCCCTGATACAGTTTTGCCGGGCGCAGGTTGCTGAATAATTTGAAGTGCTTACGCAGAGGCAGCAGCGCG
CCGCGTTCTGGTTGCTGGTCTGGTGGTAAATGTTCCCACTTCGGGCCGCCTACCGAGCCAAACAGCACGGCATCGGCTTG
CTCACAACCTTCAACCGTCGCAGGCGGCAGTGGTTGCCCGTGGTTATCAATGGCTGCGCCGCCTACATCGTAATGGCTGG
TGGTGATGCGCATCGCAAAGCGGTTGCGCACGGCATCCAGCACTTTCAGCGCCTGGGTCATCACTTCCGGACCAATACCG
TCCCCCGGCAATACGGCAATATGGTAATTCTTCGACATCACACGGTTTCCTTGTTGTTTTCGTTGTGTTGAGCTTTGCGT
TGCAACTCTTTTTCGACTTCTGCGGCACGCCAGATATTGTTCAGAACGTGCACCATGGCTTTGGCAGATGACTCGACAAT
ATCGGTAGCCAGGCCGACGCCGTGGAAGCGGCGACCGTTGTAGTTAGCGACGATATCCACCTGACCCAGCGCATCTTTAC
CGTGGCCTTTGGCGGTCAGGCTGTATTTCACCAGTTCGACGTTATATTCAGTGATGCGGTTAATTGCCTGATAGACGGCA
TCGACCGGACCGTTACCGTTGGCGGCTTCTGCTTTGACTTCTTCGCCACAGGCCAGTTTGACGGCGGCGGTGGCGATATC
GTTAGAGCCAGACTGCACGCTGAAGTAATCCAGACGGAAATGCTCCGGCTCTTCTTGCTGCTTACCGATGAAGGCCAGCG
CCTCCAGATCGTAATCAAACACCTGACCTTTTTTGTCCGCCAGCTTCAGGAAAGCATCGTACAAATTGTCTAAATTATAT
TCACTTTCTTTATACCCCATCTCATCCATGCGATGTTTCACCGCCGCACGCCCCGAACGAGAGGTCAGATTCAGCTGGAT
TTGGTTCAGACCAATAGATTCTGGTGTCATGATTTCGTAGTTTTCGCGGTTTTTCAGCACGCCATCCTGGTGTATACCGG
AGGAGTGTGCGAATGCGCCGCTGCCAACAATGGCTTTGTTTGCCGGGATCGGCATATTACAAATCTGGCTAACTAACTGG
CTGGTGCGCCATATCTCCTGGTGATTAATGGCGGTGTGGACGTTGAGAATATCCTTACGAACTTTGATCGCCATGATGAC
TTCTTCCAGGGAACAGTTTCCGGCACGCTCGCCGATCCCGTTCATTGCGCCTTCCACCTGGCGTGCACCGGCATGTACCG
CCGCCAGTGAGTTTCCGACCGCCAGGCCCAAATCGTCGTGGGTATGTACGGAGATAATGGCTTTGTCGATGTTAGGCACG
CGTTCATACAGGCCGCTGATGATTCCGGCGAACTCAAACGGCATGGTGTAGCCCACGGTGTCCGGAATGTTGATGGTGGT
GGCACCGGCATTAATCGCCGCTTCGACCACTCGCGCCAGATCGGCAATGGGTGTACGCCCGGCATCTTCGCAAGAAAATT
CAACATCATCGGTGTAATTACGGGCGCGTTTCACCATATAGATAGCGCGTTCGATCACCTCGTCCAGCGTGCTGCGCAGC
TTGGTGGCGATGTGCATTGGCGAAGTGGCAATAAAGGTATGAATACGGAAGGCTTCGGCGACTTTCAGGGATTCGGCCGC
CACGTCGATATCTTTTTCCACGCAGCGAGCTAACGCACATACGCGGCTGTTTTTAACCTGGCGGGCGATGGTTTGCACCG
ATTCAAAATCGCCCGGCGAAGAGACGGGGAAACCGACTTCCATCACGTCAACACCCATACGCTCAAGGGCCAGCGCAATT
TGCAGTTTTTCTTTCACACTCAAGCTTGCCTGTAACGCCTGTTCACCGTCGCGCAATGTGGTATCGAAAATAATGACTTG
CTGGCTCATGGTTTGGGTCCTTGTCTCTTTTAGAGCGCCTCGCTTCGGGCATAAAAAAACCCGCGCAATGGCGCGGGTTT
TTTGTTTGACTGCGTGCTGGCTTAATGCTGGATGCCGCTCACTCGTCTACCGCGCAAAGAAGATGCGTTTAGTAGTAGTA
GACCGATAAAGCGAACGATGTGAGTCATTAAATCAGCTCCAGATGAATGCGATATGCTTTTAGAGTTACTGGATACAAAA
ACGGATGTCAACCCTGACGCAATAAAAACGTCCCGCCAGCGTGAGTTCTGCATCCGTAAAATTAGCTAATTGTGCTGCGG
TGGTTAAAGTAAGCGATATTAATTTCTGCTTAACTACCGACGCTTTTCATCGGTTGACATATTTCAGCATAAATTTTTGC
ATCTAATCAACGAGGAAAAAGGGGACAAAATGCACGCGTTGCAAAACCTATCCTGATGATTTGTATTGAATTATATGTTT
TGCGATTTTTTTTGATATTGATTTGGTGAATATTATTGATCAATTAATGTTAAGAATTAATGCATTAAATATATAAATTA
ATTATTAAATAAGCACATTTAATCCATTTTGTAGATGATTGAGTATTCGCGGTAGTTATGATTAGATTGTTTTCGCAACA
AAAACATTATGGATTATTATGCTGTGGTAAATGACTCATTCCACGGCAATGGATTCTGTTTTTATCAGAACCCGTATCTT
TATGTTTTCCGAATTTTACTCATTTTGCTTTTTCTTATTTTATATGCATGATAAATCATATTCTTCAGGATTATTTCTCT
GCATTCCAATAAGGGAAAGGGAGTTAAGTGTGACAGTGGAGTTAAGTATGCCAGAGGTACAAACAGATCATCCAGAGACG
GCGGAGTTAAGCAAACCACAGCTACGCATGGTCGATCTCAACTTATTAACCGTTTTCGATGCCGTGATGCAGGAGCAAAA
CATTACTCGTGCCGCTCATGTTCTGGGAATGTCGCAACCTGCGGTCAGTAACGCTGTTGCACGCCTGAAGGTGATGTTTA
ATGACGAGCTTTTTGTTCGTTATGGCCGTGGTATTCAACCGACTGCTCGCGCATTTCAACTTTTTGGTTCAGTTCGTCAG
GCATTGCAACTAGTACAAAATGAATTGCCTGGTTCAGGTTTTGAACCCGCGAGCAGTGAACGTGTATTTCATCTTTGTGT
TTGCAGCCCGTTAGACAGCATTCTGACCTCGCAGATTTATAATCACATTGAGCAGATTGCGCCAAATATACATGTTATGT
TCAAGTCTTCATTAAATCAGAACACTGAACATCAGCTGCGTTATCAGGAAACGGAGTTTGTGATTAGTTATGAAGACTTC
CATCGTCCTGAATTTACCAGCGTACCATTATTTAAAGATGAAATGGTGCTGGTAGCCAGCAAAAATCATCCAACAATTAA
GGGCCCGTTACTGAAACATGATGTTTATAACGAACAACATGCGGCGGTTTCGCTCGATCGTTTCGCGTCATTTAGTCAAC

CTTGGTATGACACGGTAGATAAGCAAGCCAGTATCGCGTATCAGGGCATGGCAATGATGAGCGTACTTAGCGTGGTGTCG
CAAACGCATTTGGTCGCTATTGCGCCGCGTTGGCTGGCTGAAGAGTTCGCTGAATCCTTAGAATTACAGGTATTACCGCT
GCCGTTAAAACAAAACAGCAGAACCTGTTATCTCTCCTGGCATGAAGCTGCCGGGCGCGATAAAGGCCATCAGTGGATGG
AAGAGCAATTAGTCTCAATTTGCAAACGCTAACTGATTGCAGAATAGGTCAGACATGAATGTCTGGTTTATTCTGCATTT
TTTATTGAATGTAGAATTTTATTCTGAATGTGTGGGCTCTCTATTTTAGGATTAATTAAAAAAATAGAGAAATTGCTGTA
AGTTGTGGGATTCAGCCGATTTATTATCAATTTAATCCTCTGTAATGGAGGATTTTATCGTTTCTTTTCACCTTTCCTCC
TGTTTATTCTTATTACCCCGTGTTTATGTCTCTGGCTGCCAATTGCTTAAGCAAGATCGGACGGTTAATGTGTTTTACAC
ATTTTTTCCGTCAAACAGTGAGGCAGGCCATGGAGATGTTGTCTGGAGCCGAGATGGTCGTCCGATCGCTTATCGATCAG
GGCGTTAAACAAGTATTCGGTTATCCCGGAGGCGCAGTCCTTGATATTTATGATGCATTGCATACCGTGGGTGGTATTGA
TCATGTATTAGTTCGTCATGAGCAGGCGGCGGTGCATATGGCCGATGGCCTGGCGCGCGCGACCGGGGAAGTCGGCGTCG
TGCTGGTAACGTCGGGTCCAGGGGCGACCAATGCGATTACTGGCATCGCCACCGCTTATATGGATTCCATTCCATTAGTT
GTCCTTTCCGGGCAGGTAGCGACCTCGTTGATAGGTTACGATGCCTTTCAGGAGTGCGACATGGTGGGGATTTCGCGACC
GGTGGTTAAACACAGTTTTCTGGTTAAGCAAACGGAAGACATTCCGCAGGTGCTGAAAAAGGCTTTCTGGCTGGCGGCAA
GTGGTCGCCCAGGACCAGTAGTCGTTGATTTACCGAAAGATATTCTTAATCCGGCGAACAAATTACCCTATGTCTGGCCG
GAGTCGGTCAGTATGCGTTCTTACAATCCCACTACTACCGGACATAAAGGGCAAATTAAGCGTGCTCTGCAAACGCTGGT
AGCGGCAAAAAAACCGGTTGTCTACGTAGGCGGTGGGGCAATCACGGCGGGCTGCCATCAGCAGTTGAAAGAAACGGTGG
AGGCGTTGAATCTGCCCGTTGTTTGCTCATTGATGGGGCTGGGGGCGTTTCCGGCAACGCATCGTCAGGCACTGGGCATG
CTGGGAATGCACGGTACCTACGAAGCCAATATGACGATGCATAACGCGGATGTGATTTTCGCCGTCGGGGTACGATTTGA
TGACCGAACGACGAACAATCTGGCAAAGTACTGCCCAAATGCCACTGTTCTGCATATCGATATTGATCCTACTTCCATTT
CTAAAACCGTGACTGCGGATATCCCGATTGTGGGGGATGCTCGCCAGGTCCTCGAACAAATGCTTGAACTCTTGTCGCAA
GAATCCGCCCATCAACCACTGGATGAGATCCGCGACTGGTGGCAGCAAATTGAACAGTGGCGCGCTCGTCAGTGCCTGAA
ATATGACACTCACAGTGAAAAGATTAAACCGCAGGCGGTGATCGAGACTCTTTGGCGGTTGACGAAGGGAGACGCTTACG
TGACGTCCGATGTCGGGCAGCACCAGATGTTTGCTGCACTTTATTATCCATTCGACAAACCGCGTCGCTGGATCAATTCC
GGTGGCCTCGGCACGATGGGTTTTGGTTTACCTGCGGCACTGGGCGTCAAAATGGCGTTGCCAGAAGAAACCGTGGTTTG
CGTCACTGGCGACGGCAGTATTCAGATGAACATCCAGGAACTGTCTACCGCGTTGCAATACGAGTTGCCCGTACTGGTGG
TGAATCTCAATAACCGCTATCTGGGGATGGTGAAGCAGTGGCAGGACATGATCTATTCCGGCCGTCATTCACAATCTTAT
ATGCAATCGCTACCCGATTTCGTCCGTCTGGCGGAAGCCTATGGGCATGTCGGGATCCAGATTTCTCATCCGCATGAGCT
GGAAAGCAAACTTAGCGAGGCGCTGGAACAGGTGCGCAATAATCGCCTGGTGTTTGTTGATGTTACCGTCGATGGCAGCG
AGCACGTCTACCCGATGCAGATTCGCGGGGGCGGAATGGATGAAATGTGGTTAAGCAAAACGGAGAGAACCTGATTATGC
GCCGGATATTATCAGTCTTACTCGAAAATGAATCAGGCGCGTTATCCCGCGTGATTGGCCTTTTTTCCCAGCGTGGCTAC
AACATTGAAAGCCTGACCGTTGCGCCAACCGACGATCCGACATTATCGCGTATGACCATCCAGACCGTGGGCGATGAAAA
AGTACTTGAGCAGATCGAAAAGCAATTACACAAACTGGTCGATGTCTTGCGCGTGAGTGAGTTGGGGCAGGGCGCGCATG
TTGAGCGGGAAATCATGCTGGTGAAAATTCAGGCCAGCGGTTACGGGCGTGACGAAGTGAAACGTAATACGGAAATATTC
CGTGGGCAAATTATCGATGTCACACCCTCGCTTTATACCGTTCAATTAGCAGGCACCAGCGGTAAGCTTGATGCATTTTT
AGCATCGATTCGCGATGTGGCGAAAATTGTGGAGGTTGCTCGCTCTGGTGTGGTCGGACTTTCGCGCGGCGATAAAATAA
TGCGTTGAGAATGATCTCAATGCGCAATTTACAGCCCAACATGTCACGTTGGGCTTTTTTTGCGAAATCAGTGGGAACCT
GGAATAAAAGCAGTTGCCGCAGTTAATTTTCTGCGCTTAGATGTTAATGAATTTAACCCATACCAGTACAATGGCTATGG
TTTTTACATTTTACGCAAGGGGCAATTGTGAAACTGGATGAAATCGCTCGGCTGGCGGGAGTGTCGCGGACCACTGCAAG
CTATGTTATTAACGGCAAAGCGAAGCAATACCGTGTGAGCGACAAAACCGTTGAAAAAGTCATGGCTGTGGTGCGTGAGC
ACAATTACCACCCGAACGCCGTGGCAGCTGGGCTTCGTGCTGGACGCACACGTTCTATTGGTCTTGTGATCCCCGATCTG
GAGAACACCAGCTATACCCGCATCGCTAACTATCTTGAACGCCAGGCGCGGCAACGGGGTTATCAACTGCTGATTGCCTG
CTCAGAAGATCAGCCAGACAACGAAATGCGGTGCATTGAGCACCTTTTACAGCGTCAGGTTGATGCCATTATTGTTTCGA
CGTCGTTGCCTCCTGAGCATCCTTTTTATCAACGCTGGGCTAACGACCCGTTCCCGATTGTCGCGCTGGACCGCGCCCTC
GATCGTGAACACTTCACCAGCGTGGTTGGTGCCGATCAGGATGATGCCGAAATGCTGGCGGAAGAGTTACGTAAGTTTCC
CGCCGAGACGGTGCTTTATCTTGGTGCGCTACCGGAGCTTTCTGTCAGCTTCCTGCGTGAACAAGGTTTCCGTACTGCCT
GGAAAGATGATCCGCGCGAAGTGCATTTCCTGTATGCCAACAGCTATGAGCGGGAGGCGGCTGCCCAGTTATTCGAAAAA
TGGCTGGAAACGCATCCGATGCCGCAGGCGCTGTTCACAACGTCGTTTGCGTTGTTGCAAGGAGTGATGGATGTCACGCT
GCGTCGCGACGGCAAACTGCCTTCTGACCTGGCAATTGCCACCTTTGGCGATAACGAACTGCTCGACTTCTTACAGTGTC
CGGTGCTGGCAGTGGCTCAACGTCACCGCGATGTCGCAGAGCGTGTGCTGGAGATTGTCCTGGCAAGCCTGGACGAACCG
CGTAAGCCAAAACCTGGTTTAACGCGCATTAAACGTAATCTCTATCGCCGCGGCGTGCTCAGCCGTAGCTAAGCCGCGAA
CAAAAATACGCGCCAGGTGAATTTCCCTCTGGCGCGTAGAGTACGGGACTGGACATCAATATGCTTAAAGTAAATAAGAC
TATTCCTGACTATTATTGATAAATGCTTTTAAACCCGCCCGTTAATTAACTCACCAGCTGAAATTCACAATAATTAAGTG
ATATCGACAGCGCGTTTTTGCATTATTTTGTTACATGCGGCGATGAATTGCCGATTTAACAAACACTTTTCTTTGCTTTT
GCGCAAACCCGCTGGCATCAAGCGCCACACAGACGTAACAAGGACTGTTAACCGGGGAAGATATGTCCTAAAATGCCGCT
CGCGTCGCAAACTGACACTTTATATTTGCTGTGGAAAATAGTGAGTCATTTTAAAACGGTGATGACGATGAGGGATTTTT
TCTTACAGCTATTCATAACGTTAATTTGCTTCGCACGTTGGACGTAAAATAAACAACGCTGATATTAGCCGTAAACATCG

GGTTTTTTACCTCGGTATGCCTTGTGACTGGCTTGACAAGCTTTTCCTCAGCTCCGTAAACTCCTTTCAGTGGGAAATTG
TGGGGCAAAGTGGGAATAAGGGGTGAGGCTGGCATGTTCCGGGGAGCAACGTTAGTCAATCTCGACAGCAAAGGGCGCTT
ATCAGTGCCTACCCGTTATCGGGAACAGCTGCTTGAGAACGCTGCCGGTCAAATGGTTTGCACCATTGACATTTATCACC
CGTGCCTGCTGCTTTACCCCCTGCCTGAATGGGAAATTATCGAGCAAAAATTATCGCGTCTGTCGAGCATGAACCCGGTT
GAGCGCCGTGTGCAGCGCCTACTGTTAGGTCATGCCAGCGAATGTCAGATGGATGGCGCAGGTCGATTGTTAATCGCGCC
AGTACTGCGGCAACATGCCGGGCTGACAAAAGAAGTGATGCTGGTTGGACAGTTCAACAAGTTTGAGCTGTGGGATGAAA
CAACCTGGCATCAACAGGTCAAGGAAGATATCGACGCAGAGCAGTTGGCTACCGGAGACTTATCGGAGCGACTGCAGGAC
TTGTCTCTATAAAATGATGGAAAACTATAAACATACTACGGTGCTGCTGGATGAAGCCGTTAATGGCCTCAATATCCGTC
CTGATGGCATCTACATTGATGGGACTTTTGGTCGCGGTGGTCACTCACGTCTGATCCTCTCGCAGCTTGGCGAAGAGGGG
CGTTTGCTGGCGATCGATCGCGACCCGCAGGCTATCGCCGTTGCGAAGACTATTGATGATCCGCGCTTCTCCATCATCCA
CGGACCTTTCTCCGCGCTGGGCGAATACGTTGCCGAGCGCGATCTTATCGGCAAGATCGACGGCATTCTCCTCGATCTTG
GCGTCTCTTCACCGCAACTTGATGATGCTGAACGTGGCTTTTCCTTTATGCGCGATGGTCCGCTGGACATGCGTATGGAC
CCAACCCGTGGGCAGTCAGCCGCTGAATGGCTACAAACCGCAGAAGAAGCCGATATCGCCTGGGTATTGAAAACCTATGG
TGAAGAGCGTTTTGCCAAACGCATTGCCCGCGCCATTGTCGAGCGTAACCGCGAACAGCCGATGACCCGCACCAAAGAAC
TGGCGGAAGTCGTGGCTGCTGCAACGCCGGTGAAAGATAAGTTTAAACATCCCGCGACCCGTACCTTCCAGGCGGTGCGC
ATTTGGGTAAACAGTGAACTGGAGGAGATAGAGCAGGCGCTAAAAAGCTCGCTCAACGTGCTGGCCCCGGGTGGGCGGCT
TTCGATCATCAGCTTCCACTCGCTGGAAGACCGTATTGTGAAACGTTTTATGCGTGAAAACAGCCGCGGTCCGCAAGTTC
CGGCAGGGTTACCGATGACTGAAGAGCAGCTCAAAAAACTGGGTGGCCGTCAGCTGCGAGCACTAGGCAAGTTAATGCCG
GGCGAAGAAGAGGTGGCTGAGAACCCTCGTGCCCGTAGTTCAGTTCTGCGTATTGCAGAGAGGACGAATGCATGATCAGC
AGAGTGACAGAAGCTCTAAGCAAAGTTAAAGGATCGATGGGAAGCCACGAGCGCCATGCATTGCCTGGTGTTATCGGTGA
CGATCTTTTGCGATTTGGGAAGCTGCCACTCTGCCTGTTCATTTGCATTATTTTGACGGCGGTGACTGTGGTAACCACGG
CGCACCATACCCGTTTACTGACCGCTCAGCGCGAACAACTGGTGCTGGAGCGAGATGCTTTAGACATTGAATGGCGCAAC
CTGATCCTTGAAGAGAATGCGCTCGGCGACCATAGCCGGGTGGAAAGGATCGCCACGGAAAAGCTGCAAATGCAGCATGT
TGATCCGTCACAAGAAAATATCGTAGTGCAAAAATAAGGATAAACGCGACGCATGAAAGCAGCGGCGAAAACGCAGAAAC
CAAAACGTCAGGAAGAACATGCCAACTTTATCAGTTGGCGTTTTGCGTTGTTATGCGGCTGTATTCTCCTGGCGCTGGCT
TTTCTGCTCGGACGCGTAGCGTGGTTACAAGTTATCTCCCCGGATATGCTGGTGAAAGAGGGCGACATGCGTTCTCTTCG
CGTTCAGCAAGTTTCCACCTCCCGCGGCATGATTACTGACCGTTCTGGTCGCCCGTTAGCGGTGAGCGTGCCGGTAAAAG
CGATTTGGGCTGACCCGAAAGAAGTGCATGACGCTGGCGGTATCAGCGTCGGTGACCGCTGGAAGGCGCTGGCTAACGCG
CTCAATATTCCGCTGGATCAGCTTTCAGCCCGCATTAACGCCAACCCGAAAGGGCGCTTTATTTATCTGGCGCGTCAGGT
GAACCCTGACATGGCGGACTACATCAAAAAACTGAAACTGCCGGGGATTCATCTGCGTGAAGAGTCTCGCCGTTACTATC
CGTCCGGCGAAGTGACTGCTCACCTCATCGGCTTTACTAACGTCGATAGTCAAGGGATTGAGGGCGTTGAGAAGAGTTTC
GATAAATGGCTTACCGGGCAGCCGGGTGAGCGCATTGTGCGTAAAGACCGCTATGGTCGCGTAATTGAAGATATTTCTTC
TACTGACAGCCAGGCAGCGCACAACCTGGCGCTGAGTATTGATGAACGCCTGCAGGCGCTGGTTTATCGCGAACTGAACA
ACGCGGTGGCCTTTAACAAGGCTGAATCTGGTAGCGCCGTGCTGGTGGATGTCAACACCGGTGAAGTGCTGGCGATGGCT
AACAGCCCGTCATACAACCCTAACAATCTGAGCGGCACGCCGAAAGAGGCGATGCGTAACCGTACCATCACCGACGTGTT
TGAACCGGGCTCAACGGTTAAACCGATGGTGGTAATGACCGCGTTGCAACGTGGCGTGGTGCGGGAAAACTCGGTACTCA
ATACCATTCCTTATCGAATTAACGGCCACGAAATCAAAGACGTGGCACGCTACAGCGAATTAACCCTGACCGGGGTATTA
CAGAAGTCGAGTAACGTCGGTGTTTCCAAGCTGGCGTTAGCGATGCCGTCCTCAGCGTTAGTAGATACTTACTCACGTTT
TGGACTGGGAAAAGCGACCAATTTGGGGTTGGTCGGAGAACGCAGTGGCTTATATCCTCAAAAACAACGGTGGTCTGACA
TAGAGAGGGCCACCTTCTCTTTCGGCTACGGGCTAATGGTAACACCATTACAGTTAGCGCGAGTCTACGCAACTATCGGC
AGCTACGGCATTTATCGCCCACTGTCGATTACCAAAGTTGACCCCCCGGTTCCCGGTGAACGTGTCTTCCCGGAATCCAT
TGTCCGCACTGTGGTGCATATGATGGAAAGCGTGGCGCTACCAGGCGGCGGCGGCGTGAAGGCGGCGATTAAAGGCTATC
GTATCGCCATTAAAACCGGTACCGCGAAAAAGGTCGGGCCGGACGGTCGCTACATCAATAAATATATTGCTTATACCGCA
GGCGTTGCGCCTGCGAGTCAGCCGCGCTTCGCGCTGGTTGTTGTTATCAACGATCCGCAGGCGGGTAAATACTACGGCGG
CGCCGTTTCCGCGCCGGTCTTTGGTGCCATCATGGGCGGCGTATTGCGTACCATGAACATCGAGCCGGATGCGCTGACAA
CGGGCGATAAAAATGAATTTGTGATTAATCAAGGCGAGGGGACAGGTGGCAGATCGTAATTTGCGCGACCTTCTTGCTCC
GTGGGTGCCAGACGCACCTTCGCGAGCACTGCGAGAGATGACACTCGACAGCCGTGTGGCTGCGGCGGGCGATCTCTTTG
TAGCTGTAGTAGGTCATCAGGCGGACGGGCGTCGATATATCCCGCAGGCGATAGCGCAAGGTGTGGCTGCCATTATTGCA
GAGGCGAAAGATGAGGCGACCGATGGTGAAATCCGTGAAATGCACGGCGTACCGGTCATCTATCTCAGCCAGCTCAACGA
GCGTTTATCTGCACTGGCGGGCCGCTTTTACCATGAACCCTCTGACAATTTACGTCTCGTGGGCGTAACGGGCACCAACG
GCAAAACCACGACTACCCAGCTGTTGGCGCAGTGGAGCCAACTGCTTGGCGAAATCAGCGCGGTAATGGGCACCGTTGGT
AACGGCCTGCTGGGGAAAGTGATCCCGACAGAAAATACAACCGGTTCGGCAGTCGATGTTCAGCATGAGCTGGCGGGGCT
GGTGGATCAGGGCGCGACGTTTTGCGCAATGGAAGTTTCCTCCCACGGGCTGGTACAGCACCGTGTGGCGGCATTGAAAT
TTGCGGCGTCGGTCTTTACCAACTTAAGCCGCGATCACCTTGATTATCATGGTGATATGGAACACTACGAAGCCGCGAAA
TGGCTGCTTTATTCTGAGCATCATTGCGGTCAGGCGATTATTAACGCCGACGATGAAGTGGGCCGCCGCTGGCTGGCAAA
ACTGCCGGACGCGGTTGCGGTATCAATGGAAGATCATATTAATCCGAACTGTCACGGACGCTGGTTGAAAGCGACCGAAG

TGAACTATCACGACAGCGGTGCGACGATTCGCTTTAGCTCAAGTTGGGGCGATGGCGAAATTGAAAGCCATCTGATGGGC
GCTTTTAACGTCAGCAACCTGCTGCTCGCGCTGGCGACACTGTTGGCACTCGGCTATCCACTGGCTGATCTGCTGAAAAC
CGCCGCGCGTCTGCAACCGGTTTGCGGACGTATGGAAGTGTTCACTGCGCCAGGCAAACCGACGGTGGTGGTGGATTACG
CGCATACGCCGGATGCACTGGAAAAAGCCTTACAGGCGGCGCGTCTGCACTGTGCGGGCAAGCTGTGGTGTGTCTTTGGC
TGTGGTGGCGATCGCGATAAAGGTAAGCGTCCACTGATGGGCGCAATTGCCGAAGAGTTTGCTGACGTGGCGGTGGTGAC
GGACGATAACCCGCGTACCGAAGAACCGCGTGCCATCATCAACGATATTCTGGCGGGAATGTTAGATGCCGGACATGCCA
AAGTGATGGAAGGCCGTGCTGAAGCGGTGACTTGCGCCGTTATGCAGGCTAAAGAGAATGATGTGGTACTGGTCGCGGGC
AAAGGCCATGAAGATTACCAGATTGTTGGCAATCAGCGTCTGGACTACTCCGATCGCGTCACGGTGGCGCGTCTGCTGGG
GGTGATTGCATGATTAGCGTAACCCTTAGCCAACTTACCGACATTCTCAACGGTGAACTGCAAGGTGCAGATATCACCCT
TGATGCTGTAACCACTGATACCCGAAAACTGACGCCGGGCTGCCTGTTTGTTGCCCTGAAAGGCGAACGTTTTGATGCCC
ACGATTTTGCCGACCAGGCGAAAGCTGGCGGCGCAGGCGCACTACTGGTTAGCCGTCCGCTGGACATCGACCTGCCGCAG
TTAATCGTCAAGGATACGCGTCTGGCGTTTGGTGAACTGGCTGCATGGGTTCGCCAGCAAGTTCCGGCGCGCGTGGTTGC
TCTGACGGGGTCCTCCGGCAAAACCTCCGTTAAAGAGATGACGGCGGCGATTTTAAGCCAGTGCGGCAACACGCTTTATA
CGGCAGGCAATCTCAACAACGACATCGGTGTACCGATGACGCTGTTGCGCTTAACGCCGGAATACGATTACGCAGTTATT
GAACTTGGCGCGAACCATCAGGGCGAAATAGCCTGGACTGTGAGTCTGACTCGCCCGGAAGCTGCGCTGGTCAACAACCT
GGCAGCGGCGCATCTGGAAGGTTTTGGCTCGCTTGCGGGTGTCGCGAAAGCGAAAGGTGAAATCTTTAGCGGCCTGCCGG
AAAACGGTATCGCCATTATGAACGCCGACAACAACGACTGGCTGAACTGGCAGAGCGTAATTGGCTCACGCAAAGTGTGG
CGTTTCTCACCCAATGCCGCCAACAGCGATTTCACCGCCACCAATATCCATGTGACCTCGCACGGTACGGAATTTACCCT
ACAAACCCCAACCGGTAGCGTCGATGTTCTGCTGCCGTTGCCGGGGCGTCACAATATTGCGAATGCGCTGGCAGCCGCTG
CGCTCTCCATGTCCGTGGGCGCAACGCTTGATGCTATCAAAGCGGGGCTGGCAAATCTGAAAGCTGTTCCAGGCCGTCTG
TTCCCCATCCAACTGGCAGAAAACCAGTTGCTGCTCGACGACTCCTACAACGCCAATGTCGGTTCAATGACTGCAGCAGT
CCAGGTACTGGCTGAAATGCCGGGCTACCGCGTGCTGGTGGTGGGCGATATGGCGGAACTGGGCGCTGAAAGCGAAGCCT
GCCATGTACAGGTGGGCGAGGCGGCAAAAGCTGCTGGTATTGACCGCGTGTTAAGCGTGGGTAAACAAAGCCATGCTATC
AGCACCGCCAGCGGCGTTGGCGAACATTTTGCTGATAAAACTGCGTTAATTACGCGTCTTAAATTACTGATTGCTGAGCA
ACAGGTAATTACGATTTTAGTTAAGGGTTCACGTAGTGCCGCCATGGAAGAGGTAGTACGCGCTTTACAGGAGAATGGGA
CATGTTAGTTTGGCTGGCCGAACATTTGGTCAAATATTATTCCGGCTTTAACGTCTTTTCCTATCTGACGTTTCGCGCCA
TCGTCAGCCTGCTGACCGCGCTGTTCATCTCATTGTGGATGGGCCCGCGTATGATTGCTCATTTGCAAAAACTTTCCTTT
GGTCAGGTGGTGCGTAACGACGGTCCTGAATCACACTTCAGCAAGCGCGGTACGCCGACCATGGGCGGGATTATGATCCT
GACGGCGATTGTGATCTCCGTACTGCTGTGGGCTTACCCGTCCAATCCGTACGTCTGGTGCGTGTTGGTGGTGCTGGTAG
GTTACGGTGTTATTGGCTTTGTTGATGATTATCGCAAAGTGGTGCGTAAAGACACCAAAGGGTTGATCGCTCGTTGGAAG
TATTTCTGGATGTCGGTCATTGCGCTGGGTGTCGCCTTCGCCCTGTACCTTGCCGGCAAAGACACGCCCGCAACGCAGCT
GGTGGTCCCATTCTTTAAAGATGTGATGCCGCAGCTGGGGCTGTTCTACATTCTGCTGGCTTACTTCGTCATTGTGGGTA
CTGGCAACGCGGTAAACCTGACCGATGGTCTCGACGGCCTGGCAATTATGCCGACCGTATTTGTCGCCGGTGGTTTTGCG
CTGGTGGCGTGGGCGACCGGCAATATGAACTTTGCCAGCTACTTGCATATACCGTATCTGCGACACGCCGGGGAACTGGT
TATTGTCTGTACCGCGATAGTCGGGGCAGGACTGGGCTTCCTGTGGTTTAACACCTATCCGGCGCAGGTCTTTATGGGCG
ATGTAGGTTCGCTGGCGTTAGGTGGTGCGTTAGGCATTATCGCCGTACTGCTACGTCAGGAATTCCTGCTGGTGATTATG
GGGGGCGTGTTCGTGGTAGAAACGCTTTCTGTCATCCTGCAGGTCGGCTCCTTTAAACTGCGCGGACAACGTATTTTCCG
CATGGCACCGATTCATCACCACTATGAACTGAAAGGCTGGCCGGAACCGCGCGTCATTGTGCGTTTCTGGATTATTTCGC
TGATGCTGGTTCTGATTGGTCTGGCAACGCTGAAGGTACGTTAATCATGGCTGATTATCAGGGTAAAAATGTCGTCATTA
TCGGCCTGGGCCTCACCGGGCTTTCCTGCGTGGACTTTTTCCTCGCTCGCGGTGTGACGCCGCGCGTTATGGATACGCGT
ATGACACCGCCTGGCCTGGATAAATTACCCGAAGCCGTAGAACGCCACACGGGCAGTCTGAATGATGAATGGCTGATGGC
GGCAGATCTGATTGTCGCCAGTCCCGGTATTGCACTGGCGCATCCATCCTTAAGCGCTGCCGCTGATGCCGGAATCGAAA
TCGTTGGCGATATCGAGCTGTTCTGTCGCGAAGCACAAGCACCGATTGTGGCGATTACCGGTTCTAACGGCAAAAGCACG
GTCACCACGCTAGTGGGTGAAATGGCGAAAGCGGCGGGGGTTAACGTTGGTGTGGGTGGCAATATTGGCCTGCCTGCGTT
GATGCTACTGGATGATGAGTGTGAACTGTACGTGCTGGAACTGTCGAGCTTCCAGCTGGAAACCACCTCCAGCTTACAGG
CGGTAGCAGCGACCATTCTGAACGTGACTGAAGATCATATGGATCGCTATCCGTTTGGTTTACAACAGTATCGTGCAGCA
AAACTGCGCATTTACGAAAACGCGAAAGTTTGCGTGGTTAATGCTGATGATGCCTTAACAATGCCGATTCGCGGTGCGGA
TGAACGCTGCGTCAGCTTTGGCGTCAACATGGGTGACTATCACCTGAATCATCAGCAGGGCGAAACCTGGCTGCGGGTTA
AAGGCGAGAAAGTGCTGAATGTGAAAGAGATGAAACTTTCCGGGCAGCATAACTACACCAATGCGCTGGCGGCGCTGGCG
CTGGCAGATGCTGCAGGGTTACCGCGTGCCAGCAGCCTGAAAGCGTTAACCACATTCACTGGTCTGCCGCATCGCTTTGA
AGTTGTGCTGGAGCATAACGGCGTACGTTGGATTAACGATTCGAAAGCGACCAACGTCGGCAGTACGGAAGCGGCGCTGA
ATGGCCTGCACGTAGACGGCACACTGCATTTGTTGCTGGGTGGCGATGGTAAATCGGCGGACTTTAGCCCACTGGCGCGT
TACCTGAATGGCGATAACGTACGTCTGTATTGTTTCGGTCGTGACGGCGCGCAGCTGGCGGCGCTACGCCCGGAAGTGGC
AGAACAAACCGAAACTATGGAACAGGCGATGCGCTTGCTGGCTCCGCGTGTTCAGCCGGGCGATATGGTTCTGCTCTCCC
CAGCCTGTGCCAGCCTTGATCAGTTCAAGAACTTTGAACAACGAGGCAATGAGTTTGCCCGTCTGGCGAAGGAGTTAGGT
TGATGCGTTTATCTCTCCCTCGCCTGAAAATGCCGCGCCTGCCAGGATTCAGTATCCTGGTCTGGATCTCCACGGCGCTA

AAGGGCTGGGTGATGGGCTCGCGGGAAAAAGATACCGACAGCCTGATCATGTACGATCGCACCTTACTGTGGCTGACCTT
CGGCCTCGCGGCGATTGGCTTTATCATGGTGACCTCGGCGTCAATGCCCATAGGGCAACGCTTAACCAACGATCCGTTCT
TCTTCGCGAAGCGTGATGGTGTCTATCTGATTTTGGCGTTTATTCTGGCGATCATTACGCTGCGTCTGCCGATGGAGTTC
TGGCAACGCTACAGTGCCACGATGCTGCTCGGATCTATCATCCTGCTGATGATCGTCCTGGTAGTGGGTAGCTCGGTTAA
AGGGGCATCGCGTTGGATCGATCTCGGTTTGCTGCGTATCCAGCCTGCGGAGCTGACAAAACTGTCGCTGTTTTGCTATA
TCGCCAACTATCTGGTGCGTAAAGGCGACGAAGTACGTAATAACCTGCGCGGCTTCCTGAAACCGATGGGCGTGATTCTG
GTGTTGGCAGTGTTACTGCTGGCACAGCCAGACCTTGGTACGGTGGTGGTGTTGTTTGTGACTACGCTGGCGATGTTGTT
CCTGGCGGGAGCGAAATTGTGGCAGTTCATTGCCATTATCGGTATGGGCATTTCAGCGGTTGTGTTGCTGATACTCGCCG
AACCGTACCGTATCCGCCGTGTTACCGCATTCTGGAACCCGTGGGAAGATCCCTTTGGCAGCGGCTATCAGTTAACGCAA
TCGCTGATGGCGTTTGGTCGCGGCGAACTTTGGGGGCAAGGTTTAGGTAACTCGGTACAAAAACTGGAGTATCTGCCGGA
AGCGCACACTGACTTTATTTTCGCCATTATCGGCGAAGAACTGGGGTATGTCGGTGTGGTGCTGGCACTTTTAATGGTAT
TCTTCGTCGCTTTTCGCGCGATGTCGATTGGCCGTAAAGCATTAGAAATTGACCACCGTTTTTCCGGTTTTCTCGCCTGT
TCTATTGGCATCTGGTTTAGCTTCCAGGCGCTGGTTAACGTAGGCGCGGCGGCGGGGATGTTACCGACCAAAGGTCTGAC
ATTGCCGCTGATCAGTTACGGTGGTTCGAGCTTACTGATTATGTCGACAGCCATCATGATGCTGTTGCGTATTGATTATG
AAACGCGTCTGGAGAAAGCGCAGGCGTTTGTACGAGGTTCACGATGAGTGGTCAAGGAAAGCGATTAATGGTGATGGCAG
GCGGAACCGGTGGACATGTATTCCCGGGACTGGCGGTTGCGCACCATCTAATGGCTCAGGGTTGGCAAGTTCGCTGGCTG
GGGACTGCCGACCGTATGGAAGCGGACTTAGTGCCAAAACATGGCATCGAAATTGATTTCATTCGTATCTCTGGTCTGCG
TGGAAAAGGTATAAAAGCACTGATAGCTGCCCCGCTGCGTATCTTCAACGCCTGGCGTCAGGCGCGGGCGATTATGAAAG
CGTACAAACCTGACGTGGTGCTCGGTATGGGAGGCTACGTGTCAGGTCCAGGTGGTCTGGCCGCGTGGTCGTTAGGCATT
CCGGTTGTACTTCATGAACAAAACGGTATTGCGGGCTTAACCAATAAATGGCTGGCGAAGATTGCCACCAAAGTGATGCA
GGCGTTTCCAGGTGCTTTCCCTAATGCGGAAGTAGTGGGTAACCCGGTGCGTACCGATGTGTTGGCGCTGCCGTTGCCGC
AGCAACGTTTGGCTGGACGTGAAGGTCCGGTTCGTGTGCTGGTAGTGGGTGGTTCTCAGGGCGCACGCATTCTTAACCAG
ACAATGCCGCAGGTTGCTGCGAAACTGGGTGATTCAGTCACTATCTGGCATCAGAGCGGCAAAGGTTCGCAACAATCCGT
TGAACAGGCGTATGCCGAAGCGGGGCAACCGCAGCATAAAGTGACGGAATTTATTGATGATATGGCGGCGGCGTATGCGT
GGGCGGATGTCGTCGTTTGCCGCTCCGGTGCGTTAACGGTGAGTGAAATCGCCGCGGCAGGACTACCGGCGTTGTTTGTG
CCGTTTCAACATAAAGACCGCCAGCAATACTGGAATGCGCTACCGCTGGAAAAAGCGGGCGCAGCCAAAATTATCGAGCA
GCCACAGCTTAGCGTGGATGCTGTCGCCAACACCCTGGCCGGGTGGTCGCGAGAAACCTTATTAACCATGGCAGAACGCG
CCCGCGCTGCATCCATTCCGGATGCCACCGAGCGAGTGGCAAATGAAGTGAGCCGGGTTGCCCGGGCGTAATTGTAGCGA
TGCCTTTTGCATCGTATGAATTTAAGAAGTTAATGGCGTAAAGAATGAATACACAACAATTGGCAAAACTGCGTTCCATC
GTGCCCGAAATGCGTCGCGTTCGGCACATACATTTTGTCGGCATTGGTGGTGCCGGTATGGGCGGTATTGCCGAAGTTCT
GGCCAATGAAGGTTATCAGATCAGTGGTTCCGATTTAGCGCCAAATCCGGTCACGCAGCAGTTAATGAATCTGGGTGCGA
CGATTTATTTCAACCATCGCCCGGAAAACGTACGTGATGCCAGCGTGGTCGTTGTTTCCAGCGCGATTTCTGCCGATAAC
CCGGAAATTGTCGCCGCTCATGAAGCGCGTATTCCGGTGATCCGTCGTGCCGAAATGCTGGCTGAGTTAATGCGTTTTCG
TCATGGCATCGCCATTGCCGGAACGCACGGCAAAACGACAACCACCGCGATGGTTTCCAGCATCTACGCAGAAGCGGGGC
TCGACCCAACCTTCGTTAACGGCGGGCTGGTAAAAGCGGCGGGGGTTCATGCGCGTTTGGGGCATGGTCGGTACCTGATT
GCCGAAGCAGATGAGAGTGATGCATCGTTCCTGCATCTGCAACCGATGGTGGCGATTGTCACCAATATCGAAGCCGACCA
CATGGATACCTACCAGGGCGACTTTGAGAATTTAAAACAGACTTTTATTAATTTTCTGCACAACCTGCCGTTTTACGGTC
GTGCGGTGATGTGTGTTGATGATCCGGTGATCCGCGAATTGTTACCGCGAGTGGGGCGTCAGACCACGACTTACGGCTTC
AGCGAAGATGCCGACGTGCGTGTAGAAGATTATCAGCAGATTGGCCCGCAGGGGCACTTTACGCTGCTGCGCCAGGACAA
AGAGCCGATGCGCGTCACCCTGAATGCGCCAGGTCGTCATAACGCGCTGAACGCCGCAGCTGCGGTTGCGGTTGCTACGG
AAGAGGGCATTGACGACGAGGCTATTTTGCGGGCGCTTGAAAGCTTCCAGGGGACTGGTCGCCGTTTTGATTTCCTCGGT
GAATTCCCGCTGGAGCCAGTGAATGGTAAAAGCGGTACGGCAATGCTGGTCGATGACTACGGCCACCACCCGACGGAAGT
GGACGCCACCATTAAAGCGGCGCGCGCAGGCTGGCCGGATAAAAACCTGGTAATGCTGTTTCAGCCGCACCGTTTTACCC
GTACGCGCGACCTGTATGATGATTTCGCCAATGTGCTGACGCAGGTTGATACCCTGTTGATGCTGGAAGTGTATCCGGCT
GGCGAAGCGCCAATTCCGGGAGCGGACAGCCGTTCGCTGTGTCGCACAATTCGTGGACGTGGGAAAATTGATCCCATTCT
GGTGCCGGATCCGGCGCGGGTAGCCGAGATGCTGGCACCGGTATTAACCGGTAACGACCTGATTCTCGTTCAGGGGGCTG
GTAATATTGGAAAAATTGCCCGTTCTTTAGCTGAAATCAAACTGAAGCCGCAAACTCCGGAGGAAGAACAACATGACTGA
TAAAATCGCGGTCCTGTTGGGTGGGACCTCCGCTGAGCGGGAAGTTTCTCTGAATTCTGGCGCAGCGGTGTTAGCCGGAC
TGCGTGAAGGCGGTATTGACGCGTATCCTGTCGACCCGAAAGAAGTCGACGTGACGCAACTGAAGTCGATGGGCTTTCAG
AAAGTGTTTATCGCGCTACACGGTCGCGGCGGTGAAGATGGTACGCTGCAGGGGATGCTCGAGCTGATGGGCTTGCCTTA
TACCGGAAGCGGAGTGATGGCATCTGCGCTTTCAATGGATAAACTACGCAGCAAACTTCTATGGCAAGGTGCCGGTTTAC
CGGTCGCGCCGTGGGTAGCGTTAACCCGCGCAGAGTTTGAAAAAGGCCTGAGCGATAAGCAGTTAGCAGAAATTTCTGCT
CTGGGTTTGCCGGTTATCGTTAAGCCGAGCCGCGAAGGTTCCAGTGTGGGAATGTCAAAAGTAGTAGCAGAAAATGCTCT
ACAAGATGCATTAAGATTGGCATTTCAGCACGATGAAGAAGTATTGATTGAAAAATGGCTAAGTGGGCCGGAGTTCACGG
TTGCGATACTCGGTGAAGAAATTTTACCGTCAATACGTATTCAACCGTCCGGAACCTTCTATGATTATGAGGCGAAGTAT
CTCTCTGATGAGACACAGTATTTCTGCCCCGCAGGTCTGGAAGCGTCACAAGAGGCCAATTTGCAGGCATTAGTGCTGAA

AGCATGGACGACGTTAGGTTGCAAAGGATGGGGACGTATTGACGTTATGCTGGACAGCGATGGACAGTTTTATCTGCTGG
AAGCCAATACCTCACCGGGTATGACCAGCCACAGCCTGGTGCCGATGGCGGCACGTCAGGCAGGTATGAGCTTCTCGCAG
TTGGTAGTACGAATTCTGGAACTGGCGGACTAATATGTCGCAGGCTGCTCTGAACACGCGAAACAGCGAAGAAGAGGTTT
CTTCTCGCCGCAATAATGGAACGCGTCTGGCGGGGATCCTTTTCCTGCTGACCGTTTTAACGACAGTGTTGGTGAGCGGC
TGGGTCGTGTTGGGCTGGATGGAAGATGCGCAACGCCTGCCGCTCTCAAAGCTGGTGTTGACCGGTGAACGCCATTACAC
ACGTAATGACGATATCCGGCAGTCGATCCTGGCATTGGGTGAGCCGGGTACCTTTATGACCCAGGATGTCAACATCATCC
AGACGCAAATAGAACAACGCCTGCCGTGGATTAAGCAGGTGAGCGTCAGAAAGCAGTGGCCTGATGAATTGAAGATTCAT
CTGGTTGAATATGTGCCGATTGCGCGGTGGAATGATCAACATATGGTAGACGCGGAAGGAAATACCTTCAGCGTGCCGCC
AGAACGCACCAGCAAGCAGGTGCTTCCAATGCTGTATGGCCCGGAAGGCAGCGCCAATGAAGTGTTGCAGGGCTATCGCG
AAATGGGGCAGATGCTGGCAAAGGACAGATTTACTCTGAAGGAAGCGGCGATGACCGCGCGGCGTTCCTGGCAGTTGACG
CTGAATAACGATATTAAGCTCAATCTTGGCCGGGGCGATACGATGAAACGTTTGGCTCGCTTTGTAGAACTTTATCCGGT
TTTACAGCAGCAGGCGCAAACCGATGGCAAACGGATTAGCTACGTTGATTTGCGTTATGACTCTGGAGCGGCAGTAGGCT
GGGCGCCCTTGCCGCCAGAGGAATCTACTCAGCAACAAAATCAGGCACAGGCAGAACAACAATGATCAAGGCGACGGACA
GAAAACTGGTAGTAGGACTGGAGATTGGTACCGCGAAGGTTGCCGCTTTAGTAGGGGAAGTTCTGCCCGACGGTATGGTC
AATATCATTGGCGTGGGCAGCTGCCCGTCGCGTGGTATGGATAAAGGCGGGGTGAACGACCTCGAATCCGTGGTCAAGTG
CGTACAACGCGCCATTGACCAGGCAGAATTGATGGCAGATTGTCAGATCTCTTCGGTATATCTGGCGCTTTCTGGTAAGC
ACATCAGCTGCCAGAATGAAATTGGTATGGTGCCTATTTCTGAAGAAGAAGTGACGCAAGAAGATGTGGAAAACGTCGTC
CATACCGCGAAATCGGTGCGTGTGCGCGATGAGCATCGTGTGCTGCATGTGATCCCGCAAGAGTATGCGATTGACTATCA
GGAAGGGATCAAGAATCCGGTAGGACTTTCGGGCGTGCGGATGCAGGCAAAAGTGCACCTGATCACATGTCACAACGATA
TGGCGAAAAACATCGTCAAAGCGGTTGAACGTTGTGGGCTGAAAGTTGACCAACTGATATTTGCCGGACTGGCATCAAGT
TATTCGGTATTGACGGAAGATGAACGTGAACTGGGTGTCTGCGTCGTCGATATCGGTGGTGGTACAATGGATATCGCCGT
TTATACCGGTGGGGCATTGCGCCACACTAAGGTAATTCCTTATGCTGGCAATGTCGTGACCAGTGATATCGCTTACGCCT
TTGGCACGCCGCCAAGCGACGCCGAAGCGATTAAAGTTCGCCACGGTTGTGCGCTGGGTTCCATCGTTGGAAAAGATGAG
AGCGTGGAAGTGCCGAGCGTAGGTGGTCGTCCGCCACGGAGTCTGCAACGTCAGACACTGGCAGAGGTGATCGAGCCGCG
CTATACCGAGCTGCTCAACCTGGTCAACGAAGAGATATTGCAGTTGCAGGAAAAGCTTCGCCAACAAGGGGTTAAACATC
ACCTGGCGGCAGGCATTGTATTAACCGGTGGCGCAGCGCAGATCGAAGGTCTTGCAGCCTGTGCTCAGCGCGTGTTTCAT
ACGCAAGTGCGTATCGGCGCGCCGCTGAACATTACCGGTTTAACGGATTATGCTCAGGAGCCGTATTATTCGACGGCGGT
GGGATTGCTTCACTATGGGAAAGAGTCACATCTTAACGGTGAAGCTGAAGTAGAAAAACGTGTTACAGCATCAGTTGGCT
CGTGGATCAAGCGACTCAATAGTTGGCTGCGAAAAGAGTTTTAATTTTTATGAGGCCGACGATGATTACGGCCTCAGGCG
ACAGGCACAAATCGGAGAGAAACTATGTTTGAACCAATGGAACTTACCAATGACGCGGTGATTAAAGTCATCGGCGTCGG
CGGCGGCGGCGGTAATGCTGTTGAACACATGGTGCGCGAGCGCATTGAAGGTGTTGAATTCTTCGCGGTAAATACCGATG
CACAAGCGCTGCGTAAAACAGCGGTTGGACAGACGATTCAAATCGGTAGCGGTATCACCAAAGGACTGGGCGCTGGCGCT
AATCCAGAAGTTGGCCGCAATGCGGCTGATGAGGATCGCGATGCATTGCGTGCGGCGCTGGAAGGTGCAGACATGGTCTT
TATTGCTGCGGGTATGGGTGGTGGTACCGGTACAGGTGCAGCACCAGTCGTCGCTGAAGTGGCAAAAGATTTGGGTATCC
TGACCGTTGCTGTCGTCACTAAGCCTTTCAACTTTGAAGGCAAGAAGCGTATGGCATTCGCGGAGCAGGGGATCACTGAA
CTGTCCAAGCATGTGGACTCTCTGATCACTATCCCGAACGACAAACTGCTGAAAGTTCTGGGCCGCGGTATCTCCCTGCT
GGATGCGTTTGGCGCAGCGAACGATGTACTGAAAGGCGCTGTGCAAGGTATCGCTGAACTGATTACTCGTCCGGGTTTGA
TGAACGTGGACTTTGCAGACGTACGCACCGTAATGTCTGAGATGGGCTACGCAATGATGGGTTCTGGCGTGGCGAGCGGT
GAAGACCGTGCGGAAGAAGCTGCTGAAATGGCTATCTCTTCTCCGCTGCTGGAAGATATCGACCTGTCTGGCGCGCGCGG
CGTGCTGGTTAACATCACGGCGGGCTTCGACCTGCGTCTGGATGAGTTCGAAACGGTAGGTAACACCATCCGTGCATTTG
CTTCCGACAACGCGACTGTGGTTATCGGTACTTCTCTTGACCCGGATATGAATGACGAGCTGCGCGTAACCGTTGTTGCG
ACAGGTATCGGCATGGACAAACGTCCTGAAATCACTCTGGTGACCAATAAGCAGGTTCAGCAGCCAGTGATGGATCGCTA
CCAGCAGCATGGGATGGCTCCGCTGACCCAGGAGCAGAAGCCGGTTGCTAAAGTCGTGAATGACAATGCGCCGCAAACTG
CGAAAGAGCCGGATTATCTGGATATCCCAGCATTCCTGCGTAAGCAAGCTGATTAAGAATTGACTGGAATTTGGGTTTCG
AGGCTCTTTGTGCTAAACTGGCCCGCCGAATGTATAGTACACTTCGGTTGGATAGGTAATTTGGCGAGATAATACGATGA
TCAAACAAAGGACACTTAAACGTATCGTTCAGGCGACGGGTGTCGGTTTACATACCGGCAAGAAAGTCACCCTGACGTTA
CGCCCTGCGCCGGCCAACACCGGGGTCATCTATCGTCGCACCGACTTGAATCCACCGGTAGATTTCCCGGCCGATGCCAA
ATCTGTGCGTGATACCATGCTCTGTACGTGTCTGGTCAACGAGCATGATGTACGGATTTCAACCGTAGAGCACCTCAATG
CTGCTCTCGCGGGCTTGGGCATCGATAACATTGTTATCGAAGTTAACGCGCCGGAAATCCCGATCATGGACGGCAGCGCC
GCTCCGTTTGTATACCTGCTGCTTGACGCCGGTATCGACGAGTTGAACTGCGCCAAAAAATTTGTTCGCATCAAAGAGAC
TGTTCGTGTCGAAGATGGCGATAAGTGGGCTGAATTTAAGCCGTACAATGGTTTTTCGCTGGATTTCACCATCGATTTTA
ACCATCCGGCTATTGATTCCAGCAACCAGCGCTATGCGATGAACTTCTCCGCTGATGCGTTTATGCGCCAGATCAGCCGT
GCGCGTACGTTCGGTTTCATGCGTGATATCGAATATCTGCAGTCCCGTGGTTTGTGCCTGGGCGGCAGCTTCGATTGTGC
CATCGTTGTTGACGATTATCGCGTACTGAACGAAGACGGCCTGCGTTTTGAAGACGAATTTGTGCGTCACAAAATGCTCG
ATGCGATCGGTGACTTGTTCATGTGTGGTCACAATATTATTGGTGCATTTACCGCTTATAAATCCGGTCATGCACTGAAT
AACAAACTGCTGCAGGCTGTCCTGGCGAAACAGGAAGCCTGGGAATATGTGACCTTCCAGGACGACGCAGAACTGCCGTT

GGCCTTCAAAGCGCCTTCAGCTGTACTGGCATAACGACATTTATACTGTCGTATAAAATTCGACTGGCAAATCTGGCACT
CTCTCCGGCCAGGTGAACCAGTCGTTTTTTTTTGAATTTTATAAGAGCTATAAAAAACGGTGCGAACGCTGTTTTCTTAA
GCACTTTTCCGCACAACTTATCTTCATTCGTGCTGTGGACTGCAGGCTTTAATGATAAGATTTGTGCGCTAAATACGTTT
GAATATGATCGGGATGGCAATAACGTGAGTGGAATACTGACGCGCTGGCGACAGTTTGGTAAACGCTACTTCTGGCCGCA
TCTCTTATTAGGGATGGTTGCGGCGAGTTTAGGTTTGCCTGCGCTCAGCAACGCCGCCGAACCAAACGCGCCCGCAAAAG
CGACAACCCGCAACCACGAGCCTTCAGCCAAAGTTAACTTTGGTCAATTGGCCTTGCTGGAAGCGAACACACGCCGCCCG
AATTCGAACTATTCCGTTGATTACTGGCATCAACATGCCATTCGCACGGTAATCCGTCATCTTTCTTTCGCAATGGCACC
GCAAACACTGCCCGTTGCTGAAGAATCTTTGCCTCTTCAGGCGCAACATCTTGCATTACTGGATACGCTCAGCGCGCTGC
TGACCCAGGAAGGCACGCCGTCTGAAAAGGGTTATCGCATTGATTATGCGCATTTTACCCCACAAGCAAAATTCAGCACG
CCCGTCTGGATAAGCCAGGCGCAAGGCATCCGTGCTGGCCCTCAACGCCTCACCTAACAACAATAAACCTTTACTTCATT
TTATTAACTCCGCAACGCGGGGCGTTTGAGATTTTATTATGCTAATCAAATTGTTAACTAAAGTTTTCGGTAGTCGTAAC
GATCGCACCCTGCGCCGGATGCGCAAAGTGGTCAACATCATCAATGCCATGGAACCGGAGATGGAAAAACTCTCCGACGA
AGAACTGAAAGGGAAAACCGCAGAGTTTCGTGCACGTCTGGAAAAAGGCGAAGTGCTGGAAAATCTGATCCCGGAAGCTT
TCGCCGTGGTACGTGAGGCAAGTAAGCGCGTCTTTGGTATGCGTCACTTCGACGTTCAGTTACTCGGCGGTATGGTTCTT
AACGAACGCTGCATCGCCGAAATGCGTACCGGTGAAGGAAAAACCCTGACCGCAACGCTGCCTGCTTACCTGAACGCACT
AACCGGTAAAGGCGTGCACGTAGTTACCGTCAACGACTACCTGGCGCAACGTGACGCCGAAAACAACCGTCCGCTGTTTG
AATTCCTTGGCCTGACTGTCGGTATCAACCTGCCGGGCATGCCAGCACCGGCAAAGCGCGAAGCTTACGCAGCTGACATC
ACTTACGGTACGAACAACGAATACGGCTTTGACTACCTGCGCGACAACATGGCGTTCAGCCCTGAAGAACGTGTACAGCG
TAAACTGCACTATGCGCTGGTGGACGAAGTGGACTCCATCCTGATCGATGAAGCGCGTACACCGCTGATCATTTCCGGCC
CGGCAGAAGACAGCTCGGAAATGTATAAACGCGTGAATAAAATTATTCCGCACCTGATCCGTCAGGAAAAAGAAGACTCC
GAAACCTTCCAGGGCGAAGGCCACTTCTCGGTGGACGAAAAATCTCGCCAGGTGAACCTGACCGAACGTGGTCTGGTGCT
GATTGAAGAACTGCTGGTGAAAGAGGGCATCATGGATGAAGGGGAGTCTCTGTACTCTCCGGCCAACATCATGCTGATGC
ACCACGTAACGGCGGCGCTGCGCGCTCATGCGCTGTTTACCCGTGACGTCGACTACATCGTTAAAGATGGTGAAGTTATC
ATCGTTGACGAACACACCGGTCGTACCATGCAGGGCCGTCGCTGGTCCGATGGTCTGCACCAGGCTGTGGAAGCGAAAGA
AGGTGTGCAGATCCAGAACGAAAACCAAACGCTGGCTTCGATCACCTTCCAGAACTACTTCCGTCTGTATGAAAAACTGG
CGGGGATGACCGGTACTGCTGATACCGAAGCTTTCGAATTTAGCTCAATCTACAAGCTGGATACCGTCGTTGTTCCGACC
AACCGTCCAATGATTCGTAAAGATCTGCCGGACCTGGTCTACATGACTGAAGCGGAAAAAATTCAGGCGATCATTGAAGA
TATCAAAGAACGTACTGCGAAAGGCCAGCCGGTGCTGGTGGGTACTATCTCCATCGAAAAATCGGAGCTGGTGTCAAACG
AACTGACCAAAGCCGGTATTAAGCACAACGTCCTGAACGCCAAATTCCACGCCAACGAAGCGGCGATTGTTGCTCAGGCA
GGTTATCCGGCTGCGGTGACTATCGCGACCAATATGGCGGGTCGTGGTACAGATATTGTGCTCGGTGGTAGCTGGCAGGC
AGAAGTTGCCGCGCTGGAAAATCCGACCGCAGAGCAAATTGAAAAAATTAAAGCCGACTGGCAGGTACGTCACGATGCGG
TACTGGAAGCAGGTGGCCTGCATATCATCGGTACCGAGCGTCACGAATCCCGTCGTATCGATAACCAGTTGCGCGGTCGT
TCTGGTCGTCAGGGGGATGCTGGTTCTTCCCGTTTCTACCTGTCGATGGAAGATGCGCTGATGCGTATTTTTGCTTCCGA
CCGAGTATCCGGCATGATGCGTAAACTGGGTATGAAGCCAGGCGAAGCCATTGAACACCCGTGGGTGACTAAAGCGATTG
CCAACGCCCAGCGTAAAGTTGAAAGCCGTAACTTCGACATTCGTAAGCAACTGCTGGAATATGATGACGTGGCTAACGAT
CAGCGTCGCGCCATTTACTCCCAGCGTAACGAACTGTTGGATGTCAGCGATGTGAGCGAAACCATTAACAGCATTCGTGA
AGATGTGTTCAAAGCGACCATTGATGCCTACATTCCACCACAGTCGCTGGAAGAAATGTGGGATATTCCGGGGCTGCAGG
AACGTCTGAAGAACGATTTCGACCTCGATTTGCCAATTGCCGAGTGGCTGGATAAAGAACCAGAACTGCATGAAGAGACG
CTGCGTGAGCGCATTCTGGCGCAGTCCATCGAAGTGTATCAGCGTAAAGAAGAAGTGGTTGGTGCTGAGATGATGCGTCA
CTTCGAGAAAGGCGTCATGCTGCAAACGCTTGACTCCCTGTGGAAAGAGCACCTGGCAGCGATGGACTATCTGCGTCAGG
GTATCCACCTGCGTGGCTACGCACAGAAAGATCCGAAGCAGGAATACAAACGTGAATCGTTCTCCATGTTTGCAGCGATG
CTGGAGTCGTTGAAATATGAAGTTATCAGTACGCTGAGCAAAGTTCAGGTACGTATGCCTGAAGAGGTTGAGGAGCTGGA
ACAACAGCGTCGTATGGAAGCCGAGCGTTTAGCGCAAATGCAGCAGCTTAGCCATCAGGATGACGACTCTGCAGCCGCAG
CTGCACTGGCGGCGCAAACCGGAGAGCGCAAAGTAGGACGTAACGATCCTTGCCCGTGCGGTTCTGGTAAAAAATACAAG
CAGTGCCATGGCCGCCTGCAATAAAAGCTAACTGTTGAAGTAAAAGGCGCAGGATTCTGCGCCTTTTTTATAGGTTTAAG
ACAATGAAAAAGCTGCAAATTGCGGTAGGTATTATTCGCAACGAGAACAATGAAATCTTTATAACGCGTCGCGCAGCAGA
TGCGCACATGGCGAATAAACTGGAGTTTCCCGGCGGTAAAATTGAAATGGGTGAAACGCCGGAACAGGCGGTGGTGCGTG
AACTTCAGGAAGAAGTCGGGATTACCCCCCAACATTTTTCGCTATTTGAAAAACTGGAATATGAATTCCCGGACAGGCAT
ATAACACTGTGGTTTTGGCTGGTCGAACGCTGGGAAGGGGAGCCGTGGGGTAAAGAAGGGCAACCCGGTGAGTGGATGTC
GCTGGTCGGTCTTAATGCCGATGATTTTCCGCCAGCCAATGAACCGGTAATTGCGAAGCTTAAACGTCTGTAGGTCAGAT
AAGGCGTTTTCGCCGCATCCGACATTCGCACACGATGCCTGATGCGACGCTGGCGCGTCTTATCAGGCCTAAAGGGATTT
CTAACTCATTGATAAATTTGTTTTTGTAGGTCGGATAAGGCGTTCACGCCGCATCCGACATTTGCACAAGATGCCTGATG
CGACGCTGTCCGCGTCTTATCAGGCCTACGTGCGGCATCAGACAAATGTCACTGCTTTGGTTCTTCGCTCCAGTCATCGC
TTTCGGAAAGATCGCCACTGCTGGGGATTCGTTTTTCTTCAGCAGCCCATTCTCCGAGGTCGATCAGCTGACAACGTTTG
GAGCAAAATGGCCGAAACGGGCTGATTTCACCCCACACCACCGTTTTCCCGCAGGTTGGGCAATTCACCGTAATAGTTTC
TGACATTTTTACTCCTTAGCAACAGGCCAGTTCGAAATCCAGACGTTCCGGTACCTGTCCGTTTTCAGTGTCCAGCGGCA

TAAAACGAATGGCAAAACGGCTCTTATGTCCGGAAATTTGCGGATAAAGCTGTGAATCGAGCGACAGATTCAGGCGCAGC
AAGTCGGCATCGCCACCGTTATCCTGATAAAAACCATTCAGGCTGGTTTGTTTACGGAAGGGGGCCGACTGGCGAATTAA
ATCCAGCACCATGGTAAGTGCCTGGGTGAGCGGGTTCAGGCTGGCAATCCAGGTTTCTACCTGGCTGTCGCGCTGCGCCT
GGGGTAGATGCAGCCAAATGTGCAATGTAGGTAAATCAAAGCTGCAACAGCCGCCTGGGATGCTCAGTCGCTGACGCACC
AGAGCAATCAAACGATCTTCACGCAGAAATTGCCCGATACGCGGCGCGGAAATTAATACGCTCCCCGCCGCTTTTAACTG
CTGAATTAATGCTTCAATACGGCTCTGGTCCACGCCAGGCACGCCAATCCAGGTCTGGAGTTTACGTTGCTGCCGGTCAA
GTTCTTTCAACAGCTCAGTGCGGACTTCGCCGCGCTCGAAAACATCCAGTAATTCACTGACATTACGGAAGAAATGCAGC
GCGCCAGCGTGGTCAACGATGGGTAAATTAACGGTGAGTTGCTGAATCAAAAACTCAATGCGCAGCCATGTACGCATTTT
TTCATTTAGTGGATGTTCAAAAAGGACCTGGGTCTGCATTACGGTTTTTCCTGTGAGACAAACTGCGACGCAAGCTGCAA
ATAGTGTGCGTGCAGGCGGGCAACATCCGATGCGATAGCATCCGGTGCGCCGTTATTATCAATGACGTCATCTGCCACGG
CAAGGCGGGCTTCGCGCGTTGCCTGAGCAGCAAGGATTTGTTCGACATGCTCGCGAGTTACATCATCGCGCTGCATGGTG
CGCTTAAGTTGCGTTTCTGGGCTGACATCCACCACAAGCACTCGATTCGCTTTTTTATACAGTGAGTTTTCTACCAGCAA
TGGCACAACCCACAGTACATAGGGGGAAGTAGCTTGCTGGATCTGGTGTTGCGTCTCTTGCTGAATCAGCGGATGCAGCA
GGGCGTTAAGCCAGTTTTTCTCTTCCGGGTTGGCGAAGATCCGCTCGCGCAAGGCCCGGCGCTGCAATGTTCCATCAGCA
GCAATCATGTTAGCGCCAAAGTGATCAGCAATGGCATGTAGCGCAGGTGCACCTGGTTCAACCACCTGACGCGCAATAAT
ATCGGCATCAATGACGTTAATTCCGAGATCAGCAAACGCATTGGCAACGGTACTCTTGCCACTGCCAATGCCTCCCGTTA
AGGCAACTATATACCTCATAATTTATATTCCCGGGAATTCATCATGATTATCAAAACGTTAAAAATGAGTGCACGAAAGC
GAAATTGATGAAACGTTCGCTCACTATTTACCAGGTAAATTTATGGGATTGTAGCGTAAAAAAAGACAATTTCGCAGTCT
TGCGCCGCATTGATTAGTGCGTATGATAGCGTCACTGGAGTTGCGCTCTTACCCTTATAGCCATTAACCCCAGGAATCCG
CACATGCGTATTGAAGAAGATCTGAAGTTAGGTTTTAAAGACGTTCTCATCCGCCCTAAACGCTCCACTCTTAAAAGCCG
TTCCGATGTTGAACTGGAACGTCAATTCACCTTCAAACATTCAGGTCAGAGCTGGTCCGGCGTGCCGATTATCGCCGCAA
ATATGGACACCGTAGGCACATTTTCTATGGCCTCTGCGCTGGCTTCTTTTGATATTTTGACTGCTGTGCATAAACACTAT
TCTGTCGAAGAGTGGCAAGCGTTTATCAACAATTCTTCCGCTGATGTGCTGAAACATGTGATGGTTTCTACCGGTACGTC
TGATGCGGATTTCGAAAAAACTAAACAGATTCTCGACCTGAACCCGGCATTAAACTTCGTTTGTATTGACGTGGCGAATG
GTTATTCCGAACACTTCGTGCAGTTCGTTGCGAAAGCGCGTGAAGCGTGGCCGACCAAAACCATTTGTGCTGGTAACGTA
GTGACTGGTGAAATGTGTGAGGAGCTTATCCTCTCAGGTGCCGATATCGTTAAAGTTGGCATTGGCCCAGGTTCTGTTTG
TACAACTCGCGTCAAAACAGGCGTCGGTTATCCGCAACTTTCTGCGGTAATCGAATGTGCCGATGCTGCGCACGGTCTGG
GCGGAATGATCGTCAGCGATGGTGGCTGCACCACGCCGGGCGATGTGGCGAAAGCCTTTGGCGGCGGTGCCGATTTCGTC
ATGCTTGGCGGCATGCTGGCGGGCCACGAAGAGAGCGGCGGTCGCATCGTTGAGGAGAACGGCGAGAAATTTATGCTGTT
CTACGGCATGAGCTCCGAGTCTGCGATGAAACGTCACGTTGGCGGCGTTGCGGAATATCGCGCAGCAGAAGGTAAAACCG
TTAAGCTGCCGCTGCGAGGCCCGGTTGAAAATACCGCGCGAGATATTTTGGGCGGCCTGCGTTCAGCTTGTACATACGTT
GGGGCTTCACGCCTGAAAGAGCTGACCAAGCGCACCACGTTTATTCGTGTGCAGGAACAAGAAAACCGCATCTTCAACAA
CCTGTAATCTCCCAACGCTGGCGTGGAGCAACACGCCAGCGTTATCCCATCCCACTCATCGCATCGCCTAAATGGAAAAT
TGGCAGATACATTGCCACCACCAGCGTACCAATAATTCCTCCCGTTATGATCAGCAACGCCGGTTCCAGTAAGGCTGCGA
GGTTATCCGCCAGCGCCATTGTGTTTTCCCGATGATGATGGGCGAGGTTGTCTAACATGAGATCCAGAGAGCCGGATGCC
TCTCCTGTTCTCACTAATTGCAAACAGAGCGGGCTAAACTCACCGGTATTTTTTAGCGCCAGCCAGATGGGTTGACCGTT
ACTGATATCGTGCTGGATTTGTGTCAGAAGTTGCACCCAGTACGGGCAGCGCATTGTTTCTCTGACGCTCTCTACGCCCT
GTAAAAAAGTAATGCCTGCACTTTGTGTCAGCGCCAGAATCGTAAAGATCTGCGTGAGTTTTTGTCCCCGCATCAGTGAA
CCCATAATCGGGATGCGTAACAGCAATTTCTGCCGCACTATAAGCCAGGTCGGTCGGCGCATCAGCAACTTATTGGCTAT
CGCCAGCAGAAAGCCGAACAACACCAGCAGCCAGCTCCATTCGCCACTAAAGTCTGCCAGCGTCATGATCCCCTGCGTTA
GTGCCGGTAGTGGGGTGTTGAAGGTCTTATAGATAGCGGCAAACTCCGGCAGAACAAAATGCAGCATTGCCACAACCACC
ATGATTGCCATCGCTAAAATGATGATGGGATAACGTAACGCTGATTTCACTTTGTCGGTCAACTGACGCTGGGCTTTTTG
CTGACGCGCCAGTTCAAAGCAGCATTCATCCAGCTTACCGGTCAGTTCACCCGTGCGGATCATCGCCTGATAGAGCGGCG
GAAATACCTCTGACCAGGGTAATAAGGCATTGGAAAAAGCAATGCCCTGTTCGAGATCGTGCGCCAGCGATTGCAGCAAC
GCTTGCCATTGCTTACTGGGATGCTGTTCCGCCAGCAGAGCCAGCCCTTCAGAAAGCGTTAACCCTGCTTTGAGTAGCGT
CGCCAGTTGATGAATGACTTCCGCGCTTTTATCTCCTCGCCACTGCGCAGAATTGATGGCGATTCGCTTCAGGCTTAGTG
GGGTAACCATCTGTTGCTGTAGTGCCATAAGCAGTAAAGTACGGCTCTCTGCCCATAGCATCCCATCTTGCGCATTGCCG
TCGCCGGTTATGCCATGCCAGCGCCAGAGTTGCTTACTCGCCATGCGGCATCCCCAGTACGCGGATTAACTCTTCAAAGG
TGGTTAAGCCTTGCTCTACGGCCAGGCAGCCGTTTTCAAAAAGCGTACGCATACCCGCCTGTCGTGCGTGCGTTTCCAGC
GATTCAACGTCGGTATTAGCGGAAATAAGCTGACGAATGACCGGCGTTATGGGCAGAACTTCAAATAAGGCCGTACGACC
ATAAAAACCGTGGTAGCAATGTACACAACCGGGTGCCTGCCAGTGGGGCAGCGGCGATGGCCATACATTGTCTGGAATGT
GGATGGGCTCCCCTTGCTGCCGGCGACAATGTGGGCAAAGTTTGCGTACCAGACGCTGGGCTATTACCAGCGTAAGCGCC
GATGATAGCATCCAGCGGGCGACCCCCATTTGCTGTAAACGTACCAGCGTTTCGCAGGTGGAATTAGTGTGTAGGGTAGA
CAACACCAGGTGACCAGTTTGCGCCGCTTTAATAGCGATCTCTGCTGTTTCGCCATCGCGGATCTCTCCGATCATGATGA
CGTCAGGATCCTGGCGCAATAACGCACGCAAAACGCCCTGAAAGGTGAGTCCGGCACGCGGATGGATTTGCGTCTGGTTT
AGTCCGGCTATGGGGATCTCAACTGGATCTTCGACGCTACAAATATTAATGTCAGCGGTATTCAGCTTTTGCAGGGCACT

ATAAAGCGTGACCGTTTTGCCGCTGCCGGTAGGGCCAGTTACCAGCACCAGTCCCTGTGGTTGTTGCAAGGCATGAGCAA
AGTCCGCCAGTTGTAACGGCTGCATTCCAAGCGTGTTGACATCCAGTGCCTGACCCACCTGCTGTAACAACCTTAATACC
ACCTTTTCACCACCCCGACATGGTAAGGTCGCAATACGAAATGAGACGGCGTTTCCTGCCAGTTCGACAGTGAATTGCCC
GTCCTGCGGCAGGCGATGTTCCGCAATATCCAGGTTTCCCAGCACTTTTAATCTGGCGGTTAATGCGACTCCGGCATCCG
GTGAAACATCCGGTAAAGGATGCAATACGCCGTCGATACGCAAGCGGATGCGGTAGGCATTGTCCGCTGGTTCGATATGA
ATATCAGACGCGCGTTGTTCCAGCGCAGATTGTAACGTTCGAGTTAGCAACTCTGCTTTGGGCTGATGCTTCTCCTGAAC
AGCTACGGGCAATGTCTGTTGTGTGCGACTGGCGTGACCTTCCATTTGTTGGCGCGTCCAGCAGGTGATCTCAATACGTT
TGGTGGTAGCGAAATGCAATGCGTCCAGTAGCTCATGCGAAGGTGCATCGACTACCGCAACATGAACCACCTCTTCGCTG
GCATCCAGCAAGACTCCATGATAACGCAGACACAGGGCAGTGAGCTGTGGAATATTCATTGCCGCTCCTTAGTTGGCGTC
ATCAAAGCGGAAGACATCTTCGCAGGCTTGCTGCAATGCGCTGTCACTTTGAATATTGCAGTTGCGCGTCCAGCCGGTGA
CGCCGTTTGCGTTATCCCAACCCGGTGTCATGACGACGCTTAGCCCATTGAGACTTTCTTGCCCGGTCAGCGACACCACG
CCCTTTGCCACACTCATGGCTGAAACATAGCGGGTGGTGGTAGGCGAGGGAATGCCATTGCTGCCACCGTCGCAGGTATC
TAATCCACCATGTTCCAGCGCGCACAACTCTACGGCGGTACGGTAAGGCACAAAGGTTTGTAGCATGTCGGTGAGTGCGG
CTTTGCGCAGGTAGTTTTGATAAGCGGGAATACCAATGGCGCTTAAAATGGCAATGATGCCAATAACCACCATCAGTTCG
ATAAGTGTAAAACCGCGTTGCTTGTCCATCTGTTTCGCTCCTTGATTTGGTTGGCGCTACTTTGGCAAACGCCATCAGCA
CAGGCGAGGGGCAAAAAACGAAACGGGAAAGCAGATTCCGAGGTTTTTTATTTCGTTGCAGCGAAAGACAAGAAATTTGC
GAGGCGTTACGAAGAAAGTTGGGGAAGGGGAGATTATCCGCCCGCGATGGAGCGGATAAATCTGTCAACTATTAGCGAAA
ACGCATTGAAAGGTCGAGTGCTTGTACGTGTTTAGTTAGCGCACCGACGGAGATAAAGTCCACGCCCGTTTCGGCAAATT
CACGCAGTGTTTTGTCAGTGACGTTGCCAGACACTTCCAGTAGCGCCTTGCCGTTGGTGCGTTTGACGGCTTCGCGCATC
TGTTCTGTTTCGAAGTTATCCAGCATGATGATATCGGCTCCTGCTTTCAGGGCTTCATCAAGTTCTTCCAGATTCTCTAC
TTCGACTTCTACTGGCGCATCCGGGTGCAGCCAGGACGCTTTTTCGACCGCCTGGCGCACTGAGCCGGAGGCAATAATAT
GGTTTTCTTTGATCAGGAAGGCATCAGAAAGCCCCAGACGGTGATTCGCTCCGCCGCCGCAAAGTACCGCGTATTTCAGA
GCTGAACGCAGGCCGGGTAAGGTTTTGCGCGTATCCAACAACTGCGTGTTGGTGCCTTCCAGCAATTCGACATAGTGGCG
TACCTTACTGGCAACTCCTGAAAGGGTTTGCACAAAATTAAGCGCAGTGCGTTCGCCCGTTAACAGCACGCGGGATGGGC
CTTCAAGTTCGAACAAGGATTGATTGGCATTGATGACATCGCCGTCATCCACATGCCAGATTATGGTGACATCGTCGCCT
GCCAGTTGAATAAACACCTCTTCAACCCAGCGTTTGCCGCAAAAGACGCCATTCTCGCGGGTGATCACCGTGGCATGAGA
GCGAGAATTTTCCGGTAAAAGTTTTGCCGTAATATCATTGTTGGCATCGACTGTTCCGCCTAAATCTTCCCGCAGCGCCT
GGGCCACCGCGCCGGGGATATCGAGATTAATGCGTTCCAGCAGCTCGTCACGTCGGGTGTCAGGGTTATAGCGGCGAGGC
GGCATGTTAAAACTCCAGATAGCTAACGAATCATAAGGTAGAAACATGCTACTCTGAACCGGGTATTAGCACCACATATA
AGGAGATCCTGCATGTTGTTAGAACAGGGGTGGCTGGTTGGCGCGCGCCGCGTTCCCTCACCACATTACGATTGCCGCCC
GGATGACGAAACACCCACCCTGCTGGTGGTGCACAATATTAGCCTGCCGCCAGGCGAGTTTGGCGGTCCGTGGATCGACG
CATTATTCACTGGAACTATTGATCCGCAGGCACATCCTTTCTTTGCTGAGATCGCCCATTTGCGCGTCTCCGCTCACTGT
TTGATTCGCCGTGATGGTGAAATAGTCCAGTATGTTCCTTTCGATAAACGTGCATGGCATGCGGGAGTCTCTCAGTATCA
GGGGCGCGAACGCTGCAATGATTTTTCTATTGGGATTGAGCTTGAAGGCACCGATACGCTGGCGTATACCGATGCGCAGT
ATCAACAGCTTGCGGCGGTTACGCGGGCACTGATTGATTGCTATCCGGATATCGCTAAAAACATGACGGGCCATTGTGAT
ATTGCGCCGGATCGGAAAACCGATCCCGGTCCTGCATTTGATTGGGCACGGTTTCGTGTGCTGGTCAGCAAGGAGACAAC
ATGACGCTATTTACAACCTTACTGGTGTTAATTTTCGAGCGCCTGTTTAAGTTGGGCGAGCACTGGCAGCTTGATCATCG
TCTTGAAGCGTTCTTTCGGCGGGTGAAACATTTTTCTCTCGGGCGCACGTTAGGCATGACCATTATTGCGATGGGCGTGA
CTTTTTTACTGTTACGCGCATTGCAGGGAGTATTGTTCAACGTTCCCACGCTACTGGTGTGGCTGCTGATTGGTTTGCTG
TGTATTGGCGCAGGTAAAGTTCGTCTTCATTATCATGCTTATCTGACAGCTGCTTCACGTAATGATAGCCATGCCCGTGC
CACGATGGCTGGCGAACTCACCATGATTCACGGCGTCCCGGCAGGCTGCGACGAACGTGAGTATTTGCGTGAGCTGCAAA
ATGCATTGCTGTGGATTAACTTTCGTTTTTATCTTGCACCGCTGTTCTGGCTGATTGTGGGGGGAACCTGGGGACCCGTT
ACGCTGATGGGGTATGCGTTTTTGCGTGCATGGCAATACTGGCTGGCGCGATATCAGACGCCGCATCATCGTTTACAGTC
CGGCATTGATGCCGTGCTTCATGTACTGGATTGGGTGCCGGTTCGTCTTGCGGGTGTGGTATATGCCTTGATCGGTCATG
GTGAGAAAGCGTTACCGGCCTGGTTTGCTTCGCTGGGTGATTTCCATACTTCGCAGTATCAGGTGTTAACGCGTCTGGCG
CAGTTCTCTCTGGCGCGTGAACCGCATGTCGATAAGGTGGAGACGCCGAAGGCAGCGGTTTCAATGGCGAAGAAAACCTC
GTTCGTGGTCGTGGTGGTGATTGCACTACTGACGATTTACGGGGCGTTGGTGTAAAGATTATTGCCCTCACCCTGTACGG
GTGAGGGCGTAGAGAGATTAATGCGCTTTTACGGCTTTGGCGGTTTTCTCTTTAAACAGATAGCCGATACCTAACACGAT
CAGCCATACCGGGATCAGGTATACCGAAATCGCCATTCCTGGGGTCATCAGCATAATCACCAGTACCGCCGCCATAAACA
GCAGGCAGATCCAGTTACCCAGCGGATAAAGCAGAGCAGGGAAGCGAGTTACCACGCCTTGTTCCTGCTTGGCGCGACGG
AATTTCATATGCGCCAGGCTAATCATCGCCCAGTTGATTACCAGTGCAGATACCACCAGCGCCATTAACAGTCCGAAAGC
GGACTCTGGGGCAAGGTAGTTAATCAGTACGCACAACGCCGTTACCAGTGCAGACACCAGAATGGTATTTACTGGTACAC
CACGTTTATCGACAGACGCCAGCGCTTTTGGCGCATTACCCTGTTGTGCCAGACCAAACAGCATACGGCTGTTGCAATAT
ACGCAGCTGTTGTACACGGAGAGCGCCGCAGTCAGTACCACGATGTTCAGCGCATTCGCCACAAAGGTATCGCCTAACTC
GTGGAAGATCAGCACAAACGGACTGGTATCGGCGGTAACGCGGGTCCACGGCATCAGTGAGAGCAGAACGGCTAACGAAC
CAATATAGAAAATCAGGATGCGGTAGATAACCTGGTTAGTTGCTTTCGGTATACTTTGCTCCGGGTTATCAGCTTCTGCT

GCGGTGATCCCCACCAGTTCCAGACCACCGAACGAGAACATGATAATCGCCATCATCATCACCAGCCCGGTGAAGCCGTG
CGGCAGGAAACCACCCTGATCCCACAGGTTGCTAACGGTCGCCTGCGGGCCGCCGTTGCCACTGAATAGCAGCCAGCCGC
CGAAGATGATCATCGCTACCACCGCGATAACTTTGATAATGGCAAACCAGAACTCCATCTCGCCAAACACTTTAACGTTG
GTCAGGTTGATGGCGTTAATCACCACAAAGAATACGGCGGCAGAAACCCAGGTGGGGATTTCCGGATACCAGAACTGAAT
GTATTTACCCACGGCAGTCAGCTCAGCCATGGCAACTAAAACGTACAGTACCCAGTAGTTCCAGCCAGAGGCGAAACCGG
CAAAACTGCCCCAGTATTTATAAGCAAAGTGGCTAAAGGAGCCTGCGACAGGTTCTTCGACCACCATTTCACCCAGCTGA
CGCATGATCAGAAAGGCGATAAAACCAGCAATGGCGTAACCCAGGATAATCCCTGGCCCTGCGGACTGTATTACGGAGGC
GCTACCCAGGAATAACCCGGTCCCTATCGCGCCACCCAGCGCGATAAGCTGAATATGGCGGTTTTTAAGGCCGCGCTTTA
GCTGCTCGCCGTGCTGTTGACCTTCCATCATGAAACCTCGTGCGGTGGTTGTTTTTTTGATCTACGCAGTGATGCGTGTG
TAAGTTTGCAATTCCGTTTGTTGTATTAATTTGTTTACATCAAAGAAGTTTGAATTGTTACAAAAAGACTTCCGTCAGAT
CAAGAATAATGGTATGCGGCAGCGAATGCACCCGCTTTATGCATGGTTGAAGATGAGTTGCTTAAAAAGAAACCGTTTGT
AAAGCTCAGCCTCAACCCCTCTCAATATGTAGAATGAATTTAAATTCGTTTTAATTGAATTAAAAATCACAAAATTGGTA
AGTGAATCGGTTCAATTCGGATTTTTATAGTTTAATAATCGTTAAAAAACTCCTTTCCTACGTAAAGTCTACATTTGTGC
ATAGTTACAACTTTGAAACGTTATATATGTCAAGTTGTTAAAATGTGCACAGTTTCATGATTTCAATCAAAACCTGTATG
GACATAAGGTGAATACTTTGTTACTTTAGCGTCACAGACATGAAATTGGTAAGACCAATTGACTTCGGCAAGTGGCTTAA
GACAGGAACTCATGGCCTACAGCAAAATCCGCCAACCAAAACTCTCCGATGTGATTGAGCAGCAACTGGAGTTTTTGATC
CTCGAAGGCACTCTCCGCCCGGGCGAAAAACTCCCACCGGAACGCGAACTGGCAAAACAGTTTGACGTCTCCCGTCCCTC
CTTGCGTGAGGCGATTCAACGTCTCGAAGCGAAGGGCTTGTTGCTTCGTCGCCAGGGTGGCGGCACTTTTGTCCAGAGCA
GCCTATGGCAAAGCTTCAGCGATCCGCTGGTGGAGCTGCTCTCCGACCATCCTGAGTCACAGTATGACTTGCTCGAAACA
CGACACGCCCTGGAAGGTATCGCCGCTTATTACGCCGCGCTGCGTAGTACCGATGAAGACAAGGAACGCATCCGTGAACT
CCACCACGCCATAGAGCTGGCGCAGCAGTCTGGCGATCTGGACGCGGAATCAAACGCCGTACTCCAGTATCAGATTGCCG
TCACCGAAGCGGCCCACAATGTGGTTCTGCTTCATCTGCTAAGGTGTATGGAGCCGATGTTGGCCCAGAATGTCCGCCAG
AACTTCGAATTGCTCTATTCGCGTCGCGAGATGCTGCCGCTGGTGAGTAGTCACCGCACCCGCATATTTGAAGCGATTAT
GGCCGGTAAGCCGGAAGAAGCGCGCGAAGCATCGCATCGCCATCTGGCCTTTATCGAAGAAATTTTGCTCGACAGAAGTC
GTGAAGAGAGCCGCCGTGAGCGTTCTCTGCGTCGTCTGGAGCAACGAAAGAATTAGTGATTTTTCTGGTAAAAATTATCC
AGAAGATGTTGTAAATCAAGCGCATATAAAAGCGCGGCAACTAAACGTAGAACCTGTCTTATTGAGCTTTCCGGCGAGAG
TTCAATGGGACAGGTTCCAGAAAACTCAACGTTATTAGATAGATAAGGAATAACCCATGTCAGAACGTTTCCCAAATGAC
GTGGATCCGATCGAAACTCGCGACTGGCTCCAGGCGATCGAATCGGTCATCCGTGAAGAAGGTGTTGAGCGTGCTCAGTA
TCTGATCGACCAACTGCTTGCTGAAGCCCGCAAAGGCGGTGTAAACGTAGCCGCAGGCACAGGTATCAGCAACTACATCA
ACACCATCCCCGTTGAAGAACAACCGGAGTATCCGGGTAATCTGGAACTGGAACGCCGTATTCGTTCAGCTATCCGCTGG
AACGCCATCATGACGGTGCTGCGTGCGTCGAAAAAAGACCTCGAACTGGGCGGCCATATGGCGTCCTTCCAGTCTTCCGC
AACCATTTATGATGTGTGCTTTAACCACTTCTTCCGTGCACGCAACGAGCAGGATGGCGGCGACCTGGTTTACTTCCAGG
GCCACATCTCCCCGGGCGTGTACGCTCGTGCTTTCCTGGAAGGTCGTCTGACTCAGGAGCAGCTGGATAACTTCCGTCAG
GAAGTTCACGGCAATGGCCTCTCTTCCTATCCGCACCCGAAACTGATGCCGGAATTCTGGCAGTTCCCGACCGTATCTAT
GGGTCTGGGTCCGATTGGTGCTATTTACCAGGCTAAATTCCTGAAATATCTGGAACACCGTGGCCTGAAAGATACCTCTA
AACAAACCGTTTACGCGTTCCTCGGTGACGGTGAAATGGACGAACCGGAATCCAAAGGTGCGATCACCATCGCTACCCGT
GAAAAACTGGATAACCTGGTCTTCGTTATCAACTGTAACCTGCAGCGTCTTGACGGCCCGGTCACCGGTAACGGCAAGAT
CATCAACGAACTGGAAGGCATCTTCGAAGGTGCTGGCTGGAACGTGATCAAAGTGATGTGGGGTAGCCGTTGGGATGAAC
TGCTGCGTAAGGATACCAGCGGTAAACTGATCCAGCTGATGAACGAAACCGTTGACGGCGACTACCAGACCTTCAAATCG
AAAGATGGTGCGTACGTTCGTGAACACTTCTTCGGTAAATATCCTGAAACCGCAGCACTGGTTGCAGACTGGACTGACGA
GCAGATCTGGGCACTGAACCGTGGTGGTCACGATCCGAAGAAAATCTACGCTGCATTCAAGAAAGCGCAGGAAACCAAAG
GCAAAGCGACAGTAATCCTTGCTCATACCATTAAAGGTTACGGCATGGGCGACGCGGCTGAAGGTAAAAACATCGCGCAC
CAGGTTAAGAAAATGAACATGGACGGTGTGCGTCATATCCGCGACCGTTTCAATGTGCCGGTGTCTGATGCAGATATCGA
AAAACTGCCGTACATCACCTTCCCGGAAGGTTCTGAAGAGCATACCTATCTGCACGCTCAGCGTCAGAAACTGCACGGTT
ATCTGCCAAGCCGTCAGCCGAACTTCACCGAGAAGCTTGAGCTGCCGAGCCTGCAAGACTTCGGCGCGCTGTTGGAAGAG
CAGAGCAAAGAGATCTCTACCACTATCGCTTTCGTTCGTGCTCTGAACGTGATGCTGAAGAACAAGTCGATCAAAGATCG
TCTGGTACCGATCATCGCCGACGAAGCGCGTACTTTCGGTATGGAAGGTCTGTTCCGTCAGATTGGTATTTACAGCCCGA
ACGGTCAGCAGTACACCCCGCAGGACCGCGAGCAGGTTGCTTACTATAAAGAAGACGAGAAAGGTCAGATTCTGCAGGAA
GGGATCAACGAGCTGGGCGCAGGTTGTTCCTGGCTGGCAGCGGCGACCTCTTACAGCACCAACAATCTGCCGATGATCCC
GTTCTACATCTATTACTCGATGTTCGGCTTCCAGCGTATTGGCGATCTGTGCTGGGCGGCTGGCGACCAGCAAGCGCGTG
GCTTCCTGATCGGCGGTACTTCCGGTCGTACCACCCTGAACGGCGAAGGTCTGCAGCACGAAGATGGTCACAGCCACATT
CAGTCGCTGACTATCCCGAACTGTATCTCTTACGACCCGGCTTACGCTTACGAAGTTGCTGTCATCATGCATGACGGTCT
GGAGCGTATGTACGGTGAAAAACAAGAGAACGTTTACTACTACATCACTACGCTGAACGAAAACTACCACATGCCGGCAA
TGCCGGAAGGTGCTGAGGAAGGTATCCGTAAAGGTATCTACAAACTCGAAACTATTGAAGGTAGCAAAGGTAAAGTTCAG
CTGCTCGGCTCCGGTTCTATCCTGCGTCACGTCCGTGAAGCAGCTGAGATCCTGGCGAAAGATTACGGCGTAGGTTCTGA
CGTTTATAGCGTGACCTCCTTCACCGAGCTGGCGCGTGATGGTCAGGATTGTGAACGCTGGAACATGCTGCACCCGCTGG

AAACTCCGCGCGTTCCGTATATCGCTCAGGTGATGAACGACGCTCCGGCAGTGGCATCTACCGACTATATGAAACTGTTC
GCTGAGCAGGTCCGTACTTACGTACCGGCTGACGACTACCGCGTACTGGGTACTGATGGCTTCGGTCGTTCCGACAGCCG
TGAGAACCTGCGTCACCACTTCGAAGTTGATGCTTCTTATGTCGTGGTTGCGGCGCTGGGCGAACTGGCTAAACGTGGCG
AAATCGATAAGAAAGTGGTTGCTGACGCAATCGCCAAATTCAACATCGATGCAGATAAAGTTAACCCGCGTCTGGCGTAA
GAGGTAAAAGAATAATGGCTATCGAAATCAAAGTACCGGACATCGGGGCTGATGAAGTTGAAATCACCGAGATCCTGGTC
AAAGTGGGCGACAAAGTTGAAGCCGAACAGTCGCTGATCACCGTAGAAGGCGACAAAGCCTCTATGGAAGTTCCGTCTCC
GCAGGCGGGTATCGTTAAAGAGATCAAAGTCTCTGTTGGCGATAAAACCCAGACCGGCGCACTGATTATGATTTTCGATT
CCGCCGACGGTGCAGCAGACGCTGCACCTGCTCAGGCAGAAGAGAAGAAAGAAGCAGCTCCGGCAGCAGCACCAGCGGCT
GCGGCGGCAAAAGACGTTAACGTTCCGGATATCGGCAGCGACGAAGTTGAAGTGACCGAAATCCTGGTGAAAGTTGGCGA
TAAAGTTGAAGCTGAACAGTCGCTGATCACCGTAGAAGGCGACAAGGCTTCTATGGAAGTTCCGGCTCCGTTTGCTGGCA
CCGTGAAAGAGATCAAAGTGAACGTGGGTGACAAAGTGTCTACCGGCTCGCTGATTATGGTCTTCGAAGTCGCGGGTGAA
GCAGGCGCGGCAGCTCCGGCCGCTAAACAGGAAGCAGCTCCGGCAGCGGCCCCTGCACCAGCGGCTGGCGTGAAAGAAGT
TAACGTTCCGGATATCGGCGGTGACGAAGTTGAAGTGACTGAAGTGATGGTGAAAGTGGGCGACAAAGTTGCCGCTGAAC
AGTCACTGATCACCGTAGAAGGCGACAAAGCTTCTATGGAAGTTCCGGCGCCGTTTGCAGGCGTCGTGAAGGAACTGAAA
GTCAACGTTGGCGATAAAGTGAAAACTGGCTCGCTGATTATGATCTTCGAAGTTGAAGGCGCAGCGCCTGCGGCAGCTCC
TGCGAAACAGGAAGCGGCAGCGCCGGCACCGGCAGCAAAAGCTGAAGCCCCGGCAGCAGCACCAGCTGCGAAAGCGGAAG
GCAAATCTGAATTTGCTGAAAACGACGCTTATGTTCACGCGACTCCGCTGATCCGCCGTCTGGCACGCGAGTTTGGTGTT
AACCTTGCGAAAGTGAAGGGCACTGGCCGTAAAGGTCGTATCCTGCGCGAAGACGTTCAGGCTTACGTGAAAGAAGCTAT
CAAACGTGCAGAAGCAGCTCCGGCAGCGACTGGCGGTGGTATCCCTGGCATGCTGCCGTGGCCGAAGGTGGACTTCAGCA
AGTTTGGTGAAATCGAAGAAGTGGAACTGGGCCGCATCCAGAAAATCTCTGGTGCGAACCTGAGCCGTAACTGGGTAATG
ATCCCGCATGTTACTCACTTCGACAAAACCGATATCACCGAGTTGGAAGCGTTCCGTAAACAGCAGAACGAAGAAGCGGC
GAAACGTAAGCTGGATGTGAAGATCACCCCGGTTGTCTTCATCATGAAAGCCGTTGCTGCAGCTCTTGAGCAGATGCCTC
GCTTCAATAGTTCGCTGTCGGAAGACGGTCAGCGTCTGACCCTGAAGAAATACATCAACATCGGTGTGGCGGTGGATACC
CCGAACGGTCTGGTTGTTCCGGTATTCAAAGACGTCAACAAGAAAGGCATCATCGAGCTGTCTCGCGAGCTGATGACTAT
TTCTAAGAAAGCGCGTGACGGTAAGCTGACTGCGGGCGAAATGCAGGGCGGTTGCTTCACCATCTCCAGCATCGGCGGCC
TGGGTACTACCCACTTCGCGCCGATTGTGAACGCGCCGGAAGTGGCTATCCTCGGCGTTTCCAAGTCCGCGATGGAGCCG
GTGTGGAATGGTAAAGAGTTCGTGCCGCGTCTGATGCTGCCGATTTCTCTCTCCTTCGACCACCGCGTGATCGACGGTGC
TGATGGTGCCCGTTTCATTACCATCATTAACAACACGCTGTCTGACATTCGCCGTCTGGTGATGTAAGTAAAAGAGCCGG
CCCAACGGCCGGCTTTTTTCTGGTAATCTCATGAATGTATTGAGGTTATTAGCGAATAGACAAATCGGTTGCCGTTTGTT
GTTTAAAAATTGTTAACAATTTTGTAAAATACCGACGGATAGAACGACCCGGTGGTGGTTAGGGTATTACTTCACATACC
CTATGGATTTCTGGGTGCAGCAAGGTAGCAAGCGCCAGAATCCCCAGGAGCTTACATAAGTAAGTGACTGGGGTGAGGGC
GTGAAGCTAACGCCGCTGCGGCCTGAAAGACGACGGGTATGACCGCCGGAGATAAATATATAGAGGTCATGATGAGTACT
GAAATCAAAACTCAGGTCGTGGTACTTGGGGCAGGCCCCGCAGGTTACTCCGCTGCCTTCCGTTGCGCTGATTTAGGTCT
GGAAACCGTAATCGTAGAACGTTACAACACCCTTGGCGGTGTTTGCCTGAACGTCGGCTGTATCCCTTCTAAAGCACTGC
TGCACGTAGCAAAAGTTATCGAAGAAGCCAAAGCGCTGGCTGAACACGGTATCGTCTTCGGCGAACCGAAAACCGATATC
GACAAGATTCGTACCTGGAAAGAGAAAGTGATCAATCAGCTGACCGGTGGTCTGGCTGGTATGGCGAAAGGCCGCAAAGT
CAAAGTGGTCAACGGTCTGGGTAAATTCACCGGGGCTAACACCCTGGAAGTTGAAGGTGAGAACGGCAAAACCGTGATCA
ACTTCGACAACGCGATCATTGCAGCGGGTTCTCGCCCGATCCAACTGCCGTTTATTCCGCATGAAGATCCGCGTATCTGG
GACTCCACTGACGCGCTGGAACTGAAAGAAGTACCAGAACGCCTGCTGGTAATGGGTGGCGGTATCATCGGTCTGGAAAT
GGGCACCGTTTACCACGCGCTGGGTTCACAGATTGACGTGGTTGAAATGTTCGACCAGGTTATCCCGGCAGCTGACAAAG
ACATCGTTAAAGTCTTCACCAAGCGTATCAGCAAGAAATTCAACCTGATGCTGGAAACCAAAGTTACCGCCGTTGAAGCG
AAAGAAGACGGCATTTATGTGACGATGGAAGGCAAAAAAGCACCCGCTGAACCGCAGCGTTACGACGCCGTGCTGGTAGC
GATTGGTCGTGTGCCGAACGGTAAAAACCTCGACGCAGGCAAAGCAGGCGTGGAAGTTGACGACCGTGGTTTCATCCGCG
TTGACAAACAGCTGCGTACCAACGTACCGCACATCTTTGCTATCGGCGATATCGTCGGTCAACCGATGCTGGCACACAAA
GGTGTTCACGAAGGTCACGTTGCCGCTGAAGTTATCGCCGGTAAGAAACACTACTTCGATCCGAAAGTTATCCCGTCCAT
CGCCTATACCGAACCAGAAGTTGCATGGGTGGGTCTGACTGAGAAAGAAGCGAAAGAGAAAGGCATCAGCTATGAAACCG
CCACCTTCCCGTGGGCTGCTTCTGGTCGTGCTATCGCTTCCGACTGCGCAGACGGTATGACCAAGCTGATTTTCGACAAA
GAATCTCACCGTGTGATCGGTGGTGCGATTGTCGGTACTAACGGCGGCGAGCTGCTGGGTGAAATCGGCCTGGCAATCGA
AATGGGTTGTGATGCTGAAGACATCGCACTGACCATCCACGCGCACCCGACTCTGCACGAGTCTGTGGGCCTGGCGGCAG
AAGTGTTCGAAGGTAGCATTACCGACCTGCCGAACCCGAAAGCGAAGAAGAAGTAATTTTTCGTTTGCCGGAACATCCGG
CAATTAAAAAAGCGGCTAACCACGCCGCTTTTTTTACGTCTGCAATTTACCTTTCCAGTCTTCTTGCTCCACGTTCAGAG
AGACGTTCGCATACTGCTGACCGTTGCTCGTTATTCAGCCTGACAGTATGGTTACTGTCGTTTAGACGTTGTGGGCGGCT
CTCCTGAACTTTCTCCCGAAAAACCTGACGTTGTTCAGGTGATGCCGATTGAACACGCTGGCGGGCGTTATCACGTTGCT
GTTGATTCAGTGGGCGCTGCTGTACTTTTTCCTTAAACACCTGGCGCTGCTCTGGTGATGCGGACTGAATACGCTCACGC
GCTGCGTCTCTTCGCTGCTGGTTCTGCGGGTTAGTCTGCATTTTCTCGCGAACCGCCTGGCGCTGCTCAGGCGAGGCGGA
CTGAATGCGCTCACGCGCTGCCTCTCTTCGCTGCTGGATCTTCGGGTTAGTCTGCATTCTCTCGCGAACTGCCTGGCGCT

GCTCAGGCGAGGCGGACTGATAACGCTGACGAGCGGCGTCCTTTTGTTGCTGGGTCAGTGGTTGGCGACGGCTGAAGTCG
TGGAAGTCGTCATAGCTCCCATAGTGTTCAGCTTCATTAAACCGCTGTGCCGCTGCCTGACGTTGGGTATCTCGTGTAAT
GACTGGTGCGGCGTGTGTTCGTTGCTGAAACTGATTTGCTGCCGCCTGACGCTGGCTGTCGCGCGTTGGGGCAGGTAATT
GCGTGGCGCTCATTCCGCCGTTGACATCGGTTTGATGAAACCGCTTTGCCATATCCTGATCATGATAGGGCACACCATTA
CGGTAGTTTGGATTGTGCCGCCATGCCATATTCTTATCAGTAAGATGCTCACCGGTGATACGGTTGAAATTGTTGACGTC
GATATTGATGTTGTCGCCGTTGTGTTGCCAGCCATTACCGTCACGATGACCGCCATCGTGGTGATGATAATCATCATTGT
CATGATGATGATGGTCATGATCGTCGTCGTCCCAGTCGATGCTGCTGAATAGTGCGTACGTGGTAGCAACGCCCATGCTA
TAGCCGAATCCGCGTACAAAGCTGTCAACAAACGGTTCTCCGGCTGGTGGTGGCAGATAAACCGGCGGATACGCAGTATT
GGCCCAGTTCCCGTAAACCACGGTTGGGTTGTAGTTGGGAATATAGACCACATCAGGATTGGCGGGCTCAATGGAAATGA
CGGTTGTTGCCGGCTCTGTAATGACGGGGTTGGCAGTTAAAACTGTATTGGATGGTATGACGGGTGCCGTGACTGTCTGT
TTTACCGGTACAGCTTTCTTCGTTGTGGTAATAACTTTCTGTTCGGTTGATGACTTCAGCGAGCCGGTTTGTTGCGCCAG
TTGCCGCAATCGCTGTACCGAGTCCATCACGTCCTGCGGCTGGGCCAGAAAAGCATCGCCCAGGTTTTGCACCCATTGCG
GGTTTTCGCCCATCAATGCCATCAATTGTGGAAAGGCCACCAGTGATTTAACGCTGGCGTCCCACGGCTGGTCAGATACC
GCCTGAATAGCAGCATCGCCTTGTTTAAGTGGATTATCGTGCGACCATTGCACTGCTTGAGCAACGTTTGTCGGATAGGT
TGATGCCATCAGCACCTGCGAAAGTAGGGCGTCGGGATACAGCGCGACGGGCGCGACCCATTGATCTATTTGTGCAGTGC
TGAATGCGGATTTGACGACGGCGGGTGTTACGGGAGGTGCGGAAACCGTTGCAGGAAACGTAACCGCGGCAGCGTCAGAC
ACAGCCAGTTGTGTCGATTGCGGTTCCACAGGCGCTTCCACTGTGCGGCTTTTTATATATAGCCCGGTAGAGGCGGCACA
AAGCCCGGCACTGCAAATTAGCGCCAGCACATGGGGTTTAAACGGCAAAGTCATTTTCATAATTCGGATCTCAAGGAAAT
CGCAATGGTCGGCGAACTGCCACCCGCAGGTGCTGTGAATCCGAGTATAAAGAGGCGGTAGTTTAAATTTTGACTAATCT
TGGGATTCGTTGAGAAAGGTGATTATCACCATGCGAATTAACGAAGTTTTTACGGAGGGAAACAATCTCTAGACCATCCT
TAACGATTCAGCCACTTTTTTATGTTGCTTTTTTGTAAACAGATTAACACCTCGTCAAAATCCTGCTATTCTGCCCGTTG
CGGTACTGGGCATTTACCCTACAAACTGCTGTCTCACAGGAGCGTGAAGAGAATCGCCTGCCGCACTATGACAATGAGAG
CGAGGAGAACCGTCGTGCTAGAAGAATACCGTAAGCACGTAGCTGAGCGTGCCGCTGAGGGGATTGCGCCCAAACCCCTG
GATGCAAACCAAATGGCCGCACTTGTAGAGCTGCTGAAAAACCCGCCCGCGGGCGAAGAAGAATTCCTGTTAGATCTGTT
AACCAACCGTGTTCCCCCAGGCGTCGATGAAGCCGCCTATGTCAAAGCAGGCTTCCTGGCTGCTATCGCGAAAGGCGAAG
CCAAATCCCCTCTGCTGACTCCGGAAAAAGCCATCGAACTGCTGGGCACCATGCAGGGTGGTTACAACATTCATCCGCTG
ATCGACGCGCTGGATGATGCCAAACTGGCACCTATTGCTGCCAAAGCACTTTCTCACACGCTGCTGATGTTCGATAACTT
CTATGACGTAGAAGAGAAAGCGAAAGCAGGCAACGAATATGCGAAGCAGGTTATGCAGTCCTGGGCGGATGCCGAATGGT
TCCTGAATCGCCCGGCGCTGGCTGAAAAACTGACCGTTACTGTCTTCAAAGTCACTGGCGAAACTAACACCGATGACCTT
TCTCCGGCACCGGATGCGTGGTCACGCCCGGATATCCCACTGCACGCGCTGGCGATGCTGAAAAACGCCCGTGAAGGTAT
TGAGCCAGACCAGCCTGGTGTTGTTGGTCCGATCAAGCAAATCGAAGCTCTGCAACAGAAAGGTTTCCCGCTGGCGTACG
TCGGTGACGTTGTGGGTACGGGTTCTTCGCGTAAATCCGCCACTAACTCCGTTCTGTGGTTTATGGGCGATGATATTCCA
CATGTGCCGAACAAACGCGGCGGTGGTTTGTGCCTCGGCGGTAAAATTGCACCCATCTTCTTTAACACGATGGAAGACGC
GGGTGCACTGCCAATCGAAGTCGACGTCTCTAACCTGAACATGGGCGACGTGATTGACGTTTACCCGTACAAAGGTGAAG
TGCGTAACCACGAAACCGGCGAACTGCTGGCGACCTTCGAACTGAAAACCGACGTGCTGATTGATGAAGTGCGTGCTGGT
GGCCGTATTCCGCTGATTATCGGGCGTGGCCTGACCACCAAAGCGCGTGAAGCACTTGGTCTGCCGCACAGTGATGTGTT
CCGTCAGGCGAAAGATGTCGCTGAGAGCGATCGCGGCTTCTCGCTGGCGCAAAAAATGGTAGGCCGTGCCTGTGGCGTGA
AAGGCATTCGTCCGGGCGCGTACTGTGAACCGAAAATGACTTCTGTAGGTTCCCAGGACACCACCGGCCCGATGACCCGT
GATGAACTGAAAGACCTGGCGTGCCTGGGCTTCTCGGCTGACCTGGTGATGCAGTCTTTCTGCCACACCGCGGCGTATCC
GAAGCCAGTTGACGTGAACACGCACCACACGCTGCCGGACTTCATTATGAACCGTGGCGGTGTGTCGCTGCGTCCGGGTG
ACGGCGTCATTCACTCCTGGCTGAACCGTATGCTGCTGCCGGATACCGTCGGTACCGGTGGTGACTCCCATACCCGTTTC
CCGATCGGTATCTCTTTCCCGGCGGGTTCTGGTCTGGTGGCGTTTGCTGCCGCAACTGGCGTAATGCCGCTTGATATGCC
GGAATCCGTTCTGGTGCGCTTCAAAGGCAAAATGCAGCCGGGCATCACCCTGCGCGATCTGGTACACGCTATTCCGCTGT
ATGCGATCAAACAAGGTCTGCTGACCGTTGAGAAGAAAGGCAAGAAAAACATCTTCTCTGGCCGCATCCTGGAAATTGAA
GGTCTGCCGGATCTGAAAGTTGAGCAGGCCTTTGAGCTAACCGATGCGTCCGCCGAGCGTTCTGCCGCTGGTTGTACCAT
CAAGCTGAACAAAGAACCGATCATCGAATACCTGAACTCTAACATCGTCCTGCTGAAGTGGATGATCGCGGAAGGTTACG
GCGATCGTCGTACCCTGGAACGTCGTATTCAGGGCATGGAAAAATGGCTGGCGAATCCTGAGCTGCTGGAAGCCGATGCA
GATGCGGAATACGCGGCAGTGATCGACATCGATCTGGCGGATATTAAAGAGCCAATCCTGTGTGCTCCGAACGACCCGGA
TGACGCGCGTCCGCTGTCTGCGGTACAGGGTGAGAAGATCGACGAAGTGTTTATCGGTTCCTGCATGACCAACATCGGTC
ACTTCCGTGCTGCGGGTAAACTGCTGGATGCGCATAAAGGTCAGTTGCCGACCCGCCTGTGGGTGGCACCGCCAACCCGT
ATGGACGCCGCACAGTTGACCGAAGAAGGCTACTACAGCGTCTTCGGTAAGAGTGGTGCGCGTATCGAGATCCCTGGCTG
TTCCCTGTGTATGGGTAACCAGGCGCGTGTGGCGGACGGTGCAACGGTGGTTTCCACCTCTACCCGTAACTTCCCGAACC
GTCTGGGTACTGGCGCGAATGTCTTCCTGGCTTCTGCGGAACTGGCGGCTGTTGCGGCGCTGATTGGCAAACTGCCGACG
CCGGAAGAGTACCAGACCTACGTGGCGCAGGTAGATAAAACAGCCGTTGATACTTACCGTTATCTGAACTTCAACCAGCT
TTCTCAGTACACCGAGAAAGCCGATGGGGTGATTTTCCAGACTGCGGTTTAAAAAGTCAGCGCACGCGCTGCGCATAAAC
GACACAATGCCCGGTGAATGAGATTCCCGGGCATTTTTTTATTTCTAAACCATCGCCGTTCCGCTGTTTTTCTCCGGTAA

GGCTGCGATAATTACATCAATGGCGCAATGCGATTTCGGTGCATTGCCGGGAGCAGAGGAACACACTATGGATTACGAAT
TTCTGCGCGATATTACCGGAGTGGTAAAGGTGCGTATGTCCATGGGGCATGAAGTGGTCGGGCACTGGTTTAATGAAGAG
GTGAAAGAAAACCTGGCCTTGCTTGATGAAGTGGAACAAGCAGCGCACGCACTGAAAGGTAGCGAACGGTCCTGGCAACG
GGCAGGGCATGAATACACCCTGTGGATGGACGGTGAAGAGGTGATGGTTCGCGCCAATCAACTGGAATTCGCTGGCGATG
AAATGGAAGAGGGGATGAACTACTACGACGAAGAAAGCCTGTCGCTATGCGGCGTTGAGGATTTTCTGCAGGTCGTGGCG
GCTTACCGCAATTTCGTGCAGCAGAAGTAAATAAATCTGGCGGAGCCTGGGAGCTCCGCCAGAGCCGTTAAACAGCTGGC
ATATTGCGCCCGTAATAAATCTCGCGCATTTCTTTCCACAGCGCAGCGGTAATTTCCTGGCGCTCGCTGTCGGTTAAGTC
TTCCGGTTTGGTGTGGAACATGTAGTGCTTAAGGTCGAACTCTTTAAGCAACATCTTGGTATGGAAGATATTTTCCTGAT
AGACGTTCACATCCACCATGTCATACAGCGCCTTCATATCGTCAGACATAAAGTTCTGAATCGAATTAATCTCATGGTCG
ATAAAGTGCTTCATACCGTTAATGTCGCGGGTAAAACCGCGCACGCGATAATCAATGGTTACGATATCGGACTCAAGCTG
GTGGATCAGGTAATTCAGCGCCTTCAGCGGAGAAATCACGCCGCAGGTAGAGACTTCAATATCGGCGCGGAAGGTACATA
AACCGCCTTCAGGATGACTTTCCGGGTAGGTATGTACGCAAATATGACTTTTATCAAGATGGGCAACGACCGTTTCTGGC
AGTGGGCCGGGGTGTTCTGTTTTGTCGATGAGTTTCGGGTCAACCGGTTCTTCACTCACCAGAATAGTGACGCTGGCACC
CTGTGGTTCGTAATCCTGGCGGGCGATGTTAAGAATATTAGCCCCGATAATGGAACAGGTTTCTGACAGGATTTCGGTCA
GACGGTTGGCATTATAGAGTTCATCGATATAAGCAATATAACCGTCGCGCTCTTCGGCAGTTTTGGCGTAGCAGATATCG
TAAATACAAAAACTCAGACTTTTGGTCAGATTATTAAAGCCATGCAGTTTCAGTTTTTTCAATTTCTTATCTTCTCCTTA
GGACGGCTGTGAAGCCAGTGCGTCTTGCAGATACTGAGGTAAGGCAAAAGCTGCCGTATGGATTGCCGGATTGTAATAAC
GGCATTTCAGGCCAGAGGCGAGAAAACGCGCCTGAATAATTTCGGTTGAGAGATGGCGTAAGGCGTCGTTATCTGTCGCC
CATGCAAAAGTCATGATACCGCCGTAATAGGTCGGGATCGCCGCCTGATAAAAGCCAACGTCGCTGAAGTAATGGCTGAG
TTTGCGATGGCTGTCGATGGCTTCTTCCTGCTGTAAAAAGCAGACGCCGTTTTGTGCGACGAAGATACCGCCAGGATTCA
GGCAACGTTTGCAGCCTTCATAAAATGCCGAAGTGAAAAGGCTTTCGCCGGGACCGATAGGATCGGTGCAGTCGGAGATA
ATGACATCAAAGGTCTGGCTGGTTTGATTAACGAAATTGACGCCATCGTCGATCACCAGCTTAAAGCGCGGATCGTCGTA
GCTACCGGCGTTATGGTTGGGTAGATACTGACGGCAGAACGATACGACACCCGCATCGATTTCCACCATCGTGATTGACT
CAACGTTTTTATGTCGGGTTACTTCACGCAGCATGGCACCGTCGCCGCCGCCGATAATCAGCACATGTTTCGCGTGACCA
TGGGCCAGTAGCGGAACATGGGTCATCATCTCATGATAGATAAACTCGTCGCGCTCGGTGGTTTGTACTACGCCATCCAG
CGCCATTACGCGACCAAATGCAGCGTTCTCAAAAATGATCAGATCCTGGTGATCGGTCTTTTCATGATACAGAACGTTAT
CTACCGCAAAGTACTGCCCAAACTGGTCGTGTAGCGTTTCATGCCACTGTTTTTTTTCGGCCATGGGTTGATACCTCCTT
TGTTAACACCCGTAAAAAAAGGGCGCAACATAATAGCTAACATTGACCGCGGATGCACGGTCAATATTTCTACAAAAAGG
TATCAGGGATTATTTGACGTAGGCAAGCAGGCTTAAGGAATCGCGGGCCAGGGCTTTGCATTTTTTAGCGACGGGAATGC
CAATGCCGCTGAGATCGCGGTAGCTGTCTTCACCGAGGGCTTTCATGTCGAAGGTGTCGTAATTACTGAGGTCCCACTGG
TTTTGCTGAGCGAAAAAGACCAGTGCGCGACGAATTTGCCCGTTAGGTAAGTTCTGGTAACCACAATCGTTCTTCAGAAA
GACAAAAACTGCCGTTAAATCGGCCATATCTTCGGCTTCAGACTCGCTGAGCGCGTAACTGTTTGCGCAGACGGCCATCA
GGCTGCCGAATAACACTGTTCTGAAAAACGTCTTCATTGCTTCTACCAGGGGCTTAAAGAAGATAAACGTTAGCACACTG
CGAGCGAGGCGACGACCATTATTGTCGATTAAATTGTGTCTGCGGCTTGACCTTCCCGTAAGGGGAAGGACTATGCTCAA
CGTTTGATTTTGTTTCGCCTGCTTAAGAATAAGGAAATAACTATGCAACGTCGTGATTTCTTAAAATATTCCGTCGCGCT
GGGTGTGGCTTCGGCTTTGCCGCTGTGGAGCCGCGCAGTATTTGCGGCAGAACGCCCAACGTTACCGATCCCTGATTTGC
TCACGACCGATGCCCGTAATCGCATTCAGTTAACTATTGGCGCAGGCCAGTCCACCTTTGGCGGGAAAACTGCAACTACC
TGGGGCTATAACGGCAATCTGCTGGGGCCGGCGGTGAAATTACAGCGCGGCAAAGCGGTAACGGTTGATATCTACAACCA
ACTGACGGAAGAGACAACGTTGCACTGGCACGGGCTGGAAGTACCGGGTGAAGTCGACGGCGGCCCGCAGGGAATTATTC
CGCCAGGTGGCAAGCGCTCGGTGACGTTGAACGTTGATCAACCTGCCGCTACCTGCTGGTTCCATCCGCATCAGCACGGC
AAAACCGGGCGACAGGTGGCGATGGGGCTGGCTGGGCTGGTGGTGATTGAAGATGACGAGATCCTGAAATTAATGCTGCC
AAAACAGTGGGGTATCGATGATGTTCCGGTGATCGTTCAGGATAAGAAATTTAGCGCCGACGGGCAGATTGATTATCAAC
TGGATGTGATGACCGCCGCCGTGGGCTGGTTTGGCGATACGTTGCTGACCAACGGTGCAATCTACCCGCAACACGCTGCC
CCGCGTGGTTGGCTGCGCCTGCGTTTGCTCAATGGCTGTAATGCCCGTTCGCTCAATTTCGCCACCAGCGACAATCGCCC
GCTGTATGTGATTGCCAGCGACGGTGGTCTGCTACCTGAACCAGTGAAGGTGAGCGAACTGCCGGTGCTGATGGGCGAGC
GTTTTGAAGTGCTGGTGGAGGTTAACGATAACAAACCCTTTGACCTGGTGACGCTGCCGGTCAGCCAGATGGGGATGGCG
ATTGCGCCGTTTGATAAGCCTCATCCGGTAATGCGGATTCAGCCGATTGCTATTAGTGCCTCCGGTGCTTTGCCAGACAC
ATTAAGTAGCCTGCCTGCGTTACCTTCGCTGGAAGGGCTGACGGTACGCAAGCTGCAACTCTCTATGGACCCGATGCTCG
ATATGATGGGGATGCAGATGCTAATGGAGAAATATGGCGATCAGGCGATGGCCGGGATGGATCACAGCCAGATGATGGGC
CATATGGGGCACGGCAATATGAATCATATGAACCACGGCGGGAAGTTCGATTTCCACCATGCCAACAAAATCAACGGTCA
GGCGTTTGATATGAACAAGCCGATGTTTGCGGCGGCGAAAGGGCAATACGAACGTTGGGTTATCTCTGGCGTGGGCGACA
TGATGCTGCATCCGTTCCATATCCACGGCACGCAGTTCCGTATCTTGTCAGAAAATGGCAAACCGCCAGCGGCTCATCGC
GCGGGCTGGAAAGATACCGTTAAGGTAGAAGGTAATGTCAGCGAAGTGCTGGTGAAGTTTAATCACGATGCACCGAAAGA
ACATGCTTATATGGCGCACTGCCATCTGCTGGAGCATGAAGATACGGGGATGATGTTAGGGTTTACGGTATAAAAAGACC
GTATTCGGAAATATGCCCGGATCTCTCCGGGCATTAAACTGATGACAAACGCAAATCTGCCTGATGCGCTACGCTTATCA
GGCCTACGCTAGCTGTGCAATCCATTGATTTTGCACAATTTTGTAGGCTGGATAAGGCGTTCACGCCGCATCCGGCATGA

ACAACGCGCACGTTGTCAGCAATCTGAGCAAGTCTTACTTCACATCATCCGGCAGCGCATAAGCCACAATATAGTCGCCC
ATCTTCGTACCAAATGAACCGTGACCGCCTGCGGAGATCACCACATACTGCTTACCATTCACTTCATAGGTCATTGGCGT
AGCCTGACCACCCGCTGGTAAACGACCCTGCCACAGTTTTTCACCGTTGCTCATGTTGTAAGCGCGCAGGTAGTTATCTG
CCGTAGCGGCGATAAACAGCACGTTACCCGCCGTGGAGATTGGCCCGCCCAGCATCGGCATACCCATATTGAACGGCACC
GGAACCGGCATCGGGAACGGCATACTGTCCTGCGGCGTACCAATACGTTTCTTCCACACCACTTCATTAGTTTTCAGATC
CAGCGCCGAGATATAACCCCATGCTGGCTGTTTACATGGCAGACCAAATGGTGAGAGGAACGGGTTGAGCGTGACACCAT
ACGGTACACCGTACTGTGGCTGAATGCCGGATTCCGTACCCGTGCCTTTGGCATCTTTCGGCTGCTCCATCGGGTTGCCA
GGACCACGCGGGATCAGTTTCGAAACAAACGGCAGTGCCATTGGGTTGGCAATCGCCACTTCACGATTTGGATCAACGGA
AATCCCGCCCCATTCGAACATCCCCAGGTTACCCGGGAAGACCAGCGTACCCTGTTCAGATGGCGGGGTGAAAATGCCTT
CATAGCGCATCTGGTGGAACATCACGCGGCACACCAGTTGGTCAAACATGGTGGCTCCCCACATATCCGCACCGCTCAAA
TCTTTCGTCGGACGGAAGCTCAGTTCAGAAAACGGTTGAGTTGGGGTTACGTAATCGCCTTTCGCTGCACCTTGGGGAAC
CGGTTTTTCCGGTGCCGGAACCACCAGTTCGCCATTACGACGATCGAGCACAAAAATGTTGCCGGTTTTCGCCGGAGCGT
AAATAACTGGCACTTTCTGACCATTAACGGTGATGTCCGCCAGCGTCGGCTGTGCCGGAAGATCCATGTCCCACAGGTCG
TGGTGAACGGTCTGGTAGCTCCACGCCAGTTTCCCGGTAGTGGCATTCAGCGCCAGAATCGAGCTGGCATAACGTTCCTG
TTCCGGTGTGCGGTTACCGCCCCAGATATCCGGCGTGGTCACGCCCATCGGCAGATAGACCAGATCCAGCTTCGCGTCAT
AGGCCGCTGGTGCCCAGGAGTTTGGCGAGTTAAAGGTAAAGGTGTGTTCGTCAGACGGGATTGCGTTCGGATCTTTCGCG
CCGGGATCAAAAGCCCACAGCAGCTCCCCGGTGTTGACATCAAAACCACGGATCACGCCAGACGTTTCGCGGGTTGAGAA
GTTATCGGTAACTGAACCGGCCATCACGATGGTTTTATCGGTGATAATCGGTGGCGAAGTCGGTTCATACAGACCCGGTT
TGGTGTCTGGCATATTGCTTTGCAGATTGAGCACGCCTTTATTGGCGAAGGTTTCGCACAGTTTGCCGTTTTCAGCGTTA
ATCGCAATCAGTCGACCATCATTGACCGGAAGAATGATACGACGCGGGCAATCCGCCATCACTTCCGGCGAAGCGGTTTC
TGCTTTGGCTTCATGATAAGAGACACCACGGCAGGTTACGTGCTGGAAAGACTCGTTGGTTTTCAGCTCAGGATCGTAAT
GCCATTTCTCTTTGCCGCTGGCGGCATCAAGCGCAAACAGGCGCTGGTGAGCGGTACACAGGTAAAGGGTGTCGCCCACT
TTAATCGGCGTCACTTCATTGGTGATTTCACCCGGATCGTTCGGCTGCTTCACATCGCCAGTACGGAACACCCAGGCTTC
TTTCAGATTATGGACGTTATCGGCGTTAATTTGTTTCAGCGGCGAAAAGCGTTGACCTTCCTGATTACGACCATAGGCAG
GCCAGTCCTGATCGGCTACGGGGGAGATAGCTTCAGCAGGTGTGGCATCGGCGCTTAAGGTGCCGTTGATCTCCTGCGGA
TCGTTAAATCCGGCCCAGGTCAGGATACCACCGCTAATCAGCAGTGCGACCACCAGTGCGGCAACTGCGCCGCTGGCAGG
AATGACCAGGCGACGCCAGACAAACGGCAGGATCAGCCAGATGCCGAAGAAGACCAGAATGTCGCTGCGCGGAGTCAGCG
CCCAGAAGTCGAAACCAACTTCCCAGACGCCCCAAATCATGGTGCCGAGCAGCAGGGCTGCGTATAGCCAAAGCGCGGCG
CGTTTACTGCGCCACAGCATCCAGGCGACGCCGAGCATCACAAGGCCAGCGATAGGGTAGTACCAGGAGCCGCCAATCGC
GACCAGCCAGCCTCCGCCAATGAGTAGATACAGCCCGCAAAGCGCTGCAAAAAGGGCTGTTAGCGTGACGAGTAATCGTC
GCGAGCCTGTATTGTTAATTGCCATAAAGAGACACCATTTCAATTCATTAATATTTTAGTAGCAATTAATTATAGGTTTT
AACATGTGTGATCGTCATCACAATTCGAGCTTTATTAACAGATTCCGCGAATGAATAGTTTTACTGGTATACTGCGTGTC
TTGCGCTTTGTTGCGGTGCCAAAACCTGCCCGTGCGAAGTGATTTGTTTTTAAATCATATGGTTAGAGATATGAAACATA
CTGTAGAAGTAATGATCCCCGAAGCGGAGATTAAAGCGCGTATCGCCGAACTGGGTCGTCAGATTACTGAGCGTTACAAA
GACAGCGGCAGCGATATGGTGCTGGTGGGTCTGCTGCGTGGCTCATTTATGTTTATGGCGGACCTGTGCCGTGAAGTTCA
GGTATCTCATGAAGTCGACTTTATGACCGCCTCCAGCTACGGTAGCGGCATGTCCACCACCCGTGATGTGAAAATCCTCA
AAGATCTGGATGAAGATATCCGTGGCAAGGACGTGCTGATTGTTGAAGATATCATCGACTCGGGGAATACACTGTCGAAA
GTGCGTGAGATCTTAAGCCTGCGCGAACCGAAGTCGCTGGCGATTTGTACGCTGCTGGATAAACCGTCCCGTCGTGAAGT
GAACGTCCCGGTAGAATTTATCGGTTTCTCGATCCCGGATGAGTTTGTGGTGGGTTACGGCATTGATTACGCACAGCGTT
ACCGTCATCTGCCGTATATCGGCAAAGTGATTCTGCTGGACGAGTAAGTGTGAAGTTGCCGGATGTGTTGCATCCGGCAT
GGCATTTTTATTTGTGGTTGGCGTGTTTCAGCTTGAGGTTGGAAATCCCGTGACGGTAACGTTGCTCAAGGGTTTCGCGG
TTGGTGGCGGTAACATCCAGATCACGCAGCAAGCCGTCGTGAATGCCGTAGGCCCAGCCGTGAATGGTAACTTTCTGCCC
GCGTTTCCACGCTGATTGCATAATGGTGGAGTGGCCCAGGTTATACACCTGTTCCATGACGTTCAGTTCACACAAGGTAT
CCAGACGGCGCTCTTGCGGCATTTCGCCGAGCAATGAGCTATGTTTGAACCAGATATCGCGGATATGCAGCAGCCAGTTG
TTGATAAGCCCCAGTTCCGGGTTTTCAACTGCGGCTTGTACGCCGCCGCAACCGTAGTGGCCACAGATAATAATGTGTTC
AACTTCGAGTACATCCACTGCATACTGAACCACGGAAAGGCAGTTCAGGTCAGTGTGAATGACCAGGTTAGCAACATTAC
GGTGAACAAAGAGTTCGCCCGGCTCAAGACCGGTTAAACGTTCTGCAGGAACGCGACTGTCGGAACATCCAATCCATAGA
AAGCGCGGTTTTTGCGCTTGTGCCAGTTTCTCAAAAAACCCGGGATCCTCTTCCACCAGCATTTTTGACCATAGTGCATT
GTTGCTGATGAGTGTATCTATGTCTTTCATGGAGGTTAACGACCTGTAACCAAATAATTACGTTTGGCTAATATAGGGCA
ACTCCGGGACGATTTAAACCACAGATAAAGTGTAAGAACGTAAGGTAAGTAAAAATTTATGACCATTGCACTGGAACTTC
AACAGCTTAAAAAAACCTATCCAGGCGGCGTTCAGGCGCTTCGTGGGATAGATTTGCAGGTCGAAGCGGGTGATTTTTAT
GCGCTTCTCGGGCCGAACGGGGCCGGGAAATCGACCACTATCGGTATTATCAGCTCTCTGGTAAATAAAACCTCCGGGCG
GGTCAGCGTATTTGGTTACGATCTCGAGAAGGATGTCGTGAACGCTAAACGTCAGTTGGGACTGGTGCCGCAGGAATTTA
ACTTCAACCCGTTTGAAACCGTGCAGCAAATTGTGGTGAATCAGGCAGGGTACTACGGCGTGGAGCGCAAAGAAGCGTAC
ATCCGCAGCGAAAAGTATCTTAAACAACTCGATCTATGGGGAAAACGCAACGAACGTGCGCGTATGTTATCTGGCGGGAT
GAAGCGCCGTTTAATGATTGCCCGTGCGTTAATGCATGAACCTAAACTACTGATTCTCGACGAACCGACCGCAGGCGTGG

ATATTGAACTTCGCCGCTCAATGTGGGGCTTTTTGAAGGATTTAAACGACAAAGGCACCACCATCATTCTCACCACACAC
TACCTGGAAGAAGCAGAAATGCTGTGCCGCAATATCGGCATTATTCAACACGGTGAGCTGGTGGAAAATACCTCGATGAA
GGCGCTGCTGGCGAAGCTGAAATCGGAAACCTTTATTCTCGATCTCGCACCGAAAAGCCCGTTACCGAAGCTCGATGGCT
ATCAGTATCGACTGGTCGATACCGCGACGCTGGAAGTTGAAGTGCTGCGTGAGCAGGGGATCAACAGCGTATTTACGCAG
TTAAGTGAGCAGGGCATTCAGGTATTAAGTATGCGTAACAAAGCTAACCGTCTGGAAGAGCTGTTTGTTTCACTGGTTAA
TGAAAAACAAGGAGATCGCGCATGATGCATCTTTACTGGGTGGCGCTAAAAAGCATCTGGGCGAAAGAGATCCATCGCTT
TATGCGTATCTGGGTGCAGACGCTGGTGCCGCCAGTCATCACCATGACCCTTTACTTTATTATCTTCGGTAACCTGATTG
GTTCGCGTATTGGCGATATGCATGGCTTCAGCTATATGCAGTTCATCGTACCGGGGCTGATCATGATGTCGGTGATCACC
AATGCCTACGCCAACGTTGCGTCATCATTTTTTGGTGCCAAGTTCCAGCGTAATATTGAAGAGCTGCTGGTAGCGCCGGT
TCCGACTCACGTCATTATTGCCGGATATGTCGGCGGTGGCGTGGCGCGTGGTCTGTTTGTTGGCATTCTGGTGACGGCAA
TTTCACTGTTTTTTGTGCCATTTCAGGTGCATTCGTGGGTATTCGTTGCCTTAACGCTGGTGCTCACGGCGGTGTTGTTC
TCCCTTGCGGGTTTGCTGAACGGTGTGTTTGCCAAAACGTTCGATGACATCAGCCTGGTGCCAACCTTTGTGTTAACGCC
ACTCACGTATTTGGGCGGGGTCTTTTACTCACTGACTTTGTTGCCGCCGTTCTGGCAAGGGCTGTCGCACCTGAACCCAA
TCGTTTATATGATCAGTGGTTTCCGCTACGGCTTCCTCGGTATCAATGATGTTCCGCTGGTCACTACCTTTGGCGTACTG
GTGGTCTTTATTGTGGCGTTTTATTTGATCTGTTGGTCGCTGATCCAACGTGGACGTGGTTTGCGTAGCTAAGGCTATTT
CCTCTCCTCTGGATTTGGGGGAGAGGAGTTTTGACGGCTATCACCCTTTATCAACAATGGTCAGGGTAGACTGATTTTCG
GCTAAGGAGGAAGGCGATGTTAGGTTGGGTAATTACCTGTCACGATGACCGGGCGCAAGAGATACTGGATGCGCTGGAGA
AAAAACATGGGGCACTTCTTCAGTGCCGGGCCGTGAATTTCTGGCGCGGATTAAGCTCTAATATGCTCAGCCGCATGATG
TGCGATGCTCTGCATGAAGCGGACTCTGGTGAGGGTGTCATCTTCTTAACCGATATAGCCGGAGCGCCACCGTATCGCGT
GGCTTCATTATTAAGCCACAAACACTCCCGTTGCGAAGTGATTTCTGGTGTCACGTTACCGTTAATTGAACAGATGATGG
CTTGCCGTGAAACCATGACCAGTTCAGAGTTTCGCGAGCGTATTGTCGAACTGGGTGCGCCGGAGGTGAGTAGTCTTTGG
CACCAACAACAAAAAAATCCGCCTTTCGTCCTCAAACATAATTTGTATGAGTATTAACCCGCGATTCTGATGGCGCTTTT
GCTACAATAAAAGCGTTGTTTCACCCTCGGTTATTTTTTCATGTACAAACAAGCTGTTATTCTCCTGCTGATGCTGTTTA
CCGCAAGTGTCAGTGCCGCGTTACCTGCCCGTTATATGCAAACCATCGAAAATGCTGCGGTCTGGGCGCAAATTGGTGAC
AAGATGGTGACCGTGGGGAATATTCGGGCCGGACAAATCATTGCCGTGGAGCCCACTGCCGCAAGTTATTACGCATTTAA
TTTTGGCTTTGGCAAAGGTTTTATCGATAAAGGTCATCTCGAGCCGGTTCAGGGGCGACAAAAAGTTGAAGACGGTTTGG
GCGACCTCAACAAGCCGCTGAGTAATCAGAACTTAGTTACCTGGAAAGATACGCCGGTCTATAACGCGCCGAGTGCGGGA
AGTGCGCCATTTGGGGTACTGGCGGACAATTTGCGCTACCCGATTTTGCATAAACTGAAAGACAGGTTAAATCAAACCTG
GTATCAGATCCGTATTGGCGATCGACTGGCCTATATCAGCGCACTGGATGCCCAACCCGATAATGGCCTGTCGGTGCTAA
CCTATCACCATATTCTGCGCGACGAAGAAAACACCCGTTTTCGCCATACTTCGACGACCACATCGGTACGCGCTTTCAAT
AACCAGATGGCCTGGCTGCGTGACAGGGGATACGCGACACTGAGCATGGTGCAGCTGGAAGGCTACGTGAAGAATAAGAT
CAATCTCCCTGCGCGAGCGGTGGTGATTACCTTTGATGATGGCCTCAAGTCGGTGAGCCGCTATGCGTATCCTGTGTTGA
AACAATATGGCATGAAGGCGACGGCGTTTATTGTTACCTCACGCATCAAACGTCACCCGCAGAAGTGGAACCCAAAATCG
CTGCAATTTATGAGCGTTTCTGAGCTTAACGAAATTCGCGATGTATTTGATTTCCAGTCACATACCCATTTTTTGCATCG
GGTAGATGGTTATCGCCGACCCATATTACTGAGCCGTAGTGAGCACAATATTCTGTTTGATTTTGCACGTTCACGCCGCG
CTCTGGCGCAATTTAATCCGCATGTCTGGTATCTTTCGTATCCGTTTGGCGGATTTAATGACAACGCCGTGAAGGCAGCA
AACGATGCCGGATTTCACCTGGCGGTGACAACCATGAAAGGCAAAGTAAAACCGGGGGATAATCCGTTGTTACTAAAACG
ACTTTATATCTTAAGAACGGATTCGCTGGAGACGATGTCGCGGCTGGTGAGTAACCAGCCGCAGGGATAACAATCAAGCA
ACCTGTACCGGAATCGCTTTCGCGGTACGTTTCATTTCATTGTCGCCTTCAAAATAGGCGACGTTGGGTCGCCAGGTGCG
AGCTTCTTCATCTGGCATGGTAACGAAGCTGGCGATGATGACAATATCGCCGACACTGGCGCAGTGGGCCGCCGCACCGT
TAACAGAAATAATTCTCGAACCGCGTTCTGCCGCGATGGCATAAGTGGAGAAACGCTTGCCGTTGGTGACATTCCAGATA
TCAATGGCTTCGTTTTCGAGAATACCGGCTGCGTCAAGAAAATCCTGGTCAATGGCGCAAGAACCTTCATAGTGCAGGTC
CGCATGAGTCACTTTCACGCGGTGGAGTTTGCCCTGCAGCATCGTGCGAATCATAACTTCTACCTTTCTACCCTGTCGTT
AACGAAGCAGGCGATGCCTGCTTTGAGGAAATTCTCACGCAGTATTGCCCGATTTTTGTTTAGTGTCTACTCATCTGACG
GCATTTGCGTCAGCAGTTTGCGTACCGCGCCGAAGCGTGCCATGTATCGCCTGTAACGAATTGATATTTCTCTGAACGCT
GCTCGGTAAACAATAAAGAGGTGGCTGACGATAGCGCGCCTTGACTGGATACTGCCTTCACGCAAAGCCACACGGAAGAC
AGGGAAGATGGATGCACCGAGTACCACACCGCATGACGCGGTATTTAAACAATTTTTAATGCATGCGGAGACGGCTCGCG
ACTTTCTGGAGATACATTTGCCAGTGGAATTACGCGAACTTTGTGACCTCAACACGCTTCATTTAGAGTCGGGGAGTTTC
ATTGAAGAGAGCCTGAAAGGACACAGCACGGACGTGCTCTATTCCGTGCAAATGCAGGGCAATCCCGGTTATCTGCATGT
TGTGATTGAACACCAAAGCAAGCCGGATAAGAAAATGGCCTTTCGCATGATGCGTTATTCTATAGCCGCCATGCACCGGC
ATCTGGAGGCTGACCACGATAAGCTGCCGCTGGTGGTGCCGATACTGTTTTATCAGGGCGAGGCCACACCTTATCCGCTA
TCAATGTGCTGGTTTGATATGTTTTACTCGCCGGAGCTGGCGCGACGCGTCTATAACAGTCCTTTCCCGCTGGTGGATAT
CACCATCACACCGGATGACGAAATCATGCAACATCGGCGGATTGCGATTCTCGAACTACTGCAAAAACATATTCGCCAGC
GCGACTTAATGTTATTGCTTGAGCAACTGGTCACGCTGATCGACGAAGGGTACACTAGCGGAAGTCAGTTAGTTGCCATG
CAAAACTATATGCTGCAACGCGGTCATACTGAACAAGCGGATTTGTTTTACGGTGTGTTGAGAGACAGGGAAACGGGAGG
GGAGTCTATGATGACGCTGGCGCAGTGGTTTGAAGAGAAAGGGATTGAGAAGGGGATTCAGCAGGGAAGACAGGAAGTAA

GTCAGGAATTCGCCCAGCGTCTTCTGAGTAAAGGAATGTCTCGGGAAGACGTTGCAGAGATGGCAAATTTACCTCTTGCT
GAGATTGATAAGGTAATTAACCTTATTTAAGTTACCTGTGTTATGACAGATGACGTGGGGTAAATTAATAACTGGCGCCA
TCAGCCGTAGCGCCAGTTAAGTATTACGCCAGCTCGACCATTTTGTTGTCGATCAGGCGAGCATCGCCAAGCCAGGCGGC
TACCAGAATTACTGCCCGTTTGCTGGTTTCAGAAACTTCCAGCAATGTGTCGGCATCGCGAATCTGAATATCATCGGCGC
GGAAGCCTTTTTCATTCAGTTCTTGCCCCGCAATGGTAATAATTTCATCGAGATCCCGTTCCCCAGCCTGCAATTTGTCA
GCAATCGAACTTAAAACTTTGTACAGACCAGGCGCAATTTTGCGTTGTTCCGCCGTCAGATAACCGTTACGGGAACTTAG
CGCCAGACCGTCTTTGGCGCGCATAATTGGCACACCGACAATCTCAATATCGAAGCCCATATCGGCAACCATTTTGCGGA
TCAGCGCCAGTTGCTGAAAATCTTTTTCACCGAAGCAGGCGATGTCCGGCTGGACCAGGTTGAACAGCTTGCTGACAATA
GTCGAAACGCCGCGAAAATGTCCCGGACGGCTGGCACCTTCCAGCATGGTCGAAAGGCCAGGAACGTCAACGTAAGTGTG
GGTTTCAGTACCGTTCGGGTAGATCTCTTTTACCGAAGGGGCGAAAACTAAATCCACTTTACGTTTGTTTAGCTTCTCGC
AGTCCTCCTGCAAGGTCCGTGGATAACGAGCCAGATCTTCCGGGCGGTCGAACTGCATCGGGTTAACGAAAATACTGACG
ACGACCACATCGGCGCGGGCTTTGGCTTCGTCGACCAGCTTCATATGGCCATCGTGCAGGTTACCCATGGTAGGCACCAG
CGCCACGCGCTTGCCTTCCATACGCAGGCGGCGAATTTGCTGACGCAGCAGCGGCAGGGTTTCGATAATTAACACAACGT
GACTCCTTAATGGAAACTGTGTTCTTCGCCCGGATAAACGCCGGACTCCACTTCAGCCATATACTGCCGCACAGCCGCGC
GGATGTCGCCCGTTTCGGCGAGGAAATTTTTAGCGAATTTAGGAATGTGACCGCCGGTAATACCAAAGGCGTCGTGCATC
ACGAGGATCTGCCCGTCAGTGACGTTGCCTGCGCCAATGCCAATAACCGGGATCGCCAGTGCTTCGGTAATACGTTTTGC
CAGTTCAACCGGCACGCATTCCAGCACCAGCAGCTGTGCCCCAGCAGCTTCTAAGGCTAATGCATCGCTGAGCAGTTGAT
CGCCCGCTTCATCGCCGCGCCCCTGAACTTTGTAGCCACCGAAAATATTCACTGACTGTGGTGTTAAACCTAAGTGACCA
CATACAGGAACGGCACGTTCGGTCAGCATTTGTACGGTTTCTACCAGCCACTCACCGCCTTCAATTTTGACCATGTTAGC
ACCGGCACGCATAACCGTTGCGGCGTTTTCGAAGGCTTGTTCCGGCGTGGCATACGCCATAAACGGCAGGTCAGCCAGCA
GCAGGCAGTTTGGTGCGCCGCGACGTACGGCGGCAGTGTGGTAGGCGATATCGGCAACGGTAACTGGCAGGGTGGAGTCG
TGCCCCTGAACCGTCATGCCCAGCGAATCGCCCACCAGCATGACGTTAAGCCCTTCATCAGCAAAGAGTTTGGCGAAGCT
ATAGTCATAAGCGGTGATGGTCGCGAAACGTTTTTTTTCCTGTTTGTACTTCTGCAGTAAGGAGATGGTGGTCGGTTTCA
TAACGTATCCTGATAAATTGATGTTGTGCTGTCTGGCATTTTATCAGTCACATTGGTGGGGGCAATGATTTATCCGTAGC
AGCACTGCCAGAAGGTGACAGTGCTGCTACGTTATCAGGGATAGGTTACCTGGAAAGTACTGGTGGCTTTAAATTCGCCG
GGTTCGATAGCAATATTCCCGTCTTGCTTTAATGTTGCCTGGAAATGAAGAGGCTGTGATGTGCCGTTACCTTGATTAGG
GTAAACCCCATCGGAGGTGTCATCTTCGGTTTCATAATCTATATAAACAGAATTCGTATCATTAGGTTTTAATGTCATTA
GCGGATTTTTACTGGTTGCTAAACCTTCAATGAGTACGCCAACGCCTTTGGCGGCAGTGCTTCCAGTAAGCGTATTACCA
AGCAGTTGTGTGTTTTGAGTACCTACTTTTCCAGTGACGAGTTTTGTTTCAATATTACGCACACGAATACAATTCTGAAG
CGAGATATCAAAGGGAACAGGTGAAGCGCCATTTTTAATTGTCCCAGAGCTATATTCTCCCATTCTAACCGTTGAACCAT
TGACTGATGGCCCGGTAAGTATGGAGGTAAAACATGTTGGTAAGGTTAATTTGACATTATTCAGTGTGAAATTGACATAA
ATGTGGTTGCTGTGATCTACAACTTTTGTACCAGGACTGTAGGCCTTAAACGAATAAAGGTAATTTGATGAACTGGATAA
CTGGACTTGCTGATTAAGTGTAGGATCGAAATTAGTATCTGTATAAAATTCAACAGTTATGTTGTGTACTATACCACCAA
TAGCCCAAAACTTATCGTAGTCGTCTGCTTTGTTGCAACCCTTAGTTTGTAGATCGCTGTCTGTGACGGAAAAGAAAAAT
TCTTGGTTGGAAGGATCTCCGATATAGATTCCTGGCGATTGAATGTCAGTTATTGTATCGTATGCAGACCAGACCCTTGA
TATTAACATGGTGTAATACAGACCAGGAACAGATGTATTAAATAGTTTATGTCCGCCATAATCTTTACCTGAATAGACCA
TGGCATTTTCAATAGTTATTGGACCAGGATTCGCTCTGCCAGATGTATTGCAGTAAAGAATACCACCACCGGTACCTGTA
TCCAGGTATGACCAGTACATTTTTACCCCAGTCGGGCCGAGCTGCGTGGGTTTATAAAAAACCAAGTTGCTTGCTGTTGA
TGTGTAATTAAAGGTCATCTGTGCGGCAGTGGAGGGGCCTCGATAGTTATCAACTCCTGCATTATTTCCCACAATAAAAC
TTGTATATGCACTGACTGTATTACAACAAGAATACAGTGCTAAAAAAAGAATGTACCTGAAGATAGTCTTCATTTTTACT
CCAATGTTTCTTTATTGTTATGGTTTGCGCTGTTTTTGTTTGTTATTCGTAGTAAATATCCACTAACACATGGCTCATAA
GTTGACCAGATGTAACACTCAATGCAGGATCGATTTTTTGCATTCGGGTGCTAAATTTCCAGGATGAACCATTCATGTCA
TCCGACATTACTGGATAAACGGAACGAGATCCGCCTGAAGCATTAAGAACATTGAATGTATTCGCCGAATCCTGAGTAGA
AAATATAACTACTCCTACATTACTTGCTCCTGACGAAATTTCATTAGCAAATATTTGCCTAGAGCCAGCAGCCATGACAC
CGCTAAGGGCTCTAAAATCAATATGTAACTGATTTAGCACATCGCCAGTTCCCTGTAATGCACAGTCGGATACAGTGATA
GTGAACTCTTTTCCACCTGGTTGATAACTTTCAGGAGTAACATTACCTGAAAAATATCCCACCCCAACGACGCCAAGATC
AATATTACCCTGGTTACTGATTCCGCTTTTGCAGGTGCTGTTTTTTACATTGGCAACAAGGTCAATATCCTGTCCAGCAA
TGGCTGGACAGGCGATGCAAAACAGTAATGACAGAAACAGAATTATTCTCTTCATCAGCTTACGTTGAGTGGGATGCATA
TATAGTTCCTGAATCTGTAAGTCTGTTAGTTATAGGTTACTGTAAATGTGGCTGGTGCAGAAAACTTACCCGCAGTGACA
TTGTTTACGGTTTTATTTTTTTCCACGACCAGGCGTGCACTCATCGGATAATAAACGACCGCATTGGCTGCAGTGGAGAT
TGTTACCTCTTGTGATGCTGGTGTTACACAGCTAAATTGTGTTGCACTCCCTGTTGCCGGAGTTACAGTGCTCCAGACTT
CAACGGCGACAGCACTTGCTTTATCTGCGGCTGTGGAACCATTTGCAAACCCCGCACCGTCATTAGCAGTTCCCTCGCAT
GTGGCTCGCTTGGTTAATTTTATTTGCGCTTTTTTATTGGGGATACCCGCACAGTCTTTGAATTTGAGTTTGAATGTTTT
TACTTTGGTCTTGGCATTGATTTCAGAAATATATACATCACCAAAATCAACAACAGAAATGTCCTGATCAGAGTCATTTA
CTAATGTAGCGGTACAAGTACCCATTTCTACAGTAGTAATAACGGTCAGTCCAATACTGTCTGTTGCCGCATAAGAGAGT
ACGCTGAACAATGAGGCAGCCAAAACGACACTGCTGGATAATCCATAACGTAAATTTTTAAATGTCATCATAATTATCCT

TCTTATTGATAGATGAAATTAAACGTCGCCAGTGCACGAAAATTCCCCGGTACGCCTTGCCCTGCATCTGTTTCACGTAG
CGCAACGGTCATTTCAAGACCCTTATCGGGCTGCATCTCGTCTGTGCTCCAGCGTATCTTTTCCGCACTGTTAGGTTTAA
GGAAAGTGGCATCATCAGTAGTCCGTTTTTTGAAACCCATACCGATATTACTTGTCGTCGAAGATGAATCACCAGACTGC
GGTATAATAAGCTTAGGTGAGCTTGATGATGCATTTCCGGTCAGAGTGGTATCAATCCAACTGATGCCACTGCTGCACCC
ACTGGCAACCAGTTTAAAATCAGCCTGAGATTCTGTCGTTTTATTCGCGATCTTATCCAGACCCATCTTAGGGATTCTCA
ATGTGTAGTTATTATTGCCATCATTCGTGACGTTATTACCAGTAAGTGTGATGTTACAGGTTGTCGCTTTGACAGTGGCT
GTAAACTCTACATTAATATCCGTTGCAAATACTGAGGGTGATAATAATATTCCCATCAGTATCACTATTTTATGTGGCGT
TGTTTTTATCATTTGACGTCCCTTGTAGTTACTGAATCTGACACCGAATTCCATTCAGAATAATAGATTGTGCTATTTTT
TCTGCTTCTGGGCTTTGTTGATAATGCGCAAGACAACTTACGGGTTTACTTTGTTCGAGCCATTTAATGCTGATATTTCC
CTGCTGCTCAATACCACGAACAAATGCTTGTCCACCCTGTCCAACATTACCAATGACATTGCCTTGCTCATCATAAATAT
CTGCAGCAAATGGAATATTTTTACCATCACTTCGTGTGATGTTCATAATGGCTGATTGCCCTTGCACGGTTTCAAAATCA
GCAAAGACGACTGAACCCTGACGCGGTACAGCTACTGCACTGGTACTTTTTAATTCAACATCGTTCTCAAGATCGTTGAT
ATCCAGCGCGATACGGTTTTCATGATAAGGAGAAAGAGCGCTGGTGACACCATAACCCCATCGATCGATAGTACTGTTGC
CATAATTTATTCGCGCTCCTTGAGCACCTGGAGCCTGAACTACCGCCAGTGTATCGGAGTCGCTAAAACTATCATTACTG
AAAGTCAGTCCACCGCTATGCAATACAAAACCACCGTCGGTGCTGAGAGAAACTTGACGGCTGTTATCGCTATTTGCAGA
AATTGAACCTGCCAGCGTTCCCCATGGTGACTCATAGCTGGCATAACCCCCAACATAACTCAAATCTTTGCTGGCTTTAT
TCATCGTATAGCCAGTATTCACGCTATAACTGACGCGAGCGTTATCGCTATAGCCACTGCTGCTAACGTTGAGTTGGTTA
TTACCCTTAAAGTCACTGCTTATTTGAGTATCAATACTCTGGAAACCTGAAGTACGTTGTTCAGTGCCAAGTAATTTTTC
AATTGGAATGGTGAAACTAAGATAAACGCTATCGTCAGTGTCGCCGTCTTCATTCCATGAACGCTGGGCACTGACACTGT
AGCTGCCCCAGGATGTACTGTTACTGTAGCCAATAGAGTAATTGCTACGATTTTGTCCGGAAGCCCAGTAATCGGACCAA
CTTCCGGAAAGATAAAATGAACCGTAATCTTTTTTCTCAAATTTCAACGGTTGGTTAATACTGACCGTAACCTGATTTTT
CATGCGTGAGTAATTACGCATGGATTTCGGTTCAAGATCTTGTTCGGGATGTTTCACTTCATCAATTAGAGTTAGTGCAT
CATTAAGACCAAGGTAATTCTGTGTCGAATAGCGATAGGCCGCGATATTCAGTGAAGTACTTGTTTCTTCGAATAACTTG
TTCCAGGAAACACGATAACTTTGCCCCTGGTATGTTTTATCATCCGGGATACGAACATTGGAATGAGTCACATCGAAAGA
AAATGCACCAACTGAAGTATTCAGACCAAGACCTAACAAACCAGCGGTATAGTTATTATCGGTTATCTGAATACCGGTAT
AACCCGTCAGATAGTTATTCAGGCCGTAGTAGTAGCTTGCTTGAAATAAATTAGGCTCATCCTGAATATCATCTTTTAAG
ACCTGACCGCCGCTAATATCCCAACGTCCAACGCCAGGGCGTAACATTTGAACAACGGATGAGAAAGGTTGCGAGAATGT
CCGCTTTGAGCCATCGGATTCTTCGATGGTAACAATAAGATCGCTGCCGTACCCTGACGGACTCAGATCATCAATGACGA
AAGCGCCTGGCGGCACCGTCGTTTCATAAATCTTATAGCCACCTTGCGTAATAGTGACTTTGGCGTTGGTATTGGCAACG
CCATGAATGATAGGCGCAAAGCTGGCTAAAGTCGGAGGCAACATGCGGCTGTCACTGTATAAACGAATGCCTCGGATACT
GACGGAATCAAAGGTTTCGCCCGTCGTATAAGACTCACCAAGAATGAGTTGAGAACGCAGCGAGGCGATATCACGCTGAA
CATACCGATTCTTAAAATCATAATTACTGCCAGAATCGGTCATCCAGTTGTAGTTGCCCGAGGCACGCAGTCGCCATGCA
CCTAAATTCATCCCACCGTTAAATGCAGCATAAATGCTTTCATTTTTTCGACCAGGGGTTTCACTATGATATCCGTTGAG
GTTGTATGACAACATGGCCGCATTAATGCCGTTTTCCCATAACGATGGATCAACATAGTTTTGGTAATTTTTCATTACCC
AGGCTTGAGGAACGTCTATATCCAGACGTTGATCGTTAACGTCATAACGAACAGAAGCCTGAGGGATAATTTCCGTCAAA
TTGAGGCAATTGCCGAGCGTTTCATCCCTGGCAAGCAGAACGGCTTTTTCGTTATTTATATCGGGAGAATTAATATGAAA
CTGCAATAAATTCTTTAATGTGATACAAGCCTGGGCGTTCTTTTTTCCTTCAATTGCGACAAATGTAATACTTTGGTTAA
TGATTGGTTGGTCGTTTACATAAACACTGACGTCGTAAACACCCGGTATAGCGGGGTTACCTTCACTGTACCGGCTGAGA
TCAATATTAGATGCATTCTGCCCCATCAGGAAGGTATGGTCATATTCAACGAGTTCAGCACTGAAAGCAGTATTGCAATA
CAGCAGCGCGCAAAACGTGGCGATACGGGTCAGATGATGATAATTTTTAGTATATTCTATAGTCACGCTATGCTTCCTGC
GGAATTATATCCTTGCCTGATTACAGCCTGGCATTACCTTCAATTGCGCCACCAAAGTCATTAATGGCGTAAAAATGCAC
TTTTGCAGAATTCGCTTTGCCATTAAGGCCATTGACTTTCATGACCTCATCACTAAATGGTGCAATCATTTTCACATCAA
TCGGATAGCGTTTACCGCTAGCTTCTAAATCACCACTGCTAAAAGAGACGTAGTAAGGGGTTGGATTGGTCACTCGTAAT
GACGCCTTACCTTCTGAACCTGACCAGAACCACTTCAGGGCTAACGGGGCTTCAGAGGGATTTCCCTTCAATCCATCCGG
GCGATAGAAAAGTTTTATACGTGTGCGAAATGCCAGTTGCAGCAGGCTTTGATTCGCGACCTTTTCTGCATCTGGTTTTG
GTGGAACTTCCAGTACGTTAAACCAGAACACGCTCTCTCTGTCTTTAGGCAGTGAGGTGCTGGCTGTGTACATTAATTTG
ATTGTTTGCCCACGTTTGGCATCAATACGCGATACTGGCGGCGTAGCAGTAAAAGGGACTGTAATACTGCCAGGCTCAGC
GTTGTCATCGCCAGTATCTAACCAACTCTGGACAAGCAACGGGTTATTCCCTTTATTTTCCAGACGTACGTTGACACTTT
TTTGATCGCTTTTATATATTACGCGAGTACCCGAAATGACAATGTCCGCAATAGACGATGAACTAAAAGCCATACAGGTT
ACGAAGCATAAAGCTGTTGTATGTTTGGTGTTAAAAAACATAAACCATCCCTGATAGAGTTAATTAAAACAGGGAATAAT
ATAAAATAGAAATTATTATTCCCTGTATATTCATTCAATCAATTTAACTGGTGAATACTTACTGATAAGTAATGGTGTAT
GCAGTGTTAGTTTTTACATAACCAGCAGTTGCTGTTTGGTCTGCGACGGCACGAACGTAAGACGCTTTAAAATCATAAAC
AGCAGATTTTGTCGTTGCATCCAGGGCTTTAGTATACACATCGCCAGGGTTGTTAATTTGAACCTGTTTGATAGTGGAAC
CATCAATATTGTGTAGAGCGATATTAACGCCGTCAGATGGGTTGTTAATAGACATGTCGTTGTTCAAAGTCCCTTTGCTG
TTACCAAAGAAAACAGAACCGAAGGTCATTTTAGCCGTGCTACCTGGATTCGGATTTGCTTTGCTGCAGTCAACGGTGAT
GCTGAAAGGTTTAGCTTTAGCGCCAACGGTGTCATTCAGTACGCCAGCATCGATTTCACCAACGGTTGCGGTTTGCAGCA

GGATCAGACCATCCTTGTTACCGCCGTCAACGCGGGTTTCACAGGTGTTATCAACAACCAGACCACTGATATTTAACTGA
CCGCCGTCCATATCAGCAGAAGCAGTACCTGCAACCATCGCCAGCATAAGACCAGACAGGGCAAAACCTAATTTTTAGA
CATAAAAATCCTTTAATAAAATTCCATTAGTCAGACTACATGTTTGAAGAATGACTATTCATGACACAAATAGGAGAAAC
AAATGTTAGATATTAATGAGCAATGATATTTGTTACCCAAATTTACAACCATTGTTCATTAGGTCGCCTATTGTGCACTT
TAGAAGCTTTTGAACAAATTAAATTTACTTAATTCAAAATTAAGTAAAAATAAGTTCACAAGTGCAATTGGTTAGGGTAT
GGAGATGGGATTGATATTTATTTATAGGAAGTTATATTATTTCCGCAAAGAGATGTTTAAACTAAAATCGTAATTAAGGA
AAGAGATGAGATATGATTTTCAGAAAATATTTATATTCGCAATATAAATAAATTACCATTTGTTTAATTTGTCAAATGCT
CTTGTATGTAAGATTTGACGCAACATCTCCCCATCAGGAAACACCAACTCCGGCGCGATTTCAAACAGCGGCCACAGCAT
AAATCCACGATTCTTCATATCGTAGTGCGGAACGGTCAGGCGTTCAGTATTTATCACTTCATTACCAAACAGCATGATGT
CGAGATCCAGCGTGCGTGGTCCCCAGCGTTCAGCTTTGCGGACGCGACCTTGCTGCAATTCAATACGCTGTGTGTGATTG
AGTAGCTCTTCAGGTGCAAGAGAGGTTTCCAGCGCCACGGCTGCGTTTAAGTAATCGGGTTGATCTTGCGGCCCCAGCGG
TGGGGTGCGGTAAAACGAAGAAACGGTAAGAATGTGGCTTTCAGGGATATCGCCTAATGCTTTCAGGGCAGCATTGACCT
GCTCCAGCGGAGAGGCCAGATTGCTGCCTATGGCAATATACGCCACTGTCATGCGGTACCCTCACGACGTGGTGCGCGTT
TGCGTGGACGACGAGTACGACGACGCGGTGACGGTTCTTCATCCAGCTCGTTGAGCATCCCTTTTTGGTCTGGTGGCGCG
GAAACCTGGAACTCACCCCACCATTTCACCAGACGCTGCAGTTCAGCGTTACGCTCAACTTCAGCTCGCAAGGCCAACAG
GTCATAAGCCGCACGGAACTTAGGATGCTCCAGCAGTTTCCATGCGCGTTTACCCTGACGACGGGACATACGCAACTGCA
ACTGCCAGATATCGCGGGTTAATGTCGTCAGACGTTTCGGGATTGCCAGTGAACGGCAGGCTTCGTCCAGCACGTCGTTC
ATCGCCAGCGCGAAAGCGTCGTGATAGGTCAGGCCGCTTTCCTGGGCGATCTTCTGTGCCGTCTCCAGCAGTGGGTACCA
GAACATGGCGGCAAACAGGAACGCCGGGTTCACGCGCATATCGTTATGGATACGCGTATCGGTATTCTTCAGCACCTGTT
CAATGATCCGCTCCATCGGGCTGTCGCCATTTTCCGTGAAGTAGCGGGTAATGGTCGGGAACAGCGGCTGGAACAGATGA
TATTCACACAACAGCTTATAGGTTTCGTAACCGTAGCCCGCTTGTAGCAGTTTAAGCGATTCTTCAAACAGGCGTGCCGG
TGGGATATCGTTCAGCAGGGTAGCGAGGCGAGGGATCGGTTCTGCGGTTTCCGGGCTGATGCGCATACCCAATTTGGCGG
CAAAACGTACCGCGCGCAGCATACGTACCGGATCTTCACGGTAGCGCGTTTCCGGGTTACCAATCAGACGGATAACGCCG
TCCTTCAGATCCTTCATGCCGCCAACGTAATCACGGACGGTAAAATCCGCTACGCTGTAATACAGGCTGTTGATAGTGAA
ATCGCGGCGCTGGGCGTCTTCTTCGATGGAGCCGAAAATGTTGTCGCGCAGCAACATGCCGTTTTGCCCGCGTTGGGAGG
TCGTGCGGTCGCTGACGTTACCTTCGTGGTGTCCACGGAAGGTCGCAACTTCGATAATCTCCGGGCCAAACATTACATGA
GCCAGACGGAAACGGCGACCCACCAGGCGGCAGTTACGGAACAGTTTGCGCACCTGCTCAGGCGTGGCGTTAGTGGTTAC
GTCAAAATCTTTCGGCTTTTTGCCAAGTAACAGGTCGCGCACGCCGCCGCCAACCAGCCAGGCTTCGTATCCCGCTTTAT
TGAGCCTGTACATTACCTTCAGGGCATTTTCACTGATATCTTTGCGGGAAATAGCATGCTGCTCACGCGGGATCACCGTC
ACCTGTGGACGGGCGACTGCCTGTTCAGCCTCGCTTTCCTCGCGGCTTAGCACCTTGCGGCAAAAATTAGCGACTCGGGT
AAAAATAGTACACCTCGGTAGTGTCAAACATCATTCAGGACAAAAAAATAGCGGCTAATCATAGCTCAGCATGACGCATT
TGAGAATGTTGAATTTACAATTGCCGACTCGGGCACGGCGGTAAGCCGCCAGTTTTTGACGGCTGACTGAAGGATTTGCT
CGACGCTGAAATCCTGCCAGTGTGCTTCTGCCTGCTGCCCCAGAAATTGAAGTGCCGCGATTAGTACCGGGCGTGGATCG
CCTTTCGGCAACGCAGGCGCATGATTCTGCTTGGAAAGTTTAGCGCCTTGTGGATTAAGCGCCAGCGGCAGATGAATGTA
ATCTGGCACTTTCCAGCCAAAAAGCTGGTACAGCGAGATTTGCCTTACTGTTGGTTCAATCAGATCAGCCCCACGCACTA
TTTCTGTAACGCCCTGGAAATGATCATCAACCACAACAGCCAGGTTGTAGGCGAACAACCCATCACGGCGATGAATGATA
AAATCTTCCCGTGCCAGTTTTTCGTCGGCGTGAATAATGCCGCGCAGCTGGTCAGTAAATTGCGTGACCGGATGCTGCTG
GCGGATACGCACTGCGGCGTTGTCTGGTCCATGATGCAACACCCGGCAATGACCGTCGTAAATACCGCCAATGCTTTGAA
TACGCGCACGCGTACAGGTGCAGTAATAACTTAGTCCTTGTTCATGTAACCAGGCGAGTGCTTCACGATAGGCGTCGTGA
CGTTGCGATTGCCAGAGAACATCGCCGTCCCAGTGCAGACCGTAATGTTCCAGCTGGCGCAGGATAGTTTCTGCGGCACC
GGGAACTTCACGAGGCGGGTCGATATCTTCTATGCGTACCAGCCAGCGACCTTGCCGGGCGCGAGCCTGCAAATAGCTGC
CGAGCGCGGCGATCAGAGAGCCAAAATGAAGCTCGCCGGAAGGAGAGGGGGCGAAGCGGCCAATATACTGTGTGTCTGTC
ATCTCTTTGAACAAAAAATAAGGCGGGAGCATTTCCCGCCTGTGGTAAACGTGATGGAACGGCTGTAATTAGCCAGCCAT
CTGTTTTTCGCGAATTTCAGCCAGCGTTTTGCAGTCGATGCACAGATCGGCTGTCGGGCGCGCTTCCAGACGGCGAATAC
CAATTTCAACACCGCAGGATTCGCAGTAGCCGAAATCTTCGTCTTCCACTTTTTTCAGCGTCTTCTCGATCTTTTTGATC
AGCTTACGCTCGCGATCGCGGTTACGCAGTTCGAGGCTGAACTCTTCTTCCTGGGCTGCACGGTCTACCGGGTCCGGGAA
GTTGGCTGCTTCATCCTGCATATGTGTAACGGTGCGATCGACTTCATCCCTGAGTTGATTACGCCATGCTTCCAGAATAC
GACGGAAGTGCGCCAGCTGGGCTTCATTCATATACTCTTCGCCCGGCTTCTCCTGATATGGTTCCACCCCAGCGATGGCG
AGAATACTCAGGGACGATGTTTTACGGTTTTGCCCTTCTTGCATGTTGCTTCTCCTTAACACGCACTATCGATCCCCATG
TTCGGGGGAAAAATGAGGCCGCTATAAATAGCAGATGCTTTTCCGGATAGCAATTATCTAAACGTAACACTTGACAACTG
TGTGAGGAAAAGCGTATTTGCGCACGCGACCAGAATGTAAATTAACCAGTTACTTACTTTACTACAATGTAACCGGCAGT
GATTTTTTAAGAGCCATGCCTTCAGCAGAAATTTCCGCTTTGTAAGCCAGAATTTCTACCCCCCTCTGTTGAGCTTCTGA
CAATAGTTGCGCGTATTTCTCATCGATGTGGCGCGCGGGTGAAAACCGTGTAATGGCTGAATGCAGCACGGCGAAAAAGA
TAACCGCACGCTGGCCTTCAGCCGCTACGCTCATCAACTCCCGAAGGTGTTTCTGACCTCGTTCAGTGACCGCATCGGGA
AAATATCCCTGTTCGTTCTCCGCTAACGTAACCGATTTCACTTCAATATAGCAGTCTGGACGCGAATCCGCCTGCAACAT
AAAGTCAATACGGCTGCGTTCTGCGCCGTATTTTACTTCGCTTTTCAGCGAGCTATAGCCTGACAGTTCTGAAATTGATT

CATTAAGGATAGCCTCTTTCGTCAACCTGTTAGCCCAAAGCGTGTTGACGCAAATAAATGCGCCGCTCTGGCTTTGAGTT
AATTCCCAGGTGTGTGGGTATTTCCGTTTGGTGTTGTCTGAAGTCGAATACCAGACGGTATCGCCAGGCGTTGCACAACC
GGTCATCGCACCCGTATTCGGGCAGTGTAGCGTTAATTCGCGACCATCGGGTGTGATCACATCGGCTAAAAAACGTTTGT
AACGCTGAATTAGCGTCGCGCGCTGTAGAGGGGGAGAAAATTCCATTGCGACAATCCTTGTTATTGCGTTAGCGCCCAGC
GTTTTAGCGGCGTGTAGCGTGTGCGTCCACGGGCAAACGAGGAGGCGTAAAGGGTGAACTCCGTCACCGCATACGACCAG
TTAAAACCTGGCGGCGGGATTGTCACCGCCTCGCTGGCGTCGCGCAATAAGGTAATATGTGGATGAAACGGACGATTGCT
TTGAAAACAACCGCTGCGGGCAGCCTGTGAACGGAGCATATTCGCCAGCTGGATTAAGCCGCGTGGCGGCTGACGCATCC
CTAACCACACCACACGCGAACGCAGCCATTGTCCGGCGTCATCAAGCGTGAGTGTGAAACCAGGTTGACGAATCCGTCCG
GCTAAAAGAGAAAGCGCCTTCTCTTTCTCGGCGCTCACTTCGCCTAAAAATGCCAGAGTCAGATGCAAATTATCGGCGGC
GACCGGACGTCCCGCCTCAGGTGGGAAGTGTGTGGCGCGCCAATGGATAATCTGTTCGCGGATTTCTGCAGGTAAGTCGA
TAGCAAAGAACAGACGTTGCGGTTCAGACATGTGAGGCACTCGGTTATGAATTACCGCGATGCTACAATGTGGCGCGAAG
AATGTTAACCCTCTGGAGCGTTTTGTGTCGTCGTTGCCCGTTGCTGCCGTCTTACCTGAATTACTTACCGCTCTCGATTG
TGCGCCGCAGGTATTATTAAGTGCGCCGACCGGGGCCGGGAAATCAACCTGGCTGCCGCTGCAACTGCTGGCGCATCCCG
GCATTAACGGGAAAATTATCCTGCTGGAGCCGCGTCGTCTGGCGGCGCGTAACGTCGCGCAACGGCTGGCGGAGCTGCTT
AACGAAAAGCCAGGCGATACCGTTGGCTACCGGATGCGTGCGCAAAACTGCGTCGGGCCGAATACCCGCCTGGAAGTGGT
TACCGAAGGCGTGCTGACGCGCATGATCCAGCGTGACCCGGAACTGAGCGGTGTTGGACTGGTGATCCTTGATGAATTTC
ATGAGCGCAGCTTGCAGGCGGATTTGGCGTTGGCGCTGTTACTCGATGTGCAACAAGGTCTGCGTGATGACCTTAAACTG
CTGATTATGTCGGCTACCCTGGACAACGACCGCTTGCAGCAAATGCTGCCAGAAGCGCCTGTCGTCATCTCAGAAGGGCG
CTCGTTTCCGGTTGAACGCCGTTATTTACCGCTGCCCGCGCATCAGCGTTTTGACGATGCCGTTGCGGTAGCCACCGCTG
AAATGCTGCGTCAGGAAAGCGGATCATTACTGTTATTTTTACCTGGCGTCGGAGAAATTCAGCGTGTGCAGGAACAACTG
GCTTCGCGCATCGGCAGTGATGTATTGCTCTGCCCGCTGTATGGCGCGTTGTCGCTGAACGATCAGCGAAAAGCGATCCT
CCCGGCACCGCAAGGGATGCGCAAAGTGGTGCTGGCGACCAATATTGCTGAAACCAGTTTAACCATTGAAGGTATTCGTC
TGGTGGTGGATTGTGCCCAGGAGCGTGTGGCGCGTTTTGATCCGCGCACGGGGCTTACGCGACTGATTACTCAACGCGTT
AGCCAGGCATCCATGACGCAGCGTGCCGGGCGCGCCGGGCGTCTGGAGCCGGGTATCAGCCTGCATTTAATCGCCAAAGA
ACAAGCAGAACGCGCCGCGGCGCAAAGTGAACCGGAGATCTTACAAAGCGATCTTTCCGGTTTGCTGATGGAATTACTGC
AATGGGGATGCAGCGATCCGGCGCAGATGAGCTGGCTGGATCAACCGCCAGTAGTGAATCTACTGGCCGCGAAACGTCTG
TTACAAATGCTGGGGGCACTGGAGGGTGAACGGCTTAGTGCGCAAGGGCAAAAAATGGCAGCGCTGGGTAACGATCCGCG
TTTAGCGGCAATGCTGGTTAGCGCGAAGAACGACGACGAAGCTGCTACCGCGGCAAAAATTGCCGCCATTCTCGAAGAGC
CGCCACGGATGGGCAATAGTGACCTGGGCGTGGCGTTTTCGCGCAATCAACCAGCCTGGCAGCAACGTAGTCAGCAACTG
TTAAAACGCTTAAACGTACGTGGCGGTGAGGCAGACAGTTCGCTTATCGCGCCGCTACTTGCCGGGGCGTTTGCCGATCG
CATTGCTCGTCGCCGTGGGCAAGATGGACGCTATCAACTGGCAAACGGCATGGGAGCGATGCTCGATGCCAACGACGCGC
TAAGCCGCCACGAATGGTTGATCGCACCGTTATTATTGCAGGGCAGCGCCTCGCCGGATGCGCGGATTTTACTGGCGCTG
CTGGTCGATATTGATGAGTTAGTACAACGCTGCCCGCAGCTGGTACAGCAGTCTGACACTGTGGAGTGGGATGACGCGCA
AGGTACGCTGAAAGCCTGGCGTCGGCTACAAATCGGTCAGTTGACGGTGAAAGTGCAGCCGCTGGCGAAACCGTCAGAAG
ACGAGTTGCATCAGGCGATGCTTAATGGCATCCGTGATAAAGGTTTAAGCGTGCTCAACTGGACGGCGGAAGCGGAACAG
CTACGCTTGCGTTTGTTATGCGCCGCAAAGTGGTTGCCGGAATATGACTGGCCAGCGGTTGATGATGAAAGTTTATTGGC
AGCGCTGGAAACGTGGCTGCTGCCACATATGACTGGCGTACATTCACTACGCGGCCTGAAATCACTCGACATTTATCAGG
CACTACGCGGATTACTTGATTGGGGAATGCAGCAACGTCTGGATAGTGAATTGCCTGCGCATTACACTGTGCCGACGGGA
AGCCGGATCGCCATTCGTTATCATGAAGATAACCCGCCCGCGCTGGCGGTGAGAATGCAAGAGATGTTTGGCGAGGCCAC
CAATCCGACGATCGCCCAGGGGCGCGTGCCGCTGGTGCTGGAGTTGCTTTCACCTGCCCAAAGGCCATTACAAATCACAC
GAGATTTGAGCGACTTCTGGAAAGGAGCGTACCGTGAGGTGCAAAAAGAGATGAAAGGGCGTTATCCCAAACATGTCTGG
CCGGACGACCCGGCAAATACTGCACCGACGCGACGGACGAAAAAGTATTCGTAAGGTTGGTTTTCTCCCTCTCCCTGTGG
GAGAGGGCCGGGGTGAGGGCATCAGCGCGCACGTTCACCCTCACCCTAACCCTCTCCCTCAAGGGAGAGGGGACCGATCG
AGCACAAATTTTGAGAGATATCTTCTTCTGTCTTGTAACAGAAGAACAGAAAATCGGGCTTTTGCGCCTGAATATTGCGG
AGAAAAAGCATGGCCGGGAATGACCGCGAGCCAATTGGACGCAAAGGGAAACCGACGCGTCCGGTCAAACAAAAGGTAAG
CCGTCGTCGTTACGAAGATGACGATGATTACGACGATTATGATGACTATGAGGATGAAGAACCGATGCCGCGCAAAGGTA
AGGGCAAAGGCAAAGGGCGTAAGCCTCGTGGCAAACGCGGCTGGCTATGGCTACTGCTAAAACTGGCTATCGTTTTTGCC
GTGCTGATCGCCATTTACGGCGTTTATCTCGATCAAAAAATTCGTAGCCGTATTGATGGCAAGGTCTGGCAACTGCCTGC
GGCAGTTTATGGCCGAATGGTCAATCTTGAGCCAGACATGACCATCAGCAAGAACGAGATGGTGAAGCTGCTGGAGGCGA
CCCAGTATCGTCAGGTGTCGAAAATGACCCGTCCTGGCGAATTTACCGTGCAGGCCAACAGCATTGAGATGATTCGCCGT
CCGTTTGATTTCCCGGACAGTAAAGAAGGACAGGTGCGCGCGCGTCTGACCTTTGATGGCGATCATCTGGCGACGATCGT
CAATATGGAGAACAACCGTCAGTTCGGTTTCTTCCGTCTTGATCCGCGTCTGATCACCATGATCTCTTCGCCAAACGGTG
AGCAGCGTCTGTTTGTGCCGCGCAGTGGTTTCCCGGATTTGCTGGTGGATACTTTGCTGGCGACAGAAGACCGTCATTTT
TACGAGCATGATGGAATCAGTCTCTACTCAATCGGACGTGCGGTGCTGGCAAACCTGACCGCCGGACGCACGGTACAGGG
TGCGAGTACGCTGACGCAACAGCTGGTGAAAAACCTGTTCCTCTCCAGCGAGCGTTCTTACTGGCGTAAAGCGAACGAAG
CTTACATGGCGCTGATCATGGACGCGCGTTACAGCAAAGACCGTATTCTTGAGCTGTATATGAACGAGGTGTATCTCGGT

CAGAGCGGCGACAACGAAATCCGCGGCTTCCCGCTGGCAAGCTTGTATTACTTTGGTCGCCCGGTAGAAGAGCTAAGCCT
CGACCAGCAGGCGCTGTTAGTCGGTATGGTGAAAGGGGCGTCCATCTACAACCCGTGGCGTAACCCAAAACTGGCGCTGG
AGCGACGTAATCTGGTGCTGCGTCTGCTGCAACAGCAACAGATTATTGATCAAGAACTCTATGACATGTTGAGTGCCCGT
CCGCTGGGGGTTCAGCCGCGCGGTGGGGTGATCTCTCCTCAGCCAGCCTTTATGCAACTGGTGCGTCAGGAGCTGCAGGC
AAAACTGGGCGATAAGGTAAAAGATCTCTCCGGCGTGAAGATCTTCACTACCTTTGACTCGGTGGCCCAGGACGCGGCAG
AAAAAGCCGCCGTGGAAGGCATTCCGGCACTGAAGAAACAGCGTAAGTTGAGCGATCTTGAAACTGCGATTGTGGTCGTC
GACCGCTTTAGTGGTGAAGTTCGTGCGATGGTCGGAGGTTCTGAGCCGCAGTTTGCGGGCTACAACCGTGCGATGCAGGC
GCGTCGTTCGATTGGTTCCCTTGCAAAACCAGCGACTTATCTGACGGCCTTAAGCCAGCCGAAAATCTATCGTCTGAATA
CGTGGATTGCGGATGCGCCAATTGCGCTGCGTCAGCCGAATGGCCAGGTCTGGTCACCGCAGAATGATGACCGTCGTTAT
AGCGAAAGCGGCAGAGTGATGCTGGTGGATGCGTTGACCCGTTCGATGAACGTGCCGACGGTAAATCTGGGGATGGCGCT
GGGGCTGCCTGCGGTTACGGAGACCTGGATTAAACTGGGCGTACCGAAAGATCAGTTGCATCCGGTTCCGGCAATGCTGC
TGGGGGCGTTGAACTTAACGCCAATCGAAGTGGCGCAGGCATTCCAGACCATCGCCAGCGGTGGTAACCGTGCACCGCTT
TCTGCGCTGCGTTCGGTAATCGCGGAAGATGGCAAAGTGCTGTATCAGAGCTTCCCGCAGGCGGAACGCGCTGTTCCGGC
GCAGGCGGCGTATCTGACACTATGGACCATGCAGCAGGTGGTACAACGCGGTACGGGTCGTCAGCTTGGGGCGAAATACC
CGAACCTGCATCTGGCAGGGAAAACAGGGACTACCAACAATAACGTAGATACCTGGTTTGCGGGCATTGACGGCAGCACG
GTGACCATCACCTGGGTCGGCCGTGATAACAACCAGCCGACCAAACTGTATGGTGCCAGCGGGGCAATGTCGATTTATCA
GCGTTATCTGGCTAACCAGACGCCAACGCCGCTGAATCTTGTTCCGCCAGAAGATATTGCAGATATGGGCGTGGACTACG
ACGGCAACTTTGTTTGCAGCGGTGGCATGCGTATCTTGCCGGTCTGGACCAGCGATCCGCAATCGCTGTGCCAGCAGAGC
GAGATGCAGCAGCAGCCGTCAGGCAATCCGTTTGATCAGTCTTCTCAGCCGCAGCAACAGCCGCAACAGCAACCTGCTCA
GCAAGAGCAGAAAGACAGCGACGGTGTAGCCGGTTGGATCAAGGATATGTTTGGTAGTAATTAACATCTAAGCGTGAAAT
ACCGGATGGCGAGTTGCCATCCGGTAAAATAACATCCCATCTAAGATATTAACCCTTTCTTTTCATCTGGTTGTTTATTA
ACCCTTCAGGAACGCTCAGATTGCGTACCGCTTGCGAACCCGCCAGCGTTTCGAATATTATCTTATCTTTATAATAATCA
TTCTCGTTTACGTTATCATTCACTTTACATCAGAGATATACCAATGGCGCGTTCCAAAACTGCTCAGCCAAAACACTCAC
TGCGTAAAATCGCAGTTGTAGTAGCCACAGCGGTTAGCGGCATGTCTGTTTATGCACAGGCAGCGGTTGAACCGAAAGAA
GACACTATCACCGTTACCGCTGCACCTGCGCCGCAAGAAAGCGCATGGGGGCCTGCTGCAACTATTGCGGCGCGACAGTC
TGCTACCGGCACTAAAACCGATACGCCGATTCAAAAAGTGCCACAGTCTATTTCTGTTGTGACCGCCGAAGAGATGGCGC
TGCATCAGCCGAAGTCGGTAAAAGAAGCGCTTAGCTACACGCCGGGTGTCTCTGTTGGTACGCGTGGCGCATCCAACACC
TATGACCACCTGATCATTCGCGGCTTTGCGGCAGAAGGCCAAAGCCAGAATAACTATCTGAATGGCCTGAAGTTGCAGGG
CAACTTCTATAACGATGCGGTCATTGACCCGTATATGCTGGAACGCGCTGAAATTATGCGTGGCCCGGTTTCCGTGCTTT
ACGGTAAAAGCAGTCCTGGCGGCCTGTTGAATATGGTCAGCAAGCGTCCGACCACCGAACCGCTGAAAGAAGTTCAGTTT
AAAGCCGGTACTGACAGCCTGTTCCAGACTGGTTTTGACTTTAGCGATTCGTTGGATGATGACGGTGTTTACTCTTATCG
CCTGACCGGTCTTGCGCGTTCTGCCAATGCCCAGCAGAAAGGGTCAGAAGAGCAGCGTTATGCTATTGCACCGGCGTTCA
CCTGGCGTCCGGATGATAAAACCAATTTTACCTTCCTTTCTTACTTCCAGAACGAGCCGGAAACCGGTTATTACGGCTGG
TTGCCGAAAGAGGGAACCGTTGAGCCGCTGCCGAACGGTAAGCGTCTGCCGACAGACTTTAATGAAGGGGCGAAGAACAA
CACCTATTCTCGTAATGAGAAGATGGTCGGCTACAGCTTCGATCACGAATTTAACGACACCTTTACTGTGCGTCAGAACC
TGCGCTTTGCTGAAAACAAAACCTCGCAAAACAGCGTTTATGGTTACGGCGTCTGCTCCGATCCGGCGAATGCTTACAGC
AAACAGTGTGCGGCATTAGCGCCAGCGGATAAAGGCCATTATCTGGCACGTAAATACGTCGTTGATGATGAGAAGCTGCA
AAACTTCTCCGTTGATACCCAGTTGCAGAGCAAGTTTGCCACTGGCGATATCGACCACACCCTGCTGACCGGTGTCGACT
TTATGCGTATGCGTAATGACATCAACGCCTGGTTTGGTTACGACGACTCTGTGCCACTGCTCAATCTGTACAATCCGGTG
AATACCGATTTCGACTTCAATGCCAAAGATCCGGCAAACTCCGGCCCTTACCGCATTCTGAATAAACAGAAACAAACGGG
CGTTTATGTTCAGGATCAGGCGCAGTGGGATAAAGTGCTGGTCACCCTAGGCGGTCGTTATGACTGGGCAGATCAAGAAT
CTCTTAACCGCGTTGCCGGGACGACCGATAAACGTGATGACAAACAGTTTACCTGGCGTGGTGGTGTTAACTACCTGTTT
GATAATGGTGTAACACCTTACTTCAGCTATAGCGAATCGTTTGAACCTTCTTCGCAAGTTGGGAAGGATGGTAATATTTT
CGCACCGTCTAAAGGTAAGCAGTATGAAGTCGGCGTGAAATATGTACCGGAAGATCGTCCGATTGTAGTTACTGGTGCCG
TGTATAATCTCACTAAAACCAACAACCTGATGGCGGACCCTGAGGGTTCCTTCTTCTCGGTTGAAGGTGGCGAGATCCGC
GCACGTGGCGTAGAAATCGAAGCGAAAGCGGCGCTGTCGGCGAGTGTTAACGTAGTCGGTTCTTATACTTACACCGATGC
GGAATACACCACCGATACTACCTATAAAGGCAATACGCCTGCACAGGTGCCAAAACACATGGCTTCGTTGTGGGCTGACT
ACACCTTCTTTGACGGTCCGCTTTCAGGTCTGACGCTGGGCACCGGTGGTCGTTATACTGGCTCCAGTTATGGTGATCCG
GCTAACTCCTTTAAAGTGGGAAGTTATACGGTCGTGGATGCGTTAGTACGTTATGATCTGGCGCGAGTCGGCATGGCTGG
CTCCAACGTGGCGCTGCATGTTAACAACCTGTTCGATCGTGAATACGTCGCCAGCTGCTTTAACACTTATGGCTGCTTCT
GGGGCGCAGAACGTCAGGTCGTTGCAACCGCAACCTTCCGTTTCTAATTTCTCTTTTGGGGCACGGATTTCCGTGCCCAT
TTCACAAGTTGGCTGTTATGCAGGAATACACGAATCATTCCGATACCACTTTTGCACTGCGTAATATCTCCTTTCGTGTG
CCCGGGCGCACGCTTTTGCATCCGCTGTCGTTAACCTTTCCTGCCGGGAAAGTGACCGGTCTGATTGGTCACAACGGTTC
TGGTAAATCCACTCTGCTCAAAATGCTTGGCCGTCATCAGCCGCCGTCGGAAGGGGAGATTCTTCTTGATGCCCAACCGC
TGGAAAGCTGGAGCAGCAAAGCGTTTGCCCGCAAAGTGGCTTATTTGCCGCAGCAGCTTCCTCCGGCAGAAGGGATGACC
GTGCGTGAACTGGTGGCGATTGGTCGTTACCCGTGGCATGGCGCGCTGGGGCGCTTTGGGGCGGCAGATCGCGAAAAAGT

CGAGGAAGCTATCTCGCTGGTTGGCTTAAAACCGCTGGCGCATCGGCTGGTCGATAGTCTCTCTGGCGGCGAACGTCAGC
GGGCGTGGATCGCCATGCTGGTGGCGCAGGATAGCCGTTGTCTGTTGCTCGACGAACCGACCTCGGCGCTGGATATCGCC
CACCAGGTTGATGTGCTGTCGCTGGTGCACCGTTTAAGTCAGGAGCGTGGCCTGACGGTCATTGCCGTGTTGCACGATAT
CAATATGGCGGCACGCTACTGTGATTATCTGGTCGCCCTGCGCGGCGGTGAAATGATTGCTCAGGGAACGCCTGCGGAAA
TTATGCGCGGCGAAACCCTCGAAATGATTTATGGCATCCCGATGGGTATTTTGCCGCATCCGGCGGGTGCTGCACCTGTG
AGTTTTGTTTATTGATGAGCGGCTTACCTCTTATTTCGCGCCGTCGACTGTTAACGGCGATGGCGCTTTCTCCGTTGTTA
TGGCAGATGAATACCGCCCACGCGGCGGCTATTGATCCCAATCGTATTGTGGCGCTGGAGTGGTTGCCGGTGGAATTACT
GCTGGCGCTCGGCATCGTGCCTTACGGCGTGGCGGATACCATCAACTATCGCCTGTGGGTCAGCGAACCACCATTGCCGG
ACTCAGTGATCGACGTCGGTTTGCGCACAGAACCTAACCTTGAACTGCTGACCGAAATGAAACCATCGTTTATGGTCTGG
TCGGCAGGATATGGCCCTTCACCAGAAATGCTGGCTCGTATTGCGCCGGGTCGCGGATTTAACTTCAGTGACGGCAAACA
GCCGTTGGCGATGGCGCGTAAATCGCTGACGGAAATGGCAGATTTACTTAACCTGCAAAGCGCAGCGGAAACGCATTTAG
CGCAATATGAAGACTTTATCCGCAGCATGAAACCCCGCTTTGTGAAGCGTGGTGCGCGTCCGTTATTGCTGACGACGCTT
ATCGATCCGCGCCATATGCTGGTCTTCGGTCCAAACAGCTTGTTCCAGGAAATTCTTGATGAGTACGGCATCCCAAATGC
CTGGCAAGGGGAAACCAACTTCTGGGGCAGTACCGCCGTCAGTATCGATCGTCTGGCGGCGTATAAAGACGTTGATGTGC
TCTGTTTTGATCACGACAACAGCAAAGACATGGATGCGCTAATGGCAACGCCGCTGTGGCAGGCCATGCCGTTTGTCCGC
GCCGGACGCTTTCAGCGCGTACCTGCAGTCTGGTTTTATGGTGCGACGCTCTCGGCAATGCACTTTGTGCGCGTTCTGGA
TAACGCCATCGGAGGTAAAGCGTGAGTAAACGAATTGCGCTTTTCCCGGCGTTATTGCTGGCGCTGTTAGTGATTGTCGC
TACGGCGCTCACCTGGATGAACTTCTCGCAGGCGCTGCCGCGTAGCCAGTGGGCGCAGGCTGCCTGGTCGCCGGATATTG
ACGTCATCGAGCAGATGATTTTTCACTACAGCTTGTTGCCGCGTCTGGCGATTTCGCTGCTGGTGGGCGCGGGTCTGGGG
CTGGTGGGCGTGCTGTTTCAGCAAGTGCTGCGTAACCCGCTGGCGGAGCCGACGACGCTTGGCGTTGCTACAGGCGCGCA
ACTGGGGATTACCGTCACTACGCTCTGGGCGATCCCTGGTGCGATGGCGAGCCAGTTTGCTGCGCAGGCAGGGGCTTGTG
TTGTTGGCTTAATTGTCTTTGGCGTCGCGTGGGGGAAACGGCTGTCGCCGGTAACGCTGATTCTCGCGGGGTTGGTAGTG
AGCCTTTATTGCGGCGCAATCAATCAGTTACTGGTTATCTTCCATCATGACCAACTGCAAAGCATGTTTCTGTGGAGCAC
TGGAACGCTGACGCAAACCGACTGGGGCGGCGTTGAGCGTTTATGGCCGCAGCTGCTGGGCGGTGTGATGCTGACGTTGC
TGCTACTTCGTCCGTTAACCCTGATGGGGCTTGATGATGGCGTGGCGCGCAATCTCGGGCTGGCCTTGTCGCTTGCGCGT
CTGGCAGCGCTGTCGCTGGCGATTGTCATCAGTGCGCTGCTGGTGAACGCTGTGGGGATTATCGGCTTTATCGGGTTGTT
CGCGCCGCTGCTGGCAAAAATGCTGGGGGCGCGGCGTCTGCTGCCACGACTGATGCTGGCGTCGTTGATTGGTGCGCTGA
TCCTCTGGCTTTCCGATCAAATCATCCTCTGGCTGACTCGCGTGTGGATGGAAGTGTCCACCGGTTCGGTCACTGCGTTG
ATCGGTGCGCCGCTGCTACTGTGGCTGTTGCCGCGTTTACGCAGCATTAGCGCGCCGGATATGAAGGTCAACGATCGTGT
CGCGGCTGAACGCCAACATGTGCTGGCGTTTGCCCTCGCGGGCGGCGTGCTGCTGTTGATGGCTGTGGTGGTGGCGCTGT
CGTTTGGTCGTGATGCGCACGGCTGGACGTGGGCGAGCGGGGCGTTGCTCGAGGATTTAATGCCCTGGCGCTGGCCGCGA
ATTATGGCGGCGCTGTTTGCGGGCGTCATGCTGGCGGTGGCGGGCTGTATTATTCAGCGACTGACCGGAAACCCGATGGC
AAGCCCGGAAGTGCTGGGGATTAGCTCCGGCGCGGCGTTTGGCGTGGTGTTGATGCTGTTTCTGGTGCCGGGTAATGCCT
TTGGCTGGCTGTTACCTGCAGGCAGTCTCGGCGCGGCGGTGACGCTGTTGATCATTATGATCGCCGCCGGCCGCGGTGGA
TTTTCCCCACACCGTATGTTACTGGCGGGGATGGCGTTAAGCACCGCGTTCACCATGCTTTTGATGATGTTGCAGGCAAG
TGGTGACCCGCGAATGGCGCAAGTGCTGACCTGGATTTCCGGTTCGACCTACAACGCGACCGATGCGCAGGTCTGGCGCA
CCGGAATTGTGATGGTGATTTTGCTGGCGATTACCCCGCTGTGCCGCCGCTGGCTGACCATTTTACCGCTGGGTGGTGAT
ACCGCCCGAGCCGTAGGAATGGCGCTGACGCCGACGCGAATTGCGCTGCTGCTGTTAGCGGCTTGCCTGACGGCGACCGC
GACGATGACTATTGGACCGTTGAGTTTTGTTGGTTTAATGGCACCGCATATTGCGCGGATGATGGGCTTTCGACGGACGA
TGCCACACATCGTAATTTCGGCGCTGGTGGGTGGTTTACTGCTGGTGTTCGCTGACTGGTGTGGGCGGATGGTGCTGTTT
CCATTCCAGATCCCGGCGGGGCTGCTGTCAACCTTTATCGGCGCGCCATATTTTATCTATTTGTTGAGAAAGCAGAGCCG
TTAATTTTTCCATATAACGGCCCTGTACGCCTGGACGGATAAAGCGTTTACGCGACAACGGATGCCCGATGCGACGCTGG
CGCGTCTTATCGGGCCTACAAACGGCCCCGAATCGTAGGTCGGATAAGGCGTTCACGCCGCATCCGACAAACCATGCTGG
ATCACAACTTCGCAAACACCCGACGTGCAGCATCGATGGTGTTATTGATATCTTCCATGCTGTGCGCCACGGACATAAAG
CCCGCTTCAAACGCTGACGGTGCCAGGTAAACACCTTCGTCCAGCATCATATGGAAGAAACGCTTAAAGCGTTCCACGTC
ACAGGCCATCACATCCTGATAGCACGTCACGGACTCGGCGTCGGTAAAGAAAATACCGAACATGCCGCCAACGTGGTTAA
CGACCAGCGGAATTCCGGCTTCTTCTGCCGCTTCCAGCAGACCTTCTGCCAGACGTGTTGTCAGCTCATCCAGCGTTTCG
TGAACGCCCGGCTGCGCGACTTCATTCAGACAGGCGAAACCCGCTGCCATCGCAATCGGGTTACCGGAAAGCGTACCCGC
CTGATAGACCGGACCCGTCGGGGCCAGCGCATCCATTACATCACGACGACCACCGAATGCGCCTACCGGCATTCCACCGC
CGATGATTTTGCCGAGGCAGGTTAAATCTGGCACTACGCCGTAATAATCCTGTGCGCCAGCTAGCGCTACGCGGAAACCG
GTCATCACTTCATCGATGATCAGCAACGCGCCAAATTCGTCGCACAGCGCGCGCAGACCTGGCAGGAACTCTGGCAGCGG
CGGAACACAGTTCATATTGCCTGCCACCGGCTCGACGATAATACAGGCAATCTCTTGCGGGTATTGCTCAAATGCGGCGC
GTACAGAAGCCAGATCATTATAAGTACAGGTTAAGGTATATTTGGCGAAATCTGCCGGAACGCCCGGCGAGTTTGGCTGG
CCTAACGTGAGTGCGCCAGAACCGGCTTTCACCAGCAGGCAGTCAGCGTGACCATGGTAACACCCTTCAAATTTAATAAT
TTTGTCGCGACCGGTAAAACCACGGGCCAGGCGGATGGCGCTCATGGTCGCTTCAGTGCCGGAGTTCACCATGCGCACCA
TATCCATGGTCGGGACCAGTTCGGTCACCAGTTGCGCCATTTTCACTTCCATTTCGGTTGGTGCACCAAAGCTTAAACCA

CGCTCGGCGGCTTCAATCACGGCATTGCGGATTGCCGGATGGTTATGGCCCAGCACCATCGGCCCCCAGGAACCGACATA
ATCGATATAGGCTTTGCCATCAACATCGTACAGATAAGCGCCGTCCGCTTTTTCGATAAACAGTGGAGTGCCGCCCACGC
CAGTAAAGGCGCGAACAGGGGAGTTCACACCGCCAGGGATCAGCTCGCGCGCTGCGCTGTAAAGATTTTCAGACTTACTC
ATGGAGGGTTCCTGATTCGTAGAAAAAGTGAATGGCTGCTATTCTATGTTATTCATAACAAGTTAAATACTCGTCAAACA
TCAGGCTGCTTGTACTGGTGCAATCCTGATTTCTTAGAGTATAAAAGTTTTGTGCATTTGAAACATTACGCTTTGCAAAG
GATTTTCATGGAACGTGCGAGTAAAATGCCGTCATCTTATTTGTATGACCAATAAGTGATCATTGGATGAAAACTGATAC
TCCCTCTTTAGAAACACCGCAGGCCGCGCGCCTGCGACGCAGACAACTGATTCGCCAACTTCTTGAGCGCGATAAAACCC
CGTTAGCCATTTTGTTTATGGCGGCAGTCGTCGGCACGCTTGTTGGGCTGGCAGCGGTTGCTTTTGACAAAGGTGTCGCC
TGGTTGCAGAACCAACGTATGGGGGCGCTGGTACATACTGCTGATAATTATCCGCTTCTGTTAACCGTCGCTTTTCTCTG
TTCGGCGGTGCTGGCGATGTTTGGCTACTTTTTGGTGCGCAAATACGCGCCGGAAGCAGGTGGTTCGGGGATCCCGGAAA
TTGAAGGGGCGCTGGAAGATCAACGTCCCGTTCGCTGGTGGCGTGTATTGCCGGTGAAGTTCTTTGGCGGGCTGGGGACA
CTCGGCGGAGGCATGGTGTTGGGGCGCGAAGGGCCAACCGTGCAGATCGGCGGTAACATTGGCCGTATGGTGCTTGATAT
TTTCCGCCTGAAAGGTGACGAAGCTCGCCATACGCTGCTGGCAACCGGTGCTGCTGCGGGGCTGGCTGCGGCCTTTAACG
CGCCGCTGGCGGGTATTTTGTTTATTATCGAAGAGATGCGTCCGCAGTTTCGCTATACGTTAATTTCGATTAAAGCGGTA
TTTATTGGTGTCATTATGTCGACCATTATGTACCGGATTTTTAATCATGAAGTTGCGTTGATTGACGTCGGTAAACTTTC
TGATGCGCCGCTTAATACGCTGTGGCTTTATCTGATCCTCGGTATTATTTTTGGCATTTTCGGCCCTATTTTTAATAAAT
GGGTGCTGGGGATGCAGGATTTGCTGCACCGTGTGCACGGCGGCAATATTACCAAATGGGTGCTAATGGGCGGTGCGATT
GGCGGTCTGTGTGGATTGCTGGGGTTTGTGGCACCAGCAACGTCGGGCGGCGGTTTTAACCTGATTCCTATCGCTACCGC
GGGGAATTTCAGCATGGGAATGCTGGTGTTTATCTTCGTCGCGCGGGTCATTACCACCTTACTCTGCTTCTCTTCCGGCG
CGCCGGGCGGTATTTTTGCCCCGATGCTGGCGCTGGGTACTGTGCTGGGAACCGCTTTCGGAATGGTTGCCGTTGAGCTG
TTTCCGCAATATCACCTTGAGGCGGGGACGTTTGCTATTGCCGGAATGGGGGCATTACTGGCGGCATCTATTCGCGCGCC
GTTAACGGGGATCATTCTGGTTCTGGAGATGACCGATAACTACCAGCTCATTTTGCCAATGATTATTACCGGTCTTGGCG
CAACACTATTAGCGCAATTTACCGGCGGGAAACCGCTATACTCGGCGATTCTTGCGCGCACGCTGGCAAAACAGGAAGCT
GAGCAACTGGCGCGAAGCAAGGCCGCATCAGCCAGCGAGAATACTTGAACGAAATACCAGGGTATTAGATAATGGCGATT
ATTATTGGGTTAGAATTTGCCCAATTGCCGATGTCGTTTGGAGCAAAATATGAGTGATGACGTAGCACTGCCGCTGGAGT
TTACCGACGCAGCAGCCAACAAAGTTAAAAGCCTGATCGCTGACGAAGATAACCCGAATCTGAAATTACGCGTGTATATC
ACCGGTGGCGGTTGCAGCGGCTTCCAGTATGGTTTCACCTTTGATGATCAGGTGAACGAAGGCGATATGACCATCGAAAA
ACAGGGCGTTGGCCTGGTGGTTGATCCGATGAGCCTGCAATATCTGGTCGGCGGTTCCGTTGATTATACCGAAGGTCTGG
AAGGTTCTCGTTTCATCGTGACCAACCCGAACGCGAAAAGCACCTGCGGTTGCGGTTCTTCCTTTAGTATCTAATCTGTT
GTTCATGTGCCGGATAAACTATCCGGCACATCTTCTCTCTTCAACGCCCATTCTCATCCAGCGCAAACGTCGGTAGCTTA
AGATGCCAACGAATAGCCGCCAGCCGAATCAATAGCGTCACGACCATGCCCATCATACTGGCTGTTTCCAGTGGTACGGA
AAATGTGTAATAAGCCGTAGCGTGGACAATACCGCCGATAATACAGGCAGTTGCGTAGATTTCTGTACGTAAAATCATGG
GGATTTCGCGGGCCAGAACATCACGAATGATCCCGCCGCCAACGCCAGTAATGACGCCCATACAAACCGCGATTAACGGA
CCGGCTTCCGCATTAAAGGCTTTATTCACGCCAATGCCGACAAACACCGCCAGACCAACGGCGTCCAGCACCGGCAACAT
CCATTTTGGTAAGCGTCTTGGCTGGCGCACCAGCACGATGGTCAGCATGCTGGTGACCATTGCAACGACCAGATCGGTGG
GATCTTTCACCCAAAATACCGGGCCGTGATCCAGCGCCATGTCGCGAATTGTCCCGCCGCCTACTGCGGTTACCACGCCC
AGTACCAGAACACCAAAAGGGTCCATACGCAATTTTCCGGCTAACAAAACGCCGGAGATGGCAAATACCGCTGTGCCGAC
TATATCCAGCCAATAGACGAGCATTGTTCAATCCCCACTGAGCACCTGAAAAGGTCAGGCGCTAATCTACCTGTGAAAGC
GCATTACAGAGCTGTTGTGCAGCGAGGATAATACGTGGGCTTGCACGTTCAAACCAGTCACTCGTGAGAGGAATAACGGG
AATTTTGAGCTGTTCACCCCAGTATTGTTTGATTTTAGGAATTTGGTCCGGTCCGCCTGTAATGACAATCGCCTGTGGCG
AGCGTGCTAACACCTGTTCGCGGCTAACTTGCGGCCAGGGAACCCGGCTGTCTTTAAAGATGTTTTCTCCGCCACAAACT
TCGAGTACCTGGTTCTGAATCGACTCTTTTCCACTGGTAAATGGCGGATTAATGCCGAATTGCAGAAAAACACGTTTTTT
AGGTTTATCAGCATATTGCGCTTTCAATTGCGCGTACTGATCCAGCAGGGATTGCGCGGCTTGTTCGGCCTTGTCTGGTT
GCGGACTCCAGGGGGCCAGTTGACGTAACGCATTGGCAATTTGTTCAATGCTTGTCGCATCGACCCACATCACTTTTATT
CCCAGCGAAGCCAGCTGGTCAACCTGCCGCTCGGCATTACCTCCACGCCAGGCAATCACCAGATCGGGTTTCAGCGCGAC
AATGCGTTCCAGATTCATCCCCTGCCAGGTGGAAACCTGCTCAATCTTTTGCGCTTGTGGAGGATAGTCGGAATAGCTGC
TGACCCCAACCGGCGTGATCCCGGCGGCAAAGGCAAGTTCAGTGTTGGCGGGAGAAAGCGTGATGACGCGCGGCGCGGCG
TTGAGCCACAGTGGCGCAAGAAAAGACAGGGCGACCAGCGCCCTGAACAGTGACTTAGCCATGTGCAAGTTTCTGCACCA
GTGACTCAACCATCAGGCTGGACTGTTTAGCGGCAACAGCCAGGAACTCATCGAAGCTAAGATGAGACTGTTGATCGGCC
ACGTCGGAGATGGCGCGTACGACAACAAACGGGACGTTGAAATTGTGGCAGACATGGGCGATTGCCGTCGCTTCCATCTC
TACAGCAATGGCCTGTGGGAAGTTGTGGCGGATTTTCGCCAGACCAACAGAACCGTTGATGAAAGCGTCGCCGCTAACAA
TCAGGCCACGTACAGCGTTAAGATTCAGTTCGGCAATGCAGGCCTCAGCGGCAGCGATCAGTTTATCGTCAGCTTTAAAG
CCTGCCGGACAGCCTGGTAACTGACCGTATTCATAACCAAATGCCGTGACATCCGCGTCGTGATAACGTGCTTCGTCCGA
GACAACGATATCGCCCACTTTCAACGTTGGTGCCAGGCCACCGGCAGAACCGGTGTTAATAATCACATCTGGCTTGCAGT
GTTCCAACAGCAAAGTGGCACCCAGCGCCGCAGCGACTTTACCGATGCCCGATTTCAGAAGCGCAACCTCGGTTCCATTC
AGTTGGCCGGTATAGATTTCGCAACCGCCGAGACTGATAGTTTGACGGTTTTCGATTTTGTCACGCAGCAGCGTAACTTC

TTCTTCCATTGCACCAATGATGCCGATTTTCATAGATTTACTCGCGATAAGCCCGATTTGAAGGCATAGTTTACCATGCG
CTTACGGGGAAGCGTATTTCTCACGCGGGAGAGGACATGGCACAGATTGATTTCCGAAAAAAAATAAACTGGCATCGTCG
TTACCGTTCACCGCAGGGCGTTAAAACCGAACATGAGATCCTGCGGATCTTCGAGAGCGATCGCGGGCGTATCATCAACT
CTCCGGCAATTCGTCGTCTGCAACAAAAGACCCAGGTTTTTCCACTGGAGCGCAATGCCGCCGTGCGCACGCGTCTTACC
CACTCGATGGAAGTCCAGCAGGTGGGGCGCTACATCGCCAAAGAAATTTTAAGCCGTCTGAAAGAGCTTAAATTACTGGA
AGCATACGGCCTGGATGAACTGACCGGTCCCTTTGAAAGCATTGTTGAGATGTCATGCCTGATGCACGATATCGGCAATC
CGCCGTTTGGTCATTTTGGCGAAGCGGCGATAAATGACTGGTTTCGCCAACGTTTGCACCCGGAAGATGCCGAAAGCCAG
CCTCTGACTGACGATCGCTGCAGCGTGGCGGCACTACGTTTACGGGACGGGGAAGAACCGCTTAACGAGCTGCGGCGCAA
GATTCGTCAGGACTTATGTCATTTTGAGGGGAATGCACAAGGCATTCGCCTGGTGCATACATTGATGCGGATGAATCTCA
CCTGGGCACAGGTTGGCGGTATTTTAAAATATACCCGTCCGGCGTGGTGGCGTGGCGAAACGCCTGAGACACATCACTAT
TTAATGAAAAAGCCGGGTTATTATCTTTCTGAAGAAGCCTATATTGCCCGGTTGCGTAAAGAACTTAATTTGGCGCTTTA
CAGTCGTTTTCCATTAACGTGGATTATGGAAGCTGCCGACGACATCTCCTATTGTGTGGCAGACCTTGAAGATGCGGTAG
AGAAAAGAATATTTACCGTTGAGCAGCTTTATCATCATTTGCACGAAGCGTGGGGCCAGCATGAGAAAGGTTCGCTCTTT
TCGCTGGTGGTTGAAAATGCCTGGGAAAAATCACGCTCAAATAGTTTAAGCCGCAGTACGGAAGATCAGTTTTTTATGTA
TTTACGGGTAAACACCCTAAATAAACTGGTACCCTACGCGGCACAACGATTTATTGATAATCTGCCTGCGATTTTCGCCG
GAACGTTTAATCATGCATTATTGGAAGATGCCAGCGAATGCAGCGATCTTCTTAAGCTATATAAAAATGTCGCTGTAAAA
CATGTGTTTAGCCATCCAGATGTCGAGCGGCTTGAATTGCAGGGCTATCGGGTCATTAGCGGATTATTAGAGATTTATCG
TCCTTTATTAAGCCTGTCGTTATCAGACTTTACTGAACTGGTAGAAAAAGAACGGGTGAAACGTTTCCCTATTGAATCGC
GCTTATTCCACAAACTCTCGACGCGCCATCGGCTGGCCTATGTCGAGGCTGTCAGTAAATTACCGTCAGATTCTCCTGAG
TTTCCGCTATGGGAATATTATTACCGTTGCCGCCTGCTGCAGGATTATATCAGCGGTATGACCGACCTCTATGCGTGGGA
TGAATACCGACGTCTGATGGCCGTAGAACAATAACCAGGCTTTTGTAAAGACGAACAATAAATTTTTACCTTTTGCAGAA
ACTTTAGTTCGGAACTTCAGGCTATAAAACGAATCTGAAGAACACAGCAATTTTGCGTTATCTGTTAATCGAGACTGAAA
TACATGAAAAAAACCACATTAGCACTGAGTGCACTGGCTCTGAGTTTAGGTTTGGCGTTATCTCCGCTCTCTGCAACGGC
GGCTGAGACTTCTTCAGCAACGACAGCCCAGCAGATGCCAAGCCTTGCACCGATGCTCGAAAAGGTGATGCCTTCAGTGG
TCAGCATTAACGTAGAAGGTAGCACAACCGTTAATACGCCGCGTATGCCGCGTAATTTCCAGCAGTTCTTCGGTGATGAT
TCTCCGTTCTGCCAGGAAGGTTCTCCGTTCCAGAGCTCTCCGTTCTGCCAGGGTGGCCAGGGCGGTAATGGTGGCGGCCA
GCAACAGAAATTCATGGCGCTGGGTTCCGGCGTCATCATTGATGCCGATAAAGGCTATGTCGTCACCAACAACCACGTTG
TTGATAACGCGACGGTCATTAAAGTTCAACTGAGCGATGGCCGTAAGTTCGACGCGAAGATGGTTGGCAAAGATCCGCGC
TCTGATATCGCGCTGATCCAAATCCAGAACCCGAAAAACCTGACCGCAATTAAGATGGCGGATTCTGATGCACTGCGCGT
GGGTGATTACACCGTAGCGATTGGTAACCCGTTTGGTCTGGGCGAGACGGTAACTTCCGGGATTGTCTCTGCGCTGGGGC
GTAGCGGCCTGAATGCCGAAAACTACGAAAACTTCATCCAGACCGATGCAGCGATCAACCGTGGTAACTCCGGTGGTGCG
CTGGTTAACCTGAACGGCGAACTGATCGGTATCAACACCGCGATCCTCGCACCGGACGGCGGCAACATCGGTATCGGTTT
TGCTATCCCGAGTAACATGGTGAAAAACCTGACCTCGCAGATGGTGGAATACGGCCAGGTGAAACGCGGTGAGCTGGGTA
TTATGGGGACTGAGCTGAACTCCGAACTGGCGAAAGCGATGAAAGTTGACGCCCAGCGCGGTGCTTTCGTAAGCCAGGTT
CTGCCTAATTCCTCCGCTGCAAAAGCGGGCATTAAAGCGGGTGATGTGATCACCTCACTGAACGGTAAGCCGATCAGCAG
CTTTGCCGCACTGCGTGCTCAGGTGGGTACTATGCCGGTAGGCAGCAAACTGACCCTGGGCTTACTGCGCGACGGTAAGC
AGGTTAACGTGAACCTGGAACTGCAGCAGAGCAGCCAGAATCAGGTTGATTCCAGCTCCATCTTCAACGGCATTGAAGGC
GCTGAGATGAGCAACAAAGGCAAAGATCAGGGCGTGGTAGTGAACAACGTGAAAACGGGCACTCCGGCTGCGCAGATCGG
CCTGAAGAAAGGTGATGTGATTATTGGCGCGAACCAGCAGGCAGTGAAAAACATCGCTGAACTGCGTAAAGTTCTCGACA
GCAAACCGTCTGTGCTGGCACTCAACATTCAGCGCGGCGACAGCACCATCTACCTGTTAATGCAGTAATCTCCCTCAACC
CCTTCCTGAAAACGGGAAGGGGTTCTCCTTACAATCTGTGAACTTCACCACAACTCCATACATCTTCATCATCCTTTAGG
CATTTGCACAATGCCGTACGTTACGTACTTCCTTATGCTAAGCCGTGCATAACGGAGGACTTATGGCTGGCTGGCATCTT
GATACCAAAATGGCGCAGGATATCGTGGCACGTACCATGCGCATCATCGATACCAATATCAACGTAATGGATGCCCGTGG
GCGAATTATCGGCAGCGGCGATCGTGAGCGTATTGGTGAATTGCACGAAGGTGCATTGCTGGTACTTTCACAGGGACGAG
TCGTCGATATCGATGACGCGGTAGCACGTCATCTGCACGGTGTGCGGCAGGGGATTAATCTACCGTTACGGCTGGAAGGT
GAAATTGTCGGCGTAATTGGCCTGACAGGTGAACCAGAGAATCTGCGTAAATATGGCGAACTGGTCTGCATGACGGCTGA
AATGATGCTGGAACAGTCGCGGTTGATGCACTTGTTGGCGCAGGATAGCCGTTTGCGGGAAGAACTGGTGATGAACCTGA
TTCAGGCAGAGGAGAATACTCCCGCACTTACTGAATGGGCGCAACGGCTGGGGATCGATCTCAATCAACCGCGAGTGGTG
GCTATTGTTGAGGTCGACAGCGGTCAGCTTGGCGTGGACAGCGCAATGGCGGAGTTACAACAACTGCAAAACGCGCTGAC
TACGCCCGAGCGTAATAATCTGGTGGCGATTGTCTCGCTAACCGAAATGGTGGTGTTGAAACCGGCGTTGAACTCTTTTG
GGCGCTGGGATGCAGAAGATCATCGTAAGCGAGTTGAACAACTGATTACCCGCATGAAAGAGTACGGCCAGCTGCGTTTT
CGCGTTTCACTGGGCAACTATTTTACCGGTCCTGGCAGTATTGCCCGATCCTATCGTACGGCGAAAACGACGATGGTGGT
GGGTAAACAGCGGATGCCAGAAAGTCGCTGCTATTTTTATCAGGATCTGATGTTACCTGTGTTACTCGACAGTTTGCGTG
GCGACTGGCAGGCCAACGAACTGGCGCGACCGCTGGCGCGGCTGAAAACGATGGACAATAACGGCTTGCTGCGACGAACG
CTGGCGGCGTGGTTTCGCCACAATGTGCAACCGCTGGCAACGTCAAAGGCGTTGTTTATTCATCGTAATACCCTGGAGTA
TCGGCTTAATCGTATATCGGAACTGACCGGGCTTGATTTGGGCAATTTTGATGACAGGTTGCTGCTGTATGTGGCGTTAC

AACTGGATGAAGAGCGGTAGGTTATGCGTTAAGGTTGAGGCGGCGCTGGCTCATTCCCCTCAGCCCGAAGGGTAGAGGGG
AATGCCCGAATTAACCGTCAGTTCGGGATTATTTACGCGTTAGTTTTTCCAGATCGGCTTCAATCTCGCTGATCTTATTG
GTTACGACTGACTCCAGGTGACGTAAGTCGTCGAGGATCTTACGCTTAAGATCAACTTCGCTGCGGTCACGCTGGCAGAT
TTGATCAAGCTCATCAATGATATACCGTAGATTCGGGCTGATTTCCTGGACTTCTTTATAACCCTGACCCACACCATCAG
CGACGACCGTTTTACGCTGACGCGGATATTTAAACTTAACGCTCTTGGCGAAAAACTCGCCTTTGTCTTTCTGGAAATAG
ATTTTCAGAATATCGTTGTTGGCTTCCTGCCGGAGGCTGTAACGATCAATTTCTTCAGGATTGGTAATACCCAGACTTTT
CAGATTGTCGTACATAGCGTTACCTCAAAATGAGTCAGTAAATTGTGCTTATTTTAGCATTTGGCCTGGCCCGCCCGGCT
CGACTGTGATCGCAGAAAGCCTTTTTCTTCCAGACTTCTATCATGGCGTAAAAATCAAAAAATTACCTGCTTTATTCTGG
TGATAAAATTCACGATCTACACCAGTTCCAGCATCGTTACTTCCGGGCGGCAGTTCAGACGCAAACCATACAAACTGCCC
ACGCCACGGGTTGTGTAGATGTGTCTTTCGCCAAAGGCATTTAATCCGGCGACGTAACGTTTATCTTCGACAGGGGCAAA
AGGTTCACCAACCAACGGTACGCGCAGTTGCCCGCCGTGAGTATGGCCGCACAGCATCAGATCCCAGGGTTCATCACGCA
TGACTTCTTTGCTGTCGGGATTATGCGCCAGCACCAGTCTTGGCAGATTGGCTTCGCTGGCGGGAGGCGGTTTGCATTGT
CCGGCCCATAAGTCACCAGTGCCTACCAGTTCGAATTGCCTGTTCGGCGTGGCGATCACCGTAGCCTGGTTAAACAACAC
CGTGATGCCCGCTGATTTCAACGTCTCGCCAATTAAGTGATTTTTTTCTGTACCAACAGGGCGATCGTGGTTGCCGAAGC
AGGCAAACGTCGGCGCACATTCGGCAAGGGGGGAGAGTACGTCACTAAACGCCGAAAAATTCAGCGACATATCAAATAAT
ACGTAATCGCCGCCCAGTAATATCAAATCGGGCTTTTGTTCTATGCCAAGAGCAATCGCGTCAGAAATCAGGCTTAAAGG
AACAAAACGAGAGTAATGGAGATCGGCCAGAAAAAGAATTTTGAATGGTGCTGCGTTGTCTTTAAAAAAGGCGAGGCGGT
GACGGATTAATTCAAACCAGCCTGGCTCACAGTAATGCATATAACCGAAACCTGAGCTCGTGGCTATCGTCGCGGCAGTA
GCCTGCAAAAATCGGCGGCGTGAAATCATCGCTCATCCCTGCAAAAAAATCGGGCAGCGTCGTGCTGCCCGTGTGCATAC
TTTTAGTCGATGGTACGCAGCAGTTCGTTAATGCCGACTTTGCCGCGAGTTTTCGCGTCAACTTTCTTAACGATAACCGC
ACAGTAGAGGCTGTATTTGCCATCTTTTGACGGCAGATTACCTGAAACAACCACAGACCCCGCCGGAACGCGACCGTAGT
GGATTTCGCCGGTTTCACGGTCGTAAATACGGGTGCTCTGACCAATGTATACGCCCATGGAAATGACGGAACCTTCTTCG
ACAATCACCCCTTCAACCACTTCAGAGCGCGCGCCGATGAAGCAATTATCTTCAATGATGGTTGGGTTAGCCTGCAGCGG
TTCCAGCACGCCGCCGATGCCCACGCCACCGGAAAGGTGGACGTTTTTACCAATCTGCGCACAAGAACCGACGGTCGCCC
AGGTATCAACCATGGTGCCTTCATCAACATATGCGCCGATGTTGACGTAAGACGGCATCAGCACGGTGTTACGGGCAATA
AACGCACCCTGACGTACCGCCGCTGGTGGCACAACGCGGAAGCCTTCTTTCTGGAAACGTGCTTCGTCGTAGTCGGCGAA
TTTCATCGGCACTTTGTCGAAGTAGCGGCTTTCTGCCCCTTCGATCACCTGATTATCATTAATACGGAAAGAGAGCAGCA
CCGCTTTTTTCAACCACTGATGCGTCACCCACTGACCGTCAATTTTTTCCGCTACACGCAGTGCGCCGGAATCCAGCAGG
GCGATCACCTGATTTACCGCTTCGCGGGTAACGGTGTCTGCATTGGCTGGCGTGATCTCGGCACGGCGTTCAAAAGCGGT
TTCAATAATGTTCTGTAACTGCTGCATTGTTAAACTCTTTTCATATCAGTAAACACATCACCCTTTATCGTTTGGATTGA
GGGCCTCTGTCAACCGCTGATGCACTTCCTGCTGCAACTCGTTATTAAGCGCACGCCGGTCAGCGGTGGCAATTATGAAT
AAATCTTCTACTCGCTCGCCAATGGTTGTAATTCGGGCACCATGAAGCGAAATTCCCAGATCGGCAAAAATTTTCCCGAC
TCGCGCCAGCAGTCCAGGTTGGTCGAGGGCGATCAGTTCGAGGAACGATTTGCGGTCGGTATGGGTCGGCAAAAACGTTA
CTTCGGTTTCAACAGTAAAATGGCGTAATTTGGCGGGTTGGCGACGGGGCTGCGGTGGCTGCCAGCTACTTTGCGTCAGT
ACTTGCTCCAGACCAAACCGAATAACCTCATGACGATCTGCGGACAGCGGGTTGCCATCGGGTTCCAGCACGATAAAGGT
ATCCATCGCCATACCGTCGCGAGTGGTGAAAATTTGTGCGTCGTGAACACTTAAATTGCGGCGGTCTAATTCGGCACAGA
CGGCGGCAAACAGATAAGGGCGGTCCGGGCTCCAGATAAAAATCTCGGTGCCTCCACGCGTAGCCTGCGGGCTAAGCAAT
ACCAGCGGTTTGCTTAAATCATGCTGTAATAAATGGCGGGCATGCCAGGCCAGTTGATTTGGGCTATGGCGGACAAAATA
GTTAGCACGACAGCGTGACCAAATTTGGTGCAGCGCCTCTTCGTCGATGTTATCCATGCGCAGTAGTGCCAGTGCCTGGA
GTTGGTGATGGCGAACCCGTTCGCGCATATCCGGCGTGTTTTGCATTCCGCGTCGTAGCTGCTTTTCGGTGGCAAAGTAG
AGCTCACGCAACAGACTTTGCTTCCAGCTATTCCACAGCGTTTCGTTGGTGGCGCAAATGTCAGCCACAGTCAGGCATAC
CAGATAGCGCAGACGATTTTCCGTTTGCACTTCTTCGGCAAACTGCTTGATGACTTCCGGGTCCTGAATATCGCGGCGTT
GGGCGGTCACCGACATCAACAGGTGCTGGCGAACCAGCCAGGCGACCAGCTGTGTTTCGCGTGAGTTCAGCCCGTGGAGT
TCGGCAAAATGCACTACATCCTGAGCACCGAGAATGGAGTGGTCGCCGCCGCGTCCTTTGGCGATATCGTGAAACAGCGC
GGCGATGAAAATCAGCTCAGTTGACGGCAGGCGCGGCCAGACGTCCACACACAACGGATGGCGCTGGCGCGTTTCTTCAC
TGGCAAAACTCTCCAGTTTCAGCATCACGCGGATAGTATGTTCATCCACCGTGTAGGCGTGGAACAGATCAAACTGCATC
TGCCCGACGATATGCGACCATTGCGGCATATACGCGCCGAGCACGCTATGGCGATGCATTGGCAATAGCCCGCGCCGCAC
CGCTCCGGGGTGACGCAGAATGCTCAAAAACAGTTTTCGTGCTTCCGGAATATTACACAGCGGTTGTTGCAGATGGCGAC
GGGCATGGCGTAACTGGCGCAGCGTGGTGGAGTAAATGCCGGTGATCGCACTGTTGTGCACCATGGTGTAGAACATACGC
AAGATGGCTTCCGGCTGGCGCATAAATAGTGTTTCATCACGCAGGTCGATTAGCGTACCGCGTAGCTGAAACTCATCGTC
GATTGGACGTGGTTTTTCGTCGGCGGGAAGGGCGAGGATGGCTTCATCGAACAGTTGCAGCAGCATCTGGTTGAGTTCAC
TGACGCGGCGTGTAACGCGGAAGTAATCCTTCATCATCCGCTCGACCGGTTCGTTACCTTCACCACTGTAATTCAGACGC
TGGGCGACGCTAAGCTGGCGATCGAATAACAGGCGATTATCGTAACGGCTGACGACCAGATGCAGGGCAAAGCGAATACG
CCACAATATATGCAGACATTCGTTTAATTCCGCCCGCTCCGCTGAGGTTAAGAAGCCAAACCCGACCATTTCATCCAGCG
ATGTTGCGCCAAAATGACGGCGGGCCACCCATTGCAGAGTGTGGATATCGCGCAAGCCGCCAGGGCTGCTTTTGATGTCT
GGTTCAAGGTTGTAGCTGGTGCCATGGTAACGCTGATGGCGCTGGTTCTGTTCTTCAACTTTCGCCGCGTAGAACTTGTC

GGAAGGCCAGAATCCTTCGCTGAAAATATGTTTTTGCAGTTCGAGGAACAGCGCAACATCGCCAATTAATAAGCGGGATT
CGATTAAATTGGTGGCGACGGTTAAATCCGATAACCCTTCCAGCATGCACTCTTCAAGCGTGCGCACGCTATGACCGACT
TCCAGCTTTACATCCCAGAGCAGCGTTAACAGCTCGCCCACTTTTTGCGCCTGATCGTCCGGGAGCTTTTTACGGCTTAA
AATCAGTAAATCGACGTCTGAAAGTGGATGCAGCTCGCCACGACCGTAGCCACCGACGGCGACCAATGCCAGGTCGGCAA
TCTGGCTGAATCCCGCTTCAATCCATAATCGTTGCAGGAGCTGGTCGATAAACTCGGTGCGCGCCTCAATCAACTGTTCT
GCAGAGATCCCATTGTCAAAGGCATCACCCAGCCAACGCTGGAAAGTATCGATATGGGCTTTTATCCCACCGACGGTTAA
TTCATCACGGGGCCAGACGCATGGATTTTGCGGTTGACCGGGCAGGGTGGGGAGAGCGGTGTTTGCGTACTGTTCTGGAA
GGGTATTCATTGTGCGCCACCCATAAGATTAAATTATCGCATTAAAAAAGCCGGCATTCGCCGGCTTCATCTTATTCGTC
GTGCGAGATTATCGCCGGGATGGTGTCATCCTTGCGTAGCGTCAGAATTTCGCAGCCGTTATCAGTCACCACAATAGTAT
GCTCATATTGTGCAGACAAGCTGCGATCTTTGGTTTTTACCGTCCAGCCATCTTTCATGGTGCGGATCTCTTTTTTACCC
GCGTTGACCATTGGCTCGATGGTGAACGTCATCCCAGGTTTCAGTACGACGTTGGTTTCACGGGAGTCATAGTGCAGCAC
CTGCGGTTCTTCATGGAAGCCGCGACCAATACCGTGTCCGCAATATTCACGAACGACGGAGAAGCCTTCTGCTTCGACAA
ATTTCTGAATCGCCGCACCGATTTCGCGCAGATTAATGCCTGGTTTTACCATGCGTAGCGCCAGGTACAGGCTTTCTTGC
GTGATGCGGCACAGACGTTCGCCCATGATGGTCGGCTTACCGACGATAAACATTTTCGAGGTATCGCCGTGGAAACCATC
TTTGATTACGGTGACATCAATGTTAACGATATCGCCATCTTTCAGCAGCTTAGCATCGTCCGGGATACCGTGGCACACCA
CTTCATTAATAGAGATGCAAACGGATTTCGGATAGCCGTGATAGCCGAGGCAGGCAGAAACCGCGTGTTGTTCATTAACA
ATGTAATCATTACAGATGCGATCCAGCTCGCCGGTGCTGACGCCCGGTTTAACATACGGTTCGATCATCTCCAGCACTTC
GGCAGCCAGTCGGCCAGCGACGCGCATTTTTTCGATATCTTCTGGGGTCTTGATTGAGATAGCCATTAATTCTGTCCATC
AGCGTCGGTGATACCGACAATATATATGTAAGTGCCGTCAATGGTATCACACCCGGGCAAATTGAGAATCATTCTGAATT
TCGCCAAACGTGCCACTGAAGGTTTTCTATAATAGAAAATTCGACGTCTGATGCTGTACACAGCGCCAACAATTATTGGT
GTCCACGACGTATTTGTGGTATAAAGCGCGCCGGACTTCCGATCCATTTCGTATACACAGACTGGACGGAAGCGACAATC
TCACTTTGTGTAACAACACACACGTATCGGCACATATTCCGGGGTGCCCTTTGGGGTCGGTAATATGGGATACGTGGAGG
CATAACCCCAACTTTTATATAGAGGTTTTAATCATGGCAACTGTTTCCATGCGCGACATGCTCAAGGCTGGTGTTCACTT
CGGTCACCAGACCCGTTACTGGAACCCGAAAATGAAGCCGTTCATCTTCGGTGCGCGTAACAAAGTTCACATCATCAACC
TTGAGAAAACTGTACCGATGTTCAACGAAGCTCTGGCTGAACTGAACAAGATTGCTTCTCGCAAAGGTAAAATCCTTTTC
GTTGGTACTAAACGCGCTGCAAGCGAAGCGGTGAAAGACGCTGCTCTGAGCTGCGACCAGTTCTTCGTGAACCATCGCTG
GCTGGGCGGTATGCTGACTAACTGGAAAACCGTTCGTCAGTCCATCAAACGTCTGAAAGACCTGGAAACTCAGTCTCAGG
ACGGTACTTTCGACAAGCTGACCAAGAAAGAAGCGCTGATGCGCACTCGTGAGCTGGAGAAACTGGAAAACAGCCTGGGC
GGTATCAAAGACATGGGCGGTCTGCCGGACGCTCTGTTTGTAATCGATGCTGACCACGAACACATTGCTATCAAAGAAGC
AAACAACCTGGGTATTCCGGTATTTGCTATCGTTGATACCAACTCTGATCCGGACGGTGTTGACTTCGTTATCCCGGGTA
ACGACGACGCAATCCGTGCTGTGACCCTGTACCTGGGCGCTGTTGCTGCAACCGTACGTGAAGGCCGTTCTCAGGATCTG
GCTTCCCAGGCGGAAGAAAGCTTCGTAGAAGCTGAGTAATAAGGCTTGATAACTCCCCAAAATAGTTCGAGTTGCAGAA
AGGCGGCAAGCTCGAGAATTCCCGGGAGCTTACATCAGTAAGTGACCGGGATGAGCGAGCGAAGATAACGCATCTGCGGC
GCGAAATATGAAGGGGGAGAGCCCTTATAGACCAGGTAGTACACGTTTGGTTAGGGGGCCTGCATATGGCCCCCTTTTTC
ACTTTTATATCTGTGCGGTTTAATGCCGGGCAGATCACATCTCCGAGGATTTTAGAATGGCTGAAATTACCGCATCCCTG
GTAAAAGAGCTGCGTGAGCGTACTGGCGCAGGCATGATGGATTGCAAAAAAGCACTGACTGAAGCTAACGGCGACATCGA
GCTGGCAATCGAAAACATGCGTAAGTCCGGTGCTATTAAAGCAGCGAAAAAAGCAGGCAACGTTGCTGCTGACGGCGTGA
TCAAAACCAAAATCGACGGCAACTACGGCATCATTCTGGAAGTTAACTGCCAGACTGACTTCGTTGCAAAAGACGCTGGT
TTCCAGGCGTTCGCAGACAAAGTTCTGGACGCAGCTGTTGCTGGCAAAATCACTGACGTTGAAGTTCTGAAAGCACAGTT
CGAAGAAGAACGTGTTGCGCTGGTAGCGAAAATTGGTGAAAACATCAACATTCGCCGCGTTGCTGCGCTGGAAGGCGACG
TTCTGGGTTCTTATCAGCACGGTGCGCGTATCGGCGTTCTGGTTGCTGCTAAAGGCGCTGACGAAGAGCTGGTTAAACAC
ATCGCTATGCACGTTGCTGCAAGCAAGCCAGAATTCATCAAACCGGAAGACGTATCCGCTGAAGTGGTAGAAAAAGAATA
CCAGGTACAGCTGGATATCGCGATGCAGTCTGGTAAGCCGAAAGAAATCGCAGAGAAAATGGTTGAAGGCCGCATGAAGA
AATTCACCGGCGAAGTTTCTCTGACCGGTCAGCCGTTCGTTATGGAACCAAGCAAAACTGTTGGTCAGCTGCTGAAAGAG
CATAACGCTGAAGTGACTGGCTTCATCCGCTTCGAAGTGGGTGAAGGCATCGAGAAAGTTGAGACTGACTTTGCAGCAGA
AGTTGCTGCGATGTCCAAGCAGTCTTAATTATCAAAAAGGAGCCGCCTGAGGGCGGCTTCTTTTTGTGCCCATCTTGTAA
ATTCAGCTAACCCTTGTGGGGCTGCGCTGAAAAGCGACGTACAATGTCGCTAGTATTAATTCATTTCAATCGTTGACAGT
CTCAGGAAAGAAACATGGCTACCAATGCAAAACCCGTCTATAAACGCATTCTGCTTAAGTTGAGTGGCGAAGCTCTGCAG
GGCACTGAAGGCTTCGGTATTGATGCAAGCATACTGGATCGTATGGCTCAGGAAATCAAAGAACTGGTTGAACTGGGTAT
TCAGGTTGGTGTGGTGATTGGTGGGGGTAACCTGTTCCGTGGCGCTGGTCTGGCGAAAGCGGGTATGAACCGCGTTGTGG
GCGACCACATGGGGATGCTGGCGACCGTAATGAACGGCCTGGCAATGCGTGATGCACTGCACCGCGCCTATGTGAACGCT
CGTCTGATGTCCGCTATTCCATTGAATGGCGTGTGCGACAGCTACAGCTGGGCAGAAGCTATCAGCCTGTTGCGCAACAA
CCGTGTGGTGATCCTCTCCGCCGGTACAGGTAACCCGTTCTTTACCACCGACTCAGCAGCTTGCCTGCGTGGTATCGAAA
TTGAAGCCGATGTGGTGCTGAAAGCAACCAAAGTTGACGGCGTGTTTACCGCTGATCCGGCGAAAGATCCAACCGCAACC
ATGTACGAGCAACTGACTTACAGCGAAGTGCTGGAAAAAGAGCTGAAAGTCATGGACCTGGCGGCCTTCACGCTGGCTCG
TGACCATAAATTACCGATTCGTGTTTTCAATATGAACAAACCGGGTGCGCTGCGCCGTGTGGTAATGGGTGAAAAAGAAG

GGACTTTAATCACGGAATAATTCCCGTGATGGATAAATAAGGGTAAGATTCCGCGTAAGTATCGCGGGGGCGTAAGTCTG
GTTATAAGGCGTTATTGTTGCAGGCAGTTTGGTCACGGCCAGCGCGCAGCAACCGGAGCGTACAAAAGTACGTGAGGATG
GCGAGCACTGCCCGGGGCCAAAATGGCAAATAAAATAGCCTAATAATCCAGACGATTACCCGTAATATGTTTAATCAGGG
CTATACTTAGCACACTTCCACTGTGTGTGACTGTCTGGTCTGACTGAGACAAGTTTTCAAGGATTCGTAACGTGATTAGC
GATATCAGAAAAGATGCTGAAGTACGCATGGACAAATGCGTAGAAGCGTTCAAAACCCAAATCAGCAAAATACGCACGGG
TCGTGCTTCTCCCAGCCTGCTGGATGGCATTGTCGTGGAATATTACGGCACGCCGACGCCGCTGCGTCAGCTGGCAAGCG
TAACGGTAGAAGATTCCCGTACACTGAAAATCAACGTGTTTGATCGTTCAATGTCTCCGGCCGTTGAAAAAGCGATTATG
GCGTCCGATCTTGGCCTGAACCCGAACTCTGCGGGTAGCGACATCCGTGTTCCGCTGCCGCCGCTGACGGAAGAACGTCG
TAAAGATCTGACCAAAATCGTTCGTGGTGAAGCAGAACAAGCGCGTGTTGCAGTACGTAACGTGCGTCGTGACGCGAACG
ACAAAGTGAAAGCACTGTTGAAAGATAAAGAGATCAGCGAAGACGACGATCGCCGTTCTCAGGACGATGTACAGAAACTG
ACTGATGCTGCAATCAAGAAAATTGAAGCGGCGCTGGCAGACAAAGAAGCAGAACTGATGCAGTTCTGATTTCTTGAACG
ACAAAAACGCCGCTCAGTAGATCCTTGCGGATCGGCTGGCGGCGTTTTGCTTTTTATTCTGTCTCAACTCTGGATGTTTC
ATGAAGCAACTCACCATTCTGGGCTCGACCGGCTCGATTGGTTGCAGCACGCTGGACGTGGTGCGCCATAATCCCGAACA
CTTCCGCGTAGTTGCGCTGGTGGCAGGCAAAAATGTCACTCGCATGGTAGAACAGTGCCTGGAATTCTCTCCCCGCTATG
CCGTAATGGACGATGAAGCGAGTGCGAAACTTCTTAAAACGATGCTACAGCAACAGGGTAGCCGCACCGAAGTCTTAAGT
GGGCAACAAGCCGCTTGCGATATGGCAGCGCTTGAGGATGTTGATCAGGTGATGGCAGCCATTGTTGGCGCTGCTGGGCT
GTTACCTACGCTTGCTGCGATCCGCGCGGGTAAAACCATTTTGCTGGCCAATAAAGAATCACTGGTTACCTGCGGACGTC
TGTTTATGGACGCCGTAAAGCAGAGCAAAGCGCAATTGTTACCGGTCGATAGCGAACATAACGCCATTTTTCAGAGTTTA
CCGCAACCTATCCAGCATAATCTGGGATACGCTGACCTTGAGCAAAATGGCGTGGTGTCCATTTTACTTACCGGGTCTGG
TGGCCCTTTCCGTGAGACGCCATTGCGCGATTTGGCAACAATGACGCCGGATCAAGCCTGCCGTCATCCGAACTGGTCGA
TGGGGCGTAAAATTTCTGTCGATTCGGCTACCATGATGAACAAAGGTCTGGAATACATTGAAGCGCGTTGGCTGTTTAAC
GCCAGCGCCAGCCAGATGGAAGTGCTGATTCACCCGCAGTCAGTGATTCACTCAATGGTGCGCTATCAGGACGGCAGTGT
TCTGGCGCAGCTGGGGGAACCGGATATGCGTACGCCAATTGCCCACACCATGGCATGGCCGAATCGCGTGAACTCTGGCG
TGAAGCCGCTCGATTTTTGCAAACTAAGTGCGTTGACATTTGCCGCACCGGATTATGATCGTTATCCATGCCTGAAACTG
GCGATGGAGGCGTTCGAACAAGGCCAGGCAGCGACGACAGCATTGAATGCCGCAAACGAAATCACCGTTGCTGCTTTTCT
TGCGCAACAAATCCGCTTTACGGATATCGCTGCGTTGAATTTATCCGTACTGGAAAAAATGGATATGCGCGAACCACAAT
GTGTGGACGATGTGTTATCTGTTGATGCGAACGCGCGTGAAGTCGCCAGAAAAGAGGTGATGCGTCTCGCAAGCTGAGGA
TAATCCGGCTACAGAGAGTCGCGCTATTTGTTAGCGTAGGGCTTCAGTGATATAGTCTGCGCCATCTGATCGTAAGTAGT
TGGCTTTATAAGGTCAGATATGCCGTGGTTTTACACGGCTTTTTTTTGTATAGGCTTCAGTATTCCTGAGTACCGTAAAC
CCTGTCAGGGAATAAAAAACGCGTGATGTTGTCTGCTACTCAACCACTTAGCGAAAAATTGCCAGCGCATGGCTGCCGTC
ATGTTGCGATCATTATGGACGGCAATGGCCGCTGGGCAAAAAAGCAAGGGAAGATTCGTGCCTTTGGGCATAAAGCCGGG
GCAAAATCCGTCCGCCGGGCTGTCTCTTTTGCGGCCAACAACGGTATTGAGGCGTTAACGCTGTATGCCTTTAGTAGTGA
AAACTGGAACCGACCAGCGCAGGAAGTCAGTGCGTTAATGGAACTGTTTGTGTGGGCGCTCGATAGCGAAGTAAAAAGTC
TGCACCGACATAACGTGCGTCTGCGTATTATTGGCGATACCAGTCGCTTTAACTCGCGTTTGCAAGAACGTATTCGTAAA
TCTGAAGCGCTAACAGCCGGGAATACCGGTCTGACGCTGAATATTGCGGCGAACTACGGTGGACGTTGGGATATAGTCCA
GGGAGTCAGGCAACTGGCTGAAAAGGTGCAGCAAGGAAACCTGCAACCAGATCAGATAGATGAAGAGATGCTAAACCAGC
ATGTCTGTATGCATGAACTGGCCCCTGTAGATTTAGTAATTAGGACTGGGGGGGAGCATCGCATTAGTAACTTTTTGCTT
TGGCAAATTGCCTATGCCGAACTTTACTTTACAGATGTTCTCTGGCCCGATTTCGATGAACAAGACTTTGAAGGGGCGTT
AAATGCCTTTGCTAATCGAGAGCGTCGTTTCGGCGGCACCGAGCCCGGTGATGAAACAGCCTGATGGGGGTCGCTTTTGC
TGAAGTATCGCCTGATATCTGCTTTTGTGTTAATACCCGTCGTCATCGCGGCGTTGTTTCTGTTGCCGCCGGTGGGGTTC
GCCATTGTAACGCTGGTGGTCTGCATGCTGGCAGCGTGGGAATGGGGACAGCTTAGCGGTTTTACCACTCGTTCGCAGCG
AGTATGGTTGGCGGTGTTATGCGGGTTATTGTTGGCGCTGATGCTTTTTCTGTTGCCGGAATATCACCGAAATATTCATC
AACCGCTGGTTGAAATCTCACTTTGGGCTTCGCTGGGTTGGTGGATTGTCGCGCTATTGCTGGTGCTGTTTTACCCAGGT
TCCGCAGCAATCTGGCGTAACTCTAAAACATTGCGCCTTATTTTTGGCGTGCTAACCATTGTTCCCTTCTTCTGGGGCAT
GCTGGCGTTACGGGCCTGGCACTATGACGAGAATCATTACAGTGGCGCAATATGGCTGCTCTATGTCATGATCCTGGTAT
GGGGCGCTGACTCCGGCGCATATATGTTTGGCAAATTGTTTGGTAAACATAAGCTGGCACCGAAGGTTTCTCCGGGTAAA
ACCTGGCAAGGCTTTATCGGTGGACTCGCTACTGCAGCGGTAATCTCATGGGGTTATGGCATGTGGGCGAATCTCGACGT
CGCTCCCGTCACCTTACTCATTTGCTCTATTGTCGCAGCGTTAGCCTCAGTGCTCGGCGATCTGACCGAGAGTATGTTTA
AGCGTGAAGCAGGAATTAAGGACAGCGGTCATTTAATTCCAGGACACGGTGGTATTTTAGATCGTATTGATAGCCTGACG
GCTGCGGTACCGGTCTTTGCTTGCTTGTTGTTACTGGTATTCAGGACGCTTTAACGGAAGGTAATATGCTGAGTTTTCTC
TGGGATTTGGCTTCGTTCATCGTTGCACTGGGTGTACTTATCACCGTGCATGAATTTGGTCATTTCTGGGTTGCCCGGCG
TTGTGGTGTTCGCGTTGAGCGTTTCTCAATAGGGTTTGGTAAGGCGCTCTGGCGGCGAACTGATAAGCTCGGCACCGAAT
ATGTTATCGCCCTGATCCCGTTGGGCGGTTATGTCAAAATGCTGGATGAGCGCGCAGAACCGGTCGTTCCGGAACTCCGC
CACCATGCCTTCAATAATAAATCTGTCGGCCAACGAGCGGCGATTATTGCCGCAGGTCCGGTTGCAAACTTCATTTTTGC
TATCTTTGCCTACTGGCTGGTTTTTATTATTGGTGTGCCTGGCGTACGTCCGGTGGTTGGCGAAATAGCAGCCAATTCGA
TAGCTGCGGAAGCACAAATTGCACCAGGTACGGAACTAAAAGCCGTAGATGGTATCGAAACGCCTGATTGGGATGCCGTG

CGTTTGCAGTTGGTCGATAAAATTGGCGATGAAAGCACCACCATTACAGTAGCGCCATTTGGCAGCGACCAACGGCGGGA
TGTAAAGCTCGATTTACGTCACTGGGCGTTTGAGCCTGATAAAGAAGATCCGGTATCTTCGCTGGGGATTCGTCCTCGTG
GGCCGCAAATTGAACCTGTACTGGAAAATGTGCAGCCAAACTCGGCGGCAAGCAAGGCAGGTTTGCAAGCAGGCGACAGG
ATCGTTAAAGTCGATGGTCAGCCCTTAACGCAGTGGGTGACCTTTGTGATGCTTGTCCGGGATAACCCGGGTAAATCCTT
AGCGTTAGAAATCGAAAGGCAGGGGAGTCCCTTGTCTTTGACATTAATCCCGGAGAGTAAACCGGGTAATGGTAAAGCGA
TTGGTTTTGTCGGTATTGAGCCGAAAGTCATTCCTTTGCCAGATGAGTATAAAGTTGTACGCCAGTATGGGCCGTTCAAC
GCCATCGTCGAAGCCACGGACAAAACGTGGCAGCTGATGAAGCTGACGGTCAGTATGCTGGGAAAATTGATCACCGGTGA
TGTGAAACTGAACAACCTCAGTGGGCCGATCTCTATCGCCAAGGGGGCTGGGATGACAGCGGAACTCGGGGTTGTTTATT
ACCTGCCGTTTCTTGCGCTTATTAGCGTGAACTTAGGGATAATTAACCTGTTTCCGTTGCCCGTACTTGACGGGGGGCAT
CTGCTGTTCCTTGCGATCGAAAAGATCAAGGGCGGACCGGTATCCGAGCGGGTTCAAGACTTTTGTTATCGCATTGGCTC
GATTCTGCTGGTGCTGTTAATGGGGCTTGCACTTTTCAATGATTTCTCTCGGTTATGAGAGTTAGTTAGGAAGAACGCAT
AATAACGATGGCGATGAAAAAGTTGCTCATAGCGTCGCTGCTGTTTAGCAGCGCCACCGTATACGGTGCTGAAGGGTTCG
TAGTGAAAGATATTCATTTCGAAGGCCTTCAGCGTGTCGCCGTTGGTGCGGCCCTCCTCAGTATGCCGGTGCGCACAGGC
GACACGGTTAATGATGAAGATATCAGTAATACCATTCGCGCTCTGTTTGCTACCGGCAACTTTGAGGATGTTCGCGTCCT
TCGTGATGGTGATACCCTTCTGGTTCAGGTAAAAGAACGTCCGACCATTGCCAGCATTACTTTCTCCGGTAACAAATCGG
TGAAAGATGACATGCTGAAGCAAAACCTCGAGGCTTCTGGTGTGCGTGTGGGCGAATCCCTCGATCGCACCACCATTGCC
GATATCGAGAAAGGTCTGGAAGACTTCTACTACAGCGTCGGTAAATATAGCGCCAGCGTAAAAGCTGTCGTGACCCCGCT
GCCGCGCAACCGTGTTGACCTAAAACTGGTGTTCCAGGAAGGTGTGTCAGCTGAAATCCAGCAAATTAACATTGTTGGTA
ACCATGCTTTCACCACCGACGAACTGATCTCTCATTTCCAACTGCGTGACGAAGTGCCGTGGTGGAACGTGGTAGGCGAT
CGTAAATACCAGAAACAGAAACTGGCGGGCGACCTTGAAACCCTGCGCAGCTACTATCTGGATCGCGGTTATGCCCGTTT
CAACATCGACTCTACCCAGGTCAGTCTGACGCCAGATAAAAAAGGTATTTACGTCACGGTGAACATCACCGAAGGCGATC
AGTACAAGCTTTCTGGCGTTGAAGTGAGCGGCAACCTTGCCGGGCACTCCGCTGAAATTGAGCAGCTGACTAAGATCGAG
CCGGGTGAGCTGTATAACGGCACCAAAGTGACCAAGATGGAAGATGACATCAAAAAGCTTCTCGGTCGCTATGGTTATGC
CTATCCGCGCGTACAGTCGATGCCCGAAATTAACGATGCCGACAAAACCGTTAAATTACGTGTGAACGTTGATGCGGGTA
ACCGTTTCTACGTGCGTAAGATCCGTTTTGAAGGTAACGATACCTCGAAAGATGCCGTCCTGCGTCGCGAAATGCGTCAG
ATGGAAGGTGCATGGCTGGGGAGCGATCTGGTCGATCAGGGTAAGGAGCGTCTGAATCGTCTGGGCTTCTTTGAAACTGT
CGATACCGATACCCAACGTGTTCCGGGTAGCCCGGACCAGGTTGATGTCGTCTACAAGGTAAAAGAGCGCAACACCGGTA
GCTTCAACTTTGGTATTGGTTACGGTACTGAAAGTGGCGTGAGCTTCCAGGCTGGTGTGCAGCAGGATAACTGGTTAGGT
ACAGGTTATGCTGTTGGTATCAACGGGACCAAAAACGATTACCAGACCTATGCTGAACTGTCGGTAACCAACCCGTACTT
CACCGTAGATGGCGTAAGCCTCGGTGGTCGTCTCTTCTATAATGACTTCCAGGCAGATGACGCCGACCTGTCCGACTATA
CCAACAAGAGTTATGGTACAGACGTGACGTTGGGCTTCCCGATTAACGAATATAACTCGCTGCGTGCAGGTCTGGGTTAT
GTACATAACTCCCTGTCCAACATGCAGCCTCAGGTTGCGATGTGGCGTTATCTGTACTCTATGGGTGAACATCCGAGCAC
CTCTGATCAGGATAACAGCTTCAAAACGGACGACTTCACGTTCAACTATGGTTGGACCTATAACAAGCTTGACCGTGGTT
ACTTCCCGACAGATGGTTCACGTGTCAACCTGACCGGTAAAGTGACCATTCCTGGATCGGATAACGAATACTACAAAGTG
ACGTTAGACACGGCGACTTATGTGCCGATCGATGACGATCACAAATGGGTTGTTCTGGGGCGTACCCGCTGGGGTTATGG
TGATGGTTTAGGCGGCAAAGAGATGCCGTTCTACGAGAACTTCTATGCCGGTGGTTCCAGCACCGTGCGTGGCTTCCAGT
CCAATACCATTGGTCCGAAAGCAGTTTACTTCCCGCATCAGGCCAGTAATTATGATCCGGACTATGATTACGAATGTGCG
ACTCAGGACGGCGCGAAAGACCTGTGTAAATCGGATGATGCTGTAGGCGGTAACGCCATGGCGGTTGCCAGCCTCGAGTT
CATCACCCCGACGCCGTTTATTAGCGATAAGTATGCTAACTCGGTTCGTACTTCCTTCTTCTGGGATATGGGTACCGTTT
GGGATACAAACTGGGATTCCAGCCAATATTCTGGATATCCGGACTATAGTGATCCAAGCAATATCCGTATGTCTGCGGGT
ATCGCATTACAATGGATGTCCCCATTGGGGCCGTTGGTGTTCTCCTACGCCCAGCCGTTCAAAAAGTACGATGGAGACAA
GGCAGAACAGTTCCAGTTTAACATCGGTAAAACCTGGTAAGTGTTCTCCACAAAGGAATGTAGTGGTAGTGTAGCGATGA
CTTTAGGCGATCAATATAAGATCGCCGGGCCACGCAAAGAACTGCACCCTCCGGTGCAAATGGGATGGTAAGGAGTTTAT
TGTGAAAAAGTGGTTATTAGCTGCAGGTCTCGGTTTAGCACTGGCAACTTCTGCTCAGGCGGCTGACAAAATTGCAATCG
TCAACATGGGCAGCCTGTTCCAGCAGGTAGCGCAGAAAACCGGTGTTTCTAACACGCTGGAAAATGAGTTCAAAGGCCGT
GCCAGCGAACTGCAGCGTATGGAAACCGATCTGCAGGCTAAAATGAAAAAGCTGCAGTCCATGAAAGCGGGCAGCGATCG
CACTAAGCTGGAAAAAGACGTGATGGCTCAGCGCCAGACTTTTGCTCAGAAAGCGCAGGCTTTTGAGCAGGATCGCGCAC
GTCGTTCCAACGAAGAACGCGGCAAACTGGTTACTCGTATCCAGACTGCTGTGAAATCCGTTGCCAACAGCCAGGATATC
GATCTGGTTGTTGATGCAAACGCCGTTGCTTACAACAGCAGCGATGTAAAAGACATCACTGCCGACGTACTGAAACAGGT
TAAATAAGTAATGCCTTCAATTCGACTGGCTGATTTAGCGCAGCAGTTGGATGCAGAACTACACGGTGATGGCGATATCG
TCATCACCGGCGTTGCGTCCATGCAATCTGCACAAACAGGTCACATTACGTTCATGGTTAACCCAAAATACCGTGAGCAT
TTAGGCTTGTGCCAGGCGTCCGCGGTTGTCATGACCCAGGACGATCTTCCTTTCGCGAAAAGTGCCGCACTGGTAGTGAA
GAATCCCTACCTGACTTACGCGCGCATGGCGCAAATTTTAGATACCACGCCGCAGCCCGCGCAGAACATTGCACCCAGTG
CGGTGATCGACGCGACGGCGAAGCTGGGTAACAACGTATCGATTGGCGCTAACGCGGTGATTGAGTCCGGCGTTGAACTG
GGCGATAACGTGATTATCGGTGCCGGTTGCTTCGTAGGTAAAAACAGCAAAATCGGTGCAGGTTCGCGTCTCTGGGCGAA
CGTAACCATTTACCATGAGATCCAGATCGGTCAGAATTGCCTGATCCAGTCCGGAACAGTGGTAGGCGCAGACGGCTTTG

GTTATGCCAACGATCGTGGTAACTGGGTGAAGATCCCACAGATTGGTCGCGTAATTATTGGCGATCGCGTGGAGATCGGT
GCCTGCACAACCATCGATCGCGGCGCGCTGGATGACACTATTATTGGCAATGGCGTGATCATTGATAACCAGTGCCAGAT
TGCACATAACGTCGTGATTGGCGACAATACGGCGGTTGCCGGTGGCGTCATTATGGCGGGCAGCCTGAAAATTGGTCGTT
ACTGCATGATCGGCGGAGCCAGCGTAATCAACGGGCATATGGAAATATGCGACAAAGTGACGGTTACGGGCATGGGTATG
GTGATGCGTCCCATCACTGAACCAGGCGTCTATTCCTCAGGCATTCCGCTGCAACCCAACAAAGTCTGGCGCAAAACCGC
TGCACTGGTGATGAACATTGATGACATGAGCAAGCGTCTGAAATCGCTTGAGCGCAAGGTTAATCAACAAGACTAACGTT
CCATCTTTTGTTCGCCAAACTTTACGGCCTGTCTCATTCTTACGATTGCGGCAGGCCGTGTTATTATTGTCGTTTCTTAT
ATTTTGACAGGAAGAGTATCTTGACTACTAACACTCATACTCTGCAGATTGAAGAGATTTTAGAACTTCTGCCGCACCGT
TTCCCGTTCTTACTGGTGGATCGCGTGCTGGATTTTGAAGAAGGTCGTTTTCTGCGCGCAGTAAAAAATGTCTCTGTCAA
TGAGCCATTCTTCCAGGGCCATTTCCCTGGAAAACCGATTTTCCCGGGTGTGCTGATTCTGGAAGCAATGGCACAGGCAA
CAGGTATTCTGGCGTTTAAAAGCGTAGGAAAACTGGAACCGGGTGAGCTGTACTACTTCGCTGGTATTGACGAAGCGCGC
TTCAAGCGCCCGGTCGTGCCTGGCGATCAAATGATCATGGAAGTCACTTTCGAAAAAACGCGCCGCGGCCTGACCCGTTT
TAAAGGGGTTGCTCTGGTCGATGGTAAAGTAGTTTGCGAAGCAACGATGATGTGTGCTCGTAGCCGGGAGGCCTGATACG
TGATTGATAAATCCGCCTTTGTGCATCCAACCGCCATTGTGGAAGAGGGCGCGTCAATTGGCGCGAACGCACACATTGGT
CCTTTTTGTATCGTTGGACCCCATGTCGAAATTGGTGAGGGTACCGTACTGAAATCTCACGTTGTCGTGAATGGTCATAC
TAAAATTGGCCGCGATAATGAGATTTATCAGTTCGCCTCCATCGGCGAAGTTAACCAGGATCTGAAATATGCTGGCGAAC
CGACCCGTGTGGAAATCGGCGATCGTAACCGCATTCGCGAAAGCGTCACCATTCATCGTGGCACAGTCCAGGGCGGTGGA
TTGACGAAGGTGGGCAGCGACAACTTACTGATGATCAACGCGCACATTGCGCACGATTGTACGGTAGGTAACCGCTGTAT
TCTCGCCAACAACGCAACGCTGGCGGGTCACGTATCGGTTGACGACTTCGCGATCATCGGCGGCATGACCGCAGTCCATC
AGTTCTGCATCATTGGTGCGCACGTGATGGTTGGCGGCTGCTCCGGTGTGGCGCAGGACGTCCCTCCTTATGTCATTGCG
CAGGGTAACCACGCAACGCCGTTCGGTGTCAATATCGAAGGGCTGAAGCGCCGCGGATTCAGCCGTGAGGCGATTACCGC
TATCCGCAATGCGTATAAGCTGATTTATCGTAGCGGTAAAACGCTCGATGAAGTGAAACCGGAAATTGCTGAACTGGCGG
AAACATATCCGGAAGTGAAAGCCTTTACCGATTTCTTTGCACGCTCAACGCGCGGTCTGATTCGTTAATGACTGAACAGC
GTCCATTAACGATTGCCCTGGTCGCCGGAGAAACCTCCGGCGATATCCTGGGGGCCGGTTTAATCCGCGCTCTGAAAGAA
CATGTGCCCAACGCCCGCTTTGTTGGTGTTGCCGGGCCACGAATGCAGGCTGAAGGCTGCGAAGCCTGGTACGAAATGGA
AGAACTGGCGGTGATGGGCATTGTTGAAGTGCTCGGTCGTCTGCGTCGCTTACTGCATATTCGTGCCGATCTGACAAAGC
GTTTTGGCGAACTGAAGCCAGATGTTTTTGTTGGTATTGATGCGCCTGACTTCAATATTACTCTTGAAGGTAACCTCAAA
AAGCAGGGTATCAAAACCATTCATTACGTCAGTCCGTCAGTCTGGGCGTGGCGACAGAAACGTGTTTTCAAAATAGGCAG
AGCCACCGATCTGGTGCTCGCATTTCTGCCTTTCGAAAAAGCGTTTTATGACAAATACAACGTACCGTGCCGCTTTATCG
GTCATACCATGGCTGATGCCATGCCATTAGATCCAGATAAAAATGCCGCCCGTGATGTGCTGGGGATCCCTCACGATGCC
CACTGCCTGGCGTTGCTACCGGGGAGCCGTGGTGCAGAAGTTGAAATGCTTAGTGCCGATTTCCTGAAAACGGCCCAGCT
TTTGCGCCAGACATATCCGGATCTCGAAATCGTGGTGCCACTGGTGAATGCCAAACGCCGCGAGCAGTTTGAACGCATCA
AAGCTGAAGTCGCGCCAGACCTTTCAGTTCATTTGCTGGATGGGATGGGCCGTGAGGCGATGGTCGCCAGCGATGCGGCG
CTACTGGCGTCGGGTACGGCAGCCCTGGAGTGTATGCTGGCGAAATGCCCGATGGTGGTGGGATATCGCATGAAGCCTTT
TACCTTCTGGTTGGCGAAGCGGCTGGTGAAAACTGATTATGTCTCGCTGCCAAATCTGCTGGCGGGCAGAGAGTTAGTCA
AAGAATTATTGCAGGAAGAGTGTGAGCCGCAAAAACTGGCTGCGGCGCTGTTACCGCTGTTGGCGAACGGGAAAACCAGC
CACGCGATGCACGATACCTTCCGTGAACTGCATCAGCAGATCCGCTGCAATGCCGATGAGCAGGCGGCACAAGCCGTTCT
GGAGTTAGCACAATGATCGAATTTGTTTATCCGCACACGCAGCTGGTTGCGGGTGTGGATGAAGTCGGACGCGGGCCGTT
AGTTGGCGCGGTCGTCACCGCTGCGGTGATCCTTGACCCGGCGCGCCCGATTGCCGGGCTGAATGATTCCAAAAAGCTGA
GCGAAAAACGCCGTCTGGCGCTCTATGAAGAGATCAAAGAGAAAGCGTTGAGCTGGAGTCTGGGCCGCGCGGAACCCCAC
GAAATCGACGAGCTGAACATTCTTCATGCGACCATGCTGGCGATGCAGCGTGCCGTCGCTGGGCTGCATATTGCGCCGGA
ATATGTGTTGATTGATGGTAACCGCTGCCCGAAATTACCGATGCCTGCGATGGCTGTGGTGAAAGGCGATAGCCGCGTAC
CGGAAATCAGTGCCGCGTCTATCCTGGCGAAAGTGACGCGTGACGCCGAAATGGCGGCGCTGGATATTGTTTTCCCGCAA
TATGGTTTTGCCCAACACAAAGGGTACCCAACCGCTTTTCATCTGGAAAAACTGGCTGAACACGGCGCGACCGAACACCA
TCGGCGCAGCTTTGGGCCTGTCAAACGCGCACTGGGACTTGCGTCCTGATTCTTGTGTCGAGATTAAGTAAACCGGAATC
TGAAGATGTCTGAACCACGTTTCGTACACCTGCGGGTGCACAGCGACTACTCGATGATCGATGGCCTGGCCAAAACCGCA
CCGTTGGTAAAAAAGGCGGCGGCGTTGGGTATGCCAGCACTGGCGATCACCGATTTCACCAACCTTTGTGGTCTGGTGAA
GTTCTACGGAGCGGGACATGGCGCAGGGATTAAGCCTATCGTCGGGGCAGATTTTAACGTCCAGTGCGACCTGCTGGGTG
ATGAGTTAACCCACCTGACGGTACTGGCGGCGAACAATACCGGCTATCAGAATCTGACGTTGCTGATCTCAAAAGCGTAT
CAGCGCGGGTACGGTGCCGCCGGGCCGATCATCGATCGCGACTGGCTTATCGAATTAAACGAAGGGTTGATCCTTCTTTC
CGGCGGACGCATGGGCGACGTCGGACGCAGTCTTTTGCGTGGTAACAGCGCGCTGGTAGATGAGTGTGTCGCGTTTTATG
AAGAACACTTCCCGGATCGCTATTTTCTCGAGCTGATCCGCACCGGCAGGCCGGATGAAGAAAGCTATCTGCACGCGGCG
GTGGAACTGGCGGAAGCGCGCGGTTTGCCCGTCGTGGCGACCAACGACGTGCGCTTTATCGACAGCAGCGACTTTGACGC
ACACGAAATCCGCGTCGCGATCCACGACGGCTTTACCCTCGACGATCCTAAACGCCCGCGTAACTATTCGCCGCAGCAAT
ATATGCGTAGCGAAGAGGAGATGTGTGAGCTGTTTGCCGACATCCCCGAAGCCCTTGCCAACACCGTTGAGATCGCCAAA
CGCTGTAACGTAACCGTGCGTCTTGGTGAATACTTCCTGCCGCAGTTCCCGACCGGGGACATGAGCACCGAAGATTATCT

GGTCAAGCGTGCAAAAGAGGGCCTGGAAGAGCGTCTGGCCTTTTTATTCCCTGATGAGGAAGAACGTCTTAAGCGCCGCC
CGGAATATGACGAACGTCTGGAGACTGAACTTCAGGTTATCAACCAGATGGGCTTCCCGGGCTACTTCCTCATCGTTATG
GAATTTATCCAGTGGTCGAAAGATAACGGCGTACCGGTAGGGCCAGGCCGTGGCTCCGGTGCGGGTTCACTGGTGGCCTA
CGCGCTGAAAATCACCGACCTCGATCCGCTGGAATTTGACCTGCTGTTCGAACGTTTCCTTAACCCGGAACGTGTCTCCA
TGCCTGACTTCGACGTTGACTTCTGTATGGAGAAACGCGATCAGGTTATCGAGCACGTAGCGGACATGTACGGTCGTGAT
GCGGTATCGCAGATCATCACCTTCGGTACAATGGCGGCGAAAGCGGTGATCCGCGACGTAGGCCGCGTGCTGGGGCATCC
GTACGGCTTTGTCGATCGTATCTCGAAACTGATCCCGCCCGATCCGGGGATGACGCTGGCGAAAGCGTTTGAAGCCGAGC
CGCAGCTGCCGGAAATCTACGAAGCGGATGAAGAAGTTAAGGCGCTGATCGACATGGCGCGCAAACTGGAAGGGGTCACC
CGTAACGCCGGTAAGCACGCCGGTGGGGTGGTTATCGCGCCGACCAAAATTACCGATTTTGCGCCGCTTTACTGCGATGA
AGAGGGCAAACATCCGGTCACCCAGTTTGATAAAAGCGACGTTGAATACGCCGGACTGGTGAAGTTCGACTTCCTTGGTT
TGCGTACGCTCACCATCATCAACTGGGCGCTGGAGATGATCAACAAGCGGCGGGCGAAGAATGGCGAGCCGCCGCTGGAT
ATCGCTGCGATCCCGCTGGATGATAAGAAAAGCTTCGACATGCTGCAACGCTCGGAAACCACGGCGGTATTCCAGCTTGA
ATCGCGCGGCATGAAGGACCTGATCAAGCGTCTACAACCTGACTGCTTCGAAGATATGATCGCCCTAGTGGCACTGTTCC
GCCCCGGTCCGTTGCAATCAGGGATGGTGGATAACTTTATCGACCGTAAACATGGTCGTGAAGAGATCTCCTATCCGGAC
GTACAGTGGCAGCATGAAAGCCTGAAACCGGTACTGGAGCCAACCTACGGCATTATCCTGTATCAGGAACAGGTCATGCA
GATTGCGCAGGTGCTTTCTGGTTATACCCTCGGTGGCGCGGATATGCTGCGTCGTGCGATGGGTAAGAAAAAGCCGGAAG
AGATGGCTAAGCAACGTTCTGTATTTGCTGAAGGTGCAGAAAAGAACGGAATCAACGCTGAACTGGCGATGAAAATCTTC
GACCTGGTGGAGAAATTCGCTGGTTACGGATTTAACAAATCGCACTCTGCGGCCTATGCTTTGGTGTCATATCAAACGTT
ATGGCTGAAAGCGCACTATCCTGCGGAGTTTATGGCGGCGGTAATGACCGCCGATATGGACAACACCGAGAAGGTGGTGG
GTCTGGTGGATGAGTGCTGGCGGATGGGGCTGAAAATCCTGCCACCAGATATAAACTCCGGTCTTTACCATTTCCACGTC
AACGACGACGGCGAAATCGTGTATGGTATTGGCGCGATCAAAGGGGTCGGTGAAGGTCCGATTGAGGCCATCATCGAAGC
CCGTAATAAAGGCGGCTACTTCCGCGAACTGTTTGATCTCTGCGCCCGTACCGACACCAAAAAGTTGAACCGTCGCGTGC
TGGAAAAACTGATCATGTCCGGGGCGTTTGACCGTCTTGGGCCACATCGCGCAGCGCTGATGAACTCGCTGGGCGATGCG
TTAAAAGCGGCAGATCAACACGCGAAAGCGGAAGCTATCGGTCAGGCCGATATGTTCGGCGTGCTGGCCGAAGAGCCGGA
ACAAATTGAACAATCCTACGCCAGCTGCCAACCGTGGCCGGAGCAGGTGGTATTAGATGGGGAACGTGAAACGTTAGGCC
TGTACCTGACCGGACACCCTATCAACCAGTATTTAAAAGAGATTGAGCGTTATGTCGGAGGCGTAAGGCTGAAAGACATG
CACCCGACAGAACGTGGTAAAGTCATCACGGCTGCGGGGCTCGTTGTTGCCGCGCGGGTTATGGTCACCAAGCGCGGCAA
TCGTATCGGTATCTGCACGCTGGATGACCGTTCCGGGCGGCTGGAAGTGATGTTGTTTACTGACGCCCTGGATAAATACC
AGCAATTGCTGGAAAAAGACCGCATACTTATCGTCAGCGGACAGGTCAGCTTTGATGACTTCAGCGGTGGGCTTAAAATG
ACCGCTCGCGAAGTGATGGATATTGACGAAGCCCGGGAAAAATATGCTCGCGGGCTTGCTATCTCGCTGACGGACAGGCA
AATTGATGACCAGCTTTTAAACCGACTCCGTCAGTCTCTGGAACCCCACCGCTCTGGGACAATTCCAGTACATCTCTACT
ATCAGAGGGCGGATGCACGCGCGCGGTTGCGTTTTGGCGCGACGTGGCGTGTCTCTCCGAGCGATCGTTTATTAAACGAT
CTCCGTGGCCTCATTGGTTCGGAGCAGGTGGAACTGGAGTTTGACTAATACAGGAATACTATGAGTCTGAATTTCCTTGA
TTTTGAACAGCCGATTGCAGAGCTGGAAGCGAAAATCGATTCTCTGACTGCGGTTAGCCGTCAGGATGAGAAACTGGATA
TTAACATCGATGAAGAAGTGCATCGTCTGCGTGAAAAAAGCGTAGAACTGACACGTAAAATCTTCGCCGATCTCGGTGCA
TGGCAGATTGCGCAACTGGCACGCCATCCACAGCGTCCTTATACCCTGGATTACGTTCGCCTGGCATTTGATGAATTTGA
CGAACTGGCTGGCGACCGCGCGTATGCAGACGATAAAGCTATCGTCGGTGGTATCGCCCGTCTCGATGGTCGTCCGGTGA
TGATCATTGGTCATCAAAAAGGTCGTGAAACCAAAGAAAAAATTCGCCGTAACTTTGGTATGCCAGCGCCAGAAGGTTAC
CGCAAAGCACTGCGTCTGATGCAAATGGCTGAACGCTTTAAGATGCCTATCATCACCTTTATCGACACCCCGGGGGCTTA
TCCTGGCGTGGGCGCAGAAGAGCGTGGTCAGTCTGAAGCCATTGCACGCAACCTGCGTGAAATGTCTCGCCTCGGCGTAC
CGGTAGTTTGTACGGTTATCGGTGAAGGTGGTTCTGGCGGTGCGCTGGCGATTGGCGTGGGCGATAAAGTGAATATGCTG
CAATACAGCACCTATTCCGTTATCTCGCCGGAAGGTTGTGCGTCCATTCTGTGGAAGAGCGCCGACAAAGCGCCGCTGGC
GGCTGAAGCGATGGGTATCATTGCTCCGCGTCTGAAAGAACTGAAACTGATCGACTCCATCATCCCGGAACCACTGGGTG
GTGCTCACCGTAACCCGGAAGCGATGGCGGCATCGTTGAAAGCGCAACTGCTGGCGGATCTGGCCGATCTCGACGTGTTA
AGCACTGAAGATTTAAAAAATCGTCGTTATCAGCGCCTGATGAGCTACGGTTACGCGTAATTCGCAAAAGTTCTGAAAAA
GGGTCACTTCGGTGGCCCTTTTTTATCGCCACGGTTTGAGCAGGCTATGATTAAGGAAGGATTTTCCAGGAGGAACACAT
GAACATCATTGCCATTATGGGACCGCATGGCGTCTTTTATAAAGATGAGCCCATCAAAGAACTGGAGTCGGCGCTGGTGG
CGCAAGGCTTTCAGATTATCTGGCCACAAAACAGCGTTGATTTGCTGAAATTTATCGAGCATAACCCTCGAATTTGCGGC
GTGATTTTTGACTGGGATGAGTACAGTCTCGATTTATGTAGCGATATCAATCAGCTTAATGAATATCTCCCGCTTTATGC
CTTCATCAACACCCACTCGACGATGGATGTCAGCGTGCAGGATATGCGGATGGCGCTCTGGTTTTTTGAATATGCGCTGG
GGCAGGCGGAAGATATCGCCATTCGTATGCGTCAGTACACCGACGAATATCTTGATAACATTACACCGCCGTTCACGAAA
GCCTTGTTTACCTACGTCAAAGAGCGGAAGTACACCTTTTGTACGCCGGGGCATATGGGCGGCACCGCATATCAAAAAAG
CCCGGTTGGCTGTCTGTTTTATGATTTTTTCGGCGGGAATACTCTTAAGGCTGATGTCTCTATTTCGGTCACCGAGCTTG
GTTCGTTGCTCGACCACACCGGGCCACACCTGGAAGCGGAAGAGTACATCGCGCGGACTTTTGGCGCGGAACAGAGTTAT
ATCGTTACCAACGGAACATCGACGTCGAACAAAATTGTGGGTATGTACGCCGCGCCATCCGGCAGTACGCTGTTGATCGA
CCGCAATTGTCATAAATCGCTGGCGCATCTGTTGATGATGAACGATGTAGTGCCAGTCTGGCTGAAACCGACGCGTAATG

CGTTGGGGATTCTTGGTGGGATCCCGCGCCGTGAATTTACTCGCGACAGCATCGAAGAGAAAGTCGCTGCTACCACGCAA
GCACAATGGCCGGTTCATGCGGTGATCACCAACTCCACCTATGATGGCTTGCTCTACAACACCGACTGGATCAAACAGAC
GCTGGATGTCCCGTCGATTCACTTCGATTCTGCCTGGGTGCCGTACACCCATTTTCATCCGATCTACCAGGGTAAAAGTG
GTATGAGCGGCGAGCGTGTTGCGGGAAAAGTGATCTTCGAAACGCAATCGACCCACAAAATGCTGGCGGCGTTATCGCAG
GCTTCGCTGATCCACATTAAAGGCGAGTATGACGAAGAGGCCTTTAACGAAGCCTTTATGATGCATACCACCACCTCGCC
CAGTTATCCCATTGTTGCTTCGGTTGAGACGGCGGCGGCGATGCTGCGTGGTAATCCGGGCAAACGGCTGATTAACCGTT
CAGTAGAACGAGCTCTGCATTTTCGCAAAGAGGTCCAGCGGCTGCGGGAAGAGTCTGACGGTTGGTTTTTCGATATCTGG
CAACCGCCGCAGGTGGATGAAGCCGAATGCTGGCCCGTTGCGCCTGGCGAACAGTGGCACGGCTTTAACGATGCGGATGC
CGATCATATGTTTCTCGATCCGGTTAAAGTCACTATTTTGACACCGGGGATGGACGAGCAGGGCAATATGAGCGAGGAGG
GGATCCCGGCGGCGCTGGTAGCAAAATTCCTCGACGAACGTGGGATCGTAGTAGAGAAAACCGGCCCTTATAACCTGCTG
TTTCTCTTTAGTATTGGCATCGATAAAACCAAAGCAATGGGATTATTGCGTGGGTTGACGGAATTCAAACGCTCTTACGA
TCTCAACCTGCGGATCAAAAATATGCTACCCGATCTCTATGCAGAAGATCCCGATTTCTACCGCAATATGCGTATTCAGG
ATCTGGCACAAGGGATCCATAAGCTGATTCGTAAACACGATCTTCCCGGTTTGATGTTGCGGGCATTCGATACTTTGCCG
GAGATGATCATGACGCCACATCAGGCATGGCAACGACAAATTAAAGGCGAAGTAGAAACCATTGCGCTGGAACAACTGGT
CGGTAGAGTATCGGCAAATATGATCCTGCCTTATCCACCGGGCGTACCGCTGTTGATGCCTGGAGAAATGCTGACCAAAG
AGAGCCGCACAGTACTCGATTTTCTACTGATGCTTTGTTCCGTCGGGCAACATTACCCCGGTTTTGAAACGGATATTCAC
GGCGCGAAACAGGACGAAGACGGCGTTTACCGCGTACGAGTCCTAAAAATGGCGGGATAACTTGCCAGAGCGGCTTCCGG
GCGAGTAACGTGCTGTTAACAAATAAAGGAGACGTTATGCTGGGTTTAAAACAGGTTCACCATATTGCGATTATTGCGAC
GGATTATGCGGTGAGCAAAGCTTTCTACTGCGATATTCTTGGTTTCACGCTGCAAAGCGAAGTCTATCGCGAAGCGCGCG
ACTCATGGAAAGGGGATTTGGCGCTTAATGGGCAATATGTGATTGAGCTTTTCTCATTTCCGTTCCCGCCGGAACGACCC
AGCCGACCGGAAGCTTGCGGTCTGCGTCATCTGGCTTTTAGCGTTGATGACATCGATGCGGCAGTGGCGCACCTTGAAAG
CCATAACGTGAAGTGTGAAACCATCCGTGTCGATCCATACACGCAAAAACGCTTCACCTTCTTTAACGATCCGGACGGGC
TGCCGTTGGAACTGTATGAGCAGTAAGGCTTGTCATCGCCGCATTTGCCCGGTAACGTGCCGGGCATTGCTACTGTAAAA
TCGCACCATCATGACACTCACGCTCAATAGACAACTTCTCACCTCACGCCAGATTCTGGTGGCCTTTAGCGGCGGGCTTG
ACTCCACCGTTCTGCTGCATCAGTTGGTGCAGTGGCGGACGGAAAATCCGGGTGTCGCTCTGCGCGCTATTCATGTGCAT
CACGGTTTAAGTGCCAATGCCGATGCCTGGGTTACGCATTGCGAAAACGTCTGCCAACAGTGGCAGGTGCCGCTGGTGGT
CGAACGCGTACAACTTGCGCAAGAAGGACTGGGCATTGAGGCCCAGGCGCGGCAGGCACGTTATCAGGCATTTGCCCGCA
CCTTGTTGCCCGGTGAAGTGCTGGTCACCGCGCAACATCTCGACGATCAATGTGAAACCTTTCTGCTGGCGCTAAAACGC
GGCAGTGGCCCTGCCGGGCTTTCGGCTATGGCGGAAGTCTCGGAGTTTGCCGGAACGCGGCTTATTCGCCCGTTGCTCGC
CCGCACGCGGGGGGAACTGGTGCAGTGGGCGCGTCAGTATGATTTACGCTGGATTGAAGACGAAAGTAATCAGGACGACT
CATACGATCGTAACTTTCTGCGCCTGCGCGTAGTGCCGTTATTGCAGCAGCGTTGGCCGCATTTTGCCGAAGCAACGGCC
CGCAGCGCCGCACTTTGTGCTGAACAAGAGAGCCTGCTGGATGAACTGCTGGCAGATGATTTAGCACACTGTCAATCGCC
GCAGGGGACGCTGCAGATTGTGCCAATGCTGGCGATGAGTGATGCCCGCCGCGCGGCGATTATCCGCCGCTGGCTGGCAG
GGCAGAATGCACCGATGCCTTCCCGCGACGCGTTGGTGAGGATCTGGCAGGAAGTGGCGCTGGCGCGGGAAGATGCCTCA
CCCTGTTTACGTTTGGGCGCGTTTGAAATCCGACGCTATCAGTCGCAACTGTGGTGGATTAAATCCGTCACCGGGCAAAG
CGAAAACATTGTGCCGTGGCAGACGTGGCTTCAACCGCTGGAATTACCGGCGGGGCTGGGAAGTGTACAGCTTAATGCGG
GAGGCGATATTCGCCCTCCGCGTGCAGACGAAGCGGTCAGCGTGCGTTTCAAAGCGCCAGGATTGCTGCATATTGTCGGG
CGTAACGGCGGACGTAAGCTAAAGAAAATCTGGCAAGAGCTGGGCGTGCCGCCGTGGCTACGTGACACCACGCCACTGCT
GTTTTATGGCGAAACGCTGATTGCGGCGGCAGGGGTATTTGTGACGCAAGAAGGTGTGGCTGAAGGTGAGAATGGCGTAA
GTTTTGTCTGGCAGAAAACGCTTAGTTAAGTGAAAGCCGGATAAGACGCATCAAACGTCGCATCCGGCGAAAGTCAATCA
GGATTCGCTTACCACCACCGTACCGATTTCCGGGTGGCTAAAGCTGGTAATTTTATCCAGACGCAGCTCGCGGGTTTCGC
CAGCGGCCTCGACGACCAGGTACTCCACATTTTTGCGGGAGACTAAATCACTGGCTTTTGCCTGCAATTTTTCGCCATCT
TTCAGCTCAAGTGTCAGCATTAAATGATGCTGGCAGGCGAGCTCAAGATTATCGTAATCATCACAATTGATTGGTTGATA
CGTATCATTCATTGACATAATCGCTCACCAGTAAGTTTGCCGCAGCGTATGCTGCTTTTTCCCTGACAGCCTCAGAAAGG
GCGTCGTCGGCAGCCATTTCATTCAGCACTTTCAAAACGCAGCCCAGCGCGTCCGGAACGTATCCTAAGTCTCCGCTGGC
GATTTCCGCGTACCGCTTGCGTATTAACTCACAATATTTTTCCACATGCCCTCCTGTCAGCACTCTGACTTAACCGTGGA
TGCAAGTCTAAGCCTACGAAGATAAACTCTGTTTCGCAAGGTGACTATACCACACTCATTTCTGCAATATCAGCGCCGCA
ACTGCACGTATTCCGTTACAATGGCCTCCTGATTCGAAAGGAGTTTTCTTATGGCGCTTAAAGCGACAATTTATAAAGCG
ACGGTTAATGTGGCCGATCTCGACCGCAACCAGTTTCTCGATGCCTCTCTGACGCTGGCGCGCCATCCTTCAGAAACCCA
GGAGCGTATGATGCTGCGCTTGCTGGCGTGGCTGAAATATGCTGATGAACGTCTGCAATTTACCCGTGGTTTGTGTGCCG
ATGATGAGCCGGAAGCGTGGCTGCGTAACGATCACCTGGGCATTGATTTGTGGATTGAGCTGGGGCTGCCGGATGAGCGG
CGGATTAAGAAAGCCTGCACCCAGGCCGCAGAAGTGGCGCTGTTTACCTATAATAGTCGGGCGGCGCAAATCTGGTGGCA
GCAAAATCAGAGCAAATGTGTGCAGTTTGCCAATCTTTCCGTCTGGTATCTGGACGATGAACAACTGGCGAAAGTAAGCG
CCTTTGCCGATCGTACCATGACGCTGCAGGCAACGATTCAGGATGGCGTGATCTGGTTATCGGATGATAAGAATAATCTG
GAAGTGAACTTAACCGCCTGGCAACAACCTTCATGATTGTGATTTCCCGACATGTTGCTATTCCCGATGGTGAGCTTGAG
ATCACCGCCATTCGTGCGCAGGGCGCGGGCGGGCAGCATGTTAATAAGACCTCAACGGCTATTCATCTGCGTTTTGACAT

TCGGGCGTCCAGCCTGCCAGAGTATTACAAAGAGCGTCTGCTCGCCGCCAGCCATCATTTGATCAGCAGTGATGGCGTGA
TTGTCATTAAGGCACAGGAATACCGCAGTCAGGAACTGAACCGCGAAGCAGCTCTGGCCCGGCTGGTGGCTATGATTAAA
GAATTAACAACAGAAAAAAAAAGCCCGACGACCCACGCGGCCCACCCGTGCATCGAAAGAGCGCAGGCTGGCATCGAAAGC
ACAAAAATCAAGCGTGAAGGCGATGCGCGGCAAAGTGCGCAGCGGTCGGGAATAAAAAGAAGGAATGGATGGTGAAAAAA
GCGATAGTGACAGCGATGGCTGTAATCAGCCTCTTTACTCTGATGGGATGTAATAATCGGGCCGAAGTCGATACGCTTTC
TCCGGCGCAGGCTGCCGAACTGAAACCGATGCCGCAAAGTTGGCGCGGCGTGCTGCCGTGTGCCGATTGCGAAGGAATCG
AAACCTCTCTGTTCCTCGAAAAAGACGGAACATGGGTGATGAATGAGCGTTATCTCGGTGCTCGTGAAGAACCTTCCTCC
TTCGCTTCCTACGGTACATGGGCGCGAACCGCTGACAAGCTGGTATTAACCGACAGCAAAGGTGAAAAGTCATATTATCG
GGCGAAAGGCGATGCGCTGGAGATGCTCGATCGTGAAGGCAATCCGATTGAATCGCAGTTCAACTATACGCTGGAAGCGG
CACAATCCAGTTTACCTATGACGCCGATGACCCTGCGGGGCATGTATTTTTATATGGCTGATGCGGCGACCTTCACTGAT
TGCGCGACCGGAAAACGTTTCATGGTAGCGAATAACGCAGAGCTGGAGCGTAGCTACCTGGCTGCGCGCGGTCACAGTGA
AAAACCGGTGTTACTGTCAGTAGAAGGTCACTTTACGCTTGAGGGTAATCCGGATACCGGTGCGCCGACTAAAGTATTGG
CACCCGATACGGCAGGGAAATTTTACCCCAACCAGGATTGCAGTAGTTTGGGGCAGTAACCCGTCTTGAGACAGAAACAA
ACGCAAAACAGGCCAGAAGGATATATTTCAACATTTTGAATTTGCACGTTTTTTGTAGGCCGGATAAGGCGTTTACGCCG
CATCCGGCAATGGTGCTCAACGCCTGATGCGACGCTGGCGCGTCTTATCATGCCTACAACCCCCCTCATACCTTAAGGCG
GATAAGGCATTTACTTTATCACCGGGTCAAACCAACAAAGCGACCCGCCTTGATATAAATCCCAGGCTTCAGGTGCCCAA
CATACTGTAACTGCGTTTCTGGCTTAAACGCAGAGACATCACCAATACGCATATGCATCAGATCGGCAGGGCTAATCCAG
CCCGATTGCGCCAGTGTAAGTGGATGCCCGGCTTTGGCAAAGGCATCCGTGACAAATTCCGAACAAAACCACGACTTTTT
GTCTCCTTCGCCCACACTGCTTAACTGCGCTTTCGCCAGGCCGCTGACGCACTGTTGGCGAAAATCCTCGGAGAACGGAT
TCAGTGAGCACATCTGGCGAGTCACCATAAAGGGAATAAATTCGACAATGCCGCGATAGTTATAACCGCTATCTTTGATT
TTATTGGCAAACGCGGTGATATCTGTGGCTTGTTGCGGGGTAAGATCCGGGACTCGTAAGACGAAAAGCTTATCACTATG
CTTCATCGCTTTTTTAAGGGAAACGATCTGGACGCCAGCGCCTGTCGCTTCTGCAACGTTATTATCACCCAGAAAGATTG
CAACGTGACTCACAGAGGAAGTGCTGAAGACGCGGATTCCAAATGAGGTTACCCCAAGGCTTGAGGAGAACAGCAAATCG
CCGGGTTTGAGATCAGGTGCCGTTATTTCTTTTATTGATTGTTCGGTGAAAGAGCTTTGATGCTGGAATTTGACAGCCCA
TGTTTTTGCCTCGGCATCTACTGCTGTGGCTGATGGGTCTGGCTGGCTGATATCAACGGTACAAGCCGAAAGTAAAAGAA
AGCTGGGGAGAAACAGGCGGCAGTACGCCTTTGGTTTATCCATTTTATACAATCCATGTAAAAAAAGGGCCCTGAAATTC
AGGACCCTTTCTGGCATCAGCCTTTAATCTGTTTCACCAGATATTCGACGATGTCACCAGTCTTAATTAACTGTTTCTCG
CCGTTGCGACGATATTTATATTCGATATCGTCGTTGTCGAGGTTACGGTCGCCCAGCACAATAGTGTGCGGAATACCGAT
CAGTTCCATATCAGCAAACATCACGCCCGGACGCTCTTTGCGGTCATCCAGCAGCACTTCGATACCTTGTGCACGCAGTT
CGCTGTACAGTTTCTCAGCAAGCTCTTGTACGCGGAAGGATTTGTGCATGTTCATCGGCAGAATCGCCACCTGGAACGGC
GCGATAGCGTCAGGCCATACGATGCCTCGTTCGTCGTAGTTCTGCTCAATCGCCGCAGCTACCACACGCGTTACCCCGAT
ACCGTAGCAACCCATCGTCAGGATTTGGTTACGGCCATCTTCACCCTGTACGGAGGCTTTCAGTGCTTCGGAGTACTTGG
TACCCAGCTGGAAGATGTGACCAACTTCGATACCACGTTTGATCAGCAGCCTACCCTGGCCATCCGGGCTTGGATCGCCA
GCCACCACGTTACGGATATCTGCAACTTCCGGGGTAGCGACATCGCGATCCCAGTTGATGCCGAAGTAGTGTTTACCATC
GATGTTAGCACCAGCAGCGAAATCACTCATCGCCGCAACGGTACGGTCAATCACCACCGGAATCGGCATGTTTACCGGAC
CCAGTGAACCCGGACCGGCTTTAACCACGGCACGAATTTCTTCTTCGGTCGCGAAAGTCAGCGGGCTTGCAACCTGCGGC
AGTTTTTCTGCTTTAACTTCGTTCAGCTCGTGATCACCGCGCACCAGCAGCGCAACCTGCGGGAAGCTGCTGCCTTCAAC
CGCTTTAACCAGCAGAGTCTTAACCGTTTTCTCAATCGGCAGATTGAACTGTTCAACCAGTTCCGCGATGGTTTTCGCGT
TCGGCGTATCAACCAGCGTCATTTCCTGGGTAGCAGCAGCGCGCGGTTCTTTCGGCGCGATAGCTTCTGCCAGTTCAATG
TTCGCTGCATAGTCAGAGGTGTCGGAGAAGACCACATCGTCTTCACCGCTCTGCGCCAGCACCTGGAATTCGTGAGAGGC
GCTGCCGCCGATAGAACCGGTGTCGGCTTGTACGGCGCGGAAATCCAGCCCCATGCGGCTGAAGATTTTGCTGTAGGCCG
CATACATTGCATCGTAGGTTTCCTGCAGGGATTCCTGAGAAGTATGGAAAGAGTAAGCATCTTTCATCAGGAATTCGCGG
GAACGCATGACGCCGAAACGCGGACGCACTTCGTCGCGGAACTTGGTCTGGATCTGATAGAAGTTCAGCGGCAGCTGTTT
GTAAGAGCTAAGCTCGTTACGAATCAGGTCAGTGATAACTTCTTCATGAGTTGGGCCGAGTACGAACGGACGCTCGCCAC
GGTCAACAAAACGCAGCAGTTCCGGACCGTACTGTTCCCAACGACCACTCTCTTGCCACAAATCGGCTGGCTGAACCACC
GGCATCGACACCTCGATCGCACCGGCGTTGTTCATCTCTTCACGCACGATGTTTTCGACTTTTTTCAGAACGCGCACGCC
GGTCGGCAGCCAGGTATATAACCCGGAGGCCAGCTTGCGGATCATCCCGGCGCGCAGCATCAGCTGATGGCTGATCACCT
CGGCGTCGGCAGGTGTCTCCTTGAGAGTGGAGAGCAGGTATTGGCTAGTACGCATGTTGTTACGGTTCCAGTTGGAAGGT
AGAACAGGCTCAAGGCGAGCCTGGGACAAAAAAAAGTGATTTAGTTTACCAGTGCAAAAGAAATGTCAAAAGAGAAGGGC
GTGAATTTAACGCGGTTCCAGCGCAAAGACTTCAAAACCTGCGTCGGTGACGCGCCAGCGAACGTTAAAATCATGTAGCC
AGACGGCATAGGTTTTGCCCGTTTCCTCACCTTTACGATAGGCCGGGCGCGGGTCCTGCGCCAGTACTTCGCGGATAAAC
AGCGTTAACTGCGGATAACGCTTCTCCAGCGTCAAAAGCTGCTTTTCGACCTCTGCGGTAAAACTCACCGCCATCTCTGC
AGCTGGCGCACTTTGCGCATAGCTGGCACTGGCATCGGGAAGCGATTCGGCAAAGGGGAGATACGGTTTGATATCCACTA
CCGGCGTACCATCGACCAGATCCAGACTGCCGAGCTTCAGAATCACGCTGTCTTTATGGCAAACAACCTCTTTCAGCTCT
ACCAGCGACATGCCAATTGGGTTAGGGCGGAAAGTAGAGCGTGTTGCGAAAACCCCCATTCTGGCGTTACCGCCGAGGCG
CGGTGGACGCACAGTCGGACGCCAGCCGCCTTCCATCGTTTGATGAAAGACGAAAAGGATCCATAAATGGCTGAACGCTT

CCAGGCCGCGAACGGCGTCGGCCTGGTTGTAGGGAGCAATGAGATGCAGTTCGCCGTTGGCGCTTTTTACCAGACCTGGC
TGGCGCGGAACGGCGAACTTTTCTTTATAGGGCGAGCGAATAACGCCTATTTGCTCGAACTGGAAACTGCTCATTTCGCC
GTAATGTTAAGCGCAGAACCGATACATACAGCCTGACGATAGCAGCCTGGCGTACCGCTGGTGACTTCGCAGCTATGCAG
TAATACAGCATTGGCTTTCATTTTAGAGGCGTTGATTTGCATCCGCTTACGTGCGGTTGGAATGCTCGGCGGAGAGTCCT
GATTAGAGGCCTGGCAAGAGTCGCCACTGACTTCACCGAGATCGCGGAACGGTTTGCCGACTAATTCTTCTGCATTGGTA
TAAATTCGGACCGGCGTGGCGCGCGGCGCTTTCGGTTTTGCAGGCTCCGCTTTCGGCTGGGGTGCAGTGCTTTGAACGGG
TTCGACAGGGGATCTGCTTAACATGGAACAGCCGCTTAGCATGAGTGCTACTAAACAGATCGGTAAAGCACGCATAGTAT
TTCCTCAATGTATGATCAAAACGTCAATATTGAATCAGGAGCTTGTAAAAATGACAAGACGGGCAAGCGCCCGTCCTGAA
TGATATTACAAATTGTGGAAACAGCCTAAAAATTACCAGCCTTTAACAGCTCCGCCGTTAAACACTTTGTTTGCTGCTTC
GTAAACTTCGTCAGACTGATAAGCCTGGACGAATTTCTTCACGTTCTCGGCGTCTTTGTTATCTTCACGCGTCACGATCA
GGTTTACGTACGGGGACTCTTTATCTTCAACAAAGATACCGTCTTTCGCCGGAGTCAGGCCAATCTGGCTGGCATAGGTG
GTATTGATAACTGCCAGAGCGATTTGCGCGTCGTCCAGAGAACGCGGCAGTTGCGGTGCTTCCAGTTCAACAATTTTCAG
ATTTTTGGGGTTCTCAACAACATCAAGAACGGTCGGCAGCAGGCCAACGCCATCTTTCAGTTTGATCAAGCCCACTTTTT
GCAGCAGCAGCAGTGAACGACCAAGGTTAGTTGGGTCGTTTGGCACGGCAACCTGCGAACCATCCTGCAGTTCATCCAGT
GATTTGATTTTCTTGGAGTAACCAGCAATCGGATAAACAAAAGTGTTGCCTACTGCGACCAGTTTGTAGCCACGATCTTT
CAGTTGCTGATCAAGGTACGGTTTATGCTGGAAGGCGTTGGCGTCGATATCGCCTTTGCTCAATGCTTCGTTTGGCAGAA
CATAGTCGTTGAAGGTTACCAGCTCAACGTCCAGGCCATATTTGTCTTTCGCAACTTTCTGCGCGACTTCTGCAACCTGC
TGTTCGGCACCAACAATCACGCCGACTTTAATGTGGTTTGGATCTTTTTCATCCTGACCGCAGCCTACCAGTGCCAGTGA
TCCGATCAGGGCTCCCACTGCCGCAAAGGTTTTGAATTTGAACGCCATACCTTATTCCTTCTTCAATTATTTATGTTGTG
TTGAACGTTACTTGCGAGTGACAGCCCGGACGATGCGGTCGCCTGCGAACTGAATTAAATAAACCAGAATGACCAGCAAT
ACCAGTACCGTATTCATCACCGTCGCGTTATAGCCGATGTAGCCATACTGATAGCCAATCTGACCTAAACCACCGGCACC
GACTGCACCACCCATCGCGGAATAACCGACCAGGGTAATCAGGGTGATAGTTGCCGCATTCACCAGACCCGGCAGCGCTT
CCGGTAACAGCACCTTACGGACGATCTGCATCGGCGTGGCACCCATTGCGCGGGAAGCTTCAATTAACCCGGTTGGGATC
TCCAGCAGAGCGTTCTCGACCATACGGGCAATAAACGGTGCTGCACCAACGGTTAACGGAACAATCGCTGCCTGCAAACC
AATCGATGTACCGACAATAACGCGGGTAAACGGAATCATCCATACAAGCAAGATAATGAACGGGATGGAACGGAAAATGT
TCACAATCGCAGAAACGGTACGATACAGCTTCGCGTTAGCAATAATTTGCCCCGGACGCGTGACATAAAGCAGAACGCCA
ACCGGCAGACCAATCACAAAGCCAAAAAAACCGGATACGAAGGTCATTGCCAGCGTTTCCCATACGCCACGAACCAGCAG
CCACATCATCGGCTCAGACATAACCCAGTACCTCTACTTTTACATGGTGTTCCTGCAGCCAGGCAATGGCGGCTTGCGTA
TCTTGTTGTGTGCCGTGCATTTCAGTCAGCATGATGCCGAACTTAACGCCACCGGCGTAATCCATCTGCGCGCTAATAAT
GTTGTTGTTGACGTTGAAACGACGCGCGGTTTCAGAAAGCAGTGGGGCATCGACCGATTGACCGGTAAACTCCAGACGCA
GCATCGGCACGCAGTCAGTAAATGGCTCCGCTTGCAGACGTTCCTGGTAATCTTCCGGGATATCCAGATGCAGGGTCGAC
TGAATAAACTTCTGCGCCAGCGGCGTTTTCGGATGCGAGAACACTTCACTTACCGTGTCCTGCTCGATCAGTTCTCCATT
GCTGATGACCGCCACGCAATCACAAATGCGCTTCACAACGTCCATTTCGTGGGTGATCAACAGAATCGTCAACCCCAGAC
GGCGGTTGATGTCTTTCAGCAGTTCGAGAATAGAACGTGTCGTTGCCGGGTCCAGCGCGCTGGTGGCTTCATCACACAGC
AATACTTTGGGATTGCTGGCTAACGCACGGGCAATTGCCACACGTTGTTTCTGCCCACCGGAAAGATTCGACGGGTAGCT
ATCATGCTTATCGCCAAGACCAACCAATGACAGCAATTCCGTCACGCGACGTTTGACCTCGTCTTTCGGTGTGTTGTCCA
GCTCCAGCGGCAGAGCCACGTTGCCAAAAACAGTACGCGAAGAGAGCAGGTTAAAATGCTGGAAAATCATACCAATCTGG
CGGCGAGCTTTGGTCAACTCGGATTCTGACAGCGTGGTCAGTTCCTGGCCATCGACCAGCACGCTACCCTCGGTTGGGCG
CTCCAGCAGGTTTACACAACGTATAAGCGTACTCTTACCCGCGCCTGAGGCACCGATAACGCCATAAATTTGTCCAGCTG
GCACATGCAGGCTGACGTTGTTCAACGCCTGGATGGTGCGGGTGCCCTGGTGGAACACTTTGGTGATATTCGAAAGTTTT
ATCATTGATTATTTATTATCGTCATTAAGTTAGTCGTGGCATCTCGAATGCCTGAAACGGGCAACGCCGTCAATGAAATG
GATGTTAAGGCATCCAGACGTCTAAATCAATCAGGTTTATGCGAAGAGCACTTTCTTGCAGGTCGAAACATGCGATACTA
GCGTCACATGCCTTATTAAGGAGCTATAAAAGGTGGCGAAGAGCGTACCCGCAATTTTTCTTGACCGTGATGGCACCATT
AATGTCGATCACGGCTATGTCCATGAGATCGACAACTTTGAATTTATCGACGGTGTTATTGACGCCATGCGCGAGCTAAA
AAAAATGGGCTTTGCGCTGGTGGTAGTAACCAACCAGTCTGGCATTGCTCGCGGTAAATTTACCGAAGCACAGTTTGAAA
CGCTGACCGAGTGGATGGACTGGTCGCTGGCGGACCGAGATGTCGATCTGGATGGTATCTATTATTGCCCGCATCATCCG
CAGGGTAGTGTTGAAGAGTTTCGCCAGGTCTGCGATTGCCGCAAACCACATCCGGGGATGCTTTTGTCAGCACGCGATTA
TTTGCATATTGATATGGCCGCTTCTTATATGGTGGGCGATAAATTAGAAGATATGCAGGCAGCGGTTGCGGCGAACGTGG
GAACAAAAGTGCTGGTGCGTACGGGTAAACCTATTACGCCTGAAGCAGAAAACGCGGCGGATTGGGTGTTAAATAGCCTG
GCAGACCTGCCGCAAGCGATAAAAAAGCAGCAAAAACCGGCACAATGATTAAAAGATGAGCGGTTGAAATAAAAATGCAT
TTTTCCGCTTGTCTTCCTGAGCCGACTCCCTATAATGCGCCTCCATCGACACGGCGGATGTGAATCACTTCACACAAACA
GCCGGTTCGGTTGAAGAGAAAAATCCTGAAATTCAGGGTTGACTCTGAAAGAGGAAAGCGTAATATACGCCACCTCGCGA
CAGTGAGCTGAAAGCCGCGTCGCAACTGCTCTTTAACAATTTATCAGACAATCTGTGTGGGCACTCGAAGATACGGATTC
TTAACGTCGCAAGACGAAAAATGAATACCAAGTCTCAAGAGTGAACACGTAATTCATTACAAAGTTTAATTCTTTGAGCA
TCAAACTTTTAAATTGAAGAGTTTGATCATGGCTCAGATTGAACGCTGGCGGCAGGCCTAACACATGCAAGTCGAACGGT
AACAGGAAGAAGCTTGCTTCTTTGCTGACGAGTGGCGGACGGGTGAGTAATGTCTGGGAAACTGCCTGATGGAGGGGGAT

AACTACTGGAAACGGTAGCTAATACCGCATAACGTCGCAAGACCAAAGAGGGGGACCTTCGGGCCTCTTGCCATCGGATG
TGCCCAGATGGGATTAGCTAGTAGGTGGGGTAACGGCTCACCTAGGCGACGATCCCTAGCTGGTCTGAGAGGATGACCAG
CCACACTGGAACTGAGACACGGTCCAGACTCCTACGGGAGGCAGCAGTGGGGAATATTGCACAATGGGCGCAAGCCTGAT
GCAGCCATGCCGCGTGTATGAAGAAGGCCTTCGGGTTGTAAAGTACTTTCAGCGGGGAGGAAGGGAGTAAAGTTAATACC
TTTGCTCATTGACGTTACCCGCAGAAGAAGCACCGGCTAACTCCGTGCCAGCAGCCGCGGTAATACGGAGGGTGCAAGCG
TTAATCGGAATTACTGGGCGTAAAGCGCACGCAGGCGGTTTGTTAAGTCAGATGTGAAATCCCCGGGCTCAACCTGGGAA
CTGCATCTGATACTGGCAAGCTTGAGTCTCGTAGAGGGGGGTAGAATTCCAGGTGTAGCGGTGAAATGCGTAGAGATCTG
GAGGAATACCGGTGGCGAAGGCGGCCCCCTGGACGAAGACTGACGCTCAGGTGCGAAAGCGTGGGGAGCAAACAGGATTA
GATACCCTGGTAGTCCACGCCGTAAACGATGTCGACTTGGAGGTTGTGCCCTTGAGGCGTGGCTTCCGGAGCTAACGCGT
TAAGTCGACCGCCTGGGGAGTACGGCCGCAAGGTTAAAACTCAAATGAATTGACGGGGGCCCGCACAAGCGGTGGAGCAT
GTGGTTTAATTCGATGCAACGCGAAGAACCTTACCTGGTCTTGACATCCACAGAACTTTCCAGAGATGGATTGGTGCCTT
CGGGAACTGTGAGACAGGTGCTGCATGGCTGTCGTCAGCTCGTGTTGTGAAATGTTGGGTTAAGTCCCGCAACGAGCGCA
ACCCTTATCTTTTGTTGCCAGCGGTCCGGCCGGGAACTCAAAGGAGACTGCCAGTGATAAACTGGAGGAAGGTGGGGATG
ACGTCAAGTCATCATGGCCCTTACGACCAGGGCTACACACGTGCTACAATGGCGCATACAAAGAGAAGCGACCTCGCGAG
AGCAAGCGGACCTCATAAAGTGCGTCGTAGTCCGGATTGGAGTCTGCAACTCGACTCCATGAAGTCGGAATCGCTAGTAA
TCGTGGATCAGAATGCCACGGTGAATACGTTCCCGGGCCTTGTACACACCGCCCGTCACACCATGGGAGTGGGTTGCAAA
AGAAGTAGGTAGCTTAACCTTCGGGAGGGCGCTTACCACTTTGTGATTCATGACTGGGGTGAAGTCGTAACAAGGTAACC
GTAGGGGAACCTGCGGTTGGATCACCTCCTTACCTTAAAGAAGCGTACTTTGCAGTGCTCACACAGATTGTCTGATGAAA
ATGAGCAGTAAAACCTCTACAGGCTTGTAGCTCAGGTGGTTAGAGCGCACCCCTGATAAGGGTGAGGTCGGTGGTTCAAG
TCCACTCAGGCCTACCAAATTTGCACGGCAAATTTGAAGAGGTTTTAACTACATGTTATGGGGCTATAGCTCAGCTGGGA
GAGCGCCTGCTTTGCACGCAGGAGGTCTGCGGTTCGATCCCGCATAGCTCCACCATCTCTGTAGTGGTTAAATAAAAAAT
ACTTCAGAGTGTACCTGCAAAGGTTCACTGCGAAGTTTTGCTCTTTAAAAATCTGGATCAAGCTGAAAATTGAAACACTG
AACAATGAAAGTTGTTCGTGAGTCTCTCAAATTTTCGCAACACGATGATGGATCGCAAGAAACATCTTCGGGTTGTGAGG
TTAAGCGACTAAGCGTACACGGTGGATGCCCTGGCAGTCAGAGGCGATGAAGGACGTGCTAATCTGCGATAAGCGTCGGT
AAGGTGATATGAACCGTTATAACCGGCGATTTCCGAATGGGGAAACCCAGTGTGTTTCGACACACTATCATTAACTGAAT
CCATAGGTTAATGAGGCGAACCGGGGGAACTGAAACATCTAAGTACCCCGAGGAAAAGAAATCAACCGAGATTCCCCCAG
TAGCGGCGAGCGAACGGGGAGGAGCCCAGAGCCTGAATCAGTGTGTGTGTTAGTGGAAGCGTCTGGAAAGGCGCGCGATA
CAGGGTGACAGCCCCGTACACAAAAATGCACATGCTGTGAGCTCGATGAGTAGGGCGGGACACGTGGTATCCTGTCTGAA
TATGGGGGGACCATCCTCCAAGGCTAAATACTCCTGACTGACCGATAGTGAACCAGTACCGTGAGGGAAAGGCGAAAAGA
ACCCCGGCGAGGGGAGTGAAAAAGAACCTGAAACCGTGTACGTACAAGCAGTGGGAGCATGCTTAGGCGTGTGACTGCGT
ACCTTTTGTATAATGGGTCAGCGACTTATATTCTGTAGCAAGGTTAACCGAATAGGGGAGCCGAAGGGAAACCGAGTCTT
AACTGGGCGTTAAGTTGCAGGGTATAGACCCGAAACCCGGTGATCTAGCCATGGGCAGGTTGAAGGTTGGGTAACACTAA
CTGGAGGACCGAACCGACTAATGTTGAAAAATTAGCGGATGACTTGTGGCTGGGGGTGAAAGGCCAATCAAACCGGGAGA
TAGCTGGTTCTCCCCGAAAGCTATTTAGGTAGCGCCTCGTGAACTCATCTCCGGGGGTAGAGCACTGTTTCGGCAAGGGG
GTCATCCCGACTTACCAACCCGATGCAAACTGCGAATACCGGAGAATGTTATCACGGGAGACACACGGCGGGTGCTAACG
TCCGTCGTGAAGAGGGAAACAACCCAGACCGCCAGCTAAGGTCCCAAAGTCATGGTTAAGTGGGAAACGATGTGGGAAGG
CCCAGACAGCCAGGATGTTGGCTTAGAAGCAGCCATCATTTAAAGAAAGCGTAATAGCTCACTGGTCGAGTCGGCCTGCG
CGGAAGATGTAACGGGGCTAAACCATGCACCGAAGCTGCGGCAGCGACGCTTATGCGTTGTTGGGTAGGGGAGCGTTCTG
TAAGCCTGTGAAGGTGTACTGTGAGGTATGCTGGAGGTATCAGAAGTGCGAATGCTGACATAAGTAACGATAAAGCGGGT
GAAAAGCCCGCTCGCCGGAAGACCAAGGGTTCCTGTCCAACGTTAATCGGGGCAGGGTGAGTCGACCCCTAAGGCGAGGC
CGAAAGGCGTAGTCGATGGGAAACAGGTTAATATTCCTGTACTTGGTGTTACTGCGAAGGGGGGACGGAGAAGGCTATGT
TGGCCGGGCGACGGTTGTCCCGGTTTAAGCGTGTAGGCTGGTTTTCCAGGCAAATCCGGAAAATCAAGGCTGAGGCGTGA
TGACGAGGCACTACGGTGCTGAAGCAACAAATGCCCTGCTTCCAGGAAAAGCCTCTAAGCATCAGGTAACATCAAATCGT
ACCCCAAACCGACACAGGTGGTCAGGTAGAGAATACCAAGGCGCTTGAGAGAACTCGGGTGAAGGAACTAGGCAAAATGG
TGCCGTAACTTCGGGAGAAGGCACGCTGATATGTAGGTGAAGCGACTTGCTCGTGGAGCTGAAATCAGTCGAAGATACCA
GCTGGCTGCAACTGTTTATTAAAAACACAGCACTGTGCAAACACGAAAGTGGACGTATACGGTGTGACGCCTGCCCGGTG
CCGGAAGGTTAATTGATGGGGTTAGCGCAAGCGAAGCTCTTGATCGAAGCCCCGGTAAACGGCGGCCGTAACTATAACGG
TCCTAAGGTAGCGAAATTCCTTGTCGGGTAAGTTCCGACCTGCACGAATGGCGTAATGATGGCCAGGCTGTCTCCACCCG
AGACTCAGTGAAATTGAACTCGCTGTGAAGATGCAGTGTACCCGCGGCAAGACGGAAAGACCCCGTGAACCTTTACTATA
GCTTGACACTGAACATTGAGCCTTGATGTGTAGGATAGGTGGGAGGCTTTGAAGTGTGGACGCCAGTCTGCATGGAGCCG
ACCTTGAAATACCACCCTTTAATGTTTGATGTTCTAACGTTGACCCGTAATCCGGGTTGCGGACAGTGTCTGGTGGGTAG
TTTGACTGGGGCGGTCTCCTCCTAAAGAGTAACGGAGGAGCACGAAGGTTGGCTAATCCTGGTCGGACATCAGGAGGTTA
GTGCAATGGCATAAGCCAGCTTGACTGCGAGCGTGACGGCGCGAGCAGGTGCGAAAGCAGGTCATAGTGATCCGGTGGTT
CTGAATGGAAGGGCCATCGCTCAACGGATAAAAGGTACTCCGGGGATAACAGGCTGATACCGCCCAAGAGTTCATATCGA
CGGCGGTGTTTGGCACCTCGATGTCGGCTCATCACATCCTGGGGCTGAAGTAGGTCCCAAGGGTATGGCTGTTCGCCATT
TAAAGTGGTACGCGAGCTGGGTTTAGAACGTCGTGAGACAGTTCGGTCCCTATCTGCCGTGGGCGCTGGAGAACTGAGGG

GGGCTGCTCCTAGTACGAGAGGACCGGAGTGGACGCATCACTGGTGTTCGGGTTGTCATGCCAATGGCACTGCCCGGTAG
CTAAATGCGGAAGAGATAAGTGCTGAAAGCATCTAAGCACGAAACTTGCCCCGAGATGAGTTCTCCCTGACTCCTTGAGA
GTCCTGAAGGAACGTTGAAGACGACGACGTTGATAGGCCGGGTGTGTAAGCGCAGCGATGCGTTGAGCTAACCGGTACTA
ATGAACCGTGAGGCTTAACCTTACAACGCCGAAGCTGTTTTGGCGGATTGAGAGAAGATTTTCAGCCTGATACAGATTAA
ATCAGAACGCAGAAGCGGTCTGATAAAACAGAATTTGCCTGGCGGCCGTAGCGCGGTGGTCCCACCTGACCCCATGCCGA
ACTCAGAAGTGAAACGCCGTAGCGCCGATGGTAGTGTGGGGTCTCCCCATGCGAGAGTAGGGAACTGCCAGGCATCAAAT
TAAGCAGTAAGCCGGTCATAAAACCGGTGGTTGTAAAAGAATTCGGTGGAGCGGTAGTTCAGTCGGTTAGAATACCTGCC
TGTCACGCAGGGGGTCGCGGGTTCGAGTCCCGTCCGTTCCGCCACTTATTAAGAAGCCTCGAGTTAACGCTCGAGGTTTT
TTTTCGTCTGTATATCTATTATTGCCAGAATCGCAAAAATCCTCTGCATTTTACGCTCTTTTTCCTCAACAGTCTGAAGC
CCATAATCACCTCAGTTAACGAAAATAGCATTAAAAGAGGCATATTATGGCTATCCCTGCATTTGGTTTAGGTACTTTCC
GTCTGAAAGACGACGTTGTTATTTCATCTGTGATAACGGCGCTTGAACTTGGTTATCGCGCAATTGATACCGCACAAATC
TATGATAACGAAGCCGCAGTAGGTCAGGCGATTGCAGAAAGTGGCGTGCCACGTCATGAACTCTACATCACCACTAAAAT
CTGGATTGAAAATCTCAGCAAAGACAAATTGATCCCAAGTCTGAAAGAGAGCCTGCAAAAATTGCGTACCGATTATGTTG
ATCTGACGCTAATCCACTGGCCGTCACCAAACGATGAAGTCTCTGTTGAAGAGTTTATGCAGGCGCTGCTGGAAGCCAAA
AAACAAGGGCTGACGCGTGAGATCGGTATTTCCAACTTCACGATCCCGTTGATGGAAAAAGCGATTGCTGCTGTTGGTGC
TGAAAACATCGCTACTAACCAGATTGAACTCTCTCCTTATCTGCAAAACCGTAAAGTGGTTGCCTGGGCTAAACAGCACG
GCATCCATATTACTTCCTATATGACGCTGGCGTATGGTAAGGCCCTGAAAGATGAGGTTATTGCTCGTATCGCAGCTAAA
CACAATGCGACTCCGGCACAAGTGATTCTGGCGTGGGCTATGGGGGAAGGTTACTCAGTAATTCCTTCTTCTACTAAACG
TAAAAACCTGGAAAGTAATCTTAAGGCACAAAATTTACAGCTTGATGCCGAAGATAAAAAAGCGATCGCCGCACTGGATT
GCAACGACCGCCTGGTTAGCCCGGAAGGTCTGGCTCCTGAATGGGATTAAGCCTCTCTGACAGCTCCTCCGGGAGCTGTT
TTTACATGCTCGCTAAGGAAATCGATAAAAGCCCGGATGCGCGTACTTACCGCACGGTCGCTGTAATAGACGGCACTGAA
TGGCATTTCCACTGGCAACACTTTATCTGCCATTAACTCCACCAATTCTCCGCGAGCGATTTCTTTGTCGATCATGTAGT
CGGACAAACACGCAATCCCGTTGCCACTCAGGCAAAGCTGTTTCAGTGTTTCCCCACTATTGGATGACAAACCGTACTTC
ACCTCATGTAATTGTCCATCGCTACGGGCTATCGGCCAGGTATTGAGGGAAGCGGGTTCAGTGAATCCCAGGCAAATATG
TTGCTTTAAATCGTCGATCGTTTCTGGCTTCCCGTAGCGGGAAATATAATCGGGGGAGGCGATAATTTTTCGATAACTGT
TAAATAACGGCCTGGCACGTAAGCTGGAATCCGTTAACGTACCAGCGCGTATCGCGACATCCACTTTTCTTTCGATCAAA
TTAATAATCGTTTCGGAGGAGACTAGCGACAAAGTGACTTCCGGATAGCGTTCACGGAAAGGCTTAATTAACGGCATCAG
AAAGTGCAGCACCACTGGAGTTGCGGCATCGATCCGTAACAGTCCACGCGGTGTATTACGCGTCTCCATAATTTCTGATT
CTGCCGCTGCCATCTCCTGCAAAATTGACTGTACGCGACGAAAATAACGCTCGCCTTCTTCCGTCAGGCTAAGTTGTCGC
GTGGTCCGATTAAGCAGGCTAACGCCAAGTTTCATCTCCAGCTTTTTCACCGCCCGGCTTACCGCTGAGTTTGCTTGCCC
TAATTGTTCCGCTGCCCGGCTAAAGCTGCCGCTTTCGACGACCGAAACAAAAATGGCGAGTTCTTCCGACGTGGCTTTCA
TTTTTGCTCCTGTTGCAAAATAGAAGAGATATTTTGAATTTATTTGTCATTAAACCATCAGGATGCGTGATATGTCATGC
GATTTAATGTTCTCCATAATGAGCAAAATTCTGACCGGTGTAAGCACTTGCTTACATAACAATATACAATTGCTCGTTGA
AAGAGTGAGCTAAAATCCCTATAACAGTAGAACCCTCCCGAGTGCGGAAGGGTTGACGTAATAGAGGTTTCAAAGTCAAA
AGTGCGAAAAAACACCTATGCCATGCGCTATGTTGCCGGACAACCTGCGGAAAGGATCTTACCGCCGGGGTCTTTTGCGA
GCATCGGCCAGGCATTACCACCTGGGGAACCGTTAAGTACCGAAGAGCGTATTCGGATCCTGGTGTGGAACATATACAAA
CAGCAACGCGCTGAATGGTTGTCGGTATTAAAGAACTACGGCAAAGATGCACATCTGGTGTTATTGCAGGAAGCGCAGAC
AACGCCAGAGTTAGTACAGTTTGCGACCGCTAACTATCTTGCCGCCGATCAGGTACCCGCTTTCGTGCTGCCACAACATC
CTTCTGGCGTAATGACCCTTTCGGCGGCACATCCAGTGTATTGCTGCCCGTTACGTGAACGAGAACCCATTTTGCGTCTG
GCGAAGTCGGCACTGGTGACGGTCTATCCATTACCTGACACCCGCCTGTTGATGGTGGTTAATATACACGCCGTCAACTT
CAGTCTGGGCGTGGATGTCTATAGTAAGCAGTTACTTCCTATTGGCGATCAGATAGCTCACCACAGCGGCCCGGTCATTA
TGGCGGGAGATTTCAATGCCTGGAGCCGTAGAAGGATGAACGCGTTATATCGCTTTGCGCGGGAAATGTCGCTGCGCCAG
GTGCGTTTTACCGATGATCAGCGCCGTCGGGCGTTTGGTCGCCCGCTCGATTTTGTTTTCTACCGTGGTCTGAACGTCAG
TGAAGCTTCTGTACTGGTTACGCGCGCTTCCGATCACAATCCGCTACTCGTTGAATTCAGTCCCGGCAAGCCTGATAAAT
AAGGTATGTCAGGTCTGCCACAGGGCAGACCAACGTTTGGCGCTGCGCAAAACGTGAGCGCGGTGGTGGCGTATGACTTA
TCTGCCCACATGCTGGATGTCGTGGCACAAGCTGCCGAAGCCCGGCAACTGAAAAATATCACCACCCGCCAGGGATATGC
CGAAAGTCTGCCATTTGCCGATAACGCATTTGATATTGTTATCAGCCGTTATTCTGCCCATCACTGGCATGATGTTGGTG
CAGCACTGCGAGAAGTGAATAGGATATTGAAACCTGGCGGTAGGCTGATTGTGATGGACGTAATGTCTCCGGGTCACCCA
GTGCGCGACATCTGGTTACAGACGGTAGAAGCATTACGCGATACCTCTCACGTACGAAACTACGCCAGCGGTGAGTGGTT
GACGTTAATCAATGAAGCCAATCTGATAGTTGATAATTTAATTACAGATAAGTTACCGCTGGAATTTTCTTCATGGGTCG
CGAGAATGCGTACGCCAGAAGCGTTAGTAGACGCTATTCGCATTTACCAACAGAGCGCATCGACAGAGGTGAGAACGTAT
TTTGCCTTGCAGAATGATGGCTTTTTCACCAGTGATATCATCATGGTAGATGCACATAAAGCGGCATAAATAAAAAAGGC
ACCGGGGGAATCGGTGCCTTTTTATTATCTGGTTTGTCAGGAATCTGGCATGTTGTTGTTTTTCACAAACAACGTCAGCT
TATCGCCTGGTTGCAGATTCGCAGTGTCGCTGTTCCAGCGCATCACATCTTTGATGTTCACGCCGTGGCGTTTAGCAATG
CTTGAAAGCGAATCGCCTTTGCGCACACGATACGTAATGCTATCGCTGTTGTTTGCCAGTCGCTGTGCGCTACTGCCTGC
ACCAATCGTCAAACTTTGGCCTGGCTTCAGCTTAGATCCGCGCAGTTTGTTCCACTGCTGCAAATCTTTGGTGCTTACGC

CGAGACGTGAAGCGATACTTGAAAGCGTGTCGCCAGAGCGTACGGTGTAAACACGGCTGTTAAGCGGCGTATTGTCGGCA
ACCAGCGTCGACTGTACAGCAGCAATTTCGCCTGAAGCCAGAGATTCACGCAGTTGATCTGCATGCTTCTTTGGCACCAT
CACGTACTGCGGGCCACTTGCGCCCAGCGTGGAGCCTTTTACGCCAGCGTTGAATGTCTTCAGCTTGCTGACGGAAATCC
CCGCCATATCTGCTACCTTCGCCATTTCAACCGGGCTGCTCAGGTGCACACGCGCCAGAGCACGGCTTTCATCGGTCGTT
GGCAGACGTACGCCATAACGCTTGCTGTTTTTGAGAATATCACTCAATGCCAGCATTTTAGGCACGTACTGCTTCGTTTC
CTGCGGCAACGGTAACGACCAGAAGTCCGTGGATTTCCCACGCGCTTTGTTCGTTTTAATTGCCTTCATGACCCGACCTT
CGCCGCTGTTATAAGCCGCTACGGTCAGAAGCCAGTCGCCGTCAAACATTTTGTTCAGACGCTGCATCATATTCAGCGCG
GCAGTTGTTGAAGCAACAACATCGCGACGCGCGTCATAATTGCGGGTCTGTTTCAAACCATAATTGCGCCCCGTGCTCGG
AATGATCTGCCAGATGCCTGCGGCATTGGCGCCAGACGTTGCGTGAGGATCAAAAGCGCTCTCCACTATGGGTAGTAGTA
CCAGTTCCATAGGCATGTTACGTTTTTTAACTTGCCCTGCTATCCAGTACATATACGGCTCTGCCCGTAAAGTTACATCG
TGGAGATAGCTCTTATTGCGTAAATATTTCTGTTTCTGTTCGCGAATCCGGTCATTTTCCGGAATTCCCATCTTTAGCTC
GTCGCCAATGAAAGCCCACAAGTCACCATCTGGCGCGATAGACGTCCCATCGTCCATCCATCGTGCCTGACTTGTAAACT
TTGCTGCTTCCCCTTGACCAGCTGCAGAAAGGCTCTGTGCGTGCTGTTGAACGTTGCCGGTACTCTGGCAACCCACGAGC
AGGACAGAGGCGAGTAATATCGCTTTTGCCTTCATGTGTGTGTCAATAGTTGCTTAAAAGACGACCGATCATAACGGCGA
ACGGAGCCGATGACAAGAAAGTTTTATCAGAACCTATCTTTCTTTGACCTTAACCATGCAAAACGCTCTTCAGGTTGTTG
CAATAATGTTTCTTCATTAATTACATTAATTAAATCAATATCTTCCGTTCTTAAAAAAACATTAATTTGCCGCTCATTTT
TCAGAATTACGGGTAGTGTTATTTGATTTTTTGCCCGTAACTCCTTAACTTTACGATAATAATCATTTATGGACAAATCG
TGCGGAAGAATACTCAAAGCAAACTTCATATTTGATAAGGTATATTCATGAGCACAACATACCAATGTATCGTCAGGTAA
CGCACTTAACTTTTTAAGTGATTGATACATTTGTGATGCTGTCCCTTCAAACAACCGACCACACCCACCAGAAAACAGTG
TGTCGCCGCAAAATAGATAAGGTTTACTGAAGTAACAGATATGTCCTAAAGTGTGACCCGGCGTAGCAATTACACTAAAT
TCATGCCCCAAAACGAAGGCAGTTTCGCCATCTTTGACTACCTGTGTTGTTCCCTTATCTTGTGTCTCTTGTGGACCATA
CACCACAATTTGTGGAAACTTTTCCACCAGTTCTTTTACGCCGCCAACGTGATCGTGGTGATGGTGGGTGAGAAATATGG
CCTCCGGTTGCCAGTTATTGGCGGCAATGGCGTTTAATACTGGCTCTGCGTCTCCGGGATCGACAATCAGGCAGCGACCT
GCTTCATCATTCAAAACCCAGATGTAATTGTCATCAAAGGCGGGAATACTGTTAAGATTCATAGATTACCTCTCAGTGTG
AAACGGAAGGTTGTGATGAAACCGGCAAGAGTCCCTCAAACTGTCGTGGCTCCTGATTGCTGGGGCGATTTGCCCTGGGG
AAAGCTTTATCGCAAGGCGCTGGAGCGCCAGCTCAACCCGTGGTTCACTAAAATGTATGGTTTTCATCTGCTTAAGATTG
GCAATTTAAGCGCAGAAATCAATTGCGAAGCGTGCGCGGTTTCTCATCAAGTGAATGTTTCTGCGCAAGGAATGCCCGTC
CAGGTACAGGCGGACCCACTTCATCTTCCTTTTGCCGATAAATCCGTTGATGTTTGTCTACTGGCACATACATTGCCGTG
GTGCACCGATCCGCATCGTTTATTGCGTGAAGCCGATCGGGTATTGATTGATGATGGCTGGCTGGTCATTAGTGGCTTCA
ATCCCATCAGTTTTATGGGATTACGCAAACTTGTGCCGGTATTGCGCAAAACCTCGCCCTATAACAGCCGGATGTTTACT
CTGATGCGGCAGCTGGACTGGCTCTCTTTGTTGAATTTTGAAGTGCTACACGCCAGCCGTTTCCACGTTCTCCCGTGGAA
CAAACACGGAGGAAAACTATTGAATGCGCATATTCCTGCGCTTGGTTGCTTACAACTTATTGTTGCCCGGAAACGGACTA
TTCCTTTAACGCTAAATCCGATGAAACAGAGTAAAAACAAGCCACGAATTCGCCAGGCGGTTGGAGCCACCCGGCAATGT
CGTAAACCACAGGCTTAAACTTCAACTTGGTAGCCTGTATCTTCCAGTGTGGGATTCATCGCCGCGGCACGAGCCAGTTC
ATCACAGCGTTCGTTTTCCGGGTGTCCGGCATGGCCTTTAACCCATTCCCATTTGATTTGATGCTGCCCCAATGCAGCAT
CAAGACGTTGCCAGAGATCGACATTTTTTACTGGTTTTTTGTCTGCGGTTTTCCAGCCACGTTTTTTCCAGTTATGGATC
CACTGGGTGATACCCTGGCGGACATACTGGCTGTCGGTACTCAAAATGACTTCGCAATGTTCTTTTAACGCCTCCAGCGC
GACAATAGCGGCCATCAACTCCATACGGTTGTTGGTGGTGCGGGTGTAGCCAGCGCTAAAGGTTTTCTCGCGTCCGCGAT
AGCGTAAAATAGCGCCGTAACCCCCAGGTCCTGGATTGCCCAGACACGAACCATCGGTGAAAATTTCTACCTGTTTAAGC
ATCTCTGGTAGACTTCCTGTAATTGAATCGAACTGTAAAACGACAAGTCTGACATAAATGACCGCTATGAGCACTGCAAT
TACACGCCAGATCGTTCTCGATACCGAAACCACCGGTATGAACCAGATTGGTGCGCACTATGAAGGCCACAAGATCATTG
AGATTGGTGCCGTTGAAGTGGTGAACCGTCGCCTGACGGGCAATAACTTCCATGTTTATCTCAAACCCGATCGGCTGGTG
GATCCGGAAGCCTTTGGCGTACATGGTATTGCCGATGAATTTTTGCTCGATAAGCCCACGTTTGCCGAAGTAGCCGATGA
GTTCATGGACTATATTCGCGGCGCGGAGTTGGTGATCCATAACGCAGCGTTCGATATCGGCTTTATGGACTACGAGTTTT
CGTTGCTTAAGCGCGATATTCCGAAGACCAATACTTTCTGTAAGGTCACCGATAGCCTTGCGGTGGCGAGGAAAATGTTT
CCCGGTAAGCGCAACAGCCTCGATGCGTTATGTGCTCGCTACGAAATAGATAACAGTAAACGAACGCTGCACGGGGCATT
ACTCGATGCCCAGATCCTTGCGGAAGTTTATCTGGCGATGACCGGTGGTCAAACGTCGATGGCTTTTGCGATGGAAGGAG
AGACACAACAGCAACAAGGTGAAGCAACAATTCAGCGCATTGTACGTCAGGCAAGTAAGTTACGCGTTGTTTTTGCGACA
GATGAAGAGATTGCAGCTCATGAAGCCCGTCTCGATCTGGTGCAGAAGAAAGGCGGAAGTTGCCTCTGGCGAGCATAAAT
ACCTGTGAAAGGCGCTAAAAATAGCGACTTGGGCGATTTTTGCAGCAAACGATTCAAAAGATGAGAAAAACCGTTGACGA
AGGTCGAGGCAATCCGTAATATTCGCCTCGTTCCCAACGGAACACAACGCGGAGCGGTAGTTCAGTCGGTTAGAATACCT
GCCTGTCACGCAGGGGGTCGCGGGTTCGAGTCCCGTCCGTTCCGCCACTATTCACTCATGAAAATGAGTTCAGAGAGCCG
CAAGATTTTTAATTTTGCGGTTTTTTTGTATTTGAATTCCACCATTTCTCTGTTCAATGATTTTACTCTGGCGTAGGTGC
GTGATTCTCGCTTGTTGTCTCATTCATTAAAATTCAATAATGATATCGAACCATTCAGCTTAAATATATTTCTAGAGAAT
AAATTTATATTGATTAAATGAATGTATATTTCAAATTGATTTTGTTTGTTATTATTTAAGTGAGGTATATAATTAGAGTC
CGTTATCAATGCTAAATATTCTAATCATTATGACAGGCGAGGGAGTGTCCAATTATGAATTCAAAAAAGCTTTGTTGCAT

ATGTGTGTTATTCTCGCTGCTTGCAGGATGTGCCTCTGAATCTTCTATTGATGAAAAGAAGAAAAAAGCTCAAGTCACAC
AAAGTAATATTAATAAAAACACTCCCCAGCAACTGACAGACAAAGATTTATTCGGTAATGAAACCACTCTGGCCGTATCC
GAAGAGGATATTCAAGCTGCGCTTGATGGAGATGAGTTCCGCGTTCCCCTTAATTCTCCTGTAATACTTGTTCAATCCGG
CAACCGCGCACCGGAAACCATTATGCAGGAGGAGATGCGAAAATATTATACTGTTTCCACATTCTCTGGTATCCCGGACA
GGCAAAAGCCTCTGACTTGTAACAAAAACAAAGATAAAAATGAGAACGAGGATGTTGCCAGTGCTGAGAATATGAACTGG
ATGCAGGCACTGCGTTTTGTGGCTGCAAAAGGACATCAGAAAGCGATTATTGTTTACCAGGATATGTTGCAGACAGGAAA
ATATGACTCTGCGCTGAAATCAACAGTATGGTCCGACTATAAAAATGACAAACTCACTGACGCTATCTCCCTGCGCTACC
TGGTACGTTTCACGCTGGTGGATGTGGCAACAGGTGAGTGGGCTACCTGGTCGCCGGTGAATTATGAATATAAAGTGCTT
CCACCATTGCCCGACAAGAATGAAGCCAGTACGACTGATATGACAGAGCAGCAAATCATGCAACTTAAGCAAAAGACCTA
TAAAGCGATGGTAAAAGATTTGGTTAACCGCTATCAATAATAAATTATATCTGCCGCCAGGAATTTCTGGCGGCAATAAT
ACAAAATTCTTTGCATAAGAAATTTTTCTTGACAAAGACAGTGCAAGAAAGGACATGGAATCCGGAGTTAAAAACTGTTT
GTTTTAGAATATTTAATTATTTAACTGCTGAATCTTCCTTGCGAGAAATATATCCATTATTACATTTCATGCCATTTTTA
ATATAGATTGCTCCTGGGAAAATTCCCGTATTATTACCGCAGGGGTGATAATTAGTATTGACATATCCTGTGACAAAAGG
AGCTATTAAAGGTGCTATTACGATAGCTATTAGTAAAAATATAAGAGTTAGCTGTATTGTTATGTCTGTGGCGAAATTGA
CTACCTTCGTTTTTTTGATTAAGAATGATTTTATTATCGTAAGTAAAATTACATGAATATTTAAAAAGGAAAACGACATG
AAACCGAAGCACAGAATCAACATTCTCCAATCATAAAATATTTCCGTGGAGCATTTTATTATTGAATATAGAGGTTTAAC
TCCGGTAAAAAACAAAGAAGCATTGAATGCAGGGAAAAATAATATGGCCATAAAAAACATCGAAAGAAACTCTTTTAATT
TAACATGTAAACGCATGGTTAATCCTCATATCACGGGTGGAGTGTTAAGAACATACATAAATGGAGTCATGTTTTCCCTT
TTCCATTTATCAAGTTCCTGTTGCCGTTTTAGTCCATCTCTAATTGCATATTTTAATTTTTCTGATAAATGGCATTGAGC
ATCGATTTCATTTAAAACAACTGTACACACTATCCCTGTCAGTAAAATTGCTCCTCCGACAAGAATTACTGGAAAGCCAA
AGCTAACACAGGCAAAAGAAGCCAGACTTCCAATAGCGGTTGCAACACCACCGGCTATAACTGCTTTGGCCACATCCATT
GAAAAATCGCCAAGAAAATTAACCAGATCTCGCTCTGAACTCATAATAAATTCTATGGTTCTGCTGATTTAACCGCAGAG
AAGTTCGCCACAGCAATCCGAAACCACTGGCACGTGGAGAATAAGCTGCACAGGCGTCTGGACGTGGTAATGAATGAAGA
CGACTACAAAATAAGAAGAGGAAACGCAGCAGAATTATTTTCAGGGATACGGCACATTGCTATTAATATTTTGACGAATG
AGAAGGTATTCAAGGCAGGGTTAAGACGTAAGATGCGAAAAGCAGCCATGGACAGAAACTACCTGGCGTCAGTCCTTGCG
GGGAGCGGGCTTTCGTAGTAATACCCCGACTCTCCCCGTCCTTAAACACAACCCCCACTCACCACAACCTAAACTCATCC
GCATCCTGCCATGCCGGAAACTTTTCTCTATATTCCCGCAATGCTGCCATCGACAGCTCCGCATCAATGCGCGTTGCCTG
ATGCGCGTCGGCAGTAGCGATAATCTCTCCTTGCGGATTAATCACCCGGCTGTCACCGCGATAATGGCAGCCGTTGCCAT
CGCTGCCGACGCGATTGCATCCCGCCACATACGCCTGATTCTCAATCGCGCGGGCCGTCAGCAATGCCTGCCAGTGCAGA
GAGCGCGGAGCAGGCCAGTTGGCGACGTACAGGGCGAGGTCATAATCGTTGAGATTGCGCGACCACACAGGAAAACGTAA
GTCGTAGCACACCAGCGGCAAAATACGCCAGCCGCGCCATTCCACAATCACTCGCGCATTGCCCGCTTTATAATGTAGAT
GCTCATCTGCCATGCGGAACAGATGACGCTTATCATAAAAATGTACCGTGCCGCCCGGCTCAACCAGCAAAAAGCGGTTA
ACCGAACCAGACTCCGTTTGTAATGCAACACTGCCTGCAATCAGCGCATTGCACTGCTGCGCCTTAGCTGTCATCCAGTT
CACTACGTCATCTTGTGCTAGCGACGAAGCTGCCGCTTCCATGGCAAAGCCGCTGGTAAACATCTCCGGTAGAACGATCA
CATCGCGCCCGGTAATACCTTCCAGTTGACGATCAAAATGACGCAGGTTGGCAGGACCATCCATCCACACCAGTGGTTGC
TGCAAAAGCGTAATCTTCAAACCAGGCACGGTGTACAACTCCTTTATGCGAAGGGTTTTATAACTTTAACACCTTATCAG
GCAGTTGCCTTAGCGCAGAATAAATTGATAACAAATGCTGATATTGGAAATATCTGATTTGCAAATTATCGTGTTATCGC
CAGGCTTTAGGAGGTTAATAACATGGGCAGGATAAGCTCGGGAGGAATGATGTTTAAGGCAATAACGACAGTCGCCGCTC
TGGTCATCGCCACCAGTGCAATGGCGCAGGATGATTTAACCATTAGCAGCCTTGCAAAGGGCGAAACCACCAAAGCTGCA
TTTAATCAGATGGTACAAGGGCATAAGCTGCCTGCCTGGGTGATGAAAGGCGGTACTTATACTCCCGCACAAACCGTAAC
GTTGGGAGATGAGACGTATCAGGTGATGAGCGCGTGCAAACCGCATGACTGTGGCTCGCAACGTATCGCTGTGATGTGGT
CCGAGAAATCTAATCAGATGACGGGGCTGTTCTCGACTATTGATGAGAAAACGTCGCAAGAGAAACTCACCTGGCTGAAT
GTGAACGATGCGCTTTCGATTGATGGTAAAACGGTGTTGTTCGCGGCGTTGACCGGCAGCCTGGAAAACCATCCGGATGG
CTTTAATTTTAAATAATTAGCGGATAAAGAAACGGAGCCTTTCGGCTCCGTTATTCATTTACGCGGCTTCAACTTTCCGC
ACTTTCTCCGGCAACTTTACCGGCTTCGTCGCCAGCTCTTCCGGATCAAAGTCATCAACGTTAATACTGCGCAGACGGCT
TTCTTCAGCTTTCACCAGAATAGCGGCTTCATCTTTATCAATCAGCCCCTTCACCAGCGCGTTGTGCGCCAGTTCATCCA
GACGGGTAAACGGCAGGTTTTTACCCAGCTCTTTACAGATCCGCTGATGAATTGGGTCGGCGGCAATCACATCCACCAGC
GCCTCTTCCAGCAAGCCAACCGGATTATGCTCGCTCGGCGTCAGGTACTGACCGCGACCAATGCGGGAACGGGTGGCGTT
CGGCACTTGTAAAATCTTCGCCACTTTATGATCCAGCTTGTCAGAAGGTGCCAGATAATGACGTCCGGTCGGGAAGATCA
CCACATTCAGCAGCCCGGCAACCACGCGGTTCGGGAAGTTTTGCAGTAAATCATCCATCGCCTGTTCAGCCTGATACAGC
GCATCTTGTACGCCCCAGTGCACCAGCGGCAGGTCGGCTTCATTACGGCCTTCGTCGTCATAACGCTTCAGCACGGCAGA
GGCGAGGTAGAGCTGGCTTAAAATATCCCCCAGACGGGCCGAGATGCGCTCGCGACGTTTCAGGCTGCCGCCCAGCACTG
CCATCGAGACATCAGAAAGCAGGGCGAGGTTGGCGCTCAGGCGGTTCAGGTGCTGATAGTAGCGTTTAGTGGCATCGCCG
GTTGGCGTGCTGCTGGTTAAACCGCGCGTCAGGCCCAGCCAGAAGCTGCGAACTTTGTTGCTACCGACGTGACCGATATG
TTTGAACAACAGTTTATCGAACGCGTTGACGTCATTGTTCTTCGCCGCTTCCATCTCTTCCAGCACGTACGGATGGCAAC
GAATCGCTCCTTGTCCGAAGATCATCATGCTGCGGGTCAGAATGTTAGCCCCTTCAACGGTGATGGCAATCGGTGCGCCC

TGGTAAGCACGCGCCAGGAAGTTGCTTTGCCCGAGCATAATGCCTTTACCGCCGGTAATATCCATCGCATCAATAATCGA
CTGCTGCCCGCGGTGGGTACAGTGATACTTAACGATAGCCGACAGCACGGCAGGTTTTTCGCCGAGCATAATGCCGTAGG
TAATCAGCGATGCCGCAGCATCCATCACGTAGGCATTACCGGCAATACGCGCCAGCGGCTCTTCAATCCCTTCCATCTTA
CCAATAGAGATTTTGAACTGACGGCGAATGTGAGCATACGCGCCGGTTGCCAGCGCTACCGATTTCACGCCGCCGGTTGA
GTTGGAAGGCAGGGTGATGCCGCGGCCTACCGAGAGGCACTCCACCAGCATCCGCCAGCCTTGCCCGGCCATTTTCGGCC
CGCCGATGATGTAATCGATCGGCACGAAGACATCTTTACCGCGCGTCGGTCCGTTCTGGAACGGTACGTTCAGCGGGAAG
TGGCGACGACCAATTTCCACGCCCGGCGTGGTGGTTGGGATCAGCGCACAGGTAATGCCTAAATCTTCTGCACCGCCGAG
TAATTTTTCCGGGTCGGAGAGTTTAAACGCCAGCCCAAGCACGGTCGCAATCGGTGCCAGCGTAATGTAGCGTTTGTTCC
AGGTCAGACGCATCCCCAGCACCTGCTGGCCCTGCCATTCGCCCATGCAGACAATCCCGGTGTCCGGAATCGCGCCCGCA
TCGGAACCCGCTTCCGGGCTGGTCAGTGCAAAGCAGGGGATCTCCTGACCACGCGCCAGACGCGGCAGATAGTGATCTTT
CTGCTCGTCAGTGCCGTAATGTTGCAACAGTTCGCCCGGGCCTAATGAGTTTGGCACGCCGACGGTAATCGCCAGGATCC
CGCTCACGCCGGAGAGTTTTTGCAGCACGCGAGACTGGGCATAAGCCGAGAACTCCAGCCCGCCGTACTCTTTTTTGATG
ATCATCGCGAAGAAACGATGCTCTTTAAGGTACGCCCACAACTCCGGCGGCAGATCCGCCAGCTCATGGGTGATCTGGAA
ATCATTCGCCATCCGGCAGGCTTCTTCTACCGGGCCGTCGAGAAACGCTTGCTCTTCGGCGGTCAGGCGCGGCTGCGGAT
AGTTATGCAGCTTTTTCCAGTCCGGCTTGCCCTGGAACAAGTCGCCCTCCCACCAGGTGGTGCCCGCATCAATCGCTTCT
TTCTCAGTGCGCGACATCGGCGGCATCACCTTACGGAAACCGCGAAATACCGGCGCGGAAATCATCGACTTACGCATAGG
CGCAAAGTTAAATGGCACGAGGATAATGGCCAGAGGCACCAGTACCCACGCCGACCACAGACCAGCAACGCCGAGGGCGG
CTGTCCAGGCGAGCAAAATCAGACTGCTGATAAATAAGCTCACGCGGTGATAGAACAACGCGCCGAGCAGGACAACCGTA
GCGAGAATACTCAAAATCATCATAACGAAAAGCCCCTTACTTGTAGGAGGTCTGACCACTTGTGATGATATGGTTGTAGT
GGATGTAAAAACATTTAGCAATATGTTTACAATATAATTACAACAAAGCTCACATTGTTGCTGTTTTTATCCGCACTTCA
GGTCAAAAAGTCCTGGTCATAGCACCTGCCCGTACTTCTCGCTTTTGGCGGTATCCGGTACACTGCATTTTGTCTATTAC
ATTTATGCTGAAGGATATCCTCATGTACCAGGATCTTATTCGTAACGAACTGAACGAAGCGGCGGAAACGCTGGCTAACT
TTTTAAAAGATGACGCCAATATTCACGCCATTCAGCGCGCGGCGGTCCTGTTAGCAGACAGCTTTAAAGCCGGTGGCAAA
GTGCTTTCCTGCGGCAACGGCGGTTCCCATTGCGACGCTATGCACTTTGCCGAAGAGTTGACCGGTCGCTACCGTGAAAA
CCGTCCGGGCTACCCGGCGATTGCTATTTCTGACGTTAGTCATATTTCCTGCGTCGGTAATGATTTCGGTTTCAATGATA
TTTTCTCCCGCTACGTTGAAGCGGTAGGTCGCGAAGGCGATGTACTGCTGGGGATCTCCACCTCCGGTAACTCTGCAAAC
GTGATCAAAGCGATCGCAGCGGCGCGTGAGAAGGGAATGAAAGTGATCACCCTGACCGGTAAAGACGGCGGCAAAATGGC
TGGCACGGCGGATATCGAAATTCGCGTACCGCACTTTGGTTATGCCGACCGCATTCAGGAGATTCACATTAAAGTGATCC
ATATCCTGATCCAGTTGATTGAAAAAGAGATGGTTAAGTAAGTCTGGCGTAGGCCGGATAAGACGTTTACGCCGCATCCG
GCATTTGTGCGCTGATGCCTGATGCGACGCTGACGCGTCTTATCATGCCTACAAATCTGTACGCGAACCGTAGGCCGAAT
AATGCGTTCACGCCGCATCCGACCTGAAAATTCTTAAATCAATCTTCGCCGGGGGCCATGCGCTCCCGCTGTTGTGGAGG
TTACCCATGTGCGAATTGCTCGGGATGAGCGCCAACGTCCCTACCGATATCTGCTTTAGTTTCACCGGGCTTGTACAGCG
TGGTGGTGGAACCGGGCCACATAAAGATGGCTGGGGCATTACCTTTTACGAAGGTAAAGGCTGTCGCACATTTAAAGATC
CACAACCCAGCTTTAATTCCCCCATCGCCAAACTTGTCCAGGACTACCCGATAAAATCCTGTTCGGTGGTGGCTCATATT
CGCCAGGCTAATCGGGGCGAGGTGGCGCTGGAAAATACTCACCCATTTACCCGCGAGTTATGGGGGCGTAACTGGACTTA
TGCCCATAACGGACAACTGACGGGCTACAAATCACTGGAAACCGGCAACTTCCGCCCGGTAGGCGAAACCGACAGCGAAA
AAGCCTTCTGCTGGCTCCTGCATAAATTAACGCAGCGTTACCCGCGCACACCGGGCAACATGGCGGCGGTATTTAAATAT
ATCGCCTCACTGGCGGATGAACTGCGGCAGAAGGGCGTTTTCAACATGCTGCTTTCGGACGGGCGCTATGTAATGGCGTA
TTGCTCGACTAATTTACACTGGATCACCCGCCGCGCGCCGTTTGGCGTGGCAACGTTGCTGGATCAGGATGTGGAAATCG
ACTTCAGCTCGCAGACCACACCGAATGATGTGGTCACGGTGATTGCAACACAGCCGCTGACGGGCAATGAAACCTGGCAA
AAGATTATGCCAGGCGAATGGCGCTTATTTTGCCTCGGGGAGCGTGTAGTTTGATGCCAGTTGTGGCTGCACAACTTCGT
GGCTTAACGGCTTGCTGACCACGTAACGACCATTGACCACAGAAACGGTTGGTGGCTTACGGGTTTGCTCAAAGTAGTCG
TAACCCGGCTTCAGTTGCTCCCAGAAGTCCTTAAAGTTGGAATATTTATGGCGCTTCATATTGGCGTCGGTCATGCGGAA
CGGGTAAATACTCACTTGCACGCTCGGCTGACCAAACACCAGCGCACCAGTAACGAACTGGAATATCTCATCAATACCCT
GATTGGTCATTGCGTAGCAGCCGATGGAAACACAATCGCCGTGGATCATCAGGTATTTCCCTTCATAACCATGCGCACGG
TCATAGGCATTGGGGAAACCAATATTAATCGCTTTGTAGTAACGGCTGTCTGGTTTTAACTGATTACGCTGGACGCTATA
AAACCCTTCCGGGCTTTTGAAATCGCCCTGACGCTGTTTTGGCCCTAAGCCGCCGGAATATTTACAGATTTTATAGCTGT
CGAGCAGTTGATATTGCTCGCCCATTTTGACGTAGAGATCGAGCGTACGTTCTTCCTTGAAGATCTGGATGTAGACAGGG
GATCCCATCAACTGCTGCTTATACTCTTTGCTCACAGGCGTGGTAGAGCTACTGCTGCCCAGCAAACCGGCAAATGAAAC
GCACGGGATCAACAACATCGCAAGAATTAATGCGATTTTACGCATACTGCTTATTCCTTGATAAAACGGTTACACACGCC
AGGACGGCAAAATGGATCCCAAATCGGAATAGTCTGGATTTGGAAGGCTCACATTATCACCAAAAGAGTTTTACGCAAGC
CTGTCGGCGCAGGGTTTACAAATTTCATCGGAAGCGGGCTTTACCCAAAGAGCGCCGCGTGAGTTCCAGTTCTCTCAAAT
CGTAAAAGTTTATCGGTAAGTTTGTAAATCAGGATCCAGTCCGGTTCGACATGAGCATCGCGATAACCTTTCCATGAACC
TTGCAGCGGGTGGTCTTTATAAACAGCTGGAAGCGGTAAAGTATTATTGATAAGAAGCGTCATAAGATATTTCAATTTAT
TCATATCCTTATGACGCTTTTGTGCAAGTTTTACATCCTTTGAATATTGTCCCGAGTATTCAATATCCCTTTGAATCATA
TTTAAATTCCTAATTTATCAAATAAATCATCGGCGTCTTTGGCCTTATGAACATCAATGCCAGCTTCGCTGTTTTTGATT

GATTGAATGGTTAATTGATTAGGCTCGCGTAAATCAAACGGCAATGCCTTTTCACGCGCGACCTTTGTGAGGGTTATGCG
AACCAGGTCAGAGATGGTCAGCCCCATCCCGGCCAGTACGTCCGCTGCCTGATTCTTCAGATCTTCATCGATTCGGGCGC
GAACAAACGCGTTAGCAGCCATTGGGGTATCTCCTGTGCTGTATATTTATTCAGCTTGAATTGTAGCTCAAATGAGCAAC
AGCGACAATCCATGAGTAATTTAATAAACGGATGTTTTTACATAATTAACTGAAATATATATGTTAATTTTATAATAATG
GTTTATTAAGTTTGTTCAGAAACGATCCGGGATACACTGCAACATTAAAGCATGACCAGCCATTATTGAGTATGTCCTTG
CCGTCGATTCCATCCTTTGTATTGTCGGGATTACTGTTGATTTGTTTGCCGTTTTCTTCATTTGCCAGCGCCACCACATC
ACATATCTCTTTCAGCTACGCCGCCCGCCAGCGGATGCAAAACCGTGCGCGTTTATTAAAACAGTACCAAACTCATCTGA
AAAAGCAGGCCAGCTATATTGTGGAAGGCAATGCCGAAAGCAAAAGGGCGCTACGCCAGCACAACCGGGAGCAGATAAAA
CAGCATCCAGAATGGTTTCCTGCTCCGCTCAAGGCGAGTGACAGACGCTGGCAGGCGCTGGCGGAAAACAACCACTTTTT
AAGCAGCGACCATCTGCATAACATTACCGAAGTGGCGATTCACCGCCTGGAGCAGCAGCTTGGCAAGCCTTACGTCTGGG
GCGGTACGCGGCCTGATAAAGGCTTTGACTGTAGCGGGTTGGTTTTTTATGCCTACAACAAGATCCTTGAGGCTAAGCTC
CCGCGCACGGCCAATGAGATGTACCACTATCGCCGGGCAACGATTGTGGCGAACAACGACCTGCGCCGGGGAGATTTGCT
GTTTTTCCATATCCACAGCCGCGAGATAGCCGATCATATGGGCGTGTATTTGGGCGATGGGCAATTTATCGAGTCGCCAC
GTACCGGCGAAACCATTCGGATAAGCCGATTAGCCGAACCTTTCTGGCAGGACCATTTTTTGGGCGCGCGCAGGATTTTG
ACGGAAGAGACGATTTTGTAGGACGGATAAGGCGTTTACGCCGCATCCGGCAGTTGTACGCAGGTGCCTGATGCGACGCT
GGCGCGTCTTATCATGCCTACGAGCCCGCGAATATTTGCGAGCCGCTTTCCCGATATAAAACAACCTCATTGCCAACCTT
TCCTTTTCTTCTTACCGTTGAGAAAAAGGAGTCGCCATGTCTGAATATCGTCGTTATTACATCAAGGGGGGAACATGGTT
TTTCACGGTGAATTTACGAAATCGTCGAAGCCAACTTTTGACCACCCAGTACCAGATGCTCCGTCACGCCATTATTAAAG
TTAAGCGAGACAGGCCTTTTGAAATCAACGCCTGGGTCGTTTTGCCAGAGCATATGCACTGTATCTGGACATTACCTGAA
GGCGATGATGATTTTTCCTCGCGCTGGCGGGAAATTAAAAAGCAATTTACCCATGCTTGTGGATTGAAAAATATCTGGCA
ACCACGTTTTTGGGAGCACGCCATCCGCAATACCAAAGATTATAGGCATCATGTTGATTATATTTATATAAATCCAGTAA
AGCATGGTTGGGTAAAGCAAGTGAGTGATTGGCCATTCTCAACGTTCCATCGCGATGTCGCGCGAGGGTTATATCCCATC
GATTGGGCGGGGGACGTAACGGATTTTAGTGCCGGGGAGCGTATCATTTCATAATTGTGCGCAGATGCCTGATGCGACGC
TAGCGCGTCTTATCATGCCTACAAACTTGTGCCGGATCGGTAGGCCGGATAAGGCGTTTATGCCGCATCCGGCAGTTATG
CGCAGATGCCTGATGCGACGCTGGCGCGTCTTATCATGCCTACAAACTTGTGCCTGAACGGTAGGACGGATAAGGCGTTC
ACGCCGCATCCGGCAGTTGTGCACCAATGCTGGATACTTACATCAACGCCCCCATAATCTTCAACTCCAGCTCATCCGGC
ACTTCGTTATACGACAGCACATGCAGCCCCGGCGCAAACAACCTTGCATAACGCGCCAGCAAAGGGCGCAGCTGCGGTGG
CACCAGCAGCACCGGGTCTTTCCCCGCCGCTTTCATCTGCTCTTTCACCTGTGGCATCGTACTCTGGAACTGGTTGAGCA
TATTCGGATCGACCGGCACGCTGTCGAGCATCACTTTCCCGCCCTGTTGCGCCTGATTCACCACGTTGGTCAGCAGATTT
TCCAGCTCATTATTCAGCGTATACACCGTCAGCTCCTGCTTGCGAACGAACGGATGGGTAATGCTGCGCCGCAATGCCAG
GCGCACATCGGCCGCCAGCAGAATATGATCTTTCGTCACCGTGCTACTGGCGACCAGCACGGTGGCGATGGTGACGATAT
CGCGCAGGGAAACGCCTTCGGTCAGCAGCGCCCGGTACACTTTCAGCAACTGGCTGTAATTGAGCGCCGCGCTCAAATCT
TCCGCCAGACGCGGTGCCGTCGACGATAAACGGTTATGCAACTGCGTAATATCATCATAGTTAAACAAATCAGGAATATA
GCTGCGCACAATCTTATTTACATGGGTAGCGATCACGCTGGCGCTGTCGATCACCTGATACCCCATATTCAGCGCCTTCG
CTTTCTGCGCCGCCTGGATCCAGGTGACCGGCATCCCGTACGCCGGGTCGTTCCCCTGCACGCCGTCAATCTCGCCGTAG
GTTTCGCTGGAGGGCAGGGCCATCAGTTTATCCGCCGGAATATCCGCTTCATCAGCCTTTATGCCGTTGATGAAAATGGC
GTACTGACTGGGCTTAAGGCGGAAGTTTTCCCGAATGCGGATCTCCGGCAGCAGCACGCCGTTGCCGTCAGAAATCACCT
GCCGCACGCCGCGAATCCGCTGGGTGAGCGGGTTACCCTGTGCTTTGTCCACCAGCGCCACCAGTTTGTAACCGAGGCTT
AAACTGATAGGCTCGATCAGCGGAATGGTTTCCCAACTGACCTGCTGCTCGCTGGTTTCGGTGATGGTGCGGGTCAGCGT
TTCGAGGCTTTTCTCTTCCGCCTCCGCCGCCAGCGGCTGTTTGCTCATCCGCCAGCCGGTAAAACCAAGCAGGGCGCTGA
ACAGCAAAAACGGCAGGTGCGGCATTCCCGGTACCACCGCCAGCACGAACATAATCCCGGTGGCGGTGTAGAGCACCGAC
GGGCTTGCCAGCAGCTGGTTACGCACGTCATGGGCGATATCGCCGTTGTCGCTGACGCGGGTGACGATAATCGCCGCCGC
GGTGGAGAGCAGCAGGGAAGGGATCTGCGCCACCAGGCCGTCGCCGATGGTCATCAGCACATACTGCTGGAAGGCAGCAT
CGGCGCTCAGGTTGTATTTGAAGATCCCGATACAGACGCCGCCGATCAGGTTGATCGCCAGAATCATCATCCCGGCGATG
GCGTCCCCGCGCACAAACTTCGATGCCCCGTCCATCGCGCCGTAGAAATCGGCCTCGCTGGCAACATCTTTACGCCGGGT
TTGCGCCTGCGCCTGGTTGATCAATCCGGCGTTAAGATCGGCGTCAATCGCCATCTGTTTGCCGGGCATCGCGTCTAAGG
TAAAGCGGGCAGAAACCTCGGAAATACGCTCGGCCCCTTTGGTGACGACAATAAAGTTGATTGTCAACAGCGTCAGTAAA
TCCGAGCGTGAAAGCATTATCGCCGCGCTGCACGGGCAGTCGATTTTTAACGGTGGTGGGCTGTCGCCGTTAAATAAAAT
CAGCCCGTCTCATCCGCCAAAACCTGCCACCGTGGCGGTGCCGGAAGAGACGGAGAAAAAAGCGCGTGATGTGAATGAGA
AAACGGCGCTGCTGAAGAAGAAATCCGCCACCGAGCTTGGTGAGCTGGCAACCAGTATCAACACCATTGCCCGTGATGCG
CATATGGAAGCGAATCTGGAGATGGAGATTGTTCCCCAGGGATTACGCGTGCTGATTAAAGACGACCAGAACCGCAATAT
GTTTGAACGCGGCAGCGCGAAGATTATGCCGTTCTTTAAAACGCTGCTGGTGGAGCTCGCGCCAGTGTTCGACTCGCTCG
ATAATAAAATTATTATTACCGGGCATACCGATGCGATGGCCTACAAAAACAATATCTACAACAACTGGAACCTTTCGGGT
GACCGCGCGCTTTCGGCTCGTCGGGTGCTGGAAGAGGCCGGAATGCCGGAAGATAAAGTGATGCAGGTAAGCGCAATGGC
GGACCAGATGCTGCTGGATTCCAAAAATCCGCAAAGCGCGGGCAACCGGCGCATTGAGATTATGGTGCTGACCAAAAGTG
CGTCCGATACGCTGTATCAATACTTTGGTCAGCATGGGGATAAAGTGGTGCAGCCGCTGGTGCAAAAGCTGGATAAGCAG

CAGGTGCTTTCGCAGCGAACGCGTTAAATGCTGAATCTTTACGCATTTCTCAAACCCTGAAATCACTGTATACTTTACCA
GTGTTGAGAGGTGAGCAATGCGTAAAATCATTCATGTGGATATGGACTGCTTTTTCGCCGCAGTGGAGATGCGCGACAAT
CCCGCCCTGCGCGATATCCCTATTGCTATTGGCGGCAGCCGCGAACGTCGGGGGGTGATCAGCACCGCCAATTATCCCGC
GCGTAAATTTGGCGTACGTAGCGCTATGCCGACAGGGATGGCGCTCAAATTATGCCCACATCTCACCTTGCTTCCGGGGC
GCTTTGACGCCTACAAAGAAGCCTCAAATCATATCCGTGAAATCTTCTCGCGCTACACCTCGCGCATTGAACCGTTGTCA
CTGGATGAGGCTTATCTCGATGTCACCGATAGCGTCCATTGCCACGGTTCTGCGACCCTCATCGCCCAGGAAATCCGCCA
GACAATCTTCAACGAGCTGCAACTGACGGCGTCTGCGGGCGTGGCACCAGTAAAGTTTCTCGCCAAAATCGCCTCCGACA
TGAATAAACCCAACGGCCAGTTTGTGATTACGCCGGCAGAAGTTCCGGCATTTTTACAAACCTTACCGCTGGCAAAAATC
CCCGGCGTCGGCAAAGTCTCAGCGGCAAAACTGGAAGCGATGGGGCTGCGGACCTGCGGTGATGTACAAAAGTGTGATCT
GGTGATGCTGCTTAAACGCTTTGGCAAATTTGGCCGCATTTTGTGGGAGCGTAGTCAGGGGATTGACGAACGCGATGTTA
ACAGCGAACGGTTGCGAAAATCCGTCGGCGTGGAACGCACGATGGCGGAAGATATTCATCACTGGTCTGAATGTGAAGCG
ATTATCGAGCGGCTGTATCCGGAACTTGAACGCCGTCTGGCAAAGGTAAAACCTGATTTACTGATTGCTCGCCAGGGGGT
GAAATTAAAGTTCGACGATTTTCAGCAAACCACCCAGGAGCACGTCTGGCCGCGGCTGAATAAAGCTGATCTAATCGCCA
CCGCGCGTAAAACCTGGGATGAACGCCGCGGCGGGCGCGGTGTGCGTCTGGTGGGGCTGCATGTGACGTTGCTTGACCCG
CAAATGGAAAGACAACTGGTGCTGGGATTATGATGTATACTATTATGTATATTCTGGTGTGCATTATTATGAGGGTATCA
CTGTATGCATCGAATTCTCGCTGAAAAATCGGTCAATATCACTGAGTTACGTAAAAACCCAGCTAAATACTTTATTGATC
AACCGGTTGCGGTTCTTTCTAATAATCGCCCCGCAGGATATCTCTTAAGTGCCAGCGCATTCGAAGCGTTAATGGACATG
CTTGCTGAACAAGAGGAGAAAAAGCCCATAAAGGCGCGCTTCCGTCCAAGTGCTGCAAGATTAGAGGAAATTACACGCCG
CGCTGAACAATATCTTAATGATATGACGGATGATGATTTCAATGACTTTAAGGAATAAGGATGCGGGTATTCAAAACAAA
ACTTATTCGCCTGCAACTTACAGCAGAGGAACTTGATGCGTTAACGGCGGATTTTATTTCCTATAAGCGTGACGGTGTTT
TGCCAGATATATTTGGTCGCGATGCACTCTACGACGACTCCTTTACCTGGCCATTAATCAAATTTGAGCGAGTTGCTCAT
ATTCATCTGGCAAATGAGAATAATCCATTTCCGCCACAGTTGCGCCAATTCAGCAGAACGAATGACGAAGCGCATTTGGT
ATATTGTCAGGGGGCGTTTGATGAGCAAGCATGGTTGCTCATTGCCATTCTGAAACCTGAACCTCATAAACTGGCTCGAG
ATAACAACCAAATGCATAAAATTGGGAAAATGGCAGAAGCGTTTCGCATGCGTTTTTGAATTTATATTATGAATAACATA
CAAATAAGAAACTATCAGCCTGGCGATTTTCAGCAACTATGCGCTATTTTCATTAGAGCGGTTACGATGACCGCCAGTCA
GCATTATTCACCACAACAAATTTCCGCCTGGGCGCAGATTGACGAATCTCGCTGGAAGGAGAAACTCGCGAAATCACAAG
TGTGGGTTGCGATCATTAATGCACAACCGGTTGGTTTTATTTCCCGCATTGAACATTATATCGATATGTTATTTGTTGAC
CCTGAATACACCCGCCGTGGGGTTGCCAGCGCTTTGTTAAAACCTTTGATTAAGTCTGAATCCGAACTTACGGTGGACGC
AAGCATAACCGCAAAACCCTTTTTTGAACGTTATGGTTTTCAGACAGTTAAGCAGCAGCGCGTTGAATGCCGGGGAGCGT
GGTTTACTAATTTTTATATGCGATATAAACCGCAACATTAAATCCAGCTTGCAATGAAAATAACGCCCGCCTGGTATGTG
CGCGTTTTCTGATTCCACAAACTGCAAGGAGGTAAATCATGACAAATCCTTTATCAATGACTCTTTGCAGACCTTTCCAG
GATTAATTCTTTTTTTCTTGCCCTGGATTCGTCTGCCATTTCCTGATTTTTATATTTATATACTCTAAATAATTCGAGTT
GCAGGAAGGCGACAAGCGAGTGAATCGCCAGGAGCTTACATAAGTAAGTGACTGGGGTGAACGAACGCAGTCGCAGTACA
TGCAACTTGAAGTATGACGAGTATAAATGGAATGGTACATGGGCAAATATATTCGTCCCTTATCCGATGCGGTATTTACC
ATCGCATCTGATGACCTGTGGATCGAGAGTTTAGCGATCCAACAATTACACACCACGGCAAATTTACCCAACATGCAGCG
CGTAGTGGGGATGCCAGATTTACACCCCGGACGCGGCTACCCGATTGGCGCAGCGTTCTTCTCTGTTGGTCGTTTTTACC
CGGCAAGACGTCGCGGTAACGGTGCTGGAAACAGAAACGGGCCGCTACTCTGACACACTGCGTTCGGCGCTGGTTTCTCT
CGATGGCGATAACGCATGGGCATTAAGCGAAAGCTGGTGTGGCACTATTCAGTGGATTTGTCCGAGTCCGTATCGGCCTC
ATCATGGGCGCAAAAACTGGTTTCTGGGCATTGGGCGTTTTACCGCTGATGAGCAGGAACAATCGGATGCAATCCGTTAT
GAGACGCTGCGTTCGTCGGGGCCGGGCGGTCAACATGTCAATAAAACCGACTCGGCGGTACGCGCCACGCATTTGGCATC
CGGTATTAGCGTGAAGGTTCAGTCAGAGCGTAGTCAGCATGCTAACAAGCGGCTGGCACGATTGCTGATTGCCTGGAAGC
TGGAGCAACAGCAACAGGAAAATAGCGCGGCGCTGAAATCGCAGCGGCGAATGTTCCATCACCAGATTGAACGTGGCAAC
CCGCGACGGACATTTACAGGGATGGCTTTTATCGAAGGATAATGAAGGATGAAACTGCCGGAAGGCGATTAAACGCCATC
CGGCAGCAAATCAAATAATTACTTCGCCGGAATTTCTTTCAGCAGTTCAGTCAGCAGTGTCCAGTAATGACCTACGCTTT
CGATGTGAACTTGCTCATCCGGAGAGTGTGGACCGGTGATAGTTGGCCCGATAGAAACCATGTCCATTTCCGGATACGGT
TTTTTGAACAGACCACATTCCAGGCCCGCGTGGATAATCTGGATGTTCGGCGTCTTGTTGAACAGGCGCTGATAGGTTTC
ACGTACCAGATGCATCACCGGAGAATTAGCGTCCGGCTGCCAGCCAGGATATGCGCCTTTCGCTTCGGTTTTCGCGCCAG
CCAGTTTACCCAGCGAATCCAGCATGCTCACCACGTAGTCTTTACCGCTGTCGATCAGTGAACGGATCAGGCAGTGAATT
TCTACGTTATTGTCAGTCATGGTCACCACACCGACGTTCAGGGAGGTTTCAACCACACCTTTGGCTACATCGGAGTTACG
AATCACACCGTTCGGGGTGGCGTTCAGCAGACGAATAAAGGTATCGCGAGATTTCGCAATCAGGGCAGCTTTATCGTTCG
CTACAGAGTCCAGCAACAAGGCCAGATTTTTCTCTTTTTCTGCCAGCTCGTTTTTCAGGATCTCCTGATAGGTATTCACC
AGAGATTTCAGGACGTCGACTTTATCAGCTGCGACAGCAATGGTCGCAAAGGCTTCACGCGGGATGGCGTTACGCAGTGT
GCCGCCGTTGAAATCGATAAGGCGCAGATCCAGTTCTTCCGCATGACCCGCCAGGAAGCGCACCAGCAGTTTGTTGGCAT
TACCCAGCCCAACGTGGATTTCCCCGCCGGAGTGACCGCCTTTCAGACCTTTTAAGGTTAACTTGAAGGTTTCAAAACCA
GCTGGAACCGCTTCACGATCTAAATGCAGGTTGGAGGTGAAGTCGATACCCCCCGCACAACCCATGTAGATTTCACCTTC
TTCTTCGGAGTCGGTGTTAATCAGAATATCAGCCTGCAACCAGTTGCCCTGTAAGCCGAACGCACCGTCCATACCGGCTT

CTTCGGTCATGGTCAGCAGCACTTCCAGCGGGCCGTGAACCACGTTTTCGTCAGCCAGAACCGCCAGCGCAGAGGCCATA
CCAATGCCGTTATCCGCACCCAGCGTGGTGCCGCGCGCTTTAACCCATTCGCCATCAATATAAGGCTGGATAGGATCTTT
CGTGAAGTCATGCACGGTGTCGTTATTTTTCTGCGGCACCATATCGAGGTGGGCCTGTAAGACGACCGGTTTACGATTTT
CCATACCTGCGGTAGCAGGTTTACGAATCAGGATATTACCTACCTGATCGCGTTCGACATGGAAACCTTTCTCTTTTGCC
CAACCAACAATGTATTCAGCGAGTTGCTCTTCATGATAGGACGGGTGAGGAATAGAACAGATTTTGGCAAAAATATCCCA
CAGCGGCTGTGGAGATAATTGAGACAGTTCAGACACGTTAAGTCTCCTTGTCGATCACCCGCAAAACAGTATTGCAGGTC
ACAGGGTTAGCAGAAAATGTTGTCAACACAAGACAGGCTTGCGAGATATGTTTGAGAATACCACTTTATCCCGCGTCAGG
GAGAGGCAGTGCGTAAAAAGACGCGGACTCATGTGAAATACTGGTTTTTAGTGCGCCAGATCTCTATAATCTCGCGCAAC
CTATTTTCCCCTCGAACACTTTTTAAGCCGTAGATAAACAGGCTGGGACACTTCACATGAGCGAAAAATACATCGTCACC
TGGGACATGTTGCAGATCCATGCACGTAAACTCGCAAGCCGACTGATGCCTTCTGAACAATGGAAAGGCATTATTGCCGT
AAGCCGTGGCGGTCTGGTACCGGGTGCGTTACTGGCGCGTGAACTGGGTATTCGTCATGTCGATACCGTTTGTATTTCCA
GCTACGATCACGACAACCAGCGCGAGCTTAAAGTGCTGAAACGCGCAGAAGGCGATGGCGAAGGCTTCATCGTTATTGAT
GACCTGGTGGATACCGGTGGTACTGCGGTTGCGATTCGTGAAATGTATCCAAAAGCGCACTTTGTCACCATCTTCGCAAA
ACCGGCTGGTCGTCCGCTGGTTGATGACTATGTTGTTGATATCCCGCAAGATACCTGGATTGAACAGCCGTGGGATATGG
GCGTCGTATTCGTCCCGCCAATCTCCGGTCGCTAATCTTTTCAACGCCTGGCACTGCCGGGCGTTGTTCTTTTTAACTTC
AGGCGGGTTACAATAGTTTCCAGTAAGTATTCTGGAGGCTGCATCCATGACACAGGCAAACCTGAGCGAAACCCTGTTCA
AACCCCGCTTTAAACATCCTGAAACCTCGACGCTAGTCCGCCGCTTTAATCACGGCGCACAACCGCCTGTGCAGTCGGCC
CTTGATGGTAAAACCATCCCTCACTGGTATCGCATGATTAACCGTCTGATGTGGATCTGGCGCGGCATTGACCCACGCGA
AATCCTCGACGTCCAGGCACGTATTGTGATGAGCGATGCCGAACGTACCGACGATGATTTATACGATACGGTGATTGGCT
ACCGTGGCGGCAACTGGATTTATGAGTGGGCCACCCAGGCGATGGTGTGGCAACAAAAAGCCTGTGCGGAAGACGATCCG
CAACTCAGTGGTCGTCACTGGCTGCATGCGGCTACGTTGTACAACATTGCCGCCTATCCTCATCTGAAAGGAGATGACCT
GGCCGAGCAAGCGCAGGCTTTGTCAAACCGCGCCTATGAAGAGGCCGCTCAGCGTCTACCGGGCACGATGCGGCAGATGG
AGTTTACCGTACCCGGCGGTGCGCCCATCACCGGCTTTTTGCATATGCCGAAAGGCGATGGCCCGTTCCCGACGGTATTA
ATGTGTGGTGGTCTGGATGCGATGCAGACGGACTATTACAGCCTGTATGAACGTTATTTTGCGCCGCGCGGCATTGCGAT
GCTGACTATTGATATGCCGTCGGTGGGCTTTTCTTCAAAATGGAAGCTCACCCAGGACTCCAGCCTGTTGCATCAGCACG
TCTTAAAGGCGCTGCCTAACGTACCGTGGGTGGATCACACTCGCGTCGCGGCCTTTGGTTTCCGTTTCGGCGCTAACGTT
GCCGTGCGTCTGGCATACCTTGAATCGCCGCGTCTGAAAGCGGTTGCCTGTCTTGGTCCGGTAGTTCATACCCTGTTGAG
TGATTTTAAGTGCCAGCAACAGGTGCCGGAAATGTATCTTGACGTTCTGGCCAGTCGTTTGGGGATGCATGATGCTTCCG
ATGAAGCGTTGCGCGTGGAGCTGAATCGCTATTCATTAAAAGTGCAAGGATTGCTGGGACGTCGCTGCCCAACGCCAATG
TTATCAGGCTACTGGAAGAACGATCCGTTCAGCCCGGAAGAGGACTCACGCTTAATCACCTCATCATCTGCTGACGGTAA
ATTATTAGAGATCCCATTTAACCCGGTGTATCGGAATTTTGACAAAGGTCTTCAGGAAATCACCGACTGGATCGAAAAAC
GCTTGTGTTAAAAATTTGCTAAATTTTGCCAATTTGGTAAAACAGTTGCATCACAACAGGAGATAGCAATGACGTTACCG
AGTGGACACCCGAAGAGCAGATTGATCAAAAAATTTACCGCACTAGGCCCGTATATTCGTGAAGGTAGGTAATGACTCCA
ACTTATTGATAGTGTTTTATGTTCAGATAATGCCCGATGACTTTGTCATGCAGCTCCACCGATTTTGAGAACGACAGCGA
CTTCCGTCCCAGCCGTGCCAGGTGCTGCCTCAGATTCAGGTTATGCCGCTCAATTCGCTGCGTATATCGCTTGCTGATTA
CGTGCAGCTTTCCCTTCAGGCGGGATTCATACAGCGGCCAGCCATCCGTCATCCATATCACCACGTCAAAGGGTGACAGC
AGGCTCATAAGACGCCCCAGCGTCGCCATAGTGCGTTCACCGAATACGTGCGCAACAACCGTCTTCCGGAGACTGTCATA
CGCGTAAAACAGCCAGCGCTGGCGCGATTTAGCCCCGACATAGCCCCACTGTTCGTCCATTTCCGCGCAGACGATGACGT
CACTGCCCGGCTGTATGCGCGAGGTTACCGACTGCGGCCTGAGTTTTTTAAGTGACGTAAAATCGTGTTGAGGCCAACGC
CCATAATGCGGGCTGTTGCCCGGCATCCAACGCCATTCATGGCCATATCAATGATTTTCTGGTGCGTACCGGGTTGAGAA
GCGGTGTAAGTGAACTGCAGTTGCCATGTTTTACGGCAGTGAGAGCAGAGATAGCGCTGATGTCCGGCGGTGCTTTTGCC
GTTACGCACCACCCCGTCAGTAGCTGAACAGGAGGGACAGCTGATAGAAACAGAAGCCACTGGAGCACCTCAAAAACACC
ATCATACACTAAATCAGTAAGTTGGCAGCATCACCTGAAGGTAAGTGCAAAGATAATCGATTCTTTTTCGATTGTCTGGC
TGTATGCGTCAACGTGAAACCGGCACCGGAAGTGCGTGAATTCTGGGGCTGGTGGATGGAGCTGGAAGCGCAGGAATCCC
GTTTTACCTACAGTTACCAGTTTGGTCTGTTCGATAAAGCAGGCGACTGGAAGAGTGTTCCGGTAAAAGACACTGAAGTG
GTTGAACGACTGGAGCACACCCTGCGTGAGTTTCACGAGAAGCTGCGTGAACTGCTGACGACGCTGAATCTGAAGCTGGA
ACCGGCGGATGATTTTCGTGACGAGCCGGTGAAGTTAACGGCGTGAGTGAAATGTGCCGGATGCATCACATCCGGCAATA
TTCATTAAAACTGATACGTCATGCCAACCGCGACAATATCATCATTATTAATATTCAATTTGTTATCGCTATCCAGTTGG
TTGATTTTATAATCAACAAACGCTGACATATTTTTGTTGAAATAATACGTAGCACCGACGTCGATATAATTGACCAGATC
TTCATCACCGATACCTTCAATATCTTTCCCTTTCGATAAGACATAACCCAGCGATGGACGCAGACCAAAGTCAAACTGGT
ATTGAGCGACCGCTTCAAAGTTCTGTGTCTTATTGGCAAAGCCGCCAGTTATTGGCGTCATTTTGCGTGTTTCAGAATAG
AAAGTTGCCAGATAAATATTATTGGCATCGTATTTCAGACCTGTTGCCCATGCTTCTGCACGCTTGCCTGTGCCACGGCT
TTGCAGGTTCTGCTCGTTGGTGCGATCTGAGTTGGTATAGGCCCCACTAATGGCGAAATCGCTGCCGCCAAAGTCATATG
TCAATGACGTGCCGAAGCCATCGCCGTTTTGCTTTTTAACGTCGCGGTTTTCGTTTTTCCCTTGATATTGCAGGGTTAAG
TTCAGGCCATCGATAACGCCGAAGAAGTCGGTGTTCCGATACGTCGCCAGACCGCTGGCGCGTTTGGTCATAAAGTTGTC
GGTCTGCGCCGAGGAATCGCCACCAAATTCCGGGAACATATCGGTCCAGGCTTCCACGTCATACAACGCCCCCAGGTTAC

GACCATAATCGAAAGAACCCAAATCTTTATATTTCAACCCGGCAAAAGCGAGACGCGTTTTTTGCTGTGCAGTATCACTC
TCTGCTTTATTACCGGCAAACTCTGCTTCCCAACGACCATAACCAGTCAGTTGATCGTTAATTTGTGTTTCGCCTTTGAA
ACCAAAACGGATATAACTCTGGTCGCCATCTTTACTGGCGTTATCACTCATATAATGCATGGCTTTAACTTTGCCATAGA
CATCCAGTTTATTACCGTCTTTATTATATATTTCTGCAGCCTGTACAGATGCAGATGCCACAATGCCCATCACCACTAAT
GCCAGAGTGCTCTTTTTCATTTTCATTCCTGATTTTAATTAACGCGCGAATATTCAGCGGGAGAGTCCCGTTGAAAACAG
GAAAGTTTTTAACCTGAGATTGTTAAAGATATATTACAGATTAATAATATTCTTAAAATGTGGTAATTTATTAAATCTGT
AATAAAAGCGTAAACAACTGCCGCTAGGCTTGCTGATCCCGCGCAACAAAACGCCATGCTTTGCTCGCAGATGGTTGGCA
ACCGACGACAGTCCTGCTAAAACGTTCGTTTGATATCATTTTTCCTAAAATTGAATGGCAGAGAATCATGAGTGACAGCC
AGACGCTGGTGGTAAAACTCGGCACCAGTGTGCTAACAGGCGGATCGCGCCGTCTGAACCGTGCCCATATCGTTGAACTT
GTTCGCCAGTGCGCGCAGTTACATGCCGCCGGGCATCGGATTGTTATTGTGACGTCGGGCGCGATCGCCGCCGGACGTGA
GCACCTGGGTTACCCGGAACTGCCAGCGACCATCGCCTCGAAACAACTGCTGGCGGCGGTAGGGCAGAGTCGACTGATTC
AACTGTGGGAACAGCTGTTTTCGATTTATGGCATTCACGTCGGGCAAATGCTGCTGACCCGTGCTGATATGGAAGACCGT
GAACGCTTCCTGAACGCCCGCGACACCCTGCGAGCGTTGCTCGATAACAATATCGTTCCGGTAATCAATGAGAACGATGC
TGTCGCTACGGCAGAGATTAAGGTCGGCGATAACGATAACCTTTCTGCGCTGGCGGCGATTCTTGCGGGTGCCGATAAAC
TGTTGCTGCTGACCGATCAAAAAGGTTTGTATACCGCTGACCCGCGCAGCAATCCGCAGGCAGAACTGATTAAAGATGTT
TACGGCATTGATGACGCACTGCGCGCGATTGCCGGTGACAGCGTTTCAGGCCTCGGAACTGGCGGCATGAGTACCAAATT
GCAGGCCGCTGACGTGGCTTGCCGTGCGGGTATCGACACCATTATTGCCGCGGGCAGCAAGCCGGGCGTTATTGGTGATG
TGATGGAAGGCATTTCCGTCGGTACGCTGTTCCATGCCCAGGCGACTCCGCTTGAAAACCGTAAACGCTGGATTTTCGGT
GCGCCGCCGGCGGGTGAAATCACGGTAGATGAAGGGGCAACTGCCGCCATTCTGGAACGCGGCAGCTCCCTGTTGCCGAA
AGGCATTAAAAGCGTGACTGGCAATTTCTCGCGTGGTGAAGTCATCCGCATTTGCAACCTCGAAGGCCGCGATATCGCCC
ACGGCGTCAGTCGTTACAACAGCGATGCATTACGCCGTATTGCCGGACACCACTCGCAAGAAATTGATGCAATACTGGGA
TATGAATACGGCCCGGTTGCCGTTCACCGTGATGACATGATTACCCGTTAAGGAGCAGGCTGATGCTGGAACAAATGGGC
ATTGCCGCGAAGCAAGCCTCGTATAAATTAGCGCAACTCTCCAGCCGCGAAAAAAATCGCGTGCTGGAAAAAATCGCCGA
TGAACTGGAAGCACAAAGCGAAATCATCCTCAACGCTAACGCCCAGGATGTTGCTGACGCGCGAGCCAATGGCCTTAGCG
AAGCGATGCTTGACCGTCTGGCACTGACGCCCGCACGGCTGAAAGGCATTGCCGACGATGTACGTCAGGTGTGCAACCTC
GCCGATCCGGTGGGGCAGGTAATCGATGGCGGCGTACTGGACAGCGGCCTGCGTCTTGAGCGTCGTCGCGTACCGCTGGG
GGTTATTGGCGTGATTTATGAAGCGCGCCCGAACGTGACGGTTGATGTCGCTTCGCTGTGCCTGAAAACCGGTAATGCGG
TGATCCTGCGCGGTGGCAAAGAAACGTGTCGCACTAACGCTGCAACGGTGGCGGTGATTCAGGACGCCCTGAAATCCTGC
GGCTTACCGGCGGGTGCCGTGCAGGCGATTGATAATCCTGACCGTGCGCTGGTCAGTGAAATGCTGCGTATGGATAAATA
CATCGACATGCTGATCCCGCGTGGTGGCGCTGGTTTGCATAAACTGTGCCGTGAACAGTCGACAATCCCGGTGATCACAG
GTGGTATAGGCGTATGCCATATTTACGTTGATGAAAGTGTAGAGATCGCTGAAGCATTAAAAGTGATCGTCAACGCGAAA
ACTCAGCGTCCGAGCACATGTAATACGGTTGAAACGTTGCTGGTGAATAAAAACATCGCCGATAGCTTCCTGCCCGCATT
AAGCAAACAAATGGCGGAAAGCGGCGTGACATTACACGCAGATGCAGCTGCACTGGCGCAGTTGCAGGCAGGCCCTGCGA
AGGTGGTTGCTGTTAAAGCCGAAGAGTATGACGATGAGTTTCTGTCATTAGATTTGAACGTCAAAATCGTCAGCGATCTT
GACGATGCCATCGCCCATATTCGTGAACACGGCACACAACACTCCGATGCGATCCTGACCCGCGATATGCGCAACGCCCA
GCGTTTTGTTAACGAAGTGGATTCGTCCGCTGTTTACGTTAACGCCTCTACGCGTTTTACCGACGGCGGCCAGTTTGGTC
TGGGTGCGGAAGTGGCGGTAAGCACACAAAAACTCCACGCGCGTGGCCCAATGGGGCTGGAAGCACTGACCACTTACAAG
TGGATCGGCATTGGTGATTACACCATTCGTGCGTAAATAAAACCGGGTGATGCAAAAGTAGCCATTTGATTCACAAGGCC
ATTGACGCATCGCCCGGTTAGTTTTAACCTTGTCCACCGTGATTCACGTTCGTGAACATGTCCTTTCAGGGCCGATATAG
CTCAGTTGGTAGAGCAGCGCATTCGTAATGCGAAGGTCGTAGGTTCGACTCCTATTATCGGCACCATTAAAATCAAATTG
TTACGTAAGATCTTATCATTCTCCCACCAAAAAATTATCTTAATGTAACAGCTGGTGTAAGTAAATTCTATCAACGAAGA
TCAATCTTATCTACTGACCAAAAAGGCCTGATAGGGCTTCGCTCACTATACATCCTTGGCTGCAGGTTTAGTTGTACACC
ACTCCTAAATTTAATGTGTTGGCAATGTGTTCAATAAAGCTCGAACAAATTAGCTCATTATGATCGGTTAATACTTCAAC
TTCTGGTTGCATGATTGTTTGTCCGTAAAAAGATAACGCGCCTGCCGGGTAGTAGCAGGCGCATTACGCAATAGGTAAAC
AAGGGAGGAAGTTCAGAAATGTAAATCGGGAAGGTTGTACGCAATGTTCATCGTACTACGTTGTTACGGCTTTGCCGCAA
CAAGCCAGTTGCCTGCCGCGCTCGCAGAATGTCTGCAGCCCGGAGATAAGGAGATTGTTCCTGCCAGCTAAATCCCTTCC
TGTCGATACGAACCAGCTCGTATTTTTCTACCAGAAAATTCACGGCATCGGCTAGGGTGATACCGGCATCGATGTGTTCC
TTAATCACAGCCTCATTGCAGAATGGCGTGTCGTTTATTGTCAGACCATAGTGCTGTTCCAGCAGACGTGTCAGTAACAT
TTGCCAGACAGCCACGGGTGACAGGCAGGGCTTCACCGCCCGCTGAGTTATTGCAGGTAAAGTTTTCATGTTTGCTCTCG
TGTAGGTAATTAACGCTGAGTGGGGTAAATGGCGATGTATACGTAGCCGCAACTGCCAAGGGTGTCGGCTTCGCAGGTTA
AATCGTTGTGGTACAGGGTAACGCAGTGGGCATGGTGGGGGCTGAGTTCACCGGTGGTCAGCATCGATTCCATCTGGAGG
ATAAAGTGCGGGAATGTTTCATCCAGCTTCCGGCATTCGATGTCACTGAACTTGCCGGTGATGCTGGCCCGGTCAGCCAG
ATAATGCAGTCGGTTGCCTTCCTGCACCAGACGGGCTCCCAGGCGCAGTGTAATCTCCCGCTGCAGGCCCCTGGTAGGGT
TGCTCATTACAGTTCTCCACTATTGTCAGTTCAGGGTGATGCTCATCAGGCAGGTATAGGGCCCATTGCGGTCCTGGCGG
CGTTCGGCGTATACCGCGAGGACTCCGGCGATATCCGGAACGTCCCTGCCGGTGTAATGACAGACGCTACCGTGCCACTG
GTATTTGCCGGTGCAGTAGCGAAAGATTCGGGACTCAGGATGCTGGCGGTATATCGTCATTGCCCTGCGTTTACTGATAA

TTTTCATGTAATACCTCAAAGCAGACCGTGTTCTGCGAACGAATAGATTTGCCTGCCACCGACAATCAGATGGTCAGGGA
CACGGATATCCACCAGCTGAAGCACCTGAACCAGTCGCTGCGTGAGGGTTTTGTCGGCCTGGCTAGGTGTCGTCTCGCCG
GAAGGATGGTTATGCGCGAGTATCACCGCCGCCGCGTTGAAGTGCAGAGCACGTTTGACCACCTCCCGGGGATGCACCTC
GGTGCGGTTAATCGTGCCGGTGAAGAGCGTTTCATGGGCAATCAACTGATTCTGGTTGTCCAGATACAACACCCGGAACT
CTTCCCGCTCAAGCGCGGCCATATGCAGTCGCAGCCATTCACGTACGGCGTGGGTAGAGGTGAAGGCTACGCCGGGCTCA
TGCAGGTGGCGGTCCAGAGCCCTGAGCGCCCGCTGAATGAGACGCCGGTCCTGTGGCGTCATCTCGCCGGGTAAAAAGGA
AAGCTGTTTCATCTGTTGCTCCTTCGGTCAGTCGATAATACGCAGAATGGCGTGAGCCTCTGGATGTTGCATGGCATACT
CCCGCAGGCGGTAATAGTGTGCGGTCATCGCGTCACATTCTGTACGGCAGGCATGGTGGCTATACGCAATCAGGCAGACA
GCAATACCTGCTGCTTCTGCACTCATTTGGGCATCGTTACCGTTCAGGCAGTTAAACAGACGCCATGTCTCATCGTTGTC
AGGCTCGGGGGACATAAATGCGCCGCCATTGCTGAGGGTGTAGAACGACCAGATACCACCGCTGTAGCCCTCACAGAAGC
GGTCCATCCAGGCGAAGATATGCGGCTCCAGGAGTAGCCACTGCGGGATAGCGCCAAAGTACTGTGGCCAGAAATCGATA
CGCTGTTCATCGGGGACCGGCGTGACGGTCAGTTCAAATTCGGGTTGGTTAGCGGGTGCGAGGTCGTGCTGCGTCTGTGT
TGTCATGGGTATGTCTCCGTCAATAAAAACGCCAGCGGCGATGGCTGGCGTATGGGGATATAAAGTGTGTTCGGGGAGGT
GAATGCGGGTAAATGCTTCGCGATCAGCGGGTGGCCGTGTCTGTACGGATGCCTGAGGTGCGGATATAGCGGTTAAGACC
TTCACCGGCATCCGGCTCAAAGTTCCATGCCCGCCAGACCATCCGGCCTTCAGTATCACGAACCACCAGACGGAAGTGAC
TGCCCTGGTCGTCTTCGAGTGTGATATTGCTGTACGTGGTAGTGACCGCTTGCGCTTGTCTCCGGGTGAAAGGCCCCGGT
GGCAGCAACACGGATTGGGTCATTTTCGGGCTCCTGATAAAAGAAAACCCCGGCAGCCTGCTAGCTGTCGGGGTGAATTT
GCTGGGGAAGATACTACTATCAGTCGTTGCTGCAGTCTCCGAGAGTGGACAGAACTTTCTCAGCGTTCTTCCGGTCCGCA
GTGAAGGTCCCTGCTTTGTGGTCATTGACGTAGACGTCGAACTGCCCGGCCTCAGAGATATTGCTGATGAAGTCAAACCA
GGCGTTATCGCCGTTACGCCAGCCCAGGCTGGACGGAATAATGTACTGCTGGTGATCCATCACTACGGTGATAGTAGTGT
CGTCATCGTGCGAACTGACCATCTTGTCATCGGCAAGGGTAAGAAAGACTGAATGCTGATAGAAACCATTCTGGTCCGGG
TTCCCTGTGCAGTTGATGGTAAACGTCTTTCCGCTGGCTTCGGTCACGCTGTATTCCGTATTGCCCTGACCGTAACCCTG
CTGCCAGAACCCCGGGATAGCAGAGGCATTAAAGCTCGCGAGCAGTACACCCGCCAGCATAAACCGACTTAGTGAAAGTA
TTGTCATTCTTGTCTCCTTTGTCGTTGTTTTATTCCTGATTGTCAGGGTTCGAGGGTATCAGTAGCTGCCCCATCAGTTT
GCCGTCATGGGCGTACTCAAAGTATTTTTCTTTGGTATACGGGTCCGTCACCTCCTGGTATTCCAGTTTGATGTTATCGG
CAATACACAGCGCATTCATCAGTGGCTGGACGGTTTTTTCCTGCATATCCACGAGGTAGTAGTAACTGCCATCCTCGCAG
CCATCGGGCGACTGGCGGGTACGTAAAACCTGCAAGGTGGGACCGGATAAGTAGTCAACCTGTGACCATTCTTCGCTGAC
ATCATCCTGGTGGCTGACCACATCACTGAAACGCGGTGGGGTGAGGTCCTTAAATTTACTGATGGCCTTCAGGTCATCAC
TTCTGTCATCGCATGCGATGAGAAACAGAGTGGTGGCAACCAGCGCCAGCAGAGGCAGTGTTTTACGTTTCATTATTTTT
TTCCTGAAATCAGACGAACCACTTTGGCAAAGACATAAATGCCCACGAAAATACCCACCGGCACGCCGACGAACGGTGTC
AGCGCGACACTGGCAGCACCGGCTGCGCCACCTCCCGTCAGCAGTGCGGCAACAGTGGCAAGGGTCAGGGCCGCGAGACT
GTCGGACACCCCGGTTTTGTTCAGAATGATGACGATGACAACAATGGCGATAATGGCAATATCCGGACTGATACTCCAGG
TCCAGATACATCAGCCGCTCACGAAAATCACGCCGCAGCGTGCGCACCGACACACCAAACTCAGCGGCAAGCTTACGCAC
ACTCAGCGTTTCCCCTGCCACCAGACGGCTGATTATCAGTGACAGCCTGACAGCCAGCCGGTCATGGCGGCGTTCTGCCT
GTGTCATGAGACGTTCTCCGTGAAAGTTAACTGACTGAAAATGATGTGATTACTTTAAAGAGGGGGTCGGACAGGGTATG
GACACCACAGAAACTATTTTTCATTTCTGCAAAAGCCAGAGGCAGCGGGGGTTACAGGCCTTCCCTCGGTGAGTGAGGAC
CGTCAGCACGGTGCGATGCGGACAGGTGGTGTCCGATATTAACAAAGCAGCAACATTAAACGGTCACTGCAGTTGCGTGA
GCAGGGCTTCCGCCATCACCCACAGTGCCCGGTTAAGTTTCACGTCCCCGTCGATACCGCGAACGGCACGGGTATGTGAC
CGTCCTCCTTTGGCATTACGGCCACTGAGCCCGCCCTTAATCAGGTTCTCCTGAATACGCTGGTACGTGGTCCACAGGTC
ATTGCTCTCATCCTGCCAGCGGCGAGGGGAGAGGATCTGCGATTCAGTCACCGGCTGGTGGTCTTCACCAAAGCGGTATG
TGAGGGCGGCTTTTGCCAGTGCCTGCTGTGCCGGGGGTGGCAACAGCAACGACTGCATGGCATCCCGTTTCTCCTCCACC
CGGTCAAAAATCCCCAGCACCTCATACGCGCCTTCAATCACCTGACTCACCACGTCCCCCTTGTGTGGCACCCGCACCTC
GCCAAACGACTCACCGCAGACGAGCCCGTTCTGACAAACCGCACGAAATAGTCCCGGCAACATCTGATACGAACTGGTTC
CATCGTGAGAGTTGAGTAGAATAATTTCCGGCACCTGTTTACCGGTGATCTGCCCTTCCCGCCGCAGACGCAGCATATGC
TTTGTATGTTCACGACGACCCGGGTCACGCACGCGGGTCTGACAGGCAAAGAATGGCTGGAAGCCTTCTCGCTGTAGGCT
GTCGAGCAGGGAGATGGTGGGTATATAGGTATAACGCTCACTACGGGACTCGTGTTTGTCCTCACTGAATACACTGGGCA
CTACGCGAAACAGCTCTTCACGGGTTAACGGACGGTCGCGACGGATAAGGTTTGCTGCGCCAAAGCGCGAAGCCAGACGG
GTCATAAGCAGACTCCTCATAACGGGAAAACAAATAAAAGGGATCCCCGTCGCATCGGCGACAGGGGCAGGGAAGTAACA
GGGATGGGTTAAATACTCAGAAGAAGAAATCCCAGACGGCGCGGGCCACTGACACCACCGTGGTGCGCACGGCCTGAATG
ACGGCCCGCACCGGGGCGGGTATCAGGGGAAAGGCACTGATGCTATCGAGTACGGCCCCGACGGTTTCACCAAAATCGCT
ACGAGCCTGTTCCCGGACTACCGTCGTGCGAAAGGAAGGATGGAGTTGCGACACCACCGGGCTGGTGGCCTCACGTGGCA
GGCACTTAATCATCCGCTCGGCCATCACCTTAAGCCCCCATTGTAGACGGACCGACACAGCACACACCGGATGGACGGGC
TGGAACAGCTCATGCAGCAGGCAGATTTTGCGGCTGATATTCTGCTTCTGCGCCGTGGACAACTGTCCCCCACCGCTGGT
AGGTTCGGCCTTATCCGACTGGCTGATAACAAACAGCACCTTATGCCGGTATACCTCTCCAATCACCTGATGATAAAAAT
GTTCATCCACCGTCAGCGCCCGGTCATCGGCCTTAATCAGCCACAGTACCAGGTCGAGGCGAGGAAGCTGTTCGCGGTAC
AGCGCAGCATACTCGGTATCGCGAGCGCCACTTTCGCCCACGCCGGGCAGATCCATCAGCGTTATATAGCGGTCGCCGAC

TTGCAGGCGAAAGCGCAGTGGCTCACGTGTACAGGCCGCCACATCGCTGACCGGTGATATATCTCCGGCAAACAGGGCAT
TGCACAGACTGCTCTTCCCAACACCGGTTTTACCCATAATGCCGATCACTGGCTCGTAGTTAGTTAACTGGTTAATTTGC
TGCAGAAGCCGCTCTGATACCCATTGCGGCAGATCAGCAAGCGATTGCTGAAACGACTTCAAACCTTCAGAATTATTCAT
CACTACTCCTCTGAAAAATAATAAAAAACGGTAGAATCGTGAGATTCCGCCGTTAATTGCGTATGTTCAGAGTGATGATA
TATATCTGAAGATTTTTTCAATCCTCTGCGCTTGAGGCAGCTGCGCGACTGCTGGCTCAGGCAATGAATGAGTTATAATA
GCAGCATTTACTAACAGGGATTTATTGAGAGTATGAGCCGCCGTGATACACCTTAAAATCTCAACCCAGCAAAGTTTCGG
AGCCGCGAGCAAAAGTGAGAGCCTTCACAAAATAATGCTTAGTAATAAAGTTACTTTGAATTCAGAGCCCCGATTAATGT
GAACATGATCAACGAATTTTACCCAGATTGAGTTCATTCAGTAGCCAGGTTTTGAACACATCAAGCGCAGAATGCATGTG
TTGGTGCTGGGGGAAAACCAAATAATGACCGATGTAATGTATTTCGCTGGTACTGTTTACAAGTGGGCATACCAGTTTAC
CGCTAGCGATTTCACGTTCAGCCAGTAGTTTTGATTCAAGCACAACTCCCAAGCCGTCAACTGCGGCAGCAATCGCCATA
AAGCTACGGTCAAATCGAAGGCCATAATTATTGGGTGGCGTCATCTTATTCGCCTCAAACCATCCTTTCCACTGGTACAA
CTGCACATCGCACTGAATCAATGTCAACGCATAGAGATCCTCTGGTTTCTTTAGCCGCTCAGCCAGTTGGGGAGAACACA
GTGGCGTAAGTTCTTCAACAGCAAGCGGGATCTTCTCATAGGGTGATGGGCGGGGTTCACCGTAGACTATATCGAGATCA
AAATCATCCTGTTCAAAACGTGCATATTCTGTGCTGGCTGAAAGTCGCAGATCGATGCTTGGGTTCTCACGTATGAACTT
ACCAAGACGTGGTAAAAGCCATTGATGGGCAAAACTCGGTGCAGTATGAAGGCGCAAAGGCCTGGATTCATCAGCCGTCA
CTAATGCCAACCCCTGCTGTAATTCGTTAAATCCCCGCTGTATGTGCTCAAGCAGTATTTCACCTTCTTTCGTCAGCGTA
ATTTCTCGCGTACTGCGCTGAAAAAGGCGTACATCAAGCAAGTTTTCCAGTTTGCGGATGGCATGGCTGATCGCACTGGG
TGACAGTTCCAACTCAGAGGCGGCCAACGCAAAAGCTCCGGTACGACCAGCCGCTTCAAAGGCGCGTAACAGATTTAGAG
GTGCTTTTGCTAAGAGCTTCATTCATGAATCCTTTTCACTAGGGATGAAGTGGTTTACTGAATTTGGCCACCTGAACAGA
GGTGATATGCTCACCTCAGAACAACACAGGTGTCATAATGAAAAAAAGAAATTTCAGCGCAGAGTTTAAACGCGAATCCG
CTCAACTGGTCGTTGACCAGAACTACACCGTGGCAGATGCAGCCAGCGCTATGGATGTCGGCCTTTCCACAATGACGCGA
TGGGTGAAACAATTACGTGATGAACGGCAGGGCAAAACACCAAAAGCCTCCCCCATTACCCCGGAACAAATTGAAATCCG
TGAGCTCAGGAAAAAGCTACAACGTATTGAAATGGAAAATGAAATATTAAAAAAGGCTACTGTAGATTCAATTGGTCAAC
GCAACAGTTATGTGAAAACATGGGGTTGCGGAGGTTTTTTGAATGAGACGAACATTTACAGCAGAGGAAAAAGCCTCTGT
TTTTGAACTATGGAAGAACGGAACAGGCTTCAGTGAAATAGCGAATATCCTGGGTTCAAAACCCGGAACGATCTTCACTA
TGTTAAGGGATACTGGCGGCATAAAACCCCATGAGCGTAAGCGGGCTGTAGCTCACCTGACACTGTCTGAGCGCGAGGAG
ATACGAGCTGGTTTGTCAGCCAAAATGAGCATTCGTGCGATAGCTACTGCGCTGAATCGCAGTCCTTCGACGATCTCACG
TGAAGTTCAGCGTAATCGGGGCAGACGCTATTACAAAGCTGTTGATGCTAATAACCGAGCCAACAGAATGGCGAAAAGGC
CAAAACCGTGCTTACTGGATCAAAATTTACCATTGCGAAAGCTTGTTCTGGAAAAGCTGGAGATGAAATGGTCTCCAGAG
CAAATATCAGGATGGTTAAGGCGAACAAAACCACGTCAAAAAACGCTGCGAATATCACCTGAGACAATTTATAAAACGCT
GTACTTTCGTAGCCGTGAAGCGCTACACCACCTGAATATACAGCATCTGCGACGGTCGCATAGCCTTCGCCATGGCAGGC
GTCATACCCGCAAAGGCGAAAGAGGTACGATTAACATAGTGAACGGAACACCAATTCACGAACGTTCCCGAAATATCGAT
AACAGACGCTCTCTGGGGCATTGGGAGGGCGATTTAGTCTCAGGTACAAAAAACTCTCATATAGCCACACTTGTAGACCG
AAAATCACGTTATACGATCATCGTTAGACTCAGGGGCAAAGATTCTGTCTCAGTAAATCAGGCTCTTACCGACAAATTCC
TGAGTTTACCGTCAGAACTCAGAAAATCACTGACATGGGACAGAGGAATGGAACTGGCCAGACATCTAGAATTTACTGTC
AGCACCGGCGTTAAAGTTTACTTCTGCGATCCTCAGAGTCCTTGGCAGCGGGGAACAAATGAGAATACAAATGGGCTAAT
TCGGCAGTACTTTCCTAAAAAGACATGTCTTGCCCAATATACTCAACATGAACTAGATCTGGTTGCTGCTCAGCTAAACA
ACAGACCGAGAAAGACACTGAAGTTCAAAACACCGAAAGAGATAATTGAAAGGGGTGTTGCATTGACAGATTGAATCTAC
AACCGCGCTCTTGATGTCAGACTCCCTGAACAGTTCTCGATAATCGGGAAACTCAGGGCGCGTTATCCTGTGGCCACTCT
CTGCCATGTGTTCGGGGTTCATCGCAGCAGCTACAAATACTGGAAAAACCGTCCTGAAAAGCCAGACGGCAGACGGGCTG
TATTACGCAGTCAGGTACTTGAACTGCATGGCATCAGCCACGGCTCTGCCGGAGCAAGAAGCATCGCCACAATGGCAACC
CAGAGAGGATACCAGATGGGGCGCTGGCTTGCTGGCAGACTCATGAAAGAGCTGGGGCTGGTCAGTTGCCAGCAGCCGAC
TCACCGGTATAAGCGTGGCGGTCATGAGCACGTTGCTATCCCGAATCATCTTGAGCGACAGTTCGCCGTAACGGAACCAA
ATCAGGTGTGGTGCGGTGATGTGACCTATAGTGTGCCCGGAGTTCAGGGCGGGCATGGATGCTTAAATGAACCGCGAGTC
TGTCTGGAATATTGAACCGGTAACTCACGATGAGAAACCCAACAATCCCACCGGGTGTGACGGTGGAGAACCTGAGCGGC
AGTGACCTGCGGCATGCCCGCAGGGTGATGTAACCCGCTGACAACGGGGATTGAGGCGAGATCACTAAGCCGAGATGATC
CTCAAGGTTAAGTACTGAAAGGTTGAAGAACATGAACCCGTTAATCCGCCTCTGTGGGTTGAAAACGTCACCACGGCCTA
CGTGATCTGACAGGCCGTGCAGGAGGAACTGGCAGTGATACGTAAGCACTGCCGGTCGAAGGTGTTTTGACATGTATGCG
AAACACCGGGGCAGCAGCGTCTATCACGCTTGCGTTGCTGACTTCTGCCAACTTGCGGCAAGCAAGGATAAAGAGTGCGA
CGGGCAGCCTCCTCAGTATGCCTGAGTCCAGGCAGGTAAACCGGGGAAGGTCAGCGACGGATGTTAAGGGGGCATGGCTC
CGATGACGCGCTGGCTGGCGGAGCTTCCGTAGTAGTCCGCGATGGGGAAAGCCCATTACATGGCGAAGGGAAGCAGTTTG
AATGTGTTTGCGACGTGAATTAACTGACCTAACGAGGTGAAGACCTTTGATAATCAGCGAAATGCAACGCAAGCTTGCCA
CATGGGCAGCCACCGATCCGTCCCTACGGATTCAACGGCTGCTGCGTCTGATAACACAACCAGAATGGCTGGCTGAAGCG
GCGCGGATCACGCTTTCATCAAAGGGGGCCCATACCCCCGGCGTTGATGGCGTGAACAAAACAATGCTACAGGCCAGACT
GGCTGTTGAGCTGCAAATCCTCAGGGATGAATTACTCTCAGGCCACTACCAGCCCTTGCCCGCCAGACGGGTTTACATCC
CTAAAAGCAACGGCAAACTGCGACCACTGGGTATCCCCGCGTTGCGGGATCGTATTGTTCAGCGGGCCATGCTGATGGCG

ATGGAGCCGATATGGGAGAGTGATTTTCATACGCTCTCATATGGCTTCCGGCCTGAGCGCAGTGTCCACCACGCGATCCG
CACGGTGAAATTACAGCTCACAGACTGTGGTGAAACCCGGGGACGCTGGGTGATTGAAGGCGACCTGTCCAGTTACTTCG
ACACCGTACATCATCGACTGCTGATGAAGGCTGTACGCCGCAGGATCAGTGACGCACGTTTCATGACTCTGCTGTGGAAA
ACCATCAAGGCGGGACATATCGATGTCGGTCTCTTTCGGGCGGCCAGTGAAGGTGTGCCACAGGGCGGTGTTATATCGCC
GCTATTATCGAACATCATGCTGAATGAGTTCGATCAATACCTGCATGAGCGCTACCTGAGCGGGAAAGCCAGAAAAGATC
GGTGGTACTGGAATAACAGTATCCAACGGGGCCGAAGTACGGCGGTCAGAGAAAACTGGCAGTGGAAACCCGCGGTGGCG
TACTGCCGCTATGCCGATGATTTTGTCCTCATCGTCAAAGGCACCAAAGCACAGGTGGAAGCCATCAGGGAGGAGTGTCG
GGGTGTGCTCGAAGGCAGTCTGAAACTCAGGCTGAACATGGATAAGACTAAAATCCCCCATGTTAATGACGGCTTTATCT
TTCTGGGGCACAGGCTCATTCGCAAACGCAGTCGTTATGGCGAGATGCGAGTGGTCTCAACGATCCCGCAGGAGAAAGCC
AGAAACTTCGCCGCATCGCTGACAGCACTGTTATGGAAGGTGCGAATAAGCGGGGAAATTCTTCTCGGCTGACTCAGTCA
TTTCATTTCTTCATGTTTGAGCCGATTTTTTCTCCCGTAAATGCCTTGAATCAGCCTATTTAGACCGTTTCTTCGCCATT
TAAGGCGTTATCCCCAGTTTTTAGTGAGATCTCTCCCACTGACGTATCATTTGGTCCGCCCGAAACAGGTTGGCCAGCGT
GAATAACATCGCCAGTTGGTTATCGTTTTTCAGCAACCCCTTGTATCTGGCTTTCACGAAGCCGAACTGTCGCTTGATGA
TGCGAAATGGGTGCTCCACCCTGGCCCGGATGCTGGCTTTCATGTATTCGATGTTGATGGCCGTTTTGTTCTTGCGTGGA
TGCTGTTTCAAGGTTCTTACCTTGCCGGGGCGCTCGGCGATCAGCCAGTCCACATCCACCTCGGCCAGCTCCTCGCGCTG
TGGCGCCCCTTGGTAGCCGGCATCGGCTGAGACAAATTGCTCCTCTCCATGCAGCAGATTACCCAGCTGATTGAGGTCAT
GCTCGTTGGCCGCGGTGGTGACCAGGCTGTGGGTCAGGCCACTCTTGGCATCGACACCAATGTGGGCCTTCATGCCAAAG
TGCCACTGATTGCCTTTCTTGGTCTGATGCATCTCCGGATCGCGTTGCTGCTCTTTGTTCTTGGTCGAGCTGGGTGCCTC
AATGATGGTGGCATCGACCAAGGTGCCTTGAGTCATCATGACGCCTGCTTCGGCCAGCCAGCGATTGATGGTCTTGAACA
ATTGGCGGGCCAGTTGATGCTGCTCCAGCAGGTGGCGGAAATTCATGATGGTGGTGCGGTCCGGCAAGGCGCTATCCAGG
GATAACCGGGCAAACAGACGCATGGAGGCGATTTCGTACAGAGCATCTTCCATCGCGCCATCGCTCAGGTTGTACCAATG
CTGCATGCAGTGAATGCGTAGCATGGTTTCCAGCGGATAAGGTCGCCGGCCATTACCAGCCTTGGGGTAAAACGGCTCGA
TGACTTCCACCATGTTTTGCCATGGCAGAATCTGCTCCATGCGGGACAAGAAAATCTCTTTTCTGGTCTGACGGCGCTTA
CTGCTGAATTCACTGTCGGCGAAGGTAAGTTGATGACTCATGATGAACCCTGTTCTATGGCTCCAGATGACAAACATGAT
CTCATATCAGGGACTTGTTCGCACCTTCCGGAGGCGTTATGAGCTGGCGGCCCTTTTTGTATCTGATTATTAATCCCCAC
CCGCTATTAAGCGCCCGGCGCGGGCATCTGCGTCTGGTGCAGGGTTGACTTTGCATTCTGTTAACAAACGCGGTATAACA
AACCTTCTTTGGATGTTTAGATGTCCATACGTTTAGAAGGTTATATGCAAACAACACAACAAAATGCGCCACTGAAGCGC
ACAATGAAAACGCGTCACCTGATTATGCTTTCCTTGGGCGGCGTGATTGGCACAGGATTATTCTTCAATACCGGGTACAT
CATTTCCACCACTGGAGCGGCGGGAACGCTGCTGGCCTATCTGATTGGTGCGCTGGTGGTCTGGCTGGTTATGCAGTGTC
TGGGCGAGCTGTCGGTCGCGATGCCGGAGACCGGAGCGTTTCACGTTTATGCCGCGCGCTATCTTGGTCCGGCTACCGGG
TATACCGTGGCCTGGCTTTACTGGCTGACCTGGACCGTGGCGCTGGGTTCGAGCTTTACCGCCGCTGGATTCTGTATGCA
GTACTGGTTTCCACAGGTGCCGGTATGGGTCTGGTGCGTGGTGTTCTGCGCGATTATTTTTGGTCTGAATGTTATCTCCA
CGCGCTTTTTTGCCGAAGGGGAGTTCTGGTTCTCGCTGGTCAAAGTGGTCACTATCATCGCCTTTATCATCCTCGGTGGG
GCGGCGATTTTCGGCTTTATTCCGATGCAGGATGGCTCGCCCGCGCCGGGGCTGAGTAATATCACGGCAGAAGGCTGGTT
CCCGCACGGTGGCTTACCGATTTTGATGACTATGGTGGCAGTGAACTTTGCTTTTTCGGGTACCGAGCTTATCGGCATTG
CCGCCGGTGAAACGGAAAACCCGCGCAAAGTTATCCCGGTAGCGATTCGTACTACCATCGCGCGACTGATTATTTTCTTT
ATCGGCACCGTGTTTGTGCTGGCAGCGCTGATCCCGATGCAGCAGGTGGGCGTGGAGAAAAGCCCGTTTGTGCTGGTATT
TGAGAAAGTAGGGATCCCGTACGCCGCTGATATTTTTAACTTCGTGATCCTGACGGCTATTCTTTCTGCAGCGAACTCCG
GGTTATATGCCTCCGGGCGCATGCTGTGGTCGTTGTCGAATGAACGTACGCTACCGGCCTGTTTTGCGCGAGTAACGAAA
AACGGCGTGCCACTGACGGCGCTGTCGGTCAGTATGCTCGGTGGTGTGCTGGCGCTGTTTTCCAGCGTGGTGGCCCCGGA
CACGGTATTTGTTGCGCTGTCGGCAATCTCCGGGTTTGCGGTGGTAGCGGTGTGGCTGAGTATCTGCGCCTCGCATTTTG
TTTTTCGTCGCCGTCATCTGCAACAAGGTAAGGCATTGAGTGAATTACATTATCGCGCGCCGTGGTATCCGCTGGTGCCA
GTATTAGGTTTTGTGCTGTGCCTGGTGGCCTGTGTTGGGCTGGCATTCGATCCAGCGCAGAGAATTGCGTTGTGGTGCGG
GTTACCGTTTGTTGCGTTGTGCTATGGTGCTTATTTCCTTACTCAACCCCGAAACGCAAAACAGGAGCCAGAACATGTCG
CAGAATAATCCGTTACGCGCTCTTCTTGATAAACAGGATATCCTGCTGCTGGATGGCGCGATGGCGACGGAGCTGGAAGC
GCGAGGGTGTAACTTAGCCGACAGCCTGTGGTCAGCCAAAGTGCTGGTAGAAAACCCGGAGCTTATCCGCGAAGTGCATC
TTGATTACTACCGGGCGGGGGCGCAATGCGCGATCACTGCCAGCTATCAGGCGACGCCGGCGGGCTTCGCCGCGCGAGGT
CTGGATGAAGCGCAGTCGAAAGCGCTGATTGGCAAAAGCGTGGAGCTGGCGCGTAAAGCCCGTGAAGCGTATCTGGCAGA
GAACCCGCAGGCGGGAACGCTTCTGGTGGCGGGATCCGTCGGGCCTTACGGCGCGTATCTGGCGGATGGCTCTGAATACC
GTGGCGATTATCATTGTAGCGTTGAGGCATTTCAGGCGTTTCATCGCCCGCGCGTGGAAGCCTTGCTGGATGCCGGGGCC
GATCTGCTGGCCTGCGAAACCCTGCCGAATTTTTCCGAGATTGAGGCGTTGGCCGAGCTGTTGACCGCATATCCGCGTGC
GCGGGCGTGGTTCTCATTTACCCTGCGCGACAGCGAACACCTGAGCGACGGTACGCCGCTGCGTGACGTGGTTGCGTTGC
TGGCGGGTTATCCGCAGGTGGTGGCGCTAGGCATTAACTGTATTGCGCTGGAAAACACCACCGCTGCGTTGCAGCATTTA
CACGGTTTAACGGTGCTGCCGCTGGTGGTGTATCCGAACTCGGGCGAGCATTACGATGCCGTGAGCAAAACCTGGCATCA
TCACGGTGAACATTGCGCGCAGCTGGCGGATTATCTGCCGCAGTGGCAGGCCGCTGGCGCACGGTTGATTGGCGGGTGCT
GTCGCACCACGCCTGCGGATATCGCCGCGTTAAAAGCGCGAAGCTGAGGGTTTATCGGGTCTACATCGTTCATTGTAGGC

CTGATAAGCGCAGCGCATCAGGCATTGCCGGATGGCGGCACAAGCGCCTTATCCGGCCTACAAAACCACAAAACGTTATG
CCGCATCCGCCAGAACAAACATGCCGTACGGATGGATTTCAAGATAATACTGCTCGCCGACGTCCGGTTGCAGACGCGTA
GCGTTGACCTGCAATAATATCTCCTGCCCGTGCCATTCCACCGTCACTTCATACTGCGGCCCCATATAGGCGACATGGCG
GATCACGCAGCGCTGGCTCTCTTCGCCGCGATCGCTGAGCGTGATCGCTTCCGGGCGCACACCGACCATCCCTTCACCCT
GTGTACCAAAGTGCAGCGGGCGCGGCAGATGATAGCCGTAGATATCAACGTATCCGTCGCTGAAGGTTGCCGGGAACAGG
TTGGCATCGCCCATAAAGCTCGCCATAAAGCGGGAGGCGGGCTGGCGGTAAAGATCCTGCGGTGAGCCGATCTGCATGAT
GTGTCCCTTGTTCATCACCAGCACAGTATCAGAAACCGCAAAGGCTTCGCTCTGATCGTGGGTGACGTACAGCGAGGTGA
TATCAAACTGCTTTTGCAACTCGCGGATCTTGTCGCGCATGCTGCGACGCAGGTTGGCGTCGAGGTTACTCAACGGCTCA
TCAAACAGCAGCACTTTCGGCTTGAGGATCAGCGCGCGGGCCAGCGCCACGCGCTGCTGCTGCCCGCCGGAGATCTGATC
GACAAAGCGGTCTTCGAATCCTTCCAGATCCACCATCGCCAACGCCTCTTTGACGCGGGCTTTCAGCTCTGCGCGCGGTA
CGCCGAGCATTTTCAGGCCATAACCGACATTCTCTCCCAGCGACATATGCGGGAACAGGGCATAGGACTGAAACACCATA
CAGATATCGCGCTGCTGAATAGAGCGATGGGTGACGTCTTCGCCATCAATGAAAATTTGCCCTTCGCTCGGTTTTTCCAG
CCCGGCAACCAGGCGCAAAATAGTGGTTTTGCCGCAGCCGGACGGGCCGAGCAGCGTCACCATTTGCCCCTGCGGGATGG
TGAGGTTGATATTGTCGATTACCGTATTACTGCCAAATCGTTTAGTGACGTTGCGCAGTTCAACGAAATTTTTCTGAGTC
ATAGTGCGCTCCATTACGCCTGGTTTTTGGCTTTTGAACGGGAGGTACGTGATTCACCGATCAGCCAGTCAAAGATGAAA
ATAATCGCCAGCATCACCACGATCAGAATGGAACCGTAGGCAATCGCTACACCGTATTCGCCATCTTCCACGCGGTTGAG
GATGTAAGCCGTCGCTACGCGGGTATCCGGCGTGACGAGGAACACAATGGCGCTGACGGTGGTAATGGCGCGCACAAAGC
TGTAAATCAGCGCCGAGAGGATCGCCGGGCGCAGCAGCGGCAGCAGGATGTGCGTAATGGTACGCAGGGAACCGGCGCGC
AGGCTGAGTGAGGCTTCATCGAGCGATTTATCGATTTGGCCCGGTAATGACTCCAACTTATTGATAGTGTTTTATGTTCA
GATAATGCCCGATGACTTTGTCATGCTGCTCCACCGATTTTGAGAACGACAGCGACTTCCGTCCCAGCCGTGCCAGGTGC
TGCCTCAGATTCAGGTTATGCCGCTCAATTCGCTGCGTATATCGCTTGCTGATTACGTGCAGCTTTCCCTTCAGGCGGGA
TTCATACAGCGGCCAGCCATCCGTCATCCATATCACCACGTCAAAGGGTGACAGCAGGCTCATAAGACGCCCCAGCGTCG
CCATAGTGCGTTCACCGAATACGTGCGCAACAACCGTCTTCCGGAGCCTGTCATACGCGTAAAACAGCCAGCGCTGGCGC
GATTTAGCCCCGACGTATCCCCACTGTTCGTCCATTTCCGCGCAGACGATGACGTCACTGCCCGGCTGTATGCGCGAGGT
TACCGACTGCGGCCTGAGTTTTTTAAATGGCGGAAAATCGTGTTGAGGCCAACGCCCATAATGCGGGCGGTTGCCCGGCA
TCCAACGCCATTCATGGCCATATCAATGATTTTCTGGTGCGTACCGGGTTGAGAAGCGGTGTAAGTGAACTGCAGTTGCC
ATGTTTTACGGCAGTGAGAGCAGAGATAGCGCTGATGTCCGGCAGTGCTTTTGCCGTTACGCACCACCCCGTCAGTAGCT
GAACAGGAGGGACAGCTGATAGAAACAGAAGCCACTGGAGCACCTCAAAAACACCATCATACACTAAATCAGTAAGTTGG
CAGCATCACCGAGAACACAAATGGGCTAATTCGGCAGTACTTTCCTAAAAAGACATGTCTTGCCCAATATACTCAACATG
AACTAGATCTGGTTGCTGCTCAGCTAAACAACAGACCGAGAAAGACACTGAAGTTCAAAACACCGAAAGAGATAATTGAA
AGGGGTGTTGCATTGACAGATTGAATCTACAGCAGCTTTTTTAATATGTCCCGTTCGTCGGTAACCCGTTTCAGCTCTT
TCTGGAGACGGCGGATCTCGGCCTGAGCATCTGACTGTTCTTTATTAGTGGAAGAATCCGGACCGTACTTCTTTATCCAG
GCATAAAGGCTGTGGGTGGTGATATCGAGACGTGTTGCAACGCTGGCAACAGAATAACCGAGATCAACAACCTGTTTGAC
TGCTTCAGTTTTAAACTCTTCGGGATAACGCTTACCGCTCATGGGCACCTCTCTTTAAGCCATTTTAAATGACTCGGAGG
TGTCTGTTAAACCCGTGGCGATTCAAAATTGCGATATATACATACCCATGACTGCCCAGCGTATCGGCTTCACCAGTCAG
CCCGTTACGGTACAGTGTGACACAGTGCGCGTACCTGGGATTCAGCTCACAGGAGAGCAGCATCAGCTCCAGATGTGCGA
CAAAATGCGGGAAGGCTTCATCCAGTTGTAGAGTCTGCACCTCACTGAATGAGCCGGTGAATCCAGCCCGGTCAGCCAGA
AAATGCAGACGATGACCTTCCTGCACCAGTCGTGCTCCGAAACACGGTGTGATATCTCGTTTAAGCCCCCAAGTTTGAGT
AGAGTCTTTATTCATAATTTTATTCCTGTTAAAGAAGACCATGTTCGGCGAATGAGAATATTTGCCTGCCGCCGACAATC
AGGTGGTCGGGGACATGGTGAACACTTTTTAACATCCTTTACCCATGGTGATCGACTTTTTCTTCAGGTCGATCACCCCC
ACTTTCGTGCTGTACCACCACACCTCGTAGCTGCCGTCTTCCTGCATCTCCTTCAGCCCGACCCGTTCTCCCCTGAACGC
CTTGCCTGCGCTCAGACTTACCCCTTTCACGCTCAGCTTTCCGCTGATATCCACTTTCCTGACCATCACCCCTTCATCGT
ATTCCGGGGGCGTTGTGTTGCCGCTGTACTGCCGCGCTGACGGCTGATACCGCGAGCCCGGTACCGCCATATCCAGCGCC
TCGTGCGGGCGTTCAAGGTTATAGACCGTCCGCCAGTGGTCGAAGGCGCGCTGCAGTTCACCGCTGTCTGCGAACCATTT
TCCCTGCAGCACTTCCGCCTTCAGGCTGCGGTGAAAACGCTCCAGCTTCCCCTGCGTCTGCGGATGATAAGGCCGGGAGT
GCCCCACCCGGATACCCAGGCGCATCAGCCACAGCTCCAGCGCCGTCCAGGTGCCGGTGGTGTCGCCCCACGGTGAGCCG
TTATCCATGGTCATCCGGTCCGGCAGGCCGTAACGCTCAAACACGCTGACCAGCTGCTGCTGCACGGTCTCGCGCCGTTC
ATCGGTACAGTGCGCCAGGCACAGGGAAAAACGGGAGTGGTCGTCCAGCAGGGTGAGCGGATGGCAGCGTCCACCGCCAA
AAGGAAAGTGGCCCTTAAAATCCATCTGCCAGAGGCGGTTCGGCGCGTCGTGTTCGAACCGGCCCGTGGCGGGAATGCCC
GGTGAAGCGCCCGGCAGCAGGCCATGGCGGGCCATCAGGTTATGGACGGTGCTGAAGGCGGGCATGGTGTGCCCCTGGTC
CTCGAGCCAGCGCTTAATCTTGCGGGCTCCCCAGCGTTCATGACGGTCATGGGCCATACGCAGCAGGGCCGTGATGTCGT
CAGATGAGCGGTTCGGGGAATGGTGCGGAATGCGCGGGCGGTCCTGAAGACCGGCGGCACCTTCCTGAGCCCAGCGCTGG
AGCCACTTGTAGCCGGTGGCAGGTGAAATGCCGAAGCGACGGCAGAGGGAACGGATGTTCGCCCCGTCCTGCGAGGCGAA
CAAAACAAACTCAGTACGTAATGACATGGTATCTCTCGCATCCCAGGGCATAAGCGACTCCATAAACGGGTTCTTATGCC
TTAGTTGTAAGTGTCTACCATGTCCCCGAACAAGTGTTCACTATGTCCCCGGACCGTACACCCCAAAGGGGAGAGGGGAC
TGCACCGAGCCATCTTTTCCCCCTCGCCCCTTTGGGGAGAGGGCCGGGGTGAGGGGCAATATGTGATCCAGCTTAAATTT

CCCGCACTCCCTCTTCCCTTCCGATTTACCTCTCCTTGTTCTGCGTCATAGTATGATCGTTAAATAAACGAACGCTGTTC
TATAATGTAGAACAAAATGATTCAGCAAGGAGATCTCATGCCGCAGTCCGCGTTGTTCACGGGAATCATTCCCCCTGTCT
CCACCATTTTTACCGCCGACGGCCAGCTCGATAAGCCGGGCACCGCCGCGCTGATCGACGATCTGATCAAAGCAGGCGTT
GACGGCCTGTTCTTCCTGGGCAGCGGTGGCGAGTTCTCCCAGCTCGGCGCCGAAGAGCGTAAAGCCATTGCCCGCTTTGC
TATCGATCATGTCGATCGTCGCGTGCCGGTGCTGATCGGCACCGGCGGCACCAACGCCCGGGAAACCATCGAACTCAGCC
AGCACGCGCAGCAGGCGGGCGCGGACGGCATCGTGGTGATCAACCCCTACTACTGGAAAGTGTCGGAAGCGAACCTGATC
CGCTATTTCGAGCAGGTGGCCGACAGCGTCACGCTGCCGGTGATGCTCTATAACTTCCCGGCGCTGACCGGGCAGGATCT
GACTCCGGCGCTGGTGAAAACCCTCGCCGACTCGCGCAGCAATATTATCGGCATCAAAGACACCATCGACTCCGTCGCCC
ACCTGCGCAGCATGATCCATACCGTCAAAGGTGCCCATCCGCACTTCACCGTGCTCTGCGGCTACGACGATCATCTGTTC
AATACCCTGCTGCTCGGCGGCGACGGGGCGATATCGGCGAGCGGCAACTTTGCCCCGCAGGTGTCGGTGAATCTTCTGAA
AGCCTGGCGCGACGGGGACGTGGCGAAAGCGGCCGGGTATCATCAGACCTTGCTGCAAATTCCGCAGATGTATCAGCTGG
ATACGCCGTTTGTGAACGTGATTAAAGAGGCGATCGTGCTCTGCGGTCGTCCTGTCTCCACGCACGTGCTGCCGCCCGCC
TCGCCGCTGGACGAGCCGCGCAAGGCGCAGCTGAAAACCCTGCTGCAACAGCTCAAGCTTTGCTGAGCCGGACGATAACG
ATGACCATTGAGAAAATTTTCACCCCGCAGGACGACGCGTTTTATGCGGTGATCACCCACGCGGCGGGGCCGCAGGGCGC
TCTGCCGCTGACCCCGCAGATGCTGATGGAATCTCCCAGCGGCAACCTGTTCGGCATGACGCAGAACGCCGGGATGGGCT
GGGACGCCAACAAGCTCACCGGCAAAGAGGTGCTGATTATCGGCACTCAGGGCGGCATCCGCGCCGGAGACGGACGCCCA
ATCGCGCTGGGCTACCACACCGGGCATTGGGAGATCGGCATGCAGATGCAGGCGGCGGCGAAGGAGATCACCCGCAATGG
CGGGATCCCGTTCGCGGCCTTCGTCAGCGATCCGTGCGACGGGCGCTCGCAGGGCACGCACGGTATGTTCGATTCCCTGC
CGTACCGCAACGACGCGGCGATCGTGTTTCGCCGCCTGATCCGCTCCCTGCCGACGCGGCGGGCGGTGATCGGCGTAGCG
ACCTGCGATAAAGGGCTGCCCGCCACCATGATTGCGCTGGCCGCGATGCACGACCTGCCGACTATTCTGGTGCCGGGCGG
GGCGACGCTGCCGCCGACCGTCGGGGAAGACGCGGGCAAGGTGCAGACCATCGGCGCGCGTTTCGCCAACCACGAACTCT
CCCTGCAGGAGGCCGCCGAACTGGGCTGTCGCGCCTGCGCCTCGCCGGGCGGCGGGTGTCAGTTCCTCGGCACGGCGGGC
ACCTCGCAGGTGGTCGCGGAGGCGCTGGGTCTGGCGCTGCCGCACTCCGCGCTGGCGCCGTCCGGGCAGGCGGTGTGGCT
GGAGATCGCCCGCCAGTCGGCGCGCGCGGTCAGCGAGCTGGATAGCCGCGGCATCACCACGCGGGATATCCTCTCCGATA
AAGCCATCGAAAACGCGATGGTGATCCACGCGGCGTTCGGCGGCTCCACCAATTTACTGCTGCACATTCCGGCCATCGCC
CACGCGGCGGGCTGCACGATCCCGGACGTTGAGCACTGGACGCGCATCAACCGTAAAGTGCCGCGTCTGGTGAGCGTGCT
GCCCAACGGCCCGGACTATCACCCGACCGTGCGCGCCTTCCTCGCGGGCGGCGTGCCGGAGGTGATGCTCCACCTGCGCG
ACCTCGGCCTGCTGCATCTGGACGCCATGACCGTGACCGGCCAGACGGTGGGCGAGAACCTTGAATGGTGGCAGGCGTCC
GAGCGCCGGGCGCGCTTCCGCCAGTGCCTGCGCGAGCAGGACGGCGTAGAGCCGGATGACGTGATCCTGCCGCCGGAGAA
GGCAAAAGCGAAAGGGCTGACCTCGACGGTCTGCTTCCCGACGGGCAACATCGCTCCGGAAGGTTCGGTGATCAAGGCCA
CGGCGATCGACCCGTCGGTGGTGGGCGAAGATGGCGTATACCACCACACCGGCCGGGTGCGGGTGTTTGTCTCGGAAGCG
CAGGCGATCAAGGCGATCAAGCGGGAAGAGATTGTGCAGGGCGATATCATGGTGGTGATCGGCGGCGGGCCGTCCGGCAC
CGGCATGGAAGAGACCTACCAGCTCACCTCCGCGCTAAAGCATATCTCGTGGGGCAAGACGGTGTCGCTCATCACCGATG
CGCGCTTCTCGGGCGTGTCGACGGGCGCCTGCTTCGGCCACGTGTCGCCGGAGGCGCTGGCGGGCGGGCCGATTGGCAAG
CTGCGCGATAACGACATCATCGAGATTGCCGTGGATCGTCTGACGTTAACTGGCAGCGTGAACTTCATCGGCACCGCGGA
CAACCCGCTGACGCCGGAAGAGGGCGCGCGCGAGCTGGCGCGGCGGCAGACGCACCCGGACCTGCACGCCCACGACTTTT
TGCCGGACGACACCCGGCTGTGGGCGGCACTGCAGTCGGTGAGCGGCGGCACCTGGAAAGGCTGTATTTATGACACCGAT
AAAATTATCGAGGTAATTAACGCCGGTAAAAAAGCGCTCGGAATTTAATTATTTTAAGAGATAAAACCGTCTGCGGAATA
TTTCCCGCAGACGGCTTTGTTGTTTTTGAAATTTATTAATTTAAACAATTAGTTGAGATATATCGTTGGCGTCACAAAAG
CAAAATAACGTAATTCGGAAATAAGATATGACCATTGCTGGTTAATTGAATAGCTCATTACACTCCATTAACACGATGTT
GTAATTCGGCACACTACATAAGGGTGTAATTCTGATGACGCAATTAACCATGAAAGACAAAATTGGCTACGGGCTGGGAG
ACACCGCCTGCGGCTTCGTCTGGCAGGCCACGATGTTCCTGCTGGCCTATTTCTACACCGACGTCTTCGGCCTGTCGGCG
GGGATTATGGGCACGCTGTTTTTGGTCTCCCGCGTGCTCGACGCCGTCACCGACCCGCTGATGGGGCTGCTGGTAGACCG
CACCCGCACGCGGCACGGCCAGTTCCGCCCGTTCCTGCTGTGGGGGGCCATCCCGTTCGGCATCGTCTGCGTGCTGACCT
TCTACACGCCGGACTTCTCCGCACAGGGCAAGATCATCTACGCCTGCGTGACCTACATTCTCCTGACCCTGGTCTACACC
TTCGTTAACGTGCCGTACTGCGCCATGCCGGGCGTCATCACCGCCGACCCGAAAGAGCGTCACGCCCTGCAGTCCTGGCG
CTTCTTCCTGGCGGCGGCGGGCTCGCTCGCTATCAGCGGCATCGCGCTGCCGCTGGTGAGCATCATCGGCAAAGGGGACG
AGCAGGTGGGCTACTTCGGCGCCATGTGCGTGCTGGGGCTGAGCGGCGTGGTGCTGCTCTACGTCTGCTTCTTCACGACC
AAAGAGCGCTACACCTTTGAGGTGCAGCCGGGCTCGTCGGTGGCGAAAGACCTTAAGCTGCTGCTGGGCAACAGCCAGTG
GCGCATCATGTGCGCGTTCAAGATGATGGCGACCTGCTCCAACGTGGTGCGCGGCGGGGCGACGCTCTACTTCGTGAAAT
ACGTGATGGATCACCCGGAGTTGGCGACCCAGTTTTTACTTTACGGCAGCCTCGCCACCATGTTCGGCTCGCTTTGCTCC
TCACGCCTGCTGGGCCGCTTCGACCGCGTCACCGCCTTCAAGTGGATCATCGTCGCCTACTCGCTGATCAGCCTGCTGAT
TTTCGTCACCCCGGCGGAGCACATCGCGCTCATTTTTGCCCTCAACATCCTGTTCCTGTTCGTCTTTAATACCACCACGC
CGCTGCAGTGGCTGATGGCTTCTGACGTGGTGGACTACGAGGAGAGCCGCAGCGGTCGCCGCCTCGACGGGCTGGTGTTC
TCCACCTACCTGTTCAGCCTGAAGATTGGCCTGGCGATTGGCGGGGCGGTGGTGGGCTGGATCCTGGCGTACGTCAACTA
TTCCGCCAGCAGCAGCGTGCAGCCGGTTGAGGTGCTCACCACCATCAAAATTCTGTTCTGCGTGGTGCCGGTGGTGCTCT

ACGCGGGCATGTTCATCATGCTGTCGCTCTACAAGCTCACCGATGCCCGCGTGGAGGCCATCAGCCGGCAGCTGATTAAG
CACCGCGCGGCGCAGGGCGAGGCCGTTCCCGACGCCGCGACAGCCGCATCCCATTAACCGGAGGCAATATGGAAATCACT
AACCCGATACTCACCGGCTTCAACCCGGACCCGTCCCTGTGCCGCCAGGGCGAGGACTACTACATCGCCACCTCGACCTT
CGAGTGGTTCCCGGGCGTGCGCATCTACCACTCCCGTGACCTGAAAAACTGGTCGCTGGTCAGCACCCCGTTGGACCGCG
TGTCGATGCTGGACATGAAGGGCAACCCGGACTCCGGCGGCATCTGGGCGCCGTGCCTGAGCTACGCCGACGGTAAATTC
TGGCTGCTCTACACCGACGTGAAGATTGTCGACTCGCCGTGGAAAAACGGCCGCAACTTCCTCGTCACCGCGCCCTCCAT
CGAGGGGCCATGGAGCGAGCCAATCCCGATGGGCAACGGCGGGTTTGACCCGTCCCTGTTCCACGACGACGATGGCCGCA
AATACTATATCTACCGCCCGTGGGGGCCGCGCCACCACAGCAACCCGCACAACACCATCGTGTTACAGGCGTTTGACCCG
CAGACCGGCACGCTCTCGCCCGAGCGCAAAACGCTGTTTACCGGCACGCCGCTCTGCTACACCGAAGGCGCGCACCTGTA
TCGCCACGCGGGATGGTACTACCTGATGGCCGCCGAGGGCGGCACCAGCTACGAGCACGCCGTCGTGGTGCTGCGTTCCA
AAAATATCGACGGGCCGTACGAGCTGCACCCGGACGTAACGATGATGACCAGCTGGCACCTGCCGGAGAACCCGCTGCAG
AAGAGCGGCCACGGCTCGCTGCTGCAGACGCATACGGGTGAATGGTACATGGCCTACCTCACCAGCCGCCCGCTGCGCCT
GCCCGGCGTGCCGCTGCTGGCCTCCGGCGGACGCGGCTACTGCCCGCTGGGGCGCGAGACCGGCATCGCCCGCATTGAAT
GGCGCGACGGCTGGCCGTACGTGGAAGGCGGCAAGCACGCGCAGCTGACCGTGAAAGGCCCGCAAGTAGCCGAGCAGCCT
GCAGCCGTTCCGGGCAACTGGCGGGACGATTTCGACGCCAGTTCGCTTGACCCGGAGCTGCAGACCCTGCGCATTCCGTT
CGACGACACCCTCGGCTCGCTCACCGCGCGCCCGGGCTTCTTACGGCTCTATGGCAACGACTCGCTCAATTCGACCTTCA
CCCAATCGACCGTGGCGCGCCGCTGGCAGCACTTCGCCTTCCGGGCAGAAACGCGGATGGAGTTCTCGCCGGTGCACTTC
CAGCAGAGCGCGGGGCTGACCTGCTACTACAACAGCAAAAACTGGAGCTACTGCTTTGTGGACTACGAGGAGGGACAGGG
TAGAACCATCAAAGTTATCCAGCTCGACCACAACGTGCCGTCGTGGCCGCTGCACGAGCAGCCCATTCCGGTGCCGGAAC
ATGCGGAGAGCGTCTGGCTGCGGGTGGACGTGGATACGCTGGTCTACCGCTACAGCTACTCGTTTGATGGCGAGACGTGG
CACACCGTGCCGGTGACGTATGAGGCGTGGAAGCTGTCGGACGACTACATCGGCGGGCGCGGCTTCTTCACCGGCGCGTT
TGTGGGCCTGCACTGCGAGGACATCAGCGGCGACGGCTGCTACGCGGACTTCGACTACTTCACCTACGAGCCGGTCTAAC
GGCTCAGGCCGGGTAGCCCAGCGCGCGCGAGAGCGCGAGCCCGGCCTGCTGAAGCTGCTCGCGGAAATTAGCCAGCTCCG
CGTCGTCCACGCGGGAGGTCAGCGTCGACAGGCTCAGGGCGGCGATGACGCGGGACTCGTGGTTCCACACCGGCACCGCC
ACGCAGCGCACGCCCTGCTCGTTCTCTTCGCTGTCCAGGGCGTAGCCTTGCTCGCGGGTCTGCGCCAGGGCGCTCATTAA
GGCTTCGCGAGACGCGAGGGTGGCGGGCGTAAAGGTAGTGTACTGATAGCCCTCCAGCAGGGCGTTCAGCTCGGCCTCGC
CCAGCCAGGCAATCAACACCTTGCCGATGGCGGTGGCGTGCACCGGCAGGCGGCGGCCGATGCGTGAATAGGCGATGGCG
GCCAGCTTGCCTTCAATCTTCTCGATATAGACCCCTTCACGCCCGTCCAGGATCCCCAGATGGGTGGTCTGCCCGGTCCG
CCGGGACAGCTCCGTCAGCCAGCCTTTTGCCTTCTGCCGAATATCGATGGAGCCCACGACAAAATGGCCGCGCTCGACCA
GCTTCATGCCGAGGCGATACTTGCCGTTCTCCGGGTTCTGATCGATATAGCCGTGAAGCTGCAGGGTTTTTAGCAGCGAG
TGGAGGGTACTCTTGCTCAGCCCCATCAGTTTGCTGATGTCGGTGATCTTAAGCTCGGTGGCCTGCTCGTTGAACAGGTC
GAGGATCTGCAACGCACGTTCAACAGACTGAATAATCGGCATAATGCTGGCATGTCCACGCTGGAATTAAGGCGAAAACG
TACCTTTTTCGGGATGAAAAATCAATGAAATGGAGCCGGTGTTCTCCCTCTCCCTGTGGGAGAGGGCCGGGGTGAGGGCA
CCAACGCGCAGCGGACCCAATCACTCCCCAAGCGTTGCCATCATCACCGCCTTAATCGTATGCATCCGGTTTTCCGCCTG
GTCGAACACGATGCTCGCCGCCGACTCAAACACCTCGTCCGTCACCTCCATCCCGCCGTGCAGATCGAACTCCTTCGCCA
TCTGCTTGCCGAGCGTAGTCTGGTCGTCATGGAACGCCGGCAGACAGTGCAGGAACTTCACGTTCGGGTTGTCGGTCAGC
GCCATCATCTGCGCGTTCACCTGATACCCGCGCAGCAGCGCAATCCGCTCTGCCCACTTCTCTTTGGCCTCGCCCATCGA
CACCCACACGTCGGTATAGATAAAGTCCGCGCCCTTAACGCCTGCCGCCACGTCTTCCGTCAGAGTAATTTTCCCGCCGT
GCTTCTCCGCCAGCGCGCTGCACTCCGCCACCAGGCTCTCTTCCGGCCAGCAGGCTTTCGGGGCCAACAGGCGCAGATCC
AGCCCGGTCAGCGCCGCCGCTTCCAGCATCGAGTTGCCCATGTTGTTGCGCGCATCGCCCGCGTAGACCAGCGTCATCTC
GTTAAACGCCTTGCCCGGCAGGTGCTCCTGCATGGTCATCAGGTCCGCCAGCAGCTGGGTCGGGTGGAACTCGTTGGTCA
GCCCGTTCCACACCGGCACGCCCGCATACTGCGCCAGCGTTTCGACCACTTCCTGGCCGTGACCGCGATACTGAATGCCG
TCATACATCCGCCCGAGAACCCGCGCGGTGTCCTTAATTGACTCTTTATGCCCAATCTGGCTGCCGCTCGGCCCTAAATA
GGTAACGCGCGCGCCCTGGTCAAATGCGGCAACTTCGAAAGAGCAACGGGTACGAGTCGAGTCTTTTTCGAAGATGAGCG
CGATGTTTTTACCGGTAAGCTTCTGTACTTCCTTGCCATTTTTTTTATCGGCTTTGAGCTGTGCGGCAAGGGTCAGCAGA
GAAGTGAACTGTGCAGGGGTAAAGTCGAGCAGTTTCAGAAAGTGTTTTTTGTATAAATCGGACATTTTATCCTCGCATGG
CGAACGCCACTTATTGAATTAAAATTCACTTTATATGTGTAATTATTCATTTGCAACCCCATTTCACAATTCTTTCTTAC
AAAGGTGGAGGCAAACCCGTCCGTGTGTGAAAATAATCGTATCTGCCTCCGATTCTCTGCAGAAGCAGAAAGACATTGGA
TCGAATTCTACAACCAGGTCGAGTCAGAAATGAGAATGATTGGCCTTCTTTATGATTTTAAGGATTATGCTTCTAAAATG
GCGGAGAACATGGCGAGGCTTGCTGCCTTACTTCATTACTTCAGCGGTGATGGAGGCGATATATCTGTTACCGGGTAATG
ACTCCAACTTATTGATAGTGTTTTATGTTCAGATAATGCCCGATGACTTTGTCATGCTGCTCCACCGATTTTGAGAACGA
CAGCGACTTCCGTCCCAGCCGTGCCAGGTGCTGCCTCAGATTCAGGTTATGCCGCTCAATTCGCTGCGTATATCGCTTGC
TGATTACGTGCAGCTTTCCCTTCAGGCGGGATTCATACAGCGGCCAGCCATCCGTCATCCATATCACCACGTCAAAGGGT
GACAGCAGGCTCATAAGACGCCCCAGCGTCGCCATAGTGCGTTCACCGAATACGTGCGCAACAACCGTCTTCCGGAGCCT
GTCATACGCGTAAAACAGCCAGCGCTGGCGCGATTTAGCCCCGACGTATCCCCACTGTTCGTCCATTTCCGCGCAGACGA
TGACGTCACTGCCCGGCTGTATGCGCGAGGTTACCGACTGCGGCCTGAGTTTTTTAAATGGCGGAAAATCGTGTTGAGGC

CAACGCCCATAATGCGGGCGGTTGCCCGGCATCCAACGCCATTCATGGCCATATCAATGATTTTCTGGTGCGTACCGGGT
TGAGAAGCGGTGTAAGTGAACTGCAGTTGCCATGTTTTACGGCAGTGAGAGCAGAGATAGCGCTGATGTCCGGCAGTGCT
TTTGCCGTTACGCACCACCCCGTCAGTAGCTGAACAGGAGGGACAGCTGATAGAAACAGAAGCCACTGGAGCACCTCAAA
AACACCATCATACACTAAATCAGTAAGTTGGCAGCATCACCGAATTTTCAGCCCGGATGTTTCACGTACCTATGTCGAAT
ATCTGGCCGATAAGGTTGCGGATCGAGAAGAGGATGCAGAGGAATATCTGGAAGCAATCATGGAGGCTCGTGTTACCGTT
GCTGGCATGGGGCTGGTGATGGAGGTGCAGGATTACTTCGATGGCGAGGCCGATAGACTGGCGAAGGCGTGGCTGGCCGA
GTACACACCGCAAATCAAGTCGTTGAAAGATGAGCGTAAAGAGGCCTATCGTCAAATCGTCGAAATGAGTACCGAACCGC
AGGATGTGGATCTGGTCAGGCCGGCGAACAAGTTTGAAATGACCAGGGTGCGTGAAGGTGAAAAGGAAGCTGACCTTCCA
GTCTGGAAACACCATTTGTTGTGTGACGAAAGCGGGAACTATCCGGCTCTGTTGAACCATTGGGAAACCAAGGTTTTTGA
GATCGAAACCAAACGTGAAGGATTTGCTTTCTGGTATCGTAATCCACAGTACACAGGGCAGTCGTCACTGGGAATCGCTT
ATGTTGAAGCTGAACAGTACAAGATTGTTCGTCCCGATTTCCTGTTCTTTGCCGAACAGGATGGCAAAATGGTTGTGGAC
CTGGTAGATCCACATAGCCTACATCTGGCTGATGCTTTGCCCAAACTGGAAGGACTTGCGCTATATGCCGAACATCATTC
TGATGCTTACAGGCGAATCGAATCTGTCGCCGAAGTAAAGGGTAAATTACGAGTGTTAGATTTGAAACGGCAGGATGTGC
AGGATGCTGTTGCTACCGCTGAAAATGCAGAAACGTTATTTAGTAGCGGACTTGCTGATGACTATCAGTAATCTATAGAA
AATTGCGTAACGAAACTGCATTTTAGTAATTTTTTGAAACAATACAAATAAGGCCTTCTCACTGAGAAGGCCATTACCGA
CTTACAGTTCGATTTGGCTACAGCCGAAGTTACGGTCTCCTTCACCAAACACTTTGCTTTCAACTTTGGTCAGGTAGTCC
AGTCGGGTTAACAGCGCCTGGTAGTTCTGCTGGAAGTCAGTACTATTGGTGTCTAGCACATACTTACAGTTTTCAGGAAA
ATGGACCAGACCAGGGTGATCGTCTCTCTCCAGTCTCAGGGCGCTGTACCAGGCTCCTGTGATCATCCCTCTAAGGTTAT
CGTCCTGATCGTAATCCCCGAGGTGGTAGTAAGCGTCTTTGTTGAACAACAGGCAAATGTGATAGTGGCATTTACCTGAT
TCAGAATACTCTTTCGCCCATATAATAAATAAAGGGCAACGGTAAATGCGTTTATCCTCACGCACCTTACGGGTACGGTC
AGCCTCCAGCTTAGCTCTGAGGGATTCACGCATCCGGGATATCACCCCAGGTTCCAGGTTAGGGAAGCAACAGATATTGT
CACCGTTATCAACTATTTTGGGATAGTGGAGATCGACACGTACAGCAATTAACCTAGGATACCGGTTAGTCAAATAACAT
AATAATTTATTAATCTTACTCTGATATTCGAGGATATGTACTCCATGAGTACCTTGATATGTTTTCATAATATATACCTG
TATTTGGTCCTACGGTCTATAGCAGACCGTACAGGGATGCCATACAGGCAGGGCTATTCCTGGTCACTGTTGTCGGTATA
TACTGGTATCAGATTTTAGTACGGACATACTGATCTGATTACTACTCTTACTGGTGAATACAGGTAGTAAGAGTACACAA
TGCCATTATATAATACCTATCCAACATCTAAATTTGAGACGAAGGTAGCCCCAGCCTGTGATGGCTGTTAAAAAGTTACT
CTGCAATTAGCTTCTGGAATTAACCGATGATTCCGTTATTCCAGTGCCGCTTCACAGTCGATAAACTGATACTCAATTTC
TTTGCAATCTCAGCCTGAGTAAATCCACCTTTCTTAAGTATTGTGATATCACGTCGGTATTTGCTTCCCTCCTTTCGTCC
TAATTTTTTCTTAACATGTCGATATCGGCTTTTAGTTGATTTCAGATTGTGAATGTCAATCGTATCCTGACGAGCAATCA
AAAAAGACAAGCAACTTTCAGCAGAAGGCTCTATCCTTGAAGCTGGATGGTAGCTGTAGATAAACATTTCTTTTTGAAGG
CAAAAAAATAAAATATTCTGTAAGTCCTCCACAGTTCTGCCAAGACAGCTTAGATCTGGAATGACAAGCGTGTCCTGCCG
GTTCATTTCATGATGAATCAATTGTCTGAGTAAACGTTGCTCTGAAACAACCTCTTTAATATCTACCTGCTCGATCACAA
CATGTTCAGGTAGAATATTAAATTTTTGGCATATCTCAGCAAGTGAGTGATGATCTGTAATGCTTTCTTGCTGTTCGCTA
CTGCATATGGTGTAAAAAAATGAACGCATCTGATGCACTCCGGTGTCATAAATTATTTTTTGATACCTGAATAATGTCAT
TTTGCATAAAAAACAACCAAAAATAGAGAGTGATTGCCAGCGATACTTTAACTGGTAAAGTAGTATTTTACCTCTCATAA
TTGTTTGTCCTTCTTTGTTACCTCTTAGGTTCTGCTCTCTTGTGAGTATGGGTTTATAATGATGTCATCCGGTACATTAA
CACGGTTGGAAACATCTGTTTTGCGGAGCACTGGCAGCATAACCACCATTTCATTGACATGGTAAATATCAATCCAACAA
ACAACCGGATAAGGCATAACAATCCCCTTTCTTCTAAGAACTTCATTTATCCCGAATATAATCAATTGGGATGTTGACAA
GAGTAGAGCCTGCCCGGCATCAAAGTAATAACCCTCAAAGGTTGTTCTATGAATAAGAATAATGTTTGAATATCCTGAGA
CACTTTGATGCTTTACATCAAAATGTCTTTGATTGTAAATGTAAATGGCTTTTTCCATATCGTAATTTTCCCTTAGCGAG
GATAGCCTTTCACGTATAACTGTTTTTTCATCTTTCCCCATGAATTCATCATTAGCGTTCAGTTGAAAGTAATCTGCAAT
GGGGATTTTTAATGGACTCATGATGTTCGTTTTCCAGATTAACGAACAGTACTCTTCCAGAGTTACTTCTGTTTTACCTC
GGCTTCGATTGATGCTTTCTTTTATAGAGAACTTTAACAGGTCATTTTTTATTTCATTTATTGACCAGTTATGACGATTG
CATTTTTCTAATATAGGGATAGCCAAATCCACAACTTCCGCTATTACGGAAGAGTGAGATTTATTTGTTAGTCTGGCATA
ATGATCAATTACTCCTAATGAAGATTGATTTTTGAACGTGACTGAGTGTTTTTTTATGGGAGTAGTCTGTACTACCTGTG
ACTCATTGTTTTTGCCTTGACAAGGTTTTTTTCCTGGTAGAACGCTGCTCATCTCAAAATTAGTAACACTATTAGACATT
TTTAAGGAAACCTCTTTGTTATCTCAATATAAAACTACTATCTGAAATTTCTCAGTGTTGCCCCATTTTTCTCGGGGCAA
CATCGCTATAAATCAATAAAATTAAATCTGTAATTTATTACAGACTTTCTCGTACAGAAAATGTATCTATAAAATCGAGG
ACATCTTTACGTAACCAGTAATGGCGGCCATTGATTTGTTTTGGGCGAGGTAGCCCCTCAGGCAATTTGTCCTGACAAAG
CCATTTACGAATGGTTTGAGATTTGTAGCTAATGATTTTAGCTAACTCAGCTGTTGAAATGAGCTGTGATTGAGATGTAT
CCGGGAGTTTGAATGCGCTCATTTTGCCACCTCAGTGATTGAGATATTGGCAAAGAAAGCTTTAAGCATTATTGAATTAG
TAGGGGTAGTAATGAAGGCAGAAGGAAACGTTTCGGGCTCGGGGTCGGGGTCACTACCATCACCTGAATCTGATGATAAG
GCACTTTTAGCTGATTTAGCCGTACTGTGATGCGATCTGGATGCCTGATTTTTACATGACATCAGACGCGCGAGAAAGTT
AGTAGCATTTTGCATTCTTTGTTTAAGATGTAAAATTTTCAGTTTGGCGCATGCGACAGACTTGCGAAGAGCTGAGTATG
TTCCTTTGAGATACTCGACAACCTCTTCCATGGACGCAGGATCGTTAAAGGATTGAGTTGTTTTACTTGAATTAATACAC
CAGCGAGCTCTGATAGCTGCCAGAGTGGGTTCTATGGATACAGTTGCTGGGGTTGCCATTGGCTTATCTCCTGTGTAGTT

GCTGTATCATTGACTAAACTGTGTATTACGATACATGAAAATCTATCTTGATCAATAGTTGATGATATTTTGTAATATGT
TGATTTTTAATGGAATCAACGCATATCAACGTTGTTTTTATGGTAATACCTGCTTCATTTAAGATCCATGTCTCAATTT
TCTCGTGCCATTTTCGTAACAGATCTAACGGACGACGGCGATAGTGTTTTTCGGCAAGAGCGCTGGGTTTGTGTCCCATA
ATTTGAGCAACAATACCAGTGGGAACTTCAACCCACTCGGCCAAAGTACCAAAACTACGACGTAAACCGTGAAGGCTGAT
ATGTGGTAACTCAGCCAGCACTAATGCTCTGTTGTGCGCTGAACGCGGCTCAATAATTTTGCCACTTTTACTGTTACTTC
TGAAAACCCAACCCTCCTTATTTACGTCAGAATTTGGGGATTGCGCTAGTACATTTAACAATTCAGAAACATAAGGAGTG
AGAGGGATGATACGTTCACCTTCGATCTTGTCTTTAATTCGCATGCTTGACCATTTGAAATCTACGTCTGACCAGCGAAG
CGACGCAATTTCTTCACGCCGAGCACCAGTGAGCAAAAGTACTTGGAGATAGGCCGATGCAATAGGATTATTGAGGCTAC
GCACGGCACTAAACCAGCTTTTTAGTTGTTCCTTTTGCAGGCAATCATCAGCTTTACTCGCTGACACGGGAACCATTTTT
CTTACGTTGTAATCTTGTGCCAGATCGCCAGGAATGATCCCTTGATATTTTTTCTGATAATTACTCCATTTGATGAAAGC
ACGTAGTAGGCGATAAGCGTGAGCAGTGACGGTAGGTCTATTTTGCCTTTCTGTACTCAGCCACGCTGCTATGTAATCTG
GGGTTAGCTCCGATAACGGCAGGTTGAGCAAACTAGCCAATGGTCCAGCCGAAGTCGGGCCTTGGCCTCTTTTTTTACTT
TCGCCTCCACGACTGGACAAGTTAATGTGATCGGCAATGTATCGAGTAGAATATGGGCGTTTAGTTTTTGCACTGATACC
GGTTCTCAATTCTTGAAGATAGTCTTCCCAGGCGACGGAGAAAGTCACTTTTGTTTTACGTGATTCTGCCTGCAGGGATT
CTGCTTCTGCGATTTTTACAGCCTTAGCAATTCGTGGATCTATCCCTGTATCGATCAATGTTTGTAACCGTCTTGCCTCG
GCTCTCGCATCATCAATCTTCCAGTCGTTAATGTTGCCAATAGTCATGCGAAGGGTTTTCCCCGCATATACGCTTTGGAA
TACAAAGGCTTTTGCTCCGCTAGTTGCTCGGCATGCCAGGGTTGTTACATCTGCATCCCAAAGAAAAGTTTGTTTTTTCC
CTTCCGGTAAGGTGAATCTGCGAAGTCTTTCGAAGGTAAATTTTTGTCTTGAAAGCGCCATTTTTCGGCTCCAGTTGAGA
ATTGCTGTCTTACAATAATGTAACTGCTGGTGTAAGTAAATGGAGGGAATAAACATCAACTTTTGTTAATTTCAATCAAC
GGGTTTATTGCTATAACTTGGTGTTTTATAATATAAAGTATGTTGTTTTGATTGATTGCTCAAGTAGTTAAAAATGCATT
AACATCGCATTCGTAATGCGAAGGTCGTAGGTTCGACTCCTATTATCGGCACCATTAAAATCAAAGAGTTACCCCATATT
TAAATACACCACGTTTCCTCCTGTGCCGTATTTGTGCCATTGTAACCTTGGCAATTCATCAAAATACTGTTCTGACATCA
GGCAGTGCAGGTGCAGACATTTAAGCCAATTGCTGCCGCCATTCTTTGACGTAGTCAATCAGGGCGCGGAGCTTTGGTGC
AATATTGCGACGCTGTGGGAAATACAGATAGAAGCCCGGAAATTGTGGAAGAAAGTCATCAAGCAGCGATACAAGCTTAC
CGCTTTCAATATATGGCCTGAAAGTTTCCTGAGTGGCAATTGTTATTCCTCCGCCGGCAAGAGCCAGCCTCAACATCAGA
CGCAGATCATTAGTCGTAATCTGCGGTTCAATCGCAAGGTCGAAAGTTCTCCCGTTTTCTTCAAATGGCCAGCGATAAGG
CGCAACCTCCGGGGACTGACGCCAGCCGATACACTTATGGGTATTTCCCCCGGAGGCGAGAAAGCACTCTCCACGCCCGG
CCGCAAGGATCAGGACGACGGGGGCAGGCATGAATCCTCCTCCTGATGGAGACGTACAGAGGCGACTTCTGCCAGCACGG
AGAGTGCCAGAGTATGCGCATCCCGGGCTTTGGGGAATATCCCGACGGGTGCCCGGATTTGCGTTGTTTCCTCCCTGGAC
CATCCCAGCTCGTGGAGCTTTTGCAGACGTAACGTGTGGGTTCGATAGCTGCCCAATGCGCCGAGATAAAAGGGTTTTGC
TTCTCGCGCGGCCTGCAACACTGGCAGCTCCCGGTTGAGATCATGGCACAGCAAAATGACCGCCGTATCGGTATCGATCT
GAGCGCTGGCTGAGGCCGGAAAAAGATCGAAGATATGGCTGTCATAGCCTGTGGCTGCTGCAAGACTCGCGGTTGCCTGC
GCCTCAAGAGAACGTCCGTAAATCATCAGCCTGACGCATGGCCTGAACCCCACCTCAAAGCCATTGAGATTCCAGCCCGT
CCGGGTTTGCGTGGGCAGGCACACCAGCGATTGTGCTTGCGGATCGTAGCGCAGCCCCACCGGTTTTCTCTGTTCCAGGC
GGTTCAGCACGGCGAGCAGAGGCTGTGCCGAGCGTAGTTTATGGAGCGTCAGCGTGATCCCACCGCCGCAGGGCAGAACG
ATGTCAAACCACGGCGAACCTTCGCCATAGCGAATTTCGCGATCGCGGCCTGAGCCCATCATCTCCAGCGCTTCAAAAGC
GGCAGCGGCCTCCACGCAGCCGCCAGAGACAAAACCGCAGTAACGACCATCTTCGCGCACCACCATCTGCGCCCCGAGCG
GGCGCGCCGCGCCGCCGCGTATCTCCACCAGCGTCACCAGCACCGCTCCTTTACCGGACATTAGCGCCTCTACGGCGAAG
CGCAGAATGGTCCGGTGGTCATCGGTGAGAAACGCCTGCTCTGGCTTATGCCAGTGTTCGTCTTTGTCAAAAAGCGGGTA
TGACATTGTTCCTCCTTAAACCACATCCGGCAGCTTATCGAGCAGCTTATCCAGAGTGATGGGATAATCCCGTACCCGAA
TACCGGTGGCGTTATACACCGCGTTGGCGATAGCCGCGCTCACGCCGCACAGGCCCAGCTCACCGACACCTTTGGCCTTC
ATCGGGGAGGATATGGGGTCGGTATCATCCAGGAAAATCACCTCCTGTTTTGGGATATCCGCATGAACCGGCACCTCATA
CCCCGCCATATCGTGATTAACGAAGTAGCCCAAACGGTCATCCACCGCCAGCTCCTCCATCAGTGCCGCGCCCATGCCCA
TAGTCATTGCGCCAATGACCTGGCTGCGCGCAGTTTTCGGATTCAGGATGCGTCCTGCAGCACACACAGCGAGCATACGC
CGGACCCGAACTTCTCCCGTCGCGCTATGCACGCCGACCTCCACAAAATGCCCGGCAAAGGTCGACTGCTGGTACTCTTT
GCTCAGTGTTCCGAATTCAATGCTCTCTTCCGCTGTCAGTCTGCCGCCTGCGGTGGCTTCATGTAGCGTGGCGCTTCGGG
TACCGTTGGTAATCTTGCCGTCGGCAAACTGCGACTGCTCAGGATCAAACCCGACTGCCGAGGCAATCATTTCGCGAAGC
TTCATACAGGCGGCGTAAACGCCGGAGGTGGAGGTATTCGCGCCCCATTGTCCACCAGAACCCGCAGAAACCGGGAAACT
GGAATCGCCGAGGTGAACCGCAACCTGCTCCAGCGGTACGCCAAGCATTTCCGCTGCCGTCTGGGCCAGAATGGTGTAGC
TGCCGGTGCCAATGTCGGTCATGTCCGTTTCTACGGTAACGGTGCCGTTTTGTTCGAGGTGAACCCGAGCACCCGATTTT
TCCAGCAGATTATTGCGAAAGCCCGCCGCAACACCGTGGCCGACTAGCCACTCCCCGTCGCGCACCTGTCCGGGTGTGGC
GTTGCGCTGCTTCCAGCCAAATTTATCCGCTCCGGTGCGCAAGCACTCGATAAGCTGACGGCGAGAGAAGCAGCGCGTCG
GGTCGGCGGGGTCAACCTGAGTGTCATTCAGGATGCGAAACTCGACGGGATCGATGCCCGCTTTTTCCGCCAGTTCGTCG
ATCGCGATTTCGAGCGCCATCAGACCGGGGGCTTCGCCGGGCGCACGCATGGCGTTCCCTTCCGGCAAATCAAGCGTGGC
GAGCCGCAGGCCGGTATGACGATTCGCCCCGGCGTAGAGTAATTCGCTTTGCTGTACCGCCGTTTCCGGCGTGCCGCCGG
GCAGGTTTCCAGACCAGCTTTCATGTGAGATAGCGGTGATTTTCCCGCTCTGGTCGGCACCGATACGCAAGTGCTGAAGG

GTGGCGGGGCGGTGCGTGGTGTTATTGGGAATAGAGGGGCGGGGGAGCATCACTTTAACCGGACGTTTCACCGCTCGGGC
GGCGAGGGCCGCCAGCAGCGCATCGCTTCTCAGGAACAGCTTGCCGCCAAACCCTCCGCCGATATACGGGGAGATAATAC
GCACATTCTCCACGGGAACTTTCAGCGTTTTTGCCAGATCGGTGCGGCACCAGTCAATCATCTGATTTGAGGTCCAGAGG
GTAAGCTTATTTCCATCCCAGACGGCCATCGAGGCATGCGGCTCCATCGCCATATGGCTCTGGTCCGGGGTCGTGTAGGT
AGCATCAATCTTCACCGCAGCGGAGGTGAAAGCCCCGTCAAAGTCACCGACGTTTTTGTCGGGCGTGTCTTCCGGCGGCT
GATTGACGGCCTGTTTTTCGTCCGCCAGGGAGTAAGCTCCTTTATTACGGCGATAGTGTGCCTGCACCAGCGAGGCCGCC
GCTCGCGCCTGTTCGAAGGTCTCGGCCACTACCAGCGCAATGGCCTGATGATAGTGCTCAATAGTGGGGCCGCCTAACAG
CCTGGCGGTGTTTTTGTCGCCTTTGCCGAGTGCCCCGGCGTTACTGGCGGTAATGACAGCCAGTACGCCCGGCGCTTTTT
GCGCGGCGTCCGTATCAAGGGCGGTGAGGCGTCCTTTGGCAATGGCGGAACCGACGATATAGCCATAGGCGGCGTTGGGG
GCTTCTTCATGCCATTCGTAGGCGTAGCGTGCCGTGCCGGTAGTTTTCAGCGGTCCGTCGATGCGGTCATGGGGACGACC
GACAACCTTCAGCTGATCGATCGGGTTTTCCCCTGCGGGTTTATCAAATTTCATGCCTGTGCCCTCGCTTCAGCCAGTAC
GGAGGCAAGCGTTCGCTTCGCCAACAGGAGTTTAAAGGTGTTTTCAGCGGTGGGATGGGCGCTGGCGAACAGCGTGTCAT
ATACGGCCTGCGCCCCCTGGGATAGCTGAGCATCCGCAGCCTCAATGCGCCAGGGCTTATGTGCTACTCCGCCCAGCGCG
ACGCGCCCGCTGCCGTCAGGCTGAATAATCGCCGCGACCGATACCAGGGCAAAGGCGTAGGAGGCGCGATCGCGCACCTT
ACGGTAGATATGTTTTCCGCCGAGCGGTGGAGGTAACGTCACCGCAACGATAAGCTCACCGGGAAGCAGGGCGGTTTCAA
TGTGCGGCGTTTTTCCCGGAGGGTGATAAAAATCAGCCAGTGTGATACTGCGAGTCTTTCCCTCCGGCGTGATGGTTTCC
ACCACCGCATCCAGCAACCGCATTGCGACCGCCATATCGCTCGGATGGGTGGCAATGCAGGCTTCGCTTACGCCTACCAC
CGCGTGCTGACGGCTAAAGCCTTCAAGCGCCGCGCAGCCGCTCCCGGGCAGGCGCTTATTGCAGGGCTGATTGGTGTCGT
AAAAATAGGGGCAGCGCGTGCGCTGGAGCAGATTACCTGCGGTGGTTGCCTGATTACGTAACTGACCAGACGCGCCAGCG
AGCAGGGCGCGGGAGAGTACCGCGTAATCACGACGCACGCGCTCGTGAGCCGCCAGGTCGGTGTTCCGTACCAGTGCGCC
GATGCGCAGCCCACCCGCGTCGGTCACTTCAATCTTATCGAGCCCGAGGCCGTTCACATCGATAAGGTGGGTGGGCGTTT
CAATTTCCAGCTTCATCAGGTCCAGCAGATTGGTCCCGCCCGCGATAAATTTTGCGCCGGGTACGCGCTGAGCGCTAAGT
GCCGCCTCGGCTGGGGTATTCACTCGTTCATAGGTAAACGCCTTCATGATTTTATCTCCCCCGCAGCATCTTCAATGGCG
GCAAGGATGTTAGCGTATGCACCACAGCGACAGATGTTGCCGCTCATACGTTCACGGATCTCATCGGCAGTTGTTTCTGG
AGCGGAAACCAAATCGACCGTGACGTGACTGGGAATGCCGTCCTGAATCTCTTTTAGCACCGCTACTGATGAGCAAATTT
GCCCGGAGGTGCAGTAGCCGCACTGGAAGCCATCATGCTTGATAAAGGCCGCCTGCATGGGGTGAAGATTATCTGGCGAA
CCCAGGCCTTCAATGGTGGTGATCTCGGCCCCCTGATGCATGACTGCAAGCGTCAGGCAGGCATTAAGCCTGCGACCATT
GACCAGCACGGTACAGGCTCCGCACTGTCCGTGATCGCAACCTTTCTTGGTACCGATCAAATGCAGATTTTCACGCAAAG
TGTCCAGTAGCGTGGTTCGGGTATCCACCTCAAGCTGCTCGGTTTTGCCGTTCACCTTCAGTGTCAGGGGCATTATCTCT
GGCGCGGGTGTAGCTGCCGGAACGCTTGCCGCCAGCGTAGAATGAGGATAAACCACGGCGGTCGCCGCTGTTGCGGCGCT
CACTTTAATCAGATCGCGACGGGTCAAACTTAAATCGTGCGGCTCGTGCTTCCCAACCCGATTATCTTCGGGGTATTCGC
CTTGGTTGCTCATACCAGGCCTCCGGTATTCTAAAGGGGAAAATAAGAGTGCGTTGCGAGAGTGCTGATGCTTTTTTAAA
CGTTAAGCATAGTCGGCGACAGCAGGAGGATTATTCTTAAAACGCGAATGCAGCTATGAGCCCGGCTAATAAATTCATGT
TCGCCGGGATGTTGATGATGATGGGAGCTGGTTTATTGCTTTAGTTGTACGATGCAAAAACCAATAAGGAAACCTGTGAT
TTTCAGCTCTACATCACCCTGCAAATCTCTGTCACTTCTAATATAAAAATAGGGAGAAATGATGGAGCTTATATTCATTG
GCGATTAGGAAACTATCTTGTTATACAAAACAATACAGTTCTTTACATTTGCCTTGTTTTATGAATACTCCTGAAGAGGT
GTATAACATAATGGTACAAGCAGGGTAGATATGAATATATTTGAACAAACTCCACCGAACCGCAGACGTTATGGTCTTGC
TGCATTCATTGGGCTGATTGCTGGCGTTGTTTCCGCATTCGTGAAGTGGGGGGCTGAAGTTCCATTGCCGCCACGTAGCC
CGGTGGATATGTTTAATGCAGCGTGTGGCCCGGAATCATTAATCAGGGCTGCAGGCCAAATTGATTGCTCGCGTAATTTT
CTCAATCCACCGTATATTTTTCTTCGAGACTGGTTGGGGCTGACAGATCCCAATGCGGCTGTTTATACCTTTGCCGGGCA
TGTCTTTAACTGGGTTGGTGTTACGCACATTATCTTTTCGATAGTGTTTGCTGTCGGTTATTGTGTGGTCGCTGAAGTAT
TTCCAAAAATTAAACTCTGGCAGGGCTTACTGGCAGGTGCTTTAGCCCAACTTTTTGTTCATATGATTTCATTCCCTCTC
ATGGGACTGACGCCACCTCTGTTTGATCTCCCGTGGTATGAGAATGTTTCTGAAATTTTTGGACATTTAGTCTGGTTCTG
GTCTATTGAAATTATTCGCAGAGATTTACGAAACAGAATTACTCATGAGCCAGACCCTGAGATCCCTTTAGGCTCAAACA
GATAATGCATTGAATGATAAAAATGGCGCAAATACAGCGCCATTTTTATAGGTTAAAAACATTGCTTTTTATATTCTGAT
GCAGATAGTCAGTGAGTATATCGCGCTACTTCAGGATGATGTAGATCCGAAGAACGCTACAGAAGAGAGGCATTGTTGCT
GGCAAATAGAAGAAGTATCGGGTTTTGTTACCCCTGAAAAACGAAGCCCCGCTATTATCGCTGGCGGGGCAGTGCAATTA
ATTATTGTATGTTTATAACGGTGTCAGCCCGTATTTAGCCCTTGCACGATTGCAAGCCTCATTGACGACTCCGTTCTCAC
CAGACATGGCGAATTCTCTGCATGTGGACGATCGATTTTTATATATCGTGCAGCAGGCATTTTTGCCCGGGGTTCCTGCA
AGGGCAATACATCGGGGGTTTTTCTGATTGGTACCGCTCATGCATCGGTGAAAAGGGGATATTTGTTCAGTGAGCCTGGC
GGGAATAGTACCGCCAGCATCGTCGGCTTCGGCCCAGTAAAAAGAGACGCGGAAAAAATGCACAACAGGCACCACACGTCA
TGCATGGATTCAGATTGCTCATAATTCACCAGTACAGCTAAAAATCGTAAAGAGAACAGCGGTACATCGTATGTAAGAAC
GTACCGCGGTTGGCTGGGGAACTTTCGATAGTGTGAGTATTGAATGATTTCCAGCCGTTACCGATTTTACGTACTAATTC
GTGATTAAACCACTCGTTAGCAGGCTGCCTGATACCTTTCCGTGTCTCCTGAACACTATGGTGTGCATATGAGAAAGCCC
ATCGTTACAGGTGATATTGACGATCGCTTCCACATCATTTAAACGATTTTTGGGGCTCTGAGGTGGAATTCTTCCCTCGA
CCGATGGATAAGTCTTAGTCCGCCGAAGGGGGCTTAGCCGGACAGGAATCGCTAATCTTAATGAATTTGTCGTTATAGAC

CAGATAGTGATTCCCCGGCTGACGTAACTCAGGCTGATGAACCACGTCTCCCGGACGTAGATACCAGGCATCGCCTTCCT
CTTCGGTCGAATCACATCCTGGCTTAATCAGTAGCTTAAACCATGTGTTGCCAGTGTTACTTACCGTTCCTGTCACCTTG
TCGAAGGACCATTTAAACTGAACCTGACGTGGTCGTACTACCAGAATGGTATCCATCACCACCACCGGCTCCGTGCTGAC
CACGCCGCCGGTTGGGCTGCGTCTTGTATGGTTACGAGTGGGGACCTCGCGAAATGAGACCCGGTAGTAGCGCTCGCGGT
TATCCCGTGGACCATGATAGTAAAATTTAAAATACTCGCTCTCACCAGCCTGCAACGCCAGCTGGCGGGGGGCGAAAAGC
AGTTCACCATCCACCGGTCGGGTGCGCAATTCACTGCTGCCCGGGCTATCAATAGCACTGATGGCAATACGGTATATCCG
TGCGCTTTTGTTGTTATTCACTACACGTTTGCTGACAAAGTCAGTCTCGGACGGCAGCGAAAATGTCAGATTGCCGACCG
AAATCGCCTGTGTCAAAGGTGCCGCCAGCATACAGACCGCTGTCAGCAGGGCCTTAGTTAATGTTACGCCACGTCGCCTG
AACATGAATCTCTCCTGAAGCGCTGACTTCGCCAAACCAGCCGTTATCATCTACCGTACGCAGCGAAATGGCGTTATCCA
TTGGAATCGAAAATTTGACTGTGGTCTTATCCATCTGCCCCACTTCGCCAGAGATATCAGTCCACGGTGTGGTCAACCAC
AGCGCATCGGTCATATCCCGCCAGCTATCATCGCACCCGGCATCGTAGGTTTTTGTAGCTCCGTTGCGGGTAATAAAGGA
AAGTGTCGCCGGGAACGGTACTTTCGCCTTACCGTCATCGGAGCTGAAGACACAATAGGAGCGCCCGCCAATCACCTGCG
CGGGTCCGGTCACCTTGATCAGCACTTCGTCGGCAGCTGTTTTACCGCTGGTGGTTACGATATAGCCGAAATCGAGTGCC
GACTCGCCGCTGCCAACATATCCCTCCCGCGACGGTGTCTGAGTATATTCATCGGAGATAATACTGATGCTGAAATCACG
GGGTTTGATAATCAGCGTGTTGGAGGTAGAAAATTCATACCAGCCAGACTCCGGTGATGTGGTGTTCTGAAAGCGAAAGT
TGAATAGATCTTTGGTGTTGATATCGCTGATCCCGAGGTTCACCATCTGCTTAAAGAAGTTGCTCGAGAAGAAAACATAG
ATCGAGTCTGCGCTCTTCATCTCGTTGAAGGTGTAGTAATAGGCGGTATTGCTCACCGGTTTCCATGAACTGCCATTCAG
ACTGAACTGCATATCGTACGCCCCGACGGCCGAGGCTAACGACGAGTTGGCGATCGCCGGGAATATATGGATTGAGGTGT
TGCTGAGTCCGTTTGTTTGCAGGGTATAGTTAACCATCTTACAACTTAATCCTGAACGGCTGCCGATGGTTTGCGTCCGG
CAGTCGGCGTTCCCTTCGCCCAGAGTCGGTACTCCGTCGCTGTTGATAAATACTTCCGCCAGCGAGTGGGTATTTATCAA
CCGTAGATTTGCTGCTTTCGTATGGGTGACGTTGCGAACATACCAGTTGCCGGAGGCCTGATCCTTACAGCGTGCGCCGC
TGCTGGCGTCATAGTTCACAGAGGTCTGGCAGGCATTGATGGTCATTGTAAAGCTGCTGCCGACGGGCATTTGTTGCATA
TACTGGTAAAACGCGTCTGACAACATGCCGTGCATCCACTTCGCGCCGCCGCTGGTCACCGTCGCGCCATAAAATCCACT
GGCGTCGGTGGTTTGCGGCAGGATAAGACTGGTGGTCATATTACACCCAGCGTACCAGTTGATGCAGCGCAAGCCAGTTA
AAGGAGATGAAACTGGTGAATTTTCCAGCCACATATCAAACTTCCAGTTGGTATAAAGGCCGGTGTTGTAACCGTTATCA
ATGTAGCCGAGGCTTTGCTGATAGATGGTTCCTGAACCAGTGTATTTTAAACCGGTCCAGCGGTTGGCACCGGTCAGGCG
CGGATCCAGCGCCCCGCCAGGAGTGACGAAAAAGTTGTCGTCTGAGTTGTTTTCGACAAACACAAACTCGCGTGCCGGTG
CATCCGCCCAGGTTGTTTTGCTCACTGCCGCTCTGAGCGCAGTTACTGGCCAGATTAGCGCGAAAATTATCATCGTTATT
AGTAGGTTAACTCTCATTCTCTTCTCCTGTCTGCGTCACCGCCGCCCACGATGAGAGGCCGCTGCAGACCACATCACCGA
CCCAGACGGCACCGCGCGCCTGGTTGAGTTCCAGAGCCACTTCGCAGGTTTTATTGCCACTGTAGCGAAAATCGATAGTG
GGGTATTTCTTATCCACGTCCATGACAAACTCGCCGTTTTCATCGGTTCGGGTTCGGCCGATATGGTTGTTAATCCGTGC
GTTAGCCAGCAGTGTGCCGTCTTCCGCACGGATACGACCGGAGACGGTAACCATCTGCTTCACCTCTGGCTCAATGACAG
CGACATTGCCTGGATAGAGAGTCAGACGACTTTTGCGGCCGCTGACGATATCGTAACTGTCGAGTGAGTTTTTGCTGTTC
TGTAACTCCACCTCATATCTTCCATAGGGAGAGAGCGGGAGATAGTTACGCTTGCCGTTAAGCGGGAAAATCCGCCCGTT
GATTTTGGCGCTGATCTGACCGTCGTCCTCCAGCCCGGTGTTGAATATCACCCCAGCGTTGCCATCAGTCCGCCCGCTGG
CAGCAATGTTTTTACCCTGCCAGCCGACGCTGCCATTGGCGGTCAAGTTGGTATTGACGTAGCCGTCCGCCGCGCTATTG
ACGTTCAGCGTTCCGCTGGCGTAACGAGCGTCGAATTGTGCATACGCCCCGCCGCTGAGGGTTTTGTCATCACCGGTATC
GCCGGAGATGGCACGTGACAGATTGGCACCAACAGTGCGAATGGTTCCTTCATCAAACTGTTTGCGTGCTGACAGGTTTG
CCATGGTGTAGCCGTTTTGATGAGTCATCCCTGCGCTAAACCAGTTGCCCAGTGGTAGCGAGAGATCGAGAGCGATATAT
TTTCCTGTATTGGCGTTGCTGTCGCCGTTGTTATAGCGCTGAATACCGGCCCGCAGGCCAAGCGAACCAAAGGTACCGCT
GTAGACATTTTGATAGTAATCTGCCGTGTAATAATGGCTGTTGTAACGGCGGTCATCATTGTAGCTGATGCTGAATGTGC
CCAGCTTCGACCACAGTGAGTTCAGGTTGAGTGTGCCGCCGATTGCACGGTTGTCGGCATCGCTACGTCGCAATTGATTG
CCAATGCGGGTTTTTTCCTGATTAACCCACAGCGAACTAAAGCCTCCCGGTAGAGTGGCGCTGATGCTGCCGATGCTGCT
CCATGAGCTGTCACTGGCCAGCATATTTTGCAGGTTAACGTTGATCGCCCCCCCAAGCGGCAGCGTCAGACGGGTTTCAC
CCACCGCCTGATTATCGTATCCATATCCCGTTGCCGCCCAGCTAAGCGTACTCAATGAGCCGGAGGTCGAGGCACCTGCT
AGCCAACTCTCTTTAGCTGGTCGCGTCTTTTTCCCGTTTTCCGACCAGCGATCCATATGAAAGCTACCGCCCCATACCTG
CCACGCCAGTGGTGCACCGACGCCGCGCCCCCGGCTAAACAGCTTATTGACCCGCTGGGTGCGTTTGCTGATCACGCGAC
CGTTAACGATCACCTCAACTTCCACATCGTAAATCCCGTACGGTAGACCCCGGGTATCCACTTCATGATTGCCCATAGTG
AAGTTCTGAACGCTTAGTAGCCGCCCATCACGGGTGAGATGTACTTCGCCCGCCGCCGGTAAAAAGGCGATCACTGGCGT
GGCTGACTGGCTGCTGTCGAAGATGGTGGAGCTGGCCTGGTTTCCCCAGGAAAGGCCGTAAATCTTCCCTGCTGAAATGG
CGGTCATCGGCCCTAAGGACTGCAAGTTCCAGGTGTCGAGCATTCCACCGGCAAATCGGTGACCGGCAAAATCGCGTTCA
TACATCGCTTTATATAATTCACTGTCCTGTTGACCGCTACCGATCCCGTACAGCGAGCCGTCGAGCACCACATGATGTTC
GCGCAGTGCAGTAACGTTATTCAGCGACAGATAGCTGGATGTGTTGCTCCCGCCGTTACGCAACTGGTTGTTATAGACGC
CCAAGTTATAGCTCAGATTACTGCTGAGGGTGTTAACACTGGACTGCCCGATGTCTTCGCTACGTGAGCGTAGTACGGTG
CCCAGCGCTTCGCGCTTGACCACTAGCTGCAGCAGCAGTTGGCGCAAGCTGAGATCCAGCTGCGCGTTGTCAGTCAGGGG
GATGGTAAGGGCCTCATTGAACGGGGCGTTGGCCAGAGCCATCAGCTGCTGTCGAGTTTGTTCGCTGACACTGGCGTTAT

CTTCACTCTCTTCCAGCTGTATTTTCCGGATGCGTAGCTGTCCATCGTCCAGCCAGATAAAAGCGCTGCCGATTCGCTGA
TCGTCCTGGCGACCCTGGCTACCGGCGAGATGAATATAGAGCGGGACGCTCATGCCGTCCTGAAGCGCCTGACTGAAGGC
CTGCGGAATAATCACCCCACCTATTTGCTGCGCACTTATGTCAGCAGCGCTGGCATCGGGCTGAACGAACAAAAAGACCA
TGCCGAAGGCAAACTGGGCTTTCAGTCCTGGGGAGAACCGTCGTAAAGGCATTGTCGTTATCCATCTGCACAGCTATCTA
TTTCACGGGAATGAACTTATCACCCTGCCAAAGTGCAACCCGTCCTTTGTTATCCGCAGTGTCCACGCGGGTAAAACGAC
GCGACTTGCCCGGCATCAGGTAGTAATTCTCTTTACACTCCTTACCGTTGGCGGCTTTCAGGCAAGGTCCGTAGGCGAGG
ATCCGCAGCGTCGCATTTCCTGTATTTGTCAGGGAGCCGTTGGCGTACTGAAAGTGGTAGTTCGCCTGACGAGGGGCGAC
GACCAGAATGGTGCCGATGCGGGCGGAAGCAGTGGCCACAGCGCTGCGGTTGGCATTATCGCGCTGCGCATCACTGAGGG
CCTGATCAAACCAGACAATGCGGTAGTAGCGCTCTTTTTCATCTGCGGGTCCCTTATAGAAGAAGCGGATCACTTCGCTG
GCTTGGGCGGGTAGCAGCAAGCTGGCGGGAGTGAGTAGCAACTCATCCGGCTTGTCCATTGAGATAACCTGCCCGTCGTC
AAGCGGTGAAGAGAGCCGTTCGAGACGGATATTGATAAGGCGACCACTGTCGGTACTGTTTTTGATCGTTTTGCTCAGCG
TGCTGCTGTCACTGTTCATAAACGATGATATATCGCCGACATCCAGCGCCTGGGCCGGAGATATTCCGGAAAACAGCAGA
GCGAGAGGCAGAAGGTGCTTTTTCATAACAATTCCGTCCAGGAATAAAGCAGGGGGGCTACCCCTGCTGGTACATCAGAG
AGATTAACTGGTCCAGGTCGCGTCGAACTGTACGCTAACGTCGCCGCTCCAGATGCCTTCCGGTAGAGTGCTGTAATCTG
TTACTGCGGTGGTACCATTGGTGGTACCGCTGATGATGGAGAAGGTGAAACCATCCTGTGCGGTGGTACGATTGCTGGCA
TTGTAACCGTTAGCCAGCGGGCTAAGGTTGCCGCCCAGTACGCCGTTGGCGGTATCGATCATCACGGTATCGCCAGTTTT
TTCGACTGCCGCGCCGTTATAATCCACGCCCACATTCAGTGTGGAACCTGAGGTATCCAACTGGGTTAATGTGTTGGTGA
TAAGACGTGAGGTCAGTTTAAAGGCGGTAGCCGTTGAGTCACCCTCGATAGCCACGTCAAATAGACCTTTCTGTGAGTTA
AAACCTTTAATGCCTTCGGCATACTGGAACGCCAGGCTACCGAGTGGCGTCACAACCAGCTTACTGGTGGTGTCTTTTTT
GGCTGTTGCCGACCAGGTCGCTACAGCCTGAGCTGTTACGTCAGCAGCCTGCGCCACACCCATGCCGGTAAACACCGTTA
CCAGAGCTATTGCCAGAACCTTTTTTTTCATTGCATTTCTTCCCGAGTTGAATTGAGGACGTGATGTCCCAGTGAGTCAT
TTTTTAAAACTAACTTGCCTGGAGTTTACTGAACCAACTTATATATTTTTGAGTACAGCTTGGCCTCAGCATTACGCCGA
TGGGTATACACTGTCTTCACACTACAATTTTCAATTCTGGCAATCGATTTAGGTTGCATTCCCTGGGCCGTCATGCGGAT
AATTTCCATTTCCCGGTCCGTGATTTTATCTTTCTTAATATCTTTTCTCAGGAACCTGCCATTAATCACATAGACCAGTT
CTTTTCTAATATCACGACTCAGTCCAGCGTATACCACGCCCCTAATATTGCTATTGTAATACCAATAATTTGCTAAGGCC
TCTGACTTTCTGGCCGCAATCAATACGACCTGTTTCCCCTTTACCGCGGATAACCATTCATTATCACGGTTTATAAATTC
AGATAAAGAATCCGTACTGAGATTGATAAACACGAAATCTTTTCTAATTTTCTCCAACGATAGATAAATTAATCTGTCGA
TATCCACAATCAGTTCAGATAGTCCTTTATAGAAGTAGGCGTCATGGGGAGACCAGATGTATTTATCAGAACGGTTTTGA
CATTCCATATGGTTTTTAACCTCATAGTCCCTACTGTAATCACTTTGCCATGTCACTACTTTCCAAACCTGTAATTTTTA
CAGGTGCATTCAGTTTCCTTAGTAAACCGGAAGTAAATAAGATACGTAGTACATATCAATAAAGGTTAAACGAGATTAAG
TTTAATTATTTTCGATTGGGCTTAAATTTATGTTTTTTTTCCCGATGAGTGTCAATATGTGAACGGAAAAGGAATTCAAT
GGTTAAGTCAATATTTAGAAAAACTGAAAATATTGCTTTTTTGATAAGAAGAAAAAGACATTTGGTTTCATAAGGTTATT
GGCAATATAACGTTCAATTCTTACTTGCATTTTATATTGGTCAGGAACTTTGCCCTGAAGTGTTAGGAATAGTCTTAATG
CGCTGTGTGATGGACTAGTAAAAAAACAAGTTTTAAGGGTAAGGGATAGAATATAAAACTATGATGAGCTGTCATTGAAG
ATGATTTGCCAATCAGTAAGATATTTTAATGGATTAACATGCCGCACAAAATGGTTACAGGTGTTCATTCCAATAAGTAA
TGGAAAATTGAACGGGCGTATGTACGTCATCTGTTCGGTAAGCCCCCAAGAGCCAGATTAAGTGAAGTTAAACGAAGGCT
GCCAGCTGTCACAGGAACGATGGCCTTTTTGTCAGGGCACTGCATTCAGTTATCTGTGCGCGTTACTCTAACTGGCACTC
GAACGTCGGCGACGGGGTTACCGGTGGATCAGACTGTTCCTGTGGCGTAAACGTTTTTATGTTAGTACCAACAGATTTAC
CACATTTATCATCTTAACAGTTTGAGTGAAAATGCAGGCTGCGCCGCAAAGGGAGGAGACTGAGTGGCGTGTTCAGTCTA
AAAGAGGGTTGATGCCAGCATACAGGGGCGAAGCTGGTCAACAAGTCAACATTAAGATAATGGAATATTCGGAGAGAAAC
GTCAGGCAGTTGGCCTCAAATGAACAGGAAGAATATATCCCCAGGAAAATTAATGTTGGTGTTATAAACACACCAACATT
AATCCGATCTGATTATTAATCAGACAAATATCGTTAATCCGTCGAATTTTGAATCAACGTTTTTTCTTACGACCCTGAAC
GGCCTTAAAACGTGGATTAGATTTACAAATCACATATAGCCGTCCTTTTCGCTTCACAATCTGACAGTCTGGATGGCGTT
CTTTTGCGGTACGCAGAGAGTTAAGAACTTTCATCACGCCCCCTTTTTCGTGCTAACAAAACGACCAAAACGTTGGGTGA
ATCGTGCAACATTTCCTTCTGATGCCACTGTTCTCAGCTTCCCTGTATAGAACGGGTGCGATTTAGAAGAGACATCAATT
GTCACGTATGGATACGTTACGCCATCCAGCTCAATCTCACGGTCTGTTTTGATAGTCGAGCCGATTTTAAAGTACTCATC
AACACTGGTGTCGTGGAACACCACAGTACGATACTCAGGATGGATATTGGGCTTCATCATTTTTACCTGTTATGTTATAA
CATAACCATAAAGATACATGCTCATCCGGATCATTTCAACACTCAATCCCTGTGACTCTTTGTGGATGTGGAATTTTCGC
AACGGGGAACTCTGGCATTGTTAGCAGCCATTGGTGACGCTCCCATAAACGCCTGGATGAGTGATTGATGATGGTTTTGC
TGAGAGTATGGATTTTTCGAGAATGGAGGTTATCTCCACTTTACTGGCCGATAATTTATCGCAGTATGATTGATTACCTG
ACAGCGTGTATTCCTCTCCCACGGATATAATAATTACTTACAAAAAAGGGAGAGGATGCATATTTTAAATATCACTGAAG
TGAACAGTTTATTTCCGTTATTAATAGAAATGGAGAAATAAATAGGCGTATTCTACAATTGCGACAAAAACAACGATATT
AATCAGTTTATGACTGATTTGCTGTACTTTATTCTCTTTCATTGGTACTTCCTCGCTTTAAAAAAGAGTGCACTTCGTAA
GTGCCCTTATATAAATAACGAGTTTGGTCAACCAATTTTTTGACATGTATCACAAATTTGAATAGATGTATTACATCAAC
TATCTTTTATTGCACCAACGTCATTGATATATGTCGCCTGAAGTCAGTTCCGGGAATGAGTCTGATCTCAAGACTGGCCC
AGTCCGGGCGTTGATTGGTGCTGAGGAGCATATCGCATCTCATCATAATGTCGTATCTCCTGGGGTGTTATACAAGATAT

CGTTGTTGGTGACCTGGGAGAGGAATTGAGTTCTATTAAACCGTCAACTATGCCGGATACATACTGGATTACACTGCAGG
CACGCCTTATGAGAGAACGTGCCGCAGTGACGGGTTAATTATCTGAAAGAATTTGTGAGGCTGTATCGGTTACTCATTGA
TTTGATAGTTTTACTCTCGGGAGAATAATAGATATTTAATCCATTAACGGAAACCAGCCAGTTCCTTTCGATGCCTGAAT
TTGATCCCATAGTTTATCCGGAATGGTTAATTCATGAACAAGTTGTGTTATCGTTCATGAGAAGCATAACGTAAAGGGAA
AAGCTCGATTAGACGGCAGAATTTGTCAGGGGTTATGAACGAAATTCATAAATCTGTTTGAGTGTTGCGATGGGTAGTGC
AAGTTCGATATCTCCGCAATTTACAGTCCGATGAAGGAAAATGAATATCCATAAAAAATATATTGGTTTATCCTGGCATA
TATACCTATTTCGACGTATTTCCAATAGTTTTAATTAAAGGCAGGTCATTGTTATTCACTCTGAATAGTGAATTATTCAC
TGTCCGCAGAGTAAGAAATATAACTTAGGTATCTATTTAATGACTTGCACAAAAAGCTAAATTTTCCCCCATAAATAAAA
ATATAATCCCGCGCCCAACCACCTGATGAGTGGCTATAGGCACTGGATATATTAGGTGGCGGTGCACTTTCTTACATAAA
GGTATTTCCTTTTCTGCGGAAAAGGAAATCGGGAAATCCCCGGTTTTTCTGACAAGCAGACGCCATTATTTGTGTCTGCC
TATGTTCGTTAATTCGTTCATCAGGAAATTATCTCAATGTCACATTATAAAACAGGTCATAAACAACCACGATTTCGTTA
TTCAGTTCTGGCCCGCTGCGTGGCGTGGGCAAATATCTCTGTTCAGGTTCTTTTTCCACTCGCTGTCACCTTTACCCCAG
TAATGGCGGCACGTGCGCAGCATGCGGTTCAGCCACGGTTGAGCATGGGAAATACTACGGTAACTGCTGATAATAACGTG
GAGAAAAATGTCGCGTCGTTTGCCGCAAATGCCGGGACATTTTTAAGCAGTCAGCCAGATAGCGATGCGACACGTAACTT
TATTACCGGAATGGCCACAGCTAAAGCTAACCAGGAAATACAGGAGTGGCTCGGGAAATATGGTACTGCGCGCGTCAAAC
TGAATGTCGATAAAGATTTCTCGCTGAAGGATTCTTCGCTGGAAATGCTTTATCCGATTTATGATACGCCGACAAATATG
TTGTTCACTCAGGGGGCAATACATCGTACAGACGATCGTACTCAGTCAAATATTGGTTTTGGCTGGCGTCATTTTTCAGG
AAATGACTGGATGGCGGGGGTGAATACTTTTATCGATCATGATTTATCCCGTAGTCATACCCGCATTGGTGTTGGTGCGG
AATACTGGCGCGATTATCTGAAACTGAGCGCCAATGGTTATATTCGGGCTTCTGGCTGGAAAAAATCGCCGGATATTGAG
GATTATCAGGAACGCCCGGCGAATGGCTGGGATATTCGTGCTGAGGGCTATTTACCCGCCTGGCCGCAGCTTGGCGCAAG
CCTGATGTATGAACAGTATTATGGCGATGAAGTCGGGCTGTTTGGTAAAGATAAGCGCCAGAAAGACCCGCATGCTATTT
CTGCCGAGGTGACCTATACGCCAGTGCCTCTTACCCAGCAATAGTGGACACGCGGCTAAGTGAGTAAACTCTCAGTCAGA
GGTGACTCACATGACAAAAACAGTATCAACCAGTAAAAAACCCCGTAAACAGCATTCGCCTGAATTTCGCAGTGAAGCCC
TGAAGCTTGCTGAACGCATCGGTGTTACTGCCGCAGCCCGTGAACTCAGCCTGTATGAATCACAACTCTACAACTGGCGC
AGTAAACAGCAAAATCAGCAGACGTCTTCTGAACGTGAACTGGAGATGTCTACCGAGATTGCACGTCTCAAACGCCAGCT
GGCAGAACGGGATGAAGAGCTGGCTATCCTCCAAAAGGCCGCGACATACTTCGCGAAGCGCCTGAAATGAAGTATGTCTT
TATTGAAAAACATCAGGCTGAGTTCAGCATCAAAGCAATGTGCCGCGTGCTCCGGGTGGCCCGCAGCGGCTGGTATACGT
GGTGTCAGCGGCGGACAAGGATAAGCACGCGTCAGCAGTTCCGCCAACACTGCGACAGCGTTGTCCTCGCGGCTTTTACC
CGGTCAAAACAGCGTTACGGTGCCCCACGCCTGACGGATGAACTGCGTGCTCAGGGTTACCCCTTTAACGTAAAAACCGT
GGCGGCAAGCCTGCGCCGTCAGGGACTGAGGGCAAAGGCCTCCCGGAAGTTCAGCCCGGTCAGCTACCGCGCACACGGCC
TGCCTGTGTCAGAAAATCTGTTGGAGCAGGATTTTTACGCCAGTGGCCCGAACCAGAAGTGGGCAGGAGACATCACGTAC
TTACGTACAGATGAAGGCTGGCTGTATCTGGCAGTGGTCATTGACCTGTGGTCACGTGCCGTTATTGGCTGGTCAATGTC
GCCACGCATGACGGCGCAACTGGCCTGCGATGCCCTGCAGATGGCGCTGTGGCGGCGTAAGAGGCCCCGGAACGTTATCG
TTCACACGGACCGTGGAGGCCAGTACTGTTCAGCAGATTATCAGGCGCAACTGAAGCGGCATAATCTGCGTGGAAGTATG
AGCGCAAAAGGTTGCTGCTACGATAATGCCTGCGTGGAAAGCTTCTTTCATTCGCTGAAAGTGGAATGTATCCATGGAGA
ACACTTTATCAGCCGGGAAATAATGCGGGCAACGGTGTTTAATTATATCGAATGTGATTACAATCGGTGGCGGCGGCACA
GTTGGTGTGGCGGCCTCAGTCCGGAACAATTTGAAAACAAGAACCTCGCTTAGGCCTGTGTCCATATTACGTGGGTAGGA
TCATAAAGTCCTCGGGTCGTTGTCCACTCTTTTGGCATGTCAGCATATTCACATACCGTACCAGCAAAAGTTTCAACCGT
GAGCAAATCGACCGATTTTGTTGACGGTTCAAACGTGGAAACGAAATAGATATCCGTCTTAAAATTCTTCATACATCTGG
CTAAATGATGACGTCGTAGTTTGACTTCTTCATCCGGTGATTTATTCGACACTTCAATGTCATATTTAAATGAGTTGCCA
AAAATAGGTGTCTCAGGAAAGTTGATTAATCTACACTCCGTCTTTTGCTGTTGACGTATTAAAAAAGAAGGGAAGATATT
TGCGAGATCCCAATAATCACGGTGGCGGTATTCACGGGGAGAGCAGCCGAATAACTTTTTAAATTCGCGGCTGAATGACT
GCTGTGAATCAAAATGCAAAGAGAGTGCAATATCAAGCATAGATTTCGCGGTGAGCCGGACAAGAATGGCGGCACGACAA
AGCCTTCGTTTGCGAATGTATTCTCCCAAAGGCACATGCATGAAATTTCGGAACAGAAGCTGGATGTTGCGTCTGCTGTA
GCCAGATTTCTGTGCGATATCTTCGATTGAAATAGGGTGCTCAAGATTGCACTCAATCCACTCCAGGAGCTGCTGTAGAA
TCTTCTGCCTGATCATCTCATTCTCCGTTAATTCTTTGTCATATCGCACATTTTCGTTATACCCTGGGCCCTAAGTGTAT
CAAAAATAGCAAGGGCGAAGGCAAAAGCACTGGTAGCGTCTGCTTCGTTGTCCCCGAAAATAGAGAGGTTTCAGTCCTAC
ATTATTAATGAATTTTTTGCATAAGTGATATCAAAATCCACATACTACTTTGAGGTTACGTTTTAACGTAGACTCATTGT
TCATGCCTAATGGAGGGACTGACAGTGGAATTTTCCGTATAGTCTGCTTCAGAGTATTGCAGATGCCCATCAAAAATCGA
TTTCTCACTGACAGATAAAGAAATGGCACAAATTAGTTCGCTTGATTTGGGTTATGTTGGGGAGTCGGTAAAACATTTTA
ATCCTGAATTTGTTCGTGGTTGTCTTGCTGTAAAAATACATGATTGATATTAATCATGTATTTTACCTAAGACGACAAAA
ACCTTTTAACAGGGAGTGATATTGATCTTCACTCTGTCATATCTCCGGTAATATGGCGTCAGGCTTCATCATCAGAAAGG
GGGCCTGACGCCTGAAAAAGTGAACAACAGACAGTGTTCGGATTATCAATATTCAGTTTTTAACGTTGAACTGGATTCAT
TACTGCGTTGTTTAAGAATTTCCCGCGCCGAATCTGCCATTATCATGACTGCACCTGCCAGCATCAGAGTATCTTTCAAT
ACCAGGCGACCAGCACCGGATAAATAAGGGAAACCATGATGAGCGTCACCCAATGCGGGTACCCATGCCTCCGGGGTGGT
GATTAAAAATGAGAGTGTTACCAGCGGCGTGGTAAATGCCATCAGTCCTCCCAATAAACCTAACCAGCGATTGACAGGAT

TAGCCAAAACCAGCAGAGCAATAATCACCTCCACGACGCCAAGACCGTTGGAAAAACCATAGGTATTATTGGCCGTTTGC
CATGCCCTTGCTTCTGGTTTGTATTCGCCTTCGTGAGTCAGATACTGTTTATAGTCTTCCGGGTGTTCATAAAAGAACGA
CATTAGTGGACTGTTTGCGACGAATGGTGTAATGCTGTCTGCCTCGTAAGGGACAAACTTTAATAACCCAATCCACATAA
AAACAATTGCAATACTCAGACGAATCAATGTCAGGCCAATTTTATCTCCCCGACTTAACAGGTGCAGGTATTTTTCCATT
GTGAACATCCTTATTTATACAATACTGCTGTTGCGGTTGAATTATTATCAATTCGCATCGATGTGACTTTCCAGGATGAA
GCATTATATTTTCTGGCTATATCAGCAATGGTATCCTCCAGAACTGTCGGTGATGCTGATACTGACGAGACGCTGATATG
TTTCAGAATTATTTTATCATCTGCATTGGTGGAAAATGCCATAACGCCAGATAATAGTGTTGCAAATAAAACAGATTTTT
TAAACATATAAAATACCATCAATGAAGTGATTAAGAAGTTATAGAGTAACAGAGAATTAATAAGATTCTTTTCTCTGAGA
CGCCAGAATATTTGTTCTGGCGTCTGATTTTGAGTTTATTTGACTAATGAAAATAGATCATTGAGTGATTCGCTCATCGA
CGGATGAGTAAATATCTGATCGCGTAATATGCTATAAGGCAGCCCGGCATCCATCACCATTTTCACTATATTGATCATCT
CGTGGGAGTCAACACACAGCAGTGATGCCCCTAACATACGTTGGGTTTTATTATCAACAATCGCTTTTAATACCCCACGA
GTATCATTCATCACTCTGGCACGCGGAATTGCAGCTACAGGCAATGTCACCACCTGAATATCAGCACCACTCTCTCTGGC
TTGTTCTTCTGTCATACCAACCCTGGACAGGGGCGGTGTCATAAATACGGAATAAGGCACATTTTTCCGATCATCAGTAC
TACGTTTGCCTTCACCCAGTAACTCATCACGTACAATGCGGTAATCATCCAGTGATATGTAAGTAAATTGCAGCCCGCCG
GTAACATCTCCCATCGCCCAAATATTGTCTGCGGTGGTATGTAATCGCTTGTCAACGACAATTGCCCCGCGCTCGTTTAC
TGCGATACCGGCATTTTCTGGATGTAACGAAGCGGTAGCCGGTTGACGACCGGAAGCTATTAACAGTGCATCCACCGCCA
GTTGGGCGTGCTCGCTATGCACTTGCACTTGATTTTCATGGTGACTGATTCGCTCCACATGGGCATTGAGGATAATATCG
ACGCCCTGATCGCGTAAAATCGTCGCGATATTATCAGCAATATCCCGATCTTCCCGAGGCAAAAACAGCGAAGCTGCTTC
TAAAATGGTTACCTTGCTGCCAAAATTAGCGAACATAGAGGCGAACTCAACGCCAATATATCCGCCGCCCAAAATACCTA
AATGCCCAGGCAATTCTTTTAGATTAAGTAATCCGGTGCTGTCATATACTCCTGGCGTGGTGGTAATTCCAGGAATTGGC
GGAACCACGGTTTGTGCACCGGTATTAATAAAAATTTTCTCGCCATGAATTTCCAGATTTCCCTCAGGCCGATGAACACG
CAGGCTATGATTATTGATAAACTCCGCCTGGCCGTCGATCACGTCGATATTGGGCATATCCGCAAGATTATGAAAATTCT
TATTACGTAAAAAATTAACCACTTCATTTTTACGCTGTATGGCACGGACAAAATCTGTGTGCTGCTGTGCGTCATGAACC
AATGTTTTGGTTGGGATGCAGCCGATATTAATACAGGTCCCGCCATACATTGCATTTGATTGTTCGATGAGAGCCACTCG
CCAACCTGCTTTTGCCAGCGTGACGGCTAATGTTTTTCCAGCCTTGCCAAAACCAATAATCACTGCCTGATATTTATTCA
TGATGATTTCCTGTCGAAACGCGTTCATTTGACATCATGACTATAGACGCAAAAGAGCAAATAACATTTCTTCACAACGC
TTGATATTTTGTCTTATCGTCTGATATTTTCTTTTTCTTAATCAGAATCACAAAACAAAAATCATGATTAACTATTTGAT
GGTATTAGATTAGTTATTTACTAAGATTGTTGGTGTTTGTAATCAAAAACCACTCAGGAGTCTGATATGGATGCCCTTAG
CCGTTTGTTGATGCTTAACGCTCCACAAGGAACGATCGATAAGAATTGCGTGTTAGGAAGTGACTGGCAGCTTCCACATG
GTGCCGGGGAATTATCGGTTATTCGTTGGCATGCGTTAACGCAAGGAGCGGCGAAGCTGGAAATGCCGACGGGGGAGATT
TTTACATTACGCCCGGGAAATGTGGTCCTGCTACCACAAAATTCAGCTCATCGCCTGAGTCATGTCGATAATGAATCGAC
CTGTATTGTCTGCGGCACTCTTCGGTTGCAACATTCGGCGCGTTATTTTTTAACGTCTTTGCCGGAAACGCTGTTTTTAG
CACCGGTTAACCACAGCGTGGAATATAACTGGCTGAGGGAGGCGATCCCGTTTTTACAACAGGAATCCAGATCGGCAATG
CCGGGAGTGGATGCACTGTGTAGCCAGATCTGCGCTACATTCTTTACCCTCGCGGTGCGTGAGTGGATTGCACAGGTTAA
TACTGAGAAAAACATTCTCAGTTTGCTTCTGCATCCACGTCTTGGTGCGGTAATACAGCAAATGCTGGAAATGCCAGGAC
ACGCCTGGACCGTCGAATCGCTGGCCAGCATCGCTCACATGTCCCGGGCAAGTTTTGCCCAGCTTTTCCGTGATGTTTCC
GGAACCACGCCGCTGGCTGTATTAACAAAGTTGCGTCTACAAATAGCGGCCCAGATGTTTTCCCGGGAAACGCTCCCTGT
TGTGGTGATCGCTGAGTCAGTAGGCTATGCCAGTGAATCATCTTTTCACAAGGCGTTTGTCCGCGAGTTTGGTTGTACCC
CGGGAGAATATCGGGAAAGGGTCAGACAGCTTGCACCCTGAATAAAACCGCCAGAAATCAGGGCAAAGATAATCCGCATT
CCGGGAGTTGTGAGCCTTTCAACTATTTCTATTTCCAACGGTGGTTTGGGCTTTATATATTTTTTCTGATGGACTCTAGC
TCAGTTTTTAAATAAAAATGCAAACTAAATTGCTTCAATTAAATAAAATCCCGACTATTACTTGATACGTGATTATTGTC
GGTGATTTTTTTGTACACCATCAGTGTATATCTCAATAACCCCTGAATAAGTAGCTCTGAATAGGTATAAGGGATGTAGC
CATTTTTAAATGGATTTCTTATGCCAAAAATGATCGTCGATTTCCCCATAAAATGTGAGCGATGCCGAAAGAAATAAAAT
TAGTTATTGCATTTGACGTTTGGATGAAAGATTATCATTTGTCATACAAATGAGGGGTGGTATGTTGCTAGCCAATTAAA
AAAGAACGCCATATTTATTGATGATTGATCCCCCGGTGGGGGGGAAACATCGGGCATAAATGGGCATGAAGTAATGGAGT
ATTAGTTGTGAATGTCAATTTCTTTGTCACCTGTATTGGTGACGCCCTGAAATCAAGAATGGCACGAGACTCCGTGCTGC
TACTGGAAAAACTCGGCTGTCGCGTAAATTTCCCGGAGAAACAGGGATGCTGCGGTCAGCCTGCGATCAATAGCGGTTAT
ATCAAAGAAGCGATTCCAGGGATGAAAAATCTGATCGCCGCACTGGAGGATAACGACGATCCCATTATTTCACCGGCTGG
CTCTTGCACCTATGCCGTAAAAAGTTACCCGACGTATCTGGCGGATGAACCTGAATGGGCATCACGTGCCGCAAAGGTTG
CCGCGCGTATGCAGGATCTCACCTCTTTTATTGTTAATAAATTAGGGGTAGTCGATGTAGGTGCCAGTTTGCAAGGGAGA
GCGGTGTATCACCCATCTTGTAGCCTGGCCCGTAAGCTGGGAGTGAAGGACGAGCCACTTACGCTGCTGAAAAATGTGCG
TGGACTGGAGCTGTTGACCTTTGCTGAACAGGATACCTGCTGCGGATTTGGCGGCACGTTCTCGGTCAAAATGGCCGAAA
TATCCGGCGAGATGGTGAAAGAAAAGGTTGCGCACCTGATGGAAGTCCGCCCTGAGTATTTAATTGGTGCTGACGTGAGT
TGCCTGCTAAACATCAGTGGGCGATTACAACGGGAAGGGCAGAAAGTCAAAGTGATGCATATTGCTGAAGTGTTGATGAG
CCGCTGAGGATATAAAGATGTCGATCAAAACCAGTAATACAGATTTTAAGACACGCATCCGTCAGCAAATTGAAGATCCG
ATCATGCGCAAAGCGGTGGCAAACGCGCAGCAGCGTATTGGGGCAAATCGGCAAAAAATGGTCGATGAATTGGGGCACTG

GGAGGAGTGGCGCGATCGGGCCGCCCAGATACGTGATCATGTTCTGAGTAATCTCGACGCTTATCTGTACCAGCTCTCAG
AAAAAGTGACGCAAAACGGCGGTCACGTCTATTTTGCAAGAACCAAAGAAGACGCTACCCGCTACATTTTACAGGTTGCC
CAACGCAAAAATGCCCGGAAGGTGGTGAAATCTAAATCGATGGTGACCGAAGAGATTGGTGTCAATCATGTGTTGCAGGA
TGCTGGCATTCAGGTGATTGAAACCGATCTGGGTGAATATATTCTCCAGCTGGATCAAGATCCGCCATCTCATGTTGTGG
TCCCGGCAATTCATAAAGATCGCCATCAGATCCGTCGAGTGCTACACGAACGTCTGGGCTATGAGGGGCCGGAAACGCCT
GAAGCGATGACCTTATTCATCCGGCAAAAAATCCGCGAAGATTTCCTCAGTGCTGAAATAGGTATTACCGGCTGTAATTT
CGCGGTGGCAGAGACCGGTTCGGTATGCCTGGTGACCAATGAAGGTAATGCGCGAATGTGTACCACGCTGCCTAAAACGC
ATATTGCAGTGATGGGAATGGAGCGTATTGCCCCCACGTTTGCCGAGGTAGATGTATTGATCACCATGCTGGCGCGCAGT
GCCGTTGGTGCACGTTTGACGGGATACAACACCTGGCTGACAGGACCGCGCGAAGCTGGGCACGTTGATGGTCCTGAAGA
GTTTCATCTGGTTATTGTCGATAACGGGCGTTCTGAGGTGCTGGCCTCTGAATTTCGGGATGTGCTGCGCTGTATTCGCT
GCGGGGCTTGTATGAATACTTGTCCGGCATATCGCCATATTGGCGGTCATGGATATGGCTCTATTTATCCAGGGCCAATT
GGTGCGGTGATTTCTCCGCTACTTGGCGGCTATAAAGATTTTAAAGATTTACCCTACGCCTGCTCTTTATGCACAGCTTG
TGACAACGTGTGTCCGGTGCGTATTCCGCTGTCAAAACTGATTTTGCGTCATCGTCGGGTGATGGCTGAAAAAGGGATCA
CCGCAAAAGCAGAGCAACGGGCGATAAAAATGTTCGCTTATGCCAATAGTCATCCAGGATTGTGGAAAGTCGGGATGATG
GCCGGTGCTCATGCGGCAAGCTGGTTTATCAATGGCGGCAAAACACCACTCAAATTTGGCGCGATTAGCGACTGGATGGA
AGCACGCGATCTTCCTGAAGCTGACGGAGAGAGTTTCCGTAGTTGGTTTAAGAAACATCAGGCGCAGGAGAAAAAGAATG
GATAATCGAGGCGAATTTTTGAATAACGTTGCTCAGGCACTGGGTCGCCCGCTGCGACTTGAACCGCAAGCAGAAGATGC
GCCGCTTAACAACTATGCTAACGAGCGGCTTACCCAACTTAACCAACAGCAGCGCTGTGACGCGTTTATTCAGTTTGCCA
GCGATGTTATGTTGACGCGCTGTGAGCTGACCAGCGAGGCGAAGGCGGCAGAAGCTGCAATACGTCTGTGTAAAGAGCTG
GGAGATCAGTCGGTCGTGATTAGCGGTGACACGAGGCTGGAGGAATTGGGGATTAGCGAACGTTTGCAGCAGGAATGCAA
TGCCGTTGTTTGGGATCCGGCGAAAGGTGCCGAGAATATCTCGCAGGCAGAGCAGGCTAAAGTGGGTGTTGTGTATGCTG
AATATGGTTTAACCGAATCGGGAGGCGTGGTTCTTTTTTCCGCCGCCGAGCGCGGGCGTTCATTGAGCCTGCTCCCGGAA
TATTCTCTTTTTATCCTGCGTAAAAGCACTATCCTGCCGCGTGTAGCGCAACTCGCAGAAAAATTGCATCAGAAAGCGCA
GGCCGGTGAACGAATGCCTTCCTGCATTAACATCATTAGCGGCCCCAGTTCAACGGCGGATATTGAGCTTATCAAAGTCG
TCGGAGTTCATGGCCCGGTGAAAGCGGTGTATCTGATTATTGAGGATTGTTGAGAGAGATATGAAGTCTGGACATAAACC
TGAAGAAGGCATACGAGGTTGAAAATAGCGTTTAGGACAACCTGACTTAACCCCGTTCCGTCATGGTTATGCTGCTCTTC
TGTAAATATCCTGGTATTACCAATGCCAGGATATTTACAGTGAAAAAGACAGGCATCCATTCCTGAAAACGGGTTCCCTT
CCCGGTCATTGTCACTTCAGCTTTATACAGGCACTCTCTATCAGAATGTTGTTTATTACTGCCCAGAGTTTTGTTGGTAT
TCATCTACCATTTTTTTGACAAGGCAAAACATTACAGAAATTACAATGCTTAGAAAAAATCCCAGAGCCAGGCAAATGGC
AGCAGGGTATTTATTTTTTATTTCTGGTGAGCTTATAGTTACACAGCCATCACCGTTCGGATACAATGACCAGTTCCTGG
TCAGGTATATCATTTTTGTATAAAGCTCACTGTTAATTGCTGAAGGCGTTTGTATCTCATCGAGAACTTGCCTGCCATTA
TGATATTCTGACGCATACCATTTGATAATAGAGGCCTGCACGCGAGATATTTCCTCTTCAGCAGTTGGTAAAGCTCTCTT
ATCTCCTGACCATTCCAGCCTTATTTCTCTGTTCTTGACATCTGGGCTGGTGTGAAAAAATGTAGTAATTCGCTGAATAA
CATCTTCAGTCATTGTAGTCTGACAGGCAAAATTATTATTGCTATCACAGCTAACAAGTATACCTGGCGTTGTCTGGTTT
ATTTTTAGGCTGTAAATCACTTTTATATTGTCTTTATTGATATACAGAAACAACAGCACCATAGCCATACATATAGCCAT
AATAAAAAGGATAACACGAATCTTTTTCTTCAGATAAAATAAAATCTCAATCAGATCGATATCCTGTTTGATTTGTTCAC
GCATAATATATCCAGAGAATAAAATCTGTCGCAGATAAGGTTGTATTAATAGTCTGTATCAGGAATGTTCGGGTTAAATA
TCAGCAAAAAGCCCGCATCATGAATACTGGATATGAAGCATGAGAGTTACCTCAGTGTTTATATAAGGATTCGGTCCCCC
TCTCTGGAACGGTAACTCTCAATCTGATCGGTTCCTGCGTTAGTTCACATCACGACTCATTTTTTCGCTCTCACCGGCAT
CCCATTTGCCACAAAATATCCCGCCGTGCTCCTCGGCAGCGCTTCCTGTCCACGAATCATATCCGCTATTTTCTCGCCAA
TCATAATTGTCGTGGCGTTCAAATTCCCGGTGATAATCTGCGGCATAATCGACGCATCCACCACACGCAGGCCTTCTAAC
CCGTGTACGCGGCCTTCGCCGTCAACCACGGACATCTCGTCGTAACCCATTTTGCAGGTACCGCACGGATGGAAGGCGGT
TTCGGCGTGGTTACGCACGAACTCATCGAGCTGTTCATCCGTCTGGCATTCGACACCGGGGCTGATTTCGCGGCCACGAT
ACTGATCCAGCGCGGGTTGATGCATGATCTCGCGGGTGATGCGAATTGCGTCGCGGAACTCCTGCCAGTCCTGCTCGTGC
GACATGTAGTTAAACAGAATCGCCGGATGCTGGTGCGGGTCGCGGGATTTAATCCGCACATGCCCACGGCTTGGCGAGCG
CATTGAGCCGACGTGGCACTGGAAACCGTGCTCTTTCACTGCATTCGAGCCGTTATAGTTAATCGCTACTGGCAGGAAAT
GGTACTGAATATTCGGCCACGCAAATTCCTCACGGCTGCGAATAAATCCACCTGCTTCAAAGTGGTTGCTGGCACCAACG
CCAGTGCCGCCAAACAGCCACTCCGCACCGATTTTCGGCTGGTTCCACCACTGCAGGGCAGGGTAGAGGGAAACCGGTTC
TTTGCACTCATATTGCAGATACATCTCCAGATGATCCTGAAGATTTTCGCCGACGCCGGGTAATTCATGCACCAGCGGAA
TATCAAACTCCGCCAGCAGTTCAGCGTTGCCGACGCCGGAGCGTTGCAGGATCTGCGGTGAGGCAATCGCGCCTGCACAT
AACAGCACTTCTTTGTTGGCCGTTGCGCGGGTTGGGATGGTGCTGTCGCCTTCCAGCCATTCGACGCCCACCGCGCGTTT
GCCGTCAAAAATGATGTGATCGGTCATAGCGTGAGTACGAATGGTCAGGTTAGGACGCGATTTGGCCTGATCGAGATAGC
CACGCGCGGTGCTGGCGCGACGGCCCTGCGGCGTGACGGTGCGATCCATCGGACCAAAACCTTCCTGCTGATAACCGTTG
AGATCGTCCGTGCGCGGGTAGCCCGCCTGCACGCCCGCTTCAATCATCGCTTCAAACAGCGGATTGACGCCGGGTTTGGA
GGTAGTGACGCTCACCGGGCCATCACCGCCGTGATAGTCGTTTTCACCCATATCGCGAGTCTCGGCCTTGCGGTAGTAGG
GCAGGCAGTCGAGGTAGCTCCAGTTCTCCAGACCGGGTTCTTGCGCCCAGTTATCGAGATCCAGCGCATTGCCACGGATG

TAGCACATGCCGTTGATCAGCGACGATCCACCCAGACCTTTACCGCGTCCGCACTCCATGCGGCGGTTATTCATAAACGG
TTCAGGTTCCGTTTCATAGGCCCAGTTGTAGCGTTTACCCTGTAGCGGGAATGCCAGGGCAGCGGGCATCTGGGTGCGGA
AGTCAAAGCGATAGTCCGGGCCGCCCGCTTCAAGCAGCAGCACGGAGGTATTCGGATCTTCAGTCAGACGGGTAGCGAGA
ACGTTGCCGGCTGAGCCGGCACCAATAATGATGTAGTCAAATTGCAAATAAACCTCCTGGTTAGAATATGGACTGGAATT
TAGCCATCTCAACCTGGATGGACTTCACCTGGGTGTAACTCTGGAGCGTCATCACGCCGTTCTCGCGACCAATGCCGGAG
TGTTTGTAGCCGCCAACGGGCATCTCTGCCGGGGATTCGCCCCAGGTGTTGATCCAGCAAATACCCGCTTCCAGCTGATG
AATGACGCGATGCGCGCGGTTCAGGTCCGCTGTCACGATGCCCGCCGCCAGGCCGTAGTCGGTATCGTTAGCGCGGCGAA
TGACTTCGTCTTCCGACTCGTAGGTCAGAATGGACATCACTGGCCCGAAGATCTCTTCACGCACGATGGTCATATCGTCG
CTGCAATCGGTGAACACTGTCGGTGCAACCCATGCGCCGTTATCGAAGCCATCGCCTTTCAGTACATCGCCGCCGCACAG
TACGCGCGCGCCTTCCTCTTTGCCTTTGGCGATATAGCGCAGCACGTTATCGCGATGCGGGAAGCTGACCAGCGGGCCGA
AGTTAGTTTGCGGATCGAAAACGTCGCCCGCGCGAATGCGCTCAACGCGCGCCAGAATTTTCTGCTCAAATGCGGCTTTG
CATTTCGCCGGAACGAAGACGCGGGTGCCATTGGTACACACCTGACCGGAGCTGAAGAAGTTTGCCATCATGGCGATATC
GGCGGCGAGATCGAGATCCGCATCATCGAAAACGATCAGCGGTGATTTACCGCCCAGTTCCATGGTCACTTCTTTCAGGG
AAGAGGCCGCCGAGTTAGCCATCACTTTTTTGCCGCTGGCGACACCGCCGGTAAATGACACTTTGGCAATGCCCGGATGC
TCGGTCAGATATTGCCCGGTCTCCGCGCCCACGCCCGGCAACACGTTAAATACGCCGTCCGGCAGGCCCGCTTCGCTGTA
AATTTCAGCCAGCTTTAACGCGGTAAGCGGGGTAACTTCGCTCGGTTTGAAAATCATTGCGTTGCCTGCCGCCAGCGCCG
GGGCGGATTTCCACAGGGCAATCTGGATCGGGTAGTTCCATGCGCCAATCCCTGCCACTACGCCCAGCGGTTCGCGGCGG
GTATACACAAAGGACGTTTCACGCAACGGGATCTGGCTGCCTTCCAGCGCCGGGATCAGCCCGGCGTAGTACTCCAGCAC
GTCCGCACCGGTAACGATATCGACGGTTGAGGTTTCCGAATATGCTTTTCCGGTGTCGAGGGTTTCCAGTTTTGCGAGTT
CGTCATTGCGTTCACGCAGAATATCAACGGCCCGACGCAGAATACGCGAGCGCTCCATGGCGGTCATCGACGCCCAGATT
TTTTGCCCCTGCTGGGCGCTTTTCACGGCGCGATCGACATCCTCGCGCCCGGCGGCCTGCACGGTCGCCAGCACGTTACC
GTTGGCCGGGTTAATGGTCTCGAAGGTGCGACCGCTGGTGGCGGAGGTATAACCACCATGTATATAAAGCTGCTGTTCTG
CCATTCGGGACATCACGTCTCCTCGGTTAATCGGTGGGTAGATGCTGAGTGATAAAGTGGCGGGTCAGGGAATTAGCGCG
GGTTTTATCCAGCGGTTTGCCGCTCAGAGCCGCGCGCAGCCATAATCCATCAATCAGCGCGGCCAGGCCGTAGCCCGCTT
CCTGTGCCTGTTCGCGCGGCAATTCGCGACGAAACTCGCTCACCAGATTCGACAGCAAGCGGCGGCTGCTGACCTGCTGT
AAACGATAGAGCATCGGCTGATGCATACTGCTGGCCCAGAACGCCAGCCAGGCTTTCATCGCCGCACTGCTCACCTGCGT
TTCATCGAAGTTTCCGCCAACAATCGCCTGTAATCGCTGCTCTGCACTGCCCTGCGGAAGTGCATGTAATCGATTCAAAA
CCGCGTCACGCAGCTGACTGGTGATATCGCGCATGGTTGCTTCCAGCAGACCATTTTTGTCCCTGAAATAGTGGCTGATG
ATCCCCGTAGAAACGCCTGCACGGCGGGCGATCTGCGCGATCGTTGCATCGTGCATGCCCACTTCATTTATTGCTTCCAG
TGTGGCGTCGATCAGTTGTCTGCGCCGGATCGACTGCATCCCCAATTTGGGCATTTTCGCCACTCCATTCATCAGCGGTG
TTTATCTATTAAAGCGGTTATTGATTGGACGTTCAATATAAAATGTGTCTTAATTGTTACGAATTTGATTTTAAATAGTA
ACAATAACAGTGGGGATACTGGATGACAGACCTTTCACACAGCAGGGAAAAGGACAAAATCAATCCGGTGGTGTTTTACA
CCTCCGCCGGACTGATTTTGTTGTTTTCCCTGACAACGATCCTGTTTCGCGACTTCTCGGCCCTGTGGATTGGCCGCACG
CTGGACTGGGTTTCTAAAACCTTCGGTTGGTACTATCTGCTGGCGGCAACGCTCTATATTGTCTTTGTGGTCTGTATCGC
TTGTTCGCGTTTTGGTTCGGTGAAGCTCGGGCCAGAACAATCCAAACCGGAATTCAGCCTGCTGAGTTGGGCGGCGATGC
TGTTTGCTGCCGGGATCGGTATCGACCTGATGTTCTTCTCCGTAGCCGAACCGGTAACGCAGTATATGCAGCCGCCGGAA
GGCGCGGGACAGACGATTGAGGCCGCGCGTCAGGCGATGGTCTGGACGCTGTTTCACTACGGCTTAACCGGCTGGTCGAT
GTATGCGCTGATGGGCATGGCGCTCGGATACTTTAGCTATCGTTATAATTTGCCGCTCACCATCCGCTCGGCGCTGTACC
CGATCTTCGGTAAACGGATTAACGGGCCGATAGGTCACTCAGTGGATATTGCAGCGGTGATCGGCACTATCTTCGGTATT
GCCACTACGCTCGGTATCGGTGTGGTGCAGCTTAACTATGGCTTGAGCGTACTGTTTGATATTCCCGATTCGATGGCGGC
GAAAGCGGCACTGATCGCCTTGTCGGTGATAATCGCCACGATCTCTGTCACCTCCGGTGTCGATAAGGGCATTCGCGTGT
TATCGGAGCTTAATGTCGCGCTGGCGCTGGGATTGATCCTGTTCGTATTGTTTATGGGCGACACTTCGTTCCTGCTTAAT
GCACTGGTGCTGAATGTTGGCGACTATGTGAATCGCTTTATGGGCATGACGCTCAACAGTTTTGCCTTCGACCGTCCGGT
TGAGTGGATGAATAACTGGACGCTCTTCTTCTGGGCATGGTGGGTGGCATGGTCGCCGTTTGTCGGCTTGTTCCTGGCGC
GTATCTCGCGTGGGCGTACCATTCGCCAGTTCGTGCTGGGCACGTTGATTATTCCGTTTACCTTCACGCTGTTATGGCTC
TCGGTGTTCGGCAATAGCGCGCTGTATGAAATCATCCACGGCGGCGCGGCATTTGCCGAGGAAGCGATGGTCCATCCGGA
GCGCGGCTTCTACAGCCTGCTGGCGCAGTATCCGGCGTTTACCTTTAGCGCCTCCGTCGCCACCATTACTGGCCTGCTGT
TTTATGTGACCTCGGCGGACTCCGGGGCGCTGGTGCTGGGGAATTTCACCTCGCAGCTTAAAGATATCAACAGCGACGCC
CCCGGCTGGCTGCGCGTCTTCTGGTCGGTGGCGATTGGCCTGCTGACGCTCGGCATGCTGATGACTAACGGGATATCCGC
GCTGCAAAACACCACGGTGATTATGGGGCTGCCGTTCAGCTTTGTGATCTTCTTCGTGATGGCGGGGTTGTATAAATCTC
TGAAGGTAGAAGATTACCGCCGTGAAAGTGCCAACCGCGATACCGCACCGCGACCGCTGGGGCTTCAGGATCGCCTGAGC
TGGAAAAAACGTCTCTCGCGCCTGATGAATTATCCGGGCACGCGTTACACTAAACAGATGATGGAGACGGTCTGTTACCC
GGCAATGGAAGAAGTGGCGCAGGAGTTGCGGTTGCGCGGCGCGTACGTGGAGCTAAAAAGCCTGCCACCGGAAGAGGGAC
AGCAGTTGGGTCATCTGGATTTGTTGGTGCATATGGGCGAAGAGCAAAACTTTGTCTATCAGATTTGGCCGCAGCAATAT
TCGGTGCCGGGCTTTACCTACCGCGCACGCAGCGGTAAATCGACCTACTACCGGCTGGAAACCTTCCTGTTAGAAGGCAG
CCAGGGCAACGACCTGATGGACTACAGCAAAGAGCAGGTGATCACCGATATTCTTGACCAGTACGAGCGGCACCTTAACT

TTATTCATCTCCATCGTGAAGCGCCGGGCCATAGCGTGATGTTCCCGGACGCGTGATTGTTATTGCATAAAACCGCGCCA
TGTCTGCATATGGCGCTGTTTCTCCTACCTCTTGATACGTTATATCTATACGGTTAAGCCCTTAGTATCTATTGATGATT
ACCAGACAACAGATAATAAAAAAGAAAAGAACTATTGCAGCCCAAAACCTACATTTGGGCTGTTGCGAATGTTCAATAAG
TTTAGTCTTATTTAATGTAAATATTGCTGATCATTTGAAATGACGCATTATTCATGAGAAATGTGTATCGTAAATCAACT
GAAATTAACGCAACCATTTGTTATTTAAGGTTTAATTATCTGTGTGTGATATTTTATTGAATGTTTTAAATATTGTTTTT
ATTGGCATTGCTATAATATTGGTTATCATTTGCTGAATGGATTCAGTCTTAATGAGTGGGTTTTTAAGGGACAGGCATAG
AGTAATGATACGTATGCATAACCAACATCTTTACTCATTATGTCATTGAATGTTGACGCTATGTGTTTATGAGGGAGAGG
TATTTTCAGTTGATCTGGATTGTTAAATTCATATAATGCGCCTTTGCTCATGAATGGATGCCAGTATGTAGTGGGAAATT
ATAAATATTGAAATAGTCCAACTACTTCTTTATTACCAAAAATGAGTATCTGAATTTTAATATTGCATTCTTGCGTGATT
ATCTCCTGAGTTTGACTTGTGATTACCTTTTTAAGGTATTTAGCGTAACTGTTTTTGAGCGAGCATCAGAGGTAAAGATA
ATCTTCTTGATAGTGATGTGGGATGTTATACGTATGGCATCGCTGATGTTTATGGTTACCCCTTATGTGTGCTCAGGAAT
CGACAGGTAATCACTCATACTGAACAGCGATAAAAGATAAAGGTGTGTTCATGAATTCATGTGATTTTCGTGTTTTTCTG
CAAGAGTTCGGTACAACGGTTCATTTGTCATTGCCTGGTAGCGTATCCGAGAAAGAACGACTGCTACTCAAGCTGCTGAT
GCAGGGAATGTCTGTAACAGAAATATCACAGTACAGAAATCGCAGTGCAAAGACAATTTCACATCAAAAGAAACAGCTCT
TTGAGAAACTGGGGATTCAGAGCGATATTACTTTCTGGCGCGATATTTTCTTTCAGTACAATCCGGAGATCATATCCGCC
ACGGGGAGTAATAGTCACAGATATATTAATGATAATCACTATCACCATATCGTCACGCCTGAAGCCATCAGTCTGGCGTT
GGAAAACCACGAATTCAAACCGTGGATCCAACCGGTTTTCTGCGCGCAGACTGGCGTACTGACGGGCTGTGAGGTGCTTG
TCCGCTGGGAACATCCACAAACGGGAATTATCCCACCGGATCAGTTTATTCCTCTGGCGGAGTCATCCGGTCTTATTGTC
ATAATGACCCGCCAACTGATGAAACAGACTGCGGATATTCTGATGCCGGTAAAACATTTGCTGCCGGACAATTTCCATAT
TGGCATCAACGTCTCGGCGGGTTGTTTTTTGGCAGCGGGATTTGAAAAAGAGTGTCTGAACCTGGTTAATAAATTAGGTA
ACGATAAAATCAAGCTGGTTCTCGAGCTAACGGAACGTAACCCTATTCCGGTAACGCCAGAAGCCAGAGCGATATTTGAC
AGCCTTCATCAGCACAACATTACCTTTGCGCTGGATGACTTTGGTACGGGTTATGCGACCTATCGTTACTTGCAGGCGTT
CCCGGTCGATTTTATTAAGATCGATAAGTCATTTGTGCAAATGGCGAGTGTCGACGAAATCTCCGGTCATATTGTGGACA
ATATTGTCGAACTAGCGCGTAAGCCTGGTCTGAGTATCGTGGCGGAAGGGGTAGAAACCCAGGAGCAGGCGGATTTAATG
ATCGGTAAAGGCGTTCACTTTTTGCAGGGCTATTTGTACTCTCCGCCAGTACCGGGTAATAAATTTATCTCTGAATGGGT
AATGAAAGCAGGTGGTTGATGTAAACCGCTATTCACAGCGCATCGGGAGGTTGGCAGCGATTAATTCTCCCGATGCAGTA
AATCCTGATAGATCCCCGTTAATATGCCATTTGGCGCAAATTGTTTTTTAATCCACTGCGTCACCTGACCCGTAGCAGAA
TGCTGAGTCGCCAGTAACATGCGCGAATCCTGGCGCGGATTATGGATTTGTCGGGTAACCAGCAGGGATTGCGTCATCGC
CTCACGGACCATGTAATCCGGCAAAAAACCAATTCCTTCACCTAAAATCTGGCACTGACATTTGGTGTTGAAATCTGGCA
CCAGAATTGACTCCTGCCCGTGCAGCAACCAGCCCACTTTTTTATTAATCGTATGCGCGGTGTCCTCCACCATAATATTA
GGGTAGAGACGCAGTTGGCTTTCGGCGATGGGTTCCGGCACAAAGGCTAACGGGTGATCCGGGGCGATAGCAAAAGCCCA
GCGAATCGCGCCAATTTCAGTGTAATCAATACCGCCGCCGTCCAGTAGTGTATCGGGTGCGCCGATGGCGATATTGGCCT
GATTATTAATAATCGCATCCCAGACGCCGTTATACACTTCGGTGGTGACGGTAATCTGGCAGGTAGGAAACTGCTTTTTC
AGCACCTGTAACAAGCGTGCGGTGTGTTTGGGCGTATAAAGCAGCTGATTAATACAAATGCGCACACGCGCTTCTATGCC
CTGCGAAATAGTATCGATTCTGCGTTTGATGGCATAAAAATCATTCAGCAGGTCGGTAGCTTTGCGGAAAAAAATAACGCC
CGGACTCCGTTAACTCAATGCTGCGGGTACTGCGAGTGAACAGCACCACATCCAGCCCCGTCTCCATACGCTTAATGGTG
TAGCTAATGGCGGAAGTGGTTAAACCCAACTCTTCTGCGGCTTTACTGAAGCTGCCAAAACGCGCAGCGGTAGTAAATGC
CAACAGATTCTCTTCGGTAAAAATTGAGTTCATTAAACCATTCTCGCCATTATCTACTATGAATTTCTCCAGCTATGACT
GTAGAGGTATCGGTTAAAGATAGATAATCATTTTTGAATAACTTTTAATACCCGTCGCGTTTAGTCGTCGCTTCGCTTAA
TTTTTGTCCACCATATTCTGAAATTACCCCCACGCAGTTACCCGCCAGAAAACCCAGTAACGTCACCGTCCAGTTCATCC
CGCTGGCAAAAAACAGCGTCATGCCGAGAAAGCCACCGGGAATAAAGGACAGTAACCAGAAACGCCCTTGCCAGACCACT
ACCGCCGAGAAGGGTACGGTAGTAATTACGCTCGCCCAGAACGCGGACAGTCCACTTACAGAGGCCAGCCAACCAGCAGT
CAGCGCGGCGAAAAACGCCCACACCATCCCGGAATAATTAACCGCCAGACTTCTGATAAACCCCGATTTACCGCCGCCTG
CGGCATAAAAACTGCAAAAGGCGACGAAGCCAATGGTTCCTAATAATTCCCATCCTTGAGAGAGCCCGACATGTGAAGAC
ACTAACTGCCAAAGCCCTGCACAAATACCGACGGTGACACCCGTTGCCGTGAGTCCGTTCATCTTGTTCCCCTCTGGTTA
GACATGGACGAAAGCTACTCTTACATGTGCAGACTATAGAGAAATAAAACCTGATTGAGTGAAAGGGTATCGGGTCAAAG
AAACAAATATTGCACTACCGCACACTGCGAAAAGATTGTTGAATATTATTCAACAAAAAGGCGAGATACGGCGAATTAAA
TTTAAGCGGGTTCTTTTATCAGATTATCGTTGCTATTCTCAATTCATCGAAAACAGCCACAAAAACCAGGGGAATGTGAA
TGAGTATAAAAAATCTACCTGCCGACTATTTATTGGCTGCACAACAGGGTGATATCGATAAAGTAAAAACCTGCCTTGCG
CTGGGTGTCGATATTAATACCTGCGATCGTCAGGGGAAAACGGCAATTACGCTGGCAAGTTTATATCAGCAATATGCTTG
CGTTCAGGCATTAATTGATGCCGGAGCGGATATTAATAAGCAAGATCATACCTGTTTAAATCCTTTTTTAATTAGCTGTC
TGAACGATGATTTAACGCTACTACGAATTATTTTACCGGCTAAACCCGATCTTAATTGCGTAACCCGTTTTGGCGGTGTC
GGCCTGACGCCTGCCTGTGAAAAAGGCCATTTAAGTATTGTAAAAGAGCTTTTGGCGCATACGGAGATTAACGTTAACCA
GACCAACCATGTAGGCTGGACGCCGCTACTGGAAGCGATTGTGCTTAATGATGGTGGTATTAAACAGCAGGCGATTGTGC
AGTTATTGCTGGAACACGGTGCCAGCCCGCATCTGACCGATAAATATGGCAAAACGCCACTGGAACTGGCGCGGGAACGG
GGCTTTGAAGAGATTGCGCAGTTACTGATTGCCGCAGGTGCATAAACCGGGAGGCTTGCTATCAACACACCAGAAAGACG

GTGTGTGTGGGCGCTAACTGCGGATGCGGATTTTCTGGCGCAGCGGGGGCAAGGACAGGTTGAACAGGTCTTTGCCAGAG
CGGTAAATATCGCACTCCCGGCTCGCCAGCAGTTGCTGACGCTGCTTTGTGAAGAGTACGACAATGCGCCAAACAGTTGT
CGGTTGGCACTCACTCACTTTGATGATCTGTTCCGGCATGGTGATAAGGTTCAGTTTGACGATCAAGGTATTACGGTTGG
TCAACATCTTCATATAGAGATGAGTCGTTGTCGGCGTTGGCTGTCCCCAACCTTGCAAATGACCGCTGTGAATTTTCACC
TTATCGCCTGGCTACAGTGGCACGACATTATTCATCAGCACCTGGGGGAAAATGAAACCCTGTTTAATTATCGCGGCGAT
AATCCGTTTTATCAGGCGTTAAATAAAGAATTACATATTAAACGACGGGCAGTTATTCAGGCCGTAAACGATAAACAAAA
TATCGCCTCAGCGGTCGCCAGTATGATGGGGTTAGGGATTGGCCTTACGCCATCAGCCGACGATTATTTAACAGGTCTGG
CGCTTATTTTATTTATTCCCGGGCATCCGGCGGAAAAATACAAAGAGGAATTTTATCTCGGTCTGCAACGCGGCAAAAAT
AATACCACATTATTAAGTGCCATAACGCTGGAAGCCGCATTACAACAACGCTGCCGGGAAAATATTCATCGTTTTATTCA
CAACATTATTTATGACATCCCTGGGAACGCAACTCAGGCAATAGAAAAAATTAAACATATTGGCTCCAGTTCCGGCTGCG
ACATGCTGTATGGCATGGCCGATGGTTGTGCGCTGAGCCAAACCTACGGAGGGAATTATGTCAGTTAAAATAGTCATTAA
ACCGAATACCTATTTTGATTCTGTCTCGCTGATGTCTATCTCCACGCGTGCAAATAAACTCGACGGCGTCGAGCAGGCAT
TTGTGGCGATGGCGACCGAAATGAATAAAGGCGTGCTGAAGAATTTAGGACTGCTGACGCCGGAGCTGGAGCAGGCGAAA
AACGGCGACCTGATGATTGTCATCAATGGTAAATCGGGTGCGGACAACGAGCAGTTACTGGTGGAGATTGAAGAACTGTT
CAACACCAAAGCGCAAAGCGGCTCGCACGAGGCGCGTTACGCCACTATTGGCAGCGCCAAAAAGCATATCCCGGAAAGTA
ACCTGGCGGTGATTTCGGTCAACGGTCTGTTTGCCGCTCGCGAAGCGCGTCAGGCGCTGCAAAACGATCTCAACGTGATG
CTGTTTTCCGATAACGTCTCAGTTGAAGATGAACTGGCGCTCAAGCAACTGGCCCACGAAAAAGGGCTGCTGATGATGGG
GCCAGACTGTGGCACGGCGATTATCAACGGCGCGGCGCTCTGTTTTGGTAACGCCGTGCGTCGCGGCAACATCGGTATTG
TTGGCGCATCCGGCACCGGCAGTCAGGAGTTGAGCGTCCGCATTCATGAATTTGGCGGCGGCGTTTCGCAACTGATTGGC
ACCGGCGGGCGCGACCTGAGCGAGAAAATCGGCGGCCTGATGATGCTCGACGCCATCGGGATGCTGGAAAACGATCCGCA
AACTGAAATCATTGCGCTTATCTCCAAACCGCCTGCGCCTGCGGTGGCCCGCAAAGTGCTGGAACGTGCGCGCGCCTGCC
GCAAGCCGGTGGTCGTCTGCTTCCTCGATCGTGGCGAAACGCCAGTGGATGAGCAGGGGCTACAGTTTGCCCGCGGCACC
AAAGAGGCAGCGCTAAAAGCGGTGATGCTCTCCGGCGTGAAACAGGAAAATCTCGACCTGCATACGCTTAACCAGCCGTT
GATTGCGGATGTGCGTGCGCGTCTGCAACCGCAGCAGAAATACATTCGTGGCCTGTTCTGCGGCGGCACGCTGTGCGACG
AAACCATGTTCGCGGTGATGGAAAAACATGGCGATGTCTACAGCAACATTCAGCCCGATCCGGAATTCCGCCTGAAAGAT
ATCAACCGCAGCATCAAACACACCTTCCTCGACTTTGGCGATGACGACTTCACCAATGGCAAGCCGCACCCGATGATTGA
CCCCACCAACCGCATCAGTCGCTTGATCGAAGAGGCGCGCGATCCAGAAGTGGCGGTGATCGTGATGGATTTTGTGCTCG
GATTTGGATCGCATGAAGATCCGGTCGGCTCCACCATCGAGACGATCAAAGAAGCGAAAGCGATCGCCGCTGCCGAAGGA
CGCGAGTTGATCATTCTCGCCTATGTGCTGGGTACCGATCTTGATACGCCATCGTTAGAACAACAAAGCCAGATGCTGCT
TGATGCCGGAGTGATTCTGGCGAGCAGCAGCACCAATACCGGATTGCTGGCGCGTGAATTTATCTGCAAAGGGGAGGAAG
CCTGATGAGCCAGTCACTGTTTAGCCAACCATTGAACGTTATTAACGTCGGCATCGCCATGTTTAGCGATGACCTGAAAA
AGCAGCATGTAGAAGTGACTCAACTCGACTGGACGCCGCCGGGGCAGGGCAATATGCAGGTGGTGCAGGCGCTGGATAAC
ATTGCCGATTCGCCGCTGGCGGACAAAATCGCCGCCGCTAACCAGCAGGCGCTGGAGCGTATTATCCAGTCGCATCCGGT
GCTGATTGGTTTTGATCAGGCGATTAACGTGGTGCCGGGCATGACGGCGAAAACCATTCTTCACGCCGGGCCGCCGATCA
CCTGGGAAAAAATGTGCGGCGCGATGAAAGGCGCGGTCACCGGAGCGCTGGTGTTCGAAGGACTGGCGAAAGATCTCGAC
GAGGCGGCTGAACTGGCGGCTTCCGGGGAGATCACCTTCTCGCCGTGTCACGAGCACGACTGCGTGGGATCGATGGCGGG
TGTTACCTCGGCCTCGATGTTTATGCACATCGTAAAAAACAAAACCTACGGCAACATCGCTTATACCAACATGAGCGAGC
AGATGGCGAAGATTTTGCGTATGGGCGCTAACGACCAGAGCGTGATTGACCGCCTGAACTGGATGCGTGATGTGCAGGGA
CCAATACTGCGCGACGCGATGAAAATTATCGGCGAAATCGATCTGCGCTTAATGCTGGCGCAGGCGCTGCATATGGGCGA
TGAGTGCCATAACCGCAATAACGCCGGGACGACACTGCTGATTCAGGCGCTGACGCCGGGGATTATTCAGGCGGGTTATT
CCGTCGAGCAACAGCGCGAAGTGTTTGAGTTTGTCGCCAGCAGCGACTACTTCTCCGGCCCGACGTGGATGGCGATGTGT
AAAGCGGCGATGGATGCGGCGCATGGCATCGAATACAGCACCGTGGTCACCACCATGGCGCGTAACGGCGTCGAGTTCGG
CCTGCGGGTCAGCGGCCTGCCGGGGCAATGGTTTACCGGCCCGGCGCAGCAGGTGATCGGCCCGATGTTTGCCGGTTATA
AGCCGGAAGATTCGGGGCTGGATATCGGCGACAGCGCCATCACCGAAACCTACGGTATTGGCGGATTTGCTATGGCGACC
GCGCCCGCTATCGTCGCGCTGGTGGGCGGCACGGTGGAAGAAGCTATTGATTTCTCCCGTCAGATGCGCGAAATCACCCT
CGGTGAAAACCCCAACGTCACCATTCCGCTGCTCGGTTTTATGGGCGTGCCGTCGGCAATCGACATCACCCGCGTGGGTA
GCAGCGGCATTCTGCCGGTGATCAACACCGCCATCGCCCATAAAGATGCGGGCGTCGGCATGATTGGCGCGGGCATTGTG
CATCCACCTTTTGCCTGCTTCGAGAAAGCCATTCTTGGCTGGTGCGAACGTTACGGCGTCTGACCTACATGTTCATGCCG
GATGCGGCGTGAACGCCTTATCCGGCCTACGAATGGCGCAAGAATCTGTAGGCCTGATAAGCGTAGCGCATCAGGCATTT
GTCACCATTGCCGGATGCGGCGTGAACGCCTTATCCGGCCTACGAATGGCGCAAGAATCTGTAGGCCTGATAAGCGTAGC
GCATCAGGCATTTGCCACCACTGCCGGATGCGGCGTGGACGCCTTATCCGGCCTACGAGTGGCGCGAGAATCTGTAGGCC
TGATAAGCGTAGCGCATCAGGCATTTGTCACCATTGCCGGATGCGGCGTGAACGCCTTATCCGGCCTACGAGTGGCGCGA
GAATTGTAGGCCTGATAAGCGTAGCGCATCAGGCAGTCTGGCGTTGGTCATAACCCCATCACCCTCTGTAGCGGACATAA
CAACATGAAAGAGCTTGTGGTCGTTGCCATTGGTGGCAACAGCATTATCAAAGATAACGCCAGCCAGTCGATTGAGCATC
AGGCGGAGGCGGTGAAAGCCGTCGCCGATACGGTGCTGGAAATGCTGGCTTCCGATTACGACATTGTGCTGACCCACGGC
AACGGGCCGCAGGTCGGGCTGGATTTACGCCGTGCGGAGATTGCCCACAAGCGCGAAGGGCTGCCCTTAACGCCGCTGGC

GAACTGTGTGGCAGATACGCAGGGCGGCATCGGCTATCTGATCCAACAGGCGCTGAATAACCGGCTGGCGCGTCACGGCG
AGAAGAAAGCCGTCACCGTGGTGACTCAGGTGGAAGTGGATAAAAACGATCCAGGTTTTGCCCATCCCACCAAGCCCATC
GGCGCATTCTTTAGTGACAGCCAGCGTGACGAATTACAAAAGGCAAACCCTGACTGGTGTTTTGTTGAAGATGCCGGGCG
GGGCTATCGCCGCGTGGTCGCCTCGCCGGAACCGAAACGTATTGTCGAAGCACCTGCCATTAAAGCGCTGATCCAACAAG
GTTTTGTCGTAATTGGCGCGGGCGGCGGTGGAATTCCGGTAGTGCGTACTGACGCGGGAGATTACCAAAGCGTGGACGCG
GTTATCGACAAAGATCTCTCTACCGCGCTGCTGGCCCGTGAAATTCACGCCGACATTCTTGTGATCACCACTGGCGTCGA
AAAAGTGTGTATTCACTTTGGCAAACCGCAGCAGCAGGCGCTCGATCGGGTGGATATTGCCACCATGACCCGCTATATGC
AGGAAGGGCATTTCCCGCCCGGCAGCATGTTGCCAAAAATCATCGCCAGCCTGACATTTTTAGAACAAGGCGGCAAAGAA
GTGATTATCACCACGCCGGAATGCCTGCCTGCGGCGCTGCGCGGCGAAACGGGCACTCATATTATTAAAACGTAAGGACG
TAAGATGAAAGAAAGCAATAGCCGCCGTGAATTTCTGAGCCAGAGCGGTAAGATGGTCACCGCCGCCGCGCTGTTTGGTA
CCTCTGTGCCGCTCGCCCATGCGGCGGTAGCTGGCACCCTAAACTGCGAAGCGAACAACACCATGAAAATCACTGACCCG
CATTACTATCTCGATAACGTGCTGCTGGAAACCGGTTTTGACTACGAAAATGGCGTGGCGGTGCAGACCCGCACGGCGCG
CCAGACCGTGGAGATTCAGGACGGCAAAATTGTCGCCCTGCGCGAGAACAAGCTGCATCCGGACGCCACGCTGCCGCACT
ATGACGCTGGCGGTAAGCTGATGCTGCCCACCACCCGCGACATGCATATTCATCTCGACAAAACCTTTTACGGCGGGCCG
TGGCGCTCGCTCAATCGTCCGGCAGGCACCACCATCCAGGACATGATCAAACTCGAGCAGAAAATGCTGCCGGAACTGCA
ACCGTACACTCAGGAGCGGGCAGAAAAACTGATTGATTTATTGCAGTCGAAAGGCACCACCATTGCCCGCAGCCACTGCA
ATATCGAACCGGTTTCCGGCCTGAAAAATCTGCAAAATTTGCAGGCGGTGCTGGCGCGACGTCAGGCGGGCTTTGAGTGT
GAAATCGTCGCCTTCCCGCAGCACGGTTTGCTGCTGTCGAAATCTGAACCTTTAATGCGTGAAGCGATGCAGGCGGGGGC
GCATTACGTCGGCGGCCTGGACCCGACCAGTGTTGATGGCGCGATGGAAAAAATCCCTCGACACCATGTTCCAGATTGCGC
TGGACTACGACAAAGGCGTCGATATTCACCTGCACGAAACCACTCCGGCAGGCGTGGCAGCCATCAATTATATGGTTGAA
ACGGTAGAGAAAACGCCACAGCTGAAGGGCAAGCTGACCATCAGTCACGCCTTTGCGCTGGCAACGCTCAACGAGCAACA
GGTAGATGAACTGGCGAACCGGATGGTGGTGCAACAAATTTCTATCGCCTCGACGGTGCCGATTGGCACGCTGCATATGC
CGCTCAAACAGTTGCACGACAAAGGCGTAAAAGTGATGACTGGCACTGACAGCGTTATCGACCACTGGTCGCCTTATGGT
CTGGGCGACATGCTGGAAAAAGCCAATCTGTACGCGCAGCTCTATATTCGTCCTAACGAACAGAACCTCTCCCGCTCGCT
GTTTTTAGCCACTGGCGATGTATTGCCGCTGAATGAAAAAGGCGAGCGTGTATGGCCAAAAGCGCAGGATGACGCCAGCT
TTGTGCTGGTGGACGCCTCCTGTTCCGCCGAGGCGGTGGCGCGTATCTCGCCGAGAACCGCAACGTTCCATAAAGGGCAA
CTGGTGTGGGGGAGTGTGGCAGGTTGATGCGCATTTGAAACGCCTGCAACGGTGAATAGTAAGAGATTTAAGCCCCAGGG
AGTGGGGCAATCTGAATATGAGTGTGTCGGCAGAAATGGACATTATCTTTGAGGAATATGCCCTTATTGACGTTGTGTAG
ATAACTATTTGTCTACGTGAAAAGATCATCAGTTTTGCCGATTTTAGTCTTTTACAGATAGCAAATATCACACTTACAGG
CGCTCGCCTTATGGGGAACGTTGCTGCTCTGTTTCATTCAGGCTCGCGGCATGTTGGGGCTGGATCGGGTGGTTTAAATA
TTGGCTATATTCAATGGACGCGTTTTGCCGCGATGACATATCAGGCGTTGCCAAATACACATAGCTAATCAGGAGTAAAC
ACAATGAAGATCAAAGCTGTTGGTGCATATTCCGCTAAACAACCACTTGAACCGATGGATATCACCCGGCGTGAACCGGG
ACCGAATGATGTCAAAATCGAAATCGCTTACTGTGGCGTTTGCCATTCCGATCTCCACCAGGTCCGTTCCGAGTGGGCGG
GGACGGTTTACCCCTGCGTGCCGGGTCATGAAATTGTGGGGCGTGTGGTAGCCGTTGGTGATCAGGTAGAAAAATATGCG
CCGGGCGATCTGGTCGGTGTCGGCTGCATTGTCGACAGTTGTAAACATTGCGAAGAGTGTGAAGACGGGTTGGAAAACTA
CTGTGATCACATGACCGGCACCTATAACTCGCCGACGCCGGACGAACCGGGCCATACTCTGGGCGGCTACTCACAACAGA
TCGTCGTTCATGAGCGATATGTTCTGCGTATTCGTCACCCGCAAGAGCAGCTGGCGGCGGTGGCTCCTTTGTTGTGTGCA
GGGATCACCACGTATTCGCCGCTACGTCACTGGCAGGCCGGGCCGGGTAAAAAAGTGGGCGTGGTCGGCATCGGCGGTCT
GGGACATATGGGGATTAAGCTGGCCCACGCGATGGGGGCACATGTGGTGGCATTTACCACTTCTGAGGCAAAACGCGAAG
CGGCAAAAGCCCTGGGGGCCGATGAAGTTGTTAACTCACGCAATGCCGATGAGATGGCGGCTCATCTGAAGAGTTTCGAT
TTCATTTTGAATACAGTAGCTGCGCCACATAATCTCGACGATTTTACCACCTTGCTGAAGCGTGATGGCACCATGACGCT
GGTTGGTGCGCCTGCGACACCGCATAAATCGCCGGAAGTTTTCAACCTGATCATGAAACGCCGTGCGATAGCCGGTTCTA
TGATTGGCGGCATTCCAGAAACTCAGGAGATGCTCGATTTTTGCGCCGAACATGGCATCGTGGCTGATATAGAGATGATT
CGGGCCGATCAAATTAATGAAGCCTATGAGCGAATGCTGCGCGGTGATGTGAAATATCGTTTTGTTATCGATAATCGCAC
ACTAACAGACTGAAAAAATTAATAAATACCCTGTGGTTTAACATATTAACTTCGCTCTCCACTTAACTTTTTAGTTAAGG
AGAGCGAAATAATATCAAAGTAGCAGTAAAACCTATAACGTAAATTTAAATTGTTAAATTAACGCCCTCCAGTACACAAT
ACTTCACACGTTAGTTATGAGCGATTTCTGATAGTGCCTGGTTTAATCAGAGCTTTATTATCTGCGACGTTTATTTTTAT
TTAAGAGAGATCGCGATGATATCATTAAAAGCTCCGCACAATAATTTGATGCCATATACGCAACAAAGCATACTTAATAC
GGTTAAAAACAATCAGTTACCAGAGGATATCAAAAGCTCCCTGGTTTCCTGTGTCGATATATTCAAGGTTTTGATTAAAC
AATATTATGATTATCCTTATGATTGTCGTGATGATTTAGTCGATGACGACAAACTCATTCATCTCATGGCTGCCGTACGA
GACTGTGAGTGGTCAGACGATAACGCACTCACCATAAATGTGCAGTTTAATGATTTCCCTGGATTTTATGACTGGATGGA
TTACCCTGATCACCCGGTTAAGTTTGTTTTTCACATACTTGAAAATCAAAAAGGGACAGTTTGGGTATATGACCAGGATG
ATGCGTTTCTTGATATTAAAGCAAACGTGCAGGCTGGACGCTTTACCGGGCTTAAGAAATTAGTGCAGTTTATCGATTCA
GTGCGAACAGATTGCAAATGTATTCTTCTTGAATACCATATGCCTTTGCTGAGAATTTTCCCTAAAGGGAAAGAGTGTAT
GCATGTGGAAAAAATGGCTTCGCGAAATGTCGTCTATTCCGGAGACAGACGCGCCCATTAAGCAGGCGCTTGCGCATGGAT
TATTATTACACTTAAAAAATATCTACCCTGTTTTTCCTGAATCACTGGTGATGTTATTGCTCTCTGTTCTGGACGTAAAA

ACATACAGAGATGATGCGCGATTAAATGAATGGATATCTAATAGAGTGCAGGAATTAGGTGACAGATATTATCCTGTAAA
TAAGCACGTTAAAATCCGCTACACTCTTTAGTTCAGAAAGGGTTCCACCTGCAAGTTGTTATTCCACCTGAGAGTGAATC
GCAATGCAGGTAATCATTTCATCTGTATATCTGTATGCCAACTGGCATTCCATCTCTTTTTTCGCCGATATTTTGTTGTG
ATCGGCGACACTTCGCTTAAAAAAGCACCAGTAGTGGTTTCGCAGCCATGCGGTGTATAAAAAATGATCTCATGCAGATG
TTTTGTGAATGTGTTGGTTGACATTCATATGAAAAAAATCATAATTCCATCATGTTTGTGTGGGGTCTTTTCTGTATCTT
ACGCATCGCACTCAAGCCTGACAGAAAATATGCTGTAAGGCTCATATCAAAAACCGCCATTAGCTCATCAGGAAGAGCAG
ACGACAACCAGGATTGTTGTATGGTACGGGGTTCGAGGCCTCGATGGCGGTCCAACTTTTCAAAACTCTTCTGAATCAGA
TCCCCTTATTATCATCCCTGCAAAGTGGAACATTGCCATTGTTTGGCTATTCAGGATGGGGCAGACCAATGAAAAAAGCC
CACACAGGGGAGAGTGGGCTGAAATGGGAAGCTAAAGACTCAAGTAAACTTATCGGAAATAAGGACCACGCATTACGGGG
GCTATCATCGCCGATGGCCGTGATTCGGCAAATCAGGTTGATTACGTAGAGAGTAAATTATCTGCTCACCGCTGCGTCAC
CCCTTCGTAAATCAGGCGTAGCGCGAATACACCAATAATTGCACCAATAACCCGACTGGCAACGCGTTGCATACGCCCAT
AAGCACGACGCACAGCGGGCAAAGAAAACGCCTGACTAAGAAAAACTCGCCAGATAATTGATGCGAGCACAATCCCCGCC
CAGGCCATTAAACGTGCCCATGTTGGTGTTTCGGCATTTAATGTTACTGAGAAAATACTGATAAAAAATAAAACGGTTTG
CGGGTTAGAGAGATCGGTAATTAATCCGCGGCGAAAAAAGACATACCAGGGGGCGCTAATCGGTTGTTGTAGTGTGCTCA
TTTGCGGTGTTGACTGGCGGCGCATGCTGCACCACGCAAACCATAAGAGATAAGCGCCGCCGACGATTCTGATAAGCGAA
AAAATCTCCTCACACTGCGTAATTAGCGTTGCAAGACCAAACAAACCCAACCCGGAATAAAATGCATCGCCCAGCGCCAC
GCCCAGCCCGGTCAGCACCCCTGCGCGTCGACCGGAAGCCAGGCTGGTTTGTACTACCACAAAGAGATTGGCTCCCGGAT
TAAAAAAAGTAATCACGAACAGTCCTACGGTCAGGTAAACGGCATGCAAAGGATCCATAGTGATTTCATCCATAAATAAG
TGAACTAACTGCATCATATTTCTACCAAAAATAATCGGGTGCGAGAGAGATCACAAAGTGTCTTATTTCCGGTTACTGGC
GTTTATGCCCTGACTGAACTAATTATTAATCAACCCAATAATGTGGGTGGGTGATAGTGTGATAACAACTCTGGAGCCGT
AATATGAAAATAATCTCTAAAATGTTAGTCGGTGCGTTAGCGTTAGCCGTTACCAATGTCTATGCCGCTGAATTGATGAC
CAAAGCGGAATTTGAAAAAGTTGAATCGCAGTATGAAAAAATAGGTGATATTTCAACCAGCAATGAAATGTCGACTGCAG
ATGCAAAAGAAGATTTGATCAAAAAAGCGGATGAAAAAGGGGCTGATGTGTTGGTACTGACCTCCGGTCAAACTGACAAT
AAGATCCACGGCACGGCAAATATTTATAAGAAGAAGTAATTCTGAATCCTATGTAAACATCTCCGATGCGTAAGTTTATC
GGTGATCATCTATTGAAATTTATGCCGGATAAAGCGTTCGCGCTGCATTCGGCAGTTCAGCTTTTCAGCCGCCGCCAGAA
CGTCGTCCGGCTGATGCCTAAATAATTCGCCGCTGCTGTTTTATCGCCATTAAATTTCTCCAGTGCCTGTTGTGGTGTCA
GTAAGCGTGGAGCGGGAGTTTTCGCCGACTCGCGCGCCAGTTCCGGCAGTAGCAGTTGCATAAACTGCGGCGTTAAATCC
GGCGTCGGTTCCACACTTAAAAACAGCGCCAGTCGTTCCATCATATTGCGCAGTTCACGAATATTGCCTGGCCAGTCGTA
GTGCAGCAGCACAGTTTCACTTGCCTGTAACCCCTGGCGTAATGCAGCAGAAAATGGGGCGGAGAGCGCCGCCAGAGACA
CTTTCAAAAAGCTTTCCGCCAGCGGAAGAATATCCGCCACCCGCTCGCGCAGTGGTGGCAATTGCAGACGCAAAATACTC
AGCCGATAAAACAGATCACGGCGAAAACGTCCTTGCTGCATATCTTCTTCCAGATTGCAGTGAGTGGCGCTAATGACCCG
TACATCTACCGGAACAGGCTGATGCCCGCCGACGCGGGTGACCTCTTTTTCTTCCAGCACCCGCAGCAGCCGGGTCTGCA
AAGGTAGCGGCATTTCGCCAATCTCATCCAGAAACAGCGTACCGCCGTGGGCAATTTCGAACAGCCCGGCGCGACCTCCG
CGTCGCGAGCCGGTAAACGCCCCTTCCTCATAGCCAAACAGTTCTGCTTCCAGCAGCGATTCGGCAATCGCCCCGCAGTT
GACGGCAACAAACGGATGCGACTTTTTGCCCTGTCGCGCATCGTGGCGGGCAAAATATTCCCGATGAATCGCCTGGGCCG
CCAGCTCTTTGCCCGTCCCCGTTTCCCCCTCAATCAACACCGCCGCACTGGAGCGGGCATACAGCAAAATAGTCTGCCGT
ACTTGTTCCATCTGTGGTGATTGACCGAGCATATCGCCCAGCACGTAACGAGTACGCAGGGCGTTGCGGGTGGCATCGTG
AGTGTTATGGCGTAACGACATGCGCGTCATATCCAGCGCATCGCTGAACGCCTGGCGCACGGTGGCGGCGGAATAGATAA
AAATTCCGGTCATTCCGGCTTCTTCTGCCAAATCGGTAATCAGCCCTGCGCCGACCACCGCTTCGGTGCCGTTAGCTTTT
AGCTCGTTAATCTGCCCGCGTGCGTCTTCCTCGGTAATGTAGCTACGTTGGTCGAGGCGCAAATTAAAGGTTTTTTGAAA
CGCCACCAGCGCTGGAATGGTTTCCTGATAGGTGACAACGCCGATAGAAGAGGTGAGTTTTCCGGCTTTTGCCAGTGCCT
GTAACACATCGTAGCCGCTCGGTTTAATCAAAATAACTGGCACTGACAGGCGGCTTTTCAGGTACGCGCCGTTAGAGCCA
GCCGCGATGATGGCGTCACAGCGTTCGTTTGCCAGTTTCTTGCGGATGTAGGTCACTGCTTTTTCAAAGCCAAGCTGAAT
AGGGGTAATGTTCGCCAGGTGATCAAACTCGAGGCTGATATCGCGAAACAGCTCGAACAGGCGCGTTACAGATACCGTCC
AGATAACCGGTTTGTCGTCATTAAGCCGTGGTGGATGTGCCATAGCGCACCGCAAAGTTAAGAAACCGAATATTGGGTTT
AGTCTTGTTTCATAATTGTTGCAATGAAACGCGGTGAAACATTGCCTGAAACGTTAACTGAAACGCATATTTGCGGATTA
GTTCATGACTTTATCTCTAACAAATTGAAATTAAACATTTAATTTTATTAAGGCAATTGTGGCACACCCCTTGCTTTGTC
TTTATCAACGCAAATAACAAGTTGATAACAAAGGATGGGCTATGTCTCTACACTCTCCAGGTAAAGCGTTTCGCGCTGCA
CTGACTAAAGAAAATCCATTGCAGATTGTTGGCACCATCAACGCTAATCATGCGCTGTTGGCGCAGCGTGCCGGATATCA
GGCAATTTATCTTTCTGGCGGTGGCGTGGCGGCAGGTTCGCTGGGGCTGCCCGATCTCGGTATTTCTACCCTTGATGATG
TGCTGACCGACATTCGCCGTATCACCGACGTTTGTTCGCTGCCGCTGCTGGTGGATGCGGATATCGGTTTTGGTTCTTCG
GCCTTTAACGTGGCGCGCACCGTGAAATCGATGATTAAAGCCGGTGCGGCAGGATTGCATATTGAAGATCAGGTTGGTGC
GAAACGCTGCGGTCATCGTCCGAATAAAGCGATCGTCTCGAAAGAAGAGATGGTGGATCGGATCCGCGCGGCGGTGGATG
CGAAAACCGATCCTGATTTTGTGATCATGGCGCGCACCGATGCTCTGGCGGTAGAGGGGCTGGATGCGGCGATCGAGCGT
GCGCAGGCCTATGTTGAAGCGGGTGCCGAGATGTTGTTCCCGGAGGCGATTACCGAACTCGCCATGTACCGCCAGTTTGC
CGATGCGGTGCAGGTGCCGATCCTCGCCAACATCACCGAATTTGGTGCCACGCCGCTGTTTACCACCGACGAATTACGCA

GCGCCCATGTCGCAATGGCGCTGTACCCACTTTCAGCGTTCCGCGCCATGAACCGCGCCGCTGAACATGTCTACAACGTC
CTGCGCCAGGAAGGCACGCAGAAAAGCGTCATCGACACCATGCAGACCCGCAACGAGCTGTACGAAAGCATCAACTACTA
CCAGTACGAAGAGAAGCTCGACAACCTGTTTGCCCGTAGCCAGGTGAAATAAAAAACGCCCGTTGATTGTATTCGACAGC
CGATGCCTGATGCGTCGCTGACGCGACTTATCAGGCCTACGAGGTGCACTGAACTGTAGGTCGGATAAGACGGATGGCGT
CGCATCCGACAACCGATGCCTGATGCGCCGCTGACGTGACTTATCAGGCCTACGGGGTGCACTGAACTGTAGGTCGGATA
AGACGCATAGCGTCGCATCCGACAACCGATGCCTGATGCGCCGCTGACGCGACTTATCAGGCCTACGGGGTGCACTGAAC
TGTAGGTCGGATAAGACGCATAGCGTCGCATCCGACAACCGATGCCTGATGCGCCGCTGACGCGACTTATCAGGCCTACG
GGGTGAACTGAACTGTAGGTCGGATAAGACGCATAGCGTCGCATCCGACAACAATCTCGACCCTACAAATGATAACAATG
ACGAGGACAACATGAGCGACACAACGATCCTGCAAAACAGTACCCATGTCATTAAACCGAAAAAATCTGTGGCACTTTCT
GGCGTTCCGGCGGGCAATACGGCGCTCTGCACCGTGGGTAAAAGTGGCAATGACCTGCATTACCGCGGCTACGATATTCT
TGATCTGGCGAAACATTGCGAATTTGAAGAAGTGGCGCATCTGCTGATCCACGGCAAACTGCCGACCCGTGACGAACTCG
CCGCTTACAAAACGAAACTGAAAGCCCTGCGCGGTTTACCGGCTAACGTGCGTACCGTGCTGGAAGCCTTACCGGCGGCG
TCGCACCCGATGGATGTTATGCGCACCGGTGTTTCCGCGCTCGGCTGCACGCTGCCAGAAAAAGAGGGGCATACCGTCTC
TGGCGCGCGGGATATTGCCGACAAACTGCTGGCGTCGCTTAGCTCGATTCTCCTTTATTGGTATCACTACAGCCACAACG
GCGAACGCATCCAACCGGAAACCGATGACGACTCCATCGGCGGTCACTTCCTGCATCTGCTGCACGGCGAAAAGCCATCG
CAAAGCTGGGAAAAGGCGATGCATATCTCGCTGGTGCTGTACGCCGAACACGAGTTTAACGCCTCCACCTTTACCAGTCG
GGTGATTGCGGGCACCGGCTCTGATATGTATTCCGCGATTATTGGCGCGATTGGCGCACTGCGCGGGCCAAAACACGGCG
GGGCGAATGAAGTGTCGCTGGAGATCCAGCAACGCTACGAAACGCCGGACGAAGCCGAAGCAGATATCCGCAAGCGCGTG
GAAAACAAAGAAGTGGTCATTGGTTTTGGTCATCCGGTTTACACCATCGCTGACCCGCGCCACCAGGTGATTAAACGTGT
GGCGAAGCAGCTCTCGCAGGAAGGCGGCTCGCTGAAGATGTACAACATCGCCGATCGCCTGGAAACGGTGATGTGGGAGA
GCAAAAAGATGTTCCCCAATCTCGACTGGTTCTCTGCTGTTTCCTACAACATGATGGGCGTTCCCACCGAGATGTTCACA
CCACTGTTTGTTATCGCCCGCGTCACCGGCTGGGCGGCGCACATTATCGAACAACGTCAGGACAACAAAATTATCCGTCC
TTCCGCCAATTATGTTGGACCGGAAGACCGCCCGTTTGTCGCGCTGGATAAGCGCCAGTAAACCTCTACGAATAACAATA
AGGAAACGTACCCAATGTCAGCTCAAATCAACAACATCCGCCCGGAATTTGATCGTGAAATCGTTGATATCGTCGATTAC
GTCATGAACTACGAAATCAGCTCTAAAGTGGCCTACGACACCGCACATTACTGCCTGCTCGACACGCTCGGCTGCGGTCT
GGAAGCTCTCGAATACCCGGCCTGTAAAAAACTGCTGGGGCCAATTGTTCCCGGCACCGTCGTACCCAACGGCGTGCGCG
TCCCCGGAACTCAGTTCCAGCTCGACCCCGTCCAGGCGGCATTTAACATCGGCGCGATGATCCGCTGGCTCGATTTCAAC
GATACCTGGCTGGCGGCGGAGTGGGGCCATCCTTCCGACAACCTCGGCGGCATTCTGGCAACGGCGGACTGGCTTTCGCG
CAACGCGGTCGCCAGCGGCAAAGCGCCGTTGACCATGAAACAGGTGCTGACCGCAATGATCAAAGCCCATGAAATTCAGG
GCTGCATCGCGCTGGAAAACTCCTTTAACCGCGTCGGCCTCGACCACGTTCTGTTAGTGAAAGTGGCTTCCACCGCCGTG
GTCGCCGAAATGCTCGGCCTGACCCGCGAGGAAATTCTCAACGCCGTTTCGCTGGCGTGGGTGGACGGTCAGTCGCTGCG
CACCTATCGCCATGCGCCGAACACCGGCACGCGTAAATCCTGGGCGGCGGGCGATGCCACTTCCCGCGCGGTACGTCTGG
CACTGATGGCGAAAACGGGCGAAATGGGTTACCCGTCAGCCCTGACTGCGCCGGTGTGGGGCTTCTACGACGTCTCCTTT
AAAGGTGAATCGTTCCGCTTCCAGCGCCCGTACGGTTCCTACGTTATGGAAAATGTGCTGTTCAAAATCTCCTTCCCGGC
GGAGTTCCACTCCCAGACGGCAGTTGAAGCAGCGATGACGCTCTATGAACAGATGCAGGCAGCAGGCAAAACGGCGGCGG
ATATCGAAAAAGTGACCATTCGCACCCACGAAGCCTGTATTCGCATCATCGACAAAAAAGGGCCGCTCAATAACCCGGCA
GACCGCGATCACTGCATTCAGTACATGGTGGCGATCCCGCTGCTATTCGGGCGCTTAACGGCGGCAGATTACGAGGACAA
CGTTGCGCAAGATAAACGCATTGACGCCCTGCGCGAGAAGATCAATTGCTTTGAAGATCCGGCATTTACCGCTGACTACC
ACGACCCGGAAAAACGCGCCATCGCCAATGCCATTACCCTTGAGTTCACCGACGGCACACGATTTGAAGAAGTGGTGGTG
GAGTACCCCATTGGTCATGCTCGCCGCCGTCAGGATGGTATTCCGAAACTGGTCGATAAATTCAAAATCAATCTCGCGCG
CCAGTTCCCGACTCGCCAACAGCAGCGCATTCTGGAGGTTTCTCTCGACAGAGCTCGCCTGGAACAGATGCCGGTCAATG
AGTATCTCGACCTGTACGTCATTTAAGTAAACGGCGGTAAGGCGTAAGTTCAACAGGAGAGCATTATGTCTTTTAGCGAA
TTTTATCAGCGTTCGATTAACGAACCGGAGCAGTTCTGGGCCGAGCAGGCCCGGCGTATTGACTGGCAGACGCCCTTTAC
GCAAACGCTCGATCACAGCAATCCGCCGTTTGCCCGTTGGTTTTGTGAAGGCCGAACCAACTTGTGCCACAACGCCATCG
ACCGCTGGCTGGAGAAACAGCCAGAGGCGCTGGCGCTGATTGCCGTCTCTTCGGAAACAGAAGAAGAGCGCACCTTTACC
TTTCGTCAGCTGCATGACGAAGTGAACGCGGTGGCCTCAATGTTGCGTTCATTGGGTGTGCAGCGCGGCGATCGGGTGCT
GGTGTATATGCCGATGATTGCCGAAGCGCATATTACTCTGCTGGCCTGCGCGCGCATTGGCGCTATTCACTCGGTGGTGT
TTGGTGGATTTGCCTCGCACAGCGTGGCGGCGCGAATTGATGACGCTAAACCGGTGCTGATTGTCTCGGCTGATGCCGGA
GCGCGCGGTGGCAAAATCATTCCCTATAAAAAATTGCTCGACGATGCGATAAGTCAGGCGCAGCACCAGCCACGCCATGT
TTTGCTGGTGGATCGCGGGCTGGCGAAAATGGCGCGCGTCAGCGGGCGGGATGTCGATTTCGCGTCGTTGCGCCATCAAC
ACATCGGCGCGCGGGTACCGGTGGCGTGGCTGGAATCCAACGAAACCTCCTGCATTCTCTACACTTCCGGCACGACCGGC
AAACCTAAAGGCGTGCAGCGTGACGTCGGCGGATATGCGGTGGCGCTGGCGACCTCGATGGACACCATTTTTGGCGGCAA
AGCGGGCAGCGTGTTCTTTTGCGCATCGGATATCGGCTGGGTGGTGGGGCATTCGTATATCGTTTACGCGCCGCTGCTGG
CGGGGATGGCGACTATCGTTTACGAAGGATTGCCGACCTGGCCGGACTGCGGCGTGTGGTGGACAATCGTCGAGAAATAT
CAGGTTAGCCGGATGTTCTCAGCGCCGACCGCCATTCGCGTGCTGAAAAAATTCCCTACCGCTGAAATTCGCAAACACGA
TCTCTCGTCGCTGGAAGTGCTCTATCTGGCTGGAGAACCGCTGGACGAGCCGACCGCCAGTTGGGTGAGCAATACGCTGG

ATGTGCCGGTCATCGACAACTACTGGCAGACCGAATCCGGCTGGCCGATTATGGCGATTGCTCGCGGTCTGGACGACAGG
CCGACGCGTCTGGGAAGCCCCGGTGTGCCGATGTATGGCTATAACGTGCAGTTGCTTAATGAAGTCACCGGCGAACCGTG
TGGCGTCAACGAGAAAGGGATGCTGGTGGTGGAAGGGCCGCTGCCGCCGGGGTGTATTCAGACCATCTGGGGCGACGACG
GCCGCTTTGTGAAGACTTACTGGTCGCTGTTTTCCCGCCCGGTGTACGCCACCTTTGACTGGGGCATCCGTGACGCTGAC
GGTTATCACTTTATTCTCGGGCGCACTGACGATGTAATTAACGTTGCCGGGCATCGGCTGGGGACGCGCGAGATTGAAGA
GAGTATCTCCAGCCATCCGGGCGTTGCCGAAGTGGCGGTGGTTGGGGTGAAAGATGCGCTGAAAGGGCAGGTGGCGGTGG
CGTTTGTCATTCCGAAAGAGAGCGACAGTCTGGAAGATCGTGATGTGGCGCACTCGCAAGAGAAGGCGATTATGGCGCTG
GTGGACAGCCAGATTGGCAACTTTGGCCGCCCGGCGCACGTCTGGTTTGTCTCGCAATTGCCAAAAACGCGATCCGGAAA
AATGCTGCGCCGCACGATCCAGGCGATTTGCGAAGGACGCGATCCTGGAGATCTGACGACCATTGATGATCCTGCGTCGT
TGGATCAGATCCGCCAGGCGATGGAAGAGTAGGTTATTGTCGGATGCGTCGCGCGGTGCATCCGGCACTGTGTGCCGATG
CCTGATGCGACGCTGACGCGTTTTATCATGCCTACGGACCTGAACCGTAGGTCGGATAAGGCGCTCGCGTCGCATCCGAC
ACCATGCTCAGATGCCTGATGCGACGCTGACGCGTCTTATCAGGCCTACCCACTGTTTTTACACCGATAATTTTTCCCCC
ACCTTTTTGCACTCATTCATATAAAAAATATATTTCCCCACGAAAACGATTGCTTTTTATCTTCAGATGAATAGAATGCG
GCGGATTTTTTGGGTTTCAAACAGCAAAAAGGGGGAATTTCGTGTCGCAAGATAACAACTTTAGCCAGGGGCCAGTCCCG
CAGTCGGCGCGGAAAGGGGTATTGGCATTGACGTTCGTCATGCTGGGATTAACCTTCTTTTCCGCCAGTATGTGGACCGG
CGGCACTCTCGGAACCGGTCTTAGCTATCATGATTTCTTCCTCGCAGTTCTCATCGGTAATCTTCTCCTCGGTATTTACA
CTTCATTTCTCGGTTACATTGGCGCAAAAACCGGCCTGACCACTCATCTTCTTGCTCGCTTCTCGTTTGGTGTTAAAGGC
TCATGGCTGCCTTCACTGCTACTGGGCGGAACTCAGGTTGGCTGGTTTGGCGTCGGTGTGGCGATGTTTGCCATTCCGGT
GGGTAAGGCAACCGGGCTGGATATTAATTTGCTGATTGCCGTTTCCGGTTTACTGATGACCGTCACCGTCTTTTTTGGCA
TTTCGGCGCTGACGGTTCTTTCGGTGATTGCGGTTCCGGCTATCGCCTGCCTGGGCGGTTATTCCGTGTGGCTGGCTGTT
AACGGCATGGGCGGCCTGGACGCATTAAAAGCGGTCGTTCCCGCACAACCGTTAGATTTCAATGTCGCGCTGGCGCTGGT
TGTGGGGTCATTTATCAGTGCGGGTACGCTCACCGCTGACTTTGTCCGGTTTGGTCGCAATGCCAAACTGGCGGTGCTGG
TGGCGATGGTGGCCTTTTTCCTCGGCAACTCGTTGATGTTTATTTTCGGTGCAGCGGGCGCTGCGGCACTGGGCATGGCG
GATATCTCTGATGTGATGATTGCTCAGGGCCTGCTGCTGCCTGCGATTGTGGTGCTGGGGCTGAATATCTGGACCACCAA
CGATAACGCACTCTATGCGTCGGGTTTAGGTTTCGCCAACATTACCGGGATGTCGAGCAAAACCCTTTCGGTAATCAACG
GTATTATCGGTACGGTCTGCGCATTATGGCTGTATAACAATTTTGTCGGCTGGTTGACCTTCCTTTCGGCAGCTATTCCT
CCAGTGGGTGGCGTGATCATCGCCGACTATCTGATGAACCGTCGCCGCTATGAGCACTTTGCGACCACGCGTATGATGAG
TGTCAATTGGGTGGCGATTCTGGCGGTCGCCTTGGGGATTGCTGCAGGCCACTGGTTACCGGGAATTGTTCCGGTCAACG
CGGTATTAGGTGGCGCGCTGAGCTATCTGATCCTTAACCCGATTTTGAATCGTAAAACGACAGCAGCAATGACGCATGTG
GAGGCTAACAGTGTCGAATAACGCTTTACAAACAATTATTAACGCCCGGTTACCAGGCGAAGAGGGGCTGTGGCAGATTC
ATCTGCAGGACGGAAAAATCAGCGCCATTGATGCGCAATCCGGCGTGATGCCCATAACTGAAAACAGCCTGGATGCCGAA
CAAGGTTTAGTTATACCGCCGTTTGTGGAGCCACATATTCACCTGGACACCACGCAAACCGCCGGACAACCGAACTGGAA
TCAGTCCGGCACGCTGTTTGAAGGCATTGAACGCTGGGCCGAGCGCAAAGCGTTATTAACCCATGACGATGTGAAACAAC
GCGCATGGCAAACGCTGAAATGGCAGATTGCCAACGGCATTCAGCATGTGCGTACCCATGTCGATGTTTCGGATGCAACG
CTAACTGCGCTGAAAGCAATGCTGGAAGTGAAGCAGGAAGTCGCGCCGTGGATTGATCTGCAAATCGTCGCCTTCCCTCA
GGAAGGGATTTTGTCGTATCCCAACGGTGAAGCGTTGCTGGAAGAGGCGTTACGCTTAGGGGCAGATGTAGTGGGGGCGA
TTCCGCATTTTGAATTTACCCGTGAATACGGCGTGGAGTCGCTGCATAAAACCTTCGCCCTGGCGCAAAAATACGACCGT
CTCATCGACGTTCACTGTGATGAGATCGATGACGAGCAGTCGCGCTTTGTCGAAACCGTTGCTGCCCTGGCGCACCATGA
AGGCATGGGCGCGCGAGTCACCGCCAGCCACACCACGGCAATGCACTCCTATAACGGGGCGTATACCTCACGCCTGTTCC
GCTTGCTGAAAATGTCCGGTATTAACTTTGTCGCCAACCCGCTGGTCAATATTCATCTGCAAGGACGTTTCGATACGTAT
CCAAAACGTCGCGGCATCACGCGCGTTAAAGAGATGCTGGAGTCCGGCATTAACGTCTGCTTTGGTCACGATGATGTCTT
CGATCCGTGGTATCCGCTGGGAACGGCGAATATGCTGCAAGTGCTGCATATGGGGCTGCATGTTTGCCAGTTGATGGGCT
ACGGGCAGATTAACGATGGCCTGAATTTAATCACCCACCACAGCGCAAGGACGTTGAATTTGCAGGATTACGGCATTGCC
GCCGGAAACAGCGCCAACCTGATTATCCTGCCGGCTGAAAATGGGTTTGATGCGCTGCGCCGTCAGGTTCCGGTACGTTA
TTCGGTACGTGGCGGCAAGGTGATTGCCAGCACACAACCGGCACAAACCACCGTATATCTGGAGCAGCCAGAAGCCATCG
ATTACAAACGTTGAACGACTGGGTTACAGCGAGCTTAGTTTATGCCGGATGCGGCGTGAACGCCTTATCCGGCCTACGTA
GAGCACTGAACTCGTAGGCCTGATAAGCGTAGCGCATCAGGCAATTCCAGCCGCTGATCTGTGTCAGCGGCTACCGTGAT
TCATTCCCGCCAACAACCGCGCATTCCTCCAACGCCATGTGCAAAAATGCCTTCGCAGCGGCTGTCTGCCAGCTGTAGTT
TATGCCGGATGCGGCGTGAACGCCTTATCCGGCCTACGTAGAGCACTGAACTCGTAGGCCTGATAAGCGTAGCGCATCAG
GCAATTCCAGCCGCAGACCTGTGTCAGCGGCTACCGTGATTCATTTCCGCCAACAACCGCGCATTTATCCAACGCCATGT
GCAAAAATGCCTTCGCGGCGGCTGTCTGCCAGCTATTTTTCCGCCGCAACAAAACCGCCGTTCTCTCCAGTAGTGGCGGG
GCAAGAGAAATAGCTTTAAGCCCGTCATGTTGTGTGGCAATCGCTGCTGGTAACAATGTGGAAAGGGAAGTGCGGCGAAT
CAGCTCCAGAACCGCGCTAATTGAGTTCGCCTCAATGACCACCTGTGGATGTAGCCCCGCTTTCTCGCAGTAGTGGTCAA
TTTGCTCTCTGGTGGCAAATTCCGCGCTGAGCAGGACCAGTTTTTCATCATGCAAGCGACTCAACGCCACCTGTTCATGG
ACGGCCAGCGGATGATGTTGCGCCACGACTAACGCTAAACTTTCTGTCAGTAAAGGAATTGCCTCCAGCTCCGGCGAATG
CACAGGCGCGAAGGCAATCCCAACGTCCAACTCGTCGCGGCAAAGCATATCCTCGATTTTCTCCTGCGACATTTCCTGTA

GCTGGAGCGTGATGCTGGGATAGCGCGCATAGAAATCCGCCATTAAGGGGCCGATAAAGTAGCTCGTAAAGGTGGGGGTG
ACGGCGATACGCAGCGATCCTCGCGTCAGATCGGCAACATCATGAATCGCCCGTTTACCCGCCCCCAGTTCCTGTAACGC
CCGGCTGGCGTACTGTCGCCAGACTTCTCCTGCATCAGTGAGACGAATCGTTCGCCCGCTACGGTCAAACAGCGGCACGC
CTAAACTCTCCTCTAACTGGCGAATCTGCTGGGAAAGCGCAGGTTGGGAGACGTGCAACGCACTGGCGGCACGGGTGAAG
CTGCCATGTTCAGCCACGGCAAGAAAATAATTGATATGTCGAGAGAGCATTCGCAACCTATAAGTAAATCCAATGGAACT
CATCATAAATGAGACTTTTACCTTATGACAATCGGCGAGTAGTCTGCCTCTCATTCCAGAGACAGACAGAGGTTAACGGT
GAAAGAGATTATTGATGGATTCCTTAAATTCCAGCGCGAGGCATTTCCGAAGCGGGAAGCCTTGTTTAAACAGCTGGCGA
CACAGCAAAGCCCGCGCACACTTTTTATCTCCTGCTCCGACAGCCGTCTGGTCCCTGAGCTGGTGACGCAACGTGAGCCT
GGCGATCTGTTCGTTATTCGCAACGCGGGCAATATCGTCCCTTCCTACGGGCCGGAACCCGGTGGCGTTTCTGCTTCGGT
GGAGTATGCCGTCGCTGCGCTTCGGGTATCTGACATTGTGATTTGTGGTCATTCCAACTGTGGCGCGATGACCGCCATTG
CCAGCTGTCAGTGCATGGACCATATGCCTGCCGTCTCCCACTGGCTGCGTTATGCCGATTCAGCCCGCGTCGTTAATGAG
GCGCGCCCGCATTCCGATTTACCGTCAAAAGCTGCGGCGATGGTACGTGAAAACGTCATTGCTCAGTTGGCTAATTTGCA
AACTCATCCATCGGTGCGCCTGGCGCTCGAAGAGGGGCGGATCGCCCTGCACGGCTGGGTCTACGACATTGAAAGCGGCA
GCATCGCAGCTTTTGACGGCGCAACCCGCCAGTTTGTGCCACTGGCCGCTAATCCTCGCGTTTGTGCCATACCGCTACGC
CAACCGACCGCAGCGTAACCTTATTTTTAAACCATCAGGAGTTCCACCATGATTCAGTCACAAATTAACCGCAATATTCG
TCTTGATCTTGCCGATGCCATTTTGCTCAGCAAAGCTAAAAAAGATCTCTCATTTGCCGAGATTGCCGACGGCACCGGTC
TGGCAGAAGCCTTTGTAACCGCGGCTTTGCTGGGTCAGCAGGCGCTTCCTGCCGACGCCGCCCGCCTGGTCGGGGCGAAG
CTGGATCTCGACGAAGACTCCATTCTACTGTTGCAGATGATTCCACTGCGTGGCTGCATTGATGACCGTATTCCAACTGA
CCCAACGATGTATCGTTTCTATGAAATGTTGCAGGTGTACGGTACAACCCTGAAAGCGTTGGTTCATGAGAAATTTGGCG
ATGGCATTATTAGCGCGATTAACTTCAAACTCGACGTTAAGAAAGTGGCGGACCCGGAAGGTGGCGAACGTGCGGTCATC
ACCTTAGATGGTAAATATCTGCCGACCAAACCGTTCTGACAGCCATGCGCAACCATCAAAAGACGTTCACGATGCTGCTG
GTACTGGTGCTGATTGGTCTTAATATGCGACCACTGCTCACCTCCGTCGGGCCACTGCTACCGCAATTGCGCCAGGCGAG
CGGAATGAGCTTTAGCGTGGCTGCCCTGTTGACCGCTCTGCCGGTGGTTACCATGGGCGGGCTGGCGCTGGCCGGAAGCT
GGCTTCATCAGCATGTCAGCGAACGTCGCAGTGTCGCCATCAGTCTGTTGCTGATTGCCGTCGGTGCATTGATGCGTGAG
CTTTACCCGCAAAGTGCGCTGCTGCTTAGCAGCGCACTGCTTGGTGGGGTGGGGATCGGCATCATTCAGGCGGTGATGCC
TTCGGTGATTAAACGGCGGTTTCAGCAGCGCACGCCACTGGTGATGGGGCTGTGGTCCGCGGCTCTGATGGGCGGCGGTG
GGCTTGGTGCCGCCATAACGCCCTGGTTAGTTCAACATAGCGAAACCTGGTATCAAACACTCGCCTGGTGGGCGCTGCCT
GCCGTTGTTGCGCTCTTTGCCTGGTGGTGGCAAAGCGCCCGCGAGGTCGCCTCTTCCCACAAGACAACAACCACTCCGGT
TCGCGTGGTATTCACTCCCCGCGCGTGGACGCTGGGTGTTTACTTCGGTCTGATTAACGGCGGTTACGCCAGCCTGATTG
CCTGGTTACCCGCTTTCTATATTGAGATTGGTGCCAGCGCGCAGTACAGCGGTTCCTTACTGGCATTGATGACGCTTGGG
CAAGCCGCAGGAGCTTTGCTGATGCCTGCTATGGCTCGCCATCAGGATCGGCGCAAACTGTTAATGCTGGCGCTGGTGTT
ACAACTGGTGGGGTTCTGCGGCTTTATCTGGCTGCCGATGCAATTGCCGGTATTGTGGGCGATGGTGTGTGGGTTAGGTC
TGGGCGGCGCGTTTCCGCTCTGTTTGCTGCTGGCGCTCGATCACTCTGTGCAACCGGCTATTGCTGGCAAGCTGGTGGCG
TTTATGCAGGGAATCGGTTTTATCATCGCCGGGCTTGCCCCGTGGTTTTCTGGCGTGCTGCGTAGTATCAGCGGCAATTA
CCTGATGGACTGGGCATTTCATGCGCTGTGCGTCGTTGGGCTGATGATCATAACCCTGCGTTTTGCACCAGTACGTTTTC
CGCAGCTGTGGGTCAAAGAGGCATGATGCGACGCTTGTTCCTGCGCTTTGTTCATGCCGGATGCGGCTAATGTAGATCGC
TGAACTTGTAGGCCTGATAAGCGCAGCGTATCAGGCAATTTTTATAATTTAAACTGACGATTCAACTTTATAATCTTTGA
AATAATAGTGCTTATCCCGGTCGTTTATTTCGCGAATAACCCGACAAGGAACGCCAGCCGCCACGACGTTTGGTGGAATG
TCTTTTGTGACGATACTACCCGCGCCAATAACAGAATTATCCCCGATGGTGACGCCTGGATTAATAACCACATGACTTCC
GATCCAGACGTTATTGCCAATCGTTATCGGAAAAGAGTACATCTCGCCGTTTTTTCTCAATTCATGGTGTACAGGGTGTC
CCGTAACGGAAAGAGTAACGTTGGGTGCAATCAGTACGTTATCACCGATTGTTACCGTGTAGTCATCGACAATGGTTAAA
TTGAAATTTGCATAAAAATTGCGGCCTATATGGATGTTGGAACCGTAAGAGAAATAGACAGGCGGTTCTACCCAGGCGTT
TTCCCCTACCGTGGCAAACATTTCTTTAATCAGGCTTTCTCTTTTTTCAACTTCTGATGGATGCGAGTGATTAAACTCAT
ACATTAACGTTTTCCCACGAAGTCTTTTTTCCGGTAAGCCTTCGCACATATCGGTAAATAGCTTGCCTGCTCTTATTCTT
TCGGTCATTGGCATGTTCAATGCGATCACTCCGTTATGATATGTTGGTCGGATAAGGCGCTCGCGCCGCATCCGACATTG
ATTGCTTAAGCGACTTCATTCACCTGACGACGCAGCAGGGAAAGCGGGCCGGGGCCGCTAAGCGTGAACACGGAAATTAA
GGTGAAGCCCAGCGCCACCAGACCCAGCACCAGATAAGCGCCCTGGAAACCGATGCTTTCATACATATTGCCCGCCAGTA
CAGACATAAAAATCATCGCCAGTTGCTTAAAGAAGCAGAAACAGACCAGATAAATCGTCGCTGAAAAACGCACTTCAAAC
TGGCTGGTAATATATTTAAAGCAGCCCACCAGCAGGAACGGTACTTCAAACATATGCAGCGTTTTCAGAATAACCACTTC
CAGCGCTGAGGTGGCGAACGATGAGCCAATAATACGTACAGACATAATAGTGCCAGCCAGCAGCAGGGCGTTTTTCCCAC
CGATGCGATTAATGATCAGTGGCGCAAAGAACATAATCGAGGCGTTAAGTAATTCGCCCATTGTCGTTACGTAGCCAAAT
ACCCGCGTACCCTGTTCACCGGTAGCAAAGAACGAAGTAAAGAAATTAGCAAACTGTTGGTCAAAAACATCGTAGGTGCA
GGAAACGCCAATAACATACAGTGACAAAAACCACAGTTTTGGCTGTCTGAACAGTTCCAGTGCCAGCTTAAGGCTAAATG
CCGAATGGTTGGCACCTACCGCATTGGCAACCGTGGCAGAAGAGGGCGCATCCGTTTTGGCGAAAAAGAGTAAAACGGCG
AGGATGAGTGCACAGCCAGAGCCCAGCCAGAAAACAAACTGATTATTGATGGTGAACATGATGCCGACAATCGAGGCACA
CAGCGCCCAGCCAACACAGCCAAACATCCGCGCGCGACCAAATTCGAAATTACTGCGACGGCTGACTTTCTCAATAAATG

CCTCTACTGCTGGCGCACCGGCGTTAAAACAAAAGCCTAGATAAATACCACCAACAATCGATCCTACTAAAATGTTGTAT
TGTAACAGTGGCCCGAAGATAAAAATAAAGAACGGCGCAAACATCACTAACATGCCGGTAATAATCCACAGCAGGTATTT
GCGCAGCCCGAGTTTGTCAGAAAGCAGACCAAACAGCGGTTGGAATAATAGCGAGAACAGAGAAATAGCGGCAAAAATAA
TACCCGTATCACTTTTGCTGATATGGTTGATGTCATGTAGCCAAATCGGGAAAAACGGGAAGTAGGCTCCCATGATAAAA
AAGTAAAAGAAAAAGAATAAACCGAACATCCAAAAGTTTGTGTTTTTTAAATAGTACATAATGGATTTCCTTACGCGAAA
TACGGGCAGACATGGCCTGCCCGGTTATTATTATTTTTGACACCAGACCAACTGGTAATGGTAGCGACCGGCGCTCAGCT
GGAATTCCGCCGATACTGACGGGCTCCAGGAGTCGTCGCCACCAATCCCCATATGGAAACCGTCGATATTCAGCCATGTG
CCTTCTTCCGCGTGCAGCAGATGGCGATGGCTGGTTTCCATCAGTTGCTGTTGACTGTAGCGGCTGATGTTGAACTGGAA
GTCGCCGCGCCACTGGTGTGGGCCATAATTCAATTCGCGCGTCCCGCAGCGCAGACCGTTTTCGCTCGGGAAGACGTACG
GGGTATACATGTCTGACAATGGCAGATCCCAGCGGTCAAAACAGGCGGCAGTAAGGCGGTCGGGATAGTTTTCTTGCGGC
CCTAATCCGAGCCAGTTTACCCGCTCTGCTACCTGCGCCAGCTGGCAGTTCAGGCCAATCCGCGCCGGATGCGGTGTATC
GCTCGCCACTTCAACATCAACGGTAATCGCCATTTGACCACTACCATCAATCCGGTAGGTTTTCCGGCTGATAAATAAGG
TTTTCCCCTGATGCTGCCACGCGTGAGCGGTCGTAATCAGCACCGCATCAGCAAGTGTATCTGCCGTGCACTGCAACAAC
GCTGCTTCGGCCTGGTAATGGCCCGCCGCCTTCCAGCGTTCGACCCAGGCGTTAGGGTCAATGCGGGTCGCTTCACTTAC
GCCAATGTCGTTATCCAGCGGTGCACGGGTGAACTGATCGCGCAGCGGCGTCAGCAGTTGTTTTTTATCGCCAATCCACA
TCTGTGAAAGAAAGCCTGACTGGCGGTTAAATTGCCAACGCTTATTACCCAGCTCGATGCAAAAATCCATTTCGCTGGTG
GTCAGATGCGGGATGGCGTGGGACGCGGCGGGGAGCGTCACACTGAGGTTTTCCGCCAGACGCCACTGCTGCCAGGCGCT
GATGTGCCCGGCTTCTGACCATGCGGTCGCGTTCGGTTGCACTACGCGTACTGTGAGCCAGAGTTGCCCGGCGCTCTCCG
GCTGCGGTAGTTCAGGCAGTTCAATCAACTGTTTACCTTGTGGAGCGACATCCAGAGGCACTTCACCGCTTGCCAGCGGC
TTACCATCCAGCGCCACCATCCAGTGCAGGAGCTCGTTATCGCTATGACGGAACAGGTATTCGCTGGTCACTTCGATGGT
TTGCCCGGATAAACGGAACTGGAAAAACTGCTGCTGGTGTTTTGCTTCCGTCAGCGCTGGATGCGGCGTGCGGTCGGCAA
AGACCAGACCGTTCATACAGAACTGGCGATCGTTCGGCGTATCGCCAAAATCACCGCCGTAAGCCGACCACGGGTTGCCG
TTTTCATCATATTTAATCAGCGACTGATCCACCCAGTCCCAGACGAAGCCGCCCTGTAAACGGGGATACTGACGAAACGC
CTGCCAGTATTTAGCGAAACCGCCAAGACTGTTACCCATCGCGTGGGCGTATTCGCAAAGGATCAGCGGGCGCGTCTCTC
CAGGTAGCGAAAGCCATTTTTTGATGGACCATTTCGGCACAGCCGGGAAGGGCTGGTCTTCATCCACGCGCGCGTACATC
GGGCAAATAATATCGGTGGCCGTGGTGTCGGCTCCGCCGCCTTCATACTGCACCGGGCGGGAAGGATCGACAGATTTGAT
CCAGCGATACAGCGCGTCGTGATTAGCGCCGTGGCCTGATTCATTCCCCAGCGACCAGATGATCACACTCGGGTGATTAC
GATCGCGCTGCACCATTCGCGTTACGCGTTCGCTCATCGCCGGTAGCCAGCGCGGATCATCGGTCAGACGATTCATTGGC
ACCATGCCGTGGGTTTCAATATTGGCTTCATCCACCACATACAGGCCGTAGCGGTCGCACAGCGTGTACCACAGCGGATG
GTTCGGATAATGCGAACAGCGCACGGCGTTAAAGTTGTTCTGCTTCATCAGCAGGATATCCTGCACCATCGTCTGCTCAT
CCATGACCTGACCATGCAGAGGATGATGCTCGTGACGGTTAACGCCTCGAATCAGCAACGGCTTGCCGTTCAGCAGCAGC
AGACCATTTTCAATCCGCACCTCGCGGAAACCGACATCGCAGGCTTCTGCTTCAATCAGCGTGCCGTCGGCGGTGTGCAG
TTCAACCACCGCACGATAGAGATTCGGGATTTCGGCGCTCCACAGTTTCGGGTTTTCGACGTTCAGACGTAGTGTGACGC
GATCGGCATAACCACCACGCTCATCGATAATTTCACCGCCGAAAGGCGCGGTGCCGCTGGCGACCTGCGTTTCACCCTGC
CATAAAGAAACTGTTACCCGTAGGTAGTCACGCAACTCGCCGCACATCTGAACTTCAGCCTCCAGTACAGCGCGGCTGAA
ATCATCATTAAAGCGAGTGGCAACATGGAAATCGCTGATTTGTGTAGTCGGTTTATGCAGCAACGAGACGTCACGGAAAA
TGCCGCTCATCCGCCACATATCCTGATCTTCCAGATAACTGCCGTCACTCCAGCGCAGCACCATCACCGCGAGGCGGTTT
TCTCCGGCGCGTAAAAATGCGCTCAGGTCAAATTCAGACGGCAAACGACTGTCCTGGCCGTAACCGACCCAGCGCCCGTT
GCACCACAGATGAAACGCCGAGTTAACGCCATCAAAAATAATTCGCGTCTGGCCTTCCTGTAGCCAGCTTTCATCAACAT
TAAATGTGAGCGAGTAACAACCCGTCGGATTCTCCGTGGGAACAAACGGCGGATTGACCGTAATGGGATAGGTCACGTTG
GTGTAGATGGGCGCATCGTAACCGTGCATCTGCCAGTTTGAGGGGACGACGACAGTATCGGCCTCAGGAAGATCGCACTC
CAGCCAGCTTTCCGGCACCGCTTCTGGTGCCGGAAACCAGGCAAAGCGCCATTCGCCATTCAGGCTGCGCAACTGTTGGG
AAGGGCGATCGGTGCGGGCCTCTTCGCTATTACGCCAGCTGGCGAAAGGGGGATGTGCTGCAAGGCGATTAAGTTGGGTA
ACGCCAGGGTTTTCCCAGTCACGACGTTGTAAAACGACGGCCAGTGAATCCGTAATCATGGTCATAGCTGTTTCCTGTGT
GAAATTGTTATCCGCTCACAATTCCACACAACATACGAGCCGGAAGCATAAAGTGTAAAGCCTGGGGTGCCTAATGAGTG
AGCTAACTCACATTAATTGCGTTGCGCTCACTGCCCGCTTTCCAGTCGGGAAACCTGTCGTGCCAGCTGCATTAATGAAT
CGGCCAACGCGCGGGGAGAGGCGGTTTGCGTATTGGGCGCCAGGGTGGTTTTTCTTTTCACCAGTGAGACGGGCAACAGC
TGATTGCCCTTCACCGCCTGGCCCTGAGAGAGTTGCAGCAAGCGGTCCACGCTGGTTTGCCCCAGCAGGCGAAAATCCTG
TTTGATGGTGGTTAACGGCGGGATATAACATGAGCTGTCTTCGGTATCGTCGTATCCCACTACCGAGATATCCGCACCAA
CGCGCAGCCCGGACTCGGTAATGGCGCGCATTGCGCCCAGCGCCATCTGATCGTTGGCAACCAGCATCGCAGTGGGAACG
ATGCCCTCATTCAGCATTTGCATGGTTTGTTGAAAACCGGACATGGCACTCCAGTCGCCTTCCCGTTCCGCTATCGGCTG
AATTTGATTGCGAGTGAGATATTTATGCCAGCCAGCCAGACGCAGACGCGCCGAGACAGAACTTAATGGGCCCGCTAACA
GCGCGATTTGCTGGTGACCCAATGCGACCAGATGCTCCACGCCCAGTCGCGTACCGTCTTCATGGGAGAAAATAATACTG
TTGATGGGTGTCTGGTCAGAGACATCAAGAAATAACGCCGGAACATTAGTGCAGGCAGCTTCCACAGCAATGGCATCCTG
GTCATCCAGCGGATAGTTAATGATCAGCCCACTGACGCGTTGCGCGAGAAGATTGTGCACCGCCGCTTTACAGGCTTCGA
CGCCGCTTCGTTCTACCATCGACACCACCACGCTGGCACCCAGTTGATCGGCGCGAGATTTAATCGCCGCGACAATTTGC

GACGGCGCGTGCAGGGCCAGACTGGAGGTGGCAACGCCAATCAGCAACGACTGTTTGCCCGCCAGTTGTTGTGCCACGCG
GTTGGGAATGTAATTCAGCTCCGCCATCGCCGCTTCCACTTTTTCCCGCGTTTTCGCAGAAACGTGGCTGGCCTGGTTCA
CCACGCGGGAAACGGTCTGATAAGAGACACCGGCATACTCTGCGACATCGTATAACGTTACTGGTTTCACATTCACCACC
CTGAATTGACTCTCTTCCGGGCGCTATCATGCCATACCGCGAAAGGTTTTGCGCCATTCGATGGTGTCAACGTAAATGCA
TGCCGCTTCGCCTTCCGGCCACCAGAATAGCCTGCGATTCAACCCCTTCTTCGATCTGTTTTGCTACCCGTTGTAGCGCC
GGAAGATGCTTTTCCGCTGCCTGTTCAATGGTCATTGCGCTCGCCATATACACCAGATTCAGACAGCCAATCACCCGTTG
TTCACTGCGCAGCGGTACGGCGATAGAGGCGATCTTCTCCTCCTGATCCCAGCCGCGGTAGTTCTGTCCGTAACCCTCTT
TGCGCGCGCGCGCCAGAATGGCTTCCAGCTTTAACGGTTCCCGTGCCAGTTGATAGTCATCACCGGGGCGGGAGGCTAAC
ATTTCGATTAATTCCTTGCGGTCTTGTTCCGGGCAAAAGGCCAGCCAGGTCAGGCCCGAGGCGGTTTTCAGAAGCGGCAA
ACGTCGCCCGACCATTGCCCGGTGAAAGGATAAGCGGCTGAAACGGTGAGTGGTTTCGCGTACCACCATTGCATCAACAT
CCAGCGTGGACACATCTGTCGGCCATACCACTTCGCGCAACAGATCGCCCAGCAGTGGGGCCGCCAGTGCAGAAATCCAC
TGTTCGTCACGAAATCCTTCGCTTAATTGCCGCACTTTGATGGTCAGTCGAAAACTATCATCGGAGGGGCTACGGCGGAC
ATATCCCTCTTCCTGCAGCGTCTCCAGCAGTCGCCGCACAGTGGTGCGATGCAGGCCGCTGAGTTCCGCCAGCAGCCCGA
CGCTGGCACCGCCATCAAGTTTATTTAACATATTTAATAACATTAGACCGCGGGTTAAGCCGCGCACGGTTTTGTATTCC
GTCTGCTCATTGTTCTGCATATTAATTGACATTTCTATAGTTAAAACAACGTGGTGCACCTGGTGCACATTCGGGCATGT
TTTGATTGTAGCCGAAAACACCCTTCCTATACTGAGCGCACAATAAAAAATCATTTACATGTTTTTAACAAAATAAGTTG
CGCTGTACTGTGCGCGCAACGACATTTTGTCCGAGTCGTGAGGTACTGAAATGGCAATACAACACCCTGACATCCAGCCT
GCTGTTAACCATAGCGTTCAGGTGGCGATCGCTGGTGCCGGCCCGGTTGGGCTGATGATGGCGAACTATCTCGGCCAGAT
GGGCATTGACGTGCTGGTGGTGGAGAAACTCGATAAGTTGATCGACTACCCGCGTGCGATTGGTATTGATGACGAGGCGC
TGCGCACCATGCAGTCGGTCGGCCTGGTCGATGATGTTCTGCCGCACACTACGCCGTGGCACGCGATGCGTTTTCTCACC
CCGAAAGGCCGCTGTTTTGCTGATATTCAGCCAATGACCGATGAATTTGGCTGGCCGCGCCGTAACGCCTTTATTCAGCC
GCAGGTCGATGCGGTGATGCTGGAAGGGGTGTCGCGTTTTCCGAATGTGCGCTGCTTGTTTTCCCGCGAGCTGGAGGCCT
TCAGTCAGCAAGATGACGAAGTGACCTTGCACCTGAAAACGGCAGAAGGGCAGCGGGAAATAGTCAAAGCCCAGTGGCTG
GTAGCCTGTGACGGTGGAGCAAGTTTTGTCCGTCGCACTCTGAATGTGCCGTTTGAAGGTAAAACTGCGCCAAATCAGTG
GATTGTGGTAGATATCGCCAACGATCCGTTAAGTACGCCGCATATCTATTTGTGTTGCGATCCGGTGCGCCCGTATGTTT
CTGCCGCGCTGCCTCATGCGGTACGTCGCTTTGAATTTATGGTGATGCCGGGAGAAACCGAAGAGCAGCTGCGTGAGCCG
CAAAATATGCGCAAGCTGTTAAGCAAAGTGCTGCCTAATCCGGACAATGTTGAATTGATTCGCCAGCGTGTCTACACCCA
CAACGCGCGACTGGCGCAACGTTTCCGTATTGATCGCGTACTGCTGGCGGGCGATGCCGCGCACATCATGCCGGTATGGC
AGGGGCAGGGCTATAACAGTGGTATGCGCGACGCCTTTAACCTCGCATGGAAACTGGCGTTGGTTATCCAGGGGAAAGCC
CGCGATGCGCTGCTCGATACCTATCAACAAGAACGTCGCGATCACGCCAAAGCGATGATTGACCTGTCCGTGACGGCGGG
CAACGTGCTGGCTCCGCCGAAACGCTGGCAGGGTACGTTACGTGACGGCGTTTCCTGGCTGTTGAATTATCTGCCGCCAG
TAAAACGCTACTTCCTCGAAATGCGCTTCAAGCCGATGCCGCAATATTACGGCGGTGCGCTGATGCGTGAGGGCGAAGCG
AAGCACTCTCCGGTCGGCAAGATGTTTATTCAGCCGAAAGTCACGCTGGAAAACGGCGACGTGACGCTGCTCGATAACGC
GATCGGCGCGAACTTCGCGGTAATTGGCTGGGGATGCAATCCACTGTGGGGGATGAGCGACGAGCAAATCCAGCAGTGGC
GCGCGTTGGGCACACGCTTCATTCAGGTGGTGCCGGAAGTGCAAATTCATACCGCACAGGATAACCACGACGGCGTACTA
CGCGTGGGCGATACGCAAGGTCGCCTGCGTAGCTGGTTCGCGCAACACAATGCTTCGCTGGTGGTGATGCGCCCGGATCG
CTTTGTTGCCGCCACCGCCATTCCGCAAACCCTGGGCAAGACCCTGAATAAACTGGCGTCGGTGATGACGCTGACCCGCC
CTGATGCCGACGTTTCTGTCGAAAAGGTAGCCTGATATGCACGCTTATCTTCACTGTCTTTCCCACTCGCCGCTGGTGGG
GTATGTCGACCCGGCGCAAGAGGTGCTCGATGAGGTCAATGGCGTGATTGCCAGCGCCCGCGAGCGTATTGCGGCATTCT
CCCCTGAACTGGTGGTGCTGTTTGCGCCAGATCACTACAACGGCTTTTTCTATGACGTGATGCCACCGTTCTGTTTAGGC
GTTGGAGCGACGGCAATTGGTGATTTCGGCAGTGCGGCAGGAGAGCTGCCCGTGCCTGTGGAGCTGGCGGAGGCCTGTGC
GCATGCCGTCATGAAGAGCGGGATCGATCTTGCCGTTTCTTACTGTATGCAGGTGGACCACGGGTTCGCCCAGCCGCTGG
AGTTCCTGCTCGGTGGGCTGGATAAGGTGCCAGTTCTGCCTGTGTTCATCAACGGTGTCGCCACGCCGCTGCCCGGTTTC
CAGCGTACCCGCATGTTGGGTGAAGCCATTGGACGTTTCACCAGCACTCTCAATAAACGCGTGCTGTTCCTGGGTTCCGG
TGGGCTTTCCCATCAGCCGCCGGTGCCCGAACTGGCGAAAGCCGATGCCCATATGCGCGACCGTCTGTTGGGGAGCGGGA
AAGATTTACCCGCCAGTGAGCGCGAATTGCGTCAGCAACGGGTGATTAGCGCCGCTGAGAAGTTTGTTGAGGATCAGAGA
ACGCTGCATCCGCTCAACCCGATTTGGGATAACCAGTTCATGACTTTGCTGGAGCAGGGACGCATACAGGAACTGGATGC
CGTCAGTAACGAAGAGCTTTCCGCCATTGCCGGAAAGTCGACACATGAAATCAAAACCTGGGTCGCCGCTTTTGCCGCTA
TTTCTGCGTTTGGCAACTGGCGTAGCGAAGGGCGTTATTACCGCCCAATCCCGGAGTGGATTGCCGGATTTGGCTCGTTA
AGCGCCAGAACAGAGAACTGAATATGCAGGAGAAGATGATGAGTTATCAGCCACAAACCGAAGCCGCCACCAGCCGTTTT
CTGAATGTAGAAGAAGCGGGTAAAACGCTGCGCATCCATTTTAATGACTGCGGACAAGGCGACGAAACCGTTGTCCTGCT
GCATGGTTCCGGCCCGGGTGCTACTGGCTGGGCGAACTTCAGCCGCAATATCGATCCGCTGGTAGAGGCGGGCTATCGGG
TGATCCTGCTGGATTGTCCGGGTTGGGGCAAGAGCGATTCGGTCGTTAATAGTGGTTCGCGATCGGATCTTAATGCACGA
ATCCTGAAAAGCGTGGTGGATCAACTGGATATCGCCAAAATCCACCTGCTGGGCAACTCGATGGGGGGCCATAGTTCTGT
GGCGTTCACCCTTAAATGGCCGGAGCGCGTCGGCAAACTGGTGCTGATGGGCGGCGGTACGGGCGGCATGAGTTTGTTTA
CGCCGATGCCAACCGAAGGTATTAAGCGACTGAATCAGCTTTATCGTCAGCCGACTATCGAAAACCTGAAGCTGATGATG

GATATCTTCGTTTTTGATACCAGCGATTTGACCGACGCCCTGTTTGAAGCGCGCCTGAATAATATGCTGTCGCGCCGCGA
TCACCTGGAAAACTTCGTTAAGAGCCTGGAAGCTAATCCGAAACAGTTCCCGGATTTTGGCCCACGTCTGGCGGAAATCA
AAGCGCAAACCCTGATTGTCTGGGGGCGCAACGACCGCTTTGTGCCGATGGATGCGGGTCTGCGTCTGCTGTCCGGCATT
GCCGGTTCTGAACTGCATATCTTCCGCGACTGTGGTCACTGGGCGCAGTGGGAACATGCCGACGCTTTCAATCAACTGGT
GCTGAATTTCCTCGCACGCCCTTAAGGAATGGTCATGACGAAGCATACTCTTGAGCAACTGGCGGCGGATTTACGCCGCG
CCGCAGAGCAGGGCGAAGCGATTGCACCGCTGCGCGATCTGATTGGTATCGATAACGCTGAAGCGGCTTACGCCATTCAG
CACATAAATGTGCAACATGACGTTGCGCAGGGGCGTCGCGTGGTAGGGCGTAAAGTGGGCCTGACACATCCGAAAGTGCA
ACAACAACTGGGCGTTGATCAACCGGATTTTGGGACGTTATTTGCCGACATGTGTTATGGCGATAACGAAATCATTCCTT
TTTCCCGTGTTCTGCAACCCCGCATTGAAGCGGAGATCGCACTGGTGTTGAACCGCGATTTGCCCGCAACCGATATCACC
TTCGACGAATTGTATAACGCCATTGAATGGGTACTTCCGGCGCTGGAAGTGGTGGGGAGCCGCATTCGCGACTGGTCGAT
TCAGTTTGTCGATACCGTGGCAGATAACGCCTCCTGTGGGGTGTATGTCATCGGCGGTCCGGCGCAACGTCCGGCGGGGT
TAGACCTGAAAAACTGCGCCATGAAGATGACGCGTAATAACGAAGAGGTTTCTAGCGGGCGCGGCAGCGAATGCCTGGGA
CATCCGCTTAATGCGGCCGTCTGGCTGGCACGCAAAATGGCCAGTCTGGGTGAACCGCTGCGCACCGGAGATATCATTCT
TACCGGGGCATTAGGTCCGATGGTGGCGGTGAATGCGGGCGATCGTTTTGAAGCCCATATTGAAGGCATAGGTTCAGTTG
CTGCGACATTTTCAAGCGCAGCCCCAAAAGGAAGTCTGTCATGAGTAAGCGTAAAGTCGCCATTATCGGTTCTGGCAACA
TTGGTACCGATCTGATGATTAAAATTTTGCGTCACGGTCAGCATCTGGAGATGGCGGTGATGGTTGGCATTGATCCTCAG
TCCGACGGTCTGGCGCGCGCCAGACGTATGGGCGTCGCCACCACCCATGAAGGGGTGATCGGACTGATGAACATGCCTGA
ATTTGCTGATATCGACATTGTATTTGATGCGACCAGCGCCGGTGCTCATGTGAAAAACGATGCCGCTTTACGCGAAGCGA
AACCGGATATTCGCTTAATTGACCTGACGCCTGCTGCCATCGGCCCTTACTGCGTGCCGGTGGTTAACCTCGAGGCGAAC
GTCGATCAACTGAACGTCAACATGGTCACCTGCGGCGGCCAGGCCACCATTCCAATGGTGGCGGCAGTTTCACGCGTGGC
GCGTGTTCATTACGCCGAAATTATCGCTTCTATCGCCAGTAAATCTGCCGGACCTGGCACGCGTGCCAATATCGATGAAT
TTACGGAAACCACTTCCCGAGCCATTGAAGTGGTGGGCGGCGCGGCAAAAGGGAAGGCGATTATTGTGCTTAACCCAGCA
GAGCCACCGTTGATGATGCGTGACACGGTGTATGTATTGAGCGACGAAGCTTCACAAGATGATATCGAAGCCTCAATCAA
TGAAATGGCTGAGGCGGTGCAGGCTTACGTACCGGGTTATCGCCTGAAACAGCGCGTGCAGTTTGAAGTTATCCCGCAGG
ATAAACCGGTCAATTTACCGGGCGTGGGGCAATTCTCCGGACTGAAAACAGCGGTCTGGCTGGAAGTCGAAGGCGCAGCG
CATTATCTGCCTGCCTATGCGGGCAACCTCGACATTATGACTTCCAGTGCGCTGGCGACAGCGGAAAAAATGGCCCAGTC
ACTGGCGCGCAAGGCAGGAGAAGCGGCATGAACGGTAAAAAACTTTATATCTCGGACGTCACATTGCGTGACGGTATGCA
CGCCATTCGTCATCAGTATTCGCTGGAAAACGTTCGCCAGATTGCCAAAGCACTGGACGATGCCCGCGTGGATTCGATTG
AAGTGGCCCACGGCGACGGTTTGCAAGGTTCCAGCTTTAACTATGGTTTCGGCGCACATAGCGACCTTGAATGGATTGAA
GCGGCGGCGGATGTGGTGAAGCACGCCAAAATCGCGACGTTGTTGCTGCCAGGAATCGGCACTATTCACGATCTGAAAAA
TGCCTGGCAGGCTGGCGCGCGGGTGGTTCGTGTGGCAACGCACTGTACCGAAGCTGATGTTTCCGCCCAGCATATTCAGT
ATGCCCGCGAGCTCGGAATGGACACCGTTGGTTTTCTGATGATGAGCCATATGACCACGCCGGAGAATCTCGCCAAGCAG
GCAAAGCTGATGGAAGGCTACGGTGCGACCTGTATTTATGTGGTGGATTCTGGCGGTGCGATGAACATGAGCGATATCCG
TGACCGTTTCCGCGCCCTGAAAGCAGAGCTGAAACCAGAAACGCAAACTGGCATGCACGCTCACCATAACCTGAGTCTTG
GCGTGGCGAACTCTATCGCGGCGGTGGAAGAGGGCTGCGACCGAATCGACGCCAGCCTCGCGGGAATGGGCGCGGGCGCA
GGTAACGCACCGCTGGAAGTGTTTATTGCCGCCGCGGATAAACTGGGCTGGCAGCATGGGACCGATCTCTATGCGTTAAT
GGATGCCGCCGACGACCTGGTGCGTCCGTTGCAGGATCGACCGGTACGAGTCGATCGCGAAACGCTGGCGCTGGGATACG
CTGGTGTTTACTCGAGCTTCCTGCGTCACTGTGAAACGGCGGCGGCGCGTTATGGCTTAAGTGCGGTGGATATTCTCGTT
GAGCTGGGCAAACGCCGGATGGTTGGCGGCCAGGAGGATATGATCGTTGACGTGGCGCTGGATCTGCGCAACAACAAATA
ATGATGACTGCCGAGAACGTGCATTTTGTATGCCCATCCCAGCACGACTGCCGGATGCGGCGTGAACGCCTTATCCGGCC
TACGGATGGCGTGAGAATTTGTAGGTCTGATAAGACGCGTTAGCGTCGCATCAGGCATCTGCGCACGACTGCCGGATGCG
GCGTGAACGCCTTATCCGGCCTACGGATGGCGCGGGAATTTGTAGGCCTGATAAGACGCGTTAGCGTCGCATCAGGCATC
TGCGCACGACTGCCGGATGCGGCGTGAACGCCTTATCCGGCCTACGGGTGGCGCGAGAATTTGTAGGCCTGATAAGACGC
GTTAGCGTCGCATCAGGCATCTGCGCACGACTGCCGGATGCGGCGTAAACGCCTTATCCGGCCTACGGATGGCGCGGGAA
TTTGTAGGCCTGATAAGACGCGTTAGCGTCGCATCAGGCATCTGCACACGACTGCCGGATGCGATAAACGTCTTGTCCGG
CCTACATTTCGCCCGTAGGCAGTCATTAAATAGTTCTGATTACGCGGCTGGCAATGTATCAGTCGCGATTCACCTGCACT
CGCAACGAGGTTCATCTTATGTCGACTCGTACCCCTTCATCATCTTCATCCCGCCTGATGCTGACCATCGGGCTTTGTTT
TTTGGTCGCTCTGATGGAAGGGCTGGATCTTCAGGCGGCTGGCATTGCGGCGGGTGGCATCGCCCAGGCTTTCGCACTCG
ATAAAATGCAAATGGGCTGGATATTTAGCGCCGGAATACTCGGTTTGCTACCCGGCGCGTTGGTTGGCGGAATGCTGGCG
GACCGTTATGGTCGCAAGCGCATTTTGATTGGCTCAGTTGCGCTGTTTGGTTTGTTCTCACTGGCAACGGCGATTGCCTG
GGATTTCCCCTCACTGGTCTTTGCGCGGCTGATGACCGGTGTCGGGCTGGGGGCGGCGTTGCCGAATCTTATCGCCCTGA
CGTCTGAAGCCGCGGGTCCACGTTTTCGTGGGACGGCAGTGAGCCTGATGTATTGCGGTGTTCCCATTGGCGCGGCGCTG
GCGGCGACACTGGGTTTCGCGGGGGCAAACTTAGCATGGCAAACGGTGTTTTGGGTAGGTGGTGTGGTGCCGTTGATTCT
GGTGCCGCTATTAATGCGCTGGCTGCCGGAGTCGGCGGTTTTCGCTGGCGAAAAACAGTCTGCGCCACCACTGCGTGCCT
TATTTGCGCCAGAAACGGCAACCGCGACGCTGCTGCTGTGGTTGTGTTATTTCTTCACTCTGCTGGTGGTCTACATGTTG
ATCAACTGGCTACCGCTACTTTTGGTGGAGCAAGGATTCCAGCCATCGCAGGCGGCAGGGGTGATGTTTGCCCTGCAAAT

GGGGGCGGCAAGCGGGACGTTAATGTTGGGCGCATTGATGGATAAGCTGCGTCCAGTAACCATGTCGCTACTGATTTATA
GCGGCATGTTAGCTTCGCTGCTGGCGCTTGGAACGGTGTCGTCATTTAACGGTATGTTGCTGGCGGGATTTGTCGCGGGG
TTGTTTGCGACAGGTGGGCAAAGCGTTTTGTATGCCCTGGCACCGTTGTTTTACAGTTCGCAGATCCGCGCAACAGGTGT
GGGAACAGCCGTGGCGGTAGGGCGTCTGGGGGCTATGAGCGGTCCGTTACTGGCCGGGAAAATGCTGGCATTAGGCACTG
GCACGGTCGGCGTAATGGCCGCTTCTGCACCGGGTATTCTTGTTGCTGGGTTGGCGGTGTTTATTTTGATGAGCCGGAGA
TCACGAATACAGCCGTGCGCCGATGCCTGACGTGCCTTATTAGGCAGGGGCGAAAGGGACTTCACCCCAATACACCTACG
GCGTTTACAGGTATACTCGCTAAAAATTATTCAGCGGGTTTGGAAACAAAGATGGCAAAACTTACCTTACAAGAGCAGTT
GCTCAAAGCAGGATTAGTCACCAGCAAAAAAGCGGCGAAGGTGGAGAGAACGGCGAAAAAATCGCGCGTTCAGGCGCGTG
AAGCTCGGGCGGCGGTAGAAGAAAATAAAAAGGCACAGCTTGAGCGTGATAAACAGCTTAGCGAACAGCAAAAACAAGCG
GCGTTGGCGAAAGAATATAAAGCTCAGGTGAAGCAGCTTATTGAAATGAACCGAATCACCATTGCCAATGGCGATATTGG
TTTTAACTTCACTGACGGCAATCTGATTAAGAAGATTTTTGTCGATAAGCTCACACAGGCGCAGTTGATTAATGGTCGTC
TGGCGATTGCCCGCTTGTTGGTCGATAACAATAGCGAAGGTGAATACGCCATTATTCCCGCCAGCGTTGCCGATAAAATT
GCTCAGCGCGATGCCAGCAGTATAGTGTTGCACAGCGCGCTCAGCGCCGAAGAGCAGGATGAAGACGACCCGTATGCCGA
CTTCAAAGTGCCTGATGATTTGATGTGGTAAACATAGATAAGTCGTGCGCAGATGCCTGATGCGACGCTATGCGCGTCTT
ATCAGGCCTACGGTTTATGGGCGAAGTGTAGACCGGATAAGGCGTTCACGCCGCATCCGGCAGTCGTGCGCCATGCCTGA
TGCGACGCTTTGCGCGTCTTATCAGGCCTACGGCTTACGGGCGAAGTGTAGGCCGGATAAGGCGTTCACGCCGCATCCGG
CAGTCGTGCTATTATCAACGCATATTCAGTTTATTGGCGTGGTAGGCAATATGCTCGCCAATAAAACTGGAGACAAAATA
ATAGCTGTGATCATAACCCTCGTGATAACGGATTAACGTCTTGATATTCATCTCCTGGCAGATCTTTTCAAGATTTGGAG
TCCGCAGCTGTTCTGCGTAAAAATCATCACTCAACCCCTGATCAACCATGATTTCCGCAACGCGTTGACCTTGTGAAATA
AGACTCACCGGGTCGTAATCCAACCAGGCATCTTTATTTTCAGCAAGATATGCAGCAAAGGCTTGCTGTCCCCACGGCAC
TTGCGATGGGGAGACAATGGGCGAAAACGCCGAGACGCTGACATATTCATCTGGGTTACGTAACGCCAGCACCAGCGCGC
CCAGCCCGCCCATAGAATGACCAGAGATAGACTTTTTGGCCGTTGCCGGAAAATGATGCATCACTAAATCCGGCAGCTCG
TTGCGGATATAGTCATACATTTTGTAATGTTCATTCCACGGCGCTTGCGTCGCGTTCAGGTAAAACCCGGCACCTTGCCC
GAGATCGTAACGGTCAGCATCTGCGACATGACTGCCTCGCGGACTGGTGTCCGGCGCAACAACAATAATGTTGTGCTCAG
CCGCGTAACGCTGCATCCCCGATTTAGTAATGAAATTCTGCTCGTTGCAGGTCAGGCCTGAAAGCCAGTACAGCACCGGC
AATTTTTCATTCGCGGCTTTTGGTGGGAGATAGACGCCGACATTCATTTCACATTTCAGTGATTGGGAATAATGCCGATA
CATATTTTGCCAGCCGCCAAAGCTGACATGTTTTTCAATGAGTTCCATCAGTCATTATCTCAGGGTTAATCCCCCGGACG
GCTGCCCGGGGAGAGTATTAAGAACCCCCGCCTGAAGTGGACGGGGTAAACCTGCGGGAAATCAGTAACGAATTACGGTT
CGAATGGATTTGCCTTCATGCATCAGGTCGAAGGCGTCATTAATTTCATCCAGGCTCATGGTATGCGTGACAAACGGTTC
CAGATCGATATCACCTTTCATCGCATCTTCAACCATGCCCGGTAACTGGGAACGACCTTTCACGCCGCCAAACGCGGAAC
CTTTCCATACGCGACCAGTGACCAACTGGAATGGACGGGTGGAGATTTCCTGACCGGCAACCGCGACCCCGATGATCACC
GACTGACCCCAGCCGCGGTGCGCACTTTCCAGCGCCGCACGCATCACGTTGACGTTACCGATGCATTCAAAGGTATGGTC
GATACCCCATTTGTTGATATCCAACAGGACATCTTTTATCGGTTTGTCGTAGTCATTCGGGTTAATGCAGTCGGTAGCAC
CGAAGCGACGCGCCAGATCGAATTTCTTCGGGTTGGTATCGATAGCGATAATCCGACCCGCTTTCGCCTGACGCGCGCCC
TGAACCACTGCCAGACCAATCGCGCCAAGACCAAACACGGCAACAGAATCACCTGGCTGGACTTTAGCTGTGTTGTGTAC
CGCGCCAATACCGGTGGTCACGCCACAGCCCAGCAGGCAGACGTGTTCATGGTTTGCTTCTGGATTAATTTTTGCCAGAG
ACACTTCCGCGACTACGGTGTATTCACTGAATGTAGAGCACCCCATGTAGTGATAAAGCGGCTGCCCGTTGTAAGAAAAA
CGGGTGGTGCCGTCTGGCATCAGGCCTTTACCCTGGGTTTCGCGAACCGCAACACAGAGGTTAGTTTTGCCAGAACGACA
GAACTCACACTCGCCGCACTCCGCGGTGTAAAGCGGGATCACATGGTCGCCAGGTTTGACGCTGGTTACGCCTTCACCGA
CTTCAACCACAACGCCGGCCCCTTCGTGACCGAGAACCACCGGGAATACACCTTCCGGGTCATCGCCGGAGAGGGTAAAT
GCGTCGGTATGGCAAACGCCGGTATGGGTGACTTTAATTAGCACTTCACCTTTTTTCGGTGGTGCAACGTCAATTTCAAC
GATTTCCAGCGGTTTACCGGGAGCAAATGCAACGGCAGCACGTGATTTCATCTCTCGCTCTTCCTCAATATGGTAATAGA
TTCAGCTATTTAAGATAGGCACGAACCAGTTCAATAGTGTCGTCAACGGATTGGCTGACTTCGCGGCTGTAGCAGTCATT
TCGGTCAAACGTTTCCCGGATATGGCTTTCAAGCACTTCTGCCATCAGCCCATTAGCCGCGCCCCGAACGGCAGCGATCT
GTTGGAGTATGGCACGGCATTCGGCATCACCCTCCAGCGACCGTTCCAGAGCATCAATCTGCCCCCGAATACGACGAACT
CGAGTAAGGACCTTTTTCTTCTCTTCCGGAGTACTGGGCATTTCGCACCTCATCATCTGCATGCAATATACTATAGGGGG
GTATTCTATATGTCAATGCATACCCCCCTATAGTATATAACACTGGAGAATAAAATTTATCCGGTGAATGTGGTCGGAAA
ACAAAGAGGAAAGGGGGGGGGCTAATCGGCAGGGAAGGCCGCCCCGGATAGCGGGCGGCAGAAGGAATCAGAATTTCCA
GGTCAGACGGGCTGCAAGTTGCAGACCGTTAAAATCATCGGTTGGGGTGTCGTACCACACTTTACCTGCCGTCAGCCCGA
GATTAAGTTGCTCAGTCAGCGGTTGAATACGTTGCAGACTGACGCTCTTCATGCTGCCGTAGCGTGTTTCTGGCGTCCAG
TCATAGGTGTAAGCACCTGTTCCGCGGCTTAGCCATAGTTGCGTATAACCAGTGCCGCGCGGGTCATTCAGACGCACGGA
AATCATATTGCTATAACTACCACCTGCATCGCTGGAGTCATAATGGGTATAGCGGTAGCTGGTGATCACCGGGCCAGTAT
AGAGTGATACGCCGCCTTGCCAGGCATCGACTTCGACATCATCGTAATATTTAGTGTAGCGATAACCGGTCGTGAAAAGC
GTTTTTGGTAACAGGGCCAGGTTAATATCCTGACGAAAATCCTGGCGGGCAAAGACCGGCGTATTATCCGCAAAGGCAAT
ACCCGTTCGGGTTGTCAGCCAGTTATTCCATTTATACCAGACCGCACCCTGACCGCGAGTGGCATTGAAATGTTCAGTTT
CATAATCACGACGCCCCTGGGAAAGATTAAAAAGCAGCGTTGCGTTTTCAACTTTCGCCACCAGTTCAGCATACGCTAAA

TTACGGTTGCCATGATCGCCAGAATAATCGGTAAAATCGTAGCCCGCGGTAATGGAAGTTAACCCGGCATAAGCGGGCAA
TGCGCTAAAAATGGCCGCCGCTAATAACGTCCGTTTTATCATTTTAATCTCCTGTACGGATAAGTTCTTGTCGGAGTGAA
TAAGTTCCGGTAGGTAAATTACGTTCTACAATCACGCGCGGGCCAAGTTGCGTGTTCGGGGAAATAATTCGCCCAGGCAA
AATAATAACTTGTACGCCAAGGCGTGAACGCTGCCCGATATAACAACCTAATTTATCGCATCCGGTAGCGATAATTCCCT
CTGGAGTTCGAACAGACACGGGCTGTTCATCCAGACGATGATTACTGGTACGTACTTGTGCGCCTAAATATGCCTGGTTT
GCAACTACCGAGTCGGCAATAAAACATTGCGGACCAATCGTTGCTTCCGCTTCAATAACCGCATTTTTAATCTCGGTGGC
AAAACCAATTTTTACGCCATTGCTGATTATTGTGCCAGGACGAATAAACGCATAATTACTAGACTGGCCCCCTGAATCTC
CAGACAACCAATATCACTTAAATAAGTGATAGTCTTAATACTAGTTTTTAGACTAGTCATTGGAGAACAGATGATTGATG
TCTTAGGGCCGGAGAAACGCAGACGGCGTACCACACAGGAAAAGATCGCAATTGTTCAGCAGAGCTTTGAACCGGGGATG
ACGGTCTCCCTCGTTGCCCGGCAACATGGTGTAGCAGCCAGCCAGTTATTTCTCTGGCGTAAGCAATACCAGGAAGGAAG
TCTTACTGCTGTCGCCGCCGGAGAACAGGTTGTTCCTGCCTCTGAACTTGCTGCCGCCATGAAGCAGATTAAAGAACTCC
AGCGCCTGCTCGGCAAGAAAACGATGGAAAATGAACTCCTCAAAGAAGCCGTTGAATATGGACGGGCAAAAAAGTGGATA
GCGCACGCGCCCTTATTGCCCGGGGATGGGGAGTAAGCTTAGTCAGCCGTTGTCTCCGGGTGTCGCGTGCGCAGTTGCAC
GTCATTCTCAGACGAACCGATGACTGGATGGATGGCCGCCGCAGTCGTCACACTGATGATACGGATGTGCTTCTCCGTAT
ACACCATGTTATCGGAGAGCTGCCAACGTATGGTTATCGTCGGGTATGGGCGCTGCTTCGCAGACAGGCAGAACTTGATG
GTATGCCTGCGATCAATGCCAAACGTGTTTACCGGATCATGCGCCAGAATGCGCTGTTGCTTGAGCGAAAACCTGCTGTA
CCGCCATCGAAACGGGCACATACAGGCAGAGTGGCCGTGAAAGAAAGCAATCAGCGATGGTGCTCTGACGGGTTCGAGTT
CTGCTGTGATAACGGAGAGAGACTGCGTGTCACGTTCGCGCTGGACTGCTGTGATCGTGAGGCACTGCACTGGGCGGTCA
CTACCGGCGGCTTCAACAGTGAAACAGTACAGGACGTCATGCTGGGAGCGGTGGAACGCCGCTTCGGCAACGATCTTCCG
TCGTCTCCAGTGGAGTGGCTGACGGATAATGGTTCATGCTACCGGGCTAATGAAACACGCCAGTTCGCCCGGATGTTGGG
ACTTGAACCGAAGAACACGGCGGTGCGGAGTCCGGAGAGTAACGGAATAGCAGAGAGCTTCGTGAAAACGATAAAGCGTG
ACTACATCAGTATCATGCCCAAACCAGACGGGTTAACGGCAGCAAAGAACCTTGCAGAGGCGTTCGAGCATTATAACGAA
TGGCATCCGCATAGTGCGCTGGGTTATCGCTCGCCACGGGAATATCTGCGGCAGCGGGCTTGTAATGGGTTAAGTGATAA
CAGATGTCTGGAAATATAGGGGCAAATCCAATTACCTATCAGGCAGTTTGCGCCAATCACTACCGGACCCTGAATAACGG
CACCATGACAAATACGGGTATTCGCGCCGATAACAACTTCACCTGCAGTTTCATCAATAATTACCTGGTCTGCAATCTGA
ATATTTTTCTCTTTAACATTAAGCTTCAGATAGTTTTTCAGTTCCTGTGACAGAAGGTTGGGAATAAGCGGTTGACGCTT
CCACCAGTGCGGAATAGCGGGTGGGTTTGTCGCGCTGAGGTTCGCGTCCAGTAAAAAAGGCAATAAGTCCATAAATAATC
CAGATGGCATAAGCTAATAATACATACACAACGGAAAGCGGCGCTAAAGGCACCAACAACCAGCAACGATGAAACCAGGC
GCTAAAAGCACCAATAACACAAACTACGCCGACCCAGATAAGCGGAAACATTGCCAACACCACTCCATGCGGCCCGGTGG
TGATGAATTCATTAAACCAGGTAGTGAGATAAATCCCAACGCCATAAAGCACAACCAACAGCATAGGAAATATACTGAAG
ATACCGAAGCGGCTAAATAAAAGTCTTTTATGCAGGCGCATACAGACCGCGTATCCCACAATCCAACGCCGCCAGCGACG
CCACTCCTCACGCGGGCTGTTGCATTCCTGTGGGTATACGATGCAGCGATTCGCCTGCCGAATACGGTAGCCGTTTGCCA
CCAATGTCCAGGTGAGATCAAGGTCTTCGACTTTAGTACGATCCGAGAAACCGAACTTACGCAATACATCAGTACGGAAC
ATCCCGCAGGCACCGCTGATAATAAACGGTGCGCCACCCAGGAGCTGCTGTAGCGTGCGCTTCATAACAATCATCGGCAA
CTTTACGGTCGCGCGGATGTGCGGTAACAGACCCGCGCCTTTCAACGCAGTAGAGGGAATGCCGCCTACGGCATCGGCAC
CGCGCTCAATTTCTGCCAGCATATAGCCCATTCCGTCTTGATCGGGCGGAACATAGGTGTCGGCATCACTTAAAAAAACC
TGGTCGCAGGTGGCGTAATTGAGGCCATTCATCAGCGCACCACCTTTACCGGTATTTTTTTGCGTGACGGCAACAAAGCG
GTCGCCCCATTTGCGTTTGACTTCCGCCATGACCGCTTCGGTATTGTCCGTGGAGCCGTCGTTAACGCAAATTACCCGGC
AAAAATAAGGGTTACGCAGTAGATTATCCAGTGACTGCGCCAGACACGGGCCTTCGTTATACGCAGGTATAATGGCGTCA
ATACAGCCTTTCTTTTGACTGGGTTTACGTCTTAGCGTACTTAAAAACCATAGCAACATTGCTATGGACATACAGATAAA
TATCCAGGTTTTCATGCAATTTGCTCAAAGAATCATTTTATGAATTACAAAGCCTTCACCCAGATCGCTATTGACCTGTT
GTCCGCGAAATTGTGCAACTGCACGCAGGCGATCATGACGCATATAATCGCGTCGTTCCTAGCTTTTATGTTTTTTTAAT
GCAGCAAGTTTGACTGTGAAATATTCTTCTTTAACGGATTCAAAAACCTGAGGCATAAATGAAAGCCAGGTACTTGGGGT
TTCGTAGCCGAGAATTTGCGGAATAGTTCGGCAGGCAACCATTGAAGCTTGATAAACAGCAAGATGATCCTGATGGCGGT
CGGCATCATGCATGGTATATACCCGCATGATTTCAACATCAGAAGGAATTTGATTTTTAATGATGTCTTCCAGGGCGGAA
ATCATATCATTGAGCTGTAAATGAGCGCGGGTGTCAGCAAAATTAAGATGAATAGTTTGGTGGCACCCTAATATCTTTAG
GGCGTTGCGCGATTCTTCATGGCGATCGATTATTCCATCTGTGCCAGAGTTGCCGGTAGTCATCACCACGGCTGCGATAT
AAATTCCTTTTTGCGCAAGACGAGCAAGCGATGCGCCACAGCCTAATTCTATATCGTCAGGATGTGCGCCAATAGCAAGG
ATACCCTTTCTTTTATTTGCCGAAGAAAGGAGGGCTGAATCTAAAACCTTATCCACTTAATGACACTCCATTTTATTTAT
TATACTACAAGCACAACGATGCACTCAGAGACGTAATCTCTGGCGCAGCCAGATGTTAATTTATAAATGTTAAATGTTGC
TAATAACTGAAAAGCAAGGGCTTTCAGACCCGTGGGTTCGACTGGGTAATGTTTCTATGCAATTCATATGTTAAGTGTTT
GTATGTTTGGTATGTATAGTTATTTTGTTTTATACATTGTATTTAGATGTGATTTAGTTTGTCAATTAATTGCATTTAAA
AAATATGTTCTGTGAACAAGCATTGTTTATATACATTATGTGAATGTAATATGCGAGTGATTGAGAATGATACAGTGATA
TATACAATGCGAATATAATAGTTTTATTATATGTATTGATATTGATAGAAATAATGAAGTAATAAATCTCGTAATGTGGT
TGTTTATGCATCACTAAAATGAAGTGTAGTAATTTTCCCAATTGTTAGAACGGAGTAATTGCATATTTAATCTTTCCTTA
GCCGTTTTTTTGCTAAGAATAAAATCATCTGTGCGATAACGACTAATTCTTTTAATGAATGTTTTTATTCCTGAATACTG

CTCCCATAACAAGACAGGGGAGCAGACAATCATGGCAATTTCATCGCGTAACACACTTCTTGCCGCACTGGCATTCATCG
CTTTTCAGGCACAGGCGGTGAACGTCACCGTGGCGTATCAAACCTCAGCCGAACCGGCGAAAGTGGCTCAGGCCGACAAC
ACCTTTGCTAAAGAAAGCGGAGCAACCGTGGACTGGCGTAAGTTTGACAGCGGAGCCAGCATCGTGCGGGCGCTGGCTTC
AGGCGACGTGCAAATCGGCAACCTCGGTTCCAGCCCGTTAGCGGTTGCAGCCAGCCAACAGGTGCCGATTGAAGTCTTCT
TGCTGGCGTCAAAACTGGGTAACTCCGAAGCGCTGGTGGTAAAGAAAACTATCAGCAAACCGGAAGATCTGATTGGCAAA
CGCATCGCCGTACCGTTTATCTCCACCACCCACTACAGCCTGCTGGCGGCACTGAAACACTGGGGCATTAAACCCGGGCA
AGTGGAGATTGTGAACCTGCAGCCGCCCGCGATTATCGCTGCCTGGCAGCGGGGAGATATTGATGGTGCTTATGTCTGGG
CACCGGCGGTTAACGCCCTGGAAAAAGACGGCAAGGTGTTGACCGATTCTGAACAGGTCGGGCAGTGGGGCGCGCCAACG
CTGGACGTCTGGGTGGTGCGCAAAGATTTTGCCGAGAAACATCCTGAGGTCGTGAAAGCGTTCGCTAAAAGCGCCATCGA
TGCTCAGCAACCGTACATTGCTAACCCAGACGTGTGGCTGAAACAGCCGGAAAACATCAGCAAACTGGCGCGTTTAAGCG
GCGTGCCTGAAGGTGACGTTCCGGGGCTGGTGAAGGGGAATACCTATCTGACGCCGCAGCAACAAACGGCAGAACTGACC
GGACCGGTGAACAAAGCGATCATCGACACCGCGCAGTTTTTGAAAGAGCAGGGCAAGGTCCCGGCTGTAGCGAATGATTA
CAGCCAGTACGTTACCTCGCGCTTCGTGCAATAAAAGGAGGCGCAGATGCTGCAAATCTCTCATCTTTACGCCGATTATG
GCGGCAAACCGGCACTGGAAGATATCAACCTGACGCTGGAAAGCGGCGAGCTACTGGTGGTGCTGGGGCCGTCCGGCTGC
GGTAAAACCACCCTGCTGAATCTGATTGCCGGTTTTGTGCCTTATCAGCATGGCAGCATTCAACTGGCGGGTAAGCGTAT
TGAGGGACCGGGAGCAGAGCGTGGCGTAGTTTTTCAGAATGAAGGGCTACTACCGTGGCGCAATGTACAGGACAACGTGG
CGTTCGGCCTGCAATTGGCAGGTATAGAGAAAATGCAGCGACTGGAAATCGCGCACCAGATGCTGAAAAAAGTGGGGCTG
GAAGGCGCAGAAAAACGCTACATCTGGCAGCTTTCCGGTGGTCAACGTCAGCGGGTGGGGATTGCTCGTGCGCTGGCGGC
GAATCCCCAGCTGTTATTACTCGACGAACCGTTTGGTGCGCTGGACGCCTTCACCCGCGACCAGATGCAAACCCTGCTGC
TGAAACTCTGGCAGGAGACGGGCAAGCAGGTGCTGTTGATTACCCACGATATAGAAGAAGCGGTGTTTATGGCGACTGAA
CTGGTTCTGCTTTCATCCGGCCCTGGCCGTGTGCTGGAGCGGCTGCCGCTCAACTTTGCTCGCCGCTTTGTTGCGGGAGA
GTCGAGCCGCAGCATCAAGTCCGATCCACAATTCATCGCCATGCGCGAATATGTTTTAAGCCGCGTATTTGAGCAACGGG
AGGCGTTCTCATGAGTGTGCTCATTAATGAAAAACTGCATTCGCGGCGGCTGAAATGGCGCTGGCCGCTCTCGCGTCAGG
TGACCTTAAGCATTGGCACGTTAGCGGTTTTACTCACCGTATGGTGGACGGTGGCGACGCTGCAACTGATTAGCCCGCTA
TTTTTGCCGCCGCCGCAACAGGTACTGGAAAAACTACTCACCATTGCCGGACCGCAAGGCTTTATGGACGCCACGCTGTG
GCAGCATCTGGCAGCCAGTCTGACGCGCATTATGCTGGCGCTATTTGCAGCGGTGTTGTTCGGTATTCCGGTCGGGATCG
CGATGGGACTTAGCCCTACGGTACGCGGCATTCTGGATCCGATAATCGAGCTTTATCGTCCGGTGCCGCCGCTGGCTTAT
TTGCCGCTGATGGTGATCTGGTTTGGTATTGGTGAAACCTCGAAGATCTTACTGATCTATTTAGCGATTTTTGCACCGGT
GGCGATGTCGGCGCTGGCGGGGGTGAAAAGCGTGCAGCAGGTTCGCATTCGTGCCGCCCAGTCGCTGGGTGCCAGCCGTG
CGCAGGTGCTGTGGTTTGTCATTTTGCCCGGTGCGCTGCCGGAAATCCTCACCGGATTACGTATTGGTCTGGGGGTGGGC
TGGTCTACGCTGGTGGCGGCGGAGCTGATTGCCGCGACGCGCGGTTTAGGATTTATGGTTCAGTCAGCGGGTGAATTTCT
CGCAACTGACGTGGTGCTGGCGGGGATCGCGGTGATTGCGATTATCGCCTTTCTTTTAGAACTGGGTCTGCGCGCGTTAC
AGCGCCGCCTGACGCCCTGGCATGGAGAAGTACAATGAGTGAACGTCTGAGCATTACCCCGCTGGGGCCGTATATCGGCG
CACAAATTTCGGGTGCCGACCTGACGCGCCCGTTAAGCGATAATCAGTTTGAACAGCTTTACCATGCGGTGCTGCGCCAT
CAGGTGGTGTTTCTACGCGATCAAGCTATTACGCCGCAGCAGCAACGCGCGCTGGCCCAGCGTTTTGGCGAATTGCATAT
TCACCCTGTTTACCCGCATGCCGAAGGGGTTGACGAGATCATCGTGCTGGATACCCATAACGATAATCCGCCAGATAACG
ACAACTGGCATACCGATGTGACATTTATTGAAACGCCACCCGCAGGGGCGATTCTGGCAGCTAAAGAGTTACCTTCGACC
GGCGGTGATACGCTCTGGACCAGCGGTATTGCGGCCTATGAGGCGCTCTCTGTTCCCTTCCGCCAGCTGCTGAGTGGGCT
GCGTGCGGAGCATGATTTCCGTAAATCGTTCCCGGAATACAAATACCGCAAAACCGAGGAGGAACATCAACGCTGGCGCG
AGGCGGTCGCGAAAAACCCGCCGTTGCTACATCCGGTGGTGCGAACGCATCCGGTGAGCGGTAAACAGGCGCTGTTTGTG
AATGAAGGCTTTACTACGCGAATTGTTGATGTGAGCGAGAAAGAGAGCGAAGCCTTGTTAAGTTTTTTGTTTGCCCATAT
CACCAAACCGGAGTTTCAGGTGCGCTGGCGCTGGCAACCAAATGATATTGCGATTTGGGATAACCGCGTGACCCAGCACT
ATGCCAATGCCGATTACCTGCCACAGCGACGGATAATGCATCGGGCGACGATCCTTGGGGATAAACCGTTTTATCGGGCG
GGGTAATACGAGAGTGGACGGTCCCCTCGCCCCCTTGGGGAGAGGGTTAGGGTGAGGGGGCGTTCACCGTACTTTCAACA
GGTTAACTCCCCCTTTCTGAGAGGAAACAAAATTAACGCAGAATCTTCTTCTCAGCCAAATCCAGCGCAAAGTAGCTGAA
AATCAGATCCGCACCCGCACGCTTAATCGAACCTAAGCTTTCGAGCACGACTTTCTCTTCATCTATAGCACCCGCCAGCG
CGGCGAACTTAATCATCGCATACTCACCGCTCACCTGATACGCGCCAATCGGCAATTCAGTACGTTCACGCAGCTCACGC
ACGATGTCGAGGTACGCTCCAGCAGGTTTAACCATCAGGCAGTCTGCGCCCTGGGCTTCATCCAGCAGTGATTCACGAAT
CGCCTCACGACGGTTCATTGGGTTCATCTGATAGCTTTTGCGGTCGCCTTTTAATGCGCTTCCGGCAGCTTCACGGAACG
GGCCATAAAAGGAGGAGGCGAACTTGGTCGAATACGACATAATCGCCGTATCTTTAAATCCCGCAGCGTCCAGCGCCTGA
CGAATCGCCTGTACCTGGCCGTCCATCGCGGCGGAAGGGGCGATGAAGTCTGCACCTGCAGCAGCTGCAACCACGGCTTG
CTTGCCTAAATTTTCCAGAGTCGCGTCGTTGTCGACGCCATGCTCGCACAGCACACCGCAGTGACCGTGAGAAGTGTATT
CACAGAAGCAGGTGTCTGACATAACGATCATTTCTGGCACGGTCTGCTTGCAGATGCGCGACATACGCGCCACCAGTCCA
TCTTCCCGCCAGGCATCGCTGCCGGTTTCATCGGTATGGTGAGAGATGCCAAAAGTCATCACGGAACGAATACCGGCGTT
GGCGATGCGTTCAATTTCGCGTGCCAGATGTTTCTCTGGAATGCGCATCACGCCTGGCATGGCTTCAACGGCTTTGTAGT
CGTCAATTTCTTCTTCAACAAAGATCGGCAACACCAGGTCGTTAAGGCTAAGTGTTGTCTCTTCAAACATAGCGCGCAGC

GCAGGAGATTTGCGCAGGCGACGAGGGCGTTGGATTAAGTCTGTCATGGTCTGCCTGATGTTTGTGGAATCGAGGGGCAT
AGTATACCTGAAGCAGGGTAGGGATGTTTTACGAAAGTTGTCGCGATGTTGACAAGAAGAGAATGGAAGAGAGGCCAGGA
CATCTTTTTAATAGAAACAGCAATAATTTTATATTCACTGAAAATATTTTTAATCTTTATTTATAGCTGTTGGTTATTAT
TTTTTGGAGTTTGGTTGCGCTGCTATAAATTGAATAATTAAAACTTTGTTGCATATTCATAGGATATTTATCTGGTTTGT
GTTTGTGATATTTATTATGCGTATGCTTCAAAAACAAAATTATCTGCAACATGTTGAAATACTGTGCTTTTATGAATTTG
ATGCGTGTTTTTCTCCATAAATTATATATGTCCACATTCGGACTTAGGGGAAAGAATAATTGAACCATTCGTCTGTAACG
CAGCATAATCGTTAGCGCGAAACATAATATGTTTTCTATGCATTGATAATTGATGGATCAACTTATTACGTCCCTGAGGA
GGGATGACAAATGCACTCCTGGAAAAAGAAACTTGTAGTATCACAATTAGCATTGGCTTGCACTCTGGCTATCACCTCTC
AGGCTAATGCAGCAAACTATGATACCTGGACTTATATCGATAATCCCGTTACAGCACTTGATTGGGATCATATGGATAAG
GCAGGCACTGTAGATGGCAACTATGTAAACTATAGTGGTTTTGTCTATTACAACAACACCAATGGTGATTTCGATCAGTC
CTTTAACGGCGATACCGTTAACGGCACGATCTCAACCTATTATTTGAACCATGATTATGCAGACAGTACTGCTAATCAGC
TTGATATCAGTAATTCAGTGATTCACGGTTCGATTACTTCTATGCTGCCTGGCGGTTATTATGATCGTTTTGATGCAGAT
GGTAATAATCTGGGTGGATATGATTTTTACACTGATGCGGTTGTTGATACACACTGGCGTGATGGTGATGTTTTCACTTT
GAACATTGCTAACACTACTATTGATGATGATTATGAAGCTCTTTACTTCACTGATTCTTATAAAGATGGTGATGTAACCA
AGCACACAAATGAGACATTTGATACAAGTGAAGGCGTTGCTGTTAATCTTGATGTAGAAAGTAACATCAATATTTCCAAT
AACTCCCGCGTTGCAGGTATTGCATTATCTCAAGGTAATACTTACAACGAAACCTACACTACCGAATCTCATACTTGGGA
TAACAATATCTCTGTAAAAGATTCCACAGTGACTTCGGGTTCAAATTATATCCTGGATAGCAATACTTATGGCAAAACTG
GTCACTTTGGCAATTCTGATGAACCGAGTGATTATGCTGGCCCGGGTGATGTTGCAATGTCCTTTACTGCTTCAGGTTCC
GACTATGCGATGAAGAACAATGTATTCCTCAGCAATTCAACGCTGATGGGTGATGTTGCCTTTACCAGCACCTGGAATAG
TAATTTTGATCCGAATGGTCATGATTCCAACGGTGACGGGGTGAAAGATACCAACGGGGGTTGGACTGATGATAGCCTCA
ACGTTGATGAACTAAATCTCACTCTCGATAACGGAAGCAAGTGGGTTGGTCAGGCAATTTATAACGTTGCTGAAACGTCA
GCAATGTATGATGTTGCTACAAACAGCCTTACTCCTGATGCAACATATGAAAACAATGACTGGAAACGTGTTGTTGATGA
CAAGGTCTTCCAGAGCGGTGTATTTAACGTAGCGTTGAATAACGGTTCTGAATGGGATACTACAGGTCGTTCCATCGTTG
ATACCTTGACAGTTAATAATGGTTCTCAGGTTAATGTTTCGGAATCTAAATTAACTTCAGATACTATCGATTTAACTAAC
GGTTCTTCGCTGAACATTGGTGAAGATGGCTACGTTGATACCGATCATCTGACTATTAACTCCTACAGTACTGTTGCGTT
GACCGAATCTACTGGGTGGGGGGCTGATTGATCCTACCCACGTAATATGGACACAGGCCTAAGCGAGGTTCTTGTTTTCA
AATTGTTCCGGACTGAGGCCGCCACACCAACTGTGCCGCCGCCACCGATTGTAATCACATTCGATATAATTAAACACCGT
TGCCCGCATTATTTCCCGGCTGATAAAGTGTTCTCCATGGATACATTCCACTTTCAGCGAATGAAAGAAGCTTTCCACGC
AGGCATTATCGTAGCAGCAACCTTTTGCGCTCATACTTCCACGCAGATTATGCCGCTTCAGTTGCGCCTGATAATCTGCT
GAACAGTACTGGCCTCCACGGTCCGTGTGAACGATAACGTTCCGGGGCCTCTTACGCCGCCACAGCGCCATCTGCAGGGC
ATCGCAGGCCAGTTGCGCCGTCATGCGTGGCGACATTGACCAGCCAATAACGGCACGTGACCACAGGTCAATGACCACTG
CCAGATACAGCCAGCCTTCATCTGTACGTAAGTACGTGATGTCTCCTGCCCACTTCTGGTTCGGGCCACTGGCGTAAAAA
TCCTGCTCCAACAGATTTTCTGACACAGGCAGGCCGTGTGCGCGGTAGCTGACCGGGCTGAACTTCCGGGAGGCCTTTGC
CCTCAGTCCCTGACGGCGCAGGCTTGCCGCCACGGTTTTTACGTTAAAGGGGTAACCCTGAGCACGCAGTTCATCCGTCA
GGCGTGGGGCACCGTAACGCTGTTTTGACCGGGTAAAAGCCGCGAGGACAACGCTGTCGCAGTGTTGGCGGAACTGCTGA
CGCGTGCTTATCCTTGTCCGCCGCTGACACCACGTATACCAGCCGCTGCGGGCCACCCGGAGCACGCGGCACATTGCTTT
GATGCTGAACTCAGCCTGATGTTTTTCAATAAAGACATACTTCATTTCAGGCGCTTCGCGAAGTATGTCGCGGCCTTTTG
GAGGATAGCCAGCTCTTCATCCCGTTCTGCCAGCTGGCGTTTGAGACGTGCAATCTCGGTAGACATCTCCAGTTCACGTT
CAGAAGACGTCTGCTGATTTTGCTGTTTACTGCGCCAGTTGTAGAGTTGTGATTCATACAGGCTGAGTTCACGGGCTGCG
GCAGTAACACCGATGCGTTCAGCAAGCTTCAGGGCTTCACTGCGAAATTCAGGCGAATGCTGTTTACGGGGTTTTTTACT
GGTTGATACTGTTTTTGTCATGTGAGTCACCTCTGACTGAGAGTTTACTCACTTAGCCGCGTGTCCACTATTGCTGGGTA
AGATCAGATTACAACCTGTACGCCAATACTATCACCGTAACTAACGGCGGTGTATTGGATGTGAACGTTGATCAGTTCGA
TACTGAAGCTTTCCGTACTGACAAACTGGAACTGACCAGCGGCAACATCGCTGACCATAACGGTAACGTAGTATCTGGTG
TGTTCGATATCCATAGCAGCGATTACGTTCTGAACGCTGATCTGGTGAACGACCGTACCTGGGATACTTCCAAGTCTAAC
TACGGTTACGGTATTGTTGCTATGAACTCTGATGGTCACCTGACTATCAACGGTAACGGCGACGTAGACAACGGTACTGA
ACTGGATAACAGCTCTGTAGACAATGTTGTTGCTGCAACCGGTAACTACAAAGTTCGTATCGACAACGCAACTGGCGCTG
GCGCTATCGCTGATTACAAAGATAAAGAAATTATCTACGTAAACGACGTCAACAGCAACGCGACCTTCTCTGCTGCTAAC
AAAGCTGACCTGGGTGCATACACCTATCAGGCTGAACAGCGCGGTAACACCGTTGTTCTGCAACAGATGGAGCTGACCGA
CTACGCTAACATGGCGCTGAGCATCCCGTCTGCGAACACCAATATCTGGAACCTGGAACAAGACACCGTTGGTACTCGTC
TGACCAACTCTCGTCATGGCCTGGCTGATAACGGCGGCGCATGGGTAAGCTACTTCGGTGGTAACTTCAACGGCGACAAC
GGCACCATCAACTATGATCAGGATGTTAACGGCATCATGGTCGGTGTTGATACCAAAATTGACGGTAACAACGCTAAGTG
GATCGTCGGTGCGGCTGCAGGCTTCGCTAAAGGTGACATGAATGACCGTTCTGGTCAGGTGGATCAAGACAGCCAGACTG
CCTACATCTACTCTTCTGCTCACTTCGCGAACAACGTCTTTGTTGATGGTAGCTTGAGCTACTCTCACTTCAACAACGAC
CTGTCTGCAACCATGAGCAACGGTACTTACGTTGACGGTAGCACCAACTCCGACGCTTGGGGCTTCGGTTTGAAAGCCGG
TTACGACTTCAAACTGGGTGATGCTGGTTACGTGACTCCTTACGGCAGCGTTTCTGGTCTGTTCCAGTCTGGTGATGACT
ACCAGCTGAGCAACGACATGAAAGTTGACGGTCAGTCTTACGACAGCATGCGTTATGAACTGGGTGTAGATGCAGGTTAT

ACCTTCACCTACAGCGAAGATCAGGCTCTGACTCCGTACTTCAAACTGGCTTACGTCTACGACGACTCTAACAACGATAA
CGATGTGAACGGCGATTCCATCGATAACGGTACTGAAGGGTCTGCGGTACGTGTTGGTCTGGGTACTCAGTTTAGCTTCA
CCAAGAACTTCAGCGCCTATACCGATGCTAACTACCTCGGTGGTGGTGACGTAGATCAAGACTGGTCCGCGAACGTGGGT
GTTAAATATACCTGGTAATATTCTTCACTCCGAAGAAATACTGGTAATTTAATCTAAATAATGCCCGTCAAGGATTTGAC
GGGCATTACTGCAAAGGACGCGCAAATGTTATCTGTAGTTAAACCTCTTCAGGAATTTGGTAAGCTCGATAAATGTTTGT
CCAGATACGGTACGCGCTTCGAGTTTAATAATGAAAAGCAAGTTATATTTTCCAGTGATGTCAATAACGAAGATACTTTC
GTTATTTTAGAGGGAGTTATCTCTCTGCGTAGAGAAGAAAACGTACTTATCGGTATTACCCAGGCTCCTTATATTATGGG
GCTGGCTGATGGTTTAATGAAAAACGATATACCATACAAATTAATATCAGAAGGAAATTGTACGGGATATCATCTACCAG
CCAAACAAACCATTACGCTTATTGAACAAAATCAACTCTGGCGAGACGCTTTTTACTGGTTAGCCTGGCAAAATAGAATT
CTGGAATTACGCGACGTGCAGCTCATTGGGCATAATTCCTACGAACAAATCCGCGCAACATTATTATCAATGATTGACTG
GAATGAAGAATTGCGATCACGTATTGGTGTGATGAATTATATCCATCAACGTACACGCATATCGCGTTCTGTCGTCGCAG
AAGTTCTCGCTGCTTTGCGTAAAGGCGGCTATATCGAAATGAATAAAGGCAAACTGGTCGCTATCAACCGTTTGCCTTCA
GAGTATTAATCAGGACGCGGGGATAACCAACGGTTTATTCCCGCTTAGCTCGGTCACCAGGTCATTGATGCCATCGCTCA
TATTTTTAAAGCGCGTCAACGGCGAACGAGTGACCACCACAAACGCGCCAATATTCTTCTGCGGGATCATCGCCATATAG
GTAATGAAACCGCCACCACCGCCTGTCTTCTGAATAATCCCCGGACGACCCTCTTTCGGGGCCATATATACCCAACCTAA
ACCAAGCGCATCCGCTTTGCCGGGGACATCCATGCCAATCACTTTCGTAAACTGCGCGCGCTGATAAATCAACGTCTGCA
TGCGATCTGCCTGGTTACTGCGCTGATAAAAATCAGATGACAAATACTGCTGCATCCAGCGCATCATATCGCCAGGCGTG
GAATAGACGCCGCCGCTGCCAATTGCCGCCAGCGTGTTATTGCACGGACTGGCACCGCGCTCAGCAACCATTAAACGGCG
GCACTGATCCGGTGAGGGGGTGTAGGTGGTGTCTTTCATCCCCAATGGACGGGTAATCTGCTCTTCAAACAACTGGGTAT
AAGGCTTGCCGGACGCATTCGCCAGTGCATCTGCCAGCAGGTCGAACGCAAGGTTAGAGTACGCCGCCTGACTGCCTGGC
GCGGCTTTCAGCTTCGCCGTAGAAAGGTATTTCCAGCGTTGCTCGCGCGTTGGCCAGACAAACACCGGACGATGTGCCGC
GCCACCGGGCTGTTCACGGGGCAGGGCGCTGGTATGGGTTGCCAGATTGACCAGCGTAATGGGCGTCCCATTGTAGGTTG
GCACGCGTGCGCCTGGCGGAGCATATTTACTTAGCGGATCGTTTAACTTCACGGTCCCCTGGTCGAGCAATTTCACCAGC
ATCTCACTGGTCATTAGCTTGGTGAGGGAAGCAATACGCACGACCGAATCCAGCTGCGGGCGAACGTTATTACCAGGTCG
CGTTTCGCCATAACTGCGAAAGACGCGCTGGTTACCGTCGATAACAACCAGAGCCATCCCCGTGGCACCGCTGCCGTAAA
AAATATGATCGGCATAACGATCGACAATATCAGAGGCAAACTCCGGTTCAGTGATCGGCTGCGCCGCATGGACGGAGGTC
AATGACGCCGCACACAGCACGGCAGAAAAAAGCAGACTACGTTTCAACGGTGGTGTCCATAGATTGAATGAAGGTAACTG
AGCGTATTTATACTACTCATTGATAGATTGCAAAGGGCGAATTAGTGCAATAATAGTGTTAAAAACGTAACGGCGCGTAA
ACACGATCTTTTTCCTGCTCACCAATGCCTGCATTTGTGATCCTGCGCGTGTTAATCCGCTTATGTAAACAATCTTTGGT
TTGTGAAATCCGTCGAAACAATTCTTATGGTCAGGCTGGCGAACTAAGCGCCTTGCTATGGGTCACAATGGGCGGTTTCA
TTTGGCTGACGCTTTGTAGCGGTCATGCGGTTAATACACAGCAGTTGTTAAAACGATAAGAAGTTAGCAGGAGTGCATAT
GTTTAAGTCTTTTTTCCCAAAGCCGGGAACGTTTTTTCTCTCGGCCTTCGTTTGGGCATTGATTGCCGTTATCTTCTGGC
AAGCCGGTGGGGGTGACTGGGTGGCGCGTATCACCGGCGCTTCCGGGCAGATCCCGATTAGCGCCGCGCGTTTCTGGTCG
TTGGATTTCCTGATTTTTTACGCTTACTACATTGTTTGCGTAGGACTTTTTGCATTGTTCTGGTTTATCTACAGCCCGCA
TCGTTGGCAATACTGGTCAATACTCGGTACTGCACTGATCATCTTCGTCACCTGGTTTTTGGTGGAAGTCGGGGTCGCCG
TCAACGCCTGGTATGCGCCGTTCTATGATCTGATTCAAACCGCGCTAAGTTCGCCGCATAAAGTCACCATCGAACAATTT
TACCGCGAAGTGGGCGTCTTTCTGGGGATTGCGCTGATCGCTGTGGTGATCAGTGTGCTGAACAACTTCTTTGTCAGTCA
CTACGTGTTCCGCTGGCGTACAGCGATGAACGAATATTACATGGCGAACTGGCAACAACTGCGTCATATCGAAGGGGCCG
CACAGCGTGTGCAGGAAGACACCATGCGTTTTGCTTCAACGCTGGAGAATATGGGCGTCAGTTTTATCAACGCCATCATG
ACGTTGATCGCCTTCCTGCCGGTGCTGGTAACGCTCTCCGCGCATGTGCCGGAGCTGCCGATTATCGGGCACATTCCGTA
TGGTCTGGTGATTGCCGCAATCGTCTGGTCGCTGATGGGGACCGGATTGCTGGCAGTGGTAGGGATCAAACTGCCGGGGC
TGGAGTTTAAAAACCAGCGTGTAGAGGCTGCCTACCGTAAAGAGCTGGTTTATGGTGAAGACGATGCCACGCGCGCGACG
CCGCCTACGGTACGCGAGCTGTTTAGCGCCGTACGGAAAAACTATTTCCGCCTCTATTTTCACTATATGTATTTCAACAT
CGCCCGCATTCTCTATTTGCAGGTCGATAACGTTTTCGGTTTGTTCTTGCTGTTTCCGTCAATTGTTGCCGGTACGATTA
CGCTCGGCCTGATGACGCAGATTACCAACGTTTTTGGTCAGGTTCGCGGTGCTTTCCAGTACCTGATTAACTCATGGACC
ACACTGGTTGAGTTGATGTCTATCTACAAACGTCTGCGCAGCTTTGAACATGAGCTGGATGGTGACAAAATTCAGGAAGT
AACCCATACCTTGAGCTAAAAAGGAGAAGTAATGTCGCGCGTCAATCCTCTATCTTCGCTGTCATTGTTGGCGGTTCTGG
TTTTAGCGGGGTGTAGTAGTCAGGCTCCGCAACCGTTGAAAAAGGGCGAAAAAGCGATAGATGTAGCGAGCGTCGTGCGG
CAGAAGATGCCTGCGAGCGTGAAAGACCGCGATGCCTGGGCGAAAGATCTCGCCACCACCTTTGAAAGTCAGGGGCTGGC
ACCGACGCTGGAAAACGTCTGCTCGGTGCTGGCGGTGGCGCAGCAGGAGTCAAATTATCAGGCAGATCCGGCCGTTCCGG
GCTTAAGCAAGATCGCCTGGCAAGAGATTGACCGTCGTGCTGAACGGATGCACATTCCTGCTTTTCTGGTTCATACCGCG
CTAAAAATCAAATCGCCAAACGGCAAAAGTTATAGCGAACGGTTAGATTCGGTGCGTACAGAAAAGCAATTGAGCGCGAT
TTTTGACGATCTGATCAACATGGTGCCAATGGGGCAGACGCTGTTTGGTTCGCTCAATCCGGTGCGCACCGGTGGGCCGA
TGCAGGTCAGCATTGCTTTTGCCGAACAGCATACCAAAGGGTATCCGTGGAAAATGGACGGTACAGTCCGTCAGGAAGTC
TTCAGTCGCCGCGGCGGGTTGTGGTTTGGTACTTACCATTTACTGAATTATCCCGCCAGTTATAGCGCACCGATATACCG
TTTTGCTGATTTTAACGCTGGCTGGTACGCCAGCCGTAATGCCGCGTTTCAGAACGCGGTCAGTAAGGCCAGCGGCGTGA

AGCTGGCACTGGACGGTGATTTAATTCGTTACGACAGTAAAGAACCCGGGAAAACCGAACTGGCAACGCGCAAACTGGCT
GCAAAACTGGGGATGAGCGACAGCGAAATTCGCCGTCAGTTAGAGAAAGGTGACAGCTTCTCTTTTGAGGAGACGGCGCT
GTACAAGAAAGTTTATCAACTTGCCGAAACGAAAACGGGTAAATCACTCCCCCGCGAAATGTTGCCTGGCATTCAACTGG
AAAGCCCGAAAATCACCCGCAACCTGACTACGGCCTGGTTTGCGAAGCGCGTAGACGAACGGCGGGCGCGTTGTATGAAA
CAGTGATCAAACAGGAATGTCAGGCCAGATAAGGCGTTTCAGGCCGCATCTGACAATGTAAAACTTACTGGCGGCGATGT
CGCCAGTGCAGCCATAGCGCCACCACTGCAAAAATGAGGCAGCCTACTAAAAACGGGATCAGCCCGAAAATGGTGCCGAC
ACCTAAACCAATTTCCACCCGTGGGCGACCTGTTTCCTGGACCTGCATTAAACGCTCATAGACGCCGGGCGCATGCACTA
ACAGGTTCAGCAATTGCGCCCCAGCCCAAAAGCAAAACAGCACAAACAACGCATAGGCAATGTTGCCAGGCGTTGAAGAG
GCATTGCGATATTTTCCGCTAAACAGCGATTTTGACAGGGTGAAATCAGCCATAAAAACCTCCGGAAGTCATCTGCTTCT
CTTCTGGTGTGATACCAGCGGCTTATGGCAAGTCTGACAGGTCATTTAGATTGTCAATATCAGAATCATGGTAAATTGAT
GTTGGGAATATTCCCGAAGCGTGAATCTTCGTTGTGAGTCACAAAATGGCAACCTTAAGTTAAATTTATTAACAATTACG
AAAATGTTGTCTACACCACAGACGGTTGTTGCAGAATATGCAAGGATGTTGTTTTTCGTTAACGGAGCTGCCATGAATCT
GCCTGTAAAAATCCGCCGTGACTGGCACTACTATGCGTTCGCCATTGGCCTTATATTCATTCTTAATGGCGTGGTGGGGT
TACTGGGATTTGAAGCAAAAGGTTGGCAGACCTATGCCGTCGGTCTGGTGACGTGGGTGATTAGTTTCTGGCTGGCGGGG
TTGATTATTCGTCGTCGCGATGAAGAAACTGAAAACGCCCAATAAGAATATTATCGGGCGTTAAAATATTACATTGTGGT
TTTCAATGCGTTATCCGCAGCGTGACGCTCCAGCGCCAGTTCAATCAAACGTGTGATCAGATCGGTGTAACCCAGACCGC
TGGCTTGCCACAGCTTCGGATACATACTGATATTAGTGAAGCCAGGCAGGGTGTTGATCTCGTTGATCACCACTTCGTTC
TCTGGGGTTAAAAACACGTCTACACGCGCCATGCCTGCGCATCCCAACGTTTGATAAGCCTGAACGGCAATCGCCCGGAT
CTTATCGTTGATTTCTGGCGCAATGGCTGCCGGAACTACCACTTTCGCGCCATCTTCGTCAATGTACTTGGTGTCGTAGG
CATAGAAATCGCTGGTGAGTACGATCTCTCCACAGGTGCTGGCTTGCGGATTGTCGTTGCCCAGAACTGCGCATTCGATC
TCACGACCTTTGATCCCTTGCTCAACGATCACTTTATGATCGAACTCGAACGCCAGATCGACGGCAATTGCGTACTGTTC
TTCACTGGTTACTTTGCTGACACCAACAGAAGAGCCCTGATTAGCCGGTTTTACAAACAGTGGTAACCCCAGTTTAGACT
CCACTTCGGCAAAACTAATGTTGTGACGATTGGCGCGCGTCAGGGTAATAAATGGCGCAATGTTCAGCCCGGCATCGCGT
AGCAGACGTTTGGTGACATCTTTGTCCATACAGGCTGCTGAAGCCAGAACATCAGAACCTACAAACGGTAAATTGGCGAC
CCGCAGCATTCCCTGCAAGGAACCATCTTCGCCCAGCGTACCGTGGACAATCGGGAAAATGACATCCACCGTCGGCAACG
GCTGACCGTTTTGCGCGTCGATAAGCTGATGCTCATGTTTACCTGGCACCTGCGCAAGGCTGGTCGCCGAAGGGCGCAAC
GCAATATGGGCAGGATCGTCTGCATTTAGCAGATAATTGCTGGCATCGCTGACGTGCCATTGCCCTTGTTTATCAATGCC
CAGCAGCACAACGTCGAAGCGACTTTTATCAATGGCATCGACAATGTTTTTTGCCGATTGCAGAGACACTTCATGTTCCG
CTGATTTACCACCAAAAACGATTCCTACCCGCAGTTTTTCCATCTTAAAAACCTATCCCGTCTAACACAAAGTGCATACA
TTACCACGACAAAACGGGGGATTCGCGGCCTTCTGAAAGATTGTTGCAATCTTCTGCTGACAAAGCGTGCAACGTACTGG
TGAAGAAAGTGCGTTATCTCAAAGATGTGCGCAAGATCACAAAAATGATGAACGGGAAGCTAATTTATTCCTGGCTTAAA
TGGCCATGCGGTGAGTTTTTTTCTCTTAATTATAAGTTAACGAAGAGAATATATTTCATAACTTTTATTTATAATAAAGG
TTGATAATTAAAAGCCTATATTTTGTGTGGGTAATTATTTAAATAAGAGAAACGTTTCGCTGGTAATCAAACAAAAAATA
TTTGCGCAAAGTATTTCCTTTGTCATAAAAATAATACTTCCAGACACTATGAAGTTGTGAAACATAATGTTAACTTCTCC
ATACTTTGGATAAGGAAATACAGACATGAAAAATCTCATTGCTGAGTTGTTATTTAAGCTTGCCCAAAAAGAAGAAGAGT
CGAAAGAACTGTGTGCGCAGGTAGAAGCTTTGGAGATTATCGTCACTGCAATGCTTCGCAATATGGCGCAAAATGACCAA
CAGCGGTTGATTGATCAGGTAGAGGGGGCGCTGTACGAGGTAAAGCCCGATGCCAGCATTCCTGACGACGATACGGAGCT
GCTGCGCGATTACGTAAAGAAGTTATTGAAGCATCCTCGTCAGTAAAAAGTTAATCTTTTCAACAGCTGTCATAAAGTTG
TCACGGCCGAGACTTATAGTCGCTTTGTTTTTATTTTTTAATGTATTTGTACATGGAGAAAATAAAGTGAAACAAAGCAC
TATTGCACTGGCACTCTTACCGTTACTGTTTACCCCTGTGACAAAAGCCCGGACACCAGAAATGCCTGTTCTGGAAAACC
GGGCTGCTCAGGGCGATATTACTGCACCCGGCGGTGCTCGCCGTTTAACGGGTGATCAGACTGCCGCTCTGCGTGATTCT
CTTAGCGATAAACCTGCAAAAAATATTATTTTGCTGATTGGCGATGGGATGGGGGACTCGGAAATTACTGCCGCACGTAA
TTATGCCGAAGGTGCGGGCGGCTTTTTTAAAGGTATAGATGCCTTACCGCTTACCGGGCAATACACTCACTATGCGCTGA
ATAAAAAAACCGGCAAACCGGACTACGTCACCGACTCGGCTGCATCAGCAACCGCCTGGTCAACCGGTGTCAAAACCTAT
AACGGCGCGCTGGGCGTCGATATTCACGAAAAAGATCACCCAACGATTCTGGAAATGGCAAAAGCCGCAGGTCTGGCGAC
CGGTAACGTTTCTACCGCAGAGTTGCAGGATGCCACGCCCGCTGCGCTGGTGGCACATGTGACCTCGCGCAAATGCTACG
GTCCGAGCGCGACCAGTGAAAAATGTCCGGGTAACGCTCTGGAAAAAGGCGGAAAAGGATCGATTACCGAACAGCTGCTT
AACGCTCGTGCCGACGTTACGCTTGGCGGCGGCGCAAAAACCTTTGCTGAAACGGCAACCGCTGGTGAATGGCAGGGAAA
AACGCTGCGTGAACAGGCACAGGCGCGTGGTTATCAGTTGGTGAGCGATGCTGCCTCACTGAATTCGGTGACGGAAGCGA
ATCAGCAAAAACCCCTGCTTGGCCTGTTTGCTGACGGCAATATGCCAGTGCGCTGGCTAGGACCGAAAGCAACGTACCAT
GGCAATATCGATAAGCCCGCAGTCACCTGTACGCCAAATCCGCAACGTAATGACAGTGTACCAACCCTGGCGCAGATGAC
CGACAAAGCCATTGAATTGTTGAGTAAAAATGAGAAAGGCTTTTTCCTGCAAGTTGAAGGTGCGTCAATCGATAAACAGG
ATCATGCTGCGAATCCTTGTGGGCAAATTGGCGAGACGGTCGATCTCGATGAAGCCGTACAACGGGCGCTGGAATTCGCT
AAAAAGGAGGGTAACACGCTGGTCATAGTCACCGCTGATCACGCCCACGCCAGCCAGATTGTTGCGCCGGATACCAAAGC
TCCGGGCCTCACCCAGGCGCTAAATACCAAAGATGGCGCAGTGATGGTGATGAGTTACGGGAACTCCGAAGAGGATTCAC
AAGAACATACCGGCAGTCAGTTGCGTATTGCGGCGTATGGCCCGCATGCCGCCAATGTTGTTGGACTGACCGACCAGACC

GATCTCTTCTACACCATGAAAGCCGCTCTGGGGCTGAAATAAAACCGCGCCCGGCAGTGAATTTTCGCTGCCGGGTGGTT
TTTTTGCTGTTAGCAACCAGACTTAATGGCAGATCACGGGCGCATACGCTCATGGTTAAAACATGAAGAGGGATGGTGCT
ATGAAAATAACATTACTGGTTACCTTGCTTTTCGGTCTGGTTTTTTTAACCACCGTCGGCGCTGCCGAGAGAACTTTAAC
CCCACAACAACAGCGTATGACCTCCTGTAATCAGCAGGCGACGGCGCAGGCGTTGAAAGGGGATGCTCGTAAGACCTACA
TGAGTGATTGCCTGAAGAACAGCAAGTCTGCGCCTGGCGAAAAAAGTTTGACGCCACAGCAGCAAAAGATGCGCGAATGC
AATAATCAAGCAACACAACAATCTCTGAAAGGTGATGATCGTAATAAGTTTATGAGTGCCTGCCTCAAGAAAGCCGCCTG
ATACCTGATAGTGCTAACGGGTGAGCTACGAAAATGGCTCACCCGAAATATCATACTTCTGCCTTTAGCTCCGTCTCTAT
AATTTGGGAAAATTGTTTCTGAATGTTCCCAAAAATAATGAATGATGAAAACTTTTTCAAAAAAGCGGCGGCGCACGGGG
AGGAACCTCCTTTAACTCCTCAAAACGAACATCAGCGGTCCGGGCTGCGCTTCGCCCGTCGCGTCAGACTACCCCGTGCG
GTTGGCCTGGCTGGCATGTTCTTACCGATTGCTTCAACGCTGGTTTCACACCCGCCGCCGGGCTGGTGGTGGCTGGTGTT
GGTCGGCTGGGCGTTCGTCTGGCCGCATTTAGCCTGGCAGATAGCGAGCAGGGCCGTCGATCCGCTTAGCCGGGAAATTT
ACAACTTAAAAACCGATGCAGTATTAGCGGGAATGTGGGTAGGCGTAATGGGCGTAAACGTGCTGCCTTCCACCGCGATG
TTGATGATTATGTGTCTGAATTTGATGGGGGCAGGCGGCCCCCGTCTGTTTGTCGCGGGTCTGGTGTTGATGGTGGTTTC
CTGCCTTGTCACCCTCGAGCTGACGGGCATTACCGTGTCGTTCAATAGTGCGCCGCTGGAATGGTGGCTCTCCCTTCCCA
TTATTGTCATTTATCCTCTGCTGTTTGGCTGGGTCAGCTACCAGACGGCAACCAAACTGGCGGAACATAAACGCAGGTTG
CAGGTCATGAGTACCCGCGACGGCATGACGGGCGTGTATAACCGACGTCATTGGGAAACTATGTTACGCAATGAATTTGA
TAACTGTCGGCGGCATAATCGCGATGCAACGTTACTGATTATCGATATCGACCATTTCAAGAGCATCAACGATACCTGGG
GCCATGATGTGGGCGATGAAGCGATTGTGGCGCTTACCCGACAGTTACAAATTACCCTGCGCGGTAGCGATGTGATTGGT
CGGTTTGGCGGCGATGAGTTTGCAGTAATCATGTCCGGTACGCCAGCTGAGAGCGCCATTACCGCCATGTTACGGGTGCA
TGAAGGGCTAAATACATTACGTTTGCCGAATACGCCACAGGTAACTTTACGGATTAGTGTGGGGGTTGCGCCGCTGAACC
CACAAATGAGTCACTATCGTGAGTGGTTGAAATCGGCAGATTTGGCGCTTTACAAAGCAAAGAAAGCCGGACGTAACCGC
ACCGAAGTGGCGGCCTGACGTCCGGCGAAAGTCATCAGGATTTGCTGAGTTTTTCTGATTTTTCCATACACTTCGTCATC
GCTTCGATCACTGCAGCACGGAAGCCTTTCTCTTCCAGTACGCGTACCGCTTCAATGGTGGTGCCTCCCGGTGAGCAGAC
CATATCTTTCAGTGCCCCCGGATGTTCTCCCGTTTCCAGCACCATTTTTGCGGAACCCATTACCGCCTGAGCGGCAAATT
TATACGCCTGGGCGCGTGGCATCCCGCCCAGCACGGCGGCGTCGGCCATCGCTTCGATAAACATAAATACGTAGGCTGGC
GAAGAACCGCTCACACCGACCACCGGGTGGATCATCGGCTCAGCAATTACTTCCGCTTCGCCAAAGCAGCGGAAAATATT
CAGCACATCAGCGGTATCTTCTGGGGTTACCAGCGCGTTTGGCGTTACGGAGGTCATCCCGGCATTAACCAGTGCGGGAG
TGTTCGGCATGGCGCGGATAATTTTCCGGTCATGGCCCAGCGCGCGGGCAAGCTGGTCGAGCGTGACACCTGCAGCAATA
GAAACGACCAGAGAGTCTTTATTCAGGCTGGAGGTGATTTCGCTAAGCACTTTAATCATGATGCCAGGTTTAACGGCAGC
AAAAATGATGTCGGCGATTTGCGCCACTTCTTGCGCCGATTCTGCGGCGTTGATGCCGAACTGGTCATGCAGGGCGGCGA
CTTTATCCGGGGAGGGGGTGTATACCCAGATTTGCCCTGGAAGCACCTGACCGCTGGCAATCAGACCGCCGAGAATGGCT
TTTCCCATATTGCCGCAGCCAATAAAACCGATTTTCTTTTCCATTGCCTCACTCCTGCCGTGAAATTCATTGTTTTGATA
ATCGCTGGCAGAAGCATAAACAGAACTATGCCGGAAGGCAAAAGCGCGACACAATAGAGGATTACCCAACAAAGGATGAC
TTTATGACAATTTGGGTGGATGCCGACGCGTGTCCCAATGTAATTAAAGAGATTTTGTATCGCGCGGCGGAACGTATGCA
GATGCCGCTGGTACTGGTAGCAAACCAGAGTTTACGCGTGCCGCCATCGCGATTTATTCGTACGCTGCGCGTCGCGGCAG
GTTTCGACGTTGCCGATAACGAAATTGTCCGGCAGTGTGAAGCGGGCGATTTGGTGATCACCGCAGATATACCTTTGGCT
GCTGAAGCCATCGAGAAAGGCGCTGCGGCGCTTAATCCGCGCGGCGAACGTTACACGCCAGCGACCATTCGTGAGCGCCT
GACGATGCGCGATTTTATGGATACCTTACGTGCCAGTGGGATCCAGACCGGCGGACCAGATAGCCTTTCACAACGTGACC
GCCAGGCCTTTGCCGCGGAGCTGGAGAAGTGGTGGCTGGAAGTGCAACGTAGTCGTGGCTAAATGTAATTTATTATTTAC
ACTTCATTCTTGAATATTTATTGGTATAGTAAGGGGTGTATTGAGATTTTCACTTTAAGTGGAATTTTTTCTTTACAATC
GAAATTGTACTAGTTTGATGGTATGATCGCTATTCTCATGACACCGGCTTTCGCCGCATTGCGACCTATTGGGGAAAACC
CACGATGACACAACCTCTTTTTCTGATCGGGCCTCGGGGCTGTGGTAAAACAACGGTCGGAATGGCCCTTGCCGATTCGC
TTAACCGTCGGTTTGTCGATACCGATCAGTGGTTGCAATCACAGCTCAATATGACGGTCGCGGAGATCGTCGAAAGGGAA
GAGTGGGCGGGATTTCGCGCCAGAGAAACGGCGGCGCTGGAAGCGGTAACTGCGCCATCCACCGTTATCGCTACAGGCGG
CGGCATTATTCTGACGGAATTTAATCGTCACTTCATGCAAAATAACGGGATCGTGGTTTATTTGTGTGCGCCAGTATCAG
TCCTGGTTAACCGACTGCAAGCTGCACCGGAAGAAGATTTACGGCCAACCTTAACGGGAAAACCGCTGAGCGAAGAAGTT
CAGGAAGTGCTGGAAGAACGCGATGCGCTATATCGCGAAGTTGCGCATATTATCATCGACGCAACAAACGAACCCAGCCA
GGTGATTTCTGAAATTCGCAGCGCCCTGGCACAGACGATCAATTGTTGATTTTCGAGCGCCTATACTTAACGTTCATCCC
GTGAAATAAGGAAGAACGATGCCAACGAAACCGCCTTATCCTCGTGAAGCATATATAGTGACGATTGAAAAAGGAAAGCC
AGGACAGACGGTAACCTGGTACCAACTCAGAGCCGATCATCCTAAACCAGACTCGTTGATCAGTGAACATCCGACCGCTC
AGGAAGCGATGGATGCGAAAAAACGCTATGAGGACCCTGACAAAGAGTGACCGCATCAGACTGCTCGGAAGGGATTCTGA
GTGCCACTACAAGGGATCTGCGTCACATTTTTCATAATTCATGTTTTTCTAATAATTAGAATATTAAACAATAACAATCC
ATTACTGGAATCATTTGGAATCTTTACATTATGCCGTGCACGTCTGCTGCTACGCTTTTTGTCATTTGTAGCACAAGTAA
GTGTCAGCAGTGGTGCTTCACACTTGCCCGGTAATTAACGACGAAAGAAAAGTAAGGTGGATGAACAATGAGTGCGTCGT
TGGCGATCCTGACCATCGGCATTGTACCTATGCAGGAAGTTTTGCCGCTCCTGACGGAATACATTGACGAAGATAATATT
TCCCATCATAGCCTGCTGGGGAAGTTAAGTCGTGAAGAAGTGATGGCGGAGTACGCGCCAGAAGCAGGCGAAGACACCAT

TCTCACATTATTAAATGACAACCAGCTGGCCCATGTTTCGCGTCGCAAAGTGGAGCGTGACCTGCAAGGTGTGGTTGAAG
TGCTCGATAATCAGGGTTATGACGTCATTTTATTAATGAGTACAGCAAACATTAGTAGTATGACTGCGCGTAATACGATC
TTTCTTGAGCCGTCGCGAATATTGCCTCCACTGGTTTCCTCTATTGTTGAAGATCATCAGGTGGGGGTTATCGTTCCGGT
TGAGGAGATGCTGCCCGTTCAGGCGCAAAAATGGCAAATTTTGCAGAAATCGCCGGTATTTTCATTGGGTAACCCCATTC
ATGATTCAGAACAAAAAATCATTGATGCCGGGAAAGAATTACTGGCAAAAGGGGCTGATGTCATCATGCTGGATTGTTTA
GGATTTCACCAACGTCATCGCGATTTACTGCAAAAACAGCTCGATGTTCCTGTCTTGCTGTCTAACGTATTGATTGCACG
GCTGGCTGCGGAATTACTGGTGTAATTTTGCGTGACAGCCAGCGCCTCTGGCCCCTATAGTGAAGTAGATGTTCAACTAC
CAAACAGGGCCAGTTTATGCTTCAAAGTAATGAGTACTTTTCCGGCAAAGTGAAATCAATCGGCTTTTCCAGCAGCAGCA
CTGGTCGCGCCAGCGTGGGTGTTATGGTTGAAGGCGAATACACCTTCAGCACCGCTGAGCCGGAAGAGATGACGGTAATC
AGTGGCGCGCTGAATGTGTTACTGCCTGACGCGACCGACTGGCAGGTGTATGAAGCCGGTTCGGTGTTTAATGTTCCCGG
TCACAGTGAGTTTCATCTGCAAGTTGCCGAACCCACCTCTTATCTGTGCCGCTATCTGTAATTCCTCGCCTTCCCCTTGA
ACGGGAGGGCATTTTTCTGAAATATCCTTTCTTTAGCCCATAATAATATTTCCTTTGCTGCGATTTTTTCAATTTCCGAT
ATATTCATAATTTATCAAGGTTGATATAAATATCAGTGAAGATCTCCAGATATTGTTGCGGAACTGGCTACGATAAAAGA
TAAATCAGATGATGAATGGTGGCGTGCATTGCTGCAAAGTCGCAATGATCATTTACGCCAAACGGCATTGCGCAACGCAC
ATACCCCGGCGTTGTTGTTAACGACATTGACTGAGCCTCAGGATCGGTCGCTGGCTATTAATAATCCACAGCTGGCTGCC
GATGTGAAAACGGCGTGGTTAAAAGAGGATCCATCATTACTCTTATTTGTCGAACAACCCGATCTTTCGCTGTTACGTGA
TTTAGTGAAAACCGGGGCAACGCGGAAAATTCGCAGTGAAGCGCGTCACCGGCTTGAGGAAAAACAATGACACAACGTCC
ATGGAGCAAGTTACAGCGTAAAACGCATAATATTGCTGCGCTAAAAATTATTGCTCGCCGTAGCGAATAATTATATGCCT
GGTGTGGCTTCGTACGCCGGATAAGACGCGGCAGGCGTCGCATCCGGCATTAAAGGAAAATCAGCAATTAACGTTGTGCT
TCGCCACCTAATCCTTCAATCAGGTTTTGAATTAACGCTGCCAGTTCACCAGTCATCAGGATGAAATCGGCATCAAAACG
CTGGGCGAAATCTTCACGGTCGATATCTTCGTTTTGATCGCGCAGCTCGTCGCAGAACTTCAGACGCTTGAGCGAACCAT
CGTCGCACATCACAAACTGAATGCGCTGCTGCCAGTCGAGCGCCAGTTTAGTCACCACTTTTCCGGCTTCAATGTGATTG
GTGATCTCTTCGCTGGTCAGATCTTGTTTCTTCGCGCGGATCACGCCGCCATCTTCCAGCAACGATTTCAGCTCGGCTTC
ATCAAGCAGCTGGAAGCCCTGTGCCGCACTACCGGAGCGAACCCATTCGGTCAGCGTCAGTTCAATCGGGTTTTCCATGC
TCAATGGTACAACCGGTAACGACCCCAGGCTTTTACGCAGTAATGCCAGCGTATCTTCCGCTTTTTTGGCACTGGCGCAG
TCCACCATAATCAAACCGTTAACCGTGTCGATCCACATCATTGTCTGGCTAAAACGGCTGAAAGCACGCGGCAGCAGAGA
GTGCAGCACTTCGTCTTTCAGCGAATCTTTTTCGGTTTTCTTCAGCTTACGCGCCTGTTCCGCTTCCAGTTTGGCGATTT
TCGCTTCCAGCGCCTGTTTAATCACCGGAGACGGGAGGATTTTTTCTTCTTTGCGCGCGCAGATAACAATTTGACCATTG
GCAACGTGCGTTAACGCATCGCTGTGCGATCCCATCGGAGGAACCCAGCCCATCTTCGCCATGTCCTGGCTGCCGCATGG
GGTAAATGCCATCGAGGCTAGCTGTTTTTCCATCTCTTCTGCACGCAGCGAAATCTCGCGGCTAAGACGGTAAACCATTA
AATTTTTGAACCACAGCATGATAATTTCCACGGCCTTGTCGTTAAATTTAGCGGGCATGATAACGAATTGTCGGCGGCCT
TGCATTGCCAATCCGGTTGTCCGTCTCTACGCTATTGATATTGAAAAAAATAAGGAGAGTACCGTGCGTATAGGTATCGA
TTTAGGCGGCACCAAAACTGAAGTGATTGCACTGGGCGATGCAGGGGAGCAGTTGTACCGCCATCGTCTGCCCACGCCGC
GTGATGATTACCGGCAGACTATTGAAACGATCGCCACGTTGGTTGATATGGCGGAGCAGGCGACGGGGCAGCGCGGAACG
GTAGGTATGGGCATTCCTGGCTCAATTTCGCCTTACACCGGTGTGGTGAAGAATGCCAATTCAACCTGGCTCAACGGTCA
GCCATTCGATAAAGACTTAAGCGCGAGGTTGCAGCGGGAAGTGCGGCTGGCAAATGACGCTAACTGTCTGGCGGTTTCAG
AAGCAGTAGATGGCGCGGCAGCGGGAGCGCAGACGGTATTTGCCGTGATTATCGGCACGGGATGCGGCGCGGGCGTGGCA
TTCAATGGGCGGGCGCATATCGGCGGCAATGGCACGGCAGGTGAGTGGGGACACAATCCGCTACCGTGGATGGACGAAGA
CGAACTGCGTTATCGCGAGGAAGTCCCTTGTTATTGCGGTAAACAAGGTTGTATTGAAACCTTTATTTCGGGCACGGGAT
TCGCGATGGATTATCGTCGTTTGAGCGGACATGCGCTGAAAGGCAGTGAAATTATCCGCCTGGTTGAAGAAAGCGATCCG
GTAGCGGAACTGGCATTGCGTCGCTACGAGCTGCGGCTGGCAAAATCGCTGGCACATGTCGTGAATATTCTCGATCCGGA
TGTGATTGTCCTGGGGGGCGGGATGAGCAATGTAGACCGTTTATATCAAACGGTTGGGCAGTTGATTAAACAATTTGTCT
TCGGCGGCGAATGTGAAACGCCGGTGCGTAAGGCGAAGCACGGTGATTCCAGCGGCGTACGCGGCGCTGCGTGGTTATGG
CCACAAGAGTAAAAAACGTAGGCAATTGGCGCATCATGCCTGATGCGACGCTTGCCGCGTCTTATCAGGCCTACAAAAGG
TGCCAGAACCGTAGGCCGGATAAGGCGTTCACGCCGCATCCGGCAATAAGTGCTCCGATGCCTGATGCGACGCTTGCCGC
GTCTTATCAGGCCTGCAAAATGTGCCAGAACCGCGTAGGGCGGATAAGGCGTTCACGCCGCATCCGGCAATAAGTAATGA
GCACCGAGACTATAACCTACCCCAGTGGTTTCGCCAGCACCGGAGTATCCGCCGCTTGCTGGCGCTTATAGCGACCATAC
AGCAGCAACGACGACATCGCAGCAAACGAAAGCAGGGCGGCAGGCAGCGCCACGTAATTATATGCCAGCCCCAGCGTCAG
CATCATACCTCCGCAATATGCGCCGACGGCGCTACCGAGGTTAAACGCTATTTGCCCACCTGCGGCACCTAATAACTCTC
CGCCTTTGGCGTTTTGTAGTAACAATATTTGTAGCGGTGCTGAAAGGGCAAATAATCCCGCGCAACAAATAAAAGCAAAA
ATAAGCGACGTTGTTTTCATGCCGCCGCAGAAAAAGAGCATCAGCAGTGCCAGTACAATTATAAAGTCAGTCACTGCTGC
AATGCGCAGTGGTGAATAACGTCCTGAAATCCTGCCACTTAGCATATTTCCCAGCACCATCCCTAGCCCAACTAACATCA
TAATAAAGGTCATCGCCGTTTCCGAAAAACCGGAAATAAACATCATGTATGGCTTTACGTAGCTGAACCAGGCAAACACA
CCTGCGTTGCCAAACATCGTGGCGGCGAAAATTAACCACGGGGCCGGGCTGCGCAAAAAGTGAAATTGTTCGCGCAGATT
TCCTTTCGCCTCGTCGCGAATATCTGGCACCCAAAAATAGACCGATGCCATCACCGCAATATTAAAAACAGCGATCAATA
AAAAGGTGTAACGCCAGCTAAATTCCTGACTTAAATACGTTCCCAGCGGAATGCCCAGCAAATTGGCGACTGTCATCCCG

GAAACCATCCCCGCCACGGCGGCGGTGACTTTTCCGGGTTTGATAATTTTTGATAACACGATCGCTCCGACGCCAAAAAA
TGCGCCATGCGGAAAGCCGGATACCAGCCGACCAATGGCGAGCATCAGGTAAGACGAAGAGAGCGTGAACATGGCGTTGC
CAATGACGCACAACGCCACCAGAAACAACAAGATATGTTTGAGTGAGTAGCGGCTGGAAAAGAGTGCGATGATTGGCGCA
CCGACCACCACCCCCAGTGCATAATACGAGATCATATGCCCGGCGGCAGGAATCGAAATTCCTACGTTATGCGCCAGCTC
CGTGAGCACGCCCATAATGCCAAATTCGGCCATCCCCAAACCAAACGTGCCCAGAGCCAAAGATAAAATGACTTTTTTCA
TACCACCATCACAACCACCAGAACGGCTACCAATCACCGTAGCCAATAAAGTGATAGCTTGCAGGATAACGACCAGCAAG
GCCAAGAGATAATTCGCCCCTCTGTATTCATTATCCTGCTGAATAGTTATTTCACTGCAAACGTACTTTCCAGTTTGCTG
TAGCCCAGGCCGTTGATCTTTTTCACTTTGATCTGCACCGGAATACGCTCTTTCATCGCTTCTACGTGGCTAATCACACC
GATGGTTTTGCCACTGGCGTTCAGGGCATCCAGCGCATCAAGGGCGGTATCCAGCGTTTCGCTATCCAGCGTGCCAAAAC
CTTCATCAAGGAACAGCGAGTCAATACGTGTTTTATGGCTGACCAGATCCGAAAGCGCCAGCGCCAGCGCCAGACTAACG
AGGAAACTTTCGCCGCCGGAAAGGGTACGGGTATCGCGTACCGCATCTGCCTGCCAGGTATCAACAACCTCGACTTCCAG
CGCCTCGCTGGCTTTGCGCTGTAACAGATAGCGCCCGTGCAGCCGGGTAAGTTGCTGATTAGCGAGATGGACTAAATTAT
CCAGCGTCAGCCCCTGGGCAAACTTGCGGAATTTATCGCCCTCTTTGGAACCTATTAGCGAATTCAGATATCCCCAGTCC
TCAACCTGCTGCGTCATTTGAGCAATTTGCTGCATTAAGGTTTGTTGTTGCTGACGGTTATCTGCATCCTGCTTCAGCTG
CTGGCGAATCTCGCCTTGACTCGTGGTGTTTTCACGCAACTTTTGGTGAGTTTGCGCTAACTCTTGCTGAATCTGCTCCA
CCGTCACAGTGAGAGCCAACCCGTCGTCAGGTCGGTGTTGTTGATGCTGTGCCAGCGTTTCTGCTGTCTGAGTGACCAGA
GTTTGCGCCTGACGGCGCTGGTTTTCCAGATTCTGCTTGAGCTGTTCCAGCTGCGTTAGTGTTTGTTCATCCATTAGCGC
CGCAAGGAACGCCTGCTGATCGTCAAAGACGCTGGCCTGTAGCGCGGTGTCAAACTGCGCCTGGGCTTTTTGCAGACTTT
GCGCCGCCAGAACATCCTGTTGCTGTAACGTCTGCTGCTGGCTGTGTAATGCGAGACATTGTTCATGTACCTGCCGCCAG
TTTTCCAATACCACAGTTTCTTCGCAGTGCGGGAGTTCATCACTTTGCGGCAACGTTTCCAGAATCGGCGTCAGCTGCTG
AATACGGTTTTGCAGCGCGGTTAATTCGTTCTGGCGTTGCTGCCAGCTCTGCGCTTCTTGCTGACGTGTCGCCAACCAGC
TCTCTTCTTCATCTTCCTGTGGCAATGTCAGTGCATAACCCGTCAATGTCGTTAAAAGTAGTTGCTGGCGTTGTTCAATT
TGCTGTTGATACTGGATAATTTGCTGATTATGCGCGGCAATCTGCCCTTGTAATTCATGCCGTTGGCTGAGTAACCGCAG
CTGGCGTTCGTGCTCATCTTGTGCATCCAGCCACGGTTGAATATCGTCCAGTGGCTGCAAGGTGATATTGAGGCTGGCCG
TGACGGCTTGCCATTGTTGAGTAAGTGCTTGCTCATCTTGTCGGAGGCTTTGCGCTTCGTTTTCATCACGCTGAAGCTGC
TTTGTTATGGCGTCCAGTTGCCCACGTAGCGTCGCACCTTCTTCACCGAGCTTTTTAACTTCGTTTTCCAGCGCCAGTAA
TCGAGACTGATTAACGCCAGGCTCCAGCGCCTGATACGCCTCGACCGCCGGGTGGCTGGTGGAACCACAAAGTGGGCAAG
GCTGACCCGCCTGTAACTGTGCACGTTGAGCTTCCAGCGTTTTGATGCGCGCTTCCTGCTCGCAAATGGTTTTCACATCG
GCAAGTTGCTGCGTCTTTTCTTTATAACGCTGGCGCATTTCGTTAAGTGCGGCGTTACGTTGCGTCTGTTCTTGCGTGAC
ATTCTGGATAGCGACCTGTAACTGCGCCAGACGTTTTTGTTGGGGAACAATCTGTCCATGCAGCGCGACCAGGTGCTGAC
GCAGTGGGCGTTGCTCAGCATGTTGCGCCAGGGCGGTAGCAACTTCATCGGCGGTTAACGTCAACGTGATCGCCGCAAGC
GCATTAAGTTTTTGCTCAGCATGGGTTAACTGTTGCTGCCATTGCCGCAGATGCTCGCGATCGCTGGTTTGTTGGGAGAA
CTGCGCACGCCAACCCGCCGGTTCGTTGTTCCACTGACGGAAGCGGTCGTGTTCCTGTAACCAGGTATTCAGGCTTTGTT
GCTGCTGCTGTAATTCTGCTGACTGCTTCGCCGCGTGGTGGCGAATGCTCGCGCGAAGCGCCATTGTGCTCTGTAAGCGA
GTATTTACTTCTTCAATCTGCTGGCGAATATGCGCCAGCGCCGCGCTGTGTTCTGCGATGCGTTCCCAGTGTGGACGAAG
ATTTCGTGCCGGTTGTGCCAGACTAAGCGCCGCCAGTTGAGGTTGCGCTTTTTCTTCTTCGGCTAACGCCTGTTGCAAGG
CCTGCTGACGGCGGCTGGCTTCTTGCTGCAATTCGTCCTGACGCGTTAACCAGTTTAGCGATTGTTGTTCTTGCTGCTGC
GCGGTAATTAACTGTTTTTCTTCGTCAGTAAGTACCTGCAAACTCGCTGTCAGCGATTGCACTTGTTCCGGCGTGAGCAA
CGTGACGCCGCTGGCCTGCGCTTGCAGCTTCTCCAGCTCTGTGCGGGCCGATTTGTGCTGCTCAAAAACCATCGCCGAGA
TTTGCCCGTAGATTTCAGTGCCGGTTAACTCCTCGAGCAATTCCGCGCGTTCTTTGGGTTTGGCATTCAGGAAGGCAGCA
AATTGCCCCTGCGAAAGCAGCATCGAACGGGTGAAGCGCCCGTAATCCAGCCCGGTTAACGTCGCTGTCAGTTCCAGCTT
ATCTTTCACTTTGTCGGCGAGAATTTTGCCGTCGGCGCAGCGCGCCAGCTCTACGCGTGGCACCTGCAAATTACCGTCGG
GTTGGTTACGCGCCCGATTCTGGCTCCAGAATGCACGGTACGCTTCACCTTTCACTTCAAACTCCACCTCCGCCAGACAT
TCGGCGGTATCGCGGGTCATGAGATCATTTTGCGATTGTGAAACGTTAGAGAGACGCGGAGTTTCGTGATACAGCGCCAG
ACAAATGGCGTCCAGCAGGGTGGTTTTCCCCGCACCTGTTGGGCCGGTAATAGCAAACAGCCCGTTGCTGGCGAACGGCT
CGCGGGTGAAATCAATCTTCCATTCGCCTTTTAATGAGTTCAGGTTTTTCAGGCGCAGGCTGAGAATTTTCATGCTTCGT
GTTCTCCGGCGAGGGTATGCAACGTCGTGGTGAAAAGATGCTGCAGACGTTGCTGCTGCGATTCATCCAGTTCTTCCAGT
GCCAGACGGCGATTGAACACCTCTTCGACGCTGAGTTCGCTGAGGGTTTCACGCTGTTGGCTGGCTAACACACGCTCGCG
CTGTTCACGACTCCGACGTACCAGCAATACTTCGACAGGCAATGATTCGGTTAATGCCTGGATTTTGCGCTGAATATCAT
GCAGATACTCATCAGTAGTGATTTCGATATCCAGCCAGACAGGTGGCTCCTGCGATACATCGCGCCACTGTTCCAGCTGT
GCGGTAATCGACGCCAGATCGCCTTTCAGCACTGCCATGGGTTGCGTTACCGGTACGTTCAGGTTTTCCACGCTCTCTAA
TTTGCCGTTTGAAAATGTCACCAGATGGACATATTTACTCTTACCGCATTCATCAAAACTCAGTGGAATGGGGGAGCCGC
AATAGCGAACATGTTCCATGCCGCCAATAATCTGTGCGCGGTGAATATGCCCGAGCGCGATGTAGTCGGCTGGTGGAAAG
TTTTGTGCCGGAAACGCGTCCAGCGTGCCAATATAAATGTCACGCACGGCGTCACTTTTACTGGCCCCCACGGTCGTTAA
ATGTCCCGTGGCGATGATGGGCAGAGGCTGATCGCCGCGCAGTTTGCAGGCATCGGCATAGTGTTGTTGGTAATAATCGG
TAATCGCTGCCAGTAAATGCTGCTGTTTTTCAATACCGTTAAGCCCCGCCTGGCTGGTAATAATGTCACGCGGACGTAAA

AACGGAATGGGGCACAGCACTGCGCCTGGCGTCCCGTCGCGACGAGGCAAGATTTGCGGCGCATGTCCGGCGCTGGCGAC
CACGGTAGTATTGAGGAACGCCATGATATCGCGCGATTCATTCAGCGTGGCGACCGAGTCATGGTTTCCTGCCAGTACCA
CCAGATGACAGCCAGTTTGCTGTAAATTGACAACAAAACGGTTGTATAACGTGCGGGCGTAACTGGGCGGCGAGCCGGTA
TCGAAAACATCACCGGCAACAATAATCGCATCCACCTGATGGGTTTGTGCTGTCTCCAGCAGCCAGTCAAGAAAAGCCTG
ATGTTCAGCTTCGCGGCTTTTACTGTAGAAGTTCTGGCCGAGATGCCAGTCTGAGGTGTGAAGGATGCGCATAACGGTTC
CCTGGCGAAAAAGCATGGGCGCGATTATACCCAAACAGATGTGCCATTTGCTTTTTTCTGCGCCACGGAAATCAATAACC
TGAAGATATGTGCGACGAGCTTTTCATAAATCTGTCATAAATCTGACGCATAATGACGTCGCATTAATGATCGCAACCTA
TTTATTACAACAGGGCAAATCATGGCGAGACGTATTCTGGTCGTAGAAGATGAAGCTCCAATTCGCGAAATGGTCTGCTT
CGTGCTCGAACAAAATGGCTTTCAGCCGGTCGAAGCGGAAGATTATGACAGTGCTGTGAATCAACTGAATGAACCCTGGC
CGGATTTAATTCTCCTCGACTGGATGTTACCTGGCGGCTCCGGTATCCAGTTCATCAAACACCTCAAGCGCGAGTCGATG
ACCCGGGATATTCCAGTGGTGATGTTGACCGCCAGAGGGGAAGAAGAAGATCGCGTGCGCGGCCTTGAAACCGGCGCGGA
TGACTATATCACCAAGCCGTTTTCGCCGAAGGAGCTGGTGGCGCGAATCAAAGCGGTAATGCGCCGTATTTCGCCAATGG
CGGTGGAAGAGGTGATTGAGATGCAGGGATTAAGTCTCGACCCGACATCTCACCGAGTGATGGCGGGCGAAGAGCCGCTG
GAGATGGGGCCGACAGAATTTAAACTGCTGCACTTTTTTATGACGCATCCTGAGCGCGTGTACAGCCGCGAGCAGCTGTT
AAACCACGTCTGGGGAACTAACGTGTATGTGGAAGACCGCACGGTCGATGTCCACATTCGTCGCCTGCGTAAAGCACTGG
AGCCCGGCGGGCATGACCGCATGGTGCAGACCGTGCGCGGTACAGGATATCGTTTTTCAACCCGCTTTTAACGCCTTGCT
CATCGGACGCAGAGCAGGGCTTATGATTTCTTAACTGGAGTATCTTACGTGCTGGAACGGCTGTCGTGGAAAAGGCTGGT
GCTGGAGCTGCTACTTTGCTGCCTCCCGGCTTTCATCCTGGGTGCATTTTTTGGTTACCTGCCCTGGTTTTTGCTGGCAT
CGGTAACAGGACTGCTTATCTGGCATTTCTGGAATTTATTGCGCCTTTCATGGTGGCTGTGGGTGGATCGCAGTATGACC
CCGCCACCGGGGCGTGGTAGCTGGGAACCGCTACTATACGGCTTACACCAGATGCAGCTGCGAAATAAAAAACGCCGCCG
TGAACTGGGCAATCTGATTAAACGCTTTCGTAGCGGCGCGGAGTCGCTGCCCGACGCGGTGGTGCTGACCACGGAAGAGG
GCGGTATTTTCTGGTGTAACGGTCTGGCGCAACAAATTCTTGGTTTGCGCTGGCCGGAAGATAACGGGCAGAACATCCTT
AACCTACTGCGTTACCCGGAGTTTACGCAATATCTGAAAACGCGTGATTTTTCTCGCCCGCTCAATCTGGTGCTCAACAC
CGGGCGGCATCTGGAAATTCGCGTCATGCCTTATACCCACAAACAGTTGCTGATGGTGGCGCGTGATGTCACGCAAATGC
ATCAACTGGAAGGGGCGCGGCGTAACTTTTTTGCCAACGTGAGCCATGAGTTACGTACGCCATTGACCGTGTTACAGGGT
TACCTGGAGATGATGAATGAGCAGCCGCTGGAAGGCGCGGTACGCGAAAAAGCGTTGCACACCATGCGCGAGCAGACCCA
GCGGATGGAAGGACTGGTGAAGCAATTGCTGACGCTGTCGAAAATAGAAGCCGCACCGACGCATTTGCTCAATGAAAAGG
TTGATGTGCCGATGATGCTGCGCGTTGTTGAGCGCGAGGCTCAGACTCTGAGTCAGAAAAAACAGACATTTACCTTTGAG
ATAGATAACGGCCTCAAGGTGTCTGGCAACGAAGATCAGCTACGCAGTGCGATTTCGAACCTGGTCTATAACGCCGTGAA
TCATACGCCGGAAGGCACGCATATCACCGTACGCTGGCAGCGAGTGCCGCACGGTGCCGAATTTAGCGTTGAAGATAACG
GACCGGGCATTGCACCGGAGCATATTCCGCGCCTGACCGAGCGTTTTTATCGCGTTGATAAAGCGCGTTCCCGGCAAACC
GGCGGTAGCGGATTAGGGTTAGCGATCGTGAAACATGCTGTGAATCATCACGAAAGTCGCCTGAATATTGAGAGTACAGT
AGGAAAAGGAACACGTTTCAGTTTTGTTATCCCGGAACGTTTAATTGCCAAAAACAGCGATTAATCCGCCTTTGTCATCT
TTTATTGCCATAAGCCAGTCGATGCTGGCTTATTTTCTTTGCAGTCAAAATACGGGCGTTAGATTTTACAACGATTGGTG
ATTTTTTGTTCGCATGATTAGCCATGTCTTTTTCACGGAAATAGTGTTTTATACTGGTTGGTGATTTCTTATCGCTATAT
ACCTCTGGTTTTTAGATCCCTCCTTGCTTTAAAACGTTATAAGCGTTTAAATTGCGCTTCAGGTGCTGTCATACTGACTG
CATTAACGCGGTAAATCGAAAAACTATTCTTCGCCGCGCCTGGTTGGGAGTATTTCCCGCTAAAATTGTTTAAATATACC
GCTGTATCATCCCCAGGGATTGGCACAAAAATTTAACGTTACAACACCACATCCACAGGCAGTATGATTTATGACCCATC
AATTAAGATCGCGCGATATCATCGCTCTGGGCTTTATGACATTTGCGTTGTTCGTCGGCGCAGGTAACATTATTTTCCCT
CCAATGGTCGGCTTACAGGCAGGCGAACACGTCTGGACTGCGGCATTCGGCTTCCTCATTACTGCCGTTGGCCTGCCGGT
ATTAACGGTAGTGGCGCTGGCAAAAGTTGGCGGCGGTGTTGACAGCCTCAGCACGCCAATCGGTAAAGTCGCTGGCGTAC
TGCTGGCAACGGTTTGTTACCTGGCGGTGGGGCCGCTTTTCGCTACGCCGCGTACAGCTACCGTTTCCTTTGAAGTGGGG
ATTGCGCCGCTGACGGGTGATTCCGCGCTGCCGCTGTTTATCTACAGCCTGGTCTATTTCGCTATCGTTATTCTGGTTTC
GCTCTATCCGGGCAAGCTGCTGGATACCGTGGGCAACTTCCTTGCGCCGCTGAAAATTATCGCGCTGGTCATCCTGTCTG
TTGCCGCTATTGTCTGGCCGGCGGGTTCTATCAGCACGGCGACTGAGGCTTATCAAAACGCTGCGTTTTCTAACGGCTTC
GTTAACGGCTATCTGACCATGGATACGCTGGGCGCAATGGTGTTTGGTATCGTTATTGTTAACGCGGCGCGTTCTCGTGG
CGTTACCGAAGCGCGTCTGCTGACCCGTTATACCGTCTGGGCTGGCCTGATGGCGGGTGTTGGTCTGACTCTGCTGTACC
TGGCGCTGTTCCGTCTGGGGTCAGACAGCGCGTCGCTGGTCGATCAGTCTGCAAACGGCGCTGCTATTCTGCATGCTTAC
GTTCAGCACACCTTTGGCGGCGGCGGTAGCTTCCTGCTGGCGGCGTTAATCTTCATCGCCTGCCTGGTAACGGCAGTTGG
CCTGACCTGTGCTTGTGCAGAATTCTTTGCCCAGTACGTACCGCTCTCTTATCGTACGCTGGTGTTTATCCTCGGCGGCT
TCTCGATGGTGGTTTCTAACCTCGGCTTAAGCCAGCTGATCCAGATCTCCGTACCGGTGCTGACCGCTATTTATCCGCCG
TGTATCGCACTGGTTGTATTAAGTTTTACACGCTCATGGTGGCATAATTCGTCCCGCGTGATTGCTCCGCCGATGTTTAT
CAGCCTGCTTTTTGGTATTCTCGACGGGATCAAAGCATCTGCATTCAGCGATATCTTACCGTCCTGGGCGCAGCGTTTAC
CGCTGGCCGAACAAGGTCTGGCGTGGTTAATGCCAACAGTGGTGATGGTGGTTCTGGCCATTATCTGGGATCGCGCGGCA
GGTCGTCAGGTGACCTCCAGCGCTCACTAAATCACTGAACATTTGTTTTAACCACGGGGCTGCGATGCCCCGTGGTTTTT
TATTGTGTTGATGGGTTAGGAATTGATGGAAAGTAAGAACAAGCTAAAGCGTGGGCTAAGTACCCGCCACATACGCTTTA

TGGCACTGGGTTCAGCAATTGGCACCGGGCTGTTTTACGGTTCGGCAGACGCCATCAAAATGGCCGGTCCGAGCGTGTTG
TTGGCCTATATTATCGGTGGTATCGCGGCGTATATCATTATGCGTGCGCTGGGGGAAATGTCGGTACATAACCCGGCCGC
CAGCTCTTTCTCGCGTTATGCGCAGGAAAACCTCGGCCCGCTGGCAGGTTACATTACCGGCTGGACCTACTGCTTTGAAA
TCCTTATTGTCGCCATCGCCGATGTGACCGCTTTTGGTATCTATATGGGTGTCTGGTTCCCGACGGTGCCGCACTGGATT
TGGGTACTGAGCGTGGTGCTGATCATTTGCGCCGTAAACCTGATGAGCGTGAAGGTATTCGGTGAGCTGGAATTCTGGTT
CTCGTTCTTTAAAGTCGCCACCATCATCATCATGATTGTCGCCGGTTTCGGCATCATCATCTGGGGGATTGGCAACGGCG
GGCAACCGACCGGTATTCATAACCTGTGGAGCAACGGCGGCTTCTTCAGTAACGGCTGGCTTGGCATGGTAATGTCGTTG
CAAATGGTGATGTTTGCTTACGGTGGGATCGAAATTATCGGGATTACCGCCGGTGAAGCGAAAGATCCTGAGAAATCGAT
ACCGCGTGCGATTAACTCCGTGCCGATGCGTATTCTGGTGTTCTACGTCGGTACGCTGTTCGTCATTATGTCTATCTACC
CGTGGAATCAGGTTGGCACTGCCGGTAGCCCGTTCGTGCTGACGTTCCAGCATATGGGCATTACCTTTGCCGCCAGCATT
CTTAACTTTGTTGTGCTGACTGCTTCGCTGTCGGCAATTAACAGTGATGTATTTGGCGTAGGCCGTATGCTCCACGGTAT
GGCAGAGCAGGGCAGCGCGCCGAAAATTTTCAGCAAAACGTCGCGTCGCGGTATTCCGTGGGTTACGGTGCTGGTGATGA
CTACCGCGCTGCTGTTTGCGGTGTATCTGAACTACATCATGCCGGAAAACGTCTTCCTGGTGATCGCTTCGCTGGCAACC
TTCGCCACGGTGTGGGTGTGGATTATGATCCTGCTGTCGCAAATTGCCTTCCGTCGCCGTTTGCCGCCAGAAGAAGTTAA
GGCGCTGAAATTTAAAGTGCCGGGTGGGGTAGCAACGACCATCGGCGGGCTGATTTTCCTGCTCTTTATTATCGGGTTGA
TTGGTTATCACCCGGATACGCGTATCTCGCTGTATGTCGGTTTCGCGTGGATTGTTGTGCTGTTGATTGGCTGGATGTTT
AAACGCCGCCACGATCGTCAGCTGGCTGAAAACCAGTAATCCTTGCGTTCTGTAAGCCGGTTCAGGAGGAAATCCTGATC
CGGCTATTCCGAAAGTTATCCGCCCCCGTCCTCCTCCCCCAAATATCCTTCAGATGATGAGTGATCCTGCATTAGGCTAT
GGCAAGGTGATCAGATTTTCATCACAGGGGAATTATGATGTTAAATGCATGGCACCTGCCGGTGCCCCCATTTGTTAAAC
AAAGCAAAGATCAACTGCTCATTACACTGTGGCTGACGGGCGAAGACCCACCGCAGCGCATTATGCTGCGTACAGAACAC
GATAACGAAGAAATGTCAGTACCGATGCATAAGCAGCGCAGTCAGCCGCAGCCTGGCGTCACCGCATGGCGTGCGGCGAT
TGATCTCTCCAGCGGACAACCCCGGCGGCGTTACAGTTTCAAACTGCTGTGGCACGATCGCCAGCGTTGGTTTACACCGC
AGGGCTTCAGCCGAATGCCGCCGGCACGACTGGAGCAGTTTGCCGTCGATGTACCGGATATCGGCCCACAATGGGCTGCG
GATCAGATTTTTTATCAGATCTTCCCTGATCGTTTTGCGCGTAGTCTTCCTCGTGAAGCTGAACAGGATCATGTCTATTA
CCATCATGCAGCCGGACAAGAGATCATCTTGCGTGACTGGGATGAACCGGTCACGGCGCAGGCGGGCGGATCAACGTTCT
ATGGCGGCGATCTGGACGGGATAAGCGAAAAACTGCCGTATCTGAAAAAGCTTGGCGTGACAGCGCTGTATCTCAATCCG
GTGTTTAAAGCTCCCAGCGTACATAAATACGATACCGAGGATTATCGCCATGTCGATCCGCAGTTTGGCGGTGATGGGGC
GTTGCTGCGTTTGCGACACAATACGCAGCAGCTGGGAATGCGGCTGGTGCTGGACGGCGTGTTTAACCACAGTGGCGATT
CCCATGCCTGGTTTGACAGGCATAATCGTGGCACGGGTGGTGCTTGTCACAACCCCGAATCGCCCTGGCGCGACTGGTAC
TCGTTTAGTGATGATGGCACGGCGCTCGACTGGCTTGGCTATGCCAGCTTGCCGAAGCTGGATTATCAGTCGGAAAGTCT
GGTGAATGAAATTTATCGCGGGGAAGACAGTATTGTCCGCCACTGGCTGAAAGCGCCGTGGAATATGGACGGCTGGCGGC
TGGATGTGGTGCATATGCTGGGGGAGGCGGGTGGGGCGCGCAATAATATGCAGCACGTTGCCGGGATCACCGAAGCGGCG
AAAGAAACCCAGCCGGAAGCGTATATTGTCGGCGAACATTTTGGCGATGCACGGCAATGGTTACAGGCCGATGTGGAAGA
TGCCGCCATGAACTATCGTGGCTTCACATTCCCGTTGTGGGGATTTCTTGCCAATACCGATATCTCTTACGATCCGCAGC
AAATTGATGCCCAAACCTGTATGGCCTGGATGGATAATTACCGCGCAGGGCTTTCTCATCAACAACAATTACGTATGTTT
AATCAGCTCGACAGCCACGATACTGCGCGATTTAAAACGCTGCTCGGTCGGGATATTGCGCGCCTGCCGCTGGCGGTGGT
CTGGCTGTTCACCTGGCCTGGTGTACCGTGCATTTATTACGGTGATGAAGTAGGACTGGATGGCAAAAACGATCCGTTTT
GCCGTAAACCGTTCCCCTGGCAGGTGGAAAAGCAGGATACGGCGTTATTCGCGCTGTACCAGCGAATGATTGCGCTGCGT
AAGAAAAGTCAGGCGCTACGTCATGGCGGCTGTCAGGTGCTGTATGCGGAAGATAACGTGGTGGTATTTGTCCGCGTGCT
GAATCAGCAACGTGTACTGGTGGCAATCAACCGTGGCGAGGCCTGTGAAGTGGTGCTACCCGCGTCACCGTTTCTCAATG
CCGTGCAATGGCAATGCAAAGAAGGGCATGGGCAACTGACTGACGGGATTCTGGCTTTGCCTGCCATTTCGGCTACGGTA
TGGATGAACTAACGTTTTATAACGCCTTGCGTGACGCCTGCGCCATCATCCGCGGATAAAACTGCCAGAAGCGGGTTTCG
AGGGCGTCATAATGAGCGTCTAAATCGTACCAGGAGTCACGCAGGGCATCCAGACGTGGGCGGCGGCTTGCCATGCCGTT
TAACACGTTCTGGATGAAATCCATATCGCGATAGCGCACCAGCCACTGCTCTGACCACAAGTAATTGTTCAGATTGATAA
AACGTGGCGGTGAGTCCGGCAAAATCGTCATCACTTGCTCGCGGGCATAACAGACAAATTCCTGTAGCGGAAAGTCCGGC
GACAGCTGCGACCAGTGGCGGGAAAGAAAGTGATCCCACATGACATCCAGCGTAATAGGCGCAACGCGGCGCGTTTCACT
ACGAAACCACTCCCGTGCTTCGCGGACTTCCGGCAGATTGTCAGTCAATACGTCGATACGTCGATGCATATGAATGCCAG
CCACGACGTCGGGCGGAAAACTTTCTTCAGGATTTCCGCGTACGAAATCAGCCAGTAAATTGCCGGAAAGCGAGCTTTCC
GCGAGATGGGCTAAATGCAGGTGAGCTAAAAAATTCATCGATTATATTCTATCCAAAAGGGGGTAAAGGTTGCAGGGAGA
GCGCCCCGGCACTAGACTACCCGCCTCTTATTTTAGTCTGAGTCAGTGTCATGCGCGTTACCGATTTCTCCTTTGAATTG
CCCGAATCCCTGATTGCCCACTATCCCATGCCTGAACGCAGTAGCTGTCGTTTACTGTCGCTGGACGGGCCGACGGGCGC
GCTGACGCACGGTACTTTCACCGATTTACTTGATAAGCTCAACCCCGGCGATCTTCTGGTTTTTAATAATACCCGCGTGA
TCCCGGCGCGCCTGTTTGGGCGTAAAGCCAGCGGCGGCAAGATTGAAGTGCTGGTTGAACGGATGCTCGACGACAAACGC
ATTCTTGCGCATATTCGCGCCTCGAAAGCGCCAAAACCTGGCGCAGAACTGCTGCTGGGCGATGACGAAAGTATTAACGC
AACAATGACCGCGCGCCACGGCGCACTGTTTGAAGTCGAATTTAATGATGAACGCTCGGTGCTGGATATTCTCAACAGCA
TCGGCCATATGCCGCTGCCGCCGTATATCGACCGTCCGGACGAAGACGCTGACCGCGAACTTTATCAAACCGTTTATAGC

GAAAAACCGGGCGCGGTTGCAGCCCCGACCGCAGGTCTGCATTTTGACGAGCCTTTGCTGGAAAAATTGCGCGCCAAAGG
CGTGGAGATGGCGTTTGTGACGTTGCACGTTGGTGCGGGCACCTTCCAGCCGGTGCGCGTCGACACCATTGAAGATCACA
TCATGCACTCGGAATACGCTGAAGTACCGCAGGATGTGGTAGACGCGGTACTGGCGGCGAAAGCGCGCGGTAACCGGGTG
ATTGCGGTTGGCACCACTTCAGTACGTTCGCTGGAAAGCGCGGCTCAGGCAGCGAAAAACGATCTCATTGAACCGTTCTT
CGACGATACCCAAATCTTTATCTATCCGGGCTTCCAGTACAAAGTGGTCGATGCGCTGGTGACGAACTTCCACTTGCCAG
AGTCGACGCTGATTATGCTGGTTTCGGCCTTTGCCGGTTATCAACACACCATGAACGCCTATAAAGCAGCGGTAGAAGAG
AAATATCGCTTTTTTAGTTACGGTGATGCGATGTTTATCACGTACAATCCGCAGGCAATTAATGAGCGCGTCGGGGAGTA
ATTCCGCGGCGCTGGTTTAAAACGTTGGACTGTTTTTCTGACGTAGTGGAGAAAAAATGAAATTTGAACTGGACACCACC
GACGGTCGCGCACGCCGTGGCCGCCTGGTCTTTGATCGTGGCGTAGTGGAAACGCCTTGTTTTATGCCTGTTGGCACCTA
CGGCACCGTAAAAGGGATGACGCCGGAAGAAGTTGAAGCCACTGGCGCGCAAATTATCCTCGGCAACACCTTCCACCTGT
GGCTGCGCCCGGGCCAGGAAATCATGAAACTGCACGGCGATCTGCACGATTTTATGCAGTGGAAGGGGCCGATCCTCACC
GACTCCGGCGGCTTCCAGGTCTTCAGCCTTGGCGATATTCGTAAAATCACCGAACAGGGCGTGCACTTCCGTAACCCGAT
CAACGGCGATCCGATTTTCCTCGATCCTGAAAAATCAATGGAGATTCAGTACGATCTTGGTTCGGATATCGTCATGATCT
TTGATGAGTGTACGCCGTATCCTGCTGACTGGGATTACGCAAAACGCTCCATGGAGATGTCTCTGCGTTGGGCGAAGCGT
AGCCGTGAGCGTTTTGACAGTCTCGGAAACAAAAATGCGCTGTTTGGTATCATCCAGGGCAGCGTTTACGAAGATTTACG
TGATATTTCTGTTAAAGGTCTGGTAGATATCGGTTTTGATGGCTACGCTGTCGGCGGTCTGGCTGTGGGTGAGCCGAAAG
CAGATATGCACCGCATTCTGGAGCATGTATGCCCGCAAATTCCGGCAGACAAACCGCGTTACCTGATGGGCGTTGGTAAA
CCAGAAGACCTGGTTGAAGGCGTACGTCGTGGTATCGATATGTTTGACTGCGTAATGCCAACCCGCAACGCCCGAAATGG
TCATTTGTTCGTGACCGATGGCGTGGTGAAAATCCGCAATGCGAAGTATAAGAGCGATACTGGCCCACTCGATCCTGAGT
GTGATTGCTACACCTGTCGCAATTATTCACGCGCTTACTTGCATCATCTTGACCGTTGCAACGAAATATTAGGCGCGCGA
CTCAACACCATTCATAACCTTCGTTACTACCAGCGTTTGATGGCGGGTTTACGCAAGGCTATTGAAGAGGGTAAATTAGA
GAGCTTCGTAACTGATTTTTACCAGCGTCAGGGGCGAGAAGTACCACCTTTGAACGTTGATTAATATTAATAATGAGGGA
AATTTAATGAGCTTTTTTATTTCTGATGCGGTAGCGGCAACGGGTGCACCGGCGCAAGGTAGCCCGATGTCTTTGATTTT
GATGCTGGTGGTATTCGGTCTGATTTTCTATTTCATGATCCTGCGTCCACAGCAGAAGCGCACCAAAGAACACAAAAAGC
TGATGGACTCCATTGCCAAAGGTGATGAAGTTCTGACGAACGGTGGCCTGGTTGGTCGCGTAACCAAAGTAGCGGAAAAC
GGCTACATTGCTATCGCGCTGAATGACACCACTGAAGTAGTTATTAAACGTGACTTCGTAGCTGCCGTCCTGCCGAAAGG
CACCATGAAGGCGCTGTAATTAAAATTTTTCCCTAAGGGAATTGCCGTGTTAAACCGTTATCCTTTGTGGAAGTACGTCA
TGCTGATCGTGGTGATTGTCATCGGTCTGCTGTATGCGCTTCCCAACCTGTTTGGTGAGGATCCGGCTGTTCAGATCACT
GGTGCGCGCGGAGTCGCCGCCAGTGAGCAAACGCTGATCCAGGTCCAGAAAACGTTACAAGAAGAAAAAATAACTGCTAA
GTCTGTGGCACTGGAAGAGGGCGCTATTCTTGCGCGCTTCGACTCCACTGACACCCAGTTGCGCGCTCGTGAAGCATTAA
TGGGCGTTATGGGTGACAAATACGTCGTGGCGCTTAACCTTGCCCCGGCAACGCCGCGCTGGCTGGCAGCTATTCACGCT
GAGCCGATGAAGCTCGGCCTTGACCTGCGTGGCGGCGTTCACTTCCTGATGGAAGTGGATATGGACACCGCGCTTGGCAA
ACTCCAGGAACAAAATATCGATAGCCTACGCAGTGACCTGCGCGAAAAGGGCATCCCGTATACCACTGTTCGTAAAGAAA
ACAACTACGGCCTGAGCATCACTTTCCGCGATGCTAAAGCTCGTGATGAAGCCATTGCGTATCTGAGCAAGCGCCATCCG
GACCTGGTGATTAGCAGCCAGGGCAGCAACCAGCTGCGTGCGGTAATGAGCGATGCTCGTCTGAGTGAAGCGCGTGAATA
TGCGGTGCAGCAGAACATTAATATCCTGCGTAACCGTGTAAACCAACTTGGCGTGGCGGAGCCGGTGGTTCAGCGTCAGG
GTGCTGACCGTATCGTTGTTGAACTGCCAGGTATTCAGGACACTGCGCGTGCGAAAGAGATTCTGGGTGCGACGGCAACG
CTGGAATTCCGTCTGGTAAACACCAACGTTGACCAGGCCGCTGCGGCATCCGGTCGCGTACCGGGCGACTCTGAAGTGAA
ACAGACCCGCGAAGGTCAGCCAGTTGTGCTGTACAAACGCGTAATTCTGACCGGTGACCATATCACCGACTCCACTTCCA
GCCAGGACGAATACAACCAACCGCAGGTTAACATCTCGCTCGATAGCGCTGGTGGTAACATCATGTCTAACTTCACTAAG
GACAACATCGGCAAACCGATGGCAACCCTGTTTGTGGAGTACAAAGACAGCGGTAAGAAAGATGCAAATGGTCGTGCGGT
TCTGGTGAAACAGGAAGAGGTGATTAACATCGCCAACATCCAGTCTCGTCTGGGTAACAGCTTCCGTATCACCGGCATCA
ACAACCCGAACGAAGCCCGTCAGCTGTCACTGCTGCTGCGTGCCGGTGCGTTGATCGCGCCGATTCAGATTGTTGAAGAA
CGTACCATTGGCCCAACCCTGGGTATGCAGAACATTGAACAGGGGCTGGAGGCTTGCCTTGCCGGTCTGCTGGTTTCTAT
TCTGTTCATGATCATCTTCTATAAGAAGTTTGGTCTGATTGCGACCAGTGCTCTGATTGCCAACTTGATCTTAATCGTCG
GCATTATGTCGCTGTTGCCAGGCGCAACGCTGAGTATGCCAGGTATCGCGGGTATCGTCTTAACCCTTGCGGTGGCGGTC
GATGCGAACGTACTGATCAACGAACGTATTAAAGAAGAGTTGAGCAACGGACGTACTGTTCAACAGGCAATTGATGAAGG
TTATCGTGGCGCATTCAGTTCTATCTTCGATGCGAACATCACCACGCTGATTAAAGTCATCATCCTGTACGCAGTGGGTA
CCGGGGCAATTAAAGGGTTCGCGATTACTACCGGTATCGGTGTGGCGACGTCGATGTTTACCGCGATTGTCGGTACGCGT
GCCATCGTAAACCTGCTATATGGCGGCAAGCGCGTCAAGAAGCTGTCAATCTGAGGAGTGCGATGTGGCACAGGAATATA
CTGTTGAACAACTAAACCACGGCCGTAAAGTCTATGACTTTATGCGCTGGGACTACTGGGCTTTCGGCATCTCTGGTCTG
CTGTTAATCGCTGCTATCGTTATTATGGGCGTGCGCGGCTTTAACTGGGGGCTGGATTTCACCGGTGGTACGGTTATTGA
AATTACGCTCGAAAAACCGGCTGAAATTGACGTAATGCGTGATGCATTGCAAAAAGCCGGTTTTGAAGAGCCGATGCTGC
AAAACTTTGGTAGCAGCCATGACATCATGGTCCGTATGCCGCCTGCTGAAGGCGAAACCGGCGGTCAGGTGTTGGGCAGC
CAGGTTCTGAAGGTGATTAACGAATCCACCAATCAGAATGCAGCAGTGAAGCGTATTGAGTTCGTCGGTCCGAGCGTGGG
GGCAGACCTTGCGCAAACCGGTGCGATGGCGTTGATGGCAGCGCTGCTGTCTATCCTCGTGTACGTAGGTTTCCGCTTTG

AGTGGCGACTGGCGGCAGGGGTGGTTATTGCGCTGGCGCACGACGTTATCATTACGCTGGGTATTTTGTCGTTATTCCAT
ATCGAGATTGACCTGACCATTGTGGCATCGTTGATGTCGGTTATCGGTTACTCGCTTAACGACAGTATCGTGGTATCGGA
CCGTATTCGTGAAAACTTCCGCAAGATCCGTCGCGGTACGCCTTACGAAATCTTTAACGTGTCCTTGACCCAGACGCTGC
ACCGTACCTTGATCACATCCGGTACTACCTTGATGGTTATCCTGATGCTGTACCTCTTCGGTGGTCCGGTACTGGAAGGC
TTCTCGCTGACCATGCTTATCGGTGTTTCCATCGGTACTGCATCTTCCATCTATGTGGCATCTGCGTTGGCTCTGAAACT
GGGTATGAAGCGCGAACACATGTTGCAGCAGAAAGTGGAAAAAGAAGGGGCGGATCAGCCGTCAATTCTGCCGTAATCAA
GTTCCCGTTGATGTTGAAAATCCCGGTCAGAAGATCGGGATTTTTTTTGATGTATGGAGATTGCGAAAATCCCCGCATCT
TGGGAAACTGCGCGTAACCCTACATTTCATCCAGGTAACTTTTCATGGCTATCATCCCAAAAAACTATGCGCGGTTAGAA
AGCGGCTATCGCGAAAAAGCATTAAAAATCTATCCGTGGGTCTGCGGTCGCTGTTCCCGCGAGTTTGTTTATTCCAACCT
GCGTGAACTTACCGTTCACCACATTGATCACGACCATACCAATAACCCGGAAGATGGCAGTAACTGGGAATTGTTGTGTC
TCTATTGCCACGATCATGAGCATTCGAAATATACCGAAGCGGATCAGTATGGTACGACCGTTATCGCAGGGGAAGATGCG
CAGAAAGATGTCGGTGAAGCGAAGTACAACCCATTCGCTGACCTGAAAGCGATGATGAACAAGAAGAAGTGATTAAAACG
TAAAATTGCCTGATGCGCTACGCTTATCAGGCCTACGTTATTTCAGCAATATATTGAATTTACGTGCTTTTGTTGGCCGG
ACAAAGCGTTTACGCCGCATCCGGCATGAACAAAGCACACGTTGTTAACAATCAGAAATGCCGGGAATAAATCCCGGCAT
TTTCATAATCAGAAGTTGTAACCTACTACCAGGTAACCACCCCAGCCGGTAGAGCGAACGTTGAAGTTGCCGTTGCCGAA
GTTCAGTTCTGCATCGTCGTTCCACTGACCACCGTCGTGCCAGTAACGAGCTACGACAGAGTAGTGCCAGTGATCGTAGT
TCAGAGCCAGAATATGGCTGGAAGCGATAGAGTTATTAGTACGGGTCTTAATACCGTTGATTGCGTTACCGCTGTCATCC
CCTAAATCGGAACCCCAGTCGAAGTTGGTGAAGCCGATGTAGCTCAGCTGACCGCCCCACAGATCGGTAATCGGCACAAA
GTATTTAATTTTGAAACGGTAACCGTCCCACTCGTTTTCGTTCGCTGCGCCATAGTTCTGCCACTGGTATTTCGCATAGA
CGTTCATGGACAGGCTCATCGGCAGGCCAGTGTCGATATCGGTACCCAGACCCATGTACCAGGTGCTCTGGCGACCATCT
TTATTACGACCCATGTCGTAAATGTAGTTGTTCGCGAAGTACCACTCTTTGAACGGACCGAAGCTAAGGTCAGTATTGGT
CAGCTTGTCGATGGAGAAACGTGGTTCGATTTCCATAAACAGCGGAGAACCGTGGTTCCAGATACCTTTAGCATCGGAGT
TACCGCCGAAGAATACCGGCGCATCCGCATAACCATAGAAGTCGAACCAGTCTTTTTTAGCGAATGCTTCGTACTCAAGG
TAGGTATCGTTGCGGATCTGCGGTCCGAAACGGGTGTGATAGCTTCCGACAACGTTAACGCTCTGGTGCCACCAGTCGGA
AAGATACTGCGGTTTGTCGTTTTCAGCTGCGTTGACAGTAAAAGACGAAGAGAGCGCCAGTACCGCACCGGCTGCCAGTA
ATGTTTTTTTCATATGTATGCCACTGTTTGAAAATCCCTTGCGGGAGTGAAAAAGGCGCAAATTGCGTTTCTAAATATTT
CGTATACAGAGGAGCCTATTATAAAAATCATTGCTCACAAAAATATGTTTCGTTTCACAGTTCTATCATTTACGTAATCG
ATTGCGTTCACGTTTACACACATTCGGGCGGGGATTGTACTGACTTTCGCCCTTGTTGCAAACTTTTACTATCAATCCAG
GTGATGTAAGCAGAGTAACAAAATGACGGGGAAAGATGTTGCGGGACGCACAAAATCGTCCCGAAATACGTTTACTGCAT
AACAGGTTGAATGTCGTGGATACGTACAAAGCCTAACTGATCCACAGAAATCCCCTTTAACTGTAACGGAATATCAACAT
CGCTGGGGGCGAGGACGCTGGCAGGGGCATTAATCAATTGATTCTGCACATTGATTTCCTGAAAGTTGTCTATTGTTCCC
TGAATTTGCCCATATTCAACAGTTCCGCTGAATGCGGGCAAGGGATCATTCGACTCACCCTGAATACGTAGTGTAAGGGT
GGTGCCATCTGCATCAGGCGTAATATTCACCAGCGACATACGTAAAGTACCGATCTGGCTTTCCAGTCTTGCCGGTGTTT
TCGCTCCAGGCAGAAGGTAAACCCCACTGCTGGATTTTGCATTCAGCCTGTTTTGCTGAGTGATTTTGACTGTTTCTTGA
TTGAGTTGCGTCATCTCCTGGTTCAGCGTGCTGACGCTGTGTTTCATCTGTCGCACTTCACTTTGCTGCACACAGGCGCT
AAGGCTGAACAGGCTTCCCAGCAGGAGCAGTCGAAAAACGTTAGTATTCATAGGTCGTCCCTTTCTCGTCGCAATTAGCC
TAATGGTAGAGGGTAAAAGCAGCGAAAGCATCGATCCTTTATCTCAAAAGCGTTGCGCCTTTGTTGTATCGTCAGTTCAG
GGTAAAATAGATTTCCGTTAACCACCTGGTCAGGACGCCGTATGCATTGCCCATTCTGTTTCGCCGTGGACACTAAGGTA
ATTGACTCTCGTCTCGTGGGCGAGGGTTCATCCGTACGCCGCCGTCGGCAGTGTCTGGTGTGTAATGAACGTTTCACCAC
CTTTGAAGTGGCGGAGCTGGTTATGCCGCGTGTTGTAAAAAGCAACGACGTGCGTGAACCGTTTAATGAAGAGAAATTGC
GTAGCGGAATGCTGCGGGCGCTGGAAAAACGTCCGGTGAGTTCCGATGACGTCGAAATGGCAATCAATCATATTAAATCG
CAGCTGCGCGCCACCGGTGAGCGCGAAGTGCCGAGCAAGATGATTGGCAATCTGGTGATGGAGCAATTGAAAAAGCTCGA
TAAAGTCGCCTATATCCGTTTTGCCTCTGTCTACCGCAGTTTCGAAGATATCAAAGAATTTGGCGAAGAGATCGCGCGCC
TGGAGGACTAAGCCGTGCAGGACGAGTATTACATGGCGCGGGCGCTAAAGCTGGCGCAACGAGGACGTTTTACCACGCAT
CCCAACCCGAATGTCGGGTGCGTCATTGTCAAAGATGGCGAAATTGTCGGTGAAGGTTACCACCAACGTGCGGGTGAACC
ACATGCCGAAGTACACGCGTTGCGTATGGCGGGTGAAAAAGCCAAAGGTGCGACCGCCTATGTCACACTCGAACCCTGTA
GCCATCATGGTCGTACGCCACCGTGCTGTGACGCACTCATCGCCGCTGGCGTAGCGCGCGTGGTTGCCTCGATGCAAGAT
CCTAACCCGCAGGTCGCTGGGCGTGGACTTTACCGTCTGCAACAGGCTGGCATTGACGTCAGCCACGGCCTGATGATGAG
TGAAGCCGAGCAATTGAATAAAGGCTTTCTCAAGCGGATGCGCACCGGCTTTCCTTATATTCAGTTAAAACTTGGCGCAT
CGCTTGATGGTCGCACGGCGATGGCGAGCGGCGAAAGCCAGTGGATCACTTCGCCCCAGGCGCGGCGTGATGTACAACTA
CTGCGCGCGCAAAGTCATGCCATTTTAACCAGCAGCGCCACGGTGCTGGCGGATGATCCTGCCTTAACGGTGCGTTGGTC
TGAACTGGATGAACAAACTCAGGCGCTCTATCCGCAACAAAATCTCCGTCAGCCGATACGTATTGTGATTGATAGCCAAA
ATCGCGTGACGCCGGTACATCGCATTGTGCAGCAGCCCGGCGAAACCTGGTTCGCGCGTACGCAGGAAGATTCTCGTGAG
TGGCCGGAAACGGTGCGTACCTTGCTGATTCCAGAGCATAAAGGTCATCTGGATCTGGTTGTACTGATGATGCAACTGGG
TAAACAGCAAATTAACAGCATCTGGGTGGAAGCGGGGCCAACGCTCGCTGGCGCATTGCTGCAGGCGGGTTTAGTCGATG
AGCTGATTGTCTATATCGCACCTAAACTATTAGGCAGCGACGCCCGCGGATTATGCACGCTGCCAGGGCTTGAGAAATTA

GCCGACGCCCCCAATTTAAATTCAAAGAGATACGTCATGTAGGCCCGGATGTTTGCCTGCATTTAGTGGGTGCATGATC
TCCCGGCTCGAAAGGGAAGCAGCGCACGAAATATTATGCTAAAATCCGCCCCCCTGCGGGGCCATACTCGAACCCGAAGG
AAGAAAATGAACATTATTGAAGCTAACGTTGCTACCCCGGACGCTCGCGTCGCCATCACCATCGCGCGTTTCAACAACTT
TATCAATGACAGCCTGCTGGAAGGTGCAATTGACGCACTGAAACGTATCGGTCAGGTAAAAGATGAAAACATTACCGTTG
TTTGGGTGCCTGGTGCCTATGAGCTGCCGCTGGCGGCGGGTGCACTGGCTAAAACCGGTAAATACGACGCGGTGATTGCG
CTGGGTACGGTTATTCGTGGTGGCACTGCCCACTTTGAATATGTCGCTGGTGGTGCAAGCAACGGCCTGGCGCATGTTGC
CCAGGACAGCGAAATTCCGGTTGCTTTTGGGGTTCTGACCACTGAAAGCATTGAACAAGCGATCGAACGTGCTGGCACCA
AAGCTGGCAACAAAGGTGCAGAAGCTGCACTGACCGCGCTTGAAATGATTAATGTATTGAAAGCCATCAAGGCCTGAAAT
TAGTAAGGGGAAATCCGTGAAACCTGCTGCTCGTCGCCGCGCTCGTGAGTGTGCCGTCCAGGCGCTCTACTCCTGGCAGT
TGTCCCAGAACGACATCGCTGATGTTGAATACCAGTTCCTGGCTGAACAGGATGTAAAAGACGTTGACGTCCTGTACTTC
CGTGAGCTGCTGGCCGGGGTGGCGACTAATACCGCATACCTCGACGGACTGATGAAGCCATACCTGTCCCGCCTGCTGGA
AGAACTGGGACAGGTAGAAAAAGCAGTACTGCGCATTGCGCTGTACGAACTGTCTAAACGTAGCGATGTGCCATACAAAG
TGGCCATTAACGAAGCGATCGAACTGGCGAAATCGTTCGGCGCAGAAGACAGCCATAAGTTCGTCAACGGCGTACTCGAT
AAAGCAGCACCTGTGATTCGCCCTAACAAAAAGTGATATCCAGGCCGGTAGATTCACGGAAGACCGTTCCATGATCGCCG
GCCTTTTCTTTTTTACCTGCTGAGGCATAACGTATGGCATGTGGCGAGTTCTCCCTGATTGCCCGTTATTTTGACCGTGT
AAGAAGTTCTCGTCTTGATGTCGAACTGGGCATCGGCGACGATTGCGCACTTCTCAATATCCCCGAGAAACAGACCCTGG
CGATCAGCACTGATACGCTGGTGGCGGGTAACCATTTCCTCCCTGATATCGATCCTGCTGATCTGGCTTATAAAGCACTG
GCGGTGAACCTAAGCGATCTGGCAGCGATGGGGGCCGATCCGGCCTGGCTGACGCTGGCATTAACCTTACCGGACGTAGA
CGAAGCGTGGCTTGAGTCCTTCAGCGACAGTTTGTTTGATCTTCTCAATTATTACGATATGCAACTCATTGGCGGCGATA
CCACGCGTGGGCCATTATCAATGACGTTGGGTATCCACGGCTTTGTTCCGATGGGACGAGCCTTAACGCGCTCTGGGGCG
AAACCGGGTGACTGGATCTATGTGACCGGTACACCGGGCGATAGCGCCGCCGGGCTGGCGATTTTGCAAAACCGTTTGCA
GGTTGCCGATGCTAAAGATGCGGACTACTTGATCAAACGTCATCTCCGTCCATCGCCGCGTATTTTACAGGGGCAGGCAC
TGCGCGATCTGGCAAATTCAGCCATCGATCTCTCTGACGGTTTGATTTCCGATCTCGGGCATATCGTGAAAGCCAGCGAC
TGCGGCGCACGTATTGACCTGGCATTGCTGCCGTTTTCTGATGCGCTTTCTCGCCATGTTGAACCGGAACAGGCGCTGCG
CTGGGCGCTCTCTGGCGGTGAAGATTACGAGTTGTGTTTCACTGTGCCGGAACTGAACCGTGGCGCGCTGGATGTGGCTC
TCGGACACCTGGGCGTACCGTTTACCTGTATCGGGCAAATGACCGCCGATATCGAAGGGCTTTGTTTTATTCGTGACGGC
GAACCTGTTACATTAGACTGGAAAGGATATGACCATTTTGCCACGCCATAAAGATGTCGCGAAAAGTCGCCTGAAGATGA
GTAATCCGTGGCATCTACTTGCTGTCGGATTCGGAAGTGGATTAAGCCCGATCGTTCCTGGGACGATGGGCTCGCTGGCA
GCGATTCCGTTCTGGTATCTGATGACCTTTTTGCCCTGGCAGCTCTACTCGCTGGTGGTGATGCTGGGGATCTGTATCGG
CGTCTATCTTTGTCATCAAACGGCGAAAGACATGGGTGTGCACGATCATGGCAGCATTGTCTGGGACGAATTTATTGGTA
TGTGGATCACGCTCATGGCGCTGCCGACCAATGACTGGCAGTGGGTTGCCGCCGGGTTTGTGATTTTCCGTATTCTGGAT
ATGTGGAAGCCGTGGCCGATCCGCTGGTTTGATCGCAATGTGCATGGCGGCATGGGGATCATGATCGACGATATTGTCGC
CGGGGTGATTTCCGCAGGCATCCTGTATTTTATCGGTCATCACTGGCCGCTGGGTATTCTGTCGTAGTTGTGCACCGATG
CCTGATGTGACGCTTGTCACGTCTCATCAGGCCTGGACTCTTATTTAAATCCTACGACAGGATGCGGTTTATACGGCGTT
TCCAGTTCGGCAATCTGTTCCGGCTTCAAAGTGATATCCACCGCGTTCAATAGCTCATCAAGCTGTTCTTCGCGCGAAGT
TCCGATAATCGGTGCGGCAATGCCCGGTTTACTCAACAACCAGGCCAGCGCAACTTGTGCTCGTGTCGCCCCCAGTTCTT
CACTGACGCCTGTTAACCGCTCTGCGATCTGCGCGTCATTTTCATCGCTTTCTTTATAGAGATTTTTCCCCACCTCATCA
GACACCAGTCGTGCGGTAGTTTCTCCCCACGGACGCGTCAGACGGCCCCTTGCCAGCGGGCTCCATGGAATTACCGCCAC
GCCCTCCTGATAACACAGTGGTAGCATCTCGCGCTCTTCTTCACGATAAATCAGATTGTAGTGATCCTGCATACTGACAA
ACTGCGCCCAGCCGTGCTGTTTTTGGAGTTCCAGTGCCTGAGCAAACTGCGAAGCGTGCATTGATGACGCGCCGATATAA
CGCGCTTTCCCGGCTTTTACCACGTCGTTGAGGGCTTCCAGCGTCTCTTCGATCGGCGTGTTGTAATCCCAGCGATGAAT
TTGCAGGATATCGACATAATCCATGCCGAGACGTCGCAGGCTGTCGTCGATAGAGCGCAAAATTTGCGCACGGGATAATC
CTTCCGGTAAATCACCAACGCGATGGAACACTTTGGTCGCAACGACCACGTCTTCACGACGGGCGAAATCCCGCAGTGCG
CGACCGACGATCTCTTCGCTGCTGCCGTCAGAATAACTGTTGGCGGTATCAAAGAAATTTATGCCGCCTTCCAGTGCACG
TTTAATTATGGGACGGCTGCTTTCTTCCGGCAGTGTCCATGCGTGATTACCGCGATCTGGCTCGCCAAAGGTCATACAGC
CGAGGCAAAGTCGGGAAACGCGAAGGTCGGTTTTTCCTAAGGGGTTGTATTGCATGCTGCCACTCCTGCTATACTCGTCA
TACTTCAAGTTGCATGTGCTGCGGCTGCATTCGTTCACCCCAGTCACTTACTTATGTAAGCTCCTGGGGCTTCACTCGTT
TGCCGCCTTCCTGCAACTCGAATTATTTAGAGTCTATGAATAATTTCTTAAGCATAGCAGGAGTGGAGTAGGGATTATGC
CAGCCAGGCCTTGATTTTGGCTTCCATACCAGCGGCATCGAGGCCGAGTTCGGCGCGCATTTCTTCCTGAGTTCCTTGCG
GAATAAAGAAGTCCGGCAGGCCAATGTTCAGCACGGGTACTGGTTTACGATGGGCCATCAGCACTTCGTTCACGCCGCTG
CCTGCGCCGCCCATAATGGCGTTTTCTTCTACGGTGACCAGCGCTTCATGGCTGGCGGCCATTTCCAGAATTAACGCTTC
ATCAAGCGGTTTCACAAAACGCATATCGACCAGCGTGGCGTTCAGCGATTCGGCGACTTTCGCCGCTTCTGGCATCAGCG
TACCAAAGTTAAGGATCGCCAGTTTCTCGCCACGACGCTTCACAATGCCTTTGCCAATTGGTAGTTTTTCCAGCGGCGTC
AGTTCCACGCCGACCGCGTTGCCACGCGGGTAGCGCACCGCTGACGGGCCATCGTTATAGTGATAGCCGGTATAGAGCAT
CTGGCGACATTCGTTTTCATCGCTCGGGGTCATAATGACCATTTCCGGTATGCAGCGCAGGTAAGAGAGATCAAAAGCAC
CCTGATGGGTTTGACCGTCAGCACCAACAATGCCCGCGCGGTCGATGGCGAACAGGACCGGAAGCTTTTGAATCGCCACG

TCATGCAGCACCTGATCATAGGCGCGTTGCAGGAAAGTGGAGTAAATCGCGACAATGGGTTTGTACCCACCAATCGCCAG
ACCCGCAGCAAAGGTCACCGCGTGTTGCTCGGCAATTGCCACGTCGAAGTAGCGATCCGGGAATTTACGTGAAAACTCGA
CCATGCCGGAACCTTCACGCATCGCCGGAGTAATCGCCATCAGCTTGTTGTCTTTCGCTGCCGTTTCGCACAACCAGTCG
CCAAAGATTTTTGAATAGCTCGGCAAACCGCCGCTACTTTTCGGCAAACAACCGCTGGAGGGATCAAATTTAGGCACGGC
GTGGAAAGTGATCGGGTCTTTTTCTGCCGGTTCATAACCACGACCTTTTTTGGTCATGATATGCAGGAACTGCGGGCCTT
TCAGGTCGCGCATGTTCTTTAGCGTGGTGATAAGCCCCAGCACATCGTGACCGTCCACCGGGCCGATGTAGTTAAAGCCC
AGCTCTTCAAACAACGTGCCAGGCACTACCATGCCTTTAATATGTTCTTCGGTGCGTTTGAGCAGCTCTTTAATTGGCGG
CACGCCAGAGAAAACTTTTTTCCCGCCTTCGCGCAGTGAAGAGTAAAGCTTACCGGAAAGCAGCTGTGCCAGATGGTTGT
TGAGCGCGCCGACATTTTCGGAAATCGACATTTCATTGTCGTTGAGAATCACCAGCATATCAGGACGGATATCGCCCGCG
TGATTCATCGCTTCAAACGCCATGCCTGCGGTAATCGCGCCATCGCCAATGACACAGACGGTGCGGCGATTTTTGCCTTC
TTTTTCGGCAGCAACCGCAATACCAATTCCGGCACTGATGGAGGTTGATGAATGCCCGACGCTTAATACGTCATATTCGC
TTTCGCCGCGCCACGGGAACGGGTGCAGACCGCCTTTCTGACGGATGGTGCCGATTTTGTCGCGGCGTCCGGTCAAAATT
TTATGCGGATAAGCCTGATGCCCCACATCCCAAATCAATTGGTCAAACGGGGTGTTGTAGACATAGTGCAGCGCCACGGT
CAGTTCGACCGTGCCCAGCCCGGAGGCGAAGTGCCCGCTGGAACGGCTCACGCTGTCGAGTAAATAGCGGCGCAGTTCGT
CGCAGAGTTTCGGTAAACTCTCTTTCGGCAACAGTCGTAACTCCTGGGTGGAGTCGACCAGTGCCAGGGTCGGGTATTTG
GCAATATCAAAACTCATCAGGGGCCTATTAATACTTATTGTTTATTTATTACGCTGGATGATGTAGTCCGCTAGCGCTTC
CAGTGCCGAGGTATCGAGTGACTGTTCAGCCAGTTGTTTCAGCGACTGACGGGCATCGTCGATCAGATCCCGGGCTTTCT
TCCGGGCTTGCTCAAGACCCAGAAGTGCAGGGTAGGTACTTTTACCAAGTTGCTGGTCGGCACCCTGGCGTTTTCCCAAC
GTTGCAGTATCTCCCACCACATCCAGGATGTCATCCTGAACCTGGAAGGCAAGGCCGATGCTCTCTGCATACTTGTCGAG
TACCGGCAGAGCACGACGTCCTTTATCTCCGGCGCTTAATGCACCAAGGCGAACGGCGGCGCGAATCAATGCGCCGGTTT
TATGACGATGAATACGCTCAAGCGCGTCCAGAGGTACGTGTTTGCCTTCCGCGTCTAAATCTAATGCCTGACCACCGCAC
ATTCCGGCAATACCACTGGCGCTCGCCAGTTCAGAAATCATCGAAATTCTGTCGCGGTCCGACACTTCCGGCATATCGGC
ATCGCTTAAAATCGAGAACGCCAGCGTTTGTAAAGCGTCGCCAGCGAGAATCGCGTTTGCTTCGCCAAACTTCACATGGC
AGGTTGGCAAACCGCGACGCAGATCGTCATCATCCATTGCCGGTAAATCATCATGAATTAATGAGTAAGCGTGGATACAC
TCAACGGCGGCAGCGGGTGCGTCCAGCGTGTTTGTGCTAACGCCGAACATATGACCGGTGGCATAAACCAGGAAAGGTCG
CAGGCGCTTACCACCTAATAATGCGCCATACTGCATGGTTTCGACCACGGGAGTGTTCTGAAAGGGCAGTGGGGCGATAA
AACGGCTCAGCGCCTGGTTGGCCTGCTTAACGCAGGCTTCGAGTTGCTGCGGAAAGTCCATTACTCATTGTCCGGTGTAA
AAGGGGTTAGAGAGGCGTCTTCATTGTCAGACAGCAGAATTTGTACGCGCTGTTCGGCTTGTTGTAATTTGGCCTGCCCC
TGACGTGCCAGCTGCACGCCGCGTTCGAACTCGTTCAGCGCCTCTTCCAGCGGCAGGTCGCCACTTTCCAGACGGGTTAC
AATCTGTTCCAGCTCGCTCAGCGCCTTTTCAAAGCTGGCGGGCGCCTCATTTTTCTTCGGCATAATGAATGTCTGACTCT
CAATATTTTTCGCCCCGTCATGGTAACGGACTCAGGGCAAATAGCAAATAACGCGCAATGGTAAGGTGATGTGCACAGCA
AAGCGATGTTAGTGGTATACTTCCGCGCCTGGATGCAGCCGCAGGTGTGGGCTGCTGTATTTTTCCCTATACAAGTCGCT
TAAGGCTTGCCAACGAACCATTGCCGCCATGAAGTTTATCATTAAATTGTTCCCGGAAATCACCATCAAAAGCCAATCTG
TGCGCTTGCGCTTTATAAAAATCCTTACCGGGAACATTCGTAACGTTTTAAAGCACTATGATGAGACGCTCGCTGTCGTC
CGCCACTGGGATAACATCGAAGTTCGCGCAAAAGATGAAAACCAGCGTCTGGCTATTCGCGACGCTCTGACCCGTATTCC
GGGTATCCACCATATTCTCGAAGTCGAAGACGTGCCGTTTACCGACATGCACGATATTTTCGAGAAAGCGTTGGTTCAGT
ATCGCGATCAGCTGGAAGGCAAAACCTTCTGCGTACGCGTGAAGCGCCGTGGCAAACATGATTTTAGCTCGATTGATGTG
GAACGTTACGTCGGCGGCGGTTTAAATCAGCATATTGAATCCGCGCGCGTGAAGCTGACCAATCCGGATGTGACTGTCCA
TCTGGAAGTGGAAGACGATCGTCTCCTGCTGATTAAAGGCCGCTACGAAGGTATTGGCGGTTTCCCGATCGGCACCCAGG
AAGATGTGCTGTCGCTCATTTCCGGTGGTTTCGACTCCGGTGTTTCCAGTTATATGTTGATGCGTCGCGGCTGCCGCGTG
CATTACTGCTTCTTTAACCTCGGCGGCGCGGCGCATGAAATTGGCGTGCGTCAGGTGGCGCATTATCTGTGGAACCGCTT
TGGCAGCTCCCACCGCGTGCGTTTTGTCGCTATTAATTTCGAACCGGTCGTCGGGGAAATTCTCGAGAAAATCGACGACG
GTCAGATGGGCGTTATCCTCAAACGTATGATGGTGCGTGCCGCATCTAAAGTGGCTGAACGTTACGGCGTACAGGCGCTG
GTCACCGGCGAAGCGCTCGGCCAGGTGTCCAGCCAGACGCTGACCAACCTGCGCCTGATTGATAACGTCTCCGACACGCT
GATCCTGCGTCCGCTGATCTCTTACGACAAAGAGCACATCATCAACCTGGCCCGCCAGATTGGCACCGAAGACTTTGCTC
GCACGATGCCGGAATATTGTGGTGTGATCTCCAAAAGCCCGACGGTGAAAGCAGTTAAATCGAAGATTGAAGCGGAAGAA
GAGAAGTTCGACTTCAGCATTCTCGATAAAGTGGTTGAGGAAGCGAATAACGTTGATATCCGCGAAATCGCCCAGCAGAC
CGAGCAGGAAGTGGTGGAAGTGGAAACCGTCAATGGCTTCGGCCCGAACGACGTGATCCTCGATATCCGTTCTATCGATG
AACAGGAAGATAAGCCACTGAAAGTCGAAGGGATTGATGTGGTTTCTCTGCCGTTCTATAAACTGAGCACCAAATTTGGC
GATCTCGACCAGAACAAAACCTGGCTGCTGTGGTGTGAGCGCGGGGTGATGAGCCGTCTGCAGGCGCTCTATCTGCGCGA
GCAGGGCTTTAACAATGTGAAGGTATATCGCCCGTAATTTGTGGTTTTTACGTCGCATCTGGTCAGATGCGACGTTTGCC
GCATCCGACACTACTCGTAATAATTATAAATCCCTGCCGCCATCACCAGTTGTGATGCCACTTCATGGGCTTTTTCACGC
CCAACCAACAGGTCGATAATTTTCAGACCAAAGTCGATAGCTGTACCCGGCCCCTGGCTGGTCAGCAATTTTACCCGTGC
ATCCCAGACGACGCGCTTGTCCAGCCATTGTTCGGCGGGAATTTTGTCTTTCAGCGTCGGGAAGCCGGTCATATTACCAA
TCGGGAAGATATCGTGCGGCACCAGCACGGTGGCTGGCGCGGCGCAAATAGCCGCGACGATACGCCCGGAACGGTGGAAC
TGTTTAACGGTTTCAACCAGCAGAGTGCTATCGCGAAAACACTCCGCGCCTTTAATGCCACCAGGCAGCACGATCACGTC

ATATTCGCCATCAGCCACTTCGACCAGCGGCGCATCCGCCAGCAGCTTCACGCCGCGCGAGCAGGTAATCGCCAGGTTAC
CATCGCTGGCGACGCTGGCAGTGGTGACTTTGATACCGCCGCGAACCAGCAGATCGATAGTGGTGACGGCTTCAGTCTCT
TCACTACCAGGGGCGAGGCAAACCAGTGCCGATGCGCTCATATTCACTCTCCTTTCTTTTTACCATTTCAAACAGGCGGG
TGTTTTCCGGTACGGCAATCCCATGCGCGCGGGCGCGGCGTAAGAGAAAACCATTGATATAGTCGATTTCAGTGTGGCGC
AGCGCGCGGATATCCTGCAACATCGACGAGATATTTTCCGCTGTGGCATCAATCACCTGCATCACGTAATCACGCAAATC
TTCTGCTGAAGTATGATGCCCTTCGCGTTCGATCACCGCCGCGACTTCTTCGCATATCTGCATAATTTCTTGCGGATGAT
GACGTAATTCACCGTTCGGGCAATTCCAGATGGCAGTCAGTGGATTAATCACGCAGTTGACTGCCAGCTTGCGCCACAGC
TCGGCGCGAATATTGTTATGCCAGGCAACGTCAGGCAACACGGTTTGCAAAATATCCGCCAGATAACTGTAATCCCCGTC
CTGTTGCCGTGCCGGGCCAATATGCGTGATACCGTTTGCCACATGAATAATGACATTGCCGTCGCGGCGGGCTGCATGGG
TGGTGGTGCCCATCAGTAATGGCTGCTGAATGTTTTGCAACTCTTCGATGGTGCCCATGCCGTTGTGAATTAACAGTATT
GGCGTAGTTACAGGCAGTGTGGACGCGAGGCTTTTGACGGCATCGGAAACCTGCCATGCTTTCAGCGTCACCAGGAGCAG
ATCGCTGGTGGCGAGAAAATCGGGATCGTTGGCGGTCAGCGATTCGTTAAATATCGAACCATCTGTCTCAACCAGATTCA
CGCTACAATAAGGTTGCGGTACGCGCAGCCAGCCCTGAACTTCATGACCCTGTTTGCAAAGTGCTGTAAGCCATAATTGC
CCTAAGGCACCGCATCCCAATACGGTAATTTTCATTGTTCCTCCTCACCCGCAACCACTCCGGGTGTTCAATAAGGCTAT
CCCTTAATTGTGCATGCTGTTGCGACTATGCACAATTAAGGGATACGTCCTGGTGCAGGACTGTCGGTTATTTAACTTTG
CGGGTATTATGCTTCGCATCAAAAATGAAGGGAGAGGAAAAGATGCCATCTTTCGATATTGTCTCTGAAGTTGATCTTCA
GGAAGCACGTAACGCGGTCGATAACGCGAGCCGCGAAGTGGAGTCCCGTTTTGACTTCCGTAACGTTGAAGCCTCATTTG
AGCTGAACGACGCCAGCAAAACCATCAAAGTGTTGAGCGAGTCCGATTTCCAGGTCAATCAGTTGCTGGATATTCTGCGT
GCCAAGCTGCTGAAGCGCGGCATTGAAGGCAGTTCGCTGGATGTACCGGAGAATATCGTTCATAGCGGTAAAACCTGGTT
TGTGGAAGCGAAACTGAAACAGGGCATTGAGAGCGCGACCCAGAAGAAAATCGTCAAGATGATCAAAGACAGCAAACTGA
AAGTGCAGGCGCAAATTCAGGGCGATGAAATCCGCGTAACGGGCAAATCTCGTGATGATTTGCAGGCTGTCATGGCGATG
GTACGTGGTGGCGATCTCGGTCAGCCGTTCCAGTTCAAAAACTTCCGCGATTAATCGCGACTATGTGCGTTTTGTTCATG
TCAGATGCGGCATGAACGCCTGATTCGGCCTACAAAGGTTTGTAATTCAGAAACTTTGTAGGCCTGATAAGCGTAGCGCA
GCAGGCAATTCGCCGCGTTTTTTATGCCTGACGAATTGCCTGTTCTATCTCAAAGCGATTCGTCACTTTGCTGTCGATTT
TCACATAAGCTGAATGTTCTTCTTCTGCAATCAACACTTCTTTGATGCCTTCAGTTTCTAGCAAACGCACTTTTAACGCC
TCGTTTGCGGCAATGTTCGCCGGGATTTCAATGCGCAAACTGCTGACATACGGCGGTTCTTTCATGGTACTGGCGACTGT
CAGCCACACTGCGGCCAGCATTGCGCCAGCGAGAAATACCCCCTGACCGTCAAACATGCCGTTAATCCAGCCGCCCAGCG
AACCGCCAATCGCCACGCCAAGAAACTGGCTGGTGGAGTAAACACCCATCGCCGTACCTTTGTAACCTGCTGGCGACTCT
TTACTGATAAGTGAGGGCAGGAGGGCTTCCATCAAATTAAACGCCACAAAGAAAAGCTGCACGCCGACCACCAGTTGCCA
GAACTGCGTTTGCGCGTTCCACAACACAATTTCCGCAACCACGATCAACCCGACGCAGAAGACAAAGACTTGCTTCATTT
TGCGCTTAACTTCAGCGTAGATAATGAAAGGCACGACCGAGCCAAAGGCGATTAGCATTGTCGCCAGATAGACCTTCCAG
TGTTCAGCCGCCGGGAACCCTGCATCAGCCAGTTGTCCGGGCAGGGCAACAAACGTCGACATCAGCAAAATATGCAGACA
CATAATGCCAAAGTTGAGTTTCAGCAGCCGCGGTTCCGCCAGCACTTTACTGAAACTGCCTTTCACCATTCCGGACTCAC
GATTAAGTACGTGAGTGCTACTGTTGGGCACAACCCAAATGGTCAACGCAATGCCGGTCGTTGCCAGAATAGCGATCATC
CAGAACAGCGCGTGCAGCCCAAGTTTGTGAGTGATGATCGGGCCAAGCACCATCGCAATGGCAAAGGTAATGCCAAAGCT
CACGCCGATAAACGCCATCGCTTTGGTGCGGTTTTGTTCGCGCGTGAGATCGGAAAGCAGCGCCATAACGGCGGCGGCAA
TTGCTCCAGAGCCTTGTAGCGCCCGGCCCAGAATAATTCCCCAGATGGAGTCGGAAAGCGCAGCGATAACGCTACCGGCG
GCAAACACCGCCAGCCCACCGACAATTAATGGTTTGCGACCAATGCGGTCGGAAAGCAGGCCAAACGGAATCTGAAAAAC
GGCCTGAGTCAGACCATAAATACCAATGGCAATACCGATTAATGCTTCGCTGGCACCTTGCAGAGCCATGCCGTACGTGG
TCAGAACCGGCAGAACCATAAACATGCCCAGCATGCGCAACGAGAATACGGTCCCTAAACCCCAGGTCGCGCGCCTCTCA
CCTGGCGTCATTTTATAATCGTTCATTACCACCTCTGTTTTAAATTCGCGACTAGTGTAAAGCGGCAAACGAATGGGGTA
AACATGCGGATTTTTTAGTAAATATTTCGAGAGGGATATGTTTCTAATGCTAAGAAAAAAGGTGCCGTAGCACCTTTTTA
ATAGAGAGGTTTTGTTACCACACAGCAGCCAGCAGCGTATGCGAGTCCGGTACCATAAAATCAACGGACATCATCACCGA
GAGGGCAGTGATGGCGATGATAGAGAAGCCGAACAGCTTGCGCGCCCAGATTCTGTCATCAGCAACTTTATAACCGCGCA
GAGCCATACCTAACCACCAGACGCTAACCGCCGCGGCGACCACCAGATATTTATACCCAGCGTAACCGCCAAGAGAGAGC
ATCAGCGTGGCAACGGCAAAGGCGATGATATACAGCGTGATGTGATTCTTCGCCACCGAAATGCCTTTTACCACTGGCAA
TACCGGAATGTTTGCCGCCTGGTAATCCTTAAAGCGGAAAATGGCGATGGCATAGGAGTGAGGCATCTGCCACAGGCTGA
AGATAGCCAGCAGGATCGCTGCGCCGCTATCGAACTCACCGGTTACCGCACAGTAGCCGATCACCGGCGGCGCAGCGCCG
GAGAGCGAACCAATCAACGTGCCGTAGACAGAGTGGCGTTTCATGTACAGGCTATAAACGCCGACATAAACCACAAAGCC
CATCACCCCCAGCCAGCAGGCCAGCGGATTCGCGCCAAACCACAGCAGCATAAAGCCAGCAATACCCAGCAACGTGGCGT
ACACCAGCGAGACAGCAGGAGAGATCAGGCCTTTCACCAGCACCCGATTCTTCGTCCTTTCCATCTTTCTGTCGATATCC
CTGTCGATGTAGTTGTTAAACACACAACCCGACGCCACAACCAGTGACACCCCAACCAGCGTGTAGATAAACAGGGGATA
ATCAATGCTGCCCTTTGAGGCCAGCAGGAATCCCCCAATCACCGAGATCAGGTTGCCAAAGATGATGCCTGGTTTCGTTA
CTTGCAGGTATTGCTTAAACATCATAACCGCCGCTCTTAGTGCATCATCATGTTGTAGTTGAGGTTCCACATAATCCAGA
TGGAGCCTACAACCAGGATAGCGATGATTAGCACGGTGAAGACAAACGCCGTCATGTTCCAGCCTTCATCTGATTTGGTA
TTCATGTGCAGGAAGCACACCAGATGCACCAGAACCTGTACCACTGCCATTGCCAGGATTGTTCCCAGAATTACGGCCGG

AGAGGCAGCTCCTGTCATCACCATCCAGAACGGAATCACCGTCAGAATGATCGACAGGATAAAGCCTGTCATGTAGGTTT
TTACGCTGCCATGGGACGCGCCGCTGTGATCGGTAGAATGACTCATTACATCGCCCCCATCAGATAAACAACAGTGAACA
CACAGATCCAAACCACATCCAGGAAGTGCCAGAACAGGCTCAGGCACATGATGCGGGTACGGTTAGTGCTGGTCAGGCCG
CGACGGGCGATTTGCACCATCAGCACCGCCATCCAGATAAGACCAGAAGTGACGTGCAGACCGTGCGTGCCGACCAGCGC
AAAGAACGCTGACAGGAAGCCGCTGCGATCCGGACCCATGCCGTTAACAATCAGGTGATGGAATTCATAGATTTCCATCC
CGATAAATCCGGCACCAAACAACCAGGTCAACGCCAGCCAGGAGATAACCTGGCTTTTGTTGTTTTTGTACATGGCGATA
GCCGCCATGCCGTAGGTGATGGAGCTGAACAACAGCAAGAAAGTTTCAACCAGAACGAACGGCAGTTCGAAAATGTCCTT
ACCTGTCGGGCCGCCTGCGGTGCCGTTCACCAGAACGGCATAGGTAGCAAACAAGATAGAGAACAGAATGCAGTCGCTCA
TCAGGTAGATCCAAAATCCGAAGATTTTGGTTCCGCCTGCATCGTGGTGCCCGTGTTCGTGCGCGTGGGCAGTCGCGTGC
GTCAAAGTATCAGTTGCCATTTTTCAGCCCTGCCTTAGTAATCTCATCGAAATGCTGGTTTTCCAGTTTTTCGATTTCTG
CCACCGGCACGTAGTAATCCACGTCCTCGTCGAAGCTTTTCACGATCCAGGTGATGATCATGCCTGCGAAGCCAACAATC
GCCAGCCACCAGATATGCCAGATCATGGCGAAACCGAAGATGGTGGAGAAAGCTGCAATGACGATACCTGCACCGCTGTT
TTTCGGCATATGAATTTCTTCATAGTGGTCAGGCTTTTTATACGCTTCGCCTTTCTCTTTCATTTCCCAGAATGCATCAC
GTTCGTGAACGTGCGGCACTACGGCAAAGTTATAGAACGGAGGCGGGGAAGAGGTTGCCCACTCCAGCGTACGGCCACCC
CACGGGTCGCCAGTCAGGTCACGGTTCTGGTCGCGGTCGCGAATAGAAACGTACATCTGAATAACGAGGCAGAGAATACC
CAGCGCAATCAGTACTGCACCGCTGGCTGCAATCATCAGCATGGTGTGGAACTGCGGGTCAATCTGCTGGCTCAAACGAC
GGGTCATGCCCATGAAGCCCAGCGCATACAGTGGCATAAAGGCAACGAAGAAGCCGATGATCCAGAACCAGAACGCGCGT
TTACCCCAGGTTTCGTTCAGTTTGAAACCGAACGCTTTAGGCCACCAGTAGGTCATCCCTGCGAAGCAGCCGAAGACCAC
GCCGCCGATGATCACGTTATGGAAGTGCGCAATCAGGAACAGGCTGTTATGCAGAACGAAGTCCGCGCCCGGTACGGCCA
GCAGCACGCCAGTCATCCCGCCCACCGAGAAGGTGACGATAAAACCGATGGTCCACAGCATCGCAGAATGGAACACGATG
CGGCCCTGATACATGGTGAACAGCCAGTTGAAGATCTTCACCCCGGTCGGGATGGCGATAATCATTGTGGTGATACCAAA
GAAGGCGTTTACGTTCGCGCCCGCACCCATCGTAAAGAAGTGGTGCAGCCAAACGATGAACGACAGCACGGTGATACAGA
CGGTTGCCCATACCAGCGAGGTATAACCAAACAGACGTTTACGCGAGAAGGTTGCCGCAATTTCGGAGAACACACCGAAA
ACAGGCAGGATCAGGATGTAAACTTCCGGGTGGCCCCAGGCCCAAATCAGGTTGATGTACATCATCATGTTGCCACCCAT
ATCGTTGGTAAAGAAATGGGTGCCCAGATAGCGATCCAGGGTCAACAACGCGACGGTAACCGTCAGAATTGGGAAGGAAG
CAATAATCAGTACGTTCGCGCACAGTGATGCCCAGGTAAATACTGGCATCTTGAACATGGTCATGCCCGGTGCGCGCATC
TTCAGAATGGTAACGAAGAAGTTGATACCGGTAAGCGTCGTACCTATACCGGATAGCTGGAGACTCCATATCCAGTAATC
GACACCGACTCCCGGACTGTACTCTATTCCCGATAGCGGTGGATAGGCCAGCCAGCCGGTCTGCGCAAATTCGCCCACGC
CGAGAGAAACGTTAACCAGAATCACACCAACAACGGTAAACCAGAAGCTTAAGTTGTTGAGGAACGGGAACGCAACGTCA
CGCGCGCCGATCTGCAGCGGAACCACCAGGTTCATCAGACCGATAACGAAAGGCATCGCCACGAAGAAGATCATAATCAC
GCCGTGCGCGGTAAAGATCTGATCGTAGTGGTGAGGTGGCAGGAAGCCCGCTTCGCCCGCCGAGGCAAGAGCCTGCTGGC
TACGCATCATAATGGCGTCAGCAAAACCACGCAGCAACATCACAATCGCCACGATGATATACATGATACCGAGGCGTTTA
TGGTCGACGGAGGTCAGCCACTCTTTCCACAGGTAGGTCCACTTACCGAAGTAAGTGATCAGGCCAACGAGCGCCAGACC
TCCCAAAATAATGCCAGCGATCGTAACCATGACGATAGGTTCATGGAACGGGACTGCATCAAGTGATAATTTTCCGAACA
TCTTTATTCTTCCTCAACCCCTTTAATGGGCGGATTCCGCGTGGCTCATGTCCATGCCTTCCATACCTTCGTGTGCGCTG
TGCTCACCTTCTGGCTGGGTCATGTCCATGCTCTTACCGTGAGCCATAAACTTGTTAATTACATCGGCAAACAAGTCTGG
TTTCACGTTGGAGAAATATTCCACCTGGTTGTATTCGCTAGGCGCGGCCAGTTTTTCGAACGCAGCCATGTCAGACATGG
TGTTCGGCGACTGCTTCGCTTTTGCGACCCACTGGTCGAATGCGGCGCGATCCGGTGTTGCAATAGCTTTGAACTTCATG
CCTGAGAAGCCCGGGCCGCTGTAGCTGGCGGAGATACCGTCATAAGTGCCGGGTTCGTTGGCGATCAGATGCAGGCGAGT
CTGCATACCGGCCATGGCATAAATCTGGCTACCCAGACGCGGAATGAAGAAGGAGTTCATCACGGAGTTGGAGGTCACTT
TGAAGTACACCGGAGTGTTCGCCGGGAAAGCGATTTCATTCACGGTAGCAATGCCCTGTTCCGGGTAGATGAAGAACCAT
TTCCAGTCCATGGAAACCACTTCGATGGTAATGGGCTTCTCGTCGTGTGCCAGCGGCTTGCTAGGCTCAAGAGCGTGAGT
GGTTTTCCAGGTCAGTACTGCAAGGAAGATGATGATTAAGATAGGTACCGTCCAGACCACAGCTTCCACTTTATTGGAGT
GTGACCAGTTCGGGCTGTACTTAGCATCTTTATTGCTCGCACGGTACTTCCAGGCGAAACCAACAGCCATCAAGATTGCG
GGAATAACGACAATCAACATCAGGCCAAATGCCGTCAGTATCAGTGAACGTTGCTCCAGACCAATCTGTCCTTTGGGATC
TAACAGCGCAGAATTACAGCCACTGAGCAATACAGTGCCTGCAAATAATGACAACCATCCCAAACTTTTATTGTATTTCC
TGAGTCTCATTTAACGACCTCAATTCCACGGGATCTGGTGGCGTTTAAAGTGTGTGGGCATTTTACGGGAAGGTTACATT
ACTGTAAACATGATTAAATTCCTGTTACTTGGATTTGGCTGCCAGGTCACATATGTTGCAAAACATATCAGGTTGATTTT
AAGAAGCTGTTGCAAAAGGGCGTTATAACAAAAGGGGAGTGCTGAAGGAGTCTGGGCGGGCAATTGGTATAACCAATGTG
AAATAAAACAATTATTTAACAAATAATTATCATTAGAGTGACAACTGGGTGATGAGAAAATAAAGACTCATTAGCTGAAT
CGTGTAAGAAAAATAGAATTTATAAATGGCGCCAATAATTTCCAAACGCTAAGCCGCACAAAAGAACAAATATTAATAAG
CGATACTGATGTTTATTTAGCCGTAATAATTACGGCGAGTGATTACTACAGCTAAATAATATTTACAGATTACGTCAGAT
GCGTTTTTCGTAGCGCCAGATAATCCAGCAAACCACCAAGCACGACGCCAGAAAGGGCGACTAAAACCCCGACTTCCAGC
AGAGCAGGCAGGAAAGAGAAGTGCGTCAAATCCAGCGCGTCCATCGTCAATAGCAGTAACCACACGGCCAACAGGCTGAC
GCCCGCCGCCAGTGTCCACATGGCAAAGGCATAACCTGCCGGATATGCGGTACGGGAGATAAAGTTGTCATTTACTCGTG
TATATTCAAGCGTCTGGCGGCAAACCAGCAGCAAAATAAGCCCTGGTACGGCAGCGGCGACGGAGAATAGATAGAATGTC

GACCAGCCGTGTGCTTCAACAAACCAACCCGCCACGGGGCCGACATAAACTCGCCCTACAGCAGAAAGCGCTGAGAGCAG
GGCAAATTGAGTAGCGGAAAATGACTTATTACATAGCGTCATTAACAGCGCGACAAAGGCTGATGTGCCCATCCCGCCAC
AGAGGTTTTCGAAAAAGACGGCTGCGCCCATGCTGTAGAGATGCTTATCAGTAATCGACAGCAGCCAGTAACCGGCGTTA
GACGCACCTTGTAAAATGCCGAAAATCAGCAGTGCCCGGAACAGTGACAGGCGCTGCATCAAAATCCCACCGTACAATGC
GCCAACAATGGTCGCTAGTAAGCCAAGCGTTTTGTTAACCACGCCTACTTCACCCGCATCAAACCCGACGCCGCGAATCA
AAAACGTGGTTGTCAGGCTCATGGCGAATGCGTCGCCCAGCTTATACAGCACGATAAGAAGCAAAATAAGCCAGGCATTA
TTGCGACCAAAGAAATCTCGCAGAGGTGCAACAACCGCTTGTTCCAGCGTTTTGGGCACAGGAATGGTGTCGGTTGGTTC
TGGTGCAAGCAACGTCGCGATAATACAGGGGATCAACAGTGCCGCCATTAACCAGTACATGCCCTGCCAGCCCAGCCATT
TATCTGCCAGCCACAGGGCTAGGCCGCCGGAAACCAGCATCCCTAAACGGTAACCCAGCACGCTGATTGCCGCGCCCGCA
CCACGTTCTTCTGCCGGAAGCACATCGGTTTTCCACGCATCGAAGACTATATCCTGGGAAGCAGAGCAAAAAGCGATCAC
CACTGCCAGCGCCGCCATCCAGCGGAGTTGGGTGCCTGGTTCGAGAAAACCCATCGCCGCAATGGCGACTAATAACAGGA
TTTGCGTGGCGAGCAGCCAACCGCGCCGCCGCCCAAAAAATGGAGGCGTGTAGCGGTCCATCAGCGGTGACCAGAGGAAT
TTAAAAACGTAAGCCTGGCCTACCAGAGAGAAGAAACCAATGGTTTTGAGATCGATATTCTCGACCGTCATCCAGGCCTG
TAAGGTACCGGATGTCAGGGCGAGCGGTAGCCCGGAAGCAAAACCCAGGATCAGCAATATGGCTGAACGCGGCTGTTGAA
AAATACGTAAATATTGACTGGACATGGGCGTCTACAGGCCCGGCTTGCACCGGGCCAGAGGGCAGAAATTAACGCGCGTT
CTGCTTGATGAATTCGTGGATGCTGGTGTCCTGAGACATATCAGCGATGGTATCGGTCAGCACACTGTTAACCGCATCGG
CGATATTTTTGTTGGAGGCCTGGAACGCACCTTCAACGTTGTAGCTGGCACGATAGTTTTTGGTCATTTTGTTGCCATTC
TGCGCGGTAGCGATGATGGCGATATCCGCTTTGGTCGCGATGTTGTAGCGCACGTTGCCCTGGGACACGTCAGCATACAG
TTGGCTAACGATGATTTGCAGATTAACCGGGCCATTCGGACCAACCATGTAACCACGCGCGGTCATCTGTTTTTCCAGCA
CTTCTTGCAGCAGGAAACGCAGATCGCGGGAGGCGGTCAGGGTAACGATTTGATTATCGCGGGTGACTTTTGCCAGCGCC
TGATCGGTACGCTGATCGGCACCATTAATGCTTACGGTGACGCCCATCAGGCTTGGATCCTGCTGTGGCAGTGTAATCGT
CGGGGAAACTTCAATAGTTGTTGGCGGTTTTGCGCATCCTGCAAGCATAAACAGAGCAACTAACGGGAAGAGGATTTTTT
TGAACATGTTCGGGCTCTCAGAGACTCTTAAGCGTGTTTGGTAAAAATTCCCGCCATCATAACATTGCCAACGGCGAGGG
GAAGTGGGTAAGGCATGTAAATTCATCATGTTGACGAAATAATCGCCCCTGGTAAAAGAAACACTGATGCGAGGCCTGTG
TTTCAATCTTTAAATCAGTAAACTTCATACGCTTGACGGAAAAACCAGGACGAAACCTAAATATTTGTTGTTAAGCTGCA
ATGGAAACGGTAAAAGCGGCTAGTATTTAAAGGGATGGATGACATCTCAGCGTTGTCGGAGGAGATATTTCATGATGATA
CGTGAGCGGATAGAAGAAAAATTAAGGGCGGCGTTCCAACCCGTATTCCTCGAAGTAGTGGATGAAAGCTATCGTCACAA
TGTCCCAGCCGGCTCTGAAAGCCATTTTAAAGTTGTGCTGGTCAGCGATCGTTTTACGGGTGAACGTTTTCTGAATCGTC
ATCGAATGATTTACAGTACTTTAGCGGAGGAACTCTCTACTACCGTTCATGCGCTGGCTCTGCATACTTACACTATTAAG
GAGTGGGAAGGGTTGCAGGACACCGTCTTTGCCTCTCCTCCCTGTCGTGGAGCAGGAAGCATCGCGTAAAAACGCATTTG
CAACTGTCGGCGCTTTTCCAGTATGTTGCTAAAGATTTTATGAAAAACGGCCTGCGGGCCGTTTTGTTTTGTCTGGATTT
TGCGCTTTTTGCCCAGCATTCAGACGAAAATTGCCCGGGAATTGTGAAAAAATACGCGACAGCGCGCAATAACCGTTCTC
GACTCATAAAAGTGATGCCGCTATAATGCCGCGTCCTATTTGAATGCTTTCGGGATGATTCTGGTAACAGGGAATGTGAT
TGATTATAAGAACATCCCGGTTCCGCGAAGCCAACAACCTGTGCTTGCGGGGTAAGAGTTGACCGAGCACTGTGATTTTT
TGAGGTAACAAGATGCAAGTTTCAGTTGAAACCACTCAAGGCCTTGGCCGCCGTGTAACGATTACTATCGCTGCTGACAG
CATCGAGACCGCTGTTAAAAGCGAGCTGGTCAACGTTGCGAAAAAAGTACGTATTGACGGCTTCCGCAAAGGCAAAGTGC
CAATGAATATCGTTGCTCAGCGTTATGGCGCGTCTGTACGCCAGGACGTTCTGGGTGACCTGATGAGCCGTAACTTCATT
GACGCCATCATTAAAGAAAAAATCAATCCGGCTGGCGCACCGACTTATGTTCCGGGCGAATACAAGCTGGGTGAAGACTT
CACTTACTCTGTAGAGTTTGAAGTTTATCCGGAAGTTGAACTGCAGGGTCTGGAAGCGATCGAAGTTGAAAAACCGATCG
TTGAAGTGACCGACGCTGACGTTGACGGCATGCTGGATACTCTGCGTAAACAGCAGGCGACCTGGAAAGAAAAAGACGGC
GCTGTTGAAGCAGAAGACCGCGTAACCATCGACTTCACCGGTTCTGTAGACGGCGAAGAGTTCGAAGGCGGTAAAGCGTC
TGATTTCGTACTGGCGATGGGCCAGGGTCGTATGATCCCGGGCTTTGAAGACGGTATCAAAGGCCACAAAGCTGGCGAAG
AGTTCACCATCGACGTGACCTTCCCGGAAGAATACCACGCAGAAAACCTGAAAGGTAAAGCAGCGAAATTCGCTATCAAC
CTGAAGAAAGTTGAAGAGCGTGAACTGCCGGAACTGACTGCAGAATTCATCAAACGTTTCGGCGTTGAAGATGGTTCCGT
AGAAGGTCTGCGCGCTGAAGTGCGTAAAAACATGGAGCGCGAGCTGAAGAGCGCCATCCGTAACCGCGTTAAGTCTCAGG
CGATCGAAGGTCTGGTAAAAGCTAACGACATCGACGTACCGGCTGCGCTGATCGACAGCGAAATCGACGTTCTGCGTCGC
CAGGCTGCACAGCGTTTCGGTGGCAACGAAAAACAAGCTCTGGAACTGCCGCGCGAACTGTTCGAAGAACAGGCTAAACG
CCGCGTAGTTGTTGGCCTGCTGCTGGGCGAAGTTATCCGCACCAACGAGCTGAAAGCTGACGAAGAGCGCGTGAAAGGCC
TGATCGAAGAGATGGCTTCTGCGTACGAAGATCCGAAAGAAGTTATCGAGTTCTACAGCAAAAACAAAGAACTGATGGAC
AACATGCGCAATGTTGCTCTGGAAGAACAGGCTGTTGAAGCTGTACTGGCGAAAGCGAAAGTGACTGAAAAAGAAACCAC
TTTCAACGAGCTGATGAACCAGCAGGCGTAATTTACGCAGCATAACGCGCTAAATTCGCACAAAGGCCCGTCACCGCCAG
GTGGTGGGCTTTTTTTTGTCATGAATTTTGCATGGAACCGTGCGAAAAGCCTCTTTCGGTGTTAGCGTAACAACAAAAGA
TTGTTATGCTTGAAATATGGTGATGCCGTACCCATAACACAGGGACTAGCTGATAATCCGTCCATAAGGTTACAATCGGT
ACAGCAGGTTTTTTCAATTTTATCCAGGAGACGGAAATGTCATACAGCGGCGAACGAGATAACTTTGCACCCCATATGGC
GCTGGTGCCGATGGTCATTGAACAGACCTCACGCGGTGAGCGCTCTTTTGATATCTATTCTCGTCTACTTAAGGAACGCG
TCATTTTTCTGACTGGCCAGGTTGAAGACCACATGGCTAACCTGATTGTGGCGCAGATGCTGTTCCTGGAAGCGGAAAAC

CCAGAAAAAGATATCTATCTGTACATTAACTCCCCAGGCGGGGTGATCACTGCCGGGATGTCTATCTATGACACCATGCA
GTTTATCAAGCCTGATGTCAGCACCATCTGTATGGGCCAGGCGGCCTCGATGGGCGCTTTCTTGCTGACCGCAGGGGCAA
AAGGTAAACGTTTTTGCCTGCCGAATTCGCGCGTGATGATTCACCAACCGTTGGGCGGCTACCAGGGCCAGGCGACCGAT
ATCGAAATTCATGCCCGTGAAATTCTGAAAGTTAAAGGGCGCATGAATGAACTTATGGCGCTTCATACGGGTCAATCATT
AGAACAGATTGAACGTGATACCGAGCGCGATCGCTTCCTTTCCGCCCCTGAAGCGGTGGAATACGGTCTGGTCGATTCGA
TTCTGACCCATCGTAATTGATGCCAGAGGCGCAACTGTGCCGCTATACTTATCCAGGGCGGCACAACGCTGTAAGCGGCT
TGCGCCTGAGAATGGCATTTGCGTCGTCGTGTGCGGCACAAAGAACAAAGAAGAGGTTTTGACCCATGACAGATAAACGC
AAAGATGGCTCAGGCAAATTGCTGTATTGCTCTTTTTGCGGCAAAAGCCAGCATGAAGTGCGCAAGCTGATTGCCGGTCC
ATCCGTGTATATCTGCGACGAATGTGTTGATTTATGTAACGACATCATTCGCGAAGAGATTAAAGAAGTTGCACCGCATC
GTGAACGCAGTGCGCTACCGACGCCGCATGAAATTCGCAACCACCTGGACGATTACGTTATCGGCCAGGAACAGGCGAAA
AAAGTGCTGGCGGTCGCGGTATACAACCATTACAAACGTCTGCGCAACGGCGATACCAGCAATGGCGTCGAGTTGGGCAA
AAGTAACATTCTGCTGATCGGTCCGACCGGTTCCGGTAAAACGCTGCTGGCTGAAACGCTGGCGCGCCTGCTGGATGTTC
CGTTCACCATGGCCGACGCGACTACACTGACCGAAGCCGGTTATGTGGGTGAAGACGTTGAAAACATCATTCAGAAGCTG
TTGCAGAAATGCGACTACGATGTCCAGAAAGCACAGCGTGGTATTGTCTACATCGATGAAATCGACAAGATTTCTCGTAA
GTCAGACAACCCGTCCATTACCCGAGACGTTTCCGGTGAAGGCGTACAGCAGGCACTGTTGAAACTGATCGAAGGTACGG
TAGCTGCTGTTCCACCGCAAGGTGGGCGTAAACATCCGCAGCAGGAATTCTTGCAGGTTGATACCTCTAAGATCCTGTTT
ATTTGTGGCGGTGCGTTTGCCGGTCTGGATAAAGTGATTTCCCACCGTGTAGAAACCGGCTCCGGCATTGGTTTTGGCGC
GACGGTAAAAGCGAAGTCCGACAAAGCAAGCGAAGGCGAGCTGCTGGCGCAGGTTGAACCGGAAGATCTGATCAAGTTTG
GTCTTATCCCTGAGTTTATTGGTCGTCTGCCGGTTGTCGCAACGTTGAATGAACTGAGCGAAGAAGCTCTGATTCAGATC
CTCAAAGAGCCGAAAAACGCCCTGACCAAGCAGTATCAGGCGCTGTTTAATCTGGAAGGCGTGGATCTGGAATTCCGTGA
CGAGGCGCTGGATGCTATCGCTAAGAAAGCGATGGCGCGTAAAACCGGTGCCCGTGGCCTGCGTTCCATCGTAGAAGCCG
CACTGCTCGATACCATGTACGATCTGCCGTCCATGGAAGACGTCGAAAAAGTGGTTATCGACGAGTCGGTAATTGATGGT
CAAAGCAAACCGTTGCTGATTTATGGCAAGCCGGAAGCGCAACAGGCATCTGGTGAATAATTAACCATTCCCATACAATT
AGTTAACCAAAAAGGGGGGATTTTATCTCCCCTTTAATTTTTCCTCTATTCTCGGCGTTGAATGTGGGGGAAACATCCCC
ATATACTGACGTACATGTTAATAGATGGCGTGAAGCACAGTCGTGTCATCTGATTACCTGGCGGAAATTAAACTAAGAGA
GAGCTCTATGAATCCTGAGCGTTCTGAACGCATTGAAATCCCCGTATTGCCGCTGCGCGATGTGGTGGTTTATCCGCACA
TGGTCATCCCCTTATTTGTCGGGCGGGAAAAATCTATCCGTTGTCTGGAAGCGGCGATGGACCATGATAAAAAAATTATG
CTGGTCGCGCAGAAAGAAGCTTCAACGGATGAGCCGGGTGTAAACGATCTTTTCACCGTCGGGACCGTGGCCTCTATATT
GCAGATGCTGAAACTGCCTGACGGCACCGTCAAAGTGCTGGTCGAGGGGTTACAGCGCGCGCGTATTTCTGCGCTCTCTG
ACAATGGCGAACACTTTTCTGCGAAGGCGGAGTATCTGGAGTCGCCGACCATTGATGAGCGGGAACAGGAAGTGCTGGTG
CGTACTGCAATCAGCCAGTTCGAAGGCTACATCAAGCTGAACAAAAAAATCCCACCAGAAGTGCTGACGTCGCTGAATAG
CATCGACGATCCGGCGCGTCTGGCGGATACCATTGCTGCACATATGCCGCTGAAACTGGCTGACAAACAGTCTGTTCTGG
AGATGTCCGACGTTAACGAACGTCTGGAATATCTGATGGCAATGATGGAATCGGAAATCGATCTGCTGCAGGTTGAGAAA
CGCATTCGCAACCGCGTTAAAAAGCAGATGGAGAAATCCCAGCGTGAGTACTATCTGAACGAGCAAATGAAAGCTATTCA
GAAAGAACTCGGTGAAATGGACGACGCGCCGGACGAAAACGAAGCCCTGAAGCGCAAAATCGACGCGGCGAAGATGCCGA
AAGAGGCAAAAGAGAAAGCGGAAGCAGAGTTGCAGAAGCTGAAAATGATGTCTCCGATGTCGGCAGAAGCGACCGTAGTG
CGTGGTTATATCGACTGGATGGTACAGGTGCCGTGGAATGCGCGTAGCAAGGTCAAAAAAGACCTGCGTCAGGCGCAGGA
AATCCTTGATACCGACCATTATGGTCTGGAGCGCGTGAAAGATCGAATCCTTGAGTATCTTGCGGTTCAAAGCCGTGTCA
ACAAAATCAAGGGACCGATCCTCTGCCTGGTAGGGCCGCCGGGGGTAGGTAAAACCTCTCTTGGTCAGTCCATTGCCAAA
GCCACCGGGCGTAAATATGTCCGTATGGCGCTGGGCGGCGTGCGTGATGAAGCGGAAATCCGTGGTCACCGCCGTACTTA
CATCGGTTCTATGCCGGGTAAACTGATCCAGAAAATGGCGAAAGTGGGCGTGAAAAACCCGCTGTTCCTGCTCGATGAGA
TCGACAAAATGTCTTCTGACATGCGTGGCGATCCGGCCTCTGCACTGCTTGAAGTGCTGGATCCAGAGCAGAACGTAGCG
TTCAGCGACCACTACCTGGAAGTGGATTACGATCTCAGCGACGTGATGTTTGTCGCGACGTCGAACTCCATGAACATTCC
GGCACCGCTGCTGGATCGTATGGAAGTGATTCGCCTCTCCGGTTATACCGAAGATGAAAAACTGAACATCGCCAAACGTC
ACCTGCTGCCGAAGCAGATTGAACGTAATGCACTGAAAAAAGGTGAGCTGACCGTCGACGATAGCGCCATTATCGGCATT
ATTCGTTACTACACCCGTGAGGCGGGCGTGCGTGGTCTGGAGCGTGAAATCTCCAAACTGTGTCGCAAAGCGGTTAAGCA
GTTACTGCTCGATAAGTCATTAAAACATATCGAAATTAACGGCGATAACCTGCATGACTATCTCGGTGTTCAGCGTTTCG
ACTATGGTCGCGCGGATAACGAAAACCGTGTCGGTCAGGTAACCGGTCTGGCGTGGACGGAAGTGGGCGGTGACTTGCTG
ACCATTGAAACCGCATGTGTTCCGGGTAAAGGCAAACTGACCTATACCGGTTCGCTCGGCGAAGTGATGCAGGAGTCCAT
TCAGGCGGCGTTAACGGTGGTTCGTGCGCGTGCGGAAAAACTGGGGATCAACCCTGATTTTTACGAAAAACGTGACATCC
ACGTCCACGTACCGGAAGGTGCGACGCCGAAAGATGGTCCGAGTGCCGGTATTGCTATGTGCACCGCGCTGGTTTCTTGC
CTGACCGGTAACCCGGTTCGTGCCGATGTGGCAATGACCGGTGAGATCACTCTGCGTGGTCAGGTACTGCCGATCGGTGG
TTTGAAAGAAAAACTCCTGGCAGCGCATCGCGGCGGGATTAAAACAGTGCTAATTCCGTTCGAAAATAAACGCGATCTGG
AAGAGATTCCTGACAACGTAATTGCCGATCTGGACATTCATCCTGTGAAGCGCATTGAGGAAGTTCTGACTCTGGCGCTG
CAAAATGAACCGTCTGGTATGCAGGTTGTGACTGCAAAATAGTGACCTCGCGCAAAATGCACTAATAAAAACAGGGCTGG
CAGGCTAATTCGGGCTTGCCAGCCTTTTTTTGTCTCGCTAAGTTAGATGGCGGATCGGGCTTGCCCTTATTAAGGGGTGT

TGTAAGGGGATGGCTGGCCTGATATAACTGCTGCGCGTTCGTACCTTGAAGGATTCAAGTGCGATATAAATTATAAAGAG
GAAGAGAAGAGTGAATAAATCTCAATTGATCGACAAGATTGCTGCAGGGGCTGATATCTCTAAAGCTGCGGCTGGCCGTG
CGTTAGATGCTATTATTGCTTCCGTAACTGAATCTCTGAAAGAAGGGGATGATGTAGCACTGGTAGGTTTTGGTACTTTT
GCCGTTAAAGAGCGTGCTGCCCGTACTGGCCGCAACCCGCAGACCGGTAAAGAGATCACCATCGCTGCTGCTAAAGTACC
GAGCTTCCGTGCAGGTAAAGCACTGAAAGACGCGGTAAACTAAGCGTTGTCCCCAGTGGGGATGTGACGAAGTTCAAGGG
CGCATCTACTGATGTGCCTTTTTTATTTGTATTCGGTGACTTTCTGCGTCTTGTGGGCTGACAATTGCCCCCGTTTCTTG
TCACAATAGGCCTTTGCGCGCATCGATACGTTGCGTGAGGTACACAGTCATCTACAGCGGAGTGTTGTTACACCATGATG
GACAGCTTACGCACGGCTGCAAACAGTCTCGTGCTCAAGATTATTTTCGGTATCATTATCGTGTCGTTCATATTGACCGG
CGTGAGTGGTTACCTGATTGGCGGAGGCAATAACTACGCCGCAAAAGTGAATGACCAGGAAATCAGCCGTGGGCAATTCG
AGAACGCCTTCAACAGCGAGCGTAATCGCATGCAGCAACAGCTGGGCGATCAATACTCCGAGCTGGCAGCGAACGAAGGC
TATATGAAAACCCTGCGTCAACAGGTGCTGAATCGTCTGATCGACGAGGCGCTGCTGGATCAGTACGCACGTGAGCTGAA
ACTGGGTATCAGCGATGAGCAGGTTAAACAGGCGATTTTCGCGACCCCAGCCTTCCAGGTTGACGGCAAATTTGATAACA
GCCGCTATAACGGTATCCTCAACCAGATGGGGATGACCGCCGATCAGTACGCCCAGGCGCTGCGTAACCAGCTCACTACC
CAACAGCTGATTAACGGCGTTGCCGGTACCGATTTTATGCTGAAAGGTGAAACCGACGAGCTGGCGGCACTGGTCGCGCA
ACAACGCGTGGTGCGTGAGGCGACTATCGATGTTAACGCGCTGGCGGCGAAGCAGCCTGTGACCGAACAGGAGATTGCCA
GCTACTACGAACAAAACAAAAACAATTTCATGACGCCGGAACAATTCCGCGTGAGTTACATCAAGCTGGATGCCGCAACG
ATGCAGCAACCGGTTAGCGATGCGGATATCCAGAGCTACTACGACCAGCATCAGGATCAATTCACCCAGCCGCAGCGTAC
CCGCTACAGCATCATCCAGACCAAAACTGAAGATGAAGCGAAAGCGGTACTTGATGAGCTGAATAAAGGCGGTGATTTTG
CTGCATTAGCCAAAGAAAAATCTGCCGATATTATCTCTGCTCGTAACGGCGGCGATATGGGTTGGTTAGAAGATGCCACT
ATCCCGGACGAACTGAAAAATGCTGGTCTGAAAGAAAAAGGCCAACTGTCTGGTGTCATCAAATCTTCGGTCGGTTTCCT
GATTGTACGTCTGGACGACATTCAGCCAGCGAAAGTGAAATCGTTAGACGAAGTACGTGACGACATTGCGGCGAAAGTGA
AACACGAAAAAGCCCTCGATGCGTACTACGCGCTGCAGCAGAAAGTGAGCGATGCGGCAAGCAACGACACCGAGTCTCTG
GCCGGTGCAGAGCAAGCTGCCGGCGTTAAAGCCACTCAGACGGGTTGGTTCAGCAAAGATAACCTGCCGGAAGAGTTGAA
CTTCAAGCCGGTTGCCGACGCTATCTTTAACGGCGGTCTGGTAGGTGAAAACGGCGCGCCGGGCATCAACTCTGACATCA
TCACCGTAGACGGCGACCGCGCATTCGTGCTGCGCATCAGCGAGCACAAACCGGAAGCGGTGAAACCGTTGGCAGATGTT
CAGGAACAAGTTAAGGCATTGGTTCAGCACAACAAAGCTGAACAACAGGCGAAAGTGGATGCTGAGAAACTGCTGGTTGA
TTTGAAAGCCGGCAAAGGTGCGGAAGCTATGCAGGCTGCCGGTCTGAAATTTGGCGAGCCGAAAACCTTAAGCCGTTCCG
GTCGTGACCCGATTAGCCAGGCGGCGTTTGCACTGCCACTGCCAGCGAAAGACAAACCGAGCTACGGTATGGCGACCGAT
ATGCAAGGTAATGTGGTTCTGCTGGCGCTGGATGAAGTGAAACAAGGTTCAATGCCGGAAGATCAGAAAAAAGCGATGGT
GCAGGGTATCACCCAGAACAACGCACAAATCGTCTTTGAAGCTCTGATGAGTAACCTGCGTAAAGAGGCGAAAATCAAAA
TTGGCGATGCGCTGGAACAGCAATAATCCTGAAGCCGCCTCGCAAAAAAATGCGTTGCAGCTGTAACAAGAAAAGGTCGC
TTTCGCGGCCTTTTCCATTTCTGAACATTGCCATTTGTTTACTGTTTTCACTGCCGTTAAGGTGATTCCACTGTTAACAA
ACAAGGAGAAAACAGTATGAAACACGGAATTAAAGCACTGCTCATTACCCTGTCCCTGGCCTGTGCCGGAATGTCTCATA
GCGCGCTGGCGGCAGCTTCTGTGGCGAAACCGACGGCGGTAGAAACCAAAGCGGAAGCTCCTGCAGCACAAAGTAAAGCA
GCAGTACCGGCGAAAGCCAGTGACGAAGAAGGCACCCGGGTCAGCATTAATAATGCCAGCGCGGAAGAGCTAGCCCGCGC
GATGAATGGCGTTGGCCTGAAGAAAGCGCAGGCGATTGTCAGTTATCGCGAAGAGTACGGTCCGTTTAAAACTGTGGAGG
ATCTAAAGCAGGTGCCGGGGATGGGCAATTCGCTGGTGGAACGTAATCTGGCGGTATTAACCCTGTAATTAATTTGCATA
GTGGCAATTTTTTGCCAGACTGAAGAGGTCATACCAGTTATGACCTCTGTACTTATAACAACAACGTAAGGTTATTGCGC
TATGCAAACACAAATCAAAGTTCGTGGATATCATCTCGACGTTTACCAGCACGTCAACAACGCCCGCTACCTTGAATTTC
TCGAAGAAGCCCGCTGGGATGGGTTGGAAAATAGCGACAGTTTTCAGTGGATGACGGCCCATAACATCGCCTTCGTCGTG
GTCAATATCAATATTAACTATCGTCGCCCAGCGGTATTAAGTGACCTGTTAACTATTACCAGTCAGTTGCAGCAATTAAA
CGGTAAAAGCGGCATCTTAAGCCAGGTCATTACACTGGAGCCGGAAGGGCAGGTGGTAGCGGATGCGCTTATTACGTTTG
TTTGTATTGATCTTAAAACGCAGAAAGCATTAGCTCTGGAAGGGGAATTGCGCGAAAAGCTGGAGCAGATGGTTAAGTAA
ACGTTTTGTGGTGCCGGATGCTCAAGCCGCATCCGGCGACACCCGGAATAATTACCTCAACCCGGTTTTCTGCTTCATCG
CTGCCATCACCGTCGGTTTATCGGCCAGATAATGATTCAAACCGTTGGCGCGTAAATTACATGCCGCACAATGACCGCAA
CCGTCGCCTTTAAAGCCGTTATAGCAGGTCAACGTTTCGTTACGGACTAAATCCAGTTTGCCGTAATAATCTGCCAGCGC
CCAGGTTTCCGCTTTATCAATCCACATCAGCGGCGTTTCAAAACGAATATCTTTCGCCATGCCCAAACTGACGGCATGGT
TTAGTGCTTTCACAAACTCATCGCGGCAATCCGGGTAGCCGGAGAAATCCGTTTCGCAGACGCCAGTAATTACGGCTTCT
GCTTTTACCTGATACGCATATATTGCCGCCAGCGTCAGGAACAAAATATTACGCCCTGGGACAAACGTATTCGGGATACC
ATCGGCTTCAGGTTCATAATCAGGCACCGGAATGCTGTCACGCGTCAGGCTACTGACCGCCAGCTCGTTGAGCAGGGTGA
CATCCAGCACCTTATGCGCGCGTGCCCCCAGTTTCAGCGCCAGTTCGCGTGCCACGTCGATTTCTGCGCGATGCCGCTGA
CCGTAATCGAACGTCACGCAATGGACTTCATCATATTGTTGTAATGCCTGCACCAGACAGGTGGTGGAATCCTGACCTCC
ACTGAACACAACGACAGCACGTTTCATAGATAATCCTGCTTGAACAATAAAAGCGTTATGGTAACGCCTGCGATTAACCC
GGACCAGCTATTCATTGCGATGGCGCGGGAAGCCAGGCTTCGGTAAATTCAAACCAGCCGCGCGGTGTCAGTCGCACACC
GTTCACGCCGGGAGGGGCACTAATGCGATAGTGATAGTTGAACAGCGGCGTCAGCGTCGCATCTGTCATTAACTGGCTAA
AAACCGCTTTCAGGGCATTAAATCGGTTTTCTTCATCAGGCATTATTTGCACGGCATCCAGTGTCGATTGTAGATGTGCG

TATGCTGGAGCGTCGAAAACATGTGGCCACAGCGGATCGCAGCGCAGCCATTGCTCCAGAGTATATTCCGGTGCTTCGCC
AATTAATCTGTCGCCCATCATGAGGTCTGCGTGTGCCTGTAGGGTCGTGTCGTCCCAGTTTTTTGCGTTATGAAAAATAA
TTGTGAGTTCACAGCCTTCCGCTGCCAGTGTCGCCTGTAGGCGTTCTGCCATGGTATGAAGTTCTATCGGTAGGTGATAA
ACCAGCGTCAAGGTTTTCGGTAGTTTGACTTCATCCGGTACCTGCCAATGCGGAATAGTCCAGCCTGGCAGTAATGCATG
ACTGGCGGTGATCAGGTTTTCTCCGACTTCTAACGTTTGTAATAAACCGGATTGATGAATAATGGAGATCACTTTTCGCG
CCTGCCAGAGGGAGAGTCGGGGACTTTTGCGCAACGTCAAATAGCAAAAACCTAAACTGATGCCGCTACTGACCTGGCTG
ACCCGTTGCAGCTCCTCCGGTTTGCCGATGGTGATTTGCACGGGATGCCGACAACTGGTTCCCAAATCTTTTTCGAAAAG
CGGCGGAGTTATCCAGTACTCAACCGCTTTAAGCAGCGGATGACGTAAATGGTAATAATCATGGCTTTCCAGGCGCACCA
GCTCTGCTGTGAATTGTGTTAAGCGAAAAGGACCCGTGCCGATCAGTGGGAATTGCGGATGCGCCAGATGGCTGCAATAG
CTCGCCAGCCGGTGCGCAAGCCAGTAATCAGGGCGATGTAAAAAGAAGGTCAGACACTGCGGATGGGTGACTTCAATACG
CTTCACGCTAATAAATAATTGATCCAGTGCTGGCAGTTGTAACAGCATCAATAATCGCTGGTGTAAGTGTGAGGCTTTTA
CTGCATCGCCGTTATGCCAGTGTAGGGTTGAACGAAGATAAAAGTCCCAGCGTAACCCGTCAGTAGAGGTTTCCCAGTGA
TGCGCTAAATCGCCAATCGGGCGCTGAGTATTATTATCGAAGCGGGTCAGGCCGGAAAATATCTGCCCGGCGAGATGCTG
CTCGGCACGGCCGGGCAAAAAGCCTGGTTGTAGCGGTTCGAGCGGGCGATAGTAGGGAATACGCAACGTGGGTGTATCGT
TTTGCCATTGTCCGCCCATAAACGGCTGTAACAGAGTGCGCAGCTCACCTGGGGCCAGTTGCGCCAGCTCCAGCACATCT
TGCTGCTTTCCGGTTTCCAGTGCCTGTTCCATCATCGCATTGCGTAGCGATTCCGGCGTGACCAGAAAGCGTAATTGTCC
GCGCTTTCCGCGTCCTGACTGCGCCTGCCACTCCAGCCATCCCGCCTCCTGTGCCTGACGCAACAGCGTACGAACATGGC
GTTCGCTGCAAAAACAGCGTTCGGCCAGTTCGCTGACGGTGACGGTTTGCGGCTTTCCGGCGGAAGGTTGCCACAGACGT
TGATACTGGTTAAGACGGTTGAGCAATCGCATATAAACCCGGAACAATATTATTTAACTATTCACTATTACTTCCGTATA
TATCAGGTGATACTCAATCACCATTAACCGTGTCACAGAGTGGAGAAAGAAATGGCTCGTCTGGCAGCATTTGATATGGA
TGGCACTTTATTGATGCCCGACCATCATTTAGGTGAGAAAACCCTCTCTACTTTGGCGCGACTGCGTGAACGCGACATTA
CCCTCACTTTTGCCACGGGGCGTCATGCGCTGGAGATGCAGCATATTCTCGGGGCGCTATCGCTGGATGCGTATTTGATT
ACCGGCAACGGAACGCGCGTGCATTCTCTGGAAGGTGAACTTTTACATCGTGATGATTTACCTGCGGATGTCGCGGAGCT
GGTGCTGTATCAGCAATGGGATACCCGAGCCAGCATGCATATCTTCAATGACGACGGTTGGTTTACCGGGAAAGAGATCC
CTGCGTTGTTGCAGGCATTTGTCTATAGCGGTTTTCGTTATCAGATAATCGATGTCAAAAAAATGCCACTCGGCAGCGTC
ACCAAGATCTGCTTCTGTGGCGATCACGACGATCTTACACGCTTGCAGATCCAGCTATACGAAGCATTAGGCGAGCGTGC
ACATTTGTGTTTTTCCGCCACGGATTGCCTCGAAGTGCTGCCGGTGGGCTGCAATAAAGGCGCTGCATTGACGGTGCTGA
CCCAACATTTAGGTTTATCGTTGCGCGATTGCATGGCCTTTGGTGATGCGATGAACGATCGCGAAATGTTAGTCAGCGTC
GGTAGCGGATTTATTATGGGCAATGCGATGCCGCAACTGCGCGCGGAGCTCCCGCATTTACCGGTGATTGGACATTGCCG
AAATCAGGCTGTCTCTCACTATTTGACGCACTGGCTGGACTATCCACATCTACCTTATTCCCCCGAATAACGAGATCCCT
TCCAGCACCGGGCAATTGCCCGGTTTTTTTTGCGTTGAATTTGTCATTTTGTGCCGTGGTGTTTAAACCGCACAGAATAA
ATTGTCGTGATTTCACCTTTAAAATAAAATTAAAAGAGAAAAAAATTCTCTGTGGAAGGGCTATGTTAGATAAAATTGAC
CGTAAGCTGCTGGCCTTACTGCAGCAGGATTGCACCCTCTCTTTGCAGGCACTGGCTGAAGCCGTTAATCTGACAACCAC
CCCTTGCTGGAAGCGCCTGAAACGGCTGGAGGACGACGGTATCCTTATCGGCAAAGTCGCCCTGCTGGATCCGGAAAAAA
TAGGCCTCGGCCTGACCGCTTTTGTGCTGATAAAAACGCAACATCACAGCAGCGAATGGTATTGCCGCTTTGTCACGGTG
GTTACCGAAATGCCAGAAGTGCTGGGGTTCTGGCGCATGGCTGGTGAATACGATTATCTGATGCGCGTCCAGGTTGCCGA
CATGAAACGCTACGACGAGTTTTATAAGCGTCTGGTAAACAGCGTGCCGGGGCTGTCGGACGTCACTTCCAGCTTCGCGA
TGGAACAGATTAAATACACCACTTCTTTACCCATCGAATAAATATCCAGAATCAGGTCAGGACACAACGCGTGCGATTAT
TTGCTCAATTAAGCTGGTATTTCCGTCGGGAATGGCGTCGCTATCTCGGGGCTGTCGCCTTGCTTGTCATTATCGCGATG
CTGCAACTGGTTCCGCCAAAAGTGGTTGGTATTGTTGTCGATGGCGTGACAGAACAACACTTTACTACCGGGCAGATCCT
GATGTGGATCGCCACCATGGTGCTGATTGCCGTTGTGGTTTATCTCCTGCGTTACGTCTGGCGGGTATTGCTGTTTGGTG
CGTCTTATCAACTGGCTGTTGAACTGCGTGAAGATTATTACCGTCAGCTAAGCCGGCAGCATCCTGAGTTTTACCTGCGT
CATCGCACCGGTGACCTCATGGCTCGTGCGACCAATGACGTCGATCGCGTCGTGTTTGCCGCCGGAGAAGGGGTGCTGAC
GCTGGTGGATTCACTGGTGATGGGCTGCGCTGTGTTGATTATGATGTCTACGCAAATTAGCTGGCAGTTGACCTTATTTT
CCCTGTTGCCGATGCCAGTGATGGCGATCATGATTAAGCGCAACGGCGATGCTTTGCATGAACGCTTTAAGCTGGCACAG
GCGGCGTTTTCCAGTCTTAATGACCGCACCCAGGAAAGCCTCACCAGTATCCGCATGATCAAAGCCTTTGGTCTGGAAGA
TCGCCAGTCGGCGTTATTTGCCGCGGATGCCGAAGATACCGGCAAAAAAAACATGCGGGTGGCGCGTATTGATGCTCGTT
TCGACCCGACCATCTATATCGCGATTGGTATGGCGAACTTGCTGGCGATTGGCGGCGGTAGTTGGATGGTGGTGCAGGGC
AGTTTAACGCTGGGCCAGCTCACCAGTTTTATGATGTATTTAGGTCTGATGATTTGGCCAATGCTGGCGCTGGCATGGAT
GTTTAACATTGTGGAACGTGGTAGTGCTGCGTACAGCCGTATTCGCGCGATGCTGGCGGAAGCGCCGGTGGTGAACGATG
GTAGCGAACCCGTGCCGGAAGGGCGTGGCGAACTGGATGTAAATATTCACCAGTTCACGTATCCGCAGACTGACCATCCT
GCGCTGGAAAACGTCAATTTCGCCCTGAAACCCGGTCAGATGCTGGGTATCTGCGGGCCGACTGGTTCCGGCAAAAGTAC
CCTGTTGTCGCTCATTCAGCGTCATTTCGACGTCAGCGAGGGGGATATTCGCTTTCATGATATTCCTCTGACGAAGTTAC
AACTCGATAGCTGGCGTAGCCGCCTGGCGGTAGTTAGCCAGACGCCATTCCTTTTTTCTGACACTGTGGCGAATAACATC
GCGCTGGGTTGCCCGAATGCCACCCAGCAAGAGATTGAGCATGTCGCGCGGTTAGCCAGCGTACATGACGATATTTTGCG
TCTACCGCAAGGTTACGATACAGAGGTGGGCGAGCGCGGTGTGATGCTTTCCGGCGGGCAAAAACAGCGTATCTCCATTG

CTCGTGCGTTATTAGTCAACGCGGAAATCCTCATCCTTGATGATGCGCTTTCGGCGGTGGACGGACGCACTGAGCACCAG
ATCCTGCATAACCTGCGTCAGTGGGGGCAGGGAAGAACGGTAATCATCAGTGCCCATCGCCTTTCTGCACTGACGGAAGC
CAGTGAAATTATTGTGATGCAGCACGGACATATCGCCCAGCGTGGCAATCATGATGTGCTGGCACAACAAAGCGGCTGGT
ATCGCGATATGTATCGCTATCAACAACTGGAGGCGGCGCTCGACGACGCTCCGGAAAATCGCGAGGAGGCCGTCGATGCG
TAGTTTTAGCCAACTGTGGCCGACTCTCAAGCGCCTGTTAGCGTACGGTTCGCCGTGGCGTAAACCGCTGGGGATTGCGG
TCCTGATGATGTGGGTTGCGGCGGCGGCAGAAGTCAGTGGGCCGCTGCTTATCAGCTATTTTATCGACAATATGGTAGCG
AAAAATAACCTGCCGTTGAAAGTGGTTGCAGGGCTGGCTGCGGCGTATGTTGGGCTGCAACTGTTTGCCGCCGGGCTACA
TTACGCGCAGTCGCTGCTGTTTAATCGGGCGGCAGTAGGCGTAGTGCAACAGTTGCGTACCGACGTGATGGATGCTGCGT
TACGCCAGCCATTAAGCGAGTTTGATACCCAACCCGTCGGGCAGGTGATTTCCCGCGTCACTAATGACACTGAAGTGATC
CGCGATCTCTACGTTACCGTAGTGGCAACTGTCCTGCGCAGTGCCGCGCTGGTGGGCGCGATGCTGGTGGCGATGTTCAG
CCTCGACTGGCGAATGGCACTGGTGGCGATAATGATTTTCCCGGTGGTGCTGGTGGTAATGGTGATATACCAGCGTTACA
GCACGCCGATTGTCCGTCGTGTGCGCGCCTATTTGGCGGATATCAACGACGGCTTTAACGAAATCATCAATGGCATGAGC
GTTATCCAGCAGTTTCGTCAGCAGGCGCGATTTGGCGAACGTATGGGGGAGGCCAGTCGTTCACACTATATGGCGAGGAT
GCAAACCCTGCGCCTCGACGGTTTTCTGCTGCGTCCGCTGCTGAGTCTGTTTTCATCGCTCATTCTTTGTGGCTTGTTGA
TGCTGTTTGGCTTCTCCGCCAGCGGCACCATTGAAGTGGGCGTGCTGTATGCGTTTATCAGCTATCTTGGGCGACTTAAC
GAACCATTAATCGAACTGACCACGCAACAGGCGATGCTGCAACAGGCTGTTGTTGCTGGTGAGCGCGTGTTTGAACTGAT
GGACGGACCGCGCCAGCAATATGGCAATGATGATCGCCCGTTACAGAGTGGCACCATCGAAGTCGATAACGTGTCATTTG
CTTATCGCGATGACAATCTGGTGCTAAAGAACATTAATCTCTCTGTGCCTTCGCGCAATTTTGTGGCGCTGGTCGGGCAT
ACCGGCAGTGGCAAAAGCACCCTCGCCAGTTTATTGATGGGCTATTACCCGCTAACGGAAGGTGAGATTCGCCTTGATGG
TCGTCCATTAAGTTCGCTAAGTCACAGCGCGCTGCGCCAGGGCGTGGCAATGGTGCAGCAAGATCCGGTGGTGCTGGCGG
ATACCTTCCTCGCCAACGTGACGCTGGGGCGGGATATCTCCGAAGAACGCGTCTGGCAGGCGCTGGAAACCGTGCAACTG
GCGGAGCTGGCGCGTAGCATGAGCGACGGTATTTACACGCCGCTGGGCGAGCAGGGGAATAATCTCTCAGTTGGGCAAAA
GCAACTGCTGGCACTGGCGCGCGTGCTGGTCGAGACGCCGCAAATCCTGATCCTTGATGAGGCAACCGCCAGCATTGACT
CCGGTACTGAACAGGCGATTCAACATGCTCTGGCGGCGGTGCGTGAACATACCACGCTGGTAGTGATTGCTCACCGCTTA
TCGACCATTGTTGATGCCGACACCATTCTGGTGCTTCATCGTGGGCAAGCCGTGGAGCAGGGCACTCACCAGCAACTGCT
GGCGGCCCAGGGACGCTACTGGCAGATGTATCAACTGCAACTTGCGGGCGAAGAGCTGGCAGCCAGCGTGCGTGAAGAGG
AATCATTGAGCGCCTGAATAGCGCAATATTTCATCGTTGGTGCAAAAATGTAACGCACTGTGCACTGTCATAGTGCGTTT
TCATTTTCAAACTTCTTAACTTCCTGCTCTCTTTCTCGTTTTTCATTTCTGGCACACCGCTTGCAATACCTTCTTCGTGT
AGCAGAACCATTACCGAATTCTGACCGGAGGGGATCTATGAAGCTGGTGACCGTGATAATCAAACCATTCAAGCTGGAAG
ACGTTCGTGAAGCGTTATCTTCCATTGGTATTCAGGGCCTGACCGTCACCGAAGTGAAAGGTTTCGGGCGTCAGAAAGGG
CATGCCGAGCTGTACCGGGGGGCGGAATACAGCGTCAATTTCCTGCCAAAAGTAAAAATTGATGTGGCGATTGCTGATGA
CCAACTCGATGAAGTGATCGATATCGTCAGTAAGGCGGCTTACACCGGAAAAATTGGCGACGGCAAAATCTTCGTCGCTG
AATTGCAACGCGTCATTCGTATTCGTACCGGCGAAGCCGACGAAGCGGCGCTGTAATCTCTGGCACACAGCAACAGGAAC
GAAAAATGAAGATAGCGACGATAAAAACTGGGCTTGCTTCACTGGCGATGCTTCCGGGACTGGTAATGGCTGCACCTGCG
GTGGCCGATAAAGCCGACAATGCGTTTATGATGATTTGTACTGCGCTGGTGCTGTTTATGACTATTCCGGGGATTGCCCT
GTTTTACGGTGGGTTGATTCGCGGCAAAAACGTGCTGTCGATGCTGACGCAGGTGACGGTGACATTTGCACTGGTCTGTA
TTCTCTGGGTGGTTTACGGTTACTCGCTGGCGTTTGGTGAGGGCAACAACTTCTTCGGCAACATTAACTGGTTGATGCTG
AAAAACATCGAACTGACGGCGGTGATGGGCAGCATTTATCAGTATATCCACGTGGCGTTTCAGGGATCGTTTGCCTGCAT
TACCGTCGGCTTGATAGTTGGGGCGCTGGCGGAACGAATCCGCTTCTCAGCTGTGTTGATTTTCGTGGTGGTATGGCTGA
CGCTCTCTTACATTCCGATTGCGCATATGGTGTGGGGCGGTGGTTTGCTGGCTTCTCACGGTGCGCTGGATTTCGCGGGT
GGCACCGTGGTGCACATTAACGCCGCAATCGCCGGTCTGGTGGGCGCGTATCTGATAGGAAAACGCGTGGGCTTCGGTAA
AGAGGCGTTTAAACCGCACAACCTGCCGATGGTCTTCACCGGGACTGCCATTCTCTATATCGGTTGGTTTGGCTTTAACG
CCGGGTCAGCGGGCACGGCGAATGAAATCGCGGCACTGGCATTTGTGAATACTGTGGTCGCAACGGCGGCGGCAATTCTT
GGCTGGATCTTCGGTGAATGGGCGCTGCGTGGTAAGCCTTCACTGCTGGGGGCGTGTTCTGGCGCGATTGCCGGTCTGGT
CGGCGTGACGCCAGCCTGCGGCTACATTGGGGTTGGCGGCGCGTTGATTATCGGCGTGGTAGCTGGTCTGGCGGGCTTGT
GGGGCGTTACCATGCTCAAACGCTTGCTGCGGGTGGATGATCCCTGCGATGTCTTCGGTGTGCACGGCGTTTGTGGCATT
GTCGGCTGTATCATGACCGGGATTTTTGCCGCCAGCTCGCTGGGCGGCGTGGGCTTCGCTGAAGGTGTGACGATGGGCCA
TCAGTTGCTGGTACAGCTGGAAAGCATCGCCATTACGATCGTCTGGTCCGGTGTTGTGGCATTTATCGGCTACAAATTGG
CGGATCTGACGGTTGGTCTGCGTGTACCGGAAGAGCAGGAGCGAGAAGGGCTGGATGTCAACAGCCACGGCGAGAATGCC
TATAACGCGTAACAAGCACTGCAAAAAACAGCCGGACGGTTTTCACCTCCGGCTATTTTTTTAATTGTGATTACGCATCA
CCCCTTCCTGAACGGTCGAGGCAACCAGTACGCCGTCTTGGGTATAAAACTCACCGCGCACAAAGCCACGTGCGCTGGAC
GCCGAGGTGCTCTCCACGCTATACAGCAGCCATTCATTCAAATTAAACGGGCGATGGAACCACATGGAATGGTCAATGGT
GGCAATCTGAATCCCCGGTTCGAGAAAACCGATGCCGTGCGGCTGTAGAGCTACCGGCAGGAAGTTAAGATCAGAAGCGT
AACCGAGCAGATACTGATGAACGCGCAGGTCATCCGGCACGCTACCATTTGCGCGGATCCACACCTGACGATGTGGTTCT
GCGACGTGACCTTTCAGTGGGTTATGAAACTCCACCGGACGGACTTCCAGCGGACGATCGCAGATGAATTTATCTTTCAG
CACTGGCGGCAGCAGGTGCGCCAGCGATTGGGCGATTTGCGTTTCCGAAGGGAGGCCATCAGGCGCTGGCGCGGACGGCA

TTGTTTTTTGATGTTCGAAACCCGCTTCTGGTGCCTGGAAAGAGGCAGTCATATAAAAAATCGGTTTGCCGTTTTGAATA
GCAGCAACCCGGCGGGCGCTGAAGCTGTTACCGTCACGCAGCGTTTCGACATCATAAATAATCGGCTTCTTACTATCGCC
AGGGCGAAGAAAGTAGCTGTGAAACGAATGTACCAGCCGCTCTTCAGGGACGGTCTCTTTTGCAGCATACAAGGCCTGAC
CCACGACCTGGCCGCCAAACACCTGGCGTAAACCTAAATCTTCACTCTGGCCGCGAAAGAGTCCTTCCTCAATTTTTTCC
AGATTTAACAATGTCAGTAAATTTTTTAGCGCCTGACTCATATAACTCTCCAGTAACAAAGCTGCCGCAGCAAGCCAAAG
TGAGTTGAGTATAACGCAAATTTGCTACTGGTCCGATGGGTGCAATGGTCTGAATTACGGGCTAATTACAGGCAGAAATG
CGTGATGTGTGCCACACTTGTTGACGTTACTATTTTGTTAACCACTCTTCCGGCGAGGAAAGTTAGCCCGCTGGTGCATT
GATAATAAGGAGAAATGAATGAAACTCGTGCACATGGCCAGTGGTTTAGCGGTTGCGATTGCGTTGGCGGCTTGCGCAGA
TAAAAGCGCGGATATTCAGACGCCAGCACCGGCTGCAAATACGTCTATTTCAGCAACACAACAACCAGCTATCCAGCAAC
CGAATGTCTCCGGTACCGTCTGGATCCGTCAGAAAGTCGCACTGCCGCCTGATGCTGTGCTGACCGTGACACTTTCTGAC
GCGTCGTTAGCCGATGCACCGTCAAAAGTGCTGGCGCAGAAAGCGGTGCGTACTGAAGGTAAACAGTCACCATTCAGCTT
TGTTCTGTCATTTAACCCGGCAGATGTTCAGCCGAACGCGCGTATTCTGTTGAGTGCGGCGATTACCGTGAATGACAAAC
TGGTATTTATCACCGATACCGTTCAGCCGGTGATCAACCAGGGCGGAACTAAAGCCGACCTGACATTGGTGCCGGTACAG
CAAACCGCCGTGCCGGTTCAGGCCAGCGGTGGCGCAACGACTACCGTACCTTCGACTTCACCAACTCAGGTGAATCCGTC
TTCGGCAGTTCCAGCTCCTACGCAATATTAAGCGACGTTAACCCTCTCCGCCCGGAGAGGGTTAGTAGTTCCAGCGATAA
CGCTGCAAGTCGATTTGCCCGCTTCCCGATACCATCACACCTTCTGCCAGTAATGCCTGTCGCTGACGCTGTAAATCCGG
TCCGGTTAGCGAAATTGTGCCGTGGCGATTAACCACCCGGTGCCAGGGTAAGGTGCTGCCTTCAGGGAGACGCTTTAACA
CACCGCCCACCTGGCGCGCGGCGCGGGGCGATCCCGCCAGTTTCGCCACATCACCGTAAGTGGTGACATAGCCTTCGGGA
ATAGCGGCGACGATTTGCCAGACGCGTTGGGGAAATGAATCTTCTTTTTCCATCTTTTCTTCCTGAGGTAATTTTTCAGC
ATAATCTGGAAAAACGCCCGAGTGAAGTCGCATTGCGCAAGAAACCAGCATCTGGCACGCGATGGGTTGCAATTAGCCGG
GGCAGCAGTGATAATGCGCCTGCGCGTTGGTTCTCAACGCTCTCAATGGGGGCTCTGTTGGTTCTCCCGCAACGCTACTC
TGTTTACCAGGTCAGGTCCGGAAGGAAGCAGCCAAGGCAGATGACGCGTGTGCCGGGATGTAGCTGGCAGGGCCCCCACC
CATTTCTGCCTCCCACCGTTTCGTCAAAAAATCCCAACATGGCTAAACTTTAACCACAACTGACGTCGCAAGAATTGTCT
GGCTGCGCAGTACGCTTCGGAGGTATGTCTGATGAAGTATGTTGATGGTTTTGTGGTTGCCGTTCCTGCCGATAAAAAGG
ATGCCTATCGGGAAATGGCCGCTAAGGCTGCGCCATTGTTTAAAGAGTTTGGCGCGCTTCGTATTGTCGAATGCTGGGCC
AGCGATGTACCGGATGGCAAAGTGACCGATTTTCGTATGGCGGTGAAAGCGGAAGAGAATGAAGAGGTTGTCTTTAGCTG
GATTGAATACCCTTCAAAAGAGGTCCGCGACGCTGCTAATCAAAAGATGATGTCGGACCCACGGATGAAAGAGTTCGGCG
AGTCCATGCCGTTTGACGGCAAGCGAATGATCTATGGCGGATTCGAGTCAATCATCGACGAATAGGTAGCGTGACGGGCT
GTGCGGCGTGCCGTCAGCCCAAAAATTCACAAATGTTGCTCGGCCCAGCGTAAGAAATCTTCTTTCGGTAATGCCTTGCT
GTAGAGCCAGCCCTGACCGTAGTGCACGCCATGCTGGCGTAACCACTCTTCTTGTTTACTGGTTTCGATTCCCTCCGCTA
CCATTTTCAGTTTCAGTGTTTTTGCCATTTCGATGATATGCGGCGTGACATTTTTATATTCCAGCGCATCAACGAAAGAT
TTATCGATCTTCAGAATGTCGACATCCAGATCCTGTAAATAACTTAAACTTGAATACCCCGTACCAAAATCATCAAGATA
AATTTCATGGCCCGCCTCCCGGTAGCGAGAAATTATCGGGGCGCTGGTTTTCGGATCGGCAAACTCGCGTTCAGTGAGTT
CAAGCGCGATCTGTCTGGGATTAACCTGATAGTGATTGATCATGTCACGCAGCAATTGCGGGATTTTTTCCGAGGTGAGC
ACGGGGGATTCAAGATTGATCGAAATATGCTGCTGTGGATGCTGACGCAGCCAGTCGCCCATATCTTCAAAGACGCTTCT
TATAATCAGTAGCGTCAATGGCTCAGAAAGGCCCGTTTGCTGTGCCAGCGGAATAAAACTATCTGGTGACAACCAACTAC
CGTCTGTCTGCGGCCAGCGCGCCAGTGCCTCAGCACCGACAATTTTGCCATTGGCTAAGGAGACAATCGGCTGATAGTGC
ACGCAAATATCACGATTTTCGATAGCATCCTGCAGCCGATGATGCGGTGACTGAATACGGCGCAAAATACGCAGCACAAA
CATCGCTGCCAGCAGGCCAATCACCAACCCGAGCGGTAACCAAATAAAGACTTGCCGATGCCAGCCTTTCTGCAACATTT
TCGTTGAAGCCCATGTGATGATCGAAATATTCATCTCCGGTAAGGGCAGGATATCGTAAATGATTCCATTATTTTCGATA
TGCTCACCGGGTGTTTTTTGTAGCCTGGTAATAATTCCCTGAGCAATTTCATCGCTGCTGGTTATGACAACGTTATGGGC
ATTGCCAATAATGGCGGCATCAATTTGCCATGAGCTATAGGGAATGACATCAATAAAGGAAGCGGGGTCGATCATGACGA
CATAATGTGCCGTTCCCATGGCGACCATGTAACGGATAATGCCTAAATCGTTATGCGATGTTAACCAGACACGATAACCA
TCTTTCGAAATTTTACCTGGCTCGGGGAAGGTATCGGGCGGACTTTCATGCTCCAGAGACGAACACTGGGGAACGTTGTT
ATCGATATAAGCCACTTCCTGAATATAGCGATAACTGTAAGATACCCGACGCATTTCCATGAGATGGGCTTCGCTACAGG
CAGCGCCTTGCCATCTTTCCAGCTCCTGCAGCGCATCTTTCCCTTGTGTCGCCACCTTATTGGCTCGAATAGCGACGCGG
GAGGAATAGGTATCCAGCTCTTCAATAAACGATGTTTCTACCTGCTGATGGGCCAGCCAGATGCTTAAGCCGACAGGCAG
CAATACTGAAAGAATCAGTACTCCCGAAATAAGGCCGACCAGATGTCGTGTTCTCACCAGCATATCCTTATTAACACAGT
GTAGGGAAAGTATATCTGATTGTGGAAAAAGGCTGGCTGGAGATAAGGAGGTAGAGCTGGTATTTCATGAAGTGATGGGT
GGAAACGATCATTGTCATTTGACCAGGCGCAGAGCTTTACGCCTGGTATGTAACCCTAACTTAAGATGTTGATTCGGCGC
GAGCGAGGGTAAAAATATCGTAAAAAGACAGTTCACCTTTACGGACGATCATTTTTTGCAGTTGTTCCTTATGAACATCC
GCGACACGCGGAGAGTTCAAGATGGCTTCAACCAGCGCGGCAGGTACAAAGCGCGTGTTGTACCATTCGCCGTTATAGCA
AACGCGGAAATCCAGCACGTCGATATCTTCATAGCGGTAGCGTGGGTAGACATCGAAAAAGAAGAAACCATTAAGCAGGA
TAAATAATACAACCAGTAGCCAGACAGAGCCTGACAGCGTTTCGGACTGCAACATCACCGCCAGGGTGGCAAAAAAAGCA
ACGTACATACCGATAAACAGCCCCGGATGTTTACGGATAAAACTGATACTAAAGCGGGGTTTGTTGTCGCGTTTTTCGCG
GGTATTCAGAGACTCAATCGTTTCGGTCAGCAGGCGTTGTATTTCGGTCATCGTTTGCCTTCGTGGTATCTGCAACTTTC

CAGGGAACACCCTATTTTAACGTGGGAATAGTAGAGGGGAAGTAACAGATCTACCACAATTTTGCATAACAGTTGCGAAA
AACCGATTACAATTTTTTAATTATTCTGGCTGGATTACCGCCCACGACAACGTTGTCCGGGACATCTTTTGTGACAACTG
CACCTGAGGCTACCACGACGTTATCACCAATGGTCACACCAGGGTTAATGACCGCGCGTCCGCCAATCCAGACGTTATTA
CCGATGGTGACGGGTTTCCCCAGTTCAGCACCGCTATTACGTGCTACAGGGTCGATGGGATGTGTTGCCGTGTAGATATG
AACGCCTGGTGCCAACATACAGTTATCACCGATGCGAATAGGGCAGACATCAAGCATCACGCAATCGAAGTTGGCGAAAA
AATTATTACCGAGAAAAATGTTATAGCCATAGTCACAGCGAAACGTTGGCTCAATATAAGCCTCTGTCACCTGACCGAAT
AGATCAGCGAGAATTTGCTGGCGTAATGTGTGCTCTTCCGCCAGGGAATGATTGTATCGGTGAATAAGCTGACGAGCGCG
CAGGCGATCGCGAGATAACGTCTCATCTGCCGAGCGATACAACTCACCAGCAATCATCTTTTCTTTTTCTGTGCTCATTA
CTGACCTCCGGTTGCTGAAAATGCGGCAACACAGTAAGACGAAAAGTCAGTGAAGAGAACGTTCCCATTACATCTGTGTG
ATAAAGATCACATAGGGTATGTCTTCATGGCGAAAAAGTATAAAATTCTTAATAAACAGCCGGTTATAGCTCCGAAAGCG
AATTATTTATTAGCGAATAAATTTCCATACTGAGGAAGGGATCTTGTCGTACAGTTTATTCATGGTCAATTCGGCGAGGC
GGTGATCTGCGGCTGAGTAAAATACCGCCAGTTCATTATCTGATAATTCGTATTTATTTTTCTCGATAACACGCTCCAGC
GTGTCAATTGTCTGGCAACGACGTAAACGCATTAAATAATCGGTTTTCGTTAAAGGTTTTTCGGACATACTTCTACCTAT
GGTTGTAATAATTCTAACAAGATAAACTCGCAGGATTCTCTTTCGTCGCATTGACAAAACAACGGAATAGTCGATTACCT
GACTTCCGCCATTTCTGAAGATCCTGCATATTAATACCATAACTACTGAACAACATAAAGGTGTCATCCAGATATTCGTC
GATCTGCTCAATGAGCTTATTGTCTTCATTATACTTAATTTTGTAATTAAGTGCGAAGGTCGCAATATGCTCAATCAGTT
CATTCAACTGGAGGTTGATCGCCGAGGTTGGGTCGTTTACCCAGCCATGATTGCTTTCTTCAAGGTTTGCAAGGCAGTCA
TGATACAGGGTTTCACAGAGAAACTTAAGCTGTGCGATATCATGTCTTTTGGGTGAGTATTCATCCATAACGCGTCCCCT
TCTTAGCGGTTGAACTAACGGACACCTTTCGGGATGGAAAAAACTTACTGACCTGGACTTGCCCTCTCGTTTGTTAGCTT
AACTATAAGCCACTCTTTGCAGGTTTTCATCGCATTATTACGAAAAATTACAATTAGGAAGGAAGCGGGACAGGACTCAC
TTTTTCATACTAGCACTAACCCGCAGCAGGTAAAAGCAGTTTGCTCGAACTATTCATAACATTTCAGGATTATCGCCAGA
TGAAATTGGTGACACTAATACCAGGATTGCTCTGAATATGACGTAATAACCGAGGAATGAATAAAGAATTACCGCAAATA
ATTAAGAATAGCCTCTAAATGATTATGGATTATTAAATCTATTAGCGCTACTTAATATAAATTAACTAACAATCAGTATT
TTTTATGAATTTTATCCGTGGTTAATACTGGTTTTCGTATGAGATCCTGAGTTGGTGGTTCAATTACTCCTTAATGTTCG
TAGGTTATGCATAAAAAAGGCCGCTTACGCGGCCTTAGTGATTACACGTTGTATCAATGATGATCGACAGTATGGCTGTG
CTCGATATCTTCATTCTTGCGGCTAAAGCGGCGGCGAACCACCACAAAGAATACCGGAACGAAGAAGATTGCCAGTACCG
TTGCGGTCACCATCCCGCCCATTACACCGGTACCTACTGCGTTCTGCGCGCCGGAACCAGCACCAGTACTGATAACCAGC
GGCATAACGCCGAGGATAAACGCCAGCGAGGTCATCAGGATCGGACGTAAACGCATCCGCACCGCATCAAGCGTCGCTTC
AATCAGACCTTTACCTTCTTTATCCATCAAGTCTTTGGCGAATTCGACGATAAGGATCGCGTTCTTCGCCGACAACCCAA
TGGTTGTGAGCAGGCCTACCTGGAAGTAAACGTCATTGGTCAGGCCACGGAAGGTGGCAGCCAGCAACGCACCGATAACC
CCCAGCGGAACGACCAGCATAACGGAGAACGGAATCGACCAGCTCTCGTACAGCGCCGCCAGACACAGGAACACGACAAT
CAACGAAATCGCGTACAGTGAAGGTGCCTGGTTGCCGGAGAGACGTTCCTGATAGGACATCCCCGTCCAGTCATAGCCAA
CACCGGTAGGCAGTTTGCTCGCCAGTTGTTCCATCAGCTCCATTGCTTCACCGGTACTTTTACCCGGTGCCGCCTGGCCT
AAGATTTCCATGGATGGCAGGCCGTTGTAACGTTCCAGACGCGGCGAACCGTACTCCCAACGAGAAGAGGAGAACGCCGA
GAATGGCACCATCTGACCATCAGCAGCACGAACATACCAGTCGCCGATATCATCCGGCAGCATACGGTATTTCGCTTCTG
ACATGACATAAACTTTCTTCACACGACCGCGGTCGATAAAGTCGTTCACATAGCTGCCGCCCCATGCAGCGCCCAGAGTG
GTGTTAATGTCGTTGATAGAAACACCCAGCGCCTGCGCTTTTTCCTGGTCGATATCAATCTTAAACTGCGGGGTATCTTC
CAGACCGTTTGGACGTACGCTGGTCAACATATCAGGGTGCTTCGCTGCTTCTGCAAGCAACTGGTTACGCGCCTGAGTCA
GTTTTTCGTGACCAAGGCCAGCCTGGTCAATCAGCTCAAAGTCAAAGCCGGTTGCAGTACCCAGTTCCACGATTGCGGGC
AGGTTAAAGGCGAAAACCATCGCATCTTTGATTTGCGAGAAAGCGCGTGTTGCACGCATGGTAATCGCTTCAACTTTGTT
TTCTTCGCCCGGACGATCGGCCCAGTCCTTCAAGGAAACGAACGCAATACCGGTATTCTGACCACGTCCCGCAAAGCCGA
AGCCGTTAACGGCGAACACCGACTCAACGTTGTTCTTTTCTTTGGTCAGATAGTAATGCGTTACCTCATTGAGCACTTTC
TGTGTACGTTCCTGCGTTGCACCTGCTGGCAGCTGAACCATGGTCATAAACACGCCCTGGTCCTCATCTGGCAAGAAGGA
GCTTGGCAGACGCACGAACAGATAGGCCATGCCGACCACGATGATCAGATACAGCACCAGGTAACGCCCCGTACTGCGCA
GAATACCGCCTACGCTGTCGGTGTAGTGGTGCGTGCTCTTCTCGAACATGCGGTTAAACCAGCCGAAGAAGCCTTTTTTA
CCTTCCCCGTGATCGCCTTTGGCAATCGGTTTCAGCATGGTGGCACAAAGAGCTGGAGTCAGGATCAACGCCACCAGTAC
CGACAGCGCCATTGCTGAAACAATGGTAATAGAGAACTGACGATAGATAGCACCAGTAGAACCGCCAAAGAAGGCCATCG
GTACGAATACCGCCGACAGTACCATCGCGATACCGACCAGAGCGCCCTGAATCTGCCCCATCGACTTACGGGTAGCTTCT
TTTGGCGGCAAACCTTCTTCCGCCATAACACGCTCAACGTTTTCTACCACAACGATGGCGTCATCCACCAACAGGCCGAT
GGCGAGCACCATCCCGAACATTGTTAGCGTGTTTATCGAGAAGCCAAAGGCGGCAAGGACGGCAAAGGTCCCGAGCAATA
CCACCGGTACGGCAATGGTCGGAATCAACGTCGCGCGGAAGTTCTGCAGGAACAGATACATAACCAGGAACACGAGGATG
ATCGCTTCGACCAGCGTTTTAACCACTTCGTGAATAGAGATTTTCACGAACGGCGTGGTGTCGTATGGGTAAACAATTTT
CAGACCCGACGGGAAGAACGGTTCCATCTTCGCCAGTTCAGCACGGATTGCCGCAGCGGTATCCAGCGCGTTTGCACCGG
TCGCCAGCTTGATCCCCAGACCGGAAGCCGGTTGGCCGTTAAACTCTGCGATGATGTCGTAGTTCTCACCACCCAGCTCA
ATCTTCGCGACGTCACGCAGCAGCACGCGGGAACCATCCTGATTCACTTTCAGCAGGATTTTGCCGAACTCTTCAGTAGA
GGTCAGACGCGTCTGAGCAATAATAGAGGCGTTAAGCTGTTGGCCTTTCACCGGCGGCGTACCACCGAGCTGACCCGCCG

CAACCTGGGCGTTCTGCGCTTTGATGGCGGTAATGACATCAACCGGCGTTAGCTGGAATTTGTTCAGCTCATTCGGGTTC
ATCCAGATACGCATCGCGTACTGTGAACCGAACAACTGAACATCACCCACGCCCGACGTACGGCTGATGGCATCTTTCAT
ATTCGCCGCCACGTAGTCGGAGATATCCTCCTGCGTCATGGTGCCATCGGTGTTGATAACGCCGACAACCATCAGGAAGC
TGCTGGATGATTTCTCAACGCTCACCCCTTGCTGCTGAACTTCTTGCGGCAGCAACGGCATCGCCAGCTGCAGTTTGTTC
TGCACCTGAACCTGCGCGATATCCGCATCAGTACCAGACTCAAAGGTCAGGGTGATCTGCACGGTACCCGTGGAGTCACT
GTTAGAGGACATGTACATCAGGTTATCGATACCGTTCATATTCTGTTCGATAACCTGTGTCACCGTGTCCTGCACTGTTT
TCGCATCAGCGCCGGGGTAGGAGGCGGAGATCGTTACTGCCGGCGGTGCAATCGTAGGATATTGCGCCACCGGCAGTTTG
AGGATCGCCAGCCCCCCTGCCAACATGATGATAATGGCGATCACCCACGCAAAAATCGGGCGATCGATAAAGAAATTAGG
CATGTCTTAACGGCTCCTGTTTAAGTTAAGACTTGGACTGTTCAGGCTGAGCACCGCTTGCGGCTTGCTGGTTATTATCA
GCGGTAACTTCTTGTGCTTTTACCTGGACACCAGGACGCACTTTCTGCAGCCCACTTATTACTACGCGATCGCCTGCTTT
CAGACCTTCTGTCACCAGCCACTTATCGCCAATAGCCTGGCTTGCAACGATCGGACGGGTTTCCACTTTGTCATCCGCGC
CAACTACCAGTACGGTGGCATCGCCACGCGGCGTACGGGTTACGCCCTGTTGCGGGACTAAAATAGCGTTTGGATTAAGC
CCTTCTTCCAGACGTGCGCGCACGAACATACCCGGCAGCAGAGTGTGATCCGGGTTCGGGAAGATAGCGCGTAGGGTGAT
AGACCCAGTGGTCTGATCAACGGTAACGTCAGAGAATTCCAGCGTACCGTCCTGCGGGAACTTAATGCCGTCACTGGTGA
TCAGTGACACTTTGGCTTTGCCGTTCTCTTGTTTCAGCGTGCCATTCGCCAGTTCCTGTTTCAGGCGCAGGAAGTCGTTG
CTGGACTGGGTCACATCAACGTAGATCGGATCAAGTTGCTGCACGGTTGCCAGCGCAGTCGCCTGACCGTTCTGTACCAA
TGCGCCTTCCGTCACGTTCGACTTACCAATGCGACCGCTAATCGGAGAGGTGACTTTGGTGTAAGCCAGATTGATCCGCG
CAGTTTCAACGGCAGCTTTCGCCGCAGTTACCGCAGCATTCGCCTGTTGCGCATCAGCCAGAGCCTGATCGTACTCTTGC
TTACTGATGTACTGAGTACCGAGCAGTTTCTGATAACGATTCACCGTCAATTGCGCGATATTGGCTGCAGCCTGGGCTTT
CGCCAGATCACCTTTCGCACTGTCGTATGTCGCCTGATAGGTCGCAGGATCAATCTGATAGAGAGAGACACCTGCTTCGA
TGTCGCTACCTTCTTTGAAATTACGCTTCAGGATAATCCCGCTAACTTGAGGACGAACTTCTGCGATCCGGTAGGCACTG
GTGCGACCCGGAAGCTCGGTTGTGATCTGCAGAGGTTCAGTTTTGACTGTTACTACGCCAACGGCGGGCATCTGCTGGCC
ACCTTGTTGGGCCTGTTTGTCGTCACATCCTGTTAGGGCTAAGCTGCCTGAGAGCATCAGAACGACCGCCAGAGGCGTAA
ACCCTCTGTTTTTGTTCATATGTAAACCTCGAGTGTCCGATTTCAAATTGGTCAATGGTCAAAAGTTAATAAACCCATTG
CTGCGTTTATATTATCGTCGTGCTATGGTACATACATTCACAAATGTATGTAAATCTAACGCCTGTAAATTCACGAACAT
ATGGCACGAAAAACCAAACAAGAAGCGCAAGAAACGCGCCAACACATCCTCGATGTGGCTCTACGTCTTTTCTCACAGCA
GGGGGTATCATCCACCTCGCTGGGCGAGATTGCAAAAGCAGCTGGCGTTACGCGCGGTGCAATCTACTGGCATTTTAAAG
ACAAGTCGGATTTGTTCAGTGAGATCTGGGAACTGTCAGAATCCAATATTGGTGAACTAGAGCTTGAGTATCAGGCAAAA
TTCCCTGGCGATCCACTCTCAGTATTAAGAGAGATATTAATTCATGTTCTTGAATCCACGGTGACAGAAGAACGGCGTCG
ATTATTGATGGAGATTATATTCCACAAATGCGAATTTGTCGGAGAAATGGCTGTTGTGCAACAGGCACAACGTAATCTCT
GTCTGGAAAGTTATGACCGTATAGAACAAACGTTAAAACATTGTATTGAAGCGAAAATGTTGCCTGCGGATTTAATGACG
CGTCGCGCAGCAATTATTATGCGCGGCTATATTTCCGGCCTGATGGAAAACTGGCTCTTTGCCCCGCAATCTTTTGATCT
TAAAAAAGAAGCCCGCGATTACGTTGCCATCTTACTGGAGATGTATCTCCTGTGCCCCACGCTTCGTAATCCTGCCACTA
ACGAATAACCCTGAATCTGACTCCAGGATTTTTCCTGGACATTTTCGTCGTTGCTATTCTGGTTCACTGCGTCGTGATAT
TCTTGCGGTTTGACTTTTTCAGGTCGTTCTTCAGGTTCAGAAACCTTCATTCATCATGACTATGTTCCAGTATTACAAAC
GATCACGGCATTTTGTTTTTCAGCATTTATTGCTTTTGTTTTTGTCTTGTTATGCCAGAACACGGCGTTTGCGCGGGCG
TCATCGAATGGTGATCTGCCGACAAAAGCGGACCTGCAGGCGCAACTTGACTCACTAAATAAACAAAAAGATCTTTCTGC
TCAGGACAAACTGGTGCAGCAGGATCTGACAGATACATTAGCCACCCTCGATAAAATCGATCGCATAAAAGAAGAGACAG
TTCAGCTACGGCAAAAAGTCGCTGAAGCGCCGGAAAAAATGCGCCAGGCGACCGCGGCGTTAACAGCACTTAGCGATGTC
GATAACGACGAAGAAACGCGCAAAATTCTGAGCACGCTGTCGTTGCGCCAGCTGGAAACTCGCGTTGCCCAGGCGCTGGA
CGATTTGCAAAACGCACAAAACGATCTGGCGTCTTATAACAGCCAGCTGGTTTCGTTACAGACGCAGCCCGAACGCGTGC
AAAATGCGATGTATAACGCTTCGCAGCAGCTGCAACAAATTCGCAGTCGTCTGGATGGGACTGATGTCGGCGAGACAGCC
TTACGTCCCAGCCAGAAAGTGTTAATGCAGGCCCAGCAGGCGTTGCTGAATGCGGAGATTGACCAGCAGCGTAAAAGCCT
GGAAGGGAACACCGTCTTGCAGGATACCTTGCAAAAGCAACGTGATTACGTGACGGCGAACAGCGCTCGTCTGGAGCACC
AGTTACAACTGTTGCAAGAAGCGGTAAACAGCAAGCGCCTGACTTTAACCGAAAAAACGGCGCAGGAAGCCGTCTCCCCG
GATGAAGCCGCGCGTATTCAGGCTAATCCGCTGGTGAAGCAGGAACTGGAAATTAACCAGCAGTTAAGTCAGCGTCTGAT
TACCGCGACTGAAAACGGTAATCAGTTGATGCAGCAAAACATTAAAGTCAAAAACTGGCTGGAGCGGGCGCTGCAATCGG
AACGCAATATTAAAGAGCAGATTGCCGTCCTGAAGGGCAGCCTGCTGTTGTCTCGTATCCTTTACCAGCAACAACAAACG
CTGCCCTCGGCGGATGAACTGGAAAACATGACCAACCGCATCGCGGATTTGCGTCTCGAACAGTTTGAAGTTAACCAGCA
GCGTGATGCACTCTTCCAGAGCGATGCGTTCGTCAACAAACTGGAAGAAGGTCACACCAACGAAGTCAACAGCGAAGTTC
ACGATGCGTTATTGCAAGTGGTTGATATGCGTCGCGAATTGCTGGATCAACTCAACAAACAGTTGGGTAACCAGCTGATG
ATGGCCATTAACCTGCAAATCAACCAGCAGCAGTTAATGAGTGTGTCGAAAAACCTGAAATCCATCCTGACTCAGCAAAT
CTTTTGGGTGAACAGTAACCGTCCAATGGACTGGGACTGGATCAAAGCGTTCCCGCAAAGCCTGAAAGATGAATTTAAGT
CGATGAAAATCACGGTGAACTGGCAAAAAGCCTGGCCCGCCGTTTTTATCGCTTTCCTCGCTGGTTTGCCGCTGCTGTTG
ATTGCCGGGCTGATCCACTGGCGTCTGGGCTGGCTGAAAGCGTATCAACAAAAACTGGCTTCCGCTGTGGGTTCCCTGCG
TAACGACAGCCAGCTCAACACACCAAAAGCGATCCTTATCGACCTGATCCGTGCGCTGCCGGTGTGCCTGATTATTCTCG

CGGTTGGCCTGATTCTGTTGACCATGCAGCTCAACATCAGCGAACTGCTATGGTCGTTCAGCAAAAAACTGGCGATATTC
TGGCTGGTGTTTGGCCTGTGCTGGAAGGTACTGGAGAAAAACGGCGTTGCCGTACGTCACTTCGGCATGCCGGAACAGCA
GACCAGCCACTGGCGTCGGCAAATTGTCCGCATCAGTCTCGCATTGCTGCCTATCCATTTCTGGTCTGTGGTGGCAGAAC
TTTCCCCGCTGCATCTGATGGATGATGTGCTGGGGCAAGCGATGATTTTCTTCAACCTGCTGCTGATTGCCTTCCTGGTA
TGGCCGATGTGCCGCGAAAGCTGGCGTGATAAAGAGTCGCACACCATGCGACTGGTCACCATTACCGTGCTGTCGATAAT
CCCGATTGCGCTGATGGTGCTGACTGCTACAGGCTACTTCTACACTACGCTGCGTCTGGCAGGACGCTGGATTGAAACCG
TTTATCTGGTGATCATCTGGAACCTGCTGTACCAGACGGTACTGCGTGGCTTAAGCGTAGCGGCGCGGCGTATCGCCTGG
CGTCGTGCGCTGGCGCGTCGGCAGAATCTGGTGAAAGAGGGCGCAGAAGGTGCTGAACCGCCGGAAGAACCCACCATTGC
ACTGGAGCAAGTTAACCAGCAGACGCTGCGTATTACCATGTTGCTGATGTTTGCGCTGTTCGGTGTCATGTTCTGGGCAA
TTTGGTCCGATTTGATCACCGTGTTCAGCTATCTCGACAGCATCACGCTCTGGCATTACAACGGCACTGAAGCTGGCGCT
GCGGTGGTGAAAAACGTCACCATGGGCAGTCTGTTGTTTGCGATTATCGCCTCAATGGTGGCCTGGGCGTTGATTCGCAA
CCTGCCTGGTTTACTGGAAGTGCTGGTGCTCTCGCGACTGAATATGCGCCAGGGCGCGTCGTATGCCATTACTACCATCC
TTAACTACATCATTATTGCTGTTGGTGCGATGACGGTGTTCGGATCGCTGGGCGTCTCTTGGGATAAACTCCAGTGGCTG
GCCGCAGCATTATCCGTAGGTCTTGGTTTTGGTTTACAAGAAATTTTCGGTAACTTCGTCTCCGGTTTGATCATTCTATT
CGAACGTCCGGTGCGTATTGGCGATACGGTAACCATTGGTAGCTTCTCGGGGACGGTAAGTAAGATCCGTATTCGTGCGA
CAACGATTACCGATTTCGATCGCAAAGAAGTGATCATCCCGAACAAAGCGTTTGTTACCGAGCGTCTGATCAACTGGTCG
TTGACTGACACTACTACGCGTCTGGTGATCCGTCTCGGCGTGGCCTATGGCTCCGATCTGGAAAAAGTGCGTAAAGTGTT
ACTGAAGGCGGCGACTGAGCACCCAAGGGTGATGCACGAACCAATGCCGGAAGTCTTCTTTACGGCATTTGGTGCCAGCA
CGTTGGATCATGAGCTGCGTCTGTATGTGCGTGAACTGCGTGACCGTAGTCGTACTGTCGATGAGCTGAACCGTACTATC
GATCAGCTGTGCCGTGAAAACGACATCAACATTGCCTTTAACCAGCTTGAAGTGCATCTGCACAACGAGAAGGGCGATGA
GGTGACGGAAGTAAAACGCGACTACAAAGGCGATGACCCGACGCCAGCGGTAGGGTAAAAACGAAAGGGGGCAACATTTA
GTTGCCCCGAGATTGCTAACAAAGTGCGCGTTGTTCATGCCGGATGCGGCGTGACCGCCTTATCCGGCCTACGAAACCGC
AAGAATTCAATATATTGCAGGAGCGGTGTAGGCCTGATAAGCGTAGCGCATCAGGCAGTTTTGCGTTTGCCCGCAACCTT
AGGGGACATTTAGCGACCCCATTTATTTCTCACTTTTCCGCCTCATCATCGCGCGTTAATTTCTTTTCATAATCACGCTT
TACAATATCCAGCGCGCGCAGCACGGTACTGGCAGGGATCTGATTTTCCTCCAGCAGCACAATCAAATCGACAGCCAGTT
TGACATCGTCAGGGGCATTTTCCAGTGACATATTCTCTCCATTGCTAGCGGGTTAAACGCGCTAACCTGTTTTCGATTTT
TTCCAGCGCATGGCGGCAGCGTGCCAGGCGCGCTTCATAGGCTTCCACTTCACGATGCAGCGTTTGCTGTTCCACGAGAT
CGGTCACCCGCGCCAGACGGGCTCTGCGTTCGGCAACCATCTCACGTAGCCGCCGCTCAAAATCCTGATGCTGAATACGT
TTACGCTGCCAGCGGGCAATTTTCGGTGGTGCACTGTCCCACTCGCGCAATGACCAGGCGGAGGCTTCACGCGCGATGGC
TTCCAGTTGTGCCGCCAGATGTTCCGCCAGCCAGGCCACTTGCGGCAGCTGTTGCTGCTCAACTGCATGACGAAGCGCAG
CCAGATTATCGCCCGCCTCGTCGAGACAAGCCTGTAGTGTTGTCGCACGAGTCTGAAAAAGATGCCTGTCGAAACGAGCA
CTTAGCGTGGCGAACTGTGACACCGGGGCACAACGCTGACGCAGCGTAGCGAGCTGTCCTTCCAGTTTTTCCAGCAGCAG
GGCGGTTTTCACGATAAGACCTCAATGAAAATGATAATTGTTATGCTAAAGTAGCCACTCTGTAAGCTGACAACACTCAA
TATGCAACGAATCATTTTAATCATCATTGGCTGGCTGGCGGTAGTGCTGGGTACGCTGGGCGTGGTATTACCGGTATTAC
CGACGACGCCGTTTATCCTGCTGGCGGCCTGGTGCTTTGCCCGTTCTTCCCCGCGCTTTCACGCCTGGTTGCTGTACCGC
TCATGGTTTGGCAGCTATCTACGTTTCTGGCAGAAACATCATGCGATGCCGCGCGGCGTCAAACCGCGGGCGATTTTGCT
TATTTTGCTCACGTTTGCCATTTCTCTGTGGTTCGTCCAGATGCCATGGGTGCGCATCATGTTGCTGGTAATTCTCGCCT
GTTTGCTTTTCTATATGTGGCGAATTCCGGTGATTGATGAAAAGCAAGAAAAGCACTGAAGCACAACAATCGCAGTTGCA
ATTATTGCGTACAGCCAGTACATTCTGGCGTTTTCGAGCACAGGCGCAGGCGGTCAAAGGTTAAACAACTGTTACTTTTG
ATACGTTTAAAACGCGCCGTGAGTACCACCGTAACAAGCAGGCATACACTTATGACCGCGACTGCACAGCAGCTTGAGTA
TCTCAAAAATAGCATCAAAAGCATTCAGGACTACCCAAAACCCGGCATTCTTTTCCGCGATGTCACCAGCTTACTGGAAG
ACCCGAAAGCTTACGCTCTCAGCATCGACTTGCTGGTTGAGCGTTACAAAAATGCGGGCATTACCAAAGTTGTCGGCACC
GAAGCGCGTGGCTTCTTGTTTGGCGCTCCGGTAGCTCTGGGTCTGGGCGTTGGCTTTGTACCGGTCCGTAAACCGGGCAA
ACTGCCGCGTGAAACCATCAGTGAAACTTACGACCTGGAATACGGCACCGATCAGCTGGAGATCCACGTTGATGCCATCA
AACCGGGCGACAAAGTTCTGGTGGTGGACGACCTGCTGGCAACCGGCGGCACTATCGAAGCGACCGTTAAACTGATCCGT
CGTCTGGGTGGTGAAGTGGCTGACGCTGCGTTCATTATCAACCTGTTCGATCTCGGCGGCGAACAGCGTCTCGAAAAACA
GGGCATTACCAGCTACAGCCTTGTCCCGTTCCCGGGCCATTAATTATCGCCAGTCTTGTGCTGCCCACGCTACGGACAGC
ACAAGATGTGCATTCAGCCTCGCCGTTCTGACGGGGCTGTGTTAGCATTACCCCTTCGTGAATCCACCTTCCAGCGTTTC
AGAGCCTGCCAATGAGTTATCAGGTCTTAGCCCGAAAATGGCGCCCACAAACCTTTGCTGACGTCGTCGGCCAGGAACAT
GTGCTGACCGCACTGGCGAACGGCTTGTCGTTAGGGCGTATTCATCATGCTTATCTTTTTTCCGGCACCCGTGGCGTCGG
AAAAACCTCTATCGCCCGACTGCTGGCGAAGGGGCTAAACTGCGAAACCGGCATTACCGCGACGCCGTGCGGCGTGTGCG
ATAACTGTCGTGAAATCGAGCAGGGGCGCTTTGTCGATCTGATTGAAATCGACGCCGCCTCGCGCACCAAAGTTGAAGAT
ACCCGCGACCTGCTGGATAACGTCCAGTACGCTCCGGCGCGTGGTCGTTTCAAAGTTTATCTGATCGACGAAGTGCATAT
GCTGTCGCGCCACAGCTTTAACGCACTGTTAAAAACCCTTGAAGAGCCGCCGGAGCACGTTAAGTTTCTGCTGGCGACGA
CCGATCCACAGAAATTGCCGGTGACGATTTTGTCACGCTGTCTGCAATTTCATCTCAAGGCGCTGGATGTCGAGCAAATT
CGCCATCAGCTTGAGCACATCCTCAACGAAGAACATATCGCTCACGAGCCGCGGGCGCTGCAATTGCTGGCACGCGCCGC

TGAAGGCAGCCTGCGAGATGCCTTAAGTCTGACCGACCAGGCGATTGCCAGCGGTGACGGCCAGGTTTCAACCCAGGCGG
TCAGTGCGATGCTGGGTACGCTTGACGACGATCAGGCGCTGTCGCTGGTTGAAGCGATGGTCGAGGCCAACGGCGAGCGC
GTAATGGCGCTGATTAATGAAGCCGCTGCCCGTGGTATCGAGTGGGAAGCGTTGCTGGTGGAAATGCTCGGCCTGTTGCA
TCGTATTGCGATGGTACAACTTTCGCCTGCTGCACTTGGCAACGACATGGCCGCCATCGAGCTGCGGATGCGTGAACTGG
CGCGCACCATACCGCCGACGGATATTCAGCTTTACTATCAGACGCTGTTGATTGGTCGCAAAGAATTACCGTATGCGCCG
GACCGTCGCATGGGCGTTGAGATGACGCTGCTGCGCGCGCTGGCATTCCATCCGCGTATGCCGCTGCCTGAGCCAGAAGT
GCCACGACAGTCCTTTGCACCCGTCGCGCCAACGGCAGTAATGACGCCAACCCAGGTGCCGCCGCAACCGCAATCAGCGC
CGCAGCAGGCACCGACTGTACCGCTCCCGGAAACCACCAGCCAGGTGCTGGCGGCGCGCCAGCAGTTGCAGCGCGTGCAG
GGAGCAACCAAAGCAAAAAAGAGTGAACCGGCAGCCGCTACCCGCGCGCGGCCGGTGAATAACGCTGCGCTGGAAAGACT
GGCTTCGGTCACCGATCGCGTTCAGGCGCGTCCGGTGCCATCGGCGCTGGAAAAAGCGCCAGCCAAAAAAGAAGCGTATC
GCTGGAAGGCGACCACTCCGGTGATGCAGCAAAAAGAAGTGGTCGCCACGCCGAAGGCGCTGAAAAAAGCGCTGGAACAT
GAAAAAACGCCGGAACTGGCGGCGAAGCTAGCGGCAGAAGCCATTGAGCGCGACCCGTGGGCGGCACAGGTGAGCCAACT
TTCGCTACCAAAACTGGTCGAACAGGTGGCGTTAAATGCCTGGAAAGAGGAGAGCGACAACGCAGTATGTCTGCATTTGC
GCTCCTCTCAGCGGCATTTGAACAACCGCGGTGCACAGCAAAAACTGGCTGAAGCGTTGAGCATGTTAAAAGGTTCAACG
GTTGAACTGACTATCGTTGAAGATGATAATCCCGCGGTGCGTACGCCGCTGGAGTGGCGTCAGGCGATATACGAAGAAAA
ACTTGCGCAGGCGCGCGAGTCCATTATTGCGGATAATAATATTCAGACCCTGCGTCGGTTCTTCGATGCGGAGCTGGATG
AAGAAAGTATCCGCCCCATTTGATCGTAAGCACAGCTTACGTTCGTCATCCTTAACGTGATTGAGAGAGAAACCTATGTT
TGGTAAAGGCGGTCTGGGTAACCTGATGAAGCAAGCCCAGCAGATGCAAGAAAAAATGCAGAAAATGCAGGAAGAGATCG
CGCAGCTGGAAGTCACCGGCGAATCTGGCGCAGGTCTGGTAAAAGTGACCATCAACGGTGCACACAACTGCCGTCGCGTA
GAGATCGACCCGAGCCTGCTGGAAGACGACAAAGAGATGCTGGAAGACCTGGTGGCTGCAGCATTCAACGACGCAGCACG
TCGTATTGAAGAAACGCAGAAAGAAAAAATGGCCTCTGTATCCTCCGGAATGCAGCTGCCGCCTGGCTTTAAGATGCCGT
TCTGATGCAAACCAGCCCGCTGTTAACACAGCTTATGGAAGCACTGCGCTGTCTGCCGGGCGTTGGCCCGAAGTCGGCGC
AGCGTATGGCGTTCACGCTGCTTCAGCGCGATCGTAGCGGCGGGATGCGTCTGGCGCAGGCGCTCACCCGGGCGATGTCG
GAAATCGGCCACTGCGCCGATTGCCGCACTTTCACCGAACAGGAAGTCTGTAACATCTGTTCGAATCCGCGTCGTCAGGA
AAACGGTCAAATCTGCGTGGTGGAGAGTCCGGCGGACATCTACGCCATTGAGCAGACGGGGCAGTTTTCAGGTCGTTATT
TTGTGTTGATGGGGCATCTGTCACCGCTGGACGGCATCGGTCCGGATGATATCGGGCTTGATCGTCTGGAACAGCGTCTG
GCAGAGGAAAAAATCACTGAAGTGATCCTCGCCACCAACCCCACGGTTGAAGGTGAAGCTACCGCTAACTACATTGCCGA
GCTTTGCGCGCAATATGACGTGGAAGCCAGCCGAATCGCTCATGGCGTTCCGGTTGGCGGCGAGCTGGAAATGGTCGACG
GCACCACGTTGTCACACTCCCTTGCCGGGCGTCATAAGATTCGTTTTTAAGCAAACGAGAGCAGGATCACCTGCTCTCGC
TTGAAATTATTCTCCCTTGTCCCCATCTCTCCCACATCCTGTTTTTAACCTTAAAATGGCATTATTGAGGTAGACCTACA
TGAAAGGACAAGAAACTCGTGGTTTTCAGTCAGAAGTGAAACAGCTTCTGCACCTGATGATCCATTCTCTCTATTCCAAT
AAAGAAATCTTCCTGCGTGAGCTTATCTCTAACGCCTCCGATGCGGCGGACAAGCTGCGTTTCCGTGCGCTCTCTAACCC
GGACCTGTACGAAGGTGATGGCGAACTACGCGTTCGTGTCTCTTTCGATAAAGACAAGCGTACGCTGACCATCTCCGATA
ACGGCGTGGGGATGACCCGCGACGAAGTGATTGACCATCTGGGGACTATCGCTAAATCCGGTACCAAATCATTCCTCGAA
TCCCTGGGTTCTGACCAGGCGAAAGACAGCCAGCTGATCGGTCAGTTTGGTGTTGGTTTCTACTCTGCGTTTATCGTGGC
CGACAAAGTGACCGTGCGTACTCGCGCGGCAGGCGAAAAACCAGAAAATGGCGTCTTCTGGGAATCGGCTGGCGAAGGTG
AATACACCGTTGCCGACATCACCAAAGAAGATCGTGGTACTGAAATCACCCTGCATCTGCGTGAAGGCGAAGACGAGTTC
CTCGATGACTGGCGCGTGCGTTCCATCATCAGCAAATACTCCGACCATATCGCGCTGCCGGTAGAGATCGAAAAACGCGA
AGAGAAAGACGGCGAAACCGTTATCTCCTGGGAGAAAATCAACAAAGCGCAGGCGCTGTGGACTCGTAACAAGTCGGAAA
TCACCGATGAAGAGTACAAAGAGTTCTACAAACACATCGCCCACGACTTTAATGATCCGCTGACCTGGAGCCACAACCGT
GTTGAAGGTAAGCAGGAGTACACCAGCCTGCTGTACATCCCGTCCCAGGCTCCGTGGGATATGTGGAACCGCGATCATAA
ACACGGCCTGAAACTGTATGTTCAGCGTGTGTTCATCATGGACGACGCAGAACAGTTCATGCCGAACTATCTGCGCTTCG
TGCGTGGTCTGATTGACTCCAGCGATCTGCCGCTGAACGTTTCCCGTGAAATCCTCCAGGACAGCACGGTAACGCGTAAC
CTGCGCAATGCGCTGACCAAGCGTGTGCTGCAAATGCTGGAAAAACTGGCGAAAGACGACGCGGAAAAATACCAGACCTT
CTGGCAACAGTTTGGCCTGGTACTGAAAGAAGGTCCGGCGGAAGATTTCGCTAACCAGGAAGCGATCGCCAAACTGCTGC
GTTTTGCTTCTACCCATACCGATTCTTCTGCGCAGACCGTATCTCTGGAAGACTACGTTTCCCGCATGAAAGAAGGGCAG
GAGAAAATCTACTACATCACCGCAGACAGCTATGCGGCAGCGAAGAGCAGCCCGCACCTGGAACTGCTGCGTAAGAAAGG
CATCGAAGTTCTGCTGCTTTCCGACCGCATCGATGAGTGGATGATGAACTATCTGACTGAGTTCGACGGTAAACCGTTCC
AGTCGGTGTCTAAAGTTGACGAGTCGCTTGAAAAACTGGCTGACGAAGTTGATGAGAGCGCGAAAGAAGCGGAGAAAGCA
CTGACTCCGTTCATCGACCGTGTGAAAGCCCTGCTCGGCGAGCGCGTGAAAGATGTCCGTCTGACTCACCGTCTGACCGA
TACGCCAGCGATCGTTTCGACCGACGCGGACGAAATGAGCACTCAGATGGCGAAACTGTTCGCTGCGGCGGGCCAGAAAG
TGCCAGAAGTGAAATACATCTTCGAACTGAACCCGGATCACGTACTGGTGAAACGTGCGGCAGATACTGAAGATGAAGCG
AAGTTCAGCGAGTGGGTAGAACTGCTGCTGGATCAGGCGCTGCTGGCAGAACGCGGCACGCTGGAAGATCCGAACCTGTT
TATTCGTCGTATGAACCAGCTGCTGGTTTCCTGATGTAATGCCGGATGACCTTCGTGTCATCCGGCATTTTTCTTTTCAT
CATCTGCACTTTCCGCAAATTATCTCGCCATTAACCGTTTCAGCCCCAGGTGCCTTTCTTGAGGCAATCGCCTGTTGGTG
GTATCGTTTATCGCTTTTTCAAAAAATTCGACACATTTTAAGGGGATTTTCGCAATGCGTATCATTCTGCTTGGCGCTCC

GGGCGCGGGGAAAGGGACTCAGGCTCAGTTCATCATGGAGAAATATGGTATTCCGCAAATCTCCACTGGCGATATGCTGC
GTGCTGCGGTCAAATCTGGCTCCGAGCTGGGTAAACAAGCAAAAGACATTATGGATGCTGGCAAACTGGTCACCGACGAA
CTGGTGATCGCGCTGGTTAAAGAGCGCATTGCTCAGGAAGACTGCCGTAATGGTTTCCTGTTGGACGGCTTCCCGCGTAC
CATTCCGCAGGCAGACGCGATGAAAGAAGCGGGCATCAATGTTGATTACGTTCTGGAATTCGACGTACCGGACGAACTGA
TCGTTGACCGTATCGTCGGTCGCCGCGTTCATGCGCCGTCTGGTCGTGTTTATCACGTTAAATTCAATCCGCCGAAAGTA
GAAGGCAAAGACGACGTTACCGGTGAAGAACTGACTACCCGTAAAGATGATCAGGAAGAGACCGTACGTAAACGTCTGGT
TGAATACCATCAGATGACAGCACCGCTGATCGGCTACTACTCCAAAGAAGCAGAAGCGGGTAATACCAAATACGCGAAAG
TTGACGGCACCAAGCCGGTTGCTGAAGTTCGCGCTGATCTGGAAAAAATCCTCGGCTAATTCGAAAGCGCGCACGGACAG
TCCCCTCGCCCCCTCGGGGAGAGGGTTAGGGTGAGGGGAACAGGCCCGCACAAGCAAACTTATCAGCAATCTCAGGCCGG
ATATTCATTCGGCCTTTTACAAAAACCTCAATCTGCGCTATTCCTTTTTCTGATTTGACCTCTCACAGCAATTAGTTCTT
CTTCCTCACTTTTCCGCTACAATTATCAACAAGTTGAATCGATAAGAGGCGGTAATGCGTCAGACTAAAACCGGTATCCT
GCTGGCAAACCTGGGTACGCCCGATGCCCCCACACCTGAAGCGGTAAAACGCTATCTGAAACAATTTTTAAGCGACAGAC
GCGTGGTTGATACCTCACGGTTGTTATGGTGGCCATTGCTGCGCGGCGTGATTTTGCCGCTGCGCTCGCCGCGTGTGGCG
AAGCTGTATGCCTCTGTCTGGATGGAAGGTGGCTCGCCGCTGATGGTTTACAGCCGCCAGCAACAGCAGGCGCTGGCACA
ACGTTTACCGGAGATGCCCGTAGCGCTGGGAATGAGCTACGGCTCGCCATCACTGGAAAGCGCCGTAGATGAACTCCTGG
CAGAGCATGTAGATCATATTGTGGTGCTGCCGCTTTATCCGCAATTCTCCTGTTCTACGGTCGGTGCGGTATGGGATGAA
CTGGCACGCATTCTGGCGCGCAAACGTAGCATTCCGGGGATATCGTTTATACGTGATTACGCCGATAACCACGATTACAT
TAATGCACTGGCGAACAGCGTACGCGCTTCTTTTGCCAAACATGGCGAACCGGATCTGCTACTGCTCTCTTATCATGGCA
TTCCCCAGCGTTATGCAGATGAAGGCGATGATTACCCGCAACGTTGCCGCACAACGACTCGTGAACTGGCTTCCGCATTG
GGGATGGCACCGGAAAAAGTGATGATGACCTTTCAGTCGCGCTTTGGTCGGGAACCCTGGCTGATGCCTTATACCGACGA
AACGCTGAAAATGCTCGGAGAAAAAGGCGTAGGTCATATTCAGGTGATGTGCCCGGGCTTTGCTGCGGATTGTCTGGAGA
CGCTGGAAGAGATTGCCGAGCAAAACCGTGAGGTCTTCCTCGGTGCCGGCGGGAAAAAATATGAATATATTCCGGCGCTT
AATGCCACGCCGGAACATATCGAAATGATGGCTAATCTTGTTGCCGCGTATCGCTAAAGCTGAGCGGTAAAGAACTGAGC
GCCGTCGCGAAGAGCCTCGTCGGCGGTTTTCATCATCCGTGAATAATGCAAAAAGGCGTGCAGCGTGCCTGGGTAGAGTT
TGAACTCACAGGGCTGCTGATGCGCCGCTAACGTCTGGTAAAGCAGACGGCTGTCATCCAGCAGCGGATCGAACTCCGCC
CCGGCAATAAAACAGGGCGGAACTTCGCGAGTGAGATCATTATTAAACAGACAGTAATACGGCGACTCGCGGTCCGCGTC
GTTGCTTAAATATGCCTCTTCGTACATCTGCAAATCCTGTTGCGTTAAGCCATCCCAGACACCGCCCAACAGACGACGAG
TCACGGAATCCCGTAATCCGTAAAGCCCATACCACAGCAAAACGCCCGCAACTTTACCGCAATCGATCTGTTTATCACGC
AACCACAACGCACTGGCGAGCGCCAGCATGGCACCTGCGGAATCACCGGCAAAGCCAATGCGGGACATATTGATTTGATA
ATCCTCCGCCTGCTGGTGGAAATAACAACAAGCAGCCACAATTTCCTCTATCGCTTGCGGAAAACGCGCTTCAGGTGAAA
GGGTGTAATCAATACCAATCACCGTACATTGGCTGTAGCTTGCCAGCAGGCGCATGATGCGATCGTGGGTATCGAGATTG
CCGAGAATAAAACCGCCTCCATGCAAATAAAATAGCGTCGCTGGGCTATCTGGCTGCGGACAAAAGAGACGTGTTTCCAC
CTGCCCATATTTTGTTGGAACCATGTAAGCTCTGGTTGCCATTTCTGGAGCGCCCGCATTCCAGAATCGGCGCTCAAGCG
TGTAATACTGTCGTTGCTCAGCAATCGTTCCCGTTGCGGGCCAGGGCGGTAAATCCGGCTGAAGAGTATTCACAACGGTC
TTCATTTCAGCAGAAATAAGGTCCAGAACAGGTAGTTTGTTTTCCGGCTTCATACAAAAACTCCTTTCAAATTACGTCAT
TGTAAGGAACCACTGCCATGAAAATGCGATCCCGCCTGCTGATATTGAAACTGGCTGCGTCTCGCGCGCTCCCGTCAGAT
TGTGTTAACATTCGCCGCTCAGTTAACCACCCGTAAAAACAACCATGAAATTTCCCGGTAAACGTAAATCCAAACATTAC
TTCCCCGTAAACGCACGCGATCCGCTGCTTCAGCAATTCCAGCCAGAAAACGAAACCAGCGCTGCCTGGGTAGTGGGTAT
CGATCAAACGCTGGTCGATATTGAAGCGAAAGTGGATGATGAATTTATTGAGCGTTATGGATTAAGCGCCGGGCATTCAC
TGGTGATTGAGGATGATGTAGCCGAAGCGCTTTATCAGGAACTAAAACAGAAAAACCTGATTACCCATCAGTTTGCGGGT
GGCACCATTGGTAACACCATGCACAACTACTCGGTGCTCGCGGACGACCGTTCGGTGCTGCTGGGCGTCATGTGCAGCAA
TATTGAAATTGGCAGTTATGCCTATCGTTACCTGTGTAACACTTCCAGCCGTACCGATCTTAACTATCTACAAGGCGTGG
ATGGCCCGATTGGTCGTTGCTTTACGCTGATTGGCGAGTCCGGGGAACGTACCTTTGCTATCAGTCCAGGCCACATGAAC
CAGCTGCGGGCTGAAAGCATTCCGGAAGATGTGATTGCCGGAGCCTCGGCACTGGTTCTCACCTCATATCTGGTGCGTTG
CAAGCCGGGTGAACCCATGCCGGAAGCAACCATGAAAGCCATTGAGTACGCGAAGAAATATAACGTACCGGTGGTGCTGA
CGCTGGGCACCAAGTTTGTCATTGCCGAGAATCCGCAGTGGTGGCAGCAATTCCTCAAAGATCACGTCTCTATCCTTGCG
ATGAACGAAGATGAAGCCGAAGCGTTGACCGGAGAAAGCGATCCGTTGTTGGCATCTGACAAGGCGCTGGACTGGGTAGA
TCTGGTGCTGTGCACCGCCGGGCCAATCGGCTTGTATATGGCGGGCTTTACCGAAGACGAAGCGAAACGTAAAACCCAGC
ATCCGCTGCTGCCGGGCGCTATAGCGGAATTCAACCAGTATGAGTTTAGCCGCGCCATGCGCCACAAGGATTGCCAGAAT
CCGCTGCGTGTATATTCGCACATTGCGCCGTACATGGGCGGGCCGGAAAAAATCATGAACACTAATGGAGCGGGGGATGG
CGCATTGGCAGCGTTGCTGCATGACATTACCGCCAACAGCTACCATCGTAGCAACGTACCAAACTCCAGCAAACATAAAT
TCACCTGGTTAACTTATTCATCGTTAGCGCAGGTGTGTAAATATGCTAACCGTGTGAGCTATCAGGTACTGAACCAGCAT
TCACCTCGTTTAACGCGCGGCTTGCCGGAGCGTGAAGACAGCCTGGAAGAGTCTTACTGGGATCGTTAAGTTATCGTCGG
TTCGTAGGCCAGATAAGGCGTTCACGCCGCATCTGGCATTTGGCTCTCGATGCCTGATGCGACGCTGGCGCGTCTTATCA
TGCCTACATATTTTTCATATTTACATCCGGCAACCACCGTTTACCCCGTCACCACCTCACCCGCCGGTGGCGTTTCCAG
CAGTTCCAGCATGGTACGGGCGATTTCACGCTCGCCCATCACTACCTGATTCGCACCACGTTCGGTGATATACGCCACTT

CATCGTCATAATGGGCGCGGGCAATAATCTCAATATCCGGATTTTTCGCGCGGGCAGATGCCACAATCTCACCCGCTTCA
TAACCGTTGGGAATCGTCAGGATCAGCCATTTTGCACATTCCAGATGCGCCAGTTGCATAATTTCTTCGTTCGCCGCATT
GCCCAATACTGCGCGGACCCCGCGCTCTCGCAGCTCATCAACACGGGTTCGTGACGTCTCAATCACCACCAGCGGAATAT
CAGAGGCGAGCAATTTCTCCCCCAGCAGGCTGCCTACACGACCGTAACCCACCAGTAGCGCATGGTTGCAAATATCCACT
GGGATCTGCTTCTCTTCTTCGATTGCCTCTTCCAGCGTCTGCTCTTCCAGCGTTTCGGTCTTCGCCAGATATTTCTCCAG
TAGTGCGAACAGTACCGGGTTGAGCATAATCGACAGGATCGCCCCTGCCAGTACCAGGTTTTGTCCGGCCTGCGGCAGTA
AATTCAATGCCATTCCCAGTCCCGCCAGGATAAACGCGAACTCACCAATCTGCGCCAGGCTGGCGGCGATGGTTAATGCC
GTACGTTGGGAGTGACCAAACAGTCGCACCAGGAAAAATGCGGCTAACGACTTACCAAACAGAATAATCGCCAGCGTCGC
CAGCACTGCCAGCGGTTGCTGAATCAGAATTAACGGATCAAACAACATCCCGACGGAGACAAAAAACAGCACCGCAAACG
CGTCGCGCAATGGCAGCGTATCGTGGGCGGCACGGTGACTCAGTTCAGACTCGTTCAGTACCATCCCGGCAAAGAACGCA
CCGAGTGCAAAGGAGACATCAAACAGCTCTACCGCACCAAAGGCAACCCCTAACGCCAGCGCCAGCACCGACAGGGTAAA
CAGCTCGCGAGAACCGGTTGCCGCGCTGCGTGCCATAATCCACGGCACCAGACGGCGACCTACCAGCATCATAATGGCGA
TAAATGCGATCACTTTGCCGATGGTGATCCCCATATCGACTGCAAGAGTGGCAAAGCCCACATCGCCCTGTTCCATCATT
CCTGCCACTGCGGGCAGCAACACCAGCGTCAGAACCATTACCAGGTCTTCCACAATCAACCAACCGATGGCGATTTGCCC
ACGCTGACTGTCAATTAATTGCCGTTCTTCAAGTGCGCGCAGTAACACCACGGTACTGGCGGTGGAAAGACATAAACCGA
ACACGATACCGGTCATTAACGACCAGCCCAGCACGGCAGAGAGCGCCATACCCAGCAGCGTCGCCACGGCTATCTGGGCG
ATCGCACCGGGAATGGCGATGGCCTTTACCGCCATCAAATCCTTCAGCGAAAAGTGCAAACCGACGCCAAACATCAACAG
AATGACGCCCAGTTCAGCCAGTTCCGGGGCAAGCTTGGTATCGGCAACAAAGCCCGGAGTGAATGGTCCTGCCAGCACAC
CCGCTAACAGATATCCCACCAGAGGAGAAATACGTAGTTTATTGGCCAGCATGCCGAGGATAAAGGCGAGCACAAGGCCG
CCAACAATGGTGGTGATAAGCGGGGTGGCGTGATGCATTCCGTCTCCTTTTCCTGGTGGTTATTGTCCATTTTTGGCCGG
GAAAACCAAAATTACAGGTAATAGTTTATGACAATTTCATTGATGATGTTCATGAATAATTATTGAATTTTGCAGAAAAA
TGGAATTAGCTGCAAAAAAAGCACGGATCGGAAAACAGAAGGCGGTTTAACAGAGGAAAGGCTTATGGCGACTGTAAAAC
GATGCAGCCAAAGTTTGCCTTTGGCTGCAATGAAATCAGTCTTTATGCCGGTTATCAGGCAGGAATATGGTCAACATCCC
CAATAGTGGCAGGAAAGCACAGATTTTATAGACTAACTCGATGCTGGTGTGATCGGCGATAAGCCCCAGAACTGCCGCTC
CCAGACCTCCCATGCCAAAAGCAAAACCGAAAAAGAGTCCAGAAACCATACCGATACGTCCTGGAAGCAGCTCCTGAGCG
TAGACCAGAATGGCAGAGAATGCCGAAGCGAGGATAAATCCAATAATCACCGTTAAAACCCCCGTCCAGTGCAGGCTGGC
GTAGGGTAAAATCAGCGTAAACGGCGCAACGCCGAGGATAGAGCCCCAAATCACATATTTCCGCCCAATTTTATCCCCTA
CAGGCCCGCCGATCACCGTACCTGCCGCAACGGCAAACAGGAAGGCAAACAGATGAAGCTGAGCATTCTGGATAGATAAT
CCGAATTTTTGCATCAGATAAAAGGTGTAATAGCTGCTGATGCTCGCCATATAGAAATATTTCGAGAAAATGAGGATTAA
CAGAATGCTGACCGCCAGTACAACTTTATTGCGCGGCAGTGGATTGATAATCGTCGCTTTGGGTTTTCCTTTATTCATTC
GGTGCTGTGCCGAGTACCAACGGCTGATTTGCGCCAACACCACGATCGCCAGCAGTGCCGCAAGCACAAACCAGGCAACG
TTGCCTTTGCCATAAGGCGCGATAATCACCGCCGCCAGCAAGGGTCCCAGGGAACTGCCAAAGTTGCCGCCGACCTGAAA
GATAGATTGCGCCAGGCCATGCCGCCCGCCGGAAGCCATACGGGCCACGCGAGAAGATTCCGGATGAAAGACCGATGAAC
CGGTACCGACCAGCGCCGCCGCCAGCAGAACTGCGCCAAAACTGCCCGCCAGCGCAAGCAGCACCAGACCGCTTAAGGTA
AAGCACATGCCAATTGGCAACGACCATGGCATCGGATATTTATCGGTCCAGTAGCCGACCACTGGTTGCAGTAGCGAAGA
GGCGAGCTGGAAGGTGAGGGTTATCATGCCAATCTGCATAAATGTCAGAGAAAATTCTGACTGAAGCAGCGGATAAATCG
CCAGAATCAGCGATTGGATCATGTCGTTCAGCAGATGTGAGAGGCTGATAGCACCTAAAATACCAAACGATGTTCGGGCC
TTGGTCGTTGACGCAGCCGCGCCCGCCACAGGCTGGGGTTGTTCACTCATTGCCATAGGAAAGTCACTTTTTCAGGGTTG
CGATGTAAAGAATGATCTTATTTGTGATTATTACCAGACTAACATACCTGTATGCGTCGTCTGAAGGAAGTCTCAACGCC
GAATACAGAATTTCTAATCTGGATGCAGATTTATCTTCACCGGACGCAGACTTGTCTATGATGTCGCGTCATACTATTTT
TCAACACGTTGAAATCAGGTCAGGGAGAGAAGTATGAAATTATTGCAGCGGGGCGTGGCGTTAGCGCTGTTAACCACATT
TACACTGGCGAGTGAAACTGCTCTGGCGTATGAGCAGGATAAAACCTACAAAATTACAGTTCTGCATACCAATGATCATC
ATGGGCATTTTTGGCGCAATGAATATGGCGAATATGGTCTGGCGGCGCAAAAAACGCTGGTGGATGGTATCCGCAAAGAG
GTTGCGGCTGAAGGCGGTAGCGTGCTGCTACTTTCCGGTGGCGACATTAACACTGGCGTGCCCGAGTCTGACTTACAGGA
TGCCGAACCTGATTTTCGCGGTATGAATCTGGTGGGCTATGACGCGATGGCGATCGGTAATCATGAATTTGATAATCCGC
TCACCGTATTACGCCAGCAGGAAAAGTGGGCCAAGTTCCCGTTGCTTTCCGCGAATATCTACCAGAAAAGTACTGGCGAG
CGCCTGTTTAAACCGTGGGCGCTGTTTAAGCGTCAGGATCTGAAAATTGCCGTTATTGGGCTGACAACCGATGACACAGC
AAAAATTGGTAACCCGGAATACTTCACTGATATCGAATTTCGTAAGCCCGCCGATGAAGCGAAGCTGGTGATTCAGGAGC
TGCAACAGACAGAAAAGCCAGACATTATTATCGCGGCGACCCATATGGGGCATTACGATAATGGTGAGCACGGCTCTAAC
GCACCGGGCGATGTGGAGATGGCACGCGCGCTGCCTGCCGGATCGCTGGCGATGATCGTCGGTGGTCACTCGCAAGATCC
GGTCTGCATGGCGGCAGAAAACAAAAAACAGGTCGATTACGTGCCGGGTACGCCATGCAAACCAGATCAACAAAACGGCA
TCTGGATTGTGCAGGCGCATGAGTGGGGCAAATACGTGGGACGGGCTGATTTTGAGTTTCGTAATGGCGAAATGAAAATG
GTTAACTACCAGCTGATTCCGGTGAACCTGAAGAAGAAAGTGACCTGGGAAGACGGGAAAAGCGAGCGCGTGCTTTACAC
TCCTGAAATCGCTGAAAACCAGCAAATGATCTCGCTGTTATCACCGTTCCAGAACAAAGGCAAAGCGCAGCTGGAAGTGA
AAATAGGCGAAACCAATGGTCGTCTGGAAGGCGATCGTGACAAAGTGCGTTTTGTACAGACCAATATGGGGCGGTTGATT
CTGGCAGCCCAAATGGATCGCACTGGTGCCGACTTTGCGGTGATGAGCGGAGGCGGAATTCGTGATTCTATCGAAGCAGG

CGATATCAGCTATAAAAACGTGCTGAAAGTGCAGCCATTCGGCAATGTGGTGGTGTATGCCGACATGACCGGTAAAGAGG
TGATTGATTACCTGACCGCCGTCGCGCAGATGAAGCCAGATTCAGGTGCCTACCCGCAATTTGCCAACGTTAGCTTTGTG
GCGAAAGACGGCAAACTGAACGACCTTAAAATCAAAGGCGAACCGGTCGATCCGGCGAAAACTTACCGTATGGCGACATT
AAACTTCAATGCCACCGGCGGTGATGGATATCCGCGCCTTGATAACAAACCGGGCTATGTGAATACCGGCTTTATTGATG
CCGAAGTGCTGAAAGCGTATATCCAGAAAAGCTCGCCGCTGGATGTGAGTGTTTATGAACCGAAAGGTGAGGTGAGCTGG
CAGTAATCCGAAAGTGCCGGATGTTTGCATCCGGCACAATGCTTAATCGCGGCGGGCGATATCAGCAAATTTGGCATCGA
GGATCTTTGCCAGATCGCCTGCCGCCAGTTCGATATCCAGTCCGCGCTTGCCGCCGGAAACATAAATAGTGGCAAATTCT
TGTGCGGGGGCGTCGATAATCGTTGGCAGACGTTTTTTCTGCCCCAGTGGGCTAATCCCCCCAACCAGGTATCCCGTCGA
ACGCTGCGCGACCATCGGATCGGCCATCTCAACTTTCTTGGCACCCAGCGCTTTTGCTACTTTTTTAAGATCCAGTTGAC
CTGCGACCGGCGTAACGGCCACGGCAAGGTGTTTCATATCACCGTTCACTGCCACCAGCAGCGTTTTGTAGACCTGATCC
GGATTCAAACCTAATTTTTTGACGACTTCATCGCCAAAATTGGTTTCAGCCGGATCGTGCTCGTAGGTATGGATTTGAAA
CGAAATCTTGTTTTTTTCGAGTAATTTAACTGCGGGTGTCATGGTAATTTTCCTGCCTTAAACAAAAAAACGAAGCAAGC
ATACGCCTGATGGCGGTCCAAAAAAAAGAGTCATCTTGCCTAAGAGTATTGGCAGGATGGTGAGATTGAGCGACAATCGA
GTTACACCGTCGCTTAAAGTGACGGCATAATAATAAAAAAATGAAATTCCTCTTTGACGGGCCAATAGCGATATTGGCCA
TTTTTTTAGCGCAACATTTGCGGCAAATTCCCTTCTCCATACAGGTGTAGTGCACCGACCGCGACCACATATCGCCCCGG
CGGCATGGCGCGTAATTTATCCCGCCAGGCGAGATTTCGCTGATGCATCAGCACATCGTACAGCGACTGACTGAACGTAT
TGGGCAGCGTTATATCATTATTTTGCGGCGGTGCATTCAGCCACCAGCTCATCATTTGTTGCAGCAACCGTGCGTTGGTA
TGCCAGTGGGTCAGCGTATCGTCCAGCAGCGCCAGTCCTTTGTCAGGGAGCTGGAGCAACATGGCAATCTGGTTTTCAGC
CCCTTCCAGTTCAATCACGGGTTTATGTTGTTGCTTCGCCGCCTGCAATAGCTGGTAATCGATACCGTATTCTGCCCGCA
GCCCCAGTTTTTGTGCCTGCGTCGCCTGAAGAACCATCGCGATTTGCCACAGCGGTTGGGTAGAAAAGAGTGAGGGAGAA
ATGCCCATCTCCTGGCTAATGTGCTGCAGGTTTTGGAGTTGCTCCTCGCTAATGCGCTCTTCCAGCGCCTCGCAGGCAGG
CAAATTAGCAAAAGGCGTATCGCTGGTGGAAACATCCGCCTCGACGATCAGCGCATCGGCGTTTTTGAGCTTTTTGAGCA
AACGGGTGGGCAGGGGAGCCATATCGTGGCTACCCATATGAATACTGCCAATCAGATGAAAATGGCGATTGCCGGGGAGG
GTGATATCGATGGCAGGCCAGGTGTAATGATTACCGCGCAGCGCGGCCCAAAGCGTTTTTACCCGGTACAACAGATCCAT
ACGACCTCCCTTTGTGAAATATCATGCTAGCGCGCGGTGAGGGATGGCGCAAATGGAGTTGGAGACTTGTTTAATGTGTT
TGTATGATTCAGTATGTTCTTGCATCGCTATTCACAAGGAAGCAACAGTTAAAAACCATGAAACAGGCAACAAGAAAACC
GACGACACCCGGAGATATTCTTCTCTATGAATATCTGGAACCGCTCGATTTGAAAATCAATGAGTTAGCAGAGTTGCTGC
ATGTTCATCGTAATAGCGTCAGTGCACTGATCAATAACAATCGTAAACTCACTACTGAGATGGCATTTCGTCTGGCGAAA
GTTTTTGATACCACAGTCGATTTTTGGCTAAACCTCCAGGCGGCGGTTGATCTTTGGGAAGTTGAAAACAACATGCGCAC
CCAGGAAGAATTGGGACGGATTGAAACAGTGGCTGAATATTTGGCACGCCGTGAAGAGCGTGCAAAAAAGGTCGCGTAAG
GCCAAAACATCGCTTCATCTCGTAGGCCGGATAAGGCGTTCACGCCGCATCCGGCATCCGAGCACCATTGCCTGATGCGA
CGCTTACCCGTCTTATCAGGCCTACAAACTTGTGCCTGAACCGTAGGCCGGATAAGGCGTTTACGCCGCATCCGGCATCC
GAGCACCATTGCCTGATGCGACGCTTACGCGTCTTATCAGGCCTACAAACCTGTGCCTGAACCGTAGGCCGGATAAGGCG
TTTACGCCGCATCCGGCAGTCATGCGTCGATGCCAAATGCGCCACCCTAAAGCAGCGCATCCGCAATGATGTACTTATTC
CTTCGGTTTAAACCGCAGCAACCGGTTGGCGTTACTCACTACGGTAATCGACGAGAGCGCCATTGCCGCTCCGGCAACTA
CCGGGTTAAGCAGTGTTCCAGTGAACGGCCACAAAATACCGGCGGCGACCGGAATACCGATACTGTTGTAGATAAACGCA
CCGAGCAGGTTCTGCTTCATGTTGTGCAGCGTTGCGCGGGAAATAGCGAGCGCATCCGCAACGCCCATCAGGCTATGGCG
CATCAGGGTAATCGCCGCGGTTTCAATGGCAACATCACTGCCGCCACCCATCGCAATGCCGACATCCGCCTGAGCCAGCG
CTGGCGCGTCGTTAATGCCGTCGCCCACCATTGCCACCTGACGTCCTTCACTTTGCAGATGTTTGATCGCTTCGGCTTTA
CCGTCCGGCAGCACCCCGGCGATCACCTCATCAATCCCTGCTTCTTTGGCGATCGCATTGGCGGTGGTTGGGTTATCCCC
GGTCAACATCACCAGACGATATCCCGCTTTATGCAGGCGTTGCAGCGCCGCCACGCTATCACTACGCAACGGATCGCGTA
CTGCCAGCAGGGCTACCGCTTTCCCGTCAACCGCCAGCAGCACAGGCGTTGCCCCTTGCGATGCCTGAGCAGTAATCTCC
GCTTCGATAGCTTTGGTACCAACCTGTTGCTCATTTAACAGCGCCTGATTGCCCAGCAATAACGCATGACCTTCAGCTTC
ACCGCTCACGCCCAGCCCGCGCAATGTGCGGAAACCGTTGACCTGCGGTAGCTGCATATCACCTGCTTTATCGAGGATCG
CTCGTGCCAGCGGATGGCTGGAACCTTGCTCCAGTGCCGCCGCCAGACGCAATGCCTGCGCTTCATCAACATCAGCAAAT
GTTTTCACTGCGACAACCTGCGGCTTCCCTTCAGTCAGCGTCCCGGTTTTATCGAACACTACAGTGTCGAGTGTACTGGC
GCGTTGCAGCGCGTCAGCGTCCCGCACCAGCACGCCAAACTCAGCCGCCCGCCCGACGCCGGAAATAATCGACATCGGCG
TCGCCAGCCCCAGCGCACACGGACAGGCAATAATCAGTACCGTGGTGGCAATCACCAGGGTATAGACAATCTGCGGTGCC
GGACCAAAGAAATACCAGATTGCCGCACTGACAAGCGCAATAACCACCACTACCGGCACAAATACGGCTGAGATTTTATC
CGCCAGCTGACCGATTTCTGGCTTGCTGCTCTGGGCCTGGCGCACCATGCGAATGATTCGTGACAGCGTAGTATGGCTGC
CAACCGCACTGGCACGAAACAGCACACTGCCGTCCTGTACCACTGTCCCGGCATGGACGCTATCGCCTTCGCCTTTTTGC
TGCGGGATTGGTTCGCCCGTCAGCATCGCTTCATCCAGCCATGCTTCGCCCTGGGTAATCTCGCCATCTACCGGCACGCG
ATCGCCGGTCGTCAGGCGCAGCAACATACCTGGCTGCACTTCTGCCAGAGGCACGCTTTTTTCACCTTCGTCAGTAACCA
GGCGTGCCGTCGGCGGGGTTAAATCGAGTAACTTTTCCAGCGCCTTAGAAGAACGCTGGCGTGCGCGCGCTTCCAGCATA
TGGCCGAGATTGATCAGACCGATAATCATCGCGCTGGCTTCGTAATAAAGATGTCGCGCTTCCATCGGGAACCACTGCGG
CCACAGGTTGACGCTCATCGAATAGAGCCACGCCACGCCAGTACCCAGCGCCACCAGCGTATCCATCGTCGCCGCACCGT

TCAGCAGGCTTTTCCATGCACTGCGGTAAAAATGGCCGCCGGCGAAAACCATCACTGCCAGGGTTATCAGGCCGATAACC
AACCACAGGCTGCGGTTGTCAGCGGTGACCATCATGTTATCGCCGATCATCCCCCAGACCATCACCGGGATACCCACCGC
CAGTGCGACAATTGCCTGCCAGCGGAAGCGCTTCATCGTAGCGACGGCGGTTTCTTGCTGGCGCTCGCGGCGTTTAGCGT
CATCTTCAATCGCTTCCGCGCCGTAGCCCGCTTTTTCCACCGCCTGCACTAAATCTTGTGGGGAGGCACTGCCCATCACC
AGCGCAGTACGCTCCGCCAGGTTTACCCGTGCCTGAGTGACGCCCGGTACGCTTTGCAGCGCATTTTGTACGCGGGTGAC
ACAGCTGGCGCAGCTCATGCCGCTCAGCAGCAACTGCTGGCTGTCATCGTCATCGGCGGTCGCTGCCGGAAGCGCCTCAG
AAACCGCTGTCAGTGCTTCCGACGGGATTGATGACTCCGCCAGCGGTTTAGCCTTTGGGTGGCTTACAGATGCGTCATAA
CCCGCTTGTTTGATGGTTTCGATCAGCTGTTCTGCACTGGCAGTCCCGGTAACGTGCGCTTCAGTGATAGACACATCCGC
CTGCTCAACATCCGGACGCTGTTCAAGACTTTCTTTCACGCGTTTAACGCAGTGACCGCAGGACAGGCCGTCCAGGGTCA
GGTCGATAGTTTGTGACATAAAACACTCCTTTAAGACAGTTTTGACTGGCTGTGATAAAGGTTAAACCTTCCAGCAAGGG
GAAGGTCAAGAAATTAATAAACCAGGCGGGTAAAAGTCCGTAAAGATTAAAAAATCGGCTCGATTTGCATCAGGATTAGA
CATTTATCTCTTTGTTTTCCTGTAGTTAAGTTGCGGGTGCTAAGTTAAATCAGGATGCCTGAAAATCGGCACCGGGGTGA
GGAATTACCTCCCGCATCTATAAAAAGGAGTTAACAAAAGATGTTAGATGCAAACAAATTACAGCAGGCAGTGGATCAGG
CTTACACCCAATTTCACTCACTTAACGGCGGACAAAATGCCGATTACATTCCCTTTCTGGCGAATGTACCAGGTCAACTG
GCGGCAGTGGCTATCGTGACCTGCGATGGCAACGTCTATAGTGCGGGTGACAGTGATTACCGCTTTGCACTGGAATCCAT
CTCGAAAGTCTGTACGTTAGCCCTTGCGTTAGAAGATGTCGGCCCGCAGGCGGTACAGGACAAAATTGGCGCTGACCCGA
CCGGATTGCCCTTTAACTCAGTTATCGCCTTAGAGTTGCATGGCGGCAAACCGCTTTCGCCACTGGTAAATGCTGGCGCT
ATTGCCACCACCAGCCTGATTAACGCTGAAAATGTTGAACAACGCTGGCAGCGAATTTTACATATCCAACAGCAACTGGC
TGGCGAGCAGGTAGCGCTCTCTGACGAAGTCAACCAGTCGGAACAAACAACCAACTTCCATAACCGGGCCATAGCCTGGC
TGCTGTACTCCGCCGGATATCTCTATTGTGATGCAATGGAAGCCTGTGACGTGTATACCCGTCAGTGCTCCACGCTCCTC
AATACTATTGAACTGGCAACGCTTGGCGCGACGCTGGCGGCAGGTGGTGTGAATCCGTTGACGCATAAACGCGTTCTTCA
GGCCGACAACGTGCCGTACATTCTGGCCGAAATGATGATGGAAGGGCTGTATGGTCGCTCCGGTGACTGGGCGTATCGTG
TTGGTTTACCGGGCAAAAGCGGTGTAGGTGGCGGTATTCTGGCGGTCGTCCCTGGAGTGATGGGAATTGCCGCGTTCTCA
CCACCGCTGGACGAAGATGGCAACAGTGTTCGCGGTCAAAAAATGGTGGCATCGGTCGCTAAGCAACTCGGCTATAACGT
GTTTAAGGGCTGATCATGATGAACACGGAAGGTAATAACGGTAACAAACCTCTCGGTCTATGGAACGTCGTTTCCATCGG
CATTGGGGCAATGGTGGGGGCGGGGATCTTCGCGCTGCTGGGGCAGGCTGCATTGCTAATGGAAGCCTCGACCTGGGTCG
CCTTTGCTTTTGGCGGTATTGTGGCGATGTTTTCCGGTTATGCCTATGCGCGTCTGGGGGCGAGCTATCCCAGCAATGGC
GGCATTATCGACTTCTTTCGTCGCGGATTAGGCAACGGCGTCTTTTCGCTGGCGCTCTCGTTACTGTACCTGTTGACGCT
GGCGGTGAGCATCGCCATGGTCGCCCGTGCTTTTGGCGCTTATGCCGTGCAGTTTTTGCATGAAGGCAGCCAGGAGGAGC
ACCTTATTTTGCTCTACGCGTTGGGGATCATTGCGGTGATGACGCTTTTCAACTCCTTAAGCAACCATGCGGTAGGGCGG
CTGGAAGTGATCCTCGTCGGCATTAAAATGATGATCCTGTTATTGCTGATTATTGCCGGTGTCTGGTCGCTGCAACCGGC
GCATATTTCCGTCTCTGCGCCCCCCAGCTCCGGTGCGTTCTTCTCCTGTATTGGGATAACTTTCCTTGCCTATGCGGGCT
TTGGCATGATGGCGAACGCGGCGGATAAAGTGAAAGATCCGCAGGTCATTATGCCACGGGCGTTTCTGGTGGCGATTGGC
GTTACCACGTTGCTTTATATCTCGCTGGCACTGGTTTTGCTTAGCGATGTATCGGCATTAGAGTTAGAAAAATATGCCGA
TACCGCCGTAGCGCAGGCTGCTTCTCCGCTGCTCGGGCATGTGGGTTATGTGATCGTCGTCATCGGCGCTTTACTGGCGA
CGGCTTCAGCCATTAACGCGAACCTGTTCGCCGTGTTTAACATCATGGACAACATGGGCAGCGAACGCGAACTGCCGAAG
CTAATGAATAAATCCCTGTGGCGGCAGAGTACCTGGGGCAACATTATTGTCGTGGTGTTGATTATGCTGATGACGGCGGC
ACTGAATTTAGGCTCACTCGCCAGCGTTGCCAGCGCCACCTTTTTGATTTGCTACCTGGCGGTGTTTGTGGTGGCGATCC
GCCTGCGTCATGATATTCACGCCTCGTTGCCGATTCTTATCGTTGGTACGTTGGTGATGTTGTTGGTGATCGTTGGCTTT
ATCTACAGTCTGTGGTCCCAGGGTAGCCGTGCGTTGATATGGATTATTGGCTCACTCTTACTCAGCCTTATTGTGGCAAT
GGTCATGAAGCGCAATAAAACCGTATAACATCTCTCTGTGCGCAGTACTTCCTGTATTATTGTGGTGGCGGTCGATATTC
GCACTGGCAAAAAAACGTGCTTGAATATCTGTTGAAACCCTTTAACAAAGCACAGGAGGCGTTGCGCGAACGATGAACAT
CAGCGATGTAGCAAAAATTACCGGCCTGACCAGCAAAGCCATTCGCTTCTATGAAGAGAAGGGGCTGGTGACGCCGCCGA
TGCGCAGCGAAAACGGTTATCGCACCTACACGCAGCAGCATCTCAACGAACTGACCTTACTGCGCCAGGCACGGCAGGTG
GGCTTTAACCTGGAAGAGAGCGGCGAGCTGGTGAATCTGTTTAACGACCCGCAGCGGCACAGCGCCGACGTCAAACGGCG
CACGCTGGAGAAGGTGGCGGAGATCGAACGACACATTGAGGAGCTGCAATCCATGCGCGACCAGCTGCTGGCACTGGCGA
ATGCCTGCCCTGGCGATGACAGCGCCGACTGCCCGATTATCGAAAATCTCTCCGGCTGCTGTCATCATCGGGCAGGGTGA
TTAAGACGAGACGGCTCGGATATGTAGGGTTATCCCTTCTATCGCAATGACTTCAACATGCGTACCTGCACCGAGATCCT
CGCTGGCGCTGACAGGCCATGAACTGTCACCGACGCGCATATGACCGCGCCCGTTGACCAGCGGAGATTCCAGCACAAAA
CGTCGGCCAATCAGCTGCTGCCCGCGCTGGTTTAAATGACTATCGCTGTGCTTTTGTTCGCGCACCCGCCGCGACAACCA
TTTCCACCACAGCCAGGCGGCGAGCAGCGTCAGGATGGCAAACATTACCCCTTGCCACTCCCAACCCAGCGGCACCAGCC
AGACCACCAGGCCGGTAATCACCGCTGCCACGCCGCTCCACAACAAATAACCATTTCCGCCCAGCATCTCGGCTGCCAGC
AGCAAACCGCCGAGACTGAGCCAGAAAATATGTGGATGAACGACCATTAACTCCATCATGGCTGAGTCCGTTTGTTGGCG
CTGTCTTTCACCAGCTCGGCAATCCCGGCAATCGACCCCATCAGGCTGCTGGCCTCTAATGGCATCATCACTACTTTGCT
GTTACTGGAGGAACCGATCTGCTGTAACGCTTCGGTGTATTTCTGCGCTACGAAGTAGTTCACCGCCTGAATATCACCGG
AGGCGATAGCTTCAGACACCATTTTGGTGGCGCGGGCTTCTGCTTCGGCGGAACGTTCACGCGCTTCAGCCTGTAAAAAC

GCCGACTGACGTTCGCCTTCCGCTTTCAGGATTTGCGACTGTTTTTCACCTTCGGCTTTGAGGATTTCCGCCTGACGGAT
CCCTTCCGCTTCAAGAATGTAAGCGCGTTTGGTACGTTCCGCTTTCATCTGCGCGTTCATTGAAGAGATAAGCTCTGCCG
GTGGGCGCACGTCGCGAATTTCAATACGGGTGACTTTAATCCCCCACGGGTTGGTGGCTTCATCGACAATACGCAGCAGG
CGTGAGTTGATGCTGTCGCGCTGAGAGAGCATTTCGTCAAGTTCCATTGAACCCAGCACGGTACGGATGTTAGTCATGGT
CAGGTTGATGATCGCCAGCTCCAGATTGCTGACTTCATAAGCCGCGCGCGGCGCGTCAATCACCTGAATAAAGCACACGG
CGTCGATGGTAACGTTGGCGTTATCTTTCGAGATAACTTCCTGGGAAGGGATATCGAGCACTTGCTCCATCATATTGATC
TTGCGACCAATGCGATCCATAAACGGCACCACCAGACTGAGCCCCGGCTGTAACGTTTTGGTATAGCGACCAAAGCGTTC
TACTGTCCACTGATAGCCCTGCGGTACGATTTTGACACCCGCGCCGACAATGACCAGCGCGACAAAAATGAGAATCGGGA
TAAAGATAAGCATCGGAAAAACCTCCTGTTGTACCGTCCATAATCAGCAAAATTGCTGCTTGATTAAACAAATTATACCT
GATTACTGAAAGAGAGTTCCCCCTTATTCCTGCGAAGGATAAACTGTTTTTAGTAAAAATCAGAAAAAGGGAACAGCGAT
GCAGGAAAATAGTCCTTTGCTTCAGCTACAAAACGTAGGATATCTGGCGGGTGATGCGAAGATTCTTAATAACATCAATT
TTTCGCTGCGTGCTGGCGAATTTAAGTTAATTACCGGTCCTTCTGGTTGTGGCAAAAGTACGCTGCTAAAAATAGTTGCT
TCATTGATCAGCCCAACCAGCGGAACGTTACTGTTTGAAGGTGAGGATGTCAGCACACTAAAGCCAGAAATCTACCGCCA
ACAAGTCTCTTACTGCGCCCAGACACCGACGCTGTTTGGCGATACGGTATACGATAATCTGATCTTTCCCTGGCAGATCC
GTAACCGGCAGCCTGACCCAGCCATTTTTCTCGATTTTCTCGAACGCTTCGCCTTGCCGGACAGCATTTTGACGAAGAAT
ATCGCCGAGCTATCTGGTGGTGAAAAACAACGCATCTCATTGATTCGTAACCTGCAATTTATGCCGAAGGTTTTATTGCT
GGATGAAATAACCAGTGCGCTGGATGAAAGTAATAAACATAACGTCAATGAGATGATCCATCGTTATGTGCGCGAGCAAA
ATATTGCCGTGCTGTGGGTGACACACGATAAAGACGAAATTAATCATGCGGATAAAGTGATTACACTGCAACCGCATGCC
GGAGAAATGCAGGAAGCACGCTATGAACTCGCATAATATTACTAACGAATCATTAGCACTGGCATTAATGCTGGTGGTGG
TGGCAATCTTAATTAGCCATAAAGAAAAACTGGCGCTGGAGAAAGATATTCTCTGGAGCGTCGGGCGAGCGATAATTCAG
CTGATTATTGTCGGCTATGTGCTGAAGTATATTTTCAGCGTGGATGATGCCAGCCTGACATTATTGATGGTGTTATTTAT
CTGCTTTAATGCGGCGTGGAACGCGCAAAAACGCAGTAAATATATTGCTAAAGCTTTTATCTCATCGTTTATTGCCATTA
CGGTCGGGGCGGGAATTACCCTGGCGGTGCTGATTCTCTCAGGGTCGATTGAATTTATCCCGATGCAGGTGATCCCTATC
GCCGGGATGATTGCCGGTAACGCCATGGTAGCGGTGGGGTTGTGTTACAACAATTTAGGGCAACGGGTCATTAGCGAACA
GCAACAGATTCAGGAGAAACTGAGTCTTGGTGCGACGCCGAAGCAGGCTTCAGCGATATTGATTCGCGACAGTATTCGCG
CGGCTTTAATTCCGACGGTCGATTCAGCAAAAACGGTTGGCTTAGTGAGTTTACCAGGAATGATGTCCGGGCTGATATTT
GCCGGGATTGATCCGGTGAAGGCGATTAAATATCAGATTATGGTGACCTTTATGCTGCTCTCAACCGCCAGCTTGTCGAC
CATTATTGCCTGCTATTTAACCTATCGTAAGTTTTATAATTCGCGCCACCAGTTGGTGGTGACGCAATTGAAGAAGAAAT
GATGCGATGCCGGATGCGCAGCATCGCATCCGGCGTTGTGGTTCATGTGCCGATCAACGGAATATCAATACAACAATGCA
TACAGCTGGCGGCGATACTTCGACGCCAGTGCATCACCCGTACCCAGCGCAGCGAGGATCTCCTGGAACGTTTTACGCGT
CTGACCGTCTGCGGCGGTGAGATCTTTACGCAGATGCCCGAACAGCAACTCCAGCGCCTCTTCATTGCGCCCAACCTGAT
GCAGTTGCAGCGCCAGTTGCGTCGCCAGTGCGGCATCTTCTGGATTCTCCGCCACCTGCTGTTGCAACTGTTGAATTTCC
GGCGTATCAGCCGCCTGCTTCAGCAGTTCGATTTGCGCCACCAGCCCCTGGTAGCGGGTGTCCTGATCCTGCAACGGAAT
GGTTTTCAGCACCGCTTCCGCATCTTCAGAACGGTTCAGCGCAATCAGCGTTTCTGCCAGCAGCAGGCCGATCTCCCCGT
TCTGATTCGACAACTGCCAGGCGTCTTTCAGCAATGGCAGGGCATCGGTGTAATTGCTTTCCTGCATCAGTTGCATCGCC
TGCTGCGCTTTCAGCTCTTCTTCGCGCGGCAGCACTTTATCCAGCAGGGCGCGGATCGCCTCTTCCGGTTGCGGCCCCTG
GAAGCCATCTACCGGTTGCCCGTTCTGGAACAGATACACGGTCGGAATCGCACGCAGACCAAACTGCGCGGCAATCATCT
GCTCCGCGTCGCAGTCCAGCTTCGCCAGAATAAATTGCCCGTTGTACTGCGCCGCGAGGCTTTCCAGAATTGGGGTTAAC
TGCAAACAGTGCTGGCTACGTTCAGACCAAAAATAGAACAGCACCGGAGTGGTCATCGACTGTTCAAGAACCTGTTGCAG
GTTAGATTCGTTAATGTTGACAATATTTTCTACGGACATGGAGTCGCTCTCTGTTGTCGATATTTTCTTTGACATGGGGG
CTTAAGCGCGCGCTTCAACTCACCCCTGCAATATTTTGTCCATCACGCGCCCCGGCAGCAGGCGCTTAAGCACCATTACC
GCCCAGGTCACCAGCGTCACCGGATAGCGCATCTTCGGCTTCTCGCTAATAAAAGCATGGCGTACTTTGTCCACCACCGC
TTCCGGTCCCAACGTAAAGCGGGCGGCGATGCCGGGATTTTCGACTGGTTTATCACTTTGCGTCTGGTTGACGTTGTCGG
TGAAGCGAGTACGAATGGGACCGGGTTCGATCAGGCTGACTTTAATTCCGCTGTGGCGCAGCTCCATGCGCAGTGCATCT
GACCACGCCTCCAGCGCATATTTACTGGCCGCGTAAGCGCCACGACCCGGCGTGGAGATTAATCCCATCACCGATGATGT
CATCACAATACGCCCTTCACCGTGCGGTAACATCGCGGGTAACAGGCGCATGGTGAGCTGGTGTGCGCCGAAAAAGTTGG
CGGAAAACTGCTGTTCCATCTGCGCACGGCTGATGGTGGAAAGGGGGCCATACATGCCGAATCCGGCATTGTTAAAGATC
CCATACAGACAATTATCGGTCAGGGCGATCACCTCGTCGGCTGCGCGATCAACACTTTCTGGTGAATCCAGATCGATCAA
CACGCCGGTAAATCCCATGCTGTTCATGCGCTCAACATCATCCGGTTTCCGGCAACCTGCCAGCACATGAAAACCCTGGC
GTTTTAATTCGAGCGCGCTTTCCAGGCCAATTCCACTGGAACATCCGGTAATTAAGACCGATTTTTGCATAACTTTACCT
GTCAGGATCTCCGTTGCTTTATGAGTCATGATTTACTAAAGGCTGCAACTGCTTCGCCATCCAGTCGGCAATAAACGGCT
GGGCGTCGCGGTTGGGATGAATACCGTCATCCTGCATCCATTGTGGCTTGAGGTAGACCTCTTCCATAAAAAAGGGCAGC
AGCGGAACATCAAACTCTTTGGCGAGTTTGGGGTAAATGGCGCTAAAGGCTTCATTATAACGGCGACCATAGTTTGCAGG
CAGACGTATTTGCATTAACAATGGTTCAGCGTTGGCGGCTTTGACATCCTGCAAAATCTGGCGCAGCGTTTGCTCGGTTT
GCTGTGGCTGAAAACCACGCAAACCGTCATTGCCGCCCAGTTCAACCAGCACCCAACGCGGCTGATGCTGTTTCAGCAGA
GCCGGAAGGCGCGCCAGTCCTTGTTGCGAGGTGTCGCCGCTGATGCTGGCATTAACTACCGACGTTTTACTCTGCCACTT

ATCATTCAACAAGGCAGGCCAGGCCGCGCTGGCAGACATTCGATACCCGGCGCTCAGGCTATCACCCAGAATCAATAACG
TGTCCGCTGCGGCGGCACGGAAGGTTAACAGGACCAGGAACAGGAAGGGCAAATGCCAGCGGAAAACATTGTTGAAGTTC
ATCATCTTAAGAAGTCCGTCGGTCAGGGGGAGCATGAACTCTCCATCCTCACCGGAGTTGAGCTGGTTGTCAAACGTGGC
GAGACCATCGCACTGGTGGGCGAGTCGGGATCGGGTAAGTCAACCTTGCTGGCGATCCTCGCCGGGCTTGATGACGGCAG
CAGTGGCGAAGTGAGTCTGGTGGGACAACCGCTACATAATATGGACGAAGAAGCGCGGGCAAAGTTGCGCGCGAAGCACG
TCGGCTTTGTTTTTCAGTCATTTATGTTAATTCCTACCCTTAACGCGCTGGAAAACGTCGAGCTTCCGGCTCTGCTGCGC
GGTGAGAGTAGCGCGGAAAGTCGTAACGGGGCGAAAGCGTTGCTCGAACAGTTAGGGCTGGGTAAACGTCTGGATCATCT
TCCGGCACAGCTTTCCGGCGGTGAACAGCAACGAGTGGCGCTGGCACGAGCCTTTAATGGTCGACCTGATGTGCTGTTTG
CCGACGAACCCACCGGCAACCTTGACCGCCAGACGGGCGATAAAATTGCCGACCTGCTGTTTTCCCTCAACCGTGAACAT
GGCACCACGTTGATTATGGTGACCCACGACCTGCAACTGGCGGCACGCTGCGACCGCTGCTTACGGCTGGTGAACGGGCA
GTTGCAGGAGGAAGCATGATTGCACGTTGGTTCTGGCGCGAATGGCGTTCGCCGTCGCTATTAATTGTCTGGCTGGCGCT
AAGCCTGGCGGTGGCCTGCGTGCTGGCGCTGGGCAATATCAGCGATCGCATGGAGAAGGGCTTAAGCCAGCAAAGCCGTG
AGTTTATGGCGGGCGATCGGGCGTTGCGCAGTTCACGCGAAGTGCCGCAAGCGTGGCTGGAGGAAGCGCAAAAGCGCGGC
CTGAAAGTCGGCAAGCAGCTGACTTTCGCCACAATGACCTTTGCAGGCGACACACCGCAGCTGGCGAACGTCAAAGCGGT
GGATGATATCTACCCGATGTATGGCGATCTGCAAACTAATCCCCCTGGCCTGAAACCGCAGGCGGGCAGCGTATTGCTGG
CCCCACGCCTGATGGCACTGCTTAACCTGAAAACGGGCGACACCATTGACGTGGGCGATGCCACCTTGCGGATTGCCGGA
GAAGTGATTCAGGAACCGGATTCCGGTTTTAACCCCTTCCAGATGGCTCCGCGTCTGATGATGAATCTGGCGGATGTCGA
TAAAACCGGAGCCGTGCAGCCGGGGAGTCGGGTCACCTGGCGTTATAAATTCGGCGGCAACGAGAACCAGCTCGACGGCT
ATGAGAAATGGTTGTTACCTCAGCTTAAACCCGAACAACGCTGGTACGGTCTGGAACAGGACGAAGGCGCGCTGGGGCGA
TCGATGGAACGCTCGCAACAGTTCCTGCTGCTTTCGGCGCTTCTGACCTTGCTGCTGGCAGTGGCAGCGGTGGCGGTAGC
GATGAATCATTACTGTCGCAGTCGCTACGATCTGGTGGCGATCCTCAAAACGCTGGGGGCAGGGCGAGCGCAACTGCGTA
AGCTAATCGTCGGTCAGTGGTTGATGGTGCTGACGCTTTCAGCCGTTACCGGTGGGGCCATAGGCCTGTTGTTCGAAAAC
GTGTTGATGGTGCTGCTCAAGCCCGTTCTGCCTGCTGCACTACCGCCAGCCAGCCTCTGGCCGTGGCTGTGGGCGCTTGG
CACCATGACGGTCATCTCGCTGCTGGTGGGGCTACGACCATATCGCTTGTTGCTGGCAACGCAGCCTTTACGCGTATTAC
GTAATGATGTGGTAGCGAACGTCTGGCCGCTGAAGTTTTATCTGCCGATTGTCAGTGTGGTGGTTGTGCTGCTGCTCGCC
GGATTAATGGGTGGCAGCATGCTGCTTTGGGCGGTGCTGGCGGGCGCGGTAGTACTGGCTTTGCTGTGCGGTGTGCTGGG
CTGGATGCTGCTGAATGTACTTCGCCGCATGACGCTGAAATCGCTGCCTCTGCGCCTGGCGGTTAGCCGCCTGTTACGTC
AGCCGTGGTCAACGTTAAGTCAGCTTTCGGCATTTTCGCTCTCCTTTATGCTGCTGGCACTGCTGCTGGTGTTGCGTGGC
GATCTGCTCGACCGCTGGCAGCAGCAGCTACCTCCAGAAAGCCCGAACTACTTTTTAATTAACATCGCCACAGAACAGGT
AGCACCGCTAAAAGCGTTCCTCGCGGAACATCAGATAGTCCCGGAATCGTTTTATCCGGTGGTGCGGGCGCGGCTGACGG
CGATTAACGATAAGCCGACAGAAGGTAATGAAGATGAGGCGCTTAACCGCGAACTCAATCTTACCTGGCAAAATACGCGG
CCCGATCATAATCCGATTGTCGCCGGTAACTGGCCGCCAAAAGCCGATGAAGTGTCGATGGAAGAGGGGCTGGCGAAACG
CTTAAACGTTGCCCTCGGTGATACCGTGACTTTTATGGGCGATACCCAGGAGTTCCGCGCTAAAGTGACCAGCCTGCGCA
AAGTGGACTGGGAAAGTCTGCGGCCTAATTTCTATTTTATTTTCCCTGAAGGGGCATTAGACGGGCAACCGCAGAGCTGG
CTTACCAGTTTCCGCTGGGAGAATGGCAACGGCATGTTGACACAACTCAACCGCCAGTTCCCGACCATTAGCCTGTTAGA
TATTGGCGCGATTTTAAAACAGGTCGGTCAGGTGCTGGAGCAGGTAAGTCGGGCGCTGGAAGTGATGGTGGTACTGGTCA
CCGCCTGCGGTATGTTGCTGTTGCTGGCACAGGTGCAGGTGGGAATGCGTCAGCGTCATCAGGAGCTGGTGGTGTGGCGC
ACACTCGGTGCGGGGAAAAAACTGCTGCGTACCACGTTGTGGTGTGAGTTCGCCATGCTTGGGTTTGTTTCCGGCCTGGT
GGCCGCAATTGGTGCGGAAACGGCGCTGGCAGTGTTGCAGGCGAAAGTGTTTGATTTTCCGTGGGAGCCAGACTGGCGAT
TGTGGATTGTTCTGCCGTGCAGCGGAGCGCTGCTGCTGTCGCTTTTCGGCGGCTGGCTGGGTGCGCGACTGGTTAAGGGT
AAGGCGCTGTTCAGGCAGTTTGCGGGGTGATGAGAGTAAATAAGGGGCGTTCTGGTTGAATGGAACGCCTTGTTTAAATT
AATTCTACAATATGTTTCATTGATGATGTTATTGAATTGGTGATTTCCTATCTTTCTATTGCTGATATTAATCTGAATCG
GTGGAGTTTATTGATTTTAAATATAGCCCTACAAAAATAACTCAGACTAATAAAATCATAAATCATATGCGTTGAATGGA
TATTATCCATATAGTGAATTTGTTGATGATGAATTCATCTGTGCTAAAAATGTTAGTTTAATAAAATATTGAAAGTGACC
TGTAATAACAGTTGTTGTTGATTGAGAACAAATAAGTTTATGTGAAAAATATATAAATACATTAGCTGGTCTTGTGTGTC
ATTTTATTTTTTTTTGTTGCTAACACAGGGATATGAACAATAACTAAAAGGGCACTTTATATGAGCGGAAAACCAGCGGC
GCGTCAGGGAGATATGACTCAGTATGGCGGTCCCATTGTCCAGGGTTCGGCAGGTGTAAGAATTGGCGCGCCCACCGGCG
TGGCGTGCTCGGTGTGTCCGGGCGGGATGACTTCGGGCAACCCGGTAAATCCGCTGCTGGGGGCGAAGGTGCTGCCCGGC
GAGACGGACCTTGCGCTGCCCGGCCCGCTGCCGTTCATTCTCTCCCGCACCTACAGCAGCTACCGGACGAAGACGCCTGC
ACCGGTGGGCGTTTTCGGCCCCGGCTGGAAAGCGCCTTCTGATATCCGCTTACAGCTACGTGATGACGGACTGATACTCA
ACGACAACGGCGGGCGGAGCATTCACTTTGAGCCGCTGCTGCCGGGGGAGGCGGTGTACAGCCGCAGTGAGTCAATGTGG
CTGGTGCGCGGTGGTAAGGCAGCACAGCCGGACGGCCATACGCTGGCGCGGCTGTGGGGGGCGCTGCCGCCGGATATCCG
GTTAAGCCCGCATCTTTACCTGGCGACCAACAGCGCACAGGGGCCGTGGTGGATACTGGGGTGGTCTGAGCGGGTGCCGG
GTGCTGAGGACGTACTGCCAGCGCCGCTGCCGCCGTACCGGGTGCTTACCGGGATGGCGGACCGCTTCGGGCGGACGCTG
ACGTACCGGCGTGAGGCCGCCGGTGACCTGGCCGGGGAAATCACCGGCGTGACGGACGGTGCCGGGCGGGAGTTCCGTCT
GGTGCTGACCACGCAGGCGCAGCGTGCGGAAGAGGCCCGCACCTCTTCGCTATCTTCTTCTGACAGTTCCCGCCCTCTCT

CAGCCTCAGCGTTCCCCGACACACTGCCCGGTACCGAATACGGCCCCGACAGGGGTATCCGCCTTTCGGCGGTGTGGCTG
ATGCACGACCCGGCATACCCGGAGAGCCTGCCCGCTGCGCCACTGGTGCGGTACACGTATACGGAAGCCGGTGAACTGCT
GGCGGTATATGACCGCAGCAATACGCAGGTGCGCGCTTTCACGTATGACGCGCAGCACCCGGGCCGGATGGTGGCGCACC
GTTACGCGGGAAGGCCGGAGATGCGCTACCGCTACGACGATACGGGGCGGGTGGTGGAGCAACTGAACCCGGCAGGGTTA
AGCTACCGCTATCTTTATGAGCAGGACCGCATCACCGTCACCGACAGCCTGAACCGGCGTGAGGTGCTGCATACAGAAGG
CGGGGCCGGGCTGAAACGGGTGGTGAAAAAAGAACTGGCGGACGGCAGCGTCACGCGCAGCGGGTATGACGCGGCAGGAA
GGCTCACGGCGCAGACGGACGCGGCGGGACGGAGGACAGAGTACGGTCTGAATGTGGTGTCCGGCGATATCACGGACATC
ACCACACCGGACGGGCGGGAGACGAAATTTTACTATAACGACGGGAACCAGCTGACGGCGGTGGTGTCCCCGGACGGGCT
GGAGAGCCGCCGGGAATATGATGAACCGGGCAGGCTGGTATCGGAGACATCGCGCAGCGGGGAGACAGTACGCTACCGCT
ACGATGACGCGCACAGTGAGTTACCGGCGACGACAACGGATGCGACGGGCAGCACCCGGCAGATGACCTGGAGCCGCTAC
GGGCAGTTGCTGGCGTTCACCGACTGCTCGGGCTACCAGACCCGTTATGAATACGACCGCTTCGGCCAGATGACGGCGGT
CCACCGCGAGGAAGGCATCAGCCTTTACCGCCGCTATGACAACCGTGGCCGGTTAACCTCGGTGAAAGACGCACAGGGCC
GTGAAACGCGGTATGAATACAACGCCGCAGGCGACCTGACTGCCGTTATCACCCCGGACGGCAACCGGAGCGAGACACAG
TACGATGCGTGGGGAAAGGCGGTCAGCACCACGCAGGGCGGGCTGACGCGCAGTATGGAGTACGATGCTGCCGGACGTGT
CATCAGCCTGACCAACGAGAACGGCAGCCACAGCGTCTTCAGTTACGATGCGCTGGACCGGCTGGTACAGCAGGGCGGCT
TTGACGGGCGGACGCAACGTTATCATTATGACCTGACCGGAAAACTCACACAGAGTGAGGATGAGGGACTTGTCATCCTC
TGGTACTACGATGAATCGGACCGTATCACTCACCGCACGGTGAACGGCGAACCGGCAGAGCAGTGGCAGTATGATGGCCA
CGGCTGGCTGACAGACATCAGCCACCTGAGCGAAGGCCACCGTGTTGCCGTCCACTATGGCTATGACGATAAAGGCCGCC
TGACCGGCGAATGCCAGACGGTGGAGAACCCGGAGACGGGGGAACTGCTGTGGCAGCATGAGACGAAACACGCATACAAC
GAGCAGGGGCTGGCAAACCGCGTCACGCCGGACAGCCTGCCGCCGGTGGAGTGGCTGACGTATGGCAGCGGTTACCTGGC
GGGAATGAAGCTGGGCGGGACGCCGCTGGTCGAGTATACGCGGGACAGGCTGCACCGTGAGACGGTGCGCAGCTTCGGCA
GCATGGCAGGCAGTAATGCCGCATACGAACTGACCAGCACATACACCCCCGCAGGCCAGTTACAGAGCCAGCACCTGAAC
AGCCTGGTATATGACCGTGACTACGGGTGGAGTGACAACGGCGACCTGGTGCGCATCAGCGGCCCGCGACAGACGCGGGA
ATACGGCTACAGCGCCACGGGCAGGCTGGAGAGTGTGCGCACCCTCGCACCAGACCTGGACATCCGCATCCCGTATGCCA
CGGACCCGGCGGGCAACCGGCTGCCGGACCCGGAGCTGCACCCGGACAGTACACTCACAGTGTGGCCGGATAACCGCATC
GCGGAGGATGCGCACTATGTCTACCGCCACGATGAATACGGCAGGCTGACGGAGAAGACGGACCGCATCCCGGCGGGTGT
GATACGGACGGACGACGAGCGGACCCACCACTACCACTACGACAGCCAGCACCGCCTGGTGTTCTACACGCGGATACAGC
ATGGCGAGCCACTGGTCGAGAGCCGCTACCTCTACGACCCGCTGGGACGGCGAATGGCAAAACGGGTCTGGCGGCGGGAG
CGTGACCTGACGGGGTGGATGTCGCTGTCGCGTAAACCGGAGGTGACGTGGTATGGCTGGGACGGAGACAGGCTGACGAC
GGTGCAGACTGACACCACACGTATCCAGACGGTATACGAGCCGGGAAGCTTCACGCCGCTCATCCGGGTCGAGACAGAGA
ACGGCGAGCGGGAAAAAGCGCAGCGGCGCAGCCTGGCAGAGACGCTCCAGCAGGAAGGGAGTGAGAACGGCCACGGCGTG
GTGTTCCCGGCTGAACTGGTGCGGCTGCTGGACAGGCTGGAGGAAGAAATCCGGGCAGACCGCGTGAGCAGTGAAAGCCG
GGCGTGGCTTGCGCAGTGCGGGCTGACGGTGGAGCAACTGGCCAGACAGGTGGAGCCGGAATACACACCGGCGCGAAAAG
CTCATCTTTATCACTGCGACCACCGGGGACTGCCGCTGGCGCTTATCAGCGAAGACGGCAATACGGCGTGGAGCGCGGAA
TATGATGAATGGGGCAACCAGCTTAATGAGGAGAACCCGCATCATGTGTATCAGCCGTACCGTCTGCCAGGGCAGCAGCA
TGATGAGGAATCAGGGCTGTACTATAACCGTCACCGGTACTACGATCCGTTGCAGGGGCGGTATATTACTCAGGACCCGA
TGGGGTTGAAAGGGGGATGGAATTTATATCAGTATCCTTTAAATCCACTACAACAAATTGACCCTATGGGATTATTGCAG
ACTTGGGATGATGCCAGATCTGGAGCATGTACGGGGGGAGTTTGTGGTGTTCTTTCACGTATAATAGGACCAAGTAAATT
TGATAGTACTGCAGATGCTGCGTTAGATGCTTTGAAAGAAACGCAGAATAGATCTCTATGTAATGATATGGAATACTCTG
GTATTGTCTGTAAAGATACTAATGGAAAATATTTTGCATCTAAGGCAGAAACTGATAATTTAAGAAAGGAGTCATATCCT
CTGAAAAGAAAATGTCCCACAGGTACAGATAGAGTTGCTGCTTATCATACTCACGGTGCAGATAGTCATGGCGATTATGT
TGATGAATTTTTTTCAAGTAGCGATAAAAATCTTGTAAGAAGTAAAGATAATAATCTTGAAGCATTTTATCTCGCAACAC
CTGATGGACGATTTGAGGCGCTTAATAATAAAGGAGAATATATTTTTATCAGAAATAGTGTCCCGGGATTGAGTTCAGTA
TGCATACCGTATCATGATTAATTTTAGTGCTTTTATTAGTGGGGCCTATAAGGAGATTCAATGAAATATAGTTCAATATT
TTCGATGCTTTCATTTTTTATACTATTTGCCTGTAATGAGACAGCTGTTTACGGTTCTGATGAAAACATTATTTTTATGA
GGTATGTGGAAAAATTACATTTAGATAAATACTCTGTTAAAAATACGGTAAAAACTGAAACAATGGCGATACAATTAGCT
GAAATATATGTTAGGTATCGCTATGGCGAACGGATTGCAGAAGAAGAAAAACCATATTTAATTACGGAACTACCAGATAG
TTGGGTTGTTGAGGGAGCAAAGTTACCTTATGAAGTTGCGGGTGGTGTATTTATTATAGAAATTAATAAGAAAAATGGAT
GTGTTTTGAATTTCCTACATAGTAAATAATGCTGGCGCTGATGGATGCGGATGGAAACATTGCGTGGAGCGGGGAGTATG
ATGAGTGGGGCAACCAGCTGAATGAAGAGAACCCGCATCACCTGCACCAGCCGTACCGGCTGCCGGGGCAGCAGTATGAT
AAGGAGTCGGGGCTGTACTACAACCGGAACCGGTACTACGATCCGTTGCAGGGGCGGTATATCACTCAGGACCCGATAGG
GCTGGAGGGGGGGATGGAGTCTGTATGCGTATCCGCTGAATCCGGTGAATGGTATTGATCCATTAGGGTTAAGTCCCGCAG
ATGTAGCGCTAATAAGAAGAAAAGATCAACTAAACCATCAAAGAGCATGGGATATATTATCTGATACTTATGAAGATATG
AAGAGATTAAATTTAGGTGGGACTGATCAATTTTTCCATTGTATGGCATTTTGTCGAGTGTCTAAATTAAATGACGCTGG
TGTTAGCCGATCGGCGAAAGGGCTGGGTTATGAAAAAGAGATTAGAGATTACGGGTTAAATCTGTTCGGTATGTACGGCA
GAAAAGTAAAGCTATCCCATTCTGAAATGATTGAAGATAATAAAAAAGACTTGGCTGTAAATGACCATGGGTTGACATGT

CCATCAACAACAGATTGCTCAGATAGATGTAGTGATTATATTAATCCAGAGCATAAAAAAACGATAAAGGCTTTACAAGA
TGCTGGCTATCTCAAGTAATCTATCAAAGATGATAATATTTATTTTTGCTATTATAATCATTGTTGTTTTATGCGTAATT
ACTTATCTTTATTTATACAAAGATGAATCTCTTGTAAGTAAACATTACATAAACTATATGGCAATACCAGAAAATGATGG
AGTTTTTACATGGCTCCCAGATTTTTTTCCGCACGTAGCGGTGGATATATCAATATACACAAATGTAGAAGATGATTATT
TTTTTCTTATTTTTCCCTAACAAATGATGATGGGGGTAGGTTTAAGAAAACATTGACAGTGAGGGCCAGGGAACAAGTGG
CGAAAATCGTATCAAAGAATGATCCAGATACAAAAAAAGTGTGGTGTAAATATGGTAAGATACCAGGGCAAGGGGATGGT
GTAAACCTTTTTTTTGTTGGTGAAATTAATGTTACGCATTATTTATAACAAATATTGGAGCTGGATTGCCTGATGCTTG
TGCAGAGTAATTGCTTGAATTAAGAGTCTATCCCATATCGAAGTCGTCAACTTCGTAGTGAGGAAAAGTAAAATTCCTGA
CTGAGAAAAGACATGTCGGCTATTGTGTAAAGCCATATAGCTCAGACGATGAATATCTACTCGTATTCAGTTGTTTATTG
AGGGTGAGTTCCGACCCTGAAACAACAAATAAAATGAACAGTCAGAGAGTTTACATAGAATTGCACTGGTCTTTTACGAT
ATCTGACATTGTGTAATACATATTCAGCCATGCATTAATTAAAATGTTACGTGTTTAATGTGAGTCCCTATCTGAAAATA
AATAATCCTTCCGGATTAAAATAAATTCTTGCCGGGAAAGAAAGAGGAAATAAACCATTAGCGGAAAACCAGCGGCACGC
CAGGGTGACATGCCCCAGTACGGCGGCCCGATAGTCCAGGGTTCAGCGGGGGTACTGATAGTCGCGCCGACTGCATCGAG
AACTCCATACAACTTTTTACGGAACGTCACCTGATAAAATTTGTTCAGTATCGTCTTTTGAGATTCTCAAGGTTATGGAG
TAACCAGACAGAACGGACACCACTGTCGGAGATAAAAACCCCCTGTTTACGCAGCTCGTTGCTGGCCCGGTGCTGACCAT
GTGTCGGGAAAGCGACGGCGTAATCAACAACAGCCTGTTCAGTTGCCTCATCGGTACGGTTCTTAAGGTTAGGAGCGCGG
CGACTACGATTAATCTGCGCATCCACACCGCCTTCAGCGACCAGTTCGCGGTAACGATAAAACGTATCACGCGAAACGCC
CATGATTTTACAGGCTTTTGATACGTTGCTGAGTTCTTCAGCCAGATTGAGCAAACCGGCTTTGTGTTTGATGACGGGAT
TGGCAGTATGAAGCATGAGAGTTACCTCTTGTTTTGGATAAGGATTCGACACTCATATCAAAACCGGTAACTCTCAACCT
TTCAAGGCCATGTGTCAGATCAAGTCGCGACTAATACAAATACGTCCCTCATTACCGCGCCTTAACCCATTCCGCCACTT
CCGCCCACTCACCGCGAAAGACAACTTTTTCCGCTTTTTTCTCAAGCTGATAGCGATACATCGGGTCGTAATATTCTTCA
AGTAACGGCACCAGCCAGGCCAGATGACCGTCGGTGCTGCCGGTGGTGAGTTGCGTTGTCAGTGCTGCATCCAGCCTTGC
AGCCAGTTCGTTATAGCGCTGTAGCCCCAGCCGACGCTTAATCGCCGAAAGTCCGTGATGCAGGTATTCGCAATACTCCT
GCCAGCCCTGTTCGTCGCCGTACGCGTGGGTAAAATCATGATGCATACGCAAGAAATACTCTTCGTTCAGGCGCTCAAGA
CGGATCTCAAACGGATCTTCTACCACCGCAATCGCCGCCTGAGTCATTCGCTCGCGCAGGCATTCCGGCAGGTGATTCGA
ACCGATCATCCGGCTTTCGTCTTCCAGCACCCACAGGCGCAAATTCTGACGGGCGTCGGTTTTTAGCATTTCGGCAGCCA
GCAGGTTTTCAAAACTCGCCTGGCTAAGTTGTGGTTGTAACGTGCGACCAAACGCCGAACCGCGATGACGCGCCAACCCT
TCCAGATCAACACCGTTCGGCTGTTGCTGCACTAACAGCGTTTTACCGCTGCCGGTACAACCGCCAATCAGCACTATCGG
TTTTTGTGCCAGTTCAATAGTCGCCTGAATCGCGGTCTGGCGCAGTGCCTTATAACCGCCTTCCACCAGCGGATAATCAA
TCCCCGCTGCATGCAACCAGCTTTGCACAATATGTGAGCGCTGACCGCCACGGGCGCAGCAGAGAATACCTTGCGGATTT
TGCAGGCACGCTGCCCGCCAGGCGTCCATGCGCTGCTGACGAATTTCACCCGCCACCAGTTTATGTCCCAGCGCCAGCGC
TGCGTCTGAGCCTTGCTGTTTATAGCAGGTGCCAACGGCGGCGCGTTCATCGTTATTCATTAACGGCAGATTGATAGCGG
CGGGCATTGCGCCGTGCTCAAACTCGATAGGGGCGCGAACATCAATAATGGGCGTATCAGCAATCAGCAGGGCACGATAG
TCCTGTTCCGTGTGTCTCTCTTGCATAGTTAAAAGTGAACCTCAAATCAGCTTGCGCGCTATTTTACGCGCCAACGCGCA
AGGAAACTTGATTTTTAACTGCGTGGGTTGCCGAAAATTTCTAAAAATCCGCTGATTTCCGGCCTGCGCTGGGTAAACAA
GGTCACAATATCTTCTACCGCTTTGCCGCTGCCAAATTTGCGCCATGCCAGACTCAATGGCGAAGGAGGGCGCATCGTTG
GGATTACCCGGCTGACCAGTTGTTGATTATCGATCATTGACTGGCAAAGCGATTTTGGCAAAAAACCAATGCCAACGCCC
GCCAGATGGGCGGCGATTTTCGTTTCCATATCGGGAACAATAATCTCTTTTTGCCCTGGCAATCGCCAGGCGACGCGTTT
GGTTAAGGTGCGGGCGCTGTCTTCAATATTGACCGCCGGAAAGCGCCGCAACTGCGCTTCTGTTAGCGGCTCTTCAACGT
TCGCCAGCGGATGATCCGCCGCCATGACAAAGCGCCATTGCACCGATCCTAAGGGATCAAGACTAAAGGTATTTGCCAGC
GCCTCAGTTCCCGTGACGCCGATAGCCAGCGAAAAACCTTCGTACAATAGCGAGTCCCAGACGCCCATATAGATTTGTCG
GGAGATGTGAAACTGGGTAAAGGGGTAACGCTCATTCAGCCACGCCAGCAACTGGGCGACGGCCTGGGGGTTGTAGAGCA
GGTTGTTGATGACAATATTCACCTGGCGTTCCACGCCATCATTCACCTGTTGCAGCTCGCTTGGCATACTTTCCAGCCAG
CTCAGCCAGTCTCTGGCCTGGGAAAGTAGATGCTCGCCAGCCGCTGTCAACGTCACGCTGCGAGTCGTACGGAAAAACAG
CGCTACTCCGGTATTCTCTTCCAGAAGTTTAATGCGATAACTGATCGTCGCCGTGGTTTTACATAATCGTTCTGCCGCTT
TTGAAAAACTTCCTGTTTCAGCAACCGCAATGAAAGTCCGCAAGGTTTCTGGATCGAACATCTTCAGGTATCCCCTTTTA
AATCCGCAAGTTGCGTGATTTTCTTATCCTCTGATTTATCAGTATTTTTACATGATAACCCTGTTCAATTTGTGGACTAA
ATCTAGTTTTGGAAAAAATATTCCAACTTTTGTATTGATGTTGTTCTCTTAAGGTTTTAGATTGCCTGTTATTGAAACCAA
GCTGACCGGTCGGCGGTGGTTGAACGGAATTATGTTACAAGGACAAAAAGATGAAACTTCAGGTATTACCGTTAAGTCAG
GAAGCCTTTAGTGCTTATGGCGACGTAATCGAAACGCAGCAACGGGATTTTTTTCCATATTAACAATGGCCTGGTGGAGCG
TTACCACGATTTGGCGCTGGTTGAGATTCTTGAGCAAGACTGTACGCTTATCAGCATTAACCGCGCGCAACCGGCGAATC
TGCCGCTGACCATTCACGAACTCGAACGTCATCCGCTGGGTACTCAGGCCTTTATCCCGATGAAAGGTGAGGTGTTTGTG
GTGGTCGTGGCGTTAGGTGACGACAAACCAGACCTGTCAACGCTGCGGGCGTTTATCACCAACGGCGAACAGGGAGTGAA
TTACCATCGTAACGTCTGGCATCACCCACTTTTCGCCTGGCAGCGCGTCACCGATTTTCTGACCATCGATCGCGGCGGCA
GTGACAACTGTGATGTTGAAAGTATTCCTGAACAGGAACTCTGTTTTGCGTGACGCCTGCAACCGACTTGCATAAGATAA
ACTAATTGTTCATTGTTTATGCTCACTTGTAGGTCGGAGTTAACGTAGGTATGACGGAAGTTAGACGGCGCGGCAGGCCA

GGACAGGCGGAGCCTGTGGCACAGAAGGGCGCACAGGCGTTAGAGCGGGGAATTGCGATTCTGCAATATTTGGAAAAAAG
TGGGGGAAGTTCGTCGGTTAGCGATATTTCTCTCAATCTGGATTTGCCGCTCTCCACGACCTTTCGCTTGCTGAAGGTTT
TACAGGCAGCGGATTTTGTCTATCAGGACAGTCAATTAGGCTGGTGGCATATAGGATTAGGTGTCTTTAACGTCGGTGCG
GCGTACATCCATAACCGCGATGTCCTCTCCGTCGCCGGGCCGTTTATGCGCCGCCTGATGTTACTTTCCGGCGAAACGGT
CAATGTCGCGATCCGTAACGGCAATGAAGCGGTATTAATTGGTCAGTTAGAGTGTAAATCGATGGTCAGGATGTGTGCGC
CACTGGGCAGTCGTCTGCCACTGCATGCTTCCGGTGCGGGCAAAGCGCTGCTTTATCCGCTGGCGGAAGAGGAGTTGATG
AGCATCATTCTGCAAACCGGTTTGCAGCAGTTTACGCCAACTACGCTTGTGGATATGCCCACCTTGCTGAAGGACCTGGA
ACAAGCGCGTGAACTGGGCTATACCGTAGATAAAGAAGAGCATGTTGTAGGTCTGAATTGCATAGCTTCAGCAATTTACG
ATGATGTCGGTAGTGTTGTTGCCGCTATCTCCATCTCCGGGCCTTCATCAAGACTGACAGAAGATCGTTTTGTCAGTCAG
GGTGAGCTGGTCAGAGACACCGCCCGCGATATCAGCACGGCGTTGGGACTGAAAGCACATCCATAATGTCTGTCGCATCC
CGCTCTGCGGAGCGGGTTTTTTTGACAAAATTTGAAAGTTGGAAAAATTTTCCAATAAATAGAGGTAGGAATAAAATGGC
AAAAATGAGAGCCGTTGACGCGGCAATGTATGTGCTGGAGAAAGAAGGTATCACTACCGCCTTCGGTGTTCCGGGAGCTG
CAATCAATCCGTTCTACTCAGCGATGCGTAAGCACGGCGGTATTCGTCACATTCTGGCGCGTCATGTGGAAGGTGCTTCG
CACATGGCGGAAGGTTATACCCGCGCAACGGCAGGGAATATCGGCGTATGTCTGGGGACTTCCGGTCCTGCGGGCACGGA
CATGATCACCGCGCTCTATTCCGCTTCTGCTGATTCCATTCCTATTCTGTGCATTACCGGCCAGGCACCGCGCGCCCGTC
TGCATAAAGAAGATTTTCAGGCCGTAGATATTGAAGCAATTGCTAAACCGGTCAGCAAAATGGCGGTTACAGTTCGTGAA
GCGGCGCTGGTGCCTCGCGTGCTGCAACAGGCATTTCACCTGATGCGTTCTGGTCGTCCGGGTCCGGTACTGGTGGATTT
ACCGTTCGACGTTCAGGTTGCGGAAATCGAGTTTGATCCTGACATGTACGAACCGCTGCCGGTCTACAAACCTGCTGCCA
GCCGTATGCAGATCGAAAAAGCTGTAGAAATGTTAATCCAGGCCGAACGTCCGGTGATTGTTGCCGGGGGCGGGGTAATT
AATGCTGACGCAGCTGCACTGTTACAACAGTTTGCTGAACTGACCAGCGTTCCGGTGATCCCAACGCTAATGGGCTGGGG
CTGTATCCCGGACGATCATGAACTGATGGCCGGGATGGTGGGTCTGCAAACCGCGCATCGTTACGGTAACGCAACGCTGC
TGGCGTCTGACATGGTGTTTGGTATCGGTAACCGTTTTGCTAACCGTCATACCGGCTCGGTAGAGAAATACACCGAAGGG
CGCAAAATCGTTCATATTGATATTGAGCCGACGCAAATTGGTCGCGTGCTGTGTCCGGATCTCGGTATTGTCTCTGATGC
TAAAGCGGCGCTGACACTGCTGGTTGAAGTGGCGCAGGAGATGCAAAAAGCGGGTCGTCTGCCGTGTCGTAAAGAATGGG
TCGCCGACTGCCAGCAGCGTAAACGCACTTTGCTGCGCAAAACCCACTTCGACAACGTGCCGGTGAAACCGCAGCGCGTG
TATGAAGAGATGAACAAAGCCTTTGGTCGCGATGTTTGTTATGTCACCACCATTGGTCTGTCACAAATCGCTGCGGCACA
AATGCTGCATGTCTTTAAAGACCGCCACTGGATCAACTGTGGTCAGGCTGGTCCGTTAGGCTGGACGATTCCGGCTGCGC
TAGGGGTTTGTGCCGCTGATCCGAAACGCAATGTGGTGGCGATTTCTGGCGACTTTGACTTCCAGTTCCTGATTGAAGAG
TTAGCTGTTGGCGCGCAGTTCAACATTCCGTACATCCATGTGCTGGTCAACAACGCTTATCTGGGGCTGATTCGTCAGTC
ACAACGCGCTTTTGACATGGACTACTGCGTGCAACTCGCTTTCGAGAATATCAACTCCAGTGAAGTGAATGGCTACGGTG
TTGACCACGTAAAAGTAGCGGAAGGTTTAGGTTGTAAAGCTATTCGGGTCTTCAAACCGGAAGATATTGCGCCAGCCTTT
GAACAGGCGAAAGCCTTAATGGCGCAATATCGGGTACCGGTAGTCGTGGAAGTTATTCTCGAGCGTGTGACCAATATTTC
GATGGGCAGCGAACTGGATAACGTCATGGAATTTGAAGATATCGCCGATAACGCAGCGGACGCACCGACTGAAACCTGCT
TCATGCACTATGAATAAGGGAGATAAATAATGTTACGTTTCTCTGCTAATTTATCGATGTTATTTGGAGAATATGATTTT
CTCGCCCGTTTTGAGAAAGCTGCGCAGTGTGGTTTTCGCGGCGTTGAATTTATGTTTCCTTATGACTACGACATTGAAGA
ATTAAAACATGTGCTGGCGAGTAATAAACTCGAACATACGCTGCACAATTTACCGGCGGGTGACTGGGCGGCGGGGGAGC
GCGGTATTGCCTGTATTCCTGGCCGTGAAGAAGAGTTTCGGGATGGCGTAGCAGCAGCGATTCGTTATGCCCGTGCGCTG
GGTAATAAAAAAATTAACTGTCTGGTCGGTAAAACGCCGGCTGGTTTCAGCAGTGAACAGATTCACGCAACGCTTGTAGA
AAACCTGCGTTATGCCGCGAATATGCTGATGAAAGAAGATATTTTATTACTGATTGAACCTATTAACCATTTTGATATTC
CTGGTTTCCATCTCACCGGAACTCGGCAGGCGCTGAAATTGATTGATGATGTTGGTTGCTGCAATTTGAAAATTCAGTAT
GACATTTATCATATGCAGCGGATGGAAGGTGAATTAACCAACACCATGACTCAGTGGGCTGATAAAATTGGTCACCTGCA
AATTGCCGATAATCCGCATCGCGGCGAACCGGGAACCGGAGAAATTAATTATGATTATCTCTTTAAGGTAATCGAAAATT
CTGACTACAACGGTTGGGTTGGGTGTGAATATAAACCCCAAACCACCACGGAAGCCGGTTTACGCTGGATGGATCCGTAC
CGTTAAAACGTAACGCTATTCAGACAATGCTTTTTTAGGCCGCTAAGTTGGCAGGGGATCGTGTTGTCTGAATTCAGGAA
AAGCGAAATTTAAAAGAGGTTAATTATGAAACTGGGATTTATTGGCTTAGGCATTATGGGTACACCGATGGCCATTAATC
TGGCGCGTGCCGGTCATCAATTACATGTCACGACCATTGGACCGGTTGCTGATGAATTACTGTCACTGGGTGCCGTCAGT
GTTGAAACTGCTCGCCAGGTAACGGAAGCATCGGACATCATTTTTATTATGGTGCCGGACACACCTCAGGTTGAAGAAGT
TCTGTTCGGTGAAAATGGTTGTACCAAAGCCTCGCTGAAGGGCAAAACCATTGTTGATATGAGCTCCATTTCCCCGATTG
AAACTAAGCGTTTCGCTCGTCAGGTGAATGAACTGGGCGGCGATTATCTCGATGCGCCAGTCTCCGGCGGTGAAATCGGT
GCGCGTGAAGGGACGTTGTCGATTATGGTTGGCGGTGATGAAGCGGTATTTGAACGTGTTAAACCGCTGTTTGAACTGCT
CGGTAAAAATATCACCCTCGTGGGCGGTAACGGCGATGGTCAAACCTGCAAAGTGGCAAATCAGATTATCGTGGCGCTCA
ATATTGAAGCGGTTTCTGAAGCCCTGCTATTTGCTTCAAAAGCCGGTGCGGACCCGGTACGTGTGCGCCAGGCGCTGATG
GGCGGCTTTGCTTCCTCACGTATTCTGGAAGTTCATGGCGAGCGTATGATTAAACGCACCTTTAATCCGGGCTTCAAAAT
CGCTCTGCACCAGAAAGATCTCAACCTGGCACTGCAAAGTGCGAAAGCACTTGCGCTGAACCTGCCAAACACTGCGACCT
GCCAGGAGTTATTTAATACCTGTGCGGCAAACGGTGGCAGCCAGTTGGATCACTCTGCGTTAGTGCAGGCGCTGGAATTA
ATGGCTAACCATAAACTGGCCTGATACCCGCAATAAAAATGGCCGATATCAGAAAATGAATCGGCCAGCAATATTAAAAA

AGAAAGCAGCCAAAGATGTTGCTTCAGTATTAAAAATAATATTTTTATTTTATTTGTTCCTCATAGCTAGATTAAAACAA
CGTTATTCGATACGTGAAATTAAGAGGGATTTATGGAACATCAGAGAAAACTATTCCAGCAACGCGGCTATAGCGAAGAT
CTATTGCCGAAAACGCAAAGCCAGCGGACCTGGAAAACATTTAACTATTTTACCTTATGGATGGGTTCGGTTCATAACGT
TCCCAATTATGTGATGGTCGGCGGCTTTTTTATTCTCGGCTTGTCTACCTTTAGTATTATGCTGGCAATTATCCTCAGCG
CCTTTTTCATTGCCGCGGTAATGGTATTAAACGGTGCTGCGGGCAGTAAATACGGTGTGCCTTTTGCCATGATCCTGCGT
GCTTCTTACGGTGTACGTGGTGCACTGTTTCCCGGATTATTAAGGGGCGGAATTGCCGCCATCATGTGGTTTGGTTTGCA
ATGTTACGCGGGGTCACTGGCCTGCTTGATTCTGATTGGCAAAATCTGGCCGGGATTTTTAACTCTCGGTGGTGATTTCA
CTCTGTTAGGCCTTTCTCTACCGGGCTTAATTACTTTCTTAATCTTCTGGCTGGTCAACGTTGGTATAGGTTTTGGCGGT
GGCAAAGTTTTAAATAAATTCACTGCCATTCTTAACCCGTGCATCTATATCGTTTTCGGCGGTATGGCGATTTGGGCGAT
TTCACTGGTCGGGATCGGTCCAATCTTTGACTACATTCCGAGCGGTATTCAGAAAGCAGAAAACGGTGGCTTCCTGTTCC
TGGTGGTGATTAACGCGGTAGTTGCGGTCTGGGCGGCACCGGCGGTGAGCGCATCCGACTTTACGCAAAACGCCCACTCG
TTTCGTGAGCAGGCGCTGGGGCAAACGCTGGGTTTAGTTGTGGCCTATATTCTGTTTGCGGTCGCCGGGGTATGTATTAT
TGCCGGAGCCAGTATTCACTACGGCGCTGATACCTGGAACGTGCTGGATATTGTTCAGCGTTGGGACAGCCTGTTCGCCT
CGTTCTTTGCGGTACTGGTTATTCTGATGACAACTATCTCCACTAACGCGACCGGTAATATTATTCCAGCCGGTTATCAG
ATTGCCGCCATTGCACCGACAAAACTGACCTATAAAAACGGCGTACTGATTGCCAGTATTATCAGCTTGCTGATCTGCCC
GTGGAAATTAATGGAAAATCAGGACAGCATTTATCTTTTCCTCGATATTATCGGCGGAATGCTTGGTCCGGTAATTGGTG
TCATGATGGCGCATTATTTTGTGGTGATGCGCGGACAAATTAATCTTGATGAACTGTATACCGCACCTGGCGATTATAAA
TATTACGATAACGGTTTTAACCTCACTGCGTTTTCAGTAACTCTGGTGGCCGTTATTTTATCTCTTGGCGGTAAGTTTAT
TCACTTTATGGAACCGTTATCGCGTGTTTCATGGTTTGTCGGCGTCATCGTCGCCTTTGCGGCCTACGCCTTATTAAAGA
AACGTACAACAGCAGAAAAAACAGGAGAGCAAAAAACCATAGGTTAATTAATCCCGATATTGAACATTGAGTTAAAAACC
AATCTGTATTTTACAAGGAGTTTGTTATGTCTTTTGATTTAATCATTAAAAACGGCACCGTTATTTTAGAAAACGAAGCT
CGCGTTGTAGATATCGCCGTTAAAGGCGGAAAAATTGCTGCTATCGGTCAGGATCTGGGCGATGCAAAAGAAGTTATGGA
TGCGTCTGGTCTGGTGGTTTCGCCGGGCATGGTTGATGCGCACACCCATATTTCTGAACCGGGTCGTAGCCACTGGGAAG
GTTATGAAACCGGTACTCGCGCAGCGGCAAAAGGTGGTATCACCACCATGATCGAAATGCCGCTCAACCAGCTGCCTGCA
ACGGTTGACCGCGCTTCAATTGAACTGAAGTTCGATGCCGCTAAAGGCAAGCTGACTATTGATGCGGCACAACTCGGTGG
CCTGGTGTCTTACAACATCGACCGTCTGCATGAGCTGGATGAAGTGGGCGTTGTCGGCTTCAAATGCTTCGTTGCGACCT
GTGGCGATCGCGGTATCGACAACGACTTCCGTGATGTAAACGACTGGCAGTTCTTCAAAGGTGCGCAGAAGCTGGGCGAA
CTGGGTCAGCCGGTGCTGGTGCACTGCGAAAACGCGCTGATTTGTGACGAACTGGGCGAAGAAGCGAAGCGTGAAGGTCG
CGTAACCGCTCATGACTATGTGGCTTCGCGTCCGGTATTTACCGAAGTGGAAGCAATTCGCCGCGTACTGTATCTGGCGA
AAGTTGCTGGTTGCCGTCTGCACGTTTGCCACGTCAGCAGCCCGGAAGGTGTTGAGGAAGTGACTCGTGCACGTCAGGAA
GGTCAGGACGTTACTTGTGAATCCTGCCCGCATTACTTTGTACTGGATACCGATCAGTTCGAAGAAATCGGTACTCTGGC
GAAGTGTTCACCGCCGATCCGCGATCTGGAAAACCAGAAAGGCATGTGGGAAAAACTGTTTAACGGTGAAATCGACTGCC
TGGTTTCCGACCACTCTCCATGCCCGCCGGAAATGAAAGCCGGTAACATCATGAAAGCATGGGGCGGTATCGCCGGTCTG
CAAAGCTGCATGGACGTGATGTTCGATGAAGCGGTACAGAAACGCGGTATGTCTCTGCCAATGTTCGGCAAATTAATGGC
GACTAACGCAGCAGATATTTTCGGTCTGCAGCAAAAAGGCCGTATCGCCCCAGGAAAAGATGCCGACTTCGTCTTCATTC
AGCCGAATAGCAGCTATGTTCTTACCAATGACGATCTGGAATATCGCCACAAAGTCAGCCCGTATGTTGGCCGTACCATT
GGCGCGCGTATCACGAAAACCATCTTACGTGGTGATGTGATTTACGACATTGAACAGGGCTTCCCTGTTGCGCCGAAAGG
TCAATTTATCCTTAAACATCAGCAGTAATCTGGCCCCTGCAATGCCCGTCCTTGCGGCGGGCATTCTCCGGTTAAGGTGT
GTTTATGTTCAATTTTGCAGTCAGCCGCGAAAGCCTGTTATCAGGATTTCAGTGGTTTTTCTTTATTTTTTGCAACACGG
TTGTGGTTCCTCCTACGCTACTTTCTGCTTTTCAGTTGCCGCAAAGTAGCCTGCTTACGCTCACGCAATATGCTTTTCTT
GCTACCGCACTGGCCTGCTTCGCTCAGGCGTTTTGCGGTCATCGTCGCGCTATTATGGAAGGGCCAGGTGGCCTGTGGTG
GGGAACCATCCTTACTATCACCCTTGGTGAAGCATCGCGCGGGACACCGATCAACGATATCGCCACCAGCCTGGCAGTGG
GGATTGCACTCTCCGGCGTGCTGACGATGTTGATTGGTTTTAGCGGATTAGGCCATCGCCTGGCACGGTTATTTACGCCG
TCGGTGATGGTCTTGTTTATGTTGATGCTGGGCGCGCAGCTGACCACTATCTTTTTCAAAGGTATGCTCGGGCTGCCGTT
TGGCATAGCCGACCCGAATTTTAAAATTCAGTTACCGCCGTTCGCGCTCTCGGTGGCGGTGATGTGCCTGGTACTGGCGA
TGATTATCTTCCTGCCGCAACGTTTTGCCCGTTATGGCCTGCTGGTCGGCACCATAACCGGCTGGTTGTTGTGGTACTTT
TGCTTTCCTTCTTCGCACTCGCTCTCCGGTGAGTTGCACTGGCAGTGGTTCCCGCTCGGCAGTGGCGGTGCTTTGTCGCC
GGGAATTATTCTGACGGCGGTGATTACAGGTCTGGTAAATATCAGCAATACCTACGGTGCGATTCGGGGCACGGATGTTT
TTTATCCGCAGCAGGGCGCAGGGAATACGCGTTATCGTCGTAGCTTTGTGGCGACCGGATTTATGACGCTGATAACCGTA
CCGCTGGCGGTAATTCCATTTTCACCGTTTGTTTCATCCATTGGTTTATTAACCCAGACTGGCGATTACACGCGGCGTTC
GTTTATTTATGGCAGCGTTATTTGCCTGCTGGTGGCGCTGGTTCCTGCACTCACGCGACTGTTTTGCAGTATCCCTTTAC
CCGTGAGTAGTGCGGTCATGCTGGTTTCTTATCTGCCTTTACTCTTTTCCGCGCTGGTGTTTAGCCAGCAAATAACGTTT
ACCGCTCGCAATATTTATCGACTCGCATTGCCGTTATTTGTCGGCATATTTTTAATGGCATTACCGCCTGTGTATCTGCA
AGACCTTCCATTAACGCTTCGTCCTCTGCTCAGTAACGGCTTATTGGTCGGGATTTTACTGGCTGTTCTTATGGATAACC
TTATTCCGTGGGAACGCATCGAATAATTTGTTGAAAAAGGATTGATAATGAAGATTGTCATTGCGCCAGACTCTTTTAAA
GAGAGCTTAAGTGCAGAAAAATGTTGTCAGGCAATTAAAGCCGGGTTTTCGACCCTCTTTCCCGATGCGAACTATATCTG

TTTGCCGATAGCGGATGGCGGCGAAGGGACGGTGGATGCGATGGTCGCCGCGACGGGCGGCAACATCGTGACGCTTGAAG
TCTGCGGGCCGATGGGCGAAAAAGTGAATGCTTTTTATGGCCTTACCGGCGACGGGAAAACGGCGGTGATTGAGATGGCG
GCAGCAAGTGGCCTGATGCTGGTCGCGCCTGAAAAGCGTAATCCGTTGCTGGCCTCCAGTTTTGGTACGGGGGAGTTAAT
TCGTCATGCGCTGGATAACGACATTCGCCATATTATTCTCGGCATTGGCGGCAGTGCGACGGTCGACGGCGGTATGGGCA
TGGCGCAGGCGCTCGGTGTGCGTTTCCTTGATGCCGACGGTCAGGCGCTGGCGGCAAACGGTGGTAATTTAGCGCGCGTG
GCAAGCATTGAGATGGATGAATGCGATCCGCGTCTGGCGAATTGCCATATTGAAGTAGCATGTGACGTTGATAACCCGCT
GGTAGGGGCACGCGGCGCGGCGGCGGTGTTTGGCCCGCAAAAAGGGGCAACGCCGGAGATGGTCGAAGAACTTGAACAGG
GGCTGCAAAATTACGCCCGTGTTTTACAACAGCAAACTGAAATTAATGTCTGCCAGATGGCGGGCGGCGGCGCTGCGGGC
GGTATGGGTATTGCGGCGGCGGTATTTCTCAATGCGGATATTAAACCGGGCATTGAAATTGTGTTGAATGCGGTCAATCT
TGCGCAGGCAGTGCAGGGCGCAGCACTGGTGATTACCGGGGAAGGGCGCATCGACTCGCAAACGGCAGGCGGTAAAGCGC
CGCTGGGTGTGGCGTCGGTGGCGAAGCAGTTTAATGTACCGGTGATTGGGATTGCTGGCGTATTGGGTGATGGCGTGGAA
GTGGTGCACCAGTACGGCATTGACGCGGTATTCAGCATTTTGCCTCGTCTGGCACCTTTAGCCGAAGTGCTCGCCAGCGG
TGAAACCAATCTCTTCAACAGCGCGCGAAATATTGCCTGCGCCATTAAAATAGGTCAGGGAATTAAAAACTAACCCTTAC
CTTTAAAGCGGATGCGATTTATATCGCATAAGAGTGCAGTACTCATGCCGGATGCGGCATGAGTACCATATCCTTCCTGA
AAATCGCGCAAATTCTATATATTGCAGAGATCATGTAGGCCTGATAAGCGAAGCGCATCAGGCAATGTTACAAAAAAAGC
CACGGTATAAACCGTGGCAAAATCCAACATAGCTAAAAATAATCAGGCGAGTGGTATGACTTAAATCTCTACGTCGCGGT
TACAATCTTTCGAGTAAATATAGCTGAACGCTTCACCACGCCCTACACCATAACCAGCCTGTAAAGAATAAGCGCCCATA
AAGATGTAATCGCCTTTTTTCACCGGGATCCAGTTATTGTCGAGGTTATAAACCCCCTGACCGGAAAGAATATAGGCACC
GTGTTCCTGAACGTGTGTTTCGATATAACCGTGGCTGGCACCTGGTGCAAAAGAGAGGATATGCATGTTCATATCAAAAC
CTAACTCTTTGGGCAGAAAATCCAGCAGAATAACATCGTCCATGCCTTCATAATGAATGCGTTCCAGTTCGCTGGCATTG
CCAGAAACCAGCCACGGTGCATAGCCTTCTACCGGAACATAGCGGCGCTTATATAAAAAGATTTGGCTGTCTTCGGCCTG
GGCGTTAACAAACGTCATTAAGGAGCCTGGCGGGCAATAAAGATAGCCACCTTCGCTTAAGGCAAATGTTTTGCCTTCGG
CTTTGGCAGTGATATTTCCAGAGATCACATACAGGAACGTTTCAATGCCTTCGCCACCGAAGCCCTGTTGGTTGCCACCG
TTTTGATGCAGTGTGACCAGATAATCAACAAAAGAGGCACCCAGCTTTGGCGTGGAGAGGATTGTCGCGTCACAATTTTC
AAAGCCCGGAATAATATTTTTTACCAGACCATCCGGGGTTAACAGTGCGAAATTACCGTGTTTAACAATCGCACGGTTAG
CCAGTAAATCTTCGCGGTAACCGGTGACGTTATTTAAATATCCCATTTATGACTCCTTATTTCTGCCAGGCAAGTTGATA
AAGCATGAGTGCCAACGTTTTGACCCCTTCGGCAAGGTCGGTAATATTGGTGCGTTCCGCCGGGTTATGGCTGATCCCGT
TGATGCTGGGGATAAAAATCATGCAGGTTGGTACGCGAGGCGCGAAAATTTGCGCGTCGTGCCCGGCACCACTGTGCATC
ACCCGGTAATTCAGTTTTTCTCTTTCACACAATTCTGTCAGGGTGGCGACCAGCTCCTTATTCATCGGCACGGGTTCTTC
GTCCATCCATAAATCGATATCAATACCAATGTCCATTTCATCGCAAATCGCCCGCATGTCGTTTTCTAACTGTTGGGTGA
AATCGCGCAGCACGGCAGCGTCGGTATGACGACAATCAATGGTGAACGTGGTTTTACCCGGCACCACATTTACCGTATTC
GGGCGCGGCTCTACTTTGCCAAAGGTCAGAACCAGCGGATCGCCCATCCTTTTCGCTTTTTCGACCGACTGATGGCAAAT
GCGACTGAAAGCGTAAACTGTATCACGACGATAACCCATCGGCGTGGTGCCTGCATGGTTTGATTCGCCGTTCAGCGTTA
CCGTATAACGACGCTGCCCGACAATTGCATTCACCACGCCAATTGATTGCCCATTACTTTCCAGCACACAGCCCTGTTCA
ATATGCAGTTCAACAAAGGCTTTAATATCCTGACGCGGAGTTAGTGGGGCGTTCGGAAGAGTAAATCCGCAAGCCTTCAT
CGCATCGACAAAACTATTTCCTTTGGCATCACAGATATTCCGCACGTCGTCAGGATTCGCCAGCCCAAAAATATTTTTAC
TGCCCCAGAAGACATACGGGAAGCGGCTGCCTTCTTCTTCTGCCATCGCCACCACTTCGACCGTACGTAGCGGCGCGCCG
TATTGCGTTTTCAGCCAGTCAATTGCCAGCCACGCCGCCAGCGCGCCGAATTGCCCGTCAAGGTTACCGCCGTTAACCAC
GGTATCGATATGCGAACCGCTCAGAACCACTTCCTGTGGATATTCGGTGCCATTCAGGCGACCGTATAAATTCCCCACTT
CATCGAAACGTGTTTCCAGCCCGCTTGCTGCCATTCTTTTTTTAAATTGCTGCTGGGTTTCCAGCCATTCCGGCGAATAA
AGTAAACGGGTCATCCCACCCGCTGGGTCAGCGCCAAAAGAGGAAAGCCAGGGCAGCGTTTCTTCTATAGCTTGACGGAA
ATGTGTAATCATAAGAAAGTCCTGTCTCAATAATTATTGCGCAAAGGGATTTTTCGTTTCGTATGACGTGTTATAAAGCG
CGTCGGAAATTAAATACTGGTAAATATCATCAACAATTTCGATGCCTTCGACGGCGGCTTTGCGTTGTTTAATATCCTGA
TCCTGTCCGGGATAATAAACCTGATTAAAACCGGGCGCGGGGGTAATGGCATTTAATTCGCGCATGGTCTGGCTAAGATG
TTGACGGAATAATTCGCTGGAGGAGAAAAAGTTCGGATTAATAACTATATGTAATTGCCCCAAATTACGCCCTGCGTGTA
AATCGTCATACATCGAACTAACCTGTCGCCCGAACGGTAAGCCGAGTAAGACGCCTGAGAGGACGTCAATCATCATCATC
AGGCCATACCCTTTTGGCCCGGCGGCGGGGAGCAGAGCATGTACCGCGAACGGATCGGTTGTTGGTACACCGTTTTTATC
GACCGCCCAGGTATCCGGGATAGACATATTACGCGAGCGGGCGTCGAGCACTTTTCCCCATGCCTGTACGGTAGTCGCCA
TATCAAAGGTAAGGATCTCGTCGCCTTCTCCCGGCGCGGCAAAGGCCAGGGGGTTAGTACCGTAGTAAATTTCCGCGCCG
CCAAACGGCACCACCATTGGATCGGACTGGCACATCGAAATGCCAATGAATCCGGCGCGGGCTGCCTGCTGCACAAAATA
AGAGATTGCGCCGCTGTGACCCATCCGGCTGATACCGACCACCGCAACGCCATTTTGCTGGGCGGTTTTGATGGCATGTT
CCATACCCATTTTCGCCGCGACCTGTCCGGCGGCATTGTCGGCATGTAAAATTGCCGAGCACGGCCCGGTTTCCTCAAGA
CGAAACTCCGGTTCGCGGTTGGTGCCGCCTTTTGAAATGCGTTCCGCGTAGTATTCCACGCGCACCGCGCCATGAGAGTG
GATCCCTCTGGCATCGGCGTAAACCAATACTTCAGCCACGGTTGCAGCGTGCTCACGTTTTAACCCAGCCTGGCAGAGTT
TATTCTCAATTAGCTGGTGGAGTGTTTCCCGACTGATTTTCATCTGTCTTCCTTTTTAACGACGGTGTGAAGCATGACTG
CAATTAACATACAGGGAAAATATCTGGATTATGTGATCCAGACAGGCAAAAAAATATAGTTAGAATTTATTTGATAATCC

GCTCACTTTTAACCTGATTTTTAAAACAACAACGCTTATTAAAAAATAATGAGTAATAGCCTGGTGGTTATTTGAATTCT
TTTGTTAATAATTCCTGTGTGATATTCATCACCTTATTTACTCGTTGTCATCGATACCGTAATCGCCACATTAACACTGC
TCGTGCAATTGCCATGGGTGCAATTTTTAAGGAGTTGTTATGATCCACGCCTTTATTAAAAAAGGGTGTTTTCAGGATTC
GGTCAGTTTAATGATTATTTCACGAAAACTCAGCGAATCAGAAAATGTTGATGATGTTTCCGTAATGATGGGTACGCCCG
CCAATAAAGCGTTATTAGATACCACAGGTTTCTGGCATGACGATTTTAATAACGCCACGCCGAACGATATTTGCGTGGCA
ATTCGTAGCGAAGCGGCGGATGCGGGGATCGCGCAGGCGATTATGCAGCAGCTTGAAGAGGCGCTAAAACAACTGGCGCA
GGGGTCAGGCAGCAGCCAGGCGTTGACGCAGGTGCGTCGCTGGGACAGTGCCTGTCAGAAATTACCCGATGCCAATCTGG
CGCTGATTTCAGTGGCTGGCGAGTATGCGGCGGAGCTGGCAAACCAGGCGCTGGATCGCAACCTCAACGTGATGATGTTC
TCCGATAACGTCACGCTGGAAGATGAAATCCAACTTAAAACCCGCGCGGGAAAAAGGCTTGCTGGTGATGGGGCCGGA
CTGCGGTACGTCGATGATTGCCGGCACACCGCTGGCTTTTGCTAACGTGATGCCGGAAGGCAATATTGGCGTCATTGGCG
CTTCCGGTACCGGGATTCAGGAGCTGTGTTCGCAGATTGCGCTGGCAGGGGAGGGAATTACTCACGCGATTGGCCTTGGC
GGGCGCGACCTCAGCCGTGAAGTGGGCGGCATCAGTGCGCTAACAGCGCTGGAAATGCTCAGTGCAGACGAGAAAAGCGA
AGTGCTGGCATTTGTTTCAAAACCACCTGCCGAAGCTGTGCGTCTGAAAATTGTTAATGCCATGAAAGCAACCGGCAAAC
CGACGGTGGCGCTGTTTTTAGGTTATACCCCGGCGGTGGCCCGCGACGAGAATGTCTGGTTTGCCTCCTCGCTGGATGAG
GCCGCACGCCTGGCTTGCCTGCTTTCACGCGTCACGGCGCGACGTAACGCAATAGCGCCTGTCAGCAGCGGATTTATTTG
CGGTTTGTATACCGGCGGTACGCTGGCTGCCGAAGCGGCGGGATTACTTGCCGGACACCTTGGCGTGGAAGCCGACGATA
CCCATCAACATGGCATGATGCTGGACGCCGATAGCCACCAGATTATTGACCTCGGCGATGATTTCTACACCGTCGGGCGT
CCCCATCCGATGATCGACCCAACCTTACGCAACCAGTTAATTGCCGATCTCGGCGCTAAACCGCAAGTGCGCGTGTTGCT
GCTTGATGTCGTGATTGGCTTCGGTGCGACCGCCGATCCTGCCGCCTCGCTGGTGAGCGCCTGGCAAAAAGCCTGTGCCG
CGCGTTTAGATAATCAACCACTGTATGCCATTGCCACGGTGACAGGCACTGAACGTGACCCGCAATGCCGCTCGCAGCAA
ATCGCCACGCTGGAAGATGCGGGGATTGCGGTCGTGAGTTCGCTACCGGAAGCCACCTTGCTGGCGGCAGCGTTAATTCA
TCCGCTCTCGCCTGCCGCACAGCAACACACACCGTCATTACTGGAAAACGTCGCCGTGATTAACATCGGATTACGCAGCT
TTGCGCTGGAGCTACAAAGCGCCAGCAAACCGGTTGTGCATTACCAATGGTCGCCAGTCGCCGGTGGCAATAAAAAACTG
GCTCGTTTATTAGAACGTTTGCAATAAGGGGTTCCCATGTTTACATCAGTGGCGCAAGCCAATGCTGCGGTTATCGAACA
AATTCGTCGCGCTCGTCCACACTGGCTGGATGTGCAACCGGCTTCTTCACTTATCAGCGAACTAAACGAGGGCAAAACAC
TGCTTCACGCCGGGCCGCCAATGCGCTGGCAGGAGATGACCGGACCCATGAAAGGGGCGTGCGTGGGCGCATGTCTGTTC
GAAGGTTGGGCGAAAGATGAAGCGCAGGCGCTGGCAATACTGGAGCAGGGGGAAGTGAACTTCATTCCTTGTCACCATGT
GAATGCCGTCGGGCCAATGGGCGGTATTACTTCTGCCAGTATGCCGATGCTGGTGGTTGAGAACGTGACCGACGGCAACC
GGGCGTACTGCAACCTCAACGAAGGTATCGGCAAAGTGATGCGTTTTGGCGCTTACGGCGAAGATGTCCTGACTCGCCAT
CGCTGGATGCGCGATGTGTTAATGCCAGTATTAAGCGCGGCGCTGGGGCGCATGGAGCGCGGTATCGATCTCACGGCGAT
GATGGCGCAGGGCATTACGATGGGCGATGAGTTCCATCAACGCAATATTGCTTCCTCTGCACTGTTAATGCGTGCGCTGG
CCCCACAAATTGCTCGCCTCGATCATGATAAACAGCACATCGCCGAAGTGATGGATTTCCTCAGCGTGACCGATCAGTTC
TTCCTCAACCTCGCGATGGCTTACTGCAAGGCGGCGATGGATGCTGGCGCGATGATCCGCGCAGGCAGCATCGTCACGGC
AATGACCCGCAACGGCAATATGTTCGGGATTCGGGTAAGCGGGCTGGGCGAACGCTGGTTTACTGCGCCTGTAAACACTC
CGCAAGGTCTGTTTTTCACCGGCTTCTCGCAGGAGCAGGCGAACCCGGATATGGGCGATAGCGCGATTACCGAAACCTTT
GGTATCGGAGGTGCGGCAATGATCGCAGCGCCTGGCGTAACGCGCTTTGTCGGTGCGGGTGGCATGGAAGCGGCAAGAGC
GGTATCTGAAGAGATGGCGGAAATTTACCTTGAACGCAATATGCAGTTGCAGATCCCAAGCTGGGATTTTCAGGGCGCGT
GCCTGGGGCTGGACATTCGTCGCGTGGTAGAAACCGGCATTACGCCACTCATCAATACCGGTATCGCCCATAAAGAGGCG
GGGATCGGGCAGATTGGCGCAGGCACCGTGCGGGCACCGCTGGCGTGCTTTGAACAGGCGCTGGAAGCACTGGCTGAAAG
CATGGGTATTGGTTGAGGAACGCGCAATGACGATCATCCATCCTCTGCTTGCCAGTAGTAGCGCACCGAATTATCGCCAG
TCCTGGCGGTTAGCGGGAGTGTGGCGGCGGGCGATTAACCTGATGACGGAAAGCGGCGAACTGTTAACGTTGCATCGTCA
GGGTAGTGGTTTCGGCCCCGGAGGATGGGTGCTTCGCCGTGCGCAATTCGATGCGTTATGCGGTGGATTATGCGGCAATG
AACGACCACAGGTTGTGGCTCAAGGGATTCGCCTCGGGCGTTTCACGGTTAAACAGCCACAGCGTTATTGTTTGCTGCGT
ATTACGCCGCCTGCGCATCCTCAACCACTTGCAGCTGCATGGATGCAACGCGCGGAGGAAACCGGGCTTTTCGGGCCACT
GGCGTTGGCGGCAAGCGATCCGCTGCCTGCTGAGTTACGCCAGTTTCGTCACTGTTTTCAGGCCGCGCTCAATGGCGTTA
AGACCGACTGGCGGCACTGGCTGGGTAAAGGCCCCGGATTAACGCCGAGTCATGATGACACGCTGAGCGGAATGCTGCTG
GCGGCCTGGTATTATGGCGCTTTAGATGCGCGCTCCGGTCGTCCGTTTTTTGCCTGTTCCGACAATCTGCAACTCGTTAC
CACAGCGGTGAGCGTCAGTTATTTACGTTATGCCGCGCAAGGATATTTCGCCTCGCCACTCCTGCACTTTGTTCATGCTC
TGAGTTGCCCGAAACGTACCGCTGTTGCGATTGATTCGCTGCTGGCGCTGGGGCATACGTCAGGGGCAGATACGCTGCTG
GGGTTCTGGCTTGGCCAACAATTATTACAAGGAAAACCATGAAAACACTGGTTGTGGCTCTTGGGGGCAACGCCTTACTC
CAGCGCGGTGAGGCGCTGACGGCAGAAAATCAATATCGCAATATCGCCAGTGCTGTACCCGCGCTGGCACGCCTGGCCCG
TTCTTATCGGTTGGCGATTGTTCACGGCAACGGGCCGCAGGTGGGGCTGCTGGCATTGCAGAATCTGGCGTGGAAAGAGG
TAGAACCGTATCCGCTGGATGTGCTGGTTGCGGAAAGCCAGGGGATGATTGGCTATATGCTGGCGCAGAGTTTGAGCGCA
CAGCCGCAGATGCCGCCCGTGACGACGGTGCTGACGCGCATTGAGGTTTCGCCTGATGATCCGGCGTTTTTGCAGCCAGA
GAAATTTATTGGTCCGGTTTATCAGCCAGAAGAACAAGAGGCACTGGAAGCGGCTTACGGCTGGCAGATGAAACGTGATG
GTAAATATTTGCGCCGGGTGGTGGCGTCTCCGCAACCGCGTAAAATTCTCGACAGCGAAGCCATCGAGTTGTTGCTCAAA

GAGGGGCATGTGGTGATTTGCAGTGGCGGCGGCGGTGTGCCTGTGACGGATGACGGAGCAGGGAGTGAAGCAGTGATTGA
TAAAGATCTCGCCGCTGCGTTGCTCGCCGAGCAGATTAATGCAGATGGACTGGTGATCCTCACCGATGCTGATGCGGTAT
ATGAAAACTGGGGAACGCCGCAGCAACGTGCCATTCGCCATGCCACACCGGATGAGTTAGCGCCATTTGCCAAAGCCGAT
GGTTCGATGGGGCCGAATGTAACGGCGGTGAGTGGTTATGTCAGAAGCCGTGGTAAACCCGCGTGGATTGGGGCGTTATC
GCGAATTGAAGAGACGCTGGCGGGCGAAGCGGGGACCTGTATTTCGCTGTAGTCGTAGGCATTAGACATTTGTGCCTGAT
GCGACGCTTGACGCGTCTTATCAGGCCTACAACCGGTGCCGCATCCGGCAATTGGTGCACAATGCCTGATGCGATGCTTG
ACGCATCTTATCAGGCCTACAATGGGTACCGGATCGGTAGGCCGGATAAGGCGTTTACGCCGCATCCGGCAAGAATAGAG
CACCAGTTAACCGAACTTACTCTGCGCCCAAATCACGCCGCTGGCATATTCCGGCGGCAGCAGCGGGATTAAGGCTTCCA
GCGTCGCAGTCAGACGCGATGTGTCGCTGTCGGTCAAATTCAGATGCCCCACTTTACGCCCCGGACGGACTTCTTTGTCG
TACCAGTGCAGATGCACCAGCGGCAGTTTCAGCCAGTCATAATTCACATCGCTACCAATCAGATTGATCATCACCGACGG
ATTATTCACCACTGGTTGCGGTAACGGCAGATCGGTAATCGCCCGCAGATGCAGCTCAAACTGGCTGATGCTGGCACCGT
TTTGTGTCCAGTGACCGCTGTTATGCACACGCGGTGCCAGTTCGTTGATCAACAGACCTTGCGGGGTGACAAAACACTCC
ATCGCCATCACGCCCACATAGCCCAGCTCCTGCATAATCGCCGACAGCATCTCTTCGGCTTGCGCCTGCTGCTGTGCGTT
GGCCTGCGGAAAAGCGACGCTGGTGCGCAAAATACCGTCCTGATGCAGGTTATGCGTCAGCGGATAAAACACGGTGCTGC
CATCAAAGCCGCGCGCGCCAACCAGCGACACTTCACCAGAGAAGTTAATGCCCTGCTCGACAATACATTCGCCGTAACAC
TCTGCCGGTAACTGTTCGGTTTCATTTGCGCGTAAACGCCATTGACCGCGACCGTCATAACCACCAGTGCGACGCTTAAC
AATCGCCAGCTCACCTAAACGATCAAACACCGCAGGCCACTCGCTGCGTTCGGCAAGTAACTGCCACGGTGCAGTCGGCA
GGTGGAGCTTATCGAAAAGCTGCTTCTGAGTCAGACGGTCAGCAATAATCGGGAACACATCGCGGTTCACAAAGGCCGGA
TGGCGCGCCAGCTCGCGGGTTAATGCGGTTTCCGGCCAGCGTTCTATCTCAGCGGTAATCACGCTTTGTTGAAAAGGCAC
CGCCGCCGGTTCAGCGTCCAGCCCGACTGGCCAGACAGCAATGCCTAACGGTTCGCCTGCCTGACGCAGCATACGGCCTA
ACTGCCCGTTACCGAGGACGCAAACCTGTTTCATGCCGCACCTCGCGGGTCCGGGTTTTCCAGCACTTCGTCGGTCTGGG
CTTTGCGCCAGTCATTCAGACGCTGGTGCAGTTCTTTATCATGAGTCGCAAGAATTTGTGCTGCCAGTAACGCCGCGTTT
GCCGCGCCAGCTTTACCAATCGCCAGCGTACCCACCGGAATGCCGCGCGGCATTTGTACGATGGAGTAGAGGCTATCGAC
ACCGCTCAGTGCGGCGCTCTGTACTGGCACGCCCAGCACCGGCACCAGCGTTTTGGCGGCAATCATGCCTGGCAGATGCG
CTGCGCCGCCTGCGCCCGCAATAATCACCTGATAACCGTTCTCTTCGGCGCTTTCGGCGAAGCTGAACAGTTTATCGGGG
GTGCGGTGAGCAGAAACCACTTCAACGTGGTGCGGGACATTCAGGATTTCGAAGATTTCGGCGGCGAACTGCATGGTAGC
CCAGTCGCTTTTGGACCCCATCACGATGGCGACACGCGCCGGATTATTGCGGGAAGACATGCGTCTTAAAACTCCTGTGG
TGCACAACTCTCGGCTTTAGAGGGCACAGAGAATAGCACGGAAAGAGAGCAAGGAAAACGGTTGCGTGGCTGTGAAATCA
GCAAAGTTGCGGGTTTTTTAAAACGGAAAATGAATCAGCTCAACGTCATCCGCCGTGACTTTCACCATTGAACCTTCCGT
ATGCCAGGCACCCAGTACCACGCGAAAAGCAGGTTGCTGATTGGCGATAAGTTCATGCACCGCCGGGCGATGGGTATGCC
CGTGGATCAGCCATTGCACCTGATGTTTTTCCATCGCACTGACCACCGCGTTTTGGTTAACGTCCATGATCGCCAGCGAT
TTACTGCTGTTGGCTTCTTTGCTGTTCGCGCGCATTCGCGCGGCAATGCGTTTGCGCACAAACAACGGCAGGGCGAGGAA
TAGCGTCTGCAGCCAGGGTTTGTGGACCTTGGCGCGAAAAGCCTGATAACCCGCGTCATCGGTGCACAGCGTGTCGCCAT
GCATAATCAACACCCGGCGACCATAAAGTTCGAGCACCTTTTCTTCCGGCAATAACGTCATGCCACTTTCACGGGCAAAG
CGTTTGCCGAGCAGAAAATCACGGTTGCCATGAATGAAATAACAGGGAACGCCGGAATCGGACACCGCTTTGATCGCCGC
CGCCATCTTGCGATGGAGTGGGTTGGGATCGTCGTCGCCAATCCATGCTTCAAACAGATCGCCAAGAATATACAGCGCGT
CGGCCTTGCGGGCTTCCCCCGCTAAAAAACGCAGAAAACCGGCGGTGATCGCCGGTTCTTCCACGCAGAGATGAAGATCT
GCAATAAAGAGTGTCGCCACGATTACTCGCTAACGGTCACGCTTTCAATGATAACGTCTTCTTTTGGCACGTCCTGGTGC
ATACCGCTACGACCGGTTGCAACACCTTTGATTTTGTCTACCACGTCCATGCCGTCAACCACTTCAGCAAACACGCAGTA
GCCCCAACCTTGCAGGCTTTCGCCAGAGAAGTTCAGGAAGTCGTTATCAACCACGTTGATGAAGAACTGTGCAGTTGCAG
AGTGCGGAGCCTGAGTACGTGCCATTGCCAGCGTACCACGGGTATTTTTCAGGCCGTTGTTGGCTTCGTTTTTGATCGGT
TCTTTGGTGGCTTTTTGTTTCATGCCCGGTTCAAAACCGCCGCCCTGAATCATAAAGCCGTTGATAACACGGTGGAAAAT
GGTGTTGTTGTAAAAACCTTCGCGGCAGTAGTCCAGGAAGTTTTTAACTGTTTCAGGTGCTTTATCGTCAAAAGTTTTGA
TGACAATATCGCCGTGATTGGTGTGGAAAGTAACCATTTTTGCATCCTGTTCCGTTTGATTGGTGCTTCAACCCAGTTCG
GGTCATATATAGGGTGGTGTTATAGCATAACCGCACGATCGGATCATCACGCAATGTATGCTGATTCGCGCGGGAAATAT
GGGTATTATACGCAACTCAATTACCCACACATGTCTAAACGGAATCTTCGATGCTAAAAATCTTCAATACTCTGACACGC
CAAAAAGAGGAATTTAAGCCTATTCACGCCGGGGAAGTCGGCATGTACGTGTGTGGAATCACCGTTTACGATCTCTGTCA
TATCGGTCACGGGCGTACCTTTGTTGCTTTTGACGTGGTTGCGCGCTATCTGCGTTTCCTCGGCTATAAACTGAAGTATG
TGCGCAACATTACCGATATCGACGACAAAATCATCAAACGCGCCAATGAAAATGGCGAAAGCTTTGTGGCGATGGTGGAT
CGCATGATCGCCGAAATGCACAAAGATTTTGATGCTTTGAACATTCTGCGCCCGGATATGGAGCCGCGCGCGACGCACCA
TATCGCAGAAATTATTGAACTCACTGAACAACTGATCGCCAAAGGTCACGCTTATGTGGCGGACAACGGCGACGTGATGT
TCGACGTCCCGACCGATCCAACTTATGGCGTGCTGTCGCGTCAGGATCTCGACCAGCTGCAGGCAGGCGCGCGCGTTGAC
GTGGTCGACGACAAACGCAACCCAATGGACTTCGTTCTGTGGAAGATGTCGAAAGAGGGCGAACCGAGCTGGCCGTCTCC
GTGGGGCGCGGGTCGTCCTGGCTGGCACATTGAATGTTCGGCAATGAACTGCAAGCAGCTGGGTAACCACTTTGATATCC
ACGGCGGCGGTTCAGACCTGATGTTCCCGCACCACGAAAACGAAATCGCGCAGTCCACCTGTGCCCATGATGGTCAGTAT
GTGAACTACTGGATGCACTCGGGGATGGTGATGGTTGACCGCGAGAAGATGTCCAAATCGCTGGGTAACTTCTTTACCGT

GCGCGATGTGCTGAAATACTACGACGCGGAAACCGTGCGTTACTTCCTGATGTCGGGCCACTATCGCAGCCAGTTGAACT
ACAGCGAAGAGAACCTGAAGCAGGCGCGTGCGGCGCTGGAGCGTCTCTACACTGCGCTGCGCGGCACAGATAAAACCGTT
GCGCCTGCCGGTGGCGAAGCGTTTGAAGCGCGCTTTATTGAAGCGATGGACGACGATTTCAACACCCCGGAAGCCTATTC
CGTACTGTTTGATATGGCGCGTGAAGTAAACCGTCTGAAAGCAGAAGATATGGCAGCGGCGAATGCAATGGCATCTCACC
TGCGTAAACTTTCCGCTGTATTGGGCCTGCTGGAGCAAGAACCGGAAGCGTTCCTGCAAAGCGGCGCGCAGGCAGACGAC
AGCGAAGTGGCTGAGATTGAAGCGTTAATTCAACAGCGTCTGGATGCCCGTAAAGCGAAAGACTGGGCGGCGGCGGATGC
GGCGCGTGATCGTCTTAACGAGATGGGGATCGTGCTGGAAGATGGCCCGCAAGGGACCACCTGGCGTCGTAAGTAATTGC
GCTATTGCCGGATGCGAGTTTTCGCATCCGGTTATCGTCTGCGCCACCACAACATTCCCATCAGTAGCATCCCCGGCAAC
CACACCCACATCAATTCAGAAATAATCACCTGATGCCCGTACGGCGTGGTGTAACGAGACAATGCAAACGGCGCGACTTT
TATCACCTGCCAGGGAGCGAAAAAGCGTTCATCTGACCACGGCCACAGCCAGCCAACGCCTTTACCGCCAGTGGTTACCG
AATCCAGCAAGCTGTGCGATAGCAACGAGACGGTTAAAAACAGCCAGCAGCGAATCAGCCCAGCCCTGAACCATCGGCGT
CCAATAAACACACATAACAGCGGGACAACAAACGCAAACACCAGCGAATGGGTAAACCCGCGATGACCAAAAACATTGCC
GTAAGCAACGCCAAATTTAAACGACAATACGTCGGCGTCGGGCAGCATCGCCAGGATGATTCCGGCAAATAACAGACGCG
GAGGGATGACTTTCGAACCCAACCCTAAACCAATGCATAGGGGAACGGCGGCGTGCGTAATAACGGTTGGCATGATGGTC
GCTTCGGCAAAATGTCGATGCTATCAGCATGGATGAACGGGGCGTAGAGGGCAAAAGTCTGAAAAGAGAACCGGCCTGTT
GATACAGGCCGGGAAAGGGATCAGGCAACAACCTGTACGCTGTGACCTGCAAAACTCACTGTCTGACCGGCGACGATTTT
GCAGCGTTTGCGCGTTTCAACCGCACCGTCGACTTTCACCTGGCCTTCGGCAATCGCGATTTTCGCCTGCGCGCCGCTTT
CGCTCCAGCCTTCCAGTTTCAGCAAGTCGCACAGCTCAACGTGCGGATGTTTACCTAAAGAAAATGTCGCCATGTTACTC
ATCCTGTGGATCATGATATTCAACGCACGCCTGTAGCGTGTTTTCAATCAGCGTGGCAACCGTCATCGGGCCAACGCCGC
CGGGAACAGGCGTAATGTATGAGGCGCGTTTAGCCGCGTCTTCAAACACGACGTCGCCCACAACTTTGCCATTTTCCAGA
CGGTTGATGCCGACATCAATCACAATTGCGCCTTCTTTGATCCAGTCACCGGGAATAAAGCCTGGCTTGCCAACGGCAAC
GATCAATAGATCGGCATTTTCTACGTGATGACGCAGATTTTTAGTGAAGCGGTGAGTCACTGTAGTGGTGCAACCTGCCA
GCAGCAGTTCCATGCTCATCGGGCGGCCAACGATATTCGATGCGCCAATCACCACGGCGTTGAGGCCGAAGGTATCAATG
TTGTAACGCTCAAGCAGCGTGACGATACCGCGCGGGGTGCAGGGACGCAGACGCGGCGCGCGCTGGCACAGACGACCGAC
GTTGTAAGGATGGAAACCGTCCACGTCTTTGTCCGGATGAATACGTTCCAGCACTTTGACGTTATCAATACCCGCCGGTA
ACGGCAGTTGAACCAGAATGCCATCGATGGTGTTGTCGGCATTCAGCGTATCGATAAGCTCCAGCAGCTCCGCTTCGCTG
GTGGTTTCCGGGAGGTCATAAGAGCGGGAGACGAACCCGACTTCTTCACAAGCCTTGCGTTTGCTTGCGACATAAATTTG
CGATGCAGGGTTACTACCCACCAGCACAACGGCCAGTCCTGGTGCCCGCAGTCCGGCTGCAATACGCGCCTGAACTTTTT
GAGCAACTTCAGAGCGCACCTGCTGCGCAATCGTTTTACCGTCAATAATCTTTGCTGCCATCAGAGAGAGGATTCCATCT
GTTACGTAGATCGAAGGGGATGCGCCTATTTTGTCAGAAGCGGGGCGCGCTGTCAGGTTTCGTTTCAGATTTATCGCGTG
AAGCGACCTCTTGCGAAGGTGAGGCGCACCGTCGCTGAGACTGAAAGCTTCATTTTTCGTCCATGATGGCGTTGTAAATC
TGGAACTGATTTATTTCCTTGTCTAAGGATTAAGATAATTTAAGAAATACCTGACAATATAAAAAGAATTTTCAGCCTGG
TAATTTACCGCTTCAGGTCTATATTTGTGTTGAATATATTTTGCGCGGAAGTATTCATCTAACGGGGCTCTCTATTTTTT
AGAATAGAGTGCATATTTTCAATTAAGACATTCTTAGAGGATAAAAAGGAATTTACTACTATCAGTGTCTTAAATAAAGT
AATCGGTTATATACGGATGTGGAGTCGATAAATGAGATTGAAGGAATATATATGAAATTAAGATTTATTTCGTCTGCGCT
GGCTGCCGCACTATTCGCCGCTACGGGTAGTTATGCTGCCGTTGTAGATGGCGGTACAATTCACTTTGAAGGCGAACTGG
TGAATGCTGCCTGTTCAGTGAATACTGACTCGGCAGACCAGGTTGTCACACTCGGTCAATATCGTACCGATATTTTCAAT
GCTGTTGGTAATACCTCTGCATTAATTCCATTCACCATTCAGTTGAACGACTGCGATCCTGTTGTTGCCGCTAATGCTGC
CGTTGCATTTTCTGGTCAGGCTGATGCAATCAATGATAATTTATTGGCCATTGCATCCAGTACCAATACAACAACAGCAA
CGGGTGTCGGTATTGAAATACTTGATAATACATCCGCAATTCTCAAACCTGATGGGAATAGCTTCTCAACCAACCAGAAC
TTGATCCCCGGGACCAACGTTCTTCATTTTTCTGCACGTTATAAAGGCACCGGTACAAGTGCATCAGCAGGGCAAGCAAA
TGCTGACGCGACTTTTATTATGAGATATGAATAATCAAAACCACGTTGTTTTGAATTATATATCACGTCTTATAACAAAG
TAATGTACCGGTTGTCTGAAGCGGTATGGTGGCAATGTAAATCGAAATCATGTTCACTTTGTATCATGCCGCTTTATTAA
ATGAAAAGGGAATGATGTGTTGTAAGAAACCAAAGCAATCATTTCTTTATATTCCTTATTTTTGCCGTCAGGAATACACA
AGGCGTATTAACTATGATGACTAAAATAAAGTTATTGATGCTCATTATATTTTATTTAATCATTTCGGCCAGCGCCCATG
CTGCCGGAGGGATCGCATTAGGTGCCACGCGTATTATTTATCCCGCTGATGCTAAACAGACTGCGGTATGGATTAGAAAT
AGCCATACCAATGAGCGCTTTCTGGTCAATTCGTGGATTGAAAACAGCAGCGGTGTAAAAGAAAAGTCATTCATCATTAC
ACCGCCACTGTTTGTTAGTGAACCCAAAAGCGAAAATACTTTGCGTATTATTTACACCGGTCCACCGCTGGCAGCAGATC
GTGAGTCTCTGTTCTGGATGAATGTTAAGACGATCCCTTCGGTAGATAAAAATGCATTGAACGGCAGGAATGTTTTGCAA
CTGGCGATTTTATCGCGCATGAAATTATTTCTCCGTCCAATTCAATTACAAGAATTACCCGCAGAAGCGCCGGACACACT
CAAGTTTTCGCGATCCGGTAACTATATCAATGTTCATAATCCATCACCTTTTTATGTCACCCTGGTTAACTTACAAGTGG
GCAGCCAAAAGTTGGGGAATGCTATGGCTGCACCCAGAGTTAATTCACAAATTCCCTTACCCTCAGGAGTGCAGGGAAAG
CTGAAATTTCAGACCGTTAATGATTATGGTTCAGTAACTCCGGTCAGAGAAGTGAACTTAAACTAACCGAATCATCTGAC
AATATCAGAGCTAATTATGAAAATACCCACTACTACGGATATTCCGCAGAGGTATACCTGGTGTCTGGCCGGAATTTGTT
ATTCATCTCTTGCCATTTTACCCTCCTTTTTAAGCTATGCGGAAAGTTATTTCAACCCGGCATTTTTATTAGAGAATGGC
ACATCCGTTGCTGATTTATCGCGCTTTGAGAGAGGTAATCATCAACCTGCGGGCGTGTATCGGGTGGATCTCTGGCGTAA

TGATGAGTTCATTGGTTCGCAGGATATCGTATTTGAATCGACAACAGAAAATACAGGTGATAAATCAGGTGGGTTAATGC
CCTGTTTTAACCAGGTACTTCTTGAACGAATTGGCCTTAATAGCAGTGCATTTCCCGAGTTAGCCCAGCAGCAAAACAAT
AAATGCATCAATTTACTGAAAGCTGTACCTGATGCCACAATTAACTTTGATTTTGCAGCGATGCGCCTGAACATCACTAT
TCCTCAGATAGCGTTGTTGAGTAGCGCTCACGGTTACATTCCGCCTGAAGAGTGGGATGAAGGTATTCCTGCTTTACTCC
TGAATTATAATTTCACCGGTAACAGAGGTAATGGTAACGATAGCTATTTTTTTAGTGAGCTCAGCGGGATTAATATTGGC
CCGTGGCGTTTACGCAACAATGGTTCCTGGAACTATTTTCGCGGAAATGGATATCATTCAGAACAGTGGAATAATATTGG
CACCTGGGTACAGCGCGCCATTATTCCGCTGAAAAGTGAACTGGTAATGGGAGACGGCAATACAGGAAGTGATATTTTCG
ATGGCGTTGGATTTCGTGGTGTACGGCTTTATTCTTCTGATAATATGTATCCTGATAGCCAGCAAGGGTTTGCCCCAACG
GTACGTGGGATTGCCCGTACGGCGGCCCAGCTAACGATTCGGCAAAATGGTTTTATTATCTATCAAAGCTATGTTTCCCC
CGGCGCTTTTGAAATTACAGATTTGCACCCGACATCTTCAAATGGCGATCTGGACGTCACCATCGACGAGCGCGATGGCA
ATCAGCAGAATTACACAATTCCGTATTCAACAGTGCCAATTTTACAACGCGAAGGGCGTTTCAAATTTGACCTGACGGCG
GGCGATTTTCGTAGCGGTAATAGTCAGCAATCATCGCCTTTCTTTTTTCAGGGTACGGCACTCGGCGGTTTACCACAGGA
ATTTACTGCCTACGGCGGGACGCAATTATCTGCCAATTACACCGCCTTTTTATTAGGGCTGGGGCGCAATCTCGGGAACT
GGGGCGCAGTGTCGCTGGATGTAACGCATGCGCGCAGTCAGTTAGCCGACGCCAGTCGTCATGAGGGGGATTCTATTCGC
TTCCTCTATGCGAAATCGATGAACACCTTCGGCACCAATTTTCAGTTAATGGGTTACCGCTATTCGACACAAGGTTTTTA
TACCCTTGATGATGTTGCGTATCGTCGAATGGAGGGGTACGAATATGATTACGACGGTGAGCATCGCGATGAACCGATAA
TCGTGAATTACCACAATTTACGCTTTAGCCGTAAAGACCGTTTGCAGTTAAATGTTTCACAATCACTTAATGACTTTGGC
TCGCTTTATATTTCTGGTACCCATCAAAAATACTGGAATACTTCGGATTCAGATACGTGGTATCAGGTGGGGTATACCAG
CAGCTGGGTTGGCATCAGTTATTCGCTCTCATTTTCGTGGAATGAATCTGTAGGGATCCCCGATAACGAACGTATTGTCG
GACTTAATGTTTCAGTGCCTTTCAATGTTTTGACCAAACGTCGCTACACCCGGGAAAATGCGCTCGACCGCGCTTATGCC
TCCTTTAACGCCAACCGTAACAGCAACGGGCAAAATAGCTGGCTGGCAGGTGTAGGTGGGACCTTACTGGAAGGCCACAA
CCTGAGTTATCACGTAAGCCAGGGTGATACCTCGAATAATGGGTACACGGGCAGCGCCACGGCAAACTGGCAGGCCGCTT
ACGGTACGCTGGGGGGCGGGTATAACTACGACCGCGATCAACATGACGTTAACTGGCAGCTGTCTGGCGGTGTGGTCGGG
CATGAAAATGGCATAACGCTGAGCCAGCCTTTAGGGGATACCAATGTTTTGATTAAAGCGCCTGGCGCAGGCGGTGTACG
CATTGAAAATCAAACTGGCATTTTAACCGACTGGCGCGGCTATGCGGTGATGCTGTATGCCACGGTTTATCGGTATAACC
GTATCGCGCTTGATACCAATACGATGGGGAATTCCATCGATGTTGAAAAAAATATTAGCAGCGTTGTGCCGACGCAAGGC
GCGTTGGTTCGTGCCAATTTTGATACCCGCATAGGCGTGCGGGCGCTCATTACCGTTACCCAGGGCGGAAAACCGGTGCC
GTTTGGATCACTGGTACGGGAAAACAGTACCGGAATAACCAGTATGGTGGGTGATGACGGGCAAGTTTATTTAAGTGGTG
CGCCATTGTCTGGTGAATTACTGGTTCAGTGGGGAGACGGCGCGAACTCACGCTGCATTGCGCACTATGTATTGCCGAAG
CAAAGCTTACAGCAAGCCGTCACTGTTATTTCGGCAGTTTGCACACATCCTGGCTCATAAAGGAAATTATCAATAAGATA
ATCTGCAGATTATTATTGGCGATGGCATGTTTGTGTCTGGCAAACATATCCTGGGCTACTGTTTGTGCAAATAGTACTGG
CGTAGCAGAAGATGAACACTATGATCTCTCAAATATCTTTAATAGCACCAATAACCAGCCAGGGCAGATTGTTGTTTTAC
CGGAAAAATCCGGCTGGGTAGGTGTCTCAGCAATTTGTCCACCCGGTACGCTGGTGAATTATACATACCGTAGTTATGTC
ACCAACTTTATTGTTCAGGAAACTATCGATAATTATAAATATATGCAATTACATGATTATCTATTAGGTGCGATGAGTCT
GGTTGATAGTGTGATGGATATTCAGTTCCCCCCGCAAAATTATATTCGGATGGGAACAGATCCTAACGTTTCGCAAAACC
TTCCATTCGGGGTGATGGATTCTCGTTTAATATTTCGTTTAAAGGTTATTCGTCCCTTTATTAACATGGTGGAGATCCCC
AGACAGGTGATGTTTACCGTGTATGTGACATCAACGCCTTACGATCCGTTGGTTACACCTGTTTATACCATTAGTTTTGG
TGGCCGGGTTGAAGTACCGCAAAACTGCGAATTAAATGCCGGGCAGATTGTTGAATTTGATTTTGGTGATATCGGCGCAT
CGTTATTTAGTGCGGCAGGGCCGGGTAATCGACCTGCTGGTGTCATGCCGCAAACCAAGAGCATTGCGGTCAAATGTACG
AATGTTGCTGCGCAGGCTTATTTAACAATGCGTCTGGAAGCCAGTGCCGTTTCTGGTCAGGCGATGGTGTCGGACAATCA
GGATTTAGGTTTTATTGTCGCCGATCAGAACGATACGCCGATCACGCCTAACGATCTCAATAGCGTTATTCCTTTCCGTC
TGGATGCAGCTGCGGCAGCCAATGTCACACTTCGCGCCTGGCCTATCAGTATTACCGGTCAAAAACCGACCGAAGGGCCG
TTTAGCGCGCTGGGGTATTTACGCGTCGATTATCAATGAGGTACGGAGAATGAGAAGAGTACTCTTTAGCTGTTTCTGCG
GGCTACTGTGGAGTTCCAGTGGATGGGCAGTTGACCCTTTAGGAACGATTAATATCAATTTGCACGGTAACGTTGTTGAT
TTCTCCTGTACCGTAAACACAGCGGATATTGATAAGACGGTAGATTTAGGCAGATGGCCTACGACACAACTACTGAACGC
TGGCGATACCACGGCACTCGTCCCTTTTAGCCTGCGGCTGGAGGGATGTCCTCCGGGTTCAGTTGCGATTTTATTTACGG
GAACGCCGGCATCCGATACCAACCTGCTGGCTCTGGATGATCCCGCAATGGCACAAACCGTCGCCATCGAATTACGTAAT
AGCGATCGCTCCCGGCTCGCACTGGGGGAGGCGAGCCCGACTGAGGAAGTAGATGCAAATGGCAATGTCACACTAAACTT
TTTTGCCAATTATCGAGCGTTAGCCAGCGGTGTTCGGCCAGGTGTGGCGAAAGCGGATGCGATATTTATGATCAATTATA
ATTAATATTATATTAATTCGTATAATTTGGCGTAGTCGATAAGCTCTACAATTGAATGCAAACCTAGCTTGCCATAAATA
TTAGATTTATGCGCACTAACTGTTTTATTGCTAAGTAATAACTTATCGGCAATTTCTTTATTAGATAATCCGCTAACCAG
ATAACGTAATATGGTCACTTCACGATTAGATAGCACAGTGACCGTTGAACTATTCGTACTACATTTATTGCTTTTTATAT
AGTTAAGCGTTTCGCTGGGAAAAAACGTGTATCCGGAGAGGATCATCTGAACGGCATGAAAAATATCATTCTGATCATTG
CATTTACTGACAAAACCGTTAGCACCAGCTTGTATCGCTCTGCCAGCATAAAAGCATTCTGATTTCGATGATAAAAATAA
CACTTTCACTGTGCTCTGGATTTGTTTGATCCTTTTCAGGAAGGTAAAACCGTCTGTTCCGGGCAAGTCTATATCCATAA
TGATTAAATCAACAGGACGGGTTCGGAGATAATCGATGGTTATGCGATAATCATCCGTTTTCAGGACAATCTGCAATTCA

CTGTTTTTTTGCAACAGAACTTCAATAGACATTCTGATGATAGGATGAGTATCCATAATGATCACCGACGTTGGTTTCAT
AGTTACCAGTCTCATAGGAGCGGACAATTTTCCGTTAGGTCGGGAAATTGTACTTTGATACATGAAAATACGGGTTTTCT
TGATTCAGACGCGCAGCGGTGTGCGTTTGTTTGCCGCTATAGCGAAATAAATCAGAAAATCAGACGCGGTCGTTCACTTG
TTCAGCAACCAGATCAAAAGCCATTGACTCAGCAAGGGTTGACCGTATAATTCACGCGATTACACCGCATTGCGGTATCA
ACGCGCCCTTAGCTCAGTTGGATAGAGCAACGACCTTCTAAGTCGTGGGCCGCAGGTTCGAATCCTGCAGGGCGCGCCAT
TACAATTCAATCAGTTACGCCTTCTTTATATCCTCCATAATTTCAGAGTGGGACATATTTGGGACATTATCACCAAAAAT
GTCGTCTATTTTCCTCGCATGCTCTGTCAAATGATTAGGCGCAAGGTGAGCATACCTACGAACCATTTCTATGGACTCCC
ATCCGCCCATTTCCTGAAGCACTGATAATGGGACGCCTGACTGAATCAGCCAGCTTGCCCAGGTGTGTCTGAGGTCATGG
AAACGGAAATCTTCAATTCCTGCACGACGACAAGCTGATAGCCATGATGTCTTGCTGTCGATGCGCATCTTCCTGACCGC
AGGCGTTGATGTTCCATCTGCTCGCTTAGCCGCCTTGGTATGTACAAACACCCATTTGTGATGCTTGCCTATTTGATCAC
GCAACACTTTACAGGCGGTATCGTTCAGCGCCACACCAATGGCGCGGTTTGATTTGCTCTCTTCTGGATTCACCCAGGCA
ACTCGTCGCTGCATGTCGATTTGTTGCCATTCCAGATTTATGATGTTCGACTTTCTCAGACCAGTTGCCAGCGCAAACTT
GACGACAGATTTCAGTGGTTCGGGGCACTCATCAATAAGGCGTTTTGCTTCCTCCTTTTCCAGCCATCTGACTCGCTTGT
TTCTGACCGCTGGTATCTTGATGACAGGCGCTTTTTCCAGCCACTTCCAGTCGCGTTCTGCAGCACGGAGAATGGCCTTT
ATCATGGCAAGATGCTTTGCCTTTGTCTGAGTTGATACTGGCTTTGGTTCATAAACAGGCAGTTCTTTACCTTTCCTGAT
GGCGGCCTGAACTTTCTGTTTCCATATTTCTTTCGTCTTTCTGTTATGCATTCTGCTTACAGCAGAGTAAATCTTTGCCT
CCGAGATATCTTTAAGCCTTATACCCTCAAAATGTTCAAGCCAGAACTCAATCCGGCTTTTATCTGAATCGAGAGATTTT
TTATCAGCTTTTTCCTCAAGCCATCTTAGGCAGGCCTCTTCAAAAGTGACATCAGGTAAATCCCCTAGCTTTTCTACTCG
CCAGAGTTCTGCTTTTCGCTTGTCGTGCAACTCCTGAGCTTGCCGTTTGTCCTTTGTGCCAAGAGATTCCTTAATTCGTT
TCCCGCCCGGGAGCGAATACGAGGCATACCATATTTCATTTCTGCGGAAGAGTGACATTTTCTTTCCTCTGTTATGCCAT
CACCCGCGCTCACCTGGACAGTATGCAGCGGAGACTGAAGCGCCGCAATGCAGGCTTGCCGTGTTGTGAGGTACCCGGAT
ATTATCGTGAGGATGCGTCATCGCCATTGCTCCCCAAATACAAAACCAATTTCAGCCAGTGCCTCGTCCATTTTTTCGAT
GAACTCCGGCACCATCTCGTCAAAACTCGCCATGTACTTTTCATTCCGCTCAATCACGACATAATGCAGGCCTTCACGCT
TCATGCGCGGGTCATAGTTGGCAAAGTACCAGGCATCTTTTCGCGTCACCCACATGCTGTACTGCACCTGGGCCATGTAA
GCCGATTTTATTGCCTCGAAACCACCGAGCCGGAATTTCATGAAATCCCGGGAGGTACGAGTATTGCCGGAAGCGTGGCC
TGTATCCGGATGCAGAGTCTTATCCGTGGAAATCGAACGCGCATTACTGGTTGGTTACCAACTTGTACCAGAACATGCGG
GCCAATGCGCTGGCTGACGCGGAATTACGGCGCAAGGCTGCCGATGAACTGACCTGTATGACAGCGCGAATTAACCGTGG
TGAGACGATACCTGAACCAGTAAAACAACTTCCTGTTATGGGCGGTAGACCTCTAAATCGTGCACAGGCTCTGGCGAAGA
TCGCAGAAATTAAAGCTAAGTTCGGACTGAAAGGAGCAAGTGTATGACGGGCAAAGAGGCAATTATTCATTACCTGGGGA
CGCATAAGAGCTTCTGTGCACAGGACGTTGCCGCGGTAACAGGCGCAACCGTAATCTGATCTTACCCAGCAATAGTGGAC
ACGCGGCTAAGTGAGTAAACTCTCAGTCAGAGGTGACTCACATGACAAAAACAGTATCAACCAGTAAAAAACCCCGTAAA
CAGCATTCGCCTGAATTTCGCAGTGAAGCCCTGAAGCTTGCTGAACGCATCGGTGTTACTGCCGCAGCCCGTGAACTCAG
CCTGTATGAATCACAACTCTACAACTGGCGCAGTAAACAGCAAAATCAGCAGACGTCTTCTGAACGTGAACTGGAGATGT
CTACCGAGATTGCACGTCTCAAACGCCAGCTGGCAGAACGGGATGAAGAGCTGGCTATCCTCCAAAAGGCCGCGACATAC
TTCGCGAAGCGCCTGAAATGAAGTATGTCTTTATTGAAAAACATCAGGCTGAGTTCAGCATCAAAGCAATGTGCCGCGTG
CTCCGGGTGGCCCGCAGCGGCTGGTATACGTGGTGTCAGCGGCGGACAAGGATAAGCACGCGTCAGCAGTTCCGCCAACA
CTGCGACAGCGTTGTCCTCGCGGCTTTTACCCGGTCAAAACAGCGTTACGGTGCCCCACGCCTGACGGATGAACTGCGTG
CTCAGGGTTACCCCTTTAACGTAAAAACCGTGGCGGCAAGCCTGCGCCGTCAGGGACTGAGGGCAAAGGCCTCCCGGAAG
TTCAGCCCGGTCAGCTACCGCGCACACGGCCTGCCTGTGTCAGAAAATCTGTTGGAGCAGGATTTTTACGCCAGTGGCCC
GAACCAGAAGTGGGCAGGAGACATCACGTACTTACGTACAGATGAAGGCTGGCTGTATCTGGCAGTGGTCATTGACCTGT
GGTCACGTGCCGTTATTGGCTGGTCAATGTCGCCACGCATGACGGCGCAACTGGCCTGCGATGCCCTGCAGATGGCGCTG
TGGCGGCGTAAGAGGCCCCGGAACGTTATCGTTCACACGGACCGTGGAGGCCAGTACTGTTCAGCAGATTATCAGGCGCA
ACTGAAGCGGCATAATCTGCGTGGAAGTATGAGCGCAAAAGGTTGCTGCTACGATAATGCCTGCGTGGAAAGCTTCTTTC
ATTCGCTGAAAGTGGAATGTATCCATGGAGAACACTTTATCAGCCGGGAAATAATGCGGGCAACGGTGTTTAATTATATC
GAATGTGATTACAATCGGTGGCGGCGGCACAGTTGGTGTGGCGGCCTCAGTCCGGAACAATTTGAAAACAAGAACCTCGC
TTAGGCCTGTGTCCATATTACGTGGGTAGGATCAACCAGCATAAATCAGGCTGCGGCTAAAATGGCGCGGGCAGGAATCC
TGGTCGTTGATGGTAAGGTCTGGCGAACGGTGTATTACCGGTTCGCTACCAGAGAAGAATGGGAAGGAAAGGTGAGCACG
AATCTGATTTTTAAGGAGTGTCGCCAGAGTGCCGCGATGAAACGGGTATTGAGGGTATATAAAAGAACATCAATGGGAAC
ACAATGATGAAACAGGTGAGTTGAGTTCAAACTGTAGTACAATTCTCTCCAGTTTGAACAGGAAAGAATATGCTATGAAC
CCTTATATTTATCTTGGTGGTGCAATACTTGCAGAGGTCATTGGTACAACCTTAATGAAGTTTTCAGAAGGTTTTACACG
GTTATGGCCATCTGTTGGTACAATTATTTGTTATTGTGCATCATTCTGGTTATTAGCTCAGACGCTGGCTTATATTCCTA
CAGGGATTGCTTATGCTATCTGGTCAGGAGTCGGTATTGTCCTGATTAGCTTACTGTCATGGGGATTTTTCGGCCAACGG
CTGGACCTGCCAGCCATTATAGGCATGATGTTGATTTGTGCCGGTGTGTTGATTATTAATTTATTGTCACGAAGCACACC
ACATTAAAATAATTTGTTTCTAAACGACTAAAATATGGAGGCTCTTATATTTATATGAGCCTCGTTTTATGCTTTTTGTT
AATGTCTTTATTTTTTATGTATTCTTTTGTGCTTTCAAGATTATGGCGTAAGAAAATTGCAATACGATTATTGTTGTATA
TTCAAGATAATGTGACCTTAATTGTCTTTTTAAATAAAAAATAAACAAAAATTATATCCCACCACTAAGGTTTATAAAAG

CATACGTTAGCAGGTGTCACCATGAAAAAAGCCATAGCATATATGCGATTTTCATCACCAGGTCAGATGTCTGGCGACTC
ATTAAACCGACAGAGAAGACTTATTGCTGAATGGTTAAAGGTAAATAGTGATTATTATCTTGATACCATAACATATGAAG
ATTTAGGATTAAGTGCATTCAAAGGAAAGCATGCACAATCAGGAGCTTTTTCGGAATTTTTAGATGCTATAGAGCATGGT
TATATATTGCCAGGAACTACATTGTTAGTTGAAAGTCTGGACAGACTTTCAAGAGAAAAAGTCGGTGAAGCGATTGAACG
TCTGAAATTGATTTTGAATCACGGTATTGATGTTATAACTCTTTGCGACAATACAGTCTATAATATTGACTCTTTGAATG
AGCCATATTCATTAATAAAAGCCATACTTATAGCACAAAGGGCAAATGAAGAAAGCGAGATAAAGTCAAGTCGGGTTAAA
TTATCATGGAAGAAAAAACGGCAGGATGCACTGGAATCAGGTACGATTATGACGGCGTCTTGTCCGAGATGGCTCTCCTT
AGATGACAAAAGAACGGCTTTTGTTCCAGACCCCGACAGGGTGAAAACTATTGAGCTAATTTTTAAACTCAGGATGGAAA
GGCGCTCATTGAATGCAATAGCCAAGTATTTAAATGATCATGCTGTAAAGAATTTCTCAGGAAAAGAAAGTGCATGGGGA
CCTTCTGTAATTGAAAAATTATTAGCGAATAAAGCTCTGATAGGTATTTGCGTACCTTCATATCGTGCAAGAGGGAAAGG
GATAAGTGAAATCGCTGGCTATTATCCCAGAGTCATATCAGATGATTTGTTTTACGCTGTACAGGAAATTCGGTTGGCAC
CTTTTGGTATTAGCAATAGTAGCAAGAATCCTATGCTAATAAATCTACTTCGAACAGTTATGAAGTGTGAGGCTTGTGGT
AATACCATGATTGTTCATGCGGTATCTGGAAGTTTGCATGGCTATTATGTTTGTCCGATGAGAAGATTACATCGATGTGA
CAGGCCATCAATAAAAAGAGATTTGGTTGATTATAATATCATTAATGAATTGCTTTTTAATTGTAGCAAAATTCAACCAG
TTGAAAACAAGAAAGATGCTAATGAAACTTTAGAGTTAAAAATTATTGAGCTTCAGATGAAAATTAATAATTTAATCGTT
GCATTGTCTGTCGCGCCTGAAGTTACCGCTATAGCAGAGAAAATAAGACTATTAGATAAGGAATTACGAAGGGCTTCGGT
ATCATTGAAAACTTTGAAGAGTAAAGGTGTAAATTCATTCAGTGATTTTTATGCTATTGACTTAACCAGTAAAAATGGAC
GAGAGTTATGCCGTACACTTGCCTATAAAACATTCGAAAAAATCATAATTAATACGGATAATAAAACCTGTGATATCTAT
TTTATGAATGGCATTGTTTTTAAACACTATCCTTTAATGAAAGTAATATCCGCCCAGCAGGCGATAAGTGCTCTCAAATA
TATGGTTGATGGTGAGATTTATTTCTAAATAATGATCTCGGATTTTAAGTTATGCTATGGTGATAAAGTGCAAGACAGAA
TTAATTATCTTTGACGAAACTTAATGGGTAATTACTTTGTTTGCTCCCACAAGCGAGTTTTGTACGGCTGTATTGGGGTA
GTAAATGAGCTATACAATCTTAATCATTTGTTAGGTGAGAACTCTTGGTCGCAGATTCAAATACTGAAAATACGTGACAA
ATTATTATGAGCAAAATGGTGTATGTCACGTATTTTGAATGGTAGGTTAAAAAATAACACCGACTTTCGTAGGTGTTACT
AATAATAAAGCAGAGTTTTTAGATAGTATCAATGTGCTTTGTGTATATTGTGGCAAATAATTGGGTTGGGGGTACAATTG
TGATTGCTTTTGCATGAACATTGCGCCTTTATGCATAATGAGATAAAGGAATATCAAATAAAATAACGATAGGTCATAAC
AAAGAGGTTTTTATGAAAACACTTATCGTTTCAACTGTATTGGCATTCATAACATTTTCTGCGCAGGCTGCAGCATTTCA
GGTCACTAGTAATGAAATAAAAACAGGAGAGCAACTTACAACGTCTCATGTCTTTTCTGGATTTGGGTGTGAAGGTGGTA
ATACATCGCCCTCATTAACCTGGTCTGGTGTTCCTGAAGGTACCAAAAGCTTTGCCGTAACTGTATATGATCCAGATGCA
CCTACAGGCAGTGGTTGGTGGCATTGGACTGTTGTTAATATTCCAGCAACAGTAACATATTTGCCCGTTGATGCAGGGAG
ACGTGATGGAACAAAACTGCCGACTGGTGCTGTTCAAGGCCGAAATGATTTTGGCTATGCTGGGTTTGGTGGCGCATGTC
CTCCTAAAGGAGATAAACCACATCATTACCAGTTTAAAGTATGGGCTCTAAAAACTGAAAAGATTCCTGTAGATTCTAAC
TCCAGCGGAGCGTTAGTTGGTTATATGCTTAATGCTAATAAAATCGCAACCGCTGAGATAACACCAGTTTATGAGATAAA
GTAGGGTGAGAGTATGCTGGCAAGAGGTAAGACTAACTTAAAGATCGAAGAAATACGGATGCATAAACATCATGAGATTC
ATAGGGTTAAGCCTCTTATGCCAGCTTTGTGTCGTATCCGTCAGGGAAAGAAAGTTATCAATTGGGAGACGCATACTTTA
ACTGTTGATAATAATCAAATAATATTATTTCCTTGTGGTTATGAATTTTATATTGAGAATTATCCTGAAGCAGGGCTTTA
TCTTGCAGAAATGCTTTACTTACCCATTGATTTAATTGAGAGTTTCCAAAAACTTTATACGGTAACTGATCAAATACGTA
ACAAAACAAGTTTCTTTTTACCTCAGAATCCTGAGTTAATATATTGTTGGGAGCAACTAAAAACATCTGTTTCCCGAGGC
TTCTCAACTAAAATTCAGGAGCACTTAGCAATGGGCGTTCTACTTTCGTTAGGAGTGAATCATGTTAATCATTTACTTTT
ATCATATAGTAAACAATCATTGATAAGTCGTTGTTATAACCTGCTGCTATCCGAACCCGGCACAAAATGGACAGCAAACA
AGGTTGCTCGATATCTCTACATTTCTGTTTCTACATTACATCGCCGTCTAGCAAGCGAGGGGGTAAGTTTCCAAAGTATA
CTGGACGATGTGAGGTTAAATAATGCGTTGTCTGCTATACAAACGACGGTAAAACCTATAAGCGAGATTGCCAGAGAAAA
TGGTTATAAGTGTCCTTCTCGTTTTACTGAAAGATTTCATAATCGTTTTAATATAACACCAAGAGAGATAAGAAAAGCTT
CCAGAGAGTAAAAGTGTTTTAAGAAGGAGCAATTCTATCGATTTTGATTTTGGGAAATCAACACGGCATAATTATGTCAC
CGGAGCCTGAACAACTCCGGTGACTTCTGCGCTAAACGGGGACGTTTATGCGCACATACAATCCAAACTCTCTTCTCCCT
TCACAGATGCAGAAATGCACCTGCAATTCTTTGCATCTAGCGTTTGACCTCTGCGGAGGGGAAGCGTGAACCTCTCACAA
GACGGCATCAAATTACATCGCGGCAACTTCACCGCTATCGGTCGGCAGATCCAGCCTTATCTGGAGGAGGGCAAATGCTT
TCGCATGGTGCTTAAACCGTGGCGTGAGAAACGCAGTCTTTCCCAGAATGCACTCAGCCACATGTGGTACAGCGAAATCA
GTGAATACCTCATCAGCAGGGGTAAAACGTTCGCCACTCCAGCTTGGGTAAAAGATGCTCTCAAACACACATATCTCGGT
TATGAAACCAAAGACCTGGTTGATGTCGTAACCGGTGATATCACCACTATCCAGTCGTTACGCCATACCTCCGATCTTGA
TACCGGAGAGATGTATGTCTTCCTGTGTAAGGTTGAAGCCTGGGCGATGAATATTGGTTGCCACCTGACTATTCCACAGA
GCTGCGAGTTCCAGCTGCTGCGCGACAAGCAGGAGGCGTAATGGCTACACCGCTTATTCGTGTCATGAACGGACACATCT
ACAGAGTACCAAATCGTCGTAAGCGTAAACCTGAGCTGAAGCCATCCGAAATACCAACACTGCTCGGATATACCGCCAGC
TTGGTTGATAAAAAATGGTTGCGACTGGCAGCAAGGAGGAGTCATGGCTGATTTGAGAAAAGCAGCGCGTGGTCGGGAAT
GCCAGGTAAGAATCCCTGGCGTATGTAATGGCAACCCTGAAACGTCTGTACTGGCACATATCCGGCTGACTGGATTGTGC
GGCACCGGTACGAAACCGCCAGACCTGATTGCCACCATTGCATGTTCTGCCTGCCACGACGAAATCGACCGCCGCACGCA
TTTTGTTGACGCTGGATATGCAAAAGAATGCGCGCTGGAAGGTATGGCGAGAACACAGGTTATCTGGCTGAAAGAGGGGG

TTATTAAGGCGTGAATACCTACAGCATCACATTACCCTGGCCTCCGAGCAATAATCGCTATTACCGCCATAATCGCGGGC
GCACGCACGTCAGCGCAGAGGGGCAGGCATACCGCGATAACGTCGCCCGAATCATTAAAAACGCAATGCTGGATATCGGC
CTGGCTATGCCTGTGAAAATCCGCATTGAGTGCCACATGCCGGATCGCCGTCGCCGTGACCTGGATAATCTGCAAAAAGC
CGCTTTTGACGCACTCACTAAAGCAGGTTTCTGGCTGGATGATGCTCAGGTCGTTGATTACCGCGTTGTGAAGATGCCTG
TTACCAAAGGTGGAGGCTGGAACTGACCATCACCGAAATGGGGAATGAATGATGTTTGAGTTTAATATGGCAGAACTTC
TTCGCCACCGCTGGGGGCGTCTGCGCTTATATCGTTTCCCCGGTTCTGTTTTGACCGATTACCGAATACTGAAGAATTAC
GCCAAAACCCTGACAGGAGCAGGAGTATGAAGTCAGAGATAACAATCAACTAATACTGTTTTGTTGATTTTTGCTTGTAA
TTGGCGTTCTGGTCTGATTTTTGTGGAGTAAGTTGATGCGTGATATTCAGATGGTTCTTGAGCGTTGGGGAGCGTGGGCG
GCTAATAATCATGAAGATGTGACCTGGTCGTCCATTGCCGCCGGTTTTAAGGGATTAATTACTTCAAAAGTAAAATCTCG
CCCGCAATGTTGTGACGATGACGCGATGATCATTTGCGGGTGCATGGCCCGTCTGAAAAAGAACAACAGCGATTTGCACG
ATTTATTAGTAGATTATTATGTAGTCGGTATGACATTCATGTCACTGGCAGGTAAGCATTGCTGCTCTGATGGTTATATC
GGGAAAAGGTTACAGAAGGCTGAGGGCATAATTGAAGGGATGTTAATGGCATTAGATATCCGGTTAGAGATGGATATCGT
TGTTAATAACTCTAATTAATATGCCAATTGTTTACTAAAAATTATTAAAAATGGGGCGTTGAGACGCCCCCAAAAATAAA
GGGTAATATATAACAGAAGGTTTATATAGTTAGAAGCAAGGTTGTGCTTCTAAAGGAAGTGGCTTGAGGGAGCCACTTAT
ATGTTGGGGAGGCAACGCCTCCCGCAACATATCTTTTTCGTAATCAGATTAGAACTGGTAAACCAGACCTACAGCAACGA
TGTCATCAGTGCTTACACCGAGTGCTTTAGGGAAGGTGCGAATAAGCGGGGAAATTCTTCTCGGCTGACTCAGTCATTTC
ATTTCTTCATGTTTGAGCCGATTTTTTCTCCCGTAAATGCCTTGAATCAGCCTATTTAGACCGTTTCTTCGCCATTTAAG
GCGTTATCCCCAGTTTTTAGTGAGATCTCTCCCACTGACGTATCATTTGGTCCGCCCGAAACAGGTTGGCCAGCGTGAAT
AACATCGCCAGTTGGTTATCGTTTTTCAGCAACCCCTTGTATCTGGCTTTCACGAAGCCGAACTGTCGCTTGATGATGCG
AAATGGGTGCTCCACCCTGGCCCGGATGCTGGCTTTCATGTATTCGATGTTGATGGCCGTTTTGTTCTTGCGTGGATGCT
GTTTCAAGGTTCTTACCTTGCCGGGGCGCTCGGCGATCAGCCAGTCCACATCCACCTCGGCCAGCTCCTCGCGCTGTGGC
GCCCCTTGGTAGCCGGCATCGGCTGAGACAAATTGCTCCTCTCCATGCAGCAGATTACCCAGCTGATTGAGGTCATGCTC
GTTGGCCGCGGTGGTGACCAGGCTGTGGGTCAGGCCACTCTTGGCATCGACACCAATGTGGGCCTTCATGCCAAAGTGCC
ACTGATTGCCTTTCTTGGTCTGATGCATCTCCGGATCGCGTTGCTGCTCTTTGTTCTTGGTCGAGCTGGGTGCCTCAATG
ATGGTGGCATCGACCAAGGTGCCTTGAGTCATCATGACGCCTGCTTCGGCCAGCCAGCGATTGATGGTCTTGAACAATTG
GCGGGCCAGTTGATGCTGCTCCAGCAGGTGGCGGAAATTCATGATGGTGGTGCGGTCCGGCAAGGCGCTATCCAGGGATA
ACCGGGCAAACAGACGCATGGAGGCGATTTCGTACAGAGCATCTTCCATCGCGCCATCGCTCAGGTTGTACCAATGCTGC
ATGCAGTGAATGCGTAGCATGGTTTCCAGCGGATAAGGTCGCCGGCCATTACCAGCCTTGGGGTAAAACGGCTCGATGAC
TTCCACCATGTTTTGCCATGGCAGAATCTGCTCCATGCGGGACAAGAAAATCTCTTTTCTGGTCTGACGGCGCTTACTGC
TGAATTCACTGTCGGCGAAGGTAAGTTGATGACTCATGATGAACCCTGTTCTATGGCTCCAGATGACAAACATGATCTCA
TATCAGGGACTTGTTCGCACCTTCCTTAGTGAAGTCATTTTTGTCAAGCAGGTTGATTTTGTAATCAACGAAAGTAGACA
TATTTTTGTTGAAGTAATAGGTTGCACCTACATCAACATATTTGACTAAGTCCTGATCGCCCCATACTCCAAGATCCTTA
CCTTTAGATTGCAGGTAAGCAACGGACGGACGCAGACCGAAATCGAACTGATATTGTGCAACAGCTTCGAAGTTTTGGGC
TTTATTAGCAACGAAGTGATCAGCAAATACAGTCATATTCTGGGTTTCAGAATAGGTAGTGGCCAGGTAAATGTTGTTAG
CGTCATATTTCAGACCTGCGGCCCAAACTTCTGCATTTTTACCGGAAGCAAATACTTCAGGAAGAACTTTCCCTGCATTA
ACTTGAGTGTCGGTACGATCAGATTTCGCATAAGTTGCACCGATACCGAATCCTTCGTATTCATAGGTAGCAGAGAAACC
GAAGCCATCACCGTTACCTTCAGTGTAGTTATCGAAATCGCTACGATCGTTTTTGCCTTGGTACTGAGCAGCAAAGTTCA
GACCATCAACCAGACCAAAGAAGTCGTTGTTACGATAGGTTGCAACACCAGTTGCACGTTGAGTCATGAACACGTCGGTT
TGAGTCCAAGTGTCACCACCGAATTCTGGCAGGACGTCAGTCCACGCACCGATGTCGTATGCTACACCGTAGTTACGGCC
GTAATCGATGGAGCCGTAGTCACCGAATTTCAGGCCAGCGAAGGCAAGACGGGTTTTATCTTTGGAGGAACCTTGAGATT
CAGCGCGGTTGCCTTTGAATTCATATTCCCACTGACCGAAACCAGTCAGTTGATCGTTGATTTGGGTTTCACCTTTGAAG
CCAAGACGGGCATAAGTAGTATCACCATCATCTGCATCATTAGAGGAGAAGTAGTGCTTAGCATTAACTTTCCCGTACAG
ATCCAGCTTGTTACTGTCTTTATTATAAATTTCAGCTGCCTGAGCAGACATCGCCATTAGTACTGATGCAGCTACAGCAG
AAATTGCCACTGTTAATTTTTTCATCGTGAGCCCTTTTTTTGAACTATTATTAAAAAATGATGTCACTGCGCGATAAATA
TTCATCTAATCAATGTGATTATTTCAAGATGTAAGTTTTGGTTTCTCGTTTGATTTGTGAAGTAGATCTCTATTTTTATC
TGAACTTTTTTCTATCGAATCCTATTCATGGCTCTTGGCTGAATAAAAATAAATCTATTAGCCAATTTATATTAACGGCT
GTTATTTATAAGTGCTCTATAATTTGAAGGTTCAATTTAAACCGGCTAAAAATAACACTGGAAATTATTTTTTGGTTATT
TGTTGAGATTTGCTTATGTATTTGTAGTGGTGTTTTCAATACTCGGTAGCATTCTCTCAAATATCATTTAGTGGTTTACG
TACGTAAAAAATTGGTTATGCTGTTAAGAGTGGTTACTTCGTCACACAGCTTAAACCCGCCGTCGAGCGGGTTTTTCCAT
TTTTTGAGTCTCGATATTAGCTGATAACCCAATACCTGAGTTATTCACTGACTCCGAGTCTGTTACGTTTCGTAGTATTC
CCTCAATTTACACCCGCTTTGTCTGCGAGGTGGGGTTATGAAATCCATGGATAAGTTAACAACGGGTGTCGCCTATGGCA
CCTCAGCAGGTAGTGCCGGGTACTGGTTTTTACAGCTGCTAGATAAAGTCACTCCCTCACAGTGGGCAGCAATAGGTGTG
CTGGGTAGCCTGGTATTTGGCCTGCTGACGTACCTGACAAACCTTTATTTCAAGATTAAAGAAGATAAGCGCAAGGCTGC
GAGAGGTGAATAATGCCTCCATCATTACGAAAAGCCGTTGCTGCTGCTATTGGTGGCGGAGCAATTGCTATAGCATCAGT
GTTAATCACTGGCCCAAGTGGTAACGATGGTCTGGAAGGTGTCAGCTACATACCATACAAAGATATTGTTGGTGTATGGA
CTGTATGTCACGGACACACCGGAAAAGACATCATGCTCGGTAAAACGTATACCAAAGCAGAATGCAAAGCACTCTTGAAT

AAAGACCTTGCCACTGTCGCCAGACAAATTAACCCGTATATCAAAGTCGATATACCGGAAACAACGCGCGGCGCTCTTTA
CTCATTCGTTTACAACGTGGGTGCTGGCAATTTTAGAACATCGACGCTTCTTCGCAAAATAAACCAGGGCGATATCAAAG
GCGCATGTGATCAGCTGCGTCGCTGGACATACGCTGGCGGTAAGCAATGGAAAGGCCTGATGACTCGTCGTGAGATTGAG
CGTGAAGTCTGTTTGTGGGGGCAACAGTGAGCAGAGTAACCGCGATTATATCCGCTCTGATTATCTGCATCATCGTCAGC
CTGTCATGGGCGGTCAATCATTACCGTGATAACGCAATCGCCTACAAAGTCCAGCGCGACAAAAATGCCAGAGAACTGAA
GCTAGCGAACGCGGCAATTACTGACATGCAGATGCGTCAGCGTGATGTTGCTGCGCTCGATGCAAAATACACGAAGGAGT
TAGCTGATGCGAAAGCTGAAAATGATGCTCTGCGTGATGATGTTGCCGCTGGTCGTCGTCGGTTGCACATCAAAGCAGTC
TGTCAGTCAGTGCGTGAAGCCACCACGGCCTCCGGCGTGGATAATGCAGCCTCCCCCCGACTGGCAGACACCGCTGAACG
GGATTATTTCACCCTCAGAGAGAGGCTGATCACTATGCAAAAACAACTGGAAGGAACCCAGAAGTATATTAATGAGCAGT
GCAGATAGAGCTGACCATATCGATGGGCAACTCATGCAATTATTTTGAGCAATACACACGCGCTTCCAGCGGAGTATAAA
TGCCTAAAGTAATAAAACCGAGCAATCCATTTACGAATGTTTGCTGGGTTTCTGTTTTAACAACATTTTCTGCGCCGCCA
CAAATTTTAGCTGCATCGACAGTTTTCTTCTGCCCAATTCCAGAAACGAAGAAATGATGGGTGATGGTTTCCTTTGGTGC
TACTGCTGTCTGTTTGTTTTGAACAGTAAATGTCTGTTGAGCACATCCTGTAATAAGCAGGGCCAGCGCAGTAGCGAGTA
GCATTTTTTTCATGGTGTTATTCCCGATGCTTTTTGAAGTTCGCAGAATCGTATGTGTAGAAAATTAAACAAACCCTAAA
CAATGAGTTGAAATTTCATATTGTTAATATTTATTAATGTATGCCAGGTGCGATGAATCGTCATTGTATTCCCGGATTAA
CTATGTCCACAGCCCTGACGGGGAACTTCTCTGCGGGAGTGTCCGGGAATAATTAAAAACGATGCACACAGGGTTTAGCG
CGTACATGTATTGTATTATGCCAACACCCCGGTGCTGACACGGAAGAAACCGGACGTTATGATTTAGCGTGGAAAGATTT
GTGTAGTGTTCTGAATGCTCTCAGTAAATAGTAATGAATTATCAAAGGTATAGTAATATCTTTTATGTTCGTGGATATTT
GTAATCCATCGGAAAACTCCTGCTTTAGCAAGATTTTCCCTGTATTGCTGAAATGTGATTTCTCTTGATTTCAACCTATC
ATAGGACGTTTCTATAAGATGCGTATTTCTTGAGAATTTAACATTTACAACCTTTTTAAGTCCTTTTATTAACACGGTGT
TATCGTTTTCTAACACAATGTGAATATTATCTGTGGCTAGATAGTAAATATAATGTGAGACATTGTGACGTTTTAGTTCA
GAATAAAACAATTCACAGTTTAAATCTTTTCGCACTTGATCGAATATTTCTTTAAAAATGGCAACCTGAGCCATTGGTAA
AACCTTCCATGTGATACGAGGGCGCGTAGTTTGCATTATCGTTTTTATCGCTTCAATCTGGTCTGACCTCTTTGTGTTTT
GTTGATGATTTATGTCAAATATTAGGAATGTTTTCAATTAATAGTATTGGTTGCGTAACAAAGTGCGGTCCTGCTGGCAT
TCTGGAGGGAAATACAACCGACAGATGTATGTAAGGCCAACGTGCTCAAACCTTCATACAGAAAGATTTGAAGTAATATT
TTAACCGCTAGATGAAGAGCAAGCGCATGGAGCGACAAAATGAATAAAGAACAATCTGCTGATGATCCCTCCGTGGATCT
GATTCGTGTAAAAAATATGCTTAATAGCACCATTTCTATGAGTTACCCTGATGTTGTAATTGCATGTATAGAACATAAGG
TGTCTCTGGAAGCATTCAGGGCAATTGAGGCAGCGTTGGTGAAGCACGATAATAATATGAAGGATTATTCCCTGGTGGTT
GACTGATCACCATAACTGCTAATCATTCAAACTACTTAACCTGTGACAGAGCCAACACGCAGTCTGTCACTGTCAGGAAA
GTGGTAAAACTGCAACTCAATTACTGCAATGCCCTCGTAATTAAGTGAATTTACAATATCGTCCTGTTCGGAGGGAAGAA
CGCGGGATGTTCATTCTTCATCACTTTTAATTGATGTATATGCTCTCTTTTCTGACGTTAGCCTCCGACGGCAGGCTTCA
ATGACCCAGGCTGAGAAATTCCCGGACCCTTTTTGCTCAAGAGCGATGTTAATTTGTTCAATCATTTGGTTAGGAAAGCG
GATGTTGCGGGTTGTTGTTCTGCGGGTTCTGTTCTTAGTTGACATGAGGTTGCCCCGTATTCAGTGTCGCTGATTTGTAT
TGTCTGAAGTTGTTTTTACGTTAAGTTGATGCAGATCAATTAATACGATACCTGCGTCATAATTGATTATTTGACGTGGT
TTGATGGCGTAGATGCACGTTGTGACATGTAGATGATAATTATTATCATTTTGTGGGTCCTTTCCGGCGATCCGACAGGT
TACGGGGCGGCGACCTCGCGGGTTTTCGCTATTTATGAAAATTTTCCGGTTTAAGGTGTTTCCGTTCTTCTTCGTCGTAA
CTTAATGTATTTATTTAAAATACCCCCTGAAAAGAAAGGAAACGACAGGTGCTGAAAGCGAGCTTTTTGGCCTCTGTCGT
TTCCTTTCTCTGTTTTTGTCCGTGGAATGTGCAATGGAAGTCAACAAAAAGCAGCTGGCTGACATTTTCGGTGCGAGTAT
CCGTACCATTCAGAACTGGCAGGAACAGGGAATGCCCGTTCTGCGAGGCGGTGGCAAGGGTAATGAGGTGCTTTATGACT
CTGCCGCCGTCATAAAATGGTATGCCGAAAGGGATGCTGAAATTGAGAACGAAAAGCTGCGCCGGGAGGTTGAAGAACTG
CTGCAGGCCAGCGAGACAGATCTCCAGCCAGGGACTATTGAGTACGAACGCCATCGACTTACGCGTGCGCAGGCCGATGC
ACAGGAGCTGAAAAATGCCAGAGACTCCGCTGAAGTGGTGGAAACCGCATTCTGTACTTTCGTGCTGTCGCGGATCGCAG
GTGAAATTGCCAGTATTCTCGACGGGATCCCCCTGTCGGTGCAGCGGCGTTTTCCGGAACTGGAAAACCGACATGTTGAT
TTCCTGAAACGGGATATCATCAAAGCCATGAACAAAGCAGCCGCGCTGGATGAACTGATACCGGGGTTGCTGAGTGAATA
TATCGAACAGTCAGGTTAACAGGCTGCGGCATTTTGTCCGCGCCGGGCTTCGCTCACTGTTCAGGCCGGAGCCACAGACC
GCCGTTGAATGGGCGGATGCTAATTACTATCTCCCGAAAGAATCCGCATACCAGGAAGGGCGCTGGGAAACACTGCCCTT
TCAGCGGGCCATCATGAATGCGATGGGCAGCGACTACATCCGTGAGGTGAATGTGGTGAAGTCTGCCCGTGTCGGTTATT
CCAAAATGCTGCTGGGTGTTTATGCCTACTTTATAGAGCATAAGCAGCGCAACACCCTTATTCCAGCTGGCTTCGTGGCT
GTTTTCAACAGTGATGAGTCATCGTGGCATCTCGTTGAAGATCATCGGGGTAAAACGGTTTATGACGTAGCGTCAGGGGA
CGCGTTATTTATTTCTGAACTCGGTCCGTTACCGGAAAATGTTACCTGGTTATCGCCGGAAGGGGAGTTTCAGAAGTGGA
ACGGTACAGCCTGGGTGAAAGATGCAGAAGCAGAAAAACTGTTCCGGATTCGGGAGGCGGAAGAAACAAAAAACAGCCTG
ATGCAGGTAGCCAGTGAGCATATTGCGCCACTTCAGGATGCTGTAGATCTGGAAATCGCAACGGAGGAAGAAACCTCATT
GCTGGAAGCCTGGAAAAAATATCGGGTGTTGCTGAACCGTGTTGATACATCAACTGCACCTGATATTGAGTGGCCTACGA
ACCCTGTCAGGGAGTAATCATTGGGATTATGCCGCAGCACGTCTTAAGCAAGAACATGCTGCGGTTGGATGCTATTTTTT
TCCTGAAGCGGAAAACATTACTACAGTACCTTGAACCTTGGTTTTAACATTCTCGAAATGCTCTGAGAGTATATGTGTTA
AGCCTTCTTCGGAATCTTTTGTGTTTGAAAAGATGCCTTTCTGATTGTAAATGCGCATCAGTTTTTGACCGAAGCTATTG

TGCACAACTCCATCGCCAAGAATTGTGGCTCCGTATAGAGTTCCATCGTCAGTTAAGGCCTGCGCCGCATTGCGTATTAC
ACAGCTTTTTGTAGATATATTTCCAGGCAGGCAGTGAAGAAGGTAAGACATGGAAATGGAATCAAATTGACCATGTAACG
CCGCGGGATAAGGTTCAAAAACATCATGGCTAATTTTATGTTTAATTTTTGATTCCCCAGCCCTTGTAGATGCCGCGTTC
AGGCTAGCTTCGTTCAAATCCATTAAAGATATCAGACTACTCTCAGGTACGTGAGTAAGGTAAAACCCAGTTCCAACACC
AATATCCAGATGGTTGTTACCTAAATGTTCCAGAAAGTGTGGAAGAAGGTGTTCCTTTGTAGGACATCCCCATGCAAGCC
GATTTGATACTCCCAAAACCCACCAGTCATAAAGCTTTAGGGTAAGTGGTGTGTAAATTCTAGCCCCATCATCTGTGTTT
TTTTATTAATTTCACCATGTTATAGTTTTATTTGTGAATTAAATCAATTATGGCAATGAATTACAAGGGGTTAAATGCTG
CCGCAGCATAGCGATATTGAAATAGCCTGGTATGCTTCAATACAGCAGGAGCCGAATGGCTGGAAGACCGTCACCACACA
GTTCTACATCCAGGAATTCAGTGAGTATATTGCGCCACTGCAGGATGCTGTAGATCTGGAAATCGCAACGGAGGAAGAAA
GATCGTTGCTGGAAGCCTGAAAAAAGTATCGGGTGCTGCTAAACCGTGTGGACACTTCCGTAGCACCAGATATCGAGTGG
CTTATTCAACCATAATAAACAGTATGTATATCATAGGTTATTAATTGTGAGTTTTTTCGGTGTGTTATTTGTTTGTTTGA
TGTTATGCTTTTGCGCCCCAAAAGGTTGTTTAGATGTATTTTATCAATTGATTTTCAATATCGTTTAATAAAGAAAAATT
AAGCAAGCTGGATGTTGGTTTTTTGTTAATTGAATGGTTCTAATAATGTTTTTTTACTGTTGTTGAATGTGACTTGATAA
GAAATGCAAGTAAAAATGATACTCTTTTTATTTTAAATTCAAACGGTTGACATATATATAGCAAGAGGTTTCAGGTGCGT
TGTAGTGAGTTTATGTTAATAAAAAGCATAGTAAGCGTTGAAAAATGTAACTTTGAAATAAGTTAGAATAAAAAACAACA
TACATATAATAATTTAATCTTAAATGAAATTTATTAAAATTTGCAAACTATAATTTTGTGTATAAAAATATAAATGCACA
TCATCCTGATTATGATTGTGTATTTAATTGGTTGTTATTTGACTACTATCAACTTGTTTTAATTTTATGATAGGTGCAAG
ATGGATTATGTTTGCTCCGTAGTTTTCATCTGTCAATCATTTGATTTAATTATAAACAGGAGAGTTATCTCGTTCAAAAA
AAATTCATTGTTTATTGTAAGCGACAAAATTAGAAGGGAGTTACCAGTATGCCCCTCTAAACTAAGAATTGTTGATATAG
ATAAGAAAACATGTTTATCCTTTTTTATCGACGTGAATAATGAGCTGCCTGGCAAATTTACTCTTGATAAGAATGGCTAT
ATTGCTGAAGAGGAACCTCCATTATCGCTTGTTTTTTCTCTGTTTGAAGGGATTAAAATAGCAGACTCACACTCCCTTTG
GTTAAAAGAAAGACTATGTATATCCTTACTTGCCATGTTCAAAAAACGCGAAAGTGTAAATTCATTTATACTAACAAATA
TAAATACATTTACCTGTAAAATTACTGGAATAATCAGTTTTAATATTGAGCGGCAATGGCATTTAAAAGATATTGCGGAA
TTGATTTATACGAGTGAAAGTTTAATAAAAAAAAGATTAAGGGATGAAGGAACGTCATTTACTGAAATATTGAGAGATAC
TAGGATGAGGTATGCAAAAAAACTCATAACTTCAAACTCTTATTCTATCAATGTCGTAGCCCAGAAATGTGGCTATAACA
GTACTTCATATTTCATATGTGCATTTAAAGATTATTATGGTGTCACGCCATCTCATTATTTTGAGAAAATAATCGGCGTC
ACAGATGGAATAAACAAAACAATTGACTGATAATGTTTATTACAAGTTGTCTACATGTTAATTATAATATTATACAGCGT
TTTTTTTGATGTGATATTCTGGAACCATTAATTTGTAATTGGGTTGCTGTCGCCTATTTTATACATACTATAATTGATGG
TTTTCTATGTGATTTAGTTAATAACCTTCTGGGTTTATTTTAAGGGTTAATTGTTACATTGAAATGGCTAGTTATTCCCC
GGGGCGATTTTCACCTCGGGGAAATTTTAGTTGGCGTTCTTAAAATGTGTACTTAAGACCAGCAGTAGTGATGAAGTTAT
AGTTTTCTATACCTGCTCCATTTTTGCTGTAGTCTGAAGTGTTATTATTGTGATCATAAAGTGAAGTATTACCTTTTTTA
TTCGTAACCCGATTCCATGCGCCTTCAACATAAACTTTTGCGTTAGGTGTGACGTAATAACCTGCATTGACTGCAACAGA
ATAGTAATTTTGGTCTTTGACCTTACTGCGATAAGTGATTCTTTTTCCCGGGTCATAGTGTTCATCGTTATCAGATGATT
CCACCCAGCCGCTGTATTTAAATGTGCCACCGAGTTCAAAATCTTCATAACGATAACTTCCAGTCAAGCCAATGTAGGGC
ATTTTAAAACGTTGTTTGTAGCCGATTGCTCTTTCTCCATTCGGGAAGGAGCCGATATCATCTCTGAATCCCTCCTCAGA
ACTGTAGATATAGGAACCACCTCTGGCTGTAAAGCTATAACGGCTTTCCTGATATCCGGCCATGAGTCCCAGGCGGTAAT
TGGGTTCGTTGAGGAGCCAGCCTTTGATATTCAGATCAAATTCGTTGGCATAATTGAGTTGTGTATCAGGGTGTCTACTT
TCATCCGTCCAGGTTCCGGGGTTACTGGAATCCATCCAGTCCTGATCGACCATATTGCCACCTCGGCTGCCGAGAGTTGT
CCAGCCAGCAGCCCCGATAGATATCTGGGGCATCAAATCCCAATTAATTGCACCTTTAATAATTGCAGCGTTATTGAATT
TCCAGTCGAGTTGACTGACTTTTCGGCCTCCTTCTTCGGCTAGATAAACACGCTCTTTTGTTTTTCCGCTCAGAGTTCCA
AGACTAATGTCCGCATTTATGTTGTCAGGAGTAAACGATAAAGTCTCGGTAGAAGCAAAAGAGCTGATCGCAATAGGGGT
TGTCAGGACTATTCCCAGAAGTTTCGCCCGCATAAAAGTTCTCCATTCAATCGTTTTAATGATTGAATATGTATTTTTTA
TATCTAACTTAATGAGTCAATTACATATTGCTCCACTGTTTATATTTTGTTTAGTATTGAATGATTATCACAATGCGCTA
TCTGTTTTTGGTTTAATTATCTGTTATTGTTTCATATTTCGGTTTTACTGTGTGGTTTTTTTATGCTTTTGTGGTGCTTT
TATCTATTTAAGTGCCATGCCTTTAGAGGCATATAAGCGAAAATAGCATGAGGTTTATCCTCAATTACTATGTTTTTTAG
TACAAAAAAGAGGGACAAAACTGAGACACATAAGGCCTCGCAATGGCTTGCAAGGCTTTACATGTTTTGAGGTAGTGGGA
CGTGTGAGCGCAGAGATGGCGCGGTAAGTTGTTGACTTAAAATGTCGTTCTAGGAACTTCTAAGTCGTGGGCCGCAGGTT
CGAATCCTGCAGGGCGCGCCATTTCTTCCTCATTTATGCCCGTCTTATCCGTTTCCGCTTTGCCCTTCACCACATCACTT
TTTGTCGCTGTTTGGCGAGATAATTCAACGGTGTCAGGCCGTAAAACGCCTTAAAAACAGAGATAAAGTACGACGTGCTG
CTATAGCCACATAATTGCGCCACCTGAGTGATATTTTTGTTATCCATCAATAACATCTGTACGGCGTAACGCATACGACA
CTCTGTGACAATCTGGCTATAGCTGGTATTTTCGTTTTTTAATTTCTTTTTGAGCAGGCTGGGGCTTAAACATAGCGAAC
TGGCGACAATTCGCAGATTCCAGTAATGCTGAATATCGCTTTGAATAATGCGGCAGACGCTGTCGCGGACGCTGCTGCGT
AAGATATACATCAGTAGGGCAATAAACCGCGATTGCTCAAGAAAGTTAGACAATACGGTAAAAAGCAATGCGCGCGTCAA
CTCCGCTTCGCAGGGTCTGCTGGGTTGCTGCTTGCTGTGGTTGGCGGCTAGCCGGAACACTTCCGGTGTACGGCTATGAC
AAGCGATAACCGGGGTTGCCAGCCGCGACCAGGGCTTTACACAGGTTAAGTTCTTATTTAAAAACAACAGATAATCGTTG
ATGATGTTACGGTTGATGTGGGTGATTAATACGTGATTTAACGTTGAGAGTTCAATGACGTTATTGTTGCACGCGAAGGC

GGCCAGGTAGTTTTTCGGAAGGGTAAACGTAGCATGGTTATTGACGCTTACCTCTACCTCTTTTTCGGTCAGGATCACCA
CGCAAGGTTCACTGCTGCTCAATTGCATTTCGCACTCCTCAGATATCAGAAACTCCGCTCAAAGGATCTATGCTTCCTGC
ATGAGTGATCGGCCCGTTCGCCGATAACGATCTTCTTTCTTTAGCACGCTTTTTAGCAATTAATCTTGATGGAATTCTGA
TGAGAGCGAAAGAGGTAAGCCAGGTCGTACCCGACTTACCTGGAGGAGATTTAATACTCGAGAATGCCGTGCGCGACGGG
CAAATAGCGCAGAGAGAAATAGAGAGTGTCTTTACCGCAAGAGGAACGTTTCGCTAACAGCGAGGCAATTTCTGCGTCCG
GTTTCGGTTTCGCTGTAAAGGTTTTGCCTTTGCCGGCGACAAAAAGTTCATAAACAACGCCGCTGGCAGGGGCGGGACTT
TGGCAATCGGCGCTGGCCTGTAAATAACGTTCACGCTGTTCAGCGGTCAGTTTATCCACGCCGCTGCCATCCTGTACCCA
GACATTAACGCCGGTTGCTTTAACGTGTTCCAGCAGTTGGCGATAGCCATCGGGCGACATGTTTCCGGCGAAAAAACTAC
TGATATAAACCGGTTTTGCTGAAACATCGCTAATCAGCCGCTGCGCGTTGTTTAACCATGTTAGCAAAGGCTGACGGGCG
GCTTCGCTGCGCCAGTTCAGGTCGTCAATTTCCGCGCTGATGTACCAGCCATCCGGCGTTATGCCAGGCGCGGCGCTCCA
TAATCTGGCTTGCTGGAGATCGGCAGCCAGCAGGCGATTAAGATAGCTTTCCAGCGCTGCGGACGACTGTTTCTGGTGCA
TAAAAAATTCCGGATCGGCGTTCAGCCCGACAATAAGCTTCAGACCAGCCTGTTGCGCAGCTGCGGCCCGCTTAAACAAT
AACGTGCGCTGTTCTGGCTGGGTAAATGCATCGCCGTAACGGGTCCATTGCAAAACAAGGGTATCGAAGCCTTGCAAACG
TAACTGACTCATCAGCCCCTGCCACTGGGTATCGGTAACCTGACTATCTCGGTTTTGTGGTTGCCAGATAATACCTTTCA
TCGCAAAGGAAAAAGGGCTGACCAAAAGCAGTGTCAGCAATACGAAAATGAACTTACGCATTTACCAGTGCACTCCAATG
GTGAGAAACGCGTTGTTGCGCTCTCCGTTACGTTGATTAATCGCCTTAAAGGTATGTTGATACTCGACGCCGAGACTGAC
TTTGTGCGGCCAGGCGTCGTAGTGCGTCTCGCCGGTCCAGATATTCCAGCGGACCCCGACTCCGCCAAGCTGCGCGCCCT
GAGTGCCTTTATCACGATAGCCGTTGTCCTGAACGTGAGCGTAAGGCTCAATAGTCTGTCCGTTAGCTACCTTCTGATGC
CAGCTGACGCGATAATCTGCCGTCCACGCCTGAATATCCTGGCGGATATATTGCGCCGCATCGAGGTACAGGTTTTGGGC
AAACCAGCCTGAACCGTTCGGGTGCCATTCGTCGCTGTATTTGCCGCCATTAAAGAATGAGGCGCTGGCGCGCAGCATGG
TATCGGATGCGCCATTTTGGCCGTTCAGCGGCAACTGCTGTTCGACGGCGATGAAAAAGATCTGATCGCGCAGCGGCTTC
CAGCGCAGACCGGTGCCGGACATCGGATTTTTCACCGGCATCATCACCCCGTTTTCTCCGGTATCGGCAAAGACGCGGCT
ATAAACTGAGAGCAGGTCGCCTTCCAGCAGCATATTGCGTCCGATGCGGTACTCGGCTTCCAGTTGTCCGTAGCTACGAT
AGCTTTTCCCTGGCGCTGCGCCGCCGACATTATTGTTAGCGGTACTCATTGCGCCGGAACGCAAGCCGATGGAAGAATCG
AAACTGAACGTCCAGCGGCGACCGACCTCCTCATGCAAACGGCGGAAATTGAAGCGTTGTTGATTTTGTTCTGGGGTCAG
TGGGGTTATCAGCGCCTGATTATCAATGTCATCAATCACCAGCCGGGCGTAGTGCTGCGTCGCAGGCATGTCATCCAGAC
GCTGGTTCACGTAGGCCAGTTGTCGGATCAGTGCCGGATCGTCCGGAAGCCCTTTATGCGCCGGTTCGAGCATTTCCCGC
GACTGTGCGATATCACCGCTATCCCACAAGGCGTAACCAAGCGCTGCCTGGGTGTTGCTATTATTCGGTTCCAGTTCCAG
CGCGGCGCGCAAATCACTCACCGCGGCCGGGACATTATGACGTTGGCGATAAATTGTCGCCCGCGCAACGTAAGCGTTGG
CAGAAGGCGCAATATTGATTGAGCGCGTGAGATCGTTCAGTGCGAGTTCCGGCTGACCAGGAATGTAACGTTGCGCATGC
AGCCACCAGTAGAGGGCATTGCTTCCCAGTCCACGTTTTTCTGCCTGTTGTAGCCAGCGATCGCGAGCCGCACCATTTCC
TGCCGCCTGGGCGGTATTGGCAGCAGCAAGCAGATCCTCATTGCTCATGTCGTGAAGACTGATTTTCTGCCAGGCCGCCA
GTGCGGTGGCGTAGTCCTCAACCTGATACGCCTGATAGGCTACCGCACGATGTTGCCAGGCGCTCGGTTGTCGTTGTTCG
GCCTGAAGCCATGCATACAACGCCACACCGGGTAGCGTGTCCCGATAACACTTTGCCAGACGGTTCCAGGCGGCGGCATC
GTAGGAAGGCGACATATCGCCCAGCAAGCGAACTATTGCCGGGCAATTATCTGCAATACCCGGCAACTGACTTTGCCACT
GACGTTGCTCCGCCAGCGGTAAGGGTTTCGATAAAATCGCCACCTTCGCCGGCGTTGCCAGGTAAGGATGACTTTCCAGC
AGAGACGCCAGTCGCGCCATTAAAGTCTGGCTGACACGCGCATCGCCCTGGAAAGGATAGCGTTGCAGCAATAAATCGGC
AGCTTCGCGTGACTGCTCGTTCTGCATCAGTTGCCAGGTTAGTTGATCCAGGCGGGTAAGATTTGCCGGTTCTTGCTGAT
ACAGCAATCGTGCCAGACGCAGAGCTTCAGCCTTGTTACGGGTCGCCACGCTGACAGCATAACGCTCCTCAAGCATTTCA
TTGGCGGGGAGGGTGGCGAGCAGTTTTTGCGCTGCGTCGTACTGACCTTCTTTTAACAGCACCGGTAGCGTCGCGCCAAC
AACATACTGGCGGTTGTCGGCAAACTGTACCGTATAATTCGCCAACGCCTGAACGGGGTTAGCGCTGTATTTAGATAACA
GATAGAGCCAACTTTTCTCTTGTGCGTCCGTGGTAAATAGTGGCTTATTTTCAATGAGATAATGCTGGAGGCGTGCTTTT
TCGCCACGATAAGCCAGCGCGGTCGCGTAAGTAATATATGACTGAGGATCGGTGAAGATCCCCTGTGATTGCAGTGCCAG
GATCCGATCGTCCAGCTGCCCGGCAAGAAGCACGTCAAACCACTGACGGCGTTCTGCCGCGCTTAATGTGTTCTGCTGGC
GTGCTTCATTGTATAGCGTATCTGCCTGGGACCATTGTTTCAGGTAGATTGCCCGTTGCAGCAGATCGGTTCGCAGCGTT
TTTCCTTCCGGCGATGCAGCAAACGTCGCATCGTTCAGTTGCGCTCTGGCGACAGGTAACTGTGCCAGCCGCAGGGCATT
CTGCCCGACTTCACTGCGACAACGCAGGGTCGGCGCAGCATCGCACGCTTTTTGCTGGGCAAGCAGTTCTTCAACTGTCG
TAACGCTTTTCACTTCAACCGGAATAGCCGCCAGACTGCGCTCAAGTCGGGCATCTCCTGGGTGACGTTTCAGTTGATCC
TCAAGCAACAGCCGCGCCCGGTCATCATGACCAAAATGGCGATAGGCTTCCGCAAGGTATAAAGTCAGCGGAATATTATC
CGGCACCTGCTGGTGTATATATTCAAATTCGCGGATGGCGGTTGCTTCGTCGTTATTTTTCTGTGCCTTCAGCGCTTTAT
CGAGACGGGGATAAATAACAAAATGGCGATAATCGCTCAGCCCCAGCTCTTCTGCGCTGGTGCCGATATTGTCTGCGAGT
GCGCTGGTACTCAATAAAGACGTCAGCAGTAAACCAGACCATCCGATGACGCGATTAAGGTTATTCTCCTTCATTTTCGG
ACTCCAGTTGCGCAACCTGTTCTGTGTTTAAACCTGCTTTGAGTAATAGTGATTGCATCGAAACTTGTAATTCGCGTTGA
ATTGTCAGGACGCGATCCAACGTTTCCTGGCTGATAACGCCTTCGGTGACCAAAAACTTGCCGAGCGGCAGAGAACTGCG
TTCATGGCGCAATAACAACACGTTAATTGCTGAACGATTAATATGACCGAGCGTGGTCAGTATTTCGGCGAACAGGAACT
GATGCGGCACATATTGCCGCCAGATTTCACCGGCCTGCTGTTCCGTGAGCCACTGATGCTGAACCGCATTGTACAACATT

GCCCGCGGATCGTGACCGCGTCGGCGTGCATACCAGTGACGTAACCCTGTGACAATTTGTCCCCGCAGAACAATGACGTA
ACGCACTTTGCGTCCGACTTTACGCGTCAGGGCCGCCAGCGAAACCGGGTCAATACCATCTTCACTGCCGACAATTAACT
CGTCATTTTCCAGACGCAGCGGCAGTACCGCATAATGCAGCGCCACGGAGGCCGGCATTTCGGCAATCAGCGAGGAAGGG
ATCTGCCAGGCATCGATGGATTCCCACGCCACGCCGTTTTGCTCTGCCAGCGCCTGTGCCAGCTGCTCGGCGCTAATCAG
CCCCTGCATCAGCATTGAACCGCCCAGGCGTAGACCTTCGACGCGATTACGCAGTGCTGTATCGAGTTGTTCTTCAGTGA
TGACCTGATTTTCCAGCAGAATTTGACCTAACGGGCGCAACGAGCGGGTATCGCCAGTCACGCTGGGGAAGTCATGCGTT
GTTTTATCCCACGCCACGCGACGTGGATCGCCGTGTTGAAGTACCTGTTTTAGCGCGCGCCAGTTGGCCATGAAGTTAAT
CAGGTTGCCCCAGAAAAGACGCAGGACGGAAAGCAGCCCCTGCGTCAGGCCGTAGTAGCCAGTAACGAAAATCACCCGCT
GCACGATGCGGTTAACCATCAAACCAAAGTTTAGCCACAGCAGGGTCATTAACCATGCGCTGCCGCTGAAAATAGAAAGG
AAATGCCAGGCATCGGGCCACAAACTTTCATACGCCAGCAACAGCAAAAGCTGGATCATCACCAGCATCGCGAGGAAGCT
GACAAAGTTACTGATTGCCCCTTTGCGGTCGCGCCAGAGAAAGTAGTTCAGCGTCAGGCTGGAGGTCCATTTATGGGTTT
TAAAGCCTTGGAAAACAATGCCGATGATCCAGCGGGATTTTTGTCGAACCGCAGTCGAAAAGGTATCGGGGAAATATTCG
CGCACGCAGATCATGTTTGATGTCCGCGCGTGCTGTAAAAATTTACGCTGCTCGCGTTCTTTGGCTTCGTCCACCACCGG
AAAACGGACAAAAATTTCCGTCATACCTTTTTCTTTCAGGCGGAAGCCAATGTCGTAATCTTCAGTAAGACTCTGCACGT
CGAAAGCAATACCGTCACCGTCAGCTAACAGTGCGGTCACGGCGCGGCGGCTGAAACAGGTGCCGACGCCTGCGCTGGGC
ACTTGTCCGGCGAGGGCTTCACGCACCGGAACATCTTTGCCATGCAGCTCTGAAAACTCATCAATGTAAGTCATGCTGGT
GAAGTGCGTCCATTCGCGTTCGAACGGATACACCGGGATCTGAATCAGATCTTTACGCTCGACCAGATAGTTGAACAGAC
GCAATTCCATCGGTGAAATCACATCTTCGGCGTCATGCAGAATAAAACCAGCAAAAGCGAAATTGGCGCTACGCTCAAAT
TGGGTGATGGCGTCCAGCACGTTGTTCAGACAGTCGGCTTTGCTGGTGGGGCCAGGACGCGCGCAGACTACCTTATGCAC
ATTCGGGAAGCGAGCGCACACTTCGTCAACATCACGCTGAGTATCGGGGTCGTTGGGGTAGGTGCCAACAAAGATATGAT
AGTTTTCGTAGTCGAGCGTGGTCGCCGCCAGCTCGGCCATATTGCCGATGACGCCCGTTTCATTCCACGCCGGAACCATA
ATCGCTAACGGTTTTTCATCTGGTTTATACAGTTCGCGGTAACTCATTCGCGGGTAGCGGCGATAAACACTCAACTTGCG
TTTAATGCGGCGTACCCAGTAGACGACATCAATAAAAAAATCGTCCAGCCCGCTGATGAACATGATGACCGCTAACGTTA
TCGCGATTACTTTTAAGCCGTAGAGCCAGGTAGCAAAAACATCAAGAAGCCAGTCCACACAAAAACCTTACATTAACGCT
GGTTATGTTTAGGGTGGCGTATATTAAGGTTTTTTATGAATTGTGACAGCTTTTTACCATTAATAGGTATGACTATTGCG
GCACGTTATTTTTACACTGGTTATAAAAGTTGCCGTTTGCTGAAGGATTAAGCGGGTAATGTGATAACAAACCTTGTCCC
CCGCGCATCCGACGTTACCGCAACCGTGCCTTTATGCGCGACAACAATCGATTTCACTATCGCCAGGCCAATACCGCTAC
CTTCACCTTTTCGCTGGCGGGAGGGGTCAACGCGATAGAAACGGTCAAACAATCGCGGTAAGTGCTCGGGCGCAATGGGC
GTACCGGGGTTTTCGACGATAACTTGCACCAGGTGATCGACCGTCTGACAGCGCACTACAATTGTCTCTCCGGTTGGCGT
ATAACGCAGGGCGTTCGAAAGCAGGTTGCTTAGCGCCCGACGCAGCATCAGCGGATCGCCCGCGACCTGACACTTGTCGC
CAACAAACCGCAACTCCACGCCGCGATCTTCCGCTAACGCCTCGAAAAAATCGAACACTTTGCCGACTTCATCCGCCAGG
TTGAGCATTTTCTTTTCGGGGATTAGCTGGTTGTTATCGGCCTGAGCGAGAAACAGCATATCGCTGACCATTTTCGCCAT
TCGCGTCAGCTCTTCGAGATTAGAGTAGAGCACATCTTCCAGCTCCTTCTGGCTGCGCGACTGGCTGAGGGCGATTTCGG
TTTGCGTTATGAGATTCGTAATTGGTGTGCGAATTTCGTGAGCGATATCCGCTGAGAAATTGGACTGGCGGGTAAAGACA
TCCTCAATACGCTCGATCATATGGTTGAACGACAGTACCAGCTGTTCCAGCTCAATGGGCACGGTCTGCGGGTCGAGGCG
AACGTCGAGATCTTTCGAGGTAATATTCTGGATTTGACGGCTGACGCTGCGGATCGGCGCGTGACCTTTATGTACCGCCA
ACAGTACGATAAAGACGATCAGGATGCTGATTACCGATGCGGTCATAATAAGTTTATTCATCAAATCATTTATGTAATGA
AGATGAAAATCGATCGAAAGCGCGATGTAGAGCGTATAAATCGGTTTGCCGTCCACCAACGGGCCAACCGGCAAGTTAAT
CATCCGCCAGTTGCTGTGTTCCATATGCCCGTGACCGTGGCCTGGCATCATCATCGTCGGGCCGGAAAGGAGATACACCT
CGCCACCCTGAGCGTCTTTATCGGGTATGGCGTCACGCGTAAACTCGCGGATATCCGGCGCACCGGGGGAGTGATACACC
GTTTTACCCTGACTATCTGCCAGGGAAATCAACACATTGGAATAACCACTGACGATATCTTCCAGCGTCATTAAGCGTCG
GGCTTGCGTTTCGTCAGGGTGATTTAGTACCCGTTCAAGGGTGGCGCTAATCTCTTTTAAATCATTAATATCCTGCTCGG
CAAAATGAACTTTTACTGAGTGGATCATGATCCATGCAAAGGCGAAAAACGCCGCGATGGTGGCCAGGCTGATAAAAAAG
GTCAGGCGGGTTGCCAGCGAAAACGGGCGCTGAAATGGCTTACTGACCATCCGGCACCTCAAGCATGTAACCCACGCCGC
GCACGGTCTGAATTAGCTTCGGCTCAAAGTCGTTGTCGATTTTGCCGCGCAGCCGCTTCACCGCCACATCAATAGCATTG
GTATCGCTGTCAAAATTCATGTCCCATACCTGCGAGGCGATAAGCGAGCGGGGCAGCACTTCGCCCTGATGGCGAAGGAA
GAACTCCAGCAGAGTAAACTCTTTACTGGTCAAAGTGATGCGCGTGCCGCTGCGGGTGACTTTGCGGCTGACGAGATCGA
CCATCAAATCGGCAACCTGAAACTGACTTTCGATAATCACCGCCGCCCCGCGCCGCAGTAATGTGCGCACCCGCGCCAGC
AGTTCAGCAAAAGCGAATGGCTTCACCAGGTAGTCATCTGCCCCCAACTCCAGCCCCTTGACGCGATGTTCAATGGTGCC
AAGCGCGGTAAGCAACAGAATCGGCATCCCTTTATTGGCGGAGCGTAACATGCGCACGATATCCCAGCCGTTCACGTCCG
GCAGCATAATATCGAGGATTATCAGATCATAATCACCGGTCATCGCCAGATGGTAGCCATTCAGCCCGTTGTCGGCCAAA
TCGACCACAAAACCGGCTTCGGTTAACCCTTTGGTCAAGTATTCTCCGGTTTTCTTTTCATCTTCGACAATCAACAGTTT
CATATTTCCTCCGCATGTTGCCCGGGCAATTCTAGAGTAGCGGGATCAGATGGCAATCGCTTATTGGCAAAATGACAATT
TTGTCATTTTTCTGTCACCGGAAAATCAGAGCCTGGCGAGTAAAGTTGGCGGCATAAAATCACCAGAAATTATGAGCCTA
TGTCTCCTTGTAAACTTCTGCCATTTTGTGTGGCCCTTGCGCTAACCGGTTGTTCACTGGCACCGGATTATCAGCGTCCG
GCAATGCCCGTGCCGCAGCAGTTCTCACTCAGCCAGAACGGCCTGGTTAACGCAGCAGATAACTATCAGAACGCGGGCTG

GCGCACCTTTTTTGTTGATAATCAGGTGAAGACGCTGATTAGCGAGGCGCTGGTGAATAATCGGGATTTGCGCATGGCGA
CGCTGAAAGTGCAGGAGGCACGGGCGCAATATCGTCTGACCGATGCCGACCGCTACCCACAGCTCAATGGCGAGGGCAGC
GGCAGCTGGAGCGGTAATCTTAAAGGGAATACCGCCACGACACGGGAGTTTTCGACCGGCCTTAACGCCAGCTTTGACCT
CGATTTTTTCGGTCGCTTAAAGAACATGAGCGAAGCCGAGCGACAAAATTATTTAGCCACTGAGGAAGCTCAGCGCGCGG
TGCATATTCTGCTGGTTTCTAATGTCGCGCAAAGCTATTTCAATCAGCAACTGGCGTATGCGCAATTGCAAATAGCCGAA
GAAACGCTGCGTAATTATCAGCAGTCATATGCGTTTGTCGAAAAACAACTGTTGACAGGTAGCAGTAATGTTCTGGCGCT
GGAACAGGCTCGCGGGGTGATAGAAAGTACCCGCAGCGACATCGCTAAACGTCAGGGGGAACTGGCGCAGGCGAATAATG
CATTGCAACTGTTGCTGGGAAGCTACGGCAAGCTGCCGCAAGCGCAGACAGTAAACAGCGACAGCCTGCAAAGCGTTAAA
TTACCGGCGGGATTGTCGTCGCAAATCTTATTGCAGCGCCCGGATATTATGGAAGCTGAACACGCGTTAATGGCGGCTAA
TGCCAATATTGGCGCTGCACGCGCGGCATTTTTTCCGTCTATAAGCCTGACCAGCGGAATATCGACCGCCAGTAGCGATC
TATCGTCATTATTTAACGCCAGCAGCGGGATGTGGAATTTTATACCCAAAATTGAGATCCCCATTTTTAATGCCGGACGC
AACCAGGCCAATCTGGATATCGCCGAAATTCGCCAGCAGCAGTCGGTGGTGAATTATGAACAGAAAATCCAGAACGCCTT
TAAAGAAGTGGCAGATGCGCTGGCATTACGTCAAAGCCTGAACGATCAAATTAGCGCCCAGCAGCGTTATCTGGCATCGC
TGCAAATTACTTTGCAACGGGCGCGGGCATTATATCAGCACGGCGCAGTAAGTTATCTGGAAGTGCTGGATGCCGAGCGT
TCTTTATTTGCAACCCGACAAACTTTACTTGATCTGAATTATGCCCGTCAGGTTAACGAAATTTCTTTGTATACCGCACT
TGGTGGCGGTTGACAGCAATATACTCGTCATACTTCAAGTTGCATGTGCTGCGTCTGCGTTCGCTCACCCCAGTCACTTA
CTTATGTAAGCTCCTGGGGATTCACTCGCTTGTCGCCTTCCTGCAACTCGAATTATTTAGAGTATGACTTTTAACTCCAG
GAGAGAATAAATGAAAAAAGCACTGCAAGTCGCAATGTTCAGTCTGTTTACCGTTATTGGCTTTAATGCCCAGGCTAACG
AACATCATCATGAAACCATGAGCGAAGCACAACCACAGGTTATTAGCGCCACTGGCGTGGTAAAGGGTATCGATCTGGAA
AGCAAAAAAATCACCATCCATCACGATCCGATTGCTGCCGTGAACTGGCCGGAGATGACCATGCGCTTTACCATCACCCC
GCAGACGAAAATGAGTGAAATTAAAACCGGCGACAAAGTGGCGTTTAATTTTGTCCAGCAGGGCAACCTTTCTTTATTAC
AGGATATTAAAGTCAGCCAGTAACCCAGGTTTAATGAGATGAAAAAAATCGCGCTTATTATCGGCAGCATGATCGCGGGC
GGTATTATTTCTGCGGCAGGTTTTACCTGGGTTGCAAAGGCGGAACCGCCTGCAGAAAAAACGTCGACCGCAGAACGTAA
AATCTTATTCTGGTACGACCCAATGTATCCCAATACGCGGTTCGATAAACCAGGTAAATCGCCGTTTATGGATATGGATC
TGGTGCCGAAATATGCCGATGAAGAGAGTTCTGCGTCTGGTGTGCGCATTGACCCGACTCAGACGCAGAATCTGGGGGTG
AAAACGGCTACCGTCACGCGCGGACCGCTGACTTTTGCCCAGAGTTTCCCGGCGAATGTCAGTTACAACGAGTATCAGTA
TGCCATTGTGCAGGCTCGCGCTGCCGGGTTTATCGACAAGGTGTATCCGCTTACCGTGGGCGATAAAGTACAAAAGGGCA
CACCGCTTCTCGACCTGACCATTCCTGACTGGGTGGAAGCGCAGAGTGAGTATTTACTGCTGCGCGAAACCGGCGGTACG
GCGACCCAGACTGAAGGCATTCTTGAGCGGCTGCGACTGGCGGGAATGCCGGAGGCGGATATTCGCCGCCTGATCGCCAC
GCAAAAAATCCAGACTCGCTTTACGCTCAAAGCGCCCATTGATGGCGTGATCACCGCGTTTGATCTGCGCGCGGGAATGA
ATATCGCCAAAGATAACGTGGTAGCGAAAATTCAGGGTATGGACCCGGTGTGGGTCACTGCTGCGATCCCGGAGTCTATC
GCCTGGCTGGTGAAAGATGCCTCGCAGTTTACCCTCACCGTTCCGGCGCGACCGGATAAAACACTCACCATCCGCAAATG
GACGCTGCTACCTGGCGTGGATGCCGCGACCCGCACGCTGCAGCTGCGTCTGGAAGTCGACAACGCCGACGAGGCGCTAA
AACCGGGCATGAACGCCTGGTTGCAACTCAACACCGCCAGCGAACCGATGCTGCTCATTCCGTCACAAGCGCTGATTGAT
ACCGGCAGCGAACAGCGGGTGATTACCGTTGATGCCGACGGGCGCTTTGTACCGAAACGCGTTGCTGTTTTCCAGGCATC
GCAAGGCGTCACCGCATTACGCTCTGGTCTGGCGGAAGGTGAAAAGGTGGTTTCCAGCGGCCTGTTCCTGATTGATTCCG
AAGCCAATATTTCTGGCGCACTGGAGCGGATGCGCTCTGAAAGTGCTACCCATGCGCATTGAGGGAATAACCAATGATTG
AATGGATTATTCGTCGCTCGGTGGCGAACCGTTTTTTGGTGCTGATGGGCGCGTTGTTTCTGAGCATCTGGGGCACCTGG
ACCATCATTAATACGCCAGTGGATGCGCTGCCGGATCTCTCCGATGTGCAGGTTATTATTAAAACCAGCTATCCCGGTCA
GGCACCACAAATCGTTGAAAATCAGGTGACTTATCCGCTAACCACCACCATGTTGTCGGTGCCTGGCGCGAAGACTGTGC
GCGGTTTCTCACAGTTTGGCGACTCTTATGTGTATGTCATTTTCGAAGATGGCACCGATCCGTACTGGGCGCGTTCGCGG
GTGCTGGAGTACCTCAACCAGGTACAGGGTAAGCTGCCTGCGGGAGTCAGCGCCGAGCTGGGGCCAGATGCCACGGGTGT
TGGCTGGATCTATGAATATGCACTGGTGGATCGCAGCGGTAAGCACGATCTGGCCGATTTACGCTCATTACAGGACTGGT
TTCTCAAATATGAGCTGAAAACCATCCCTGACGTTGCGGAAGTGGCGTCGGTGGGCGGTGTGGTGAAAGAGTATCAGGTG
GTTATCGATCCCCAGCGCCTGGCGCAGTATGGCATCAGTCTCGCCGAAGTAAAAAGCGCGCTGGATGCTTCAAACCAGGA
AGCGGGCGGTTCGTCGATCGAACTGGCGGAAGCGGAATATATGGTGCGCGCCAGCGGCTATCTGCAAACGCTCGACGACT
TTAATCACATCGTTTTAAAAGCCAGTGAAAATGGCGTGCCCGTTTATCTGCGCGATGTTGCGAAGGTCCAGATTGGCCCG
GAGATGCGCCGGGGCATTGCCGAACTAAACGGCGAAGGCGAAGTGGCGGGCGGGGTGGTGATCCTGCGATCCGGCAAAAA
CGCCCGAGAAGTGATCGCCGCCGTGAAGGACAAACTGGAAACGCTGAAAAGTAGTCTGCCGGAAGGCGTGGAGATAGTTA
CAACATACGATCGCAGCCAGCTCATTGACCGCGCCATCGACAACCTCAGCGGCAAGTTGCTGGAAGAGTTTATTGTGGTG
GCGGTAGTCTGTGCGCTGTTTCTCTGGCATGTGCGCTCGGCGCTGGTGGCGATTATTTCGTTGCCGCTGGGGTTGTGTAT
TGCTTTTATTGTCATGCACTTCCAGGGACTGAATGCCAATATTATGTCGTTGGGTGGCATTGCGATTGCCGTCGGGGCGA
TGGTCGATGCTGCTATCGTCATGATCGAGAATGCGCATAAACGGCTGGAAGAGTGGCAGCACCAGCATCCTGACGCCACG
CTGGATAATAAAACGCGCTGGCAGGTGATCACCGATGCGTCTGTTGAAGTGGGGCCGGCGCTATTTATCAGTCTGCTGAT
TATCACGTTGTCGTTTATCCCGATCTTCACCCTGGAAGGGCAGGAAGGGCGTCTGTTTGGCCCGTTGGCGTTCACCAAAA
CGTATGCGATGGCGGGTGCGGCGCTGCTGGCGATCGTAGTGATCCCGATCCTGATGGGCTACTGGATCCGTGGCAAAATT

CCGCCGGAAAGCAGTAACCCGCTCAATCGCTTTTTGATTCGTGTTTATCATCCGCTGTTGCTGAAAGTACTGCACTGGCC
GAAAACCACGCTGCTGGTGGCGGCGCTTTCGGTGCTGACGGTTCTCTGGCCGCTCAATAAAGTTGGCGGGGAATTTTTAC
CGCAGATCAATGAAGGCGACTTGTTGTATATGCCATCGACGCTGCCGGGGATTTCCGCAGCAGAGGCGGCGAGTATGCTG
CAAAAAAACCGACAAGCTAATTATGAGCGTACCTGAAGTGGCGCGGGTATTTGGCAAAACCGGGAAAGCGGAAACCGCCAC
CGATTCTGCTCCGCTGGAGATGGTAGAAACGACCATCCAGCTTAAGCCGCAGGAGCAGTGGCGGCCAGGCATGACGATGG
ACAAAATCATTGAGGAACTGGATAACACCGTGCGGCTGCCGGGGCTGGCGAATCTGTGGGTGCCGCCAATTCGTAACCGT
ATCGATATGCTCTCAACCGGCATTAAAAGCCCCATCGGCATTAAAGTTTCCGGCACTGTGCTGGCGGATATCGACGCGAT
GGCTGAGCAAATTGAAGAAGTAGCGCGAACGGTGCCAGGCGTAGCTTCTGCGCTTGCCGAGCGGCTGGAAGGTGGGCGCT
ATATCAACGTTGAGATTAACCGTGAAAAAGCCGCACGTTACGGTATGACGGTGGCGGATGTGCAGTTGTTTGTGACTTCT
GCGGTGGGCGGGGCGATGGTTGGCGAAACGGTGGAAGGGATTGCCCGTTATCCAATTAATCTGCGTTATCCGCAAAGCTG
GCGCGATAGTCCGCAGGCACTGCGCCAGCTGCCGATCCTGACGCCGATGAAGCAGCAAATCACCCTGGCAGACGTGGCCG
ACATTAAAGTCTCTACCGGACCGTCGATGCTGAAAACCGAGAATGCGCGCCCGACGAGCTGGATTTATATCGATGCCCGC
GATCGTGACATGGTGTCGGTGGTTCACGATTTGCAAAAAGCGATAGCTGAAAAAGTGCAGTTAAAACCGGGCACCAGCGT
GGCATTCTCCGGGCAGTTCGAGCTACTGGAGCGCGCCAACCATAAGCTTAAACTCATGGTGCCGATGACGTTGATGATTA
TCTTCGTGCTGTTGTATCTGGCGTTCCGTCGGGTGGGCGAAGCGTTGCTGATTATCAGCAGCGTACCGTTTGCGCTGGTG
GGCGGCATCTGGTTGCTGTGGTGGATGGGCTTTCATCTTTCCGTGGCGACGGGCACTGGCTTTATCGCCCTCGCCGGGGT
CGCCGCCGAATTTGGCGTGGTGATGCTGATGTATTTACGTCACGCCATAGAGGCCGTGCCGTCGTTGAATAATCCACAAA
CATTCAGCGAGCAGAAGCTGGATGAGGCGTTATATCACGGCGCGGTCCTGCGCGTGCGCCCGAAAGCGATGACGGTGGCG
GTGATTATCGCCGGTCTGCTGCCGATTCTGTGGGGAACGGGGGCTGGTTCAGAGGTGATGAGCCGGATTGCCGCGCCGAT
GATTGGCGGCATGATCACCGCACCTTTGCTGTCGCTGTTTATTATCCCGGCGGCGTATAAGCTGATGTGGCTGCACCGAC
ATCGGGTACGGAAATAAAAGCAGGATACCCCGTTTAACCGTGTGGATTGTGTCTTGCGACGATGGGCACTAAATGTTAAA
AGGTGCCCCTCAACAAAAAAGACACACAGGGGAAAGGCGTGAAAAACGCGTCAACCGTATCGGAAGATACTGCGTCGAAT
CAAGAGCCGACGCTTCATCGCGGATTACATAACCGTCATATTCAACTGATTGCGTTGGGTGGCGCAATTGGTACTGGTCT
GTTTCTTGGCATTGGCCCGGCGATTCAGATGGCGGGTCCGGCTGTATTGCTGGGCTACGGCGTCGCCGGGATCATCGCTT
TCCTGATTATGCGCCAGCTTGGCGAAATGGTGGTTGAGGAGCCGGTATCCGGTTCATTTGCCCACTTTGCCTATAAATAC
TGGGGACCGTTTGCGGGCTTCCTCTCTGGCTGGAACTACTGGGTAATGTTCGTGCTGGTGGGAATGGCAGAGCTGACCGC
TGCGGGCATCTATATGCAGTACTGGTTCCCGGATGTTCCAACGTGGATTTGGGCTGCCGCCTTCTTTATTATCATCAACG
CCGTTAACCTGGTGAACGTGCGCTTATATGGCGAAACCGAGTTCTGGTTTGCGTTGATTAAAGTGCTGGCAATCATCGGT
ATGATCGGCTTTGGCCTGTGGCTGCTGTTTTCTGGTCACGGCGGCGAGAAAGCCAGTATCGACAACCTCTGGCGCTACGG
TGGTTTCTTCGCCACCGGCTGGAATGGGCTGATTTTGTCGCTGGCGGTAATTATGTTCTCCTTCGGCGGTCTGGAGCTGA
TTGGGATTACTGCCGCTGAAGCGCGCGATCCGGAAAAAAGCATTCCAAAAGCGGTAAATCAGGTGGTGTATCGCATCCTG
CTGTTTTACATCGGTTCACTGGTGGTTTTACTGGCGCTCTATCCGTGGGTGGAAGTGAAATCCAACAGTAGCCCGTTTGT
GATGATTTTCCATAATCTCGACAGCAACGTGGTAGCTTCTGCGCTGAACTTCGTCATTCTGGTAGCATCGCTGTCAGTGT
ATAACAGCGGGGTTTACTCTAACAGCCGCATGCTGTTTGGCCTTTCTGTGCAGGGTAATGCGCCGAAGTTTTTGACTCGC
GTCAGCCGTCGCGGTGTGCCGATTAACTCGCTGATGCTTTCCGGAGCGATCACTTCGCTGGTGGTGTTAATCAACTATCT
GCTGCCGCAAAAAGCGTTTGGTCTGCTGATGGCGCTGGTGGTAGCAACGCTGCTGTTGAACTGGATTATGATCTGTCTGG
CGCATCTGCGTTTTCGTGCAGCGATGCGACGTCAGGGGCGTGAAACACAGTTTAAGGCGCTGCTCTATCCGTTCGGCAAC
TATCTCTGCATTGCCTTCCTCGGCATGATTTTGCTGCTGATGTGCACGATGGATGATATGCGCTTGTCAGCGATCCTGCT
GCCGGTGTGGATTGTATTCCTGTTTATGGCATTTAAAACGCTGCGTCGGAAATAAGGCATTCACGCTACATCCGACAAAA
CGATGTCAACCATCCGAAACCGCTCTCATCCATTCGATGAGAGCGGTTTTTTTAATTACTGCTTAAATGCACCCGCCAGA
GAGCGAATATCATTGCCGGTTGGCGACTGATGAAGTCGCAGACCAAACTCTTCGACAATCGCAAATATGTGATCGAATAT
ATCAGCCTGAATGCTTTCATATTCCAGCCACACCACGGTGTTAGTAAACGCGTAGATCTCGAGCGGTAAACCGTTATCAC
CTGGAGCCAGTTGGCGTACCATTAAGGTCATATCTTTACGAATCCGCGGATGGTTACGCAGATATTCGTTCAGATAGGCA
CGAAAGGTTCCAATATTGGTCATTCGGCGCAGATTTAATACCGACTCCGTAGAACCTTGCTGGCGATTCCACTCATTAAT
TTCCTGATGGCGGCTGGTTAAATAAGGCTTTAACAAATGCGCTTTATTCAGACGTTGCATTTCGTCTTCATCAAGAAAAC
GAATACTGGTGACATCAATACTGATACTGCGCTTAATACGTCGCCCGCCAGATGCTGACATCCCGCTCCAGTTTTTAAAG
GAGTCAGAAACCAGAGACCAGGTGGGAATAGTGGTAATGGTATTGTCCCAGTTACGCACTTTGACGGTGGTTAACCCAAT
ATCGATCACCGCGCCATCCGCGCCGTATTTCGGCATCTCCAGCCAGTCGCCCAGTTTCAGCATATCGTTCGCGGAAAGCT
GAATACCTGCCACCAGACCAAGAATCGGATCTTTAAATACCAACATCAGCACGGCAGCCATTGCACCAAGACCGCTGATC
AGAATCGCTGGCGACTGACCAATCAGCAGCGAGATCATCAAAATGCCGACCAGAATCGCGCCGATCAGTTTAATCCCCTG
AAATATCCCTTTCAGCGGTAACTGAGATGCTGCCGGGAATTTCTGCGCCAGATTCAAAATAACATCCAGCAACGAGAAGA
CTGAAAGCAGCGCATACATCATGATCCACAACTGCGCGCAGGTAGTCAGAATATCTGCCGCTTCGGTGCCTTTTTGCAGC
CAGAATACCGCCTGAATATTGACGATAATCCCCTGCAGGGTAAAAGCTAAACGGTGGAAGAGTTTATTCTGGGTAATGAT
TTGCAACCAAAGCCGTGAACTGGCGATGGCACGTTTTTCGAAGGTCCGCAGTACCACCCAATGCAAAATAATATGCACCA
CGACGGCGGTCAGAAAAATAATACCGAAAATCATCACCATCGAGGTGGTGTGATCGATCTCAATACCCGCTAAATCTTCA
ACCTGGGATATTAAATCCTGCATAACGTCTCCTTTATACAACAGCAGCCTATGATGACGGCTGAAACAGGGTTATGCAAA

TCAGGAGAATCTGAGAGGAAATAGCCGGGCAGATGCCCGGCAAGAGAGAATTACACTTCGGTTAAGGTGATGTTTTGCGG
CAGACGAGATTTCGGCAGCGTAGCGTTAAAATCTTCAACGCTGTGATGACCTACCGGAACAACCACCAGACTGGTGTAGC
CTTTCTCTTTCAGACCAAATTCTGCATCGAGGATGGCGGCGTCAAAACCTTCGATGGGTACCGCGTCCAGACCCAGAGCC
GCCACGCCGAGCAGGAAGTTACCGACGTTGAGATAAACCTGTTTTGCCATCCACTCTGCATCATCATGCAGATCTTTACG
GTGCATATCAGCGAAGAACTTGCGACCTTTATCGTTCGCGGCTTTCGCTTCCGGCGTGGCAAAGCGGCCATCGGCATCTT
CCTGGTCAACAACCAGCTTCAGCCAGACATCGTCCATCGCGGTTTTTGCACAGAACACCACGACGTGCGAGGCATCAAGC
ATTTTACGCTCGTTGAACACGTAATTACCGGCAGCGGATTTGGCAACACGCGCTTTACCTTCTTCCGTGCTGGCAACAAT
AAAATGCCACGGCTGGGAGTTGGTGCTGGATGGGCTGTATTGCAGTAGCGTTTTGATCTGCTCGGCCTGTTCCGGGGTAA
GTTTTTTGCTGGCATCAAATGCCTTAGTGGAATGACGCTTTAAGGCGACAGAAATGATATCCATAAAGACTCCATGTGAA
AGTAATTTTGCGTGCCAGCAGATTACAAGGTTCAACGAGAAATGGTAAGCGAGAAAAATGCGCTATAGATTTCCGCTTTA
GCCTGGACGCACTCTTTTTTGATCGCGTTTAGCCAGACCATCAACCACCAGATTCCACGAATCGTTGATCAGATCGCGAA
GTAACGCTTCGGAGATTTCTTCGCCGGGATACACTGAAATCCAGTGCTTTTTATTCATGTGATACCCTGGCTTAATGCTT
GGGTATATTTGCTGATTTAACAGGGATTTTTTGTGGATCGGACTTCAGATTGATAAAGGGGACGCCGCGTAGCTCCGACGA
CAGCATAAAAATCTTGCCGCCAATTTTAAAAACATCGAACTCCGGGCCAAAAGGCCAGCAAAGCTCGACAAAGGGTAACT
CAAGGGCCAGGCGTTTCGCCGTTTCGTGCAGTGATTGCTTATCCATAAACGTTCCTTTAGGCGAAGGAGAATAAGCAAAG
TATGCCGCGAAGTACGGCGATAATCGACGTTTAATCCGCCAGCGAGAACCAGCGTCGCCAGATAAAGCGCAGAACAAAAT
ACTCAATAGCGCCCAGCACTAAAAACCACAGACAAAACAATAAAGTGTAAAGCTGACTAAGATCCATCAGATGGAACATG
GTCACCAGTTTTTGTGCCAGCGCCAGCCCCAGTGCGGGGGCGGGCAGCAGCAGGCAAGAGAGGAAAGCCATCAGCAAAAT
GCCTGCTGCGGTGGTCAAGGTTTCTAAAGGGTGTTTCATAACATTGTTAAATGTAAGTTAAAACACCATTGTCAGGGATA
TTCTTCTGTAAGGCAATTCCCGGCTTAGTCACCGGCCCAGATCTCACAATGCTTTTTCACCAGCCCAATCAGCGAGCCGC
CATCGGCGACGAAATCGCGCATTAGCTGCGCTTCATTCAGACCGCTGACGACCTGGCGATGCAGGGCCTCAATCGCGCTC
GATGCACCAATTTTATGTGCGGAAGGGGCGATTTTTTCCAGCAATCGCAAGGTATCTTCCGTTAGCGGTCGACGATCTCC
AGTGTGCGGATCGGTGATGACGCCTTCAAGCCCATAGCGACAGGCCTGGAAACGGTTGAATTTATACAGCAGGTAATCTT
TTTCCTGATGTTTAAACGGGCGTTCCGTCAGTAACCAGTGGGCGGTAGCCTGAATTAATCCCGCCATATTTACTGCGTGG
CTAAGGGTTAACGGGGTATCCATCACCCGAACCTCCACCGTGCCAAAATGAGGACTGGGGCGAATATCCCAGTGCAGATC
TTTAATGCTGTCGATCATCGTGGTGTAACTCAGACAGCGAAACAGGGCTTCAAATTGTTGCCAGTTACTGACCCACGGCA
TCGGGCCATTATCAGGAAAGGCGGAAAAAATATTCGGTCGTGAGGAGGCAAAACGCGTATCCGTTCCCTGCATATATGGC
GACGCGGCGGAAAGGGCGATAAAGTGCGGCACAAATCGTGACAAGCCGTGCAGCAAATAAATGGCGTCATCGCCACTGGC
GCAGCCAACATGGACATGCTGACCAAAAACGGTCGCCTGCTGAATGAGATAACCAAAGTTTTCCAGCGTGCGTTGATAGC
GTTCGTTATCGCATACCTCCTGACGCTGCCATTTCTGAAACGGGTGCGTGCCACCGCCGCAAATTTCCAGATGATGGTCT
GTGGCTGCCTGCAATACGACTTTCTGCATCGCTGAAAACTGCCCGGCAGCCTGGTTGATATCACGGCAAACATCCGTCGC
CAGCTCCAGCATACTTTCGGTGATATCGTGCTTTACCTCTCCGGCCGTGATCTTATTTTTAACCGCGTCAATCAGCATTG
AAGAGTCCTGGCTTAAGTCATAGCCCGGCGGATTAACCACCTGCATTTCCAGTTCAATACCGAGGGTAAAAGGTTCAGAA
ACATGAAAATCGGGTAATGGCATAGGTTTCTCTTATGTTGGCGTTTTCTATTCAGTATAGAAGTCGGAGCGGCTGGGCGA
GATGCGGAAGTTCTGGAATGTTTCTTTTTTTTGGTGATGGTGAACTGATGGTGCCTGAAGCAATTTGGCTACTTTTGCAA
TGTGACAAGTTATGGCAGTGCTGACTGGTGGCGAAGAATTTTGACGATTGAGTCATGCAGAAAAAAACGGGTTCAGCTTC
AGTTGATTAAATGAGGAGTGAGAAGTCCGAAACAGGACTCACTGTATAAATAAACAGCTATTTTGTTGAGGAAGGGTAAG
ATAACGGCGGGTGCCTGAAGCTTTCCGGTTTCAGGTTTACTCTGAGGTCTGGAAAGATGAAGCCCCAGGAGATATTTCTA
TCAACCCTGGGGCTGCCACTCCAAACCCGAACAATTTGGATGGTAGTCCCTTCTTCGCATGGAGGCAATATAAACATGCT
GACGAAATATGCCCTTGCGGCAGTCATAGTGCTGTGTTTAACGGTGCTGGGATTTACGCTTCTGGTCGGAGACTCGCTGT
GTGAGTTTACGGTGAAGGAACGTAATATTGAGTTTAAGGCTGTTCTCGCTTACGAACCGAAGAAGTAGCCGTTGTGCGGG
GAGTAATCCCATAAGCGCTAACTTAAGGGTTGTGGTATTACGCCTGATATGATTTAACGTGCCGATGAATTACTCTCACG
ATAACTGGTCAGCAATTCTGGCCCATATTGGTAAGCCCGAAGAACTGGATACTTCGGCACGTAATGCCGGGGCTCTAACC
CGCCGCCGCGAAATTCGTGATGCTGCAACTCTGCTACGTCTGGGGCTGGCTTACGGCCCCGGGGGGATGTCATTACGTGA
AGTCACTGCATGGGCTCAGCTCCATGACGTTGCAACATTATCTGACGTGGCTCTCCTGAAGCGGCTGCGGAATGCCGCCG
ACTGGTTTGGCATACTTGCCGCACAAACACTTGCTGTACGCGCCGCAGTTACGGGTTGTACAAGCGGAAAGAGATTGCGT
CTTGTCGATGGAACAGCAATCAGTGCGCCCGGGGGCGGCAGCGCTGAATGGCGACTACATATGGGATATGATCCTCATAC
CTGTCAGTTCACTGATTTTGAGCTAACCGACAGCAGAGACGCTGAACGGCTGGACCGATTTGCGCAAACGGCAGACGAGA
TACGCATTGCTGACCGGGGATTCGGTTCGCGTCCCGAATGTATCCGCTCACTTGCTTTTGGAGAAGCTGATTATATCGTC
CGGGTTCACTGGCGAGGATTGCGCTGGTTAACTGCAGAAGGAATGCGCTTTGACATGATGGGTTTTCTGCGCGGGCTGGA
TTGCGGTAAGAACGGTGAAACCACTGTAATGATAGGCAATTCAGGTAATAAAAAAGCCGGAGCTCCCTTTCCGGCACGTC
TCATTGCCGTATCACTTCCTCCCGAAAAAGCATTAATCAGTAAAACCCGACTGCTCAGCGAGAATCGTCGAAAAGGACGA
GTAGTTCAGGCGGAAACGCTGGAAGCAGCGGGCCATGTGCTATTGCTAACATCATTACCGGAAGATGAATATTCAGCAGA
GCAAGTGGCTGATTGTTACCGTCTGCGATGGCAAATTGAACTGGCTTTTAAGCGGCTCAAAAGTTTGCTGCACCTGGATG
CTTTGCGTGCAAAGGAACCTGAACTCGCGAAAGCGTGGATATTTGCTAATCTACTCGCCGCATTTTTAATTGACGACATA
ATCCAGCCATCGCTGGATTTCCCCCCAGAAGTGCCGGATCCGAAAAGAAGAACTAACTCGTTGTGGAGAATAACAAAAA

TGGTCATCTGGAGCTTACAGGTGGCCATTCGTGGGACAGTATCCCTGACAGCCTACAAAACGCAATTGAAGAACGCGAGG
CATCGTCTTAACGAGGCACCGAGGCGTCGCATTCTTCAGATGGTTCAACCCTTAAGTTAGCGCTTATGGGAGTAATCCCC
GCATATCCGGTTGTCAGGTCAGGATGGTAAGGCACCTGCTTTACACTTTCGCCCGTGGTCAGTGATGGCTGCGGGCGAAT
CGTACCAGATGTTGTCAATTAATCGTGTTGGCACAGCGTTATGACTATCTTTTCTTTTATCTGCCAGTGCACAGCAAACA
TCTCATTCTCACGATGAATGATGACCTGCTGTTTATTCCAGCTAATTATCTGATAGTCCAGAAAACCTGCATCAGTTTGG
ATCTCACTTGCCTTAAATGCGCTCTCTTTGGCGGAAAATGCCAGTGTCAGCGCCAGAGAAAAGGCTAAACCGCAGTCTGC
GAGTCGCTCGTGTTCCGCTGGTGTAATAATGTTGTCTGTCAATTCTCTTGCGGTTTGTACAGAAAAAATTTCTTCTATAT
CAATGCCAATCGGTTGACGAGATACCACGGCTAATGCCGTAGTCCCACAGTGGCTAATACTGCCGTATACCTCCGCAGGC
CAGACAGGTTGGCGTAGCTCGCCGATTGCGGGCACACATTTATAGCCATATTCCCGCAAAGCATAAACAGCAGCGATCCG
TCCGGCTAAATGCTCTGTTTTACGTTTACGTCCAGCGTGTTGCAGTTGTGCGTAGTGCGGCAGCCAGAGTAAATCCTGCT
CACAAAAATTCGCCGGATCGAACTCAACAAAATGCAGCGTATGTCCGGCAAAGGGGAGGGAGGTATGCGTAGTTTTCATA
TCGACCATATTCGAGACTGATGACAAACGCAAAACTGCCTGATGCGCTACGCTTATCAGGCCTACATGGTCCTGCAATAT
ATTGAATTGGCAAGATTTTTGTAGGCCGGATAAGGCGTTCACGCCGCATCCGGCATGAACGACGCGCACTTTGTCAACAA
TCTGACGTTAGCATCAGAAGTGGGTGTTTACGCTCATATACCACGTACGTCCCGGCTCGTTATAGGTATACGCCCCGGCA
CCGGCGATATAGTTGGCCCCTGCCAAATCGCCCGTGGTCTGGGCATTACCCGCACGCCACAAACGTTTGTCGAACAGATT
GTCCACGCCGCCGGTCAGACTGACATTCTTCGTCACATCCCAGGTCGCGCTCAGGCCAACAATGCTGTAAGGACTAATTT
CTTTGGTTTCCGGTCCAACCGCTGGCTGACCTTTATAGTTGTACTTCTTCGGCTGCTGCTTGCCGTACCAGGTGAAGGTC
GTTTGCATCGACAAATCTTCCCGTGCCTGCCAGCTCAGCGTTGAGTTCAACGTATACTCCGGGATGATCGACAAACGGTC
GCCCGTGGTTTTGTTTTCACTCTTCAGCATATAAGTGATGTTATTGGTCCACATCACCGTTTCGCTAACCGGTACGTTTA
ACGATCCTTCCAGACCTTCAACCACCGCTTTCGGCACGTTATCCCACTGATAGAGATCGGTGCCGACTGCGTTTTGCCCT
ACAGCCACATAGCCCGCTTCAATCTTATTGCGATAATCGTTACGGAACCAGGTGACGCCCGCCAGCCACCCGTCGCGTTT
GAACTCCAGACCAATCTCTTTGTTGATGCTGGTTTCTGCTTTCAGGTCATCGTTACCTTGCAGATAGCAGCCGCCCGCGC
TGGCATAGCAACCCTGACCTTTACTGTAGAGAATGTAGTTCGGGTTAGTCTGGTACAGGCTCGGCGCTTTATAAGCACGG
GCGATGCCCATTTTCAGCGTGAAGTCATCGCCTAAACCTTGCGATATGTTCAGCGCCGGGCTCCAGTTATTGCCGACAAT
ACTGTGATGATCGAAACGCAGCCCCGGCGTTACGATGGTGCTGTCAGTCAGCTCCATGTTGTTTTCGGCAAACAGCGAGA
AAATTTCTGCTTTTGAATACGGGCTACGGTCGGTGGTACTCACGCCATCAATAGCGCCACCGGTATTCGTTCCGGTCAGT
GCCTGGGTGTTGGAACTTAAGTCCTTCATCCGTTGCTGATTCCACTCCGTACCCAGCGTCAGCGTCTGGTTAACGAGGAA
ATCAATCGGCAGGTTAACTTCGCTGTGCAGCATCACGTCATCAAGATCGATATCGACGAAATCCTGTGTCGCTTTTTCGT
TAAATTTCCCTTCGGTACCGCCCGCCAGACCTTCCGGAATACGCGAGTTACGGGTGTGTTCGTACTGCACCCAGTTGCTG
GTGGTCACGCCGTTATCCCAGCCACCGTTCCAGGTCAGCGCGTAGTTCTGGCGATACAGACGGTTGGTTTCATCGCCATA
TTTCGAGCGGGTATAGGAATCGGAGTTGGTATTCTGGGTGTCGCCCGCATACAGGTTACCCTGGCGGCTGTAACCTGCTT
CCAGTTCCAGCGATTGCAATGGCGCGAAATCCCAGCGCACCACGCCATTAATATCTTTGTTGATTACCCCTTCGCGCCCG
GCTGGTAACGTCGTGGCATACGTTCCGGCACGCGCGGACTGATGGCCCTGGTTGATATCCCACGCGTCAGCCTGGGTTTT
GTCGAGGTTGCCATACAAACGGAAGCTGAATTCGTCGCCCAGCGGACCGGTCAGGCTAAAGTTAGTGCGTTTGGTGGCAC
CTTCCTCTTTATGTTCTGGCGCATTGAAATATGCGTCCCAGGAGCCGTGCCACTCGCCGCTGCCTTTTTTGGTAATGATG
TTAACCACGCCGCCCGCCGCGCCGTTGCCATAACGCGCAGCTGCCGGACCACGCAGAACTTCAATACGTTCAATCATTTC
AGGTGGCACCCAGGAAGTATCACCACGGGTATCGCGCTCGCCACGCCAGCCCTGACGCACCGAGTTACGGCTGCTTACCG
GCTTGCCGTCAATCAAAATCAGCGTGTTTTCCGGACCCATACCGCGAATATCAATCTGTCGGTTATTCCCACGCTGACCA
CTGGTGGAGTTACCGGTCAGGTTAACGCCTGGCATGGTACGGATGATCTTCGACACATCGCGGGCAACCGGGTTTTTGCG
GATTTCATCTGCGGTGATGGTCGAAACGCCAGGCGCCTGTAAGTTCTGCTCGGCGGCGGTAACGACAATAGTATCGTCAT
GTGAAACAGGAGTATCGGTCGGCTCTTGTGCCTGCGCTACCCCATAAATCCCCAGATTGACCAACAAGGCCAGGGAATGA
ATCTTCTTGTTCATTGTTTTATTCCTGCATTTTTGCCACGAATTGCAACTGTCGGGCATGGTCGTCATCAACACGACGCA
TCCCGCTACCGCGAAAACCTTTGATCCTGAAAGACACGCAGTGCAGTTGGTTAATTAATGTCCGCGCTTCCCACGGCGCG
CCATTACGCTATTGCAAATGCAAATAGTTATCAATAATATTATCAATATATTTCTGCAATCAATGAAAAATTGCACAGTA
AACATGGGGTTATGGTGTGACGGCGTTAAAAGTAGGAAGTGAGAGCTGGTGGCAGTCGAAACATGGCCCGGAATGGCAGC
GTCTGAATGACGAAATGTTTGAGGTCACTTTCTGGTGGCGTGATCCCCAAGGTTCTGAAGAATACTCGACGATAAAGCGC
GTATGGGTCTACATCACTGGTGTGACCGATCACCATCAGAACAGCCAGCCCCAGTCGATGCAGCGAATTGCAGGCACTAA
CGTCTGGCAGTGGACGACACAACTCAATGCCAACTGGCGCGGCAGCTACTGCTTTATTCCCACCGAACGCGATGACATTT
TTTCTGTACCATCCCCCGATCGCCTCGAATTGCGCGAAGGCTGGCGAAAACTATTACCCCAGGCGATAGCCGATCCGCTG
AACCTACAAAGCTGGAAAGGCGGGCGAGGGCACGCTGTTTCTGCACTCGAAATGCCGCAAGCGCCTCTGCAACCGGGATG
GGATTGTCCGCAAGCGCCAGAAATACCTGCCAAAGAAATTATCTGGAAAAGTGAACGGTTGAAAAAGTCACGGCGTGTAT
GGATTTTTACCACCGGCGATGCAACAGCAGAAGAACGCCCGCTGGCAGTTTTGCTCGATGGCGAATTTTGGGCGCAAAGT
ATGCCCGTCTGGCCAGTGCTGACTTCGCTGACCCATCGTCAGCAACTTCCTCCCGCCGTGTATGTGTTGATCGACGCTAT
CGACACCACGCACCGCGCCCACGAACTGCCGTGTAATGCGGATTTCTGGCTCGCAGTACAGCAAGAGTTATTACCCCTGG
TGAAAGCTATTGCCCCTTTTAGCGATCGTGCCGATCGCACCGTGGTTGCCGGGCAGAGTTTTGGTGGGCTTTCCGCGCTG
TATGCCGGACTGCACTGGCCTGAACGCTTTGGCTGTGTATTAAGCCAGTCAGGATCGTACTGGTGGCCGCATCGGGGCGG

GCAGCAAGAGGGCGTGTTACTTGAAAAGCTAAAAGCTGGTGAAGTTAGCGCCGAAGGTCTGCGCATTGTGCTGGAAGCGG
GTATTCGCGAGCCGATGATCATGCGGGCCAATCAGGCGCTGTATGCGCAATTACACCCCATAAAAGAATCCATTTTCTGG
CGTCAGGTTGACGGCGGACATGATGCGCTTTGTTGGCGCGGTGGCTTGATGCAGGGGCTAATCGACCTCTGGCAACCACT
TTTCCATGACAGGAGTTGAATATGGCATTCAGTAATCCCTTCGATGATCCGCAGGGAGCGTTTTACATATTGCGCAATGC
GCAGGGGCAATTCAGTCTGTGGCCGCAACAATGCGTCTTACCGGCAGGCTGGGACATTGTGTGTCAGCCGCAGTCACAGG
CGTCCTGCCAGCAGTGGCTGGAAGCCCACTGGCGTACTCTGACACCGACGAATTTTACCCAGTTGCAGGAGGCACAATGA
GCCAGCATTTACCTTTGGTCGCCGCACAGCCCGGCATCTGGATGGCAGAAAAACTGTCAGAATTACCCTCCGCCTGGAGC
GTGGCGCATTACGTTGAGTTAACCGGAGAGGTTGATTCGCCATTACTGGCCCGCGCGGTGGTTGCCGGACTAGCGCAAGC
AGATACGCTGCGGATGCGTTTTACGGAAGATAACGGCGAAGTCTGGCAGTGGGTCGATGATGCGCTGACGTTCGAACTGC
CAGAAATTATCGACCTACGAACCAACATTGATCCGCACGGTACTGCGCAGGCATTAATGCAGGCGGATTTGCAACAAGAT
CTGCGCGTCGATAGCGGTAAACCACTGGTCTTTCATCAGCTGATACAGGTGGCGGATAACCGCTGGTACTGGTATCAGCG
TTATCACCATTTGCTGGTCGATGGCTTTAGTTTCCCGGCCATTACCCGCCAGATCGCCAATATTTACTGCACATGGCTGC
GTGGCGAACCAACGCCTGCTTCGCCATTTACGCCTTTCGCTGATGTAGTGGAAGAGTACCAGCAATACCGCGAAAGCGAA
GCCTGGCAGCGTGATGCGGCATTCTGGGCAGAACAGCGTCGTCAACTGCCGCCGCCCGCGTCACTTTCTCCGGCACCTTT
ACCGGGGCGCAGCGCCTCGGCAGATATTCTGCGCCTGAAACTGGAATTTACCGACGGGGAATTCCGCCAGCTGGCTACGC
AACTTTCAGGTGTGCAGCGTACCGATTTAGCCCTTGCGCTGGCAGCCTTGTGGCTGGGGCGATTGTGCAATCGTATGGAC
TACGCCGCCGGATTTATCTTTATGCGTCGACTGGGCTCGGCGGCGCTGACGGCTACCGGACCCGTGCTCAACGTTTTGCC
GTTGGGTATTCACATTGCGGCGCAAGAAACGCTGCCGGAACTGGCAACCCGACTGGCAGCACAACTGAAAAAAATGCGTC
GTCATCAACGTTACGATGCCGAACAAATTGTCCGTGACAGCGGGCGAGCGGCAGGTGATGAACCGCTGTTTGGTCCGGTA
CTCAATATCAAGGTATTTGATTACCAACTGGATATTCCTGATGTTCAGGCGCAAACCCATACCCTGGCAACCGGTCCGGT
TAATGACCTTGAACTGGCCCTGTTCCCGGATGTACACGGTGATTTGAGTATTGAGATCCTCGCCAATAAACAGCGTTACG
ATGAGCCAACGTTAATCCAGCATGCTGAACGCCTGAAAATGCTGATTGCCCAGTTCGCCGCGGATCCGGCGCTGTTGTGC
GGCGATGTCGATATTATGCTGCCAGGTGAGTATGCGCAGCTGGCGCAGCTCAACGCCACTCAGGTTGAGATTCCAGAAAC
CACGCTTAGCGCGCTGGTGGCAGAACAAGCGGCAAAAACACCGGATGCTCCGGCGCTGGCAGATGCGCGTTACCTGTTCA
GCTATCGGGAAATGCGCGAGCAGGTGGTGGCGCTGGCGAATCTGCTGCGTGAGCGCGGCGTTAAACCAGGGGACAGCGTG
GCGGTGGCACTACCGCGCTCGGTCTTTTTGACCCTGGCACTCCATGCGATAGTTGAAGCTGGAGCGGCCTGGCTACCGCT
GGATACCGGCTATCCGGACGATCGCCTGAAAATGATGCTGGAAGATGCGCGTCCGTCGCTGTTAATTACCACCGACGATC
AACTGCCGCGCTTTAGCGATGTTCCCAATTTAACAAGCCTTTGCTATAACGCCCCGCTTACACCGCAGGGCAGTGCGCCG
CTGCAACTTTCACAACCGCATCACACGGCTTATATCATCTTTACCTCTGGCTCCACCGGCAGGCCGAAAGGGGTAATGGT
CGGGCAGACGGCTATCGTCAACCGCCTGCTTTGGATGCAAAATCATTATCCGCTTACAGGCGAAGATGTCGTTGCCCAAA
AAACGCCGTGCAGTTTTGATGTCTCGGTGTGGGAGTTTTTCTGGCCGTTTATCGCAGGGGCAAAACTGGTGATGGCTGAA
CCGGAAGCGCACCGCGACCCGCTCGCTATGCAGCAATTCTTTGCCGAATATGGCGTAACGACCACGCACTTTGTGCCGTC
GATGCTGGCGGCATTTGTTGCCTCGCTGACGCCGCAAACCGCTCGCCAGAGTTGCGCGACGTTGAAACAGGTTTTCTGTA
GTGGTGAGGCCTTACCGGCTGATTTATGCCGCGAATGGCAACAGTTAACTGGCGCGCCGTTGCATAATCTATATGGCCCG
ACGGAAGCGGCGGTAGATGTCAGCTGGTATCCGGCTTTTGGCGAGGAACTGGCACAGGTGCGCGGCAGCAGTGTGCCGAT
TGGTTATCCGGTATGGAATACGGGTCTGCGTATTCTTGATGCGATGATGCATCCGGTGCCGCCGGGTGTGGCGGGTGATC
TCTATCTCACTGGCATTCAACTGGCGCAGGGCTATCTCGGACGCCCCGATCTGACCGCCAGCCGCTTTATTGCCGATCCT
TTTGCCCCAGGTGAACGGATGTACCGTACCGGAGACGTTGCCCGCTGGCTGGATAACGGCGCGGTGGAGTACCTCGGGCG
CAGTGATGATCAGCTAAAAATTCGCGGGCAGCGTATCGAACTGGGCGAAATCGATCGCGTGATGCAGGCGCTGCCGGATG
TCGAACAAGCCGTTACCCACGCCTGTGTGATTAACCAGGCGGCTGCCACCGGTGGTGATGCGCGTCAATTGGTGGGCTAT
CTGGTGTCGCAATCGGGCCTGCCGTTGGATACCAGCGCATTGCAGGCGCAGCTTCGTGAAACATTGCCACCACATATGGT
ACCGGTGGTTCTGCTGCAACTTCCACAGTTACCACTTAGCGCCAACGGCAAGCTGGATCGCAAAGCCTTACCGTTGCCTG
AACTGAAGGCACAAGCGCCAGGGCGTGCGCCGAAAGCGGGCAGTGAAACGATTATCGCCGCGGCATTCTCGTCGTTGCTG
GGGTGTGACGTGCAGGATGCCGATGCTGATTTCTTCGCGCTTGGCGGTCATTCGCTACTGGCAATGAAACTGGCAGCGCA
GTTAAGTCGGCAGGTTGCCCGCCAGGTGACGCCGGGGCAAGTGATGGTCGCGTCAACTGTCGCCAAACTGGCAACGATTA
TTGATGCTGAAGAAGACAGCACCCGGCGTATGGGATTCGAAACCATTCTGCCGTTGCGTGAAGGTAATGGCCCGACGCTG
TTTTGTTTCCATCCTGCGTCCGGTTTTGCCTGGCAGTTCAGCGTGCTCTCGCGTTATCTCGATCCACAATGGTCGATTAT
CGGCATTCAGTCACCGCGCCCCAATGGCCCCATGCAGACGGCGGCAAACCTGGATGAAGTCTGCGAAGCGCATCTGGCAA
CGTTACTTGAACAACAACCGCATGGCCCTTATTACCTGCTGGGGTATTCCCTTGGCGGTACGCTGGCGCAGGGTATTGCG
GCGCGACTGCGTGCCCGTGGCGAACAGGTGGCATTTCTTGGCTTGCTGGATACCTGGCCGCCAGAAACGCAAAACTGGCA
GGAAAAAGAAGCTAATGGTCTGGACCCGGAAGTGCTGGCGGAGATTAACCGCGAACGCGAGGCCTTCCTGGCAGCACAGC
AGGGAAGTACTTCAACGGAGTTGTTTACCACCATTGAAGGCAACTACGCTGATGCTGTGCGCCTGCTGACGACTGCTCAT
AGCGTACCGTTTGACGGTAAAGCGACGCTGTTTGTTGCTGAACGCACACTTCAGGAAGGTATGAGTCCCGAACGCGCCTG
GTCGCCGTGGATAGCGGAGCTGGATATCTATCGTCAGGATTGTGCGCATGTGGATATTATCTCTCCAGGGACGTTTGAAA
AAATTGGGCCGATTATTCGCGCAACGCTAAACAGGTAAATTAATATTATTTATAAACCCATAATTACAGAAAATAATTAT
GGGTTTTTTATTTGTTTGATTTATAGGTTTGATGAATATTTCTCTTAAATAGAGTGAATGTTGCATTCAGTGGAAGGAAT

TTACAAACTTCAGCTTCGGATAATTATCACCAACTGGTAAAGTGAGCGCCGTGGAGATTTCCCCCTATTGGACTCATTTT
CAGGTTATACCCCATGTCATCACTGAATATTAAACAGGGAAGTGACGCTCATTTTCCCGATTATCCTCTGGCGTCGCCCA
GTAATAATGAAATTGATTTACTTAATCTAATCTCAGTTTTATGGCGGGCCAAAAAAACGGTCATGGCGGTCGTTTTTGCG
TTTGCCTGCGCAGGCTTGCTGATCTCTTTCATCCTGCCGCAAAAATGGACCAGCGCGGCGGTTGTCACGCCTCCAGAACC
TGTTCAGTGGCAAGAGTTGGAGAAATCATTCACTAAGCTTCGTGTGCTGGATCTGGATATCAAAATTGATCGTACAGAAG
CATTTAACCTGTTTATCAAGAAGTTTCAGTCGGTTAGCTTGCTGGAAGAGTACCTGCGTTCATCACCTTATGTGATGGAC
CAATTAAAAGAGGCGAAAATCGACGAACTGGATTTGCATCGCGCAATTGTCGCATTGAGCGAAAAAATGAAAGCGGTTGA
TGACAATGCCAGTAAGAAAAAAGATGAACCGTCACTGTATACCTCCTGGACGCTAAGTTTTACCGCGCCAACCAGTGAAG
AGGCGCAGACCGTTTTGAGCGGGTATATCGATTATATCTCTACGTTGGTGGTGAAAGAGTCGCTAGAAAACGTCCGTAAT
AAACTGGAGATCAAAACCCAGTTTGAAAAAGAAAAACTGGCTCAGGATCGCATTAAAACGAAAAATCAACTTGATGCAAA
CATTCAGCGCCTCAATTATTCACTCGACATTGCCAACGCGGCAGGAATTAAAAAGCCCGTTTACAGTAATGGTCAGGCCG
TTAAAGATGACCCCGATTTTTCTATTTCTCTCGGTGCAGACGGTATTGAACGCAAACTGGAAATAGAAAAAGCGGTCACT
GACGTTGCGGAACTGAACGGTGAATTACGTAATCGGCAGTATCTTGTCGAGCAATTAACAAAAGCACATGTCAACGATGT
GAATTTTACGCCGTTTAAATATCAGTTAAGCCCGTCATTGCCAGTGAAAAAAGACGGTCCGGGTAAGGCGATTATTGTGA
TCCTTTCCGCGTTGATCGGCGGGATGGTGGCTTGTGGTGGCGTGCTGTTGCGCTATGCGATGGCATCCAGAAAACAGGAT
GCCATGATGGCAGACCACTTAGTTTAACTATTTGCGGTTGAAGGTGCCGTTCGTCCGAGCGGCACCACAAGCGGCGTTCC
GGCAACTGGATCGTCAATGATCATGCAGCGCAGACCATAAATGCGCTCAATCAGTTCAGCAGTGACAATCTCCTTCGGCG
CTCCCTGAGCAACAATTTTCCCTTCCCGCAATGCAATCAAATGGCTGGCGTAACGACAGGCCTGATTAAGATCGTGCAGC
ACCGCCGCCAGGGTATAGCCTTTTTCGCGGTTCAGTTCGCTTAACAACTCCAGCAAATCAATCTGATGACTGATATCCAG
CCAGGTCGTGGGTTCGTCGAGCAGCATAATTGCCGTTTCCTGGGCCAGCACCATCGCGATCCACGCTCGCTGGCGTTGTC
CGCCAGAAAGGGTATCCACGCTTTGATCTGCCAGATGAGTTATTCCCGTGGCCTGCATTGCTTTCGTTACCGCTTCTTCA
TCCTCTTTGCGCCAGCGGGTAAACAGCGGTTGATGCGGATAACGTCCACGCGCCACCAGCTCCTGCACGGTGATATCGCC
CGGCGTGGTAGCATTTTGCGCCAACAGACCAATCCGGCGTGCAACCTCTTTACTGGCGTAATGTTGAATGTGCTCGCCAT
CCAGCCAGACATGCCCATGAGCAGGCGTCATCAGGCGGCTTAAGGTACGCAGTAACGTGGATTTACCGCAGCCATTTGGC
CCGATAATTGCCGTGAAGTGACCATCAGGTATTTCTACAGTCAGATTTTCCGCAACGGTATATTTGCCATATCCCAGGGT
TAACTGTTCGCCGCGCAAACGGGCTACTGATTCGGTCATTTTTTGCGAGACTCCTGAATTAACAAGACGATAAGGTAAAT
ACCGCCGAGGCTGACGGTAACGACGCCAACCGGAAGTTGATACGGCATAAACAGTTGTTGGGCGCAGAGATCGGCCGCCA
GCAGTAACAGCGCCCCGCATAGCGCCGCCTGGGTTAGCCCCCAGCGAGCGGTGCCGCTAATGCGTCGGGCAATGTGCGGT
GCGACTAAAGCAATAAAGGAAATCGGCCCGGCAAGCGCTGTTGCCGCAGCGGTAAGCACCACTGCAACCAGCATCATTAA
CAGACGCGAACGTTCGACGCTGACGCCCAGCGCACACGCGGTATCATCGCCCATTTCCAGCAAGCGCATCCGGCGTACCA
GTAAGGCGGCGGCAATGAGCATCAATATAATGATGGGTGCGGAAGGCGAGGTTTTTGCCCACGTCAGGCCGTTGAGCGAT
CCGGCATTCCACAAACCTGCTGTTAGCGCCGTTTCTAAAGACGCTTTCAGCAACAGCCAGGTATTAAAGGCCACCAGCAT
GGCGCGAACGCCGATACCGATAATAATCAACCGAAAGGTGTCGATGCCGTTGCGCCAGGCGAGCAGCCAGACCAGCAGCG
AAGTGACAATGCCGCCCACCATTGCTGACAGCGCGATAGCCGTCAGGTCCTGACCAAACAGCACCATCGCCACCAGCACG
CCGCTCCACGCCCCGGTGTTAAAGCCCATTACGTCAGGGCTGCCGAGCGGGTTACGCATCAGCGACTGAAAAATCGCGCC
ACTGACGCCCAGTGCTGCGCCAATCAACAGCGCCATCAGCACGCGTGGTAAACGCCATTCGGTGACCACCATCGTCATAC
TGCGCGGCGCATCGCCCATCAGCGCGGCGAATACCTGCGAGGTTTCCAGCGTGACGGCACCGCTGCGTAATCCCCAGATA
CCTGCAACCACACAGGCGGAAACCAGCAGCAAACAGGTGATGAGTAATCGGCGAGAGACGTAAATCATGCACCACCTCGC
GTTTTACGTCGCACGAGGAAGATCAGCACCGGTGCACCAATAAACGCACTGACCACAGAAACGCGCAGTTCGCCGGGCAC
AATCACCCGCCCGATGATATCGGCAAACAGCAGCAGGGCAGGGGTAGCAAGTAGCGTGACGGGCAGCGACCAGCGATGAT
CGGCACCCACCAGCCAACGCGCCATATGCGGCATCATCAGGCCAATAAAGGCAATCGGGCCAACTATTGCCGTCGCACTA
CCACAAAGCACGGTAATCGCCAGCAGACCAATCAACTGTGTGCGCGCCACGCGACTGCCCAGCGCCGTCGCGGTGTCGCT
GCCGAGGCTCAAACTGTTCAGCGCGCGACTCAGTAATAGCGCAGTTGCTCCGGCGATCAGCACCGGGATCAGCACCACTT
TTAAGGTATGTAGATTGCGAATATCCAGCGAACCGGCTTGCCAGAAACGCAACTGATCGTAGACGTCAGGATTAAGCAGG
GCGATGCCGCTGGTCAGTCCTTCCAGCACCGCCGCCAGCGCCACGCCCGCCAGGGTTAAACGCACCGGACTTAACTGCCC
GCCGCCCTGACTGCCGGTAAAGGCAACAATCAATGAGGCCACCAGCGCCCCGGCGAAGGCCATCGCCAGTTGTTCCTGCG
CGGAAGAGTAACCAAACAGCGCCGCACCCAGCACAATGGCAAAGCTGGCTCCGGCGTTCACGCCAAGCAAGCCGGGGTCG
GCAAGTGGGTTTCGGGTGAGGGTTTGCATTAACGCCCCGGCAAGGCCAAGCGCGCCGCCTGCCAGTAAACCGGCAAGGGT
ACGCGGCAGCCGCGCGTCGAGCACGATGGTGCAGTCGGCGCTCTGGCAGGTGCCGGAGAAGGCCTCCAGCACTACGGAAG
CGGGGAGTGATTTTGCCCCAATGAGCAGGCTTAATGCCGTCGCGATAATCAGTAATAACAGCAATCCGGGCACGGCAATG
GCGCGTGTCACGGCAACAGAACCAGACATACGAACTTCCATGATAATGAAATTAATTATCGTTATCGATCTTATTTGGAT
ATGTTAGCATGTGCAGCCTAAGAATAGGTATTTAAAATATTTGATGGCAAGGCATTGTAATGAATAAACAATCCTGGCTG
CTTAACCTCAGCCTGTTGAAAACGCACCCGGCGTTTCGCGCAGTATTCCTCGCTCGTTTCATCTCAATTGTGTCTCTGGG
TTTGCTCGGCGTCGCGGTGCCGGTGCAGATCCAGATGATGACACATTCCACCTGGCAGGTGGGGCTTTCGGTGACGCTGA
CCGGCGGCGCGATGTTTGTTGGCCTGATGGTCGGCGGTGTGCTGGCGGATCGCTATGAGCGCAAAAAAGTGATTTTGCTG
GCGCGCGGCACCTGTGGCATTGGCTTCATTGGACTGTGCCTTAATGCACTGCTGCCGGAGCCGTCATTGCTGGCAATCTA

TTTACTTGGTTTATGGGATGGTTTTTTCGCATCGCTTGGCGTTACGGCGCTATTGGCGGCGACACCAGCACTGGTAGGGC
GTGAAAACTTAATGCAGGCCGGGGCGATCACCATGTTGACCGTGCGTCTGGGGTCGGTGATTTCGCCCATGATTGGCGGT
TTATTGCTGGCGACCGGTGGCGTAGCCTGGAACTACGGGCTGGCGGCGGCGGGCACGTTTATTACCTTGCTACCGTTGTT
AAGCCTTCCGGCGTTGCCACCGCCACCGCAGCCGCGTGAGCATCCGTTGAAATCATTACTGGCAGGATTTCGTTTTCTGC
TTGCCAGCCCGCTGGTGGGCGGGATTGCGCTGCTGGGTGGTTTATTGACGATGGCGAGCGCGGTGCGGGTACTGTATCCG
GCGCTGGCTGACAACTGGCAGATGTCAGCGGCACAGATTGGTTTTCTCTACGCGGCGATCCCGCTCGGCGCGGCTATTGG
TGCGTTAACCAGCGGGAAGCTGGCACATAGTGCGCGACCAGGGTTATTGATGCTGCTCTCCACGCTGGGATCGTTCCTCG
CCATTGGTCTGTTTGGCCTGATGCCGATGTGGATTTTAGGCGTGGTTTGTCTGGCGCTGTTCGGCTGGTTGAGTGCGGTC
AGCTCGTTGCTGCAATACACAATGCTGCAAACGCAAACCCCGGAAGCGATGTTAGGGCGGATTAACGGTTTGTGGACGGC
GCAGAACGTGACGGGCGATGCCATAGGCGCGGCGCTGCTGGGTGGTTTGGGCGCGATGATGACACCGGTTGCTTCCGCAA
GCGCGAGCGGTTTTGGTTTGTTGATTATCGGCGTGTTGTTATTGCTGGTGCTGGTGGAGTTGCGACATTTTCGCCAGACG
CCGCCGCAGGTGACAGCGTCCGACAGTTAATGCTTAAAACAGCGCCTTAAGCCTATCCAGCACTTGCATGGCGCTGTAGT
AATCCAGACGGAACGTCTCGGTTCCCAGCGCATAAACCTGCTTGTTTTGTACTGCAGGCAGGTGCGCGAGCAGCGGATTA
GCATAAATAGCATCGGCATCTTTCTGATCACCGGCGAACAGGAATAGTGACTCGCCATTTAACCCTGCAGCCAGATTTTC
CCCACCAAGCTGAATGATGTCATGGCGTTTACCCTGACTTTGGCTGGCATTTAACCCTGCGGGTAACTTCGCCAGCGTAA
AGCCGAGTTGTTCCAGCATCTGCCCTTGTGCTGATTCTGGCGTCCAGAGATTGGCACTGTGTGCAGCGGCAGTATAGACA
ATGGCAGTGACCGGCTGCGGCGGTAATTTGATTTGCTCTTTCGCCGCCGCCAGTTGCTTATCAAACTGCGCAATCCGCTC
TGCCGCTTGTTTCTCATGCCCGGTAATTTCGCCAAGTTGCGTTAACAGCGACTGCCAGCTTTTGTCGTCGTAATTGATGA
TTAATGTCGGGGCGATGGTGGAAAGCTGATCATACAGTGCCAGCGCCGAATCCCCGCCGGTTGCGCTAATTAAAATCAGA
TCCGGCATTTGCGCGGCAACGGCTTCGGCGCTCGGTTCGCCGATATAGAGCCGTTGCAGTTTGCGTTCTTTCGCCACCTT
GCTCCACTGGCGTAAAAAGCCCTGGTCATCCGCGACGCGGTTATTCGGCGTGGTCGCGCCGCTGGCGATCACCGGAGCAT
CAATCGCCAGCAGTGAGCCGGTCAGGGTGACGCTGGTGGAAACAATACGCTGCGGCTGGCTTTCCAGTGTATGTGTGCCA
CGGCTGTCAGTAATCTGACGCGGCCAGTCAGCGGCCTGAACTGCGGCTATTCCTGAAAGCAAAAGTCCTGTTAATAGAAG
GGCGTTGCGGTAGAGCGGGGCGAGTCTCACAAATCAGCTTCCTGTTATTAATAAGGTTAAGGGCGTAATGACAAATTCGA
CAAAGCGCACAATCCGTCCCCTCGCCCCTTTGGGGAGAGGGTTAGGGTGAGGGGAACAGCCAGCACTGGTGCGAACATTA
ACCCTCACCCCAGCCCTCACCCTGGAAGGGAGAGGGGGCAGAACGGCGCAGGACATCACATTGCGCTTATGCGAATCCAT
CAATAATGCTTCTCATTTTCATTGTAACCACAACCAGATGCAACCCCGAGTTGCAGATTGCGTTACCTCAAGAGTTGACA
TAGTGCGCGTTTGCTTTTAGGTTAGCGACCGAAAATATAAATGATAATCATTATTAAAGCCTTTATCATTTTGTGGAGGA
TGATATGGATACGTCACTGGCTGAGGAAGTACAGCAGACCATGGCAACACTTGCGCCCAATCGCTTTTTCTTTATGTCGC
CGTACCGCAGTTTTACGACGTCAGGATGTTTCGCCCGCTTCGATGAACCGGCTGTGAACGGGGATTCGCCCGACAGTCCC
TTCCAGCAAAAACTCGCCGCGCTGTTTGCCGATGCCAAAGCGCAGGGCATCAAAAATCCGGTGATGGTCGGGGCGATTCC
CTTCGATCCACGTCAGCCTTCGTCGCTGTATATTCCTGAATCCTGGCAGTCGTTCTCCCGTCAGGAAAAACAAGCTTCCG
CACGCCGTTTCACCCGCAGCCAGTCGCTGAATGTGGTGGAACGCCAGGCAATTCCGGAGCAAACCACGTTTGAACAGATG
GTTGCCCGCGCCGCCGCACTTACCGCCACGCCGCAGGTCGACAAAGTGGTGTTGTCACGGTTGATTGATATCACCACTGA
CGCCGCCATTGATAGTGGCGTATTGCTGGAACGGTTGATTGCGCAAAACCCGGTTAGTTACAACTTCCATGTTCCGCTGG
CTGATGGTGGCGTCCTGCTGGGGGCCAGCCCGGAACTGCTGCTACGTAAAGACGGCGAGCGTTTTAGCTCCATTCCGTTA
GCCGGTTCCGCGCGTCGTCAGCCGGATGAAGTGCTCGATCGCGAAGCAGGTAATCGTCTGCTGGCGTCAGAAAAAGATCG
CCATGAACATGAACTGGTGACTCAGGCGATGAAAGAGGTACTGCGCGAACGCAGTAGTGAGTTACACGTTCCTTCTTCTC
CACAGCTGATCACCACGCCGACGCTGTGGCATCTCGCAACTCCCTTTGAAGGTAAAGCGAATTCGCAAGAAAACGCACTG
ACTCTGGCCTGTCTGCTGCATCCGACCCCCGCGCTGAGCGGTTTCCCGCATCAGGCCGCGACCCAGGTTATTGCTGAACT
GGAACCGTTCGACCGCGAACTGTTTGGCGGCATTGTGGGTTGGTGTGACAGCGAAGGTAACGGCGAATGGGTGGTGACCA
TCCGCTGCGCGAAGCTGCGGGAAAATCAGGTGCGTCTGTTTGCCGGAGCGGGGATTGTGCCTGCGTCGTCACCGTTGGGT
GAGTGGCGCGAAACAGGCGTCAAACTTTCTACCATGTTGAACGTTTTTGGATTGCATTAAGGAGCGAGGATGAGCATTCC
ATTCACCCGCTGGCCGGAAGAGTTTGCCCGTCGCTATCGGGAAAAAGGCTACTGGCAGGATTTGCCGCTGACCGACATTC
TGACGCGACATGCTGCGAGTGACAGCATCGCGGTTATCGACGGCGAGCGACAGTTGAGTTATCGGGAGCTGAATCAGGCG
GCGGATAACCTCGCGTGTAGTTTACGCCGTCAGGGCATTAAACCTGGTGAAACCGCGCTGGTACAACTGGGTAACGTCGC
TGAATTGTATATTACCTTTTTCGCGCTGCTGAAACTGGGCGTTGCGCCGGTGCTGGCGTTGTTCAGCCATCAGCGTAGTG
AACTGAACGCCTATGCCAGCCAGATTGAACCCGCATTGCTGATTGCCGATCGCCAACATGCGCTGTTTAGCGGGGATGAT
TTCCTCAATACTTTCGTCACAGAACATTCCTCCATTCGCGTGGTGCAACTGCTCAACGACAGCGGTGAGCATAACTTGCA
GGATGCGATTAACCATCCGGCTGAGGATTTTACTGCCACGCCATCACCTGCTGATGAAGTGGCCTATTTCCAGCTTTCCG
GTGGCACCACCGGCACACCGAAACTGATCCCGCGCACTCATAACGACTACTACTACAGCGTGCGTCGTAGCGTCGAGATT
TGTCAGTTCACACAACAGACACGCTACCTGTGCGCGATCCCGGCGGCTCATAACTACGCCATGAGTTCGCCAGGATCGCT
GGGCGTCTTTCTTGCCGGAGGAACGGTTGTTCTGGCGGCCGATCCCAGCGCCACGCTCTGTTTCCCATTGATTGAAAAAC
ATCAGGTTAACGTTACCGCGCTGGTGCCACCCGCAGTCAGCCTGTGGTTGCAGGCGCTGATCGAAGGCGAAAGCCGGGCG
CAGCTTGCCTCGCTGAAACTGTTACAGGTCGGCGGCGCACGTCTTTCTGCCACCCTTGCGGCGCGTATTCCCGCTGAGAT
TGGCTGTCAGTTGCAGCAGGTGTTTGGCATGGCGGAAGGGCTGGTGAACTACACCCGACTTGATGATAGCGCGGAGAAAA

TTATCCATACCCAGGGTTACCCAATGTGTCCGGATGACGAAGTATGGGTTGCCGATGCCGAAGGAAATCCACTGCCGCAA
GGGGAAGTCGGACGCCTGATGACGCGCGGGCCGTACACCTTCCGCGGCTATTACAAAAGTCCACAGCACAATGCCAGCGC
CTTTGATGCCAACGGTTTTTACTGTTCCGGCGATCTGATCTCTATTGATCCAGAGGGTTACATCACCGTGCAGGGGCGCG
AGAAAGATCAGATTAACCGTGGCGGCGAGAAGATCGCTGCCGAAGAGATCGAAAACCTGCTGCTGCGCCACCCGGCGGTG
ATCTACGCCGCACTGGTGAGCATGGAAGATGAGCTGATGGGCGAAAAAAGCTGCGCTTATCTGGTGGTAAAAGAGCCGCT
GCGCGCGGTGCAGGTGCGTCGTTTCCTGCGTGAACAGGGTATTGCCGAATTTAAATTACCGGATCGCGTGGAGTGTGTGG
ATTCACTTCCGCTGACGGCGGTCGGGAAAGTCGATAAAAAACAATTACGTCAGTGGCTGGCGTCACGCGCATCAGCCTGA
AGGAGAGAACACGATGGCTATTCCAAAATTACAGGCTTACGCACTGCCGGAGTCTCACGATATTCCGCAGAATAAAGTTG
ACTGGGCCTTTGAACCGCAACGTGCCGCGTTGTTAATCCATGATATGCAGGACTATTTTGTCAGCTTCTGGGGCGAGAAC
TGCCCGATGATGGAGCAGGTGATCGCGAATATTGCTGCGCTGCGCGACTACTGCAAACAGCACAATATCCCGGTTTATTA
CACCGCCCAGCCGAAAGAGCAGAGCGATGAAGATCGGGCGCTGTTGAATGATATGTGGGGGCCGGGCCTGACCCGCTCGC
CGGAACAGCAAAAGGTGGTGGATCGCCTGACGCCAGATGCCGACGACACGGTGCTGGTGAAGTGGCGCTACAGCGCGTTT
CATCGTTCTCCGCTGGAGCAAATGCTGAAAGAGAGTGGACGTAACCAGCTGATTATTACCGGGGTATATGCCCACATTGG
CTGTATGACCACCGCAACCGACGCATTTATGCGCGATATTAAACCGTTTATGGTGGCGGATGCGCTGGCCGATTTCAGCC
GTGACGAGCATTTGATGTCGCTGAAATATGTGGCCGGACGTTCTGGCCGGGTGGTGATGACTGAAGAATTACTGCCAGCA
CCTATCCCCGCCAGCAAAGCGGCGCTGCGTGAGGTGATCCTGCCGTTGCTGGACGAGTCCGATGAACCGTTCGATGACGA
CAACCTGATCGACTACGGTCTGGATTCGGTGCGCATGATGGCGCTGGCGGCGCGCTGGCGCAAAGTGCATGGTGATATCG
ACTTTGTCATGCTGGCGAAAAACCCGACCATCGACGCCTGGTGGAAGCTACTCTCCCGCGAGGTGAAATAATGGATTTCA
GCGGTAAAAATGTCTGGGTAACCGGCGCAGGTAAAGGTATCGGCTACGCCACGGCGCTGGCGTTTGTTGAGGCGGGAGCG
AAAGTTACAGGTTTTGATCAAGCGTTCACTCAGGAGCAATATCCCTTTGCGACCGAAGTGATGGATGTTGCCGACGCTGC
GCAGGTCGCGCAAGTGTGTCAGCGACTGTTAGCTGAAACGGAGCGACTGGACGCGCTGGTCAATGCGGCGGGAATTTTAC
GCATGGGCGCGACCGATCAGCTCAGTAAAGAGGACTGGCAGCAGACTTTTGCGGTTAACGTCGGCGGTGCGTTTAACCTG
TTCCAGCAAACCATGAACCAGTTTCGCCGTCAGCGGGGCGGGGCGATTGTCACTGTGGCGTCCGACGCCGCGCACACGCC
GCGTATTGGCATGAGTGCTTATGGCGCATCGAAAGCGGCGCTGAAAAGCCTGGCGTTGAGCGTCGGGCTGGAACTGGCGG
GTAGCGGCGTGCGCTGTAATGTGGTTTCGCCTGGCTCCACCGACACCGATATGCAACGCACGCTGTGGGTGAGCGATGAC
GCCGAAGAACAGCGTATTCGCGGCTTTGGCGAGCAGTTTAAACTCGGCATTCCGCTGGGGAAAATCGCCCGTCCACAAGA
GATCGCCAACACGATTTTGTTCCTCGCCTCTGACCTCGCCAGCCATATTACCCTACAGGATATTGTGGTCGATGGCGGCT
CAACGCTGGGGGCATAAGCATGATCTGGAAACGCCATTTAACGCTCGACGAACTGAACGCCACCAGCGATAACACAATGG
TGGCGCATCTGGGAATTGTGTATACCCGTCTGGGCGATGATGTGCTGGAAGCCGAAATGCCGGTTGATACCCGTACTCAT
CAGCCGTTCGGTTTACTACATGGCGGCGCGTCGGCGGCGCTGGCGGAAACGCTGGGATCGATGGCCGGATTTATGATGAC
CCGCGACGGACAGTGTGTGGTAGGCACAGAACTTAATGCAACACACCATCGCCCGGTGTCTGAGGGAAAGGTACGCGGCG
TCTGCCAGCCGCTGCATCTTGGTCGGCAAAATCAGAGCTGGGAAATCGTCGTTTTCGATGAACAGGGGCGGCGTTGCTGC
ACTTGTCGGCTGGGTACGGCAGTTTTGGGATGAACCCGACAGAATTAGATGAGATTGCAGGAAAACTCGGTTAACGGAGT
GATCGAGTTAACATTGTTAAGTTAAATATTGGTTTCAACTCCGATTTACATGGTTGCTGTGTTGTTAAATTGTACAAAGA
TGTTATAGAAACAAAATGTAACATCTCTATGGACACGCACACGGATAACAACTATGAACAAATCAGGGAAATACCTCGTC
TGGACAGTGCTCTCTGTAATGGGAGCATTTGCTCTGGGATACATTGCTTTAAATCGTGGGGAACAGATCAACGCGCTGTG
GATTGTGGTGGCGTCGGTCTGTATCTATCTGATCGCTTACCGTTTTTATGGGCTGTATATCGCCAAAAATGTGCTGGCGG
TTGACCCGACGCGTATGACGCCAGCGGTGCGCCATAACGACGGGCTGGACTATGTGCCGACGGACAAGAAAGTGCTGTTC
GGTCACCATTTTGCGGCCATTGCCGGAGCAGGTCCGCTGGTGGGGCCGGTACTGGCGGCGCAAATGGGCTACCTGCCGGG
GATGATCTGGCTGCTCGCTGGGGTGGTTCTCGCCGGTGCGGTGCAGGATTTCATGGTGCTGTTTGTTTCTACGCGCCGTG
ACGGTCGCTCGCTGGGTGAGCTGGTCAAAGAAGAGATGGGGCCAACCGCCGGGGTGATTGCGCTGGTGGCCTGCTTTATG
ATCATGGTCATTATCCTTGCAGTGCTGGCGATGATCGTGGTGAAAGCCCTGACTCATAGCCCGTGGGGAACATATACCGT
TGCGTTCACCATTCCGCTGGCGCTGTTTATGGGGATCTACCTGCGCTATCTGCGTCCGGGGCGTATTGGCGAAGTGTCGG
TCATCGGTCTGGTATTCCTGATTTTCGCCATTATCTCTGGCGGCTGGGTGGCAGAAAGTCCGACCTGGGCACCGTACTTT
GACTTTACCGGCGTGCAGTTGACCTGGATGCTGGTGGGTTACGGTTTTGTGGCGGCGGTGCTGCCGGTGTGGTTACTGCT
GGCCCCGCGTGACTACCTCTCTACCTTCCTGAAAATCGGGACTATCGTCGGTCTGGCGGTAGGCATTTTGATTATGCGCC
CGACGCTGACCATGCCTGCGCTGACCAAATTTGTCGATGGCACTGGCCCGGTATGGACCGGTAACCTGTTCCCGTTCCTG
TTTATCACCATCGCCTGTGGCGCGGTGTCTGGCTTCCATGCGCTGATCTCTTCTGGGACCACGCCGAAGATGCTGGCGAA
CGAAGGGCAGGCGTGCTTTATCGGCTACGGTGGGATGTTAATGGAATCCTTCGTGGCGATTATGGCGCTGGTTTCCGCCT
GTATCATCGATCCGGGCGTGTATTTTGCCATGAACAGCCCGATGGCGGTGCTGGCTCCGGCAGGGACGGCGGATGTGGTC
GCTTCTGCCGCGCAGGTGGTGAGTAGCTGGGGCTTTAGCATTACACCAGATACGCTAAACCAGATTGCCAGCGAAGTGGG
TGAACAGTCGATCATTTCCCGTGCGGGCGGTGCGCCGACGCTGGCGGTGGGGATGGCCTACATTCTGCACGGCGCGCTGG
GCGGCATGATGGATGTGGCGTTCTGGTATCACTTCGCCATTTTGTTTGAAGCACTGTTTATTCTGACGGCGGTGGATGCA
GGTACGCGTGCTGCGCGCTTTATGTTGCAGGATCTGCTGGGCGTGGTGTCTCCTGGCCTGAAGCGGACCGATTCACTGCC
TGCTAACCTGCTGGCAACAGCGCTGTGCGTACTGGCGTGGGGCTACTTCCTCCATCAGGGCGTGGTCGATCCGCTGGGCG
GCATTAACACTCTGTGGCCGCTGTTTGGTATTGCCAACCAGATGCTGGCAGGGATGGCGCTGATGCTCTGTGCCGTGGTG

TTGTTCAAGATGAAACGTCAACGTTACGCCTGGGTGGCGCTGGTACCAACGGCCTGGCTGCTGATTTGTACCCTGACCGC
AGGCTGGCAGAAAGCGTTTAGCCCGGATGCGAAAGTCGGCTTCCTGGCCATTGCTAATAAGTTCCAGGCAATGATCGACA
GCGGCAATATTCCGTCGCAGTATACTGAGTCACAGCTGGCGCAACTGGTGTTCAACAACCGTCTGGATGCCGGGTTAACC
ATCTTCTTTATGGTGGTCGTGGTGGTTCTGGCACTGTTCTCGATTAAGACGGCACTTGCGGCATTGAAAGATCCGAAGCC
AACGGCGAAAGAAACGCCGTATGAACCAATGCCGGAAAATGTCGAGGAGATCGTGGCGCAGGCAAAAGGCGCACACTAAA
GTCAGAGTGAGGGGGGGATGTTGGCGAATGTTGGCTTAGTGCCCAGGGTTCCCTCTCACCCTAACCCTCTCCCCGGTGGG
GCGAGGGGACTGACCGAGCGCGTTGATAGCATTTGTAGGCCGGATAAGGCGTTCACGCCGCATCCGGCACTCTTTCAGCA
ACATGGTTAGCGGAGGCCAAGATGTTTGATTCACTGGCAAAAGCCGGAAAATATTTAGGTCAGGCGGCGAAGCTGATGAT
TGGTATGCCTGATTACGACAACTATGTCGAACATATGCGGGTTAACCATCCCGATCAAACGCCGATGACCTACGAAGAGT
TTTTCCGTGAGCGGCAGGACGCGCGCTACGGTGGAAAAGGCGGCGCGCGCTGCTGCTAAATTTGTACGTCAGGCTTTAAA
CGATTCCACTTTTTTGAACGCTGCACGCAACGTATCTGGTGTCAGCGTGACTGGCAGGTAATGAATGGACTCCACCGGAC
GCAGGGTGTGGGCAATCACTTTGTCGATCTCCGCCTGATTATTGATATCCACTTCCAGCTCCGCCAGTGTAGTCGGCAGA
TGAAAACGCTGATACGCTCCAGTTAATTGCGCCAGCACATCATCCTGACCCAGCAAGGCGCTTTGCACCAGAATTCCGTA
GGCGACTTTGGTGCCGTGGAGAAACTTCTCGGTTTGCGGCAGCACGGTCAGACCGTTATGCACGGCATGAGCTGCCGCCA
CACGCGTAAAACGATCGCCCAGACCACCAACCATCCCACCACCAGCAATAATAGCATCCACCACATCGCAAAATGATTGC
GTTAACTGTTGATTTTGCTGATCGCTCAGCGCCTGTTCGCTACTGTTTAACAAGACGTCGCGAATGGCTTGCGCATTATT
GATCCCCAGTCGCACGGTTAGCGGCAACGTTTCTGGTTGCGGAGCCAGCACCACCGCTTCATACCATTTCGCCAGCGTGT
CACCGATCCCCGCCAGCAGATATTGTTGCGGTGCATTGAGGATAATCTCCGGTTCCACCAGCACCATAAAATTGGCGTCG
TCGAAAATCTCATAATGCAGCGCCTGTCCGGCATCATTATACCAGACGGAGAGCGGTGTCCAGGCGGCGCAGGTGGCGGC
GATCGTCGGAACGGCAACAAACGGCAGACCGAGACGGCGGGCGAGGGCTTTCGCGGTGTCGAGCAGTGCACCGCCACCGA
CGCCAATCACCACGCTGCGGTCGTCACCGGACTCAGCCGCCAGTTGTTGTACATCGCTTTCGCTGCAATGACCGCGAAAC
AAAATATGCTTTGCCCCTGGCAGTCCAAACGCTGGCGGAAGTTTGGTTTGCGCCGCAGCAATGGCGCGTTTGCCGTAGAT
CCACACCGCGCGAGAAAGTTGTTCATCAGTGAAAAAATCGTGCAGGTGATTGAAACTTCCTGGATGTGAAAAGTAGTTAG
CCGGGCCGACGACCACGCGGATAGGATTGTGAGGCATGTTGTGTTGTCCTTATTGTTTTATTTAGACATCTAAACGTCTT
GATTGCCAAATACTAGCATCGTGTTATAGTGCCTTCAACACGCAACTTCGTCAGGTACAATAAAAATGACAAATAACCCT
CTGATTCCACAAAGCAAACTTCCACAACTTGGCACCACTATTTTCACCCAGATGAGCGCGCTGGCGCAGCAACACCAGGC
GATTAACCTGTCGCAAGGCTTTCCTGATTTTGATGGTCCGCGCTATTTACAGGAGCGGCTGGCGCACCACGTTGCACAGG
GGGCAAACCAATACGCGCCCATGACCGGCGTGCAGGCCTTGCGCGAGGCGATTGCTCAGAAAACGGAACGTTTGTATGGC
TATCAACCAGATGCCGATAGCGATATCACCGTAACGGCAGGGGCGACGGAAGCGTTATACGCGGCGATTACCGCACTGGT
GCGCAATGGCGATGAAGTGATTTGTTTTGATCCCAGCTATGACAGTTACGCCCCCGCCATCGCGCTTTCTGGGGGAATAG
TGAAGCGTATGGCACTGCAACCACCGCATTTTCGCGTTGACTGGCAGGAATTTGCCGCATTATTAAGCGAGCGCACCAGA
CTGGTGATCCTCAACACTCCGCATAACCCCAGTGCAACTGTCTGGCAGCAGGCTGATTTCGCCGCTTTGTGGCAGGCGAT
CGCCGGGCACGAGATTTTTGTCATTAGCGATGAAGTCTACGAGCACATCAACTTTTCACAACAGGGCCATGCCAGTGTGC
TGGCGCATCCGCAGCTGCGTGAGCGGGCAGTGGCGGTTTCTTCATTTGGCAAGACCTATCATATGACCGGCTGGAAAGTG
GGTTATTGTGTTGCGCCAGCGCCCATCAGCGCCGAAATTCGCAAGGTACATCAGTATCTGACCTTTTCGGTGAATACCCC
GGCACAGCTGGCGCTTGCTGATATGCTACGTGCAGAACCTGAGCATTATCTTGCGTTACCGGACTTTTATCGCCAGAAGC
GCGATATTCTGGTGAATGCTTTAAATGAAAGCCGGCTGGAGATTTTACCGTGTGAAGGTACATACTTTTTGCTGGTGGAT
TACAGCGCGGTTTCTACCCTGGATGATGTTGAGTTTTGCCAGTGGCTGACGCAGGAGCACGGCGTAGCGGCGATTCCGCT
GTCGGTGTTTTGCGCCGATCCCTTCCCACATAAACTGATTCGTCTCTGTTTTGCCAAGAAGGAATCGACGTTGCTGGCAG
CAGCTGAACGCCTGCGCCAGCTTTAGCTATTTAACCGTCCAGGCGCGGGAATATTGACGGTCGGCAAACAACTCTTGCAG
GCCGTTAATTTGCTTCAGGCGCAGCACTTCGTCGCTGTCCATTCCCAGCTCTTTGCCAATTTTATTATCGTCCCATCCCA
ACTGGCTAAGTTCACGGACGATTTCCGACATGGCGGTGATTTGATGGCGTCCACGAGCGCGATTATGACGAATAGTCGCG
GCGATGCGTTGATTACGCGTACCTTCCAGACAGGTCACCGGCAAATAACCTTTTAAGCGTAATTTTAATGATGATGAGCC
TTTACCAATTTCATGGCGGTGAAAACCATCCACGATTTCCATGGCATTTTTATCCGTATGTGTGACCACAATGGGTTGGG
TAAAACCATCTATTTCAATGGATTTTTGCAGCAGTTTTTTTTCGGGTGGTGCCACGTTGTTTGGGTTGTAATCATTAGGC
ATAAGTTGGCTGTTTTTAACCCACAGAACGCAATCCACTGGCTCTTCACGAAAGGGGCTAACCTGATGGATCGCCATGCG
GATTTCATTAATCGCTTTGATGCGATCATCTTCCGGCAAGCTGGCGAGAAATTGCGTTAAATCCTGCGTTAATCGTTGTT
GCATAGTATCCCCCATTCCTGACGTTTTCCTTTTATCCGTTCGTTATAACGCTGATAGTTCTTCGATTTTGTGGGGCTAA
ATGATAATGCCCGACACCAGTAATCGTTATTGAGTAAAACTTTGCATATCCGCCGCCAGGAGGGGATATCTTTCGCGCCA
ATGTCCCCTTGCTGGGTTTGTGGAACCTCGATGCCTTTTTTCTGATACCAGTGCAAATAAATGGCGATTTTATTGCGGTA
ATGCTCAGCTGTTTTTTCCGGCATGCTATTGAGCAGCAATAATGCATATTCTTGCCAGTCTAAATGTTCTGGCTTGAGGA
TTTTCCGATGCCCATAGAAATGATTGTCATGTCCGGCGTAAATGCCGCCACTTTTTACCCCGCTGACTCGTGCGCACATA
GCAGCCCAGCGATCCGGTTCGATAACGTGATAGAGCCATAATCCTTGTCGTTGCTCCGGGCCAAAAGGTTCGCAAATTCG
CATATGACGCAGAGGAACGCCTGCCTGATACATTAGGTTATACAGTGGGTTGCAGAGACTTTGATGATTTGCATACCAGG
TCCAGATATCAGCCACTTTCCAGTCGTAAATGGGGTAAATGTACCAGCTATGACCGCCTGGTGCGGCCGTAGTCCAGGGT
TTATCGTCGGCAAAACGTTGTTTATTTAAACTGGCGATGGCGACAAAACGGTTGTAGGACTCATCCGCACGGATGCCGAT

CATCATCGCCGCCGGACGTTTTTGTGAAAACCATTCGGCAAACTCACGTACAAATTGTTCGAAGGTCATGCCTGGCTGGT
AAAAGCAGAAAAAGTCAGGGTCGGTTATCGCATCTTGGGGAGGTTGACGTACCCATTCGACATCAGGTTCCCAGCACTGC
CATTCGGGTTGGTATTGTGAAAGGGAATTTTGCGTCGTAAGCGGGAGCGCAACCCAGTAAAACTCTTCGATGACATCGGT
GTACAACTCGCGCAGGGACTGAACATAGTTAATGGTGCAAGAGAATTGCGCCTCCCAGTCGATAAACAAAACGCAGATTT
TTTTGCCCATTTGTCGGGCAAGTTCGGCTGTCAGATGCAGCATTAAACCAGAGTCTTTGCCGCCAGAAAATGAGACACAA
ACGCGGGGTAGGGTGTTGAGTGTCCAGGTAATACGCTCTCGTGCAGCTTCCAGAATATTGAGCGGAAGAGGAATTTTATA
AATAGACATCTGAAGACGCTCTCTGAACGTTACAGGGTGATCAGTGAGATAAGTCTGTTAATTATAAAGTAAACACTGTA
AAGCAGAGTGTTTTATTTTTCATTCGCGCTGTGAATAAATAGATGAAAGTTATAGTAGCTGTCTGTAGTTATATATCAAA
AACTCTCATTTAGACGGTCAATAAATCGGGAGAACACCAAATTATTTAAAGAAGATTTATTATAGGAAATGTAAAGCTTT
ATTGAAGGTAACGGATGTTCTAGTTTTATCTCTTTTAAGTTAAGAAAGTCACGGTAGGAATTATAAAGTTCATAGGGGAC
TATTCCCAGTAAATCTGTAACTGCAATGCTATTAATGATTGTCAGCAAAGAGGAACCACGAAATGAGATTTTGCGATTCA
TAAATCTCTCGTCTATTTCCATTTGAATATCATCAACCCCTGCTGATTTAGATATTAATTGGGTGAACTCTTCTGCCATA
ATTTGTTCATAAGTGCTATTGTCAGTAATTCGTGGATGCCTATTACTACAAATTAGAGTATTGCGAATCGTATGCAATGG
CATGCAAATGACGGAACGGCTGATCACTGGCATTTGGGTGATCACCAGATCGGCTTTGCGGTGAACCAACAGTTCTTCAG
CATTCTCTGCTGACATGAGGATATCATGGCACTCAATCTCGACAGAGGAATCTTGCCGTAGGCAGCGGATCAGCATACTA
TTATTAGAGCAAGAAATGAGCTGAGGGCCGTAGATAATAAAGTTTTTCTTTAGTTCGGACTTATTGACTATATTAATTGT
TTGTTCAAGGCCTCTGAGATTTTTCTCAAGATGGTGATGCAGATTTAACCCTGTTGTCGTCGGCGCGATACCTTTTCCTG
AGCGAATGAATAAAGGGTCATTAAATTGCGCACGTAAGCGTTGTAACGACTGGCTCACGGCTGATGGGGTGATATATAGT
GATTCAGCAGCTTTGCTAATGCTTAAATGCTGGTAAATACACTCAAATATGACAAGAAGATTTAAGTCGAACTTTTTCAA
GTCGTAGAGATTGGCCATACACTACTCCGGTTATATTTTTCTTCCATGATATATTCACTTAATCAATGTTTTTTGCGTAT
ATATTTTTATTGATTATGTTTTTTTGCTTAACTCAGGATTAAGTTTTTCTAAAATAGCACAGCCACTTAGGGCAGAGCGA
GCAATTCATGCTTTTTTAGGATGGTTGAAAAGCAGGGATATAGTTCAACCATCCATGAATTACCTTTTATTTATTCCCCA
TAATGATATTAAGCGTTTTCTGATCGGGCAACCCCACGGCCTGTTGTAGCGTATTTTCCTTACTCATGTAATAGATAGCC
GGCGTGACATTTGCCCCCAGATCGTCCATCAGTTTCTCATTGTCACTTAACACTTTCATTTGCTCTGTACTTACGTTTGC
AGGCACGTTTAGCTTAAGCTTGCCACCAGAGGCTTCATATTGTTGCCAGGTTTTTGCGGGATCTTTGGAGGCAAGAATTG
CCGCTGCTGTCGCCGGGCTTTCTGGCTTGATAACCCCAACCAACAATGTTCTTAATTGCACTTTGCCAGAATCTACCCAC
GGGCGCGCCTGCTGCCAGAACTGTTTACAATATGGGCAGAACGGATCGGCGAAGACGTAGACAATGACCGGCGCATCTTT
TTTACCGTCGAGGAGCCAGTGGGATTGTTCCATCCGTTGCCACATTTCGCGTCCGGCTGGTGCGTAAATTTCTTTTTCGA
TAAGTGTGTTACTCAGGTTTTCACCTTTCTCGTTGTACATGTAACCAGAGATAGCGTGCTTACCATCTGGAGTCAGGTAG
ATGGTGACGCCCATATCCTGATACTTTCCGAGATAACCTTTCATTCCTCCGGGGGCATCGAATGTTTTGATGATTGTAAT
GCCCTGTTTTTCAATCGCTTTTACTGGAGCAGGAAGTTCCTCTGCGAAGGCGATTGCAGGAAGCAGAGCCAGTAAAAGTA
TCTTTTTTAACATTAATTTGTCCTTTTCAGTCAGTGCAAAAGTCGAGTAAAAGGCATAACCTATCACTGTCATAGGTAAG
AGCTTAGATCAGGTGATTGCCCTTTGTTTATGAGGGTGTTGTAATCCATGTCGTTGTTGCATTTGTAAGGGCAACACCTC
AGCCTGCAGGCAGGCACTGAAGATACCAAAGGGTAGTTCAGATTACACGGTCACCTGGAAAGGGGGCCATTTTACTTTTT
ATCGCCGCTGGCGGTGCAAAGTTCACAAAGTTGTCTTACGAAGGTTGTAAGGTAAAACTTATCGATTTGATAATGGAAAC
GCATTAGCCGAATCGGCAAAAATTGGTTACCTTACATCTCATCGAAAACACGGAGGAAGTATAGATGTCCTTGATTAACA
CCAAAATTAAACCTTTTAAAAACCAGGCATTCAAAAACGGCGAATTCATCGAAATCACCGAAAAAGATACCGAAGGCCGC
TGGAGCGTCTTCTTCTTCTACCCGGCTGACTTTACTTTCGTATGCCCGACCGAACTGGGTGACGTTGCTGACCACTACGA
AGAACTGCAGAAACTGGGCGTAGACGTATACGCAGTATCTACCGATACTCACTTCACCCACAAAGCATGGCACAGCAGCT
CTGAAACCATCGCTAAAATCAAATATGCGATGATCGGCGACCCGACTGGCGCCCTGACCCGTAACTTCGACAACATGCGT
GAAGATGAAGGTCTGGCTGACCGTGCGACCTTCGTTGTTGACCCGCAGGGTATCATCCAGGCAATCGAAGTTACCGCTGA
AGGCATTGGCCGTGACGCGTCTGACCTGCTGCGTAAAATCAAAGCAGCACAGTACGTAGCTTCTCACCCAGGTGAAGTTT
GCCCGGCTAAATGGAAAGAAGGTGAAGCAACTCTGGCTCCGTCTCTGGACCTGGTTGGTAAAATCTAAATTTCCTTCGTC
TTTCACGCCATAGCGGCGTTGGCGTCGCCCGCTCACCCCGGTCACTTACTTGTGTAAGCTCCCGGGGATTCACAGGCTAG
CCGCCTTGCTCTGACGCGAAATACTTCGGAAATTCACCTAATTCTTCGGGTGCTGCGGCACCCGATTTTCTTCCCCGCAC
CATGATGCAAGCTGCATCCAGGTAGCCGCAGAGGCCGCTTGCATGATGATGTTTAAAGCCCAGGAGATAAACATGCTCGA
CACAAATATGAAAACTCAACTCAAGGCTTACCTTGAGAAATTGACCAAGCCTGTTGAGTTAATTGCCACGCTGGATGACA
GCGCTAAATCGGCAGAAATCAAGGAACTGTTGGCTGAAATCGCAGAACTGTCAGACAAAGTCACCTTTAAAGAAGATAAC
AGCTTGCCGGTGCGTAAGCCGTCTTTCCTGATCACCAACCCAGGTTCCAACCAGGGGCCACGTTTTGCAGGCTCCCCGCT
GGGCCACGAGTTCACCTCGCTGGTACTGGCGTTGCTGTGGACCGGTGGTCATCCGTCGAAAGAAGCGCAGTCTCTGCTGG
AGCAGATTCGCCATATTGACGGTGATTTTGAATTCGAAACCTATTACTCGCTCTCTTGCCACAACTGCCCGGACGTGGTG
CAGGCGCTGAACCTGATGAGCGTACTGAACCCGCGCATCAAGCACACTGCAATTGACGGCGGCACCTTCCAGAACGAAAT
CACCGATCGCAACGTGATGGGCGTTCCGGCAGTGTTCGTAAACGGGAAAGAGTTTGGTCAGGGCCGCATGACGTTGACTG
AAATCGTTGCCAAAATTGATACTGGCGCGGAAAAACGTGCGGCAGAAGAGCTGAACAAGCGTGATGCTTATGACGTATTA
ATCGTCGGTTCCGGCCCGGCGGGTGCAGCGGCAGCAATTTACTCCGCACGTAAAGGCATCCGTACCGGTCTGATGGGCGA
ACGTTTTGGTGGTCAGATCCTCGATACCGTTGATATCGAAAACTACATTTCTGTACCGAAGACTGAAGGGCAGAAGCTGG

CAGGCGCACTGAAAGTTCACGTTGATGAATACGACGTTGATGTGATCGACAGCCAGAGCGCCAGCAAACTGATCCCAGCA
GCAGTTGAAGGTGGTCTGCATCAGATTGAAACAGCTTCTGGCGCGGTACTGAAAGCACGCAGCATTATCGTGGCGACCGG
TGCAAAATGGCGCAACATGAACGTTCCGGGCGAAGATCAGTATCGCACCAAAGGCGTGACCTACTGCCCGCACTGCGACG
GCCCGCTGTTTAAAGGTAAACGCGTAGCGGTTATCGGCGGCGGTAACTCCGGCGTGGAAGCGGCAATTGACCTGGCGGGT
ATCGTTGAGCACGTAACGCTGCTGGAATTTGCGCCAGAAATGAAAGCCGACCAGGTTCTGCAGGACAAACTGCGCAGCCT
GAAAAACGTCGACATTATTCTGAATGCGCAAACCACGGAAGTGAAAGGCGACGGCAGCAAAGTCGTTGGTCTGGAATATC
GAGATCGTGTCAGCGGCGATATTCACAACATCGAACTGGCCGGTATTTTCGTCCAGATTGGTCTGCTGCCGAACACCAAC
TGGCTCGAAGGCGCAGTCGAACGTAACCGCATGGGCGAGATTATCATTGATGCGAAATGCGAAACCAACGTGAAAGGCGT
GTTCGCAGCGGGTGACTGTACGACGGTTCCGTACAAGCAGATCATCATCGCCACTGGCGAAGGTGCCAAAGCCTCTCTGA
GTGCTTTTGACTACCTGATTCGCACCAAAACTGCATAAGAAGAAGTAAGATTCACCTGCAATTGCTTAGCCGCCGGGGTC
AAACCTGGCGGCTTTTTTATGGCATTAAAAAGCCCCGCCGGGATGGCTCCGGGCAGGGCGGGATACTTATTCTGGCAATT
AACGCACAACCAGCACCGGCAGATTGGCGTGGCGGATCACGCTCGAGGCGTTAGAACCTAACAGATGGGTCGAAATCGAT
GGGTTGCGAGAACCAATAACTACAACATCAGCCCCCAGTTCTTCTGCCAACTCATTGACTTCATCCCGCACGCTACCAAA
ACGGACATGTTGTTTAATGCGGGAAGGATCGATGGTGAAGTGGCTGACCATCGTTTGCAGACGTTCTTGTGCTTCATGTT
GCAGATGCTCTTCAAAACGACGCACATCAGCGGCAAAACGGTGCAGGCTCAGGCTGGCTGACCCGGGTAGTACGTGAAGT
AGATGAATAACTCCGTCATCCTGGGCGAGGAATTCAGCGTGGCGAACAGCTTTGTCGCTCAATTCCATTTCAAATACATC
AACTGGCATAATGATTGTCTTATACATAACCCTTTCTCCCTGTTAATCATGAACAAATCATTCGCCATGATTATAATATT
TATCCCTGATATTTGTCTGGTTCTTTTTCCTTACGAACTGTTTCTGTGATGAATATATTCTCACTGAACACCAGGAATTC
TCCCAAAACCTGTGGTACCGCCCGTTTTCCCGCTGTGATAGCTACCCTTAAAGACTGACTCTTTTTTGAACTGTCTCTGG
AGGTTGCATGAAAGCATTGACTTATCACGGCCCACATCACGTTCAGGTAGAAAATGTTCCCGATCCGGGCGTTGAACAGG
CAGATGATATTATTCTGCGTATTACGGCAACGGCGATCTGTGGCTCTGACCTCCATCTTTATCGAGGCAAAATACCTCAG
GTTAAACATGGCGATATTTTTGGTCATGAATTTATGGGGGAAGTAGTTGAAACCGGAAAGGACGTAAAAAATTTGCAAAA
AGGCGACCGAGTGGTAATTCCGTTCGTCATTGCTTGTGGCGACTGTTTTTTCTGTCGATTGCAACAATATGCCGCCTGCG
AAAATACCAATGCGGGTAAAGGCGCTGCGCTCAATAAAAAACAGATACCAGCTCCAGCGGCATTGTTTGGTTATAGTCAC
CTGTATGGCGGCGTTCCTGGTGGGCAGGCGGAATATGTCCGCGTCCCTAAAGGGAATGTGGGGCCGTTTAAAGTACCGCC
TTTGCTTTCAGATGATAAAGCGCTTTTCCTTTCTGATATTCTGCCAACGGCATGGCAGGCAGCAAAAAATGCGCAGATCC
AACAAGGTTCAAGCGTTGCAGTCTATGGTGCTGGTCCTGTGGGATTGTTGACAATCGCCTGTGCACGGTTGCTCGGTGCG
GAACAGATTTTTGTTGTTGATCATCATCCCTACCGCTTGCATTTCGCCGCCGACCGCTACGGCGCGATCCCGATTAATTT
TGATGAAGACAGCGATCCGGCACAGTCAATTATTGAACAAACGGCAGGTCACCGGGGCGTGGATGCAGTAATAGACGCCG
TCGGTTTTGAAGCGAAAGGCAGCACCACGGAAACGGTGCTGACTAACCTGAAACTGGAGGGCAGCAGCGGTAAAGCGTTG
CGTCAGTGTATTGCGGCGGTCAGGCGTGGCGGCATTGTTAGCGTACCGGGCGTCTACGCTGGATTTATTCACGGTTTCCT
GTTTGGCGACGCCTTTGATAAAGGGTTGTCGTTTAAAATGGGACAGACCCACGTTCACGCATGGCTGGGAGAATTATTAC
CGTTAATTGAGAAAGGATTACTGAAACCAGAAGAAATTGTTACCCACTATATGCCGTTTGAAGAGGCCGCCCGGGGATAT
GAGATTTTCGAAAAACGTGAAGAGGAGTGCCGTAAGGTGATTCTGGTACCCGGTGCACAAAGCGCAGAGGCGGCGCAGAA
GGCGGTTTCAGGTCTGGTGAATGCGATGCCGGGGGGAACAATATGATCGTCAGGAGTGGTTTTCGAGGTAAAGGACAGCC
ATGACGATAATCGCCGCCATAATCAGAAATCCTATCAGGATGTAAAATGCTTCTGCCATGGTTATTCCCACAAACGAAAC
GGAATAATTTTGCAGCAAAAGTGAACAGTGAGAACCAGGAAAAATTGCTGATTTTGCGTAAAGAGGATGCGAGTGCATCC
TCTGGGCAAAGCGAGTTATCGCTTGTGCAGATGGGATTAAAGCAGGTAGTCGCCAGCAGCTTCTGGCTGGTATTCGAGTT
CCAGCACTTCAAGGTGGGTTGCAACGCCGCCCGGAAGTTCCCAGTGAATGGAATCGCCAACGCGCAGCCCCAGCAGTGCG
GCACCTACCGGAGCCATAACGGAAAGCTGAGTATTGCTATCGGTCATTTTTGCCGGATACACCAGCGTGCGCACACGCAC
TTCGCCATCGCTAAGATTGCGGAATTTAACCCGGCTGTTCATTGTCACCACGTCGTGTGGCATCTCTTCTGGCGAACACA
TTTGGGCGCGATCCAACTCTGCGTTTAACGCGTCGGCGATTGGCAAACCAGCATAGGCGGGTTGCTCCAGCAGAATATCG
ATGCGTTCGGCATCCAGGTCGTTAATGATGATAGTTGGTCTGGACATTTTTACTCCATGTCGTCGGTGCTGCGAGTGTCG
CAGATAAACATACCCAAAAGAAAACCCTCACCGTCAGGCGGCGAGGGTTTAACTCACATGATGATACTGACTGTTGCTCA
CTCTTTGAAGTGATTTGCGTCACATTCAGGGAATTCCTCAATGCAGCGCATTATGTATAAATCTTAATCGCCTTGGTTTA
TGGAAGACGAATTAGCGTGTTTTGTAAATCAGATGATTAATAACCCGCTTTATCAATCACAAAGGTTTTGCCACAGTTAC
CTGGGTGAGGTTGTGGCAAGAATGAGTTTGCAGAAAGCGGAGCGTTGATGGCGTCAGCTTTGATCGAGATCTGAATTTCA
GTCAAATACGCAGGGTTACCCTGGCACGTTAGCTTAACTGCTTTCACGTTCTCTTTTCCCCAGCTTTTGGCAAAGGCGGC
GTCAAAGTCACGGCGGCTCACTGTTTTACCGTAATTATCCGCAAGGAATTTTCCGGCTTCGCTCTCTTTAATTTCTTGAT
TCAGGCGTACCATCGTACCGAAGTATGCGTCCGGATCAAAACCAAAGCAGGCACCGTGTTTGGCATATTCGTAGCGTTCC
AGGCAGGAACGTCCGCCAGCTCCTGGCATGACTTCACTTAGTTTAGCGGCCGTTTCCAGTGATAATCCGGTTTCCGGCGA
TGAACACATTCGGCTGGCGCGCGCTTCTGGTAGATTCGGGATTGGGCGAGTAGCGCAACCGAAGCGCATCCAGCGGCGTT
CATCAACACCACGGGCAGCAACCGATTTAGGCAATCCTGGCCACAGACCATGTACGGTCAGAAAATCAGCTTTGTTGGTC
GTTTCGGTTTGCAGGCGACATTCATCTCGTTCGTTACGATTTCGATCGTGTTGACTCTGGCAAAATCCGGTTTGCCAGGA
GAGGGCCAGGACATAGCGATCAAAATCGCCATACTGTTTTGCCTGCAACGCTAAGGCGTTGGCAGAAGAGAAGGGAAGCA
GAGAAACCGCGAGCAACGCGGCGTTACGCCAGAATGCTTTCATAATGGGTGTGGAACTCATACATACACTGAATACTATC

TATTAAATCATAAAAAAGCCCGCCAGGTGGGCTTACCTGGCGGGCGTGATGATTTATTCAGCGTTTGGCGAACGTATTAG
TTCCACATGGCGAGAATCGGCCAGCCAACCAACAGCAACATAGAGATGTAAATCACCCCGAAGATTGCGCCAAGACGCCA
GTAATCTTTTGATTTCACATAGCCACAGCCGTAAATAATCACCCCAGGACCGGTTGCATACGGCGTCAGACAGCCCATGA
TACCGATAGACAGCACCAGCAGGATACACAGTTGTTCCATTGGTACGCCCGGAATACCTTTACCGACGGCCAGAATAACC
GGCAGCATGGTTGCGGTGTGCGCAGACAGGCTGGCAAACAGGTAGTGTGCAAAGTAGAACACCAGAACCAGTACAATCAC
CGTTGCGTTTGGTGAGAATCCTTCCAGGTGCGTACTCATGGTACCGGCGAACCAGTCAATAAAACCAGAACGAGTCAGGC
CGTTAGCCATCACAACCAGAGTTGCCAGGTTGACCAGTGTGTTCCATGCGCTGTTATAGCGGGTAATGTCTTTCCAAGGC
ACAACGTGCAGGGCCAGCATTAGCGAAACTGCCAGCAGACCAACCGCAGTAGCATTAATGACTTCACTGCCAAATACCCA
CAAACCTAAGCTGAGCAATACAAGGCCAATCAGTGTCCACTCTCTGCGTGTCAGCGCACCCATGGTTTTCAGTTCATCAC
CCGCCCAGGTTGCCACTTCTTCACTGTGTGTGATTTCCGGTTTGTACAGCACGTAGGAAAGCCACGGCGCAATGATAAGC
AAGATAACCCCAACCGGCAGGAAGCAGAGGAACCACTGCAACCAGCTAATCTGGATACCGGCAATTTTGCTGACGAACTC
CAGACCCAGCACGTTTGGTGCCGCACCGGTGACAAACATGGACGAACTCAGACTGGTACTAATGACCATCATCCACATCA
AATAGCCGCCAATACGACGCGCGGACGGATCGTTCGGGAATGATTTAAACAACGGCGGCAGGTTTTTAATGACCGGAAAA
ACCGTACCCCCGGTACGCGCGGTGTTGGAAGGTGTAAACGGTGCCAGCAGAATGTCGATAATGACAATCGCATAACCCAA
CGTCAGCGTGCGTTTGCCCATGAATTTCACCAGGAAAAGGGCAATGCGACGACCTAACCCGGAAACTTCATACCCTAATG
CAAAAATAAATGCGCCAAATACCAGCCATACCGTGGTGCTGGAAAAACCAGCCAGGCCCCATTTCAGCGCCTGTTTTTGC
GCATTAAACGCTGGGTCAGCTAATTCTTTGGCATCAAAGAGCAGGTAATTACTGCCAATAACGCAAATAGTAACCGCAAT
AAAACTGATCGCTGTTGCCGGAATTGGCTCGAGGATCATGCCGACAATCATTGCCACAAACACAGCGAAGTAATGCCATG
CCTGCGGCGGCATACCGTCGGGGACAGGGATAAGAAACATGACACCCATCACCACCAGTGGGGCCAATAGTTTCCATATA
TTATCTTTTGCTAAAGACATACGGGTTCTCCGAAAATTAATATTTCCAAATTTATCAAGTGCTTAAATAATTAAATCTGT
GCTAAAAACCAGGTAAGGATCAGTAGGTCAGCACTGCCGCCTGGACTGAGATTTCGTTCGATACACTCCCTGTCGAACTG
CCGGAGATAATCGAGATCGGCGGGGGTTCGAATGCCCCCTTTTTGCAATAATGTTTGCGCCTCGCGCTGTAGCCAGCGCA
GGCCCCCCTCGCCACCGCGCGATGCAACGTTGGTATCGCCGTTGATCGCCATCAGTAGGAGCAAGGTATCGAGCAATGCC
AGTTCAGGATCTAACCCCTGATCCAGCAGAGTGAGGTAATGCGGCAAGGCGTGATTGATCACCAGTGGATAACCCGCTTC
GGCTTCACCGCGTGCGCCGGTAAGGCCAAGCTGTTGGTACAACCGTTGACCTGCCGTCAGTTGTGAATTATTGGTACGCA
GTTCGCGATCGGTCAGGCCACGGCAGAAACTTGCCGCCGTAGAACAAACGGTTGTTGGCGTTACCGGTTGGTTGAGTTGA
AGCAAACGGCCAATTGCCGCACATAGCAGCCCTAAAGAAAAAATGCTGCCTTTATGCGTGTTTACGCCCGCAGTGGCGCG
GAACATATCACCTTCGCAAGCCATACCAATTGGGCGTAATCCGTGGAGTACCGCTTCTGGTGCCATTTCCGCACTACAGG
CACCAAATTCAATGAAACGGGGTAGCCAGCCCTGAATCGCCAGCGCGCTGCGGTGGAAATCTTCCAGCGCCATATCTTTG
TGCGCACCGCAGTTAATGCGATCCACGAGGCCTGGTTTCGGTGACAGATTGACTTCAGTCAGCATGGCGCGCCAGCCCAG
CAGGGCGTACTCATCGATTAATGACGTCGCAAGCTTTGTGGTTTTAGTTGACGTTGCAGGCATCGACATCGTTCAGCAGT
GCCTCCATGCGGTTGAGTAAATCGGTCAGTTGATGGGTTTTTCCACGCGCGCAGACGGCTGCGCTTTGTTCGCACAACAG
GCAGCGGCGAGGCGGCAGTGAATAGTCGCGGCGGGAGAGAATTTCGCCTTCGGGCGTCAGGACATCGATATCCCATAACC
GCCCGAGAGGATGACTATGTTCAAGCTCAATGGTGGCGAGCTTGAGGTCGCGAGCCGGGGCGGCAATGCTCAACATGCCC
TCCGGCCCGCTGGCGGAAACCAGTGCAGCCTGCTCCTGAATTTGCCAGCCCTGTTTTGCGGCTAAGGCACGCAAGGCTGT
CACGCCATGATTAAAAATTCGGCGTGTGACCTCGCTGTCTTTAATCGGCCCAGGCGCAACCACGGTAAAGGAGACCAGTG
GAACAGGATGGCGCTTGAGCCAGACGTGTTGCCGTGCTTGCCTTTCATCCCGGCTGACGAGCAGCTCGGGAATTGATACC
GCATGGTGGCTGGCGAGTTCAGGAAGCAGGTGCATGGCTTATTCCTTCACCTGATGCACAACATCGATCACCGAGCCATC
GCGGTAACGCACAACGGCAACGACGCGGTCTGTGAATTCAATCGGCTGTGGTTCACCGGTCAGCAGACGCGCACGTTCGC
GCAGCCACTCAATGGAAACCACTTTAATGCCCGCTTCCTGCAGACGTTCTGCCAGTTCCGGACGTGCCGGGTTAACTGCG
ATACCGTGGTCTGTGACCAGAATATCGACACTGGAGCCTGGGGTGATGCAGGTCAGTACGTTATCCACCAGAGTCGGAAT
ACGACCGCGTACCAGCGGCGCGACGATGATGGAAAGCGCAGAGGCAATCGCGGTATCGCAGTGACCACCGGAAGCACCAC
GCAGTACGCCGTCAGAGCCGGTCAGCACGTTAACGTTGAACTGGGTGTCAATTTCCAGCGCGCTCAGTACCACCACGTCG
AGACGATCAACCGATGCGCCTTTCGAACCCCAGTTAGCGTACTGGTTGGCGCTGATTTCGATGTGATTGGGGTTACGGGC
CAGCGATTGCGCAGCATGGCTGTCAAAGCTCTGCACATCCAGCAGTTTGCGGATCAGACCTTTTTCGTGCAGGTCAACCA
TCGTCGCGGTAATACCGCCAAGGGCGAAGTCGGCGCGAATATCGCGGCTACGCATTTTGTCTTCCAGGAAACGGGTTACC
GCCAGCGATGCGCCGCCGGTGCCGGTTTGCATGGAGAAACCTTCTTTGAAGTAGCCAGAGTTGACAATCACATCCGCAGC
GCTACGGGCAATAAGCAGTTCGCGCGGGTTAGTGGTCATACGGGTCGCGCCAGCGCCGATTTTTGCAGCATCGCCAACGC
GGTCAACTTTGACGATCAAATCAACCTGATCTTGCTCAATGCTTGCCGGATTATGCGGATAAGGCAGCAGTTCTTCGGTA
AGCATCACGACCTGTTTTGCGTTGTCGGCATCAACTATTGCATAGCCGAGGGAGCCGCAGCAGGCTTTACCGGTGTAGCC
GTTGGCATTACCGAATTCATCACAGGACGGGACGCCGAGGAAAGCCACGTCGATATTCAGTTCGCCGCTCTGTACCAGAT
GCACACGACCGCCGTGAGAGTGGATCTGCACCGGTTCTGCCAGCAGACCACGGGAGATCTCTTCCGCCAGTGGACCACGC
AGGCCGGAGGTATAAATGCGGGTAACCACGCCCTGGCGAATGTGTTCTACCAGCGGCGCATGGCAATCACTCAGGGAGCT
GGACGCCAGGGTCAGGTTTTTAAAGCCCATCTTCGCGATGACGTCCATCACCATATTGACGGTCAGGTCACCGCCACGGA
AAGCGTGATGGAAGGAAACCGTCATGCCGTCCTGTAAACCAGAGCGACGAATCGCTTCTTCCAGGTTGGCGCACAGTTTG
CGATCGCGCGCTTTTTCAGCCTGGTAGGTTTGCTTTGGCGAGTTCTGGAAAGCGGCAAGATCGCATTCAGCGCGACGATT

CCAGGCCGCTACCCGTTCTTGTCGTTGAGATTGTTCAATTTTCTGCGTCATTTTGATTGCCTTATTCTTCGCGGATGCCG
GAAAGTTCTGCACGGGAGAGCACCAGACGGGCGCGATCGATAACCGGACCGTCCACCATCTTGCCGTTCAGGGAAACCAC
GCCGAGGCCTTCGCGAGCGGCGGCTTCAGCGGCTTCTACGACGCGGCGGGCGTGATCCACTTCTTTCTGGGTCGGTGCGT
AGAGGTTGTGCAGCAGATCAATCTGACGCGGGTTGATCAGCGATTTGCCGTCAAAGCCCAGCTGTTTGATGTGGGCGGCT
TCTTGCAGAAATCCGGCTTCGTTGTTAGCGTCGGAATAGACGGTATCGAACGCCTGAATACCCGCAGAGCGCGCGGCCTG
CAAAATGGAACAGCGTGCGAACAGCAGTTCAGTTCCTTCCGGGGAGCGTTCTGTACGCAGGTTGCGCACATAGTCTTCTG
CACCGAGGGCGATACCGATCAAACGCTCGGAAGCGTGAGCGATTTCCACTGCGCGGGTAATCCCCAGCGGAGATTCAATC
GCCGCCAGCAGGCCGGTGCTGCCGGGTTCACGACCACAGGCTTTTTCGATACGCAGGATCTCTTTTTCAATATCCAGAAC
ATCCTGAGCGGTATCGGTTTTCGGCAGACGCACAACGTCCGCACCACCGCGAACGACGGCTTCCAGGTCGTTAACACCCC
ATTCGGAATCCAGCGCGTTGACACGCACAATGGTTTCAATATCGCGATACAGCGGATGTTGCAGCGCGTGGTAAACCATG
CGGCGGGCGGTGTCTTTTTCACGCAATGCTACGGAGTCTTCGAGGTCAAACATCAGGGCATCAGCCGGGTAGATGAAGGA
GTTGCTGACCATCGCGGCATTGGCACCAGGCACAAACAACATGCTGCGGCGGGTGCGAGTTTTACGTTGTTGCAGCGAAG
CGGAAATCATTGGCAATCCTCCCATGGCAGAGCCGGGATACCGCTGGCGCGTGCCAGCAGGGCTTCCAGTCGTGCACGTA
AAATGCAGTCCAGTGCGCCTTTGTCATCGACATTCAGCTGTACGCCGCGCACGTTGTAGCGGGCGAGAACGTCCAGAATG
GTGGTGCGAATTGCATCGCCAAACTGTTTCTCAACGCTGCTATTGATTTGCAGGTCGATATCCTGCGTATCGAGTGGGGC
GATGCGTATCATCACATCCCCAGACTCAAGGGTGCCTGCAACGGCGGGCTGGTTTATTTTCATTTTTCACCTGTTTCTCA
TGCGGGGGTCTTTTGACGAGCTGCCGCGTCCTGGCGGGAGTGCTCAAGCAGGTTCTGCAAATAATGCAGCGTGACTGCAG
GGACCAGCGGCGCGATAGCCGTGAGATCGTTTTTCGCCAGCAGTTGACGTACCCGGGAAGCGGATATCGGCATCTCCTGG
TAACGCAGCCGCTCAATTTCAACCAGTTCGATGGGCGGTGCGGAGATAGTCGGCGTTTCCAGCCAGTAGCGCATATCCTG
GTTGTACTGGGCGGTAACGCGACAAAAGGGTTCAGTACCGACAAAGCGGTGAGTTACACCCAGCGCGGGAGCGAGGTACT
GACGGAAAATCTTCAGATCAATTTCGGTGTAACAATGGTTAATGACGCTCTGTTCTTTAATGAAGTAGCAAGGGAACGTA
GCGCGGGAGATGATGTATTCGGAGCCACGATGCACAGTCAGGCGTGGAATATCGGCGGTGCCTTTTAACACCAAATCCAG
CCGGTCTTCATAGGGGAAGCGTGAAGAATCTTCTTTGACTAAAAACAGATGCAACCAGTCGCACTGTGCCGCAGCCTGTT
GAATCAGATAACGGTGACCATTCGTAAAGGGATTGGCGTTCATCACAATGCAGCCAATCTTGTTCCCTGGATGACGAAAT
TTTTTCAGCGATTCGGCATAGCGTTTCAGTCGCGTGGCGCTGTTTTCCATCAGCACCATCACGCCGGGTACGCTGGTCAG
CGTGGAAAAACCGCACTGGCGGAACAGCGCCTCGTATTCGGTTTTGGTATAAATAAACAGATGCGTGCTGTGCCGCTCAT
AGGCGAGGTTTATCAATTCAGTGGCTAATGTCAGCGCCAGTCCTTCACCGCGGACGGATTCACTGATAGCAACGCATTTA
ATAATATTTCCGGCAATTCCACCGCACGCGATAAGCTTTTCATCGCGGGTTACGGTAATAAATACTTCGACTGTGGTGTC
AACGCTCAAATCATTTTCATGCAGGAATTGGGCGATTTCCGCCATTTTTTTATTTTCTGAACGTTTTACGCGGGTGAAAA
TATCATTGCCGAACATAATAAATAGTATCCTGAAGGTGCATGTTGTTATCGATTTGCAACGAATGTTGTTCAATGTTGCA
AACTGATAACCTTTTATTTTCACTTGGGAGAAAGGGGGTGATCGAGGTATATCTTTTTCTCCTTTCGCTATACATCCTAA
GGAGTATTTCGGCGTGAAATTTTGATTTATTTCACATAGAGTTAGTGGTTTTTTATTTATTTAATGATTTTAAGTTTTTT
AATTAATGTAATTACGAAATGACTCGCAGGTTTAAGTGATTTAATTGATTTAATGAATAAAATTTGCCACGATCATAATT
AATATCTATGTATTTTGATTCAACATTTTAATTACATCCGTCAAAGAGGCTCGGGACAACCCGCAAGGAAAACAATGTTG
CAGCTTAACGAGAATAAACAGTTTGCATTTTTCCAAAGACTGGCATTTCCGCTGCGTATCTTTTTGCTGATTCTGGTGTT
CTCAATATTTGTCATTGCAGCCCTGGCGCAATATTTACGGCCAGTTTTGAGGACTATTTAACGCTTCATGTACGCGACA
TGGCAATGAATCAGGCGAAAATTATTGCCTCCAATGACAGTGTCATCTCTGCGGTGAAAACGCGTGACTACAAACGGCTG
GCGACCATCGCTAACAAATTACAAAGAGATACCGATTTTGATTATGTGGTGATTGGGGACCGGCACTCGATCCGCCTTTA
CCATCCTAATCCGGAGAAAATTGGTTATCCTATGCAGTTCACCAAACAGGGCGCGCTGGAGAAAGGGGAGAGCTACTTCA
TTACCGGGAAAGGGTCAATGGGGATGGCGATGCGCGCCAAAACGCCAATCTTTGATGACGATGGAAAAGTCATCGGCGTG
GTGTCGATTGGCTACCTGGTGAGTAAAATCGATAGCTGGCGGGCTGAGTTTTTATTACCGATGGCAGGTGTGTTTGTCGT
GCTGTTAGGGATTCTGATGTTGCTGTCGTGGTTCCTGGCCGCGCATATCCGTCGGCAGATGATGGGCATGGAGCCAAAGC
AAATCGCACGCGTGGTCCGTCAGCAAGAGGCGCTGTTTAGTTCGGTTTATGAAGGGCTGATTGCGGTGGATCCGCATGGT
TACATTACCGCCATCAATCGTAACGCAAGAAAGATGCTGGGGCTGAGTTCCCCCGGACGGCAATGGTTGGGTAAACCCAT
TGTTGAAGTGGTCAGGCCCGCCGATTTCTTTACCGAACAGATTGATGAAAAACGTCAGGATGTGGTGGCGAACTTTAACG
GTCTGAGCGTTATTGCCAACCGGGAAGCTATTCGTTCAGGTGATGATTTGCTGGGGGCCATTATCAGCTTTCGTAGTAAA
GACGAAATTTCCACCCTCAATGCGCAACTGACGCAAATAAAACAATACGTTGAGAGCCTTCGTACATTGCGACACGAGCA
TCTCAATTGGATGTCGACGCTCAATGGTCTGTTGCAGATGAAAGAGTATGATCGCGTGCTGGCGATGGTGCAGGGGGAGT
CTCAGGCCCAGCAACAGCTTATTGACAGCCTGCGCGAGGCGTTTGCCGATCGCCAGGTGGCGGGGCTGCTTTTTGGTAAA
GTGCAGCGCGCCCGGGAACTGGGGCTAAAAATGATCATTGTCCCCGGTAGCCAGCTTTCGCAACTGCCGCCAGGACTGGA
TAGCACCGAGTTTGCAGCCATTGTGGGCAATTTACTTGATAACGCCTTCGAAGCCAGCCTGCGTAGCGATGAAGGAAACA
AGATCGTTGAATTATTCCTCAGCGATGAAGGCGATGATGTGGTGATTGAAGTCGCCGATCAGGGCTGCGGCGTTCCAGAG
TCTCTACGAGACAAAATATTTGAGCAGGGGGTCAGTACGCGTGCTGACGAGCCCGGTGAACATGGCATTGGGTTGTACTT
GATTGCCAGCTACGTAACGCGCTGCGGTGGTGTTATCACTCTCGAAGATAATGATCCCTGCGGTACCTTATTTTCAATCT
ATATTCCGAAAGTGAAACCTAATGACAGCTCCATTAACCCTATTGATCGTTGAGGACGAAACGCCGCTGGCAGAGATGCA
TGCGGAATATATTCGTCACATTCCCGGATTCAGTCAGATATTACTGGCGGGAAATCTGGCGCAGGCCCGAATGATGATCG

AGCGTTTTAAGCCGGGGCTAATCTTGCTCGATAACTATCTTCCTGACGGTAGAGGGATTAATTTACTGCATGAACTGGTG
CAGGCGCATTATCCCGGCGACGTGGTGTTTACCACTGCAGCCAGCGATATGGAAACGGTGTCTGAAGCCGTACGTTGTGG
TGTATTTGATTATCTCATTAAGCCCATTGCCTATGAACGGCTGGGGCAAACGCTAACCCGTTTCCGCCAGCGTAAACATA
TGCTGGAAAGTATTGATAGCGCCAGCCAGAAGCAAATTGATGAGATGTTTAATGCTTATGCTCGCGGTGAACCTAAGGAC
GAGCTACCGACCGGCATTGACCCCTTGACGCTAAACGCGGTGCGAAAACTGTTTAAAGAGCCTGGTGTGCAACATACGGC
AGAAACGGTGGCGCAGGCACTGACCATCAGCCGCACCACTGCCAGGCGTTATCTTGAATATTGCGCCAGCCGCCATCTGA
TTATTGCTGAAATTGTTCACGGCAAAGTTGGCAGACCACAACGCATATACCACAGTGGGTGACATGATAAAGCCGGAGGA
AACTTGCCTCCGGCATTACTATTTACTTGCCTGTGACCGCTGCTGCCGTTCCTGGCACCATCAGCTCTGTAGCAACGATA
ACAATCACCAAACCAACAAGCACCGGTACCGAGGTGCGTTTTACGACTTCAAACGGCGAGATCTTCGCCATCCCGGCAAC
CGCAACGACTACGCCAGAAACGGGCGAAAGGGTACGGCCAAGGTTTGACGCCTGCAGCATCGGGATAGTCAAATACGCCG
GGTTAATGCCGGAAGAGTGCGCCAGTTTCGGGATCATCTCAACAAACGCATAAAACGGCGCATTGCCTGAACCGGTCGTG
ACTGCCGCCAGCATTGTCAGAATCACCAATACCAGCATCAGGATGATACTCGCCGAACCAAACGAGGTAGCGATAGAAAT
CAGACTTTGAATAAAGCCGATGGTGCTAAGCCCCTGAGCGAATACCCCAGCGGCAACCAGCAGCATCACCACGTTAGCAA
ACGCATCTGCCATCCCGCGATAAGCCACTTCCAGACCAGAGAAAACTTTCTGGGTATTAAAGCTGCGGAGGAACTCCAGA
ATGGAGGCAATCAGCATACAAATCACCAGAATAGTGATGATGTGTAATTGCGGACCCCATTTACCGTCAAAAATCAGTAC
ACCGATGATCGGCGTGAACGGCAAAATGGCATAAAACGCAGGAGCAGTGGTGGTGATTTCACTGACATCTAACATTTCAT
GAGAGATGTGCTCTTTTTTATCCAGATAACGTTGCCAGAAGAAGTGGGCGATCGCCATGCCGATAATTGCAGCAATTGAG
ATAGGCAGCGTCGTTTTGAAGGCGAAGTCAATCAGCGACATTTCGGAAGCTTGCGCCGCCAGCACCACATCCCCTGAAGT
CGGTGCGAGAATAATCGCCGCCGGGGAGGCACAAATGGCAGCAGCTGCGCCACGACTGATACCAACGTTTACCATCACCG
GAAATAGGGTTGCCATCAGCAAAACACCCAGACCGGTTGCGGAAGAGACGGCCAGAGACATCAGACAGGCGACAAAATAG
GCGGCAATCATCAGCAGGTAAGGGGAGTTAATATACTGCAATGGTTTTGACGCCAGCTTGACCACCATATCATTCGCGCC
GATATGGGTCATGTAAGCGGCAAATCCACACAGCATCATAATCATCATGCCGAGGTCGCCGCCGCGGCTCATTAGTAATA
TTTTAACGTATTCAACGATATCCGTGGCGCTGTAGCCTGTTGAAGCCTGGCTGGACGGTAACACTTTGTGCCCCATAATG
GCACTGATAATCAGCAATAACAGGCCACCGACAAATAACACACCAGTGGCGGAATACCCTTTAATGATGTAGCGAGCTAC
ACCCACAATAACCACAACCCCAATAAGGAGCTCAATGAATGTCAGCATAATTTTTCCTGTCTCCAGGCCCCAAAGTAAAT
AATAAAAAATTCCTAAAGCTTAAGGAAAAAATATGCCCAATAAATTGGCGATGAATGCTGATTAAAATCAAGAAAAACTG
CCATTAAGACATTGAAGTTGCTGTTTTTATACATAGATAACAAAACGCATATTTAGCGGTTGGCAATTATCACATAATAA
TGTGTGCAAATTGATGGTGGATTGATGCAAATTTGTTAATTGCAATGGTGTTAGCTGCGGCTGTGCGCTCAAAAAATAAT
CTAATATGAGCATAGGTTGACGATATATATTATATACTTCGTTAAGATGATTGTTGTATCTCGTTAAAAAATAAAATAAT
TTTTCCTTGATTGCATTTTGTCATCAAAAAAGACTTGGTTTTTCTTTTTTGACTATTCCCATCGCAGAAAACGACGCATC
ATCTTTAATCGATGCGCGGAAATATTTAACTTGAACAAGCGGAAATAAATAGAGCAGCTATTCAGATTATTCTTTATGTT
GGGTCTATTAAGGTTATGTTAATTGTAGCTTTGCTATGCTAGTAGTAGATTTTTGATAAATGTTTTATGGTCACAAATGA
ACGTGAGTAAATATGTCGCTATCTTTTCCTTTGTTTTTATTCAGTTAATCAGCGTTGGTAAAGTTTTTGCTAACGCAGAT
GAGTGGATGACAACGTTTAGAGAAAATATTGCACAAACCTGGCAACAGCCTGAACATTATGATTTATATATTCCTGCCAT
CACCTGGCATGCACGTTTCGCTTACGACAAAGAAAAAACCGATCGCTATAACGAGCGACCGTGGGGTGGCGGTTTTGGCC
TGTCGCGTTGGGATGAAAAAGGAAACTGGCATGGCCTGTATGCCATGGCATTTAAGGACTCGTGGAACAAATGGGAACCG
ATTGCCGGATACGGATGGGAAAGTACCTGGCGACCGCTGGCGGATGAAAATTTTCATTTAGGTCTGGGATTCACCGCTGG
CGTAACGGCACGCGATAACTGGAATTACATCCCTCTCCCGGTTCTACTGCCATTGGCCTCCGTGGGTTATGGCCCAGTGA
CTTTTCAGATGACCTACATTCCGGGTACCTACAACAATGGCAATGTGTACTTTGCCTGGATGCGCTTTCAGTTTTGAGAC
AAATGAAGTTTTAGTAACTTCTTTAAAATCAATAGCTAAAATAAGTAACATCAAAAATAACGCGACTTTTATCACTTTTT
AGTAAAGTTACACTGGACAAAGCGTACCACAATTGGTGTACTGGTAACCGACACAGCATTTGTGTCTATTTTTCATGTAA
AGGTAATTTTGATGTCTAAGATTAAAGGTAACGTTAAGTGGTTTAATGAGTCCAAAGGATTCGGTTTCATTACTCCGGAA
GACGGCAGCAAAGACGTGTTCGTACACTTCTCTGCAATCCAGACTAATGGTTTTAAAACTCTTGCTGAAGGTCAGCGCGT
AGAGTTCGAAATCACTAACGGTGCCAAAGGCCCTTCTGCTGCAAACGTAATCGCTCTGTAAGATACGTCAGCAAGAATTC
AAAACCCGCTTAATCAGCGGGTTTTTTTTGGTCTTTAGTGTGCGGTTGAGGCCGAAAACAGCCAGAATGCCAGTGCGGTC
ATGGCAAAAGACCCCAGAAGGTTGACGAAAACGTTCAGTAATGCCCAGCCAAAGCGGCCCTCTTGTAACAAAAACACCAC
TTCTGCCGAAAATGTTGAGAAGGTTGTTAGACCGCCACAAAATCCGGTGGTGATTAATACTTTCCACACTGGATCAATGT
TCGTCATCCTGCTGAACCATGCGAATCCTATTCCTATGATGAATGCCCCAATCAGGTTTGCTGTCAGCGTCCCCAACGGA
ATCGCCTGATGCAGTGGGTTAAATCGCATACTTAACAGCCATCTCGCCACGCTTCCCGTACCACCGCCAATAAAAACTGC
TAAAAGAAGTTGTAACACTGCAAAATCCTGCTATTTGATTTGTATGAGTGATAAGTGTAACGCCGAATAATCGTCGTTGG
CGAATTTTACGACTCTGACAGGAGGTGGCAATGCTGGTTGCCGCAGGACAGTTTGCTGTTACATCTGTGTGGGAAAAGAA
CGCTGAGATTTGTGCCTCGTTGATGGCGCAGGCGGCGGAAAACGACGCATCGCTGTTTGCCCTGCCGGAAGCATTGCTGG
CGCGCGATGATCATGATGCAGATCTATCGGTTAAATCAGCACAGCTGCTGGAAGGCGAATTCCTCGGACTTTACGGCGAG
AAAGTAAACGTAACATGATGACGACAATTCTGACGATTCATGTTCCTTCAACGCCGGGGCGCGCATGGAATATGCTGGTG
GCACTTCAGGCAGGAAACATCGTCGCCCGTTATGCCAAACTGCATCTCTATGATGCATTTGCCATTCAGGAATCACGCCG
TGTTGATGCTGGTAATGAAATCGCTCCGTTACTGGAGGTGGAAGGGATGAAGGTCGGTCTGATGACCTGTTATGACTTAC

GCTTTCCAGAGCTGGCGCTGGCACAGGCATTACAGGGAGCTGAAATCCTGGTACTTCCTGCCGCCTGGGTTCGCGGGCCG
CTCAAAGAGCATCACTGGTCAACGTTGCTTGCCGCTCGTGCGCTGGATACCACCTGTTATATGGTGGCGGCGGGGGAGTG
CGGGAACAAAAATATCGGTCAAAGCCGGATTATAGATCCCTTTGGCGTCACCATTGCGGCAGCGTCAGAAATGCCTGCAC
TCATTATGGCGGAAGTGACGCCCGAACGTGTGCGTCAGGTGCGCGCGCAACTGCCCGTCTTAAACAACCGTCGCTTTGCG
CCGCCGCAATTATTATGATGTTTTTTTACTCGGCGCTTGATTCACCTTGTTACAGATTGCTATTGTGTGCGCGCGTCGAA
TGACCGTTAATATTCTCTGGTTTTTAAGGCGCGTTCTGTTGCCGGTTATATGTCAAGAAGGTATCTATGGGTGAGATTAG
TATTACCAAACTGCTGGTAGTTGCGGCGCTGGTCGTTCTGCTGTTTGGGACTAAGAAGTTACGTACGCTGGGCGGAGACC
TTGGAGCGGCCATTAAAGGGTTCAAGAAGGCGATGAATGATGACGATGCTGCGGCGAAAAAAGGCGCAGACGTTGATCTT
CAGGCTGAAAAGCTCTCTCATAAAGAGTGACGTGGCGAGCAGGACGCTCCCTCAATATCTTGTTCGATACAAAAAACCCG
CTTCAAAAAGCGGGTTTTTTATCAGACAGATGTAAGTAATTATTACAGGATTACTTAACTTCCATCCCTTTCGCCTGCAA
ATCGGCGTGGTAAGAAGAGCGGACAAACGGACCGCATGCAGCATGGGTAAAGCCCATCGCCAGCGCTTCGGCTTTCATTT
CGTCGAACTCATCCGGGCTAACGTAACGTTGAACCGGCAGGTGATGGCGGCTTGGCTGCAAATATTGCCCCAGCGTTAAC
ATCGTCACACCATGACGGCGCAGGTCGCGCATTACCTCAATAATTTCTTCATTGGTTTCACCCAGTCCCACCATCAGACC
AGACTTGGTCGGGATTTCCGGATGCGCTTCTTTAAAGCGTTCCAGCAGCTTCAGCGACCAGTTGTAATCTGCACCAGGCC
GTACCTGACGGTAAATACGCGGTACGTTTTCCAGGTTATGGTTGAACACATCTGGTGGCGTTGCAGTCAGAATATCCAGA
GCACGATCCATACGACCGCGGAAATCCGGCACCAGAGTTTCAATTTTGATTTGCGGGCTTTTTTCCCGAATGGCAGTAAT
GCAATCCGCAAAGTGCTGGGCACCGCCATCGCGCAGGTCATCACGGTCAACGGAGGTGATAACCACATAACGCAGCGCCA
TATCGGCAATGGTCTGCGCCAGTTTCACTGGTTCATTGGCATCAGGAGCTACCGGGCGACCGTGGGCAACATCACAGAAC
GGACAACGGCGGGTACAAATAGCGCCGAGGATCATAAACGTTGCTGTGCCGTGGTTGAAGCATTCCGCCAGGTTAGGGCA
GGAGGCTTCCTCGCAGACAGAATGCAGGCCATTTTTGCGCATTGCGGCTTTGATGCCCTGGATACGTGTAGAGTCCGCTG
GAAGCTTGATTTTCATCCATTCCGGCTTGCGCAGCAGGGCTTCGCGCTCTGTTGCCACGTTTTTAACCGGGATAAGGGCC
ATCTTATCGGCATCGCGGTATTTAACACCGCGTTCCATCACAATGGGTTTACTCATAGCGTGCGTGTTCCAGTTGCGAAT
TACGAAGGAAAGCGTTTCAATTCAATAGATTGTTGTAATTATCAACTATTTTTGAATTAACGACTGGCAGTATATCATTG
AAACGGACCTGAAAGCAGCCAAAGCGGTCGCGAAAATGTAAAATTGTTGTTGGATTGTGCCATTTTATCGTTCTGCGCTG
TGATCTGGCTGTAACAATACTTTTTCAAAATGCATTACGGATAACATCTATTACTCCGTGCAGGATGGGATCACGCAGGC
TGAATTTGTTGTAGTGGATAGAGAAATCTATTTGCTCCTCATTTAAGGACGGAAAAGGCAGTTTTTCCAGCGGCCAGCAG
CGGCTAAACAGGTTATAAAAACGGCTGGGAATAATCGCTAACATGTCACTGTTGGCAACCAGTGCAGCGATTGTCAAAAT
GTTGTAGCTGGTGAAATTGATTTGCCGGTCCGGAAACATTTCTTGAACTCTCTGCCGCAGACCGCTGAAATTTTGCTCTT
CCGGTAACAGAAGTACATGCGCAGCGTTGTCGATAGTCTCTCTGTCATCTTCTAAAGAGAGTAGTGGATTTCCCTCGCGG
CAAATTAACACCATATTGTCGGTGAACAGAACATGATGTTGCACCGTACGATTGGTGCAAAACATGTTATCGATGATGAG
ATCGGTTTGAAACTGACTGAGTTGGTTTTCCGCGTCGCTGATGGGCGGGTTGCGCAGTAAAAGCTGCGGATAGTGAGTTT
TAATCGCCCGATAGATGACAGGAAGGACCAGTGCTCCGACCGAGGGAGTGGTCGCAATAGTTATCGTTCGTTGCTTATCA
TAGCTTCCTTCGATATCCAGCGCGCCAAGAATGGACTCAAGGCCCTGACTGATATACTCATGTAGATGCATCGCAAATGC
GGTAGGAGTGACACCCTGGCCTTTGCGAATAAACAATGGGTCAGGGAATATAACGCGCAGTTTCTGAATAGACTGACTGA
TTGCCGAGGGGGTCAGATTAAGCACTTTCGCTGCATTAACGATCCCTTTATGTACATATACAGCTTCAAAAATAGTCAGA
AGGTTAAGATCAATATTTCGTAAGGTTGTAAATATTTGTGGTTTACCTTCTGACGACTTTCTGCTTAAACAGGGTTCAAT
TTGATTATTACTATCCACGCACTTACTCCAATTTTATTCATGGAAAAATAATATTTAAAAAATTACAATAATCTTATGTC
TAATTGGAACGGAACGCTTTTGCTCACCATAATCAACTATTTCAATAGGTTAATCAATGGGGTGAGTTACGGAGCAAGGC
GTGGCACCGCATTTCTGCAAGTGATAAAGAAAATATACAAACGCGAAGGAGATGTAAAGCATTAGCAGATATTATGAGTA
ATGACCCAGTGTAAATTGGGCCATTGATGTATGGAATTAAGCGGTAATATATTCGAAGTCCGGATTGTTTAGTAGCGCTA
AAATATTTTCCAGTAAACGTGGAGCAATATTATTAGTCGTCGCTTCGGGTTTCCATTGTGATATTTTAGCCATTTCCATT
CCGGCATACCCACAAGGATTAATACGTAAAAATGGTGAAAGATCCATATTGACGTTTAATGCCAGACCGTGGAATGAACA
ACCGCGTCGAATACGTAAACCCAGTGAGCAAATTTTCTTTTCCCCAACATAGACACCTGGCGCGTCAGCCCGAGGATGCG
CTTCTATACCCAGTTCAGCCAGGGTATTCACCACTGTTTGCTCAAGCAAGGTCACCAGTTCACGCACACCGAGTTTACGG
CGTTTCAGGTTAAGCAACACATACATCACCTGTTGCCCCGGCCCGTGATAAGTCACCTGCCCACCGCGATCGCTCTGGAT
CACCGGAATATCACCCGGCATTAAAATGTGCTCCGCTTTTCCTGCCTGACCTTGGGTGAATACCGGATAGTGCTCGACCA
GCCAGATTTCATCAAGGGTACTATCATCGCGGGTATCGGTGAATTCATGCATAGCCTGGGAGATTGGCTCGTAAGGCTGA
AGACCGAGCTGGCGGACAAGAATTTTATCCTGATACAAAACGGCATCTCCGTGGAGAATGAGTAAAAGTGGGGGAAAAGT
ATATCACAGCGAGGAGAGGGGAGTTACCCGACCAGGAGCCGGGTAACGGAGAAGCGAGTTACAGAACCATGCGGACAATA
TCGATTTTGCCCAGTTCTTCATACAGTGTTTCAACCTGCTCGATATGAGTGGCGTTGATAGTGATAGATACCGAGTGGTA
GTTGCCTTTGCTGCTTGGTTTTACCGTTGGGGTGTAGTCACCTGGCGCATGGCGCTGTACCACTTCAACCACCTGATCAA
CCAGCTCAGGTAACGCCTGCCCCATAACTTTGTAAGTAAAAGGAGTAGGGAATTCAAGCAGTTCGTTAAGTTTGGTTTTC
ATGTCAGCTCCGGCGTAACGTAATTAAATAGCAACTCCCGCCAGAAGGCGGGAGTTTTTTACTGATGCTTAGTATATGGG
GACGGAAATTACACTTTCAAGTGTTTAATTTTTAACCAAACCAGTGATGGAACATTAATTTAATGTAATCAATGATTTTG
CCGAAGAAGTTACCTTCCGGGATTTCTTGCAACACAACCAGCGGGCGTTGCTCGATCGTTTTGCCATCAAGCTGGAAGTT
GATAGTTCCGACGACCTGATTCTTTTGCAGCGGCGCATGCAATTCACTGCTGTTCAGCACATAGCTGGCTTTCAGATCTT

TCATGCGACCACGCGGAATGGTCAGGTACACGTCTTTATCAACCCCTAACGAAGCGCGATCAGAATCACCAAACCAAACC
GGTTCAGAGGCGAACTCTTTACCTACTTTCAGTGGGTTAACGGTTTCAAAGAAACGGAAGCCCCAGGTTAGCAGTTTTTT
ACTTTCGGCTTCACGGCCTTTAAAAGTACGTCCGCCCATTACCGCAGAAATCAAGCGCATCTGGCCTTCAGTCGCAGAAG
CAACAAGGTTGTAACCTGCTTTGTCAGTGTGTCCGGTTTTGATGCCGTCGACATTCAGGCTGTTATCCCATAACAGGCCG
TTACGGTTCAGCTGGCGAATACCGTTAAACGTAAATTCTTTTTCTTTATAGATCGAGTATTCATTCGGTACGTCACGGAT
CAATGCCTGGCCGATCAGCGCCATATCTCGCGCGGAGCTGTACTGACCATCAGCATCCAGACCATGTACCGTCTGGAAGT
GGGTATTTTTCAGGCCCAGTGCGTTAACGTAGCTGTTCATCAAGCCAACAAAAGCGTCCTGGCTACCAGCGGCAAAATCG
GCCATGGCGACACAAGCATCGTTACCCGATTGCAGGTTAATACCGCGGATCAGCTGAGAAACCGGAACCTGCATGCCCGG
TTTGAGGAACATCAGCGAAGAACCTTTAAACACCGGGTTACCGGTGGCCCATGCGTCGTTGCCGATAGTGACTAAATCAG
TTTCTTTAAATTTACCGGCTTTCATTGCCTGGCCGATAACGTAACTGGTCATCATTTTGGTCAGGCTGGCAGGATCGCGG
CGGACATCTGCGTTCTGTTCGGCGAGCACTTTGCCGGAGTTATAGTCAATCAGGATGTAGGACTCCGCATCGATCTGCGG
TACACCCGGGATCATAGTTTTGATATTCAGGTCATCGGCATGTGCAGCAGAGATAAAGGCTGTGCAAAGAGCCGTGGTGA
GCGCCAGGCGCTTCATGATACGAGCGGAAAAAATGGTATTCATGGTCTGAACTACGACATCCGTGATGGAATTAAAAAAG
TGCCCTACTATAGCAAATGCACTACCGGCAGGCATCTGACTTTCCGCGTGACTTTGTTAACGTCATTTACAGAAATTGAC
ACATCAGATGCCTGCTTTACGCTACTGCGCGGTAGTAATAAATGACTGTAATTGGGCTTCGGTTTGCAAACGTTGCTGCA
AGGTACTGGCTTCGGCTTTGCTGGCGAATGGGCCAAGCTGGATCCGCCAGACCGCGCCATTTTGAGTTACGCGACCGGGG
ACGCCGAACTTCTGTCCCAGTTGCTGTTGGTACTGTTGCGCACGAGCCTGATCGCTTACGGCCCCGACTTGCACCATAAA
GTTGCCGCTGGCGCTTTGCGAGACGGCTTGCGGTGTCACCATTGCAGGCGAGGTTGCAGGCGTTGTCGACGGAGCTGTAA
CAACGGGCTGTGGCGCAGGCGTCGGTTCGCTGCCTTCCAGTACACCAGGCGCTAAGGTCGTTGGTGCGCCGAGGAAACCG
CTACTGGTTACCGGCGCGCCGGTCGGATCTTCGCTTTTTAGCGTCGAATTACTGACCGGAAGAATGTCACCCTGCGGGCC
AGACACTGAACTTGTTCCCGCGCCACCGCTTAAATCGGGAGGTGCAGGCAGGGCGTAAGTCTGTTTGGCGACTGTGGTAC
AAGCCATACCAGGACCAGAAAGCGAACCATCCTGGGCAACAATAATCGGATCGATACGAACTTTGGTGTTGTTTGACGTG
TTAAGACGGTCAGCTGCCGCGCGAGAAAGTGAAATAACGCGGTCGTTGCCGTAAGGGCCGCGATCATTAATGCGCACCAC
GATCATTCGCCCGTTAGCCAGGTTAGTGATTCTGGCGTAGCTGGGGATCGGAAGCGTTGGATGGGCTGCCGTCAGCTGTG
TCGGATCGAATGCTTCGCCAGAGGCCGTCAGGTTACTGCCGGGTTCGGCATCATAGATTGCCGCCAGTCCCGCCTGGCTA
AATCGAGACGGATCCTGCACGATTTTGTAGCTTTTACCGTCGCGCTGGTAATCCTGATTTGCCGTCGCGTTCAGTGGTTC
GAAACGCGGGTCCGCCCCGCTAATTTCAACTATAGGGCCGTTACATACCGCAGGCTGCGGTACACTTACCGTCTGTTGCT
GACCATCATCGCTTGTACATGCCGCGAGCATTCCTGCCGCGATGCAGATCCCGAGCCACTGCTTACGCATTGCGCACCTC
TTACACGCTTTTCGACAACATTTTCCTGTGGGTGTGGATTGACATTACAATCCCGAACCCAGCCATCAGCACAATTAGCG
CCGATCCTCCATAACTGACCAGTGGGAGCGGAACCCCTACAACCGGCAGAATACCGCTTACCATACCAATATTTACGAAG
ACATAAACGAATAATATCAGCATTAAGCCGCCAGCCATGACGCGACCAAAGGTGGTTTGCGCTCTGGCGGCTATCCACAG
CCCGCGCATGATCAGCAGAATGTAGAGAGCGAGCAGAATCAGAATGCCCACTAATCCCAGCTCTTCCGCCAGTACCGCGA
AGATAAAGTCAGTATGGCGTTCGGGGAGAAATTCAAGCTGTGACTGAGTGCCGTGCAGCCAGCCTTTGCCGCGTAATCCG
CCGGAGCCAATAGCAATTTTAGACTGAATAATGTGATAGCCCGCGCCGAGTGGGTCTGATTCCGGGTCCAGGAGCATCAT
TACGCGCTGGCGCTGGTAATCATGCATCAGGAAGAACCACAGAATCGGAATGAACGCCGCTACCAGCACTACTGCGACGC
CAATCAGACGCCAGCTAAGGCCAGAGAGGAACAGTACAAACAGACCGGAAAGCGCAACGAGGATTGATGTTCCCAGGTCA
GGCTGTGCAGCCACCAGCAGCGTGGGCATAAATATCAGCACCAGCGCGATGCCAGTGTTCTTCAACGATGGCGGGCAAAC
GTCGCGGTTGATAAAGCGCGCAACCATCAGTGGTACGGCTATTTTGGCAATTTCCGACGGCTGAAAACGAACAATACCGA
GGTCCAGCCAGCGTTGAGCACCTTTAGAGATGGCACCGAAAGCATCTACCGCCACCAGCAAAATAATACAGATGATATAG
AGATAGGGGGCCCAGCCTTCATAAACGCGTGGAGGAATTTGCGCCATCACCACCATGATGACCAGACCCATCGCGATTTG
GCCGATTTTACGCTCCATCATGCCAATATCCTGACCGCTGGCGCTCCAGATAACCAGGGCGCTGTAAACCAGCAATGCCA
GTAAGATCAGCAGCATTGTGGGATCGAGATGGACTTTATCCCAGAATGTTTTTTTATTCGGATTATCCGTCATGATTAAT
GGTCCTCCGCTGCGGCAACCGCTGGATTTTCCGCAGGCAGATCGGTGTTGTTATCACCCAGCATAATGTGGTCGAGGATC
TGGCGCATCAGTGTACCAACCGCCGGACCCGCACCACCGTTCTCCAGAATCATGGCGACAGCCACTTGCGGATTGTTGTA
TGGCGCAAAGGCGGTCATCAGTTTGTGGTCACGTAAACGCTCGGCAATTTTGTGCGCATTATAGGTTTCGTTCGCTTTCA
GACCGAAGACCTGAGCGGTACCGGATTTCGCCGCAATTTTGTACGGTGCGCTAGCAAAGTATTTATGCGCCGTACCGTTA
GGGCGGTTAGCAACACCGTACATACCGTCTTTCGCCAGCTCCCAGTAACCGGAATGAATATCGCCGACGGGCGGTTCATG
CGGCTGTACCCATGGCACCTGTTTGCCGTCTTCGGCGGTGCTCATCAGCAAATGAGGAACCTTCACGATACCGTCATTAA
TCAGGATCATCAGTGCCTTACTCATCTGGATTGGGGTCGCTGTCCAGTAACCCTGACCGATACCAACCGGAATGGTGTCA
CCCTGATACCACGGTTTTTTAAAGCGTTTCTGTTTCCATTCGCGGGTAGGCATGTTGCCGGAACGTTCTTCCGCCAGGTC
GATACCGGTGTAATGACCATAACCGAATTTACCCATCCATTCGGAGAGGCGATCGATCCCCATATCGTAGGCCACCTGAT
AGAAGAAGGTATCCGCAGATTCTTCCAGCGATCTTGTGACATTCAGACGCCCGTGGCCCCATTTTTTCCAGTCACGATAA
CGTTTTTCCGAACCTGGCAGTTGCCACCAGCCTGGGTCAAACAGCGTCGTATTGCGCGTGATCACCCCGGCGCTCAATGC
CGAAACCGCCACATAGGGTTTAACTGTAGACGCGGGAGGATAAACCCCCTGTGTGGCGCGGTTCACCAGCGGTGTATTCG
GATCGTTCAACAAGGCGGAATAATCTTTGCTGGAGATACCGTCAACAAACAAGTTTGGGTCATAACTAGGCGTGGAAACC
AGCGCCAGCACCCCACCTGTACGCGGATCGGTGACTACCACAGCTGCGCGGCTACCCGCCAGCAGCGTTTCAATATATTG

CTGGAGTTTGAGATCCAGCGTCAGGTAAATATCGTGTCCGGCTTGCGGTGGTACTTCTTTTAACTGGCGAATAACACGCC
CACGGTTGTTAACTTCAACCTCTTCATAACCGGTCTGACCGTGCAGCACATCTTCATAGTAACGCTCAATGCCCAGCTTA
CCGATATCATGCGTTGCCGCATAGTTGGCCAGTTTGCCGTCATTATTCAGGCGTTCGACGTCTTTATCGTTGATTTTCGA
CACATAGCCGATGACGTGGGTCAACGCCGAACCGTAAGGATAGTAACGACGTTTATAGCCTTTAACTTCGACACCCGGAA
AACGGTACTGATTGACGGCAAAGCGAGCTACTTGTACTTCGGTCAGGTTAGTTTTCACCGGAATAGAGGTGAAACGGTGT
GAACGTGCGCGCTCTTTTCGGAATGCAGCAATATCGTCATCGGTCAGATCTACCACGCTGCGCAAAGCGTCCAGCGTTTG
CTGCACGTTATCGACTTTCTCCGGCATCATTTCTATCTGGTAGATAGTGCGGTTGAGGGCCAGAGGGATACCGTTACGAT
CGTAGATAATGCCGCGGCTGGGCGCGATAGGCACCAGCTTAATGCGGTTTTCATTAGAGCGGGTCTGGTAGTCGGTAAAG
CGAACAATTTGCAGATTATACAGGTTGGCGATAAGCACGCCGGTCAGCAGCAAAATCCCCAAAAAGGCGACCAGCGCCCG
GCGCACAAACAGCGCGGACTCTGCCGTATAGTCGCGAAAAGAGTTCTGTAGTTTCATCCGCTGCGTTTTCTACTCAAAGC
TCCCTTATCACTCACGGTGATAAGGATGGTTGGTGGTGATGCTCCACGCCCGGTACAGACTCTCTGCGACCAGCACGCGA
ACCAGCGGATGGGGGAGGGTAAGCGCCGACAGCGACCAGCTCTGCTCAGCCGCCGCTTTACAGGCAGGCGACAACCCTTC
AGGCCCGCCAATCAGTAGACTGACGTCGCGACCATCCAGCTTCCAGCGTTCCAGCTCAGCGGCTAACTGCGGCGTATCCC
AGGGCTTGCCTGGAATATCGAGGGTGACAATGCGGTTTTTGCCTGCGGCCGCCAACATCTGCTCACCCTCTTTGTCGAGT
ATGCGCTTGATGTCCGCATTCTTGCCGCGTTTTCCGGCCGGAATTTCAATCAGCTCGAAGGGCATATCTTTCGGAAAACG
ACGCAGGTACTCGGTAAAACCGGTTTGTACCCAGTCCGGCATTTTCGTTCCCACGGCGACAAGTTGCAGCTTCACGCATT
AACTCCAGAGTTTTTCCAGTTCATACAGGCGACGGCTCTCTTCCTGCATGACATGGACAATCACATCGCCCAAATCCACG
ACAATCCAGTCGGCGCTGTTTTCACCTTCTACGCCGAGCGGTAACAGGCCCGCTGCGCGAGACTCCTGCACAACGTGGTC
AGCAATGGACATAACATGACGGCTGGACGTACCCGTACAGATGATCATGCAGTCGGTGATGCTGGATTTGCCCTGAACGT
CTAAGGCGATGATGTCCTGACCTTTGAGGTCATCAATTTTGTCGATAACAAAATCCTGGAGTGCTTTACCCTGCAAGTTT
TCCCCCTGGGTGAATCAAATAGATAAAAATGGTCTGTCAGTATACCTGAACCAGAGGCGATTTCGGGACAATTGTCGCCG
AATCGGCTTTCGAAAGTGGGCTATCATCCCACCCCGCGCCGCAGATTGCATCGCCATTTTTGTAAAACAATTTCTACAAA
GTCGTGTCTGGCGGAAAAAGTCTGGCTGCGGAGAATATCAGCCTGCCCGGGTCTGTCAATGGTCGTTGCGGCTTACCCGT
AAAAAAACAGAAAAGTCATGCATTCTCGACCCCGATGGCACGGCTATTGAGGACGCGTAGCGTCGCGAATTTTTGGTTGA
TATCAATGGCGCTCCAACACCCCTGGTCAACGCGAAAATGCCACATAGCTTCGGCAGGCATGCCAATTAAACGGGCGATT
AACAGACTCAGTACACCCTGATGGCTGACGACTAAAATATTCTGATAGTGCTGAAATTCACTAAGCCTTGCGATAAAGCG
TTCCACACGTTGCGAAAATGCCTGAAATCCTTCACCGTTCGTGGGGATTGCATGCTGCCAGTCATTGCACCACGCGCTAT
AGTTTTCGGCATCTTCTTGCATGAGGTCGCGATGATGTCGCATCTCCCAGTCGCCAAAAAACATTTCGTTGAGTTCAGGT
ATGATTTGCACGGGGAGCTGGCGGTCACTGAGAACCAGTCGCGCGGTATGCTGTGCCCGTTCCAGTTCACTGCATAAAAC
CAGATCAAAGGAAACACCATGTAGCAGCGTATGCAGATTTTGCGCTTGCTCAATACCGCGCGCGGTCAGGGGGGTGGGCG
CATGACCGCTGTAAAGACCATCGATATTCGCTTGCGTTTCACCATGACGAATTAACCACAGTCGCATCATGCCCTCCGTA
ACGACAGGTATCAGCGATACAAGCCTTGTTGGTTAATGTAAGTCAGTACCGGTTCCGGCAATAAATCCTCACATGATTCA
CCGTTTTGCAAACGTTCGCGGATGATGGTCGCCGAGATGTTAAACCACGGCGTTTCAGCCAGATAAATTTTACCGGCAGG
CTGAAGGTGAAGATCTTCCGGGTTATGTGTCAAATGATCTTCCAGCCATTGCTGGTATTGCGGTTGCGCCATTTCAAGTG
GGTAACCTGGACGCCGACAGACGATCAAATGTGCATTGTCGAGTATCGTTTCGTATTCGTACCAGGTCGGAAAGGTCAGC
AGTGAATCCTGACCAATAATAAACGCCAGCGGCACGTCCGGTCCTTGTTCCTGCCGCCACTCTTTCAGTGTTTGCGCAGT
GTAAGAGGGGGCATTGCGCTTTAGCTCGCGTTCATCAAGAGTAAATAATGGCTTGTCGGCAATCGCCAGTTCAAGCATGT
GTTTACGCTGCACGCTGTTCGCTTCCGGCTGGGGACGATGCGGAGGAACATTATTAGGGATGATTGTGACCCGCGTCAGA
CCAATCAAATTCGCCAGCGTTTCCACGGGTTTTAGATGACCATAGTGCACCGGATCAAAGGTGCCGCCAAACAGAGCCTG
TAAAGATTTCATATCAACCGTCGATAAATACGTCCGCCAGGGGTTTATGGCACAACAGAAGAGATAACCCTTCCAGCTCT
GCCCACACTGACTGACCGTAATCTTGTTTGAGGGTGAGTTCCGTTCGTGTCAGGAGTTGCACGGCCTGACGTAACTGCGT
CTGACTTAAGCGATTTAACGCCTCGCCCATCATGCCCCGGCGGTTCTGCCATACCCGATGCTTATCAAACAACGCACGCA
GTGGCGTATGGGCAGACTGGCGTTTCAGGTTAACCAGTAACAACAGTTCACGTTGTAATGTGCGCAACAAAATAACCGGT
TCGCTGCCTTCCAGACGCAGTTGCTGAAGAATATGCAATGCGCGCTTACTTTTTCCCATCAACAAAGCATCAACCCAATG
AAAAGGGGTGAAATGCGCGGCATCATTCACCGCCTGTTCAACGCGCGGTAATGTCAATTTGCCGTCTGGCCAGAGCAGCG
ATAAACGCTCCAGTGCCTGAGCCAGCGCCAGCAGGTTACCTTCATAACAGTAGCAGAGCACCTGATTTGCCGCGTCATCC
AGTTCTAAGTTGAGCTGTTTTGCGCGCGCAGCAACCCAGCGGGGAAGCTGAGCCTGCTCCGGTGTCTGACAGGTCACCTG
CACGCTGCGATTCGCAAGCGCAGTAAACCAGGCGGCATTTTCTTGCGCTTTGCTTAATTTATTACCGCGGACGATCAACA
GCAGGTCGTCATGCAGAAGTCCGGTGAGTGTGAGAAGTTGCTCATTGATCGCCGCATTCGGTCCGTTTTCTGGTAACAAC
AGCAATAGCGTTTGTCGACTGGCAAACAGACTCATAGCCTGGCATAACGAAAAGATCGCATTCCAGTCAGTGTTGGGATC
AATGGAAAAAGTGTGGTGTTCTTCGAATCCTTGTGCCGCAGCTACCTGACGAACAGCGTCCTGGCTTTCCTGCAATAACA
GAGGATCGTTACCAAGTAAAAGATACGCCGCGCGCAGCCCTTCATTGAGCTGCGCGCGGAGTTGTTCCGGGTACAACCGA
ATCATCAGTTACCCAGCGTGGTGGAGACGCGTGCAGGCGTTGCCGGAGTATCCGTTGTGGTCGACGTCTGTTCTTCGTCG
GAACGAATATCCGCAGCACGGATGCTTGGCAGCTTACGAATCAGCTGTTCGGCAGCACGGTCGTACATCTCTTTTACGAT
CATGTCTTGTTCGTTATCTTTCGCTAACGCCATTTGCGGGTTATCGAAGAACGAACGGAAGACTTTGGCGCTAATCGGGT
AGATATCACGGCCGGGGATCAACACGGTCGCATTAACCGTCATGATCATCTGATACTCTGCTGTTTGACCGTTACGGAAT

ACCGATGCGGTATCTTTCGCGATGCTCACTTTACCCAAACGCAAGGATGGAACGTCCTTACGCGTGGTTTCTTTATCAAG
CAACTCGACACCATTCAGACGTAACTGGTTACGCACCGCACGGCTTAATGGCCCGTTCGGATCGCCTGAGTCCAGGATCA
TGACCTTCATAGTGGAAGGAACCTGCGTGGTATCACGCAGATGCCAGCCACACCCGGCGGTGATTAACACCGCCAGAGAT
AACAACAATGTTGCCAGATATCGCACGCTTCCTCCCGCGCTTAGCCAACGACCAGATTGAGGAGTTTACCTGGTACGTAA
ATCACTTTACGTACAGTAACGCCATCAAGATATTTTGCTACCAGATGTTCCTGGCCAGCACGTTCGCGAACCTGTTCTTC
CGTTGCGTCCACCGGAACGGTGATTTTGGCACGGACTTTACCGTTAACCTGCACCACGACCAGCGTGGAGTCTTCCACCA
TCGCTTTTTCGTCAGCAACCGGCCACGGCGCGTTGTCGATATCGCCTTCGCCTTTCAGTTCCTGCCACAGCGTGAAGCAG
ATGTGCGGGGTGAACGGGTTAAGCATACGGACAACGGCCAGCAGTGCTTCCTGCATCAGAGCGCGATCCTGCTCGCCATC
GGTTGGTGCTTTCGCCAGTTTGTTCATCAGCTCCATAATCGCCGCAATTGCGGTGTTGAAGGTCTGACGACGGCCGATAT
CATCGGTCACTTTAGCGATCGTTTTATGCACATCGCGACGCAGCGCTTTCTGATTTTCAGTCAGCGCATCAACGTTCAGT
GCCGCAACATCACCTTTTGCTGTGTGCTCGTAAACCAGTTTCCAGACACGTTTCAGGAAGCGGTTAGCCCCTTCCACACC
GGATTCCTGCCATTCGAGAGTCATATCAGCCGGAGAAGCAAACATCATAAACAGACGAACGGTGTCCGCGCCGTAACGTT
CAACCATCACCTGCGGGTCGATACCGTTGTTCTTCGACTTGGACATTTTGCTCATGCCGGTATAAACCAGTTCATGGCCT
GCCGCATCTTTCGCTTTCACGATACGGCCTTTCTCGTCACGTTCAACGATAGCATCAACCGGGGAAACCCAGTTACGTTC
GCCGTTTTCGCCAACATAGTAGAAGGCATCTGCCAGCACCATACCCTGACACAGCAACTGTTTCGCTGGTTCGTCAGAGT
TCACCATGCCTGCATCACGCATCAGTTTGTGGAAGAAGCGGAAGTAGAGCAGGTGCATAATGGCGTGTTCAATACCACCA
ATGTAGATATCCACCGGCAGCCAGTAGTTAGCCGCTTCGGAATCCAGCATACCTTCTTTGTACTGCGGGCAAGTGTAGCG
CGCATAGTACCAGGAGGACTCCATAAAGGTGTCGAAAGTGTCGGTTTCACGCAGTGCTGGCATACCGTTAACGGTAGTTT
TCGCCCACTCCGGATCTGCTTTAATCGGGCTGGTAATGCCGTCCATTACCACATCTTCCGGCAGGATCACCGGCAGCTGG
TCGTCCGGGGTCGGCATTACGGTACCGTCTTCCAGCGTCACCATCGGAATCGGCGCGCCCCAGTAACGCTGACGGGAAAC
ACCCCAGTCGCGCAGGCGGTAGTTCACTTTACGCTCGCCAACGCCCATCGCAGTCAGTTTATCGGCGATGGCGTTGAAGG
CCGCTTCATGGTCAAGACCGTTGAACTCGCCAGAGTTGAACAGCACGCCTTTTTCAGTCAGGGCTTGCTGAGAAAGATCT
GGCTCAGAGCCGTCAGCTGCCAGGATAACCGGTTTGATGTTCAGGCCGTATTTAGAGGCAAACTCGTAGTCGCGCTGGTC
GTGCCCCGGTACCGCCATAACTGCGCCCGTGCCGTACTCCATCAATACGAAGTTTGCTGCCCAAACGGGAATTTCTTCGC
CCGTTAATGGGTGAACCGCTTTAAAGCCAGTATCGACGCCTTTTTTCTCCATCGTCGCCATTTCAGCTTCGGCAACTTTG
GTGTTACGGCATTCGTCAATAAAGGCCGCCAGTTCAGGATTATTTTCCGCCGCTTTCTGCGCCAGCGGATGACCCGCAGC
TACCGCCAGGTAGGTACAACCCATAAAGGTGTCCGGGCGGGTAGTGTAAACGGTCAGCGTGTTGTCATAGTCGTTAACGT
TGAAGGTGATCTCCACGCCTTCGGAACGACCGATCCAGTTACGCTGCATGGTTTTAACGGTGTCTGGCCAGTGATCCAGT
TTATCCAGATCGTTGAGCAGCTCGTCAGCGTAAGCAGTGATTTTGATAAACCACTGCGGGATCTCTTTACGTTCAACTTT
GGTATCGCAGCGCCAGCAGCAGCCGTCGATAACTTGTTCGTTCGCCAGTACGGTCTGGTCGTTCGGGCACCAGTTGACCG
CAGAAGTCTTCTTATATACCAGGCCTTTTTTATACAGCTCGGTGAAGAATTTCTGTTCCCAACGGTAGTATTCCGGCGTA
CAGGTTGCCAGCTCGCGGCTCCAGTCATAACCAAAGCCCAGCATTTTGAGCTGGTTTTTCATATACGCGATGTTGTCGTA
CGTCCACGGTGCCGGAGCGGTGTTGTTTTTCACCGCCGCGCCTTCCGCAGGCAGACCAAACGCGTCCCAGCCGATCGGCT
GCAGGACGTTTTTGCCCAGCATACGCTGGTAGCGGGCGATCACGTCACCGATGGTGTAGTTACGTACGTGGCCCATGTGT
AGTCGACCAGAAGGATAGGGAAGCATAGACAGGCAGTAATACTTCTCTTTGCTCTCGTCTTCGGTTACTTCAAATGTGCG
CTTCTCATCCCAATGAAGCTGTACTTTGGATTCTATCTCTTCCGGGCGGTATTGCTCTTGCATGGCAGCCAGTGGTCCTG
TTTTCAATACGGCTACAAATGTAGCGTTGAGGTGGTTTTTCAGATCCGCATAGCATAGCCCAAACGTCCGCGTCAAAACA
GCCTTTCGCGCACTCGACGTTGAAATGATGCCCGGATTATTCATACATTAATTTACAGAGTTTGTGGGCGTATTAGCAAA
GCAAGGAACAAAGAACGTCTATTATTATAGTCAGTTAACGACCCGGGAGATGAAACGATGAACAAGGTTGCTCAATATTA
CCGTGAACTGGTTGCGTCACTGAGCGAACGCCTGCGCAATGGCGAACGTGATATCGACGCACTGGTGGAACAGGCGCGCG
AGCGCGTAATAAAAACAGGGGAGTTAACGCGAACCGAGGTCGATGAGCTGACGCGAGCTGTCAGACGTGACCTGGAAGAG
TTCGCCATGAGCTATGAAGAGAGCCTGAAAGAAGAATCTGACAGCGTCTTTATGCGGGTGATTAAAGAAAGCTTGTGGCA
GGAGCTGGCAGACATCACCGATAAAACGCAGCTTGAATGGCGCGAAGTTTTCCAGGACCTCAATCATCATGGGGTTTATC
ACAGCGGAGAAGTGGTCGGGCTGGGAAATCTGGTCTGCGAGAAATGTCACTTCCATCTCCCGATCTACACACCGGAAGTG
CTGACGCTATGCCCGAAATGTGGTCATGACCAGTTCCAGAGACGCCCGTTTGAGCCGTAATAGTCTGATTTCGCACAGCA
ATAAAGAGAATATGGGCCGAATGAAATTCCCCGGCCCTTGTTTTTATCTCTACAGTAAATCTTCAGTCTCACGAATTTCC
TGACTGACATCTCTGGTTCCGGATGACTGAACTTTACGATACAGGTCGAGAGCCTGTCGGAGATCGACAGGTCTTCCTTC
TCCGTAGTGATACATAATGGCCAGGTTATACCAGGCAGTGGCGTCATTGCATTCAGCGGCTTTCGTAAACCATTCAAAGG
CAGCCTGATAATCTTTCTCAACGCCTTGTCCGTGTTTATACATATAACCGATGTTGACGTAAGCGCCGACGCTCCCCTGA
GCGGCAGCCTTCAGATACCAGTACATCGCTTGCTTATAGTCCTGGTCCACCCCTTCGCCAGCGTTATAATCCCATGCAAT
TTGAAACTGGGCGTGCCGATTACCCTGCTGTGCGCTTTTCAAATACCAGAATGCGGCGAGTGTCTTGTTTTGAGCAACGC
CTTTTCCGTCTTCATAAAGATCGGCCAGATTGTTTTGCGCGTCACTATGGCCTTGTAATGCAGCTTGTTTGTACCAGAAA
AATGCCAGCGCATAGTCTTTTGCGACTCCGTTGCCATTTCTGTACATCCACCCAAGATTATTTTGCGCATAAGACATTCC
GCTCTCCGCGGCTATCTGATACCAGACAACCGCTTCTTTATAATTGGGGGCTTCTCCACGATCCAGTGTCCAGCCAAGGG
CGTTGGAAGCGAAAGTATGGCCTTGTAGGGCAGCTTTTTTCAGCCAGAAGATGGCTTGCTCATTATCTTTATGACGGCTT
TTATCCTCGGTATACTTTTGCCCTAAGGAATACTGTGCTTCACAATGACCTTGCTCAGCGGCCAGCTTCAGCCAGTAAAA

GGCTTTTTCGTCGTCCGGAGAATCAATTGCGCTATCGCGATTATAATAAAACCCAACAATATATTGAGCCTCGCAATCGC
CTTTTTCAGCACGTTCGATAATCTCATCTATTGATAAATTATCGCAGCAACTTGACGTGAAAATCATTATCATTTCCTTA
TGGTTGCAGATAAAACATATTATATTTTTATGTGTTCCATTTTTGCGTAAGCCTTATTTTATGCGTATTATGTCGCGTCA
GAAAAGTATAAATAAACGAGCCTCGCAATGCGGCTAATATTCATTTAATGAATATTTAAGGATAAATTATATGGACATGG
AGTCGCAAAAAATATTGTTTGCACTTTCCACTCCGATGGAAATACGCAATGAATGCTGTTTACCTTCGCATTCATCACCC
AAAATGTATTTAGGTACATGCTTTTTTGACCTTTCTTCCTCATGGGGAATTGATGACCGTGATGACCTGCTGCGCACAAT
TCACCGAATGATCGATAACGGTCATGCCGCTCGGTTGGCAGGGTTTTATCACCGCTGGTTTCGTTATTCGCCATGTGAAT
GGCGTGACTATCTTGCTGAACTAAATGAGCAAGGTCAGGCTTATGCGCAATTTGTCGCCAGCACCGCCGAATGCTGTGGC
GAAGGGGGAATCAAAGCCTGGGATTATGTCCGGATGGGTTTTTTAAGCCGAATGGGCGTACTTAATAACTGGTTGAGCGA
AGAAGAAAGTCTATGGATCCAGTCGCGCATTCATCTACGGGCTCTACGTTATTATAGTAACTGGCGGCAATACTTTGCCG
GTTATACCTTTGGTCGGCAATATTGGCAGTCTCCCGAAGATGATCATCTGCCTTTATTACGCGAATTCTTAGCGCGTAAA
GAATACGACGATTCCGGCAATGATATGTTTTATCAATTATTTGCCAGTGATGATGCGTATTACCCTACCTTGTCCTGGCA
ACCATTGGCTTACTATTCTGCATGCCCGGAAACGCTTAAGGATATGAGCGACCTATGAAAAATTGCTGGAAGATCCTCGA
TATAGAGGAAACGACTGACGTCGATATTATCCGCCGCGCTTATCTGGCGCTGTTACCGTCCTTTCATCCAGAAACCGATC
CGCAGGGTTTTAAACAACTTCGTCAGGCGTATGAGGAGGCGCTACGGATTGCGCAGTCGCCTGCTAAATCTGTTTGGCAA
CCAGAAGAATATGAGGTAGCAGAACATGAAATTCTGCTCGCCTTTCGTGCGTTACTTGCCTCTGATAGTGAACGTTTTCT
GCCCTCCGCCTGGCAGCGATTCATTCAGCAATTAAATTATTGCTCGATGGAGGAGATTGATGAATTACGCTGGTCGCTGT
GCACAATCGCCATGAACACTGCCCATTTATCCTTCGAGTGCGTGGTGTTATTAGCAGAAAGATTGCGGTGGTTACAGGAG
GAAAATACCGGGGAAATAGACGAAGAAGAACTGGAATCCTTTTTATATGCCATTGCGAAGGGGAATGTTTTTAACTTCCA
GACCATTCTGCATCTGCCCGTTGCCGTGCAAAATGACACCATCGATTTTTACCAAATGTTCGCTCGGATTTGGTCATCGC
ATCCACAATGGCTGACATTGTATTTAGCGCAACATCGCGCAGTGATTATCCCCGATGATGCAAAACTGCACAGAAATTTA
CTCCGCTGGTATAGCGCAGGTCGCCTGGATATCCCCGAACTTCTGGATTACGCCCAGTCATGGCGGGAAACTGAACCTGA
TAATGAAGATGCGCCTTATTATGAATACGCGCAACGCGTCTATTGTGGAGAAGGCGAAAGCCTGTTGGCAGAACTTTGTG
ACTACTGGCGCGAGTATCCCTCCACCCAGGCGGATGCTTTAATGTTGCAATGGTGCCGTCAGCATCGGGTCGATTATTAC
CCATTACTGGTGATGATGATTGAAGCGCGTGATCTGGTTAACGATCAGGGAAAACCGCTACTTTATGTCCCCGGCGACAG
CGCCCGTACGCGTTTTCATTTATACGAAATACTCAGCGATGAAAAACTCTCTGCGCTGGGGCGTTCACTGGTCGAGATGG
TTTTGCACAAAGGACGTAAGCCGCGGATCTCACTCACGCGTGATACAGAACATACCTTATGGCCATTATATCTAGTTGCC
AAACAATTAGTGCAGGCCTGCCAACCTACAGAAGAATCATTAATGCCGATTGTGAGCCGCCTTGATGCAGAAAATCGTTG
TCCACTGGAAGCATTAATTATTCGTCGATTATTAATTCAGGCGGCGAATTTTACCGAGAAGCAAACTGTTGAACCGGAGC
CGCAACCGCAGCCAATGCCCGTTGACGATGGTGGGCCAGGCTGTCTGGGCATCATTAAAATTATTTTCTATATTTTTATC
TTTGCTGGTTTGATAGGGAAAATACTCCATCTGTTCGGGTGACAGTCGACGTTAATGTGCGGGAAGCCCGGCAAGTAATC
TTTCCGCATTTTTGTAATGTTGTTTGGCGGTCAGTTTTAACCATTTTTGAGATTCATAATGGTTAACATTAATACCTGAG
CCATTTCTGTAGATCAAACCTAAAGTATATTGAGCATGAGCATACCCTTGTTCAGCTGCTTCCTTATACCAGAGAATTGC
TTTACGGGTGTCGGGTTTGACCCCCAGACCTTTGAGGTAGATCCACCCCAGTTTACTTTGCGCGCGTGGATTTTCCTGTT
CCGCTGATTGCTCATACCAGTATCTCGCCTGTGTATAATCCAGTGGTTTACCGTCGCTACCATGAAGATAAAATTCTCCA
AGGCGATATTGCGACGTTGAGTGACCATGAATCGCGGAAAGCTCCGTGGCTGCCGTGGTGAATGGAAGACTGCCCGGTGA
GCTTAAATAATCAAAGGCATAATTCACCATGGCTTTATCACGCAACGTCGTTTTCTTGGGTTGGCTAATACAGGTAATTG
CTGCAACTATAAAAATTGCGAATATATACATTAATTTTTTATTCATGTGTGAATGCTTCGATTTTCTTTTTATTTTGAAA
GCGTCAAGTTTATACTCATCAATAATAAAAAAGTAGTCAGTTAATGCACTTAAGGGATTATTAACAAATGAATAAGGAAG
AACAATATCTTCTTTTTGCGCTTTCTGCGCCCATGGAAATACTTAATCAGGGTTGCAAACCCGCGCATGACTCGCCAAAA
ATGTATACTGGGATAAAAGAATTCGAGCTCTCATCATCCTGGGGAATAAATAATCGTGATGATTTAATTCAGACTATTTA
TCAGATGACTGATGATGGTCATGCCAATGATTTAGCTGGCTTATATCTGACATGGCATCGCTCTTCCCCTGAAGAGTGGA
AAGCATTAATTGCTGGTGGCTCGGAGAGAGGGTTGATTTATACCCAATTCGTTGCCCAAACCGCTATGTGCTGTGGGGAA
GGGGGAATCAAAGCCTGGGATTATGTTCGAATGGGGTTTCTGTCACGGGTCGGTGTGCTCAACAAGTGGCTGACGGAAGA
AGAGAGTTTATGGCTGCAATCGCGCGTCTATGTACGAGCTCACCATTACTATCACAGCTGGATGCACTATTTTTCCGCCT
ATTCGTTAGGACGTCTCTACTGGCAATCTTCTCAGTGCGAAGACAACACTTCGCTACGCGAGGCGCTAACCCTGTACAAA
TACGATAGCGCCGGGAGTCGTATGTTCGAAGAGCTGGCAGCGGGAAGCGATCGTTTCTATGCCACGCTTCCCTGGCAGCC
ATTAACTGTCCAGTCTGAATGCCCGGTAACGCTTAAGGATGTGAGCGACCTATGAAAACATGTTGGCAAATCCTCGAAAT
TGAAAGCACGACGCAAATAGACATTATCCGCCAGGCTTATCTTGCTCGCTTACCGTTGTGTCATCCCGAAACCGATCCGC
AAGGGTTTAAAGCATTACGCCAGGCCTATGAAGAGGCCCTGCGACTGGCGGTAAATCCTGTCGAGGAAGCAGATGATGAA
GAAAAAGATGCTGCCGCTGAACATGAAATACTACGTGCATTCAGGACATTACTGGATTCAGAAAGTGATCGTTTTCAGCC
TTCCGCCTGGCAGAAATTTATTCAGCAATTAAATACCTGGAACATGGAGGATGTCGATCAATTACGCTGGCCGCTGTGTG
CAATCGCCATAGAAGCGCGATATCTTTCATTAAATTGTGCTTCTTTGCTGGCAGAGCGTTTGAACTGGCATTCATTTAAT
GACAGCGAAGGAATGGATGAGGAAGAAAGGGAGGCTTTTCTTGAGGCCATTCAGGCTGGTGATTGTTTCGATTTCCTTAG
CCTTCTGGAATATCCCATTGCGTTGCAGAACCAGACTGTTGAGTATTACTTCGCGCTGGAACGTTGCTGCCGTTACCATC
CTGACTATGTCACTGCGTTTTTGGCGATGGAAGGTCCGTGGTTAATTCCTGATGATGCAAAGTTACATCGCAAACTGTTG

CGCTGGTACAGCTCGGTGCAAACAGGTATGGCGGAACTCATTCCTGTCGCTCAACAGTGGCAAACGGAAGAACCAGAAAG
CGAAGATGCCCGGTATTACTTGTGTGCACAACGTTTGTACTGCGGCGAGGGGGAAAGCCTGCTTGCCGATCTCTGCGCGT
ACTGGGAAAGTTACCCATCTACACAAGCTGATAATTTGTTGTTGCAGTGGAGCAAGCGTCATTGCCCGGATTATTTCGCG
TTATTAGTGATGGTTATCGAAGCGCGGAGCATGGTAGATGCGCAAGGTCAACCGCTGAAATATGTTCCTGGTGAGAGCGC
CCGGACGCGGCTGTTATGGGCGGAGATTTTACATAGCGGAAAATTATCGCCGTTAGGTCAATCGTTTATTGAGTCGTTAT
TCTTCAAGCGCAAAGCATGGGCGTGGTGGAAATCGAGAGTCGGTAGCGAGACAGAGCAAGATTCACCGTTCCTGGATTTG
TATCGGGTAGCGGAACAGGTAGTACTTGAAGCGTTTCCGAAACAAGAGATGCTGGCCCGTCTTAATACAAGGCTGGAAGG
CGGAGATGCTCATCCATTAGAGGCCATTGTCACCCGGATGCTTTTGACGAAAGTGAAACTCGAGCCGGAGGATGAAGATG
TCGATGAGCCAACACCTGAAAATCATGAAGAAAAAAATGATGAGGGTGAAAAACCACAGAGCATTACCAGCATTATCAAA
ATCAGTTTAACGGTGCTGGTGATAGGTTATGCTCTCGGCAAAATCGCGATGTTGTTTAGCTGACGATGTGTTTTTTGCGC
CGATGCCGGATGCAGCGTTTACGCCGCATCCGGCAGCCGTGTTTATGGGATCTCAATGGCTAAATAATCGCTCGCCCGCC
GCCGAACTTCATCCACGCGGGCCATATCGTTTGACTGCAACGCCGCATCAAAATCGGTGATCACCCGGCCGATCTCTTCT
CGCTCGTCACCCAGCGCCCGCGCCCACAACTCTTCCAGTTTGGCTTTAAAGGTGCGATTGATGAGCATATCGCGCGGATA
AATTTTCAATGCGGATAACCGCGTCCGACTCTCTTCAATCTGCTGCGCGCTCAATGTTACCGGGCTGTGGTTAATCACTC
TGGACTTAACGCTGCCGTCTTCCAGAAGCACGTCAACTTCAAGCAACCCGTTGATATCATAACTAAAGCGAATATCAATC
GACTGATAAGCCCCGGTTTTCTTCAACGGCACATCGAAGGATTCCACCAGAATGTTGTTTTTAACTTTGTGGTTTTCTCC
CTGATAGACGTTAACCGTAATTGAATCCTGTTCCGGGTGCATGGTTGAATAAGTTTCTACACGCGACACGGGCACAGTGG
TGTTTCGTTCAATAATCGGCGAGAAAATGCCGGAAACGCCCTGGCGGTTAACTTCAACGCCCAACGAGTAAGGGCAAATA
TCAGTGAGGATTACCTCTTCAATATCTTCACTGCGTAAGCGGCAGGCGGCCTGGATTGCTGCGCCCAGCGCGACAATGGT
GCTCGGATCGTAACTTTGATACGGTAATTTGCCAAACAGACGCACGGCGATTCGCTGCACCAGCGGCATTTGTGACGCGC
CGCCAACCAGCACCAGACTGTCGATTTGACTCGGCTTCAGACGCGCATCGCGCAACGCCTGTTCAATCGGCACTCGCAAG
CGATTGAGCAGCGGCAACCACAAATCTTCCAGTTCGTTCTCGTAAAATTCGCATTCCCGCGTTTCTTCCTGATACTGCCA
GCGAATGTGCAATGGCGATTGATTGCTACATTTTGCCGCTTCCACACAGGCGTACAAGGCTGCCAGTTCACTCTCGTTAA
GCGTGGTCCTGGCGACATCCGCGCGTTTTAAAACCTCATCGACCAGCATATGGGTAAAATCTTCGCCACCAAGAAAGTTG
TCGCCAGCGGAGGCGTGAACTTCAATCACCGGCGTGGCGTACTCAAGCACCGTAACGTCAAACGTGCCGCCACCGAGATC
AAACACCAGCGAACGGGTATTTTGTTGGGTATGCAGGCCATACGCCATCGCAGCTGCTGTGGGTTCATTAATTAAGCGTA
CCGCATTTAACCCGGCTAACTCCGCTGCTAAACGGGTATGCTTGCGTTGTTCATCGCTGAAATAAGCCGGAACGGAGATC
ACCACATCTTTAATCGGACGTTGCAGAAATTCTTCGGCATCTTCTTTTAATGAGCGTAATACCAAAGAGGACAGTTCTGG
CGCGTTAAATGTGTCGCTGCCTAACCGCCAGTTGGTATTACTGCCCATTGCACGTTTAAATAACGCTGCCGTTTTATCCG
GATGCGAAGTACGCCGTGATACAGCCGGTTTTCCGACTAAAATATGATTATTTTCATCCATGCTAATTATGGATGGTGTT
AAATATTCACCGAACTTATTTGGAATTAATTGCGCGGCACCGTCTTTCCAGACGGCAATTAAACTATTGGTAGTACCGAG
ATCGATACCAATGGCGAGTTCTGCATTATCCATTGCATTTATATCTTGAAAGAAAAAGAGATAAACAGATTAAGACCACC
CAGGTAACGCGTGGTGATCTTAATCAATGACGTGTGTTAAGCGTAAAATTTCAGACGATCAGCCAGTAAATCAACAAAGC
CCTGACGATCAACATCGACCATTACGGTGGCATTCGGTTTATTGCCTGTCAGATAATAATAATCAACAACCGTCATACCC
TGGGTATATTTCCCCTGTGTTTCCACGCCAACCCAGCGCTCAACAGAGGTAAATAACTCCGGTTTCAACAGCCAGGCGAT
GGTGCATGGGTCATGCAGTGGTGCGCCGACAAAGCCCCATTTTTCGTCTTTATGATATTCGAGGAAGAAATCCAGCAGTT
CGGCAACAATGGTTGAAACAGGGTTACCAATCGCGCGGAAACGCTCGGTGTCTTCAACGTGGATTTGTGCTTTATGAGTA
ACATCCAGACCGGCCATCACCACCGGGATCCCTGACTGGAAGACAATTTCTGCCGCTTCCGGGTCAACGTAAATATTAAA
TTCAGCCGCAGGCGTCCAGTTACCCAGCCCCATAGCGCCACCCATAATCACGATACGGGCAATTTTGCTATGCAGTTCCG
GATGGCTATTGAGCAGCAAGGCAACGTTAGTTTGCGGTCCGGTAGACACAATGGTGACAGGTTCCGCACTTTCACGCAGC
GTTTTCGCCATCAGCTCTACCGCCGTACAGTTTTGCGGTGCGAATGTCGGTTCCGGTAATGCCGGGCCGTCGAGACCGCT
TTCGCCGTGCACATTGTCCGCGATAATCAACTCACGCATTAACGGTTTTACCGCGCCGCCTGCTACCGGAATATCGGTGC
GATTAAGCAAGGTCAGCATACGCAGAACATTGCGTAAGGTTTTTTCTGGTGTCTGGTTTCCGGCGGAAGACGTAATTGCT
TTGACATCAAGCTCTGGTGAGGCGAGGGCGAGAACTATTGCGATAGCGTCGTCATGACCTGGGTCGCAATCTAACAGAAT
TGGCAGTGCCATTGTTGCTCCTTGTTGTGTGCTTCTTTGCGACAAGGGTAACGCCAGGATGTAACAGATACGAGGGGCGA
AACGATAAAGCGTGAGATGGCGCGCAATTGGGTATGCGCGCCAGAGTGATTAATGCAGGATTTTCGCGAGGAAGTCTTTT
GCGCGGTCCGATTTCGGATCATCGAAGAAAGCGTCTTTCGGCGAGTCTTCGACAATTTTACCCTCGTCCATAAAGATCAC
CCGATTCGCCACTTTACGGGCAAAGCCCATTTCGTGGGTCACCACCATCATGGTCATTCCTTCGTTCGCCAGTTCCACCA
TCACGTCCAGTACTTCGTTGATCATCTCCGGATCCAGCGCCGATGTCGGTTCGTCAAACAGCATCGCAATAGGATCCATA
CACAACGCGCGAGCGATTGCCACACGCTGCTGCTGACCGCCGGAAAGCTGCGCCGGAAACTTATTGGCGTGAGCAGAAAG
CCCGACACGCTCCAGCAGTTTCAGGGCTTTTTCACGAGCCGGCGCTTTATCGCGTTTAAGCACTTTCACCTGCGCCAGGG
TCAGGTTTTCGATAATCGACAGATGAGGGAACAGCTCGAAATGCTGGAATACCATCCCGACGCGGGAACGCAGCTTTGCC
AGATCGGTTTTCTTGTCGTTAACCACGATACCATCGACGGTGATTTCACCTTGCTGCACCGGTTCGAGGCCGTTGACGGT
TTTAATCAGCGTTGATTTGCCGGAACCAGACGGGCCGCAAACCACCACCACTTCGCCTTTTTTCACTTCGGTTGAGCAGT
CGGTCAGCACCTGAAAGTGACCATACCATTTTGAAACATTTTTCAGGGTAATCATTATGCTGTCCTTCTTTTCAAGTAGC
TGACCAACAACGACGCGCTAAGACTAATAACGAAATAAACAAATCCGGCAAACAGGATCATCTCAACCTGCGTACCATCA

CGCTCACCAATGGTTGAGGCGGTACGGAAGAAATCGGCCAGGGATAACACATACACCAGTGAGGTATCCTGGAACAGTAC
GATGCCCTGAGTGAGCAGCAGCGGCACCATCGCGCGGAACGCCTGCGGCAGAATAATCAGTTTCATCGACTGCCAGTGAG
TCATTCCCAACGCCAGCGCGGCGCTCGATTGACCACGAGAAATACTTTGAATACCAGCACGGATAATCTCTGAATAGTAG
GCCGCTTCAAACATCGAAAACGCCACCATCGCCGAAATTAAACGGATATCATTTTTTGGCGATAATCCCAGCACGTTTTG
CAGAAAACCCGGCACGATCAGGTAAAACCACAGCAAAACCATAACTAAAGGAATCGAGCGGAATACGTTAACGTAGGCTT
TGGCAAACCACGCCACGGGCGCAAAGCTGGATAAACGCATCACCGCCAGCATCGTGCCCCACAAAATACCAATCACTACC
GCCGTGACGGTGATTTTCAGGGTGATCACCAGCCCGTCGAGCAGATATGGCAGGGAAGGGACAATGGAACTCCAGTCAAA
CTCGTACATTATTTGCCCCCCATGTTGCCAGGCAGGCGAACTTTACGTTCAACCAGCGTCATCACCAGCATGATAAAAGC
GTTAATCAACACATACGCCAGCGTAATGGCGGTAAACGACTCCCAGGCATGGGCTGAGTAATCGAGCAATTTACCCGCCT
GCGCCGCCATATCCACCAGACCGATAGTCGAGGCGATGGCGGAGTTTTTCACCAGGTTCATCATCTCTGAGGTCATCGGC
GGGACGATAACGCGATAAGCATTAGGCAGCAGTACGTATCGATAAGCCTGCGGTAGCGTCAGGCCCATCGCCAGCGCGGC
ATTTTTTTGCCCTCGCGGCAGCGACTGAATCGCGGCGCGTACCTGTTCGCAAACACGGGCGGCGGTAAACAGCCCCAGGC
AGAGCATGGATGAAAGGAAAAACTGAATATTGGGATCCAGCTCGGCCTTAAACCACATGCCGATTTTCTCCGGCAGCAGC
TCCGGGATCACCAGATACCAGGTAAAGAATTGCACAATCAACGGCACGTTGCGGAACAGTTCGACATACAACGTACCCAG
ACCAGAGAGAAAACGGTTTGGAACGGTACGTAAAATGCCGAAAAATGAACCGACGAGGAAAGCGATAATCCAGGCGCAGA
TCGACAAAGCGATCGTCACCTGAAAACCACTCCAGATCCAACCAAGATAGGTGGTGTTGCCGAACGGGGCTTGTTGTAAA
AAAATACCCCAGTTCCAGTCTATAGACATAAATCTACTCCAGAAAAAAGAGGGTAGCAGCGTTAACTGCTACCCTCGAAG
ATTGTTACCCAGCGTATTGCGGTTTTCAGGCCCGATGGGGAACGACCATCAGGCGTATAGTCTGTCCGTGCTACGTAACA
ATCGAGAGGGCTGGAATTTCCGCCCCTGGTTCTTGTAATTAGTTCAGTGCCTTGTCATTCGGTTCTTTGAACAGTGCTTT
CATTTCGTCTGACAGTTCGAAATTCATGTTCAGGTTTTTCGGCGGAATTGGATTTTTGAACCACTTATCAAACCATTTTT
CCGCTTCACCGGAGGTCTGCACCTGAGCGATGGTGTCATCCATCAGCTTTTTGAACTGCGGATCATCTTTACGCAACATA
CAACCGTAGGCCTCCTGAGACTGCGGCTTGCCGACGATTTCCCAGTTGTCTGGTTTCTTCGCTTTCGCACGTTCACCGGC
CAGCAGAGCGTCATCCATCATAAAGGCAACGGCACGACCGCTTTCCAGGGTGCGGAAAGAGTCACCGTGATCTTTGGCGC
TGATGATGCGCATATTCATTTTTTGCTCTTCATTCAGTTTGTTGAGCAAAACTTCAGAGGTAGTGCCGGAAGTGACGACT
ACGGCTTTGTCTTTCAGGTTGGCAAAATCTTTGATATCGCCACCCTTTTTGGTCAACAGGCGCGTACCGACCACGAAAAT
AGTGTCAGAGAAAGCCGCCTGTTTTTGGCGTTCGACGTTGTTGGTGGTAGAACCACATTCAAAATCGAAAGTGCCGTTTT
GCAGCAGTGGAATACGGTTTTGTGAGGTAATCGGAATCAGTTTTACCTGCAAGTCCGGTTTGTTGAGTTTCTTTTTCACT
GCTTCAACAATGGCGTTGGAGTAATCCTGCGAGTAACCCACCACTTTTTGCTGATTGTCGTAATAAGAGAAAGGCACTGA
AGATTCACGGTGACCGACGACAATCACACCGTTTTTGGCGATTTTGTCCAGAGTACTGCCCGCTGCCGGGGCGGCGTCAT
CTGCCTGTGCCAGTCCTGCGGAAAGCGCCAGGGCGAGGATTGCTGTGGCAGGTTTACGTAATTGCATATCCAACTCCTTT
ATCTTCTGCGTTAAGAACGCATGGATACCCGTTGTGAGTGTTTGTGTTGTTATCGTCTGCAACTTTATTGTGCAGTGTTG
TGCCTGTTAGGGAAGGTGCGAATAAGCGGGGAAATTCTTCTCGGCTGACTCAGTCATTTCATTTCTTCATGTTTGAGCCG
ATTTTTTCTCCCGTAAATGCCTTGAATCAGCCTATTTAGACCGTTTCTTCGCCATTTAAGGCGTTATCCCCAGTTTTTAG
TGAGATCTCTCCCACTGACGTATCATTTGGTCCGCCCGAAACAGGTTGGCCAGCGTGAATAACATCGCCAGTTGGTTATC
GTTTTTCAGCAACCCCTTGTATCTGGCTTTCACGAAGCCGAACTGTCGCTTGATGATGCGAAATGGGTGCTCCACCCTGG
CCCGGATGCTGGCTTTCATGTATTCGATGTTGATGGCCGTTTTGTTCTTGCGTGGATGCTGTTTCAAGGTTCTTACCTTG
CCGGGGCGCTCGGCGATCAGCCAGTCCACATCCACCTCGGCCAGCTCCTCGCGCTGTGGCGCCCCTTGGTAGCCGGCATC
GGCTGAGACAAATTGCTCCTCTCCATGCAGCAGATTACCCAGCTGATTGAGGTCATGCTCGTTGGCCGCGGTGGTGACCA
GGCTGTGGGTCAGGCCACTCTTGGCATCGACACCAATGTGGGCCTTCATGCCAAAGTGCCACTGATTGCCTTTCTTGGTC
TGATGCATCTCCGGATCGCGTTGCTGCTCTTTGTTCTTGGTCGAGCTGGGTGCCTCAATGATGGTGGCATCGACCAAGGT
GCCTTGAGTCATCATGACGCCTGCTTCGGCCAGCCAGCGATTGATGGTCTTGAACAATTGGCGGGCCAGTTGATGCTGCT
CCAGCAGGTGGCGGAAATTCATGATGGTGGTGCGGTCCGGCAAGGCGCTATCCAGGGATAACCGGGCAAACAGACGCATG
GAGGCGATTTCGTACAGAGCATCTTCCATCGCGCCATCGCTCAGGTTGTACCAATGCTGCATGCAGTGAATGCGTAGCAT
GGTTTCCAGCGGATAAGGTCGCCGGCCATTACCAGCCTTGGGGTAAAACGGCTCGATGACTTCCACCATGTTTTGCCATG
GCAGAATCTGCTCCATGCGGGACAAGAAAATCTCTTTTCTGGTCTGACGGCGCTTACTGCTGAATTCACTGTCGGCGAAG
GTAAGTTGATGACTCATGATGAACCCTGTTCTATGGCTCCAGATGACAAACATGATCTCATATCAGGGACTTGTTCGCAC
CTTCCTTAGGTAACATTTAGTTTGGCTAAATGTAAAGATATTGCTGTTTTATTGTTTGTTTTTGCGAGATGCGCCGCACC
ATTCCGAAGCAAAATTCTTAAAATGCACTCTTTTAGTGCTACCGCTGGATTACTGTGGTGCAACTAGGTTGTACTGATGC
TGTTTCAGGGTTTGCCTTGTATAACAAAGCAATAGATGTGCCAAAGTTGGATAGGAGAATATTGTTATCCGGATAATGCA
CTGATGCCGCATCCGGTGAGCGTGCCGAAATATGGGATGTATTCCGGCACGATAAGAAGGGATTATTTACGTCGCTGACG
CAGACTCATCAACACAGCAGCAAAACCAAACAATGCCGTCAGCACCCACAGCGGCCAGTTGCCGGTACGTGCGTATGGTG
TGAGTCCGGTGGTCGGCGTCACGTTAGTGGTTAACACCTCGCGGGTGAACTGCGGGATCATCGCCTGAATCTCACCCTGC
GGGCCAATCACCGCCGTAATGCCGTTGTTGGTGCTGCGCAACAGTGGGCGCGCCAGCTCCAGCGCACGCATTCGCGCCAT
CTGGAAGTGTTGCCATGGACCAATAGATTTACCAAACCACGCATCGTTGGAGATAGTCAGCAGATAGTCGGTATCCGGGC
GGAAGTTATCGCGCACTTGCTCGCCGAGAATGATCTCGTAGCAAATAGCCGCAGTAAGCTCAATACCATTTGCCGACAGC
GGCGGCTGGATATATGGCCCACGGCTGAACGACGACATCGGCAGATCAAAGAACGGTGCTAACGGACGCAGAATCGACTC

CAGCGGGACAAACTCGCCAAACGGCACCAGATGGTTTTTGTTATAGCGATCGGCTGATTCGTAGCTGTACGGCGCACCTT
TACCCAGCGTGATGATGGTGTTGTAGGTATCGTAGCGGTTCTGCTTATTGAGACGCGCGTCGACAATCCCGGTTACCAGC
GAGCTACCTTTATCACGCAACTCACCGTCCAGTGCTTTGAGGAACGGTTGCTGATTAATTTCCAGATCGGTTATCGCCGA
CTCCGGCCAGATAATCAACGATGATTTGCCCATCAGCGGTGCCGTTGCGTTGTAGTAAATCTTCAGCGTATTAAGAAGCT
GGCCTTCGTCCCATTTCAGCGATTGCGGAATATCGCCCTGAACCATCGAAACCTGAATGGTTTTCTCCGGTTGTGGGGTA
AACCACTGGATGTAACGCAGCGGGAAGGGAAGGGCAAACAGCACGACGGCCACCACCAGCGGACGCCAGTTGCGTTTGAC
CAACGCCAGTGCCAGCAGGCCACTAACCATCATCAGCAGGAAGTTAATGGCTTCCACGCCCATTATCGGTGCCAGCCCTT
TTAACGGGCCATCAATCTGGCTATAGCCGAACTGTAACCACGGGAAGCCGGTCAGTACCCAACCGCGCAGAAACTCGGTC
ACTTGCCAGAGGGCAGGGGCGGCAATCGCTACGCGCAGCCAGGTGGTTTTCGGCCACAGACGCGACAGCACGCCAGCAAA
CAGTCCGGTATACAGCGACAAATACGCCGCCAGCAGCACCACCAGGAAGATGTTAACCGGGCCAGGCATTCCGCCAAAGG
TCGCGATGCTGACATAGACCCAGTTAATACCGCTGCCAAAGAGGCCAAATCCCCAGCAAAAGCCAATAGCGGCAGACTGG
AGTGGACGGCGGTTAAAGGTCAACGCCTGAAGCCCCATCAGCGAAATAATCGCCGCAGGCCAGACGTCGTAAGGAGAGAA
GGCCAGCGTTCCGCAGGCACCGAATAATAACGCCAGCAGCAGGCGAATGCGCTGGCGTTCAATTAATGAGGCAAAAGCCA
TGTAGTTATCTATCCAGTTTCGGTTTTATTCATCCAGCTTCGGCTGGGGTGAGTCATCCGGGATTTTGACATGAACCTGA
ATAATACGCCGACTGTCGGCCATCGCCACTTTGAACTGGTAACCGTCGATGTCGATAGTTTCGCCACGCGCCGGAAGATG
CCCAAATGCCTGCATCACCAGACCACCGATAGTGTCGACTTCTTCATCGCTAAAGTGGGTGCCGAACGCTTCGTTGAAGT
CTTCAATGGAAGCCAGTGCGCGTACGGTCCAGGTATGACGACTCAGCTGACGGAAGTCGATATCATCTTCTTCGTCATAC
TCGTCTTCAATCTCACCAACAATCAGTTCCAGGATGTCTTCAATGGTCACCAGACCGGAAACCCCACCGAATTCGTCAAT
AACGATCGCCATGTGGTAACGCTGAGAGCGAAACTCTTTCAGCATCCGGTCTACGCGCTTACTTTCAGGAACGACAACCG
CCTGACGTAACACTTTGTCCATGCTGAAGGCTTCAGCATCGCTGCGCATAAACGGCAGCAAGTCTTTCGCCATCAGAATC
CCTTCAATGTGATCTTTGTCTTCGCTAATCACCGGGAAACGTGAGTGGGCGGACTCGATGATGACATCAAGACATTCGTC
CAGCGTCTGGTTGCGTTTCAGGGTAATCATCTGGGAGCGGGGGATCATGATGTCGCGAACGCGTTGGTCTGCGATGTCCA
TCACCCCTTCGAGCATATCGCGCGTATCTTCGTCGATAAGGTCGTTCTGCCCGGAATCACGGATCAGCGCCAGCAGTTCG
TCACGGTTTTTCGGTTCACCGTGGAAAAGTTGGCTGAGTAACAGGGAGAAAAATCCCTTCTTGTTGCTTATCGTGTCACT
ACTGTGTGAATTGTCGTCGCTCATGGCGTCGTATGGGTTCTCATGTTAGTTAATCAAAACGCCGTCGTTAATCACCAACG
GCGGGGACGTCTGCCAGTCAAATGCCTGGCAAATTATTCTTTCTCGGCAATGTACGGATCCTCATAGCCCAGAGCAAGCA
TAATCTCTGTTTCGAGGGCTTCCATTTCTTCTGCTTCGTCATCTTCGATGTGATCGTAACCTAACAAATGCAGACTGCCG
TGCACCACCATATGCGCCCAGTGCGCCTCCAGTGGTTTGCCTTGCTCCTGAGCTTCCTTCTCAACCACCTGACGGCAGAT
AACCAGATCGCCCAGTAGCGACATTTCCATGCCAGGCGGCACTTCAAACGGGAAGGAGAGCACGTTGGTCGGCTTATCCT
TACCGCGATAGGTCAGATTCAGACTGTGGCTTTCGGCGGTATCGACCACGCGAATCGTCACTTCCGATTCTTCCTGAAAC
TGCGGGATCACCGCATTCAGCCATGTCTGAAACTGGCTCTCTTCCGGTAACCCGGAATTATCTTCACATGCCAGTTGTAA
ATCGAGGATCACCTGACTCATTTTTGTTCCTGTTCTTCGCGCTTGCGTTCTGCTGCCAGCGCCGCTTTTCGTTTTTGTTC
GGCTTCTTCCCAGGCTTCATAGGCGTTAACGATACGCGCCACCACAGGGTGACGAACCACGTCTTCGCTGTGGAAGAAGT
TAAAGCTGATCTCTTCGACATCGGCCAGCACTTCGATGGCGTGACGTAAGCCTGATTTAGTATTACGCGGCAGGTCGATC
TGTGTGACGTCGCCGGTGATAACCGCTTTTGAGTTAAAACCGATACGGGTCAGGAACATCTTCATCTGTTCGATGGTGGT
GTTCTGGCTCTCATCGAGAATGATAAACGCGTCGTTCAGCGTACGACCACGCATATAGGCCAGCGGTGCGACTTCAATAA
CGTTGCGCTCAATCAGTTTCTCGACTTTCTCAAAGCCCAGCATTTCAAACAGCGCGTCGTACAGCGGGCGCAGATACGGG
TCTACTTTCTGGCTTAAATCGCCAGGCAGGAAGCCCAGTTTCTCACCGGCTTCTACTGCCGGACGAGTCAGCAGAATACG
GCGAATTTCCTGACGCTCCAGGGCATCAACTGCCGCAGCCACTGCCAGGTAGGTTTTACCCGTACCCGCCGGGCCAACGC
CGAAGGTAATGTCATGGTCGAGAATATTGGCGATGTACTGCGCCTGGTTTGGCGTGCGCGGCTTAATTACGCCGCGTTTG
GTTTTGATATTGACCGCTTTGCCGTACTCCGGCACGCTCTCCGCGCTTTGCTCCAGTACCCGCGCTTCTTTAATCGCAAG
GTGGATCTGTTCCGGTTCGATATCCTGAATCTGACCGCGCATCGGGGCAGTATCGACATACAGGCTACGCAGAATGTCTG
CCGCAGCGGTGACGCAAATCGGACGGCCGGTCAGTTTAAAGTGGTTATCGCGGCGATTGATCTCGATGCCGAGACGGCGT
TCGAGCTGCTTGATGTTGTCATCAAACGGGCCGCACAGGCTCAACAGACGCGCATTGTCTGCTGGCTCCAGGGTGATTTC
GCGAGTGTCTATGTTCAAACCGTTCCTCTTATCTGTATGCCGCCGGAAGCTGAACATTCACCGGCCTATAAGGAAATTAT
TCACGCCACAGGAAAAAGGCGCAAGCATTGCAATAAAGATGGGGATAAAGAGAGAAAAAACAAGGCCCACCGGAACGGCA
GGCCTGAGAATTACGGCTGATAATAACCCACGCCAAGGTCGTTTTCTTTGCGGGTACGGGCAATCACTGATTCCGGTGTT
TCTGCCACGCGCAGACCCATTTCATCTTCAGTACGCACCACTTTACCGCGCAGAGAGTTCGGGTAGACGTCGGTAATTTC
TACATCGACGAATTTACCGATCATATCCGGCGTGCCTTCGAAGTTGACCACGCGGTTATTTTCCGTACGCCCGGAAAGCT
CCATGATGCTCTTACGCGATGTACCTTCTACCAGAATACGCTGGGTGGTGCCGAGCATCCGGCGGCTCCACGCCATCGCT
TGCTGATTAATGCGCTCTTGCAGAATATACAGACGCTGCTTCTTCTCTTCTTCCGGAACATCATCAACCATATCGGCGGC
TGGTGTACCCGGACGTGCAGAGAAGATAAAGCTGTAGCTCATGTCGAAATTGACGTCGGCAATCAGCTTCATCGTTTTCT
CGAAGTCTTCGGTGGTTTCGCCAGGGAAGCCAACGATGAAATCAGAACTGATCTGAATATCTGGACGCGCCGCACGCAGT
TTACGGATGATCGCTTTGTACTCCAGCGCCGTATGGGTACGGCCCATCAGGTTCAGAATGCGATCGGAACCGCTCTGTAC
CGGCAGATGCAGGAAGCTCACCAGCTCCGGCGTGTCGCGATACACTTCGATGATATCGTCGGTGAATTCGATCGGATGGC
TGGTGGTAAAGCGAATACGATCGATCCCGTCGATCGCAGCAACCAGACGCAGCAGATCGGCAAACGATCCGGTGGTGCCG

TCGTAGTTTTCACCACGCCAGGCGTTCACGTTCTGACCGAGCAGGTTGACTTCACGCACGCCCTGAGCCGCAAGCTGGGC
AATCTCAAACAGAATATCGTCGGACGGACGGCTTACCTCTTCACCACGGGTGTAAGGCACCACGCAGTAGGTGCAATATT
TATTGCAGCCTTCCATGATGGAGACAAACGCGGTCGGCCCTTCGGCGCGCGGTTCCGGTAGACGGTCAAACTTCTCGATT
TCCGGGAAGCTGATATCTACAACCGGGCTGCGGTCGCCACGCACGGAGTTGATCATCTCCGGCAGACGGTGCAGCGTTTG
CGGCCCAAAAATAATATCGACATAGTGGGCGCGCTGGCGAATGTGCTCGCCTTCTTGCGATGCCACGCAGCCACCGACGC
CGATAATCAGGTCTGGATTCTTCTCTTTTAACAGTTTCCAGCGACCCAACTGATGGAAGACTTTTTCCTGAGCCTTCTCG
CGGATTGAGCAGGTGTTCAGCAGCAGCACATCCGCTTCTTCCGCCACGTCGGTCAGTTGATAGCCGTGGGTGGCATCCAG
CAGATCGGCCATCTTCGATGAATCGTACTCGTTCATCTGACAGCCCCAGGTTTTAATATGGAGTTTTTTGGTCATCGACT
TGCTCTTGCGAAATAGTAGCCAGGAATGCAGGGCGCATAGTGTAATGCTTTGCTGCCGTTGTGACCAGTATGAGCGTTAT
CAGCCCTTAGGGGTAAAAATCCTGTAAACTTAAAGCATATTGCTAACAGGATGATTGACCATGACAAATCAACCAACGGA
AATTGCCATTGTCGGCGGAGGAATGGTCGGCGGCGCACTGGCGCTGGGGCTGGCACAGCACGGATTTGCGGTAACGGTGA
TCGAGCACGCAGAACCAGCGCCGTTTGTCGCTGATAGCCAACCGGACGTGCGGATCTCGGCGATCAGCGCGGCTTCGGTA
TCATTGCTTAAAGGGTTAGGGGTCTGGGATGCAGTACAGGCTATGCGTTGCCATCCTTACCGCAGACTGGAAACGTGGGA
GTGGGAAACGGCGCATGTGGTGTTTGACGCCGCTGAACTTAAGCTACCGCTGCTTGGCTATATGGTGGAAAACACTGTCC
TGCAACAGGCGTTGTGGCAGGCGCTGGAAGCGCATCCGAAAGTAACGTTACGTGTGCCAGGCTCGCTGATTGCGCTGCAT
CGCCATGATGATCTTCAGGAGCTGGAGCTGAAAGGCGGTGAAGTGATTCGCGCGAAGCTGGTGATTGGTGCCGACGGCGC
AAATTCGCAGGTGCGGCAGATGGCGGGAATTGGCGTTCATGCATGGCAGTATGCGCAGTCGTGCATGTTGATTAGCGTCC
AGTGCGAGAACGATCCCGGCGACAGCACCTGGCAGCAATTTACTCCGGACGGACCGCGTGCGTTTCTGCCGTTGTTTGAT
AACTGGGCATCGCTGGTGTGGTATGACTCTCCGGCGCGTATTCGCCAGTTGCAGAATATGAATATGGCACAGCTCCAGGC
GGAAATCGCGAAGCATTTCCCGTCGCGTCTGGGTTACGTTACACCGCTTGCCGCTGGTGCGTTTCCGCTGACGCGTCGCC
ATGCGTTGCAGTACGTGCAGCCAGGGCTTGCGCTGGTGGGCGATGCCGCGCATACCATCCATCCGCTGGCGGGGCAGGGA
GTGAATCTTGGTTATCGTGATGTCGATGCCCTGATTGATGTTCTGGTCAACGCCCGCAGCTACGGCGAAGCGTGGGCCAG
TTATCCTGTCCTCAAGCGTTACCAGATGCGGCGCATGGCGGATAACTTCATTATGCAAAGCGGTATGGATCTGTTTTATG
CCGGATTCAGCAATAATCTGCCACCACTGCGTTTTATGCGTAATCTCGGGTTAATGGCGGCGGAGCGTGCTGGCGTGTTG
AAACGTCAGGCGCTGAAATATGCGTTAGGGTTGTAGCCTTACAACATTGCCGGATGCGTGCCAACCGTAGGTCGGATAAG
ACGCGGCAGCGTCGCATCCGACATTGAAGGATAAGACGTGTCAACATCGCATTCGACATTGAATGAACGCAGAAAAGCAA
AAAGCTCGCCGAAGCGAGCTTTTTTAATGTGGCTGGGGTACGAGGATTCGAACCTCGGAATGCCGGAATCAGAATCCGGT
GCCTTACCGCTTGGCGATACCCCAACTGGGTGCACTTACAAGGTAAGCGTCTTGAATAAATTGGCTGGGGTACGAGGATT
CGAACCTCGGAATGCCGGAATCAGAATCCGGTGCCTTACCGCTTGGCGATACCCCAACAAATTGGTTTTGAATTTGCCGA
ACATATTCGATACATTCAGAATTTGGTGGCTACGACGGGATTCGAACCTGTGACCCCATCATTATGAGTGATGTGCTCTA
ACCAACTGAGCTACGTAGCCAGATTGTTTCTTCGATGGCTGGGGTACCTGGATTCGAACCAGGGAATGCCGGTATCAAAA
ACCGGTGCCTTACCGCTTGGCGATACCCCAATAACCGGGCGGTGAACCGCTTACTCGAAGAAGATGGCTGGGGTACCTGG
ATTCGAACCAGGGAATGCCGGTATCAAAAACCGGTGCCTTACCGCTTGGCGATACCCCATCCGTACAACGCTTTCTGGTG
AATGGTGCGGGAGGCGAGACTTGAACTCGCACACCTTGCGGCGCCAGAACCTAAATCTGGTGCGTCTACCAATTTCGCCA
CTCCCGCAAAAAAAGATGGTGGCTACGACGGGATTCGAACCTGTGACCCCATCATTATGAGTGATGTGCTCTAACCAACT
GAGCTACGTAGCCATCTTTTTTTTCGCGATACCTTATCGGCGTTGCGGGGCGCATTATGCGTATAGAGCCTTGCAGCGTC
AACCTCTTTTTCAAGGAAAATTGCTCGAAAGTGACTGTTTGGTTAGGTTGCGAACAGCGTGGCGCTATATTCGTCAATTA
TTGTTTACTTTGTGTTTTTTCCCACCCTACAGCCATTCTTTTGTCATACAGGATGAAATTCGGAATTTAACAATAGTGGT
GGGAAATTAATCTATGAAATACTGGCCTACAGTGATGAGTTGTCAAACAGTGATGTGGCAAACCCGGAACATTTCCTTAC
TGCATATCAGAATCAACAAGCACCTCAATAACTGAAACAGCCCCGGATTTCACCGGGGCTGTTTCGCATTTCTTACTTAT
ACGCCGACTGGTGAACACCAACCGCGCGACCAGACGGATCGTCCATTTTCTTGAACGCTTCATCCCATTCGATCGCTTTA
GCGGAAGAACAAGCGACGGAAGGACCGCCCGGCACGCACTCAGCGGCGCTCGGAAGCGGGAATAGTTCTTCAAAGATCTC
CCGATACAAGTACGCTTCTTTAGAGGTTGGCGTGTTGTACGGGAAGCGGAAGCGGGCAGTTTCCAGTTGCTGATCAGAAA
CCTGCTGCGCAGCCACTTCTTTCAGGGTGTCGATCCAACTGTAACCGACGCCATCGGAGAACTGCTCTTTCTGCCGCCAG
GCCACGCTTGCAGGCAGATACGCTTCAAAACATTCACGCAGGATGTGTTTTTCCATTTTGCCGTTACCGCACATTTTATC
CTGTGGGTTAATACGCATCGCCACATCAAGGAATTTTTTGTCGAGGAACGGAACGCGTGCTTCCACGCCCCAGGCTGACA
TCGCTTTGTTGGCACGCGCGCAGTCATACATATGCAGGGCCAGCAGTTTACGCACCGTCTCTTCATGCAGTTCTTTGGCA
TTCGGTGCTTTGTGGAAGTAAAGATAACCGCCGAACACTTCATCAGAACCTTCACCGGACAGCACCATTTTAATGCCCAT
CGCCTTGATCTTACGCGACATTAAATACATCGGTGTTGAAGCGCGAATAGTGGTCACATCATAAGTTTCGATGTGGTAAA
TCACGTCGCGGATGGCATCCAGACCTTCCTGTACAGTGAAGTGAATTTCGTGATGCACCGTGCCCAGATGGTTTGCCACT
TCCTGGGCTGCTTTCAGATCCGGTGAACCCGGCAGACCTACAGCAAAGGAGTGTAACTGCGGCCACCAGGCTTCAGAGCG
TTCCTGATCTTCCACGCGACGGGCTGCGTATTTCTTGGTGATAGCGGAAATAATTGAGGAATCCAGACCACCAGAAAGCA
GCACACCGTAAGGCACATCAGACATCAGATGGCTTTTAACTGAATCTTCCAGTGCCTGACGCAGCTCGTTTTTGTCGGTC
ACGTTATCTTTCACCGCATCGTAGTCGAACCAGTCGCGATGATAGTAAGAACGGATTTCGCCGTCCTGGCTCCACAAATA
GCTCCCCGCCGGGAACTCTTTAATCGTGCGGCAAACTGGCACCAGCGCTTTCATTTCTGAGGCCACATACAGCTGACCGT
GTTCGTCATACCCCATATACAGTGGGATGATCCCCAGATGGTCGCGACCAATCAGGTAGGCATCTTTTTCGCTGTCGTAC

AGTGCAAAGGCAAACATGCCCTGCAAGTCGTCGAGAAATTCCGGCCCTTTTTCCTGATACAGCGCGAGGATCACTTCACA
GTCAGACCCGGTCTGGAACTGGTAACGATCGCCATATTCGGCGCGCAATGCCTGGTGGTTGTAGATTTCACCGTTTACCG
CCAGTACGTGGGTTTTTTGTTGGTTGTAGAGAGGTTGCGCCCCCGCGTTAACGTCAACAATTGACAGACGTTCGTGGGCG
AGAATGGCGTTATCGCTGGCATAAATACCGGACCAGTCCGGGCACGATGACGCATCAGGCGTGACAGCTCGAGGGCTTT
CTTACGCAGCTCAACTGCGTCTGTTTTGATATCGAATACGCCAAAAATTGAACACATAACCTTCTCCGTTAACCTGGTAT
TTGTTGCTTGTTGTGTTTGCTTGTTTAAAAAAATGCCGCAAAGCAGCACTGTGCGCAAGCGATTTGGCGGTGAAAAAATA
AAAAACGTAATGGTGATTGTCGATTGGTGAAAAAAGGTCTGGTGTGAGGATATATTTATTGATGAATCGATAATTTTTAG
CGGGTTTTATTGAATGTTTATATTTTACGGGGGCCAAATTGCTGACAAAGTGCGATTTGTTCATGCCGGATGCGGCGTGA
ACGCCTTATCCGGCCTACAAAAGCATGAAAATTCAATATATTGCAGGAGCTGCGTAGGCCTGATAAGCGTAGCGCATCAG
GCAGTTTGGCGTTTGTCATCAGAGCCAACCACGTCCGCAGACGTGGTTGCTATTCAGATAACGTCGATTTCAGCGACTGA
CGGGTAAATCCAGCTGGGGCGGAAAGGCATACTGTCGATATCGTCGAGCGACGAAACACCAGAAAGCACCAGAATCGTCT
CCAGACCTGCCTGGAAGCCGGCCAGAATATCGGTACGCAGGTTATCGCCGACAATCACCGTTTCTTCCGAATGCGCCTGC
ATTTTGTTTAATGCTGCGCGGATGATCCACGGGCTGGGCTTACCAACATAGAACGGTTTGCGCCCGGAGATTTTCTCAAT
CCCTGCACACAACGCGCCACAAGCGGGATAAAAACCGCGCCCGTGGGTGTCCGGATTGGTGGCGATAAAACGTGCACCGT
TAGCGACGAAATAGGCTGCTTTATGCATCATGTCCCAGTTGTAGGAACGCGTTTCGCCAACAATCACGAAATCAGGGTTC
ACATCGGTAATAGTGAAACCGGCTTTGTACAGTTCATGAATCAGTGCGCCTTCGCCCACCACATACGCTTTCTTGCCTTC
CTGGCGACGCAGGAAATCGGCAGTCGCCATCGCAGAGGTATAAAACACGCTGTCAGGTACATCGACACCTGCGGTGGCAA
AGCGGTTCGCCAGATCTTGCCCAGTCTGCGAAGGATAGTTGGTCAGCAACACCAGCGGCAGGCCTTTATCCATAATCCCG
TGCAAAAATTCCGCTGCACCCGGTACGGCGACGTTATCGTGCATCAGCACGCCGTCGATATCGCAAATTACATTTTTAAT
GGTCATGGACTACCCAGAATATTGACAACAATAAGCGCCACTATAAAAGCACATTAATTTTCCAGCAAATGCTGGAGCAA
AATACCGTTGAGCATGGCGCGTTTTACCAGCGCAAAAGCGCCGATTGCCGAGCGGTGATCCAGCTCAGAACGTACCACCG
GCAGATTAGTGCGAAACGCCTTCAGCGCCTGGGTATTAATGCAGCTTTCAATAGCAGGGAGCAGCACTTTATCGGCTTCG
GTGATTTCACCGGCAATAACAATTTTTTGCGGATTAAATAAGTTGATAGCAATGGCGATGGTTTTACCCAGATGACGACC
GACATACTCAATTACTTCCGACGCCAGACTATCGCCTTTGTTCGCGGCTTTGCAGATAGTTTTGATGGTGCAGTCGTCCA
GCGGCACGCGGCTCTGGTAGCCCTGCTTTAACAGATTCAACACCCGTTGTTCAATGGCAGCGTTGGCAGCGATAGTTTCC
AGGCAGCCAAAGTTGCCGCAGTGGCAGCGTTCACCCAGCGGTTCGACCTGAATATGGCCAATTTCACCGACGTTGCCGTT
GCGGCCAATAAAAATGCGCCCGTTAGAGATAATCCCGGCCCCGGTTCCGCGATGGACACGCACCAGAATGGAGTCTTCGC
AATCCTGACTTGCACCGAAGTAGTGCTCCGCCAGCGCCAGACTACGGATATCGTGACCAACGAAACAGGTCACTTTAAAA
CGTTCTTCCAGAGCTTCTACCAGCCCCCAGTTTTCTACCTGAATATGCGGCATGTAATGAATTTTGCCGCTGTCCGGGTC
AACAAGCCCTGGCAGGATCACCGAAATCGCGATCAGCTCGCGCAGTTTGCGCTGGTAGCTATCAATAAACTGAGCAATGG
CATTCAACAGGGCATGTTCCAGCGTTTGCTGGGTACGTTCCGGCAGCGGGTAATGTTCTTCTGCCAGCACTTTGCTGCTG
AGATCAAACAGAGTGATGGTGGCGTCATGACGACCAAGCCGTACGCCGATTGCGTGGAAATTGCGGGTTTCGGTGACGAT
GGAGATAGCGCGGCGGCCCCCGGTGGAGGCCTGCTGATCAACTTCTTTGATCAGCCCGCGTTCGATAAGCTGACGCGTAA
TTTTGGTTACGCTGGCGGGGGCAAGCTGGCTTTGCTCGGCAATCTGAATCCGCGAGATTGGCCCGTACTGGTCAATCAGG
CGATAAACCGCCGCGCTGTTAAGCTGTTTTACGAGATCAACATTACCTATCTGAGCTTGTCCGCCTGGTGTCATACTTTC
TCTTATTGAGTTACGACCTCGTTACCGTTAACGATGGTCTTGGTGATTTTAAAATCAGGTGTGAATGCAGTCAGGTTGGC
TACTTTACCTGCGGCGAGTGTGCCGAGACGTTTCTCAACGCCAATCGCACGCGCCGGATAGAGCGTCGCCATACGTAGCA
CTTCATCCAGTGCGATACCGCAATGTTCGACCAGATTACGCACGCCTTCAATCATGGTTAAGGATGAACCGCTTAACGTA
CCGTTCTCATCCACACAAAGTCCGTTACGGTAGTATATTGTTTTACCCGCAAAAATGAACTGTTCAATGTTGGCACCTGC
TGGCGCGGTGGCGTCAGTAACCAGACACAGTTTGTCGCCTTTCAGACGTTTAGCGTTGCGAATGTTGGCGTAATCAACAT
GCAGGCCATCAGCAATAATACCGCAATAAATGTCAGCTTCGTCGAGGATCGCGCCCGCCAGGCCAGGTTCACGACCGGTA
ATATACGGCATCGCGTTGTACAGATGGGTGGCAAAGGTAATCCCCGCGCGGAAACCGGCTTTTGCTTCTTTCAACGTCGC
GTTGGAGTGACCGGCAGAAACCACAATCCCGGCATTTGCCAGTTTGCTGATGACTTCCGCAGGAACCATTTCCGGTGCCA
GGGTCACTTTGGTAATGACGTCGGCGTTTTCACACAGGAAATCGACCAGCGCGGCATCAGGCTTACGCACAAAATTCGGA
TTATGGGTGCCTTTTTTTACCAGATTCAGCCACGGACCTTCCAGATGCAGACCTAACGCCTGATTCGGATGTTTTGCCAG
GTACTCGCGCATAACGCGCACGCCCTGTTTCATCAGCTCATCGCTGGTGGTGATAAGCGTCGGCAGATAGTTAGTACAGC
CTGATTTCTCATTGGCTTTCTGCATGATTTCCAGCGTTTCCACGCTGACCGCTTCAGCGGTGTCGTTAAACTGTACGCCG
CCGCAGCCGTTTAACTGCACATCGATAAAACCGGGGGAGAGAATGGCCCCGTTCAGTGAACGTTGTTCGATCTCTGGCGG
CAGTTCCGCTACCGGACAGACGCTTTTAATCAGGCCATCAGCGATAACAACCGCGTGGTCATCAAGAAATTCGTGGCCGG
TAAAGATCCGGCCCTGGGTTAATGCATACATTCTGACCCCCGATTTTAAAAAATATTGCCCTGAGCAAGGAGCCAGGGCA
GGGATAACAATTACAGACCTTTGATATTTTCTGCTTCTAATTCATTGAAATATCTTAAAGTCTTAACTTTCAGCTCCATG
GTGGAAGGTTCATCGCACACCATGATCGCTTTCGGATGCAGTTGCAGACAGCTGATGGTCCACATATGGTTCACGCAACC
TTCAACGGCGGCCTGCAGCGCCAGTGCTTTCTGGCTACCCAGCACCAGAATCATCACTTCTTCGGCATCCAGCAGTGTAC
CAACACCGACAGTCAGGGCATATTTTGGCACCTGATTAACATCGTTATCAAAGAAACGAGAGTTTGCGACGCGAGTGTCA
TGAGTCAGGGTTTTGATACGAGTACGAGAAGCCAGAGAAGACGCCGGTTCGTTAAATGCAATATGACCGTCGTTACCTAC
ACCGCCCATAAACAGATGAATTTTTCCGTAAGAACGGATTTTTTCTTCATACTGGCGGCACTCGGCGTCGATATCCGGGG

CGTTGCCGTTGAGAAGGTTGATGTTTTCTGCTGGAATATCAACGTGATCGAAGAAATTACGGTGCATAAAGCTGTAGTAG
CTTTCCGGATGCTCTTTCGGCAGACCGACATATTCGTCCATGTTGAAGGTGACAACGTGCTTAAAGCTGACCTGGCCTGC
TTTATGCATTTCGACTAACGCTTTATAGGTGGTCATCGGCGTGCCGCCAGTCGGCAGGCCCAGTACAAACGGACGATCGG
CAGTCGGTTTGAACGCATTGATACGATTGACGATATGGCGAGCAGCCCATTTGCCGACCTGTTCAGCGGTAGTCAGGGGG
ATCAGTCTCATTATTCACCTCAATAAGTAAAATGTAAGCCGTTGGCGGATTAGGCATCTTTAAGCGTAACCTGGATTTGC
GCAGACAGGCGTCAATCCGACCTGATTTTTTGAATGATAAAATAAGTTTTCTGGTTTAGCCAGTAAAAGGGAGTGATAAT
AACGATATTTGGTGACAAAACTCACAAAAGACACGCGTTTAATTTGCGATACGAATTAAATTTTCACACACTCTGTAGCA
GATGATCTAACAATCTGATTACAGAACATCGGCAGTACAATTTGCAGCAAAATAAAAATACGGCTTGAAACGAGCCAAAT
AGGGTTCTCGTAGGGGGAATAAGATGAATATTTTAGGTTTTTTCCAGCGACTCGGTAGGGCGTTACAGCTCCCTATCGCG
GTGCTGCCGGTGGCGGCACTGTTGCTGCGATTCGGTCAGCCAGATTTACTTAACGTTGCGTTTATTGCCCAGGCGGGCGG
TGCGATTTTTGATAACCTCGCATTAATCTTCGCCATCGGTGTGGCATCCAGCTGGTCGAAAGACAGCGCTGGTGCGGCGG
CGCTGGCGGGTGCGGTAGGTTACTTTGTGTTAACCAAAGCGATGGTGACCATCAACCCAGAAATTAACATGGGTGTACTG
GCGGGTATCATTACCGGTCTGGTTGGTGGCGCAGCCTATAACCGTTGGTCCGATATTAAACTGCCGGACTTCCTGAGCTT
CTTCGGCGGCAAACGCTTTGTGCCGATTGCCACCGGATTCTTCTGCCTGGTGCTGGCGGCCATTTTTGGTTACGTCTGGC
CGCCGGTACAGCACGCTATCCATGCAGGCGGCGAGTGGATCGTTTCTGCGGGCGCGCTGGGTTCCGGTATCTTTGGTTTC
ATCAACCGTCTGCTGATCCCAACCGGTCTGCATCAGGTACTGAACACCATCGCCTGGTTCCAGATTGGTGAATTCACCAA
CGCGGCGGGTACGGTTTTCCACGGTGACATTAACCGCTTCTATGCCGGTGACGGCACCGCGGGGATGTTCATGTCCGGCT
TCTTCCCGATCATGATGTTCGGTCTGCCGGGTGCGGCGCTGGCGATGTACTTCGCAGCACCGAAAGAGCGTCGTCCGATG
GTTGGCGGTATGCTGCTTTCTGTTGCTGTTACTGCGTTCCTGACCGGTGTGACTGAGCCGCTGGAATTCCTGTTCATGTT
CCTTGCTCCGCTGCTGTACCTCCTGCACGCACTGCTGACCGGTATCAGCCTGTTTGTGGCAACGCTGCTGGGTATCCACG
CGGGCTTCTCTTTCTCTGCGGGGGCTATCGACTACGCGTTGATGTATAACCTGCCGGCCGCCAGCCAGAACGTCTGGATG
CTGCTGGTGATGGGCGTTATCTTCTTCGCTATCTACTTCGTGGTGTTCAGTTTGGTTATCCGCATGTTCAACCTGAAAAC
GCCGGGTCGTGAAGATAAAGAAGACGAGATCGTTACTGAAGAAGCCAACAGCAACACTGAAGAAGGTCTGACTCAACTGG
CAACCAACTATATTGCTGCGGTTGGCGGCACTGACAACCTGAAAGCGATTGACGCCTGTATCACCCGTCTGCGCCTTACA
GTGGCTGACTCTGCCCGCGTTAACGATACGATGTGTAAACGTCTGGGTGCTTCTGGGGTAGTGAAACTGAACAAACAGAC
TATTCAGGTGATTGTTGGCGCGAAAGCAGAATCCATCGGCGATGCGATGAAGAAAGTCGTTGCCCGTGGTCCGGTAGCCG
CTGCGTCAGCTGAAGCAACTCCGGCAACTGCCGCGCCTGTAGCAAAACCGCAGGCTGTACCAAACGCGGTATCTATCGCG
GAGCTGGTATCGCCGATTACCGGTGATGTCGTGGCACTGGATCAGGTTCCTGACGAAGCATTCGCCAGCAAAGCGGTGGG
TGACGGTGTGGCGGTGAAACCGACAGATAAAATCGTCGTATCACCAGCCGCAGGGACAATCGTGAAAATCTTCAACACCA
ACCACGCGTTCTGCCTGGAAACCGAAAAAGGCGCGGAGATCGTCGTCCATATGGGTATCGACACCGTAGCGCTGGAAGGT
AAAGGCTTTAAACGTCTGGTGGAAGAGGGTGCGCAGGTAAGCGCAGGGCAACCGATTCTGGAAATGGATCTGGATTACCT
GAACGCTAACGCCCGCTCGATGATTAGCCCGGTGGTTTGCAGCAATATCGACGATTTCAGTGGCTTGATCATTAAAGCTC
AGGGCCATATTGTGGCGGGTCAAACACCGCTGTATGAAATCAAAAAGTAATCTGCTTTATGCCTGATGCGACGCTTGAGC
GTCGCATCCAACAATGACAAGCGGTGGAGATCTTCTCTGCCGCTTTTTTTTTCATCAATCATCCCCATAATCCTTGTTAG
ATTATCAATTTTAAAAAACTAACAGTTGTCAGCCTGTCCCGCTTATAAGATCATACGCCGTTATACGTTGTTTACGCTTT
GAGGAATCCACGATGAGTGAGGCAGAAGCCCGCCCGACTAACTTTATCCGTCAGATCATCGATGAAGATCTGGCCAGTGG
TAAGCACACCACAGTACACACCCGTTTCCCGCCGGAGCCGAATGGCTATCTGCATATTGGCCATGCGAAATCTATCTGCC
TGAACTTCGGGATCGCCCAGGACTATAAAGGCCAGTGCAACCTGCGTTTCGACGACACTAACCCGGTAAAAGAAGATATC
GAGTATGTTGAGTCGATCAAAAACGACGTAGAGTGGTTAGGTTTTCACTGGTCTGGTAACGTCCGTTACTCCTCCGATTA
TTTTGATCAGCTCCACGCCTATGCGATCGAACTGATCAATAAAGGCCTGGCGTACGTTGATGAACTGACGCCGGAACAGA
TCCGCGAATACCGCGGCACCCTGACGCAACCGGGTAAAAACAGCCCGTACCGCGACCGCAGCGTTGAAGAGAACCTGGCG
CTGTTCGAAAAAATGCGTGCCGGTGGTTTTGAAGAAGGTAAAGCCTGCCTGCGTGCGAAAATCGACATGGCTTCACCGTT
TATCGTGATGCGCGATCCGGTGCTGTACCGTATTAAATTTGCTGAACACCACCAGACTGGCAACAAGTGGTGCATCTACC
CGATGTACGACTTCACCCACTGCATCAGCGATGCCCTGGAAGGTATTACGCACTCTCTGTGTACGCTTGAGTTCCAGGAC
AACCGTCGTCTGTACGACTGGGTACTGGACAACATCACGATTCCTGTTCACCCGCGCCAGTATGAGTTCTCGCGCCTGAA
TCTGGAATACACCGTGATGTCCAAGCGTAAGTTGAACCTGCTGGTGACCGACAAGCACGTTGAAGGCTGGGATGACCCGC
GTATGCCGACCATTTCCGGTCTGCGTCGTCGTGGTTACACTGCGGCTTCTATTCGTGAGTTCTGCAAACGCATCGGCGTG
ACCAAGCAGGACAACACCATTGAGATGGCGTCGCTGGAATCCTGCATCCGTGAAGATCTCAACGAAAATGCGCCGCGCGC
AATGGCGGTTATCGATCCGGTGAAACTGGTTATCGAAAACTATCAGGGCGAAGGCGAAATGGTTACCATGCCGAACCATC
CGAACAAACCGGAAATGGGCAGCCGTCAGGTGCCGTTTAGCGGTGAGATTTGGATTGATCGCGCCGATTTCCGCGAAGAA
GCTAACAAGCAGTACAAACGTCTGGTGCTGGGTAAAGAAGTGCGTCTGCGTAATGCTTATGTGATTAAGGCAGAACGCGT
CGAGAAAGATGCCGAAGGTAATATCACCACCATCTTCTGTACTTATGACGCCGATACCTTAAGCAAAGATCCGGCAGATG
GTCGTAAAGTCAAAGGTGTTATTCACTGGGTGAGCGCGGCACATGCGCTGCCGGTTGAAATCCGTTTGTATGACCGTCTG
TTCAGCGTGCCTAACCCAGGTGCTGCGGATGATTTCCTGTCGGTGATTAACCCGGAATCGCTGGTGATCAAACAGGGCTT
TGCTGAACCGTCGCTGAAAGATGCGGTTGCGGGTAAAGCATTCCAGTTTGAGCGTGAAGGTTACTTCTGCCTCGATAGCC
GCCATTCTACGGCGGAAAAACCGGTATTTAACCGCACCGTTGGGCTGCGTGATACCTGGGCGAAAGTAGGCGAGTAATTT

TAAGTTTCGCTATGCCGGATGGGGCGTTTACGTCGCATCCGGCAAGGAACAGACAAACAGTTTCAAACGCTAAATTGCCT
GATGCGCTACGCTTATCAGGCCTACATGATCTCTGCAATATATTGAGTTTGCGTGCTTTTGTAGGCCGGATAAGGCGTTC
ACGCCGCATCCGGCAAGAAACAGCAAACAATCCAAAACGCCGCGTTCAGCGGCGTTTTTTCTGCTTTTCTTCGCGAATTA
ATTCCGCTTCGCAATTTATCCATAAAATAAATTTAAAATAACAAAACATAATTAAATAAAATGTAACCGCTTTCATCTTG
CTGGAATTTCACGCTTTTATTCTTCTGCAAGCCTTTCAACCGCAAACTTAAGCCTTGTAACAAAAATCATCAAAATATGT
GCGGTTGCTCATGTTCTTACATTCTGGTTACAGAAAGAGATTGATAATTCGCGTCGCGAAAAATAGTCTGTTCCTGTAGT
CAGCGAGACTTTTCTCAACGCTACTTTTTTAATTTTTATTTTTTCGCTGTTCACCTTTGGTGCAGCAATTTATACGTCAA
AGAGGATTAACCCATGCGTACGTTTAGTGGCAAACGTAGTACGCTGGCGCTGGCTATCGCCGGTGTTACAGCAATGTCGG
GCTTTATGGCAATGCCGGAGGCTCGCGCCGAAGGATTCATCGACGATTCAACCTTAACCGGCGGTATCTATTACTGGCAG
CGTGAACGCGACCGTAAAGATGTTACCGACGGCGACAAATACAAAACCAACCTTTCTCACTCCACCTGGAATGCCAACCT
CGATTTTCAGTCCGGTTATGCTGCTGATATGTTCGGCCTTGATATTGCTGCGTTTACGGCGATTGAAATGGCGGAAAACG
GCGACAGCTCCCACCCGAACGAAATCGCGTTTTCAAAAAGTAATAAAGCCTATGACGAAGACTGGTCCGGCGACAAAAGC
GGTATAAGCCTGTATAAAGCTGCGGCCAAATTTAAATACGGTCCGGTTTGGGCGAGGGCAGGTTACATTCAGCCAACTGG
TCAAACGCTGTTAGCGCCGCACTGGAGCTTTATGCCAGGTACTTATCAGGGGGCGGAAGCCGGGGCGAATTTTGATTACG
GCGATGCTGGTGCGTTGAGTTTCTCCTACATGTGGACCAACGAATACAAAGCGCCGTGGCATCTGGAAATGGATGAGTTT
TATCAGAACGATAAAACCACCAAAGTTGATTATCTGCACTCCTTTGGGGCGAAATACGACTTCAAAAATAACTTCGTACT
GGAAGCGGCATTTGGTCAGGCGGAAGGGTATATCGATCAATATTTTGCCAAAGCCAGCTACAAATTTGATATCGCCGGTA
GCCCGTTAACCACCAGCTACCAGTTCTACGGTACCCGAGATAAAGTTGACGATCGCAGCGTCAACGACCTTTATGACGGC
ACCGCCTGGCTGCAAGCGTTGACCTTTGGTTACCGGGCGGCTGACGTAGTGGATTTGCGCCTCGAAGGCACCTGGGTTAA
GGCTGACGGTCAGCAGGGATACTTCCTGCAACGTATGACTCCAACCTACGCTTCCTCAAACGGTCGCCTGGATATCTGGT
GGGATAACCGTTCTGACTTCAACGCCAACGGCGAAAAAGCGGTCTTCTTCGGTGCGATGTATGACCTGAAAAACTGGAAT
CTTCCAGGCTTCGCCATCGGCGCTTCCTACGTTTACGCATGGGATGCTAAACCTGCGACCTGGCAGAGCAATCCGGATGC
GTACTACGACAAAAACCGGACTATTGAAGAGTCTGCCTACAGCCTGGATGCGGTCTATACCATTCAGGACGGTCGCGCCA
AAGGCACGATGTTCAAACTGCATTTCACCGAATACGACAACCACTCCGACATCCCAAGCTGGGGCGGTGGTTACGGCAAC
ATCTTCCAGGATGAGCGTGACGTGAAATTTATGGTAATCGCACCATTCACCATCTTCTGATGCCCGACGCGGCAGGTTTT
GTGCCTGCCGCACGTTTGAGGAATTTGCTATGAAAAAACTGATTCTCATCGCCATAATGGCATCGGGGCTGGTAGCTTGT
GCGCAATCAACCGCGCCACAGGAAGACAGTCGTCTGAAAGAGGCATACAGCGCCTGTATCAACACCGCACAAGGTTCGCC
AGAAAAAATTGAAGCCTGCCAGAGCGTGTTAAACGTGCTGAAGAAAGAGAAACAACATCAGCAGTTTGCCGACCAGGAAA
GTGTACGCGTACTGGATTATCAGCAGTGTCTGCGTGCAACGCAAACCGGAAATGATCAGGCGGTGAAAGCCGATTGTGAT
AAAGTCTGGCAGGAAATACGCAGTAATAACAAATAAGTGAGAGCTGTAACTCTCGCTTTTCTTATTTCCCTTGCATAAAA
AAGCCAACCCGCAGGTTGGCTTTTCTCGTTCAGGCTGGCTTATTTGCCTTCGTGCGCATGTTCATCTTCGCGGCAATCGC
CTTCGGCACAGTGACCGTAAAGATAGAGACTGTGGTTAGTCAGGCGAATGCCATGTTTTGCGGCAATTTCACGCTGACGC
GCTTCGATGGAATCATCACTAAATTCGATAACCTTGCCGCAGTCGAGGCAGATCAGGTGATCGTGGTGATGTTGCTGTGT
CAGTTCAAATACGGATTTACCGCCTTCAAAATTGTGGCGGGTGACGATACCAGCGTCGTCAAACTGGTTCAGTACGCGAT
ATACCGTAGCCAGACCAATTTCTTCACCCATATCGATCAGACGTTTGTATAAATCTTCCGCACTGACGTGATGGTTGTCC
GGCTCCTGAAGAACTTCCAGGATTTTTAAACGAGGAAGCGTTACTTTCAGGCCAGCTTTCTTTAGGGCGGTATTGTTATC
AGTCATGCGGAATCTGTCCTGTTACTAAGCGGTTCACTTCATTAGAAGAAGTGACAGAATTTGCTCTTGAGATAATGCGT
ATCATTATAGAATTGCCACGCCTAAATGAAAACCACAAGTCCCTGGCAAATATTGTTAATAAAAACGTGGCACAGCTTAC
ATTTACAACGGCAAGGCCACTTGAACACCGGGACATTGTACAGGTACAACAGCAAAAGTTACAAATTTGTAGCAATTATT
TTGATTGGCATTATCTATTAATACGGCGTAGACATGAGTCTACGCCGCATCACATCAGGCATTGAGAATTTCGTCGAGAT
GCAACTCTTCAGAAATCTGTTTAACCCATTTTTCTACACGTTCAGCGGTCAGTTCCGGCTGACGGTCTTCGTCGATAGCC
AGACCGACAAAGTGGTCGTCATCTGCCAGACCTTTTGATGCTTCGAAATGATAGCCCGCAGTTGGCCAGTGACCAACGAT
GGTTGCACCGCGCGGTTCAATGATGTCGCGGATGGTGCCCAATGCGTCGCAGAAATATTCGGCGTAATCTTCCTGGTCAC
CACAACCAAACAGCGCAACCAGTTTGCCGTTGAAATCAATCTCTTCGAGAGTCGGGAAGAAGTCATCCCAGTCACACTGC
GCTTCGCCGTAATACCAGGTTGGGATGCCCAGCAGCAGAATGTCATAAGCTTCCAGATCTTCTTTGCTGCTTTTTGCAAT
GTCATGGACATCGGCAACGTCTTTACCAAGCTGTTTTTGAATCATTTTTGCGATATTTTCGGTATTACCGGTGTCGCTGC
CGAAAAAGATGCCAGTGATAGCCATGAGTGAAATAACCTCTTGAAACTTATTGAAATGGGGGTGGAAAATTGCCCACGGA
TAAAGGCAATCATAGCAGAACAGGCAGTCTTGCGGAATCAGCAAACGAGCAGGACTGCACACTGTGCTACATGAAAGTGG
AAATTTAAACGATGCCCTGACTACGCAGCGCCGCCAGTTGCTGCATTAACATCTCTTCGATCAGTTCGCTACGGCTCATA
TTGCGCGACTCCGCCAGCTCGTTCAGCGCCTCGACAGCTTCCGCGTTCAGCTTCAGTTCGACACGCTTAAGGCCACGTAC
TTTGTCGCGTTTTAGCTGGTTGCGTTTATTAATACGCAGCTGTTCATCGCGCGAAAGCGGATTAGTTTTCGGTCGTCCCG
GTCGACGCTCGTGCGCGAACAGATCTAATGTCGTACGGTCCGTTTGTTCTTTGGCCATGATCTTGGTGACTTCGGGGGAA
ACAATCAGCCAGGCCTCTGCCCGGATGGATAGCGCGCCATAATACATCAGCGCGATGAGTCACGCCAACGCCCACGCGCG
GAAAGCGACGCGGACGCTGGGTTTTTAATCAGTTGCGTTAATCATTGAGATAGCGACGGATAGCGCGTAATACCGCATCC
GGTTTTTCAGCATGGACCCAGTGACCCGCGCCTGCAATCACATGCGCCCGTGCCTGTGGAAATTGAGCCAGTAAATCATC
ACGGTACTGCTCGCTAACATACGGAGAATTGCCGCCAGGGATAAACAGGGCAGGGTGATCCCATGCCGGGATTTTCTCCC

AACCTACAATATGCGGATACTGATCCCACAATACCGGCACGTTAAAGCGCCACTCCCCGTCAACAAAAGATTTCAGCAGA
AACTGAATCACCCCTTCTTCATTAAGATGCTGGCGCATTATTGCTGCTGCTTGCTGGCGAGTTTGTGCGTCCGATTCACT
GACCGCGTTGATAGCCGCAAAAATCTCATCATGACGGCGTACGTGATAGTCGACCGGCGCGATATCGATCGCCACCAGTT
TATCGATGCGATCGGAGGCTAGTGCAGTAAGTGCCATTACCGCTTTACCGCCCATGGAGTGACCGATAAATGTTGCTTTG
TCGATCTGCTGTGCATCCAGAGTATCAACAAGATCCTGCGCCATCGCCGGGTAATTCATTACCGGATCTCTCGGTGAAAG
ACCGTGGTTACGCATATCAACCTGGATGATATTGTGATCGTTTACCAGATCGCGAGCCAGTACGCCAAGGTTGTCGAGGC
TGCCAAACAGACCGTGGACAAGAACGATGGGAGAATTATTGTGCTGGTTTTGTGCAGTTTGCGCGCGGATATTCAATTTC
ATGGCAAAGTTCTTTTTTTCGCGTTGTCGGGTTAGGGTATTATGTTGACCATTGTGCCACAGGGCTGCAACAAATAAGGT
TTATTCCGAGTTTTTCTGCAAGCCAGGCTTGACGCTATCCGCTGCCGGGATTTATTCATATACTCCTGGCGACTTGTATT
CAGCTAAGACACTGCACTGGATTAAGATGAAAACGATTGAAGTTGATGATGAACTCTACAGCTATATTGCCAGCCACACT
AAGCATATCGGCGAGAGCGCATCCGACATTTTACGGCGTATGTTGAAATTTTCCGCCGCATCACAGCCTGCTGCTCCGGT
GACGAAAGAGGTTCGCGTTGCGTCACCTGCTATCGTCGAAGCGAAGCCGGTCAAAACGATTAAAGACAAGGTTCGCGCAA
TGCGTGAACTTCTGCTTTCGGATGAATACGCAGAGCAAAAGCGAGCGGTCAATCGCTTTATGCTGCTGTTGTCTACACTA
TATTCTCTTGACGCCCAGGCGTTTGCCGAAGCAACGGAATCGTTGCACGGTCGTACACGCGTTTACTTTGCGGCAGATGA
ACAAACGCTGCTGAAAAATGGTAATCAGACCAAGCCGAAACATGTGCCAGGCACGCCGTATTGGGTGATCACCAACACCA
ACACCGGCCGTAAATGCAGCATGATCGAACACATCATGCAGTCGATGCAATTCCCGGCGGAATTGATTGAGAAGGTTTGC
GGAACTATCTAAAACGTTGCAGACAAAGGACAAAGCAATGGCAATCCACAATCGTGCAGGCCAACCTGCACAACAGAGTG
ATTTGATTAACGTCGCCCAACTGACGGCGCAATATTATGTACTGAAACCAGAAGCAGGGAATGCGGAGCACGCGGTGAAA
TTCGGTACTTCCGGTCACCGTGGCAGTGCAGCGCGCCACAGCTTTAACGAGCCGCACATTCTGGCGATCGCTCAGGCAAT
TGCTGAAGAACGTGCGAAAAACGGCATCACTGGCCCTTGCTATGTGGGTAAAGATACTCACGCCCTGTCCGAACCTGCAT
TCATTTCCGTTCTGGAAGTGCTGGCAGCGAACGGCGTTGATGTCATTGTGCAGGAAAACAATGGCTTCACCCCGACGCCT
GCCGTTTCCAATGCCATCCTGGTTCACAATAAAAAAGGTGGCCCGCTGGCAGACGGTATCGTGATTACACCGTCCCATAA
CCCGCCGGAAGATGGTGGAATCAAATACAATCCGCCAAATGGTGGCCCGGCTGATACCAACGTCACTAAAGTGGTGGAAG
ACAGGGCCAACGCACTGCTGGCCGATGGCCTGAAAGGCGTGAAGCGTATCTCCCTCGACGAAGCGATGGCATCCGGTCAT
GTGAAAGAGCAGGATCTGGTGCAGCCGTTCGTGGAAGGTCTGGCCGATATCGTTGATATGGCCGCGATTCAGAAAGCGGG
CCTGACGCTGGGCGTTGATCCGCTGGGCGGTTCCGGTATCGAATACTGGAAGCGTATTGGCGAGTATTACAACCTCAACC
TGACTATCGTTAACGATCAGGTCGATCAAACCTTCCGCTTTATGCACCTTGATAAAGACGGCGCGATCCGTATGGACTGC
TCCTCCGAGTGTGCGATGGCGGGCCTGCTGGCACTGCGTGATAAGTTCGATCTGGCGTTTGCTAACGACCCGGATTATGA
CCGTCACGGTATCGTCACTCCGGCAGGTTTGATGAATCCGAACCACTACCTGGCGGTGGCAATCAATTACCTGTTCCAGC
ATCGTCCGCAGTGGGGCAAAGATGTTGCCGTCGGTAAAACGCTGGTTTCATCTGCGATGATCGACCGTGTGGTCAACGAC
TTGGGCCGTAAACTGGTAGAAGTCCCGGTAGGTTTCAAATGGTTTGTCGATGGTCTGTTCGACGGCAGCTTCGGCTTTGG
CGGCGAAGAGAGTGCAGGGGCTTCCTTCCTGCGTTTCGACGGCACGCCGTGGTCCACCGACAAAGACGGCATCATCATGT
GTCTGCTGGCGGCGGAAATCACCGCTGTCACCGGTAAGAACCCGCAGGAACACTACAACGAACTGGCAAAACGCTTTGGT
GCGCCGAGCTACAACCGTTTGCAGGCAGCTGCGACTTCCGCACAAAAAGCGGCGCTGTCTAAGCTGTCTCCGGAAATGGT
GAGCGCCAGCACCCTGGCAGGTGACCCGATCACCGCGCGCCTGACTGCTGCTCCGGGCAACGGTGCTTCTATTGGCGGTC
TGAAAGTGATGACTGACAACGGCTGGTTCGCCGCGCGTCCGTCAGGCACGGAAGACGCATATAAGATCTACTGCGAAAGC
TTCCTCGGTGAAGAACATCGCAAGCAGATTGAGAAAGAAGCGGTTGAGATTGTTAGCGAAGTTCTGAAAAACGCGTAAAC
ACATTTAATAAAAAAAGGGCGGTCGCAAGATCGCCCTTTTTTACGTATGACAAACACAGAATTGCCTGATGCGCTACGCT
TATCAGGCCTACGAGGATGGTGCAATATATTGAATTTAAGCGATTTTGTAGGCCGGATAAGGCGTTCACGCCGCATCCGG
CAAAAACAACGAACACTTTGTCAACAAACTGAGTAGCTCAAGGAAATCCCAATGAAGACCAACAGGAGTCTGGTCGTCAT
AGTGTCACTAATTACCGCCACATTACTGCTGACTGCATGCGCCCAACCTGAACAATCATCATTAGCTGGCGACTGGTTAC
TGACCCCTAAAGATAAAACCAGAGGATTAACGGGTAGCATTGCGGTAAATATTGCACCTTTCCGGTGTAAGACAAATTGC
CGTGGTGATAACTTACCTGATAATACCCGCCGCTGGCAGTTGTCAGGCGGGAACGAAAAAGAACTTACTTATCTTCATAA
TATGTCAGCACAAGAAAAGGTCGGTTTAAATCCCGGTTGGCAATGCTACACCTCATTTTTTATGCGGGTTTGCCAGGGCA
AGCCCGGCACTCGACCGATAGTCAATGAGGATTATGTCAGCGAAAGCGGATTTTTTGGCTCGATGATGCATGTCGGGATT
ATTGAGTTACGACGCTGCCAGTCAGAAAATTGCCAGCAAGAATTGAAAGCGATAAATACACATTAAAATAGTGAGTCCGG
GAATGAAACCCGGACCTGAACATGTCTTATTGCGTGAATGTTTCTTTCATTTGATACTGCATTTCCCAGAGTTCAGTAAG
CGAAATAACTTTTCTTGTTCTTGCTTCTTCACTTTGTACCCATAAGAAAGGGTAGAACGTGAAGGCTTCCGTTGCCGATA
ATTTTATTACGTCTTCTCGCCATTGCTGCCATCGTACATTTTCATAGAAGGTATCTAAATCACCGGATAATGCCCACTGA
AGAAACTCAGAATATCCAACTTCCAGACTTTCCCAATTCAGCGTATCTGGCGCAAAATAATAAACGCAGCCAGTATCTTC
CCCAAGATCGCCACCATTAAGAGCAAAAATTACCCCTGCGACATCGTCAGCGATGGGTAAAGCCCGCACGCCTGCGAATT
CAGGGCGTTGTGTCCAACTTACTGGATCTCGTGGCAACCGGGGGTGACCAGATCCTGCTATGCGTAACTAACCATTATCA
ATCAGCAGACCTCCGGAGCCATAGACAAGCGCACCCATCGGTGATTTTGTTGTCACCTGTAATTGATAAAGTGCGGTTTC
GGCTAAATCTTTATTAACCGGTAAGAGTTCGGTATGGTTTGTGGCGTCTTTTAACCAGTTCTGGATTATTGGCCAGGCTG
AATCTTCAGTATTTACTAACTGATTCATTTCACGCATCGCTACTTGCTCCTTGTGTGCTACGGTGTCATTTATGATGAGG
AATTGTATGGAACATTTTTTAAATATTAAGTTCCTTATATTTGAATAAAAAATGACTTTATCCATACATTGCCACTTTTT

TCAAAGCATAAACCGATACCCAATACCGGTTTCAGTAATAAAATGGCGAGGCCGGGCCGGATCCTGTTCCAGTTTTTGTC
GCAGATGCCCCATATAATTACGCAAATAGTTGCTGTGTTCGATCGCGTTTAGACCCTACATCTGCCGGATGCGGCATAAA
TAACGAACTATTTGCCAACAAACAGAAAGGGCGATCATTCAATCGCCCTTTTTTACTTTGTTTTATTAACCGTGTTTATT
TTTCAGTTCAAAGCGTGGTGATACCAGACCATACAGTGTCCAACCAAGGAAGGTCACGATGGAACCGTACAGCATGGCTT
CTTCCCCGGATGAGTACAGCGCATAAAAGCTATACATCGCGCCAACAAAAGCAACAAAGTTTGCAACTTTCGCTTTTGAG
GGGGGCACATTAGCGACCTTCTGAATAATGACTAATGCCGCCATTGACAGAATATACGGAATGATATTGGTCACCACGGC
CAGGTTAACCAGCACGTTGAACTGACTGTTTAGCGACGGGCTAATGGTCATCAGTGCCAATCCACTCTGAATAATCACAA
TGGTCAACATTCCCTGCACCGGTGCATCCACTTTGGTTACACGGGAGAAAATTTTAGGGAAGTAGCCTTCATCAGATGAA
GATTTAAACACCTGGGCAATGGTGAACTGCCAGCCAAGTAGCGAACCGCAGCAGGACATCACCATCAGCGCCATAATGAC
TTTACCCACTTCCGGCGTGAACATCTGCGCGAAGGCCAGACCAAATGGTGCCGTTGAATTTGCCAGCTCCATATTTGGCA
CAATCCCGGCAATCACGTTGGTGGAGACGATATAAATCACCGCCGCACCTAACGTACCGCCGAGTACCGCGATTGGCACA
TTACGTTCCGGGTTTTCCACTACATCAGTATTCGCACACGCAGACTCCAGACCAAGAAAAGCCCACAGCGTCATAGCGAT
GGAAGAACCTACCGCACTGAAGAACGGTGCATGATGCGGATTCCAGGAATCAACGTACAGCGTCGGGCTAAACCAGAACC
AGCCAATAATGCACAGACCGACGACCGGAATAATGACCCCCCACACGGTAATGCTACTGATTTGCCCGGTAATGCGCGCA
CCACCAAAGTTAGCCACGGTACAAATCCACAGCACCCCGATGGTCGCAAGACCAATCTGCACTGGCGACAAACTCGCGCC
GAGCAGTTCGGTGCCGTAACCAACCGCCGAAATAGCAATCGCGACGTTAGCAATCAGCAGTGAGACGCCGTAGGTATAGT
TCGCCATAAAGTTACCGGATTTACCGAAAGCATACTCGGCATAACCGCCCATACCGCCTGATTTACGGCTGAACATACCG
CATTTTGCGAATGCCCATGCCAGTGCCATTGAGCCTACGGCTGTCACCAGCCAGGAGATAATTGAGATTGTCCCGACTTC
GGCAAGCTTTGTCGGCAGCATGATGATACCGGAGCCCATCATGTTGACCATCGTCAGTATGGTTAACTGAACGACGCCCA
TTTTGTTCGATTTAGCCTGACTCATAATTTTTCCCCTTTCAACAGGGTGCTTTGCGCATCACGAGGCTTGATGACATAGC
ACCAAACTTGCTTACGACCATCATGTTCTTCGATATAGACACCCTGCAGCTCCGGTGCAAAACCTGGCAGCAGGTTGATC
CCTTCTTCCAGAGCGCTGAAGTAACGCAGAACAGCACCACCCCAGATTTCACCCGGAACAACACACAGCACACCCGGAGG
ATAAGGAAGCGCACCTTCGGCAGCGATACGGCCTTCTGCATCCGGCAGACGAACCAGTTCCACTTCACCGCGTAAATAGG
CGTAGTTGGCTTCTTGCGGATTCATGCTGACGCGTGGGAAGTGCTCCTTACGGAACATCTCTTTTTGCAGTTGTTTCACG
TTGTGGCGGGCATACAAATCATGCATTTCCTGACACAACTGACGCAGGGTATAACCGGCGTAGCGCTCTTCATGCTGTTT
GTAGATGGAAGGTAGCACTTCTGCTAATGGCGCGTCGGACTCAAGCAGTTTTTCGAAGCGTACCAGCAGGGCAACAAGTT
GCTGAAGTTTGGCCATATCTTCTGCCGGAGTCAGCAGGAAGAGGATGGAGTTAAGATCGCATTTTTCCGGCACTACGCCA
TTTTCGCGCAGGAAGTTAGCAAGAATCGTCGCGGGTACACCGAACGCTTCATATTCGCCGTTACGTGCATCAATACCTGG
GGTTGTCAGCAACAGTTTGCATGGATCGACAAAGTATTGATTCTCTGCATAGCCTTCAAAAGAATGCCAGTGTTCCCCTG
GTACAAACTGGAAGAAGCGCAGATCAACCGCAATTTGCGCTGTTTCATACGACTGCCAGGGTTTACCATCCACCAGTTCA
GGTACGAACGGACGAATATGCTGACAGTTATCGAGGATCAGTTTGCGGGCATTAATGCCATTTACCACACAATCCATCCA
CATATTACGACCGCTGACACCTTCATGCATTTTGGCGTTGATATTCAGTGCGGCAAACAGCGGATAGAACGGGCTGGTGG
AGGCGTGCATCATAAAGGCGTTGTTCATGCGTTTGTGCGGTACATAACGCTGTTGCCCTTTGATGTGGCTGTCTTTTTTA
TGAATTTGTGAAGTCTGAGAAAAACCAGCCTGTTGTTTATGCACAGATTGCGTAACCAGAATACCCGGATCGTTCTCATT
AAGATCCAGCAACAGCGGCGAACAGTCCGCCATCATCGGAATAAACTGTTCATAGCCGACCCATGCTGAGTCAAACAGGA
TGTAGTCACACAGATGACCAATTTTATCCACCACTTGGCGGGCGTTATAAATCGTACCGTCGTAGGTGCCTAACTGAATC
ACAGCGAGGCGGAAAGGACGAGCCTCTTTTGCCCGCTGCGGTGCGACTTCCGCGATCAGCTCACGCAGGTAACTTTCTTC
AAAACAGTGCGCATCAATGCCACCGATAAAGCCATACGGGTTACGTGCCGTTTCCAGATAAACCGGTGTTGCACCAGCCT
GTAGCAACGCTCCGTGGTGGTTAGATTTGTGGTTATTGCGATCAAACAGCACCAGATCACCCGGTGTTAGCAGGGCGTTT
AAAACCACTTTGTTAGAAGATGAAGTGCCATTTAAAACGAAGTAGGTTTTATCGGCATTAAACACTTTTGCCGCATGTTG
CTGTGCAATGCATGGCGCGCCTTCGTGAATCAGCAGATCGCCCATCGCTACGTCGGCGTTGCACAAGTCGGCACGGAACA
GCGCCTCACCAAAGTATTCCACAAACTGATTCCCCGCCGGATGGCGACGGAAAAATTCGCCGCCCTGATGACCAGGGCAA
TCAAACGCGCTGTTACCTTGATTGACATAATCGACCAGTGCGCGGAAGAAAGGTGGGCGCAGTTGAGTTTCATAATGGCT
GGCAGCGGTTTCTAACTGGCGACCATAAAATTCTCGTCGCGATTCGCAATTCTCAAAGACACCCGAAATACGGGGCAAAT
ACTCTGCGGGTACACGTTCTTGATTTTCAGTAGCAATAAATACCGGGATGCCATAACCCGTGGCATCGATTTCATCGAGT
TTTCCGCATGTAACATCGTTGAGCGATAAAACTATTGCCGCCACATCGATATTACGACTTTCATTAATGTAGATACATTC
TCGCTGCGTGGTAAAACAGTCCGGGCAAGAATCACTAACCGCAATTTTTAATTTTGACATTTTTCATCTCTTTATTTTAG
GTAATAGCAGGTCCTCAATTTCTCGAATGAGAAATCGATCGTGCCGGAAAAAAGCAAAGTTACCCCGCTGATAAATAAAT
CAGGTAGATTGCTTTTTTCAGAATGCAGAAACCCTTACCAGTCAGGTGGCTGATAATACAAGGGTTAACAGAGAATGAGC
ACTGCGGAATACAGGCATATGCCTGTAATACAGAGAGTCAGGAAGACCTGTACGCTGAAGTGCCCTTAGTCGGTAGACTA
ACGGGCATTGAAGAAGTGAAAGTCGAAGCTATTGCGATGAGCAAACTTCATAATATGAGTTGTCCGCCTTATATGGGGCA
TAGTGCGGCTGTTATTTTCCATTTAATTTACCTTTCCTGATGTGAATGAAGGTGAAGATATTCTATCCAGGAAATACGGT
TGAACTGTGAAGGTGATCAATAAAAAATGATCAATCTTAATTTATTTAATGATGAGCTTTTTACTCAGTAATATAAAATA
TTGAATTGTTATTTTTGTGTGTTGTTTAAGATAAAAAGCCGTATTTATTATTACGGCTTTAATTAATAAAAGGCAGGCTG
TATTAAAATTAATATTCAAAGCATAAACCGATAGCCAATACCGGTTTCAGTAATGAAATGGCGTGGGCGGGCGGGATCCT
GTTCCAGTTTTTGTCGCAGATGTCCCATATAAATACGCAAATAGTGACTGTGTTCGACCGCGTTTGGCCCCCACACCTGG

TTAAGGAGCTGGCGCTGGGTGAGTACTTTTCCGGCATTGTTGAGCAGCACCGCCAGCAGGCGGAACTCAATTGGTGTGAG
ATGCACCTCTTCCTCACCCCGGTGAATCACGCGGGCGGCTAAATCGACGGTAACATCGGAAAATTTACCAGCGGATCGG
GCGCGGTGGTGGCAGAGTGGCGGCGTAATGCGACGCGCAGACGGGCCTGCAATTCGCCAATGCCAAACGGCTTACTCAGA
TAATCATCCGCTCCGGCATCCAGCGCGGCGATTTTGTCGCTCTCTTCGCTGCGTGCGGAAAGCACAATCACCGGCACCGC
GCTCCACTGGCGCAGGTCGCGGATAAACTCAATCCCATCACCATCGGGCAGGCCGAGATCGAGAATAATCAAATCTGGCT
TACGGGTTGCCGCTTCCAGCAAGCCGCGTTGCAGCGTTTCGGCCTCAAAGACGCGCATCCCGTCGCCCTCCAGCGCCGTG
CGCAGAAAGCGACGAATAGCCTGTTCATCTTCAACAATCAGAACGTTTGTCACATATCCTCATGAAATTCTTCAAGTTCA
GGGGCAGTTTGCTGGGGAAGTGTAACACGAAAACAGGCACCACCTTCCGGTCGGTTGAACGCGGTAATAGTGCCCCCGTG
TACATCCACTATCGCCCGACAAATTGCCAGTCCAAGCCCTACCCCCGGTACTGCCGACTCTTTATTCCCGCGAGCAAACT
TATCAAATATCGTCTGCTCCTGGCCTGGCGGAAGACCGGGGCCGTTATCCCAGACATCCAGTTGTAGATTTTCGCCCTCA
ACGTGGGCATCGATACCAATTTCGGCCTGCGCACCCGCATATTTCACCGCGTTCTCCAGCAGATTAATCAGCACCCGTTC
AAAGAGTGGCCCGTCAACGTGGATTAAGGTCAGCGGTTCTGGCAGAGAAAGATTGATGGGCGACGATAAACCCGGTTCCA
GCATTTGCAGCGCGCTGCCGACTACTTCTTCCAGCGTTAACCACTCTTTCTTCAAATTAAAGCCGCCGGACTGAATTCGC
GCCATATCCAGTAGATTATTCACCAGTCGGGTAGTGTTCAGCACATGCTGACGGATCTCGCTGGCCTGGCGGGCGTGGGG
TGATCCTTCGCTTGCCAGATCGAGCGTTAAGATTTCTGCCTGACCAAACAGCACCGTAAGCGGCGTGCGTAAATCATGCG
AAAGCGCCGCCAGCAGGGCGTTGCGGATCTGTTCACGTTCGCTTGCCATCCGCGCCTGTTCTTCGCTGGCGGTTAGCGTC
AGCCGCTCAAGGGCATTGGCGACTAACAGCGTAAACGTCTCCAGCAGGCGCTGCTGTTCCGGGATCATCAACTGGCGCAG
ATTCCCCGGCTCCACCACCACCAGCCCGTAGGTTTTCTCGCCGCTTTTTAGCGGCAAAATCTGGTACGGTACACCGGGTA
ACGTGTCGGTGCCCGCGCCCGCAGGCAGGCCTTTATCAAAACTCCACTGCGCGATGGCATCGTCCCACGGCGTCATTCCT
TGCGGATGTGTTAACGGCTGCAATTTACCGTTGTCATCGGGCAACAACACCTGACTGCGGGCATGAAACGTGGAGGCAAT
AAATTGTTCGCTGGTGGCAGCGATATCCTGCGGACTGCGGCCCACCGCCAGAGCTTTCGACATTTCATATAAGTGCCGTG
TGCGTTGCTCGCGGTAACGGGCTACCCGCGCCTGATAACGCACGCCAGCAGTAAGGTTCCCGATCACCAGCCCGACGGTT
AACATCACCGCGAAGGTCAGCAGATATTGCACATCAGAGACGGCGAGCGTGCCGCGTGGGGCGATAAAAAAGAGATCGAA
ACTCACTACATTAATGACGGTGGCAACCACTGAAGGCCAGCGTCCATAAAATAGCGCCACCACCACCACGCCAAGCAGAT
ACAGCATCACCAGGTTGGCGGCATCAAACGCCATCAGCCACTGCATGGCAATTAAGGTGATAACGGCGCATAACGCGGCG
GCAACCACGCATCCCTGAATTTGTACACGCCACTTGTCTTTAAAAGAGCGGTTATCCGGCGCGTTGTTAATCGTGCGGGC
GGGTGGTTCATCAAGCGCGACCAGCACCTGATCGAGATCGGGGGCGATGCGCGCCAGTCGGTCAGCAAACGTTTCCCGAC
GCCACCAGCGGCGCGAGGCCGGGCGACCGAGAATAATCTTGCCGAGATTATGTTCACGGGCATAACGCACTACCGCTTTC
TCTTCCGCTGGATCAGAAAGTGTTGCCGTCTCCGCGCCCAGTTCCTGCGCCAGACGTAAGGCGCTGAGAATTGCCCGACG
TTTTTTTTCCGGTAAGCGGTGCAGGGCAGGGGTTTCAACATACACCGCGTGCCAGACGCTACCCAGCCGTGACGCCAGCC
GCGCCGCTGCGCGGACCAGTTTTTCGCTGCCGGTGTTATGGCCGATGCATAAAAGGATCGCGTCGCGCGTGTGCCACACT
TTCTCTTCGCCAGGATGCCCCCGCCAGGCGCGCATTTGCTCATCAACGCGATCGGCAGTACGGCGCAGTGCCAGTTCGCG
CAGGGCGATCAGATTACCTTTGCGGAAAAAAATGTTCAATGGCGCGCTCCGCCTGCCCGGCAATATAGACTTTGCCTTCTT
TCAGCCGCTGGCGCAGATCGTCCGGGGGCAAGTCCACCAGCACCACGTCGTCGGCGGCATCGAAAAAAGGATCGGGCACG
GTTTCCCGTACCTGAATTCCGGTGACGCCGCTGACCACATCATTCAGACTTTCCAGATGCTGAACGTTGACGGTAGTGAA
AACATCAATGCCAGCTTCCAGCAGTTCTTCGATATCCTGCCAGCGTTTGGGATGACGGGAACCTGGCGCATTACTGTGCG
CCAGTTCGTCCATTAAGATCAGCGCCGGGCGGCGGGCGAGGGCGGCATCGAGATCAAACTCGCTGATATGCCGCCCACGG
TACGCCTGGCGTTTTAACGGCAGAACAGCCAGCCCTTCCAGCATGGCGGCGGTATCTTTTCGCCCGTGGGTTTCTACCAC
GCCAACCACAATATCCAGCCCTTGCGCCCGCAGTCGCTGGGCTTCTGCCAGCATCGCCCAGGTCTTCCCGACGCCTGCAC
AGGCACCGAAGAAAACTTTCAGCTTCCCCCGATGCGGCGCGGCAGTTTGTTCCAGCAGACGATCGGGGTCGGGACGTAAG
GGTTCGTTATTCATCAAGTTTATCCAGCGCCAGATTGAGTTCAACAATGTTGACAACCGGCTGGCCGATATATTTCACCA
GCGGTTGTTGGCTGTATTTTGCGATCAGTTGCGTGAGCTGTTCAACGCTGAGATTACGCGCTTTCGCCACGCGTGGGATT
TGCCAGGCCGCCGCTTGCGGGGTGATATTATTGTCCAGCCCGCTTGCCGATGCCGTCACCAGTTCAACCGGAACGCTCGC
GCTGGCATCCGGGTTAGCGGCCCGTAATGCAGCAACGCGTGCGGCTATTAGTTTATCCAGCTCAGGGTTACTGACCGCCA
GATTGCTCCCGCCAGAAGCCTGTGGATTATAGGGCATTTCTGCCGTTGCCGACGGGCGACCATGAAAATAGCCGTTGCCG
GTAAAATTCTGCCCGATTAATGCCGAACCGCGCACCGTATCACCTTCACGAATCAACGAACCATTGGCCTGCCAGGGAAA
CCACCATTGCCCCAGTACGGTGGTCAGCAGCGGGTAAACGCCGCCAGTAATCAATAACAGAAAGATAAATGTTGATAATG
CCGGACGTAATCCACTCATGGTAAACCTCACACCAGACCGCAAACGGTCAGCAGTAAATCAATGACTTTGATACCGATAA
ACGGCACCAGCAGCCCACCCAGACCGTAAATCCATAAGTTACGGCGCAACATGGCAGAAGCGGTAAGCGGTTTATAACTC
ACGCCTTTTAACGCCAGGGGAATCAAAAAGACGATAATCAAGGCGTTGAAAATCACCGCACTGAGGATTGCGGAGTCGGG
CGAATGCAGGCACATGATGTTCAGCGCATTTAACTGCGGATACGTTGCCGCGAATGCCGCCGGAATAATGGCGAAGTATT
TCGCCACATCGTTGGCAATGCTGAAGGTGGTCAGCGAGCCACGGGTCATCAGCATCTGTTTGCCAATGTGCACCACCTCG
ATCAACTTGGTCGGGTTAGAGTCGAGATCGACCATATTGCCCGCCTCTTTCGCCGCCTGGGTGCCGGAGTTCATCGCCAC
CGCGACATCTGCCTGCGCCAGCGCCGGAGCATCGTTGGTGCCGTCGCCGGTCATCGCTACCAAACGACCTTCCGCCTGAT
ACTGACGGATCAATGCCAGCTTGGCCTCCGGTGTCGCTTCGGCGAGAAAATCATCGACACCCGCTTCCGCAGCAATCGCG
GCGGCAGTCAGACGGTTATCGCCGGTAATCATCACCGTTTTAATGCCCATTTTGCGCAGCTGGGCGAAGCGCTCTTTAAT

ACCGCCTTTGACGATATCTTTCAGCGCAATAACGCCCAGCACACGAGAACCTTCCACCACCACCAGCGGCGTGGCTCCCT
GACGCGCAACCTGATCGACTTTTTGATCAACATCGGTAGGGAAGTGACCACCGTTAGCCTCAACATGGCGACGAATGGCA
TCGACAGAACCTTTACGGATCATGCGGTTGTCGATGTTGATCCCGCTCATCCGGCTTTGCGCAGTAAACGGTACAAAGGT
GGCATGGAGCGACTGCACATCGCGCTCGCGCAGGTTAAAACGCTGCTTGGCGAGGATCACAATACTGCGGCCTTCCGGCG
TTTCATCAGCCAGCGAAGCCAGTTGTGCGGCGTCAGCCAGCGTTTTTTCATCCACGCCCTGCGCGGGGATAAACTCCGAC
GCCTGACGGTTACCGAGTGTGATGGTGCCGGTTTTATCCAGTAGCAGAACGTCAACGTCACCTGCCGCTTCAACTGCACG
TCCGCTGGTGGCAATCACATTCGCGCCTAGCATCCGGCTCATCCCGGCGACGCCGATCGCTGACAACAGGCCGCCAATAG
TGGTTGGGATCAGACAGACCAGCAGCGCCACCAGTACCGTTACGCTGACTGCATTACCGCCCCACGCGGAAAACGGCCAC
AGCGTGGCGGTTGCCAGTAAAAAGACGATAGTCAGGGCAATCAGCAGAATGGTCAGGGCAATTTCGTTCGGCGTTTTGCG
TCGCTGTGCGCCTTCCACCATCGCGATCATCCGATCCAGAAATGTCTCGCCGGGGTTAACGCTACACTCAATCACCAGCC
AGTCAGAAAGAATACGCGTGCCGCCGGTGACGGAGGCAAAATCGCCGCCGGATTCACGGATCACCGGTGCCGATTCCCCG
GTGATGGCGCTTTCATCGACCGATGCACCCCCTTCAATAACTTCACCATCGCAGGGGATAATATCGCCAGCTTCTACCAG
TACGATATCGCCTTTACGAAGTTGGTCGGCAGGAACTTTGTCCGCCGCAGCGCCATATTTCGGCTCACGCAGCTTGCGGG
CAAAGGCAGTTTTTTTCACCCCTTTCAGACTGTTGGCCTGCGCTTTACTGCGGCCTTCTGCCAGCGCCTCGGCGAAATTA
GCGAACAGTACGGTGATCCACAGCCAACCGCTAATGGCCGCGCTAAACAGCGCATTGCCGGGCATCGCACCGCTTGCCAT
CGCGATGCTAATACAGGTGGTCAGCAGACTGCCGATCCAGACGATAAACATCACCGGATTGCGCCATTGCGCCTGCGGGT
TTAATTTTTTCACCGCTTCTTTCAGCGCCTGAACGACAAGTGTTGGTTCGAATAGCGCCAGTTGTTTACGACTCATATTC
AGTGCTCACTCAATATCATCAGGAGAGATATTCCGCCACCGGACCAAGCGCCAGGGCAGGGATAAAGGTCAGTGCGCCAA
CCAGCAACACGGTGCCGATTAACAGGCCAACAAACAGCGGGCCGTGCGTTGGCAGCGTGCCGGAGCTGGCGGCTTGGCTC
TTTTTACTCACCAGCGAACCGGCAATTGCCATCACCGGGATAATCACCCCGAAGCGACCGACAAACATGCAGAACGCCAG
TAAACAGTTCCAGAACGGAGAGTTGGCGCTTAATCCGGCAAAGGCGCTGCCGTTGTTGTTAGCGGCGGATGACACGGCGT
ACAGCACTTCGCTAAAACCATGCGGGCCAGGGTTGAGCATGGCGCTACGTCCGGCGTCGGTCATCATCGCCAACGCCGCG
CCCATCAGCACCAGCGTCGGGGTAACCAGAATTGCCAGTGCAGTCAGTTTCATCTCGCGTACGTCGATTTTTTTACCCAG
ATATTCCGGTGTACGACCAATCATCAGCCCGGCAATAAACACCGCCAGCAGGACAAACAGCATCATGCCGTAAAGACCAG
AACCGACACCGCCGAACACCACTTCACCAATTTGCATCAGCCACATCGGCACCATGCCACCGAGAGCGGTAAACGAATCA
TGCATCGCAATCACCGCGCCACAGGAAGCCGCCGTCGTCACGACCGCAAACAGGCTACTGACCAGCACGCCGAAACGGCT
CTCTTTACCTTCCATATTGATGCTGCTGTCCGTGCCCAGTGCCAGCAGATGAGGATTACCCTGAACTTCTGCCCACATCA
CCACGCCTACGCAGATGACAAAAATCACTGACATCGCCCACAGCAACATGCGCCCCTGGCGGCGATCGCCCATCACTTCA
CCAAAGGCAAAGCACAGCGCCGTTGGGATCAAGAAGATCGCCAGCATCTGCACGAAGTTGGTCAGTGCGGTTGGGTTTTC
AAACGGATGCGACGAGTTGGCATTAAAGAAGCCACCGCCGTTAGTACCGAGCATCTTGATCGCTTCCTGAGAAGCTACAG
GCCCCATGGGTAACAGCTGTTGCGCTCCTTCAACGGTATTCACAGCCTGATAAGGCAGAAAGTTTTGCAGCGCACCTTGT
TGAATAAAAAACAGTGCAATCAACAACGCCACAGGGACTAGCACCCATAACGTGATGCGTAGCAGATCGACCCAGGCATT
CCCGAGCGTGCTCATGCTCTGGCGGGTAAACGCACGGATGAGGGCAAAAATCACCGCAATCCCGCTGGCGGCAGAAAGAA
AGTTTTGCACCGTTAAGCCCGCCATCTGGCTGAAATAGCTCAACGTGGTTTCACCGCTATAAGATTGCCAGTTGGTATTG
GTGACAAAGCTGACGGCGGTATTCAGCGCCAGATCCCACGACAGCCCTGGCAACTGCTGTGGATTAAGCGGCAGATAGTG
CTGACCGAGCAACATAAAAAACAGCACCGCCAGCCCCAGCATGTTCAGGCCGAGAATGGCACAAAGATATTGCTTCCAGT
TCATCTCACGGTCAGAGACGCCAAGTGCGCGAAAAAGTACGCGCTCAACGCCCGTTGTACCGGGAAGAGGAATGTCATTA
ATCAGCCGCGCCAGCCCGCTGCCTAAAGGACGCGCCAGCACCATTAACACCAGTAAAAACGTGGCGATCAGTAAGAACCC
TTGCGCAGCCATCAGAACGCCTCCGCATTGATCAGGGCATAAACCAGATAACCCAGTAATAAAAACACCAGCAATACGCC
GGTTATCACGCCTGCACTCACAGTGCACCTCCAGTGGCCTAAAAGTGATACCGGAAGGGTAGAATTCTGGCTGCAAAGAT
TTCGCAAAAATCTGCGGGCGGGGTGTAAAAAAAGTATAAAAATGGCAAAAGCCATGATTTAACTAATGTTTAGTATTAAT
TTAACTTTTGTGTAACTTAATTACAGGATGAATGTAAATAAACCATCAATAAGCAAAAATAAGTGGTCGGATGAGTAGTA
AAATTACACAAAAGGCGGTACTATTTTCATCAGATAAACAAATTCATTTTTCCGGTGCCATTCACCGGGTAGATACAAAA
GGGGGAGAAAAGTATGGAACTCTACAGAGAATATCCTGCATGGCTTATCTTTTTACGCCGTACTTATGCGGTTGCAGCGG
GCGTTCTGGCGCTGCCTTTCATGCTTTTCTGGAAAGACCGCGCCCGCTTTTACAGCTACCTGCATCGCGTCTGGTCGAAA
ACCAGCGATAAACCGGTGTGGATGGATCAGGCCGAAAAGGCAACTGGTGATTTTTATTGATTGCTGAGTTTTGAATACAT
TGAAAACCGCCAGCGGAAACGCTGGCGGTTTTTTGTATCAGGAAGATGTTCAGAGGAATATTTAGACATCGTTATACCAA
TCGAAACATATATTCATGAAATATATATAAATATTTTCCTAATTGTTCTTATCTGACAGATATCTCACTTAAGGCTTTCT
TATAAATCTGTAGGGTTTCGCCTGTCAGCAGACAAATAACCCGATAAAACAAGGATGAGCAGATGAGCGGAAAACCGGCG
GCGCGTCAGGGCGACATGACGCAGTATGGCGGTAGCATTGTCCAGGGTTCAGCCGGGGTACGCATCGGTGCCCCCACCGG
CGTGGCCTGTTCGGTGTGCCCCGGCGGGGTGACGTCCGGCCATCCGGTCAATCCGCTGCTCGGTGCAAAGGTCCTTCCCG
GTGAAACCGACATCGCCCTGCCCGGCCCGCTGCCGTTCATTCTCTCCCGCACCTACAGCAGTTACCGGACAAAAACGCCC
GCGCCGGTGGGGAGCCTCGGCCCCGGCTGGAAAATGCCTGCGGATATCCGCTTACAGCTGCGCGATAACACACTGATACT
CAGTGATAACGGCGGCAGAAGCCTGTATTTTGAGCACCTGTTTCCCGGTGAGGACGGTTACAGCCGCAGCGAGTCACTGT
GGCTGGTGCGCGGCGGCGTGGCGAAACTGGATGAAGGTCACCGGCTGGCCGCACTCTGGCAGGCGCTGCCGGAAGAACTC
CGCTTAAGTCCGCATCGTTATCTGGCGACAAACAGTCCGCAGGGGCCGTGGTGGCTGCTCGGCTGGTGTGAGCGGGTGCC

GGAAGCGGATGAGGTGCTGCCTGCGCCGCTGCCGCCGTACCGGGTACTGACCGGGCTGGTGGACCGCTTCGGGCGCACAC
AGACGTTCCACCGCGAAGCCGCCGGTGAATTCAGCGGCGAAATCACCGGCGTGACGGATGGTGCCGGGCGTCACTTCCGG
CTGGTACTGACCACGCAGGCGCAGCGGGCAGAAGAAGCCCGGCAGCAGGCCATTTCCGGCGGGACGGAACCGTCCGCTTT
TCCTGATACCCTGCCGGGTTACACCGAATATGGCCGGGACAACGGCATCCGTCTGTCTGCCGTGTGGCTGACGCACGACC
CGGAATACCCGGAGAATTTACCTGCCGCGCCGCTGGTGCGCTATGGCTGGACGCCCCGCGGCGAACTGGCGGCGGTGTAT
GACCGCAGCAATACACAGGTGCGCAGCTTTACTTACGATGATAAATACCGGGGCCGGATGGTGGCGCACCGTCACACGGG
CCGACCGGAAATCTGTTACCGTTACGACAGCGACGGGCGGGTGACAGAACAGCTAAACCCGGCAGGCTTAAGTTACACGT
ATCAGTATGAGAAGGACCGCATCACCATCACCGACAGCCTGAACCGCCGTGAAGTCCTGCACACGCAGGGTGAAGGCGGG
CTGAAGCGGGTGGTGAAAAAGGAACACGCGGACGGCAGCGTCACGCAGAGTCAGTTTGACGCGGTGGGCAGGCTCAGGGC
ACAGACGGATGCCGCAGGCAGGACAACAGAATACAGTCCGGATGTGGTGACGGGCCTCATCACGCGCATCACCACGCCGG
ATGGCAGGGCATCGGCGTTTTACTATAACCACCACAGCCAGTTAACGTCAGCCACCGGGCCTGACGGGCTGGAAATACGC
CGGGAATATGATGAATGGGGCCGTCTGATTCAGGAAACTGCCCCTGACGGCGATATCACCCGCTACCGTTATGATAATCC
ACACAGTGACTTACCCTGCGCAACGGAAGATGCCACCGGCAGCCGGAAAACCATGACGTGGAGCCGTTACGGTCAGTTGC
TGAGCTTCACTGACTGTTCCGGTTATGTAACCCGCTATGACCATGACCGTTTTGGTCAGGTGACGGCGGTGCACCGCGAG
GAAGGGCTGAGTCAGTACCGCGCATACGACAGCCGTGGACAGTTAATTGCCGTGAAAGACACGCAGGGCCATGAAACGCG
GTATGAATACAACGCCGCCGGTGACCTGACCACCGTCATTGCCCCGGACGGCAGCAGAAACGGGACACAGTACGATGCGT
GGGGAAAAGCCATCTGTACCACGCAGGGCGGTCTGACGCGCAGTATGGAATACGATGCTGCCGGACGGGTCATCCGCCTG
ACCAGTGAAAACGGCAGCCACACCACCTTCCGTTACGATGTACTCGACCGGCTGATACAGGAAACCGGCTTTGACGGCCG
CACACAGCGTTATCACCACGACCTGACCGGCAAACTTATCCGCAGCGAGGATGAGGGGCTGGTCACCCACTGGCACTATG
ACGAAGCAGACCGCCTCACGCACCGCACCGTGAATGGCGAAACCGCAGAGCGGTGGCAGTATGACGAACGCGGCTGGCTG
ACAGACATCAGCCATATCAGCGAAGGGCACCGGGTGACGGTGCACTATGGTTATGACAGTAAAGGCCGCCTCGCCAGTGA
ACACCTGACGGTGCATCATCCGCAGACGAATGAACTGCTCTGGCAGCATGAGACCAGACATGCGTACAACGCACAGGGAC
TGGCGAACCGCTGTATACCGGACAGCCTGCCCGCCGTGGAATGGCTGACCTACGGCAGCGGCTGGCTGTCAGGCATGAAA
CTCGGCGACACACCGCTGGTGGAGTACACCCGCGACCGCCTGCACCGGGAAACGCTGCGCAGCTTCGGCCGTTATGAACT
CACCACCGCTTATACCCCTGCCGGGCAGTTACAGAGCCAGCACCTGAACAGCCTGCTGTCTGACCGCGATTACACCTGGA
ACGACAACGGCGAACTCATCCGCATCAGCAGCCCGCGCCAGACCCGGAGTTACAGCTACAGCACCACCGGCAGGCTGACC
GGCGTTCACACCACCGCAGCGAATCTGGATATCCGCATCCCGTATACCACAGACCCGGCAGGTAACCGCCTGCCCGACCC
GGAGCTGCACCCGGACAGCGCCCTCAGCATGTGGCCGGATAACCGTATCGCCCGTGACGCGCACTATCTTTACCGGTATG
ACCGTCACGGCAGGCTGACAGAGAAAACCGACCTCATCCCGGAAGGGGTTATCCGCACGGATGATGAGCGCACCCACCGG
TACCATTACGACAGTCAGCACCGGCTGGTGCACTACACGCGGACACAATATGCAGAGCCGCTGGTCGAAAGTCGCTATCT
TTACGACCCGCTGGGCCGCAGGGTGGCAAAACGGGTATGGCGGCGTGAACGGGACCTGACGGGCTGGATGTCGCTGTCAC
GGAAACCGCAAGTGACCTGGTACGGCTGGGACGGCGACCGCCTGACCACGATACAGAACGACAGAACCCGCATCCAGACG
ATTTATCAGCCGGGGAGCTTCACGCCACTCATCAGGGTTGAAACCGCCACCGGTGAGCTGGCGAAAACGCAGCGCCGCAG
CCTGGCGGATACCCTTCAGCAGTCCGGTGGCGAAGACGGTGGCAGTGTGGTGTTCCCGCCGGTGCTGGTGCAGATGCTCG
ACCGGCTGGAAAGTGAAATCCTGGCTGACCGGGTGAGTGAGGAAAGCCGCCGCTGGCTGGCATCGTGCGGCCTGACGGTG
GCGCAGATGCAAAGCCAGATGGACCCGGTATACACGCCGGCGCGAAAAATTCACCTGTACCACTGCGACCATCGCGGCCT
GCCGCTGGCCCTTATCAGCACGGAAGGGACAACAGCGTGGTACGCAGAATACGATGAATGGGGTAACCTGCTGAATGAAG
AGAACCCGCATCAGCTGCAGCAGCTTATCCGCCTGCCGGGGCAGCAGTATGATGAGGAGTCCGGCCTGTATTACAACCGC
CACCGCTATTATGACCCGCTGCAGGGGCGATATATCACTCAGGATCCGATTGGGCTGAAAGGGGGATGGAATTTTTATCA
GTATCCGTTGAATCCGATCTCAAATATAGATCCATTAGGATTAGAAACACTAAAATGCATTAAGCCACTGCATTCAATGG
GCGGAACTGGTGAAAGAAGCGGTCCAGATATATGGGGGAATCCGTTCTATCATCAATATCTTTGTGTCCCAGATGGTAAA
GGGGACTATACTTGTGGTGGCCAAGACCAACGGGGAGAATCAAAAGGAGATGGTCTATGGGGGCCAGGTAAAGCAAGTAA
TGATACAAAAGAAGCTGCTGGCCGTTGTGACCTCGTTGAAACCGATAATAGTTGTGTGGAGAACTGTTTAAAAGGGAAGT
TTAAAGAGGTAAGGCCGCGTTATTCTGTATTGCCTGATATATTCACACCTATAAATTTAGGGCTATTTAAAAACTGCCAA
GACTGGTCTAATGATTCTTTAGAAACATGTAAGATGAAGTGCTCCGGAAATAACATTGGACGTTTTATTAGATTTGTATT
CACCGGAGTGATGTAATGAAATATATTATTTTTTTATTCAGAGCTATATGGCTGGCTTTATCGCTTTTAATACTATTCTT
TTCCATGCATAGACTTTCACTATTAGACTCCACTCGTGATGTGAGCGAGTTAATTAGTCTCATGTCTTATGGAATGATGG
TAATATGTTTTCCAACAGGCATAGTATTCTTTATTGCTCTAATATTTATAGGGACTGTATCAGACATTATTGGCGTAAGG
ATTGATAGTAAGTATATTATGGCGATAATAATATGGCTTTATTTTCTGTCAGGAGGGTATATTCAATGGTTTGTATTAAG
TAAGCGCATTATAAACAAATAAATTAAGTTAATTGCTCTTATTATTATATGTAACCTGGGCATTGATATCCCGTATGCCA
CAGACCCGGCAGGTAACCGCCTGCCCGACCCGGAGCTGCACCCGGACAGCACCCTCAGCATGTGGCCGGATAACCGTATC
GCCCGTGACGCGCACTATCTTTACCGGTATGACCGTCACGGCAGGCTGACGGAGAAAACCGACCTCATCCCGGAAGGGGT
TATCCGCACGGATGATGAGCGCACCCACCGGTACCATTACGACAGTCAGCACCGGCTGGTGCACTACACGCGGACACAAT
ATGCAGAGCCGCTGGTCGAAAGCCGCTATCTTTACGACCCGCTGGGCCGCAGGGTGGCAAAACGGGTGTGGCGACGTGAA
CGGGACCTGACGGGCTGGATGTCGCTGTCACGGAAACCGCAAGTGACCTGGTACGGCTGGGACGGCGACCGCCTGACCAC
GATACAGAACGACAGAACCCGCATCCAGACGATTTATCAGCCGGGGAGCTTCACGCCACTCATCAGGGTTGAAACCGCCA

CCGGTGAGCTGGCGAAAACGCAGCGCCGCAGCCTGGCGGATACCCTTCAGCAGTCCGGCGGCGAAGACGGTGGCAGTGTG
GTGTTCCCGCCGGTGCTGGTGCAGATGCTCGACCGGCTGGAAAGTGAAATCCTGGCTGACCGGGTGAGTGAGGAAAGCCG
CCGCTGGCTGGCATCGTGCGGCCTGACGGTGGAGCAGATGCAAAACCAGATGGACCCGGTGTACACGCCGGCGCGAAAAA
TCCACCTGTACCACTGCGACCATCGCGGCCTGCCGCTGGCGCTTGTCAGCACGGAAGGGGCAACAGAATGGTGCGCAGAA
TACGATGAATGGGGCAACCTGCTGAATGAAGAGAACCCGCATCAGCTGCAGCAGCTTATCCGCCTGCCGGGGCAGCAGTA
TGATGAGGAGTCCGGCCTGTATTACAACCGCCACCGCTATTATGACCCGCTGCAGGGGAGGTATATCACTCAGGATCCGA
TTGGGCTGAAGGGGGGATGGAATTTTTATCAGTATCCGCTGAATCCGGTTCAGTATATAGATTCAATGGGACTGGCATCA
AAATATGGACACTTAAATAATGGCGGATATGGAGCGAGACCCAACAAACCGCCTACGCCCGATCCAAGTAAATTGCCGGA
CATAGCGAAACAATTAAGACTGCCATATCCTATTGACCAGGCCAGTAGTGCGCCTAATGTTTTCAAAACATTCTTCAGAG
CATTAAGCCCTTACGACTACACACTGTATTGCAGGAAGTGGGTAAAACCAAATCTGACTTGTACGCCACAGGATGATTCC
CAGTATCCAGGGATGGATACAAAGACAGCAAGTGATTACCTGCCACAGACAAATTGGCCAACAACTCAATTACCACCAGG
ATATACTTGTGCAGAACCCTATTTATTCCCAGACATTAATAAACCCGATGGGCCAGCAACAGCAGGGATAGATGATTTGG
GTGAAATTTTAGCTAAGATGAAACAGAGAACATCGAGAGGAATAAGAAAATGAAAAGAGTTTTGTTCTTTTTGCTGATGA
TATTTGTTAGTTTTGGTGTTATAGCTGATTGCGAAATACAAGCTAAAGATCATGATTGTTTTACTATTTTCGCTAAGGGT
ACTATTTTTTCCGCGTTTCCTGTCTTAAATAATAAAGCCATGTGGAGATGGTATCAGAATGAGGACATCGGTGAGTATTA
TTGGCAAACAGAACTGGGTACATGTAAAAATAATAAATTCACACCAAGCGGGGCGAGACTCTTAATTCGCGTCGGGTCAC
TACGCCTTAATGAAAATCATGCCATCAAAGGAACGTTGCAGGAGTTGATAAATACAGCAGAAAAAACGGCGTTTCTCGGC
GATCGGTTTAGAAGTTATATAAGAGCGGGTATCTATCAAAAAAAGAGCAGTGATCCAGTTCAGTTGTTGGCAGTCCTTGA
TAATTCAATCATGGTAAAATATTTTAAAGATGAGAAACCAACTTATGCTCGAATGACAGCACATCTCCCGAACAAAAATG
AGTCTTACGAATGTTTAATAAAGATACAGCATGAACTAATTCGTAGTGAAGAGAAATAAAATCTCTTCCTGAGAAAACAA
ATTAATCATTGTGAAAAATTATATAACTCATGGAATAGGTGTTTATTTTGCGTTCCATTTGCAGGGAAAGATCACGTAAC
GCTACTTTTTTGTACTAAATAATTCGAATTTTAAGTTCAACAATTGAGATACTACTTATTGTCTAAAGCTGTTTTTCATA
GCTTATACATGATCAAATACTCCTTACATAAATAAGGTGAACAAATGGAACTTAAAAAATTGATGGAACATATTTCTATT
ACACCCGATTACAGACAAGCCTGGAAAGTGGTGCATAAATTGTCAGATATTCTACTGTTGACTATTTGTGCCGTTATTTC
TGGTGCAGAAGGTTGGGAAGATATAGAGGATTTCGGGGAAACACATCTCGATTTTTTGAAGCAATATGGTGATTTTGAAA
ATGGTATTCCTGTTCACGATACCATTGCCAGAGTTGTATCCCAGGGAAAGATCACGTAACGCTACTTTTTTGTACTAAAT
AATTCGCATTTTATGTTTAAAAATTGAGATATTCCTTATTACCTAAAGCTGTTTTTCATAGCTTATACATGATCAAATAC
TCCTTACATAAATAAGGTGAACAAATGGAACTTAAAAAATTGATGGAACATATTTCTATTATTCCCGATTACAGATAAGC
CTGGAAAGTAGAGCATAAATTGTCAGGCATCCTACTATTGACTATTTTTGCCGTTATTTCTGGTGCAGAAAGTTGGGAAG
ATATAGAGGATTTCGGGGAAACACATCTCGATTTCTTGAAGCAATATGGTGATTTTGAAAATGGTATTCCTGTTCACGAT
ACTATTGCCAGAGTTGTATCCTGTATCAGTCCTGCAAAATTTCACGAGTGCTTTATTAACTGGATGCGTGATTGCCATTC
ATCAAATGATAAAGACGTCATTGCAATTGATGGAAAAACGCTCCGGCACTCTTATGACAAGAGTCGCCGCAGGGGAGCGA
TTCATGTCATTAGTGCGTTCTCAACAATGCACAGTCTGGTCATCGGGCAGATCAAGACGGATAAGAAATCCAATGAGATC
ACAGCTATCCCTGAACTTCTTAACATGCTGGATATTAAAGGAAAAATCATCACAACTGATGCGATGGGTTGCCAGAAAGA
TATTGCAGAGAAGATACAAAAACAGGGAGGTGATTATTTATTCGCTGTAAAAGGAAACCAGGGGCGGCTTAATAAAGCCT
TTGAGGAAAAATTTCCGCTGAAAGAATTAAATAATCCAGAGCATGACAGTTACGCAATGAGTGAAAAGAGTCACGGCAGA
GAAGAAATCCGTCTTCATATTGTTTGCGATGTCCCTGATGAACTTATTGATTTCACGTTTGAATGGAAAGGACTGAAGAA
ATTATGCGTGGCAGTCTCCTTTCGGTCAATAATAGCAGAACAAAAGAAAGAGCCAGAAATGACGGTCAGATATTATATCA
GTTCTGCTGATTTAACCGCAGAAAAGTTCGCCACAGCAATCCGAAACCACTGGCACGTGGAGAATAAGCTGCACTGGCGT
CTGGACGTGGTAATGAATGAAGACGACTGCAAAATAAGAAGAGGAAACGCCGCAGAATTATTTTCAGGGATACGGCACAT
CGCTATTAATATTTTAACGAATGATAAGGTATTCAAGGCAGGGTTAAGACGTAAGATGCGAAAAGCAGCCATGGATAGAA
ACTATCTCGCGTCAGTCCTTGCGGGGAGCGGGCTTTCGTAATCTTTCCCTGCTTTTTGTACTAAATAATTCGCATTTTAT
GTTTAAAAATTGAGATATTCCTTATTACCTGAAGCTGTTTTTTATTGCTTATACATGATCAAATACTTCTTACATAATTA
AGGAGAACAAAATGGAACTTAAAAAATTGATGGAACATATTTCTATTATCCCCGATTACAGACAAGCCTGGAAAGTGGAA
CATAAATTATCGGATATTCTACTGTTGACTATTTGTGCCGTTATTTCTGGTGCAGAAGGCTGGGAAGATATAGAGGATTT
TGGGGAAACACATCCCGATTTTTTGAAGCAATATGGTGATTTTGAAAATGGTATTCCTGTTCACGATACCATTGCCAGAG
TTGTATCCTGTATCTGTCCTGCGAAATTTCATGAGAGCTTTATTAACTGGATGCTTGACTACCATTCTTCAGATGATAAA
GACGTCATCGCAATTGATGGAAAAATACACCGGCATTCTTATGACAAGAGTCGCCGTAAGGGAGCGATTCATGTCATTAG
TGCGTTCTCAACAATGCACAGTCTGGTCATCGGACAGATCAAGACGGATAAGAAATCCAATGAGATCACAGCTATCCCTG
AACTTCTTAACATGCTGGATATTAAAGGAAAAATCATCAAAACCGATGCGATGGGTTGCCAGAAAGATATTGCAGAGAAG
ATACAAAAACAGGGAGGTGATTATTTATTCGCTGTAAAAGGAAACCAGGGGCGGCTAAATAAAGCCTTCGAGGAAAAATT
TCCGCTGAAAGAATTAAATAATCCCAAGCATGACAGTTACGCAATTAGTGAAAAGAGTCACGGCAGAGAAGAAACCCGTC
TTCATATTGTTTGCGATGTCCCTGATGAGCTTATTGATTTCACGTTTGAATAGAAAGGGCAGCTATGGTTAGAAACTACC
TGACGTCAGTCCTTGCGGGGAGCAGGCTTTCGTAAATTTGTCCTGCTACAACAGGATTAACTTCACAAATATCATTTCTC
AACGTCTACACTTACTCCTGTAAACCGCTCAGGAGCAGTAATGAATCTACAACGATTTGATGACAGCACCCTAATCCGTA
TCTTTGCCCTTCATGAGTTACATCGACTGAAAGAACATGGCTTAACGCGCGGGGCGCTTCTCGATTATCACAGCCGCTAT

AAACTCGTCTTTCTGGCGCATTCTCAGCCGGAGTACCGCAAACTTGGCCCGTTCGTGGCTGATATTCACCAGTGGCAAAA
TCTGGATGACTATTACAACCAGTACCGCCAACGCGTAGTTGTTTTGCTTTCTCACCCCGCCAACCCGCGCGATCACACCA
ATGTTTTGATGCACGTTCAGGGTTATTTTCGCCCGCATATTGATTCCACAGAACGCCAGCAGCTGGCTGCGCTTATCGAC
AGTTATCGCCGTGGCGAGCAACCACTTCTTGCGCCGCTGATGCGTATCAAACACTATATGGCGCTTTATCCTGACGCCTG
GCTTTCAGGGCAGCGTTATTTCGAACTTTGGCCGCGTGTGATTAACTTGCGCCATTCAGGAGTTTTATGACTACCCATCT
GGTCTGGTTTCGCCAGGATTTACGTCTGCACGATAATCTCGCACTGGCTGCCGCCTGCCGCAATTCGTCTGCACGCGTGC
TGGCGTTGTATATCGCTACACCACGCCAGTGGGCGACGCATAACATGTCGCCGCGTCAGGCTGAACTTATCAATGCTCAA
CTGAATGGGCTACAAATAGCGCTTGCGGAAAAAGGTATTCCTTTATTGTTCCGTGAAGTGGATGACTTTGTCGCCAGTGT
CGAAATAGTTAAACAGGTGTGCGCGGAAAACAGCGTTACCCACCTGTTTTATAACTATCAGTATGAAGTGAATGAGCGGG
CGCGGGATGTGGAAGTTGAAAGAGCGCTGCGTAACGTGGTGTGTGAAGGATTTGATGACAGCGTGATCCTGCCGCCTGGC
GCGGTGATGACCGGTAATCACGAGATGTACAAAGTCTTTACGCCTTTTAAGAATGCCTGGCTGAAACGGCTGCGGGAAGG
GATGCCGGAGTGCGTCGCTGCGCCAAAAGTTCGTAGTAGCGGATCGATAGAGCCCTCGCCATCCATTACGCTGAATTATC
CTCGTCAGTCTTTCGATACTGCGCATTTTCCGGTGGAAGAAAAAGCGGCGATTGCGCAATTACGCCAGTTTTGCCAGAAC
GGTGCCGGAGAATATGAGCAACAACGAGATTTTCCGGCAGTGGAAGGCACCAGCCGTTTGTCGGCCAGCCTGGCAACGGG
CGGGTTATCGCCTCGCCAGTGCTTGCATCGCTTGTTGGCTGAACAGCCGCAGGCGCTGGACGGTGGGGCCGGTAGTGTCT
GGCTTAATGAGCTGATCTGGCGCGAGTTTTACCGTCACCTGATAACGTATCACCCCTCGTTGTGTAAACATCGTCCATTT
ATTGCCTGGACGGATCGTGTACAGTGGCAGAGCAATCCCGCACATTTACAGGCCTGGCAGGAAGGCAAAACGGGATACCC
GATTGTTGATGCCGCTATGCGTCAGCTTAACAGCACTGGCTGGATGCATAACAGGCTACGGATGATTACAGCCAGTTTTC
TGGTGAAAGATTTATTGATCGACTGGCGCGAAGGCGAGCGATATTTCATGTCGCAGCTGATTGATGGTGATTTGGCAGCC
AATAACGGTGGCTGGCAGTGGGCCGCTTCAACCGGAACCGATGCAGCGCCGTATTTTCGTATTTTCAACCCGACAACCCA
GGGCGAGAAATTTGATCATGAGGGCGAGTTTATCCGCCAGTGGCTACCGGAACTGCGCGATGTGCCAGGGAAAGTGGTGC
ATGAGCCGTGGAAGTGGGCGCAGAAAGCAGGTGTGACGCTGGATTATCCGCAACCGATAGTCGAGCACAAAGAAGCGAGA
GTACAAACGTTGGCAGCGTATGAGGCGGCGCGGAAGGGGAAATAACAGACGCGTCAGGCAATCGAGCCCAGATGCCGGAT
GCGGCGTGAACGCCTTATCCAGCCTACAAATTTGTGCAAATTCAATAAATTGCAGGAAATACGTAGGCCTGATAAGACGC
GTCAAGCGTCGCATCAGGCATCGGTGCTGGCCTATTAAGACTCCAGCGCCAGCGCGCGGTTTCTGAATTTCAGCGCCTGA
TACAGCCAAATCATCAGTACCAGTCCTACACACGCCAGTGCGCCCCAGGTAATTTGATCAAATACTTCAATATATGCATT
GATGGAGTAGTTGATCGCCCCGGAAGCATCAAACGAAGCCTGCGATGTCTGATCGGCAATCACGCCCGCCAGATAGTTCG
CGATCGCGCCAGAAAGCAGCATGTAGATGCCGGTTAATACGCCGGTCACACCGGGGATTTCAATGCGCGTAATTTGCGAC
ATGGCAACCGGGTCGATAAACAGTTCCGCAAAGCCCATCACCGCCAGGCCTAATACCATCAGTGGCAGAGAAGAGTGACC
ATACATTGCGGACCAGCGGGCGCTTAAGGTCAGAATGCAGAATCCGGCGCTCATCAGGCCAAGGCCAAGAGCAAATTTCC
CCCAGATGCGCACGGTACGATTACCCGCGACACTCTCTTTTACCACCCACGCCAGGAACACACCGCACAGCATAACTGCG
AAGGCATTAATCGACTGGAACATCGCGGTCGGAACGGTATAACCAAACATATCGCGGTTAACGAAGCGGTCGATATAAAG
GCTAATCGAGCTACCGCCCTGTTGTGCGAAGGCCCAGAACAACATACTGAAGAAGGTGAGCGTCACAATCAGCCCCAGCT
CCTTCCGCTGTTTCTGGTTTTCTGCTTTGCGATAAATTTTTGCCAGTACACCCAGGCCAATAATAGTCGCGACAATTAAG
GCGTATACCGACCACTCTTTCCAGAACAGTATGGTAATCAGTGCAGGCGTTGCCACCAGCAGAACCAGCAGCCATCCCCA
GTTCGGCAGGAGAAAGTTTGTCGCACGCAGTACTTTTTTGTTAACGCCGCGGGTATGAGTGAAATGACGATTGCCACATA
AGAAAATGACCAGACCCGCGATCATGCCAACCGCCGCCAGGCCAAAGCCCATCGCCCAGCTGTACTCTTCCTGGGCGTAA
CCACAGGCGATAGGTGCGATAATAGACCCCACGTTACCCGCCGCATACATCAGCGAGAAACCGCCATCACGACGCGGATC
GGTTGGCTCATACAGCTCGCCGAGCAGACAGCTTACGTTAGATTTAAACAGGCCATAGCCGCAGACGATAATCGCCAGGG
ACAGATAGAGGAATGACGGATGGATCTCACTGGCACCCAGCACCACATGACCGATCGCCATCAACAACGCCCCCAGCATC
ACCGCCATGCGATTGCCGAGAACTTTATCCGCCAAAAAGCCACCGAGGATTGGCGTGACATACACCAGCGAACAGTAGGC
GCTAAATAACTCGTAGGCGTGAGTATCGTTGTATTTTAGTTGATTGGTGAGATAGAGAATCAGCAGGGCACGCATGCCGT
AAAAGCTAAAATATTCCCAGATTTGCAGCGCAACGACATAGTAAATAGCGCGCGGCTGTGATGCGTGTTTATTCATAATA
ATTCCACAATTGACGGCAACGTCACGTAATAGTTGCGCGGCGTGCTTGTGTTTCCTTAAAGTTAAAACTTTGATACAGAT
CTGATTATTTATGCAATATGCTGTCTGATTGCATAAATATACATTAGCTGAAGCGTGATGATATAAACAATTTGCGTCTT
TTGGCGTCGGGTTTGTTTCTTAATATGTCAAAAGATTGGCTATCGACGAACTGGCGAGGTAACGCTATGTTAACGGTGTA
GCTATCAGAATACGGAAGGCGCGAAGATGAAAAACACCGAACTGGAACAACTGATTAACGAAAAACTGAACAGCGCGGCG
ATTAGTGATTACGCGCCGAATGGTTTGCAGGTGGAAGGCAAAGAGACGGTGCAAAAAATTGTTACCGGTGTCACCGCCAG
CCAGGCTTTGCTCGATGAGGCAGTGCGTCTGGGCGCGGATGCGGTCATTGTGCATCATGGCTACTTCTGGAAAGGAGAGT
CTCCGGTCATTCGCGGAATGAAGCGTAACCGTTTAAAAACGTTGCTGGCGAATGATATCAACCTGTATGGCTGGCATTTG
CCGCTTGACGCACATCCTGAGCTGGGCAATAACGCACAGCTGGCGGCGTTACTGGGGATCACGGTCATGGGCGAAATTGA
GCCGTTGGTGCCGTGGGGCGAACTGACCATGCCTGTGCCGGGACTGGAGCTGGCTTCCTGGATTGAAGCGCGTCTGGGAC
GTAAGCCGTTATGGTGTGGCGATACCGGACCTGAGGTGGTCCAGCGCGTCGCCTGGTGCACGGGCGGCGGGCAAAGTTTT
ATCGATAGCGCCGCGCGTTTTGGCGTGGATGCTTTTATTACTGGCGAAGTTTCTGAACAGACCATTCATTCAGCCCGCGA
GCAGGGATTGCATTTTTATGCTGCAGGTCACCATGCCACTGAACGTGGTGGTATTCGCGCATTGAGCGAGTGGCTGAATG
AAAATACCGATCTTGATGTGACCTTTATTGATATTCCTAATCCTGCATAACGAATAATCAGAGGGATCGAAAGTGCAACG

AGCGCGTTGTTATCTGATAGGTGAAACGGCGGTAGTGCTGGAACTGGAACCGCCGGTGACGCTGGCTAGCCAGAAACGGA
TCTGGCGACTGGCGCAGCGTCTGGTGGATATGCCGAATGTGGTTGAAGCCATTCCCGGCATGAACAATATCACGGTGATT
TTGCGTAATCCTGAGTCGCTGGCGCTGGATGCCATAGAGCGTTTGCAACGCTGGTGGGAGGAGAGCGAGGCGCTGGAGCC
GGAGTCTCGCTTTATTGAAATTCCGGTGGTTTACGGTGGTGCAGGCGGACCGGATTTGGCGGTGGTCGCGGCGCATTGCG
GGTTGAGCGAAAAACAGGTTGTTGAATTGCACTCCTCCGTGGAATACGTGGTCTGGTTTTTAGGTTTTCAACCGGGCTTC
CCGTATCTCGGGAGTTTGCCGGAACAACTACACACGCCACGGCGCGCTGAACCGCGCTTACTCGTTCCGGCAGGTTCTGT
CGGGATCGGCGGGCCGCAGACTGGTGTTTATCCGCTGGCAACGCCGGGTGGCTGGCAGTTGATTGGTCATACCTCACTCA
GCCTGTTTGATCCGGCGCGTGACGAACCCATCTTATTACGTCCGGGAGACAGCGTGCGCTTTGTACCGCAGAAGGAGGGA
GTATGCTGAAGATTATTCGTGCGGGCATGTATACCACTGTGCAGGATGGCGGTCGTCACGGTTTTCGCCAGTCGGGTATC
AGCCACTGCGGCGCACTGGATATGCCCGCGTTACGCATTGCTAACCTACTGGTGGGTAATGACGCCAATGCCCCCGCGCT
GGAGATCACGCTCGGTCAGTTAACTGTTGAGTTCGAAACTGATGGGTGGTTTGCTCTGACGGGTGCCGGTTGCGAAGCGC
GGCTGGATGATAATGCCGTCTGGACCGGCTGGCGATTGCCGATGAAAGCAGGCCAGCGTTTAACGCTTAAACGCCCGCAG
CACGGGATGCGCAGTTATCTGGCGGTCGCGGGTGGTATTGATGTTCCGCCGGTAATGGGCTCATGCAGCACCGATCTCAA
AGTGGGGATTGGCGGGCTGGAAGGCCGTTTACTGAAGGATGGTGACCGACTCCCGATTGGCAAATCGAAGCGTGATTCTA
TGGAAGCGCAGGGCGTTAAACAGCTGCTGTGGGGCAACCGCATTCGCGCCTTGCCGGGGCCGGAATATCATGAGTTCGAT
CGCGCCTCGCAGGATGCATTCTGGCGTTCGCCCTGGCAGCTTAGCTCGCAAAGTAACCGCATGGGCTATCGCTTACAGGG
GCAAATTTTAAAACGCACCACCGATCGCGAACTGTTATCTCACGGTTTGTTACCGGGCGTGGTGCAGGTGCCACATAACG
GGCAGCCGATTGTGTTGATGAACGACGCACAGACCACCGGTGGTTACCCGCGTATTGCCTGTATCATTGAGGCTGATATG
TACCATCTGGCGCAAATTCCGCTCGGTCAGCCGATTCATTTTGTCCAGTGTTCACTGGAAGAGGCACTAAAAGCACGGCA
AGATCAGCAACGTTATTTTGAACAATTAGCGTGGCGGCTGCACAATGAAAATTGACCTGAACGCCGATCTGGGCGAAGGC
TGCGCCAGCGACGCAGAGCTATTAACGCTGGTTTCCTCTGCCAATATTGCCTGTGGATTTCATGCAGGCGATGCGCAAAT
CATGCAGGCTTGCGTGCGTGAAGCAATAAAAAATGGTGTCGCGATTGGCGCTCACCCGAGTTTTCCCGACAGGGAAAATT
TTGGTCGCAGCGCCATGCAGCTGCCGCCAGAAACCGTTTACGCCCAGACGCTGTATCAAATTGGCGCGCTGGCAACGATT
GCCCGTGCGCAAGGCGGCGTAATGCGTCATGTCAAACCGCACGGCATGTTGTACAACCAGGCGGCGAAAGAAGCACAACT
GGCAGACGCCATCGCCAGAGCGGTATACGCTTGCGATCCAGCATTGATTCTCGTCGGGCTGGCGGGAAGCGAGCTGATTC
GTGCAGGCAAGCAATATGGTCTGACAACGCGCGAGGAAGTGTTTGCCGATCGCGGTTATCAGGCTGACGGCTCGCTGGTG
CCGCGAAGCCAGTCAGGCGCGTTGATTGAAAACGAAGAACAGGCGCTGGCGCAAACGCTGGAGATGGTGCAACACGGCAG
AGTCAAAAGTATCACCGGCGAATGGGCAACGGTCGCGGCGCAAACGGTCTGCCTGCATGGCGACGGCGAGCACGCACTGG
CGTTCGCCCGCCGACTACGCTCTGCATTTGCCGAAAAGGGGATTGTTGTCGCAGCATAACCCCGATTAATAAAGAATGAA
AAAAGGATATCACCATGCCTGAAGGCCCGGAGATCCGCCGTGCAGCGGATAACCTGGAGGCGGCGATCAAAGGCAAACCA
CTAACTGATGTCTGGTTTGCCTTCCCGCAGTTAAAACCTTATCAATCACAACTTATCGGTCAACACGTTACCCATGTGGA
AACGCGTGGTAAGGCGTTGTTAACTCATTTTTCCAACGACTTAACGCTCTACAGCCATAATCAGCTTTACGGCGTCTGGC
GCGTGGTTGATACCGGCGAAGAGCCGCAGACCACGCGAGTATTGCGGGTAAAACTGCAAACGGCTGACAAAACCATTCTG
CTTTATAGCGCCTCGGATATTGAGATGTTGACCCCGGAACAACTGACCACGCATCCGTTTTTACAACGCGTTGGTCCCGA
TGTGCTGGATCCGAATCTGACGCCGGAGGTGGTGAAAGAACGATTATTGTCGCCGCGCTTTCGTAACCGTCAGTTTGCTG
GATTACTGCTCGATCAGGCGTTTCTGGCTGGGCTTGGCAATTATTTGCGGGTGGAGATCCTCTGGCAGGTTGGGTTGACT
GGAAATCATAAAGCGAAAGATCTCAATGCGGCGCAACTGGATGCACTCGCACACGCGTTACTGGAGATTCCTCGATTTTC
CTACGCTACGCGGGGGCAGGTGGATGAGAATAAGCATCATGGGGCGCTGTTTCGCTTTAAGGTTTTTCATCGAGATGGCG
AACCGTGCGAACGTTGTGGCAGCATCATTGAGAAAACCACGCTGTCATCTCGCCCGTTTTACTGGTGCCCTGGCTGCCAG
CACTAGGCCGACCGCTTCGGCGCATAGGTTGAAATAAACCGCGCAATGGCAGGCCCTGTCAGCAAAATACTGAACAGGCG
TAGGGTTTGCATCGCCATAATGAGCGCCATATCGGCATTGCTCCCTGCGGCGATGACCGCCACGGTATCAAGCCCGCCGG
GGCTGGTGGCGAGGTAGGCAGTCATAAAATCAATGTGCATAAACCGGGTCAGCCCCCACGCCATACCCGCACAAATAGCC
AGCAGAGCAAAAATCGACAGCAGGATTTGCGGTAGCGGGCGTAGTGCCCGCAGTAAGATTTGTTTATCGAAACCAAGACC
AATCCGCCAGCCAATTGCCATATACGCCATCGCCAGCAGCCATTCCGGTAGTTCGATGGTGATGAGCTGACCAGACTGGA
GCACTGCGCCCGCCAGCATCGGGATGAGCATCGTGCCGGAAGGAAGACGCAGCAGGCATCCCACTGTACCGGCAACGACC
GCGAGCAAAATAGTAAGCAGGAGATTAATGCTCACTGGCGGGAACCAGACGATATGCTGGTTAACCGCTTCAGCGTTATC
GCCCAGCATCATGCGGGTGACCAGAACAGCGGCCCCCGCGACGAACAGCACTCGCAGATATTGCATAAACGCCACCAGGC
GAATATCTGCGCCGTAATCTTGCGCCATAGCGACCATTGCCGCCGCGCCGCCAGGAGAGGAGCCCCAGGCACCGGTATTT
CCAGGCAATGAGCTATAGCGCACCAATAACCAACCCACAATGGCGCTGGAAAGCAGCGTTACCAGTAAAATTGCAAGCAC
GATCGGCCAGTTGACGGCCAGGGTGGTGAGAATCGAACCAGTGAGGTTTTGCGCAATCATGCAGCCAAGAATGGCCTGGG
CGGCGAGAAAAGCAGAGCGGGGAAGTTGCAGAGTAATTCCGCGCATACTAAAGATGATCCCGGCGATCATCGGCCCAAGT
AATAGCGCCGCCGGGAGATGTACCGCGAGGAAACCAATAGAAAGGAGAAGTGATAGCACACATAACATTCCCCACTGCAA
AACTGGCATCCTTCGCTCCCTCATTATTTTCTCTTTGGTGTATCGATACAGCATAAAGAAAGCTGGGCCAGGAAAAAATA
ACAGCAATCAAGGAAAAGGGGAAAATCAGCAATTTTCTGAAAGAGATGCCCTTTCCGGCGGCAAAGGGCATCATGGTAAA
TCAGTTATAGTTAATCTTAAAAAGCACCACTGTATCGAAGGGGCCAGCCTCGATGGGGGTGCCTGGAATGGACGAAAGTT
CAGCCGTATAGGTTTTTTCGTAGACCAACATGTCACTGGTGAAATCGGCGTATTCAGCATATTTATTGAAGGTATAAGGC

GAGGCGTTTTCATCCAGTAAACGTAGCTTCAGACCATTACCAATCAACAATGCTTTATCTTCTTCAACCAACGTTTCTTC
GGTATAAAAGGAGGAACTGACTTTAAATCCGTCGGTGCATTGATCGTTTTGGCTCTTGGTCGTTTTAATACTGAACGTTT
TCGACATTGTGTGGCGACGTATATCCAGCAAATTAAATTTACCAAAATCAATCACCTGTGTTTCCGGTGAAATGGAAAAA
TTGACGCTGCAATCCAGCACGCGAATGTTTTCCAGACCAGTAATATGATATTTCAGGTTTTGGGCAGTAGGGTCTTCGTT
AACGCTACCGGCACCGTCAAATTGCACGACGATATAGTCGCTAAGCGTACTTTGATAATCATGCGGCGGCATTTCTCGTA
TTTTGACGTATAACCGCATGCGAGCGAGAAAGGTACGCGACATATGAATATTAGCGGGATCGCCCGAGCAAATTTTGTTC
TGCCATCCCATCGCGATGATCTGCTCAGGGGTATAAATATCAATGTTCTTACTGTCGATACACTGGTTAGTGTCGATGCG
ACTTTTTCCCAGACTGGCATCGTAATCGACACCGTTGTATGTCACGCCGAGTTGATAATAACGATCCTGCACCCCCGGAT
AGGGGTTTACCCAGGCGTAGACATGCTCGCTTTCAAAGTTGCCGTTGGTATTGTTGTCACAATAGACGGGAATTTTAATA
TCGTCAGATTCCCAGATCTTATCACCGGGTTTGGCATTGCCTGGTACAGCAAACGGTTGAATCGCTTCTGATTTTTCTAC
TGCGCCACCAGATGTACCAAAATAGCAGTTCAGCGCCATTGCAGACTTCAACGGCAGAAATAACAGGCAGATGACGAGCA
ACAATCCCTTGCCAGCACTCATTTCGCCTCCACTGCTTGTAAACGGCATTGTCCGCTGCAGTTGAGCGTCGTCATGCGTA
ATGCGCCGTAATCATCCATGTAACCGAGATAAAACTGGCTGCCGCTATAACTTCCGGTGTTTGTGTTGACCGTACTAAAA
GGTGCAACCATCACGGTTTTGAAGCCTGGGAGAACGCCTTTTTCATTTCTCCCCAGATAGGCAATGGTCAGATAATACGC
GGTAGGATTTTTCAGTGTCAGTTGGTTGCCCTGCTGGCTGACCTGTAACTGCAGTTCGACCTTTTCTCCTGCTTTCTTGC
GTAATGCAGCCGGTCGCCAGAACAATTTAATACGACTTTGAATAGCGACCTGAAGTATCGCATGGTCGCTGCTTTTATCC
GGAGCGGGAGGAATTTCGCGCATATTGTAGAAAAATAGCGTTTCGCGATCGCCAGGTAATTGTGTGGTTGAGGCTTGTTT
CACAACCCGCACCTGCGATGTCGCTTTCGGTTCCAGACGTTGAATAGGGGGCAAAGCGACCAGAAGAGCATCGCTTTTCT
CCCCTTTTTCATTTTCTATCCAGGAATAAGCAAGATAAGGCAGTTTGTCACTTTGGTTTTCGATACGCAGGCTGGTGGCT
TTGTCATTGGCGTTAAACACAATGCGTGTGCGATCGGGTTGCACCGCAGCATTGCAACCCAAGCTGATGGTTAACAACAT
TAAGGGTAATCCTTTAATAAATGTCATTCTCTTTTCCTTAATTAATGACAAGGTAAAATCAGGCGTTTGGTCGTGTCTTC
CAGACGTTCTGGCAGATGAATAATGCAGCTTTGCTCACCCCAGACCACGGTAAACAGTTGATTTTCAGCGACACCGCTTA
ACCAGGCATGTCCTTCCTCGCCAACCATACCCACGCTAATGCCGCTGTCATCCTGGCGGATATCCGCACCGAGGGGAGGG
AACTGACCGCTGGCGTTGCGTATGATGACGTTAACGTCTTTACCGGAACGGGAAGCCAGTGATTTGTAACCTATCGCGCC
TTCAATCCATGTTTCCTTGATGACGTTTTCTGCAACTGTTACGCCGTCGGGTAAGTCATTCATGTTCACCGCCACGGTGG
AAGGCTGGTAACTGGAAATCAACGGCACCACCGCAATGCCAAAATGGTTGGTGTAGTCGAGATTGCCCTGAACCGGAATA
TCTGCCACGCCATCGGTGCTGACCATCAGGCGTGGTTCATTGGTGGAGCTGCGGCGATGAAATGCTGCACCATATTGGGT
TGCGGTGAAAGAACCGCTCCAGCTGCTGCTGACGGAACTGTAATCACTGGCGGCATAGGTACCAGAAATATCCCACTCAC
CCGCTGAACTCAGGTGCTGATAGTTACCGCTCACCTGGGCTCCATTGTCCGGACGATCGGATTGCAGTCCGGCAGACATG
CCCCAGCTATTACGTTCATCCAGCGTATCGTTCCACGACATGCGGTGTATGGTGCTGTGGCTACTGTTTTGCATGTCATA
GCCAACCCGACCACCGTTACCGAAGGGGAGCGAAATCGACAGATAAATCTGGTTGTCGCGATCTTTATCTTCGTAGTGGG
TCGTATTGAATGACGTCGAAATCGAGATATCTCTCCAGTCACCAATATCAACATTAAAACCTGCTGTGATGTTGGCGGTC
GTGGAGGCGTCTGCATTCCACCAGGTTTGATGTAGCAGGTTGGCGTAAAGATTGAGGTTTAGTGGGGTAATCGGTTGGCC
CACGGATAAGCTGATCGTCTGTTTTTCGTCCTGCGCATCGCTGTCGTTGTATTTGTGATCCAGATAGTTGGCGTAGCTGT
GAAAATGACGATCTGAGAAGCGATAAGCGGCGAGCGAAATCGTGCTGTTGGTGGCATCCACTTGTTTGCTGTAATTAAAA
CGGTAGCTTAAGCCCCGCTCGTCCTGCTGAGTATCAAAATGGCTACTGGCCCAGGTGACATCAAAGGACAGTGCGCCAAG
CCACAGCATATTTTGCCCGATACCCATTGCGGCAGAATGGTAGTCATCATCAGAAATCAGCAGGCCGCCGTACAGCGAGG
TGTTTGAGAGCATCCCCCAGGAAACTTCATTGCTAAAAAAGGTTTCATTTTCAGTTTGATGTGACATGGAAGGCCGTGGC
TGACCCGCGGCCAATTTATAGCGAACCTGTCCCTGGCGAGTCAGGAAGGGCGTCGATGCTGCCGAAACCTGGAAATTGTT
CACCCGACCATCTTCTTCCGTCACTTTGACATCCAGCGTGCCCTGAACAGACTGATTGAGGTCATCAATAATAAACGGGC
CTGGCGGGACTTTTTTCTGGTAAATGACGCGGCCTGATTGACTGATCGTCACCGTGGCATTGGTCTGTGCAATACCGCTA
ATTTGTGGGGCGTAGCCACGCAGCTCCCACGGTAACATTCGATCGTCACTCGCCAGTGCCGCGCCGGTATAAGAAAAACC
ATCGAAAATATTGGAACTGAAATCGGTTTCGCCGAGGGTTAACTTAGAGCCTAATTGCGGTAATGGACGAAAAAGATAGG
TGCGCGATATTCCGCCTGACTGGTCATGGTTATCTTCGCTATCGGTCTTATTAAGCTGGTAATCACTGCGTAAGCGCCAT
GCCCCGGCGTTAATTCCGGCGGTACCGTAGGCATTCAGGTTAGTGCTGCTGCTGCCGTCCTGTGGGCGGTAGCTGCTGGC
AAACAAGTTGTAATCCATCAGGACACCGGCAACACCTTCTTTCCATGTAGAGGGGGGAGCCCAGTTTTCTGAGTGCCACG
CCAGCCAGGCTTGCGGAATACTAATATTCAGTTGCTGATTGGCTTGATCGAAATTGAAGAGCATTTCAGGTCGGGAACTG
AAATCAATACACCGATCTATCTGTGGCAAGGACTGACGGATATCTGGTTTTAAACCAAATTTATCGACCAGTGAATCATT
GATGCATGGAATGGTTTTGTCACCCTTTTTTTGCCAGTTAATTTTTTGCCCATTACTGATTTTGTTGTTATTCACCGCAA
CGCTAACAAAATATTCACCGGGAGCAATGACTCCTTTTTCCTTTAATAATGAAATATCAATGCGGTCGCGCATTGATTTA
TCGAGAACATTCAGATTGAATTCGACCGCCATTGCGCAAGGCATCGCCATGACCAGGCAGGATACAAAAGAGAGTCGATA
AATATTCACGGTGTCCATACCTGATAAATATTTTATGAAAGGCGGCGATGATGCCGCCAAAATAATACTTATTTATAATC
CAGCACGTAGGTTGCGTTAGCGGTTACTTCACCTGCCGTGACATCGACTGCATTATCAATTTGTTCCATCCAGGCGAAAA
AGTTCAGCGTCTGTTCTGATGAGCTTGCATCCAGGTCAAGATCTGGCGCGGCTGAACCTAATACGATGTTACCGTCATTT
TTGTCCATCAGTCGTACACCGACCCCAGTTGCTTCGCCTGCACTGGTGTTGCTCAACAAAGGCGTAGCACCAGTTGTCTT
AGCCGTGCTATCGAAGGTTACGCCAACTTTGGATACCGGCATTCCGCTACCGTTGTCAGAAGCAGGCAGATCACAGTTGA

TCAAGCGAATGTCGACGGCCACTTTATTGCTATGATGCTCCCGGTTTATATGGGTTGTCGTGACTTGTCCAAGATCTATG
TTTTTATCAATATCTTCTGGATGAATTTCACAAGGTGCTTCAATAACCTCCCCCTTAAAGTGAATTTCGCCAGAACCTTC
ATCAGCAGCATAAACAGGTGCAGTGAACAGCAGAGATACGGCCAGTGCGGCCAATGTTTTTTGTCCTTTAAACATAACAG
AGTCCTTTAAGGATATAGAATAGGGGTATAGCTACGCCAGAATATCGTATTTGATTATTGCTAGTTTTTAGTTTTGCTTA
AAAATATTGTTAGTTTTATTAAATGCAAAACTAAATTATTGGTATCATGAATTTGTTGTATGATGAATAAAATATAGGGG
GGTATAGATAGACGTCATTTTCATAGGGTTATAAATGCGACTACCATGAAGTTTTTAATTGAAAGTATTGGGTTGCTGAT
AATTTGAGCTGTTCTATTCTTTTTAAATATCTATATAGGTCTGTTAATGGATTTTATTTTTACAATTTTTTGTGTTTAGG
CATATAAAAATCAACCCGCCATATGAACGGCGGGTTAAAATATTTACAACTTAGCAATCAACCATTAACGCTTGATATCG
CTTTTAAAGTCGCGTTTTTCATATCCTGTATACAGCTGACGCGGACGGGCAATCTTCATACCGTCACTGTGCATTTCGCT
CCAGTGGGCGATCCAGCCAACGGTACGTGCCATTGCGAAAATGACGGTGAACATGGAAGACGGAATACCCATCGCTTTCA
GGATGATACCAGAGTAGAAATCGACGTTCGGGTACAGTTTCTTCTCGATAAAGTACGGGTCGTTCAGCGCGATGTTTTCC
AGCTCCATAGCCACTTCCAGCAGGTCATCCTTCGTGCCCAGCTCTTTCAGCACTTCATGGCAGGTTTCACGCATTACGGT
GGCGCGCGGGTCGTAATTTTTGTACACGCGGTGACCGAAGCCCATCAGGCGGAAAGAATCATTTTTGTCTTTCGCACGAC
GAACAAATTCCGGAATGTGTTTAACGGAGCTGATTTCTTCCAGCATTTTCAGCGCCGCTTCGTTAGCACCGCCGTGCGCA
GGTCCCCACAGTGAAGCAATACCTGCTGCGATACAGGCAAACGGGTTCGCACCCGAAGAGCCAGCGGTACGCACGGTGGA
GGTAGAGGCGTTCTGTTCATGGTCAGCGTGCAGGATCAGAATACGGTCCATAGCACGTTCCAGAATCGGATTAACTTCAT
ACGGTTCGCACGGCGTGGAGAACATCATATTCAGGAAGTTACCGGCGTAGGAGAGATCGTTGCGCGGGTAAACAAATGGC
TGACCAATGGAATACTTGTAACACATCGCGGCCATGGTCGGCATTTTCGACAGCAGGCGGAACGCGGCAATTTCACGGTG
ACGAGGATTGTTAACATCCAGCGAGTCGTGATAGAACGCCGCCAGCGCGCCGGTAATACCACACATGACTGCCATTGGAT
GCGAGTCGCGACGGAAAGCATGGAACAGACGGGTAATCTGCTCGTGGATCATGGTATGACGGGTCACCGTAGTTTTAAAT
TCGTCATACTGTTCCTGAGTCGGTTTTTCACCATTCAGCAGGATGTAACAAACTTCCAGGTAGTTAGAATCGGTCGCCAG
CTGATCGATCGGGAAACCGCGGTGCAGCAAAATACCTTCATCACCATCAATAAAAGTAATTTTAGATTCGCAGGATGCGG
TTGAAGTGAAGCCTGGGTCAAAGGTGAACACACCTTTTGAACCGAGAGTACGGATATCAATAACATCTTGACCCAGCGTG
CCTTTCAGCACATCCAGTTCAACAGCTGTATCCCCGTTGAGGGTGAGTTTTGCTTTTGTATCAGCCATTTAAGGTCTCCT
TAGCGCCTTATTGCGTAAGACTGCCGGAACTTAAATTTGCCTTCGCACATCAACCTGGCTTTACCCGTTTTTTATTTGGC
TCGCCGCTCTGTGAAAGAGGGGAAAACCTGGGTACAGAGCTCTGGGCGCTTGCAGGTAAAGGATCCATTGATGACGAATA
AATGGCGAATCAAGTACTTAGCAATCCGAATTATTAAACTTGTCTACCACTAATAACTGTCCCGAATGAATTGGTCAATA
CTTCCACACTGTTACATAAGTTAATCTTAGGTGAAATACCGACTTCATAACTTTTACGCATTATATGCTTTTCCTGGTAA
TGTTTGTAACAACTTTGTTGAATGATTGTCAAATTAGATGATTAAAAATTAAATAAATGTTGTTATCGTGACCTGGATCA
CTGTTCAGGATAAAACCCGACAAACTATATGTAGGTTAATTGTAATGATTTTGTGAACAGCCTATACTGCCGCCAGGTCT
CCGGAACACCCTGCAATCCCGAGCCACCCAGCGTTGTAACGTGTCGTTTTCGCATCTGGAAGCAGTGTTTTGCATGACGC
GCAGTTATAGAAAGGACGCTGTCTGACCCGCAAGCAGACCGGAGGAAGGAAATCCCGACGTCTCCAGGTAACAGAAAGTT
AACCTCTGTGCCCGTAGTCCCCAGGGAATAATAAGAACAGCATGTGGGCGTTATTCATGATAAGAAATGTGAAAAAACAA
AGACCTGTTAATCTGGACCTACAGACCATCCGGTTCCCCATCACGGCGATAGCGTCCATTCTCCATCGCGTTTCCGGTGT
GATCACCTTTGTTGCAGTGGGCATCCTGCTGTGGCTTCTGGGTACCAGCCTCTCTTCCCCTGAAGGTTTCGAGCAAGCTT
CCGCGATTATGGGCAGCTTCTTCGTCAAATTTATCATGTGGGGCATCCTTACCGCTCTGGCGTATCACGTCGTCGTAGGT
ATTCGCCACATGATGATGGATTTTGGCTATCTGGAAGAAACATTCGAAGCGGGTAAACGCTCCGCCAAAATCTCCTTTGT
TATTACTGTCGTGCTTTCACTTCTCGCAGGAGTCCTCGTATGGTAAGCAACGCCTCCGCATTAGGACGCAATGGCGTACA
TGATTTCATCCTCGTTCGCGCTACCGCTATCGTCCTGACGCTCTACATCATTTATATGGTCGGTTTTTTCGCTACCAGTG
GCGAGCTGACATATGAAGTCTGGATCGGTTTCTTCGCCTCTGCGTTCACCAAAGTGTTCACCCTGCTGGCGCTGTTTTCT
ATCTTGATCCATGCCTGGATCGGCATGTGGCAGGTGTTGACCGACTACGTTAAACCGCTGGCTTTGCGCCTGATGCTGCA
ACTGGTGATTGTCGTTGCACTGGTGGTTTACGTGATTTATGGATTCGTTGTGGTGTGGGGTGTGTGATGAAATTGCCAGT
CAGAGAATTTGATGCAGTTGTGATTGGTGCCGGTGGCGCAGGTATGCGCGCGGCGCTGCAAATTTCCCAGAGCGGCCAGA
CCTGTGCGCTGCTCTCTAAAGTCTTCCCGACCCGTTCCCATACCGTTTCTGCGCAAGGCGGCATTACCGTTGCGCTGGGT
AATACCCATGAAGATAACTGGGAATGGCATATGTACGACACCGTGAAAGGGTCGGACTATATCGGTGACCAGGACGCGAT
TGAATATATGTGTAAAACCGGGCCGGAAGCGATTCTGGAACTCGAACACATGGGCCTGCCGTTCTCGCGTCTCGATGATG
GTCGTATCTATCAACGTCCGTTTGGCGGTCAGTCGAAAAACTTCGGCGGCGAGCAGGCGGCACGCACTGCGGCAGCAGCT
GACCGTACCGGTCACGCACTGTTGCACACGCTTTATCAGCAGAACCTGAAAAACCACACCACCATTTTCTCCGAGTGGTA
TGCGCTGGATCTGGTGAAAAACCAGGATGGCGCGGTGGTGGGTTGTACCGCACTGTGCATCGAAACCGGTGAAGTGGTTT
ATTTCAAAGCCCGCGCTACCGTGCTGGCGACTGGCGGAGCAGGGCGTATTTATCAGTCCACCACCAACGCCCACATTAAC
ACCGGCGACGGTGTCGGCATGGCTATCCGTGCCGGCGTACCGGTGCAGGATATGGAAATGTGGCAGTTCCACCCGACCGG
CATTGCCGGTGCGGGCGTACTGGTCACCGAAGGTTGCCGTGGTGAAGGCGGTTATCTGCTGAACAAACATGGCGAACGTT
TTATGGAGCGTTATGCGCCGAACGCCAAAGACCTGGCGGGCCGTGACGTGGTTGCGCGTTCCATCATGATCGAAATCCGT
GAAGGTCGCGGCTGTGATGGTCCGTGGGGGCCACACGCGAAACTGAAACTCGATCACCTGGGTAAAGAAGTTCTCGAATC
CCGTCTGCCGGGTATCCTGGAGCTTTCCCGTACCTTCGCTCACGTCGATCCGGTGAAAGAGCCGATTCCGGTTATCCCAA
CCTGTCACTACATGATGGGCGGTATTCCGACCAAAGTTACCGGTCAGGCACTGACTGTGAATGAGAAAGGCGAAGATGTG

GTTGTTCCGGGACTGTTTGCCGTTGGTGAAATCGCTTGTGTATCGGTACACGGCGCTAACCGTCTGGGCGGCAACTCGCT
GCTGGACCTGGTGGTCTTTGGTCGCGCGGCAGGTCTGCATCTGCAAGAGTCTATCGCCGAGCAGGGCGCACTGCGCGATG
CCAGCGAGTCTGATGTTGAAGCGTCTCTGGATCGCCTGAACCGCTGGAACAATAATCGTAACGGTGAAGATCCGGTGGCG
ATCCGTAAAGCGCTGCAAGAATGTATGCAGCATAACTTCTCGGTCTTCCGTGAAGGTGATGCGATGGCGAAAGGGCTTGA
GCAGTTGAAAGTGATCCGCGAGCGTCTGAAAAATGCCCGTCTGGATGACACTTCCAGCGAGTTCAACACCCAGCGCGTTG
AGTGCCTGGAACTGGATAACCTGATGGAAACGGCGTATGCAACGGCTGTTTCTGCCAACTTCCGTACCGAAAGCCGTGGC
GCGCATAGCCGCTTCGACTTCCCGGATCGTGATGATGAAAACTGGCTGTGCCACTCCCTGTATCTGCCAGAGTCGGAATC
CATGACGCGCCGAAGCGTCAACATGGAACCGAAACTGCGCCCGGCATTCCCGCCGAAGATTCGTACTTACTAATGCGGAG
ACAGGAAAATGAGACTCGAGTTTTCAATTTATCGCTATAACCCGGATGTTGATGATGCTCCGCGTATGCAGGATTACACC
CTGGAAGCGGATGAAGGTCGCGACATGATGCTGCTGGATGCGCTTATCCAGCTAAAAGAGAAAGATCCCAGCCTGTCGTT
CCGCCGCTCCTGCCGTGAAGGTGTGTGCGGTTCCGACGGTCTGAACATGAACGGCAAGAATGGTCTGGCCTGTATTACCC
CGATTTCGGCACTCAACCAGCCGGGCAAGAAGATTGTGATTCGCCCGCTGCCAGGTTTACCGGTGATCCGCGATTTGGTG
GTAGACATGGGACAATTCTATGCGCAATATGAGAAAATTAAGCCTTACCTGTTGAATAATGGACAAAATCCGCCAGCTCG
CGAGCATTTACAGATGCCAGAGCAGCGCGAAAAACTCGACGGGCTGTATGAATGTATTCTCTGCGCATGTTGTTCAACCT
CTTGTCCGTCTTTCTGGTGGAATCCCGATAAGTTTATCGGCCCGGCAGGCTTGTTAGCGGCATATCGTTTCCTGATTGAT
AGCCGTGATACCGAGACTGACAGCCGCCTCGACGGTTTGAGTGATGCATTCAGCGTATTCCGCTGTCACAGCATCATGAA
CTGCGTCAGTGTATGTCCGAAGGGGCTGAACCCGACGCGCGCCATCGGCCATATCAAGTCGATGTTGTTGCAACGTAATG
CGTAAACCGTAGGCCTGATAAGACGCGCAAGCGTCGCATCAGGCAACCAGTGCCGGATGCGGCGTGAACGCCTTATCCGG
CCTACAAGTCATTACCCGTAGGCCTGATAAGCGCAGCGCATCAGGCGTAACAAAGAAATGCAGGAAATCTTTAAAAACTG
CCCCTGACACTAAGACAGTTTTTAAAGGTTCCTTCGCGAGCCACTACGTAGACAAGAGCTCGCAAGTGAACCCCGGCACG
CACATCACTGTGCGTGGTAGTATCCACGGCGAAGTAAGCATAAAAAAGATGCTTAAGGGATCACGATGCAGAACAGCGCT
TTGAAAGCCTGGTTGGACTCTTCTTACCTCTCTGGCGCAAACCAGAGCTGGATAGAACAGCTCTATGAAGACTTCTTAAC
CGATCCTGACTCGGTTGACGCTAACTGGCGTTCGACGTTCCAGCAGTTACCTGGTACGGGAGTCAAACCGGATCAATTCC
ACTCTCAAACGCGTGAATATTTCCGCCGCCTGGCGAAAGACGCTTCACGTTACTCTTCAACGATCTCCGACCCTGACACC
AATGTGAAGCAGGTTAAAGTCCTGCAGCTCATTAACGCATACCGCTTCCGTGGTCACCAGCATGCGAATCTCGATCCGCT
GGGACTGTGGCAGCAAGATAAAGTGGCCGATCTGGATCCGTCTTTCCACGATCTGACCGAAGCAGACTTCCAGGAGACCT
TCAACGTCGGTTCATTTGCCAGCGGCAAAGAAACCATGAAACTCGGCGAGCTGCTGGAAGCCCTCAAGCAAACCTACTGC
GGCCCGATTGGTGCCGAGTATATGCACATTACCAGCACCGAAGAAAAACGCTGGATCCAACAGCGTATCGAGTCTGGTCG
CGCGACTTTCAATAGCGAAGAGAAAAAACGCTTCTTAAGCGAACTGACCGCCGCTGAAGGTCTTGAACGTTACCTCGGCG
CAAAATTCCCTGGCGCAAAACGCTTCTCGCTGGAAGGCGGTGACGCGTTAATCCCGATGCTTAAAGAGATGATCCGCCAC
GCTGGCAACAGCGGCACCCGCGAAGTGGTTCTCGGGATGGCGCACCGTGGTCGTCTGAACGTGCTGGTGAACGTGCTGGG
TAAAAAACCGCAAGACTTGTTCGACGAGTTCGCCGGTAAACATAAAGAACACCTCGGCACGGGTGACGTGAAATACCACA
TGGGCTTCTCGTCTGACTTCCAGACCGATGGCGGCCTGGTGCACCTGGCGCTGGCGTTTAACCCGTCTCACCTTGAGATT
GTAAGCCCGGTAGTTATCGGTTCTGTTCGTGCCCGTCTGGACAGACTTGATGAGCCGAGCAGCAACAAAGTGCTGCCAAT
CACCATCCACGGTGACGCCGCAGTGACCGGGCAGGGCGTGGTTCAGGAAACCCTGAACATGTCGAAAGCGCGTGGTTATG
AAGTTGGCGGTACGGTACGTATCGTTATCAACAACCAGGTTGGTTTCACCACCTCTAATCCGCTGGATGCCCGTTCTACG
CCGTACTGTACTGATATCGGTAAGATGGTTCAGGCCCCGATTTTCCACGTTAACGCGGACGATCCGGAAGCCGTTGCCTT
TGTGACCCGTCTGGCGCTCGATTTCCGTAACACCTTTAAACGTGATGTCTTCATCGACCTGGTGTGCTACCGCCGTCACG
GCCACAACGAAGCCGACGAGCCGAGCGCAACCCAGCCGCTGATGTATCAGAAAATCAAAAAACATCCGACACCGCGCAAA
ATCTACGCTGACAAGCTGGAGCAGGAAAAAGTGGCGACGCTGGAAGATGCCACCGAGATGGTTAACCTGTACCGCGATGC
GCTGGATGCTGGCGATTGCGTAGTGGCAGAGTGGCGTCCGATGAACATGCACTCTTTCACCTGGTCGCCGTACCTCAACC
ACGAATGGGACGAAGAGTACCCGAACAAAGTTGAGATGAAGCGCCTGCAGGAGCTGGCGAAACGCATCAGCACGGTGCCG
GAAGCAGTTGAAATGCAGTCTCGCGTTGCCAAGATTTATGGCGATCGCCAGGCGATGGCTGCCGGTGAGAAACTGTTCGA
CTGGGGCGGTGCGGAAAACCTCGCTTACGCCACGCTGGTTGATGAAGGCATTCCGGTTCGCCTGTCGGGTGAAGACTCCG
GTCGCGGTACCTTCTTCCACCGCCACGCGGTGATCCACAACCAGTCTAACGGTTCCACTTACACGCCGCTGCAACATATC
CATAACGGGCAGGGCGCGTTCCGTGTCTGGGACTCCGTACTGTCTGAAGAAGCAGTGCTGGCGTTTGAATATGGTTATGC
CACCGCAGAACCACGCACTCTGACCATCTGGGAAGCGCAGTTCGGTGACTTCGCCAACGGTGCGCAGGTGGTTATCGACC
AGTTCATCTCCTCTGGCGAACAGAAATGGGGCCGGATGTGTGGTCTGGTGATGTTGCTGCCGCACGGTTACGAAGGGCAG
GGGCCGGAGCACTCCTCCGCGCGTCTGGAACGTTATCTGCAACTTTGTGCTGAGCAAAACATGCAGGTTTGCGTACCGTC
TACCCCGGCACAGGTTTACCACATGCTGCGTCGTCAGGCGCTGCGCGGGATGCGTCGTCCGCTGGTCGTGATGTCGCCGA
AATCCCTGCTGCGTCATCCGCTGGCGGTTTCCAGCCTCGAAGAACTGGCGAACGGCACCTTCCTGCCAGCCATCGGTGAA
ATCGACGAGCTTGATCCGAAGGGCGTGAAGCGCGTAGTGATGTGTTCTGGTAAGGTTTATTACGACCTGCTGGAACAGCG
TCGTAAGAACAATCAACACGATGTCGCCATTGTGCGTATCGAGCAACTCTACCCGTTCCCGCATAAAGCGATGCAGGAAG
TGTTGCAGCAGTTTGCTCACGTCAAGGATTTTGTCTGGTGCCAGGAAGAGCCGCTCAACCAGGGCGCATGGTACTGCAGC
CAGCATCATTTCCGTGAAGTGATTCCGTTTGGGGCTTCTCTGCGTTATGCAGGCCGCCCGGCCTCCGCCTCTCCGGCGGT
AGGGTATATGTCCGTTCACCAGAAACAGCAACAAGATCTGGTTAATGACGCGCTGAACGTCGAATAAATAAAGGATACAC

AATGAGTAGCGTAGATATTCTGGTCCCTGACCTGCCTGAATCCGTAGCCGATGCCACCGTCGCAACCTGGCATAAAAAAC
CCGGCGACGCAGTCGTACGTGATGAAGTGCTGGTAGAAATCGAAACTGACAAAGTGGTACTGGAAGTACCGGCATCAGCA
GACGGCATTCTGGATGCGGTTCTGGAAGATGAAGGTACAACGGTAACGTCTCGTCAGATCCTTGGTCGCCTGCGTGAAGG
CAACAGCGCCGGTAAAGAAACCAGCGCCAAATCTGAAGAGAAAGCGTCCACTCCGGCGCAACGCCAGCAGGCGTCTCTGG
AAGAGCAAAACAACGATGCGTTAAGCCCGGCGATCCGTCGCCTGCTGGCTGAACACAATCTCGACGCCAGCGCCATTAAA
GGCACCGGTGTGGGTGGTCGTCTGACTCGTGAAGATGTGGAAAAACATCTGGCGAAAGCCCCGGCGAAAGAGTCTGCTCC
GGCAGCGGCTGCTCCGGCGGCGCAACCGGCTCTGGCTGCACGTAGTGAAAAACGTGTCCCGATGACTCGCCTGCGTAAGC
GTGTGGCAGAGCGTCTGCTGGAAGCGAAAAACTCCACCGCCATGCTGACCACGTTCAACGAAGTCAACATGAAGCCGATT
ATGGATCTGCGTAAGCAGTACGGTGAAGCGTTTGAAAAACGCCACGGCATCCGTCTGGGCTTTATGTCCTTCTACGTGAA
AGCGGTGGTTGAAGCCCTGAAACGTTACCCGGAAGTGAACGCTTCTATCGACGGCGATGACGTGGTTTACCACAACTATT
TCGACGTCAGCATGGCGGTTTCTACGCCGCGCGGCCTGGTGACGCCGGTTCTGCGTGATGTCGATACCCTCGGCATGGCA
GACATCGAGAAGAAAATCAAAGAGCTGGCAGTCAAAGGCCGTGACGGCAAGCTGACCGTTGAAGATCTGACCGGTGGTAA
CTTCACCATCACCAACGGTGGTGTGTTCGGTTCCCTGATGTCTACGCCGATCATCAACCCGCCGCAGAGCGCAATTCTGG
GTATGCACGCTATCAAAGATCGTCCGATGGCGGTGAATGGTCAGGTTGAGATCCTGCCGATGATGTACCTGGCGCTGTCC
TACGATCACCGTCTGATCGATGGTCGCGAATCCGTGGGCTTCCTGGTAACGATCAAAGAGTTGCTGGAAGATCCGACGCG
TCTGCTGCTGGACGTGTAGTAGTTTAAGTTTCACCTGCACTGTAGACCGGATAAGGCATTATCGCCTTCTCCGGCAATTG
AAGCCTGATGCGACGCTGACGCGTCTTATCAGGCCTACGGGACCACCAATGTAGGTCGGATAAGGCGCAAGCGCCGCATC
CGACAAGCGATGCCTGATGTGACGTTTAACGTGTCTTATCAGGCCTACGGGTGACCGACAATGCCCGGAAGCGATACGAA
ATATTCGGTCTACGGTTTAAAAGATAACGATTACTGAAGGATGGACAGAACACATGAACTTACATGAATATCAGGCAAAA
CAACTTTTTGCCCGCTATGGCTTACCAGCACCGGTGGGTTATGCCTGTACTACTCCGCGCGAAGCAGAAGAAGCCGCTTC
AAAAATCGGTGCCGGTCCGTGGGTAGTGAAATGTCAGGTTCACGCTGGTGGCCGCGGTAAAGCGGGCGGTGTGAAAGTTG
TAAACAGCAAAGAAGACATCCGTGCTTTTGCAGAAAACTGGCTGGGCAAGCGTCTGGTAACGTATCAAACAGATGCCAAT
GGCCAACCGGTTAACCAGATTCTGGTTGAAGCAGCGACCGATATCGCTAAAGAGCTGTATCTCGGTGCCGTTGTTGACCG
TAGTTCCCGTCGTGTGGTCTTTATGGCCTCCACCGAAGGCGGCGTGGAAATCGAAAAAGTGGCGGAAGAAACTCCGCACC
TGATCCATAAAGTTGCGCTTGATCCGCTGACTGGCCCGATGCCGTATCAGGGACGCGAGCTGGCGTTCAAACTGGGTCTG
GAAGGTAAACTGGTTCAGCAGTTCACCAAAATCTTCATGGGCCTGGCGACCATTTTCCTGGAGCGCGACCTGGCGTTGAT
CGAAATCAACCCGCTGGTCATCACCAAACAGGGCGATCTGATTTGCCTCGACGGCAAACTGGGCGCTGACGGCAACGCAC
TGTTCCGCCAGCCTGATCTGCGCGAAATGCGTGACCAGTCGCAGGAAGATCCGCGTGAAGCACAGGCTGCACAGTGGGAA
CTGAACTACGTTGCGCTGGACGGTAACATCGGTTGTATGGTTAACGGCGCAGGTCTGGCGATGGGTACGATGGACATCGT
TAAACTGCACGGCGGCGAACCGGCTAACTTCCTTGACGTTGGCGGCGGCGCAACCAAAGAACGTGTAACCGAAGCGTTCA
AAATCATCCTCTCTGACGACAAAGTGAAAGCCGTTCTGGTTAACATCTTCGGCGGTATCGTTCGTTGCGACCTGATCGCT
GACGGTATCATCGGCGCGGTAGCAGAAGTGGGTGTTAACGTACCGGTCGTGGTACGTCTGGAAGGTAACAACGCCGAACT
CGGCGCGAAGAAACTGGCTGACAGCGGCCTGAATATTATTGCAGCAAAAGGTCTGACGGATGCAGCTCAGCAGGTTGTTG
CCGCAGTGGAGGGGAAATAATGTCCATTTTAATCGATAAAAACACCAAGGTTATCTGCCAGGGCTTTACCGGTAGCCAGG
GGACTTTCCACTCAGAACAGGCCATTGCATACGGCACTAAAATGGTTGGCGGCGTAACCCCAGGTAAAGGCGGCACCACC
CACCTCGGCCTGCCGGTGTTCAACACCGTGCGTGAAGCCGTTGCTGCCACTGGCGCTACCGCTTCTGTTATCTACGTACC
AGCACCGTTCTGCAAAGACTCCATTCTGGAAGCCATCGACGCAGGCATCAAACTGATTATCACCATCACTGAAGGCATCC
CGACGCTGGATATGCTGACCGTGAAAGTGAAGCTGGATGAAGCAGGCGTTCGTATGATCGGCCCGAACTGCCCAGGCGTT
ATCACTCCGGGTGAATGCAAAATCGGTATCCAGCCTGGTCACATTCACAAACCGGGTAAAGTGGGTATCGTTTCCCGTTC
CGGTACACTGACCTATGAAGCGGTTAAACAGACCACGGATTACGGTTTCGGTCAGTCGACCTGTGTCGGTATCGGCGGTG
ACCCGATCCCGGGCTCTAACTTTATCGACATTCTCGAAATGTTCGAAAAAGATCCGCAGACCGAAGCGATCGTGATGATC
GGTGAGATCGGCGGTAGCGCTGAAGAAGAAGCAGCTGCGTACATCAAAGAGCACGTTACCAAGCCAGTTGTGGGTTACAT
CGCTGGTGTGACTGCGCCGAAAGGCAAACGTATGGGCCACGCGGGTGCCATCATTGCCGGTGGGAAAGGGACTGCGGATG
AGAAATTCGCTGCTCTGGAAGCCGCAGGCGTGAAAACCGTTCGCAGCCTGGCGGATATCGGTGAAGCACTGAAAACTGTT
CTGAAATAAATATCTGTAATAAGAAATAGCCCTCGCCGCTTCCCTCTACAGGAATGGCGAAGGGCTGTCGGTTTCGACAT
GGTTGGCCATCGTATGATGGCCTTTTTTGTGCTTATCGCGATGATTTTCGCTGCGCTATCAGGGTAAATTTATAGTCATC
GGTATTAAAAGCGTTGCGGCTATATTCAAACACCCGACCATCAACTAAATATCCACGCGATACTTTTTCAAGAATCGGCT
TTGTCTGGCTGATATTAAGCAGACGGCTCATCTCTTCGGTTGGCATCAGAGGAATGATTTCCTGTTCGCTACGATCGATA
ACCATTTTCTTCACTTCTTCGATAAAGTGATATTTCGAATTTTCCATGACCTGCCAGGTGAGATCCGGGAACAACGCAAG
CGGCATCCAGGTTTCTTCCAGCGCCATTGGCTTTTGCTTGCGATAGCGCACGCGCTTCACATGCCACACACGATCCTGCG
GGGTGATTTGTAGCTGTTGCTGAAGAAAATCGTCAGCCGGAATCACTTCGAATATCAGAACTTCACTGTGTGTATCGACG
TGACGGTCCGACAGTTTTTCATCAAAACTGGTTAACTGAAAAATATCGTAATTGACCCGCTCTTCTTTGACGTAAGTCCC
GCTGCCCTGAATGCTTTCGAGGATCTGCTGCTCGACTAGCTGGCGCAAAGCCTGACGCACCGTAACCCGGCTGACGCCAA
ACTCTGTTTGTAGCGCTGATTCAGTGGGTAACGCATCGCCAGGTTTAAGCTCGCCACGCGCAATTTGTTCACGAATGCGA
TCGGCAATCTGCCGGTATAAGGGCTTGTGTCCCATTTTTAGTATCTCATTAATACGAATTTAACCATTATGCCCGATAAA
TTCATCCTGTAAATAATACAAATACAATACAAATAATTTCAATCAAGTGAAATTGATCACATAATGGTATTGTTTTATCG

GGCACACTGGCGCGACTATAAAAACGATCAAGTGAGGATCATGATGAATCTGACGACTCTGACCCACCGCGATGCGTTGT
GTCTGAATGCGCGCTTTACCAGCCGTGAAGAGGCCATCCACGCGTTGACTCAACGTCTTGCTGCTCTGGGGAAAATTTCC
AGTACTGAGCAATTTCTGGAAGAAGTGTATCGCCGTGAAAGCCTTGGCCCGACCGCCTTAGGTGAAGGGTTGGCTGTGCC
GCATGGCAAAACTGCTGCGGTAAAAGAAGCGGCGTTTGCGGTCGCCACACTCAGCGAGCCGCTTCAGTGGGAAGGCGTTG
ATGGCCCGGAAGCAGTTGATTTAGTGGTGCTGCTGGCGATTCCCCCCAATGAAGCGGGTACTACGCATATGCAACTGCTG
ACAGCGCTGACCACGCGCCTTGCGGATGATGAGATTCGGGCGCGTATACAGTCGGCGACGACGCCTGATGAGTTGCTCTC
GGCGCTGGATGACAAGGGAGGCACGCAACCTTCTGCCTCTTTTTCCAACGCGCCAACTATCGTCTGCGTAACGGCCTGTC
CGGCGGGTATTGCTCACACCTATATGGCTGCGGAATATCTGGAAAAAGCCGGACGCAAACTCGGCGTAAATGTTTACGTT
GAAAAACAAGGCGCTAACGGCATTGAAGGGCGTTTAACGGCGGATCAACTCAATAGTGCAACCGCCTGTATTTTTGCGGC
TGAAGTCGCCATCAAGGAGAGTGAGCGTTTTAATGGCATTCCCGCGCTTTCAGTGCCTGTTGCCGAGCCGATTCGCCATG
CAGAAGCGTTGATCCAACAAGCGCTTACCCTCAAGCGTAGCGATGAGACGCGTACCGTACAGCAAGATACGCAACCGGTG
AAAAGTGTCAAAACGGAGCTGAAACAGGCACTGTTGAGCGGAATCTCTTTTGCCGTACCGTTGATTGTCGCGGGGGGCAC
GGTGCTGGCGGTCGCGGTATTACTGTCGCAAATCTTCGGGCTACAAGATCTGTTTAATGAAGAAAACTCCTGGCTGTGGA
TGTACCGCAAGCTGGGCGGCGGGCTGCTCGGAATTTTGATGGTACCGGTGCTCGCGGCCTATACCGCCTATTCTCTGGCA
GATAAACCGGCGTTAGCGCCAGGCTTTGCGGCTGGACTTGCCGCCAACATGATCGGCTCCGGGTTTCTCGGCGCGGTCGT
TGGCGGATTGATAGCCGGTTACTTGATGCGCTGGGTGAAAAATCACTTGCGTCTTAGCAGTAAATTCAATGGATTCCTGA
CTTTTTATCTCTACCCGGTGCTCGGTACGTTGGGAGCGGGCAGTCTGATGCTGTTTGTGGTGGGGGAACCTGTCGCCTGG
ATCAATAACTCGCTTACCGCCTGGCTGAACGGTCTGTCAGGAAGTAACGCGCTGTTGCTGGGTGCCATTCTCGGTTTTAT
GTGTTCCTTTGACCTTGGAGGGCCAGTGAATAAAGCCGCTTATGCATTCTGCCTGGGCGCAATGGCGAACGGCGTTTACG
GCCCGTATGCCATTTTCGCCTCCGTCAAAATGGTTTCGGCATTTACCGTAACCGCTTCCACGATGCTCGCACCGCGCCTG
TTTAAAGAGTTTGAAATTGAGACCGGGAAATCCACCTGGCTGTTAGGGCTGGCAGGTATTACCGAAGGGGCGATCCCGAT
GGCGATTGAAGATCCGCTGCGGGTTATTGGTTCGTTTGTGCTGGGCTCTATGGTAACGGGCGCTATTGTCGGTGCGATGA
ATATCGGCCTTTCGACACCCGGTGCCGGCATTTTCTCGCTCTTTTTACTTCATGATAATGGCGCGGGCGGTGTTATGGCG
GCAATTGGCTGGTTTGGCGCGGCATTGGTGGGGGCTGCAATCTCGACTGCAATTCTCCTGATGTGGCGGCGTCACGCGGT
TAAGCATGGCAACTATCTGACTGATGGCGTAATGCCATAAACAAAAAGGAAACGACGATGAAAGCAGTATCTCGCGTTCA
CATCACCCCGCATATGCACTGGGATCGAGAGTGGTATTTCACCACCGAAGAGTCACGTATTCTGCTGGTCAATAATATGG
AAGAGATCCTGTGCCGACTGGAACAGGACAACGAATACAAATATTACGTACTCGACGGGCAAACGGCGATCCTCGAAGAT
TATTTCGCGGTGAAACCGGAAAACAAAGACCGTGTGAAGAAACAGGTAGAAGCCGGCAAGTTGATTATCGGCCCCTGGTA
TACCCAGACCGATACCACGATTGTTTCTGCGGAATCCATCGTCCGTAATCTGATGTACGGAATGCGTGACTGCCTCGCGT
TTGGCGAGCCGATGAAAATAGGTTATTTACCAGATTCCTTTGGCATGTCCGGGCAACTGCCGCATATCTACAATGGATTT
GGCATTACCCGCACCATGTTCTGGCGCGGATGTTCGGAGCGCCACGGTACTGATAAAACCGAGTTTTTGTGGCAAAGCAG
TGACGGTAGCGAAGTGACGGCGCAGGTGCTGCCGCTGGGCTACGCCATCGGTAAGTACTTACCTGCCGACGAAAACGGAT
TACGTAAACGCCTCGACAGTTATTTTGACGTGCTGGAAAAAGCGTCTGTAACCAAAGAGATTTTGCTGCCGAATGGGCAT
GACCAGATGCCATTGCAGCAAAATATCTTCGAAGTGATGGATAAGCTACGTGAGATCTACCCTCAACGTAAGTTTGTGAT
GAGCCGCTTTGAAGAGGTATTTGAGAAGATCGAAGCGCAGCGAGATAATCTGGCAACCCTGAAAGGGGAATTTATTGATG
GCAAATATATGCGCGTGCATCGCACCATCGGTTCTACGCGTATGGATATCAAAATTGCCCACGCGCGTATTGAAAATAAG
ATTGTTAATCTGCTGGAACCGCTGGCAACACTGGCCTGGACGTTGGGTTTTGAATACCACCACGGCTTGCTGGAGAAAAT
GTGGAAAGAGATCTTAAAAAATCATGCCCACGACAGTATCGGCTGCTGCTGTAGTGACAAAGTTCATCGCGAAATCGTCG
CCCGCTTCGAACTGGCTGAAGACATGGCGGATAATCTGATTCGTTTCTACATGCGCAAAATTGCCGACAACATGCCGCAG
AGCGACGCCGACAAACTCGTCCTGTTTAACCTGATGCCCTGGCCGCGTGAAGAAGTTATCAACACCACTGTGCGGCTGCG
CGCCAGCCAGTTTAATTTGCGGGACGATCGCGGTCAGCCTGTACCGTATTTTATTCGCCATGCCCGTGAGATCGATCCAG
GCCTAATCGATCGGCAAATAGTTCATTACGGTAATTACGATCCCTTTATGGAGTTTGATATACAGATCAACCAGATTGTC
CCTTCTATGGGCTATCGCACGCTTTATATCGAAGCGAATCAGCCTGGCAACGTAATTGCGGCAAAAAGTGACGCTGAAGG
GATACTGGAAAATGCTTTCTGGCAAATTGCGCTCAATGAGGATGGTTCTCTGCAACTGGTAGATAAAGACAGCGGTGTGC
GCTATGACCGGGTATTGCAAATTGAAGAAAGCTCTGATGATGGTGATGAATATGACTATTCACCCGCAAAAGAAGAGTGG
GTAATTACCGCAGCGAACGCGAAACCGCAATGCGATATTATTCATGAAGCCTGGCAGAGCAGGGCTGTTATCCGCTATGA
CATGGCAGTGCCGCTCAATTTGTCAGAACGCAGCGCCCGGCAATCCACTGGCAGAGTAGGGGTGGTGTTGGTTGTCACTC
TTAGTCATAACAGCAGGCGTATTGATGTGGATATCAATCTTGATAACCAGGCTGACGATCATCGCCTTCGTGTCCTGGTC
CCTACACCTTTTAACACCGACAGTGTTCTGGCAGATACGCAGTTTGGTTCGCTAACGCGCCCCGTGAACGACAGTGCAAT
GAACAACTGGCAGCAAGAAGGCTGGAAAGAAGCGCCGGTTCCGGTATGGAATATGCTCAACTATGTTGCCTTACAGGAAG
GGCGTAACGGCATGGCTGTCTTTAGCGAAGGGTTACGTGAATTTGAAGTCATCGGTGAAGAGAAGAAAACCTTTGCCATT
ACGTTGCTGCGTGGCGTGGGCTTACTGGGCAAAGAAGATCTGCTTTTAAGGCCTGGGCGGCCTTCGGGAATTAAAATGCC
AGTCCCGGACTCACAACTACGTGGTCTGCTTTCTTGTCGCCTAAGTTTATTGAGTTATACCGGTACGCCAACCGCCGCTG
GTGTAGCTCAGCAGGCGCGAGCATGGCTGACTCCAGTACAGTGTTACAACAAAATCCCATGGGATGTGATGAAGCTCAAC
AAAGCCGGATTCAACGTGCCGGAAAGTTATAGTTTGTTGAAAATGCCCCCAGTGGGATGCCTGATAAGCGCACTTAAGAA
AGCTGAAGACCGACAAGAAGTGATTTTACGGCTGTTTAATCCGGCTGAATCAGCAACCTGTGATGCGACTGTTGCTTTCA

GTCGCGAGGTGATTTCTTGCTCAGAAACGATGATGGATGAACACATTACCACCGAGGAAAATCAAGGTTCAAATCTATCG
GGGCCTTTTTTACCCGGCCAGTCACGGACGTTCAGTTACCGGCTTGCCTGAATAGCAATCAAACCGAAGCCACATATGCG
CGGCCAGATTGTTGACAAAGGGCGCTTTGTTCATGCCGGATACGGCATGAACGCTTTATTCGGTCTACAAAAGCAGGCAA
ATTCAATATATTGCAGAGATGATGTAGACACTGGCAAGCGTAGCGCATCAGGTAATTTTGCGTTTATCTTCACTCTCAAG
CCACGTATATGTGGCTTTATTTTTAACAAAATAATAACCCTGGGTGAGTTAATTATAATATAATTATAAGTTAACTAAAT
GTTAATATTGGCGGGGTGGATTTATGCCTTTATTAGTAATCCTGAAACTCTGCGTCGTATTAGCCAGTGACCAAAAAAAG
AATTAAGGTCAACCGTGCTGTTTTTGCTTCGTCTCTTTTTATCTTTAATTGCCAACCGAAACTAATTTCAGCCTTATAAC
TCACACATTTTAAACATAAATGTCACTAAAGTTACCTTATTGAAACATGATTAACATAATTTGTAGGAATTGATATTTAT
CAATGTATAAGTCTTGGAAATGGGCATCAAAAAGAGATAAATTGTTCTCGATCAAATTGGCTGAAAGGCGGTAATTTAGC
TATAAATTGATCACCGTCGAAAAATGCAAATTTGCTTCAACAAAAACCTGTTTATTGTAAGGATTTTGCGGCGTAATATA
TACGTGGGATCAATTTGAGTTTTTATTAACATGTTTGCAACCTTTCTTTACGCCGTTTTTGTGTGCATTCACATGGTATG
ATGAAAGTGTTCAAACAAATTTCTATTGGGGCATGCGTGTGACCCTTTCTAACGGGGTTCACTCTCGGAGTCTTCATGCG
ATGAGCAAGGAGTCATGATGTTAGATATAGTCGAACTGTCGCGCTTACAGTTTGCCTTGACCGCGATGTACCACTTCCTT
TTTGTGCCACTGACGCTCGGTATGGCGTTCCTGCTGGCCATTATGGAAACGGTCTACGTCCTCTCCGGCAAACAGATTTA
TAAAGATATGACCAAGTTCTGGGGCAAGTTGTTTGGTATCAACTTCGCTCTGGGTGTGGCTACCGGTCTGACCATGGAGT
TCCAGTTCGGGACTAACTGGTCTTACTATTCCCACTATGTAGGGGATATCTTCGGTGCGCCGCTGGCAATCGAAGGTCTG
ATGGCCTTCTTCCTCGAATCCACCTTTGTAGGTCTGTTCTTCTTCGGTTGGGATCGTCTGGGTAAAGTTCAGCATATGTG
TGTCACCTGGCTGGTGGCGCTCGGTTCAAACCTGTCCGCACTGTGGATTCTGGTTGCGAACGGCTGGATGCAAAACCCAA
TCGCGTCCGATTTCAACTTTGAAACTATGCGTATGGAGATGGTGAGCTTCTCCGAGCTGGTGCTTAACCCGGTTGCTCAG
GTGAAATTCGTTCACACTGTAGCGTCTGGTTATGTGACTGGCGCGATGTTCATCCTCGGTATCAGCGCATGGTATATGCT
GAAAGGTCGTGACTTCGCCTTCGCTAAACGCTCCTTTGCTATCGCTGCCAGCTTCGGTATGGCTGCTGTTCTGTCTGTTA
TTGTTCTGGGTGATGAATCCGGCTACGAAATGGGCGACGTGCAGAAAACCAAACTGGCTGCTATTGAAGCCGAGTGGGAA
ACGCAACCTGCGCCTGCTGCCTTTACTCTGTTCGGCATTCCTGATCAGGAAGAGGAGACGAACAAATTTGCGATTCAGAT
CCCTTACGCACTGGGCATCATTGCAACGCGTTCCGTGGATACCCCGGTTATCGGCCTGAAAGAGCTGATGGTGCAGCATG
AAGAACGCATTCGTAACGGGATGAAGGCGTACTCTCTGCTCGAACAACTGCGTTCTGGTTCTACCGACCAGGCGGTTCGT
GACCAGTTCAATAGCATGAAGAAAGACCTCGGTTACGGTCTGCTGCTGAAACGCTATACGCCAAACGTGGCTGATGCGAC
TGAAGCGCAGATTCAACAGGCAACCAAAGACTCCATCCCGCGTGTAGCGCCGCTGTACTTTGCGTTCCGTATCATGGTGG
CGTGTGGCTTCCTGCTTCTGGCAATCATCGCGCTCTCTTTCTGGAGTGTCATCCGCAACCGCATTGGCGAGAAAAAATGG
CTTCTGCGCGCCGCGCTGTACGGTATTCCGCTGCCGTGGATTGCTGTAGAAGCGGGCTGGTTCGTGGCTGAATATGGCCG
CCAACCGTGGGCTATCGGTGAAGTGCTGCCGACAGCTGTGGCGAACTCGTCACTGACCGCAGGCGATCTCATCTTCTCAA
TGGTGCTGATTTGCGGCCTGTATACCCTGTTCCTGGTGGCAGAATTGTTCTTAATGTTCAAGTTTGCACGCCTCGGCCCA
AGCAGCCTGAAAACCGGTCGCTATCACTTTGAGCAGTCTTCCACGACTACTCAGCCGGCACGCTAAGACAGGAGTCGTCA
AATGATCGATTATGAAGTATTGCGTTTTATCTGGTGGCTGCTGGTTGGCGTTCTGCTGATTGGTTTTGCAGTCACTGACG
GTTTCGACATGGGGGTGGGCATGCTCACCCGTTTCCTCGGTCGTAACGACACCGAGCGTCGAATTATGATTAACTCCATT
GCACCACACTGGGACGGTAACCAGGTTTGGCTGATCACCGCGGGCGGCGCACTCTTTGCTGCCTGGCCGATGGTCTATGC
CGCTGCGTTCTCCGGCTTCTATGTGGCGATGATCCTCGTGCTGGCGTCTTTGTTCTTCCGTCCGGTCGGTTTTGACTACC
GCTCCAAGATTGAAGAAACCCGCTGGCGTAACATGTGGGACTGGGGCATCTTCATTGGTAGCTTCGTTCCGCCGCTGGTA
ATTGGTGTAGCGTTCGGTAACCTGTTGCAGGGCGTACCGTTCAACGTTGATGAATATCTGCGTCTGTACTACACCGGTAA
CTTCTTCCAGTTGCTTAACCCGTTCGGCCTGCTGGCAGGCGTGGTGAGCGTAGGGATGATCATTACTCAGGGCGCAACCT
ATCTGCAAATGCGTACCGTGGGCGAACTGCACCTGCGTACCCGTGCAACGGCTCAGGTGGCTGCGCTGGTGACACTGGTC
TGTTTCGCACTGGCTGGCGTATGGGTGATGTACGGTATCGATGGTTATGTCGTGAAATCGACAATGGACCATTACGCAGC
CTCTAACCCACTGAATAAAGAAGTGGTTCGTGAAGCTGGCGCATGGCTGGTTAACTTCAACAACACGCCAATTCTGTGGG
CTATTCCGGCACTGGGTGTGGTTCTGCCGCTGCTGACCATCCTGACTGCACGTATGGATAAAGCCGCGTGGGCGTTTGTG
TTCTCCTCCCTGACGCTGGCCTGCATCATCCTGACAGCCGGTATCGCAATGTTCCCGTTTGTGATGCCGTCCAGCACCAT
GATGAACGCAAGTCTGACAATGTGGGATGCAACTTCCAGCCAGCTGACGCTTAACGTCATGACCTGGGTTGCGGTGGTTC
TGGTACCGATCATTCTGCTCTACACCGCCTGGTGTTACTGGAAAATGTTCGGTCGTATCACCAAAGAAGATATTGAACGT
AACACCCACTCTCTGTACTAAGTAAGGAGCTAAAAATGTGGTATTTCGCATGGATTCTGGGAACGCTTCTTGCCTGTTCG
TTTGGGGTAATCACCGCGCTGGCGCTTGAACACGTCGAATCAGGCAAAGCCGGTCAAGAAGACATCTGATGAGTAAGATT
ATCGCGACTTTGTATGCGGTAATGGACAAGCGCCCCCTGCGGGCGCTTTCCTTCGTGATGGCGCTTCTGTTAGCAGGATG
TATGTTTTGGGACCCATCTCGTTTTGCCGCGAAGACCAGTGAACTGGAAATCTGGCATGGTTTATTGCTGATGTGGGCCG
TCTGTGCTGGTGTGATTCACGGCGTGGGCTTTCGTCCGCAGAAGGTTCTTTGGCAAGGGATTTTTTGCCCATTGCTTGCC
GATATTGTTCTCATTGTCGGGCTGATTTTCTTCTTCTTTTAAATCAGAATTCTCTTTAAAAAATTATGGGCCGCTCCAGG
CCCATAAATTTTTACGCTCCCTTAACTTGCCCTCATTCCCAAACCTCAATCGCGCGCGTATAGTAGCAGCGTTTAAAAGC
TCTAACTTTTGTTGCATTACCGGGATGTAAAGTGAATACAACGCTGTTTCGATGGCCGGTTCGCGTCTACTATGAAGATA
CCGATGCCGGTGGTGTGGTGTACCACGCCAGTTACGTCGCTTTTTATGAAAGAGCACGCACAGAGATGCTGCGTCATCAT
CACTTCAGTCAGCAGGCGCTGATGGCTGAACGCGTTGCCTTTGTGGTACGTAAAATGACGGTGGAATATTACGCACCTGC

GCGGCTCGACGATATGCTCGAAATACAGACTGAAATAACATCAATGCGTGGCACCTCTTTGGTTTTCACGCAACGTATTG
TCAACGCCGAGAATACTTTGCTGAATGAAGCAGAGGTTCTGGTTGTTTGCGTTGACCCACTCAAAATGAAGCCTCGTGCG
CTTCCCAAGTCTATTGTCGCGGAGTTTAAGCAGTGACTGACATGAATATCCTTGATTTGTTCCTGAAGGCTAGCCTTCTG
GTTAAACTTATCATGTTGATTTTGATTGGTTTTTCAATCGCATCTTGGGCCATTATTATCCAGCGGACCCGTATTCTTAA
CGCAGCGGCGCGCGAAGCCGAAGCGTTTGAAGATAAATTCTGGTCTGGAATCGAACTCTCTCGCCTCTATCAAGAGAGCC
AGGGGAAACGGGATAATCTGACTGGTTCGGAACAAATCTTTTACAGCGGGTTCAAAGAGTTTGTGCGCCTGCATCGTGCC
AATAGCCATGCGCCGGAAGCCGTAGTGGAAGGGGGCGTCGCGTGCTATGCGTATCTCCATGAACCGTGAACTTGAAAATCT
GGAAACGCACATTCCGTTCCTCGGTACGGTTGGCTCCATCAGCCCGTATATTGGTCTGTTTGGTACGGTCTGGGGGATCA
TGCACGCCTTTATCGCCCTCGGGGCGGTAAAACAAGCAACACTGCAAATGGTTGCGCCCGGTATCGCAGAAGCGTTGATT
GCGACTGCAATTGGTCTGTTTGCCGCTATCCCGGCAGTTATGGCCTACAACCGCCTCAACCAGCGCGTAAACAAACTGGA
ACTGAATTACGACAACTTTATGGAAGAGTTTACCGCGATTCTGCACCGCCAGGCGTTTACCGTTAGCGAGAGCAACAAGG
GGTAAGCCATGGCCAGAGCGCGTGGACGAGGTCGTCGCGATCTCAAGTCCGAAATCAACATTGTACCGTTGCTGGACGTA
CTGCTGGTGCTGTTGCTGATCTTTATGGCGACAGCGCCCATCATCACCCAGAGCGTGGAGGTCGATCTGCCAGACGCTAC
TGAATCACAGGCGGTGAGCAGTAACGATAATCCGCCAGTGATTGTTGAAGTGTCTGGTATTGGTCAGTACACCGTGGTGG
TTGAGAAAGATCGCCTGGAGCGTTTACCACCAGAGCAGGTGGTGGCGGAAGTGTCCAGCCGTTTCAAGGCCAACCCGAAA
ACGGTCTTTCTGATCGGTGGCGCAAAAGATGTGCCTTACGATGAAATAATTAAAGCACTGAACTTGTTACATAGTGCGGG
TGTGAAATCGGTTGGTTTAATGACGCAGCCTATCTAAACATCTGCGTTTCCCTTGCTTGAAAGAGAGCGGGTAACAGGCG
AACAGTTTTTGGAAACCGAGAGTGTCAAAGGCAACCGAACAAAACGACAAGCTCAAGCGGGCGATAATTATTTCAGCAGT
GCTGCATGTCATCTTATTTGCGGCGCTGATCTGGAGTTCGTTCGATGAGAATATAGAAGCTTCAGCCGGAGGCGGCGGTG
GTTCGTCCATCGACGCTGTCATGGTTGATTCAGGTGCGGTAGTTGAGCAGTACAAACGCATGCAAAGCCAGGAATCAAGC
GCGAAGCGTTCTGATGAACAGCGCAAGATGAAGGAACAGCAGGCTGCTGAAGAACTGCGTGAGAAACAAGCGGCTGAACA
GGAACGCCTGAAGCAACTTGAGAAAGAGCGGTTAGCGGCTCAGGAGCAGAAAAAGCAGGCTGAAGAAGCCGCAAAACAGG
CCGAGTTAAAGCAGAAGCAAGCTGAAGAGGCGGCAGCGAAAGCGGCGGCAGATGCTAAAGCGAAGGCCGAAGCAGATGCT
AAAGCTGCGGAAGAAGCAGCGAAGAAAGCGGCTGCAGACGCAAAGAAAAAAGCAGAAGCAGAAGCCGCCAAAGCCGCAGC
CGAAGCGCAGAAAAAAGCCGAGGCAGCCGCTGCGGCACTGAAGAAGAAAGCGGAAGCGGCAGAAGCAGCTGCAGCTGAAG
CAAGAAAGAAAGCGGCAACTGAAGCTGCTGAAAAAGCCAAAGCAGAAGCTGAGAAGAAAGCGGCTGCTGAAAAGGCTGCA
GCTGATAAGAAAGCGGCAGCAGAGAAAGCTGCAGCCGACAAAAAAGCAGCAGAAAAAGCGGCTGCTGAAAAGGCAGCAGC
TGATAAGAAAGCAGCGGCAGAAAAAGCCGCCGCAGACAAAAAAGCGGCAGCGGCAAAAGCTGCAGCTGAAAAAGCCGCTG
CAGCAAAAGCGGCCGCAGAGGCAGATGATATTTTCGGTGAGCTAAGCTCTGGTAAGAATGCACCGAAAACGGGGGGAGGG
GCGAAAGGGAACAATGCTTCGCCTGCCGGGAGTGGTAATACTAAAAACAATGGCGCATCAGGGGCCGATATCAATAACTA
TGCCGGGCAGATTAAATCTGCTATCGAAAGTAAGTTCTATGACGCATCGTCCTATGCAGGCAAAACCTGTACGCTGCGCA
TAAAACTGGCACCCGATGGTATGTTACTGGATATCAAACCTGAAGGTGGCGATCCCGCACTTTGTCAGGCTGCGTTGGCA
GCAGCTAAACTTGCGAAGATCCCGAAACCACCAAGCCAGGCAGTATATGAAGTGTTCAAAAACGCGCCATTGGACTTCAA
ACCGTAATCGCGATGTTGACTGTTCGGACGGTCAACATCAGGCACCGGTTGCCACGGGGTTCTGGTAGTTTTGTGTATTT
TAGTTTGTTAACATTCTGCTAAATTATCGTGGGCCATCGGTCCAGATAAGGGAGATATGATGAAGCAGGCATTACGAGTA
GCATTTGGTTTTCTCATACTGTGGGCATCAGTTCTGCATGCTGAAGTCCGCATTGTGATCGACAGCGGTGTAGATTCCGG
TCGTCCTATTGGTGTTGTTCCTTTCCAGTGGGCGGGGCCTGGTGCGGCACCTGAAGATATTGGCGGCATCGTTGCTGCTG
ACTTGCGTAACAGCGGTAAATTTAATCCGTTAGATCGCGCTCGTCTGCCACAGCAGCCGGGTAGTGCGCAGGAAGTACAA
CCAGCTGCATGGTCCGCACTGGGCATTGACGCTGTAGTTGTCGGTCAGGTCACTCCGAATCCGGATGGTTCTTACAATGT
TGCTTATCAACTTGTTGACACTGGCGGCGCACCGGGTACTGTACTTGCTCAGAACTCGTACAAAGTGAACAAGCAGTGGC
TGCGTTATGCTGGTCATACCGCCAGTGATGAAGTGTTTGAAAAACTGACCGGCATTAAAGGTGCGTTCCGTACTCGTATT
GCCTACGTTGTTCAGACCAACGGCGGTCAGTTCCCGTATGAACTGCGCGTATCTGACTATGACGGTTACAACCAGTTTGT
CGTTCACCGTTCACCGCAGCCGCTGATGTCTCCGGCGTGGTCACCAGACGGTTCTAAACTGGCTTATGTGACCTTCGAAA
GCGGTCGTTCCGCGCTGGTTATTCAGACGCTGGCAAATGGCGCTGTACGTCAGGTGGCTTCATTCCCGCGTCACAACGGT
GCACCTGCATTCTCGCCAGACGGCAGCAAACTGGCATTCGCCTTGTCGAAAACCGGTAGTCTGAACCTGTACGTAATGGA
TTTGGCTTCTGGTCAGATCCGCCAGGTGACTGATGGTCGCAGTAACAATACCGAACCGACCTGGTTCCCGGATAGCCAGA
ACCTGGCATTTACTTCTGACCAGGCCGGTCGTCCGCAGGTTTATAAAGTGAATATCAACGGCGGTGCGCCACAACGTATT
ACCTGGGAAGGTTCGCAGAACCAGGATGCGGATGTCAGCAGCGACGGTAAATTTATGGTAATGGTCAGCTCCAATGGTGG
GCAGCAGCACATTGCCAAACAAGATCTGGCAACGGGAGGCGTACAAGTTCTGTCGTCCACGTTCCTGGATGAAACGCCAA
GTCTGGCACCTAACGGCACTATGGTAATCTACAGCTCTTCTCAGGGGATGGGATCCGTGCTGAATTTGGTTTCTACAGAT
GGGCGTTTCAAAGCGCGTCTTCCGGCAACTGATGGACAGGTCAAATTCCCTGCCTGGTCGCCGTATCTGTGATAATAATT
AATTGAATAGTAAAGGAATCATTGAAATGCAACTGAACAAAGTGCTGAAAGGGCTGATGATTGCTCTGCCTGTTATGGCA
ATTGCGGCATGTTCTTCCAACAAGAACGCCAGCAATGACGGCAGCGAAGGCATGCTGGGTGCCGGCACTGGTATGGATGC
GAACGGCGGCAACGGCAACATGTCTTCCGAAGAGCAGGCTCGTCTGCAAATGCAACAGCTGCAGCAGAACAACATCGTTT
ACTTCGATCTGGACAAGTACGATATCCGTTCTGACTTCGCTCAAATGCTGGATGCACATGCAAACTTCCTGCGTAGCAAC
CCGTCTTACAAAGTCACCGTAGAAGGTCACGCGGACGAACGTGGTACTCCGGAATACAACATCTCCCTGGGTGAACGTCG

TGCGAACGCCGTTAAGATGTACCTGCAGGGTAAAGGCGTTTCTGCAGACCAGATCTCCATCGTTTCTTACGGTAAAGAAA
AACCTGCAGTACTGGGTCATGACGAAGCGGCATACTCCAAAAACCGTCGTGCGGTACTGGTTTACTAAGAGAATTGCATG
AGCAGTAACTTCAGACATCAACTATTGAGTCTGTCGTTACTGGTTGGTATAGCGGCCCCCTGGGCCGCTTTTGCTCAGGC
ACCAATCAGTAGTGTCGGCTCAGGCTCGGTCGAAGACCGCGTCACTCAACTTGAGCGTATTTCTAATGCTCACAGCCAGC
TTTTAACCCAACTCCAGCAACAACTTTCTGATAATCAATCCGATATTGATTCCCTGCGTGGTCAGATTCAGGAAAATCAG
TATCAACTGAATCAGGTCGTGGAGCGGCAAAAGCAGATCCTGTTGCAGATCGACAGCCTCAGCAGCGGTGGTGCAGCGGC
GCAATCAACCAGCGGCGATCAAAGCGGTGCGGCGGCATCAACGACGCCGACAGCTGATGCTGGTACTGCGAATGCTGGCG
CGCCGGTGAAAAGCGGTAATGCAAACACGGATTACAATGCAGCTATTGCGCTGGTGCAGGATAAATCCCGCCAGGATGAC
GCAATGGTGGCATTTCAGAATTTCATCAAAAATTACCCTGATTCAACTTACCTGCCAAACGCCAATTATTGGCTGGGTCA
GTTAAACTACAACAAGGGTAAAAAAGATGATGCGGCGTACTATTTTGCTTCGGTAGTGAAAAACTATCCGAAGTCACCAA
AGGCTGCAGATGCGATGTTTAAAGTCGGCGTCATCATGCAGGACAAAGGTGACACCGCAAAAGCGAAAGCCGTATACCAG
CAGGTTATCAGTAAATACCCTGGTACTGATGGCGCTAAACAGGCACAAAAACGTCTGAACGCGATGTAATGCATAACACA
CGACCAGAAGTCGCATTATTTCTGGTCGTGTCGTGCGAATCATAAGCAGTTGAGTGATCTACATCGAAATTTTTGTTGCG
CTCAAGTCTGAAATCAGTAATATATGCCGCCGTTGCCACGGGATATCAAACAAACCGAAAGCAACGAAAAAGTGGGTCGT
TAGCTCAGTTGGTAGAGCAGTTGACTTTTAATCAATTGGTCGCAGGTTCGAATCCTGCACGACCCACCAATCGCTAAGGT
GGAAGCGGTAGTAAAACGTGAAGGATAACGTTGCATGAGCAACGGCCCGAAGGGCGAGACGAAGTCGAGTCATCCTGCAC
GACCCACCACTAACATAGTTAGTTGTAGTATCCAGCGTAGTATCGGGTGATTAGCTCAGCTGGGAGAGCACCTCCCTTAC
AAGGAGGGGGTCGGCGGTTCGATCCCGTCATCACCCACCACCGGGTCGTTAGCTCAGTTGGTAGAGCAGTTGACTTTTAA
TCAATTGGTCGCAGGTTCGAATCCTGCACGACCCACCAGTTTTAACATCGAAGACAGATGTTAAGCGTGTAGGATAACGT
TGCGTCAGCAACGGCCCGTAGGGCGAGCGAAGCGAGTCATCCTGCACGACCCACCACTAATGACGGTGGGTTCGGTGGAA
GTAGTTTGTAGTATCCAGCGCAGTATCGGGTGATTAGCTCAGCTGGGAGAGCACCTCCCTTACAAGGAGGGGGTCGGCGG
TTCGATCCCGTCATCACCCACCACTCGGGTCGTTAGCTCAGTTGGTAGAGCAGTTGACTTTTAATCAATTGGTCGCAGGT
TCGAATCCTGCACGACCCACCAGTTTTAACATCAAACTCAGATGTTAAGCGTGAAGGATAACGTTGCGCCAGCAACGGCC
CGTAGGGCGAGCGAAGCGAGTCATCCTGCACGACCCACCAATCTTAAAGATTGGCCCCGAGTAAAAATCTTTCAGGTAAC
ACCCGTATGGGTCGTTAGCTCAGTTGGTAGAGCAGTTGACTTTTAATCAATTGGTCGCAGGTTCGAATCCTGCACGACCC
ACCAATTTAAAGGTGGTTACTGGTAGAGAACGTGAAGGATAACGTTGCGTTAGCAACGGCCCGAAGGGCGAGACGAAGTC
GAGTCATCCTGCACGACCCACCATCCTGAATGATTAAGGCAGCATAATCCCGCAAGGGGTCGTTAGCTCAGTTGGTAGAG
CAGTTGACTTTTAATCAATTGGTCGCAGGTTCGAATCCTGCACGACCCACCAATGTAAAAAAGCGCCCTAAAGGCGCTTT
TTTGCTATCTGCGATACTCAAAGATTCGAACCTGCAGCAGGTTTGAGTTGAGCGCAGCGAAACAACGGAGCCGCTCGCGG
CGACGGCCCGAAGGGCGAGCGAAGCGAGTCATCCTGCACGACCCACCAATGTAAAAAAGCGCCCTAAAGGCGCTTTTTTG
CTATTCAGGCATCCTCAATTTCACTTTGTAAACCTGATGACATCGTCAGAGCTTACTGTGCAAGCAACTCTATGTCGGTG
GAATTAGGCGTAAAATGACGCATCCTGCACATTAGGCGTAATTCGAGTGACTTTTCCCCACCATTCGACTATCTTGTTTA
GCATATAAAACAAATTACACCGATAACAGCGAATATTACGCTAATGTCGGTTTTAACGTTAAGCCTGTAAAACGAGATGG
TAAGATGAGCGTAATGTTTGATCCAGACACGGCGATTTATCCTTTCCCCCCGAAGCCGACGCCGTTAAGCATTGATGAAA
AAGCGTATTACCGCGAGAAGATAAAACGTCTGCTAAAAGAACGTAATGCGGTGATGGTTGCCCACTACTATACCGATCCC
GAAATTCAACAACTGGCAGAAGAAACCGGTGGCTGTATTTCTGATTCTCTGGAAATGGCGCGCTTCGGTGCAAAGCATCC
CGCTTCTACTTTGTTAGTCGCTGGGGTGAGATTTATGGGAGAAACCGCCAAAATTCTCAGTCCGGAAAAAACAATTCTGA
TGCCGACACTTCAGGCTGAATGTTCACTGGATCTCGGCTGCCCTGTTGAAGAATTTAACGCATTTTGCGATGCCCATCCC
GATCGTACTGTCGTCGTCTACGCCAACACTTCTGCTGCGGTAAAAGCGCGCGCAGATTGGGTGGTAACTTCAAGCATTGC
CGTCGAACTTATTGATCATCTTGATAGTTTGGGTGAAAAAATCATCTGGGCACCCGACAAACATCTGGGGCGTTACGTGC
AAAAACAGACGGGTGGAGACATTCTATGCTGGCAGGGTGCCTGTATTGTGCATGATGAATTTAAGACTCAGGCGTTAACC
CGCTTGCAAGAAGAATACCCGGATGCTGCCATACTGGTGCATCCAGAATCACCACAAGCTATTGTCGATATGGCGGATGC
GGTCGGTTCCACCAGTCAACTGATCGCTGCTGCGAAAACATTGCCACATCAGAGGCTTATTGTGGCAACCGATCGGGGTA
TTTTCTACAAAATGCAGCAGGCGGTGCCAGATAAAGAGTTACTGGAAGCACCAACCGCAGGTGAGGGTGCAACCTGCCGC
AGCTGCGCGCATTGTCCGTGGATGGCCATGAATGGCCTTCAGGCCATCGCAGAGGCATTAGAACAGGAAGGAAGCAATCA
CGAGGTTCATGTTGATGAAAGGCTGCGAGAGAGGGCGCTGGTGCCGCTCAATCGTATGCTGGATTTTGCGGCTACACTAC
GTGGATAACGAATAATAAGGCGTAACGTTACGCTTTGGGGGAAAGATGGATTTTTTTAGTGTGCAGAATATCCTGGTACA
TATACCAATAGGGGCAGGCGGTTATGATCTCTCATGGATCGAAGCGGTAGGCACGATCGCCGGGTTGCTGTGTATTGGCC
TTGCCAGTCTGGAGAAGATCAGCAACTACTTCTTTGGCCTGATCAACGTCACCTTGTTTGGCATTATTTTCTTTCAGATT
CAGCTGTATGCCAGCCTGCTATTACAGGTGTTTTTCTTTGCCGCGAATATTTACGGTTGGTATGCGTGGTCGCGACAAAC
CAGTCAGAACGAGGCGGAGTTGAAAATTCGCTGGTTGCCATTGCCGAAGGCACTCAGCTGGTTGGCGGTTTGCGTTGTTT
CGATTGGTCTGATGACGGTATTTATCAATCCGGTGTTTGCATTTTTGACCCGCGTGGCAGTCATGATCATGCAAGCATTA
GGATTACAGGTTGTGATGCCTGAACTGCAACCGGACGCTTTCCCGTTCTGGGATTCATGCATGATGGTGTTATCTATCGT
GGCAATGATTCTGATGACGCGTAAGTATGTGGAAAACTGGCTGTTGTGGGTGATTATTAACGTGATTAGCGTCGTTATTT
TTGCACTTCAGGGCGTTTACGCCATGTCTCTGGAGTACATCATCCTGACCTTTATTGCGCTCAACGGCAGCCGGATGTGG
ATCAACAGCGCACGTGAAAGAGGCTCACGCGCGCTGTCCCATTAATGGTGATGATGTGAATGGCCAGACACCCCTTCATT

CAGGTGGCAGTCTGGCCCATGACAAGGTTGATATTCCATCTGAATGGTGGCGTGCTCAATCTGATAGTGATCCATCAGGT
AGTGTTGGATCTGATCCAACAAGGCATCGTGATCGTGTGGCGGGATCACCTGCACATGCAACGTCATCACCGGCTTCTCG
CCTACCATCCATACATGTACATGGTGCACATTGCGGACTTCCGGGATTTCCCGGCACATACGACGCTTCAGTTCAGCGAT
ATCCAGCGATACCGGTGCACCTTCAAGTAATTCATTCACGCTATCTTTCAACAGTCGCCACGCGCTACGCAGAACCAGAA
GCGACACCAGTATCGAGAGAATGGGGTCAGCAGGTGTCCAGCCAGTCCAGATAATAATCAACGCGGCAATAATAGCCCCA
ACCGAACCCAGCAGGTCCCCGAGCACATGCAGTGCCGCTGCTCGTACGTTGAGGTTTTTCTCTTCGCTGCCGTGATGAAG
TAACCAGAAAGAAAGTATATTTGCCAGCAACCCAGCCACGGCAATTGCCATCATCATGCCGCCCTCGACCGGACGCGGCG
TGCGGAAGCGTTCTATCGCCTCCCAGACAATCAAAATGGTAATTACCACGAGGGCTATGGCATTCACAAACGCCGCGAGC
GTGGTCAGTCTTAGCCAACCGAAAGTGTGGCGAATGGTGGGAGGACGACGTGAAAATTGCACGGCGAGTAGGGCGAAAAG
CAGGGCGGCAGTATCGGTCAACATATGACCCGCATCGGCCAGCAATGCCAGAGAACCAGAAAGAAAACCACCAACGACTT
CTACCAGCATAAACCCGGCAGTCACGCCGAAAGCATACAACAAGCGACGAGCATTATTATCTTCTGGCAGGTGTGAGGAC
GTGTGTGAGTGTGAGTGCGCCATGACGAGTCATCCCTTAATTATTCTCACTTAACTTTATGACATCATACTGCTTTTAGA
AGTGAAAAATTAAAAGGGAGAGACTCCGCTCTCCCATTATTGGCTATTTTGCAGGGTTACTGCGTGGTACCGTCGGTTTT
GGTATCGACATCATTATTGATGCCATCACCGGTTTGTACCTTTTTATTGATATCCGGACAGCGACCATCTTTGCACATGG
TGTTCTTGTGCTCTTCATCTTTGGTCATTCCGTCATTGTTCATTGAAGAACCATCCGAATGCAGCATTGTGCCGCCAGAA
CCGGTATTTACCCCGTTATTGTCGACGTTATTTGGCGCGACATTTTCACGGGCGTCAGGGGCTACCTGGCCCGCATCAGC
TGCGGCGTTTGCCTGGCCGTTATTAGTTTGCGCTCCGCTATCGGCAGCCAGTGCGGCACCGCTGGCAAGGCTTAGAGTGG
CAGTCAGAAATAATGTGGCCAGTTTTGTCATTTTCATAGGATGCTCCTGTTATGGTCGTTATGTCGGATAACCTCTTCCA
ACAGTGCATTTGCAGGTGAATATAAGGCATTGGTTTAAGATTTCAGCCAGGTTATGAAACGCAGCAGAGAATCTTGAAAT
AATTAACAAACAAAGGAGTTACAGTTAGAAATTGTAGGAGAGATCTCGTTTTTCGCGACAATCTGGCGTTTTTCTTGCTA
ATTCCAGGATTAATCCGTTCATAGTGTAAAACCCCGTTTACACATTCTGACGGAAGATATAGATTGGAAGTATTGCATTC
ACTAAGATAAGTATGGCAACACTGGAACAGACATGAATTATCAGAACGACGATTTACGCATCAAAGAAATCAAAGAGTTA
CTTCCTCCTGTCGCATTGCTGGAAAAATTCCCCGCTACTGAAAATGCCGCGAATACGGTTGCCCATGCCCGAAAAGCGAT
CCATAAGATCCTGAAAGGTAATGATGATCGCCTGTTGGTTGTGATTGGCCCATGCTCAATTCATGATCCTGTCGCGGCAA
AAGAGTATGCCACTCGCTTGCTGGCGCTGCGTGAAGAGCTGAAAGATGAGCTGGAAATCGTAATGCGCGTCTATTTTGAA
AAGCCGCGTACCACGGTGGGCTGGAAAGGGCTGATTAACGATCCGCATATGGATAATAGCTTCCAGATCAACGACGGTCT
GCGTATAGCCCGTAAATTGCTGCTTGATATTAACGACAGCGGTCTGCCAGCGGCAGGTGAGTTTCTCGATATGATCACCC
CACAATATCTCGCTGACCTGATGAGCTGGGGCGCAATTGGCGCACGTACCACCGAATCGCAGGTGCACCGCGAACTGGCA
TCAGGGCTTTCTTGTCCGGTCGGCTTCAAAAATGGCACCGACGGTACGATTAAAGTGGCTATCGATGCCATTAATGCCGC
CGGTGCGCCGCACTGCTTCCTGTCCGTAACGAAATGGGGGCATTCGGCGATTGTGAATACCAGCGGTAACGGCGATTGCC
ATATCATTCTGCGCGGCGGTAAAGAGCCTAACTACAGCGCGAAGCACGTTGCTGAAGTGAAAGAAGGGCTGAACAAAGCA
GGCCTGCCAGCACAGGTGATGATCGATTTCAGCCATGCTAACTCGTCCAAACAATTCAAAAAGCAGATGGATGTTTGTGC
TGACGTTTGCCAGCAGATTGCCGGTGGCGAAAAGGCCATTATTGGCGTGATGGTGGAAAGCCATCTGGTGGAAGGCAATC
AGAGCCTCGAGAGCGGGGAGCCGCTGGCCTACGGTAAGAGCATCACCGATGCCTGCATCGGCTGGGAAGATACCGATGCT
CTGTTACGTCAACTGGCGAATGCAGTAAAAGCGCGTCGCGGGTAAGGTTTAATTGTCGGATGCGCCGTCAGAGTGGCGTA
TCCGATGAATCACCACAGGCCTGATAAGTCGCGCAGCGTCGCATCAGGCAATGTGCTCCATTGTTAGCAACAAAAAAGCC
GACTCACTTGCAGTCGGCTTTCTCATTTTAAACGAATGACGTTTACTTCGCTTTACCCTGGTTTGCAACCGCCGCTGCTT
TCGCTGCGATCTCGTCAGCATTACCCAGATAATAGCGTTTCAGCGGTTTGAAATTCTCGTCGAACTCATACACCAGCGGC
ACGCCAGTCGGGATATTAAGCTCAAGAATCTCTTCTTCGCTCATGTTATCAAGATATTTCACCAGCGCACGTAAAGAGTT
ACCGTGTGCAGCGATGATCACGCGCTCACCGCTCTTCATACGCGGCAGAATAGTTTCATTCCAGTAAGGGATCACGCGGT
CAATGGTCAGCGCCAGGCTTTCCGTCAGCGGCAGTTCTTTCTCGCTCAGTTTCGCGTAACGCGGATCGTGACCCGGATAA
CGCTCATCATCTTTAGTCAGTTCCGGCGGAGTCACTGCAAAACCACGACGCCACTGTTTCACCTGCTCGTCGCCATACTT
TTCAGCAGTTTCCGCTTTGTTCAGACCCTGCAACGCACCGTAGTGACGTTCGTTCAGTTTCCAGGATTTCTCAACGGGCA
GCCATGCCTGATCCAGTTCGTCCAGCACATTCCACAGGGTATGGATAGCGCGTTTCAGCACAGAAGTGTAAGCAAAGTCA
AAGCTGTAACCTTCCTCTTTCAGCAGCTTACCTGCTGCTTTTGCTTCGCTTACGCCTTTCTCAGACAGATCCACGTCGTA
CCAACCGGTGAAACGGTTTTCTTTGTTCCACTGACTTTCGCCATGACGAACCAGAACCAGCTTAGTTACAGCCATATACT
TACTCCTCAAATCATCTTTTAATGATAATAATTCTCATTATATTGCCGCGACGAAGCAACAGCAATGCTTACGCATAACC
ATAGCGAAAATAGTGGCGCAGTGTAAGGTTGTTGTGAATATTGAGTTGCAAATATGTCGGTGTTTGCTGGTGATTTGAAC
AATATGAGATAAAGCCCTCATGACGAGGGCGTAACATTACTCAGCAATAAACTGATATTCCGTCAGGCTGGAATACTCTT
CGCCAGGACGCAGGAAGCAGTCCGGTTGCGGCCATTCAGGGTGGTTCGGGCTGTCCGGTAGAAACTCGCTTTCCAGAGCC
AGCCCTTGCCAGTCGGCGTAAGGTTCGGTTCCCCGCGACGGTGTGCCGCCGAGGAAGTTGCCGGAGTAGAATTGCAGAGC
CGGAGCGGTGGTGTAGACCTTCAGCTGCAATTTTTCATCTGCTGACCAGACATGCGCCGCCACTTTCTTGCCATCGCCTT
TGGCCTGTAACAAGAATGCGTGATCGTAACCTTTCACTTTGCGCTGATCGTCGTCGGCAAGAAACTCACTGGCGATGATT
TTGGCGCTGCGGAAATCAAAAGACGTTCCGGCGACAGATTTCAGGCCGTCGTGCGGAATGCCGCCTTCATCAACCGGCAG
ATATTCGTCCGCCAGAATCTGCAACTTGTGATTGCGCACGTCAGACTGCTCGCCGTCAAGATTGAAATAGACGTGATTAG
TCATATTCACCGGGCAAGGTTTATCAACTGTGGCGCGATAAGTAATGGAGATACGGTTATCGTCGGTCAGACGATATTGC

ACCGTCGCGCCGAGATTACCCGGGAAGCCCTGATCACCATCATCTGAACTCAGGGCAAACAGCACCTGACGATCGTTCTG
GTTCACAATCTGCCAGCGACGTTTGTCGAACCCTTCCGGCCCGCCGTGCAGCTGGTTAACGCCCTGACTTGGCGAAAGCG
TCACGGTTTCACCGTCAAAGGTATAACGGCTATTGGCGATACGGTTGGCATAACGACCAATAGAGGCCCCCAGAAACGCG
GCCTGATCCTGATAGCATTCCGGGCTGGCACAGCCGAGCAGCGCCTCGCGGACGCTGCCATCGGAAAGCGGAATACGGGC
GGAAAGTAAAGTCGCACCCCAGTCCATCAGCGTGACTACCATCCCTGCGTTGTTACGCAAAGTTAACAGTCGGTACGGCT
GACCATCGGGTGCCAGTGCGGGAGTTTCGTTCAGCACTGTCCTGCTCCTTGTGATGGTTTACAAACGTAAAAAGTCTCTT
TAATACCTGTTTTTGCTTCATATTGTTCAGCGACAGCTTGCTGTACGGCAGGCACCAGCTCTTCCGGGATCAGCGCGACG
ATACAGCCGCCAAATCCGCCGCCGGTCATGCGTACGCCACCTTTGTCGCCAATCACAGCTTTGACGATTTCTACCAGAGT
GTCAATTTGCGGCACGGTGATTTCGAAATCATCGCGCATAGAGGCATGAGACTCCGCCATCAACTCGCCCATACGTTTCA
GGTCGCCTTGCTCCAGCGCGCTGGCAGCTTCAACGGTGCGGGCGTTTTCAGTCAGTATATGACGCACGCGTTTTGCCACG
ATCGGGTCCAGTTCATGCGCAACAGCGTTGAACTCTTCAATGGTGACATCACGCAGGGCTGGCTGCTGGAAGAAACGCGC
ACCGGTTTCGCACTGTTCACGACGGGTGTTGTATTCGCTGCCAACCAGGGTACGTTTGAAGTTACTGTTGATGATGACGA
CAGCCACACCTTTGGGCATGGAAACTGCTTTGGTCCCCAGTGAGCGGCAATCGATCAGCAAGGCATGATCTTTCTTGCCG
AGCGCGGAAATTAGCTGATCCATGATCCCGCAGTTACAGCCTACAAACTGGTTTTCTGCTTCCTGACCGTTAAGCGCGAT
TTGTGCGCCGTCCAGCGGCAGATGATAAAGCTGCTGCAATACGGTTCCGACCGCGACTTCCAGTGAAGCGGAAGAACTTA
ACCCGGCACCCTGCGGCACATTGCCGCTGATCACCATGTCCACGCCGCCGAAGCTGTTGTTACGCAGTTGCAGATGTTTC
ACCACGCCACGAACGTAGTTAGCCCATTGATAGTTTTCATGTGCGACAATGGGCGCATCGAGGGAAAACTCGTCGAGCTG
ATTTTCATAATCGGCTGCCATCACGCGAACTTTACGGTCATCGCGTGGTGCACAACTGATCACGGTTTGATAATCAATCG
CGCAGGGCAGAACGAAACCGTCGTTGTAGTCGGTGTGTTCACCAATCAAATTCACGCGGCCAGGCGCCTGAATGGTGTGA
GTGGCAGGGTAGCCAAATGCGTTGGCAAACAGAGATTGTGTTTTTTCTTTCAGACTCATTTCTTACACTCCGGATTCGCG
AAAATGGATATCGCTGACTGCGCGCAAACGCTCTGCTGCCTGTTCTGCGGTCAGGTCTCGCTGGGTCTCTGCCAGCATTT
CATAACCAACCATAAATTTACGTACGGTGGCGGAGCGCAGCAGAGGCGGATAAAAGTGCGCGTGCAGCTGCCAGTGTTGA
TTCTCTTCGCCATTAAATGGCGCGCCGTGCCAGCCCATAGAGTAGGGGAAGGAGCACTGGAAGAGGTTGTCATAACGACT
GGTCAGCTTTTTCAACGCCAGCGCCAGATCGCTGCGCTGGGCGTCGGTCAAATCGGTGATCCGTAAAACGTGGGCTTTGG
GCAGCAGTAGCGTTTCGAACGGCCAGGCAGCCCAGTAAGGCACGACGGCTAACCAGTGTTCGGTTTCGACAACGGTACGG
CTACCGTCTGCCAGCTCGCGCTGAACATAATCCACCAGCATTGGTGATTTCTGTTCGGCAAAATATTCTTTTTGCAGGCG
GTCTTCGCGCTCAGCTTCGTTAGGCAGGAAGCTATTTGCCCAAATCTGACCGTGCGGATGCGGGTTAGAGCAGCCCATCG
CCGCGCCTTTGTTTTCAAAAACCTGCACCCATGGGTACGTTTTCCCCAGTTCTGCGGTTTGCTCCTGCCAGGTTTTGACG
ATTTCCGTCAATGCTGCAACGCTGAGCTCTGGCAGCGTTTTACTGTGATCCGGTGAAAAGCAGATCACCCGGCTGGTGCC
GCGCGCGCTCTGGCAACGCATCAGCGGATCGTGACTTTCTGGCGCATCTGGCGTGTCAGACATCAAAGCCGCAAAGTCAT
TAGTGAAAACGTAAGTCCCGGTGTAATCGGGGTTTTTATCGCCTGTCACCCGCACATTACCTGCGCAGAGGAAGCAATCT
GGATCGTGCGCAGGTAACACCTGTTTGGCTGGCGTTTCCTGCGCCCCCTGCCAGGGGCGCTTAGCGCGGTGCGGTGAAAC
CAGAATCCATTGCCCGGTGAGCGGGTTGTAGCGGCGATGTGGATGATCAACGGGATTAAATTGCGTCATGGTCGTTCCTT
AATCGGGATATCCCTGTGGATGGCGTGACTGCCAGTGCCAGGTGTCCTGCGCCATTTCATCGAGTGTGCGCGTTACGCGC
CAGTTCAGTTCACGGTCGGCTTTGCTGGCGTCCGCCCAGTAGGCCGGAAGGTCGCCCTCGCGACGCGGTGCAAAATGATA
ATTAACCGGTTTGCCGCAGGCTTTGCTGAAGGCATTAACCACGTCCAGCACGCTGTTGCCTACGCCAGCGCCGAGGTTGT
AGATGTGTACGCCTGGCTTGTTCGCCAGTTTTTCCATCGCCACGACGTGACCGTCCGCCAGATCCATTACGTGGATGTAA
TCGCGTACGCCAGTACCATCTTCGGTCGGATAATCGTTACCAAAAATCGCCAGCGAGTCGCGACGGCCTACAGCAACCTG
GGCGATGTATGGCATCAGGTTATTCGGAATGCCTTGCGGATCTTCGCCCATATCGCCCGACGGATGCGCGCCAACCGGGT
TGAAGTAGCGCAGCAGGGCAATGCTCCAGTCCGGCTGGGCTTTTTGCAGATCGGTGAGGATCTGTTCCACCATCAGCTTG
CTTTTGCCGTAAGGGCTTTGCGGTGTGCCGGTCGGGAAGCTTTCAACGTATGGAATTTTGGGCTGATCGCCATAAACGGT
GGCGGAGGAGCTAAAAATAAAGTTTTTGACGTTAGCGGCGCGCATGGCGCTAATCAGGCGCAGAGTGCCGTTGACATTGT
TGTCGTAATATTCCAGCGGTTTTTGTACCGATTCGCCCACGGCTTTCAGCCCGGCGAAGTGGATCACGGTGTCGATAGCG
TGATCGTGCAGGATCTCGGTCATCAACGCTTCGTTACGAATATCGCCTTCAACAAACGTTGGATGTTTGCCGCCTAAACG
CTCGATAACAGGCAGTACGCTGCGCTTACTGTTACAGAGGTTATCAAGAATGATGACATCATGACCGTTTTGCAGTAATT
GCACACAGGTATGACTTCCAATGTAACCGCTACCACCGGTAACCAGAACTCTCATAATTCGCTCCATTAGGCTTATGGTA
TGAAATAACCATAGCATAACAAAGATGCGAAAAGTGTGACATGGAATAAATTAGTGGAATCGTTTACACAAGAATTTAGC
CGTTTTTTATGCGCGATTAAGTGATTATAAAACAGAGGGTTTATGAATGATTGCGCTTTTTATCTGAAAAAAGACGCGGT
TTCATGCCTGCATGCGTCGAACCGTTGGCCGGAGAGGGTGCTAAGGCCGCCTCCGGCAAGGTCAGCACTACCGACTCAAT
ATATTTTTGTCAGCACATAGCGATAGAGTCCACCGTCCGGCACGAACTCAAGACGATGGGTAATACAGGCAGGCGCATCT
TCAGCGTGGTGCGAAACAAACAACAATTGCGTTTCACCTTCGCTAATCAGCACATCAACAAAACGGCGGATAAGCTGGCG
ATTCAGCGGATCAAGCCCCTGTAGTGGTTCATCGAGAATAAGCAACGTCGGATGTTTCACCAGTGCGCGGACAATCAGCG
CCAGACGCTGCTGTCCCCAGGAAAGACTATGGAACGGAGCGTCAGCCGTGCGTTTATCAATGCCGAGAATATCCAGCCAC
TGCTGCACCAGTTTTTGCTGGCGATCCGAAACGGCCTGATAAATGCCAATCGAATCAAAATAGCCAGAAAGAATCACATT
ACGCACGGTAGTGCTGACCCGGTAATCCAGATGCAAACTACTGCTGACGTAACCGATATGCTTTTTGATATCCCAGATGG
TTTCGCCGCTGCCGCGACGTCGTCCGAAAAGCGTCAAATCGTTGCTGTAACCTTGCGGATGATCGCCAGTAACCAGGCTT

AATAACGTCGATTTTCCTGCACCATTTGGCCCGACAATTTGCCAGTGTTCGCCTGGATTCACCTGCCAGCTAAGGTTATT
AAGAATGGGGCGATCGTTATAAGAAACCACGCCATTGTTCAGCACAATGCGCGGTTCGTTGGCGGGTAAGGCGTGACGTG
CTGAAGGTTCATCCGGCTCCGGCAGTTGCACACCTTCAAGCTGTTCACTATGCGCCAGTTGCGCGACGAGTGCTTGTTGG
AGCAGTTCCTCTTTAGCGCCAGTTTCCGCTAACGTGCAATCCGCCAGCACGCCAGCAAACTGGACAAACTCCGGGATCTC
ATCGAAGCGATTGAGCACCAGTACCAGAGTAATACCGGACTGATGTAACGAGGCGAGTCGCTCAGCCAGCTGCTGACGTG
AGGCAACATCCAGGCCATCGAACGGCTCATCAAGAATCAACAAGTCAGGCTCCGACATCAGCGCCTGACACAGCAGGGTT
TTTCGCGTCTCGCCAGTGGAAAGGTATTTAAAGCGTCGGTCGAGGAGGGCGGTAATACCGAACTGCTGCGCCAGTTGCAT
GCAACGCGGTGCATCCTTTACTTCATCCTGAATGATCTCAGCCGTAGTGCGTCCGGTGTCATCTTCGCCAGGGCCGAGCA
TATCGGTGTTATTCCGCTGCCATTCGTCGCTGACGAGTTTTTGCAATTGCTCGAAGGAGAGACGAGTGATGTGGGAAAAC
TGGCTTTGCCGTTCACCTTTCAAAAGCGGAAGTTCCCCCGCCAGCGCGCGGGCCAGGGCCGATTTCCCGCTTCCATTCGA
ACCGACAAACGCCCAACTATCACCCGCGTTTAACGTTAGCTGAGGCAATTGCAGCGTTTTTGTGTCGCTAAGACGAAACG
TGCCTTGCAAAATTTGCAACGATGACATTTTATATCCCACTTTGTGCAGCGATTACTGACAGGGATACGTGTTTCATAAC
AAATTGTCAACACGCTTAGCACAGCGTGGCGATAATCACGCTGTCGGCATTAAAGTAGGCCGTGACATTCTGTCCTTGCT
GAAGAGAAGTCGCTTCATTTACCGGCACTGTGGCGCACAGTGTTTGCCCGTCGGGTAGCGCCATTAATACTTCGCACTGC
TCTGCGCCGCGCTCAATATGACTAATAATACCCGGTAATTGGTTGTCAGCGTTTTGCGCGACCGCCTCGTCCTGAGTAAT
ACCTACCCACGGCGCTTTTAGCAATATCAACACTTCTTTGCCTTCATCCAGCCCCAGACGCGCGCCGCTTTGTGCGGTAA
TTGCGACTTTCAGGCGTGTTTTTCCGTCAGCCAGTAAGACATCAACATGCTGTTGAACGTCATCATGATCGCGGGCGGTG
ATGGTACCGAACCACTGGTTACGGGCGCTGGTTTGCAGTGAAAAACGTGAGATCGCGGCCAGCAGGCTGTTCAGCGGCAG
GGCGTCATCGTCACTTAACACATCAAAGGCTTTTTGCTGGATTTGCGCCAGTAAGTCATAGAGCTGAATCAGTCGCTGAC
CATAGCGGGTCAGTACTGCGCCGCCGCCACCTTTACCGCCTGTTGCGCGCTCGACCAGAATATGCTCACTTAACTGATTC
ATCTCGTTAATGGCATCCCAGGCGCTTTTATAGCTAATACCGGCATCTTTCGCTCCCTGGCTAATGGAACCGGAAAGCGC
AATGTGTTTTAGTAGCGAAATGCGGCGCGGGTCGGCGAATAATTTTTGTTGGAGCTTAAGGGTGAGAAGGATTTCGGCCT
GCATAACAATGTCCTGGCAAAAGTCTTATTGTGACGGAAAACGAACGCCACGCAAAGCTGACCGCACAAAAGGGGAGTGC
TTTTCTGTGCTTAGCGGTTAGAATAGTCTCATGACTATATCTGGAGTTGACCATGTTAGAGTTATTAAAAAGTCTGGTAT
TCGCCGTAATCATGGTACCTGTCGTGATGGCCATCATCCTGGGTCTGATTTACGGTCTTGGTGAAGTATTCAACATCTTT
TCTGGTGTTGGTAAAAAAGACCAGCCCGGACAAAATCATTGATTCCCTGAATGCCCGCTTAGTCGGGCATTTTCTTTTTC
TCAACTTCCTGCTTTTCCTGCCGATATTTTTTCTTATCTACCTCACAAAGGTTAGCAATAACTGCTGGGAAAATTCCGAG
TTAGTCGTTATATTGTCGCCTACATAACGTTACATTAAGGGGTTACCAATGGCTCGTAAATGGTTGAACTTGTTTGCCGG
GGCGGCACTCTCTTTCGCTGTTGCTGGCAATGCACTGGCAGATGAAGGGAAAATCACGGTGTTCGCCGCCGCATCACTGA
CTAACGCAATGCAGGACATTGCTACGCAGTTTAAAAAAGAGAAAGGCGTGGATGTGGTTTCTTCTTTCGCTTCGTCATCT
ACTCTCGCCCGTCAGATTGAAGCGGGTGCGCCTGCGGATCTGTTTATTTCTGCCGATCAGAAATGGATGGATTATGCGGT
TGATAAAAAAGCGATCGATACAGCTACGCGTCAGACACTGCTCGGCAATAGCCTGGTCGTTGTAGCACCGAAAGCCAGCG
TGCAGAAAGATTTCACCATCGACAGCAAAACCAACTGGACTTCACTGCTGAATGGCGGTCGCCTGGCGGTTGGCGATCCG
GAACATGTTCCCGCTGGCATTTATGCAAAAGAAGCACTGCAAAAACTGGGCGCATGGGATACGCTCTCTCCGAAACTGGC
CCCAGCGGAAGATGTTCGTGGGGCGCTGGCGCTGGTCGAACGTAACGAAGCGCCTCTGGGCATTGTCTACGGTTCTGACG
CAGTTGCCAGCAAAGGGGTAAAAGTGGTTGCCACCTTCCCGGAAGATTCACATAAAAAAGTGGAATATCCGGTTGCTGTT
GTGGAAGGGCATAACAATGCGACAGTGAAAGCATTTTATGATTATCTGAAGGGACCGCAGGCAGCGGAAATCTTTAAACG
TTACGGATTTACAATCAAGTAATGATACTGACCGATCCAGAATGGCAGGCAGTTTTATTAAGCCTGAAAGTTTCTTCCCT
GGCTGTGCTGTTTAGCCTGCCGTTTGGGATCTTTTTTGCCTGGTTACTGGTGCGTTGCACGTTTCCGGGCAAAGCTCTGC
TCGACAGCGTACTGCATCTACCGCTGGTGTTACCGCCCGTGGTCGTCGGTTACTTATTATTAGTTTCGATGGGACGGCGC
GGATTTATCGGTGAACGTCTGTATGACTGGTTTGGTATTACCTTCGCCTTTAGCTGGCGCGGCGCGGTTCTCGCTGCCGC
CGTCATGTCGTTTCCGCTGATGGTGCGGGCAATTCGTCTGGCGCTGGAAGGGGTTGATGTCAAACTGGAACAGGCCGCAA
GAACACTGGGGGCCGGGCGCTGGCGCGTTTTCTTTACTATCACGTTACCGCTGACCTTACCGGGAATTATTGTTGGTACG
GTACTGGCTTTTGCTCGTTCTCTCGGTGAGTTTGGTGCAACCATCACCTTTGTGTCGAACATTCCTGGTGAAACGCGAAC
CATTCCTTCTGCCATGTATACCCTGATCCAGACCCCCGGCGGGGAAAGTGGAGCGGCGAGACTGTGCATTATTTCTATTG
CGCTGGCGATGATCTCCCTGTTGATTTCAGAATGGCTGGCCAGAATCAGCCGTGAACGGGCGGGGCGCTAATCATGCTGG
AACTGAATTTTTCCCAGACGTTGGGCAACCATTGCCTGACTATTAATGAAACGCTGCCCGCCAATGGCATCACTGCTATC
TTTGGCGTCTCCGGTGCCGGAAAAACTTCGCTGATTAACGCCATCAGTGGACTGACGCGCCCGCAAAAAGGGCGGATTGT
CCTCAATGGGCGGGTACTAAATGATGCCGAAAAAGGTATCTGCCTGACGCCGGAAAAGCGTCGCGTTGGCTATGTTTTTC
AGGATGCGCGGCTGTTCCCGCATTACAAAGTGCGTGGCAATCTGCGCTACGGCATGTCGAAAAGTATGGTCGATCAGTTC
GATAAGCTGGTGGCGCTTTTAGGCATTGAACCGTTGCTTGACCGTTTACCAGGCAGCCTGTCCGGAGGCGAAAAACAGCG
CGTGGCGATTGGTCGGGCTTTGCTGACAGCACCGGAATTGCTGTTGCTGGATGAACCGCTGGCGTCACTGGATATTCCGC
GTAAACGCGAACTGTTGCCTTATCTGCAACGGCTGACACGGGAAATCAACATTCCGATGTTGTATGTCAGCCATTCGCTG
GATGAGATCCTCCATCTGGCAGACAGAGTGATGGTACTGGAAAACGGTCAGGTGAAAGCCTTTGGCGCGCTGGAGGAAGT
GTGGGGCAGTAGCGTGATGAATCCGTGGCTGCCGAAAGAGCAACAAAGTAGCATTCTGAAAGTGACGGTGCTTGAGCATC
ATCCGCATTACGCGATGACCGCGCTGGCGCTGGGCGATCAGCATTTGTGGGTCAATAAGCTGGACGAACCGCTGCAAGCT

GCGCTACGTATCCGCATTCAGGCTTCCGATGTTTCTCTGGTTTTACAACCGCCGCAGCAAACCAGCATTCGTAACGTATT
GCGGGCAAAAGTTGTTAATAGTTATGACGACAACGGCCAGGTGGAAGTGGAACTGGAAGTCGGCGGTAAAACGCTGTGGG
CGCGTATCAGCCCGTGGGCCAGGGATGAACTGGCGATCAAACCTGGCCTGTGGCTGTACGCGCAAATTAAAAGTGTGTCG
ATAACCGCCTGATTAAATCAGGTGGCTATAAATGAACTGGGCAATGCTGTCGGTGGTGTTATCACCAATCACAATGTTGG
CGCGCGCTTTTACCGCGTCATCGGCGTTGCCCATCGCCACGCCTGTACCAGCGGCTTCCAGCATACTGATATCATTAAAG
TTATCGCCGAATGCCACGACGTTTTCCATCGACCAACCTTGCGCCTCAACCCATTTCGTCAAACGTTTACCTTTGCTGTT
GCCGCCGCGTGCAATATCAACCTGATCGTGCCAGGACCATTCACACTCCAGTCCCAGTTCATGTTCGACATGCTTACCAA
AATGCTGCAATTGCGGCAGGTCATCGTGCGTCAGGGCGAACTTCCATACGGCGTTAACTTGTTGCGCCGTTTCAGCCAGA
GAAGCGACTTGTGTGAAAGTCGGACGCTGTTCCGGCGGCAGGGTTTGCGCCCAGTTAGATGTGCGAATGACATGCCCGGT
CGGGTGCTCATAGACCATTGCATCATCGACATACATCAGACCGTGAATGTGGTGTTCATTCAGCATCTCAATGAGTTGCA
GGGCTTTAATAACGGGCATTGGGTCCGCTTCCAGCACGGTTTTTGCATGATAATCATACAAATAGGTGCCATTACAGCAA
ATAGCAGGTGTATCCAGCGCCAGCGCCTGATAAAAAGGATGAATAGCGACGTGATGGCGACCTGTGACGATGATTAATTG
ATAGCCTGCTTCGCGAGCGCGGGCCAGGGCTTCTATCGATGAAGGAAGCAGGGTCTTTTTCGGGGTCAATAAGGTGCCGT
CTAAGTCGAGAGCAATCACGCGTGTGGTCATGGCGTATTCCAGATTAAGGTTAAGAATTTTCGTCTGCGCGAATGGTACA
CCGATACCACTATCGGACAAAATTCTGCGTTTTAATTCAGCATTCACCGCCAAAAGCGACTAATTTTAGCTGTTACAGTC
AGTTGCTAAATGCAAAGGAGCATTCATGAAGCAAACAGTTTATATCGCCAGCCCTGAGAGCCAGCAAATTCACGTCTGGA
ATCTGAATCATGAAGGCGCACTGACGCTGACACAGGTTGTCGATGTGCCGGGGCAGGTGCAGCCGATGGTGGTCAGCCCG
GACAAACGTTATCTCTATGTTGGTGTTCGCCCTGAGTTTCGCGTCCTGGCGTATCGTATCGCCCCGGACGATGGCGCACT
GACCTTTGCCGCAGAGTCTGCGCTGCCGGGTAGTCCGACGCATATTTCCACCGATCACCAGGGGCAGTTTGTCTTTGTAG
GTTCTTACAATGCGGGTAACGTGAGCGTAACGCGTCTGGAAGATGGCCTGCCAGTGGGCGTCGTCGATGTGGTCGAGGGG
CTGGACGGTTGCCATTCCGCCAATATCTCACCGGACAACCGTACGCTGTGGGTTCCGGCATTAAAGCAGGATCGCATTTG
CCTGTTTACGGTCAGCGATGATGGTCATCTCGTGGCGCAGGACCCTGCGGAAGTGACCACCGTTGAAGGGGCCGGCCCGC
GTCATATGGTATTCCATCCAAACGAACAATATGCGTATTGCGTCAATGAGTTAAACAGCTCAGTGGATGTCTGGGAACTG
AAAGATCCGCACGGTAATATCGAATGTGTCCAGACGCTGGATATGATGCCGGAAAACTTCTCCGACACCCGTTGGGCGGC
TGATATTCATATCACCCCGGATGGTCGCCATTTATACGCCTGCGACCGTACCGCCAGCCTGATTACCGTTTTCAGCGTTT
CGGAAGATGGCAGCGTGTTGAGTAAAGAAGGCTTCCAGCCAACGGAAACCCAGCCGCGCGGCTTCAATGTTGATCACAGC
GGCAAGTATCTGATTGCCGCCGGGCAAAAATCTCACCACATCTCGGTATACGAAATTGTTGGCGAGCAGGGGCTACTGCA
TGAAAAAGGCCGCTATGCGGTCGGGCAGGGACCAATGTGGGTGGTGGTTAACGCACACTAACCGCTGATTTACCCGGCGC
AGTCTCTCCTGCGCCGGTGTATTAACCTATCTCCTGTAACGCGTGTCTCTGGCGTTCGACGATATTGGTCCACAAATTGT
CTTTATCGTCAGTCCATAAATTAATCAGCAAGGCAAAAAAGCGTTCTGCTGCTGGAGAAAGTACGGCATCTTTACGGCGA
ATTATTCCCAATGTTCGACGTATAACGGGTTCCACCAATGGGATACCAATAATCGAAGAATAGGGCGCATGAGGCATTGC
CAGGCCAGGAAGCGCCGAAATCCCCAGTCCCGCCTCCACCAGTCCTAATGACGTCGAAAGATGACGCACTTCGTAAAACC
AGTCCAGCTTCCAGGGCTTGTCGGCCAGCTGTTGTTCTATCAACAGTCGGTTGCCGCTGGAGGAGCGTACGCCAATCATT
TTGTAACCCACTAACTCCTGCCATTCTACAAGCTGCTTTTTGGCCAATGGATGGTCACGCCGACAGGCCAGCACGAACGG
CTCGTTGACCAGTGGGGTAAAATCAATGGATGAATTTGTGACGTTGTTCATGTTTATGCCAAAGTCTGATTCGTTACACA
GCACCGATTCCATGCAATTATTCGTGCCTTGTTCCAGAATCCGCACTTTAATATTGGGATACAGCTCATTAAATTTACCG
ATTGCCAGCGGTAAAAAATAAAATACTGCGGTCGGAATACACGCTAACGTCACCATACCACGATGATAAGCATTCATATC
ACGAATATTAAAAAGCGTTTCATCGAATTTCTTTATTAATTCCCGCGCCTCCGGGAGCAACCTTTTTCCTGCTTTTGTTA
ACGTAACCTTGCGAGTTGTACGCTCAAATAGTTGAACATGTAAATCTTCCTCCATCTTTTTTATTCTTCGCGTTAATGCA
GGTTGCGTAATATTGAGTAATTTAGCGGCATTGTTAAACGAACTGGACTCTGCCAGTATGACAAATGCCTTCATACTTGA
TAATTCATGCTTCATTATTACTCCGGAAAATGGAAGCGACGATTTTGGGTGGCTGGCCGTTAAAAATTTTAACTGCATTT
AGCCAACTTAAATTAATGAAAAAATGTTATTAATCGTTGAGCTAAAGTCATTAGAGATGCTTTGCCCTTAATGTAACCAT
ATCGCAATAAGTTATGTTTTTAAATTGAGGGCATTATTATGAAAAAAATACCCTGCGTGATGATGCGAGGTGGAACCTCG
AGGGGCGCGTTCCTGTTAGCGGAACATTTACCCGAAGATCAAACGCAGCGCGATAAAATATTGATGGCAATTATGGGTTC
CGGTAACGATCTGGAAATTGACGGTATTGGCGGCGGTAATCCACTGACCAGTAAAGTCGCCATTATTAGCCGTTCCAGCG
ATCCGCGTGCTGATGTCGATTATCTGTTTGCGCAGGTAATCGTTCATGAGCAACGTGTCGATACCACGCCTAACTGCGGC
AATATGCTGTCTGGTGTTGGGGCATTCGCCATTGAAAATGGTTTGATTGCAGCGACTTCGCCAGTTACTCGCGTACGTAT
CCGCAACGTCAATACGGGTACGTTCATCGAAGCTGATGTGCAAACGCCAAATGGTGTTGTCGAGTACGAGGGTAGCGCCA
GAATTGACGGCGTACCGGGTACTGCCGCACCGGTTGCGCTCACTTTCCTGAATGCCGCTGGAACCAAAACCGGAAAAGTT
TTCCCGACTGATAATCAGATTGATTATTTTGACGATGTCCCGGTGACCTGTATCGATATGGCGATGCCAGTCGTCATTAT
TCCGGCTGAATATCTGGGAAAAACAGGTTATGAATTACCGGCGGAACTGGATGCCGACAAAGCATTATTAGCCCGCATTG
AATCTATCCGTCTACAAGCGGGTAAAGCAATGGGCTTAGGTGATGTCAGTAATATGGTTATCCCTAAACCTGTGCTTATT
TCTCCAGCGCAGAAAGGCGGGGCAATTAATGTGCGTTATTTTATGCCGCATTCTTGCCATCGCGCGCTGGCGATAACCGG
TGCTATTGCTATTTCCAGTAGTTGTGCATTGGAAGGCACCGTCACCCGACAAATCGTCCCTTCTGTAGGATACGGCAATA
TCAATATTGAACACCCCAGTGGTGCGCTCGACGTTCATTTAAGTAATGAAGGTCAGGATGCCACGACGTTACGCGCATCT
GTTATTCGGACGACCAGAAAAATATTTTCCGGTGAAGTTTATCTTCCCTGAAAAAATTCGTTGTCAGGATAAGGACAATC

AATAAAGGACTTCTGTATGAGTCATACAGAAAGAACAGGATTTTAAATGAATAAGAAATCGTTATGGAAGCTAATTCTGA
TATTAGCGATCCCATGTATTATTGGTTTTATGCCAGCTCCGGCAGGATTAAGCGAACTGGCGTGGGTGCTTTTTGGTATT
TACCTGGCGGCCATTGTGGGGCTGGTTATCAAGCCTTTCCCGGAACCTGTCGTACTGTTAATTGCCGTTGCTGCCTCAAT
GGTGGTGGTCGGTAACTTATCCGACGGTGCGTTTAAAACCACCGCCGTATTAAGCGGTTACTCTTCAGGTACCACCTGGC
TGGTGTTCTCGGCGTTTACCTTAAGCGCCGCATTTGTGACCACCGGTTTAGGTAAACGTATTGCCTATCTGCTGATTGGT
AAAATCGGTAACACCACGCTGGGTCTGGGTTACGTTACGGTATTCCTCGATCTGGTACTGGCTCCGGCAACACCGTCTAA
CACCGCGCGTGCGGGCGGCATTGTGTTACCGATCATCAACAGCGTGGCGGTGGCTTTGGGGTCCGAACCGGAAAAAAGTC
CGCGTCGTGTCGGACATTACCTGATGATGTCCATTTACATGGTCACCAAAACCACCAGCTATATGTTCTTTACCGCAATG
GCGGGGAACATTCTGGCGCTGAAAATGATCAACGACATTCTGCACCTGCAAATTAGCTGGGGTGGATGGGCGCTGGCAGC
CGGATTGCCGGGCATCATTATGCTGCTGGTCACCCCGCTGGTGATTTACACCATGTATCCACCAGAAATTAAGAAGGTGG
ATAACAAAACCATCGCTAAAGCGGGCCTTGCCGAACTAGGACCGATGAAAATCCGCGAAAAAATGCTGCTCGGTGTCTTT
GTGCTGGCGCTGCTGGGCTGGATTTTCAGTAAGTCTCTGGGGGTTGATGAATCCACCGTGGCAATCGTTGTTATGGCAAC
CATGCTGCTGCTGGGTATCGTTACCTGGGAAGACGTGGTTAAAAATAAAGGCGGCTGGAATACCTTAATCTGGTACGGCG
GTATTATCGGCTTAAGCTCCTTATTATCGAAAGTTAAATTCTTCGAATGGTTAGCTGAAGTCTTTAAAAATAACCTGGCA
TTTGATGGTCACGGTAACGTTGCTTTCTTCGTTATTATTTTCCTCAGCATTATCGTGCGTTATTTCTTCGCTTCCGGTAG
TGCCTATATCGTTGCTATGTTACCGGTATTTGCCATGCTGGCGAACGTCTCCGGCGCACCGTTAATGTTAACCGCGCTGG
CACTGTTGTTCTCCAACTCCTATGGCGGCATGGTTACTCACTATGGCGGCGCGGCAGGTCCGGTCATCTTTGGCGTGGGT
TATAACGATATTAAATCCTGGTGGTTGGTCGGTGCGGTACTGACGATATTAACCTTCCTGGTGCATATCACCCTCGGCGT
GTGGTGGTGGAATATGCTGATCGGCTGGAACATGCTGTAAATATTCTCGTCATACTTCAAGTTGCATGTGCTGCGTCTGC
GTTCGCTCACCCCAGTCACTTACTTATGTAAGCTCCTGGGGATTCACTCGCTTGTCGCCTTCCTGCAACTCGAATTATTT
AGAGTATATCCATTTATTATCTTTCTGCGCACTTCACGGTGCGCAGATATCTGGAGCATTTGATGATCAAGTTATCTGAA
AAAGGCGTGTTTCTCGCCAGTAATAACGAAATAATTGCCGAAGAACATTTCACCGGCGAAATTAAAAAAGAAGAAGCCAA
AAAAGGCACTATTGCCTGGTCTATTCTCTCTTCGCATAATACGTCCGGAAATATGGATAAACTTAAAATTAAGTTTGATT
CATTAGCCTCTCACGATATTACCTTTGTTGGTATTGTACAGACCGCTAAAGCGTCCGGTATGGAACGTTTCCCGCTGCCG
TATGTGCTGACCAACTGCCATAACTCACTCTGCGCCGTCGGCGGCACTATTAACGGTGATGACCATGTTTTTGGTTTATC
GGCGGCCCAGCGTTATGGCGGTATTTTTGTGCCTCCGCATATTGCGGTCATCCATCAATATATGCGTGAGATGATGGCAG
GCGGCGGCAAAATGATCCTCGGGTCAGACAGCCACACCCGTTACGGTGCATTAGGGACAATGGCAGTCGGTGAGGGCGGC
GGTGAGTTGGTAAAACAGCTGCTTAATGACACCTGGGATATCGACTATCCGGGCGTGGTTGCGGTGCATCTGACCGGAAA
ACCTGCGCCGTATGTGGGGCCACAGGATGTGGCGCTGGCTATCATTGGCGCGGTGTTCAAAAACGGTTACGTCAAAAACA
AAGTCATGGAGTTCGTTGGACCGGGCGTTAGCGCGCTCTCTACCGATTTCCGTAACAGCGTTGACGTGATGACCACTGAA
ACGACCTGTTTAAGTTCTGTCTGGCAAACCGATGAAGAAGTCCATAACTGGCTGGCGCTGCACGGTCGCGGCCAGGATTA
CTGCCAGCTTAACCCTCAACCGATGGCGTACTACGATGGCTGCATCAGCGTTGATTTAAGCGCCATCAAACCAATGATTG
CGCTGCCGTTCCACCCGAGCAACGTGTATGAAATCGACACACTGAACCAGAACCTGACCGACATTCTGCGTGAGATTGAA
ATTGAGTCCGAACGCGTGGCGCACGGTAAAGCCAAACTCTCGCTGCTGGATAAAGTGGAAAATGGTCGCCTGAAAGTGCA
GCAGGGGATTATCGCGGGCTGTTCTGGCGGTAACTACGAAAACGTCATCGCGGCGGCGAATGCACTGCGCGGTCAATCCT
GTGGCAATGACACCTTCTCGCTGGCAGTTTACCCGTCATCACAGCCGGTGTTTATGGATCTCGCCAAAAAAGGTGTGGTA
GCAGATTTGATTGGCGCAGGCGCAATCATCAGAACCGCGTTCTGCGGCCCATGCTTTGGCGCGGGCGATACGCCAATCAA
CAACGGTTTGAGTATTCGCCACACCACGCGTAACTTCCCGAACCGCGAAGGCTCTAAGCCAGCTAATGGGCAGATGTCAG
CGGTGGCGTTGATGGACGCTCGTTCTATCGCTGCGACTGCGGCAAACGGTGGCTATTTAACCTCTGCCAGCGAACTTGAT
TGCTGGGACAACGTGCCGGAGTACGCCTTCGATGTAACGCCGTATAAAAACCGTGTTTATCAGGGCTTTGTGAAAGGGGC
AACTCAGCAACCGCTGATTTACGGGCCGAACATTAAAGACTGGCCGGAATTGGGTGCGCTGACTGACAATATCGTCCTGA
AAGTGTGCTCGAAGATCCTCGACGAAGTGACCACCACCGACGAACTGATTCCTTCCGGTGAAACCTCTTCTTATCGTTCA
AATCCGATTGGTCTGGCGGAGTTTACCCTGTCTCGCCGCGATCCCGGTTATGTTAGCAGAAGTAAAGCGACTGCTGAGCT
GGAAAATCAGCGTCTGGCGGGGAATGTCAGCGAGCTGACAGAGGTGTTTGCGCGCATTAAGCAGATTGCTGGTCAGGAGC
ATATTGATCCGCTGCAAACTGAAATTGGCAGCATGGTCTATGCGGTGAAACCAGGCGATGGTTCTGCGCGTGAACAGGCG
GCGAGCTGCCAGCGTGTGATTGGCGGTCTGGCGAATATTGCCGAGGAGTACGCGACTAAACGCTATCGTTCTAACGTCAT
CAACTGGGGGATGTTACCGCTGCAAATGGCGGAAGTACCAACCTTTGAAGTGGGGGATTACATTTACATCCCTGGCATTA
AAGCGGCGCTGGATAATCCGGGTACGACGTTTAAAGGTTATGTGATCCATGAAGATGCGCCGGTAACGGAAATTACGCTC
TATATGGAAAGTCTGACTGCTGAAGAGCGCGAGATTATCAAGGCGGGTAGTTTGATTAACTTCAATAAAAACCGTCAGAT
GTAAAAAGCGCCATGTGAATGTAGGTCGCATTCGGCACTTATTGTCGGATGCGATGCTTGCGCATCTTATCCGACCTACG
AATCGCATCGAATCTGTAGGCCAGATAAGGCATTTTCGCAGCATCCGGCACTTATTGTCGGATGCGATGCTTGCGCATCT
TATCCGACCTACAAATCGCATCGAACCGTAGGCCGGATAAGGCGTTTACGCCGCATCCGGCAAATAGTTAATTGCTCTTA
CTTCTTCGCCTCTGCAACCACTTTACTACCCACGCCGCGGTTATTGTATTCCCACATGCGGTTGTAGTTAGTGTCATTCA
GATTGCGCTGTATTTCGTCGTTATCATCTACGCTGCCGGTATTACCCGCAAACGGACGATTAGAGATCACCGCATCGGCC
CACGGTTTAGCCGTGTTAAAACCTTCGTTGATGGCGCTATCACGGATCACCACCTGACCGTTGGTATTGGCATCAACATC
CAGCGAGCGGCCCAGTTGCGCCACACCATCACCGAAAGCATTGAAACGGCTGTTTACGGCGAGGAAACCGTAGTAAATGT

TGGACAGCGTAGCCGGTGCAAACACATACGCTTCTTGCTGAGTACGTGAGTTCACCACGCGGAATTCGGTGTTATCGAAC
ACCACTGCGCCGCGACCAGAAACGATATCCACATCCCCTTCAATGTAGCTGTTGGTCACCAGCGTACGCGGCTGACGATT
CGTTTCCAGACGGTTCTGCACACCGCTGTTGGTGACAAAGAAGGTGTTCTGACGACCGAGAATGTTAACGTTGTTAATCT
GTACCTGGTCACCATCAGTACGCAGTGCCACCGCCGGATGGTTACCTGCATCTACGCTATCGCCCAGCGTGTTTTCGATG
GTCAGATTTTGCAGTTGCAGGCCATTGTTTTGTGACCAGAAGACCGCAGAGCAGAGAACACCGATACTGTCGCTGCGTTT
GCTCTGGCAGCTATCGTACATATACCACGCTGGTTTACCTGGCATATATTTGCCGCGCGGGTTGACGTCGTGACGCCAGT
CGGCAGGGCTCATGCCACCATCAAGGGAAAGCCCAATCTTCACATCAATCGGTTTTTCACCTGTACCGTACAGAGTAATT
CCACCCGGAGCGGCAGGGACATATACCGTTCCCTGATACTCACCAGGCATCACGGCAATATACTGGCGCTTGTTGGTACG
CTTGATAATTGCCGCATCTACCGCCGCCTGAATCGTGGTATGCGTTACACCTTGAGTGCCCGCCGGGCCGACAACAAAGT
CAGGTTGCGCAGGCAGGGTAATCGGGGAAGGATTCCACGCTGCAGCACCTGGTGTCAGGGATGCAAAATAGTGTTGAGCA
TCGAAATTCTGCGCTTCTTTTGCCGACAGAATCGGGCGAGAAGAGGTACCAGGCGCGGTTTGATCAGAAGGACGTTGATC
GGGCGGGGTTGAGCTACAGGCGGTCAGCGTCACGCCAAAAGCCAATGCCAGCGCCAGACGGGAAACTGAAAATGTGTTCA
CAGGTTGCTCCGGGCTATGAAATAGAAAAATGAATCCGTTGAAGCCTGCTTTTTTATACTAACTTGAGCGAAACGGGAAG
GTAAAAAGACAAAAAGTTGTTTTTAATACCTTTAAGTGATACCAGATGGCATTGCGCCATCTGGCAGAGTGATTAACTAA
ACATCGCAGTAATCGAGGCGCTTGCCAGAGAGTGGAAATGAACGTTAAACCCGACCATCGCGCCGCTGGCACCTTCATCG
ACATCAATACGTTCTATATCCAGCGCGTGAACGGTAAAAATGTAGCGATGAGTTTCGCCTTTCGGCGGTGCTGCGCCATC
GTACCCGGTTTTACCAAAGTCGGTACGCGTCTGCAAAACGCCGTCTGGCATTGCTACCAGACCAGAGCCAAACCCTTGCG
GTAATACGCGGGTATCAGCGGGTAAGTTAACAACTACCCAGTGCCACCAGCCGGAGCCGGTTGGCGCATCCGGGTCGTAG
CAGGTGACAACAAAACTTTTCGTTCCCGCAGGAACATCATCCCACGCCAGATGCGGTGAAATATTATCGCCATCGTAACC
CATGCCGTTAAAGACATGACGATGCGGCAATTTATCGCCATCGCGCAGATCGTTACTGATGAGTTTCATGAACCCTCCTT
TCTTGTTTGCAGAAAGTGTAGCCAGAAACCCTCACGCGGACTTCTCGTTATTGGCAAAAAAATGTTTCATCCTGTACCGC
GCGGTTAACCGCTGCGGTCAGACGCTGCAACTGTTGCGGGAGAATAATATAGGGCGGCATCAGGTAAATCAGTTTGCCAA
AAGGCCGGATCCAGACACCCTGTTCGACAAAGAATTTTTGCAGCGCCGCCATATTCACCGGATGAGTGGTTTCGACCACG
CCAATGGCCCCCAGTACGCGCACATCGGCAACCATTTCGGCATCACGGGCGGGGGCAAGTTGCTCGCGCAGCTGTACTTC
AATATCCGCCACCTGTTGCTGCCAGTCGCCAGATTCGAGAATCGCCAGGCTGGCGTTTGCTGCCGCGCAGGCCAGCGGAT
TGCCCATAAAAGTTGGCCCATGCATAAAGCAACCGGCTTCACCGTTACTGATGGTTTCTGCAACCTCGCGCGTGGTGAGT
GTGGCGGAAAGGGTCATTGTGCCGCCGGTTAAGGCTTTACCGAGGCACAAAATGTCCGGCGCGATTTCTGCATGTTCACA
GGCAAACAGTTTCCCGGTACGACCAAATCCAGTGGCGATCTCGTCGGCAATCAGCAAGATACCTTCGCGATCGCATATTT
TGCGGATTCGTTTTAACCATTCCGGATGGTACATGCGCATCCCGCCTGCGCCCTGGACAATCGGCTCAATGATCACCGCC
GCGATTTCATGACGATGCGCCGCCATCAGGCGGGCAAAGCCCACCATATCGCGCTCATCCCATTCGCCATCCATGCGGCT
TTGCGGGGCGGGAGCAAACAGGTTTTCTGGCAGGTAGCCTTTCCACAGACTGTGCATTGAGTTATCCGGATCGCACACCG
ACATCGCGCCAAAGGTATCGCCATGATAACCATTGCGGAAGGTCAGAAAACGCTGGCGCGCTTCGCCTTTGGCTTGCCAG
TACTGCAACGCCATTTTCATCGCCACTTCCACCGCTACGGAACCGGAGTCCGCGAGAAAAACGCACTCCAGCGGTTGCGG
CGTCATCGCCACCAGTTTGCGGCACAGCTCAATGGCTGGCGCATGGGTGATACCGCCAAACATCACATGCGACATGGCAT
CAATTTGCGACTTCATCGCCGCATTAAGCTGCGGGTGATTGTAGCCGTGGATCGCCGCCCACCAGGACGACATACCGTCA
ACCAGGCGTCTGCCGTCAGACAAAATCAGCTCGCAACCTTCGGCGCTCACCACCGGATAAACCGGCAGAGGGGAGGTCAT
GGATGTGTATGGGTGCCAGATATGGCGTTGGTCAAAGGCAAGATCGTCCGTTGTCATAATCGACTTGTAAACCAAATTGA
AAAGATTTAGGTTTACAAGTCTACACCGAATTAACAACAAAAAACACGTTTTGGAGAAGCCCCATGGCTCACCGCCCACG
CTGGACATTGTCGCAAGTCACAGAATTATTTGAAAAACCGTTGCTGGATCTGCTGTTTGAAGCGCAGCAGGTGCATCGCC
AGCATTTCGATCCTCGTCAGGTGCAGGTCAGCACGTTGCTGTCGATTAAGACCGGAGCTTGTCCGGAAGATTGCAAATAC
TGCCCGCAAAGCTCGCGCTACAAAACCGGGCTGGAAGCCGAGCGGTTGATGGAAGTTGAACAGGTGCTGGAGTCGGCGCG
CAAAGCGAAAGCGGCAGGATCGACGCGCTTCTGTATGGGCGCGGCGTGGAAGAATCCCCACGAACGCGATATGCCGTACC
TGGAACAAATGGTGCAGGGGGTAAAAGCGATGGGGCTGGAGGCGTGTATGACGCTGGGCACGTTGAGTGAATCTCAGGCG
CAGCGCCTCGCGAACGCCGGGCTGGATTACTACAACCACAACCTGGACACCTCGCCGGAGTTTTACGGCAATATCATCAC
CACACGCACTTATCAGGAACGCCTCGATACGCTGGAAAAAGTGCGCGATGCCGGGATCAAAGTCTGTTCTGGCGGCATTG
TGGGCTTAGGCGAAACGGTAAAAGATCGCGCCGGATTATTGCTGCAACTGGCAAACCTGCCGACGCCGCCGGAAAGCGTG
CCAATCAACATGCTGGTGAAGGTGAAAGGCACGCCGCTTGCCGATAACGATGATGTCGATGCCTTTGATTTTATTCGCAC
CATTGCGGTCGCGCGGATCATGATGCCAACCTCTTACGTGCGCCTTTCTGCCGGACGCGAGCAGATGAACGAACAGACTC
AGGCGATGTGCTTTATGGCAGGCGCAAACTCGATTTTCTACGGTTGCAAACTGCTGACCACGCCGAATCCGGAAGAAGAT
AAAGACCTGCAACTGTTCCGCAAACTGGGGCTAAATCCGCAGCAAACTGCCGTGCTGGCAGGGGATAACGAACAACAGCA
ACGTCTTGAACAGGCGCTGATGACCCCGGACACCGACGAATATTACAACGCGGCAGCATTATGAGCTGGCAGGAGAAAAT
CAACGCGGCGCTCGATGCGCGGCGTGCTGCCGATGCCCTGCGTCGCCGTTATCCGGTGGCGCAAGGAGCCGGACGCTGGC
TGGTGGCGGATGATCGCCAGTATCTGAACTTTTCCAGTAACGATTATTTAGGTTTAAGCCATCATCCGCAAATTATCCGT
GCCTGGCAGCAGGGGGCGGAGCAATTTGGCATCGGTAGCGGCGGCTCCGGTCACGTCAGCGGTTATAGCGTGGTGCATCA
GGCACTGGAAGAAGAGCTGGCCGAGTGGCTTGGCTATTCGCGGGCACTGCTGTTTATCTCTGGTTTCGCCGCTAATCAGG
CAGTTATTGCCGCGATGATGGCGAAAGAGGACCGTATTGCTGCCGACCGGCTTAGCCATGCCTCATTGCTGGAAGCTGCC

AGTTTAAGCCCGTCGCAGCTTCGCCGTTTTGCTCATAACGATGTCACTCATTTGGCGCGATTGCTTGCTTCCCCCTGTCC
GGGGCAGCAAATGGTGGTGACAGAAGGCGTGTTCAGCATGGACGGCGATAGTGCGCCACTGGCGGAAATCCAGCAGGTAA
CGCAACAGCACAATGGCTGGTTGATGGTCGATGATGCCCACGGCACGGGCGTTATCGGGGAGCAGGGGCGCGGCAGCTGC
TGGCTGCAAAAGGTAAAACCAGAATTGCTGGTAGTGACTTTTGGCAAAGGATTTGGCGTCAGCGGGGCAGCGGTGCTTTG
CTCCAGTACGGTGGCGGATTATCTGCTGCAATTCGCCCGCCACCTTATCTACAGCACCAGTATGCCGCCCGCTCAGGCGC
AGGCATTACGTGCGTCGCTGGCGGTCATTCGCAGTGATGAGGGTGATGCACGGCGCGAAAAACTGGCGGCACTCATTACG
CGTTTTCGTGCCGGAGTACAGGATTTGCCGTTTACGCTTGCTGATTCATGCAGCGCCATCCAGCCATTGATTGTCGGTGA
TAACAGCCGTGCGTTACAACTGGCAGAAAAACTGCGTCAGCAAGGCTGCTGGGTCACGGCGATTCGCCCGCCAACCGTAC
CCGCTGGTACTGCGCGACTGCGCTTAACGCTAACCGCTGCGCATGAAATGCAGGATATCGACCGTCTGCTGGAGGTGCTG
CATGGCAACGGTTAATAAACAAGCCATTGCAGCGGCATTTGGTCGGGCAGCCGCACACTATGAGCAACATGCAGATCTAC
AGCGCCAGAGTGCTGACGCCTTACTGGCAATGCTTCCACAGCGTAAATACACCCACGTACTGGACGCGGGTTGTGGACCT
GGCTGGATGAGCCGCCACTGGCGGGAACGTCACGCGCAGGTGACGGCCTTAGATCTCTCGCCGCCAATGCTTGTTCAGGC
ACGCCAGAAGGATGCCGCAGACCATTATCTGGCGGGAGATATCGAATCCCTGCCGTTAGCGACTGCGACGTTCGATCTTG
CATGGAGCAATCTCGCAGTGCAGTGGTGCGGTAATTTATCCACGGCACTCCGCGAGCTGTATCGGGTGGTGCGCCCCAAA
GGCGTGGTCGCGTTTACCACGCTGGTGCAGGGATCGTTACCCGAACTGCATCAGGCGTGGCAGGCGGTGGACGAGCGTCC
GCATGCTAATCGCTTTTTACCGCCAGATGAAATCGAACAGTCGCTGAACGGCGTGCATTATCAACATCATATTCAGCCCA
TCACGCTGTGGTTTGATGATGCGCTCAGTGCCATGCGTTCGCTGAAAGGCATCGGTGCCACGCATCTTCATGAAGGGCGC
GACCCGCGAATATTAACGCGTTCGCAGTTGCAGCGATTGCAACTGGCCTGGCCGCAACAGCAGGGGCGATATCCTCTGAC
GTATCATCTTTTTTTGGGAGTGATTGCTCGTGAGTAAACGTTATTTTGTCACCGGAACGGATACCGAAGTGGGGAAAACT
GTCGCCAGTTGTGCACTTTTACAAGCCGCAAAGGCAGCAGGCTACCGGACGGCAGGTTATAAACCGGTCGCCTCTGGCAG
CGAAAAGACCCCGGAAGGTTTACGCAATAGCGACGCGCTGGCGTTACAGCGCAACAGCAGCCTGCAGCTGGATTACGCAA
CAGTAAATCCTTACACCTTCGCAGAACCCACTTCGCCGCACATCATCAGCGCGCAAGAGGGCAGACCGATAGAATCATTG
GTAATGAGCGCCGGATTACGCGCGCTTGAACAACAGGCTGACTGGGTGTTAGTGGAAGGTGCTGGCGGCTGGTTTACGCC
GCTTTCTGACACTTTCACTTTTGCAGATTGGGTAACACAGGAACAACTGCCGGTGATACTGGTAGTTGGTGTGAAACTCG
GCTGTATTAATCACGCGATGTTGACTGCACAGGTAATACAACACGCCGGACTGACTCTGGCGGGTTGGGTGGCGAACGAT
GTTACGCCTCCGGGAAAACGTCACGCTGAATATATGACCACGCTCACCCGCATGATTCCCGCGCCGCTGCTGGGAGAGAT
CCCCTGGCTTGCAGAAAATCCAGAAAATGCGGCAACCGGAAAGTACATAAACCTTGCCTTGTTGTAGCCATTCTGTATTT
GGTTAAATTGCGAGCGAGATCGCGTCTTCGATTGACTGCAATTTAACCAATTAAATTCTAAAATAATCACGAAAAAAATT
TTACTTCCGCCTCATGCGGCGAATGTGGGAATTGCCCAGGCGGCGGGGGATAGGGGCTGGAGACAGTTATCCACTATTCC
TGTGGATAACCATGTGTATTAGAGTTAGAAAACACGAGGCAAGCGAGAGAATACGCGGCTTGCACGCGAATTGGCGTTAA
AGACGGCTCAAAGAAATATCTTTTATTTTTTAACTGGTTAGATAAATGCAATGGCAGTCACTGAACAGGCATCTCTTGCC
ATAAAACTGTCATCACTCATCTTGACAAATGTTAAAAAAGCCGTTGCTTTGGGGATAACCCGGTAAGGCCGGAGTTTTAT
CTCGCCACAGAGTAAATTTTGCTCATGATTGACAGCGGAGTTTACGCTGTATCAGAAATATTATGGTGATGAACTGTTTT
TTTATCCAGTATAATTTGTTGGCATAATTAAGTACGACGAGTAAAATTACATACCTGCCCGCCCAACTCCTTCAGGTAGC
GACTCATGAGTAAACCGTTCAAACTGAATTCCGCTTTTAAACCTTCTGGCGATCAGCCAGAGGCGATTCGACGTCTCGAA
GAGGGGCTGGAAGATGGCCTGGCGCACCAGACGTTACTTGGCGTGACTGGCTCAGGGAAAACCTTCACCATTGCCAATGT
CATTGCTGACCTTCAGCGCCCAACCATGGTACTTGCGCCCAACAAAACGCTGGCGGCCCAGCTGTATGGCGAAATGAAAG
AGTTCTTCCCGGAAAACGCGGTGGAATATTTCGTTTCCTACTACGACTACTATCAGCCGGAAGCCTATGTACCGAGTTCC
GACACTTTCATTGAGAAAGATGCCTCGGTTAACGAACATATTGAGCAGATGCGTTTGTCCGCCACCAAAGCGATGCTGGA
GCGGCGTGATGTGGTTGTGGTGGCGTCTGTTTCCGCGATTTATGGTCTGGGCGATCCTGATTTATATCTCAAGATGATGC
TCCATCTCACGGTCGGTATGATTATCGATCAGCGCGCGATTCTGCGCCGACTGGCGGAGCTGCAATACGCTCGTAATGAT
CAAGCATTCCAGCGTGGTACTTTCCGCGTTCGTGGCGAGGTGATAGATATCTTCCCGGCAGAATCGGATGACATTGCACT
TCGCGTGGAACTGTTTGACGAGGAAGTGGAACGATTGTCGTTATTTGACCCGCTGACCGGGCAGATTGTTTCCACTATTC
CACGTTTTACCATCTACCCGAAAACGCACTACGTCACACCGCGCGAGCGCATCGTACAGGCGATGGAGGAGATCAAAGAA
GAGCTGGCCGCCAGACGCAAAGTGCTGTTGGAAAACAACAAACTGCTGGAAGAGCAGCGGCTGACCCAGCGTACCCAGTT
TGATCTGGAGATGATGAACGAGCTGGGCTACTGTTCGGGGATTGAAAACTACTCGCGCTTCCTCTCCGGTCGTGGACCGG
GTGAGCCACCGCCGACGCTGTTTGATTACCTGCCTGCCGATGGGCTGCTGGTCGTCGATGAATCTCACGTCACCATTCCA
CAAATTGGCGGCATGTATCGCGGTGACCGGGCGCGTAAAGAGACACTGGTGGAGTACGGCTTCCGCCTGCCATCAGCGCT
GGATAACCGTCCGCTTAAGTTTGAAGAGTTCGAAGCATTAGCGCCGCAAACCATCTATGTTTCGGCGACGCCGGGTAATT
ACGAGCTGGAAAAATCCGGCGGCGATGTGGTGGATCAGGTGGTGCGTCCAACCGGATTGCTTGACCCGATTATCGAAGTG
CGGCCGGTGGCGACACAGGTTGATGATCTTCTTTCGGAGATTCGTCAGCGAGCGGCAATTAACGAACGCGTACTGGTCAC
CACACTGACCAAGCGGATGGCGGAAGATCTTACCGAATATCTCGAAGAACATGGCGAGCGCGTGCGTTATCTTCACTCAG
ATATCGACACCGTCGAACGTATGGAGATTATCCGCGACTTGCGTCTGGGTGAGTTCGACGTGCTGGTAGGGATCAACTTA
CTGCGCGAAGGTCTGGATATGCCGGAAGTGTCGCTGGTGGCGATCCTCGACGCTGACAAAGAAGGCTTCCTGCGTTCCGA
ACGTTCGTTGATCCAGACCATTGGTCGTGCGGCACGTAACGTTAACGGTAAAGCGATTCTCTACGGCGATAAGATCACCC
CATCAATGGCGAAAGCGATTGGCGAAACCGAACGTCGCCGTGAGAAACAGCAGAAGTACAACGAGGAACACGGAATTACG

CCGCAAGGCTTGAACAAGAAAGTGGTCGATATCCTGGCGCTGGGGCAGAACATTGCCAAAACCAAAGCGAAGGGCAGAGG
AAAATCGCGCCCGATTGTTGAGCCGGATAATGTGCCGATGGATATGTCGCCTAAAGCGTTGCAGCAGAAAATCCATGAGC
TGGAAGGGTTGATGATGCAACACGCGCAGAATCTGGAGTTCGAAGAAGCGGCGCAAATTCGTGACCAGTTGCATCAGCTG
CGTGAGCTGTTTATCGCGGCATCGTAACAGGATAGCGAAGAAGACTGATGACAAACGGAAAACAGCCTGATGCGCTACGC
TTATCAGGCCTACATTTTCTCCGCAATATATTGAATTTGCGCGGTTTGTAGGCCGGATAAGGCGTTCACGCCGCATCCGG
CATAAACAACGCGCACTTTGTCTTCAATAGGAAGCCGGAATTTTCCTTCGGATTTCCGTTAACCTAAAGCCTGTAACGCC
TTTTCCAGCGCGTTATGTAACAACTGGCGGTCATGACGATACGGAATATCGCTGGCCTCCAGTACCTCCTGGATCACAAT
CCGCTCTTTCACCGCCGAGACATCCACTTTTGGCCCGACGATGACCGCATCAATGACCTTTTTACCAACATACTGCTCCA
TAATTGCCAGCTTGCTTTCCAGCTTCAAATTAGCCGCAGGTAAACTCAACTCACGCCCCAGATTGCCGATATAAACCATC
GGCGCTGGCGTGCGGCGTAATGCCTGGGCGATTTCCTTCAGCAGCAGAATTGGCATCAGGCTGGTATAAAAACTGCCAGG
CCCAATAATGATGAGATCCGCTTCATTGATAGCGTGAACCGCCTCACGCGTTGCGGGTACATTAGGCGTTAACAATAACT
CTTGAATCGGCGTAGTTAACTGGTCGATATTGACCTCGCCGTAAACTTCATGCCCCTGATCGTCAATCGCCATCAGATCA
ACAGGATGCTCTGACATTGGAATCAAATGCGTATCCACTTTCAGCAGATTACGAATTAAATTGATGGCTTCCAGAGGCCG
CACGCTAAGGTGATCCAGCGCCTTTAACATCAAGTTTCCGAGATTATGACCGGAAAGTTCGCCATTGCCACCAAAACGGT
ATTCAAACATCGCGGAGGCGACGCTCGGTTCCGTTATCAGCTGGTTGAGGCAGTTGCGCATATCGCCCCAGGCAATGCCG
CCTTCTGAACGGCGAATACGCCCCGTCGAGCCACCATTATCGGTGGTGGTGACGATACCCGTTAAACGAGAACCCAAAGA
CGAAAGTGATGAGAGAACGCGTCCCAGTCCATGCCCTCCGCCGAGAGCAACGACACGATCAAGATCAGCCAGCGTACGAT
TGCGCATATAGTTTCCTGGAGTCAGATTATCCGCGCTACAGTAGCGCAAATTGCGGGGAAACAGCAATTAACCTGCCAAT
ATTGAGGATACAAACTATTTTCTTCTACCTCTAAAGGACGATGCACGCTATGCCTCCCTGATGATGTATATCAAAGTAAA
ACCGCCATTTTCCCTTATTCTGTAGCGAAATAGCACGATCATGACGCTATATACATGATTACATAGCGAAAGTGTGGATG
GTAAAAATCTCATTTACACGCTAGTATCGGCATAACCACTAAACACTCTAGCCTCTGCACCTGGGTCAACTGATACGGTG
CTTTGGCCGTGACAATGCTCGTAAAGATTGCCACCAGGGCGAAGGAAGAAATGACTTCGCCTCCCGTATCTGGAAAGGTG
TACATGGCTTCACAACTGACTGATGCATTTGCGCGTAAGTTTTACTACTTGCGCCTGTCGATTACCGATGTGTGTAACTT
TCGTTGCACCTACTGCCTGCCGGATGGCTACAAACCGAGCGGCGTCACCAATAAAGGCTTTCTTACCGTCGATGAAATTC
GCCGGGTTACGCGCGCCTTCGCCAGACTGGGCACCGAAAAAGTGCGCCTGACAGGAGGAGAGCCGTCTTTACGCCGCGAC
TTTACCGATATCATCGCCGCTGTGCGGGAAAACGACGCTATCCGCCAGATTGCGGTCACAACCAATGGTTACCGTCTGGA
ACGCGATGTGGCGAGCTGGCGCGATGCGGGACTTACTGGCATTAACGTCAGTGTCGACAGTCTGGACGCCCGCCAGTTTC
ACGCTATTACCGGGCAGGATAAATTCAACCAGGTCATGGCAGGGATTGATGCTGCATTTGAGGCCGGTTTTGAGAAGGTC
AAAGTCAATACCGTGCTGATGCGTGATGTTAATCATCACCAGCTCGACACCTTTCTGAACTGGATCCAGCATCGCCCTAT
CCAGCTGCGTTTCATCGAACTGATGGAAACGGGCGAGGGCAGCGAGCTCTTCCGTAAGCATCACATCTCTGGTCAGGTTC
TGCGTGACGAGCTACTGCGTCGCGGCTGGATCCACCAATTACGTCAACGCAGCGACGGTCCCGCGCAAGTCTTTTGCCAT
CCAGATTACGCCGGAGAGATTGGCCTTATCATGCCGTATGAAAAAGACTTCTGCGCCACTTGCAACCGCCTGCGCGTTTC
CTCCATTGGTAAACTCCATCTCTGCCTGTTTGGTGAAGGCGGCGTTAACCTGCGCGATCTGCTGGAAGACGATACCCAGC
AACAGGCGCTGGAAGCGCGTATTTCAGCGGCGCTGCGGGAGAAGAAACAGACCCATTTCCTGCATCAAAACAACACCGGT
ATTACGCAAAACTTATCGTACATTGGCGGCTAAAACGTCAAAAGGAGAGATCAGATGAGTCAGGTAAGCACTGAATTTAT
CCCGACCCGTATTGCTATTCTTACGGTTTCTAATCGTCGCGGTGAAGAAGACGATACCTCCGGTCACTATCTGCGCGATT
CGGCGCAAGAAGCGGGCCATCACGTTGTCGATAAAGCCATTGTGAAAGAAAACCGCTACGCTATTCGCGCTCAGGTATCT
GCGTGGATCGCCAGCGACGATGTACAAGTGGTATTGATTACGGGTGGTACTGGCCTGACGGAAGGTGATCAGGCTCCCGA
AGCATTGCTGCCGTTGTTCGACCGTGAAGTTGAAGGTTTTGGTGAAGTGTTCCGGATGTTGTCGTTTGAAGAGATTGGCA
CTTCCACGTTGCAATCTCGTGCGGTAGCGGGCGTCGCCAACAAAACGCTGATTTTCGCCATGCCGGGTTCGACCAAAGCG
TGCCGTACCGCATGGGAAAATATCATCGCGCCGCAGCTGGATGCCCGTACGCGTCCGTGTAATTTCCATCCACATTTGAA
GAAATAAGTATGTCGCAACTGACCCATATCAACGCCGCTGGCGAAGCGCACATGGTGGATGTCTCCGCCAAAGCGGAAAC
CGTGCGTGAAGCGCGGGCGGAAGCCTTTGTCACCATGCGCAGCGAGACGCTGGCGATGATTATTGATGGTCGCCACCACA
AAGGCGACGTATTTGCCACTGCGCGTATTGCCGGTATTCAGGCGGCAAAACGCACCTGGGATCTGATCCCGCTCTGTCAT
CCGCTGATGCTCAGCAAAGTTGAAGTCAATTTACAGGCCGAGCCGGAGCACAATCGGGTGCGTATAGAAACCTTATGCCG
CCTGACCGGGAAAACCGGTGTCGAAATGGAAGCATTAACCGCGGCCTCCGTGGCGGCGCTGACCATTTATGACATGTGCA
AAGCGGTGCAAAAAGATATGGTGATTGGTCCGGTACGTTTGCTGGCGAAGAGCGGCGGCAAGTCGGGTGACTTTAAGGTG
GAAGCGGATGATTAAAGTTCTTTTTTTCGCCCAGGTGCGCGAGTTGGTGGGAACAGATGCAACCGAAGTGGCTGCGGATT
TCCCAACTGTTGAAGCGTTACGCCAGCACATGGCTGCGCAGAGCGATCGCTGGGCGCTGGCGCTGGAAGATGGCAAATTA
CTGGCTGCCGTCAACCAGACGCTGGTGAGTTTTGACCATCCGCTGACTGACGGCGACGAAGTAGCTTTCTTCCCGCCGGT
AACCGGAGGTTAAGATGGCAGAAACCAAAATTGTTGTTGGTCCGCAGCCGTTCAGCGTAGGAGAAGAGTACCCGTGGCTG
GCGGAGCGTGACGAAGACGGTGCGGTAGTCACCTTTACTGGTAAGGTGCGCAACCATAACCTGGGCGACAGCGTCAACGC
ATTAACCCTCGAACACTATCCGGGGATGACTGAAAAAGCACTGGCAGAAATTGTTGATGAAGCGCGTAACCGCTGGCCGC
TGGGGCGCGTCACTGTGATTCACCGCATCGGGGAATTATGGCCGGGCGATGAAATCGTTTTTGTCGGTGTCACCAGTGCG
CATCGCAGCAGTGCGTTTGAAGCCGGGCAGTTTATTATGGATTATCTCAAAACCCGCGCACCGTTCTGGAAGCGCGAAGC
CACGCCGGAAGGCGACCGCTGGGTTGAAGCTCGGGAGAGCGATCAGCAGGCGGCAAAACGCTGGTAGTTTTTTGTTAGCC

GGATAAGGCACCGCTGCGTCCGGCAATTTCACCGCTCAGTACATACGTTTACGCTGCGTCGATGCACAGCCTCATCATTT
TGCAGTATCCTTAAGATATTCCTTATATCTTCAGGAGATCGTCATGGACAGATTCCCACGTTCTGATTCAATCGTACAAC
CCCGGGCTGGCTTGCAAACTTATATGGCTCAAGTCTATGGCTGGATGACCGTTGGCTTGTTGCTGACCGCATTTGTTGCC
TGGTATGCGGCTAATTCCGCGGCCGTGATGGAGCTGTTGTTCACTAACCGTGTCTTTTTAATCGGTCTGATCATCGCGCA
ATTAGCATTGGTTATTGTGTTATCAGCGATGATTCAAAAGCTGAGCGCAGGTGTAACGACGATGCTCTTTATGCTTTATT
CGGCGCTGACGGGTCTTACGCTTTCCAGTATATTCATTGTCTATACCGCTGCTTCTATCGCCAGTACTTTCGTCGTTACT
GCCGGGATGTTCGGCGCAATGAGCCTGTACGGTTACACCACGAAGCGCGATTTAAGTGGCTTTGGCAATATGCTGTTTAT
GGCGTTAATCGGCATTGTGCTGGCATCGCTGGTCAACTTCTGGTTGAAAAGCGAAGCATTGATGTGGGCAGTTACCTACA
TCGGCGTGATTGTCTTTGTCGGATTGACGGCGTATGACACGCAGAAACTGAAAAATATGGGTGAGCAGATTGATACCCGC
GACACGTCGAACCTGCGCAAATATTCCATTCTTGGCGCGTTAACCTTGTATCTGGACTTCATCAACCTGTTCCTGATGTT
GTTGCGGATCTTCGGCAACCGCCGTTAATTCTTTTTCACCATCACTATCCCGGCCATGTGTCGGGATTTTTTTCTTACTA
AAAACGACACTTAACAAGTACCAGGTATAAAATATGCCTTATGGACGAGCGGAAGCGTTTTCTGTATCCATAAATGCAAA
AGTATTTTGTATGCGTGGTTTTAAATTTATTGATGGTGAATATTAATATTTTTTCTAAGGATAACTAAAAGAATGGAATC
ATACTCGCAAAACAGCAATAAATTAGATTTTCAGCACGAGGCCAGGATATTAAACGGTATATGGCTCATTACCGCTTTAG
GTTTGGTGGCAACCGCAGGACTAGCCTGGGGAGCTAAGTATATCGAAATTACGGCAACCAAATATGATTCACCACCAATG
TATGTCGCCATAGGGTTATTATTACTTTGTATGTATGGCTTAAGTAAGGATATCAACAAGATAAATGCCGCCATCGCGGG
CGTAATATATCTGTTTTTACTCTCTTTGGTGGCGATTGTCGTTGCAAGTTTAGTTCCTGTATATGCCATTATCATCGTGT
TCAGCACTGCGGGCGCGATGTTTTTAATCAGTATGCTGGCCGGTTTATTATTTAATGTTGATCCTGGTTCTCACCGTTTT
ATCATTATGATGACGTTGACAGGGTTGGCCCTGGTAATCATCGTGAATGCGGCATTAATGAGTGAACGGCCCATTTGGAT
AATAAGTTGCTTAATGATTGTGTTATGGTCAGGCATTATCTCGCATGGACGAAATAAGCTCCTTGAATTGGCGGGGAAAT
GCCATAGTGAAGAGTTGTGGAGTCCGGTTCGTTGCGCTTTTACAGGTGCATTAACACTCTATTACTATTTTATCGGCTTC
TTTGGGATACTTGCCGCGATAGCTATAACGCTTGTCTGGCAAAGGCATACGCGTTTTTTTCATTAGCCGTAAATGCACAT
ACTAAAGAACTTAACTAAACTTCACATCGCCGCTTCATTTTTCGCCCGTAGCTTCTTCGCCTGGCTTTCCAGCAACAGGT
AACAGATCAGCGCCAGCAGCAGTGGGATAAAGTAATACAGCACACGGTAAGCGAGTAGGGCGGCGATAATTGTACCTTTG
GAGGTATGCTCCCCAGCCAGTAGTGCGATAAACACCGCTTCCAGCACACCGATCCCCGCCGGAATATGCACGATGACGCC
AGCAATACTACTAACCAGTAACACGCCCAGTACAAAGAAATAGTTCACGCTTTGACCAAGTAACAGCCAGATAATCGCCC
CCATTACCATCCAGTTAACGCTGGAAATCAGCATCTGAGCGAGGGCGAATTTCCATGAAGGCAGCACCAGCTTTTGCCCT
TTGATGGTCATATGGCGGTGCTTCGCGAAAGCGCAAAACCACAAATAAACCGCGATAATCATCAGTAAGCCAATGCCGAG
AATACGCAGCGTAGTTTGATCGACATACCAGTGATCCGGCAACTCCACCACGCCTGCGGTAAAGATAATCCCTGCCAGTA
AAATGTAGCCCAGCCAGTTGGTGGTAATACTGAGCGAGAAAATCCGCGTAATAGTGCTACCGGGCAACCCCAGACGAGAG
TACAAACGATAGCGCATACCAATGCCGCCGACCCAGGTACTGAGCGTCAGGTTGAAGGCGTAGCAGATAAACGACACCAG
CATCACCTGGCGCTTCGCCAGTTTGTGACCGCAGTAAAAGCGGGCGAGCAGGTCATAGCAGCCGTAAATCAGATAGCTGA
CGACCACCAGCCCGACCGCACTAAGCAGCGCAACGCGATTGTAGTCGCGGATGACCTTCCAGACCTCTTCCCAGTCCACT
TTTTTGGCGTAGACCACCAGCAACACAATCACCGCGATAAAAAACAGCCAGGTGAGGATCTTCTTTGCTAAGCGCCAGCG
CGGGTGTGATTTACTCATCAGGGTTTTACCCCCGTGTTTTCAGTTTCTACCCGATCCTGCGTTTCCATTGTCGGTTGTGC
GGGCGGATCAACCTGCGCCAGACGTGGCGTGTGTGCCGGAAGCCAGCCAACCAGCGCCGGGAAGTGGCGTAAAAAGTGGA
ACGCCAGCACGCTTTTGGTCAGGTTCCACCAGGTGCGTTTGGGCAGCATGGTTTCATCCACCTGCTGACAATCTGCGGCA
ATAATGCCGTTCAGATTATCGCGCAGCGTCTGGTTAAAATGACGATCGTGGATGATGACATTTGCTTCGAGATTCAGTGA
CAAACTGAGCGGATCGAGATTACTGGACCCTACTGTCGCCCAGTGATCGTCCATCAATGCCACTTTGCCGTGGAGCGGGC
GGCGGCGGTACTCAAAAACCTGAACGCCGCCTTTAACCAGATAGTTATACAGCAAGCGCGCACCGACTCTGACAATCGGC
ATATCCGGTTCGCCCTGAATGATCAGTTTGATCCGCACCCCGCGCCGTGCCGCTTTACGCAAGGCGTGTAAAAATCGATA
GCCGGGGAAGAAGTAGGCGTTGGCGATAATCACTTCCCGCCGCGCCTGAGTGAGCATTTTCAAATAATGGCGTTCAATAT
CATCGCGATGTTCTTCGTTATCGCGCCAGACCAGCAATACCTGCGCTTCTCCCGGCTGGCGGTTCTCTTCCGCTTTGTGA
TGACGTCGCCACCAGCGTCGTGCCGCGCTCTGTCCAGGCAGGTTTTCCAGCTCAAACTGGAGAATATCTTCGACAATCGG
CCCTTCAAGGCGTACCGCGTAATCCTGTTTAGCCTCTGGACCGTAGCTGGACATATGCTCGGCGGAGTAATTCAGCCCGC
CAATAAAGGCTATACGCGCGTCGATCACCACAATTTTGCGATGCATCCGGCGAAACACATTGGTGCGCATACCAAAAAGG
CGAGGGCGGGGATCGTAGTAGCGGAACACTACGCCAGCTGCCGTCAGTTCATTGACAAACTCATCGCTGAGATCCGGCGA
ACCGTAGCCATCCAGCAAGACTTCCGCTTTAACCCCGCGTTGCGCTGCTGCCAGTAGTGCCGCATGCAGTTGTTTGCCGA
CGTCATCCTCAAACCAGATAAACGTTTCAAGAATGATGCGTTCTTGTGCCTCGCCAATCGCCTTAAACACCGCGGGATAA
TATTGCTCGCCGTTTTCCAGCAACTGGATCTTATTGCCTTCGCGCCAGCTACATTTCATAAATGAATCTCCGCACTTAAA
GGGGCATGATCAGAAAGGTGTCGCCATGTCCGCAGCGGCAACGCGGTTGGCGCGCTGGCGCTGGCATTTTTGACGTAGAT
CCTGTCCAGTCGTAGTAGAGGAAATTGCACCGGAAACGTGCGCGCCGGGCGTCCGTGGGCGCGGGTAAAAATCTCATCCA
GTCCGGCCTGCACTTTTAACGGATGATTAGCTTTTTGCCGCCAGTCATTGAAATCACCCGCCACCAATACCGGTTCGCCG
TCCGGTAGCTCATTCACCCATTCGGCGAGCATCGCAAGCTGCGCCTGACGGTGCGCCTCACGCAGGCCCAGATGTACGCA
CATCACATGAATCGCTTTTCCGGTCATCGGCGGCACAATGCGGCAGTAGAGCACGCCGCGCTTTTCCGCACCATCGACCG
AAACATCGCGATTCTCATAATGTTCAATGGGATAACGCGACAGTACGGCGTTGCCGTGATGCCCTTCCGGGTATACGGCA

TTGCGACCGTAGGCAAAATCGCTCCACATAGTGTCGGCGAGAAACTCGTAGTGCGAGGTATCGGGCCAGTTTTCCACATG
CAGCGGATGAACTTCGTGCGCGCCCATCACTTCCTGCAGGCAAACAATATCGGCGCTGACGGTACGCACGGCGTCGCGAA
GTTCCGGCAAAATGAAGCGTCGGTTAAACGCGGTAAAGCCTTTGTGAATATTGATGGTGAGCACCTTGAACGAAAATTGT
TGTGTTTGATCGGGCATAATTTTCCTGTCTTTGCCTCTTATCTCATTGAAATAGTGTAGTCGGCGTCACAAAAAGGTGCG
GTCTTACGGAATTTTCCGTAAAGTTCGGTACTCTGAGTAAGTAGAGATAAATTCTTCAGGAGAGAAGCCATGAAGTGGCA
ACAACGTGTTCGTGTCGCAACGGGTCTAAGTTGCTGGCAGATTATGTTGCATTTACTGGTAGTGGCGCTGCTGGTGGTGG
GCTGGATGAGTAAGACTCTGGTTCACGTCGGCGTGGGATTATGCGCACTGTATTGTGTCACGGTAGTGATGATGCTGGTG
TTTCAGCGCCACCCCGAGCAACGCTGGCGTGAGGTGGCAGACGTGCTGGAAGAGCTGACCACGACCTGGTATTTTGGCGC
AGCGCTGATTGTGCTGTGGCTGTTGTCCCGCGTTCTGGAAAACAACTTTTTGCTGGCAATTGCAGGGCTGGCAATCCTTG
CCGGCCCGGCGGTAGTGTCTTTGCTGGCGAAAGATAAGAAGTTACATCACCTTACGTCTAAACATCGCGTACGCCGCTGA
CCCTGTCGTGGCCGTTATCACCAGTAGCGGCCACAAACTATTCCACACAATATCCAGACTCGCATCCTTCAAATAAATCT
GCTTGGTAATGTCCGTAAAGTGGCGAATAGGGTTAATCCACGTCAGGTTTTGCAGCCATACCGGCATGTTTTCCACCGGA
GAAACGTAACCGGAAAGGAGAATGGCGGGCATCATAAAGACAAACACGCCGATAAACGCCTGCTGTTGTGTTGAACAGAG
TGATGAAATCAACAGACCGAATCCCACCAGCGATAAACCATAAATCACCATCGTAAAGTAGAACAGCGCCAGCGATCCGG
CGAAGGGGATTTGATACGCCCAGATACCAATCGCCAGCACAATGGTGGCCTGGAAGGTGGCGACAATTAACGCCGGTACG
GCTTTGCCGATGAAGATCTGCCAGGTGGTGAGCGGCGAAACCAGTAGCTGATCGAGCGTACCTTGTTCACGTTCGCGGGC
GACGGAAAGTGAAGTGACGATCATTACGCCGATAGTGGTGATCATGGCGATCAGTGACGGCACCACAAACCATTTGTAGT
CGAGATTCGGGTTATACCAGTTGCGTACCACCAGCTCGCTGTTGTTAGGTTTCGGTTTTCCTTCCAGCAGCTCCTGCTGA
TAATTTTTGACGATCTGTTGCAGGTAGTTGGCGGCAATTTGCGCACTGTTGGAGTTACGCCCGTCGAGGATCAACTGCAA
AGGCGCGGTCTGGAAGGTATCCAGTTTGCGCGAGAAGTCAGCCGGGAAACGCACCAGTAGTAACGCCTTTTGTGTGTCGA
TGGTTGGGCGGATCTCCTGTGGGCTTTTCAGCAGCAGCACATGAGTAAAGGCGCTGGCGCGGGCAAAACGTTGGGTCAGC
TCCACCGAATGCTCGCCGTTATCTTCATCGTAGATGGCGATGGTGGCGTTAGTCACTTCCAGCGTGGCGGCGAACGGGAA
CAGGATCACCTGAATTAGCACGGGTAAAATCAGAATCGCGCGGGTTTGCGGTTCGCGCAGCAACGACTGCAACTCTTTGC
GGATTAACGTCCATAAGCGATGAAACATGCTCTTCTCCCTAATCCAGCCGACGTTTGGTTTTCAGCCACGTCAGGCCGAT
AAACATCACCGCCGAAGCGATCAAAAACAGCACGTTTACCACCAGCACCACTGGAATATTCCCGGCGAGGAACAGGCTTT
GCAGGGTGCTGACGAAATAACGAGCGGGAATAATGTACGTCACCGCGCGGATCACCGCGGGCATACTGTCGATCTGAAAA
ATAAAGCCGGAAAGCATAATCGACGGCAGAAAAGCGGCGTTCAGGGCGACCTGAGCGGCATTGAACTGGTTGCGGGTAAT
CGTGGAAATCAGCAGCCCCATCCCCAGGGTACTGAGTAAAAACAGGCTGGAGATAAAAAACAGAATCAGCAGCGACCCGC
GATACGGCACGCCGAGAATAAACACTGACACCAGCATACACAGCAACATCGCCAGCATCCCGAGAAAGTAATAAGGGATC
AGCTTACACAGCAGCAGTTCCGTGCGGGTAATCTCCGTAGAGAGCAGAGCCTCCATGGTGCCGCGTTCCCATTCTCGCGC
CACCACCAGCGAGGTGAGAATCGCGCCGATGACCGTCATGATAATGGTCACCGCACCGGGGATAATGAAGTGCTGGCTAA
TCGCCGCCGGGTTAAACCAGTAGCGGGTTTGTACATCAATAAGCGGTTCAAAAGTCTGCCCGTTGTCCTCCGCTCGCTGC
ATTTGCCAGATCTGCCAGATCCCTTCGACATACCCCTGTACAAAGTTAGCGGTATTCGGCTCACTGCCGTCGGTGATCAC
CTGAATCGGTGCGGTGGCGTTGGCGCGCTCCATCTGTTCCGCAAAATCCACCGGAATAACCACCAGACCGCGAATTTTCC
CCGCCTGCATTTTGGCGATCAGTTCCTGACGGTTATCGCTGATGGTGGCGTCGATGTAGGGCGAACCGGTCATGGTGTGG
GTGAAATCCAGCGCCGCTTCGCTACGCTGTTCCAGTAAAATCCCGACCCGCAGCTTGCTGGAGTCGAGGTTAATGCCGTA
ACCAAAAATAAACAGCAGTAGCAGCGGGATCACTACCGCAATCAGCCAGCTACTCGGATCGCGAACGATCTGCCGCGTCT
CTTTAACGCACAGCGCCCGTACGCGACGCCAGGACAGGATCGGGTTACTCATTGCTATGCTCCTTATCCCAGTCGTGGAT
CAACTGAATAAAGGCTTGCTCCATGGTGGGATCGGGTTGCTCATCGTTAGCCGACTGTGCTTTCAAATCGTCCGGCGTGC
CGCTGGCGATTAATTTCCCGCGGTACACCAGGCCGATGCGGTCGCAATATTCCGCTTCATCCATAAAGTGGGTGGTGACC
ATCACCGTGACGCCTTTCTCTACCATGCTGTTGATGTGCAGCCAAAATTCACGGCGGGTGAGGGGGTCAACGCCGGAAGT
CGGTTCGTCGAGAAACAGAATGTCCGGTTCATGCATCAGCGAACAGGCCAGCGCCAGCCGCTGTTTAAAACCTAATGGCA
GTTCATCGGTGGCGTGGGAGGCGATACTTTTCAGGCCGAACGCCTCGCTCATGCGGGAGATTTTTTCGTTCTGCGCCCGA
CCGCGTAAGCCATACACACCAGAGAAAAAGCGTAAATTCTGTTCGACCGTCAGGTTACCGTAGAGCGAAAATTTTTGCGC
CATATAGCCGAGATGCTGGCGCGCTTTACCGGAACTCTCTTTCAGATCCATCCCCAGCACCAGCGCCTGGCCGGAAGTCG
GCACCAGCAAACCGCACATCATCTTAAAGGTGGTCGATTTACCCGCGCCGTTTGGCCCCAGCAAACCAAAAATCTCCCCA
CGTTTAACGGCAAAGTTGACGTGATCGGTGGCGGCAAAATCCCCAAATTTCTTGGTCAGTTCTTTCGCTTCGATCACCGT
CTCGCCGGGTGTGCCTTCTACCGTATGTAATATTGCGCCCAGCGGCGATTCCGAGGTTCCGGCACCGCCCAGCAAATCAA
TAAACGCATCTTCAAAACGCGGCGTAGTTTCGTTGATGTTGATTTCCGGCATCCCGTCGGCATGGCGAATATCGTCTGGT
GTGGCCTCTTTTTTGAGGATCAGACGTACCGATTTCCCCTGAATCATGCCGTCGCTGACCTGCGGCAGTTTCAAGGCGCG
TTGCAACAGTTTGCGGTTGCCCTCGTGTGGACTGGTCATCAGAAAGCTGCGTCCGGCCATGGTTTGTGTCAGGGCTTTTG
GTTCTCCCTGATACAGCAACTCGCCTTCGTTCATCAGTAACACGTCACGGCACTGCTCGGCTTCGTCGAGATACGAGGTA
CTCCAGAGGATTAACATCCCTTCGCCCGCCAGCTCATGCACCATCTGCCACAGTTCGCGCCGTGAGATAGGGTCAACGCC
GACGCCGGGTTCATCGAGCAGCAACACTTTCGGTTCGCCCACCAGGGTACAGGCCAGACCGAGTTTTTGTTTCATCCCAC
CGGAGAGCTTGCCCGCCAGGCGTCCGGTAAACGGCCCAAGAGACGTAAACTCCAGCAGGCGAGCAAAAGTTTGCTTACGT
GCCTCGCCGGTGACGCTGCGCAAATCCGCGTACAGATTGAGGTTCTCCATCACCGTGAGATCTTCATACAGACCAAATTT

CTGCGGCATATAACCGAGCACGGCGTGCAGCGCGCCGTCGTTTTTGATCGGATCAAAGCCAATCACCGTGGCACTGCCGC
TGTCGGGTTTCAGTAATCCCGCCAACATCCGCATCAGCGTGGTTTTACCTGCACCGTCCGGCCCCACCAACCCCGTCACA
TAACCGGCGTGAATGGTACAATCGAGCGGCGCGACGGCGGGCTTGTCCATGCCCGGAAAGCGTTTTTCCAGGCCGTTCAG
CGTGATAACGGCATCATTCATGTCCTGCCTCGTCACCGAATTGTACCGTCACTGGCATTCCCTGGCGTAACGCATCATCG
GCGTCGGTCACCACAATACGCAGGCGATAGACGAGGTCGGTACGCAGATCCGGCGTTTCGACGGTTTTCGGGGTAAATTC
AGCAGTCGGCGAAACGAAACCAATCTGCCCGTGATACGGCTTGTCCGGGCGACCATCGGTATAAAGCAGCACTTTGCGCC
CCGGCTGGGCCTGGTCAAGATTACGTTCATCAACATAAGCGCGCACCCACACCGGACGCGTTAGTGAAACGGTAAACACC
GTGCCACCTTCATTGAGGACCGTGCCTGGCTCCACCGCGCGCGTTAACAGCGTGCCATCAGACGGGGCTATCAACGTTGA
GTCCTGTAAATTCAACTCCGCCTGCGCCAGTTGCGCCTGCGCCTGTTCGAGGCTGGCTTTCGCCTGAGCGATGTCCTGTT
CACGGTTACCGGAACGGTACTGACGCAATTTATCCTGTGCTGATTTCAGCGTTGCCTGCGCCTGGTCGCGCGAGGAGCGG
GCATTTTCCAGGTCATTTGCCGAAATAGTGCGGCTTTTCCACAACCCTTGCTGGCGGTTATAGAAGTTCTGCGCATAGTC
ATAGGCGGCTTGCGCCTGTTTCACCGCTGCGGCGGCCTGAGCGATTTCTTCATTGCGATACCCGGCAAGCATCAGGTCAT
ACTGCGCCTGTGCCACCGAAACACCCGCTTTCGCCTGCATCAGGGCAATCTCATACGGCTTGTGATCCAGTTCGCCCAGC
ACCTGGCCCGCTTTGATAGCATCACCTTCGTCCACCGCCAGCGATTCAACGCGCCCCCAACACGGAAACTAAGATTTAC
CGTACGAATATCCACGTTGCCATACAGCGTCAGGCCGTTATCCTGGCGGCTTTGATACCACCAGTAGCCTCCGGCAACCA
CGGCGGCAAGTACCACTACCGCCAATCCGATCACGACAGGTTTTTTCATCACTACAAACTCCTTTGCGATAATCCTTGCA
AAATCAGGTCGATATGACAGGTCACCGTCTGGTTGATCAGTTCGGTCTTTTCTTCATCGAACGCGGTCCAGCCGGTACGT
AACAGAATTGTTTCTTTGCCAAGACGGAACGCCAGAATCTCACCAATCAACGCATGGGTATGAAGGATCATGCGGGTGTC
ATTGGCGTCGCAGCCGGTCCAGGCGGCAATCAGGCGTGTCAGGTGGCTGTGTAGCGGACTAATCACCTGTTCGTGCACCA
GGTGGTAGGCTGCCGTGGGAGAGAGCTGCTCACGGGAGATAAACTTGCTGAGGTTGACGGTGTCATCCTGGGTGAGCAGC
TTAATCATGTTCCTGCAGGCGCGAAGGATCAGTTCACGGATGGCAGCCCGATCAGGCTGTGGTTGCGCGAACAAGCGTTC
GGCTTCCTCGGCATGCGGACGGAACTGCTCGCCAATAAAATCGGCAATCCACTGGGCGCAGGCGAGGTACAAATCTTCTT
TCGAACCGAAGTAGTAGGTGATGGCAGCGATATTCTGCCCGGCCTGGGCGGCTATCTCGCGAGTGGTGGCGTTCATTCCA
TATTCACCAAACTGCGCCAGTGCGGCAGCAATCAGCTGTTTTTTCGCCTGTTCACCCTTGATTGTCATGGCAGGATTATT
CATCGCACAGTCCATTCTTAATCAAATGATTGATTAAGATTATGACTCCATAGGGGAGTTGTCCAGTATGGCTAAGAATT
TTAGCAACGCCAGTCACAGGGATAATTTATGCGCTGCGTCACAAAAACTGCTACACTCCGCTCCCTCATGACATTGTGGT
TTTTGTCATTTTCCTTTTCAGTATCTCCCTGAAAACTACACCGGTAACGGTCGGGGCGGTTCGGAGTAGTTATGTCTTTC
GATTCTTTGGGTTTAAGCCCTGATATCCTGCGCGCCGTTGCCGAGCAGGGTTACCGTGAACCCACCCCTATTCAGCAGCA
GGCGATCCCTGCGGTGCTGGAAGGCCGCGACCTGATGGCTAGCGCCCAGACCGGCACCGGCAAAACAGCGGGCTTTACGC
TGCCGCTGTTGCAACACCTGATCACTCGCCAGCCGCACGCCAAAGGGCGTCGTCCGGTACGTGCGCTCATTCTTACCCCG
ACCCGTGAACTGGCGGCGCAGATTGGCGAAAACGTCCGTGATTACAGCAAATACCTGAACATTCGTTCGCTGGTGGTGTT
TGGTGGTGTGAGTATTAACCCGCAGATGATGAAACTGCGTGGCGGCGTTGATGTGCTGGTGGCAACCCCGGGACGTTTGC
TGGACCTGGAACATCAGAATGCAGTGAAGCTGGATCAGGTTGAAATCCTCGTCCTCGATGAAGCTGACCGCATGCTCGAC
ATGGGCTTTATCCACGATATCCGTCGCGTGTTAACAAAACTACCTGCGAAGCGCCAGAACCTGTTATTCTCCGCGACCTT
CTCTGACGATATTAAAGCCCTGGCGGAAAAACTGTTGCACAACCCGCTGGAAATCGAAGTGGCACGCCGCAATACCGCGT
CTGATCAGGTGACCCAGCACGTTCACTTTGTCGATAAGAAACGCAAACGCGAATTGCTGTCGCACATGATTGGGAAAGGG
AACTGGCAGCAGGTGCTGGTGTTTACCCGTACCAAACACGGCGCTAACCATCTGGCTGAACAGCTCAATAAAGATGGCAT
CCGTAGTGCGGCGATCCACGGCAATAAATCGCAAGGTGCGCGTACTCGTGCGCTGGCTGATTTTAAATCGGGCGATATTC
GTGTACTGGTGGCAACTGACATCGCTGCGCGCGGCCTGGATATTGAAGAGCTGCCGCACGTCGTCAACTATGAACTGCCA
AACGTACCTGAAGATTATGTCCACCGTATCGGGCGTACCGGTCGTGCGGCTGCTACCGGTGAAGCGTTGTCGCTGGTGTG
TGTTGATGAACACAAACTGCTGCGTGATATCGAAAAACTGCTGAAAAAAGAGATCCCGCGCATTGCGATTCCGGGCTATG
AGCCGGACCCGTCAATCAAAGCCGAACCGATCCAGAACGGTCGCCAGCAACGTGGCGGCGGCGGTCGTGGGCAAGGTGGT
GGTCGCGGTCAACAGCAACCACGCCGTGGGGAAGGTGGCGCAAAATCTGCAAGCGCGAAACCTGCAGAAAAACCGTCTCG
CCGCCTCGGCGATGCCAAACCGGCAGGCGAACAACAACGTCGCCGCCGTCCGCGTAAACCTGCCGCTGCGCAGTAATCTT
TTATGCCGGGCTATGCCCGGCATCAGGCTGATGAACAAACGCAAAACTGCCTGATGCGCTACGCTTATCAGGCCTACGTG
AACTCTGCAATATATTGAATTTGCATGCTTTTGTAGGCCGGATAAGGCGTTTACGCCGCATCCGGCATTTCACAACAAGC
ACTTGTCAGCAATTTGAGAACACGGGAAAAAATTTATCTGTTTTACCGCCCATAAGCCACCGAGACGTTACTTCTCTATA
GCCAATTGTTCGCGCAACTCCATTAAAAGGTAGCCCAGTCGATTCTTGCCCTTACCATGACCACCGTCTCCCCAGTAAGC
ATCGTTTTCCGTATGCTCAACCAGTTTTGCGGGCGCGGTTGCCAGCAAGAGCGCACGCAGTTCTGCATGCTGTTCGAATT
TAGCGCGAAGTGCTTTTCGCATCACCTGTTCTTTGACCGACTCCCAGTTTTTACGCAGAGGCTTAGAACGATCGCGCCCC
ATGCGTGCGGCGACCATAGGGGAAGAAACCCGACGGATCTCTTCGCGGTATTTTTCATCAAGGAATTTTTGTGCCTGAAA
ATAGTGTTCTGAGGTAGGCCAGGTTTTCCCGTCAACCTTGATGGGCCAGGCGGCAAAGTTAGAAAAATCACCGTAGTCAT
CGCTGGTGCTGTAGAAATTTATGATGGTGTCTTGCATGACGTGTTGGATCCTTTGTGCTCGAACGGGCATTAAACCGCAT
TATGTTGGTGGTTATTGCGAGCCGCTTTCCAGAAACAGAAAAACCATTACCCCTGAAAACCGAAAAATGCCACAATATTG
GCTGTTTATACAGTATTTCAGGTTTTCTCATGGCATTAACCGCCGCGCTTAAAGCGCAAATTGCCGCCTGGTATAAGGCG
CTTCAGGAACAGATCCCCGACTTTATTCCCCGTGCGCCGCAGCGGCAGATGATTGCGGACGTCGCCAAAACGCTGGCCGG

AGAAGAAGGGCGACATCTGGCGATTGAAGCCCCCACCGGCGTTGGGAAAACGCTCTCTTATTTGATTCCCGGCATCGCCA
TTGCCCGCGAAGAGCAAAAAACGCTGGTGGTGAGTACCGCCAACGTGGCATTGCAGGATCAGATTTACAGCAAAGATTTA
CCGCTGCTGAAAAAGATCATTCCCGATCTTAAATTCACTGCCGCTTTTGGGCGTGGGCGCTACGTTTGTCCGCGTAATCT
GACGGCGCTCGCCAGTACTGAACCCACGCAACAGGATCTGCTGGCGTTTCTTGACGACGAACTGACGCCGAACAATCAGG
AAGAGCAAAAACGTTGTGCGAAGCTGAAGGGCGATCTCGATACTTATAAATGGGATGGTCTGCGCGATCATACTGATATC
GCTATAGATGACGATCTCTGGCGTCGTTTAAGTACCGACAAAGCCAGCTGCCTCAACCGCAATTGTTACTACTATCGTGA
ATGCCCGTTTTTTGTCGCTCGTCGGGAGATTCAGGAAGCGGAAGTGGTGGTGGCAAACCATGCGCTGGTGATGGCGGCGA
TGGAAAGCGAAGCGGTATTGCCTGACCCGAAAAATTTACTGCTGGTGCTGGACGAAGGCCATCACCTGCCGGATGTGGCG
CGGGATGCGCTTGAGATGAGCGCCGAAATCACCGCGCCGTGGTATCGGCTACAGCTGGACTTGTTCACGAAACTGGTCGC
TACCTGCATGGAGCAGTTTCGCCCGAAGACCATCCCACCGCTGGCGATCCCTGAACGTTTGAATGCGCATTGTGAAGAGT
TGTATGAGCTTATCGCCTCGTTAAACAACATTCTCAATCTCTACATGCCTGCCGGGCAGGAGGCAGAGCACCGTTTTGCG
ATGGGCGAACTGCCAGATGAAGTGCTGGAGATCTGCCAGCGGCTGGCAAAACTCACCGAGATGCTGCGTGGCCTGGCGGA
GTTATTTCTTAACGATTTAAGTGAGAAAACCGGCAGCCATGACATTGTACGTCTGCATCGGTTGATTTTGCAGATGAACC
GCGCGTTGGGGATGTTCGAGGCGCAAAGCAAACTCTGGCGGCTGGCTTCGCTGGCGCAATCTTCCGGTGCACCGGTGACC
AAATGGGCGACGCGGGAAGAGCGCGAAGGGCAGCTACACCTCTGGTTTCACTGCGTGGGAATACGTGTCAGCGATCAGCT
GGAAAGGCTGCTGTGGCGCAGTATTCCGCACATTATTGTCACCTCCGCAACCTTGCGTTCGCTGAACAGTTTTTCGCGTT
TGCAGGAGATGAGTGGTCTGAAAGAGAAAGCGGGCGACCGTTTTGTGGCGCTGGATTCCCCCTTTAACCACTGCGAACAG
GGCAAAATTGTTATTCCCCGGATGCGCGTTGAGCCTTCCATCGACAACGAAGAGCAGCATATTGCCGAAATGGCGGCCTT
TTTCCGTAAGCAGGTGGAGAGCAAAAAACATCTCGGTATGTTGGTACTGTTTGCCAGCGGACGGGCGATGCAGCGCTTTC
TCGACTATGTGACGGATTTACGTCTGATGTTGCTGGTTCAGGGCGATCAGCCGCGTTACCGTTTAGTTGAACTGCACCGC
AAACGCGTCGCCAACGGTGAGCGCAGCGTGCTGGTGGGCTTACAGTCATTTGCCGAAGGGCTTGATTTGAAAGGTGATCT
GCTCAGCCAGGTGCATATCCACAAAATCGCTTTTCCGCCCATCGACAGCCCGGTGGTGATCACCGAAGGGGAATGGCTGA
AAAGCCTCAACCGCTATCCGTTTGAGGTGCAAAGCCTGCCGAGCGCCTCGTTTAACCTGATTCAGCAGGTTGGGCGACTG
ATTCGAAGCCACGGTTGCTGGGGCGAAGTGGTTATCTACGACAAACGCTTGCTGACCAAAAATTATGGCAAGCGACTACT
GGATGCATTACCGGTATTTCCGATAGAGCAACCGGAAGTCCCTGAAGGTATAGTTAAAAAGAAAGAAAAAACGAAATCCC
CACGCCGTCGGCGGCGTTAATGATGTGAGTCAGGTAAGGAGTCGTAAATGGACTATCGCAAAATCATTAAAGAGATCGGG
CGCGGGAAAAACCACGCGCGCGATTTAGACCGGGATACTGCGCGCGGTCTGTATGCTCATATGCTCAATGGTGAAGTCCC
TGACCTTGAGTTGGGCGGCGTATTGATTGCGCTGCGTATCAAAGGAGAAGGGGAAGCAGAGATGCTCGGCTTTTACGAAG
CCATGCAAAATCACACCATCAAGCTGACGCCGCCAGCAGGCAAGCCGATGCCGATTGTCATCCCCAGTTACAACGGCGCG
CGTAAACAGGCCAACCTGACGCCGTTGTTGGCGATTCTCCTGCACAAACTCGGTTTTCCTGTGGTGGTTCACGGGGTTAG
CGAAGATCCAACCCGCGTGCTGACTGAAACCATTTTTGAATTGATGGGTATTACGCCAACGTTGCACGGCGGGCAGGCGC
AGGCGAAGCTCGACGAACATCAACCGGTGTTTATGCCAGTCGGCGCGTTTTGCCCGCCGCTGGAAAAACAACTGGCGATG
CGCTGGCGGATGGGCGTGCGTAACAGTGCACATACCCTGGCGAAACTGGCGACGCCATTTGCCGAAGGTGAGGCGCTGCG
TCTTTCCAGCGTTTCGCATCCGGAATACATTGGACGCGTCGCGAAGTTCTTTAGCGATATCGGCGGGCGGGCGCTACTAA
TGCATGGTACTGAAGGTGAAGTGTATGCTAATCCGCAGCGCTGCCCGCAGATCAATCTCATTGACCGTGAAGGGATGCGG
GTGCTGTATGAAAAACAGGACACTGCTGGTAGCGAGTTACTGCCACAAGCAAAAGATCCGGAAACCACGGCGCAGTGGAT
TGAGCGTTGCCTTGCTGGCAGCGAACCGATTCCCGAATCGCTGAAAATCCAGATGGCTTGCTGCCTGGTGGCTACGGGTG
AAGCGGCAACTATCAGCGACGGCCTGGCGCGCGTTAATCAGGCATTTTAATTCTTTTCTCAGCCGGATGAGCCATGTCAT
CCGGCTTTTACCCCACCGCAATATGAAATTCCTGCATCTTTATTGACCTTCCCACGCCCGGCGTGCAGCATAAAAATACA
ACAAACACATAACATAAACAGGAGTTAACCATGGAAAGTGGTCATCGCTTTGATGCTCAGACGCTGCACAGTTTTATTCA
GGCTGTATTTCGTCAGATGGGTAGCGAGGAACAAGAAGCGAAATTAGTTGCCGATCATTTAATCGCGGCAAACCTGGCAG
GGCATGATTCACATGGTATTGGCATGATCCCAAGCTATGTACGCTCCTGGAGTCAGGGGCACCTGCAAATTAACCATCAT
GCCAAAACCGTTAAAGAGGCGGGGGCGGCGGTCACGCTCGATGGCGATCGCGCATTTGGTCAGGTCGCGGCACATGAAGC
GATGGCGCTGGGGATTGAGAAAGCGCATCAGCACGGTATTGCCGCCGTGGCGCTACATAACTCGCATCATATCGGCCGTA
TCGGTTACTGGGCGGAGCAGTGTGCAGCGGCGGGGTTTGTCTCTATCCACTTTGTTAGCGTGGTCGGTATTCCAATGGTC
GCGCCGTTCCACGGTCGCGACAGCCGCTTTGGCACCAATCCGTTCTGTGTGGTTTTCCCTCGTAAAGATAATTTCCCGCT
GTTGCTTGATTACGCCACCAGCGCCATTGCATTTGGCAAAACCCGCGTCGCCTGGCATAAAGGCGTCCCCGTGCCGCCAG
GTTGCCTGATTGACGTTAACGGCGTGCCGACGACCAATCCGGCGGTAATGCAGGAGTCGCCGTTGGGTTCGCTGTTGACC
TTTGCCGAACATAAAGGCTACGCCCTTGCAGCGATGTGTGAAATTCTTGGCGGGGCGCTTTCCGGCGGTAAAACGACGCA
TCAGGAAACGTTACAAACCAGTCCCGATGCCATTCTTAACTGCATGACCACTATCATCATCAACCCGGAACTCTTCGGCG
CGCCGGATTGTAACGCGCAGACCGAAGCCTTTGCCGAGTGGGTGAAAGCCTCGCCGCATGATGATGATAAGCCGATTTTG
CTACCGGGCGAGTGGGAAGTGAACACGCGTCGCGAACGGCAGAAGCAGGGGATTCCACTGGATGCGGGAAGCTGGCAGGC
CATTTGTGATGCAGCGCGGCAGATTGGTATGCCGGAAGAGACGTTGCAGGCTTTCTGTCAGCAGTTAGCCAGCTAAAAAA
AAGCCCGTCCAGTGGCGGACGGGCAAACAAGGGTAACATAGGATCAATGAGGGTTAGAGCATATGCGTCTGTCGGCAAAC
AGACAGGGAAATACTTGTGCTGGACGTAGCGTAAACGCCTGATCCGTCCAACGGTTCGGATTTTGTAGGCCTGATAAGAC
GCGGTAGCGTCGCATCAGGCATCTGTTGCCGGATGCGGCGTAAACGCCTTATCCGTCCTACGAATTACTTATAAATTACC

GCAGTACCGCTTAATTTATTGTTGTTGGTGGCGGAAGTGATGCTGTATCCACTGGCACCGGCTGCCGCGGCTTTCTCAGC
CAGTTTCGCTTCCAGGGCATCGAGGGTGGATGCGCCATCGGCAGAAACCACGCCGATTTTATTCATGTTCTGTGCCTGGG
ATGCCGTTACCGGTTCCGCCGCGAACACGCCAAATGACAGAGTTGAAAGAGCCATAGCAGCAACAACAGTATTGATAGTT
TTCATAATTAATCTCTCGCAGGTGATTTTTGTATAAGTGACGTTGTTTCGTCGATGTGATGAGTATCACGTTTTTTTACG
AGAGATAAAATCGAAGAGAATTGACCGCCTTGTTCAAATAAATTGATTGATATCTAACCTACTGATAAACCATTTATTTG
TTGTACTTATGAACTTGTTTTATACCGCGTGGCAGTCACAGCAGCGTTAAAGGCACGGAATGACACGCAAAATACAAATT
ATACTCGCTATGCATCTCGCCAGTTGTAGTTAACGTAACTGGCTGTCTTTCGAACCTCTGCGATTATATCCTGTATAAGC
TGGTTTTTGTAAATCTTTCTCCTGCTGACAATGAATACATAAGCGCACGCCAGGAATGGCTTCCCGACGGGCCTGCGGGA
TGGGGGCACCGCACTCTTCACATTCATCCAGGCTTTCGCCGCGCGGAATTTCACCCCGAGCGCGGGCAATCGCATCTTCA
ATTGTACTGTTGATCTGTTCGTTGACGGCGTCATCGTTAGCCCAACCGGATGCCATATCGACCTCCCCATATCAATACTT
GTACAGTTAAGTGTAGCTAATCCAGGGACGAACTCGGGCAGTTCAAGCATCAGATCTCCGACCATTCCCGCAGCAGATTA
TGATAAAGATTAAGCAGCGACAGGATCTCTTCACTTTCGCCGTAGCGGCTTTTCAGCGACTGAATATTGTTGTCCAGTTC
AAACAGCATGGCGCGCTTTTTATCATCGCGGATCATCGACTGGATCCACATAAATGATGCCACTCGTACGCCGCGGGTTA
CGGGTGTCACGCAATGCAGGCTGCTGGAGGGATACAACACGAGATCGCCTGCCGGGAGTTTTACCCGATGTTGTCCGAAG
GTGTCATTAACGACCAGTTCGCCGCCGTCGTAGCTTTGTGGATCGCTTAAAAACAGCGTGGCAGAAAGGTCAGTACGCAT
CCAGCCGTTTTGTGGATGGCTGCGTACTGCGCCATCCACATGGAAACCATAGGTTTCATTGTTCTGATAGCGATTAAACA
GCGGCGTGGAAAGGGTACGCGGCAAGGCCGCGGCAAAGAATAAAGCATGTTGGTTAACCGCGTTCAGCACCTCATTTTGC
AGGGCGGCGTATAACGTGCTGCGGGTGTCGACCTGTTGATTGTTCTTAACTTGCGCACCTTGTGCGCCGGTGGTGACGCG
TCCATCCACCCATTCGGCTTGTTCCAGTTGTTCGCGAAAACGAGCGACGTCCTGTGGCGATAACACGCCGGGAATGTGGT
ACATCATACAATTTCTCCAAAAAGTGGGGCCTGCGCCCCACATCTGAATCAGAAATGCATATTGGCTGTGAGCAAGAAGG
TTCTTGGCTCGCCCGGGTGATAACGGTAGCCGCTCTTGTTGATTGAGGCGACGTAATCGGTATCAAACAGGTTGTAAACG
TTTAGCTGGAAGTCGAGATTGCGATTAACTCGATACCCCAGTTTGGCATCGGCGACCCAGTAACCTTCGGTAAACGCTGG
CGTTCCCACCGCGCCGTCTGAACCTTTATGCATACTGCCGATATAGCGTGCGCCCGCGCCAACAGAGATATCGTCGGTTG
CCTGATATTGGCTCCATAAGGTGAAGGCGTGCTCCGGGGTATACGGCAGCGATGAGGAACCATCCTGGGCAACATCTTTG
CCGTTTTTGATGGTTGCTTTTTGCTGGGTATAGCCGCCAATCACCTGCCACGCGGGAGTGATATTCCCGGCCACGGATAT
CTCATAGCCTTCGACGCGTTTCTTACCGTATTGCGAGTAAGTTCCGTCATCATTTTGCTCAACTTCATTTTCGATATCAG
TGCGGAACAGCGCGGCGGTGAGCAACAGACGTTTATCCAGAACCTGCCATTTGGTGCCAATCTCGCTGGTGTTGGCTTTT
TGCGGTTTAAAATCGGTGCGGTTGGCACTGTTACCGCTGCCAGACTGCGCAAGGGCGAAGTTGTTGCCGCCCGGAGGCTG
CTGGGAAACGGCATAGTTAATATAGACATTGCCGTTTTCCGTCAGGTGATACAGCGCCCCGGCTTTCCAGTTCATCAGAT
TGCCCGACTTGGCGGTGTCGACGGTGGTGACCGGAGAACCTTTTGCCACACCAGTTGGGCAGGTGATGGCACCGCGTCCG
CTGCCGCCGCAGGCGGTGGCACTGTCATATTCAGTATGATAATTATCCAGACGGATCCCGCCGTTCAGCTCAAAATCACG
GGTGATTTGCAGCGTATCAAAGGCGTAAATTGCGAAGGTATCCGTCTGACCATTGGCGTTTGCGCCGTTGCGCGTCAGGC
CGCCGGGATGAATGCTGCTGTCAGGATGATAAATATTTACCGCGGGTAACGTCACCGGATTAACGCCGTAGTTAGTCTGC
GTTTCACGGGTAAATTCCACGCCGGTACTGACATCATGACCGATAGAACCGGTATAGAACGTCGAGGTCAGGTTGGTCTG
GTTGGTGAGAATTTTATTACTCACATCTTTGGTATTCGCCGTGCGTGACCAGGTCCAGCTATTCACATCGCTGGTGGGCT
GAGTAATATTCGACGCCCCGCCCATAATCGCCGTCATCAGGTAATCCTGCTTTACGCGCGACCAACGGGTAGTATTGCGA
ATGGTGGTGTTATCGTTGATGTCGTGCTCAAAACGCATGGTGGCGGTGTCGGTGGTCGAATCGTCGTAATCGGAATCCGT
GCCGTAAAAGTTATGAGTATCAACTTTTCCGGAATGATTCAGGGCCGCCGTTCCCGCAGATGGGGCAGAATAGCCCGGCA
AACCGATGGTCGGAATGCCGCCGTCTGGCGTGTTGTGCTGGGTGACATGCAGATAATTAAGATACAAACGATTCGCTGTA
CCAAGGCCAAAAGCGACAGAAGGGGCGACGCCGTAACGCTCATTTTTGACTTTGTCGCGTCCGGCATCGTGCGTTTTTTC
GCCCATTACATTCAGGCGCACCGCGGTGGTATCACCAATGACCTGATTGACGTCCAGCGTGCCGCGGCGGAACCAGGCGC
TGCCAATACTGGCGGAGGCGTCAATGCCGGAATCATTGCGCGGCTGCTTGCTGATCATATTGATCGAGCCTGTCGGTGCG
CTGCGCCCGTAGTCGGTGCCGGACGGCCCTTTAATCACTTCGACCTGCTCGGTATTGAAGGTGTCGCGCGAGACGCTGCC
GATATCGCGAATGCCATCAATATAAATACTGTTAGAGGTATCGGCACCACGCATATAAATGGCGTCGCCAGTGGTGGAGT
TACCGTTCTCACCCGCAAAAAACGCACCCACGCCGGGGACGTTTTTGAGCGCGTCGGTAAGGTTGGTTGCGCCCTGATCT
TTAATCACTTGTTCAGAAATTACCGTCATCGTGCGGGTAGTATCCGCTACCGGACGCGAGAATTTCGGATCGGCAGATTG
TTGTGGCGCATAAAGCGAAGGCGTTGATGCTTCGACAACCAGCGTGTCATCCGCGTTAGTTTGCCCTTCGGCGGCGAGTG
CCTGAGCCACAGGCGTGATGCCAATACAAAGACCGGCAAAGAACGTGAGCGAATGAAATTGTCTGGCAGGGAAATTGCGA
TTGTTTTCCATTTTGCAGGTGACTTTTTCTTATATGTTGAAATTTTCACTGCCACGAGAAAGCCTCTCTGCGGATGCGAT
GCCACTGTTAATCCCGTGATAGTGCTCGTGAAAGTGATAATAATTTTGATAAGCATTATCACTTGGAGCGATTTTCTATC
ACCCATTGCGCGAAAAAGAAATACATTTATTTACATTGAGTTCATATTTGATGTTTTTTGATTACAAAACAGACGAAAAC
TGCATCCAGAGGCGAATGCAGTTACGTTGGTGGGGTTATTTGTAGATGGTTGCAGTACCGAACATCTGGTCATTTCCGCC
TGCGGAATTAATGACATAACCTTTCGCGCCTTGTTCGCGCGCTTTTTCTGCCAGTTTATCCTCCAGATCGCTTAGGTTTG
ATGCGCCAGTTGCCGAAACGGTACCGGCGGGCCGTAGCTGACCGCTGTCCAGGTTACTCATCGGTTGCGCGGCATAAGCC
GTGAGAGAGATTCCGCTCAGGACGGTGGCAATCAGTAGTGTGAGGCACTTTTTCATAATGACATCCTCATAAATACAACG
GGTAGTGCCTGATAAGTGTAGGTCGCCTGCGCGATGCAAATGACGCAAAAAATTGACAGAGATGTGCTTATTTTTTGAAC

GATATATTTTTACAAAATAAGACAAATCAATGACATGCATAATGCTGATTTTTATGATGAATTGCCATAAACCATAGCCA
TGTTAAGGTTTTGGGGAATGATAAACGCAGCCAGATATTTACCCTGTCGCGTTATGCGGGTATCTTACGCCGCTGCTTAA
AGGAGAATGCTATGTCCGCCCAGAAACCGGGGTTGCATCCGCGCAACCGTCATCACAGCCGCTACGATCTCGCCACGCTT
TGTCAGGTCAATCCTGAACTCAGGCAATTCCTCACGCTTACACCCGCCGGGGAGCAAAGCGTAGACTTTGCCAATCCGCT
GGCGGTGAAGGCGCTCAATAAGGCGTTGCTGGCCCATTTTTACGCCGTAGCGAACTGGGATATCCCCGACGGTTTTCTCT
GCCCACCAGTACCGGGCCGGGCGGATTATATTCATCACCTTGCCGATTTACTGGCAGAAGCAAGCGGAACAATTCCAGCG
AATGCCAGCATTCTGGATATCGGCGTTGGTGCGAACTGTATTTATCCGCTGATTGGCGTACATGAATATGGCTGGCGTTT
TACCGGTAGTGAAACCAGCAGCCAGGCGTTAAGCAGTGCGCAGGCGATTATCAGTTCTAATCCGGGGCTTAACCGCGCCA
TTCGTCTGCGTCGGCAAAAAGAGAGTGGGGCGATTTTTAACGGCATCATCCATAAAAACGAGCAATACGACGCGACCTTG
TGTAACCCGCCATTCCACGATTCCGCCGCTGCGGCACGGGCAGGTAGTGAGCGTAAACGCCGTAACCTGGGGCTGAACAA
AGACGATGCACTGAACTTTGGCGGCCAGCAACAGGAGTTGTGGTGTGAAGGCGGTGAAGTCACCTTTATCAAAAAGATGA
TTGAAGAGAGCAAAGGCTTCGCGAAGCAGGTGATGTGGTTTACATCACTGGTATCTCGTGGTGAAAACTTACCGCCGTTG
TATCGTGCCCTGACGGACGTGGGCGCGGTGAAGGTGGTTAAAAAAGAGATGGCCCAGGGGCAAAAGCAGAGTCGCTTTAT
TGCCTGGACCTTTATGAACGACGAGCAGCGCCGCCGTTTTGTCAATCGCCAGCGTTAAAGCGTTGGTTCCCCCGGCGGTA
ACGGTTCAGCCGGGGTCGCGCCCGGAGCAGGCAGCACCTGATAAGTCTGCACTGGCGCGCGAACGCCCGCCAGGTCGAAA
TGTTTTTTCACCTGGCTGTCGAGGGCAAAGCGTACCGTCCACTGTTTGAGTGGCAGCGTGGTGAACGAAACACGCAGTGT
AAACGCGGTATTGCTTAAGCCGACAATCCCGGCAAAATTCGGTTCACCAATAATCAGCCCGCGAATTTCTTCGTTTTCCA
TTAATTCCGCTACCGCATCTTTCAGTGCCTGATTGGCTTTATCAGCATCTTCATGGCGATCAACATCATAATTTGCCACC
ACCGAACCAATGCCGCGGACAAAGTTAGCAAAGGTGGTTATCGAAGACCACGGAATGATGTGATACGCCCCGGTATCCTG
TCGTACGCCCACGGAGCGAATCGACATCCGTTCCACTGTGCCGGTCAACGGCCCGATAGTCACCAAATCTCCAGTGTTCA
TGCCGTTTTCAAACTGAATAAATACCCCGGTGATAATATCTTTCACCAGCGTTTGCGAACCAAACGAGATAGCCAGACCT
AATGCCCCGGCACCTGCCAGCAATGGCGCGATATTGACGCCGATTTCCGACAACACAATCATGATGGTGATGGTACTGAT
AATCACCGCCAGCGCGTTACGAAACAGCGTAAGCAGGGTACGCGTGCGGGCGCTGGGTAGCGGGCGGCCATGAATATCCG
AAGCCAGCCGGTTTTCGATCAAACTGGCGAGCACCGTCCAGCCAACCGCCGAGAAGAAAAGAATGAGTGCGATACGGATC
AGGATATCTACGGTTTTCTGCCCCGCGCCGTTTTGCAGCCAGTTCCAGAAATCGAACAATCCCCATGCGCTCAACAGCAA
CATTACCGCCACGCAGACTGTCAGAATACGCGCCGTTTTCAGCGCCGCCGACAGCCAGCCATTCAACCGTTTTTGCAGCT
CCGGATAGTTACGCTGAGTATGTGGCGAGAGAGTGATGGTTTTCGCCAGCCAGCGGGAAAACATACCGGAAACAAACGCT
GCGATACCAATAATCGCCAGGCTGCGCACCGTTGCACCCATCATAAATTTCAGGCTGTTGCCCGGATCGAACAACGAAAA
GAAAAACAGCACGATAAAATAGGCGCTTGCCAGCCAGTGCCACACCAGCGCAAAGGCGCGGATAAACAGGCTGAAAAAGG
CCAGCGAATGCTCCGCGAAGTTGAGCAAATGCTGGGTAATCTCTTTTTTATTACGAAAGATCAGGTACAACGCCCAGACG
GTCATGCACAGCATAATGATGACGTTCGCCAGCGCACCTATCTGTACATTCACCTGATTAGAGATAATCGGCACGGCCAC
AATCAGGCCATAACCTATCAAACTGCTTAACCAGCTCAGGCGACGACTCCAGTAACGGGCGCTCTCGTCCTGAATCGTGA
ACGGGCGCAGCTCCGCCACGTTTGGGCAAAAAATCAGGCGTAGTACGGCTTTGAAAAATTCAATGAGGGCAAAGGCGTTG
AGAAACAAACTTTGTTGGAAAGCGATGGTGCGACTGCCTGCATTCAGGTTGTCGCTTAATACCTGGCCGACAAATAATGT
CAGGGCCAGTAACAGCAGGTCGATAATAAACGCCCCGATAATCATCGCCGGAAGCTGCAACCAGTTGCTGCGCTCACGAT
TTTTTTGCCGCGCCCACTGACCCATTTTGCGATACAGCGGCAGTGCGCACAGGCGAATCAGCCAGTAAAAACCAAACACT
AATACCGCTAACATTGAAAAATGGGTCAGCGCATTGCTGAAGGTTTGTGGATTAAACGGCTTATGCGGGGAGCCGGTGAT
ATTGCGATAAAGTTGCCCGAAGCGGGCGGAAAGGGCTTCACCATAATGGCGGCTGACTTCGGTGACCTTTTGCAGCACGG
TTTGCTCTTCGACCAGCGTCGGCGGCACTATTTTTGGTACCGGTTCAGCAGGGGGCGTAGCGGCAACGGTGCGCAACTGG
TCGATCAACTCTTTACGCGAGGTGTCATTATCCAGCACATCCGCCAGTGCGCCATAGGCCGCTTTTTTTTGTTCGATATC
CGGTTCCGGGGCCGGTTCAGTCGTTGAGTCCGTTGTCGTTGTGGTTGTAACGCCGGGTATGGATACCGCGTGGGCAGGTG
CGCCCAGCAGGCAGAAGAGGATGAACAGGATCCACCGCATGACTCCTCCAGTGAGAAAATAGAGCAAAAAGATAAGTATA
GATGCTGGAGGAGGGGCGATTTTAAATGAGAGGAATCTGGTGTGCCTCCCTTTCGGGTGAAAGGGAGGAAGGGATATTAA
GCAGGTTGCTGACAACGTGCGGGCTTTTTTGCCGGATGCGGCGTGAACGCCTTATCCGTCCTACAAGTTCATGCAATTTT
AATAAGTTGCCAGATATCCTGTAGGCCTGATAAGCGTAGCGCATCAGGCAATGTGTCTTATTAAGAGACGTGCTGCAAAA
ATTCCTGCAAGCGCTGGCTCGGCGGGTTCTTGATCAACACCTGCGGATTGCCATCTTCCGCAATCCGGCCTTTGTCGATA
AAGATCAGCCGCGAAGCTACTTTCTCGGCAAAACCGATTTCGTGGGTCACGATCACCATCGTCATCCCTTCTTCAGCCAG
ATCCTGCATAACCTTCAGCACTTCATGGCGCAGTTCCGGGTCAAGAGCGGAAGTCGGTTCATCAAACAGCATCATTTTCG
GCTTCACCGCCAGCGCGCGGGCAATCGCCACACGCTGCTGTTGACCACCAGAAAGTTCGGAAGGGTAGTGATGTGCACGT
TCTGCCAGACCGACTTTCGCCAGCAGCTCACGTGCCAGTTTTTCCGCCTCTTCTTTGTTCGCGCCACGCACGCGTAGCGG
GCCAAACATGACGTTTTCCAGCGCTGTCAGATGCGGGAAGAGGTAAAACTGCTGGAACACCATACCTGCTTCCTGGCGAA
TCAGGCGCTCGTCAACTTTCGGATCGTTAACCTTCAGGCCATCGACAATCAGATCGCCGGAGGTGATTTCTTCCAGTTTG
TTGATGCAGCGCAGCAGGGTCGATTTACCGGAACCGGACGGCCCGATAATCACCACGACTTCGCCCTGGGCAATGTTCAA
ATCGATATTGTGCAGCACCTGGGTTGGGCCAAAGTGCTTGGAGACGTTTTTAAATTCAATCACAGGATTTTCATCCTTCT
TTCCAGACGACGCAGAATAAAGCTCAGCACCAGGGTAATAATCAGATAGAACACCGCCACGGCGCTCCAGATCTCAAGGG
CGCGGAAGTTACCGGCAATAATTTCTTGCCCCTGACGGGTCAGTTCCGCCACGCCGATCACAATAAACAGCGAGGTGTCT

TTAATGCTGATGATCCACTGGTTACCCAGCGGCGGCAGCATACGACGCAGTGCCAGCGGTAAAATGACGTAGCGAATGGT
TTCCCAACGTGAAAGACCGAGCGCCAGTCCTGCTTCACGAAAACCTTTGTGGATAGACAGCACCGCACCACGCGTGATTT
CCGCAATATACGCGCCGGAGTTGATCATGATGGTGACCACCGCCGCAGTAAATGGGTCGATGCGTAAGTCGTTAAACGCC
ATCGGCAGGGCGAAATAAATAAACATCACCTGGACGACGATAGGTGTGCCGCGGATCACTTCAATAAAGACCAGCGCGAC
GTGGTTGGCTATCCAACCTCCGAAGGTGCGTGCAAAACCTGCCAGCAATCCGATTACCAGACCGCCTGCCAGACCGAGGA
CCGAAATCCACAGGGTCATTTTGGCACCTTCAATCAGAAGCGGAATGGCAGGCCAGATGGCACTCCAGTCAAACTGCATA
TGTTGTTCCTGTTACCGTGGTGTAAATAATCAAATTCAGGGTAATTAATGGCCCCGGAGCGGGTTTGCGCCCCTCACCCT
AATCCTCTCCCCATAGGGGAGAGGGAACTGCCAGTGCGTTTTACAGGTGTAGCGTTATTATTTCGGTTCAGTACCGAACC
ATTTTTTGTAGATTTCGTTGTAAGTTCCGTTCTCGCGCAGGGTTTTCAACGCGCCGTTGACTTTGTCACGCAGCTCGTCG
CTACCTTTCGGGAACGCAATACCGTATTGCTGCGCTTCCAGAGAGTCACCTACCGCTTTGAACTGACCGTTACCGGCGGT
TTTGATGAAGTACAGAATGTTTGGCGTATCGTGCAGAACGGCGTCTGCGCGGTTGGTGCCCAGTTCCATATAGGCGTTAT
CGATGTTCGGGAACTGACGCAGATCTTTAGTTTTGATGTTTGCTTTCGCGTAATCAACGGAGCCAGTACCGCTCTTCACA
GCAACCACTTTCCCGTCGAGATCTTTCACGCTTTTCACATCGTTATTGTTAGCTTTCACCATCACTAACAGGCCGCTTTT
GTAGTAGCCGTCAGAGAAATCGATCGCTTTTTTACGCTCGTCGGTGATGGTAATGCCCGCCAGCGCCAGATCGACGTTTT
TGGTTTGCAGTGCCGGAATGATCCCACTGAAATCCATCGGCTTCAGTTCGTAATCCAGCTTCAGCTCTTTAGCGATGGCA
GCCCACAGATCAACGTCAAAGCCCACATATTTATCGCCCTGTTTAAATTCAAACGGAACGAAGGCGGTATCCGTCGCGAC
AACTAATTTTTTATCCGCGGCATGAGAAGAAACCGCAAAAGCCAGGGTCAGTGCAGCCAGTGAAACTTTTAATACAGACT
TCATAGCATTTCCTTTTTTCTATCCACGGGACGATCCCCTGCGTGACATTCACATATATGAAAAAATCGTGCCAGTTTTG
CAACTCCTTGATTTTTCGAGATGCGGCAAAAACGTGATGCACGATTTATAGGGCAATACCCTGAAGATGCACCATTCTGG
GGCACCAATCTGGTGCGCTAAAATTGTGCACTCAACACAGTGATTATTTAGCGTAAAACCTGATGAAAAAACAATCTTTA
TGTAACGATTGTGTGATGATGTGGATACAAAAAATTTACTTAATCAGCTGGAGATAGCAGATGGATGCACTAAATAAGTG
CGTTGAGGTGGGCTGCAAAACAAAACGGCCTCCTGTCAGGAAGCCGCTTTTATCGGGTACTAAAGTTCTGCACCATCAGC
GATGGATTTATTCGATGTTAGACTCGATAAACCACAGGAATTTATCCAGGTCGCGAGACGCGGCGGTCAGGATATCTGCG
GTGTCGTCATCTTTCGCTTCGCCAATCGCTTTGCGTACGTCATTAGCGACGATTGCGTAACGGTCAGCCAGTTCTTTCAG
GTGATCCTGAACGTTGTGGATGTCCAGCGGGTAACTTTTCAGCGGGGTTTTGCTGTTGATAACTTGAGTGGTCCCCAGAG
CTACACCGCCCAGCTGCACTGCACGTTCTGCCATGGTATCCAGATGATCGATCAGTGCGGTGCGGAAGCCATCCAGCATT
TCATGTACGGCAATGAAGTTAGCGCCGCGCATGTTCCAGTGCGCTTGTTTGGTAATCAAAGAAAGATCAATAAACTGGAT
AACCTGGCGATTCAGCAACTCTACTGTTGCTTTTTTCTCGCTGTCGGAGACATCGTTGCGGGTATAAAGCAGATTGGTCG
CTTTTGATTTAACTAATTTAGCGGTACTCATAATTTCATATCCTCTTGATGTTATGTCCCAGTAATTAACGAGATTAAGT
ATAGCACCGGCTATGTGTTCCGCTATTCTGGCTGTTCCTATCACACTAATAGTGGTAACAAGCGTGAAAAACAAAACTAA
TAAAGATTCAATGAGTTAGATATATTGATAAGAACAATTCTTATTTATCTGCTCGCCAGGAATTTTCGGAATAAATATGC
TTTTGCATAATTCGATATATAAAGACGGTGTAGAGGAAAAGTAGCGAGAAATTCTGCATGGTTATGCATAACCATGCAGA
AATGTAAATTTAATTAATGTCTAATTCTTTTATTTTGCTCTCTTTGCGTACTGTCAGCGTAGACCCCATTGAAGCGGCGA
TGATAGCGCCGAGCGCCAGTAGCTGTATGGGTGTCAGTGTTTCTCCGAGGAAAATCATCCCGGAAACGGCAGCCAGCGCC
GGTTCCATGCTCATCAGCGTACCAAATGTCCGTGTTGGCAAACGGGTGAGGGCAATCATTTCCAGCGAATAAGGCAGAGC
GGTCGAGAGAATAGCGACAGCCAGACCCAATGGAATAACCGACCAGTGCCAGAGTGCTTCACCAGCCTGAAGCGCTCCAA
TTGGCACGAAAATTAACGCTGCAATCAACGAACCAATTGCCACCGTCGCAGGGCCATGTTCCGCTCCTGCGCGTTGCCCA
CTTAAAATGTAAATAGCCCAACAAGCCCCGGCCCCCAGTGCCAGCGCACAGCCGGTTAAATCGACATGGGAAACGTCTTG
CCCCAGCGGTAGCAGGAACCACAGACCAAGAACCGCCAGCACAACCCAGACGAAATCTACCGGGCGACGAGAAGAGAACA
GCGCCACCGCCAGTGGTCCGGTGAACTCCAGCGCCACCGCAATACCCAGCGGTACTGTCTGAATAGAAAGATAAAAAAGA
TAATTCATCCCACCCAGCGAAACGCCGTAAAACAACAGCGGTAACCGTTGCTCTTTGGCAAAGCGCAGTCGCCATGGCTT
AAAGAACGCGATGAGGATCAGCGTTCCTAATGCCAGACGCAGCGCAGTGACACCCGGTGCGCCCACCAGAGGAAAAAGTG
ACTTAGCTAACGAGGCTCCACCCTGAATAGACGCCATGGCAACGAGCAATATGACTATTGGTAACCAGACCGGCATTTTA
CGTAATGAACCAGGCATCCTTTCTCCCACAAATATCTAGACTTAAGTAAAGCGTGGAGTGTACTGGATATACCCAATGCT
GGTTGAGCATTTGTTGAAAAAATTTTCCCCCGTTTTGACTAAAATGCGCCAGGATTGATGGAATCATTAGTCTGGTGATT
AGGAATAATCTGGATGAATGACAGGGAAAACATGCGTAATACTTACGCAGTTCTCTGAAAAAGTGATTTAAATTTAGATG
GATAGCGGTGTATGGAAACGTTCTGTTACATGAAATGGCCCGTTAGACATCACAAATCGCGAAGAGTTTCCCATTAATTT
TTGATATATTTAAAACTTAGGACTTATTTGAATCACATTTGAGGTGGTTATGAAAAAAATTGCATGTCTTTCAGCACTGG
CCGCAGTTCTGGCTTTCACCGCAGGTACTTCCGTAGCTGCGACTTCTACTGTAACTGGCGGTTACGCACAGAGCGACGCT
CAGGGCCAAATGAACAAAATGGGCGGTTTCAACCTGAAATACCGCTATGAAGAAGACAACAGCCCGCTGGGTGTGATCGG
TTCTTTCACTTACACCGAGAAAAGCCGTACTGCAAGCTCTGGTGACTACAACAAAAACCAGTACTACGGCATCACTGCTG
GTCCGGCTTACCGCATTAACGACTGGGCAAGCATCTACGGTGTAGTGGGTGTGGGTTATGGTAAATTCCAGACCACTGAA
TACCCGACCTACAAACACGACACCAGCGACTACGGTTTCTCCTACGGTGCGGGTCTGCAGTTCAACCCGATGGAAAACGT
TGCTCTGGACTTCTCTTACGAGCAGAGCCGTATTCGTAGCGTTGACGTAGGCACCTGGATTGCCGGTGTTGGTTACCGCT
TCTAATCACTTTGGTGATATAAAAAATCCGCCTCTCGGGGCGGATTTTTGTTTTTAAGGTTTCGGGTCGAAAATATCGGT
TCCGAGATGGTTGTAATCCACCTTCTGTAACTGGAAGTTGGTGATGTATATCGGCCCGGCTTTCTTCTCAGATATAAACG

GGTATTTGATGTTAATTTCCTTCGCTTTAATCCCCGTCCACTGGGAGAAAAAGCCTAAGAAGTCATTGGCTGAGCGGCGG
GCTTTAATCACACGATGCGCTTTATCGTCGCTGGAAATGACCATAAAAGGCACCTGGAAATTTTGCTGATATTTATCATC
ATGGGCAAGGTATTGCACGTCTTTACCGCGCTCTTTAAAGGCCAGACCGTGGTCAGAAAAGTAAACCAGCGAGAAGCTGC
TGCCGCTGTTGCGTAACTGATCGTACAGCTTGCGCAGTAAATCGTCCGTTTGCGTCATGGTATAGAGATAGCACGACGTT
TCTTTCGATTGCACAAAGGTTTCGTATTTTCCTTGTGTCCTGTCGCAGGCCTGCGGATGTGAGCCCATCAGATGTAGAAC
AATCAGCTGCGGTTGCGAGTGCTCTTGCGCCAGCACTTGAGCGGTCATATCCAGTAACGCTTCGTCTTTGGTGTTTTTAT
CTGCTTCAAAATTACCTTCTTTCAGGAAGTACACTTCATCTGCTCGTTTGGCGATGCTGGCGATAGCGGTATCGTATTCG
CCGATTTGACCCTGGTTGGAAAACCACCAGGTCTGGAAGCCCGCGCGATTTGCCAGGGTGACAAAGTTATCCTGAAACTG
TGGTTTGCCATCGACAACGCGATTGAGCGTTAAGCCAAGCGATTTCTGCGTGGAGCCACTGGCGGCAATGTAGTCAGCAA
ATATCAAACCGTTAACGCTGCTGGCAAACGGGGTATTGTCCCAGTGACCGCCAAAGGCACCGAGGGCATCGCGACGCGCG
CTTTCACCGATCACCACCACATAGGTCTGATACTTCGGCTTAACGGCAGTGACCGTCCAGGTATCTTTCATTCCGGAAAG
TTTTGCCATCCGTTCTTGCTCTTCAATCACCTCGTTATTATTGACGATGACATCCTTAGCAAAACGAAATACCGGATAGC
CAGTATCTTTCAGTTTAAATACGCCACCCCAGGCCAGGTTTTGCACGGGAGCAACAAAAAATGTCGCCACACTGAATACC
AGACACAGGCTGTCGAAGGTATTCCAGCGCGCTTTTTCATTCTCTTTTTTTCGCCTGATTGCTATTACGCCGAGCGCAAA
AATAAATAAGCCGACCAGGTAGCTGTACCACGGAAAAATCGTCAGGATTTCGGTCGACTCTTCCATATTGGTGGAGTGCA
ATGCCAGCAATGTATTGAAATTAGGCGCGCCGTAGGCCTGAGCAAAAGGGAAATAACAAGCCGCCACCAGCGAACTGACA
CCGACCAGAACTTTTTGTACGCGAGGCAATGTTCGCCATAGCAAAAGCAAAATAGCCGTAAACGCAGCGGTGTAGAGCAA
ACTGAAGGGGTAACCTAAGCCGAGGTTAATTAATAGCGACTGTAAAAAGTAGAACGCAGTCCACGGGCTAAATACCCGGC
TACGGGTAACAAGCGATTCTTTGAGGGTTAAATTCATATGCCACTATCGTAACAAACGCCATGTGCTTACCCTGGCGTCA
AGGGTCAATACCTGCAAGAGTGCGTGAAGAGTGGAAAGGGGTCCGCATCCGCGAGCCGCAATATACGCAGGGCTGCAAGA
AGATAGAGCGAGCCGCCGTTCAGGTCAACTACTGACGAACAAATCTTTTGCGAAGGGGATTGCAAATCCGACAAAAAGTG
GGGTTTTTCAGACCAAAACGCGTTATGCCTGCATGAAAGCAGGAGTGAAGCGGCGTACCGCGACGCCGCTATTTATCGGA
AGGTTTATCTTGCTGCGGTTTGTTGTTGACCATATCGCACAACATAGAGAGCAGCATTAACCGTACTTTAAAGGGAGAAT
GACTAAACACGCGCATACACCTCTTGAACTCATTCATAAGACCTCCTGACTTGCTAATCCCGTCGATCCTTGAGGGATGA
TTGCATTACATACAGATATAGCACAGGCTATATTATATAGCTATTGCTAAAACGTTAATTTTTTGTACCTTCGCAACTCT
GGTTTACAATGTGCGCACGAAATGAGAACGCTATGTATGCGTCACCATAATGAGGAAGCACAATGAGTCGTCGCGCAGGT
ACGCCAACAGCAAAAAAAGTGACGCAGTTAGTGAACGTGGAAGAGCACGTTGAAGGGTTCCGCCAGGTCAGAGAGGCGCA
TCGGCGCGAGCTTATTGATGATTACGTTGAGCTGATTTCTGACTTGATCAGGGAAGTGGGGGAAGCTCGTCAGGTGGACA
TGGCTGCTCGTCTGGGAGTTTCGCAACCGACGGTGGCTAAAATGCTTAAGCGGCTGGCAACCATGGGGCTGATTGAAATG
ATCCCCTGGCGTGGCGTGTTTTTAACGGCAGAAGGAGAGAAGCTGGCGCAGGAAAGCCGCGAGCGACATCAGATAGTCGA
AAATTTCTTGTTGGTGTTGGGCGTCAGTCCGGAAATCGCCCGTCGCGACGCGGAAGGCATGGAGCACCATGTTAGTGAAG
AGACGCTCGACGCTTTTCGTTTGTTTACCCAGAAACACGGTGCCAAATGAGCCTGCCTTTTTTACGCACGCTGCAAGGCG
ATCGTTTTTTTCAGTTATTAATTCTTGTTGGTATCGGATTAAGCTTTTTCGTGCCCTTTGCACCGAAATCCTGGCCTGCT
GCTATCGACTGGCACACCATCATCACCTTAAGCGGCCTGATGCTGCTGACCAAAGGTGTGGAGTTAAGCGGTTATTTTGA
TGTGCTGGGGCGCAAAATGGTGCGCCGCTTTGCTACGGAGCGTCGGCTGGCGATGTTTATGGTGCTGGCGGCGGCGCTGC
TTTCTACCTTTCTGACCAACGATGTCGCGCTGTTTATTGTTGTTCCGCTGACTATCACGCTAAAAAGACTGTGTGAGATC
CCGGTTAATCGGCTGATTATTTTTGAGGCGCTGGCAGTCAACGCTGGTTCGCTACTGACGCCAATTGGCAACCCGCAAAA
TATTCTTATCTGGGGACGTTCTGGTCTTTCGTTTGCCGGATTTATTGCCCAAATGGCACCGCTGGCTGGCGCAATGATGC
TGACGCTCCTGCTCCTGTGCTGGTGTTGTTTCCCTGGAAAGGCGATGCAATACCATACGGGGGTGCAAACACCGGAGTGG
AAACCGCGGCTGGTGTGGAGTTGTCTGGGGCTGTATATCGTCTTTCTGACGGCGCTGGAGTTCAAACAAGAGCTGTGGGG
ACTGGTGATTGTGGCGGCAGGCTTTGCGCTGCTGGCACGTCGCGTGGTGCTCAGTGTGGACTGGACGCTGCTGCTGGTGT
TTATGGCGATGTTTATCGACGTCCATTTACTGACCCAGCTTCCAGCGTTGCAAGGCGTGTTGGGTAACGTGAGTCATCTA
TCTGAACCCGGGTTATGGTTAACGGCAATCGGTTTATCGCAGGTGATCAGTAACGTGCCGAGTACCATATTGTTGCTGAA
CTATGTGCCGCCGTCTTTATTACTGGTATGGGCGGTAAACGTAGGTGGCTTTGGGTTATTACCCGGATCGCTGGCAAATT
TGATTGCGCTACGTATGGCGAACGATCGCCGCATCTGGTGGCGTTTCCATCTCTATTCAATACCGATGCTGTTGTGGGCG
GCGTTGGTGGGATATGTTTTGTTAGTTATACTCCCGGCCAACTAGGGTCTGGCAAATAAAAAAGGCGGATTATGAGTCCG
CCTTTTGCTTTAAGTTTTGAAGATTAATTCAGACGAACCGGCATCCCGGAGCGGTTTTTAATCGCTTCATCAAGAACAAC
CTGGTCAACATCTGGCTGACCGGTCACTGTCTGCACGCTCTTCGTCAGGGTAATTGGCACAATTTCCTGACCTTCAAACT
GGGCTTCGGTGGTAGACAGCGGGTTATGGACTTCAATATAACGGCTGCCGTCTGGCTCGGTGGTCGCTTTTACCGGCTCA
TCAATAAACTGTACGCGGGTACCGACCGGTACTTTCTCGAACAGGAATTTGATGTCTTCGTTACGCAGACGCACACAACC
ATGACTTACACGCAGGCCGATACCGAAGTTGGCGTTGGTGCCATGGATAGCATACAGGCGACCGATATAGAGTGCATACA
GCCCCATCGGGTTATCCGGACCTGCCGGAACGACAGCCGGAAGCGGTTCGCCCGCAGCGCGGTACTCTGCGTGCATTTTG
GCGGTCGGCGTCCAGGTCGGGCCTGCTTTTTTACGCTCAACTTTGGTGGTCCAGTTGATAGGCGTATCTTTGCCTAACTG
ACCAATGCCGATCGGCAGCACGATAACGGTGTTGGTCCCTTTCGGATAGTAATAAAGACGCATCTCAGCACTGTTAATGA
CGATGCCTTCATGAACGGTATCCGGCAGGATCAGCTGCTGCGGAATGTTCAGTACAGTACCGCCTTTCGGCAGGAAGGTA
TCCACACCCGGGTTCGCTTCCATCATATTGGAAAGCCCCATCTGGTACTCGGCGGCAAAATACTCCAGCGGCTGAGTGTT

ACCTTCAGGAATGGTGATCACCTGATTCTGACCAACCAGGCGACTCCCGTCGGTTGGCAGAGGATAAGTTACCGCAGAGG
CGGTACTGCAAAAGCCGACAACAGCGAAGGCCGCTGCGAATAATGTTTTCAATTTCATATTCATGTTAAGCGAGATTTTG
TGCCTGGCAGGCCATTGGGTTGAGAATATTAGAGTATTGGAAGCGCATTATAAGTTCATTCCAGCTCACAGTGAAATCAG
ATGTGTACGAAATCACATTTTTTGCCTTTGGCTTGAGTGTAGACCTTAAGCGAGGAGCAGGATCTTCTTTCAGACTTATG
GCATAATGCGCGGTTTGTCATATCTCTTTTCAGGATACGCCTGTGTTAGTTTCCAGTAACGTCACCATGCAGTTCGGCAG
TAAGCCGTTGTTTGAAAACATTTCCGTCAAATTTGGCGGCGGCAACCGTTACGGCCTGATTGGCGCGAACGGTAGTGGTA
AATCCACCTTTATGAAGATCCTCGGCGGCGACCTTGAGCCGACGCTGGGTAACGTTTCCCTCGATCCCAACGAGCGCATT
GGTAAACTGCGTCAGGATCAGTTTGCCTTTGAAGAGTTCACTGTGCTGGATACGGTGATCATGGGGCATAAAGAGTTGTG
GGAAGTGAAGCAGGAGCGCGACCGCATCTATGCTTTGCCGGAAATGAGTGAAGAAGACGGCTATAAAGTGGCCGATCTGG
AAGTTAAATACGGCGAAATGGACGGTTACTCTGCGGAAGCTCGCGCCGGTGAACTGTTGCTTGGCGTGGGAATTCCAGTG
GAACAGCACTACGGCCCGATGAGTGAAGTTGCTCCTGGCTGGAAGCTGCGTGTGCTTCTGGCGCAGGCGCTGTTTGCTGA
TCCGGATATTCTCCTGCTCGACGAACCGACCAACAACCTCGACATCGACACCATTCGCTGGCTGGAACAGGTGCTGAACG
AGCGTGACAGCACCATGATCATCATCTCGCACGACCGTCACTTCCTTAACATGGTCTGTACCCACATGGCGGATCTGGAT
TACGGCGAGCTGCGCGTTTATCCGGGTAACTACGATGAGTACATGACGGCGGCGACCCAGGCGCGTGAACGTCTGCTGGC
CGATAACGCCAAGAAGAAAGCGCAGATTGCTGAGTTGCAATCTTTCGTTAGCCGCTTTAGCGCCAACGCCTCGAAATCTC
GCCAGGCAACTTCGCGCGCGCGCCAGATTGATAAAATCAAACTGGAAGAGGTGAAAGCCTCCAGCCGTCAGAACCCGTTC
ATCCGTTTTGAACAGGATAAGAAACTGTTCCGTAACGCGCTGGAAGTGGAAGGTCTGACCAAAGGGTTTGATAACGGTCC
GCTGTTTAAAAATCTCAACCTGCTGCTGGAAGTGGGTGAAAAACTGGCGGTACTGGGTACCAACGGCGTCGGTAAATCAA
CGCTGCTGAAAACGCTGGTGGGCGATCTGCAACCGGACAGCGGCACCGTAAAATGGTCTGAGAACGCGCGCATTGGTTAC
TATGCTCAGGACCACGAATATGAGTTTGAAAATGATCTGACCGTGTTCGAATGGATGAGCCAGTGGAAGCAGGAAGGCGA
TGACGAGCAGGCGGTACGCAGTATTCTCGGTCGTTTGCTGTTCAGCCAGGACGACATCAAAAAGCCAGCTAAAGTGCTTT
CCGGTGGGGAAAAAGGGCGGATGCTGTTTGGTAAGTTAATGATGCAGAAGCCGAACATTCTGATCATGGACGAACCGACC
AACCACCTGGATATGGAATCCATTGAGTCGCTGAACATGGCACTGGAACTGTATCAGGGCACGCTGATCTTTGTTTCACA
CGACCGTGAGTTCGTAAGCTCCCTGGCGACCCGCATTCTGGAAATCACCCCGGAACGCGTGATCGACTTTAGCGGTAATT
ACGAAGATTACCTGCGTAGTAAAGGGATCGAGTAAGTGATTATCCGTCACCCCGATACTGTCGGGGTGACGGCGGCTGAT
GACAAATGCAAAATAGCCTGATGCGCTACGCTTATCAGGCCTACGCAGTACCTGCAATATATTGAATCTGCAGGACTTTG
TAGGCCGGATAAGGCGTTAACGCCGCATCAGGCATTAACAAAGCGCATTTGTCAGCAATCTGAGTAACGTCGAATCGATT
TTCTTAATCCCACATCAATTCACTTCCATTAATTCTCAAACATCCATGCCCAACGCTCGCTTACCGTGAATGTTGAGGTC
GGCAAGCGTAAAGCGTCCTTCCCAGTTTGTTTCATAGTCCTCGCGCGGGAAGTCGCCCGGCGATGCGCCTTTTTCCAGTG
CGGCCTTCACCTTGTGCGCGTAGGCAAGATTTTTCTCGCACATCGGTGCCGCAGGAATGTACATCACGTTGCCCCAACCT
TGTTGATTTTCAACGGGGGCAACGGAATGGATGACGTCGCAGTGCCACCAGACGGAGTCTCCGGCTTCGAGTTTTGGAAT
GCTGGTTAACGCCTCAATCAACAGTGGATGCCATTGCTCTGATACCGGCAATACTCTTCCGGGCGCTACGCCGCACAGTT
CATCCTCCGGCACATCATCAAGCAGCGGACGTAACAGTACGTACGCCATAGCTTCAGGAATGGGCACGACGTGCAGCAGC
CCCTGACCAGGCAGCATATCAGAGAGCGCTGTCCAGCCCTGGAATGTCCGAAACACGGAACATTTGGTGGTGTTGTCCAC
CGTGTACTCTTCAACTTCCGTACGATGTGCCGCATGCCAGGGATCATATTGCGCCAGATTGCCATTAAAGACGTTGGCGA
AAACGCGCTGATACGCTGGAAGCAGCCAGCGTTCCAGTGCCCCGGAGTCGGTATGCGCTCCAAGACCTTTGGAGGTCGTT
CCGGGCGGACGGCGGCGGATACGGTCAGGGTAGATGACGCTCACATCCGGGTTAAACCATTGCTTTCCATCACTTTCAAA
TGTCCACAGACGATTGAGAAACGACTGCGCATTCGCCATTTCTTCACTCTGGCGGGCCTGCATTTGCGCCTGCGACCAGT
AGATGGGGTAAATCTCGGGACGTGAAGCGCTGAGCGTCCCGAAGAAATTATCGCCGGGGCCTTTGTAGACCTCGTCAAAG
CGGTTGCGGTCCAGATAGTCCAGCATCGACTGATCCCAGCCTAGCGCTTGTTCGCGGGGGAAATGGCCTTTTATCACCGC
ACAACCGCGACGTTTAATCTGTTCGCGCTGCTCTGCAGTAACATGACCTGCTTTGATATCGGCATAAGACAGCACCGGCC
AGACGGCATCGCCCTGTGCTTTGAGTGCGTTGATTTCAGCCACTCGCGTGGCAATGTCATCGCTTAGCTGATTAAAGATC
TGCTGGACGTCGCCAAGCTGCGCCCGCAGCGCGTGCTTCATCTGACGGATAGCTGCTTTGTGATCGGCAGGCAATGTGTC
GCTGGTAAAAGTAGAAGCCATAACCACCTCTCATTAACTTTCATTCGAAACCATTCTGATTTGCATGTGACAATATAAGT
TATAAATAAGTTAATGCAAGTTTAATGATTTGATGTGATGTACAGGATGTAAAAAGAAGGGAAAACCGGAGCGCACGGCT
CCGGTAGAGAGGTCAGCTGTTAAAAGGGGATGTGTTATCCAGCACCGCCTGAATCACATTCAGCGCGCCTTCATGATTAT
TATCATCGGTAGCGTAACGGGCGATTTGTTTAATGTTTTCCGCAGCATTGCCCATCGCAAAGGAATAACGCGCCATTTTC
AGCATCTCCGCATCGTTACCGCTGTCGCCAATCGCTACCACATTTTGCGGTGACAGATCCCAGCGTTTCAGTAACCGCGA
AATACCGTTTGCTTTATGTAGACCGGGAATAATCAGGTCGATAAAGCCAAAACCACTGGTAACGGGTTTCATAATGCCAT
CGAGCGCTACGTGCAGTTTGTCGATCACTAACGGGATTTGTTCATCCGGCAGGTTGAGCGAAAACTTGAACAGTACGTCG
TCAATCTCCTGATAATCTTTTACAGGTTTCAGGCGATGGTAGTGTTTTGCCATCAGTGCGACAAATGCTTCGGGGGCATT
TTCGCTGACATATGCACTTTGCAGACCGCAGGCGACAAAATTGAGTTGCTTATCTTTTAGCAACTCGCCAATAACAATCC
GCGATTCATGTCGGGTCAGTTCGCCGTGGAACAACTGCTTGCCATGTTCGTAAACCAGTGCGCCGTTTTCCGCGACAAAA
GAGATCTCATCCTTTAGCTCAGGAAAGAATGAAATAAGCTGGTAATACTGATTACCGCTGGCAACAACGAACTTAATGCC
GCGCTTTTTCAGTTCCTGATATTGCGCCATAAAACGTGGTTGGTTGTACGTTTTGGCGTCGTTAAGAAAAGTACCGTCCA
TGTCTGTGACGATAACTTTTACGCTCATAGGTGTGCTCCTGGCTCGAAAATGAAACCGTAACAGTGTAATAACAATGTGA

CGCAGAGCACAAATTATATTTCGAATGAAAGTAAGGATGAAATTGATGATGTGAATGATTTAGCCCGGCGACGACGCCGC
CGGGCCGAGGAGATTACAGCATATGTTCAGTACGGGCGATGATATCGTCCTGAGCGTCTGGAGAGAGCGCGGTGAAGAAC
GCGGAATAGCCCGCGACACGCACTACCAGATCGCGATACTGATCGGGATGTTTTTTCGCATCCAGCAGCGTTTCGCGGGA
AACGATGTTGTACTGAATATGCCAGCCTTTATGCACTTCAAAGAAGGTACGCAGCAGGATCATCAGTTTCTGCTTGTCAG
ATTCGTTCTCCAGCGTTGCCGGATTCAGTTTCTGGTTGAGCAACACGCCGCCGAGAATCGCTGCCGTAGGCAGTTTACCC
ACTGAGCCAATGACCGCAGTAGGGCCAAGATGGTCAGTACCGGAGGCCGGGCTTGCGCCTTCTGCCAGCGGGGTGTGGGC
TTTACGCCCGTCCGGTGTTGCCATAGTCTGCGCGCCAAACGGTACGTTAGCGGAGATTGATGACGTACCCGCGTAATAGT
TGCCGCCAACCGGACCACGACCGTAGCGCGGATTATGGTACTGTTTCAGTTCGTCGATATAGGTCTGATAAGCGCGAGCC
AGCAGCGTATCGACAGTATCATCGTCGTTGCCGTACTTCGGCGCACCGTTAATCAGCCGCTGACGCAGCTGCTCGTGAGT
CAGGCCGTCGAAGTCATCTGCCAGTGCGGCAGCAAGCTGTTGCTGACCAATCGCACCTTGTTCAAACACCAGTTTCTTCA
CTGCCGCCAGGCTGTTGCCGAGGTTGGCAATGCCGACCTGCAGGCCAGAAACCCAGTCATATTTCGCGCCGCCTTGCTTG
ATACTTTTCGCTCGCTCAATACAGTCATCCACCAGCGCCGAGCAGAGAATATCGTGCACGTTCTCTTCCAGCATGGTGTC
GACGACATATTCGATTTCGATTGATTTGCGGGTGTAGTAACGGATTTGCGTATCCCACGCGTCCATCACTTCATCGAAGT
TGTTGAAGTTACCTGCCGACAACGCTTTTTCTTGTGGCAGGAACACTTTGCCGCTGGTGGCATCATGCCCGCCTTCCAGC
GCCGCCAGCATCACGCGGGCGAAGTTGATAAAGCTCATGCCGGTACAGCGATAGCCCCATTTGCCACCGACGGCGGTTTC
TATACAACCAATCGCTGCGTAGTCATAAGCGTCCTGCGGTTCAATACCGAGTTTAATAAATTCCGGGATCACGATTTCGT
CGTTGTTGAACGCCGGCATCCCGAAGCCGCAACGGATCACCTGTACGCAGGCGTCGAGGAAATCGTTGCTCATTCCTGCA
TGGTAACGCACGCTGAGGTTAGGCTGAGTGGAACGCAGGCGACCGCAGGATTCGAGGATCGCGTAAGAGAGTGGATTCAC
CGCGTCCATTGGTTGACCATCAACCAGATTTTGCCCGCCAATAGTGACGTTCTGATACAGCGGACTTCCCGCAGAGGCTT
TTGAGTGTGAGCCGGAGCGGATCTTGTTCACTTCCAGCAGTTTCAGCCAGCAGCTATGCAGCATCTCGATGGCGTGTTCG
CGATCCAGCGTCTGGTTGAGTTCAACGTCGCGGCGATAGTACGGGTAGAGATACTGGTCCATACGACCAAACGATACTGA
GTGACCGTTAGATTCGATCTGCAAAATCAACTGGATGAAGTAACACAGTTGCAGCGCCTGCCAGAAAGTCTGCGGCGGCT
GGTGGGCGATAAGATCGCAGTTTTCTGCCATCGCCAGCAGTTCATCGCGACGGCTTTCGCGGGTTTCGGTCGCGGCCATT
TCACGCGCCAGGGCAGCGAAACGTTCAATGTGTTCACTGACTGCCACCAGCACGATATCAATCGCTTTCAGGAATTGCTC
ACCGTGTAAATCTTCCAGCACCGTCAGGTTGATGCGCGAGCGACGTTCCGCTACTTCCTCGCGCAGACCATCAAGCCCTT
TTTCCAGCAGCAGCGGGAAATTCACCGCCAGGTGCGCATCGCCGGAGGTCATATTGCCTTCCGCTTTAATGATTCCGGTC
GCCAGCAGACCTTTTTGCTCATCGGTAAACATGCCGTAGCAGCGATCCTGTACGGTCTGACCGCGCCACCACGGGCACAC
TTCATGCAGAACGCGTTTGTTCTCTTCGCTCACCGCAAAGCCAGCACCGGGACGATCTGCCAGATCATCAATCTCTTTTT
CGATCCACGAGACAGTATATTCCGGGAAGATCGGCGCGGCGCGAACTTCGCTTGCCTGGTTGCCAATGATCAACTCATCG
TGTTTGATCCAGATGGTGCGATTCGCCAGGTGATGCGCCAGTGCCAGCGCGCGACGTACCGGGATCGGCTTATCGAGATG
TTGTTGATACATCTCGGTATAGTGCTGCGCGCGCTCGGTACAGACTGGCGGTTTCACAATATGCACCAGCGCATTTTTGT
GCGCTTTAATGCGGTCGCTGAGCGTGTCCAGTTTCAGTGTGGTCATGGTTGTTATCCTCGTAAGGTCGCGGTTAACCCTT
TCTGGCAGGCATACTGCTGGGCAAAGTCGAGCAGTTCTGGCGCATCAAGCGGTTTTTCCGGGGCGTCATAGGGCAGATTA
AGTAAGTGATATTTGTTGATGCCCAGCGTGTGGTAGGGCAGAAAATGAATTTCGCCAACGTGCAGCTCGTCGGCGGCAAA
ATCGGTAATGGCTTTTACAGAGGTTTCGTCGGCATTAAAGCCCTGAATCAGCGGCACGCGGATAATGATTTTTTTGCCCG
CTGCGGCGAGTTTTTTCAGGTTATCCAGCACTCTGGCGGCGTTACCGTCGGTCCACTGTTTAAACGGCGCGTCGGCAACG
TGTTTTAAATCGGCAAGAAACAGATCGATATAGGGCAGAGAAGGGGCGATATATTTCCACGGCACATGCAGACAGGTTTC
TACCGCAGTATGAATGCCTGCCTCGTGGCTGGCTTGCAGTAGCGCCATCGCCATTTCCGGCTGCATAAAGGGCTCACCAC
CCGAAAGCGTTAAACCGCCGCCGCTGCGATCGTAAAACGGTTTATCGCGCAGAACGGTCGTCATGATCTCCTCAACGCTT
TTCACTTCACCACACACGGTTAATGCCTGTGTCGGACAGCAGTCGGTTAACGCCGTCAGATGCTCCGGGGTTAACTTTTC
CCGATGAATAAGCAAACCATTCAGCGCGCGCTCAATCACTTCCGGCGCGGCCTTAGCGCACAGCTCGCAGCCTTCCAGAC
ACAGTCGTGCGTCATACAGCAGATCCTGCGTGCGGGCGCGGCTTTCCGGGTTCTGACACCAGCGGCAGCCCAGCGAACAG
CCTTTAAGAAATACGACCGTGCGGATACCGGGGCCATCATGGGTCGAGTAGCGCTGAATATTGAAAATCATAGTTGCCTC
TCTATTTCGTTCAAGCATTAAAATACTTTCGAATGAAAGTTAGATTGATGTGCGTCAACTGTTCAGAGAGTTTTCCCGTG
ATAGTCTACATTCAGACAAAAAGTACATTTTGAGGATGGTTATGGAACTGTATCTGGATACTTCAGACGTTGTTGCGGTG
AAGGCGCTGTCACGTATTTTTCCGCTGGCGGGTGTGACCACTAACCCAAGCATTATCGCCGCGGGTAAAAAACCGCTGGA
TGTTGTGCTTCCGCAACTTCATGAAGCGATGGGCGGTCAGGGGCGTCTGTTTGCCCAGGTAATGGCTACCACTGCCGAAG
GGATGGTTAATGACGCGCTTAAGCTGCGTTCTATTATTGCGGATATCGTGGTGAAAGTTCCGGTGACCGCCGAGGGGCTG
GCAGCTATTAAGATGTTAAAAGCGGAAGGGATTCCGACGCTGGGAACCGCGGTATATGGCGCAGCACAAGGGCTGCTGTC
GGCGCTGGCAGGTGCGGAATATGTTGCGCCTTACGTTAATCGTATTGATGCTCAGGGCGGTAGCGGCATTCAGACTGTGA
CCGACTTACACCAGTTATTGAAAATGCATGCGCCGCAGGCGAAAGTGCTGGCAGCGAGTTTCAAAACCCCGCGTCAGGCG
CTGGACTGCTTACTGGCAGGATGTGAATCAATTACTCTGCCACTGGATGTGGCACAACAGATGATTAGCTATCCGGCGGT
TGATGCCGCTGTGGCGAAGTTTGAGCAGGACTGGCAGGGAGCGTTTGGCAGAACGTCGATTTAACCGGACGTTCTGCATC
CTCATAAATTGCTGATGACGTGGCGGAGTGCCGCGTCTTATCAGGCCTGGAGGTGGCAATTACTGCCCACACACCTCACA
CCCCGGATTACGCATCAGTTTCATTTCGCGAAACTGACAGGTCATCGCATCGTACATGACGATTTTCCCGCTGGCCGGTT
TGCCATAACCTGCCAGCATTTTGATCGCTTCCATTGCCTGCAACGAACCAATTACGCCGATCAACGGTGCCATTACGCCT

GCTTCCACGCAGGTTAATGCATTTTCACCAAACAAACGGCTAAGGCAGCGATAGCACGGTTCACCGTCCTGATAAGTAAA
GACGGTGATTTGACCTTCCATACGAATTGCCGCGCCGGAAACCAGCGGTACCTTCGCGGCAAAACAGCCTGCGTTCAGTT
GATTACGTACCGCAACGTTATCCGTACAGTCGAGCACCAGATCGTGTTCAGCAATCAATGCTGCAAGTTCTGCGTCATCC
AGCAGTGCATTGACTGGCGTAATCGCGATATGTGGGTTGATCCGCGTCAGGGCGTCACGGGCGGATTCCACCTTCGGTTG
CCCGACCGTGGCATCACTGTGCAGTGTCTGGCGTTGCAGATTCGAGAGCGAAACCGTGTCGAAGTCGAGCAGCGTCAGGT
TACCGACACCGGCGCTTGCCAGATACTGCGAGGCTGCACAGCCGAGGCCGCCCAGGCCCACTATCAGCACGCGAGAATCT
TTCAGCGCCTCCTGGCCGTCAAAATCAAAGCCGCGCAGAATGATTTGCCGGTTGTAGCGCAGCATCTCCTGATCGCTGAG
TTCCGCCATTACAGGCCTCCGAACAACGCGTTAAACGGTTCTACTTCCACCCATTCGCCCACTTCCACATTGCCGCGATC
GCGTTCCAGCACGATAAAGCAGTTGCCGAGGCTAAAGGAGCTAAATATATGTGAACCCTGATGTCCGGTGGTCGTCACTT
CCAGTTCGCCATCGGCGTTGCGTTGCAGCACGCCGCGCTGGAAATCAAGACGTCCTGGCGTTTTTTTCAGGCGGGACGCT
GTGCGTACGCGCTGGCGCGCGGGCAGGCCGCTGGCGGTGTTACCGCTTAGTTTTGCCAGCAAAGGCTGTACCAGTTGATA
GAAGGTCAGCGTCGCTGAAACCGGGTTGCCCGGCAGGCCGCAGAACCAGCTATTGCTGAGTTTACCGAACGCGAACGGTT
TACCTGGTTTAATCGCCAGCTTCCAGAAGGCGATCTCCCCCAGCTCTTCAAGAATCGTTTTGGTGTAATCCGCCTCACCC
ACTGAAACACCGCCGGAACTGATCACCACATCCGCCTGGCTGTCGGCTTCAATAAATGCGGCGCGCAGGGCATGGGGATC
GTCGCGGATAATCCCTAAGTTAATTACCTCGCATCCCAACTGTTCTAACATCAGGTGTACGGCGAGACGGTTGGTATCGT
AGATTTGGCCGTCGCCCAGCGGCTGACCGGGCAACTGGAGTTCATCACCGGTAGAAAAAAGCGCTACACGCACTTTACGA
ATCACCGGAACTTCGGCAATCCCCAGTGAAGCAATTACTGGCAGCTCTGCGGTAGTCAGGCGAGTTCCCGCCGGGAAAAC
AACCGCACCTGCAGAGATATCTTCACCGCGACGGCGAATATTTTGCCCGCTACGCACTTCAGCAGTAAAACGCACGCCAT
TGTCCATTTGTTCAGTCTGCTCCTGCATCACCACCGCTTCGCAGCCTTCCGGCACCGGCGCACCGGTCATAATACGAATG
CAGGTACCCGCAGGCCATTCACCATGGTATGGCTGACCGGCAAAGGATTTACCGGCAACGGGCAGCGGTTGCCCGGAGGC
AATATCGGCTAAACGCACCGCGTAGCCGTCCATTGCGGAGTTATCAAACCCCGGAACATCAAGCGGCGAAACGACATCGC
TCGCCAGAATACGACCAAAACACTGTACCAGTGGCAGCGTTTCCTGGGCGGTCAGTGGGGTGACGCGAGAAAGCATCTCA
TTAAGCGCGGTGTCGAGCGACATCAATCCGGTGGTAAATTCCATGAAAACACTCCTGCGGAGGCAAAATCGAATTTGCCT
ATTATGTCAGAAAAACGCCACAGACTGTATGCCACCTCGGGCGTAGCGCTGGGTCCTGCCTTTACATGCCATATCCATCT
TTCTATATTCAAAAATTGAATGAGTAATTCATAAAAATTCTGATATTTATAGCAAAAGTGGCGAACCACCCTTAATGGAC
GAATACTATGGGCAAAGCAGTCATTGCAATTCATGGTGGCGCAGGTGCAATTAGCCGCGCGCAGATGAGTCTGCAACAGG
AATTACGCTACATCGAGGCGTTGTCTGCCATTGTTGAAACCGGGCAGAAAATGCTGGAAGCGGGCGAAAGTGCGCTGGAT
GTGGTGACGGAAGCGGTGCGTCTGCTGGAAGAGTGTCCACTGTTTAACGCCGGAATTGGCGCTGTCTTTACGCGTGATGA
AACCCATGAACTGGACGCCTGTGTGATGGATGGTAACACCCTGAAAGCCGGTGCGGTGGCGGGCGTTAGTCATCTGCGTA
ATCCGGTTCTTGCCGCCCGGCTGGTGATGGAGCAAAGCCCGCATGTGATGATGATTGGCGAAGGGGCAGAAAATTTTGCG
TTTGCTCGTGGCATGGAGCGCGTCTCGCCGGAGATTTTCTCCACGTCTTTGCGTTATGAACAACTACTGGCAGCGCGCAA
GGAAGGGGCAACCGTCCTCGACCATAGCGGTGCGCCACTGGATGAAAAACAGAAAATGGGCACCGTGGGGGCCGTGGCGT
TGGATTTAGACGGCAATTTGGCGGCAGCCACGTCCACAGGCGGAATGACCAATAAATTACCCGGACGAGTTGGCGATAGT
CCCTTAGTGGGTGCCGGATGCTACGCCAATAACGCCAGTGTGGCGGTTTCTTGTACCGGCACGGGCGAAGTCTTCATCCG
CGCGCTGGCGGCATATGACATCGCCGCGTTAATGGATTACGGCGGATTAAGTCTCGCGGAAGCCTGCGAGCGGGTAGTAA
TGGAAAAACTCCCTGCGCTTGGCGGTAGCGGTGGCTTAATCGCTATCGACCATGAAGGGAATGTCGCGCTACCGTTTAAC
ACCGAAGGAATGTATCGCGCCTGGGGCTACGCAGGCGATACGCCAACCACCGGTATCTACCGTGAAAAAGGGGACACCGT
TGCCACACAGTGATGAACTTGATGCCGGTAATGTGCTGGCGGTTGAAAATCTGAATATTGCCTTTATGCAGGACCAGCAG
AAAATAGCTGCGGTCCGCAATCTCTCTTTTAGTCTGCAACGCGGTGAGACGCTGGCAATTGTTGGCGAATCCGGCTCCGG
TAAGTCAGTGACTGCGTTGGCATTGATGCGCCTGTTGGAACAGGCGGGCGGTTTAGTACAGTGCGATAAAATGCTGTTGC
AGCGGCGCAGTCGCGAAGTGATTGAACTTAGCGAGCAGAACGCTGCACAAATGCGCCATGTTCGCGGTGCGGATATGGCG
ATGATATTTCAGGAGCCGATGACATCGCTGAACCCGGTATTTACTGTGGGTGAACAGATTGCCGAATCAATTCGTCTGCA
TCAGAACGCCAGTCGTGAAGAAGCGATGGTCGAGGCGAAGCGGATGCTGGATCAGGTACGCATTCCTGAGGCACAAACCA
TTCTTTCACGTTATCCGCATCAACTCTCTGGCGGGATGCGCCAGCGAGTGATGATTGCGATGGCGCTGTCATGCCGCCCG
GCGGTGCTGATTGCCGATGAGCCAACCACCGCGCTGGATGTCACTATTCAGGCGCAGATCCTGCAATTAATCAAAGTATT
GCAAAAAGAGATGTCGATGGGCGTTATCTTTATCACTCACGATATGGGCGTGGTGGCAGAGATTGCCGATCGGGTACTGG
TGATGTATCAGGGCGAGGCGGTGGAAACGGGTACCGTCGAACAGATTTTTCATGCACCGCAACATCCTTACACCCGTGCG
CTGTTAGCTGCTGTTCCGCAACTTGGTGCGATGAAAGGGTTAGATTATCCCCGACGTTTCCCGTTGATATCGCTTGAACA
TCCAGCGAAACAGGCCCCCCCCATCGAGCAGAAAACGGTGGTGGATGGCGAACCTGTTTTACGAGTGCGTAATCTTGTCA
CCCGTTTCCCTTTGCGCAGCGGTTTGTTGAATCGCGTAACGCGGGAAGTGCATGCCGTTGAGAAAGTCAGTTTTGATCTC
TGGCCTGGCGAAACGCTATCGCTGGTGGGCGAGTCTGGCAGCGGTAAATCCACTACCGGGCGGGCGTTGCTGCGCCTGGT
CGAATCGCAGGGCGGCGAAATTATCTTTAACGGTCAGCGAATCGATACCTTGTCACCCGGCAAACTTCAGGCATTACGCC
GGGATATTCAGTTTATTTTTCAGGACCCTTACGCTTCGCTGGACCCACGTCAGACCATCGGTGATTCGATTATCGAACCG
CTGCGTGTACACGGTTTATTGCCAGGTAAAGACGCGGCTGCACGCGTTGCGTGGTTGCTGGAGCGCGTGGGCCTGTTACC
TGAACATGCCTGGCGTTACCCGCATGAGTTTTCCGGCGGTCAGCGCCAGCGCATCTGCATTGCTCGCGCGTTGGCATTGA
ATCCAAAAGTGATCATTGCCGACGAAGCCGTTTCGGCGCTGGATGTTTCTATTCGCGGGCAGATTATCAACTTGTTGCTC

GATCTCCAGCGTGATTTCGGCATTGCGTATCTGTTTATCTCCCACGATATGGCGGTGGTAGAGCGGATTAGTCATCGTGT
GGCGGTGATGTATCTCGGGCAAATTGTTGAAATTGGTCCACGGCGCGCGGTCTTCGAAAACCCGCAGCATCCTTATACGC
GTAAATTACTGGCGGCAGTTCCGGTCGCTGAACCGTCCCGACAACGACCGCAGCGTGTACTGCTGTCGGACGATCTTCCC
AGCAATATTCATCTGCGTGGCGAAGAGGTGGCAGCCGTCTCGTTGCAATGCGTCGGGCCGGGGCATTACGTCGCACAACC
ACAATCAGAATACGCATTCATGCGTAGATAACATTCAGGCGGAGAATAAAATGGCAAGAGCTGTACACCGTAGTGGGTTA
GTGGCGCTGGGCATTGCGACAGCGTTGATGGCATCTTGTGCATTCGCTGCCAAAGATGTGGTGGTGGCGGTAGGATCGAA
TTTCACCACGCTCGATCCGTATGACGCAAATGACACGTTATCTCAGGCCGTAGCGAAATCGTTTTACCAGGGGCTGTTCG
GTCTGGATAAAGAGATGAAACTGAAAAACGTGCTGGCGGAGAGTTATACCGTTTCCGATGACGGCATTACTTACACCGTG
AAATTGCGGGAAGGCATTAAATTCCAGGATGGCACCGATTTCAACGCCGCGGCGGTGAAAGCGAATCTGGACCGGGCCAG
CGATCCGGCGAATCATCTTAAACGCTATAACCTGTATAAGAATATTGCTAAAACGGAAGCGATCGATCCGACAACGGTAA
AGATTACCCTCAAACAGCCGTTCTCAGCGTTTATTAATATTCTTGCCCATCCGGCGACCGCGATGATTTCACCGGCAGCG
CTGGAAAAATATGGCAAGGAGATTGGTTTTTATCCGGTGGGAACCGGACCGTATGAACTGGATACCTGGAATCAGACCGA
TTTTGTGAAGGTGAAAAAATTCGCGGGTTACTGGCAGCCAGGATTGCCCAAACTGGACAGCATAACCTGGCGTCCGGTGG
CGGATAACAACACCCGCGCGGCAATGCTGCAAACCGGTGAAGCGCAGTTTGCTTTCCCCATTCCTTACGAGCAGGCCACA
CTGCTGGAGAAAAACAAAAATATCGAGTTGATGGCCAGTCCGTCAATTATGCAGCGTTATATCAGTATGAACGTGACGCA
AAAGCCGTTCGATAACCCGAAGGTCCGTGAGGCGCTGAATTACGCCATTAACCGTCCGGCGCTGGTGAAAGTTGCCTTTG
CGGGCTATGCAACGCCAGCTACTGGTGTGGTACCGCCAAGTATCGCCTACGCGCAAAGTTATAAACCGTGGCCTTACGAT
CCAGTGAAAGCGCGCGAATTACTGAAAGAGGCGGGATATCCCAACGGTTTCAGTACCACGCTGTGGTCGTCACATAACCA
CAGCACCGCGCAGAAAGTGCTGCAATTTACCCAGCAGCAGTTAGCGCAGGTCGGGATTAAAGCCCAGGTGACTGCGATGG
ATGCCGGACAGCGGGCGGCAGAAGTTGAAGGTAAAGGGCAAAAAGAGAGCGGCGTGCGGATGTTCTACACTGGCTGGTCG
GCTTCAACCGGCGAAGCGGACTGGGCACTATCGCCGCTGTTTGCCTCGCAGAACTGGCCACCGACGCTGTTTAATACCGC
GTTTTACAGCAATAAACAGGTGGATGACTTCCTGGCTCAGGCACTGAAAACTAATGATCCGGCGGAAAAGACCCGCTTAT
ATAAGGCGGCGCAGGATATCATCTGGCAAGAATCGCCGTGGATCCCGCTGGTGGTAGAAAAACTGGTGTCGGCACACAGT
AAAAACCTGACCGGTTTTTGGATCATGCCAGACACCGGCTTCAGCTTTGAAGACGCGGATTTGCAATAAGCAACGCAGGG
AGTGGAATGCTTAATTACGTTATCAAACGCTTACTGGGGTTGATTCCGACGCTGTTTATCGTCTCGGTGCTGGTGTTTTT
ATTTGTCCATATGCTGCCCGGCGATCCGGCGCGATTGATTGCCGGGCCCGAAGCTGATGCGCAGGTTATAGAACTGGTGC
GTCAGCAGCTGGGGTTGGATCAGCCGCTGTATCACCAGTTCTGGCACTATATCAGCAATGCTGTGCAGGGGGATTTTGGC
CTGTCGATGGTGTCGCGTCGTCCGGTTGCCGATGAGATTGCCAGCCGCTTTATGCCAACGCTGTGGCTGACCATAACCAG
TATGGTCTGGGCGGTTATATTTGGTATGGCGGCGGGAATTATCGCCGCCGTCTGGCGTAACCGTTGGCCGGATCGATTGA
GTATGACCATTGCGGTGTCGGGGATCTCGTTTCCGGCATTTGCTCTGGGGATGCTTTTAATTCAGGTATTCTCCGTTGAA
CTGGGCTGGCTGCCTACCGTGGGAGCAGACAGTTGGCAGCACTACATTTTACCCTCCCTGACGCTCGGCGCGGCAGTGGC
CGCCGTGATGGCGCGCTTTACCCGCGCGTCGTTTGTCGATGTTTTAAGCGAAGATTATATGCGTACCGCGAGGGCGAAAG
GGGTGAGCGAAACCTGGGTTGTCCTCAAACACGGGCTACGTAACGCGATGATCCCGGTAGTGACCATGATGGGCTTACAG
TTTGGCTTTTTGCTCGGTGGTTCCATCGTTGTGGAGAAAGTTTTCAACTGGCCGGGACTTGGACGCTTACTCGTTGACTC
CGTAGAAATGCGTGATTACCCGGTGATTCAGGCGGAAATTCTGCTTTTCTCGCTGGAATTTATTCTTATCAACTTAGTGG
TGGATGTGCTTTACGCCGCCATTAACCCGGCTATCAGGTACAAGTAAGGATGCGACTATTTAACTGGCGACGTCAGGCGG
TGTTAAACGCCATGCCACTGGTCAAACCTGACCAGGTACGTACACCGTGGCATGAATTCTGGCGACGATTTCGCCGTCAG
CATATGGCGATGACCGCCGCATTATTCGTTATTTTATTGATTGTGGTGGCCATTTTTGCACGCTGGATCGCTCCCTATGA
CGCCGAAAATTATTTTGATTATGACAATCTGAATAACGGACCTTCTTTGCAGCACTGGTTTGGCGTCGATTCACTGGGGC
GTGACATTTTCAGCCGTGTCCTGGTTGGTGCGCAAATCTCGCTGGCGGCGGGCGTGTTTGCCGTGTTTATCGGTGCGGCG
ATCGGGACGTTGCTGGGCTTGCTCGCTGGATATTATGAAGGCTGGTGGGATCGGCTGATCATGCGCATTTGCGATGTGCT
GTTTGCCTTCCCGGGTATTTTACTGGCGATCGCTGTTGTTGCGGTGTTGGGAAGCGGCATTGCTAACGTGATTATTGCAG
TCGCCATTTTTTCCATCCCCGCGTTTGCCCGCCTGGTGCGCGGCAACACGCTGGTGTTGAAACAGCAAACCTTTATTGAG
TCAGCACGCAGTATTGGTGCCAGCGATATGACCGTTTTGTTGCGTCATATCCTGCCTGGGACCGTCTCTTCTATCGTGGT
GTTTTTCACCATGCGCATTGGTACCTCGATTATCTCTGCCGCCAGCCTCTCATTTCTCGGCCTCGGTGCGCAGCCGCCGA
CACCAGAGTGGGGAGCAATGCTCAATGAGGCTCGAGCGGATATGGTTATCGCGCCGCATGTCGCTGTTTTTCCGGCCCTG
GCTATTTTTCTGACCGTACTGGCGTTCAATTTGTTGGGCGATGGTTTACGCGATGCGCTGGATCCGAAAATTAAAGGATA
GTTACGTTTGAATATTGCTTGAAAGGGTAATCACCTCACAGGAAATTATTGCCCTAAGCAAGTGTTGTAACTTTCTGCTG
ATTTTGTAGAATCGGGTAATTTGGTTAAAAAGCCGCAGCAAGGGACAATTTTTGCAGCGGCACAGCGTTCAGATAGTTAT
TTTGTTAAATGTATTAACATGCTGAGTTTATACGAAAAGATAAAGATAAGGCTGATAATTTTATTTTTATTGGCAGCACT
GTCATTTATTGGTCTTTTTTTCATCATTAACTATCAACTGGTATCGGAGCGCGCGGTAAAACGTGCCGATAGCCGCTTTG
AACTTATTCAGAAAAACGTTGGCTATTTCTTTAAAGATATTGAACGTTCGGCCCTGACATTAAAGGACTCACTGTATTTA
TTAAAAAATACAGAGGAGATTCAACGCGCCGTGATTCTTAAAATGGAAATGATGCCATTTTTAGACTCGGTGGGACTGGT
ACTTGATGATAATAAATATTATCTTTTTTCGCGGAGGGCGAATGATAAAATCGTTGTTTATCATCAGGAACAAGTAAATG
GACCGCTTGTCGACGAGTCAGGGCGGGTTATTTTTGCCGATTTTAACCCATCGAAACGACCGTGGTCGGTGGCTTCAGAT
GACTCTAACAACAGCTGGAATCCGGCATACAATTGCTTTGATCGTCCGGGTAAAAAATGTATCTCTTTTACGCTACACAT

CAACGGCAAAGATCACGATTTGTTAGCGGTGGATAAAATTCATGTCGATTTAAACTGGCGATATCTGAACGAGTATCTTG
ATCAAATCAGCGCTAATGATGAAGTTCTATTTTTGAAACAAGGCCATGAGATCATTGCCAAGAATCAACTCGCTCGTGAA
AAACTGATTATTTATAATAGCGAAGGTAATTATAATATTATTGATTCTGTCGATACTGAATATATCGAAAAAACATCAGC
GGTGCCAAACAACGCATTATTCGAAATCTATTTTTATTATCCTGGCGGTAATTTATTGAACGCATCAGATAAACTTTTTT
ATCTGCCGTTTGCGTTCATTATTATCGTATTGCTGGTGGTTTATTTAATGACCACTCGTGTGTTCCGTCGGCAATTTTCT
GAAATGACAGAGCTGGTTAATACGCTGGCGTTTTTGCCTGACTCAACGGATCAAATCGAGGCTCTGAAAATTCGTGAAGG
CGATGCGAAAGAGATTATCAGCATCAAAAATTCGATCGCGGAAATGAAAGATGCCGAAATTGAACGGTCAAATAAATTGC
TCTCACTGATCTCTTACGATCAGGAAAGTGGTTTTATTAAAAATATGGCGATTATTGAGTCTAACAATAATCAGTATCTG
GCTGTGGGGATCATCAAACTGTGTGGTCTGGAAGCCGTGGAAGCGGTGTTTGGTGTTGATGAGCGCAATAAAATCGTCAG
GAAATTGTGTCAGCGAATTGCCGAGAAATATGCGCAATGCTGCGATATCGTGACATTCAATGCCGATCTCTATTTACTTC
TGTGTCGGGAAAATGTACAGACATTTACCCGTAAAATAGCGATGGTAAACGATTTTGACAGCAGCTTTGGCTACCGCAAT
CTGCGCATCCATAAGTCTGCCATTTGTGAACCTTTGCAGGGGGAAAACGCCTGGAGTTACGCAGAAAAACTGAAACTGGC
GATTTCCAGTATCCGTGACCATATGTTCTCAGAGTTTATTTTCTGTGATGACGCGAAACTCAACGAAATAGAAGAGAATA
TCTGGATTGCGCGTAATATTCGCCATGCAATGGAAATTGGCGAACTATTCCTCGTCTATCAACCGATCGTTGATATTAAC
ACCCGCGCCATTCTGGGCGCGGAGGCGTTGTGCCGTTGGGTGTCTGCGGAGCGGGGGATCATTTCACCGCTGAAGTTCAT
TACCATTGCTGAAGATATCGGGTTTATCAATGAGCTGGGTTATCAGATTATTAAAACGGCAATGGGTGAATTCAGACATT
TTAGTCAGCGTGCGTCGCTGAAGGATGATTTCTTACTGCATATTAATGTTTCGCCCTGGCAGTTAAACGAACCACACTTT
CATGAGCGTTTTACCACCATCATGAAAGAAAATGGCCTGAAGGCGAACAGCCTCTGTGTTGAGATCACTGAAACCGTGAT
CGAGCGAATTAATGAACATTTTTATCTCAATATTGAACAACTGCGTAAACAAGGGGTACGGATATCGATTGATGACTTTG
GCACCGGTTTGTCAAACCTGAAACGTTTTTATGAAATTAATCCAGACAGCATAAAGGTGGACTCGCAATTCACCGGCGAT
ATTTTCGGTACTGCGGGAAAAATTGTGCGCATTATTTTCGACCTGGCACGCTATAACCGGATCCCGGTGATTGCGGAAGG
CGTAGAGAGCGAAGACGTTGCGCGCGAATTAATCAAATTAGGATGTGTTCAGGCTCAGGGGTATCTGTACCAGAAACCCA
TGCCATTCTCCGCCTGGGATAAAAGTGGAAAATTAGTAAAAGAGTAGTTTACGTATGTCCAGAATCAATAAGTTCGTACT
TACAGTCAGTCTGCTGATTTTTATCATGATTTCAGCAGTTGCCTGCGGGATCTACACTCAAATGGTAAAGGAACGGGTGT
ATAGCCTGAAACAGTCCGTTATTGATACTGCTTTTGCGGTGGCAAATATTGCTGAATATCGGCGTAGCGTGGCAATTGAT
CTTATCAACACGCTAAATCCCACGGAGGAACAGCTGTTGGTTGGTTTGCGCACAGCTTACGCCGACTCGGTTTCCCCCTC
TTATTTGTACGATGTCGGTCCTTATCTGATTTCCAGTGACGAATGTATTCAGGTAAAGGAGTTCGAGAAAAATTATTGTG
CAGATATTATGCAGGTTGTGAAGTATCGACATGTCAAAAATACAGGGTTTATCTCTTTTGACGGTAAAACCTTCGTCTAT
TACCTCTATCCGGTAACTCACAATCGTAGTCTGATATTTTTGCTTGGTCTGGAGCGTTTTTCTTTACTGTCAAAATCGCT
GGCGATGGACAGCGAGAACCTGATGTTCTCTCTATTTAAGAACGGTAAACCGGTGACCGGTGATGAATATAATGCTAAAA
ACGCCATCTTCACCGTTTCGGAAGCGATGGAGCACTTCGCCTATTTGCCGACCGGATTGTATGTATTTGCGTATAAAAAA
GATGTTTATTTGCGGGTTTGTACATTGATTATTTTCTTTGCCGCATTGGTGGCAGTGATATCGGGTGCCAGTTGCCTCTA
TCTGGTACGCAGAGTGATTAATCGTGGTATTGTGGAGAAAGAAGCCATCATTAATAACCATTTTGAACGCGTACTGGATG
GCGGGCTTTTCTTTTCGGCTGCCGATGTCAAAAAACTCTACAGTATGTATAACTCGGCGTTCCTGGACGACCTGACCAAA
GCAATGGGCAGAAAATCCTTTGACGAAGATTTAAAAGCGCTGCCGGAAAAAGGCGGTTATTTGTGCCTGTTTGACGTCGA
TAAATTCAAAAACATTAACGACACCTTCGGTCATTTGCTGGGCGATGAAGTGTTGATGAAAGTGGTGAAAATCCTTAAAT
CACAGATCCCGGTAGATAAAGGTAAAGTCTACCGCTTCGGCGGTGACGAATTTGCGGTGATTTATACGGGTGGAACGCTG
GAAGAGTTGCTATCGATTCTAAAAGAAATCGTTCATTTCCAGGTGGGAAGCATTAATTTAAGTACCAGTATCGGTGTAGC
ACATTCAAATGAATGTCCTACCGTCGAACGCTTGAAAATGCTGGCGGATGAGCGGCTGTATAAGAGTAAGAAAAACGGCA
GGGCACAGATTAGCTGGCAGTAATCATTATTCGCAGGCCGGACAAATGATTTTGCCCGGCCTGAATTAATTAAACCCGGC
TACCCCACAAATCGTACTCATCGGCGTGCTCGACTTTCACACGCAGGATATCACCCGGCTTAACGTTGGTTTCACCATTG
AGATAAACCGCGCCGTCGATTTCCGGTGCATCTGCCATGCTGCGACCAATCGCGCCTTCTTCGTCCACTTCGTCGATAAT
CACCAGAATTTCACGGCCCACTTTCTCTTGCAGGCGCTCGGCGGAAATCTGCTGCTGCAGCTGCATGAAACGGTTCCAGC
GTTCTTCTTTAACTTCTTCCGGAACCTGGTCAGGCAGGGCATTGGCGTCTGCACCTTCAACCGGGCTGTATTTAAAGCAG
CCAACGCGATCCAGACGCGCTTCTTTCAGGAAGTCGAGTAGCATCTGGAAATCTTCTTCTGTCTCGCCAGGGAAGCCGAC
AATAAAGGTTGAGCGTAGGGTCAGTTCCGGGCAGATTTCGCGCCACTGTTTGATGCGCGCCAGTTGGCGATCTACAGAAC
CCGGACGCTTCATCAGTTTGAGAATGCGCGGGCTGGCGTGCTGCAACGGAATGTCCAGATACGGCAGGATTTTGCCTTCT
GCCATCAGTGGGATGACGTCGTCCACATGCGGATAAGGGTAAACGTAGTGCAGACGTGTCCAGATCCCCAGTTTCGATAA
CTGTTCGCACAGGCTGACCATGCTGGTTTTTACCGGCTCGCCGTTGTGGAAGCCAGTACGATGTTTAACATCAACGCCAT
AGGCGGAAGTATCCTGCGAGATCACCAGAATCTCTTTAACGCCCGCATCTACCAGACGTTTCGCTTCACTTAACACTTCG
CCAATCGGACGGCTCACCAGGTCGCCGCGCATAGACGGAATAATGCAGAAGGTGCAGCGGTGATTACAGCCTTCAGAAAT
TTTCAGATAGGCATAATGACGCGGCGTCAGTTTCACACCTTGTTCTGGCACCAGGCTCAGGAATGGGTTGTGTTTCGGTT
TTGGCACGTAGTGATGAACGTGCTCCAGAACCTGCTCATAGCTATGAGGCCCGGTGATTTCCAGCACTTTCGGGTGGACT
TCGCGGATCTGATCTTCTTTTGCCCCCAGACAACCGGTCACAATAACCTTGCCGTTTTCATTCAACGCTTCACCAATGGC
TTCCAGTGATTCTTGTACCGCGCTGTCAATAAAGCCGCAGGTGTTGACGATCACCATGTCCGCATCGTCATAGCTCGGTA
CCACGTCATAACCTTCAGTGCGGAGTTCGGTGAGAATACGCTCTGAATCAACAAGGTTTTTCGGACAGCCAAGGGAAACA

AAGCCGATTTTCGGCTGGGGAGTTACTTTGCTCATAGCTTAAAAAATATTCAGTTACAGGAAAGGTCAGGGCAGGGATTC
TACAGAGTTCTGGATAAAATTTGTATCGCAATCTCATTCGCTGGCGGAGGCGAAGGAAATGTAAATTTTGTTAATTCGGC
GTGAAGAATTGATCCTGGACAGCATTTTGCTCAAAAAATAGCCATACTATTTAATTGCAACAAGGCTGGGAAGAGGAGGA
TCGAAGTATGTTCGTTGACAGACAGCGAATCGATCTGCTGAACCGGTTGATCGACGCACGCGTTGACCTCGCCGCATACG
TGCAACTGAGGAAGGCAAAAGGATACATGTCCGTCAGCGAAAGCAATCATCTACGAGATAACTTTTTTAAACTGAATCGC
GAACTGCACGATAAATCGCTGCGGTTGAATCTTCATCTGGATCAGGAAGAGTGGAGTGCTCTTCATCATGCTGAAGAAGC
ATTAGCGACAGCCGCAGTATGTTTGATGAGTGGGCACCATGATTGCCCGACTGTTATTACCGTCAACGCCGATAAGCTTG
AAAATTGTCTGATGAGCTTAACGCTGAGTATCCAGAGCCTGCAGAAGCACGCCATGCTTGAGAAGGCCTGAAAACTAAGG
GGGAGAAAGCGTCTCCCCCTTCATGTTTAAGTTTTTGTAAAAATGAATTTGTTATCTCCTCCACTGACTACGCTTTAAGC
CAGAGTCAATCCGGAGGCGTTATGCATCGACAATCCTTTTTCCTTGTGCCCCTTATTTGTCTTTCTTCCGCTCTCTGGGC
GGCTCCTGCAACGGTAAATGTCGAAGTACTGCAAGACAAACTCGACCATCCCTGGGCACTGGCCTTTTTACCCGATAATC
ACGGTATGTTAATCACTCTGCGCGGCGGCGAGTTGCGTCACTGGCAAGCAGGAAAAGGATTATCTGCGCCGCTTTCCGGA
GTTCCGGACGTTTGGGCGCACGGGCAGGGCGGCCTGCTGGACGTGGTTTTAGCGCCTGATTTTGCTCAGTCTCGCCGCAT
CTGGTTAAGTTATTCCGAAGTTGGCGATGATGGCAAAGCCGGAACTGCTGTGGGTTATGGCCGCTTAAGTGATGATCTCT
CAAAAGTGACCGACTTCCGCACCGTCTTTCGCCAGATGCCAAAACTGTCTACCGGCAACCATTTTGGCGGGCGGCTGGTA
TTCGACGGTAAAGGTTATCTTTTTATTGCTCTGGGCGAAAACAATCAGCGCCCGACGGCGCAGGATCTGGATAAATTACA
GGGCAAACTGGTGCGTCTGACCGACCAGGGCGAAATCCCGGATGATAATCCTTTTATAAAGGAATCCGGTGCGCGCGCCG
AGATCTGGTCTTATGGCATTCGTAATCCGCAAGGAATGGCGATGAATCCGTGGAGTAATGCACTGTGGCTGAATGAACAT
GGCCCGCGCGGTGGTGATGAAATTAATATCCCGCAAAAAGGCAAAAACTACGGCTGGCCGCTGGCAACCTGGGGAATCAA
CTATTCAGGCTTTAAGATACCGGAAGCGAAAGGGGAGATCGTCGCCGGGACCGAGCAACCTGTTTTTTACTGGAAAGATT
CGCCCGCTGTGAGCGGCATGGCCTTCTATAACAGCGATAAATTCCCCCAGTGGCAGCAAAAATTATTTATTGGCGCGCTG
AAAGATAAAGATGTCATTGTGATGAGCGTCAACGGCGACAAAGTGACAGAAGATGGCCGTATTTTAACGGACAGAGGGCA
GCGAATTCGTGATGTTCGCACTGGACCCGACGGTTATTTATACGTTCTCACCGACGAGTCCAGTGGGGAATTACTTAAAG
TTAGCCCACGCAATTAGCTAACGGGAATCATCACCACTTTGCGCACTGCGGGGCGTTCAGTGAGTTGCTGATACCAGCGT
TGCAGATTTGGACGCGGTGTCCAGGTCAGGCCAACGTTAAACAAATTGTAGATGAACGGTGCGATAGCGATATCGCCCAC
ACCAAACTCGTCGCCAGAGAACCATTTTACTTTTGCCAGTTCCGCATCGAGCAGGGCAAACAGGGCGTCGCACTCTTTGC
AACTGGCATCAATGGCGGCCTGATCGCGCTCTTCCGGTGGTGTTCTGACTAATCCCATCAGGATCCCGCGATGAGCATTA
CTGAGCGTCTGGTTTGCCCAGTCCATCCATTTTTCCGCTTCCGCACGACGTGCCGGTGAGTCGATCCACAGGCGTTTTTG
CCCGTACTGTGCCGCCAGATAGCGGACAATGGCGTTTGATTCCCAAAGAATGAGATCACTTTCGTCGTCACGCAACAACG
GCACCAGCCCGTTAGGGTTCATCGCCAGAAAATCAGCATCGTGATTTATCCCAAACTCACGGCCCGCGAGAATTTGCTCA
TAAGGTAGTTCCAGTTCTTCGAGCGTCAGCAATACTTTTTTTACGTTAGTTGAATTATTCCGACCCCACAGCGTAATCAT
ACTCACCCCTTTTCCGACAGGCAGCCACAGGCTGAACAGGACTCCGATGGTGAAGTAAAGTTAACTTTTAAGCAACAGCT
GGCAAAAAAATGACACCAGAAGCAGCGAAGCGGCAGGATATTGCATAAACTTTAAAAACTTTACCAACTTACGGTTTCTT
TAAGTTTGTGTGTGCGTTATTAATCACCAAACTTATCATACGGCGATATAACGTATTTTTTTTGAATGGATACTCGGGTG
GCATTTATGACGCAATACTCCTCTCTCCTTCGTGGTCTTGCAGCGGGTTCTGCATTTTTATTCCTTTTTGCCCCAACGGC
ATTCGCGGCGGAACAAACCGTTGAAGCGCCGAGCGTGGATGCGCGTGCATGGATTTTAATGGATTACGCCAGCGGTAAAG
TGCTGGCAGAAGGCAATGCGGATGAGAAACTGGATCCCGCGAGCCTGACTAAAATCATGACCAGCTATGTGGTTGGGCAG
GCGCTTAAGGCCGATAAGATTAAACTCACCGATATGGTGACGGTCGGTAAAGATGCCTGGGCGACGGGAAATCCGGCACT
GCGTGGTTCATCGGTAATGTTCCTCAAACCGGGCGATCAGGTTTCGGTGGCAGACTTGAACAAAGGTGTGATTATCCAGT
CCGGTAATGACGCCTGTATTGCGCTGGCTGATTACGTTGCCGGGAGCCAGGAGTCATTTATTGGTCTGATGAATGGTTAT
GCCAAAAAACTGGGTCTGACCAACACTACCTTCCAGACGGTGCACGGCCTGGATGCGCCGGGGCAGTTCAGCACCGCGCG
CGATATGGCATTGCTGGGTAAAGCATTGATCCACGATGTGCCGGAAGAGTACGCCATTCATAAAGAGAAAGAGTTCACCT
TCAACAAAATTCGTCAGCCTAACCGTAACCGTCTGCTGTGGAGCAGCAATCTGAATGTTGATGGCATGAAGACAGGAACC
ACGGCAGGCGCGGGATATAATCTGGTTGCTTCGGCTACCCAGGGCGATATGCGTTTAATCTCCGTAGTGTTGGGGGCGAA
AACCGACCGTATCCGTTTTAATGAGTCTGAGAAATTATTGACCTGGGGTTTCCGCTTCTTTGAAACCGTGACGCCAATTA
AACCTGATGCCACCTTTGTGACTCAGCGCGTCTGGTTTGGTGATAAGAGCGAAGTGAATCTCGGGGCAGGTGAAGCGGGC
TCAGTGACCATACCGCGTGGGCAGCTGAAAAACCTGAAAGCGAGTTATACGTTAACGGAACCGCAGCTTACCGCACCGCT
GAAAAAAGGTCAGGTTGTCGGGACCATTGATTTCCAGCTTAACGGTAAATCCATTGAGCAGCGTCCGCTGATCGTGATGG
AAAATGTGGAAGAGGGCGGATTCTTTGGTCGGGTGTGGGATTTCGTGATGATGAAATTCCATCAGTGGTTCGGCAGCTGG
TTCTCTTAATCTTCTGATAACCGGATGGCGCGAAACGTCATCCGGTTATACGTCATTAATACATCAACTTAATGCGCTGC
GTCTGCGCGTACTTCACATACTCATCTTCCGGGCAACAATCGCTCACCACAATATCAAAGCGTTTCAGGTCACCCATGCG
CGCCGGACGCACCTTGCCAAATTTACTGTGGTCGACAACCAGCACATGCTTTTGCGCCATCGACATGGCCCAGTGTTTTA
CCGGCAACTCTTCAAGATTAAAACAGGTAGCGCCTTTACTGACATGCACGCCCGCCGCAGAATAAAAAGCGATATCCGGG
CAAAAATTATTCAGCGTTTGCTGAAAATCGATGGGTTTGAAAATGGCGTTGCTGGCGTGAAATTCACCACCGCAAAGAAA
CGCGCGGCAATGGGGTTTCTCTTTCAGCGCCAGAAAGGTATTTAGCGAATAACAAACGGCGGTAAAAGGGATTTCATTAT
CAATCGCTTCAATAATCCACGGCGTGGTGGTGCCACAGTCAAAAAAGAGGGTCTGATCGGGTTCTACCAGCGTCGCAGCC

AGTTTTGCCGCCCGGCGTTTTTCTTCCACCAGGCGGGATTTTTGATCGCTTAACAGGTAATGGCTGGCACTGCGCGGTTC
CAGAACAATATAGCCGCCGAGCAAAACGACGGGCGCACTGTGGTTGTTCAGATCGCGACGAATCGTCATCTCCGAAACCC
CAAGCAGGGCGGCGGCGTCTTTAAGATGTAACTTATCGCTGCGTTTTAATTCTTGCAGCAGCTGCCCGATACGCTCTTCG
CGACGTGTTTCCATAATCCCTCTGAATAGTTATTGAAGCGAGCCGCTCAATACTACACTTTTTAGCAGAGATCAGTCACG
CACCCAGCCTTTGCGGATCGGTAATGCAAAACAGACGCGATACCACGATTGCAGACGTTGATAGAGTTTATGCCCCATCG
CTTGCCAGATAATCTGGGCACTAAGGCAGCCAATCATACCTGCCAGCAATCCACCGAGCATATCCAGCGGCCAGTGGACG
CCAAGATAAACGCGCGACCAGGCAATGACGACGGCCAGCACCATTAAAAGTGAGCCGGACCACAGGCGATGCCAGCATAA
AAATGCCAGTGCAAAGGTGAAAATCACCGTACCGTGATCGCTTGGGAATGAGTCATCCGCCGCATGATGCAGGAAGTTAT
AGCCGATATTTTCGACAAAGGGTCGGTCGTGCGGAAAAAGATGTCCCATCGTCCAGGACACAAACAGGCTGACCGCCAGC
GCGATAGCGATTTTTATCACCAGTTGCCGTTGTGCTGTAAGCCCCCACAACCAAAGTACCACGGCCAGCAACGGCACCAC
GGTAATCAAATCTTTAGCAATAAAAATCGCCAACGAGATCATCCACGGAGCCGAGTCTGGCGTCGCGTTAATAAGAGAGA
ATAGAGAGAGATTCAAATTTTCCAGCATAACTTCCCGACGCAAAGTGATTAAAAGGGAGCCAATACAGGCAAGTCGTTGA
GAATAAAGTGCAGGTTAAACTGGGTAAAGCGGCATCGTCTTATTTCCCTCAAGCGGCCTGTTTACGGTGGGTGATTGTAA
CGGGCATAGGTTAAATAAAACTTAAAGAAAGCGTAGCTATACTCGTAATAATGTAAGAATGTGCTTAACCGTGGTTTCAG
CTACAAAATTCGCTTTCTCGTTAGCTGCGCTTTTATTAAACTCTGCGCGATTATTATTGGCGAAGAAATTGCATGCAAAA
TAAATTAGCTTCCGGTGCCAGGCTTGGACGTCAGGCGTTACTTTTCCCTCTCTGTCTGGTGCTTTACGAATTTTCAACCT
ATATCGGCAACGATATGATTCAACCCGGTATGTTGGCCGTGGTGGAACAATATCAGGCGGGCATTGATTGGGTTCCTACT
TCGATGACCGCGTATCTGGCGGGCGGGATGTTTTTACAATGGCTGCTGGGGCCGCTGTCGGATCGTATTGGTCGCCGTCC
GGTGATGCTGGCGGGAGTGGTGTGGTTTATCGTCACCTGTCTGGCAATATTGCTGGCGCAAAACATTGAACAATTCACCC
TGTTGCGCTTCTTGCAGGGCATAAGCCTCTGTTTCATTGGCGCTGTGGGATACGCCGCAATTCAGGAATCCTTCGAAGAG
GCGGTTTGTATCAAGATCACCGCGCTGATGGCGAACGTGGCGCTGATTGCTCCGCTACTTGGTCCGCTGGTGGGCGCGGC
GTGGATCCATGTGCTGCCCTGGGAGGGGATGTTTGTTTTGTTTGCCGCATTGGCAGCGATCTCCTTTTTCGGTCTGCAAC
GAGCCATGCCTGAAACCGCCACGCGTATAGGCGAGAAACTGTCACTGAAAGAACTCGGTCGTGACTATAAGCTGGTGCTG
AAGAACGGCCGCTTTGTGGCGGGGGCGCTGGCGCTGGGATTCGTTAGTCTGCCGTTGCTGGCGTGGATCGCCCAGTCGCC
GATTATCATCATTACCGGCGAGCAGTTGAGCAGCTATGAATATGGCTTGCTGCAAGTGCCTATTTTCGGGGCGTTAATTG
CGGGTAACTTGCTGTTAGCGCGTCTGACCTCGCGCCGCACCGTACGTTCGCTGATTATTATGGGCGGCTGGCCGATTATG
ATTGGTCTATTGGTCGCTGCTGCGGCAACGGTTATCTCATCGCACGCGTATTTATGGATGACTGCCGGGTTAAGTATTTA
TGCTTTCGGTATTGGTCTGGCGAATGCGGGACTGGTGCGATTAACCCTGTTTGCCAGCGATATGAGTAAAGGTACGGTTT
CTGCCGCGATGGGAATGCTGCAAATGCTGATCTTTACCGTTGGTATTGAAATCAGCAAACATGCCTGGCTGAACGGGGGC
AACGGACTGTTTAATCTCTTCAACCTTGTCAACGGAATTTTGTGGCTGTCGCTGATGGTTATCTTTTTAAAAGATAAACA
GATGGGAAATTCTCACGAAGGGTAAAAAAATGCCTGACTGCTTTGTGCGATCAGGCATTCTCGAATTAATGGTGATGGTC
GTCAATCTGGTGTTCGATAACCATCCCTTCACCTACGCTGGCAAGATGGCGAACATAAGGATGCGGGCGGTAAGCCGGAG
CTGGCGCAGGAGCCACATAGACGGTTTGCGGCACAGTCGCGACGCTGACCGCTTGTGGAACGCTGACTGAATCAGGAACC
ACAACCACTTTGTATCCACTCGGCACATCGACGGTGACACTTTGCGCCATCGCCACACCAGTAAAGCCCATTAAAAGCGC
GCCCAACAGTAGACAATTTTTCATAATCATTTCGCCTGAAATAATGCAGCGGGTTAATTTCCGCGAATTATGCAGCCTTC
ATTCAGGCATTGTGTTGCCGGGCGTGCTGCCGGTTTTATTGGTCAAATGGCGCTTCGTGTTTAAGAACTTTATCGATCAC
ATCCAGTACGCCTTCACGGTTATTGGAGCCTGCGCGGTATTTTGCCGCTGCGACGACCGCGCTACCGGCATTTTCCATTG
CAAAACTAAAGCCTGCCTGACGCAGCATCTCAATATCGTTACCGCCATCGCCAAAGACCACCACTTCGCTGTCGTCTATT
CCCCATAATTTCTGCAGTTGGCGAAGGCCATTGGCTTTATGTACGCCGGGGATAATCAGATCGATGCTGCCGTTGCCGGT
GTGGACCGACACCATAATATCGCCGATGGCCTCATGTAATGCTTTTTGTACTTGTGGAATCAGTTCATCGGAAAGATTCA
GGCCAAACTTAAAGAAGATATCCTCTAAGTTGTCAAAGTTATCGACGTATTCCAGACGGTGATAATACATTTCCGCCACC
GTTTTCATGGCATCGTCATATTTTTTGAGTGTATAGGCACTATTTTTTCCGCAGGCAATAATTTCCACTTCCGGGCGCGT
CAGCAAATGTTCCACGACAGTAGCAAACGCATCCTTCGATAGCTCGCCATTAAAAACATCTTTGCCTTCGCTCACTACCC
AGCCGCCGTTTTCAGCCACAAAGGCAATTTCATTAGCAATTTCAGGGAAGAAAGAGATCAACTGATAATATTGATTCCCG
CTGGCGACTACAAAGCGAATTCCTTGTGCTTTCATTTGCTGATACTGAGCCATAAACCGCTCACGGTTATAGGTTTTTTG
ATCGCTTAAGAAAGTACCATCCATGTCTACCGCAATTAATTTAATGCTCATCAACTATTCTCCATCGCCGTCTGCGTTTT
GGTATCGGGTTTGGCGACGGCTTTCGCGACAATAGCCGCGAGAATAACCAGCGCCAGTACAACCAGCATTGCACTACGTA
ATCCATAATGTTCGCCGAGATAGCCCAGCAGCGGCGGCCCGACGAGGAAAGCCAGATAACCGGTCGTTGCTACCACACTG
ACGCGGGTTGGTGCATCGGGGCCGGTATCGCTGGCGGCAGAAATGGTCAGCGGGAAGCCCAGCGAGGCACCCAGTCCCCA
GAGTACAACAGACACCCCAGCGACCCAGGCGCTATCGACAAAAATAATCAGCCCAATACCCAACGCCCCCATTAGTGCAC
TGGCCCGAACCACGGCAACGCGACTGTAACGGTCGATGAACCAACCGCCGGTGAAGCGTCCAACGGTCATCCCCAGGGTA
AAACCGGCATAAATCAGCGAGCCGGAAGTAGGGCTAAAACCGTGACCGTCAACCATTAATAAGGGTAACCAGTCGTTGGC
AGAACCTTCGGCAAAGGCCATCGCCAGCACCACAACACCTATCAGCAGCAACTGGATATCGCGATAAAAAGGTACGCCTT
TTTCGCCATGCTGGGTGCCATCGGCAGCATTTTTGCCCGTACCGTCAGGGATTGCCTGAATGGCGATATAAATAGGTGCG
ATACCTACCAGCGCCGCCAATAAAATGTGCACCGTTGCCGGAACGCCAAAGGCCGTCAGTGCCATCCCGACACCTGCGCC
TGCCAGCGTGCCCAGGCTATAAAAACCGTGCATCATCGGCAAAACCGTCTTATTCATTTCTCGCTCAACGGCAGCACCTT

CAACGTTTATCGCCACTTCCGCAGAACCAAAACTTGCCCCAAAGACGCCGAGACCAACGGCAAAGAGCAGGGGCGATGTC
AGCCAGAGTGCCAGACTTAATATCATCATCCCGATCAATGCGCAGGACATCGTGACCAGGATGACATTACGTGTCCCAAA
GCGTTTCACTAACCACGCCGAGCAGAGAATACCGCTCATCGAACCGATCGACAGACCAAAGAGAACACCGCCCATTTCAG
CGATCGAGACAGAGAGAATATCGCGGATAGCAGGCGTACGGGTTGCCCAGGACGCCATTAACAGGCCTGGCAAAAAGAAG
AACATAAACAGCGCCCAGGTTCGGCGTTTCAATGCATTACGTGAAGAATTTACGGTCATAGATCACGTCAAAATAAGAAG
AGGGAAGACAACATTAGCAAGGTTGTGTACATTTGTACACAATTGCAGAGAGAGGAAATGACATGCGTCGCGCTAACGAT
CCGCAACGGCGAGAAAAAATTATCCAGGCCACGCTGGAGGCGGTGAAACTTTACGGAATACATGCTGTTACGCACCGCAA
AATTGCTACCCTTGCCGGGGTACCGTTGGGATCGATGACCTACTATTTTTCAGGAATTGATGAGTTGTTACTGGAGGCGT
TCAGCAGTTTTACTGAGATCATGTCCCGGCAATATCAGGCATTTTTTAGCGATGTTAGTGATGCTCCGGGCGCATGCCAG
GCTATCACCGATATGATCTACAGCTCACAGGTTGCAACGCCGGATAACATGGAGCTGATGTACCAGCTCTACGCGCTGGC
TAGCCGAAAACCGCTATTAAAAACGGTAATGCAAAACTGGATGCAGCGCAGTCAGCAAACGCTCGAACAATGGTTTGAAC
CCGGAACCGCCCGCGCGCTTGATGCGTTTATTGAGGGGATGACGCTGCATTTTGTCACCGACCGTAAGCCGCTATCGCGC
GAGGAGATTTTGAGGATGGTTGAGAGGGTTGCAGGGTAGTAGATAAGTTTTAGATAACAAAAAACCCATCAACCTTGAAC
CGAAATGGCGGGGTTGATGGGCTCCACAAAATGGGGACATCAAAGAAAAGCAGTGGCACTAATTAAGACTGATGCCCTGC
GGAAAAGTTCTGCGGTTGTGCAAAAAAATTTCATTTTCAGGGCAACTTCAGTTTTATCCTAATCCTGGCCATACCATGAC
GATGATTGTCCCTGCCAGCGTCAGCAGGACGTTGGCGATTGCATAGGTGCCCGCATAGCCCAGCGCCGGGATGTTACTGC
GAGCTGTATCACTGATGATCTCCATTGCCGGCGCGCAGGTACGTGCGCCCATCATTGCGCCGAACAACAGCGCGCGGTTC
ATTCGCAATACATAAGCACCGAACAAGAAACAGATAACCACGGGCACCAGACTGACAATCAATCCGGCAATCAACATCTG
ACCGCCAATCGCGCCCAGGCCGTTATTAATACCGCTACCGGCGCTCAGACCAACGCCTGCCATAAACACCATCAAGCCGA
ACTCTTTCACCATGCTTAATGCACCCTGCGGAATGTAACCGAAGGTCGGGTGGTTAGCACGCATAAAGCCCAGCATAATT
CCGGCGAATAACAACCCGGCAGCGTTCCCCATGCCGAAACTGAATGTGCTGAACTGGAAGGTGATCATCCCGATCATCAG
CCCAATAACAAAGAAGGCGCAGAATGCCAGCAGGTCAGTGACCTGGCTGTGAATCGAGATAAAGCCGATGCGATCGGCGA
TGGTTTTTACGCGGCGGGCATCGCCGCTGACTTGTAAAACGTCACCTTTGTTAAGCACGACGTTGTCATCTATCGGCATC
TCAATCTGGCTACGAATGACGCGGTTAAGGAAGCAACCGTGATCGGTCAACTTCAGTTGTGCGAGACGTTTACCTACAGC
GTTATGGTTTTTAACGACCACTTCTTCAGTGACGATACGCATGTCGAGAAGGTCACGATCGAAAACTTCTTTACCGTTAC
GGAAGCTGGGATCGAGTCGGGCATGGGCGTCGGGATAGCCTACCAACGCTATTTCATCGCCCATTTGTAGCACGGCATCA
CCGTCTGGATTTGCCAGAATCCCGTTACGTCGAATACGTTCAATGTAGCAGCCGGTTTGTCGATAAATACCCAGTTCACG
CAGATTTTTGCCGTCGGTCCAGGCCACCAGTTCCGGGCCGACGCGATAGGCGCGGATCACCGGTAAATAAACCTTACGGT
TGGCATCAGTGTCCAGGCCACGTTCGCGGGCGATTTGCTGGGCGCTGGTCTGTAAGTCCTGATGCTGCAATTTCGGCAAG
TAACGCGCACCAACAATCAAACTCACCAGACCGATTAAATAGGTTAAGGCATACCCGAGGCTCAGATTATCCAGTGCCAG
TGAGAGCTGCCTGCTTTCCATGCCGGAATGACGCAGTGTATCGCCAGCACCGACCAGAACCGGTGTCGACGTCATAGAGC
CTGCTAACATACCGGCCGTCAGGCCAATATCCCAGCCAAACAGCTTACCTAACCCTAAGGCGATCACCAGCGCACTGCCA
ACCATCACCAGTGCTAACATTAGGTAATTTTTCCCATCGCGAAAAAAAATGGAAAAAAAGTTCGGTCCGGCTTCGACCCC
GACGCAGAAAATAAACAGCATAAAGCCAAGATTAAGCGCATCGGTGTTAATGCTGAAATGTTGTTGGCCTAATAACAGCG
ATACGACTAAAACGCCAATGGAATTACCCAGTTGGATCGAACCAAGTCGTAACTTTCCGAGACATAGCCCAAGCGCGAGG
ACCACAAATAATAACAGAATGTAATTCCCATTTAACAATTCGGCGACGTTTATATTCACGGAGGCTAACTTCTTGTTTAC
TAGTAAGCTGTTGAAAGAAATGGTAATTTACGATAATGTTTTTTACCAGAATTCAGGGCGCAGATTCATTCAGCGCACCT
AAACGATAGTAAAGTAACAATATATTTTACTAGTGTAATCACATTAGGTATCAACGGCTATATGAATTGCGTTGGCCTAT
ATTAGCATGGAATGCGAAGCGGCTTTATCTTACTGAACGCCACACTGGCGAAAAATGTGTTCGATAGACGCAGTGTCAGG
AGGAACGAGTGAAACATAAACAACGTTGGGCGGGGGCAATCTGCTGTTTTGTCCTCTTCATTGTGGTGTGCCTTTTTCTG
GCGACGCACATGAAAGGCGCTTTTCGGGCTGCCGGGCATCCTGAAATCGGCTTGCTATTTTTCATTCTTCCTGGAGCAGT
CGCCAGCTTCTTTTCACAGCGTAGAGAAGTCCTGAAACCTCTGTTTGGCGCAATGCTGGCGGCACCCTGTTCGATGCTCA
TTATGCGGCTGTTTTTTTCACCGACGCGCTCATTCTGGCAAGAGCTGGCATGGTTACTAAGCGCGGTGTTCTGGTGTGCG
CTGGGGGCACTGTGTTTCTTATTTATCAGTAGTTTGTTTAAACCACAGCACAGAAAAAATCAGTAAAGCCCTCAACGCGA
GGGCTTGTCAGACGATCAGGCGTCCAGATTTTCTTTCACCCATGCAGCAAAATCGGTATAGCCGCCGATATGTTGCTGAT
CGACAAAAATCTGCGGCACGGTTTCTACGGGTTTACCTGCCTTTTGTTGTAGATCTTCTTTAGTGATCCCTTCCGCACGA
ATATCTACATACTGATACTGAAAATCATCGCGTTCATTGCTCAATTTCTCAGCCAGATCTTTTGCACGCACACAGTAAGG
GCAACCCGAACGACCAAAAATAACGGTTTGCATTATTTCTCTCCTCATAGATTTATGCCTGTAATGATCACGCTAAAATG
TATTCGCTGAAAGTAGGTTTAACCTGTTGCATTAATTGCTAAAAGCTATAACTGTTAAACACAATACAGTGAAAAGTTTT
AGACTGAAGGCTCACTTTGCAGAGGGAAGCGTATGCGCGCGATCGGTAAATTGCCTAAAGGCGTGTTGATACTGGAATTT
ATCGGAATGATGCTACTGGCGGTGGCGCTGCTGTCGGTAAGCGACTCCCTGTCGCTGCCTGAGCCATTTTCTCGGCCAGA
AGTGCAGATTCTGATGATTTTTCTCGGTGTTTTGCTCATGCTTCCCGCTGCGGTGGTGGTTATTCTTCAGGTGGCAAAAC
GTCTTGCCCCACAGCTGATGAACCGTCCACCGCAATATTCACGTTCAGAAAGAGAAAAAGATAATGACGCCAACCATTGA
ACTTATTTGTGGCCATCGCTCCATTCGCCATTTCACTGATGAACCCATTTCCGAAGCGCAGCGTGAGGCGATTATTAACA
GCGCCCGTGCGACGTCCAGTTCCAGTTTTTTGCAGTGCAGTAGCATTATTCGCATTACCGACAAAGCGTTACGTGAAGAA
CTGGTGACGCTGACCGGCGGGCAAAAACACGTAGCGCAAGCGGCGGAGTTCTGGGTGTTCTGTGCCGACTTTAACCGCCA

TTTACAGATCTGTCCGGATGCTCAGCTCGGCCTGGCGGAACAACTGTTGCTCGGTGTCGTTGATACGGCAATGATGGCGC
AGAATGCATTAATCGCAGCGGAATCGCTGGGATTGGGCGGGGTATATATCGGCGGCCTGCGCAATAATATTGAAGCGGTG
ACGAAACTGCTTAAATTACCGCAGCATGTTCTGCCGCTGTTTGGGCTGTGCCTTGGCTGGCCTGCGGATAATCCGGATCT
TAAGCCGCGTTTACCGGCCTCCATTTTGGTGCATGAAAACAGCTATCAACCGCTGGATAAAGGCGCACTGGCGCAGTATG
ACGAGCAACTGGCGGAATATTACCTCACCCGTGGCAGCAATAATCGCCGGGATACCTGGAGCGATCATATCCGCCGAACA
ATCATTAAAGAAAGCCGCCCATTTATTCTGGATTATTTGCACAAACAGGGTTGGGCGACGCGCTAAAACCGCCACGTCGA
TGTATGATACGCGGGCTTTTGACCAGGTCTGACAGAGAGGTGCAGGGTGAAAATTGCCATATTGTCCCGGGATGGAACGC
TCTATTCGTGTAAGCGGCTGCGTGAAGCCGCTATACAGCGCGGTCACCTGGTTGAAATTCTTGATCCGCTTTCTTGCTAC
ATGAACATAAATCCTGCGGCGTCTTCTATTCACTACAAAGGCCGCAAGTTACCCCATTTTGACGCAGTGATCCCGCGTAT
TGGCACCGCCATTACCTTTTATGGGACGGCGGCACTGCGCCAGTTCGAGATGCTGGGGAGCTATCCGCTCAATGAGTCGG
TCGCCATTGCCCGGGCGCGTGACAAATTGCGTTCCATGCAACTGCTGGCGCGTCAGGGCATCGACCTGCCTGTCACGGGC
ATTGCGCATTCGCCGGATGATACCAGCGATTTAATCGACATGGTCGGTGGTGCGCCGCTGGTGGTCAAGTTGGTTGAAGG
CACGCAGGGAATTGGCGTCGTGCTGGCGGAGACGCGTCAGGCGGCGGAAAGCGTGATTGACGCTTTCCGCGGTCTGAACG
CGCATATTCTGGTGCAGGAATATATCAAAGAGGCGCAAGGGTGCGATATCCGCTGTCTGGTTGTTGGCGATGAAGTGGTC
GCTGCGATTGAACGGCGGGCGAAAGAGGGCGATTTTCGTTCCAATTTGCATCGTGGCGGCGCGGCAAGCGTCGCCAGTAT
CACACCACAGGAGCGTGAAATCGCGATAAAAGCCGCGCGAACGATGGCGCTGGACGTTGCTGGTGTGGATATTCTGCGTG
CTAATCGCGGGCCGTTGGTGATGGAGGTGAATGCGTCGCCGGGGCTGGAAGGAATAGAAAAAACCACCGGTATCGACATC
GCGGGTAAAATGATCCGCTGGATCGAACGCCACGCTACGACAGAATATTGCCTGAAAACGGGTGGTTAGTCGCAATCACA
TTACTGATCATGGTTTTGCCTGCGCTTTTTGCGTAAGCTGTGCCGGTCTTTTTATCGAAAGAGGTTGTACAAAATTATGA
CATCGCTGGTCGTTCCTGGTCTGGATACGCTGCGTCAATGGCTCGATGACCTGGGGATGAGTTTTTTTGAATGTGATAAC
TGTCAGGCTCTGCATCTGCCCCATATGCAGAATTTCGACGGTGTCTTTGATGCCAAAATCGATCTGATCGATAACACGAT
CCTGTTTTCTGCCATGGCGGAAGTCCGACCTTCAGCCGTATTGCCGCTGGCGGCGGATTTATCTGCCATCAATGCCAGTT
CGCTGACCGTGAAAGCATTTCTTGATATGCAGGATGATAATCTGCCAAAGCTGGTGGTTTGCCAGTCTTTATCCGTTATG
CAGGGCGTAACCTATGAGCAGTTTGCATGGTTCGTGCGTCAGAGCGAAGAGCAGATTTCGATGGTCATTCTTGAAGCTAA
TGCCCATCAACTGCTGTTACCGACTGATGATGAAGGGCAAAACAACGTTACCGAAAACTATTTCCTCCACTGATAACTCC
TTTCGAGCACGCAGTCGCTGGTGCAGTGGCTGCGCGCTGCAAAATTATCTGCTGTTTTTAACCTTTTCTTAAAGATTATT
TCACTTCTCTTGTGTCGATTTGGCTTTATCACATAGAGCAAATATGCATAAAAATTTGTTAAATACCGTTTTTAATCCG
AGCTATAGTCTCAAACCCTGGCTAAAGTTATTCTTGCGATGCTTTTATATAGCGAGCAGTGCTGGCCGGGAGAAAGTTCT
CTTTTCTTACACCGCGCCGATAAAAAATATGCACGTTTATTGCATATCTTTCAGTGTGACAACTTTTGTTCGTTTGTTAA
CGAACTTTCAGAAGGAAAGAGATATGACCGCCTTAAATAAAAAATGGCTATCGGGTCTGGTTGCGGGTGCTCTGATGGCC
GTCTCTGTCGGCACGCTCGCGGCTGAACAAAAAACACTCCACATTTATAACTGGTCTGATTATATCGCCCCGGACACGGT
GGCCAATTTTGAAAAAGAAACCGGTATTAAAGTCGTCTACGATGTTTTCGACTCTAACGAAGTACTGGAAGGCAAATTAA
TGGCCGGGAGTACCGGCTTTGATCTGGTGGTTCCATCTGCCAGCTTTCTGGAGCGCCAGTTGACTGCGGGAGTTTTCCAG
CCGCTGGACAAAAGCAAATTGCCGGAGTGGAAGAATCTCGACCCGGAACTGCTGAAGCTGGTCGCCAAACACGATCCCGA
CAATAAATTTGCTATGCCCTATATGTGGGCGACGACCGGGATTGGCTATAACGTTGATAAAGTTAAAGCGGTGCTGGGCG
AAAACGCGCCCGTCGATAGCTGGGACTTGATCCTCAAACCTGAAAATCTGGAAAAACTGAAAAGCTGCGGTGTCTCTTTC
CTGGATGCGCCAGAAGAAGTTTTTGCTACCGTGTTGAATTATCTCGGCAAAGATCCCAACAGCACTAAAGCGGATGATTA
CACCGGACCGGCAACAGATCTGCTGTTAAAGCTGCGCCCGAACATTCGTTATTTCCATTCATCTCAATACATTAACGACC
TGGCAAACGGCGATATTTGCGTCGCTATCGGCTGGGCAGGTGATGTCTGGCAGGCGTCAAACCGCGCGAAGGAAGCGAAG
AATGGCGTGAATGTCTCGTTCTCGATTCCAAAAGAAGGGGCGATGGCGTTCTTTGATGTATTCGCCATGCCTGCGGATGC
CAAAAACAAAGACGAAGCCTATCAGTTCCTGAATTACCTGCTGCGCCCGGATGTAGTAGCGCATATTTCCGACCATGTGT
TCTATGCCAACGCCAATAAAGCAGCCACGCCGCTGGTGAGTGCGGAAGTCCGTGAGAACCCAGGTATTTATCCGCCTGCG
GATGTTCGTGCGAAGCTGTTCACTCTGAAAGTGCAGGATCCGAAAATCGACCGTGTGCGCACCCGCGCGTGGACCAAAGT
GAAGAGCGGAAAATAATCCGCAGTCGTAGATGCCGGAGGGGCGCACCACACCCGCCGGCAATTCGCACCATTATGGTGCG
CTTGCACACATTCAATGCCGGAGAGCAGCCGTGAATGACGCTATCCCTCGCCCGCAGGCGAAAACCCGTAAGGCGCTGAC
GCCGCTATTAGAAATCCGCAACCTGACCAAATCCTACGATGGTCAACATGCGGTGGATGATGTCAGCCTGACCATCTACA
AAGGTGAAATCTTCGCGCTGCTGGGCGCATCCGGCTGTGGCAAGTCCACGCTGCTGCGTATGCTGGCAGGTTTCGAACAA
CCTTCTGCCGGACAGATAATGCTTGATGGCGTCGATTTGTCACAGGTTCCGCCTTACCTGCGCCCCATCAATATGATGTT
TCAGTCTTACGCGCTGTTTCCGCATATGACCGTGGAACAGAACATCGCTTTTGGCCTGAAACAGGACAAACTACCGAAAG
CGGAAATTGCCAGCCGGGTCAATGAGATGCTCGGCCTGGTGCATATGCAGGAGTTCGCCAAACGCAAACCGCATCAGCTT
TCCGGTGGTCAGCGACAACGTGTGGCCCTGGCCCGAAGCCTTGCGAAGCGCCCGAAACTATTACTGCTCGATGAGCCGAT
GGGCGCGCTGGATAAAAAGCTGCGTGACAGGATGCAGCTTGAAGTGGTGGATATTCTGGAGCGCGTCGGTGTGACTTGTG
TGATGGTCACCCACGATCAGGAAGAGGCGATGACCATGGCGGGGCGCATCGCCATTATGAATCGTGGGAAATTTGTCCAG
ATTGGCGAACCGGAAGAGATCTACGAGCATCCGACTACCCGCTATAGCGCCGAATTTATTGGCTCGGTAAACGTCTTTGA
AGGCGTACTCAAAGAGCGTCAGGAAGATGGCCTGGTGCTTGATTCGCCGGGGCTGGTGCATCCACTGAAAGTCGACGCGG
ATGCCTCGGTGGTCGATAACGTGCCGGTACATGTGGCGCTGCGCCCGGAAAAAATCATGCTTTGCGAAGAGCCGCCCGCC

AATGGTTGTAACTTCGCGGTGGGGGAGGTGATACACATTGCCTATCTCGGCGATCTTTCGGTGTATCACGTTCGTCTGAA
AAGTGGGCAGATGATTAGCGCCCAGCTACAAAACGCCCATCGTCATCGTAAAGGGTTACCGACCTGGGGCGACGAAGTGC
GTTTGTGCTGGGAAGTGGACAGCTGTGTGGTGCTGACGGTTTAAGGAGCAAAGATGAGTACACTTGAACCTGCTGCCCAG
TCGAAACCGCCGGGCGGATTTAAGCTGTGGTTGTCGCAGCTGCAAATGAAGCATGGGCGCAAACTGGTCATTGCGTTGCC
ATATATCTGGTTGATCTTGCTGTTTCTGCTGCCATTTCTGATTGTCTTTAAAATAAGCCTGGCGGAGATGGCGCGCGCTA
TTCCACCTTATACCGAACTGATGGAGTGGGCTGACGGGCAACTGTCCATCACTCTTAATCTCGGTAATTTTCTGCAACTG
ACCGACGATCCGCTCTATTTCGATGCTTATCTCCAGTCGCTACAGGTGGCTGCGATTTCGACATTTTGCTGTTTACTGAT
CGGCTATCCGCTGGCGTGGGCGGTGGCGCACAGTAAGCCTTCGACCCGTAATATTTTATTACTACTGGTGATCCTGCCGT
CGTGGACCTCGTTTCTGATCCGCGTTTATGCCTGGATGGGAATATTAAAAAACAACGGTGTGCTGAATAATTTTCTGCTG
TGGCTGGGGGTTATCGATCAACCGCTGACCATTCTGCATACCAATCTGGCCGTTTATATCGGCATTGTTTACGCTTACGT
GCCGTTTATGGTACTGCCGATTTATACCGCGTTGATTCGTATTGATTATTCGCTGGTGGAAGCAGCGCTGGATCTCGGTG
CACGACCGCTGAAAACGTTCTTTACTGTGATCGTGCCGCTGACTAAAGGTGGGATTATTGCCGGATCGATGCTGGTGTTT
ATCCCGGCTGTGGGCGAGTTTGTGATCCCGGAACTGCTCGGTGGCCCGGACAGCATCATGATCGGGCGCGTGCTATGGCA
AGAGTTTTTCAATAACCGCGACTGGCCGGTGGCCTCGGCGGTAGCGATCATCATGTTGCTGCTGCTAATTGTGCCGATAA
TGTGGTTCCACAAACACCAGCAAAAAAGCGTGGGAGAACACGGATGAATAATTTACCGGTAGTTCGTTCGCCCTGGCGGA
TTGTGATTTTGCTGCTGGGCTTTACCTTTCTCTACGCGCCAATGCTGATGCTGGTTATCTATTCGTTTAACAGCTCGAAG
CTGGTGACGGTGTGGGCCGGCTGGTCAACGCGCTGGTATGGTGAGTTATTGCGCGATGACGCGATGATGAGTGCGGTTGG
TTTAAGTCTGACAATTGCGGCCTGTGCGGCGACGGCGGCGGCGATCCTCGGAACTATTGCGGCGGTGGTGCTGGTGCGCT
TTGGCAGGTTTCGCGGATCAAATGGCTTTGCCTTTATGATCACCGCGCCGCTGGTAATGCCAGATGTCATCACGGGCTTG
TCGCTGTTGTTATTATTCGTCGCGCTTGCTCATGCCATTGGCTGGCCTGCGGACCGCGGTATGCTCACCATCTGGCTGGC
GCATGTCACTTTTTGTACGGCTTATGTGGCGGTCGTTATTTCTTCAAGATTGCGGGAACTGGATCGCTCGATAGAAGAAG
CAGCGATGGATCTCGGTGCGACGCCGCTGAAAGTGTTTTTCGTCATTACGCTACCGATGATCATGCCCGCGATCATTTCT
GGCTGGTTACTGGCTTTTACTTTGTCGCTTGATGATCTGGTGATCGCCAGCTTTGTTTCCGGGCCGGGAGCCACCACGTT
ACCGATGCTGGTCTTTTCCAGCGTGCGGATGGGGGTGAATCCGGAAATCAACGCCCTGGCAACATTGATCCTCGGCGCGG
TCGGAATTGTCGGATTTATCGCCTGGTATCTGATGGCTCGCGCAGAAAAACAGCGGATACGCGACATCCAGCGTGCAAGA
CGTGGCTGAAGACACTAAAATTTGCCAACCTGGCTACATAATGCCGCGCATGTCGCGGCATTGTTTTCATGGAAGACGAA
ACGTTGGGATTTTTTAAGAAAACATCTTCATCTCATGCTCGCCTGAATGTGCCTGCGCTGGTGCAGGTGGCGGCGCTCGC
CATTATTATGATCCGTGGCCTCGACGTGCTGATGATTTTCAATACGCTGGGCGTGCGCGGTATTGGCGAGTTCATTCATC
GCAGCGTACAAACCTGGAGTTTAACGCTGGTCTTTTTAAGCAGTCTGGTGCTGGTTTTCATTGAGATCTGGTGTGCGTTT
TCACTGGTGAAAGGGCGTCGCTGGGCGCGCTGGCTATATCTGCTGACACAAATCACCGCCGCAAGTTACTTGTGGGCGGC
TTCGCTGGGGTACGGTTATCCGGAGCTGTTCAGCATTCCCGGTGAATCAAAACGTGAAATCTTCCATAGCCTGATGCTGC
AGAAGCTGCCGGATATGCTCATCCTGATGCTGCTGTTCGTTCCCTCGACCAGTCGGCGGTTCTTCCAGTTGCAATAATGT
GTATAATCGTCGCCCCTGATGATGTGAAGGTCAATGTATGCAGTGCGCACTTTACGACGCGGGTCGCTGTCGTTCCTGTC
AGTGGATAATGCAGCCGATTCCAGAGCAACTCTCCGCTAAAACCGCCGATCTTAAAAATCTGCTCGCCGACTTTCCGGTT
GAGGAATGGTGCGCGCCGGTGTCAGGCCCGGAACAAGGGTTTCGTAATAAAGCCAAAATGGTGGTGAGTGGTAGCGTTGA
AAAACCACTGCTCGGTATGCTGCATCGCGATGGCACACCAGAAGACCTTTGTGACTGCCCGCTTTATCCTGCCTCATTTG
CGCCCGTTTTTGCGGCGCTAAAACCGTTTATCGCCCGAGCGGGGTTAACGCCCTACAACGTGGCGCGTAAGCGTGGCGAA
CTGAAATACATTCTGCTGACTGAAAGCCAGAGTGATGGAGGCATGATGCTGCGCTTTGTACTGCGTTCTGATACCAAGCT
GGCGCAACTGCGTAAGGCGCTGCCGTGGTTACACGAACAACTACCGCAGCTGAAAGTTATTACCGTCAATATTCAGCCGG
TACATATGGCGATTATGGAAGGGGAGACGGAGATCTACCTGACCGAACAACAGGCACTGGCGGAGCGTTTTAATGACGTA
CCGCTGTGGATCCGTCCGCAAAGTTTCTTCCAGACTAATCCGGCGGTCGCCAGCCAGTTGTACGCCACCGCGCGCGACTG
GGTACGACAGCTGCCGGTTAAACATATGTGGGATCTGTTCTGCGGTGTGGGGGGCTTTGGTTTACACTGCGCGACGCCTG
ACATGCAGTTAACCGGGATCGAAATTGCATCAGAGGCCATTGCCTGTGCAAAGCAGTCAGCCGCTGAACTGGGCTTAACG
CGTTTGCAATTTCAGGCGCTGGACTCCACTCAGTTTGCCACCGCTCAGGGGGATGTGCCGGAGCTGGTGCTGGTTAACCC
GCCGCGCCGCGGCATTGGTAAACCGCTGTGTGATTATCTCTCAACGATGGCACCGCGTTTTATCATCTACTCCAGCTGTA
ACGCCCAAACTATGGCGAAAGATATCCGCGAACTGCCTGGGTTTCGTATTGAACGGGTGCAGCTTTTCGATATGTTCCCG
CATACCGCGCACTATGAAGTGCTGACGCTGCTGGTGAAGCAATAAAAAAGCCGCATGTGCGGCTTCAGATTGCTGACAAA
GTGCGCGTTGTTTATGCCGGATGCGGCGTAAACGCCTTATCCGGCCTACAAAAGCGTGCAAATTCAATACATTGCATGGG
CCATGTAGGCCTGATAAGCGTAGCGCATCAGGCAATTTTACCTTTGTCATCAGTCTCAAGCCGCGGTTGCGGCTTTCTGA
ATCTTACTGTGGGAACCACTGGTCACTGATTTTTTGATAAGTACCGTCAGCTTTAATTGCTGCCAGCGCGTTATTCAGTT
TTTCCAGCAGGGCTTTGTTATCCGGACGTACAGCGATGCCCAGGCCGGTGCCAAAATATTGCGGATCGGTCACTTTCTCA
GTAGCAACACCAAGTTGTGGATTGGTTTTCAGCCATTCGTTTACCACCGCTGTGTCACCAAATACCCCATCAATACGACC
ATTTTTCAGATCGATAAAGGCATTCTGATAACTGTCATAAGAGACAGTTTTCACTTCCGGGTGCTGATCCTGAATATATT
TCTGGTGCGTAGTACCGTTTTCCATCCCAATACGTTTGCCTTTCAGATCGGCAAACGTTTTGTAGGTATCTTTTTTGGCA
ATCACGACGGCTGAGTTTTCATAGTAGGGCGTGGTAAACGATACCTGTTTGCTACGCTCCGGGGTGATATCCATACCGGA
GATTACGGCGTCATATTTTCTGAATTTCAGGGACGGATCAGGCTGTCGAACGCGTGATTAGTAAAAGTACATTCTGCCT

GCATTTGTTTGCACAAGGCTTTTGCCAGATCGATATCAAAGCCGACAATCTCATTATTAGCACCTATAGATTCAAAGGGT
GGATAGGTGGCTGAAACGCCAAAATTGATTTTCTCTGCGGCAGAAGCACCGAAAGTAAAGGAAGCAAGTAAAGCGGCAAG
AACTAACTTTTTCATGATGGAACTCCCGTCTGTCAATCTTATGATTTTTGGCCGTGTCTGCGGCATGGGATAACAATGCC
ATCAAGTGAATTTATATGCAATAAACATGATTAAATAATTTAAATGAAATAAAAAAGACGGACAACTTAGTGGGTTGTCC
GTCTTCATTATAAGAATTTATGCACTATGTAGGCCGGATAAGGCGTCCCCGCCGCATCCGGCACAGGCACCGTGCTGATG
TCTGATGCGACGCTGGCGCGTCTTATCAGACCTACAAAACCCCCCGGCGAATGTACGCAGCCACATTAATTTCGCCGTTC
GAATGCCAGCGCTTTGCGCTCGATCAGACGCATCATCAGCGTCAGCAGGCCGTTAACGACCAGGTAAATAATCCCTGCCG
CACCGAACACCATTACATCGTAGGTGCGTCCGTACAACAACTGGCTGTATCCCATCACTTCCATCAGCGTAATGGTGTAT
GCCAGAGAGGTACTTTTGAATACCAGCACCACTTCGTTGGAATAAGAAGAGAGCGAGCGTTTAAAGGCATACGGCAGCAG
GATCGCCAGCGTATCTTTTTTGCTCATTCCCAGGGCGCTACAGGACTGCCACTGACCTTCCGGGATCGCACGAATTGCAC
CGTAAAACAGCTGCGTGGTATACGCCGCACTATTCAGCGACAACGCAATCAGCGCACATAACCACGGTTCTGACAACAAA
TGCCACAGTGCCGGATACTCCTGCAAAGTCGGAAACTGGCCCGGCCCGTAATAAATCAGGAAGATCTGCACCAGCAGCGG
CGTACCGGTAAACAGCGTGATATAACCCCGCACCAGCCACACCAGCACCGGCGTTTTCAGCGTCAGGATGATGGTAAAAA
TCAATGCCAGAATCAGTGCCACAATCAGCGAGGCAACGGTTAGCGTCAGGCTGGTGTGTAGCCCTTTCATCAGTTCGGGT
AAATACTCAAACATTAGCTGGGCCTCCGCTCAAAACGTGTCGCGCGCAGGTCAATGCGTTTGAGAATGTACTGACTGAGC
AGGGTGATCACCAGGTAAATCGCCGCCGCCACAATGTACCAGGTAAATGGTTCCTGGGTACGAGTAGCGATGCTTTTTGT
TTGCAGCATTAAATCATTCACACTAATCAAACTGACCAGCGCGGTATCTTTCAGCAGCACCAGCCACTGGTTACCGAGGC
CAGGCAGCGCATGACGCCACATCTGCGGCATCACCAGACGGAAAAAGATAGCCGATTTCGACAGCCCCAGCGCCTGACCA
GACTCCCACTGACCCACCGGCACCGCTTTCAACGCGCCCCGAAGCGTTTGCGAGGCATAGGCGGCATACAGCAGTGACAG
AGCGATGACACCACAAAGGAACGGGCTAACGTCGAAGTTCTCAATGTCCATCTGCACTGGGATCTGCACGAACCCAAGAT
TGATAGTGAAGCCATCCGAAAGCGTCAGCAGCAGCTGCGAGGAGCCAAAATAGATAAACAGCACCACCAGAATTTCTGGC
AGGCCACGCAGAATGGTTACCAGCGCTGAACCTGCCCACGCGACAGGACGCCATTTTGCCGACTCCCATACCGCAAAGAA
CATCGCCAGCGCCAGCCCGACAATCAATGCACAAACGGCAAGGCCGACGGTCATCCCGGCGGCGCTTGCTAAAGGAAAAA
ATTCATTCATCAGGAATTACTTCTGGAACCATTTGTTGTAGATGGTTTCGTAAGTGCCATCTTTCTTCACTTTTTCCAGC
GCAGTGTTGAGTTTCTGCTGCAGCTCAGTGTTGCCCTGACGTACCGCGATGCCGAGGCCAGTGCCGAAGTAATCTTTATC
GGTCACTTTGTCGCCCACCGCCGCCAGTTTCGGGTTATCTTTCAGCCACTCAGTGACCACTGCGGTGTCACCGAAGACGC
CGTCGATACGCCCGTTTTGCAGATCCAGTTTTGCGTTCTGGTAGCTGTCATACGGAACGGTAGTGATTTCCGGGTGCTTA
TCCATAATGAATTTCTGGTGTGTCGTCCCGTTCTGTACGCCGACTTTTTTGCCTTTCAGCTGATCAACACTGGTGTATTT
GCCTTGCTGACCCACAAACAGGGCAGAGTTGTCATAGTACGGGGTGGTAAACAGCACCTGCTTTTCACGCTCCGGAGTGA
TATCCATGCCCGCCATCACGGCTTCTACGCGACGGAATTTCAGGCTTGGGATCAGGCTGTCAAACGCCTGGTTAGAGAAA
GTGCAGGTTGCATCAATCTCTTTACACAGCGCTTGTGCCAGGTCGACGTCAAAACCAACGATCTGGTTGTTTGCATCAAT
CGATTCAAACGGAGGATAGGAGGCTTCGGTAGCAAAACGAATGGTTTCGGCAGCTGTGGCGGAAAGACTAAAACCTGCAA
TTAACGCGGCAATCAGAACTTTTTTCATTGTTGTTATCCCGAATCTTAGTGAGAGAGATAGTTTTTAAATGCTTCGGTTT
GCGGCTCGGTAAAGCAGCTCGCGTCGCCTTGTTCTACGATATGACCATTTTCCATATACACCACTCGGCTGGCGGTTTTA
CGCGCCACTTCAACTTCGTGGGTGACGATCACCTGGGTAATATTCGTTTCTGCCAGCTCACGAATGATGCTGACGATTTG
TGCCGTAATTTCCGGGTCCAGTGCGGCGGTCGGTTCATCGAACAGCAGTACCTGCGGTTCCATCATCAACGCACGGGCAA
TAGCAACACGCTGCTGCTGACCACCAGAAAGATGCAGCGGGTAACGATCGCTATAAGGTTTGAGACGCAGACGTTCCAGC
AGTTTTTCTGCACGGGCCAGCGCCTGATCTTTACTCAACCCCAGTACACGGCAGGGCGCTTCAATCAGGTTTTGCTGCAC
GGTCAGATGCGGCCACAGGTTGTATTGCTGAAACACCATGCCAACGTTACGACGCAAATCGCGAATCGCTTTGTCAGAGG
GTGTTTTGGTGAAATCGAAATGGTTGCCTGCAATGTTGAGCGTACCGGAGCGCGGCATCTCAAGCAGATTGAGTACACGC
AGCAGCGAGCTTTTACCCGCGCCGCTGGGGCCAAGTAACACCAGCGTTTCGCCCTGTGGGCAATCCAGCGTGATATCGAA
CAGCGCCTGATGCGCGCCGTAGAAGCAATTAATGCCGTTTAATTGAATACTCATTGACACTCGTATACTGGCAGTCTGAT
AGCTATTGAGGTCGAAGATAGTACCTTTGACAGAATAATTATGCAATATTTCTGCTTTAAAAGTTAAAAGCAAAGCGCAT
TATTCAATAAACATAGCACAAAATAACGGGGGCGGTGGTCGGCGAGCATAAATGTCGGCATTCCTCACGAAATGCCGGAC
AATTTACGGGGTTTATTGGTTGATCAAGGCGTTAGCGATTCTCGATGGACTGACGGAGCGTACCCGCCGTGGCATGAACG
CTACCGCCTAAGTAACGCACATCGTCGATGACCCAACACTGGCCTTCCTGAATCATTAACACTTCATCCTGCCAACCCTG
GTCACCCTGTTTGAGATCCACGCGCAATGGAATGTTACGGGCATCACGATTAGGGATAGTCGATGCACTGGCAACGTGGG
CGCTATCTGGCAAGGTGGTTCGACTGGAGAATGGATCGTTGGTCAGTAGTTCCCGATGGTTATTATCCCGGGAGGCATCG
CTAAGCAGTGTCGCCAGTTTGTCGCTCAGATAAGGGCGCAAGGCGGTGATGTCGTTGCTGCGGTGCAAAATGCGGTAGTC
ATAAAATTGCTGGGCCACGTTATCCGGGCCTCCTTCAACGCAAGGACCACTGCGTGTGCCGTTATCTTTATAAGCTGGAG
TGACTGTGGTGCAGGCACTGAGGAGCAGTGCGCAGGGGATAAGCATTGTCAATTTGCTGTAGCGCATAATGATTTCCTTA
TAAGCGATCGCTCTGAAAGCGTTCTACGATAATAATGATATCCTTTCAATAATAGCGTATCAGTCTGATAATGCTTTTGA
GATCGAAGGCTTAGCAAACAAGGAGATCGATCATGCAATTTTCTACAACCCCAACTCTGGAAGGCCAGACCATCGTTGAA
TATTGCGGTGTGGTGACCGGCGAAGCGATTTTAGGTGCCAATATTTTCCGTGATTTCTTTGCCGGTATCCGCGATATCGT
TGGCGGACGTTCCGGTGCGTATGAAAAAGAACTGCGTAAAGCACGGGAGATCGCCTTTGAGGAATTAGGCTCCCAGGCGC
GGGCGCTGGGGGCCGATGCCGTCGTCGGTATTGATATCGACTACGAAACGGTCGGGCAAAACGGCAGTATGCTGATGGTT

AGCGTCAGCGGTACGGCGGTGAAAACGCGTCGATGAGAAGATTCTTCTGGCTGGTCGCTGCCGCTCTGTTATTGGCAGGG
TGTGCAGGCGAAAAAGGCATTGTCGAGAAAGAGGGATATCAGCTTGATACCCGACGCCAGGCGCAGGCGGCGTATCCGCG
CATTAAAGTGCTGGTGATCCACTACACCGCAGATGATTTTGATAGCTCGCTGGCGACACTGACCGATAAGCAGGTCAGCT
CGCATTATCTGGTCCCTGCGGTACCACCGCGATACAACGGTAAACCGCGCATCTGGCAACTGGTGCCGGAACAAGAACTG
GCCTGGCATGCGGGGATTAGCGCCTGGCGCGGGGCAACGCGCCTTAACGACACCTCTATTGGCATTGAGCTGGAAAACCG
TGGCTGGCAAAAATCGGCCGGAGTGAAATATTTTGCCCCGTTTGAACCGGCACAGATTCAGGCGCTTATTCCACTGGCGA
AAGATATTATTGCCCGTTATCACATCAAGCCGGAAAACGTAGTGGCACATGCGGATATCGCACCGCAGCGCAAAGACGAT
CCGGGGCCATTATTTCCCTGGCAGCAACTGGCGCAGCAGGGGATTGGTGCCTGGCCGGATGCGCAGCGGGTTAACTTTTA
CCTTGCCGGGCGCGCGCCGCACACACCGGTAGATACCGCGTCATTGCTGGAGCTTTTGGCGCGCTACGGTTATGACGTTA
AACCTGATATGACACCGCGCGAGCAGCGGCGCGTGATTATGGCATTCCAGATGCATTTCCGCCCGACGTTATATAACGGC
GAAGCGGATGCAGAAACTCAGGCGATTGCCGAAGCATTGCTGGAGAAATACGGGCAGGATTAGCGCGGCAGTTTTCCGTG
GTCGCGTAGCCAGGCGGCAGTTTTCTCGATACCTTCATCCAGGGTGATGACCGGCTGATAACCTAACTCTTCCTGCGCAC
GCGTAATATCCAGCGTAAAGTCAAAATTCAACTTGGAGACGCCGTAGTGGGTCAGCGGCGGCTCTTTTGCTGACTTGCGG
CCTAAACGCTCCATGCTGCGGGCGATCATATCCAGCATCGGGTAGGGGACGGAACGAATACGACAGTCAATATTCAACTC
GTCGATCAGCTTCTGCACGATGCTGCGCAGTGTGCGATGCTCGCCGTTGGTGATGTTGTACACACGCCCGGAAGGTAGCT
TATCGCAGGCTTCCTGGCTTGCCAGCCACATTGCGTGCACGGCATTTTCATAGTAGGTCATATCGACCAGCGCACTGCCG
CCATGCGGTAACAGAATACTGCCGTAGTGGTGCATCATATGCGCCAGACGGGGAATAAAGACTTTATCGTGCGGTCCGAA
CAGACTTTGTGGGCGCAGAATAGTAAAGCGCGTTTGTGGATTCGCCTGCGAAAGCATATTGATCACTTCTTCGCTGGCTG
CTTTGCTGCGGGCAAACTCGTTGGCGAAGCGGTGAGGGCGAAAATCTTCTTTAATATCGCGATGGTGGTGATAATCGAAG
TACAGGGAGGGGGAAGAGATATGAATAAAGTTACGCACACCCCAGGCGACAGCCCATTCACCCAGGCGGCGAGTGGCGCG
AACGTTAGCCAGATCGAAAGCCTGTTGTGTCCCCCAGGGTGAGGTAAAGCTGGAGCAGTGCCACAGCGTATCAATGCCCG
CGAGCATCACTTTAGCTTGTGATGAAACCAGCTCGGTCAGATCCGCCGGAACAAACTCTGCGCCCATTTTTTCCAGCAAT
TTGCCCATTGCCTCGTTGCGACCGGTCGCTCGCACGCTGATGCCTTTCTGGCATAAAAACTCTACCGCGTTTCGACCTAA
GCCGCTGGTGGCGCCGGTAACCAGTACCTTCATATCAATCCACTGTTGTTGAGAAAATAACGTGCGCATTCTTCCGTGAT
TTCCCCCATGATGCAATGGGAAACATGAAAGAATAACGCAGGTTTTGTCGATTAATCTGTGCTTTGTTCTGCCAGTCTGG
CGATTTGTTTTGCCATTCCGCGAAAAATAAACAGATGCGCGGGGATCATCAATAACCAGTAAAACAGCCCCGGCATACCG
TGCGGATGCCAGAAAGCGCGGACATCGATAGTACGATAGTCGCCTTTATCTTCCAGGCTAAAACACAGTCGTCCCAGCCC
CGGCGCTTTCATGCCAAATAACAACGTAAGTTGTTTTTCCGGTTCAACGACAATCACTTTCCAGCTATCCACCGCATCGC
CAGTCTGTAAATATTCGCGCTCCGGGCGGCCTTTCGCCAGCTTATGACCGATCGCGCGGTCCATCAACGCCCGTGTCTGC
CACAAAATATTGCCAAAGAAATAACGCTCTTTACCGCCGATTTGGTTCACTACCTGCCATAAAGCAGCAAGGCTGGCGGA
CGTTTTAACGGTAAACCCCGCCTGTTTGGCAAAATAACCGTACTCCGGTCGCCAGCGGGCAAAGGCCTGAGCGTCGTAGC
CCCAGTCGCTGGAGTTGACCAGTTTTTCCTCCTCTTTCAACGTGCTACGTACCGCGTCATCGAAAGCGATCAGCCGTTGT
GGGATGAGTGCACGTAGCGCGGTATCATCCGCCAGCAGATCGTGTTTCAGCCCCTGAATCAACGCCCTGGCGGTGGTGGG
CGGTACGGAAGTAATCACATTGAGAAACCACACCGAAATCCAGCGGGTGGGGAGGGGGATGGGGATCAACCAGCGGCGCT
TACCGCTCACCGCCATAAAATGTTCAAACTGTTGCTGATAACTGAGCACCTCTGGTCCGGCGGCTTCGAAGATGCGGTGT
TCGCTGGCCGGATGATCTAACAACGCAACCAGATAGTGCAGCAAGTTTTCCAGCGCAATGGGCGTGGTGCGTGAACGAAC
CCAGCGCGGAGGCGTTAAGACTGGCAGGTTGTAGACCATATCGCGCATGACTTCGAACGCCGCGGAACCTGCGCCAACGA
TAATTCCGGCCCGCAGTTCGGTCACGGGTACATTCGCTTCACGCAGAATGTCCGCCGTAGCCTGACGAGCACGCAGATGA
TCCGACTGCTCATGTGGCGGGGCCTGCAACGAACTAAGAAAGATTAATTGCTTAACTGGTACTTCACGTAGCGCATCGCG
GACGTTGAGAGCCACCTGGCGCTCCTGAGCGATAAAATCGCCGCCTTCGCCCATGCTGTGCACCAGAAAATAGACCGTAT
CGATATCCTGCAACAGGGCCGGTAGGTTATCCGGCCAGCTGAGATCGACTTTATGGCAACTGACGTTTGCCAGTTGCAGC
TTTGCAAGCCTGTCGACATGACGTGCCGCCGCCAGGATCTGATGCCCTTGCTGGCTGAGTGTGCGCACCAGATGCTGACC
AATGTAGCCACTGGCACCGAGAACTAAAATGCGTTGCGGCACGTCTCTCTCCTTAACGCGCCAGGAATGCACGCCAGTGG
GCGGCGACTTCCGCCAGTTGTTCGCGCGAGACGTCAAGATGCGTCACCAGGCGGACAATCGGCGAGGCGTTAATCAGCAC
GTTTCTCGCTTTCATGTATTCGCCTAACGCGGCAGCATTTTCTTCCCCGACGCGAACAAACAGCATATTGGTGTCCTGAC
GCATCACATCCGCGCCTGCTTCACGCAGCTGCTCCGCCATCCAGGCAGCGTTGTCGTGGTCTTCCTGCAAGCGCGCAACG
TTATTTTTCAGGGCATATATCCCGGCGGCAGCCAGAATGCCGGACTGGCGCATCCCGCCACCTGTCATTTTCCGCCAGCG
AATGGCACGTTTAATGTAATCACGATTACCGACGAGTAATGAACCGACTGGCGTCCCAAGACCTTTCGACAGGCAAATGG
TGAACGAATCACAATATTGCGTGATCTCTTTCAGTTCGCAGCCGTAAGCCACCACGGCATTAAAGATGCGCGCACCGTCA
ACATGCAGCGCCAGATTGCGCTCGCGGGTAAATTCCCATGCTTCTTTCAGGTATTCCCGCGGCAACACTTTGCCGTTGTG
GGTGTTTTCCAGACTGAGTAATTTGGTGCGGGCGAAATGGATATCGTCGGGTTTGATTTTCATCGCCACTTTATCCAGCG
GTAGCGTGCCGTCGGCAGCCGCGTCTATGGGTTGCGGTTGAATACTGCCCAGCACCGCCGCGCCACCGGCTTCAAACAGA
TAGTTATGCGCGGCCTGACCGACAATATACTCTTCGCCGCGTTCGCAGTGACTGAGCAGAGCGACCAGGTTGGCCTGAGT
GCCGGTAGGCAGAAAAATGGCGGCTTCTTTACCGGAAAGCTCTGCTGCGTAGTCCTGCAGAGCATTAACGGTAGGGTCGT
CTCCGTAAACGTCGTCCCCAACCGGGGCGGCCATCATCGCTTCGAGCATGGCGCGGCTTGGTCGGGTAACGGTATCACTG
CGTAAATCAATCATGGCATGTCCTTATTATGACGGGAAATGCCACCCTTTTTACCTTAGCCAGTTTGTTTTCGCCAGTTC

GATCACTTCATCACCGCGTCCGCTGATGATTGCGCGCAGCATATACAGGCTGAAACCTTTGGCCTGTTCGAGTTTGATCT
GCGGTGGAATGGCTAACTCTTCTTTGGCGACCACCACATCCACCAACACCGGACCGTCGATGGAGAAGGCGCGTTGCAGG
GCTTCATCAACTTCAGACGCTTTTTCTACACGGATACCCGTAATGCCGCACGCTTCGGCAATGCGGGCAAAGTTTGTGTC
GTGTAGTTCGGTGCCGTCAGTCAAATAGCCACCAGCTTTCATCTCCATCGCCACAAAGCCCAGCACGCTGTTGTTAAAGA
CGACAATTTTCACTGGCAGTTTCATCTGCACTACTGAGAGGAAATCGCCCATCAACATGCTAAAACCGCCATCGCCGCAC
ATGGCGACCACCTGACGTTCTGGCTCTGTCGCCTGCGCACCCAGCGCCTGCGGCATGGCGTTAGCCATCGAACCGTGGTT
AAACGAACCTAACAGGCGACGCTTGCCGTTCATTTTTAGATAACGTGCCGCCCACACCGTTGGCGTACCAACGTCACAGG
TGAAAATAGCGTCATCGGCGGCAAAATGACTAATTTGCTGCGCCAGATATTGCGGGTGAATGGCTTTCTCGCTCGGTTTA
GCTAAATCGTCCAGCCCTTTGCGGGCGTCGCGGTAATCTTCCAGCGCTTTATCCAGAAACTTGCGATCGGCTTTTTCTTC
CACCAATGGAAGCAATGCACGCAGAGTCGACTTGATATCGCCGACCAGTGCCATATCCACCTTGCTGTGAGCGCCGATGC
TGGCTGGGTTGATATCAATCTGAATGATTTTGGCATCGGTCGGGTAGAAGGCGCGGTAGGGAAATTGCGTGCCGAGTAGC
ACTAACGTGTCGGCGTTCATCATGGTATGGAAACCTGACGAGAAGCCGATTAACCCGGTCATTCCAACATCATACGGATT
ATCGTATTCGACATGTTCTTTACCGCGCAGGGCATGAACAATAGGCGCTTTAATTTTCCCGGCAAACTCAACTAACTCTT
TATGCGCCCCCGCGCAGCCGCTGCCACACATCAGGGCGATATTGCTGGAATAACGCAGCAGTTGCGCCAGTTTGCGTAAC
TCTTCTTCTTCCGGCGTCACGACTGGTTGTGGCGCATGATACCAGTGCATGGTTGCCCCTTCTGGCGCAGGTTTTAACGC
CACGTCGCCTGGTAACACGACAACCGAAACGCCACGGTTAAGCACCGCTTTGCGCATGGCAATCGCCAGTACTTGTGGGA
TCTGCTCCGGGCTGGAAACCAGCTCGCAATAGTGACTACATTCGCGGAATAGCTCTTGTGGGTGGGTTTCCTGGAAATAG
CCGCTGCCAATTTCGCTGGAGGGAATATGAGCGGCAATCGCCAGTACCGGAACGTGATTGCGGTGGCAATCGAACAGGCC
GTTGATTAAGTGCAGGTTGCCGGGGCCGCACGATCCGGCGCAGACCGCCAGTTCTCCGCTAAGTTGTGCTTCAGCGCCAG
CGGCAAAGGCCGCCACTTCTTCGTGGCGGGTGGACATCCACTCGATGGTGCCCATGCGATTAAGACTGTCACTAAGACCG
TTCAGAGAGTCGCCTGTGACTCCCCAGATGCGTTTCACCCCTGCCGATTCGAGTGTTTTGGCGATATAAGCTGCAACCGT
TTGTTTCATGGTTCTCCATCTCCTGAATGTGATAACGGTAACAAGTTTAGTTCATCTGACGGAGGGGGAAGGGATGGGAG
AGAAAGGAGGCACTAACGGTTAAATAGCCCGATGAAAGGAATATCATCGGGCATAAGGCGATTATGCGAGAACCAAATCC
CCCTGCGGATGGCAGGAGCAGGCCAGTACGTAACCTTCAGCGATTTCGGCGTCGGTCAGCGTCATTGTGCTGCTCACCGT
ATATTCACCGGAAACCACTTTTGTCTTACAGCAGCCGCAAACACCCGCACGGCAGGCAGCGACAACCGGAACGTTATTGC
TTTCCAGCGCCTCCAGTAGCGTGGTGCCAACCGGGGCGTAAAATTCTCGTGCCGGTTGCAGTTTGGTGAATTTCAGACCG
CTGGTCGCCGCTTCTGCTACTGGGGTGAAGAATTTCTCTTTAAAGAAACGCGTCACGCCGAGCGCTTTCACTTCCTGCTC
TACCCAATCCATATACGGAGCCGGGCCGCAGGTCATCACGGTACGTGAAGCTAAGTCAGGTACACCTGCCAGCAGTTCGC
GAGTGAGACGACCAGCGATAAAGCCTTCGGTAACGTTATTTTCTGCCACCAGCGTTACCGGATAGTTACGCCACTCATCG
GCGAAAATAACATCCTGCGGCGTACGCACGTTGTAGATCACCCGCACATCGGCCTGTGGACGGTTCTTCGCAAGCCAGCG
ACGCATCGACATAATCGGCGTGACGCCGCAGCCTGCCGCCAGCAACAGGAATTTATCTTCTGCTTTATCGTCGCAGGTAA
ATTCCCCCATCGCGTCCGAAAGCCAGAGATAATCACCGCGTTTTACATCGCGCGTCAGCCACTGGGAGCCGACACCGTCA
TCAATCCGCCGCACGGTCAGGGTGATATATTCACTCACGCCTGGCGTGGAGGAAATGGTGTAAGCACGCAGCGTTTCCGC
TGAGTTTCGCACGCTGACCAGTGCATATTGCCCGGCGCGATATGGGTAGTAATCGTGGCAAATCAGGGAAATCGTCCACA
CATCCGGCGTTTCTTGCGTAATGTGATGAACCTGCATCCGCCACGGGCATTGATTCGTTGGCATCGTCATCGACAAACTC
CTTACGCGCTCAACAGTTGCTTCATGTCTTCTTCAACAGTGGTGATAGAACGCAGGCCGAATTTCTCGTTCAGCACCGCC
AGCAGGTCTGGTGTCAGGAAACCAGGTGCAGTCGGGCCGGTGACGATATTTTTCACGCCCAGAGAAAGCAGCGTCAGCAG
AATGACGATCGCTTTCTGTTCAAACCAGGAGAGCACCAGCGACAGCGGCAGATCGTTGACACCGCAGCCCAGTTTCTCTG
CCAGAGTGACAGCCAGAATAATCGCTGAGTAAGCATCGTTACATTGACCTGCATCTACCAGACGCGGCAGACCTTCGATA
TCGCCAAACTCAAGTTTGTTAAAGCGATATTTACCACAGGCGAGGGTCAGGATCAGGCAGTCATCCGGCACGCTGGTGGC
GAAATCGGTGAAGTAGTGGCGCTCGCCGCGTGCGCCGTCACAGCCACCAAGCAGGAAGATATGACGCAGTTTTTCACGGC
TCACCAGATCAATCAGCGTATCAGCAGCGCCAAGCAGCGTCTGGCGACCAAAACCCACGGTGATAAGGTGCGGAATTTCG
CTGTACGGGAAGCCTGCCATCTGTTGCGCCTGGGTGATAACCGCAGAGAAATCATCACCGTCCAGATGACGCACGCCAGG
CCAGCCAACAATGCTGCGGGTCCAGATACGATCGTCATAAGCGCCTACGGTTGGGTCGATGATGCAGTTCGAGGTCATCA
CGATGGGGCCAGGGAAACGAGCGAACTCCACTTGCTGATTCTGCCAGCCGCTGCCGTAGTTACCGACCAGATGCTTGAAT
TTACGCAGCTCCGGGTAGCCATGCGCAGGCAGCATTTCGCCGTGGGTGTAGACATTAACGCCCGTGCCTTCGGTCTGTTC
CAGCAGGTTGTAGAGATCTTTGAGATCGTGACCGGAAATCAGAATGCATTTACCCGCCGTCGCTTTGACGTTGACCTGGG
TTGGCGTCGGGTGACCGTATTTACCGGTTTCGCCTGCATCCAGAATGCTCATCACTTTGAAGTTCATCTGGCCGATTTCC
ATTGAACACTCAAGAAGCGCGTTCATATCGGCAGGCCAGGTCCCCAGCCACGCCATGATTTTATGGTACTGGGCATAAAT
ATCGTTGTCGTATTGACCGAGAACATGCGCGTGTTCCATATAGGCCGCCGCACCTTTCAGGCCATACAGGCACAGCAGAC
GCAGGCCGAGAATGTTTTCGCCAATCGCCGCTTTATCTTTGTTAGGGGTAAATTCTGCTGCCTGACGTTGCAGCTCGCCG
AGATCGTCGCTCACCAGTTGCAGGTCAGCCATCGGGTTATCGACGCGCGCGTTGGCATCTACAGCCAGGCATTGCGCTTT
CAGCGCCTCGCGCAGGGCAATCGCTTCACGAGCGTAGCCGACAATACGCGGAGAATCGAAGTTAACGTTGGTCAGGGTTG
AGAAAAAGGCACGTGGCGCGAAGCTGTCAACATCGTGGTTGATGATGCCGTATTCACGCGCTTTTACCGCCCAGGCAGAA
AGCCCTTGCAGCGCCGCGATGAGTAAATCCTGAAGGTCAGAAGTTTCCGCCGTTTTACCACACATCCCCTGCGCGTATGA
GCAGCCGTTTCCTGCCGGAGTACGGATAGTTTGTTCACATTGCACACAAAACATGATCACACCTTTTAAAGTTATATTTA

ATATACATGTTTAAGGTTAAGACGCTTAACGCGGGGATAAAAGGGATTTTTCATGCAACTTTAAGGGAGATTGATTTAGC
GCAATTTTGGCGGCAGGGATCTACCGCCAGAGAGGTATTACGCAGAGAAAAAGGCGATGAGGATCGGCACTAACAGGCTA
AGAATAAAACCGTGAACAATTGCCGCCGGGACCATATCCAGCCCGCCAGTACGTTGAAGAACGGGCAGGGTGAAATCCAT
TGATGTGGCACCGCATAAGCCCAGTGCAGTAGAGCGGCTGCGGCGAATCAGCCCAGGGATCAACATAATAGCAATCAGTT
CACGGGCCAGATCATTAAAAAACGCCGCGCTCCCGATTACCGGACCAAAAGATTCGGTCAATAAAATACCGGAAAGAGAA
TACCAGCCGAAACCGGAGGCCATTGCCAGCGCGGTATTGATGGGGAGATCAAGAATAAAGGCGTTAATTAAACCACCAAT
TAATGAACTGACAACCACCACCACGGCGACAATCATTCCCCGGCGATTAAGGACAATCTGCTTTAAGGTCATGCCATTAT
TGCGCAACTGAATACCAACGAGGAAAAGTAGCAAAATTAACGTGTATTCACTGGCTTCGGTCGCGTGTTGTAAGAAAGCC
AGTCCACTTAGACCAATGGCAAAACCAATCACTACTACGCCGCACAGTTTTAGCGACTCCAGCGCCATCGCAATACGCGA
CGGGAGTTTTTCTTGCTGATGGTGGTTGCGCCACGGCAGGCCTCGCTCCAGCCACATCAGGGCGGCAATATTACACAGTA
AAATAACGGTAATACTGACGGCAGAATAATGCAGAATCGCCAACAGGTTACTGGCGAGGTTATCGAGAAACGCCAGACTG
ATACCCATAAAAAAGAGAATAAGGTAAACCATCCAGCTTAATAGCTGATTAATAACTTTTAACGCAGCTTGTTGGCGAAG
CGGAATGAGGTAACCCACAATCAGGGGAACCAGAATGATTAACAGCCCAGAAAACATGAAAACCCAGTCCTTGCAAAGAT
GAAGTCGAAATGCGCGATGACACACTACTGAAAGCGGAAGGACGAGTAAAGTTGCAATTAAAGGAAATGTTATGCATAA
GGAGCAGTAGAGTATTCGTTTTCATTTAAAGATATTCTTGCGCTTTAATTACAAACTGCACCGATGTTGGTGGCGTCAAA
ATCGCCGAGGCGTTCCCTGAAGGCCGGGGCAGCCCACATGGATGTGGGCTGAGGGCGCGTTTTACAGGGATGTTACCTCG
CGCCCGACCCGGTAGCCGTAAGGGATAAGTCGAGGGCACCGCGCAGCGGCGATTTTGTTCGCCAGAGCCCGGGGGTGCAG
GGGGCGGCGGCGATTGGCCGCCCCCTGCGCGCTCCTTGCGCCAGTGGCAATATGTTGCTTAGCTCATGAAAGGAGCGCAA
CAAGATGATGAATCAACATATAACAACATCTTAAAAAAAGGCCTGACATTACGCCAGGCCTTCTGCGTTAATTAATCACG
CTTTTCCAGCAGGGTCCGGTAAATCAGACCACCGATAATGCCGCCGACAATTGGCACCACCCAGAAGAACCACAGTTGTT
CTAATGCCCAGCCGCCCTGGAAGATAGCAACCGCGGTGCTGCGCGCCGGGTTAACAGAAGTGTTAGTCACCGGAATACTA
ATTAAGTGAATCAGGGTTAAGGCCAGACCAATAGCGATCGGCGCAAAACCTGCCGGCGCGAATTTGTCGGTTGCGCCGTG
GATCACCAACAGGAAACCTGCACTCAATACCAGTTCAACTACCAGCGCGGAAAGCATGGAATAACCGCCTGGTGAATGCT
CGCCATAACCGTTAGAAGCAAAACCGCTGGCTGCCGCGTCAAAACCCGTTTTACCACTGGCAATTAAATACAGCAGCGCC
GCTGCAACAATACCGCCGACAACCTGGGCAATTACGTAGCCAACGACTTCTTTTGCCGGAAAACGTCCGCCAGCCCATAA
ACCAATAGTGACCGCCGGGTTAAAATGACCACCAGAAATATGACCAACAGCAAAGGCCATCGTCAGAACGGTCAGACCGA
ACGCCAACGCCACGCCGGCAAAACCAATGCCTAATTCCGGGAAGCCTGCGGCCAGTACAGCACTACCACAGCCACCAAAA
ACAAGCCAGAAAGTACCAAAACATTCAGCTGCTAATTTTCTGAACATATCCACCACAATTAAAAATTGACCCTGTGAAAA
ATATGGTCGTTTTATAGGGTCGTCGTAAAAAAGTGACGACGGAAATAATGCGCGGCTATTTTAAAAACGAAGGCGAGTCA
TTCACCAGATAAATAAATCCAGTAAATTTGATTTAGGGCAACAGCGGGTTGCCCCATATAGTCATTTGTCTGATTGACAG
TGTAGTGCACGCAAAAGATTTAATCCTTTAGGCGTAATAAAAAATAATTTATCATGCTAATTATTTGATTTTGTTGTTTT
TGCAGACTTATCAGCAAGAGGGAGTATAACGCGATTATTCGCTCATTTTTCAGACATTTGCCATGCTTAAATGTGATGTC
ATCACGTATTAGCAAGGCCTTTCCCGTTATACTGCCAGCGTAAAGGATAAGTCACATATTTCTGGAGGGGATATGATTCT
TGAGCGCGTTGAAATTGTGGGTTTTCGCGGTATCAACCGTTTGTCGTTGATGCTGGAACAAAACAACGTCCTGATTGGGG
AGAACGCGTGGGGTAAATCCAGCTTGCTGGACGCCTTAACTCTGCTGCTATCGCCAGAATCAGATCTCTACCATTTTGAG
CGCGACGATTTCTGGTTCCCGCCGGGAGATATCAACGGGCGAGAACATCATCTGCATATTATTTTGACCTTCCGCGAATC
GCTGCCAGGCCGACATCGGGTTCGCCGTTATCGGCCGCTGGAAGCGTGCTGGACGCCATGCACCGATGGCTATCACCGTA
TTTTTTATCGTCTGGAAGGGGAGAGTGCGGAAGACGGCAGCGTGATGACACTGCGCAGTTTTCTCGATAAAGACGGACAT
CCGATTGATGTCGAGGATATTAACGATCAGGCACGCCATCTGGTGCGTTTAATGCCGGTGCTGCGCTTGCGTGATGCCCG
TTTTATGCGCCGTATTCGTAACGGCACGGTGCCAAATGTCCCTAATGTGGAAGTCACCGCGCGCCAGCTCGATTTCCTCG
CCCGTGAGTTATCCTCACATCCGCAAAATCTCTCTGATGGGCAGATTCGTCAGGGACTTTCCGCAATGGTACAGCTGCTT
GAGCATTATTTCTCTGAGCAGGGGGCCGGACAGGCGCGATATCGTTTAATGCGGCGGCGAGCCAGCAATGAGCAACGAAG
CTGGCGCTATCTGGATATCATCAACCGGATGATTGACCGACCTGGTGGGCGCTCGTATCGGGTTATTTTGCTCGGCCTAT
TTGCTACTTTGTTGCAGGCAAAAGGCACATTGCGACTGGATAAAGACGCCCGTCCATTGTTGCTGATCGAAGATCCAGAA
ACCCGTTTACACCCCATTATGCTTTCAGTTGCCTGGCATCTGTTGAATCTTCTGCCATTGCAGCGCATTGCCACCACCAA
CTCGGGTGAGTTGCTTTCGTTAACGCCGGTAGAGCATGTTTGCCGACTGGTACGTGAGTCCTCGCGCGTTGCCGCCTGGC
GTCTGGGGCCGAGTGGCTTGAGTACCGAAGATAGCCGACGCATATCCTTTCACATTCGTTTTAACCGTCCGTCATCGCTG
TTTGCACGCTGCTGGTTGCTGGTGGAAGGGGAAACGGAAACCTGGGTTATCAATGAACTGGCGCGTCAGTGCGGACATCA
TTTTGATGCCGAAGGGATCAAGGTCATTGAGTTTGCCCAGTCCGGGCTAAAGCCACTGGTTAAATTTGCCCGCCGAATGG
GGATTGAATGGCATGTACTGGTCGATGGCGATGAAGCAGGGAAGAAATATGCCGCTACGGTACGCAGCCTGTTGAATAAC
GATCGGGAAGCCGAACGAGAACATTTAACGGCGTTACCGGCGCTGGATATGGAACATTTTATGTATCGCCAGGGATTTTC
CGATGTGTTCCACCGCATGGCGCAAATCCCGGAAAATGTACCGATGAATCTACGCAAAATTATCTCGAAAGCGATCCATC
GCTCTTCCAAACCCGATCTTGCCATTGAAGTGGCAATGGAGGCAGGACGTCGTGGTGTGGACTCCGTACCGACGCTGCTG
AAAAAAATGTTCTCACGCGTGCTGTGGCTGGCGCGCGGTCGCGCGGATTAACCGCGAAACATCGTGGCCATTTGTGGCTG
AATAGCGTCGAGCATCTCATAGCGCCGACGGTATTCAGCCCGTTTTTTACTGGCGATTTCGGCAATCTCTTTTCGTGCTA
TCTGTGCTGGAAGGCGGTAATGGCGTTCAGCATCACATACGCCGCCAACCGATTCCCAGAAAGCGTTGTAATCAGCGTGG

ATCTTGCCTTCTTTATCGCGATAACGCAGGCTGCGGTAAATATGCGTTTCATTGCTGACGGCAATAATCTGCTCTACCTG
CAAACGTTGGGCAAACAGACAGGCCGCTTCCATCACGAGGCGTTTGGGAAATAGCCCGTGGCAGGCTTTCGTCGCATTCT
GGATTTCCTGATGTGGAATTTCCCATTTTGCGCCTTGCAGTCCGCCAATAAACATCGTTCTTTTCCCCTGATATTCACAC
AGGGTAAACGTGATCTCTGCCAGAGGAATACCTTCGCTGTTGCGGAACAGGATTGTGCTGTCACCTTCTTTATCCATTGA
GATCATCATGGTCAGCTCAAGCGTGAACTGCTCGCCGTTTTTGCCTTCCAGCTTCGCCAGTTGCAGCCCGGGGGTATTCA
AATATAAGCTGAATTCTTCCGCCGACATACATTCGCGGAGTAACGCATAATGGTAACGTAACGCCTCCAGCAATTGCTTA
CGGCTAAGATTCGCCGCAAGGTAAGGGCGATGCAGACGCACAGGCAGTCGCGGCTGGCGCGTTAACAATACATTGAGATT
AGGCCAGTGGGAAAGTTCGTTCATCCACTCAACGCTTAAACGCGGCATAATCAACGAGCGCAGCAAAAATTTCTGGCGAA
AACTACGGCGATGCCAGAATTTACCCGGCCGACACTGTCCACGTGCCAGACTAAGAAAAAGTGACAGGCTGCTGAGAGAT
TCAGATGGCGTAAAGGTCCGTTCAGTTAGCTGCGACATATTCATGAAATCAATGGTTATACATGGCGTCGATTTCACCAT
TGCGTATCTTAACCAAACATCAATAGTGTGATTACTAACGTAAATTTTAGGGTTTTGTTGATATTTCGTTGAAGTTAATG
ACCCGGATTGGCATATGGAGTATTCAGAAAATTTATGAAAAAGCGGAAAACCGTGAAGAAGCGTTACGTTATTGCGCTGG
TGATAGTCATCGCCGGACTGATTACGTTATGGAGAATTCTTAACGCACCCGTGCCGACTTATCAGACGCTGATTGTGCGC
CCCGGTGATTTACAGCAAAGCGTGCTGGCGACCGGAAAGCTGGACGCGCTGCGTAAGGTTGATGTGGGCGCGCAGGTCAG
CGGTCAGTTGAAAACTCTGTCGGTGGCGATTGGCGATAAAGTAAAAAAAGACCAGCTTTTAGGGGTTATTGATCCTGAAC
AGGCTGAAAACCAGATTAAGGAGGTCGAAGCAACGCTGATGGAGCTACGTGCGCAGCGGCAGCAGGCGGAAGCGGAGCTG
AAACTGGCGCGGGTGACGTATTCCCGTCAGCAACGTCTGGCACAAACGAAGGCTGTTTCACAGCAGGATCTCGACACCGC
CGCGACGGAGATGGCTGTGAAACAGGCGCAAATTGGCACCATTGACGCGCAAATCAAGCGCAATCAGGCTTCTCTCGATA
CGGCTAAAACCAATCTCGATTACACTCGCATCGTTGCCCCGATGGCCGGGGAAGTCACGCAAATCACCACTCTGCAAGGC
CAGACGGTGATTGCCGCACAACAAGCACCGAACATTCTGACGCTGGCAGATATGAGCGCCATGCTGGTAAAAGCGCAGGT
TTCTGAAGCGGATGTAATCCACCTGAAGCCGGGGCAAAAAGCCTGGTTTACGGTGCTTGGCGATCCACTGACGCGCTACG
AGGGGCAAATCAAGGATGTACTACCGACGCCGGAAAAGGTTAACGACGCTATTTTCTATTACGCCCGTTTTGAAGTCCCC
AACCCCAATGGTTTGCTGCGGCTGGATATGACTGCGCAAGTGCATATTCAGCTCACCGATGTGAAAAATGTGCTGACGAT
CCCTCTGTCGGCGTTAGGCGATCCGGTTGGCGATAATCGTTATAAAGTCAAATTGTTGCGTAATGGTGAAACACGCGAGC
GTGAAGTGACGATTGGCGCACGTAACGATACCGATGTTGAGATTGTCAAAGGGCTTGAAGCGGGCGATGAAGTGGTGATT
GGTGAGGCCAAACCAGGAGCTGCACAATGACGCCTTTGCTCGAATTAAAGGATATTCGTCGCAGCTATCCTGCCGGTGAT
GAGCAGGTTGAGGTGCTGAAGGGCATCAGCCTCGATATTTATGCGGGTGAGATGGTCGCGATTGTTGGCGCTTCGGGTTC
CGGTAAATCGACCCTGATGAATATTCTCGGCTGTCTGGATAAGGCCACCAGCGGCACCTATCGCGTCGCCGGTCAGGATG
TTGCCACGCTGGACGCCGATGCGCTGGCGCAACTGCGCCGCGAGCATTTCGGCTTTATTTTCCAGCGTTACCATTTGCTT
TCGCATTTAACCGCCGAGCAGAACGTTGAAGTACCCGCCGTCTATGCTGGTCTTGAGCGGAAACAGCGACTGCTTCGTGC
CCAGGAGTTGCTGCAACGGCTGGGGCTGGAAGACCGTACAGAGTATTATCCGGCACAGCTTTCGGGTGGTCAGCAACAGC
GCGTCAGCATCGCGCGGGCATTGATGAACGGTGGTCAGGTAATTCTTGCCGATGAACCAACCGGCGCACTGGACAGCCAT
TCTGGCGAAGAGGTGATGGCGATCCTGCATCAGCTGCGCGATCGTGGGCATACGGTGATTATCGTCACCCACGATCCGCA
GGTCGCTGCTCAGGCCGAGCGGGTGATCGAAATTCGCGACGGCGAAATTGTGCGCAATCCTCCCGCCATTGAAAAAGTGA
ATGTTACTGGCGGGACGGAACCTGTTGTCAACACGGTGTCTGGCTGGCGGCAGTTTGTCAGCGGTTTTAACGAGGCGCTG
ACGATGGCATGGCGGGCGCTGGCAGCGAATAAAATGCGTACTTTACTGACCATGCTGGGGATTATTATCGGTATTGCGTC
GGTGGTTTCCATTGTCGTGGTGGGTGACGCCGCCAAACAAATGGTGCTGGCGGATATTCGTTCTATTGGTACGAATACTA
TTGATGTCTATCCCGGGAAAGATTTTGGCGATGACGATCCGCAATATCAGCAGGCGCTGAAGTACGACGACTTAATCGCC
ATCCAAAAACAACCGTGGGTCGCCTCAGCCACACCTGCCGTCTCGCAAAACCTGCGCCTGCGTTATAACAATGTTGATGT
TGCTGCCAGTGCCAATGGCGTGAGCGGCGATTATTTTAATGTCTATGGCATGACCTTCAGTGAAGGAAACACCTTTAATC
AGGAGCAGCTGAACGGTCGTGCGCAGGTCGTGGTTCTCGACAGTAATACTCGCCGCCAGCTTTTCCCCCATAAAGCAGAT
GTGGTTGGCGAGGTGATTCTGGTCGGCAATATGCCCGCCAGAGTCATTGGTGTGGCGGAAGAAAAACAGTCGATGTTTGG
TAGCAGTAAAGTGCTGCGTGTCTGGCTACCTTACAGCACGATGTCCGGGCGAGTTATGGGCCAGTCGTGGCTTAACTCCA
TTACTGTCAGGGTGAAAGAAGGATTTGACAGCGCCGAGGCGGAACAGCAACTCACGCGTTTACTTTCACTGCGCCACGGA
AAGAAGGATTTCTTTACCTGGAACATGGACGGCGTCTTGAAAACTGTTGAAAAGACCACACGTACTTTACAACTGTTTCT
GACGCTGGTGGCGGTGATTTCGCTGGTGGTGGGCGGTATTGGTGTAATGAATATTATGCTGGTGTCAGTGACCGAGCGGA
CGCGGGAAATTGGCATTCGCATGGCTGTAGGTGCGCGAGCAAGCGATGTTTTGCAACAGTTCCTGATCGAAGCCGTACTG
GTTTGCCTGGTCGGTGGCGCGTTGGGAATAACACTGTCACTGTTAATTGCTTTCACCTTGCAGCTTTTCTTACCCGGCTG
GGAGATTGGTTTTTCACCGTTGGCGCTGCTGCTGGCGTTTCTCTGCTCGACGGTCACCGGGATTTTATTTGGCTGGTTAC
CCGCACGAAATGCGGCACGACTGGATCCAGTAGATGCTCTGGCACGAGAGTAATTTTTGAGATAAAAATGCCAGCCGATC
GGGCTGGCATTTTGCCTTTAGGATGTACACAATGAGACAGAAGAGCTATGCGACTGCCGCTTCTACTTCGACGGGCACAA
TAACACTGGCGTGATTGCCTTTTGGCCCCTGGTGGACATCAAACTGAACGGATTGTCCAGCTTTTAGCGTTCTGTAACCA
TCCATCTGAATGGTGGAATAATGAGCGAAAATATCTTCGCCGCCGCCTTCAGGGCAGATGAAACCAAACCCTTTGGCATT
GTTGAACCACTTAACAGTACCCTTTTCCATGCTTCGACATCCTTCGCAAATCTTATACAAGTAAGATGGAATAAACCGGG
GTCAGAGAGGGGGCTGTTCAAAACCTCGCCAACTCTAGAAATACAATTTAGAGAATTAGGGCGAGCCGTCAAGCATTTGA
CAGGGGACAAGGGGCAGGTATGAATCAAAAATTTGAAGCAGTTAACGCTATTGACAGGAATGTGACAGATGTCGCTGATG

CCAACGATAGATGATAGTTATCTATCATGTGGAGTAGATTGGTCAGGCAAATAAGCTCTTGTCAGCGGCAGGGCGTTCTG
CCGATAACCGTAACCGAAGATGATAACTGACAATGGGTAAAACGAACGACTGGCTGGACTTTGATCAACTGGCGGAAGAA
AAAGTTCGCGACGCGCTAAAACCGCCATCTATGTATAAAGTGATATTAGTCAATGATGATTACACTCCGATGGAGTTTGT
TATTGACGTGTTACAAAAATTCTTTTCTTATGATGTAGAACGTGCAACGCAATTGATGCTCGCTGTTCACTACCAGGGGA
AGGCCATTTGCGGAGTCTTTACCGCCGAGGTTGCAGAAACCAAAGTGGCGATGGTGAACAAGTACGCGAGGGAGAATGAG
CATCCATTGCTGTGTACGCTAGAAAAAGCCTGAATGCAGGCATAAAAATTGGGGGAGGTGCCTATGCTCAATCAAGAACT
GGAACTCAGTTTAAATATGGCTTTCGCCAGAGCGCGCGAGCACCGTCATGAGTTTATGACCGTCGAGCACTTGTTACTGG
CGCTGCTCAGTAACCCATCTGCCCGGGAGGCGCTGGAAGCGTGTTCTGTGGATTTGGTTGCGCTCCGTCAGGAACTGGAA
GCCTTTATTGAACAAACCACACCCGTTCTGCCTGCCAGTGAAGAGGAGCGCGACACACAGCCGACGCTGAGTTTTCAGCG
TGTACTGCAACGTGCGGTCTTCCATGTCCAGTCCTCCGGTCGCAATGAGGTTACCGGTGCAAACGTTCTGGTCGCTATCT
TTAGCGAACAGGAGTCGCAGGCGGCATATCTGTTGCGTAAACATGAAGTCAGCCGTCTCGATGTGGTGAACTTTATCTCT
CATGGCACGCGTAAAGACGAGCCGACACAGTCTTCTGATCCTGGCAGCCAGCCAAACAGCGAAGAACAAGCTGGTGGGGA
GGAACGTATGGAGAATTTCACGACGAACCTGAATCAGCTTGCGCGCGTGGGCGGAATCGACCCACTGATTGGTCGTGAGA
AGGAGCTGGAGCGTGCTATTCAGGTTCTCTGCCGTCGCCGTAAAAACAACCCGCTGCTGGTGGGGGAATCTGGTGTCGGT
AAAACCGCGATTGCGGAAGGTCTTGCCTGGCGAATTGTTCAGGGCGATGTGCCGGAAGTGATGGCTGACTGTACGATTTA
CTCTCTCGATATCGGTTCTCTGTTAGCGGGCACAAAATATCGCGGCGACTTTGAAAAACGTTTTAAAGCGTTGCTCAAGC
AGCTGGAGCAGGACACTAACAGCATCCTGTTTATTGATGAGATCCACACCATTATCGGTGCGGGTGCAGCGTCTGGTGGT
CAGGTCGATGCGGCTAACCTAATCAAACCGTTGCTCTCCAGCGGTAAAATTCGTGTAATTGGTTCGACAACCTATCAGGA
GTTCAGCAACATTTTCGAGAAAGACCGTGCTCTGGCGCGTCGCTTCCAGAAAATTGATATTACTGAACCGTCGATCGAAG
AAACTGTTCAAATCATCAATGGCCTGAAACCGAAGTATGAAGCGCACCACGACGTGCGTTATACCGCAAAAGCGGTGCGT
GCGGCGGTAGAGCTGGCGGTGAAATACATTAACGATCGTCATCTGCCGGATAAAGCCATTGATGTTATCGACGAAGCGGG
CGCTCGCGCACGCCTGATGCCGGTAAGCAAACGCAAGAAAACCGTTAATGTGGCGGATATTGAGTCCGTGGTGGCCCGTA
TTGCACGCATTCCAGAGAAGAGTGTTTCTCAGAGTGATCGTGATACCCTGAAAAACCTCGGCGATCGCTTGAAAATGCTG
GTCTTCGGTCAGGATAAAGCCATTGAGGCGCTGACTGAAGCCATTAAGATGGCGCGTGCAGGTTTAGGTCACGAACATAA
ACCGGTTGGTTCGTTCCTGTTTGCCGGCCCTACCGGGGTCGGGAAAACAGAGGTGACGGTACAGCTTTCGAAAGCTTTGG
GCATTGAGCTTCTGCGCTTTGATATGTCCGAGTATATGGAACGCCATACCGTCAGCCGTCTTATTGGTGCGCCTCCGGGA
TACGTTGGTTTTGATCAGGGCGGTTTGCTGACTGATGCGGTCATCAAGCATCCACATGCGGTGCTGCTGCTGGACGAAAT
CGAGAAAGCGCACCCGGACGTGTTCAATATTCTGTTGCAGGTGATGGATAACGGTACGCTGACCGATAACAACGGACGCA
AAGCAGACTTCCGTAACGTGGTGCTGGTGATGACCACCAACGCCGGGGTACGGGAAACTGAGCGCAAATCCATTGGTCTT
ATCCACCAGGATAACAGCACCGATGCGATGGAAGAGATCAAGAAGATCTTTACACCGGAATTCCGTAACCGTCTCGACAA
CATTATCTGGTTTGATCATCTGTCAACCGACGTGATCCATCAGGTGGTGGATAAATTCATCGTCGAGTTGCAGGTTCAGC
TGGATCAGAAAGGTGTTTCTCTGGAAGTGAGCCAGGAAGCGCGTAACTGGCTGGCCGAGAAAGGTTACGACCGGGCAATG
GGCGCTCGTCCGATGGCGCGTGTCATCCAGGACAACCTGAAAAAACCGCTCGCCAACGAACTGCTGTTTGGTTCGCTGGT
GGACGGCGGTCAGGTCACCGTCGCGCTGGATAAAGAGAAAAATGAGCTGACTTACGGATTCCAGAGTGCACAAAAGCACA
AGGCGGAAGCAGCGCATTAATCTGATTGTTAGGTAGGTTGGTCAAGTCCGTAATCTCGAAAGAGGTTACGGACTTTTTGT
TTATGGGGTGGAGGAGGTTCAGACCCTTTTTTTAATGATGATGGTAAGTTGTTGATAATTAGTGCTGCGGGAAGGTAAGG
ATAAAAAAGGGTGCTGCAGGAGAATGGGATGGTTTTGCTTTATTAACAACGGGCTAAACGTGTAGTATTTGAGTTCACTG
CCGTACAGGCAGCTTAGAAATTCACAGGTAACATACTCCACCCGCCCACCATGTTCACTGCCGTACAGACAGATAAAATG
CGAAAAAAAAGCTCGCACTTTCGTACGAGCTCTTCTTTAAATATGGCGGTGAGGGGGGGATTCGAACCCCCGATACGTTG
CCGTATACACACTTTCCAGGCGTGCTCCTTCAGCCACTCGGACACCTCACCAAATTGTTTTGCTACCAAACCTCATGGGT
GGCAACGGGGCGCTACTATAGGGAGTTGGAGTAAAACGGTCAAGAAGAATTTTAATGATAATTATTGTTTGCTCATACTG
TAAACAAGTTGTGCAGTATATCTACATCGAGACAAGTTACGGACTTATACTTCCAAAGTACTTCATACATATCACAAAAT
AAAAAGGCCGGTTAAACCGACCTTTTACTCGTTCTTTCTCTTCGCCCATCAGGCGGTAAAACAATCAGCGACTACGGAAG
ACAATGCGGCCTTTGCTCAGGTCGTACGGGGTCAGTTCAACAGTCACTTTGTCGCCCGTCAGGATGCGGATGTAGTTTTT
GCGCATTTTACCGGAGATGTGTGCAGTAACCACGTGACCGTTTTCTAACTCTACGCGGAACATGGTATTAGGCAACGTTT
CAAGAACGGTACCTTGCATTTCAATATTGTCTTCTTTGGCCATCTAATCCTCTGGGGTATCACTACCGTAATTTGAACCG
GCAAGATAATGCCGAAGTTCTGTAAATAAGTAAAGATTTGCGCGCTAAATCGCAACAAACAGGTTCGGCACATTACTCCG
AAAACACACGGCTAAGCCGCACCAAAAGCGCAACGTATAAGGGAGCGGTGAGATAAACGATGGGCGTTACCTGACGCGAA
AAATTCCTTATCGGCAGCGGGGTAATGAGCGTAACCAACTCTGCGACCGCAATTATAACACTCTGGGGAGAAATGTGCCG
AAAACATTCATTCTTGTGGTGAAAACAAGCATCGTGGTACCCAGAAATTATTCGGCAATCGTCCGAGGCGCATTTGATTG
AGATAATTAAGGTAATCCCGGCGGGGAATTTCGCAGGCACCAAGCGATGCTGTGTGATCGTTAAGGACCTGGCAGTCGAT
AAGCTTACCGCCATGACCGATAAATTCCTCACAGAATACCAGAAGCGCCGTTTTAGACGCATTTTCCATCCGGCTGAACA
TGGACTCGCCACAAAATAGCGTTCCCTGGGCCACGCCGTACATACCGCCGACAAGCTCATCTTCACGCCAGACTTCAATG
GAGTGGGCATGGCCGAGTTCGTGAAGGCGATGGTAGGCTTCGACCACGCCACGCGTGATCCAGGTTCCTTCTTCGCGATC
GCTGGCACAGCCTTCAATGACCTGACCAAAAGCGTAATTCATCGTGACACGATAGGGCGAGCGTTTATGAAATCGCTTCA
TACTACGGCTGATATGCAGTGATTCTGGCCATAGCACCGCGCGGGGATCGGGCGACCACCAGAGGATGGGGTCGCCTGGA

GAAAACCACGGAAAAATACCACGCTGGTAAGCCATTAACAGGCGCGCAGGGCTAAGATCGCCCCCAAGTGCCAGCAGGCC
GTTAGGCTCACGTAATGCGCCTTCCGGGGAAGGGAAGGCTATTGAATGGCGAGAAAGCTGAACCAGGCGCATGACCGCAA
AACTCCACGCAAGTCGGATCGTTCAATAATAGCTTACAAACCCTGCTTGAACTGGTAATAACGCCCCTGTCTGGCAAGCA
GTTCTGCGTGAGTACCTTGCTCAATAATTTGCCCGTTGTCCATCACTATTATTTGTTGGAAACGAGAGAGTCCGCGAAGT
CGATGGGTGACCATTAACACCGTTTTCTCACGCATCATTTCTGCAAGCAATTCAAGGATCTGGCTTTCGGTTGTGGCATC
TAAGCCTTCGGTAGGTTCATCCAGCAACACCAGTGGCGCATCATGTAACAGCGCACGGGCGATAGCCAGACGGCGCAGTT
CACCACCGGAGAGCTGGCGTCCGCCTTCACCTAACCAACTGTTGAGACCTGCATCCTCGAGCAGCTTTTCCAGGCCAACG
CGACGCAAGATCTCCGACAGAGCCTCATCACTACTGCCAGGCGAGGCGAGTAAAAGATTATCACGCAGCGTGGCGCTAAA
CAGATGCACTCGCTGAGGAACAACGCTGATGGTCTGTCGTAGAGCCGCTTCATTCAGGCTGGCTATGGGGCTATCGTTAA
GCAAAATCTCGCCCTGTTGCGGGTCCCATGCGCGGGTCAGCTGTTGTAACAGTGTTGATTTGCCGCATCCGGTTCGCCCG
AGAATCGCTATATGTTCCCCGGCGTTTACCTGAAGAGAAATCCCTTTAAGTGCCTGTTGAGATTGCTCCGGATAAGTGAA
CTGAACATCCCGTAACGTCAGCGAAACGCGATCGGCAACACGAGTTTGGGTATCAGGAAAGGTGACCTCCGGTTTTTGAT
CCGTTAAGTCAGAGATACGTACGGCAGAGGCAATGACTTGCCCCAGATGCTGAAATGCACCCGTTACTGGTGCCAGTGCT
TCAAACGCGGCTAACGCGCAGAAGACAAACAGGGCAATTAACGCGCCGGGTTGAGCATTGCCGCCAACGCCGCCAGACGC
CATCCACAGCATCAGGATCACCGCTAACGCGCCAATGAGCAGCATTATCGCTTGCGACAATGCGGTCAGTTCAGATTGAC
GGCGTTGCGCTTCCAGCCATTGAATTTCTGTATTCTCTAGTTGCGTGCGATAACGATCGCTGGCACCAAAAATGGTCAGC
TCAGCTTGCCCTTGCAGCCAGGCCGTCAGTTGTTGGCGATACTGTCCGCGAAGATGAGTCAGATTTTGCCCGGTGCTTTT
TCCCGCACGATAAAACAGCGGTGGCATCAGGAAAAGCGTCAGTAACATAATGCCGCCCAGCGTAAAGGCGAGGGTGAAAT
CAAGGAAACTTAACCCGATTGTCACCACCATAATCACCACAAAAGCGCCCACCAGCGGCGAGATAACGCGCAGGTAAAGA
TGATCGAGCGTATCAACATCCGCCACCACGCGATTGAGCAATTCGCCCTGACGATAGCGCGCCAGTCCGGCAGGGGAGAG
GGGCAGCAATTTGCTGAAGGTGTAAATGCGCAGATGCTGCAACACGCGGAAAGTCGCGTCGTGACTTACCAGACGTTCAA
AATAGCGCCCGGCAGTACGGGTGATTGCTGCGCCACGCACGCCCGCAGCGGGTAGCATATAGTTGAAGCTGTACAGTCCG
GCAACCCCCGCAACCGCTGAGGCCGAGAGGAACCAGCCGGAAAGTGTCAACAGACCGATACTGGCGAGCAGCGTCACAAT
TGCCAGCACAATACCAAGACTTAACATCCATTTATGACGTTTATACAGTGCCAGATAGGGTAGCAAAGCGCGCATTTAAA
TCTCCTCCTGACGATGGGCCAGTAATGTGGCGAATGGGCCACCAGCCACACTTAATTCCGCGTAACGTCCTTGCTCAATA
ATCCGGCCATCCTGCATAACCCAAATGACATCCCAGTCAGCAAGATCTTCTAACTGGTGGGTGACCATTAACGTTGTCTG
GCGCAGAGAGGCGGCATTCAGCGCCTCCATTACGCGCTGTTCACTGTGAGCATCAAGGCTGGCAGCGGGTTCATCCAACA
GTAATAGCGAACAGGGATTTAGTAACGCACGGGCCACCGCCACGCGCTGCGCCTGCCCCACGGAAAGGCGGGCAGCCTGG
TCGCCAACAGGCGTATCAACGCCTTGTGGGAGGAGCGGTAGAAACTCGCTGACCCAGGCGTTATCCAGCGCTGCTTGTAA
TTCTTGTTCGCTGGCATCAGGTCGCGCCAGTAGTACGTTATCCCGCAATGTTGCTGCCGGTAATTGTGGGTTTTGCCCAA
CCCAGGAGAGATGTTTACGCCATGATTCTGGTGATAAATCGCGTAATTCTATCCCGTTGATTCGTAGCGATCCCTGATAT
GAGAGAAAACCAGAAAGCGCGTTCAGCAGTGAGCTTTTACCTGAACCGCTGCGACCAACCAACACCGCACGTTGGCCTGC
TGGCAAAGTAAAGTTCAGCGGTCCGGCCAGCGTTTTACCTTCCGGCGACGTGATAAACAGCTCCTCGGCCTCAATGGTCA
CCGGATCGGTCGATGCTAATTCCGCCTCACCACGTTGCGGATGGGCGAGCGGGGTTTCCATAAACGTTTTCAGACTGTCA
GCTGCGCCAACAGCCTGGGCTTTAGCATGATAAAACGTACCGAGATCGCGTAATGGCTGGAAAAACTCTGGCGCAAGGAT
CAGGGCCAGAAAACCCGCAGCCAGCGTCACACCGGTATCGTAGTGACCAAAATCCAGCTCGCCGAGATAGGAAAAACCAA
AGTAGACCGCCACCAGAGCAATTGACAGCGAGGTAAAAAATTCGAGAATGCCGGAGGATAAAAACGCCAGCCGTAGCACT
TCCATTGTCCGTTGGCGGAAATCTTCCGAAGCAGAACGAATACTTTCAATTTCAGCTTCACCACGACCAAAAATACGCAA
TGTTTCCATGCCGCGCAGGCGATCGAGGAAATGCCCACTTAAGCGAGCAAGAGCGAGAAAGTTACGTCGGTTAGCATCGG
CAGCCCCCATTCCAACCAGCGCCATAAACAACGGAATTAACGGTGCAGTGCCCAGCAGAATGAGCGCCGCAGCCCAGTTA
GAGGGGAAGATTGCCACCACAATCAGCAACGGCACCGACACTGCCAGCGCCATTTGCGGCAGATAGCGTGCATAGTAATC
ATGCATATCGTCAATTTGCTCGAGTACCAGCGTCGCCCAGCTCCCCGCAGGTTTACCCTGAATCCACGCTGGCCCTGCTT
GTTGCAGACGGTCGAGAACCTGACGGCGGATGGCAAAGCGGATATGCTGCCCGGCGTGATAACCCACCCGTTCGCGTAAC
CAGACCACCCATGCGCGCAGTACAAAGGTCAGAACCAGTAACGTAAAGGGAAGCAGCAGGGCTTCACGGGGAATATTCTC
CATAATCATATGTTGCAGAATTCGCGCCATGAACCAGGCCTGGGCAATGATCAATATGCCGCTCACAAAGCCCAGCAGAC
GAGAAATATTCAGCCAACGTTGGGAGATGACGCTTTGCTGTTTTAACCAGCGGGTTAACTCTTTTTGACGAGATTTATTC
ATTGCACGCTTAGCAGGTGAGTTATCAGAATTATTTGCAGAGCAATGTTACAACGGGGAAAAAATAAAGGCGACCCATAG
TCGCATGGTGTCGCCTTCTTTACTTTTGTTACTGATTTGTAAAATTATTTTGCGTCAGCTAAACCATCGAGGTAGCGTTC
CGCATCAAGTGCTGCCATGCAGCCTGTACCGGCCGAAGTAATGGCCTGGCGATAAATGTGATCCATCACGTCGCCTGCGG
CAAAGACGCCAGGAATGCTGGTCTGGGTGGCATTACCATGAATACCCGACTGTACTTTGATGTAGCCGTTTTCCAGTTCC
AGCTGCCCTTCGAAAATCGCAGTATTCGGGCTGTGACCGATAGCAACAAACAGACCGGCAACGTCGAGTGACTCGATGTT
ATCGCTGTTTTGCGTATCGCGCAGACGAACGCCAGTGACACCCATTTGATCGCCGGTCACTTCTTCCAGCGTACGGTTGG
TGTGCAGAATGATGTTGCCGTTCTCCACTTTATCCATCAGGCGCTTAATGAGGATTTTTTCCGCGCGGAAACCGTCACGG
CGGTGAATCAGATGCACTTCCGAAGCGATGTTAGACAGATACAGCGCCTCTTCAACCGCGGTATTGCCGCCGCCGATGAC
CGCAACTTTCTGGTTGCGATAGAAGAAACCGTCGCAGGTTGCACAAGCAGAAACCCCACGGCCTTTAAAGGCTTCTTCAG
AGGGCAGGCCGAGATAGCGTGCAGAAGCTCCGGTGGCAATAATCAGCGCGTCGCAAGTGTATTCGCCGTTATCGCCATTC

AGACGGAACGGACGGTTTTGCAGATCCACCTTGTTGATATGATCAAAAATGATCTCAGTTTCAAACTTGGTGGCATGTTC
GTGCATGCGCTCCATTAATAACGGACCGGTCAGATCGTTTGGATCGCCAGGCCAGTTTTCCACTTCCGTGGTGGTGGTCA
GTTGGCCGCCTTTTTCCATGCCGGTAATCAGCACAGGTTGCAGGTTGGCGCGCGCCGCGTAGACAGCAGCGGTGTATCCC
GCCGGGCCTGAACCCAGGATAAGCAGTTTACTGTGTTTGGTCGTGCCCATGAGATCCCCATAGTTGTTGGCAGACAATGG
GCAGGATTGTAGGGAATTTACAGACGTAAAAAAAGAGTATGACGATTTTGTTAACAATTTGTGCAATCGGCAGCATCGAT
AAGCAGGTCAAATTCTCCCGTCATTATCACCTCTGCTACTTAAATTTCCCGCTTTATAAGCCGATTAAATGATGAATAAA
CGCCCCTGTTAATGAATATCTGGCATGTTGTACTAAAAATCGATGTTTTGCTTTGACAATCCCCTGGTGTTTTGCGAAAA
CATTCGAGGAAGAAAAAAAACAGTATTCTTATATGCGCATAACCATGCATGTAAATACCATGTTTACCGTGCTAGTGAAA
TCTACGTATGGCGTGGACAGACGCCATTCGTGATGTCGATAGCTGCCACAAGGCAACGGTCTTCTCACCGTAGACCCAGG
CATTGCGCGCCGTGAATCTTCATGATTTCGGTCTATCGTGACGGGTAGCGACTCTGAACAGTGATGTTTCAGGGTCAGAC
AGGAGTAGGGAAGGAATACAGAGAGACAATAATAATGGTAGATAGCAAGAAGCGCCCTGGCAAAGATCTCGACCGTATCG
ATCGTAACATTCTTAATGAGTTGCAAAAGGATGGGCGTATTTCTAACGTCGAGCTTTCTAAACGTGTGGGACTTTCCCCA
ACGCCGTGCCTTGAGCGTGTGCGTCGGCTGGAAAGACAAGGGTTTATTCAGGGCTATACGGCGCTGCTTAACCCCCATTA
TCTGGATGCATCACTTCTGGTATTCGTTGAGATTACTCTGAATCGTGGCGCACCGGATGTGTTTGAACAATTCAATACCG
CTGTACAAAAACTTGAAGAAATTCAGGAGTGTCATTTAGTATCCGGTGATTTCGACTACCTGTTGAAAACACGCGTGCCG
GATATGTCAGCCTACCGTAAGTTGCTGGGGGAAACCCTGCTGCGTCTGCCTGGCGTCAATGACACACGGACATACGTTGT
TATGGAAGAAGTCAAGCAGAGTAATCGTCTGGTTATTAAGACGCGCTAACACGGAACAGGTGCAAAATCGGCGTATTTTG
ATTACACTCCTGTTAATCCATACAGCAACAGTACTGGGGTAACCTGGTACTGTTGTCCGTTTTAGCATCGGGCAGGAAAA
GCCTGTAACCTGGAGAGCCTTTCTTGAGCCAGGAATACATTGAAGACAAAGAAGTCACATTGACAAAGTTAAGTAGCGGC
CGCCGCCTTCTGGAGGCGTTGCTGATCCTTATTGTCCTGTTTGCCGTCTGGTTGATGGCTGCCTTACTAAGCTTTAACCC
TTCGGACCCCAGCTGGTCGCAAACGGCCTGGCATGAACCTATCCATAATTTAGGTGGGATGCCCGGTGCGTGGTTGGCAG
ATACGCTGTTCTTTATTTTTGGCGTGATGGCTTACACCATTCCCGTCATTATTGTCGGCGGTTGTTGGTTTGCCTGGCGT
CATCAGTCCAGCGACGAATACATTGATTATTTTGCCGTTTCGCTACGCATCATTGGCGTTTTGGCGCTCATCCTTACCTC
CTGTGGTCTGGCGGCAATCAACGCTGACGATATCTGGTATTTTGCCTCCGGTGGCGTCATTGGCAGCTTACTAAGCACTA
CGCTACAACCACTGCTACACAGTAGCGGGGGAACTATTGCGCTGCTCTGCGTTTGGGCAGCGGGCCTGACGTTGTTCACC
GGTTGGTCATGGGTGACCATTGCTGAAAAACTCGGCGGCTGGATTTTAAACATTCTCACCTTCGCCAGTAATCGTACCCG
TCGCGATGATACCTGGGTCGATGAAGATGAGTATGAAGACGACGAAGAGTATGAAGATGAAAATCACGGCAAACAGCATG
AATCACGCCGTGCCCGTATTCTTCGCGGCGCGCTAGCGCGTCGTAAACGGTTGGCGGAAAAATTCATTAATCCGATGGGG
CGGCAAACAGACGCTGCGTTGTTCTCCGGTAAGCGGATGGATGATGACGAAGAGATTACCTACACTGCACGCGGTGTGGC
TGCCGACCCGGACGACGTCCTATTTTCGGGCAATCGTGCAACGCAGCCAGAATATGACGAATACGATCCATTATTAAACG
GTGCGCCAATTACCGAACCTGTCGCTGTAGCAGCTGCTGCTACCACGGCGACACAAAGCTGGGCTGCGCCGGTTGAACCT
GTGACTCAGACGCCGCCTGTTGCCTCTGTTGATGTTCCACCTGCGCAACCTACAGTAGCCTGGCAGCCTGTACCGGGTCC
ACAAACGGGAGAGCCGGTTATTGCTCCTGCACCGGAAGGTTACCCACAGCAGTCACAATATGCGCAGCCTGCAGTGCAAT
ATAATGAGCCGCTGCAACAACCAGTACAGCCGCAGCAGCCGTATTATGCACCTGCAGCTGAACAACCTGCGCAACAGCCG
TATTATGCCCCTGCGCCAGAACAACCGGTGGCAGGTAACGCCTGGCAAGCCGAAGAGCAGCAATCCACTTTTGCTCCACA
GTCTACATACCAGACTGAGCAAACTTATCAGCAGCCAGCCGCTCAGGAGCCGTTGTACCAACAGCCGCAACCCGTTGAAC
AGCAGCCTGTTGTGGAGCCTGAACCCGTTGTAGAAGAGACAAAACCCGCGCGTCCGCCGCTTTACTACTTTGAAGAAGTT
GAAGAGAAGCGAGCCCGTGAACGTGAACAACTTGCGGCCTGGTATCAACCGATTCCAGAACCGGTTAAAGAACCAGAACC
GATCAAATCTTCGCTGAAAGCACCTTCTGTTGCAGCAGTACCTCCAGTAGAAGCCGCTGCCGCTGTTTCCCCGCTGGCAT
CTGGCGTGAAAAAAGCGACACTGGCGACGGGGGCTGCCGCAACCGTTGCCGCGCCAGTCTTCAGTCTGGCAAATAGCGGT
GGACCGCGTCCTCAGGTCAAAGAGGGGATTGGTCCGCAGTTGCCACGACCGAAACGTATCCGCGTGCCAACTCGTCGTGA
ACTGGCGTCTTACGGTATTAAGCTGCCCTCACAGCGTGCGGCGGAAGAAAAAGCCCGTGAAGCCCAGCGCAATCAGTACG
ATTCTGGCGATCAGTACAACGATGATGAAATCGATGCGATGCAGCAGGATGAACTGGCACGTCAGTTCGCCCAGACACAG
CAGCAACGCTATGGCGAACAGTATCAACATGATGTGCCCGTAAACGCAGAAGATGCAGATGCTGCGGCAGAGGCTGAACT
GGCTCGTCAGTTTGCGCAAACTCAACAACAACGTTATTCCGGCGAACAACCGGCTGGGGCGAATCCGTTCTCGCTGGATG
ATTTTGAATTTTCGCCAATGAAAGCGTTGCTGGATGATGGTCCACACGAACCGTTGTTTACGCCAATTGTTGAACCTGTA
CAGCAGCCGCAACAACCGGTTGCACCGCAGCAGCAATATCAGCAGCCGCAACAACCAGTTCCGCCGCAGCCGCAGTATCA
GCAGCCACAACAGCCGGTTGCGCCGCAGCCACAATATCAGCAGCCGCAACAACCGGTTGCGCCACAGCAGCAATATCAGC
AGCCGCAACAACCGGTTGCGCCGCAGCAGCAGTATCAGCAGCCACAACAGCCAGTTGCGCCACAACCGCAGGATACCCTG
CTTCATCCGCTGTTGATGCGTAATGGCGACAGCCGTCCGTTGCATAAACCGACGACGCCGCTGCCTTCTCTGGATTTGCT
GACACCGCCGCCGAGCGAAGTGGAGCCGGTAGATACCTTTGCGCTTGAACAAATGGCTCGCCTGGTGGAAGCGCGTCTGG
CTGATTTCCGTATTAAAGCCGATGTCGTCAATTACTCTCCGGGGCCGGTTATCACTCGCTTTGAATTGAACCTGGCACCG
GGCGTAAAAGCGGCGCGCATTTCTAACTTGTCACGGGACCTTGCCCGTTCACTTTCGACGGTGGCGGTGCGTGTCGTTGA
AGTTATTCCTGGCAAACCCTATGTAGGTCTGGAGTTACCGAATAAAAAACGACAAACCGTTTATCTGCGCGAAGTTTTGG
ATAACGCCAAATTCCGCGATAATCCGTCGCCATTAACCGTGGTGCTGGGTAAAGATATCGCCGGTGAGCCGGTGGTTGCC
GATCTGGCGAAAATGCCGCACTTGTTGGTTGCGGGGACTACCGGTTCCGGTAAATCTGTCGGTGTGAACGCGATGATCCT

GAGCATGCTTTATAAAGCACAGCCAGAAGATGTGCGTTTCATCATGATCGACCCGAAAATGCTGGAGCTTTCGGTTTATG
AAGGCATTCCGCATCTGTTAACGGAAGTCGTTACTGATATGAAAGATGCCGCCAACGCGCTGCGCTGGTGTGTTAACGAG
ATGGAGCGTCGGTATAAACTGATGTCTGCGCTGGGTGTGCGTAATCTGGCGGGTTATAACGAAAAAATTGCTGAAGCCGA
TCGCATGATGCGTCCGATTCCAGACCCGTACTGGAAGCCGGGTGACAGTATGGATGCCCAGCATCCGGTGCTGAAAAAAG
AACCATACATTGTGGTGTTGGTTGACGAATTTGCCGACCTGATGATGACGGTAGGTAAAAAAGTGGAAGAGCTGATAGCA
CGTCTGGCGCAAAAAGCCCGTGCCGCGGGTATCCACCTCGTACTGGCAACTCAGCGTCCATCGGTTGATGTTATTACTGG
TCTGATTAAAGCGAATATTCCGACCCGTATCGCCTTTACCGTATCCAGTAAGATTGACTCACGTACCATTCTTGATCAGG
CTGGCGCGGAATCACTGCTGGGTATGGGGGATATGCTCTACTCTGGGCCGAACTCCACGTTGCCGGTACGTGTCCATGGT
GCTTTTGTTCGCGATCAGGAAGTTCATGCCGTGGTGCAGGACTGGAAAGCGCGTGGTCGCCCACAGTATGTTGATGGCAT
CACCTCCGACAGCGAAAGCGAAGGTGGTGCGGGTGGTTTCGATGGCGCTGAAGAACTGGATCCGTTGTTCGATCAGGCGG
TGCAGTTTGTCACTGAAAAACGCAAAGCGTCAATTTCTGGCGTACAGCGTCAGTTCCGCATTGGTTATAACCGTGCAGCG
CGTATTATCGAACAGATGGAAGCGCAGGGGATTGTCAGCGAACAGGGGCACAACGGTAATCGTGAAGTGCTGGCCCCACC
GCCGTTTGACTAACTAATGCATCGTATGCCGGATAAGGCGCGGTAGCGTCGCATCCGGCACTCTATCAACTGAAAATTCA
GTATTTTCTTCTTTCCTCAAGCTGATTATTAGCCTGGAATAGAGAGTAGAGGGAACTCCCGATCGGGAGTGACGTAATTT
GAGGAATAATGATGAAAAAAATTGCCATCACCTGTGCATTACTCTCAAGCTTAGTAGCAAGCAGCGTTTGGGCTGATGCC
GCAAGCGATCTGAAAAGCCGCCTGGATAAAGTCAGCAGCTTCCACGCCAGCTTCACACAAAAAGTGACTGACGGTAGCGG
CGCGGCGGTGCAGGAAGGTCAGGGCGATCTGTGGGTGAAACGTCCAAACTTATTCAACTGGCATATGACACAACCTGATG
AAAGCATTCTGGTTTCTGACGGTAAAACACTGTGGTTCTATAACCCGTTCGTTGAGCAAGCTACGGCAACCTGGCTGAAA
GATGCCACCGGTAATACGCCGTTTATGCTGATTGCCCGCAACCAGTCCAGCGACTGGCAGCAGTACAATATCAAACAGAA
TGGCGATGACTTTGTCCTGACGCCGAAAGCCAGCAATGGCAATCTGAAGCAGTTCACCATTAACGTGGGACGTGATGGCA
CAATCCATCAGTTTAGCGCGGTGGAGCAGGACGATCAGCGCAGCAGTTATCAACTGAAATCCCAGCAAAATGGGGCTGTG
GATGCAGCGAAATTTACCTTCACCCCGCCGCAAGGCGTCACGGTAGATGATCAACGTAAGTAGAGGCACCTGAGTGAGCA
ATCTGTCGCTCGATTTTTCGGATAATACTTTTCAACCTCTGGCCGCGCGTATGCGGCCAGAAAATTTAGCACAGTATATC
GGCCAGCAACATTTGCTGGCTGCGGGGAAGCCGTTGCCGCGCGCTATCGAAGCCGGGCATTTACATTCTATGATCCTCTG
GGGGCCGCCGGGTACCGGCAAAACAACTCTCGCTGAAGTGATTGCCCGCTATGCGAACGCTGATGTGGAACGTATTTCTG
CCGTCACCTCTGGCGTGAAAGAGATTCGCGAGGCGATCGAGCGCGCCCGGCAAAACCGCAATGCAGGTCGCCGCACTATT
CTTTTTGTTGACGAAGTTCACCGTTTCAACAAAAGCCAGCAGGATGCATTTCTGCCACATATTGAAGACGGCACCATCAC
TTTTATTGGCGCAACCACTGAAAACCCGTCGTTTGAGCTTAATTCGGCACTGCTTTCCCGTGCCCGTGTCTATCTGTTGA
AATCCCTGAGTACAGAGGATATTGAGCAAGTACTAACTCAGGCGATGGAAGACAAAACCCGTGGCTATGGTGGTCAGGAT
ATTGTTCTGCCAGATGAAACACGACGCGCCATTGCTGAACTGGTGAATGGCGACGCGCGCCGGGCGTTAAATACGCTGGA
AATGATGGCGGATATGGCCGAAGTCGATGATAGCGGTAAGCGGGTCCTGAAGCCTGAATTACTGACCGAAATCGCCGGTG
AACGTAGCGCCCGCTTTGATAACAAAGGCGATCGCTTTTACGATCTGATTTCCGCACTGCATAAGTCGGTACGTGGTAGC
GCACCCGATGCGGCGCTGTACTGGTATGCGCGAATTATTACCGCTGGTGGCGATCCGTTATATGTCGCGCGTCGCTGTCT
GGCGATTGCGTCTGAAGACGTCGGTAATGCCGATCCACGGGCGATGCAGGTGGCAATTGCGGCCTGGGATTGCTTTACTC
GCGTTGGCCCGGCGGAAGGTGAACGCGCCATTGCTCAGGCGATTGTTTACCTGGCCTGCGCGCCAAAAAGCAACGCTGTC
TACACTGCGTTTAAAGCCGCGCTGGCCGATGCTCGCGAACGCCCGGATTATGACGTGCCGGTTCATTTGCGTAATGCGCC
GACGAAATTAATGAAGGAAATGGGCTACGGGCAGGAATATCGTTACGCTCATGATGAAGCAAACGCTTATGCTGCCGGTG
AGGTTTACTTCCCGCCGGAAATAGCACAAACACGCTATTATTTCCCGACAAACAGGGGCCTTGAAGGCAAGATTGGCGAA
AAGCTCGCCTGGCTGGCTGAACAGGATCAAAATAGCCCCATAAAACGCTACCGTTAATGTTATCGTTGCGGTAATGTTGT
TACTGTATCCCTGTGGTCGCAGGCTGTGGCCACATCTCCCATTTAATTCGATAAGCACAGGATAAGCATGCTCGATCCCA
ATCTGCTGCGTAATGAGCCAGACGCAGTCGCTGAAAAACTGGCACGCCGGGGCTTTAAGCTGGATGTAGATAAGCTGGGC
GCTCTTGAAGAGCGTCGTAAAGTATTGCAGGTCAAAACGGAAAACCTGCAAGCGGAGCGTAACTCCCGATCGAAATCCAT
TGGCCAGGCGAAAGCGCGCGGGGAAGATATCGAGCCTTTACGTCTGGAAGTGAACAAACTGGGCGAAGAGCTGGATGCAG
CAAAAGCCGAGCTGGATGCTTTACAGGCTGAAATTCGCGATATCGCGCTGACCATCCCTAACCTGCCTGCAGATGAAGTG
CCGGTAGGTAAAGACGAAAATGACAACGTTGAAGTCAGCCGCTGGGGTACCCCGCGTGAGTTTGACTTTGAAGTTCGTGA
CCACGTGACGCTGGGTGAAATGCACTCTGGCCTCGACTTTGCAGCTGCAGTTAAGCTGACTGGTTCCCGCTTTGTGGTAA
TGAAAGGGCAGATTGCTCGCATGCACCGCGCACTGTCGCAGTTTATGCTGGATCTGCATACCGAACAGCATGGCTACAGT
GAGAACTATGTTCCGTACCTGGTTAACCAGGACACGCTGTACGGTACGGGTCAACTGCCGAAATTTGCTGGCGATCTGTT
CCATACTCGTCCGCTGGAAGAAGAAGCAGACACCAGTAACTATGCGCTGATCCCAACGGCAGAAGTTCCGCTGACTAACC
TGGTGCGCGGTGAAATCATCGATGAAGATGATCTGCCAATTAAGATGACCGCCCACACCCCATGCTTCCGTTCTGAAGCC
GGTTCATATGGTCGTGACACCCGTGGTCTGATCCGTATGCACCAGTTCGACAAAGTTGAAATGGTGCAGATCGTGCGCCC
AGAAGACTCAATGGCGGCGCTGGAAGAGATGACTGGTCATGCAGAAAAAGTCCTGCAGTTGCTGGGCCTGCCGTACCGTA
AAATCATCCTTTGCACTGGCGACATGGGCTTTGGCGCTTGCAAAACTTACGACCTGGAAGTATGGATCCCGGCACAGAAC
ACCTACCGTGAGATCTCTTCCTGCTCCAACGTTTGGGATTTCCAGGCACGTCGTATGCAGGCACGTTGCCGCAGCAAGTC
GGACAAGAAAACCCGTCTGGTTCATACCCTGAACGGTTCTGGTCTGGCTGTTGGTCGTACGCTGGTTGCAGTAATGGAAA
ACTATCAGCAGGCTGATGGTCGTATTGAAGTACCAGAAGTTCTGCGTCCGTATATGAACGGACTGGAATATATTGGCTAA

TACCCAATTTTTCTGAATCTAAAAAGCGCCTGCGGGCGCTTTTTTTGTCTCCCTTTGATACCGAACAATAATTACTCCTC
ACTTACACGTAATACTACTTTCGAGTGAAAATCTACCTATCTCTTTGATTTTCAAATTATTCGATGTATACAAGCCTATA
TAGCGAACTGCTATAGAAATAATTACACAATACGGTTTGTTACTGGAATCAATCGTGAGCAAGCTTGAGTGAGCCATTAT
GAAAACGAAAATCCCTGATGCGGTATTGGCTGCTGAGGTGAGTCGCCGTGGTTTGGTAAAAACGACAGCGATCGGCGGCC
TGGCAATGGCCAGCAGCGCATTAACATTACCTTTTAGTCGGATTGCGCACGCTGTCGATAGCGCCATTCCAACAAAATCA
GACGAAAAGGTTATCTGGAGCGCCTGTACAGTTAACTGTGGTAGTCGCTGCCCGCTACGTATGCACGTCGTGGACGGTGA
AATCAAATATGTCGAAACGGACAATACCGGCGATGACAATTACGACGGCCTGCACCAGGTTCGCGCCTGCCTGCGTGGGC
GTTCCATGCGTCGCCGTGTCTACAATCCGGACCGCCTGAAATATCCGATGAAACGAGTCGGGGCGCGCGGTGAAGGCAAA
TTCGAGCGCATTAGCTGGGAAGAAGCCTACGACATCATCGCGACCAATATGCAGCGCCTGATCAAAGAGTACGGCAACGA
GTCTATCTATCTGAACTATGGCACCGGTACGCTGGGCGGCACCATGACCCGCTCCTGGCCGCCGGGAAATACCCTGGTCG
CGCGGCTGATGAACTGCTGCGGCGGCTATCTGAACCATTACGGCGACTACTCCTCCGCGCAAATTGCGGAAGGTTTGAAC
TATACCTACGGCGGCTGGGCAGATGGCAACAGCCCGTCGGATATCGAAAACAGTAAGCTGGTAGTGCTGTTTGGTAATAA
CCCTGGCGAAACGCGAATGAGTGGCGGTGGGGTGACTTACTATCTTGAACAGGCACGCCAGAAATCTAATGCCCGCATGA
TCATCATCGATCCGCGCTATACCGACACCGGTGCCGGGCGCGAAGATGAGTGGATCCCTATTCGTCCGGGAACAGATGCC
GCACTGGTTAACGGTCTGGCGTACGTCATGATCACTGAAAACCTGGTGGATCAGGCATTCCTCGATAAATATTGCGTTGG
CTACGATGAGAAAACCCTGCCAGCCAGTGCGCCGAAAAATGGCCACTATAAAGCTTATATTCTGGGTGAAGGGCCAGATG
GCGTGGCTAAAACGCCGGAATGGGCCTCGCAAATCACTGGTGTTCCGGCAGACAAAATCATCAAATTGGCTCGTGAAATC
GGTAGTACCAAACCGGCGTTTATCAGCCAGGGATGGGGCCCGCAGCGTCACGCTAACGGTGAAATCGCAACCCGTGCTAT
CTCGATGCTGGCGATTCTGACCGGTAACGTTGGTATTAACGGAGGCAACAGCGGCGCGCGTGAAGGTTCATACAGCTTAC
CGTTTGTCCGTATGCCGACCTTGGAAAACCCGATCCAGACCAGCATTTCGATGTTTATGTGGACCGATGCCATTGAACGT
GGCCCGGAAATGACGGCGCTGCGTGATGGTGTACGCGGGAAAGATAAGCTGGATGTGCCGATCAAAATGATCTGGAACTA
TGCCGGTAACTGCCTGATTAACCAGCATTCTGAAATCAACCGTACCCATGAAATCCTTCAGGATGATAAGAAGTGCGAGC
TGATTGTGGTTATCGACTGCCACATGACCTCATCGGCGAAATATGCTGACATCCTGCTGCCTGACTGCACCGCTTCCGAA
CAGATGGACTTTGCGCTGGATGCATCCTGCGGGAATATGTCTTACGTGATTTTCAACGATCAGGTGATTAAACCGCGCTT
TGAATGTAAGACCATCTATGAAATGACCAGCGAACTGGCAAAACGTCTTGGCGTTGAGCAACAGTTTACTGAAGGCCGTA
CCCAGGAAGAGTGGATGCGGCATCTGTATGCCCAGTCGCGGGAAGCGATTCCTGAACTGCCAACGTTTGAAGAGTTCCGC
AAGCAGGGGATCTTTAAAAAGCGCGACCCACAAGGGCATCACGTTGCTTATAAAGCCTTCCGTGAAGATCCGCAGGCAAA
CCCACTGACTACGCCATCGGGCAAAATTGAGATTTATTCGCAGGCGCTGGCTGACATTGCCGCTACCTGGGAATTGCCTG
AAGGCGATGTGATCGATCCACTGCCGATCTACACGCCGGGCTTTGAAAGTTATCAGGATCCGCTGAACAAACAGTATCCG
CTGCAGCTTACAGGTTTCCACTATAAATCTCGCGTTCACTCAACTTACGGCAACGTTGATGTGCTGAAAGCGGCTTGCCG
TCAGGAAATGTGGATCAACCCGCTTGATGCCCAAAAACGCGGTATCCACAACGGCGATAAAGTCAGGATCTTTAACGATC
GTGGTGAGGTTCATATTGAGGCGAAAGTGACGCCACGAATGATGCCGGGTGTGGTCGCACTGGGTGAAGGTGCCTGGTAT
GACCCGGATGCAAAACGTGTCGATAAGGGTGGTTGTATTAACGTACTGACCACTCAACGTCCGTCTCCTCTCGCTAAGGG
GAATCCGTCACATACAAACCTTGTTCAGGTTGAAAAGGTGTAAGGAGTAACCGATGACAACCCAGTATGGATTTTTTATT
GATTCCAGCCGTTGCACCGGTTGCAAAACCTGCGAGCTGGCCTGTAAAGACTACAAAGATTTGACGCCAGAAGTCAGCTT
CCGCCGCATTTATGAATATGCTGGCGGCGACTGGCAGGAAGATAACGGTGTCTGGCACCAGAACGTGTTTGCCTACTATC
TGTCGATTTCATGTAACCACTGCGAAGATCCGGCTTGTACTAAAGTCTGCCCGAGCGGTGCGATGCATAAACGTGAAGAT
GGTTTTGTTGTGGTCGATGAAGATGTGTGCATTGGCTGCCGCTACTGCCATATGGCTTGCCCGTATGGCGCACCGCAATA
TAACGAAACGAAAGGCCATATGACCAAATGCGATGGTTGTTATGACCGTGTTGCGGAGGGTAAAAAGCCGATCTGTGTTG
AATCTTGTCCGCTGCGGGCGCTGGATTTCGGCCCTATCGACGAGCTGCGTAAAAAACATGGCGATCTGGCGGCCGTTGCG
CCGTTGCCGCGAGCTCACTTTACCAAACCGAATATTGTGATCAAACCCAATGCCAATAGCCGCCCGACCGGGGATACCAC
TGGCTATCTGGCAAACCCGAAGGAGGTGTGAGATGGGAAGTGGATGGCATGAATGGCCGCTGATGATCTTCACGGTCTTC
GGGCAATGTGTAGCAGGTGGTTTTATCGTTCTGGCTTTGGCGCTGCTCAAAGGCGACCTGCGAGCAGAAGCCCAGCAGCG
TGTTATCGCCTGCATGTTTGGTTTATGGGTGCTGATGGGCATTGGCTTTATCGCCTCTATGCTCCATCTTGGTTCACCAA
TGCGCGCTTTTAACTCGCTCAACCGGGTAGGGGCTTCAGCACTCAGTAACGAAATCGCCAGCGGTTCGATCTTTTTTGCC
GTAGGCGGCATCGGCTGGTTGCTGGCAATGCTGAAAAAGCTGTCACCGGCATTGCGTACGCTGTGGCTGATAGTGACGAT
GGTTCTTGGCGTCATCTTTGTCTGGATGATGGTGCGTGTGTATAACAGCATTGATACCGTTCCGACCTGGTACAGCATCT
GGACGCCGATGGGCTTCTTCCTGACGATGTTTATGGGCGGCCCGTTACTGGGTTACCTGTTGTTGAGTCTGGCTGGCGTC
GATGGCTGGGCGATGCGTCTGCTGCCAGCAATTTCTGTACTGGCACTGGTAGTGAGTGGCGTGGTGTCAGTGATGCAGGG
CGCAGAGCTGGCGACTATTCATAGTTCTGTGCAGCAGGCCGCAGCGCTGGTGCCGGACTATGGTGCGCTGATGTCCTGGC
GGATCGTGCTTTTGGCCGTTGCCCTGTGCTTGTGGATTGCACCACAGCTAAAAGGTTATCAGCCTGCGGTTCCGCTACTT
TCTGTATCATTCATTCTGCTTCTGGCAGGGGAATTAATCGGTCGCGGCGTATTCTATGGTTTGCATATGACCGTGGGGAT
GGCCGTCGCAAGCTAATCATAACAACCGGGGTTTCGGCCCCGGTTCTCTTTTATTTCTGCTTCGTTAACGTGTCATAACT
GGTCATCAAATTACGATAATCAGGAATGTGGTTAGAGAACAACGTCGCCAGTCCTTCAATATCATTACGCCAGTCGCGAT
GCAGCTCGCACGCCACGCCGAACCATGTCATCAATTGCGCACCAGCTTGCGACAAGCGATCCCATGCCGAATGCCGGGTA
ATTTCATTAAAGGTCCCTGACGCGTCAGTCACGACAAAGACGTCAAACCCTTCTTCAATGGCTGAAAGCGCCGGGAATGC

CACGCAAACTTCGGTTACCACACCGGCAATAATTAACTGTTTTTTACCTGTCGCTTTGACAGCTTTTACAAAATCTTCGT
TATCCCAGGCGTTAATATTTCCCGGGCGAGCAATGTAAGGTGTATCGGGGAATTGTGCTTTCAGTTCTGGAACTAATGGG
CCGTTAGGACCGGTTTCAAAACTGGTGGTAAGAATAGTTGGTAAGTTGAAATACTTAGCCAGGTCACCCAGCGCCAGCAC
GTTATTTTTAAACTTATCGGGTTCGATATCCCGTACAAGGGAAAGTAAACCAGCCTGGTGATCAACAAGCAAAACGGCAG
CATCATTTTTATCAAGACGAACATACGGTTTGGTCATCCTCTTCTCCTTTCGAGAGTCAGTGGTACGTCTGAAATCGATC
AGACGCGATGCATTGCTCTGAAAGCATAGACGGGAAATATGAGTTTGCTGTGACCATGAAATTTTTCGACTGAACGAAGC
GTATGAAATTTGTGTTAGTTCAATAAAAACAATCAGATGAGACTAATCATCTCTTCAGCATTAGCGTTATGAATGAGTAA
TATAAATCGATAATACCGCCGCTGGGATATCGCGTATTTTCACCCATTGACAATGTTTTTGGCGGTGGCATGATGCGCAT
GAAATTTGAACTTCCTCACGGTTTTAATTCATGTCCACGTATACCCAGCCTGTCATGCTTTTGCTGTCTGGCCTGCTTTT
GTTGACTCTGGCGATTGCGGTGTTAAATACACTCGTGCCGCTTTGGCTCGCCCAGGAACACATGTCCACATGGCAGGTAG
GCGTTGTCAGCTCATCCTATTTTACCGGCAACCTTGTCGGTACATTGCTGACAGGGTATGTCATTAAGCGCATTGGCTTT
AACCGCAGCTATTATCTGGCCTCCTTCATTTTTGCCGCTGGCTGTGCCGGCCTTGGCCTGATGATTGGATTCTGGAGCTG
GTTGGCTTGGCGTTTTGTCGCGGGCGTCGGCTGTGCCATGATTTGGGTGGTTGTTGAGAGCGCGCTGATGTGCAGTGGGA
CGTCACGTAACCGTGGGCGTTTGCTTGCTGCGTATATGATGGTTTATTACGTGGGAACGTTTTTAGGCCAGTTACTGGTC
AGCAAAGTTTCAACCGAGCTGATGTCCGTATTGCCGTGGGTTACAGGTTTGACGTTGGCAGGGATCTTACCGCTGTTGTT
TACGCGTGTGCTGAATCAGCAGGCTGAAAACCATGATTCGACGTCAATTACGTCAATGCTAAAACTCCGTCAGGCGCGGC
TTGGCGTGAATGGCTGCATTATCTCAGGAATCGTTCTGGGATCTCTATATGGCCTGATGCCGCTGTACCTCAATCACAAA
GGGGTGAGCAATGCCAGCATTGGTTTCTGGATGGCGGTACTGGTCAGTGCGGGTATCCTTGGACAATGGCCGATTGGACG
TCTGGCGGATAAGTTTGGTCGACTGCTGGTGTTGCGTGTTCAGGTCTTTGTCGTCATTCTCGGCAGTATCGCGATGCTTA
GCCAGGCGGCGATGGCCCCAGCGTTATTCATCCTCGGTGCCGCTGGCTTTACGCTATATCCGGTGGCGATGGCATGGGCT
TGCGAGAAAGTTGAACATCATCAACTGGTGGCGATGAACCAGGCCTTACTGTTGAGCTATACTGTGGGAAGTCTGCTTGG
CCCGTCATTTACCGCTATGCTAATGCAGAATTTCTCCGATAATTTATTGTTTATCATGATCGCCAGCGTATCGTTTATCT
ATTTGCTGATGCTGCTGCGCAACGCCGGTCATACGCCGAAACCCGTTGCTCACGTGTAAATGAATTCAAGCAGAGTGTGA
ACTTACTGTTTCACACTCTGCTTTTTTGTTTCTTCTATCTGACTTGCTTTATTCCAAATTTTATTCGTTTAAAAATAAAA
TGTGCAGCAGGTTATAATTTTGCATTTCGCTATTTCCGCACTTCTTATTTGCCGCGCATAATCCCTCGTTTTACCGATGC
CCCTTTAATTTTGGCGAAGGATTTGTCTATGGCTGGGAATGTTCAGGAAAAACAGTTGCGATGGTACAACATTGCGCTGA
TGTCTTTTATCACTGTCTGGGGTTTTGGCAACGTTGTTAATAACTATGCCAACCAGGGGCTGGTGGTTGTTTTTTCATGG
GTGTTTATCTTTGCACTCTATTTCACACCTTATGCGCTAATTGTTGGTCAGTTAGGCTCGACCTTCAAAGATGGGAAGGG
CGGGGTCAGTACCTGGATTAAACACACGATGGGACCCGGACTGGCTTATCTCGCCGCGTGGACCTACTGGGTGGTGCATA
TTCCCTATCTGGCACAAAAACCCCAGGCAATTCTGATTGCGCTCGGTTGGGCGATGAAAGGCGACGGTTCGCTAATCAAA
GAATATTCAGTCGTAGCGTTACAGGGGTTAACGCTGGTGCTGTTTATCTTCTTTATGTGGGTTGCTTCACGCGGTATGAA
ATCGCTGAAAATCGTCGGTTCTGTGGCAGGGATTGCTATGTTTGTTATGTCACTCCTGTATGTGGCGATGGCGGTAACCG
CGCCTGCAATTACTGAAGTGCATATTGCGACCACAAACATTACCTGGGAAACGTTCATTCCTCATATCGACTTTACCTAC
ATTACCACTATTTCAATGCTGGTTTTCGCGGTTGGCGGAGCAGAGAAGATTTCTCCTTACGTTAATCAAACGCGCAACCC
AGGAAAAGAATTTCCAAAAGGGATGTTATGCCTGGCGGTGATGGTTGCGGTTTGTGCCATTCTGGGCTCGCTGGCGATGG
GGATGATGTTTGATTCGCGTAATATCCCGGATGACTTAATGACCAACGGTCAGTATTACGCCTTTCAGAAGCTGGGCGAG
TATTACAACATGGGTAATACTTTAATGGTGATTTACGCCATTGCGAATACCCTGGGACAAGTAGCGGCGCTGGTATTCTC
GATTGATGCCCCGCTTAAAGTGCTATTAGGTGATGCTGACAGCAAATATATTCCAGCCAGTTTATGTCGTACCAACGCTT
CTGGTACGCCCGTTAATGGCTATTTTCTGACCCTGGTACTGGTGGCGATTCTGATTATGCTGCCGACTCTCGGCATTGGT
GATATGAACAATCTCTATAAATGGCTGTTGAACCTTAATTCGGTAGTGATGCCGCTGCGTTATTTATGGGTATTTGTTGC
ATTTATTGCAGTCGTTCGCTTGGCGCAGAAATATAAACCAGAATATGTCTTTATTCGTAATAAGCCTCTGGCAATGACCG
TCGGGATTTGGTGTTTTGCCTTTACCGCCTTTGCCTGTTTGACGGGGATCTTCCCGAAAATGGAAGCCTTCACTGCAGAG
TGGACCTTCCAGTTGGCGCTGAATGTTGCAACGCCGTTTGTGCTGGTAGGATTAGGACTGATATTCCCGCTGCTGGCGCG
TAAAGCGAATAGTAAATAATTATTGTGGTCGGTGACGCTGACTCTGGCAGATCTGACCCGTTTTTACATAATCGAGAAAT
GCGCGCAAGCCGCAGGACATATACTGGCGGTTTGGATAATAGATCTGGAAGCCTGGACGCTCTGTGCTCCAGTCTTCCAG
TACACATTCTAAACGACCAGTATCAAGGTACTCCTTGATCTGTTCATAGAGCAGATACCCGATACCAGCACCCATCAAAA
CGGCCTCCAGTTCAGCATCAACATCATCCAGAATGATATTTCCTGCGACAGCAATTTCCAGCTCCTTTGCAAATTGCCAG
TGGAAAGGTTTGCCGCTGGGATAACGAAAAACAACACATTGATGATTAAGCAGATCATGAGGATGGCGGGGTTTGCCATA
TCTTGCAAAGTACTCTGGCGTTGCTGCTACACATAATTTCACTGGCGGGCCGATCGCCACGCTGATCATATCTTTTTCGA
CGATGCAACTAAGTCGAACGCCAGCGTCAAAGCCCTGTTGGACGATATCGACAAGAGAGTCGTCAGTTGTGAGCTCGACT
TTAATATCAGGATATTCACGTGTGAAACCAACCAGTAAAGACATTAAAAATATACGTGCAGCTACCCGGGCGGCATTGAT
TTTGAGCGTTCCTGTCGGTGTCAGACGAAAATCATTCATCTCATCTAACATGATCTGAATTTCGTCGAAAGCTGGGCGCA
ATCGTTCATAGAGATTAGAACCCGCTTCCGTTAACGATACGCTTCGGGTTGTCCGATTGAATAAGCGAATTTTAAGACGT
TGTTCCAGTGTTTTAATACTATGGCTAATGGCGGACGAGGATAAGCCTAACTCATCGCCCGCTGCACGAAAGCTTTGATT
ACGGGCCACGGCAAAGAAAGTGGCAAAGTCAGACATATTCATCCGCATTAGTGAAAATCCTTCATTAACTCATCCTGAAT
CATCACGTTTACAGGATTATGCCGCCTGCGTAAAGTGCGCTCCAGAACTTAACGTGGAGGTAAAATTATGCAGTCTGAAC

GTATTTATTTGGTATGGGCCCATCCTCGTCATGATTCATTGACCGCACATATTGCTGATGCGATCCATCAGCGGGCAATG
GAGCGGAAAATACAGGTGACGGAACTCGATTTATATCGGCGTAATTTCAACCCAGTGATGACGCCGGAAGATGAACCAGA
CTGGAAGAATATGGATAAACGTTATTCTCCAGAGGTTCATCAGCTTTATTCAGAGCTGCTTGAACATGACACGTTAGTGG
TGGTTTTTCCTCTCTGGTGGTACAGCTTCCCGGCAATGCTAAAAGGATATATTGACAGAGTATGGAATAATGGGCTGGCT
TATGGAGATGGGCACAAATTACCATTCAATAAAGTTCGTTGGGTGGCGCTGGTTGGAGGAGACAAAGAATCATTTGTCCA
GATGGGCTGGGAAAAAAATATAAGCGATTATTTAAAAAATATGTGCAGTTATCTTGGTATTGAAGATGCCGATGTCACTT
TCTTGTGTAATACAGTGGTATTCGATGGGGAAGAACTTCACGCGAGCTATTATCAGTCGTTATTATCTCAGGTACGGGAT
ATGGTAGATGCACTATAAGATGTGTTAAAAACGCTGTAGCAGAATGAAGCGCGGAATAAAAAAGCGGCAACTCAATAAAG
TTGCCGCTTTACGGGGAAATTAGAACATTACCTTATGACCGTACTGCTCAAGAATGCCTTTCACGCGTTCCATGGTCTCT
TTCTTCGGTGGTTTAACACCGTCGAGTTTGTACTCTTCACCCATTGCCACCCATTTGTGTTTGCCCAGCTCGTGGTAGGG
GAGAAGCTCGATTTTCTCAACGTTGCCCATATCACGGGTAAATTCACCGAGGCGATGCGCTGAATCGTCATCGTCAGACC
AGCCTGGGACAACAACGTAGCGGATCCACACCTTCACATTTTTGTTCGCCAGATATTTAGCGAACTCCAGCGTGCGGTGG
TTGGAAACTCCAACCAGATTTTGGTGGATCTCGTCGTTCATCTGTTTGAGATCGAGCATTACCAGGTCGGTTACTTCCAG
CAGTTCATCAATCACCGGATCGTAACGACGAACAAAACCGTTGGTGTCCAGACAGGTATGAATGCCTTCTTTTTTGCAGG
CGCGGAACCAGTCACGAACAAACTCAGCTTGCAGGATTGCTTCACCGCCGGATGCGGTAACGCCGCCGCCGGAAGCGTTC
ATAAAGTGGCGATAGGTCACCACTTCCTTCATCAAATCTTCAACGGTAACTTCTTTACCGCCATGCGTGTCCCAGGTGTC
GCGGTTATGACAATACAGGCAGCGCATCAGGCAGCCCTGGAAAAAGGTGATAAAGCGAATACCTGGGCCGTCTACGGTTC
CACAGGATTCAAAGGAGTGAATGCGACCAATAACTGACATTGCGGTGTTTCTCCAGATGTGGCCCATCTGAGGCCGTGTT
GGTGCGCAGCTCGAAGGCTACGTCGAGTCTGTTTTGGCAGTCACCTTAAAGTATAGATAGCTGACAAAAAAGGCTCTCGC
GCTAAAAAAGGCCCCACTTTCGTGGAGCCTTTATTGTACGCTTTTTACTGTACGATTTCAGTCAAATCTAATTACATAGA
TTGAGTGAAGGTACGAGTAATAACGTCCTGCTGCTGTTCTTTAGTCAGCGAGTTGAAACGTACTGCGTAGCCAGATACAC
GGATGGTCAGCTGCGGATATTTTTCCGGGTTTTCCATCGCGTCGAGCAGCATTTCACGGTTCATCACGTTAACGTTCAGG
TGCTGACCACCTTCGATGGATGCTTCGTGGTGGAAGTAACCATCCATCAGACCAGCCAGGTTGGTCTTACGAACTTCGTC
GTCTTTACCCAGTGCGTTCGGAACGATAGAGAAGGTGTAGGAGATACCATCTTTAGCGTAAGCAAACGGCAGTTTAGCAA
CGGAAGTCAGAGAGGCTACTGCACCTTTCTGGTCACGACCGTGCATCGGGTTAGCACCCGGTCCGAACGGCGCGCCAGCA
CGACGACCGTCTGGGGTGTTACCCGTTTTCTTACCATACACAACGTTAGAAGTGATGGTCAGAACAGACTGAGTCGGGAT
AGCGTCACGGTAGGTGTGCAGTTTCTGAATTTTCTTCATGAAACGTTCTACCAGGTCAACAGCCAGGTCATCTACACGCG
GATCATTGTTACCAAACTGCGGGTATTCGCCTTCGATTTCGAAGTCGATAGCCAGACCGTCTTCGTCACGAATCGGTTTA
ACTTTCGCATATTTGATTGCAGACAGGGAGTCAGCAGCAACGGACAGACCAGCGATACCACACGCCATGGTGCGGATAAC
GTCACGGTCGTGCAGCGCCATCAGAGAGGCTTCGTAGCTGTACTTGTCGTGCATGTAGTGGATGATGTTCAGTGCAGTGA
TGTACTGTTTAGCCAGCCAGTCCATGAAGTGATCCATGCGCTCCATCACTTCATCATAGTTCAGGACATCGCCTTTGATC
GGTTCAGACTTCGGACCAACCTGCATTTTCAGTTTTTCGTCAACGCCGCCGTTGATTGCGTACAGCATGGTTTTCGCCAG
GTTTGCACGCGCACCGAAGAACTGCATTTGTTTACCAACGATCATCGGGCTTACGCAGCAAGCAATAGCGTAGTCATCGT
TGTTGAAGTCCGGACGCATCAGGTCATCGTTCTCATACTGCAGAGAAGAGGTGTCGATGGACACTTTAGCGGCGAATTTC
TTGAAGTTCAGCGGCAGTTTTTCAGACCACAGAATGGTCATGTTCGGTTCCGGAGACGGACCCATGGTGTACAGGGTGTT
CAGGAAACGGAAGCTGTTTTTGGTAACCAGGGTACGACCGTCGAGGCCCATACCACCGATAGATTCGGTTGCCCAGATCG
GGTCGCCAGAGAACAGTTCATCGTATTCCGGAGTACGCAGGAAGCGAACCATACGCAGTTTCATGACCAGGTGGTCAACC
ATTTCCTGCGCTTCTTGTTCGGTGATCTTGCCAGCTTTCAGGTCACGTTCGATGTACACATCCAGGAAGGTGGAGGTACG
ACCGAAGGACATTGCAGCACCGTTCTGAGACTTAACAGCAGCCAGGTAGCCGAAGTAAGTCCACTGGATAGCTTCCTGAG
CGTTGGTAGCCGGACCAGAGATGTCGTAGCCGTATTTCGCAGCCATTTCTTTCATCTGACCCAGAGCGCGGTGCTGTTCA
GCGATTTCTTCGCGCAGACGGATAGTCTGTTCCAGGTTTACGCCGTTTTCCAGATCAGCCTGCAGAGAAGTGAACTGTGC
CAGTTTGTCTTTCATCAGGTAGTCGATACCGTACAGCGCAACGCGACGGTAGTCACCGATGATACGGCCACGGCCATATG
CATCTGGCAGACCGGTCAGAACACCAGATTTACGGCAACGCAGGATGTCCGGAGTGTAAACGTCGAACACGCCCTGGTTG
TGAGTTTTACGGTATTCAGTGAAGATTTTTTTGATCATCGGATCCAGTTCGCGGTTGTACGCTTTGCAGGAACCTTCGAT
CATTTTGATACCACCGAACGGGATAAGAGCACGTTTCAGCGGAGCTTCAGTCTGCAGACCAACGATTTTCTCAAGCTGCT
TGTTGATGTAGCCAGCGTCGTGAGAGGTGATGGTGGAAGCAACAGCGGTGTCAAAGTCAACTGGCGCGTGAGTGCGGTTT
TCCAGTTTAACGCCTTCCATTACTTTGTCCCACAGGGTGGTGGTCGCTTCAGTAGCGCCAGCCAGGAAGGACTCGTCACC
CTCGTACGGAGTGTAGTTTTTCTGAATGAAGTCACGGACGTTTACTTCATTCTGCCAGTCACCTTTGGTAAAACCTTCCC
AGGCTGTGGCTAACTTTTCATTAAGCTCGGACATGTAACACCTACCTTCTTAAGTGGATTTTTTATTTACTGCGTACTTC
GACAACCATTAATGGTGGTCGTTTTCACGCAGGTAAATGACCCAGTATGTCAACCCAACCAACAAACCACCACCGATAAT
GTTGCCGATCGTAACCGGAATCAGGTTATCAGTGATGAAATTCATCACGGTCAGGTGAGAAAAATTTTCCGGTGCAGAAC
CGACTGCGGTCCAAAATTCCGGGGATGCGAAGTCGCGGATTACAATACCCATCGGGATCATAAACATGTTTGCGATACTG
TGCTCAAAACCGCTGGCAACAAACATCGCGACCGGCAGCACCATAATGAACGCTTTGTCCATCAGGCTGCGGCCAGAATA
ACTCATCCATACTGCCAGACATACCATCAGGTTTGCCAGGATACCAAGACAGACGGCCTCAATAAAAGTATGGTGCACTT
TGTGGTCGGCGGTTTGTAGGACGTTTAGTCCCCATTGACCATTTGCGGTCATATACTCGCCGGAAAGCCACATTAAAAGT
ACAAACAGCAGTGCGCCGACCAGGTTGCCAAAATAGACATTTAGCCAGTTTTTCGCCAACTGACCCCAGGTGATGCGCCC

ACTCGCCTTAGCAACAACAATCAACACGGTGGAAGTAAAGAGATCGGCTCCGCAGACAACACAAAGAATCAGCCCCAGAG
AGAAGCAAATGCCGCCAACCAGTTTTGCCATGCCGAAGGGCATTGTGCCTGTGCCAGTGGTTGCTGTGATATAGAAGACG
AATGCGATTGAGATGAAAACACCGGCGGTAATCGCCAGATAGAAAGTCTTAAGCGGATGTTTCGTTGCTTTATAGACACC
CGCCTCTTCGGCCACTTTGGCCATTGCAGCAGGAAGTAAAAGATCAAAAGGGTTGTCAGCTTTCACACTAACTCTCTCTT
TATTAAGTCGGCGACGAGATACTAACAAAGCATTATAGATGAGAAATTGATATAGATCATATCTCGCCTGGCTTATAGGC
CCGTAACTCGCATGGTTTTTATGCAAATACGGAGTAAATATTTGATTATCCAAATAAAAATAAATTTTAAAAATTAACAA
ATGAGTTGAATTTTTTCCGCATCCTCCGCTAAAACAGTTAATTAAAAGGGAGCATCAGGCGAATAAAGTAACAATATCGA
TCGTATTTATTAAATACAAATTACCGATATTTAACCTTATAATTACAATTATTTTATTAATGCAAATATATGTAAAGCGG
GGCATTAAAAAAACGCCCCGTAATATAACTCAGACTAATCATTAAGCCTACATTGCGTAGGCTATTTGATTTTATTTTGC
CCAGAATGCTGCTTTGGCGCGCTGCAGCTTTTCGTAGGCCTTCAACAACGACTGATGTGCAGCAAACGCGTGCAGATCGC
TATCTACCGGTTGCAGGCCGTAAAACGCCGCTTCGCCGCTCATTGCCGCACTGGCGGCTTCTACGGCATCTGCGCCGTAC
ATGCGAACAAACGCATTCAGATATTGCAGCGGCTGGCGATCTTCTTCCTGTGCCAGTAATAACAACGTTTGCAGGCAGCG
ATAATAGTTGGCGCGTTCCGGACTAAATACTGATGAGTTAAACTCCATCGTCCATTCGGTCCAGACCAGAGCCTGTTCCA
GATCGCCACCAGCCAGCGCCAGCATGGCTTTTAATTCACCGATACGCAGGGTGTACCAACCGTTATCCGACCCGGTCGCC
AGACCCAACAGCTCACGCACGCGGGTAAAGTCATCAAAACCTTCTTCATCCAGTTGCTCGATGAGGTTCAGGTAATCTTC
TTTTTCCCACTCGCTGCCTGGTAGCGAAAGAATCGTTTCACGTAAATGGCTGCCCATACTGTTATTCGCGAGCCACAGAT
CTTCAGCCGGATAAATATCGGACATGCCAGGCACGATAATACGGCAAGCATAAACGCCCAGATGCTCGTAATCGGCAATA
TAAACTTCTTTATCTTCTTTGTTGAAGATAGCCATCAGTGTGGCGAACTCTTCTTCCGTGGTGCCGGAGAAATTCCAGTC
CACAAACGGATAATCGGCATCCTGCTTGAACAGGTCCCAGGAGATTAAACCGCTGGAATCGATAAAGTGCGTTTCGAGGT
TGGTATGTTCAGCGACTTCTTCATCATCGAAGGTTGGCGGAGTAAACACATCCAAATCTTTCAGGCCACGACCTTGCAGC
AGCTCGGTCACGGTACGTTCCAGTGCTACGCCAAAATCAGGATGCGCACCGAAAGAGGCAAAGCAGGTACCGTTAGCAGG
ATTGAACAGTACCACGCAAATCACCGGATACTGGCCGCCAAGCGAACCGTCATAAGCGAAGATTGGGAAACCCTCCGCTT
CCAGCGTTTCGATGGCTTCAACTACTGCTGGGTAACGCGCCAGCACGTCTGCCGGGATCTCTGGCAGGCTGATGCTTTCA
GCAATAATGCGGTTTTTCACGTAGCGTTCGAAAACTTCAGACAATCCCTGAACGCGTGCTTCGTTGCGGGTATTACCTGC
GGACATACCGTTGGAGACGTACAGGTTACCAATGATATTCATCGGAATATAAACGGTCTGATTGTCGGACTGACGCGTAA
ACGGCAGGCCGCAAATACCACGATCTTCGTTACCGGATTGTAGGTCAATCAGCATGCTGCCGGTCAGTTCATTCTCCGGA
TCATAAAACGCGCGCAGACGGTCATCGAGCAGCCCTTCTGGCACATCGTCATTTTCGGTCAGTGGGAACCATTTTTCGTT
GGGATAATGCACGAACGGACCGTTGGCGATGGTTTCGCCCAGCCAGAAGTCCGCAAAAAAGTAGTTGGTTGAGAGACGCT
CGAAATATTCACCGAGTGCAGAAGCCAGCGCCGCTTTCTTGGTTGCGCCTTTACCGTTGGTAAAACACAGTGCGCACTCT
TTGTCGCGAATATGTACAGACCAGACGTTAGGCACGGGATTCAGCCAGGAGGCCTCTTCAATCTGAAAGCCGAGGTCTGA
AAGTTTTTGCTGGAAGCGAGCGATGGAATCTTCCAGAGCGGCATCTTTGCCGGGGATAAATGTTTGCGTCATGAAAATCA
CTTTAGTCGTACGGAAAGCGCGCAATAATACGGGTTTTATCTCAAAGGCGCTATCACCGCCGCCATGCCGATGAACTGTT
GACTATGCTTTTAGCGGATAAACCACGTTAAGAGCATAAAAATGAAGGCGTTCGATCTCCACCGTATGGCATTTGATAAA
GTGCCTTTTGATTTCCTTGGCGAAGTTGCACTACGTAGTCTTTATACCTTTGTACTGGTCTTTTTGTTCCTCAAAATGAC
CGGAAGACGCGGTGTGCGGCAGATGTCGCTGTTTGAAGTTTTAATCATTCTGACGCTGGGATCAGCGGCGGGAGATGTGG
CGTTTTATGATGATGTGCCGATGGTCCCGGTACTTATCGTCTTTATTACTCTGGCGTTGTTATACCGCCTGGTAATGTGG
TTGATGGCGCACAGTGAAAAACTGGAAGATCTTCTGGAAGGCAAGCCAGTTGTCATTATTGAAGATGGCGAGCTGGCCTG
GTCGAAACTCAATAACTCCAACATGACGGAATTTGAGTTCTTTATGGAGCTACGATTGCGTGGCGTGGAGCAGCTGGGGC
AGGTACGTCTGGCGATTCTCGAAACCAACGGGCAAATCAGTGTCTATTTCTTTGAAGATGACAAGGTGAAACCGGGTTTA
CTTATTTTACCCAGTGATTGTACGCAGCGTTACAAAGTGGTGCCGGAGTCGGCGGACTATGCCTGTATTCGTTGTAGTGA
AATCATTCATATGAAAGCGGGGGAAAAACAATTATGTCCGCGCTGTGCAAATCCAGAATGGACGAAGGCAAGTCGGGCAA
AACGGGTGACCTGACAGTAAAAACATCGGCTTTTTGCTAATAATCCGAGAGATTCTTTTGTGTGATGCAAGCCACATTTT
TGCCCTCAACGGTTTTACTCATTGCGATGTGTGTCACTGAATGATAAAACCGATAGCCACAGGAATAATGTATTACCTGT
GGTCGCAATCGATTGACCGCGGGTTAATAGCAACGCAACGTGGTGAGGGGAAATGGCTCAAATCTTCAATTTTAGTTCTG
GTCCGGCAATGCTACCGGCAGAGGTGCTTAAACAGGCTCAACAGGAACTGCGCGACTGGAACGGTCTTGGTACGTCGGTG
ATGGAAGTGAGTCACCGTGGCAAAGAGTTCATTCAGGTTGCAGAGGAAGCCGAGAAGGATTTTCGCGATCTTCTTAATGT
CCCCTCCAACTACAAGGTATTATTCTGCCATGGCGGTGGTCGCGGTCAGTTTGCTGCGGTACCGCTGAATATTCTCGGTG
ATAAAACCACCGCAGATTATGTTGATGCCGGTTACTGGGCGGCAAGTGCCATTAAAGAAGCGAAAAAATACTGCACGCCT
AATGTCTTTGACGCCAAAGTGACTGTTGATGGTCTGCGCGCGGTTAAGCCAATGCGTGAATGGCAACTCTCTGATAATGC
TGCTTATATGCATTATTGCCCGAATGAAACCATCGATGGTATCGCCATCGACGAAACGCCAGACTTCGGCGCAGATGTGG
TGGTCGCCGCTGACTTCTCTTCAACCATTCTTTCCCGTCCGATTGACGTCAGCCGTTATGGTGTAATTTACGCTGGCGCG
CAGAAAAATATCGGCCCGGCTGGCCTGACAATCGTCATCGTTCGTGAAGATTTGCTGGGCAAAGCGAATATCGCGTGTCC
GTCGATTCTGGATTATTCCATCCTCAACGATAACGGCTCCATGTTTAACACGCCGCCGACATTTGCCTGGTATCTATCTG
GTCTGGTCTTTAAATGGCTGAAAGCGAACGGCGGTGTAGCTGAAATGGATAAAATCAATCAGCAAAAAGCAGAACTGCTA
TATGGGGTGATTGATAACAGCGATTTCTACCGCAATGACGTGGCGAAAGCTAACCGTTCGCGGATGAACGTGCCGTTCCA
GTTGGCGGACAGTGCGCTTGACAAATTGTTCCTTGAAGAGTCTTTTGCTGCTGGCCTTCATGCACTGAAAGGTCACCGTG

TGGTCGGCGGAATGCGCGCTTCTATTTATAACGCCATGCCGCTGGAAGGCGTTAAAGCGCTGACAGACTTCATGGTTGAG
TTCGAACGCCGTCACGGTTAATGCCGAAATTTTGCTTAATCCCCACAGCCAGCCTGTGGGGTTTTTATTTCTGTTGTAGA
GAGTTGAGTTCATGGAATCCCTGACGTTACAACCCATCGCTCGTGTCGATGGCACTATTAATCTGCCCGGTTCCAAGAGC
GTTTCTAACCGCGCTTTATTGCTGGCGGCATTAGCACACGGCAAAACAGTATTAACCAATCTGCTGGATAGCGATGACGT
GCGCCATATGCTGAATGCATTAACAGCGTTAGGGGTAAGCTATACGCTTTCAGCCGATCGTACGCGTTGCGAAATTATCG
GTAACGGCGGTCCATTACACGCAGAAGGTGCCCTGGAGTTGTTCCTCGGTAACGCCGGAACGGCAATGCGTCCGCTGGCG
GCAGCTCTTTGTCTGGGTAGCAATGATATTGTGCTGACCGGTGAGCCGCGTATGAAAGAACGCCCGATTGGTCATCTGGT
GGATGCGCTGCGCCTGGGCGGGGCGAAGATCACTTACCTGGAACAAGAAAATTATCCGCCGTTGCGTTTACAGGGCGGCT
TTACTGGCGGCAACGTTGACGTTGATGGCTCCGTTTCCAGCCAATTCCTCACCGCACTGTTAATGACTGCGCCTCTTGCG
CCGGAAGATACGGTGATTCGTATTAAAGGCGATCTGGTTTCTAAACCTTATATCGACATCACACTCAATCTGATGAAGAC
GTTTGGTGTTGAAATTGAAAATCAGCACTATCAACAATTTGTCGTAAAAGGCGGGCAGTCTTATCAGTCTCCGGGTACTT
ATTTGGTCGAAGGCGATGCATCTTCGGCTTCTTACTTTCTGGCAGCAGCAGCAATCAAAGGCGGCACTGTAAAAGTGACC
GGTATTGGACGTAACAGTATGCAGGGTGATATTCGCTTTGCTGATGTGCTGGAAAAAATGGGCGCGACCATTTGCTGGGG
CGATGATTATATTTCCTGCACGCGTGGTGAACTGAACGCTATTGATATGGATATGAACCATATTCCTGATGCGGCGATGA
CCATTGCCACGGCGGCGTTATTTGCAAAAGGCACCACCACGCTGCGCAATATCTATAACTGGCGTGTTAAAGAGACCGAT
CGCCTGTTTGCGATGGCAACAGAACTGCGTAAAGTCGGCGCGGAAGTGGAAGAGGGGCACGATTACATTCGTATCACTCC
TCCGGAAAAACTGAACTTTGCCGAGATCGCGACATACAATGATCACCGGATGGCGATGTGTTTCTCGCTGGTGGCGTTGT
CAGATACACCAGTGACGATTCTTGATCCCAAATGCACGGCCAAAACATTTCCGGATTATTTCGAGCAGCTGGCGCGGATT
AGCCAGGCAGCCTGAATGAACAACGGGCAATAAATAGCCAAATCTTTCTTTATCAAAACGTCGGCACATTGTCGGCGTTT
TTTTTCGGACCTTGTGAGTCATTTTGATTAATGGTAGCGTCGCTTGTCAATGTAAGTTGTTGATACATAATATTTATATA
TGATTAATCAACGGATGATTCACATGAAGAATACTAAATTACTGCTGGCGATTGCGACCTCTGCAGCATTACTGACAGGG
TGTCAAAATACCCACGGTATTGATACCAATATGGCTATCAGCTCCGGTTTAAATGCCTATAAAGCAGCAACATTAAGCGA
TGCCGATGCAAAAGCGATTGCCAATCAGGGCTGTGCCGAAATGGACAGCGGCAATCAAGTCGCAAGTAAATCCAGCAAGT
ACGGTAAACGTCTGGCAAAAATCGCCAAAGCATTGGGTAACAATATTAATGGCACGCCGGTCAACTATAAGGTTTATATG
ACCAGCGACGTCAACGCATGGGCGATGGCGAACGGCTGTGTTCGTGTCTACAGTGGCCTGATGGACATGATGAATGACAA
CGAAATTGAAGGCGTTCTGGGCCATGAACTGGGCCACGTCGCGTTGGGTCACTCGCTGGCTGAAATGAAAGCTTCTTATG
CGATCGTTGCCGCACGCGATGCCATTTCAGCTACCAGCGGTGTGGCTTCCCAGCTTTCCCGCTCACAATTAGGTGATATC
GCAGAAGGCGCTATCAATGCTAAATACTCCCGTGATAAAGAGTCCGAAGCAGATGATTTCTCCTTTGATCTGTTGAAGAA
ACGTGGCATCAGCACCCAGGGGCTGGTTGGCAGCTTTGAAACACTGGCTAGCCTGGATGGCGGTCGCACCCAGTCCATGT
TTGACTCTCACCCACCATCAACAGAGCGTGCGCAACACATCCGTGATCGTATCGCCTCTGGTAAGTAAATCATTGTCATC
TTTCGGGCTGGTCTTCTGCCAGCCCGCTATAATTGCGCAATAAATCCCCATCTGAATACAGACAAAACTGGTTTTTGCAC
ACAACGTTAACGATTTGTGGCGTCGGCGCGTATAATGCGCGCGGTTATGTTAACGGTACGCCTGTTTTAAGGAGATAAAG
ATGACGGCAATTGCCCCGGTTATTACCATTGATGGCCCAAGCGGTGCAGGGAAAGGCACCTTGTGTAAGGCTATGGCGGA
AGCGTTGCAATGGCATCTGCTGGACTCGGGTGCAATTTATCGCGTACTGGCATTGGCGGCATTACATCACCATGTTGATG
TTGCGTCGGAAGATGCGCTGGTACCGCTGGCATCCCATCTGGATGTACGTTTTGTGTCGACCAATGGCAATCTGGAAGTG
ATCCTCGAAGGGGAAGATGTCAGCGGCGAAATTCGTACTCAGGAAGTGGCGAATGCAGCTTCACAAGTCGCGGCATTCCC
ACGCGTTCGTGAAGCATTATTGCGTCGCCAACGCGCGTTTCGCGAATTACCAGGTCTGATTGCCGATGGCCGCGACATGG
GAACGGTGGTATTCCCTGATGCACCAGTGAAAATTTTCCTTGACGCCTCCTCGGAAGAACGTGCGCATCGCCGCATGCTA
CAGTTGCAGGAGAAGGGCTTTAGTGTTAACTTTGAGCGCCTTTTGGCCGAGATCAAAGAACGCGACGACCGCGATCGTAA
CCGAGCGGTAGCGCCACTGGTTCCGGCAGCCGATGCTTTAGTGTTGGATTCCACCACCTTAAGCATTGAGCAAGTGATTG
AAAAAGCGCTACAATACGCGCGCCAGAAATTGGCTCTCGCATAAGCGACCGAATTTGCAGTACCCCCGTTGCAATGGAAT
GACAGCGGGTATGTTAAACAACCCCATCCGGCATGGAGCCAGGTGGACGTTAAATATAAACCTGAAGATTAAACATGACT
GAATCTTTTGCTCAACTCTTTGAAGAGTCCTTAAAAGAAATCGAAACCCGCCCGGGTTCTATCGTTCGTGGCGTTGTTGT
TGCTATCGACAAAGACGTAGTACTGGTTGACGCTGGTCTGAAATCTGAGTCCGCCATCCCGGCTGAGCAGTTCAAAAACG
CCCAGGGCGAGCTGGAAATCCAGGTAGGTGACGAAGTTGACGTTGCTCTGGACGCAGTAGAAGACGGCTTCGGTGAAACT
CTGCTGTCCCGTGAGAAAGCTAAACGTCACGAAGCCTGGATCACGCTGGAAAAAGCTTACGAAGATGCTGAAACTGTTAC
CGGTGTTATCAACGGCAAAGTTAAGGGCGGCTTCACTGTTGAGCTGAACGGTATTCGTGCGTTCCTGCCAGGTTCTCTGG
TAGACGTTCGTCCGGTGCGTGACACTCTGCACCTGGAAGGCAAAGAGCTTGAATTTAAAGTAATCAAGCTGGATCAGAAG
CGCAACAACGTTGTTGTTTCTCGTCGTGCCGTTATCGAATCCGAAAACAGCGCAGAGCGCGATCAGCTGCTGGAAAACCT
GCAGGAAGGCATGGAAGTTAAAGGTATCGTTAAGAACCTCACTGACTACGGTGCATTCGTTGATCTGGGCGGCGTTGACG
GCCTGCTGCACATCACTGACATGGCCTGGAAACGCGTTAAGCATCCGAGCGAAATCGTCAACGTGGGCGACGAAATCACT
GTTAAAGTGCTGAAGTTCGACCGCGAACGTACCCGTGTATCCCTGGGCCTGAAACAGCTGGGCGAAGATCCGTGGGTAGC
TATCGCTAAACGTTATCCGGAAGGTACCAAACTGACTGGTCGCGTGACCAACCTGACCGACTACGGCTGCTTCGTTGAAA
TCGAAGAAGGCGTTGAAGGCCTGGTACACGTTTCCGAAATGGACTGGACCAACAAAAACATCCACCCGTCCAAAGTTGTT
AACGTTGGCGATGTAGTGGAAGTTATGGTTCTGGATATCGACGAAGAACGTCGTCGTATCTCCCTGGGTCTGAAACAGTG
CAAAGCTAACCCGTGGCAGCAGTTCGCGGAAACCCACAACAAGGGCGACCGTGTTGAAGGTAAAATCAAGTCTATCACTG

ACTTCGGTATCTTCATCGGCTTGGACGGCGGCATCGACGGCCTGGTTCACCTGTCTGACATCTCCTGGAACGTTGCAGGC
GAAGAAGCAGTTCGTGAATACAAAAAAGGCGACGAAATCGCTGCAGTTGTTCTGCAGGTTGACGCAGAACGTGAACGTAT
CTCCCTGGGCGTTAAACAGCTCGCAGAAGATCCGTTCAACAACTGGGTTGCTCTGAACAAGAAAGGCGCTATCGTAACCG
GTAAAGTAACTGCAGTTGACGCTAAAGGCGCAACCGTAGAACTGGCTGACGGCGTTGAAGGTTACCTGCGTGCTTCTGAA
GCATCCCGTGACCGCGTTGAAGACGCTACCCTGGTTCTGAGCGTTGGCGACGAAGTTGAAGCTAAATTCACCGGCGTTGA
TCGTAAAAACCGCGCAATCAGCCTGTCTGTTCGTGCGAAAGACGAAGCTGACGAGAAAGATGCAATCGCAACTGTTAACA
AACAGGAAGATGCAAACTTCTCCAACAACGCAATGGCTGAAGCTTTCAAAGCAGCTAAAGGCGAGTAATTCTCTGACTCT
TCGGGATTTTTATTCCGAAGTTTGTTGAGTTTACTTGACAGATTGCAGGTTTCGTCCTGTAATCAAGCACTAAGGGCGGC
TACGGCCGCCCTTAATCAATGCAGCAACAGCAGCCGCTTAATTTGCCTTTAAGGAACCGGAGGAATCATGACCAAGTCAG
AATTGATAGAAAGACTTGCCACCCAGCAATCGCACATTCCCGCCAAGACGGTTGAAGATGCAGTAAAAGAGATGCTGGAG
CATATGGCCTCGACTCTTGCGCAGGGCGAGCGTATTGAAATCCGCGGTTTCGGCAGTTTCTCTTTGCACTACCGCGCACC
ACGTACCGGACGTAATCCGAAGACTGGCGATAAAGTAGAACTGGAAGGAAAATACGTTCCTCACTTTAAACCTGGTAAAG
AACTGCGCGATCGCGCCAATATTTACGGTTAAGTTTTTTACTCAAACTTGAACGAGAGAAAAGCACCTGTCGGGTGCTTT
TTTCATTTCTCTAATCTGGAACTGGAAGCTGCCTCGCAGAGTTTTGAACAGTTTTCACCCTTTCGTTAAATTCTTCTGAA
TATGCCTCGGGGAACGCAAAATTCCCACCAGACAACCGCTCAACAAAGTTGCACACTTTCCATAAACAGGGAGGGGTGCA
TGAAAATAACGACAGTCGGTGTATGCATAATTAGCGGAATTTTTCCGTTGCTGATTTTGCCCCAATTGCCTGGGACATTA
ACCCTTGCGTTTCTGACTCTCTTCGCCTGCGTACTGGCATTTATCCCTGTTAAAACCGTCCGTTATATCGCGCTGACGTT
GCTGTTTTTCGTTTGGGGCATATTATCAGCAAAGCAAATTTTGTGGGCAGGAGAAACCTTAACTGGCGCGACGCAGGATG
CAATTGTTGAGATCACTGCAACTGACGGCATGACCACTCATTACGGTCAAATTACTCATCTACAAGGTCGACGTATATTC
CCTGCGTCAGGTCTCGTGATGTATGGCGAATATCTTCCGCAAGCGGTTTGTGCTGGACAACAATGGTCAATGAAACTCAA
AGTTCGTGCAGTTCATGGCCAACTTAATGATGGCGGCTTTGATAGCCAGCGTTATGCCATTGCCCAGCATCAGCCGCTCA
CCGGCCGTTTTCTGCAGGCAAGTGTCATTGAACCGAATTGTAGCCTGCGTGCACAGTATCTGGCGTCACTACAAACAACG
CTGCAACCCTATCCGTGGAATGCGGTTATTCTTGGTTTAGGTATGGGGGAACGGTTATCCGTCCCCAAAGAAATCAAAAA
TATCATGCGCGATACTGGAACGGCGCATTTAATGGCGATATCGGGATTGCACATCGCTTTTGCGGCGTTGCTGGCTGCCG
GACTCATTCGCAGTGGACAAATTTTTCTGCCTGGGCGCTGGATCCACTGGCAAATACCATTAATTGGCGGAATCTGCTGT
GCTGCTTTTTATGCCTGGTTGACGGGAATGCAACCTCCTGCATTGCGTACCATGGTGGCGCTTGCTACGTGGGGAATGCT
TAAGTTAAGTGGGCGACAGTGGAGTGGCTGGGATGTATGGATATGTTGTCTGGCGGCAATTTTGCTGATGGATCCTGTTG
CCATTCTCTCGCAAAGTTTATGGCTCTCTGCCGCTGCGGTCGCGGCATTGATATTTTGGTATCAGTGGTTTCCCTGTCCT
GAGTGGCAACTGCCGCCGGTATTGCGTGCAGTTGTTTCCCTCATCCATCTGCAACTGGGAATCACACTTCTGCTTATGCC
CGTGCAAATCGTCATATTTCATGGCATTAGTCTGACCTCGTTTATTGCAAATCTATTAGCAATTCCCTTGGTGACATTTA
TCACGGTTCCGTTGATCCTCGCCGCGATGGTTGTGCATTTAAGCGGGCCGTTAATCCTGGAGCAAGGGTTATGGTTTCTT
GCCGACCGGTCTTTGGCTTTACTTTTCTGGGGGTTAAAGAGTTTGCCGGAAGGGTGGATCAACATTGCTGAATGTTGGCA
ATGGCTATCATTTTCCCCATGGTTCTTACTGGTGGTATGGCGATTAAACGCCTGGCGAACGTTGCCAGCAATGTGTGTGG
CTGGAGGCTTGCTGATGTGCTGGCCGCTGTGGCAAAAACCTCGACCTGACGAGTGGCAGCTGTACATGCTTGATGTCGGG
CAAGGGCTGGCAATGGTGATAGCCAGAAACGGCAAAGCGATTCTCTATGACACAGGACTGGCCTGGCCTGAAGGGGATAG
TGGGCAACAACTGATTATCCCCTGGCTCCACTGGCATAATCTTGAACCGGAAGGCGTTATTCTGAGCCATGAACATCTGG
ATCACCGGGGAGGGCTGGATTCAATATTGCATATATGGCCGATGTTATGGATCAGAAGTCCGTTAAACTGGGAACATCAT
CAGCCCTGTGTGCGTGGCGAAGCGTGGCAATGGCAAGGATTGCGTTTCAGCGCGCACTGGCCTTTACAAGGTAGCAACGA
TAAAGGAAATAACCATTCCTGTGTGGTTAAGGTTGATGACGGGACGAATAGCATTCTTCTAACCGGTGATATTGAAGCCC
CAGCTGAACAAAAGATGCTAAGCCGTTACTGGCAGCAAGTGCAGGCAACATTGCTTCAGGTACCTCACCATGGCAGTAAT
ACCTCATCATCGTTGCCATTAATTCAGCGAGTGAATGGAAAAGTGGCACTCGCATCGGCATCGCGCTATAACGCATGGCG
ACTGCCCTCTAACAAAGTTAAGCATCGCTATCAACTGCAAGGATATCAATGGATTGATACTCCACATCAAGGTCAAACAA
CGGTCAATTTTTCAGCGCAAGGCTGGCGGATTAGCAGCCTCAGGGAGCAAATTTTACCTCGTTGGTATCATCAGTGGTTT
GGCGTGCCAGTGGATAACGGGTAGAATATGCGGCTATTTCAACAAATGCTGGTTTTTTGAATGCATAACGACAAAGATCT
CTCTACGTGGCAGACATTCCGCCGACTGTGGCCAACCATTGCGCCTTTCAAAGCGGGTCTGATCGTGGCGGGCGTAGCGT
TAATCCTCAACGCAGCCAGCGATACCTTCATGTTATCGCTCCTTAAGCCACTTCTTGATGATGGCTTTGGTAAAACAGAT
CGCTCCGTGCTGGTGTGGATGCCGCTGGTGGTGATCGGGCTGATGATTTTACGTGGTATCACCAGCTATGTCTCCAGCTA
CTGTATCTCCTGGGTATCAGGAAAGGTGGTAATGACCATGCGTCGCCGCCTGTTTGGTCACATGATGGGAATGCCAGTTT
CATTCTTTGACAAACAGTCAACGGGTACGCTGTTGTCACGTATTACCTACGATTCCGAACAGGTTGCTTCTTCTTCTTCC
GGCGCACTGATTACTGTTGTGCGTGAAGGTGCGTCGATCATCGGCCTGTTCATCATGATGTTCTATTACAGTTGGCAACT
GTCGATCATTTTGATTGTGCTGGCACCGATTGTTTCGATTGCGATTCGCGTTGTATCGAAGCGTTTTCGCAACATCAGTA
AAAACATGCAGAACACCATGGGGCAGGTGACCACCAGCGCAGAACAAATGCTGAAGGGCCACAAAGAAGTATTGATTTTC
GGTGGTCAGGAAGTGGAAACGAAACGCTTTGATAAAGTCAGCAACCGAATGCGTCTTCAGGGGATGAAAATGGTTTCAGC
CTCTTCCATCTCTGATCCGATCATTCAGCTGATCGCCTCTTTGGCGCTGGCGTTTGTTCTGTATGCGGCGAGCTTCCCAA
GTGTCATGGATAGCCTGACTGCCGGTACGATTACCGTTGTTTTCTCTTCAATGATTGCACTGATGCGTCCGCTGAAATCG
CTGACCAACGTTAACGCCCAGTTCCAGCGCGGTATGGCGGCTTGTCAGACGCTGTTTACCATTCTGGACAGTGAGCAGGA

GAAAGATGAAGGTAAGCGCGTGATCGAGCGTGCGACTGGCGACGTGGAATTCCGCAATGTCACCTTTACTTATCCGGGAC
GTGACGTACCTGCATTGCGTAACATCAACCTGAAAATTCCGGCAGGGAAGACGGTTGCTCTGGTTGGACGCTCTGGTTCG
GGTAAATCAACCATCGCCAGCCTGATCACGCGTTTTTACGATATTGATGAAGGCGAAATCCTGATGGATGGTCACGATCT
GCGCGAGTATACCCTGGCGTCGTTACGTAACCAGGTTGCTCTGGTGTCGCAGAATGTCCATCTGTTTAACGATACGGTTG
CTAACAACATTGCTTACGCACGGACTGAACAGTACAGCCGTGAGCAAATTGAAGAAGCGGCGCGTATGGCCTACGCCATG
GACTTCATCAATAAGATGGATAACGGTCTCGATACAGTGATTGGTGAAAACGGCGTGCTGCTCTCTGGCGGTCAGCGTCA
GCGTATTGCTATCGCTCGAGCCTTGTTGCGTGATAGCCCGATTCTGATTCTGGACGAAGCTACCTCGGCTCTGGATACCG
AATCCGAACGTGCGATTCAGGCGGCACTGGATGAGTTGCAGAAAAACCGTACCTCTCTGGTGATTGCCCACCGCTTGTCT
ACCATTGAAAAGGCAGACGAAATCGTGGTCGTCGAGGATGGTGTCATTGTGGAACGCGGTACGCATAACGATTTGCTTGA
GCACCGCGGCGTTTACGCGCAACTTCACAAAATGCAGTTTGGCCAATGATCGAAAAAATCTGGTCTGGTGAATCCCCTTT
GTGGCGGCTATTGCTGCCACTCTCCTGGTTGTATGGCCTGGTGAGTGGCGCGATCCGTCTTTGCTATAAACTAAAACTGA
AGCGCGCCTGGCGTGCCCCCGTACCGGTTGTCGTGGTTGGTAATCTCACCGCAGGCGGCAACGGAAAAACCCCGGTCGTT
GTCTGGCTGGTGGAACAGTTGCAACAGCGCGGTATTCGCGTGGGGGTCGTATCGCGGGGATATGGTGGTAAGGCTGAATC
TTATCCGCTGTTATTGTCGGCAGATACCACAACAGCACAGGCGGGTGATGAACCTGTGTTGATTTATCAACGCACTGATG
CGCCTGTTGCGGTTTCTCCCGTTCGTTCTGATGCGGTAAAAGCCATTCTGGCGCAACACCCTGATGTGCAGATCATCGTA
ACCGACGACGGTTTACAGCATTACCGTCTGGCGCGTGATGTGGAAATTGTCGTTATTGATGGTGTGCGTCGCTTTGGCAA
TGGCTGGTGGTTGCCGGCGGGGCCAATGCGTGAGCGAGCGGGGCGCTTAAAGTCGGTTGATGCGGTAATCGTCAACGGCG
GTGTCCCTCGCAGCGGTGAAATCCCCATGCATCTGCTGCCGGGTCAGGCGGTGAATTTACGTACCGGTACGCGTTGTGAC
GTTGCTCAGCTTGAACATGTAGTGGCGATGGCGGGGATTGGGCATCCGCCGCGCTTTTTTGCCACGCTGAAGATGTGTGG
CGTACAACCGGAAAAATGTGTACCGCTGGCCGATCATCAGTCTTTGAACCATGCGGATGTCAGTGCGTTGGTAAGCGCCG
GGCAAACGCTGGTAATGACTGAAAAAGATGCGGTGAAATGCCGGGCCTTTGCAGAAGAAAATTGGTGGTATTTGCCTGTA
GACGCACAGCTTTCAGGTGATGAACCAGCGAAACTGCTTACGCAACTAACCTTGCTGGCTTCTGGCAACTAGTTACGCCG
CGGCAGCGTTCGATTGATGGAGTCATGAATGTCGCTGCCGCACCTCTCCCTTGCTGATGCGCGTAATCTTCACCTTGCCG
CACAAGGCCTGTTAAACAAACCCCGCCGTCGAGCGTCGTTGGAGGATATTCCGGCAACGATCTCCCGCATGTCCTTGCTG
CAAATCGATACCATCAATATTGTTGCCCGTAGTCCATATCTGGTGCTTTTCAGTCGTCTGGGAAATTATCCTGCCCAGTG
GCTGGATGAGTCTCTGGCGCGTGGCGAATTAATGGAATACTGGGCGCATGAAGCCTGCTTTATGCCGCGTAGCGACTTTC
GTCTTATTCGCCACCGCATGCTGGCACCTGAAAAAATGGGCTGGAAATACAAAGACGCCTGGATGCAGGAACATGAGGCG
GAAATTGCACAGTTAATTCAGCATATTCATGATAAGGGGCCGGTACGTTCAGCCGATTTTGAGCATCCTCGTAAAGGTGC
AAGCGGCTGGTGGGAATGGAAGCCGCATAAACGGCATCTGGAAGGTTTATTTACTGCCGGAAAGGTGATGGTGATTGAAC
GGCGCAACTTCCAGCGCGTTTATGATTTAACCCACCGTGTCATGCCTGACTGGGATGATGAGCGCGATCTCGTTTCGCAA
ACAGAAGCAGAAATCATCATGCTGGATAACAGTGCGCGTAGCCTGGGAATATTCCGCGAACAGTGGCTGGCAGATTACTA
TCGGCTGAAACGTCCGGCACTGGCGGCGTGGCGCGAAGCGAGGGCTGAACAGCAGCAAATCATTGCTGTGCATGTTGAAA
AATTGGGCAATCTTTGGCTGCATGATGATTTGCTGCCGCTACTCGAGCGAGCGCTGGCCGGAAAGCTCACTGCAACGCAC
AGCGCGGTACTTTCGCCTTTTGATCCTGTTGTCTGGGATCGCAAACGCGCAGAGCAGCTTTTTGATTTAGCTACCGGCT
GGAGTGCTATACCCCAGCGCCGAAACGCCAGTATGGCTATTTTGTTCTGCCGTTATTACATCGTGGGCAATTAGTTGGGC
GAATGGATGCCAAAATGCATCGCCAGACAGGCATCCTTGAAGTTATCTCTCTGTGGTTACAGGAAGGTATTAAACCAACG
ACAACGCTGCAAAAAGGGCTACGTCAGGCGATTACTGATTTCGCTAACTGGCAGCAGGCAACGCGGGTGACATTAGGATG
CTGCCCGCAAGGCCTCTTTACTGATTGCCGCACCGGCTGGGAAATAGACCCCGTCGCATAAATGATTATGATAAGATTTA
TGCATTCATAACCCGATCCATCTGGAGGAACTATGGATCATCGTCTGCTTGAAATCATTGCCTGCCCGGTTTGCAACGGA
AAACTTTGGTATAACCAGGAAAAACAAGAGCTTATTTGCAAACTGGACAACCTCGCTTTCCCGCTGCGTGATGGCATTCC
AGTGTTGCTGGAAACGGAAGCCCGCGTGCTGACTGCTGATGAGAGTAAATCATGAGTTTTGTGGTCATTATTCCCGCGCG
CTACGCGTCCACGCGTCTGCCCGGTAAACCATTGGTTGATATTAACGGCAAACCCATGATTGTTCATGTTCTTGAACGCG
CGCGTGAATCAGGTGCCGAGCGCATCATCGTGGCAACCGATCATGAGGATGTTGCCCGCGCCGTTGAAGCCGCTGGCGGT
GAAGTATGTATGACGCGCGCCGATCATCAGTCAGGAACAGAACGTCTGGCGGAAGTTGTCGAAAAATGCGCATTCAGCGA
CGACACGGTGATCGTTAATGTGCAGGGTGATGAACCGATGATCCCTGCGACAATCATTCGTCAGGTTGCTGATAACCTCG
CTCAGCGTCAGGTGGGTATGGCGACTCTGGCGGTGCCAATCCACAATGCGGAAGAAGCGTTTAACCCGAATGCGGTGAAA
GTGGTTCTCGACGCTGAAGGGTATGCACTGTACTTCTCTCGCGCCACCATTCCTTGGGATCGTGATCGTTTTGCAGAAGG
CCTTGAAACCGTTGGCGATAACTTCCTGCGTCATCTTGGTATTTATGGCTACCGTGCAGGCTTTATCCGTCGTTACGTCA
ACTGGCAGCCAAGTCCGTTAGAACACATCGAAATGTTAGAGCAGCTTCGTGTTCTGTGGTACGGCGAAAAAATCCATGTT
GCTGTTGCTCAGGAAGTTCCTGGCACAGGTGTGGATACCCCTGAAGATCTTGAGCGCGTTCGCGCTGAAATGCGCTAATT
TCACTTCACGACACTTCAGCCAATTTTGGGAGGAGTGTCGTACCGTTACGATTTTCCTCAATTTTTCTTTTCAACAATTG
ATCTCATTCAGGTGACATCTTTTATATTGGCGCTCATTATGAAAGCAGTAGCTTTTATGAGGGTAATCTGAATGGAACAG
CTGCGTGCCGAATTAAGCCATTTACTGGGCGAAAAACTCAGTCGTATTGAGTGCGTCAATGAAAAAGCGGATACGGCGTT
GTGGGCTTTGTATGACAGCCAGGGAAACCCAATGCCGTTAATGGCAAGAAGCTTTAGTACGCCCGGAAAAGCCCGACAAC
TGGCATGGAAAACCACCATGCTGGCAAGAAGTGGGACTGTCCGTATGCCGACTATTTATGGTGTGATGACGCATGAAGAA
CACCCCGGCCCCGATGTCCTGCTACTGGAGCGGATGCGTGGTGTTTCGGTGGAGGCACCAGCCCGAACACCAGAACGCTG

GGAACAACTCAAAGACCAAATCGTTGAAGCCTTACTGGCCTGGCACCGTCAGGACAGTCGCGGTTGCGTCGGCGCGGTCG
ACAATACTCAGGAAAATTTCTGGCCCTCATGGTACCGGCAACATGTTGAAGTGCTATGGACCACGCTCAATCAGTTCAAT
AACACCGGTCTGACGATGCAGGATAAGCGGATCCTGTTTCGCACTCGCGAATGTCTCCCGGCATTATTTGAAGGCTTTAA
CGACAATTGTGTGCTGATTCACGGTAACTTCTGTTTACGCAGCATGTTGAAAGATTCGCGCAGCGATCAGTTACTGGCGA
TGGTCGGGCCGGGACTAATGCTTTGGGCACCGCGAGAATACGAACTGTTCCGACTAATGGATAATTCTCTGGCGGAAGAT
TTGCTCTGGAGTTACCTGCAACGCGCGCCAGTGGCGGAGTCGTTCATCTGGCGGCGTTGGTTGTATGTGTTATGGGATGA
AGTTGCGCAACTGGTTAATACCGGACGATTTAGTCGGCGCAACTTCGATCTGGCATCAAAATCACTCTTGCCGTGGCTCG
CCTGACGAACCTTTTAGCCACTGCCAGATACGGCCAAGCGTTTCATAGCCAACACGATCGCTATGCATCAACCAGACCGG
AGAAGGGATTGCCCGTTCCCACGGGTTAAGCGGCGAGTCGATGGCCAACTGATTCGCCGGGGCTGGTAGCGGATTTAACC
CTTCCTGCTGAAAAAAGATCATCGCCCGCGGCAGATGTGATGCGGAGGTCACCAGCAGGAAAGGGGCGTCACCAATCGCC
TGTTTCACTGCTGCAGCTTCTTCTTCGGTATCTTTTGGCAAATCCAGGGTGATAATTTGCTCGCGCGGCACGCCCAGCGA
TTGCGCAACTCTGGCACCTACTTCCGCTGTACTCACCGTATTGGTTTTTGCTACGCCTCCCGTGAAGATCAGTTTTGATC
CCGGATTTTCGCGCCATAAGCGAATACCTTCATTCAGGCGTGGCAGACTGTTATTGATTAAATTAGAGCTCGGTGCCCAC
TGCGGGTTCCAGGTATAACCACCTCCGAGCACCACGATATAGTCCACTTTTTGCGAATTATTCCATGTCGGATAGGTGCT
TTCGATGGGACGCAGCAGGCGGTCGGCTACCGGTTGCAAACTCAATAACAAGAGCGCCAGCCACCCAATGCTAATAAAAA
TCTTCCCGGTTTTTTGAAAACGGCTAAACCACAGCAGCGCCAGGCCAGCGCCAATAATCAACAACATCAGCGGAAGTGGC
AGCAACATATTGCCAATCACTTTTTTCAGTGTAAAAAGCATCCTTTCTGGTTCCTTTTTTAACCATATAGCAAGGGATCG
CCAGCGATATTACACCAGACGGGTTCATTCTCGCCGTGGCTGTGACAAAATAGCGTCTTTATCAGAAAACGTCTTAGCCA
ACCGGTGGAGAAAGCAATGCAGGATCGCAATTTTGATGATATTGCGGAAAAGTTTTCCCGTAACATTTACGGCACCACCA
AAGGGCAGCTTCGACAGGCTATTCTGTGGCAGGATCTCGATCGCGTGCTGGCGGAAATGGGCCCGCAAAAACTGCGTGTG
CTGGATGCTGGCGGTGGAGAAGGGCAGACCGCAATCAAAATGGCCGAGCGTGGGCATCAGGTCATTTTATGCGATCTTTC
TGCGCAGATGATCGACCGCGCAAAACAGGCGGCAGAAGCAAAAGGTGTGAGCGACAACATGCAATTTATACATTGCGCCG
CTCAGGATGTTGCTTCGCATTTGGAAACGCCCGTTGATCTGATATTGTTCCATGCGGTGCTCGAGTGGGTGGCTGATCCC
CGCAGCGTATTGCAGACCCTCTGGTCAGTGTTGCGTCCAGGCGGCGTGTTGTCGTTAATGTTCTACAATGCGCATGGTTT
GTTGATGCATAACATGGTCGCCGGGAATTTTGATTACGTGCAGGCGGGAATGCCGAAAAAGAAAAAACGGACGCTTTCGC
CAGATTATCCACGCGACCCGGCGCAGGTTTATCTGTGGCTGGAAGAAGCTGGTTGGCAAATTATGGGTAAGACAGGCGTT
CGCGTGTTTCATGATTATCTGCGCGAGAAACACCAGCAGCGCGACTGCTATGAAGCATTACTTGAATTAGAAACGCGTTA
TTGCCGTCAGGAACCGTATATTACCCTGGGGCGTTATATTCATGTCACCGCGCGCAAACCGCAGAGCAAGGATAAAGTAT
GAGTGAATTTTCCCAGACAGTCCCCGAACTGGTTGCCTGGGCCAGAAAAAATGACTTCTCCATCTCGCTGCCGGTAGACC
GACTCTCTTTTCTGCTGGCGGTTGCCACGCTGAACGGCGAGCGTCTGGATGGTGAGATGAGTGAAGGCGAGCTGGTGGAT
GCATTCCGCCATGTGAGTGATGCGTTTGAGCAAACCAGCGAAACCATCGGCGTGCGCGCCAATAACGCGATCAACGACAT
GGTGCGTCAACGTCTGCTGAACCGCTTTACCAGCGAGCAGGCGGAAGGGAACGCAATTTACCGTCTGACGCCGCTCGGCA
TCGGCATTACTGACTACTACATCCGTCAGCGCGAGTTTTCTACGCTGCGTCTTTCTATGCAGTTGTCGATTGTGGCGGGT
GAGCTCAAACGCGCAGCAGATGCCGCCGAAGAGGGCGGTGATGAATTTCACTGGCACCGTAATGTCTATGCGCCACTGAA
ATATTCGGTAGCAGAAATTTTCGACAGTATCGACCTGACGCAACGTCTGATGGACGAACAGCAGCAGCAGGTGAAGGACG
ATATCGCCCAGTTGCTGAACAAAGACTGGCGGGCGGCGATTTCCAGCTGTGAATTGTTGCTTTCGGAAACTTCCGGAACG
CTGCGTGAATTGCAGGATACGCTGGAAGCGGCAGGCGACAAATTGCAGGCTAATCTGTTGCGCATTCAGGATGCGACGAT
GACCCATGACGATCTGCATTTCGTCGATCGTCTGGTGTTCGATCTGCAGAGCAAACTCGATCGTATTATCAGTTGGGGCC
AGCAATCCATCGACTTGTGGATTGGCTACGACCGCCACGTACACAAATTTATTCGTACCGCGATCGATATGGATAAAAAC
CGCGTCTTTGCTCAGCGGTTACGTCAGTCGGTACAAACCTATTTTGATGAGCCGTGGGCGCTAACTTATGCCAATGCCGA
TCGTCTGCTGGATATGCGTGACGAAGAGATGGCACTGCGCGATGAAGAAGTGACTGGGGAACTTCCTGAGGATCTGGAAT
ACGAAGAGTTTAACGAGATCCGCGAACAGCTGGCGGCGATCATCGAAGAACAACTTGCCGTGTACAAAACCAGACAAGTG
CCGCTGGATCTTGGTCTGGTGGTACGCGAATATCTGTCACAGTATCCGCGTGCACGTCACTTTGACGTTGCGCGTATTGT
TATTGATCAGGCGGTACGTCTTGGCGTAGCGCAAGCAGATTTCACCGGACTGCCAGCGAAATGGCAGCCGATTAATGATT
ACGGAGCCAAGGTACAGGCGCATGTCATCGACAAATATTGAACAAGTGATGCCGGTTAAGCTGGCGCAGGCGCTGGCGAA
TCCGTTATTTCCGGCGCTGGACAGCGCCTTACGTTCAGGACGCCATATTGGCCTCGACGAACTGGATAATCATGCATTCC
TGATGGATTTTCAGGAATATCTGGAAGAGTTTTACGCGCGTTATAACGTTGAGCTTATTCGCGCACCAGAAGGGTTCTTC
TATTTACGCCCACGTTCCACCACGCTGATCCCTCGTTCCGTCTTGTCGGAACTGGATATGATGGTCGGGAAAATCCTCTG
TTATCTCTATCTCAGCCCGGAACGGCTGGCGAATGAGGGGATTTTCACCCAGCAGGAACTGTACGACGAACTGCTCACCC
TGGCCGATGAAGCAAAACTGCTGAAACTGGTGAACAACCGTTCAACCGGTTCAGACGTTGACCGTCAGAAGTTGCAGGAG
AAAGTACGTTCTTCGCTCAACCGTCTGCGTCGTTTAGGCATGGTGTGGTTTATGGGCCACGACAGCAGCAAGTTTCGCAT
TACCGAATCGGTGTTCCGCTTCGGAGCCGATGTGCGTGCTGGCGACGATCCCCGTGAAGCACAGCGTCGCCTGATTCGTG
ATGGCGAAGCAATGCCGATTGAAAATCATCTGCAACTCAACGATGAAACCGAAGAGAATCAGCCAGATAGCGGAGAGGAA
GAATAATGATTGAACGCGGTAAATTTCGCTCACTGACGCTGATTAACTGGAACGGCTTTTTTGCCCGAACTTTTGACCTT
GACGAGCTGGTCACGACGCTTTCTGGCGGTAACGGGGCGGGTAAATCCACCACCATGGCGGCGTTCGTTACGGCGCTGAT
CCCCGACCTGACCCTGCTGCATTTCCGTAACACTACGGAAGCCGGGGCCACCAGCGGTTCGCGCGATAAAGGTCTGCACG

GTAAGCTGAAAGCGGGTGTCTGTTATTCGATGCTCGACACCATTAACTCGCGCCACCAGCGCGTGGTGGTCGGTGTGCGT
CTGCAACAGGTTGCCGGACGCGATCGTAAAGTGGATATCAAGCCGTTTGCCATTCAGGGACTGCCGATGTCGGTGCAGCC
GACACAGCTGGTGACCGAAACCCTGAACGAACGCCAGGCGCGCGTGCTGCCGCTTAACGAGCTGAAAGACAAGCTCGAGG
CGATGGAAGGCGTGCAGTTTAAACAGTTCAACTCCATTACTGATTACCACTCGCTGATGTTCGATCTGGGCATCATCGCG
CGTCGTCTGCGCTCCGCATCTGACCGTAGCAAATTCTATCGTCTGATCGAAGCTTCGCTGTATGGCGGGATCTCCAGTGC
CATTACCCGTTCTCTGCGCGACTACCTGTTGCCAGAAAACAGCGGCGTGCGTAAAGCGTTCCAGGACATGGAAGCGGCGC
TGCGTGAAAACCGTATGACGCTGGAAGCGATTCGTGTCACCCAGTCGGACCGCGACCTGTTTAAGCATCTGATCAGCGAA
GCCACCAACTACGTGGCGGCGGACTACATGCGTCACGCCAACGAGCGCCGTGTCCATCTCGACAAAGCCCTGGAGTTTCG
TCGCGAGCTACATACTTCGCGTCAGCAACTGGCGGCTGAGCAGTACAAACACGTCGATATGGCGCGTGAGCTGGCAGAGC
ACAACGGTGCCGAAGGCGATCTGGAAGCGGATTATCAGGCGGCCAGTGATCACCTGAACCTGGTGCAAACCGCACTGCGT
CAGCAGGAGAAGATCGAACGCTACGAAGCGGATCTCGATGAGCTGCAGATCCGTCTGGAAGAGCAAAATGAAGTGGTGGC
AGAAGCCATCGAACGCCAGCAAGAGAATGAGGCTCGTGCGGAAGCTGCCGAACTGGAAGTGGACGAGCTGAAAAGCCAGC
TTGCTGACTACCAGCAGGCGCTGGACGTCCAGCAAACGCGCGCGATCCAGTATAACCAGGCGATTGCTGCGCTTAATCGT
GCCAAAGAACTGTGCCATCTGCCGGACTTAACCGCCGACTGCGCCGCCGAATGGCTGGAAACCTTCCAGGCGAAAGAGCT
GGAAGCGACTGAAAAAATGCTCTCTCTTGAGCAGAAAATGAGCATGGCGCAAACCGCGCACAGCCAGTTTGAGCAGGCTT
ATCAGCTGGTGGTGGCAATCAACGGCCCACTGGCGCGTAACGAGGCGTGGGATGTCGCTCGCGAACTATTGCGCGAAGGG
GTCGATCAGCGTCACCTGGCAGAGCAGGTTCAGCCGCTGCGGATGCGATTAAGCGAACTGGAACAGCGTCTACGCGAGCA
GCAAGAAGCTGAGCGTCTGCTGGCAGATTTCTGCAAACGTCAGGGCAAGAATTTTGATATCGACGAACTGGAAGCCCTGC
ATCAGGAACTGGAAGCACGCATTGCCTCTCTTTCCGATAGCGTGTCTAACGCCCGTGAAGAGCGCATGGCACTGCGCCAG
GAGCAGGAACAGCTGCAGTCTCGCATTCAGAGTTTGATGCAGCGTGCGCCGGTTTGGCTGGCAGCGCAAAACAGTCTCAA
CCAGTTGAGCGAACAGTGCGGCGAAGAGTTTACCTCCAGCCAGGACGTCACCGAATATCTGCAACAGTTGCTGGAGCGTG
AGCGAGAGGCGATTGTTGAACGCGATGAAGTGGGCGCGCGCAAAAACGCCGTCGATGAAGAGATCGAACGTTTAAGCCAG
CCTGGCGGCTCTGAAGATCAGCGTCTGAACGCGCTGGCGGAGCGTTTTGGTGGTGTGCTGCTGTCAGAAATTTATGACGA
CGTTAGCCTGGAAGATGCGCCGTACTTCTCAGCGCTGTATGGCCCGTCACGCCACGCCATCGTGGTGCCAGATCTGTCAC
AGGTAACTGAACACCTGGAAGGCTTGACCGATTGCCCGGAAGATCTCTATCTGATCGAAGGAGATCCGCAGTCATTCGAT
GACAGCGTGTTCAGCGTTGATGAGCTGGAAAAAGCGGTAGTGGTGAAAATCGCCGATCGTCAGTGGCGTTATTCACGTTT
CCCGGAAGTGCCGCTGTTTGGTCGTGCTGCGCGTGAAAGCCGTATTGAAAGCCTCCATGCCGAGCGTGAAGTGCTTTCCG
AACGCTTCGCCACGCTCTCCTTTGATGTACAGAAAACTCAGCGTCTGCATCAGGCGTTCAGCCGCTTTATCGGCAGTCAT
CTGGCGGTTGCGTTTGAGTCTGACCCGGAAGCAGAAATCCGTCAACTGAACAGCCGTCGCGTCGAACTGGAGCGGGCGTT
AAGTAATCATGAAAATGATAACCAGCAGCAGCGTATTCAGTTTGAGCAGGCGAAAGAGGGCGTTACGGCGCTGAACCGCA
TTCTGCCGCGTCTCAACCTGTTGGCTGATGACAGCCTGGCGGATCGCGTCGATGAAATCCGCGAACGTCTGGATGAAGCC
CAGGAAGCCGCGCGTTTTGTTCAGCAGTTTGGCAATCAACTGGCGAAACTGGAACCGATTGTTTCGGTATTGCAGAGCGA
CCCGGAACAGTTCGAACAGTTAAAAGAAGATTACGCGTACTCTCAGCAGATGCAGCGCGATGCCCGTCAGCAGGCGTTTG
CCCTGACGGAAGTGGTGCAGCGTCGTGCGCACTTTAGCTATTCTGACTCGGCAGAAATGCTTAGCGGTAACAGCGATCTC
AACGAAAAACTGCGTGAACGTCTGGAACAGGCGGAAGCGGAGCGTACCCGCGCTCGCGAAGCGTTGCGCGGTCACGCAGC
GCAGTTGAGTCAGTACAACCAGGTGCTGGCTTCGCTGAAAAGTTCTTACGACACCAAAAAAGAGCTACTCAACGATCTGC
AACGTGAATTGCAGGATATCGGCGTGCGTGCTGATAGCGGGGCAGAAGAGCGGGCGCGTATTCGCCGTGACGAGCTGCAT
GCGCAACTGAGCAATAACCGTTCACGCCGCAATCAACTGGAAAAAGCGCTTACCTTCTGCGAAGCGGAGATGGACAACCT
GACCCGCAAACTGCGCAAGCTGGAGCGGGATTACTTTGAGATGCGCGAGCAGGTAGTGACCGCCAAAGCGGGCTGGTGTG
CGGTGATGCGCATGGTGAAAGATAACGGCGTTGAGCGCCGCTTACACCGTCGTGAGCTGGCTTATCTCTCCGCTGATGAT
TTGCGTTCCATGTCGGATAAGGCGTTAGGTGCGCTGCGTCTGGCGGTGGCGGATAACGAACATCTGCGCGACGTGCTGCG
CATGTCGGAAGATCCGAAACGTCCGGAGCGTAAAATTCAGTTCTTCGTGGCGGTTTATCAGCATCTGCGTGAACGTATTC
GTCAGGATATTATTCGTACCGATGATCCGGTGGAAGCTATCGAACAGATGGAGATTGAACTTAGCCGTCTGACCGAAGAA
TTAACCTCCCGTGAACAGAAACTGGCGATCAGTTCCCGCAGCGTGGCGAACATCATTCGCAAAACCATTCAGCGCGAGCA
GAACCGTATCCGTATGCTCAACCAGGGGTTGCAGAACGTATCGTTTGGTCAGGTGAACAGCGTGCGTCTCAACGTGAACG
TGCGTGAAACGCACGCCATGCTACTGGATGTGCTCTCTGAACAGCACGAGCAGCATCAGGATCTGTTTAACAGCAACCGT
TTGACCTTCTCGGAAGCGCTGGCGAAACTGTATCAACGTCTTAACCCGCAGATTGATATGGGGCAGCGCACGCCGCAGAC
CATCGGTGAAGAACTGCTGGATTACCGCAACTATCTGGAAATGGAAGTTGAGGTTAACCGTGGTTCCGATGGCTGGCTGC
GCGCAGAGTCTGGTGCATTGTCGACCGGTGAGGCGATTGGTACCGGTATGTCGATTCTGGTGATGGTGGTACAAAGCTGG
GAAGATGAATCTCGCCGCCTGCGCGGTAAAGATATCTCTCCTTGCCGCCTGCTGTTCCTCGATGAAGCAGCGCGACTGGA
TGCTCGTTCTATCGCCACGCTGTTTGAATTGTGTGAGCGTTTGCAAATGCAACTCATCATCGCAGCGCCGGAAAATATCA
GCCCGGAGAAAGGCACCACCTATAAACTGGTGCGTAAAGTCTTCCAGAATACCGAACACGTTCATGTCGTCGGCCTGCGA
GGATTTGCGCCGCAACTCCCTGAAACGCTTCCAGGAACTGACGAAGCGCCTTCTCAGGCGAGTTAAAATTAAGGCGGCAG
CAATGCCGCCTTTTCTTTTTCCGAAAACTCCGTTTCTGCACTAAAAAAGTGGCACATTACGGCGCGCTTTTCTTTAAACT
TCTTTACATTAGGTTATGTAAAAACGTATCGGCGTTTATATACTGAAGATAAGCCTGATGAGTAACAGGCTTGCTCGTCA
TACTTTCGTGAGTATTGGCGTTGTACAGGCAAGTCGTAAAATAACAGCCTGGCTATTCAGAGTATGATAAAAACAGGGGG

CAAGGGATGTTGCTTAATATGATGTGTGGTCGTCAGCTGTCGGCAATCAGTTTGTGCCTGGCCGTAACATTCGCTCCACT
GTTCAATGCGCAGGCCGATGAGCCTGAAGTAATCCCTGGCGACAGCCCGGTGGCTGTCAGTGAACAGGGCGAGGCACTGC
CGCAGGCGCAAGCCACGGCAATAATGGCGGGGATCCAGCCATTGCCTGAAGGTGCGGCAGAAAAAGCCCGCACGCAAATC
GAATCTCAATTACCCGCAGGTTACAAGCCGGTTTATCTTAACCAGCTTCAACTGTTGTATGCCGCACGCGATATGCAACC
CATGTGGGAAAACCGTGATGCTGTTAAAGCCTTCCAGCAACAGCTGGCAGAGGTGGCGATTGCCGGTTTCCAGCCGCAGT
TTAATAAATGGGTAGAGTTACTGACCGATCCTGGTGTTAACGGGATGGCACGCGACGTGGTGCTCTCTGATGCGATGATG
GGCTATCTCCATTTCATTGCAAATATTCCGGTCAAAGGCACTCGCTGGCTATATAGCAGTAAACCTTATGCACTTGCAAC
GCCGCCGCTCTCGGTGATTAACCAATGGCAGCTGGCGCTGGATAAAGGTCAATTGCCTACGTTTGTTGCAGGACTGGCAC
CGCAGCATCCGCAATATGCGGCGATGCATGAATCGTTACTGGCCTTACTCTGTGACACCAAACCGTGGCCCCAACTGACC
GGCAAAGCAACGTTGCGCCCAGGGCAGTGGAGTAACGACGTACCGGCGTTGCGCGAAATATTGCAACGCACAGGCATGTT
GGACGGGGGGCCGAAAATTACTCTACCTGGCGATGACACGCCAACTGACGCGGTAGTCAGCCCATCCGCTGTTACTGTTG
AAACAGCAGAAACTAAGCCGATGGATAAGCAAACGACGTCTCGTAGTAAACCTGCGCCTGCCGTTCGCGCCGCCTACGAT
AATGAACTGGTGGAAGCCGTTAAACGTTTTCAGGCATGGCAAGGATTGGGGGCAGATGGTGCTATTGGCCCGGCAACACG
TGACTGGTTAAACGTAACGCCCGCCCAGCGTGCTGGTGTGTTGGCTCTCAACATCCAGCGATTGCGCTTGCTGCCAACAG
AGCTTTCTACCGGGATCATGGTTAACATTCCGGCCTATTCGCTGGTCTACTATCAGAACGGCAATCAGGTGCTGGATTCG
CGAGTCATTGTCGGTCGCCCCGATCGCAAAACGCCGATGATGAGCAGTGCCCTTAACAACGTAGTGGTAAACCCGCCGTG
GAACGTACCTCCAACTCTGGCACGCAAAGATATTCTGCCAAAAGTGCGCAACGATCCGGGATATCTCGAAAGCCATGGCT
ATACGGTGATGCGCGGCTGGAACAGCAGAGAAGCGATTGACCCATGGCAGGTTGACTGGTCTACAATCACGGCCTCGAAT
TTACCGTTCCGCTTCCAGCAGGCTCCAGGCCCACGGAACTCGCTGGGGCGCTATAAATTCAATATGCCGAGTTCAGAGGC
CATTTATTTGCATGACACGCCGAACCACAATCTGTTCAAGCGTGATACACGCGCATTGAGCTCAGGCTGTGTACGAGTGA
ATAAAGCTTCCGATCTGGCGAATATGCTGTTGCAGGATGCAGGCTGGAATGACAAACGTATTTCTGATGCGCTGAAGCAG
GGTGATACACGTTACGTCAATATTCGGCAGTCGATTCCGGTGAATCTCTACTACCTGACGGCCTTTGTTGGTGCAGATGG
TCGTACCCAGTATCGTACAGATATTTACAATTATGATCTGCCTGCGCGATCCAGCTCGCAAATCGTATCGAAAGCGGAAC
AATTAATCAGGTAAATGAAGTAGTTCGGGGAAATCAGTTGTCGTAATAATTAGCATGATTGGGGGCGATTCTCTGCAGCC
CCCGTCACTGCTGGGGTTGAGTCATCTTGACGTCTGCTTTACGGGCGGTTAAGGTGCCTCTTGTGCGCCAGAAGTGCATA
TAAACGATAACATTGACCTGTAGACTTGATTATCATGGACAAATTCGACGCTAATCGCCGCAAATTGCTGGCGCTTGGTG
GCGTTGCACTCGGTGCCGCCATCCTGCCGACCCCTGCGTTTGCAACACTCTCTACCCCACGCCCGCGCATTTTGACACTC
AATAATCTTCATACCGGAGAGTCAATCAAAGCGGAGTTTTTCGATGGCAGAGGCTATATTCAGGAAGAATTGGCAAAACT
TAACCATTTTTTCCGCGATTACCGCGCGAACAAAATAAAGTCCATCGACCCAGGATTATTCGACCAGTTGTATCGCCTGC
AAGGGTTGTTAGGCACGCGCAAACCGGTGCAACTCATTTCCGGTTATCGTTCTATTGATACCAACAATGAACTACGCGCC
CGCAGCCGTGGAGTAGCGAAGAAAAGCTATCACACTAAAGGCCAGGCGATGGATTTCCATATTGAAGGTATCGCGTTAAG
CAATATTCGCAAAGCCGCGTTATCTATGCGCGCAGGTGGTGTAGGATATTATCCACGTAGTAACTTTGTGCATATTGATA
CCGGGCCAGCACGGCACTGGTAGTAATTGCTTAACGAAACAGGGGCAGTATGAACTATCGTATTATTCCGGTCACCGCAT
TCTCCCAGAACTGTTCATTAATCTGGTGTGAACAAACCCGTCTGGCCGCACTGGTCGATCCTGGCGGCGATGCGGAAAAA
ATCAAACAGGAAGTTGATGACAGCGGCCTGACACTGATGCAGATCCTGCTGACGCATGGTCATCTGGACCACGTTGGCGC
AGCGGCGGAACTGGCGCAACATTACGGCGTGCCGGTTTTCGGCCCGGAAAAAGAAGATGAGTTCTGGCTGCAAGGCTTGC
CTGCGCAAAGTCGTATGTTTGGTCTGGAAGAGTGCCAGCCGCTGACGCCAGATCGTTGGCTGAACGAAGGCGATACCATC
AGCATAGGGAATGTGACTTTACAGGTGTTACATTGCCCTGGGCATACGCCGGGTCATGTCGTGTTTTTTGATGATCGGGC
AAAGCTGCTGATTTCTGGCGATGTTATTTTCAAAGGCGGAGTAGGGCGCAGTGACTTCCCGCGTGGCGATCATAATCAAC
TGATTTCTTCAATCAAAGATAAATTGCTGCCACTGGGGGATGACGTGATATTTATTCCGGGTCACGGACCATTATCCACA
CTTGGTTATGAACGCCTGCATAATCCCTTCCTGCAAGACGAAATGCCCGTCTGGTAAGGCACATAAAAAAGCCCGCTTTT
AATGCTGGCCTGGATTTCTGGCAAAGTGCGCTTTGTTTATGCCGGATGCGGCACGAGCGCCTTATCCGGCCTACAAAATC
GTGCAAATTCAAAATATTGCAGGGGACGCGTAGGCCTGATAAGCGTAGCGCATCAGGCAATGTTGCGTTTGTCATCAGTC
TCAGCCCGCTTTTCAGCGGGCTTCATTGTTTTTAATGCTTACAGCACTGCCACAATCGCTTCGCACAGCGGAGCCATGTT
ATCTGGTGTCATCCCGGCCACATTTACGCGACCAGAAGCAACCGCATATACGCCAAACTCTTCGCGCAGACGCAGCACTT
GTTCTTTTGTCAGGCCACTGAAGGAGAACATGCCGTTCTGTTTGATGATAAAGCTGAAGTCGCGGTTTGCGCCTTTTTCC
TGCAGCGTATTGACGAACAACTGACGCATACGCTGAATACGCTGGCGCATATCAGTCAGCTCTTGTTCCCAAATCGCACG
TAACGCATCGTTGCTCAGGATGGTGGCAACAACAGAAGCGCCGTGTGCTGGTGGGTTAGAGTAGTTAGCGCGAATCGCCG
CTTTCATTTGGCTGAATGCGCGATCAACGGTTTCACTGTCGGCAGCAACCAGAGTACAAGCGCCAACACGCTCGTTGTAC
AGGCCAAAGTTTTTAGAGTAGGAACTGGCAACAATCAGCTCTTTATGCATAGCCGCGAAAGCGCGCAGTCCTTCAGCATC
TTCTTCCAGACCACGGGCAAAACCCTGGTAAGCGAAGTCAAACAGCGGTAACCAGCCTTTCTCAACGGAGAGTTGTGCCA
GTGTTTGCCATTGTTCCAGCGTAGGGTCGATACCGGTTGGGTTATGGCAGCAGCCATGGAACAGCACTACGTCGCCAGCC
TGAGCTTCATTCAGGCTGTTAATCAGTGCATCGAAGTCAAGAGTGTGATTTTCCGCATCATAATAAGCGTATTCACGAAC
TTCCAGACCTGCAGAGTTAAAGACGCTCTTATGGTTCGGCCAGCTTGGGTTGCTCACCCACACACGCTTAACGCTGGTAT
TTTTTGCCAGGAAATCGGCAGCCACGCGTAGTGCGCCAGTGCCCCCCGGAGTCTGTGCCGTGCGAGCACGTTTGTCATTG
ATCAGGGCGCTACCTTTACCAAACAGCAGTTCCTGAGTGCAGCGACCAAATTCAGGGATGCCGTCAATGCCGAGGTAATT

TTTGGTGGTTTCATTTTCGAGCAGATACTGTTCAGCCTTTTTCACGCTGGTCAGTACCGGGGTTTTGCCCGTCTCATCTT
TATAGACACCAATCCCGAGGTTAATTTTGCCGGGACGTTCATCGGCACGAAACAGATCGGCCAGGCCCAGAATCGGGTCG
GCAGGAGCGGCGGTAATGTTCTCAAACATGACGAGGTTCCATTATGGTTACAGAAGGGAAGTCCGCTATCAGGGTAACGG
GAGATTTACAAAATTCCAACTATTACTGATGAAAACGCAGGCTGTTTTTGCAAGACGTGAGATTGCTCTGGAAGGTATAA
AAAAAACAGGACCAAAGTCCTGTTTTTTCGGCATTTAACAAAGAGGTGTGCTATTAGAACTGGTAAACGATACCCACAGC
AACGGTGTCGTCTGAACCTACGCCCAGTTTGTTGTCAGAATCGATCTGGTTGATGATGTAGTCAACATAGGTGGACATGT
TTTTGTTGAAGTAGTAGGTTGCGCCCACTTCAAAGTAGTTCACCAGATCAACATCACCGATACCTTCTACGTCTTTCGCT
TTAGATTTGGTGTAAGCGATGGACGGACGCAGACCGAAATCGAACTGGTATTGCGCAACTAACAGAACGTCTTGCGTTTT
GTTGGCGAAGCCGCTGGTGTTTGTAAATTTATTAGTGATCGGCGTAGCGTTACGGGTTTCACCGTAGTTCGCTGCCAGGT
AGATGTTGTTCGCGTCGTACTTCAGACCAGTAGCCCACTGTTCAGCTTTTTTACCGTTGCCAAGAGGTTGAGCTTCTTGC
AGGTTGGTACGGTCAGCTGCACCATAAGCACCAACGATACCAAAGCCTTCGTATTCGTAGCTGATAGAACCGCCAACACC
GTCGCCGTTAGAACGGCGTGCAGTGTCACGCTCGTTTTTACCCAGGTACTGAACAGCGAAGTTCAGGCCATCAACCAGAC
CAAAGAAGTTGGAGTTACGATAGGTAGCAACGCCGCCAACACGACCAACGAAGAAGTCATCGCTGTATGCAGTATCACCA
CCAAATTCTGGCAGCATATCGGTGTAACCCAGTGCATCATAAACCACACCGTAGTTACGGCCGTAATCGAAAGAACCAAC
GTCAGCGTATTTAAGACCCGCGAATGCCAGACGCGTTTTGTTACCAGTTTGAGCGTCAGCGCCTTCAGAGTTGTTACCCT
GGAAGTTATATTCCCACTGACCATAACCGGTCAGATCGGAATTGATTTGAGTTTCCCCTTTAAAACCAAGACGGGCATAG
GTCATGTCGCCATTGCCACCGTAACTGTTTTCACCGTTACCCTTGGAAAAATAATGCAGACCAACAGCTTTACCGTACAG
ATCTACTTTGTTGCCATCTTTGTTATAGATTTCTGCAGCGTTTGCAGTACCTGCTACTAACAGAGCAGGGACGATCACTG
CCAGAATATTGCGCTTCATCATTATTTATTACCCTCATGGTTTTTTTATGACACCTGCCACTGCCGTCAATAAGTTCTG
TCAATAAAAATTTACGGAACTATTGATGAGAGTTTGGTGTCTTTATGTGTCTGCAGGCATCTTTCCATTCAAACTAACGT
TTCGCTACCGTGAAAGTGCTACAAAGATAAAGATTTGGTTTCAAAAAGAAAAAATATGTAACCAAAAGTAAAATTTAAGG
AACTTTGTGAACACCGTCATATTTCCATAGAGACGTGATGATATTTACAGCAATTTTAATCTATTTATATGATTTCCTTA
TATTTAAATTAACTAAACGGAAATTTTGTTTCTGATGGAAACTTTATCGACCTGGCACAAAATCTTCTTTCAGACATCCA
GAATGCCAAAAAATAGTATGAAAATTGTGCTATTAGCTAAGAAAAAACTAATCCGCAATAAGAATGCGGATTAGTTTTTG
CGCGTAAATGTTGCAATGTAATATTCCGTAACAGGATGATCGTTATGGCGGGGTTGCAAATAGATTGCTTGACTTAACAA
ACAGATAACTTGACAGAAAAGATAAAAAAAGAGCCAGCGGGCGCTGGCTCTTGAAGACGCTAAATTAGAAGCTGGCGTTA
CGCGGAGTACGTGGGAACGGAATCACATCACGTACGTTTTGCACGCCAGTTACGTAAGCAATCAGACGTTCAAAACCAAG
ACCGAAACCTGAATGCGGAACAGTACCGTAGCGACGCAGATCGCGATACCACCAGTAATCTTCTTTATTCAGGCCCATTT
CCAGCATACGCTCGTCCAGCACGTCCAGACGTTCTTCACGCTGGGAGCCACCAATGATCTCACCGATGCCCGGAGCCAGA
ACGTCCATAGCCGCAACGGTTTTACCGTCTTCGTTAAGGCGCATATAGAACGCTTTAATATCTTTCGGATAGTTTTTAAC
CACTACCGGTGCTTTAAAGTGTTCTTCCGCCAGATAACGCTCATGCTCAGAAGAGAGATCGACTCCCCAGTAAACCGGGT
TTTCAAACTTCCTGCCGCAGTTTTCGAGAATGGTCACTGCGTCGGTATAATCCACCTGCGCAAAATCGGCTTCAATGAAG
CGTTCCAGACGTGAAACGGCATCTTTATCTACGCGTTCAGCGAAGAATTTCATGTCGTCAGCGCGTTCTTCGAGAACCGC
TTTGAAGACATATTTCAGCATGGCTTCAGCCAGACCCGCAATATCGTTCAGGTTAGCAAACGCCACTTCCGGCTCCAGCA
TCCAGAATTCCGCCAGGTGACGGCTGGTGTTGGAGTTTTCAGCACGGAAAGTCGGGCCGAAGGTATAAATTTTGGACAAT
GCGCAAGCGTAGGTTTCGCCGTTCAACTGGCCAGATACGGTCAGGAAAGACTCTTTACCAAAGAAGTCTTTGTCGAAATC
CACTTTGCCCTGATCGTTACGCGGCAGGTTTTCCAGATCCAGCGTAGAAACGCGGAACATTTCGCCTGCACCTTCGGTAT
CAGATGCGGTAATCAGTGGCGTTGAAACCCAGAAGAATCCCTGCTCGTTAAAGAAGCGATGCAGCGCCTGCGCCAGCGTA
TGGCGAACGCGCGCGACGGCACCAATCAGGTTTGTGCGCGGACGCAGGTGAGCGACTTCACGCAGATACTCAATGCTGTG
GCGTTTTGCCGCCATCGGGTAAGTGTCTGGATCTTCAACCCAACCAGCAACTTCAACCTTGCTGGCCTGAATTTCAAATT
GTTGCCCCTGGCCCGGCGACGCCACGACTTTACCCGTCACAATGACCGAGCAGCCGGTGGTCAGACGCAGGACGTCTTCA
TTGTAATTGGGCAGAGAATTATTGATGACAGCCTGTACAGGATCAAAGCAGGAACCGTCATAAACGGCGAGGAAGGAGAT
GCCAGCTTTTGAATCTCGGCGGGTACGTACCCATCCGCGCACGGTGACTTCGCTGTCAACGGCTACACGGCCCTGGAGTA
CGTCGGCTACAGGCACAACGCTCATAATATTCTCTCTGTTAATAGTCGGAAAAAATAAACACTTGTCCACCCGTAAATGG
GGGGATACCTATGTTACCTGGCATCTGCAATCAGACAAGCAGAATTCGCAAATGCAGCGAAAGATTTCGGAATTAAAGGT
AAAAAAAGGGAGCCGATTAGCTCCCTGATGATATTAACTGGCTTTTTTAATATGCGGAAGGTCGAACGCTTTGCGCAGCG
CCCGAACAAACGCTTTATCATGGCAGATAGTTTTGCCAGGGCTGTCAGAAAGTTTCGCCACCGGTTTACCGTTACACTCT
ACCAACTTAATGACAATATTCAGGGGTTTTACCTGGGGGATATCGCAGGTCAGGCGAGTCCCAATACCAAAACTTAATTG
CACGCGGGAAGAGAAGTGGCGGTATAGCTCAACCGCTTTGCGTAAATCCAGATTGTCAGAGAAAACCAGCGTTTTACTCT
GTGGATCAATTCCCAGCTTTTCATAATGTGCAATGGCTTTTTCACCCCATTCAACCGGGTCGCCAGAGTCATGACGCAGG
CCCTGATACCGACTAGCGAACTCGACACCGAAATCACGCAGGAAAGCATCCATAGTGATGCAGTCGGTTAATGCAATGCC
AAGTTGGTCGGGATACTCTTCCAGCCAGGCAGCAAGTGCAGCTCGCTGGCTGTTGGCTAGATCCGGGCTGATTTGCTGAT
GTGCCTGGAACCATTCGTGTGCCTGTGTTCCCATCGGCGTGAGGGAAAGCCGACGCGCCAGATCGTAGTTGCTGGTGCCC
ACAAACCAGGATTCCTGTTGCAGACGCTTAACGATGGTTTCTTGTACTTCGCGAGAAAAACGGCGACGGGTGCCAAAATC
CATCAGATGGAAGCGCGACATATCAAGACCGGCGGTTAACGCCGAGAAGTCGACTAATTTGCTTTCCAGCGTGTCGAGGG
CTTGCGCAACGTCGGCCTGCGGTGAGCGATAGCGATGTACCATTTCACTGATAACCGCCAGCAAAGGAACTTCCCAGAGG

ATGACTTCACGCCACGGGCCGCTTAAACGAATATCCAGCTTGCCATTATCGTTGGACACGGTGACTTGTTCCGGGTTAAA
GCGGAACTCGCGTAACCAGTTAAGATAGTCGGCCTTAAAGAAAGGCAGGGCAGAAAGCCACTGATATTCATCATCCTGCA
GGCGCAGGTGCTGCATCGCCTGAACCTGTTCACGAATAGCATCGGCATAAATACCCAGCAGATCGTCACCTCGGCAACGA
AACTCCGCCGCGACATGCACATCGTAATAGTGATGAAACACGGCTTGCTGCATATGCAACTTATAAGCATCTGTATCCAG
CAACGAGTGCAGAACAGGAGAAGCGAATTGTGTCATAGGTGCGCAGTAGCGTCCTCGTACAGGAGCGTTTAGTACAATAA
ACATCTTCAGGAACTGCTGGAGTATACCTTGTTTCGCAATTTATTGAACCCCGATCACACCATATGCCACCTTTCTGGTC
GATGGCATTACGCGGTGTATGTTATAAAAATGTAGCAATAAAGGCGTTTGTACCTGAAAAGATGAAGATTCTGCATAGCG
CGATTTACGCAACAGGAATAGACTGAACACCAGACTCTATAAAAGATGCTAAAGGTTATTTATGACTCAACAGCCACAAG
CCAAATACCGTCACGATTATCGTGCGCCGGATTACCAGATTACTGATATTGACTTGACCTTTGACCTCGACGCGCAAAAG
ACGGTCGTTACCGCGGTCAGCCAGGCTGTCCGTCATGGTGCATCAGATGCTCCCCTTCGTCTCAACGGCGAAGACCTCAA
ACTGGTTTCTGTTCATATTAATGATGAGCCGTGGACCGCCTGGAAAGAAGAAGAGGGCGCACTGGTTATCAGTAATTTGC
CGGAGCGTTTTACGCTTAAGATCATTAATGAAATAAGCCCGGCGGCGAATACCGCGCTGGAAGGGCTTTATCAGTCAGGC
GATGCGCTTTGCACCCAGTGTGAAGCCGAAGGTTTCCGCCATATTACGTATTATCTCGACCGCCCGGACGTGCTGGCGCG
TTTTACCACCAAAATTATTGCCGATAAAATCAAATATCCCTTCCTGCTTTCCAATGGTAACCGCGTTGCGCAAGGCGAAC
TGGAAAACGGACGCCATTGGGTACAGTGGCAGGACCCGTTCCCGAAACCGTGCTACCTGTTTGCGCTGGTGGCAGGCGAC
TTTGATGTACTGCGCGATACCTTTACCACGCGTTCTGGTCGCGAAGTAGCACTGGAGCTGTACGTCGATCGCGGCAACCT
TGATCGCGCGCCGTGGGCGATGACCTCGCTGAAAAACTCCATGAAATGGGATGAAGAACGCTTTGGCCTGGAGTATGACC
TCGACATCTATATGATCGTCGCGGTGGATTTCTTCAATATGGGCGCAATGGAGAATAAGGGGCTGAATATCTTTAACTCC
AAATATGTGCTGGCCCGCACCGACACCGCCACCGACAAAGATTACCTCGATATTGAACGCGTTATCGGCCATGAATATTT
CCATAACTGGACCGGTAACCGAGTGACCTGTCGCGACTGGTTCCAGCTCAGCCTGAAAGAAGGTTTAACCGTCTTCCGCG
ATCAGGAGTTCAGCTCTGACCTTGGTTCCCGCGCAGTTAACCGCATCAATAATGTACGCACCATGCGCGGATTGCAGTTT
GCAGAAGACGCCAGCCCGATGGCGCACCCGATCCGCCCGGATATGGTCATTGAGATGAACAACTTCTACACCCTGACCGT
TTACGAGAAGGGCGCGGAAGTGATTCGCATGATCCACACCCTGCTTGGCGAAGAAAACTTCCAGAAAGGGATGCAGCTTT
ATTTCGAGCGTCATGATGGTAGTGCAGCGACCTGTGACGACTTTGTGCAGGCGATGGAAGATGCGTCGAATGTCGATCTC
TCCCATTTCCGCCGTTGGTACAGCCAGTCCGGTACACCGATTGTGACCGTCAAAGACGACTACAATCCGGAAACCGAGCA
GTACACCCTGACCATCAGCCAGCGCACGCCAGCCACGCCGGATCAGGCAGAAAAACAGCCGCTGCATATTCCGTTTGCCA
TCGAACTGTATGATAACGAAGGCAAAGTGATCCCGTTGCAGAAAGGCGGTCATCCGGTGAATTCCGTGCTGAACGTCACT
CAGGCGGAACAGACCTTTGTCTTTGATAATGTCTACTTCCAGCCGGTGCCTGCGCTGCTGTGCGAATTCTCTGCGCCAGT
GAAACTGGAATATAAGTGGAGCGATCAGCAACTGACCTTCCTGATGCGTCATGCGCGTAATGATTTCTCCCGCTGGGATG
CGGCGCAAAGTTTGCTGGCAACCTACATCAAGCTGAACGTCGCGCGTCATCAGCAAGGTCAGCCGCTGTCTCTGCCGGTG
CATGTGGCTGATGCTTTCCGCGCGGTACTGCTTGATGAGAAGATTGATCCAGCGCTGGCGGCAGAAATCCTGACGCTGCC
TTCTGTCAATGAAATGGCTGAATTGTTCGATATCATCGACCCGATTGCTATTGCCGAAGTACGCGAAGCACTCACTCGTA
CTCTGGCGACTGAACTGGCGGATGAGCTACTGGCTATTTACAACGCGAATTACCAGAGCGAGTACCGTGTTGAGCATGAA
GATATTGCAAAACGCACTCTGCGTAATGCCTGCCTGCGTTTCCTCGCTTTTGGTGAAACGCATCTGGCTGATGTGCTGGT
GAGCAAGCAGTTCCACGAAGCAAACAATATGACTGATGCGCTGGCGGCGCTTTCTGCGGCGGTTGCCGCACAGCTGCCTT
GCCGTGACGCGCTGATGCAGGAGTACGACGACAAGTGGCATCAGAACGGTCTGGTGATGGATAAATGGTTTATCCTGCAA
GCCACCAGCCCGGCGGCGAATGTGCTGGAGACGGTGCGCGGCCTGTTGCAGCATCGCTCATTTACCATGAGCAACCCGAA
CCGTATTCGTTCGTTGATTGGCGCGTTTGCGGGCAGCAATCCGGCAGCGTTCCATGCCGAAGATGGCAGCGGTTACCTGT
TCCTGGTGGAAATGCTTACCGACCTCAACAGCCGTAACCCGCAGGTGGCTTCACGTCTGATTGAACCGCTGATTCGCCTG
AAACGTTACGATGCCAAACGTCAGGAGAAAATGCGCGCGGCGCTGGAACAGTTGAAAGGGCTGGAAAATCTCTCTGGCGA
TCTGTACGAGAAGATAACTAAAGCACTGGCTTGATAAATAACCGAATGGCGGCAATAGCGCCGCCATTCGGGGAATTTAC
CCCTGTTTTCTCAGGCGAGTTTCAGATTCACCACGTTGCATCACCCGTTGCAACACCTCCGCTTCCAGCTCCGCTAATCG
CACCGACCCTAATCGACGCGGACGGGGGATATCCACTGTCAAATCCAGACCAATTTTTCCCTCTTCAATTAACAGCACCC
GGTCAGCCATCGCTACCGCTTCGCTCACATCATGCGTCACCAACAGTACGGTAAAGCCGTGCTCCTGCCAAAGTGACACA
ATCAAATCCTGCATCTCGAGTCGCGTTAAGGCGTCCAGCGCCCCGAGCGGTTCATCAAGCAACAATAATCCCGGTCGATG
AATCAACGCTCTTGCCAGCGCCACTCGCTGTTTCTGCCCGCCAGAAAGTGCGGCAGGCCATTCCCCTGCGCGATTCTCCA
GTCCTACCGCAGCCAACGCTCGACGTGCGGCATCGCGCCACTGGCCTTTAAGGCCTAACCCAACGTTATCAATCACCGAT
TTCCACGGTAGCAGACGCGCATCCTGAAACATCATTCGCGTGTCTTCCTGAATTTCAGCCAGCGGTGTGGTGCCCGCTAA
CACATCGCCTGCGGTTGGCGTTTCCAGACCTGCCAGCAGGCGCAGCAGGGTACTTTTGCCACCACCGCTGCGGCCCACCA
CCGCCACAAACTGACCTGCCGGAATATGTAAATCCAGTTGGTTCAGGACGATATTTTCCGCGTAATGTTTGCTTACTGCA
TTGAGCAACAATGGCGTGCCCTGGTTCAGACGAGCAGTATTCATACCGTGGCCTCCTTCAAATGATAAGCCGGGTTCCAG
CGCAACCAGAGGCGCTCTAACAGCTGCGCGCTGACGTCAGCCAGTTTGCCGAGCAGGGCGTAAAGAATAATAGCGACTAC
CACCACGTCCGTTTGCAAAAACTCCCGCGCATTCATCGCCAGATAACCAATGCCTGAATTGGCAGAAATGGTTTCGGCAA
CAATCAGCGTCAGCCACATCAGGCCCAACGCAAAACGCACGCCGACCATAATTGAGGGCAGGGCACCAGGCAGGATCACA
TGGATAAACAGCGGTATGCCGGATAATCCATAGCTACGCGCCATCTCCACCAACCCGCGATCGATATTACGGATGCCATG
CCAGGTGTTGATATAAATGGGGAATAGCGTACCGAGCGCCACCAGAAAGATTTTTGCGGATTCATCAATGCCAAACCACA

AAATCACCAGCGGGATCAGCGCCAGATGCGGCACGTTGCGCAACATCTGAATTGAGGTATCCAGCAGCCGTTCTCCCCAG
CGAGATAGCCCGCTAATCAGTCCCAAAATCAACCCCAGCGATCCGCCAATTGAAAAGCCAATCAGCGCCCGCCAGGAGCT
GATCGCCAGATGCTGCCACAGTTCGCCGCTGGCGGAGAGCGTCCAGAACGCCGTCACCACTCCTTCCGGTGAAGGCAAAA
TACGCGTCGACAGCCAGCCAACCGAGGAGGCCAGTTGCCACACCGCCACGATGCCCACCGGTAAAAACCAGGGGGCAACG
CGCAATAACCACTTCTTCACTGGCGTTGCCATTATTTCTTCCTTAGCTTTGCGCGACTTTACGGGGGATAAAATCATTCG
CCACCGCTTCGCCTTGCGGATTCAGCGGCTGCGGCTGGGGAATTTCCGGGATGGCGACATCCAGAAGCGGGAACAGCAAC
TCGCCAACCCGATACGCTTCTTCCAGATGCGGATAGCCCGAAAGCACAAAACTGTCGATGCCAAGCGCGGCATATTCGTT
GATTCGCGCAGCGACCGTAGGACCATCGCCCACCAGCGCCGTCCCGGCACCGCCGCGCACTAAGCCAACGCCCGCCCATA
AATTGGGGCTGATCTCCAGATTGTCGCGCTTGCCGTTATGTAACGCCGCCATTCGCTGTTGCCCTACGGAATCCGTCCGG
GCGAATGCGGCCTGTGCTTTGGCGATAGTTTCATCATCAAGATGCGAGATTAACCGCTCGGCGGCCTGCCACGCTTCGTC
GTTAGTTTCACGAACAATCACATGCAGACGAATACCGAAACGAATTTTGCGTCCATGCGCGGCAGCTTTCGCCCGCACTT
GTTCGATTTTCTCTTTAACCAGTTCCGGCGGTTCGCCCCAGGTGAGGTAGAGATCAACCTGTTCTGCCGCCAGCTCCTGG
GCGACATCTGACGATCCGCCAAAGTAAAGTGGCGGATACGGCTGTTGAATCGCCGGGAAGAGCAGTTTTGCTCCGCGCAC
ATGAATATGTTTACCGTTGAAATCGACGGTTTCTCTCTGCAATAAACGCCGCCAGACCTGGGTAAATTCCGCCGAGGCTT
CGTAGCGCTCGCTATGATCAAGGAACACTCCGTCGCCTGCCAGCTCTTGTGGATCGCTGCCTGTGACCAGGTTAAACAAC
GCACGTCCATTTGAGAGACGGTCAAGCGTGGCGGCCTGGCGGGCGGCAACGGTAGGTGAGGTTACGCTGGGACGCAGGGC
GACAAGAAACTTCAGCCGCTGCGTCACCGGGATCATCGATGCGGCAACCAGCCACGCATCTTCGCAGGAGCGCCCCGTTG
GAATTAGCACACCGGTATAGCCAAGACGATCCGCCGCTTGCGCAATTTGTTGCAGATAACCGTGATCAACCGGGCGTGAA
CCTTCTTCCGTTCCCAGATAATGCCCGTCACCGTGGGTCGGTAAAAACCAGAACATATTCAGACTCATAATTGTTTTCCT
TCCAGTTGAGTGGGCTGCCAGATGCGCTGGCGAATATCGACTTTTTTCGGCACCAGACGATTTTCATAAAACAGATCTGC
CGTTTGCTGCTGTAAGGCGGCAACCTCGGCGTTAACCGGTTTGATGGTGGTAGGAGGGCGATGATCTAAGTAAGAGGCAA
TCACCGGTGCCGGTAAGCCCATCGTTTTTGCCAGTAAAGCGATGCTTTGCTCGCGCTGGCTGCGGGTTAACGCATCGGCC
TCACTAAAGGTTGCCAGTACGCCCTGAATAAAAGCGCCGTTTTTTTCTGCATAGGGACGAGCTGCCAGATAAAACGATCC
AGTTTGATTGAGATCGGTGCCGTCTTTCAGCACCCGCACGCCGCCCTGTAATAATGCAGCAGAGTAGTAGGGATCCCAGA
TAGCCCAGGCGTCAACGTTACCTTGCTGGAACGCGGCGCGGGCATCAGCGGGCGTCAGGTAAGTGGGTTGGATATCGGTA
AACTTAAGTCCGGCCTGACGCAGTGCACGCAGTAAAAGGTTGTGTGAACTGGAACCTTTCTGAAAGGCAACTTTGTGACC
TTTAAGATCGGCTACGGTTTTGATCGGGCTGTTTTCTGCCACCAGAATCACTTCGGCTTTGGGCTTCGGTGGCTCGACGC
CCACGTACACCAAATCAGCCCCGGCAGCCTGGGCAAAGATTGGCGGAATATCCCCGGTACTGCCGAGATCAATACTGCCA
ACGTTTAACGCTTCCAACATTTGCGGACCCGCGGGGAACTCCACCCAGGAGATTTTTGATTCCGGATAGCGTTTTTCCAG
TAACTGGTGGCTTTTTGCCAGCACCATACCAATACTGCCTTTCTGATAGCCTATACGTAACGCTTCAGGCGAGGATTCTG
CAGCAACCGCAAAAGTAGAGACGCTAAGCAATCCCGCCAGCGCCAGTTTAATGATGTTACGCATGGGCATTACCTCGCAG
AGACAGAAGGTCAGGAACCTGAACATCGCGGCGGTGCAATGCCTGCCAGAAAGTTTCTAGCGCGGTATCAAGACGGGTTT
GCAGATTTGGCGTGAACTGGGGTCTGTGATGGTAATCAATTACTTGTGAGTCATCGGCAAACACGCCGTGCAGGATCTCC
TGAGCTTTCAGTGCGCTTAAAACTGGTTTAAGGGCATAATCGACCGCCAGCAGATGGGCCACGGTACCGCCCGTCGCCAG
CGGTAGCACCACTTTGCCTTGCAAAGCGCGTTCTGGCAGCAGGTCGAGCAGGGTTTTCAACGCACCGGAATAGGCGGCTT
TATACACAGGCGTGGCGACAATCAGCCCATCGGCCTGTTGCAGCTGTTCGGTGAAGGTCTTGAGTGCCGGACTATCGAAA
CGAGCATAAAGTAGATCTTCCGGGGCGAAGTTTTGCAGATTCCAGTGATAAACCTCTACATCCAGGCCATTTAGTTTTTC
CCGCGCATATTCCAGCAAGGAGCTGGAGCGAGAAGGAAAGCGAGGACTACCCGCCAGGGTGATGACACGCATACTCTCTC
CTTATAACCAATTGTTCTTTTTTTGTTAACATTGATAACAATTCGGTCAGTCTGTCGGAGAGACAAGAAAATTCCAAATA
TAAATTTTGTGTATCTTTTTCTGAAAATAAACTAAAGAAAGGGCTTTATATATCACGCATATTTATTTATTGGATAGTCA
TTAGATGTTGAATCAATGAATTACTCGTTCAGTTAATATTTAAATATTCATGAAATCTATAAATTAAAGATTTGTCACTT
ATTGGATTTAGTATGCTTGATTTAAATGGCGGACAATTATTGTTATTATTAAGTCTAATGTCATTTAAAGGCATTATCAT
ATTTACGAAGAGATCAGGATGATAACGATGAAAAAAAGTGTATTGACGGCGTTTATAACTGTGGTATGTGCAACGTCCAG
CGTTATGGCTGCTGATGATAATGCTATCACGGATGGCTCAGTAACATTTAATGGTAAAGTTATTGCTCCAGCTTGTACCC
TGGTAGCTGCGACGAAAGATTCCGTGGTGACTTTGCCAGATGTTAGTGCCACGAAGTTGCAAACCAATGGTCAGGTTTCT
GGCGTGCAAATTGATGTGCCAATTGAATTAAAAGATTGTGATACTACCGTAACAAAAAATGCAACGTTCACCTTTAATGG
CACTGCGGATACTACTCAGATTACAGCGTTTGCTAACCAGGCCTCATCTGATGCTGCTACAAACGTGGCCCTGCAAATGT
ATATGAATGATGGTACAACGGCCATCACGCCAGACACAGAAACCGGGAACATTTTGTTGCAGGATGGAGATCAGACGTTG
ACTTTTAAAGTTGATTATATCGCTACGGGGAAAGCGACTTCAGGTAATGTGAATGCGGTAACAAATTTCCATATTAACTA
TTATTAATAGAACTCATTAATTGTTTTATTAATTAGTACCCCTCCAGTGTTCTGGAGGGGATATTCATATTTTTTAAGAG
TGACTATTTATGAAAACTTGCATAACAAAGGGAATTGTGACCGTAAGTTTAACGGCAATATTACTCAGCTGCTCATCCGC
ATGGGCGGCAGGTAAAGGCGGGATTGGACTTGCAGCAACACGTCTTGTTTATTCAGAAGGTGAAGAGCAAATTTCACTGG
GTGTACGTAACACCAGTCCGGATGTTCCTTATCTTATTCAGTCATGGGTGATGACCCCAGATAATAAAAAATCAGCAGAC
TTTATTATTACACCACCGCTATTTGTGCTGAATCCGGCAAATGAGAATCTGTTACGCATTATGTACATTGGAGCGCCGTT
GGCGAAAGACAGAGAAACCCTTTTCTTCACTAGCGTACGGGCAGTCCCTTCAACAACGAAGCGGAAAGAGGGAAATACCC
TGAAGATTGCCACACAAAGCGTCATCAAACTTTTCTGGCGACCAAAAGGTTTAGCGTATCCCTTAGGCGAGGCTCCGGCG

AAACTGCGTTGCACTTCGTCAGCTGACATGGTTACGGTCAGTAACCCAACACCTTATTTCATTACCCTGACAGACCTGAA
AATAGGTGGAAAAGTAGTTAAAAATCAAATGATTTCCCCCTTTGATAAATACCAATTTTCTCTGCCAAAGGGGGCCAAAA
ATAGCAGCGTAACGTATCGAACCATCAATGACTACGGGGCGGAAACGCCGCAACTCAACTGTAAATCGTAAGCCGTCTTC
AGTTAAGAGAGCGAGATGTATAGAACTCATCGACAACACAGCCTGTTAAGCTCTGGTGGAGTGCCATCGTTTATTGGTGG
GCTGGTGGTGTTTGTGTCGGCAGCGTTCAATGCACAAGCTGAAACCTGGTTCGATCCTGCTTTTTTCAAAGATGATCCCT
CAATGGTGGCCGATTTGTCTCGTTTCGAAAAAGGACAAAAAATAACGCCAGGGGTTTATCGTGTCGATATTGTTCTGAAT
CAGACAATTGTAGATACGCGCAACGTCAATTTTGTTGAGATAACGCCAGAGAAGGGGATTGCCGCCTGTTTGACGACTGA
AAGCCTGGATGCAATGGGTGTTAATACTGATGCGTTTCCGGCTTTTAAACAACTGGACAAACAAGCGTGTGTGCCATTGG
CGGAGATTATTCCGGATGCCAGCGTAACGTTTAATGTGAATAAACTCCGTCTGGAAATTTCAGTACCGCAAATCGCCATC
AAAAGTAATGCCCGTGGTTATGTCCCCCCTGAACGTTGGGATGAAGGGATCAACGCGCTATTACTGGGATATTCGTTTAG
CGGGGCTAACAGTATTCATAGCAGCGCAGACAGTGATTCTGGCGACAGCTATTTTCTGAATTTAAACAGTGGCGTTAATT
TAGGCCCATGGAGATTGCGCAACAATTCAACATGGAGTCGTAGTAGTGGCCAAACCGCAGAATGGAAGAATCTCAGCAGC
TATTTGCAACGGGCGGTTATTCCTCTGAAAGGCGAACTGACCGTAGGTGATGATTATACTGCAGGCGATTTTTTCGATAG
TGTCAGCTTTCGTGGTGTGCAGCTGGCGTCAGATGACAACATGCTGCCAGACAGCCTGAAAGGGTTTGCGCCTGTGGTGC
GTGGTATCGCCAAAAGCAATGCCCAGATAACGATTAAGCAAAATGGTTACACCATTTACCAAACTTATGTATCGCCTGGT
GCTTTTGAAATTAGTGATCTCTATTCCACGTCGTCGAGCGGTGATTTGTTAGTTGAAATCAAAGAAGCGGACGGCAGCGT
CAATAGCTACAGCGTACCGTTCTCCAGTGTGCCATTACTCCAGCGTCAGGGGCGAATCAAATACGCGGTGACACTGGCGA
AATACAGAACCAATAGTAATGAACAGCAGGAGAGCAAATTTGCCCAGGCCACGTTGCAGTGGGGCGGACCGTGGGGAACG
ACCTGGTATGGTGGTGGACAATATGCTGAATATTACCGTGCCGCCATGTTTGGTCTGGGATTTAACCTCGGCGATTTCGG
AGCAATTTCGTTCGATGCGACCCAGGCGAAGAGTACGCTAGCAGATCAAAGCGAACATAAAGGTCAGTCATATCGTTTTC
TGTATGCCAAAACGCTCAACCACTTGGGCACTAACTTTCAATTGATGGGCTATCGCTATTCGACGTCGGGTTTCTACACC
CTTTCCGACACCATGTATAAACATATGGATGGCTACGAATTTAATGACGGTGATGATGAAGATACGCCGATGTGGTCGCG
TTATTACAATTTGTTTTACACCAAACGTGGCAAACTGCAGGTCAATATCTCCCAGCAATTAGGCGAGTACGGTTCGTTTT
ATTTAAGTGGTAGCCAGCAAACTTACTGGCATACGGATCAACAGGATCGGCTATTACAGTTTGGCTACAACACGCAAATT
AAAGATCTTTCGCTGGGGATTTCCTGGAACTACAGTAAGTCCCGTGGTCAACCTGATGCTGATCAGGTGTTTGCTCTAAA
TTTTTCCCTGCCGCTCAATCTGTTGCTCCCCAGAAGTAATGATAGCTATACCAGGAAAAAAAATTACGCCTGGATGACCT
CTAACACCAGTATCGATAACGAAGGGCACACTACACAAAACCTGGGTTTAACGGAGACATTACTCGATGACGGTAACCTG
AGCTACAGCGTGCAACAGGGATATAACAGCGAGGGGAAAACGGCTAATGGTAGCGCCAGCATGGATTACAAAGGGGCGTT
TGCGGATGCCCGAGTGGGCTACAACTACAGCGATAACGGCAGTCAACAACAACTGAACTACGCTCTTTCAGGCAGTTTAG
TTGCCCATTCACAGGGCATTACCCTGGGGCAATCGCTGGGGGAAACTAACGTTCTGATTGCAGCACCAGGCGCAGAGAAT
ACTCGTGTGGCGAACAGCACCGGGCTGAAAACTGACTGGCGCGGATATACCGTTGTTCCTTATGCCACTTCTTATCGGGA
AAATCGAATCGCACTTGATGCGGCGTCGTTAAAACGTAACGTGGATCTTGAAAATGCAGTAGTCAACGTGGTTCCCACCA
AAGGGGCGTTGGTTCTGGCGGAGTTCAATGCCCATGCGGGTGCAAGGGTATTAATGAAAACATCAAAGCAGGGTATACCG
CTGCGTTTTGGCGCGATAGCGACGCTGGACGGCGTACAGGCTAATAGCGGCATAATTGATGATGATGGCTCGCTCTATAT
GGCGGGTTTACCGGCGAAGGGAACAATAAGCGTGCGCTGGGGCGAAGCTCCCGATCAAATTTGTCATATCAATTACGAGC
TTACCGAACAACAAATTAACTCTGCGATTACGCGAATGGATGCCATATGCAGATAATCTTTGGAGAAAAATGCGTGTCAT
TACTACGACTATTTTTTGCCGCCGTCTTAATGTTATGGTGCGCTCAAACCGCTGCTTATAGCGGGCAGTGTCATACTACT
CAGGGGAATCCGTATATTGGCGTCAATTTTGGCGTTAAAACCCTGGAGGAAGAAGCAAATACGGCAGGGGTAGTTAAAGA
CAAATTTTATCAGTGGAACGAATCGAATGATTATTATGTTTCCTGTGATTGCGATAAAGACAATGTCAGAAGTGGCCGAT
GGGCATTCGCCGCGGATTCACCGTTAGTCTATTTAGGCGACAACTGGTACAAAATTAATGACTATCTTGCCGCCAAAGTT
TTATTGCAGGTTAAAGGCAGTTCTCCTACTGCGGTTCCTTTCGAAAACGTGGGCACAGGGGGGGGATACCCGATGGCATAT
TTGCGACCCTGGCGGTCAACGTTTAGGTGGGCAGGGGGCAAGCGGTAATAGCGGTAGCTTTTCCCTGAAAATATTGCAGC
CGTTCGTTGGCTCGGTCGTCATTCCTCCTATGGCGCTGGCGCGATTATATGAATGCTACAACATACCCGCAGGTGATTCC
TGCACGACTACAGGTACACCGGTTTTAGTGTATTACCTGTCTGGTACGATCAATTCACTTGGCTCATGTTCCGTCAATGC
CGGAGAGACAATTGAAGTTGATTTAGGTGATGTCTTCGCTGCCAATTTCCGTGTTGTAGGGCATAAACCTCTTGGGGCCA
GAACGGCAGAACTTGCAATTCCAGTCAGGTGTAACACGGGAAACGCGGGATTAGTTAATGTCAACCTGAGTCTGACGGCA
ACCACAGACCCCAGCTATCCCCAGGCGATTAAGACGTCACGTCCTGGCGTGGGCGTGGTGGTGACCGATAGCCAGAACAA
CATTATTTCCCCTGCTGGTGGAACATTACCGCTCTCTATTCCTGATGATGCAGACAGTATCGCGCGAATGAATGTCTATC
CAGTCAGCACGACAGGTGTACCACCAGAAACCGGGCGATTTGAAGCCACGGCAACGGTGAGAATAAATTTTGATTAACGC
GTGAACGTATGAAGAAAAAAACGATATTTCAGTGCGTTATCTTGTTCTTTAGCATTCTTAACATCCATGTCGGGATGGCT
GGGCCTGAACAAGTTAGTATGCATATTTATGGGAATGTGGTCGATCAGGGCTGTGATGTCGCCACCAAAAGTGCATTACA
AAATATTCATATTGGTGATTTTAATATCAGTGATTTTCAGGCCGCGAATACCGTAAGCACTGCTGCTGATTTGAATATCG
ATATCACCGGTTGTGCCGCTGGTATTACTGGCGCGGACGTCCTTTTTAGCGGCGAGGCTGACACCCTTGCGCCGACACTG
CTCAAACTAACTGACACAGGCGGAAGCGGTGGTATGGCAACGGGGATTGCCGTGCAAATTCTTGATGCGCAAAGTCAGCA
AGAAATCCCGCTCAATCAGGTCCAGCCTCTTACGCCCTTAAAAGCCGGGGATAACACACTCAAATATCAACTTCGTTATA
AGTCCACAAAGGCGGGAGCAACGGGCGGTAATGCGACGGCGGTTCTCTATTTTGATCTGGTTTACCAGTGAAGGAGTGAT

GTTGAAACGGATAATCTGGATTCTGTTCTTATTGGGATTAACGTGGGGCTGTGAGCTATTTGCCCATGATGGCACGGTCA
ACATTAGCGGATCGTTTCGCCGTAATACATGCGTGCTGGCACAGGATAGCAAGCAAATCAATGTGCAGTTAGGCGATGTC
TCGCTGACACGTTTTTCTCATGGGAATTATGGCCCGGAGAAATCTTTCATCATTAATTTGCAGGATTGCGGAACAGATGT
CAGCACGGTTGACGTCACCTTTTCAGGAACCCCCGACGGAGTGCAGAGCGAAATGTTGTCCATTGAAAGTGGTACGGATG
CAGCCAGCGGGCTGGCAATTGCGATTCTGGATGATGCGAAGATATTGATTCCGCTTAACCAGGCGAGTAAAGACTACAGC
CTACATAGCGGTAAGGTGCCATTAACTTTTTATGCGCAATTGCGACCTGTCAATAGCGATGTGCAGTCGGGTAAAGTGAA
TGCCAGCGCGACATTTGTACTTCATTATGACTAATACGTGGAACAGATTGGCGCTCTTGATTTTCGCCGTTTTATCGCTG
CTGGTGGCGGGTGAATTGCAGGCAGGAGTGGTGGTCGGCGGAACGCGATTTATCTTTCCGGCAGACAGAGAATCGATATC
TATTTTACTGACTAATACCAGTCAGGAATCCTGGCTTATTAACAGTAAAATCAACCGCCCAACGCGTTGGGCCGGGGGGG
AAGCGTCGACAGTGCCAGCACCATTACTGGCCGCTCCGCCACTTATTCTCCTGAAGCCCGGTACGACAGGCACGTTGCGC
TTGCTGAGAACGGAAAGCGACATCTTGCCTGTGGATCGCGAAACGCTATTTGAGTTAAGCATTGCCAGCGTGCCATCCGG
CAAAGTTGAAAATCAGAGCGTAAAAGTGGCGATGCGCTCGGTATTTAAACTGTTCTGGCGACCCGAAGGTTTGCCGGGCG
ACCCGCTGGAAGCTTATCAACAATTACGCTGGACACGGAATTCGCAGGGTGTACAACTCACTAACCCAACGCCTTATTAC
ATTAACCTGATTCAGGTGAGTGTAAATGGTAAAGCGTTAAGTAATGTGGGAGTTGTGCCGCCTAAAAGCCAGCGTCAGAC
AAGCTGGTGTCAGGCAATCGCACCGTGTCATGTCGCCTGGCGGGCGATAAATGATTACGGCGGGTTGAGTGCAAAGAAGG
AGCAAAATCTGCCCTGAAACAGGTTCGGAAAACGTTTGCGTTTTTTTTGCCGCAGGTCAATTCCCTTTTGGTCCGAACTC
GCACATAATACGCCCCCGGTTTGCACACCGGGAATCCAGGAGAGTTCATGTACTACCCCTTCGTTCGTAAAGCCCTTTTC
CAGCTCGATCCAGAGCGCGCTCATGAGTTTACTTTTCAGCAATTACGCCGTATTACAGGAACGCCGTTTGAAGCACTGGT
GCGGCAGAAAGTGCCTGCGAAACCTGTTAACTGCATGGGCCTGACGTTTAAAAATCCGCTTGGTCTGGCAGCCGGTCTTG
ATAAAGACGGGGAGTGCATTGACGCGTTAGGCGCGATGGGATTTGGATCGATCGAGATCGGTACCGTCACGCCACGTCCA
CAGCCAGGTAATGACAAGCCGCGTCTCTTTCGTCTGGTAGATGCCGAAGGTTTGATCAACCGTATGGGCTTTAATAATCT
TGGCGTTGATAACCTCGTAGAGAACGTAAAAAAGGCCCATTATGACGGCGTCCTGGGTATTAACATCGGCAAAAATAAAG
ATACGCCAGTGGAGCAGGGCAAAGATGACTATCTGATTTGTATGGAAAAAATCTATGCCTATGCGGGATATATCGCCATC
AATATTTCATCGCCGAATACCCCAGGATTACGCACGCTGCAATATGGTGAAGCGCTGGATGATCTCTTAACCGCGATTAA
AAATAAGCAAAATGATTTGCAAGCGATGCACCATAAATATGTGCCGATCGCAGTGAAGATCGCGCCGGATCTTTCTGAAG
AAGAATTGATCCAGGTTGCCGATAGTTTAGTTCGCCATAATATTGATGGCGTTATTGCAACCAATACCACACTCGATCGT
TCTCTTGTTCAGGGAATGAAAAATTGCGATCAAACCGGTGGCTTAAGTGGTCGTCCGCTTCAGTTAAAAAGCACCGAAAT
TATTCGCCGCTTGTCACTGGAATTAAACGGTCGCTTACCGATCATCGGTGTTGGCGGCATCGACTCGGTTATCGCTGCGC
GTGAAAAGATTGCTGCGGGTGCCTCACTGGTGCAAATTTATTCTGGTTTTATTTTTAAAGGTCCGCCGCTGATTAAAGAA
ATCGTTACCCATATCTAATTATTTCTTCGAATTCCTCTTTATAAGATAATCAGGGCTTTATTTTCAGCCCTGGTTGTTTT
ATATTCATCGCTGTTGCTTATTTAGACATTTTGTACTTTTATTGTTGAGGTTATTAAGCGAAGCGACAATGGATTGTGTT
GCTGCGGTTTTATAGGGTAGGGGAGAGGCAGATGCGAATTAAACCAGACGATAACTGGCGTTGGTATTACGATGAAGAGC
ACGATCGAATGATGCTCGATTTAGCCAATGGTATGCTATTTCGCTCACGTTTTGCGCGCAAGATGTTGACTCCAGATGCC
TTTTCTCCCGCAGGCTTTTGCGTTGACGACGCCGCGCTCTATTTCTCTTTTGAAGAAAAGTGCCGCGATTTTAATTTATC
TAAAGAACAAAAAGCCGAACTGGTGCTGAATGCACTGGTAGCAATTCGCTATCTCAAACCGCAAATGCCGAAAAGCTGGC
ATTTTGTTTCCCATGGTGAAATGTGGGTGCCCATGCCGGGCGATGCGGCCTGCGTTTGGTTGAGCGATACTCACGAGCAG
GTCAATTTGCTGGTCGTTGAATCTGGCGAAAATGCCGCACTATGCCTGCTGGCACAACCCTGCGTTGTTATTGCGGGTCG
CGCGATGCAGTTGGGCGACGCCATTAAAATCATGAACGACAGGCTGAAACCGCAGGTTAATGTTGACAGCTTCAGCCTCG
AACAGGCAGTCTAACGCGCCAACTTAAGTGCAGTCTTCGGTACACAGCTACAGCAAAGAATGGTGCCATCATCGCCCATT
GCTGATTTTTTCAGCGGCGTGACTTCGCCTTCTAAAAGCTGAACACGGCAACTTCCACAAATGCCCGCGCGGCAAGAATA
AGGGATACGAATTCCCTGATTTTCTAATTGCTCCAGCAACACCTGTTGGTTATTTCCACGAAATGCCTGTCCCTGCCAGT
CAATATCTACATTTGCGTCCGGTTGTTGCGTGATGTTGGCGGTATCATCAGCGGCAGCTGCGCCGTAAATTTTTGCCGGA
GCCGTTGCCAGAATTTCCACCTCATCGCCAACGCGAATCACGCCGCTATTACGGGCAATTAAATTCTGACCAAAATCGAC
ATCGCCGTTATCCTGGGCAGTGCGGAAAGATTGCAATGTTTTTAATGGTTCGCCTGCCGGATGTTTTTGCCCTTTTTCTG
GGCTGACGGTGGTGAAAATACAGCGGCTACAAGGTTTAACCACATCAAACACCACATCACCAATGCGAATCACTTTCCAG
CGATCTTCTTCCCAGGCTGACGCGCCGGAAACCACCAGATTGGGGCGGAATTGCTCCATTTTTACACTGGCCGGACAACG
TTGTTGGAGATCACGTAACGAGGCTTCGTTAGCAAGAAGGTAAGGATAGCCATCAGCAAATGACAGAGGTACAGTGTTGT
GGCGTTTCACGCGCCGGGTCATTTGTGGCCCCACCCAGCGTAATTGCACTTCGCGGGAGAAAAATCCACTTAGCCATTTG
TTGATCGCGTCTGGCGCAATTCGCGCGGTAAAATGTGTGCCCCAAACTTCGGTTGGTGCGTCTTGTGTGGCGAAATCAGC
AAAACGAACATATGCGCTACTGCCATCTGGTGCGGTGAGATGCAAGCCATCATGCACAGGTGAAGGAGTAAACCGTACCA
TCTGGGGAAACTGGCGAGCGGTAATAAACGTACCGTCAGGTTCCGTGATCATAAAGATGCGATCGAAGGCCAGACCACTG
ACATCTGCCAGAGCATGTGTAAGACCAATGCCGCGCATCGATTTAACAGGATGAATAAAAAGCCGGATTAATGTCGCCAC
GGTGCGGTCCTCAAATGAAAATAAGCCCTCAACTTTATGACATGACGCGCTTATTAGCTATAATGCGCAACAATTTTCTT
AGCGCCTGTCCCATTAGGCTATTTTATTTGTCAGTTTGGCCCTGGGTTATGCTCGAAATCCTCACGTACTTGTGTACGCT
CCGGTTTCTCCGCGCTGTCCATGTCCAGACTGACTGCAACAATTACGCCTACTGCGGTAGGCTCTTAGAGTAAAAGTGAC
GATATGAATTCTCTGTTTGCCAGTACGGCCCGTGGGCTGGAAGAGCTGTTAAAAACTGAACTGGAAAACCTGGGGGCCGT

TGAATGCCAGGTGGTTCAGGGTGGGGTCCATTTCAAGGGCGACACACGGCTTGTTTACCAGAGCCTGATGTGGAGCCGCC
TGGCCTCGCGTATTATGTTGCCGCTGGGCGAGTGTAAGGTTTACAGCGATTTAGACCTCTATCTCGGTGTTCAGGCGATC
AACTGGACAGAGATGTTTAATCCTGGCGCGACCTTCGCTGTCCACTTCAGTGGTTTGAATGACACCATCCGCAACAGTCA
GTACGGCGCGATGAAAGTGAAAGACGCGATCGTCGATGCTTTCACGCGGAAAAATCTGCCGCGTCCAAATGTTGATCGCG
ATGCGCCGGATATCCGCGTTAACGTCTGGCTGCATAAAGAAACCGCCAGTATCGCTCTTGATCTGAGTGGTGATGGTTTA
CATCTGCGTGGCTATCGCGATCGTGCTGGTATTGCGCCGATCAAAGAAACCCTGGCAGCCGCTATTGTGATGCGATCCGG
CTGGCAGCCAGGAACACCGCTGCTCGATCCGATGTGTGGTTCCGGTACGTTGCTGATTGAAGCAGCGATGCTGGCGACCG
ATCGCGCACCAGGCTTGCACCGTGGGCGTTGGGGCTTTAGCGGCTGGGCGCAGCATGATGAAGCTATCTGGCAGGAAGTG
AAAGCGGAAGCGCAAACTCGCGCCCGTAAAGGCCTGGCTGAGTATAGCTCTCATTTTTACGGTTCGGACAGCGACGCACG
GGTGATTCAACGTGCACGCACTAACGCCCGTCTTGCGGGGATTGGTGAACTGATCACCTTTGAGGTGAAAGATGTCGCGC
AACTGACCAATCCGCTGCCGAAAGGGCCGTACGGCACAGTGTTGAGCAACCCGCCATACGGGGAACGTCTGGACAGCGAA
CCGGCGCTGATTGCGCTGCATAGCCTGCTGGGTCGGATCATGAAAAACCAGTTTGGTGGCTGGAATCTCTCTTTGTTTAG
TGCCTCGCCGGATCTGCTAAGCTGCTTGCAGCTGCGTGCAGACAAACAGTACAAGGCGAAAAACGGCCCGCTGGACTGCG
TACAGAAAAATTACCACGTTGCCGAAAGCACTCCAGACAGCAAACCGGCGATGGTAGCGGAAGACTACACCAACCGCCTG
CGTAAGAACCTCAAAAAATTCGAGAAGTGGGCTCGCCAGGAAGGGATTGAATGTTACCGCCTGTATGACGCCGATCTGCC
AGAATATAACGTTGCCGTTGACCGTTATGCCGACTGGGTGGTGGTGCAGGAGTATGCGCCGCCAAAAACTATTGATGCTC
ACAAAGCGCGTCAGCGTCTGTTCGATATTATCGCTGCAACCATTTCGGTACTGGGGATTGCGCCAAACAAACTGGTGCTG
AAAACCCGTGAACGCCAGAAGGGCAAAAACCAATACCAGAAACTGGGCGAGAAGGGCGAGTTTCTTGAAGTTACCGAATA
TAACGCTCACTTGTGGGTGAACCTGACGGATTATCTCGATACTGGTCTGTTCCTCGATCACCGCATCGCCCGTCGTATGC
TCGGTCAGATGAGCAAAGGCAAAGATTTCCTCAACCTGTTCTCTTATACCGGCAGCGCCACCGTGCATGCGGGATTAGGC
GGTGCACGCAGCACCACCACCGTGGATATGTCGCGTACTTATCTGGAATGGGCAGAACGCAACCTGCGTCTGAATGGCCT
GACCGGGCGTGCGCATCGCCTGATTCAGGCCGATTGCCTGGCGTGGCTGCGTGAGGCAAATGAACAGTTCGATCTGATCT
TTATCGATCCGCCAACCTTCTCTAACTCAAAACGAATGGAAGATGCGTTTGATGTTCAGCGCGATCATCTGGCGCTGATG
AAAGATTTGAAACGTCTGCTGCGTGCAGGTGGGACGATCATGTTCTCGAACAACAAACGTGGCTTCCGTATGGATCTCGA
CGGCCTGGCAAAACTGGGACTGAAAGCACAAGAAATTACGCAAAAAACGCTCTCCCAGGATTTCGCCCGTAACCGCCAGA
TCCACAACTGCTGGCTGATTACCGCAGCCTGAAAGGAATAGTAATGTCATTAATCAGTATGCATGGCGCATGGCTGTCGT
TCAGCGACGCGCCGCTTCTCGATAACGCAGAACTGCATATCGAAGATAACGAACGTGTTTGTCTGGTGGGCCGCAACGGC
GCAGGCAAATCGACGTTAATGAAAATCCTCAACCGTGAACAAGGGCTGGATGACGGTCGCATTATTTACGAGCAAGATTT
GATTGTAGCGCGTCTGCAACAGGATCCGCCGCGTAACGTTGAGGGTAGCGTTTATGATTTCGTTGCCGAAGGCATTGAAG
AACAAGCGGAATATCTGAAACGCTATCACGATATTTCGCGCCTGGTGATGAACGACCCGAGCGAGAAAAATCTCAACGAA
CTGGCGAAGGTTCAGGAACAGCTGGATCACCACAACCTGTGGCAGCTGGAAAACCGCATCAACGAAGTGCTGGCGCAACT
GGGGTTAGATCCTAACGTTGCGCTGTCGTCGCTTTCCGGCGGCTGGTTGCGTAAAGCGGCATTAGGACGCGCGCTGGTGA
GTAATCCGCGCGTGCTGTTGCTTGATGAACCGACAAACCACCTGGATATTGAAACCATCGACTGGCTGGAAGGGTTTTTG
AAAACTTTCAACGGGACGATTATTTTCATCTCCCACGACCGTTCGTTTATCCGCAATATGGCGACGCGCATTGTTGATCT
CGATCGCGGCAAGCTGGTGACCTATCCAGGGAATTACGACCAGTACCTGCTGGAAAAAGAAGAAGCCCTGCGCGTGGAAG
AATTACAAAATGCCGAGTTCGATCGCAAACTGGCGCAGGAAGAGGTGTGGATCCGCCAGGGGATCAAAGCACGCCGTACC
CGTAATGAAGGCCGCGTACGCGCCCTGAAAGCGATGCGTCGCGAACGTGGTGAACGTCGCGAAGTGATGGGTACCGCAAA
GATGCAGGTGGAAGAGGCCAGCCGCTCCGGTAAAATCGTTTTCGAAATGGAAGACGTTTGCTACCAGGTTAACGGTAAGC
AACTGGTGAAAGATTTTTCTGCCCAGGTTCTACGTGGCGACAAAATTGCCCTGATTGGTCCGAATGGGTGCGGCAAAACC
ACGCTGCTAAAACTGATGCTCGGTCAGCTTCAAGCGGACAGCGGGCGTATTCACGTTGGCACCAAACTGGAAGTGGCTTA
TTTCGATCAGCACCGCGCGGAACTGGATCCCGATAAAACGGTGATGGATAACCTTGCCGAAGGTAAGCAAGAGGTGATGG
TTAACGGCAAGCCACGCCACGTATTGGGCTATTTGCAGGACTTTCTGTTCCATCCGAAACGGGCGATGACGCCGGTACGT
GCGCTTTCTGGCGGTGAGCGGAACCGCTTGCTGCTGGCGCGTTTGTTCCTCAAACCAAGCAACTTATTGATTCTTGACGA
ACCGACCAACGATCTTGATGTCGAAACGCTGGAACTGCTGGAAGAACTGATCGACAGCTATCAGGGCACGGTATTGCTGG
TTAGCCACGATCGTCAGTTTGTCGATAACACCGTTACAGAATGTTGGATCTTCGAAGGCGGCGGTAAAATTGGTCGTTAT
GTCGGCGGTTATCATGATGCCCGTGGTCAGCAAGAGCAGTATGTGGCGCTCAAACAGCCTGCGGTGAAAAAAACCGAAGA
AGCCGCCGCGGCAAAAGCAGAAACTGTAAAACGCAGCAGTAGCAAACTAAGCTATAAATTGCAGCGCGAACTGGAGCAGC
TACCGCAATTGCTCGAAGATCTGGAGGCGAAGCTGGAAGCCCTACAGACGCAAGTGGCGGATGCTTCCTTCTTCAGTCAG
CCGCATGAGCAGACGCAAAAAGTGCTTGCTGATATGGCTGCTGCAGAGCAGGAGCTGGAGCAAGCCTTTGAACGCTGGGA
GTATCTTGAAGCGTTAAAAAATGGTGGCTGATCGCAAAATAGTCGATTTAAACAGAGACAGCTGACAACGGTAAAATTGC
CTGATGAACTGGGTTTATCAGGCATGAAAACCAACACTTTGTTAGTCATCTGTAAAATAGCGCATCATTAAGGAGTACCA
ATGTGCGAACATCATCATGCCGCGAAGCACATCCTGTGCTCGCAGTGTGACATGCTGGTGGCGTTACCGCGCCTTGAGCA
TGGTCAGAAAGCGGCATGTCCCCGGTGTGGCACAACGTTAACCGTGGCGTGGGATGCCCCTCGGCAGCGTCCCACCGCCT
ATGCGTTGGCTGCACTGTTCATGCTGTTGCTGTCCAACTTGTTTCCTTTTGTGAATATGAACGTTGCAGGAGTTACCAGT
GAAATTACATTACTGGAAATTCCCGGCGTGCTTTTTTCTGAGGACTACGCCAGCCTCGGCACCTTTTTCCTGTTGTTTGT
GCAACTGGTTCCCGCGTTTTGTCTGATAACCATTCTGTTACTGGTGAATCGCGCGGAATTACCGGTCCGTTTAAAAGAGC

AACTGGCACGGGTGCTTTTTCAACTCAAAACCTGGGGAATGGCGGAGATTTTCCTCGCGGGTGTGCTGGTCAGTTTCGTT
AAACTGATGGCTTACGGCAGCATTGGGGTAGGCAGCAGCTTTCTCCCCTGGTGTTTATTTTGTGTCCTGCAACTGCGCGC
TTTTCAGTGCGTTGATCGTCGCTGGTTATGGGACGACATCGCCCCGATGCCAGAACTGCGCCAGCCGCTAAAACCAGGCG
TCACGGGGATACGTCAGGGGCTGCGTTCGTGCTCCTGTTGTACGGCAATCCTTCCTGCTGATGAACCCGTGTGCCCGCGT
TGTAGTACCAAAGGGTACGTTCGGCGTAGAAACAGCCTGCAGTGGACACTCGCGCTGCTTGTAACGTCCATCATGCTGTA
TCTTCCGGCTAATATTTTGCCCATCATGGTGACGGATTTATTAGGCTCGAAGATGCCGTCGACGATTCTCGCTGGGGTCA
TTCTGTTATGGAGCGAAGGCTCTTATCCCGTCGCTGCGGTGATCTTTCTGGCCAGTATTATGGTGCCAACGTTAAAGATG
ATCGCCATCGCGTGGCTGTGTTGGGATGCCAAAGGGCATGGCAAGCGCGACAGTGAAAGAATGCATTTGATTTATGAAGT
TGTTGAGTTTGTAGGCCGCTGGTCGATGATTGACGTTTTCGTTATCGCGGTGCTCTCGGCGCTGGTGCGTATGGGAGGTT
TAATGAGTATTTATCCGGCAATGGGTGCATTAATGTTTGCTTTAGTCGTCATAATGACAATGTTTTCTGCTATGACGTTT
GACCCGCGTTTGTCGTGGGATCGTCAACCTGAATCAGAGCATGAGGAGTCCTGACAGTATGGAATCTAATAATGGGGAAG
CCAAAATCCAGAAAGTGAAGAACTGGTCTCCCGTGTGGATATTTCCTATCGTCACGGCGCTCATTGGGGCCTGGGTTCTT
TTTTATCATTACAGCCATCAGGGACCGGAAGTGACCCTGATCACCGCGAATGCGGAAGGAATTGAAGGTGGCAAAACCAC
CATTAAAAGCCGTAGCGTTGACGTCGGCGTGGTTGAAAGCGCCACACTGGCTGATGATTTGACGCACGTTGAAATCAAAG
CGCGGCTGAATTCCGGTATGGAAAAATTGCTGCATAAAGACACCGTCTTTTGGGTGGTGAAACCGCAGATTGGTCGCGAA
GGGATTAGCGGCCTGGGAACGCTGCTGTCTGGAGTTTATATCGAACTGCAGCCAGGCGCGAAAGGCAGCAAAATGGATAA
ATACGATTTGCTGGACTCGCCACCGTTGGCCCCGCCTGATGCGAAAGGTATCCGTGTGATTCTCGATAGCAAAAAAGCCG
GGCAGCTCTCGCCAGGAGATCCGGTGCTGTTCCGTGGCTATCGGGTAGGTTCGGTTGAAACCAGCACCTTCGATACACAA
AAACGCAATATCAGCTATCAACTGTTCATCAATGCACCTTATGACCGACTGGTGACCAACAATGTTCGCTTCTGGAAAGA
TAGTGGCATTGCGGTTGATCTGACGTCAGCAGGGATGCGTGTGGAGATGGGCTCATTGACAACGCTGCTGAGTGGCGGTG
TCAGCTTTGATGTGCCGGAAGGTCTGGATTTAGGGCAGCCAGTGGCACCGAAAACAGCTTTCGTTTTGTATGATGATCAG
AAGAGCATTCAGGATTCGTTGTACACCGATCACATTGATTATCTGATGTTCTTTAAAGATTCGGTACGCGGTCTGCAACC
GGGAGCTCCGGTAGAGTTCCGGGGTATTCGCCTGGGTACCGTAAGCAAAGTGCCATTCTTTGCGCCGAATATGCGTCAGA
CATTTAACGATGATTACCGTATTCCGGTACTGATTCGTATCGAGCCAGAGCGGCTGAAAATGCAGCTTGGCGAAAATGCG
GATGTTGTTGAGCACCTTGGCGAATTGTTGAAACGTGGTTTACGCGGATCGCTGAAAACCGGAAACCTGGTCACTGGTGC
ACTGTATGTTGATCTCGATTTCTATCCAAATACGCCTGCAATAACCGGTATTCGTGAATTTAATGGTTATCAGATTATCC
CGACCGTTAGCGGCGGCCTGGCGCAAATCCAGCAACGACTGATGGAAGCGTTGGATAAGATCAACAAACTGCCATTGAAT
CCGATGATTGAACAGGCAACCAGTACGCTTTCTGAAAGTCAGCGCACAATGAAAAACCTGCAAACGACGCTGGATAGCAT
GAACAAGATCCTCGCTAGCCAGTCGATGCAGCAGTTGCCGACGGATATGCAGTCAACGTTGCGTGAATTGAATCGCAGCA
TGCAGGGCTTCCAGCCTGGCTCCGCAGCCTACAACAAGATGGTGGCGGATATGCAGCGCCTTGATCAGGTGTTGCGAGAA
CTGCAACCGGTGCTGAAAACGCTCAATGAGAAGAGTAACGCGCTGGTATTTGAAGCGAAGGACAAAAAAGATCCAGAGCC
GAAGAGGGCGAAACAATGAAAAAGTGGCTAGTGACGATTGCAGCACTGTGGCTGGCCGGATGCAGCTCCGGCGAAATTAA
TAAAAACTATTACCAGTTACCTGTGGTGCAGAGCGGTACACAAAGTACCGCCAGCCAGGGCAATCGTCTGTTATGGGTAG
AGCAGGTCACTGTTCCTGACTATCTGGCGGGGAATGGTGTGGTTTATCAAACCAGTGATGTGAAGTATGTGATTGCCAAC
AACAACTTGTGGGCCAGCCCGTTGGATCAACAGTTGCGCAACACCCTGGTTGCCAACCTGAGTACGCAACTGCCCGGCTG
GGTGGTTGCCTCCCAGCCTCTGGGAAGCGCCCAGGACACGCTCAATGTTACCGTAACGGAGTTTAACGGTCGCTATGATG
GCAAGGTCATTGTCAGTGGTGAGTGGCTGTTGAACCACCAGGGACAACTGATCAAACGTCCGTTCCGTCTGGAAGGAGTG
CAAACTCAGGATGGTTACGATGAGATGGTTAAAGTGCTGGCCGGTGTCTGGAGTCAGGAAGCCGCTTCTATTGCACAAGA
GATAAAGCGTCTACCTTAATTATAAAGATTTGTAAATATAACCGTCTCCGGTATGTTGCCTGAGGCGGTTTTTTTGTCTC
TAACGTGCGGAAAAAATTTGTTCCTCTTCACATTTTTTGTACAACCGACATGCCCGTGTAGCTCACAAATATGACAGTGGC
GTGAATTTTGCGCATTGACGGCAGTTATGATTCGCGGTATTGCTTAACTGTGATTGCACATTTAGTAATCACTGTTTTCT
TTTCCACCAGAAACCAGTATGAGGGAAACGAGGCATGAAGAGACAAAAACGAGATCGCCTGGAACGGGCACATCAACGTG
GTTATCAGGCCGGCATCGCCGGACGCTCAAAAGAAATGTGTCCCTATCAGACGCTGAATCAAAGGTCACAATGGCTGGGA
GGCTGGCGAGAAGCCATGGCGGACAGGGTAGTAATGGCCTGATTCTGTCTCTTTAAAAAGAAACCTCCGCATTGCGGAGG
TTTCGCCTTTTGATACTCTGTCTGATTATAATCAGAAGGCAGACGTATCCTGGAACAGACCGACTTTCAGGTCGCTGGCG
GTATAGATCAGACGACCATCAACCAGCACTTCGCCATCCGCCAGGCCCATAATCAGACGACGGTTAACAATGCGTTTAAA
GTGAATACGGTAGGTCACTTTTTTCGCTGTCGGCAGTACCTGACCAGTGAATTTCACTTCGCCAACGCCCAGCGCGCGGC
CTTTACCTTCGCCGCCCAGCCAGCCGAGGTAGAACCCTACCAGCTGCCACATTGCGTCCAGGCCCAGGCATCCCGGCATA
ACCGGATCGCCAATAAAGTGGCATCCGAAGAACCACAGATCCGGATTGATATCCAGTTCTGCTTCAACATACCCTTTGTC
GAAGTTACCACCCGTTTCGGTCATTTTGACCACACGGTCCATCATCAGCATGTTCGGTGCTGGCAATTGCGGGCCTTTAG
CGCCAAACAGTTCACCGCGACCAGAGGCAAGAAGGTCTTCTTTTGTATAGGATTCGCGTTTATCTACCATGTTCTCTGTA
AGCCTTATTTTATTGAAGCACGCAGGATAGCTAACACGTGTACGCTGAACAAGTCCGATCAGTTCGGAATAAACCAGTTC
AGCCAACGTAATGGCCATGGAAAACGGTGACGTCCTTCCTGTTGCGATGCTTGCGCGATACGTTCCTGGATGGTTTGCAT
CAGCGTCGTTTGGCCTTCGCCATCCCACACCAGATTTAATAATAACGGCAGTGCGTCAGTCACATCGTCTACTGCCCAGA
TGGTGAATTTGCCTTCTTCTACCGCTTTCACCAGTTCACTGTGAAGACTTAAATGGCGAACGTTAGCTGTGGGGATAATG
ACACCTTGTTTCCCGGTTAACTCACGTTGCTGGCAAATAGCAAAGAAGCCTTCGATTTTCTCATTTAAACCACCGACCGG

CTGGGCGCGACCGAACTGATCGACTGAACCTGTGATAGCGATACTCTGATTCACCGGCACATCGGCGAGGGCGCTTATCA
GGGCGCAGAGTTCAGCCATCGAGGCACTATCTCCATCAACTTCACTGTATGACTGCTCAAATGTCAGCGATGCTGAGAAG
GGGATCTGTTGCTCAAGCTGTAGTTCCGACATCAGGAACGCTTGCATGATCATCATCCCTTTCGCATGGATATTGCCGCC
AAGCTCCGCTTTGCGTTCGATGTCGGTGAATTCACCATCGCCAATATGCACAACGCAGCTAATGCGAGAAGGTTCGCCAA
AAGCGCGTGGATGACCCGGAAATTCAATGACCGAAAGGGCGTTAATTTGCCCGATGCGTTCGCCTTCGGTTTCAATCAGG
ATTTGCTCCTGAAGGATCTCATCCTGCATACGTTCAGCGAGGAAACCTTCGCGCCATTCACGCTGCTGCAGCATTAAGTT
TAGCTGCTCGCCGGAGAAGGTGTCGCCATCACACAGGGAGGCGACCTCTTTACACTGGCGGAGGATCCACTGCGGGCTAA
GCGGAAGTGTTTCTTGTTCACCGGTGTAGCGTGCTGCTTCGCGGATAAGTATCGGCCAGGCATCCGCTCCCGGTGCAGGT
AAGTGATTATGTCTGGCGGTAAATGTCACCCAGCGACACCACTGGGTTACTGACTCCGCATCGACAATCTGCAGAGTATC
TTCAAATTCGCTATAAATAGCCTGCTCTGAAAGCTCTGGCTCCATCTCCTGGAAATCAGCCAATGATTCGCGTTCGCCTA
CCAGAATGACTTTCAGCTTCAATGGCATCGAAGGCACAGAGACGGGGAGAGGGCGCGACTCATCAAACGCAACCCAGTCA
AAACGCTCGCGGTTAACGATATTTTTCAGCCGCATCCACAGCAGAGGTTGCGCCAGCAGTGTACGCAAAGAGATAATGAG
AATACCGCCATTTGCCTGATGCACCAGACCAGGCTGCAGGGTAATGTCGCCATTAAACTGGCGCAGGCAGCCAAAGAGTT
GCTCCGCTTCTACCCAGTCGGCAGCGACAACTTGCGTTAAAGTCGCAAAATTATCATCTGCACTCACTGCGTGACGTAAG
CGGATGGAGTGGCCGGAAACCTCATAGTGACCGCCCACCAGTTGGCCTGCATCGCTTTGTAGCGTACGCGCGGCATTGGC
AATAAGATTCAGATACTCAGACTCTTCCGGGGCCTTCGCCAGCATAAAAGAGGAGGATGCTCGCGTATGCAGCAACTGCT
CCAGCGCAAATTGCAGCCGCGGTTGAGTATCACTGAATAAAGGATCGTTTTCGTCAATCAAATGTGGCTGAGCAAATATT
TCCTGATAGCTATCGGTATCAGGAACCAGGTCACGCCATGCAAGTTTCGTAATGGTCAAAGTTGATGTTTTTTAGTCTGT
TGTCAAAGCCGCGATTATACCGTAACCGGCACTACAGCACACGTAGAAAGCACCGACAATACTCCTGGCATGGGCGTTAA
AGCTCACAGGATGGAGATTCTTTTCTTCACTGGCCTAAAAAGCTGATATTCTGTAAAGAGTTACACGGTAACATTGAGAT
CGCTATGAAATATCAACAACTTGAAAATCTTGAAAGCGGTTGGAAATGGAAGTATCTGGTTAAGAAGCATCGCGAAGGGG
AGTTAATCACCCGTTACATAGAAGCCAGTGCCGCCCAGGAAGCCGTCGATGTGTTGCTCTCGCTGGAAAATGAACCCGTG
CTGGTAAATGGCTGGATTGACAAGCATATGAATCCGGAACTGGTCAATCGCATGAAGCAAACCATTCGGGCAAGACGAAA
GCGCCATTTTAATGCGGAACACCAGCATACGCGCAAAAAATCGATCGATCTGGAATTTATTGTCTGGCAACGTCTGGCTG
GTCTTGCGCAGCGTCGCGGTAAAACGCTTTCTGAAACGATTGTTCAGCTGATTGAAGATGCGGAAAACAAAGAGAAATAC
GCGAATAAAATGTCTTCTCTGAAGCAGGATCTGCAGGCATTGCTGGGTAAGGAATAACTGACGAAAGTCAGTTCAATTTA
CTAAAGGCAAAAAAAACCCCGCAGCAGCGGGGTTTTTCTACCAGACGAGAACTTAAGCCTGCGGCTGAGTTACAACGTCT
TTGATACCTTTAACTTCGATCTCTACGCGACGATCCGGAGCCAGGCAGTCGATCAGTGCAGCACGCTGTTTCACGTTGTC
ACAGGTGTTGCCAGTAACCGGGTTGGATTCGCCCATACCACGTGCGGAGATCTTGTCTGCCGGGATACCTTTGGAGATCA
GGTAATCAACAACAGACTGAGCACGGCGCTCGGACAGACCCTGGTTGTAAGCGTCAGAACCGATGCGGTCGGTGTAACCC
AGAACAACTACGGAACCGTCTTTCGGATCCAGGTTGCTCAGCTGGCTGTACAGCTGATCCAGAGCAGCCTGACCTTCCGG
TTTCAGGGTTGCTTTGTTGAAGTTGAACAGAACGTCAGACTTCAGAGTGAAGTGCTTGGTCTGTACTTCCGGTGCCGGAG
CTGGAGCCGGAGCAACTACTGGAGCTGCTTCGCCCTGACCGAAACGGTAGGAAACACCCAGGCTCAGCATGCCGTTGTCC
GGACGAGTGCCGATGGTGTGTGCGTCACCGATGTTGTTGGTCCACTGGTATTCCAGACGGGTAGCGATTTCAGGAGTGAT
CGCGTACTCAACACCGCCAGCGAAGACCGGAGAAACGCCGGTGTCGTGGTTTTTACCATAAACGTTGGATTTAGTGTCTG
CACGCCATACCATGCCACCCAGACGAGTGTAGATGTCCAGGTCGTCAGTGATTGGGTAACCCAGTTTAGCGGTCAGTTGA
ACGCCCTGAGCTTTGTATGCACCGTTTTCAACGCTGCCTTTGTACGGCATACGACCTAACCAGTCGTAACCCATTTCAAA
GCCAACATACGGGTTAACCTGGTAACCACCAAAAGCACCAGCGCCCAGTTGGTTTTCATGGGTCGGCCATTGTTGTTGA
TGAAACCAGTGTCATGGTACTGGGACCAGCCCAGTTTAGCACCAGTGTACCAGGTGTTATCTTTCGGAGCGGCCTGCGCT
ACGGTAGCGAAACCAGCCAGTGCCACTGCAATCGCGATAGCTGTCTTTTTCATTTTTTGCGCCTCGTTATCATCCAAAAT
ACGCCATGAATATCTCCAACGAGATAACACGGTTAAATCCTTCACCGGGGGATCTGCTCAATATTAACTCTACCGATATC
TTCGGCTTATGCCGAGCACCCCTGGCGATGTAAAGTCTACAACGTAGTTGAAAACTTACAAGTGTGAACTCCGTCAGGCA
TATGAAAAAAAAGTCTTGTATAAGGTATGTTTAATCTTTTTTGTCAGCGACAATTTACAGAAGAGAATCGCGGAAACCGC
TTCAGACAAGCCTCCGCAAGGAAAATTAGTCACGACTGAAAGCATTGGCTGGGCGACAAAAAAAGTTCCAGGATTAATCC
TAAATTTACTTAATGATACAAATTAGAGTGAATTTTTAGCCCGGAAAGTTGTCTCGTGGCGTGAGAGGATGCGCTTACCG
GACGCATAATAAACCCCATAGCGTTACCTTCATTTGCCGCATCAACAAGTTCAGCATGCTCTTCTTCAGTCAAATCATCT
GCCAACCAACCGATCACCACACTGTAATTGCCCGTGCGTAAAGCGCGAACCATTGACTCCACAGTGTGGCAAGGGGAGAG
CTGGCTAATCTGCATTACTTTCGTTAAGGGTAGCCCAGATGCCTGAACCCATTCCCGACTCAGTTTTTGTTGCGGTGTTA
ACCAGAGTTGCCAGCGCGATTGCTGACCGAGTTGCTGTAACAATGGCAACAGTAGAAGTTGCGTCATCATGGGCTGATCT
TCGCGATAGACAACTTCACTGATAAGCCCGGCTGTAGTGTTTTCCGTAGAGACACGCGCAATTTTACTTGCTGCGGATGA
GAACGACGAAGAACGATGTGCATAGCCTGAAGTGTACATAATCAATCCAGCCCCTGTGAGTTACTGTATGGATGTACAGT
ACATCCAGTGACAACAAAGATCAACCCTATTTTCGGAAAGAGCCTCGCAAATTTTGTCGTTGGTGACGGGAAAACATAAA
TTAATCTTGCCCCTTAAGAATAAGTTGCCTATTTTCGTAGTTAACGGATCCGTTAATGTGAATCATTCTTTTATGTTATG
ATTTTAAAAGGAATTTTATGAAAAGCCTCTCCTATAAGCGGATCTATAAATCACAAGAATACCTGGCAACGTTGGGCACA
ATTGAATACCGATCATTGTTTGGCAGTTACAGCCTGACCGTTGACGACACGGTGTTTGCGATGGTTTCTGATGGTGAGTT
GTATCTTCGGGCTTGTGAGCAAAGTGCACAGTACTGTGTAAAACATCCGCCTGTCTGGCTGACATATAAAAAGTGTGGCC

GATCCGTTACCCTCAATTACTATCGGGTTGATGAAAGTCTATGGCGAAATCAACTGAAGCTGGTGCGTCTGTCGAAATAT
TCTCTCGATGCAGCGCTGAAAGAGAAAAGCACGCGCAATACCCGGGAAAGACTGAAAGATTTGCCCAATATGTCTTTTCA
TCTGGAAGCGATTCTCGGGGAGGTGGGGATTAAGGATGTACGGGCGTTACGTATACTTGGGGCAAAAATGTGTTGGTTGC
GACTGCGGCAGCAAAACAGTCTGGTGACAGAAAAGATTCTGTTTATGCTTGAAGGTGCCATTATCGGCATTCATGAAGCT
GCGCTCCCGGTGGCACGCCGCCAGGAGCTTGCAGAATGGGCTGACTCTCTTACGCCGAAACAGGAGTTTCCTGCGGAACT
TGAGTAATCTGGCGTTGCAGACGACCAATCTCAGGCAGTAATGCAATCAATAATCCAACTTGTTGTACGACCAGAGGTTC
TTTGCTGTCTGCCCGTGGTTCAAGTTGCTGCATCCGCTGTTTCAGGCTAGCTAATGCCTCATTGACGCGTTCTTCATCAG
CAGGTTGATGATGTAACGCGTCATCAACATAGCAAACTGCGTCATCAAGAAACGCCAGAATTTCAGGATTAGTTAACTGC
TCCCGGTGAGCACCGAGGGCTGAGATATAGCTGGTAAACGTATGGTTAAGGCACAGCAACCGAAACGCGGCTTCGCGAAT
TTGCGGGGTAACGTTCGGCTCGCTGGACATATTTGATACCACCGACGCCAGCTCAGCATCACGGTTGTGTGCATCGCGGC
GGGCAATACGATACGCCAGACGGTTATCACGCCCCTGATGGTATTGCTCCAGTATGGCATCGAGATACCGACAGTTGGCC
TCTGTGGCGCGTTCGAGCATGCGCGGCAGATTGCGAAACTGCCAGTCAGGCCAGATGTAGCTCACTGCCGCCCACGCAAT
GGCACAACCAATCAGCGTATCGATTACGCGAGGTAACGCTACTTCAAAACCTTCACCCAGTAAGTTAAAACACAGTAGCA
CCAAAAGTGTGATGAACATCGTTGCATGAGCGTATTGCACGTTACGGAAGGCAAAAAAGAGCACGCCGGTAATAACCAGC
AGCACCAGCTGCCCTTCCAGTGATGGCACAAACCACAGCACAGGAATGCCAATGGCGATACCTACCAGCGTACCAATAAT
CCTTAACTTCAGGCGGTGGCGCGTGGCGTTATAGTTTGGCTGGCAGACAAACAAACTTGTCAGCAAGATCCAATACCCGT
GATGCATTCCGGTTATCTGAATGATGGCGTAGCCGAAGCACAACACCAGCGACATTCTTACCGCATGACGGAAGAGGGCG
GATTCCGGCGTGAAGTGACGGCTAAGACGCAGCCAGATATCACTCAACCCGTGCGGGCTGTCATCAGCGAGCTCATTTTC
GTCATTATTATGGGGTAGTGCCTGGGCCTGTTCTGATTCAATTGTTGCCAGTTGGGCATCAATGGCGCGTAAATTGTTCA
GCAAAAATCCCAGTGTTTTGAGTAAATCGGCGGGTGCGCCGTTATCGCGCATCCGCTCCAGCGCAGCATCAATATGCGTA
AAAGCGCGCTCAAAATGCGGATCATGTTGATAAGGCTGACGCAACAAAATACAGCGTGACAGTTGCTGGCACGCCTGGCC
CTGCATCGACATCAGCCGCTGAAAACGGAACAGCACGTCGCTGTGGCGAAAATGTTCACGCAATGTTTGATACTGAATAT
GAGAAGAGCTGGCACGCTCGTGAATATCCTGTGCGACAAAGTAATAATGCAGCGTGCGACGCGTTCCCCGTTGACCACGA
TCGCCACGTAAGCGGGTCAGCAGCGAGAGTTTCGTCTGATTCAATGTCGCCATCAGCAGACCGTTGGCGAGAGCCAAATC
GTACAGCGGTGCCTGGCTTTGATCTTCAATATCAGGATCAAACATGCGCGACTTGAGCTCAAGATAACGCGCCAGTTGTT
CATAGCAACGCGCCAGGTTGTCCTGCAGCGGGCGGACCGGGAACAGCAGATGACCAATAAGTGTCAGGACGTTGTACCAG
ACGGCACCGGCCAGCAGATACATCGGCTGCTGATACCAGTGCTCATACAGTGATGTTCCCAACATAGTGTAAATGGCGAT
CAGCAATGCACCGAAGGCAATTGTTGCATAGCGTTGACCCAGACCGCCGAGCAAAATGAAGCCGCTGGTAGAGAGCGTTA
AGCCAATCGCAAATAGCCAGGGCCAGGGAAACAGCAATTCTACTGAGGCCGAGGCGATAAAAAAGCAGAACAGCGTAATG
ATGAGGTTACGCAAACGTCCCGCCAGTCGGTCATCGAGATCGGTCAGCGCCGCTGCCACCATCCCCAGCGTTAGCGGAAT
CGTCAGTTTTACATCACCCAGCCACCACGGAAACGCTGTGGTTCCACAAAGCGCAATAAAAATACGCGCGTAATACAGCC
AGGCGCTGTTCCAGGTATAGCGTTTGAGCAAAGGACTTAGCATAAAGGCCATATTAGTGGATTATTCAAAACGACGACGT
GCATTGGCTTCACGCGCAGCTTGCGCTGTTTCTACCGATACCACGCGACGACCAACCGGCCATAGTGCAATAGCGGCAAT
TTTAAAGTTCGCAATGCCGACAGGAATGCCAATGATTGAAATACATTGTGCGATGCCCGTTGCAATGTGCATCAGGCATA
ACCACCAGCCAAAGAAAATCAGCCAGAAAATATTCAATACCGTACCGCCAGTATTCAGCAGCACATTTTTGCCAGCCGGG
TTCAGTTCATCGACATGAATAGCTTCATTGCCATAAGGCACCAGAGACAGTTTAGTGATCTCCCAGCAGGATCGTGTCAG
CGGTAAGGTAAAAATCAGCACAATACTGACCAGAGTCGCCAACAGCCAGCCCAGAGTGGTGGCAAATCCGCCAAGCACAA
AGTTCAGAATGTTCAAAACGGTACGCATAAAACCTCGCTTTACTGTGGTTTTCAGTAATGGGCGGCAATTGTAACGTTTT
TTTGGGCTGGAGCACGTTTTCTCTGACGGTTACACTGATAAGAAATAATTTCGTGTGGATCTACAGAGTCATGGAACTGA
AAGCGACAACGCTTGGAAAACGTCTGGCACAGCACCCTTACGATCGGGCGGTGATCCTCAATGCCGGGATTAAAGTCTCC
GGCGATCGCCACGAATACCTTATTCCTTTCAATCAATTACTGGCGATTCACTGTAAGCGCGGTCTGGTATGGGGCGAGCT
GGAATTTGTACTGCCGGACGAAAAAGTGGTGCGTCTGCACGGCACCGAATGGGGCGAGACGCAGCGTTTTTACCATCATC
TTGATGCTCACTGGCGGCGGTGGAGTGGCGAGATGAGCGAAATTGCGTCTGGTGTTTTACGCCAGCAACTGGATTTGATT
GCCACGCGCACTGGGGAAAATAAATGGCTGACGCGTGAGCAAACCTCTGGCGTGCAGCAACAAATCCGCCAGGCTTTGTC
GGCGTTGCCGTTGCCGGTTAACCGACTGGAAGAATTCGATAACTGCCGTGAGGCGTGGCGTAAATGTCAGGCCTGGTTGA
AAGATATTGAAAGCGCTCGGTTGCAGCATAACCAGGCGTATACCGAAGCCATGCTTACCGAGTATGCGGATTTTTTCCGC
CAGGTCGAGTCTTCACCGCTGAATCCGGCGCAGGCCCGGGCAGTCGTTAATGGCGAGCATTCTCTGTTAGTGCTGGCAGG
TGCAGGAAGCGGAAAAACGTCGGTGCTGGTGGCCCGTGCAGGCTGGTTGCTGGCGCGTGGTGAAGCGTCCCCTGAGCAAA
TTTTATTGCTGGCGTTTGGTCGCAAAGCCGCTGAAGAGATGGACGAGCGGATTCGCGAACGGCTACATACCGAAGACATT
ACCGCACGCACGTTTCATGCGCTGGCGCTGCATATTATTCAGCAGGGCAGCAAAAAAGTTCCGATAGTCAGCAAACTGGA
AAATGATACCGCTGCCCGTCATGAACTCTTTATTGCTGAGTGGCGCAAGCAATGCAGCGAAAAGAAAGCGCAGGCGAAGG
GCTGGCGGCAATGGCTGACGGAAGAAATGCAGTGGTCAGTGCCAGAAGGTAACTTCTGGGATGATGAAAAATTACAGCGT
CGCCTTGCCTCTCGCCTCGATCGCTGGGTAAGTCTGATGCGGATGCACGGTGGTGCACAGGCAGAAATGATTGCCAGTGC
ACCCGAAGAGATTCGCGATCTGTTCAGTAAACGTATCAAGTTGATGGCCCCGTTATTAAAAGCCTGGAAAGGTGCGCTGA
AGGCAGAAAACGCTGTCGATTTTTCGGGCCTTATTCATCAGGCGATTGTGATTCTGGAGAAAGGTCGCTTTATCAGCCCG
TGGAAGCATATTCTGGTTGATGAATTTCAGGATATCTCGCCGCAGCGGGCAGCGTTGTTAGCGGCATTACGCAAGCAAAA

CAGTCAGACGACGTTGTTCGCTGTTGGTGATGACTGGCAGGCGATTTACCGATTCAGCGGTGCGCAAATGTCGCTCACCA
CCGCTTTCCATGAAAACTTTGGTGAAGGCGAACGCTGTGATTTAGACACGACTTACCGTTTTAACAGTCGTATCGGTGAG
GTGGCAAACCGGTTTATTCAGCAGAACCCAGGCCAGCTGAAAAAGCCGCTAAACAGCTTAACCAATGGAGACAAAAAAGC
CGTCACGTTATTGGATGAGAGTCAACTTGACGCTTTGCTGGATAAGCTCTCTGGTTATGCCAAACCGGAAGAGCGCATTC
TGATCCTGGCGCGTTACCATCACATGAGGCCTGCCAGCCTGGAAAAAGCGGCAACACGCTGGCCGAAGTTGCAAATCGAC
TTTATGACCATTCATGCCAGCAAAGGGCAACAGGCGGATTACGTCATCATCGTTGGCTTGCAGGAGGGAAGTGATGGTTT
TCCGGCTGCGGCGCGGGAGTCGATTATGGAAGAGGCGCTACTGCCACCGGTTGAGGATTTCCCGGACGCTGAAGAACGGC
GGTTAATGTACGTGGCGCTGACCCGGGCACGCCATCGGGTATGGGCACTGTTTAACAAAGAGAATCCCTCTCCCTTTGTG
GAAATACTGAAAAATCTGGATGTGCCGGTGGCGAGAAAACCGTAAGAAACAGGTGGCGTTTGCCACCTGTGCAATATTAC
TTCAGACGGTCCGCGAGATAACGCTGATAATCGGGGATCAGAATATCGACCGCGTCGTTGAAATGCGGCGACTGGATTAT
GAAGTCTGCCGTTGCCACGTTGGTGGCGACCGGAATGTTCCATACCGTCGCCAGACGCAGCAAGGCTTTCACGTCAGGAT
CGTGCGGCACGGCATTTAGTGGATCCCAGAAGAAAATCAATACATCAATTTTCCCTTCTGAGATCAATGCGCCAACCTGC
TGGTCACCCCCCATTGGGCCACTCAACATCGCGTTGACGTTCATGCCGGTCGCGCGGGAAATTAAGTTACCGGTAGTGCC
TGTTGCATACAGTACGTGTTGTTCCAGTAACGGTTGATGCCGTTCCACCCAGCTCATCAGCATTTGTTTGCAGTGATCGT
GTGCCACCAGCGCAATATGTTTCCGCGCAGGTAAAGTGCGAGTCGTCAGTTCCATAATGTACATCCGTAGTTAACTTTCC
TACAGATTACTGTAAGCACTTATCGCTGCAAGATAAAGACCGAAAAAGCCTGCGCACAGGCACAAAAATCTCAGGAAGAT
GGTTGTTTTTCCGCCCACTGCAGGAAAGTATTTCGCGTTTGTGGGTCAGCCAGTTTAAACCAATACTTCAGCCGTTGTTC
TGTGAGCACCTGAGACTGCGGTGGAATAGCATCCAGCTCGGAAACACCCGAAAGCAGCGTACTGTCATCTGCCATCATCG
TGGCAAATTGCGGTAGTGAAGCGCGTTTCGCGGATTTGTTATAGCGTTCAACCTCGACCTCATAATCAATCGTTTTTGCC
GTTGAGGTAATGGCGAGAATATCCAGCTTTACCGGAATCGGCGTCGCATCGCCATCCAACAATTCAAGGCGCGGCGCGGC
ATCAAAATGGTTAGCTTCCCGCTCATTCTCCAGGCGAGGCAGGCGAAAATTGACCTGGTTGATGAGCTGGGTATTAAAAC
TGACCACCAGCGGTGGGGAGATATACAGCCGTTCTTCACTATTGGAAAGATGAATCGTCTTCTCAACGCGAAACACTAAC
TGATGCGGTCCATTATCCAGTTCAATGCTGTCCGCACCGCGCAGCAGAGAGCTGGAAACCTTCTTGCCGTCGAGTACCAG
AAGATCGACATCGGTTGAAAGCCGCAGCGTGGTGGCAAAAACGGATACCGGCAAACATAATGCAATCAAGGTGGTCACGA
TGCCGGTTTTCATAGGTAGCTCCTGTCAAAAGACCGCCCGAATAATAATGTGTCAAAATTTTTCTTTAATCATACTTACT
AACATATAGACATATTTCCCGCTTTTGCTCTCATTCATTCGTATTAGCTGCATGGTTGGCATGTCGGCTTTGTCGTACAC
TTTGTTAAACAGTCTGGAGGAAATAATGAAAGAAACCGATATTGCAGGCATTTTAACGTCCACTCACACCATTGCGCTGG
TGGGGGCAAGCGACAAACCCGATCGTCCCAGCTATCGCGTGATGAAATATCTACTCGACCAGGGGTATCACGTCATTCCG
GTTTCGCCAAAAGTTGCCGGCAAAACGCTGCTGGGCCAGAAGGGGTATGGCACGCTGGCAGACGTTCCGGAAAAAGTCGA
TATGGTCGATGTTTTCCGTAATTCAGAAGCGGCGTGGGGCGTGGCACAGGAAGCTATTGCCATTGGCGCGAAAACTCTCT
GGATGCAACTGGGGGTGATTAATGAGCAAGCGGCAGTACTGGCACGGGATGCCGGGTTAAATGTGGTGATGGATCGCTGC
CCGGCTATTGAGATCCCTCGCCTGGGCCTGGCCAAATAAAAAATCCCCGGAAGGCAAAAACCTTCCGGGGATTTGTTCAG
GGATTAGTTACGCAGACGCGGGGCCTGGAGTTGTTTGCGGATGGTCTGCGCCAGCTCATCCATGGATGGCTGTTCAGGAT
GTTCATCCTGCAATTCGCTACTTAGCTGGGCTTCAGCCAGGTAGGTATGAACCGGTAGGCCGTTATCGTCCTCCATCACT
ACGTGATACCACGGAGCGGCGCGAAGCTCGTCATTCACCGCCAGCTCATCAGGCGACGGTTCAGAGAGCGAATAAACCGG
GTCGATATCCACGACCACTCCGAGATAACCTAACAGGGAATGGCGGACCTGCTGGCCGATACCGAATTTGCTGGCAATCA
TAGTCACCTCCCGGGAAATCTTTACCTTTGATATAAGGGTAAAATTCCACATTTCAAGTTACATGACGCGACAGGCAAAC
CCTTTCAGATATAGCCCTTCCGGATAGGTAGCGATCACCGGATGATCGGCTGCCTGACGGAACTGCTCTATAAATTGTAC
ATCACGACCGGCATCAATTGCGGCATCCGCGATGATTTTCTGAAATAAATCGCTGGTCATCAGACCGGAACAGGAGAAAG
TCAGGAGAATACCGCCTTCATTCAGCAGCTGAATCGCCAGCATGTTGATGTCTTTATAACCACGACACGCGCCCATCAAC
TGGCTTTTATTCTCAACAAACTTCGGCGGGTCCATCACGATAACGTCAAATTTTTCACCGCGATCGCGATAAGTACGCAG
CAATTTAAAGACATCATCACGGACAAACTCAGCCTTGCTCAGATCCAGTTTGTTCAGCTCAACGTTCTGCCGTGCAATAT
CCAGCGCTTCCTGGGAGGTATCAACGCTGACAACCTGGCTGCAACCGCCCATCAGTGCCGATACGGCGAAACCACCGGTA
TAGGAGAAACAGTTCAGCACACGTTTATTTTCAACGTAGCGGCGGGTAGCCAGGCGGCTATCACGCTGGTCCAGGTAGTA
GCCCGTTTTGTGTCCGTGCTGAATATCCACCAGCAGTTTCATTCCGTGTTCTTCAATCGGCAGCAGGGCAGGTGGCAACT
CGCCGGTGACGGGGCCCTGGGTCAGCTCCATTCCTTCTTTTTTACGTACCGCGACGTCGCTGCGATCGTAAATCGAACAT
TCCGGGTACAGCGTTTGCAGGGCACTAATTAATGCCGCGCGCTGATATTCTGCCCCAGCACTCAGCAGTTGCAGCACCAG
AAAATTACCGAAACGATCGATAGTAATACCCGGCAGGCCATCAGATTCTCCGGCGATTAAACGATAGCTGTCGAGGCCAT
CTTTTTGCGCCAGCCAGTCACGCCATTTTTGTGCTTGTTGCAAACGGCGGGAAAAAAAAGCAATGTCGATAGACTCAGAC
GGGTCAAACGTCCAGACGCGCGCCCGGATTTGCGAAGCTGGCGAATAAGCGCCGCGTGCTAACCATTTTCCCTGATGATC
AACAATATCGATGGTTTCACCGAGGCTGGCTTTACCTTCCATGCGGGCAACGGCCCCGGAAAAGACCCACGGATGGCGAC
GAAGTAATGATTTTTCGCGCCCTTTGGCTAACACTAAACGTACACTCATAATTTACTTTTCTGTCGATGCAAAGAAATGG
GCGTCATTGTCCGGAGTTTTCCATGGATTTGCAACGCGTCCATTAAGGATAAGGAGAGCAGCAATGTCGAAAGTCTGCAT
AATTGCCTGGGTTTACGGGCGAGTTCAGGGCGTAGGATTTCGCTACACCACACAGTACGAAGCGAAAAGACTGGGTTTAA
CCGGGTACGCCAAAAATCTTGACGATGGCAGCGTGGAAGTGGTTGCTTGCGGTGAAGAAGGGCAGGTGGAAAAATTAATG
CAGTGGTTAAAGAGTGGCGGCCCGCGTTCTGCGCGTGTAGAACGGGTGCTCAGCGAGCCGCATCATCCCTCGGGGGAATT

AACCGATTTTCGTATCCGTTAAATACATTTTACCGGTTTTGGCAGGCCAGCAATTTTGGTGGCTTGCTTAGCCGGACCTT
TCGGAAACAGTCGGTATAAATAGCGGCTGTTACCTTTTTCTTCGCCAAATTTATTCGCCATCGCTTTTACCAGCATACGA
ATCGCCGGAGAAGTATTGAATTCCAGATAGAAATCACGCACAAAACGCACCACTTCCCAGTGTTCCGGTGACAGCGAAAT
CCCTTCGTTCTCTGCAATCACCACAGCCAGCGGCTCACTCCACTGGCTGCTTTCTTTGAGATAGCCTTCGGTATCCGTTT
CTATCTCTTTACCTTCGAAGATCAGCATAATTATTACTACCTTAATCAGACTGGCGGCAGTGTAACAAAAAACAAAGCCC
CGCATAAAGCGAGGCTATGAAAGTGTTAGCGGGTGAGATTAATCGCGGCTAGCGAAGCCCAGAATGCTCAGCAGGCTGAC
GAAGATGTTGTACAGCGAAACATACAGGCTAACCGTGGCACGAATATAGTTCGTCTCACCGCCATGAATGATGTTGCTGG
TTTCAAACAAGATAGCGCCAGAGGAGATCAGAATGAAGACCGCGCTGATCGCCAGATGCAGAGCAGGCAGCTGCAGGAAG
ATATTCGCAACCATACCAATCAGCACCACCACAATACCCGCCATCAGCATACCGCCGAGGAACGACATATCTTTGCGGGT
GGTCAGCACATATGCAGAGCAGCAGAAGAACACTAACGCCGTTCCGCCCAGTGCCATAGCGATTACGTCACCCATTCCGG
CAGACAGATAGGTGTTCAGAATAGGTCCGAGGATATAACCCAGAAAACCGGTAAAGGCGAATGCGGAGATAATCCCGGTC
GGCTTATTCGCCGTTTTATAGGTCAGGAACATCAAACCATACATACCCACCAGCGTCAGAATCAGACCCGGAGATGGCAG
CATCAGCACCGTACTGGCAGTTGCGGTAATCGCCGAAAAGGCCAGCGTCAGGCTCAGCAGAAAATAGGTATTACGCAGCA
CCTTATGGGTGCTAAGCAGTGATGTACGGTCATGTGAAGAACTAACAATACGATCCATTGAGTCACTCTCTATGACAGAT
GTAATTAATTAAGCAGCATAATGATAATGCGTAAGGGCACCCAGAAGTTTTACCCATCTTTACGCATTTGATCTGGAACA
GGTTTAACAGCGGATTATCAGGTCATTAAGCAAATATAACGCCCTGAGAATTTCGACAGGCAAAAGAAAAAGGGGTTAGC
ATTTAGCTAACCCCTTATCTTATTTGGCGGAAGCGCAGAGATTCGAACTCTGGAACCCTTTCGGGTCGCCGGTTTTCAAG
ACCGGTGCCTTCAACCGCTCGGCCACACTTCCGGAATGACGCGCACTATAAACATCCCGATGCGGCGTGTAAACCCCTAA
TTTGTTTGTTTGCCTGAAAAACAGCCAAAAGTGCATTGATAGCGTGAAATAACAGCAGATTGATCATTTCATCACCATGA
ATTCCTTCTCTTTTACTCGTTTAGCAACCGGCTAAACATCCCCACCGCCCGGCCAAAAGAAAAATAGGTCCATTTTTATC
GCTAAAAGATAAATCCACACAGTTTGTATTGTTTTGTGCAAAAGTTTCACTACGCTTTATTAACAATACTTTCTGGCGAC
GTGCGCCAGTGCAGAAGGATGAGCTTTCGTTTTCAGCATCTCACGTGAAGCGATGGTTTGCCTTGCTACAGGGACGTCGC
TTGCCGACCATAAGCGCCCGGTGTCCTGCCGGTGTCGCAAGGAGGAGAGACGTGCGATATGAATAACGAGGAAACATTTT
ACCAGGCCATGCGGCGTCAGGGCGTTACCCGGCGCAGCTTTCTCAAATATTGTAGTCTGGCTGCCACGTCGCTGGGATTA
GGCGCGGGAATGGCACCAAAGATTGCCTGGGCGCTGGAGAACAAACCGCGCATTCCGGTGGTATGGATCCACGGTCTGGA
ATGCACCTGCTGTACCGAATCTTTTATCCGCTCCGCTCACCCACTGGCGAAGGACGTCATCCTTTCCCTGATTTCCCTCG
ATTACGACGATACTTTGATGGCTGCCGCCGGAACCCAGGCGGAAGAAGTCTTTGAAGACATCATCACGCAATACAATGGC
AAATATATCCTCGCAGTAGAAGGTAATCCGCCGCTGGGCGAGCAGGGGATGTTCTGTATCAGCAGCGGTCGACCGTTTAT
TGAGAAACTCAAACGTGCCGCTGCCGGAGCCAGCGCGATTATCGCCTGGGGAACCTGCGCGTCCTGGGGCTGCGTGCAGG
CCGCGCGACCCAATCCGACGCAGGCAACGCCTATCGACAAAGTCATCACCGACAAACCCATTATCAAAGTACCTGGCTGC
CCGCCGATCCCGGATGTGATGAGCGCCATCATTACTTACATGGTGACCTTTGATCGCTTGCCAGATGTCGACAGAATGGG
CCGTCCGCTGATGTTCTATGGTCAGCGAATCCACGATAAATGCTATCGCCGCGCCCACTTCGACGCCGGAGAGTTCGTCC
AGAGTTGGGATGATGACGCTGCCCGCAAAGGTTACTGCCTGTACAAAATGGGCTGCAAAGGGCCTACCACCTATAACGCC
TGTTCCTCCACACGCTGGAATGATGGCGTTTCTTTCCCAATCCAGTCTGGTCACGGCTGCCTGGGCTGTGCGGAAAATGG
TTTCTGGGATCGCGGTTCGTTCTACAGCCGCGTGGTCGATATTCCGCAAATGGGTACTCATTCCACCGCCGATACCGTCG
GTTTAACCGCGCTTGGCGTGGTGGCAGCGGCTGTTGGTGTGCACGCAGTCGCCAGCGCCGTTGACCAGCGCAGACGTCAT
AACCAGCAACCTACAGAAACCGAACATCAGCCAGGCAATGAGGATAAACAGGCATGAGCACTCAGTACGAAACTCAGGGA
TACACCATCAATAATGCCGGACGCCGCCTGGTGGTCGACCCGATTACGCGCATCGAAGGCCACATGCGCTGCGAAGTGAA
TATTAACGATCAGAATGTGATCACCAATGCCGTCTCCTGCGGCACCATGTTTCGCGGGCTGGAGATCATCCTACAAGGGC
GCGACCCGCGCGATGCGTGGGCGTTCGTTGAACGTATCTGCGGCGTCTGTACTGGCGTACACGCCCTGGCTTCGGTTTAC
GCCATCGAAGATGCTATCGGTATTAAAGTGCCGGACAACGCCAATATCATCCGCAACATTATGCTGGCAACGCTCTGGTG
CCACGATCATCTGGTGCACTTCTATCAGCTTGCCGGGATGGACTGGATCGATGTGTTAGATGCGCTGAAAGCCGACCCGC
GGAAAACCTCCGAACTGGCGCAAAGTCTCTCCTCTTGGCCGAAATCATCCCCTGGCTATTTCTTCGACGTACAAAACCGC
CTGAAAAAATTTGTTGAAGGCGGGCAGTTGGGGATCTTCCGCAATGGCTACTGGGGGCACCCGCAGTACAAACTGCCGCC
AGAAGCTAACCTGATGGGCTTTGCCCACTATCTCGAAGCTCTCGATTTCCAGCGTGAAATTGTCAAAATCCACGCGGTCT
TTGGCGGTAAAAACCCGCATCCAAACTGGATTGTCGGCGGGATGCCTTGCGCCATCAACATTGACGAAAGCGGCGCGGTC
GGGGCAGTCAATATGGAACGCCTGAACCTGGTGCAGTCAATTATCACCCGCACGGCGGACTTCATTAACAACGTGATGAT
CCCCGACGCCTTAGCCATCGGTCAGTTCAACAAACCGTGGAGCGAAATCGGCACTGGTCTTTCTGATAAATGCGTTCTCA
GCTACGGCGCATTCCCGGATATTGCCAACGACTTTGGCGAGAAAAGTCTGCTGATGCCTGGCGGCGCGGTGATTAACGGC
GACTTCAACAATGTGCTGCCAGTGGATTTGGTTGATCCGCAGCAGGTGCAGGAGTTTGTCGACCACGCCTGGTATCGATA
TCCCAACGATCAGGTCGGGCGTCATCCGTTCGATGGCATCACCGACCCGTGGTACAACCCCGGCGATGTCAAAGGCAGCG
ATACCAACATTCAGCAGCTGAATGAACAGGAACGCTACTCGTGGATCAAAGCGCCACGCTGGCGCGGTAACGCGATGGAA
GTGGGGCCGCTGGCGCGCACGTTAATCGCTTATCACAAAGGCGATGCTGCGACCGTTGAGTCGGTCGATCGCATGATGTC
GGCGTTGAACCTGCCGCTTTCCGGTATCCAGTCAACGTTAGGCCGCATTTTGTGCCGCGCGCACGAAGCGCAGTGGGCCG
CAGGTAAGTTGCAGTATTTCTTCGACAAGCTGATGACCAACCTGAAAAACGGCAATCTCGCCACTGCTTCCACGGAAAAA
TGGGAACCTGCAACCTGGCCGACAGAGTGCCGTGGTGTCGGTTTTACCGAAGCGCCGCGCGGGGCGTTAGGCCACTGGGC

CGCCATTCGCGATGGCAAGATTGATCTCTACCAGTGCGTGGTGCCGACCACCTGGAACGCCAGCCCGCGCGATCCCAAAG
GGCAGATTGGCGCTTATGAAGCGGCGCTGATGAACACCAAAATGGCGATCCCCGAGCAACCGCTGGAGATCCTGCGTACT
CTGCACAGCTTTGACCCGTGCCTCGCCTGTTCAACACACGTGCTGGGCGACGACGGTAGCGAGCTGATCTCCGTGCAGGT
GCGTTAACAGCGAAGGAGAATCATCATGCAACAGAAAAGCGACAACGTTGTCAGCCACTATGTCTTTGAAGCGCCAGTGC
GCATCTGGCACTGGTTGACGGTGTTATGCATGGCGGTGTTGATGGTCACCGGATACTTTATCGGCAAGCCGCTACCTTCC
GTCAGCGGCGAGGCGACGTATCTGTTCTATATGGGCTACATCAGGTTAATTCACTTCAGCGCCGGGATGGTTTTTACCGT
GGTTTTGCTGATGCGGATCTACTGGGCTTTTGTTGGCAATCGATACTCCCGCGAGCTGTTTATCGTGCCGGTATGGCGTA
AAAGCTGGTGGCAGGGCGTGTGGTATGAAATCCGCTGGTATCTGTTTCTGGCAAAACGTCCGAGTGCCGATATAGGCCAT
AATCCCATCGCCCAGGCGGCGATGTTCGGCTATTTCCTGATGTCGGTCTTTATGATCATCACTGGTTTTGCGCTGTACAG
CGAACACAGCCAGTACGCTATTTTTGCGCCGTTCCGTTATGTGGTGGAATTTTTCTACTGGACGGGTGGCAACTCAATGG
ACATTCACAGCTGGCATCGGCTGGGGATGTGGCTGATTGGCGCGTTTGTGATCGGTCATGTCTACATGGCGCTGCGTGAA
GACATCATGTCCGACGACACGGTGATCTCCACCATGGTCAACGGCTACCGTAGCCACAAATTTGGCAAAATAAGTAACAA
GGAGCGTTCATGAGCGAGCAACGCGTGGTGGTCATGGGGCTGGGCAACCTGCTGTGGGCCGATGAAGGCTTCGGCGTGCG
GGTGGCGGAACGGCTGTATGCCCATTACCACTGGCCCGAGTATGTGGAGATTGTCGATGGCGGTACTCAGGGACTGAACT
TGCTGGGGTATGTCGAAAGCGCCAGCCATCTGTTGATTCTCGATGCCATTGACTACGGGCTGGAACCTGGAACGCTGCGA
ACCTATGCCGGAGAACGCATTCCGGCTTATCTCAGCGCGAAGAAAATGAGCCTGCATCAGAACAGTTTCTCCGAAGTGTT
GGCGCTGGCGGATATCCGCGGACATCTGCCAGCACATATTGCCCTCGTCGGTCTGCAACCCGCAATGCTCGACGACTACG
GCGGTAGCCTGAGCGAACTGGCACGGGAGCAACTGCCCGCTGCGGAACAGGCGGCGCTGGCGCAGCTTGCTGCGTGGGGA
ATTGTGCCGCAACCGGCTAATGAATCGCGCTGTCTCAATTATGACTGTCTGTCGATGGAAAATTACGAAGGCGTTCGCTT
GCGCCAGTACCGGATGACACAGGAGGAGCAGGGATGAGCAACGACACGCCATTTGATGCGTTGTGGCAACGAATGCTGGC
GCGCGGCTGGACGCCAGTCAGTGAATCCCGTCTTGACGACTGGCTTACGCAAGCGCCAGACGGCGTGGTGTTATTAAGCA
GTGACCCGAAACGCACGCCAGAGGTCAGCGATAATCCGGTAATGATTGGCGAATTACTGCGCGAGTTTCCCGACTATACA
TGGCAGGTGGCGATTGCTGACCTTGAGCAGAGCGAAGCCATCGGCGATCGCTTTGGCGTCTTTCGCTTTCCTGCCACTTT
AGTGTTTACCGGCGGAAACTATCGCGGCGTGCTGAATGGTATTCACCCGTGGGCGGAACTGATAAACCTGATGCGCGGGC
TTGTCGAACCGCAGCAGGAGCGTGCCTCATGAGCGAAACTTTTTTCCATCTGCTGGGGCCAGGAACGCAACCGAACGATG
ACAGTTTCAGCATGAATCCACTGCCGATCACCTGTCAGGTGAATGATGAACCGAGTATGGCGGCCCTGGAGCAATGTGCT
CACAGCCCGCAGGTGATTGCGCTGTTAAACGAGTTACAACATCAACTAAGCGAACGCCAACCGCCGTTGGGCGAGGTGCT
GGCAGTCGATCTGTTAAATCTCAACGCCGACGATCGTCACTTTATCAATACGCTTCTCGGGGAAGGGGAAGTGTCAGTGC
GCATTCAGCAGGCTGACGACAGTGAAAGTGAAATACAGGAGGCGATCTTCTGCGGATTATGGCGGGTGCGCAGACGTCGC
GGCGAAAAGTTGCTGGAGGACAAACTGGAGGCTGGCTGCGCGCCGCTGGCGTTGTGGCAGGCGGCAACGCAAAATCTCTT
GCCGACAGATTCGCTGTTACCGCCGCCCATTGATGGCCTGATGAATGGCCTACCGTTGGCGCATGAGTTACTGGCACATG
TACGTAACCCCGACGCGCAGCCGCACAGCATTAATCTGACGCAATTACCCATCAGCGAGGCTGATCGGCTTTTTCTCTCA
CGTCTCTGTGGGCCGGGAAATATTCAGATTCGTACCATTGGCTATGGCGAGAGCTATATCAACGCCACGGGGTTACGCCA
TGTCTGGCATTTACGCTGTACGGACACCTTAAAAGGCCCGTTACTGGAAAGTTATGAAATCTGCCCAATACCGGAAGTGG
TGCTGGCAGCGCCAGAAGATTTGGTCGACTCTGCGCAGCGGCTTAGCGAGGTATGTCAGTGGCTGGCGGAAGCTGCACCG
ACGTAAAAAGACGGTAAGTATCGCTTTCAGTCTTATGAATATCGCAATCGGCGAATACCTCTGGTCGTAGAGTTTCAGGA
TAAAGAGGGAGATCTACCATTATCGGGTTATTTTTCTCTCTTCGCCTACAGGAGTGCGCATGTGGGATGTCATTGATTTA
TCGCGCTGGCAGTTTGCTCTGACCGCGCTGTATCACTTTTTATTTGTACCCCTTACCCTGGGGCTGATTTTTTTGCTGGC
TATTATGGAAACCATTTACGTGGTCACCGGCAAAACAATCTACCGCGATATGACGCGCTTCTGGGGTAAGCTCTTCGGTA
TCAATTTTGCTCTTGGCGTGGCTACCGGCCTGACCATGGAGTTTCAGTTTGGTACTAACTGGTCATTCTATTCCAACTAT
GTGGGCGATATTTTTGGCGCACCGCTGGCGATGGAAGCATTAATGGCCTTCTTCCTCGAATCCACCTTTGTCGGGCTGTT
CTTCTTCGGCTGGCAACGGCTGAATAAATACCAGCACCTGCTGGTGACGTGGCTGGTGGCGTTCGGTTCAAATCTCTCTG
CGTTGTGGATATTGAATGCCAACGGTTGGATGCAATACCCGACCGGTGCGCATTTTGATATCGACACCCTGCGTATGGAG
ATGACCAGCTTCAGCGAGCTGGTCTTTAATCCGGTCAGCCAGGTGAAATTTGTGCACACCGTAATGGCGGGCTACGTGAC
CGGGGCCATGTTTATTATGGCGATCAGCGCCTGGTATTTACTGCGCGGACGGGAGCGCAATGTCGCATTACGCTCGTTTG
CCATCGGTTCCGTCTTCGGTACTCTGGCGATTATCGGTACCCTGCAACTCGGAGACAGTTCTGCGTATGAAGTCGCGCAA
GTACAACCGGTAAAACTGGCGGCGATGGAAGGGGAGTGGCAAACGGAACCTGCACCTGCACCGTTCCATGTGGTTGCCTG
GCCGGAACAGGATCAAGAGCGTAACGCCTTTGCCCTCAAAATTCCCGCGCTGCTAGGGATCCTCGCCACTCACTCATTAG
ATAAACCCGTGCCGGGTCTGAAGAATTTGATGGCTGAAACCTACCCACGCTTGCAACGCGGACGTATGGCCTGGCTGTTA
ATGCAGGAAATATCGCAAGGCAATCGTGAGCCGCATGTGTTGCAGGCATTCCGGGGACTGGAAGGTGACCTGGGCTACGG
CATGTTGCTCTCCCGCTATGCGCCGGATATGAATCATGTCACAGCCGCACAGTACCAGGCGGCGATGCGTGGCGCGATAC
CTCAGGTTGCGCCGGTATTCTGGAGTTTCCGCATCATGGTGGGCTGTGGTTCCCTGCTGCTACTGGTGATGCTGATTGCG
CTTGTCCAGACGCTGCGTGGCAAAATCGACCAGCATCGCTGGGTGCTGAAAATGGCGCTCTGGAGTTTGCCGTTGCCGTG
GATTGCGATTGAAGCCGGGTGGTTTATGACCGAGTTTGGTCGTCAGCCGTGGGCGATACAGGACATCTTACCGACATACT
CCGCGCACTCCGCTTTAACCACAGGACAACTGGCTTTCTCACTGATCATGATCGTAGGGCTTTACACCCTGTTCTTAATC
GCCGAAGTCTACCTGATGCAGAAATATGCCCGTCTGGGGCCGAGCGCGATGCAGAGTGAACAACCGACGCAGCAACAGGG

GTAAAGGAGAAAATCATGTTTGATTATGAAACATTGCGCTTCATCTGGTGGCTGCTGATTGGCGTGATCCTGGTGGTCTT
TATGATCTCCGACGGATTTGACATGGGGATCGGCTGTCTGCTGCCGCTGGTGGCGCGTAATGATGATGAACGCCGGATAG
TGATAAACAGCGTTGGTGCACACTGGGAAGGCAACCAGGTCTGGTTGATCCTCGCTGGTGGGGCATTATTTGCCGCCTGG
CCCAGAGTGTATGCAGCGGCGTTTTCCGGCTTTTATGTGGCGATGATCCTGGTGCTGTGCTCACTGTTCTTCCGCCCGCT
GGCCTTTGATTATCGCGGAAAAATCGCCGATGCACGCTGGCGTAAAATGTGGGACGCCGGTCTGGTCATCGGCAGTCTGG
TGCCGCCGGTAGTCTTCGGTATCGCCTTCGGCAACTTGTTGCTCGGCGTGCCGTTTGCCTTCACACCGCAATTACGCGTG
GAGTATCTCGGCAGCTTCTGGCAACTGCTGACGCCATTCCCTTTATTGTGCGGATTGCTCAGCCTTGGGATGGTGATTTT
GCAAGGTGGCGTCTGGTTACAACTGAAAACTGTTGGTGTGATTCATCTGCGTTCACAGCTGGCGACCAAACGCGCTGCAC
TGTTGGTGATGCTGTGCTTTTTGCTGGCGGGTTACTGGCTGTGGGTCGGTATTGATGGCTTTGTACTGCTCGCCCAGGAT
GCTAACGGTCCTTCCAATCCGTTAATGAAACTGGTGGCAGTGCTACCTGGTGCCTGGATGAATAATTTTGTCGAGTCGCC
CGTTTTGTGGATCTTCCCGCTGCTGGGATTCTTCTGCCCATTGCTGACGGTGATGGCGATTTATCGTGGTCGCCCGGGTT
GGGGATTTTTGATGGCATCATTGATGCAATTTGGCGTGATTTTCACGGCAGGCATCACGCTGTTCCCCTTTGTCATGCCG
TCAAGCGTGAGTCCGATCTCCAGCCTGACGTTGTGGGACAGTACTTCCAGTCAGCTGACGCTGAGCATTATGTTGGTAAT
CGTGCTGATATTTTTGCCCATTGTGTTGCTCTACACTCTCTGGAGCTACTACAAAATGTGGGGGCGCATGACAACAGAAA
CTCTCCGCCGTAACGAAAACGAGTTGTACTAAGGAGCAGAAACAATGTGGTATTTACTTTGGTTCGTCGGCATTTTGTTG
ATGTGTTCGCTCTCCACCCTTGTGTTGGTATGGCTGGACCCGCGTCTGAAAAGTTAACGAACGTAGGCCTGATGCGGCGC
ATTAGCATCGCATCAGGCAATCAATAATGTCAGATATGAAAAGCGGAAACATATCGATGAAAGCGATCTTAATCCCATTT
TTATCTCTTCTGATTCCGTTAACCCCGCAATCTGCATTCGCTCAGAGTGAGCCGGAGCTGAAGCTGGAAAGTGTGGTGAT
TGTCAGTCGTCATGGTGTGCGTGCTCCAACCAAGGCCACGCAACTGATGCAGGATGTCACCCCAGACGCATGGCCAACCT
GGCCGGTAAAACTGGGTTGGCTGACACCGCGCGGTGGTGAGCTAATCGCCTATCTCGGACATTACCAACGCCAGCGTCTG
GTAGCCGACGGATTGCTGGCGAAAAAGGGCTGCCCGCAGTCTGGTCAGGTCGCGATTATTGCTGATGTCGACGAGCGTAC
CCGTAAAACAGGCGAAGCCTTCGCCGCCGGGCTGGCACCTGACTGTGCAATAACCGTACATACCCAGGCAGATACGTCCA
GTCCCGATCCGTTATTTAATCCTCTAAAAACTGGCGTTTGCCAACTGGATAACGCGAACGTGACTGACGCGATCCTCAGC
AGGGCAGGAGGGTCAATTGCTGACTTTACCGGGCATCGGCAAACGGCGTTTCGCGAACTGGAACGGGTGCTTAATTTTCC
GCAATCAAACTTGTGCCTTAAACGTGAGAAACAGGACGAAAGCTGTTCATTAACGCAGGCATTACCATCGGAACTCAAGG
TGAGCGCCGACAATGTCTCATTAACCGGTGCGGTAAGCCTCGCATCAATGCTGACGGAGATATTTCTCCTGCAACAAGCA
CAGGGAATGCCGGAGCCGGGGTGGGGAAGGATCACCGATTCACACCAGTGGAACACCTTGCTAAGTTTGCATAACGCGCA
ATTTTATTTGCTACAACGCACGCCAGAGGTTGCCCGCAGCCGCGCCACCCCGTTATTAGATTTGATCAAGACAGCGTTGA
CGCCCCATCCACCGCAAAAACAGGCGTATGGTGTGACATTACCCACTTCAGTGCTGTTTATCGCCGGACACGATACTAAT
CTGGCAAATCTCGGCGGCGCACTGGAGCTCAACTGGACGCTTCCCGGTCAGCCGGATAACACGCCGCCAGGTGGTGAACT
GGTGTTTGAACGCTGGCGTCGGCTAAGCGATAACAGCCAGTGGATTCAGGTTTCGCTGGTCTTCCAGACTTTACAGCAGA
TGCGTGATAAAACGCCGCTGTCATTAAATACGCCGCCCGGAGAGGTGAAACTGACCCTGGCAGGATGTGAAGAGCGAAAT
GCGCAGGGCATGTGTTCGTTGGCAGGTTTTACGCAAATCGTGAATGAAGCACGCATACCGGCGTGCAGTTTGTAATGCAT
AAAAAAGAGCATTCAGTTACCTGAATGCTCTGAGGCTGATGACAAACGAAGAACTGTCTAATGCGTAGACCGGAAAAGGC
GTTCACGCCGCATCCGGCCACTTTCAGTTTTACTCTTTCTCGGAGTAACTATAACCGTAATAGTTATAGCCGTAACTGTA
AGCGGTGCTGGCGCGTTTAATCACACCATTGAGGATAGCGCCTTTAATATTGACGCCTGCCTGTTCCAGACGCTGCATTG
ACAAACTCACCTCTTTGGCGGTGTTCAAGCCAAAACGCGCAACCAGCAGGCTGGTGCCAACAGAACGCCCCACGACCGCG
GCATCACTCACCGCCAGCATCGGCGGCGTATCGACAATCACCAGATCGTAATGGTCGTTCGCCCATTCCAGTAATTGACG
CATCCGATCGCGCATCAGCAGTTCAGACGGGTTAGGTGGCACCTGACCGCGAGTAATCACATCAAAGCCTCCTTTGCCAA
AATGCTGGATCACTTTGTTGAGCTCATCTTTACCTGCCAGATATTCCGACAAGCCATGTTCATTACTCACGGTAAACAGG
TTATGCGAATAACCACGGCGTAAGTCGGCATCAATAAATAACACTTTTTGATCGGACTGGGCGATCACCGCTGCCAGAGT
TGAACTGACAAACGTTTTACCACTGTCTGGCGTCGCACCGGTGATCATTAGAATGTTATTCTCCGTCTCCATCATAGCGA
AATGCAGACTGGTTCGTAGCGCACGTACGGCTTCCACAGCAGAATCCGCCGGGTTATCCACCGCCAGGAAGGGGATATTT
TTAGTACGATGGCGCTGCTGATTAGAAAATAAATTTTTCTTACGCAGACGGGTGCGTTTATCCAGCCACTCGGACATTGG
GATAGTGGCATAAACGCTGATGCCGTGCTCTTCCAGTTGTTCCGGGGCTTCTACACCACGACGCAACATCGCACGCGCCA
GCACGGCACCCACAGAAATAAACAGGCCAAGAATAAAACCAAGCACCACGTTCAACGCTTTTTTCGGTTTCACTGGCTGC
GGCTGAGTGACTGCCGGGTCGATAATCCGCACGTTACCAATGGCACTGGATTTCGAAATACTCAACTCCTGCTGGCGGTT
AAGTAATTGCAGATATACCGCACGGCCCGCTTCTACGTCACGACTTAAACGCAACACTTCCTGTTGGGTGGAAGGCATTG
CCGATACCCGCTTATTCAGGCGTTTGCGTTCTTGCTCCAGCGTCTGGCGTTTTTCCAGCAGCGCACGATAAGTTGGGTGA
TCTTTCTTATACAGCTGGGAGATCTCTGCCTCGCGGAAGGTCAGCTCATTGAGTTGATTATCAACGTTCACAATCTGCTC
AAGAACGGCTTTGGCTTCCAGGTTAAGGTCAACCGAATCGCGCTGCTGGCGATAAACGTTGAGTTTTTCTTCCGCTTGGT
CCAGCTCGCTGCGCACTTCAGGTAACTGGCGCTGTAAGAATTCAAGGCTTTGTGAATCCTGCGCCGCCTGGCGAGCGATA
TTCTGTTGCAAATAGTTGTTAGCGATGCTGTTCAGAATACGAGTAATCAACTGGGGATCATCACCAGTCATGGTAAGTTC
CAGCATCCCGCTTTCTTTACTGCGTTCGCTAACGGTAAAGGTTTCCTGCAATGCGTTAATCGCTTCCAGTTCGGTACGCT
GGCTCAGGACAAACTGTGTTCCTGGTTTGGCCTTAATGTCCGCGATAGTCAGCGCAACGCCATCTTTTTCCAGACGCTGG
CCGACCATACCATTGACGGTGAACTCTTCACCTTCCAGTGTATAGTGGCCGTTTTCCCCAACCGTGAGTGTCAGTTGCTG

ATCCTGACCATTCAGTTGTGGAATATGCATCCAGCTGATCGCCAGCTCACCTGGTTTTTCTTTGGTTAATCTCGCCCAGC
CGCGACCCACAATCGGAAAATACTTCTGCTCAACTATGTCGCGCAGATTCAGTTCAGCAATGGTTTTACCGAGAATCATG
CGCGATTGCAGCAGTTGGATCTCCGGTGCAGACTCGGGCGATGAGTTAGGGATCATATCGCTCAGGCCGCTGAGAATGGC
GTTGCCCTGTTTTTGCTCAACCTGGACCAGAGTATCTGCCTGATAAATTGGTGTGCTTAACAGCGAGTAAGCGACAGCGA
TCAGCGTGAATAACGCGGTCACGCTGATAATAAACTTACGGTGATCCCATAACTCGCCGACCAGACGAAGCAGATCGATC
TCATTTTCCTGAGTGCTGCCTGGTGGCGTATTCATATTTTTAGTTGTCATCGTATTTTATAAACTCAAATTACCGGCTGA
GGCGCTTCGCCCATTCCTGACTGGCGCGCTCCAACATACCGTAGACATGTTCAAATGCGTCCTGACTTTTACGATAGGGA
TCCGGGATCTCTTTCTGTTCCAGCCATTGCCCAAACAGCATTGTCTTCCCGCGGACCTCGGGAGCAATTGCGGTAACCTG
AGCAATATGTTCCGACTCCATTGCCAGAATCAGATCGTAATTTCGTGCCATCTCAGCAGTGAGCTTACGTCCGGCATGTC
CTTCAAGAGATACGCCATGATTAGCGGCAACATCTGCCGCTGTCGCATCTGCAGGGTGTTTTACCAGACCATGAACACCT
GCCGATTTGACTTTTACGCCCGGCAGACGTTTACGCAGCAAGCGCTCGCCAATGGGCGAACGGCAAATGTTGCCGGTACA
AACCACCAGGATTGAGTTAAATTTTAGTTGGGCCATGTTTGAATCCACCGTGAAGTCTCTGTCAGGTCATGCACCCCGGA
AATCGTCGGTACCAGCAGAGAAATCACACGGTTCCAACGAGCCAGCGGCGCGGTAGTGACATAAACAATGTCGTAAGGTT
CAAGCTGGAATTCCGTTCCGAGTATCATTGCCGATGCGTCCTTCGCATTCAGCTGATAAATATTGGCGATTTTGCCATTC
TGCTTGTTTTGCGTTGCACGAATAACAAAAATCCCCGTCGCATCAGCGACATCCTGATTCATACCCTCAGCGTTACCCAG
CGCTTCCGCCAGGGTCATACCGCTGCGGTCCATTTTCAGCGTGCTCTGTTTACCCACTTCACCCATCACGAAGACTTTCA
GATCATCGTTGCGCGGAATAAATAAAATATCGCCCGGGTGCAGCAGTTTGTTCTGGCGCAAGTCGCCACGCTGCATCAGC
GCATAGAGGTTCACTTTGGTCTTTACGCCATTCTGGGTGAGCACCACGTTGCGCCAGTCTGCATCTGCGGTCAGACCGCC
CGCGGCGTTAATCGCATCCATGATGGTCAGAGGAATATTGGTAATCGGCTGCTGACCCGATTTGCTGACTTCGCCGGTGA
CGTACGCTTTTTGCGATCGGAACGCCGCGACGCTGACATCGACTTGCGGGCTTTCAATGACTGAATCGAGACGCGCTGTA
ATCTCGTTGCGTACCTGGGTAAGTGTTTTCCCGGCCACTTTCAGGCGACCAATATAGGGGTAGAAAATCGCACCATCGGC
GTTAACCCAGTTGCCGGTATCACTGGCGCTACGGTATTGCCCTGCGGGGGTGGTTAGTTCCGGGTGATCCCAGACCGTAA
CCATCAACACATCACCAATCCCGATACGATATTCATAGTTTGCGATTTGCTGCTCCAGCTCCGGGTTAGCCTGCGACATA
ATCGGTTTGGCGCGCAACTGATCAATCAATCCTGGCGTGACGGGATAGATATTGACCATTTTGTCCAGGTCATACTGATT
GTCAGGTAGTTCAATGACATCCTTGTTACTGGTCGATAAATTCTGACCAGGAACAAGGGTACATGCTGTCAAAGACAGAA
CAGCCAATGTCAGTACCGAAAACTTAAAAATGTTCTTTTTCATTGGTTTATATTTATCAGTCAATGTAATGTTATGAATT
ACGTCAGGTTATTGTCCAACCGGTACTTCTCGCTCTGCCGTCATTGGATAGAGCTGGTATTTGCGTGACAGCATTTGTCC
ACCATCACGCGTCAATGGTGTCCACGAAACCACCGCGCGGTTGCGGTTAGGTCCAATGGTCATCAAGTCGAATGGAATCG
AGATATAAAAACCTTTACTAAAGCCGCCTTCGCCGTAGTCATCTTTAGATACGTTACTGATTGCCGCCCATACCCCTACC
GCCACGCCGCTGTCAAAGCGTTTGGCGACGTCGATCGTTGCGCCTTTATCTTTTGCCAGATATTGCCCAACGCTAAGTTT
CATCAGTACGCCATTGAGCGTCGGCGGGTTCCAGTAAGCCGTCACGAAACCTGTTGGCGTGGAATAATCGGTGAAACGCA
TCATGTTGTCCCAGTCACGTTGCTTCACGTAGTTAACGTCCACACCCAGCGCCCAGCAGGCATCTAGCGGGCGATAAAGC
AGCTCGGAACCGACACCGGCGTACATCGTTTCCAGATAACCGCCATACACCTGGCCATAGAAACCATTGCCTAAGTCAGC
AAAATAGTTCGCCTGCAAGTTGTTGAGATAAACGTCATTGCGAACGTAGTCACGGATATGCGTGCGCACGCGGGGCAGGG
TAGAGTCCGCGGGCAACAGCGAAGACTTAAACTTGTCGTAGTTGTTGTAAATATTGGTGAAAATACCGCCATCAAGCAGC
AGGTGGTCGGTAAACCAGTAGCGGGCACTGGACATCAGCCCCAGCTGGAACATATAGAAATCTTCCGGGCCGCCCAGCGA
CTGTGAAAGTGTTGGGTTGAAAGAGTAGCTAAAGCGATCTTCACGAATACGGTAGCCCCGACCAAAGGCAGAAAGATCTT
CAGCTTCAACACGTTGTTGTTGCAGTGGCTCTGATTGACCAGGCGCTGTACCTGCCAGCTGTTTGCGCAGGCTGGCTACG
TCGGTTTCGGTAGTCACCATCGCCATATGCTCGCGCTTTTGCGTCACGCTAATCTTCTCAACGCCTTGCGGCAGGTTATT
CACCAGAATCCGGTTGGCACGATCGACCGCTTCACGAGAGTCACGGTATTTGTATTGCTGACCAGACATATACAGTGTCT
TATCGCGTAGCTGAATTTCTGGCGCGTCAAAGCCCGCGTTATACTTCAGGGCGGTAAGTTGGTTTGCTACCGTGGTGTAC
TGCAATCCTTCAGATTCAGGCGCAGGTTGATATGCCGGTTTTGGCGTATCGCGCAGGGCAGGGCGCAGATCGTTGAAATT
GGTCCGTAACGTGAAGCCAAACATCAACGTGTTACCGCGTTCATAACTCAGGTTGAGATCTGCCCAGCTGGCAGCGCGAT
AAACTGCGCCGACGTTGAAATGGCTTGCCTGAGGCAGTTTGCCAGCGAAATCATTCTGGTAATTGTTTCCATCGTATTCG
AGTTTCAGACGCAGGGGATTCCACGGCGTTTGATACTCAATGCCGCCAAAGATGGAAGCCGGGCCACGAAAGATATCGCT
AAAGCTGATATCGCCCGCATCGTGAGACTCTGCGCGATGACAATATTTATCGCTCACCCGGCAAAACGGGTTGGTAATAT
TGCCCGCATTACCGGCGTAGCCCCATGCCATCCCGAGGGTGAAATCAAATGGCCCCGCTTGCTTGCTGGCCACCAGATAC
TCACCGTCAAACAGACCCGTACCAGCAATATCACGTTTACCAAACGCCACTTGCGGTAGCCAGTAACCTTCTTCCCACAG
GCGAAGTTTAAAATCGAATGATTTGTCTTTGTATGACTGATCGCCGCTGAAATCTTCCCACTGGCTATATTTGCGTGTGC
GCACATCCGTATAACGAATGGTGCCTTCCAGCCATGGGAACAGCGCCACGGAGGTGGAGTAGAACCGGTATTGATCGTTA
TCCCGGTAGTTGACGCTGAATTCACCTTCCGGTGCGATGCGCGCATTTGGCATCTGCAACAATCCTGTGCCGCCAAAGTC
TGATTGCGACGAACCCAGCGGATCCGGGTAGGTTAATACTTCAGCATGACAGGCACTGGAGACGGCAATGGCCAGGCAGC
TTAAAAGATAAGAATTCTTCTTCATCAGTCAGGAACACGTTGCGTTAAAACAGACACAATCTGGTCATTTAAATCAGCAT
ATTTTTCGGGCAGTACATGCGCAGAAAAGCCCAACCAAAGCTGGCTGCCAGGCGGTGGTTCCACATGTCTTTTATTCCAC
AGGGCAACAGGGGCGACGACAGTCTCACCCTCAGGCGTAATCACCATAACGTTATTCTTGTCCGCGCCTGCAAGGCGGGG
GTGATCTTGCAGATAATCGGTAACGCTACGGCCTGCCTGCCAGGGCAATTGCCCGGCCCCAGAAACCGCACCCAGCAGAG

TAATAGTCACCGGACGCTGTACGGTATAGAGCGTATAGTCCCCGACCAACGGGGGATTGCTGTTCTCATCCACTCGTACA
AAATCAGGGTCCAGTTTGACCGGTAAGCGACCAGTGATATTGAGATTGAGTAACTGCTGGCGCACGGATTTAATCGTTGC
CGCCACATCGTCGTCAGCTTCTGCTTCCCAGGAAGCAAGCTGTGCCATGACATGTTGGTAGTCTTTAAGCGCTTTAGCTT
TCGCCGCAGAGTCGGTCAGCAAGGCACCAGGCCACCAGAGGCGATCGCGCAGTTGCGGTTGTGTCACCAACTGGACAACA
TTTTCCACGGGGCCAACGGAAAGTGTCTGTTGTTCGCCAGGCAGATAAATAGTCACCGTTCCTTGCGCAAAGGCATGGGG
TGTCATTACGTAAAGTACGCTGGCAATGAAATACGACTGTAATTTATTCATGGTGCGGGTTTAAGAAACGTCATTTCTAC
GGGAATAACCCCTGCGCCTAACATTTGCCGACTTTGACGAACCTGGCCAGTAGCCGAATCGACCCAGAATGTGTTTTGCC
ACTGTTTTTCTGGCCGCGTGGAAGTCACCTCTTCCTGCCAGACATTACACAGCACATTGCGACCCGCAATATTCAGCGTC
TCTAATCCAGCAAAGGAAAAAGTAGAACTCAGGGTCGCAGAGCGAAAATGGTTGTCTTCACTCCAGAGAATATCGCGCGT
CCAGCGGCTGCCATCTTTTATTGCCAGCGCGTTACGCAGAGGGTCCTGCCCGGAATTTGTCACTTCCAGCAGATTATTAT
TAAGTTTGACGGTTTTTAAAAGACGTCCATTGTGGGTAACCAGCATGGCGTTATCCTGGGATAACCATTTGCTTTGTTCT
TGTTCTATATATCCCAGTACCACAAAGATTCGTTGCCCTTCATTAAGGCGTAAATACATAGTGGAATAAGGCAACGCCTG
GATCTGCTGATCCGCTACGGTGATATCCTGATTATCGAAAAGGCTGGCGCGAAATGTATCGACCATACTTTGCTGGCTGT
GCGTACATCCCGCAAGAAATAGTGCGAAAATCGATAAAATAAGAGGGCGCACCTTTAATCCTTGAAAGGTTAATTAAAAA
AATGCAAATCTCTAAAGCCCGGTACGAATATCGCAACCGGGTATCATTTAACTTTAATTTCGACGCGTTAAAGCAGTATT
AGCGTGTAGTGGACGTGGTGGTTGAGGTGTTATGAGAACCGCCATCGCCACTGTCATTTGCTGCAACAACTGCCAACGTC
GCCACACCAACAGCCGTTGCCACACCGACAACTGTCGCCGTGCCGACAGCGCCAGCAGCCACGCCACCAGTGGTAGTAGC
AGTCGTGGTTGCGCCGGTAGTGGTCGTTGTGGTGCCAGTAGTACCCGTCGTTGCCTCGGTCCCGTTTGTTGCTTCAGGGG
CAGCAAACGCAGCTGGCGTCGTCAACATGAAGGCCATAAGGATTGCAGAAAGCTTGTGTTTCATAACTTTTCCTTTATTC
ATCGCATGGACAATACGGGTGATGCTGCCAACTTACTGATTTAGTGTATGATGGTGTTTTTGAGGTTCTCCAGTGGCTTC
TGTTTCTATCAGCTGTCCCTCCTGTTCAGCTACTGACGGGGTGGTGCGTAACGGCAAAAGCACTGCCGGACATCAGCGCT
ATCTCTGCTCTCACTGCCGTAAAACATGGCAACTGCAGTTCACTTACACCGCTTCTCAACCCGGTACGCACCAGAAAATC
ATTGATATGGCCATGAATGGCGTTGGATGCCGGGCAACAGCCCGCATTATGGGCGTTGGCCTCAACACGATTTTCCGCCA
TTTAAAAAACTCAGGCCGCAGTCGGTAACCTCGCGCATACAGCCGGGCAGTGACGTCATCGTCTGCGCGGAAATGGACGA
ACAGTGGGGATACGTCGGGGCTAAATCGCGCCAGCGCTGGCTGTTTTACGCGTATGACAGGCTCCGGAAGACGGTTGTTG
CGCACGTATTCGGTGAACGCACTATGGCGACGCTGGGGCGTCTTATGAGCCTGCTGTCACCCTTTGACGTGGTGATATGG
ATGACGGATGGCTGGCCGCTGTATGAATCCCGCCTGAAGGGAAAGCTGCACGTAATCAGCAAGCGATATACGCAGCGAAT
TGAGCGGTATAACCTGAATCTGAGGCAGCACCTGGCACGGCTGGGACGGAAGTCGCTGTCGTTCTCAAAATCGGTGGAGC
TGCATGACAAAGTCATCGGGCATTATCTGAACATAAAACACTATCAATAAGTTGGAGTCATTACCGACAATACGTACGCT
TGAGATCAGGATTGTCTACTTTGAGTATAACATCCTTGTGAAATCTCCCTTTCCTCATACCGCTTGCACTATATACTGCG
GGTAGGAAAGGCGCGCAGAGGGAAATATAAGATTGTTAACTATCCCCCTCTGGAAATACGACTTGTGAGCATAAGATAAA
TCTTAGCAAAATACAAATTAAAGATAAGGAAATTGTTACGAAAGCTATTAATTATTGTTGGTAATATAGTTTCAAGTGGT
ACTATAACTGGCTGTTAAATGGCATTTATAACTATTAGGTGCAGAGATATTCGCTTAAATGGATTAGTTTCTTTGTGGAA
AAGTAACTGATGTTATAATGTATGATGTTGACTATTTCACTATTCCAATAAAACCAGTCAGCTTTAAACAAGCAGCGTCA
TATTAAGAGAGATAAACATTTGCCGCTGTTGGTCCTCGCAGGCCATTTACGCGGCAAAATTCCACACGTAATCCTGGTAT
AAGCACTTCTGCGTCGCGGGGAGTGAATGCGGAAATATGGACCTGAACTTCTTTACGACCGTCGGAGGGGATAATGAATC
CTTTGCCGCTTTTGCGATCAAAGGTTTTGACAATTCCTGTCATTTTACGGGACAAACAAATTCCTTACTGAAAATACTGC
GCTGCACTATACGGGGTTAATAAAATAAAGCCAGCGATATTTAAGACCGCCGGACGGCTAAAATAAAATTTGCTTAATCT
CAATTATCATGCGTTAATAGCTGCGTCGGTTTGAAAGACAGACAGCATACAAAGTAGTTTACTAAAGCAGTTCTCATTAT
CAGGCATTATCCCCTTCTTTTGAGTCTCTCTCCTGAACACTAAGTAGTTTCTGTATTAAAGCCCTGTTTGCCGAAAGGCC
CAAAATGAAGGAAGTAAAATATGTCTAATAAAATGACTGGTTTAGTAAAATGGTTTAACGCAGATAAAGGTTTTGGCTTT
ATCACTCCTGATGATGGCAGCAAAGACGTTTTCGTCCATTTCACCGCCATCCAGAGCAATGAATTCCGCACGCTGAACGA
AAATCAGAAAGTTGAATTTTCTATTGAGCAGGGGCAACGTGGCCCCGCGGCAGCGAACGTTGTTACGCTCTAAGGTTGCC
ATTATTACTCAACATCTCCATTTCCGCTGTCCATGTTGTCATGGTTCACAGTACCGCACATCGGCATTCGATGTGACGGA
GCGAAACCCTTTGGGCGCTAAGTGTATTTTTTGTAAATCGACGATGATCACCTTTGATAACGTCGCGCTGCAAATACGCA
CTGACCATGCGCCGCTGGATTTCACAAAATAATATCAGGCTCCCTCGTGGAGCCTTTTTTATATCTGCCTTATTTTTCTT
CAACGCTGTATGTATAGTAAGCGATAACCTGTTGATTATTGAATCTTTCGGGGAGATGGCTTATAACATTTCTTACCTGA
CCAGGGTACCGGGAACCAACACCTTACTGGCGTGTTGCTGTCTTTTAAGACCAGAAGAGGTTAACAGTGAATATTGAAGA
GTTAAAAAAACAAGCCGAAACGGAAATCGCCGACTTTATCGCGCAAAAAATCGCCGAGCTGAACAAGAATACAGGGAAAG
AAGTCTCTGAAATTCGCTTCACCGCACGAGAAAAAATGACCGGGCTTGAAAGTTATGATGTCAAAATCAAAATAATGTGA
TTTTGTGAACATCACCCCGTGCGAGGTGATGTTCCGCTTGTTGCTAATTTAGTGACCAATCATTGGCGCTTGTGGAATTA
AGCGTCGGTACAATTCCTCCGGCACCGGGCTTTGCCATACTCCCGCATACATTGCGTAACCAATCACCGCAAACATAATC
CCCAGAACCAGTAGCGTCATTAACCAGCCAGACAACGCAAAGGCTTTTTTATTTGCCGCAGGTTTTTGCAGTGAAAAGGT
CAATGTTGAGGCTACCGGACATGACTCTACGCAAGTCATACAGCCGGTACATTCCACTGTTCGTACCTGAATTAATTTAT
CGACCGGGATCCGTGATGGGCAATTTTTTGCGCATTTGCCACAGTCGATACAACTTTCGGCATTGCGACGAATCTTAAAC
GGCGACAATAGCGAAACCACGCCCATCAGCGCGCCATATGGGCAAAGATAACGACACCAGGCATGGCGAATAAACAGGCT

GGCAATCAGCAAAACGGTCACGCTGATTAATGTCGCGGTCCCCATATGACGAAAGAAATCGAGCATTTTAACGTCCATCA
CCACGCTGTAGGGCGACAACATAAAATAGTGAATCGCCTGAGCGGGCATCAATAACGCGATATAGATAAAAAAACTCAAC
AGCAAATACTTCACGCCGCGCAGAGGAATATCCAGCCAGCGGGGAAGGACACATTGCCGACCAAACAGTTTGTTACCGAG
ATCGCCGATTAATTCAGAAAGCGTACCAACCGGGCATAACCATGAGCAAAAGGCCTTTTTGAGTAATAGACTGATGACGA
TAAAAGCGACCAATAACAGCATCGCGGCGGCGTGGACGGACGGTAACTGACCTGTTACAAGGCTATATTTCAGATTCATC
AGCCCGGCAATCGGTAGCCAGCCTTCGATACCTCCCGGTCTGGCGACAAATGTCGTGCTACTTGCCGTTTCGTAATAGCG
CACCCAATACCAGAACGTGATGGCAATATAAATATTCATTGCCAACAGTAATAATTGCGTCGCTTTACGCCAGGTCGTGG
CATTACGCCAGTCATTCCACGGTAATTTGCCGCCCGTCGTGCCTGGCCGCCGCTGCCAGCGGGTTCTTTTATTCTCTGCC
ATGATTTTGCCAGTCCGTTAAGTTGTATACCAAATGCCACTATTCTAGTTGTTCTTAACTGGCTGATATTGATTCAAATC
GCGTTCAGGTCTTTCTTATGCAACCATGCTTCCAGAGCGGCAACACTGCGTGTAATTTCTTCGTGTGGAAGGGGGGCAGA
TAATGGCTGCTGCTCCAGTTGTGCGCATAGCTGGCTGGCGATATGCATTCCCAGACTCGAGCAACTGCTTTTTAGCTGAT
GCGCGGCACGCTTTATTTTCTCGCTATCCTGACTGGCGCGGGCAATGTCGATTTCATCGAGAAGCGGCAGGGCATGTTGT
GTAAATAATACCAGCCATTCGTGGATCTTCTCCGTCCCCATTAACTGAGCATCTTCATTGAGTTGCGATACATCCAGCGA
TTGATCATTATTGACTTGCAGTTGGAGATAGTGCGCCAGTAACTGACCGAGCACTTCACGCGGCACCGGTTTAGGGATAA
TCCCGCGGAATAATGAACTGGTACGCTGGCGCAGCGTTTCGTCAATGACATGGGCGCTAAAGCCAATCAAAACCAGCGAC
GGATATTGCTGTGCCAGTTGTCGGGCAAGCGTAATGCCGTCGATATCCGGCAGATCAAAATCCACCAGTGCGGCAGCAAA
CGGTTCGCTATTTTGCAGTGTCTCTAAAGCCTGCGCGGCATTGCCAACAGCAACAATCTGCGCACCACTGGTTTTCAGCA
TCTCAATGGTAATTCGCTGGGTTAGCGGGTTATCTTCAATTAACAGCAAACGTAAACCGTCAAGACGCACCGCCTGATTG
ACTGTTTTTGGCACGGGTGCCGTGGCAACACGTAACGGCAAGCGTAAACAAAAACAGCTTCCAACCTCCGGCGTGCTGGT
GGCGCTCAGTTCGCCGCCCATCGCCTGGGCCAGACGGCTACTGATAGTCAGTCCCAGCCCGGTGCCGCCGCGTTTGCCGC
TTACCTGCACAAATGGCTGGAAGATTTCTGCCAGTTTCGCGGGATCAATACCGCAGCCGCTGTCTTCCACTTCGACCAGC
CATTGCTCGCCATCAGTGCGACTACGCAGGATAATGTACCCTTCGTCAGTAAAACGCAGGGCGTTGCTCAACAGGTTGGT
TATAACCTGACGAATACGTCGTGGATCGCCCATTAACGCGCACGGCATATCATCGGCAATTGCCGTTGCCAGGCGAATCG
GGCGACCTTTCACCCGTCCGCTCATTAATTGCAGGGTACTTTCCAGCAGCGGGCGCGGTTCAAAGGGCTCATCGCTGACC
GAAACATTCTTGCCACCTGCTTCGATAGCGGAATAATCGAGAATATCGTTGAGGATGGTCAGCAACGATTCACCAGAGTC
AGTAATTGCCCGCAAATCATCACGCTGGGCGTTAAGTGCGGGGTTATCTGCCAGCAGTTGAGCAGTGCCGAGAATACCGT
ACAGCGGTGTGCGGATCTCATGGCTCATCGCCGCCAGAAACGCCGATTTTGCCTGGCTGGCTTTTTCTGCTTCCGCCCGT
GCCTGTCGGTGTTCTATCACCAGTTCCTGCAATTCAGCTGTACGCGCTTTGACCTGCGCCGCCAGCTGTTCGCGGTGGCG
ATTCAGTGCATGAACATTGCTGCGAAACGCATCCATCAGCCGCCCGATGGTATCCAGCTCCCGTACGCCAGCGGTTTCCG
GGAAAGGGGAGTCAATATCACCGTCCAGCAGCCGTTGCAGCGCCTGCGTTTGTTCGGCAAGTGGACGCGTGACTGAGCGA
TAAACCACGCGCCAGAGGATCAGAATCAGTGCGCAAAGTGAAACCATCCCCAGCAATAACAGGCTGTATTGCCCGCGTGC
ACTCGCTTTTTCCAGATGCGCCAGTCCGTGCTGATTACGCAGCTCAATGGTGTCGACCAGCTGACTGACTTCGCTACTAA
ACTGCGCGAACTGGGCGATGTTATTTTGTGCGAGAGTTTGTAGGTGATTGCTGATTTCACTGTCCTGCTGATACAGCGCC
AGCAAATCGCTATATTGGCTAACGGTAGTTAACGTTGTTGCGACCTGCGCACGAACACCCGGATCTTCAATGCGTATTTG
CCGACGTTGCAGAATTTTCACCGCATTATTGAGCTGCTTTTCCAGCGTTGGTGCATTTTTCTGGATCTGCTCCAGCCCCA
GATTCATCACCATTTGCTGCACCCGCAGAGCGCTAAGGCGCAGTTCATTCATCTGGTTAACATACTCAAGATCGATATCA
ATCAGCCGATCGAGTGCACTTTCAGCAGCCTGACGCTGATCTTGTTCGATCAAATCGTAAATCCCGGCCTGGGTCGCTCC
AGCGGAAGTTGTCGCATTATTCGCCTGACCTTGCGCCAGGCGTGCGATCTCATCGGCGGCAGCGACTATCTGCTGACTGA
GTTGCTGTTGTTGCTGGCGCAGTTGCAAACGCTGCCCCACCAGTTCCCCTTGCTGACGTAACGAACGGGAGATCTCCTGC
TCCTGTTGTTCAATAGCGGTGGTATCAAAACCTTGTTCCCGTAACGCTTGCAGCAACGCATTAATCTTCAGGCTTTGTGC
GGTGAGCATTCGCCCCTGCGCCTGCCACATCTTTTCGTTATCGGCACTGGTCAGGTTCTGCGCGGCGAAAAGTTCCCAGG
CGCTGGCTTCGCTCAACTGGCGCGCCATATTCATGGTAGGAATCAATGCCTGAGTGTTGTCTTTTTCCACCTGGCTGATA
AAGCGCAGGTTGTACCATCCCACCAGGGTACTGGTCAGGGTTAACAGCGCCATCAGGGCAAAGCCCATCCAGAGTCTTCG
GGTCAGGGTTAAATTCACGGTCGGTGCACTTTAGGTGAAAAAGGTTGAGTCGCAAAGCGGAATGCATCTAGCATAAAGCC
TTATTATTGATGAGGCTATCATGCGCGTACTGCTATTTTTACTTCTTTCCCTTTTCATGTTGCCGGCATTTTCGGCTGAT
AACCTGTTGCGCTGGCATGATGCGCAGCATTTCACGGTGCAAGCCTCTACGCCGCTTAAAGCCAAACGCGCATGGAAACT
GTGCGCGCTTTATCCCAGCCTGAAAGATTCATATTGGTTATCGTTGAACTATGGTATGCAGGAGGCTGCTCGCCGCTACG
GTGTGGATTTAAAAGTGCTGGAGGCAGGCGGCTACAGCCAGTTGGCTACCCAGCAAGCACAAATCGACCAGTGTAAACAG
TGGGGCGCAGAGGCCATTTTGCTCGGTAGTAGCACGACCTCATTTCCCGACCTGCAAAAGCAGGTAGCAAGTCTGCCGGT
GATCGAACTGGTAAATGCTATTGATGCTCCCCAGGTGAAAAGCCGCGTTGGTGTGCCCTGGTTTCAGATGGGCTATCAAC
CGGGGCGATATCTGGTGCAATGGGCGCACGGTAAACCACTGAATGTGCTGTTGATGCCCGGACCCGATAACGCCGGGGGC
AGTAAGGAGATGGTCGAGGGTTTTCGCGCAGCCATTGCCGGAAGCCCGGTGCGTATTGTTGATATTGCGCTTGGTGATAA
CGATATTGAAATCCAGCGTAACCTGTTGCAGGAGATGCTGGAACGCCATCCAGAAATCGACGTCGTTGCCGGAACGGCCA
TTGCGGCAGAGGCGGCAATGGGGGAAGGGCGTAACCTGAAAACGCCGCTTACCGTGGTGTCGTTTTATCTTTCACATCAG
GTGTATCGCGGGCTGAAGCGGGGAAGAGTGATTATGGCTGCCAGCGATCAAATGGTCTGGCAGGGGGAACTGGCGGTTGA
GCAGGCCATCAGGCAATTACAGGGGCAATCGGTTTCTGATAATGTCAGCCCACCGATTTTAGTTCTGACGCCGAAAAATG

CCGACCGTGAACATATTCGCCGCTCGCTGTCACCAGGGGGATTTCGTCCGGTCTATTTTTATCAGCACACATCAGCGGCT
AAGAAATAACCTTCACCATGTTGCGTCACCAGTAAATCCGCGCTGAGTTTATGACGTAAACGACGAATTAACACATCGAC
GGTGCGCAGGTCAGGGTTTTCCACCCGACGCGCAGAAAGCATACGTAGCAGACGTTCACGGCTGAGAATTTCGCCCGGAT
TCGTCACAAATGCCACCAACATTTCATACTCTGCGCGGGTCAGTTTAATCGGCTCGCCATCCCGCTCCAGCGTATGGCGC
GACACATTCAGGCAATAACCGGCAAAGCGATAGCAGTTGTCCTGAGTGTGCGGTTGAGCTTGTCGCGCGAGGTCGATTCG
CCAGAGCAGATTTTTCACCCGTACTACCAGTTCGCGCAGTTCCAGCGGTTTGGTGACGTAATCGTCTGCGCCCATTTCCA
GCCCAACAATACGGTCAATCCGATCGCTGCGTCCGGTAACCAGAATAATCCCCACCGTTGAGCGTTCTCGCAGGGCGCGG
GTTAACATCAGGCCATTTTCATCGGGTAAGTTGATATCCAGCAGAATTAAATCTACCGACTGATTCTGCATAATTTCCCG
TAGCCCAGCACCGCTCGCCGTAACGGAAACGGTATACCCCTCCTGAGTGAAGTAGGATTGTAATCGCGCCTGGGTAACCG
GCTCATCTTCAACAATAACAATGTGATGTGGCATCAGAGGGTTTTACTCATTCTGTTCATATCTGTTCATATTCTGCCGT
AAGCCGTTCATCCTGACCAGTGCCGCTGTTCATATTTGCTCATTAAGATCGCTTCACTAAACCATAATTCTACAGGGGTT
ATTATGCGGAAACTCTGGAACGCGCTACGCCGACCCAGTGCTCGTTGGTCGGTACTGGCGCTGGTCGCAATTGGGATTGT
GATTGGCATTGCGCTGATTGTATTGCCACACGTTGGGATCAAAGTCACCAGCACAACCGAATTTTGTGTCAGTTGCCACA
GTATGCAACCGGTGTATGAAGAATATAAACAGTCGGTGCATTTCCAGAACGCCTCCGGCGTGCGAGCTGAATGCCATGAC
TGTCATATCCCGCCGGATATTCCAGGCATGGTGAAGCGCAAACTGGAAGCGAGCAATGATATCTACCAGACCTTTATTGC
TCACTCCATTGATACACCTGAAAAATTCGAAGCCAAACGCGCGGAACTTGCCGAGCGTGAATGGGCGCGAATGAAAGAAA
ACAACTCGGCAACCTGCCGCTCCTGCCATAACTACGATGCGATGGATCATGCGAAGCAGCATCCTGAAGCAGCACGTCAG
ATGAAGGTGGCAGCGAAAGATAATCAATCCTGCATCGACTGTCATAAAGGTATTGCCCACCAGTTACCGGATATGAGTAG
CGGCTTCCGTAAGCAGTTCGATGAGCTGCGCGCCAGTGCTAATGACAGTGGTGACACGCTGTACTCTATTGATATTAAGC
CAATTTATGCGGCGAAAGGCGATAAAGAAGCCTCTGGTTCTCTGCTGCCTGCTTCGGAAGTGAAAGTCCTTAAACGTGAC
GGCGACTGGCTGCAAATTGAAATTACCGGCTGGACGGAAAGCGCCGGACGTCAGCGTGTACTCACCCAATTCCCAGGTAA
ACGCATCTTTGTTGCCTCGATTCGTGGTGATGTGCAGCAGCAGGTAAAAACGCTGGAGAAAACCACCGTTGCCGACACCA
ATACCGAGTGGAGCAAGTTGCAGGCCACTGCGTGGATGAAGAAAGGCGACATGGTGAACGATATCAAACCGATCTGGGCT
TATGCGGATTCGTTGTACAACGGCACCTGTAACCAGTGCCACGGCGCACCGGAAATCGCCCACTTTGACGCTAACGGTTG
GATCGGCACGCTCAACGGCATGATTGGCTTTACCAGTCTCGATAAACGTGAAGAACGCACCTTGTTGAAATATCTGCAAA
TGAATGCGTCTGACACCGCAGGTAAGGCTCACGGCGATAAGAAGGAAGAAAAATAATGAACAATAACGATCTCTTTCAGG
CATCACGTCGGCGTTTTCTGGCACAACTCGGCGGCTTAACCGTCGCCGGGATGCTGGGGCCGTCATTGTTAACGCCGCGA
CGTGCGACTGCGGCGCAAGCGGCGACTGACGCTGTCATCTCGAAAGAGGGCATTCTTACCGGGTCGCACTGGGGGGCTAT
CCGCGCGACGGTGAAGGATGGTCGCTTTGTGGCGGCAAAACCGTTCGAACTGGATAAATATCCGTCGAAAATGATTGCCG
GATTGCCGGATCACGTACACAACGCGGCGCGTATTCGTTATCCGATGGTACGCGTGGACTGGCTGCGTAAGCGCCATCTC
AGCGATACCTCCCAGCGCGGTGATAACCGTTTTGTGCGCGTGAGCTGGGATGAAGCCCTCGACATGTTCTATGAAGAACT
GGAACGCGTGCAGAAAACTCACGGGCCGAGTGCCTTGCTGACCGCCAGTGGTTGGCAATCGACGGGGATGTTCCATAACG
CTTCGGGGATGCTGGCGAAAGCTATTGCCTTGCATGGTAATAGCGTTGGTACGGGCGGAGATTACTCTACCGGTGCTGCG
CAGGTGATCCTGCCGCGCGTAGTCGGTTCGATGGAAGTGTATGAACAGCAAACCTCCTGGCCGCTGGTATTGCAGAACAG
CAAAACCATTGTGCTGTGGGGCTCCGATTTGCTGAAAAACCAGCAAGCGAACTGGTGGTGCCCGGATCACGATGTTTATG
AATATTACGCGCAGCTAAAAGCGAAAGTCGCCGCCGGTGAAATTGAGGTCATCAGCATCGATCCGGTTGTCACATCCACC
CATGAGTATCTGGGGCGCGAGCATGTGAAGCACATTGCGGTTAACCCGCAAACTGACGTGCCGCTGCAACTGGCGCTGGC
ACATACGCTGTACAGTGAAAACCTGTACGACAAAAACTTCCTTGCTAACTACTGTGTGGGTTTTGAGCAGTTCCTGCCGT
ATCTGCTGGGTGAGAAAGACGGTCAGCCGAAAGATGCCGCATGGGCTGAAAAACTGACCGGCATTGATGCCGAAACCATT
CGTGGGCTGGCGCGGCAGATGGCGGCGAACAGAACGCAAATTATTGCTGGCTGGTGCGTGCAGCGTATGCAGCACGGTGA
ACAGTGGGCGTGGATGATTGTGGTTCTGGCGGCGATGCTGGGGCAAATTGGCCTGCCAGGTGGTGGTTTTGGTTTTGGCT
GGCACTACAACGGCGCAGGCACGCCGGGGCGTAAAGGCGTTATTCTGAGTGGTTTCTCCGGCTCTACGTCGATTCCGCCT
GTTCACGACAACAGTGACTACAAAGGCTACAGCAGCACTATTCCGATTGCCCGTTTTATCGATGCGATCCTCGAACCGGG
GAAAGTGATCAACTGGAACGGTAAATCGGTAAAACTGCCGCCGCTGAAAATGTGTATTTTTGCCGGAACTAACCCATTCC
ATCGCCATCAGCAGATCAACCGCATTATTGAAGGCTTGCGCAAGCTGGAAACGGTTATCGCCATAGATAACCAGTGGACC
TCAACCTGCCGCTTTGCCGATATCGTACTGCCTGCGACCACGCAGTTTGAGCGTAACGATCTCGACCAGTACGGCAATCA
CTCCAACCGTGGCATTATCGCCATGAAACAGGTGGTGCCGCCGCAGTTCGAGGCGCGCAACGACTTCGATATTTTCCGCG
AGCTGTGCCGTCGCTTTAATCGCGAAGAAGCCTTTACCGAAGGGCTGGACGAAATGGGCTGGCTGAAACGCATCTGGCAG
GAAGGTGTACAGCAAGGCAAAGGACGCGGCGTTCATCTGCCAGCGTTTGATGACTTCTGGAATAACAAAGAGTACGTCGA
GTTTGACCATCCGCAGATGTTTGTTCGCCACCAGGCATTCCGCGAAGATCCGGATCTCGAACCGCTGGGCACGCCGAGTG
GCCTGATTGAGATCTACTCGAAAACTATCGCCGATATGAACTACGACGATTGTCAGGGGCATCCGATGTGGTTTGAGAAA
ATCGAACGCTCCCACGGTGGGCCTGGCTCGCAAAAGTATCCGTTGCATCTGCAATCTGTGCATCCGGATTTCCGACTTCA
CTCGCAGTTATGTGAGTCGGAAACGCTGCGTCAGCAATATACGGTAGCGGGTAAAGAGCCAGTATTCATTAACCCGCAGG
ATGCCAGCGCGCGCGGTATTCGTAACGGTGATGTGGTACGCGTCTTTAACGCTCGCGGTCAGGTGTTGGCAGGGGCAGTG
GTTTCTGACCGCTATGCACCCGGCGTGGCACGAATTCACGAAGGGGCATGGTACGATCCAGATAAAGGCGGCGAGCCTGG
TGCGCTGTGCAAATACGGTAACCCCAACGTGTTGACCATCGACATCGGTACATCGCAGCTGGCGCAGGCGACCAGTGCGC

ACACTACGCTGGTGGAAATTGAGAAGTACAACGGAACAGTGGAGCAGGTGACGGCGTTTAACGGCCCCGTGGAGATGGTG
GCGCAGTGCGAATATGTTCCCGCGTCGCAGGTGAAATCATGACCACGCTGACAGCACAACAGATTGCCTGTGTTTACGCC
TGGCTAGCGCAGTTGTTCTCCCGTGAGCTGGACGATGAACAACTGACGCAAATCGCCAGTGCGCAGATGGCTGAATGGTT
TTCGTTGCTGAAAAGCGAACCGCCGCTCACTGCGGCGGTGAACGAGCTGGAAAACCGTATTGCCACGCTGACAGTACGTG
ACGATGCCCGTCTGGAACTGGCCGCGGACTTTTGCGGCCTGTTTCTGATGACCGACAAACAAGCGGCGCTGCCGTATGCA
TCGGCCTACAAACAGGACGAGCAAGAGATTAAACGCTTGTTAGTTGAGGCAGGGATGGAAACCAGCGGCAATTTCAACGA
ACCGGCAGATCATCTGGCGATCTATCTCGAATTGCTCAGCCATCTGCATTTTTCGCTGGGAGAGGGGACCGTTCCTGCGC
GAAGAATCGACAGTTTGCGGCAAAAAACACTGACGGCGCTGTGGCAATGGTTACCAGAGTTTGTTGCGCGTTGTCGTCAG
TATGACAGCTTTGGTTTTTACGCGGCACTAAGCCAGTTATTGCTGGTGTTAGTGGAGTGCGACCACCAAAACAGATAACG
TCGTTTGTGCGCCTGAAAAGACGCGTTTAGCGTCGCATCAGGCATTATGGCGCAGTTGCCGGATGCGGCGTGAACGTCTT
ATCCGGCCCACAGGAACTGTAATCTTTGTAGACCGGTTAAGATGCGTCATCGCATCCGGCAAACACACATCACGGATGAG
CTACAAACCGGGAAAGCCGCTGGCGCAGCAGGCGGTTTTCCTGCTTCAGGTGCGCAATATCATCCATTAACGTCAGCGCC
ACCGCGATCCCCGGCCAGTCCAGAGCCAGTTCATGACGCAGGCGTACCGCGCGTTGCACCACAATGGCGGCATGGTCGTC
AAATACCCAGGTTGTTTCCTGGATCTCACGCGGTTCAACCACCCCCAAACCGACAATTTCATTCAACTCCTCTTCAGAGA
TGCCGGTATGCAGGCAAAATTCGGTAATAGTAAAAGTCACCGTAACATTAGCCATTATGCTTTCCCCCAATCTTTACGTG
GATCAAAAGACGACTGGGCGTCTGCCAGTTGCTGCCACAGCGCGGCAGTGTTTTCATCCGGTTTCGGCGGCATCACGATT
TTCAGTACCGCATACAGATCGCCGGTCTGTTTTTTGCTCACCAGACCTTTGCCTTTAACGCGCAATCGTTGCCCGGCCTG
GCTGCCTGGCGGGATAGTCAGCAAAATGCTTTCTTTCAGTGTTGGAACGGTGACTTTAGCACCCAGCGCCGCTTCCCACG
GGCTAACCGGCACCACAATTTCCAGATCCTGGCCGACAATATCAAACAGCGGATGTGGCGCAATATGAATCACCAGCCAC
AAATCGCCATTTGGACCGCCGTTTTCGCCCGGCGTCCCCTGGCCTTTCAGACGGATGCGTTGACCATTGCCGACGCCCGC
CGGGATCTTCACATTCAGCGTTTTCGGAATTTCCTGTTCGATCATGCCAAAGGCGTTATAAACCGGCAGGTTATAGCTGA
TGGTACGCTTATGCTCAGTAAGCGTTTCTTCGAGGAATACCGCCACTTCGATTTCAATATCGTGGCCGCGTGTGGCGGGG
CGTTGACGGCTCTGGCGGGCATGCTGACCGAAAATTGACGAGAAGATATCGTCAAAATCTTCGGCGTTAAAACTCTGACC
GTCGCCATGGTGGAACTGACGGTTAAATTGCGGATCGTTGCGATGTTGCCACATCTGATCATACTCAGCGCGACGTTGTT
CATCACTTAACACTTCCCAGGCTTCAGCGACCTCTTTGAAGCGGGCTTCGGCATCCGGTTCTTTGCTGACATCAGGATGG
TATTTGCGGGCAAGTCGACGATAGGCGGTCTTGATTGTCTTGAGATCGTCCGTCGGTTTCACGCCCATGATGGCGTAATA
ATCCTTTAATTCCATAGCGTTATCTCGCGTAAATCAACACAAATTGAAGGAACCCCTGTAAGGTAACTCCTATAAGTGTA
GGGTAATCCTCAAAATTTCATATGCCAACACAGAATATGTTATTGAAATCATCGCGGAGAGGAGGTCGCCATCAAGATGG
GTTGCTGAACATATTTTAAACAGGTGAAAAAGGGTGAGCGATTTTTGATAGTTGAACCAGGCACTTTAAGTTTAACTAGG
GCGTCATTATTTATTAAATTTTATAGACGCTATATATGGGTAGTAATATACATGGAATTAGTTGCACTGCAAATAATTAT
TTGAAACAGGCCTGGAACGATATAAAAAATGAGTACGAAAAAAATCAAACATATTCAATCACGCTTTTTGAAAACACACT
GGTGTGTTTTATGCGGTTATACAATGAACTCAGACGTAAAGTAAATGAAGAGGATACTCCATGTCTGGAATGTGAATCAC
TAGAAAAAGAATTTGAGGAAATGCAGAATGATAATGATCTATCATTATTTATGAGAATATTGCGTACTAATGATACACAA
ATTTATTCAGGGGTTTCAGGAGGTATTACATATACTATACAATATGTTCGAGATATTGATATTGTTAGAGTGTCCTTGCC
GGGCAGAGCTTCAGAGTCTATCACAGATTTTAAAGGTTATTATTGGTATAACTTTATGGAGTATATTGAAAACATTAATG
CGTGTGATGATGTTTTTTCTGAGTATTGTTTTGATGATGAAAATATAAGTGTCCAGCCAGAGCGGATAAATACGCCGGGA
ATATCTGATTTGGATTCTGACATTGATTTGTCTGGTATATCTTTTATTCAGCGTGAAACTAACCAGGCATTAGGATTAAA
ATATGCTCCTGTAGATGGCGATGGATATTGTCTGTTAAGAGCTATACTGGTTTTAAAACAACATGATTATTCATGGGCGC
TGGTCAGTTATAAGATGCAAAAGGAAGTTTACAACGAATTCATTAAAATGGTTGATAAAAAAACGATCGAGGCTCTTGTT
GATACGGCATTCTATAATCTCAGGGAAGATGTAAAGACGTTATTTGGCGTTGATCTACAATCTGACAACCAAATTCAGGG
GCAGAGTAGTCTTATGTCATGGAGCTTTCTGTTTTTTAAAAAACAATTCATTGATAGTTGCTTGAATAACGAAAAATGTA
TCCTGCATTTACCCGAGTTTATATTTAATGATAACAAGAACTTGCTTGCTTTAGATACCGACACGTCGGATAGGATTAAA
GCGGTGAAGAATTTTCTTGTTGTTCTTTCAGATAGCATTTGCTCATTATTTATTGTTAATAGTAATGTGGCATCAATCTC
CTTGGGGAATGAATCCTTTTCAACAGATGAAGATCTTGAGTATGGTTATTTAATGAACACTGGCAATCATTATGACGTTT
ACCTCCCTCCTGAACTTTTTGCTCAGGCTTACAAGTTAAACAATAAGGAAATGAATGCGCAACTCGACTATTTAAATCGT
TATGCAATTTAATGGCAAAGGCATATGCTAAAAACCATTGTTATTAGTCTCACACTTTTTTATTGGTAAATATTGTCTCT
GTATTGGTAACGCCGCAGATATTCTGTTTAGCCACAGGTGCAATTATCAGCGGCGTACGCGAGGCAGGGGCTAATCAGGC
ATAGTTTGCGTCAAACCTTGCCTGTTTTTGAAGATGTATATAGAAAAACAGGCGTTCAACAAGCCATTTTGCGAACCTGT
TCCCGGAAAAAAAGTCATATTTCTGTCACACTCTTTAGTGATTGATAACAAAAGAGGTGCCAGGAATGAACAAAACGCTAA
TCGCCGCAGCTGTGGCAGGGATAGTTTTACTCGCTTCAAACGCTCAGGCACAAACCGTACCGGAAGGCTATCAGCTACAG
CAAGTGCTCATGATGAGCCGCCATAACTTACGTGCGCCGCTGGCGAACAATGGCAGTGTGCTGGAGCAGTCGACGCCGAA
TAAATGGCCAGAATGGGACGTCCCCGGTGGGCAACTCACCACCAAAGGTGGCGTGCTCGAAGTGTATATGGGCCATTACA
TGCGTGAATGGCTGGCAGAGCAGGGGATGGTGAAATCGGGGGAATGCCCGCCGCCGTACACCGTTTATGCCTATGCCAAT
AGTCTGCAACGTACCGTTGCGACCGCACAGTTCTTTATTACCGGCGCATTCCCGGGGTGTGATATTCCTGTGCATCACCA
GGAAAAAATGGGCACTATGGACCCAACCTTTAACCCGGTGATCACCGATGATTCCGCCGCATTCAGTGAACAGGCGGTGG
CGGCAATGGAGAAAGAGCTCAGCAAACTCCAGCTTACCGACAGCTACCAGCTACTGGAAAAAATCGTTAACTATAAAGAT

TCCCCTGCCTGTAAAGAGAAACAACAGTGTTCGCTGGTGGATGGCAAAAATACCTTTAGCGCCAAGTATCAACAAGAACC
AGGTGTTTCCGGGCCGCTGAAAGTCGGCAACTCGCTGGTAGATGCGTTTACTTTGCAATATTACGAAGGTTTTCCGATGG
ATCAGGTGGCCTGGGGAGAAATCAAATCTGACCAGCAGTGGAAGGTGTTGTCGAAGCTTAAAAACGGCTACCAGGACAGC
CTGTTTACCTCACCGGAAGTGGCGCGCAATGTTGCGAAACCGCTGGTCAGTTATATCGACAAAGCTCTGGTCACCGATCG
CACCAGCGCACCGAAAATTACAGTGTTGGTTGGGCACGACTCCAACATTGCCTCTCTGTTAACGGCGCTGGATTTCAAAC
CGTATCAGTTGCATGACCAGAACGAACGCACGCCGATTGGCGGCAAAATCGTTTTCCAGCGTTGGCATGACAGCAAAGCC
AATCGCGATTTGATGAAAATTGAATATGTGTATCAGAGTGCGGAACAGTTACGTAATGCCGATGCGTTAACCCTGCAGGC
ACCTGCGCAGCGTGTGACGCTGGAATTAAGCGGTTGCCCGATAGACGCTGATGGTTTCTGCCCGATGGATAAGTTTGATA
GCGTGTTGAATGAAGCGGTGAAATAACAGAAAACTCCCCCGCGAGAAGCGGGGGAGTCGCTGGTTAAACGTTTTTACGTT
CGATGGTCTGTTCGCCCCAAAAAAGCGAATCTTTATCGGTCTTAGCAAAGGCTTTGACTAACACTTCATCACTACCTTCT
TCCCAAATCTTTTCCGCCATTTTTTCGTCGTACCCGGCGACTTCGAAAATGGCCTCGGCTATTTCCGGCGACGTATTGCG
CAGAGATGCCCATTCACCGACGTGATGAGCTTTCGCTTCTTGAGTTGGCATGCGTATCCTCCTGTTGAAGATTAGCCGTT
AAGTTTAACTGCCAGACCTGCGACATATTCCCCTTGATAACGAGCAATAGACAGTTCTTCCTGGCTGGGCTGGCGTGAAC
CGTCACCGCCTGCGATGGTGGTTGCGCCGTACGGCGTACCGCCGCGAACCTGTGAAACGTCAAATAATTCCTGCGCTGCG
TAGCCAATAGGGACAATTACCATGCCGTGATGCGCAAGGGTCGTCCAGGTGGATGTGATGGTTTGTTCCTGACCGCCGCC
AGTACCGGTGGAACTAAAGACGCTCGCCAGTTTTCCGTATAGTGCGCCGGAAGCCCACAGGCCGCCCGTCTGGTCGAGGA
AGGTACGCATTTGACCGGACATGTTGCCAAAGCGGGTAGGTGTACCAAAAATAATGGCGTCGTAATCGGCCAGTTCTTGC
GGGGTTGCAACCGGTGCAGTTTGCGTTTTACCGCCTGCTTTTTCAAATAATTGCGGCGGCATGGTTTCCGGTACACGCTT
AACGACAACTTCAGCGCCATCCACTTTGCTTGCACCCTCAGCGACTGCGCGTGCCATCGTTTCAATATGTCCGTACATGG
AATAATAAAGCACCAGAACTTTAGCCATTTCTAACCACTCCTCGTGTTATCTCTATTCCGTAGCGATTCGCTACCACTTA
TTTAAAGATAAGACGTCCTTTTCAGAGTGCAAATTTCACAACCACTTATTTGATTTATAACAACTTTCACAAGCACGTAA
TTTTGTCGCAAAATGACACATTTTTATCTCATCGCGTTTTTTTAATCATAAGAGCGGCTTATGGATAATTATTGGAGATG
ATATCTATTCTCGCTAAGAAGCTGTTGCAGGATATTACCAAACGCGGGTCTGCCCGCGTCAGTTCACTAAGCTTAGTCCC
ACGTAGCGAAAATATGGCAGCCGCCATACGCCGCGTTAATTCTATGCAATATGATGTCTATACCCAGACGGAGGTCAGTA
ATGGCAAACCATCGAGGCGGTTCCGGCAATTTTGCAGAAGACCGCGAAAGAGCATCAGAAGCAGGTAAAAAAGGTGGACA
GCACAGCGGGGGTAATTTCAAGAATGACCCGCAGCGCGCATCTGAAGCAGGTAAAAAAGGTGGTAAGAGCAGTCACGGCA
AAAGCGACAACTAGCCGGGCTAATCAATGACGAATGCATTTTTGTCTGTAGCTCGTCAAAAAGCCATCACCGCCGGTTAC
CCGGTGGTTGATACTGATGACAAATGTAAGCTTGCCTGATGCGCGATGCTTATCAGGCCTACCAGAAGATTGCAATATAT
TGAATTTGCACTGTTTTGTAGGCCGGATAAGGCGTTTACGCCGCATCCGGCATGAACAATGCGTACGTTGTCAACAATCT
GCACCGCCGGTAACCCCGGCGGTTTTCTGTTTATGGCTCCTGATGAACAACTTCTGGCGGTGGAACGTCAACCAACTTTC
TGCTTAACAACGCATTGAGTAAAATCGCGCCAAAGGTTGCTGTACCAATCCCTCCCAACGTAAAACCGCCCAGCGTGAGA
GCAAAATCACCCGCGCCCAGCACTAAGGTTACTGCGACCATAATCAAATTACCGTTCTGGCTTAAATCGACACGGTTTTG
TACCCATATCCTTGCGCCTGCGACGGCAATCAGCCCGAACACAACAATTGATGCACCACCAATAACCGCGGCCGGAATGG
TATGAATCAGCGCACCAAATTTCGGTGAAAAGCCCAACAGCATGGCGATGACGGCAGCAGCAACAAACACCAGCGTCGAG
TAGACTTTGGTCACGGCCATCACACCGATATTTTCAGCATAGGTGGTCACGCCGCTACCGCCGACAGAGCCGGAAAGCAT
CGTTGCCAGACCATCGCCTACGAATGCCCGCCCCATATACGGGTCCATATTGCGTCCGGTCATCCCGGCGACTGCCTTGA
GATGACCTAAGTTTTCCGCCACCAGAATCACCGCCACGGGCGCAATCAGCATCATTGCCTGACCATTAAAAGCAGGAGTG
GAAAAATGTGGCAGACCGAACCAGGCAGCATGGCTGACGAGAGTAAAATCGACGGCTTTTCCCAGCCCTAAAACGTTGGT
CATCACGCCATACAGCAGACAGGCGACAATTAATCCTACGAGAATCAATAACCGCTGGATCATGCCACGGGTAAACACCG
CCACCAGCCCAATACACAGCACCGTCATTACCGCCATCCAGCTATCAAAGGCCGAAGCCGATACACTTTTCACTGCGATA
GGCGCTAAGTTCAGGCCAATCGCCATCACCACCGCACCCGTCACCACCGGCGGCATCAGTCGTTCAATCCAGCGCGTACC
GATTTTCATCACCACCAGGCCAATGACGGTATAAACCAGCCCACAGGCGATAATCCCGCCCAGCGCAATGCTGATATTCG
GGTTAATGCCCTGACCGTTAAAGCCCGTCGCGGCGATCACCACGCCGACAAAAGCCGCGCTGGAGCCGAGATAACTGGGG
ACGCGCCCGCCGGTAATAAAGAAAAACAGTAACGTGCCGATCCCCGACATTAAAATGGAAAGATTGGGATCCAGCCCCAT
CAGAATCGGCATTAACACCGTCGCGCCAAACATCGCCACCGCGTGTTGAACGCCCATTACTGCCGTCTGAGCAAACGGCA
ATCGTTCATCCGGCGCGACCACGCCGCTCTCTGTAGAGGTCGATTTTAACTGCCAGTGAGGAAAACCGAACATTGCCATC
AGCTGTCTCCTTAAGGAGGTTAACAAGCAGGGCGCATCAGCGCGTGATAACTGCGATCGAACCACACCAGCCCGTAGGGT
GTGGTGTGACGATGAATCGCTTCGATGGCGCAAAACAGAATGTCGTGGGTGCCGACGCTCACCACCTGGCTGATACGGCA
GTCAAACGAAACCAGAGCCTCTTCCAGTTGCGGGCATCCGGTCACCCCCGTCTGCCAGCGGGCGGCGGCAAAGCGGTGTT
CCATGGGCGTTTTGCCGCCAAAAAGGTTTGAAAGCGGCTCCTGCCCGGCGCTAAGTGTATTTACACACAGCGTTCGATTT
TCATTGAATGCCGGCCAGACGGACGCCCCACGATTCAGGCACACCAGTAATGTGGGCGGCGTATCGGTCACACTGCAGAC
GGCGCTGGCGGTGAACCCGGCGCGCCCGGCTGGACCGTCCGTGGTGATAATATTGACCGCCGCGCCCATGCAGGACATCG
CATCGCGAAAAGTTTGTTGATCGACAATGTTCATAGTTTGCTCCTTACAACAGCCCGCAGGCTTCTTCAAAGGACAGACG
TGGCAGGCGCGCATAAAGCTTGCTGCTATCGCCATAGCCGATATTAATCAGCAGATTGCTCTTCAGCGTGCTGCCCGTAA
AAAAGGCGTCGTCCACGTGTTGACGGTCAAAGCCCGACATCGGGCCGGTATCCAGTCCCAGCGCCCGGCAGGCGACGATC
AGATAGGCCGCCTGCATGGAACTGTTGCGAAACGCTGTTTCTTCGGCAAGTTGTGGGCTGGAGGTAAACCAACTGCGGGC

ATCACCGTGGGGAAACAGTAGTGGTAACCGTTCATAAAATTCACTGTCCCAGGCGACGATAGCGGTGACGGGCGCGGTCA
GGGTTTTTTGCAGATTGCCGCTGGAAAGTGCCGGGCGCAGACGTTCTTTTCCTTCTGCCGTGCGGGTAAACACGATCCGT
GCCGGAGAACAGTTAGCTGATGTCGGCCCCCATTTCATCAGGGCATAAATCTCCCGTAACGTCTCATCGCTGACGGGTGT
CTCCCGCCAGCCGTTGTGAGTGCGGGCATCGGTGAACAGGGTGCTAAGCGCACCTGGGCTAACGGCTTCGTTCATAGCAA
TTCCTTACAGGGCGGCTTCACGGTGATGTAACAGGCTGGCAAGCCCGTTGAGTAACAGAGCATTAAACGTTTCGGGATCG
GTCACGTTGCAGGCGTGTCCGCCATAGGGCATCACCATTTTCTGGCTATCGGGCAGGGCGGCATGAAGTTCACTGGAACA
TGCTGTTGGCACCAGCAGATCATCACTGGCGCAGATGATTTGCACCGGGCAGCGGATGCGATCCGCATGGTGACTAAAGT
CAGCGCGTTTGAGGGCGTTAAGTCGACGCAGTAAATTATTTTTGCCCTGAAAATGCGCCAGTGCCAGCGCGTCTTCTGCC
TCCAGGCGAGGTGCGCGGGCCGCCATCCAGTCGGCGGGATAGAGGAACAACGGCTGCGCTTCCACCCATGCCTGCGCGCC
GCCGCTATACAGTAATCGTTCGCGAACCTGAAAACAGCGGCGCGTATGGGCGTTTATTCGTAGCCAGCCGTTAACGCTGA
TCAGCACAGTTACCGACGCGGGATAATCCAGCGCCAGCTGCATTCCCACCAGCGCACCGAGCGCATGGCCGACCACTGCG
TAATGCTCAATCCCTGCGGCTACCAGCGCCTGATGCAGTTCCGCTGCCATCTGGGCGATACTGTAATCTTCTGCCAGCGT
GTCGGGATTATTGCCGGTGCCGCGCTGGTCGTAACAGACTACCTGATACTCCTGCTCCAGCACCGCCAGTTGCGGTAACC
AGTAACTGCCGCTACCCCCAAGACCCGAAATCAACACCACTACGGGCGCATCAGCATAAGGGGGAGGTGAGAGTGAAAGT
TTCATCGCGGCCTCACTTGGCGATATGCGCAATTGTGGCGATTTCCACCAGCGCGTCAGGTTTTACCAGTCCGCACTGAA
TGCAGAATCGCGCCGGTTTATCACCCGGAAAAAACTCGGCGTAGATTTCGTTAATCGCGGCGTAATTTTTCCAGTCGGTA
ATAAAGATGCTGTTGAAGGTCACATCCGCCATCGTGCCACCCGCCGTCTCGATCACCTTGCGGATAGTTTCCAGAACGTG
GCGGGTTTGCGCCTTTGGGTCATCGGCAAACAGCACGTTATTATGTTGATCAAAAGCCAGCGTACCGGAGACATACACCA
CGCCATCAGCCAGCGTGCCGGGAACGAAGGGGGCCAGCGGTGCGCTGCTGCCAGCGGGAATAATTACGGATTTTGGCATC
GTTAAACTCCTTAAGCGATATGAGCAAAGGACGTGGGAGAAAGCGCGTCGCAGAATGTTTCGACGTCGCTGACCCAGCCA
AAAAAGGTTTCGATATTGAACAACGCGGCTTTCTGCGCAAATTTCGGCCCCGCCTGGTGAGTTGCGTCTTCAAGCACCAC
GCCGAAATACTCCAGAAAAAAGCCGTCGCGTAGCGTCGATTCGACGCAGACGTTGGTAGCGATGCCGGTGAAAACCAGAT
GGCGTATTCCGCGGCTGCGCAAAATGCTGTCCAGCGGCGTATTGAAGAAACCGCTGTAGCGCGGCTTCGGCAGCACAATA
TCGCCAGGCTGCGGCACCAGTTCATCCACCAGTTGATAATCCCAGGAGCCTTTCGCCAGCAATTTCCCCTGCAGCTGCGG
CTGCTTACGCATGGTTTTCAGGGCGTTCGATTTATGAAAATTCGGTGAGCCGGGTCCGCCAGCCTCGACATACTGTTCAT
CCCAGCCATTTTGAAACCAGATGATCAGCATCCCTGCCGCTCGCGCTGCGGTCACGGCGGTTTGAATGTTGGCAATGACC
GGGCGAGTGGTTGAGACATCAAACCCGGCGAGATCTAAGTAGCCGCCTGGCGTGGCATAAGCGTTTTGCATATCCACCAC
GATCAGCGCACTTTGCTGCGGATCGAAGGTAATGGCTTCCGGTCGAGCGGTTAAGGTCGTCATCATGCCACCTCCTGAGT
CAGCGCAGGTAGATGGGCGCGGCATTGCATCAGTGGTTGAATGCGCTCGCCGAAGGTTTCGATTCCCGACAGAAAATCGT
CGAAGGTTAACAGCACGCCTTCGGCACCAGGCACGCTTGCGACTTCATCTAACATGCGCGCGACACTGGCGTAAGAACCG
ACTAACGTCCCCATATTGATGTTTACCGCCGAAGTGGGATCGGCCATCTGACGAACGTTGGTGTCAGTACCGGAGCGGGT
ATCTTTCTGACTTTGTTCGGTTAGCCAGCTTAACGCCTCTTCATCCGCGCCCGCTTTGTAGTGTTCCCATTTGGCGCGAG
CGGCATCGTCGGTTTCATCGGCAATCACCATAAACAACACATAAGAGCCAACGTCGCGTCCGGTTTGCTCTGCGGCCTGT
TTCATCCGCGCAGCGGTCGGGGCGAAAGCCGTGGGTGTATTTACGCCTTTGCCGAAACAGAAGTTGAAATCGGCATACCG
GGCGGAGAACGCCATGCCAGCGTCGCTTTGCCCGGCGCAGATCACTTTCATGGGGACACTCGGTTGCGGACTCACGCGAC
AATCATTCATGGTGAAAAAATCGCCTTTAAAATCGCTTTTCCCCGTGCCCCACAGGTCGCGCAGCACCTGAACATATTCG
GTGAGATAGTCGTAACGACGGGAGAAATAGTCATCGCCAGGCCAGATACCCATCTGCTCATACTCGGGCTTTTGCCAGCC
AGTCACGAGGTTGACGCCAAAACGCCCGCCAGAGATGGAGTCGATGGTTGCGGCCATACGGGCGACGATTGCCGGAGGTA
ACGTTAAGGTGGCAGCAGTGGCGTAAATCTGAATGCGCGAGGTCACGGCCGCCAGCCCCGCCATCAAGGTGAACGACTCA
AGGTTGTGATCCCAGAACTCAGTTTTGCCGCCAAAGCCACGCAGTTTGATCATCGACAGGGCGAAATCGAAATGGTAGTG
CTCCGCTTTTTGCACAATGGCTTTATTCAGTTCAAAGGTCGGCATGTACTGCGGCGCGTGGGTCGAAATGAGCCAGCCGT
TGTTGCCAATAGGTACGAATACGCCAATTTTCATCATCAACCTCTCTTCGTCTCGTAAAGTGAAAGTCAGACGGGGCGCT
GCATCCTGCATATCCTTTTCAGCCGCGTATTGGCTTGTTTGCAAAGCGGATGCCAGTTTTTAAAAAGTTAATGTTATTAA
TCTGTTAACATTACGTTATCTAAAATATCTGGTAAAAAGTGGACTAAACGGTCAAAACAGTTGCACATAAAACATGCATC
TGTGCGCGATGAGAGTGCAGAAGGTCGAGGCCGGGCGGGGGTTTTGCTATCCTGTTGCCAATCTACAAGAGGGGAGAGCG
CATGACGCAAGGCGCAGTGAAAACAACGGGTAAACGTTCGCGCGCAGTAAGCGCGAAGAAAAAAGCGATTCTTAGCGCAG
CACTGGACACTTTTTCACAATTCGGTTTTCACGGCACAAGGCTGGAGCAGATCGCAGAGTTGGCGGGTGTTTCAAAAACC
AATCTGCTGTATTACTTTCCGTCAAAAGAGGCGCTGTATATTGCCGTGCTGCGGCAGATTCTCGATATCTGGCTGGCACC
GTTAAAAGCGTTTCGTGAAGATTTCGCCCCGCTGGCGGCGATCAAAGAGTACATCCGTCTGAAGCTGGAAGTCTCACGCG
ATTATCCGCAGGCTTCGCGCCTGTTCTGTATGGAGATGCTGGCAGGCGCGCCGCTGTTAATGGATGAACTGACGGGCGAT
TTGAAGGCATTAATTGATGAGAAATCGGCGCTGATTGCCGGTTGGGTCAAAAGCGGCAAACTCGCGCCGATTGATCCGCA
GCATTTGATTTTTATGATTTGGGCTTCCACTCAACATTACGCCGATTTCGCCCCTCAGGTGGAGGCGGTGACAGGCGCGA
CGTTGCGCGATGAGGTATTTTTCAATCAAACGGTTGAAAACGTGCAGCGGATTATTATTGAGGGGATTCGACCACGTTAA
AGATGCCGGAGGAGGTTGTAACATCCTCCGGCTACCTGTTTAACCTATAGTCATTAAGCTGGCGTTACCGCCAGCGGCAG
CGGTATTCACACTCAGCGAACGCTCGATATACAGCCGTTCCAGAAGGATATTGCTTTCGCCACGGGCAAAACCCTGCACC
GAAACAATTGTGCCATCCCGCGCGGCAACTGCTTCACACAATGCGCGAAGCTGATCCGAATCACCGTGGAAGATCACCGC

ATCAAACGGTTGAGCGGTTATATTTTCCGCTTTCGCCAGTTGAATACGTTCGCTGACTGCCGATGGCAATGCCTTCACTA
ACTGACGATGCAGCGCGTCATCCGGCCACAGTACCTGGCTGCCCACCGCCAGCACGGCGGCGAGCTGAGTCAGCGCATCC
TGCTCATCATCGGCAATACACAACACGCGCTCACGCGGCAGCAGCGTCCAGGTGTTGCGTTCACCCGTCGGCCCCGGCAG
CAATCGTTGTGTTCCTGCCTGCGCCAGCTCGCCATATTGCGTACATAACGCCTGCAATTCTGGACGATTTGCTGCCCATT
CCCGCAGTGCATTTAGCGGCTGAGTCAATGCGGCTTTCAACTGCGCATCGACCGGATACTTTGCATCCTGACGCGCGAGC
GTCACTGCCAGCGCACTTTCCGGGCGATTCGCCAGCAGACGGTAGAGATAGAGCGGACCGCCTGCTTTCGGCCCGGTACC
GGACAACCCTTCGCCGCCGAACGGCTGCACACCAACCACTGCGCCCACCATATTACGGTTAACATACAGGTTACCAACAT
GGGCCGAGCCAGTGACCTGGGCGATGGTTTCATCAATGCGCGTATGGACGCCAAGCGTCAGACCATAACCGGAAGCGTTA
ATCTGCTCGATCAGCTCTGGTAGCTGGTTACGGTTGTAACGCACCACATGCAGCACCGGACCAAAGACCTCTTTTTGCAA
TTCGGCAAAGTCATCCAGTTCGATCAGCGTCGGGGCGACAAAGGTGCCGCTTTGCCATTCACGGGCATCTTCGCTGTTTT
CCCGCACCGCCTGGAACACCGGACGGCCTTTGCTACGCATGGTCTGAATATGGCGCTCAATATTGGCTTTCGCTTCGCTA
TCAATCACTGGACCGATATCGGTGGTCAGGCGACCCGGATTACCCATCCGGCATTCGGCCATTGCGCCGCGCAGCATTTT
CAACGTGTGGTCGGCAATCTCATCTTGCAGGCACAGCACGCGCAGCGCCGAACAACGCTGACCCGCACTGTCGAACGCCG
AGGCCAGTACATCCACGACGACCTGTTCGGTCAGTGCTGAAGAATCGACAATCATCGCGTTCATGCCGCCGGTTTCAGCG
ATGAGCGGAATAGGGCGACCCTGAGCGTCCAGGCGGCTGGCGATATTGCGCTGCAGTAACGTAGCGACTTCGGTTGAACC
GGTAAACATCACCCCGCGCACGCGATCATCACCCGTCAGTTGCGCGCCCACGGTTTCACCCCGACCTGGCAGCAATTGCA
CCACGCCTGGCGGTACACCCGCTTCCAGCAAAATGGCGATCCCTTGCGCGGCAATCAGCGGCGTTTGTTCTGCCGGTTTT
GCCAGCACGCTGTTACCTGCCGCCAGTGCGGCGGCGATCTGCCCGGTGAAAATAGCCAGCGGGAAGTTCCACGGACTGAT
ACACACCACAGGCCCTAATGGACGGTGGGTTTCGTTAGCGAAATCATCCCGCACCTGTCCGGCGTAGTAGTGGAGAAAAT
CGACCGCTTCGCGCACTTCGGCAATGGCGTTACTGAAGGTTTTTCCGGCCTCACGCACCAGAATACCAATCAGTTGCTGC
ATCTGGCTTTCCATCAGCACGGCAGCGCGGTGCAAAATCGCTGCGCGTTCAGCCGGAGGCGTGGCAAACCAGATTGGCGC
GTTATTAACCGCACTTTCCAGCGCCTGTTCTACTTCACGCGGCGTGGCTTCACGCACATAGCCCACAATATCTTTCGGTT
CCGCAGGGTTAATAACGGGCGACATCTCACCTGCCGCTACCGGTTGTTCCAGCATTGGCAAGGCCTGCCATTTTTGCAGT
GCACTATTGAGCAGGGCAGAGGAGAGCGAGGCCAGGCGGTGTTCGTTAGCGAGATCCAGCCCTGCCGAGTTGTCGCGCCC
GTGACCGTAAAGATCGCGCGGCAGGGGAATTTTCGGATGCGGTAATCCAGTTTGCCCTTCCTGTTGCGCCAGTTTTTCTA
CAGCAGTGACCGGATCGGCGACCAGTTCATCCAGTGGCAAAGAGGTGTCGGCAATACGGTTAACAAACGAGGTGTTAGCA
CCGTTTTCCAGCAGGCGACGCACCAGATACGCCAACAGCGTTTCATGTGTGCCAACCGGAGCATAAATACGACACGGACG
GTTAAGTTTGCCGTCGGCAACTTTCCCGGTGACCTGCTCATACAGTGGCTCGCCCATACCATGCAGGCACTGGAACTCGT
ACTGACCCGGGTAGTAGTTCTGCCCCGCCAGTTGATAAATCGCCGCCAGCGTATGGGCGTTGTGCGTCGCGAACTGCGGG
TAGATTAGATTCGGCACCGCCAGCAGCTTTTTCGCACAGGCGAGATAAGAAACGTCGGTATACACCTTGCGGGTATAAAC
CGGATAACCTTCAAGGCCGTCCATCTGCGCACGCTTAATTTCACTATCCCAGTACGCGCCTTTCACCAGGCGAATCATCA
GACGGCGACGGCTGCGGGTGGCGAGATCAATCAGGTAATCGATCACCAACGGGCAGCGTTTTTGATAAGCCTGAATAACA
AAACCGATGCCGTTCCAGCCTGCCAGTTCCGGCTCGAAACAGAGTTTTTCCAGCAGATCGAGGGAGATCTCCAGGCGATC
GGACTCTTCGGCGTCAATGTTGATACCAATATCGTACTGACGCGCCAGCAGGGTGAGTGATTTCAGACGCGGGTAAAGCT
CTTCCATTACCCGGTCATACTGGGCGCGGCTATAACGCGGATGCAGCGCCGACAGTTTGATTGAAATGCCCGGCCCTTCA
TAGATGCCACGACCGTTAGACGCTTTACCGATGGCGTGAATCGCCTGCTGATAGGAAACCATATACGCCTGTGCATCTGC
GGCGGTCAGCGCGGCTTCGCCCAGCATATCGTAAGAGTAACGGAAACCTTTCTCTTCCAGCTTGCGGGCATTGGCTAACG
CTTCCGCGATGGTTTCGCCAGTGACGAACTGCTCACCCATCAGGCGCATCGCCATATCCACACCTTTGCGGATCAGCGGT
TCACCGCTTTTACCGATAATGCGGTTCAGCGAGCGGGAGAGGCTGGCTTCGTTATGGGTGGAAACCAGTTTGCCAGTAAA
CAGCAGCCCCCAGGTGGCGGCATTAACAAACAGTGACGGGCTACGACCAATGTGTGACTGCCAGTTACCGTTGCTGATTT
TGTCGCGAATTAACGCGTCGCGGGTGGCTTTGTCGGGAATACGCAACAACGCTTCCGCCAGACACATCAGCGCCACGCCT
TCCTGCGATGACAGCGAAAACTCCTGCAATAACCCCTGGACCATACCTGCGCGACCACTGGCATTTTTTTGATTACGCAG
TTTATCGGCCAGCTGATACGCCAGTTTGTGCGCCTGTTCAGCAACTGGCTGCGGCAGGCGGGCTTGTTCCAGCAGCATAG
AAACCGCTTCGGTTTCCGGGCGGCGATAGGCCGCGGTGATCGCGGCGCGGGAAACCGACTGGGGCAATATTTGCTCGGCA
AAGTCGAGGAATGGCTGGTGTGGTTCCTCTGCCGGAGTCGGTGCTTCATCGCTCTCATTGGCCGCGCCAGAAAGCAGCGC
AGGTAGCTCCGGCAGAGTATCGCTGTTTTCCAGTTGTTCGAGATAAGAAAAAATCGCCTGCTTAATTAACCAGTGTGGTG
TGCGATCGATACGTGTCGCGGCAGACTTAATACGCTCACGCGTCGCGTCGTCCAGCTTAACCCCCATGGTGGTGGTTCCC
ATGCCATTACTCCTGTTGTTCAGAAAGGTGCAACTTAACGTTATCGTGAAATATCCATGATGTTGCAACTTTGTGCAACC
ATGTTAAATGTGACATGCGTAGCAAGCTTAAAAATGAATGAAATGTTAATAAAAGAAATCGATATGACAGGGATTAAAAA
AATAACTCAGACTTTTTCTCTGCGGCAGTTAACATTTTTGAAAGGTGCAACCGCAAAAAATGTGAGAGAGTGCAACCTGA
TGAAAAATAGTGTCGCTGAGCACTAAAATTTAATGTAAATGGTGTGTTAAATCGATTGTGAATAACCAGCGCTTCCGGCA
GGATACGGTCGCCCTGGTAAAACATAAACTCTGTTACCCCGTTCCGGTGGCAGATATAACGGCAAGTTTCGACATTGCCG
ATAATAATTTTTTGGAGACTTTAGATGGCTATTAGCACACCGATGTTGGTGACATTTTGTGTCTATATCTTTGGCATGAT
ATTGATTGGGTTTATCGCCTGGCGATCAACGAAAAACTTTGACGACTATATTCTGGGCGGTCGTAGTCTTGGGCCATTCG
TGACGGCATTATCGGCGGGTGCGTCGGATATGAGCGGCTGGCTGTTAATGGGGTTGCCGGGCGCTGTTTTTCTTTCCGGG
ATTTCCGAAAGCTGGATCGCCATTGGCCTGACATTAGGCGCGTGGATTAACTGGAAGCTGGTGGCCGGGCGGTTGCGTGT

GCATACCGAATACAACAATAACGCCTTAACACTGCCGGATTATTTCACCGGGCGCTTTGAAGATAAAAGCCGCATTTTGC
GCATTATCTCTGCGCTGGTTATTTTGCTGTTCTTCACCATTTATTGCGCTTCGGGCATTGTGGCAGGCGCGCGTCTGTTT
GAAAGTACCTTTGGCATGAGCTACGAAACGGCTCTGTGGGCGGGCGCTGCGGCGACGATCCTTTACACCTTTATTGGCGG
TTTCCTCGCGGTGAGCTGGACTGACACTGTACAGGCCAGCCTGATGATTTTTGCCCTGATCCTGACGCCGGTTATCGTCA
TTATCAGTGTCGGTGGCTTTGGTGACTCGCTGGAAGTGATCAAACAAAAGAGCATCGAAAACGTTGATATGCTCAAAGGT
CTGAACTTTGTTGCCATTATCTCACTGATGGGTTGGGGGCTGGGTTACTTCGGGCAGCCGCACATTCTGGCGCGTTTTAT
GGCGGCGGATTCTCACCACAGCATTGTCCATGCGCGTCGTATTAGTATGACCTGGATGATCCTCTGCCTGGCAGGGGCGG
TGGCTGTCGGCTTCTTTGGGATTGCTTACTTTAACGATCATCCGGCGTTGGCTGGTGCGGTAAATCAGAACGCCGAGCGT
GTGTTTATCGAACTGGCGCAAATTCTGTTTAACCCGTGGATTGCCGGGATTCTGCTGTCGGCAATTCTGGCGGCGGTAAT
GTCAACCTTAAGTTGCCAGCTGCTGGTGTGCTCCAGTGCGATTACCGAAGATTTGTACAAAGCGTTTCTGCGTAAACATG
CCAGCCAGAAAGAGCTGGTGTGGGTAGGGCGTGTGATGGTGCTGGTGGTGGCGCTGGTGGCGATTGCGCTGGCGGCAAAC
CCGGAAAACCGCGTGCTGGGCTTAGTGAGCTACGCGTGGGCAGGCTTTGGCGCGGCGTTTGGTCCAGTGGTGCTGTTCTC
GGTGATGTGGTCACGCATGACGCGTAACGGTGCGCTGGCGGGGATGATCATCGGTGCGCTGACGGTTATCGTCTGGAAAC
AGTTCGGCTGGCTGGGACTGTACGAAATTATTCCGGGCTTTATCTTCGGCAGTATTGGGATTGTAGTGTTTAGTTTGCTG
GGTAAAGCGCCGTCAGCGGCGATGCAAAAACGCTTTGCCGAGGCCGATGCGCACTATCATTCGGCTCCGCCGTCACGGTT
GCAGGAAAGCTAAGGGACTTAGCCTGCGGCGGTTTTGTTTGGCTTCAGCAGCGGGTTGCGCTCCCTTAATGTGCCTCGCC
ATATAAATTGAATGGTGCAGGGAGCGCGCAGGGGGCGGCCAATCGCCGCCGCCCCCTGCTGTCCCGGCCTTCGGGGAACG
CTTCAGCGATTTTGACGCCACCAACACCCGAGCTGTTATTATGTTCCGGGCAAAAAGTTAGATTTGATAATCGCGGATGG
ACGAAATTGCTTGATACACCCGCTTATCAGTTTTACATGGAAGCTCTGATGCATTGAGTCTGGACAGTTTTGTCGGCTGG
ATACGGCGTTTACGCGGCATCCGGCAAGAACACATGGTTCTTTGCAAACAATCCCATCTTTCTACCCTGGAATAATCGTT
TATATCCCTTGGCATTACCTCTCTTTGTTTACATTCCAACATCATTTTATAAACATTCCGCTTGTGTTTTTCTTTGCCGT
AATGATAATCGCTATCACTGCGATTTACTTTTCTTTGCATAGATTGACTCAGAAAAACGTTTAAGGGTGGGTGGCATGTT
TGTTCCGTTTCTCATTATGTTGCGCGAAGGACTTGAAGCCGCGCTGATTGTCAGTTTGATTGCCAGCTATCTTAAGCGTA
CCCAGCGAGGCCGATGGATTGTGTGATGTGGATTGGCGTGTTGCTTGCCGCTGCGTTGTGCCTGGGCTTGGGTATCTTCA
TTAACGAAACCACCGGCGAATTTCCGCAAAAAGAACAGGAACTGTTTGAAGGTATCGTGGCGGTGATCGCCGTGGTGATC
CTTACCTGGATGGTTTTCTGGATGCGCAAAGTGTCGCGCAACGTCAAAGTGCAACTGGAACAGGCAGTCGATAGCGCATT
GCAGCGTGGAAATCATCATGGCTGGGCGCTGGTGATGATGGTCTTTTTTGCCGTTGCAAGGGAAGGGCTGGAGTCGGTCT
TTTTCCTGCTGGCGGCATTTCAACAAGATGTCGGGATCTGGCCGCCGCTGGGTGCAATGCTCGGTCTTGCTACTGCCGTG
GTGCTAGGCTTCCTGCTCTACTGGGGCGGTATTCGCCTCAATCTTGGTGCATTTTTTAAATGGACCAGCCTGTTTATTCT
CTTCGTCGCCGCAGGGCTGGCAGCTGGTGCCATTCGCGCATTTCATGAAGCCGGATTGTGGAACCACTTTCAGGAAATCG
CCTTCGATATGAGTGCGGTGCTCTCAACTCACTCGCTGTTTGGCACGCTGATGGAAGGGATTTTTGGCTATCAGGAAGCG
CCGAGCGTCAGCGAAGTCGCCGTCTGGTTTATTTATCTCATCCCGGCGCTGGTGGCATTTGCTCTGCCACCACGCGCAGG
GGCGACAGCGTCTCGCTCCGCGTAACAAATACGACGCAAACTCTTGCTTAGTTACAACATACTTTAAAGGGATAGTCTCG
TCATGACCATTAACTTCCGCCGTAACGCATTGCAGTTGAGCGTGGCTGCGCTGTTTTCTTCTGCTTTTATGGCTAACGCC
GCTGATGTGCCGCAGGTCAAAGTGACCGTGACGGATAAGCAGTGCGAACCGATGACCATTACGGTTAACGCCGGGAAAAC
ACAGTTCATTATTCAGAACCACAGCCAGAAGGCGCTGGAGTGGGAGATCCTCAAAGGCGTGATGGTGGTGGAAGAGCGGG
AAAATATCGCCCCTGGCTTTAGCCAGAAAATGACGGCGAATTTACAGCCTGGCGAATACGATATGACCTGCGGTCTGCTG
ACTAACCCGAAAGGGAAGTTGATCGTCAAAGGTGAGGCAACGGCGGATGCGGCGCAAAGTGATGCGCTGTTAAGTCTTGG
TGGTGCAATTACTGCATATAAAGCGTATGTCATGGCGGAAACCACGCAGCTGGTGACCGACACCAAAGCCTTTACCGACG
CGATTAAAGCAGGCGATATCGAAAAAGCGAAAGCACTGTATGCACCGACGCGCCAGCACTATGAGCGTATTGAACCGATT
GCTGAACTGTTCTCCGATCTGGATGGCAGCATTGACGCCCGTGAAGATGATTACGAGCAAAAAGCCGCCGACCCAAAATT
CACTGGTTTCCACCGTCTGGAAAAAGCATTGTTTGGCGACAACACCACCAAAGGGATGGATCAGTACGCTGAGCAGCTTT
ATACCGATGTGGTCGATTTGCAAAAACGCATCAGTGAACTGGCTTTCCCACCTTCAAAAGTGGTCGGCGGCGCAGCCGGA
CTGATTGAGGAAGTGGCAGCCAGCAAAATTAGCGGTGAAGAAGATCGCTACAGCCACACCGATCTGTGGGATTTCCAGGC
TAACGTTGAAGGCTCGCAGAAAATTGTCGATTTGCTGCGTCCACAACTGCAAAAAGCCAACCCGGAACTGCTGGCAAAAG
TCGATGCCAACTTTAAAAAGGTCGATACCATTCTGGCGAAATACCGTACTAAAGACGGTTTTGAAACCTACGACAAATTG
ACCGATGCCGACCGGAATGCACTGAAAGGACCGATTACTGCGCTGGCGGAAGATCTGGCGCAACTTCGCGGTGTGCTGGG
ACTGGATTAAGCGTTATGCAGTATAAAGATGAAAACGGCGTGAATGAACCGTCACGCCGACGTTTACTGAAAGTGATAGG
TGCACTGGCGCTGGCGGGAAGTTGTCCGGTCGCTCATGCACAAAAAACGCAAAGTGCGCCGGGTACGCTTTCACCGGATG
CTCGCAATGAGAAACAGCCGTTTTATGGTGAGCATCAGGCAGGGATCCTGACGCCACAACAGGCCGCAATGATGCTGGTG
GCGTTTGATGTGCTTGCCAGCGATAAAGCCGATCTTGAGCGGTTGTTTCGCTTGTTGACTCAGCGTTTTGCTTTTCTGAC
TCAGGGCGGAGCAGCACCAGAAACGCCAAATCCGCGCCTGCCACCACTCGATTCCGGCATTCTTGGCGGCTACATTGCGC
CCGATAATCTCACCATCACGTTATCGGTGGGTCACTCATTGTTTGATGAGCGCTTTGGCCTTGCGCCACAGATGCCAAAA
AAGCTGCAGAAGATGACGCGTTTCCCCAACGACTCGCTGGATGCGGCGTTATGTCATGGTGATGTGTTGCTACAGATTTG
CGCCAACACCCAGGACACGGTTATCCATGCGCTGCGCGATATCATCAAACACACGCCGGATTTGCTCAGTGTGCGCTGGA
AGCGGGAAGGGTTTATTTCCGATCACGCGGCGCGTAGTAAAGGCAAAGAGACGCCGATTAATTTGCTGGGTTTCAAAGAC

GGCACTGCCAATCCCGATAGCCAGAATGATAAGTTGATGCAAAAAGTGGTGTGGGTAACGGCAGATCAGCAGGAGCCTGC
GTGGACAATCGGTGGCAGCTATCAGGCAGTACGCTTGATTCAGTTTCGAGTGGAATTTTGGGACAGAACGCCGCTGAAAG
AACAGCAGACGATTTTTGGCCGTGATAAGCAAACCGGTGCGCCGCTGGGAATGCAGCATGAGCATGATGTGCCTGATTAC
GCCAGCGACCCGGAAGGGAAGGTGATCGCGCTGGACAGCCATATCCGGCTGGCGAATCCCCGCACGGCGGAGAGTGAGTC
CAGCCTGATGCTGCGTCGTGGCTACAGTTATTCACTGGGCGTCACCAACTCCGGGCAACTGGATATGGGGTTGCTGTTTG
TCTGCTACCAACACGATCTGGAAAAAGGCTTCCTGACAGTACAAAAAAGGCTCAATGGCGAAGCGCTGGAGGAATACGTT
AAACCTATCGGCGGCGGTTATTTTTTTGCGCTGCCGGGGGTGAAGGACGCGAACGATTATTTCGGAAGCGCGTTATTGCG
GGTTTAATGTTTTTAGGCGGATAAGGCATTTGTGCGCAGATGCCTGATGCGACGCTTGCGCGTCTTATCATGCCTACAAT
CAGTGCGGGTTTGGTAGGCTGGATAAGGCGTTCACGCCGCATCCGGCGATCGTGCACTGATGCCTGATGCAAATCCTGCT
GAAAGCACACAGCTTTTTTCATCACTGTCATCACTCTGTCATCTTTCCAGTAGAAACTAATGTCACTGAAATGGTGTTTT
ATAGTTAAATATAAGTAAATATATTGTTGCAATAAATGCGAGATCTGTTGTACTTATTAAGTAGCAGCGGAAGTTCCCGG
CAGTGATAGTCAGTCACTATGGAGATCGCGGATGGTAACGTCCTGTACTGGACATGTTTTAGATAATCAACGCGCCACCA
CTCGCGGAGTTTTCTCCTCCGGTAGTCATCTCGTCACTTTGCATTTTCAACCTCATCCTTTCTTTTCATGTGTTACCGAC
GCCGTAAACGGTGCGCGTAGCCGTTTTTCGGCATTTTATCCAAAAGCAAACTATGGCTTACAAGGAAGCCAACCCTCTGA
TGTTCGTGCGCATAATCGCGCTGCCAACGGCGCGTGTGATGAATACAAACAACTCAAGGTGCTCTCCATGGGAAGACAAA
AAGCAGTGATCAAAGCTCGTCGCGAGGCAAAACGTGTGCTGAGACGGGATTCGCGCAGCCATAAGCAGCGTGAAGAAGAA
TCGGTCACCTCGCTTGTGCAGATGGGCGGCGTAGAAGCCATTGGTATGGCCCGCGACAGTCGCGATACTTCGCCCATCCT
CGCGCGAAATGAAGCGCAATTGCACTATCTGAAGGCTATTGAGAGTAAGCAGCTGATATTCGCCACGGGCGAAGCCGGGT
GCGGAAAAACCTGGATCAGCGCAGCAAAAGCGGCAGAGGCCCTGATACATAAGGATGTCGACAGGATTATCGTCACCCGT
CCAGTTCTGCAAGCCGATGAAGATCTTGGCTTCTTACCTGGAGATATCGCAGAAAAGTTTGCTCCCTATTTTCGCCCGGT
CTACGACGTGCTGGTCCGGCGCTTAGGGGCTTCCTTTATGCAGTACTGCCTGCGACCGGAAATAGGGAAGGTGGAAATTG
CGCCGTTCGCCTATATGCGTGGACGTACCTTTGAAAATGCAGTCGTCATTCTTGACGAGGCGCAGAATGTGACTGCCGCG
CAAATGAAAATGTTTTTAACCCGCCTCGGGGAGAACGTGACGGTTATCGTCAACGGTGATATCACGCAATGCGATTTGCC
TCGCGGCGTGTGCTCCGGATTAAGTGACGCGCTGGAACGTTTCGAAGAAGATGAAATGGTTGGGATCGTCCGGTTCGGTA
AAGAGGACTGCGTACGTTCGGCACTTTGCCAACGTACGCTGCATGCCTACAGTTAAGTGTGTTATCGGTGCAGAGCCCGG
GCGAACCGGGCTTTGTTTTGGGTGTTTATGCCCGGACTAGCGCTTTTTCTGAAACAACCATTTTTATTTGCCCCTGGCTG
GTGAAGTGTACGCTCATCCTGTGGCTTTTTTGTAGTTGCTGATAGAGCTCATCAGGTATTGCTAAGCTCTCTGCATATTC
TTGCGGCGTATATTGGTAGGCTGCATGATGCTGCTGTTTTTGAAAACGCAGCTTATTGTACAGCGCCCAGACAATTAACA
CGACGGCATTCGCCACTGCCAGCAAAAAATAGAACTGAAGTCGGCTTCTGGCCTCGCTTTGCCAGTAATAACCCGTCAGC
AGATCCATGGCGAATAAGAATATGAACAACGCAAATAATGTCCACAAGATGGTTGTGGCAACATAATCAACCAGTAAACG
TACTGGTGATTGTCGGGTCGTAATAATTAAATTGTTCATATTAACCTCTCAGAATCCCGCGATCGGGACTTACCCAACGG
GCGCGTTGCTTTTTAGGCATCAACATGACTCGTGTAAATGATACCAATGTCGTTGCCAGGCTCAGCATCCAGAAAATAAC
CGGGAACCAAATAATCCAGAAAAGCGATGAAGTCAGATTATGCTCATAGCGATTCTCGATCATCAGGCTGACAATAAATT
GCAGTAAACATAACGTACACAATAATATTCCGGCAGTATGTGTCGCAGCGATATGTGTCAATTCAATATTTAACGGTACA
CCGGCAAGTTGGACTGCGTAAATAATGAAACCGACCAGGCAGGTGAAGGCCCATATTGTCGTCAGGCAGTATTCAAAAAA
CAGCGGCCACATTCGAAAGTTTTCTTTGCGCCACAACCTTGTCATATTTTTGAGGAATACTTCTGCACCGCCCTGAGCCC
AGCGCAGGCGCTGTTTCCACAGCCCTTTTAACGTTTCAGGCATTAATATCCAGCACAGTGCCCGTGGCTCGTAAAAAATC
GTCCACTGATTCAACTGCAGCTTCCAGCTAATATCAATATCTTCGGTGATCATATCGTCACTCCAGTAACCCACTTCTGC
CAGGGCGCTGCGACGAAATGCGGCAATAACACCGGAAACGGTAAATACGTTTCCATAGATACGCTGGGTTCGCTTGATCA
AACCAATAATTGAGGAATACTCGCCAACCTGAATTTTACCCACCAGGGTAGAACGTGTTCGAATACGAGGATTACCGGTT
ACGGCACCCACACGCGGGTTGTACAACATCGGTTCCACAATATATGCCGCCGCATCGCGGTCTAATAACGCATCGCCATC
AATGCACACCAGATATTCACTTTTCGCCGCGGCAGCTCCGGTTTTAAGCGCAATGGCTTTCCCCTGGTTTTGCGCCAGAT
GAATGACCCGCAAATGGGGAATTTGTGCAGCCATGCGATCCAGGATGGCACGGGTTTTATCTGTTGAACCGTCATTTACG
GCAATAACTTCAATGTTCTCATAACGCTGTGCTAAAGCGGCGTGTATGGTTTCCTCAACGTTTTTCTCCTCATTAAAACA
GGGAATGATAATGGAGATAGACGGATTATCTTTCAACTGGGGAGCTGGTGCGTTTTCTCCCCACGGCCAGTGGCGTTCAC
GATAGACCCAGAAATAGACGCCGCCAACAATCCACATAATGGACATAAAAAACGGCCAGAAGAAAACGAACCTCATCATC
AGTTCACCAGAGTGAAAGTACGCTACGCATAGGGGTATGCATAACACCAGACATAATATAAAAAACGATACGATGCGATT
AATCATTTTTCGGATACCAGGCTGTTGAAAACTCAGGACGAATAAGGTCTATTTCAGGTTGGTTATGCAGAAAATTGTCG
GGATAATAACCATAGTTTTTCACTCCATTCAGTTGTAATAGGCTCATCCAGTGAGCGAGTTGTTGCGAAGAAATAGCCTG
ATGCTGACCATTTTTCTGCCAGTTTTGTGCCTGTAATTCTAAAATAGATTTGTCTTTAGCCTGAGGGATGTTTTTAATTT
GATTGGTCAATTGTATTAACCATTGGTCAGCCGATTTTTCTGCGACACCTTCCAGATAAGGCATAGCCATAATAGCGGTC
CAGTCATAGCTTTTTAGGAAATCAGCATAATTCTGTGCAAACCAGGCTTCACTTTCAGGTTGTATTACCGGAAGTGCAAA
AATATTTCGTGCAGTTTTAATATGTGGACCGCGAATGGCTTTTACGCGCGCACTAAGTTCTAAAGTGAAGTCAGTTAACG
CACGACTTTTAAAGCGGGCCCACTGTTTAAATTGCTCCGGGTTTTGTCGAATTTCGCTCAGACTCCCGCTAAAGCCTGCT
TGCTGATAAGCCGTGATAGCCGGTGCACTGGCATCTTCATAATCTGAAAGCAAAGCATCATCGTGGAACAATATGCCATC
AAAAGCAGCATGTCCGGCAAGATCTTCATATAACATGCCAACTTGTGCTCTGACTCTGTCATCGAAAGGAGAGAGACGGT

GATATTGTTCAGGATGAATTTGTGCTTTTTTCTCCCCTGTTGGTAAGTATTTTACTCGCGTTAATGTGGGATCTAAATCC
CAGCTTAATACCGGCATCCACGCATAGATGTTTACACCTGAGCGGGTACGTAATTGCCAGGCAACCCGACTAAAAATATC
TGCTTTCATTGGTAGCAAACGATTTGGAAACCAGACCTCTTTGACCAGCCCATCACCATCGGGATCAGCAAATGCCTGCA
AATACACGGTTGATATTTGCATATCTTTCACCCGCTGAATTAGCACATCAATATTGCGATCCATTTGCTGGAGGTTTTCG
TCATAAACGTAATCAAGATCGATATGCATTATCCGTTGTGGTGATTTTTCCTGTACGGTAATAATTTGCTGGGCAAACTC
TTTTAATGAGGGATTATTGGCGATTAATACCCGCGGAATGGAATCCAATTGCGACGCATTTGCCAAACCTGATTCAAGGG
TGAAGAACATGTCATAACCGAGTTTTTTTAATTCCTCTATCGCTATGCCATTCGCTTCGCCATAAGGCCAAACAAAAACG
TGTGGATTTACCTCAACCTTTGTACGCAGGTATTCCGTCATTTTTACAGCATCCAGACGAATTCTTTCCCGGTATTCTGC
TGCGGTTTCATACCGTGCGTGGTCAGTAAAATATGCACGATTTACATATACAGGCAATAAGCTGCCGGTGGCATTAGCCT
GAATACCGTAGTGAGAATTCCATGTATGAGAAGCGAGCTCAACGAGCCGGGAACGCGCAACTTCTCGCACTTGTTGCCAC
GTGGCAAAATATTCTCGATCGACCAACTCATCGCCAAATTTTACTTGTTTATCCGCTGGCGTATCGACCCAACTGCCGAC
GGGGGCCCATACAGCAGGCCACTGGAAGGCCTGAAGAATTGGGAAGACGCGGGTATAAAAACTCTGGTAGCCGTCATCAA
AAGTCAGCACTACAGCTTTTTCCGGTAGCGGTTTTCCTCCTCGATGTGCTTCACGAATTTGAGCAATACTGACCGGTTGA
TAACCGTTCTCGCGCAGCCAGGCAAATTGTTCACGCAGTGCTGATGTCCGCACTGACATAAAACGCTGGTCGGCAGCTTC
GTCTTCAACGTTATGCCATGAAATCGCTACAAAACCATTATGCGGCCACGGTTGCTCGGCGAGTAAAGATTCGCGATCCT
GTGGCGGTATAAATGATGTTCTTGACTGGCTAATGCACGCGGTGAGCATAATTATACTCACCAGCATCAGGAGATATTTA
TTTCCATTACGTAACATATTTATCCTTAAAATCTGAATGTCATATCGAATTCAACGTATAAGTTGTGTTCTCTGTCACCG
TCATAAGGTCGTTTTTCCCAGCGTAGCGTTGCGCCAGCATCAATCACGTCATTCCAACTAATGCGTTGCCCGTAGCCGAG
TTGGGTGACGACATCCGTGCCATAATGTTTTTGCCAGGAGGCACCAACACCTGCGCTGAATATTTGCTCCCAGCTATTTT
CATAGCTTCGCCATAACAAATGGCTTGCCTCAAATGCCGGAACAATATCGAACGTTTTTATAGGGTTGTAGTATGGGGTA
TCGTGTTCTGTATTTTGTTCGTAATACAGACTGGGTAGGAAATCGACAATCAAATATGGTGAAGACCAGATGCGTTCCTG
ACCCTCAAGTGAGACTTCATGACGCTGGTTACTGTCGGAAAAATCAGTGAAAGCCCAGGAGACACCGTACTTACGCCGCT
CATTTTGATACCAGCGAACATAAGCCTGAGCACTGTTGCCTGTAACACCATTTTTCATTGCCCGTAATGGAACGCGGTGA
GAGAGGCGTTCCAGTTGCGAACCAATACGCCAGTTATCATTAAAATCATACCAGCCAGACAGGCGCGCGCCGGGTTTATG
CTCATGATTGAAAACGCGTTCAGCGTACTCTGCCTCGAGCCAGATATTACGTGACCGCCACTCAACACCCGCAAGCCAGT
CGCGAACAATCCCTTTTCCTTCGCTAAATTGTCCATCGGCATAACCGAATCCAGCAAAACCGCGCCAGTTATCCTTCAGC
GGTGGTGAATAAACGATGGTGGTTAAGTCGACATCATGTTTACCACTATCCGGGCCTTCGGCATCAATTCCTGTTGAGCC
AGCGATACGAAGCTCTGCAAGATTATGTACATCAACCGCACGTTTTAATCGTACAACGCCGGGATCTTGCGGTTCACGTT
CGACAACATCGTGCGTTAAGACAGCTGCCTGCTGCCATTCTTGTAACGTTAATGCTGTCCAGGCTTGTTCAACCTCCAGA
TTAATATTACGTGGCTCGATCACTTCTGCTTTTTTTAATTCATTTTCTGCTGCACGAGGCCAACCGCGGGCTTGTAACAC
ACTCGCGTAATCAATGCGCAGTCCCTGATTTCCTGGTGCGTTATAAGCAAGCTCTCTGGCTGTCATTTCAGCCTGAGGAA
GATCATTACTATATTTTGCTACGGTTGAGAGAAACGAATGCCCCTGTAACCAGGTATCATTCGGGATGCTCGTAGGCGTG
CCCATTAACCGAAGGAAAGGCGGCGAAGTATTAATGGTATGTTGGGTGACAGTTAGCGCGCCCGGATAATTTTCACTCTC
CAGGTGGCTGTAAAAGAGATCCGCAAGTTCTTCATCGGATAAATCCGGGGCAATGGTCTCCTTGTGATAAAAGAGCTCGG
TCATTATTGACTGTGCTTTTTTCGGCTGATGATCTTTGAGATAAGCCGATGCAACCCAATATTGCCCCCAGGGCGGAATA
ATTTGCCCCGTCTTTTTTAATCGCTGATAGTGAGAAATAACGTCTTTATAACGATCGCGAGTTAATAACGCGCCAAGATG
ATCAACCTGAATACGCTGGTACTGGGCAGTGCGGTCTGGGTTATCGTGCCACAGAATTTCTAATGCAGCGTATTGGGCGA
GGGCGCGATCGGCAATGGCATAACGTTCACTTTCACTGCGCGTAGGCATAAACGACAGTCTGACCAGTTCGGCATGAATA
TCAGCGCGAATATCTGGCGTTAAATTGGCATCGTCAATCGCGGCAGCAAGTTGATTATTACGTAATGCCTGCACGTATTC
TGTGGGATATTGTTGCGTAGATGCATTTTCAGGTAATGACTCTGTCATCGCCCGTAATTCATCCTGATGACGCCCCGCCA
GTTTATAGATATAGGCTTCTGCGAGTAAATTGGCTTTGTCCGGTGCTCCAGAGTTAAGCTGCTTAAGTTTAACCAGCGCA
GTATCATAGTGACCAGCATCTGCCAGGGTTAAAATTTGTCCCCGTTGATAATCCTTATTTTGCGGCTCCAGAGAGAGCGC
CTTTTGCCACAGTGTAAGCGAGTTTTGCCATTGTTGCAGGTTACGATAAGCGACGGCGACAGCTGCATAACCACGCGCTG
GTAATTGCTGATGACGGTAGCGGTTGTAAACGGTAATAACCTGTTTATCTTGCCCGGCCCATAAGGCAATCTGTAACCAG
TCAGCAATTTGGTTATTGCTGAGTGCTGATTTTAGTGCAAACCATGACAAAGCTGGCTGAGTATTACCCTTGCGAGCTTC
AATAATCAATGCATCATAGGCGTTATTAACAGCACTCTTCGCCGCGGGACTCGCTGCTAAAAATGCGGCAGTAAGAAGTT
TCAAAGCCCATTTGGTTTTCGGGCACCTTTTTCTGCTACTTGAATACATCCTGTATTACTCCATGTATTGCCAAAATCTC
TCTCTGTATCTAATTACAGGTAACTGAAAAGAAAGATATTTTTGCACCTCATAATCCGTTATTAAACGCGGAAGAGAGAC
GTGAATTGTTGATGATGAGAAGAAGAAATGATGAGCAGAGTGTCCATATAAAATCCTTTTCTCGCCCGAAAATCCATTCC
AATGATGAGGATCTTCAGGAATACGGCATAAATCCCAATGCCTTTTTCAAAATAAATTAGGATTAAAATAATTAAATCAG
TAAATTCCGATGCATGATTTCGGATTTTTCGAAAGTCCTGATGAAAGGCTGCGCTACGGCACTCACGGATTTAATTGTTA
TTCCTAATGTATCTATGAATTAATGTTTTATAAGGATTTTCTATAACCATTTATAAGTATTTTCAAGCCTGGCTTGTTGC
AAACAATGTATAAAGCACTTAGGCAATAATAATTACATTCAGCAACTATCATCATCGGTATTGTTTGTGGGCGGAATTTC
ATATTGACAACAGTACAGTTCTTATATCTATTAATAATAGAAAGGGATCTACAACCTACAGATTGGTGTAGCTTTATGGA
AAAAGACTATTTGAGAATTAGTAGTACTGTATTAGTGAGCTTATTGTTTGGGCTTGCTTTGGTTTTGGTGAATAGTTGGT
TTAATCAGCCAGGCGTTGAGGAAGTTGTTCCACGATCAACCTATCTGATGGTAATGATTGCTTTATTTTTTATCGATACT

GTTGCATTTATTTTTATGCAGTTGTATTTCATTTATGACCGTAGGCAATTTTCAAACTGTGTACTTAGTTTGGCTTTTCT
GAGTTGTTTGATTTACTTTGTTATAACCGTCATTATCATTCAGCAAATTATTGAGGAGCGTTTGACAAGCAGTGTTGTCC
AAAATGACATTGCAATCTATTATTTGTTTCGTCAGATGAGTTTGTGCATATTAATATTTCTGGCATTGGTGAATAAAGTT
AGTGAAAACACAAAACAGCGCAATTTATTTTCCAAAAAAATGACTTTGTGCATAAGTTTGTTTTTTGTTTTTGGGGGGCC
GATAGTTGCTCACATACTTTCTAGTCATTATGAGAGCTACAATTTACACATTGCCGAGTTAACCAATGAAAATGGTCAGG
TGGTTTGGAAAGCCTCATATGTTACCATAATGATTTTCATGTGGTTAACCTTGCTTTCGGTTAATCTATACTTTAATGGT
TTACGCTATGATATCTGGAATGGAGTGACGGTGATTGCTTTTTGTGCCGTACTGTACAATATCTCTCTGTTATTTATGAG
CAGGTATAGCGTGTCCACTTGGTATATTAGTCGCACTATTGAAGTAGTCAGTAAGTTAACCGTTATGGTAATATTTATGT
GCCATATATTCAGTGCGCTACGAGTAACAAAGAACATTGCACATCGCGATCCCTTAACCAATATATTTAACAGAAATTAT
TTTTTTAATGAACTGACAGTTCAATCAGCATCAGCCCAAAAAACGCCTTATTGCGTCATGATTATGGATATCGACCACTT
CAAAAAAGTCAACGACACCTGGGGGCATCCGGTTGGCGATCAGGTGATAAAAACAGTGGTGAATATCATTGGGAAAAGCA
TACGACCAGATGACCTTTTAGCGCGCGTCGGCGGCGAAGAGTTTGGCGTCTTGCTGACGGACATCGATACTGAACGTGCG
AAAGCTTTAGCGGAAAGGATTCGGGAAAATGTTGAGCGTTTAACTGGCGATAATCCTGAATACGCTATACCACAAAAAGT
GACGATTAGTATTGGCGCTGTTGTGACTCAGGAAAATGCGTTAAACCCAAATGAGATCTATCGACTGGCTGATAACGCAC
TTTACGAGGCGAAAGAGACCGGGCGTAATAAGGTGGTTGTGAGGGATGTGGTGAATTTTTGTGAGTCACCATAAAGCGGC
ATTTTGATCCTACCCACGTAATATGGACACAGGCCTAAGCGAGGTTCTTGTTTTCAAATTGTTCCGGACTGAGGCCGCCA
CACCAACTGTGCCGCCGCCACCGATTGTAATCACATTCGATATAATTAAACACCGTTGCCCGCATTATTTCCCGGCTGAT
AAAGTGTTCTCCATGGATACATTCCACTTTCAGCGAATGAAAGAAGCTTTCCACGCAGGCATTATCGTAGCAGCAACCTT
TTGCGCTCATACTTCCACGCAGATTATGCCGCTTCAGTTGCGCCTGATAATCTGCTGAACAGTACTGGCCTCCACGGTCC
GTGTGAACGATAACGTTCCGGGGCCTCTTACGCCGCCACAGCGCCATCTGCAGGGCATCGCAGGCCAGTTGCGCCGTCAT
GCGTGGCGACATTGACCAGCCAATAACGGCACGTGACCACAGGTCAATGACCACTGCCAGATACAGCCAGCCTTCATCTG
TACGTAAGTACGTGATGTCTCCTGCCCACTTCTGGTTCGGGCCACTGGCGTAAAAATCCTGCTCCAACAGATTTTCTGAC
ACAGGCAGGCCGTGTGCGCGGTAGCTGACCGGGCTGAACTTCCGGGAGGCCTTTGCCCTCAGTCCCTGACGGCGCAGGCT
TGCCGCCACGGTTTTTACGTTAAAGGGGTAACCCTGAGCACGCAGTTCATCCGTCAGGCGTGGGGCACCGTAACGCTGTT
TTGACCGGGTAAAAGCCGCGAGGACAACGCTGTCGCAGTGTTGGCGGAACTGCTGACGCGTGCTTATCCTTGTCCGCCGC
TGACACCACGTATACCAGCCGCTGCGGGCCACCCGGAGCACGCGGCACATTGCTTTGATGCTGAACTCAGCCTGATGTTT
TTCAATAAAGACATACTTCATTTCAGGCGCTTCGCGAAGTATGTCGCGGCCTTTTGGAGGATAGCCAGCTCTTCATCCCG
TTCTGCCAGCTGGCGTTTGAGACGTGCAATCTCGGTAGACATCTCCAGTTCACGTTCAGAAGACGTCTGCTGATTTTGCT
GTTTACTGCGCCAGTTGTAGAGTTGTGATTCATACAGGCTGAGTTCACGGGCTGCGGCAGTAACACCGATGCGTTCAGCA
AGCTTCAGGGCTTCACTGCGAAATTCAGGCGAATGCTGTTTACGGGGTTTTTTACTGGTTGATACTGTTTTTGTCATGTG
AGTCACCTCTGACTGAGAGTTTACTCACTTAGCCGCGTGTCCACTATTGCTGGGTAAGATCAGTGCCTGGCACACGCGTT
TGATGGAAGACGGCGTTCAGGCAATGCGCGATTATCTTGCCGGACTGGATATCGCTTCGCCAGAGCATCAGGTTCTGATG
AACGTAACAGCAAAAAGCGAGGTCGCTCCGTCAATTATCAAAGAAAATCTCTCATTACACTTAACTCATACGGTGAAATG
GACTGAATCTCTCGATACATTTCTGAATATGCCAACCCCGGTAGCTTTCCTTGAAATAAGTAATAAGCCCTATTTGGGGA
ATATGTTAAATGATTTTGCTGGCGTGGATCAGCAACGAGTTATGCATTGTCGTAAAGCATTTAGTGATGCAAAGGTATTC
AAATGATTCCTGATTATTTAACTTTTATTCGCTTTCAGGATAAACGAAATCTGATATACATTTATGCTATTGGACTTATT
CTGATAGGCTTTTATTGGAAGAATGCAGGGTTTACTTTTCCATCAGAGGATATTGGTGTAGTTAGTGGGATTCTGGCTCT
GGTGCTGTATAATTTTATTTTTGATCTCAAGGCGTACTGGGCTTATAAATGCGTCACGAAGAATATCGATTTTTCGTGGT
TTAAGAAAAAGCAGAACCACAAAATAGAATTATTTCTTACACAACCTCTGGTGGCAGGATTTCTGTCGTTAATCATGTTG
AGTGCAATGAGTTGGGGGCTATACCAGCTTCTACCCTCGTTATATGCGCTGTTCCTGATTTCGTTACTTGGGCCGTTGGT
CATCTTTCTGCTGTTTCGGATGATCCGCACCAGTTATGTCAAGCAGGTCGCTATTTCAGTAGCGAAAAAAGTAAAATATA
AAAGTCTGACTCGCTATGTGCTGCTTTCGGTGTGCATCTCAACGGTTGTTAACCTGCTTACTATCAGCCCGTTGCGTAAC
AGTGATTCTTTTGTGACAGAGGGGCAGTGGTTAACGTTTAAATCGATAATTGCATTGCTCATTCTTTGTGGCGTAGTGTT
GGCGATTAATCTGTTTTTTCTGCGCTTCTCCAAGCGGTACGCTTTTCTGGGCAGGCTTTTTTTGCAGGAAATCGATCTGT
TTTTCTCCAGTGAAAATGCGTTGTCGACCTTTTTTGCCAAGCCGCTTTGGCTTCGGTTATTCATATTGCTGGTTATTGAA
GTGATGTGGATTACGCTGGTGTCGGTATTGGCAACGCTTGTAGAATGGCGGATTTGGTTTGAAGCCTATTTTTTACTCTG
CTATGTACCGTGCTTAATTTACTATTTTTTCTATTGTCGATTCCTCTGGCATAACGATTTTATGATGGCATGTGACATGT
ATTTCCGTTGGGGGCATTTTAATAAGTGAGGAAGTGATAGGAAGTGACCAGATAATACATATATGTTCTGTACTCTCTTG
CGCATTTTGATTGTTGACTGAGTAACCAGACAGTTGATGTGCACGATTTCCCCTCGCCCTAACAGACGTGGGCGGGGGCA
CCCGGTGTGTGCAATATACGAAAAAAAAGCCCGTACTTTCGTACGAGCTCTTCTTTAAATATGGCGGTGAGGGGGGGATT
GACTCGCTTCGCTCGCCCTGCGGGCAGCCCGCTCACTGCGTTCACGGTCTGTCCAACTGGCTGTCGCCAGTTGTCGACCC
CCGGTCGGGGCTTCTCATCCCCCCGGTGTGTGCAATACACGAAAAAAAAGCCCGTACTTTCGTACGAGCTCTTCTTTAAA
TATGGCGGTGAGGGGGGGATTGACTCGCTTCGCTCGCCCTGCGGGCAGCCCGCTCACTGCGTTCACGGTCTGTCCAACTG
GCTGTCGCCAGTTGTCGAACCCCGGTCGGGGCTTCTCATCCCCCCGGTGTGTGCAATATACGAAAAAAAAGCCCGTACTT
TCGTACGAGCTCTTCTTTAAATATGGCGGTGAGGGGGGGATTGACTCGCTTCGCTCGCCCTGCGGGCAGCCCGCTCACTG
CGTTCACGGTCTGTCCAACTGGCTGTCGCCAGTTGTCGAACCCCGGTCGGGGCTTCTCATCCCCCCGGTGTGTGCAATAT

ACGAAAAAAAAAGCCCGTACTTTCGTACGAGCTCTTCTTTAAATATGGCGGTGAGGGGGGGATTCGAACCCCCGATACGTT
GCCGTATACACACTTTCCAGGCGTGCTCCTTCAGCCACTCGGACACCTCACCAAATTGTCGTTCCTGTCTTGCTGGAACG
GGCGCTAATTTAGGGAAATCATGACCTGAGGTCAACAAACTTTTTGAAAAAATCGCGCGTTTATTCAAACTTCAATCAAT
GTGTGGTTTTAATAAGCGAAATCTGCTTTTTTTGCCACCGACCACGGATTTGTTATGCTGGTGGCCTTTGTAGATCATAA
CGATAAGTGCGAATAAATTTCGCACAACGCTTTTCGGGAGTCAGTATGGATATCATCTTTTATCACCCAACGTTCGATAC
CCAATGGTGGATTGAGGCACTGCGCAAAGCTATTCCTCAGGCAAGAGTCAGAGCATGGAAAAGCGGAGATAATGACTCTG
CTGATTATGCTTTAGTCTGGCATCCTCCTGTTGAAATGCTGGCAGGGCGCGATCTTAAAGCGGTGTTCGCACTCGGGGCC
GGTGTTGATTCTATTTTGAGCAAGCTACAGGCACACCCTGAAATGCTGAACCCTTCTGTTCCACTTTTTCGCCTGGAAGA
TACCGGTATGGGCGAGCAAATGCAGGAATATGCTGTCAGTCAGGTGCTGCATTGGTTTCGACGTTTTGACGATTATCGCA
TCCAGCAAAATAGTTCGCATTGGCAACCGCTGCCTGAATATCATCGGGAAGATTTTACCATCGGCATTTTGGGCGCAGGC
GTACTGGGCAGTAAAGTTGCTCAGAGTCTGCAAACCTGGCGCTTTCCGCTGCGTTGCTGGAGTCGAACCCGTAAATCGTG
GCCTGGCGTGCAAAGCTTTGCCGGACGGGAAGAACTGTCTGCATTTCTGAGCCAATGTCGGGTATTGATTAATTTGTTAC
CGAATACCCCTGAAACCGTCGGCATTATTAATCAACAATTACTCGAAAAATTACCGGATGGCGCGTATCTCCTCAACCTG
GCGCGTGGTGTTCATGTTGTGGAAGATGACCTGCTCGCGGCGCTGGATAGCGGCAAAGTTAAAGGCGCAATGTTGGATGT
TTTTAATCGTGAACCCTTACCGCCTGAAAGTCCGCTCTGGCAACATCCACGCGTGACGATAACACCACATGTCGCCGCGA
TTACCCGTCCCGCTGAAGCTGTGGAGTACATTTCTCGCACCATTGCCCAGCTCGAAAAAGGGGAGAGGGTCTGCGGGCAA
GTCGACCGCGCACGCGGCTACTAATAAAGCATCAGGATTCCTGCTATCCTTGGCGGGAATTGAATACAGGAGAGAGTTAT
GTATCCCGTCGACCTTCATATGCATACCGTTGCCAGCACACATGCATATAGCACATTAAGTGATTACATTGCCCAGGCCA
AACAAAAGGGCATTAAACTTTTTGCGATCACCGATCATGGCCCGGATATGGAAGATGCGCCGCATCACTGGCACTTCATT
AACATGCGTATCTGGCCGCGAGTGGTTGATGGGGTAGGGATCCTGCGCGGCATCGAAGCTAACATTAAAAATGTTGATGG
TGAAATTGACTGCAGCGGTAAAATGTTTGACTCGCTGGATCTAATTATTGCCGGTTTTCATGAGCCGGTTTTTGCGCCAC
ATGACAAAGCGACCAATACACAAGCGATGATCGCCACTATCGCCAGCGGCAATGTGCATATAATAAGCCATCCCGGAAAT
CCCAAATATGAAATAGATGTGAAAGCCGTTGCTGAAGCAGCCGCGAAACATCAGGTGGCGCTGGAAATCAATAATTCCTC
ATTTTTACACTCACGTAAGGGCAGTGAAGACAACTGTCGTGAGGTAGCCGCAGCGGTACGTGATGCTGGAGGTTGGGTGG
CATTAGGCTCGGATTCTCACACCGCGTTTACCATGGGGGAATTTGAAGAGTGTCTTAAAATCCTCGACGCGGTAGATTTT
CCGCCAGAGCGCATTTTGAATGTTTCTCCGCGCCGCTTACTGAACTTCCTTGAATCTCGCGGTATGGCACCGATTGCGGA
ATTTGCAGACCTTTAATTACTAGTAATGGAAATATATTAATGAACGAGTTTTCTATCCTCTGTCGTGTGCTGGGTTCGCT
CTATTACCGCCAACCACAAGATCCTTTACTGGTGCCGCTGTTTACCCTGATTCGTGAGGGGAAACTGGCTGCGAACTGGC
CACTGGAGCAGGATGAGTTGCTGACACGTTTGCAGAAAAGCTGTGATATGACGCAAGTCTCTGCCGATTACAATGCGTTG
TTTATCGGCGATGAATGTGCTGTGCCGCCATATCGTAGCGCATGGGTTGAGGGCGCGACGGAAGCGGAAGTGCGCGCTTT
TCTTTCTGAGCGAGGGATGCCATTAGCGGATACGCCAGCCGATCACATCGGCACATTGCTGCTCGCAGCTTCCTGGCTGG
AAGATCAGTCAACGGAAGATGAGAGCGAAGCACTGGAAACACTGTTCAGTGAGTATCTGTTACCCTGGTGTGGTGCGTTC
CTTGGCAAAGTGGAGGCCCATGCAACCACGCCTTTCTGGCGCACCATGGCACCGCTAACCCGCGATGCCATTAGTGCAAT
GTGGGACGAGCTGGAAGAAGATTCTGAAGAATAAGTGTGATCTACGTCACTCATAACTGCAACGGATAATTTGTTGTTGC
ATAAAATGTGTGCTCGATCTCATTCATGGCCGCGTTTTCTGCTATCATGCGCGGCATGAACATACTTCTCTCTATTGCAA
TCACAACAGGCATTCTCTCCGGTATCTGGGGATGGGTGGCTGTTTCCCTGGGCTTACTAAGCTGGGCGGGCTTCCTGGGC
TGTACGGCCTACTTTGCCTGCCCGCAAGGTGGGCTGAAAGGGCTGGCGATCTCCGCTGCAACCCTGCTTAGTGGCGTGGT
GTGGGCGATGGTCATTATTTACGGTAGTGCACTGGCACCACATCTGGAAATTCTCGGTTATGTCATAACCGGTATTGTCG
CGTTTCTGATGTGTATTCAGGCCAAACAGCTGCTGCTTTCATTTGTTCCGGGGACGTTTATAGGCGCATGCGCGACGTTT
GCCGGGCAGGGTGACTGGAAACTGGTGTTACCTTCGCTGGCGCTTGGGCTGATATTTGGTTACGCAATGAAAAACAGTGG
CCTGTGGCTGGCGGCGCGTAGTGCAAAGACGGCGCACCGTGAGCAGGAAATCAAAAATAAAGCGTGAGGGGCACTCACGC
TTTCGCTTAAACAGTAAAATGCCGGATGATAATTCCGGCTTTTTTATCTGTCAGGATTCCGGTGGAACCGACATATGGCG
GTATTTCACCAGAATGTCATTCTGCCGTTCTGCTTTATTTTGCAAATCCCACAGACCACGGTCGATACCATCATTAATCA
GGAAAATGACCCCTGTTTCGATAGCCGACATCAGGCACAGCATAACAGGTTCGTTCGAGGTGTAACCCACTTCCCCTTCA
AGCAAGCGCTGGTAGTCAATAAAGCGGAAAACCCCGGCCTGAACTTCATAGGAAAGTATCGTCTTACTGGTGTTCACCGA
AGAAAGGATCTCGCCGGTACTCACATTGACGACGCGCAGGTTCACGGCAATCTGATCGAGCTGGTATTGCGTGTCGGCAC
CGATGCCAAAATATCTTGCCCCAACCCCGCCAGATTTGACGTTGCTTTCATAACCGATAATCGAACCTTCAACCATGATA
TTTGCCGCCGTTAAAGATTGCAGCGGGATTCGGTTATTAATGGCAACCGTGCCGTTTTCTTGTGCCGCACGAATAATCTT
GCGCTCGTTAAGCAGGTTTTGTAAGCCCTGGCGCTCCAGCGGTATAAACCAGCGAGAATCTTTCAGTGCCGTGACCAGCA
TTGCCGTGGCGCTTTGCGGAACAGCAGTGGAGAAGTTACTTGCCGGGTAGGGTTTAAATTGCCCGGTTTCGTCCTGAATG
TTGTATACCGAAACAAAGATTTTACCCGTCGGCGCTGGCAGATGGGTCAAATCTTTGTAGCTCTGAGCACGAGGCATTAA
TGTCGGTCTGGCGGCTTCTTTAGGCGGGGCGGTTAAGCATCCGCTCAGTAACATGACGGCAACCAAAAGAAATAAGCGCT
GCATGATTATTTTCCTTATGAAGCTGGGGCTTAAAAATCGGTTGAGTTATTTTGTAAACCCGAAACCTGGATGGTCGAGG
TTTGTCCGGTTTTACGATCTGTCACGTTCAACTGCAATTGACCATCGCGGTTGGCAATATCGACAATATAATCGTTGGTC
ACCATGCGGCCCGGTTTACCGGTATTAATATTCGACAGTAGCCCACCTAAAATTTGTGACTGGATGGCCTGAGTAAAGTT
ATCTAACGCTGAGGGTGTTTCAATACCAAAGTCATCGTTATAGCTCGGATCTTTATAAGAGTTTTGGGCCTGAGCGCTAT

TTAATAAAAAAGCGCCATTATTTGGGTTACCACCAAAGTTTGGATTACGGAACTGGAAAGTCATGGTTCCAGCCCAACTT
AATGGCGAAATAAGCATGAGTAGAACTACTGCATGTTTGACACGCATTGCAGCCTCCGAACAATTTTTTATTTAGAATTC
ATCATGCGCCAAATCGCCCGTACTTAATAACGCCTGATTTATCTGGCGACGATTTAGTGCTTCTTCAGTTTGAATCAGTG
CAAAGACGACAGTTTTCTCGAAGTCTCTTTTCAACGGAAATAAAAAAGTCTGGAAAATAACGTCCTGATTGACCGTTATA
GTGATCCAGCTTCCCCATCGTGCACTGGGCCTTTCATTAATCGTTAAGTTACCCGTATAGTCACTTTCCCATTTATCACT
AAAGGCTCGGTAAAAATCATGGCCAATAGATGAAACAGTATGGTCAGTTAGCAATCCCGGGACTTCTACCTCAACGGCGT
GAAGATTCCCTGCGGCGAACAGAAATTCTGCCGCCACAATCCAGCGTAAATAACGTTTCATGGCTTTATCGCCTGAGGTT
ATCGTTTGCCCAGGAAACCGCTTGTGTCCGGTTTTTTACGGCTATCTTCTTGAAAAGATTATAAAGATGCGTTTTAACCG
TATTTTCGCTGATGAACAACGAACGAGCGATCTCGTTATTAGACGCGCCGATACGCAGCTTATTCAGGATCTCTTTTTCC
CGATGAGTAAGGAGGGCTGATTCCGTGCTGTTATAACGATAGTTACCTGAATGCGTAATCAGGTAGCTGGCAAGCTTTTG
CGTAAAGTAGCATTCGCCGCGCAGGACGCCTTGCAACCCATTGACAACACGTTCTTGATCCTCCATGGAATAAAAAACGC
CGTTGATATGAGGCCAGTTTTCAATGTCGCGGTACGGGTAATCTTCAGGCGTATTTAGCAACAATATTTTGATATTGTTG
TTTTTCCTGCTCAAAGTATCCTGCCAATAATGGATAAGCTTTTTATCCGCTTCCATCATATCCAGAAGAATAATAGAGCC
TGAAGAGATATCGTCCAGAGAACGTTGAATATTATGTAATTTTCCTGTAATTGCCAGCGATTGTTTAAGGTGCTGCAAGA
GAGCTGTCGCCTGCAAAGAAGATTTAGTGATCAACAATAATGTATGACCATGAATACTATGGACTTCATTAAACATGATG
AAACCCCGCTTTTTTTATTGATCGCACACCTGACAGCTGCCTCTAAAATAGAAGCACCAGAAGTACTGACAGATGTTGCA
CTGCTGTGTGTAGTAATAAATCAGCCCTAAATGGGTAAAATATAAAACTAATGGATTACATCTGATTTCAATCTAGCCAT
TACAAATCTTAAATCAAGTGTTAAACATGTAACTAAATGTAACTCGTTATATTAAAATGTTAACCTTAAGGTTTTATTAA
GTTTAGAAATGATAGAAAAGTTGTACATTTGGTTTTTATTGCACAATTTTAAAAAATCATACAAATGGTGATAACTTACT
AATAATGCATATAAAAAATATTTCGGTGTAGTCCTTTCGTCATGTAAAACGTTCTTGTTTTTTCTCCACACCTCCGTGGA
CAATTTTTTACTGCAAAAAGACGAGGTTTGTCACGGCTTGTGCGCAAGACATATCGCAGCAATCAGCGACGGGCAAGAAG
AATGACTGTCTGGTGCTTTTTGATAGCGGAAAACGGAGATTTAAAAGAAAACAAAATATTTTTTTGCGTAGATAACAGCG
TATTTACGTGGGTTTTAATACTTTGGTATGAACTAAAAAAGAAAAATACAACGCGCGGGTGAGTTATTAAAAATATTTCC
GCAGACATACTTTCCATCGTAACGCAGCGTTAACAAAATACAGGTTGCGTTAACAACCAAGTTGAAATGATTTAATTTCT
TAAATGTACGACCAGGTCCAGGGTGACAACATGAAAAACAAATTGTTATTTATGATGTTAACAATACTGGGTGCGCCTGG
GATTGCAGCCGCAGCAGGTTATGATTTAGCTAATTCAGAATATAACTTCGCGGTAAATGAATTGAGTAAGTCTTCATTTA
ATCAGGCAGCCATAATTGGTCAAGCTGGGACTAATAATAGTGCTCAGTTACGGCAGGGAGGCTCAAAACTTTTGGCGGTT
GTTGCGCAAGAAGGTAGTAGCAACCGGGCAAAGATTGACCAGACAGGAGATTATAACCTTGCATATATTGATCAGGCGGG
CAGTGCCAACGATGCCAGTATTTCGCAAGGTGCTTATGGTAATACTGCGATGATTATCCAGAAAGGTTCTGGTAATAAAG
CAAATATTACACAGTATGGTACTCAAAAAACGGCAATTGTAGTGCAGAGACAGTCGCAAATGGCTATTCGCGTGACACAA
CGTTAATTTCCATTCGACTTTTAAATCAATCCGATGGGGGTTTTACATGAAACTTTTAAAAGTAGCAGCAATTGCAGCAA
TCGTATTCTCCGGTAGCGCTCTGGCAGGTGTTGTTCCTCAGTACGGCGGCGGCGGTAACCACGGTGGTGGCGGTAATAAT
AGCGGCCCAAATTCTGAGCTGAACATTTACCAGTACGGTGGCGGTAACTCTGCACTTGCTCTGCAAACTGATGCCCGTAA
CTCTGACTTGACTATTACCCAGCATGGCGGCGGTAATGGTGCAGATGTTGGTCAGGGCTCAGATGACAGCTCAATCGATC
TGACCCAACGTGGCTTCGGTAACAGCGCTACTCTTGATCAGTGGAACGGCAAAAATTCTGAAATGACGGTTAAACAGTTC
GGTGGTGGCAACGGTGCTGCAGTTGACCAGACTGCATCTAACTCCTCCGTCAACGTGACTCAGGTTGGCTTTGGTAACAA
CGCGACCGCTCATCAGTACTAATACATCATTTGTATTACAGAAACAGGGCGCAAGCCCTGTTTTTTTTCGGGAGAAGAAT
ATGAATACGTTATTACTCCTTGCGGCACTTTCCAGTCAGATAACCTTTAATACGACCCAGCAAGGGGATGTGTATACCAT
TATTCCTGAAGTCACTCTTACTCAATCTTGTCTGTGCAGAGTACAAATATTGTCCCTGCGCGAAGGCAGTTCAGGGCAAA
GTCAGACGAAGCAAGAAAAGACCCTTTCATTGCCTGCTAATCAACCCATTGCTTTGACGAAGTTGAGTTTAAATATTTCC
CCGGACGATCGGGTGAAAATAGTTGTTACTGTTTCTGATGGACAGTCACTTCATTTATCACAACAATGGCCGCCCTCTTC
AGAAAAGTCTTAATTTGTTGAAATATCGAGCATAAGATGAATCTGGAGAGAATGGTCTGCTGCGAATCAGCCAACCTGAA
AGTATGGATAACACAACCCTCAAGGATGACTAATCATTGAGGAAATAGAATAAATGTTCAGACCTTTTTTAAACTCTCTT
ATGCTCGGCAGTTTGTTTTTTCCTTTTATTGCCATTGCTGGAAGCACCGTGCAAGGGGGGCGTGATCCATTTTTATGGCCA
AATTGTGGAACCGGCATGTGACGTCAGCACCCAGTCATCACCCGTAGAAATGAACTGCCCACAGAATGGTTCTATTCCGG
GCAAAACCTACTCCAGCAAAGCGTTAATGAGCGGCAATGTCAAAAACGCGCAAATAGCCTCAGTAAAAGTGCAGTATCTT
GATAAACAGAAAAAGCTGGCTGTAATGAACATCGAATATAACTAAGTTTCTGAACAACTCACGTTGCTGAGCAGAAAAAT
GCGATTTAACCAAAAAGCCTGCTGTACACTTAAGAAACAAGAAGGTGTAAGGAGGCGTTATGAAAACGCGTATTCATGTT
GTGCAGGGTGATATTACCAAACTGGCCGTTGATGTGATTGTGAATGCGGCTAATCCGTCATTAATGGGAGGCGGCGGCGT
CGATGGGGCCATTCATCGCGCAGCGGGTCCGGCCCTGCTGGATGCTTGTTTAAAAGTCAGGCAACAGCAGGGCGATTGCC
CTACGGGGCATGCCGTTATTACGCTTGCAGGCGATCTTCCCGCTAAAGCCGTAGTGCACACCGTCGGGCCAGTCTGGCGT
GGTGGTGAACAAAACGAAGACCAGCTTTTGCAGGATGCCTATCTCAATAGCCTACGACTGGTGGCGGCAAACAGCTATAC
GTCAGTGGCTTTTCCTGCAATCAGTACTGGGGTTTATGGTTACCCTCGTGCGGCAGCGGCTGAAATCGCAGTAAAAACCG
TTTCAGAATTTATTACCCGTCACGCTTTACCCGAACAGGTATACTTTGTCTGTTATGATGAAGAAAACGCCCACCTCTAC
GAAAGACTCCTTACCCAACAAGGAGATGAATGATTTGCCCCGGCTGGCGAGCGCGGTGCTGCCACTGTGTTCGCAACATC
CCGGTCAGTGTGGCCTTTTTCCTCTGGAGAAAAGTCTGGATGCGTTTGCCGCCCGGTATCGTCTGGCCGAAATGGCAGAG

CATACGCTCGATGTTCAGTATTACATCTGGCAGGACGATATGTCGGGTCGGTTACTGTTTTCCGCCCTGTTAGCCGCAGC
AAAGCGTGGCGTTCGCGTCCGTTTGTTGCTGGACGACAACAATACGCCCGGACTTGACGACATTTTACGCTTGCTTGACA
GTCATCCACGCATTGAAGTCCGGCTTTTTAATCCTTTCTCGTTTCGCTTGCTGCGTCCGCTTGGTTATATCACCGACTTT
TCCCGTCTTAATCGCCGTATGCACAATAAAAGTTTCACTGTCGATGGCGTGGTGACCCTGGTGGGAGGACGAAATATTGG
TGATGCCTATTTTGGAGCAGGGGAGGAGCCACTTTTTTCGGATTTAGATGTCATGGCAATAGGACCCGTGGTAGAGGACG
TTGCCGATGATTTCGCCCGCTACTGGTATTGCAAATCGGTTTCACCCTTACAGCAGGTGCTGGATGTCCCGGAGGGTGAA
ATGGCGGATCGCATCGAGTTACCCGCCTCCTGGCATAACGATGCCATGACGCATCGTTATTTACGCAAAATGGAATCCAG
TCCATTTATAAATCATCTGGTTGATGGAACATTGCCGCTTATCTGGGCGAAGACACGTTTATTAAGTGATGATCCGGCGA
AAGGGGAGGGCAAGGCAAAACGGCATTCACTGTTACCGCAGCGCCTGTTCGATATCATGGGCTCACCCAGTGAACGCATC
GATATTATCTCTTCCTATTTTGTACCGACACGCGCAGGTGTGGCGCAACTCTTACGGATGGTGAGAAAAGGGGTAAAGAT
TGCGATCCTAACCAATTCTCTTGCCGCTAACGATGTTGCTGTCGTCCATGCCGGATACGCGCGCTGGCGCAAAAAATTGC
TCCGCTATGGCGTGGAATTATATGAACTCAAGCCGACGCGTGAACAAAGTAGTACGTTACACGATCGCGGCATAACCGGT
AATTCCGGAGCCAGCCTGCATGCTAAAACCTTTAGCATCGATGGTAAAACGGTGTTTATCGGCTCTTTCAATTTCGATCC
GCGTTCAACATTGCTCAATACTGAAATGGGCTTCGTGATAGAGAGCGAAACGCTGGCACAGTTAATTGATAAACGCTTTA
TTCAGAGCCAGTATGATGCGGCCTGGCAGCTCCGTCTGGACAGGTGGGGACGGATCAACTGGGTTGATCGTCATGCAAAG
AAAGAGATTATTCTCAAAAAAGAACCCGCCACCAGTTTCTGGAAGCGGGTTATGGTCAGACTGGCGTCGATATTGCCCGT
GGAATGGTTATTGTAAATGTGGCTTAACGGGCTGGTGCTTTATCGTTCTCACGCTTAACAACCGGTTTACCAGAGAACAA
AAACTTCAGTAACGGGATGCGCAAATGAATTTCATACAGAATTATCGCAATCCCTACTACAAATATCAGGCCACAGAGAA
AACCAAGCCAGTTGGAGGTGATGTGCGGTGTAATGTATGCGCCGAAAAACAGCGTTAACGGGTGGTGAACCAGATAGATA
AACAGCGATGCGTTAACAAAATAAGTCACCCGCGCTGACTGGAAGTTAAGCAAACGGTGGCCAAAGGAGAAGACCACATT
CACCATCCACAGACCGAGGACCATGGTGATCACCGACTCGGTTTCGTACATCCAGGCATCGCCACTGCCATAGCGCTGGT
TGAGTAAATAAGCGACAAACGCCAATGCTGCTGCAAGGGTACAGCCACGAGACGGCGTGGTAAACAAGGCTTTAAGATGA
GGGAAAATGAAAGCCAGTGCGCCGAGGATAAAGAACGGCAAATAAAACAGCGTTTGCATGACAATAAAATTGAACATGCC
ATTACTCAGAATGGGCGGATACACAATAAAAATCGTTCTTCTTATTACCGCATAACCGATGCCGAGGCATAAAAAAATCA
CCGATAGTTTTACCATCGAGAATTTTTTATTCGTTTTATCAGAATTTTCTAAATTATTTCTGATGCGCTTAAATATCCAT
ACGCACAGCGTCGTCATGACCACTAACACCAGTAAAAACCACAGGTGTGATATTAATTCCCAGGCCAACGTATTATATTT
GTCATACAATGACAGCCCAGGCCAACTTTCCGCTTTTCCTTTGACATATTGCAGCATAATAAATTGCGGTAATGTCAGTA
GGGGGATGGCTGTTAACATCGGGATACCTACACGTTCGACACGTACTTTCCACCATTTTTTCAAGGGATAGCGTAAAAAA
AGCATGTAGGAAAAGTAGCCGGATATAACGAAAAATACCTGCATGCGGAACGAGTGGATGAAGTCATTAAAAAGGGTCAG
CCACAATGATGATTCGGCGCTATTCACATGCCATGTATGGCTCGAATAGATTAAAGAAATATGAAAAGGTATCCCTAACA
ACATCAGCCAGGCGCGGATGGAGTCGAGGAAATATTCACGTTGCGCGGGTACTGGGTTCATATATGGTTAACTAATCTCG
GATTTTTCGTCTTATCCCTGTCGGGTTATGCCTTTAGGCTTGTTGCCATAGTGACACCGACCTGACCGCGCCAGGCGCAG
GCTTCAAGGTTTTTATGCATAGCATCATCGCTACCACTAACCAGAATGGAAGCGTCTGTAAGACGGTTGATAAATAAATT
TGCTGGCAAACCCTACACGAAGTCGATGCTTCTGTCTTTAGGAGAAGCACGGAAAGTGAAAACGGTTGCAATCAGGTGCT
TAATCCATGAGCCAGCGTGCTGAACGATACCGGGATTCTGTTGTCGGAATGGCTGGTTATCCATTAAAATAGATCGGATC
GATATAAGCACACAAAGGGGGAAGTGCTTACTAATTATGAAACATAAACTACAAATGATGAAAATGCGTTGGTTGAGTGC
TGCAGTAATGTTAACCCTGTATACATCTTCAAGCTGGGCTTTCAGTATTGATGATGTCGCAAAGCAAGCTCAATCTTTAG
CCGGGAAAGGCTACGAGACGCCCAAAAGCAACTTGCCCTCCGTTTTCCGCGATATGAAATACGCGGACTATCAGCAGATC
CAGTTTAATCATGACAAAGCGTACTGGAACAATCTGAAGACCCCATTCAAACTCGAGTTCTACCATCAGGGTATGTACTT
CGATACCCCGGTCAAAATAAATGAAGTGACTGCCACCGCAGTCAAACGAATCAAATACAGCCCGGATTATTTCACTTTCG
GCGATGTTCAGCATGACAAAGATACGGTAAAAGACCTTGGCTTTGCCGGTTTTAAAGTGCTTTACCCGATCAACAGCAAA
GATAAAAACGATGAAATCGTCAGCATGCTCGGGGCCAGCTATTTCCGCGTGATTGGTGCAGGTCAGGTTTATGGCCTTTC
TGCCCGCGGCCTGGCAATTGATACCGCCTTGCCATCGGGTGAAGAATTTCCGCGCTTCAAAGAGTTCTGGATCGAGCGTC
CAAAACCGACTGATAAACGTTTAACCATCTATGCATTGCTTGACTCGCCGCGTGCGACAGGTGCTTACAAATTCGTGGTT
ATGCCAGGGCGTGACACGGTTGTGGATGTGCAGTCGAAAATCTATCTGCGCGATAAAGTCGGCAAACTGGGGGTTGCACC
GTTAACCAGTATGTTCCTGTTTGGGCCGAACCAACCGTCGCCTGCAAATAACTATCGTCCGGAGTTGCACGACTCTAACG
GTCTCTCTATCCATGCCGGTAATGGCGAATGGATCTGGCGTCCGTTGAATAACCCGAAACATTTAGCGGTCAGCAGCTTC
TCCATGGAAAACCCGCAAGGCTTTGGTCTGTTGCAGCGCGGTCGTGATTTCTCCCGCTTTGAAGATCTCGATGATCGTTA
CGATCTCCGTCCAAGCGCATGGGTGACTCCGAAAGGGGAGTGGGGTAAAGGCAGCGTTGAGCTGGTGGAAATTCCAACCA
ACGATGAAACCAACGATAACATCGTCGCTTACTGGACGCCGGATCAGCTGCCGGAGCCGGGTAAAGAGATGAACTTTAAA
TACACCATCACCTTCAGCCGTGATGAAGACAAACTGCATGCGCCAGATAACGCATGGGTGCAACAAACGCGTCGTTCAAC
GGGGGATGTGAAGCAGTCGAACCTGATTCGCCAGCCTGACGGTACTATCGCCTTTGTGGTCGATTTTACCGGCGCAGAGA
TGAAAAAACTGCCAGAGGATACCCCGGTCACAGCGCAAACCAGCATTGGTGATAATGGTGAGATAGTTGAAAGCACGGTG
CGCTATAACCCGGTTACCAAAGGCTGGCGTCTGGTGATGCGTGTGAAAGTGAAAGATGCCAAGAAAACCACTGAAATGCG
TGCTGCGCTGGTGAATGCCGATCAGACGTTGAGTGAAACCTGGAGCTACCAGTTACCTGCCAATGAATAAGACAACTGAG
TACATTGACGCAATGCCCATCGCCGCAAGCGAGAAAGCGGCATTGCCGAAGACTGATATCCGCGCCGTTCATCAGGCGCT

GGATGCCGAACACCGCACCTGGGCGCGGGAGGATGATTCCCCGCAAGGCTCGGTAAAGGCGCGTCTGGAACAAGCCTGGC
CAGATTCACTTGCTGATGGACAGTTAATTAAAGACGACGAAGGGCGCGATCAGCTGAAGGCGATGCCAGAAGCAAAACGC
TCCTCGATGTTTCCCGACCCGTGGCGTACCAACCCGGTAGGCCGTTTCTGGGATCGCCTGCGTGGACGCGATGTCACGCC
GCGCTATCTGGCTCGTTTGACCAAAGAAGAGCAGGAGAGCGAGCAAAAGTGGCGTACCGTCGGTACCATCCGCCGTTACA
TTCTGTTGATCCTGACGCTCGCGCAAACTGTCGTCGCGACCTGGTATATGAAGACCATTCTTCCTTATCAGGGTTGGGCG
CTGATTAATCCTATGGATATGGTTGGTCAGGATTTGTGGGTTTCCTTTATGCAGCTTCTGCCTTATATGCTGCAAACCGG
TATCCTGATCCTCTTTGCGGTACTGTTCTGTTGGGTGTCCGCCGGATTCTGGACGGCGTTAATGGGCTTCCTGCAACTGC
TTATTGGTCGCGATAAATACAGTATATCTGCGTCAACAGTTGGCGATGAACCATTAAACCCGGAGCATCGCACGGCGTTG
ATCATGCCTATCTGTAACGAAGACGTGAACCGTGTTTTTGCTGGCCTGCGTGCAACGTGGGAATCAGTAAAAGCCACCGG
GAATGCCAAACACTTTGATGTCTACATTCTTAGTGACAGTTATAACCCGGATATCTGCGTCGCAGAGCAAAAAGCCTGGA
TGGAGCTTATCGCTGAAGTCGGTGGCGAAGGTCAGATTTTCTATCGCCGCCGCCGTCGCCGCGTGAAGCGTAAAAGCGGT
AATATCGATGACTTCTGCCGTCGCTGGGGCAGCCAGTACAGCTACATGGTGGTGCTGGATGCTGACTCGGTAATGACCGG
TGATTGTTTGTGCGGGCTGGTGCGCCTGATGGAAGCCAACCCGAACGCCGGGATCATTCAGTCGTCGCCGAAAGCGTCCG
GTATGGATACGCTGTATGCGCGCTGTCAGCAGTTCGCGACCCGCGTGTATGGGCCACTGTTTACAGCCGGTTTGCACTTC
TGGCAACTTGGCGAGTCGCACTACTGGGGACATAACGCGATTATCCGCGTGAAACCGTTTATCGAGCACTGCGCACTGGC
TCCGCTGCCGGGCGAAGGTTCCTTTGCCGGTTCAATCCTGTCACATGACTTCGTGGAAGCGGCGTTGATGCGCCGTGCAG
GTTGGGGGGTCTGGATTGCTTACGATCTCCCGGGTTCTTATGAAGAATTGCCGCCTAACTTGCTTGATGAGCTAAAACGT
GACCGCCGATGGTGCCACGGTAACCTGATGAACTTCCGTCTGTTCCTGGTGAAGGGTATGCACCCGGTTCACCGTGCGGT
GTTCCTGACGGGCGTGATGTCTTATCTCTCCGCTCCGCTGTGGTTTATGTTCCTCGCGCTCTCTACTGCATTGCAGGTAG
TGCATGCGTTGACCGAACCGCAATACTTCCTGCAACCACGGCAGTTGTTCCCAGTGTGGCCGCAGTGGCGTCCTGAGCTG
GCGATTGCACTTTTTGCTTCGACCATGGTGCTGTTGTTCCTGCCGAAGTTATTGAGCATTTTGCTTATCTGGTGCAAAGG
AACGAAAGAATACGGCGGCTTCTGGCGCGTTACATTATCGTTGCTGCTGGAAGTGCTTTTTTCCGTGCTGCTGGCTCCGG
TACGCATGCTGTTCCATACGGTCTTCGTTGTCAGCGCGTTCCTTGGCTGGGAAGTGGTGTGGAATTCACCGCAGCGTGAT
GATGACTCCACTTCCTGGGGTGAAGCGTTCAAACGCCACGGCTCACAGCTGCTGTTAGGGTTAGTGTGGGCTGTTGGGAT
GGCGTGGCTGGATCTGCGTTTCCTGTTCTGGCTGGCACCGATTGTCTTCTCGTTGATCCTGTCACCGTTTGTTTCGGTGA
TTTCCAGCCGTGCCACCGTTGGTCTGCGCACCAAACGCTGGAAACTGTTCCTGATCCCGGAAGAGTATTCGCCGCCGCAG
GTGCTGGTTGATACCGATCGGTTCCTTGAGATGAATCGTCAACGCTCCCTTGATGATGGCTTTATGCACGCAGTGTTTAA
CCCGTCATTTAACGCTCTGGCAACCGCAATGGCGACCGCGCGTCACCGCGCCAGTAAGGTGCTGGAAATCGCCCGTGACC
GCCACGTTGAACAGGCGCTGAACGAGACGCCAGAGAAGCTGAATCGCGATCGTCGCCTGGTGCTGCTAAGCGATCCGGTG
ACGATGGCCCGTCTGCATTTCCGTGTCTGGAATTCCCCGGAGAGATATTCTTCATGGGTGAGTTATTACGAAGGGATAAA
GCTCAATCCACTGGCATTGCGTAAACCGGATGCGGCTTCGCAATAAAAACGTAGTTGCCTGATGCGCTACGCTTATCAGG
CCTACATCGTTCCTGCAATTTATTGATTTTGCAAGACTTTGTGGGTCGGATAAGGCGTTCACGCCGCATCCGGCAAAAAC
AACGAGCCAATAAAAATACCGGCGTTATGCCGGTATTTTTTTACGAAAGAGGTATCAAATGCGTTTAATTGTGGTGAGCA
TCATGGTGACCTTGCTGAGTGGCTGTGGCAGCATTATTAGTCGCACTATACCGGGGCAGGGGCATGGCAACCAATATTAT
CCCGGTGTGCAATGGGATGTGCGTGACTCCGCCTGGCGTTATGTCACGATCCTTGATCTGCCATTCTCTCTGGTTTTTGA
TACTTTACTGCTGCCGATCGACATTCATCATGGCCCGTATGAGTGATTAACGTTCATCCCACTCATCAGCTGCTGCGCGT
CCTTCCTCGGTATCCAGCGGTGGCTCAAGCTGAAATTCCCCCTCGTCCCATTCATGTAATGTATTCTCTTCCTGCCACTC
CTGGCGTATCTCTATCTCATCATAGTCGCCATCAAAAACACTTTGCGCGGCTTCACCGCTAAGCATAGGTAAACATTCAC
CTTCTTCCCCTTCGTCGGCAAAAAACTCAACTTGCCACATGATGTCGCCGTCCTGCAAAACGTATTTTTGGGCATTGAAC
TGTTGCACATTCGCATCTTCGGCGTCGATGCCGGGGTTGTCTGCAAGAAATTCTTCGCGTGCAGCGTCAATGGCTTCTTC
AAGCGTTGCGTACATGGTCATCACGATTCTCCCTTTGAGTTGATGAGGTTTCAGGGAAAAGGATAGCTGATTCTCCGCTT
TTGCAAGTATGAAAGGCGAAAAATCAGTTCGATACCTGGGGTATTCGACGACGACGTAGACTGTTCCATGAATAGACTGC
GTTGAATAACACTACGCCAGCGGTGACGAGAAATACCGCTCTGAAACCGTAGTTCGCTGAAATCGCTGCTCCCATCAATG
GTCCGGTAACGTTGCCAATATCACGAAACGATTGGTTATAGCTGAAGATACGCCCGGCGATCTGGTTGCTCGAGTTGTAA
ACCAACAGTGTCTGTACGGCGGGGAGTAGTGCACCATCGGCGGCACCGAGCAAAAAACGTAAAATCCCAAGTTGCAATGG
CGTCTGAACGTAAGACATTGGGATCAACAGCAGTACAGAAAAGATCAGCGCTGTAATCAGGATCTTTTCGGGTCCGATTC
GATCGCCAAGTTTGCCGAGTCGTGGTGCACTTAGCAGAGCCGCCACGCCTGGCACCGAGGCGATCATGCCACTGATAAAG
GCGACGTTACTGACGTTACCCGCCAGTTCGCGGACATACAGCGTCAGAATGGGGGCAATTGAGCCCGTCGCCACCTGGAT
GATTAACGTAGTGACAAACAGGCTGAGTACCAGTTTCGGGTTTTTAAGTGATGTCACCACTTCCCGCATGTGCAGCATCT
CTTTTTTGCTGACCGGCTGGAATTTTTCTCTGATGCAAAACAGGGTGACGAAAAAGCAGAGTATGAGCACACTGGCGGTA
ATAAAGAATACCGGACGTAAGCCGTAGCTATCGGCGAGCAGGCCGCCAGCCATTGGGCCGAGCAACGCACCACTAACGCC
GCCTGTGGAGAGCGTACCCAGCGCCCAGCCGCTTTTATTACGCGGTACTTGTGTGGCGATAAGAGCATTAGCGTTGGGGA
CAAATCCGCCAAGTAACCCAAGAAGCGCCCGCAGGATCAAAAACTGCCAGATATTTTGTGCCAGCCCCATCAACACCATC
ACGATGCCCATGCCGAGGGCAGAGCGTAATAGCATGAGTTTTCGGCCTTTACGGTCGGCGAGTCCACCCCAAAACGGTGA
GGCGATGGCCGAAAATAAAAATGTAATGCTGAAGACAATACCGGACCACATATTCAGGGCGGAGTGACCGGTAACGCCAA
GCTGCTCAACGTAGAGGGGTAAGAAGGGCATTACCAGACTGAAGGCGGCACCGGTAAGAAAACAGCCTAGCCAGGCGACG

ATCAGGTTTCGTTTCCAGTTTATAGGGGTGTCATTTTCACAGGGTGACATAGCAATCCGCTGTTGGTGCGCCAGGCGCGG
TGAACATAAGAAGAAAAGATAAGCACACTAATTATGCGCCCGACTTCCAGGGGGCGCAATCCAGAGAGCTTTTATCGCTA
AATCAGGGGGATTTGCTGTGGTAATGCCGGATGCCATTCTGAAGCATCCGGCATGGGAGATTTAATAGCGTGAAGGAACG
CCTTCCGGGCGTGTTTTAAAGCGACGGTGTAACCACATATACTGCTCTGGTGCCATCATGATGCATTTTTCGACCACTTT
GTTCATCCACGCGGCGGTAGTTTCGGCATCATCCAGTGGCGGAGAACACTCTGGCGGCAGCATAATCAATTGATACCCTT
TGCCATCTGGCTTACGGCGTGGAACGAAGGGCACCAGACATGCGCCGGACATCCGTGCCAGCATCCAGGTTCCGGTCGTG
GTCGCAGCCTGCTCAACGGCAAACAACGGGACGAAAACGCTTGAGCGCGGGCCGTAATCATGATCCGGTGCGTACCAGAC
CACTTCGCCTTTTTTCAGGGCTTTAATCATGCCTTTTAAATCTTTGCGGTCGAGCATCGATTTATTTGAGCGCAAACGGC
CCCAGGTTTGTAGCCAGTCAATCAGTGGATTATCGTTCGGGCGATAAACGCCAATACCCGGTTCCTGCATACCAAACTGC
CGCGCACCCAGCTCCAGTGTCAGAAAATGGATGCCAACTAACAGGATGCCGCGTTTTTGCGCCTGCACGTCACGAATGTG
TTCCATGCCGATCACTTCCGTCCAGCGGGCGATTCGGCGGTCCGGCCAGAACCACGCCATGCCGGTTTCCATCAGGCCCA
TGCCAACGGATTCGAAATTCTTCACCACCATTTTACGGCGTTCTTGTTCGCTCATTTCCGGGAAGCACAGTTCCAGGTTG
CGATGCACAATTTTTGCGCGTCGTTTCATAAAACGTAACGCCAGTTTTCCTAATCCACAACCGAGGCGGTAGATAACCGG
GTAGGGCAATTGCACGACTAACCAAAGTACGCCAATACCCAACCAGGTTAACCAATAACGCGGATGAAGCAGTGCGGTGG
AGAACTTGGGTAGATTCGTCATATCAATCCTGTTTTTCAACCTATTCGGGCAATTGTATGTATTGTCGCATTTTTTCGCC
CGCAACCAAAATTTGTGGCTGAAGACTGGGCGAAATTGCCGCGCTTGTAAATAACAAATAATTTTTAATGCGCAAATGTA
GCGTAAAATGTGTGGATGTTAATTATCGATAATTGCTATATCATGCCGCGGATTTTTACTTTCCCATCTCGCAGGAACCG
TACACCATGCCAGTGTTACACAACCGCATTTCCAACGACGCGCTAAAAGCCAAAATGTTGGCTGAGAGCGAACCGCGAAC
CACCATTTCGTTTTACAAGTATTTCCACATCGCCGATCCTAAGGCGACCCGTGACGCTTTATATCAGCTGTTTACCGCGC
TGAATGTTTTTGGGCGAGTGTATCTGGCGCATGAGGGCATTAACGCGCAAATCAGCGTACCTGCGAGCAATGTTGAAACA
TTTCGCGCGCAGCTCTATGCCTTCGACCCGGCTTTAGAGGGTTTACGCCTGAATATCGCGTTGGATGATGACGGGAAATC
CTTCTGGGTACTGCGCATGAAGGTACGCGATCGCATTGTTGCCGACGGTATTGACGATCCTCACTTTGATGCCAGCAATG
TTGGTGAGTATCTGCAAGCGGCGGAAGTGAACGCCATGCTTGACGATCCCGATGCACTATTTATCGACATGCGTAACCAC
TATGAGTATGAAGTGGGGCACTTTGAAAACGCGCTCGAAATTCCGGCAGATACCTTCCGTGAGCAGCTGCCAAAAGCAGT
CGAGATGATGCAGGCACATAAAGATAAAAAAATCGTCATGTACTGCACCGGCGGCATTCGTTGTGAAAAGGCCAGTGCCT
GGATGAAACATAACGGATTCAACAAAGTCTGGCATATCGAGGGCGGAATTATTGAATACGCCCGTAAGGCGCGCGAGCAG
GGCTTGCCGGTGCGTTTTATTGGCAAAAATTTTGTTTTTGACGAGCGGATGGGCGAACGTATATCGGATGAGATTATCGC
GCATTGCCACCAGTGCGGCGCGCCGTGCGACAGCCATACCAACTGTAAAAATGATGGCTGCCACCTGCTGTTTATTCAGT
GTCCAGTATGCGCGGAAAAATACAAAGGTTGTTGTAGTGAGATTTGCTGCGAAGAAAGCGCGTTACCGCCAGAAGAACAG
CGACGCCGTCGGGCAGGACGTGAAAATGGCAATAAGATCTTTAATAAGTCTCGTGGACGTCTGAATACAACACTGTGCAT
TCCTGATCCAACAGAATAAATATCATTGCCGGATGCGTGCCATCCGGCAACATTTCACGCTTACTTCTGCTGTACGCCTT
CCACTGAAATAATCAGATCCACTTCCTGAGAAGCTGGACCTAAATCTGTCTTGATATTGAAGTCCTTGAGCTTAATTTTG
CCTTCGGCCTCGAAGCCTGCACGTTTACCACCCCATGGGTCGTCGCCCTGACCAATTAATTTCGCTTCCAGCGTGACGGG
TTTGGTTACGCCATTCAGCGTCAGATCGCCGGTAATATCCAGTTCGTCACCGTCTTTCTTCACGCTGGTGGAGGTGAATG
TTGCCTGTGGATATTTTGCGGTATTGAGGAAATCTGCACTGCGAAGATGTTTATCGCGTTCGGCGTGATTAGTATCGACG
CTGGTGGTGTTAATTGTCACATTCACTTTATCGGCAGCCGGATTTTTTTCGTCAAAGGTAAAAGTACCGTCGAAATCTTT
AAAGGTGCCGTATAACCAGCTATAGCCAAGGTGCTGGATGCGGAAATTAACAAAGGCGTGCTGACCTTCTTTGTCAATTT
TGTAATCGGCGGCAACCGCTGAACCGGCAGAGAACATCAGGGACGCGAAGGTTAAACCAAGCAGGCTTTTTTTCATTTTT
TATACTCCATAGTCAGATGACGACTTTCCCAGCATGCGCTTCAGAGTGTCGTCTTTATCGATGAAATGATGCTTCAGGGC
CATAAATCCGTGCATAACGGACAGTACTACGACGCTCCACGCGAGCCAAAAATGCAGGGCACCGGCAAAGTCTGCCTGTG
CGCCAGCGTCGGCAAGGGTCGCGGGGACGTCAAACCAGCCAAAAACGCTGATCGGTTTACCATCGGCAGTTGAGATCAGA
TAGCCGCTGATGCCGATGGCGAAAAGCAATAGGTACAATGCGAGATGTCCAGCTCTTGCGGCAAGACGAGTCATGGGCGA
ATAACTCGGCAGCGGACCCGGCGGTGGAGATATGACACGCCACAGAACGCGAATAACCAGCCCCATCATTAACAAAATAC
CGATGCTTTTATGCAGTTCGGGTGCTTTGTGATACCAGCCATCGTAATAACTGAGCGTGACCATCCACAGACCTAAAGCA
AACATGCCGTAGACAATAATTGCGCTTAGCCAATGGAAGGCCGCAGATATAACACCATAGCGTTCAGGGGTATTTGTGAA
TGACATAAGTGCATCCAAAACGAATTTGCGAGAGAAAGAAAATGGCGTGTCGGAGAATGAATTGCAACACAAAAATAACA
AATTGAATGATATTTATTTTTATTTCAATAATTTTGAATTAATAATGAAAGAATCTTCATAAGTTTAGTGGGTTCAGCAT
CAGAGATGTCTGGTGAGTGCTGTAGAAGCAGGAATAATCTCATTCAATTTTTCACGCTAAATGAAAGGGAGATTAAATAT
TTGTCAGTTTATGTCAGTGCGGAAAACTAAAAATAAAACACATAGATCAGATCCATAATTGCCAACAATCCCCAGAGAAA
AAGATAAAGCATCAGATGCTCGCGAATATTATTGATGAGATAGTGCAACAGCCGACGCATTAAATACTCCTGTAAAAACG
GCCTCATTCTGGAGGCCGTAATGATGATTATTGGAAGCGGGAAAGCCTGAATGGCGTCAAATCAAAATCGCTTTTTTTGT
CTTGCGCAAAATCGGCAGCTATTTCCCCTAAAACTGACGCAAATTTAAAACCGTGCCCACTCAGGCCGGTAATGAGCAGT
GTATTATCGTGGCCGGGTAGGGTATCGATAATAAAATCTTCGTCAGGCGAATTATCATAGGTGCAGGCAGCGCCGTACAG
GCAGCAACCGATACCCGGCAATACATTGCGCAAGAACGGGAAGGCTTCTGACCCATCACTGGCCACTTCCGCAAACGGAA
CACGTTCATCCGCTGAATGGATAACCTGGCCTCCGTTATGTTTGCCAATCTTCAACGCGTCGTTTTCTGCCGGAAAACCA
TAATATTGATCGCCATTGGGCAGTTCACCGGTAAACGCCGGGAATTTATTCTTCACGCTATAGCGGCCATCGGCCTGATA

CCAGGCAAATACTTTGCGTACGGGCTGGACAGGCAGCTCCGGGAGCAGGTCTTTTACCCATGTTCCCGCGCAGACAATCG
CTTTTTTCGCCTGATACTCACCGTCAGCCGTTTCAATAGTTACGCCATCATCGTCATGACGAATTGCGGTGACCGGGCAG
TTGAACAGTTGCGCACAGCCCGCTTCCTTCGCCAGTTGGATCCAGGTTTTAATCGCCAGTTCGCTGCGCAAAAAAACCGGA
ATCAGTTTCAAATAAGCCGATGTAGTTGTCCGGGACGCGTATTTCCGGCCAGCGGGCCATAATCCCTTGCGCATCGAGCT
TTTCAACGTTGAGTTGCCATTGTTCGGCGCTGTGGGCGACGTTGGCGAGAAATGTGGAATCAGCCGGGCCAAGGTTAATG
ACGCCAGAGCGTACAAAAATGGGATCTTCTTCGTTGTGGCGGGAGAGTTCATCCCACAGCGTTTGCGCGCGGAGGACCAG
CGGGACATACTTTTCGCCTTCACCATAAGCATGGCGAATTAATCGCGTATCGCCGTGGTGGCTGCCGTGTTGATGCGGTG
GCATATGGGCGTCGGTCATTAGCACGTTTAAACCGGCGCGGGTTGCATAATACCCGGCGGCAGCGCCTACGGAACCGCTG
CCAATAATGATGAGATCGTATTTCATTTTTTTCTCTCTGCTCTCATGGTTTAAGCAGAGTAATTAACTGAATGCAGATAT
ACAACCCAGAAATGGTAAAGGCACCGGTGAGGTGCCTTTTGGGTGGATGGTCATGTCATGTTAATGACGCCGATACTCGT
TTACCTGGAAATCACCGGATTCAATTTTGGCGATTCCTGCTTCTAATATCGAAATAAATTGTCTGGCAACATCAGTGGTT
AACCAGAGCGTCTGACCAACTTCAGTCCCTTCCTGCTCGGACTTATTTGGGGTCTGGTAGTGCAAACGCAACATCAGCGC
ATCATAGCTATCAACGGTGCTGATGTCCCACCCTACGAGCGGATGAGTCTGAATGACTTCATTATTTTTTTCCATCATGG
CCCCCTAATTCGTGTTACTAGACAACGTTATTCGAGGTTCAATGCGTTTTTATCTGAAGCAACTTCAGTATACCAATAAG
TAAGGGTATTCACCGAATTTTTAAAGGGGGAAACAGATAAATTTCTGCTTTTAAGATTTATATGAACAATAAAACAGCAT
GTCATTCATATTTTTTTAGCATATTGTGCAATTATTTTGAGGAAGTGTAGAAATTTTGTACTCAAAATTCGTAAGTAAAA
TAAAAAAGCCGGGGCGACCCGGCAAAAAAAATCACTGCATATTATTCGCTGACAAACCAGTCATCGGCGCTTTCCCACGT
TTCCTGGAGAATTTCACTAATGCGCTGTTTATCTTCTTTTGTCGCGCCAATAACCGATAAATTATTCGCTGCGGCATAAC
GTACCGATACGTGGCCTTCATTATCAGGAAACGCATACTGAATACGGCGGGAAAGTTCGCCAGCCAGGGCGTCAATAGCC
CCAGCTGGCAATGGAGAAGTTTTCGCTATGGTGACTTCAATTCGCATAATAGCCCCCTGTTGAATATACTGGTTATTTAT
ACAGGTAAAATAACCTAATGACAACAGGAAGCTACGATTTTTATTGTTTAACGGACCAGCGTACCGTTTCCCCGGCGAGG
AATGGCACCAGCGTGTCATCAGTCAGTGCGATGCTTTCAGCAACCTGTTGCTCTTCACGTACCAGTTCGATGAATGTGTC
GTTGACCGGCAACCCATAGAACTGCGGGCCGTTTACAGAACAGAATGCTTCAAAGTGCTGCAAAGCATTCATTTCTTCAA
AGACGGTAGCGTAACTGCCCAGCGCGGTTGGGGCGTTGAAGCAGCCCGCGCAGCCGCAACTGCTCTCTTTGCGATGACGT
GCATGTGGCGCAGAATCCGTACCGAGGAATACTCGATTAAAACCGCTGGCGACCAGTTCACGCAATGCCTGTTGGTGAAT
ATTACGTTTGAGGATGGGTAGACAATACAGGTGCGGACGCACGCCTCCAACCAGCATATGGTTGCGGTTAAACATCAGAT
GCTGCGGAGTGATGGTGGCAGCCAGCCGTTCATTTCCGTCACGGACATAGTCGGCAGCATCTTTGGTGGTGATGTGCTCA
AAAACGACTTTCAGCGCAGTCAGGCGCTGGCGCAGAGGTTCCATCACGCTTTCTATAAAGCGCGCTTCACGATCAAAAAT
GTCGATATCTGCATGTGTCACTTCACCATGCACCAGTAGCGGCATACCGATTTTTTCCATGCGCTCAAGTACCGGCATGA
TTGCGTCAATTGACGTCACGCCGTGGCTGGAGTTAGTGGTTGCGTTTGCCGGGTAAAGTTTTGCAGCGGTGAACACGCCT
TCGTTAAATCCGCGCTCCAGCTCATTAGGATCCAGCGAATCTGTTAAATAACAGGTCATCAATGGGGTGAAATCGTGCCC
GGCAGGTACGGCGTCAAGAATACGCTGGCGATACGCCACGGCAGCCTCAACGGTGGTCACGGGCGGAGCCAGATTGGGCA
TTACGATAGCCCGTCCATAAATTTCGCTGGTATATGGCACGACAGTTTTTAACATGTCGCCATCGCGGAGGTGAAGGTGC
CAGTCGTCTGGGCGGCGGATCTTTAATACCTGGGATGGTGCAGTCATTAATCTCTATGCTCCGGCTGAAGGGATGTTTTT
GCCGGACACAAAGGATAAGCGGAAACGTTTTCCTTTGCACGAAAAATAAAGGGCGCGAATGCGCCCTCGTGATTAATCAG
TAAATGGAATGACAATTTCGCCTGGCTTCACTTCAATGCCTTTCGCCAGTTTTTTCGCCATTGCTTCGCCCTGGCTGCCA
TCTTCGCGCAGGACGTAAGCAGGTTGCTGGTTAAAGTAATTGCGTAATGCCTGGTTCAAATAGGGAAGCAACGTTTGCAT
CACCGTTTGCATTTTTTCCGGTTGTACCGTCGCATCGACCACTTCCATCTCTTTCAGGAAGATCGCACCTTTTTCTTTAT
CAAACACGGGCAGCGCTTTCAGCTTCAGTTTCATGGTCGCTTTCTGACTACCGAACAGGGAGTTCATGTCCAGATTGGCG
TCTCCGGTTAGGGTAACCTTATTCGGCTCTTCGCGACCAATTTGGCTGGTCAGGTTTGTCAGAACAATATGGGCGTCAGC
CACGCCGGGTAAACCGATATCTTTTGAGAAATTATTATGTTTCGCAAGCGACTGGTTAATTTCTTGTTCGGTGATGGTGT
ATTGGGTGAGTTGATTACAGCCAACGAGCAGGCCACTGACGATCAATGCAGCGGCAAATAAAAACTTGTTCATGGTAGTC
CTCGACATGAAATCTGCGTCAATATCCTGACACAACGCAGCATGTGTCACCAGCGATAAACTCGCCAGCAGAAAAAACTG
AAAACGGCGGCAACCCGCGAATACAGGCTGCCGCGGCGGGTCAGGATTAAATCGCCATTGATGATAACAAATTGATTTGT
GTCTGTTTCGCCATATTATCGCGGTAATCAGCAACGCGGCTTGGCCAGTTAATTCCGGCTACCAGCGTCAGATTACGCAG
TAGCGGGAATAGCTGAATATCATCTTCCGAAAGTTCGCCATTCACGGCGTTCGGTTTGACGATCAGTTTGTCCAGCGCAC
GTAAATCATCGCTGATATTCTTAATCAGACCGTCAGAGTGGGCCAGCAGGTCGGCAAAATTACCCGCGCTGGCCTCTTTC
TTGTCGACGAAATATTTGCGCGCGGCGGGAGTAGAAAACTCATCAAATGCCGATTTGGCAAAACGCGGCAACAGCAGTTT
GTTGGCGTAGCCATTGACCTTGCGCAGCCACTCTTCAATGGCAGGGGAACGTTTGCCGGTCAGTAACGGTTTGCCGTCGA
GTTTATCGACATAGTGAACGATGTCCATGCTTTCTGGCATATAGCGGCTGTCATCTTTTTGCAGAATGGGAACCTGTTTT
TGACCGACCATCCGGGTGGGTGTTTCTGCGTCGTCGTTGAGCAGAACATGTAATTCGACGGGGATATTTTTCAGGCCGAA
AATCATGCGGGCTTTGAGGCAGTAAGGGCAGTGATCGTAAATGTATAGCTTCACGTGACTCCTCCATTTGGCTGTCAGTT
CACTTTCAGTATGAAGGAGTCAGGGGGGAGTATCAAATCAGGCGTCGCGTTCAAGCAAACGACGCGCGGCGCGTTTCTGG
CTAAACTGCCAACCCAGCGCAAGGAAAGTGAAGATGCCAATAATGCCCAGCATCATCCACGGAAGCTCTGGCTGGTGCGC
CGATTTGCCCAGGTCAAACAGCCAGCCGCCACCGATATAACCAATAGCGCCGCCAATCGCCAGACCCAGACGGCTAAACC
CCATATAGCTGCCGCGAGCTCTTGCGTCCGCCAGCGAAGCACTTAAGGTTTCACGCGCAGGCTCGGCAATGATCGACCCG

ATATAAAACAGACAAATCAGGGTGAAAAGTTGTTGCAGGCCGCTGACCATGCCCACCGGCATCATGCTTAATGACATTAT
CAACAGCCCAGCCATCAACCGGTGTTCCAGACGAAAATGCTTTTCACTCCAGCGGGCGATAGGGTAGAGCAACGTTAACG
ACAGACACGCTTCAATGGCATACATCCATTTAACGGCAGAGGGCGCGCCAGCCACGTCGTTGACCATAATTGGCAGCATC
AGCATCACTTGTACAGCCAGCATGTAGTAACCCGCCAGCGTCAGAACATAGGTGACAAAACGCTTGTCACGCATCACGCG
GGTCATGCCTTCGCGAACGGGCGTGCGTACGGTGGAGAGTTTCCATGCTGGTAACAACCACGCATTGAACGCCGCACATA
GCACAAATAGAACTGCCCCTGTGGCGCAGACCAGGCGAAAGTCGTATTGCAACAGCCAGCTCCCCAACAATGCGCCAATG
ACCGCACCGGCACTGTCCTGCATCATCAACAGCGAGAAAAAACGACCACGCTGCTGTGGACGGATTAATTTCACCACCAG
CGCCGAACGCGGCGGATCAAACAACGTGCCACCGAGTCCCGAGAGCAGGCATGAAAACCACAATAGCCACGGTTCGTGGG
CGATACCCATTGTGGCGAATCCGGCGGCGCGCATCAGCATACCGGTAACAATCATCGGTTTGGCACCAAAGCGGTCGGCA
ATTGCACCGCCGAAAATACCCAGACCTTGCTGAATAAATTGGCGTAGACCGAGAGCAATACCGACCATGACGGCGGCCCA
GCCCATTTGATCAACGAAGCGGATAGAGATCAGCGGGAAGACAACAAAGAACCCCAGCACGACCAGCATATTATCGATGA
GCAGGAAATATTTACCCAGGTTCCTCGCCTGCGACACGCGGGACATTTCCCCTCCCGGGAAATAAAAGATGAGCACTTTC
TATTCTGCATTGCCGCTAAGACTTTTCCCATCACTTCGGGGACAATATTTTTTTATCAAAACGTCGCTTTGATAGAGAGT
TTTTATCAAATATGTGAATAATGCAGAAAATGGCATTTTGGACTTTTCACAGGGTCTGGTTGCGCAGGTATAGTAATACT
TACAGCGTATTAAAGACGTTACGGGAAGGAGTAGGTATAGAATGTTTGGCTATCGCAGTAACGTGCCAAAAGTGCGCTTA
ACCACAGACCGACTGGTCGTGCGTCTGGTGCATGATCGTGATGCCTGGCGTCTTGCGGATTATTACGCAGAGAATCGCCA
TTTCCTCAAGCCCTGGGAGCCAGTGCGCGACGAAAGCCACTGTTATCCATCAGGCTGGCAGGCCAGGCTGGGGATGATTA
ACGAATTTCATAAACAAGGTTCAGCTTTCTACTTTGGCTTATTCGACCCGGACGAAAAAGAGATTATTGGCGTTGCCAAT
TTTTCCAATGTTGTTCGTGGCTCTTTTCATGCCTGCTATCTCGGTTATTCGATTGGGCAAAAATGGCAGGGCAAAGGACT
CATGTTTGAAGCCCTGACCGCAGCCATTCGTTATATGCAGCGCACCCAACATATTCATCGCATTATGGCTAATTATATGC
CGCACAATAAACGCAGCGGTGATTTACTGGCGCGACTGGGTTTTGAAAAAGAAGGCTATGCGAAAGACTATCTGTTGATT
GATGGACAATGGCGCGATCACGTACTGACGGCATTAACTACCCCAGACTGGACGCCCGGCCGCTAAGGAGAATCTCATGA
AATATCAACTTACTGCCCTTGAAGCTCGCGTGATTGGTTGCTTGCTGGAAAAGCAGGTGACCACGCCGGAGCAATACCCG
CTCTCAGTCAATGGTGTAGTCACGGCCTGTAATCAGAAAACGAACCGTGAACCGGTCATGAATCTGAGTGAATCCGAAGT
GCAGGAACAGCTGGATAATCTGGTCAAACGTCATTATCTACGCACAGTGAGCGGTTTTGGTAATCGGGTCACCAAATATG
AGCAACGTTTTTGTAATTCAGAATTTGGCGATCTGAAACTGAGCGCAGCGGAAGTGGCGTTAATCACCACGTTGTTATTG
CGTGGTGCCCAGACGCCAGGTGAACTGCGCAGCCGCGCCGCGCGAATGTATGAATTCAGCGATATGGCGGAAGTGGAGTC
GACGCTGGAACAACTGGCAAATCGCGAAGATGGTCCTTTTGTGGTGCGTCTGGCCCGCGAACCGGGTAAACGCGAAAACC
GCTACATGCATCTTTTCAGTGGTGAGGTTGAAGATCAGCCGGCGGTGACGGATATGTCGAACGCGGTTGACGGTGATTTA
CAGGCCCGCGTCGAAGCCCTGGAAATCGAAGTGGCAGAACTGAAACAGCGTCTTGATTCGTTGCTGGCCCATCTGGGAGA
TTAAAGTGAAAAAATTACGTATCGGCGTAGTGGGATTAGGTGGCATTGCGCAAAAAGCGTGGTTACCGGTGCTGGCGGCA
GCGTCTGACTGGACGTTACAAGGAGCCTGGTCGCCTACGCGCGCGAAAGCCCTGCCAATTTGTGAAAGCTGGCGCATTCC
TTATGCCGATTCGTTATCCAGCCTTGCCGCCAGTTGCGATGCGGTTTTTGTGCATTCCAGCACCGCCAGCCACTTTGACG
TGGTCAGTACGTTACTCAATGCGGGGGTACATGTCTGTGTCGATAAACCGCTGGCAGAAAATCTGCGCGATGCTGAACGG
CTGGTGGAACTGGCGGCGCGGAAAAAACTGACGTTGATGGTCGGTTTTAACCGTCGTTTCGCACCACTCTACGGTGAGTT
AAAAACGCAACTCGCCACCGCAGCCTCGCTAAGAATGGATAAACATCGTAGCAATAGCGTCGGGCCACACGATCTTTATT
TCACGTTGCTGGATGATTATCTGCATGTGGTGGATACCGCGCTGTGGTTGTCGGGCGGCAAAGCCTCTCTGGATGGCGGT
ACGCTACTGACTAACGACGCTGGCGAAATGCTGTTTGCCGAGCACCATTTTTCGGCTGGTCCTTTGCAGATCACCACCTG
TATGCATCGCCGTGCCGGAAGTCAGCGTGAAACCGTGCAGGCCGTGACTGACGGTGCGCTCATCGACATTACGGATATGC
GCGAATGGCGTGAGGAGCGCGGGCAGGGCGTAGTGCATAAACCGATTCCTGGTTGGCAGAGTACGCTTGAGCAACGTGGG
TTTGTCGGCTGTGCGCGGCACTTCATTGAATGTGTGCAAAACCAGACAGTTCCGCAAACCGCCGGCGAACAGGCCGTGCT
GGCGCAACGTATCGTTGACAAGATCTGGCGCGATGCGATGAGTGAATAAACCCTGTAACATCTGGCGGTAGCAATTCACC
ATAATCCAGGTAGACTATTCGCCTCTTTCAGCGCCTGCCTTGCAGGCGTTTTGCCCGTGGGTCTGGATTAGAACACCGAT
GAATTTATTAAAATCGCTGGCCGCCGTCAGCTCGATGACCATGTTTTCGCGTGTGCTTGGCTTCGCACGAGACGCAATTG
TCGCCAGAATCTTTGGCGCAGGGATGGCAACCGACGCCTTTTTCGTCGCTTTTAAACTTCCTAACTTGTTACGCCGTATC
TTTGCCGAAGGGGCATTTTCCCAGGCATTTGTACCGATTCTGGCGGAATATAAAAGTAAGCAGGGTGAAGACGCCACGCG
GGTCTTTGTCTCTTATGTTTCTGGCCTGCTGACACTTGCGCTGGCGGTTGTGACGGTCGCTGGCATGCTCGCCGCACCGT
GGGTGATCATGGTGACCGCGCCAGGCTTCGCTGACACAGCTGACAAATTTGCCCTGACCAGCCAGCTACTAAAGATTACC
TTTCCCTATATCTTGCTGATCTCCCTGGCGTCGCTGGTGGGAGCGATTCTGAATACGTGGAACCGCTTCTCGATTCCGGC
GTTTGCTCCAACACTGCTTAACATCAGCATGATTGGTTTCGCGCTGTTTGCCGCACCGTACTTTAACCCACCGGTGCTGG
CGCTGGCGTGGGCTGTTACGGTCGGCGGCGTCCTGCAGCTGGTGTATCAGCTACCGCACCTGAAGAAGATCGGCATGCTG
GTCCTGCCGCGCATTAACTTCCACGATGCCGGAGCAATGCGCGTGGTGAAACAGATGGGACCGGCGATCCTTGGCGTCTC
TGTGAGCCAGATCTCCTTAATCATCAACACCATTTTTGCCTCGTTTCTTGCTTCCGGTTCGGTGTCTTGGATGTATTACG
CCGACCGCTTAATGGAGTTTCCGTCCGGTGTGCTGGGCGTGGCGCTTGGCACCATTTTGCTGCCGTCGCTGTCGAAAAGT
TTTGCCAGTGGCAATCATGATGAATACAACCGTTTGATGGACTGGGGGTTGCGTCTTTGTTTCCTGTTGGCGCTGCCGAG
TGCGGTTGCGTTGGGCATTCTTTCCGGTCCGTTGACCGTTTCGCTGTTCCAGTACGGTAAATTTACCGCGTTTGATGCGC

TGATGACCCAGCGGGCGTTAATTGCCTACTCGGTGGGTTTGATTGGCCTGATTGTAGTGAAAGTGTTGGCTCCTGGCTTT
TATTCCCGCCAGGACATTAAAACGCCAGTGAAAATTGCCATCGTTACGCTGATTTTAACGCAATTGATGAACCTGGCGTT
TATTGGTCCGTTGAAACATGCCGGGCTGTCACTTTCTATTGGTCTGGCGGCGTGTCTGAATGCTTCGCTGCTTTACTGGC
AGTTGCGTAAGCAGAAAATCTTTACCCCGCAACCCGGCTGGATGGCGTTTCTGTTGCGTCTGGTGGTGGCGGTACTGGTG
ATGTCTGGCGTGCTTTTAGGTATGTTACATATCATGCCGGAGTGGTCATTGGGTACCATGCCCTGGCGTTTACTGCGTTT
AATGGCGGTCGTGCTGGCGGGGATTGCCGCGTACTTCGCTGCACTGGCGGTACTGGGCTTCAAAGTTAAAGAATTTGCCC
GCCGGACGGTGTAACAATGCATTCCGGCCTGCAGTGCAGGCCGGAGATAATCTTCAGATCGAAATCTTTTTACCGCCGCG
ATGGGTTGTTGAGGTCTGACCGTTCGCCCCATATAGCGTCGGTTCCTGATGCGGTTTCAACATTTCCAGCGCCTGTTGAT
TGCGCTCAATCTGTCCTTCCAGTAACCAGCCGTTATGTTGATTCATTTGGCGTAGTTGCTGCGTTTTCACAGTAATTTCC
TGCCAGCGTTGACTAATATCAACGCTATTTGCTGTATTGGGTTCTTTCCTGCGTAACTGTTCGAGGTAATCCAGCGTCGC
CAGCAGTGAGCTTTTTTGTTCTGTAATCCATTGCAACTGGCTGCCGTTGATCTGCCCCATAGAGAGATGTTGCTGCTCTT
GATCCATTACCGTTTTGAGATCGTTAAGCACAGCGGACATCTGGTCGAGGATCTCTGCAAGACGTGTCATACGATCAGTT
ACTCTGCAAGTCTTGCTGCGCTTCGTTGATCAGCGCATCGGCAATTTTGCCGGTGTCCATTTTTAGTTCACCGTTACGAA
TCGCCAGTTTTAACGCTTCGACACGTTCAAGATTGATATCACTGCTGCCGGGTTGCATCAGTTTTGCTTGCGCGTCGCTT
AACGTCACACTGGTGCTGGTGGAGGCGGTTGTTTTTGCCGCCCGGCTGTTCGTTACCGGCGCGTCAGTGGTTTCGCGCGG
TTGAACGGTGCTTACAGGCTTCAGAGGCGAAGTGCGATCAATACTCATGGTTTATTCCTCATTGAGGGCGCTTTTATCAT
GTGTTGCTTATTTATCGGCAAGGGACGGGTAATCTTTAACAGCTTACAGGTTTATAAGAATATTCCCATCTGCATCAACA
ACGCCGCTGACTACCTGTCCCGATACCATGCGCACCCGCGCATTCTGTGCGACGGCTGCATTGTTCAGCGCCTGACCTTC
TGCGTTGGCGCTAAACCCATCACCGCTGGCGATCACATTGACGCGTTGTCCCGCTTTTACCCGCCATGCCTGGCGAAACT
GGGTTAACTGGATAGGTTGATCGGGTGATAGATCGCGCAGGCTAATGGCATCAACAAGTTGATTGATATCCAGCACCGTA
CGCGGTGGCAGGGTATCCAGCCGTCCGCGTTTCAGTTTGACATTGCCAGCTTCCAGCTTTCCTCCCCGCGCAATGGGCAT
CGCGGCAACCACATAATTTCCTGTGGCCTGTACATTAACCTGTAAATATCGTTTGTCGTTACCGCAGCGTGCCAACACAT
TCACATTGCCCCACAGGCGGGAATTATTGCTCATCGAAAGCAATGGCTGCTCGCATGGCGGTAGTAGATTGGGCGCTGTA
CGAATAGAAACACGAACCTCATCACTTACCCCCGCGAGTTGGGCGCTAAAAAAGTTGTGCAATTGCGATGTGAGATTGCT
CGCCGTACTTAACGGACTGAACAGTATCGCGATGATCGCCACGCTACGTTTTATTATCAGCATTTTCGCCCCCAGCCATT
TCTACAACGTGAATTGTACCTGTCCGCAATGACCATCAACGGCATAAATAGCGACCCATTTTGCGTTTATTCCGCCGATA
ACGCGCGCGTAAAGGCATTTAAGCTGATGGCAGAATTTTGATACCTGCGGAGGAGATATGCTCGATAAGCTCGACGCCGC
CTTACGTTTTCAACAAGAGGCGCTCAATCTGCGCGCCCAGCGTCAGGAAGTGCTGGCAGCAAACATCGCCAATGCCGATA
CCCCTGGTTATCAGGCGCGCGATATCGATTTTGCCAGTGAACTTAAAAAAGTCATGCAACGTGGACGGGATGCAACCAGT
GTGGTTGCACTGACGATGACCTCAACGCAACACATTCCGGCGCAGGCGCTGACGCCTCCTACCGCAGAACTGCAATACCG
TATTCCGGACCAGCCTTCGCTTGACGGTAATACCGTCGATATGGATCGCGAACGCACCCAGTTTGCCGATAACAGCCTGC
AATACCAGATGAGCCTTAGCGCGTTGAGCGGGCAAATCAAAGGCATGATGAACGTTTTACAGAGCGGAAATTAACGGATG
GCACTGCTGAATATTTTTGATATCGCCGGGTCGGCGTTAACTGCCCAGTCCCAGCGCCTGAACGTGGCGGCCAGTAATCT
GGCGAATGCTGATAGCGTGACCGGTCCCGATGGACAGCCATATCGGGCAAAACAGGTGGTATTCCAGGTTAACGCTGCAC
CAGGTGCTGCGACAGGCGGCGTAAAGGTTGCCGATGTTATAGAAAGTCAGGCCCCGGACAAACTGGTTTATGAACCGGGT
AATCCGCTGGCAGATGCAAAGGGCTACGTAAAAATGCCGAACGTTGATGTTGTCGGAGAGATGGTTAACACCATGTCGGC
GTCACGCAGCTATCAGGCCAATGTTGAAGTGCTCAACACGGTGAAAAGCATGATGCTGAAAACCCTTACGCTCGGTCAAT
AAAGGAGAAAGCTATGTCCATTGCGGTAACCACCACCGATCCGACAAATACCGGCGTCAGTACCACCAGCAGTAGTTCGC
TCACGGGCAGCAACGCCGCAGATTTACAAAGCAGTTTTCTGACTTTGCTGGTGGCGCAGCTGAAAAACCAGGACCCGACC
AATCCAATGGAAAACAACGAGCTGACGTCGCAATTGGCACAAATCAGCACGGTCAGCGGGATTGAAAAACTCAATACCAC
GCTCGGATCTATTTCCGGACAGATTGATAACAGCCAGTCGTTACAGGCCAGTAACCTGATCGGTCACGGCGTGATGATCC
CCGGCACCACTGTTCTTGCGGGAACCGGCAGTGAAGAAGGGGCTGTGACCACGACCACGCCGTTTGGTGTTGAGCTGCAA
CAGGCGGCAGACAAAGTTACGGCCACCATCACCGATAAAAATGGCGCGGTTGTGCGCACCATTGATATTGGTGAACTGAC
CGCCGGAGTTCACAGTTTCACCTGGGACGGTACGTTGACTGATGGCAGCACTGCGCCGAACGGTTCTTACAATGTAGCGA
TTAGCGCCAGTAACGGTGGTACACAACTGGTTGCCCAGCCGCTGCAGTTTGCTCTGGTGCAGGGTGTGATCCGCGGCAAC
AGCGGTAATACGCTGGATCTCGGCACTTACGGCACCACCACCCTCGACGAAGTACGGCAGATAATTTAAGCCTTCACATT
TCAGGAGTCAGTCATGGCCTTTTCTCAAGCGGTTAGCGGATTAAACGCTGCCGCCACCAACCTCGATGTTATTGGCAACA
ATATCGCCAACTCCGCCACCTACGGCTTTAAATCAGGCACGGCCTCTTTTGCCGATATGTTTGCCGGTTCGAAAGTGGGA
CTGGGGGTAAAAGTTGCCGGTATCACTCAGGACTTTACCGATGGCACGACCACCAACACCGGGCGAGGTCTGGACGTTGC
TATCAGCCAGAACGGTTTTTTCCGTCTGGTAGACAGCAACGGTTCGGTGTTCTACAGCCGTAACGGACAATTTAAGCTGG
ATGAAAACCGTAACCTGGTGAATATGCAAGGTTTACAGCTGACGGGTTACCCGGCAACCGGTACGCCGCCGACTATTCAG
CAAGGGGCGAATCCGACCAATATTTCGATCCCGAATACCCTGATGGCAGCGAAAACTACCACCACGGCATCGATGCAGAT
CAACCTGAATTCCAGTGATCCGCTTCCTACTGTTACGCCATTCAGCGCCAGCAATGCGGATAGCTATAACAAAAAAGGTT
CGGTGACTGTTTTCGACAGTCAGGGTAATGCTCATGACATGAGCGTCTACTTTGTGAAGACCGGGGATAATAACTGGCAG
GTCTACACCCAGGATAGCAGTGATCCAAACAGCATTGCGAAGACAGCGACAACACTGGAATTTAATGCTAATGGCACATT
AGTGGATGGTGCGATGGCGAATAATATCGCAACCGGCGCAATTAACGGTGCAGAACCCGCCACGTTTAGTCTGAGCTTCC

TCAACTCCATGCAGCAAAATACCGGCGCTAACAATATTGTGGCAACCACCCAGAACGGCTACAAACCGGGCGATCTGGTG
AGTTATCAAATCAATGATGACGGTACGGTTGTCGGCAACTATTCCAACGAACAAACCCAACTGCTGGGGCAGATTGTACT
GGCGAACTTTGCCAACAACGAAGGTCTGGCATCCGAAGGCGACAACGTCTGGTCTGCGACGCAATCTTCTGGCGTGGCGC
TGTTGGGGACAGCCGGGACGGGAAACTTTGGCACCCTGACCAACGGTGCGCTGGAAGCGTCCAACGTCGATCTCAGTAAA
GAACTGGTCAATATGATCGTTGCCCAGCGTAACTATCAGTCTAACGCCCAGACCATCAAAACCCAGGACCAGATCCTCAA
CACGCTGGTTAACTTACGCTAATCGCTGACGGGATAGCTCAATGGATCACGCAATTTATACCGCGATGGGAGCAGCCAGC
CAGACACTGAATCAACAGGCGGTAACCGCCAGTAATCTGGCCAATGCCTCAACGCCCGGTTTTCGCGCGCAGTTGAATGC
TTTACGCGCGGTGCCAGTGGAAGGGCTTTCTCTGCCCACGCGCACGTTGGTCACGGCGTCAACGCCGGGCGCAGATATGA
CGCCCGGCAAAATGGATTACACCTCGCGCCCGCTGGACGTCGCGTTGCAGCAGGATGGCTGGCTGGCCGTGCAGACCGCT
GACGGCAGCGAAGGGTATACGCGTAATGGCAGCATTCAGGTTGATCCCACCGGGCAACTGACAATTCAGGGGCATCCGGT
GATAGGCGAGGCTGGGCCAATTGCTGTGCCGGAAGGGGCGGAAATCACTATTGCTGCCGATGGCACAATCTCGGCGCTCA
ATCCGGGCGATCCGGCAAATACGGTTGCGCCAGTAGGGCGTCTTAAACTGGTGAAAGCCACGGGCAGCGAAGTGCAGCGC
GGTGACGACGGCATTTTTCGTTTAAGCGCAGAAACCCAGGCCACGCGTGGGCCGGTACTGCAGGCAGATCCAACCTTGCG
TGTGATGTCGGGGGTTCTGGAAGGCAGTAACGTCAATGCCGTTGCGGCAATGAGCGACATGATTGCCAGCGCGCGGCGTT
TTGAAATGCAGATGAAGGTGATCAGCAGCGTCGATGATAACGCAGGCCGTGCCAACCAACTGCTGTCGATGAGTTAATTG
AAAGGATACATGACAAGTATAAGTTGCCCGATGCGCAAGTTTATCGGGTCTATGGGGGCAATCGCAATTTATCGATTTTG
CGAGCACTTGTAGGCCGGATAAGGCGTTTACGCCGCATCCGGCAAGAAGACATATGCACTTTGTCACTAATCCACTACAG
GACATTTTATGATCAGTTCATTATGGATCGCCAAAACGGGCCTTGACGCCCAGCAAACCAATATGGACGTCATTGCCAAC
AACCTGGCAAACGTCAGTACTAACGGTTTTAAGCGTCAGCGCGCGGTGTTTGAAGATCTGCTTTATCAAACCATTCGCCA
GCCGGGGGCACAGTCTTCCGAACAAACCACCTTACCCTCCGGATTACAAATCGGCACGGGGGTACGCCCGGTCGCCACTG
AACGCTTACACAGCCAGGGAAACCTGTCGCAGACCAACAACAGCAAAGATGTCGCGATTAAAGGGCAGGGCTTTTTCCAG
GTGATGTTGCCAGATGGTTCATCAGCCTATACCCGTGACGGCTCTTTCCAGGTGGATCAGAACGGGCAGCTGGTGACGGC
TGGTGGTTTTCAGGTGCAGCCAGCGATCACCATTCCGGCGAATGCGTTAAGTATCACCATCGGTCGTGATGGCGTGGTCA
GCGTAACCCAACAAGGCCAGGCAGCTCCGGTTCAGGTTGGGCAGCTCAATCTCACCACCTTTATGAATGACACCGGGCTG
GAGAGCATTGGCGAAAACCTCTACACCGAAACGCAATCCTCTGGTGCACCGAACGAAAGCACGCCGGGCCTGAACGGCGC
GGGACTGCTGTATCAAGGGTATGTTGAAACGTCTAACGTCAACGTGGCGGAAGAACTGGTCAATATGATTCAGGTGCAAC
GCGCTTACGAAATCAACAGTAAAGCGGTGTCCACCACCGATCAGATGCTGCAAAAACTGACGCAACTCTAAGGCTTAACC
GGTGGCAGGTTCACCGGTTTACTGATTTTTGAAGATGATAGCCATGCAAAAAAACGCTGCGCATACTTATGCCATTTCCA
GCTTGTTGGTGCTTTCACTAACCGGCTGCGCCTGGATACCCTCCACGCCGCTGGTGCAGGGGGCGACCAGTGCACAACCG
GTTCCCGGTCCGACGCCCGTCGCCAACGGTTCTATTTTCCAGTCTGCTCAGCCGATTAACTATGGCTATCAACCGCTGTT
TGAAGATCGTCGACCACGCAATATTGGCGATACGCTGACCATCGTGTTGCAGGAGAACGTCAGCGCCAGCAAAAGCTCCT
CTGCGAATGCCAGCCGTGACGGTAAAACTAATTTTGGCTTTGATACTGTGCCGCGCTATTTGCAGGGGCTGTTTGGTAAC
GCTCGTGCCGATGTCGAAGCCTCCGGTGGTAACACGTTCAACGGAAAGGGCGGGGCCAATGCCAGCAATACCTTTAGCGG
CACGTTGACGGTGACGGTTGACCAGGTACTGGTCAACGGCAACCTGCATGTGGTGGGTGAAAAACAGATTGCCATTAATC
AGGGTACCGAATTTATTCGCTTCTCTGGCGTGGTTAATCCACGCACTATCAGCGGCAGCAATACCGTACCGTCTACTCAG
GTGGCGGATGCGCGCATTGAATACGTAGGCAATGGCTACATTAACGAAGCGCAAAATATGGGCTGGTTGCAGCGTTTCTT
CCTTAACCTGTCGCCAATGTAAGTGAGGCTGTGGTGATTAAATTTCTCTCTGCATTAATTCTTCTACTGGTCACGACGGC
GGCTCAGGCTGAGCGTATTCGCGATCTCACCAGTGTTCAGGGGGTAAGGCAAAACTCACTGATTGGCTATGGTCTGGTGG
TGGGGCTGGATGGCACCGGTGACCAGACAACCCAGACGCCGTTTACCACACAAACGCTTAATAACATGCTCTCACAGCTG
GGAATTACCGTTCCGACGGGCACCAATATGCAGCTAAAAAACGTCGCTGCGGTAATGGTGACAGCGTCACTTCCTCCGTT
TGGACGTCAGGGGCAAACCATCGATGTGGTGGTTTCTTCCATGGGAAATGCCAAAAGCTTGCGTGGAGGTACGTTGTTGA
TGACACCGCTTAAGGGCGTTGACAGTCAGGTGTATGCGCTGGCGCAGGGCAATATTCTGGTTGGCGGCGCAGGAGCCTCC
GCTGGCGGTAGCAGTGTTCAGGTTAACCAACTGAACGGTGGACGGATCACCAATGGTGCGGTTATTGAACGTGAATTGCC
CAGCCAGTTTGGCGTCGGGAATACCCTTAATTTGCAACTTAACGACGAAGATTTCAGCATGGCGCAGCAAATCGCTGACA
CCATCAACCGCGTGCGTGGATATGGCAGCGCCACCGCGTTAGATGCGCGGACTATTCAGGTGCGCGTACCGAGTGGCAAC
AGTTCCCAGGTCCGCTTCCTTGCCGATATTCAGAATATGCAGGTTAATGTCACCCCGCAGGACGCTAAAGTAGTGATTAA
CTCGCGCACCGGTTCGGTGGTGATGAATCGCGAAGTGACCCTCGACAGCTGCGCGGTAGCGCAGGGGAATCTCTCAGTAA
CAGTTAATCGTCAGGCCAATGTCAGCCAGCCAGATACACCGTTTGGTGGTGGACAGACTGTGGTTACTCCACAAACGCAG
ATCGATTTACGCCAGAGCGGCGGTTCGCTGCAAAGCGTACGTTCCAGCGCCAGCCTCAATAACGTGGTGCGCGCGCTCAA
TGCGCTGGGCGCTACGCCGATGGATCTGATGTCCATACTGCAATCAATGCAAAGTGCGGGATGTCTGCGGGCAAAACTGG
AAATCATCTGATGATCAGCGACAGCAAACTACTGGCAAGTGCGGCCTGGGATGCGCAATCACTCAACGAACTAAAGGCGA
AAGCGGGCGAAGATCCGGCGGCAAATATCCGTCCGGTGGCCCGTCAGGTGGAAGGGATGTTCGTGCAGATGATGTTGAAA
AGCATGCGCGACGCTTTACCAAAAGATGGCCTGTTCAGCAGCGAGCACACTCGCCTGTATACCAGTATGTATGACCAGCA
GATTGCCCAACAGATGACGGCGGGCAAAGGTCTGGGGCTTGCAGAGATGATGGTTAAACAGATGACGCCAGAACAACCAT
TGCCAGAGGAGTCCACGCCAGCAGCACCGATGAAATTCCCGCTCGAAACTGTGGTGCGTTATCAAAATCAGGCGCTTTCG
CAGCTGGTGCAAAAGGCCGTGCCACGTAACTACGATGATTCGCTGCCGGGTGACAGTAAAGCATTCCTCGCGCAACTCTC

GCTGCCCGCCCAACTGGCAAGCCAGCAAAGCGGTGTGCCACATCATTTGATCCTCGCTCAGGCGGCACTGGAATCTGGTT
GGGGGCAACGGCAAATCCGCCGCGAAAACGGCGAGCCGAGCTATAACCTGTTTGGTGTCAAAGCCTCTGGCAACTGGAAA
GGGCCAGTTACTGAAATCACCACGACTGAATATGAAAACGGCGAAGCGAAGAAAGTAAAAGCGAAGTTTCGCGTCTACAG
CTCGTATCTGGAAGCCTTGTCGGATTACGTTGGGCTGTTAACGCGTAACCCGCGCTACGCCGCCGTGACGACCGCCGCGA
GTGCGGAACAGGGGGCGCAGGCCCTACAGGACGCGGGCTATGCCACCGATCCTCACTATGCCCGCAAACTCACCAACATG
ATTCAGCAGATGAAATCGATAAGCGACAAGGTGAGCAAAACCTACAGTATGAACATTGATAATCTGTTCTGAATAACTCA
AGTCCGGCGGGTCGCTGCCGATAATACTCTGTAATTGAAGGCTTATAAGGAACCTCCATGTCCAGCTTGATTAATAACGC
CATGAGCGGACTGAACGCGGCCCAGGCGGCGTTAAATACGGCAAGTAATAATATCTCCAGCTATAACGTTGCCGGATATA
CCCGCCAAACCACTATTATGGCGCAGGCCAATAGCACGTTGGGCGCTGGCGGCTGGGTTGGCAATGGTGTCTACGTTTCT
GGTGTGCAGCGTGAGTATGATGCGTTTATTACCAACCAGTTACGTGCGGCGCAGACGCAAAGTAGCGGTCTGACTGCCCG
CTATGAGCAGATGTCGAAAATCGACAATATGCTCTCCACCAGTACCTCTTCGCTGGCAACACAGATGCAGGATTTCTTCA
CCAGCCTGCAAACGCTGGTGAGTAACGCGGAAGACCCGGCAGCGCGCCAGGCGCTGATTGGGAAATCAGAAGGATTGGTG
AATCAGTTTAAAACCACCGATCAATATCTGCGCGACCAGGACAAACAGGTCAATATCGCGATAGGTGCCAGCGTTGATCA
GATCAACAACTACGCTAAACAAATTGCCAGCCTGAACGATCAAATCTCGCGCCTGACAGGCGTGGGGGCAGGGGCGTCAC
CTAACAATCTGCTGGATCAACGCGATCAACTGGTGAGCGAATTAAACCAGATTGTTGGTGTAGAAGTCAGCGTTCAGGAT
GGCGGCACTTATAACATCACGATGGCCAATGGTTACTCACTGGTTCAGGGAAGTACGGCGCGGCAACTGGCGGCAGTTCC
TTCCAGCGCTGACCCTTCTCGTACGACTGTCGCTTATGTTGATGGGACGGCAGGCAATATTGAGATCCCGGAGAAATTAC
TGAATACCGGGTCGCTGGGCGGCATTCTGACATTCCGTTCTCAGGATCTGGACCAGACGCGTAATACGCTTGGACAACTG
GCGCTGGCATTTGCCGAGGCTTTCAACACCCAACACAAAGCCGGATTTGATGCTAACGGCGATGCCGGTGAAGATTTCTT
TGCTATCGGTAAGCCCGCGGTTCTGCAAAACACGAAAAACAAAGGTGACGTTGCGATCGGTGCCACGGTAACTGATGCCT
CCGCGGTACTGGCGACAGATTACAAAATCTCGTTCGATAATAATCAGTGGCAGGTCACCCGCCTTGCCAGCAATACCACT
TTTACGGTGACGCCGGATGCCAACGGTAAAGTGGCATTTGATGGTCTGGAGTTGACGTTTACAGGAACGCCTGCCGTTAA
CGACAGCTTCACGCTGAAACCAGTAAGTGACGCCATCGTCAACATGGATGTATTAATCACCGACGAAGCGAAAATAGCGA
TGGCGAGCGAAGAAGATGCGGGTGATAGCGATAACCGCAACGGTCAGGCCCTGCTGGATCTGCAAAGCAACAGTAAAACG
GTGGGCGGTGCGAAATCCTTTAACGACGCTTATGCCTCGTTAGTGAGTGATATCGGTAATAAAACCGCGACGTTGAAAAC
CAGTAGCGCCACGCAAGGTAATGTGGTGACGCAGCTTTCCAATCAGCAGCAGTCGATTTCCGGTGTCAATCTCGATGAGG
AGTACGGAAATCTGCAACGTTTTCAGCAGTATTACCTGGCGAATGCGCAGGTTCTGCAGACGGCAAACGCGATTTTTGAT
GCGCTGATTAACATTCGCTAAGGGGAGATAAGATGCGTTTCAGTACACAGATGATGTACCAGCAAAACATGCGTGGTATC
ACCAATTCTCAGGCAGAATGGATGAAGTACGGCGAACAGATGTCGACGGGTAAGCGAGTCGTTAACCCTTCTGACGATCC
CATTGCTGCATCACAAGCCGTAGTTCTCTCCCAGGCACAGGCGCAAAACAGCCAGTACACGCTGGCGCGTACTTTCGCCA
CTCAAAAAGTGTCACTGGAAGAGAGTGTACTTAGCCAGGTCACCACTGCTATCCAGAATGCTCAGGAAAAAATTGTCTAC
GCCAGCAATGGCACCTTGAGTGACGATGACCGGGCCTCGCTGGCTACGGATATTCAGGGGCTTCGTGACCAGTTGCTGAA
TCTGGCAAACACCACTGACGGTAACGGGCGCTACATTTTTGCCGGTTATAAAACAGAGACTGCGCCGTTTAGCGAAGAGA
AAGGGAAATACGTCGGTGGAGCAGAAAGTATTAAACAACAGGTCGATGCTTCGCGTTCGATGGTGATAGGGCACACGGGT
GACAAAATTTTCGACAGTATTACCAGCAACGCGGTAGCGGAACCAGACGGTAGCGCTTCTGAAACCAATCTTTTTGCCAT
GCTGGATAGTGCCATCGCAGCCCTGAAAACGCCGGTCGCGGATAGCGAAGCGGATAAAGAAACCGCCGCTGCGGCGTTAG
ATAAAACCAACCGCGGACTGAAAAACTCGCTGAACAATGTGCTGACTGTTCGCGCGGAATTAGGCACGCAGCTGAACGAA
CTGGAGTCGCTGGATTCATTAGGTAGCGATCGCGCTTTAGGGCAAACGCAGCAGATGAGCGATCTGGTTGATGTGGACTG
GAATGCAACTATTTCATCTTACATCATGCAGCAAACGGCATTGCAGGCATCGTATAAAGCATTTACCGATATGCAGGGAT
TGTCGCTCTTCCAGCTCAGCAAATAATTTCGCTTTAAAACATATCATGAAACTGGGTATGTTTTGTCTGCCTGCTCTGGG
ATCGCTGGGGCGGGCATTTTTTTGCCTATTTTGCATTGTTGGTTAGCAAGGATGCCATTCGATGAATTTTAATATGTTGA
TTCAAAGATGAAATAAAAAAGCCCTGGCAGTTACCAGGGCTTGATTACTTTGAGCTAATTATTACTCAACAGGTTGCGGA
CGCGCAGGAGCGGCAGAGGCATGATGTGTTGCCGTATGACCACCTGCGGCACCTTTACCTTCGAAGGCAAAAGTAGGGCG
CTGCCAGTCACTGTGACGCGGTGCCTCCGGAACATATTCCGGTGCTGGAGCGCGCGTCATTGGCGCGGTAGCGTGGTTAT
GCTCAACGGTGACCTCAGGTTCGACCGCAGCTACCGTTTCAACTTCTGCTGCGACTTCAGCGACTACTGGCGCGGCAGGT
TGAGCAACAACTTCAGGTTCAGCAACTACAACCTCAGCAGTTTCGACAACTTCTTCAATATCTGCCGTCTCTTCCTGCGG
TTCAACCACCGGTTCTGCTTGTTCTGCAACTTCCTGGGCTACGGCAACATCAGACTCGGTAATCACCTGCGGCTGTTCAG
TTACCGCGGCAGCGATCACTTCAGGATGCGTCGTTTCAACCACTTCCGGTTGCGGTTCGGCAACCTGAACGGGGGCTTCT
ACGACACCGGCCACTTCTTCAACAACTGGCGCGCTAACAACCGGTTCGATAGCGGCGGCGACAGGGACCTCAGTCACCAT
CGGCTGCACATGTACTTCTTCCTGTTCGCGCTGCTCTTCAACCTGTACATCTTGCGGACGTACAATTGGATAGCGGATCC
AGACTTTGCCAGAGGCCAGTTCCGGAGACGCGCACGCTACGGTCAACGGCATTGGCGACTGGGTTGGATAACGCTCGTCA
CGATAGCGACGACGACGCTGACCACTTACGCGCAGGTGACGAGGCGAGCGGCGAGAACGACGCGGCATGCCACCGTTGTC
ACGGTTATCAGCATTGTTCTCTTCTTGCTGTTCTGGTGCAGTTTGCGCTACGACTGGCAGCGGGACTTTCACCAGTTCTG
TGCGTGGAGCTGGCGCTTCCTGAACAATTGGTTCGGCAGCGACAGTTTCTTCAACCACCGGTGCGACTACCGCTTCTTCG
GCTACGCTTTGCTCGTAACGCACTTTCTGATTGAGCTGACGCTGTTTACGACGCGGCTGAACCGGACGTACACGTTCTTC
CTGTTCGGTTTCCTGAACAGATTGCTCTTCAACATTCAGCGCCTTCGCTTCTTGTTGCGCCTGACGTTTATCATCATTAC

GGCGGCGGCTACGTTCACGACGCGGCGCTTGCTGCTCGTCGGCGGTACGCGCTTTTTCCGTTACCTCAGCCTGCTGACGG
CTCTCACGCGTCTCGGCAGTCTGCTGCTGTGCCTGGCGACGATTACGACGGTTTTCTTCGCGATTATCGCTGCCTTCAGT
ACGTTCACTACGGGTGTCGCGGCGCTCATTACGGTCACGGCGGTTGTTCTGACGAGGCTTGCGACGATCCTGTTGACGTT
CCGGTTTCGCTTCTGCTTTCGGTGCTGGTTGCTCGGTCGGTTTGGTTTCTTCACCACCGCTGAACAGCGCTTTCAGTGCG
CCGAAGAAGCGGCTCAACAGCCCAGGTTGTGCAGGAGCTGCTGGTGTTGCCGGTGCAGCTTTCGGTGCTGGAGCTACAAC
AGGCGCGGCAGGTTCAGCTGGCGTTGGCGCAGGCGGCACATCCGGCATGGCAAAGGTTGCCAGCGCAGGTTGTTCCGGAC
GCTTACGTTCAGCGAACTCTTCTTCAGACGGCAGCGCCATCGCTTCTTCATGCAGCTTCGGCAGCATGTAGCTTAAGGTT
GGGGTTTCTTCCCCTTTACGCACGCGCAGCACGTGGTAGTGCGGGGTTTCCATCTGATCGTTTGGCACAATTACACAGCG
CACACCGTCCTGACGAGTTTCAATGGCATTTACCGCAGAACGTTTTTCATTCAGCAGGTAAGAAGCGATTGGCACAGGAA
CAATGGCGTGAACTTCCTGGGTGTTCTCTTTCAGCGCTTCTTCTTCGATCAGACGCAGAATAGAGAGCGACAGCGATTCG
TTGTCACGCACGGTGCCAGTACCAGAACAACGCGGACAAACGTGATGACTGGATTCACCCAGTGATGGGCTCAGGCGCTG
ACGGGACATTTCCAGCAGGCCAAAGCGAGAAATATGGCTGATTTGAATACGCGCACGGTCCTGACGCACCGCTTCACGCA
GACGGTTTTCTACCGCACGCTGGTGGCGTACTGGCGTCATGTCGATGAAGTCGATAACAATCAGGCCGCCGAGGTCACGC
AGGCGCAGCTGACGAGCAATCTCATCGGCAGCTTCGAGGTTAGTGTTAAACGCGGTTTCTTCGATATCGCCGCCGCGGGT
CGCGCGTGCGGAGTTGATGTCGATGGCCGTTAACGCTTCGGTGCTGTCGATAACAATGGAACCACCAGACGGCAGACGAA
CTTCACGCTGGAAGGCGGACTCGATCTGTGACTCGATCTGGTAGTGGCTGAACAGCGGGATCTCGCCGGTGTACAGTTTG
ATTTTGCTGCTGAAATCCGGGCGACCTAATGCAGCGATATGCTGACGTGCCAGTTCGAGCACTTTCGGGTTATCGATAAG
GATTTCGCCGATGTCCTGACGTAAGTAATCGCGGAATGCGCGAACGATTACGTTGCTCTCCTGATGAATCAGGAACGGGG
CCGGGCGGCTTTCAGCGGCTTTTTTGATGGCTTCCCAGTGTTTCAGACGGAAGCTTAAATCCCATTGCAGCGCCTCAGCA
GATTTGCCGACGCCAGCGGTGCGCACGATAAGCCCCATGCCTTCCGGCAGTTCAAGGCTTGCCAGTGCTTCTTTTAATTC
GGTACGGTCGTCGCCTTCGATACGGCGAGAAATGCCACCCGCGCGCGGGTTGTTCGGCATCAGAACCAGATAGCTACCCG
CCAGACTGATAAAGGTGGTTAATGCCGCGCCTTTGTTGCCGCGCTCTTCTTTATCGATCTGAACAATGACTTCCTGACCT
TCACGCAACACATCTTTAATGTTGGGACGACCATGAGCACTGTAGTTAGCAGGGAAATATTCGCGGGCAATTTCTTTTAG
TGGGAGGAAACCGTGACGTTCAGCGCCGTAATCAACAAAAGCAGCTTCCAGACTCGGTTCAATGCGGGTGATTTTACCTT
TGTAGATGTTTGCCTTTTTCTGCTCGTGCCCTGGACTTTCGATATCCAGGTCATACAGACGCTGCCCATCTACAAGGGCA
ACGCGCAACTCTTCCTGCTGAGTTGCGTTGATTAACATTCTTTTCATCGTAACTTACTCATTATTCTTACATTGACGACT
AAGCTGCGGGCAAAGTAACGCCTTTCCGGGTGTGAACCGATGGCCTCGTGTCTAGTCGCGTCGCCAACCTCACGGTTATC
GTCAGCTCAAAGAGGCGCAGAGTGTCGGTTGCCCGTTTTTCATGCGGAAAAACAGCGCAATTATCAAAGAAACAGACTGG
GTATTACTCTCCAGAAATATTTCCATCTACCGGTAAGGACTGCAACCCGCAGCCCGCTAACTGCCTGAAAGATCAATACG
TCTTACGCCATTGCTGCGTGGATGATCGGTCGGGCAAAATGGGTTATTCCGTAAAATTTCTTGTTTTAACAAGGATGGAC
ACGGAAACGGCCTCATTATTCCACTGCAAGCCTTGTTATAGCAAGATGACTTTTACCATTTATCACCCGCTTACTCACAG
TTTTTTCACTTCTTGCTGGTGATTGGTTTAATAACCATCAATAAAGTGATCACGCAGTGAAGTAATAAGGTGCAAAAGTA
AATATAAGCATAGAAAAATGAGTGGCGCGAATCCTGTTGGCTAATTAGAATCGCCAACCATGAAAACAGAGACTCCATCC
GTAAAAATTGTTGCTATCACCGCTGACGAAGCGGGGCAACGTATCGATAACTTTTTGCGTACCCAATTGAAAGGCGTACC
AAAAAGTATGATTTACCGTATTTTGCGTAAAGGCGAAGTGCGGGTGAACAAAAAACGTATTAAGCCTGAATATAAACTCG
AAGCGGGTGATGAGGTGCGTATTCCACCGGTTCGCGTTGCTGAGCGGGAAGAAGAGGCGGTTTCGCCACATCTGCAAAAG
GTGGCGGCGCTGGCGGACGTCATCTTATATGAAGATGATCACATCCTGGTGCTGAATAAACCTTCCGGTACGGCGGTACA
TGGCGGCAGTGGTTTAAGCTTCGGCGTTATTGAAGGTTTGCGGGCGTTGCGCCCGGAAGCGCGGTTCCTTGAACTGGTTC
ATCGTCTTGACCGGGACACCTCAGGTGTTTTGCTGGTAGCGAAAAAACGCTCGGCGTTGCGTTCTCTGCATGAGCAATTA
CGTGAAAAAGGGATGCAAAAAGATTACCTGGCGCTGGTGCGCGGTCAGTGGCAGTCGCATGTGAAGAGCGTTCAAGCACC
GTTACTGAAAAATATTCTGCAAAGCGGCGAACGTATCGTGCGTGTGAGTCAGGAAGGCAAACCGTCGGAAACACGCTTTA
AAGTGGAAGAACGCTATGCATTTGCCACCCTGGTGCGTTGTAGCCCGGTAACAGGGCGTACTCACCAGATCCGTGTGCAT
ACACAATATGCGGGTCATCCGATTGCCTTTGACGATCGCTACGGTGACCGTGAATTTGACAGGCAGCTCACTGAAGCAGG
CACGGGATTAAATCGCCTGTTCCTGCACGCTGCAGCGTTGAAGTTTACTCATCCGGGGACCGGTGAGGTGATGCGTATCG
AAGCTCCGATGGATGAAGGTTTGAAGCGTTGTTTGCAAAAGCTGCGTAACGCGCGCTAATACAAGCATATAAACCTGATA
ATGGGCGGTTGGCCTGATAAGGCGTTTACGCCGCATCAGGCCGCCAGCACCGATTGCCGGATGCGACGTAACCACATCCG
GCGCAAAAAACTATCCCATCAGCGGGTTTTTCCCTTCCCGACGTAACATCTGACACAGCGCGATTAGCGGTAAACCAACC
AGCGTGTTAGGATCACGCCCCTCTAAACGCTCAAACAGCGTAATGCCAAATCCTTCACTCTTAAAGCTACCCGCGCAGTG
CAGGGGATGCTCTTTACGCACGTAATTATCAATCTCCGCCTCGCTCAGGTGACGGAAATGGACGTCAAAAGGCTCCACTT
CTGTTTGCAGATGCCCATTCGCCGAATTAAACAACGCCAGTCCAGTATAGAAGGTGACGATATTGCCGCTGGCTTTGCGT
AATTGCAGACGGGCATTTTCTTCCGTTAACGGTTTACCGGTGATTTCGCCATCAAGAACACATACCTGGTCTGAACCAAT
AATTAAATGATCCGGATAACGTGACGCCAGAGATTGCGCTTTTTCTTGTGCCAGTCGAAGCACCAACTGTCGCGGTGATT
CGTCGCTGCGTGGGGTTTCGTCGACCTCTGGTGCTGCACATTCAAAAGAGATTTGCAGTTTTTCCAGAAGAGCGCGACGC
CATGGCGAGGTGGAGGCTAAAATAAGTTTAGGCATATTTTTTTCCATCAGATATAGCGTATTGATGATAGCCATTTTAAA
CTATGCGCTTCGTTTTGCAGGTTGATGTTTGTTATCAGCACTGAACGAAAATAAAGCAGTAACCCGCAATGTGTGCGAAT
TATTGGCAAAAGGCAACCACAGGCTGCCTTTTTCTTTGACTCTATGACGTTACAAAGTTAATATGCGCGCCCTATGCAAA

AGGTAAAATTACCCCTGACTCTCGATCCGGTTCGTACGGCTCAAAAACGCCTTGATTACCAGGGTATCTATACCCCTGAT
CAGGTTGAGCGCGTCGCCGAATCCGTAGTCAGTGTGGACAGTGATGTGGAATGCTCCATGTCGTTCGCTATCGATAACCA
ACGTCTCGCAGTGTTAAACGGCGATGCGAAGGTGACGGTAACGCTCGAGTGTCAGCGTTGCGGGAAGCCGTTTACTCATC
AGGTCTACACAACGTATTGTTTTAGTCCTGTGCGTTCAGACGAACAGGCTGAAGCACTGCCGGAAGCGTATGAACCGATT
GAGGTTAACGAATTCGGTGAAATCGATCTGCTTGCAATGGTTGAAGATGAAATCATCCTCGCCTTGCCGGTAGTTCCGGT
GCATGATTCTGAACACTGTGAAGTGTCCGAAGCGGACATGGTCTTTGGTGAACTGCCTGAAGAAGCGCAAAAGCCAAACC
CATTTGCCGTATTAGCCAGCTTAAAGCGTAAGTAATTGGTGCTCCCCGTTGGATCGGGGATAAACCGTAATTGAGGAGTA
AGGTCCATGGCCGTACAACAGAATAAACCAACCCGTTCCAAACGTGGCATGCGTCGTTCCCATGACGCGCTGACCGCAGT
CACCAGCCTGTCTGTAGACAAAACTTCTGGTGAAAAACACCTGCGTCACCACATCACTGCCGACGGTTACTACCGCGGCC
GCAAGGTCATCGCTAAGTAATCACGCATCTGCGTGATGAAGCTTAGTGAGGATTTTCCCCAGGCAACTGGGGAAAGACCA
AACCGGGCGGCGACGATACCTTGACACGTCTAACCCTGGCGTTAGATGTCATGGGAGGGGATTTTGGCCCTTCCGTGACA
GTGCCTGCAGCATTGCAGGCACTGAATTCTAATTCGCAACTCACTCTTCTTTTAGTCGGCAATTCCGACGCCATCACGCC
ATTACTTGCTAAAGCTGACTTTGAACAACGTTCGCGTCTGCAGATTATTCCTGCGCAGTCAGTTATCGCCAGTGATGCCC
GGCCTTCGCAAGCTATCCGCGCCAGTCGTGGGAGTTCAATGCGCGTGGCCCTGGAGCTGGTGAAAGAAGGTCGAGCGCAA
GCCTGTGTCAGTGCCGGTAATACCGGGGCGCTGATGGGGCTGGCAAAATTATTACTCAAGCCCCTGGAGGGGATTGAGCG
TCCGGCGCTGGTGACGGTATTACCACATCAGCAAAAGGGCAAAACGGTGGTCCTTGACTTAGGGGCCAACGTCGATTGTG
ACAGCACAATGCTGGTGCAATTTGCCATTATGGGCTCAGTTCTGGCTGAAGAGGTGGTGGAAATTCCCAATCCTCGCGTG
GCGTTGCTCAATATTGGTGAAGAAGAAGTAAAGGGTCTCGACAGTATTCGGGATGCCTCAGCGGTGCTTAAAACAATCCC
TTCTATCAATTATATCGGCTATCTTGAAGCCAATGAGTTGTTAACTGGCAAGACAGATGTGCTGGTTTGTGACGGCTTTA
CAGGAAATGTCACATTAAAGACGATGGAAGGTGTTGTCAGGATGTTCCTTTCTCTGCTGAAATCTCAGGGTGAAGGGAAA
AAACGGTCGTGGTGGCTACTGTTATTAAAGCGTTGGCTACAAAAGAGCCTGACGAGGCGATTCAGTCACCTCAACCCCGA
CCAGTATAACGGCGCCTGTCTGTTAGGATTGCGCGGCACGGTGATAAAAAGTCATGGTGCAGCCAATCAGCGAGCTTTTG
CGGTCGCGATTGAACAGGCAGTGCAGGCGGTGCAGCGACAAGTTCCTCAGCGAATTGCCGCTCGCCTGGAATCTGTATAC
CCAGCTGGTTTTGAGCTGCTGGACGGTGGCAAAAGCGGAACTCTGCGGTAGCAGGACGCTGCCAGCGAACTCGCAGTTTG
CAAGTGACGGTATATAACCGAAAAGTGACTGAGCGTACATGTATACGAAGATTATTGGTACTGGCAGCTATCTGCCCGAA
CAAGTGCGGACAAACGCCGATTTGGAAAAAATGGTGGACACCTCTGACGAGTGGATTGTCACTCGTACCGGTATCCGCGA
ACGCCACATTGCCGCGCCAAACGAAACCGTTTCAACCATGGGCTTTGAAGCGGCGACACGCGCAATTGAGATGGCGGGCA
TTGAGAAAGACCAGATTGGCCTGATCGTTGTGGCAACGACTTCTGCTACGCACGCTTTCCCGAGCGCAGCTTGTCAGATT
CAAAGCATGTTGGGCATTAAAGGTTGCCCGGCATTTGACGTTGCAGCAGCCTGCGCAGGTTTCACCTATGCATTAAGCGT
AGCCGATCAATACGTGAAATCTGGGGCGGTGAAGTATGCTCTGGTCGTCGGTTCCGATGTACTGGCGCGCACCTGCGATC
CAACCGATCGTGGGACTATTATTATTTTTGGCGATGGCGCGGGCGCTGCGGTGCTGGCTGCCTCTGAAGAGCCGGGAATC
ATTTCCACCCATCTGCATGCCGACGGTAGTTATGGTGAATTGCTGACGCTGCCAAACGCCGACCGCGTGAATCCAGAGAA
TTCAATTCATCTGACGATGGCGGGCAACGAAGTCTTCAAGGTTGCGGTAACGGAACTGGCGCACATCGTTGATGAGACGC
TGGCGGCGAATAATCTTGACCGTTCTCAACTGGACTGGCTGGTTCCGCATCAGGCTAACCTGCGTATTATCAGTGCAACG
GCGAAAAAACTCGGTATGTCTATGGATAATGTCGTGGTGACGCTGGATCGCCACGGTAATACCTCTGCGGCCTCTGTCCC
GTGCGCGCTGGATGAAGCTGTACGCGACGGGCGCATTAAGCCGGGGCAGTTGGTTCTGCTTGAAGCCTTTGGCGGTGGAT
TCACCTGGGGCTCCGCGCTGGTTCGTTTCTAGGATAAGGATTAAAACATGACGCAATTTGCATTTGTGTTCCCTGGACAG
GGTTCTCAAACCGTTGGAATGCTGGCTGATATGGCGGCGAGCTATCCAATTGTCGAAGAAACGTTTGCTGAAGCTTCTGC
GGCGCTGGGCTACGACCTGTGGGCGCTGACCCAGCAGGGGCCAGCTGAAGAACTGAATAAAACCTGGCAAACTCAGCCTG
CGCTGTTGACTGCATCTGTTGCGCTGTATCGCGTATGGCAGCAGCAGGGCGGTAAAGCACCGGCAATGATGGCCGGTCAC
AGCCTGGGGGAATACTCCGCGCTGGTTTGCGCTGGTGTGATTGATTTCGCTGATGCGGTGCGTCTGGTTGAGATGCGCGG
CAAGTTCATGCAAGAAGCCGTACCGGAAGGCACGGGCGCTATGGCGGCAATCATCGGTCTGGATGATGCGTCTATTGCGA
AAGCGTGTGAAGAAGCTGCAGAAGGTCAGGTCGTTTCTCCGGTAAACTTTAACTCTCCGGGACAGGTGGTTATTGCCGGT
CATAAAGAAGCGGTTGAGCGTGCTGGCGCTGCCTGTAAAGCGGCGGGCGCAAAACGCGCGCTGCCGTTACCAGTGAGCGT
ACCGTCTCACTGTGCGCTGATGAAACCAGCAGCCGACAAACTGGCAGTAGAATTAGCGAAAATCACCTTTAACGCACCAA
CAGTTCCTGTTGTGAATAACGTTGATGTGAAATGCGAAACCAATGGTGATGCCATCCGTGACGCACTGGTACGTCAGTTG
TATAACCCGGTTCAGTGGACGAAGTCTGTTGAGTACATGGCAGCGCAAGGCGTAGAACATCTCTATGAAGTCGGCCCGGG
CAAAGTGCTTACTGGCCTGACGAAACGCATTGTCGACACCCTGACCGCCTCGGCGCTGAACGAACCTTCAGCGATGGCAG
CGGCGCTCGAGCTTTAAAAGAGGAAAATCATGAATTTTGAAGGAAAAATCGCACTGGTAACCGGTGCAAGCCGCGGAATT
GGCCGCGCAATTGCTGAAACGCTCGCAGCCCGTGGCGCGAAAGTTATTGGCACTGCGACCAGTGAAAATGGCGCTCAGGC
GATCAGTGATTATTTAGGTGCCAACGGCAAAGGTCTGATGTTGAATGTGACCGACCCGGCATCTATCGAATCTGTTCTGG
AAAAAATTCGCGCAGAATTTGGTGAAGTGGATATCCTGGTCAATAATGCCGGTATCACTCGTGATAACCTGTTAATGCGA
ATGAAAGATGAAGAGTGGAACGATATTATCGAAACCAACCTTTCATCTGTTTTCCGTCTGTCAAAAGCGGTAATGCGCGC
TATGATGAAAAAGCGTCATGGTCGTATTATCACTATCGGTTCTGTGGTTGGTACCATGGGAAATGGCGGTCAGGCCAACT
ACGCTGCGGCGAAAGCGGGCTTGATCGGCTTCAGTAAATCACTGGCGCGCGAAGTTGCGTCACGCGGTATTACTGTAAAC
GTTGTTGCTCCGGGCTTTATTGAAACGGACATGACACGTGCGCTGAGCGATGACCAGCGTGCGGGTATCCTGGCGCAGGT

TCCTGCGGGTCGCCTCGGCGGCGCACAGGAAATCGCCAACGCGGTTGCATTCCTGGCATCCGACGAAGCAGCTTACATCA
CGGGTGAAACTTTGCATGTGAACGGCGGGATGTACATGGTCTGACCGCGATTTGCACAAAATGCTCATGTTGCGCGCAGT
CTGCGTGGTTATGAGTAATAATTAGTGCAAAATGATTTGCGTTATTGGGGGGTAAGGCCTCAAAATAACGTAAAATCGTG
GTAAGACCTGCCGGGATTTAGTTGCAAATTTTTCAACATTTTATACACTACGAAAACCATCGCGAAAGCGAGTTTTGATA
GGAAATTTAAGAGTATGAGCACTATCGAAGAACGCGTTAAGAAAATTATCGGCGAACAGCTGGGCGTTAAGCAGGAAGAA
GTTACCAACAATGCTTCTTTCGTTGAAGACCTGGGCGCGGATTCTCTTGACACCGTTGAGCTGGTAATGGCTCTGGAAGA
AGAGTTTGATACTGAGATTCCGGACGAAGAAGCTGAGAAAATCACCACCGTTCAGGCTGCCATTGATTACATCAACGGCC
ACCAGGCGTAAGTGAACATCTCCAGGCGGTCGTTCGACCGCCTGAGTTTTATCTTTTTGTCCCACTAGAATCATTTTTTC
CCTCCCTGGAGGACAAACGTGTCTAAGCGTCGTGTAGTTGTGACCGGACTGGGCATGTTGTCTCCTGTCGGCAATACCGT
AGAGTCTACCTGGAAAGCTCTGCTTGCCGGTCAGAGTGGCATCAGCCTAATCGACCATTTCGATACTAGCGCCTATGCAA
CGAAATTTGCTGGCTTAGTAAAGGATTTTAACTGTGAGGACATTATCTCGCGCAAAGAACAGCGCAAGATGGATGCCTTC
ATTCAATATGGAATTGTCGCTGGCGTTCAGGCCATGCAGGATTCTGGCCTTGAAATAACGGAAGAGAACGCAACCCGCAT
TGGTGCCGCAATTGGCTCCGGGATTGGCGGCCTCGGACTGATCGAAGAAAACCACACATCTCTGATGAACGGTGGTCCAC
GTAAGATCAGCCCATTCTTCGTTCCGTCAACGATTGTGAACATGGTGGCAGGTCATCTGACTATCATGTATGGCCTGCGT
GGCCCGAGCATCTCTATCGCGACTGCCTGTACTTCCGGCGTGCACAACATTGGCCATGCTGCGCGTATTATCGCGTATGG
CGATGCTGACGTGATGGTTGCAGGTGGCGCAGAGAAAGCCAGTACGCCGCTGGGCGTTGGTGGTTTTGGCGCGGCACGTG
CATTATCTACCCGCAATGATAACCCGCAAGCGGCGAGCCGCCCGTGGGATAAAGAGCGTGATGGTTTCGTACTGGGCGAT
GGTGCCGGTATGCTGGTACTTGAAGAGTACGAACACGCGAAAAAACGCGGTGCGAAAATTTACGCTGAACTCGTCGGCTT
TGGTATGAGCAGCGATGCTTATCATATGACGTCACCGCCAGAAAATGGCGCAGGCGCAGCTCTGGCGATGGCAAATGCTC
TGCGTGATGCAGGCATTGAAGCGAGTCAGATTGGCTACGTTAACGCGCACGGTACTTCTACGCCGGCTGGCGATAAAGCT
GAAGCGCAGGCGGTGAAAACCATCTTCGGTGAAGCTGCAAGCCGTGTGTTGGTAAGCTCCACGAAATCTATGACCGGTCA
CCTGTTAGGTGCGGCGGGTGCAGTAGAATCTATCTACTCCATCCTGGCGCTGCGCGATCAGGCTGTTCCGCCAACCATCA
ACCTGGATAACCCGGATGAAGGTTGCGATCTGGATTTCGTACCGCACGAAGCGCGTCAGGTTAGCGGAATGGAATACACT
CTGTGTAACTCCTTCGGCTTCGGTGGCACTAATGGTTCTTTGATCTTTAAAAAGATCTAAGTTGTCATTTTCCACCCTTA
TAAAAGGTCCGCTTGCGGGCCTTTTTTCTTAGCTTTTATTCCGACTTGTTCCGTAGTGAACATGCTGCCACACTAACAAT
TCTCTGATAAGGAGCCGGTATGTTCTTAATTAACGGTCATAAGCAGGAATCGCTGGCAGTAAGCGATCGGGCAACGCAGT
TTGGTGATGGTTGTTTTACCACCGCCAGAGTTATCGACGGTAAAGTCAGTTTGTTATCGGCGCATATCCAGCGACTACAG
GATGCTTGTCAGCGGTTGATGATTTCCTGTGACTTCTGGCCTCAGCTTGAACAAGAGATGAAAACGCTGGCAGCAGAACA
GCAAAATGGTGTGCTGAAAGTCGTGATCAGTCGCGGTAGTGGCGGGCGAGGGTACAGCACATTGAACAGCGGACCGGCAA
CGCGGATTCTCTCCGTTACGGCTTATCCTGCACATTACGACCGTTTGCGTAACGAGGGGATTACGTTGGCGCTAAGCCCG
GTGCGGCTGGGGCGCAATCCTCATCTTGCAGGTATTAAACATCTCAATCGTCTTGAGCAAGTATTGATTCGCTCTCATCT
TGAGCAGACAAACGCTGATGAGGCGCTGGTCCTTGACAGCGAAGGGTGGGTTACGGAATGCTGTGCGGCTAATTTGTTCT
GGCGGAAGGGCAACGTAGTTTATACGCCGCGACTGGATCAGGCAGGTGTTAACGGCATTATGCGACAATTCTGTATCCGT
TTGCTGGCACAATCCTCTTATCAGCTTGTCGAAGTGCAAGCCTCTCTGGAAGAGTCGTTGCAGGCAGATGAGATGGTTAT
TTGTAATGCGTTAATGCCAGTGATGCCCGTATGTGCCTGTGGCGATGTCTCCTTTTCGTCAGCAACGTTATATGAATATT
TAGCCCCACTTTGTGAGCGCCCGAATTAGTCATGAAAAAAGTGTTATTGATAATCTTGTTATTGCTGGTGGTACTGGGTA
TCGCCGCTGGTGTGGGCGTCTGGAAGGTTCGCCATCTTGCCGACAGCAAATTGCTTATCAAAGAAGAGACGATATTTACC
CTGAAGCCAGGGACCGGACGTCTGGCGCTCGGTGAACAGCTTTATGCCGATAAGATCATCAATCGTCCACGGGTTTTTCA
ATGGCTGCTGCGTATCGAACCGGATCTTTCTCACTTTAAAGCCGGGACTTACCGCTTTACACCGCAGATGACCGTGCGCG
AGATGCTGAAATTGCTGGAAAGCGGTAAAGAAGCACAGTTCCCTCTGCGACTGGTAGAAGGGATGCGTCTGAGCGATTAC
CTCAAGCAATTGCGTGAGGCCCCGTATATCAAGCATACGCTGAGCGATGATAAGTACGCCACCGTAGCGCAGGCACTTGA
ACTGGAAAACCCGGAGTGGATTGAAGGTTGGTTCTGGCCAGACACCTGGATGTATACCGCCAATACCACCGATGTCGCGT
TACTCAAGCGAGCGCACAAGAAAATGGTGAAAGCGGTCGATAGCGCCTGGGAAGGGCGTGCGGACGGTCTGCCTTATAAA
GATAAAAACCAGTTGGTGACGATGGCATCAATTATCGAAAAAGAAACCGCCGTTGCCAGTGAACGCGATAAGGTTGCCTC
AGTATTTATCAACCGTTTACGCATTGGTATGCGCCTGCAGACCGACCCGACCGTGATTTACGGGATGGGAGAGCGTTATA
ATGGCAAACTTTCTCGTGCAGACCTGGAAACGCCGACAGCGTATAACACCTATACCATTACCGGTCTGCCGCCAGGTGCG
ATAGCGACGCCGGGGGCGGATTCGCTGAAGGCTGCTGCGCATCCGGCAAAAACGCCGTATCTCTATTTTGTGGCCGATGG
TAAAGGTGGTCACACGTTTAATACCAATCTTGCCAGTCATAACAAGTCTGTGCAGGATTATCTGAAAGTGCTTAAGGAAA
AAAATGCGCAGTAAGTATATCGTCATTGAGGGGCTGGAAGGCGCAGGCAAAACTACCGCGCGTAATGTGGTGGTTGAGAC
GCTCGAGCAACTGGGTATCCGCGACATGGTTTTCACTCGGGAACCTGGCGGTACGCAACTTGCCGAAAAGTTAAGAAGCC
TGGTGCTGGATATCAAATCGGTAGGCGATGAAGTCATTACCGATAAAGCCGAAGTTCTGATGTTTTATGCCGCGCGCGTT
CAACTGGTAGAAACGGTCATCAAACCAGCTCTGGCTAACGGCACCTGGGTGATTGGCGATCGCCACGATCTCTCCACTCA
GGCGTATCAGGGCGGCGGACGTGGTATTGACCAACATATGCTGGCAACACTGCGTGATGCTGTTCTCGGGGATTTTCGCC
CCGACTTAACGCTCTATCTCGATGTTACCCCGGAAGTTGGCTTAAAACGCGCGCGTGCGCGCGGCGAGCTGGATCGTATT
GAGCAAGAATCTTTCGATTTCTTTAATCGCACCCGCGCCCGCTATCTGGAACTGGCAGCACAAGATAAAAGCATTCATAC
CATTGATGCCACCCAGCCGCTGGAGGCCGTGATGGATGCAATCCGCACTACCGTGACCCACTGGGTGAAGGAGTTGGACG

CATGAGATGGTATCCATGGTTACGACCTGATTTCGAAAAACTGGTAGCCAGCTATCAGGCCGGAAGAGGTCACCATGCGC
TACTCATTCAGGCGTTACCGGGCATGGGCGATGATGCTTTAATCTACGCCCTGAGCCGTTATTTACTCTGCCAACAACCG
CAGGGCCACAAAAGTTGCGGTCACTGTCGTGGATGTCAGTTGATGCAGGCTGGCACGCATCCCGATTACTACACCCTGGC
TCCCGAAAAAGGAAAAAATACGCTGGGCGTTGATGCGGTACGTGAGGTCACCGAAAAGCTGAATGAGCACGCACGCTTAG
GTGGTGCGAAAGTCGTTTGGGTAACCGATGCTGCCTTACTAACCGACGCCGCGGCTAACGCATTGCTGAAAACGCTTGAA
GAGCCACCAGCAGAAACTTGGTTTTTCCTGGCTACCCGCGAGCCTGAACGTTTACTGGCAACATTACGTAGTCGTTGTCG
GTTACATTACCTTGCGCCGCCGCCGGAACAGTACGCCGTGACCTGGCTTTCACGCGAAGTGACAATGTCACAGGATGCAT
TACTTGCCGCATTGCGCTTAAGCGCCGGTTCGCCTGGCGCGGCACTGGCGTTGTTTCAGGGAGATAACTGGCAGGCTCGT
GAAACATTGTGTCAGGCGTTGGCATATAGCGTGCCATCGGGCGACTGGTATTCGCTGCTAGCGGCCCTTAATCATGAACA
AGCTCCGGCGCGTTTACACTGGCTGGCAACGTTGCTGATGGATGCGCTAAAACGCCATCATGGTGCTGCGCAGGTGACCA
ATGTTGATGTGCCGGGCCTGGTCGCCGAACTGGCAAACCATCTTTCTCCCTCGCGCCTGCAGGCTATACTGGGGGATGTT
TGCCACATTCGTGAACAGTTAATGTCTGTTACAGGCATCAACCGCGAGCTTCTCATCACCGATCTTTTACTGCGTATTGA
GCATTACCTGCAACCGGGCGTTGTGCTACCGGTTCCTCATCTTTAAGAGAGACATCATGTTTTTAGTCGACTCACACTGC
CATCTCGATGGTCTGGATTATGAATCTTTGCATAAGGACGTGGATGACGTTCTGGCGAAAGCCGCCGCACGCGATGTGAA
ATTTTGTCTGGCAGTCGCCACAACATTACCGGGTTATTTACATATGCGGGATCTGGTAGGCGAACGTGACAACGTCGTAT
TTTCTTGTGGCGTGCATCCACTCAACCAGAATGATCCCTACGATGTAGAAGATTTACGCCGTCTGGCGGCAGAAGAGGGT
GTTGTAGCGCTGGGTGAAACCGGGCTGGATTATTATTACACGCCGGAAACTAAAGTACGTCAGCAAGAGTCCTTCATCCA
TCATATCCAGATTGGTCGTGAACTGAACAAGCCGGTTATCGTCCATACCCGTGACGCCCGTGCCGATACGCTGGCAATTC
TGCGCGAAGAAAAAGTGACGGATTGCGGTGGCGTACTACACTGTTTTACAGAGGACAGAGAAACGGCGGGTAAATTACTG
GATCTCGGATTTTACATCTCCTTTTCCGGCATTGTGACCTTCCGTAATGCGGAGCAACTGCGCGATGCTGCGCGTTATGT
CCCCCTGGATCGGTTACTGGTGGAAACTGACTCACCTTACCTTGCGCCGGTACCGCATCGAGGAAAAGAGAATCAACCTG
CGATGGTTCGTGACGTTGCAGAATACATGGCTGTGTTGAAAGGTGTTGCCGTTGAAGAACTGGCGCAGGTAACCACCGAT
AACTTCGCCCGTCTGTTTCACATCGACGCTTCCCGCCTTCAATCCATCCGTTGAATGAGTTTTTTTAAAGCTCGTAATTA
ATGGCTAAAACGAGTAAAGTTCACCGCCGAAAATTGGGCGGTGAATAACCACGTTTGAAATATTGTGACATATGTTTTGT
CAAAATGTGCAACTTCTCCAATGATCTGAAGTTGAAACGTGATAGCCGTCAAACAAATTGGCACTGAATTATTTTACTCT
GTGTAATAAATAAAGGGCGCTTAGATGCCCTGTACACGGCGAGGCTCTCCCCCCTTGCCACGCGTGAGAACGTAAAAAAA
GCACCCATACTCAGGAGCACTCTCAATTATGTTTAAGAATGCATTTGCTAACCTGCAAAAGGTCGGTAAATCGCTGATGC
TGCCGGTATCCGTACTGCCTATCGCAGGTATTCTGCTGGGCGTCGGTTCCGCGAATTTCAGCTGGCTGCCCGCCGTTGTA
TCGCATGTTATGGCAGAAGCAGGCGGTTCCGTCTTTGCAAACATGCCACTGATTTTTGCGATCGGTGTCGCCCTCGGCTT
TACCAATAACGATGGCGTATCCGCGCTGGCCGCAGTTGTTGCCTATGGCATCATGGTTAAAACCATGGCCGTGGTTGCGC
CACTGGTACTGCATTTACCTGCTGAAGAAATCGCCTCTAAACACCTGGCGGATACTGGCGTACTCGGAGGGATTATCTCC
GGTGCGATCGCAGCGTACATGTTTAACCGTTTCTACCGTATTAAGCTGCCTGAGTATCTTGGCTTCTTTGCCGGTAAACG
CTTTGTGCCGATCATTTCTGGCCTGGCTGCCATCTTTACTGGCGTTGTGCTGTCCTTCATTTGGCCGCCGATTGGTTCTG
CAATCCAGACCTTCTCTCAGTGGGCTGCTTACCAGAACCCGGTAGTTGCGTTTGGCATTTACGGTTTCATCGAACGTTGC
CTGGTACCGTTTGGTCTGCACCACATCTGGAACGTACCTTTCCAGATGCAGATTGGTGAATACACCAACGCAGCAGGTCA
GGTTTTCCACGGCGACATTCCGCGTTATATGGCGGGTGACCCGACTGCGGGTAAACTGTCTGGTGGCTTCCTGTTCAAAA
TGTACGGTCTGCCAGCTGCCGCAATTGCTATCTGGCACTCTGCTAAACCAGAAAACCGCGCGAAAGTGGGCGGTATTATG
ATCTCCGCGGCGCTGACCTCGTTCCTGACCGGTATCACCGAGCCGATCGAGTTCTCCTTCATGTTCGTTGCGCCGATCCT
GTACATCATCCACGCGATTCTGGCAGGCCTGGCATTCCCAATCTGTATTCTTCTGGGGATGCGTGACGGTACGTCGTTCT
CGCACGGTCTGATCGACTTCATCGTTCTGTCTGGTAACAGCAGCAAACTGTGGCTGTTCCCGATCGTCGGTATCGGTTAT
GCGATTGTTTACTACACCATCTTCCGCGTGCTGATTAAAGCACTGGATCTGAAAACGCCGGGTCGTGAAGACGCGACTGA
AGATGCAAAAGCGACAGGTACCAGCGAAATGGCACCGGCTCTGGTTGCTGCATTTGGTGGTAAAGAAAACATTACTAACC
TCGACGCATGTATTACCCGTCTGCGCGTCAGCGTTGCTGATGTGTCTAAAGTGGATCAGGCCGGCCTGAAGAAACTGGGC
GCAGCGGGCGTAGTGGTTGCTGGTTCTGGTGTTCAGGCGATTTTCGGTACTAAATCCGATAACCTGAAAACCGAGATGGA
TGAGTACATCCGTAACCACTAATCCGTAAGACGTTGGGGAGACTAAGGCAGCCAGATGGCTGCCTTTTTTACAGGTGTTA
TTCAGAATTGATACGTGCCGGTAATGCTGAAATTACGCGGTGTGCCGTAGACGATAGAACCTTCCACGTTGGTATCGTAG
GTTTTGTCGAACAGGTTATTGACGTTCCCCTGTAACGAGAAGTTTTTCGTCACCTGGTAGCGGGTGAAGAGATCCACCAG
CGCGTAGCTACCTTGCTCGGCGCGGAAGGTGCCATACGGCGTCACGGTGTCGGTATACACGCGATTTTGCCAGTTAACAC
CACCGCCGACCGTCAACTCTGGCATGACAGGCAACCGATAGCTGGTGAACATTTTAACCGTGGTGCGTGGCAGATTAGGA
TTAACGGCGTTTCCTTCGTTATCCTCTGCAATATAGCGCGTTGCGCCAAATGTCAGCTGCCAGTTGTCGGTAATTGCGCC
GTTGAGTTCAAATTCCACCCCTTTACTGACTGTCCCATCCACCGCTTTATAGGCGGTTTCGCCGTTGCTGCCGGGGATAG
GTGTACCGGTGGACTGAGCGACATTATCCTGCTCAATACGGAAGATGGCTAACGTGGTGGTCAGACGGCTATTCATCCAG
TCCGATTTCAGACCCAGCTCGTAGTTGTTACCGGTGATTGGAGCCAGATATTTGCCTGAACTGTCACGATCATTTTGCGG
CTGGAAAATAGAGGTATAGCTGGCGTAGGTCGACCAGTTGTCATTGATGTCAAACACCAGACCAGCGTAAGGCGTGGTGT
GGTTTTTCTCCATGCTGTAAGTCAGCGTATCAACCCGCCAGTTGGTATAACGTGCGCCGAGGATCAGATGCAGCGGATCG
GCAAGGGTGACACGAGTGGCAGCATATAACGATTTCATATGTGTGGTATCGTCCTGCGCCAGGCTCTGTGGTGACCAGTC

GGTTTGTGGGAAATTGCCATTAAAGTTGTAGAAACTGCCAATTTCATCCGGGAAGATGTTGGCCCATGAACTGAAGTAAC
GATTGTTTTGTTTGCTGTAACTGCCACCAAACATTAGATTGTGCTGACGACCAAACAATTCATAACTACCGTCAGCGAAC
AAATCCAGCGCATCAACTTTACGTTTGCCACTGTTCCAACCGGTGCCGCCGACATAATCAAAGCCAGGTCCATAATTACT
GTATGGCCCAACCAGCATACCATCCGCTTTGTTTACATAGGCATCGACATACATCATTTTGCTGTCAAATTCGACTTCAG
AGTGGGTGGCATTCAGTGTCGCTTGCCAGGTATCAGCAAACTGCTGCTTCAGGGTCATAAAGACCTTGTTGATCTCTTTA
TCGTTGTACGCCCAGTCAGGTGCGGTACTGCGTGCGCGATCGTAACTGTTGCTGCTGCCATCAGTATTCCAGCGCGGTAA
ACCGCCCCAGGTAGGGCTATTAACATCAATGCGCTGATATTCGTAACCGGCTGAAAGCGTCGTAAGATCGCCTAAATCAG
CATCGACAATGCCCGAGAAGAAGGTCTTTTCACTGTTGTAGCGGTCCAGCCATGAGTCGTTATTCTGGTAGCCGCCGACA
ATTCGCGCGCGGATTTTACCGTCTTCGGTGAGTGGGCTTTGTAAATCCGCCACATACCGTTCTTTGTTCCAGCTACCGTA
TTCCGCCGAGACATCGCCTTTAAATTCACGACTGGTCGCGTGTTTTCGAACCATATTAATTGCCGCAGATGGATTACCCG
TCCCGGTCATGAGTCCTGTCGCGCCACGCACTACTTCTACGCGTTCAAAGAGTGCCATATCAGAAAGTGCGTCGCCCAGA
TTCCAGCGCGATTCAAAATAGGTGGGGATACCATCAACCATATAGTTATCGATCTGGAATCCGCGGGAATAATAAAGAGC
ACGATCGGAATCCGCCTGACTTTTGCTGATCCCCAGCGTGTTTTCCATCACTTCGCCCAGCGTTTGTAACTGCTGATCTT
CCATCCGCTGCTGGCTAACAATAGTGACCGACTGAGGAATATCACGTTGAGTCATCTGCATTTTGGTACCCGCAGAGGTA
GACGTTACGCTGTAATCATTTTCGCCATCATCTGGAGCTGTGGCTGAACCCTCAACAATCACCGTTTCTTCAGTGGCTGG
TGCAGCAAAAGCGGCAGAAGGTAATAGTGCCAGTGCTATGCAACCGGCAAGTAGTGACGGTTTGGTGATGGCTTGATATT
GATTATCCCTGTTAAATTGTGTTGAAAGCATTTGAAATCTCTTGGTTTATGTTGTCGTCAGGCTCATGCCGGAGGAGGGT
GATGAGCGATTATATTTAGTGTGCGAATAATTTTGTTTGTAAATGCAAATGAGAAATATACGCATTCATATTTGTCATGT
AAACCAAACAGAGAATGTCTTTTCAGCGCATTCGCAGGCAGAAATGGGAATAATGGCGATATATACGGCAGCAAAACGAT
TTTTTGCTTAAGAAATCGTGAGTTAAGGTTGAAAGAGCAGGTTTAACTCGACCATACTCTATACTCGCAGTGTGGCGCGG
CGTAGCATGGCGCAACGCATGGCTATTTGAAAAAGGAAAATGTCGTGGCAGAAGAAACTATATTCAGCAAAATTATTCGT
CGTGAGATCCCCTCCGATATCGTCTACCAGGATGATCTGGTAACGGCGTTTCGCGATATTTCGCCGCAAGCGCCAACGCA
TATTCTGATCATTCCGAATATCCTCATTCCGACTGTGAACGACGTCTCAGCTGAGCATGAGCAGGCGCTGGGACGCATGA
TCACCGTAGCGGCAAAAATTGCTGAGCAAGAAGGTATTGCCGAAGATGGCTATCGTCTGATCATGAATACCAACCGCCAT
GGCGGACAAGAGGTTTACCACATCCATATGCACTTGTTGGGTGGCCGTCCGCTGGGACCAATGCTGGCGCATAAAGGTCT
GTAACGATGAGAAAAGGATGCTTTGGGCTGGTGTCTCTGGTGTTGTTACTGCTGGTGGGCTGTCGTTCACATCCGGAAAT
TCCGGTGAATGATGAGCAATCGCTGGTGATGGAGTCATCTTTATTGGCTGCGGGCATCAGTGCAGAAAAGCCCTTCCTTT
CGACGTCTGATATTCAACCTTCAGCATCCTCAACGCTTTATAACGAAAGGCAAGAACCCGTTACCGTTCATTATCGTTTT
TACTGGTATGACGCCAGAGGGCTGGAGATGCATCCTCTGGAAAGGCCACGCAGCGTTACCATTCCCGCACATTCGGCGGT
AACGCTGTACGGCAGCGCCAATTTTCTGGGGGCGCACAAAGTCAGACTTTATCTATATTTGTAAGGGGTGAATCTTGATG
ACAAAAATGAGTCGCTACGCCTTGATTACCGCGCTGGCGATGTTTCTCGCCGGGTGTGTGGGGCAACGTGAACCTGCACC
GGTAGAAGAAGTGAAACCAGCGCCGGAACAACCAGCCGAGCCACAACAGCCTGTCCCCACAGTGCCCTCGGTGCCGACGA
TCCCGCAGCAGCCAGGCCCAATTGAGCACGAAGATCAAACTGCACCGCCTGCGCCGCATATTCGCCATTATGACTGGAAT
GGCGCAATGCAGCCGATGGTCAGTAAGATGCTTGGGGCTGACGGGGTGACTGCGGGTAGCGTCCTGCTGGTTGATAGCGT
TAACAACCGTACTAACGGTTCGCTGAATGCCGCAGAAGCGACCGAAACGCTGCGAAATGCGCTGGCTAATAACGGGAAAT
TTACCCTGGTTTCCGCCCAGCAACTGTCGATGGCGAAGCAACAGTTAGGTTTGTCGCCGCAGGACAGTTTAGGCACCCGT
AGTAAAGCCATAGGCATTGCCCGCAATGTCGGCGCTCATTACGTGCTGTACTCCAGCGCCTCTGGCAACGTTAACGCTCC
GACCCTACAAATGCAGCTGATGCTGGTGCAGACGGGCGAAATTATCTGGTCAGGTAAAGGTGCCGTTTCGCAGCAATAAT
CCCATCACGCGCGACGAATTGCTGTCGCGCTTTTTCCCGCAGTATCATCCCGTCACGACGTTTAATAGTGGGCTTAGTGG
CGGGAGTTTTCTCATTGAACATCAGGGCCAGCGTTTTGTTGTGCGTCAGCCGCACGATCCTGATGCGCCGCAGTCCGCGT
TCTTGCGCCAGTATCGGGCTTTATCACAACTACCCGCATGCATTGCACCGAAGCCGCATTTATATCTCCGTGACTGGATG
GTAGTCGACTATCTGCCCGGCGCGGTAAAAACGTATTTGCCGGATACCAACGAACTGGCAGGCTTGCTGTATTATCTACA
TCAACAACCACGTTTTGGCTGGCGAATAACGCTGTTGCCGTTACTGGAACTGTACTGGCAGCAAAGCGATCCGGCGCGGC
GGACAGTGGGTTGGCTGCGAATGTTAAAACGTCTGCGCAAAGCGCGGGAACCACGGCCTTTACGCTTAAGTCCATTGCAT
ATGGATGTCCACGCCGGAAATTTAGTGCATAGCGCGTCAGGGTTAAAACTCATCGACTGGGAGTATGCCGGAGATGGTGA
TATCGCGCTGGAACTGGCGGCGGTGTGGGTGGAAAATACTGAACAGCACCGGCAATTGGTCAATGACTATGCCACTCGCG
CGAAGATTTATCCGGCGCAATTATGGCGTCAGGTCAGGCGATGGTTTCCCTGGCTGCTGATGCTCAAAGCAGGGTGGTTT
GAGTACCGCTGGCGACAAACCGGCGATCAACAATTTATCAGGCTGGCCGATGACACCTGGCGGCAGCTATTAATAAAACA
ATAAGGAGAGCAGTGTGGGTCCAGTAATGTTGGATGTCGAAGGTTACGAACTGGACGCGGAAGAGCGTGAAATACTGGCG
CATCCGCTGGTGGGAGGGCTGATTCTCTTTACGCGTAACTATCATGATCCTGCCCAGTTACGTGAACTGGTGCGCCAGAT
CCGCGCAGCTTCGCGCAATCGTCTGGTGGTGGCGGTTGATCAGGAAGGTGGACGCGTGCAGCGTTTTCGTGAAGGTTTTA
CCCGCTTGCCAGCGGCGCAATCATTCGCTGCGCTGTCAGGAATGGAAGAGGGTGGCAAACTGGCGCAGGAGGCAGGTTGG
TTGATGGCCAGCGAAATGATCGCTATGGATATTGATATCAGCTTTGCGCCTGTGCTGGATGTCGGGCATATCAGCGCGGC
GATTGGCGAGCGTTCTTATCATGCCGATCCACAAAAAGCCCTGGCAATTGCCAGCCGGTTTATTGATGGTATGCATGAAG
CCGGAATGAAAACGACCGGGAAACACTTCCCAGGACACGGTGCAGTAACGGCAGACTCACACAAAGAAACACCGTGCGAT
CCACGTCCACAAGCGGAGATTCGCGCTAAAGATATGTCGGTCTTCAGTTCCTTAATCCGCGAAAATAAACTCGACGCCAT

TATGCCTGCGCATGTGATCTACAGTGATGTTGATCCGCGTCCGGCGAGCGGTTCTCCCTACTGGCTGAAAACCGTTTTGC
GTCAGGAACTGGGTTTTGACGGCGTGATTTTCTCTGACGATTTATCGATGGAAGGTGCCGCGATTATGGGCAGTTATGCC
GAACGCGGGCAGGCTTCACTGGATGCGGGTTGCGATATGATCCTGGTCTGCAATAATCGTAAAGGGGCCGTCAGCGTGTT
AGATAATCTGTCACCGATCAAGGCAGAACGTGTTACACGTTTGTATCATAAAGGTTCATTTTCGCGACAGGAACTGATGG
ACTCGGCTCGCTGGAAAGCGATCAGCACCCGTCTGAATCAGTTACATGAACGCTGGCAGGAAGAGAAAGCAGGTCACTAA
CCCTGGCTTATGTGAGGAAGCGATGATTATCTATTTACACGGTTTTGACTCTAACAGTCCGGGTAACCACGAGAAAGTCT
TACAATTGCAGTTTATTGACCCGGATGTACGCTTGATAAGCTACAGTACGCGGCATCCGAAACATGATATGCAGCATCTG
CTTAAAGAAGTGGACAAAATGTTGCAACTGAACGTTGACGAGCGTCCGCTAATTTGCGGCGTTGGCTTGGGCGGATACTG
GGCGGAACGGATTGGTTTTCTTTGCGACATCCGCCAGGTGATCTTCAACCCTAATTTGTTCCCTTATGAGAACATGGAAG
GCAAGATTGATCGCCCGGAAGAGTATGCCGATATTGCCACTAAGTGTGTGACCAACTTCCGTGAGAAGAATCGCGATCGT
TGCCTGGTGATTTTGTCGCGTAATGATGAAGCGCTTAACAGCCAGCGGACATCTGAAGAGTTGCATCATTATTACGAGAT
TGTCTGGGACGAAGAGCAGACGCACAAATTCAAGAATATCTCCCCGCATTTACAGCGCATTAAAGCGTTCAAAACCCTCG
GGTAAATGCCCTCGTCGCATCAGGTAACCTTGCCGGTACCTGATGCGCTCCGAATTCTGTGGGTCGGATAAGGCGTCCAC
GCCGCATCCGACAGTCGAGCATCAATGCCTGATGCGCTTCTTATCAGGCCTACCGAACGCCCTGCATACACCCCTCACTC
TATATCACTCTCACAAATTCGCTCAAATAATAAACAATAAACTCTGTTTTTTGATCTCACCCGGTAAAGTCGCCTATCTT
TTCAGCAACAAAACTTGATTAACATCAATTTTGGTATGACCAATGCACCATTCATGTTATTCTCAATAGCGAAGAACATT
TTCATTGCTGTAACCTGTTGTTAATTAAGAGCTATGTTAATAACCATTAATTAACAATTGGTTAATAAATTTAAGGGGGT
CACGTTGACTACGCCATTGAAAAAGATTGTGATTGTCGGCGGCGGTGCTGGTGGGCTGGAAATGGCAACACAGCTGGGGC
ATAAGCTGGGACGCAAGAAAAAAGCCAAAATTACGCTGGTCGATCGTAACCACAGCCACCTGTGGAAACCGCTGCTGCAC
GAAGTGGCGACTGGCTCGCTTGATGAAGGCGTCGATGCGTTGAGCTATCTGGCCCATGCGCGCAATCATGGTTTCCAGTT
CCAGCTGGGTTCCGTCATTGATATTGATCGTGAAGCGAAAACAATCACTATTGCAGAACTGCGCGACGAGAAAGGTGAAC
TGCTGGTTCCGGAACGTAAAATCGCCTATGACACCCTGGTAATGGCGCTGGGTAGCACCTCTAACGATTTCAATACGCCA
GGTGTCAAAGAGAACTGCATTTTCCTCGATAACCCGCACCAGGCGCGTCGCTTCCACCAGGAGATGCTGAATTTGTTCCT
GAAATACTCCGCCAACCTGGGCGCGAATGGCAAAGTGAACATTGCGATTGTCGGCGGCGGCGCGACGGGTGTAGAACTCT
CCGCTGAATTGCACAACGCGGTCAAGCAACTGCACAGCTACGGTTACAAAGGCCTGACCAACGAAGCCCTGAACGTAACG
CTGGTAGAAGCGGGAGAACGTATTTTGCCTGCGTTACCGCCACGTATCTCTGCTGCGGCCCACAACGAGCTAACGAAACT
TGGCGTTCGCGTGCTGACGCAAACCATGGTCACCAGTGCTGATGAAGGCGGCCTGCACACTAAAGATGGCGAATATATTG
AGGCTGATCTGATGGTATGGGCAGCCGGGATCAAAGCGCCAGACTTCCTGAAAGATATCGGTGGTCTTGAAACTAACCGT
ATCAACCAGCTGGTGGTGGAACCGACGCTGCAAACCACCCGCGATCCAGACATTTACGCTATTGGCGACTGCGCGTCATG
CCCGCGTCCGGAAGGGGGCTTTGTTCCGCCGCGTGCTCAGGCTGCACACCAGATGGCGACTTGCGCAATGAACAACATTC
TGGCGCAGATGAACGGTAAGCCGCTGAAAAATTATCAGTATAAAGATCATGGTTCGCTGGTATCGCTGTCGAACTTCTCC
ACCGTCGGTAGCCTGATGGGTAACCTGACGCGCGGCTCAATGATGATTGAAGGACGAATTGCGCGCTTTGTATATATCTC
GCTATACCGAATGCATCAGATTGCGCTGCATGGTTACTTTAAAACCGGATTAATGATGCTGGTGGGGAGTATTAACCGCG
TTATCCGTCCGCGTTTGAAGTTGCATTAATCGACGTACACTGGCGGATGTGGCATAAACGCCTCATCCGCCCTTGAGGAA
CAGCGCGATCGGCAGCCGCGTTGTATCAGGCATCCTTTCAGACTCCTCCGAATCCTTAAGTATTTCCAGCCATTCCCGCG
CTTTTCATCTTCTGTCTGATAGCTGCTTTTCTCCTTCGCTTGCATGATTGGCATAACTGCAAAGAAGGAGGTGTTCCCGT
GAATAAATCAATGTTGGCGGGTATCGGGATTGGTGTCGCAGCTGCGCTGGGCGTAGCGGCAGTGGCCAGTCTGAACGTGT
TTGAACGGGGCCCGCAATACGCTCAGGTTGTTTCTGCAACCCCAATCAAGGAAACGGTTAAAACACCGCGTCAGGAGTGT
CGCAACGTCACAGTGACCCATCGTCGACCGGTGCAGGATGAAAATCGCATTACCGGGTCGGTGCTCGGCGCTGTTGCTGG
CGGCGTGATAGGGCATCAGTTTGGTGGTGGTCGCGGTAAAGATGTCGCCACTGTTGTGGGGGCGCTGGGTGGTGGATATG
CCGGTAACCAGATCCAGGGCTCTCTCCAGGAAAGCGATACTTACACGACTACGCAACAGCGTTGTAAAACGGTGTATGAC
AAGTCAGAAAAAATGCTCGGTTATGATGTGACCTATAAGATTGGCGATCAGCAGGGCAAAATCCGCATGGACCGCGATCC
GGGTACGCAGATCCCGCTAGATAGCAACGGGCAACTGATTTTGAATAACAAAGTATAACAAGGCTGTACTCTGCAATTTG
GCCCCTCATTCGCTCAGGCTGAGGGGCTTTTTTTGCGACTTATTTCACCAGTTCGGGCCATAAACGCAAAGTCGTTCCGG
CAATCTGCATCAGTTTTTCCAGCGATGCACCTTCGCGTGCGCTGATCGACATCCCCTGAATAATACAATTAAGGAATTCT
GCCAGATGTGTCACGTCACAGTGGGGCGGGATTTCCCCGCGCGCTTGTCGTTGACATAAAAACTGCTGCAAAGTGCGCTC
TTGCATCGCATGGCGTGATTTCAACGTATTGGCGATATCTCCTGAATCTGGGGAGAGGGTGGTGCAGTTGTTGATCATGA
AGCAGCCTGCCGGGGTGTCTTTGCTGGTAAAGCAGTTGGCGATGGCAGCAAAATAGTCAGCCAGCGCAGACTCCACGCTT
TTCTCTTCACAAAACAGCTGTGCTTCATGCTTAGCGGCAAAACGATCGATATAGCGGTCGAGAACGGCACGAAATAACCC
CTCTTTGTTGGTAAATTCCGCGTATAGCGTGGGCGCTTTGGCTCCGGTCGCTTCGACGAGGTCCGCAAGAGAAGTCGCTT
CATAACCGTGTTGCCAGAACAATTTCATGGCCTTATCAAGCGCGGCATCCCTGTCGAACACTTTTGGGCGGCCACGGCTT
TTTTTTACACATTGTGTTGAGTCAGTTGCCATGATGCCGTTGTACCTGGTGACTGTGAATGAAAGGTTATTATAAAAATA
ATCACCTCCGTTCACCAGTCCAGATCCCATAAAAATAATTGCTTTCTATTTAACTGAAATTTAAAGATTTTTAAATTAAT
TAATGATTGTTATAAAAAATATCTTGTATGTGATCCAGATCACATCTATCATTTAGTTATCGATCGTTAAGTAATTGCTT
GCGACGTCATTCATCTGCATAAGGCCACTATTATGAAAAACGTAAAAACCCTCATCGCTGCGGCGATTTTAAGCTCCATG
TCATTTGCCAGCTTTGCGGCTGTCGAAGTTCAGTCAACGCCAGAAGGCCAACAAAAAGTCGGTACAATCAGTGCTAACGC

GGGGACAAATCTGGGATCGCTGGAAGAGCAGCTGGCGCAAAAAGCGGATGAGATGGGCGCAAAATCTTTCCGTATTACTT
CTGTAACCGGTCCGAATACCCTCCATGGAACAGCAGTAATTTATAAATAAGCATTAACCCTCATTAATGCCTGCTACTGC
TGATTTTTTCCCCGCGACATGCCGTGTCGCGGGGATTTTTTTATCCGGGCTTTACAGCGTTTGTGGGCTCACTTGATGAC
GGCGGACATCCACCGGCATCCCGGAACGGACATCCATCACATGTTGCATCACTTCAGCGTCAGTTTGTGCTGCATCTTTA
AATGATTGCATTGCGCTATTCAGCGTAATTGGCAGCAGCTGCGGATCGTCATCAATCTTCTCTGACAGCGGCTGATGTAC
TTCAACCAGACGCGCACCGTTTGGTTCGGCAGAGACTTTTATCGGAGTGTTGATGATATTCACTTTGGTGCCTGGGGTGA
CCTGGCTAAAGAGTGTTTTGATATCGTCATCCCGCAGACGAATACAGCCAGAACTTACCCGCATGCCAATGCCGAAATCG
GCGTTCGTACCATGAAGCAAATAAACGCCGCCATAGGCCGCCAGACGAATCGCATGATGGCCCATTGGGTTATCCAGTCC
AGCCGGCACTACCGCAGGCAACTCAATTCCCTGTGCTTTATAACGGGCGCGGATGTTTGCCGTTGGCGTCCAGGTTGGGT
TTGCACGTTTGTCTGAAACGGTGGTCACCATTGTCGGTGTCAGCGTGTCACCACCTAACTGACCAATACCTATTGGATAC
ACGGTTACCGAATTTTTACCCGGCGGGTAGTAATAGAGACGCAGCTCCGCAATGTTGATCACAATGCCTTCGCGCGGCGC
ATCTGGAAGTAGGGTTTGCAACGGGATCGTTAACACGCTGCCCGCGCGCGGTACGTAAGGATCAACGCCGGGGTTAGCCT
GTAACAGAGCGAGAAAGCCGACGTTGTATTTTTTGGCGATGGCTTCCAGAGAACCACCGTCATTTTCCACCACATGAAAT
TTGTTTTCGCCAACCAGACGACTGCCCGCTGGCGGCAGCGGCCAGGTGTTGGCTTTTGCCGGTAGCGCCAGCGCCACGGC
AGCGGCGAACGTAAAAAACGTTAGCCAGCGAGAAAAACGCGTTTTGATCATCACCAAAAATCCATAATAAATATAAGGTT
ATTGTAATAAACAGATAAGCCTGAATTATGGATGGTGACAGTGTCGGATAGTGCAGGGAAGTGCAAAGAATTTGTAAATG
TTGCAGATGGGGGCGCAGAAACGCCCCCGATTTACCATTAAGCGATCGCGTTCTCTTCCAGTTCACGCATAAACTGGCGT
ACCCATTCGATACGCGTTTTCCGCTCACTCAAATCCTGAATAAATTTCAGGCGCGTCGGACCATCAAGGCGGTAATGCTG
CGGCTGTTTTTGCAGCAAACCAATCAACCAGGCCGGATTAACGTGATTCTTCTCGGCAAATTCGATCACCCCGCCTTTCT
CATTACCTTCCAGCTTCCTGATCCCCAGTTTCTGCGCTTGCTGGCGCAGACGGGCAATATCCAGCAGGGTACGCGCCGGA
TCTGGCAGCAGGCCGAAGCGATCGATAAGCTCGACTTTGATCTCTTCCAGTTCGTTTTCCGTTTTGGCGCTGGCAATACG
TTTGTAGAACGACAGACGCGTGTTCACGTCAGGGATGAAATCATCTGGCAATAGCGACGGCATCCGCAGCTCGACTTCTG
TTTGCTGGCTGGTGAGATCTTCCAGCGACGGCTCGCGTCCGGCTTTCAGTGCATCGACGGCGTTTTCCAGCAACTCCATA
TACAGCGAGAAACCGATGGTTTCCATTGAGCCGCTTTGTTCTTCGCCAAGCAGTTCACCCGCGCCGCGAATCTCCAGATC
GTGCGTTGCCAGCGCAAAACCTGCCCCGAGATCTTCCAGCGAGGCAATTGCTTCAAGACGTTTTTGTGCATCGGTAGTCA
TCGCTTTTGGATGCGGTGTCAGCAACCATGCATATGCCTGATGATGCGAACGTCCGACGCGACCGCGTAACTGGTGCAGC
TGCGCCAGACCGAAGTGATCCGCGCGTTCAATGATAATAGTGTTGGCTGTCGGGATGTCGATCCCGGTTTCGATAATGGT
TGTACAAACCAGCACGTTGAAACGTTGATGATGGAAATCATTCATCACCCGTTCCAGTTCGCGCTCGCGCATCTGCCCGT
GACCGATGGCGATCCGCGCTTCTGGCACCAGTTCTGCCAGCCGTTCGGCGGCTTTCTGAATGTTTTCCACATCATTGTAG
AGATAATAAACCTGTCCTCCGCGCAAAATTTCACGCAGGATCGCCTCCCGGACCACCATGCTGTCATACTCACGGACAAA
GGTTTTAACTGCCAGACGACGGGCGGGCGGCGTGGCGATAATCGACAGGTCACGCATTCCGCTCATTGCCATATTCAGCG
TACGTGGGATCGGCGTTGCAGTAAGCGTCAGAATATCCACGTTCGCGCGCATCGCTTTAATGCGCTCTTTATGACGCACC
CCGAAGCGGTGTTCTTCATCGACAATCAGCAGGCCTAAATCTTTAAACTTGACGTCACTTTGCAGCAGTTTGTGCGTACC
GATCAGAATATCGATTTTCCCTTCCGCCACTTCCGCAAGGATTTGCGTCTGCTCTTTGGCGCTGCGGAAACGGGAGATCA
TTTCGATACGTACCGGCCAGTTGGCGAAACGGTCGCGGAAGTTGTCGTAATGCTGCTGCGCGAGAAGGGTGGTAGGCACC
AGCACCGCCACCTGCTTGTGGTTATCTACTGCCAGGAAAGCTGCGCGCATCGCCACTTCTGTTTTACCAAAGCCAACATC
GCCGCACACCAGACGATCCATTGCCAGCGGCTGACACATGTCGCTAAGTACCGCATTAATGGCCTGCGCCTGATCCGGCG
TGGTTTCAAACGGGAAGCTGTCGCAGAACAACTGATACTGCTCACGATCGTGTTTAAACGCGAAGCCCTCTTTGGCGGCG
CGTTGCGCGTAGATATCCAGCAATTCCGCCGCCACATCACGCACTTTTTCCGCCGCTTTCTGCCGCGCGCGTGACCACGC
ATCGCCGCCAAGTTTATGCAGCGGGGCGTTTTCTTCCGCGCCACCTGCGTAACGGCTAATCAGATGCAGTGACGACACCG
GAACATACAGTTTGGCGTCGTTGGCATAGGTGAGCATCAGATACTCGCCAGTAATGCCACCCGCTTCCAGCGTGGTCATT
CCGGCATAACGACCGACGCCGTGCTCCAGATGGACCACCGGCTGACCAATATGCAGTTCCGCAAGGTTACGGATCAGTGT
ATCGGGGTTGATGGTGCGGCGAGAATCCTGACGACGACGGGCAACGCGTTCACCGAGCAGATCGCTTTCGCAAATCAGCG
CCAGATTACGCACCGTATCGACAAAACCATGTTCGGCAGCGCCAATCATCAGATAACGCCCACGGTCGCTGGCTTCATCA
AGACGCATAATGCGTTGCGGAGCAATTTTAATTCGTGCGAGCAGTTCACCCAGCGCTTCACGGCGACCTTCACTTTCTAC
CGAGAACACCACCGGACCGTCGAAAGTCTCGAGGAACTTACGCAGCGCATCCAGCGGCGCTTTTTGTTGTGCCTGAACGG
CCAGGTCTGGCAGTTTCTGGAAACCTAAATTGGCATTCGCGGCTTTTGTCGGTAAATGTTCAGTTTTTAGCTGCACCCGG
GGCCAGTTTTTCAGCTCTGAGAAGAGCTCGTCCACCCGCAGCCAGAGCGATTGTGGTGGCAACAGCGGGCGCATCGGATC
GACGCCGCGATTCTCAAAACGCGCCAGCGTGTCAGCCTGGAAACGTTCGGCACTGGTTTCCAGATCGCCAGTATTCACGA
GCAAGGTATTGGCAGGGAAATAACTGAACAGCGGCGGCAGTGGTTCGCTGAAGAACAATGGCTGCCAGTACTCGATCCCG
GCAGGTAATGTGCCTTTACTCACTTGCTGGTAAATATGTTCTGGATCGCGCTTCACTTCGAAGGTATCGCGCCACTGGCT
GCGGAACAGTTCAATTGCCGCTTTATCGGTCGGAAATTCGTGCGCGGGCAGCAGATTGATCGCTTCTACTTCCTCCAGCG
TGCGCTGGCTGTCGACGTCAAACACCCGCAGGCTGTCGATTTCATCATCAAAGAAATCAAGACGATAAGGCAGCTCACTC
CCCATCGGGAAGAGATCCAGCAACGCGCCGCGCGTGGCGTATTCGCCGTGCTCCATCACCTGGTCAACATGGCGATAACC
GGCGCTGTCCAGTTGGGTTCGTAATGCATCTCGTGACAGGCGCTGACCTTTTTTCATCACCAGCGCATGACCGTGGAGAA
AACTGTGTGGGCAAACGCGCTGCATAAGCGTATTCACCGGAACAATCAGTACGCCACGCTGCATCGTCGGTAGCTGGTAA

AGGGTGGAAAGGCGCGAGGAGATAATGTCCTGATGAGGCGAAAAACTGTCGTAGGGAAGAGTTTCCCAGTCCGCCAGATT
CATCACCATTTGATCGGTAAACTGGCTGATTTCATCATGCAAACGCAGAGCATTTTGCATATCTGGTGCAATGAGTACCA
CCGGACCGGCGTGACGTTCGGCAATTTCCGCTACCAGCGTTGCACAGGCTGCGCCGGTTAACTCGCCCAGCAGACGCTGC
TCACCCGCTTTGACGGGCAGCGTATAACGATATTGTTCAGGCATAACGGTTGTCAGATTCTCGTTAGGATATGCCTCAAC
ATATGGGGGCATATCTCTGATAAGCAATGCTGTTCATTATCCGTAATCGACATGGTTTAGCAAATTGAATCGCCCCGGCT
GGGGCGATGGCTTAGCGGGAGTAACTGACGGCAGCGGGCAGTGAAGGCGGGGAGAACAACAGATCGCTGAATGCACGCTG
TGAAAGTTTTCTTACCAGCAAGCCCGCAACAGTACAGATGAACAAACTGACAAAGGGATAAACCAGCAGGAGTGTCAGTT
CGACTTGCGGCGACCAGCGTGCTGCGTTCATTTGCGCAAGCAGAGTTAAGCTGAATATTTCAACCAGAATGCGATGGGTG
GTGTAGATAGCAATGGTGTTGGAGCCAATCACATTCAGCAGGCTGGTGGAGCGCATACCGAATCGTTGCTCGTATTGATA
AAACAGCTTCATGATCACCACAATCGATACCAGCGACAACAGCAGCGAGATAGTAAACAACCAGGCACCGACCGCCAGAA
CGGTAAGCAAAGAAGCCATCAGCAGATGGCGGCGCAACGGCACCTCTTTAACACAGGTCATTATTGTTGCGCCAAACCAT
GCGCCAAGGCTGTAATAAGGCAAATTGCGGATCACACTGTTCATTCCCCACCACGGCGTGGGAACGAAATTAACCGCCAC
ACTCAGCAGTACAAACAAGGCGAATAGTGGCAGCGCCAGACGGCTAAAAATTTTACATACCACGAAATAGACAATTAACG
CATACAGATACCACAAGCTGGTGCTGGCGGTGATCATCCCGTGCAGGAACTCACCGGTGGAATCGGCATAAGCGGCATTG
GAGGCATTGCTTAAATCGCGCTCAGGTGCCAGCCACTGGTTCAGCGCACTTAACGCCAGCCACTGCACCACGCCCCAAAG
CGCCAGCACCCAGAAGATGTTCCAGATCCGTTTATCGAGACAATTTCCCCACGGTACGCTGTCGATATAGCGGCGAATCA
AATAGCCGGATATAAAGAAAAAAACCGGCATACGAAAGGGGGCAAGGTAAAGATTGAAATAGATCCAGCATTTGCTCAGG
ACTTCCGATAACGGATGCTGAAAAGTGGTCAGATGCGGATAAAAGGTAATGACCGAGTGATAAATCACCACCAGACAAAT
ACATAACCCTTTGATCTGGTTAATCCATAGCTCTTTTTGTTTCATCAGGACAACACGCTCTTTTTTAATTTACATGCGGT
TGATCCTGCGGGTTATGACCCGAGGTGTAATGGGTATCTGTCTGTATTGGATGATTTTTCAGATTAAGATCAGGCGGCAA
GATTGATGATAAAACATGGCAATTTAGCCGATTGATTTACGGGGGCTTTTCAGATTAGCCCTGACGATCACTTACAGTTC
AGACGTTTACCCATCTTGCTTTCGCTTATATACTCGTGTCTTTGCTACAGCAACCAGACGGATTTCATGTACCAACCTGT
CGCTCTATTTATTGGCCTGCGTTACATGCGTGGGCGTGCAGCGGATCGCTTCGGTCGTTTCGTCTCCTGGCTTTCTACCA
TCGGCATTACCCTCGGGGTGATGGCGCTGGTCACAGTATTGTCAGTGATGAACGGCTTTGAGCGCGAGCTGCAAAACAAC
ATCCTTGGCCTGATGCCACAGGCAATTCTCTCTTCTGAGCATGGCTCTCTTAACCCGCAGCAACTCCCAGAAACGGCAGT
CAAACTGGACGGCGTTAATCGCGTCGCACCTATTACTACCGGTGATGTGGTACTGCAAAGCGCGCGCAGCGTGGCGGTCG
GGGTGATGCTCGGTATCGACCCGGCGCAAAAAGATCCACTTACACCGTATCTGGTCAATGTGAAACAAACTGACCTCGAG
CCGGGGAAATATAATGTCATCCTCGGCGAACAACTTGCCTCACAGCTAGGCGTTAATCGCGGTGATCAAATCCGCGTGAT
GGTACCATCTGCCAGCCAGTTCACGCCGATGGGGCGTATTCCAAGCCAGCGCCTGTTCAATGTGATTGGCACTTTCGCCG
CCAACAGTGAAGTCGATGGCTATGAAATGCTGGTGAATATTGAGGATGCCTCGCGTCTGATGCGTTATCCGGCAGGCAAT
ATTACCGGCTGGCGTTTGTGGCTGGATGAGCCGCTGAAAGTCGACTCATTAAGTCAGCAAAAACTGCCTGAAGGCAGCAA
ATGGCAGGACTGGCGTGATCGTAAAGGCGAGTTGTTCCAGGCCGTACGCATGGAAAAAAATATGATGGGTTTACTGCTGA
GCCTGATTGTCGCCGTTGCGGCGTTTAACATTATTACCTCACTAGGGCTGATGGTAATGGAGAAGCAGGGCGAAGTAGCG
ATCCTGCAAACGCAAGGCTTAACTCCGCGACAAATCATGATGGTCTTTATGGTGCAAGGGGCCAGCGCCGGGATTATCGG
TGCGATCCTCGGAGCGGCGCTTGGCGCCCTGCTTGCCAGCCAGTTAAATAATCTGATGCCGATAATCGGCGTCCTGCTTG
ATGGCGCGGCGCTGCCGGTGGCTATCGAACCTTTACAGGTCATTGTTATTGCGCTGGTGGCGATGGCTATCGCGCTGCTG
TCTACGCTTTACCCTTCATGGCGCGCTGCCGCCACTCAACCCGCTGAGGCTTTACGTTATGAATAAGATCCTGTTGCAAT
GCGACAACCTGTGCAAACGCTATCAGGAAGGCAGTGTGCAAACCGATGTGCTGCACAATGTCAGTTTCAGCGTCGGCGAA
GGTGAAATGATGGCGATCGTCGGTAGCTCTGGTTCCGGTAAAAGTACCTTGCTGCACCTGCTGGGCGGGCTGGATACGCC
AACCTCCGGCGATGTGATTTTTAATGGTCAGCCAATGAGCAAACTGTCGTCGGCAGCGAAAGCTGAACTGCGCAACCAGA
AGCTGGGCTTTATTTATCAGTTTCACCACCTGCTGCCGGATTTTACCGCCCTGGAAAACGTGGCTATGCCGCTGCTGATT
GGCAAGAAAAAGCCCGCTGAAATCAACAGCCGTGCACTTGAGATGTTAAAAGCGGTGGGGCTGGATCATCGTGCGAATCA
CCGCCCATCTGAACTTTCTGGCGGCGAACGCCAGCGTGTGGCGATTGCCCGTGCGCTGGTGAATAACCCGCGCCTGGTAC
TGGCGGATGAACCTACCGGTAACCTCGATGCGCGTAACGCCGACAGCATCTTCCAGTTGCTTGGGGAATTGAATCGCTTG
CAGGGCACCGCCTTCCTGGTGGTTACTCACGACCTGCAACTGGCGAAACGTATGAGCCGCCAGTTAGAAATGCGTGATGG
TCGTCTGACGGCGGAACTGAGCCTGATGGGGGCGGAGTAATGGCGATGCCTTTATCGTTATTAATTGGCCTGCGTTTTAG
TCGCGGACGGCGGCGCGGCGGCATGGTGTCGCTGATCTCCGTTATTTCTACCATTGGCATTGCCCTCGGCGTGGCGGTAT
TGATCGTCGGCTTAAGCGCGATGAACGGCTTTGAACGCGAACTGAACAACCGTATTCTGGCGGTGGTGCCGCATGGTGAA
ATCGAGGCGGTGGATCAGCCGTGGACTAACTGGCAGGAAGCACTGGATCACGTGCAAAAAGTGCCGGGTATTGCCGCTGC
CGCGCCGTATATCAATTTCACCGGGCTGGTGGAAAGTGGCGCGAATCTTCGCGCAATCCAGGTGAAGGGCGTTAACCCGC
AACAGGAACAGCGTCTGAGCGCATTACCCTCGTTTGTCCAGGGCGATGCGTGGCGCAATTTTAAAGCGGGCGAACAGCAA
ATTATCATCGGCAAAGGCGTGGCGGATGCGCTGAAAGTGAAGCAGGGGGATTGGGTGTCGATTATGATCCCCAACTCGAA
TCCCGAGCATAAACTGATGCAGCCAAAACGTGTGCGTTTGCACGTTGCCGGTATTTTGCAGTTGAGTGGTCAACTCGATC
ACAGTTTTGCCATGATCCCGCTGGCGGATGCCCAACAATATCTTGATATGGGTTCCAGCGTGTCAGGTATTGCCCTTAAA
ATGACGGATGTTTTCAACGCCAATAAGCTGGTACGCGATGCGGGTGAAGTGACCAACAGCTATGTTTATATTAAAAGCTG
GATTGGTACTTACGGCTATATGTATCGCGATATCCAAATGATCCGCGCCATTATGTATCTGGCGATGGTACTGGTGATTG

GCGTGGCCTGTTTCAACATCGTCTCCACCTTAGTGATGGCGGTGAAAGACAAGAGTGGCGATATCGCAGTATTAAGAACG
CTGGGGGCGAAAGATGGTTTAATTCGCGCCATCTTTGTCTGGTATGGATTGCTGGCAGGGCTGTTCGGCAGCCTGTGTGG
TGTGATTATCGGCGTAGTGGTTTCACTGCAACTTACCCCGATTATTGAGTGGATTGAAAAGTTGATCGGTCATCAGTTCC
TCTCCAGCGATATCTATTTTATTGATTTCCTGCCATCGGAATTGCACTGGCTGGACGTCTTCTACGTACTGGTCACAGCA
TTGTTGCTGAGTCTTTTGGCAAGTTGGTATCCGGCGCGGCGCGCCAGTAATATTGACCCTGCGCGAGTCCTTAGCGGCCA
GTAAAGGCAGTACATTAAAACAAGGAGCGGCAATGTATTACGGGTTTGATATTGGTGGAACAAAAATTGCGCTTGGCGTG
TTTGATAGCGGTCGGCAGTTGCAGTGGGAAAAGCGGGTGCCGACACCGCGTGACAGCTATGACGCATTTTTAGATGCAGT
GTGTGAGCTGGTAGCTGAAGCTGACCAACGTTTTGGCTGTAAAGGTTCTGTCGGCATCGGTATTCCGGGAATGCCGGAAA
CAGAAGATGGTACGCTGTATGCCGCCAATGTCCCCGCTGCCAGCGGTAAACCGCTGCGTGCCGACCTGAGCGCACGTCTT
GATCGCGATGTACGCCTTGATAACGATGCCAACTGTTTTGCCCTTTCAGAAGCCTGGGATGATGAATTTACTCAATATCC
ACTGGTGATGGGGTTGATTCTCGGCACCGGTGTTGGCGGCGGGCTGATTTTCAACGGCAAACCGATTACCGGTAAAAGCT
ACATTACCGGCGAGTTTGGCCATATGCGTCTGCCGGTTGATGCGTTAACCATGATGGGGTTGGATTTCCCGTTACGCCGC
TGCGGCTGTGGTCAGCATGGCTGCATTGAAAATTATCTGTCTGGTCGCGGTTTTGCGTGGCTGTATCAACACTATTATCA
TCAACCGTTGCAGGCTCCCGAAATTATTGCGCTTTATGATCAAGGCGATGAGCAGGCAAGGGCGCACGTTGAGCGTTATC
TGGATTTATTAGCGGTTTGTCTGGGAAATATCCTGACCATCGTTGACCCTGACCTGGTCGTCATTGGTGGTGGCTTATCG
AATTTCCCGGCAATCACAACGCAACTGGCGGACAGGCTGCCTCGTCATCTCTTACCTGTAGCTCGTGTTCCGCGCATTGA
ACGCGCGCGCCACGGTGATGCGGGAGGAATGCGTGGTGCGGCCTTCCTACATCTAACCGATTAAACAACAGAGGTTGCTA
TGCTGTCGCGTCGGGGTCATCGGTTAAGTCGTTTTCGTAAAAATAAACGCCGCCTGCGCGAGCGTTTGCGTCAGCGTATT
TTTTTCAGAGATAAAGTGGTGCCGGAAGCAATGGAAAAACCAAGAGTACTCGTACTGACAGGGGCAGGAATTTCTGCGGA
ATCAGGTATTCGTACCTTTCGCGCCGCAGATGGCCTGTGGGAAGAACATCGGGTTGAAGATGTGGCAACGCCGGAAGGTT
TCGATCGCGATCCTGAACTGGTGCAAGCGTTTTATAACGCCCGTCGTCGACAGCTGCAGCAGCCAGAAATTCAGCCTAAC
GCCGCGCATCTTGCGCTGGCTAAACTGCAAGACGCCCTCGGCGATCGCTTTTTGCTGGTGACGCAGAATATCGACAACCT
GCATGAACGCGCAGGTAATACCAATGTGATTCATATGCATGGGGAACTGCTGAAAGTGCGTTGTTCACAAAGTGGTCAGG
TTCTCGACTGGACAGGAGACGTTACCCCAGAAGATAAATGCCATTGTTGCCAGTTTCCGGCACCCTTGCGCCCGCACGTA
GTGTGGTTTGGCGAAATGCCACTCGGCATGGATGAAATTTATATGGCGTTGTCGATGGCCGATATTTTCATTGCCATTGG
CACATCCGGGCATGTTTATCCGGCGGCTGGGTTTGTTCACGAAGCGAAACTGCATGGCGCGCACACCGTGGAACTGAATC
TTGAACCGAGTCAGGTTGGTAATGAATTTGCCGAGAAATATTACGGCCCGGCAAGCCAGGTGGTGCCTGAGTTTGTTGAA
AAGTTGCTGAAGGGATTAAAAGCGGGAAGCATTGCCTGATGCACTACGCTTATCAGGCCTGCAAGGGGACGCAATTAATT
GAATTTGCGGGTTTTGTGGGCCGGATAAGGCGTTCGCGCCCCATCCGGAATAATACACGCGCACTTTTCCGGCCTCTTTT
ATCGTTTACTCGTCAATAACAATGCGGCAATAAACGTACAGACACCAATTAGCCCTGCCCAGAGTGGCGATATTCTGGTA
ATCGCAAAGCTATTATCGATATACAAACATAAAGCCTGATAACCCATATTGGCAATAGTCATCGCCACTGCAAATTTTAA
CGCATAAATAAAACGAGTCGTGTAGTAAAAGATCCTGGAAAATAAGATAAACGTCGCAGCCCAGGCGATAAAACCTAATG
TGTTGGTGCTGCCGGGGGGAGCATTTTCATGCTGTTGTCCTGGATGCGGATAATACGTTAGTGGCGGCGTCGATTGTTTT
AATATCTGTGCTTGTAGAACAGCCAATGCATTATTGATTCCCTGATACCAGAGATCTGCATTTAAATAAGCCAGCGTGTG
ATTTTCTATCAGAAGGCCGAGATGTTTATCGTCCAGACCACTTTCAACCTGGCCGTTGGTGATCATAATTAACCGTGGTT
CGCGGGTAGTAAATAGTAACAGAACACTTCCTTTTACATCACTCAATGAAGATGACGCATGCGCCAGCATCGCTTTACCA
TTTTGTCTGAGACTGGCTTCATTATTAATGTCGGGTAAAACCACAATAAACGGTGCAATTCCCGTTTGTTGGCGAAAAAG
CTCGGCTTTATGCGCGATCTCTTCAGCATCTGGTACAGTACCGGTAACGTCCGTAAAAAGTGTCTTCATTATTGGAATTA
ATGGCTTGCTGGCTGCTGTCAGAGGCAGGAGTATCAGTAAACTGAGCAGTATAATGAATTTTTTCATGGGCGGGACTTCC
GTTTCCTGCGTGATTTTTTAGAGGAGGGCATACTTATTAATAAACCGACGGCGATAAAAATAACGCCCATAATGCCGATA
AACCCCCATTTAAAAAAATTAACCCACGGGTTATTTATCTCAGCCTTTTCAGGATGCCCGGGAGAATAATAAACATTAAC
TTTATCGCCTTCGCTGTCTTCATAAAAATCGCTGCCGATACTCGAATTGAAAATTAGCGTGTAATCTGGCGTCGGTCGAA
AGGCAACGACGGGATACCACGAACCATCTTTACCCGTGCGATGAGAATGACTGGAGTGCCAGACGGTATCAACAATGACG
CCTTCGGTATGAATCGCGTCTTTGCTATAGGTGAACTGGTTATAAAAGACAACAACAGAAATGAGGATCAGTACCAGACC
GATACCGAGGAATATACGAAAGAAAACTTTACTGTCCTGACTCATTAGCCACATCCTTGCTAACTAAAAAACGGGCGGTA
ATACCACCGCCCGCTTGCTGAATTAACGTCCTGCTTTCAGCTTCTGATAATACTCTTCATAAATGCTGCTGGCTGCGCCA
ACGTCATTCTGCCATTCGCCATTTTTAATGGTTTCAGCATCCGGGTAGAGTGTTTTATCGTTCGCCACTTCTGGACTTAA
CAGCTTACGCGCCGCAAGGTTTGGCGTTGGATAACCGATAGTTTCAGCAACCTGTTTTGCCACATCCGGGCGCAGCAGGA
AGTTGATCAATTTCAGCGCGCCTTCTTTGTTTTTGGCATTTGCCGGGATCGCCAGGCTGTCCATCCAGAAAATGCCGCCT
TCTTTCGGCCACACCACGTCAATTGGCGTACCCGCCTGGCGTGCAACGAAAGCAGAACCGTTCCAGATCATGCCGAGGTT
AACTTCGCCTTCCATGTACGGGTTCGCCGGGTTATCGGAGTTAAAGGCTGCCACGTTTGGCATCAGTTTTTTCAGCTCGT
TATATGCAGCTTCAATCTCTTTCGGATCGGTGGTGTTACCGGAGTAGCCCAGCTTACGCAGCGCCATCTGGAACACTTCA
CGGGCATCGTCGGTCAACAGCAGGCTGCCTTTGTACTCTGGCTTCCACAGATCGGCCCAGCTGGTGACAGATTTCGGATC
CACCGCATCACCGTTAACACCAATCGCCGTCGCACCCCAGATATACGGAATGGAGTAGTCGTTATTCGGGTCAAAAGGCT
TGTTGAGCATGTCTGGATCGAGATTGCTGAAGTTTGTTAACTTCGACTTGTCGATCTTCTGGATCATCCCTTCTTTACGC
ATTTTATCAACGTAATAGGTTGAAGGAACCACCAGATCATAGGCACCGTCTTTGTATGTTTTCAGCTTGGCGTACATGGT

TTCGTTCGACTCGTAAGTCGAATAGATAACCTTAATACCGGTTTCTTTGGTGAACTGTTCAAGCAGTCCTGGCGGCACGT
ACTCGGTCCAGTTGTAGAAATACAGCGTGTTGTTGTCATCGGCGTGAGCGGCGCTCATGCCCAGTGCCAGAGCACCCGCC
GCGAGCAGGTGGCGTGACCATTTTTTCATTTAACGTCCCCTGTGTTACCTTTCGTTTTATCACGAGCAATAAGCTGGCTG
GCAATAACCATCACCAGCGACAGCACCAGTAATATGGTTGCCAGCGCGTTAACTTCCGGCGATACGCCGACTTTGACCAT
CGAATAAATTTTTAACGGCAGAATTTCATAACTTGGTCCGGTGACGAACGAAGAAACTACCACATCGTCCATCGACAGGG
TAAAGCTTAACACCCAGCCCGCCGCCACCGCTGGCATTGCCAGTGGCAGAATGATTTTTCGCAGAATGGTAAATTCGCTG
GCACCGAGATCTTTCGCCGCTTCCAGCATTCGCACGTCAAAACCTTTCAGGCGCGAATACACCGTCACCACCACAAATGG
CAGGCAGAAGGTGATATGCGAGAACAGCAGCGACCAGAAGCCAAGCTGAATACCCAGCAGCATAAACAGCACCAGCAGCG
AAATCGCCATCACGATATCTGGTGACATCATCACCACAAACAGCATTCCGCTAACGAACGGCTTACCACGAAAGCGATAA
CGGTACAGCGCAACTGCCGTCAGTGAACCGATAAGCGTAGCAAACGTCGCCGAAAACACCGCCATTGTTAGTGAATGCTG
CGCTGCCTGTAACAGGCTGTCGTTGTTCATCAGCAGGCTATACCATTTGGTGGTAAAGCCCTGCCAGTTGATGCCAAAGC
GCGAGCTGTTAAAGGAGTTCACAATCAAAATAATGATTGGGATATACAGGTACGCGTAGATAGCGGTCATAAAACCGCCG
CGAAGCAGTCGACCGATCATTCGAGTTCCACCTTCTTATTCAGCAAACGAGAAGCGCGCCAGTAAACCAGCAACATCAGG
CCCATTACGATAGTCAGCGTAATGCTGGTAGCTGCACCAAACGGCCAGTCACGAATATTAAGGAACTGGACCTTGATGAC
GTTACCGATCAGCAGGTTTTTCGCACCGCCCATCAGGTCGGATACATAGAACAGGCCCATCGCTGGCAGCATCACCAGCA
GACATCCGGCAATAATTCCCGGCATCGTCAGTGGAATAATGATACGGATAAAAGTCTGTAACTTGCTGGCACCGAGATCG
CGCGCCGCCTCAAGCAACGGCTTATCCAGTTTTTCGATACTGGAGTACAGCGGCATCACCATAAACGGCAGCAGAATGTA
AACCAGACCGATAATCACCGCACTGGGCGTGAACATGATACGGATTGGTGTGTCGATAACGCCCAGCCAGAGCAAAAACT
CGTTGAGATAGCCTTTGGTGCTGAGGAAAATTTTCAGCCCGTAGATACGAATTAATGAGTTGGTCCAGAACGGAACAATC
AGCAGAAACAGCAGCAGCGGACGCACCTTGTGTGGCAACTTCGCCAGAAACCAGGCAAACGGGTAGCCCAGCACCAGGCA
GGCGAGGGTGGCGATCAGCGCCATATTCAGCGAGTGCAATAGCACTTCAAAATAGAGCGGATCGAGCAGACGCGTGTAGT
TATCCAGCGTAAAGACCATTTTGACAAAACTGGCGTCGTCGCGGGTCAAAAAGCTGGTGCCAATGATCATCAGGTTGGGC
AGAAAGACAAACAACACAAGCCAACCGACAATAGTGACAATCACTACATTCTGGAACTTACTTGTGTTCTTCATCAGCCA
GTACGACCTCCCAGCTTTCTACCCAATTAATGGCCATTTTTTGGTCGAGAGAGTGGTCAAAGTCAGGATCGTCTTCATTG
AAGAATTCGCTGACCATCACCATCTTGCCATTTTCCAGTTCAACAACCGACTCCAGCGTCATGCCTTTGTAGTTACGCTC
GCGAACGTAACCAATCAGCCCTTCAGCGTGGTTGTCGTCGTTAATCTCTTCAACACGTAAGTCTTCCGGGCGCAGCAGAA
CATGCAGTTTTTGCCCCGGTTCAACGGCGAAGTTAACGTAGATATTACATTCGCGGCCTTCAACGTTGGCGCGTACGCGC
TGCTCGTCTAGACGTTCGATGACAGTGGCGTTAAACATATTGATTTCGCCAATGAAGCCGGCAACAAACAGGTTTTTCGG
CTCTTCGTAGATTTCACGCGGCGTGCCGTCTTGCTCAATGCGACCATCGCGCATCACCACAATCCTGTCTGACATGGTGA
GTGCTTCTTCCTGGTCGTGAGTCACAAAGACGAATGTAATGCCAAGCTTACGCTGTAACGCTTTCAGCTCGTTCTGCATT
TGCTTACGCAGTTTGTAATCCAGCGCTGAGAGCGACTCATCCAGCAACAACAGACGAGGCTTGTTAACCACCGCGCGAGC
AATGGCGACGCGTTGCTGTTGACCACCAGAGAGCTGATGCGGTTTGCGTTGAGCGAAGGTTTCCAACTGCACCATCCGCA
GGGCTTCCATCACGCGGGGCGTAATTTCAGCAGCGGGGGTTTTTTGCATGCGCAACCCAAAGGCCACATTTTCGAACACG
GTCATGTGGGGGAAAAGTGCGTAGCTTTGGAAAACAGTGTTCACATAGCGGTTTTCCGCCGGAACGTGGGTGATGTCCTC
GTTATCCAGCATGATGCGTCCGGAATCAACAGTTTCCAGACCTGCAATCAGGCGAAGAACGGTTGTTTTACCGCAGCCAG
AAGGGCCAAGCAGCGTGAGGAACTCGCCATTGTTGATAGTCAGATCCAGCTGGGGAATGACCTCTTTACCATCAAAGCAT
TTGCGAATTCCCGCCAATTGCACCAGCGGTGAAAGCGAACTCGGTTGTTTATTCAATTTTTTACTCTGTCCCATGTAAAC
GCAACGGATGGCTTACCGATGCGGGGTTTGTGGTTAACCACCTTGGTGACTCTTAATGAGGGCGGTAATTCTACGGCAAA
CCGCTTGAATCGCCAATCTTTGTTGTGAATTACTGGCTTAGCTTTATATTCATTAAGGTAATGCTGATAAATATTCCCGC
TTGCAGGGGTAAAAGTGACCTGACGCAATATTTGTCTTTTCTTGCTTCTTAATAATGTTGTCACAAAAAGTGAGGGTGAC
TACATGGATAAACTACTTGAGCGATTTTTGAACTACGTGTCTCTGGATACCCAATCAAAAGCAGGGGTGAGACAGGTTCC
CAGCACGGAAGGCCAATGGAAGTTATTGCATCTGCTGAAAGAGCAGCTCGAAGAGATGGGGCTTATCAATGTGACCTTAA
GTGAGAAGGGCACTTTGATGGCGACGTTACCGGCTAACGTCCCTGGCGATATCCCGGCGATTGGCTTTATTTCTCATGTG
GATACCTCACCGGATTGCAGCGGCAAAAATGTGAATCCGCAAATTGTTGAAAACTATCGCGGTGGCGATATTGCGCTGGG
TATCGGCGATGAAGTTTTATCACCGGTTATGTTCCCGGTGCTGCATCAGCTACTGGGTCAGACGCTGATTACCACCGATG
GTAAAACCTTGTTAGGTGCCGATGACAAAGCAGGTATTGCAGAAATCATGACCGCGCTGGCGGTATTGCAACAGAAAAAA
ATTCCGCATGGTGATATTCGCGTCGCCTTTACCCCGGATGAAGAAGTGGGCAAAGGGGCGAAACATTTTGATGTTGACGC
CTTCGATGCCCGCTGGGCTTACACTGTTGATGGTGGTGGCGTAGGCGAACTGGAGTTTGAAAACTTCAACGCCGCGTCGG
TCAATATCAAAATTGTCGGTAACAATGTTCATCCGGGCACGGCGAAAGGAGTGATGGTAAATGCGCTGTCGCTGGCGGCA
CGTATTCATGCGGAAGTTCCGGCGGATGAAAGCCCGGAAATGACAGAAGGCTATGAAGGTTTCTATCATCTGGCGAGCAT
GAAAGGCACCGTTGAACGGGCCGATATGCACTACATCATCCGTGATTTCGACCGTAAACAGTTTGAAGCGCGTAAACGTA
AAATGATGGAGATCGCCAAAAAAGTGGGCAAAGGGTTACATCCTGATTGCTACATTGAACTGGTGATTGAAGACAGTTAC
TACAATATGCGCGAGAAAGTGGTTGAGCATCCGCATATTCTCGATATCGCCCAGCAGGCGATGCGCGATTGCGATATTGA
ACCGGAACTGAAACCGATCCGCGGTGGTACCGACGGCGCGCAGTTGTCGTTTATGGGATTACCGTGCCCGAACCTGTTCA
CTGGCGGTTACAACTATCATGGTAAGCATGAGTTTGTGACTCTGGAAGGTATGGAAAAAGCGGTGCAGGTGATCGTCCGT
ATTGCCGAGTTAACGGCGCAACGGAAGTAAGCGAAAAGGGATGCGGCATGTGATGCCGCATCCGGCTTAAATCCAAACTT

ACCCTTCGAAGAACCAATACCCGCTATTGACCAGCGCCGCGAGCATCGCGAGGAATGACGGATCTTCCAGCGCATCGCCA
AAATTCTCCGCAGTCAGCGCAATGTTGCTGGCGAGTGCATCCAGTGCCGGACGGTGCGGGGAATCGATCTTCTCACCATT
GGCATACACGTCGTCGCCAATGCGCAATACGCGCAGACCACCCAGGCGCACCAGCACTTCACCTTGTTTCAGCGCATCGT
AGATTTCATCCGGCTGATAAGGCGGCTCTGGCGGCGCGATATCCAGTTCATGACGTGACTGGGATATAAACTCGCCAAAC
CATTGCTTAAAGTGTTCCGGCTGGTTGATCAATTCGAGCATCATCTCACGCAGTTTATCCATCTCTTGCGGCAGAACATC
CGCAGGATGAGCGCGAGGTGGAACATCCGGATCGCTGTAGTAGTTGCCGCCCAGTTCACGTTGCAGCACATAATCGGCAA
ATCCACTAATCAGTTCCCGCGTATTTGGCGCGCGAAAGCCCACGGAATAGTTCATCGCATTTTCCAGCGCGTAGCCTTCA
TGCGGGAATCCTGGCGGAATATAAAGAATATCACCAGGCTCCAGCTCTTCATCGATGATGGCTTCGAACGGATCGACCTG
TAACAGATCCGGATGTGGGCAGTGCTGTTTCATTTGCAGCTTTTCGCCCACTCGCCAGCGACGACGTCCGGTACCCTGAA
TGATAAACACGTCGTACTGATCGAGATGCGGGCCGACGCCGCCGCCGGGTACAGAAAAAGAAATCATCAGATCATCAATA
CGCCAGTCCGGTAGTTCACGGAACGGTCGCATCAGCGCGGCGGTCGGCTCATGCCAGTGGTTCACTGCCTGTACCAGTAA
TGACCAGTTGGTTTCACCGAGATGATCGTAGCTTTCGAACGGGCCGTGGCTGACCTGCCATTTGCCATCCTGGTGACTGA
CCAGTCGACTGTCAACTTCGCTTTCCATCGCCAGACCCGCCAACTCGTCTGGAGAGATCGGGTCAATAAAATTATTAAAG
CCGCGTTTTAACACCACCGGGCGTTTCTGCCAGTGACGTTCAAGAAAATCGGGCCAGTTAAGAGTGAGTTGGTATTCCAT
GTTAAGCATCCACAGGCTGGTATCTGCAACCGATTATAACGGATGCTTAACGTAATGCGTGAAGTATGGGCATATTTATT
CATCTTTCGGCGCAGAATGCTGGCGACCAAAAATCACCTCCATCCGCGCACCGCCCAGCATGCTCTCTCCGGCGACGATT
TTACCCTCATATTGCTCGGTGATTTCGCGGGCTACCGCCAGCCCTACACCTTGCCCAGGGCGTAAAGTATCAACCCGTTG
ACCACGGTCGAAAATGACCTCTCGCTTGCTTAATGGAATACCGGGGCCATCATCCTCGACCACAATATAGAGATGCTCGT
CGGTTTGCCTTGCAGAAATTTCGACAAACTCGAGGCAATATTTACAGGCATTATCCAGCACGTTGCCCATCACCTCGACA
AAATCGTTCTGCTCACCGACAAAGCTGATCTCTGGCGAAATATCGAGAGAGATATTGACCCCTTTGCGTTGATACACTTT
GTTCAGCGCTGAGGTGAGATTGTCCAGCAGTGGGGCGACCGGATGCAGCTCGCGGCTGAGCAATGTCCCGCCGCGCATAC
TGGCACGATGCAGGTAGTAGCCAATTTGCTGTGAAATGCGGCTGATTTGCTCCAGCATTACCGGCTCAGCATCACTGACG
CTCATCTTTTCACTACGCAGAGAACGCAGCGTACTTTGCAGCACCGCCAGTGGCGTTTTCAGACTATGGGTCAGGTCGGT
GAGCGTCGTACGGTATTTGTCGTAACGTTCGCGTTCACTTTTTAACAATCGGTTCAGGTTTCGTACCAGACTGGTCAGTT
CTCGCGTTGTGGCTGGATTGAGCAATTCGCGGTTATGTTCTTCCAGTTCGCGGACTTCTTTTGCCAGGGCTTCGATGGGG
CGTAAACTCCACCAGGCGGCGACCCACAGCAGCGGGATCACTAACAGCAGATTGGCTGAGAGCACATAGATAAACCAGCT
CCAGACCATATAGGAACTTTTTAGCTCCACCGGAATGGTATCCACCACCACAATGGTTAATTTTGGCATCCGCGATGTTG
CCGGGTAGACGTTTACTGCCACCGAGTGGGTCATCTCCGCGTCGTCATCATCTTCCCGCACTTCCTGCAACTGTTGCTGT
ATCGAATGATCTCCACTCAGCAAGAGGCTGGTATCGTTAACATCCGCTTCAATTTCATGAAAACCATTCGATTTCAGCCA
GTCAGGCTGGATCATCTTCATCAGCCAGGGCACGTCACGTTGCGCCCATAAAAGCTGCCCGTTCTCATCATAAATTAGCG
TCATGGTGGGGCTTTGCTTGTCGATATTTTCGGGTAACTCGACATGCAACTTATTGTTTTCCCACTTCGCAAGGGTATAG
AACAGATTGCTCTCGCCACGTAACAGCCGAAACGTAGTTTTATCGAAACTGACGCTATAACCGATCAGCGCGACCATTCC
GTAGGCAAGCGAAAGCACCAGTACTACCGCTGCCGTTGCCAACAGAAAACGTACCCGCAGCGAGAGCGGGAAAAAAAGAC
GCAGTAATTTTTTCATCAGCGCAATTCGAACAGATAGCCCTGGCCGCGAACGGTGGTAATCACTTCTTGGGGATATTGTG
CCTGAATTTTTTTGCGCAGACGTCCCATCAGTACATCAATGGTATGGCTTTCCCGCAGCTCCGCATCCGGATAGAGTTGG
AGCATTAACGAATCTTTGCTGACCACTTTGCCATTATTGCGTATCAACGTTTCCATAATAGTGTATTCGAACGCGGTCAG
TTTGATCACTTCGTCATTAATAGATAATTCACGGCGAGAGAGATCAACCTGAAACGGGGGGAGCGAAATGACCTGTGAAG
CCAGACCGCTATTACGCCGCATTAATGCCTGCATTCGCGCCATCACCTCTTCAATATGAAACGGTTTAGTCACATAATCA
TCAGCACCGGCACTTAATACTTCGACTTTGTCCTGCCAGCTTTCACGGGCGGTTAATACCAGAATCGGCAGTGAAACATC
GTTGCTACGCCAGCGGCGAATCAGTGACAGACCGTCCTCGTCTGGCAATCCGAGATCGACAATCGCAATATCCGGTATAT
GTTCATTGAGATAATAATCGGCTTCTTTGGCATCTTCTGCGTCATCGACCTGATGACCAGCATCCTGAATCTGAACTTTA
AGGTGGTGACGTAACAACGCATTGTCTTCAACAACCAGTACGCGCATTTTTATTTCTCCCTGTCTTAATTATTAAAATAG
TGTAACGCGATTATGTGGTTATGGGGGTAAACATTAAATAAACCAGCGGGGAGGGGAGGTAAAGTGAAAAAATAAAAAGC
GGATAATCTTAATAAGCAGGCCGGACAGCATCGCCATCCGGCACTGATACGAGGTTTATTTCAGCTCATCAACCATCGTG
ATAGCTCGACCAATATAGTTAGCCGGCGTCATCGCTTTCAGGCGGGCTTTCTCTTCTTCTGGCAACGCCAGACCATCGAT
AAACTGCTTCATGCCTTCGGCGTCAACGCGCTTACCGCGAGTCAGCTCTTTCAGCTTCTCGTACGGTTTTTCGATGCCAT
AGCGACGCATAACTGTCTGGATTGGTTCAGCCAGCACTTCCCAGTTGTGATCCAGTTCATCCAGCAGATGGTCACGGTTC
ACTTCCAGTTTGCTCACGCCTTTCAGGGTGGATTGATATGCAATCAAGGCATAACCGATACCCACGCCGAGGTTACGCAG
CACGGTAGAGTCGGTCAGGTCACGCTGCCAGCGGGAAACCGGCAGTTTGCTTGCCAGATGCTGCAATACCGCGTTGGAAA
GGCCCAGATTCCCTTCGGAGTTTTCGAAGTCGATCGGGTTAACTTTATGCGGCATGGTGGAAGAACCAATCTCACCAGCA
ATGGTTTTCTGTTTGAAGTGGTTAAGGGCGATATAACCCCAGACGTCACGGTCAAAGTCGATCAGAATAGTGTTGAAGCG
CGCAACGCAATCAAACAGTTCGGCAATGTAGTCGTGCGGTTCGATCTGGGTGGTGTACGGGTTCCACTGAATACCCAGCG
AGGTGACGAACTCTTCGCTGAACTGATGCCAGTCAACTTCCGGGTAAGCGGCGATGTGGGCGTTATAGTTACCGACCGCG
CCGTTGATTTTGCCGAGGATCTCCACCTGGTTAAGCTGGCGGTACTGGCGCTCCATACGGTAGGCGACGTTTGCCATCTC
TTTACCGATGGTTGACGGCGTGGCTGGCTGACCGTGGGTACGAGACAGCAGCGGGATATCGCGATACTGAACGGCGAGAT
CTTTAATGCCATCAATCAGTTGACGCCAGTATGGCAGGATCACTTCATCACGCGCGGTTTTCAGCATTAATGCGTGGGAG

AGGTTATTGATATCTTCCGAAGTACAGGCAAAGTGGATGAATTCAGAAACCGCGTGCAGTTCCGGGATCTCCGCCACTTT
TTCTTTCAGGAAATACTCAACCGCTTTAACGTCGTGGTTAGTGGTACGCTCGATAGTTTTGATGCGCGCCGCATCTTCTT
CGCTGAAACTGGCGACGATTGCATCAAGGTAACCGATTGCGTCGGCAGCAAAAGCAGGAACTTCCTTGATCGCTGCGTGC
GCGGCCAGTTTTTGCAGCCAACGTACTTCAACTTGTACACGGAATTTCAGCAAACCATATTCGCTGAAAATCCCGCGCAG
CGCGCTGACTTTATCGCCGTAGCGTCCATCGACAGGGGAAACGGCGGTCAGTGAGGATAATTCCATAGATCACAACTCCG
GGGTTAAATGAGCAAGAATTTGTTTTGCCTGAGTGGTCAGGCGATTACGAGAAAACATCAGTTGCAGACGTCCGCCGCCG
ACCTGGTGCCAGAGCACGGCGGCGCGAATGCCTGCCAGCAGGGTTGCGCGAACTTTCGCCTGCACTTGTGGGCTTTGCAG
TACAGCAGGGGAACCGGTGACCTGAATGCGCGGGCCAAGCGGGCTAATCACATCAACATAGATAGCAGCCATCGCGCTCA
TCAGCGTTTCGGACTGTAAATCGAAGTGTTCGAGCTGGCGTTGCAGGCCGTTGATCCGGTTGCCCAGAGTGTCGAGCGCG
CCTTTCGCTGAGGAGAGTTTGCGCTCAAGCACCATCAAGCTGAGTGTGTAGCGGGTTAATTCGGCGTTTAAGCCCTGGCG
ACTGCTGGCATTGAGCACGCCGAGCAGGGTTTCCAGCCCGACGCGCAGGTTGGCTTCGCTACCGCCAAAAACCGCCAGCG
TCGAGCTGGGGTTCATATCAATAATACTGTTGAGTGAGACGTGTAGCGCATCGGCATCACAATGCCCCTGGTGAGCGAGT
TGTTGCACCAGGCGTGCCGACTGACAAATACCGGCCAGGGCGAGGGTGATGTCATAGTAATTCTTTGCCACGTTCACTGC
TTCCTTGTTTTAAGTAAAGATAATAATCAGACCGGCAGCGGCAGACGCTGCTCAATAATACCGCCACCGAGGCACACTTC
ACCGTTATAGAAGACGGCAGACTGGCCCGGCGTCACGGCGGCAACCGGTTCATCGAAAATCACTTCAATGCGATCATCGT
CCAGCGCCTTGACGGTGCAAGGGATATCGGTCTGGCGATAGCGGGTTTTTACCGTGCAACGCATAGTGCCGGTGAATGGT
TCGCGATCGACCCAGTGCAACTGCTGGGCAATCAACCCGACAGACATCAGCCGCGGGTGTTCATGGCCCTGAGCGACAAC
CAGAATGTTGTTTTCGACGTCTTTGTCCACCACATACCACGGTTCTTCGGTACCTTCTTTGGTGCCACCGATACCCAGAC
CTTTACGCTGACCGAGAGTGTGATACATCAGCCCCTGGTGCTCACCAATTTCATCGCCATCGACGGTAATGATTTTGCCC
GGTTGCGCCGGGAGATAACGGCCCAGGAACTCGCGGAATTTACGTTCGCCAATGAAGCAAATGCCGGTAGAGTCTTTTTT
CTTCGCGGTGACCAGACCAAGATCTTCAGCAATCTTACGCACCTGCGGTTTTTCCAGTTCGCCGACCGGGAACAGGCTTT
GCGCAATCTGCTCATGGCTGAGCGTATAAAGGAAGTAGCTCTGGTCTTTATTGCTGTCCAGACCACGCAGCAGGCGGCTC
TTGCCATCGACATCGGCCCGACGTACGTAATGACCGGTAGCGATATAATCGGCACCTAAATCTTCGGCGGCAAATTCGAG
GAAGGCTTTAAATTTGATCTCTTTGTTGCACAGAATATCCGGATTCGGCGTGCGACCGGCTTTATATTCGGCAAGGAACA
GTTCGAAGACGTTGTCCCAGTACTCGGCAGCAAAGTTAACGGTGTGCAGTTCAATGCCGAGCTTGTCGCAGACAGCCTGG
GCATCAGCCAGATCCGCTGCCGCTGTGCAATATTCCTCACCGTCGTCTTCTTCCCAGTTCTTCATAAACAGGCCTTCGAC
CTGATATCCCTGTTGTTGCAACAGCCAGGCAGAAACGGAGGAATCGACACCGCCGGACATGCCGACGATTACTTTTTTTG
CGGTTTCAGACATTGGATCACTCACGACATTGAACTTCAAGGCGGCGTATTCTATCACGCAGCCTTTTAGATGACACCCT
TTGTAAAAGGCCAGTTAAAATCGCCAATCATCTCCAGCGGATAACGTTGCCCGCTTTGATAACAACGAATACTTTCCGCC
ACCAGCGGCGAACGAAGATTTGACGCCTGTAAAATTTCTTCGGCGCTGACCCAACGGCAGCAGTCGATATCGCTGTCATG
AGGCTGGGTCGGGCATATTTGCTCAAGCTCAATGGCAAAGAGGAAACGCAAAAACGGCGTTTTATCTGGCGCAATCCACT
GATGCATACGAATAAAGTGTTGCGGCTGCGCGCTGATGCCGGTTTCTTCCCACAGTTCACGGGCGGCGGCTTCCACTAAG
GTTTCATCGGCTTCCAGATGCCCGGCAGGTTGGTTCCATAACGCTTTACCATTAATCGTCTCTTCAACGACTAAAAATTT
GCCTTCTGCGTGCACCACGCAAGCAACGGTAACGTGCGGTTTAAACATTTTGCATCCTTAATCTGTCACTTCTCGCCATT
CACCATTGGCAAGATTATCCAAAGAGTAATCACCCATCGCATAGCGAATCAGTCGCAGCGTGGGGAAGCCAACATGGGCG
GTCATGCGGCGCACCTGGCGATTACGTCCTTCATATAAGGTGATCTTCAGCCAGCTGGTGGGAATACTTTTGCGTTCACG
AATTGGTGGATTCCGCGGCCATAACCACGCGGGTTCGTCAACCAGCTCCGCGCCGGCGGGCAGGGTAGGGCCATCATTTA
AGGTTACGCCATTGCGCAAGGCTTCAAGTGCGTCTTGTGTGGGAATACCTTCCACCTGCACATAATAGATTTTTCCGGTG
CGTTTACCCGGCTGGGTTAAACGCGCCTGCAACGCGCCGTTATTGGTCAGCACCAGCAACCCTTCGCTATCGCGGTCAAG
GCGACCTGCTGCATAAACACCCTGAACCGGGATGAATTCTTTTAATGTTTTGCGTCCGGCTTCATCGGTGAACTGCGGAA
GAACATCGTAGGGTTTATTGAACAGGATCACACGCGTGGGCTGGTTTTCAGGTTTACGCCTGGTAGAACGTTGCGAGCTG
AATCGCTTAACCTGGTGATTTCTAAAAGAAGTTTTTTGCATGGTATTTTCAGAGATTATGAATTGCCGCATTATAGCCTA
ATAACGCGCATCTTTCATGACGGCAAACAATAGGGTAGTATTGACAAGCCAATTACAAATCATTAACAAAAAATTGCTCT
AAAGCATCCGTATCGCAGGACGCAAACGCATATGCAACGTGGTGGCAGACGAGCAAACCAGTAGCGCTCGAAGGAGAGGT
GAATGGAAAGTAAAGTAGTTGTTCCGGCACAAGGCAAGAAGATCACCCTGCAAAACGGCAAACTCAACGTTCCTGAAAAT
CCGATTATCCCTTACATTGAAGGTGATGGAATCGGTGTAGATGTAACCCCAGCCATGCTGAAAGTGGTCGACGCTGCAGT
CGAGAAAGCCTATAAAGGCGAGCGTAAAATCTCCTGGATGGAAATTTACACCGGTGAAAAATCCACACAGGTTTATGGTC
AGGACGTCTGGCTGCCTGCTGAAACTCTTGATCTGATTCGTGAATATCGCGTTGCCATTAAAGGTCCGCTGACCACTCCG
GTTGGTGGCGGTATTCGCTCTCTGAACGTTGCCCTGCGCCAGGAACTGGATCTCTACATCTGCCTGCGTCCGGTACGTTA
CTATCAGGGCACTCCAAGCCCGGTTAAACACCCTGAACTGACCGATATGGTTATCTTCCGTGAAAACTCGGAAGACATTT
ATGCGGGTATCGAATGGAAAGCAGACTCTGCCGACGCCGAGAAAGTGATTAAATTCCTGCGTGAAGAGATGGGGGTGAAG
AAAATTCGCTTCCCGGAACATTGTGGTATCGGTATTAAGCCGTGTTCGGAAGAAGGCACCAAACGTCTGGTTCGTGCAGC
GATCGAATACGCAATTGCTAACGATCGTGACTCTGTGACTCTGGTGCACAAAGGCAACATCATGAAGTTCACCGAAGGAG
CGTTTAAAGACTGGGGCTACCAGCTGGCGCGTGAAGAGTTTGGCGGTGAACTGATCGACGGTGGCCCGTGGCTGAAAGTT
AAAAACCCGAACACTGGCAAAGAGATCGTCATTAAAGACGTGATTGCTGATGCATTCCTGCAACAGATCCTGCTGCGTCC
GGCTGAATATGATGTTATCGCCTGTATGAACCTGAACGGTGACTACATTTCTGACGCCCTGGCAGCGCAGGTTGGCGGTA

TCGGTATCGCCCCTGGTGCAAACATCGGTGACGAATGCGCCCTGTTTGAAGCCACCCACGGTACTGCGCCGAAATATGCC
GGTCAGGACAAAGTAAATCCTGGCTCTATTATTCTCTCCGCTGAGATGATGCTGCGCCACATGGGTTGGACCGAAGCGGC
TGACTTAATTGTTAAAGGTATGGAAGGCGCAATCAACGCGAAAACCGTAACCTATGACTTCGAGCGTCTGATGGATGGCG
CTAAACTGCTGAAATGTTCAGAGTTTGGTGACGCGATCATCGAAAACATGTAATGCCGTAGTTTGTTAAATTTATTAACG
GGAGCGTAACGCTCCCGTTGTTTTTTGTTAGGCTGCTAACGGTTATCAAAATTTTATCAAAAAAAGTTATCAAAACCCCT
CGGTAGTTTTGGGGTAGGCTGGCCGGTCAGGTGGTAGTTCTACTACTAGTCTCCCACATAGATATTCCTTAGCTTTTTAT
TATTGCTGGCGGACGCTCGTTAATATTTAAGGTCTTCATTGATTAAGACATCCCCAAAGTTAGTTATGTATTCACTGTTA
TTAGGACAATTATGAATTACCACTCCTTACACCCGCTCAAATATTGTTAAATTGCCGGTTTTGTATCAACTACTCACCCG
GGACTCGCCAGGGGACAGCCAACAGGCATTGGGTGCAATCACCTTAGCGTTCAGGTACATGCGGAATGTAAAAAAGGCCG
CGAGCGCGGCCCCTTCACATACATCTTTAGTACTGAGACTGTTTAACCTAGGGAATTATTATCGTATTATATTGCATTAC
AACTCCCAACAGTGACATATGAACTTCCTGCTTTACTCCACGACTTTAATATTTCAAGGCCATGTGAAGAACATATTTCT
TGCACTGCGAGTTCATCGAGTAATCCATAGTAAGAAACACTTTTTGAATTTTTGTAAATGTATCCGTAAAGATGTTTTCT
TCCTGTTTCGTATTTTTTGAAGTATGAGCTTTTATATTGATTTACAATAAGAGTCTCACCTCCTGATTTTAGTAATCTCT
TGATGCTAAGAACTATTTTGTCGATTGTATCCCGACAAGGAACGGCAGAGAGAACATTAGAGCAAAGGATGAAATCGTAA
CCCCCAATTATTTTGTCGACATCCTCGAAAGCAACTGTATTTGCATTTTTATAATATCGTGGGACATAGTCAATAATTTT
AGTTTTAATTCCTCTAATAATTTGCTCTCTTTCAAGTTGCCTTTTCGAGTCTAGAAAAGTAACTTCATCAAATTTACTGA
TTAATTCATCAGAATATCTAAGTTTTCCGCAGCCAAAATCAAGGGCGTGGCCATTTTTCTCAATGCTTCTAATATACTCG
CAAAGATATCTAGAGGGCATCGTATGAGGTTTTGCTGCATTCTCTGAGCGAATATTAACTCCGTGCATATTATAGTTCAA
AGCAAGTACCATTCAATTAATGTTATTTTTAGTGAAAAATTCTTTTATTTTATCGTCCGGGATTTTTCCGACTTGACTTT
CAGTTCTGGGCTTAACTGTCAAAGCAAAGATAATACCACTCACTGAGAATGATAAAATTACAAAAAAGAATAAAGACAGC
AAAGAACCTTCAACATGAAAAATATCCATTTGTTTGCAAAAAAAGATTATTAGGAAGGAAATTAATGCAATTATCGAAAA
TTCAAAAAATATCCAAAAATAGTATACTTTATTCCAGAAGAGTTCAATATAATGTTTGTCTTCAATTTTTCTTACTTCAG
GGTAATATAGATTGCTCATTACATTGTGAGCTTCATCTTTATTTAATTTTCTGTTGACTCCAGCTCTCCGTGATAACGGT
TTTATAATTAGATGCTTATCCCAAAGATATCGCACCCGAAGTAGTTTGGCTGCATTGTTATGTAGGTCTAACGCACCGCT
AATTAAATATGCAAAAATCGCAAAAGCACAAGGTAATATACCAAAAGATAGTGCCCAATTAATAAAGCTCTCATGATCTT
TAATAGGTGGGACATACTTGGTTGGTAATGTTATTAAAGGGATGTATTCGTAAATAACTAGAAGTATGCAATATATTAGC
GTATTTAAGAAAGTTGCTTTATGAAGTTTAGGCAACATTTCATTATAATTTTTGGGGGGTTCGAACATATTATTCACCTG
AGTCACGCTTTAGGATAGTGTAATGGTAATATTTAATTAAGTGTCATTATATACTTTTCAGTAGGTTAGTTACAATTTTT
TGTATCTGTTCAGGCTGACCTAGCTTCGCTGACAGACAATATTGTGATCAGTAGCACGTATCGAGGAGGAGTAGCGCTAC
AAATTTGACGCTGGGTGAGAATCTGAAATTGATAGAAATGAAATAATGAAATAATGAAATGATGAAATGATGAAATGATG
AAATGATGAAATGATGAAATGATGACAGAGTGTCCAGTGGGCACGGATGGTGTCTTACGACATGCTTACCTTAATCGTTC
CCAGTGTGCCTATAGCAGATATTCTAAACATGTCGATAATTCATTACGCATATAGTATCGAACATAGAAAAAACTGAAGA
TTCATCTTATTTTGTATATACTACCTAGCCCAACAATGTAGAGGTTAACGAAAAATGCGCTCACCAATTTGTCATCTTTT
CTCAGCAATTAATTCATCACCATTTAAGATTGCACCAGAGAAGGAGCAAGATCTTAAAACGATAGTTGACGACAAAAAAA
TTATAATTTCAGTTGTGAGTGAACCTGGTTTTAATATCCGAGTCAGGAAGAATGAGAGTAATAATTCACATGAAATAGTT
CTAACAGTAGCTTCACTTGAATATATTTGGGCATTTTCCAATTTCTTTTGGGTTTTTACGCAAGAGTACTCCAAATCTCA
GAAAAATAATGATGAGCACTTTGATTTAACAGGAAAAAATAGGCTTAAAAAGTCTGATGAACTTCTTAAATGGGCAAGGA
AAAACTTGCAAACAACAGGTTGCGAATCATGGCCTAAAAAATGTCCCAAGCCAGAAGCATATTTACAAGGAAGCGAAGAC
TCACAAGTTGCTAGCGAGATATTTCTTTGTGCTATTGCTTGGATTCTTCATCATGAAATAAGTCATGTTGTTTTACAGCA
TCCATTGGTCACTACAGCATTCTCCACTCAAGAGGAGCGTGAAGCAGATTCACATGCTACAAAATGGATATTAGGCAACC
TGTATGAATCCGCTCCTGAATTAAAGAAACGTGCACTTGGCATTGCTACGGCAGTGCTTTGTATACAAAGCTTAGAAGTT
GAAAATTACTTCTGTTTACAAAATACACACCCAGCTGCATATGAGCGTATATATTCGAATATTTCATGCTACCCTGTCGG
AAATGAAGAGTTGATTGAAGCTCTATGTACAGTGATGCTTCAATATCTTTTCCATGGCAAAAATATCAATGTGAATCTAG
ATGGGGAGTCCTTTTCATCGATTTTAGGTGATCTTCTCTGTGATATTTCACGTCTTACCAGTAACTGATATGGCTGTCCG
CCGCTCGCTTAAAGTGGACTTTTTAGTTTTTATCATGTGCGGTGAGAAATTCAATGTGGCGTTGAGATGCTTAAAGGTTC
ACAACGCTACTTTGCTCCATCCTTTACCTCGATCATCATGATAACGATCGGTTTGTTGTTGTGTTTTATGACCAAGTAGT
TTTTGTGTGTCTAACCCCTGTTCTTTATACAGACGTTCAGATAAAGACCTTTGCTCATGGAATGTCGCAGGTGAACCCTC
TCCCCAGTCAATTCTTGCTAAATCTCTCGCTTTACTAAAATTCATCGTCAATGTATTGGCTTTAACCTGCGCTCCGCGCT
CTGCTTGTGAAGTTGAACGAAAAAAATGCACTAAGTATGCACTGACTGCATAGTCACGGCAGCGGGCTACTACATCGCGT
AAACTCCAGTTAATCGCATTGAGGCGAAGAGAAAGAGGAATTGCGATTTTGCTCCCGGTCTTTTCCTGAATGACATGAAG
ATGATCATCCCAAATATCGCTAAATTTCATACGCGAAATATCACCTAACCGCTGACCAGTAACCAGCGCTAACAGCATGG
CATTTCCCATGTAACGATGAGTAGCGTCTGCGATATCGAAGATTTTTTTCCATTCTTCAAGGCTCAGCCGTTGTCGGGTA
ATTTTTCTTCTTGGTTGTTTAGTGGCTAATGCTGGGTTATAGCCAGGAGGTACTTCTCCGTAGTGCTGCGCCTCTTTGAA
AACATCAATCAGGACGGAGCGAACTACTTGTGCCATTCTTGGCCGCCCAGCGGCGATATACTCATCAAGCAATTGTGCTA
TATCTCTGACATCAACGGCTGAGATCAACTTCATTCCTGCTCGTTCTCTGAGCAAGGATACTGGTTTAGCTTTTTGTTTA
TAGGTGTTGAGTCTTATATCACCACTTTTAAGCCTGTCATCCTGGATCGCTTGATAGCGATCTAACCAGGTTGACGTTGT

GATAGCCTTTCCTTTGCTGGTTGCGATCCTGTCACTGATAGCCAGAATCTGCCGGGTTCTTTGTTCAGCCAGGCGAGTGT
TGGCCTCAGTGGCAATAGCGATAGCTTCAGCTTCGTTTGTTCCCAAAGCATGGAATTTTCCTGTCACTGGATGCTTATAC
CGCCAATAGACTTTATTTACCTTCCTACTATAAAGCGGATATAAGTTAGGGACTGAAACATTATTCTTACGCGGTCTGGC
TGCCATTACTCAAAATCCGTTGCAAAAGTAATGAGTCATTTTTCTTGATTACAGGTGTTACCAACTCCCCAACTAACTCG
GCGTCCTCACGCACTCGCCATAACCGGCCTTGTTTCATGGCCGGTGGACAAAATAAATTCTGCTTAGCATAACGACGCAA
TGTGGACACACTTGGAGGATTACTTCTGTATTTTTCAGCAGCCCATTCTTCAAGAGTTAACATTTGAAGCATATGCGATC
ACCTTATTACTACACTAACTGCTTAGTCTCAGCATATCGACCCTGCACGGTCGGTTAGTTTCTCCACAAAACAGAGAAGA
GCACCTGTGGCCACAGCTATCAGGATGGGTCGGGTTATTAACCCGTCATCCGGGGATACTCTTCTCTGTTTTGTAAAAAG
GGCGGTACCAGAAAGGACTAAGGAAAAAACTGGTACCGCCAAGACTACACACAGCATAAAGTTGTGGTGTCGGGTGCCCC
CGGTGCCTGGCGAAGGTTGCACACCAGGCGGGTGGGTATCCACAGAAGGTCGATTGTCAGCCTCAACCTTAACCCGCGTG
CGCTGAGCCGCATTCACCACAACGCTAAGGATTCTCTCTGGTTGAAAATACTTAGCTGTTATGTGCCTGTCTTTTCACCA
CTTCAGGCTCGGTGGTATGCTGGAGTTCTCACACAGCCAGCAAGCAAGGAAACTTAATGAACCAGTTTTATGTTCACGTT
CGTCTATTTGAAGACACAGCCGAACAGACCAAAAAATTTGAAGAATTAATGCTTAACTTTCTGTACCAGAAAACAGTTAA
AGAGTCTGACGATAGCTGCTGCAGACTGATTCCAGAGGGATATATCCTCAAAAGTACAATGAACTGCCAACAAATCCTTG
ATCAAACATTTTCAATTGCTAACAGTGCCGGTGTTGACGCAAATATATTTGTCTGTAAATTTGAACAAAGCGCATGCTTA
CTTCCGTCTGCTTCCTTAGTTGGTAACGATTTCGTTCATTACGATCTTACGCCTAAGCCCATCAAGCTCGATTCTTAAAG
CCTTAACCATTGTGTCGTGATAAACACGGCTCACCTTCTCTCCATTGCATGGCAGAGGGGTGAGTGTGTTAGCCATGAAA
TTCATGAACTCGGTTCGACCAGGGGCTTGCGCCCCGCAAGTCTTTAATGCCTGTTTTGCTAACAAAATGCGGGCCTCAGT
GCCTGCATTTGGCTCTATCTGCTGCAAACGTTTAGCGTCTTCCAGCAACAATGCGATCACATGCTTCAAATTCTGCTCAT
TCATCTATTCTCTCCACTGAAATCATCCGCTAACGAATCATCCCGGTCTTCGTACGTACCGGGCGGGCTACTTCGTGGGC
GTCCTGCCTGTTTGTTGTTTCTCTTGGGTACATTATGTATCTCAAAGGTACATTGTCAAGTATAAAAAAACCTGCCGAAG
CAGGTTCATAAACATTGATTAGGCTTTGATTTTGTATCTTCTTGGTTTTCCTGAGAAAATCACAGTACCAATTATAGAGC
AATTACCGTTGATCTTAATGTAAGGCTCAGGCCAGTTTGGGTTTAACGCTTTGAGATAACGCTGTGTCCCATCTTCTATC
AACCTTTTGAAGGTGGTTTCACCTGTATCGTGCATCAATGCAATAACGTCGTCACCGTGGCAGGCAGGTACTTCAGGATC
GACAAAAATCATGTCTCCCGGGCGGTACTCATCAATCATTGAATCACCTATTACCCGCAAGATATAAGTCATTTCCCCAC
AGGGTACAGGGCAGGGATACGTTTCTGCTGTGCTCAAATCAACCTCAGAATATCCAACTTCTTTCCATGCTCCGGCCTGT
ACCCATGATATGACAGGGACTAATGTGATTTGTTTATTAGTGATTGAAACATCAGGTTTTTTTGTGATGTTCGTTGTCTG
GTGTTCTTGATCGAGCCATCCTACAGGCAGGTCGAAACATTTTTCGATGTGTCGTGCCATGCTGTCACCGATATTTTTAG
TAGCACCATCTCCCATAAACCTGCTGGTCTGGGTTGGCTCGCGATCAATCATAGTGGCAAAGGAAGAATTCCCGCCAACA
CCATCTCTCAGTTTTCTGGCGTTAGACCGCCGGATGTCATGGATTGTTTTCATAACGAAATTAAAACCCTTGTACCGTTA
AGGTACAAGTATCTTGAAGGTTCATTTCAATCATGTAATATGTACACCGGAGGTACATATTGTATGAAAGCGTATTGGGA
CTCTTTAACCAAAGAACAGCAGGGCGAGTTGGCCGGAAAAGTTGGCTCAACACCTGGCTACTTACGGCTGGTTTTCAATG
GCTATAAAAAAGCCAGTTTTGTGCTGGCTAAAAAACTTGAGCAATACACATCAGGTGCAATTACGAAATCTGACTTAAGA
CCGGATATCTATCCGAAAGATTAGCAGAACACTTTCAATTTTTAACCACAGAACGATGAGGCTAATCGTGGGTAAGCATC
ACTGGAAAATAGAAAAACAGCCTGAGTGGTACGTGAAAGCTGTCAGAAAAACTATCGCGGCGTTGCCGAGTGGTTACGCT
GAAGCGGCTGACTGGCTCGATGTAACAGAAAACGCTTTATTCAACCGCCTTCGTGCAGATGGCGATCAGATTTTCCCGCT
GGGATGGGCAATGGTTTTACAGCGTGCTGGTGGCACTCACTTCATTGCTGATGCTGTGGCGCAGTCTGCAAATGGCGTCT
TTGTGTCTCTTCCTGACGTCGAGGATGTGGACAACGCCGATATTAACCAGCGTCTGCTGGAAGTCATTGAACAGATCGGC
AGTTATTCAAAACAGATTCGTTCAGCAATCGAAGACGGTGTAGTGGAACCGCATGAGAAGACAGCAATTAACGACGAGCT
GTATCTCTCAATTTCGAAGCTGCAGGAGCATGCAGCACTTGTCTACAAAATTTTTTGCATTTCAGAAAGTAATGACGCCC
GCGAGTGTGCAGCTCCGGGCGTCGTGGCGTCGATTGCTTCTGGTTGTGGAGAAACTAACGCATGAACAGTTTAACAACAC
ACTACCGTCGCTCGCAACTGATTGCGCTTCCTGTACCGGGTGGAAAAGCGAAGGTGGAATATTGCTATGCAGTGAATGTA
CCAGGTGACAGGGAAATTGTAACCCACAGCTTTGCAGAGTGGGCTGTGGGTGATTTCAACCGGCAGAAGGAGACAGTCCT
TTGCGACAAGTTAACCGCTGGTTCAAAGATCACTACGGAGTGCCCGTCAGAGTCATTCGTTGGGAGCCGGAAACACAACG
GGTTATCTACCTCCGCGAAGGTTATGAGCATGAATGCTTCAGCCCGCTCGAACAGTTTCGTCGTAAATTCAGGGAAATAG
AGGTCGGTCATGAGCACTAAATTAACCGGCTATGTATGGGATGGTTGCGCTGCATCAGGCATGAAGTTATCCAGCGTGGC
AATTATGGCCCGCCTGGCTGATTTCAGTAATGACGAAGGTGTGTGCTGGCCATCAATTGAAACCATTGCCCGTCAGATTG
GCGCGGGGATGAGTACCGTCAGAACGGCTATCGCACGGCTGGAAGCAGAAGGCTGGTTAACGCGTAAGGCGCGTCGCCAG
GGTGATGGTTCATCACCCCACTGTGCCGTGGTGGATGAATATCACGAGCACGCCACAGATGCGCTTTACACCACGATGCT
TACCGGGATGGGGGCGCGACGCCAGCCACTGATGTGGGCCATTACCACCGCCGGGTACAACATTGAGGGGCCGTGCTACG
ACAAACGGCGGGAAGTCATCGAGATGCTCAACGGCTCGGTGCCAAACGATGAACTGTTCGGGATCATCTATACCGTTGAT
GAAGGTGACGACTGGACCGACCCGCAGGTGCTGGAAAAAGCCAATCCAAATATTGGCGTGTCGGTTTATCGCGAATTTTT
GTTAAGTCAGCAGCAGCGTGCGAAAAATAACGCCCGTCTGGCAAACGTCTTTAAAACAAAACACCTCAATATCTGGGCGT
CGGCGCGTTCGGCGTATTTCAACCTGGTGAGCTGGCAGAGCTGCGAGGATAAATCACTGACCCTTGAGCAGTTCGAGGGG
CAGCCGTGCATTCTGGCCTTTGACCTGGCGCGTAAGCTGGATATGAACAGCATGGCGCGACTTTATACCCGCGAGATTGA
CGGTAAAACGCATTACTACAGTGTGGCCCCGCGTTTCTGGGTACCGTATGACACGGTGTACAGCGTCGAGAAAAATGAAG

ATCGCCGGACAGCCGAACGCTTTCAGAAATGGGTGGAAATGGGCGTTCTGACCGTTACCGATGGTGCGGAGGTGGATTAT
CGCTACATCCTCGAAGAGGCCAAAGCGGCGAACAAAATCAGCCCGGTCAGTGAGTCACCCATCGACCCCTTCGGGGCGAC
CGGGCTGTCACATGACCTTGCTGATGAAGACCTGAACCCCGTCACCATCATTCAGAACTACACCAACATGTCCGATCCGA
TGAAAGAGCTGGAAGCGGCGATTGAATCGGGGCGCTTTCATCATGACGGCAATCCCATCATGACCTGGTGTATCGGCAAC
GTGGTCGGCAAAACCATTCCGGGTAACGATGATGTGGTGAAGCCCGTCAAGGAGCAGGCGGAAAACAAAATCGATGGTGC
AGTTGCGCTGATTATGGCGGTTGGCAGAGCCATGCTGTACGAGAAAGAAGACACGCTGTCTGATCACATTGAGTCCTACG
GGATCCGCTCGCTTTAACTGAGGTAATTATGATCATGCTGATTCTCGCGCCTCTGGTGGGCGTGCTGGGTGCGCTTTTGC
TGGCGTATGGTGCCTGGCTGATTTATCCCCCGGCGGGTTTTGTTGTTGCCGGGGCGCTGTGCCTGTTCTGGTCGTGGCTG
GTGGCGCGATATCTCGACCGTACACAGTCGTCTGTCGGCGGAGGTAAATAGTGTTCTTTTCGGGATTATTTCAACGAAAA
AGTGACGCACCGGTGACCACGCCAGCAGAGCTGGCGGATGCCATCGGGCTGTCGTATGACACCTATACCGGAAAGCAGAT
CAGCAGTCAGCGGGCTATGCGACTGACGGCGGTTTTTTCCTGCGTCAGAGTGCTGGCAGAGTCGGTCGGGATGTTGCCCT
GCAATCTGTATCACCTGAACGGCAGCCTGAAGCAGAGAGCCACCGGCGAACGTCTGCATAAACTGATCTCCACGCATCCC
AATGGCTATATGACGCCGCAGGAGTTCTGGGAGCTGGTGGTCACCTGTCTGTGCCTGAGGGGAAACTTTTACGCCTACAA
AGTGAAAGCATTTGGCGAAGTGGCTGAACTGCTGCCCGTCGATCCCGGCTGTGTGGTATATGCGCTGGGAAGGTGTCAGC
GATGGCCTGAAGGTGACCGCCGGGAGTGTTATTCAGCGCGATGACCTGGTGCAGTACACGACAACTGACGATGCAACCAG
CTCCGGTGGTGTCCTGCGCGTGCCGATCGCCTGCTCAAGTGCAGGTGCGGTCGGTAACGCTGACGACGGTACGGCATTAA
TCCTGGTCACGCCGGTGAATGGTCTGCCGTCTTCCGGTGTGGCTGACACCCTGACAGGCGGATTTGATACTGAAGAGCTG
GAAACGTGGCGCGCCCGCGTCATTGAGCGGTATTACTGGACGCCGCAGGGCGGGGCTGACGGGGACTATGTCGTCTGGGC
TAAAGAAGTGCCCGGCATTACCCGCGCATGGACATACCGTCACTTGATGGGAACGGGAACTGTCGGTGTGATGATTGCCA
GCAGTGACCTGATTAATCCCATTCCGGAAGAATCAACGGAAACGGCGGCAAGACAACATATCGGGCCACTGGCCCCGGTG
GCAGGCTCTGATTTGTATGTGTTCAGGCCGGTGGCACATACGGTGGATTTTCATATCCGCGTGACGCCGGACACACCAGA
AATACGGGCTGCCATTACCGCGGAGTTGCGTTCGTTCCTGCTGCGTGATGGTTATCCGCAGGGAGAACTCAAGGTATCGC
GTATCAGTGAGGCGATTTCCGGTGCGAACGGGGAATACAGCCATCAGTTGCTTGCACCGGTGGACAATATCTCCATTGCG
AAAAACGAACTGGCGGTACTGGGGACGATTTCATGGACGTGACAAACGATGATTACATCCGCCTGTTATCGGCACTGTTG
CCGCCCGGTCCGGTGTGGTCAGCCAGCGATCCGGCGATTGCCGGTGCGGCACCGTCATTAACCCGTGTTCATCAGCGTGC
GGATGCCCTGATGCGGGAGCTGGATCCGCGCACCACCACTGAACTGATAAACCGCTGGGAGCGTCTGTGCGGTCTGCCGG
ATGAATGTATTCCGGCGGGAACGCAGACCCTTCGCCAGCGTCAGCAACGGCTGGATGCGAAGGTTAACCTGGCGGGCGGC
ATCAACGAGGATTTTTATCTTGCACAGCTTGCTGCCCTGGGCAGACCAGATGCCACCATCACGCGATACGACAAAAGCAC
TTTCACCTGCTCATCGGCCTGTACTGACGCGGTGAATGCGCCGGAATGGCGGTATTACTGGCAGGTCAACATGCCAGCCA
CCACCAACTCCACCTGGATGACATGTGGCGATCCCTGTGATTCCGCACTGCGTATCTGGGGTGACACCGTTGTCGAGTGT
GTGCTTAACAAACTCTGCCCGTCGCATACCTACGTAATTTTTAAATATCCGGAGTAATCCATGCATCGTATAGACACGAA
AACCGCGCAGAAGGATAAGTTCGGCGCGGGTAAGAACGGTTTTACCCGTGGTAACCCCCAGACCGGCACGCCTGCCACCG
ATCTGGATGATGACTACTTTGACATGTTGCAGGAGGAACTTTGCAGCGTGGTGGAGGCATCCGGTGCCAGCCTGGAGAAG
GGGCGGCACGACCAGTTACTTACCGCACTTCGCGCGCTGCTGTTAAGCCGCAAGAATCCGTTTGGCGATATCAAATCGGA
TGGCACTGTGCAAACGGCTCTCGAAAACCTTGGTTTGGGAGAAGGAGCAAAACTCAATGCAGCAACGGCTACATTAGGAC
GCACCGGTTTCATAGCTATACCGGTTATGATTGGTGGTATTGAGCAATCAGTAATCATTCAGTGGGGGTGGAATGCCGCA
AAAGCATCTGCCTCTGGGGGGGATGGAAATACAGTTGTATTCCCGGTTGCGTTTAATAATGCCTGTGTTGCCGTTGTTGC
AAATTATGACAATGTCAGCGCACCTATCAATGCAGTGGCAACGGGGGGATATACAACCACTTCGTTTTTATTACGGTGCG
CAGCTCAAACGGGTAGTTATTACTATAACTGGATTGCTATTGGGTATTAAGATGAAAATATACTGTTGCTTAAATACCGT
TGGTTTTTTTATGGATGGCTGTGGCGTCATTCCGCCAGATTCTAAAGAAATAACGGCAGAACACTGGCAGTCATTATTAA
AATCTCAAGCTGAAGGAGGCGTGATCGATTTTTCTGTTTTTCCTCCTTCTATTAAAGAGGTTATCCGTACTCATGATGAT
GAAGTCGCAGATGCGAACTTTCAAAAGCAGATGCTTATCTCTGATGCAACTGATTTTATCAATAGCAGACAGTGGCAGGG
TAAGGCTGCATTGGGAAGACTTAAAGAAGATGAGCTGAAACAATATAATTTGTGGCTGGATTATCTGGAAGCACTGGAAC
TGGTTGATACATCCAGTGCGCCAGATATTGAATGGCCTACGCCTCCGGCAGTTCAGGCCAGATGACATCCGGCGCGGTGC
TGGTATCTGTTGCCGTCACCGCGTCAATGTAATCCAGCACAGCGTTAAGTCTGGTTGTTTCTGCCTGCGTCAGTTTACGT
CCGGCCTGCAATTTCAGTTGAATCAGACTAATGGAAGCCATTGCAGCATCAATCAGTGACTGGCGCTGTGCTTCTGCCGC
GTCTACTGCGGCGCTATGCTGTGCTTCAGTATCGGTCACCCATTTCTCACCATCCCATTTATCGTATGGAGATAAAGGGG
CGATAGTGGTTGTATTTTCAGGGTAATCACCCGGAGCTTTGATTTCTTTTGATTCTCCAGTTTTGGTGCTATAGACCGTT
TCACCGCGATGGTCTGGCACATATTCCCATGAGTTAAAATCTGCAGAACGGCAGATTGCATAACCAGCCTTATGTGTACC
AGGGGCATCTAAACAGGAACATGCCGGAATGCCGACACCAACGGCAAGATATTCATTTGAAGTGGAAATATATTCCCGTG
TTTCACCATCGTAGTTATAAACGGTAACATCCCCTGCCTTTGTTGCAATAAGGTCACTATTTAATATTGCTTTATGCATC
AGGCTGCCCTCACGATATAGTTAAATGCAATATTACGCGGACGCGTTTCTGAGGCTGCGGCACCTAAACCATCCACTGAT
TGTTTATATGTTTTAAAGGTTCCATAATCCGGGGCTGGTAATCCGGCATCGTTTGTGTTTCCTCTTTTGATAATGTCAGT
GCCACTATTTACCCATATTTCATCAAAATAGAAATTAATCGTTGCATCAGTCACAATCGTGGATCTTGACGGTAATCCAT
GAGCATGATCCTCCGTTGCATACCCCTGAATACTTAAAATAGAGCGACCTGTATCAATCCCCCGCCCGTCATCCCAGCCA
CGAATAAACTCACCACGTAAATCAGGCAATTTATTTGTCGGATAAGCCTTTGCCAGTTCCGGGTATTCTTCAGCAGAAAA

AGCGGCACCATTGCATTTCAGCCAGCCTGTTGGCGGAGTGGCTGAAGGCCACGGAACCGGGACACCAACAGGTAATGCAG
AGCCTTCTCCCAAACCAACGTTTATGAAAATGAAGAAATAACAAGCAAATGGCATCATTCCTGCTTTTACCAGGGGGATT
TAACATGCTTATTGGCTATGTACGCGTATCAACAAATGACCAGAACACAGATCTACAACGTAATGCGCTGAACTGTGCAG
GATGCGAGCTGATTTTTGAAGACAAGATAAGCGGCACAAAGTCCGAAAGGCCGGGACTGAAAAAACTGCTCAGGACATTA
TCGGCAGGTGACACTCTGGTTGTCTGGAAGCTGGATCGGCTGGGGCGTAGTATGCGGCATCTTGTCGTGCTGGTGGAGGA
GTTGCGCGAACGAGGCATCAACTTTCGTAGTCTGACGGATTCAATTGATACCAGCACACCAATGGGACGCTTTTTCTTTC
ATGTGATGGGTGCCCTGGCTGAAATGGAGCGTGAACTGATTGTTGAACGAACAAAAGCTGGACTGGAAACTGCTCGTGCA
CAGGGACGAATTGGTGGACGTCGTCCCAAACTTACACCAGAACAATGGGCACAAGCTGGACGATTAATTGCAGCAGGAAC
TCCTCGCCAGAAGGTGGCGATTATCTATGATGTTGGTGTGTCAACTTTGTATAAGAGGTTTCCTGCAGGGGATAAATAAA
GTTAAAGACACTTTGTGTACAAAAGAAAGTAAAACAACAGCAACTTGTTGCAATTTTATCAATAAAAGTAGTATTGTCGT
GAAAAATTGATTAAAGATTAATATTATGCATGTTTTTGATAATAATGGAATTGAACTGAAAGCTGAGTGTTCGATAGGTG
AAGAGGATGGTGTTTATGGTCTAATCCTTGAGTCGTGGGGGCCGGGTGACAGAAACAAAGATTACAATATCGCTCTTGAT
TATATCATTGAACGGTTGGTTGATTCTGGTGTATCCCAAGTCGTAGTATATCTGGCGTCATCATCAGTCAGAAAACATAT
GCATTCTTTGGATGAAAGAAAAATCCATCCTGGTGAATATTTTACTTTGATTGGTAATAGCCCCCGCGATATACGCTTGA
AGATGTGTGGTTATCAGGCTTATTTTAGTCGTACGGGGAGAAAGGAAATTCCTTCCGGCAATAGAACGAAACGAATATTG
ATAAATGTTCCAGGTATTTATAGTGACAGTTTTTGGGCGTCTATAATACGTGGAGAACTATCAGAGCTTTCACAGCCTAC
AGATGATGAATCGCTTCTGAATATGAGGGTTAGTAAATTAATTAAGAAAACGTTGAGTCAACCCGAGGGCTCCAGGAAAC
CAGTTGAGGTAGAAAGACTACAAAAAGTTTATGTCCGAGACCCGATGGTAAAAGCTTGGATTTTACAGCAAAGTAAAGGT
ATATGTGAAAACTGTGGTAAAAATGCTCCGTTTTATTTAAATGATGGAAACCCATATTTGGAAGTACATCATGTAATTCC
CCTGTCTTCAGGTGGTGCTGATACAACAGATAACTGTGTTGCCCTTTGTCCGAATTGCCATAGAGAATTGCACTATAGTA
AAAATGCAAAAGAACTAATCGAGATGCTTTACGTTAATATAAACCGATTACAGAAATAAAATTATTTATTAAAGTCACAT
TTAAGACGTAATACCCTACAGGGTAAAAATTTTCTCTGATCTTAACTTCTGCAAATGTTAACTGCTATTTTTATGCTAAA
AATGGTTATCAAAACTCAAAAACACATGTTTATAATCAATGAGTTATAGAAATGCTAAGGGCTAATGAGTTATATGCAAA
TTAGTAAAATTATGTTGCTATGTCAGATAGTTACGATTTAGTCATCTAACTAATGCTGCGCCATATGGGTTGGACTGAAG
CGGCTGACCTGATTGTTAAAGGTATGGAAGGCGCAATCAATGCCAAGACCGTAACTTATGACTTCGAACGTCTGATGGAA
GGCGCTAAGCTGCTGAAATGTTCAGAGTTTGGTGAAGCGATCATCGAAAACATGTAATCTCTCCATGTGTTAAATATTGA
AACGGGCGTATAACACGCCCGTTGTTTTATTTATGTGGATATTATTAATAGCATATCGAGCATATTTATATGAAGCCCAT
TACTTGAGCCCATATGGGCATATTTTTATAATGCAACTATTATGTAAACATTTATTTGTTATTTTGCTTTCTCCTGGAGG
ACACTCTTGACTGCTTTTGAGTAAACTCCATAAATCCTTGTTGAATGGTGCGATGTGATAAATAGTAATAGGATATTCTT
TATCCTTAAGGATAATACCAGACTTAACCGGTGTAAATATACTGCCAGGAGGGAGAAATATAGTAGATTGATACCAGATG
ATCATTTTCATATTACCCCATATGGCTGAAAAAGATATACCACATGTAGGTTGAATTACCGTGTCAATTACTATCCACTT
CATTTGTTATGTCTTATCCCACGGTATTTAATATGGTTCATTAGGATGTTTATTTCTTGATTTTGCATATGAGTATATTA
CCCCCCCCTCAAAAAAATAAATTAATTAAAATGATGGCTTATATAAAATAAAATTTAAAGCAAGGAATCTCAATGGATGT
TAAACAAAATGAGATTTTGTGAAAGCAATAAATTATTGACTTCGTTTTAGATTTGTTTAGCTATAATGTTATACATTCAA
ATGACTGAACATCCTGTAATTAAAACATAGCCTTTATGCTACTTTGTGCCAATTTGCTAAACATTATGGTTGCCTTTTTA
TATAACGATAATAATGAATATAAGCATGACATGAGAATAAGGTTTCAATTTTTGAGTTATATAGGAATGATTTAACCTGT
TCCTGGCTAAAATACATATAACCGGATGATGACTAAACCAAAATACATGTGCGTTAAGTATTGAAACGGACGTGTGGCAC
GGCCGTTGTTTTTATAAATATGTTAACCGTTATAAAATAACGTATCAAAAGTCAAGTGATCACATTTCAAATATCAAGTT
GATAGTATTAGTCTGGTGATTATTTATGGGTGACAATAAAAAGACAGTATTAATCATCCATAGAGATAGTCTCTGCACTT
TTATTTCCATTATGCTAATGCCTTACTGAATTATGAAGCATTTCTTAAGTATCCAACTTTAGCTAGATTAATGGTTTATT
ATTTTCTACATCTTCAATATATAAAAGCGTATTATCAATGGCGTAGTAACTGCGTTTGTTATGATTAACATCAGTAACCC
ACCGGAAAACGCCCGCGCCTGCCAGTGTTGAACAGTATTCCCGAAATGTAGATTTTCCGCAAATATGAAGCAATGCGGCC
TCTTTTATTTTAGCAGGGTTCTTGGTCGTACTAACTTTTAACAGGTTCCTGGTTCCTCTTAATAACAAAACCGTATCATC
GTGAGTAATAATTCTGATGTTATCCGTAGCCAGATAATAAATGTAATGTGCAATACGGTGATGTTTTAATTCTGAATAAA
ACCAGGAGAAGTTTTGCTCTTTTCTCACTTGCTCAAACATCTTTTGAAAAACAACGACCTGATCCATCAGGATAATAACC
TCTTGTTAGTTGTGAGACTGCGTAGTGTGCACGATCGGTTTTACCACTTCAATCTGGTCTGTCCTTTGGCTGTGATATGT
ACAGAGTGTGATAGAGGGAATATCTGAATTCTCCCGGTGAGCATTTTGCAACGGACCAGCTCCGGTACAAACGCTGTTGT
GGGTTCAGATTATAACATTCTGTCTAAGGGGCGGGATAAAGGTGAAATTAGGGGGCATGAAAGATGACTTTATAACCTTG
CTCACCCCAGTGTTGTAAAAGTTCGTTTTGCCTTCTCGTTGGTGCCATGCCTGTCCAGACAATCAATGTTTGCGTCGGGA
ACAGTTCCGGGCGCGGCGATTCAATGGGATCAGCAAGAACAGAAATGTGCCATCCTGACAATGATAAACGCCATGCTTCC
AGCCACAATCGTGCTCTCTCTTCAACATTCCACGCCATCAGAATGGCTTCTTTACCGGGCTTACGGCGCATTTCGAAAAG
CGAGGTTGCTGCGTATTCAATTAATGCGCCGTCAAACATACTGCTCATAATGCGGGAGGTGTTGTGATCAAGCACGAGAC
GCTGGCGAACAGGAAGGTAAACATGATTAATCAATTGATCTACTGGGTACTCTCGACCCAGTGAAATAATTCTCGCGCGT
AGTTTGGCAGGATTAGCCATGCGAAGAATTGACATCATCTCTTCTTGCAGGCGGCTCCAGTCATCTTCCGTATCCTGGCT
GGTGGTTTCCAGTAATGCTTTAACTTTGCCTACAGGGACGCCATTACTTATCCAACGCTTGATCTCTTCGATGCGTTGTA
TGTCTTCTTCATCAAAGAGTCGGTGTCCGCCTTCACTGCGCTGTGGTTTTAACAAACCGTAGCGGCGTTGCCAGGCCCGG

AGAGTGACAGGATTAATCCCGCAACGTTCAGCAACATCACCAATGCTGTAATAAGCCACAATTCCTCCTTGCGGTCACAA
ATCTCCGTCGCCTGTACACGACCCAATAATACTTTGTACAATATACGCTAAAATTGTACAAAGTATAAATAAGATTTCAG
CTAAATTGGATGAAACATTATTTTTAATGTGGATTAAATTTAAACGTAACGTATTCATTTTCACGATGATTTACTGAAAT
CATGTGAAAGAATGTGCTGAAAATTATTTTTTCTCTGGCCACGCTATGGAAGGGATACCATTCAATTTAGCTTTAGCAAA
CAGATCTCCCTGAAACATCTCAATTCCTGCGGATTCAAGCCACATCCACTCTTCTGGTGTTGCCACGCCCATAGCACTGA
CTTGAATTTCAAGTGATGTACAGCATTTTATGATCGCCTGAATAATTGCCTGCCGTGGCCCACTTTTATGAACATTGGTA
ATCAATTCCTGACTGATTTTAATTCTGTCAGGCTGGAAGCGTGACAGGAGTAACAAACCAGCAAAACCTGCGCCAAAATG
ATCAATTGCTACACTGATACCAGCAGCCTTTAGCGATTTAATCGCTTCGGCAAACTCATCAAACCGAGATATGACTTCAC
TTTCAGTAAATTCAACGATGATTTGTTCAGGCACCAGAGCATTGGCCTTTATTTCATTAAGTAAAAAAGAGACTGCGTCA
GGTTCGTTAACCAGGGTCATAGGTAATAGATTGATTGAAATCATTTTATCACCGAGCTCAAGTGCGTGTGCCATCGTGAA
TGCAAGCGCCTTACTTTTGAGATCCGCTGTGTAGATTTCCCCGTCTTTACGCTGCCCAACCGCTATGGCTGATGGGCTAT
CTTCATTTTTTTGCACAATGGCTTCAAAAGCGATTATCCGCCGCGATAAGGGATCGACAATAGGATGAAAGGCAAAGTGG
TCGTTTATAGTTGGTGAAAGGGCACAGGAATCAAGTTCTTTATCAGATCCGTCAGCGATAAAAAGCCAGGAGTCTTCGGC
AGGGATCTCGAAATAGGTTGATTGTTCGGTTGCAAGGACAAAAGTACGAAAAAATTGTAGCGCTCTGTCATCATAAGTTA
GCTGAAATTTTGATGTGCCTTTGTCGAATACGGCCTGTAAAACGTCATCTCGCTCGTGCAGGCGCAAATCAAATAATTCC
ATTCCCGCTTTGCCAAAACGGCGAGCAGGCGCGTAATCGCACAGCAGCTCAACAATATTATAGTGCCGTGGATCCTGGCA
TATAGCCCGATATATCATTTTAACCTGTTCTTCCGGACCTTCCAGAAGCTGGAAAAAATGAGAACCATTAAACAGTAAGA
TCCCTGTTACGTCAGACTGCATGTTCCTGCGATTTGCTATCGAAACCATTTCTTCGATTTTTTTGACAGGTTCGTCGTCA
CGTATATGGCTACGATAAATAAGGGTGGTAAGCATTAACAATCCAGGGTAATGGGTGAGGCGAGAGTAAGACGGTAACAG
ACATATCTTCTTGTGTCTTTCTTTTAATACCAAAACATAACCGTTTCTTTACATTGATAAAAAATGGAAAAAGTTGAACA
CTAGTTGGCGAAAAATCTTGTATAGATTGTCAGTTAAATGATGCAATATGTTTTATCATAACACATTGTTTTATATGCAT
TAGCACTAATTGCAAAAAATTAATTTATCATTCTGTACACATATTTCGTACAAGTTTGCTATTGTTACTTCACTTAACAT
TGATTAACATTTTTAACAGAGGCGTAGCATGCATCAAAATTCAGTGACTTTAGATTCTGCGGGAGCAATCACTCGTTACT
TTGCAAAAGCTAACTTGCATACTCAGCAGGAAACTCTCGGGGAAATTGTGACTGAGATTTTGAAAGATGGACGTAATCTG
AGTCGAAAGTCGCTTTGTGCCAAACTTCTTTGCCGACTGGAACACGCGACTGGGGAAGAGGAACAGAAACATTATAATGC
ACTAATTGGGTTGCTTTTTGAATAATGCAACAGCCTGATTATTTAACAGGTTAGCTATGAAGTCGTTATGAAGACATCTG
ATAATGAACGTATAAAATATGAAATTACTGGCCAGGCGGTGCTCCAGATACTGCGCATGAAGATAAATTTTTCATTGCAG
ACTCTTATTAAGCAATTACTCGTAATGAAATCGGCTGAGGAAGATGCTTTCCGACGCGATTTAATTGACAGCATAATTCG
TGATTTTAGCAATAGTGATTCAGGAGGGCCAAACCGAAGAACAGCGACAGCTGACAATAAAAGTATGTTCAATGGTAAGA
AAATAAACAGAATACATTAAAATTTCATAAGTAAGATGAGAGGTTACCATGCTTGAAGATACTACAATTCATAATGCAAT
AACTGATAAAGCGTTAGCAAGTTACTTTCGCAGTTCGGGTAATTTGTTAGAAGAAGAATCAGCAGTGTTAGGGCAGGCTG
TCACCAATTTAATGCTTTCAGGCGATAATGTTAATAATAAAAATATTATCTTAAGTCTGATACACTCCTTGGAAACAACA
AGTGATATTCTCAAAGCTGATGTGATTAGAAAAACACTGGAAATCGTGTTGCGATACACAGCTGATGATATGTAACTTCT
AATTATTAAGTATAAGTTTATAGAAAACTCATTCATCTTATTTTTGTCTGTCGCTTTAGACTATACACAGCATAATTTTA
TTGGGTTAATATTTCTACGAGGCTGACATGAATAATTCAATCCCAGAGAGATTTATTTTTCAATGTGCTTTGTTTAAAAA
TCTCGAGAGGGAGGTGTTCATGACGCATGGGTATGTTGATAGTCATATTATTGATCAGGCTTTACGCTTGCGTTTAAAAG
ATGAAACCAGTGTGATTCTTTCTGATCTCTATCTGCAAATATTGCAGTACATTGAAATGCATAAGACTACACTAACGGAT
ATCATTATTAATGACAGGGAATCCGTGCTCTCTTAGAACTTGAGTAAGGTTTTAGAATAAACATCAGCTGTATCACCATG
CTGATGCAAAGTGAGCATTTCAGGCGTTATGCTTTCTTATTATGTCCGCAATATCAGGTGTCAAGAATGGAGAGTTCTCG
CTCTCCATTCTTGACGCCTGATATCCCGCCTAACTTATTTTGTACTGCCTACATTATGCTCAATAATTAATGGGTTAGAT
CAAGATGGAGAAAATTTTATCTTCGGCGTGGATATTTCTTATGCTCTCTACCATGCTCTCTGTAGATGCCAGCGCGAGCG
AATGAATGGCATTTTCATGTATTAAACCCTTTGGCCGGGACAAGCAAATGCGCAATACACTCATACCCATCCTCGTTGCG
ATATGCTTGTTTATTACCGGTGTCGCTATTTTGAACATCCAGCTCTGGTATTCCGCAAAAGCAGAGTACCTGGCGGGAGC
GAGATATGCCGCCAACAATATCAATCATATACTTGAAGAAGCGTCACAAGCGACTCAAACAGCGGTTAACATTGCCGGGA
AGGAATGCAACCTCGAGGAGCAATATCAGCTTGGCACTGAAGCAGCTCTGAAACCTCACCTGCGCACAATCATCATTCTC
AAACAGGGAATAGTCTGGTGTACATCCCTGCCTGGGAATCGGGTCCTGTTGTCTCGTATTCCTGTTTTCCCGGACAGTAA
TTTACTGTTGGCTCCAGCAATCGACACCGTTAATAGATTACCTATCCTGCTCTATCAGAACCAATTTGCAGATACGCGCA
TTTTGGTTACGATAAGCGATCAGCATATTCGTGGGGCACTTAATGTACCCTTGAAAGGGGTAAGGTATGTATTACGCGTG
GCGGATGACATTATTGGACCAACGGGTGATGTGATGACGCTTAATGGACATTATCCCTATACCGAGAAGGTTCACTCCAC
AAAATATCATTTCACTATTATCTTTAACCCGCCGCCACTCTTTAGCTTCTACAGACTTATCGATAAAGGCTTTGGGATTT
TAATATTTATTCTGTTAATCGCCTGCGCCGCTGCCTTCCTGCTTGATAGATATTTCAATAAAAGCGCAACGCCTGAAGAG
ATCCTGCGACGGGCAATAAATAATGGGGAGATCGTCCCTTTTTACCAACCTGTGGTAAATGGTCGGGAAGGGACATTGCG
GGGAGTTGAGGTGTTAGCCCGCTGGAAACAACCTCACGGTGGATATATATCACCCGCGGCATTTATTCCACTTGCTGAAA
AATCCGGATTAATCGTTCCGCTTACGCAAAGCCTGATGAATCAGGTTGCCAGACAGATGAACGCTATCGCGAGTAAATTG
CCGGAAGGTTTTCATATTGGAATTAATTTTAGCGCTTCGCATATTATTTCGCCGACGTTTGTCGACGAGTGTTTAAATTT
CCGTGACAGTTTTACCCGCCGCGATTTAAACCTTGTTCTGGAAGTCACCGAGCGTGAGCCATTGAATGTTGATGAAAGTC

TGGTTCAGCGGTTGAACATACTGCATGAAAATGGTTTTGTCATCGCACTGGATGATTTCGGTACTGGCTACTCAGGGCTT
TCTTATCTTCATGACCTGCATATTGATTATATCAAAATTGATCACAGTTTCGTTGGCCGCGTAAACGCAGACCCAGAATC
AACCCGAATTCTGGATTGTGTATTGGATCTGGCGCGTAAACTTTCGATCAGTATCGTCGCTGAAGGTGTCGAAACGAAAG
AACAACTTGACTATCTGAACCAAAATTATATCACATTTCAGCAGGGTTATTATTTCTATAAACCTGTTACATACATCGAC
CTGGTCAAAATTATCCTTTCTAAACCGAAGGTGAAGGTTGTGGTTGAGTGAAAATTGATCAGTAAGGCCATAGTGCGGTG
TAATTATAGACAGCTAATTAGCTCGTTGCCTCTTGTTACTATTGTTCATTATTTTGTTTGCTATAATTGTTTGAAAGTTT
TGACAGGATTGCCATTAGTAGCATGAACAATAGTAATAATCTGGATTATTTCACTCTCTATATCATATTTTCCATTGCAT
TTATGCTGATCACCCTCCTGGTCATCCTTATTGCAAAACCCAGTACCGGGCTGGGAGAAGTGCTTGTGACGATAAATTTG
CTTAATGCCCTTGTTTGGCTGGCGATCAATCTGGTTAATCGATTAAGAGAAAGACTCGTCAACCACAGGGATCAGCAATA
ATCTTTCAGTTTCTCACTGTCAGTATGCGGCTGAATGGGTTGCTGGCAGTGAACGCCTGGATCATTGAAGGAAAGGCATT
ATTGCGCAAATAGTTGTCAACCCTGGTGTTATCACGGTTGTTTTTATATATCACCGAAATAATCCTCATCGCAACTATTA
ACAATTTTGATGTCGAAGAGTTATTTGTTAAACAAAATCGTCACCTCAAAGTGATCAATGTCATGAAAATAAGGTGAAAA
ATGATAATGCCGACTTATTTATCATTTATATATTGTCGCTGTTTATCTATTTAAATTTTTTGTGCTTTTGTTTTTGTCTT
TACCCTCCGTTTTGGTAATCTGAGCTCATAGGTTATTTGACCAGGAATGTTTGTTAATCCAATGAATGGATGGTTAATCT
ATGAAACTGAAAAAACTCCCCGGGTTTAGTTTGGGACTTATTGCTCTGGCGGTGGGTAATGCATATGCAACACAATTGTT
GGATGATTATAGTATAATTTCCTATATGACTGATGAAGAATCGCCGATTGAAATCAAAGATAATAATCCGATAAGTAATG
GAGAGTATCTAACCACTGAAGACGAAAGCCATGCTGTGAAAGTGGATGACGGTGTAACTGGATATATAAATAATGCCAGT
GTGATGACTAGTGGTGATGGATCTTATGGTATTTCTGTTGATAGTCAAAACAAAGTATTATATATAAGCGATAGCGATAT
TAAGACCTCTGGAAGCGTATCTGACAAAGAAAATGGAGGGATAACAGCCAGCGCAGTAGTCAGTGAATTTGGTGGCACCA
TCTTTATGAATGGTGATAATTCAGTCGAGTCGGGTGGGGCATATTCAGCGGGACTTTTAAGCCAGGTTAATGATTCTGAA
AAGATGGTAAATAACACCCGTCTTGAAACCACAGATAAAACGAACATTGTTACCTCTGGGGAAAATGCAGTAGGTGTTCT
TGCATGTTCAAGTCCTGGAGAGTCTCGAACATGTGTCGATGCTGTAGATGATGAAGTTAGTGATTCTAACAGTTACGAAG
TTATTAGCCGTGCTGATTTAAAAATGAATGGTGGTTCCATAACAACTAATGGCATTAATAGCTATGGTGCTTATGCTAAT
GGGAAAAAAAGCATATATTAATTTAGATTATGTGGCACTTGAAACTGTGGCTGATGGAAGTTATGCAGTTGCTATTCGACA
AGGTAACATTGATATAAAAAATAGTTCTATTACAACAACAGGCACTAAAGCCCCCATTGCAAAAATATACAATGGTGGAG
AGTTATTTTTTTCCAATGTCACCGCGGTATCAAAACAAGATAAAGGAATATCAATTGATGCATCAAATATCGATTCTCAA
GCCAAAATAGCACTATTAAGTGTTGAACTTTCAAGTGCTTTGGATAGTATTGATGTTAACAAAACTACAACGGATGTAAG
TATCCTTAATCGAAGTATTATCACACCTGGTAATAATGTTCTGGTTAATAATACTGGAGGTGACTTAAACATAATTTCGT
CCGACTCTATTCTAAATGGAGCGACTAAACTCGTCAGCGGCACAACCACGCTGAAGCTTTCAGAAAATACAATCTGGAAT
ATGAAAGATGACTCCGTTGTTACCCATCTGACTAATTCAGACAGTATTATCAATCTTTCGTATGATGATGGTCAAACATT
TACCCAAGGAAAAACATTAACCGTAAAAGGTAATTATGTCGGTAATAATGGTCAGCTTAATATCCGCACCGTATTAGGTG
ATGATAAATCGGCTACGGACAGACTTATTGTTGAGGGTAATACTTCGGGTTCAACTACCGTCTATGTGAAAAATGCTGGA
GGAAGCGGCGCGGCCACGCTAAACGGGATCGAACTCATAACTGTGAATGGCGATGAATCTCCAGCAGATGCCTTCAGATA
AGGTGATGCAAGGATTGCAGCTGGAGCTTTCGAATATCAACTAAAACAACAAGGCAAAAACTGGTATTTGACAAGTTATC
AGTCAGTGAATGAGGAGGATAACAGCTCAGAGGGAAATTCAGAATCAACAGAAACGCCTACCCCCGTCCTGCGCCCCGAA
GCTGGAAGTTACGTCGCTAACCTGGCAGCTGCTAACACTCTTTTTGTTATGCGTCTGAACGACCGTGCGGGTGAAACGCG
CTACATCGATCCTGTAACTGAACAGGAGCGTTCAAGCCGACTTTGGCTACGTCAAATTGGCGGGCATAATGCCTGGCGTG
ACAGCAACGGACAGTTGAGAACGACCTCGCATCGCTACGTCTCGCAGTTAGGGGGCGATCTGTTAACCGGTGGTTTTACC
GATAGTGACAGTTGGCGTTTGGGAGTGATGGCTGGTTATGCCCGCGACTACAACTTAACTCATTCCAGCGTGTCGGATTA
TCGTTCGAAAGGGAGTGTCAGAGGCTATAGCGCAGGGCTGTATGCCACCTGGTTTGCCGATGACATCAGTAAAAAAGGCG
CATACATTGACTCCTGGGCGCAATATAGCTGGTTTAAAAACTCGGTGAAAGGGGATGAATTAGCCTATGAATCCTATAGC
GCGAAAGGTGCAACCGTCTCGCTGGAAGCGGGTTACGGTTTTGCCCTGAATAAATCCTTTGGTCTGGAAGCGGCGAAATA
TACGTGGATCTTCCAGCCACAGGCACAGGCTATCTGGATGGGCGTCGATCATAATGCGCACACGGAAGCCAATGGCTCAC
GTATTGAGAATGACGCAAATAACAACATCCAGACCCGACTCGGCTTCCGCACCTTTATTCGTACTCAGGAGAAAAACAGC
GGTCCGCACGGTGACGACTTTGAACCTTTTGTTGAAATGAACTGGATCCATAACAGTAAAGATTTTGCTGTCTCAATGAA
CGGTGTGAAAGTCGAACAAGATGGGGCGAGTAATTTGGGGGAAATTAAACTTGGCGTAAATGGCAACCTGAATCCAGCGG
CCAGCGTCTGGGGAATGTGGGCGTGCAGCTGGGTGATAATGGCTACAATGACACCGCAGTGATGGTGGGCCTGAAATATA
AGTTCTGATCCCGCCGTTAGCTAAAAAACCGCGTCGTATTCATCGACGCGGTACATATGAAATATTATTTTTTGCCGATA
GCACGCATGGTGTCATCAATTGCCGTGATCAACAGCATTTGCGGGTCTTTAGCGCAAACCTGATTCAGTTTTTCTACCAC
TTTGGCGCTCAGTTCCGGAGATTGCTCAATTTTTAAATCACGGCTGGCAACGCTGGCATTACCCATTACCGCAACAATTT
CTGCAACCTGTGCGCTGTCAGTTTTTGCCATTTCGTTGGCTTCTGCGCAAGTAATATAGGTTTCTGACGGCAAACCGTTT
TTAATATTGTAGTCCTGCGCCCAGGTCATTGGTGCGAAAACAAACAGGCCCGCCAGTAAAGCAAATTTTTTCATCATCAT
TCCTTATTTCATTTTACCCAGAATTGCACCACCCGTACCGCCAATCACGGCACCTTTAATCGCCCCTTCGAGGCCATTGC
CGGTCAGAACGCCAGTGACAGCACCAACGGCGGCACCCACTTTTGCACCTTTACGCGCATTTTTACCGTCGCGGCCTTTT
TCTGTTACTGCACCAACACCAGCGCCAACAGCTGCACCTTTCAGTACGCCATTAACACCATTGCCAGTAAGTAAACCAAC
GCCTGCGCCTAGCAATGCACCTTTCGTGGTGCGGTTCATATCCGCCATCGCTGGCGTGGAGCAGAACAATGCTGAGATAA

GCCCGAAGGCAAGTATTTTTTTCTTCAACTTAGATGTCCGGTATTAAGTAAGTTGCACACACAATAATTTCGTCTTCAAT
TAAGATCTGCTTAACTAAAGAACGCTCGCTATTATTCAGATAATTCAAAATGAGCGTGGCTGTGATGATAGGAATTATGT
TTTTTACGTGAATGAGAATAATCTTAAATGAGGAATAACTCATTGATTGACAATATTTTTATTCAAGAAGTGTCATTGAC
TGTTAACGCAATGTTGTAAAGGTAAGATAATCTGATTTATCAATATTATTGTGTGATTTTTATGTGAGCAGAAGATATTC
ATCAGCAACGATTACATTAGTCATTTTATTTTGCCGACGGCCTCATTGTCGAAAGATAAGCGTACGACAGTATTATCAGA
AAAGAGTGATTTTTTATCCAACTACACTTCAGCGCACTGCGTGTAAAAAATGCCTCTTTCTTATGCGGGATATCATCATT
TCATCATGATGTCTTTGGTGAGCGGTGAACACAATACACCTGCGCTGTCTCTTCAGGATGAATCCCCTGGTCTGGTGCCT
GCGGGCTGATGTTGCAGCAGAGCTTAGGTCACTTAGACGCTACTATCATTTATCCAATGGCATGGAATCGAAATCAGTCG
ATACCCGCAGTATATATCGTGAACTGGGTGCAACGCTGAGTTACAACATGCGCCTGGGGAACGGTATGGAAATGAACCCT
GGCTGAAGGCGGCTGTGCGCAAAGAATTTGTCGATGATAACCGGGTGAAGGTGAATAATGACGGTAATTTCGTCAATGAT
TTGTCGGGCAGACGTGGAATATACCAGGCAGCTATTAAAGCCTCATTCAGCAGTACGTTTAGCGGACATCTCGGGGTGGG
GTATAGCCATGGTGCCGGTGTGGAATCCCCGTGGAACGCGGTGGCTGGTGTGAACTGGTCGTTCTGACCATCAACGATTA
AACTGCGCTTCGGCGCAGTTTTCGTTTACAGGATGTTGAAAGGGAAAATTCTGGGGCAAAAAAAGCCCGCCAGTTACGGC
GGGAAACCTCATCCTATGGGAGAACAATGAATAATGAAATTGCGGGGTTATCATCTCCCAGTATATCCATACTAACAATA
AGGTTATTTACTCAACCAGGCATAAACATTTTGTTTTGTGCGTGGGAACAGCCTTAAGGTGTAAAGGGGGAGGTGGAAAT
AGCAATGAGGAGTATCAGCAAGAATACTCGCCGCTTTACCACAACGTGGATGAGAGGGATGAAAAACTCAAGGCAGAGAT
AACTCTGCCTTGAAGATAAATGCGCTTTTACAGCGGGCTTATTTCAGCTCTTCTGCTTCCGGTAAGGTCACGTTCAGCTC
AAGAATAGAAATATCGCCATCTTTTTGCTCAAGCTGTACGGTTACCATCTCAGGATCAATTTGTACGTATTTACAAATGA
CCTCAAGAATATCTTTACGCAACTGCGGCAGATAATGCGGTTCTGCATCGCTGCGACGGCGTTCAGCAACAATAATCTGC
AGCCGTTCTTTTGCAATGTTGGCTGTGTTTTTCTTCCGCGAGAGAAAGAAATCGAGTAATGCCATAACTTATCCTCCGAA
CAAGCGTTTGAGGAAGCCTTTCTTCTCTTCTTCAATGAAGCGGAAAGGACGTTCTTCTCCCAACAGACGTTCTACGGTAT
CTGCGTAGGCTTTACCCGCATCGGCGTTAATGTCGAGAATGACCGGTTCACCCTGGTTAGAGGCGCGCAATACTGATTGA
TCCTCTGGGATCACGCCGACGAGTTTGATGCGCAGGATCTCCAGCACATCTTCCATGCTCAGCATGTCACCTCTGCTTAC
GCGGCCTGGGTTATAGCGCGTTAACAGCAGGTGCTCTTTAATAGGCTCTTCGCCATTTTCTGCGCGGCGTGATTTCGACG
CCAGAATGCCTAAAATACGGTCAGAGTCGCGTACTGAGGAGACTTCCGGGTTGGTGGTAATAATGGCTTCGTCTGCAAAA
TAGAGTGCCATTAACGCACCGGTTTCAATCCCTGCCGGGGAGTCACAAACGATAAATTCAAAATCCATCGCTTTCAGATC
ATCAAGAACTTTGGCGACCCCTTCACGGGTGAGGGCATCTTTATCGCGTGTTTGCGATGCCGGCAGAATATAGAGATTTT
CAGTACGCTTATCTTTAATTAACGCCTGATTTAGCGTTGCATCGCCCTGAATGACGTTGACGAAATCGTAAACGACCCGG
CGTTCACAACCCATAATCAGGTCGAGATTACGCAGGCCGATATCAAAATCTATCACGACAGTTTTCTTTCCCTTCTGGGC
CAAACCAGTGGCGATGGCCGCGCTGGAGGTTGTCTTACCAACACCCCCTTTGCCCGAAGTAACAACAATAATGCGTGCCA
TAGAAATTCCTTGTTAAAAAGGGATCAATTTAACGGTTGAACGGTCAAAGCGTTTTCGACTAACTGCAGTCGCGCCGCTT
TGCCATAAAATTCTGCTGGGATTTGATCACTCAGCCAGTATTCACCTGCGATGGACACCAGTTCCGCCATCAGGTTCGTA
CAAAATATTTGCGTTTCCCGGTCACCACTTGCCCCTGCCAGCGCACGACCGCGCATCATGCCATAGACATGAATGTTCCC
ATCGGCAATCAATTCGGCCCCAGCGCTAACGTGGCTTGTAACAATCAGATCACATTGTGGAGCATAAATACGCTGACCGG
AACGCACCGGGGTATCTATTAAACGCGTTTTTGTGACCGGCGTTGTATTTTGCGCTGGAGCCTGCGGTGTGGGAGCTGGA
CGTGGCGCTTTTTCCTTTCCTTCCGTCAGGATAGGCAGCCCCATCTTTTCAATTTCGGCTTTAAGTTGCGCATCTTTGCA
GCCACTTACGCCAATAACCCGCAAACCGGTTGCCGAAACCGCCTTATGCATCGCTGACCAGTTTACCGGGTCTTCCAGTG
CACTGACGTTGAGTACAACGGGGGCATGTTTTAAAAATGCGGGGGCCTGAGCGATTTTGTCTTCCAGCGCCTGATGGATA
ACCTTAGGTTCTGCCTCATGCAGATGAACCACAGATAAAGTGAAGCTACTGCCTTTAAGCTCGATTGGCGTGTTTGACAT
CCTGGCCTTACTCAATTAGCTATTAATCATCGCCAGCGCGCGATGATGTTCCGAAGACTATAAGGCATGTTATAGTCTGG
ATTATATTGAGGCAAGTCACCCTCCCATTTATTCAGAGTAAAAGTCTATTCTGTGATAAATGGCGCTGATTCATAGCTTA
AAAAATACCCTTGTCAATCAACCCATTGCCGTCGTACTTTTGATTGTTCTTATTTACGCTTCCTTTTTCCGCACCCTAAT
CGTGACAGCATCTCCAGACCTGTTTAAATATCCAGTATTGAGGCAAGTGTATTTTTTCACCACGCCAGGCTTTCTTAACT
TTCCATTTTTTGCGGCATCATTAAAGAACAGATACCCGTTATCTTAATATCCGCCACAGAATTGCTCTATCATAGAAACC
GCAGAAGACGTCTGATACTTCTGTCAGGTCATCGTAACTCGTCCAGGTCAGCGGTTTTGATTACGGATTGATGATGAAAT
GAGCATAAGTGTTGTACGGTTGGATTATCAGGATGTGCTATGAATATGATGCGTATTTTTTATATCGGATTGTCAGGTGT
GGGGATGATGTTCTCATCAATGGCGTCTGGAAATGACGCTGGCGGACTTCAATCTCCGGCGTGCGGCGTTGTTTGCGACC
CGTATATTTGTGTGAACTCAGATGGCATTTCCCCAGAGTTAACAAGGAAATATCTCGGCGAAAAAGCCGCTGAAAACTTA
CAATCATTACAAGGCTACGATCCCAGCGAATTTACATTCGCTAACGGTGTATTTTGCGATGTTAAAGAAAAATTATGTCG
TGATGATCGTTATTTTGGTGTGGATGGTAAGCGGAGTGGAAAAATCAATCAAACCACCACAAAGATGTTATTTATGTGTC
GTGAGTGATGTCTTGTTTACATTATGTTCTATTCGTCATTAACCTCTTGAGCGACAGAGAGGAGTGGGGCATTAACGTTT
ATGACGGGGAGAGTCCCCGTCGGTTGACATTTACTTAATTTGAATATCGACGTTATATTTTTTAGTTTTGTTTTTACGGG
CGTCGTTTCGGGTTTGCAACACTCGCAGCTCATATTTGCCAGAAGCAGGGAGTGAGTATTGTCCGTGACTATCGAGTTCT
GGGGAATATCTGGATAAATCAACTGAATCATCGATTCCTGGCCCGAAGAGGTAAGTATCAGCGCCTTCATTAGAGATACT
CACATGCACTTTCTGACCTTTTTTGGCATAGAAGGTATATGTATCGTAATCGTATCCCTTTATTTCGCCGGAGTACTGAG
CGCTGCTATGGCCTTTTCTGAACTCAACATTGACATTCTTACCCGCAGCAAAGCTCGTGGAGGTTAGCAGTGCAAGCAGC

AATACAGCCTTCCTGATGCTCTTGATTTTCATTGGTAGTCTCCTGTTCAGAATGAGTACGATGAACTGTTATAATATAAC
AATCCCTAACGGGAGAATTCCAGTGGCTTTCCTGCGTTCGGTAAAATCACGTTGCGGCATTTTTGCCGGGGGGATTACTT
ATAAAGGGGGCATGGACTCAACAAACCGTAGAAATCTACAGCTGGCTGGCTTGTGGCGCGGGTTTCATTGATGTTAGCTT
ATGCCAAAACCTGGCATCTTAAAGAGTAAAAGTATGTTTTGTGTGATTTATCGAAGCAGCAAGCGTGACCAGACCTATTT
ATATGTCGAAAAAAAAAGACGATTTTTCGCGTGTTCCTGAGGAACTGATGAAAGGTTTTGGTCAGCCTCAGTTAGCGATGA
TTCTGCCGCTGGATGGGCGGAAGAAACTGGTCAATGCCGATATTGAAAAGGTTAAACAGGCATTAACCGAGCAAGGTTAC
TATTTGCAGTTACCGCCACCACCCGAAGATTTGCTGAAGCAACATCTTTCCGTCATGGGGCAGAAAACAGACGACACTAA
CAAATAACCGATATCCGGCGGTGGCATTATCTTTGTCGGCGCGGGTTTTCATATCCACGATAAGGTGAGGGGAACGTTAT
GTATCAACATCACAACTGGCAAGGTGCGCTGCTGGATTATCCGGTGAGTAAAGTAGTCTGTGTTGGCAGTAACTATGCCA
AACATATTAAAGAGATGGGCAGCGCAGTGCCCGAAGAGCCAGTGCTGTTTATTAAACCAGAAACGGCACTGTGCGATCTG
CGGCAGCCATTGGCGATCCCATCCGATTTCGGTTCAGTTCATCATGAAGTCGAACTGGCGGTGTTGATTGGCGCGACACT
GCGTCAGGCTACGGAAGAGCATGTCCGCAAAGCCATTGCCGGTTATGGCGTGGCGCTCGATCTGACATTGCGTGATGTTC
AGGGAAAAATGAAGAAAGCCGGGCAGCCGTGGGAAAAGGCTAAAGCGTTTGATAACTCTTGTCCGCTTTCCGGGTTTATT
CCCGCGGCGGAATTTACCGGCGACCCGCAAAATACAACGCTGAGCCTGAGCGTAAACGGCGAACAACGCCAGCAAGGTAC
GACTGCGGACATGATCCATAAAATCGTTCCGCTGATCGCTTATATGAGCAAGTTTTTTACCCTCAAGGCCGGTGACGTTG
TGTTGACAGGCACGCCTGATGGCGTCGGCCCGTTGCAAAGCGGTGATGAGCTGACAGTCACTTTCGATGGGCATTCTTTG
ACAACTCGCGTTTTGTAATACTTTTTGCCGCCTGAAAGCGGCGGCAAAACTTGCATCGCTGTGCCAGACTGGTTATAAGG
TGCGTTTTAACGTAATGGCGGAACACCTGATGAGCGATGTACCTTTCTGGCAAAGTAAAACCCTGGACGAAATGAGCGAT
GCGGAATGGGAGTCGTTGTGTGATGGTTGCGGTCAGTGTTGCCTGCATAAACTGATGGATGAAGACACCGACGAAATCTA
CTTCACTAACGTCGCCTGTCGCCAGCTCAATATTAAAACCTGTCAATGTCGGAACTACGAACGTCGTTTCGAGTTTGAAC
CCGACTGCATTAAATTAACCCGTGAAAATCTGCCAACATTCGAATGGCTGCCAATGACCTGCGCTTATCGTTTGCTGGCG
GAAGGTAAAGATTTACCTGCGTGGCATCCGCTACTTACTGGTTCGAAAGCGGCAATGCATGGTGAACGTATCTCTGTGCG
TCATATCGCAGTGAAAGAATCAGAAGTGATTGACTGGCAGGATCATATCTTAAATAAACCTGACTGGGCGCAGTGATAAA
TTTAATAGATGCTGATGGGGGCTTTCTTAATATACGTCTGGATGCTCGCCATTTCGTAGCCCTGTTTTTGAAAGCGCTCT
GTCATAATGAGAGTTACCCGATACCTTATTATCAACAGGTAACTCTCAATCTTTAAAAAACGTCTGTGTTTTTAAATGTG
ATAAACCTTATACCTTGAGTACATGGAGAGAATAATCGCTTATCAGACTTCAGGTACCTCAAAGAGTGTCTTTTTACCGT
GTCTTTTCTGATACTCATTACAGGTGTTAATCATTTTTTTGGCCGCTTCTTTTAGCAAAGAAAGCATTAAATCATCATAA
TCAACGTAGAATCTGGTTGTTTCAGTTTCCGTTTTTATCTCACCGATGGCGGCTATTTCGGTGGTTAATTTCAATTTGGC
GGCATCGATATCTTTATTCGCTTGTTTAACCGTGTTAGACAGGGTGGTAAAGAAATTCTGCACAGATTTTAACTTGTTCT
TCAATTCTGGAATCAGTTTTCCTTCAACTACGCCCGCAGCAATAGAATAGGAAATGATTAATCCAAATGGACCGGCGACG
ACACCGGCTGCGGCACCGGCATATGCTTCCTTCCTGATTTTATCTACCTGTGACTGGAAATAGCTGCTTTTTTCTGAAAA
ATCATTGGTTAACTGGCTATCTAACGCCAGCAGTTTCCCGGAAGCGTTGTTGAAACTTTGTGAGCTTACCAGCAGGGATT
TTTGCGCTTCATTCAGCTTCGTGATGCCGTCATCCAGTACCTTAATGAGAATGTCTTTCTGGGCGGATGCTTTCTTCTCA
TTGTACTCATCAAATAGCAAAATATACGCTGCGAGCAATTGCGTCGCAACACCACACCATTCATACACTGTTTGGGTTGC
TTCAAAATACTTATCCTGGCTATCCATAAGTAAGGTTTTAATATCGCCGACTAAAACGGAGGCTGCCTGTGAATACTCCT
GTTTAAAGCGACTTAACTCTTTTATGGTTTCATCAAAGGTCTGCCAGGGGATGACCTGATCGAGATATTTATTATAAAGA
TCTAATGCTCCATCTGCGGTTTCGATTGCGTTTTTAACTACTTCTACCGTTTTATCTGCAACGATTTCAGTCATAATCAT
TCGCCTCTTTAAATATATAAATTGTAATGAAACTCCTGTTTTACAACTATTAATAAATTTTACTTCATCTAATTCATAGT
TAGCCGGGCGGGATGCGTCAATGTCTTTATTTCTATTAATATGATAAATATCAAACAATGTTTAATGTCATTATGGCGAA
TGCTTCTATTCTATTTTTTAGCCGGGTGATATTTTTCATTTCTGCTGGATGAGCGTCGTCGCCAGAAGGCCACGTGAGCA
CAAGATAAGAGAACGAAAAATCAGCAGCCTATGCAGCGACAAATATTGATAGCCTGAATCAGTATTGATCTGCTGGCAAG
AACAGACTACTGTATATAAAAACAGTATAACTTCAGGCAGATTATTATGTTGTTTATCAAGCCTGCGGATCTCCGCGAAA
TTGTGACTTTTCCGCTATTTAGCGATCTTGTTCAGTGTGGCTTTCCTTCACCGGCAGCAGATTACGTTGAACAGCGCATC
GATCTGAATCAACTGTTGATCCAGCATCCCAGCGCGACTTACTTCGTCAAAGCAAGTGGTGATTCTATGATTGATGGTGG
AATTAGTGACGGTGATTTACTGATTGTCGATAGCGCTATTACCGCCAGCCATGGTGATATTGTCATCGCTGCTGTTGACG
GCGAGTTTACGGTGAAAAAATTGCAACTACGCCCGACGGTACAGCTTATTCCCATGAACAGCGCGTACTCGCCCATTACC
ATCAGTAGTGAAGATACGCTGGATGTCTTTGGTGTGGTGATCCACGTCGTTAAGGCGATGCGCTGATGTTTGCCCTCTGT
GATGTAAACGCGTTTTATGCCAGCTGTGAGACGGTGTTTCGCCCTGATTTATGGGGTAAACCGGTGGTTGTGCTATCGAA
TAATGACGGTTGCGTTATCGCCCGAAACGCTGAGGCAAAGGCGCTTGGCGTTAAAATGGGCGATCCCTGGTTCAAACAAA
AAGATCTGTTTCGTCGCTGTGGCGTGGTTTGCTTTAGCAGCAATTATGAGCTTTACGCAGACATGAGCAATCGGGTGATG
TCGACGCTGGAAGAGCTATCGCCCCGCGTCGAGATTTACAGTATTGATGAGGCATTCTGCGATCTGACAGGTGTGCGTAA
TTGTCGCGATCTGACTGATTTTGGCAGAGAAATTCGCGCAACGGTGCTACAACGTACCCATCTTACTGTTGGTGTGGGGA
TCGCCCAGACCAAAACGCTGGCTAAGCTTGCCAATCATGCGGCAAAAAAATGGCAGCGGCAGACGGGTGGGGTGGTGGAT
TTATCAAATCTGGAACGCCAGCGTAAATTAATGTCTGCTCTCCCCGTGGATGACGTCTGGGGGATTGGACGGCGGATCAG
CAAAAAACTGGACGCGATGGGGATCAAAACCGTTCTCGATTTGGCGGATACAGATATCCGGTTTATCCGTAAACATTTTA
ATGTCGTGCTCGAAAGAACGGTGCGTGAACTGCGCGGCGAACCCTGTTTGCAACTGGAAGAGTTTGCACCGACGAAGCAG

GAAATTATCTGTTCCCGCTCGTTTGGTGAACGCATCACGGATTATCCGTCGATGCGGCAGGCCATTTGTAGTTACGCTGC
CCGGGCGGCGGAAAAACTTCGCAGCGAGCATCAATATTGTCGGTTTATCTCCACGTTTATTAAGACGTCACCATTTGCGC
TCAATGAACCTTATTACGGCAATAGCGCGTCGGTAAAACTGCTGACGCCCACTCAGGACAGCAGGGATATCATTAACGCT
GCTACGCGATCTCTGGATGCCATCTGGCAAGCGGGCCATCGTTACCAAAAAGCGGGCGTGATGCTGGGGGATTTCTTCAG
TCAGGGAGTCGCGCAGCTCAATTTATTCGATGACAACGCACCGCGCCCCGGGAGTGAGCAATTGATGACGGTAATGGATA
CACTGAATGCTAAAGAGGGCAGAGGAACACTCTATTTTGCCGGGCAGGGGATCCAGCAACAATGGCAGATGAAGCGAGCC
ATGCTTTCACCACGTTATACAACGCGAAGTTCTGATTTACTGAGGGTCAAATAAATATAGCGGCAGGAAAAAAGCGCTCC
CGCAGGAGCGCCGAATGGATTAGCGACCGAACAGATCACGTTTTTTCGCTTTAAACGGCTGGGAAATCACCACCAGCACT
GCGACAATCAGGTAAGCGATAAAAATACCGAGCAGCCACTGCGGCATTTCCAGACCTAAAAAATCCCACTGACGCTCGGC
GCAATCGCCAGAGGCGACAAACACTTGCGGCACCCACTTATCCAGCGGCAGCCATTCCGGGAAACGAACCATAAAATCAC
AGGTGGCAAACGGCGAAGGATAGAGCTGAAGCATGGTGTGCTCGTAAGTTAACTGCACACCGCGGAACGCACTATACAAC
CAGATAACCATCGCTACATAACGCAGCGGAGTTTTCGGGGCGATCGCGCCAATCAGCGCAGCACCCAGAACGCCGAATAA
CGCGCAGCGTTCATAAATACAGAGCACGCAAGGTTTCAGTAACATCACATGCTGGAACCACAGCGCCGTCAGTTCCAGTG
CCAGAGCAGTAAACGCCATCAACAGCCACGCGCCCCGGCCTTGTGAACATTGGTTCAAAAATCGCAACATAATCATTTCC
CTGCAATATGCATAGAGTGCGCAGTTTAAACCAATTCATTCGCTGCGCCACCAGGAGGCAGCAAAAATAGGACGCAATTG
GACAATTATCGGGCGAAAAGGCAAACCGGGCATTTATATGCCCGGTAAGTTGTTATCAAAGCGTTGCTATCCAGCCCATT
TGCATAAACCATTCGGTTACAGGGGCAAGCGTAAACTCGACGCAGAGCAAGCCGACGAGTGTCAGGACGAGGGTGTAAGG
CAGGGCCATCCACACCATGCGGCCATAAGAGAGGCGAATCAATGGCGCGAGTGCAGAGGTCAGCAGGAACAGGAACGCAG
CCTGACCGTTCGGCGTAGCGACGGAGGGCAGATTGGTACCGGTATTAATGGCGACCGCCAGCAGCTCGTATTGCTTCAAC
GTGATAGCGCCACTTTCCATTGCCGCTTTCGCTTCGTTGATATAAATCGTCCCCACGAAGACGTTATCCGAAATGGATGA
CAGCAGACCGTTGAAAATATAGAACAGCGACAGCTGAGCATGTTCCGATGCCTGCAACACAAACTGAATAATTGGCGAAA
ACAGTTGTTGGTCGATAATCACCGCGACTACCGAGAAAAACACCGTCAACAGTGCGGTGAATGGCAGAGATTCGGTGAAG
GCTTTACCGATAGCATGCTCATCGGTGACACCGGTCAATGATGTTGCCAGAATAATGACTGACAAACCAATCAAGCCAAC
TTCCGCCAGATGCAACGCCAGCGCAGTCACCAGCCAGACGCCAATAATCGCCTGGACAATCAGACGGATTTTATCCTGAC
GGGTACGCTGGTGGCGGCTTTGATCGTCAAACTGTTGCAACACTTCGCGGACTTTCTCCGGCAGCGTTTCACCGTAGCCA
AACCAACGCAGCTTCTCTACCAGCAGGCAGGTTAACAGGCCACAAATCAGAACCGGAACGGTCACCGGCGACATGCGCAG
GAAGAAATCGCCAAAATGCCAGCCAGCCGCTTTAGCGATGATCAGGTTCTGTGGTTCGCCCACCATGGTCATTACGCCGC
CTAATGCGGTGCCGACACCGGCATGCATCATCAGGCTACGCAGAAAGCCACGGAACTGTTCCAGAACCACTTTGTAATGC
TTGTCGATATGACTATCGTCTTGCAGGTCGGTGTCTTCGGTACGGGAAGAGGCTACGCGGTGATAAATACCATAAAAACC
GACTGCAACGCTGATCACCACCGCCACGACGGTTAAGGCATCGAGGAACGCGGAGAGGAACGCAGCCGCCACGCAAAAAG
AGAGCGACAGCAGCATTTTGGAGCGAATGCTTAACAGCAAACGGGTAAATATGAACAGCAACAGCTGTTTCATAAAATAG
ATACCCGCCACCATAAACATCAGTAACAGCAAGACTTCAAGATTTGCCGCCACCTCTTCACGGACGTGTTCCGCGCTGGT
CATGCCGATGAATACCGCTTCGATAGCCAACAGACCACCGGGGAGCAGCGGGTAGCATTTCAGGGCCATCGCCAGAGTGA
AAATAAATTCCGCTACCAGCAACCAGCCCGCGACGAAAGGGCTGATGAGGAAAATTAACGGGTTTACGATTAAGAAAATG
ATGAGGGCGAGTTTGTACCAGTCGGGGGACTGGCCCAAAAAATTGCGCCATAGCGCGCGGCCCCAGGAGATCTCCATGAT
GGTTTCCCTTACCTTACAAATAATCAATGATGTTTTTATGTTTAAACGCAAAGCTTAACGGTCAGGCAGGAGTGAGGCAA
GTCTTGATAGTCAAGGGGAAAGAGATGCGGAAAATGAAGCCTTGATCCCTTTTTCTTCTTTTTGTCTGCTATCAGCGTAG
TTAGCCCTCTGGTATGATGAGTCCAACTTTGTTTTGCTGTGTTATGGAAATCTCACTATGGTCATTAAGGCGCAAAGCCC
GGCGGGTTTCGCGGAAGAGTACATTATTGAAAGTATCTGGAATAACCGCTTCCCTCCCGGGACTATTTTGCCCGCAGAAC
GTGAACTTTCAGAATTAATTGGCGTAACGCGTACTACGTTACGTGAAGTGTTACAGCGTCTGGCACGAGATGGCTGGTTG
ACCATTCAACATGGCAAGCCGACGAAGGTGAATAATTTCTGGGAAACTTCCGGTTTAAATATCCTTGAAACACTGGCGCG
ACTGGATCACGAAAGTGTGCCGCAGCTTATTGATAATTTGCTGTCGGTGCGTACCAATATTTCCACTATTTTTATTCGCA
CCGCGTTTCGTCAGCATCCCGATAAAGCGCAGGAAGTGCTGGCTACCGCTAATGAAGTGGCCGATCACGCCGATGCCTTT
GCCGAGCTGGATTACAACATATTCCGCGGCCTGGCGTTTGCTTCCGGCAACCCGATTTACGGTCTGATTCTTAACGGGAT
GAAAGGGCTGTATACGCGTATTGGTCGTCACTATTTCGCCAATCCGGAAGCGCGCAGTCTGGCGCTGGGCTTCTACCACA
AACTGTCGGCGTTGTGCAGTGAAGGCGCGCACGATCAGGTGTACGAAACAGTGCGTCGCTATGGGCATGAGAGTGGCGAG
ATTTGGCACCGGATGCAGAAAAATCTGCCGGGTGATTTAGCCATTCAGGGGCGATAATCCCTTCCGTTTAAAGAGCAAAC
CCCTCAAACGAGGGGTTTTTTGTTGTTTTTACAGATTTCCCATTCTTGGCGGGCAACGTTCCAGCAACTCGATGCTGCCG
TCTTCGTTTTGCTGTTCGAGCATCACATCAAATCCCCACAGGCGATGCACATGCTTCAGGACTTCTTTGCGCCCCCGATC
CAGCGGTGCGCGATTATGTGGAATATAACGCAGCGTCAGCGAACGGTCGCCGCGCAAATCCACGTTCCAGATCTGAATAT
TCGGCTCCAGATTACTTAAGTTATATTGCGACGATAACCGGTTACGGATCTCCCGATAACCTTCTTCATTATGAATAGCG
GAAATCTCCAGATAATTATGCCGATCGTCATCCAGCACGGTGAAGAAGCGGAAATCACGCATCACTTTCGGTGACAGGAA
CTGGCTGATAAAACTCTCATCTTTGAAATCACGCATCGCGAAATGCAGCGTTTCCAGCCAGTCGGAACCGGCGATATCCG
GGAACCAGTATTTGTCTTCTTCCGTTGGCGACTGACAAATCCGTTTAATATCCTGGAACATGGCGAACCCGAGGGCATAC
GGGTTGATGCCGCTGTACCACGGGCTGTTATAGGGGGGCTGGAAGACCACATTGGTGTGGCTGTGCAAAAACTCCAGCAT
AAAACGTTCCGTTACTTTCCCTTCATCATACAGATGGTTAAGGATGGTGTAGTGCCAGAAGGTCGCCCAGCCTTCGTTCA

TCACCTGAGTCTGTTTTTGCGGATAAAAATACTGGCTCACCTTACGCACAATACGCAGGATTTCACGCTGCCATGATTCC
AGCAGTGGCGCATTTTTCTCCATAAAATAGAGCAGGTTTTCTTGTGGTTCGGACGGATAGCGGCGCGCTTCAGCAACCGT
TTTCTCTTCCTCGCGCTTCGGCAGGGTACGCCAGAGCATATTGACCTGACTTTGCAGATACTCTTCGCGACTTTTCTGCC
GGGCTTTCTCTTCTTGCAGCGAGATTTTTTGCGGGCGTTTGTACCGGTCCACGCCGTAGTTCATCAGCGCGTGGCACGAG
TCCAGAAGCCGTTCTACTTCATCAACGCCATAACGCTCTTCGCACTCGGTAATATATTTACGGGCAAAAATCAGATAATC
GACAATCGAACTGGCGTCGGTCCAGCTACGGAATAAGTAATTGTTTTTGAAGAAAGAGTTATGCCCATAGCAGGCATGAG
CCATCACCAGCGCTTGCATGGTAATGGTGTTCTCTTCCATCAGGTAAGCGATACACGGGTTAGAGTTAATGACGATTTCA
TAGGCCAGTCCTTGCTGACCGTGCTTATACAGCCGTTCAGTCTCGATAAACTTTTTACCGAATGACCAGTGCGGATAGTT
AATTGGCATGCCGACGCTGGAGTAGGCATCCATCATCTGTTCTGAGGTTATCACTTCAATCTGGTGCGGGTAGGTATCCA
GCCGGTAGAGTTTCGCCACCCGGTCTATCTCTGCCAGATAAACATCCAGCAGGTCGAACGTCCAGTCGGGTCCATCGCTC
AAACGTGTGGTGTCCTTATTCATAGAATCGATCGTCGCCATACGCGCACCTCATTGTTGTCGGCGCTCTCTGTGTGGAGC
ACCTCATTTCAAGCATAGAACACCTGTTAAAAACCGCGTCGCCGGAGAATTTTTTTCTTTGCGATTTCTTATTATCAGAG
TGCCACTAATCCGCTTCTGAACGGAATTTTATGCTGGATAAAAAGGGCGTTCAGCAGGAGATACTAAAGACGCCATATTG
CCGCAGAGTCAGGGAGATGTGAGCCAGCTCACCATAAAAAAGCCGCATGTTGAATAATATTTTCAACTGAGTTATCAAGA
TGTGATTAGATTATTATTCTTTTACTGTATCTACCGTTATCGGAGTGGCTATGCGAGTTGTCATACTGGGAAGTGGTGTG
GTAGGCGTTGCCAGCGCCTGGTACTTAAATCAGGCAGGACATGAGGTCACCGTCATTGATCGGGAGCCGGGGGCCGCCCT
GGAAACCAGTGCCGCGAATGCCGGGCAAATCTCCCCCGGATATGCTGCACCGTGGGCGGCACCAGGTGTGCCTTTAAAAG
CGATTAAATGGATGTTCCAGCGCCATGCGCCGCTGGCGGTTCGTCTCGACGGTACGCAGTTCCAGTTGAAATGGATGTGG
CAAATGTTACGTAACTGCGACACCAGCCACTACATGGAAAACAAAGGGCGGATGGTGCGTCTGGCGGAATACAGCCGTGA
TTGCCTGAAAGCATTGCGCGCCGAAACCAATATTCAGTATGAAGGGCGTCAGGGTGGGACGCTGCAACTGTTCCGTACCG
AACAACAGTATGAAAATGCGACCCGCGATATCGCCGTGCTGGAAGATGCCGGCGTACCGTATCAGCTGCTGGAATCCAGC
CGCCTGGCGGAAGTGGAGCCCGCGCTGGCAGAAGTGGCGCACAAACTGACGGGCGGCCTGCAGTTACCCAATGATGAAAC
CGGAGACTGTCAGCTATTTACCCAGAATCTGGCGCGGATGGCGGAGCAGGCGGGGGTTAAATTCCGCTTTAATACGCCCG
TTGACCAACTGCTTTGCGACGGCGAGCAAATCTACGGCGTGAAGTGTGGCGATGAAGTGATTAAGGCCGATGCGTATGTG
ATGGCGTTTGGTTCTTACTCGACGGCGATGCTCAAAGGCATTGTTGATATTCCGGTGTATCCGCTGAAAGGCTACTCGCT
GACCATTCCAATTGCGCAGGAAGATGGTGCGCCGGTATCCACCATTCTTGATGAAACCTACAAAATCGCCATTACCCGTT
TCGATAACCGCATTCGTGTTGGCGGAATGGCGGAGATTGTTGGTTTTAATACCGAGCTGTTGCAACCGCGTCGTGAAACG
CTGGAGATGGTGGTTCGCGATCTCTATCCACGCGGCGGTCATGTCGAGCAGGCGACTTTCTGGACTGGTCTGCGCCCGAT
GACGCCAGACGGCACGCCGGTTGTCGGGCGTACACGCTTTAAAAATCTGTGGCTGAATACCGGTCACGGCACGCTCGGCT
GGACGATGGCTTGCGGTTCCGGTCAGTTGTTAAGCGATCTGCTCTCTGGTCGCACGCCAGCGATCCCATATGAGGATCTA
AGCGTAGCGCGCTACAGCCGTGGATTTACGCCATCACGTCCGGGCCATTTACATGGCGCACACAGCTAAGGAAACGAGAT
GACCCGTCCGATACAGGCCAGCCTCGATCTGCAGGCATTAAAACAGAATCTGTCCATTGTCCGCCAGGCCGCGACGCACG
CGCGCGTCTGGTCGGTGGTAAAAGCGAACGCTTACGGGCATGGTATTGAGCGTATCTGGAGCGCGATCGGGGCCACCGAT
GGCTTTGCATTGCTTAACCTGGAAGAGGCAATAACGTTACGTGAGCGCGGCTGGAAAGGACCGATCCTGATGCTGGAAGG
ATTTTTCCATGCTCAGGATCTGGAGATTTATGACCAGCACCGCCTGACCACCTGCGTACACAGCAACTGGCAGCTCAAAG
CACTGCAAAATGCGCGGCTAAAAGCACCGTTGGATATTTATCTTAAAGTGAACAGTGGGATGAATCGGTTGGGCTTCCAG
CCCGATCGCGTGCTTACCGTCTGGCAGCAGTTGCGGGCAATGGCGAATGTTGGCGAAATGACCCTGATGTCGCATTTTGC
CGAAGCGGAACATCCTGATGGAATTTCCGGCGCGATGGCGCGTATTGAGCAGGCGGCGGAGGGGCTGGAGTGTCGGCGTT
CGTTGTCCAATTCGGCGGCGACTCTGTGGCACCCGGAAGCGCATTTTGACTGGGTTCGGCCTGGCATTATTTTGTATGGC
GCTTCGCCGTCCGGTCAGTGGCGTGATATCGCCAATACCGGATTACGTCCGGTGATGACGCTAAGCAGTGAGATTATTGG
TGTCCAGACGCTAAAAGCGGGCGAGCGTGTGGGCTACGGCGGTCGCTATACTGCGCGCGATGAACAGCGAATCGGCATTG
TCGCCGCAGGGTACGCCGACGGTTATCCGCGCCACGCGCCTACCGGTACCCCTGTTTTAGTGGACGGCGTGCGCACCATG
ACGGTGGGGACCGTCTCGATGGATATGCTAGCGGTCGATTTAACGCCTTGCCCGCAGGCGGGTATTGGTACGCCGGTTGA
GCTGTGGGGCAAGGAGATCAAAATTGATGATGTCGCCGCCGCTGCCGGAACGGTGGGCTATGAGTTGATGTGCGCGCTGG
CGCTACGCGTCCCGGTTGTGACGGTGTAACTTGTTGTAAGCCGGATCGGAGGCAACGTCTTCTGGGTGCAAAAAAATCAT
CCATCCGGCTGGTCAGCAACTGTAGTTGTTAATGTGACAGAGCCATTGCCCATGATAGTGTCCATTAAAAGGATGGACAC
TATTTCCCCGGAACCTGAACTCACCGCACAGGCGTTCTACATAAAACGCTTACGCTTCATTGTTGACTCGAACTCGACTT
CAGATAAATCACGCTTTACCCTTGATGGAGCCTGTACATAGATTTGTGTAATTGCCTGATTTTGATATGTTCAATTCAAC
ATCAAATGAAGGTTAAATTATGGACGACAAACAATTGCAGGCTCAGGCTGCGTTCAGCAAAGCATCGCAACCGGCGATAG
ATGCTTCATTAAATTTAAGATTCAGCTTCCTCTTCAGCCACCCGTACGCCAATCTTCAACACTTCATTATCTTCTTTCTC
GGCCACCGTCCAGATCATCCCGGCAAACTCTACCTGGTCACCGACAACCGGTGCCGCGCCTAACAACTGCTGGACAATTT
CACCCAGCGTTTGCTGCTTATCACGATATTCTCGCCCGTCTTCCAGGCCATATATCAGCGCAACATCAGCATATTTGGCG
CTGGCTTCGAGAATGAAGTCACCAAAGAAGCGTTGATCCAGCGCGACCGGCGGCGACTGGCTGAACAGTTTACCGAGCGC
CGGAAGATCGCGTTCCCGACCAATTACACACAACACATCGCCTTCGCGCAGTCGGGTGCTGCCGGTAGGATGAAGCAACT
GGTTATCACGAAACAGTGCCGCAATACGCGTCTCTTTTGGCATATGCAAATCGCGCAGTGCCGCGCCCACGCACCATTTA
TCGGCACTCAATTGATAAACAAACTGCTCCCACGGATTTTCCGGATGAATATCCAGGCCAACGCGTGACACCGGACGTCC

CACTGGCGGAACCACCACTTTGGCTTTTTTAGCCGCCCACGAGAGTGATGTTCCCTGCAAGAGCAGTGAAACCAGAACCA
CAAAGAAGGCGACATTAAAGAACAGACGTGCATTCTCCAGCCCCGCCATCATCGGGAACACGGCCAGGATGATTGGCACC
GCGCCGCGTAATCCTACCCAGCTGATAAACACGCGCTCACGCAGATTGAAACCGCGGAAGGGGAGCAATCCAGCAAATAC
CGAAAGGGGACGGGCGAAGAATATCATCCATGCGGACAAAATGAGCGCCGGAATGGCAATAGGCAGCAGATCGCTTGGGT
TAACCAACAGCCCCAGCACCAGGAACATGGCGATTTGCGCCAGCCAGGCGAGGCCGTCGAAATTTTGCAGGATGCCGTAG
CGGTTGCGAATAGGGCGATTACCCAGCAGAAAACCGCACAGATACACCGCCAGAATACCGCTACCTTCCAGCGCAGTAGT
TAATGAGAAAATCAGAATACCGCCACTTAATGCCAGCAATGGATATAATCCGGCGGGCAGGGCGATGCGGTTGATCATTT
GCAGTAGTAAATAACCGCCGCCAAGACCTATTACAATGCCCAGACCAAATTGTTGCAGAATATCGACAATGAACATCCAG
CTAATATTGCTCTCATGATGCTGGATCATCGCAATTAGGGTAATCGTCAGAAAGACCGCCATTGGATCATTACTGCCGGA
TTCAATTTCCAGCGTCGAGCCAACACGTTCGTTAAGCCCCTTACCACCCAGCAGAGAAAAGACCGCTGCAGCATCGGTAG
AGCCGACGATAGCGCCGATTAATAAGCCTTCAATCAAATCAAGATTAAACAGCCACGCCGCCATCATGCCGGTTAAACCA
GAGGTGATAAGCACGCCCAGCGTCGCCAGCGACAGTGCCGGTCCTAACGCCACACGAAAGGAACTGGCCTGAGTGCGCAT
CCCGCCGTCGAGCAAAATAATCGCCAGTGCCAGGTTACTTACCATGTAGGCGAAGGGGTAATTATCAAACGGGATACCGC
CGACGCCATCGACTCCTGCCAGCATGCCGATCGCCAGAAAAATAACCAGAATAGGAATGCCAAGACGGGAAGAAAATGAA
CTAAGTAATATACTGCTGGTGACAAGGATGGATCCTAAGATGAAAAGGCTAATTATTGTTGTGGCATCCAACGGTCGGTT
ACTCCTGATTACGCTGTCTCTTATATAAACCCTACCATATTAGCGGCAGAGACAGCGTTTTACTTAGCCCTGAAGCGTGA
TTTTTTTACATTTTAAGAACAGGATGACCGCTAATCGTCAACTGAGTGCCTTCCCGGGTGTTATTCAGAATGGCATGTGC
GCCCAACGGCAGCGTAACCGTGCGTTGTTCATGACCAAAATCGAGGCCGGTAATGAGTGGAATCGACAGGCGGGAACGCA
AAAACGCGTACACTGACTCCAGGTTGTAACCTGCGTCATAATCATTGGGCGTGCTGCCGCTAAAGCTACCGAGAATAATC
GCCTTCTGACGTGGCAAAATTCCCGCATGATAGAGCTGCAACAGCATACGTTCGACCCGGAAAGGGTGCTCGTTAATATC
TTCCAGCACCAGAATACCGTTCTCAATTTTTGGCATCCACGGTGTACCGATCAGTGAAATCAGCATCGCAAGATTGCCTC
CCCACAACGTGCCTTCGGCCCGACATGTCGGGCCTTCCCCTTGCCATTCAATGGTGAAGGTTTCATTGCGTAACGCCAGC
CAGAAATGGTGCTCGGTAAAGGCGTTCAGTTCATCCGCGCCAAAGTTTGCTACCAGCATGGGGCCGCTAAAGGTGATGAC
ATTGCCATGCGCCAGAAGACCGCACTGAATGGCGGTAAAATCGCTATGTCCGCAAATGAGCAACGGGTCATGTTGTTGGC
GAGCCACCAGTGCCTGCCAGTCAATATCTGCCAGTAAACGACTGGCACCGTAACCGCCGCGTACAGCCAGCACGATGGTG
TTGGGGGTAGTCAGTCTGGCAAGGGAATTGAGATCTTCCAGACGCTCTGTTTCCGTACCCGCAAAACGCTCACAACGACG
GGCAATAACCTCGACGTTATTGACTTGATGCCCCGCGTCGGTAAGGCGCTGGATACCACGCAGCGCGGCGTGCTGTTTAA
TGCAGTAACCCGATGGGGCAATTAAGTGAAACAGAGACATGGCAATTCCTTGCTGACAACAGAAACGAAATGTATATCAT
GCCGCTTAGGTGTGCCGTTGTCACCTCAACGGCGATTCCAGGCTATAAGGATAGAAGAAGTGAAATTGAGATGGTTTGCC
TTTTTGATTGTGTTATTAGCGGGTTGTTCATCAAAGCATGACTATACGAACCCGCCGTGGAACGCGAAAGTTCCGGTGCA
ACGTGCGATGCAGTGGATGCCAATAAGCCAGAAAGCCGGTGCAGCCTGGGGCGTCGATCCACAATTGATCACGGCGATTA
TCGCTATCGAATCGGGTGGTAATCCGAACGCGGTGAGTAAATCGAATGCCATTGGTTTGATGCAGTTAAAAGCTTCAACC
TCCGGACGTGATGTTTATCGCCGTATGGGCTGGAGTGGTGAGCCGACGACCAGCGAGCTGAAAAATCCAGAGCGTAATAT
TTCAATGGGGGCGGCTTACCTGAATATTCTGGAAACCGGCCCGCTGGCAGGCATTGAAGATCCGAAGGTACTGCAATATG
CGCTGGTGGTGTCATACGCTAACGGGGCAGGTGCGCTGCTACGGACTTTCTCGTCAGATCGGAAAAAGGCGATCAGCAAA
ATCAACGATTTGGATGCTGACGAGTTCCTCGAACACGTAGCGCGAAATCACCCTGCGCCGCAGGCTCCGCGCTATATCTA
CAAACTTGAGCAGGCACTGGACGCGATGTAAATCAGTCGCGCACTTTGTCCGCTTTTTCCCGGGCTTCTCGCTCGAGAGA
GAAAATAATCCGCTGTAATTGCCGCTCCACCGTCGGGCTGACGTTAAGAAAACGGAAGCTCAGACGGGGAGTGGTGATGG
TTTCATTCTTGCCATCAATCACTTTGCGCTCGCTGATGGAGATTAACTGGGCGTCAAAGTGAAAAACACCCCATTGCCCC
ATGTTGACTTCAATCTGAGCGAAGCGCATGCCTTCTTGTAATTCGGCAGGCTTTGCTGTTTCCAGTAATGCGCCCATGCC
GCCTAACGACAAATCATACAGGCGGAAACGTAACGTACTGTTATCCGCCAGTTTGGTCTGGCAAAAATAAGGCGGATGGA
GTGGGGCGGAGATGCGGAAATATCGGCGTCGTTGTACAAACCATAAGGTGGGAGGCGGTACGGTAATAAATGCCGGAAGC
TGCAAGTATTCACTCTGCTGTAGTTGTTCAACAGTAAACTCGACTTTCGCACCCTGAGTTTCGGCGGTAATGGTAATGTG
CTGTGCCTTTAGCACGGCGATGTTGTCTTCGGCTTGACTGCCGAAATCCAGCACCAGTTTATCCGGGGTTATTGCCAGTA
ATTTGCTGATCAGCTGCCCGCCATTCCAACTGAGACGCAAAGGAATTGCGGCTTTGTGCAAATCGCGTAACACGCCCAGG
ACGGCTAACGGATTTTGTTTCAGGAACTGCTCATGGTAATGACTCACGCGGAAAAACTCCTGACTGACAAACTGTCTTTG
GTTTATCGGTCACAGTTAACAAAACTTAATACAAATGCGTGAATATTTTTTACATGTTGTTCTTAAATCAGCCGACATAC
GCCCAGCATCGATCCCGCCCTTGCCTATACTTAGAGCGTTGATGTAAGCATTTCTTGCGTCCGATTCATCGAAACGAGGG
CCTGAACATGGGAATTATTGCCTGGATTATTTTTGACCTGATAGCCGGCATTATCGCCAAGCTAATCATGCCGGGGCGTG
ATGGTGGTGGATTTTTCCTGACCTGTATTCTCGGGATAGTCGGTGCGGTGGTCGGCGGCTGGCTGGCGACCATGTTTGGC
ATTGGCGGCTCCATCAGTGGTTTTAATCTGCACAGCTTCCTGGTGGCGGTGGTGGGAGCTATTCTCGTTCTGGGCATATT
CCGCCTCCTGCGAAGAGAATAAGATTTTCATAAGGCGGATAGCGATACAGATGCCGCTATCCGCTTTCACATCAGAACGT
ATACTCGACACCTGCTTTACGGGTGAAAAAAATCAATATAGCACTTCGATATCCGATAAAGTCGGTTCCCGGTAACAACA
ACAGAATCCTTTTTTAATTATTGTTTCGTTGTTTTTGGTGTGATTCAGAAAATAAATATTTTAGACAGTAGCCAGTCAGG
CGTTAAAAATGGGGCGGATAGTGAGGCGGTTTTGGCTGCGCTTATTGAACATTTAGAATTGATCAATCCTTCTGGCAGAT
TAAGCCAAAATACCCGTTCGGCAATGCTTCAGTTACGCGAAGAGGAATGGTCTGAATTCTTTTTTTGGTTGCTAAATTCT

CTGGAGTGTCTGGATTATGTGATTATTAATTTAACTCCAGAAAGCAAAAAAACGTTGATGAGTGAACACCGTAACAATAT
TCAGGTAGCAATTGATGCTTTATATAGCCAACGTCGGCGTAAATCGCCGGGTGATGAAAGTGAAACACTTACCCGTAGAA
ATGACGCTATCTTCGGCAACCACGTATGGCAAACATTTGCGCAATACTTCCCGCCAGGACTTGAAAAACCGTCTGTGTAG
AAAAAACCATAGCTATGGTGTTATCTGCCGACAACGCCATCCGGAGAAGCCGAATGACGTTGTCGGAGTAAGCGCTGGTT
AAGGTGTGGGTTGTGCCTCTTTGGTTGAGGGTTGCGTCGTTGCTGACTTAACGGTCGGACGCGTCGCCGGAACATTGTCA
CACGGTTGCTCTTTCGGGCAGATCAAATCCAGCATTTTCAGCGTCACGCCATTGGTCCAGCCAAAGCCATCCTGTAATGG
ATATTCGCCACCGCCGCCCCCCGTTCCGGTGGTGCTGACATCATATTTTTCCACCAGCTTTTTCTCCCGGTCATAGGTGT
GCTGAACATTGGTCAGGAAGTGCCAGCTAATGTCCATCGCCACCTCTTTTTGCCCGTAGTTTTGTAATCCTTCTGTCGCG
ACCCACTGTAACGGTGCCCAGCCATTTGGCGCATCCCATTGTTGCCCACTTTTCACCGACGTGGTGTTCAGGCCGCCGGG
TTGCAGCAGATGTGTTTTCGTCGCCGTCGCCATTTTGTTGGCGCGATCTTTCGCTGCCGCATTGACGTACAGCGGGAACA
GGGCGGCCGCGGTTAACTGATTGCGCACTTTATGACTTTTCAGGTCGTAATCGGCATACCAGCCTTGTTGATCGTTCCAC
AGGTATTTTTCGATCCCTTTTTGACGGGCATTTGCCAGCGTTTCGTACTGGTTTGCCATCGCGTTATCTCCGGCAGCTTT
GCTGGCGCGGGCGAGGATTTTTTCCATTTTAAACATCAGGCTGTTCAGATCGACCGGTACGATGCTGGTGGTGCGTAAGG
TATTTAACTGCTGCGGGTTGTCCATCCAGCGCGAGCTGAAATCCCAGCCAGACGCAGCGGCAGAGCGCAGGTCGCGGTAA
ATTTCAGTGGCAGGTCGATTCGGATTGCTTTTGGCGGTGGCAATATCTTCCACCCATGACTCTGGTCGTGGCGTATCGCG
ATCGTCCCAGTAGCGGTTGAGAAGGGTACCATCCTGAAGTTTGACAACGCGTTTTTCCTGTTGTCCGGCTTGCAGGTTTT
CAACACCGTCCATCCAGTAAGCATATTCTTTTTGCATTTGCGGCAGGTATTGCTTCAACGCGGCATCGCCTTCATGCTGC
GCCAGTAACTCTACCATCAGGGCAAAGAAGGGCGGTTGCGAGCGGCTTAAATAGTAACTGCGGTTGCCGTTGGGAATATG
ACCGTAAGTGTCTATTTCATGAGCAAAATTGGCCACCATATCCGCGACTTTATCCCAGTGACCGCTTTCGGCAAGTCCTA
ACATGGTGAAGTAACTGTCCCAGTAATATACCTCGCGAAAGCGTCCGCCCGGCACGACATAAGGTTCCGGCAGCGGTAAC
AGAGAATCCCATTTTTCGGTGTTTTCGGTAGAACGCGTTAATACCGGCCAAAGTCCGTCAATATGTTCGCGCAGTGACTG
CCCCTCTGGCGGAACATATTTCTCGCCTTCTTTCGGCAGGGTGAAATTGACGTTAACGAAATGGCGCAGATCAAATCCGC
TCTGGTTTTGCTGCATCCGATAATCAGCAAGGATCATCAGCGGATCGCTGTTCGGCACGGCATCGGCAAAGGTTTTTTGG
TCCGGAAAAAGTTTGGCGTTTTGCACATCATTAAACAGCGGCCCTAATAAAATATCAGGCGGCTGTGGTGTTACCGGTGT
TTCTTCTGCCTGCACCGATAGCGCAGCGAAACACAAAAAGATACAGGCTGGAATTAACGCCATTTTTTGCGGGCGAGAAG
GTGCGGGGGATTTCATCAATCATTCTCCTTTGGCGAAACCGAATAAAACGGTATTCAAGTATCAGAAAACCTTAGTTCAG
GATCGCACTAGCTGCATGATCGAAATCTCATTCTGCGAACTGTCAGGCATTTTAGAAAAGGGAATTAAAGGGATAAATAA
CGGCGAAATAGAGTGATCAAATAACGTCTGTTTATTAGCCAGCCAGCGGCGTTGGGTTCGTGATTAGCGAAGAAAAATCA
TCATAAATTGGTGAAAAATATAACAGGTAGTTAAAAACCATTAGTGCTGAGTAAATTGCCGGATGACATCAGAACGATGC
CATCCGAACAGTGGCTTAACCCTGACGGTTGAAACGTTGCGTTTTAACGTCCAGCGTTAGCGTTTCTTCTGGTTGTATCG
CATACAGTTTCTCACCCTGCTGGCAAATCCAGCCAATCCCCAGTTCACGGGCGATTAGGGCGCTGTGGGATACCGGACTT
CCGGCGCTAAGGCAGATACCTTTTACAACCGCCGGATCCAGTTGCAGTACTGTGGAAGGATAAATGTTCTCCGCCAGTAG
AATAGTTGGCGAGTTAAACTGCGGGAGTTCTTCTTTCGTTTGGGTCAGGTGGACCAGGGTGCGATGCAGAAGATCGTCCA
CATCAATATAGCGAGCTTGTAGATATTCATCATCCAGTTGCTGGTATTGCTGGCTAAGTTCTTTAAGAACTTGCTGCCAG
GCATATTCTGCCGTGCAATGTTCATGCTGAAGGAGTTCGCTTGCCGCCGCCAGCAGTTCCGGATCATCTAACAGTGTATG
GTGACCAGAAAAGATTGCGGCAATATCGTCAAGCCCGCTGGCTTCTGCTTTCGCTGTTAACGTCATCAGATCTAATAACG
TGAAGTCAATAGCCTGGCGTAATCGATCTTGTTCTTCTTCCACGGTCAGGGTTGATTTTGCCTGTACCGTACATAAAACT
GGTTGATAATAAAAGGCTTTACCCGAAACAGGCGGAACGGGACGCAGAGTAGGTGGAGCGACTTCCTCCGTTTCACCAAA
GTTATCTTCAGCCAGCTGACGGAAAGCGATCAGTGCCTCTTCAGCTTCTGGCCCTTTCGCAATCAGGCGCAGCGTATCGT
TATAGCGAACTTGTAGTAACGCAATCTGGTTAATACTCTCTGGTGTGACGCATTTGCCGTTTTTTTCCAGCAACATATCG
GCATTAAATGTCGATAAGGTATAAACCAGCCGGGAGGCCGGACGTACATGCAGGCCGTTACGGTTTTTTATGACCACCGC
CAGAGAACGGGCTTCTTCATCGTACGCAGGACATGTGTCAGAGATTTCAGTGTCGGAGGACGGTAAACCCAGTTGTTCAC
GTTTGGCTTCCAGCGCATGCATGGCGTCAAAGATAACTTTGTCGATATCCGCCCCCGAGGCCGCGCTGACCGTTGCTGCC
AGTGTACCTTCGACCAACGGCGCAGCACACAAACGTACTTTTGCGGCGATCTCGGGAGCCAGCAATTCCAGCGCAGTTTC
AGCACTCAATAATGCGCTACCCATATCCATCATGACCAGCACATGGTCGGCATCAGCAACAGATTCGATGGCCTCCATCA
CTTTGACGGCATCGGTACCAATGGGATTTTGTGGATCGTCAATTCCCGCGGCAATGGCGATTTTACAACTATCACTCATT
AACATCTGACGGGCTAATTCACCGACACCTTCTCCCAGTCGGCTGCTATGTGAAACTATGACCAGGTTTACCATCACCAA
TTCCTTACTCTTTTGCGGCTAACGCCAACATTTGCATCATAAACATCACCGAGGTCGCGCCGGGATCCTGGTGACCAATA
CTGCGTTCACCGAGATAACTGGCGCGGCCTTTGCGGGCTTGCATCGTAATCGTACTTTGTGCAGCGGATTCGGCGATGCT
ACTGGCAGCTTCGAGCGCCACCGGAACAGAGAGATTTTGCTCGCTGGACTGACGTAACGATTCCACCACCGGCACCCACA
CATCACACATGGTTTTATCGCCAGGTTCGGCTTTCCCGCGACTGATTACGCCGTCCGCGCCATCGCGGAACATCTGATAA
AGCTCTTCCAGTGTCAGGCTTTGCCGTGCCTGGGTCGCCTGTGCGGCGCGGATAAAGAAGGTACCGAACAGCGGACCACT
GGCACCACCGACGCTGGAAAGCAGCGTCATACCGGTATTCTTGAGAATGAAACCGATATCTTTATCTGCGATAGCAGGGA
GTTTTTCCACCACTTTGCTAAAGCCTCGGTTCATATTTAGCCCGTGGTCAGCATCGCCAATTTCGCGATCCAGTCCGGTA
AGATACTCGCTCTCGGTGCTGAAAATATCGCCACAACGAGTGAGCCAGTTAACAATTTGAGTTCTGCTCAGTGACATTGC
TTTCTCCTTATTTACCCCAGTTAAGGGCCGGGGTGTGGACCGGGGCGTCCCAGAGTGCCAGCGTTTCGTCATCAACTTTC

AGTAAGGTGATTGAGAAACCGGTCATATCCAGTGAGGTGCAGTACGCGCCAATTAAATTACGTTCGATAGTCAATCCCGC
TTGCTGGCAACGTGTGGTCAGGCGGTTATAGACGCCGTACAGCTCAGAAAGCGGAGTTGCGCCAAGATTGTTAACCAGCG
CAATCACCCGATCGCCAGACTGGAGCGGTTGTTTGGTTTGTTGTTCTTCCTGCCAACTGCCTTGTTGATAATCCCAGAAA
CGCAAAGTGCGATGGTATGAGCCATTTACCAGCAGGGTGTCGAACATTTCATCGACGGTTTGATCAAGGGAAGAGAAGGG
GCGGCGGTCAATACCCGGCTCACCATGAATGCCGACGCCAAACTCCATCTCATTATCCGCCAGGGTAAAAGAAGGTTTGC
CCGCGGCAGGAACGGTACAGGCACCGAGAGCGATACCTATTGAGTGGCCTTGATTATTCAGCTTACGCCCCAGTTCCGCA
CAGGCGTCCAGTGAGTCGCCACGCTCCGCCGCTGCGCCTACGAGTTTTTCAATTAATACGGTGTTGGCAACGCCGCGTCG
CCCGGCAGTATAAAGACTGTCTTTTACCGCAACGTCGTCATCAATGACCACAGTGGTCACTTTTACGCCGCTATCGTGCA
GTAACTCGGTCGCTGTTTCAAAGTTAAGAATATCGCCGGTGTAATTTTTGATAATCAACAGTACACCTTCGCCGCCATCA
ACTTGCATGGCGCATTCAAAGATTTTATCGGGCGTCGGTGAGGTGAAAATTTCGCCCGGACAGGCCCCCGAAAGCATCCC
CTGACCGATATAACCACAGTGCATCGGCTCGTGTCCGCTGCCGCCACCCGACAGCAGGGCGACTTTTCCTGCAACAGGGG
CATCAGCTCGGGTGACATACACCGGATCCTGATGCAGTGTCAGCGATGGATGCGCTTTCGCCAGTCCTGCCAGTTGTTCG
TCCAGTACGTCTTGCACATCATTGATCAATTTTTTCATTATTTTGCTCCAGCAATTACGGTAGGGCATGGATGATGTTCA
ACGACACGGCGACCACTGACTGCCGATGAATCCATTGTGCATCAGGCAAGGGAAAAGAAAATTAGCCCAAATTATGTTTC
ATAGTGAAACATATGCTTTAATGAATGTTCCATATTGAAACTTTTACGTGTATTAATACTTAAAATTGCGAGCCGGAACA
CCTTTTGTCATAAGGGATGCGGGATATGAGTGGCGCTTTTAACAACGATGGTCGGGGCATATCTCCCTTAATTGCAACCT
CCTGGGAGCGATGCAATAAGCTGATGAAACGGGAGACATGGAACGTACCACATCAGGCCCAGGGCGTGACATTTGCTTCT
ATTTATCGGCGTAAGAAAGCGATGCTGACGCTCGGGCAGGCTGCGCTGGAAGATGCCTGGGAATATATGGCACCGCGAGA
GTGTGCGCTGTTTATCCTCGATGAAACCGCCTGCATTCTCAGCCGTAATGGCGATCCGCAAACCTTGCAGCAGCTAAGTG
CACTGGGATTCAATGACGGCACGTATTGCGCCGAGGGAATTATTGGTACTTGTGCGCTATCGTTAGCGGCTATCTCTGGT
CAGGCCGTGAAAACGATGGCCGATCAACATTTCAAACAGGTACTCTGGAACTGGGCCTTTTGTGCAACGCCGTTGTTTGA
CAGCAAGGGCCGATTGACGGGAACAATAGCGCTGGCGTGTCCGGTTGAGCAAACTACCGCAGCTGATTTGCCGTTGACGT
TGGCAATCGCCCGCGAGGTCGGAAATTTACTGCTGACGGACAGTTTGCTCGCTGAAACTAACCGTCATTTAAATCAACTT
AATGCCCTGTTAGAAAGTATGGATGATGGCGTGATTAGCTGGGACGAGCAGGGTAATTTGCAATTTATTAATGCCCAGGC
GGCGCGGGTCTTGCGCCTTGACGCGACGGCAAGTCAGGGACGGGCAATCACTGAACTCTTAACGTTACCCGCCGTATTGC
AACAAGCAATAAAACAGGCACATCCGCTCAAACACGTAGAAGCAACCTTTGAAAGCCAGCACCAGTTTATTGATGCGGTG
ATAACCCTTAAACCGATAATAGAAACGCAGGGAACCAGCTTTATTTTGTTGCTCCATCCTGTGGAACAGATGCGGCAGTT
GATGACCAGTCAATTAGGAAAAGTCAGCCATACCTTCGCTCATATGCCACAGGACGATCCGCAAACCCGCCGCTTGATTC
ATTTTGGTCGCCAGGCGGCGCGCAGTAGCTTTCCTGTCCTGCTTTGTGGAGAAGAGGGCGTGGGCAAGGCACTGCTAAGT
CAGGCAATTCATAATGAAAGCGAGCGTGCTGCAGGTCCTTATATCGCCGTCAATTGTGAGTTATATGGTGATGCTGCGCT
GGCGGAAGAATTTATTGGTGGCGATCGCACGGACAATGAAAATGGCCGTCTGAGTCGGCTGGAACTGGCACACGGCGGCA
CGCTGTTTCTTGAAAAGATTGAATATCTGGCGGTGGAGTTACAGTCTGCTTTGCTTCAGGTTATCAAGCAGGGGGTTATC
ACGCGACTGGATGCGCGGCGTTTAATACCAATTGATGTCAAAGTGATTGCAACAACGACCGCGGACCTCGCAATGCTGGT
GGAACAAAATCGTTTTAGTCGCCAGCTGTATTACGCGCTGCATGCATTTGAAATTACCATCCCGCCTCTGCGTATGCGGC
GTGGCAGCATTCCGGCGCTGGTGAATAACAAATTACGCAGTCTTGAAAAACGCTTCTCTACGCGGCTGAAAATTGATGAC
GATGCCCTCGCTCGCCTGGTTTCTTGTGCATGGCCAGGCAACGATTTTGAACTTTACAGCGTCATCGAGAATCTTGCTCT
GAGTAGTGATAACGGGCGCATTCGCGTCAGTGATTTGCCGGAACATCTGTTTACCGAGCAGGCGACAGATGATGTCAGCG
CCACCCGCCTTTCCACCAGTCTGTCATTTGCGGAAGTTGAAAAAGAGGCAATTATTAACGCAGCCCAGGTCACAGGCGGT
CGCATTCAGGAAATGTCGGCTTTACTTGGGATCGGCCGCACTACGCTGTGGCGGAAAATGAAGCAACATGGCATTGATGC
AGGGCAGTTTAAGCGCCGGGTATGAAAGACAGAAACGATTTCTGATACATCAGAGTGATCTGTATTTCATTCCGGCGCAC
GCTAACAATTTTCAGCATCGTTTAAGGGCTTGTCTATCCCGCACTTAAAAGCTGAAGCGATATCCTCCGTTGACTTGCTT
CTGATCAAAGAGGTTACCCTGCGTGTAATCCGCTTCGAGATAGAATGTGTGCTGTTTGTTATACTGTGCACTGACTCCCA
CGCCGTTATTCCAGCCATTACCTTTGAAACTGTACTTCTCCCGGGAGTTGTTCAACAGATATTCGGTATCCCCTGAAAAC
TCGCGGATAGCGCCAGTCTTCACATAGACATTCAGCTGGCTGTTGCCTGCGGTGATGTCATACCCCAGTATCATGCTGGC
ACGCCCCAGCAGCGATTCGTAGTGATTCAGATGTATATTGAGGCCATTACTCGCCTTCATAGTCATCTCATTCTGGTGGC
TGTATGTAAGCTGGGTTTGCGGCTCTATATAGAACCCATAACCAGTAGGGGACAGGTTGAACCTCTGCCCGGCTTCCAGG
GAGATGCTCATTCCATTCGCAGTGCCGTTGGCGTTAACGCCGTTGTTCTGACTGTCCAGTACGTGGAAACTATTTTTCTG
GCGCGATGCTTTTATAACGAGATCGCTGTAAAAACCGTTTTGTGCCATGTAACTGGCGTACATTCCCATGTAGTCTGAAC
GTGCGGTACCGTCGCCTCCGCTATAGTCCGGCGATGCATGTGTTGAGCCAATATACAGACCGACATACAACGGCATTACA
TCAGAGAGACGTTTATCCCCACCAAACTGGATACCGCTGTAACCCATGTCAAAGCCGCTCAGTTTGCCACTGGCAAAGGA
GTCCAGGCTTCCCCCATAACTGCGCAACCAGATATTACCGTCTTTACTCTGATTTCGCAGGTCACCCATCCGTTGCATCA
GCGTACGGTTTTCAACATAGTTCAATAAGTAACCGACATTGAGATAATTGCCGCCAGCATCTGCGGTCGTGGTGGGTTTA
GGCGTGGGAGCGGGTTCAGGCGTAGGATCGGGGTTGACTATGGGAGGCTGAGCGGGAGCTGGTGTGGGTTCAGGATTAGG
AGTCGGTTCCGGAACTGTCCCGGAAGCGTAAAGCTCCCAGTTAGTGCCATTTTTACGCACATCGTACAGATATCCCCCCA
ACTCAACCTGAGAAGACGCGCTGAACGAGGCCGCGCCGTCAGTGGTTTTTACCACTGTCAGAACTTCATTTCCCGTTGTG
GCCTCGCTGCCCTGGTTGCGGATAGCCAATACGTGATTACCAGCACTACTCCCGCTGATATTCAATAAATCCCCTTTATT

ATTAACGCCATTACCCTCGCCAACAACATCAGCACGCATAATAAAGGTACTGTTACCGCTCAGGTTCTCTACGTTTAATG
TGGCAAATGTGCCGGCAGTTGACCCGTGGCTGGCAAAATCGACAGTTGAATGGCTCAGCGCCAGCGTGTCGAGATTAGAA
TTACTTGTTACGTTCCAGACGCTGTTATTCATTGCAACGTCCAGTTTTCCGCCATTGACATTATCGCTGAGGGAGGAACC
TGTCCAAACCGACCCAGGGTGCATATCCAGATTGATTAGCCCACCTTTGGAAAGAACGCTACCGTTGATAAGCATACGAC
CCGAGGCGTTGATGCGGCTGGCGGCATAACCATCGTCATGTTGCGTTGCGATGGCCATCTGGTCGGGTGTACTCATATCA
ATGACCAGATCGCTCGTGAGGTCGATCTGGCTGTTGGTCATGGCATAAATTCCCCTGGCTCCTGCCGCGCCGGTGATAGC
CAAACTGTCTCCGGTTATTCTACCGCCGCTGAGAGCCCACAAACCCAGCGCCAGACTGCCATTACGATCAACTGTAATAT
CGGTATTTTGCATGTTGACAACAGCCGTTGCCGTCTGGGCCGAGGCACCATAAGAACCGCCGGAAAAGATGCTGTTTCGT
TGCGCTGCCGTGCCAGTAAAATTGATTATCGCGTCTGAACCACTGGTGACGAGGCCACCGCCCTGCGCGGAAGAAATATG
GCTATCTGCACCGATAGTGGTTGTACCACCACGAACTTCGACGCCATTGGCCGCGGCTCCAGTTACATCAACAGTGAGTG
CATTCGCGCTAACCTGGCCAAAGCTCCAGAGGCCGTGAGCATTATCGCCATTGGTTTTAATGGTACTGTTTGTTCCGAGA
TCGACAACAGAGTTTTTCTGTACGTTTATCCCCATGGCGCTGTAGCCCTGAACATCGATTGTCAGGTCTGTAGCCGTAAA
ACGCGCAGCACCATTGGCGTTATTGCCGTTGAGACCACCGATATAAACACCTGTACTTCCATCGGTCGTGATTTTACTTC
CGCTTCCAAGATCGACACTGGTGCCATAGTCATTGATGGTTAGGCCTATACCGTTCGAGTTTTCAATGGTGAATTGAGTG
GCTGTTAGTGTTGAGCTATGCCCAATAATGATGCCGTCATCGTTACTCTTAATGGTGCTGCCTGTGCCTAAGTCAGCATG
GGTATAGTCACCAATTAAGTTAATGCCGATGGCAGAGGTTTGACCAACAACATCTACTGTGAGTCGGTTAGCTGTCAGGG
AGCTGTTTTTGCCCTGAATAATGATTCCTTTTGCATATCCAGAGGCGTCGTTGACATTGACGGTGACATCATTACCCAGG
TTGATATTGCCAGGCGTGTTTCCTGCTGGGGTCATTACACCATAGAGGTTTCCTGATGGATCGGCGGTATCTCCTGTAAT
TCTGTCACCATCATTAATAGTAATATTGGTGCCATATTGACTTCCTGTATCAGCAGCTATCAGCAGGGGGGAAAAGATAA
TTGCATTCAAACCAATAGCGCCAAATTTTATCAGTTGAATTGAGGGCGAACGGATTTTTAATTCAGCGAGACGATCTGCT
TTGGTATTCCCATTGTGTTGTTTGATGCCCATACCACTCCTATATAGTACCCAGACTATGGGTCTATGGATTTTGTCATT
TTGTTTAAATCAATGAATAATATCCTCTTATCATCATGATATTTGTATGGTGCAATCATGATGAGCTTCTCAGAGAATAA
TTCTCTATTACCGGGCAAAATCAGGAACACAGGAATTAATATATATTTAACATCCCCCTTACAAAGGTGAGTTTATATCG
AGTTATTCCCTAAGTTGTCGTTACTTATTCCAATTCGTTACTATAAATATGGCAAAAATATTACAACAAACAGCGGTTAA
AAAATTTGGTGAAGATTTTTTGTAATAATCCTAATTAGTTATGGGAATTTCGCTTGATGTATCTGCATAACAAAAACAAT
ATGCGCCATTTTTGCATTACTAATAAGAAAAAGCAAAAAACATCGATATTCATTTTTGGTCAATGGGTTTTCTGGCCTAT
CGTTGTTATTGTGCTACTGGTTATCATTTTCATCTTGCCAGCATATTGGAGCGTGATCAATTTTGATCAGCTGTGAAGGG
GGCTGATGTGTAGTGATACAAAAACTTACGTTATATCACTGCTAATGAGATATCCGGCATCTGAACACTTATGTCTTTAA
TTATTCTCGTGGTTCACTATAGGCAATAAGCACAAAAGTGTAGGATGTTACAAGAATGATTAGGACTCGGTGAAATGAAA
AATCCACGCAATTGCGTGGATTTTATATACTTTTGCGCTCTTCATGAGATTTAGCGAAACCTCATGAGACAATAAATTAA
TTAGACGTTGAAAAGGAAGTTCATCACATCGCCATCTTTCACGATGTAATCTTTACCTTCTGCACGCATTTTGCCTGCTT
CTTTCGCGCCTTGTTCACCTTTGTAAGTGATGAAATCTTCAAACGAGATGGTTTGTGCACGGATAAAGCCTTTTTCAAAA
TCAGTATGGATTTTGCCCGCTGCCTGCGGCGCGGTTGCTCCAACCGGAATGGTCCATGCACGCACTTCTTTCACCCCAGC
GGTGAAGTAAGTTTGCAGGTTCAGCAGTTTATAACCGGCACGGATCACACGGTTCAGGCCCGGCTCTTCCAGCCCAAGCT
CCTGCATAAACTCGTCACGTTCTTCGTCGTCCAGTTCGGCAATGTCTGCTTCAACAGCAGCACAAACCGGAACCACAACA
GAACCTTCTTTCGCCGCGATTTCACGCACCTGGTCAAGATATGGGTTGTTTTCAAAACCGTCTTCGTTGACGTTGGCGAT
GTACATTGTTGGTTTCAGCGTCAGGAAGCTCAGGTAACGAATAGCCGCTTTCTCTTCAGCGCTTAAATCCAGCGCGCGCA
GCATACCTGCATTTTCCAACTGGGGCAGGCATTTTTCCAGGACCGCCAGCTCAGCTTTCGCGTCTTTATCGCCACCTTTG
GCTTTCTTCTGTACGCGATGAATCGCACGTTCGCAGGTGTCGAGGTCTGCCAGCGCCAGTTCGGTGTTGATAACTTCAAT
ATCGTCAGCCGGGTTAACTTTGCCGGAAACGTGAATGATGTTGTCATTTTCAAAGCAGCGAACAACGTGACCGATCGCTT
CGGTTTCACGGATGTTGGTCAGGAACTGGTTACCCAGACCTTCGCCTTTCGATGCGCCTTTTACCAGACCGGCGATATCG
ACAAATTCCATGGTCGTGGGAAGCGTACGCTGCGGTTTTACGATTTCAGCCAGTTGATCCAGGCGAGGATCAGGCATTGG
TACGACGCCTGTGTTCGGCTCAATGGTGCAGAATGGAAAGTTGGCCGCTTCAATACCGGCTTTGGTCAGCGCGTTGAACA
GGGTAGATTTCCCGACGTTGGGCAAACCGACGATACCGCATTTGAATCCCATGATTTAAATCACCTTAATATCTTAATAA
TCAACCTGTTATTGCTAACAGATTGCAGAAATGGAAATAACTTTGCCTATTATACACGGCACTCGGCAAAAATGCCGCAG
ACAACGACTTATTGCGCTTTAAAGGCGTGCAATCGGTTCGTTGCTTTGGTCAAGCCATCTGTAAACCACATTTCAGTACA
ACGCGCCGCTTCGTCAATGGCTTCATCAATTAACTTCTGTTCACTAACAGGCGGTTTGCCTAACACAAAACCGACAACTT
TATTTTTATCGCCCGGATGACCGATTCCGATGCGTAAACGGTGAAAGTTAGGGTTATTACCCAATTTACTGATGATGTCT
TTCAGTCCATTGTGACCACCATGGCCACCGCCCAATTTAAATTTGGCGACGCCAGGAGGCAGATCCAGTTCGTCGTGGGC
CACCAGAATTTCGTCCGGATTAATGCGGAAAAAACTGGCCATCGCCGCAACGGCTTTGCCGCTGAGATTCATAAATGTAG
TCGGGACTAACAGGCGGACATCTTCGCCTCCAAGAGTGACTCGCGAAGTATAACCAAAGAATTTAGCCTCTTCGCGCAGC
GGAGCGCGCAAACGCTCTGCCAGTAAGTCAACGAACCAGGCACCAGCATTATGTCGCGTTGCGGCGTATTCAGCACCGGG
GTTCGCCAGGCCGACAATCAATTTAATCGTCACGTTTTTTTGTCCTGAGTGTGTACATAACTGGCGCGTAGTTTACTGGT
TGCGGCCCCGCTTGACAAAAAACTGCGTATCAAATGCAGATAACGTAATAATTGCCTGAGTGGACTATTAGAAAGTCAAG
GTGTTCAGGCGTTTATTTGTAAAGTTTTGTTGAAATAAGGGTTGTAATTGTGATCACGCCCGCACATAACCCACTGGGTG
TTGTCTATACTTTACACATAAGGAAGAGGGGTATTCCCTGTTACAACCCAGAAAGTTCCGGAGGTGACATATGAAACGCA

AAAACGCTTCGTTACTCGGTAACGTGCTCATGGGGTTGGGTCTGGTGGTAATGGTGGTCGGCGTGGGGTATTCAATCCTC
AACCAGTTACCACAGTTTAATATGCCCCAGTATTTCGCACATGGTGCAGTGCTAAGTATTTTCGTCGGTGCCATTCTCTG
GCTGGCGGGTGCCCGTGTTGGCGGGCATGAACAGGTGTGCGACCGTTACTGGTGGGTTCGCCACTATGACAAACGTTGCC
GCCGTAGCGATAATCGCCGTCATAGCTAACAAATAATGCAGTTTGCTGACCAGTCAGTTTGCGCTGACTGGTCAATCTCG
TACTTATAAATCCGCCATCGCCGCGCGACGATTCGGGAAGAACGCCAGGCGTCCCGGGATCGGTTGAATGCCAGCGCGAG
CCATAGTGCGCAGTGGCTGGAATTCCACGTTGCACACGCGCAGTTCACATCCCTCGGGCAGACGCTTCACAAAACGCTGG
AACGCATCAAGACCACCAGCATCAAGTACCGGAACGGCATCCCACTTCAGAATCACAATCCGTTTGCCTTCAAGACGTGA
CTCCAGGTCCGTGAATAAGCCTTCAGCAGCAGCAAAAAACAGCGGGCCAATAACGCGCAGAACCAGGACATCGTCTGGAA
CATCTACGACTACCGGTGCCAGGCGAGTCATACGTGCGATACGACGCATAAACAGCAGCGATGCCAGCACGATCCCCACG
CTGATGGCAATAACCATATCAAACAACACGGTCAGCGACATGCACAGCAGCATGACGATGATGTCATCTTTCGGCGCATG
ACGCAGCAAGTCGACCACTTTGTGCGCTTCACTCATGTTCCACGCCACCATCAACAGCAGGGCTGCCATGGCGGAAAGCG
GCAGCCAGGAGAGCAGCGGTGCCAGTACCAGCAGGGCAAGAATAACCAGAATAGAGTGGATCACCGCCGAGATAGGGGAC
GTTGCCCCGGCACGGACGTTAGCGGCAGAACGCGCGATGGCAGCTGTAGCGGTAATACCACCAAAGAACGGAGCGATAAT
ATTCCCCAGTCCCTGTCCAACCAGTTCGCTGTTCGCCTTGTGTTTCGTCCCGGTCATACCATCCAGCACCACGGCGCAGA
GCAGAGATTCGATTGCGCCGAGCATTGCCATTGAGAATGCCGCAGGCAGCAGTGTGCGAATAGAATCCCAGGTTAGCGTG
AATTCTGAATTAGGCAGATCCCACGGCAGCACCAGTTGCGGCAGCAGTTGCGGAATACCGTTACCCTGAGAACCATCGGC
CAGGACGTAGTGGAATTGCGAACCGATGGTAGCAACATGTCCGCCGAGCAGGTTAACAATCCCCATCACCGCGCAACCAG
CCAGCAAGGCCGGAAGGTGACCGGGTAAACGAATGCCCAGACGCGGCCAAAAAACAAGAATACCTAGCGTCACAATGCCA
ATGGCAGCATCACCCCACATTAATGGTCGGCAGCGCCATAAATAATGCGCCGACTTTTTGTAGATAATGTTCCGGGACATG
GGCCATTTGCAGACCGAGAAAATCTTTAATCTGCATGGTACCGATGGTGATCCCGATACCCGAGGTGAAACCTAAGGTGA
CGGAAACCGGAATATACTCAATCAGGCGACCAAAGCGTGCCAGACCCATCAGAATCAAAAAGATCCCCGACAGCAAGGTC
GCAACCAGCAGTCCTGCCAGTCCAAACTGTTGCGACACGGGATAGAGAATTACCACAAATGCCGCAGTCGGACCGGAAAC
GCTAAAGCGTGACCCACCCGTCAGAGCAATGACAATCCCCGCAACAGCTGCGGTATATAAACCGTACTGGGGTGCCACAC
CACTACCAATAGCCAACGCCATCGCCAGCGGGATAGCAATAATCCCGACGGTTATCCCGGCAATCAGGTCACGGGTAAAC
CGTGCGGCAGTATATTTTTCTTTCCAGCAAGCGTCGATCAGAGCGCGGAAAGGCATCACATGTGAGGAAAATATTTTGTT
CACAATAATGTTTCATCCGTGAGCGCATCATCTGTCAACTAAATGGCAGGTGAAGGAGGCATAGGTCATACAAATGGATA
TTACAGACAAAAAAACCCGCCGCAGCGGGTCTTTGAGCCGGGTTCGATTAGTGTTCGAACATGGCAGAGATCGATTCTTC
GTTGCTGATACGACGAATCGCTTCGGCCAGCATACCTGACAGGGTCAGAGTACGCACGTTCGGCAGTGATTTGATTTCAT
CGCTCAGCGGAATGGTATCGCAGACAACGACTTCATCAATTACAGAGTTACGCAGGTTGTTCGCCGCGTTGCCAGAGAAG
ATCGGGTGAGTCGCGTACGCAAATACACGTTTAGCACCACGTTCTTTCAGAGCTTCAGCAGCTTTACACAGCGTACCGCC
AGTGTCGATCATATCATCGACCAGTACGCAGTCACGACCTGCAACGTCACCGATGATATGCATCACCTGTGAAACGTTCG
CACGCGGACGACGTTTGTCGATGATTGCCATATCGGTATCGTTCAGCAGCTTAGCGATAGCGCGGGCACGCACAACGCCG
CCGATGTCCGGAGAAACCACAATTGGGTTATCCAGATTCAGCTGCAGCATGTCTTCCAGCAGGATCGGGCTACCAAATAC
GTTATCAACCGGAACGTCGAAGAAACCCTGAATCTGTTCAGCGTGCAGATCCACTGTCAGCACACGGTCAACACCGACGC
TGGAGAGGAAGTCTGCAACCACTTTCGCAGTGATTGGTACACGAGCGGAACGGACGCGACGGTCCTGGCGCGCATAGCCA
AAGTAGGGGATAACAGCGGTGATACGACCTGCGGAAGCACGACGCAGGGCATCAACCATAACGACTAATTCCATCAGGTT
GTCGTTAGTAGGGGCACAAGTGGACTGGATGATGAAAATATCACCACCGCGTACATTTTCATTAATTTGTACGCTGACTT
CGCCATCGCTAAAGCGACCTACAGCGGCGTCGCCGAGTGAAGTGTACAGGCGGTTGGCAATACGTTGTGCTAGTTCCGGG
GTGGCGTTACCAGCAAAAAGCTTCATATCAGGCACGAGAAGAACCTCAGGCATGCGTCCATTGGTGGAAAGAATCTGCCG
AAAACTGTGCGGGCCAGGCATGATCCTTTCCAGGCGGTGTATTAAAGAGCGCGATGCAACGTCTGGAACAAGGTGACGTT
GTCACCGAAACTCAGCTTGCCCGGCTTAAAGCATGGCTCTGTGCAATGGGGAAAGATTAGCGCCTTTCGCCACAAAGCCA
TTGAGCCATTCCGGGGCTTGCTCTAGCACCTGGCGGGCTTCAGACTCTGTATCAAATTCAGCAAAGACACAGGCCCCTGT
CCCAGTCAGGCGCGACGGGGCGTATTCTAACAGCCAGGAAAGCACCGCATCAACCTCGCGAAAACGTTTTCTTGCGATAA
CCTCGCAATCATTGCTGAATTCACATTTTAGCAACGTTTCTATTGACCTTTTTGGCGTATTGCGCGGGAGTTCAGGATCT
TTAAAAATCACCGGAGTCGGAATACTTACACCAGGGTGCGCCACCAGATACCACTTCTCTGGCGGATCCACCGGCGTTAG
TATTTCACCAACGCCTTCGGCAAACGCGGCATGCCCCCGAACAAAGACAGGAACATCTGCGCCCAGCGTCAGCCCCATTT
CCGCCAGCTCATCCATGCTTAGCCCGCATTGCCAGAGATGATTTAATGCCACCAGGACCGTCGCGGCATTGGATGAACCA
CCGCCGAGACCGCCGCCCATCGGCAAACGCTTGTCAATGCTGATATTCGCACCGCTTCCCGTCGGAAGACGCCCGCTGTC
TGCCGCAGTTTTCATCAACAATCGCGCTGCGCGAACGATCAGGTTATCTTCATGTTCCACGCCTTCAACGGGCGTTAACA
GACGAATATCCCCATCGTCACGAAGCTCAATGCTGATGGTGTCGCCGTAATCAAGAAACTGAAACAGCGTTTGCAGCGTG
TGGTAACCATCCGCACGCTGACCGGTAATGTATAAAAACAGATTAAGTTTTGCCGGAGAGGGCCACTGTGTCCGCATTAT
TTCACTATCCAGTTATCCATTTTTAACTTGATGCGTTGACCACCGTCGGTGAGTTCCATATTGGCTGGCATCGCAGGTTG
CGTTTTGGTGTCATAACCACCATAAACAACCTTCCAGTTTTTGCCATTCTGGCTGTAGGTAATTTCGCTCAGGCGGTACT
GGTCGTCCAGTTTGTAGTCGGTTGCATCACCCGGTAAACCTAAAATCCACTGGCGCAAGCTGTTGAGCGGAATTGGCATT
CCGGTCAATTTGCCAATCATCTCTTCGGCGTCATCGGCGGTATAACGCTGACCTTTATTGTCGACTAACTGCACGTTACC
CGGTTGAGCATTCAGCTCCAGTTCCGTGCTGCCCAATGGGTTAGTGAGCAGCAGACGGTAGCGATCCTGGCCGGTTTGCT

GCCAGAAAAAGCGGGCGTACACTTTTTGTTGGTCAGAAATATAAGCGAACGCGCCGCGAGTCTGATACTGATTAAGATTG
CGCACGTCTTGCTGATGCTGACGCCATTGTGGCGAATCCGGGCTTTTGCCAGGACCTTTGGGCGTGGTAACGGAACAGGC
AGTGAGCACAAGAGCAGCCAGCGGTAGCAGGCGGATAAGACGAAAATCGGGCAGGGGCATAGTGATGACAAGTCCTTGAG
ATACGTTGCAGTTATAACCCTTAATGCTAGCGTTACCGTCCGCTATCGTCTATGTTCAAGTTGTCTTAATTGCCAGAATC
TAACGGCTTTCGGCAATTACTCCAAAAGGGGGCGCTCTCTTTTATTGATCTTACGCATCCTGTATGATGCAAGCAGACTA
ACCCTATCAACGTTGGTATTATTTCCCGCAGACATGACCCTTTTAGCACTCGGTATCAACCATAAAACGGCACCTGTATC
GCTGCGAGAACGTGTATCGTTTTCGCCGGATAAGCTCGATCAGGCGCTTGACAGCCTGCTTGCGCAGCCGATGGTGCAGG
GCGGCGTGGTGCTGTCGACGTGCAACCGCACGGAACTTTATCTTAGCGTTGAAGAGCAGGACAACCTGCAAGAGGCGTTA
ATCCGCTGGCTTTGCGATTATCACAATCTTAATGAAGAAGATCTGCGTAAAAGCCTCTACTGGCATCAGGATAACGACGC
GGTTAGCCATTTAATGCGTGTTGCCAGCGGCCTGGATTCACTGGTTCTGGGGGAGCCGCAGATCCTCGGTCAGGTTAAAA
AAGCGTTTGCCGATTCGCAAAAAGGTCATATGAAGGCCAGCGAACTGGAACGCATGTTCCAGAAATCTTTCTCTGTCGCG
AAACGCGTTCGCACTGAAACAGATATCGGTGCCAGCGCTGTGTCTGTCGCTTTTGCGGCTTGTACGCTGGCGCGGCAGAT
CTTTGAATCGCTCTCTACGGTCACAGTGTTGCTGGTAGGCGCGGGCGAAACTATCGAGCTGGTGGCGCGTCATCTGCGCG
AACACAAAGTACAGAAGATGATTATCGCCAACCGCACTCGCGAACGTGCCCAAATTCTGGCAGATGAAGTCGGCGCGGAA
GTGATTGCCCTGAGTGATATCGACGAACGTCTGCGCGAAGCCGATATCATCATCAGTTCCACCGCCAGCCCGTTACCGAT
TATCGGGAAAGGCATGGTGGAGCGCGCATTAAAAAGCCGTCGCAACCAACCAATGCTGTTGGTGGATATTGCCGTTCCGC
GCGATGTTGAGCCGGAAGTTGGCAAACTGGCGAATGCTTATCTTTATAGCGTTGATGATCTGCAAAGCATCATTTCGCAC
AACCTGGCGCAGCGTAAAGCCGCAGCGGTTGAGGCGGAAACTATTGTCGCTCAGGAAACCAGCGAATTTATGGCGTGGCT
GCGAGCACAAAGCGCCAGCGAAACCATTCGCGAGTATCGCAGCCAGGCAGAGCAAGTTCGCGATGAGTTAACCGCCAAAG
CGTTAGCGGCCCTTGAGCAGGGCGGCGACGCGCAAGCCATTATGCAGGATCTGGCATGGAAACTGACTAACCGCTTGATC
CATGCGCCAACGAAATCACTTCAACAGGCCGCCCGTGACGGGGATAACGAACGCCTGAATATTCTGCGCGACAGCCTCGG
GCTGGAGTAGCAGTACATCATTTTCTTTTTTTACAGGGTGCATTTACGCCTATGAAGCCTTCTATCGTTGCCAAACTGGA
AGCCCTGCATGAACGCCATGAAGAAGTTCAGGCGTTGCTGGGTGACGCGCAAACTATCGCCGACCAGGAACGTTTTCGCG
CATTATCACGCGAATATGCGCAGTTAAGTGATGTTTCGCGCTGTTTTACCGACTGGCAACAGGTTCAGGAAGATATCGAA
ACCGCACAGATGATGCTCGATGATCCTGAAATGCGTGAGATGGCGCAGGATGAACTGCGCGAAGCTAAAGAAAAAAGCGA
GCAACTGGAACAGCAATTACAGGTTCTGTTACTGCCAAAAGATCCTGATGACGAACGTAACGCCTTCCTCGAAGTCCGAG
CCGGAACCGGCGGCGACGAAGCGGCGCTGTTCGCGGGCGATCTGTTCCGTATGTACAGCCGTTATGCCGAAGCCCGCCGC
TGGCGGGTAGAAATCATGAGCGCCAGCGAGGGTGAACATGGTGGTTATAAAGAGATCATCGCCAAAATTAGCGGTGATGG
TGTGTATGGTCGTCTGAAATTTGAATCCGGCGGTCATCGCGTGCAACGTGTTCCTGCTACGGAATCGCAGGGTCGTATTC
ATACTTCTGCTTGTACCGTTGCGGTAATGCCAGAACTGCCTGACGCAGAACTGCCGGACATCAACCCAGCAGATTTACGC
ATTGATACTTTCCGCTCGTCAGGGGCGGGTGGTCAGCACGTTAACACCACCGATTCGGCAATTCGTATTACTCACTTGCC
GACCGGGATTGTTGTTGAATGTCAGGACGAACGTTCACAACATAAAAACAAAGCTAAAGCACTTTCTGTTCTCGGTGCTC
GCATCCACGCTGCTGAAATGGCAAAACGCCAACAGGCCGAAGCGTCTACCCGTCGTAACCTGCTGGGGAGTGGCGATCGC
AGCGACCGTAACCGTACTTACAACTTCCCGCAGGGGCGCGTTACCGATCACCGCATCAACCTGACGCTCTACCGCCTGGA
TGAAGTGATGGAAGGTAAGCTGGATATGCTGATTGAACCGATTATCCAGGAACATCAGGCCGACCAACTGGCGGCGTTGT
CCGAGCAGGAATAATGGAATATCAACACTGGTTACGTGAAGCAATAAGCCAACTTCAGGCGAGCGAAAGCCCGCGGCGTG
ATGCTGAAATCCTGCTGGAGCATGTTACCGGCAGAGGGCGTACTTTTATTCTCGCCTTTGGTGAAACGCAGCTGACTGAC
GAACAATGTCAGCAACTTGATGCGCTACTGACACGTCGTCGCGATGGTGAACCCATTGCTCATTTAACCGGGGTGCGAGA
ATTCTGGTCGTTGCCGTTATTTGTTTCGCCAGCGACCTTAATTCCGCGCCCGGATACGGAGTGTCTGGTGGAGCAGGCAC
TGGCGCGGTTGCCTGAACAACCTTGCCGTATTCTCGATCTCGGGACGGGTACCGGGGCGATTGCGCTTGCGCTGGCTAGC
GAGCGCCCGGACTGCGAAATTATCGCTGTAGATCGTATGCCTGATGCTGTCTCCCTGGCACAACGTAATGCCCAGCATCT
GGCGATCAAAAATATCCACATTCTGCAAAGCGACTGGTTTAGCGCGCTAGCCGGGCAGCAGTTTGCGATGATTGTCAGCA
ATCCGCCGTATATTGACGAGCAGGACCCTCATCTTCAACAAGGCGATGTCCGCTTTGAGCCGCTCACTGCGCTGGTTGCG
GCAGACAGTGGAATGGCAGACATCGTGCATATCATCGAACAGTCGCGTAACGCGCTGGTATCCGGCGGCTTTCTGCTTCT
GGAACATGGCTGGCAGCAGGGCGAAGCGGTGCGACAAGCATTTATCCTCGCGGGGTATCATGACGTCGAAACCTGCCGTG
ACTATGGTGATAACGAGCGCGTAACGCTCGGCCGCTATTATCAATGACAAGTTTTTCTACACTGCTTAGTGTTCATCTTA
TTAGTATCGCGCTTTCTGTTGGGCTATTAACCTTACGTTTCTGGCTACGTTATCAGAAGCATCCACAGGCATTTGCTCGC
TGGACGCGCATTGTGCCGCCGGTTGTCGATACGCTGTTACTGTTAAGCGGCATTGCGTTGATGGCTAAAGCGCACATCCT
GCCATTTTCCGGGCAGGCACAGTGGCTGACTGAAAAGCTGTTTGGAGTTATCATTTATATCGTTTTGGGTTTTATTGCAC
TCGATTATCGTCGTATGCACAGTCAGCAGGCGCGCATTATTGCCTTCCCGCTGGCGTTGGTGGTGCTGTACATCATCATT
AAACTCGCCACCACAAAAGTACCGTTACTGGGGTAAGTCATGAGATCGTTAGCTGATTTCGAATTTAATAAAGCGCCATT
GTGCGAAGGCATGATCCTGGCTTGCGAAGCAATCCGCCGCGATTTTCCCTCGCAAGATGTTTACGACGAACTGGAGCGTC
TCGTTAGTCTGGCGAAGGAAGAAATCAGCCAGCTTCTGCCTTTAGAAGAGCAGTTGGAAAAACTGATCGCGCTGTTTTAC
GGCGACTGGGGATTTAAAGCCTCACGCGGTGTTTATCGTCTTTCCGATGCATTATGGCTGGACCAGGTGTTAAAGAATCG
ACAGGGCAGTGCGGTATCATTAGGTGCGGTTTTATTATGGGTCGCGAATCGTCTCGATTTGCCGCTGCTGCCGGTGATTT
TCCCTACGCAGCTGATATTGCGCATTGAATGTCCGGATGGCGAAATTTGGCTGATTAATCCTTTTAACGGTGAATCGTTA

AGCGAACATATGCTGGACGTATGGTTAAAGGGAAATATCAGCCCGTCGGCGGAACTGTTTTATGAAGACCTTGATGAAGC
TGATAACATTGAGGTAATCCGCAAATTGCTGGATACACTCAAAGCCTCGTTGATGGAAGAAAATCAGATGGAGCTGGCGT
TACGCACCAGCGAAGCTTTATTACAATTCAACCCTGAAGATCCCTATGAAATTCGCGATCGCGGGTTGATTTATGCGCAA
CTGGATTGCGAACACGTTGCGTTGAACGATTTAAGTTATTTCGTTGAACAGTGTCCGGAAGACCCGATCAGCGAAATGAT
CCGTGCGCAAATAAATAACATCGCGCATAAACATATTGTGCTGCATTAATTAATCGACATTTTACTCAAGATTAAGGCGA
TCCTATGAAACAAAAAGTGGTTAGCATTGGCGACATCAACGTAGCAAATGACCTGCCGTTCGTACTGTTTGGCGGTATGA
ACGTGTTGGAATCTCGCGATCTGGCGATGCGCATTTGCGAGCACTACGTAACTGTGACCCAGAAACTGGGTATCCCTTAC
GTGTTCAAAGCCTCTTTTGACAAAGCCAACCGCTCCTCCATCCACTCTTATCGTGGACCGGGCCTGGAAGAAGGGATGAA
AATCTTCCAGGAGTTGAAGCAGACTTTTGGCGTGAAAATTATCACCGACGTTCACGAACCAAGTCAGGCACAGCCCGTTG
CTGATGTCGTGGATGTGATTCAGTTGCCGGCGTTTCTTGCTCGCCAGACTGACCTGGTTGAAGCCATGGCGAAAACCGGT
GCGGTAATTAACGTCAAGAAACCACAGTTTGTCAGCCCGGGACAGATGGGTAATATCGTTGATAAATTCAAAGAAGGCGG
CAACGAAAAAGTGATTCTTTGCGATCGCGGTGCTAACTTCGGCTATGACAACCTGGTTGTCGATATGCTGGGCTTCAGCA
TTATGAAGAAAGTGTCTGGTAACTCGCCGGTGATTTTCGACGTGACCCACGCACTGCAATGCCGCGATCCGTTTGGCGCA
GCTTCCGGTGGTCGTCGTGCTCAGGTGGCTGAGCTGGCACGAGCCGGTATGGCGGTAGGTCTGGCGGGGCTGTTTATTGA
AGCGCATCCGGATCCGGAACATGCGAAATGTGATGGTCCATCCGCGCTGCCGCTGGCTAAACTGGAACCGTTCCTCAAGC
AGATGAAAGCGATTGATGATCTGGTGAAAGGTTTCGAAGAACTGGATACCAGCAAGTAATCTTTTTTGCTTGAAAAATAA
AGTATTAGCGTTCTGCGTTAAGACTTTTTTCATGGGTGCCGGATACAAAAAAGGCCGCAGGCTGTTACCCCTGCGGCCGG
TTTCGGGCGCATATTGCCATCACGGCAGCCTGACGCCCGTTTTCACCTTACTTCCGGTTACGCCACCAGCTGACAATCGC
TGCGGTAATAATTCCCGCCAGGATCGGTGCTGCCAGGTCGTGCCAGAAAATCATGGCAAACTGCGCGAGCGTCATATAGC
CGCCTTGTTGTAATGACAACATTTTGCGGCTATTCTTGAATTGTTCTGGTTCAAGATTAGCCCCCGTTCTGTTGTCAGGT
TGTACCTCTCAACGTGCGGGGGTTTTCTCTTTCCAGCAACCAATGCCACCAGGGATAAAGCCCCCGCAACATTGCGCCTC
ACCGGATAACGCCGGCTTGGTGTGGATACTACGACTCAATTCATCTTCACTTCATCCCTGAAATGTTTGCAATGAAGAGT
GCATTCCGGTTTTTCAACAGCTGTTACAGTCATTTCATGAGTGCTCTGGATGAGGCTTCCAGCTCGGGTTGCCAATATTT
ACTTGTGGAAGTGATAAAGACAAAAATGGCCGCAGGCTGTTACCCCTGCGGCCGGTTTCGGGCGCATATTGCCATCACGG
CAGCCTGACGCCCGTTTTCACCTTACTTCCGGTTACGCCACCAGCCGACAATCGCTGCGGTAATAATTCCCGCCAGGATC
GGTGCCGCCAGGTCGTGCCAGAAAGTCATGGCAAACTGCGCGAGCGTCATATAGCCGCCTTGTTGTAATGACAACATTTT
GCGGCTATTCTTGAAGTGTCTGGTTTCAAGATTAGCCCCCGTTCTGTTGTCAGGTTTTACCTCTCAACGTGCGGGGGTTT
TCTCTTTCCAGCAACCAATGCCACCAGGGATAAAGCCCCCGCAACATTGCGCCTCACCGGATAATTCCGGCTTGGTGTGG
ATACTACGTCTCAATTCATCTTCACTTCATCCCTGAAATGTTTGCAATAAAGAGTACATTCCGGCTTTTCAACAGCTGTT
GCAGTCGTTTCATGAGTGCTCTGGATGATGCTTCCAGCTCGGGTTGCCAATATTTACTTGTGGAAGAGATAAAGACAAAA
ATGGCCGCAGGCTGTTACCCCTGCGGCCGGTTTCGGGCGCATATTGCCATCACGGCAGCCTGACGCCCGTTTTCACCTTA
CTTCCGGTTACGCCACCAGCTGACAATCGCTGCGGTAATAATTCCCGCCAGGATCGGTGCTGCCAGGTCGTGCCAGAAAA
TCATGGCAAACTGCGCGAGCGTCATATAGCCGCCTTGTTGTAATGACAACATTTTGCGGCTATTCTTGAAGTGTCTGGTT
TCAAGATTAGCCCCCGTTTTGTTGTCAGGTTTTACCTCTCAACGTGCGGGGGTTTTCTCTTTCCAGCAACCAATGCCACC
AGGGATAAAGCCCCGCAACATTGCGCCTCACCGGATAACGCCGGCTTGGTGTGGATACTACGTCGCAATTCATCTTCACT
TCATCCCTGAAATGTTTGCAATGAAGAGTACATTCCGGCTTTTCAACAGCTGTTGCAGTGGTTTCGTGCAGGGTGTGGTA
CAGGCTCGCAATTCTGATTAACGACCTGTCCAGGTAGTATGAAGAAATACAATAAGCAGGAATTTATATTATCTCCCCCG
ATTACCGTCATCGGAGGAGATATAACTGTCAGGCAAATATCGTCATCAAATAGGCGGCAAACAGTGCCAGATGCGCTGCG
CCATTGAGCACGTTAGTACGTCCGGTGGAGAAGGAGATATGGCACAGCACTAAAGAGGCCACCATCACCACCATTTCTGG
CGCACCAAGTGCAAACTGCAATTCGTTACCCGTCATAAAGGCAATTAGCGTGACGACAGGTACGGTAAGCGAAATGGTTG
CTAACACTGAACCAAAGAACAGATTCATCGCGCGCTGAACCTGGTTGTTCAACACTGCTTTTAATGCACCTAAACCTTCC
GGCGACAGAATCAACAGTGCCACCAGGAAGCCAGTAAAGGCGACAGGGGCATTCATGCTGTCGAGCAATGTCTCCAGCGA
GCTGGCGTTCATTTTGGTCACCGCAATAACGGCAATCAGATGGATAATCAACCAGATAGCATGCCACAGGCTGCTATGGG
CAGACGGTTTACCGTGATGCGGGTCGTCATCATCACTGTCATCTTCGTGCTCGTAGACAAACAAACTTTGATGCGTTTTG
GTCTGGATCAGCAAAAATACGCCATACATTGCCGCAGAAATTAATGCTACCAGTAACGCCTGACCGGTTGAAAAATTCGC
CGCAGGCAGAGCCATTGGAAATACCAGTACGATTATCGCCAGGGGGAACAGCGCAATTAAATACTGCTTGATACCAAACA
GATTCATATATTGGGTGGCAAACTTACGACCGCCCAACAATAATGAAAAGCCAACCAGCCCACCGGTAACAATCATAATG
ATTGAATAGAGCGTATCACGCATTAGCGTTGGCGCGGCGTCGCCGGTTGCCATTAAAGCTGAAATCAAACTGACTTCAAG
AATAACCACTGAAAGGCTAAGAATAAGCGAACCGTAAGGTTCTCCCAGGCGATGGGCTAATACGTCCGCATGACGGACAA
CACTAAAGGCGCTACTTAAAATACCAATAAGCGCAAGAAGATTGATGGCAATGACCACTGGTAGTGTCTGGCTGCTTCCC
CACAGGAACAGCACTACCAGCGCCAGAACCGGGAAAATAAGCGAAGTCTCCTTGTGGCGGGTTTTTACCGCCTCTTGAGC
ATTTGACATTATGGTTATCCCTTTGCAGATGAATTTATCGAAAATGTAAAAAATAGGTAGGAAAAATAACAGAAAGTGTC
TGGATATCGGTAACATTTTACGAATTTTTACCCCGCTGTCGATTTTTTACTCATTGGGGCATAAAAATAAGTATTACGTT
TAGACAATGTTTGTTTTAGCGTCTCTTAAGAAGAGTCTGACCTGAAAATTCTTATGTTTTGGCAAGAGTAGATATTGTTG
ACCACACTTAATGTTCAACTTTGTAAAAGGAGTCAACGATGCCGTATAAAACGAAAAGCGATCTGCCGGAAAGCGTAAAG
CACGTTCTACCGTCTCATGCCCAGGATATCTATAAAGAAGCGTTCAACAGCGCATGGGATCAATATAAAGATAAAGAAGA

TCGGCGTGATGACGCCAGTCGCGAAGAAACCGCGCATAAAGTGGCCTGGGCTGCTGTGAAGCATGAATATGCCAAAGGGG
ATGATGATAAATGGCATAAAAAATCGTAAAACCGGTCGCTTAGTTAAAGCTATTCGTGCGGTGTTGCCTTGCAAGTGGTC
CGTGGATTGCATATTGTCCCGTTAGTGGTTTCAAAATGAGCAGTAAAAATGTCCGGAAGACACCAAAAAGTTGTCGCAGG
GAAGTATGCAGTGGCGGAAGTGTAAGGTGATAACGCGTGATTTCTTGATGAATGCCGATTGTAAAACGGCATTTGGTGCC
ATTGAAGAATCACTCTTATGGTCAGCAGAACAACGGGCGGCTTCGCTGGCGGCGACGCTGGCTTGTCGACCTGATGAGGG
ACCGGTGTGGATCTTCGGCTATGGATCGTTGATGTGGAATCCGGCACTGGAGTTTACCGAATCGTGCACCGGTACACTGG
TTGGATGGCATCGCGCATTTTGCCTGCGCCTGACCGCCGGGCGCGGGACTGCGCACCAGCCGGGACGGATGCTTGCACTG
AAAGAGGGCGGACGCACCACAGGCGTCGCCTATCGACTGCCAGAAGAGACGCTGGAGCAGGAACTAACCCTGTTGTGGAA
GCGAGAGATGATTACCGGCTGTTATCTGCCAACCTGGTGTCAGCTTGATCTTGATGATGGACGCACAGTAAACGCCATTG
TGTTTATTATGGACCCGCGACATCCAGAATATGAATCTGATACTCGCGCTCAGGTCATCGCGCCGTTGATTGCGGCGGCG
AGCGGTCCGCTGGGAACCAACGCACAATACCTGTTTTCACTGGAACAGGAGCTTATCAAACTGGGAATGCAGGACGATGG
GCTGAATGATTTGCTGGTATCGGTAAAAAAACTGCTGGCGGAGAATTTTCCGGATGGTGTGTTACGTCCGGGATTCGCCT
GAGTAAACTTCCCGCATAGTGGGGCGTCAGACGCCCCCTCAAACATTAAAATGTGAGCACTTTATCGGCTGACAGCGTCC
ATTGCGCCAGTTCCACAAGAGTACCGATTTCCACCCCATCAATCAGAGGAAGTGTACTAATCCCGCGCCCGTCGGTACAG
GTTTTGCACAATTTCACCGGTACATTCTGAGCGGTAAGGATCTCCAGCATTTGCTGAATGTTGTAGCCTTCCCCTGGTTT
TTGCCCGCGCAACCCGGCTGTGACCGCATCAGACATGAGGAACAGACGCAGATCCAGATTGCTCTCCTGCTCTCGTAACG
CAATGGCCAGCCGCAAGCTGTTAAACAAGGATTCGCTCCCGTAAGGTGCGCCATTGGCAACGATCACGATTTTTTGCATT
ATTTACTCCTGTATTCAGGGAATTAGACACTCATCTTCTATCTTACTGCTTCTGCAGCGTCTGACCAATCGGTCACATTT
TTAAGGATTTTCCTGAAAGCGCGAGAAAATACGACAAAAGTTGCCAGTAATCGTTATTCTTTAAGGCTATGGTTTTTCAT
TATTACCGGAAGTTACCGACGTTTTGAGCCGTTTCGTTCCTCGCATTATCCCGTTTTATTTACTCTTGCTTGTAGCAGGC
GGTACAGCTAACGCACAATCTACCTTCGAGCAAAAAGCGGCAAATCCCTTTGATAATAACAATGATGGTCTGCCGGATTT
AGGCATGGCTCCCGAAAATCATGATGGGGAAAAACACTTTGCTGAAATTGTGAAAGATTTCGGCGAAACCAGTATGAATG
ATAACGGGCTGGATACTGGCGAGCAGGCAAAAGCTTTCGCATTGGGAAAAGTCCGCGACGCGCTTAGTCAACAGGTTAAT
CAGCACGTAGAGTCCTGGCTATCACCGTGGGGAAATGCCAGTGTTGACGTCAAAGTGGATAACGAAGGACATTTCACCGG
CAGTCGTGGAAGCTGGTTTGTGCCGTTACAAGATAATGATCGTTATCTCACCTGGAGCCAGCTTGGTCTTACTCAGCAGG
ATAATGGGTTGGTGAGCAATGTGGGCGTTGGGCAACGCTGGGCGCGCGGCAACTGGCTGGTGGGTTATAACACTTTTTAT
GACAACTTGCTGGACGAAAATCTTCAGCGAGCGGGCTTTGGTGCCGAAGCGTGGGGCGAATATTTGCGATTATCGGCAAA
CTTTTATCAGCCGTTTGCTGCATGGCATGAACAGACAGCCACGCAGGAACAACGGATGGCGCGCGGGTACGACCTGACAG
CTCGGATGCGCATGCCGTTCTATCAACACCTCAATACCAGTGTCAGCCTAGAACAGTATTTTGGTGATCGTGTTGATTTG
TTTAACTCTGGTACGGGTTATCACAATCCCGTCGCGTTGAGTCTGGGATTAAATTACACCCCTGTGCCATTAGTCACTGT
GACGGCCCAGCATAAACAGGGTGAAAGTGGCGAAAATCAAAATAACCTCGGGCTGAATCTTAATTACCGCTTTGGTGTAC
CGCTCAAAAAACAACTTTCTGCGGGCGAGGTTGCCGAAAGTCAGTCGTTACGTGGTAGTCGCTATGACAATCCGCAGCGA
AATAATCTACCGACTCTTGAGTACCGACAGCGAAAAACGTTAACGGTGTTTCTGGCGACACCGCCGTGGGATCTAAAACC
TGGCGAAACAGTGCCGCTGAAATTACAAATCCGCAGTCGTTACGGTATTCGGCAACTGATTTGGCAGGGCGATACGCAGA
TATTAAGTTTGACGCCGGGCGCACAAGCCAACAGCGCGGAGGGCTGGACGCTGATCATGCCTGACTGGCAGAACGGGGAA
GGGGCAAGCAATCACTGGCGATTGTCTGTGGTGGTGGAAGATAACCAGGGGCAGCGTGTCTCCTCCAATGAGATCACGCT
AACGCTTGTCGAACCGTTCGATGCATTGTCAAACGACGAACTGCGCTGGGAACCGTAATCAGAAAATGCGCTCCTGATGC
ACCCATACCGCTGCTTCCACGCGAGACTTGAGCTTCATTTTCTTCAGCATGTGCTTGACGTGCACTTTTACTGTGCTTTC
GGTGATATCCAGGCGGCGGGCAATCATCTTGTTCGGCAAACCCTGGGCAATCAGCTTGAGAATATCGCGCTCGCGTGGGG
TTAACTGGTTAACATCGCGCTCAGTAGTGGCACGGTTAGCGCGCAAGCTGGCGGCCAGAACAGGCGTTAATGCTTCGCTT
AATACCATTTCGCCAGCAGCTGCCTGATGCAATGCTTTCAGCAGATCTTCCGGTTCCATATCTTTTAACAGATAGCCATC
CGCGCCGCGTTTCAGTGCGGTGACCACATCTTCTTCATGGTTAGAGACGCTGAATACCACAATGCGCCCTGAGAGGGACT
TTTCGCGCAGTTTATCCAGCGTTTCCAGACCGTTCATGCCGGGCATATTGAGATCTAACAGGATCAGATCGGGATCAAGA
GACTCCGCCAGTTCAATACCCTGTTCGCCATTACTCGCTTCGCCAACCACGGTGATATCTGGTGCCATACTGATAAGCTG
TTTTACGCCAGTTCGCAGCATCGGGTGATCGTCAATCAGCAGGATAGTAGCCGGTTCCTGATTACTCATGGGTATCTCCT
TGGACGTCTGTGAAAGTTTTTTCGGGAATAAAGGTGACCACCACTTCGGTGCCACCTGATTCACGACGGCGGACGCGGCA
ATCGCCTCGTAAACTTTGCGCACGATCGCGCATTATTATCATGCCGTAGTGATTGCTGCGGATGGCATTTTCAGGCACGC
CGCAGCCGTTATCCTGGACGGTCAGTTTGACCTGATTATCGTTTTGCGCCACCGTCACCACGACTTCACTCGCTTGCGAA
TGTTTGAGGGCGTTACTTAATGCCTCACGGGCAATTTGCAACAAGTGGATTGCCTGATGCGAAGGCACCAGGCGAGGCGG
CAATTGATAATCCAGCTTCACCGGGAAGCCAAATTTGGCGCTGTACTCTTCGCAACTCGCCTCCAGCGCCGGACGTAATC
CAGGCTCGGTGAGCTGCAAGCGGAATGTGGTGAGCAATTCACGCAACTGCGCCCAGGATGCATTCAGTTCGTTACGGATC
TGACTTAACAGTTCGCGGCTGCTTTCTGGCAGCGCATCGCCCTGCATCTGTAAACAACTCACCTGCATCTTCATGCAAGA
GAGAGATTGGGCAATAGAATCATGCAGTTCGCGCGCAATGGTGGCACGCTCTTCCATCACGATCAACTGTTGCTGACGTT
CCTGATGGCGATCCAGCGCCAGCGTGGCGGTGAGTTGTTCAACCAGGGTATCCACCAGTTGTTGTTGATCATGGCTAAGA
TGACGCCCCTGCGGCAGGGTCGCCAGCAAAATACCGTACTGCGTATGAGAGTCAGCCAGCCGCCACTTCAGGGTCGTGCC
GCGATCACCAACGGGTAATACGCCGCGCGGGCAGAGCTGGCAGCCTTTATCATCACAAGTCATATCTGGCTGGCAGGTAA

ACTCCTGATGATTCTCTTCATCATCAGTGTCATACACCCGCAATTCGATATCACGTAGCAGGGTTAAATTCTGTAAGCCG
TTGAGTACAGGTGACAGGCGTTCACACAGCGGGGCGCGGGAATGCAAACGGCGGTTAGCCTGCCATAAAAAAGAGAGGAT
CTGATTTTTATGCTCCAGCCCGGCGGTTTTCTCCTGAACCCGCTGCTCAAGTACGGCATAACTTTCGGCCAGTTCTGCAG
ACATATTGTTCAACGCAGTTCCAAGCATCGCCATTTCGTTGCGCCCGCTGATGTTTGCGCGTTGGGTAAAATCGCGATGA
CTGACGGCACTCGCCATTGCCAGCAGTTGCCGCCACGGTTGTAGCAGTCGCGCCCGCAACCAGATAATAGTGAACACCAG
TAAAAGTGCCATAAATACCGCCATTACCCGATGGACCAGTACCACTGTCTCGATGCGCATTTCCGTGGTGCGGTCAAAAC
CAGATACCAGTTGATCAAGCCCGGCAACAAACTGGCTGACATCCGCTGACACCGTTTCTCGGTTTTGTGCACGCATCAGC
GCAGGGATCAGTTCATTACGCCAGTAATCTTGTAAACCCTGTAATTGCGCCAGTTGTCCGTCTCGTTCTGCTGCTCGAGT
CAACTCGGCGCTAAATGCCGTTTGTTCCATCTCTTTAATTAAGGGCTTGTCTTTCTCGCTTAATGGCACTGCCGCCAACA
GACGGTAACTTTGCATGCGCAGCGATCCCGCTTTGTTGATCGCATGGGCGCTGCCCTGAACGCCTTGCACCAGCCAGCCA
GAAACCGCCATCCCTGCCAGTCCAATAGCAGTAGAAAGCAACACAATAAGCGCAACCTGATTAACCAGGGTGAGCGGAGA
GAGACAACGTTTAAGCATGTAAACCTCTTCCTTCAGGCTTTAAATGAGCAATAACCTTAATGAATGTGACGATACATTCT
GGAATGGCAGTATTCTCGGCTATTGGCTGAAGTATACCCATACCCGGAAAGAGTTACTCCTTATTTGCCGTGTGGTTAGT
CGCTTTACATCGGTAAGGGTAGGGATTTTACAGCACCGTGAAAAATCTCATAATTTTTATGAAGTCACTGTACTCACTAT
GGGTAATGATAAATATCAATGATAGATAAAGTTATCTTATCGTTTGATTTACATCAAATTGCCTTTAGCTACAGACACTA
AGGTGGCAGACATCGAAACGAGTATCAGAGGTGTCTATGAGTCACTCATCCGCCCCCGAAAGGGCTACTGGAGCTGTCAT
TACAGATTGGCGACCGGAAGATCCTGCGTTCTGGCAACAACGCGGTCAACGTATTGCCAGCCGCAACCTGTGGATTTCCG
TTCCCTGTCTGCTGCTGGCGTTTTGCGTATGGATGTTGTTCAGCGCTGTTGCGGTGAACCTACCGAAAGTCGGCTTTAAT
TTTACGACCGATCAGCTATTTATGTTGACTGCGCTGCCTTCGGTTTCTGGCGCGTTATTACGTGTTCCATACTCCTTTAT
GGTTCCTATCTTCGGTGGTCGTCGCTGGACGGCGTTCAGCACCGGTATTCTGATTATTCCTTGCGTCTGGCTGGGTTTTG
CCGTGCAGGATACCTCCACGCCTTATAGCGTCTTCATCATCATCTCTCTGCTATGCGGCTTTGCTGGCGCGAACTTCGCA
TCCAGTATGGCAAACATCAGCTTCTTCTTTCCGAAACAGAAGCAGGGTGGCGCGCTGGGTCTGAATGGTGGTCTGGGAAA
CATGGGCGTCAGCGTCATGCAGTTGGTTGCTCCGCTGGTGGTATCACTGTCGATTTTCGCAGTATTTGGTAGCCAGGGCG
TCAAACAGCCGGATGGGACTGAGCTGTATCTGGCGAATGCGTCCTGGATATGGGTGCCGTTCCTTGCCATCTTCACCATT
GCGGCGTGGTTTGGCATGAACGATCTTGCTACCTCGAAAGCCTCCATCAAGGAGCAGTTGCCGGTACTCAAACGGGGTCA
TCTGTGGATTATGAGCCTGCTGTATCTGGCAACCTTCGGCTCCTTCATCGGCTTCTCCGCGGGCTTTGCAATGCTGTCAA
AAACGCAGTTCCCGGATGTTCAGATTCTGCAATACGCTTTCTTCGGGCCGTTTATTGGTGCGCTGGCGCGTTCTGCAGGT
GGTGCATTATCTGACCGTCTGGGCGGAACTCGTGTCACGCTGGTGAACTTTATTCTGATGGCGATTTTCAGCGGCCTGCT
GTTCCTGACCTTACCGACTGACGGGCAGGGCGGAAGCTTCATGGCGTTCTTCGCGGTCTTCCTGGCGCTGTTCCTGACAG
CTGGGCTGGGTAGTGGTTCCACTTTCCAGATGATTTCAGTGATCTTCCGTAAACTGACAATGGATCGCGTGAAAGCAGAA
GGGGGTTCTGACGAACGTGCGATGCGTGAAGCGGCAACCGACACGGCGGCGGCGCTGGGTTTCATCTCTGCGATTGGCGC
GATTGGTGGCTTCTTTATCCCGAAAGCGTTTGGTAGCTCGCTGGCATTAACGGGTTCGCCAGTCGGCGCAATGAAGGTAT
TTTTGATTTTCTATATCGCCTGCGTAGTGATTACCTGGGCGGTATATGGTCGGCATTCTAAAAAATAAACCGTTACTCGT
CATACTTCGGGTTACATGTGCTGCGGCTGCGTTCATTCACCCCAGTCACTTACTTTAGTAAGCTCCTGGGATTCATTCAC
TTGCCGCCTTCCTGTAAACCGAATTATATAGAGTAAAATATTTGATTATCCTTTGCGCGGCATGATGTCGCGCTTTTTTT
ATGCGTCATTTAGTTACAACATACTAATGTTATATGGTTTATTTCGCCGGATTTCATTAAGAGCCATTAATATGTTACCC
ATGGGGAATACTCCTTAATACCCATCTGCATAAAAATCTTAATAGTTTAAATAACTACAGGTATAAAACGTCTTAATTTA
CAGTCTGTTATGTGGTGGCTGTTAATTATCCTAAAGGGGTATCTTAGGAATTTACTTTATTTTTCATCCCCATCACTCTT
GATCGTTATCAATTCCCACGCTGTTTCAGAGCGTTACCTTGCCCTTAAACATTAGCAATGTCGATTTATCAGAGGGCCGA
CAGGCTCCCACAGGAGAAAACCGATGAGTAAATTCCTGGACCGGTTTCGCTACTTCAAACAGAAGGGTGAAACCTTTGCC
GATGGGCATGGCCAGCTTCTCAATACCAACCGTGACTGGGAGGATGGATATCGCCAGCGTTGGCAGCATGACAAAATCGT
CCGCTCTACCCACGGGGTAAACTGCACCGGCTCCTGCAGCTGGAAAATCTACGTCAAAAACGGTCTGGTCACCTGGGAAA
CCCAGCAGACTGACTATCCGCGTACCCGTCCGGATCTGCCAAACCATGAACCTCGCGGCTGCCCGCGCGGTGCCAGCTAC
TCCTGGTATCTTTACAGTGCCAACCGCCTGAAATACCCGATGATGCGCAAACGCCTGATGAAAATGTGGCGTGAAGCGAA
GGCGCTGCATAGCGATCCGGTTGAGGCATGGGCTTCTATCATTGAAGACGCCGATAAAGCGAAAAGCTTTAAGCAGGCGC
GTGGACGCGGTGGATTTGTTCGTTCTTCCTGGCAGGAGGTGAACGAACTGATCGCCGCATCTAACGTTTACACCATCAAA
AACTACGGCCCGGACCGTGTTGCTGGTTTCTCGCCAATTCCGGCAATGTCGATGGTTTCTTACGCATCGGGTGCACGCTA
TCTCTCGCTGATTGGCGGTACTTGCTTAAGCTTCTACGACTGGTACTGCGACTTGCCTCCTGCGTCTCCGCAAACCTGGG
GCGAGCAAACTGACGTACCGGAATCTGCTGACTGGTACAACTCCAGCTACATCATCGCCTGGGGGTCAAACGTGCCGCAG
ACGCGTACCCCGGATGCTCACTTCTTTACTGAAGTGCGTTACAAAGGGACCAAAACTGTTGCCGTCACACCAGACTACGC
TGAAATCGCCAAACTGTGCGATCTGTGGCTGGCACCGAAACAGGGCACCGATGCGGCAATGGCGCTGGCGATGGGCCACG
TAATGCTGCGTGAATTCCACCTCGACAACCCAAGCCAGTATTTCACCGACTATGTGCGTCGCTACACCGACATGCCGATG
CTGGTGATGCTGGAAGAACGCGACGGTTACTACGCTGCAGGTCGTATGCTGCGCGCTGCTGATCTGGTTGATGCGCTGGG
CCAGGAAAACAATCCGGAATGGAAAACTGTCGCCTTTAATACCAATGGCGAAATGGTTGCGCCGAACGGTTCTATTGGCT
TCCGCTGGGGCGAGAAGGGCAAATGGAATCTTGAGCAGCGCGACGGCAAAACTGGCGAAGAAACCGAGCTGCAACTGAGC
CTGCTGGGTAGCCAGGATGAGATCGCTGAGGTAGGCTTCCCGTACTTTGGTGGCGACGGCACGGAACACTTCAACAAAGT

GGAACTGGAAAACGTGCTGCTGCACAAACTGCCGGTGAAACGCCTGCAACTGGCTGATGGCAGCACCGCCCTGGTGACCA
CCGTTTATGATCTGACGCTGGCAAACTACGGTCTGGAACGTGGCCTGAACGACGTTAACTGTGCAACCAGCTATGACGAT
GTGAAAGCTTATACCCCGGCCTGGGCCGAGCAGATTACCGGCGTTTCTCGCAGCCAGATTATTCGCATCGCCCGTGAATT
TGCCGATAACGCTGATAAAACGCACGGTCGTTCGATGATTATCGTCGGTGCGGGGCTGAACCACTGGTATCACCTCGATA
TGAACTATCGTGGTCTGATCAACATGCTGATTTTCTGCGGCTGTGTCGGTCAGAGCGGGGGCGGCTGGGCGCACTATGTA
GGTCAGGAAAAACTGCGTCCGCAAACCGGCTGGCAGCCGCTGGCGTTTGCCCTTGACTGGCAGCGTCCGGCGCGTCACAT
GAACAGCACTTCTTATTTCTATAACCACTCCAGCCAGTGGCGTTATGAAACCGTCACGGCGGAAGAGTTGCTGTCACCGA
TGGCGGACAAATCCCGCTATACCGGACACTTGATCGACTTTAACGTCCGTGCGGAACGCATGGGCTGGCTGCCGTCTGCA
CCGCAGTTAGGCACTAACCCGCTGACTATCGCTGGCGAAGCGGAAAAAGCCGGGATGAATCCGGTGGACTATACGGTGAA
ATCCCTGAAAGAGGGTTCCATCCGTTTTGCGGCAGAACAACCAGAAAACGGTAAAAACCACCCGCGCAACCTGTTCATCT
GGCGTTCTAACCTGCTCGGTTCTTCCGGTAAAGGTCATGAGTTTATGCTCAAGTACCTGCTGGGGACGGAGCACGGTATC
CAGGGTAAAGATCTGGGGCAACAGGGCGGCGTGAAGCCGGAAGAAGTGGACTGGCAGGACAATGGTCTGGAAGGCAAGCT
GGATCTGGTGGTTACGCTGGACTTCCGTCTGTCGAGCACCTGTCTCTATTCCGACATCATTTTGCCGACGGCGACCTGGT
ACGAAAAAGACGACATGAATACTTCGGATATGCATCCGTTTATTCACCCGCTGTCTGCGGCGGTCGATCCGGCCTGGGAA
GCGAAAAGCGACTGGGAAATCTACAAAGCCATCGCGAAGAAATTCTCCGAAGTGTGCGTCGGCCATCTGGGTAAAGAAAC
CGACATCGTCACGCTGCCTATCCAGCATGACTCTGCCGCTGAACTGGCGCAGCCGCTGGATGTGAAAGACTGGAAAAAAG
GCGAGTGCGACCTGATCCCAGGTAAAACCGCGCCACACATTATGGTCGTAGAGCGCGATTATCCGGCGACTTACGAACGC
TTTACCTCTATCGGCCCGCTGATGGAGAAAATCGGTAATGGCGGTAAAGGGATTGCCTGGAACACCCAGAGCGAGATGGA
TCTGCTGCGTAAGCTCAACTACACCAAAGCGGAAGGTCCGGCGAAAGGCCAGCCGATGCTGAACACCGCAATTGATGCGG
CAGAGATGATCCTGACACTGGCACCGGAAACCAACGGTCAGGTAGCCGTGAAAGCCTGGGCTGCCCTGAGCGAATTTACC
GGTCGTGACCATACGCATCTGGCGCTGAATAAAGAAGACGAGAAGATCCGCTTCCGCGATATTCAGGCACAGCCGCGCAA
AATTATCTCCAGCCCGACCTGGTCTGGTCTGGAAGATGAACACGTTTCTTACAACGCCGGTTACACCAACGTTCACGAGC
TGATCCCATGGCGTACGCTCTCTGGTCGTCAGCAACTGTATCAGGATCACCAGTGGATGCGTGATTTCGGTGAAAGCCTG
CTGGTTTATCGTCCGCCGATCGACACCCGTTCGGTGAAAGAAGTGATAGGCCAGAAATCCAACGGCAACCAGGAAAAAGC
GCTCAACTTCCTGACGCCGCACCAGAAGTGGGGTATCCACTCCACCTACAGCGACAACCTGCTGATGCTGACTTTAGGTC
GCGGTGGTCCGGTGGTCTGGTTGAGTGAAGCCGATGCCAAAGATCTGGGTATCGCCGATAACGACTGGATTGAAGTCTTC
AACAGCAACGGTGCTCTGACTGCCCGTGCGGTTGTCAGCCAGCGTGTTCCGGCAGGGATGACCATGATGTACCACGCGCA
GGAACGTATCGTTAACCTGCCTGGTTCGGAAATTACCCAACAGCGTGGTGGTATCCATAACTCGGTCACCCGTATCACGC
CGAAACCGACGCATATGATCGGCGGCTATGCCCATCTGGCATACGGCTTTAACTACTATGGCACCGTAGGTTCTAACCGC
GATGAGTTTGTTGTAGTGCGTAAGATGAAGAACATTGACTGGTTAGATGGCGAAGGCAATGACCAGGTACAGGAGAGCGT
AAAATGAAAATTCGTTCACAAGTCGGCATGGTGCTGAATCTCGATAAGTGCATCGGCTGCCACACCTGTTCAGTTACCTG
TAAAAACGTCTGGACCAGCCGTGAAGGCGTGGAATATGCGTGGTTCAACAACGTGGAAACCAAGCCGGGCCAGGGCTTCC
CGACTGACTGGGAAAACCAGGAAAAATACAAAGGCGGCTGGATCCGTAAAATCAACGGCAAACTGCAGCCGCGCATGGGT
AACCGTGCCATGCTGCTGGGTAAAATCTTCGCTAACCCGCATCTGCCGGGGATCGACGATTATTACGAGCCGTTCGATTT
TGACTATCAGAACCTGCATACCGCGCCGGAAGGCAGCAAATCGCAGCCGATTGCCCGTCCGCGTTCGCTGATTACCGGGG
AACGGATGGCGAAAATCGAAAAAGGGCCGAACTGGGAAGATGACCTGGGCGGTGAGTTTGACAAACTGGCGAAAGACAAG
AACTTCGACAACATCCAGAAGGCGATGTATAGCCAGTTCGAAAACACCTTCATGATGTATTTGCCGCGCCTGTGCGAACA
CTGCCTGAACCCGGCATGTGTGGCGACCTGCCCGAGCGGTGCGATTTACAAGCGTGAAGAAGATGGCATCGTCCTGATCG
ACCAGGATAAATGCCGTGGCTGGCGTATGTGCATCACTGGATGCCCGTACAAAAAAATCTACTTCAACTGGAAGAGCGGT
AAGTCTGAGAAGTGCATCTTCTGCTATCCGCGTATTGAAGCGGGTCAGCCGACCGTGTGCTCAGAAACCTGTGTCGGTCG
TATCCGTTATCTTGGCGTGCTGTTGTACGATGCCGACGCTATTGAACGTGCAGCCAGCACCGAGAACGAGAAAGATCTTT
ACCAGCGTCAGCTGGACGTGTTCCTCGATCCGAACGATCCGAAAGTCATCGAGCAGGCGATTAAAGACGGTATTCCGCTG
AGCGTTATTGAAGCCGCACAGCAGTCGCCGGTTTATAAAATGGCAATGGAATGGAAACTGGCGCTGCCGCTGCATCCGGA
ATATCGCACACTGCCGATGGTCTGGTACGTGCCGCCTCTGTCTCCGATTCAGTCTGCAGCAGACGCGGGTGAGCTGGGTA
GCAACGGCATTCTGCCAGACGTCGAAAGCCTGCGTATTCCGGTACAGTATCTGGCGAATCTGCTGACCGCCGGTGATACC
AAACCGGTACTGCGCGCACTGAAACGTATGCTGGCGATGCGTCATTACAAACGTGCTGAAACCGTTGACGGTAAAGTTGA
TACCCGTGCGCTGGAAGAGGTCGGTCTGACCGAAGCCCAGGCACAGGAGATGTACCGTTATCTGGCGATTGCTAACTACG
AAGATCGCTTTGTGGTGCCGAGTAGTCATCGTGAACTGGCACGGGAAGCCTTCCCGGAGAAAAATGGCTGCGGCTTTACC
TTTGGTGATGGCTGCCACGGTTCAGATACCAAATTCAATCTGTTCAACAGCCGTCGTATCGATGCCATCGATGTGACCAG
CAAAACGGAGCCGCATCCATGATCGAACTCGTGATTGTATCGCGTCTCCTTGAATATCCGGATGCTGCCTTATGGCAGCA
TCAACAAGAGATGTTTGAGGCGATTGCCGCGTCGAAAAATCTGCCAAAAGAGGATGCCCATGCGCTGGGCATTTTCCTGC
GCGATTTAACGACGATGGATCCGCTCGATGCCCAGGCGCAGTACAGCGAACTGTTCGACCGTGGCCGCGCCACGTCACTG
TTGCTGTTTGAACATGTGCACGGCGAATCCCGCGACCGCGGCCAGGCGATGGTGGACCTGCTGGCGCAGTACGAGCAGCA
CGGCTTGCAGTTAAACAGCCGCGAATTGCCGGACCATCTGCCGCTGTATCTGGAGTACCTGGCGCAGCTGCCGCAAAGCG
AAGCCGTGGAAGGTTTGAAAGATATCGCGCCGATTCTGGCATTGCTGAGCGCGCGTCTGCAACAGCGTGAAAGCCGTTAT
GCCGTGCTGTTTGATCTGCTGCTGAAACTGGCGAATACCGCTATCGACAGCGACAAAGTGGCGGAAAAAATTGCCGACGA

AGCGCGCGATGATACGCCGCAGGCGCTGGATGCTGTCTGGGAAGAAGAGCAGGTTAAATTCTTTGCTGACAAAGGCTGCG
GTGATTCAGCAATCACTGCGCATCAGCGTCGCTTTGCCGGTGCCGTCGCGCCGCAATATCTGAATATCACCACCGGAGGA
CAGCACTAATGCAATTCCTGAATATGTTCTTCTTTGATATCTACCCGTACATAGCCGGGGCGGTCTTCCTGATTGGTAGC
TGGCTGCGTTATGACTACGGGCAGTACACCTGGCGCGCGGCGTCCAGCCAGATGCTGGATCGCAAAGGGATGAACCTGGC
GTCGAACCTGTTCCATATCGGGATTCTGGGGATTTTTGTCGGTCACTTCTTCGGTATGCTGACGCCGCACTGGATGTATG
AAGCCTGGCTGCCGATTGAAGTGAAACAGAAAATGGCAATGTTTGCTGGTGGTGCCAGCGGCGTGCTGTGTCTGATTGGC
GGCGTGCTGTTGCTGAAACGTCGTCTGTTCAGCCCACGCGTGCGTGCAACCACTACCGGAGCGGATATCCTGATCCTGTC
GCTGCTCGTTATCCAGTGCGCGCTGGGCCTGTTGACCATTCCGTTCTCCGCTCAGCATATGGACGGTAGCGAGATGATGA
AACTGGTTGGCTGGGCGCAGTCGGTGGTGACCTTCCACGGTGGCGCTTCTCAACACCTCGATGGTGTGGCGTTTATCTTC
CGTCTGCACCTGGTGCTGGGGATGACGTTATTCCTGCTGTTCCCGTTCTCGCGTCTGATACACATCTGGAGCGTACCGGT
GGAGTATCTGACACGTAAGTACCAGCTGGTGCGCGCTCGTCACTAAGCGAATTTTAGTTCACATAGACCCTGCTTCGGCG
GGGTTTTTTTATGGGCACGGTGCGGGGTGAGTTGTCGGATGCGCTTCGCTTATCCGACCTACAGGGGAGGATATTGTAGG
CCCGGTAAACGTGGTGCCATCGGGCAAAATTAGCTCGGAGTAACAGGTTTTGATTATTTAAGGAAGCGATGGTGGTGGGG
GAAGGATTACTCAGCGCTGCGCGCTTCGCCCTTCGGGTCGTTGCCTGCGGCAACGCTCTCTCGCTGGCGCTCAAGTCGAA
CCTTGGTCGAAGCTTCTCATCCTTCCCCGCTTGGGCAGAATATTTGATTGCGGATTCGTTTGAGAATTCCGGGGCTTTTG
AAAGTGATGGTGGTGGGGGAAGGATTACTCAGCGCTGCGCGCTTCGCCCTTCGGGTCGTTGCCTGCGGCAACGCTCTCTC
GCTGACGCTCGAGCCGAACCTTAGTCGAAGCTTCTCATCCTTCCCCGCATGGGCAGAATATTTGATTGCGGATTCGCTTG
AGAGTTCAGGGACTTTTGAAAGTGATGGTGGTGGGGGAAGGATTACTCAGCGCTGCGCGCTTCGCCCTTCGGGTCGTTGC
CTGCGGCAACGCTCTCTCGCTGGCGCTCGAGTCGAACCTTGGTCGAAGCTTCTCATCCTTCCCCGCATGGGCAGAATATT
TAATTGCGGATTCGTTGGGAAGTTCAGGGACTTTTGAAAGTGATGGTGGTGGGGGAAGGATTCGAACCTTCGAAGTCGAT
GACGGCAGATTTACAGTCTGCTCCCTTTGGCCGCTCGGGAACCCCACCAGGGGTAATTCAAATTTTGAGGTAATGCTTGA
GATGGTGGTGGGGGAAGGATTATTCGTCGCTTCGCTCCTCACCCTTCGGGCCGTTGCCTGTGGCAACGTTCTCTCGCTTT
CGCTCGAATCGAACCTTAGTCGAAGGTTCTCACCCTTCCCCGATGAGTGCAAACTTTCACAATCTCACCGAAGTTACCAC
ATCGCTGTGGTGAATTATGGTGGTGGGGGAAGGATTCGAACCTTCGAAGTCGATGACGGCAGATTTACAGTCTGCTCCCT
TTGGCCGCTCGGGAACCCCACCACGGGGTAATGCTTTTTACTGGCCTGCTCCCTTATCGGGAAGCGGGGCGCATCATATC
AAATGACGCGCCGCTGTAAAGTGTTACGTTGAGAAAAATGAACTGGTTGCGTAATTTTCATCCGTAACGGATTAAAGGTA
ACCAGTTATTTTTGCTGGCGATTAAAGAATAATCGTTCGATTACCGTAAACAAAGACGCGCTGTGCCAGTACTTTGTATA
GTGCACGACTTAAGACGTTTTTCTCGACGTCACGACCTGCGCGCATCATATCTTCAGCTGTGTAGGTATGATCGACATGA
ATAACGTCCTGCATGATGATTGGGCCTTCGTCCAGATTGTCATTCACATAGTGAGCGGTTGCGCCAATAATCTTCACACC
ACGTTCATAGGCCTGGTGATAAGGACGTGCGCCAATAAACGCTGGCAGGAATGAATGGTGAATATTGATGATCTTATTCG
GGAAGCGTGCCACAAATTCCGGCGTTAATACCCGCATATACTTCGCCAGCACCACGTAGTCAGGTTGATAAGCATCAATG
GCATCCGCCATCTTTTGATCGTGCTCGTTGCGGGTTAACCCTTCATGGCTTACCAGCTCAAACGGAATATCAAAACGCTC
AACCAGAGAACGTAAAGTATCGTGGTTACCAATAACTGCCGCGATTTCGACATCCAGGCCGCCGTAATTGGCTTTCATCA
ACAAATCGCCAAGGCAATGCGCTTCTTTAGTGACCAGAATCACTATCCGGCGACGACCGGCAGGATTCAGCTCACGCACG
GAGCCTTCTGGCAATGCGCTATCGAGATCCGCCAGCAGGGTGGAATCATTAAAAATCCCTTCCAGTTCCGTGCGCATAAA
AAAGCGCCCGGTACGGTGATCAACAAATTCATTGTTCTGTACGATATTTAACTCGTGCTTGTAGCAAATATTGGTAATAC
GTGCGATCAGACCTTTTTGGTCCGGACAAATAGTACGCAGAACTTTACGTTGGAGTGAATGCATTGCTGGAAAAACCTTG
TTGAGAGTGTTTGCTAAACCGTACCGTCAACCATTATTGGCCGCAGCACTTTTTAAATTTTTTACCTGAACCACAAGGGC
AGGGATCGTTGCGACCAAACTGCGGACGTGTACCGTCAATATAGTACCATTGACCGTTTTCTTTCAAAAATCGCGAACGT
TCAATTATTGCACCTGTTTTACCACCTTCGGTAAATCGAGCAACAAAACTGACAAATCCGATATTGTCGGCGTCCTGCCA
ACAATGTTCAAATACGGTCAATCCCAACCATTCAGTATGCGCAAATCCGGCCATCAATTCGGCACGTAACGCCGCTGCTC
CACAAGAGGGATGCCAGGTCTTTATTAAATAATCTGCGTCTTGCATCACAAAAGCGCAGTAACGCGAACGCATGAGATGT
TCTGGATCAGGTGCAACCTTTTCACCAGACACATAAGGGTGGCAACATAGGCTATACTCGACAGCACTACCACAGGGACA
AAGCTGAGACACAAATAATCTCCCTGGAAACAATAACGGCGTATTAACCGCCTGAGTAGCACTATGTTAACCGAGCAGTA
GCGATGTGGCTACGATTGCATTCCAGGGGAATCTTGCGGGAATAATGAGAAAGATAAAAATAGGGCTGGCGCTGGGATCT
GGCGCGGCGAGAGGTTGGTCGCATATTGGCGTTATTAATGCGCTAAAAAAAGTGGGTATTGAAATTGATATCGTTGCAGG
ATGTTCAATTGGTTCGCTGGTGGGCGCTGCCTATGCATGCGATCGATTATCTGCGCTGGAAGATTGGGTGACCTCTTTCA
GTTATTGGGATGTTTTACGCCTGATGGATCTCTCCTGGCAGCGCGGTGGGTTACTGCGCGGCGAGCGTGTCTTCAATCAA
TACCGCGAAATAATGCCGGAAACAGAGATCGAAAATTGTTCCCGTCGCTTTGCGGCTGTTGCCACCAATTTAAGTACGGG
ACGTGAATTATGGTTTACTGAAGGCGATCTCCATCTTGCTATTCGCGCATCATGCAGTATTCCAGGACTCATGGCACCTG
TTGCACATAACGGCTACTGGCTGGTTGATGGAGCAGTCGTTAACCCAATTCCTATTTCCCTCACGCGTGCATTGGGGGCT
GATATTGTGATAGCGGTTGACCTGCAGCACGATGCTCATTTGATGCAACAAGATTTGCTCTCCTTTAATGTCAGTGAAGA
AAATAGCGAGAATGGTGATTCTCTGCCGTGGCATGCGCGTCTGAAAGAAAGGTTAGGCAGCATAACGACACGTCGGGCGG
TGACAGCGCCAACGGCAACAGAGATTATGACCACTTCTATCCAGGTGCTGGAGAACCGCCTTAAAAGGAACCGCATGGCA
GGTGATCCGCCCGATATTCTGATTCAACCTGTTTGCCCGCAAATATCTACGCTTGATTTCCATCGCGCGCACGCTGCCAT
TGCGGCCGGACAGCTGGCAGTGGAAAGGAAAATGGACGAACTTTTGCCGTTGGTACGCACCAACATTTGACCAGAATTTT

TATCTACACTTAAGTTAATTCTGACAGGCGCAGGTGGCAATAGCATGCCACTATTGAGTAAAGCCAGTCAGGGGAGAGAA
CATGACGCAGCCATTGGTCGGAAAACAGATTCTCATTGTTGAAGATGAGCAGGTATTTCGCTCGCTTCTGGATTCATGGT
TTTCCTCATTGGGAGCGACAACGGTACTGGCGGCTGATGGGGTGGATGCCCTTGAGTTGCTGGGAGGTTTCACTCCAGAC
CTGATGATATGTGATATCGCGATGCCACGAATGAACGGGCTTAAACTGCTGGAGCATATACGTAACAGAGGCGACCAGAC
CCCAGTTCTGGTGATATCTGCCACTGAAAATATGGCAGATATTGCCAAAGCGTTACGTCTGGGCGTTGAAGATGTTTTGC
TGAAACCAGTTAAAGATCTGAATCGCTTGCGCGAGATGGTTTTTGCCTGTCTCTATCCCAGCATGTTTAATTCGCGCGTT
GAGGAAGAGGAAAGGCTTTTTCGCGACTGGGATGCAATGGTTGATAACCCTGCCGCAGCGGCGAAATTATTACAGGAACT
ACAACCGCCGGTTCAGCAGGTGATTTCCCATTGCCGGGTTAATTATCGTCAATTGGTTGCCGCGGACAAACCCGGCCTGG
TGCTTGATATTGCCGCACTTTCGGAAAACGATCTGGCATTTTATTGCCTTGATGTCACCCGAGCTGGACATAATGGCGTA
CTTGCTGCCTTGTTATTACGCGCATTGTTTAACGGATTATTACAGGAACAGCTTGCACACCAAAATCAACGGTTGCCAGA
GTTGGGCGCGTTATTGAAGCAGGTAAACCATTTACTTCGTCAGGCCAATCTGCCGGGGCAGTTTCCGCTATTAGTTGGCT
ATTATCATCGCGAACTGAAAAATCTCATTCTGGTTTCTGCGGGTCTGAATGCGACGTTAAATACCGGCGAACACCAGGTG
CAAATCAGTAATGGTGTTCCGTTAGGCACTTTAGGTAACGCTTATTTGAATCAATTGAGCCAGCGATGCGATGCCTGGCA
ATGCCAAATATGGGGAACCGGTGGTCGACTGCGCTTGATGTTGTCTGCAGAATGAGCAAACGATAACGCGGGCTAAATTT
GCATTACCTGCTAATGTCGGCTGGTGGTACTATCGTCGCCATTCGTATAAGTAATTGTCTTAATTATGCTAACTCGCCTC
CTTTTCAGAACTTAGCCCCTTCGGGGTGCTGATATACTGGGATGCGATACAGAAATATGAACACGTTCAAAACACGAACA
GTCCAGGAGAATTTAAATGGCTGCCATTAATACGAAAGTCAAAAAAGCCGTTATCCCCGTTGCGGGATTAGGAACCAGGA
TGTTGCCGGCGACGAAAGCCATCCCGAAAGAGATGCTGCCACTTGTCGATAAGCCATTAATTCAATACGTCGTGAATGAA
TGTATTGCGGCTGGCATTACTGAAATTGTGCTGGTTACACACTCATCTAAAAACTCTATTGAAAACCACTTTGATACCAG
TTTTGAACTGGAAGCAATGCTGGAAAAACGTGTAAAACGTCAACTGCTTGATGAAGTGCAGTCTATTTGTCCACCGCACG
TGACTATTATGCAAGTTCGTCAGGGTCTGGCGAAAGGCCTGGGACACGCGGTATTGTGTGCTCACCCGGTAGTGGGTGAT
GAACCGGTAGCTGTTATTTTGCCTGATGTTATTCTGGATGAATATGAATCCGATTTGTCACAGGATAACCTGGCAGAGAT
GATCCGCCGCTTTGATGAAACGGGTCATAGCCAGATCATGGTTGAACCGGTTGCTGATGTGACCGCATATGGCGTTGTGG
ATTGCAAAGGCGTTGAATTAGCGCCGGGTGAAAGCGTACCGATGGTTGGTGTGGTAGAAAAACCGAAAGCGGATGTTGCG
CCGTCTAATCTCGCTATTGTGGGTCGTTACGTACTTAGCGCGGATATTTGGCCGTTGCTGGCAAAAACCCCTCCGGGAGC
TGGTGATGAAATTCAGCTCACCGACGCAATTGATATGCTGATCGAAAAAGAAACGGTGGAAGCCTATCATATGAAAGGGA
AGAGCCATGACTGCGGTAATAAATTAGGTTACATGCAGGCCTTCGTTGAATACGGTATTCGTCATAACACCCTTGGCACG
GAATTTAAAGCCTGGCTTGAAGAAGAGATGGGCATTAAGAAGTAACATCCGTATCGGTGTTATCCACGAAACGGCGTTGA
GCAATCGACGCCGTTTTTTTATAGCTTATTCTTATTAAATTGTCTTAAACCGGACAATAAAAAATCCCGCCGCTGGCGGG
ATTTTAAGCAAGTGCAATCTACAAAAGATTATTGCTTGATCAGGAAATCGTCGAGGGATTTACCTTGCTCATCCATTGCT
TTTTTGATTACAGCTGGAGTACGGCCTTGGCCAGTCCAGGTTTTAGTTTCGCCGTTTTCGTCAACGTAGCTATATTTTGC
CGGACGCTGAGCACGTTTAGCTTTGGTGCCAGATTTAACGGCAGCAAGGCTATTCAGCAGTTCGTTCGGGTCAATACCGT
CAGCGATCAGCATTTCGCGATATTGCTGCAGTTTACGAGTGCGCTCTTCAACTTCAGCAGCAGCCGCGCTTTCTTCTTCG
CGACGTTCGTTAACAACAACTTCTAATTTTTCCAGCATTTCTTCCAGCGTTTCAAGTGTACATTCTCTTGCCTGCGCACG
AAGAGTACGGATGTTGTTCAGAATTTTAAGTGCTTCGCTCATTGTAGTAATCTCAAACTTATATTGGGGTGGTTTGTTGA
GGTAATAATAGAGCCTTAAATTCAGTTGTGCAATAGCCAGGAATGTAAGGAATTCAAAATTGTTCTTTATTTTGTGCCGC
CAATAAATATCTTTTCATAAAATTAGCCAGAAAAGACGCGGCATATAGCCCTATTTACACCGATGATTTCGCAGCACGTG
AGGTTAAAACTTCCTGATTCATGTCACATTTTATGGGGAGATTATCGTAGGCTGACGACCTTTCAGTCTTCTGTATTAGT
TGTGTTTACGAGAATTCCCTATTAAGCGAATGATGAAAAGTAGAACAGTCGCAATAAGAGCATGGACTTAGTATTGCACT
ATCTCCTGGAGGTCAACAGAGGGCTATTACTTGCGCAACAGGTTAAAGATTGTGAATAGTTACCAGCAGTCATTTACCCG
CTTATAACAAGCGAGGCAGTTGTAATGATAGCTCAGAAGGATTATGCAAGGCTTCGTAAGGGAGAACGCATATACCCACT
TCTGTGCATACTGTTGAGCTGAAAAACTGACGAATTATGATAAACTCCAGCCAACTTTATTTCATATCATTGAGGGCCTG
TGGCTGATGGCACAGCTATATTTCTACTATTCCGCAATGAATGCGGGTAAGTCTACAGCATTGTTGCAATCTTCATACAA
TTACCAGGAACGCGGCATGCGCACTGTCGTATATACGGCAGAAATTGATGATCGCTTTGGTGCCGGGAAAGTCAGTTCGC
GTATAGGTTTGTCATCGCCTGCAAAATTATTTAACCAAAATTCATCATTATTTGATGAGATTCGTGCGGAACATGAACAG
CAGGCAATTCATTGCGTACTGGTTGATGAATGCCAGTTTTTAACCAGACAACAAGTATATGAATTATCGGAGGTTGTCGA
TCAACTCGATATACCCGTACTTTGTTATGGTTTACGTACCGATTTTCGAGGTGAATTATTTATTGGCAGCCAATACTTAC
TGGCATGGTCCGACAAACTGGTTGAATTAAAAACCATCTGTTTTTGTGGCCGTAAAGCAAGCATGGTGCTGCGTCTTGAT
CAAGCAGGCAGACCTTATAACGAAGGTGAGCAGGTGGTAATTGGTGGTAATGAACGATACGTTTCTGTATGCCGTAAACA
CTATAAAGAGGCGTTACAAGTCGACTCATTAACGGCTATTCAGGAAAGGCATCGCCACGATTAATAAGAATTTCTTTACT
GACAGGGTGAGCAGGGCACTTTTATCCTGTCAGTTCGTTTTACGCACTTCTTCCGGGCTATATACCCTTCTCGGCAGTTT
TTTAACGCCGCTATACGCCTCACAGGGCTCTTAAGCACCGACGTTGACTTGTGACCTGTAAAGTACAATATCCCTGTGTT
TAGGCGTTATACATCGTCGCAAATATGATGAAGGCTAATGCTGTCGGTTTATGGAAAAGTTGCTTTGGGTAAACAAAAAA
TACGGCCCCAGAAGGGCAATGCCGTTCACTTAAGAGGAGCGGCACTATGTTTCACAGGATAACGGGTTTTTGATATCTTA
ACCGACCTCGGCCTTGATGGTCGGGGGCGTTTTGTTATGAACACCACTTCCAGAGCACCCCGAAGATGCTCCAGTCGTTT
CGGGATGGTCCCTGGTGACGCCGTGTTTCCCAGCTCTATCATTTCTGATGCGATATTCTTCCACGCAGGCAGTAGCCAGT

GGCGGTTACAACCCTTCTGGTTCAGCGTCAGCAGCAGGTCTTCGCTGTAAAAAAGTTTATCAAACAACGTAATAGAGTTA
TCCGGGATGGTGGCGAGCATGGAGTGGGCCAGCACAGTTTCGCTCTGCCGGTAAGGTGCGGTCACGGCATTCAGCAGAAT
GTGACTTCCCAGGTTCATTAAGGCCACCAGACGCATAACCGGGTAGGCGTTCTGCCGCTTAGTGGATGTGTTGGCAGACC
CATAATATTCACGCAGCTCGGGTTTATCAGGTGTCCTGAACTGTGCGCCATCAATGGCAAAAAGTTGCAGGCCGTGCCAG
TCATCCTTCAGGTAACGTTCCGCGCCCCTGTCTGTGCGGTCTGGCGGAAGAGCCATTCCACTGGGGCGGCCCCCACGCGC
TGACGCGCCTGGGTGACAGCGCTGCGGGCCAGCAGGTTCATCCCCGCTTCGCCATCCGCGCTCAGGTTCAGACGGCGAAC
AACATCGGTAATTGGCTCATTGCACCACCATCCAGATAACCATGTCCCCCGGTAAACGACGGCGGCGAACGGTCGCATGA
GCAGAAAGCGTCAGGCAGTGTTGTATCCACTCGGTGGGAAGGTGTTCTGCAAATAGTTGTGCAGAGGGCGGAGGCATAAG
CGGATGGTCACTGAAATCGAGCAGATCATTGAGAAGTGGCATAAGAAAACGGCTCCCTGTTGTGGAAGCCGTTATAGTGC
CTCAGTTTAAGGATCGGTCAACTAATCCTTAACTGATCGGCATTGCCCAGAAGGGGCCGTTTATGTTGCCAGACAGCGCT
ACTGATTAAGCGGATTTTTTCGCTTTTTTCTCAGCTTTAGCCGGAGCAGCTTCTTTCTTCGCTGCAGTTTCACCTTCTAC
ATAATCACGACCGTAGTAGGTATCCAGCAGAATCTGTTTCAGCTCGGAGATCAGCGGGTAACGCGGGTTAGCGCCGGTGC
ACTGGTCATCGAATGCATCTTCAGACAGTTTATCCACGTTCGCCAGGAAGTCTGCTTCCTGAACGCCAGCTTCACGGATA
GATTTCGGAATACCCAGTTCAGCTTTCAGCGTTTCCAGCCATGCCAGCAGTTTCTCGATCTTAGCAGCAGTACGGTCGCC
CGGTGCGCTCAGACCCAAGTGGTCGGCAATTTCAGCATAACGACGGCGAGCCTGCGGACGGTCATACTGGCTGAATGCAG
TCTGCTTGGTCGGGTTGTCGTTCGCATTGTAGCGAATAACGTTACAAATCAGCAGGGCGTTTGCCAGACCGTGCGGAATA
TGGAACTGGGAACCCAGTTTGTGCGCCATTGAGTGACATACACCCAGGAAGGCGTTCGCAAACGCGATACCCGCGATAGT
CGCTGCACTGTGAACACGTTCACGCGCTACCGGATTTTTAGACCCTTCGTGGTAGGACGCTGGCAGATATTCTTTCAGCA
GTTTCAGTGCCTGCAGAGCCTGACCATCAGAGAACTCAGATGCCAGTACAGAAACATAAGCTTCCATGGCGTGAGTTACT
GCGTCCAGACCACCGAAAGCACACAGGGACTTCGGCATGTCCATAACCAGGTTGGCGTCGACAATCGCCATATCCGGAGT
CAGCGCATAGTCTGCCAGCGGATATTTCTGACCAGTAGCGTCGTCAGTTACAACCGCAAACGGAGTGACTTCAGAACCTG
TACCAGAAGTGGTGGTGACAGCGATCATTTTCGCTTTCACGCCCATTTTCGGGAACTTGTAGATACGTTTACGGATATCC
ATAAAGCGCAGCGCCAGCTCTTCGAAGTGAGTTTCCGGATGTTCGTACATAACCCACATGATCTTCGCGGCGTCCATCGG
GGAACCACCACCCAGCGCGATAATCACGTCTGGTTTGAAGGAGTTTGCCAGTTCTGCACCTTTACGAACGATGCTCAGGG
TCGGGTCCGCTTCTACTTCGAAGAAGACTTCAGTTTCAACGCCTGCTGCTTTCAGTACGGAAGTGATCTGATCAGCATAA
CCATTGTTGAACAGGAAGCGGTCAGTCACGATGAGCGCACGTTTGTGGCCATCAGTAATCACTTCATCCAGCGCGATTGG
CAGGGAGCCACGGCGGAAGTAGATAGATTTCGGAAGTTTGTGCCACAACATGTTTTCAGCTCGCTTAGCAACGGTTTTCT
TGTTGATCAGGTGTTTCGGACCAACGTTTTCAGAGATGGAGTTACCACCCCAAGAACCACAACCCAGAGTCAGGGAAGGT
GCGAGTTTGAAGTTATACAGGTCACCGATACCACCCTGAGACGCTGGGGTGTTAATCAGGATACGCGCCGTTTTCATTTT
CTGACCGAAGTAAGAAACGCGAGCCGGTTGGTTATCCTGGTCAGTGTACAGGCAAGAGGTATGACCGATACCGCCCATAG
CAACCAGTTTCTCTGCTTTTTCTACCGCGTCTTCGAAATCTTTAGCGCGGTACATTGCCAGAGTCGGGGACAGTTTTTCA
TGTGCGAACGGTTCGCTTTCATCAACAACGGTCACTTCACCGATCAGAATCTTGGTGTTTTCTGGTACAGAGAAGCCTGC
CAGTTCAGCAATTTTATAGGCTGGCTGACCAACGATAGCCGCGTTCAGCGCACCGTTTTTCAGGATAACATCCTGAACAG
CTTTCAGCTCTTTACCCTGCAACAGATAGCCGCCGTGGGTTGCAAAACGTTCACGTACAGCGTCATAAACAGAGTCAACA
ACAACAACAGACTGTTCAGAAGCACAGATTACGCCGTTGTCGAAGGTTTTGGACATCAGTACAGATGCAACTGCACGTTT
GATATCAGCAGTTTCATCGATAACAACTGGAGTGTTGCCCGCGCCTACACCGATAGCTGGTTTACCGGAGCTGTATGCGG
CTTTAACCATGCCCGGACCACCAGTCGCGAGGATCAGGTTGATGTCTGGGTGGTGCATCAGTGCGTTAGACAGTTCAACA
GAAGGTTGATCGATCCAGCCGATCAGATCTTTCGGAGCACCGGCAGCGATAGCAGCCTGCAGAACGATATCAGCCGCTTT
GTTGGTGGCATCTTTTGCACGCGGGTGCGGGGAGAAGATAATGGCGTTACGGGTCTTCAGACTGATCAGCGATTTGAAGA
TAGCAGTTGAAGTCGGGTTAGTGGTCGGAACGATACCGCAAATAATACCGATTGGTTCAGCGATAGTGATGGTACCAAAA
GTGTCGTCTTCAGACAGAACACCACAGGTTTTTTCATCTTTATAGGCGTTGTAGATATATTCAGAAGCAAAGTGGTTTTT
GATCACTTTATCTTCGACGATACCCATGCCGGATTCGGCAACGGCCATTTTCGCGAGTGGGATTCGAGCATCTGCAGCAG
CCAGAGCGGCGGCGCGGAAGATTTTGTCTACTTGCTCTTGAGTGAAACTGGCATATTCACGCTGGGCTTTTTTTACACGC
TCTACGAGTGCGTTAAGTTCAGCGACATTAGTAACAGCCATAATGCTCTCCTGATAATGTTAAACTTTTTTAGTAAATCA
TCTGCTCGAATACGAGAGTATAGTCAGTGCGGTGATGATTTGCTTAACCTATGAAAATCAAAAGCTTACTCGCGCTCACA
CTCACTGTGATTTACTAAAAGAGTTTAAACATTAGAGTTATTATCTCTAATGCGTCACTTCCAGGTGGCGTAAGCAAGAT
TACTCACTTCTGGGTACTGATTACGTGATCCAAATCAAATTTTTGCAAAGCTGACACCTTTCAGCATCGCTTTTCGCCAT
TATAGCTAACAGTTAATAAATTGTAGTATGATTTGGTGGCTACATTAGCATGTTTTGCACAACTAGATAACAATAACGAA
TGATAGCAATTTTAAGTAGTTAGGAGGTGAAAAATGCTGTCAAAAGGCGTATTGTCAGCGCGTCTTTTCAACCTTATTTA
TGGCTAACATTATCCGGCTTTTGCTTCGGAGCTAACCGTGATTCAGACCTTTTTTGATTTTCCCGTTTACTTCAAATTTT
TCATCGGGTTATTTGCGCTGGTCAACCCGGTAGGGATTATTCCCGTCTTTATCAGCATGACCAGTTATCAGACAGCGGCA
GCGCGAAACAAAACTAACCTTACAGCCAACCTGTCTGTGGCCATTATCTTGTGGATCTCGCTTTTTCTCGGCGACACGAT
TCTACAACTTTTTGGTATATCAATTGATTCGTTCCGTATCGCCGGGGGTATCCTGGTGGTGACAATAGCGATGTCGATGA
TCAGCGGCAAGCTTGGCGAGGATAAACAGAACAAGCAAGAAAAATCAGAAACCGCGGTACGTGAAAGCATTGGTGTGGTG
CCACTGGCGTTGCCGTTGATGGCGGGGCCAGGGGCGATCAGTTCTACCATCGTCTGGGGTACGCGTTATCACAGCATTAG
CTATCTGTTTGGTTTCTTTGTGGCTATTGCATTGTTCGCTTTATGTTGTTGGGGATTGTTCCGCATGGCACCGTGGCTGG

TACGGGTTTTACGCCAGACCGGCATCAACGTGATTACGCGTATTATGGGGCTATTGCTGATGGCATTGGGGATTGAATTT
ATCGTTACTGGTATTAAGGGGATTTTCCCCGGCCTGCTTAATTAATTCCTTTCAAATGAAACGGAGCTGCCATGCTCCGT
TTACTTCGTCATTATTTTTACTTTGTTCCCGCGCAGTTATCAAAAGCAAAAGGAATAGGTAAAAATATTCTTCTCAAATT
ACAGTTAGTTATAAGGATTTCCTTAACTGCTTCTCCTCACCATCATGTTATTTTCGCCACATCATAATCCTGGGCTTGCT
GAAGAATAATTGAAATGATATTATTAATTCCACTGCCTTTGGTAGAGGAAAGTGCTAAGGAAGGTGCGAACAAGTCCCTG
ATATGAGATCATGTTTGTCATCTGGAGCCATAGAACAGGGTTCATCATGAGTCATCAACTTACCTTCGCCGACAGTGAAT
TCAGCAGTAAGCGCCGTCAGACCAGAAAAGAGATTTTCTTGTCCCGCATGGAGCAGATTCTGCCATGGCAAAACATGGTG
GAAGTCATCGAGCCGTTTTACCCCAAGGCTGGTAATGGCCGGCGACCTTATCCGCTGGAAACCATGCTACGCATTCACTG
CATGCAGCATTGGTACAACCTGAGCGATGGCGCGATGGAAGATGCTCTGTACGAAATCGCCTCCATGCGTCTGTTTGCCC
GGTTATCCCTGGATAGCGCCTTGCCGGACCGCACCACCATCATGAATTTCCGCCACCTGCTGGAGCAGCATCAACTGGCC
CGCCAATTGTTCAAGACCATCAATCGCTGGCTGGCCGAAGCAGGCGTCATGATGACTCAAGGCACCTTGGTCGATGCCAC
CATCATTGAGGCACCCAGCTCGACCAAGAACAAAGAGCAGCAACGCGATCCGGAGATGCATCAGACCAAGAAAGGCAATC
AGTGGCACTTTGGCATGAAGGCCCACATTGGTGTCGATGCCAAGAGTGGCCTGACCCACAGCCTGGTCACCACCGCGGCC
AACGAGCATGACCTCAATCAGCTGGGTAATCTGCTGCATGGAGAGGAGCAATTTGTCTCAGCCGATGCCGGCTACCAAGG
GGCGCCACAGCGCGAGGAGCTGGCCGAGGTGGATGTGGACTGGCTGATCGCCGAGCGCCCCGGCAAGGTAAGAACCTTGA
AACAGCATCCACGCAAGAACAAAACGGCCATCAACATCGAATACATGAAAGCCAGCATCCGGGCCAGGGTGGAGCACCCA
TTTCGCATCATCAAGCGACAGTTCGGCTTCGTGAAAGCCAGATACAAGGGGTTGCTGAAAAACGATAACCAACTGGCGAT
GTTATTCACGCTGGCCAACCTGTTTCGGGCGGACCAAATGATACGTCAGTGGGAGAGATCTCACTAAAAACTGGGGATAA
CGCCTTAAATGGCGAAGAAACGGTCTAAATAGGCTGATTCAAGGCATTTACGGGAGAAAAAATCGGCTCAAACATGAAGA
AATGAAATGACTGAGTCAGCCGAGAAGAATTTCCCCGCTTATTCGCACCTTCCCTAAATAATAATCAATTGTTAAATTAT
TGTGCATTTCACTACTGGAACTGTAATCAGAAAAGATAGACATGCTTAGCCAATCTCTATTTGATTGAATTGAAAGATGT
TTGTTAAGGCATGGATGCAAGCTATAGATTCTGATACGGTCAATAAAAGAGAATTGCTTAACAATTTTGCAAAATGTATT
GGCGAGTAAGAACCGCATTTGGTACTTTCCGGGCAACCGCCAGACGATTCTTTATTGGTAATGAGAATAATTAACAATTA
AAGAGCGTCGCGAAAGAATAATGTGTCTCGACAGGGGAGACACAGTACGAATCGACATAAGGTGATCGTCTGAATCACCA
GAATAAATAAAGTCGGTGATAGTAATACGTAACGATAAAGTAACCTGACAGCAGAAAGTCTCCGAGCCTGTGCAGGGTCC
CAATCCGGGATTACACATGCTGGTTAATACCAGTAATTATAATGAGGGAGTCCAAAAAACAATGACCAACATCACCAAGA
GAAGTTTAGTAGCAGCTGGCGTTCTGGCTGCGCTAATGGCAGGGAATGTCGCGCTGGCAGCTGATGTACCCGCAGGCGTC
ACACTGGCGGAAAAACAAACACTGGTACGTAACAATGGTTCAGAAGTTCAGTCATTAGATCCGCACAAAATTGAAGGTGT
TCCGGAGTCTAATATCAGCCGAGACCTGTTTGAAGGCTTACTGGTCAGCGATCTTGACGGTCATCCAGCACCTGGCGTCG
CTGAATCCTGGGATAATAAAGACGCGAAAGTCTGGACCTTCCATTTGCGTAAAGATGCGAAATGGTCTGATGGCACGCCA
GTCACAGCACAAGACTTTGTGTATAGCTGGCAACGTTCTGTTGATCCGAACACTGCTTCTCCGTATGCCAGTTATCTGCA
ATATGGGCATATCGCCGGTATTGATGAAATTCTTGAAGGGAAAAAACCGATTACCGATCTCGGCGTGAAAGCTATTGATG
ATCACACATTAGAAGTCACCTTAAGTGAACCCGTTCCGTACTTCTATAAATTACTTGTTCACCCATCAACTTCACCGGTG
CCAAAAGCCGCTATCGAGAAATTCGGCGAAAAATGGACCCAGCCTGGTAATATCGTCACCAACGGTGCCTATACCTTAAA
AGATTGGGTCGTAAACGAACGAATCGTTCTTGAACGCAGCCCGACCTACTGGAACAACGCGAAAACCGTTATTAACCAGG
TAACCTATTTGCCTATTGCTTCTGAAGTTACCGATGTCAACCGCTACCGTAGTGGTGAAATCGACATGACGAATAACAGC
ATGCCGATCGAATTGTTCCAGAAGCTGAAAAAAGAGATCCCGGACGAAGTTCACGTTGATCCATACCTGTGCACTTACTA
TTACGAAATTAACAACCAGAAACCGCCATTCAACGATGTGCGTGTGCGTACCGCACTGAAACTAGGTATGGACCGCGATA
TCATTGTTAATAAAGTGAAAGCGCAGGGCAACATGCCCGCCTATGGTTACACTCCACCGTATACTGATGGCGCAAAATTG
ACTCAGCCAGAATGGTTTGGCTGGAGCCAGGAAAAACGTAACGAAGAAGCGAAAAAACTGCTGGCTGAAGCGGGTTATAC
CGCAGACAAACCGTTGACCATCAACCTGTTGTATAACACCTCCGATCTGCATAAAAAGCTGGCGATTGCTGCCTCTTCAT
TGTGGAAGAAAAACATTGGTGTAAACGTCAAACTGGTTAACCAGGAGTGGAAAACGTTCCTCGACACCCGTCACCAGGGT
ACTTTTGATGTGGCCCGTGCAGGCTGGTGTGCTGACTACAACGAACCAACTTCCTTCCTGAACACCATGCTTTCGAACAG
CTCGATGAATACCGCGCATTATAAGAGCCCGGCCTTTGACAGCATTATGGCGGAAACGCTGAAAGTGACTGACGAGGCGC
AGCGCACAGCTCTGTACACTAAAGCAGAACAACAGCTGGATAAGGATTCGGCCATTGTTCCTGTTTATTACTACGTGAAT
GCGCGTCTGGTGAAACCGTGGGTTGGTGGCTATACCGGCAAAGATCCGCTGGATAATACCTATACCCGGAATATGTACAT
TGTGAAGCACTAATGGCAATACGTGGGGCAGGAGTGTCCTGCTCCACGGTGTCTGATTTTTATCGCATTACAGAAGGCAC
AGGCCAGAAGGTAGGGCAATGTTAAAATTTATTCTACGTCGCTGTCTGGAAGCGATTCCGACGCTATTTATTCTTATTAC
TATTTCGTTCTTTATGATGCGCCTCGCGCCGGGAAGCCCTTTTACCGGCGAACGTACTTTACCGCCAGAAGTGATGGCCA
ATATCGAAGCGAAATATCATCTTAATGATCCGATCATGACACAGTATTTCAGCTACCTGAAACAACTGGCGCACGGTGAT
TTCGGTCCATCGTTTAAATATAAAGATTATTCGGTCAATGACTTGGTTGCATCCAGTTTTCCCGTTTCTGCCAAACTGGG
AGCCGCAGCATTTTTCCTTGCGGTAATACTGGGTGTTAGTGCTGGCGTTATTGCCGCATTAAAACAAAACACCAAATGGG
ACTATACCGTGATGGGGCTGGCAATGACCGGGGTTGTTATCCCCAGTTTTGTGGTTGCACCATTATTAGTCATGATATTT
GCGATCATTTTGCATTGGCTGCCGGGCGGTGGCTGGAATGGTGGGGCGCTTAAATTCATGATATTGCCGATGGTGGCGTT
GTCACTCGCTTATATCGCCAGTATTGCGCGTATTACCCGTGGCTCTATGATTGAAGTATTACACTCCAATTTTATTCGTA
CTGCCCGGGCGAAAGGGTTACCTATGCGGCGGATCATTTTACGTCACGCATTAAAACCTGCTCTGTTACCCGTGCTCTCT

TATATGGGCCCCGCATTTGTCGGCATTATTACCGGTTCTATGGTTATCGAAACCATTTATGGTTTGCCGGGGATTGGACA
ACTGTTCGTTAATGGCGCATTGAACCGTGACTATTCCTTAGTGTTAAGCCTGACCATCCTGGTTGGTGCTTTAACCATTT
TGTTTAATGCCATTGTCGATGTGCTATATGCGGTTATCGACCCGAAAATCCGTTACTGATACTGGAGCTCGCGATGATGT
TAAGTAAGAAAAACAGCGAGACGCTGGAAAATTTCAGTGAAAAGCTGGAGGTCGAAGGGCGCAGCTTGTGGCAGGACGCA
CGTCGACGTTTTATGCATAACCGTGCGGCGGTTGCCAGTCTGATAGTGCTGGTGCTGATCGCGTTATTTGTAATCCTGGC
ACCGATGCTTTCCCAGTTTGCCTATGACGATACTGACTGGGCGATGATGTCCAGCGCCCCGGATATGGAGTCCGGTCACT
ACTTTGGTACTGACTCATCCGGTCGCGACCTGCTTGTGCGCGTTGCGATTGGCGGGCGTATCTCACTCATGGTCGGTGTT
GCTGCGGCACTGGTGGCTGTGGTCGTCGGGACACTTTATGGTTCGCTTTCCGGTTATCTGGGCGGTAAAGTGGATTCGGT
AATGATGCGTCTGCTGGAAATCCTCAACTCCTTCCCATTCATGTTCTTCGTCATTTTGCTGGTGACCTTTTTCGGTCAAA
ACATCCTGCTGATTTTCGTGGCGATAGGCATGGTTTCCTGGCTGGATATGGCTCGTATTGTGCGTGGGCAAACCCTGAGT
CTGAAGCGCAAAGAGTTTATTGAGGCGGCACAAGTTGGCGGTGTATCGACATCGGGCATTGTTATTCGCCACATTGTGCC
GAATGTACTCGGTGTGGTGGTGGTCTACGCATCACTTCTGGTGCCCAGCATGATCCTCTTTGAATCTTTCCTTAGCTTCC
TGGGGTTGGGTACGCAAGAGCCGTTAAGCAGCTGGGGTGCATTGCTGAGTGATGGCGCGAACTCGATGGAAGTCTCTCCA
TGGTTATTGTTGTTCCCAGCGGGATTCCTCGTGGTGACGCTGTTTTGTTTCAACTTTATCGGCGATGGCTTGCGTGATGC
CCTCGACCCGAAAGATCGTTAAGGAGTGCAGCCATGAGCGTAATTGAAACTGCAACTGTGCCGCTCGCACAACAACAGGC
TGACGCACTGCTGAACGTGAAAGATTTGCGTGTCACCTTTAGTACCCCGGACGGCGACGTCACGGCGGTCAATGATTTGA
ATTTTTCCCTACGTGCCGGAGAAACGCTGGGTATTGTAGGTGAGTCTGGTTCGGGTAAATCGCAAACTGCATTTGCGTTG
ATGGGCCTGTTGGCTGCCAACGGACGTATTGGCGGATCGGCAACCTTCAATGGGCGTGAAATCCTCAATTTGCCAGAGCA
TGAACTCAATAAACTGCGCGCTGAACAAATCTCAATGATTTTTCAGGACCCAATGACTTCGTTGAATCCCTATATGCGCG
TCGGTGAGCAGTTGATGGAAGTGCTGATGCTGCATAAGAACATGAGCAAAGCTGAGGCGTTTGAAGAGTCGGTGCGGATG
CTCGATGCGGTAAAAATGCCGGAAGCGCGTAAACGCATGAAAATGTACCCGCACGAATTTTCTGGCGGCATGCGTCAGCG
AGTCATGATTGCGATGGCCTTGTTATGTCGACCTAAGCTGCTGATTGCGGATGAACCAACAACTGCGCTGGACGTCACCG
TACAGGCGCAGATCATGACGCTATTAAATGAACTGAAGCGGGAATTTAATACCGCCATCATTATGATCACCCACGATCTT
GTCGTGGTGGCGGGGATCTGCGACAAAGTGCTGGTAATGTACGCCGGGCGCACGATGGAATATGGCAACGCGCGCGATGT
CTTTTATCAACCCGTTCATCCTTATTCTATCGGTTTGCTCAACGCGGTGCCGCGTCTCGATGCGGAAGGTGAAACAATGT
TGACCATCCCGGGTAATCCGCCAAACCTGCTGCGATTACCGAAAGGTTGCCCGTTCCAGCCACGTTGTCCGCATGCGATG
GAAATTTGTAGTAGCGCACCGCCGCTGGAAGAGTTTACGCCTGGCCGTCTGCGTGCTTGCTTTAAACCGGTGGAGGAACT
GTTATGAATGCTGTAACTGAAGGAAGAAAAGTCCTCCTTGAAATCGCCGATCTTAAAGTGCACTTTGAAATCAAAGATGG
CAAACAGTGGTTCTGGCAACCGCCGAAAACGCTCAAAGCCGTCGATGGTGTCACTCTTCGCCTGTATGAAGGGGAAACAT
TAGGTGTGGTAGGGGAATCGGGATGCGGTAAGTCCACCTTTGCTCGCGCCATCATCGGTTTGGTCAAGGCGACCGACGGT
CATGTTGCCTGGTTAGGTAAAGAGTTGCTGGGCATGAAACCCGATGAATGGCGTGCCGTTCGCAGTGATATTCAGATGAT
TTTCCAGGATCCGTTGGCATCGCTAAACCCGCGTATGACCATCGGCGAGATCATCGCTGAACCACTGCGTACTTATCATC
CGAAAATGTCACGCCAGGAAGTTCGCGAGCGCGTGAAGGCGATGATGCTGAAAGTCGGGTTATTGCCTAACCTGATTAAC
CGCTATCCGCATGAGTTCTCTGGTGGGCAGTGCCAGCGTATCGGGATTGCGCGTGCTCTTATTCTTGAACCGAAGCTGAT
TATCTGTGATGAGCCGGTGTCGGCGCTGGACGTGTCAATTCAGGCACAGGTGGTCAACCTGCTCCAGCAATTGCAACGTG
AGATGGGATTGTCATTAATTTTTATCGCTCATGACCTGGCCGTGGTAAAACACATTTCCGATCGTGTGTTGGTGATGTAT
CTCGGCCATGCGGTAGAACTGGGGACCTATGATGAGGTCTACCACAATCCACTACATCCTTACACCAGGGCATTGATGTC
GGCAGTCCCCATACCTGATCCGGATCTGGAGAAGAACAAAACCATCCAGTTACTGGAAGGGGAATTACCGTCGCCGATCA
ACCCGCCTTCCGGTTGTGTTTTCCGTACCCGTTGCCCGATTGCCGGTCCAGAGTGCGCCAAAACACGTCCTGTTCTGGAG
GGGAGTTTCAGACACTCCGTTTCTTGCCTGAAAGTCGATCCGCTTTAAAAACAATAAGGGCTGACAGTTGTCAGCCCTTT
TTCACGCTAAAAGCGATTATTTATTCCCGCCAGATGATATGGCAAAGTTTGTGATCTTTTTCGCGGCATAACAGAATGCG
GGCAAAAACATCGTTGATTTCACCATCTTCACTGTCCGCCAGACCAATCACCACTTCGGCAAAAAAGTCCGGGTTCAAAT
CGAAATCAACATGTTCCTGCCAGTCTTCCGCCGGATCGAATAACTCCGCGCCGCCGCGCTCTTCAAACTGAAGATTGAAC
AGCAGAACATCAGCTGGATCGAGATTGTCGGCAGCCAGTTCGAGAAAAATATCGTAGGCCTGTTCAAGCGTTTCATCTTC
AGTCAGGCGATTGTTTAGATCCATATCCATAGTTACTACCTGTTTAACCTCTGTTGGCGACGTTTTACAGCAACGGACTG
AAGAAGTAAAACAGTCGCTCGGCGACACGTTGCCATAATGGACGTTTTAGCCATAAACGGGCATCGAGCAGACGTGAACG
CGAAATATAATCGTCCTGAACGGCGGCGAGGTCAGCACCAAAACCTTTATCGTCGATTGCCAGGGTAATCTCGAAATTTA
GCCACAGACTACGCATATCAAGGTTAACTGTGCCAACCAGACTTAGTTCGCCATCGACCAGCACGCTCTTGGTATGCAGT
AACCCGCCTTCAAACTGATAAATTTTAACCCCAGCAGCCAGCAGTTCCGTAAAGAATGCGCGACTGGCCCAGCCGACCAG
CATCGAGTCATTTTTTTCGCGGAAGGATAATACTGACATCCACCCCGCGCTGCGCCGCCGTGCAAATCGCATGAAGTAAAT
CATCGCTTGGCACAAAGTAGGGCGTGGTCATGATCAAATATTCACGCGCCGAATAAGCCGCAGTCAATAATGCCTGGTGA
ATGAGATCTTCCGGAAAGCCGGGGCCAGAAGCAATTGTGTGAATGGTGTGACCGCTGGCCTGTTCAAACGGCATAATATT
GACATCTGGTGGTGGCGGCAGAATACGTTTTCCGGTTTCAATCTCCCAGTCGCAGGAATAAATAATCCCCATCGCGGTGG
CGATAGGGCCTTCCATACGCGCCATCAGATCAATCCATTGCCCTACGCCCGCATCTTGTTTGAAGTAGCGAGGATCGACC
ATATTCATGCTGCCGGTGTACGCGATGTAATTATCGATCATGATCATCTTGCGATGTTGGCGCAGGTCCATACGGCGTAA
AAACACACGCATCAGATTGACCTTTAAGGCTTCGACCACTTCAATACCGGCATTACGCATTAGCTCGGGCCACGGGCTGC

GGAAAAAAAGCCACACTCCCGGCGGAGTCGAGCATCAATCGGCAATGAATGCCGCGTCGCGCAGCCGCCATTAATGATTCA
GCCACCTGGTCCGCCATGCCGCCGGGCTGCCAGATATAAAACACCATCTCAATATTATGGCGCGCGAGCTGGATGTCGCG
GATTAACGCCTGCATCACATCATCTGACTCGGTCATCAGTTGTAGCTGATTCCCTTTGACCCCAGCGATCCCCTGACGAC
GCTCGCAAAGCTTGAATAATGGCGCAGCGACACTGCTATTTTCTTCGGCGAAGATATGCTTACAGGCTTTAAGGTCGTTA
AGCCATTTTGCGGTGGAAGGCCACATCGCTCTGGCGCGCTCAGCGCGGCGTTTGCCTAAATGGAGCTCGCCAACGGCAAG
ATAGGCAATAATTCCGACTAACGGCAGAATGTAAATAATCAACAGCCAGGCCATCGCGGAGGGAACTGCGCGTCGTTTCA
TTAGAATGCGTAAAGTTACGCCTGCAATGAGCAACCAGTATCCCAGAATGGCCAACCAACTCACCAACGTATAAACGGTT
GTCATAGATTAGAAATCCTTTTGAAAGCGCATTGTTATGAGTTTACGCATATGGATTCATCTGGCAAATAAAAACGCGGG
AAAAGCACTGGTCAGCGTCGGGGGTGGGGGTATAATGACCATTCTGTTATTGCATAGAGTAGTTAACATGAAGCGGAGTA
GAACGGAAGTGGGGCGCTGGCGCATGCAGCGTCAGGCTAGCCGACGTAAATCGCGTTGGCTTGAGGGGCAATCGCGCCGA
AATATGCGTATCCACAGCATCAGGAAGTGCATTCTAAACAAACAGCGTAACTCGTTATTGTTTGCGATCTACAATATCTA
AATGTACTGGGCACCATTGCGGTGCCCAATAAGTTTACTCTTCCGTCTTTAAACTCTATTTTTGCGCCGATTCTTTACTG
TCAGCTTTCGTTTCTAGTTCATCGTTATCGCTGAAAATACCATGACCTGAGTTCAACAACATTGAAACGAGCATATCATT
ATTAATCTCTTCACCATTTAAAACTCGCGCCAGAATTTCGCTGCCAAACAGTTGCGGTGAGAGAATGATATCCGGATGTA
CCATCTTAATCTTATTCAGGTTTTTGCTATCACTGACGGCGAGAACTGTTTTGACATCACTGCTCATATCTTTTGCCGAG
AGTACAACAAACGCGTTATCTGCATCGTTATCACTCAGCGCCAGAATGGCCCGGCAACGATCGATTCCCGCTTTCTTTAA
TACAGAACTGTCATTACTGTCACCGGGGATAACATCAGCATTATCGCCTAAGCGTTGCTCAAGTTGCTTGATATCATCTT
CAGGCAAGTTGCTGATAACCGTTACGTTTTGTCCGCGTTGATTCAGTTGCAGGATCGTATTGATGGCGAGAATCGAATGT
CCGCAAACAATAAAATGATCTTTACGATGCATTGTATGATTGTTTCCTTTTACAAGTTTGTTGAATCCCCCGCGGATAAG
CGGACCAAAAATTGAGGTCATAGATGTGGCAAAAACGGTAATGCCGGAAATAATGACCGAAATAGTGAACAATCGTGCTG
ATTCAGAAACAGGGACAATATCGCCATAGCCGACGGTTGACATGGTTTCTATCGAAAAATAGAACGCGGTCATCAAACTT
TCTATTCGCGGATTAAAACCTTCGCTTAAATAAAGCGCACCGTAGGTTGAGTAAAACAGTAATGTCGTGAAACTAATAAA
TGCAAAAATTGTCCCGGCTGCGGCGCTACTGTGGGAGAAGTCTTTGCGCAGTATCAGCAAAAACACCAGCGTAAAAATGC
AAAATCCAATACTAAATTTCAGCCAGGGATAAAAATGCAGGGTGTAAATTAGCGCTATCAACAACAAAATGATACTGATT
GCCCATGCGAGCTTTGCCCGGAACAGTAAGCCAATGGAGTTCAGGACAAGAAAGACGCCGAGCATAAAGAGTGGGGCGTT
TGCCAGCAAGGAGAGATCCAACTCTGAAAATGCTTTGATATGGAAAATATCAAGCAAATTGACCGACATACCATAGATTG
TTTTAAAAATAAGCAATCCATTTAAAAAGACGGCCAGCGCGAGAATGTCGTGCCGAAGGGTCACCCATAAATTTGTTGCT
GTTTGTTTGAATGTAGCCCAGTGACTCACTGATACCTCTGTAACCACAATTTTGTTTAATTATCTTAGCGTGCACATTTT
CTGAACATACATGCAGCGCGTGAATGTGTTAAACAATAGTAGATGACTTTATCCGCAGTAATCAAAGGATTTGAGCGAGC
AACTGTACCTCAGGAAAGGTCATTTTTCCTGAATATGCTCACATCATATAAAGAAATACAGATAAAGTTATTATCTGCTT
GTGGTGGTGAATGCACTGACCGGCTATAAGGAAAGGCCAAACAAGAACACGGTTGCAAAAACCGTGCCCTTAAATATTGA
ATCTCTATTCAGAACACTTTCTTAAATGGTTTCACTGAAACGTGTTCATAGACTCCTGCCGCTACGTACGGGTCAGCATC
GGCCCAGGCCTGAGCTGCTTCCAGCGATTCAAATTCAGCAATAACGGTTGAGCCAGTAAATCCCGCAGCCCCTGGATCGT
TACTGTCTACCGCTGGCATTGGACCAGCTGTCAACAAACGACCTTCATCATGCAGTAACTGTAAACGTGCTAAATGTGCC
GGACGAACGGAAAGGCGTTTTTCGAGGCTATCAGCTTTGTCTTGAGCGTAAATAACATACAACACGGGCACAACTCCTTG
TTCGGGAAAGTTGTAAAGTACGTTATTTGAAAGGGCGAAGATCTGCAACGGAAAGATGATGTCTTTGTTAAGGCCATGCA
TAAAGTAAGGGTAATTACGCCAAAAATGACATTTTCACTGATCCTGATCGTCTTGCCTTATTGAATATGATTGCTATTTG
CATTTAAAATCGAGACCTGGTTTTTCTACTGAAATGATTATGACTTCAATGACCCTTGATTTACCTCGCCGCTTCCCCTG
GCCGACGTTACTTTCGGTCTGCATTCATGGTGCTGTTGTGGCGGGTCTGCTCTATACCTCGGTACATCAGGTTATTGAAC
TACCTGCGCCTGCGCAGCCGATTTCTGTCACGATGGTTACGCCTGCTGATCTCGAACCGCCACAAGCCGTTCAGCCGCCA
CCGGAGCCGGTGGTAGAGCCAGAACCGGAACCTGAGCCGATCCCCGAACCGCCAAAAGAAGCACCGGTGGTCATTGAAAA
GCCGAAGCCGAAACCTAAGCCAAAACCGAAGCCGGTGAAAAAGGTACAGGAGCAGCCAAAACGCGATGTCAAACCCGTAG
AGTCGCGTCCGGCATCACCGTTTGAAAATACGGCACCGGCACGCCTGACATCAAGTACAGCAACGGCTGCAACCAGCAAG
CCGGTTACCAGTGTGGCTTCAGGACCACGCGCATTAAGCCGTAATCAGCCGCAGTATCCGGCACGAGCACAGGCATTGCG
CATTGAAGGGCAGGTTAAAGTTAAATTTGACGTTACGCCGGATGGTCGCGTGGATAACGTACAAATCCTCTCAGCCAAGC
CTGCGAACATGTTTGAGCGTGAGGTGAAAAATGCGATGCGCAGATGGCGTTATGAGCCGGGTAAGCCAGGCAGTGGGATT
GTGGTGAATATCCTGTTTAAAATTAACGGCACCACCGAAATTCAGTAAGCAGAAAGTCAAAAGCCTCCGACCGGAGGCTT
TTGACTATTACTCAACAGGTAAGGCGCGAGGTTTTCCTTCAGGATCAACCGCGACATACTTAAATAATGCTTCTGTCGCT
TTATAGCGTTGCCCAATTGGTTCAGACGCTACTTTTTTCACCCACACTTCAATATTAATGCTGACCGATGTCGTCCCTTT
CTGGACACAGCGTGCATAGCAGCACACCACATCGCCGACCGCAACCGGCCGTAAGAAAGTCATTCCTTCAACCCGCACAG
TCACTACGCGACCGTGGGCAATTTCTTTTGCCAGAATAGCGCCGCCAATATCCATTTGTGACATTAACCAACCACCAAAG
ATGTCACCATTGGCATTGGTATCGGCGGGCATGGCTAAAGTACGTAAAACAAGATCGCCCTGAGGGACGTTATGTGTTGT
AGACATGGTAAAACCGACTTAAAAGGAAAATCACAGGCGCGATGCTACTATGATTTTCCTGAGGAGAACAGAGGAGATAC
AGCGCCAGCCCCGAAGGACTGGCAGTCTGGTTAGGATTTATCTTCCTGCGGCATGTGGCGGTAGATATAGATACCGCTTA
ACAATGTGAAGATTAAGGTAAGGGCGGTCAGGCCAAAGACTTTAAAGTTGACCCAAATATTTTGCGGCAGCCAGAATGCG
ATGTAGATGTTTGCCAGACCGCAAAGGATAAAGAAAACAGCCCAGGCCAGATTCAGCTTCGACCATACCGGTTGCGGCAG

CGTGAGTTCTTTACCCAGCATCCGCTGAATTAGCGGCTTTTTCATCACCCATTGGCTGACTAACAGGGCTCCCGCAAATA
GAGCATAAATGACAGTAACCTTCCATTTAATAAACTCATCATTGTGGAAGAACAGCGTCAAGCCACCGAAGACGACCACC
AGAACAAAAGTGATCAGGGCCATCTTCTCAACCTTACGAAAGCGAACCCAGCTATAGATAAGCACAATCGCCGTGGCGAC
GATCAGCGCCGCAGTAGCCGCATAGATGTCATAAATCTTGTAAAACGCGAAAAAGACAACCAGCGGTAAAAAATCAAGAA
ACTGCTTCATTTTACGATTCCGTAATCAAATGCTTAAGGTTATTGGCGAATCAACATATACAGGCGGAACAGATAGATGA
GCAATATGGCTGAAATCAGGTTGCTCAAGGTGTTCGCCAGTACGGCACCAATTTCCGGGGTTAATGCGGCAAAAGAAGAG
GCAAAAAGCAGCAACAGTGTTTTTGCCAGCAACCAGCTCAGTACTGCGGGTGCCACCAGACGCATATTCGCCCAAGTCAG
CCGCATACTGCTACGCATCGAGGCAAAAACGCCCATTTTGTCCTGTACCAACATCACCGGTGCCAGGGCCAGTAAAATGG
CCATGATAATTCCCGGAACGACCACCAGCATGATGCCAATCTGTACTAAAAGGGTAGTCAGAAAAATCAGAATAAATAAC
TTTGGCAATATCGGCGCACTGGCACCAATAGCTCGCAGCGCACTGACTCTCTGACCCGCAGACACCAGCTGGATAATTAA
TATTACGCCTCCGGCGAGAATGGCGTTACCGATTAATCCTGAAAAAGTGGACGCCGCTGAAGCCTGCAGCAAAATTTGTT
GCTGTTCCGGTGACATATTCTGAACCAGGTCGAACAACCCACTACTGCCGCTAACGGGCACGCCGTCATTGAGCTGCGCA
AGCTGTGCATCACTGGGTGAGAAAACATGCCCTAACACCACTGTGATAAACGCACATAGCAACGATACCAACAGAATGGT
CATAAATTGATTACGGAAGAAATTTCCGGTGTCACGGTATACGGACTGCGCCGTGATAGACATGCACTCTCCTTGAGTTT
TATGCAGGTGTTAATTAGCGGGCAATTGTACCCTGGTTAAGCCTCGGGTGGCAGCATCAGGTCTTGTATGGAAAAGCATA
TCTTTGTAAAGCGGAGGTAATTGCTGGCCTTCAGGCATGGGCAAATCGCGGCAAGTGGCGACCGAGTTAATATTTGCGTA
GCGAAAATATTTAAAAATTGATTTAAATCACATTAACCAGGATTCTCAATGCAACTTCTAAATTAATCCAGATCAATAAA
GGGTGAATTATCATATGTAATGTGATCTATGTAGGATCATTTGTTACTCCAATGTAGGTATATTCGTCACGTTTTTATAA
CCATAACGACGGAGCGGATATGAAAAAGTTAACAGTGGCGGCTTTGGCAGTAACAACTCTTCTCTCTGGCAGTGCCTTTG
CGCATGAAGCAGGCGAATTTTTTATGCGTGCAGGTTCTGCAACCGTACGTCCAACAGAAGGTGCTGGTGGTACGTTAGGA
AGTCTGGGTGGATTCAGCGTGACCAATAACACGCAACTGGGCCTTACGTTTACTTATATGGCGACCGACAACATTGGTGT
GGAATTACTGGCAGCGACGCCGTTCCGCCATAAAATCGGCACCCGGGCGACCGGCGATATTGCAACCGTTCATCATCTGC
CACCAACACTGATGGCGCAGTGGTATTTTGGTGATGCCAGCAGCAAATTCCGTCCTTACGTTGGGGCAGGTATTAACTAC
ACCACCTTCTTTGATAATGGATTTAACGATCATGGCAAAGAGGCAGGGCTTTCCGATCTCAGTCTGAAAGATTCCTGGGG
AGCTGCCGGGCAGGTGGGGGTTGATTATCTGATTAACCGTGACTGGTTGGTTAACATGTCAGTGTGGTACATGGATATCG
ATACCACCGCCAATTATAAGCTGGGCGGTGCACAGCAACACGATAGCGTACGCCTCGATCCGTGGGTGTTTATGTTCTCA
GCAGGATATCGTTTTTAATTCCGCACAAAAACGACCCCGTAATATACGGGGTCAATAAGGACATGGTATAAAGGGTATTA
TTTCTTCGCTTCTACGCCATCAGTTTCAGAGCGAATTAAAAATTTCTCAGTTGTTTGCGGAATATTCTGAATCAGCCAGT
CGGCCATTTGCTTTTCCTCATTTAAAATCGCTTCGATGGTTGGAATTGAAGCTGTATCACCGGCATTTTTTGCTGCTGCT
AATAGTGAGGTGTAACAGGCGATTTCAAATTGCTCGAAGACATATCCGCTAATAGAGCCTTTGACTATTTCATCAGAAGG
GAATATACCACCGATTGACTGCCCAAGCGCAGCCATTTTACTCATGGAATCTTTAATGACTGAACGTGAAATGTCATTAC
GATCAAGAATAGTTTCCAGTTGAACAATCTGGTTTTTGGTTTCACTAAGATGTTGTTCAATACGAGCGCGTAGTTCAGGA
TAATTATCTATACGGCTGGCCATGGATTCAAGCATAGATTCGGCTTGCTTTTCCATTGCGTGGGCGTCACGTAACCAGTC
ATGATAATGTTCAATACGATTCATGAAAATGTCCTCTTTATAAAATGACTAAAAGTTAAAATTCATATTTCAGGCTTTAT
TTTCGGCTTTCTTATTTACGTTATTAATGGCCAGATCAGTCAGTTTGATGTCGGTGGCCTTTTCTTCTTCCAGGGTTTCT
TTCAGAAGCTTCGCTGCTTTACGGTAACCTAATTGTTCAGCCAGCGTCGCTAATGTCCCGTAACTGGCAATCTCATAATG
CTCGACTTTCTGTGCTGCGGCAATCAGTGCGGCATCACGCACTTCGTTTTTCTCGGTACTTTCGATGACCTCATTAGCTT
CTTCAATAAGACCTTCCATTGCCACACATTTCATGCGCTTAATTTTCAGGTTCGATTCGGACTCCACAACTTGATCAATA
CGTTCAATCTGTCCATGAGTTTCCTCGAGGTGCGCATGAAAAGCCTGACTTAATTTTTCATTTGATGTTGCTCTTGCGAG
TTTTGCCAGTGCCCGGGTTAATTGTTTTTCTGCGCTGTAGGTATCTGAAAGCAGGTGAATAAATACATCTTCAATGGTCT
TCATATTCATATTTTTCTCCAGTGAAATCACTGCGAGCCATACGGCCCGCAGATGCAATTATCTTTATCAGCTGATATTA
ATAATTAACGGTGAATCAGGATTTGCCTGATTTATTACCACCGCTTTGTTGACCGCCTTTTTTACCCGCTTCAGATGCGC
GTTGCGGATCATTTTTAAAATTACCGCCGCTATGCTGACCGCCTTTACGGCCTGCGTCGGATGCCTTCTCACGGTCTTCG
GCGAAATTTCCTGAACCACCACGATGTTCGGCCATGTTATTTCTCCCGTTGCGTTGCATTGTTTCATTAATATGAGTGTT
GTGTGTCGACACTCATTAAAATTAGTCGCTAATGAGAATTAGTCAAATTAAGCGCAACGAGAAGATAGAGGGAAAATATA
TTTTGAGGAACATTCTGGATATATTAACAATTACCTGAGGAATAAGTGACTTAGAGAAAATTATTGATTTTACTGGTGTT
ATGTTGCGGGATTAATTTGTTTGATTATATTACTGTTGGGCGGAAAAATGACGTAAGTTGACGTTCGACCGGGGTAAGCG
AAACGGTAAAAAGATAAATATTAAATGAATTTAGGATTTTTCCGGCTTCATTAAAGAAAGTTAAAATGCCGCCAGCGGAA
CTGGCGGCTGTGGGATTAACTGCGCGTCGCCGCTTTCATCGGTTGTACAAAAACTTTCAGTGCCGCCAGCATTTTCTCTG
GCTCATTAATATGTTGCTCGATGATTTTAACAATGGCCGAACCAGAAATCGCGCCCGCAGCTCCTGCATCAATCGCTGCT
TTTACCTGATCCGGGGCGGAAATACCAAATCCCTGCAATGGAGGTGCAGCGTTGTACTCTTTCAGCTTCGCAACCAGATG
ATTGAGGGGTAACGCGGCGCGGTTTTCTGCGCCGGTCACGCCTGCTCGTGACAGCAAATAGGTGTAACCACGACCGTAAG
AGGCTATCTGGCGCAGCAGGTCGTCATCGGCATTTGGCGGGCAGATGAAGATAGGTGCGACATTATGACGCAACGCGGCC
TGGCGGAAGGGCGCGGACTCTTCAACTGGCACATCGGCAACCAGCACCGAATCGACGCCGACTTTTTCGCACTGGGCATA
AAACTCATCAATGCCTTTGTTAAACACCAGATTGGCATACATCAACAGGCCAATGGGAATGGTCGGGTGTTTCTGGCGAA
TCAGTGCCAGCATTTCAAAACATTGTGCCGGAGTCACACCTGCCGCAAAGGCGCGCAGAGTGGCGTTTTGAATCGTCGGG

CCATCCGCCAGTGGGTCGGAGAAGGGGATACCTAACTCCAGCGCGTCAGCACCGGCTTCAATTAGCGTATCGATAATTTT
CAATGACTGCTCAATGCCCGGATCACCGAGCGTGACGAAAGGAACGAATGCGCCTTCTTTGCGCTCCTTCAACTGGGCAA
ACAGAGATTCGTAGCGTTCCATCAGATTTCCCCTCGTGCTTTCAAAATATCGTGAACGGTGAAGATGTCTTTATCGCCGC
GACCGGAAAGGTTAACCACCAGTAGCTGCTCTTTATCCGGGTTTTCGCGCATCATTTTCAACGCATGGGCCAGGGCGTGG
GAGGATTCCAGCGCCGGGATGATCCCTTCGTGCAGGCACAGCGTTTTGAAGGCTTCAAGGGCTTCATCATCGGTAATAGA
CACGTAATCAGCGCGTCCAGTGCTGTTAAGATACGCGTGTTGTGGGCCGACAGACGGGAAATCCAGTCCGGCGGAGATGG
AGTAAGATTCTTCAATCTGCCCGTCTTCGGTTTGCATCATCGGCGCTTTCATACCGAAATAGATACCCACGCGACCATGT
TTTAGCGGTGCGCCGTGCTCGCCAGTTTCGATACCGTGACCACCTGGCTCCACACCAATCAGGCCGACGTTGGTTTCATT
GATGAAATCAGCAAACATGCCGATGGCATTCGAACCGCCGCCAACACAGGCGATAACGGCATCCGGCAGGCGACCTTCTC
TTTCCAGAATCTGCGCTTTGGTTTCTTCGCCAATCATCCGCTGAAACTCACGCACAATGGTCGGATAAGGATGCGGGCCA
GCTGCGGTGCCCAGCATATAGTGCGCGGTTTCGTAACTACCGGACCAGTCGCGCAGCGCCTCGTTACAGGCATCTTTCAG
CGTCGCGGAACCGCTATGCACCGGGATCACTTCCGCACCCATTAAGCGCATACGAAAAACGTTAGGCGACTGGCGTTCAA
CGTCTTTGGCACCCATATAAATACGGCATTTCAGGCCGAGCAGGGCGCTGGCAAGGGCCGACGCCACGCCATGCTGACCG
GCACCGGTTTCGGCGATGATTTCGGTTTTACCCATCCGCTTCGCCAGCAACGCCTGCCCCAGCACCTGGTTAGTTTTATG
CGCGCCGCCGTGCAGCAAATCTTCACGCTTGAGATACAGCGTGGTGTTCGTCCCGGCTGTAATGTTCTGGCATTTGGTCA
GCGCGGTTGGACGCCCGGCATAGTTTTTCAGCAGGTCGTTGAACTGAGCCTGAAATTCAGGATCTTTTTGCGCACTGACA
AAAGCTTCTTCCAGCTGGCGCAGAGCAGGCATCAGGATTTGTGGCACGTACATGCCGCCAAACTCACCAAAATAGGGGTT
AAGTAATGTTGTCATTGTTCCTTTCCTTAATATGCGCGCAGCGTCTGGAAAACCGAGGCCAAAAGACGTGCGTCTTTGAT
GCCCGGTTGCGACTCTACAGCAGAATTAAAATCAAGTCCGGCGCAGCCGGTTTGTGCCGCTTCCACGCAGTTATCTGCGC
CTAAGCCCCCCGCCAGCAGAACGTTGCCAAGCGATTGACCATTTAATAGTGACCAGTCAAAACGTTGCCCGCTTCCACCC
TGGCCGTTGTCTAAAACATATTTATCAACGTGCTGAAACTCGCGGGCGGGCAGGGTTTCACCGACGCTTAATGCTTTCCA
GATGGCAACATGTGCTGGCAGAGCTTCACGCAGCGTATCGATATACAGCTGTTCTTCATTACCATGCAGTTGCACTGCCG
CCAGCGATAACACCTTAGCTTTGTCCACCACATCGGCAATATCGTGATTGCGGAACACGCCAACATACTGCAACGGTGCC
GCAGCCATCACTTCCTGCGCCTGTTCAACGTTGACGCAACGCGGTGATGTCGCAACAAAAATCAACCCACCGTAAATCGC
GCCCGCGTCATAAGCTGCTTTAGCATCTTGCCCACGCGTCAGGCCACATACTTTATTCTCACCCAGCAACACCCGGCGCA
CGGCGGCGTGCAAATCGTCATGGGCCATCAACGCCGAACCAATCAGAAAACCGTTAGCGAAGTGGCTTAACTCGCGCACC
TGAGCGTAAGTATTGATGCCGGATTCGCTGATTACCGTCACGTTGTGCCCCAGTTTCGGCGCAAGCTCGCGGGTACGGTT
GAGATCAATCGACAAATCACGCAGATCGCGGTTGTTGATGCCAACGACCTTTGCTCCCAATGCAATGGCGCGCTCCTGTT
CCTCTTCATTACTGACTTCGGTCAGCACCCCCATCTCCAGACTGTGAGCGACGGCGGCAAGCTGGCGATATTGGTCGTCA
TCCAGTACTGAAAGCATTAATAAGCAGGCATCGGCCTGGTAATAGCGCGCCAGATAGATCTGGTAAGGGTCGATAATGAA
GTCTTTACATAAAATCGGCTGCGGGGCGATTTGGCTGACGATGGGGAGGAAATTAAAGCTCCCCTGAAAATATTTCTCAT
CAGTCAGCACCGAAATTGCCGAAGCGTAATGTTTATAAATGGCGGCAATGCGTGCTGGATCGAAATCATCACGGATCACG
CCTTTTGACGGCGACGCTTTCTTGCACTCCAGAATAAACGCCGTGCGCGCACCCTGTAGCGCATCATAAAAATGTCGCGT
GCTCGGCTGAACCTCATTCTGAAAACTGGCCAGCGGTTGCTGCTGTTTGCGGGCTTCTACCCAAATCGCCTTGTCTGCGA
CGATTTTCGCTAAAACGGTTTGCATCATTTACCCTCGTGCCGCCAGTGCGGTGACTCTGTCGTAAGCGGAACCACTGCGC
AGTACCTCAAGAACGGTTTGCGCATTGGCTTGCAGATCTTCATGGCCATGCAGGCGCATTAACATGGCGACGTTCGCAGC
GACGGCTGCTTCATGGGCGGCGTCGCCTTTACCTTGTAACAAACGTGTTAAAATGTCACGGTTTTCTTCCGGTGTTCCGC
CTGCCAGTTGCTCCTGGTGGTAGGGTGTCAGGCCAAAGTCTTCTGCGGTGAGCTGATAGCTTTTAATTTCGCCGTCATGC
AGTTCGGCAACGATTGTCGGCGCGTGTAATGAAACTTCATCCATCCCGCCGCTGTGCACCACCGCCGCGCGTTGATACCC
CAGCACGCGCAAGGTTTCGGCAATCGGCAGCACCAGTTCCGGACTATAAACACCAATTAACGCCAGCGGCGGATGCGCCG
GGTTAATCAATGGCCCCAGCACATTGAACAGGGTGCGGGTTTTCAGTTGCTGGCGAACCGGCATCGCGTGGCGGAATCCG
GTGTGATACTTCGGCGCAAAGAGGAAACATACACCTAACTCATCCAGCGCCTGGCGCGATTTATCGGCGTTCATATCAAG
ATTAATACCGAACGCCGCCAGCAGATCGGACGAACCAGATTTACTGGAGACGCTACGGTTGCCGTGTTTCGCCACTTTCA
GCCCACAGGCCGCGGCGACAAACGCACTGGCGGTAGAAATATTGATACTGTTGCTGCCGTCACCGCCAGTACCGACGATA
TCAGCAAACAGATAATCCGGGCGCGGGAACGGCGCTGCGTTTTCCAGTAGCGCGGTTGCTGCCCCGGCGATCTCGTTCGG
GTGCTCACCGCGAATTTTCATGCTCACCAGCGCCGCCGCCAGTTGTTCCGGCTTCAGCTCGCCACGCACCACCGCTGAAA
ACAGCTGGTGGCTTTCTTGTTGGCTAAGCGTCTGCGCCTGATACAGTTTTTCCAGAATCGGTTGCAGCGTGTTGGCTGGC
TCTAGTTTCTGCTGCGCCCAGGCCAGCGTTTGTTCCAGCAGGCGAGCGCCCTGGGTGGTGAGAATGGATTCCGGATGGAA
CTGGAATCCACAAACGCGATCCGCATCGTGACGTACTGCCATCACCATGCCATTAAAATGGGCGTTGATGGTTAAACCGG
CCGGAATGTTACTGCCAACCAGCGAGTGATAACGCGCCACCGGCAGCGGGTTTGTTAATCCGGCAAACATCGCCTGACCG
TCATGTTCAATGCTGGAGGCTTTACCGTGGAGAATTTCGCCCGCCTGACCGACATAGCCCCCGTAAGCTTCGACAATCGC
CTGATGTCCGAGGCAAATGCCAATAATGGGCAGCTTGCCACGCAAGCGGGTGAGGAGTTCCGGCATACAACCGGCTTCGC
TCGGCACACCGGGGCCAGGAGAAAGCATCAGCACCGGATTGCTCATGGTCGCCAGGCGTTCAATTAAGGTTTGCGCCGGA
ATATGGTTGCGGTAAATCACCACGTTATGCCCATTGCTGCGCAACTGATCTGCCAGGTTGTACGTAAAAGAGTCGATATT
ATCGAGCAGCAGAATGTCAGCCATCAGAAAGTCTCCTGTGCATGATGCGCGGTGGCAATAGCGCGCAGTACAGCGCGGGC
TTTGTTACGGGTTTCGTCGGCTTCCGACTGCGGAACAGAATCAAGGACTACACCAGCACCCGCTTGCACGGTGGCGATAC

CGTTTTCCACCAGCGCCGAGCGGATCACAATGCAGGTGTCGAGATCGCCATGCGCGGTGAAATAACCTACCGCGCCGCCG
TAGCTGCCGCGGCGACGACCTTCCGCCTCGGCAATTAACTGCATAGCGCGTACTTTCGGCGCACCGCTTAACGTCCCCAT
ATTCATACAGGCGCGATAAGCGTGCAGGGCGTCAAGATCGTGACGCAGTTCGCCGACTACGCGAGAGACGAGGTGCATCA
CATAGGAATAACGGTCAACTTTGGTGAGATCGGCGACGTAGCGGCTGCCGGGGGTGCAAATGCGTGCCAGATCATTACGG
GCGAGATCAACCAGCATCAGATGTTCAGACAGCTCTTTATGATCGGTACGCATTTCCAGTTCAATACGGCTGTCGAGATC
TCTGTCCAGTGAACCATCGGCGCGACGACCGCGTGGGCGTGTTCCGGCAATCGGGTAGATCTCAATCTGGCGGCTGGTGG
CATCATACTTGAGCGAGCTTTCCGGCGACGCGCCAAATAGGGTGAAATCATTATCCTGCATAAAAAACATGTACGGGCTG
GGATTACTCTTTTTCAGCACGTAATAGGCCGCCAGCGGTGACGGGCAGGGCAGAGAGAAACGGCGAGATGGCACCACCTG
GAAAATTTCTCCAGCGCGAATCGCTTTTTGCAACAAACGCACTACGCCACCGAACTCTTCATCGCTCTGATTACATTCAC
AACGCATATGCGGCACGGAAACCACTGGCAGCGGCGGCGCGGCTTCGGTCAGTTGCTGACGTAGTTCGTTCAGGCGAGCA
GTGAGACGTTGTTTTTCTTCTTCATTCGGAGCAAACAGGCTGGCCTGAATACGGGTGCTTTTTTTCTGATGGTCAATCAC
CATCAGCGTTTCAGCGAGATAAAAACAGAAATCAGGGCAGTTATTTTCCGCTGACAGTTGCGGTAAATCTTCAAATCCCG
CCACAAGGTCATAAGAGAACAGGCCGCCGAAGAACATGGCTTCTCGTTCTTCCTTCGGTACATTCAACAGATTCTGCAAT
AAACGGAAAGCGTCAAAAACCGAAAGGGAGCATAAGCGGGCGTCTTCATCCAGCAGTGGACTGACAGGGGGGAAGCGCAG
CACACGGCAGTTTGGTGATTGTTCACTTTCCACACCCGCAGGCAGGGCGTTATCCAGTAGTGCCAGGAGGGCTTCGCCGT
TGCCGGAAAGTGCCTGGATTGTGACAGTGTCACCTAAAGCTGTAATGCGCAGCGCACTGTCTACCAGCAGCAGGCTTTTT
AAATCATCTTTGCTGTCGATATCTGCGGATTCCAGCAGCAGCGTTGCCGGACGATCCCCACACAACTGGTGAAAAAGCGC
GGTGGGATTGTCGCGATAAGCGCCTTCGCAGGTTAGCAGTTCGAGAGTCGGTTTTTGTGTTTGCATTGTTATTCTCTAAT
TTTGTTCAAAAAAAAGCCCGCTCATTAGGCGGGCTGGGTATCTGATTGCTTTACGCATGGTGAATACACTGCCCGTTTCA
GGAAGTGCGCCACCAACCTTTCAGTACGAAAATTGCTTTCATTGTCGATACCCTTTTTACGTGAACTTGCGTACTAGTTA
ACTAGTTCGATGATTAATTGTCAACAGCTCATTTCAGAATATTTGCCAGAACCGTTATGATGTCGGCGCAAAAAACATTA
TCCAGAACGGGAGTGCGCCTTGAGCGACACGAATTATGCAGTGATTTACGACCTGCACAGCCATACCACAGCTTCCGATG
GCTGCCTGACGCCAGAAGCATTGGTGCACCGTGCAGTCGAGATGCGCGTCGGCACCCTGGCGATCACCGACCATGACACC
ACAGCTGCAATCGCGCCAGCAAGAGAGGAAATTTCACGTTCGGGACTGGCGTTGAATCTTATTCCCGGCGTGGAAATTTC
CACGGTCTGGGAAAATCATGAAATTCATATTGTCGGGCTGAATATTGATATTACTCATCCGCTAATGTGTGAGTTCCTCG
CGCAGCAGACAGAACGGCGCAATCAGCGGGCGCAGCTGATTGCCGAAAGACTTGAAAAAGCGCAAATACCTGGGGCGCTG
GAAGGCGCACAACGACTGGCGCAGGGCGGCGCAGTGACGCGCGGTCATTTTGCGCGTTTCCTTGTTGAGTGCGGCAAAGC
CAGTTCAATGGCGGATGTCTTTAAAAAGTATCTGGCGCGCGGGAAAACCGGATACGTTCCGCCACAGTGGTGTACAATAG
AACAAGCTATTGATGTCATTCATCATTCTGGCGGTAAGGCGGTGCTAGCTCATCCTGGGCGGTACAATCTTAGTGCTAAA
TGGCTGAAAAGATTGGTTGCGCATTTTGCCGAACACCACGGTGACGCGATGGAAGTCGCGCAGTGCCAGCAATCGCCCAA
TGAACGTACCCAGCTGGCGGCCCTTGCGCGTCAGCATCATTTATGGGCATCACAAGGATCTGATTTTCATCAGCCATGCC
CGTGGATCGAACTGGGTCGTAAACTCTGGCTGCCCGCAGGCGTTGAAGGCGTCTGGCAGCTATGGGAACAGCCGCAGAAC
ACCACAGAGAGGGAATTATGAGCCAGTTTTTTATATTCATCCTGATAACCCACAGCAACGTCTGATCAACCAGGCGGTG
GAGATCGTGCGTAAAGGCGGGGTGATTGTTTATCCAACTGATTCCGGCTATGCGCTCGGCTGTAAAATTGAAGATAAAAA
CGCGATGGAGCGTATTTGTCGTATTCGCCAGCTGCCGGACGGTCACAACTTTACCCTGATGTGTCGCGATCTTTCTGAAC
TCTCGACCTATTCATTTGTTGATAACGTTGCGTTTCGTTTAATGAAAAACAACACGCCGGGCAACTATACCTTCATCCTG
AAGGGGACGAAGGAAGTGCCACGCCGTCTGTTGCAGGAAAAACGCAAAACCATCGGTATGCGTGTGCCTTCTAACCCTAT
CGCTCAGGCGTTACTTGAAGCATTGGGCGAACCGATGCTTTCCACTTCGCTAATGCTGCCAGGCAGCGAATTTACCGAAT
CGGACCCGGAAGAAATTAAAGATCGTCTGGAAAAACAGGTAGATTTGATTATTCACGGCGGCTATCTCGGGCAGAAACCG
ACAACGGTTATTGATCTTACCGATGATACGCCGGTCGTGGTGCGTGAAGGCGTAGGTGATGTGAAGCCTTTCTTATAACA
AATTGCGTTCCACGGATGGAAGACTATGGCAGGGAAGTTTCGCTGCATTTTGCTGTTGATAGCAGGGCTGTTTGTATCAT
CTCTAAGTTATGCAGAAAACACGGAGATCCCTTCTTATGAAGAAGGGATCTCGCTCTTTGATGTTGAAGCCACTCTGCAA
CCAGATGGGGTGCTCGACATCAAAGAAAATATTCATTTTCAGGCGCGAAATCAGCAGATTAAGCACGGCTTTTATCGTGA
TTTACCACGACTATGGATGCAGCCTGATGGGGACGCTGCACTGCTGAACTATCATATTGTTGGCGTCACCCGTGATGGTA
TTCCTGAACCCTGGCATCTTGACTGGCATATCGGGTTAATGAGTATTGTCGTGGGCGATAAACAACGTTTCTTGCCTCAA
GGCGACTATCATTATCAAATTCATTATCAGGTTAAAAATGCTTTCCTGCGTGAGGGGGATTCTGATCTGCTAATCTGGAA
CGTGACCGGTAACCACTGGCCGTTTGAAATTTATAAGACCCGTTTTTCTCTCCAGTTCTCTAATATTGCGGGTAATCCAT
TTAGCGAAATCGATCTTTTTACCGGAGAAGAGGGCGACACATATCGTAATGGCCGCATCCTTGAGGACGGAAGAATTGAA
TCCCGCGATCCGTTTTATCGTGAAGATTTCACGGTCCTCTACCGCTGGCCTCACGCTTTACTTAGCAATGCCTCGGCTCC
GCAAACGACGAATATTTTCAGCCATCTTCTTTTACCCTCCACGTCATCGTTGTTAATTTGGTTTCCGTGTCTGTTCCTGG
TTTGTGGATGGTTATATCTCTGGAAGCGCAGGCCGCAATTTACGCCGGTAGATGTGATTGAAACCGATGTCATTCCGCCA
GATTACACGCCCGGCATGTTACGTCTCGATGCGAAGCTAGTTTACGACGATAAAGGTTTTTGTGCCGATATCGTAAATCT
GATTGTGAAAGGAAAAATTCATCTGGAAGATCAGTCTGACAAGAACCAGCAAATCCTGATTCGTGTTAATGAAGGCGCGA
CCAGAAATAATGCGGTATTACTGCCCGCAGAGCAGTTATTACTGGAAGCGTTATTTCGTAAAGGCGATAAGGTCGTTCTT
ACGGGGAGACGCAACAGAGTCTTACGCAGGGCATTTTTACGGATGCAGAAATTTTATCTGCCGCGTAAAAAGTCTTCGTT
TTACCGGTCTGATACTTTTTTGCAATGGGGTGGACTGGCAATATTGGCGGTCATTCTTTACGGTAACTTGAGTCCTGTAG

GCTGGGCAGGAATGAGTCTGGTGGGCGATATGTTTATTATGATCTGCTGGATTATTCCTTTTTTATTTTGTTCCCTTGAG
CTTTTGTTTGCCCGCGATGATGACAAGCCTTGCGTTAATCGTGTAATCATCACTTTGTTTTTACCACTGATTTGTTCAGG
CGTGGCCTTCTATTCTCTCTACATCAATGTCGGAGATGTATTCTTTTACTGGTATATGCCAGCGGGTTATTTTACCGCTG
TTTGCCTGACCGGTTATCTCACTGGCATGGGGTATATTTTTCTGCCAAAGTTTACCCAAACTGGACAGCAACGTTATGCC
CACGGTGAAGCTATCGTTAACTATCTTGCGCGTAAAGAGGCAGCAACACACAGTGGACGTCGGCGGAAAGGGGAAACACG
GAAACTGGATTACGCGTTGCTAGGTTGGGCTATATCGGCAAATTTGGGGAGGGAATGGGCGTTACGCATTGCCCCTTCGC
TTTCTTCGGCGATTCGCGCTCCAGAGATTGCCCGTAACGGCGTTTTATTCTCATTACAGACGCACCTAAGTTGCGGGGCC
AATACCAGTTTGTTGGGGCGAAGTTATTCCGGTGGTGGTGCTGGCGGCGGCGCGGGTGGCGGAGGCGGTGGTGGCTGGTA
ATTAAGCTGATGTTAATCGAAACAAGCTTTAACCATAAAGATAGTGTAGTATGTTGCGCCTCAAAGCCAGGCCGATAAAC
GTCGAGTCGTTTACTTAAGGCCTGAAGAGTTCAAACAGACTTTACTATCAGTGAGTCAGATGTAAGTTCTGACTCACTGT
TGCACAAAAGCATATCAGACGCCTGGGAAGGCGACACCAAAAGGAAGCTCTATGAGCGAAAAGCTACAGAAAGTGCTGGC
GCGTGCCGGCCACGGTTCTCGCCGTGAAATCGAATCTATTATTGAAGCCGGTCGTGTGAGTGTTGATGGCAAAATTGCTA
AACTCGGCGATCGTGTTGAAGTTACCCCTGGATTGAAAATCCGTATCGATGGTCACCTGATTTCGGTACGTGAGTCCGCT
GAACAAATTTGTCGCGTTCTGGCCTATTACAAACCGGAAGGTGAGTTGTGTACCCGTAACGATCCGGAAGGACGTCCAAC
GGTGTTTGACCGTCTGCCAAAACTGCGTGGCGCACGCTGGATTGCCGTGGGTCGCTTGGACGTTAATACCTGTGGTCTGC
TGCTGTTCACCACCGATGGTGAACTGGCAAACCGTTTAATGCACCCAAGCCGTGAAGTTGAACGTGAATATGCCGTGCGT
GTATTTGGTCAGGTTGACGACGCGAAACTGCGTGATTTGAGTCGTGGCGTGCAGTTGGAAGATGGTCCGGCAGCTTTTAA
AACCATCAAGTTCAGCGGCGGCGAAGGGATCAACCAGTGGTACAACGTGACTCTGACCGAAGGCCGTAACCGTGAAGTTC
GTCGTCTGTGGGAAGCGGTTGGTGTGCAGGTGAGCCGCCTGATCCGTGTTCGTTACGGTGATATCCCACTGCCAAAAGGT
CTGCCACGCGGTGGCTGGACGGAGCTGGACCTCGCCCAGACTAACTATCTGCGCGAACTGGTGGAGCTACCGCCAGAAAC
CAGCTCTAAAGTCGCTGTAGAAAAAGACCGTCGTCGCATGAAGGCGAATCAGATTCGTCGTGCGGTGAAACGTCACAGTC
AGGTGAGCGGCGGTCGTCGTTCTGGCGGACGTAATAATAACGGTTAATCAAAGTATGCCGGACGTCATATCCGGCATTTT
TACAGATTAATAATCGATCCCTATCTGCGCTTTTACACCGGCATCAAACGCATGTTTGACGGGGCGTAATTCACTTACCG
TATCTGCCAGTTCAAGAATATCCCGATGACAACCACGACCCGTGATAATCACCGTCTGTTGATGTGGACGTTCATTTAAC
GCCTGCACCACTTCTTCCAGTGGCAAATAGTCATACGCCACCATATACGTCAGTTCATCAAGCAAAACCATATCCAGTGA
GGAATCAGCAAGCATCCGCTTTGCATGTTGCCAGACTTCGCGGCAGGCGGCGGTATCAGACTCGCGGTTTTGTGTATCCC
AGGTAAAGCCCGTTGCCATCACCTGAAACTCAACGCCATGTGGCTCCAGCAGATTGCGTTCGCCATTAGGCCAGGTGCCT
TTAATAAACTGCACGACGCCTACTTTTTTTCCGTGACCAACTGCGCGTGTTGCCGTACCAAATGCCGCGGTGGTTTTGCC
TTTTCCATTGCCGGTAAAGACGATGATAATACCGCGTTCATCCTGGGCCTGGGCCACACGAGCATCTACTTTTTCTTTCA
CTCGCTGCTGACGCTGTTGGTAGCGTTCATCACTCATTGGGAAATTCCTGGTTTACGGCCCGGTTGGGCGTCAAAGGTCA
TGCCGGTTTTACGGCGGCTGTCATCGCCCATCAGCCAGAGGTAGAGCGGCATGATATCAGCGGGTGTTTTAAGTTTCTGT
GGATCTTCGGTCGGGAAGGCGCTGGCACGCATTGCGGTGCGCGTACCGCCTGGGTTAATGCAGTTGACACGCAGGCGCTG
CTGATATTCATCGGCCAGTACCTGCATCATCCCTTCGGTGGCAAATTTCGACGCTGCATATGCACCCCAGTTGGCTCGTC
CCTGACGTCCAACGCTTGATGAAGTAAAGACCAGTGAACCGGCGTCCGATTTGAGTAATAAAGGAAGCAGTGCCTGGGTG
AGCATAAAGGTGGCATTAACGTTGACCTGCATGACGTCCTGCCAGACCTGCGGATTTTGTTCGCTCATTGGGCAAACATC
GCCGAGCAATCCGGCATTATGCAAAACACCATCCAGACGCGGATAATTAACGGCAATGCGCTGTGCCAGTTGTTGGCAAT
TTTCGGACGTGCAGGTCAGCAAATCGAGAATAAACCACTGTGGCTGACGCCCAGTTTCTTCGTTTATGTGGCTGGCTACC
TGACGTAATTTTTCTTCATTACGGCCCAACAGAATCACTGTCGCACCATAGCGTGCATACGTCATCGCGGCTTCACGACC
AATACCATCGCTGGCTCCCGTCACCAGGATAATGCGATCATTGAGTAAATCTTGTTTTGGCTGGTAATGCATGGCTACTC
CTCAACGACGTTGTCTGTCGCGCTGCCAGTGTACTTTATGACTTTGGGGCTTTATGCCTGAAACAGAAGGGCATTTCAAT
CAGCCTGGGGCGATAACACCGCAAAATGGATACTTTGTCATACTTTCGCTGCAATAACATCTCTGCGAGACGGCTTAACA
TGCCTGTTGTAAACTGTGAGCCAAAGCGTTGTTTAACCAAGGTGGGGACTCGTGGAATTGTTGTCTGAATATGGTTTGTT
TTTGGCGAAAATCGTTACCGTTGTGCTAGCGATTGCGGCGATTGCCGCCATTATTGTCAATGTTGCTCAACGTAATAAAC
GCCAGCGTGGCGAGTTACGGGTCAACAATCTCAGCGAACAGTATAAGGAGATGAAAGAAGAACTGGCCGCGGCGCTGATG
GACTCACATCAGCAAAAACAGTGGCACAAAGCGCAGAAGAAAAAGCATAAGCAAGAAGCGAAAGCAGCAAAAGCGAAAGC
CAAACTGGGCGAGGTGGCAACTGACAGTAAACCCCGCGTCTGGGTGCTGGATTTTAAAGGCAGCATGGACGCCCATGAAG
TGAACTCGCTACGTGAAGAGATAACGGCTGTACTCGCAGCATTCAAACCGCAGGATCAGGTTGTGCTCCGTCTGGAAAGC
CCTGGTGGCATGGTGCATGGTTACGGGCTGGCGGCTTCGCAACTGCAGCGTCTGCGCGATAAAAACATTCCTTTAACTGT
TACGGTAGACAAAGTCGCTGCCAGCGGCGGTTACATGATGGCCTGTGTGGCGGACAAAATTGTTTCCGCACCGTTTGCTA
TTGTGGGTTCCATTGGCGTGGTGGCGCAAATGCCCAACTTTAACCGCTTCCTGAAAAGCAAAGATATTGATATCGAACTG
CACACTGCCGGGCAGTATAAGCGCACGCTGACGTTGCTGGGTGAAAATACCGAAGAAGGGCGGGAGAAATTCCGCGAAGA
GTTGAACGAAACGCATCAGTTGTTTAAAGATTTTGTGAAGCGTATGCGTCCTTCTCTGGATATTGAACAGGTGGCAACGG
GTGAACACTGGTACGGACAACAGGCGGTAGAGAAAGGCCTGGTTGATGAAATCAACACCAGTGATGAAGTTATTCTTAGC
CTGATGGAAGGCCGTGAAGTGGTCAATGTACGCTATATGCAGCGTAAACGACTCATTGACCGATTCACCGGCAGCGCGGC
AGAGAGCGCCGATCGATTGTTGCTACGCTGGTGGCAGCGGGGTCAAAAGCCATTGATGTAAAAGACAAACGCGAGGCTAA
GACCTCGCGTTTTGCTTTAATCAACCAGATGATATTTTTCTGAAAGCACATGGGCCAGGTGTTTGAACATATTAAACACC

GCGGTGCTTTTGGCTGTTGGCAATCCTTGTTCATCTAAAAAGTAGTCGCCGGTAAATACCAGCACGCCATTACGCTGCGT
GACGCCGGTGGCTTCAATCCCTGCGAGCGTATCCTCATGCTCACGAATGATTTTGTTGGCCTCTTTCAACAGCGTTTCGC
GGTCGATGGGTTGTGTCTCTTTGTTCATTATTTACTCCTTAAACAAGGACATTAGTCTACGCCAGGCATGGCTTGCAGAC
AAATATACCACGCTGGTGGCAAGAGCGCCTTACTGGCAACTTTGGATTTTGCATGCTAATAAAGTTGCGTATCGGATTTT
ATCAGGTACAGTGTGACGCTTTCGTCAATCTGGCAATAGATTTGCTTGACATTCGACCAAAATTCCGTCGTGCTATAGCG
CCTGTAGGCCAAGACCTGTTAACTCAGTCACCTGAATTTTCGTGAACAGAGTCACGACAAGGGGTTGATATCCGCAGAGA
GCGAGTCCATATCGGTAACTCGTTGCCAGTGGAAGGTTTATCAACGTGCGACGCATTCCTGGAAGAATCAAATTAGGTAA
GGTGAATATGGGTAAAGCTCTTGTCATCGTTGAGTCCCCGGCAAAAGCCAAAACGATCAACAAGTATCTGGGTAGTGACT
ACGTGGTGAAATCCAGCGTCGGTCACATCCGCGATTTGCCGACCAGTGGCTCAGCTGCCAAAAAGAGTGCCGACTCTACC
TCCACCAAGACGGCTAAAAAGCCTAAAAAGGATGAACGTGGCGCTCTCGTCAACCGTATGGGGGTTGACCCGTGGCACAA
TTGGGAGGCGCACTATGAAGTGTTGCCTGGTAAAGAGAAGGTCGTCTCTGAACTGAAACAACTGGCTGAAAAAGCCGACC
ACATCTATCTCGCAACCGACCTTGACCGCGAAGGGGAAGCCATTGCATGGCACCTGCGGGAAGTGATTGGGGGTGATGAT
GCGCGCTATAGCCGAGTGGTGTTTAACGAAATTACTAAAAACGCGATCCGCCAGGCATTTAACAAACCGGGTGAGCTGAA
TATTGATCGTGTTAATGCCCAGCAGGCGCGTCGCTTTATGGACCGCGTGGTGGGGTATATGGTTTCGCCGCTGCTATGGA
AAAAGATCGCTCGTGGCCTGTCTGCCGGTCGTGTGCAGTCGGTGGCGGTTCGCCTGGTGGTCGAGCGTGAGCGTGAAATT
AAAGCGTTCGTGCCGGAAGAGTTCTGGGAAGTCGATGCCAGCACGACCACGCCATCTGGTGAAGCGTTGGCGTTACAGGT
GACTCATCAGAACGACAAACCGTTCCGTCCGGTCAACAAAGAACAAACTCAGGCTGCGGTAAGTCTGCTGGAAAAAGCGC
GCTACAGCGTGCTGGAACGTGAAGACAAACCGACAACCAGTAAACCTGGCGCTCCTTTTATTACCTCTACGCTGCAACAA
GCTGCCAGCACCCGTCTTGGATTTGGCGTGAAAAAAACCATGATGATGGCGCAGCGTTTGTATGAAGCAGGCTATATCAC
TTACATGCGTACCGACTCCACTAACCTGAGTCAGGACGCGGTAAATATGGTTCGCGGTTATATCAGCGATAATTTTGGTA
AGAAATATCTGCCGGAAAGTCCGAATCAGTACGCCAGCAAAGAAAACTCACAGGAAGCGCACGAAGCGATTCGTCCTTCT
GACGTCAATGTGATGGCGGAATCGCTGAAGGATATGGAAGCAGATGCGCAGAAACTGTACCAGTTAATCTGGCGTCAGTT
CGTTGCCTGCCAGATGACCCCAGCGAAATATGACTCCACGACGCTGACCGTTGGTGCGGGCGATTTCCGCCTGAAAGCAC
GCGGTCGTATTTTGCGTTTTGATGGCTGGACAAAAGTGATGCCTGCGTTGCGTAAAGGCGATGAAGATCGCATCTTACCA
GCAGTTAATAAAGGCGATGCTCTGACGCTCGTTGAACTTACACCAGCCCAGCACTTTACCAAGCCGCCAGCCCGTTTCAG
TGAAGCATCGCTGGTTAAAGAGCTGGAAAAACGCGGTATCGGTCGTCCGTCTACCTATGCGTCGATCATTTCGACCATTC
AGGATCGTGGCTACGTGCGAGTAGAAAATCGTCGTTTCTATGCGGAAAAAATGGGCGAAATCGTCACCGATCGCCTTGAA
GAAAATTTCCGCGAGTTAATGAACTACGACTTTACCGCGCAGATGGAAAACAGCCTCGACCAGGTGGCAAATCACGAAGC
AGAGTGGAAAGCTGTACTGGATCACTTCTTCTCGGATTTCACCCAGCAGTTAGATAAAGCTGAAAAAGATCCGGAAGAGG
GTGGTATGCGCCCGAACCAGATGGTTCTGACCAGCATTGACTGCCCGACTTGTGGTCGCAAAATGGGGATTCGCACAGCG
AGCACCGGGGTATTCCTTGGCTGTTCTGGCTATGCGCTGCCGCCGAAAGAGCGTTGCAAAACCACCATTAACCTGGTGCC
GGAAAACGAAGTGCTGAACGTGCTGGAAGGCGAAGATGCTGAAACCAACGCGCTGCGCGCAAAACGTCGTTGCCCGAAAT
GCGGCACGGCGATGGACAGCTATCTCATCGATCCGAAACGTAAGTTGCATGTCTGTGGTAATAACCCAACCTGCGACGGT
TACGAGATCGAAGAGGGCGAATTCCGCATTAAAGGTTATGACGGCCCGATCGTTGAGTGTGAAAAATGTGGCTCTGAAAT
GCACCTGAAAATGGGGCGTTTCGGTAAATACATGGCCTGCACCAACGAAGAGTGTAAAAACACACGTAAGATTTTACGTA
ACGGCGAAGTGGCACCACCGAAAGAAGATCCGGTGCCATTACCTGAGCTGCCGTGCGAAAAATCAGATGCTTATTTCGTG
CTGCGTGACGGTGCTGCCGGTGTGTTCCTGGCTGCCAACACTTTCCCGAAATCGCGTGAAACGCGTGCGCCACTGGTGGA
AGAGCTTTATCGCTTCCGCGACCGTCTGCCGGAAAAACTGCGTTATCTGGCCGATGCGCCACAGCAGGATCCGGAAGGTA
ATAAGACCATGGTTCGCTTTAGCCGTAAAACCAAACAGCAATATGTCTCTTCGGAAAAAGACGGAAAGGCGACTGGCTGG
TCAGCATTTTATGTTGATGGCAAATGGGTTGAAGGAAAAAAATAACCTTTAATTCTGTCAGGTTTTTATAAACAAAGGGT
CGCGAAAGCGGCCCTTTTTTATTGCATATTATTTTTTCTTCACACCTATACACTAAGGCTATAAATGATATAGTGGTTAT
AGTTAGCACCTTTTTTATTATTAAATCGTATTAGTCACCCGCCAGGTGTGACGAAAAAACGATGTTCTGATGGCGTCTAA
GTGGATGGTTTAACATGAAATTACAACAACTTCGCTATATTGTTGAGGTGGTCAATCATAACCTGAATGTCTCATCAACA
GCGGAAGGACTTTACACATCACAACCCGGGATCAGTAAACAAGTCAGAATGCTGGAAGACGAGCTAGGCATTCAAATTTT
TTCCCGAAGCGGCAAGCACCTGACGCAGGTAACGCCAGCAGGGCAAGAAATAATTCGTATCGCTCGCGAAGTCCTGTCGA
AAGTCGATGCCATAAAATCGGTTGCCGGAGAGCACACCTGGCCGGATAAAGGTTCACTGTATATCGCCACCACGCATACC
CAGGCACGCTACGCATTACCAAACGTCATCAAAGGCTTTATTGAGCGTTATCCTCGCGTTTCTTTGCATATGCACCAGGG
CTCGCCGACACAAATTGCTGATGCCGTCTCTAAAGGCAATGCTGATTTCGCTATCGCCACAGAAGCGCTGCATCTGTATG
AAGATTTAGTGATGTTACCGTGCTACCACTGGAATCGGGCTATTGTAGTCACTCCGGATCACCCGCTGGCAGGCAAAAAA
GCCATTACCATTGAAGAACTGGCGCAATATCCGTTGGTGACATATACCTTCGGCTTTACCGGACGTTCAGAACTGGATAC
TGCCTTTAATCGCGCAGGGTTAACGCCGCGTATCGTTTTCACGGCAACGGATGCTGACGTCATTAAAACTTACGTCCGGT
TAGGGCTGGGGGTAGGGGTCATTGCCAGCATGGCGGTGGATCCGGTCGCCGATCCCGACCTTGTGCGTGTTGATGCTCAC
GATATCTTCAGCCACAGTACAACCAAAATTGGTTTTCGCCGTAGTACTTTCTTGCGCAGTTATATGTATGATTTCATTCA
GCGTTTTGCACCGCATTTAACGCGTGATGTCGTTGATGCGGCTGTCGCATTGCGCTCTAATGAAGAAATTGAGGTCATGT
TTAAAGATATAAAACTGCCGGAAAAATAATTTCTTGCGTTATTTTCGGCACCTTTTATGTAGCGAAGGTGCCGGAATATA
TTCTCTTTTGTTACTTATTTCTATACCGTGCACAATATTTTTATCCCTCATATTTATAGGGTAAATTACCTGTAAAGTAG

TGTCTAATTACCCGCCTCGCAAACTCCAGTTTTCAAATATCTATTTCAGGTCAAAAGATTGAATTATTCACTGGAGACGA
TTCGTAAATTCGCTGGATTTTTGACTAAAGTTTCTTTAGGATTTGTCTCATCGTATGATTAATTACACCAATCGTTCAAC
GTCTTGCTGATAAGTGATGGCGATTATATGAGGTTAGCAATGCCTTCTGGAAATCAGGAACCCCGCCGCGATCCTGAATT
AAAACGTAAAGCCTGGCTGGCGGTTTTTCTTGGTTCTGCACTTTTCTGGGTGGTTGTCGCACTGCTGATTTGGAAAGTGT
GGGGATAACTATGGTCGGTCAGGAGCAACTGGAGTCGTCACCATTATGCCAGCATAGTGACAATGAAACAGAAACGAAAC
GGGAATGTTCCGTCGTTATTCCAGACGACTGGCAACTAACATCGCAGCAGCAAGCCTTTATAGAACTGTTTGCTGAAGAT
GATCAGCCGAAACAATAATTATCATCATTCTTATTACCCATTTTTAATGAATTAAAGGGCTTTTAATACACCGCAGCAAT
AACAGCTTGAGTTATCTCAACACAAAATAATAACCGTTAAGGGTGTAGCCTATGATCAACACAAATATGAAATATTGGTC
CTGGATGGGCGCGTTTTCTCTGTCGATGCTCTTCTGGGCCGAACTCCTCTGGATCATTACTCACTGATCCTTGACCCCGC
TGCGGCGGGGTTGTCATTTGCTTTGCCACAAGGTTTCTCCTCTTTTATCAATTTGGGTTGTTATCAAATCGTTACGCGAT
GTTTGTGTTATCTTTAATATTCACCCTGAAGAGAATCAGGGCTTCGCAACCCTGTCATTAAGGAGGAGCTATGTCGTCAA
CCCTACGAGAAGCCAGTAAGGACACGTTGCAGGCCAAAGATAAAACTTACCACTACTACAGCCTGCCGCTTGCTGCTAAA
TCACTGGGCGATATCACCCGTCTACCCAAGTCACTCAAAGTTTTGCTCGAAAACCTGCTGCGCTGGCAGGATGGTAACTC
GGTTACCGAAGAGGATATCCACGCGCTGGCAGGATGGCTGAAAAATGCCCATGCTGACCGTGAAATTGCCTACCGCCCGG
CAAGGGTGCTGATGCAGGACTTTACCGGCGTACCTGCCGTTGTTGATCTGGCGGCAATGCGCGAAGCGGTTAAACGCCTC
GGCGGCGATACTGCAAAGGTTAACCCGCTCTCACCGGTCGACCTGGTCATTGACCACTCGGTGACCGTCGATCGTTTTGG
TGATGATGAGGCATTTGAAGAAAACGTACGCCTGGAAATGGAGCGCAACCACGAACGTTATGTGTTCCTGAAATGGGGAA
AGCAAGCGTTCAGTCGGTTTAGCGTCGTGCCGCCAGGCACAGGCATTTGCCATCAGGTTAACCTCGAATATCTCGGCAAA
GCAGTGTGGAGTGAATTGCAGGACGGTGAATGGATTGCTTATCCGGATACACTCGTTGGTACTGACTCGCACACCACCAT
GATCAACGGCCTTGGCGTGCTGGGGTGGGGCGTTGGTGGGATCGAAGCAGAAGCCGCAATGTTAGGCCAGCCGGTTTCCA
TGCTTATCCCGGATGTAGTGGGCTTCAAACTTACCGGAAAATTACGTGAAGGTATTACCGCCACAGACCTGGTTCTCACT
GTTACCCAAATGCTGCGCAAACATGGCGTGGTGGGGAAATTCGTCGAATTTTATGGTGATGGTCTGGATTCACTACCGTT
GGCGGATCGCGCCACCATTGCCAATATGTCGCCAGAATATGGTGCCACCTGTGGCTTCTTCCCAATCGATGCTGTAACCC
TCGATTACATGCGTTTAAGCGGGCGCAGCGAAGATCAGGTCGAGTTGGTCGAAAAATATGCCAAAGCGCAGGGCATGTGG
CGTAACCCGGGCGATGAACCAATTTTTACCAGTACGTTAGAACTGGATATGAATGACGTTGAAGCGAGCCTGGCAGGGCC
TAAACGCCCACAGGATCGCGTTGCACTGCCCGATGTACCAAAAGCATTTGCCGCCAGTAACGAACTGGAAGTGAATGCCA
CGCATAAAGATCGCCAGCCGGTCGATTATGTTATGAACGGACATCAGTATCAGTTACCTGATGGCGCTGTGGTCATTGCT
GCGATAACCTCGTGCACCAACACCTCTAACCCAAGTGTGCTGATGGCCGCAGGCTTGCTGGCGAAAAAAGCCGTAACTCT
GGGCCTCAAGCGGCAACCATGGGTCAAAGCGTCGCTGGCACCGGGTTCGAAAGTCGTTTCTGATTATCTGGCAAAAGCGA
AACTGACACCGTATCTCGACGAACTGGGGTTTAACCTTGTGGGATACGGTTGTACCACCTGTATTGGTAACTCTGGGCCG
CTGCCCGATCCTATCGAAACGGCAATCAAAAAAAGCGATTTAACCGTCGGTGCGGTGCTGTCCGGCAACCGTAACTTTGA
AGGCCGTATCCATCCGCTGGTTAAAACTAACTGGCTGGCCTCGCCGCCGCTGGTGGTTGCCTATGCGCTGGCGGGAAATA
TGAATATCAACCTGGCTTCTGAGCCTATCGGCCATGATCGCAAAGGCGATCCGGTTTATCTGAAAGATATCTGGCCATCG
GCACAAGAAATTGCCCGTGCGGTAGAACAAGTCTCCACAGAAATGTTCCGCAAAGAGTACGCAGAAGTTTTTGAAGGCAC
AGCAGAGTGGAAGGGAATTAACGTCACACGATCCGATACCTACGGTTGGCAGGAGGACTCAACCTATATTCGCTTATCGC
CTTTCTTTGATGAAATGCAGGCAACACCAGCACCAGTGGAAGATATTCACGGTGCGCGGATCCTCGCAATGCTGGGGGAT
TCAGTCACCACTGACCATATCTCTCCGGCGGGCAGTATTAAGCCCGACAGCCCAGCGGGTCGATATCTACAAGGTCGGGG
TGTTGAGCGAAAAGACTTTAACTCCTACGGTTCGCGGCGTGGTAACCATGAAGTGATGATGCGCGGCACCTTCGCCAATA
TTCGCATCCGTAATGAAATGGTGCCTGGCGTTGAAGGGGGGATGACGCGGCATTTACCTGACAGCGACGTAGTCTCTATT
TATGATGCTGCGATGCGCTATAAGCAGGAGCAAACGCCGCTGGCGGTGATTGCCGGGAAAGAGTATGGATCAGGCTCCAG
TCGTGACTGGGCGGCAAAAGGTCCGCGTCTGCTTGGTATTCGTGTGGTGATTGCCGAATCGTTTGAACGAATTCACCGTT
CGAATTTAATTGGCATGGGCATCCTGCCGCTGGAATTTCCGCAAGGCGTAACGCGTAAAACGTTAGGGCTAACCGGGGAA
GAGAAGATTGATATTGGCGATCTGCAAAACCTACAACCCGGCGCGACGGTTCCGGTGACGCTTACGCGCGCGGATGGTAG
CCAGGAAGTCGTACCCTGCCGTTGTCGTATCGACACCGCGACGGAGTTGACCTACTACCAGAACGACGGCATTTTGCATT
ATGTCATTCGTAATATGTTGAAGTAACAACTATTTGCTTGCCGGTTATTTTGCTTCCGGCAAGCAAATGAATTACACAAT
GCAAGAGGGTTATTTGTTCAGCAAATGGCCCATTTTCTCGGCTTTGGTATCGAGATAATGTTCGTTATTGGGGTTACGAC
CTACAATCAATGGTACGCGTTCAACAATATTAATCCCTGCTTCGGTCAGAATTTCGACTTTTTTCGGGTTATTGGTTAAC
AAGCGGACTTCATTGACGCCAAGGAGTTTGAACATATCAGCGCAAAGAGTGAAGTCGCGCTCATCAGCGGCGAAGCCTAA
CTGGTGGTTAGCCTCTACGGTATCGTAACCTTGATCCTGCAGTGCGTAAGCGCGGATTTTATTCAGCAGACCAATGTTAC
GACCTTCCTGACGGTGATACAGCAAAATACCACGGCCTTCCTCGGCAATTTGCGTCAATGCCGCTTCGAGCTGGAAGCCA
CAATCGCAGCGCAAGCTGAACAGGGCGTCACCGGTCAGACATTCGGAATGGACGCGCGCAAGTACCGGGGTATGCCCGGA
AATATCGCCATAGACTAGCGCGACATGATCGTGTCCGGTTGCCAGTTCTTCAAATCCCACCATCAGGAAATCGCCCCATG
GGGTTGGCAGTTTGGCTTCTGCCACACGTTTAAGCTGCATGAAATTCTCCAGATAATGCTGGTTCTGTATTGGCTTATTT
TGCCATAACGAGAAGGGGTTCACCTAATCACGACGCGTCGATCGTTCACGGAATGGCACAAATCTGTCAATTTTTCCTGG
AACTGGCGTTTTCAGTTATGATTGTGGGACTTATCAAAAAGGAGAGGCCATGCGTTCGATTGCCAGACGTACCGCAGTGG
GAGCTGCACTATTGCTTGTCATGCCAGTAGCCGTATGGATTTCTGGCTGGCGTTGGCAACCTGGAGAACAAAGTTGGCTA

CTAAAAGCGGCTTTTTGGGTTACTGAAACTGTCACCCAGCCCTGGGGCGTCATTACACATTTGATTTTATTCGGCTGGTT
TCTCTGGTGTCTGCGTTTTCGCATTAAGGCTGCCTTTGTATTATTTGCCATTCTGGCGGCCGCAATCCTTGTGGGACAAG
GCGTTAAATCCTGGATCAAAGACAAAGTCCAGGAACCACGACCTTTTGTTATCTGGCTGGAAAAAACACATCATATTCCG
GTTGATGAGTTCTACACTTTAAAGCGAGCAGAACGCGGAAATCTAGTGAAAGAACAGTTGGCTGAAGAGAAAAATATCCC
ACAATATTTGCGTTCACACTGGCAGAAAGAGACGGGGTTTGCCTTTCCTTCCGGTCACACGATGTTTGCTGCCAGTTGGG
CACTGCTGGCCGTTGGTTTGCTGTGGCCGCGTCGGCGAACGTTAACCATTGCTATCTTGCTGGTCTGGGCAACGGGAGTC
ATGGGAAGCCGCCTGCTGCTCGGGATGCATTGGCCACGCGATCTGGTAGTAGCTACGTTGATTTCGTGGGCGCTGGTGGC
GGTGGCAACGTGGCTTGCGCAACGAATTTGTGGGCCATTAACACCACCTGCGGAAGAAAATCGCGAAATAGCGCAACGAG
AACAAGAAAGTTAACGCTGGTTGATTTTCCGAATTTAGCCCTTAAATCATCAACAATGCGTGTGGATGCCATTTCGCAGA
CGGCGCGAAAATGGTACTTTAAAGGGCTATTGCGGTAAGTTGACCATAATTTATTCGCTCTAACCACATAACGGGAAGTA
ATGTGAAATATTTACTCATTTTCTTACTGGTGTTAGCGATCTTCGTGATTTCGGTCACGTTGGGTGCGCAGAACGATCAA
CAGGTGACGTTTAATTATCTGTTAGCGCAAGGGGAGTACCGTATTTCCACATTGCTGGCGGTATTGTTTGCTGCGGGGTT
TGCTATCGGTTGGTTGATTTGTGGCCTGTTCTGGCTGCGAGTTCGTGTTTCCCTGGCGCGCGCTGAACGTAAAATAAAGC
GACTGGAAAACCAGCTTTCACCCGCGACTGACGTGGCTGTAGTGCCGCACTCGTCAGCGGCGAAGGAATAACTTTCTATG
CTGGAGTTGTTGTTTCTGCTTTTGCCTGTAGCCGCTGCCTATGGCTGGTATATGGGCCGCAGAAGTGCGCAACAAAACAA
GCAAGATGAAGCCAACCGCTTGTCGCGTGATTACGTAGCGGGGGTTAACTTCCTGCTTAGTAATCAACAGGATAAAGCGG
TAGACCTGTTTCTCGATATGCTTAAAGAGGATACAGGCACCGTTGAAGCCCACCTTACGCTCGGAAACCTGTTCCGTTCG
CGTGGCGAAGTTGATCGCGCTATTCGCATCCATCAGACCCTAATGGAAAGCGCCTCGCTGACCTATGAACAGCGTCTGTT
GGCGATTCAACAACTGGGGCGTGATTACATGGCCGCCGGGTTATATGACCGCGCGGAAGACATGTTCAATCAGCTGACCG
ATGAAACTGACTTCCGCATTGGCGCGCTGCAACAGTTGCTACAAATCTACCAGGCTACCAGTGAGTGGCAGAAAGCAATT
GATGTTGCCGAACGCCTGGTGAAGCTGGGTAAAGATAAACAGCGCGTCGAAATTGCCCATTTCTACTGTGAGTTAGCCCT
GCAGCATATGGCCAGCGACGATCTCGATCGTGCCATGACCTTGCTAAAAAAAGGGGCGGCGGCAGATAAAAACAGCGCCC
GCGTATCCATAATGATGGGACGCGTGTTTATGGCGAAAGGAGAATACGCCAAAGCCGTCGAAAGTCTGCAACGCGTGATA
TCCCAGGACAGAGAACTGGTCAGCGAAACGCTGGAAATGTTGCAAACCTGCTATCAGCAGTTGGGTAAAACTGCCGAATG
GGCAGAATTCCTGCAGCGTGCGGTGGAAGAGAACACCGGTGCCGATGCTGAATTGATGCTTGCTGATATCATCGAAGCGC
GCGACGGTAGTGAGGCCGCACAGGTCTATATTACGCGCCAGCTTCAGCGTCATCCGACCATGCGTGTGTTCCATAAGTTA
ATGGATTACCACTTAAATGAAGCGGAAGAAGGGCGTGCCAAAGAGAGCCTGATGGTGCTGCGTGACATGGTTGGCGAGAA
GGTACGTAGTAAGCCTCGTTATCGTTGCCAGAAATGTGGTTTTACCGCATACACCCTCTACTGGCATTGTCCGTCTTGTC
GGGCCTGGTCAACCATTAAACCGATTCGCGGTCTTGATGGCCTGTAATTTTTAAAAAAAATCCGACTTTAGTTACAACAT
ACTAATTATTAATGTTCCATTGTGCTCCGGCAACGACGGCGCAGAAAAAGCCTGCCAGGGGAGAAATCGCAACTGTTAAT
TTTTTATTTCCACGGGTAGAATGCTCGCCGTTTACCTGTTTCGCGCCACTTCCGGTGCCCATCATCAAGAAGGTCTGGTC
ATGACGTTAACTGCTTCATCTTCTTCCCGCGCTGTTACGAATTCTCCTGTGGTTGTTGCCCTTGATTATCATAATCGTGA
TGACGCGCTGGCCTTTGTCGACAAGATCGACCCACGCGATTGTCGTCTGAAGGTCGGCAAAGAGATGTTTACATTGTTTG
GGCCACAGTTTGTGCGCGAACTTCAACAGCGTGGTTTTGATATCTTTCTTGACCTGAAATTCCACGATATCCCCAACACT
GCAGCGCACGCTGTCGCTGCTGCAGCTGACTTAGGCGTGTGGATGGTGAATGTTCATGCCTCTGGTGGGGCGCGTATGAT
GACCGCAGCGCGTGAGGCACTGGTTCCGTTTGGCAAAGATGCACCGCTTTTGATTGCTGTGACAGTGTTGACCAGCATGG
AAGCCAGCGACCTGGTCGATCTTGGCATGACACTGTCACCTGCAGATTATGCAGAACGTCTGGCGGCACTGACGCAAAAA
TGTGGCCTTGATGGTGTGGTGTGTTCTGCTCAGGAAGCTGTGCGCTTTAAACAGGTATTCGGTCAGGAGTTCAAACTGGT
TACGCCGGGCATTCGTCCGCAGGGGAGTGAAGCTGGTGACCAGCGCCGCATTATGACGCCAGAACAGGCGTTGTCGGCTG
GTGTTGATTATATGGTGATTGGTCGCCCGGTAACGCAATCGGTAGATCCAGCGCAGACGCTGAAAGCGATCAACGCCTCT
TTACAGCGGAGTGCATGATGAGTGATTCCAACAGCCGTCTGGTCTACTCAACGGAAACCGGACGTATTGATGAGCCCAAA
GCGGCCCCTGTACGCCCTAAAGGCGACGGTGTGGTGCGTATTCAGCGTCAGACCAGTGGACGTAAAGGTAAGGGCGTTTG
CCTGATTACCGGTGTCGATCTCGATGATGCCGAACTGACAAAACTTGCAGCGGAACTGAAGAAAAAATGCGGCTGCGGCG
GAGCAGTTAAAGATGGAGTTATTGAAATCCAGGGCGATAAGCGTGATTTATTAAAATCACTACTTGAAGCGAAAGGGATG
AAGGTAAAACTCGCAGGCGGTTAACATAAAAAGCCACGGATATATCCGTGGCTTTCGAATATTTTACTGTGCGTATTATT
CAGTTATTTCGTACTGAACAGAATCAAGCGTATTATAATTGCGAGTCGATAATCGACATTTATTTACGATTATTTACCGA
CCTGGTGACCAATAACACCTCCGACAGCTGCACCACCTAATGTACCCAACGTACTGCCATCGGTCAGTACTGCACCGCCT
AATGCCCCTGCACCCGCGCCGATTGCGGTGTTGCGGTCCCGTTTAGACCAGTTAGAACAGGCACTCAGAGACATTGCCAA
AGTAATTGCCAGAACAGCCGCGGTCATTTTTTTGCTCGTTACAAACATAATACTCTCTCCTGAATTTATGATTCACGGAA
GTAAGCTCTCTATAACTATAATAGCTAAGAATAAGTCTGGTGAAATTATTCCGTGAAATCTGCTCGCGCGCAGGGTTATA
TCACGCAGGTGATAATGACTTCCTGTTATATCGCTGATAATAATTTTATATCTTGAGAGTGTTAATAACAGGTAAATAGT
CTTAATTATCAACCAGGAATCATCTTAGAGCGGATGATTTGCCAAACTGCAAATCATCCGTAGAGAAGGGAAATGGTTAA
ATGTCAACGACGTGAATGGTGAGTTTACTGTGCTCAAGATGCATCAGATCGCTGGCGCGTATTTTCGAATCGGTAATCAC
GCGATTGAATCGCTCAACGGGACCGATGGAGTATGAATGTACAGCACCAAATTTCGAGCTGTCAGTCAGGACTATCGCTT
CGCACTCTTTTTCCAGCACGGCATTGACCACATCGGTACGCATCATATCGCGACCGGTAAATCCAGTTTCAGGTTGCCAG
CCATCAATACCAATAAATGCTTTGCTGAAATGCACCTGTTGGATGCACTGGCGTGTCAAAGGGCCAACCATACTTTCGCT

TTTTTTCTGATACACGCCACCGAGCAAAATAACTTCACAAGGCGCGTCTTTCAGCAAATGCGCGATGTAGCTGCTGACCG
TGATGATAGTGACATTTTTCTTCTGCTCGCCCAGAGTCCGGGCGAGTAGGGCATTGCTGCTGCCATTTTCGATAAAGATG
GTTTCGCCCGGTTGAACCAGCGACGCGGCAAACTCGGCGAGTTCACGCTTCAGCGTATAGTTGCTCATCATACGGGTTTC
GACGTCATCACTATCAAGCGAAACAGCAAAGCCATGTGCACGGCGGAGGTAACTCAGTTTTTCGAGTGTGTTGAGATCCT
GGCGAATGGTAACTTCAGAAACTCCAGTGGCTTTTGCCAGATCGGTTACGCTAACCTGACCCTGGTCAATGACCATCTGT
AGAATAGTTTGTTGTCGGGAATTCATCGCTGTAATTCTTATAACGTTATAATACTTAATAAAAAAATACTGACGCGCCGG
GCATCCTCAAATAGGAAAACCTGAAGGCACGATTGTTAAATCTCCCACGGTGCCTTCGGGATGTCTGGCGTAGATTCAGC
AACCTGGCGCAAGAGTTCTGCTTTAAGTATCTCGAGATTATGGATCGCAGAGTGGTAATCGCCTGCCACAAGGATATCTA
ACACAATATCAATACGTTCGGCAACGCGCTGGGCATCAAATTCAGACATCAAGGACATCCTTATAGCAAAAGGGGGAAAA
TGAATAATGCAAAAATTGCCGCTAAAAGAGAAGTGTTTAACAGCAACGGCTAATTATCATCCAGGAATACGATATATAAT
GACGGGATATAGCGCTAAGTATATATATTCATCTACTTATGCGCGCTTCAGATAGCGTTTATACCAGCGTTCGAAGGCGA
CGGCGGGCATCGGTTTGGCAAACAAAAATCCTTGCCGCTCATTGATCCCGTTCTTGGTTAAAAAAGCATCTTCCTTTGCA
CTCTCTACACCTTCGGCGATCACCTGAAGATTCAATGCCTGGGCCACAGCGACGATCGCCCGGACCAGTGACTGCGAGAC
AGGTTGTTTGTGAATATCTCGAACAAAAACCTGGTCAAGTTTGATGGCATCGATCGGAAAGCGCGCCAGTTGCGAAAGTG
AAGAGTAGCCGGTACCAAAATCATCCAGATGCACTTGCGCACCTAGTTGGCTAAATTGTTGAATAACAGACAGTGCCAGT
TCATCATTCTCAATCAGACAACTCTCTGTCAGTTCAACATCTATAGGGCAGTATTCAAAATTGAGTTCCTGGAGAACCTG
TTTCAGGGCGGTGAAAATGGTTTGATCGGCGAGCTGACGTGCAGAAATATTTACCGCCACTCGCAGGTTTATGCCTTTAT
CCCGCCACTTTGCCACCTGGCGTACGACATCGAGAATCACCCAACGGCCTAAAGGCACAATTAGCCCTGACTCTTCGGCG
TAGGAAATGAAGTCCAACGGTGGAATCAACCCACGTTCAGGTGACTGCCAACGTACTAGTGCTTCCAGACTGCGCACTTC
GCCACGCCAGGTGATTTTCGGTTGATAGTGAATAACCAACTGATCGTTTTCCAGTGCTTTACGCAAGTTGGTATCCAGCC
AGAGATATTCAAATACCCGTTGATTCATTTCTGGGGTAAAAACGCAAAATTGTCCTCGTCCGCCTTCCTTCGCTGTGTAC
ATTGCTGTGTCGGCGTGACGAATAATAGCCGTGCTGTCTGAACCATGTTCGGGAGAGAGTGCAATACCTACTGAACAGCT
GGTATAAACTTCAATTAAACCAATGCGAAAGGGGAGCCGTAAGCGGGTCAAAATTCGTGATGCCATTGCTTCCAGCGCGC
TTTGTGAGGTGTTGGATGCCAGTACCAGAAACTCATCCCCACCTGGACGCGCCAACACCTGGTCATGTTCGAGACAGCTT
AAAATAGCCAATGACACGTCGCGTAATAACTGGTCACCAAACAAATGCCCATAGGCGTCGTTGACCTTTTTGAAATTATC
CAAATCAAGATACACAACCCCAACTTTATTGTTATCTGCATGATTAATAGCGTGATCGATTAAATCCTGCATTGCGTTAC
GATTCGGCAGTCCGGTGATACTGTCGGTATTTGCCAGAATACGCAGTCGCTCCTGAGCGCGGCGCTCTTCGGTAATGTCG
GTGCCGGAACAGATTAAAAAAATCTCGTTTTTGCCACTGCCGCTGTGGACAAATTTATTGCGAAACAGAAACAGCCGCTG
GCCTTTACATGTTGGTATCCACAGTTCGACTTCATATGCATTGCCGCTTCGAAAAAATACACGGTTATTGCGCCTGGATG
CCGCAGCTTCACGACGGCTCATAAACAGTTTAAACACGCTTTGCCCAATGACGTCGTGTTCTTTCAACCCTGTGTAATCT
TCACATAACCGATTGAAGCGTTGGATATTCCCCCGGCTGTCGAGAATCACTATTGCGGAATGTGCTTCAGAAACTACCTG
CTCAGCAAAGGAAAGCCCGTGTGATAAATCACGAGCAACCGCCGGTGTGTCATGCCATGCAGACGCGTTGCCAGCCCATT
CCCGTTTATTGATTTTTCGTCCTACCAGATGCACAGAAAGATCGCTTTCATCGACGGTCAGACTCATCATCAGGCTGGAG
GTGATGACCGTCATTTCCCTGATGCGAGCAGCCTGTTCGGCAGATAACTGCAATGTACGATCAGGTTCTGTGGTTTCGGT
GGTAGAAAAGCGCAAAACATCGCTGCTTTCCGTCAACCGCCAGTATGGATTGTGCGATCCGAGAAAGTTGTACAACGTTG
TGGACTCCCTAACGGTTTTCATGATGCATATCCTCCGGTTAACAGCGGAGTCCGCGGTTAAATAAAAGGAACAACATGAC
TTACATGAAATTAACGGCGGCTAAACGCATTGTTTTTTTTATATATTTATTTGTAATCCAGTTTTGGAAAAACGCCAGTT
TTCAAACGAAAGTCAGTTAAAAAATCTGCCTGGATATAACGAAGGTAGAGCGGGGAAATAAACGGCCCATCCATGAGGAA
TGGGCCGTGAAAGGAGATTACGCGACCGGGCGCGCAATAATGCTGCGGGTTTCCATGCGGACTTCGGCAATAGTGACGTC
AATAACGTCAGTCACTTTGTAAACCGTTTCACCTTTAATTTGTACAGTGCCGTTTTCCTGGCTGCAAACCAGTTCATCGC
GCACAGCGTGTAAGAAAGGTGCCGGAATAAAGGCGATAGCGCCGTTATCAACCAAACGAACACGCATGCCGCCACGGCTG
ATATCGACAATTTCCGCTGCGAAACGGGTGTCGGTCCCGGCTTTGTCTTTCAGGAAGCGTGCGTATAACCAGTCACCAAC
ATCACGTTCTGCCATCCGGTTGAGACGGCGACGCTCGGCCATTTGGACAGTGATTTCATCCTGTGGGCGCGTCGCAGTTT
CGCCTTTGATAACCGCTTTCAGCAGACGGTGGTTGATCATGTCGCCATATTTACGGATCGGCGAAGTCCAGGTGGCGTAT
GCTTCCAGACCGAGGCCAAAGTGAGGACCGGGTTCAGTGCTAATTTCAGCAAATGACTGGAAGCGACGAATGCGGCTGTC
GAGGAAACCAGTTGGTTGCGCGTCCAGTTCACGACGCAGTTTGCAGAAACCGTCCAGCGTGAGCACTTCTTCGGCATCGA
CATGCAGACCGTGCGTTTTCAGCAACGCTGCCAGCGCGTCGGCATTCGCCGGATCAAAGCCCATATGCACGTTATAGATG
CCAAAACCGAGCTTATCGCGCAGTACGCGGGCCGCACAAATGTTAGCGGCAATCATCGCTTCTTCGACGATACGGTTGGC
AATGCGACGAGGCTCGGCGACGATATCCAGCACTTCACCTTTTTCACCGAGAATAAAGCGGTAATCCGGGCGATCTTTAA
ACACCAGTGCGTGGTTATGACGCCACTCGCCGCGGCGTTGGCAAATTTGCGCTAGCAAACGGACTTGTTCGGCAATTGCT
TCACTTTCAGGCTGCCAGTCACCGGTATTTTCCAGCCAGTCAGAAACCTGGTCATACACCAGCTTTGCTTTGGATTCGAT
GGTGGCGGCAAAGAATTCGATATTATCTTCAATGGTGCCATCAGCGGAGAGCGTCATGCGGCATGCCAGTACCGGGCGGA
CTTCATTGGCGCGCAGTGAGCAGAGATCGTCAGAAAGCTCGCGAGGCAGCATAGGGATGTTGAAGCCAGGCAGATAGTTG
GTGAATGCGCGAATTTTCGCGGCTTTGTCCAGCTTGCTGCCTTCAGCAATCCACGCGGTTGGATCGGCAATCGCCACAAT
CAGCTGAAGTTTGTCATCCGGCAACGCCTTAGCGAAAAGGGCGTCATCCATATCTTCTGTGCTGGCACTGTCGATGGTGA
CAAAATCCAGCGCGGTCAGATCTTCACGAACCAGACCTTCATCGAGCATTTCGGTAGCGACGCCGTCTGGTGCTTCTTTT

TCCAGATTATGGCGTGCAAGGGTAACCCACCACGGTACAAAGTGATCGTCACCAAAAGTGATGTATTGTGTCAGTTCTGC
ATAGAAAGAACGATCGCCTTTCAGCGGATGACGGCGCATTTCGGCAACCGCCCAGTCGCCTTCTTTAAACTCGTGGTTCA
GGCCACGGGCTGCGCGGCAAGGAATGGCGTCTTTTAAGAGTGGATGATCAGGAACGATGGCCAGACGGTCATTTTTGCCC
TGAACCTTACCCACGAAACGAGTCAGGAACGGTTCAACCAGTTCTTCTGGCTCTGCGGATTCACGTTCTTTTTCACTGTG
GATCACCGCGATAATTCGGTCGCCATGCATGACTTTTTTCATCTGCGGCGGCGGAATGAAATAACTTTTTTGCGCGTCGA
CTTCCAGGAAGCCAAAGCCTTTTTCTGTGGCTTTTACCACCCCTTCAGCGCGTGGCGTCTGGGAATGCAGTTGCTGTTTA
AGCTGCGCTAGCAGCGGGTTGTCCTGAAACATAATTGTCTATTTTGGTGGCCATTAGAGCGGCTGACAGTTTTACGCGAA
TCTGTCTGACGCGGCAAGGTTAATATGTCTCACCCAACGCGATTTTTAAGCGATTTATCCAGCCACACAGCCCGCTCCAT
ACCAGCAGATTAATAATCTGCGTTGATGATTTTTCGTGTTCGAGTAAAGGGCTGAACTGGGCGGCGCTAAAGCGATCGGG
TGCTCGGGTCAGCCATTGTATCGCCTGAACGGTAGCGCGCTCAACAGAATGACGGTCAGCCCATTGGTTAATTTCATTTT
CGCCCTTGCGCATAAGGGTGCTGATTTTTTTATATTCATTTGATGAATCCATACAGGTGGCAAAACAAGCAGGACTGCCA
TTTATACGCGCGCATAACAACGCCGCAAGCTGATTATCCGGCTGTGTATTGCCCCGTGTGCTGTTTAACAACTTTTCGAG
AAGGGAAAGTGAAATAGGGTGGAGAGAGAGTATTTCGGCCAGTTGGCAAAGTTCTGCAGTTGAACTTTTTGTGTGCTCTT
CAGGTAGTTTTTCCACCTCATAGCTGCTTCGCCAGCGTAATGATTCATCAGCAAACAGTGACGCGTCGGCGTAGTTTTGT
ATCTCCAGCCCGGGTAACCAGCGTACCGGATGCCCGAGATAAGCCTGAAATGTCGCAATTGTCCGCGCCTGAAAGCCAAT
AAAACCAATGATTTGGCTAATGATCACACAGTCCCAGGCAGTAAGACCGACGTCATTCAGATGCTGGCGCGATCGGTTGT
CGATAATAGAAGGCGAGCTAGCCAGTTGGCGGGCATATTGCGTGATTTGTGCCAACCGATGATTACTTTCCCTGGTGGAA
TCAGGCCCGGGGAGCGGCGTAAGTCGCGCCGAATAGTAGTTACATAACCGCTGGACGCCGCAAACCTGAGCAACCGTCAG
GGCCGTCGACAAGCGTTCATAAGCGGTGAAGGTCTGTGAACGGGTCACGGTAAGCTGGTCAGGGAACAAATCCACTGCAA
GAACACGCGCAGGGTCCAGCCATGAAAGGAAGGGGGGCCAGCGTTTCTTTTTCAAGGATCACGTCGAGTAGAAAGGGATCG
CTGACCTTTGCGGCTTCCGGGACCAGAGGCAGAACGTCATACTCCGTAGTACTGGATTGCGTTTCATGATACCAGTGGCT
TTTGCCGGTGATGTGGCGTTGTTCCATGGGCGCTCCTTGGTCGTAAAGGAAATCGTTATCCTGACGCAAGGCGGGAAGGG
GAGAAAGACGGATCGGCGATAACAAATATCAGAAAGGTATAACAGAGATAACGGGCGGCAGAACGCCGCCCATCTTTACC
AACAGAACGATTATTTCAGTTCGAGTTCGTTCATTGCAGCAATGCTGAAACCGCCGTCAACGTGGACCACTTCACCGGAG
ATACCGGCAGAGAGATCGGAGCACAGGAATGCCGCAGAGTTACCCACATCTTCAATAGTAACGGTACGGCGAATCGGGGT
AACGGCTTCGCAATGAGCCAGCATTTTGCGGAAGTCTTTGATACCGGAGGCCGCCAGAGTACGGATCGGACCAGCAGAGA
TGGCGTTAACACGCACACCTTCCGGACCCATCGCGTTCGCCATATAGCGCACGTTCGCTTCCAGAGACGCTTTTGCCAGA
CCCATAACGTTGTAGTTCGGGATAGCGCGCTCAGCGCCAAGGTAGGAAAGGGTCAGCAGGGCAGAACCCGGATTCAGCAT
GGAGCGGCAAGCTTTTGCCATTGCAACGAAGCTGTAGGAGCTGATGTCGTGGGCAATTTTGAAGCCTTCACGGGTAACGG
CGTTAACATAGTCACCATCCAGCTGATCGCCAGGTGCAAAACCAATAGAGTGTACGAAACCGTCAAATTTCGGCCAAACT
TTCCCCAGTTCAGCGAACATGGTGTCGATGCTGGCATCTTCTGCAACATCGCACTGCAGAACGATGTCAGAACCCAATTG
AGCGGCAAATTCTTCTACGCGGCCTTTCAGTTTGTCGTTCTGGTAGGTGAATGCCAGTTCAGCTCCTTCGCGGTGCATCG
CCTGAGCGATACCGTAGGCGATGGATAGTTTGCTGGCAACACCGGTTACCAGAATGCGCTTACCGGAAAGAAAACCCATA
GCTTTAATCCTTATTGTTGATGCTTGTTGTGCCTGAAAATCAGGCGATTCGTCGTTTTAGTAAACAGTACGAACAGATAA
ACGGTTATTATAATCAACCTGGCTGTGAGTAGCTATAGTTGCCAGGTCCGACCGGAGCAGGCTGCGGCAGGGGGGGCGCT
TTTCCCCTCACCCTAACCCTCTCCCCAGAGGGGCGAGGGGACCGTATTGTGCAAATATTGTTACCCCAGCAACAAACAGG
CTCATACAGCCCCTAACCCTTTCATGGCGATGGCTGGGACGGTTCAGACCTTGCCGAATATTCTCCAGCACCGTCTCCAT
GTTTTCACCACAACAGCTATTCGGCTCGGTCTGCCCCCTCGCTCTTTCAGGGAGAGGGTGACCGGCGGTTCAGTTCCTGC
AGAATATTCTCCAGCACCGTCTCCTCATTACAATCAATCTCGTTATTCCAGAAACGCAGCACGGTCCAGCCCTGCGACTC
AAGCCAGCTAGTGCGCCTGGAATCATAGGCAACTGCTAAATCATGCTGCCCACCATCCAGCTCAACGACTACACGCGCCG
AGCAGCAAGCAAAATCGAGAATGTAGCTCCCCACTGGATGTTGACGGCGAAATTTGAAATCACTAAAACGTCGGCTGCGA
AGATATCGCCAGAGCTTTCGTTCCTGCAAAGTGAGATTGCGTCTTAAATCACGCGCATTTGATTTAATTTTATCTATCAC
CTCATTCTGACAAGATTTAATCTTTTGTCAGAATGAGGTGAATAATCTGGAAGGAGGATTCAGAAAATTAGCGATCTTTA
CGCCACGCATCCGCCGTCAATGCCTCGCCAAAATGACCGGCAATCAGCCGTTTGGTGAGTTCATGCAGCGGCGATGCCAG
CACATCCGCGGTGCTGCCTCGCTCGACAACCTCGCCCTGATGCATCACCAGCACCTGGTCGCTAATGTGCTTCATCATTC
CGATATGCTGGGTAACATAAATATACGAAATGCCCTGTTTTTCCTGTAATTCCAGCATCAGATTAATCAACTGCGAACGC
ATCGACATATCCAGTGAAGCGAGGGCTTCATCGGCAATAATGACTTTTGGGCGCAATATCAGCGCGCGCGCCAGACCCAG
ACGCTGTTTTTGTCCGGGTGCCAACATATGCGGATAGTAACTGACGTGATCCGGTAGCAGCCCAACCATACGCATCGTTT
CAATAATCTGTTTGCGACGCTGTTCCGGTTCCAGGTCGGTGTTCAGGCGCAGTGGAAAATCCAGAATTTGCGAGATACGT
TGACGGGGATTCAACGAGGTCGAAGGATCCTGAAAAATCATGCGAATACGCTGACTGCGGAAGGAATAATCGCCAAAATG
CAGTGGATGATCGTCAATCAATAACTCGCCGCTGGTAGGCTCTATCATTCCCGCCAGCATTTTTGCCAGCGTGGATTTAC
CCGAACCATTCTCGCCAATAATCGCCAGTGTCTGGCCTTCACGTAGCGTAAAGCTCAAGGGTTTTACCGCTTCTACGGTC
TGACGACGAAACCAGCCGGTCCGGTAGCGGAACGTTTTACTTAAATTACGCACTTCAAGCAGCGTTTCGATCATCTCACT
CTTTCTCCATGTTCAGCGGGAAATGACAGGCATAGAGATGATTTTTCGCCCCCGTCAAACGTGGCGTCACAATGCATTCT
CGTTGTGCATACGGGCAACGTGGCCCCAGACGACAACCAATCGGTAACTGTTCCAGCAGTGGGATAGCGCCGGGCAGCGT
ATTGAGGCGACTTTTATGCGGCATCGCGCTGCCGAAGTCTGGTATCGCGCGGATCAGCGCCTGGGTATAAGGATGATGTG

GCATCGTCACCAACTCCTTACTCGGCGCGGTTTCCACTGTTTGACCGCAGTAAAGCACGTTAATTTTATCCGCCCATTGG
CTAAGCATTTGTAAGTCATGGCTGATAAGCAAAATAGTGGTATTGCTGTTTTGGTTGAGACGCGTCAGCAGGCGAAAGAT
TTGCGCCTGGGTTGTTGGCTCCATTGAGTTGGTCGGTTCGTCAGCAATCAGCAGACGCGGTTGATTCGCCAGTGCAATGG
CTATCATCACTTTCTGACATTCACCTTCGGTCAACTCATAGGGAAAACTGCGCATCGCATCTTTGTGATCTTTAATCCCC
ACGCGGTGCAGCAGTTCAATGGCACGGCGTTTGCGCCAGCCAAAACGCTGCCACCAACGGCCTTTATAGGTCCAGGCTGG
GATGTTTTGCATCAACTGGCGGCCCACACGTTCTGAAGGGTCAAGACACGACTGCGGTTCCTGGAAAATCATCGACACGT
TATGGCCAACCAGTTTGCGCCGTTCGCGTGCGGAGAGACGCAGCAAATCGATATCATCAAAACGCATACGGTCAGCAGTA
ACACGCCAGTTATCTTTATTCACCCCACAAATCGCTTTCGCAATCAAACTCTTGCCGGAACCGGATTCACCAACAAGACC
GCGGATTTCACCTTCGGTTAAGGTCATACTTACCCGGTCGACGGCTTTAACCCACTCATCACCGGTTTTAAATTCAATGG
TCAGGTTACGAATATCGAGTAATGGCATTATTCCACCCCCGCAATAATCGCACGACGAACGCCATCGCCAAGGAGGTTAA
CCAACAACACGCTAATCATAATTGCCGCACCTGGCAGCATGACAGTCCACGGGGCGACATAAATCAGTTCCAGCGCATCA
CCGAGCATCGCTCCCCATTCAGGCGAGGGGAGTTGTGCGCCGAGATCGAGAAAGCCCAGCGCGGCGATATCGAGAATTGC
CATCGACAGTGCGCGGGTGATCTCGGTGACCAGCCCGGCGGTGATGTTTGGCATCACAGCAAACCAGAGAATATTCAGCG
TTGATGCGCCATCCAGACGGGCGGCGATAACGTACTCTTTTTCCAGTTCGTCATGCACCATGCTGTAAATCGAACGTACC
ATACGCGGCAGCAATGCCAGCCAGACAGCAAACATAGCGTGGGACAAACTCGGTCCGGCAAACGCTACAACGATAATTGC
CAACAGCAGCGAAGGGATCGCCAGTAAGGTATCCAGAATATGGTTAAGCACCGCTGAGCGGAGGCCGTGTGTCGCCCCGG
CGAAGGTGCCCAGTACCAGGCCACAGATCGTCGCGGCAAGCGTCACCACAAATGCGCCACCCACGGTTGGCGCAGCTCCG
CTCAGTAAACGGCTTAAAACATCGCGTCCGAGGTCGTCAGTCCCCAGGAAGAAAGAAACTTCGCCATAGCGCGACCATGA
CGGCGGCAATAATTGATAACCGAGAAATTGCTGGTCGATGCCGTAAGGCGCAAACCAGCCGCCAAAAATACACAGTACAG
CCAGCCCCGCGCAGCCGTACAGGCCGACCATCGCAGAGGCGTCACTATAAAATTTGCGCCAGGCGGTACGCAGCGTGCCC
GGCGGGCGCTTTTCGCTGTATACGCTATCGTAAGGCATACCATTCCTTATGTTTCAGAGGGTTAGCCATGGCACCCAAAA
TATCAGAAATCACGTTAACAATAATGACCAGTGAGCCACACACCATCACTCCGGCGGAAATGGCTGCGTAATCCTGCTGG
CGGATGGCGTTAATTAACCAGCGCCCTAAACCCGGCCAGCTAAAAACCATTTCGGTGATCATCGCCAGCGTCAGCATGGT
GGAAAACTGTAAGCCCAGGCGAGGAATAACCGGCGGTAACGCGTTATGCAGAACGTGGCGACGCAAAATAGTAAAGCGCG
ACAAACCGCGGGTCGCCGCCGCTTTTACATAGTTCTGGTCATACACTTCGATAGTGCTGATACGCATCAGGCGGATCACT
TCAGTTGTTGGCGCAACCGACAGGGTGATCACGGGCAATATCATATGGCGGATTGCGCTCATGATCATTTCATCCCGCCA
TGGTGAGTCCGAAAGCCAGGCATCAATCAACGCAAAACCGGTAATCGGTTTCACTTCGTAGAGCAGATCGAAACGCCCTG
AAACGGGCAACCAACCTAGCGTGAGTGAACAAAACAGCGTCAACAGAAGCGCCAGCCAGAACACCGGGATTGAAAAGCCC
AGTAAGGCGATGGCGTTGATCAAGTTATCCTGCCACTTATGGCGCGTAATCCCGGCAATCATCCCCACGGGGATGCCAAC
AATCAGCGCAAAACCGAAGGCGAGGATGCACAGTTCCATCGTCGCCGGGAAGACCTCTTTTAACTGTTCGGCAATCGGTT
GACCGTTAATGCTGGAAACACCAAAATCCCAGTGGATCAGGCCGTTAAACCAGAACACCCAGGCATTCCACAGTGACGCG
CCTTGTAACGGCGCATGAGGGGTGAAATAGCTCAAGCTGAAGCCAACGAAGGTCAGCAGGAACAAGGTGACAATCAATAA
CAAAATGCGGCGTAAGGTGAAGATAATCATGGTTTTTTCACCTCATCCTGTTTCTCGCGATACACCCCAGCAAAGGAGGC
GTTACCAAACGGGCTAAGTACCAGACCTTTGATATCGTACCGATAGGCCTGCAAACGCAATGACGACGCCAGCGGCAAAA
TGGGCAATTCCTGCGCCAGAATACTCTGCGCTTCGTCATAGGCTTCAATACGCGCCGCCAGCTGCTGCGAAGAGAGCGCC
TTACGCAATACGCTGTCGAATTTCGGATCGCACCAGTGGGCGAGGTTGGTCTGCGAATGAATTGCCGCGCAGCTCAGTAA
CGGACGGAAGAAACTGTCCGGGTCGTTACTGTCCGTCGCCCAACCGGATAACGTCAGATCATGGCTCATATCCATCAACC
GCGCCTCCTGAAAGCGACCTTCTACCGGCACAATCACCACTTTTACGCCAACCTGCGCCATATCCGCCTGAATCAGTTCG
GCAGTTTTCAGTGGACTGGGGTTCCACGCCTGCGAACGTGTGGGCACCCACAGTTTCAGCGTTAAATTTTCCAGCCCCAA
CGACTTCAACTGTTCGCGCGATTTCGCCGGATTGTATTCAGTAATTTTAGCCTCGTTGTCATAGGCCCACGAGGCGCGCG
GTAAAATAGAGGCCGCCGTTTCAGCCGTACCATAATAGATGGATTGCATCAGGCGCTGGTTATTAATCGCCAGTGCCAGC
GCATGGCGGACAGCGGGATTATTTAGCGGCGGTTTGGCGGTGTTAAATGCCAGATAGGCGACGTTCATCCCAGGACGCAG
CGTTAAACGCAAGCGCGGGTCGTCACGCAAAATGGATAGCTGGCTGGCAGCAGGCCAGGCCAGAACGTCGCATTCCCCGG
TCAGGAGTTTCGACAGACGTCCGGTGCCGCCGGAGCCTAAATCCACCACTACCTGCGGCATTAACGGTTTACCGCGCCAG
AAGTCATCATGACGTTGTAGGCGAATAAATTGCCCGGCGCGGTATTCCGACAACTGATACGGACCGGTGCCGACCGGTTG
ACGGTCGAGTTGCTCCTGGCGATCTTCTTTCTCTAACTTCCGGGCATATTCTGCCGACATGACCGAAGCATAATGGGTTG
CGAGGTGCCACAAAAAAGAAGCATCCGGCTGGGCGAGTCGGAACTCAACGGTATGATTATCCAGTTTGCGGACGCTTTTC
ACGTTATCGGCAAATTGCAGGCTGTCGAAGTAGGGGAAGTTGCTGCCGTTGACGTTATGCCACGGGTTGTTGCGGTCAAA
AATTCGCTGGAAGGTAAACACCACATCGTCGGCATTCATTTTACGAGTGGGAGTAAACCAGTCGGTTTTTTGAAACGGAA
CATCGCGACGCAGGTGGAAGCGATAGGTCGCGCCGTTGTCGAGTACTTCCCAGCTTTCGGCAAGTTCCGGCATCAGGCGA
TAGGTATAGGGATCGACATCCAGCAGTCGATCATAAAACTGGGCGGCAAGGGTATCGACAATTAACCCACTGCTCGCTTT
GGATGGGTTAAAGGTGTTGACTTGCCCGCTGACGCAATAGACAAAACCGCTGTCGCGGATATCAGCATGCGGGGGAGATT
CAGGCGCGGCGATTGCCTGACCACTCACAAGTCCAGCAATCACCAAAAGAGACGATAATACCTGGCGCATAATATTAAGG
GATTTTATGTAAAGAGGCTATCTTACTAATATTTAATGACATTTGCCATTACCGTTTGTGTTCAGGGGTCGTAATGAGGG
CCGTGTGGTTGGTCGTTGAACAGGTGACGTCGCCATCTGTTCAACATTCGTACCCGTGATTTCTCTATAACTATAACTCA
CAGAACAACTTAGCGAGGAGCAGAGCGGTACGATCGTGAGAAACCCATTAAAGCACGCTGCAGTGCGTGTCATTGTTAGC

CAGATGCGGCGTGAACGCTTTATCCGGACAACGATACTGACCGATCGTCTGCAATTAAATCATTACTCATTACCCCATTG
ATTCAAAAACTCTGCGATCTCATCAATGCGTACGGGATTAATCCCCGCTTCAGCAGCCATTTCATGTTGGGCTTCTTCGC
TGATCTCTTCATTGTTCATCAAACGGGTGAGCAGTAACTGGAAGTAGTGGGCCAGCGGTGTGCGTTCAGCCTCTGCGACA
GGCTCTGCGCATTCGGTCGCGTACTCATCGGCAATATCAAAATATTTTAGCGGGATATCGTGGTTCATTATTTGCCCCTG
GGGTTAATGCGCTAAGCATAAGGGCGGATGATAGCACTCGTGATCCGGGTCATACATCTTTTTATAGTTAATGGCGCGGC
GCGTTACCCTCAGGCAGGATAATGCGCCGCGCATCCGACTATTACGTTTCACTCACCGGCGTTCTGTCACCATCATACAA
CGGCACTTTGCGATAGCGGCGGATCAGATACCATAAATATGCGCCGCCTAACGAAGCCCATACCAGACCGAGTGTCAGTG
ACGTTGACTCGAGGTTAACCCACAGCACACCCACCGTCAGCGCGCCAACCAGCGGCATCAGCAAATAGTGGAAGTGATCC
TTCCAGCTTTTATTCATTCCTTTACGCCGCCAGAAATGATTAAACACCGACAGATTCACGAAGGTAAACGCCACCAGAGC
ACCGAAGTTAATTAACGCTGTGGCGGTAACTAAATCGAAGAACAGCGCCGACAACGCGACAATCCCGACCATAATGACGT
TGAGTGCCGGAGTCCGCCATTTTGGGTGCACATAGCCAAACACGCGCTCCGGAAACACATTGTCACGCCCCATCACATAC
AGCAGACGCGACACGCTGGCATGTGAAGCCAGGCCAGAGGCTAACGTGTTCACAAACGTGGTGCAGAGGAAAATCGACTG
GAAGAGCTTGCCGCCGACGTACAGCGCAATTTCAGGCAGTGCGGCATCCGGATCTTTAAAGCGGCTGATATCGGGAAAGA
ACAGCTGCATAAAGAACGACGCCGCGATAAAGATAACGCCACCGTAGACCGCCGTCAGGAAGATGGCTTTCGGGATCACG
CGTGCGGCGTCCGGCGTCTCTTCCGAAAGCGTGGTCACCGCATCGAAACCGAGGAAGGAGAAACAGACAATCGTCGCCCC
GGTAATAATCGGGATCAGGTGCGCGTTCTCGCTGATAAACGGCTGAAGTGACCAGACGGTGCCAACGCCTTCTCCTTTAT
GCAGTCCCTGAACTACCAGGAAGATAAACACCACCATGATGGAGATTTGCACCAGTACAAACAGGGTATTGAAGTTAGCG
ACCAGGTTGACGCTCTTCAGATTCGCGGCGGTTAAAATGGCGACGAAGGTTACCACCCACACCCACGGCGGCACTTCCGG
GAAGAGGGCGGAGAGATAGATTTTCGCCAACAAGACGTTAATCATCGGCAAAAAGAGATAATCCAGCAGCGATGACCAGC
CGACCATAAATCCGACGTGCGGGTTAATCGACTTTTGCGCGTAGGTATAGGCCGAACCGGCCTCCGGAAACTGGCGAACC
AGTTTGCCGTAGCTGATAGCGGTAAACAGCACACCCGCCAGCGCCAGCAAATAGGACGCCGGAACGTGACCGTCGCTAAT
GCCGGAGACAATACCAAAAGTATCAAATACGGTCATCGGCGTGAGATAGGCCAGACCCATCATCACCACCTGCCACAATT
TCAGTGATTTTCGCAGACGGGTTTTGCCGGGTTGCGCAGCAATATTCAGTGGTGAATTAATAGCCATAATTGCTGCCTCC
CCTGCGTAATAAGGTTTGCGGTCGTGTCGACCTGAAATCAAACCTGTTTAGCAAGGGAAGCAAAGGGGGCGGAGTCCAGT
CAGTAGCAGGAGGGGCGCATACGCAGCCTCCCTCGCCATGCGGGGATTTAATCATCGGCATCATCTCATTTCCTCGTCAC
AGTCTTAATCGTTAACGGAAGCCCGTTGCCGCCCGCGCTCCATAACGCGACATCCGGGAAATGTCTGGATTACCAGCAAA
TGTCAGGCCTGGCTCCGCTCAGGCCGATGAAACAACCCCGCAAGGGGTATTACGCGTTTTTCAACATCCACTCAATTTCT
GTTTCTGTGATGAGGCGCTCAAACTGCAACAGCTCATCATTTTTACAGGCGTGATAGACATGGCAGAAGCGTTCGCCTAA
ATAGCGGCGCAGGTGATCATTCTCGATAAACTCACCCAGGGCATCGCTCTGGCGAATCGGAAAGGGTAAGCCTTCCTGTT
CCAGCCCGTTGCCTTCGACTTCTTCCTGCAACGGCAGCTCGTTATCAAGGCCATGCAAAATACCGGCAAAAATCGCTGCC
ATCACCAGATATGGGTTGGCATCGGCACCCGCCACGCGATATTCCACGCGGTGATTATGACGGTCGCCGCACGGAATACG
CAGGGCGACGGTGCGGTTGTTATGGCCCCACGACGCCTGCGTCGGCACATACATTCCCGGCTGGAAGCGGCGATACGAGT
TCACGTTTGGTGCCAGCAACGCCATCGACGACGGCATCAGGTCAATCATCCCGGCGAGCATCTTTTTCAGCAGCGGCGAA
TCTTCGCCTTCCGCGTCAGAAAGCACGTTCTCGCCACGATTATTTTGCATACTGATATGGATATGCATTCCGCTGCCCGC
GTGCTCTTCATACGGCTTCGCCATAAAAGTGGCGTGCATCTTATGCTTTTCTGCCATCAGACGCACCAGGCGTTTTAGTG
CCAGCGCATCATCGCAGGCTTCCAGCACGTTATCAGTATGGTAAAGGTTGATTTCAAACTGACCCGGCGAGGCCTCAGCG
ACCGCGCCATCTGCCGGAATCAGCTGTAACTGCGCCAGTTCATCAATATCATTGAGCACGTCGGCGAAGTGGTTGAGGTT
ATCAACGGAGTAAACCTGGCTTTGTGTATTGCGGTCATCGGTGCCAGGCGCGCAGGGCGGTTGCAGATACCCTTCAGCGT
CGCGCTGGCGATCCAGTAAATAGAACTCCAGCTCTACCGCTACGACCGGGAACAATCCGCGCTGGCGCAGCTGCTGCCAC
AGGCGGTTAAGAACGTTCCGCGGCTCAACGTCAAAGGGAGCGCCATCTTCATCGACCATGGTCAGGAGCATCTGACCGAT
AAACTCTGGATCGGCGGCAGAAGGAGTTAAGGAACCGAGAACAGGAACACAGGTACGATCCGGCTCGCCCATTTCCTGAC
CCAGACCTGCTTCTTCTACTACGTTGCCCAGAATATCCATTGCAAATACCGAGGCCGGGAAATAACACCCTTTCTCGAGC
TTCTTCAGGCTTGAAACAGGAATACGCTTTCCACGGAAGCAACCATTTAAATCGGTAAGTAAAACATCAACATATTGCGT
ATTCGGGTAGCGCTCCAGGTAGCGTTTCACTTCCTGCGTAAAGGCGCTACCCCGCCTCTCTTCTGACTGCTGAACAAAGT
TCTCTACTTCAACGATATTGGTTTCCATGATTCTTCGCCTTTGGTTTGTTTTCCGCTCGTTATCAAAGCGTAAAATATAA
TGACCACCATTCGAATCTGTATGCAAACTAAATGTTTGTCAAATGTTAAATTGAGTTTGCAAAAATGAAAACCCACTGCT
AGATTGAAAAAATATTGAACATAAAGGTCATTTAAAGCGCAGTAATGGCGATAATTTAGTCCACTTTGTGAGATTGAGCA
TGGAAAATATAATGAACAATCCGGTTATCGGTGTCGTAATGTGCAGGAACAGGCTTAAGGGTCATGCGACCCAGACTCTG
CAAGAAAAGTACCTGAATGCCATCATCCATGCAGGCGGCTTGCCTATTGCGCTACCACATGCGCTGGCGGAACCGTCATT
ACTTGAACAACTTTTGCCGAAACTCGATGGCATTTATCTTCCTGGTAGTCCCAGCAATGTGCAGCCGCACCTATATGGTG
AAAACGGCGATGAGCCTGACGCCGATCCCGGGCGTGATCTTCTGAGCATGGCGATAATTAATGCCGCACTCGAAAGGCGC
ATCCCCATTTTCGCCATCTGCCGGGGTTTACAAGAACTGGTGGTGGCAACCGGTGGTTCGTTGCATCGCAAGCTGTGCGA
ACAGCCTGAATTGCTGGAACATCGGGAAGATCCCGAACTGCCGGTGGAACAGCAATATGCACCGTCGCATGAAGTTCAGG
TTGAAGAGGGGGGATTACTGTCTGCGTTGTTACCTGAATGTAGCAACTTTTGGGTAAACTCTCTACATGGACAAGGGGCG
AAGGTCGTTAGCCCACGGTTGCGTGTTGAAGCTCGCTCGCCGGATGGCTTAGTTGAGGCGGTTAGCGTCATCAATCATCC
TTTTGCGCTGGGCGTACAGTGGCACCCGGAATGGAACAGTAGCGAGTACGCGCTTTCGCGTATATTGTTCGAGGGCTTTA

TCACCGCTTGTCAGCACCATATCGCTGAAAAACAGCGACTCTGACCACTACAGTTTAAGGAAATGCAAATATGAGTGATG
AGGGACTGGCGCCAGGAAAACGCTTGTCGGAAATCCGCCAGCAGCAGGGGCTTTCACAACGTCGTGCCGCCGAACTCTCC
GGGCTGACTCACAGTGCTATCAGTACGATAGAACAAGATAAAGTCAGCCCTGCCATCAGTACGCTGCAAAAGCTGCTGAA
GGTGTATGGTCTGTCACTCTCGGAATTCTTTTCCGAGCCGGAAAAACCTGATGAGCCGCAGGTCGTCATTAATCAGGACG
ACTTAATTGAGATGGGTAGTCAGGGTGTGTCAATGAAGCTGGTTCATAACGGTAACCCGAATCGCACGCTGGCGATGATC
TTTGAAACGTACCAGCCGGGCACAACCACTGGGGAAAGAATTAAGCATCAGGGTGAGGAAATAGGCACTGTACTGGAAGG
TGAAATTGTTCTGACGATTAATGGTCAGGATTACCACCTCGTCGCGGGGCAAAGTTATGCCATTAATACCGGCATCCCGC
ACAGTTTCAGTAATACGTCGGCAGGTATTTGCCGAATTATCAGCGCCCATACGCCCACCACGTTTTAATCTTTTTGTTCT
GTAAGCCGGGTAAGCGCAATGCGCCCGGCAATCTATACACAAAATCATTCAAGTTGCATCAAGGCGGCAAGTGAGCGAAT
CCCGATGAGCTTACTCAGGTAAGTGATTCGGGGGAGCGAACGCAGCCAAGGCAGAGGCGGCTTGAAGGATGAAGTGTATA
TAAAGAATGTCGCCAGAAATAAACGGGCATACGGCCCGGGGATCTCTGCGCCCTGACGTTCACAAACTGCATATATCTGA
TAGACGTGAAACAGGAGTCATAATGAATTTTCATCATCTGGCTTACTGGCAGGATAAAGCGTTAAGTCTCGCCATTGAAA
ACCGCTTATTTATTAACGGTGAATATACTGCTGCGGCGGAAAATGAAACCTTTGAAACCGTTGATCCGGTCACCCAGGCA
CCGCTGGCGAAAATTGCCCGCGGCAAGAGCGTCGATATCGACCGTGCGATGAGCGCAGCACGCGGCGTATTTGAACGCGG
CGACTGGTCACTCTCTTCTCCGGCTAAACGTAAAGCGGTACTGAATAAACTCGCCGATTTAATGGAAGCCCACGCCGAAG
AGCTGGCACTGCTGGAAACTCTCGACACCGGCAAACCGATTCGTCACAGTCTGCGTGATGATATTCCCGGCGCGGCGCGC
GCCATTCGCTGGTACGCCGAAGCGATCGACAAAGTGTATGGCGAAGTGGCGACCACCAGTAGCCATGAGCTGGCGATGAT
CGTGCGTGAACCGGTCGGCGTGATTGCCGCCATCGTGCCGTGGAACTTCCCGCTGTTGCTGACTTGCTGGAAACTCGGCC
CGGCGCTGGCGGCGGGAAACAGCGTGATTCTAAAACCGTCTGAAAAATCACCGCTCAGTGCGATTCGTCTCGCGGGGCTG
GCGAAAGAAGCAGGCTTGCCGGATGGTGTGTTGAACGTGGTGACGGGTTTTGGTCATGAAGCCGGGCAGGCGCTGTCGCG
TCATAACGATATCGACGCCATTGCCTTTACCGGTTCAACCCGTACCGGGAAACAGCTGCTGAAAGATGCGGGCGACAGCA
ACATGAAACGCGTCTGGCTGGAAGCGGGCGGCAAAAGCGCCAACATCGTTTTCGCTGACTGCCCGGATTTGCAACAGGCG
GCAAGCGCCACCGCAGCAGGCATTTTCTACAACCAGGGACAGGTGTGCATCGCCGGAACGCGCCTGTTGCTGGAAGAGAG
CATCGCCGATGAATTCTTAGCCCTGTTAAAACAGCAGGCGCAAAACTGGCAGCCGGGCCATCCACTTGATCCCGCAACCA
CCATGGGCACCTTAATCGACTGCGCCCACGCCGACTCGGTCCATAGCTTTATTCGGGAAGGCGAAAGCAAAGGGCAACTG
TTGTTGGATGGCCGTAACGCCGGGCTGGCTGCCGCCATCGGCCCGACCATCTTTGTGGATGTGGACCCGAATGCGTCCTT
AAGTCGCGAAGAGATTTTCGGTCCGGTGCTGGTGGTCACGCGTTTCACATCAGAAGAACAGGCGCTACAGCTTGCCAACG
ACAGCCAGTACGGCCTTGGCGCGGCGGTATGGACGCGCGACCTCTCCCGCGCGCACCGCATGAGCCGACGCCTGAAAGCC
GGTTCCGTCTTCGTCAATAACTACAACGACGGCGATATGACCGTGCCGTTTGGCGGCTATAAGCAGAGCGGCAACGGTCG
CGACAAATCCCTGCATGCCCTTGAAAAATTCACTGAACTGAAAACCATCTGGATAAGCCTGGAGGCCTGAAATGACCGAA
CATACCAGCAGTTACTACGCCGCCAGTGCGAATAAATATGCACCATTCGACACGCTGAATGAGTCAATCACCTGCGACGT
TTGCGTGGTTGGCGGCGGCTATACCGGGCTCTCCTCCGCGCTGCATCTGGCGGAAGCGGGCTTTGACGTAGTGGTTCTCG
AAGCCTCACGCATCGGCTTTGGCGCAAGCGGGCGCAATGGCGGACAGCTTGTGAACTCCTACAGCCGCGACATCGACGTG
ATCGAAAAAAGCTACGGCATGGACACCGCCCGTATGCTCGGCAGCATGATGTTCGAAGGTGGTGAGATCATCCGCGAACG
TATCAAACGTTATCAGATTGACTGCGACTACCGCCCCGGCGGCCTGTTTGTGGCGATGAATGATAAACAGCTCGCCACAC
TTGAAGAGCAGAAAGAGAACTGGGAACGCTACGGCAATAAACAGCTGGAATTGCTGGACGCCAACGCCATTCGCCGTGAA
GTAGCCAGCGATCGCTATACCGGTGCGCTGCTGGATCACAGCGGTGGGCATATTCATCCGCTAAACCTTGCCATTGGCGA
AGCGGACGCCATCCGCCTCAACGGCGGGCGCGTGTATGAACTTTCTGCCGTGACGCAGATCCAGCACACCACGCCAGCCG
TTGTGCGAACTGCCAAAGGTCAGGTGACGGCGAAGTATGTGATTGTCGCCGGGAATGCGTATCTGGGCGATAAAGTAGAG
CCGGAACTGGCGAAACGCAGCATGCCGTGCGGCACGCAGGTGATCACCACCGAACGGCTGTCGGAAGATTTAGCCCGTTC
GCTGATCCCGAAAAACTACTGTGTGGAAGACTGTAACTATCTGCTTGATTACTACCGTCTTACCGCCGACAACCGCCTGC
TGTACGGCGGCGGCGTGGTCTACGGCGCGCGCGACCCGGATGACGTTGAGCGCCTTGTGGTGCCGAAACTGCTGAAAACC
TTCCCGCAGCTGAAGGGCGTGAAAATTGATTACCGCTGGACGGGCAACTTCCTGCTGACCCTGTCGCGTATGCCGCAGTT
TGGTCGCCTCGATACCAACATCTATTACATGCAGGGCTACAGCGGCCACGGCGTGACCTGTACTCATCTAGCCGGACGTT
TGATTGCCGAACTGCTGCGCGGCGACGCCGAACGTTTCGATGCCTTCGCCAATCTGCCGCATTACCCGTTCCCCGGCGGG
CGCACGCTGCGTGTGCCGTTTACCGCGATGGGCGCGGCGTATTACAGCCTGCGCGATCGTCTGGGCGTTTAATTTCCGAT
TAACCGTGAAGAGTCAAAAGGTGTGAAACATGAGCAACAATGAATTCCATCAGCGTCGTCTTTCTGCCACTCCGCGCGGG
GTTGGCGTGATGTGTAACTTCTTCGCCCAGTCGGCTGAAAACGCCACGCTGAAGGATGTTGAGGGCAACGAGTACATCGA
TTTCGCCGCAGGCATTGCGGTGCTGAATACCGGACATCGCCACCCTGATCTGGTCGCGGCGGTGGAGCAGCAACTGCAAC
AGTTTACCCACACCGCGTATCAGATTGTGCCGTATGAAAGCTACGTCACCCTGGCGGAGAAAATCAACGCCCTTGCCCCG
GTGAGCGGGCAGGCCAAAACCGCGTTCTTCACCACCGGTGCGGAAGCGGTGGAAAACGCGGTGAAAATTGCTCGCGCCCA
TACCGGACGCCCTGGCGTGATTGCGTTTAGCGGCGGCTTTCACGGTCGTACGTATATGACCATGGCGCTGACCGGAAAAG
TTGCGCCGTACAAAATCGGCTTCGGCCCGTTCCCTGGTTCGGTGTATCACGTACCTTATCCGTCAGATTTACACGGCATT
TCAACACAGGACTCCCTCGACGCCATCGAACGCTTGTTTAAATCAGACATCGAAGCGAAGCAGGTGGCGGCGATTATTTT
CGAACCGGTGCAGGGCGAGGGCGGTTTCAACGTTGCGCCAAAAGAGCTGGTTGCCGCTATTCGCCGCCTGTGCGACGAGC
ACGGTATTGTGATGATTGCTGATGAAGTGCAAAGCGGCTTTGCGCGTACCGGTAAGCTGTTTGCCATGGATCATTACGCC

GATAAGCCGGATTTAATGACGATGGCGAAAAGCCTCGCGGGCGGGATGCCGCTTTCGGGCGTGGTCGGTAACGCGAATAT
TATGGACGCACCCGCGCCGGGCGGGCTTGGCGGCACCTACGCCGGTAACCCGCTGGCGGTGGCTGCCGCGCACGCGGTGC
TCAACATTATCGACAAAGAATCACTCTGCGAACGCGCGAATCAACTGGGCCAGCGTCTCAAAAACACGTTGATTGATGCC
AAAGAAAGCGTTCCGGCCATTGCTGCGGTACGCGGCCTGGGGTCGATGATTGCGGTAGAGTTTAACGATCCGCAAACGGG
CGAGCCGTCAGCGGCGATTGCACAGAAAATCCAGCAACGCGCGCTGGCGCAGGGGCTGCTCCTGCTGACCTGTGGCGCAT
ACGGCAACGTGATTCGCTTCCTGTATCCGCTGACCATCCCGGATGCGCAATTCGATGCGGCAATGAAAATTTTGCAGGAT
GCGCTGAGCGATTAATAGCCCAACGCAATAATGTCTGATGCGCTGCGCTTATCAGGCCTGCAAACGACGTATTGATTATG
TATGCCGAATAAAGCATTCACGCCGCATCCGGCAAGTTGTATTGCTCAACTTCGCTAAATCTGGTGCTTTTTCAACAACG
CGCGGAACTGATGATAAGTTAACCCCAGTAATTCAGCCGCGCGCTTCTGGTTATATTTCCCTTGTTGCAAACTGAGTTGC
AGCAACTCTTTTTCCTGCTGCATCTGAAACTCACGTAAATCCAGCGGCAGTGTTGGAAGCGAGGTGGTTTCTGAAACGGC
GATAGCGTCTTCAGGCGGACGCCGTTTAAAGGGATCAATAATGATGTCATCAAGCGGATAATCGCTGGTGCCGTGGCGAT
ACACTGAACGTTCCACCACGTTTTTCAATTCACGAATATTTCCCGGCCAACGATAATTCAGCAATGTTTCTCTGGCGCGC
TCCGTAAACCCCGGGAACAGAGGCAGCTTGATTTCCCGACACATCTGGATGGCAAAGTATTCTGCCATCAACATTATGTC
GCTTTCGCGCTCGCGCAGTGGTGGCAGTTGTACAACATCAAAAGCCAGTCGGTCGAGCAGGTCAGCGCGAAAAGTGCCTT
CATTGACCATCGCCGGGAGATCGGCATTCGTCGCGCATACCAACCGCACATTCACCTGCAATGGTTGGCTGCCGCCAACG
CGCTCCAGTTCACCGTACTCAATCACGCGCAATAATTTCTCCTGCACCATCATCGGTGCCGTAGCGAGTTCATCAAGAAA
TAGCGTACCGCCGTCGGCACGTTCAAATCTCCCTGGATGACGTTTTTGCGCACCGGTAAACGCCCCCGCTTCGTGACCAA
ACAGTTCGGAATCCAGCAGATTTTCATTTAACGCCGCGCAGTTAAGGGAAATAAACGGCCCTTGCCAACGGGAGGAGAGA
TAATGCAGGCGGCTGGCAATCAGCTCTTTACCGGTGCCGCGTTCGCCGATGATGAGCACCGGTTTGTCCAGCGGTGCGAG
ATGCGAAACCTGTTCCAGCACTTCGAGAAAGCTGTTCGCCTCACCAAGTAAATTATCTTTGTATTCTGCCATGATGAAAT
TCGCCACTTGTTAGTGTAATTCGCTAACTCATCCTGGCATGTTGCTGTTGATTCTTCAATCAGATCTTTATAAATCAAAA
AGATAAAAAATTGGCACGCAAATTGTATTAACAGTTCAGCAGGACAATCCTGAACGCAGAAATCAAGAGGACAACATTAT
GGGTATTTTTTCTCGCTTTGCCGACATCGTGAATGCCAACATCAACGCTCTGTTAGAGAAAGCGGAAGATCCACAGAAAC
TGGTTCGTCTGATGATCCAGGAGATGGAAGATACACTGGTTGAAGTACGTTCTACTTCGGCGCGCGCGTTGGCAGAAAAG
AAACAGCTGACTCGCCGTATTGAACAAGCGTCGGCGCGTGAGGTTGAATGGCAGGAAAAAGCCGAACTGGCGCTGCTGAA
AGAGAGAGAGGATCTGGCACGTGCAGCGTTAATTGAAAAACAGAAACTGACCGATCTGATTAAGTCCCTGGAACATGAAG
TGACGCTGGTGGACGATACGCTGGCACGCATGAAGAAAGAGATTGGCGAGCTGGAAAACAAATTGAGCGAAACACGCGCT
CGCCAGCAGGCATTGATGTTACGTCATCAGGCGGCAAACTCGTCGCGCGATGTGCGTCGTCAGCTGGACAGTGGCAAACT
GGATGAAGCAATGGCTCGTTTCGAATCTTTCGAACGTCGTATTGACCAGATGGAAGCGGAAGCAGAAAGCCACAGCTTCG
GTAAACAAAAATCGCTGGACGATCAGTTTGCCGAACTGAAAGCCGATGATGCAATCAGCGAACAACTGGCACAATTAAAA
GCCAAAATGAAGCAAGACAATCAATAATAACATCCAGGCGGCGTCCGAACGCGCCGCCGCTCATCGTCTAAGGAGTACTT
ATGAGCGCGCTATTTCTGGCTATTCCGTTAACCATTTTTGTGCTGTTTGTTTTACCGATCTGGTTATGGCTGCATTACAG
CAATCGTTCTGGTCGCAGTGAATTGTCGCAAAGTGAGCAGCAGCGATTAGCGCAACTGGCTGATGAAGCAAAACGGATGC
GCGAACGTATTCAGGCGCTGGAATCTATTCTTGATGCAGAACATCCGAACTGGAGGGATCGCTAATGGCGGGCATTAATC
TCAATAAAAAATTATGGCGTATTCCACAGCAGGGCATGGTCCGCGGCGTCTGCGCCGGGATTGCCAACTATTTTGATGTA
CCGGTAAAACTGGTGCGTATCCTGGTGGTGCTGTCGATTTTCTTCGGTCTGGCGCTGTTTACCCTGGTTGCTTACATCAT
TTTGTCATTTGCGCTTGATCCAATGCCGGACAACATGGCCTTTGGTGAGCAGCTACCTTCCAGCAGCGAATTGCTGGATG
AAGTCGACCGTGAACTGGCGGCAAGTGAAACGCGTTTACGCGAGATGGAACGTTATGTCACTTCCGATACTTTCACGTTA
CGTAGCCGTTTCCGTCAACTGTGAGGAAAGTTATGAATACTCGCTGGCAACAGGCCGGGCAAAAGGTAAAGCCTGGTTTC
AAATTAGCAGGCAAGCTGGTACTTCTTACCGCACTGCGCTATGGCCCGGCGGGTGTGGCGGGCTGGGCGATAAAATCAGT
TGCTCGCCGACCGCTGAAAATGTTGCTGGCTGTGGCGCTGGAACCGCTGTTAAGTCGGGCTGCTAATAAACTGGCACAGC
GTTATAAAAGGTGAGGGGAGTTTCGCAAAAATTGTTAAATCTCAGGCGTATAATGGATGGCAATTTTCATCCATAGAAGG
ACGCTTACATGTTTAAAAAAGGCTTACTTGCTCTGGCACTGGTGTTTTCACTGCCCGTTTTCGCCGCTGAACACTGGATC
GATGTTCGTGTTCCAGAGCAGTATCAGCAAGAGCACGTTCAGGGGGCCATCAATATTCCCCTGAAGGAAGTGAAAGAGCG
CATTGCCACCGCCGTTCCGGATAAAAACGACACCGTGAAAGTGTATTGCAATGCCGGACGCCAGTCAGGGCAAGCAAAAG
AGATCCTTAGCGAGATGGGATATACCCACGTTGAGAACGCCGGTGGCCTGAAAGACATCGCAATGCCGAAGGTCAAAGGT
TAAAAGAATTCACCATGAGCGGCGCTTATGCGCCGTTTTTTTTTCTGTCACACCTTATTTACATCCCCATAGATTATTTG
CGTCAGCTCACAAATACGCTTTTTCCCTGGTAAAAAATGATTTCCTGCGTGACTAAAACCCTTGTGCTCAATTGACAGTT
TATTTTCTGCGGAGTAGTCTCTCGTTTCATGGGACCGCTACCACGGAAAGGCAACATGAAACAGAAAATTACGGATTACC
TGGACGAAATCTACGGTGGAACATTTACCGCAACTCATTTACAGAAACTTGTAACGCGTCTTGAGAGTGCGAAACGATTA
ATTACACAGCGACGTAAAAAACACTGGGATGAAAGTGATGTCGTGTTAATTACCTATGCCGATCAATTTCACAGCAATGA
TTTAAAACCATTACCCACATTTAATCAGTTTTACCATCAATGGCTGCAAAGCATTTTTTCACATGTTCATTTGTTGCCGT
TTTATCCATGGTCATCTGATGATGGCTTTTCGGTAATTGATTATCATCAGGTCGCCAGTGAAGCGGGGGAGTGGCAGGAT
ATTCAGCAACTCGGTGAATGCAGTCATTTAATGTTTGATTTTGTCTGCAACCATATGTCGGCAAAAAGTGAATGGTTTAA
AAACTATTTACAACAGCATCCAGGTTTTGAAGATTTTTTTATTGCCGTTGACCCGCAAACCGATCTCAGCGCCGTCACTC
GCCCGCGTGCGTTACCGTTATTAACGCCATTCCAGATGCGCGATCATTCAACGCGCCATTTATGGACCACCTTTAGTGAC

GATCAAATTGACCTGAATTACCGTAGCCCTGAAGTGTTGCTGGCGATGGTGGATGTTTTACTCTGTTACCTTGCGAAAGG
TGCTGAGTATGTCCGCCTGGATGCCGTTGGCTTTATGTGGAAAGAGCCGGGAACAAGCTGCATCCATCTGGAAAAAACAC
ATCTGATTATCAAACTGTTACGGTCGATTATTGATAACGTTGCGCCAGGTACAGTGATCATTACCGAGACCAATGTTCCG
CATAAAGACAACATTGCTTACTTTGGCGCAGGCGATGACGAAGCACATATGGTGTACCAGTTCTCGCTGCCGCCACTGGT
GCTGCATGCGGTGCAAAAACAGAACGTTGAGGCGCTTTGTGCGTGGGCGCAAAACCTGACACTACCTTCCAGCAACACCA
CCTGGTTTAACTTCCTCGCCTCTCACGATGGCATCGGGCTAAACCCGCTACGGGGCTTGTTGCCTGAAAGCGAAATATTA
GAGCTGGTCGAGGCGTTACAGCAGGAAGGTGCATTAGTAAACTGGAAAAATAATCCCGACGGTACACGCAGTCCGTATGA
AATAAATGTGACCTATATGGATGCGTTAAGCCGCCGTGAGAGTAGCGATGAAGAACGTTGCGCCAGGTTTATCCTTGCCC
ATGCGATTTTGTTAAGTTTCCCCGGTGTGCCAGCGATATATATTCAAAGTATTCTTGGCTCGCGTAATGATTACGCAGGT
GTCGAAAAACTCGGATATAACCGTGCGATTAACCGTAAAAAATATCACAGTAAAGAGATAACCCGAGAACTGAACGATGA
AGCTACATTAAGGCATGCGGTATATCATGAGTTGTCGCGTTTAATTACACTTCGTCGCAGCCATAACGAGTTTCATCCGG
ATAATAATTTTACCATTGATACGATTAATTCATCCGTAATGCGTATTCCAAGAAGTAACGCTGATGGTAATTGTCTGACT
GGATTGTTTAATGTCAGTAAAAATATTCAGCATGTAAATATTACTAATCTGCATGGTCGGGATCTGATTAGTGAAGTTGA
TATATTGGGTAATGAAATAACGCTGCGCCCCTGGCAGGTTATGTGGATTAAATAAAAAGGAACATCTCATGATTAAATCA
AAAATCGTGCTGTTATCAGCACTGGTTTCATGCGCCCTGATTTCAGGCTGTAAAGAAGAAAATAAAACGAATGTATCCAT
CGAATTTATGCATTCTTCGGTGGAGCAGGAGCGCCAGGCCGTTATCAGTAAATTGATTGCCCGTTTTGAAAAAGAAAACC
CTGGCATCACCGTTAAGCAAGTGCCCGTGGAAGAAGATGCCTATAACACTAAAGTCATTACTCTTTCACGTAGCGGTTCG
CTGCCGGAAGTGATCGAAACCAGCCATGACTACGCCAAAGTGATGGACAAAGAGCAGCTTATCGATCGCAAAGCGGTTGC
CACAGTCATCAGCAACGTTGGTGAAGGCGCGTTTTACGATGGCGTACTGCGTATTGTGCGTACCGAAGATGGTAGCGCAT
GGACCGGTGTTCCTGTCAGCGCCTGGATTGGCGGTATCTGGTATCGCAAAGATGTGCTGGCAAAAGCGGGGCTTGAGGAG
CCGAAAAACTGGCAACAGCTGCTGGACGTTGCACAGAAACTGAATGACCCGGCGAATAAAAAATACGGCATTGCGCTGCC
TACAGCAGAAAGCGTGTTGACGGAACAATCCTTCTCCCAGTTTGCGTTATCCAACCAGGCTAACGTCTTTAACGCCGAAG
GCAAAATCACCCTTGATACACCAGAGATGATGCAGGCACTGACCTATTACCGCGACCTTACTGCCAACACTATGCCGGGT
TCTAACGACATCATGGAAGTGAAAGACGCCTTTATGAACGGCACCGCGCCGATGGCGATTTACTCCACCTATATCCTTCC
GGCTGTGATTAAAGAAGGCGACCCGAAAAACGTCGGTTTCGTGGTGCCAACCGAGAAAAACTCTGCGGTCTACGGCATGT
TGACCTCGCTGACCATTACCGCCGGGCAAAAGACCGAAGAGACGGAAGCAGCAGAAAAATTTGTCACCTTTATGGAGCAG
GCAGACAACATTGCCGACTGGGTGATGATGTCGCCAGGTGCTGCGCTGCCGGTGAATAAAGCGGTGGTGACTACCGCCAC
CTGGAAAGACAACGACGTTATTAAGGCGCTGGGTGAACTACCGAATCAGCTAATCGGTGAACTGCCAAATATTCAGGTTT
TTGGCGCAGTAGGGGATAAAAACTTTACCCGCATGGGTGATGTGACGGGTTCTGGCGTGGTGAGTTCAATGGTGCATAAC
GTCACCGTGGGTAAAGCCGATCTCTCTACTACGCTGCAAGCGAGCCAGAAAAAACTGGATGAACTGATCGAACAGCACTA
AACCCAGGACAGGAATCCGCAATGAACAGGCTTTTTTCAGGTCGTTCCGATATGCCCTTTGCGCTGCTGCTTCTCGCGCC
CAGCTTATTACTGCTGGGCGGTCTGGTGGCGTGGCCGATGGTGTCGAATATCGAAATCAGTTTTTTACGTCTGCCGCTCA
ATCCCAACATCGAGTCAACGTTTGTTGGGGTGAGCAACTATGTGCGTATCCTCTCCGATCCCGGCTTCTGGCATTCGCTG
TGGATGACGGTCTGGTATACCGCGCTGGTGGTGGCGGGCAGCACCGTGCTCGGGCTGGCGGTGGCGATGTTTTTCAACCG
CGAGTTCCGCCTGCGCAAAACCGCGCGTTCGCTGGTGATCCTCTCCTACGTAACGCCGTCCATTTCGCTGGTGTTCGCCT
GGAAATACATGTTCAACAACGGCTACGGCATTGTTAACTACCTCGGCGTCGATCTTCTGCATCTCTATGAGCAGGCACCG
CTGTGGTTCGATAATCCGGGCAGTAGCTTTGTGCTGGTGGTGCTGTTCGCCATCTGGCGCTACTTCCCGTATGCCTTTAT
CTCGTTTCTGGCGATTTTGCAGACCATCGACAAATCGCTGTACGAAGCGGCAGAGATGGATGGCGCTAATGCCTGGCAAC
GGTTTCGTATCGTCACGCTGCCCGCAATTATGCCGGTCCTGGCGACGGTGGTGACACTGCGCACCATCTGGATGTTCTAC
ATGTTCGCGGATGTTTATTTGCTGACGACCAAGGTCGATATTCTCGGTGTATATCTCTACAAAACCGCCTTTGCCTTTAA
TGATTTAGGAAAAGCGGCGGCGATCTCGGTGGTGCTCTTCATCATTATTTTCGCTGTCATTCTGCTGACCAGGAAACGGG
TGAACCTCAATGGCAACAAATAAACGCACACTCAGTCGCATCGGTTTTTACTGCGGGCTGGCGCTGTTTCTCATCATCAC
GCTGTTTCCATTTTTTGTGATGCTGATGACCTCGTTCAAGGGCGCGAAAGAGGCGATCTCACTGCATCCTACGCTGCTGC
CGCAGCAGTGGACGCTGGAGCATTACGTCGACATTTTTAACCCGATGATTTTCCCGTTTGTCGACTACTTCCGTAACAGT
CTGGTGGTGTCTGTGGTTTCATCCGTGGTGGCGGTATTCCTCGGCATTCTTGGGGCTTACGCGCTTTCCCGCCTGCGCTT
TAAAGGTCGGATGACCATCAACGCCAGCTTTTACACGGTGTATATGTTCTCTGGCATTTTGCTGGTGGTGCCGCTTTTCA
AAATCATCACCGCGCTTGGCATTTACGACACTGAGATGGCGCTGATCATCACCATGGTGACGCAAACTCTGCCCACCGCC
GTGTTTATGCTGAAAAGCTACTTCGACACCATCCCCGATGAGATCGAAGAAGCGGCGATGATGGATGGCCTCAACCGTCT
GCAAATTATCTTCCGCATCACCGTGCCGTTGGCGATGTCCGGGCTGATATCCGTGTTCGTCTACTGCTTTATGGTGGCGT
GGAACGACTATCTGTTTGCGTCGATTTTCCTCTCCAGCGCCAGCAATTTCACCTTACCGGTGGGCCTGAACGCGCTGTTC
AGTACGCCAGATTACATCTGGGGACGGATGATGGCGGCCTCACTGGTGACCGCATTGCCGGTGGTGATTATGTATGCGCT
TTCGGAACGTTTTATTAAGAGTGGTTTGACCGCCGGTGGCGTGAAGGGCTAAAGCGGCCAGTTTCTTTACAAGGAGTTTT
AAATGAAAAAGTTAGTAGCCACAGCACCGCGTGTTGCTGCGCTGGTTGAGTATGAAGATCGGGCGATTTTAGCTAATGAA
GTGAAGATCCGCGTGCGTTTCGGCGCACCGAAACACGGAACGGAAGTGGTCGACTTCCGCGCCGCCAGCCCGTTTATTGA
TGAAGACTTTAACGGCGAATGGCAGATGTTCACTCCGCGTCCGGCAGATGCGCCGCGCGGCATTGAGTTTGGCAAATTCC
AGCTTGGCAACATGGTGGTTGGCGACATTATCGAGTGCGGCAGCGACGTTACCGACTACGCGGTGGGCGACAGCGTATGC

GGCTACGGCCCGCTCTCCGAGACGGTCATCATTAACGCAGTGAATAACTACAAGCTGCGCAAAATGCCGCAAGGCAGCTC
CTGGAAAAACGCCGTCTGCTACGACCCGGCGCAGTTTGCCATGAGCGGCGTGCGCGATGCCAACGTGCGCGTAGGGGATT
TTGTGGTGGTGGTAGGGCTTGGCGCAATCGGTCAAATTGCCATCCAACTGGCTAAACGCGCTGGCGCTTCTGTGGTGATT
GGCGTCGATCCTATCGCCCATCGCTGTGATATTGCCCGTCGCCACGGCGCGGATTTCTGCCTTAACCCCATCGGCACTGA
TGTAGGTAAAGAGATCAAAACGCTGACCGGCAAGCAGGGTGCCGATGTGATTATCGAAACCAGCGGCTACGCCGACGCGC
TGCAATCGGCGCTCCGCGGTCTGGCTTATGGCGGCACCATCTCCTATGTCGCGTTTGCCAAGCCGTTTGCCGAAGGTTTT
AACCTCGGACGCGAAGCGCATTTCAATAACGCCAAAATTGTCTTCTCTCGCGCGTGCAGCGAACCGAACCCGGATTATCC
GCGCTGGAGCCGCAAGCGTATTGAAGAAACCTGTTGGGAACTGCTGATGAACGGTTATCTCAATTGCGAAGATTTAATCG
ACCCGGTGGTGACCTTTGCCAATAGCCCGGAAAGCTATATGCAGTATGTCGACCAGCATCCGGAACAGAGCATCAAAATG
GGCGTTACGTTTTAATTCAAAGGAATGAAAAAAATGAAAATCGGCACACAGAATCAGGCGTTCTTTCCGGAAAACATTCT
GGAGAAATTTCGTTATATCAAAGAGATGGGCTTCGATGGTTTTGAGATTGACGGCAAACTGCTGGTTAACAACATCGAAG
AAGTCAAAGCGGCGATCAAAGAAACCGGTTTACCGGTGACCACCGCCTGCGGTGGCTATGACGGATGGATTGGCGACTTT
ATCGAAGAGCGTCGTCTTAATGGCTTAAAGCAGATCGAGCGCATTCTCGAAGCGCTGGCAGAAGTGGGCGGTAAAGGCAT
CGTCGTTCCGGCTGCGTGGGGCATGTTTACCTTCCGCTTACCGCCGATGACCTCGCCGCGTAGCCTGGACGGCGACCGCA
AAATGGTGAGTGATTCCCTGCGCGTACTGGAACAGGTCGCCGCGCGTACCGGAACCGTGGTGTATCTCGAACCGTTAAAC
CGCTATCAGGATCATATGATCAACACCCTCGCCGATGCCCGCCGTTACATCGTCGAAAACGATCTTAAACATGTACAGAT
TATCGGCGATTTCTATCACATGAATATCGAAGAAGATAACCTGGCGCAGGCGCTGCATGACAACCGCGACCTGCTCGGTC
ATGTGCATATTGCGGATAACCATCGCTACCAGCCGGGCAGCGGCACCCTGGATTTCCACGCGCTGTTTGAACAGCTGCGC
GCGGATAACTATCAGGGCTATGTAGTGTACGAAGGGCGTATCCGGGCGGAAGATCCTGCCCAGGCGTACCGTGATTCGTT
GGCCTGGTTGCGTACCTGCTAAGAGGTCTTTGTGAAAAGTGCAATGACAAGCTCTCCGCTGCGGGTCGCGATAATAGGCG
CAGGCCAGGTGGCGGATAAGGTTCATGCTTCGTACTACTGCACCCGCAACGATCTGGAACTGGTGGCTGTCTGTGACAGC
CGCCTTTCCCAGGCGCAGGCGCTGGCAGAAAAATACGGGAATGCATCCGTGTGGGACGATCCGCAGGCCATGCTGCTGGC
GGTGAAACCTGATGTGGTTAGCGTCTGCTCACCTAACCGTTTTCATTACGAACATACCCTGATGGCACTGGAAGCGGGCT
GCCATGTGATGTGCGAAAAACCGCCCGCCATGACGCCAGAACAGGCGCGGGAAATGTGCGATACCGCGCGCAAACTGGGC
AAGGTGCTGGCCTACGACTTTCACCATCGTTTTGCGCTCGATACGCAACAGCTGCGTGAACAGGTGACCAACGGCGTTTT
GGGAGAGATTTACGTTACCACCGCCCGCGCCCTGCGTCGCTGCGGCGTTCCCGGCTGGGGTGTCTTTACCAATAAAGAAC
TGCAGGGTGGTGGCCCGCTGATCGACATCGGCATTCATATGCTGGATGCTGCGATGTATGTGCTGGGCTTTCCGGCGGTG
AAAAGCGTGAATGCGCATAGCTTTCAAAAGATCGGCACGCAAAAGAGCTGTGGTCAATTTGGTGAGTGGGATCCGGCAAC
TTACAGCGTCGAAGATTCGCTGTTTGGCACCATTGAATTTCATAACGGCGGCATTCTGTGGCTGGAAACGTCATTTGCAC
TCAACATCCGCGAACAGTCGATTATGAACGTCAGCTTTTGTGGTGATAAAGCTGGTGCGACGCTGTTTCCAGCACATATC
TACACCGATAACAACGGTGAATTAATGACGCTGATGCAACGGGAAATAGCAGACGACAACCGCCATTTGCGCAGCATGGA
AGCCTTTATCAATCACGTACAGGGCAAGCCCGTGATGATAGCCGACGCCGAGCAGGGGTACATCATCCAGCAACTGGTGG
CGGCGTTGTATCAATCCGCAGAAACAGGGACGCGTGTGGAATTATGACCAGGCCAGTAACGTTATCAGAACCCCATTTCA
GCCAGCATACCCTGAACAAGTATGCATCGCTGATGGCGCAGGGGAACGGCTATCTTGGGCTTCGCGCCAGCCATGAAGAA
GATTACACGCGCCAGACGCGAGGGATGTATCTGGCGGGGCTGTATCATCGGGCGGGAAAAGGTGAAATCAACGAACTGGT
GAACCTGCCTGATGTCGTGGGGATGGAGATTGCCATAAATGGTGAGGTTTTCTCGTTATCCCACGAAGCCTGGCAGCGTG
AGCTTGACTTTGCCAGTGGCGAATTACGCCGCAATGTTGTCTGGCGTACCAGCAACGGCTCAGGTTACACCATCGCCAGC
CGTCGCTTTGTTTCGGCAGACCAACTGCCGCTCATTGCGCTGGAAATCACTATTACGCCACTGGACGCCGACGCGTCAGT
GCTGATTTCAACAGGCATTGACGCCACGCAAACCAATCACGGTCGCCAACATCTCGACGAAACCCAGGTGCGGGTGTTTG
GTCAGCATCTGATGCAGGGGAGCTACACCACCCAGGATGGACGCAGTGATGTGGCCATCAGCTGTTGCTGTAAGGTGAGC
GGTGATGTGCAGCAATGCTATACCGCCAAAGAGCGCCGTTTACTGCAACATACCAGTGCGCAGCTTCATGCAGGCGAGAC
AATGACGTTGCAAAAACTGGTGTGGATCGACTGGCGGGATGACAGGCAAGCTGCTTTAGACGAGTGGGGCAGCGCGTCGC
TTCGCCAGCTTGAAATGTGCGCGCAGCAGAGTTACGACCAACTTCTTGCAGCATCAACAGAAAACTGGCGTCAATGGTGG
CAGAAACGTCGTATCACGGTAAATGGCGGCGAAGCGCACGATCAGCAAGCGTTAGATTATGCGCTTTATCATCTGCGCAT
CATGACGCCTGCCCACGACGAGCGCAGCAGCATTGCGGCAAAAGGCTTAACCGGCGAAGGCTACAAAGGCCACGTTTTCT
GGGATACAGAAGTATTTTTGTTACCGTTTCATCTGTTTAGCGATCCGACGGTTGCCCGAAGTTTACTGCGTTATCGCTGG
CACAACTTGCCAGGCGCGCAGGAGAAAGCGCGACGCAACGGCTGGCAGGGCGCGCTATTTCCGTGGGAAAGCGCGCGCAG
CGGCGAAGAAGAGACGCCGGAATTTGCCGCCATTAACATTCGCACCGGGCTGCGGCAAAAAGTGGCCTCGGCGCAGGCGG
AACATCATCTGGTGGCCGATATCGCCTGGGCGGTTATTCAATACTGGCAGACCACGGGGGATGAAAGTTTCATTGCGCAT
GAAGGCATGGCGCTACTTCTGGAGACGGCAAAGTTCTGGATTAGCCGCGCGGTGAGAGTTAACGATCGTCTGGAAATTCA
TGATGTTATTGGGCCAGACGAATATACCGAACATGTCAATAATAATGCATACACCAGCTATATGGCCCGCTACAACGTTC
AACAGGCGCTGAATATTGCCCGCCAGTTCGGCTGTAGCGACGATGCGTTTATCCATCGCGCCGAAATGTTCCTCAAAGAG
CTATGGATGCCAGAAATTCAGCCCGACGGCGTTTTGCCGCAGGATGATTCGTTTATGGCTAAGCCGGCGATTAATCTGGC
GAAATACAAAGCGGCGGCGGGGAAGCAAACCATACTGCTGGATTATTCACGCGCAGAAGTGAACGAGATGCAGATCCTCA
AACAAGCTGATGTGGTGATGCTCAATTACATGCTGCCGGAGCAGTTCTCAGCGGCATCGTGTCTTGCCAATCTGCAATTT
TATGAACCGCGCACTATTCACGACTCGTCATTAAGTAAAGCAATCCACGGCATTGTTGCCGCACGCTGTGGCCTGCTGAC

CCAAAGTTATCAGTTCTGGCGCGAGGGGACTGAAATCGATCTTGGTGCTGATCCGCATAGTTGTGATGATGGTATCCATG
CTGCCGCAACTGGCGCTATCTGGCTGGGGGCGATTCAGGGTTTTGCCGGGGTGAGCGTGCGTGACGGTGAATTGCATCTC
AATCCGGCGTTACCTGAGCAGTGGCAACAGTTGTCTTTCCCTCTGTTCTGGCAGGGCTGCGAATTACAGGTCACTCTTGA
CGCGCAGCGTATTGCGATTCGAACTTCTGCGCCCGTTTCACTGCGTTTGAACGGTCAGCTTATAACCGTGGCTGAAGAAT
CTGTTTTCTGTTTGGGTGATTTTATTTTGCCCTTCAATGGGACCGCTACCAAACATCAGGAGGATGAATGAAACTGCAAG
GGGTAATTTTCGATCTGGATGGTGTAATCACCGATACCGCGCATCTGCATTTCCAGGCGTGGCAGCAGATTGCCGCTGAA
ATTGGCATCAGCATTGATGCGCAGTTTAACGAATCCCTAAAAGGGATCAGCCGCGATGAGTCTCTGCGGCGCATTCTGCA
ACACGGGGGCAAAGAGGGCGACTTTAACTCGCAGGAGAGGGCGCAACTGGCGTATCGCAAAAATCTGCTCTATGTCCACT
CACTACGCGAGTTGACGGTCAACGCTGTTCTACCCGGCATTCGCTCTTTGCTGGCAGATCTCCGTGCACAGCAGATCTCG
GTTGGGCTGGCTTCTGTCTCCCTGAATGCGCCGACGATTTTAGCGGCGCTGGAGCTGCGCGAGTTTTTCACCTTCTGCGC
GGATGCTTCCCAACTTAAAAACTCGAAACCGGACCCGGAAATCTTTCTCGCCGCCTGTGCAGGGCTGGGCGTGCCGCCGC
AGGCATGTATCGGCATTGAAGATGCGCAGGCGGGCATTGACGCCATTAACGCCAGCGGTATGCGCTCGGTGGGGATCGGC
GCGGGCTTAACCGGGGCGCAATTACTGTTGCCTTCAACGGAATCACTCACCTGGCCGCGGTTATCGGCCTTCTGGCAAAA
CGTATAGCAAAGGAATCAACATGGCTCAGCTTTCGTTACAACATATTCAAAAAATCTACGATAACCAGGTGCATGTGGTG
AAGGACTTCAACCTGGAAATTGCCGATAAAGAGTTCATCGTGTTTGTCGGCCCGTCGGGCTGCGGTAAGTCGACCACCCT
GCGCATGATTGCCGGGCTTGAGGAGATCAGCGGCGGCGATCTGTTGATCGACGGCAAACGAATGAATGACGTTCCAGCCA
AAGCACGCAATATAGCGATGGTGTTCCAGAACTACGCGTTGTATCCGCATATGACGGTTTACGACAACATGGCGTTTGGT
CTGAAGATGCAAAAAATCGCCAAAGAGGTGATTGATGAGCGGGTGAACTGGGCGGCGCAAATTCTCGGCCTGCGTGAGTA
CCTGAAACGTAAGCCGGGGGCGCTTTCCGGCGGGCAACGTCAGCGAGTGGCGCTTGGGCGGGCGATTGTACGCGAAGCGG
GCGTGTTTTTAATGGATGAACCGCTCTCTAACCTTGATGCCAAGCTGCGCGTGCAAATGCGCGCAGAGATCAGCAAGCTG
CATCAGAAACTGAACACCACCATGATCTACGTGACCCACGATCAGACCGAAGCGATGACCATGGCGACGCGGATTGTGAT
TATGAAAGACGGGATTGTTCAGCAAGTAGGTGCGCCGAAAACCGTTTATAACCAACCCGCGAATATGTTTGTTTCCGGAT
TTATTGGATCACCAGCGATGAATTTTATTCGCGGCACGATCGATGGCGATAAATTCGTTACGGAAACGCTTAAATTAACC
ATTCCCGAAGAGAAATTAGCGGTTCTGAAAACACAGGAAAGTTTGCATAAGCCCATCGTGATGGGAATACGACCGGAAGA
TATTCATCCGGACGCGCAAGAGGAAAATAACATTTCCGCCAAAATTAGCGTGGCAGAATTAACCGGTGCGGAATTTATGC
TCTACACCACGGTTGGGGGCACGAGTTAGTGGTCCGTGCTGGTGCGTTAAATGATTATCATGCAGGAGAAAATATCACTA
TTCATTTTGATATGACGAAATGTCATTTCTTTGATGCAGAAACGGAAATAGCAATTCGCTAAATACAGGGGGAAGGCATT
CCCCCAGGATAATACAAGGAACAATAATGAAAAAGTTATTACCCTGTACCGCACTGGTGATGTGTGCGGGAATGGCCTGC
GCACAGGCCGAGGAAAGGAACGACTGGCACTTTAATATCGGCGCGATGTACGAAATAGAAAACGTCGAGGGTTATGGCGA
AGATATGGATGGGCTGGCGGAGCCTTCAGTCTATTTTAATGCCGCCAACGGGCCGTGGAGAATTGCTCTGGCCTATTATC
AGGAAGGGCCGGTAGATTATAGCGCGGGTAAACGTGGAACGTGGTTTGATCGCCCGGAGCTGGAGGTGCATTATCAGTTC
CTCGAAAACGATGATTTCAGTTTCGGCCTGACCGGCGGTTTCCGTAATTATGGTTATCACTACGTTGATGAACCGGGTAA
AGACACGGCGAATATGCAGCGCTGGAAAATCGCGCCAGACTGGGATGTGAAACTGACTGACGATTTACGTTTCAACGGTT
GGTTGTCGATGTATAAATTTGCCAACGATCTGAACACTACCGGTTACGCTGATACCCGTGTCGAAACGGAAACAGGTCTG
CAATATACCTTCAACGAAACGGTTGCCTTGCGAGTGAACTATTATCTCGAGCGCGGCTTCAATATGGACGACAGCCGCAA
TAACGGTGAGTTTTCCACGCAAGAAATTCGCGCCTATTTGCCGCTGACGCTCGGCAACCACTCGGTGACGCCGTATACGC
GCATTGGGCTGGATCGCTGGAGTAACTGGGACTGGCAGGATGATATTGAACGTGAAGGCCATGATTTTAACCGTGTAGGT
TTATTTTACGGTTATGATTTCCAGAACGGACTTTCCGTTTCGCTGGAATACGCGTTTGAGTGGCAGGATCACGACGAAGG
CGACAGTGATAAATTCCATTATGCAGGTGTCGGCGTAAATTACTCGTTCTGATAATGGGCTAAATTGCCGGATGCGGCGC
GAGTACTTTATCCGATCTATAAATGTAGGCCGGATAAGATGCGCTAGCATCGCATCTGGCATTCAGGCAAGGTAGCTGGT
ATTTATTTCAGCGTCATATGCGTGGCAACGGTAATATTCTGTGGTGACGGTTTTCCAGAAATTAAGCGGAATAATAACTC
GCAGCTTTGTTGACCTAACTCCTGCGTCGGAACATCGATGCCGCCCGGTGCAGGCGTTAAAATAAATGACAGCGTTTCAT
TGCTATAACCCACCACCGCTAACTGCTGCGGAATAGCAATATTTTTCTCTGCTGCCGCACGATAAATGCTCATTAATTTC
AGGCTGTCAGTGGCAAACACCGCCTCAGGCAACGGTGACTGGCTTAATAATTGCCGTGCTGCTTTTAATGCAGTTTCATG
GGTATAACCGCCATCAACAATCCATTCATCACGCACTGCAATATTATGCGCAGCCAGGCTCTGCTTATAACCATTAACGC
GATCAACCGAAACATGGACATCAAGCGGAGCATGCAGGCAGGCAATATTTTGATGCCCACTTTCAATTAATGCATCGGTC
AACGCAATGCTGTCGCCAAAATTATCGGTATCGACAGAATAAACATGGGCATATTGACCTTCAACTTTGCCAATCACCAC
CACCGGAATATCATATTTATCGAGTTGGGCAAAAAATGACTCATCCGCTGGCGAACTGAGCATAATAATGCCTTTAATCA
TTTTCTGCTTAATTTTGCTTTCGCATTTTTGCAGATCTTCTGCCGGATTGTGCGACGTTTGTAATATCACGTCGAAACCT
TCTTCTTCAGCTTTGGCGGTGATGGCATGCAAAACTTCAGAGAAAAACGGATTACCCGCCGTAGTTTTGGTCGAACGGGT
AGAAATCACCATAATGGCATCAAAACCCGAAGAGGTCAGCGCGCGGGCCAGCTTGTTTGGCTGATACTGTAATTCTTCAA
TGGCCCGTAACACTTTTTCGCGCGCTTCCGGGGAGATATTAGTTTGCTTATTCAGCACGCGTGATACGGTGGATTTTGAT
ACGCCTGCAACCCTGGCAATATCATAAATAGTAGGGGACATAGGTCAGGAACTCCGTCCGAACGTGAATGCTGCACATCT
TATGGAGTTCGCCGGTCGATGACAACACGGGCAGTTGATAATCAATGGCCTGGCCCCCACATTCATATCCTTACGAATGA
TTTTTTTTCTGGTCTTCAGAGCAGCACAGGACAGCAATGAAGCGACTTAAAAATGAACTCAATGCGCTGGTGAATCGGGG
TGTCGACAGACATCTGCGCCTCGCTGTAACCGGACTTAGCCGCAGCGGCAAAACAGCGTTTATCACCGCGATGGTTAATC

AGTTGCTTAATATTCACGCCGGAGCACGTTTGCCGCTGTTAAGTGCGGTGCGTGAAGAGCGCCTGCTGGGCGTGAAACGC
ATTCCCCAGCGTGACTTTGGCATTCCGCGCTTCACCTATGACGAAGGGCTGGCGCAGTTATATGGCGATCCACCCGCCTG
GCCAACGCCAACGCGCGGCGTCAGTGAAATCCGCCTGGCGCTACGTTTTAAATCGAATGATTCGCTGCTACGCCACTTTA
AGGATACCTCCACGCTGTATCTGGAAATTGTGGATTATCCCGGCGAATGGTTGCTCGACCTGCCGATGCTGGCGCAGGAC
TATTTAAGCTGGTCGCGCCAGATGACGGGCTTACTCAATGGTCAGCGCGGAGAATGGTCGGCGAAATGGCGAATGATGAG
CGAAGGGCTGGACCCGCTAGCGCCTGCCGACGAAAACCGGCTGGCGGACATTGCCGCCGCGTGGACCGATTATCTCCACC
ACTGTAAAGAGCAGGGGCTGCACTTTATTCAGCCTGGGCGCTTTGTCTTGCCGGGAGATATGGCAGGTGCGCCCGCGCTG
CAATTCTTCCCGTGGCCGGATGTCGATACCTGGGGCGAGTCCAAACTGGCGCAGGCCGATAAGCATACCAATGCCGGAAT
GCTGCGCGAGCGCTTTAATTATTACTGCGAGAAGGTGGTGAAGGGGTTCTATAAGAATCATTTTCTGCGCTTTGACCGCC
AGATTGTGCTGGTGGATTGCCTGCAACCTCTCAACAGTGGGCCACAGGCATTTAATGATATGCGTCTGGCACTGACGCAG
CTGATGCAAAGTTTTCACTACGGGCAGCGTACCCTGTTCCGGCGTTTGTTTTCGCCGGTTATCGATAAGCTATTGTTTGC
TGCCACTAAAGCGGACCATGTGACCATCGATCAGCACGCTAATATGGTTTCATTACTGCAACAACTGATTCAGGATGCCT
GGCAAAATGCGGCGTTCGAAGGGATCAGCATGGACTGCCTGGGGCTGGCGTCAGTTCAGGCGACCACCAGCGGCATTATT
GATGTTAACGGTGAGAAAATCCCGGCGCTGCGTGGTAATCGACTTAGCGATGGCGCACCGCTCACTGTTTATCCTGGCGA
AGTTCCCGCACGTTTGCCTGGTCAGGCGTTCTGGGATAAGCAAGGCTTCCAGTTTGAGGCATTTCGTCCGCAGGTGATGG
ATGTCGACAAACCACTACCGCATATTCGTCTTGATGCTGCGCTGGAATTTTTAATAGGAGATAAATTGCGATGACCGAAC
CGTTAAAACCACGTATTGATTTCGACGGTCCTCTGGAGGTCGATCAGAATCCTAAATTCAGGGCGCAGCAGACCTTTGAC
GAAAATCAGGCGCAAAATTTTGCCCCGGCCACGCTCGACGAAGCGCAGGAAGAAGAGGGGCAAGTCGAAGCGGTAATGGA
CGCAGCGTTACGTCCGAAACGCAGCCTGTGGCGCAAAATGGTGATGGGAGGGCTGGCTCTGTTTGGCGCAAGCGTTGTCG
GGCAGGGTGTACAGTGGACAATGAATGCCTGGCAAACCCAGGACTGGGTGGCGCTGGGTGGATGTGCCGCTGGGGCATTG
ATTATCGGCGCTGGCGTAGGTTCTGTGGTAACAGAGTGGCGGCGCTTATGGCGCTTGCGACAGCGCGCCCATGAACGCGA
CGAAGCGCGTGATTTATTGCATAGCCACGGCACGGGCAAAGGCCGCGCATTTTGCGAAAAACTGGCGCAGCAGGCGGGTA
TTGATCAGTCGCATCCGGCGCTGCAACGCTGGTATGCCTCAATCCATGAAACGCAAAACGACCGTGAAGTGGTCAGCCTG
TATGCGCATTTGGTCCAGCCAGTTTTAGATGCCCAGGCGCGGCGCGAAATCAGCCGTTCGGCGGCGGAATCAACGTTGAT
GATTGCGGTCAGCCCGCTGGCGTTGGTCGATATGGCGTTTATCGCCTGGCGCAATCTGCGTTTAATTAATCGCATCGCCA
CGCTGTATGGCATTGAACTGGGGTATTACAGCCGTTTGCGTCTGTTTAAGCTGGTATTGCTGAATATCGCTTTTGCCGGA
GCCAGCGAACTGGTGCGCGAAGTGGGGATGGACTGGATGTCGCAAGATCTCGCTGCTCGTTTGTCTACCCGCGCAGCTCA
GGGGATTGGTGCAGGACTTCTGACGGCACGACTCGGGATTAAAGCTATGGAGCTTTGCCGCCCGCTGCCGTGGATTGACG
ATGACAAACCTCGCCTCGGGGATTTCCGTCGTCAGCTTATCGGTCAGGTGAAAGAAACGCTGCAAAAAGGCAAAACGCCC
AGCGAAAAATAATGCAATATCGGGTGCTGACCGGATATCTTTACGCCGAAGTGCCCGTTTTTCCGTCTTTGTGTCAATGA
TTGTTGACAGAAACCTTCCTGCTATCCAAATAGTGTCATATCATCATATTAATTGTTCTTTTTTCAGGTGAAGGTTCCCA
TGCGTCTGGAAGTCTTTTGTGAAGACCGACTCGGTCTGACCCGCGAATTACTCGATCTACTCGTGCTAAGAGGCATTGAT
TTACGCGGTATTGAGATTGATCCCATTGGGCGAATCTACCTCAATTTTGCTGAACTGGAGTTTGAGAGTTTCAGCAGTCT
GATGGCCGAAATACGCCGTATTGCGGGTGTTACCGATGTGCGTACTGTCCCGTGGATGCCTTCCGAACGTGAGCATCTGG
CGTTGAGCGCGTTACTGGAGGCGTTGCCTGAACCTGTGCTCTCTGTCGATATGAAAAGCAAAGTGGATATGGCGAACCCG
GCGAGCTGTCAGCTTTTTGGGCAAAAATTGGATCGCCTGCGCAACCATACCGCCGCACAATTGATTAACGGCTTTAATTT
TTTACGTTGGCTGGAAAGCGAACCGCAAGATTCGCATAACGAGCATGTCGTTATTAATGGGCAGAATTTCCTGATGGAGA
TTACGCCTGTTTATCTTCAGGATGAAAATGATCAACACGTCCTGACCGGTGCGGTGGTGATGTTGCGATCAACGATTCGT
ATGGGCCGCCAGTTGCAAAATGTCGCCGCCCAGGACGTCAGCGCCTTCAGTCAAATTGTCGCCGTCAGCCCGAAAATGAA
GCATGTTGTCGAACAGGCGCAGAAACTGGCGATGCTAAGCGCGCCGCTGCTGATTACGGGTGACACAGGTACAGGTAAAG
ATCTCTTTGCCTACGCCTGCCATCAGGCAAGCCCCAGAGCGGGCAAACCTTACCTGGCGCTGAACTGTGCGTCTATACCG
GAAGATGCGGTCGAGAGTGAACTGTTTGGTCATGCTCCGGAAGGGAAGAAAGGATTCTTTGAGCAGGCGAACGGTGGTTC
GGTGCTGTTGGATGAAATAGGGGAAATGTCACCACGGATGCAGGCGAAATTACTGCGTTTCCTTAATGATGGCACTTTCC
GTCGGGTTGGCGAAGACCATGAGGTGCATGTCGATGTGCGGGTGATTTGCGCTACGCAGAAGAATCTGGTCGAACTGGTG
CAAAAAGGCATGTTCCGTGAAGATCTCTATTATCGTCTGAACGTGTTGACGCTCAATCTGCCGCCGCTACGTGACTGTCC
GCAGGACATCATGCCGTTAACTGAGCTGTTCGTCGCCCGCTTTGCCGACGAGCAGGGCGTGCCGCGTCCGAAACTGGCCG
CTGACCTGAATACTGTACTTACGCGTTATGCGTGGCCGGGAAATGTGCGGCAGTTAAAGAACGCTATCTATCGCGCACTG
ACACAACTGGACGGTTATGAGCTGCGTCCACAGGATATTTTGTTGCCGGATTATGACGCCGCAACGGTAGCCGTGGGCGA
AGATGCGATGGAAGGTTCGCTGGACGAAATCACCAGCCGTTTTGAACGCTCGGTATTAACCCAGCTTTATCGCAATTATC
CCAGCACGCGCAAACTGGCAAAACGTCTCGGCGTTTCACATACCGCGATTGCCAATAAGTTGCGGGAATATGGTCTGAGT
CAGAAGAAGAACGAAGAGTAAGCGCGAATATGCCTGATGGTGCAACACCATCAGGCATATTAAATTATGCTTTCAGTACA
GCCAGAGCTGCTTCGTAATCCGGCTCGGTGGTGATTTCATCCACCAGCTGGCTGAAAATCACATTGTCATTTTCGTCAAT
AACCACAACGGCACGCGCTGCCAGACCTTTCAGTGGGCCATCAGCAATTGCCACACCGTAAGCTTGCAGAAATTCAGCGT
TACGGAAAGTGGAGAGGGTGATAACGTTGTTCAGACCTTCTGCGCCGCAGAAACGAGACTGGGCGAACGGCAGATCGGCA
GAGATACACAGCACAACGGTGTTGTCGATCTCAGTTGCCAGTTGGTTAAACTTACGTACTGATGCGGCGCAAACACCGGT
ATCAATACTCGGGAAAATGTTCAGCACTTTGCGTTTACCCGCAAACTGACCGAGGGTGACGTCAGACAGATCTTTTGCCA

CGAGAGTAAAAGTCTGCGCTTTGCTACCCGCCTGCGGGATGGAATTGGCGACTGTAACCGGGTTGCCCTGGAAATGAACG
GTTTGTGACATGATTATCTTTCCTGTTTACATATAGTTAACGTCACACCTAGTTTATGCTAACTGTCAATAACACAGCAA
ACGCTATTTGCGCTTAATCCGCAGACCACCGCGACAACAAGGAGTAAAGATGAGAACCGTTAAGGTATTCGAGGAAGCCT
GGCCCTTACATACCCCGTTTGTGATTGCCCGGGGAAGTCGCAGTGAAGCGCGCGTGGTGGTGGTTGAACTGGAAGAAGAG
GGTATTAAAGGCACCGGCGAATGCACGCCGTATCCGCGTTATGGGGAAAGTGATGCCTCGGTAATGGCGCAAATTATGAG
CGTCGTGCCGCAACTAGAGAAAGGGCTGACACGGGAGGAGTTGCAAAAAATTCTCCCTGCCGGCGCAGCACGTAATGCGC
TGGATTGTGCATTGTGGGATCTGGCCGCGCGAAGACAGCAGCAATCGCTGGCTGATTTGATCGGCATAACGCTTCCCGAG
ACAGTTATCACTGCACAGACGGTTGTCATCGGTACGCCTGATCAGATGGCCAATAGTGCATCAACACTCTGGCAGGCAGG
CGCGAAATTACTGAAAGTGAAGCTGGATAACCATCTTATCAGTGAGCGGATGGTGGCAATTCGCACAGCTGTGCCCGATG
CGACGCTGATCGTTGATGCAAATGAATCCTGGCGTGCAGAAGGGTTGGCGGCGCGTTGCCAGCTATTGGCGGATTTAGGC
GTTGCGATGCTTGAACAACCGCTTCCTGCGCAGGACGATGCGGCGCTGGAGAATTTTATTCATCCGTTGCCGATTTGTGC
TGATGAAAGTTGTCATACTCGTAGCAATTTGAAGGCGCTGAAAGGGCGCTATGAGATGGTTAATATTAAGCTCGATAAAA
CCGGGGGTCTGACGGAAGCGCTGGCGCTGGCGACTGAAGCGCGTGCACAAGGTTTCAGTCTGATGCTGGGCTGCATGTTG
TGTACCTCTCGTGCCATTAGCGCCGCTTTACCGCTGGTGCCGCAGGTCAGTTTCGCCGATCTTGACGGACCGACCTGGCT
GGCGGTAGATGTGGAACCGGCGCTTCAGTTCACGACGGGCGAATTGCATCTTTAGGATGCCAGCGCAGCAAGTTTGCCAT
CGCAAAGAGATATTTTTCGCTGGCTTCGTCGGAGGAGATGGGCGGAAACTCTGCGGTGATGCAATGCAAGTTCAGATCTG
CACACCAACTGCCGAAAGAGCCAGGCGTTTCATAACCGACGCTGGTTACCAGAGGCAATTCAAACGCCTGAGCCAGCCAT
TCGCCTAATTCGCTGTGTCTGGGATCTTCAATACAGGCCAGTGGATCGTGGAATGAGACCACCCAGGCAGGTTGAATGCG
GTGTATAAGCTGGCACAGCGCCTGGGTTTCAGGTTCGGAGCCAGGTTTGTCACCTGTCAGTAATACGACATCGCGTTCTT
CAGCGGCGCTGTTCCAGCGATAAACGGTTTCACCTTCCTTCCAGTTCGCCGCCGGAAAGTTTCTGTTTAAATCCACACCA
TTAGCATTGGCCCGTAACCCCAACTGGCAGCCGTCAGGATTCACACACAGCACCACATGATGACGGCGCAAAGAAGGTGT
CAACGTCCGCAGAGCACAGGAGAGCGTGACGACCGAAGAGTTTTCATCACCGTGAGTGCCAGCCAGAATCAAACCACTTT
CGCGGCTGGCTGCAGGGGCAGGAAACCAGATTAACGGCGCGCCCAATAATGAACGTCCGTAATGTTCTGTTCCGGGCGGA
AATGCGCCGCGTTCGGCGCGTGGGCGGGTTACGGTCATGATCGTCTCTGGTTCAGGAAGTATTACAGGCAGTGTTGTGCA
AATTTTGCGGATTATCAAATCCTTTTCTTCAACGCTATGTCATCGATCTTGTATCACTTTTTTGCCGGAAGTTGTTTGCA
TTTTATTAGTTGGATAGGGTTTGTGGATTTTTGCTGGCTGACGGGCAAATAAAGTTTGTTTAATGGTTTGCTTATCTGCC
AGTTGATTAATCACATGGCGGGCTATCTGACCGCCAGCACCAAGAATCAGTACATTTTTCATGAAAATTATCCCAGATTA
TTGGCAGCAATATGCCAGTCCATATCACTATTGCGGATTTGAATATGTTTCATTACAGAGTTTCCTCAAAGAACGGAGCT
AATACTGAAATTGCTTCAGCCACATAGGCTTTGCCGTCATAAAGATCCATATGGTTTGCGCCTTCAACAATGTGATAGCG
TTTATCCTGGCTTGATGCTCGATCGTACAGGTCGTCACTCATCCATTTGCTCCCTGCCTGGCTGCCCGCCACAATCTGCG
TCGGCTGAGTCAGGTACACTTCCGCCATATGGTAAGCATCATAGGTAATAATCTGGTTAAGGCTGCGCAAAGTAGCGTAA
CCCGGTGCTGTTGGATACTGCGCGCGAGGGGTGTGGTAATACTCCCAGGCCTGACGCAGTTCTTCGTTCGGCGCATCGGA
CTCCTTCATTGGTGCCAGTGGCATAATGGCGTATTCTCCGCTGCTAATATCGCTGGTTCTGGCGTTTGAACCCGCTTCAA
CGTATGGCAGGGCATCAATAGATTTCACATTGTTTTCCCAACCATTACGGAATATCGAACCAATATTGACCGCACTAACG
GTACCGATGGCCTTGATGCGGCGATCCTGAATTGCAGCATTGGCTGTATATCCTGCACCGGCACAAATTCCCATCGCACC
AATTCGGGTATTGTCGACATAAGAAAGCGTTGTCAGGTAATCAATCACGGCACTGATGTCTTCAGTACGAATGTATGGGT
TTTCTAACTGACGCGGCTCGCCGCCACTTTCACCTTGATAAGATGCGTCATAAGCAATAGTGACAAATCCCTTTTCCGCC
AGTTTTTTGGCATAGGTTCCGGCCGTTTGTTCTTTAACGCCCCCACCTGGGTGAGATAACACAATTGCCTGATACTGACG
GGTTTCATCAAATTTTGGAGGGAAATAGATCACTGCAGACAAAGAGATGGTTGGATTATTGCTGTTAGTGAAGCTGACTT
TATTATTCATCATTCCGTTCCTCATGGAGTTGTCGGTTCGTTTTAACGGTTGGTGATATCACTATAGATATTGATCATTA
AGTTGATTAGACCCAAAATCATGATTAGACCTATCATTTAAATGATTAATAGATTGCATGGAGATACTGAATGAAGCGTG
AAGAAATCGCTGATCTGATGGCGTTTGTCGTCGTTGCAGAGGAGCGTAGCTTCACTCGTGCAGCAGCCCGCCTGAGCATG
GCGCAGTCAGCTTTAAGCCAGATAGTGCGTCGTATAGAAGAACGATTGGGATTGCGGCTTCTGACGCGAACCACGCGCAG
CGTTGTTCCAACTGAAGCGGGCGAGCATCTTTTGTCTGTTCTTGGCCCTATGTTGCATGACATAGATTCAGCCATGGCAT
CCCTGAGCGATCTGCAGAACCGCCCATCCGGGACAATACGTATTACTACTGTAGAACATGCAGCAAAAACGATATTGTTA
CCAGCAATGCGCACATTCCTGAAATCGCATCCTGAAATTGATATTCAGCTCACCATTGATTATGGTTTGACCGATGTCGT
TTCTGAACGTTTTGATGCAGGCGTCCGTCTGGGTGGGGAGATGGATAAAGATATGATCGCCATTCGAATCGGGCCAGATA
TACCAATGGCTATTGTTGGCTCACCGGATTATTTTTCTCGCCGAAGTGTTCCAACGTCAGTGTCACAATTAATAGATCAT
CAGGCAATTAATTTGTATCTTCCCACATCGGGTACAGCAAATCGCTGGAGATTAATACGCGGTGGACGTGAAGTTCGTGT
TCGCATGGAAGGTCAGCTTTTACTGAATACGATAGACCTGATCATTGATGCTGCAATTGATGGGCATGGATTGGCGTATC
TACCTTATGATCAGGTTGAGCGGGCTATTAAAGAAAAAAAACTGATACGTGTTTTGGATAAATTCACACCAGATTTACCC
GGTTATCACCTGTACTATCCACACCGTCGACATGCTGGCTCGGCATTCTCATTATTTATAGATAGGCTGAAGTATAAAGG
TGCTGTTTAGCACTACTTGCTGATACATTAATTTAATTCTTCTCTTAACGTATTCTCAGTTCCTTTCAACGTTTTGGTCA
TTTTTTATTCTTCGTACAATGGCGACAGATGCTGATTATGATACCGAAAACGGGTTTGAACGTGCGAAGCCCGAACGTAG
TGTTCGGAGTTCTATGTGCTTTACCGCATTTTGGAGACTATTATTTACACTAAATCTGATTTGATATATTGATACTTAAA
ACATTTGATGCTTCCTTTGTCACTTTTTTGATGGAAGTTGTTTGCATTTCTTTAAGGCGAAACAAATAATTACGCATCAA

TTTTAATGTCGGTTAGAGGGAAACTTATGAAGCACTCTGTTTCAGTCACGTGTTGTGCGCTGTTGGTCAGCAGCATTTCT
CTTTCGTATGCTGCAGAAGTTCCGAGCGGCACAGTACTGGCAGAGAAGCAGGAGCTGGTGCGCCACATTAAAGATGAGCC
TGCGTCGCTGGATCCCGCTAAAGCCGTGGGCCTGCCAGAGATTCAGGTCATTCGCGATCTGTTTGAAGGTCTGGTGAATC
AGAACGAAAAAGGGGAGATTGTCCCCGGCGTTGCGACTCAGTGGAAAAGTAATGACAACCGTATCTGGACTTTTACCCTG
CGCGATAACGCAAAATGGGCGGATGGCACACCGGTAACGGCGCAAGATTTTGTCTACAGCTGGCAACGTCTGGTGGACCC
AAAAACATTGTCGCCATTTGCATGGTTTGCCGCGCTGGCGGGAATCAACAACGCACAGGCGATTATTGATGGTAAAGCTA
CGCCTGACCAGCTTGGCGTCACCGCAGTTGATGCCCATACTTTGAAAATTCAGCTTGATAAACCGTTGCCGTGGTTTGTG
AATTTAACCGCTAACTTTGCCTTCTTCCCGGTGCAAAAAGCCAACGTAGAAAGCGGTAAAGAGTGGACGAAACCCGGAAA
TCTGATCGGCAATGGCGCTTATGTTCTTAAAGAGCGCGTAGTCAATGAAAAACTGGTCGTGGTACCGAATACCCATTATT
GGGATAACGCCAAAACGGTACTGCAAAAAGTGACCTTCCTGCCAATTAATCAGGAATCCGCAGCCACTAAGCGTTACCTC
GCGGGGGATATTGATATCACCGAATCCTTCCCGAAAAATATGTATCAGAAGCTGTTGAAGGATATTCCGGGGCAGGTTTA
TACGCCGCCGCAGCTCGGGACCTATTATTATGCGTTTAACACGCAAAAAGGGCCGACGGCAGATCAGCGCGTTCGTCTGG
CATTAAGTATGACGATAGATCGCCGCCTGATGACCGAAAAAGTATTAGGGACGGGCGAAAAGCCAGCGTGGCATTTTACA
CCAGATGTTACCGCGGGATTTACGCCGGAACCTTCGCCGTTTGAACAAATGAGTCAGGAAGAACTGAATGCGCAGGCAAA
AACTTTGTTGAGCGCAGCTGGTTATGGTCCGCAAAAACCGCTGAAGCTGACGCTTTTGTATAACACTTCAGAAAACCATC
AAAAAATTGCGATTGCTGTAGCATCGATGTGGAAAAAGAACCTTGGCGTAGATGTTAAATTGCAAAATCAGGAATGGAAA
ACCTATATCGATAGCCGTAACACCGGCAATTTTGATGTTATCCGCGCCTCGTGGGTGGGGGATTATAATGAACCCTCCAC
TTTCCTGACATTATTAACGTCAACGCATTCAGGAAATATTTCACGCTTTAACAATCCGGCATATGACAAAGTTCTGGCCC
AGGCATCGACGGAAAATACCGTTAAAGCGCGTAATGCCGATTACAACGCGGCAGAAAAAATCCTCATGGAGCAAGCACCG
ATTGCACCAATTTATCAATATACCAATGGACGATTAATCAAGCCGTGGCTGAAAGGTTATCCCATTAATAATCCTGAAGA
TGTGGCGTACAGTCGGACTATGTATATTGTGAAGCATTGATGTGATGGGAACTGGCGTTACCCTTGTGCATAACGCCAGT
GATGTCTGATTTAGCGACCCTGTTCCGGCGGTGTAATGTTATCCATATACAGCGTCTGGCTGGGGAAGGCAAAGTCCGCG
CCGTGTGACTGTACAATATCGATAATCTTCAAATAAACGTCTTGCTGTGCAGCAAGCCATTCAGCCCATACCGTGGTTTT
GGTAAAGCAATAAACCATAATATTCAATGAAGAGTCAGCAAACTGGTTGAAATAAACCAGTAAGGTTTGTCGCTGGTCGA
TGGCCGGGTGATTTTTCAGCATCTCACGTACAGCTTCGACAATAACGCCCACTTTTGCCGCATCCTCATAACGTAAACCA
ATGGTCGTGGTAATGCGGCGGTTGGTCATTCGTCCTGGGTTTTCTACGCTGATCGACGAAAACAGCGAGTTCGGTACGTA
CAATGGACGATTATCAAAGGTCGTAATTTTGGTAATTCGCCAGCCAATTTCCGCTACTGTACCTTCGATATTTCTGTCCG
GTGAACGGATCCAGTCGCCAATACTGAAAGGACGGTCGAAATAGAGCATAATCCCGGAAAAGAAGTTACTCAGAATATCT
TTACCGGCCATACCGACAGCCAGACCACCAATACCACCAAAGGTCAGCAAGCCAGAAAGGCTCATGCCGAAATGTTCGCC
ATAAAGCAGAACAAGCACCACAATAATGGTGATTTTGATGATACGCGACATAATCCGCGCACTGGTGATATCGCGACCTT
TTTTAATCTGCTGTTTTTCAAACTGATTAATCAGCAGAAATAGCTTAATCGTCAGAATAACCGCAATCAGGGACGTACAG
ATAAAATCGATAACGCCTGGGGTGATAAATTTGAGTTTATAGTTTTCTATAACATAATTAATAATGCTACCAACAGCACT
GATAATTATGGTGTAGATTAAAAATTGCACCGCATGGAATAAAAATCCTTTTCTTTTACGATTTCCACGGCGAAACCAAA
AGCTCATCAGAATCAATGCTGCGCAGCTACCGAAAATAATGACCAGATTAAGCGCATTATTTGTAAACAGTTCAGCGATC
ATTGTTTTATCAGGCTCCTCCAGATAATTGTCGTCATGCCGGAAACCCCTGGCGGGGCTATTTTACCGCGACAATTCATT
CAGATCATCAATAGTCAGGGAAGGAAGTAGCAACATTAGCTAAGGAAGGTGCGAACAAGTCCCTGATATGAGATCATGTT
TGTCATCTGGAGCCATAGAACAGGGTTCATCATGAGTCATCAACTTACCTTCGCCGACAGTGAATTCAGCAGTAAGCGCC
GTCAGACCAGAAAAGAGATTTTCTTGTCCCGCATGGAGCAGATTCTGCCATGGCAAAACATGGTGGAAGTCATCGAGCCG
TTTTACCCCAAGGCTGGTAATGGCCGGCGACCTTATCCGCTGGAAACCATGCTACGCATTCACTGCATGCAGCATTGGTA
CAACCTGAGCGATGGCGCGATGGAAGATGCTCTGTACGAAATCGCCTCCATGCGTCTGTTTGCCCGGTTATCCCTGGATA
GCGCCTTGCCGGACCGCACCACCATCATGAATTTCCGCCACCTGCTGGAGCAGCATCAACTGGCCCGCCAATTGTTCAAG
ACCATCAATCGCTGGCTGGCCGAAGCAGGCGTCATGATGACTCAAGGCACCTTGGTCGATGCCACCATCATTGAGGCACC
CAGCTCGACCAAGAACAAAGAGCAGCAACGCGATCCGGAGATGCATCAGACCAAGAAAGGCAATCAGTGGCACTTTGGCA
TGAAGGCCCACATTGGTGTCGATGCCAAGAGTGGCCTGACCCACAGCCTGGTCACCACCGCGGCCAACGAGCATGACCTC
AATCAGCTGGGTAATCTGCTGCATGGAGAGGAGCAATTTGTCTCAGCCGATGCCGGCTACCAAGGGGCGCCACAGCGCGA
GGAGCTGGCCGAGGTGGATGTGGACTGGCTGATCGCCGAGCGCCCCGGCAAGGTAAGAACCTTGAAACAGCATCCACGCA
AGAACAAAACGGCCATCAACATCGAATACATGAAAGCCAGCATCCGGGCCAGGGTGGAGCACCCATTTCGCATCATCAAG
CGACAGTTCGGCTTCGTGAAAGCCAGATACAAGGGGTTGCTGAAAAACGATAACCAACTGGCGATGTTATTCACGCTGGC
CAACCTGTTTCGGGCGGACCAAATGATACGTCAGTGGGAGAGATCTCACTAAAAACTGGGGATAACGCCTTAAATGGCGA
AGAAACGGTCTAAATAGGCTGATTCAAGGCATTTACGGGAGAAAAAATCGGCTCAAACATGAAGAAATGAAATGACTGAG
TCAGCCGAGAAGAATTTCCCCGCTTATTCGCACCTTCCCTAACTAATCAATGCGTTGATTGTAAATCCAGCTAAGAGGTG
AGGTTTTCAGAGCAGACAACGGTGAAATGTCATGGTATTGTTACGTTTAGGTAACAAGAAATTTGTCTGCACAAGGATTA
CATCATGATTATGGCGAAACTGAAGTCAGCGAAAGGGAAGAAATTTCTCTTTGGTTTGTTGGCGGTTTTCATTATTGCGG
CGTCGGTTGTGACTCGCGCGACCATCGGCGGCGTTATAGAACAGTACAATATTCCGCTGTCTGAGTGGACGACATCAATG
TATGTGATTCAGTCATCGATGATTTTTGTTTATAGCCTGGTCTTTACTGTGTTGCTGGCAATCCCGTTGGGAATTTATTT
CCTTGGCGGCGAAGAGCAGTAAGTAAAAAATAGGCCCGATAACTCGGGCCTTGTCAGTTATTGAAGAGTCGTTAATCGTC

TTCTTCGTCATCCAGTTCAACGGGTGTCTGATACTGGTCAGGTTTAATGACCAGCAGGTCGCAGCGAAGATGATCAATCA
CCTGTTCCGCCGTGTTGCCGAGGAATGCTGCTGAAATACCGGTGCGTCCTACCGTGCCCAGAACCACAATCCCCGCCTGT
AAGTGCTCCGCCAAATCAGGAATCACCTCTTCTGGCAGACCTTTTTCTACGTGCGTCATGTTTTCATTAATGCCGAATTT
CTGCCGCAGGGCTTTCATTGCCAGCAAATGTTGCCCACGAATGGCATCGTTATAAACGCTCGGGTCAAATTCCGGCAGTT
CAATCGCGATATTAATTGGCGTTACCGGATAAGCGCCAACCAGATGAACTTCGGTATGGTTGACTTGTTCTGCCAGTTCG
ATCGTCTCTTTGACCAGTTTTTCATTGAGCGCATTATGATACGGCTCTTCACTGGCGAGATTCACCGCCACCAGCGCCTT
GCCTCCTTCCGGCCACGGCTGGTCTTTCACCATCCACACCGGGCTTGGGCATTTGCGTAACAGATGCCAGTCCGTTGGCG
TAAAAATCACCGCTTCCAGACGGTCATGTTGGTGCGCCATTTTTAGCACCAAATCGTGTCCGCCGCTGATCACTTCCTGA
ATGATGGCTTCGAAAGGACGGTTATGCCAGACCACTTTAATTTCAATGGGAACGCCAGCATTGAGATAATATTTTGCCTG
CTCGTGGATCCAGGCTGTACGCTGGCTGATGACGCCCTGACGCATAGCGGTACGTTCGTCCGGGGAGAGCAGGGTGGTCA
TTTCGTATGAGAAGTCATAGATCGGCAAAAAGGCTTTAATTTTGCCACCAATCCGTTGATGTAAATAAACAGCTCGCCGC
AATGCTGGTTGGTCGTCCTGGTTAGGATCGATAACAACGAGCATGTTCTGATACATAGCCATACAGGGTCTCCTTACAAC
AACTGTCAACGCAGTTTGTAATTAAAAGATTAACCCATATCTGGTGAATGAAACAGTGATGAACCTTCTGCCAGATCAAT
AAATCAGAAAAATTTAATGATATGACAGAAGGATAGTGAGTTATGCGGAAAAATCAGGCAACGTTACGCGTATGACCAGC
AAGCTGGGCCAGCGCATCGTTATTTTCGATGGTGATGTATTTACCTTTGACTGCCAGCATGCCGCTTTTCTGGAAGCGAC
CCAGCAGACGGCTGATGGTTTCTACCGTCAGGCCCAGATAGTTACCGATATCGCCACGAGTCATCGTCAGGCGGAATTCA
CGAGGGGAGAAGCCGCGTTGGGCAAAACGACGGGACAGGTTGTAGATGAATGCAGCCAGACGTTCCTCGGCATTTTTCTT
CGACAACAGCAGGATCATGTCCTGATCGCCTTTGATTTCACCGCTCATCAGACGCATCATCTGCTGACGCAGATTCGGCA
TTTTACCGGACAAATCGTCCAGCGTTTCGAACGGGATTTCACATACCATCGAGGTTTCCAGCGCCTGCGCGAAGCTCGGG
TGATGGCCGCTGCCGATGGCGTCAAATCCCACCAGGTCGCCTGCTAAATGGAAACCAGTGATTTGCTCGTCGCCTTGCTC
AGTGATGGTATAACTTTTAATCGTACCGGAGCGGATGGCATAAAGCGATTTAAGTTCATCACCAGCCTTAAACAGCGTCT
GGCCTTTCTGAATAGGCTTCTTCCGCTCAATGATATTATCAAGCTGATCAAGCTCATGTTCGTTGAGTGTGAACGGGATG
CAAAGCTGGCTGATGCTGCAATCCTGGCAATGGATAGCACAACCGCCAGACTGAATGCGCCGTATAATTCGCTTTTCCGG
GATCATAGGTCTGCTCAAGCCGTAATTGATATTTGTCAATTTTAACATCTTTTTAGGGAGCAAGTAAGTCTAAGCAAACC
TTAACAGCAGAGAATTCCGATATTAGATGTAAATATATGTCTATCTATTTGAAAACCCTTAAGTTGTTAAGGGTAACTTT
ACATAAAAGTGTGAACAAGCTGGCACAAATTGTTTAATGTTTACAGCAAAAGATAACCTTCATGGCGCAATAACCACTCT
TTTCGCTGAACTCCGCCTGCATATCCGGTCATGGTGCCGTTTCGGCCAATAACCCGATGGCAAGGTACGACGATGCTGAT
GGGATTCGATCCGTTTGCCGCACCAACGGCACGCGCCGCGCCAGGACGGCCCAATTGCTCAGCCAGTTGGCCGTAATGCA
TTACCTGCCCGCAGGGGATAGTGCGTAGTGTTTTCCAGACTTCGCGCTGAAATGGCGTCCCCCCCGTAGCAGTGGGAAGC
GTATCAATAATGCTAAGATTACCGGCAAAATATTCACGAAGCTTGTCGCTTAAACCGCCTGGATTGGTGGCAGAAATGCG
CTCATAGCCTTCTTTGCGATAATGGATGTCCAGCAGCTGCACCATGCGTTCGCTGTACTCTTCCCATTCAACCGCCCGCA
GGCGAAATTGCTCATCGCAAATCACCCACAGTGGACCCAGTGGCGTGGCAATTTTTTCTTCAAGTAATCTCAGCATCCGT
TCTCTCTTAAGACAAACGTGGGTAAATACCCGGACCTATCGGCAGACCGACAAGATACCACGCCAGCAACATCAGCAGCC
ATACCACCAAAAAGATAAGTGGATAGGGCAAGACTAACGAATAGTAAGTACCCAGTTTCGCGTCTGGTTTGTAGCGTTGC
AGGAATCCAAGAAACAGTGGAACAAAAGGAGATACCGGCGCTAAAGGCAATACGGATGAGTCGGCAATACGAAAGAGGAT
TTGCGCAAATGCCGGGTGAAAGCCAAGTAGCATAAACATTGGTACGAAAATGGGGGCGAGAATCGACCAGATTGCGGAAC
CGCTGGCAATAAACATGCATAAGAAAGAGGAAAGCAACGCCAGACCGACAAACGCCGGGATGCCGCTAAGCCCTGAACTT
TCCAGTATATCGGTCAGCCCCACGGCGATGAATTTCCCCATGTTGCTCCAGTTAAACATGGCGACAAATTGGGCGAGGGG
AAAAACCATCACGATAAATCCCGCCATCTCTTTCATCGGTTCAATCATTAAATGCGGTAAATCCGCCTGACGTCGAATTG
TGCGGGTAGCGATGCCATAAGCCAGCGAGACAACAAAGAAAAAAAGAATGATCAGTGGCACGATACCTTTAATAAAGGGT
GATGGCATCACGGTGTGATTAATCGGATCGCGCAATATCCCGTTTTGCGGGATCACCATCAGCGCAATCGCAGCAATAAA
AAGTAGCGATACGACACCTGCTATGCGTAAACCAAAACGCTGACTTTCGGTCAATGTCTGCAGTTTCTCATCGCTGTTTC
CCTGCCATTGACCTAACCGTGGCTCGATGATTTTGTCGGTTATCAGGCCGCCAACAATCGTCAGTACGACTACGGAGCTG
GCCATAAAATACCAGTTATCAATTACACTGACGTGCATTTGCGGATTGAACGCAGCTGCCGCTTCCGTGCTGATCCCCGA
CAGCAACACGTCGGTTGTGACAATCAGTAAATTAGCCGTAAAGCCGCAACCTACGCCTGCAATGGCAGCCAGTAAACCTG
CAACTGGATGCCTGCCCACCGCCAGAAAAATCAGCGCACCCATCGGTGGCATGATCACTAACGCCGCATCGGAAGAAATG
TGGCTGAAAAAAGCAATAAACAGCACCATATAACTGGCGTAGCGGGCATTAACATGCGATGCCATTTTAACCATTAGTGC
TGGCAGTAAGCCGACGCGCTCCGCCAGACCGGCACCTAAAACCAGCGCCAGGATCGCACCAAGTGGAGCAAAACCGCTAA
AGTTTTTAATAACATTGGGTAAAAACCAGTGTAATCCTTCCACACTGAGCAGGTTTTTCACCACGACCGGCGTACCATCG
GTCGGGTTTTTCGCACTGACGCCAAAGGCCGACAAAATTGCCGTCGTCACCATGAGTACGATAATCAAATAGATAAAGAG
CAGAAAAGGATGGGGAACCTTGTTACCAATTCTTTCGACCCAGCCATAGAGCTTCCCGGATTGGGAGGACGACGGTATGG
ATGACATACTCATGGGCATTCCTCGGTTGTTGTGTTTGCGTTGTTGTTGTTATTTTAAAGGTGACGGTGTCACGTTTTTC
GGGATAGGGCAGTGATACGGTTGCGTGTCCGTTACTTGCTGATGCTCTTGTTGGCATTCTTGCAATAGCCCTGAATCAAG
GAAGAGATTGACTGTGGTTGCTGCCATAGTTTTCGCCGCCAGCAGCATTCCTTTATGAGCAATAGATGTTCGCCCCTGGC
TAACCAGTTGCCACGTATGTAGCGGTGTACCGACGGCAAAACAGGGGCTGAAACACTGGGCAACAGGCAGTTTCCAACTG
ACGTCGCCGACATCAGTCGATGCCGCAAGCACGTTATCGGTGGCGGCATATGGAGCGACTTCATTCGCCAGTACCGTTTC

ACGATGACGTAGTGCAAAAACCTTGCCGTTTTCGCCACCGGTTGCGGCGATATTATTCAGACTGTTTTGCCGATCGTTGG
AGGTGAGCGTAGCCTGAATTTGTTTCGCAAAAGCCAGTTCTTCGGAGTTCCATTCCGGGGTACCAAAATGGGATAGGGCC
TGGTACATGGCATTTTCTAAGGTGCGATTCGGGAGATAACTGGAACAGGCTTTGTCGAAGCGGCATTCAACCGTGGTTTC
GGTCATCAATGCCGCACCTTCGGCGATTTTGGCGACCCGATCATAAATATGCTGCACGTCGGTCATTTCGGGGGCGCGGA
TAAGATAAAGCACTTCTGCCTGCGCCTGGACCACGTTGGGCGAGATCCCGCCGCTATTTGTGATGGCATAGTGTACGCGC
GCTTTTTCAATAATATGTTCGTTGAGGAAGTTGGTGCCAGTGGTCATCAACGTTACGGCATCAAGGGCGCTGCGTCCCAA
ATGAGGGGAATTCGCGGCATGTGCTGCGATCCCTTTAAAGCGCCATGATGCCTGAATGTTTGCCAGCGTGCGGGTATTGA
ACATACCGGCAAAGGCTTCCGGGTGCCAGGTGAGTGCCGCATCCACATCATCAAATACCCCCTCGCGAACCATGAACGTT
TTACCCGAGCCGCCTTCTTCGCCAGGACAACCATAAAAGCGCACCGTGCCGCCTTGCCCATATTGTTCCAGCCATTTCTT
GACGGCTATTGCAGCGGCAAAGGCGGCGGTTCCCAGCAAATTGTGTCCGCAACCGTGACCATTTTCACCGGGCGTCACGG
ATGTAGGTTGCGCGCAACCTGCTTGCTGACTTAAACCTGCCAGGGCGTCATATTCTCCCAGCAGGGCGATAACCGGTTTG
CCTTGACCAAACGAAGCAATAAAGGCATTTGGGATATTGCCTACGTTGCGGGTAACGGTGAAGCCTGCAGATTCCAGCGC
CGAAGCCAGATGCTCCGCTGACCAGAACTCTTCAAAACGTGTTTCTGGATGATCCCAGATTTGATCGGCAATATCGGTAT
AACGTTGGCGATCGGCTTCAATCGCATCGTCGATAAAACGATAGATTTCCTGCATCAGATACCTCGCGTCCAGGGAAAAT
TGAGCGCGGTGCGCGCCAGCGTTTCGACGGCAATAGCGAGAACCTGCTCGTCAAAATCGAATTTTTCGTTGTGATGACCT
GCCGCCAGCTGTGTGCCAAACACCACGTAGGAGGCTTGCCCTTGATGTTGCTGCACGCGGGCCATCATTAATGTGGCATC
TTCGGAACCCGCAGGCGCTTCAACACGTTCAATGGCCTGATTGACCCCCGCGACCTGAGCCGCCTGACTTTGCAACCATG
CGACCCATTGCGGCGAAGGAGAACTGGCGGTAGCTGCACCCATCAGACGAGTTTCAACGCCGACACCATACATGGTTGCT
GCGCCCTGAATCGCTTGTTGTGCACGGTCAAAAACATATTGATTAATGACGTCGCTGGCCCCGCGTGTTTCCACTTTCAG
CAACGCCGAGGCAGGAACAACGTTACGACCGCTTCCTGCCTGCATAACGCCCACGTTTACTCTGGAAGCTCCTTCGCTGT
GCGGGGCGATTGCATGCAGTGCAAGAGTGGCTTGTGCTGCCGCCAACAAGGCATTGTGACCGTCTTCTGGTTTTGCGCCT
GCGTGAGCGGCGGTACCGGTGAAGTGCGCGTCAAATTTGGTGGTTGCCATAAAATTATCACTGCCGCACACCACGGTGCC
CGCAGGTACGCCAGTGCCAATGTGCACGGCAGTAAAATAATCAACATCATCTACGACACCTGCATCGACCATCGCCCGCG
CGCCACGCGTACCTTCCTCTGCAGGCTGAAAAATCAGTTTGATGACGCCATGTAGTCCGGACTCGAACTGTTTAAGGGTA
TGCGCCAGCCCAAGCCCAATGGCGGTATGTCCATCATGACCACAGGCATGCATCATTCCGGCGTTACATGACGCAAAACC
GTCGCGGTAGGGGCGATGGCTGACATCCTGCTCTTCACTGAGATCCAGCGCGTCCATATCGACACGGAAAGCCATCACCG
GACCGGGGCGACCGGTATCCAGGGTGGCGACGATACCAGTGAAACCACCTTCAAAAGCCGCAATCCATTGTGCTAGCGCA
CCCTGTTGACGAGCGCGCTCGAATTCGCGTTGTAGAGTGAATTCATCAGGTAATCCCATCCGGCTACTTTCATTAACTAC
TTCGCGACCCAGCGCCAGTGAATAGCCGAGCTGGTGCAATTCTTCCGCAACAAGGGTGGCAGTGCGGAATTCCACCCAGC
CAGACTCTGCATAGTGATGAAAATCACGTCGCCAGTGCGATAATTTTGGGGCAAGCGAATTAACAAATTGATTCAAAGAC
TCCATAACCTTTCCCGTCATCAGTAAAAAGTGTGACCCGGTTCACGTAGCGATAGTTTTTACTTATCACTAACTGATTTT
TCACAGTTTTAACCGTTCATAAATTACCCTGACACAATCATCTGCATTAAAGTAGATGCCAGTTTCTTTGGTCTGATAAA
TAACGGTTATCGGTGGCGTCATGGCTTTTCAGGTAAAAATTCATCAAATTCGGGCTTTTGTTGAAGTGGCTCGTCAGGGC
AGCATTCGCGGAGCGAGCCGAATGTTGAATATGTCGCAACCGGCACTGAGTAAATCTATTCAGGAGCTAGAAGAAGGGTT
AGCGGCGCAACTCTTTTTTCGCCGTAGTAAAGGCGTGACGTTAACTGATGCCGGTGAAAGTTTTTATCAGCACGCCAGTC
TAATTCTTGAAGAGCTGCGCGCAGCCCAAGAGGATATTCGCCAACGACAAGGGCAACTGGCAGGGCAGATTAATATCGGC
ATGGGGGCCAGTATTTCCCGCAGTCTGATGCCAGCTGTCATATCTCGTTTTCATCAGCAGCATCCGCAGGTAAAAGTACG
CATTATGGAAGGGCAACTGGTGTCGATGATTAATGAATTGCGTCAGGGAGAATTGGATTTCACCATCAATACCTATTATC
AGGGACCGTACGACCACGAATTTACTTTTGAGAAATTACTGGAAAAGCAATTCGCGATCTTTTGCCGCCCGGGACACCCC
GCCATTGGTGCCCGTTCGATCAAACAGTTACTGGATTACAGCTGGACAATGCCGACGCCACACGGCAGCTACTACAAACA
GTTGAGTGAATTGCTTGACGATCAGGCGCAAACGCCACAGGTCGGTGTAGTCTGCGAGACGTTCTCAGCCTGTATCAGTC
TGGTGGCAAAAAGCGATTTTCTCAGCAAACTGCCTGAAGAAATGGGCTGCGATCCCTTGCACGGACAGGGGCTGGTGATG
TTGCCGGTTAGCGAAATTTTACCGAAAGCGGCCTATTATTTGATTCAGCGGCGTGATAGTCGCCAGACACCACTGACCGC
GTCATTAATCACGCAATTCCGGCGAGAATGCGGCTATCTGCAAAGTTAAAACTGCATAAAAAAATAGAGTCTGTCGACAT
CCGCCAGACTCTACAGTACACACAGCAGTGCATCCGCGTCTTAAATCCGGCATTGTCTCCTCTGCGCCGGTGACTGTGTT
ATAACGGTTTTAACCTTTAGTTGCCAATTTTCTTCAGTGACAGATTTCACGAAAATATAAGTTACATTATTAATATCGTG
AATGAATAATCATGCATAAGTATTTTGCTTAAAATATCGGCAATATTTGGAACTTATTACTGGAAATTTGGGTAATACGT
TGTTGGACCGACCCGGTCTGGTTATCATATCGCGCTCTTAATTGCGGGAGGATGTAACATGAACCTTGACGACAAATCGC
TGTTTCTTGACGCCATGGAAGATGTCCAGCCGCTGAAACGTGCTACCGATGTCCACTGGCATCCAACGCGTAACCAACGT
GCGCCGCAGCGTATCGACACGCTGCAGCTTGATAATTTCCTCACCACCGGATTTCTCGACATCATCCCACTAAGTCAGCC
GCTGGAGTTTCGGCGGGAAGGGTTGCAACATGGGGTGCTGGATAAGCTGCGCAGTGGTAAATATCCGCAACAGGCGAGCC
TGAATCTTTTGCGCCAGCCGGTGGAAGAGTGCCGCAAAATGGTGTTCAGTTTTATTCAACAAGCCCTGGCGGATGGTTTG
CGTAACGTGCTGATTATTCATGGTAAAGGGCGGGATGATAAATCGCATGCCAATATTGTCCGCAGCTATGTGGCGCGCTG
GCTGACCGAATTTGATGATGTTCAGGCATATTGCACCGCGCTACCGCATCATGGCGGCAGCGGGGCGTGTTACGTCGCAC
TACGTAAAACGGCGCAGGCGAAGCAAGAAAACTGGGAGCGCCACGCTAAGCGCAGTCGTTGATCTCGAGACGCATCCGCG
GCTTATGCCGCCAGCACGCGGTTGCGTCCATCATTTTTCGCCCGATACAAAGCATCATCAACGCGTTTAAACAGTTCATC

GATGCTTTCATTTCCTTCGTGATGCGCCACACCAATGCTGACGGTAAAGCGTGGTAAGCCCGAAATACTCACTTTTGCCA
CGCTTACGCGGATAGTTTCAGCCAGCGAAAGCGCGGTATCCAGTGGGGTTCTTGGTAGCAATAAGACAAACTCTTCGCCT
CCCCAACGAAACACCAAATCGCCTTTGCGAGCGCAACTTTCGAGGGTGCGGGCGAGGGCGCATAACACCTCATCACCTTT
AGAATGCCCATAGAGATCGTTAATGTGTTTAAAACGATCGGTGTCGATGAGCAACAAGCTGTAATCCTGAGCGATGGCGA
GATGCTGCATTTGGCCTGGTTCCGTAATGTGATAAAACTGTCGCCGATTCAGTAATCCGGTCATCGCGTCATGGTGAGCA
GCATGTTCCAGCTGCTCCTCCAGCCGTTTTTGCTCAGTAATATCATGCACAATACATAACATGAGCTTGTCGCCATAAAT
TTCAATCGGTCCGGCATAGGTCTGCACATGACGAGTCGAACCATCCGCCAGTTTATGAACAAAATTCAAAGGTTTATGAC
CACCGGGTAAATGCGAGATTTCATGCATGATAGGCATGACGCGACGCCCGAGCATATTTATTTCCCAGGTATGTTTCTGG
CACATCGTTTCATGGTTATAACCATAGAAATTGAGCGCGGCGAGGTTAGCATCGACGATTTGTCCATCTCGTGACGGGTC
AATCAACAACATTGGTGCAGAGTTAGTCAGAAAAAAGCGCGCATAAAAACCTTGTTTTTTGCGCTGATAATTTGCCGAGC
GACTGGCTTTTAAACCCAGCGTTGCCGGCGCTTCGATACCTTCGAAAATAATCACCGGTTCTGTTTCTGTCAGCTTTCGC
AAAACAAGCCGACAGCTCAATGCTGTTTCCTCTTCTTTACGCTGAACAGTGAGGATTTCGATAATATCGTGTTGGTTTTG
CAGATCGGAGAGGTATTTCGGCAGTTCTTTTTGTGAGGAGACGGAATAGGGTCCGGTTCGTAGCTGACTAAACGTGAGGT
CTTGCATCAACAGTTTCGCCGCGCTATTGGCATAAATTAACTGTTCCTCAAAGGGCGAAACGATCCAGACAGGACTGGTG
AGTAAGTCCAGGGTATTGAAGTTGTGCGTAATCATTGAGATCCCGTTATTTTTATCAATTTTTGTTGCTATCCGATCGCA
AAAAAGCCACGTCATATGATCAGATAATTCTGATAATGATAGACGCTATTTAACACTTCACACGGTTTGTATACGGAAAA
GCATTTTGCTTTTTGTATTCAATTTAGACAGAATTTTATTAATCATTTCAGGGTAATGGGGTGATGAGATGTTGCGTAAC
AGGGCCAGAAGGCTAGACTACAAAATAATGCGTTGATGATGGAGGCACTGTGGAAGCGATTAAGGGATCGGACGTTAATG
TCCCGGATGCAGTATTTGCCTGGATGCTGGATGGTAGAGGCGGCGTTAAACCGCTGGAAAATACAGATGTGATTGATGAA
GCGCATCCCTGTTGGCTCCACCTTAATTATGTACACCATGATAGCGCCCAATGGCTGGCGACAACACCGCTGCTTCCCAA
TAACGTACGTGATGCGCTGGCGGGCGAGAGCACGCGTCCCCGAGTCAGCCGTCTCGGTGAAGGCACGCTGATTACATTGC
GCTGTATAAACGGCAGCACCGATGAACGCCCCGATCAACTGGTCGCCATGCGTGTATATATGGACGGGCGGTTAATTGTT
TCGACCCGACAACGCAAAGTGCTGGCGCTGGACGATGTGGTGAGCGATCTGGAAGAGGGCACGGGTCCGACCGATTGCGG
GGGATGGCTGGTGGATGTGTGCGATGCGTTGACCGATCATTCCAGTGAATTTATCGAGCAGCTGCACGATAAAATTATCG
ACCTTGAAGATAATCTCCTTGATCAGCAAATTCCACCGCGTGGATTCCTGGCTCTGCTGCGCAAACAATTAATTGTGATG
CGTCGCTATATGGCACCGCAACGTGATGTTTATGCTCGTCTTGCCAGTGAACGTTTGCCGTGGATGAGCGATGACCAACG
CCGTCGGATGCAGGATATTGCCGATCGCCTTGGGCGCGGCCTTGACGAAATCGACGCCTGTATAGCACGGACTGGCGTGA
TGGCGGATGAAATCGCTCAGGTGATGCAGGAAAATTTAGCTCGTCGTACCTATACAATGTCGTTGATGGCAATGGTCTTT
TTACCCAGTACCTTTCTGACCGGGTTATTTGGCGTCAACCTTGGTGGGATCCCTGGCGGCGGGTGGCAATTCGGATTTTC
AATTTTTTGTATTCTGTTAGTTGTTCTTATTGGTGGTGTTGCTTTATGGTTGCATCGTAGTAAATGGTTGTAACAAAAGC
AATTTTTCCGGCTGTCTGTATACAAAAACGCCGCAAAGTTTGAGCGAAGTCAATAAACTCTCTACCCATTCAGGGCAATA
TCTCTCTTGCAGGTGAATGCAACGTCAAGCGATGGGCGTTGCGCTCCATATTGTCTTACTTCCTTTTTTGAATTACTGCA
TAGCACAATTGATTCGTACGACGCCGACTTTGATGAGTCGGCTTTTTTTTGCCTGTTATTTATCAGCGTCTACCCTTTAA
GAGTCCACCCAATGACCAGAGGGAAATATGACGACACTTATTTATTTGCAAATTCCTGTCCCTGAACCGATTCCTGGCGA
TCCTGTTCCAGTGCCCGATCCGATCCCTCGCCCGCAACCCATGCCTGACCCACCACCCGATGAAGAACCGATTAAATTGT
CGCATCGTGAGCGTAGATCTGCGAGGATACGCGCCTGCTAACTTTGCGTCGATGACCACGAGAATAGATTGTGACCGCTT
TTTCTACCCTGAATGTTTTGCCTCCCGCCCAACTCACGAACCTTAATGAGTTGGGTTATTTAACCATGACGCCGGTGCAG
GCCGCCGCGCTTCCGGCGATCCTTGCCGGAAAAGATGTTCGCGTGCAGGCGAAAACCGGCAGCGGCAAAACGGCGGCTTT
TGGCCTCGGCTTGTTACAGCAAATTGATGCGTCGCTATTTCAAACCCAGGCTTTAGTGCTGTGTCCTACGCGTGAACTGG
CGGATCAGGTGGCAGGTGAATTGCGTCGGCTGGCGCGTTTTCTGCCAAATACCAAAATTTTGACGTTGTGCGGTGGTCAA
CCGTTCGGTATGCAGCGTGATTCGTTGCAACATGCGCCGCATATTATCGTGGCAACGCCGGGGCGTTTGCTGGATCACCT
GCAAAAAGGCACGGTATCACTGGATGCGTTGAATACGCTGGTGATGGATGAGGCCGACCGCATGCTGGATATGGGATTTA
GCGATGCCATTGATGATGTCATCCGTTTTGCGCCTGCATCTCGACAGACGCTTCTGTTTTCGGCAACCTGGCCGGAAGCC
ATCGCTGCAATCAGCGGACGAGTGCAACGCGATCCTTTGGCGATTGAAATTGACTCAACAGATGCTTTGCCACCCATTGA
ACAACAATTTTATGAGACATCCAGCAAAGGCAAAATTCCTCTGTTGCAACGGTTATTAAGCTTGCATCAGCCATCCTCTT
GCGTGGTGTTTTGCAATACCAAAAAAGATTGCCAGGCTGTCTGCGACGCGCTGAATGAAGTAGGGCAAAGTGCATTGTCA
TTACACGGCGATTTGGAGCAACGCGATCGCGATCAGACCCTGGTACGTTTTGCTAACGGTAGCGCCCGTGTACTGGTCGC
GACTGATGTTGCTGCGCGTGGTCTGGATATTAAATCGCTTGAGCTGGTGGTGAACTTTGAGCTGGCGTGGGACCCTGAAG
TTCATGTACATCGCATCGGTCGTACAGCTCGTGCAGGAAATAGCGGTCTGGCGATCAGTTTCTGTGCTCCGGAAGAAGCA
CAGCGGGCCAATATCATTTCTGACATGTTGCAGATAAAACTTAACTGGCAAACGCCGCCAGCTAATAGTTCCATTGCGAC
GCTGGAAGCAGAAATGGCAACGTTGTGTATCGATGGCGGGAAAAAAGCCAAAATGCGCCCGGGTGATGTATTAGGTGCAC
TGACAGGAGATATCGGGCTTGATGGCGCAGATATTGGCAAAATCGCCGTGCATCCGGCGCATGTCTATGTCGCGGTCCGT
CAGGCTGTTGCTCATAAAGCATGGAAACAGTTACAGGGCGGGAAGATTAAAGGAAAAACGTGCCGGGTGCGGTTATTAAA
ATAATGAAATGTTGAATTGCCGGGTGCAAGAGTAAACATCTTATTCGGGATTGCCGGATGCGACGCTGGCCGCGTCTTAT
CCGGCCTCCATAAGAGTAGCCCGATACGCTTGCGCATCGGGCGCTATCCTGGTTATTTCACTTCAACCACATTCAGCCGT
AACTCATCCAACTGATTTTCATCTTCTTCTGGCTGCCAGCACGCCGGTTGTAGTGGGATCTCTTCGCGATCAAACGCCAG

ATCACCCCCGTTAACCACTTCAGAACCGTGGGTGATGCCTTTGAAATCGAACAGGTTGGTATCGCACAGATGCGACGGCA
CCACATTCTGCATCGCGCTGAACATCGTCTCGATACGCCCTGGATAACGTTTATCCCAGTCACGCAACATGTCAGCAATC
ACCTGACGTTGCAGGTTAGGCTGTGAACCGCACAGGTTGCACGGAATAATCGGGAACGCTTTTGCATCGGCAAATCGCTG
AATATCTTTCTCGCGGCAGTAGGCCAGCGGACGAATAACGATATGTTTGCCATCATCGCTCATCAGTTTCGGAGGCATAC
CTTTCATCTTACCGCCGTAGAACATATTTAAGAACAACGTTTGCAGGATATCGTCACGATGGTGACCCAACGCGATCTTC
GTCGCCCCCAGTTCCGTTGCGGTACGATAAAGGATACCGCGACGAAGGCGAGAACACAGTGAGCAAGTGGTTTTGCCCTC
TGGAATCTTCTCTTTCACGATACCGTAAGTATTCTCTTCAACAATCTTGTACTCAACGCCCAGCTTTTCAAGATACTCGG
GCAGAACGTGTTCCGGGAAGCCCGGTTGCTTTTGATCGAGGTTAACAGCCACCAGCGAAAAATTGATTGGCGCGCTTTGC
TGCAAATTGCGCAGAATCTCCAGCATGGTATAGCTGTCTTTACCCCCGGAGAGGCAAACCATGATGCGATCGCCTTCTTC
AATCATATTGAAGTCAGCAATGGCTTCGCCCACGTTACGACGCAGACGTTTTTGTAATTTGTTCAGGTTGTATTGTTCTT
TCTTTGTAATTTGTTGATTTTCTTGCATTATTTCAGTTCTCTGGTACTAAATGGGGCAAATTGGGGGCAAACTTTGCAAC
TACGATAACCGCGCATTCAACATGGCTATCTGTTCGTCGTTCATGTCATCAATCCACATACCGTAAATTTCATACACCAT
CTGCGCAGTTTCATGCCCCATTTGGCTGGCTATAAATGCCGGGTTCGCTCCTGCCGTCAACAGCCAGCAGGCAAAAGTAT
GCCGCGTATGGTACGGATTACGGCGGCGAATACCAGCACGTTTTACTGCTGCATTCCACCTTGCCCCCAAACTGCTTACC
GAGTAATAAGGTTTTTGTTTTCCGTTACACACCCTGGGCATGAAAACAAAATGCAGTTTTTGCTTTTCGGTTCTGCCGTA
CTCCCGATGATAAAAGGTGATTTCGCTTTTGCGATGATGCCCGGTCAGTTTGTATTGCTCCTTCAGTGCTTCAAGAGCAG
GCTGCAGTAGTGTTACTGTTCGGATCCCGGCATTTGTTTTTGGGGGACCGAACATATCAAGTATCGTCAGGTTTCTTCTG
ACATTCACTATTCCCTTTTCGAGATCCACATCCTCCCACGCCAGAGCTGCCAGTTCCCCGTGACGAAGTCCTGAGTAAAC
GGCAAATTTCCACAAGTTCTGGCTCTGTCCTTTTTCACTTTCCATTAATGCATTGAATTCTGTTTTAGATAACGGATCAG
GCTTTATTCTGTTTCGCTGTAATTTTTTTACTCCTTCAAATGGTTTGGTTGATATAAATCCCGACTGATACGCAAAACGC
AACAGCGAACAGAGCAGGGCGATATAGTTATCAACTGTGCGCACGGTTCTTCCTTTTTTGTTGGATCTTGGATTATCCAG
GTAAAGCGTTTCTCCATGCAGCAGTTCATTCCGGTAGTTTAAGATATCGCTATAACGAATATGTGATATCGGGGTACTTT
CACAAATTATTATTCTGAGTGTTTTTAATTGTGATTTCGTTTTCTTCATTGTGTTTGTTGTTAACTCTGTCTCTTTAATT
TTTGTCCAGATATCACAAAGCTCTCCGAACGTTTTTATGACTCTCGTTGTCACCATTTTTGCCCCAGTGCTGGACTGGGG
AAAACGTCTTAAATACTCAAATTCACCGGAGTTTATTTCATGAACTATCAGCGCTCTTAAATTTCCGGCCTTTTTAATAT
TACTGTTTGTAATCTCCCAGCCTTTTAATGTTTCCCGACATCGTTTTCCTCGAAACATGAACCAGATGCGAATGTATCTA
CCTCTAATCTCGACACCTGTTGGTAATTTAGACATATCATGAGTCTTTGATAAACTGATTTATCTTTGGATAGTTGTACC
AGATAATCCCTCGTTTGCTGTCTGGCTTACCTAAAGGAGATACTCGTTTGAAGTGGAAGCCCTCCACCCAACAGTTCTGG
CGGTATGCTTCAATTTGTCTGGCCCCCAGACCAGTGCGAAGCATCAGGCCGTATTCAACCATCCACTCTTCATTAAAGAT
TACTTGTGCCATCGCATCACCTCTGGCAGGCGCCAATGTTAGACTGAAATTGACGCCCGATGTTGATTATTAATAATCAG
CTATGAAGTTTTAATTTGAATACAATGCAATTCTCGAGGACTGAAGTTTCTCGCAATTAAAATTTATCAGTTTTACTTTC
TGCTCTCTGGAAACGCCTGCTTCTTTTTTACCTGAGAGCATTTTTTCGCATTCTGATTTCGTTAGTTTAGATTTTGAATA
TCTTGTCCAGTTAGTAGGAGTGCCACCTTCCTTTTCAATAGTGGCGGTAATTTTATACATGAACACCTCCATTATTATTT
CCAGTGGTTCGTTTATTCCATCTTTCGAGTGCTTCTTTTTCACTTCCACCATAACCGGTTCGGGATTCGCATCCGTTACA
CTTCGCTCGGTAATATCCTGAAATGGCTTTCACCGTTACTGATGGACAACCACAAAATGGACATGGTTTAACATTGTCAT
ATCTCATAATTTTTCTCATAAAAAATATTTCAAGTTGGCGGTGCATTACACCGCCAGGCTGAATTATTCCTCTGAATTAT
CGATTACACTGTATTCCCCGGTTAATACAGAGGAATCTGCAGGATCGATTGTCAGTGGTTCCTTTTCATCCATTGATACT
GCACGCTGGATCTCAATTGATACGGGCAAATATTTGAACAGGCGACGAATAGCCGTTTTCTTTGCCATTTCTTCCCAGTG
AGTTACCCACGGCCCGTTATTACCAGCTTTACTCAGGCTGCGCACCAGCTCAATCTGTTTGCGCGTCATAACTTCAAACT
GAGTACCTCCGTCTTTCAGTCTTGCGACAGCATAGACGTGGGTAACCGGGGCATCTTCGTTTTCTCCCGGGCGGTGTATT
AACTTTTCATCAAGGCCAAATTCGAAGCTAAACTCGTCACCTTCACGGACAACACGGGCTGACAGGCTGGCGATTTGACC
AGAACGGCGAGCCAGATCAATCATGCCGCGATAGCCAATGATTAGCTGAACGTTCTTTTTACCGCTCTTTTCGTTTTTAT
TACCAAAAGGCAGTAAATATGCATGACCGAGGGCGCTACCTGGCTCAAGTCCGAGCTGTGAACACTGTACGATCGCACTG
ACAAAACTCATAGTGTCACAGTTTCCTAACGCCGGAACTTTACGAATTTCTGTGGTGGCGATACGGATCATACGTTCAGC
CGTCATATGGCGTGGAAGAGCTGCTGCCAGTTGCTCTTTCATTGATGGCTGGTTAATAAAACTAATCACGTCGCTATTTT
TAACTGCTGCTGGTGCACGGTTTCCCTGAGTTTTTTGCAGATCGGCTTTTGCGATTGGTGGTTGCTTAGTCATTTGCATA
TTCCTTAGCCCAGCGGGGCAGTGATAATGTCTTAATAGCTGGCCATTCATCGGTATTCAGGCAGTCAGACAGGGTTCGCA
GATTGCGGTGATATTCCTGTTGACCTGCCAGTTTTGCTTCTTCGCCCATCATGAAAATTTCAACCGGATAACGTCCGCAT
TCAATAGTTGTGCTGGCAACCAGAAAAACGAAAGTTGGCTGCACTCCAAACTGTGCTTCATAACCGTCACTGTAGAATGC
ATCCTGAACGTGATAGCGGTAGTCGTAATAAGCGGTTTTGAATCGTTGAATATCCGCCGTAGTTTTCACGTCCATGATCC
AGTGAAATTCAGGGATAATTTTGTCCGGACGGCACCGACACAAAATTCCTGTTTCAGGATCTTCCCAGTAAATTGATGAT
TCAGCGTGTCCGGCGCTTTCAACAAGCCATTGCCCCAGCGGCAAAGCCATAACGCTTTGATACATGAGTTCAATTTTCCG
GCCTTCTTCCGCAGTGATAACCGTTTTTCCTGTGCTTGCGCATTCCATCAGAAACGCTTTCTCTTCTTCTTTTCCGGCGT
TTGTACGGCGGTTAAATTCAGGTGCTACGATAAAGCGGTTACTGAATTCTTCCGGTTCAAGTACCCGGCAGTGGAAAGCA
GTTCCTAAATCGAGCGTTTTTGTCTTTGTGGTGTCCACGGGGGCATTTTTACGCCACAAATATAGTGCCGGAGTATCAGC
AATGTCATCGAGCTGAGACTTACTGATACCGGGACCCGCGTGGTAATTCTCATTCGAAATTCCGTAATAAATACCTGGCT

CTATGTCTTCTACGATTACGGGATCTGCGACTTCGCCAGTTTCATCACTGCAATCGCGATGCGGATCGCTGCCAGCATTC
TCATTGTGCGGATGTTCAGCGCCTTCCATTTCCTCCGGATCATTTTCCTTAGCTTCAACCTGACTCTCTTCATCGAATGT
TTCCTGGTATGTTGCGTCGCCCATCACCGCACCACAGTCAGGGCAGTTATCCCCGCCAGTCTGGCCGCAGGCATTGCAGG
CTATTTCCGGTTCCTGTTGCACTACTGGCTCAGGTTGATTCATATCTGGGCTGGTTTTTTCCGTTTCTGGCTGGTTCTGG
TACACACAATCGCGAGTCTGGATCCCCTTTACCCATTTCGGATCGTTCGGGTCGCTAATTCCGTCAACAAATTCACCACG
TGATGCAGCAAGCAATTTATCGTCATCGACAGGATTTTTTGATGGAATGTTTTTCCGGGCTTCATGGAGTTCTGCCCGCA
GTTCCTGATATTTCGCATCAACAGAATTTACCTGTGACTGAGCATCCAGCGGCTGCGTGTCCTGATGATGTTCAGTTGCG
TCCGGTTCCATTGTTTCAGCCTCTCCCTGTTCAACTGCCGTTGTTCCAGATGGTTGCGGTTTTTCTTCATCATCCTGTTT
TCCTTCTTCTGTTACTCGCTGCGGCATCGGGGCAGAGGAGCGACCGCAGGCAATATCCACGATTTCCGGATCAGGGTTGG
CATGATCGGTTTCAGTCAGTACTTTGTTCAGATATTCAGTGACGTGCGCGGGGATGACCTCGATCCCAATTGGTGCTTCT
TTTACGGACGCAACCACGATGGCGCGGGAATAATCCAGCCCGCCAGGCATGGTGATGAATTTGTCGCGGAAAACAGAAAA
GGGCGGTTTATTTTCAGCGATAATTTCCTCAATGCGTTTAGCGTGTGCCGGATGAAGGTTATAGATGTCCAGATCCATTG
AACGGGCCAGTACGCCAGTGGCTACGTCGCGCGCCAGTGACGTCAGATCGTGTACGAAACCTTCGCCGCGATCGGTGAGG
TTTCCGCCGCCAGCATTAGCACCGGAAGCCGTGCGAGTGATGTGTGAAACACGATTACCCTTCATCCACTCTTTTGTCAG
CAGTCCTCGATCGGTGTAGTCAGCGTTCAGGTATGCTTCGAAAAAAGCAGTTATCAGTCCCAGGTTTGAATTACCAGGAT
TAGGGAAAACTTTGTCAGTGTCACGAACCAGTTTGTGGAGTTCGCGAATTTCCAGCGGGTCGAGCAGGCTGGTTTTGTGG
GAAACAGCCAGGGCAGTAACAGCCGGTAGTTCTTCAGCCCGAGCAATGTGTAATGCCTGGAGTCCGTCGCGTGAAACGTG
CGTTACCGGTTTTTCGCTGCCGTGTTGAGCAAGCCAACGAATGGGCAGTTCCTGGCCAGAAATTGGGAGTAGCATATTCT
CCTCAATCTCAGTCATGTCTTCGCCGTTGACGTTGGTATTGCCTTGATAGTGAGCGTTGTCTGGTGCTGCTCCCGGTTTT
AGTTCCCATGTCATGGAGTCTTTGCTGAGTTGATAGCGTTCACTCCAGGTAAAATCGATCTCACCTTCAGCGGGCAGGTC
ATTAACGACAGGAAAATTCGTGGCAACAGCTTTAAAATAGCTGCTCAGTTTTTTACCTGACTTAACGATCAGGTAGTCCA
GAGTGGCACAGGTCGATTCAAAATCGTTGCTTGCCCACAGGACGACGTCAGGTTCACCGGATGATTTTTTCGCTTTCCGT
AACAGGAAGAGTGGTTTTGTGCTCATTGTTTTTTAACCTCAACTCAGATTAAAATTCGTTTTGTTCAGTGAATGATCTTG
CCGGATACACACTGTTCATAGCCTGCGCCATACGCAGGCTATTTCTTTCAGATTTCACCTTTTAATTTCATTGCAATTAG
AGTTGCCAGAAATTCGGCTTTTTTTTCTGCGGGCAGATTCTTTCCGATATGCACCAGGCACATTTTTTTGACACCTTCAT
CAAGTGTTTTTACGTTGCCTGATGGACCATCGATATCAACCACAGTGAATGGGGTTTCTTTATTTTCTGTTTTAATTACG
TAGCCAATGCGCTTTCCTTCCAGATTCACCTCGTGAACAATGTCATCGGTAGTTACAACAGTGGCTTCATAATTGGTAAT
CATGTTTTTCTCCTTAATTAAGGTTGAGCGAATACCTGCCATTTCTGGCATAAATTCAGTTTCGAATAGTCAATTAATTA
AAGTTCATGTGCCATCTGGTCTTTTTCGGCACAAGCTTCACTGCAATATTTTCTCGGTTCGTCTTTTGATAAAATCCCGT
GCATGAAGTGAAGCATTCTTTCAATAGCTTTGCTTTCTTCAACGTCTTTTTTGCAAAGGTGGTAAGCACATTTTATTTTC
TTAGTCATCACCATGACTCCGCCTTTACAGGTAAACCATCACGACCGAGGAAGACTTTAATCATGCGGTCAGTAATGAAT
GTTTTTGTGGTCAGGTTACGAATATATAGTTTTCGCTTTTTAATATTGTTTGCCGAGGCAATATATGTCCGGCCTTCATG
AAGAACATAATCGCCAGGAGTCACACACTGACGTGGTATTTCATCAGTTCCGAAGTGATGTGCAATCATAATTATCTCCA
TTTTTACAAATGAACTTTGTTGATGCGGTGTCTGGTGCCTCCAGGTGACTGCAACCAGTTAACAATTACAGTCGGCTTTC
CCACCCAAACCAATAAGGACTAACATGACTTTTAACTGTGCCACGTGCGCTTAGCCGCATTCACCGCATCACAAAATTCA
CTTTAAAAAGGGCGGACATCAGCCGAACTTCAAGAAAAAAACTGATGCCGCCAGGACTACACACAGCAATGTCGTTATTT
ACAACCGGAGGCGCACTCCCACCATTTAAATTTAACAGACAAGACCGACTCTTTATGGATATCGGAAATGCGCCTTCGTG
TTGTGCCCGGTTTTATTTCACCACCTCCGGGCTTCGGTGGTCTCGGCTATACCCCTACAGCGAGAGCTTGTGTTAACATT
TCAATACCCTTACAGTTGAGAGTTATTGATATGTTGGATGTATTTACTCCATTGTTGAAACTTTTTGCTAACGAGCCACT
CGAAAGACTTATGTATACGATTATCATTTTTGGTCTCACTCTCTGGCTGATACCGAAAGAGTTTACTGTCGCATTCAATG
CTTATACTGAAATACCTTGGCTCTTTCAGATTATCGTTTTTGCCTTTTCTTTCGTGGTCGCCATTTCCTTCTCAAGATTG
CGAGCACATATTCAAAAGCATTATTCATTACTACCAGAGCAACGAGTATTGCTTCGTTTATCTGAGAAAGAAATCGCTGT
ATTTAAAGATTTCCTTAAAACAGGAAATCTTATTATCACTTCTCCTTGCCGTAACCCGGTTATGAAAAAATTAGAACGGA
AGGGCATCATTCAACATCAGAGTGATAGCGCAAACTGTTCTTATTATCTCGTCACCGAAAAATACTCCCATTTTATGAAG
TTATTCTGGAACAGCAGGAGTAGACGTTTTAATCGTTAGCTTACTGTGTGCTTCTCCAACCATCGGCGCGCACCAGTTTC
GGTTTTAAATGTTTTGCTTTTGGTATACGTCATGGCAGTGAACGTTCCATCCTGGTTGGGGAACACGCCGCACACCAGGG
ATTCGTTGTTGCCGAGGTCGATTTTTTGCATTTTGCGAATCTCACATCTTGTTGCTACGTATAGCGACTTCTGCCTGCCA
GAGATCCCAGTCGTTGCTGCGTAAAGCCTGCACAGCCTGGTTGTAAGTGATACCGCAACAATCCATCAAATACTGAACTA
CTTCGTAATGCACCATCTTATCTCTCCCCTTAACGCCGGGTGGCGGAACTAACTGCTGCACTGCAAAATTTGAATCCCGC
CGTCATGTTCATACGCCTCGGGCTGGCTACTTAACCCCTTACCACTGCCTGGTAACTCGAAGTATTGCCCGGCGTTCTGT
GGGGCGGGGTGGGTGGTATGCTGGAACTATAGGTAATGCCTAATTGATTGTCAATAGGCTATGCCTAATGTTTTGAGCGT
AACCTAATAGGTGATGGCGACAGCAGAAAGTGATGGGGGGGTTAAATAACGGAATCCAGGAGTTTTCCGTCAGACCATAT
AAGTTTAAGTTCCAGTTTTTGTGATGTTCTGGCTTTTCCGTTCAGATTCAAGAGCTTTCAGATACTTACCCACTTTCATT
TCCATCGCTGCTATGTAGGCGCGAACATCGTGGTCAACCCAATCTGGTTCTGTAGCATTTCCAGATAACAGGAAAGCTAC
AATCGCTCTTATTTCATCAGAGGCTGCTTGATAAAGGTTGTTTATATCTAAAAGTTCACTTTTTGTATCTGAATTGGTGG
GGGTTGGTATGGGGTATTCGTTAAGCCCCCAATGCTCTGGACCAACAACATCAGAAAAGAAACGCCATAATTCTGGAAGT

TTATCTTTACTTATAGAGCCTTTCTTAATCCAGTCATAAATTGATGGTGGTTGGACTTTAAAGTGGCGTGCGACCTCCGC
CTTTGATTTGACGGATCCCGATGCGATTTTTTTGTTAATGGCCTGCTCTATCGCTCGGCCTAAGTCTTTACCACTAAGCA
TTGCTTAATATTCTCCTATGCGCATTACATTAGGCAATCCCTACCCTTACTGCATTAGGCACAGCCTATTGACAATTGCG
TTAGGCGTCGCCTAATATTTCTGTGTGTTTTTGGAGTTCATTCGATGAAAAAAGAGAACTATTCATTCAAGCAAGCTTGT
GCTGTTGTCGGTGGGCAATCAGCAATGGCTAGGCTTTTAGGTGTATCACCTCCAAGCGTAAATCAATGGATCAAAGGGGT
ACGTCAATTGCCTGCCGAGAGATGTCCAGCAATTGAACGTGCAACAAGAGGTGAGGTTCTGTGCGAAGAACTTCGTCCTG
ATATTGACTGGTCATATTTACGACGTTCGGCATGTTGTTCGCAGAATATGTCAGTGAAGCAACTAAATGACAGTAACAAA
TCCTCATTTGATCATACCTGAAACATCAAGAGGCAAATGATTCATGAAAATCAAGCATGAGCACATCGAATCAGTGTTGT
TTGCCCTAGCAGCCGAAAAAGGGCAGGCATGGGTAGCCAATGCAATTACTGAAGAATATCTGCGCCAGGGGGGCGGCGAA
TTGCCCCTGGTTCCAGGCAAGGACTGGAACAATCAGCAGAATATCTATCACCGTTGGTTGAAAGGTGAAACGAAAACGCA
AAGAGAAAAAATTCAGAAGCTGATCCCAGCAATTCTGGCAATCCTTCCGCGCGAGCTGCGTCACCGACTCTGCATCTTCG
ATACCCTGGAACGCCGTGCATTACTGGCGGCGCAGGAAGCGTTAAGTACGGCAATTGATGCGCATGATGATGCAGTCCAA
GCCGTTTACCGGAAAGCGCATTTCAGCGGCGGCGGTTCTTCCGACGATTCTGTCATTGTTCATTAAGCAAAAGTTTCCAT
GCTGTTTGTGCTTATTCTAAGCCACCGGGCAGCATCATACGGGGCAATTATGGCCGCATTACCATACATGCAACTGTACA
TAGCTGATTACCTGGCTGACACCATGCATTTGTCAGCAGAGGAGCATGGTGCGTATTTGTTGCTGATGTTCAATTACTGG
CAAACAGGAAAGCCAATACCTAAAAACAGGCTGGCAAAAATTGCCCGTCTGACTAACGAGCGATGGGCTGATGTTGAACC
ATCCTTGCAGGAGTTTTTTTGCGATAACGGCGAGGAATGGGTGCATCTTCGGATTGAGGAAGATCTGGCATCAGTCAGGG
AAAAATTAACCAAAAAATCAGCCGCAGGAAAAGCATCTGTTCAGGCCAGAAGAAGCAGAAAGGAAGCAGATGTTCAAACA
AAACAAGAGAGAAATTTAACAGGTGTTCAAACAGATGTTGAAGTGGTGTTTGAACATGATGTCAACACAAAGGCAACTAA
TAAAGATACAGATAAAGATCTAAAAACAGATCCCCCCCTAAATCCCCCCCGGGGGAATCGAGGTGTCAAAAAGTTTGACC
CTCTGGATATTACTTTGCCGAACTGGATTTCTGTCTCGCTTTGGCGTGAGTGGGTTGAATTTCGCCAGGCATTGCGAAAA
CCGATTCGAACGGAGCAGGGCGCTAACGGGGCGATACGGGAGCTGGAAAAATTCCGCCAGCAGGGTTTTTCACCTGAGCA
GGTGATTCGACACAGCATCGCCAATGAATACCAGGGCTTGTTCGCGCCGAAAGGTGTTCGACCTGAGACGTTACTCCGAC
AGGTTAACACCGTCTCGTTACCGGATAGTGCGATCCCGCCAGGCTTCAGGGGGTAACTGACCATGAAAAATATTGCGACA
GGCGATGTTCTTGAACGTATCCGCAGACTGGCCCCGTCACATGTAACCGCGCCATTCAAGACGGTAGCGGAGTGGCGCGA
GTGGCAACTTTCCGAAGGCCAGAAACGTTGTGAGGAGATCAACCGTCAGAATCGTCAGTTGCGGGTGGAAAAAATTCTGA
ATCGCTCTGGCATCCAGCCATTGCACCGCAAATGCTCGTTTTCGAATTACCAGGTGCAGAACGAAGGGCAGCGATACGCG
TTGAGTCAGGCGAAATCCATCGCTGATGAACTGATGACCGGGTGTACAAATTTTGCGTTCAGCGGAAAACCTGGTACCGG
GAAGAACCACTTAGCGGCAGCTATCGGGAATCGCCTGCTGAAAGACGGTCAGACAGTGATTGTGGTTACCGTGGCTGATG
TTATGAGTGCCCTGCACGCCAGCTATGACGATGGGCAGTCAGGCGAAAAATTTTTGCGGGAACTGTGCGAAGTGGATCTG
CTGGTTCTTGATGAAATTGGCATTCAGCGCGAGACGAAAAACGAGCAGGTGGTACTGCACCAGATTGTTGATCGCCGGAC
AGCGTCGATGCGCAGCGTGGGGATGCTGACAAACCTGAACTATGAGGCCATGAAAACATTGCTCGGCGAGCGGATTATGG
ATCGCATGACCATGAACGGCGGGCGATGGGTGAATTTTAACTGGGAGAGCTGGCGTCCGAATGTCGTCCAGCCAGGAATT
GCGAAGTAATTTTTACCGGGAGAAAAATTTAATGGAGACTGTTTTTGACGCACTGAAAGCAATGGGAAAAGCCACATCCA
TAGAACTTGCTGCGCGACTTGATATCAGTCGTGAAGAAGTGCTGAACGAACTATGGGAACTGAAAAAGGCTGGTTTTGTT
GATAAAAGCGCGTACACCTGGCGTGTGGCTGATAACAATGTTCAGCAGGAACAGCCAGCGCAGGCAGAACTGCCGGAAGA
AATCACCACAGCAACAGTAGCGAAAATCTCAGAGTGCGATTTAACCGCGACGATTGAACAACGAGGACCACAAACGGCTG
ATGAGCTGGCTACATTGTTTGGTACCACATCACGCAAAGTGGCTTCAACGCTGGCAATGGCAATCAGCAAAGGTCGTCTG
ATTCGCGTAAATCAGGGCGGTAAATTTCGTTACTGCATACCGGGCGATAATTTACCAGCAGAGCCGAAAGCAGCATCGGT
ATCTCCGCTCTGGTTATCTGCATCGTCGTCTGCCTGTCATGGGGTGTTAATCATTACCGTGATAACGCCATCGCCTACAA
AGAACAGCGCGACAAAAATGCCAGAGAACTGAAGCTGGCGAACGCGGCAATTACTGAGATGCAGATGCGTCAGCGTGATG
TTGCTGCGCTCGATGCAAAATACACGAAGGAGTTAGCTGATGCGAAAGCTGAAAATGATGCTCTGCGTGATGATGTTGCC
GCTGGTCGTCGTCGGTTGCACATCAAAGCAGTCTGTCAGTCAGTGCGTGAAGCCACCACCGCCTCCGGCGTGGATAATGC
AGCCTCCCCCCGACTGGCAGACACCGCTGAACGGGATTATTTCACCCTCCGGGAACGACTGGTAATGATGCAGGCCCAAC
TTGAAGGTGCTCAGCAATACATAACCGAGCAGTGTTTAAAGTAAAATCTTAACTACAATATGATTCATTTTGATGATTGT
TTCATAAGGAACAGTGAAGTAAGATCTAAGAGGAGTTAAATTTTATACAGTATAATCATAATATTGCAGCAAGGTGGTTA
TAATTGAAAGAATATTTAGATATGAATACATCTCATGTAAGAGTTGTTACTCATATGTGTGGGTTCCTGGTTTGGCTCTA
TAGTCTTTCAATGTTGCCACCAATGGTTGTAGCATTGTTTTATAAAGAAAAAAGCCTGTTTGTTTTCTTTATAACTTTCG
TTATATTTTTTTGCATTGGTGGCGGAGCGTGGTATACAACTAAGAAATCTGGCATTCAATTACGTACCCGTGATGGGTTT
ATTATAATTGTAATGTTTTGGATTTTGTTTTCTGTTATTAGTGCATTCCCTTTATGGATTGACTCAGAACTTAATTTAAC
GTTCATTGATGCTCTGTTTGAAGGGGTTTCTGGAATAACAACAACAGGAGCAACTGTAATTGATGATGTTAGTTCATTAC
CTCGGGCATATTTGTACTATCGGTCACAGTTAAATTTTATAGGTGGTTTAGGAGTTATTGTTCTGGCGGTTGCTGTATTG
CCATTATTGGGTATTGGTGGTGCAAAGCTTTATCAGTCAGAAATGCCGGGGCCATTTAAGGATGACAAACTCACTCCCCG
CCTGGCCGATACGTCACGGACACTGTGGATAACTTATTCTTTATTAGGTATTGCTTGTATTGTCTGTTATAGACTTGCAG
GAATGCCTTTGTTTGATGCTATTTGTCACGGGATTTCCACAGTTTCGCTTGGTGGTTTCTCAACTCATAGCGAGAGTATC
GGATATTTTAATAACTATTTGGTTGAGCTGGTGGCTGGTTCTTTTTCCCTGCTATCGGCTTTCAACTTCACTCTTTGGTA

TATTGTTATTAGCAGGAAAACGATAAAACCTTTAATCAGAGATATTGAACTTCGTTTCTTTCTGTTAATAGCCTTAGGGG
TGATCATTGTTACCTCTTTCCAGGTCTGGCATATAGGTATGTATGACTTGCATGGAAGTTTTATTCATTCGTTTTTTCTT
GCCAGCTCCATGCTCACTGATAATGGTTTAGCTACGCAGGATTATGCAAGTTGGCCCACGCACACGATAGTGTTTTTGCT
GTTGTCAAGTTTCTTTGGGGGATGTATAGGTTCAACTTGTGGTGGAATTAAGTCACTTCGATTTCTTATACTTTTCAAAC
AAAGCAAACACGAGATAAATCAGCTTTCTCATCCCAGAGCGTTGTTGAGTGTAAATGTAGGAGGGAAGATAGTTACAGAT
CGTGTAATGAGGTCTGTATGGAGTTTCTTTTTTCTTTATACTCTCTTCACGGTGTTTTTTATACTGGTGTTAAATGGTAT
GGGATATGATTTTCTTACATCATTTGCAACAGTGGCTGCATGTATTAATAATATGGGATTAGGTTTTGGGGCTACTGCAT
CGTCATTCGGAGTGCTTAATGACATTGCAAAATGCTTAATGTGCATAGCTATGATTCTTGGTCGCCTTGAAATTTATCCT
GTTATTATATTGTTTTCAGGTTTTTTTTGGCGCTCCTAATATATGGCTGATTTATAATTGTGAGTTTAATATTATGTTGA
CTCACTCATTGATCCAATACCTAACTTTACCAGCAACACCTCCGTCCCCAGTAGCACTGGCTGCTGGGGTGCGTTTTATT
CATAAAGCAAGGCTGTATGAGCGAGAAATTAAAGATAGTCTATCGCCCATTACAAGAATTGTCACCGTATGCGCACAACG
CCAGGACGCACAGTACTGAGCAGGTGGCACAACTGGTAGAAAGTATTAAGCAATTCGGCTGGACTAATCCGGTGCTGATT
GACGAAAAGGGCGAAATTATTGCGGGTCACGGTCGTGTTATGGCGGCTGAAATGCTCAAAATGGATTCTGTTCCGGTCAT
TGTTCTGTCTGGCCTGACGGATGAGCAGAAGCAGCGATAACGATCAGTATCGCTCCCGTAATGCATTAATCCGTCGCCAC
ATTGAGAAAATGGATGCCAGTTTGCACGTCGGAACGAAGGAGTTTGATATTTCAAAGGTTTCCGAGGTGGATTCTGTTGA
TGATTTACTCATTGATAATGCCGCTCGTTATCTGCTGAAAGACTGGAAAGGGGTTGGTGAACTGGTTAATGGTGTTGAGG
TTGCACTGGAATATACGGCAGAACGAGGGATCGCGCTGCTTAAGCAGAATCCAGAGTTGTACTGGCAGATCCTTGCAGAA
GCAGCCAGCATCGCCCAGGGTAAAGAGCAGCAGAAGCAGGATACGATAAAAAAGCCATAGCTGCCCAGCGGTGGTTATCG
GAGTTCGGGGGAGAAAGGGGGGAAAAGGCAAGATGGAAGCGAGAAAAACTCAGGTTGCCACCGATACCGGAACCAGAAAT
AGACCCGGTGCTTAAGGAGTTGTTGTACGCCTATTCGGTAATATCCCGTGCCCGACGTTATGCTGGAATGGCTGGGGTGC
CTTTGCCTTTATCTCTGACAGAGATAAATGAATATTTAGCCACTCATCCGGTATTGATTGAGCGCGATGAATTTGAAGCA
GTGATCTTTGCACTGGATGACCAGTATTTTCAGGAGCAGTGTGTGTAGTTGTTAATTACGTACACTCTGTTACAGAGATG
TGATGGTGTCTTTAATTAAATCGATGATGCTCCTGGAGAAAAGCATTGCGTGGCCTCGTAATCGCTATATCTACTATTAT
GTCGCCTGAAACCCACTTCGCGGTGGGTTTTTTGTTGTCAGGAGTTTTAATAAATGGCAGAGCAAACCTCGCGTCTCGCA
ATAATTATTGATAGCACTGGAGCGAAAAATAATGCTGACAATCTGACCTCCTCATTAGTCAAAATGACGCAGGCTGGGGA
AACTGCTGCAAATAGCGCAGGGAAAGTGACTAAGGCAACAGAAGATGAGAAGAACGCGCTCGCAAAATTAAAAGCAGCTA
TTGATCCAGTTGGTGCCGCAATTGATACTGTCGGTCGACGCTATTCTGAATTAAAGAAATTTTTCGATAAAGGGCTTATT
GATAAAGAAGAATATGAATTTCTTGTCCGTAAACTTAATGAAACCACAGAGGAATTGAGCGGGGTTGCGCAAGCGCAGAG
AGAAGCCGAGAAGGCCGGAAAACTTGCTGCCGCTCAGCAGGAAGCGCAGGCTCAGGCCTTTCAAAGAATGCTGGACAAGA
TCGACCCTCTGGCTGCGGCGCTAAGAAATCTTGAACAACAGCATGATGAACTTAATGCTGCGTTTGCATCCGGGAAAATA
AATGGTTCTCAGTTTGAGAATTATAGCCGAAAAATACAGGAAACACGGCGAGAGCTTACCGGAGAGGCTCAGGCAGAGCG
AGAAGCAGCAAAAGCGCATGATGAACAGGTTGTTGCTTTGCAACGTCTGATTGCTCAACTTGATCCTGTCGGAACTGCTT
TTAATCGTCTGGTAGAACAACAGAAACAGCTCAATGAAGCAAAAGCTAAGGGGATGCTTTCTCCTGAAATGTATGAGGAG
CTTTCTGGAAAACTTCGTGCTATGCGGAGTGAGCTTGAGGTTACTCAATCACAATTAAGCAAAACCGGAATGTCGGCAAA
ACAAACGGCTTTTGCTATGCGCATGTTGCCTGCACAAATGACGGATATTGTTGTTGGGTTGTCCACTGGTCAGTCGCCAT
TTATGGTGTTAATGCAGCAGGGCGGCCATTCAGCTATGCAGGAGACAAGAATCGCCAGCTTACCCGTTACAGCGATACCC
GCTGGCATGAAGATTCCGTGCGTAACCGCTGGTTCAGCGTGATGGTGGGGCCGTCTGTGCGCGTGAATGAATGGTTCAGC
GCGTATGCGATGGCGGGTATGGCTTACAGCCGTGTGTCGACTTTCTCCGGGGATTATCTCCGCGTAACTGACAACAAGGG
AAGGTGCGAATAAGCAGGTCATTTCTTCCCAAGCTGACTCGCTGATTAAAATTTCGCGGATCTGGGCCGATTTTTTTCCC
GCAAACACATCGAATCAGCCTATTTAGGCTATTTTTTCCACCATTTCTGGCGTTATTTCCGGTTTTTACTGAGATCTCTC
CCACTGACGTATCATTTGGTCCACCCGAAACAGGTTGGCCAGGGTGAATAACATCGCCAGTTGGTTATCGTTTTTCAGCA
GCCCCTTGTATCTGGCTTTCACGAAGCCGAACTGCCGCTTGATGATGCGAAACGGGTGCTCCACCCTGGCACGGATGCTG
GCTTTCATGTATTCGATGTTGATGGCCGTTTTGTTCTTGCGCGGATTCTGCTTCAAGGTTTTTACCTTGCCGGGACGCTC
GGCGATCAGCCAGTCCACATCCACCTCGGCCAGCTCCTCGCGCTGTGGCGCTCCTTGGTAGCCGGCATCGGCTGAGACAA
ATTGCTCCTCTCCATGAAGCAGATTACCCAGCTGATTGAGGTCATGCTCGTTGGCCGCGGTGGTGACCAGGCTGTGGGTC
AGGCCACTCTTGGCATCGACACCAATGTGGGCCTTCATGCCAAAGTGCCACTGATTGCCTTTCTTGGTCTGATGCATCTC
CGGATCGCGTTGCTGCTCTTTGTTCTTGGTAGAGCTGGGTGCCTCAATGATGGTGGCATCCACCAAAGTGCCTTGGGTCA
TCATGACGCCTGCTTCGGCCAGCCAGCGATTGATGGTCTTGAACAATTGACGGGCCAGTTGATGCTGCTCGAGCAGGTGG
CGGAAATTCATGATGGTGGTGCGATCCGGCAGGGCGCTATCCAGGGATAATCGGGCAAACAGGCGCATGGAGGCGATTTC
GTACAGGGCATCTTCCATGGCACCGTCGCTCAGGTTGTACCAATGCTGCATGCAGTGAATACGCAGCATGGTCTCCAGCG
GATAGGGCCGTCGGCCATTGCCCGCCTTGGGATAAAACGGCTCGATGACAGCGGTCATATTCTGCCATGGCAGAATCTGC
TCCATGCGGGAGAGGAAAATCTCTTTTCGGGTCTGACGGCGCTTAGTGCTGAATTCACTATCGGCGAAGGTGAGTTGATG
GCTCATGATGTCCCTCTGGGATGCGCTCCGGATGAATATGATGATCTCATATCAGGAACTTGTTCGCACCTTCCCAAGGG
GAAAACGCACGACGTGCTGACCGGAAGTGATGACGGTCGCCACAGCAACACGTCTCTGGCGTGGGGAGCTGGCGTGCAGT
TTAACCCGACCGAATCCGTGGCCATTGATATTGCTTATGAAGGCCCCGGCAGTGGCGACTGGCGCACTGACGGTTTCATC
GTGGGTGTCGGTTATAAGTTCTGATTAGCCAGGTAACACAGTGTTATGACAGCCCGCCGGTTCAGGCGGGCTTTTTTGTG

GGGTGAATATGGCAGTAAAGATTTCAGGTGTACTGAAAGACGGCACAGGAAAACCGGTACAGAACTGCACAATCCAGCTG
AAAGCAAAACGTAACAGCACCACGGTGGTGGTGAACACGCTGGCCTCAGAAAATCCGGATGAAGCCGGGCGTTACAGCAT
GGACGTTGAGTACGGTCAGTACAGCGTTATTCTGTTGGTGGAAGGATTCCCGCCGTCACATGCCGGGACCATTACCGTGT
ATGAAGATTCTCAACCCGGTACGCTGAATGATTTTCTCGGTGCCATGACGGAGGATGATGCCCGTCCGGAGGCACTGCGC
CGTTTTGAACTGATGGTGGAAGAGGTGGCGCGTAACGCGTCCGCGGTGGCACAGAACACGGCAGCCGCGAAGAAGTCAGC
CAGTGATGCCAGCACATCAGCCCGTGAGGCGGCAACCCATGCGGCTGATGCTGCGGACTCAGCACGCGCAGCCAGCACGT
CAGCCGGACAGGCCGCGTCGTCGGCTCAGTCAGCGTCTTCCAGCGCAGGAACGGCATCAACAAAGGCCACTGAAGCATCA
AAAAGTGCTGCCGCTGCAGAGTCCTCAAAAAGCGCGGCGGCCACCAGTGCCGGTGCGGCGAAAACGTCAGAAACGAATGC
TTCAGCGTCACTACAATCAGCAGCCACATCTGCATCCACCGCGACCACGAAGGCATCAGAAGCTGCGACCTCGGCCCGGG
ATGCGGCGGCCTCAAAAGAAGCGGCAAAATCATCAGAAACGAACGCATCATCAAGCGCCAGTAGTGCAGCTTCCTCGGCA
ACGGCGGCAGGAAATTCCGCGAAGGCGGCAAAAACGTCCGAGACGAACGCCAGGTCTTCTGAAACGGCAGCGGGACAGAG
CGCCTCGGCTGCGGCAGGCTCAAAAACAGCGGCTGCGTCGTCTGCCAGTGCAGCGTCAACAAGTGCCGGGCAGGCCTCAG
CCAGTGCCACCGCCGCCGGAAAATCGGCAGAAAGCGCCGCATCGTCTGCTTCAACAGCCACAACGAAGGCTGGCGAAGCC
ACTGAACAGGCCAGCGCAGCAGCGAGGTCTGCTTCCGCAGCGAAGACATCCGAAACGAACGCGAAAGCGTCGGAAACAAG
CGCAGAATCCTCAAAAACGGCTGCCGCATCGTCAGCCAGTTCGGCGGCGTCATCGGCATCATCGGCGTCTGCTTCAAAAG
ATGAGGCGACCAGACAAGCGTCAGCAGCGAAGAGCAGCGCCACGACGGCATCCACGAAGGCGACAGAGGCTGCTGGCAGT
GCGACGGCGGCAGCTCAGAGCAAAAGTACGGCGGAATCCGCGGCAACGCGCGCCGAGACAGCAGCTAAACGGGCAGAGGA
TATTGCATCCGCCGTGGCGCTTGAGGATGCAAGTACGACGAAAAAGGGGATAGTACAGCTCAGCAGTGCGACCAACAGTA
CGTCTGAAACGCTGGCGGCAACGCCAAAGGCAGTAAAATCAGCCTATGACAATGCAGAGAAACGTCTGCAGAAAGACCAG
AACGGCGCTGATATACCCGATAAGGGATGCTTCCTGAACAACATTAACGCGGTCAGTAAAACAGACTTTGCTGATAAGCG
TGGTATGCGTTATGTGCGGGTTAACGCTCCTGCAGGTGCAACATCTGGAAAATATTACCCTGTTGTTGTTATGCGTTCTG
CTGGCTCAGTAAGCGAACTGGCATCAAGAGTCATTATCACCACGGCAACGCGAACCGCAGGCGATCCGATGAATAACTGC
GAGTTTAACGGATTTGTTATGCCTGGTGGCTGGACTGACAGGGGGCGTTATGCTTATGGCATGTTCTGGCAATATCAAAA
CAATGAACGAGCCATTCACTCAATAATGATGAGTAATAAGGGCGATGATTTGCGCTCTGTGTTCTATGTTGATGGCGCTG
CTTTCCCTGTTTTTGCGTTTATTGAAGATGGCCTGTCAATATCCGCACCTGGTGCTGATCTCGTTGTTAATGATACGACC
TATAAGTTTGGGGCAACAAATCCGGCGACTGAATGTATCGCGGCGGACGTTATCCTTGATTTTAAGAGTGGGCGTGGTTT
TTATGAGTCTCATTCGTTAATCGTTAACGATAACTTGTCGTGCAAAAAACTTTTTGCCACAGACGAAATTGTAGCGCGTG
GTGGTAATCAGATTCGAATGATAGGTGGGGAGTATGGTGCATTATGGCGTAATGATGGCGCTAAAACTTACCTGCTGCTT
ACCAATCAAGGTGATGTTTATGGTGGCTGGAATACATTAAGACCGTTTGCTATTGATAACGCAACCGGCGAACTGGTTAT
TGGAACCAAACTGTCCGCAAGTCTGAACGGTAATGCATTAACAGCAACAAAGCTGCAAACGCCAAGACGGGTTTCTGGTG
TTGAGTTTGATGGTTCCAAAGATATTACTTTAACCGCCGCGCATGTGGCTGCTTTTGCCAGAAGGGCAACGGATACATAT
GCCGATGCGGATGGTGGCGTTCCATGGAATGCCGAATCTGGCGCTTACAATGTCACCCGCTCTGGCGACAGCTATATTCT
GGTTAACTTCTATACCGGAGTCGGAAGTTGCCGGACCCTGCAGATGAAGGCGCATTACAGAAATGGTGGTCTGTTCTACC
GTTCTTCAAGAGACGGTTATGGTTTTGAGGAAGACTGGGCAGAAGTTTATACCTCGAAAAATCTTCCACCAGAAAGCTAC
CCAGTCGGCGCACCAATCCCGTGGCCATCAGATACCGTTCCGTCTGGTTATGCCCTGATGCAGGGGCAGGCTTTTGACAA
ATCTGCTTACCCGAAACTTGCAGCCGCTTATCCGTCAGGCGTGATCCCTGATATGCGTGGCTGGACGATTAAGGGCAAAC
CTGCCAGTGGTCGGGCCGTATTGTCTCAGGAACAGGACGGCATTAAATCGCATACCCACAGCGCCAGCGCATCCAGTACA
GATTTGGGGACGAAAACCACATCGTCGTTTGATTACGGCACTAAATCCACGAATAACACCGGGGCACATACACACAGTGT
GAGCGGCTCTACAAACTCGGCTGGAGCACACACACACTCACTAGCCAACGTGAACACGGCTAGTGCTAACTCCGGTGCTG
GTAGTGCATCAACAAGATTGTCTGTTGTGCATAATCAAAACTATGCAACATCATCTGCTGGCGCACATACCCACTCACTG
TCCGGCACTGCTGCAAGCGCAGGTGCACACGCGCATACTGTCGGTATTGGTGCTCATACGCACTCCGTTGCGATTGGTTC
ACATGGACACACCATCACCGTTAACGCTGCTGGTAACGCGGAAAACACCGTCAAAAACATCGCATTTAACTATATTGTGA
GGCTTGCATAATGGCATTCAGAATGAGTGAACAACCACGGACCATAAAAATTTATAATCTGCTGGCCGGAACTAATGAAT
TTATTGGTGAAGGTGATGCATATATTCCGCCTCATACAGGTCTGCCAGCAAACAGTACCGATATTGCACCGCCAGATATT
CCGGCTGGCTTCGTGGCTGTTTTCAACAGTGATGAGTCATCGTGGCATCTCGTTGAAGATCATCGGGGTAAAACGGTTTA
TGACGTGGCTTCCGGCAACGCGTTATTTATTTCTGAACTCGGTCCGTTACCGGAAAATGTTACCTGGTTATCGCCGGAAG
GGGAGTTTCAGAAGTGGAACGGCACAGCCTGGGTGAAGGATACGGAAGCAGAAAAACTGTTCCGGATCCGGGAGGCGGAA
GAAACAAAAAAACAACCTGATGCAGGTAGCCAGTGAGCATATTGCGCCGCTTCAGGATGCTGCAGATCTGGAAATTGCAAC
GGAGGAAGAAATCTCGTTGCTGGAAGCATGGAAAAAGTATCGGGTATTGCTGAACCGTGTTGATACGTCAACTGCACAGG
ATATTGAATGGCCAGCACTGCCGTAGGGTAAAACATATAAATTCTATAATTAGATGTATCTTTCCATTTACGGCAAGGAA
GGGGGCTTGGAAGACGTAAAGCATCTCACACCGAGATTATTTTTTATATGTCAGGTGTCTGAAGTTTTGCTTTGGCTCTT
AAAATGGTTTGCCGCGAGGTTTTGAATTCCCGGGCAATGGCACTTATACTTACACCTGACTTAATTCGTTCGAATACCAC
CTGTTTCTGTTCTTCATTTAACACAGGAGGTCGACCAAAACGTTTCCCTGCGCCGCGGGCTCTTACTATCCCGGAATGAG
TGCGTTCAAGTAAAAGGTCTCGTTCAAATTCAGCGACTGCTGAAATTACTTGCATCATCATTTTTCCTGTTGGACTGGTC
AGGTCAATGCCACCCAATGCTAAGCAATGCACTCTGATACCTGTTTCGGTCAGTTGTTCCACTGTTTTCCTGATATCCAT
TGCATTACAACCAAGGCGATCCAGTTTTGTCACAATCAATTGATCACCACATTTCAGGCGAGCAAGCAACCGGTTAAAAC

CAGGACGCTCACTGGTTGCTGCTGAGCCGCTAATGTGTTCTTCGATTATTTGCTGAGGTTTGATTTTAAAACCTGCACTT
TCGATTTCCCGGCGTTGATTTTCGGTGGTCTGATCCAGCGTTGATATCCGACAGTAAGCAAAAATTCGAGACATAGTGAG
ACTCTATACGAAATTGGTGTTCATATCATAATGCATCTCAGAAAATAATTATGATTATTTTTGTGCATATTTGTATGTAC
ACGTTCGAAAATAAACGAATGCGTATGCAACCCCGTAATTTTGGTGAGACCCAAAATCGATTTTGTGAAAAATGGCTTTA
ACTCGGTTTGTTTTTCGAGTTCCGGGCGGACTCAAGGAAGAAGAATAGTGTTGCGTGTTATTTTAACCAGATTTCAAGTT
GTTTGGTCGTGGAAAAGTGGAGCAAAATGTTGTTAAAGTGGAAAAATGATAAAAAAGTAAGTTTATTATATTACATTTTA
CCATTTAAATTTTGGTTGTCTTTAAGAACTGATATCGCTGTTTGTAATAATTCTTTGTTATCCAGCCATGATGTTTTCTT
TATGTTTCCTTCAATGTAATCAAGCAATGTTCTGGTATTGATAGGTCTTCCCTGTTTTGCTACTTCCACTACAGCATCCC
CTAGGATAATTCTTACTTCAGGAAGCTGCGCAGGGAACCACTTTAGGGTGTCTTTTGATTTCATGAAGATATTCCTTAAA
ATATTATTGATTTTCATTGCGATATTGTATGTCTGATTCAGGATATGTTGACTTATACATCGGTTTTGTCTGGGTTATTG
GATATGCCAATCCCTAATTTTATTAGAGCATGACTAAAAATGCTGAATATGATAAGGAGCGAAGTGATTATCAGTATGCT
GTTCATATAGCCTCGAATTAGTAATGTGTTATATATGATATAGTTGACAATTTTTATCCTGGGTGTTCTTAAAGTTCGTA
GATAAACATTGTCGTTTCAGGTATACAGGAATGCTAACAGGTGGCGGCAAAAATCAGGCGGTTTATGGCGCAAGCTGAAG
TGGCAACTGCAAACTATCTTATGCAGAGACTCTACACGGATTGGGTTTAAAAGTATACATAGATAACAGTTTTTATCTGA
AGAAGAAAAATATCAAGGTGATATAGCCTATATGCCTTTGATGCGGAGGAATGAATGTGATGGGAGTGATGTATCTGAAT
AGTTGAAAAACCGCAGTCACGTCGTATGCAAGAACGTGCTGCGGTTGGTTTGACTTTGATTGAGACGTTTTGGAATTTTT
TTCGGTGGCAAAAATGGGGCAAAACGCTGCAAAAGGGGCAAAAAAGGGGCAAAAAAAGAGTGGATTATCGTAGCTTATTG
TTGTCGCTGATGATATTTAACACATTGAAAAATAAGTAAAATACTTATGAGTCAGAGAGTTGTGATTTTTGCCCTTACTT
GTTCAGGTTGTATTGTTCTTTCTTACTAATTTCTTGATTTTGCGACATTTAAAAGCGACTCAATTCGTTATATGGCATCA
GAAGAGTATGCGTCATGCCGGAACGCCCAGCATAAGAAATCTGATATAAAAAACTGTGGCGTGTATGGTACGGATTAGAG
GGGAAAATGTCAGCACATTTGCGAAATGAATCAAAAAGCCCGCAGCAATGTGCGGGCGTTAGTGTCAGCGCACAACCAGC
ACGGAGCACTCTGCGTGACGCACTACAGCTGCGGCGTTGGAACCGAGCAGATAAGTGGTGATATCCGGTCGATGGGAAGC
AATGATGATCATATGAGCGGGGATCTTCTTCGCCAATTCCAGAATGCGGTCTTTGGGCGAGCCTTCCTCAACATGGACAT
GCACTCTGTCGGTTGGCAGTTTAAATTTTTTAATGATCTCTTCCAGTTGCGATTTGGCTTCCGCTTTCAGGTCATCCATT
GCCGGTAATTCTGCGGAATACGCTAAACCCAGAGAGGCATAGTAGGGCAGTGAAGGTATTACCGTCAGGAAATGAACCTC
TGCATCATCAATCTTTGCCTCTTCCTCAACGTGGCTAATCACGCGTTGAGTTAATTCTGAATCGGAAATATCGATAGGGA
CAAGAATCGTTCTGTTCATAAAACCTCCTGTTTTAGTATCCGCATAAAGTGTAACGCCAGATGACACTTTTTGTGTAATG
ACGGAGTTCACATTTTTAATTTAGATCAAAGGAGGAAGAATAAGCAGAAAAAGCCCGCCATAACAGCGGGCAGGAGGATT
TAGAACTGATAAACCAGACCTAAAGCGACAATATCATCGGTAGAGATGCCATTGGCAGCGTAGAAGCTGTCATCTTCATC
CAACAGGTTGATTTTATAGTCAACGTAGGTGGACATGTTTTTATTGAAATAGTAAGTCGCGCCAATATCGGCGTATTTAA
CCAGATCTTTATCATCAACACCTGCCGGGTTGTCTGCACCACCCGCAGCGTGCAGGTCACGGCCTTTAGACATCAGGAAA
GAGACTGCCGGACGCAGACCAAAATCAAACTGGTACTGTGCAGTGACTTCAAAATTCTGGGTTTTGTTTGCCACAGCATA
ATCGCTGTCGCCAAACGGGGTCATATTACGCGTTTCTGAATACATGGTTGCCAGGTAAATATTGTTAGCATCGTATTTTA
GCCCAGCAGTCCACGCGTCTGCTTTATCACCACCCGCCGCAGTATGGTTAACCTGGTCATTGGTGCGGTCAGAAGAGGTG
TATGCCGCACCAGCGCTAAAGCCCATGCCTAAATCATATGTTGTGGAAAGACCCCAGCCGTCACCGTTTTCATGGCGAAC
ATCACGTCCGTTGTTGGTGCCTTCCTGACCATTACTGGCTCCTTCGTTGTTACCTTGATACTGCACCGCGAAGTTCAGAC
CATTTACCAGACCGAAGAAATCAGTATTACGATAAGTCGCGACGCCATTGGCTCGACCAGTCATAAAGTTGTCTGCATTG
GTATAAGAGTCACCGCCAAATTCAGGCAGCATATCGGTCCAGCCTTCGATGTCGTACATTACGCCATAATTACGTCCGTA
ATCGAAAGAACCGTAATCTGCAAATTTCAGCCCGGCAAATGCCAGACGGGTCCATGACTGGTTTTTTGAAGATTCAGTGT
TGTTTGCCTGAATATTGTATTCCCATTGACCGTAGCCAGTGAGTTGATCGTTAATTTGGGTTTCGCCTTTAAAACCCAGA
CGCGCATAGCTCTGGTCGCCATCTTTCGCTGAATTATCAGAAAAATAATGCAGGCCATCAACTTTGCCATACAGATCTAA
TTTGTTGCCGTCTTTATTATAAACTTCGGCTGCATGTGCAGCACCTGCGGCGAGCAGGGCAGGAATTAAAAGTGCCAGTA
CTTTGCTTTTCATTGAATAAATCCTTTAGTTATTTTATTTGCCTTTTATCCCCATTTCGGGGAGTGAAAACATCCTAAAG
GAGAAGTTCAGTTAGCCGATAAATATCATTTGTTACGCCTTAAGTAAAACCTTAATCAAATAATTCCTCTGAAATGATAA
TAAGTCTGGTGAATGTATCGAAGATAATACATACAAAATAAAAATTATACTTTTAATTTGCTATACGTTATTCTGCGCGG
GTTATATGCCTTTATTGTCACAGATTTTATTTTCTGTTGGGCCATTGCATTGCCACTGATTTTCCAACATATAAAAAGAC
AAGCCCGAACAGTCGTCCGGGCTTTTTTTTTAGAATTGGATAATCCTTATCCAGAGCATTTAATCGGTGTTGCTTTTTTC
CGCTTTTCCGGCCATTTGTGCCAGGAAGTCATAGCGTTTTTGCAAATCAGCTGCAGCATCTTTCCATAACTGTTCTGCCA
CTTCTGGCTGCTGCGAATTCAGCCGACGGAAACGTTGCTCGTGAAGTAACGTTTCTTCCGGTGCTTCTGACGGCGGGCGT
GAATCCAAGGCCAGCGGCAGTTTGCCTTCATCGGCACGACGCGGATCAAAGCGATATAGCGGCCAGAAGCCGGTAGCTGT
GAGTTGGCGCATCTGGTCGTGGCTGAGTGCCAGATCGTAACCATGCTCTTCACACGGGCTATAAGCAATGATCAGCGATG
GCCCCGGATACGCTTCCGCTTCCTGAATCGCTTTCACCGTCTGGTTCAGCTGCGCGCCGAGAGAAATCTGCGCCACATAA
ACATGACCGTACATCATCATACTGACGCCAAGATCTTTACGCGCTTTACGTTTGCCGTGCTCGCCAAATTTAGTTACTGC
ACCCAGCGGTGTCGCTTTCGACGCCTGACCACCGGTGTTGGAATAGCATTGCGTATCCAGCACCAGAATGTTGACGTTTT
CCGTCAAACTCAATACATGATCCAGACCGCCAAAGCCGATATCGTAAGCCCAGCCATCACCACCAATCAGCCAGATTGAT
TTTTCCACCAGTGCATCTGCATCACGTAGCAGTTCATGTGCTTCGGCAACATCGTTGAGTTGCTGGCGTAAAGCTGCAAC

CTGTTCACGACGAACCTCTGGCGTGGCGTCTGATTTCAACGCCGTCAGTAATTCCGCCGGGATTTTATCGGCAAATTGAT
CCAGCAGACGCAGCACGCGGACACGGTGTTGATCGACCGTCAGGCGGAAACCAAGGCCAAATTCGGCATTATCTTCAAAT
AGAGAGTTCGCCCATGCCGGCCCACGACCGTTGGCATCGGTGGTATACGGTGTAGAGGGCAGGTTACCGCCATAAATTGA
AGAACAGCCAGTGGCGTTAGCGATCAACATCCGGTCGCCATAGAGCTGAGTCAGTAATTTAATATACGGCGTCTCGCCAC
AACCGGAGCAAGCACCTGAATATTCAAACAGCGGTGTAATCAGCTGCGATGTACGAATATCAATACGTTCCAGTTTGCTA
CGGTCGATTTCTGGCAGGTTGAGGAAGAAATCGTAATTGATTTTCTCTTCTTCGACATGTTCCAGGCGAGACATCATATT
GATGGCTTTAATCTCTGGATTCTGACGGTCTTTCGCCGGGCAAACTTCGACGCACAGGTTACAACCGGTGCAATCTTCCG
GTGCCACCTGCAAGACATATTTCTGCCCGCGCATATCACGCGATTTCACATCCAGCGAATGCAGGCTGGCAGGGGCGTTT
TCCATCGCTTCAGGCGGCACCACTTTTGCGCGAATAGCTGAGTGTGGGCAAGCGGCAACGCAGTGGTTACATTGGGTACA
GAGTTCCTCTTTCCAGATGGGGATCTCTTCGGCGATATTGCGTTTTTCCCAGCGCGTAGTGCCCATCGGCCAGGTGCCGT
CTGGCGGCAGCGCCGAAACGGGGAGGGCGTCACCAAGCCCGGCGAGCATCGCAGCGGTTACGGTTTTCACGAAATCAGGG
GCGGCATCGGAAACCACTGGCGGTCGATTGGCGCTGTGCGGATTTACCGGTTGCAACGGAACTTCTTCTACGGATTCACG
CGCCAGCGCCAGAGCCTGCCAGTTGCGTTCCACCAGATCCTGGCCTTTGCTACTGTAACTTTTGGCAATCGCACCCTGCA
ATTCTGCGAGGGCGCTATCGCCAGGCAGAATTTGCGTCAGATGGAAAAAAGCCATCTGCATGACGGTATTAATACGGGCC
GCCAGGCCACATTCGCGGGCGATTTTCGCCGCGTTAATCACATAGAAGCGCGCTTTTTTCTGGTTTAACACGGCCTGAAC
TTCTTGCGGCAAGCGCGACCACACTTCATCTGCGCTGTACGGCGTGTTGAGCAGGAAAATGCCGCCAGGTTTTAAACGCT
CAGCCATCTGATATTTATCGATAAACTGCAACTGGTGGCAGCCAACAAAATCAGCCTGGGAAATGAGATAAGCGGAACGA
ATCGGCTGTTCGCTCACTCGAAGGTGAGAAACCGTCAGGCCGCCCGCCTTTTTGGAGTCGTAAACAAAATAGCCCTGTGC
GTACCACGGCGTGGAATTACCGATAATCTTGATATTGTTTTTGGTCGCGGAAACGCTGCCATCACTACCAAGGCCATAAA
ACAAGGCTTCCAGTTTCGCCGAGTTTGGCAGGGTGTTTTCCGGCAACGGCAGTGACAGATTGGTCACATCATCGTAAATA
CCAACCGTAAAGCGCGCTTTCGGTTTAGCCGCGTTGAGCTCGGCAAATACCGCCAGTACACAGTCTGGGCCAAATTCTTT
GGATGAAAGACCATAGCGCCCACCAATGACACGGGGCAGAGTTTCGCGCTCGCCATTATTAAAGGCTTCTGCCAGTGCGG
TCATTACATCCAGATAGAGCGGTTCTGCCTGGGCACCGGGTTCTTTGGTTCTGTCCAGTACCGCCACGCTGCGTACGGAT
CCCGGCAGAGCTTGCAGTAAATGTTTAGCGGAGAAGGGGCGGTACAGGCGAACTTTCAGCACGCCAACTTTTTCGCCACG
GGTTAGCAATTCATCAACCACTTCTTCACAGGTGCCAATGGCAGAGCCCATCAGGATAATCACCCGTTCCGCTTGCGGAT
GCCCGTAATATTCAAACGGCTGATACTGACGACCTGTCGCGGCAGAGAAATCATTCATCGCCTGTTCAACATGGTCATAG
ACCGCGTTGTACCATGGGTTGGTGGCTTCGCGAGACTGGAAATAAGTGTCAGGATTGGCGGACGTACCGCGGATCACCGG
ATGTTCCGGGTTGAGTGCCCGGGCGCGATGAGCATCAATTTCGACCTGCGGCATGAGATCAAGAATCGTGTCATCGGCCA
GCGGGACAATTTTATTGATTTCGTGGGACGTGCGGAAACCATCAAAGAAATGAATAAATGGCACGCGGCTTTTCAGCGTC
GCGATTTGCGAAATGAGAGCAAAGTCTTGCGCTTCCTGGACGTTTGCTGCACACAACATCGCGCAACCCGTCTGGCGCAC
CGCCATAACGTCGGAATGATCGCCAAAAATAGAGAGTGCATGTGTGGCAACGGTACGTGCCGCTACATGCAGGACAAACG
GTGTTAGTTCGCCTGCCAGTTTGTACAGCGTCGGGATCATCAGCAGCAAACCCTGCGATGACGTAAACGATGTTGAAAGG
GCACCCGTCTGCAAAGCGCCATGCACGGTAGCGATAGCACCCGCTTCCGACTGCATTTCAACCACGCGTGGTGTGTCTCC
CCAAACGTTCTTTAAGCCGTTTCCGGCCCAGGCATCAGCCTGTTCTGCCATCGTGGAACTGGGGGTAATAGGGTAGATGG
CGATAACTTCACTGGTGCGAAATGCGACCGAAGCAACCGCGCCATTACCGTCAATAGTAATCATATGACACCCTTACATT
GCGCAAATGAGGGGCGCACGAAATTGCTGCGCGCCCAGTAGTAATCTTTCAATTTTAGCAAATGGCTTTCTTCTGCATTT
TCGCTTTTGTGTCCCCCACATCAGCGTAATGAATGTTTTGATCAAACAGAGGGCAAAAAAATAGCCATAAAAAAGTAAAA
ATGCGGGGCAACGCGCAGGACAGCTCTCGACGCCGCCCTCTGTGCTGCCTATTATTTATGGCGGTGTCGTTTGGCTTGAA
GATCAGAGGAAGAAAAGATGCGAGCAGCGTTTTGGGTAGGGTGTGCCGCTTTATTGTTGTCGGCGTGTAGTAGTGAACCT
GTTCAGCAGGCGACTGCCGCGCACGTAGCGCCAGGTTTAAAAGCGTCGATGTCCAGTAGTGGAGAAGCAAATTGTGCAAT
GATCGGCGGTTCGCTTTCTGTTGCCCGTCAACTGGATGGTACGGCGATTGGGATGTGTGCATTACCCAACGGCAAACGCT
GTAGCGAACAGTCACTTGCCGCCGGGAGCTGTGGCAGCTATTAATTCATTAAATCCGCCAGCTTATAAGTTAATGTCTGT
TTTGCGGTCGCCAGCGTTAACTGGTTCGCGGTCAGATCCACTTGTGCACCTTCTTTCAGCATTTCGCTAATGGTGTTATC
GAGTTCATTAAGCTGCGGGTTAGCGCACATCATACGGGTCATTGCCAGCCCTTTGGCTGTCAGTTCACCATTAGACAGTT
TGCCTTCACCGCTAAAGCGGTTACACATGCTGCCGGAAATCATCATTTTTTCACCAAAGCTGATTTCTGGCGGATTTTTA
TCGCTGGTCACGGGCTTACCGTTTACGCTTTCCAGCACAAAGCGATGATGCTGTAGCTGTTCTGGCGTAACAGCAATTTT
GTCATTACTTACACATCCCGCCATCAGCAGGCTTAGCGCAACAAACGCGGCTACTTTCTTCATTGTGGTTCTCAATTACA
GTTTCTGACTCAGGACTATTTTAAGAATAGAGGATGAAAGGTCATTGGGGATTATCTGAATCAGCTCCCCTGGAATGCAG
GGGAGCGGCAAGATTAAACCAGTTCGTTCGGGCAGGTTTCGCCTTTTTCCAGATTGCTTAAGTTTTGCAGCGTAGTCTGA
GAAATACTGGTCAGAGCTTCTGCTGTCAGGAATGCCTGGTGCCCGGTAAACAGCACGTTGTGGCAGGCAGACAGGCGACG
GAATACGTCATCCTGGATCACGTCGTTGGATTTATCTTCAAAGAATAGATCGCGTTCGTTCTCATACACGTCCATACCCA
ACGAACCAATTTTCTGATTTTTCAGCGCTTCAATTGCTGCCTGAGAATCAATCAATGCACCGCGACTGGTATTGACGATC
ATCACGCCATTTTTCATCTGTTCGAAGGCGGCTTCGTTCAACAGATGATAGTTTTCCGGTGTCAGCGGGCAGTGCAGAGA
GATAACGTCTGATTCAGAGAACAGGGTTGGCAGATCGACATACTCCACACCGAGTTCCAGCGCCGCTGCACTTGGATACG
GATCGAACGCCAGCAGACGCATACCAAAACCTTTCAGAATGCGCAGCATCGCCACACCGATTTTACCGGTACCGATAACG
CCTGCCGTTTTGCCATACATAGTAAAGCCGGTCAGACCTTCCAGAGAGAAGTTAGCATCACGGGTACGCTGATACGCGCG

GTGAATACGGCGGTTCAGCGTCATCATCATACCGATGGCGTGTTCAGCAACGGCCTCTGGATCATAGGCTGGAACACGGA
CTACTTTCAGCCCCAGTTCTTTTGCCGCGTCAAGGTCGACGTTATTGAAACCGGCACAGCGCAGGGCGATATATTTAACG
CCGTGCTTTTTCAGCTCTTCCAGCACCGGGCGGCTGCCGTCATCGTTTACGAAAATACATACCGCTTCGCAGCCATTGGC
AGTTTTAGCGGTTTTTTCCGTCAGCAGAAAGTCAAAAAATTCCAGCTCAAAGCCAAAGGACTCGTTCACCTGTTGCAGGT
ACTTCTTGTCGTACTGTTTTGTGCTATAAACGGCGAGTTTCATAAGACTTTCTCCAGTGATGTTGAATCACATTTAAGCT
ACTAAAAATATTTTACAAAATTTCAAATTTAATTGAAAGCTATGGCGATATTGAAAAATTCATCAACAACTATGCTTAGT
GTAGGCGCAACCTTCAACTGAACGGTTAAACATGCCACAATACCCGTATTGAATGCTTAATTTTTCGCTAAATCAGGATA
TTAACTACCCATGCTGGGTAAATATAAAGCCGTTCTCGCGCTGTTATTACTGATTATTCTTGTGCCGTTGACGCTGCTGA
TGACGCTCGGGCTGTGGGTTCCCACGCTGGCGGGCATCTGGCTACCGCTCGGGACACGTATTGCATTAGATGAAAGCCCA
CGCATTACGCGTAAAGGTTTAATCATTCCCGATCTCCGTTATCTGGTGGGAGATTGTCAGCTTGCGCATATCACCAACGC
CAGCCTTTCACATCCCAGCCGCTGGTTATTGAACGTCGGCACGGTAGAACTTGATTCTGCTTGTCTGGCGAAATTGCCGC
AGACGGAGCAATCGCCAGCCGCTCCAAAAACACTCGCGCAGTGGCAGGCCATGCTGCCTAACACCTGGATCAATATCGAT
AAACTGATTTTTTCTCCCTGGCAGGAATGGCAGGGAAAACTCTCTCTCGCATTAACCTCTGATATCCAGCAACTGCGTTA
TCAGGGCGAAAAAGTTAAATTTCAAGGCCAGCTGAAAGGGCAACAACTTACAGTCAGCGAACTGGATGTCGTCGCGTTTG
AAAATCAGCCGCCGGTAAAACTGGTGGGGGAATTTGCTATGCCGCTCGTGCCGGATGGACTTCCTGTAAGTGGGCATGCT
ACTGCGACGTTAAACTTGCCGCAGGAACCGTCACTGGTGGATGCCGAGCTGGACTGGCAGGAAAATAGCGGGCAATTGAT
CGTGCTGGCACGGGATAACGGCGATCCGTTGCTCGATTTGCCGTGGCAAATTACTCGTCAACAATTGACCGTAAGCGATG
GTCGCTGGAGCTGGCCGTATGCAGGTTTTCCTTTGAGTGGCCGACTGGGTGTCAAAGTCGACAACTGGCAGGCAGGGCTT
GAGAACGCTCTGGTCAGCGGACGACTGAGTGTGCTGACCCAGGGGCAAGCGGGTAAGGGCAACGCGGTGCTTAATTTTGG
CCCAGGAAAATTAAGCATGGATAACAGTCAGCTGCCTCTGCAGCTGACCGGTGAAGCGAAACAGGCGGACCTCATTTTAT
ATGCCCGTTTACCTGCGCAGCTAAGTGGAAGTCTGTCTGACCCAACGCTGACCTTTGAGCCAGGCGCGTTACTTCGTTCA
AAGGGAAGAGTCATCGATTCGCTGGACATCGATGAAATCCGCTGGCCTTTAGCGGGTGTAAAAGTCACCCAACGTGGTGT
TGACGGACGTTTGCAGGCCATCTTGCAGGCGCATGAAAATGAACTGGGCGATTTCGTGCTGCATATGGATGGGCTGGCGA
ATGATTTTCTCCCTGACGCTGGCCGCTGGCAGTGGCGCTACTGGGGAAAAGGGAGTTTTACACCGATGAATGCCACCTGG
GATGTCGCAGGAAAAGGTGAGTGGCATGACAGCACGATTACGCTGACCGATCTCTCCACCGGTTTCGACCAGTTACAATA
CGGTACGATGACGGTAGAAAAGCCGCGATTAATTCTCGACAAGCCCATCGTCTGGGTACGTGACGCACAGCATCCCTCCT
TTAGCGGCGCGCTGTCACTGGACGCCGGGCAAACGCTGTTCACTGGCGGCAGTGTGTTACCGCCATCAACCTTAAAATTT
AGCGTCGATGGGCGCGATCCTACCTATTTCCTCTTTAAAGGCGATTTACATGCTGGTGAGATTGGCCCGGTTCGGGTAAA
TGGTCGCTGGGACGGTATTCGTCTGCGCGGTAACGCCTGGTGGCCTAAACAATCACTGACCGTATTCCAGCCGCTGGTGC
CACCCGACTGGAAGATGAACTTACGCGATGGTGAACTGTATGCTCAGGTTGCATTTTCTGCTGCGCCTGAACAAGGATTC
CGCGCGGGAGGACACGGCGTGTTGAAAGGCGGTAGTGCCTGGATGCCAGATAATCAGGTTAACGGTGTCGATTTTGTCCT
GCCTTTCCGTTTTGCCGATGGAGCCTGGCATCTGGGGACTCGCGGCCCCGTTACGTTGCGAATTGCCGAAGTGATTAATC
TGGTGACAGCGAAAAATATTACGGCTGATTTGCAAGGGCGTTATCCGTGGACTGAAGAAGAACCCTTGCTGTTGACTGAT
GTTAGCGTCGATGTGTTAGGCGGTAACGTACTGATGAAACAATTACGTATGCCGCAACATGACCCGGCGCTGTTGCGGCT
GAATAATCTCTCATCCAGCGAACTGGTTAGCGCCGTCAATCCGAAACAATTCGCCATGTCCGGGGCATTTAGTGGTGCAC
TGCCGTTATGGCTGAACAATGAAAAATGGATTGTGAAAGATGGCTGGCTGGCGAATAGCGGGCCGATGACATTGCGACTG
GATAAAGACACTGCGGATGCGGTGGTAAAAGACAATATGACTGCGGGTTCAGCAATTAACTGGTTGCGCTATATGGAAAT
TAGCCGTTCATCGACAAAAATTAATTTAGATAATCTCGGTTTATTAACCATGCAGGCCAACATTACAGGTACCAGTCGCG
TTGATGGTAAAAGCGGTACGGTAAACCTTAATTACCATCATGAAGAGAATATTTTTACGCTGTGGCGCAGTTTACGCTTT
GGCGATAATCTCCAGGCATGGCTGGAGCAGAACGCACGTCTGCCGGGAAATGACTGTCCGCAAGGAAAAGAGTGTGAGGA
AAAACAATGAAAATTTTACTGGCTGCGTTGACGTCATCTTTTATGCTGGTTGGCTGTACGCCTCGCATTGAAGTCGCTGC
ACCTAAGGAACCGATCACTATCAATATGAACGTTAAAATTGAGCATGAGATCATCATCAAGGCAGACAAAGATGTCGAAG
AGCTGCTTGAAACTCGTAGCGATCTTTTCTGAGGTGATGATGAAGAAAACATTACTTCTTTGTGCGTTTCTTGTTGGGCT
GGTAAGCAGCAATGTAATGGCATTGACTCTGGATGAAGCCAGAACTCAGGGGCGGGTAGGTGAAACATTTTACGGTTATC
TGGTTGCGCTGAAAACGGATGCTGAAACAGAGAAATTAGTAGCCGACATTAATGCCGAACGTAAAGCGAGTTACCAACAA
CTGGCAAAGCAAAATAATGTGTCGGTAGATGATATCGCGAAACTCGCCGGGCAAAAGCTCGTAGCTCGGGCCAAACCGGG
GGAATATGTACAAGGGATAAACGGTAAATGGGTGCGAAAATTTTAATAGCAAAACCGCAATATCGGGATAACGCTACAGG
AGAAGGCGATTGATCTATTTTCCTGAAACAAGGTGAATATTCAAAAACTCCTGTCAAATTGCCTTTTGCCCTGAAAAATG
CATAGTCAAATATCTGTTTTAACTAATTGGCGTTGCAGTACATGCAACGCCAATTAGTTAGCGGAATTTACGTCGATACT
CGCCTGGCGTCATCCCAAAGCGTTGCTTAAATACCGTTGAAAAATGACTCTGGTCAGAAAATCCCCAATGAAAGCCGATG
CCTGCCAGTTTTTCATCATCTGCGGCATGGCGAATCGCATCTGCACAAAAATCGAGACGACGGTTACGAATATATTGCGC
GACTACCAAACCTTTATCGGCAAACATTCGGTACAAACTACGTACTGACATACCTGTCTCTCCGGCTATCCACTCCGGGC
GTAATATCTCTTCGCGAATATTATCGTCTATCAACGTAACCACTTTTTGAAACTGACGTTCACGACGAGGTTGAACAGAT
TCCCGCTGATGAAGTACCGGGCGCAGCAGACACACCATCGCCTGTAGCGCAGCTTCACTTTCTGTTTCAGAAAGTGCCGG
ATTATTCATGCTCTCCTGTAACAGGCGATGACTGAGTTGCACCATGGGTAAGTCAGCGTCCAGTCTTTCTGCGCAGATAG
GTTTTTGATGGGGAAAATATTGTTCCAGCAGAGTGCGTGGCAAAAGTAATGAAATCTGTTTAGAAGACTCCTGCCAGTAA

AGCGAACAGGGGCGTGAGGCATCGAGTAACGTAATATCGCCAGCGCCAATCTGCACCTGACGCTCATCCTGCTCCATTAT
TGCCTGACCACTAAGCTGAAAAACGGTGTAAAACCAGGCATCGTCGCTGCCTTTTACTTCCTGCCAGGTGCGGGATAAAT
TCACCCCGCTGGTTGTCACGGTACTCAGCTTTAGTCCTTTGGCAAAATGCGTGTCCAGTACACCCGTGTAACGCTCAGTC
AGCAGGCGTCCGGTAAAATTTCCGCATACCTGATTGATTTGGGAAAGCCATTGCTGAAACTCATTATCCACTGCGGGGTT
CATGGCACGTTTTCGCTCTGTGAAATGTATTTTTATTGTTGCATTTGTGTTGCAATAAACGAAGCTAATGAGCCTGACTA
TAGGAAATAAGTCTTGTCAGGCATAGAGACATAAGCGGTTATTGTCACGATTTGCGGAGCTTGTCACAGCTGACAAAGCG
AATGTCACAGCGAAAAAAGTGACTTTTCTTGTCGCTGCGTACACTGAAATCACACTGGGTAAATAATAAGGAAAAGTGAT
GACAGAGCCGCATGTAGCAGTATTAAGCCAGGTCCAACAGTTTCTCGATCGTCAACACGGTCTTTATATTGATGGTCGTC
CTGGCCCCGCACAAAGTGAAAAACGGTTGGCGATCTTTGATCCGGCCACCGGGCAAGAAATTGCGTCTACTGCTGATGCC
AACGAAGCGGATGTAGATAACGCAGTCATGTCTGCCTGGCGGGCCTTTGTCTCGCGTCGCTGGGCCGGGCGATTACCCGC
AGAGCGTGAACGTATTCTGCTACGTTTTGCTGATCTGGTGGAGCAGCACAGTGAGGAGCTGGCGCAACTGGAAACCCTGG
AGCAAGGCAAGTCAATTGCCATTTCCCGTGCTTTTGAAGTGGGCTGTACGCTGAACTGGATGCGTTATACCGCCGGGTTA
ACGACCAAAATCGCGGGTAAAACGCTGGACTTGTCGATTCCCTTACCCCAGGGGGCGCGTTATCAGGCCTGGACGCGTAA
AGAGCCGGTTGGCGTAGTGGCGGGAATTGTGCCATGGAACTTTCCGTTGATGATTGGTATGTGGAAGGTGATGCCAGCAC
TGGCAGCAGGCTGTTCAATCGTGATTAAGCCTTCGGAAACCACGCCACTGACGATGTTGCGCGTGGCGGAACTGGCCAGC
GAGGCTGGTATCCCTGATGGCGTTTTTAATGTCGTCACCGGGTCAGGTGCTGTATGCGGCGCGGCCCTGACGTCACATCC
TCATGTTGCGAAAATCAGTTTTACCGGTTCAACCGCGACGGGAAAAGGTATTGCCAGAACTGCTGCTGATCACTTAACGC
GTGTAACGCTGGAACTGGGCGGTAAAAACCCGGCAATTGTATTAAAAGATGCTGATCCGCAATGGGTTATTGAAGGCTTG
ATGACCGGAAGCTTCCTGAATCAAGGGCAAGTATGCGCCGCCAGTTCGCGAATTTATATTGAAGCGCCGTTGTTTGACAC
GCTGGTTAGTGGATTTGAGCAGGCGGTAAAATCGTTGCAAGTGGGACCGGGGATGTCACCTGTTGCACAGATTAACCCTT
TGGTTTCTCGTGCGCACTGCGACAAAGTGTGTTCATTCCTCGACGATGCGCAGGCACAGCAAGCAGAGCTGATTCGCGGG
TCGAATGGACCAGCCGGAGAGGGGTATTATGTTGCGCCAACGCTGGTGGTAAATCCCGATGCTAAATTGCGCTTAACTCG
TGAAGAGGTGTTTGGTCCGGTGGTAAACCTGGTGCGAGTAGCGGATGGAGAAGAGGCGTTACAACTGGCAAACGACACGG
AATATGGCTTAACTGCCAGTGTCTGGACGCAAAATCTCTCCCAGGCTCTGGAATATAGCGATCGCTTACAGGCAGGGACG
GTGTGGGTAAACAGCCATACCTTAATTGACGCTAACTTACCGTTTGGTGGGATGAAGCAGTCAGGAACGGGCCGTGATTT
TGGCCCCGACTGGCTGGACGGTTGGTGTGAAACTAAGTCGGTGTGTGTACGGTATTAATCTGGTTCGCTCATAAGTAAAA
AACGGCACCTGGTGCCGTTTTTTTGTCTGAAACAATCACTTATCTTTCTTCAGCGCCCCTAGCGTTGGCGTTTCGTCAAA
GAAGTTCCATGGTTTCAGCAGAGTATGTACCCATTCGGTCGGCATAATCGGCCACTCTTCGGCGCGGGCCACATGTGTGG
TGCCGGTGGTCATCCAGACAACGGCGTCGGTGTTGTCCAGCGACTCGTTATCCTTACTGTATTGTCCAAGACCGGTGTCA
TGAGTAGAACGGTTCGGATATTTGCCTTCCGGGAAACGCTCGCCAGGATGATAACGCGTTACCCAGAGCTGCTTGTCCAT
AAAGCTTAAACGATGATAGATCCACTCGTCCGGCGCGAACTGGGCACCTTTTGCTACCGGGTGAGTACCACCTGCATAAG
GAATAATTTGATAGGAAACCGGATTGCCCATGCGGTTCTCTTTGTTCGGGTTACTCAACAGACGAATCGTGCCCGGATCA
AATTTCTGTGCGGCATCCTGTTCATTGCCGATGTTGTACTGATTAACTTGCATGGTACTGGTGCGTGGGCCACCGGCAGT
ATTCGGTTTTACCACTGGGTCCATCGCCACCAGGCTGTTATTCTCGCCATCTACATCCAGATCGAGGCGGAAATTATAAA
TATGTTGGTGTGTAGTACCCACGATATTGTGATCGATAAGCGTGCCGTAGCGCGTGTCATCTTTCGCCGTCTCATCGTGC
ATGGTTTTCGCTTTAACACCTTTCACCGCTTCGATGCCCGTAGCACCGGCATCGATGCCAATAGTGCCGTTTTCATGGAA
GATCCAGTCAAAAATGTAGTCATAGTTACCCACTGTACTGATCCAGCGCACCACTAACTCCCGGCGTTCGGTACTGACGT
TGGGCTGGCCCATTTCCTGATGCTTATACTCCGGCCCGGCATAACGTTCAAATACCGCGATAGCGCGAGGGATCTCCATC
GGCACGCCAGTGTAGTCGGCGATGGTTTCATTAAGGAGCACTGCGTTAGACGGGGCATCTTTACCACGAGCAATTGGTGA
GGTTAGCGTGCCCATACCGTAGTCACCAGAGTCCAGATACGCTTTAAAGTACCAGCCAATATCAGGATCACCGTAAGGCA
CAATCATGCCGCCGAGAGAACCTTCGTACATGACTTTGCGTTTGGTGCCATTGTCGTTATAAGTCACGGTGGAGATCATC
GGCCCGACGCGAGAGTTCATGCTGAGGTGAAAATCCCAGTTCCGCCAGTGAATCATATCGCCAGTAATGGTGTAATTTTT
ACCTTCAGGCTCAATGATTTGCATAGGCTTAACTGCCGGAGCAACGCGGTCACGGCCATCAAATGGGCGTGCGGTCATTG
GCACCGGAACTACCGGACCTTCTTCAATCTTAACGATTTTTTTCTGTTCTAAATCAACGACCGCCACCAGGTTTTCGATG
GGATGTGCCCAGTAGTTGCCATCACCGACATCAAGATAGCTGATGACTTTGAGCAACCGGGCATCTTGTTTCAGGCCATC
TTTACCATCGAAATAACCTACGGTCAGCGGCGTGGTAATCACTTTTTTCGCATCAGTAATACCGCGTTTCTTCACGGCAG
CGGCAAATTCTTCACTGTTGTTAATAATGTTCTGCACACTGGCGAAATCATCCAGCAACACCATACCGTGGGCGTCTTTA
ATGGGTTGCCAGGAGAGCAGTTTGTTGTTTTGCAGATCCACCACCGCTTCGATGATATGTTTGCCGTCGAGCATAATGAC
GTCGGCTTTGCGCGGCTGGTCAACCGGTTTGTTTTCCAGCGCAAACGCCCAGACAGCTTCTTTATCTGGCGGTAGCAGGG
AGATCTCAGTAAAACGGGTATTGGGTTTGAAGTCCGCGGAAGCTTTAACAATTTCAACGGCCTGTTTAATTTCGTCCGCA
GTTAGCGCATTAAGTGGGTGAGGGCGCTTTTCTACCTGAAAGGTTTGATCCAGCCCGGACTGGAAAACATCGTTAATAAA
GGTGTCAGAAACCCAGGCTTTATTGTCTTTCATCACTACCGGTACTTGCAGTGCCAGAGGCTGACCATTAACAATTGCTG
TTTGCGCACCAGGCTTCACTTTCACGTACGCGCCATCTTTAATCAGGGTAAAGAGCTGGGCGTAGTCGTCCCACTGCACA
TCGGCACCAAATTCTTTAAGCGTTTTATCCATTGGCACCATATGCGCTTCACCACCGTGGGCAAATACCGGCGCTTGCCA
GGCGAAACTTAAGGCGACTGCCAACGCCAGGGTTGTTTTACGGGCAGAATACAGAGAGGGGCTTCCCATTATTAACCTCG
TCAGATGTTGTGTTCTTGTTAGCAACCGCGCTCTGTGGGCGGTTTAGTCAGGTTCACATTATCAGTACTGATGCAAAGGG

GATTGCCTGCACCTGCCAGGTTGTTTGGCAGGTGTGCCAGCTTTTCATACAGTGGATGCCCTGAAAATAGATGTACACAT
CATGCATAATGTGACAACGTCACAAAACTTAGTGAAATAAAAGGGCAACTATTCGCCGTTGCCCTTCATTCACCGATTAA
TCGACAAAATCACCGTGCTGCCTGGCCACCAGCGTCAGAATTGAATACAGCGCCACCGGGGTTTGATGCTGATTGAATAC
CTCTACAGCCCATTCCACCACACCTGTTGGTTTTTCTTCTGCGCTACGCTGTTTTTTCAGCGTCTTGCGCTTACAGGTGA
GACGCACCTGGATGGTATCGCCTGGCTTTACGGGTTCGATAAAACGCAAGCTTTCCAGCCCGTAGTTAGCAATGACCGGA
CCGACACCGGCATCGACAAACAGACCCGCAGCCGCAGAAAGCACAAAATACCCATGCACCACCCGCTCACCGAAAATAGA
TTCGGCAGCAGCAATCTTATCCATATGTGCATAGAAATGATCGCCGCTGAGGCAAGCAAAGTTAACAATATCGGCCTCTG
TCATTGTGCGGCGGGGAGTCAACAGGCTGTCGCCTGGTTGTAGCTCCTCAAAATATTTGCGGAACGGATGAATACGATCT
TCTTCGACTTTCGCACCGCGCACCCACTGTTTACTGATAGCGGCAAGCATCGTCGGACTACCCTGAACAGCGGTTCGCTG
CATGTAATGTTTCACCGCTCGTAAACCGCCTAATTCTTCACCGCCTCCTGCGCGACCAGGCCCACCATGTACCAGTTGTG
GCAGTGGGGAGCCATGCCCGGTGGATTCTTTTGCCGACTCTTCATTGAGGATCTGAATTCGCCCATGCGTACGTGCCGCG
TCGGCAATAAACTGACGCGCAATTTGCGGATCAGCCGTCACCAGCGTTCCCGCAAGGCTACCGCCGCCTGCACAAGCCAG
TTGCAGAGCATGTCGCTGGTTTTGTGCTGGCATCAGCGTTGCGACAGGGCCAAAGGCTTCTGTTGCATGTACCGCCGGTG
TTTCATCCGGCTGCGGACAGTACAATAAGGTTGGCGGGAAGAAGGCACCCGCAGCAGATAAATCCGCCTGACCACCGAGG
CGAATCTCGCATCCTGCAGCCAGCAATATGTTCACTTTTTCCTGCACATCGGCACGCTGCTCAGCATTTACCAGTGCGCC
CATTTTCACGCCTTCCTGAGCAGGATCACCGACCACGACTTTCTGTAATCGCGCAACCAGAGCATCACTGACAGCATTAA
CCAATGCCTGCGGCACAATAATCCGCCGGATTGCCGTACATTTTTGCCCGGCTTTTGTGGTCATCTCACGCACAACTTCA
CGAATAAACAGCGCAAACTCCGGTTGATCCGGGGTGACATCTTCGCCCAGTACGCAGCAGTTCAGGGAATCAGCTTCCAT
AGTGAAGGGGATAGATTTGGCGACGATATTTGGCTGAACTCGCAGCATCTGTCCGGTCGCCGCTGACCCCGTGAAAGTCA
CCACATCCTGGCTGTCCAGATGATCCAACAAGTCGCCAGCACTACCGCAGATCAGACTAATTGCGCCTTCGGGAACAAGA
CCACTATCGACAATTGATTTCACCATCGCCTGAGTCAGTTGGGCCGTCGCGGTAGCTGGTTTGATGATGGCTGGCATTCC
GCCCAGCCACGTTGGTGCCAGCTTTTCCAGCATTCCCCAGCAGGGGAAGTTAAAGGCGTTAATATGCACTGCCACGCCTG
ACTTTGAGGTCAGTAAATGGCGCGCGGCAAATCCACCTTCTTTCGATAAGGGGATCAATTCATCTTCCGGCCACAGCGTA
TCGTCAGGCAGCTCCCGGCTACCGAGGCTGGCGTAAGTAAATAACGTCCCAATGCCACCTTCAATATCAACCCAACTGTC
TGCCCGCGTTGCGCCTGTTTGCGCAGAAAGAGCATAGAAACGCTCTTTTTCACTCAGCAGATGTTTAGCGACCGCTTTAA
GCATCGCCGCACGTTCGATAAAGGTCATAGCGCGAAGGGCGGGGGCACCTTTTTCAATGGCAAACTGGCGGGCAGCCGCC
ATATCAAGACCTTCACTGGTCACTTCCCATAACGCCTCGCCGCTAATAGCGTGGTGAATCAAACGGCTACGGCCCCGGCC
AGACTGCCAGGTACCGGATAAGAAACTGGCTAACTGCTGCATCGCTACTCTCCAGATGTTTCACATTTCTGTTGCTAATA
GTTAAATCGCGAATCATAAAAAGCAAAGGATCTTTTAACGAAATGTTAACTATGCGATCTGTATAGCAACTGCGGAAAAC
ATTAATGCACTGATAAATAATGATTTATAAAAATAGGGTGCGAAATCCGTCACAGTTCAAACATACAAAATTTGTGATTT
TACTTAACTATTGTGTAACTTTCATAAAACAATGTGATTCGTGTTTTTAATTAATTCACGAAAACTGGAATCGTAAAGGT
GATGACGTGACCCAAGAAGAACGCTTTGAGCAACGGATAGCCCAGGAAACGGCTATCGAGCCACAGGACTGGATGCCCGA
TGCTTACCGAAAGACATTGATCCGCCAGATTGGGCAGCATGCGCACTCCGAGATTGTTGGTATGTTGCCTGAAGGTAACT
GGATCACTCGCGCACCAACCTTGCGGCGTAAAGCCATTCTGTTGGCCAAAGTGCAGGATGAAGCCGGTCATGGTCTGTAT
CTCTATAGCGCCGCCGAAACACTGGGCTGCGCCAGAGAAGACATCTACCAAAAAATGCTCGACGGACGGATGAAATATTC
CTCCATCTTTAATTATCCGACATTGAGTTGGGCTGATATCGGTGTTATCGGCTGGCTGGTGGATGGCGCAGCGATCGTCA
ATCAGGTGGCGTTATGCCGCACTTCTTATGGCCCGTATGCCAGAGCAATGGTGAAAATCTGCAAAGAAGAGAGTTTTCAC
CAGCGTCAGGGGTTTGAAGCCTGCATGGCACTGGCGCAGGGCAGCGAAGCTCAAAAGCAGATGTTGCAAGACGCCATTAA
CCGTTTCTGGTGGCCAGCCTTAATGATGTTCGGGCCAAACGATGATAACTCGCCAAACAGCGCCAGAAGTCTCACCTGGA
AAATCAAACGTTTCACCAACGACGAACTCCGCCAGCGTTTCGTGGATAACACCGTTCCACAGGTTGAAATGCTCGGTATG
ACCGTTCCTGACCCGGATCTGCATTTTGACACTGAAAGCGGTCACTACCGCTTTGGTGAGATCGACTGGCAGGAGTTTAA
CGAAGTAATTAACGGTCGCGGAATTTGTAATCAGGAGAGGCTCGACGCCAAACGTAAAGCCTGGGAAGAAGGTACCTGGG
TACGGGAAGCAGCGCTGGCCCATGCACAAAAACAACATGCCCGTAAGGTCGCATAAGGAGATTCAAAATGAGTAATGTTT
ACTGGCCGTTATACGAAGTTTTCGTGCGTGGCAAACAGGGCTTATCACACCGCCATGTTGGCAGTTTACATGCTGCCGAT
GAGCGGATGGCACTGGAAAATGCCCGTGATGCTTACACCCGTCGTAGCGAAGGATGTTCAATTTGGGTGGTGAAGGCGAG
TGAAATTGTTGCCTCGCAACCGGAAGAACGCGGTGAATTTTTTGATCCGGCTGAAAGCAAGGTCTATCGCCATCCAACGT
TTTACACCATCCCTGATGGCATTGAGCACATGTGAGGTCGGAAATGAATCAGTTAACGGCTTACACCTTGCGCCTGGGCG
ATAACTGCCTGGTGCTCTCCCAGCGGTTGGGTGAATGGTGCGGTCACGCACCGGAACTGGAAATCGATCTCGCACTGGCA
AACATTGGCCTCGATTTATTAGGTCAGGCACGCAACTTCTTATCGTATGCCGCTGAATTAGCGGGAGAAGGCGATGAAGA
TACCCTGGCCTTCACCCGAGACGAGCGCCAGTTCAGCAACTTATTGTTGGTTGAACAACCAAACGGCAATTTTGCCGACA
CCATTGCACGCCAGTATTTCATCGATGCATGGCATGTGGCGCTCTTTACCCGTCTGATGGAAAGCCGTGATCCGCAACTG
GCGGCGATTTCTGCCAAAGCAATTAAAGAAGCGCGCTATCACCTGCGTTTTAGTCGTGGCTGGCTGGAGCGACTGGGCAA
TGGTACTGACGTATCAGGGCAAAAGATGCAGCAGGCCATCAACAAGTTGTGGCGTTTTACCGCCGAACTGTTCGATGCCG
ACGAGATTGATATTGCACTGAGTGAAGAGGGTATTGCGGTTGATCCACGCACTTTACGCGCAGCGTGGGAAGCCGAAGTT
TTTGCCGGGATCAACGAAGCCACATTGAACGTACCGCAAGAGCAGGCGTATCGCACTGGCGGTAAAAAAGGACTGCATAC
CGAACACCTTGGACCCATGCTGGCAGAAATGCAGTATCTCCAGCGTGTCTTGCCCGGTCAGCAATGGTAACAGAGGAGAT

GGGTATGCAACGTCTGGCCACCATTGCACCGCCACAGGTTCATGAGATATGGGCGCTACTCAGCCAGATCCCGGACCCGG
AGATCCCGGTGCTGACCATTACTGATTTAGGCATGGTGCGTAATGTGACACAGATGGGAGAAGGATGGGTGATCGGCTTT
ACGCCGACATATTCCGGTTGTCCGGCAACGGAACATTTGATTGGCGCGATACGTGAGGCAATGACAACCAACGGGTTTAC
CCCCGTTCAGGTTGTGCTGCAACTCGACCCGGCATGGACCACCGACTGGATGACCCCCGATGCCCGTGAGCGTCTGCGAG
AGTATGGCATTAGCCCGCCCGCCGGACACAGTTGCCATGCCCATTTGCCGCCAGAAGTACGTTGCCCGCGCTGCGCCAGC
GTCCATACCACACTTATCAGTGAATTTGGTTCCACGGCCTGCAAAGCATTGTACCGCTGCGATAGTTGCCGCGAACCTTT
CGATTATTTCAAATGTATTTGAGGATGCCATGACAACGTTTCATTCCTTAACAGTGGCAAAAGTCGAGTCGGAAACCCGT
GATGCGGTGACCATTACCTTTGCGGTGCCCCAGCCTTTGCAGGAGGCGTATCGCTTTCGCCCCGGTCAACATTTGACCTT
AAAAGCCAGCTTTGATGGTGAAGAATTACGCCGTTGTTACTCCATTTGCCGCAGCTATCTGCCTGGCGAAATTAGTGTGG
CGGTGAAAGCCATTGAAGGCGGACGTTTCTCCCGCTATGCCCGCGAACACATCCGCCAGGGTATGACGCTGGAGGTCATG
GTGCCGCAGGGGCATTTCGGCTATCAGCCGCAGGCCGAACGCCAGGGGCGCTATCTGGCAATTGCAGCAGGATCAGGTAT
TACGCCAATGCTGGCGATTATCGCCACCACTTTACAAACCGAGCCTGAAAGTCAGTTCACCCTGATCTACGGTAACCGTA
CCAGCCAGAGCATGATGTTTCGCCAGGCACTGGCAGACCTGAAAGACAAATATCCTCAGCGTTTACAGTTGTTGTGCATT
TTCAGTCAGGAAACCCTCGACAGCGATCTGCTTCACGGGCGTATTGACGGTGAAAAATTACAGTCACTTGGGGCCTCGCT
CATTAATTTTCGTCTTTATGATGAGGCATTTATTTGTGGTCCGGCGGCGATGATGGATGACGCGGAAACCGCCTTAAAAG
CACTGGGAATGCCAGATAAAACCATTCATCTGGAGCGGTTTAATACGCCTGGCACGCGCGTCAAACGTAGCGTTAACGTG
CAAAGTGACGGACAAAAAGTGACTGTACGTCAGGATGGGCGGGATCGGGAAATCGTGCTTAATGCCGACGATGAAAGCAT
TCTCGATGCGGCATTGCGCCAGGGGGCGGATCTGCCCTATGCCTGCAAAGGCGGCGTCTGTGCGACCTGCAAATGCAAAG
TGCTGCGTGGCAAAGTGGCGATGGAAACCAATTACAGTCTGGAACCGGATGAACTGGCCGCAGGTTATGTGTTGAGTTGC
CAGGCACTGCCGCTGACCAGCGATGTGGTGGTTGACTTTGACGCGAAGGGGATGGCATGAGCGAACTGATCGTCAGCCGT
CAGCAACGAGTATTGTTGCTGACCCTTAACCGTCCCGCCGCACGTAATGCGCTAAATAATGCCCTGCTGATGCAACTGGT
AAATGAACTGGAAGCTGCGGCTACCGATACCAGCATTTCGGTCTGTGTGATTACCGGTAATGCACGCTTTTTTGCCGCTG
GGGCCGATCTCAACGAAATGGCAGAAAAAGATCTCGCGGCCACCTTAAACGATACACGTCCGCAGCTATGGGCGCGATTG
CAGGCCTTTAATAAACCACTTATCGCAGCCGTCAATGGTTACGCGCTTGGGGCGGGTTGCGAACTGGCATTGTTGTGCGA
TGTGGTGGTTGCCGGAGAGAACGCGCGTTTTGGGTTGCCGGAAATCACTCTCGGCATCATGCCTGGCGCAGGCGGAACGC
AACGTTTAATCCGTAGTGTCGGTAAATCGTTAGCCAGCAAAATGGTGCTGAGCGGAGAAAGTATCACCGCTCAGCAAGCA
CAGCAGGCCGGGCTGGTTAGCGACGTCTTCCCCAGCGATTTAACCCTCGAATACGCCTTACAGCTGGCATCGAAAATGGC
ACGTCACTCGCCGCTGGCCTTACAAGCGGCAAAGCAAGCGCTGCGCCAGTCGCAGGAAGTGGCTTTGCAAGCCGGACTTG
CCCAGGAGCGACAGTTATTCACCTTGCTGGCGGCAACAGAAGATCGTCATGAAGGCATCTCCGCTTTCTTACAAAAACGC
ACGCCCGACTTTAAAGGACGCTAATGATGGAATTCATCCTCAGTCATGTAGAAAAGGGCGTGATGACACTAACGCTCAAC
CGCCCGGAACGCCTGAACAGTTTTAATGATGAGATGCACGCACAACTGGCAGAGTGCCTGAAACAGGTCGAGCGCGACGA
CACTATCCGTTGCCTGTTACTTACAGGTGCCGGGCGCGGATTTTGTGCTGGTCAGGATCTTAACGATCGTAACGTTGATC
CCACAGGCCCCGCACCGGATTTAGGGATGTCAGTTGAACGTTTCTATAACCCACTGGTACGTCGCCTGGCAAAACTGCCA
AAACCGGTGATCTGTGCAGTCAATGGCGTGGCGGCAGGGGCAGGCGCAACACTGGCACTGGGGGGCGACATCGTTATTGC
TGCCCGTTCAGCAAAATTCGTCATGGCGTTTAGTAAGTTAGGCTTAATACCCGATTGCGGTGGAACCTGGTTACTGCCAC
GCGTTGCCGGACGAGCGCGCGCCATGGGGCTGGCACTGCTGGGGAATCAACTGAGTGCTGAACAGGCGCACGAATGGGGG
ATGATCTGGCAGGTTGTTGATGATGAAACGCTGGCAGATACCGCGCAACAGCTGGCACGGCATCTGGCGACACAACCGAC
ATTTGGTCTTGGACTTATCAAGCAAGCGATAAATAGCGCTGAAACCAATACGCTCGATACGCAACTGGATCTGGAACGTG
ACTATCAGCGACTTGCCGGACGGAGCGCAGATTATCGTGAAGGTGTCAGTGCGTTCCTGGCTAAACGCTCACCGCAGTTC
ACGGGGAAATAGCATGATGATAAATGTGCAAACTGTGGCAGTGATTGGGAGCGGCACCATGGGGGCAGGCATTGCTGAAG
TTGCTGCCAGTCATGGACACCAGGTTTTACTGTATGACATTTCTGCTGAAGCGCTGACCCGCGCAATCGACGGGATACAC
GCGCGGCTAAATTCACGCGTGACGCGGGGAAAACTGACTGCTGAAACCTGTGAACGCACATTGAAACGCCTGATCCCGGT
GACCGATATTCACGCGCTGGCAGCTGCGGACCTGGTCATTGAAGCGGCGTCTGAACGTCTGGAAGTCAAAAAAGCGCTCT
TTGCACAGCTGGCGGAAGTTTGCCCGCCACAAACGCTATTGACCACTAACACTTCGTCAATCTCTATAACCGCGATTGCT
GCGGAGATAAAAAATCCTGAACGTGTTGCGGGGCTGCATTTTTTTAACCCGGCACCGGTGATGAAGTTGGTGGAGGTGGT
CAGTGGGCTGGCAACGGCGGCGGAAGTTGTTGAGCAGTTGTGTGAACTAACGTTGAGTTGGGGTAAGCAGCCTGTGCGCT
GTCATTCGACTCCTGGATTTATCGTTAACCGTGTTGCGCGTCCTTATTATTCCGAGGCCTGGCGGGCACTGGAAGAGCAG
GTTGCTGCACCAGAAGTGATTGACGCTGCACTTCGCGATGGCGCTGGTTTCCCGATGGGGCCGCTGGAATTAACCGATCT
GATTGGTCAGGACGTCAATTTTGCTGTCACCTGTTCGGTGTTTAACGCTTTCTGGCAGGAGCGTCGTTTTTTACCTTCGC
TGGTGCAACAGGAACTGGTGATTGGTGGACGGTTGGGCAAGAAAAGTGGGCTGGGCGTGTACGACTGGCGCGCGGAACGT
GAGGCAGTTGTTGGCCTGGAAGCGGTAAGCGACAGTTTTAGCCCAATGAAAGTAGAAAAGAAAAGTGACGGTGTCACGGA
AATTGACGATGTTTTATTGATTGAGACACAAGGCGAGACGGCACAGGCGCTGGCAATACGACTGGCACGCCCGGTGGTAG
TGATCGATAAAATGGCGGGCAAGGTGGTGACCATTGCTGCTGCAGCGGTGAACCCGGACTCAGCGACCCGCAAGGCCATT
TATTACCTGCAACAGCAGGGCAAAACAGTGCTGCAAATTGCAGATTACCCAGGAATGCTGATTTGGCGAACGGTAGCAAT
GATCATCAATGAAGCCCTTGATGCGCTTCAAAAAGGCGTGGCCTCTGAACAGGATATCGATACCGCCATGCGTCTTGGGG
TGAATTATCCATATGGCCCACTTGCCTGGGGAGCGCAACTTGGCTGGCAGCGAATATTAAGGCTCCTTGAAAATCTACAG

CATCACTATGGCGAAGAACGCTATCGCCCATGTTCATTGCTGCGCCAACGGGCGCTTCTGGAGAGCGGTTATGAGTCATA
AGGCCTGGCAAAATGCCCATGCAATGTATGAGAACGATGCCTGCGCCAAAGCGCTTGGCATCGACATTATCTCAATGGAT
GAAGGCTTTGCTGTAGTGACCATGACCGTCACTGCACAAATGCTTAACGGTCATCAAAGTTGCCACGGCGGGCAGCTATT
TTCACTGGCTGATACTGCCTTTGCCTACGCCTGCAATAGCCAGGGGCTGGCAGCCGTCGCTTCTGCCTGCACGATTGATT
TTTTGCGTCCAGGCTTTGCCGGAGACACCTTAACTGCTACTGCGCAGGTACGTCATCAGGGCAAGCAAACCGGTGTTTAC
GACATCGAAATTGTTAACCAACAACAAAAAACGGTTGCGCTGTTTCGCGGTAAATCTCACCGCATCGGCGGCACCATTAC
AGGAGAAGCCTGATGCGTGAAGCCTTTATTTGTGACGGAATTCGTACGCCAATTGGTCGCTACGGCGGGGCATTATCAAG
TGTTCGGGCTGATGATCTGGCTGCTATCCCTTTGCGGGAACTGCTGGTGCGAAACCCGCGTCTCGATGCGGAGTGTATCG
ATGATGTGATCCTCGGCTGTGCTAATCAGGCGGGAGAAGATAACCGTAACGTAGCCCGGATGGCGACTTTACTGGCGGGG
CTGCCGCAGAGTGTTTCCGGCACAACCATTAACCGCTTGTGTGGTTCCGGGCTGGACGCACTGGGGTTTGCCGCACGGGC
GATTAAAGCGGGCGATGGCGATTTGCTGATCGCCGGTGGCGTGGAGTCAATGTCACGGGCACCGTTTGTTATGGGCAAGG
CAGCCAGTGCATTTTCTCGTCAGGCTGAGATGTTCGATACCACTATTGGCTGGCGATTTGTGAACCCGCTCATGGCTCAG
CAATTTGGAACTGACAGCATGCCGGAAACGGCAGAGAATGTAGCTGAACTGTTAAAAATCTCACGAGAAGATCAAGATAG
TTTTGCGCTACGCAGTCAGCAACGTACGGCAAAAGCGCAATCCTCAGGCATTCTGGCTGAGGAGATTGTTCCGGTTGTGT
TGAAAAACAAGAAAGGTGTTGTAACAGAAATACAACATGATGAGCATCTGCGCCCGGAAACGACGCTGGAACAGTTACGT
GGGTTAAAAGCACCATTTCGTGCCAATGGGGTGATTACCGCAGGCAATGCTTCCGGGGTGAATGACGGAGCCGCTGCGTT
GATTATTGCCAGTGAACAGATGGCAGCAGCGCAAGGACTGACACCGCGGGCGCGTATCGTAGCCATGGCAACCGCCGGGG
TGGAACCGCGCCTGATGGGGCTTGGTCCGGTGCCTGCAACTCGCCGGGTGCTGGAACGCGCAGGGCTGAGTATTCACGAT
ATGGACGTGATTGAACTGAACGAAGCGTTCGCGGCCCAGGCGTTGGGTGTACTACGCGAATTGGGGCTGCCTGATGATGC
CCCACATGTTAACCCCAACGGAGGCGCTATCGCCTTAGGCCATCCGTTGGGAATGAGTGGTGCCCGCCTGGCACTGGCTG
CCAGCCATGAGCTGCATCGGCGTAACGGTCGTTACGCATTGTGCACCATGTGCATCGGTGTCGGTCAGGGCATCGCCATG
ATTCTGGAGCGTGTTTGAGCATATCAACCTGCGAGTACCCTACAATGATAACCAATACAAAGCTTGACCCGATTGAAACC
GCGTCTGTTGATGAGTTACAGGCGTTGCAAACACAGCGTCTGAAATGGACGCTCAAACACGCGTATGAAAATGTGCCGAT
GTATCGGCGCAAATTCGACGCAGCAGGCGTACATCCTGATGATTTCAGGGAACTTTCAGACCTGCGTAAATTTCCCTGTA
CCACCAAACAGGATCTGCGCGACAACTATCCCTTTGACACCTTTGCCGTGCCAATGGAACAAGTGGTGCGCATTCATGCT
TCTTCGGGAACCACAGGTAAACCGACAGTTGTCGGCTATACGCAAAACGATATTGATAACTGGGCCAATATTGTAGCGCG
TTCTTTGCGTGCGGCAGGGGGCTCGCCCAAAGACAAAATTCATGTTGCCTATGGTTACGGCCTGTTTACTGGTGGGCTGG
GTGCGCACTATGGTGCCGAACGTCTGGGCGCTACGGTGATCCCGATGTCTGGCGGGCAGACGGAAAAACAAGCGCAACTG
ATCCGTGATTTTCAACCAGATATGATCATGGTTACGCCATCTTATTGCCTTAACCTGATTGAAGAGCTGGAGCGGCAGTT
GGGCGGTGATGCCAGCGGTTGCTCGCTGCGGGTTGGAGTATTTGGTGCTGAGCCGTGGACACAGGCCATGCGTAAAGAGA
TTGAGCGTCGCCTGGGGATCACCGCACTGGATATTTATGGCCTGTCAGAAGTGATGGGGCCGGGGGTGGCGATGGAGTGT
CTGGAAACTACCGACGGCCCAACCATTTGGGAAGATCATTTCTATCCTGAAATTGTTAATCCTCATGACGGCACACCGCT
TGCCGATGGTGAACATGGCGAACTGTTATTCACCACGCTGACCAAAGAAGCATTGCCGGTCATTCGTTACCGCACGCGTG
ATTTAACCCGACTGTTACCAGGAACGGCGCGGACTATGCGCCGCATGGATCGCATCAGCGGACGCAGCGATGACATGTTG
ATTATTCGCGGTGTAAATGTCTTTCCGTCACAACTGGAAGAAGAGATTGTCAAATTCGAACATTTATCGCCGCATTACCA
ACTGGAGGTGAACCGCCGGGGGCATCTTGATTCACTTTCTGTGAAAGTGGAGTTGAAAGAAAGTAGCCTGACATTGACGC
ATGAGCAGCGTTGCCAGGTATGCCACCAGCTGCGCCATCGGATTAAGTCGATGGTGGGGATCTCTACCGATGTGATGATC
GTTAACTGTGGCAGTATCCCGCGTTCAGAAGGCAAGGCGTGTCGGGTGTTTGATCTGCGCAATATTGTTGGTGCCTGACG
TATCGTCTGGCCCTGGTGGGGTAAAGCGCCAGGGCCAGAAGTCGATACGACCTGTGCTATGATTCATAAATCACAACAAT
AACAACAGACTGAAGCGAATGAGTAAACTTGATACTTTTATCCAACATGCTGTAAACGCTGTTCCGGTCAGTGGCACATC
TTTGATCTCCTCTCTGTATGGTGATTCGCTTTCCCATCGTGGTGGTGAAATCTGGTTGGGTAGTCTGGCTGCTTTGCTGG
AAGGGCTGGGATTTGGTGAGCGTTTCGTGCGCACCGCTTTGTTTCGTCTTAATAAAGAAGGCTGGCTGGATGTTTCCCGC
ATCGGGCGACGCAGTTTCTATAGCCTCAGTGATAAAGGCTTGCGCCTGACGCGACGGGCAGAAAGTAAAATTTATCGCGC
AGAGCAACCTGCATGGGATGGTAAATGGCTCCTGTTGCTCTCGGAAGGTTTAGATAAATCAACGCTGGCTGATGTCAAAA
AGCAGTTGATCTGGCAAGGTTTTGGCGCACTGGCACCCAGCCTGATGGCATCGCCGTCGCAAAAACTGGCCGATGTACAG
ACACTTTTGCATGAAGCGGGTGTGGCGGATAACGTGATTTGTTTTGAAGCGCAAATACCACTGGCGCTTTCTCGCGCAGC
ACTGCGTGCCAGAGTAGAAGAGTGCTGGCATTTAACTGAACAAAATGCCATGTACGAAACCTTTATTCAGTCATTCCGCC
CGCTGGTGCCGCTTTTAAAAGAGGCGGCAGACGAGTTAACCCCGGAGCGGGCATTTCATATTCAGCTTTTACTGATCCAT
TTTTATCGCCGTGTCGTCCTTAAAGACCCATTGTTGCCGGAGGAGTTGCTTCCGGCACACTGGGCAGGGCATACGGCGCG
TCAGCTGTGTATCAACATTTATCAGCGCGTAGCGCCTGCTGCTTTAGCGTTCGTTAGTGAAAAAGGTGAAACCTCGGTCG
GTGAACTGCCTGCGCCGGGAAGCCTGTATTTTCAACGTTTTGGCGGCTTGAATATTGAACAGGAGGCGTTATGCCAATTT
ATCAGATAGACGGTCTGACTCCGGTTGTGCCAGAAGAGAGTTTTGTCCATCCGACAGCGGTATTGATCGGCGATGTTATT
CTCGGCAAGGGCGTTTACGTTGGGCCAAATGCCAGCCTGCGTGGCGATTTTGGTCGTATCGTGGTGAAAGATGGCGCGAA
CATTCAGGATAATTGCGTTATGCACGGTTTTCCCGAGCAGGATACTGTTGTAGGAGAAGATGGACATATTGGTCATAGCG
CTATCCTTCACGGCTGCATTATCCGCCGCAATGCATTAGTGGGAATGAACGCGGTAGTGATGGACGGTGCGGTGATTGGC
GAGAACAGCATTGTTGGTGCATCCGCATTTGTGAAAGCCAAAGCAGAAATGCCAGCTAATTACCTGATTGTCGGCAGCCC

GGCGAAAGCGATTCGTGAACTCAGTGAGCAGGAGTTGGCATGGAAAAAGCAGGGTACGCATGAGTACCAGGTGCTGGTGA
CACGCTGTAAGCAGACGTTACATCAAGTCGAGCCATTGCGGGAAATTGAACCTGGCAGGAAACGCCTGGTATTTGATGAG
AATCTGCGACCGAAACAGTAACAGATGTAAAATTATTTTGTCCCTTTAATTATAAAGCAGAGTTATGTTTAAGCTCTGCT
TTATTTATTTGAGTATTAATTCATACCGTTTTTTCATCATATAATTATTTATAATGAGAATGTGGTTTTAATTTGTAATT
TATATTATATACACAATTTATATATTTCATGGTCTTTTTTTATTCACCTGAATTATAATTGTGAATTATAGGAAAGTATG
TTTGATTAGATAATAATCTACTGGCAATATTGGATGTCTTCTATGTTTTAAATAACTAATTGGTCGGGTTAGTGCATCCG
GCTTTCTTTATATTCGCCAGAAGGATTTATTATGCAAAGGAAAACTCTATTGTCGGCCTGTATTGCATTAGCTCTGAGTG
GTCAGGGTTGGGCGGCAGATATCACAGAGGTAGAAACCACCACAGGTGAAAAGAAAAATACCAATGTGACTTGTCCGGCA
GACCCAGGAAAACTCAGTCCGGAAGAGCTTAAACGCTTACCCTCTGAATGCTCTCCTTTAGTCGAACAAAACCTGATGCC
ATGGCTTTCCACAGGCGCTGCTGCGTTAATCACGGCCTTAGCCGTAGTGGAACTAAACGACGATGATGATCATCATCATC
GCAACAATTCTCCACTCCCACCGACACCCCCTGATGATGAATCAGACGACACTCCAGTTCCCCCAACTCCTGGCGGAGAT
GAGATAATACCGGACGATCCGGATGATACGCCTACACCTCCCAAACCGGTTTCGTTTAATAATGACGTTATTCTCGATAA
AACAGAAAAAAACGTTAACTATTCGCGATTCAGTTTTTACTTATACCGAGAATGCTGACGGGACTATATCTCTGCAAGATA
GCAATGGTCGTAAGGCAACGATTAATCTTTGGCAGATTGATGAAGCGAATAACACTGTTGCCCTTGAAGGGGTGAGCGCA
GATGGCGCAACGAAGTGGCAATATAATCACAACGGTGAGCTTGTTATTACGGGTGATAATGCCACAGTAAACAACAATGG
CAAAACCACCGTTGACGGTAAAGATTCCACCGGTACGGAAATCAACGGTAATAACGGGAAAGTGATTCAGGACGGCGATC
TGGATGTCAGCGGCGGCGGTCACGGTATTGATATCACCGGTGACAGCGCGACGGTGGATAACAAGGGCACCATGACCGTC
ACCGATCCGGAGTCCATGGGTATCCAGATCGACGGTGACAAGGCCATCGTCAATAACGAAGGCGAGAGCACCATCACCAA
CGGTGGCACCGGCACCCAGATTAATGGTGATGACGCCACGGCAAACAACAACGGCAAAACCACCGTTGACGGCAAGGATT
CCACCGGTACGGAAATCAACGGTAATAACGGAAAAGTTATTCAGGACGGCGATCTGGATGTCAGCGGCGGCGGTCACGGT
ATTGATATCACCGGCGACAGCGCAACGGTGGATAACAAGGGCACCATGACCGTCACCGATCCGGAGTCCATCGGTATCCA
GGTTGACGGCGACCAGGCGGTTGTTAACAACGAAGGCGAGAGCGCCATCACCAACGGTGGCACCGGCACGCAGATTAACG
GTGATGACGCCACGGCAAACAACAACGGCAAAACCACCGTTGACGGCAAGGATTCCACCGGTACGGAAATTGCTGGCAAT
AACGGGAAGGTGATTCAGGACGGCGATCTGGATGTCAGCGGCGGCGGTCACGGTATTGATATCACCGGCGACAGCGCAAC
GGTGGATAACAAGGGCACCATGACCGTCACCGATCCGGAGTCCATCGGTATCCAGATTGACGGCGACCAGGCCATCGTCA
ATAACGAAGGCGAGAGCACTATCACCAATGGCGGCACCGGCACTCAGATCAACGGTAACGACGCCACCGCGAATAACAGT
GGAAAAACCACTGTTGATGGAAAAGATTCCACGGGTACCAAAATCGCGGGCAATATCGGCATTGTAAATCTGGATGGTAG
CCTGACTGTTACAGGCGGTGCGCATGGTGTTGAGAACATTGGTGACAACGGCACGGTTAACAACAAAGGAGATATTGTTG
TTTCCGATACTGGATCGATTGGCGTGCTCATCAACGGTGAGGGGGCAACAGTATCCAATACGGGTGATGTTAACGTTAGC
AATGAAGCGACAGGGTTCAGCATCACAACCAACAGTGGGAAGGTTTCGCTGGCAGGCAGTATGCAGGTTGGCGATTTCTC
GACCGGGGTAGATCTTAATGGCAACAATAACAGCGTGACGCTGGCGGCAAAAGATCTAAAAGTGGTCGGGCAGAAAGCGA
CGGGCATAAACGTTTCTGGCGATGCGAATACAGTGAATATCACTGGTAACGTTCTGGTTGATAAGGATAAAACCGCAGAC
AATGCGGCGGAATATTTCTTCGATCCATCCGTGGGTATCAACGTTTACGGCAGTGATAATAACGTGACGCTGGATGGAAA
GTTAACTGTTGTATCAGACAGTGAGGTTACTTCTCGTCAGAGTAATTTATTTGATGGCAGCGCAGAGAAAACGTCAGGTC
TGGTTGTGATTGGCGATGGCAATACCGTTAATATGAATGGTGGACTTGAACTGATTGGAGAGAAAAACGCGCTTGCAGAT
GGGTCGCAGGTTACTTCCTTGCGCACAGGATATAGTTATACCAGCGTTATTGTCGTTAGTGGTGAGTCGTCGGTATATCT
GAATGGAGATACGACAATCAGCGGAGAATTCCCTCTGGGGTTTGCCGGGGTTATTCGGGTACAGGATAAAGCTTTGCTGG
AAATTGGCAGTGGCGCTACGCTAACAATGCAGGATATTGACAGTTTTGAACATCATGGGACAAGAACCCTGGATTTGCCC
CTATATTTCCAGACATCTGTTATCACTTAACCCATTACAAGCCCGCTGCCGCAGATATTCCCGTGGCGAGCGATAACCCA
GCGCACTATGCGGATGCCATTCGTTATAATGCTCGAACGCCTCTGCAAGGTTCTTTGCTGCCGTTAACCCGTCTGGTTTG
GGCATGATACTGATGTAGTCACGCTTTATCGTTTTCACGAAGCTCTCTGCTATTCCGTTACTCTCCGGACTCCGCACCGC
CGTGTTCTTCGGTTCAAGTCCCAACATCCGGGCGAACTGGCGTGTTTCATTAGCCCGGTAGCATGAACCATTATCCGTCA
GCCACTCCACTGGAGACGACGGAAGATCGTTGCCGAAGCGGCGTTCCACCGCTCCCAGCATGACGTCCTGTACTGTTTCA
CTGTTGAAGCCGCCGGTAGTGACCGCCCAGTGCAGTGCCTCACGATCACAGCAGTCCAGCGCGAACGTGACACGCAGTCT
CTCTCCGTTATCACAGCAGAACTCGAACCCGTCAGAGCACCATCGCTGATTGCTTTCTTTCACGGCCACTCTGCCTGTAT
GTGCCCGTTTCGATGGCGGTACAGCAGGTTTTCGCTCAAGCAACAGCGCATTCTGGCGCATGATCCGGTAAACACGTTTG
GCATTGATCGCAGGCATACCATCAAGTTCTGCCTGTCTGCGAAGCAGCGCCCATACCCGACGATAACCATACGTTGGCAG
CTCTCCGATAACATGGTGTATACGGAGAAGCACATCCGTATCATCAGTGTGACGACTGCGGCGGCCATCCATCCAGTCAT
CGGTTCGTCTGAGAATGACGTGCAACTGCGCACGCGACACCCGGAGACAACGGCTGACTAAGCTTACTCCCCATCCCCGG
GCAATAAGGGCGCGTGCGCTATCCACTTTTTTGCCCGTCCATATTCAACGGCTTCTTTGAGGAGTTCATTTTCCATCGTT
TTCTTGCCGAGCAGGCGCTGGAGTTCTTTAATCTGCTTCATGGCGGCAGCAAGTTCAGAGGCAGGAACAACCTGTTCTCC
GGCGGCGACAGCAGTAAGACTTCCTTCCTGGTATTGCTTACGCCAGAGAAATAACTGGCTGGCTGCTACACCATGTTGCC
GGGCAACGAGGGAGACCGTCATCCCCGGTTCAAAGCTCTGCTGAACAATTGCGATCTTTTCCTGTGTGGTACGCCGTCTG
CGTTTCTCCGGCCCTAAGACATCAATCATCTGTTCTCCAATGACTAGTCTAAAAACTAGTATTAAGACTATCACTTATTT
AAGTGATATTGGTTGTCTGGAGATTCAGGGGGCCAGTCTAAACCCCAGAACTTACTTATGCTGATTCCGGTGCGAAAATT
GTTAATAAAGGTACTTGTAGATTCAATTGGTCAACGCAACAGTTATGTGAAAACATGGGGTTGCGGAGGTTTTTTGAATG

AGACGAACATTTACAGCAGAGGAAAAAGCCTCTGTTTTTGAACTATGGAAGAACGGAACAGGCTTCAGTGAAATAGCGAA
TATCCTGGGTTCAAAACCCGGAACGATCTTCACTATGTTAAGGGATACTGGCGGCATAAAACCCCATGAGCGTAAGCGGG
CTGTAGCTCACCTGACACTGTCTGAGCGCGAGGAGATACGAGCTGGTTTGTCAGCCAAAATGAGCATTCGTGCGATAGCT
ACTGCGCTGAATCGCAGTCCTTCGACGATCTCACGTGAAGTTCAGCGTAATCGGGGCAGACGCTATTACAAAGCTGTTGA
TGCTAATAACCGAGCCAACAGAATGGCGAAAAGGCCAAAACCGTGCTTACTGGATCAAAATTTACCATTGCGAAAGCTTG
TTCTGGAAAAGCTGGAGATGAAATGGTCTCCAGAGCAAATATCAGGATGGTTAAGGCGAACAAAACCACGTCAAAAAACG
CTGCGAATATCACCTGAGACAATTTATAAAACGCTGTACTTTCGTAGCCGTGAAGCGCTACACCACCTGAATATACAGCA
TCTGCGACGGTCGCATAGCCTTCGCCATGGCAGGCGTCATACCCGCAAAGGCGAAAGAGGTACGATTAACATAGTGAACG
GAACACCAATTCACGAACGTTCCCGAAATATCGATAACAGACGCTCTCTAGGGCATTGGGAGGGCGATTTAGTCTCAGGT
ACAAAAAACTCTCATATAGCCACACTTGTAGACCGAAAATCACGTTATACGATCATCCTTAGACTCAGGGGCAAAGATTC
TGTCTCAGTAAATCAGGCTCTTACCGACAAATTCCTGAGTTTACCGTCAGAACTCAGAAAATCACTGACATGGGACAGAG
GAATGGAACTGGCCAGACATCTAGAATTTACTGTCAGCACCGGCGTTAAAGTTTACTTCTGCGATCCTCAGAGTCCTTGG
CAGCGGGGAACAAATGAGAACACAAATGGGCTAATTCGGCAGTACTTTCCTAAAAAGACATGTCTTGCCCAATATACTCA
ACATGAACTAGATCTGGTTGCTGCTCAGCTAAACAACAGACCGAGAAAGACACTGAAGTTCAAAACACCGAAAGAGATAA
TTGAAAGGGGTGTTGCATTGACAGATTGAATCTACACTGTTGAAATTCAGAATTTAGGTTTTGCTTTTGTTACTGGTGAA
AATACAACAGGTATAAATAGTGGCACGATCTCGTTATTACAAAATGGTAAAGATCCGGCACCGTCTCCCATTGTTTTACT
GGCTACTAACGGAGGGAGCGCCACTAATGCAGGTACGATCACAGGTAAAGTGACGGAACAACATAGCGTATTTAACAAGT
ATTCAACGGGCACATCGAATTCATTTATTTTTAATAACGATGTCAGTAGCATAACAGGGTTAGTCGCTCAATCGAATAGC
ACAATTATCAATACTGACAGCGGCATCATTGATTTGTATGGTCGTGGTAGTGTCGGCATGCTTGCTATAGCAGATTCAAC
AGCAGAAAATCAGGGTAAAATTACACTGGATTCTATGTGGGTAGATGCAAATGACACTACCGCAATGCGAGATATAGCTA
GCAACAGCGCCATTGACTTCGGTACAGGTGTGGGAGTTGGTACTGATAGTTATAGTGGTGCAGGGAAAAATGCAACAGCA
ATTAACCAATTGGGCGGTGTTATAACTATTTATAACGCCGGCGCAGGTATGGCGGCCTATGGCGCCAGCAATACAGTTAT
TAACCAGGGGACGATTAACCTCGAAAAAAATGGTAATTATGACGATAGTCTGGCAGCAAATACTCTGGTAGGGATGGCTG
TTTATGAGCATGGTACTGCTATCAACGACCAGACGGGTGTTATCAATATCAATGTTGGTACTGGTCAGGCGTTTTATAAC
GATGGCACAGGAACAATTGTTAACTATGGTACAATCTGCACTTTCGGCGTGTGCCAATCGGGGAATGAGTACAATAATAC
AGATGATTTCACCTCACTGATCTATACCGGTGGCGATACGATTACACGAAGCGGAGAAACTGTAACGCTAAATAAATCTG
CTGCTGTGACTGATAAGCTGGCTGGGAATGTTGTTAATAGCGGAACGCTTTCCGGTGATCAAATTACGGTATCAAGCGGT
CTTCTGGAAAATACCAGCGGTGGCATCATCAATAACTTAGTAAAACTTGACAAGGGTGCCGTCATTAAAAATGCCGGGGT
GATGACGAATAACGTCGATGTTAGCGGTGGAATCCTCAATAATGCCGGAGAAATGACTGCGCAAATTACCATGAATGCTG
GTGCTGATAGTTCGTTAGTGAACAACACCGGAACCATCAATAAAATCGTGCAGAACGCGGGGGTATTCAATAATAGTGGC
AGTGTAACAGGGCGGATGATGTCGGCTGGCGGGGTCTTTAATAATCAAACTGACGGGGCGATTATGAGAGGTGCTGCGCT
GACAGGTACTGCAGTGGCAAATAACGAAGGAACCTGGAACCTCGGAAGTAGTAGTGAGGGTAACAACACCGGGATGCTGG
AAGTTAATAATAATTCAGCTTTCAATAACCGCGGCGAGTTTATTCTTGATAACGACAAGAATGCTGTGCACATCAACCAG
TCCGGTACGCTTTATAATACCGGTCACATGAACATCAGTAATTCTTCCCACAACGGAGCCGTTAATATGTGGGGCGGAAA
TGGTCGTTTTATCAATGACGGAACGATTGATGTTTCTGCGAAGTCACTGGTAGTCAGCGCTAATAATGCCGGCGATCAGA
ATGCCTTCTTCTGGAACCAGGATAACGGGGTCATCAACTTCGATCACGACAGCGCCAGTGCCGTGAAAGTCACCCACAGC
AACTTTATTGCCCAGAATGACGGCATCATGAACATCAGCGGCACCGGTGCTGTGGCTATGGAAGGTGATAAGAACGCGCA
GCTGGTTAACAATGGCACCATCAACCTCGGTACCGCAGGCACTACTGACACGGGTATGATCGGTATGCAACTCGATGCCA
ACGCCACGGCGGATGCGGTAATCGAAAACAACGGCACCATCAATATCTTCGCCAATGACTCGTTTGCATTTAGCGTACTG
GGTACAGTAGGTCATGTGGTTAACAACGGCACGGTGGTGATTGCCGATGGGGTTACGGGTTCTGGACTGATCAAGCAGGG
CGACAGCATCAATGTTGAAGGTATGAACGGTAACAACGGTAATAGCAGCGAAGTGCATTATGGCGACTATACGTTGCCGG
ATGTGCCGAAGCCCAATACGGTTAGTGTAACGTCGGGAAGTGATGAGGCTGGTGGCAGCATGAACAACCTCAACGGCTAT
GTCGTCGGTACCAACGTTAACGGCAGCGCCGGGAAGCTGAAGGTTAACAATGCCAGCATGAACGGCGTGGAGATTAACAC
GGGCTTTACCGCTGGTACGGCAGACACCACTGTGAGTTTTGATAACGTAGTGGAAGGTAGCAACCTGACCGACGCTGACG
CCATCACCTCAACGTCCGTGGTATGGACTGCCAAAGGCAGCACCGATGCCAGCGGTAACGTTGACGTCACCATGAGCAAA
AATGCCTACACCGATGTGGCAACAGATGCCTCGGTGAATGACATCGCGAAAGCACTGGATGCGGGTTACACCAACAACGA
ACTGTTTACCAGCCTGAACGTCGGCACGACTGCTGAACTGAACAGTGCTCTGAAACAGGTCAGCGGTAGCCAGGCGACCA
CGGTATTCCGCGAAGCGCGCGTGTTAAGCAACCGCTTTAGTATGCTGGCAGATGCCGCGCCGAAAGTGGGTAACGGTCTG
GCGTTCAACGTTGTCGCGAAAGGCGATCCGCGTGCCGAGTTAGGTAATAATACCGAATACGACATGCTGGCATTGCGTAA
AACTATCGACCTGAGCGAAAGCCAGACGATGAGTCTGGAGTACGGTATCGCTCGTCTCGATGGTGATGGTGCGCAGAAAG
CGGGTGATAATGGCGTTACAGGCGGTTATAGCCAGTTTTTTGGCCTGAAACATCAGATGTCGTTCGATAACGGCATGAAC
TGGAATAACGCCTTGCGTTACGACGTTCACAACCTTGACAGCAGCCGCTCGATTGCATTTGGCAACACGAACAAAACGGC
TGATACCGACGTGAAACAGCAGTACCTGGAGTTCCGCAGCGAAGGGGCGAAGACTTTCGAACCGAGCGAAGGACTGAAGG
TTACGCCATATGCGGGTGTAAAACTGCGTCACACACTGGAAGGTGGCTATCAGGAGCGCAATGCCGGAGACTTTAACCTG
AATATGAACAGTGGCAGCGAAACGGCGGTGGACAGCATCGTCGGGCTGAAACTGGACTACGCAGGTAAAGACGGCTGGAG
CGCTAGCGCTACGCTGGAAGGCGGGCCGAACCTGAGCTACGCGAAGAGCCAGCGTACGGCAAGCCTGGCAGGCGCAGGCA

GTCAGCACTTTAACGTCGATGACGGTCAGAAGGGCGGCGGCATCAATAGCCTGACAAGCGTCGGCGTGAAGTACAGCAGC
AAAGAAAGTTCGCTGAATCTGGATGCGTACAACTGGAAAGAGGATGGCATCAGCGATAAAGGCGTGATGCTGAACTTCAA
GAAAACGTTCTAATTTTTAGCATGTGATCCCTAAACCGCAACGCTGATACAGGTTGCGGTTTTTTTATTGCCGGATGTGG
TACGTGACGCGTTTTGTTTTGTGTCTTTCAGGACAATAGAGCAACTCATCCAGTAATCTTGTTTACACCTTCGCATTATT
TATCTCTTTTCGTTTCTATACTGATTTTTCTTAATCCGTTTTATTACAGGGCAGGGTGCGATGAGCAGCAATACATTTAC
TCTCGGTACAAAATCCGTTAACCGTCTTGGTTATGGCGCGATGCAACTGGCAGGTCCTGGAGTTTTTGGCCCCCCACGAG
ATCGCCACGTCGCTATAACCGTGCTGCGTGAGGCGCTGGCATTGGGCGTCAATCATATTGATACCAGCGACTTTTATGGT
CCGCACGTCACCAATCAGATTATCCGCGAAGCGCTTTATCCTTACTCTGACGACCTGACAATTGTCACTAAAATTGGTGC
GCGGCGTGGAGAGGACGCATCCTGGTTGCCCGCATTTTCTCCGGCAGAGCTGCAAAAAGCGGTGCACGATAATCTACGTA
ATCTCGGGCTGGACGTGCTGGATGTGGTTAACCTGCGCGTTATGATGGGGGATGGTCATGGCCCAGCGGAAGGATCGATT
GAGGCCAGCCTGACCGTGCTGGCAGAGATGCAACAACAAGGCCTGGTAAAACATATTGGCCTGAGCAACGTCACACCGAC
GCAGGTTGCAGAGGCGCGCAAGATTGCCGAAATTGTCTGTGTGCAAAACGAATACAACATCGCGCACCGTGCTGATGATG
CAATGATTGATGCTTTGGCCCACGATGGCATTGCCTACGTGCCGTTCTTCCCGCTCGGGGGCTTTACACCGCTGCAATCG
TCCACACTTTCCGATGTTGCTGCGAGCCTGGGTGCAACACCAATGCAGGTGGCGCTGGCGTGGCTGTTACAGCGTTCACC
GAATATTTTGCTGATCCCAGGGACGTCTTCGGTTGCGCATTTACGGGAGAATATGGCTGCTGAAAAATTGCATCTTTCTG
AGGAAGTGTTGTCTACGTTGGATGGTATTTCGCGAGAATAACGAATATACAAAAGGGAAAGATGCATTTCCCTTTTTTTC
TTTTTTAATGGCATGGAGTGCATATGTTGAAGGCCAGGAATTGCGGCTGGATACGTTTACTGCCCCTGTTTATGCTGAGT
CTACCAGTACAGGCGGAACTTCGATGTGTTGCAAATGCAGTTGATATTGAATCATTTTTTTCTGCGGCTACCGCTGAAGA
TAAACAACAAGTTGAACAAGCTATCAACAGTAGCGTGAATCTTGTCCCCTTCGGTTTATCTGCATCGAACTGGAAAGTGC
ATCGTGGCGATTTAGTGGTAGAAGGTAATATAGAGAGTAATCAAAAATTGATTGTTCTTGGGAATTTGACAGTCAAAGGT
AATATTTCCACTTTCTCTCTTTCTAATCCATGGGTTATTCTCGGTAACGTGACGGCAACGAATATAGTTGCGGATTCACC
ACTGTTAATTACCGGTTCGATAAATGCAAGTGGACTGGTATTTATCGACTCATATTACGATAATCCGTCTACGATTAAGG
GGAGTATTAATGCGCGTGGGATATTTATCAATGACATAATTGCACCTGTTGTTGCGTCTTCGACAAATAGTGAATTCATG
GTCCGTGCGAGTGACAAACATGACACTGAAAATGTCAAAAAAGCGCTGATGATAATAAATCCTGATGCATATTATTGGGG
GCTAATTAATGATGAAGATGCTCTGAAAGAAATTTTTAAGCGAAGCAATATTCGCATGGCAGGGAATGTCTGTAATCAGA
TGAAAAAAGAAGCGCTGTTTCGCCCTAAGCCTTCTCCTGAGTTAGTGCAAGAATTGCAAATGCTGGATGAAGGCAAAGTT
GCTGCATTTGAAGGACGAGACATTGCGACATTTGATCTTGCCGTAATGCGAACTCTCCCAAGGCTTAAAGGAATTTCAGC
TAACCTACGCAAACAACTTATTAATAGCAATGATGAACAAACGATTGAAAGTATGGCGAGATATATGCCTGACAATGAGA
TTCTGGAGTTGACCGATCAGCAATTGGGTTACCAGCCTGTTGTTCTGGGGTTGCTCGACCGTGAACCGCTCTCTGTCGAA
ATAATGACGCGAATGAGTCGTCTACCTGATGGTGTCGGTCCGTTGAATCTTGCGCTACGTGAAAATCTCCCTCTGGATAT
CGTCATGACTCTGGCGAAAAGAGATTGGGATATGATCATTCAGGAGCTTTATAAAGATGCGTGGTTATTGCCTGAATCCA
TTATTGATGGCTATATCCGCAGTGATGATTCCTCTATTCGTCAGGTCGGTGCTGGAGGACAACTCACCTACAATCAGGCA
ATGCAGTTGGCGAACGATTCATCAAACAATGTTGTCACAAGCTTAGCGTTCAAGCTGGCAGAGATGAAACACCATGGTCA
ATTGTTGCGGATGACGCCACAAGAGAGTGACAAAGTTGCAGGCTATTTATACCAAAAATTCGAGAATGACGATGATCTAA
TACGTGTGTTATTTTTAGCATTGCCAGATAACTTACAGTTTAATTTTGTTAAAAGGATGGAGAAAAAATCCCCGGCCTAC
TTTTGCTGTCGGGATATGCAGGTAATTCACTCTGACGCTGCTTTACAACGGTTATTGACACGTTTCAACGATCCTGAAGG
GTGGAGTAATCTGGCGAAAAATCAGTATCTGAGCACGTCGATGAAACAAAAAATTTGGCAACGTGCTTTATCGCATCGGA
AAAATAATCCGAAAGCAGATTCAGATGCATATGAAACCAGCGCTGATATGATTTTGTCTGAGCTGATTAGCCACGGCGAA
GTTGATGACCAGATGCTATTAAATGCCACCGCCCTGATACGTTCGGACGACTGGGACTTTTTAGAGAGTGCATTAATTAG
TTGGGATAATTTACCCGCTGTTGTTCTCAAGGAATTACAGCAAAACACGCCACGCAATGATATTTGGGCGAAGTTTTTTC
TGAGGCAGGAAAACAGTTCCCGTGCGCAGGTTGACGAAGCGTTACGTGTTTATTATGCACTAGACCCCGATGCGTTAGCA
CAACTGGATGTACTGGCAAAACAACCGGATCGTATATGGTGGAGTACACTGGCGAAAAGCAATCTCACATTTTTCAAGTT
CGGCGCACTTAACAACCGCCACACACCGCCTGCAGTACTGGCGGCAGAAATTGATCCCGAGTGGTGGATTGTGGCGATGA
ATAATCCCCGTTTTCCAGTTGATGTATTAAAGGCGAGGCTGAAACGTGATCCTTTGCTGGCGTTAGAGCTTGTTAACCCT
GAACTGGATTTAGTCCGCCAGCTGGCGCTTAACGGTAAGACGCGCGCAATACGGGAACAAGCGATGAGAAAACTTGATGA
GTTGTATTGATCCACAACGTAATGTTTTTTAACTATCTGATTAATTGGGGATAATCATTCCTGACAGTGAGTCCCCAATA
CCTTGATATATTCTGAATTTTTAATGAAACGGCGTGTTGCGATATCTCCGTCAGGGGAATTGATGCACCATAGCGCAAAC
CGAATTATCAAGGATTGATAATGACGCTCTACCAGATAAAACCGCTCTTTCAGTCGCTGTTAAGGCCGACGATGTTTTGG
CTTTATAAGCACCACGTTACAGCGAATCACATCACTCTTGCTGCACTGGCGCTTTCTCTTCTTACCGGATTGCTGTTGAT
GTTGGCGGCACAACCCATCCTCTTTTTGCTATTGCCCATCGTGCTTTTTATCCGTATGGCGCTCAATGCGCTGGATGGCA
TGTTGGCGCGTGAGTGCAACCAGCAAACACGTCTGGGGGCGATTTTGAATGAGACTGGCGATGTTATTTCCGATATTGCG
CTCTATTTACCATTTTTATTTTTACCGGAAAGTAACGCATCACTCGTGATACTCATGTTATTTTGCACCATATTGACCGA
GTTTTGCGGTTTACTCGCGCAGACGATTAACGGTGTTCGCAGTTATGCCGGTCCATTTGGCAAAAGCGATCGCGCGTTAA
TATTTGGTCTGTGGGGTCTGGCCGTTGCCATTTATCCACAGTGGATGCAGTGGAATAATCTTTTATGGAGCATTGCTTCA
ATTCTGCTTCTCTGGACTGCGATTAATCGTTGTCGGAGTGTGCTTCTTATGAGCGCTGAAATATAATGCTGGAAAAATCT
CTGGCAACACTTTTCGCGTTGTTAATTTTAGCAACGCTGATAAACCGTTTTCTGCTATGGCGGTTACCGGAGAGAAAAGG

GGGTGAGGTTACATTACGTATTCGTACCTGGTGGGGCATCGTCATTTGTTTTTCAATGGTGATTTCAGGTCCACGCTGGA
TGACGTTGACGTTTTTTGCGCTGATAAGTTTTCTGGCATTGAAAGAATATTGTACGCTTATATCTGTACATTTTCCGCGT
TGGTTATATTGGGGTATTCCTCTTAACTATTTGCTTATCGGTTTTAATTGCTTTGAGCTGTTTCTGTTATTTATACCTTT
GGCTGGGTTTCTGATATTAGCCACCGGGCAAGTTTTAGTGGGTGACCCCTCTGGTTTTCTGCATACCGTGAGCGCCATTT
TTTGGGGCTGGATAATGACCGTTTTCGCCTTGAGTCATGCCGCCTGGTTATTAATGTTGCCAACCACAAATATCCAGGGC
GGGGCGTTACTGGTGTTATTTCTTTTGGCATTAACCGAGTCAAACGATATTGCACAGTATTTATGGGGAAAATCCTGCGG
CAGAAGAAAAGTGGTCCCTAAAGTCAGCCCGGGAAAAACATTGGAAGGTCTGATGGGCGGCGTCATCACCATCATGATTG
CGTCACTGATTATCGGACCGTTACTGACACCGCTAAATACATTACAGGCATTATTAGCGGGTTTGTTAATTGGTATTAGT
GGTTTTTGCGGCGATGTCGTGATGTCAGCCATCAAACGAGATATTGGTGTTAAAGATAGCGGAAAACTATTGCCAGGACA
TGGCGGACTCCTTGACAGGATTGACTCATTAATTTTCACCGCTCCGGTATTTTTTTATTTTATACGCTACTGCTGTTACT
GAAGGAAAATAGAGAAAATGGAAAATTCACGCATCCCTGGGGAACATTTTTTTACCACCAGTGATAATACAGCGTTGTTT
TATCGGCACTGGCCCGCTTTACAGCCCGGGGCGAAAAAGGTCATCGTCTTATTTCATCGCGGGCATGAACATTCTGGTCG
TCTACAACATCTCGTTGATGAACTGGCGATGCCAGATACTGCTTTTTATGCCTGGGATGCCCGAGGGCATGGAAAAAGTT
CGGGGCCGCGTGGTTATAGCCCATCTCTTGCGCGTTCAGTGCGGGATGTCGATGAATTTGTCCGTTTTGCTGCCAGCGAC
AGCCAGGTTGGACTGGAAGAGGTGGTAGTGATCGCGCAAAGCGTCGGCGCAGTGCTGGTTGCCACATGGATTCATGATTA
TGCACCTGCAATTCGCGGGCTGGTGCTGGCTTCTCCGGCCTTTAAGGTTAAATTGTATGTGCCGCTGGCACGTCCTGCGC
TGGCGTTATGGCATCGTCTGCGTGGTCTGTTTTTTATTAATTCCTATGTGAAAGGACGCTATTTGACCCACGATCGGCAA
CGGGGGGCGAGTTTCAATAATGATCCGCTGATCACACGGGCGATTGCCGTTAATATCTTGCTCGATCTCTACAAAACGTC
TGAACGTATTATTAGAGATGCGGCGGCGATTACGCTCCCCACGCAACTTCTGATATCAGGCGATGACTATGTGGTGCATC
GCCAACCGCAGATTGATTTTTATCAGAGATTACGTAGCCCTCTGAAAGAGCTGCATCTGCTGCCAGGCTTTTATCACGAC
ACGTTGGGTGAAGAGAACAGGGCGCTGGCATTTGAAAAAATGCAAAGCTTTATTAGTCGTTTATATGCTAACAAATCGCA
AAAATTTGATTATCAGCATGAAGACTGCACAGGACCATCAGCGGATCGATGGCGGCTACTTTCTGGTGGACCCGTGCCAT
TATCGCCGGTTGATTTAGCGTATCGCTTTATGCGAAAGGCGATGAAATTGTTCGGGACGCACTCTTCGGGCCTGCATCTC
GGAATGAGCACCGGCTTTGATTCAGGCAGTTCGCTGGATTATGTCTATCAAAATCAACCGCAAGGTAGTAACGCATTCGG
GCGTTTAGTCGACAAAATCTACCTGAACAGTGTTGGCTGGCGCGGTATTCGCCAGCGCAAAACCCATTTACAAATACTGA
TTAAACAAGCCGTTGCCGATCTCCACGCCAAAGGTTTAGCCGTCCGCGTGGTTGACATTGCCGCAGGGCATGGGCGCTAT
GTACTGGATGCGCTGGCAAACGAGCCTGCCGTAAGCGATATTTTGTTACGTGATTACAGCGAGTTAAATGTTGCACAGGG
GCAAGAGATGATTGCTCAACGGGGAATGTCTGGGCGGGTGCGTTTTGAACAGGGCGATGCGTTTAACCCGGAGGAACTCA
GCGCGTTAACTCCGCGGCCTACGCTGGCGATTGTCTCTGGCCTGTATGAGCTTTTTCCCGAAAATGAGCAGGTAAAAAAC
TCACTCGCAGGTCTTGCCAATGCCATCGAACCGGGCGGCATTCTCATCTACACCGGGCAGCCGTGGCACCCTCAACTGGA
GATGATTGCCGGGGTGTTAACCAGTCATAAAGATGGTAAACCGTGGGTAATGCGCGTGCGTTCGCAAGGGGAGATGGATT
CACTCGTGCGTGATGCCGGATTTGATAAATGCACACAACGGATTGATGAGTGGGGTATTTTTACGGTTTCGATGGCGGTG
CGTCGTGATAACTGAACGTCGGAACGTATTGCTACAAGGCGCTGGCTGGTTATTGTTGCTGGCCCCGTTTTTCTTCTTCA
CCTATGGATCTCTTAATCAGTTCACCGCGGTTCAGGACCTTAACAGCCATGATATCCCCAGTCAGGTATTCGGTTGGGAA
ACGGCGATCCCTTTTCTTCCCTGGACTATTGTTCCTTACTGGAGTCTGGATCTTTTATATGGATTTTCGCTGTTCGTTTG
TAGCACGACATTCGAACAGCGCCGACTTGTCCACCGGCTTATTCTGGCAACGGTAATGGCCTGCTGCGGTTTTTTGCTCT
ATCCGCTGAAGTTTAGTTTTATCCGTCCTGAAGTGAGTGGGGTGACGGGATGGCTATTTTCGCAACTTGAACTGTTTGAT
CTGCCTTATAACCAGTCTCCTTCGCTGCATATTATTCTCTGCTGGCTACTTTGGCGTCACTTTCGTCAGCATCTGGCTGA
GAGGTGGCGTAAAGTCTGCGGCGGATGGTTTTTACTCATCGCCATTTCGACGCTGACGACCTGGCAGCATCATTTTATTG
ATGTCATCACAGGGCTGGCGGTAGGTATGTTGATTGACTGGATGGTGCCCGTCGACCGTCGTTGGAATTATCAGAAACCT
GATCAACGTCGAATCAAAATAGCACTGCCCTATGTCGTAGGCGCGGGCTCGTGCATTGTATTGATGGAGCTAATGATGAT
GATTCAGTTATGGTGGTCAGTCTGGTTATGTTGGCCAGTATTATCGCTACTCATAATTGGCCGTGGGTACGGTGGGCTTG
GCGCGATAACAACAGGGAAAGATAGTCAGGGGAAACTACCGCCCGCCGTTTACTGGCTGACATTGCCCTGCCGCATCGGG
ATGTGGCTGTCTATGCGTTGGTTTTGTCGTCGCCTGGAGCCGGTGAGCAAAATGACTGCTGGTGTTTATTTAGGGGCGTT
TCCACGACATATTCCGGCACAGAATGCGGTTCTGGACGTCACCTTTGAATTCCCTCGCGGACGAGCCACAAAAGATCGAC
TCTATTTTTGTGTACCGATGCTGGATCTGGTGGTTCCGGAAGAGGGGGAGCTCCGACAGGCCGTGGCGATGCTGGAAACA
TTACGCGAAGAGCAAGGCAGCGTTCTGGTCCATTGTGCATTGGGATTATCGCGCAGTGCGCTGGTGGTGGCGGCATGGTT
GTTATGTTACGGACACTGTAAAACCGTTAATGAAGCGATTAGCTATATTCGAGCCAGACGCCCGCAGATTGTGCTGACAG
ACGAGCACAAAGCGATGCTGAGATTATGGGAAAACAGGTAAGTGGATTGAGATGTGGACTGAATATCTACAGTCCACATC
AAGACCGTGTCCGGTTATGCAGAAACAATGCTGTCGATGGCTGCTTTTGCGTCAGACTGTGCTTTCGCTGCCATTTCCGG
ACCGTATGCGATCCCTTCGGCGAAGACAAATTTCACATCGGTAATGCCGATAAAGCCGAGGAACGTGGACAGATACGGCG
TCACCAGGTCCGTTGGTCCATCTTTGTGGATCCCGCCGCGGCTGGTAATAACGATGGCTTTTTTACCCGTTACCAGACCT
TCCGGACCGTTCTCGGTATAGCGGAAAGTAACGCCTGCGCGGGCAACCAGGTCAAAATAGTTTTTCAACTGAGTCGAGAT
GTTGAAGTTATACATCGGTGCCGCAATAACGATAACGTCGTGGGCTTTCAGCTCGGCAATCAACTCATCGGAAAGTGCCA
GAGCTTCCTGCTGACGCGGAGTCAGCGGCGCATCGCTCGGACGCAGAGCGCCAACCAGTTCGCCATCCAGTACCGGAATC
GGATTTGCAGCCAGGTCGCGAACGGTGATTTCATCAGCGGAGTGCTTTTCGCGCCATTGTTCAACAAAATAATCGGACAA

CTGATTAGACTGAGAGTACCCTGCCAGGATGCTGGATTTAAGAACTAATACCTTGCTCATGGTGTTTCCTTATAGATGTT
TGAATGGGCGATGCCCCGTTGCTTGTTGACACTTTATTCACAATCCTGCCACAGAGATAGCGCAATAAATCGAAGCCTAT
GTTCGAATTTATTGAACAACGCATAGAAAGCCGCGATGTGGTACTCTATATCTATCATTTAAAAGAAAATTAATCAGGCA
GACTACTGCCCACTAACGTTATGACAGAACAACAAAAATTGACCTTTACGGCCTTGCAGCAGCGGCTGGATTCGCTGATG
CTGCGTGACAGACTGCGTTTTTCTCGCCGTCTGCACGGCGTGAAGAAGGTTAAAAATCCTGATGCACAACAGGCCATTTT
CCAGGAGATGGCGAAAGAGATTGACCAGGCGGCAGGGAAAGTCCTGCTGCGTGAAGCGGCACGACCGGAAATTACTTATC
CTGACAATTTACCGGTTAGTCAGAAAAAACAGGACATTCTCGAAGCGATTCGTGATCACCAGGTGGTGATCGTCGCCGGG
GAAACGGGTTCTGGTAAAACGACTCAGTTACCGAAAATCTGTATGGAGCTGGGGCGCGGGATTAAAGGACTGATCGGCCA
TACCCAGCCGCGTCGTCTGGCGGCAAGAACAGTGGCGAACCGTATTGCGGAAGAGCTGAAAACGGAGCCGGGCGGTTGCA
TCGGTTACAAAGTGCGTTTCAGCGATCACGTAAGTGATAACACGATGGTCAAGCTGATGACCGACGGTATCCTGCTGGCG
GAGATCCAGCAAGACCGCCTGCTGATGCAGTACGACACTATCATTATTGACGAAGCGCACGAACGCAGCCTGAATATCGA
TTTTTTGCTCGGCTATTTGAAAGAGTTGCTGCCGCGGCGTCCTGACCTAAAAATCATTATCACTTCCGCGACTATCGACC
CGGAACGCTTTTCGCGCCACTTTAATAATGCGCCGATTATTGAAGTCTCCGGTCGGACCTATCCGGTGGAAGTGCGCTAT
CGCCCGATTGTTGAAGAAGCCGATGACACCGAGCGCGATCAGTTGCAGGCGATTTTTGACGCCGTAGACGAACTGAGTCA
GGAAAGCCATGGCGACATTCTGATCTTTATGAGCGGCGAGCGGGAAATCCGCGATACCGCCGATGCGCTGAACAAGCTGA
ACTTACGCCATACCGAAATCTTGCCGCTTTATGCGCGGCTTTCGAACAGCGAACAAAATAGGGTATTCCAGTCGCACAGC
GGACGGCGCATTGTGCTGGCGACCAACGTCGCGGAAACGTCGCTGACCGTACCGGGGATTAAATACGTTATCGACCCCGG
TACAGCGCGTATCAGCCGCTACAGCTATCGCACCAAAGTGCAGCGTTTGCCGATTGAGCCGATTTCCCAGGCGTCTGCCA
ATCAGCGTAAAGGCCGCTGTGGTCGTGTGTCCGAAGGGATCTGTATTCGTCTCTATTCCGAAGACGATTTCCTCTCGCGC
CCGGAGTTTACCGATCCGGAGATTCTGCGTACCAACCTGGCCTCGGTTATTTTGCAGATGACCGCGCTGGGGCTGGGCGA
TATCGCTGCGTTCCCGTTTGTCGAAGCACCGGATAAACGCAATATCCAGGATGGCGTGCGTCTGCTCGAAGAGCTGGGCG
CGATCACCACTGATGAACAGGCCAGCGCCTATAAACTGACGCCGCTCGGTCGCCAGCTCTCGCAGTTGCCTGTCGACCCA
CGTCTGGCGCGTATGGTGCTGGAAGCGCAAAAACATGGCTGCGTGCGTGAGGCGATGATTATCACGTCCGCGCTCTCCAT
TCAGGATCCGCGCGAACGTCCGATGGACAAACAGCAGGCATCGGACGAAAAACATCGTCGCTTCCACGACAAAGAGTCTG
ACTTTCTCGCGTTTGTGAATCTGTGGAATTATCTTGGCGAGCAGCAAAAGGCGCTTTCTTCCAACGCCTTCCGTCGCCTG
TGTCGTACCGATTATCTCAACTATCTGCGCGTGCGCGAATGGCAGGATATCTACACCCAGTTGCGTCAGGTGGTGAAAGA
ACTTGGCATTCCGGTTAACAGCGAACCGGCGGAGTATCGCGAAATTCACATTGCGTTGCTGACCGGTTTACTTTCCCATA
TCGGCATGAAAGATGCCGATAAACAAGAATATACCGGCGCACGTAACGCGCGTTTCTCCATCTTCCCCGGTTCTGGTTTA
TTCAAAAAACCGCCTAAATGGGTAATGGTGGCGGAACTGGTAGAAACCAGCCGCCTGTGGGGGCGCATTGCTGCGCGTAT
CGACCCGGAATGGGTGGAGCCAGTTGCTCAGCATTTGATTAAACGCACCTACAGCGAACCGCACTGGGAACGGGCGCAGG
GCGCGGTGATGGCAACGGAAAAAGTCACTGTTTATGGTTTGCCGATTGTTGCCGCGCGCAAGGTCAACTACAGCCAGATC
GATCCGGCGTTATGTCGTGAACTCTTTATTCGCCACGCGCTGGTGGAAGGTGACTGGCAGACGCGTCACGCATTCTTCCG
TGAAAACCTGAAACTACGGGCGGAAGTAGAAGAGCTGGAACACAAATCACGTCGCCGCGATATTCTGGTTGATGACGAAA
CGTTGTTTGAGTTCTACGACCAGCGCATCAGCCACGATGTAATCTCCGCTCGCCACTTCGACAGCTGGTGGAAAAAAGTC
AGCCGCGAAACGCCTGATTTGCTCAACTTTGAAAAAAGCATGTTGATCAAAGAGGGCGCAGAAAAAATCAGCAAGCTGGA
TTACCCGAACTTCTGGCATCAGGGCAATCTCAAGCTGCGTTTGAGCTATCAGTTTGAGCCCGGCGCGGATGCTGACGGTG
TGACCGTACATATTCCGCTGCCGTTACTTAACCAGGTTGAGGAAAGCGGGTTTGAATGGCAGATCCCCGGTCTGCGCCGC
GAACTGGTGATTGCTCTGATTAAATCGTTGCCGAAACCGGTACGCCGTAATTTTGTACCCGCGCCAAACTATGCCGAAGC
GTTTTTAGGCCGCGTCAAACCGCTGGAGTTACCGTTGCTCGACAGCCTTGAGCGCGAGTTACGGCGGATGACCGGCGTTA
CCGTTGACCGCGAAGACTGGCACTGGGATCAGGTGCCCGATCACCTGAAAATTACCTTCCGCGTGGTGGATGACAAAAAC
AAGAAGCTAAAAGAAGGGCGCTCGCTACAAGATCTGAAAGATGCGCTGAAAGGCAAAGTGCAGGAAACGCTATCTGCGGT
GGCGGATGACGGTATCGAGCAGAGCGGCTTACATATCTGGAGTTTTGGTCAGCTGCCGGAAAGCTACGAACAGAAGCGTG
GCAACTACAAAGTGAAGGCGTGGCCGGCGCTGGTGGATGAGCGCGACAGTGTGGCGATCAAACTGTTTGATAACCCGCTG
GAGCAAAAGCAGGCAATGTGGAACGGTCTTCGCCGTCTACTGCTGCTGAATATTCCATCGCCAATCAAATATTTACATGA
AAAGTTACCGAACAAAGCCAAGCTGGGACTGTACTTTAACCCGTATGGCAAAGTGCTGGAGCTGATCGACGACTGTATCT
CCTGCGGTGTGGATAAATTGATCGACGCCAATGGTGGCCCGGTCTGGACGGAAGAAGGCTTTGCTGCGCTGCATGAAAAA
GTGCGTGCCGAACTGAACGACACGGTGGTGGATATTGCGAAGCAGGTCGAGCAAATCCTTACGGCAGTGTTCAATATCAA
CAAACGTCTGAAAGGGCGGGTGGATATGACCATGGCGCTGGGGCTTTCTGACATTAAAGCGCAGATGGGCGGTTGGTAT
ATCGCGGTTTTGTCACTGGTAACGGCTTCAAACGGCTGGGCGACACGCTGCGATATTTGCAGGCGATTGAAAAACGGCTG
GAAAAACTGGCGGTTGATCCACATCGCGACCGTGCGCAGATGCTGAAAGTCGAAAACGTCCAGCAGGCGTGGCAGCAATG
GATCAACAAACTGCCGCCCGCACGTCGTGAGGATGAAGACGTGAAAGAGATCCGTTGGATGATAGAAGAGTTGCGCGTTA
GTTACTTCGCTCAACAACTTGGTACGCCTTATCCGATTTCAGATAAGCGTATTTTGCAGGCGATGGAGCAGATTAGCGGT
TAACCCTGCTATTTGCCTGATAAAGAAAAAACCCGGTAAGCATTTAGCGCCGGGTTTTTTATTAATTCTAAAACGGTAAG
GGTAAAAATTCAGGAATTCAGAAAAATACAATTCTCTGCTGCAAGATGAATAATGTTTATCTACAGCATTTCCTTAAAAG
ATATGTCAGGCTTGCGGAGTGGCGGTTAAGGACATACGATTTCCTCCTTTCAGAGTGCTCCGCTTCTCACTATTATCTCA
CGCAGTATTCTTAAGGGAACGATAAGGAGGAACCATGAACATTACCCCGTTTCCGACGCTTTCGCCGGCAACTATAGATG

CCATAAATGTTATCGGACAGTGGCTGGCGCAGGATGATTTCTCCGGTGAGGTGCCGTATCAGGCCGATTGCGTGATCCTT
GCAGGCAATGCGGTTATGCCGACTATCGATGCGGCATGTAAGATTGCCCGCGATCAGCAAATTCCTTTACTGATTAGTGG
TGGTATCGGTCACTCGACAACTTTTTTGTATAGCGCCATCGCACAGCATCCGCACTACAACACTATCCGCACCACTGGCA
GAGCAGAAGCGACCATCCTGGCGGATATCGCTCATCAGTTCTGGCACATTCCGCATGAAAAAATCTGGATTGAAGACCAG
TCAACAAACTGCGGTGAAAACGCACGCTTTAGCATCGCGCTATTGAATCAGGCCGTAGAACGAGTTCATACGGCTATCGT
TGTTCAGGACCCCACCATGCAGCGGCGCACGATGGCGACGTTCCGCCGTATGACTGGGGACAATCCCGATGCACCACGCT
GGTTAAGTTATCCCGGATTCGTTCCTCAGTTAGGAAATAACGCAGACAGTGTAATCTTTATTAATCAGTTACAAGGATTA
TGGCCAGTTGAGCGTTATCTCTCACTACTCACTGGCGAGCTGCCGCGTTTACGCGATGATAGCGATGGCTACGGTCCCCG
CGGGCGAGATTTTATCGTTCACGTTGATTTTCCGGCAGAAGTCATCCATGCATGGCAAACGCTGAAACATGATGCGGTGC
TCATCGAGGCGATGGAAAGTCGCTCGTTACGTTAAAAATTGCCCGTTTGTGAACCACTTGTTTGCAAACGGGCATGACTC
CTGACTTTTATTTCTGCCTTTTATTCCTTTTACACTTGTTTTTATGAAGCCCTTCACAGAATTGTCCTTTCACGATTCCG
TCTCTCTGATGATTGATGTTAATTAACAATGTATTCACCGAAAACAAACATATAAATCACAGGAGTCGCCCATGTCAGTA
CCCGTTCAACATCCTATGTATATCGATGGACAGTTTGTTACCTGGCGTGGAGACGCATGGATTGATGTGGTAAACCCTGC
TACAGAGGCTGTCATTTCCCGCATACCCGATGGTCAGGCCGAGGATGCCCGTAAGGCAATCGATGCAGCAGAACGTGCAC
AACCAGAATGGGAAGCGTTGCCTGCTATTGAACGCGCCAGTTGGTTGCGCAAAATCTCCGCCGGGATCCGCGAACGCGCC
AGTGAAATCAGTGCGCTGATTGTTGAAGAAGGGGGCAAGATCCAGCAGCTGGCTGAAGTCGAAGTGGCTTTTACTGCCGA
CTATATCGATTACATGGCGGAGTGGGCACGGCGTTACGAGGGCGAGATTATTCAAAGCGATCGTCCAGGAGAAAATATTC
TTTTGTTTAAACGTGCGCTTGGTGTGACTACCGGCATTCTGCCGTGGAACTTCCCGTTCTTCCTCATTGCCCGCAAAATG
GCTCCCGCTCTTTTGACCGGTAATACCATCGTCATTAAACCTAGTGAATTTACGCCAAACAATGCGATTGCATTCGCCAA
AATCGTCGATGAAATAGGCCTTCCGCGCGGCGTGTTTAACCTTGTACTGGGGCGTGGTGAAACCGTTGGGCAAGAACTGG
CGGGTAACCCAAAGGTCGCAATGGTCAGTATGACAGGCAGCGTCTCTGCAGGTGAGAAGATCATGGCGACTGCGGCGAAA
AACATCACCAAAGTGTGTCTGGAATTGGGGGGTAAAGCACCAGCTATCGTAATGGACGATGCCGATCTTGAACTGGCAGT
CAAAGCCATCGTTGATTCACGCGTCATTAATAGTGGGCAAGTGTGTAACTGTGCAGAACGTGTTTATGTACAGAAAGGCA
TTTATGATCAGTTCGTCAATCGGCTGGGTGAAGCGATGCAGGCGGTTCAATTTGGTAACCCCGCTGAACGCAACGACATT
GCGATGGGGCCGTTGATTAACGCCGCGGCGCTGGAAAGGGTCGAGCAAAAAGTGGCGCGCGCAGTAGAAGAAGGGGCGAG
AGTGGCGTTCGGTGGCAAAGCGGTAGAGGGGAAAGGATATTATTATCCGCCGACATTGCTGCTGGATGTTCGCCAGGAAA
TGTCGATTATGCATGAGGAAACCTTTGGCCCGGTGCTGCCAGTTGTCGCATTTGACACGCTGGAAGATGCTATCTCAATG
GCTAATGACAGTGATTACGGCCTGACCTCATCAATCTATACCCAAAATCTGAACGTCGCGATGAAAGCCATTAAAGGGCT
GAAGTTTGGTGAAACTTACATCAACCGTGAAAACTTCGAAGCTATGCAAGGCTTCCACGCCGGATGGCGTAAATCCGGTA
TTGGCGGCGCAGATGGTAAACATGGCTTGCATGAATATCTGCAGACCCAGGTGGTTTATTTACAGTCTTAATGAGTGAAA
GAGGCGGAGGTTTTTTCCTCCGCCTGTGCGCGTCAGAGTTTAGCGAATTTTTCGAGGGTGCGAATAAGCTGTGTGACGAA
GCCATATTCGTTATCGTACCAGGCGACCGTTTTCACCAGTTGTAAATCGCCCACGGCGGTAATTTCCGTTTGCGTGGCAT
CAAACACCGAACCGAAATGGCTGCCAATGATATCGGAAGAGACTATTTCTTCATCGGTATAACCAAATGACTCGTTATTG
GTGGTTGCTTGTTTAAGTGCGTTATTCACCTCTTCGGCAGTCACTTTTTTCCGAGAATCGATACCAGTTCAGTGACCGAA
CCTGTTTTCACCGGCACGCGTTGCGCATGACCTTTCAGTTTGCCGCTCAGTTCCGGGATCACCAGACCAATGGCTTTTGC
CGCCCCCGTAGTGTGGGGAATGATATTTTCTGCCGCTGCGCGTGAAGCACGTAAATCTTTACCACGCGGGCCATCCACCA
GTGACTGGGTGCCAGTATAGGCATGAATGGTCGTCATCGTGCCGACTTCTATCCCGAAACTGTCATGCAAGGCTTTGGCC
ATCGGCGCAAGACAGTTAGTGGTGCATGACGCCACGGAAACAATGGTGTCGTTGCCATCCAGAGTGTCGTCATTGACGTT
ATAAACGATAGTTTTCATTTCACCGGCAGGGGCGGAAATCAACACCTTCTTCGCACCAGCATCAAGATGCGCCTGCGATT
TCTCGGCGGAGGTATAAAAGCCAGTACATTCGACAATGATTTCTGCACCTTTCGCTTTCCACGGAATATTTTTAGCCTCT
TTTTCGGCGTAAACCGCGATACTTTTCCCATCAACGATAAGTGAATCTTCCGTAAAATCAACGCTCCAGGGGAATGGTCC
GTAGTTTGAATCATGTTTCAGCAGGTAGGCGAGAATTTATGGGGAAGTGAGATCATTAATAGCGACAACGTCTATGTTGC
TTTTGACTTCAAGTAATCGACCCAACACCAGTCGACCGATACGACCAAAACCGTTAATACCAACTTTACTCATGGTTTTC
TCCTGTCAGGAACGTTCGGATGAAAATTGATCCTTTCCAAGCTTAGACCAGGATGGCGGGATGGGCAATCTCCATTCTCA
CAGTGAAACGTAACGTACTGAAAACGGGTGAACAATATTTAATGAAATTTTGAGAAAAGCCCGTTATGTTAACGGAAAAT
TATGTTAAAGCAGGAAATGTTATGGAAAATAAATATTCAAGGTTACAAATCAGCATTCACTGGCTGGTCTTTTTACTGGT
TATCGCAGCGTATTGCGCAATGGAGTTTCGTGGTTTCTTCCCACGTAGCGATCGGCCACTCATCAACATGATTCATGTTT
CCTGTGGCATCTCAATTCTCGTGCTGATGGTCGTTCGTCTTTTGTTAAGGCTGAAATACCCAACCCCGCCGATTATACCT
AAGCCAAAACCGATGATGACGGGACTGGCGCATTTGGGACATTTGGTGATTTATCTCCTGTTTATTGCGCTGCCAGTGAT
TGGTTTGGTGATGATGTATAACCGGGGCAACCCGTGGTTTGCGTTTGGTTTGACGATGCCTTACGCTTCAGAGGCCAATT
TCGAACGGGTAGATAGCTTAAAGTCGTGGCATGAAACGCTGGCGAATCTGGGATATTTTGTCATCGGGTTGCACGCTGCG
GCTGCACTGGCACACCACTATTTCTGGAAGGACAACACACTTCTACGCATGATGCCGCGTAAACGTTCCTGAAGGATATT
TAAAGAAAACGCCTGTACTAAAACCGACCCGTGGTACAGGCGAAGAATACGGGTCTACATCGGAAGCGCCTATTATATTT
ATTTGTATGATAAATAAAACCATAATCCTTGCCCATACGTCCATCTGGCTTATTTTTAATCAATTCACCCGATCTTTGAT
CTCATCAACGGTATCAAAATAAACCAGCGTATAACGTTCAAATCGAACATAAGATTCGATAAACCATGGAGGTTATATGA
AAAAACTGGCACTTATTTTGTTTATGGGAACGCTTGTTTCCTTTTATGCCGATGCCGGGCGCAAACCCTGTTCTGGTTCG

AAAGGGGGGGATCTCACACTGTACGGCAGGCGGCAAATTTGTCTGTAATGATGGTTCTATTAGTGCATCGAAAAAAACATG
CACTAACTGAAGTGTAAAAGGGGTGCCATGAGAAAATGAATCTGCTAGTCAAATGCGCGGGGAAAATCCCCGCGCTTGCC
CTTACCTGGACGTGCAGGCCATGAGCGCAGCAACCTCCTTATCACCGTCCCGGAACCGCAGTTCGTAGAGCGTTTGTCGG
GTCAGGAGTGTGAATGTCAGAATCGTAATGCAGATAATGAGCAGACACACCACCAGAGGGTTGTGCTTCATAGCCTTTCT
CCTTGCCGGATGGCGGGTAAGAGGCTAAGATCTGAATTGCTAGGTTCATTCGTTGGCCTCGGTTGATAGAAATATCGGTC
GGGGCCTTCGTCTTTCTGATTCCCGGTTAGCCTGAAAACAGAAAGTCTCAGGCACCCGCAGGCATCCTATGAGGTTTCCT
TAGGGACGAAAATAATCACTTCACGAAATTGCGTGCTGTTTTCCAGAATTTTTCGTCATTCGGGTTAGCCAGTTTAGCCA
TTCGTTACTCTCTTCATTCCAATAGCATTAATTTTCTATGCAATAATTGTTGTAAAAATGTGACGCAAAGAGGTTTTTGG
TCATAAGTAATTACCGTCAAGTGCCGATGACTTTCTATCAGGAGTAAACCTGGACGAGAGACAACGGTAATGAATACAAC
TCCCTCACAGCGATTAGGTTTTTTGCATCACATCAGGTTGGTTCCGTTATTTGCCTGCATTCTAGGCGGTATCTTAGTTC
TATTCGCATTAAGTTCAGCCCTGGCTGGCTATTTCCTCTGGCAGGCCGATCGCGATCAGCGTGATGTTACTGCGGAGATT
GAGATTCGGACCGGGTTAGCGAACAGTTCAGATTTTTTGCGTTCAGCCCGGATCAATATGATTCAGGCCGGGGCTGCGAG
TCGTATTGCGGAAATGGAAGCAATGAAGCGAAATATTGCGCAAGCCGAATCGGAGATTAAACAGTCGCAGCAAGGTTATC
GTGCTTATCAGAATCGACCGGTGAAAACACCTGCTGATGAAGCCCTCGACACTGAATTAAATCAACGCTTTCAGGCTTAT
ATCACGGGTATGCAACCTATGTTGAAATATGCCAAAAATGGCATGTTTGAAGCGATTATCAATCATGAAAGTGAGCAGAT
CCGACCGCTGGATAATGCTTATACCGATATTTTGAACAAAGCCGTTAAGATACGTAGCACCAGAGCCAACCAACTGGCGG
AACTGGCCCATCAGCGCACCCGCCTGGGTGGGATGTTCATGATTGGCGCGTTTGTGCTTGCCCTGGTCATGACGCTGATA
ACATTTATGGTGCTACGTCGGATCGTCATTCGTCCACTGCAACATGCCGCACAACGGATTGAAAAAATCGCCAGTGGCGA
TCTGACGATGAATGATGAACCGGCGGGTCGTAATGAAATCGGTCGCTTAAGTCGTCATTTACAGCAAATGCAGCATTCAC
TGGGGATGACAGTAGGGACTGTTCGACAGGGCGCGGAAGAGATTTATCGTGGCACCAGCGAAATTTCAGCTGGCAATGCG
GACCTGTCATCTCGCACCGAAGAACAAGCGGCGGCTATCGAACAAACTGCCGCCAGCATGGAGCAACTCACTGCGACGGT
GAAACAGAATGCGGATAACGCGCATCATGCCAGCAAACTGGCGCAAGAGGCTTCTATTAAAGCCAGCGATGGCGGGCAGA
CGGTTTCCGGTGTAGTAAAAACGATGGGCGCTATCTCCACGAGTTCGAAGAAAATTTCTGAGATCACCGCCGTCATCAAC
AGTATTGCTTTCCAGACGAATATTCTGGCACTGAATGCTGCCGTTGAAGCCGCGCGAGCGGGTGAGCAAGGGCGTGGATT
TGCCGTTGTCGCCAGCGAAGTACGGACACTCGCAAGTCGCAGCGCTCAGGCGGCGAAAGAGATTGAAGGCTTGATCAGTG
AATCAGTCAGGTTAATTGACCTGGGGTCGGATGAGGTGGCAACGGCCGGGAAAACCATGAGCACTATTGTTGATGCCGTC
GCGAGTGTCACACATATCATGCAGGAAATCGCCGCCGCCTCGGATGAACAAAGTAGAGGCATAACGCAGGTTAGCCAGGC
GATTTCTGAAATGGATAAGGTGACGCAACAGAATGCTTCTCTGGTAGAAGAGGCCTCAGCGGCGGCGGTGTCCCTTGAAG
AACAGGCGGCACGATTAACTGAGGCGGTGGATGTATTCCGTCTGCACAAACATTCTGTGTCGGCAGAACCTCGCGGAGCG
GGTGAACCAGTTAGTTTCGCTACGGTGTGAAAATGTTCAAGGAGGGATCGACAGATCCCTTCACCTTTCAGAACGGCATT
GATTTTCGAATAGCGTTAATCATCAACTGGCAACCAGAAGAGAACGTCGCATCTACGCGGGTCAGTATTCCAATCGGTTC
GCCTGCACCATGTCCCGGAACAGGCAGGGCCACCAGCGTGGCATGACGCAGGTCGTCTTTTACAGCGCCAGAAGGGACAA
ACCACACGTAATCGTATTCAACCGTAAGTTGACGAGATAGCGAAGCAGACAGCGTTTCGATACAACCCGAAGGAATTTTA
CAGCCCTGGCTCTGCACTAATGCATCTGAATGCTGGCGTGGCGCAGTGCCTTCTGGTGATACAACGACCGGCCATTCCAG
CACCCGGCTTAGCGTTACGTTCTCCTGAAGTAGCGGGTGATTAGGGCGGACAACCAGCTTCAACGATTCAAGAAACAGCA
GTTCGTAATTAAGCCCGGTCATCAGTTCAGGATCTGACATCCGACCAATGCCGATATCGATTTCCCCGGTTTTTAAACCC
GCCAGAATCATAGGGTTACTCATTGTCGCAACTTGCAAGGTCGTCTCTTTTTGTTGCTGATGAAACTGACCTATAACCGA
AGGTAATATCCCCAGTGCCGCAGTAGGTAGTGCACCAACCCTGACGACATCATTATTAAGACCTTCTTTACGATGAAGCG
ACCGTCCGGCAGTGTTGATGGCGTCAAGAACTCTGACTGCATGCGTTAAAAATTGTTCGCCGGGTAAGGTAAGTTGCGCC
CCCTGACGACCACGCTCAAACAAGCGAGCGCCAGTCAGCTGCTCCAGTTCATTCAATGTCTTAGAGAGCGCAGGTTGACT
CAAATTAAGGGTTTCAGCCGCGCGCCCCAAAGTTCCTTGTTGTGCGACAGCTACGAATGTATGAAGGTGGCGCAAACGGA
TGCGCTGACTAAACAGACTATTTTTTTCCATAAGCGATGTTAAAAACGAAGCGGTGTCGCTGACAAGTGAAGTTGTTTGA
TTATGATAACTTGATTGCAAAATATTATTAACAATTAAAGCAATTATGTTACAGCAAAATGGATAATATTGATGTTTTCG
CGGCGAGATCACAGTTTGTAAATTCTTCCCGCAAGAGTGAATGCGGTTACCTACACTCCAGATTACTGACCACTGGAGGC
AGACACTATGGCGAACAGCATCACGGCGGATGAGATTCGGGAACAGTTTTCGCAGGCAATGTCAGCCATGTACCAGCAAG
AAGTTCCGCAATATGGCACGCTGCTGGAACTGGTAGCTGATGTGAATCTGGCTGTGCTGGAAAACAATCCTCAACTGCAC
GAAAAAATGGTAAATGCAGACGAGCTGGCGCGACTGAATGTTGAACGTCATGGGGCGATTCGCGTTGGGACTGCACAAGA
GCTTGCTACTCTTCGGCGGATGTTTGCCATTATGGGGATGTACCCGGTGAGCTATTACGATCTCTCGCAGGCAGGGGTGC
CGGTACATTCGACAGCATTTCGGCCCATTGATGATGCTTCTCTGGCGCGTAATCCCTTCCGCGTTTTTACCTCCTTACTC
CGCCTTGAGCTTATCGAGAACGAAATTTTGCGCCAGAAAGCGGCGGAGATTCTGCGTCAGCGCGATATCTTCACCCCACG
TTGTCGACAACTGTTAGAGGAATATGAGCAGCAGGGCGGTTTTAACGAAACACAGGCACAGGAGTTTGTGCAGGAAGCCC
TGGAAACGTTTCGCTGGCACCAGTCAGCAACGGTAGATGAAGAAACCTATCGCGCATTGCACAACGAACATCGGTTGATT
GCTGATGTGGTCTGTTTTCCTGGATGCCATATCAACCACCTGACGCCACGTACGCTGGATATTGACCGGGTGCAGTCGAT
GATGCCTGAATGCGGAATTGAACCCAAAATTCTCATCGAGGGGCCGCCGCGCCGCGAGGTACCGATTTTACTACGCCAGA
CCAGCTTTAAAGCACTGGAAGAGACGGTGTTGTTTGCGGGGCAGAAACAGGGCACGCATACCGCGCGCTTTGGTGAAATT
GAGCAGCGTGGCGTGGCATTAACGCCGAAAGGGCGACAACTGTATGATGATCTTCTGCGTAACGCTGGAACCGGGCAGGA

TAATCTCACTCACCAAATGCATTTACAGGAAACCTTCCGCACTTTTCCTGACAGTGAGTTTTTAATGCGTCAGCAAGGGT
TGGCATGGTTCCGGTACCGTCTGACGCCTTCGGGTGAGGCGCATCGTCAGGCGATTCATCCTGGAGACGATCCACAGCCC
TTAATTGAACGTGGTTGGGTAGTGGCGCAACCCATCACCTATGAAGATTTCTTGCCCGTTAGCGCGGCGGGGATCTTCCA
GTCAAATCTGGGTAATGAAACGCAGACACGCAGTCACGGTAATGCCAGTCGCGAAGCATTTGAGCAGGCGTTGGGTTGTC
CGGTTTTGGATGAGTTCCAGCTTTACCAGGAAGCGGAAGAACGCAGTAAACGTCGCTGTGGTTTGCTTTAAAATCTGACC
ATCCGCCTTTGCAAAAATTTGCCTGATTTTACAAACGAATCAGGCTCATCCCATCGACATAAAAAAAATGCCGATTTATG
CATATTCTCTCAGTTCAACAATTGGATTATTAATAAATATTGTCTAGAGTGAGCGGTCATAAATAAGCACTTTCTTGCCG
CTGAAAACGACCAGCGCGGGACCATTCACAACACCAGAAGGACTCACTTTCAGGTATGGATCGTAGACGATTTATTAAAG
GTTCAATGGCTATGGCCGCCGTGTGCGGTACCAGCGGCATTGCTTCTCTTTTTTCTCAGGCGGCATTCGCGGCAGATTCT
GATATTGCCGACGGGCAAACCCAGCGTTTTGACTTCTCCATTCTACAGTCAATGGCGCACGACTTAGCGCAAACAGCGTG
GCGTGGTGCGCCTCGTCCGTTACCTGACACGCTGGCGACAATGACGCCGCAGGCTTATAACAGTATTCAATACGACGCCG
AAAAATCGCTCTGGCATAACGTTGAGAACCGTCAACTGGACGCTCAGTTCTTCCATATGGGAATGGGATTCCGTCGCCGC
GTTCGTATGTTTTCTGTAGATCCAGCAACACATCTGGCGCGTGAAATTCACTTTCGCCCGGAGTTGTTCAAATACAACGA
TGCAGGTGTTGATACAAAACAATTAGAAGGGCAAAGCGATCTCGGCTTTGCCGGTTTTCGCGTGTTTAAAGCCCCCGAAC
TGGCGCGCCGTGATGTAGTATCATTTCTCGGCGCGAGTTATTTCCGCGCCGTTGATGATACATATCAATACGGTTTGTCG
GCCCGCGGCCTGGCGATCGACACTTACACCGACAGTAAAGAAGAGTTCCCCGACTTTACCGCCTTCTGGTTTGATACGGT
AAAACCGGGGGCAACTACCTTTACCGTTTATGCGTTGCTCGATAGCGCCAGCATTACTGGTGCCTATAAGTTCACTATCC
ATTGTGAGAAAAGTCAGGTGATTATGGATGTGGAAAATCACCTGTATGCGCGCAAAGACATTAAACAGCTGGGCATTGCG
CCGATGACCAGTATGTTCAGCTGCGGTACTAATGAACGTCGGATGTGCGATACAATTCATCCGCAAATTCATGACTCTGA
TCGTCTGTCCATGTGGCGGGGCAACGGCGAGTGGATTTGCCGTCCGCTGAATAATCCGCAAAAATTGCAGTTCAATGCTT
ACACCGACAACAACCCGAAAGGGTTTGGTTTATTGCAACTGGATCGTGACTTCTCCCATTATCAGGACATTATGGGCTGG
TATAACAAACGCCCAAGTCTGTGGGTGGAACCGCGTAACAAGTGGGGTAAGGGCACCATCGGCCTGATGGAAATCCCAAC
AACGGGCGAAACGCTGGATAACATTGTCTGCTTCTGGCAGCCAGAAAAAGCTGTAAAAGCAGGTGATGAGTTTGCATTCC
AGTATCGTCTGTACTGGAGTGCGCAACCGCCTGTTCATTGCCCATTAGCGCGCGTTATGGCGACGCGTACCGGCATGGGC
GGTTTCTCGGAAGGTTGGGCGCCAGGTGAACACTATCCCGAAAAATGGGCGCGTCGTTTTGCCGTCGATTTCGTTGGTGG
TGATCTGAAAGCTGCCGCGCCAAAAGGCATTGAGCCGGTGATTACGCTTTCCAGTGGGGAAGCGAAGCAAATCGAAATTC
TCTATATTGAACCCATCGATGGTTATCGTATTCAGTTTGACTGGTATCCGACTTCGGACTCCACTGATCCGGTCGATATG
CGGATGTATCTACGTTGTCAGGGGGACGCTATCAGTGAAACATGGCTGTATCAGTATTTCCCGCCAGCGCCGGATAAACG
TCAGTATGTTGACGACCGCGTGATGAGTTAATCGTTTTTTCTTCGGCACCTTCTTCGGGAGGTGCCGTCTGGTTAAACAC
GATCCCGCTCGCATTTTTCCCTAAGTTAAATGAGTAATCTGATGGTGTGTATTTCAGATACACCTTGTCAGCCACTAACA
GGGAGTGCGTATGTTTCCAGAATACCGAGATTTAATATCCCGTCTGAAAAACGAAAATCCTCGCTTTATGTCCTTGTTCG
ATAAACACAATAAACTTGATCATGAAATTGCCAGAAAGGAAGGTTCCGACGGTCGAGGGTACAATGCGGAAGTGGTCCGC
ATGAAAAAACAAAAGCTACAGTTAAAAGATGAGATGCTCAAAATCCTGCAGCAGGAGAGCGTCAAAGAGGTGTAAACTTT
CCTAAGCCGCCAGCCAGGCGGCTTTTTTAACAACTGCATGGATTGACTGGAGATAAGATGACTGAAACGATAAAAGTAAG
CGAATCACTTGAATTACATGCTGTTGCAGAAAATCACGTCAAACCTCTTTATCAGTTAATCTGTAAAAATAAAACCTGGT
TACAGCAGTCGCTAAACTGGCCGCAGTTTGTTCAAAGTGAAGAGGACACGCGAAAAACGGTGCAGGGTAATGTGATGTTG
CATCAACGCGGCTATGCCAAAATGTTCATGATTTTCAAAGAAGATGAACTTATCGGCGTTATCTCGTTTAATCGTATTGA
ACCACTGAATAAAACCGCTGAAATAGGCTACTGGCTGGACGAATCTCATCAGGGGCAGGGGATCATTTCTCAGGCGCTGC
AGGCATTGATTCATCATTACGCCCAGTCTGGTGAACTTAGACGCTTCGTGATCAAATGTCGGGTGGACAATCCGCAAAGC
AACCAGGTCGCTTTGCGCAATGGTTTTATCCTTGAAGGTTGCCTGAAACAGGCTGAGTTCCTGAATGATGCCTATGATGA
TGTGAACTTATACGCGCGTATTATCGATTCACAATAACCCTGCCAGCGGCGTTCGCGTAATGCGCTCCTCGCCGTTAATG
ACTTTTGGACCACGGACATGAACCGTGTCGCCATCAAAGGCTTCAACGACGGCGTGGTCGGTTAACTCCACATGGTTTTC
AATTATCACCGCACCTGTAATACGGCTCTCACCTTGTATGACAACGTGCTCGTCGAGCAGAATCGGTCCGCCACGTACCA
CGGCATTTCCGCCAACCAAGACATGATGTTTTAACACACAATTACCTTCCACAATGGCATATTCCGCCACCTGCGAACTG
TAATGAATCGTCGGAATGGCATCTTCTTCTATGCCAGCTTTCACCTGCGCGTGACCGTAGACTTTAGCGCAATCGCATAG
CCAGACATTGTTCTCTTCATTACCTTCAATACTGGCAAAATCAAAAACTTCGGCGCGATGTTCAATAAAAGCATACCGGA
CGACGGCATCGCCATAAATTTGTGCCTGGTGGACAATACGCGAGGCGCTAACCCTTGCGCGATCATAAATTTGGAGCAGG
AGTTGATGGTCGGGCGTTAAGCCTTGTGCGGCGACGATCATAGAATGTTGATCAATTAAGGCGTGACCAAATATTCTACA
TTGCCCGTAAACTAATGAGTCATGAATGGTGACGCTGTCACTTATATATGCACCCTGGCTGATTTCACTATTATCGATCC
AGACGTTATCCGTTGCGTAAACCTCTCCCCACAGCACGCTGGTGCCGGTGATACGCGTATTACCGGAAATCACCGCTCCG
CCGAACGCAATAGCATTCTGGTCGTAAATCCAGCAATTTCCTTCTTGTGCTAACACCGTTTCGCGATCGATCCAGCCGCC
AGCGGTTCCTGCTATCACATCGTTAAAATCGCTAATGGCGATAATCTGCCGTAATAACACATTTTTTTTAGTGCCATCTT
CCTGATAACTAAAGGCTCGCTGTTCTTCACTTAGACGATATTTGCGCATAGCGTTTTCCCACAGGTGACTTACTATAACC
GTAGCAAATTCTGCGGCTCTGGCTATGCTCGAGAAAATTCCATAAAATGCATTTCAAATATACTTTATAAATTAAACAAA
ATGAGTAAGAAGATGCAGAGCGATAAAGTGCTCAATTTGCCGGCAGGCTACTTTGGTATTGTGTTGGGGACGATAGGGAT
GGGATTTGCCTGGCGCTATGCCAGCCAGGTTTGGCAGGTCAGCCACTGGTTAGGGGATGGGCTGGTGATTCTGGCGATGA

TCATCTGGGGATTATTGACTAGCGCATTTATTGCCCGACTCATACGCTTTCCGCATAGCGTGCTGGCGGAAGTTCGCCAT
CCAGTGCTGAGCAGTTTTGTGAGTTTGTTTCCGGCAACGACGATGCTGGTGGCGATTGGTTTTGTTCCGTGGTTTCGCCC
ACTGGCGGTGTGCCTGTTCAGTTTTGGTGTCGTGGTTCAGTTGGCTTATGCCGCCTGGCAAACTGCGGGATTATGGCGCG
GATCTCACCCTGAAGAAGCTACCACGCCTGGACTGTATCTGCCGACAGTTGCCAACAACTTTATCAGCGCAATGGCCTGT
GGTGCGTTGGGCTACACCGACGCCGGTCTGGTGTTTTTAGGCGCAGGCGTTTTCTCATGGCTAAGCCTTGAACCGGTGAT
CTTGCAGCGTCTGCGCAGTTCGGGAGAATTACCCACGGCACTGCGGACATCACTCGGCATTCAGCTCGCTCCTGCGCTGG
TGGCTTGTAGTGCCTGGCTGAGCGTCAACGGCGGCGAGGGTGACACGCTGGCGAAAATGCTTTTCGGTTATGGACTGCTG
CAACTGCTGTTTATGCTACGTCTGATGCCATGGTATCTCTCCCAGCCATTTAATGCTTCATTCTGGAGTTTCTCGTTCGG
CGTATCTGCACTGGCAACCACCGGTTTGCATCTGGGGAGTGGCAGCGATAATGGATTTTTCCATACGCTGGCGGTGCCGC
TGTTTATCTTTACCAATTTTATTATTGCAATACTGCTCATCCGTACTTTTGCGCTTCTGATGCAGGGAAAATTGTTAGTC
AGAACCGAGCGCGCCGTTTTAATGAAAGCAGAGGACAAAGAATGATCATTCGTGACGAAAACTATTTTACTGATAAATAT
GAATTAACCCGCACACACTCTGAAGTACTGGAAGCGGTGAAAGTGGTTAAACCGGGTAAAACGCTGGATCTGGGCTGTGG
CAATGGTCGTAACAGTCTTTACCTGGCAGCCAATGGTTATGATGTTGACGCATGGGATAAAAATGCCATGAGTATCGCCA
ACGTCGAGCGCATTAAATCCATTGAAAATCTGGATAATTTACACACCCGAGTCGTTGATCTGAATAACCTCACATTTGAT
AGACAGTACGATTTTATTCTTTCGACTGTGGTGCTGATGTTCCTTGAGGCTAAAACCATCCCCGGGTTGATTGCCAATAT
GCAACGTTGCACTAAACCTGGTGGTTACAACCTGATTGTGGCGGCGATGGATACCGCTGATTATCCATGTACCGTCGGCT
TCCCGTTTGCCTTCAAAGAGGGAGAATTACGTCGATATTACGAAGGCTGGGAGAGGGTGAAATACAATGAAGACGTCGGC
GAGCTGCACCGCACCGACGCCAACGGTAATCGTATTAAACTGCGTTTCGCCACGATGCTGGCACGTAAAAAATGACCCGG
TAAGCACAAAACGCGTGAAAATTCCCCACGCTGAGATGATTTACTGTTCTTCTTTTCGGTAAGCATATTTTTTATCGAAG
GGATGTGAAATTAATCACAGTAGTCGAAGTTTTTAGCAGCTTAACTTACTGAAATTTAAGTACTGATGATTGACTTAGCC
CCTTTTTCGGCATTGACTATGTCGTCTGAAAAGGGGCTGAAAAATTTATTTCACCAACACTTTTTTTGCCACAACACGA
AGCGGCGCTTTTTGCTATAACTTAGAAAGTAATATAATCATCTCAGGAAACTATTCATGCGTACCACATCATTTGCGAAA
GTTGCAGCTTTATGCGGCTTATTGGCTCTGTCTGGTTGTGCATCTAAAATCACCCAGCCAGATAAATATTCTGGTTTTTT
AAACAATTACTCTGATTTAAAAGAAACAACCTCGGCTACAGGTAAACCTGTTTTACGTTGGGTAGACCCGAGTTTTGATC
AAAGCAAATATGACAGCATCGTCTGGAACCCAATCACTTATTATCCGGTACCGAAACCGTCGACCCAGGTAGGGCAGAAA
GTTCTGGATAAAATTTTGAACTATACCAACACCGAAATGAAAGAAGCGATAGCGCAGCGTAAACCACTGGTTACCACCGC
TGGGCCGCGTAGTCTGATTTTCCGTGGGGCCATTACCGGTGTAGATACCAGCAAAGAAGGGCTGCAATTCTATGAAGTGG
TTCCTGTTGCATTAGTGGTTGCGGGGACGCAAATGGCTACAGGCCACCGTACCATGGATACTCGCCTCTATTTTGAAGGT
GAGCTGATTGATGCAGCGACTAATAAACCGGTTATCAAAGTCGTTCGTCAGGGCGAAGGTAAAGACCTGAATAACGAAAG
TACGCCAATGGCTTTCGAAAATATTAAACAAGTTATTGATGACATGGCGACCGATGCCACCATGTTTGACGTTAACAAAA
AGTAGTTCCAGACGCGCCATCGTTAGATGGCGCTTTTTATCCGGTGCGCCGTAAAACCCCATCCTTCAGGGCGGGGATAT
AAGGCGCGGTTTTCCACCTGACCAGGTGTTTGCTGTTGTTCAATGTATTGGCGGATGACGGATATTGGCGCACCTCCGCA
GCTACTGGCAAAATAGCCAGGACTCCACAAAACGCCTTTGTAGTAATACCTGAATGCAATATCTGGTCGATCTCGTCGCA
GTCACCTACCCGATACGCCTTTGAGGCTGTTTACCAGACTGGATATCGCCAGTTTGGGAGGATAGTTAATTAACAAATGG
ACGTGATCTGGTTCGCCATCCATTTCAACCAGTTCAGCTTCAAAATCAGCACATACATTTGAAAAGTAAGTGCGTAGTTT
TTCTGTCGCGTCATGGTCAAAAATCTGGCGTCGGTATCTGGTGACAAAGAGCAGGTGAACATGCATCAGGAAAACACAAT
GCCTTCCACGCCGGATATCGGTTTCTTTTTTCACAGACCAAAGTATCATTTGACCTGTGAAACGATTACAGGCATTTAAA
TTCCAGTTAAGACCCGGTGGTCAACAGGAGTGTGAAATGAGGCGCTTCGCAGGCGCATGTCGTTTCGTTTTCAATCGTGC
TCTGGCACGTCAGAATGAGAATCATGAGGCCGGTAATAAATACATCCCTTACGGGAAAATGGCTTCCTGGCTGGTTGAGT
GGAAAAATGCCACTGAAACGCAATGGCTTAAAGATTCTCCCTCACAGCCATTGCAACAGTCACTGAAAGACCTTGAGCGG
GCTTACAAAAACTTCTTCCGGAAGCGGGCTGCTTTTCCCCGATTCAAAAAGCGGGGACAGAATGATGCATTCCGCTACCC
GCAGGGCGTTAAGCTCGATCAGGAAAACAGCCGTATTTTTCTGCCGAAACTGGGCTGGATGCGCTACCGGAACAGCCGTC
AGGTCACGGGTGTTGTGAAAAATGTCACTGTCAGCCAGTCCTGCGGTAAGTGGTACATCAGTATTCAGACAGAAAGTGAA
GTATCAACTCCGGTTCACCCTTCAGCATCAATGGTCGGGCTGGATGCTGGCGTGGCTAAACTCGCCACGCTGTCAGATGG
CACAGTCTTTGAGCCTGTAAACAGTTTTCAGAAAAACCAGAAGAAGCTGGCGAGACTTCAGCGACAGTTAAGCCGCAAGG
TCAAATTCAGCAACAACTGGCAAAAGCAGAAACGCAAAATACAGCGACTGCATTCCTGTATCGCAAATATCCGCAGGGAC
TACCTTCACAAAGTCACAACGGCCGTCAGCAAAAACCACGCAATGATAGTCATTGAGGATTTGAAGGTCAGCAACATGTC
AAAGTCAGCAGCGGGTACGGTCAGCCAGCCGGGGCGCAATGTCCGGGCAAAATCAGGTTTAAACCGTTCGATACTGGATC
AGGGCTGGTATGAAATGCGCCGCCAGCTTGCGTATAAGCAGCTCTGGCGTGGCGGTCAGGTGCTTGCTGTTCCGCCAGCG
TATACAAGCCAGCGTTGCGCGTACTGTGGTCATACAGCGAAAGAGAACCGCCTGTCACAAAGTAAATTCAGATGCCAGGT
ATGTGGATATACAGCGAACGCCGATGTAAATGGCGCTCGCAACATTTTAGCGGCGGGGCACGCCGTTCTTGCCTGTGGAG
AGATGGTGCAGTCAGGCCGCCCGTTGAAGCAGGAACCCACCGAAATGATTCAGGCGACAGCCTGAACGTAGCAGGGATCC
ACGTCCTTCAGGGCGTGGAGGATGTCAATATCGGTTTCTGTCAGCGATTAAATTCAACACCACGTAACAAACGCCTCCGG
CAATTAATCCCCAAAACGCAGAACCAATCCCGACCAGCGTCAATCCACTTGCCGTTACCAGAAATGCCACCACCGCCGCG
TCTCGCTCACGCTCATTATGCAGCGCCTGATACAAACTGCCGCCGATGGTACTTAACAGCGCCAGACCTGCCAGCATCTG
GATCCAACTTACGGGCAGGGCAGCCATCATCCCGGTAATGGCACTACCAAACAGACCTGCGAGCAAATAGAAAATGCCTG

CAACGGCAGCGGCCAGCCAACGTTGATCTTTATCCGGATGCGCTTCCGGGCTTTGGCAAATAGCCGCGGTGATTGCCGCA
ATACCGACGGAATAAACGCCGAAAGGGGAAAAAACCAGTGCCAGCAATCCAGTAAATACAATTAATGGCGAAACAGGAGC
CGAATATCCAGCTGCTTTCATTGCTGCGATACCCGGTGCGTTTTGCGATGCCATCGTCACCAGAAAAAGGGGGAGTGCAA
CGCTCAGGCTGTGAGCAAACGAAAAATCAGGGGTAATATAAGTGGGGAGAACGGGTTTAAAGACAACATCAGTTGTGACA
ACGTCACCTTGCGCGATGACGATCACGATCCCAATAATCATCGCGGCAATTACCGCATAGCGCGGCGCAACGGCCTTGGT
TGCCAGCCATACCAGCAACATACTTCCACACAACGTAAATTGACCGTCCAGACTGGCAAACGCCTGTAAACCAAAGCGTA
ATAAAATCCCGGCAAGCATTGCCGCCGCAAGCGAGTGCGGAATAATGCGCATCAGACGAGCAAAGAGTCCCGTTATGCCG
CAGAGGACTATTAGCGCGTTGGTGACAATAAAAACGCCGATGGCTTCGTTAAGTGTTAGTCCCTGCAATCCGGTGACCAA
CAAAGCCGCGCCAGGCGTTGACCATGCGGTGAGAACAGGTACGCGATACCATAATGTCAGAGTCAGCGTACTGACGCCCA
TTGCCAGCCCCAGCGCCGTCATCCAGCCAGAGATTTGTGCAGTGGTGGCTCCGGCGACAATCGCTGCTTGCCAGATTATT
GCCGCTGAACTGGCGTAGCCAATTAATACCGCCAGAAACCCCGCCAGTAGCGTGGGTGGAGGAATAGAAAACAGACGCAT
AGTCACCTCGTGCGTTATAGCGTACAGGCACCGTAGCATTTGTCCGTTATAACGCACAAGTGATAAACTTCCGTTTTGCC
GGAGGAGTCGCATGGAAAATCTCGCTCGCTTTTTATCCACCACACTTAAACAACTACGCCAGCAGCGCGGCTGGAGTCTT
TCGCGACTGGCAGAAGCGACGGGCGTTTCTAAAGCAATGTTAGGGCAGATTGAGCGTAATGAGTCCAGTCCGACGGTCGC
GACATTATGGAAAATTGCCACCGGGTTGAACGTACCATTTTCAACATTTATTTCTCCGCCGCAGTCCGCTACGCCTTCAG
TTTATGATCCACAACAGCAGGCGATGGTGATCACTTCGCTGTTTCCTTATGATCCACAACTCTGCTTTGAACACTTCTCG
ATTCAGATGGCATCTGGTGCAATAAGCGAATCGACGCCCCATGAGAAGGGGGTAATCGAACATGTCGTCGTCATTGATGG
ACAACTTGATCTGTGCGTTGATGGGGAATGGCAGACCCTTAATTGTGGGGAAGGCGTTCGATTTGCTGCAGACGTCACGC
ATATCTATCGTAATGGTGGGGAGCAAACCGTACATTTTCATTCCCTCATCCATTACCCGCGCAGTTAAGCGGGAAAACTA
TTTCGCAACGTCGCGCTTGTGGCTAAAATAGCCGCCATTTTTCAGCTACTGGATAAGAATGTGACCGTATCTTCTCATCG
ACTTGAACTGTTAAGCCCGGCACGCGATGCCGCCATTGCCCGCGAAGCTATTTTGCACGGTGCCGATGCTGTTTATATCG
GCGGCCCTGGTTTTGGTGCCCGTCATAATGCCAGTAATAGCTTGAAAGATATTGCCGAGCTGGTGCCGTTTGCCCATCGT
TATGGTGCAAAAATTTTCGTCACGCTTAACACCATTTTGCATGATGATGAGCTGGAACCCGCGCAACGGCTGATTACTGA
CCTCTACCAGACCGGTGTCGATGCGCTGATTGTTCAGGATATGGGGATTCTGGAACTTGATATTCCGCCGATTGAACTGC
ACGCCAGTACGCAGTGCGACATTCGTACAGTTGAAAAAGCGAAGTTCCTCTCTGATGTTGGCTTCACGCAGATTGTGCTG
GCGCGAGAGCTGAATCTTGATCAGATCCGCGCGATTCACCAGGCTACGGACGCGACCATTGAATTCTTTATTCATGGGGC
ACTGTGCGTGGCCTATTCGGGTCAGTGCTACATTTCTCATGCGCAAACAGGGCGTAGCGCCAACCGTGGCGATTGCTCGC
AGGCGTGCCGTTTGCCATACACATTGAAAGACGATCAGGGGCGGGTGGTTTCCTATGAAAAACATCTGCTGTCGATGAAA
GATAACGATCAGACTGCCAACCTCGGCGCGCTGATTGATGCTGGTGTACGCTCCTTCAAGATTGAAGGGCGTTACAAAGA
TATGAGCTACGTGAAGAATATCACCGCCCATTATCGCCAGATGCTTGATGCCATTATTGAAGAACGTGGCGATCTGGCGC
GCGCTTCATCAGGTCGTACTGAACATTTCTTTGTTCCATCGACGGAAAAGACTTTCCACCGTGGTAGCACAGATTATTTT
GTGAATGCCCGTAAAGGCGATATTGGCGCGTTCGATTCGCCGAAATTTATCGGCCTGCCGGTAGGCGAAGTAGTGAAAGT
GGCGAAAGATCATCTCGATGTTGCCGTTACCGAGCCACTGGCAAATGGCGATGGCCTGAACGTGTTGATTAAACGTGAAG
TCGTCGGTTTTCGTGCCAATACGGTCGAGAAAACCGGAGAAAATCAGTACCGCGTCTGGCCCAATGAAATGCCAGCAGAT
TTGCACAAAATTCGTCCACATCACCCACTAAACCGTAATCTTGATCATAACTGGCAGCAGGCACTGACAAAAACCTCCAG
CGAACGTCGGGTGGCGGTAGACATTGAACTGGGCGGCTGGCAGGAACAACTGATTCTGACCCTCACCAGTGAAGAGGGTG
TCAGCATCACGCATACGCTGGACGGGCAGTTCGACGAAGCCAATAACGCCGAAAAAGCAATGAACAATCTGAAGGATGGT
CTGGCAAAACTGGGGCAAACCCTCTATTACGCCCGCGATGTGCAAATTAATTTGCCGGGGGCGCTGTTTGTACCAAACAG
TCTGTTAAACCAGTTCCGCCGTGAAGCTGCTGACATGCTGGATGCTGCGCGTCTTGCCAGTTACCAGCGCGGCAGCCGTA
AACCGGTTGCTGATCCTGCGCCGGTTTATCCGCAAACGCATCTGAGTTTCCTCGCGAACGTATACAACCAGAAAGCGCGT
GAATTTTATCATCGCTATGGTGTGCAGCTGATTGACGCGGCGTATGAAGCACATGAAGAGAAGGGCGAAGTCCCGGTGAT
GATCACCAAGCATTGTCTGCGCTTTGCCTTTAATCTGTGCCCGAAACAGGCGAAAGGCAATATCAAAAGCTGGAAGGCGA
CGCCAATGCAACTGGTTAACGGCGATGAAGTATTAACGCTAAAGTTTGATTGCCGCCCATGCGAGATGCACGTCATTGGC
AAAATCAAAAATCACATACTGAAAATGCCGTTACCGGGAAGCGTAGTGGCATCCGTAAGTCCGGATGAGCTGCTGAAAAC
ATTGCCGAAGCGAAAAGGGTAAAACGCCAGTTTTCTGGTTACTCACAACTTATTGAATCTGCATGATATTGCCTGCCGGG
TAAGGCGTTACGCCGCATCCGGCATCAAATGACTCAACGTTGTCCCGCTTCTGGTTTGCGCGATTTTTGCCAGTAAAAAT
GCTCGCGCAAACCTTCCGCCGACTCTTCCGCCACAGCACGCAATTCATCGCTGTCCGCTTCATGACGCAGCTGATGATCC
ACATTCTTTACCCACACAAATTCATGTCCTTTGTGCCCTGCCATGAGTTGTCCTGAAAACAGAGCACACGTTAATAAGAC
AACCGATAACGCCTTCGTAAACATCCTGCCACCTTTTTCTTACCTTTTGCCGCTATGATGCCGATCGTTTCTTGAGGTTA
TTATTCAGTTTTGCAAATTAGCGCAAAGAAATTCTGGAATCTTCCTTCCTGATTTTGCATTGCATTCTGCCGTTGCGGCG
ATTTAGTGCTATTTTCGAGCAAATTACACACGGAGGTAAACACTAATGTTTACTTTGTTGATATACTCAGCGGCAGGGAG
GCGATGTGAAACAAAGCGAGTTCAGACGTTGGCTCGAATCTCAGGGCGTCGATGTAGCGAATGGCAGCAACCATTTGAAA
CTCAGGTTTCATGGGAGGCGCAGTGTCATGCCGCGTCACCCCTGCGATGAGATTAAAGAACCATTGCGTAAAGCAATCCT
GAAACAACTCGGTTTGAGTTAATCGCCAATTAAAAAGGTTAATGACATGCGAGAGACAGTCGAAATTATGCGTTATCCCG
TCACTCTTACACCCGCGCCGGAAGGCGGTTATATGGTTTCTTTTGTGGATATCCCTGAAGCGTTGACCCAGGGCGAAACT
GTCGCTGAAGCGATGGAAGCGGCAAAAGATGCTTTACTGACCGCATTTGATTTTTATTTTGAAGATAACGAGCTTATCCC

TTTACCTTCGCCATTAAATAGTCACGATCACTTTATTGAAGTACCTTTGAGCGTCGCCTCTAAGGTATTGCTGTTAAATG
CTTTTTTACAGTCAGAAATCACTCAGCAAGAGTTAGCCAGGCGAATTGGCAAACCTAAACAGGAGATTACTCGCCTATTT
AACTTGCATCATGCGACAAAAATCGACGCCGTCCAGCTCGCGGCAAAGGCGCTTGGCAAAGAGTTATCGCTGGTGATGGT
TTAATTACAGTTAACGAAAAGTTGTCATTTTTAACAACTGATATAGACTGCCGAATCATCTGCACATAATTACGATTCGA
TAATGAAAAAATACCAGCAGCTTGCAGAACAATTACGCGAGCAGATTGCGTCGGGTATCTGGCAACCCGGCGATCGTTTG
CCTTCGTTGCGTGACCAGGTGGCGCTTTCAGGCATGAGCTTTATGACTGTCAGCCATGCCTATCAGTTGCTCGAAAGTCA
GGGATATATTATCGCACGACCGCAGTCGGGTTATTACGTTGCGCCACAGGCAATAAAAATGCCGAAAGCGCCAGTCATTC
CAGTCACTCGAGATGAAGCAGTCGATATCAACACTTATATTTTTGATATGTTGCAGGCCAGTCGCGATCCGTCGGTCGTT
CCGTTTGCCTCGGCCTTTCCCGACCCGCGACTTTTCCCCCTCCAACAACTAAACCGCTCGCTGGCGCAGGTAAGCAAAAC
CGCCACAGCGATGAGCGTGATTGAAAACTTACCGCCAGGAAACGCAGAACTGCGTCAGGCTATTGCTCGTCGCTATGCCT
TACAGGGCATCACCATTTCTCCTGATGAAATTGTCATTACTGCCGGGGCGTTAGAGGCATTAAACCTCAGTTTGCAAGCG
GTAACTGAACCGGGCGATTGGGTGATAGTAGAGAATCCTTGTTTCTACGGTGCGTTGCAGGCGCTGGAGCGGCTACGGCT
GAAGGCGTTATCGGTGGCGACGGATGTTAAAGAAGGGATAGATCTTCAGGCGCTGGAACTGGCGTTGCAGGAGTATCCGG
TGAAAGCGTGCTGGCTGATGACTAATAGCCAGAATCCACTCGGATTTACCTTAACGCCGCAAAAAAAAGCACAACTGGTG
GCGTTGCTCAATCAGTACAACGTAACGCTGATTGAAGATGACGTTTACAGCGAACTTTATTTTGGACGGGAAAAACCGCT
GCCTGCGAAAGCGTGGGATCGCCACGATGGCGTTTTGCATTGCTCTTCGTTTTCGAAATGTCTGGTGCCTGGTTTTCGTA
TTGGTTGGGTCGCCGCCGGAAAACATGCACGTAAAATTCAACGCTTGCAGTTGATGAGTACGCTTTCCACCAGCTCACCG
ATGCAACTTGCGCTGGTGGATTACCTTTCCACGCGCCGATACGACGCCCATCTTCGTCGCCTGCGTCGCCAGCTTGCGGA
ACGTAAACAACGTGCCTGGCAGGCACTGCTGCGTTATCTGCCTGCGGAAGTGAAAATTCATCATAATGACAGTGGTTACT
TTCTCTGGTTGGAGCTCCCCGAGCCGTTAGATGCCGGCGAATTAAGCCTGGCGGCACTGACGCATCATATCAGTATTGCG
CCGGGTAAAATGTTTTCTACCGGTGAAAACTGGTCACGTTTTTTCCGTTTTAATACCGCGTGGCAGTGGGGAGAGCGTGA
AGAACAGGCGGTAAAACAATTAGGCAAACTTATTCAAGAACGGCTGTAATAGCGTTTAATTTAATTCCTCTTAGATTGGG
TAATATGAATTTCGAATAGCAGTCATATTTCCTAACTCCTTGACTATACTCCAGAAGATAACCTTACAGACGGCATAATG
CGCGGTAGCTCACAACCTGAATAAATTTTCTCAGGGGCGAAGGTGTGCCTGCAAGCCGCCGTCTATGGTTAAACAAGGAG
ATATTTTTACGGCACGGCGGCTGAACAATTAATTACGACAGGAGTAAGACCTTATGAGCAAGACATTTGCCCGCAGCAGC
CTGTGTGCGCTCAGCATGACAATAATGACCGCTCACGCCGCCGAACCGCCTACCAATTTAGATAAACCGGAAGGGCGACT
GGATATTATCGCCTGGCCGGGATACATCGAACGCGGACAAACTGATAAACAATACGACTGGGTAACGCAGTTCGAAAAAG
AGACAGGCTGCGCGGTGAATGTGAAAACCGCCGCGACTTCCGATGAAATGGTCAGTCTGATGACCAAAGGGGGTTACGAT
CTGGTTACGGCATCCGGCGATGCCTCGCTGCGTTTGATTATGGGTAAACGCGTGCAGCCGATTAATACCGCATTGATTCC
CAACTGGAAAACGCTCGATCCGCGCGTGGTTAAAGGCGACTGGTTTAATGTTGGCGGCAAAGTTTACGGCACACCTTACC
AATGGGGGCCGAACCTGCTGATGTACAACACTAAAACCTTCCCGACGCCGCCGGATAGCTGGCAAGTGGTTTTTGTTGAG
CAAAATCTGCCGGACGGCAAGAGCAATAAAGGCCGCGTTCAGGCTTATGATGGCCCTATCTATATTGCGGACGCTGCGTT
GTTCGTTAAAGCCACTCAGCCGCAGTTGGGCATCAGCGATCCGTATCAACTCACCGAAGAACAGTACCAGGCGGTGCTGA
AAGTGCTGCGCGCTCAACACAGTTTGATCCATCGCTACTGGCATGACACTACCGTGCAAATGAGCGATTTCAAAAACGAG
GGTGTGGTTGCTTCCAGTGCCTGGCCCTATCAGGCCAACGCCCTGAAAGCCGAAGGCCAGCCTGTTGCTACCGTTTTCCC
GAAGGAGGGTGTTACCGGTTGGGCTGATACCACCATGCTGCATAGCGAAGCGAAACATCCGGTTTGCGCCTACAAATGGA
TGAACTGGTCATTAACGCCAAAAGTGCAGGGCGATGTGGCGGCCTGGTTTGGCTCGTTACCGGTAGTGCCGGAAGGGTGT
AAAGCCAGTCCGTTATTAGGCGAAAAAGGTTGTGAAACCAACGGTTTTAACTATTTCGACAAAATCGCCTTCTGGAAAAC
GCCTATAGCAGAAGGGGGCAAGTTTGTTCCCTACAGTCGCTGGACGCAGGATTACATTGCCATTATGGGCGGTCGCTAAC
TTCGCTGGGGTGCTTTATGACGTACGCAGTGGAGTTTGACAACGTCTCGCGGTTGTACGGTGACGTGCGCGCAGTAGATG
GCGTCAGTATTGCGATAAAAGATGGTGAGTTCTTCTCTATGCTGGGGCCGTCCGGCTCCGGCAAAACCACCTGCCTGCGC
CTGATTGCTGGCTTCGAACAGCTTTCCGGCGGGGCTATCTCTATCTTTGGTAAACCCGCCAGCAATCTGCCACCGTGGGA
GCGGGACGTGAATACTGTCTTTCAGGACTACGCGCTATTTCCGCATATGTCGATTCTTGACAATGTCGCCTATGGGCTGA
TGGTCAAAGGCGTGAATAAAAAGCAGCGGCACGCAATGGCGCAAGAGGCGCTGGAGAAAGTGGCGTTGGGGTTTGTACAT
CAACGTAAACCGTCACAACTTTCTGGTGGTCAGCGCCAGCGGGTTGCTATCGCCAGAGCATTGGTGAATGAACCGCGCGT
ATTGCTGTTGGATGAACCGCTCGGCGCACTGGATCTCAAATTGCGTGAGCAGATGCAGCTGGAACTGAAAAAACTGCAAC
AGTCTCTCGGTATCACTTTTATCTTCGTTACCCACGATCAGGGCGAAGCGTTATCGATGTCCGATCGTGTGGCGGTTTTC
AATAATGGACGCATTGAGCAGGTCGATTCCCCGCGCGATCTCTATATGCGCCCGCGCACGCCGTTTGTTGCCGGGTTCGT
TGGTACATCGAATGTTTTTGATGGACTGATGGCAGAGAAACTTTGTGGCATGACGGGAAGCTTCGCCCTGCGACCGGAAC
ATATCCGCCTCAACACCCCTGGTGAACTGCAGGCCAATGGCACGATCCAGGCGGTGCAATATCAGGGCGCGGCAACTCGT
TTTGAACTGAAATTGAACGGCGGTGAAAAACTGCTTGTGAGTCAGGCCAATATGACAGGCGAAGAACTGCCTGCCACGCT
CACGCCCGGACAACAGGTGATGGTTTCCTGGTCGCGTGATGTGATGGTGCCGCTGGTTGAGGAGAGGTGAATGGCGATGA
ATGTATTGCAATCACCTTCACGTCCAGGTCTGGGTAAGGTGTCCGGTTTCTTCTGGCATAATCCGGGGCTGGGGCTGTTT
TTACTGCTGCTTGGCCCGCTAATGTGGTTTGGCATTGTCTATTTCGGCTCGCTGCTGACACTGTTATGGCAGGGATTTTA
TACTTTTGACGATTTCACCATGTCGGTAACGCCGGAACTGACGCTGGCGAATATCCGTGCGCTGTTTAATCCGGCGAATT
ACGACATCATTCTCCGCACGCTGACTATGGCTGTGGCAGTCACTATCGCCAGCGCCATTCTGGCTTTTCCAATGGCGTGG

TATATGGCGCGCTATACCAGCGGGAAAATGAAAGCGTTTTTTATATTGCGGTAATGTTGCCGATGTGGGCGAGCTACAT
TGTTAAAGCCTATGCCTGGACGTTATTGCTGGCAAAAGATGGCGTGGCTCAGTGGTTTTTACAACATCTTGGGCTGGAAC
CACTGCTGACTGCGTTCCTTACATTACCTGCGGTGGGCGGAAATACGCTGTCAACTTCCGGGCTGGGGCGCTTTCTGGTG
TTTCTCTATATCTGGTTGCCGTTCATGATCCTGCCCGTTCAGGCGGCGCTTGAGCGTTTGCCGCCGTCATTGTTGCAGGC
GTCGGCTGATCTCGGCGCACGTCCACGACAAACCTTTCGCTATGTGGTGCTGCCGCTGGCAATCCCGGGTATTGCCGCTG
GCTCTATCTTTACCTTCTCACTCACACTGGGCGATTTTATCGTCCCGCAGCTGGTTGGTCCTCCAGGATATTTTATCGGC
AATATGGTTTATTCCCAGCAGGGGGCGATTGGCAATATGCCGATGGCGGCGGCATTCACCCTGGTGCCGATTATTCTCAT
CGCACTGTACCTGGCGTTCGTGAAACGTCTGGGAGCGTTCGATGCACTCTGAACGCGCACCGTTTTTCCTCAAACTGGCG
GCCTGGGGCGGCGTTGTTTTCCTACATTTTCCCATCCTGATAATCGCCGCCTATGCGTTTAACACTGAAGATGCGGCGTT
TAGTTTTCCACCGCAGGGCCTGACGCTGCGCTGGTTTAGCGTGGCAGCACAGCGTAGTGATATTCTTGATGCCGTGACAC
TGTCACTTAAAGTGGCGGCGCTGGCGACATTAATTGCGCTGGTGTTAGGGACGCTGGCAGCTGCCGCGCTGTGGCGACGA
GACTTTTTCGGCAAAAACGCCATTTCGCTGTTACTGCTGCTGCCCATTGCGCTGCCGGGCATTGTCACTGGTCTGGCGTT
ATTAACCGCCTTTAAAACCATCAATCTGGAGCCGGGATTTTTCACCATCGTGGTCGGTCATGCGACTTTTTGTGTAGTTG
TGGTGTTTAACAATGTCATCGCCCGTTTTCGCCGCACCTCCTGGAGTCTGGTTGAGGCGTCAATGGATCTTGGGGCCAAT
GGCTGGCAAACCTTCCGCTACGTAGTGTTGCCGAATCTCAGTTCGGCGTTACTGGCAGGAGGAATGCTGGCGTTTGCCTT
GTCGTTCGATGAAATCATCGTTACGACCTTTACGGCAGGTCATGAACGAACGTTACCGTTGTGGTTGCTCAATCAGCTTG
GGCGACCGCGTGATGTACCGGTAACTAACGTGGTGGCACTGCTGGTTATGTTGGTAACAACCTTGCCGATCCTGGGGGCC
TGGTGGCTAACCCGCGAAGGCGACAATGGTCAATAACCACTGATACAGGAATATGCTATGCAACATAAGTTACTGATTAA
CGGAGAACTGGTTAGCGGCGAAGGGGAAAAACAGCCTGTCTATAATCCGGCAACGGGGGACGTTTTACTGGAAATTGCCG
AGGCATCCGCAGAGCAGGTCGATGCTGCTGTGCGCGCGGCAGATGCAGCATTTGCCGAATGGGGGCAAACCACGCCGAAA
GTGCGTGCGGAATGTCTGCTGAAACTGGCTGATGTTATCGAAGAAAATGGTCAGGTTTTTGCCGAACTGGAGTCCCGTAA
TTGTGGCAAACCGCTGCATAGTGCGTTCAATGATGAAATCCCGGCGATTGTCGATGTTTTTCGCTTTTTCGCGGGTGCGG
CGCGCTGTCTGAATGGTCTGGCGGCAGGTGAATATCTTGAAGGTCATACTTCGATGATCCGTCGCGATCCGTTGGGGGTC
GTGGCTTCTATCGCACCGTGGAATTATCCGCTGATGATGGCCGCGTGGAAACTTGCTCCGGCGCTGGCGGCAGGGAACTG
CGTAGTGCTTAAACCATCAGAAATTACCCCGCTGACCGCGTTGAAGTTGGCAGAGCTGGCGAAAGATATCTTCCCGGCAG
GCGTGATTAACATACTGTTTGGCAGAGGCAAAACGGTGGGTGATCCGCTGACCGGTCATCCCAAAGTGCGGATGGTGTCG
CTGACGGGCTCTATCGCCACCGGCGAGCACATCATCAGCCATACCGCGTCGTCCATTAAGCGTACTCATATGGAACTTGG
TGGCAAAGCGCCAGTGATTGTTTTTGATGATGCGGATATTGAAGCAGTGGTCGAAGGTGTACGTACATTTGGCTATTACA
ATGCTGGACAGGATTGTACTGCGGCTTGTCGGATCTACGCGCAAAAAGGCATTTACGATACGCTGGTGGAAAAACTGGGT
GCTGCGGTGGCAACGTTAAAATCTGGTGCGCCAGATGACGAGTCTACGGAGCTTGGACCTTTAAGCTCGCTGGCGCATCT
CGAACGCGTCGGCAAGGCAGTAGAAGAGGCGAAAGCGACAGGGCACATCAAAGTGATCACTGGCGGTGAAAAGCGCAAGG
GTAATGGCTATTACTATGCGCCGACGCTGCTGGCTGGCGCATTACAGGACGATGCCATCGTGCAAAAAGAGGTATTTGGT
CCAGTAGTGAGTGTTACGCCCTTCGACAACGAAGAACAGGTGGTGAACTGGGCGAATGACAGCCAGTACGGACTTGCATC
TTCGGTATGGACGAAAGATGTGGGCAGGGCGCATCGCGTCAGCGCACGGCTGCAATATGGTTGTACCTGGGTCAATACCC
ATTTCATGCTGGTAAGTGAAATGCCGCACGGTGGGCAGAAACTTTCTGGTTACGGCAAGGATATGTCACTTTATGGGCTG
GAGGATTACACCGTCGTCCGCCACGTCATGGTTAAACATTAAGGATAATATTGCAGATCGTAAGAGTATGAGATGATCTT
GCGTACTGTCAGAGCACTGATTTCTCTGACAGTACGTGCTGCGTGCAAAATCACTACATCAAATAAACCAGCCAAATCTC
ATAGAGATAAGAGTAAACAAACCAACAGCAGCAAAGAAATTTATCAGTACTACAGTTCTACTGGAAACATTCATTTTTGC
CACCTTTTATCATTGCCCCTATGTGTATAGTACGGGTTAAGAAAATCCGCAATAATACTCATTCTGAAGATGGATAAGGG
CAAGTTGCTGTTTGATGATTTTTTCTCAAACTGGTGAAAAAACTTGATGCACGTCAAAAAATGACGCATATTTGCGCGCG
TTTTATTCATCTGGCTGGACGCCCGTACATGTCTCTCTATCAACACATGCTTGTTTTTTATGCGGTTATGGCAGCAATCG
CATTTCTTATCACCTGGTTTCTTTCTCACGATAAGAAACGCATCCGTTTCTTAAGCGCCTTTCTGGTGGGGGCAACATGG
CCGATGAGTTTCCCGGTGGCGCTGTTGTTTTCACTGTTTTAAATCTTACTCACTGGCTTGACGGCACCACGCAGACTTAT
ATCATTTGGATGAATCGATAAATTTCACAAGTGGCTAAGGAGAAAGTATGTCGCATCTGGATGAAGTCATCGCGCGCGTG
GATGCCGCGATTGAAGAGAGCGTCATTGCCCATATGAACGAATTATTAATCGCCCTGAGCGATGACGCGGAGTTAAGTCG
GGAAGATCGCTACACCCAGCAGCAACGTCTGCGCACAGCGATCGCCCATCACGGTCGCAAGCATAAAGAAGATATGGAAG
CGCGCCACGAACAGTTAACCAAAGGCGGCACCATCCTCTGATTAAAATGAGCGTCTGGCTACCAACCAGGCGCCAACCAC
AAGTAAAATTGCGCCACAAATTGGGCCGAACAACGCCCGTAGTGGTACGCCATGACTGCGGAAGATATCCAGCATCAGAC
CGCCAATCAGCTGACTGGCGACCAGTACAGCAATAGTTGTCGCCGCTCCCACATTCTGATATCCGCTGATACTGGCGAAG
ACAAAAAACGATCCCAGCAAGCCAGGTATAAGTGTCCACCAGCGCACGCTGGACACCAGTTCGCCAAACCCCGCCATGCC
TTGTTTAAACCATAAAATAGAAACAAAGAGCACAATCCCCACCAGTGAGTTCAGCAACATGGCGATGAGAATGGTAGAGG
AGGTCTGGGTGATGCGCACCATCAGCGTGTTCTGGACAACCAGACCAATTCCGGCGGCAATCAAAAAAGCAAGGGTGAGC
GACTGATTCATCCAATCGCGTCCGGTTCAGTGCGCTCGTCGAGTTGCAACTGCATAAATGTCAGATCCAGCCAACGACCA
AATTTAGTGCCTACCTGCGGCATTTGCGCGGTGACGACAAATCCCAGCGACTGGTGGAGATGCAGCGAGGCCTGATTTTG
CGATTCGATCCCGGCGACCATGACATGCTTCCCGCAATCCCGCGCTTCATCAATCAATCGGCTTAACAATTTACGCCCCA
GACCTTTGCCCTGATGATCGGGATGGACATAAACCGAATGTTCCACGGTATGGCGAAAACCATCGAAACTACGCCAGTCG

CCAAACGAGGCATATCCCGTCACTACGCCGTTTTCCTCGCTCACCAGCACTGGATAACCTGCTAAAGTCCGCGCTTCAAA
CCAGGCAATGCGGTTATCAGCATCCACCGTTTGGTCATTCCAGATAGCCGCCGTATACAACACGGCGTGGTTATAAATTT
CCGCAATGGCAGCACAGTCGGCTTTGCGGGCAAAACGGATGGACATGTCTGGCCTCAATAAAATAATGATGATGATGTTA
TCAATACTATCGAAAACAGCCCGAAGCAACGGATTCCTCGATGAAATTATTGCGTGTTGCAGAGGGGATGTGACGGCTGC
AAACAAAACCAATCCCGTGAGCCTGCTACTATTGATGGATTCATGCCAAAAGCACAGGAGAGCATTATGGGGCAACAAAA
GCAGCGTAATCGACGTTGGGTTCTGGCCTCGCGTCCACATGGCGCACCTGTTCCGGAGAATTTCCGTCTTGAAGAAGATG
ATGTCGCCACACCGGGTGAAGGACAGGTGTTACTGCGCACAGTTTATTTGTCCCTGGACCCGTATATGCGTGGACGTATG
AGCGATGAGCCATCTTATTCACCGCCTGTTGATATTGGCGGCGTGATGGTCGGCGGTACGGTGAGCCGTGTCGTGGAGTC
GAATCATCCTGATTATCAGTCTGGCGACTGGGTGCTGGGCTACAGTGGATGGCAAGACTATGACATATCCAGTGGTGATG
ATCTGGTGAAACTTGGCGATCATCCGCAAAATCCATCGTGGTCGCTGGGTGTGCTAGGGATGCCAGGCTTTACCGCTTAT
ATGGGCCTACTGGATATCGGTCAGCCTAAAGAGGGCGAAACGTTGGTGGTAGCTGCGGCGACAGGACCAGTGGGGGCGAC
GGTGGGGCAAATCGGCAAACTTAAAGGTTGCAGAGTGGTGGGGGTAGCCGGTGGCGCGGAAAAATGCCGCCATGCTACCG
AGGTGTTAGGCTTCGATGTTTGTCTTGATCACCACGCGGATGATTTTGCCGAACAACTGGCGAAAGCGTGCCCAAAAGGT
ATTGATATCTATTATGAAAACGTGGGCGGTAAGGTATTCGATGCGGTGCTACCGTTACTTAATACATCTGCGCGCATTCC
CGTCTGCGGATTAGTGAGCAGCTATAACGCTACAGAGCTACCACCCGGTCCGGATCGTTTACCTCTGTTGATGGCTACAG
TGCTGAAAAAACGTATTCGCTTGCAAGGTTTTATTATCGCTCAGGATTATGGTCACCGCATCCATGAGTTTCAGAGGGAG
ATGGGGCAATGGGTGAAAGAGGATAAAATCCACTACCGCGAAGAAATTACTGACGGTTTAGAGAATGCGCCACAGACGTT
TATCGGCCTGCTGAAGGGTAAAAACTTCGGCAAAGTGGTGATCCGCGTGGCGGGTGATGATTAAAGTTTCAACGGCGGCG
TAAGCCGCCATAAAAATCCAGGCAAAAAGCTTATTTTCAGCTTTAATTAACTAACCACATCATTGTTCTGCTGCTAATAA
CAACAAAGGTGAGTGGTTTATGATTATTCACTTTAATACACCAGGTGAATTCCTTCTGCCATGCAGGCAGGGTTGGACAG
AAAACACGCGAAGGAATTAAAATGCCTGGAACGGGAAAAATGAAACATGTCAGTTTGACTCTGCAGGTTGAGAACGACCT
GAAACATCAGCTTAGTATTGGCGCATTAAAACCTGGCGCACGCCTGATTACTAAAAATCTGGCGGAGCAATTAGGTATGA
GTATTACACCTGTGCGTGAAGCATTATTACGTCTGGTTTCGGTGAATGCGCTTTCTGTCGCACCTGCACAAGCATTTACA
GTTCCGGAAGTGGGGAAACGTCAATTAGACGAAATCAATCGGATCCGCTACGAACTGGAATTAATGGCAGTTGCTCTGGC
TGTTGAAAACCTCACCCCGCAAGACCTTGCGGAACTCCAGGAATTGCTCGAGAAATTACAGCAGGCGCAAGAAAAGGGCG
ACATGGAACAAATCATTAATGTAAACAGGCTATTTCGCTTAGCGATTTATCATCGCTCAAATATGCCCATCCTGTGTGAG
ATGATTGAGCAACTGTGGGTCAGGATGGGGCCTGGTTTACATTATCTCTATGAAGCGATTAATCCAGCGGAATTACGGGA
GCATATAGAAAACTATCATCTGTTACTCGCTGCGTTAAAAGCAAAAGACAAAGAGGGATGCAGACATTGTCTTGCTGAAA
TTATGCAACAAAATATTGCTATTTTATACCAGCAATACAATCGTTAAAGTAAATGCCCGGCAAATACCGGGCATCAAGGT
AATTACTCAAATCTCCACGCAATATTCATGCCGACACCATAATTTCGTCCGGGCGAAGGTTCGTAATATCGCCCGTTTGA
CTCATTGACAATGACAGAACCAACGTATTCTTTATCGAATAAATTATCGACACGACCAAATAAATCCACAGTTAAATTGT
GGTAATTGTATTTATACCCGGTGAATAAGCCGACGAGAGTATAAGACGGCGCTTTTGCCGTATTTTCATCATCTGCCATA
ATATCGCCCATATAACGCGCTTCCGTGCCTGCATACCAACCATCTTCCGGTACATAACCTATCGACGCAAAGCCCATATT
ACGGGCGATCCCTGGCATCCGATTACCGTTACAATCCTGTTCATTGCAAACATTGCTGCGATAGGTCGCATCAAGCCAGG
TCCAGGACGCGTTTACGCGAAAATCTCCTGCGAAACGTTGATCCCATGCCAGTTCAGCGCCTTGACGACGGGTCTTTCCG
GCATTTTTGTAAGTCGTACGCCCACCGCTACTGCTATCGACAACAATTTCATCATCAGTGTCGGTCTGAAACAATGCGAG
ACTAAGCAGCCCATCACCAATACGCGTTTTACTGCCGATCTCAATTGTATCGTTGGTGGATGGTTTTAAACCTAAGTTCA
TACCGCTTTGCCCATCAGCACGATAAGACAGCTCATTAATCGTCGGCGTTTCAAAACCTCGCCCGGCTGCCAGATAGATA
TTCCAGGCATCGGTCATTGCATATTTTAACGAACCGGCAGGTAGCCATTTATGATAACTGGCATCACCGCTGTCATCGCC
GTTACCCGGAGTAACGTAATGGTCGTTGGAATCAAACCACACGGAGCTGTAGCGCACGCCAGCATCCAGCGACAGTTTTT
CGCTCAGCTGCCACTGCGTCTGTAAATAGGGATCGATGTTCCACATCAGATTGCGTTCGTCGCGACGCAACTCACCTTTT
TGCCCGTACTCCGGCATGCCGCTATTCAGGCGGAAGTTATTGTAGCCCTTGCGGTTTTCACTCATGTTTTCGTAGTTCAG
GCCGGTAGTGAACGTGACCGGAACGCCCAGTTCACCACGGTGTGTCCAGCGGCTGTCTATTCCCTGGTAATGGCGTTGCA
GGGTAATCACGCCGCCCGCATGTGACGGGTTAAGTTGTGGTGCCATGGGTATTGACTGGTACTGGGTCGTTTCTCGCTCT
CCGGCATACATCATCACACTCATATCATCCCGCGAACTCAGGCTACGCTCATAGCGCAACCCAGCCTGAGTTTGCTTGAT
GGTTTTTCGCGTGTCGTACTGTTCTGCACGAGGCGCTTGTTGTGGATTAGCCTTCCATTCTGCTTTGGTTAGCCCACCTG
GGTCATCTGCTTTGATATCCACACTATTGAAAATCAGACTTAATTTGCTGGCTTCATCAATGCGTACGCCCAGTTTGGCA
TTGGCTAAATTTTTCTGTGCGCCACTATGGTCACGATAGCCGTGGGTCGTAAAACGCGTGGTTGAGACGGTGTAATCGAC
ATCGCCAGGCTGTGTGCCGTCTCCCGTTGCGCCCGTTGCTTTCAGCCCATAGCGCCAGCTGCCAAAACTGCCGTAGTAAC
TACTGGCTTCAATGGTTGGTGGCTGTTGTCCGGTCTGGGTGGTGACATTCATTACCCCACCAGACGCGTTGCCATACAGG
GCAGAGAAGGGGCCACGCAGCACTTCCACATTTTGCACACTGCTTAAATCGATGTTGGATGTTTGCCCTTGCCCGTCGGG
CATGGTGGCGGGAATACCGTCCACATACAGGCGAATACCGCGAATACCGTAAGTGGAGCGGGAGCCAAATCCGCGAATCG
ACAGCTGTAAATCTTGCGCATAGTTCTGCCGGTTTTGTACCTGCAAACCAGGCACGCCGGTCAGTGATTCGGACAAGTTA
ATGCGCGGTGTTGCCAGGCGCATCTCCTCGCCATCCACCACGCTTACTGCTGCTGGGGTATCCAGTTCTGAAACCACCTG
CGGTGCGGCACTGACAATCATAGTCTGTTCATCAGCGGCAAAAACAACGGGGGAAAGGACAAGCAGTGCGGGCAAAACGG
TCTGTCGGACGGAAAAAATCTTCATGAAAAAAGCCAGGTTAAGAATGGGAAAACGCCGTCATGGTAATGAAATTGTAAAT

TTATGGAAAATGAAACGGCACAATACGTTAAGTAATTGAGAAAATTGTAGTCGTAACGGCAAGAAATGCTCCACATTTGA
GAAAATAATGATTACCATTCCCATTTATAACAAGAGCGTAACGATGATTACGCTTAGCGAAGCATTGTGAAGCAGCAAAA
ATATCGGTTCATCAAAGGGAGTCGTCATGCATTTACGTCATCTGTTTTCATCGCGCCTGCGTGGTTCATTACTGTTAGGT
TCATTGCTTGTTGTTTCATCATTCAGTACGCAGGCCGCAGAAGAAATGCTGCGTAAAGCGGTAGGTAAAGGTGCCTACGA
AATGGCTTATAGCCAGCAAGAAAACGCGCTGTGGCTCGCCACTTCGCAAAGCCGCAAACTGGATAAAGGTGGCGTGGTTT
ATCGTCTTGATCCGGTCACTCTGGAAGTGACGCAGGCGATCCATAACGATCTCAAGCCGTTTGGTGCCACCATCAATAAC
ACGACTCAGACGTTGTGGTTTGGTAACACCGTAAACAGCGCGGTCACGGCGATAGATGCCAAAACGGGCGAAGTGAAAGG
CCGTCTGGTGCTGGATGATCGTAAGCGCACGGAAGAGGTGCGCCCGCTGCAACCGCGTGAGCTGGTAGCTGACGATGCCA
CGAACACCGTTTACATCAGTGGTATTGGTAAAGAGAGCGTGATTTGGGTCGTTGATGGCGGGAATATCAAACTGAAAACC
GCCATCCAGAACACCGGTAAAATGAGTACCGGTCTGGCGCTGGATAGCGAAGGCAAACGTCTTTACACCACTAACGCTGA
CGGCGAATTGATTACCATCGACACCGCCGACAATAAAATCCTCAGCCGTAAAAAGCTGCTGGATGACGGCAAAGAGCACT
TCTTTATCAACATTAGCCTTGATACCGCCAGGCAGCGTGCATTTATCACCGATTCTAAAGCCGCAGAAGTGTTAGTGGTC
GATACCCGTAATGGCAATATTCTGGCGAAGGTTGCGGCACCGGAATCACTGGCTGTGCTGTTTAACCCCGCGCGTAATGA
AGCCTACGTAACGCATCGTCAGGCAGGTAAAGTCAGTGTGATTGACGCGAAAAGCTATAAAGTGGTGAAAACGTTCGATA
CGCCGACTCATCCAAACAGCCTGGCGCTGTCTGCCGATGGCAAAACGCTGTATGTCAGTGTGAAACAAAAATCCACTAAA
CAGCAGGAAGCTACCCAGCCAGACGATGTGATTCGTATTGCGCTGTAATAGATATGGCGAGGGTGAAAACGGTGAGACTT
GCCTGCCTTTTATCACGGCGTATCCGGCACTTTCGTCAGTAATCTGAATTTGCCCGGCACGTAGCCGGGCAATTTTGCAA
TTAACTCGCCGTTTCAGGCTTAAACACAATTTCCTGTTTTTCTTCATCTTCCTCGACGACTGGCGCAGTGCTGTGAATTT
CAGCAACGCGTTTGCGCACACCAAACCAGCCTATAACCAGCAAAATACCGATAATCGGCAGCGCCGCGATAGTATAAGTC
CCGTTCGGGTAATCGAACGCCATCAGCACAAGGACACTCAGTAAAAACAGTAATGTCAGCCAGGAAGTGAAGGGCGCGCC
AGGCAGTTTAAAACTGACATCCGCTGCTTTGCCTTCTTTAATCGCTTTACGCAGGCGCATCTGGCACACGATGATAAACG
CCCATGAAGCGATGATTCCCAGCGACGCGAAGTTCAACACAATCTCAAATACGCGCGACGGCACCAGATAGTTGAGGAAT
ACGCCGACGACATACACAACTAGTGTCGCCAGAATCCCGGCATACGGCACATGCTGACGACTCATTTTCGCCATAAAACT
CGGTGCGGAACCGCCCATCGCCATTGAGCGCAGAATACGTCCGGTGCAGTACAGACCTGAATTCAGGCTGGAGAGGGCAG
CGGTCAGCACCACAATGTTCATAATGCTGCCGATATATGGCACACCCAGTTTAGAGAAAAACGTCACGAACGGACTTTGC
CCCGCCTGATACGCGCTCCACGGCAATAACATAACCAGCAACACCACGGAGCCGACGTAAAACAGGCCAATACGCCAAAT
CACACTGTTAATGGCTTTAGGCACCATGGTCTGCGGATCTTTACATTCTCCGGCAGCTGTACCCACCATTTCAATGGAGG
CAAAAGCAAACACTACGCCCTGAATCAACACCAGCGCAGGCAGCAAACCGTGGGGGAAGAAGCCGCCATTATCGGTGATT
AAATGAAAGCCAGTGGTGTTGCCATCCAGCGGCTGACCACTACCGAGGAACACTGTACCCACGACCAAAAAGGTCACAAT
GGCGAGCACTTTAATAAGCGCAAACCAGAACTCCATCTCCGCAAACCATTTCACACCGATCATATTCATGGTGCCAACGA
TGGTAAGTGCAGCGAGCGCAAAGACCCACTGCGGCACGCCGCCAAACGCACCCCAGTAATGCATATACAGAGCGACGGCG
GTAATATCAACAATCCCCGTCATCGCCCAGTTGATGAAGTACATCCAGCCAGCAACATAAGCGGCTTTCTCACCCAAAAA
CTCACGGGCATAAGAAACAAAACTGCCACTGGAAGGGCGGTGTAGCACCAGCTCACCCAATGCACGCAGAATAAAAAACG
AAAACAAGCCACAAATTAAATAAACCAGTGCCAGTGCGGGCCCCGCCATTTGCAGTCGGGCTCCTGCACCTAAAAACAAG
CCGGTGCCAATCGCGCCGCCAATGGCGATCATCTGCACCTGGCGATTGCCCATCGCTTTGTGATACCCCTCTTCGTGGGC
ATTAAGCCAGCGGCGTTTCGCGGCGTGTTGATCTGAAGTGTCGGTGTCGTGTTTACTCATTGCTCTCCCTGATTGCTTTA
ATGAAAAAGTCATATAAGTTGCCATGAACAATGTTTATTCATCCTGGCGATGATAGTTATCACCATTATTCTTTATGGGT
ATAAAACTATCGGCGCAGCATCCTACCCGTTATCGATAAACGATGCAAAACATCCCCTTACAATCCTGAAGGGGATTAAT
ACAACTGACGAAAAAATGACAAATCCTTTTGCTGGTTAACCTGTGTACTGTCCTACACTTAATCTTTAAAAGATTGTGAG
GGGCATATGATTAAGGTGTATGGCGTACCCGGATGGGGCTCTACAATCAGTGAGCTGATGCTAACACTGGCTGATATTCC
TTATCAGTTCGTTGATGTCAGTGGTTTTGACCACGAGGGAGCTTCACGCGAGTTATTGAAAACCCTGAACCCGTTGTGTC
AGGTTCCGACACTGGCATTGGAAAATGACGAGATTATGACGGAGACAGCGGCGATTGCATTGATGGTTCTCGATCGTCGC
CCGGACCTTGCGCCACCGGTTGGGCGCGCCGAACGTCAGCTGTTTCAACGGCTATTGGTCTGGCTGGTTGCTAATGTCTA
TCCAACATTCACTTTCGCCGATTACCCTGAGCGTTGGGCTCCTGACGCACCTGAACAGTTAAAGAAGAATGTTATTGAAT
ATCGAAAATCGCTTTATATCTGGTTGAATTCGCAGCTCACTGCTGAACCTTATGCGTTTGGTGAGCAACTAACGCTGGTG
GACTGTTATCTTTGCACTATGCGCACATGGGGGCCTGGGCATGAATGGTTTCAGGATAATGCCACGAATATCAGTGCGAT
TGCTGATGCTGTATGTCAGTTACCGAAATTACAAGAAGTATTAAAAAGGAATGAAATTATTTGAGTGGGATACCAGAAGA
TTATGTTATTTATTATTCAGTAACTTTATCCACAATGATGGGCGTAATTAATTAAATTCATGGTATGTTTTTAATTTAT
ATCACTCTCCTTTTCATTCAGCGTGTCTATTTCATTTCCTCTGAAAAGAAACTAACTATTCACATCGTGCAGATGTTTCA
GTTGTTATCACAGGCATTCTATAATCTCAAAATGTTTTTAATGATGGATATGCTCGGAGTTGGAGATGCAATTAATATTA
ATACAAATAAAAATATCCGGCAGGTATGCTAAAAGGATAAATGGCGTTCTCTGTATTTATTCGCAATTATCTGTAGGACA
AATATGGAGAGCTTGATGAGTCAGAGCTAAAACAGCAATTGATTAATAAACATGTTGATTTATTCAAGAAAATGGATGTA
TTTTAAATGTTGATTCTTAATATAACCTAATCAATAGATTATCAAGTGAGAGCTACACATTATTGGAGGGGCAGGGGAAA
ACACCATTTTGGGAACAATGCATCAGGTACTCTTACCACTGAAGCAAGATCATCCGGCACCAGGTGGTGGTGCAGTGCTT
TGGAAATGGTCCCCTCGGGGGAGTTGTCATTATTTTCTATGCTATTTGCTTATTATATTCATCAGGTGAATTTAATAAAT
TCCTAATTTGAAATATCCACTATTAAGCTAGTGTTTAACGACGTTAAGATGGCAATGTGCAGATCATGTTATCAGGGTGG

GGCGGTAGCGTATATTCCTTATACAAACCATCATTCATTTCAATGATTGTTATTGAATTATTTATTGTTGTGAATAAAAA
GACATTATGAATATTGGTTTGACTTTGCATATCAGGTTTTTTATTACTCGCTGTAATGTACGAGCCTGTCGTGAATAACG
TCGAGAACCCATCCGTTGCGTTTGTGATTATTTTGTTGACTAAACAGACACCCGTTTCTCTGAAGTAAAATCCCAGACTA
AATCATCACATAACCATGACATTTTTCTGATATTCCCCGGTAACGCCAGATGTCGACTCGCTTAACCACCCTCAGCCATA
CCAGTGAAGGCCACCGCGTGTCGGTCCACTACGGCTATGACGATAAAGGCCGCCTGACCGGCGAACGCCAGACGGTGGAG
AACCCGGAGACGGGGGAACTGCTGTGGCACCATGAGACCGGACATGCGTACAACGAACAGGGGCTGGCAAACCGCGTCAC
GCCGGACAGTCTGCCGCCGGTGGAGTGGCTGACCTACGGCAGCGGCTATCTTGCGGGCATGAAGCTGGGCGGGACGCCGC
TGCTGGAGTTCACGCGCGACCGCCTGCACCGTGAGACGGTGCGCAGCTTCGGCAGCATGGCAGGCAGTAATGCCGCATAT
AAACTGACCAGCACATACACCCCCGCAGGCCAGTTACAGAGCCAGCACCTGAACAGCCTGGTGTATGACCGTGACTACGG
GTGGAATGACAACGGCGACCTGGTGCGCATCAGCGGCCCGCGACAGACGCGGGAATACGGCTACAGCGCCACGGGCAGGC
TGGAGAGTGTGCGCACCCTCGCACCAGACCTGGACATCCGCATCCCGTATGCCACGGACCCGGCGGGCAACCGGCTGCCG
GACCCGGAGCTGCACCCGGACAGTACACTCACAGTGTGGCCGGATAACCGCATCGCGGAGGATGCGCACTATGTCTACCG
CCACGATGAATACGGCAGGCTGACGGAGAAGACGGACCGCATCCCGGCGGGTGTGATACGGACGGACGACGAGCGGACCC
ACCACTACCACTACGACAGCCAGCACCGCCTGGTGTTCTACACGCGGATACAGCATGGCGAGCCACTGGTCGAGAGCCGC
TACCTCTACGACCCGCTGGGACGGCGAATGGCAAAACGGGTCTGGCGGCGGGAGCGTGACCTGACGGGGTGGATGTCGCT
GTCGCGTAAACCGGAGGTGACGTGGTATGGCTGGGACGGAGACAGGCTGACGACGGTGCAGACTGACACCACACGTATCC
AGACGGTATACGAGCCGGGAAGCTTCACGCCGCTCATCCGGGTCGAGACAGAGAACGGCGAGCGGGAAAAAGCGCAGCGG
CGCAGCCTGGCAGAGACGCTCCAGCAGGAAGGGAGTGAGAACGGCCACGGCGTGGTGTTCCCGGCTGAACTGGTGCGGCT
GCTGGACAGGCTGGAGGAAGAAATCCGGGCAGACCGCGTGAGCAGTGAAAGCCGGGCGTGGCTTGCGCAGTGCGGGCTGA
CGGTGGAGCAACTGGCCAGACAGGTGGAGCCGGAATACACACCGGCGCGAAAAGTTCATTTTTACCACTGCGACCACCGG
GGCCTGCCGCTGGCGCTCATCAGCGAAGACGGCAATACGGCGTGGCGCGGGGAGTATGATGAATGGGGCAACCAGCTTAA
TGAGGAGAACCCGCATCACCTGCACCAGCCGTACCGTCTGCCAGGGCAGCAGCATGATGAGGAGTCGGGGCTGTACTATA
ACCGTCACCGGCACTACGATCCGTTGCAGGGGCGGTATATCACCCCGGACCCGATTGGGTTGAGAGGTGGATGGAATATG
TATCAGTATCCGTTGAATCCCATACAAGTGATAGACCCAATGGGGTTAGATGCGATTGAGAATATGACATCAGGTGGACT
AATTTATGCCGTATCTGGTGTACCTGGATTGATTGCTGCAAACAGCATTACTAACAGTGCTTACCAGTTCGGTTATGATA
TGGATGCTATTGTTGGCGGAGCTCATAATGGGGCCGCCGATGCAATGAGACATTGTTACTTGATGTGTCGAATGACTAAG
ACATTTGGATCAACAATAGCTGACGTGATAGGTAAAAATCATGAGGCGGCAGGGGATAGACAAGGTCAGCCAGCTAAAGA
AAGAATCATGGATCTTAAAAATAACACTGTCGGTATTGCTTGTGGCGATTTTTCTGCCAAATGTAGCGATGCATGTATTG
AAAAATATAACACTGGGCAACTCTTCGGGTTAGATGGTATAAAAGCAGATAATCCAATAAAAGCAAAGCAAGGGAGTTCA
GATGCTTCAAATTATTAGAGGCAAACTTGTCATTTTTTTAATTACCCTTTGTTTATTTGTTGTTTACCTTGGGTTTGATA
ACAATTCAAATTCTGACATCGTATTTTATGGACATAAAACACCAAAGAGCGTTGAGATATATCTTTCTGAAAAAAATATT
ATTTATAAAATAATTAATGACCAAAAAATTAGTAGAGGGAATGGTCATTTTATAAGTATAATGGTTAATAATTACAGGAC
GCACTGTGGAGTGGTTGATATAAATCTTAATTTTTTCAATGATATTCTTTATAGCGTGCGATTAAAAAACATTAGTAAAC
TGGAGAATATGGAGTTCTGCGCTACAAAACAACGGGTGTATTTTAGTGATAAAAACAAGAAGGCTAGTTATAAAATAATT
AATTATGGAGACTATTATGATGTTGATTATTATGATAACAATTTGAAAAATGAAGTTTTTGACTGGATTGGTAAATGGTC
ATAATGAACAACACCAGAAAGAGAAAAATATAAAATACATACAGAAAGACATGAGAGCAAGGGACGGGGCAGGGCAAGAT
CATGAAAATTGTGATGTAAATCACGATTTTCATCTTTGCTTTAACGCCTACAGGTGATCAAAATTTAACCTTTGGAATAA
CTAAAAAGATAAAAAAGGACGCCAGGTGAGTATTCAAAGTTTGCTTGATTATATTTCAGTGACCCCTGATATACGACAAC
AAGGAAAGGTTAAACATAAATTATCTGCTATTTTGTTTCTCACCGTATGTGCAGTAATTGCAGGTGCCGATGAGTGGCAG
GAAATTGAAGATTTTGGACATGAAAGACTTGAATGGCTAAAGAAATATGGTGATTTTGATAATGGCATTCCGGTCGATGA
CACCATTGCACGCGTTGTGAGTAACATTGACAGTTTGGCCTTTGAAAAGATGTTTATTGAATGGATGCAGGAGTGCCATG
AAATCACTGATGGTGAAATTATAGCAATAGATGGAAAGACCATAAGAGGCTCCTTTGATAAGGGAAAAAGAAAAGGAGCA
ATCCATATGGTGAGTGCATTCTCGAACGAAAATGGTGTTGTACTGGGGCAGGTGAAAACGGAAGCCAAAAGTAATGAGAT
TACAGCCATTCCAGAGTTGCTTAACCTACTGTATTTAAAGAAAAATTTGATAACCATTGATGCTATGGGCTGTCAGAAAG
ATATCGCTTCGAAGATCAAAGATAAAAAAGCAGATTATCTTCTGGCAGTAAAAGGCAATCAGGGGAAATTACATCATGCA
TTCGAGGAAAAATTTCCTGTAAATGTGTTTTCTAATTATAAAGGCGATTCGTTTAGTACGCAGGAGATAAGTCATGGAAG
AAAAGAAACACGTTTGCATATTGTCAGTAACGTAACGCCTGAACTTTTGTGATTTTGAATTCGAATGGAAGGGATTAAAA
AAGCTTTGTGTAGCATTGTCATTCAGGCAGAAGAAAGAAGATAAATCAGCAGAAGGTGTAAGCATCCGATATTATATTTC
ATCAAAGGATATGGATGCTAAAGAATTTGCACATGCTATCAGAGCGCACTGGCTGATCGAGCACAGTCTTCATTGGGTGT
TAGATGTAAAAATGAATGAAGATGCCAGCCGGATAAGAAGAGGAAACGCAGCCTAAATAATATCTGGAATAAAGAAGATG
GCACTGAATTTATTAAGAGATTGCAAAGACATTAAGGGTGAAGAGGAAAAGAAAGAAGGCTGCGTTAAACACAGGGAAAG
ATCATCAGAAGTTCACTTTTTGTACTAAACAATTCGCATTTTATGTTTAAAAATTGAGATATTCCTTATTACCTAAAGCT
GTTTTTTATTGCTTACACATGATCAAATACTCCTTACATAATTAAGGAGAACAAAATGGAACTTAAAAAATTGATGGGAC
ATATTTCTATTATCCCCGATTACAGACAAGCCTGGAAAATGGAACATAAGTTATCGGATATTCTACTGTTGACTATTTGT
GCCGTTATTTCTGGTGCAGAAGGCTGGGAAGATATAGAGGATTTTGGGGAAACACATCCCGATTTTTTGAAGCAATATGG
TGATTTTGAAAATGGTATTCCTGTTCACGACACCATTGCCAGAGTTGTATCCTGTATCAGTCCTGCAAAATTTCACGAGT

GCTTTATTAACTGGATGCGTGACTGCCATTCTTCAGATGATAAAGACGTCATTGCAATTGATGGAAAAACGCTCCGGCAT
TCTTATGATAAGAGTCGCCGCAGGGGAGCGATTCATGTCATTAGTGCGTTCTCAACAATGCACAGTCTGGTCATCGGACA
GATCAAGACGGATGAGAAATCTAATGAGATTACAGCTATCCCAGAACTTCTTAACATGCTGGATATTAAAGGAAAAATCA
TCACAACTGATGCGATGGGTTGCCAGAAAGATATTGCAGAGAAGATACAAAAACAGGGAGGTGATTATTTATTCGCGGTA
AAAGGAAACCAGGGGCGGCTAAATAAAGCCTTTGAGGAAAAATTTCCGCTGAAAGAATTAAATAATCCAGCGCATGACAG
TTACGCAATGAGTGAAAAGAGTCACGGCAGAGAAGAAATCCGTCTTCATATTGTTTGCGATGTCCCTGATGAACTTATTG
ATTTCACGTTTGAATGGAAAGGGCTGAAGAAATTATGCGTGGCAGTCTCCTTTCGGTCCATAATAGCAGAACAAAAGAAA
GAGCTCGAAATGACGGTCAGATATTATATCAGTTCTGCTGATTTAACCGCAGAGAAGTTCGCCACAGCAATCCGAAACCA
CTGGCATGTGGAGAATAAGCTGCACTGGCGTCTGGACGTGGTAATGAATGAAGACGACTGCAAAATAAGAAGAGGAAATG
CAGCAGAATTATTTTCAGGGATACGGCACATTGCTATTAATATTTTGACGAATGATAAGGTATTCAAGGCAGGGTTAAGA
CGTAAGATGCGAAAAGCAGCCATGGACAGAAACTACCTGGCGTCAGTCCTTACGGGGAGCGGGCTTTCGTAATCTTGCCC
TGGGCATTATTGTCGTATAAACGCTTTACCGGACACATGACATTCACAATGTGTGTTAATTTTACTGACATCTTTCAAAA
GGAGCGTAATCATGCCGCACATCGACATTAAATGTTTTCCGCGTGAACTGGACGAACAACAAAAAGCAGCACTTGCTGCA
GATATTACCGACGTTATTATTCGTCATCTGAACAGTAAAGACAGTTCGATAAGCATTGCTCTACAGCAGATTCAACCAGA
ATCTTGGCAAGCTATCTGGGATGCCGAAATCGCGCCCCAAATGGAGGCTTTGATAAAGAAACCTGGTTATAGCATGAATG
CTTAACCCGCCGTAACACGCTTGCCGCTGGTAACAAACGTCCCAGCACCTAAATGATGCAACGTATTGAGCTTATCATCA
TCAAACTGCCAGCGGCCTTCGACAAATACCCGTGCGTCTGCCGCTGCTGATACTACTTCGCCAAACAGCGTGTCGTATTC
TTCTTGCGCAGAAGTCGCAGGTAGCAATCGACACTCCATCCACGCCAGACATTTTTCCTCGACCAGAGGCAAACCAAATA
CCGGGCCTCTCACAACCGGAATGCCATAGCAATTAAATTTATCTTCATCACGCCCCGACACACTTCCCACCGCCCACGTC
CAGTTAGTTGCTGCAACGCCCGGGATAACGATGCCAAATTTACCGTTATGTTCAATCAACTCCCTGGTCCATGTCGATTT
ATCTACCACAATCGCCACGCGTGGAGGTTCAAACTCCACCGGCATTGACCAGGCTGCAGCCATAATGTTACGCCGCTGGG
ATTGCTCATCGAAACTGGTGATCAAGACCGTTGGGCCATGATTTAACAGACGGCTGGCATGGTGTAATTCTATGGGGATG
AATCGGCTCATGGTATCGCCTCGCGAAGAAGGTTTTTTTAAGCGTAGTCCGTAACGCAATAAGTAACGAAATTAACGGGA
TTGGCGATTTGCGAACGTGATGCATGTCCGCGATCGCACAAAATAGCCGGTGCGGCGTCTATTCCAGGTTATAAGTTGAG
AAAACCACTAAGGGAAACGCCTGATGACGCCCATTCTGAATCACTATTTTGCCCGTATTAACTGGTCGGGAGCTGCTGCG
GTCAATATTGATACGCTTCGTGCATTGCACCTGAAACACAATTGCACCATTCCGTTTGAAAACCTCGACGTTTTGCTGCC
GAGGGAAATACAGCTTGATAATCAATCGCCGGAAGAGAAACTGGTGATAGCCCGTCGTGGCGGTTACTGTTTTGAGCAGA
ATGGCGTGTTTGAGCGGGTGTTACGCGAGCTGGGGTTTAACGTTCGCAGCTTGTTAGGGCGCGTAGTGTTATCAAATCCG
CCAGCATTACCGCCGCGCACCCATCGTTTGCTGTTGGTGGAACTGGAAGAGGAAAAATGGATTGCTGATGTCGGTTTCGG
TGGGCAGACGCTAACCGCGCCGATTCGTTTAGTTTCCGATCTCGTGCAGACCACGCCACACGGAGAGTATCGGTTGTTGC
AGGAGGGTGATGATTGGGTGTTGCAGTTTAATCATCATCAGCATTGGCAGTCGATGTACCGTTTTGATCTCTGCGAGCAG
CAACAAAGCGATTATGTGATGGGCAATTTCTGGTCGGCGCACTGGCCGCAGTCGCATTTTCGCCATCATTTGCTGATGTG
CCGCCATTTGCCGGACGGCGGCAAGCTGACACTGACCAATTTTCATTTTACCCATTATGAAAATGGGCACGCGGTGGAGC
AGCGAAATCTACCGGATGTGGCGTCATTATATGCTGTGATGCAAGAACAGTTTGGTCTGGGCGTGGATGATGCGAAACAT
GGCTTTACCGTGGATGAGTTAGCGCTGGTGATGGCGGCGTTTGATACGCACCCGGAGGCGGGAAAATAATTTATGTCAGG
TTGCCGGATGCGGCGTAAACGCCTTATCCGGCATACATTAGCCCGGTTGTAGACCTGACAGGCGAAGCGCATCAGACAAC
GGTTTCAGAGTTCAATTGCCCATTCGGCATGAAACAAAATCACCGCTGTGCCTGAAATGGTGACTTTTTCCGGTTGGTTA
TCACGAATTGTCACCGTCACTTCAATCATACCGTCACGCCCCAATGCGCGCCCCTGATGGCCTTTAACACGCAACACGTT
GCCATCGTGGGGCAATACGTTGTGATGTACCAACCATGCACCCATCGGGCCATTGGCATTGCCAGTCACCGGATCTTCCA
CAATACCAATCGCAGGCGAGAACATGCGACCATCGGTTTCGTTTTTGCCTGGGCGGATCTGGAACGGGAAGAAACCATTG
CAGCCAATCTTTTTGCTGATAGCGGTCAGCGCATTAAGGTCAGGCGAAAGGGCGTCGATATCCACTTCCGGTTTCAGTGG
GATCATCACTTTTGAGTGTCCTGTTGTTGCCACCTGAATTGGCAAGCCTGGCAGAATATCGTCCTCAGTGAGATGAAGCG
CGTTGATAATCGCCGCACGTGTTTCACCTTCCAGCGGTGGCTCAAAGCCCGGCGTACCTTGTTCCAGCGAAATACGATAA
TCATCGTTGTGCTTTTCGATAGTCACGCGATGTTTTCCTGCCAGCGATGTTTGCCAGATCGTGCAATTTCCTAAACCTAA
CACCTTCGCACGTACATAGTGCGCAGCTACTGTCGCGTGACCGCAAATCGGCACTTCAACCGTTGGCGTAAAGTAGCGGA
TGCGCACATCGCTGTCGTCGCTGTGCAGCAGAAAAGCAGTTTCCGAATGGCCTAACTCGCGGGCGATAAGCTGCATTTGC
GCTTCGCTAAGATTATCGGCGGGGAAAACAACACCGGCAGAATTGCCGCGAAACGGTTGTGAGGTAAAAGCATCGACGTG
GTACACCTGCGGTTTCATTAACGTTCTCCTGTGACTGGAGAACTATCATAGCCTGCAAGTGGCCGGAGAGCGAAGGGCTA
TCCGGCCAGGGTGAAATTATCGCCGCGAACGCACAATTTGATATCGACGAGTAAAGTACTCAAACGGCGCGCTCCACACA
TGCACCAGTCGGGTGAACGGGAAGAGCAGGAAGATGGTCATTCCCAACACCAGATGCAGGCGGAAGACGAACGCTACGCC
GTTGAGCATCTCTGACGATCCACCACGGAAAGTGACAATGCTTTGCGCCCAACCCACCAGCTTCATCATTTCGCTACCAT
CAGGATACTGTGCTGAAAACGGTATGGTACTTAGCCCCAACAGACACTGGATCAGCAAAATGCTCATGATGATGATATCC
GGCGTAGTGGAAGTGGCACGCACGCGCTGATTTGTCAGCCTGCGCCACAGCAGTCCTGCGCCGCCAATCAACGTCAACAC
ACCGCAAATACCGCCGAGCACCATCGCCATGAGTTGTTTCGCTGCCACTGGCAAAAACCACGCGTACATCCAGTGCGGCG
TTAACATGCCGAACAAGTGCCCGAAGAAAATCCCCAAAATGCCGATATGGAACAGATTCGACCATATCACCATCCCGCGT
TTATCGAGCATTTGACTTGAGGAGGCGCGCCAGGTGTACTGCCCGTAGTCGTAGCGCAGCCAGCTGCCGAGGAAAAACAC

CGTCGCACAAATGTACGGGTAGATATCGTAAAAAAAGACGTTCAGATACTGAATCATTTCCCACCTCCCGCACTGATGTC
GACATATTGCGGCGCGACATCCTGGCTAAAGCGTCGCTGATATTGATTAAGCGGCGAGCTGTCACACGCCGTGGCGTTAT
CTTCAATAAACTTCACCTGTTCCTCTTCCCACACTGCATCAAGCGCCTGGCGGGTGTCATCGCGCTCTTCGCTGTTTACT
TGTTTTGTGACACTGTCACTTGAAAGGGAGCTTCCCGCCAGTTGCAGCAGAGCATCAAACAACGCGTACCACGGTGCCTC
GCGTTGTTTTAAGCGACCGCCAAGCAGGGCGAGGATCGGCGCAACATTGAGCAATCCTTCTTTCGCCTGATCGTCAGGCA
GTACGCTTAAATACTCCAGATACAGCGGTAGATAATCGGGCAGTTCCCGACAATCCAGCTGCAGGCCGACCTTTTCATAC
TCCGCCAGCAGGTCCACCATTGCCTGGCCGCGATCGCGGGACTCGGCATGAACATGTTCGAACAGCAGCAGCGACGTGGT
GCGCCCGCGGTCAAACACTTCGCACCATTCGGCCTGTTTATCCAGCAGCGGCGCGTTAAGCAGGTTGTGGGTGAAATCCG
TAAGCATCGGCGCGTCGCGGCGGATCAACGCCAGCGCGTCCTCCTTGCATTCCCACAACAGCTCGTCCGGATACTCCATC
AACAGGCCGATCACTTTGAGGATCTGCATTATTCGCCCTCCGCTTTGTCGCGCACTTCGGTGATGTTGATGGCATCGATA
CGGCTACTGTTGAACAGGTTGAATTTACTGTCCGAACCGTGGCAACCGTCGCCAAAGGTAAAACCGCAGCCGTTGCGTTC
TGCGAAGGCATCGCCCGCCATTTCCCGATGGCTGGTCGGGATAACAAAACGATCTTCATAGTTGGCAATGGCGAGATAGC
GATACATCTCTTCGACCTGGGCGACGCTCAGGCCTACTTCGTCGATGGCACGAGTATCAGTAACGCCTTCCACGGTTTGT
GAACGCATATAGTGGCGCATCGCCATCATCCGTTTCAGCGCCCGCAGTACCGGACCGGTATCGCCGGCACTCAACATATT
GGCGAGATATTGCACCGGAATACGCAGGCTTTCGATGGCGGGCAGCACGCCTTCGCTTTTCGGCAAACCGCCCGCATCTG
CGTAGGACTGAATCGGTGACAGCGGAGGAACGTACCAGACCATCGGCAGGGTGCGGTATTCAGGGTGCAACGGTAGTGCC
AGTTTCCAGTCCATCGCCATTTTGTAGACTGGCGAACGCTGGGCAGCGTCAATCACATTTTGTGGAATACCTTGTTTCAG
GGCTTCCTCGATCACTGAGGGATCGTGTGGATCGAGGAACACTTCGCACTGGCGTTCATAGAGGTCAACTTCGCGCTCGG
TGCTCGCCGCTTCCTCGATGCGGTCGGCGTCGTAAAGCAGCACGCCCAGATACCGGATGCGACCCACGCAGGTTTCTGAG
CACACGGTCGGTTGACCGGACTCAATTCGTGGGTAACAGAAGATGCATTTTTCTGACTTGCCGCTTTTCCAGTTGAAGTA
GATTTTTTTGTACGGACAACCGCTTATGCACAAACGCCAGCCACGGCATTTATCCTGATCAATCAGCACAATGCCGTCTT
CTTCGCGTTTGTAGATAGCGCCGCTTGGGCAGGTCGCCACGCAGCTGGGATTGAGGCAGTGTTCGCACAGGCGCGGCAGG
TACATCATGAAGGTGTTTTCAAACTGCCCGTACATCTCCTTTTGCATGGCCTCGAAGTTGCGGTCGCGGGCACGTTTTTC
GAACTCACCGCCCAGCAGTTCTTCCCAGTTTGGCCCCCAGATCACTTTGTCCATCCGTTTGCCGTCAATCAGTGAACGCG
GGCGAGCAGTAGGAATATGTTTGCCTTCCGGTGCGCTATGCAAATGTTCGTAGTCGAAGGTGAAAGGTTCGTAGTAATCA
TCAATCTGCGGCACCACCGGGTTGGCGAAGATTTTGGTTATTACGCCCATCTTGTTGCCCAGACGCGGGCGTATCTTGCC
ATTCACATCACGCACCCAGCCGCCTTGCCACTCTTCCTGATCTTCCCAGTTTTTCGGATAACCAATGCCCGGTTTGGTTT
CGACGTTGTTAAACCATGCGTACTCCATGCCTTCGCGCCCGGTCCAGACGTTTTTACAGGTCACCGAACAGGTATGGCAG
CCGATACATTTGTCGAGGTTAAGCACCATGCCGACTTGTGAACGGATTTTCATTTTTTCGCCTCCTGTACCTGATCGCGA
CCTTCATCATCCAGCCAGTTAACGTTCTTCATCTTGCGGATCATGATGAACTCATCGCGGTTCGATCCGACGGTGCCGTA
GTAGTTAAAGCCCCAGGCCAGCTGCGCGTAACCGCCAATCATATGCGTTGGTTTCGGGCAAACGCGGGTAACCGAGTTAT
GAATGCCGCCGCGCATGCCAGTTACTTCCGAACCAGGAATATTCATAATGCGTTCCTGGGCGTGATACATCATGGTCATG
CCCGGCGGTACACGTTGGCTGACCACCGCGCGGGCAGTCAGCGCGCCGTTGGCGTTGAATACTTCCACCCAGTCGTTATC
GACAATGGTCAGTTCTCGGGCATCTGTTTCGCTGATCCAGACAATCGGTCCACCGCGAGAGAGCGTCAGCATTAGCAGGT
TTTCACTGTAGGTTGAGTGAATGCCCCATTTCTGGTGCGGCGTCAGGAAGTTAAGTGCTTTTTCCGGGAAGCCGTTTGGC
GGTATCTGGCGCATCTCACTGACGCTACGGGTGTCGATAGGCGGGCGATAAGCCACCAGGCTTTCACCAAAAGCACGCAT
CCACGGATGATCCTGATAGAGCTGCTGGCGTCCCGACAGCGTGCGCCACGGAATTAACTCATGAACGTTGGTGTATCCCG
CGTTATAGGAGACGTGATCGCTTTCCAGACCGGACCAGGTGGGGCTGGAGATAATTTTACGCGGCTGCGCCTGAATATCG
CGAAAGCGAATCTTCTCGTCCTCTTTGTGCAGCGCCAGATGGGTATGTTCGCGCCCGGTGATCTCGCCCAGCGCCTGCCA
CGCTTTGACTGCAACATGACCGTTGGTTTCCGGTGCCAGTGCCAGAATCACTTCTGAAGCGTCAATGGCGGTGTCAATCA
GCGGACGCCCCTGTGCCGGGCCATCACGCTTGGTGTAATTGAGTTTGCCGAGGAAATCGATTTCATCCTGGGTATTCCAC
GAAATCCCTTTACCGCCATTGCCAAGTTTGTCCATCAATGGCCCGAGCGAGGTAAAGCGTTCATACGTAGCAGGGTAGTC
GCGCTCCACCGCCACAATATTCGGCGCGGTTTTGCCTGGAATCAGATCGCATTCGCCTTTGCGCCAGTCGAGCACTTCAC
ACGGCTGTGAGAGCTCTGCCGGAGAGTCATGCAGTAGGGGTTGTAATACCACGTCGGTTTCTTTGCCAAGATGACCCACG
CACACTTGCGAAAATGCTTTGGCGATACCTTTGTAGATTTCCCAGTCGCTGCGTGATTCCCACGCCGGATCGACCGCCGC
AGAAAGCGGATGAATAAACGGATGCATATCCGAGGTGTTCATATCGTCTTTTTCGTACCAGGTGGCGGTGGGCAGAACGA
TATCGGAGAACAGGCAGGTACTGGACATGCGGAAGTCGAGCGTCACCAGCAGGTCGAGCTTGCCTTCAATCGCTGCAGTT
TGCCACTCGACTTCTTCCGGTTTGATCCCGTCGCTGGCACCGAGTTCCTCGCCCTGAATCCCGCTTTCGGTCCCCAGCAG
ATACTTCTGCATATACTCGTGGCCTTTGCCGGAGGAGCCAAGCAGGTTAGAGCGCCAGACAAACAAATTACGCGGATGAT
TGCTGCTGCTATCTGGTTGTTCGCAGGCCATACGTAAATCGCCCGATTTCAGCGCCTGGGCGGTAAATTCTGTGGGGGAT
AATCCGGCCTTGTCGGCTTCAGCTTTAATCCCGAGCGGGTTACGCCCCAGCTGCGGCGCAGAAGGTAGCCAGCCCATACG
TTCGGCGCGAACGTTGAAATCAATCAGATGACCGCTGTACTTACTGGCATCGGCGAGCGGTGAAAGTAACTCCTGAGCAG
AGACTTTTTCATAGCGCCATTGGCTGGAATGATTGTAGAAAAACGAGGTGCTGTTCATTTGGCGCGGTGGTCGGTTCCAG
TCGAGCGCAAAGGCCAGTGGCAACCAGCCGGTTTGTGGGCGCAGTTTTTCCTGGCCGACATAGTGTGCCCAGCCGCCACC
GCTTTGCCCGACACAACCGCAGAAGATCAGCATATTGATCATCCCGCGGTAGTTCATGTCCATGTGATACCAGTGGTTAA
CACCTGCGCCGAGGATAATCATCGAGCGCCCATGCGTTTTATGGGCGGTATCGGCAAATTCACGGGCGATGGTTTCAATA

TACTGGCGCGGCACGCCGGTAATTTGCTCACCCCAGGCTGGGGTGTACGGTTTGATTTCAGCGTAATCTTTCGCACTGTT
TTCATCTTCCAGCCCGCGATCGAGGCCGTAATTCGCCAGTACCAAATCATAAACGCTGACCACCGGACAGGTGTTGCCAT
CGACTAACGTCAGGTTTTTAACGGGCAATTGGCGCACCAGCACCGGATTGTGTTTTACGCTGCGAAAATGCGGATTTTCA
ATGCCGCCAAAGTAGGGGAAGGCCACGCCTGCAACAGCGTCATGTTGACCGAGCAGGGTTAACGACAATTCGGTTTCCGT
ACCGGCGGCAATGGATTCCAGATTCCATTTGCCTTTTTCTCCCCAGCGGAAACCAATCGAACCGTTCGGCACTACCAATT
CACCTGCGGTATTAACTGCTACGGTTTTCCACTGCGGATTGTTGCTTTCGCCCAGTCCATCCACCAGGTCAGATGCGCGG
ATCATGCGCCCGGGAACGTAGCTACCATCGTCGCGAGGCTCCAGCATTACCAGCATCGGCATGTCGCTGTAGCGGCGGCA
GTAGTTGATAAAGTAGTCGCTGGGATTATCGAGATGAAACTCTTTTAAAATCACATGGCCCATTGCCATCGCCAGGGCGC
TATCAGTGCCTTGTTTCGGTGCCAGCCACTGGTCGCACAATTTGGCCACTTCAGAGTAGTCAGGGGTAATGGCGATGGTT
TTAGTGCCTTTGTAGCGTACTTCGGTAAAGAAGTGGGCGTCCGGCGTACGTGTCTGCGGTACGTTAGACCCCCAGGCGAT
GATGTAGCTGGAGTTATACCAGTCGGCAGATTCCGGTACGTCGGTTTGCTCGCCCCAGGTCATCGGCGAGGCGGGCGGCA
GGTCGCAATACCAGTCGTAGAAACTTAAACAGGTGCCGCCAAGCAGCGACAGATAACGCGTTCCGGCGGCGTAAGAAACC
ATCGACATCGCCGGGATCGGCGAGAAACCGGCAACGCGATCCGGGCCGTAGGTTTTGATGGTCCAGACGTTAGCGGCGGC
AATCAGCTGGTTTAGTTCCTGCCAGTTGGAGCGGATAAACCCGCCGCGCCCACGCACTTGTTTGTAGCTCAGGCACTTTT
GCGGATCGTTCATAATCGATGCCCACGCCAGTACCGGATCGCTGTGTTGCTTGAGGGCTTCGCGCCACAGTTCAATCAGT
CGTTTACGAATGAGCGGGTATTTCAGGCGGTTAGCGCTGTAAAGATACCAGGAGTAACTTGCGCCACGCGGGCAGCCGCG
AGGTTCATGATTGGGCAGGTCAGGGCGAGTGCGCGGGTAGTCGGTCTGTTGGATTTCCCAGGTCACCAGACCATTTTTAA
CGTAGATTTTCCAGCTACAGGAGCCTGTACAGTTAACACCGTGGGTGGAACGCACGATTTTGTCGAACTGCCAACGCTGG
CGATAGCTGTCCTCCCAGTCGCGGTTGCTATGCATCACCTGTCCGTGACCATCGGCAAAGGTTTCGCCCTTTTGTTTGAA
GTAGCGAAAGCGATCCAAAAGTTTACTCATGACATGACTCCTGCTCCAGGAATGAATTGGGATCTGATGCCTTGTCGGAT
GCGACACGGTTGTACATCAGGCATCGATCTCTTATTTTTGGCTGAACTTCCGCCGACCATAAACCAGCCAGGTCAGCAGC
ACACAAACGATGTAGAAGATTAAAAACACTTTCATCGCGCCGACCGGAGAGCCGGTCATATTGAGCGACATGCCAAACGC
CTGCGGAATAAAAAAGCCGCCCACTGCGCCAATGGCTGAGATAAAGCCCAGAGCCGCCGCCGTTTCGGTGACGGCTTCTT
TATGAGCTTGCTCATCACTACCGCCTTTCATCTTTACCCGATAAATGGTTATCTGGCGAAAGATGACGGCGATCATCTGG
AAAGTAGAACCACTTCCCAGACCCGCAGTCAGAAACAGCCCCATAAATACGGCGTAAAAGGCGATGAAATTACCGGAGCC
TGTGCCCGGTAAGGTAAGGAACAGCAGGGCACTGAAAATCGCCATAAAAATAAAGTTGATCAACGTCACCCGCACGCCGC
CGAACTTATCGGAAATAGCACCACCAACCGACCGCGCGATGGCACCGATAAATGGGCCAAAGAACGCCAGGCGCAGAATA
TTCACATCCGGGAACTGGGTTTTTGCCAGCATGGCAAAACCCGCAGAAAAACCGATAAACGAACCGAAGGTGGCAAGGTA
AAGCAGGCTCAGCAGCCAGAGATGCAGGCGTTGTAAGACAGGGAGCTGGTCGGCAATTGAGGCGCGTGAACTGGCGATAT
CATTCATCCCTGACCATGCGGCGATCGTGGCAATCGCCAGTAGCGGTACCCAAATCCATGCGGCATTCGCCAGCGACATC
ACCGAACCGTCGGCCTGCGGTACGCCATTGACGCCGAGAAAGGCAAATACAGGTACAAAAATGACCAGCGGTGCAACCAG
CTGCATTACACTTACACCTAAGTTTCCTAATCCGCCATTAATCCCAAGAGCGCTCCCTTGTTTGGCTTTTGGAAAGAAGA
AACTGATATTGCCCATGCTCGAAGCAAAGTTTGCACCTGCAAAACCGCATAGCAAAGCGATAACGATAAATATCCCAAAA
GGAGTATTCGGATTTTGCACGGCAATTCCGAGCCAGACGCAAGGAATAATCAGGATTGCAGTACTAAAAACCGTCCATCG
GCGTCCACCGAATATAGGCACCATAAAGGAGTAGGGAACACGCAATAATGCGCCAGAAACGGAGGGTAATGCGGTTAATA
AAAAGAGTTGATCGGTAGTAAAATTAAAACCGATTTTATTGAGATTAACGGTAACTGCGCTAAATAGCATCCAGACACAG
AAGGCAAGAAGTAGACAACTGACTGATATCCAGAGATTTCTTCGAGCAATATGTTTTCCTTTATTTTCCCAGAAGGCCGG
ATTTTCTGGTTTCCAGTCGCGCAAAAGATAACGACTATTTTTCTCATTTTGCAGTGCCATATTGTTCCTCACATGCACAC
ATTGGTAATGAAAAAAAGACAAAACACGAGGTAAGGCGCAATAGCCAGTTATTAGAATTAAGGATGAATTGGGTGAAGTG
CTGATTGAAAGAATAGATAAGAAAGCGTAACCGCGGGGGCAGAATGGGGATTAAGTAGCCAGATATGTGTTACTAAATGT
AACTAACAAGCTAACCTTCAAACGGGGTTAATCTTTGAACGTATGTCATGTTTCAACCCTTCAGATCGTGAATCTAAAGG
GTTACATATTAACTATATTATCAACAAGACCAGACACTTATAGAATTAGTGATGATTTATTCACTAAAAAGGCCATGAAC
TGGAGGAAAACGATCTGTCCAGAAGGTCTATCGGTTCCTCTCTTATTGCCTGATTGTAATAATCGTTATTACAGTAAGTA
TTGTTTATTTCATCTAAGGTCATGTTTTTAACTTTTATATTCATCTGGTTTACAACTTCCTCTGTCTTGTAAGTGTGTAT
ATTATATGGGGTATATGCTTTTTTAATAGGATAGTGAAACAATTCGTTATCCACATCTATTCCGTCAGAGTGAACTTCAA
ATGCACCAGGCGAAAGAATTGCCAGGCCAGTATTAAGCTTAAGTGCTGGGGAAAACAAATTAAATAACATGAGCTGCATA
GTAGGGCAATTGTTCTTCCCAGAAACATTTAAAAAATTTAAGTTTTGCTCTGTTTTATTATGGGGAATAAAGTTCCAATA
TCGGATGTATTATTAGTATCAATGTGGGTTATTTTATTATTGTCCAGTCTTAAACTTTTGATAGATGGAAGATAAGTCAG
GAAATCAAGATTTATATATTTCAACTGGTTGTTAGCAGCGTTTAATGTCTGAACATTGCAGAAGTTGCTATAGAATATTT
TAATTTCAGAAAGCAAATTGTTGTTAATAAGTAACGTATTCAAGTTGGGAAATAAGTCATTGTGTAAACTTGTTAGTTTA
TTATGGGATAGATCCAGCAGTAAGAGTTCATTTTTATTACCTGCAACAATATCAGTTAATTGATTATGGCTGAGATTCAG
GTATTGCAGCCATTCGCAAGATTCTAATTGCACAAACTCTAGTTGATTATGTGCCGCACTAAAATAAGTAACTGATGAAA
GCCTATCTATATCAATATTTCTGAGAGCATTATTATTCATAGATATATGTGTAATACTTACATTTCTGCCTTGAGGAAAA
TTTATTGACTCAAGAGCATTACTATTCAGACTTAGCTTTACGAGTCTATCGTAGTTGGTTGCATCAAAATGTGTGAATTG
GTTATTATCAAGATTGATAGATGTGAAGTTTTGCAGCCCTGGTGGTTGAGTGGTTAAATTAAGTCCTGATAAATCAATGG
TCACTTCATTAGTGTCATCAATTATATTTCTTACTGCAGTCCAGTACTGCATTTGTGCAAGTGGATCATTGTTGATGCCG

TTAACTAATACATGCCAAAGATGTTTTGATGATATTAGTTGATCAATGTCATTGGTTGGCAGTAAATTGTTATTGCTTAA
GTTTAAATATTCCAGTTTTGTAGTGTTTTTGGCGTTTAAATGTGCAATATGGTTGTCGTTTAAAGTGATTGTTTTCATCC
TGGGATGATTGTGTAATATAAGGTCAGTGATCATATTGTGATGGGCACTTAACTCAATCAGAGATTCTTGCCGCGCGAGC
CGTTGGCTTCATCGTTATAGCCTTTAAAATCATATTCCCAGTGACCAAAACCAATCATTTGATCATTGATTTGCGTTTCT
CCTTTAAAGCCGAGACGGGCATAAGTTTTATCACCATCGTCACGCTTATCATCAGTAAAATAACGTAGAGCGGTAACCTT
GCCATAAAGGTCGAGTTTATTACCATCCTTGTTATAGACTTCGGCAGCGTGTGCTACGTTCGCAGCTAACAAACCAGTTA
CAACCACCGCAACTATTTTTAATTTCATTTAACAGAATCCTTTTAATTATCGTTAAACGTATTTTCTAAACGAATTTTAA
ACGGCGTCATTTATAAATGACATACTGTTTTTAAAATAGAATGGTGATTAAAAACAATGAGAAAAATAGATGAAATAATA
TTATTTATCGATATGTGATCGAAGTCGAAATGAGATATAAGGTGAATTACTGGTATTTGAAATTTATTTTTTTAATATTG
TCGGAATTTATCTGATTAACTACCGGGCCGTAGACCCGGCAGTTATTTTAACCACGACGTGTCGCCAGCCAGCAGAGCAG
GGAACCGCCGCAGACCATCAGCGCGCCTTGCCAGAACGAGAACGACAGCGGGGCGCTGAGCAGCACGGCTGCAAGCGCTG
AGGAAAGTACAGGCGTAAAATACGAACCTACCGCCATAATGGTGACATTGCCATGCAATATACCGACATTCCATGCAGCA
TAAGCAAATCCTAAGGTAAATGCCGCAGAGATGAGTTTAATCATGACGGGCGTGCTAAATATCATTTCTGGTTGTGGCGT
AAGAAAATAGTAAACCCACAGACTTGCTCCCGTTAGCAGGACAAAAACGGTAATTCCATTAAATCCGCGTGCGTATTTAT
TCGTTACTGTGCAATAGGCTGCCCAGATAAACGCACCAATGAACGCCAGGAAATAACTCAATGGGCTGGTGGTGATATTA
TTGATGATTTCATCATAATGTAACCCATTGTCACCGCCTAACACCCAACAGACGCCGACGAGGGCTAATAATAATCCAGG
TACAATCAACCAGTTGGTTTTCTGACCATTAAACAGAATGGCAAAGAGAATTGTCAGGCTGGGCCACAGATAGTTCACCA
TACCCACTTCAATCGCCTGATGATGGGTCGCCGCATACCCTAAGGAAAGCGCCAGACAGATTTCATAGCTGACGAATAAC
AGACTCCCGGCGAGTAAATAGCCTTTCGGGATTTGCCGAATACGCGGAAATCCAACCGTGAAGATTAACAGCAGCCCGCT
TAATGAATAGATAGCAGCTGCGCCGCCGACCGGGCCGAGCCCCTCACTGACACCGCGAATCAATCCTACCATCGTGCTCC
ACAGGACGATCGCTATCAGCCCTATGAGCGTTGCTTTTTGTCGTGTCATGCTCGCTGTTTTGTCTCTCTTGCCGTTAAAA
ATTAAGCTGAATTTTATAGCATTTTTTTAACTGGCCTGTCAGGCAGTGGTGCGTTTTTCTACCGCTATTGAGGTAGGTCA
ATTTGCGAAGGCGGATTATTTTGTGGCAAACAGATGTTCTTTTTGATTTCGCGCAAAAAGATTCAGAATTTTACTGTTAG
TTTCCTCGCGCAGTAATACCCCTGAAAAAAGAGGAAAGCAATGGACGTCAGTCGCAGACAATTTTTTAAAATCTGCGCGG
GCGGTATGGCTGGAACAACAGTAGCGGCATTGGGCTTTGCCCCGAAGCAAGCACTGGCTCAGGCGCGAAACTACAAATTA
TTACGCGCTAAAGAGATCCGTAACACCTGCACATACTGTTCCGTAGGTTGCGGGCTATTGATGTATAGCCTGGGTGATGG
CGCAAAAAACGCCAGAGAAGCGATTTATCACATTGAAGGTGACCCGGATCATCCGGTAAGCCGTGGTGCGCTGTGCCCGA
AAGGGGCCGGTTTGCTGGATTACGTCAACAGTGAAAACCGTCTGCGCTACCCGGAATATCGTGCGCCAGGTTCTGACAAA
TGGCAGCGCATTAGCTGGGAAGAAGCATTCTCCCGTATTGCGAAGCTGATGAAAGCTGACCGTGACGCTAACTTTATTGA
AAAGAACGAGCAGGGCGTAACGGTAAACCGTTGGCTTTCTACCGGTATGCTGTGTGCCTCCGGTGCCAGCAACGAAACCG
GGATGCTGACCCAGAAATTTGCCCGCTCCCTCGGGATGCTGGCGGTAGACAACCAGGCGCGCGTCTGACACGGACCAACG
GTAGCAAGTCTTGCTCCAACATTTGGTCGCGGTGCGATGACCAACCACTGGGTGGATATCAAAAACGCTAACGTCGTGAT
GGTGATGGGCGGTAACGCTGCTGAAGCGCATCCCGTCGGTTTCCGCTGGGCGATGGAAGCGAAAAACAACAACGACGCAA
CCTTGATCGTTGTCGATCCCCGTTTTACGCGTACCGCTTCTGTGGCGGATATTTACGCGCCTATTCGTTCCGGTACGGAC
ATTACGTTCCTGTCTGGCGTTTTGCGCTACCTGATCGAAAACAACAAAATCAACGCCGAATACGTTAAGCATTACACCAA
CGCCAGCCTGCTGGTGCGTGATGATTTTGCTTTCGAAGACGGTCTGTTCAGCGGCTACGACGCTGAAAAACGTCAATACG
ATAAATCGTCCTGGAACTATCAGCTCGATGAAAACGGCTATGCGAAACGCGATGAAACACTGACTCATCCGCGCTGTGTG
TGGAACCTGCTGAAAGAGCACGTTTCCCGCTACACGCCGGACGTCGTTGAAAACATCTGCGGTACGCCAAAAGCCGACTT
CCTGAAAGTGTGTGAAGTGCTGGCCTCCACCAGCGCACCGGATCGCACAACCACCTTCCTGTACGCGCTGGGCTGGACGC
AGCACACTGTGGGTGCGCAGAACATCCGTACTATGGCGATGATCCAGTTGCTGCTCGGTAACATGGGTATGGCCGGTGGC
GGCGTGAACGCATTGCGTGGTCACTCCAACATTCAGGGCTTGACTGACTTAGGCCTGCTCTCTACCAGCCTGCCAGGTTA
TCTGACGCTGCCGTCAGAAAAACAGGTTGATTTGCAGTCGTATCTGGAAGCGAACACGCCGAAAGCGACGCTGGCTGATC
AGGTGAACTACTGGAGCAACTATCCGAAGTTCTTCGTTAGCCTGATGAAATCTTTCTATGGCGATGCCGCGCAGAAAGAG
AACAACTGGGGCTATGACTGGCTGCCGAAGTGGGACCAGACCTACGACGTCATCAAGTATTTCAACATGATGGATGAAGG
CAAAGTCACCGGTTATTTCTGCCAGGGCTTTAACCCGGTTGCGTCCTTCCCGGACAAAAACAAAGTGGTGAGCTGCCTGA
GCAAGCTGAAGTACATGGTGGTTATCGATCCGCTGGTGACTGAAACCTCTACCTTCTGGCAGAACCACGGTGAGTCGAAC
GATGTCGATCCGGCGTCTATTCAGACTGAAGTATTCCGTCTGCCTTCGACCTGCTTTGCTGAAGAAGATGGTTCTATCGC
TAACTCCGGTCGCTGGTTGCAGTGGCACTGGAAAGGTCAGGACGCGCCGGGCGAAGCGCGTAACGACGGTGAAATTCTGG
CGGGTATCTACCATCATCTGCGCGAGCTGTACCAGTCCGAAGGTGGTAAAGGCGTAGAACCGCTGATGAAGATGAGCTGG
AACTACAAGCAGCCGCACGAACCGCAATCTGACGAAGTGGCTAAAGAGAACAACGGCTACGCGCTGGAAGATCTCTATGA
CGCTAATGGCGTGCTTATTGCGAAGAAAGGTCAGTTGCTGAGTAGCTTTGCGCATCTGCGTGATGACGGTACAACCGCAT
CTTCTTGCTGGATCTACACCGGTAGCTGGACAGAGCAGGGCAACCAGATGGCTAACCGCGATAACTCCGACCCGTCCGGT
CTGGGGAATACGCTGGGATGGGCCTGGGCGTGGCCGCTCAACCGTCGCGTGCTGTACAACCGTGCTTCGGCGGATATCAA
CGGTAAACCGTGGGATCCGAAACGGATGCTGATCCAGTGGAACGGCAGCAAGTGGACGGGTAACGATATTCCTGACTTCG
GCAATGCCGCACCGGGTACGCCAACCGGGCCGTTTATCATGCAGCCGGAAGGGATGGGACGCCTGTTTGCCATCAACAAA
ATGGCGGAAGGTCCGTTCCCGGAACACTACGAGCCGATTGAAACGCCGCTGGGCACTAACCCGCTGCATCCGAACGTGGT

GTCTAACCCGGTTGTTCGTCTGTATGAACAAGACGCGCTGCGGATGGGTAAAAAAGAGCAGTTCCCGTATGTGGGTACGA
CCTATCGTCTGACCGAGCACTTCCACACCTGGACCAAGCACGCATTGCTCAACGCAATTGCTCAGCCGGAACAGTTTGTG
GAAATCAGCGAAACGCTGGCGGCGGCGAAAGGCATTAATAATGGCGATCGTGTCACTGTCTCCAGCAAGCGTGGCTTTAT
CCGCGCGGTGGCTGTGGTAACGCGTCGTCTGAAACCGCTGAATGTAAATGGTCAGCAGGTTGAAACGGTGGGTATTCCAA
TCCACTGGGGCTTTGAGGGTGTCGCGCGTAAAGGTTATATCGCTAACACTCTGACGCCGAATGTCGGTGATGCAAACTCG
CAAACGCCGGAATATAAAGCGTTCTTAGTCAACATCGAGAAGGCGTAAGGGGGCGAACAGATGGCTATGGAAACGCAGGA
CATTATCAAAAGGTCCGCAACTAACTCCATCACGCCGCCTTCTCAGGTGCGTGATTACAAAGCAGAAGTCGCAAAACTTA
TCGACGTTTCCACCTGTATCGGCTGTAAAGCCTGTCAGGTGGCGTGTTCGGAGTGGAACGACATCCGTGATGAAGTGGGG
CACTGCGTCGGGGTTTACGATAACCCCGCCGATCTGAGCGCCAAGTCCTGGACGGTGATGCGCTTTAGCGAAACCGAACA
GAACGGCAAGCTGGAGTGGCTGATCCGTAAAGACGGCTGTATGCACTGTGAAGATCCCGGCTGCCTGAAGGCGTGCCCGT
CTGCTGGTGCAATCATTCAGTACGCTAACGGGATTGTCGATTTCCAGTCGGAAAACTGCATCGGCTGTGGTTACTGCATT
GCCGGGTGTCCGTTTAATATTCCGCGCCTCAACAAAGAGGATAACCGGGTATATAAATGCACGCTCTGCGTCGATCGCGT
CAGCGTCGGCCAGGAACCGGCTTGTGTGAAAACCTGTCCGACCGGGGCTATCCACTTCGGCACCAAGAAGGAGATGCTGG
AGCTGGCGGAACAGCGCGTGGCGAAACTGAAAGCGCGTGGTTACGAACATGCTGGCGTCTACAACCCGGAAGGGGTCGGT
GGTACGCACGTTATGTACGTGCTGCATCACGCCGATCAGCCGGAGCTGTATCACGGTCTGCCGAAAGATCCGAAGATCGA
CACCTCGGTAAGCCTGTGGAAAGGCGCGTTGAAACCGCTGGCAGCGGCTGGCTTTATTGCCACTTTTGCCGGGTTGATTT
TCCACTACATCGGTATTGGCCCGAATAAGGAAGTGGACGATGACGAGGAGGATCATCATGAGTAAGTCGAAAATGATTGT
GCGCACCAAATTTATTGATCGCGCCTGTCACTGGACCGTGGTGATTTGCTTCTTCCTGGTGGCGCTGTCCGGGATTTCGT
TCTTCTTCCCGACGCTGCAATGGCTGACGCAAACCTTCGGTACGCCGCAGATGGGACGCATTTTGCACCCGTTCTTCGGC
ATTGCGATTTTCGTCGCACTGATGTTTATGTTTGTGCGTTTTGTGCATCACAACATCCCGGATAAGAAAGATATTCCGTG
GCTGTTGAACATTGTCGAAGTATTGAAAGGCAATGAGCATAAAGTGGCGGATGTCGGTAAGTACAACGCCGGGCAAAAGA
TGATGTTCTGGTCGATCATGAGCATGATTTTCGTGCTGCTGGTGACCGGGGTGATTATCTGGCGTCCGTACTTTGCGCAG
TACTTCCCGATGCAGGTTGTTCGCTACAGCCTGCTGATCCACGCGGCTGCGGGTATCATCCTGATCCACGCCATCCTGAT
CCATATGTATATGGCATTTTGGGTGAAAGGATCGATTAAAGGGATGATCGAAGGGAAGGTAAGTCGTCGCTGGGCGAAGA
AACACCATCCGCGCTGGTATCGTGAAATCGAGAAGGCAGAAGCGAAAAAAGAGAGTGAAGAAGGGATATAATCTCTTTTG
AACTTTAAGCTGAAAATGGCGCTGTAAAAGGCGCCATTTTCATATTGTAGACAACGTAGGCTTTGTTCATGCCGGATGCG
GCGTGAACGCCTTATCCGGCATGAAAACCCTTCAAATCCAATAGATTGCAGTGAACGTGTAGGCCTGATAAGCGTAGCGC
ATCAGGCAATGTTGCGTTTGTCATCAGTTTCAAATGGCGCTGTAAAAGGCGTCATTTTCATATTGTAGACAACGTAGGCT
TTGTTCATGCCGGATGCGGCGTGAACGCCTTATCCGGCATGAAAACCCTTCAAATCCAATAGATTGCAGTGAACGTGTAG
GCCTGATAAGCGTAGCGCATCAGGCAATGTTGCGTTTGTCATCAGTTCTAAATGGCGCTTTATAAAGTGCCATTTTTTTA
TTGCGTAACCAGACGGCGTAATCGCGACACATCCACTGTTTTTTCTGCCTCTGCCAGACTCCAGGCGTTTTGCAGATTCA
GCCACATTTGCGGCGAACTGCCGATCACCACGGAAAGTTTAATCGCCATTTCTGGCGTCAAAGCTGCTTTTCCTGTCAGC
AATCGGCTTGCCGTTGAGGGAGCAATTTCCATTGCTCTGGCAAACTCGCGCAGGCTGACATTAAGTTCGTCCAGTGATTC
CTGAATAATGTCCCCCGGGCGGGGATGATTGGCCATTTTCATTAGTGTCTTTACTCTCAGTAGCGTTCCTTCACTGGGAA
CACTATCATTATTGTTCCTTCCTACGCAACAATAATCCATTTAGCCATTTCATGTTGGAGAGTGTGCGATGCTGCGACCC
GAACATGGCAGTCGCAGCAAAGGCCTCTTAGTGACGGAAATCAATCACCATGCGGCCACGGATTTTGCCTTCTTCCATCT
CAGTAAAGATGGTGTTGATGTCCGCTAACGGACGCAGGGCGACTTTCGGCACCACTTTACCTTCGGCGGCAAACTGGAAG
GCTTCAGTTAAATCCTGGCGCGTGCCGACCAGCGAACCGACCACTTCAATACCATCCAGCACAAGACGTGGGATATCCAG
GCTCATAGACTCCGGCGGTAGACCGACAGCCACAACACGACCGCCTGCACGGACAGCATCAACTGCCGAGTTAAACGCAG
CTTTAGCTACCGCTGTTACCACCGCAGCGTGAGCGCCACCAGTTTTCTCCTGCACAATTTTGGCGGCGTCTTCGGTGTGT
GAGTTAATCGCTAAATCTGCGCCCATTTCGGTTGCCAGTTTTAACTGCTCATCATTGACATCAATGGCGATCACTTTGGC
GTTAAAGACATTCTTCGCGTATTGCAGGGCGAGGTTACCCAGACCGCCAAGACCGTAGATAGCAATCCACTGCCCTGGAC
GAATTTTTGACAGCTTAACGGCTTTGTAGGTGGTGACTCCCGCACAGGTAATGCTGCTGGCCGCCGCCGAGTCCAGACCA
TCTGGCACTTTTACCGCGTAATCGGCGACCACGATGCACTCTTCCGCCATCCCGCCATCAACGCTGTATCCGGCATTTTT
AACTGAACGGCAGAGCGTTTCGTTACCACTGTTACAGTATTCGCAATGACCGCATCCTTCGTAGAACCACGCCACGCTGG
CACGATCGCCTGGTTTTAATGAGGTGACACCTGGACCCACTTCTGCCACCACACCGATGCCTTCATGGCCCAGAATTACG
CCGGTTTTGTCACCAAAATCGCCATTCTTAACATGAAGATCGGTATGACATACACCACAACACTCCATTTTCAGCAGGGC
TTCGCCATGTTTCAGTGAGCGCAGTGTTTTATACGTAACGTCAACATGATGATCCTTCGTAACAACTGCAGCCTTCATAG
TTCCTCCTTTTCGGATGATGTTCTGCATAGCAGGTGAGGCAAATGAGATTTATTCGCCACTACCCAGTATGGATGAGATC
TGAAAAAGGGAGAGGGAAATAGCCCGGTAGCCTTCACTACCGGGCGCAGGCTTAGATGGAGGTACGGCGGTAGTCGCGGT
ATTCGGCTTGCCAGAAATTATCGTCAATGGCCTGTTGCAGGGCTTCGGCAGAGGTTTTCACCGCCACGCCTTGCTGCTGC
GCCATTTTGCCAACCGCAAACGCAATTGCGCGGGAGACTTTCTGAATATCTTTCAGTTCCGGCAGTACCATACCTTCGCC
GTTCAGCACCAATGGTGAATACTGCGCCAGCGTTTCACTTGCCGACATCAGCATCTCATCGGTGATACGTGACGCGCCGG
AAGCAATAACACCCAGGCCGATGCCCGGGAAAATAAAGGCGTTGTTACACTGGGCGATAGGGTAGATTTTATCTTTCCAT
ACCACTGGATTAAACGGGCTGCCCGTGGCGACCAGCGCGTTACCTTCGGTCCAGGCGATAATGTCCTGCGGTGTGGCTTC
CACGCGTGACGTCGGGTTAGACAGCGGCATCACGATCGGACGCGGACAGTGTTTATGCATCTCACGGATGATCTCTTCCG

TAAACAGCCCGGTCTGTCCTGAGACGCCAATCAGAATATCTGGTTTTACATTGCGCACCACATCCAGCAGTGACAGCACA
TCGCTGTCGGTATCCCAGTCACTGAGGTTTTCGCGCTTCTGCACCAGTTTGGTCTGGAAAGGCAGCAGGTTCGGCATCTT
GTCAGTCAGCAAGCCAAAGCGATCGACCATAAAGACTTTCTGCCGCGCCGCTTCCTCGCTTAATCCTTCGCGCTGGGTCT
GGGAGATGATCATTTCGGCAATGCCGCATCCCGCTGAACCTGCGCCAAGGAAGACGATTTTTTTCTCGCTTAACTGACCA
CCTGCCGCGCGGCTTGCTGCGATCAGTGTGCCGACTGTTACCGCCGCAGTGCCCTGAATGTCATCGTTAAAAGAACAAAT
TTCATTGCGATAGCGGTTAAGTAACGGCATCGCATTTTTTTGAGCAAAGTCTTCAAACTGCAACAGCACGTCTGGCCAGC
GTTGTTTCACAGCCTGGATAAATTCATCAACGAATTCATAGTATTCGTCGTCAGTGATACGCGGATTACGCCAGCCCATA
TACAGCGGATCGTTAAGCAGCTGTTGGTTGTTCGTTCCGACATCCAGCACCACCGGAAGGGTATACGCCGGGCTGATGCC
GCCACAGGCGGTATAGAGCGACAGTTTACCGATCGGAATGCCCATCCCGCCGATGCCCTGGTCACCAAGCCCCAGAATGC
GTTCACCGTCAGTCACCACAATCACTTTAATATTATGGTTCGGCACGTTTTGCAGAATATCGTCCATATTGTGCCGGTTC
TGGTAAGAGATAAACACGCCGCGTGAACGGCGGTAGATCTCAGAAAAACGCTCACAGGCTGCGCCGACGGTTGGGGTATA
AATAACAGGCATCATCTCATCAAGATGATTGTTTACCAGACGGTAGAAGAGGGTTTCGTTAGTGTCCTGGATGTTACGCA
GGTAGATGTGTTTGTCGATTTCGGTTTTGAATCCCTGATACTGGATCCATGCTCGTTCCGCTTGTTCTTCGATGGTTTCG
ACCACTTCCGGCAGTAACCCCAGCAGGTTGAAGTTACGGCGTTCTTCCATGCTGAAGGCACTGCCTTTATTCAACAACGG
AAATTCCAGCAGTACAGGGCCAGCGTAAGGGATATAAAGCGAACGCTGTTTTTTTGTTTTTGGTTCCATGTCACTCACTC
TTTTTTGAATATCCATCCCTGGGGGGCTTTTATCGTCTTTGCTTTACCGCCAGGGCGTCGGCCTCAAGTATAAAGCAGAT
AAAAACAAAAACACCATTGCGCAGGCAATGGTGTTTAATCGTCATTGAGGACTGATGGTTATGAATTACTTTTCAGCGGG
GCGTTTTCTGCCGGTTGGGTTATTTACTACGCTGGATTTGTCACCTTCGGTAACTATTTTGCGCTGGTTAGAAATTTTAT
GGTCCAGTCCAAGAATATGACGTGCCTGACGGTTCGATTTCATTTTAACTCCTCAATCCTGTAGCTAGTTTTAAGGACAA
CATCGCCGTAGCGAAGAAACACGTGCTAAACCCCTAAATTAGGTTGCCGATCAAGCATAGCACCTTAAAGCGTAGGGTGC
TGGCCACTGACCACATAATTGATCGTTTGCTGGTAGATATCACTGAGGATGTCGTTATCAGAAGCTTCAACCCATTTGGT
CAGCTCCATGAGAATGTCATCTTCAGTGACAACACCATGCTGTGCCCGAAGGCCTTGCTCAATGGCATTAACCAGAGCGG
GTTCTGCTGTTGAATTTTCTGCCTGATAATAAGTAAACATAGTGATTCTCCGTGTCTGTGTATTTATGGTGTCTGCTACG
GATCGCAGATTTATAAAGCACATTCAGCATGGCAAATATTTGCCGCTTCGTTGTTAAGATTAGTCCTGGTTGATGATTTT
TATATTTTAACACCATGATATTCATAGGGATTGTGATTGGTATGATCCGATTAATATTGATACAATATCTTTTGGGTTAT
ATATTCCCGGTAATCTATTGTGGGAATTTAATTTAAGTGCAGAAGTAATATTTTCGCCGGATTTTATTCGGAATATCCTG
CTTATCCTCGTGCTGTTTCTCACGTAGTCTATAATTTCCTTTTTAAGCCCACAGGAGAGCAACAATGACAATCCATAAGA
AAGGTCAGGCACACTGGGAAGGCGATATCAAACGCGGGAAGGGAACAGTATCCACCGAGAGTGGCGTGCTGAACCAACAG
CCGTATGGATTTAACACGCGTTTTGAAGGCGAAAAAGGAACCAACCCTGAAGAACTGATTGGCGCAGCGCATGCCGCATG
TTTCTCAATGGCGCTTTCATTAATGCTGGGGGAAGCGGGATTCACGCCAACATCGATTGATACCACCGCCGATGTGTCGC
TGGATAAAGTGGATGCCGGTTTTGCGATTACGAAAATCGCACTGAAGAGTGAAGTTGCGGTGCCGGGTATTGATGCCTCT
ACCTTTGACGGCATAATCCAGAAAGCAAAAGCAGGATGCCCGGTCTCTCAGGTACTGAAAGCGGAAATTACGCTGGATTA
CCAGTTGAAATCGTAAAGCATTGCCGGATGACGCGTCAGGCGCGTGAATGCCTGATGCGTTGTTAGCATCTCTACAGCGC
CCGCCAGCACCGGAGCTCACGTCCGTCCTCCCTTATTGCTAATGATTGCCGGACTTCACATCCGTGGGTTGCCAGCGGGC
AACGTTCGTAGAAAAAAACAGCCTTGCGGCAACGTGCGGTTTCCCGGCAGATCCGTTTTACGTAATGCCCATTCTTCCTCC
AGCGGTTTATCAATGGCGGGGAGGGAATCCAGCAATAATCGGGTGTATGGATGTGCAGGTGCCGTCAGCACCTGCTGCGC
GTCGCCCAGTTCTACAATCTGCCCGAGATACATCACCGCCACCCGATCGCTCATATGACGTATCACCGAGACATTGTGTG
AAATCAGCACATAGGTCAGCCCGTGATTTTCCTGTAGCGTTACCAGTAAATTGAGGATCTGCGCCTGAACGGAGATATCC
AGCGCAGAGGTTGGCTCATCAAGCACAATCACGTCAGGCTGCGAAGAGAGTGCTCTGGCAATGGCGATACGTTGCCGCTG
CCCGCCGGAGAACGCATGAGGCAGGCGGTCGAGATACTCCGGACGAATACCCACCTGCACAGCCAACTCCTCTGCCAACG
CTCGCCGCTGTTGTTCACTACTACGCTTAGCTATCCAGAGCGGTTCTGTGATGATGCGCCACACCGGTAAGCGCGGATTA
AGCGAAGAGAGCGGGTCCTGAAACACCATCTGCATAATGCGTTGTGAGCCAGAACGGATGTACTGCCCGTGGCTCGGTTG
CAGCATACCCATTAAAAGCTGTGCGAGGGTGCTTTTGCCGCAGCCTGACTCGCCGACGATCCCTAAGGTTTCACCACGGC
GGATCTGTAAATCAATACCATTAATGGCATGAACATGTTCCGTAGTTTTACCAAGCCAGTTTTTACGGGCCGGGAAATTG
ATATGGACGTCGCGTAACGTTAATAACGTGTCAGACACTTATAACCTCCTGCTGCGGATACCAACAGGCACAGCGCTGGT
TGTTGTCACCACACGCTGTCAGCGCCGGGACGTTTTCACACTGTGCACCTGCCGCATAGCAACGATCGCGAAAAGCGCAG
CCGTCAGGCAAATGGGTGAGGTTTGGTACCGTCCCTGGAATGGCGGGTAATAGCTGGCGTGGTACTCCATGTTCTGGTGC
GCATTGCAGCAAACCAATGGTATACGGATGCCGGGGATGATGGATAACGTCTGCCGTCACGCCGCTTTCAATTACGCTTC
CGGCATACATCACGTAAACGCTATCGCACAGTTGCGACACCACGGCCATATCATGGCTGATGAACAGAACCGCAGTTCCA
CTGGCGCGGGCTTTATGTTTAAGCAAACGCAGTACCTGCAACTGCACCGTGACGTCCAGCGCCGTAGTCGGTTCGTCGGC
AATAATCAATTGCGGCTCGCAGGAGAATGCCAGCGCAATCATTACCCGCTGGCGCATACCACCTGAAAGCTCAAACGGAT
AGCGCGACATAACTTCCACGGCATCCGGGATTTGCATCTCTTCCAGCAGGTCAATCGCTTTAGCTCTGGCTTCCCGACGA
CTTATTGGTTGATGATGGCGGATCACGTCCATCATCTGAAGACCTATTCGACGTGTCGGATTGAGGGCGGTCATCGGTTC
CTGAAAGATCATCGCCACTCGTGCGCCGCGCCACTGACGAAGCTGTTTTTCCCGCGCGTTAAGAACATCTTCTCCCAGCA
GGGAAATCTGCCCCCGATGTACGCAATAACTGCCCGTCGGTAGCAGACGCATAATCAGCATTGCGGTGACTGATTTACCT
GAGCCGGATTCTCCCACCAGACCGACAATTTCACCGCGGTTAATCTGCAAGGACACATTGTTGAGCGCGTGAACATCACC

GTTAAAACCGGGGAAACTCAAATGCAGTTGTTGAATGTCCAGAACGGGTTGGGTCATGACTGCTTTCCTCCTGCTTTCGG
GTCAAGCAGATCGCGAATACCATCACCAAAGAGATTAAACCCGACGGCGGTAAGCAAAATCGCTGCCCCCGGAAATGCGC
AATACCACCACTGATCGAGTACATAGTTGCGACCATTTGCTACCATCGCGCCCCATTCAGCACTCGGTTGTTGAGCACCC
AGGCCAATAAATCCCAACGTGGCGGCCATTAAAATCGCGCTACCGATATCCAGCGATGCCTGCACGATCAGCGGCGGTAG
GGAGTTACGTAAAATATGCCAGTTGATCAGATGCCAACGAGACGCGCCAAAGGTTTTCGCCGCCTGAACATAGGTATACT
GGCGTACAACTAATGCTTGCCCCCGCGCCAGGCGCACATAAAAGGGAATTCGCACAATAGCAATAGCCAGCATGGCGTTA
AACAAACTCGGCCCGAGAGCGGCAGCCAGTGCCATTGTCAGTACCAACGAAGGAATCGACAGCATAATGTCCATGATGCG
CATGATGATGGCGTCTGCGCGTCCACCAAGCACACCGGATAGACATCCGAGTAGCGAACCAATCATACCCGCAATGGCAA
CCACGACTAATCCGGCGAGAATTGATTGCTGACTGCCGACCAGTACGCGGCTAAACAGATCGCGTCCCACTTCATCGGTG
CCAAACCAGTGCGCCGCAGAAGGCGGCAAAAGGCGGGCGGTTAAATCAATGGCGTTGGGATCATGCGTCGCCAGCCACGG
TGAAAAAATCATCATCAATAGCATTAATACAATGATGACTGCGCTGGTCACGGTGAGCGGACTGCCTTTCAGCATCCATA
CCAGTTTTGCACCGTTAAATCGCGTTTGTTTTTGTGGGCGTACGGCGGACGTTTCCTCGCTTAGCATCATTCACCACCTC
CACGTCCGATACGCGGATCAATCCACAAATAGAGCAAATCCACCACCAGGTTGACCAGCACATAAGCAAATGAAACCACG
ACGGCAAAGCCCATCACTGCCGGGAAGTCGAGCGCCTGTATTGATGTTACTACCCATGCACCCATTCCGGGCCAGGCAAA
AACGGTTTCGGTGAGCACTGCGCCATACAACAAATCGCCCAGCGCCAAACCCAATACGGTAATCGATGGGATCAACGCAT
TGGGTAGCGCATAACATAAAACGATATACCAGCCGGGCAAGCCGCTGGCCCGGGCGGTACGAATGTAGTCTTCACTCAAT
TGTTCCAGCATCGCTGAGCGGATCTGGCGAGCGACAATTCCCAGGTGAACGAACGCCAGCGTTAATGCCGGTAAGATGAG
ATGTTGCAACGCATTGAAGAAGACTTCACCGTTGCCTTCAAGCAGCGCATCGAGCAGATAAAAGCCGGTAACGTGCGTTG
GTGGATCCAGCCAGTCATCAAGCCTTCCGCCGCCGGGAAGAATTTGCAGATGACCATAAAACAGCACAATGACGCCCAGC
CCAAGCCAGAACGCAGGTGTGGAGATTCCGGTAATGGCCATTATTCGCACCAGATGATCCAGCCAGCGATTTCGCCAGAC
TGCAGAGAGTATGCCCAGCGGGATGCCAATCAGGAGTGCCAGCAGCAGGGCACCAAAAGCCAGCTCCAGCGTCGCCGGGA
AAAATATACGCAACTCTTCCAGCACCGGACGCCCGGTACGAATGGATGTTCCCAGGTCACCATGAAACAGGTCGCTGACG
TAGCGGTAAAACTGTACATACAGTGGCTGGTCCAGTCCCAGTTGCTGGCGAATATTTTCCACGATAGCATCGCTGGCGCG
GTCACCCGCCAGTAACCGCGCCGGATCACCAGGGATCAGATGCGAGATAATAAACGTAATCACGCAGACGCCCGCCACCA
CGAGCACCAGCCCCCAGCAGCGTTGGCGTAAAATACTCCAGAAGGTCACGTTATTTACTCATGGTATTGATATTGAAGAC
CTGTTCCAGCATGGGATTGAACACAAAGCCTTTCACCTCTTTGTTCATCGCCAGTTGGTAGTTTTTCTGGAACAGGTACA
CATAAGCAGCGTCATCAATGACGATTTTCTGTGCCTGCTGGTAGTCCCGGGTACGCTGCGTCTGGTCGGTGGTCGCAAGC
GCATTGCGCAGTAACTTATCGACCTCACTGTTTTCATAGAACGAGCGGTTACCCGGCAGACCTTTTTTGTCTGACTCAAA
CCAGTAATTCATAAACATATACGGGTCGGCAAAATCCGGACTCCAGTTGCCAATCGCAATGTCGTAATCACCTTTACCCA
CTCTGTCGCGCATGGTGGCGTTCGCCAGCTTTTCCAGCTTCACAATGATGCCCAGCTTGTTGAGACTGGATTGTGTCGCC
AGAGCAATAGGCTCCCAGTTCGGATCGTTATCGGAGTAGAGAAACGTCAGGCTGGTGGGTTTGCTCGTCACTTTATCCCA
TTCGGCTTTGGCTTTCGTTTCGTCATGGTTGTATTGCATTGCCGTCGCATCGTAGCCCCACATGCCTTCCGGAATCGGGC
CGCGCATCTGTTTTCCGTTACCACTCAGAATGCCGTTAACCATGCCCTGATAATCGGTAGACCAGGAAATGGCCCGACGC
AGATCCGCCTGATTAAGAGGCGCTTTGCTGTTATTCAGATACAGATAGGTAACGCGCAGTGACGGATACTCTGCCACATT
GACTTTATTTTCCTGCTTCAGGGCGTTGAGTTGATCCACCGGCAGCGCATCGGCAATGTCAATGTCGCCACGGGAGAGCT
GCAGGCGACGGGAGGCACTTTCACCAATAATTTTTACCGATACCCGTTTGAAGTTCGGTTTATTGCCTGGGTAATGCGGA
TTTGGCACCAGAACTAATTGCTGACCTTTTTGCCAGCTTTTCAGCATAAATGGTCCGGAACCGGCGGTATTTTGCGCGAG
GAAGCCGCGAGCATCATCCGCTGCATGTTCCTTTAAGACCGCCGGATTGATAATGGATGCACCGTCATTCGCCAGCGTGT
AGAGGAACGGTGCGAATGGTTGGCTAAGGGTAAACTTCACCGTATGTTCGTCGGGAGCATCAATCTTTAAATCTTTGGGA
AATGCTTCTGCTGGCCCCTGGCCGATTTTTAGTAGCCGCTCAAAAGAAAGTTTTACTGCTTCGGCAGTGACAGGTGTGCC
ATCGGCAAATTTAGCATTATCTTTCAGGGTGAACGTCCACTCTTTTTGATCGTCAGACGCTTTCCAGCTACTTGCCAGAT
CGCCTTCAACGTCGGTTGAGCCTTTATCACCGTCCGTTTTGTACTGAACCAGCCGCTGATAAGACGGGTAGGTCACTGTC
CAGTCGTTATTATCTATTGTTACCGCCGGGTCGAGGGTTTGTGGATCGGCGGCCTTACCAATCACCAGCATATCTTTTGG
TACGGCGGCGTGCGCAACCGGGAAATTTGTGGCAAGGACGAGCGCGAGCAATGTGGGACGAAACGATATCGATCTCTTCA
TGACAGTTTCCTGATTAACTGACGTGCTGTGTGCCAGGGGATATAAAACAAGAGAATTGATCAGCGAGCAGAGGGTAACT
CGCTGCCTGGGGTAATTCGAAGTGCCACCATTCGCTGGAGATACCGACAAAACCGCCACCAGTCATTATCGCATTCAGCA
ACAGCCGATTGCGCTGAGCGGCGGGCGGGACGGAAGGGTGATAGGCATGGGAGCGCTCGTGCATTTCATCGAACCCTGCG
CCCATATCGAGGATGTTCCCGTGCTCATCACGAAGCGTCAGGTCGATCGCCGTGCCACGGCTGTGATTAGAACCGACCGT
CACATCAACAACATATTGCGGGTCTGGGCAGGCTTGCCACAACATCGCCTGTGCTTGTTGTGGGCGATACGCATCGTAAA
TCACCAGTTGTAACCCTGACAGCTGGGCGATGCTGATACTTTTCGCCAGCGCGGTAATCGCATCCTTGTGTAACAGACAA
CGCGCTTGCTGATAAATAGCTTTACCTGTGATGTTATCAGCGCAGGCGTATTTCAATTCGATCTCCAGATCAGGGAAGAT
TACGGCTAAATCAACCAGTTCGGTGGTATCCGACATAAACGGTTCCTGTATAAGACAAAAATTGCTGCGCTTTCCGCTTA
TGCAGATCTCATGCCATGCCGGGATAAGCGCCAGAATGCTGGCTTAAAGTTATATTTTTTAACTTTTGATCAACATTTGT
GCAGCGTAGTGCAGTTTTGGTGCAAGAGGGGAAGTTAAGGAAGGAATCTCCCGGAATCGTAGCTGAAATCACAGTATTTA
AGTGACAGTGTCACGTTAAATGAAAACCCGCGAGTGCGGGCGAGAGGAATTTGTCAGATTTTCAGCGGTAACACGCTGCT
CATCCAGCCTGGAATTTCTTCGGCGGGTAGGGGGCGGGAAAAGAAATATCCCTGAATAACGCGACAGTGGATCTTGCGTA

GCATCTCAAATTGCTCTTTGGTTTCGACGCCTTCCGCCACGACGGTTAAATTGAGGCTTTGCCCAATGCTGGTAATGGCT
TCAAGTAAGGCAAGGATGCGTTTTTCGGTCAGACAACGATCGACAAAACTTTTGTCAATTTTGATTTCCGTTACCGGAAG
ACTGACTAAGCGGGATAATCCGGAAAAGCCCGTACCAAAATCATCTACCGATAAGCCCACGCCCATATCACGCAGGATCT
GAATGCGCTTAAAGATTTCGGTATCGTGTTCCATCATCATGCTTTCCGTGATTTCTACCGTCAGCTGGTGGCCGTCAATA
CCCCAGGCGTGCATTGCATCAGACACCTGATTAGGCAGTTGATTACTGCGAAAGTGCAGCGCCGACAAGTTCACGGATAA
CGCCGGGATATGAATATTCTGGCTACGCCATTCTGCTAACTGACGGCAAGCTTCCGCGATGACCCAGCGCCCAATATTTT
CGATTTCACCAATCTCTTCTGCGAGAGGAATAAACCGTGAAGGGGGCACATGACCATGCAGGGGATCGTGCCAGCGAGCA
AGGGCTTCGATGCCGTACAGTTCACCCGTTTCTGCGAAGATTTGCGGCTGGTAAACCAGTTTCAGTTGGTTATTGCTAAT
CGCTTCTTTCAGCGCTGCGCCTAAAACCAAACGCTCTTTTACCATTTCGTTCATCGCCGGGCTGAAGAACTGCCAGCCGT
TACCGCCATTCTTGCGAATATAATCCATTGCATTGTGAGCAGTGGAGAGCAAGTAATCGCGGTTTTTACCCAGGTCGTAG
CTGATGCCAATACTCAAGGTAAGCGGGAAGGGTTTATCGTCAATCATTATCGGCTTGCTGACCACATTCCGTAGCTCATC
GGCGATTTGGGTAATGTTACTGACGTCGTTTTCGAGGCTCACGAGGACAAACTGCGTACCTTCGATACGACAGAGATACT
GATCCGGTTTGAGTTTTTCACGAAAGCGATTGACCACTTCCAGCAATGCCTGATCGGCCCACGCATAGCCAAGGCTATCA
ATCACATCCTGAATATGGTCAACACCGATGAGATACACCACGGGAGAGACGGCTTTGTCGACCAGGTCATCGAGGTAATT
GTGCAGGTTATTGCGATTTGGCAGACCGGTCATCGGATCAAATTGGATGAGTTGTTCAATATGCTGACGGCTTTTTTCCT
GTTCCAGCGCCAGCGCGGCCATATGCTGGCTGATATCTGCCACGCGTTCGATAAAGGCGCTGGTTTCTGCTCCTGACGAG
GTTTTAATTTGCAGGATCCCCGCAGGCGCGCCATCACGCTGACGAATGGTCGCTGACCAGCTTTGCGCATTTTGAATTTC
TGCACCGTGGGAAGATGACGCCCAGTGTATCGGCATCCCGTTGCGCAGTGCGAACAGCGAAACATGCGATTCGTTGAGTA
CAGATTCGATGTTACGACAAATGATTTCCCCCATTTCATGAAATGGCGGGCTGCTGCACATGGCGGCGAGAATATTGCCT
TCAAGCTGGCGAATCTGCCGTTCTTCGGTGATATCCGAGAAAGTCATTACCAGGTTCTGCAGATGCGCGAGCACGTCATA
AACCGGGCTGATAGAGGCTTTAATCCAGATTTTTTCACCGGTGCGCGTCAACAGCAGAAATTCGTCCTGATCGCGGGCGG
TTTTCCATAGCAACTGTTGTAAACGAATGCGGTTATCGGCAGGGAATTCAGGAATGTTCAGGAGTGTATCGGGCTGCATA
CCGCTGGCTTCGCTAATGCAGTAACCAAACATTTCGGTAAATGCGCGATTGCACTGCACAATATGGCGTTCCGGATCGAG
GACAATCACCGGTCGGTCGAGATGGTCAACGGCAATAATCAATTGTCGGGTCTGTTCTTTTTGCGCCATTTCTACGCTGG
CATCCCGTACCAGCGCCAGGTAATAAACTTTCCCCTCGGCGCTCACTTTCGATAGCGCAAAACGGGTCCAGATTTTACTG
CCGTCTTTTTTCTCCAGCTGCAGCTCCCGACTCATCCCCTCAACACGCGCTTTACCGCCTTCACGGTTGTGACGAATGTA
TTCAGGATGCGCAGGACGCAAATCCCGCGGAATCAGCATATCAATGTTATTGCCAATGACTTCTTCACGTTTGTATCCCC
AGAGCTTCTCTGCGGCGGGGTTGAAAAACATCACTTCATCATTTTCGTTAATTAACACCGCACCCATCATATTTTGCTCA
AGGGCGGGGAAAAAAATGCCATCGGCGGCATTATCCGCATCGGTTAGCTTCATGATTACCTCTGCATCCTGGCGCATCTA
AAGACTGGCTTTCCAGAGTTCAACACGGTTTCTACCTCGTCTTTTGGCGATATACAGAGCTTCATCGGCTATTTGAATGA
GGCGCTCATAGTCAGGATGACCATTAAACATGGCGGCACCGATGGAAAGTGAGAGGGCAATATCTTCGCCGTTTGCGGCT
TTCAGTTTGGTTTTCTCCACCCGACTGCGAATACGTTCTGCGGTACGTAACGTTTCGTTTTCAGAAGCTTCAGTCAAAAC
AATGATAAATTCATCGCCCCCGTAGCGGAAAACATAATCACTACTGCGGACGTTGTCATAAAAGGCCTGAGAGACTTTAC
GCAGAATTTCATCACCAGTGTTATGGCCCCACGTATCGTTGATCTCTTTGAATTTATCAACGTCAATAATCAGCACTGAC
AGCGGTGTACCGGTCCGGTTGGCATGGGCAATTTCGCGTTTGAAGATAGTCGGTAGGAAACGGCGGTTAAGTAATTTCGT
CAGTACATCCATACCGACTTCGTGGCGCGATACTTCTTCAAACAATTCACGCAGCAAGGTAATAATTTGCGATACGGTAT
TTCTTATCTGTAATAAAAATTTCACCCGCAGACTTCTGTTATTCAAATTTCTGGTGTTACGCATGGTTTGATTGAAAATA
CCGTCGAAATCCTGAATCAGACGGGAGATATGGCCTACTTCGGCAATACCACTAAAATAATGTCGACCTTTATGGTTAAA
CCACAGGCCAAAATCAGCCTGGCTTAAAGGCAAACTACTGCCTAAATCAGAATCCAGCAGGATTTTATAGATAATATCTA
TTTCCCATGAAAGTATTGAGGCTATTTGCCGTTCTTTTTCTTCTTCGGCGTTTTCCAGTAACGAGAAGATACGATAGTTT
TCATCTTCCTTTGAGGCACTACTGTCACTAAAGGTAAACGCGCGAGTCATCACTTCCATCGCGATATCAATACTGTTAAT
CGAGAAATGGTAGACCTGAAGTTTTTCTGCGGCGGAATAATCCGAAGAGAAGATCACCGGATAGAGGATTTTTTTCAGCA
CCCGAAACCCCATCTCGACAATTTCTACCGGAATTCCTATGCGGGCATGCACTTCCGCGACGGTATGCTGGATTTGTATT
AGCCTTTCGACATCGTCAACCTGGGCAGAAAGCACGTTAATAATCCAGCGTTCCATCGCACTCTTCAACTGCCGCTCAAC
TTGTTCATTACTCAAGAATTCTTCGGCATGCGGGTCGATGCGGACAATTCGATAAAACTCGATACTCAGATAATGAGCAT
GCGCAACGGCAATTTCCGCGGCTTTAGCACGAATGGGCGGATCTGCCTGTTCGACAAGTCCGGTCCACTCATCTTTCATT
CTTTTAAAATACATCTCCATAATTCACACCCTTATAAGGCTGGGAAATCAGACGGAATCAAAATGAAACGCAACGTGCGA
GATCGACTAACTGCACCATATTCTCCTGAAATATGAAGATATACTGAAAAGAAATAAGCGATTTAGGACAGTTTCAATCT
ACGCTACTGTTCTTCAGAAGAGTATAGCCCATCGTAATTATTTTTCGGTGACAGCGAATATCGTATGGTTTTTCATATTC
ATACATTTTTATTAGGGATTTATGGCTGTTTAACTAAGTGTGGTTAATTTGACTTAAGTAAGCATGATTATTAGTGGGAT
AGTTTAAGAGGGTAACAAGCCGGTGGGTAAAGCACCGGCTTGTTACAAAGTAAGAATGGGAGTTTAACTGCCCCAGCGAC
TTTGCAGATAGCTGACCGCTTGTTGAGTCTGCGGTTTATTCAGATAGTCCTCACGGAACAAGATGGTGCCGCTAATTTCG
GGCACAGCATCGTTAAGATCGAGCTGCTTTTTCAGTTCCGGTACGCCGCCGTTAATCATCCAGTCTGGCTCTATCTTTGA
AGGTTCACCCACTTTATAGAAGGCGATACCGATATACAGGCGGGTCCTGGTCGGTTTAACGACATCCGCCCACCATTTTG
CCAACACGTCATAACGCGCGGCACTCCGTGAGAACGGCCAGTAAATTTGGGGAGCAATGTAATCCAGCAATCCTTGTTCC
ACCCATCGACGGGTGTCAGCGTAGGATTCGTCATAGGCTGCCGCGCCTCGGGTATCGGAACCGAGCGGATCGTGTGATCG

GTTACGCCACACGCCTGCCGGGCTAACACCAAATTCGACTCCCGGCTTAATGCTTTTAATGGTGTGCGATACCTTTGCAA
TTAACTGCTGAGTATTGTTGCGCCGCCAGTCTGCTTTTGACGCAAATGCGCCTCCGTATTTACGGTACGTTTCGTTATCA
TTTAGCCGTGAACCCGGTGACTCCGTATAGAAATAGTCGTCAAACTGCACGCCATCTACGGGATAGCGGGAAACCACTTC
TGCGACTATTGATGTGATCCAGTCCTGAACCTCAGGGATGCCCGGGTCGAGGACAAAGCGATCGCCAGACGTTCTGATCC
AGTCGCGGTGTTGCACATAGACGCTCGCCGGTTGTTGAGACAGAGTGCTATTCAGTTCCCTGATAGTACCGGGCTTCGTA
TTAACCGATACGCGATAGGGGTTAAACCAGGCGTGTACTTTCATCCCACGCTTGTGGGCTTCGTCGAGCATAAATTGCAG
CGGATCGTAACCCGGATTTTCACCAATCTTACCGGTCATAAGATCGGACCACGGCAAAATTTTCGATGGCCACAGGGCGG
TACCGTCCGGCTTGACCTGGAAAAAGACCGTGTTTATGCCGAGACGTTGCAGATGATCCAGTTTGTCGATCATCGCCTGT
TGTTGTACACGGGCCCGGCTGGTGGGGTTACTAATGTTAACCGAGGAAACCGGTGGCCAGTCGAGCCGAGAAACCGTGGC
CAGCCAGATGCCACGCATCGGTTGTGACGATTGTTGCGTCGTGGCTGGTGGCTTTGAACCTGCTGGTGGTGTCACCATGG
ACTCTGGAGGCGTGCTTTTACAACTACACAGTAAAAGTGCCAGTGCAACTAGTATCGCTGGTCTTCTAATCGTTAATTTC
TTGTTTCGGGAGCAGATATCCATATGTGCTGGTTTCCGGTAAACAGATGTGCGCTCATTCTCGTACTTATCCCCGTTAAG
TCAATACGACAGCAAGCACGAAAAAGGGAGCGATGAATTATCGCTCCCTTGTCTTATAACCATTCAGACATGGTTAGTGT
TTCTTGTCATTCATCACAATATAGTGTGGTGAACGTGCACGCGGGTGCAGGAAGAAGTGACCTTTTGGTGCGTTCTGACT
GTTGATTGGCTCCAGAGTGACGCCGGTATTTGCTTTGCCTTTACGATCATGAACAGCATAGAGAATAAAGGGCAGGGCAA
GTACCACCAGGAAACTAACAACCAGTAATTCAACATACATATCGGTAGAATCACCCTGGATGTTATCCGGCGGCAGGAAG
GAAACAATAAACGCCATAATTGAAGTCAGCAGACCGACAATTGCCACGACCAGTTTCACCCCTTTACCACCAGGGATATT
AAATGTGCGTTTTAAGTCAGGATGTTTAAGAACCAACACAATGTAGCCAATAAACAGCATGAAATAAGCACACAGATAAA
TCACCACCGTCAGCGCCAGTGCGATCAGGAAGGACATGTTGTTACCGCCACCGGTATTGGTGAGGATGATCAACGCGATA
GACGTAATCACCAGCTGCGAAATGACCAGCGTTACCGGTACGCCATTTTTGTTCATTTTAGCGAATGCCGCTGGCAGCAG
GTTTTTCTGCGCTGTTACATACATCCCGCGAGAAGGACCAACAATCCAGGAGGCGATTTCCGCCAGAACACCCAGCAACA
GCAGTGCGGAGATCACGCGAACCGTCCACTCAATTTCTGGTGCCACATGGGACATCAGAACGGTAAAGGTTTGCATTACC
CCTGCGGAGAGGTTGATTTCATTACCCGGAATGACCATCGCAATAGACAAACCACCAACAGAGCTTAAGCAGATTGCCGC
CACCATCAGCAGTAACATAGCCAACGGATAGTCGCGCCCTGGGTTGCTCATTTCATTGACGTGGGTTGCGGATGCTTCTA
CGCCCATATAACTCAAAATGAAGGCAACAAATACTACCAGGGTGCCCACTTTAGAGAAGTCAGGGAAGAAGGTCTTCGAA
TCCATTTCGATAGCAACGGGGGCACCGGAGTGCAGATAAATAGCCGCTAATGCGATCAAAATAAATGCAGGTAACAGGAT
ACCGGCGAAGAAGCCAACTTTAGCAATTCGCGCCGTGTATTTCGTGCCACCAAACTGCGTTAATGCCAGCGCCCAAAGAA
TGATGAGTGCTGCAATAGTTTTGGTAATGGGGTCTTCATTCAGCGCTGGCCATTTCAGGATGTAGGAGAGTGCCCCTAAC
ACGAAATAGAGCATCGGAATAAAACCAATGGCGATTTGCAGATAGCCAAATGAGATCGCTGCAAATCCCCATCTCGGCCC
CAGAGTATTTGATACCCAGGCGAAGACACCACCTTCTTCCCAGCCGTCGACGGTGGCCATTTCCGCAGCACAAAGTCCCA
CGGGAATAAACCATAAAATCCCGCCTAATAGCAGGAAGAAGACTAATGAAAAGCCCGATGTTGCGAAGGTAGGGTATTCA
TAAACAGCCATTACCATCGATGCCGTTATGGCAAAGAATCCAAGTAATGTGAGCTGCTTAGCTTTACCTGTCTGTACTGA
TGTAGCCATATTATCCCCCTAAAACGGTATTCCTGTCGGAACCGCACCTGTTTTGTTCTGAAGCGTATTCAGAACAATAT
TTTCCGTTGCTAATGCCAGTGAACAGACTTTGGAAATTGTCCCGAAACGGGTTCGTTTCGGGACACCGTTACCGTTAAAC
GTTATCAGGTATGTTTAAAGCTGTTCTGTTGGGCAATACCCTGCAGTTTCGGGTGATCGCTGAGATATTTCAGGGAGGCT
TTGTAGTCTTCCAGCAACAGTTCAGCAAAGTCCATTTCGAAGCCGCGACGACACATAATGCGCATCACCACGATGTCGGT
GGCTTCACCGCCGAGAGTGAAGGCCGGAACCTGCCAGCCGCGCAGACGCAGACGTTCAGAGAGGTCATACAGGGTGTATC
CCGGATCTTCACCATCTTTCAGTTTGAAGCAAACCGCCGGGATGCCTTCGTCCGGGCGACCCGTACAGATGAACTCATAC
GGCCCCAGTTTGGCGATTTCATCCGCCAGATAAGCGGCAACCTGGTAAGAGGCGTTCTGTACTTTGGTATAGCCTTCACG
ACCGAGGCGCAGGAATTCATAGTACTGTGCAATTACCTGACCCGCCGGGCGGGAGAAGTTGATGGCAAAAGTACCAATTT
GACCACCCAGGTAGTCAACGTTGAACACCAGTTCCTGCGGCAGCGCTTCTTCGTCACGCCAGATAACCCAGCCGCAGCCC
AGCGGAGCCAGACCGAATTTATGGCCTGAAGCACTGATCGATTTCACACGCGGCAGGCGGAAGTCCCAGACGATATCCGG
GGCGACGAACGGTGCCAGGAAGCCACCGCTGGCAGCGTCGATGTGCATGTCGATGTCGATACCGGTATCGGCCTGGAATT
TATCCAGCGCATCGTGCAGCGGTTGTGGGAACTCATAGTTACCAGTGTAGGTCACGCCGAAAGTCGGCACCACGCCGATG
GTGTTTTCGTCACAGGCTTCAATCATGCGTTTCGGGTCCATAAACAACTGACCGGGGCGCATAGGGATCTCACGCAGCTC
CACATCCCAGTAGCGGGCGAATTTATGCCAGCAGATTTGTACCGGACCGCACACCAGGTTTGGTTTATCCGTTGGTTTGC
CTGCAGCTTCCATACGCTTGCGCCAACGCCATTTCATCGCCATCCCGCCGAGCATACAGGCCTCGGAAGAACCAATGGTG
TTGGTGCCAACGGCCTGACCATTTTTCGGCGCAGGCGCATGCCACAGATCGGCAACCATATTTACGCAACGCAGGTCGAT
GGCTGCGGATTGCGGATATTCTTCTTTGTCGATCCAGTTTTTGTTAATGGATAAATCCATCAATTTGTGGACATTTTCGT
CGTCCCAGGTCTGGCAGAAAGTGGCCAGGTTCTGACGAGCGTTGCCATCAAGATATAATTCGTCATTGATAATCTGGAAT
GCGACATCGTCGCGCATTTCGTGCAGCGGAAAACGTTTTGATTCTGCGATAGTGGAAATAGACTTCGCACCAAAACGTGA
ATCGAGTAGTTCCGACCTTAAATCCGTTACTTGCTTCTTATCCATTTTAAACTCCTTAAAATGATTGGATCGCATTAAAA
AAGTAGGATTTATCGATAAAGTAAGCAAGTGCAAAGGACTCGTGTTTAAATAACAAAATCCTAATGTTATTTATCGTGAG
ATATTACGCGAATAATATTTTTTCATTGAAAAACAATACAATATGAAATTCTTGGGTGGTGGTAAGGTGTTTTATGCTGT
TATTTTTATGCGCATTCTGTGTCTCCTGAATTATCACGTAAAAATCAGACCTTAAAATATCACTATTAGTACTTGATTAT
TATTTTGAACGCATTTATAAAATTATTACATAAAAATAGCGAATATTGCTAAAATCCCCGCCAACGATGTGTTGACGGGG

CTGTTATTATTTTGGCAATAATACTCCGGTATAAGTATTTACCGGATGAGAAAGATATTGTTTAACGGCAGTGTTAACAT
TCTCTACCGTCATTTGTTTCAACAATTGCTCCTGCTCAGTCCATGCTGCAGGATCGTCATATTGAATAAGACTATTTACA
ATAGTGTTCGCTAATTGTTGAACGCTACGCTGTTGGATATCGAGGCTGCGCTGAACGTTTTGCTGGTATTCATTCAGTTC
TTGCTCACTGATCCCTTTAGCCAGACGCTTAACCATCACTTCATTCGCTAACGTTAACAGTTCATCATGTCGTTCTGGTT
GACAAGTAAAAGCCAGCAAATGACTGATATCTTTGGCCTGAGGATCAACCGAGAGGCGAGAAGAAACGCTGTATGCTCCA
GATGCCTGTTCACGAATATTAACACGTAGATCTTTTGCCAGTGCGACGTTAAAAGCATCGAGCGCCATACGCGTCGGCAG
ATTAACAGGTGTCCGGGAATCATAACGCTTCCACTGTGAAACCTGTGCCACAGGTTCATTTTGTTCTTTTACAGTAACCG
ATGCGTTGTCCGTCGCGCGAGTTAATGGTTTACCTGCGGCTAATGGCGAATCAGAGTGTTTGATTGATCCTAAGTAACGC
GTAATTAACGCCACGAGTTTGTCTTCTGCGACATTACCGACAATGACAAACGTGATATCCGCTGGAGATGAAAACAATTG
GCGATCGGCAGCCAGCGCATCTGCGGCAGTAAACTGTGCAATCTGATTTTCTTGCAGTAATTTCGTGCGGTCATCAGCAT
AGCGCGTCTCATACATCTGCTGGGCGAATTTCTCCGCTGGACGCTGGTCGAGCGTTTTCAACGCCTGAATTTGAGCATTT
TGTAGCGATGCCCAAATATTATCGTTAATCGTGCTGTGGGTGATTCGCTGGTTAATCAACTGAAAACCAGGTTCAGGGTT
ATTAGTCCGCGCGCTAACGGAGAGCAACGTATTCATGCCACTGACTTTACTGCTCATGGTTACCGAATTTTCCGCACTCC
AGCGTTTCAGGCTGGAAGAGGAGAGTTCGCCAACGCCGCTTCCGCTAACTGCTTTATTTGCCAGCGCGATAAGTGATTTT
TGCTGCGCAGGGAAACTTAAATCGCCTTTATTCGATACGGCAATAATTTGTAGCTTTTGCTCTTCACCCGCGGATTTTGC
CAGAATAACCCTGGCACCATTGGAAAGTGTTAATGATGTCAGATTCTCCGCCAGCGTTTCTTTGCTGCTAATTTCCGCCT
GTGGGTCTGCGTCTACTGTTAACGATAAATTTCTGCCTGGGAAGACGTAAGCCGCCAGCTTTTTGTTGGCGTACTCCTTT
TCCAGCGCCAGGATAGCCGCAGGAGACAATGCTTTTTTGGCGGCAACCTCATTGTTTACCATTTGCTCCCAAAATGCGTC
CTGGTTCTTTCTTAACTGCTGCCATTTTTCCGCCAGACTTTGCACGGTAATTTGCTGCCACAGACGTTTCGAAAGTTGAT
ATGTCTCTTCCGGCGACAAGAACGGCGTATTATTTAATGAGCTGGATGCCAGGCGACTGGTCAGCATACGTAAATCACGT
TCAGCTTGCTGATCAACCGCATTTTTCAGCCAGGTGAGGCGGGTAGATTTGACATCATCGAGTTCTTCAGCAGAAAAACC
ATGCTGATCAATGGTTGCCAACTCTGCCATTAATGCATTCGCAGCATCCTGCATATTATCGTCTCGTGCATTTACACGGA
AAAACAGCGACTGATAATCGGGTGCAATTTTAACGCTGCGCGCAGTGCCGCCAGAAATAGTCTTCAACTCGCCCGACTGT
ATGCGTTCCTGCAGACGTTGATTGAACAGCTGAACTAACATGCTCCATTCAGCTTGTTCGATAAAGCTTTGCTCATCGTT
CACTTGTACCATTGGCAGGCGATAATAGAGTGCGATGCCGTTCACCCGGTTTTCTTTATCATTAATGATATTAAAGCGCA
GGTGGTTTTCGGCTTTTGTCGGCCAGACGCGATTTTCAGCTGCTTTGTTAGCCGGAAGCTTACTTAAATTATCCTTTATC
AGCGCCAGCGCTTCTTTACTGTCGATATCGCCGACCACGATAAAGGTCATATTATTTGGTTGATACCAGCGTTGATAAAA
TTGGCGCAATTGTGCCGGTGTGACCGTGGCGACAGTATCCATCAGGCCGATAGGTTCACGGTCTAAATTACGGGTATTTG
CCAGCAGGAAAGGGCGGCGCGCCTGAGAGGTGCGCCATTTCGCATCCTGATGGGCACGCCATTCCTCAGTAATTACGCCA
CGTTCAGCGTCTACTTCGAGTTTTTCAAAGGTTGCGGCATTACTCCATTCACTGAAGATTGCCATCACTTGTTGCAGATT
TTGTTTCTGCGTAGTCGGCAAACTCACCTGATACACCGTTTCGTCATAGCTGGTATAGGCATTAACATCGCGACCAAAAC
GCAGGCCCATTGACTCAAATGTTTCGATGACTTTATTACCCGGCCATGTTTTTGTGCCGTTAAACATCATATGTTCTACA
AAATGAGCCACGCCGAGCTCATTGTCTTCTTCCTGCAATGAACCGGTATGAATTTGCAGCCATAAATTTACCTGATCCTT
TGGATGAGCATGCGGATAAATCATATATCGCAAGCCATTGTCCAGTTGCCCGGTAATTAACTTTTCATCCTGCGGTAAGG
CGGCGGCAATCAGCCGCCCGGGGAGCAACAGAGTTGCCACTAACGTCAGTAAGAAACAGAGGTTTCTCATAATTATCTCC
ATGCGAAAACCGGGCGAATTTACCCGGTTAAGTAAAATCCGAACTATTAAAATTTCATGCTGACATCAAGCCAGAAAGTA
CGGCCACTGGCGTAGGTCGCCACACCGGTGTTCGTTGTATCAACAGCGGTTTTGCTATCCAGTACATTGAGAATATCGGC
ACTGATCGTCAGGTTTTGTTGTTGCAGAAATTGTGGCGTCCAGGACAAGCGGGTGTCCCAGGTCAGGCTGCTATCCAGTT
TTTCGTCAACATACTGCTTGTAATCTGAATATTCGCTGATGTATTGCGCATTTGTCTTACCAAGGATAATGCGAGCTTTA
CGCGCTTCTTGCCAGGCCAGCGTATTCGCCCACACTAACCCGCTCGGTTGATGCGTGAAATCCATGTTTAAGGAGATCTT
TAATGGGTTATTAAAATCTGCCACTGGAACGCTATCGTAAGAGACCAGATTACCGTTATAAACCACCTGGTTATCACCGG
TATTGCTCTCCTCATAACCATTGTTTAACGACAAGTTGCCCTTGCTCTTGATATAGCTAAAGACAATTTGTGGGTTAATA
TCTACCTGGCGGATATGCAGGGGTTCGGCCAGTTCAAAACTGAGGCTGAACGAATGCGTTTTGGTTTTGCCATCGTTGTT
ATATTCAGTAATGGTGGTTTTAGTCGCGCTGTCGGTACGACTGCTTTTGCTGATTTGATCATGCGCTTCACGGTAAACAT
AGTTTGCACGCGCAATAACGTTCTTACCGATTTTCTGCTGCAATCCCATTGCCAGTTCATCGTTATAAGGCGTTTTCAAA
TCCTGATAACGCGTCAGAGTTTTATTACCTGATACCGATTCCGTCCAGCTATTGCGGATATCACGTAATCCCATATCAAG
AATATTCCCGCCATAGTAACGGTTATAACCTGCTGTAATCATTGAGGTTTGATTAGCAAAAATATCCCATTCCGTCATAA
AGCGCGGGGAGATATTGTGGTTTGACAGATAGTTGTCATAGTCATACCGCACGCCGGGCATTAATGACACATTACGCCAG
CTAATGCGATCCGCCATATACAGCGTGTAGTTGTCAATTCCCAGGCGGCCTTTACCTTTATGGTAAATGGTATGGTTAGT
TTTCTTTCCGGCAGCATTAATCACATAGGATTCAGACTGGTTATGGCGTTCAGTCCACGCATCGGAATAGATGTATTCCG
CGCCGAAGTAGGGTTGATGCGAAACATTACCCACGGCGAATTTTTGCCAGTCCAGGCGTGTTTTGAAGGTGTAATTATCT
ACAGCCTGGGAAATGTGTCCTAATCCGCCACGGGTGCAACGCCCTGTAATATCACCATATGTACATGAAAGTTCGGTGTA
CCAGATGTCATGATCGTGACGGGTATAATCACTAATATGATCCCAACCAACGGTGGTACGCAGTTTGGCCCATGCGAGCT
GCGTATCCATATCCCATGCCAGACCATAGGATTTATTACCCATTTCGCGATCAGACTGCGGGAAGGTGCTGGTATTATAA
TCACGGCTGGAGCCGGTATATTTTAAGGTTAAATCGTGGGTAAAGCGGTCGCTGGCAAACCAGGTAAATTTGCTTAATGC
AGTATCGATAACGTTTTTATACTGTGCCCGACCGGCGACAATGCCGTCATTCGAAACATAATCCGCGCGGGTGATATCAG

ACTGGCGGCGCGATAAACCGGCGGTAACGCCGAAGTTATCAGCGAGTTCCTGATTAAACGACAAGGTATAAAAGTTCTTT
TTAAAATCTGGGGAGTAATAAGTACTTCCTGAAGAGCCTTGATTAAATGCGCTCTTGTTATTCTCATCGATATGCGATGT
TAACCAGTCCGAACGCGTAGTGCGATAACCCAATTTCACCTTGCTATCATCAGCGTTGAAGCGTTTGATCTTTGCATCAA
TTACCCCGCCATTGAAGCGACCAAATTCAACCGGCACAAAACTGTCATAAAGCGTCACATTGTCCAGTAAGCTGACATCA
AGATAATACCCCTGTGACATCCCGCTAATATTGGTTGCACTACTGGCATCGGACTCATTCGCTGGGTTCAGGTTATTAGT
TGCACTAATACCGTCAATCAAATAGGCATTCTGGTAGGGCGACGCACCGTGAATAGAGATTTTCTCAGGGCGAATATCTC
CCTGGTTCAACGAGGTACTTTGCGTTGAATCCATGCGTACCGCAGGGTTGGTTCTCAGCAGATCGCTGATATTGCCATTC
CCGGTCGGTAAACGCTCGATGCTTTCACTGGTGTAATGCGTGTTGCCGTTTACGGGTACAGGGACAGGAGCATAGACCGT
CATCTCTTCAAAAAAATGCATGTACATGTTTTTGTCATCGACGGCCTGCGCCACATCAGCGCCACATAAAATGACGCCAG
GAATAAGAACTCGCTTCATTTATTTATATCTCCGTACAGCAGGGCTTTTATTGTTTTTATAAAACCGCGCTAATGTCACA
AATATCATCGGCCAGGTTCCAGACACCGGGTTGATGTGTAACCATAATGACGCCGCTTGTGGGTAGTTTTTCACGCACTA
AACGCAGTAAGCGGATAGCCTCTTGTTCCTCAAGATGAGAGGTAGTTTCGTCAAGAAATATCCATTTCGGACGTCGTAAA
ATTAATCGTGCCAGGGCGATACGTTGTTTTTCGCCGCTGGAAAGAATATCTCCCCAGCGATCGTGGTCATGAATACGCGC
AGCCAATTTCCCAAGACCAACCTGATGCAGTACTTCGCTCAACGATTTATCGTCTACGGGCAGGGGAAGTGCTTTACAAA
TAATCTCTTTCAGTAAGCCGGTTTTGATTAACGGTGTTTGTGACACATACCAACTGTCAGCAGGAGAAGAAATATCACCT
TTAAACCACGGCCAGCAGTGGGATAATGTTTTAAGCAGTGTGGTTTTTCCCGCGCCAGAGTAGCCTTTCAGTAATAGCCA
TTTGCCTGGCGAAACATGAAAGTTCAGGTTCTCTAATATGATCTTATTATCAGGCGTACGAATACTCGCATCAGCCACTT
GTACCGCATGTTGGCAATTTTTAGGCTTATTCGTAGGGCGCTGTTCAGTGAGTTGATGGAACTCATACAAGCGATCGATA
ACCGCAGCCAGTTCAGCAAGTTCGTCATATTTATAAATAAACCAGCTTAAATTGTTCGATACCAGCATAAATGCCTGGCG
CGATTTCATCAGTCCGCCCAGATTGATCTGCCCGCTAATAAACTGCGGCAATAACAGAAAGTAGGGAAGAACGCTAAGCG
AGCGCGAATAAATATTCTGCCAGTAATCAAGCCACCGCTGACGATTCATTAAACGATGCCAGTTCTCTTTAATGGTATGA
AAATTGTCGCTCAACTCCTGGCGTTGTAGAGACTCAGCATTGCTTAGCGCAATCAGTTCTGCCTGCTTATTATGCTGCAC
AAGATTTGTTCGAAATGTCGCTTCGCTACGTTGTTTTTCCACATTAAGCGGACGAATACGTTTACCCACCTTATGGGTAA
ATAAAGTTCCACCGATCACAATGAGCACGACGGTATAGACCATATACCCCTGGATATTCCATTCTGTTCCACCAACAGTG
AATGAGAGCGTACCCGCGCTTTCCCAAAGAATAACGGTAAAGGTGATCAGCATGCTAAGTGACTGGATGAAGCCAAATGA
AAGACTGAGCGTTTTGCTGATCAGTAAGAGAATGTCTTCAGCGATACGTTGGTCAGGGTTATCTGTATTTTTATGCTCGC
CGTAGATCTGCGTGAAGTAGTAGTTTTTATCTGCAAACCACCGATTAAGGTAGTAATCTGTTAACCATTCGCGCCAGCGA
ATAGTCAGTAGTTTAATTAACCATGTTTTGTTTACGGATATTAATACAAAGATCCCCAGCAGGGCAGGGAACCAAAGGAC
AAGCTGCCAAAGCTTATCGGTTTCTTTCTGGCTTAGCGCATTGAAAAAATCATTATTCCAGTCGTTTAACCAGACCTGAA
TTTTAACCACGCCAAGGATCATGGCGAGGATAATAATGATTAACAACACTGAAGTTTTATTATTCTTACGCAGCCAAAAG
GGCTTCAACAGACAGAGATACTTTGCTATCAACATACGAAGCGTAATGGGAATGGTTATCATTAGCGAAAATTGATTGTG
CCATCCAATGATTATCTATGGCAATGATTTACTGATGAAATTTATTTAAGTATCATTTAACAATATGAAATATAAAGGAA
TATTTCATTTTCAGTGGTTATGGTTATGCTATTGATAATCAAGGATATTGCTAATGCCCTGATGCATACCACGTAAGCCA
GGATTTTCGCAAGGGAAGATGATGATGACGAAACACCCGACAGGAATTTATGTCGGGTGCCTTGTTAAGGTCATAAGAAG
GAGGCTAAGAATGGAGTTAAAAGAGAGCGTTATTAATTATTCTCCATTTGTTTTGCAACATCCATAATGTGGTAAAGCGG
TACTCTGTTCTTAGCTAACTCTACCATGGCGTTCATATAAGGTACCATGGTTGAAAAAAGGATTTTATAACCTTCGCAAA
AATAAGAAACAGTCTCATTGTTTACTTTAGTAATACGATGCTTAGGACAACCGCCATTGCAGATAGGTTTATATGCACAT
TGCTGACATTTCGCTGGAATCCGTTTTTTTTGCGCTGTCAGTTGTACACTGTTCATCGTTTTGAGTTCAGATTTATTAAT
GTTTCCAATTTTGTACTGTGGATAGACAAAATGGTCGCATTCGTAAATGTCTCCATTACTTTCAACAACCAGATTATCCT
TGCAGGACTCCTGGAAAATACAACTGGTATGCCCATTCCCCAAAAAACGGCTGACAAAGCTTTCAAACTGACGGATGAAA
ATTTCACCCACATCGTTTTTAACCCATTGCATAAAAATGGTTGACATAAACTTGCCATAAGCCGTGGGAGGCACAGAAAA
ATCAATGATACGGAATGTGTTCTCACTATGACCACTGAAATCAATATTCGGCGTCCCGGTTTCTAGCAATTCGATAAATT
GCATATGTTTACTGCCGATAGATTTTAAAAAATGATAAACCTCAAGAGGGTAATGGACATTAACGTTATTAATGACGGTT
AACGTATTAAACTCTACTTGATATGATTTCAGACGCTCGATGGCTGCTATCACTTTTGCAAAAGTACCGTTACCTGAATT
ACTGCGTCTGTAACGGTCATGTAACTCCTGGGGGCCATCGATCGAGATACCAACCAGAAATTCATGTTCTTTGAGAAAGG
CACACCATTCATTATTCAATAAAATGCCATTCGTTTGTAATGCATTAAAAATACGTTTTTGGCCTGCATAGCGTTGTTGA
TAGTGAATAACTTTACGGAAAAAATCCAGGCCAGCCAGAGTGGGTTCACCGCCTTGCCAGGTAAAATAGACCTGATTGCC
AGACGCTGCGATATATTGTTTGATGAACTCTTTCAGAGTGCTGTCATCCATCCATTTTTCATGAGTAAACTGCGACTCTT
TTTCAAGGTAAAAACAGTAATCACATTTGAGATTACATTGAAAACTGGAGGGCTTGGCTGTAACGTGCATCGCTATCTCG
CTCAATAAGGCGGCGGAAAAATCCGCCGCATGAAGGTTTAGTTATTTCGCTTCGCTTAGTGCTTTCTTGATATTGTTAAA
CTTCTCCTGATTTACCTCGCTAAGCGGTGGCTGGCTGCTGTCGATAAACTCTCTTACCACGCCTTGCATCTCTTTAACGA
CCTGCGGATTGGCGGCGGCAAGGTTATCTTTTTGCTGTAGATCCGTCAGTTTGTAGAGACCTAACTGATTGTTTTCTACT
GTATAGACAAGCGAATAATCGTTATTTCTCACCGTATAAGAGAATTGGCTTAAGTCCTCAGTGTTGGGGTTATGCGGGTA
ATCGTCTGACTGATGGCGAACAAATTTGTGGTAATTATCCCAGAATGGAATATTTTCCTCGTCAAACCAGTGAGAATAAG
AGGTTATCCAGGTCAGATTTTTATGTGGCTCGCCTTGTTTCTTATCTTGCAACCAGGGCAGCAAGGAAACGCCATCCAGC
TTAAGGTCTTTTGGAATGCTGATATCGGCTGCATCAAGAGCTGTCGGGTAGAAATCCATTGCGGAAATCAGCTTGTCATA

ATTACCGGGTTGAAGTTTTCCTTTCCACCACATAAACATTGGGGTGTGAGTACCGCCAGGATAGGTCTGACTCTTATAGC
CTTTTTGCGCCCCGTTCAGCGGCAGAGGACCATCGATAACCGCACCATTATCGGAGGTAAAGAGAATAATTGTATTGTCA
TACTGTCCGTTTTTCTTCAGTTGTTCGAGAATGCGTTTTACACCCTGATCAACAGAATAAACGGAAGCGTAGTAGTTATC
TGCTGTTTGACTACCGGTATTAAATTGCTTCTGATATTGATCCGGTGCAGGATTATCATTTGGCAGGTGCGGAGCATTAT
AAGCCAGGTAAAGCATAAAAGGCTGGTCAAGTGTTTTGGCACGATCAACAACGCCAATTGCCTCATCGGTTAACTGATCG
CTGATATAACCTTTTGCGGGGACACGTTCACGATTTTTGAACAGTGAAGGGGAGTTGTAATATGCCGTTCCTGCAGCGTG
GAATCCCATAAAGTAATCAAAGCCACGGTTTTGAGGTTGCCATTCTTCCGCAGAAAATGTGGTGAAGTTGTCATGATAGT
CACGCGTTTGTTTATCTTCCGGTACCGGCACATTACTGATTTTTGACAAGTGCCATTTACCTACTGCTGCAGTGTAATAA
CCATGATTCTGGAATAATTCAGGCAAGAAAGTTTCTGTTAGCGGAATACCATCCTGAGCATCGGTATTGGAATAGACACC
AAAGCGGGCGGGAGCTCGACCGGTCATTATTGCGGCGCGGGAGGGGCCGGAAACACCGTGTGCCACATAGCCGTTAGTAA
AACGTACGCCTTCATCCATTAATGAAAGGAGCGTCGGCGTTGATTTTTGTGCAGCTTCAATGGCTTTATCTATCCCTATT
TTGTAGGTATCGACAACTTCACGATTTTCCATTGTTTTTGGGTCAAAAGATCCCTTATCAAAAGGAAGTTGTCCATAACC
AAGATCATCCATGGTCAGTACGATAATATTTGGCTTTCCTTTGGTACTGTATTCTGTCGGCGTAAAGTCTGAGAAAGCAA
CGTTTGTTTTGGTTGCTTTCAGCTTTACATCATCTGCCGCATGAGCAGCAAATGCAGCCATACCAGATGCCAGTATCAAA
GATATCGAGGTACTTACGACACTTTTCTTTAATGCAGACTTCATAAATGTTCCTCTTCTTATTGTACTTAATTCCAAAGT
AATTTTACGAAGAGAAATAAGTGGATGTAAGTGAAGTTAGTCACATAAAGAGATAGCAGATTTAGCTAAAAAAAGGGAAA
AAACAGTCCATAAAGCGTTGACATTACTTTCTGTTCTATTAAGTAATTTCTCGCCGATAAACAACTAATTTATTGATATT
TAATAAATTATTGCATTTTACTGACAAAATGCAGAATTGAGATCATAAATAATCATGCAACAGGTTATGCAAGTGCATAA
ATATGTGATGGATGTCACTTATTTATTTCAATAAATATATCGCCTAAAAACAACGCGGGGCAGGGAATGGCTGCCCCATT
TAATTCTTACGCAGCGTGTGTGGTTGACTACTCGTTAGCAAATAATCAAATAGCTAAAGCATTCATCGTGTTGCCCGTAT
TCATACCCGTGTGACTTTGACAACGGTACTCCTTAGAAACTCTCTTCGGACTGTTGCCGAAATGTTTGCGGAACGCATAA
ATAAAATAAGATGTACTGGCATAACCACATTGTTCGGCAATTTTATTGACTGAACCTTCTACGCGTATCAAATTTTTTGC
GTGCTGCATTCTTGCATCTAAAAGAATCTGTGAGAATGTCGTTTGCTCTTGCTTAAGTTTTTTCTTCAACAGGCTTTCAC
TGATGTACAGGCAGTCACAAATATCTTTCAGCTTCCATGGGTGCGCCGGCTTCATGTTGACAATATTTCTCACTTTCCCA
GAAACGGATAGCACACCGTTAGTTAATAGTGTAATGAAACCTTTGCATGCGGCAAAAATAGACAGGCAAGAAAAAAGCAA
AAGTTCTGAAAAATTATGATGATTGCGTTCTTCCGAGTTGAGATAAGCAATCATTTCATTAAGCAATCCAGTCGGTACAT
TTGAAGCACGTAAAAACTTCGCAGGAACAGGCACCGTCACTGTCTGGATGTTAGTGCATTGTAGATATTTTTTAATGGTA
TTTTCAGTAAATCCGATCTCTTTAAAATGCCGCTCATAAGGTTCAAAATGCGTACGAACAGCGTTATCTACCATCAGGAT
CTCGCCGTCAGAGAAGGCGTAATCTTTATCTAAAATGTTAGCGTTGAAGGCATGATGAATAAATATAACAGAACAAACGA
GCGACATTTTATCTCCTTAAAACAATAAAGTTTTTATCGATACTTTTTTAACATTTCATATTTATAATTTGCTGTTTGTT
TTCAGCCTTGCAAACTATTGATAATGAAATGTGTAAGATCTCTTGCAATGCGACCCATTTCTGAAATACTCAGCTGATCA
ATTGCCGAAAAAATGGCTATCAACGTAAGTGTCAGTAATAAGACAACCACAATTGCAGACATGATTTCTTTATAGTTGTC
TCTTTGCGTGATTTTGTTTTTCACTGTGGTCGCATGCATTTCAAATATGTTTATTTAGCGGATAACGTTAAAAATCGTTA
ATCAGTATGCTTAATATAACAGGCTGGAGAAAAGAGGAAATAGGACTGGTGCTTCAGTACTGAGCGGAGTTTCTTACAGC
TGTAGGCAGAAGTTTATATCTTATAGTATGTAGTTTAATTTAATCCATGTGAGCAAATTGCGAAATAATAGTCAATGAGG
AATTCTTCTGCTGTGCGGATGACAGCAGAAGAAATGAGAAGAGGCATTAATTTGATGGTTCTAATTCAACCGGAATACTT
TTATAGCCAGGAATGCCACTTAATGGATCGTGGTTATCAAGTGTTAGCATGTGATTCGATTCTGGAAAATAGGTCACCAG
TGAGCGGTCAGCCATAGGGTAAATGACCACTTTTAATCTATCCATGCGGCGTGAGCTGCGCTTACCGTCTGGCGTAAGCG
CAATAAGATTAACTCTTTCGCCGTTTTTTACACGGCAAATTTTAGCTTGTTTAGCACTCATAAAGACCACATCTCGTTGA
CCGAATACCCCTCGATAGCGATCATCCATACCATAAATCGTCGTGTTGTACTGATCGTGGCTGCGTACTGTCGCCATGAC
CAGCTTACTGTTAAACGCTGAAGAGGGATCTTCTAACAGCCCTTTGCTGGTAATGAAATTAGCCTTACCTGACGGCGTCA
TCCAGCGCCTTTCAGCAGCTGCATTTATCAGGTGAAAACCACCGGGATGACGGATGCGCTGGTTATAGTCGGCGAACTCT
GGCAGCACAGCTTCAATGTCATTGCGAATGCGATCATAATCTTCCACCAGATACTCCCAGGCTACCACGCTCTGGGGTAG
TGCTGCCTGCGCGATTCCCGCGACCACTGCACACTCTGATTTCAGCATTACACCGGCGGGTTTTAACACGCCACGCGAGG
CATGAATCATCGACATTGAATCCTCAACGGTTACCGCCTGCGCACCGTTTTTTTGCATGTCAATCTCGCTACGTCCCAGG
ACCGGCAGAATATAGCTATGCCGTGCGGTCAACAGATGAGAGCGGTTAAGCTTAGTGGCTACGTGTACCGCCAAATCTAA
TTGCGTTAACGGTACAGCGCTCGCTTCCCGATCTGGCATTGCCAGCGCAAAATTGCCCCCCATGCAGATCAATGCTCGAG
CCTGCCCCGTACATATTGCTTGCATGCTGGCAATTGCAGCATGTCCAGGTGCATGAGGTGGGGTGAAGCCATAGCGCTCA
CCCAGACGAGCCAGAAACTCTGCAGACGGTTTCTCGGTGATACCGACGGTTCGGTCGCCCTGTACATTAGAGTGTCCACG
TAGTGGGCAGATACCCGCACCAGGCTTGCCAATGTTACCTTTCATCAACAGCAGATTGACCAGTTGCTGTACGTTCTGGG
TACCATGTTCGTGCTGAGTGATCCCCATTCCGTAACAGATAATGGTGCGTTCGGCAGCGGCATATGCGTCAGCCAGTTCG
GCGATTTGTGTCTGACTTAGTCCAGAAATACGTTCGATATCTTTCCACTCGGAATTGAGAACGTCACGGCGTAGCTCGTC
AAAGCCGACGGTATGCGTTTGAATAAACTCGTCATCAAGCAATGAAGGCCGACCTGCGGCGCTTGCAGCATCATCGCGCT
CAATTAACAGGCGCATCATCCCCTTGAGCAACGCCATATCGCCACCAATGCGCACGTTATAGTAGGCACTGGCCAACTGA
GTCTCAGAGTTCGTCAGCATTTCAAACGGGTTTTGCGGTGCGGTAAATCGCTCCAGGCCACGTTCCTGTAGAGGATTGAT
GGCGATCATTTTCGCTCCCCGTTTCACTAAAGCGCGCAACGAAGTCAGCATGCGAGGGTGGTTTGTACCAGGGTTATGCC

CAATGCAAATGACTAAATCGCACTTCTCAAAGTCTTCCAGCAACACGGTCCCTTTACCTACACCGATACTCGCTGCCAAA
CCCACGCTTGTCGGTTCATGGCACATGTTGGAGCAGTCGGGAAAGTTATTGCTCCCGTATTCACGGGCAAAAAGCTGATA
AAGAAAGGCAGCTTCATTGGAAGTGCGGCCCGAAGTATAGAATTCAACCTGATTGGGATCACTATAGCTTTGAAGGCGTG
CGCCAATTTCGTCGAAAGCTTGTTGCCAGCTTAATGGCTTGTAACAGTCGCTGACGGCATCATATTTCAAAGGCTGAGTG
AGTCGCCCCGCAGCCTCAAGCTCGTGGTCTCCCCAGGTAAGTAATGATTGAACCGTATTCTCAGCAAAGAAAGAGGCGTT
TACCTGCTTATCCGTGACTTCCCAGGCGATTGCTTTTGCGCCGTTTTCACAAATGTCGAATGACGCACTGTGCTTAGGAT
CTGGCCATGCACAACCCGGACAGTCAAAGCCCTCTGGCTTATTCATGTCAAACATGGCAATAACATCCTGGCGTATATCC
ATCTGCTTACGTACTGCATTCGCTACGGATTTAACAGCACCCCAACCACCTGCAGCACCCTGGTAGGATTCAATTTTTTT
CTTCATCTACTTATCCTGTGTGGAAATGAATTATTAGGATTTGTAATAGCGATGATGCACAGTACATACAAAGGGATTAT
CGCCAACATGGATATATAAGAGTAGGGGACTACTCTCTTTCTATTTTTAATCTATTATCGTTAAACAATAGATTAAATAC
GATAAGTTAAGCATCATCTCTCATAGTGTTTAAATTAACATATTTTTCAATATAAAAAATAAGACTCTGGCTTCAATTGT
GCGCGGATTTTCTTACAGGTGTAGGCTAATAGCCTTCACAAAATAATTATCCGCGCAATTTTCGTGAATGGAGCGCATAG
ACTAAGGTCAAAATCACAATTATTCATAGATAAAAGTGACACCAATGACTGACTGGACGGTTCCGGCTGAAACCTTGTTT
CCTGTTTGTCCATAGGTTGCGCTTAAACCCAGAGGTACTTTGCTTTTATTTACTGTCCCCAGGGAGACATTTTCTCCCGT
AGCCAGTATTTTTCCATTACGCATTAGAGTGACACCCACTCCCGATGCTTTTGTTGCATCAGGTGCTGTGTTAGCAAACA
CTTGTCGGCTACTGTCAGTGGTGGCACCCGAAAGATAAAATGAGAGCTTTTGTTCGCTTGAGCAATATACGCCAAGGGGT
ATTTCTGCGCTTCCAGGAAAGTCCGGGAGATCCACGGTGACATTTCGACTATCAACGGTGCAGCCGCCTGTTGGCATAAC
AACATTGTTATTTGAAATAATATTCCACGTAAAATTACGCGGATTTCCGCTTCCTAATGTTGCAATTTTATACATATGAA
TACGGGCAATGACTTCCCCCGCTTTTATGACAACTCCACCGGCCGCCCCAACTGGGGTAATATAGAGTTTTAACGGTAAT
GGCATCGGTGTCTTATCACCAATATCCAATACGTTGGTATTTGTCGTCAACGGGAAGGGGTAGGTCACATTATTCCAGTA
CAGCGATCCTTTATATGACTGTAGGGAGCCTGCGAAAGCTGAGCCTTGTACCAGGTTTATATGATCAGTGTCGTACCAGC
CGCCGTAATCATTCCAGCAACTTATATGCTGAGACAAGTCTACAACCAGATTCTGGCCCGGCTGTATTACAGGGTCGAGA
TTAACATAAACAGAGGTTGTACCTGCTCCAATACTTGACCCGCCATCGACATTACAACTGAAGGCAAAAACTTTGCCTGC
CATTAATAGAAGATAAATACCGAACAGGACCTTTATACTGATTGTTTTACCCATGATATATCCTAAGGTTAAAAATTGAT
TTAAAAAGAAGGCTAAGAAAATCGAATCGACGTTATTGCCAGGTGTAGATCACATTGATTAGCGCCTCGATCGTTCCCTG
GCTTGCGTTTCCATTCACCGTGATAGCTCTTGCCTTAAGTGGAAACTGTGCATTACGAGTGATCTCATCAACAATAACCG
TTTTGCTATCGCCATTTTTTAACGCAGCATCCTGGTCATCCCTCAGCTCTATCTGAATATTTTCGGCAGTACCTTCATTT
TTGTAATAACCCGTATTGTCAGTTGAACCTGTCACGATTGCCGTCACTGCACTTGTTTCAACCGGACAATCGGTTAATGA
CAAAGTAATATTGTGCCAGCCAGATGCAGAACCAGGTTGTTGCAGATTGCGCGTATAAAGATCCCCGAGATTAACGTTAG
CTTCTTTGGTTTGAATAGTGCAGGGTTTAGCGACTACCCGACCATTAACAGTGATAGTGACATCGGCGGCCTGGAGAGTT
GCTGTGAATAACGCGCAAAACGTAGCTAACAGGAACCTCTTGTGAAGTCTTTTCATATCTGACTCTGATTATTGAAATTC
AAGGGTAAAGGTTGCCGAAGCCCTCACCAGTCCCGGATTGACGGACTTCTGAGTTGACTTCAGACGAGCGGAGTAAGGCA
AAATATTGTTCTGTTCTGGTACCAGTGGGATCCACTGCATCCCGGCATGAAGATCATTCAGTTTCACCGGACGCATATTT
GCGTCCAATATTTCTATACCCAAACCGGAGGCCGTATTGCTTCCTTCATCCAGTTTCAACAAAGTATTATTTTCTGCATC
CTCAATACCGTTAAATGCAACCCGAACCCCCGTTGTCCCTTTGCTGCATTCACTTAACGTAATCTGAAAAGGGACGGCTG
GACTTGTGCTACCGGTCGTTGGAAATTGTCTGGCACTGTTTTTTTGGAGATCTACGGTAAAATTAAGCGAATCCGATGAG
ACTGTGCAGCCATAATCGAGGACGCGCCCGCTAATTTTAATAACGCTATCTGCGGATAAAGCAGAATAGGTGGTTAACCC
CAGACATAAACCGAGGAAAATAATGTTATTGTATTTCATAATCTATTGTTCCTTAGCGACAGATTGCTGTCTGCTGGTTC
AGTAAGGTACCAGGAGAAACTTCAGGAAGCTTGTACTCGACAATACAGTTTGAGTTTTTATCTTTGCCCCATGAAACCTG
TAATTGCCCTGACTGTGGAAGTCCAGTCAGATAAACCTGACCATTTTCCGCGACAATGCTGCCATTTTTATTCTCTCCGT
GTGTGACAATTGCACCGAATGGAACGCTCTTATTACCGTACTTCAACGTCATTAATACTTTCCCGCCGATTTGTGCATTA
AATGTTGCTCTGGCAATAGCACCGTGAGTTGGGATGACAGTGACCACGGTTTCATCCAGTTCAACATTATCTGCAAGGGA
ATTCGCGTTAAGAGCAACACGGTTTTCTCTATATTCTGTCGCAAATGGTAATATGGCATAGCCACGCCAGTCGGTATGAA
TTCCGGTCTGGTTCTCTATTTTGACATTATCAGCACCAGGAGCCTTAACCAGAACCATTGTGTCGCCCAGCGGCTGTCCA
AAGGTGATGCCATCAGCATGAGCAATAATCCCACCACTCATTCCGTAATAAATCTGGCTGCTGTCACCACTCCGACTGTA
ACCGACATTAGTATTACCATAAGCTCCACGATAATTAAGAGAACTGTAACCACTGGTGCCAGACGATGTATTACCTCCGT
GGGTGTTACCGACCTGAACGCTATAATTCAGGTTATTATCCGGCAGCAGAGTGCCATAAACCCCCGATAGATTGGTCATG
CCGCCTTTCAAATCGTTTGACATACTGTAACTGGCGTTTGAATTACGAAATGCCGACTGACTGTCTGTACGCATCCAATG
ACTGAAGGGAACATTAAGCGTAAAAGCGAGTAAATGATCCCGATCGTTTTGCCATATATTATTGGAATAGCTGTAATTCA
GCGAAGTCGTAATATCACCAAACGGCACATTTAATCCAAATGATATTTGCTGGTCGCTGCGTGACGTGTTCCAGTAACTT
TGGCGACTGGCACTGAAAAATGTCGTACCGTAATTTCCAAGCTGCTGAGAGATGCTTATTTGTTCCTGACCACGCTTACT
GTAGAACAGATTAAAATAATCAATAAATTGTGTCTGCTCATTGGTGTCTCCGGTAGGAGGCTTGACGGTGTAACCACTCA
TTCGACTGTAGGCACTGTCGGATAAGTTATAAAAACCTTGCGTAGAATAGCGATATCCTGCGACCTGGATATTGGTTCCA
CAACAAGTTTATTGCTGCACTTGTTGAAGAGAGTATTTGCAAAAACATTTTATGTCGCTAAATAACATGACGATTTCATT
ATGCATGTTATTTATAAACCGTGACTAAACAGAATATTATTGCGAACAAGGGAAGTGTCAGGAAAGGTCAAGAGCTACCA
ATGGTTAATATGGCTATAAATCATTAGCTATGGTTAATGTTTGCTTGCTGAAACTAACACCGAATTGCTCTAAACAGATA

AGTGCAGGGTTTATTAAAGCTGTCCGACAGACTTTCTTAACCTGGTAATATCTCTCTTGGGTGACTCTCCAAACATCCGC
GAATATTCCCGACTGAAATGGGATAGCTTTCGTAACCGACAGCATAGGCTGCTGTGGTGACATCGTAATGTTCATTCAGC
ATCTGTCGCCTGACTTCATTGAGACGTAGCCATTTCTGATACCGGAGTGGACTCATTCCCGCTTGATCCGTCCTTAAATA
AGTAACAGCACCCTCTGGTGTTTTCGTATGCAGTGTCTATGCGACAGACACTGCGCTCTTAGCATGGGGCAGGGAATAAC
CAGGCTATTTCTCCATGCACAGGCCAATTATGAAAGAGGAGAGGCTTAAGTTCTTGAACCCTGAACGAATAACGACTACC
AGAAAAGCACTTCGCATCACTCAGACATGATTTAACATAATATACATTATGCGCACCAATATAAACCAAGGAAGAATCCA
GTCGTTGGCGGTCATGATTGTCATGCTCATTAACAATGACCAAACCCCATATCTCACTTACTACCGTATTCTCGGCTTAA
CCGTCCATGCAACCTCAACACATTGCTTTCAACTGCCGTCACCACGTTCTCCGGAAAATCTGTCGGTAATGAAGTCTTCA
CGTTATCCAGTGCTGCTGGAATCATTCTGGCAAAGTCACTCAGGATTTCATGCATCTGCACTTCCGGGAATCTCAGCACC
TTTGCTGTCGCCAAAAAATGTCGCGGATAAATTTTATCGATTGCCGTTTTTTTGCCTTTGGATGCGTTAAGCCCCATTGC
CAGTTTGAGATCGCTGATGTGTATTCCCGTACCGCCAAGGACCGGAAATGCTGAAATGATGTCGTAAAATGGCGTGAGTC
GATAACTGCCGCCAGCCTGAATAAATACGGAGAAGTTTTTTGCATGACCGTCCGTTGCGCCAATCAACCACTGGAAGACC
TGGAATTTCATAAAATCATAGCGATCTTTCAGCGCCTCGCTGGACCCCATCAAAAAAGCCATGATCCGCGCGATGCCTGG
GCCTCCATCTGATTCATATTTCACCGATGAAGGTAAACCGAATGTCTGACACATATCCTCCTGTGGCAAGCGAAGTAAAA
CCGTTCGCTCAGCATTCCAACGCCTGTCAAAACGTTCGACCGCTAACGCGCGCACATTTCCCGCTTTAATGATTTCTGCG
TCCGGAACATTCAACCCAAGTTCTTTCGCCAGCAGCAGACAGTAATACTCATTATCAACGCTTTGGCTGAGATCGAGCGT
CGCATTGGGCTGCCTGATTTCGCCAATCGGTAATTTAATGATGTGCGTCGTCGGCGTTATTCCTTTCGGAATGCACCAGT
CATTGCCTATTCTGAGCAGTGCTGTCTTCTCCTGTGCGCCAGCAACCGAGATGCGAAAGTCATTTTCTTCTCTAATCATG
CCTAGCGGGATATCTGCTTTATAAGCCGTTAATACTTCTTCAAGTCTGGCTTCAGTAAGCTTTTCCCATGCCATTATCGG
ATGCGTTACGGTTTCGTCTTCGGGTATTAACGTCACGGCACCAACGCTGTCTCGCCCTATTTCTGACAATAAATCAAACG
GTTGTCTGGATTTGGCATGATAACGTTTAACGATCCGGTCACGTACAATCGGGCTATCGGGTAACAGGTTATCGAAGAAG
TTAAATACGGCATCAGAGGTGATATTCCCCCTCTGCAATGGCAGCGAAAGTGACAACGGTCTGGCATAACGGCTTGCTAA
CCACTCCGGTGCATACTTAAAGGTGTGCGCGCCGTTGGCTAACTTCGTTAACTCGCCTACCCGCTGGTTGTTCATCCAAG
TGACAAGTTTAGGCATTACCACTCCAGATTTTGCTGTTCTGTTGATTCTGGCGAGGCATTTTTCGCGTCGCATAGCGTCA
TTGAGAGTTCAAGCGACTGTAAAATCTTAAAAAATGTCGTGAGCGTGGTATTGTCAGGGTTGTTTTCGAAATTGGAAATC
GTCGCCTGCTTAATACCAATTTTTTTCGCCAGCTCGCTCTGCGTCCAGCCATTTTGCTGGCGAACCAGTTTCATTGCATT
CGCCAATTGCGTTGGGCTATAGATCTTCTGAAAGCTCATCATGTCATACGTCCACAGCAAGTTTATCCGCTTAAGGGGAT
ATTATAAGTTTTATCCTTTAGTGAGGATAAGTCAATCTGGAAACACATCTTATCCGCTCTACGGGATAAACGCTGCGATA
TCCGCGATCGCGGATAAAAAGGAGGATTTAAATAAGGCGTTGATGCGCCGTTTGAAGTCGGCAATATTAAGCCGCATGCC
ATCTCGACATGCGGCTTATACGGTTTACCAGCTATATTTCACTCCCAGCATGCCCTGAGTATCGCTATAGCCTTTATCAC
CTAGTTGCACACCGACATTCCCCCACAGGCTAAGGTTGTTATTTACTTTCGCCTCAACCCCGGTACGTACTTCACCGAGA
TTACGCGCACCTTCACGGCCTACGGTTTGACCATTCATCTTCACGGCGTAGACTTTGCTATTGTTGATCCAGTTCGCTTC
AATGTAAGGCTGGAACTCACGCTGTTTACCATCGTCACGCTGGTGATGGCTGTTCAGGTAGGTTTTCACCCCAAGTCGCG
TTTGCACATTTCCGTCGCCTTCCGTTTCAATGCGCGTTCCGTCTTTCCGGGTATGGTCGGAATCTTTCACACCCATCCAG
GTGATTTGCGCCTGTGGCTGGACGTACCAGGTATTCAGCGTCCCTTCGCTGCCGCTAAATGTTCCCGCTTCAAAGGTATA
CCCACCCTCAACAGAGGCCGTCACACCGCGAGAATCATAGTCGTCAGCAGAACGGTTATCGGAACTGACGCTGTTATCAA
ACCAGTTATACAGCGCCCAGCTGTCAACATAAGCGCCGGTCTTATTCGCATCGTTCTGATACCAGGTCGCGTACAGCCCA
GCGCTGTAACCGCTGATGCGCCCATCCGATTTATAACCCACACGATTACTCTGAGTATTACTGTGCTGATTGGCGTAGCC
TGCCATCACGCCAAGATGCCAGCGATCCTGCGCGTTGCTACTCCACTGCGCCAAATCGCCGCCTAGCTGCAATACATAGC
GGTTAGCCTGAGTATTTAGCTGACCGTCACCGGCCCTTGAACGTTCGTGCCCTCCGACATGACGCATCCACATACTGCTT
GCCGACCCCTGAGAATGCAGTGAATCTGTATACTGCGGCTCACCCAGACGGTCGTGTAAACGATGGCTAAACAGCGAGTT
GGCTGCGGCAATGTTGCTGATATAGCTTCCGGCCTCCGGGCGATAAACTGATGGGCCTTCCGGATCCACAACAGGGGGAT
TATTGATGGGATCGGGTGTATCCGCTGGCGTTACGCCGTCCCATTTACTGGTCAGATACCAGTTTTTCTCGTCATTCCCC
TTCCCTTTAGCCAGCGTGTAGACGTAAGCCCCAGCTTCGACAGTTCCGGTGGTCAGCGCGAAGTTACCTGCAGAATTACC
GCCAACCTCAATGAGTTCAATACCGTTGACCGTTTGTGCACCGACGCCGCCAATGTTATCAACCCGAACGCGAGTGTTCC
CTTGGGTATCGCCTTTCACGTTCATCTTATCGGTGGGCGAATTATCGCCGCCCAGCGTCGCGTTGAACGTCATCAGGCCA
TTGTTTCCGGTATAGTTGCCGTTAACGGTCAGTACATTCCCCGGTTTCCCCCCTTCGCTATTCATTTGAACCGTGCCGCC
ATTCTCCAGGTTGCCGCCGATAGTGGTTTTAGCGACACGCAGTGCCCCCCCCAGGCATAACGTTCATATCACCTGCAGTG
GTGACATTTCCCGACATCACACCGCTGTTATGGATATTAATGTGTTGACTTGCCATATTAATGGCAGAGTCGGAACCGAA
AGCAATTTCCCCCCCCACGATATCCGTCGTACCCTGATACGCCGCGGAATCGTTGTTAAGAGTCAGGATCCCGGTACCCA
GTTTTGTCACATCACCGCTACCGCTGATACTGTGCGTCATCGCCCAGCTACGCTCAGTGGAGATGGTGAGCAGGCCATTA
TTGATAACGGAAGCATCACCCAGGTTTTTCTGCTCGTTGACGCTGAGCGCGCTGCCTGTATCAATGTTGAATTGCCCCAC
AAAGCGGCTATTATCGCCATCCAACTCTACATCTGTCCTGGCGGTTGCGCTCACGATACCCTTCCCGCTTATGCTATTAC
GCAGTTCGCCAGTCACGTTTTTTAGCGTTAACAGACCGTCATTGGCAATATTTCCTCTGCCTAACCCCTGAGTATTATCG
AGGCTGACTTCCGCATTTGGGCTAATGCTGGTTAACGCATTGTAGCGTGCATTAAGCCCCTCGATAGCCAGCGTTCCCCC
TGTAACATTCAGGTTTCCGCCACCTGTCAGTTCACCCTGACTGATCCCACCTTTATTTACCGTCAGCGCCCCCTCTTTGA

ACAAAACAGTCGAACCCATCTGCCCGGTGAATGTCTCTACCGTCTGCGTCGATCCATTAAGATCGACGATGGCCGCGTTG
CTGATGTTCAGTTCCCGGGTGTTGCCAAGTGCACCATCCGCATCGGTACGTAATGTCCCCATCTGAACGTAGGTTGCCCC
CTGATAGTCGTTCTGACCGTTGGAAAGCGAAACCTGTCGCACCGTATTGATTGCCAGATCCCCTTCACCACCGATTTTTG
CCGACATATCGGCCGTTGCGCCATAGGCTCCGCCATGTTCGGCTAACGTCAGCTTTTGCCCACCATGGATGTTCAGCGCT
TTCAGCCCATAGTTAACGTACAAACCATTACCTGGTGCGGTCGTGAGGCGGAATCCATAATCACCCTCGGCGACCACCGT
TCCGTTTTGCGCAATGTGTAACGTTTTGTCCGCTTCCACCTCGTCGCCCTGTAAATCACGTAACGTTAATGAGCCACCCG
ACCCAATAACCGTTTGCGCCTTCACCAGTTGAACGCCGACATGGCTATCGTCGTGTTCCAGCAAATTGAGCGTCGTCAGA
GGGTTATTCGCCATGGGATCATTCCACGGTTTAGGCACGTCGATAAGCACGTCGCCCGTCCCGTTTACCTGATAGTTACG
GCCTTTCCAGGTGTAGTCACCCGCGCCGACAACCAGCGTATCGACGCTGATATATCCCTCCGCGAGCGTCGCAGCAGGAA
TATCCGTATCGAAAATGATGGTACCTCCATTCATGGCCAGTCCACCAATGGATTGCTCTCCAACTTTTACCGATGTGGTA
TTTTCACTGTCAGACTGCAACATCGCGTGAGTAAGCGCAGCGGTGTTGTCGCGTTCCAGAGTGAAGGTACTGTCTTTCAG
TTGGGCAACACCGGCGAATTCAGTCCCTGTTGCATGGGTAAAGCCAAACATCTTGTCGGAGGATGACAGCTGCACTCGCA
TCAAGCCATCGCCTTTGAGCGCATTGGTCAGCGTGTAATCTCCTGCACTGTTCGTTGATGCGAGAATGTCGAGCGTACCG
GGGTTTGCAATGGTTACTTCACTCGACCCCATGCCCGTTACCCCATCTGCCGTATGAGACGCAATGGTCAGGAGGCCGTC
ATTGACCAGCGTTTTACCGCTATAAGTATTCCCTGCATTGAACACCGTATGGCCTGCATCATGGATCACTTTTCCGTCTT
TATCGTCACCTGTAATCAGCATATCGACCTGGTAGCCGGCATCACTGTTATTGGTATGATTAAAGACAAAAACGCCTTCG
CCAAGACCAAACTCCACTTTCGTCGCATTGGTGATAAATCCGGCATCTGCCGCCGCCTCACCGTGAGCAGCACCAATGTT
TAGCGTTCCTACAGCAGGCTCAAAAACACCTAAGTAAACTTCTCCACCTTCGACATTCAGCGTACCGTTATTCGTCAGGG
TTAACGTACCCGTACCAGATGTCCCTACGTACATATTGAATGTTTCAAGAAGAGAGTTCTGCCCATCCACCCTCACGTCG
CCCTTACTTTTGTCATTGAGAGCTATCTGTGTATCACGCGCTTTAACTATACCTCCGGTGGTAATATTCAGTTCACCCGT
GCCTAATACACCGACCTGTAGCAATTGTGCGTTAGTAGATGATGTCTTTAAATTCCAGAGACTGTCCGTTGAAATATTAA
CTATCCCCTTACCGGAGGCATTTCCTCCGATAAATCCATAGCCCGTTGAATTAAGGGTGGCACCATTACTGACATTTACG
ATACCGCTACCAAAATTACCGACATTGATACCCACAGTATCGAACGATGATGTCGCATCCATCAGGTTCAGTGTGCCGAT
ACCGCTTGCTTTATCGCCCAAAAAAGTAATATTTTGCGATACGAACTTACCGCCATCGCTAATATTGAGGTTTCCGACGC
CCGCAACACCAATGGTGGTATAGACATTCTTATTGACCTCCCACATTCCCCCGTGGTGATGCTGACATTCCCGACGCCG
GTTTCTCCATAACCGAGTGAACTTCCTCCGTTGCTGACAACAAGCCCCTGATTACTGATATTCATTTCGCCGTGGCCGTC
ATAACCAAGATTAGTTCCGAGATTAGTGATAACGGAGTTCTTATCCTTAACAGTAATGTTGCCTGTGCCTGTTTCTTTCA
TCCCCGCAGTGATAATTCCCGAATCTACTTTGCCTTCACTCGAGACATTAAGTTCACCGTCGCCAGCATCACCGATATAG
ATATAGCGGAAAGCTTCGCCCGTTCCGAGGAAATTCCAATGCCCTTTATCGGTCACGTTGACGACACCGTGGGAACCGTC
CTGAACGCCCACCAATGAATATTCTTTGTTATTAATCAGTCCATTATTGGAAATATTGACTGTGCCATTACCGAAATATC
CATTATAGAGTCTGCGTACCGTGATGACAGAGTCTTGATCCTGCACATTCAGGGTTCCGATACCGGTGGCATTGCCACCG
ATAATCGTATTTTCAGCCGTAACCAACCCCCCCTCGCGAATAGTCGCCTCCCCAGTTCCTTGATTACCAATTTGAAATTC
AATTGAGGAATCATTATTTTTTATTAGCCACTCGCCACCCTTTTCAACGACAACCTGGCCATTACTGCCCGCCTGATAGC
CTAAAATGGCGACGATTGAACTCGTGACGTAACCCTTATCCGTAATATTTAATGAACCTGTGCCATAGCTACCTATTTCG
AATAATTCGGTCGTCAGAACAGAGTCCTCTCCCTCAACATTGACCGTCCCGACGCCTCCTGTCGAAGAACCTAATCTTAA
ATAGCCTCCATCGACGTGACCCTTCTGTTTAATATTCAGCGTCCCCGTTCCGGATTGACCCACATTTAAAGGCCTTGCAT
TATTTCCGCTATCATACAATCGCCAGGTGCCACCCAAAACATTAACGGTGCCCTCTGAGTCCTCATTAGCGCCAATGACG
CTGGTAGTAATAGTGGTTAAGGAGGCATTACCGCCAGCCAAAATATTAAGCACTCCAGTGCCATACCAGCCAACCAGGTA
GGCATCATAAGCAACATCAGTATCAATATTGGTAATCTGATCATTATCAACTTCCAGACTTGCACCAGACGCTGAACCGC
TTAGTGCCAGCAAAACACCCAGCGTCAATCTACTGAATTTCGTTGTCAGTCCAGAGGATTTACGCAAATTAACCGTCGAT
GTTTTACCTGCCCTGCGAGTTAATTCCGAGCAGGCCTGAAATACCTGTAGAGTGCAATTCCATATCACGCGATAGATTCT
ATTCATATAAACGCTCCATATACAAACAATACAGCCACGAAAGAGATGAGTCGTCAGACATTAAAAATGAAACTTATTAA
ATTGTCAGAGGTCTGTATTGAGTGTTAGTTGGAGGTGGGAAGGTGTCAATTTGGCTAATTTGCTAATGCTTTCGAATTGA
TAGATTAAAAATTGCATAGTTCTTATTTTATTTAAATATGAACTATGAGTTATTGTTGCTTAATAAGAAAACTGTTTCTT
TCAATAGGAAAATTATCATCATCATATTTAACAAAGAATAGCACTAATTGCTAAAAATCGAAGTTTATTAAACCCCTTTT
GCATCCATTAGTGTCTTTAGGAATATTCGCTATAAAATAAGGGTTGTGCTTAATGCTTTAAGAAAAATAGCAATTTTCCC
TTGAATATCGTACTGGTGATGGAACGATGAATCTGCAAGCTGGCTTTTAACAAGCCAGCTCTAAAAGAAGGGAAATAAGA
ATAACTATACTCAAAAACTAACAGCCACGGTCATCATGATGTGGCTGTCAATGAAACTATAACCCAGGCGCTTTCCATAA
CGACGTCGTCAGTCCATGATCAACCAGCCCCAGCTGATCCTGATAAACTGCCTGCCACTTATCGCGTGAATCCTGATAAA
GTTCATGCTTTTCCGGGTCTGGTGTGTGCGTCCGTTCCCAGCGAACCAGGCGTTCTCCGGTTTCTGCCATTGATGAAAAA
ATTCCGGCACCGACGCCAGCTGCAATGGCACATCCTAATGCAGTGGCTTCTTTGACCACCGGAATATTGACGGGTAATCC
CGAGACATCAGCGAGAATTTGACTCCATAATTTCCCTTTTGAACCTCCGCCTGCAAAGACTAACGATGAAGGATGAATAT
TCGAGAAATCAGCAATTTGCTGCAAGTTACACGCTGATACAATCGCCGCATTTTCTTCCAGCGCACGGAACAATGTCGCT
TTGTTACATTTATCCGGGTCAATGGACAAGTTAATAAAGGAAGGCGCAGCGTGATACCAGGTTTTAAAGCGCATTCTGTC
GGAGAAGATCGGCATTACGCCCCACGACCCAGGCGGCACCCGACTGGCCATCTCTTCCAGCAGCGTATAGGTGTCGATGC
CTAAACGTTCGGCAATCAGTTTTTCTTCGGCACAGAAAGCATCGCGGAACCAGCGCATGGTGAGTCCGGTAAAAAAGCTT

ATAGATTCAGCTTGTACCATGCCAGGAATAACATGAGGATTAACGCGCACGTTCATTTCTGGGTCTGTCACCGGCGCGGC
TAAATTTACAACTTGCTGCCAGAATGTGCCGCCAAGAACCGCGGTTTGTGCCGGACGCACAACGCCTAACCCAAGGCAAC
CAAGCTGCACGTCGCCTCCTCCAACGACCACCGGAGTGCCCGCCTTCAGACCGCAGAGTTCCGCCGCTTGTGAACTTACC
ACGCCCAGCAATGTGCCGGTTTCTTTGACAGGAGAAAGAATATCGGCACGTAGGCCAGCCATATCCAGCAATGCAGGTTT
CCAGTCACGGGTGGTTAGATCAAGAAGTCCCGTGGTGCCAGCGTTAGAGGGATCCACCGCCAGTTCGCCGCTGAGCATAT
AGGCCAGCCAGTCGCTGATCATGGTGATGGTTGATGCCTGACGGTAAATATCGGAACGATGGTGCGCCAGCCAAAGTAAT
CTGGGGATGGCACTTAAAGCCAGTGTTTGTCCGGTCGCGCGATAAACTTCGTTTTCAAAGGTATTGTTGTGCAGTTCTTT
AAGTTCGCTAACTTCGCGTGCCGCTCTGGCATCCACATTGGCGCAGGCCCAGATCGGGGCTCCTTCATTATTATATAAAA
CAATGCCTTCACGCATCGAACATGCCGAAACGGCAGCGATATACTCCGGGGCTATGCCGGCGTTGTGCAGCGCCTGGCGC
ATACACTCACACGCCAGTTGCCAGTTTTTGTTGAGATCAAATTCCATAGAACCAGGAACGTCCGGTACTGCCAGATGCCG
CCACTCCGCCTGTCCCACTGCTATTTGATTGCCTTCCAGGTCGAATATCACAGCCCGAATACTTCCGGTGCCTGCATCCA
GCGCCATCAGGTAGTACTTTGATTCTGAAAGGGTAAAGAGTCGAGCCATTAGATTATCCTCGGCTTATGCGTTCTGTCTG
TTCCTCTATACGTTCTCCATCATTCCCGGTAATAAGGTCATGCAAATTTAACTACGTAAAATCGCCGCTGCTGTGTCCTG
ATCGGTAACCAGTGCGTTGATATAACCGCCTTTCATTGCAGCGGCAATTGCTTCGGCTTTATTTTCTCCCCCTGCCACGC
CAACCCGGACGGGTATGGTCTTCAGCGCGCTTAAAGGTAAGCCAATCAGTTCGTTATGTATTTTGATATTCGTGACAACG
TCACCTTTTGCATCAAAAAAGTAGCCTAAAATGTCGCCAACCGCCCCTTTTCGGCCAATCATTAACTGTTCGCCCTGGCT
GATATAACCGGAGCGAATGATTGTCGCATCGTCCTGTTGACTCACAGCACCAATGCCGACAATCGCCACATCCGCTGCTT
GCGCGGCTAACAGAACATCTTTGACGCAATTTTCATTTTTTAGCGTACGGGCAATGTCAGCGGAGGATGCCCGCAACGGA
GCCGGAATAATATTCACACTGCACGCCGCGTTAAGCTGCCCGATTCCCGTCATATAAGAACCGACGCCACCGGAGAGCGT
GACCAGGCGAATTTGCTGTGACGAAATAAAACCACTTAAGCGTTGCAGCGTATTCATGGTTGCCTCGCCAAAACCAATCG
CCAGCATCTGTTGTGGTTGAAGTAAACTCATCAACATATGCGCCGCGCCTATCCCCAGTCGCCCACCGACATCAGCATCC
GCAAGCCCAGGGATCACCCGGACATGTTGCAGCGAAAACTGACGACGTAATTGAGTTTCATATTCCAGACAGCCTTCAAA
GCGAGAATTAATCTGTACGCGAATAATGCCGGACTGATGCCCTTTCTCCAGCAATCGCGACACTTTCAAACGTGTCAGGC
CGAGACGATCGCTGATCTCGCTCTGGGTCAGCCCGTCGTGATAGTAAAACCACGCGATCCGCGCGACCTGTTCTTCTTCA
CACATTCCCTGTTCTGAAATTGCCGAATCGTTGATTGTCATAATTCATTCTTCACTTTGAACATATTTAAATCTTTAATG
CAATTGTTCAGTTCTTGCTCATTTATATCTGTGATGGCAACCACAGTTTGACTCTACGAGCATGAACAAACGCAACCGTG
AAAATCAAAATAGCATAAATTGTGATCTATTCGTCGGAAATATGTGCAATGTCCACCTAAGGTTATGAACAAATTAAAAG
CAGAAATACATTTGTTCAAAACTCACCTGCAAAACTGAACGGGGGAAATATGCAAACGAGTGATACCCGCGCGTTACCGC
TACTTTGCGCCCGCTCGGTTTATAAACAGTATTCAGGGGTCAATGTCCTGAAAGGCATCGATTTTACGTTGCATCAGGGG
GAGGTCCACGCCCTGCTCGGCGGCAATGGTGCCGGTAAATCGACGTTAATGAAGATTATTGCCGGTATTACCCCTGCTGA
TAGCGGTACGCTGGAGATTGAGGGCAACAACTACGTCAGATTAACGCCAGTTCATGCTCATCAGCTGGGTATTTATCTCG
TTCCCCAGGAACCGCTGCTTTTCCCAAGCCTGTCGATAAAAGAAAACATCCTGTTTGGGCTGGCAAAAAAACAGCTCTCC
ATGCAGAAAATGAAGAACTTGCTGGCGGCGCTGGGCTGCCAGTTTGATCTGCATAGTCTGGCAGGATCGCTGGATGTCGC
CGATCGCCAAATGGTGGAAATCCTCCGCGGGCTGATGCGCGACTCGCGGATTCTGATCCTCGATGAACCTACCGCCTCGC
TTACCCCTGCGGAAACCGAACGCTTGTTTAGTCGCTTGCAAGAGCTGCTTGCTACTGGCGTGGGTATTGTTTTTATCTCG
CATAAGCTGCCGGAAATTCGCCAGATTGCCGATCGAATTAGCGTGATGCGCGACGGAACCATCGCCTTAAGCGGCAAAAC
CAGCGAACTGTCTACCGACGACATTATTCAGGCCATCACCCCAGCGGTACGGGAAAAAATCGCTCTCTGCCAGCCAAAAAT
TATGGCTGGAGTTACCTGGTAACCGCCCACAACATGCCGCCGGAACGCCGGTGCTGACACTGGAAAATCTGACCGGCGAA
GGTTTCAGGAATGTCAGCCTGACGCTCAATGCCGGAGAAATTCTGGGCCTGGCTGGGCTGGTGGGGGCCGGACGCACAGA
ACTGGCCGAGACGCTCTATGGTCTGCGTACTTTGCGTGGCGGACGCATTATGCTGAATGGTAAAGAGATCAATAAATTAT
CCACTGGAGAACGTTTACTGCGCGGTCTGGTTTATCTGCCGGAAGATCGCCAGTCATCCGGACTGAATCTCGATGCTTCG
CTGGCCTGGAACGTCTGCGCCCTTACTCATAACCTTCGTGGATTCTGGGCGAAAACCGCGAAAGATAATGCCACCCTGGA
ACGTTATCGTCGGGCGCTGAATATTAAATTCAACCAACCGGAACAAGCTGCACGGACATTATCCGGTGGCAACCAGCAAA
AAATCCTCATTGCCAAATGCTTGGAAGCTTCGCCGCAAGTATTGATTGTCGATGAGCCGACGCGCGGCGTGGATGTCTCG
GCCCGTAATGATATCTACCAGCTGTTGCGCAGCATCGCCGCACAAAATGTGGCTGTGCTGCTTATCTCCTCCGACCTGGA
AGAGATCGAACTGATGGCAGATCGTGTGTATGTGATGCATCAGGGCGAAATTACCCACTCTGCACTGACCGAGCGCGATA
TTAATGTCGAGACTATTATGCGCGTTGCCTTCGGCGATAGTCAGCGTCAGGAGGCGTCATGCTGAAGTTTATTCAGAACA
ACCGTGAAATCACGGCACTGCTGGCGGTGGTGCTGCTGTTTGTATTACCCGGTTTTCTCGACCGCCAGTATTTAAGTGTG
CAAACGCTGACCATGGTTTATAGCAGCGCGCAAATCCTGATCCTGCTGGCAATGGGCGCGACGCTGGTAATGCTTACGCG
CAATATTGATGTTTCAGTGGGTTCGATTACCGGAATGTGCGCGGTGCTGTTGGGGATGTTACTGAACGCAGGATATTCAC
TACCTGTTGCTTGTGTCGCGACTTTACTGCTTGGTTTGCTCGCGGGATTTTTCAACGGTGTCCTGGTCGCGTGGCTAAAG
ATCCCTGCCATTGTTGCCACCCTTGGCACGTTAGGGTTGTACAGAGGCATCATGTTGCTGTGGACTGGCGGCAAATGGAT
TGAAGGGTTACCCGCCGAACTGAAACAGCTCTCCGCCCCGCTGCTGCTTGGCGTTTCAGCAATTGGTTGGTTGACGATAA
TTCTGGTGGCATTTATGGCCTGGCTGCTGGCAAAGACGGCGTTTGGACGCAGTTTTTATGCCACGGGCGATAATTTACAG
GGCGCTCGTCAACTGGGCGTTCGTACTGAAGCCATTCGCATTGTGGCATTTTCGTTGAACGGCTGCATGGCGGCACTGGC
GGGAATTGTGTTTGCTTCGCAGATTGGTTTTATCCCCAACCAGACCGGTACCGGGCTGGAGATGAAAGCAATTGCAGCCT

GCGTGCTGGGCGGCATTAGTTTGCTCGGTGGTTCCGGTGCGATCATTGGTGCGGTACTCGGCGCATGGTTCCTGACGCAG
ATCGATAGCGTACTGGTGCTGTTGCGCATTCCGGCATGGTGGAATGATTTTATCGCGGGTCTGGTTCTGCTGGCGGTGCT
GGTGTTTGATGGACGCCTGCGTTGTGCGCTGGAACGTAATCTACGGCGGCAAAAATATGCCCGCTTTATGACGCCACCGC
CATCCGTTAAACCCGCTTCGTCAGGTAAAAAACGGGAGGCCGCATAATGCGTATTCGCTACGGTTGGGAACTGGCTCTTG
CCGCACTGCTCGTTATTGAGATTGTCGCATTTGGTGCAATTAACCCGCGAATGTTAGATCTCAATATGTTGCTGTTCAGC
ACCAGTGACTTTATCTGCATTGGCATTGTCGCCCTACCGCTAACGATGGTGATTGTCAGTGGCGGGATCGATATTTCGTT
TGGTTCGACCATCGGCCTCTGCGCCATTGCATTGGGCGTACTGTTTCAAAGTGGTGTGCCGATGCCGCTGGCGATACTCC
TGACCTTACTGCTCGGCGCATTGTGCGGGCTGATCAACGCCGGATTAATTATCTATACCAAAGTTAACCCGCTGGTGATT
ACGCTTGGCACGCTGTATCTGTTTGCCGGAAGCGCTCTGCTGCTTTCCGGTATGGCCGGAGCGACGGGGTACGAAGGTAT
TGGTGGATTCCCGATGGCGTTTACAGATTTCGCTAACCTGGATGTGCTGGGACTCCCCGTTCCGCTGATTATCTTCCTGA
TATGTCTCCTCGTTTTCTGGCTCTGGCTGCATAAAACCCATGCCGGACGTAATGTGTTTTTGATTGGGCAAAGCCCGCGC
GTGGCGCTTTATAGCGCGATTCCAGTTAACCGTACCTTATGTGCGCTCTATGCCATGACGGGGCTGGCGTCTGCGGTCGC
CGCTGTGCTGCTGGTATCGTATTTTGGTTCAGCACGTTCCGATCTCGGTGCGTCGTTTCTGATGCCCGCCATCACCGCCG
TGGTGCTTGGCGGGGCCAATATTTATGGTGGTTCCGGTTCCATTATCGGCACCGCCATTGCGGTTTTATTAGTGGGATAT
TTGCAACAAGGTTTGCAAATGGCAGGAGTGCCAAATCAGGTGTCCAGCGCCCTTTCCGGTGCGCTACTTATCGTCGTTGT
CGTAGGTCGTTCCGTTAGCCTGCATCGCCAGCAAATTAAAGAGTGGCTGGCGCGTCGGGCCAATAACCCATTGCCATAAA
GGATATCTTCATGACACTTCATCGCTTTAAGAAAATCGCCTTACTTAGCGCTCTTGGCATTGCCGCAATCTCTATGAATG
TGCAGGCCGCAGAGCGTATTGCATTTATTCCCAAACTGGTTGGCGTGGGATTTTTTACCAGCGGTGGCAACGGCGCACAA
CAAGCGGGTAAAGAGCTGGGCGTTGATGTGACCTACGACGGGCCGACAGAACCCAGTGTTTCTGGTCAGGTACAGTTGAT
TAATAACTTCGTCAATCAAGGTTATAACGCCATTATCGTTTCTGCGGTTTCGCCTGATGGCTTGTGTCCGGCACTGAAAC
GCGCCATGCAACGTGGTGTGAGAGTGCTGACCTGGGACTCTGATACTAAACCGGAGTGCCGCTCTTACTACATTAATCAG
GGAACGCCCGCCCAGTTAGGAGGTATGTTGGTGGATATGGCGGCGCGTCAGGTGAATAAAGACAAAGCCAAAGTCGCGTT
TTTCTACTCAAGCCCCACCGTTACGGACCAAAACCAGTGGGTGAAAGAAGCGAAAGCGAAAATCGCCAAAGAGCATCCAG
GCTGGGAAATTGTCACTACGCAGTTTGGCTATAACGATGCCACTAAATCGTTACAAACCGCAGAAGGAATATTAAAAGCG
TATAGCGATCTCGACGCCATTATCGCCCCCGATGCCAACGCCCTGCCCGCTGCCGCACAAGCCGCAGAAAACTTGAAAAA
TGACAAAGTAGCGATTGTCGGATTCAGTACGCCAAATGTGATGCGCCCGTATGTAGAGCGCGGCACGGTGAAAGAATTTG
GCCTGTGGGATGTGGTTCAGCAAGGCAAAATTTCAGTGTATGTCGCGGATGCATTATTGAAAAAAGGATCAATGAAAACG
GGCGACAAGCTGGATATCAAGGGCGTAGGTCAGGTTGAAGTCTCGCCAAACAGCGTTCAGGGCTATGACTACGAAGCGGA
TGGTAATGGCATCGTACTGTTACCGGAGCGCGTGATATTCAACAAAGAGAATATCGGCAAATACGATTTCTGATGTGCAT
TACTTAACCGGAGTAAGTTATGGCAGATTTAGACGATATTAAAGATGGTAAAGATTTTCGTACCGATCAACCGCAAAAAA
ATATCCCTTTTACCCTGAAAGGTTGCGGTGCGCTGGATTGGGGAATGCAGTCACGCTTATCGCGGATATTTAATCCGAAA
ACGGGTAAAACCGTGATGCTGGCTTTTGACCATGGTTATTTTCAGGGACCGACTACCGGACTTGAACGCATTGATATAAA
TATCGCCCCGCTGTTTGAACATGCCGATGTATTAATGTGTACGCGCGGCATTTTGCGCAGCGTAGTTCCCCCTGCGACCA
ATAGGCCGGTGGTACTGCGGGCGTCAGGTGCGAACTCTATTCTGGCGGAATTAAGTAATGAAGCCGTGGCGTTATCGATG
GATGACGCCGTGCGCCTGAACAGTTGCGCGGTGGCGGCGCAGGTTTATATCGGCAGCGAATATGAACATCAGTCGATCAA
AAATATTATTCAGCTGGTTGATGCCGGAATGAAAGTGGGAATGCCGACCATGGCCGTGACTGGCGTGGGCAAAGATATGG
TGCGCGATCAGCGTTATTTCTCGCTCGCGACTCGAATCGCCGCTGAAATGGGGGCGCAAATTATCAAAACCTATTATGTC
GAAAAAGGTTTTGAACGGATTGTTGCCGGATGTCCGGTACCCATTGTTATTGCTGGCGGTAAAAAATTACCGGAGCGCGA
GGCGCTGGAAATGTGCTGGCAGGCTATCGATCAGGGCGCTTCTGGTGTGGATATGGGGCGTAATATTTTCCAGTCTGACC
ATCCGGTGGCGATGATGAAAGCCGTACAGGCGGTGGTTCACCATAACGAAACGGCTGATCGGGCATATGAACTCTATCTG
AGTGAAAAACAGTAACTGCGGATCTAAGGAGAAGAATTATGCACGTCACACTGGTTGAAATTAACGTTCATGAAGACAAG
GTTGACGAGTTTATCGAAGTTTTTCGCCAGAACCACCTGGGCTCTGTACAGGAAGAAGGCAATTTGCGCTTCGATGTCTT
ACAGGACCCGGAAGTGAATTCGCGCTTTTATATCTACGAAGCCTATAAAGATGAAGACGCAGTGGCGTTCCATAAAACCA
CGCCCCACTACAAAACCTGTGTCGCGAAACTGGAATCTTTAATGACCGGGCCGCGTAAAAAACGTCTGTTCAATGGTTTG
ATGCCGTGAGGCGAATTTATCAATTTTATCTACAATTGGGGTAACGCGCTGACGGGAGTAAAAAAATGTCTGACTGGAAC
CCCTCTTTATATCTACACTTTTCCGCTGAACGATCGCGTCCGGCGGTGGAGCTGCTTGCCAGAGTGCCGCTGGAAAATGT
CGAATATGTTGCCGATCTTGGCTGTGGCCCAGGTAACAGCACCGCCCTTCTACAACAACGTTGGCCTGCGGCCAGGATAA
CAGGCATCGACTCGTCTCCGGCGATGATTGCTGAAGCGCGCAGTGCTTTGCCAGACTGCCAGTTCGTGGAAGCAGACATC
CGCAACTGGCAACCGGTACAGGCACTCGATCTGATTTTTGCTAATGCCTCACTGCAATGGCTGCCCGACCACTACGAATT
GTTTCCTCATCTGGTTTCTTTACTTAATCCGCAGGGCGTGCTGGCAGTACAGATGCCAGATAACTGGCTGGAGCCGACCC
ATGTGCTCATGCGCGAAGTTGCCTGGGAACAAAACTACCCGGATCGCGGGCGTGAGCCGTTGGCTGGCGTTCATGCTTAC
TACGATATTTTGAGCGAAGCCGGATGTGAGGTCGATATCTGGCGAACCACCTACTATCACCAGATGCCGTCACACCAGGC
GATTATCGATTGGGTGACTGCCACTGGATTACGTCCGTGGTTACAGGATCTGACCGAGAGCGAACAGCAGCTTTTTCTTA
AGCGCTACCATCAGATGCTGGAAGAGCAGTATCCACTGCAAGAGAACGGACAGATACTGCTGGCATTTCCGCGTCTGTTT
ATTGTTGCCCGGCGTATGGAGTAAATATCACGTCAGCTGGTAATGACGATCGGGAAGAATTTTTGCTGGAATTTCGGCTT
CATCGTTCATCTGTAACAGGTCAATTTCAATAGCGTTGCAGATGGCATCCAGTGGTAAATCATTGTTTTCAGTACCGAAC

GGATCTTCCAGTTCTTCCGCCAGACAATCCAGCGAAATAAAAGTGTAGGAAATCAGCACAGAGATAAAAGGCGTCATGTA
ATGCAGGTCCACGACCAGCGCGAACGGCAGCATGATACAAAACAGATAAACGGTACGATGCAAAATCAGGGTGTAGGCAA
AGGGAATTGGCGTATAGGCAATGCGCTCGCATCCTGCCAGGACCGCTGAAATATCATTAAGCCGATCGTTGAGGCTAATA
AACAGGATATCTGAAAGCTGTCCATTGCGGCGCTGAACCGCCAACCATTCTCCCATTATTAACAAGATACGGTTAGCTGG
AGAGTTCGAAGCCAGTACACGCTGAAGATCTTCAGTCTTGAGATAATGAGCCAGCACTTCCGCCTGTGGCTGTTTGCGTA
ATGTCATGCGTAAACAGTGGGCGAAAGCGATTTGCAGCCGGGCAAACTCCCTTACACTTGCCGAATCCGGCAATGTCGTT
TTTACCTCGCGCAGTAACGACCGTGAGGCAATCATCAACTGCCCCCAAAGTTTTCGCGCTTCAACGTAACGGGCGTACCC
GGCATTATTACGAAAACCAAGAAAAATGGCGATGGCGACACCGAGAATGCTGAACGGTGCGAGGGTGAATTTGATGCCCA
GATGCGTGTACCAGGGCAGCATGAAAATAACAGCGATAGAAAAGAGAAAATTGAGTAGTAAGCGCGAGGATATCTTGGAT
AATACTGAGCCGTGCCAGACAAAAATACGGCGCAGCCAGTGTTGTTGTGGACGAACAATCATGGTTATCTTCAGGCGTGG
AAAAGTCGCCCTATTAAACGTGATTACGATCACATTCTCAAGACGCTACTTACAAATTACCTACAAGCTTACAACATTAG
CAGGCGCTGCATGTGGCACCTGTTAATGATAAAGGCATATAGGATGTTGTAACTAATATGGTGAAATAAGAAACCCGGTC
GAAACCGGGTTCAGAAGTAACGGTGTTATTAGCACAACGGACGTACAGCTTCGCGCATCCCTTTTTCGAGAATCGCATCC
AGGTCATTAGCAACCTGCTCGACCAGACCAGGCACTTGCGTCAGGTCCTGCTCCCAGTGGTCTTTTTCTGCCAAAACAAT
CGCTACCAGTTCCTGGGTGCCGATAACACGGTCACGATGCTGGCTCCAGAGTTGCTGATAACGTTCCAGCCAGTGTGCAT
CGTCCTGTACCGGATATGTTTCGCCGTTACGCTCACCACGATAGAACGCAATCAATGCCGCTAATGCGAAAGTAAGGCGC
GCCGGAAGTGTGCCGTTTGCCTTCTGCCCTGCCAGCAGCTGTGGCAGGATGCGGGTGCGGAACTTGGTCATACCGTTGAG
CGCGATAGACAGCAGCTGATGCTTAATGTACGGGTTACGGAAACGCCCGGTGACTGCACTGGCGAAAGATTCCAGTTCAT
CACGAGGCAAATCCAGTACCGGAATAATTTCTTCGTAGATAGCTTTTTCAACGAATGCGCAAATTTCAGCATCGTTCATC
GCTTCACCTACGGTATCCAGCCCTGCCTGGAACGCCACCGGCACCAGCGCGGTGTGCGCACCGTTGAGGATTGCCACTTT
GCGTTCTTTGTACGGTTTAATATCGTCAACAATCAGCACGTTGAGCGGATATTTGTCCAGACGCAGTTCAGTCGCTAAGG
ATTTCGGTCCCTGAATCACAAACAGGTAAAAGTGTTCAGCGGTGTCGAGAAAACCATCGTGATAACCCAGTTCTTCTTCC
AGTTTAGCCACTTCATCGCGCGGATAACCGGTAACGATACGGTCCACCAGCGTAGAACAGAAGCTGTTAGCCTGATCCAG
CCATTGAATAAATGCTTCTGGCAGTGCCCACTCTTGCGCATAGCGCAGCACCAGTTCACGCAACGCGTCGCCATTGTAGT
CAATCAACTCACACGGAATGATGATCCAACCTTTATCCAGCGCACCGTTGAAATGGCTAAAGCGTTCGAACAGCAGACGG
GTCAGTTTTGCCGGATAGCTTACCGCTGGCGCGTCATCGAATTTATCGCCCGCATGGTAGCTGATACCCGCTTCGGTGGT
GTTAGAGAAAACAAAGCGCATTTCCGGGTTGTGCGCCAGTTTCAGGAATTCATCGTATTCACTGTAGACGCTGATTTCAC
GATTAACCGAGCGAATCAGACGCGCGTCGCTGACCGCTTCCCCCTTCTCATTCAGGCCACGGATAATGGTGGTGTACAGA
CCATCCTGCGTGCTCAGTGACGGCGGGAATGAAGTTTCAATCGGACGAACAACGACCACGCCAGAATTCAGATCGGTGTG
CTCATTCAGGAGATCGATTTGCCAGTCAACAAAGGCGCGCAGGAAGTTACCTTCACCAAACTGAATGATACGTTCTGGAT
ACTGTGCACCGGGAAAATCGCGACGATTTAGTGTTTTCACAATGGGTTCCCTTCTGATTAGTCATACAACCTGTTTGAAT
TGGTACGACAGGTTAGCAAACTTTAATACGCCGAACCCCTGTTTTGATCAACTCCTGATGATTAATGAGCAGTTTTATGA
GAAAAGTGTGGCGCGGATCATGGTTTAATCGAGGAAAAACGCCTTTTCCTGGATCATAAAGTGGTAGAACACATTGCATT
CAAATCGCGCGTAATGAATAAAGATGTCAGACAACTTCCTCACCGTAACGCATAGTGCTGGTACGGTTGCGCCCATCTTT
CTTCGAGCGATACAGACACGTATCGGCTTCGACCATCAATTTATTAAAGTCATCGGTCAGCGTTAGGTACGATGCGCGAC
CACTACCGACGCCAATACTTACCGTGAGATAAAGCGTTTTTTGTTGCCAGGTGAATGGTTGCAGTTCAACGCCTTTACGG
ATTTTTTCCGCCATTAGCAGACCATCGACAGGATTCACCGACGGCACTGCAACAGCAAATTCTTCGCCGCCCATTCGCGC
CACCAGCCCCTTATCACCGACAATCTTCTGAATATGCCGGGCAAACACGCTTAACACTTTATCGCCACATTCATGTCCAT
AGTTATCGTTAATGCTTTTGAAATAGTCGATATCAAGCAGCATGACAGTCAGATGTTGTGTCTGTTTCAGCGATGGACTT
TTCAGCGCCTCATAAAGACCGGACCGTGAGTAAACCTGAGTCAGAAAATCAAAGTCGGCTCGCAGCGCAACTTGCTTCAT
TAGCGAATTGATCGCTGCCACGCTAAAAGAAACCATAATTGGGCATATCGCCATCGTGGCAATACCGAGACGTGCGGAGA
ACATTTGCGGAATGGAGAACGGCGAACCGACCGAGATATCAATCACCGAATTTGCCACCAGTACGATTTCCACCGCACCG
GTGACAAAGGTCAACAGACATGTTACCTGCGGCGTATAGCGCACTGCACACCAGATTAATGCAGGCAGCGGGAACGCCAG
ACTCCCCGCTCCGCCAATGACTACCGAGGCAATAACCGACACAATAAGCGCGATGGCGGGCATCATCTGTTCTGCTTTAA
AGCGCGGCAGCACTCCAGGAATAGCCAACGTCAGCATACAAGGCACGATCAACACGCCCGTTGAGAATTGCTCACTGAAC
CAGTCCGCAAGCAAAGGCCAGAAATCCAGACTATCAATACTGACCGAACCAAGATAGCATGTTCGCTGACAGCGGCGGCA
AATAAAGGTACCCTGGGAATGCTATTTTGTCGTTTTCCGCAATTCTCATGGCTGATTCATCTGTACAACCAGCCAGTGAT
AGATTACCGCCACGATATAACGTTGTTGTTCTTCGCTTAACGAAACACCCTGCGGAATGTTATGCCACTTAGCCAGACAA
CCGCGACAGCAGGTCGCGGTGGCGTGCTGAGCGATAAACACCGGATGCCCGCGCATGGGGGTTTGCTTACCGTCATTAGC
CGGTAATGCCGGAGCAAGACGTTTAGCGACAAAATCCGCCGCATGTTGTTCGATGACTGGCGCGCCTTTCTCCAGGCAAT
ACTGACGCTCTTTCATGCCCAGACGAAAGCGAGAGCGAAATTTTGAACGGGATAAACGCGCGAAGAGTGGATCGAGAGAC
TGCATTAATAAACCGAACGCCCTAACTGTTTCGTCAATTGTTCAAGAACGGCAATACCCGCAAGCGAGTTACCTGCATCA
TCCAGTTCCGGACTCCAGACAGCGATGGCCATTTCATGCGGAACAATCGCCACAATACCGCCACCAACGCCAGATTTCGC
CGGTAGCCCCACCCGCCAGGCAAACTCCCCCGCGTTCTGGTACATACCACTGGTCGCCATCAGCGCGTTAATTTGCCGCG
CCTGCATTGGCGTCACCACTGGTTCATCAATATGAATAGCTTTCCCCTGATTAGCCAGAAAGACAAACGTCCGGGCCAGC
TCTACACAGCTCATTTTCAGAGCGCAGTAATGAAAGTAGTTTTGCAGAACGGTTGTCACGTCATGATGGAAATTGCCAAA

CGACTTCATCAGCCAGGCGATAGCCGCATTTCGCGCGGAATGTTCAAATTCGGAACGCGCTACCACCGTATCGTAGGAAA
TATCAGACACACCGCTTAAGCCGCGCACGACTTCCAGCATACGTTGCCGTGGTGCGCTTAATCGCCCTTGCAACATATCG
CAGACCACCAGCGCACCGGCATTAATGAACGGATTACGCGGTATACCCTGCTCCATTTCCAGTTGCACTAAGGAATTGAA
CGGTGATCCAGACGGATCTTTGCCGACGCGTTGCCAGATTTCCTCTTCGGAGTAATGACGCATGGCGACAACGAGACTCA
GCACTTTGGAAATAGACTGAATGGAAAAACGTTCTTGCGCGTCTCCGGCCTGAAAAAGCTGTCCGTCAACGGTACAGATA
GCAATCCCCAATCGGGAACCGTCTACTGTAGCCAGCGCCGGAATATAATCCGCGACTTTACCCTGACCAATGAGCGGCCG
CACTTGCCGCAAGATGTTTTCTAAAATTGCATTATCCATGGCGACTGCCACTTTCTACTCCTGGACCGCAGGTCTGAAAA
GACCTGCGAGTATATCAGAGCTGAATATGTCGCGTCAGATCCGGTCTTTCCACACCGTCTGGATATTACAGAATTCGTGT
AAGCCGAAATGGGAAAGCTCACGACCAAAGCCACTCTTTTTCACGCCACCAAAGGCCACTCGCGCGTCGCTGGCACAATA
ACCATTGATAAACACCCCACCGCATTCCAGACGTGCCGCCATCTGTCTGGCCTGTGTTTCGTCAGTGGTAAAAATGGTCG
CTGAAAGGCCGAACTCACTATCATTAGCCAGTTCCAGTGCATGTTCTGCATCTTTCGCAATGGTGATTGCCGCAACGGGG
CCAAACATTTCTTCCCGAAACGCGGTCATTTCTGGGGTAACATTCGCCAGAACCGTTGGCGGATAGTAGTTACCTGCCCC
AGCCATCTTTTCCCCGCCCAGTAACAAACGCGCACCCTGCGCCAGGGTTTTCTCCACCTGATGATGCAGCTCATCACGTA
AATCAAAACGAGCCATTGGTCCGAGAGCGTTCTCTTCGTCACGGGGATCGCCCATTTTCAAGGCTGCCGCAGCTGCCACA
AAACGTTCGGTAAATGCCGAAGCAATTCCCTCTTCGATAATAAAGCGTTTTGCCGCTGCACATACCTGTCCGGTATTCTG
ATAACGTCCGGCTACCGCCGCTTTCACCGCCAGTTCCAGATCGGCATCGTTAAGCACAATAAACGGATCCGAACCGCCCA
GTTCCAGTACGCATTTTTTCAGTGCCGCTCCAGCCTGTGCGCCAATAGCCGCTCCCGCACGAACACTTCCGGTCACCGTG
ACAGCAGCAATGCGCGAGTCTTTAATCATCTGACTGACACCGTCGTTGTCGGCATTCAGCCAGCCATATACGCCTTGTGG
GATACCCGCATCTTTAAACACCTGGGCAATGAGCTGTGCACAGCCCATCACATTCGGCGCATGTTTAAGTAAGTAGCCGT
TACCTGCAAGAATGATGGGAACAGCGCCACGCATCACCTGCCATAACGGAAAATTCCACGGCATAATCGCCAGAATCGTC
CCCAACGGTCGATACTCAATAACCGCCTGCTGATTTTCCACCAGCGTAGGTTCCGCCTTCAGCATTGCCGGACCATGTTC
TGCATACCAGTCACACAAATTCGCCGATTTCGCCACTTCAGCGCGCGCCTGGTTGATTGGTTTGCCCATTTCGCGGGTGA
TCATTTGCGCCATTTCTTCGCTACGAGCGCGCAGAGCCTTACCGATATCACGCAGTTTTTCAGCACGATAATCTATATTT
GTCTCGCGCCAGTCGCGAAAGCCTGCTGCCGCCAGCTGAAGTGCGTTTTCGATATCGTCAGCGCCAGCCCACGGCAGCAC
AGAAAGTTGTTCACCCGTGGCAGGATTTATCGAAATTGCATGAGTTGCCGGAGTAATGGTCATCGGGGTATCTCCTTTAT
GAGTCATGGTATGAAGATACGCAGATTTACTCTTGCTTTAAAATGAATAATATTAAGCCACTTATTCACGAATCGAGAAT
GCTATGGATCTGACCCAACTGGAGATGTTCAACGCGGTTGCCGAAGCTGGCAGCATAACCCAGGCTGCAGCAAAAGTGCA
TCGCGTGCCGTCGAATCTCACTACCCGTCTGCGCCAGCTGGAAACAGAACTGGGGGTTGATCTGTTTATTCGCGAGAATC
AGCGTTTACGTCTCTCTCCTGCCGGGCATAACTTTTTACGCTATAGCCAACAAATTCTCGCGTTAGTGGATGAAGCGCGG
AGCGTTGTCGCTGGCGATGAGCCGCAAGGTTTATTTTCTCTTGGTTCGCTGGAAAGCACCGCTGCAGTGCGCATTCCAGC
CACGCTGGCGGAATTCAACCGTCGTTATCCCAAAATTCAGTTTTCACTTTCCACCGGCCCTTCCGGCACTATGCTGGATG
GTGTACTGGAGGGAAAACTGAATGCGGCGTTTATTGATGGACCCATTAACCATACTGCCATCGACGGGATACCGGTATAC
CGCGAGGAACTGATGATCGTCACGCCACAAGGATATGCGCCAGTAACCCGTGCCAGTCAGGTTAATGGCAGTAACATTTA
TGCCTTCCGCGCCAATTGTTCGTATCGTCGCCACTTCGAGAGCTGGTTTCATGCTGACGGTGCCGCTCCGGGAACTATCC
ATGAGATGGAGTCTTATCACGGAATGTTGGCCTGTGTGATCGCAGGAGCAGGCATTGCGCTTATTCCGCGCTCTATGCTG
GAAAGTATGCCGGGGCATCACCAGGTTGAAGCGTGGCCGTTAGCTGAGCAATGGCGTTGGTTAACAACCTGGCTGGTCTG
GCGTCGTGGTGCGAAAACACGTCCGCTCGAGGCATTTATTCAACTGCTGGATGTGCCTGACTCGGCAAGACAGGGATATC
AATGAGCTATTTTTGATAGTTCTTGCGGTTAATATGCTCTATATAGTGATGTTCCGATGACTTATGACTATATGGGGCAA
ATATGGTTACGCCAGTAAGCATCAGCAATTACATATCTCTTCCTGATGATTTTCCCGTACGTAATATTGCACCGCAGGTA
AAAGAAGTTTTAAAGGATTTTATTGATGCACTTAGTACAATAATATGTAATGAAGAGTGGCGCACGTCTTTAAACATCAA
CTCAGCCACAAAAAAGATATTTAATAACCTTGACAACTTATCATATATTCAGAGGACTTCTTTTCGGGGTAACGACACGC
TATACAATGAAAAGGTTCAGTTTAAACTCACTTATCCCGCCAGGAATGGAAGACACAAAGAAAATATTGAATTTCAGGTA
GTAATAAATTTAAGCCCTATTTATTTAGATAATTTTCGCCATGATGGAGAAATTAATATTTTTTGCGCTCCCAACCCAAA
GCCTGTCACTATGGGGCGCGTATTTCAGACCGGCGTCGAGCGTGTACTCTTTCTGTTTCTGAATGATTTTATTGAACAAT
TTCCAATGATCAACCCTGGTGTTCCCATCAAAAGAGCGCATACACCACATATTGAACCCCTGCCTTCGGATCACCATACC
GCTGCAGATTACTTACGCCAGTTTGATTTGCTTGTCCTGAATTTTATCTCTCGCGGTAATTTTGTCATACTCCCCCGATT
ATGGAATAACTCTGAGGTTCACAGATGGTTCGTCAATAAAGATCCTAACTTGATCACCGCCATTCTCGACATAACGGACA
GCGAATTAAAAGAGGATTTGTTGCAAAGCCTGATGGATTCATTAGGTTCTAACAAACATGTACTACCCGAAGTCTGCATC
TGCTTTTTATCCCTTTTAGCAGAACAAGAATCTCCTCATTTTCAGAACTTGTTTTTATTTTTCGCCAATATGTTATTGCA
CTATCACCAATTTATGAATCCCAATGAAAGTGATTTGAATGACGTGTTGATGCCAGCATCATTAAGTGATGATAAAATTA
TCAAACATATGGCGCGCAGGACCCTCAAACTGTTTGTAAAAAATGAAACACCGCCAAAAGTTACTCACGAAGACCTGGTG
AAAAACAGGCCTCGCTCCCCTGTCAGACCACCTATACCCGCAACCGCCAAAACGCCAGACCTCCCTGAACGTCATTAAAC
CGTGATGTTACCGACTCTCTGACGCGTGAAAGAATCAGCGTCAGAGAAACGGAAAACGCGATCCAGATCACAAATGCATT
GTATTCACATCATTAACCGTTTTAAGATCATTTCATCACTTTTTCGCAACTCACCCGATAATCTGTTATGACAACAAACA
CTGTTTCCCGCAAAGTGGCGTGGCTACGGGTCGTTACGCTGGCAGTCGCCGCCTTCATCTTCAACACCACCGAATTTGTC
CCTGTTGGCCTGCTCTCTGACATTGCGCAAAGTTTTCACATGCAAACCGCTCAGGTCGGCATCATGTTGACCATTTACGC

ATGGGTAGTAGCGCTAATGTCATTGCCTTTTATGTTAATGACCAGTCAGGTTGAACGGCGCAAATTACTGATCTGCCTGT
TTGTGGTGTTTATTGCCAGCCACGTACTGTCGTTTTTGTCGTGGAGCTTTACCGTTCTGGTGATCAGTCGCATTGGTGTG
GCTTTTGCACATGCGATTTTCTGGTCGATTACGGCGTCTCTGGCGATCCGTATGGCTCCGGCCGGGAAGCGAGCACAGGC
ATTGAGTTTAATTGCCACCGGTACAGCACTGGCGATGGTCTTAGGTTTACCTCTCGGGCGCATTGTGGGCCAGTATTTCG
GTTGGCGAATGACCTTCTTCGCGATTGGTATTGGGGCGCTTATCACCCTTTTGTGCCTGATTAAGTTACTTCCCTTACTG
CCCAGTGAGCATTCCGGTTCACTGAAAAGCCTCCCGCTATTGTTCCGCCGCCCGGCATTGATGAGCATTTATTTGTTAAC
TGTGGTGGTTGTCACCGCCCATTACACGGCATACAGCTATATCGAGCCTTTTGTACAAAACATTGCGGGATTCAGCGCCA
ACTTTGCCACGGCATTACTGTTATTACTCGGTGGTGCGGGCATTATTGGCAGCGTGATTTTCGGTAAACTGGGTAATCAG
TATGCGTCTGCGTTGGTGAGTACGGCGATTGCGCTGTTGCTGGTGTGCCTGGCATTGCTGTTACCTGCGGCGAACAGTGA
AATACACCTCGGGGTGCTGAGTATTTTCTGGGGGATCGCGATGATGATCATCGGGCTTGGTATGCAGGTTAAAGTGCTGG
CGCTGGCACCAGATGCTACCGACGTCGCGATGGCGCTATTCTCCGGCATATTTAATATTGGAATCGGGGCGGGTGCGTTG
GTAGGTAATCAGGTGAGTTTGCACTGGTCAATGTCGATGATTGGTTATGTGGGCGCGGTGCCTGCTTTTGCCGCGTTAAT
TTGGTCAATCATTATATTTCGCCGCTGGCCAGTGACACTCGAAGAACAGACGCAATAGTTGAAAGGCCCATTCGGGCCTT
TTTTAATGGTACGTTTTAATGATTTCCAGGATGCCGTTAATAATAAACTGCACACCCATACATACCAGCAGGAATCCCAT
CAGACGGGAGATCGCTTCAATGCCACCCTTGCCCACCAGCCGCATAATTGCGCCGGAGCTGCGTAGGCTTCCCCACAAAA
TAACCGCCACCAGGAAAAAGATCAGCGGCGGCGCAACCATCAGTACCCAATCAGCGAAGGTTGAACTCTGACGCACTGTG
GACGCCGAGCTAATAATCATCGCTATGGTTCCCGGACCGGCAGTACTTGGCATTGCCAGCGGCACAAAGGCAATATTGGC
ACTGGGTTCATCTTCCAGCTCTTCCGACTTGCTTTTCGCCTCCGGTGAATCAATCGCTTTCTGTTGCGGAAAGAGCATCC
GAAAACCGATAAACGCGACGATTAAGCCGCCTGCAATTCGCAGACCGGGAATCGAAATGCCAAATGTATCCATCACCAGT
TGCCCGGCGTAATACGCCACCATCATGATGGCAAATACGTACACCGAGGCCATCAACGACTGACGATTACGTTCGGCACT
GTTCATGTTGCCTGCCAGGCCAAGAAATAACGCGACAGTTGTTAATGGGTTAGCTAACGGCAGCAACACCACCAGCCCCA
GGCCAATTGCTTTAAACAAATCTAACATTGGTGGTTGTTATCCTGTGTATCTGGGTTATCAGCGAAAAGTATAAGGGGTA
AACAAGGATAAAGTGTCACTCTTTAGCTAGCCTTGCATCGCATTGAACAAAACTTGAACCGATTTAGCAAAACGTGGCAT
CGGTCAATTCATTCATTTGACTTATACTTGCCTGGGCAATATTATCCCCTGCAACTAATTACTTGCCAGGGCAACTAATG
TGAAAAGTACCAGCGATCTGTTCAATGAAATTATTCCATTGGGTCGCTTAATCCATATGGTTAATCAGAAGAAAGATCGC
CTGCTTAACGAGTATCTGTCTCCGCTGGATATTACCGCGGCACAGTTTAAGGTGCTCTGCTCTATCCGCTGCGCGGCGTG
TATTACTCCGGTTGAACTGAAAAAGGTATTGTCGGTCGACCTGGGAGCACTGACCCGTATGCTGGATCGCCTGGTCTGTA
AAGGCTGGGTGGAAAGGTTGCCGAACCCGAATGACAAGCGCGGCGTACTGGTAAAACTTACCACCGGCGGCGCGGCAATA
TGTGAACAATGCCATCAATTAGTTGGCCAGGACCTGCACCAAGAATTAACAAAAAACCTGACGGCGGACGAAGTGGCAAC
ACTTGAGTATTTGCTTAAGAAAGTCCTGCCGTAAACAAAAAAGAGGTATGACGATGTCCAGACGCAATACTGACGCTATT
ACCATTCATAGCATTTTGGACTGGATCGAGGACAACCTGGAATCGCCACTGTCACTGGAGAAAGTGTCAGAGCGTTCGGG
TTACTCCAAATGGCACCTGCAACGGATGTTTAAAAAAGAAACCGGTCATTCATTAGGCCAATACATCCGCAGCCGTAAGA
TGACGGAAATCGCGCAAAAGCTGAAGGAAAGTAACGAGCCGATACTCTATCTGGCAGAACGATATGGCTTCGAGTCGCAA
CAAACTCTGACCCGAACCTTCAAAAATTACTTTGATGTTCCGCCGCATAAATACCGGATGACCAATATGCAGGGCGAATC
GCGCTTTTTACATCCATTAAATCATTACAACAGCTAGTTGAAAACGTGACAACGTCACTGAGGCAATCATGAAACCACTT
TCATCCGCAATAGCAGCTGCGCTTATTCTCTTTTCCGCGCAGGGCGTTGCGGAACAAACCACGCAGCCAGTTGTTACTTC
TTGTGCCAATGTCGTGGTTGTTCCCCCATCGCAGGAACACCCACCGTTTGATTTAAATCACATGGGTACTGGCAGTGATA
AGTCGGATGCGCTCGGCGTGCCCTATTATAATCAACACGCTATGTAGTTTGTTCTGGCCCCGACATCTCGGGGCTTATTA
ACTTCCCACCTTTACCGCTTTACGCCACCGCAAGCCAAATACATTGATATACAGCCCGGTCATAATGAGCACCGCACCTA
AAAATTGCAGACCCGTTAAGCGTTCATCCAACAATAGTGCCGCACTTGCCAGTCCTACTACGGGCACCAGTAACGATAAC
GGTGCAACCCGCCAGGTTTCATAGCGTCCCAGTAACGTCCCCCAGATCCCATAACCAACAATTGTCGCCACAAACGCCAG
ATACATCAGAGACAAGATGGTGGTCATATCGATAGTAACCAGACTGTGAATCATGGTTGCGGAACCATCGAGAATCAGCG
AGGCAACAAAGAAGGGAATGATTGGGATTAAAGCGCTCCAGATTACCAGCGACATCACCGCCGGACGCGTTGAGTGCGAC
ATGATCTTTTTATTGAAGATGTTGCCACACGCCCAACTAAATGCTGCCGCCAGGGTCAACATAAAGCCGAGCATCGCCAC
ATGCTGACCGTTCAGACTATCTTCGATTAACACCAGTACGCCAAAAATCGCTAAGGCGATCCCCGCCAATTGTTTGCCAT
GCAGTCGCTCCCCGAAAGTAAACGCGCCAAGCATGATAGTAAAAAACGCCTGTGCCTGTAACACCAGCGAAGCCAGTCCA
GCAGGCATACCGAAGTTAATGGCACAAAAAAGAAAAGCAAACTGCGCAAAACTGATGGTTAATCCATACCCCAGCAGCAA
ATTCAGTGGTACTTTCGGTCGTGCGACAAAAAAGATAGCCGGAAAAGCGACCAGCATAAAGCGCAAACCGGCCAGCATCA
GCGGTGGCATGTTATGAAGCCCCACTTTGATGACCACAAAATTTAGCCCCCATACGACCACTACCAGTAGCGCCAACACC
CCATCTTTTCGCGACATTCTACCGCCTCTGAATTTCATCTTTTGTAAGCAATCAACTTAGCTGAATTTACTTTTCTTTAA
CAGTTGATTCGTTAGTCGCCGGTTACGACGGCATTAATGCGCAAATAAGTCGCTATACTTCGGATTTTTGCCATGCTATT
TCTTTACATCTCTAAAACAAAACATAACGAAACGCACTGCCGGACAGACAAATGAACTTATCCCTACGACGCTCTACCAG
CGCCCTTCTTGCCTCGTCGTTGTTATTAACCATCGGACGCGGCGCTACGCTGCCATTTATGACCATTTACTTGAGTCGCC
AGTACAGCCTGAGTGTCGATCTAATCGGTTATGCGATGACAATTGCGCTCACTATTGGCGTCGTTTTTAGCCTCGGTTTT
GGTATCCTGGCGGATAAGTTCGACAAGAAACGCTATATGTTACTGGCAATTACCGCCTTCGCCAGCGGTTTTATTGCCAT
TACTTTAGTGAATAACGTGACGCTGGTTGTGCTCTTTTTTGCCCTCATTAACTGCGCCTATTCTGTTTTTGCTACCGTGC

TGAAAGCCTGGTTTGCCGACAATCTTTCGTCCACCAGCAAAACGAAAATCTTCTCAATCAACTACACCATGCTAAACATT
GGCTGGACCATCGGTCCGCCGCTCGGCACGCTGTTGGTAATGCAGAGCATCAATCTGCCCTTCTGGCTGGCAGCTATCTG
TTCCGCGTTTCCCATGCTTTTCATTCAAATTTGGGTAAAGCGCAGCGAGAAAATCATCGCCACGGAAACAGGCAGTGTCT
GGTCGCCGAAAGTTTTATTACAAGATAAAGCACTGTTGTGGTTTACCTGCTCTGGTTTTCTGGCTTCTTTTGTAAGCGGC
GCATTTGCTTCATGCATTTCACAATATGTGATGGTGATTGCTGATGGGGATTTTGCCGAAAAGGTGGTCGCGGTTGTTCT
TCCGGTGAATGCTGCCATGGTGGTTACGTTGCAATATTCCGTGGGCCGCCGACTTAACCCGGCTAACATCCGCGCGCTGA
TGACAGCAGGCACCCTCTGTTTCGTCATCGGTCTGGTCGGTTTTATTTTTTCCGGCAACAGCCTGCTATTGTGGGGTATG
TCAGCTGCGGTATTTACTGTCGGTGAAATCATTTATGCGCCGGGCGAGTATATGTTGATTGACCATATTGCGCCGCCAGA
AATGAAAGCCAGCTATTTTTCCGCCCAGTCTTTAGGCTGGCTTGGTGCCGCGATTAACCCATTAGTGAGTGGCGTAGTGC
TAACCAGCCTGCCGCCTTCCTCGCTGTTTGTCATCTTAGCGTTGGTGATCATTGCTGCGTGGGTGCTGATGTTAAAAGGG
ATTCGAGCAAGACCGTGGGGGCAGCCCGCGCTTTGTTGATTTAAGTCGAACACAATAAAGATTTAATTCAGCCTTCGTTT
AGGTTACCTCTGCTAATATCTTTCTCATTGAGATGAAAATTAAGGTAAGCGAGGAAACACACCCACACCATAAACGGAGGC
AAATAATGCTGGGTAATATGAATGTTTTTATGGCCGTACTGGGAATAATTTTATTTTCTGGTTTTCTGGCCGCGTATTTC
AGCCACAAATGGGATGACTAATGAACGGAGATAATCCCTCACCTAACCGGCCCCTTGTTACAGTTGTGTACAAGGGGCCT
GATTTTTATGACGGCGAAAAAAAACCGCCAGTAAACCGGCGGTGAATGCTTGCATGGATAGATTTGTGTTTTGCTTTTAC
GCTAACAGGCATTTTCCTGCACTGATAACGAATCGTTGACACAGTAGCATCAGTTTTCTCAATGAATGTTAAACGGAGCT
TAAACTCGGTTAATCACATTTTGTTCGTCAATAAACATGCAGCGATTTCTTCCGGTTTGCTTACCCTCATACATTGCCCG
GTCCGCTCTTCCAATGACCACATCCAGAGGCTCTTCAGGAAATGCGCGACTCACACCTGCTGTCACGGTAATGTTGATAT
GCCCTTCAGAATGTGTGATGGCATGGTTATCGACTAACTGGCAAATTCTGACACCTGCACGACATGCTTCTTCATCATTA
GCCGCTTTGACAATAATGATAAATTCTTCGCCCCCGTAGCGATAAACCGTTTCGTAATCACGCGTCCAACTGGCTAAGTA
AGTTGCCAGGGTGCGTAATACTACATCGCCGATTAAATGCCCGTAGGTATCATTAACCAATTTAAATCGGTCAATATCCA
ACAACATTAAATAAAGATTCAGAGGCTCAGCGTTGCGTAACTGATGATCAAAGGATTCATCAAGAACCCGACGACCCGGC
AATCCCGTCAAAACATCCATATTGCTACGGATCGTCAGCAAATAAATTTTGTAATCGGTTAATGCCGCAGTAAAAGAAAG
CAACCCCTCCTGAAAGGCGTCGAAATGCGCGTCCTGCCAGTGATTTTCAACAATAGCCAGCATTAATTCCCGACCACAGT
TATGCATATGTTGATGGGCAGAATCCATTAGCCGAACGTAAGGTAATTCATCGTTATCGAGTGGCCCCAGATGATCAATC
CACCGACCAAACTGGCACAGTCCATAAGAATGGTTATCCGTTATTTCTGGCTTACTGGCATCTCTCGCGACCACGCTGTG
AAACATACTCACCAGCCACTGGTAGTGGGCATCGATAGCCTTATTGAGATTTAACAAGATGGCATCAATTTCCGTTGTCT
TCTTGATCATTGCCACTCCTTTTTCACAGTTCCTTGTGCGCGCTATTCTAACGAGAGAAAAGCAAAATTACGTCAATATT
TTCATAGAAATCCGAAGTTATGAGTCATCTCTGAGATAACATTGTGATTTAAAACAAAATCAGCGGATAAAAAAGTGTTT
AATTCTGTAAATTACCTCTGCATTATCGTAAATAAAAGGATGACAAATAGCATAACCCAATACCCTAATGGCCCAGTAGT
TCAGGCCATCAGGCTAATTTATTTTTATTTCTGCAAATGAGTGACCCGAACGACGGCCGGCGCGCTTTTCTTATCCAGAC
TGCCACTAATGTTGATCATCTGGTCCGGCTGAACTTCTCGTCCATCAAAGACGGCCGCAGGAATAACGACATTAATTTCA
CCGCTCTTATCGCGAAAAACGTAACGGTCCTCTCCTTTGTGAGAAATCAAATTACCGCGTAGTGAAACCGAAGCGCCATC
GTGCATGGTTTTTGCGAAATCAACGGTCATTTTTTTTGCATCATCGGTTCCGCGATAGCCATCTTCTATTGCATGAGGCG
GCGGTGGCGCTGCATCCTGTTTTAAACCGCCCTGGTCATCTGCCAACGCATAAGGCATGACAAGAAAACTTGCTAATACA
ATGGCCTGAAATTTCATACTAACTCCTTAATTGCGTTTGGTTTGACTTATTAAGTCTGGTTGCTATTTTTATAATTGCCA
AATAAGAATATTGCCAATTGTTATAAGGCATTTAAAATCAGCCAACTAGCTGTCAAATATACAGAGAATTTAACTCACTA
AAGTTAAGAAGATTGAAAAGTCTTAAACATATTTTCAGAATAATCGGATTTATATGTTTGAAAATTATTATATTGGACGA
GCATACAGAAAAAGCAAATCACCTTTACATATAAAAGCGTGGACAAAAAACAGTGAACATTAATAGAGATAAAATTGTAC
AACTTGTAGATACCGATACTATTGAAAACCTGACATCCGCGTTGAGTCAAAGACTTATCGCGGATCAATTACGCTTAACT
ACCGCCGAATCATGCACCGGCGGTAAGTTGGCTAGCGCCCTGTGTGCAGCTGAAGATACACCCAAATTTTACGGTGCAGG
CTTTGTTACTTTCACCGATCAGGCAAAGATGAAAATCCTCAGCGTAAGCCAGCAATCTCTTGAACGATATTCTGCGGTGA
GTGAGAAAGTGGCAGCAGAAATGGCAACCGGTGCCATAGAGCGTGCGGATGCTGATGTCAGTATTGCCATTACCGGCTAC
GGCGGACCGGAGGGCGGTGAAGATGGTACGCCAGCGGGTACCGTCTGGTTTGCGTGGCATATTAAAGGCCAGAACTACAC
TGCGGTTATGCATTTTGCTGGCGACTGCGAAACGGTATTAGCTTTAGCGGTGAGGTTTGCCCTCGCCCAGCTGCTGCAAT
TACTGCTATAACCAGGCTGGCCTGGCGATATCTCAGGCCAGCCATTGGTGGTGTTTATATGTTCAAGCCACGATGTTGCA
GCATCGGCATAATCTTAGGTGCCTTACCGCGCCATTGTCGATACAGGCGTTCCAGATCTTCGCTGTTACCTCTGGAAAGG
ATCGCCTCGCGAAAACGCAGCCCATTTTCACGCGTTAATCCGCCCTGCTCAACAAACCACTGATAACCATCATCGGCCAA
CATTTGCGTCCACAGATAAGCGTAATAACCTGCAGCATATCCGCCACCAAAAATATGGGCGAAATAACTGCTGCGATAGC
GTGGCGGTATAGCAGGAAGATCCATATTTTCCGCCACCAGCGCCCGCAATTCAAAATCATCGACATCCTGCATTGCTTCG
TTTTCTTCCAGGCAATGCCAGCGCATATCGAGAAGTGCGGCGCTAAGCAGTTCGCTCATCTCATACCCTTTGTTGAACAG
GCTGGCATTACGCATTTTCTGTTGCAGTTCGTCAGGCATTGCTGCCCCGCTCTGATAATGCCGGGCGTAGCGAGCGAATA
CCTGCGGATGCGTTGCCCAGTGTTCGTTGATTTGCGACGGAAATTCGACAAAATCACGCGGCGTGTTGGTGCCGGAAAGC
GTGGCATAACGCTGGCGGGCAAAAAGGCCGTGCAGCGTATGACCAAATTCATGGAATAAGGTTATGACATCATCCCAGAG
TAACAACGCAGGCTCACCGGCAGCGGGTTTCTGATAATTGCAGACGTTATAAATTACCGGATGTGTTTTATTAAGCGTTG
ATTGCTCAACAAAATTGCCCATCCATGCACCGCCGCTTTTTGAATCACGGGCGAAGAAATCACCGTAAAATAACGCCAGC

CCCACGCCATTATGATCAAAAATTTCCCACACACGAACGTCAGGATGGTAGACAGGAATATCAAAACGTTCGACAAACTT
AATACCGAAGAGCTGATTCGCGGTCCAGAATACACCTTCATTTAACACCGTGTTTAATTCAAAATATGGCTTGAGCTGCG
CCTCATCAAGATCAAATTTCTCCCGCCGTACCTGTTCCGCATAAAATGCCCAGTCCCACGGCTGCGCGCTAAACCCGCCC
TGCTGCTTATCGATAACCGCCTGTATGGAGGCTAATTCATCGCTCGCACGTTGACGCGCCGCTGGAACAATTTCCCGCAT
AAAGTTAAGTGCTGCTTCAGGTGTTTTTGCCATCTGATCGGCGATTTTCCATGCGGCATAATGAGGAAAACCAAGTAGTG
TTGCCTGTTGTGCACGGATCTCCACCAGACGTTGAATGATAGCGCGGGTATCATTGGCATCATTTTTTTCCGCTCGCGTC
CAGCCCGCAATAAACAGTTTTTCACGCGTCGCACGATCGCGCATTTCGGCAAGCGCCGGTTGTTGGGTGGTATTCAGCAG
CGGAATCAGCCATTTGTTATCCAGACCTTTCTCGCGAGCCGCCTCTGCCGCCAGCGCAATCTCTTGCTCACTCATTCCTG
CCAGCTGCGCGATATCGTTCACAACCAGACCGCCGGATTTATTTGCTGCCAGTAATCGCTGGTTAAACTGGCTGGTCAGG
GTCGCAGCTTCTGTATTCAGTACTTTTAATTTTGCTTTATCAGCTTGCGCAAGTTTGGCTCCGGCAAGGACAAAACGTTG
ATGAATCACCTCCACCAGGCGGATGGATTCACTATCAAGCCCCAGGGATTCACGGCGCTGCCAGACAGCATCTACCCGCG
CGAATAATTCACCGTTCAGATAGATATCATTAGCCAGTTCCGCCAGTTCAGCGGAAAACTGCTCGTCAAGACGCTGTAAT
TCATCATTGGTATGCGCCGCAGTCATCGCAAAAAAGACGCTGGTAACGCGGGTAAGTAATTCTCCGCTTTGTTCCAGTGC
CAGAATAGTATTGTTGAAATCAGGCATTTGCGGGTTAAGCGCGATGGCAGCAATTTCTGCCCGCTTTTGCTGCATTCCCT
CATCGAATGCCGGGCGATAGTGATGATTGGCAATTTGATCAAAATGGGGAGCCAGATACGGCAGTGTGCTTTGCACAAGG
AAAGGATTCATTGTTGTCATTTTCTTCTCCTGAACGCGAGGTGTTCCATAGCGTAGGCTTACTGATAACGGAGTGCAATC
TTGCAATCCAGTATTACCCGCTCTTAAGCATCCCGTGCTATGTTATTGACACACAAAAGCGTTGAGGAACAGTGAGATGA
TCGTTTTAGTAACTGGAGCAACGGCAGGTTTTGGTGAATGCATTACTCGTCGTTTTATTCAACAAGGGCATAAAGTTATC
GCCACTGGCCGTCGCCAGGAACGGTTGCAGGAGTTAAAAGACGAACTGGGAGATAATCTGTATATCGCCCAACTGGACGT
TCGCAACCGCGCCGCTATTGAAGAGATGCTGGCATCGCTTCCTGCCGAGTGGTGCAATATTGATATCCTGGTAAATAATG
CCGGCCTGGCGTTGGGCATGGAGCCTGCGCATAAAGCCAGCGTTGAAGACTGGGAAACGATGATTGATACCAACAACAAA
GGCCTGGTATATATGACGCGCGCCGTCTTACCGGGTATGGTTGAACGTAATCATGGTCATATTATTAACATTGGCTCAAC
GGCAGGTAGCTGGCCGTATGCCGGTGGTAACGTTTACGGTGCGACGAAAGCGTTTGTTCGTCAGTTTAGCCTGAATCTGC
GTACGGATCTGCATGGTACGGCGGTGCGCGTCACCGACATCGAACCGGGTCTGGTGGGTGGTACCGAGTTTTCCAATGTC
CGCTTTAAAGGCGATGACGGTAAAGCAGAAAAAACCTATCAAAATACCGTTGCATTGACGCCAGAAGATGTCAGCGAAGC
CGTCTGGTGGGTGTCAACGCTGCCTGCTCACGTCAATATCAATACCCTGGAAATGATGCCGGTTACCCAAAGCTATGCCG
GACTGAATGTCCACCGTCAGTAATTTTTATACCCGGCGTAACTGCCGGGTTATTGCTTGTCACAAAAAAGTGGTAGACTC
ATGCAGTTAACTCACTCACAAGCAAGAACGAATGACCGTCGAAACGCAACTTAATCCCACACAGCCTGTCAATCAGCAGA
TTTATCGTATTCTTCGTCGCGACATTGTCCATTGCCTGATTGCTCCAGGCACACCGTTGTCGGAAAAAGAAGTTTCTGTT
CGTTTCAATGTGTCACGCCAGCCGGTTCGTGAAGCCTTTATTAAACTGGCGGAAAACGGCCTGATTCAAATTCGTCCGCA
ACGTGGCAGCTACGTCAACAAAATTTCCATGGCCCAGGTGCGCAACGGCAGTTTTATCCGTCAGGCCATTGAGTGCGCGG
TGGCGCGTCGGGCGGCGAGCATGATTACCGAAAGCCAGTGCTATCAACTGGAACAAAATCTTCACCAGCAACGCATTGCC
ATTGAGCGCAAGCAACTGGATGATTTTTTTGAACTTGATGACAACTTCCATCAACTCCTGACGCAGATTGCCGACTGTCA
ACTGGCGTGGGATACCATTGAGAACCTGAAAGCGACCGTTGATCGCGTGCGCTATATGAGTTTCGACCACGTTTCTCCAC
CAGAAATGCTGTTACGCCAGCATCTTGATATTTTCTCTGCCCTGCAAAAACGTGATGGCGATGCGGTAGAACGTGCAATG
ACGCAACATTTGCAGGAAATCAGCGAATCCGTGCGCCAGATCCGCCAGGAAAACAGCGACTGGTTTAGCGAAGAGTAATT
CATTTCCTCTCATCCCATCCGGGGTGAGAGTCTTTTCCCCCGCCTTATGGCTCATGCATGCATCAAAAAAGATGTGAGCT
TGATCAAAAACAAAAAATATTTCACTCGACAGGAGTATTTATATTGCGCCCGTTACGTGGGCTTCGACTGTAAATCAGAA
AGGAGAAAACACCTATGACGACCTACGATCGTAACCGTAACGCAATCACCACTGGCAGCCGTGTTATGGTTAGCGGCACC
GGTCACACTGGCAAGATCCTGTCGATTGATACTGAAGGTCTGACCGCTGAGCAAATCCGCCGCGGAAAAACCGTAGTTGT
TGAAGGTTGTGAAGAGAAACTGGCACCACTGGACCTGATTCGTCTCGGCATGAACTAAGCGTGTGAATGCCGCCGATGGC
GGCATTGCTTTTTTACTTCACGGAATATTTTGCCACGGTCGCTTTCGCGCCATGCGCTAATAAAGACAAGTACGTTTCCG
TCACTCTTGCAGTAAACAAACTATTGTCTGGCAAATCATCACCAAAGATCGCCTTAATCGCCAGCAATGACTGGACGCGC
GCTTTCCCTTCGGCACTACTTTGTACAGCCTTCTGAATAACAGGTAACAGTGGGTCACTGATTTCTATCGGATTTCCCTG
TTCATCAACACCACCGACATAACGCATCCAACCCGCGACGCCCAGCGCCAGCAGATCGAACTTGCTGTCATGCGCCAGAT
GCCAGCGAACAGAATCCAACATCCGCTGTGGCAATTTCTGGCTACCATCCATCGCAATCTGCCAGGTTCGATGACGTAAC
GCCGGGTTGCTATAGCGTGCAATTAATCGGTTAGCGTAATCTTGCAAATCAACGCCCTGCACTTTCAACGTCGGCGCTTG
TTCCTGCAACATCAAGCCATACGCCGCATAACGATAATGTTCATCTTCCATACAGTCATTAATGTGCTGATATCCTGCAA
GATACCCCAGATACGCCAGGAATGAATGACTGCCGTTGAGCATGCGCAACTTCATCTCTTCATAAGGCAGCACATCGCTA
ACCAGTTCGGCTCCCGCTTTTTCCCATTCCGGACGTCCGGCAACAAAGTTATCTTCTATTACCCACTGGCGGAAAGGTTC
ACAGGCAACGCCCGCAGGATCGCGCACACCGGTAAGTTGTTCGATTTTCGCCAGCGTATCCTCTGTCACTGCGGGCACAA
TACGGTCCACCATTGTTGATGGGAAAGTCACGTTATCTTCGATCCATTGTGCCAGTTTTACATCAACGGCTTGTGCGTAG
GAAGTGACAACGTCACGCATAACATGACCGTTTTCTGGCATGTTGTCACATGACATGACGGTAAATGCGGGAAGTCCTGC
CGCTTTACGGCGAGCCAGCGCCTCAACAATCACCCCTGTTGCTGTTTTCGGCTGGTGGGGATTTTGCACGTCGGCAGCTA
CCATCGGGTGATCGAGCATTAACTGTCCGGTCGCCGGAGAGTGGAAATACCCTTTTTCGGTGATTGTCAGAGAGACAATC
GCGATTTGCGGTTCACACATCGCTGCCAACACGGTTTCTAAGCCATCTATCTGTACGTGCAAGGCTTTTTTAACGACGCC

AACGACGCGAGCCGTCCACACATCGGCCGACATTTCCGCAACGGTATAAAGATTATCTTGCTGTTGTAAATCGGCAATTT
GCTGTTCGCCGCCGATTAAGTTGACCTCATAATATCCCCAGTCACTGAAATGTTCCGTAGCAAGAATATCGGCATACACA
CCCTGATGCGCACGGTGAAATGCACCAAAGCCTAAATGAACAATTCTTGGAGCCAGGTTATTAAGATCATAAACAGGGAG
TGTCGCTTTTGCTGATAACAAATTATTTCCCATAACAATTCCTTAAATATAAATATGGCAAGCTATATGTTTTGTTATAT
GAATAAAAATCCCCTCTCCGGTAAGAGAAGGGATTAAGGGTTTACAGACTTCTGGAAGGTTGCGCAGCTCTTACAACACG
CGGTTGATCTTCCGCAGCGTCTTCCAGCGCACTTAAATCACGGTCTTTCACCTCTGGCATTTTCAGCGCAGAGATTAAAC
CAATCACTGAATATGCCATGATCATAATGGCGATCGGATACCAGGATTCCGTCATGGTGCAGAAAATACCCGCCAGGATA
GGACCAAAACCGGAAGCGATAAGACCACCAATTTCTTTAGAAATAGCCATCCGGGTAAAGCGGTTTTTACAGCCGAACAT
TTCTGCCATGGTAATGTTTTCCAGAGCAAATAATCCCAGCACCGCACAGTTATGAATCACAATCAGTGCAACCATAATGG
TGCTCGGGGCATAGCTTTTATCTACAATGATAGAAAGCATTGGCCATGCCAGCACAATCGCGGAGGTATTCATAATAATA
TACGGGATCCGGCGACCAATTTTATCGGATAACCAACCAAGGAACGGAATGGTCATAAAGCCGAGAATCGAACTGATCAT
CAATGCATCTGTTGGAATTGCTTTGTTAAACAATAACGTCTGCACTAAATAGCCTGCAAGGAAAGTCTGAATTAACCCGG
AGTTACCCGCCTGACCAAAACGCAGCCCTGTTGCCAGCCAGAAGGATTTGCTCTGGAACATGCTACCAGCAGGTGCAGGT
TTTGCTGTCGGTTGGTTACTGTCGTTAACCTTCTCAAAGACCGGGCTTTCTTTCAGATTCATACGCAACCAGATAGCAAA
GACCATCACGACAACACTCGCCAGGAACGGTATACGCCATCCCCACGCCAGCAGTTCCTCTTTACTGAGAATGAAGAACA
TAAAGGCCCAGATTGCCGTTGCGCTCAAGGTTCCGCAGTTAGTTCCCATAGCCACAAATGAGGAGATAATTCCGCGCTTA
CCTTTTGGCGCATATTCCGCCAGCATCGTACCGGCACCGGAAATTTCCGCACCTGCACCCAACCCCTGAATAATACGCAA
CGTCACCAGCAAGATGGGTGCAAAAACACCAATCTGTGCATAGGTCGGTAACACACCAATTAAGGTGGTACAGATCCCCA
TCATGGTGATGGTAATAAAGAGCACTTTTTTACGCCCTATTCTGTCGCCCATTTTGCCGAAAATAAATGCTCCGACAATA
CGCGCCACATAACCTGCACCGTAGGTTCCCATTGCCAGAATTAACGCCATTGCCGTTGATGATTCAGGAAAAAATATTTC
ATGAAACACTAACGCTGCGCCGAGCGAATATAACTGGAAATCCATAATTCACAGGTGTTTTTTCCCATCCTGTGGTTTCC
TTGGCGTTTTCTAGGTTTTTTCAGATAGTTGCATTTTTTTAAAAAGCATCCTAAGTTCGATCTCAGTGTCTATCTGGGGC
CTATTTCTGTCCCATATATGCCCCAAAAAAACTCCCCAACAGATAAGTAGTTTTTTCATGGATTTATGCGTAAAATCAAG
AACGGCTGGAAATCATTCAATACTCACACTATCGAAAAATTTACCAGCCAATCGCAGCACGTTCTTGCATAAGGTGTGTC
TGCGGTTTTTCAACTATTCAGATACATCACTCCCATCACATTCATTCCTCCGCATCAAAGGCATATAGGCTATATCACCT
TGATATTTTTCTTTTTCAGATAAAAACTGTTATCTATGTATACTTTTAAACCCAATCCGTGTAGAGTCTCTACATAAGAT
AGTTTGCAGTTGCCGCTTCAGCTTGCGCCATAAACCGCCTGATTTTTGCTGCCACCTGTTAGCATTCCTGTATACCTGAA
ACGACAATGTTTATCTACGAACTTTAAGAACACCCAAGATAAAAATTGTCAACTATATCATATATAACACATTACTAATT
CGAGGCTATATGAACAGCATACTGATAATCACATCTCTCCTTATCATATTCAGCATTTTTAGTCATGCCCTAATAAAATT
AGGGATTGGCATATCCAATAACCCAGACAAAACCGATGTATAAGTCAACATATCCTGAATCAGACATACAATATCGCAAT
GAAAATCAATAATATTTTAAGGAATATCTTCATGAAATCAAAAGACACCCTAAAGTGGTTCCCTGCGCAGCTTCCTGAAG
TAAGAATTATCCTAGGGGATGCTGTAGTGGAAGTAGCAAAACAGGGAAGACCTATCAATACCAGAACATTGCTTGATTAC
ATTGAAGGAAACATAAAGAAAAAATCATGGCTGGATAACAAAGAATTATTACAAACAGCGATATCAGTTCTTAAAGACAA
CCAAAATTTAAATGGTAAAATGTAATATAATAAACTTACTTTTTTATCATTTTTCCACTTTAACAACATTTTGCTCCACT
TTTCCACGACCAAACAACTTGAAATCTGGTTAAAATAACACGCAACACTATTCTTCTTCCTTGAGTCCGCCCGGAACTCG
AAAAACAAACCGAGTTAAAGCCATTTTTCACAAAATCGATTTTGGGTCTCACCAAAATTACGGGGTTGCATACGCATTCG
TTTATTTTCGAACGTGTACATACAAATATGCACAAAAATAATCATAATTATTTTCTGAGATGCATTATGATATGAACACC
AATTTCGTATAGAGTCTCACTATGTCTCAAATTTTTGCTTACTGTCGGATATCAACGCTGGATCAGACCACCGAAAATCA
ACGCCGGGAAATCGAAAGTGCAGGTTTTAAAATCAAACCTCAGCAAATAATCGAAGAACACATTAGCGGCTCAGCAGCAA
CCAGTGAGCGTCCTGGTTTTAACCGGTTGCTTGCTCGCCTGAAATGTGGTGATCAATTGATTGTGACAAAACTGGATCGC
CTTGGTTGTAATGCAATGGATATCAGGAAAACAGTGGAACAACTGACCGAAACAGGTATCAGAGTGCATTGCTTAGCATT
GGGGGGCATTGACCTGACCAGTCCAACAGGAAAAATGATGATGCAAGTAATTTCAGCAGTCGCTGAATTTGAACGAGACC
TTTTACTTGAACGCACTCATTCCGGGATAGTAAGAGCCCGCGGCGCAGGGAAACGTTTTGGTCGACCACCTGTGTTAAAT
GAAGAACAGAAACAGGCGGTATTCGAACGAATTAAGTCAGGTGTAAGTATAAGTGCCATTGCCCGGGAATTCAAAACCTC
GCGGCAAACCATTTTAAGAGCCAAAGCAAAACTTCAGACACCTGACATATAAAAAATAATCTCGGTGTGAGATGCTTTAC
GTCTTCCAAGCCCCCTTCCTTGCCGTAAATGGAAAGATACATCTAATTATAGAATTTATATGTTTTACCCTACGGCAGTG
CTGGCCATTCAATATCCTGTGCAGTTGACGTATCAACACGGTTCAGCAATACCCGATACTTTTTCCATGCTTCCAGCAAC
GAGATTTCTTCCTCCGTTGCAATTTCCAGATCTGCAGCATCCTGAAGCGGCGCAATATGCTCACTGGCTACCTGCATCAG
GTTGTTTTTTGTTTCTTCCGCCTCCCGGATCCGGAACAGTTTTTCTGCTTCCGTATCCTTCACCCAGGCTGTGCCGTTCC
ACTTCTGAAACTCCCCTTCCGGCGATAACCAGGTAACATTTTCCGGTAACGGACCGAGTTCAGAAATAAATAACGCGTCG
CCGGAAGCCACGTCATAAACCGTTTTACCCCGATGATCTTCAACGAGATGCCACGATGACTCATCACTGTTGAAAACAGC
CACGAAGCCAGCCGGAATATCTGGCGGTGCAATATCGGTACTGTTTGCTGGCAGACCTGTATGAGGCGGAATATATGCAT
CACCTTCACCAATAAATTCATTAGTTCCGGCCAGCAGATTATAAATTTTTATGGTCCGTGGTTGTTCACTCATTCTGAAT
GCCATTATGCAAGCCTCACAATATAGTTAAATGCGATGTTTTTGACGGTGTTTTCCGCGTTACCAGCAGCGTTAACGGTG
ATGGTGTGTCCATGTGAACCAATCGCAACGGAGTGCGTATGAGCACCAATACCGACAGTATGTGCATGCGCGCCTGCGCT
TGCAGCAGTGCCGGACAGCGAGTGGGTATGAGCACCATCTGATGATGTCTTCCCTGCATTACGAGTCTGGCCACTACCGC

TTGTTGTGCTCATAATCCCCGCGCTTAGATTTGAAATCGCGGTATAACCATTAGGGAAAATGCTCGTGTTCGTGCCACCA
AATGCACCGGAACTCTTGTGTTGGTGCGCACCGGCACTATTTGCGGTCCCGCTAATACTATGGGTATGCGCCCCGGTGTT
ATTCGTGGATTTGGTTCCGTAATCAAACGACGATGTGGTTTCCGTCCCCAAATCCGTACTGGATGCGCTGGCGCTGTGGG
TGTGCGATTTAATGCCGTCCTGTTCCTGAGATAATACGGCCCGACCACTGGCGGGCTTGCCCTTAATCGTCCAGCCACGC
ATATCAGGGATCACGCCTGACGGATAAGCAACTGCAAGTTTCGGGTATGCAGATTTGTCAAAAGTCTGCCCCTGCATCAG
GGCATAACCAGACGGAACGGTATCTGATGGCCACGGGATTGGTGCACCGACTGGATAAAACTCTGCAGGAGGATGAGCCG
AGGTGTAAAGCTGCGCCCACGGCGACCAGTTTGCGTCGGTCGTATCCCGTCGTGAACGAATAAATGCCGGAGCATGAGCA
CCGCTTGTACCACTCCAGCCGATGAGTAACTCACCTTCGCCAACGGCTGTCATCCCTTTCAGGTGAATGATATTTCCATA
CGCTGTTGGATATCCGTTGTTATACACCTCGTATAACTCAAGACCTGCTGCCCCCTGCGTATTGTCTGTCAGCGCGGTTA
TATTCACTCAGCAACCCCGGTATCAGTTCATCCAGCGCGGCTGCTTTGTTCATGGCTTTGATGATATCCCGTTTCAGGAA
ATCAACATGTCGGTTTTCCAGTTCCGGAAAACGCCGCTGCACCGACAGGGGGATCCCGTCGAGAATACTGGCAATTTCAC
CTGCGATCCGCGACAGCACGAAAGTACAGAATGCGGTTTCCACCACTTCAGCGGAGTCTCTGGCATTTTTCAGCTCCTGT
GCGTCGGCCTGCGCACGCGTAAGTCGATGGCGTTCGTACTCAATAGTCCCTGGCTGGAGATCTGTCTCGCTGGCCTGCCG
CAGTTCTTCAACTTCCCGGCGCAGCTTTTCGTTCTCAATTTCAGCATCCCTTTCGGCATACCATCTTATAACGGCGGCAG
AGTCATAAAGCACCTCATTACCCTTGCCACCGCCTCGCAGAACGGGCATTCCCTGTTCCTGCCAGTTCTGAATGGTACGG
ATACTCGCACCGAAAATGTCAGCCAGCTGCTTTTTGTTGACTTCCATTGTTCATTCCACGGCCAAAAACAGAGAAAGGAA
ACGACAGAGGCCCAAAAGCTCGTTTTCAGCACCTGTCGTTTCCTTTCTTTTCAGGGGGTATTTTAAATAAAAACATTAAG
TTACGACGAAGAAGAACGGAAATGCCTTAAACCGGAAAATTTTCATAAATAGCGAAAACCCGCGAGGTCGCCGCCCCGTA
ACCTGTCGGATCACCGGAAAGGACCCGTAAAGTGATAATGATTATCATCTACATATCACAACGTGCGTGGAGGCCATCAA
ACCACGTCAAATAATCAATTATGACGCAGGTATCGTATTAATTGATCTGCATCAACTTAACGTAAAAACAACTTCAGACA
ATACAAATCAGCGACACTGAATACGGGGCAACCTCATGTCAACGAAGAACAGAACCCGCAGAACAACAACCCGCAACATC
CGCTTTCCTAACCAAATGATTGAACAAATTAACATCGCTCTTGAGCAAAAAGGGTCCGGGAATTTCTCAGCCTGGGTCAT
TGAAGCCTGCCGCCGGAGACTGTGCTCAGAAAAAAGAGTTTCTTCTGAAGCAAACAAAGAAAAGAGTGACATTACTGAAT
TGCTCAGAAAACAGGTCAGACCAGATTGAAGCAATTTAGATAATCGTGCAGACTACGCCCCCTCATATCACATGGAAGGT
TTATCTATGGATCAGGTAGTCATTTTTAAACAAATATTTGATAAAGTTCGAAACGATTTAAACTATCAATGGTTTTATTC
TGAGCTAAAACGTCACAATGTCTCACATTACATTTACTATTTAGCCACAGAGAATGTTCATATTGTATTAAAAAATGATA
ATACAGTGTTATTAAAGGGCCTAAAAAACATTGTGTCTGTCAAATTTTCAAAGGATAGGCATCTTATAGAAACGACCTCT
AATAAGCTGAAATCCAGAGAGATCACATTTCAGGAATACAGAAGAAACCTTGCTAAAGCAGGAGTTTTTCGGTGGGTTAC
AAATATCCACGAACAAAAAAGATATTACTATACCTTTGATAATTCATTACTATTTACTGAAAGCATCCAGAAAACTACAC
AGATCTTACCACGCTAAACCATAACGTCCGGCTTCTCTCACTCCTGAGCCGGACTGCATTGGTTTAATAAAAACCATCAA
CAATTGTGATTTAGATATTCGGAACCATTCAAATATAACAAAACCCCGTAAAAACGAGGTTTATGGATAAATTTTATTAT
TGAATACATCAGATTAAATTAATCTTGACATCATAGCTTTCAAGACCCGTCATTTTTTCCCGTGCGGTAAACTGAATACT
GGTAACTTCTTTCCCGGTCTTTTTCTTAAGTTCAATAATTTTTTTTGTTATATATTCAGAAATATCTGCTTCTGCTTTTG
TTTTTAAGTTTTCAATATTCATCATTTCCTCTTTTAGTCTGTTATGACTTTCCAGTTACACAGTAAGTCGATTATATGGT
GCAAACGTGTAAAAGATAAGATGAAACATCGCAATAATCAACATACGATAGTCTAAATTTTACACAAACAGACAAAGAGA
ATTTTCCTGAATTATCAATGCAATAGCATCAAATCAACTCAAGAGCCTTATTGCTGCTTCCAGAATTTCTTCTGAAGTAA
CATGTCGATCCGCGGCTACATAAATGACTTTATGATCTCCGGTCAGAGATGGAAACCCTGCGGCCATTACAGTAAGGTGT
GTTTTTTCGCCATTTGGATATTCACGCATGATGGTGTTAACTCCAGTCATCGCTGGCACTACCACTGCTGGTTCAGAGTT
AAAAAAACTATGATTTTTTTCATGATGTTACCGTAGTATGTGAGTATCCATCGAATAGACACCAAGCAAAAAAGCTCCCG
AAGGAGCCTTCATTTTCACTTTTTTAAATCCAACGACAGACGGCTGGCATTTAAGTATTGTGAAATATTATCAAATGTAA
TCATCATTGATTTACAAAAGATACATTTTGCCCCGAAAGGATTCATGTCAGAAACATCAAAAGATGATGTTCTATACTGG
GAACCATGACAACACGGGCATCTAAAGTGAATATGGTTTGTAATATTGTCTACCTCAAAGCGCCACTACATGAACAGCGG
CAGGACCTTTAGGTCCGTTCTCAATACCAAATTCAACTTCCTGATTCTCAGTTAATGTTTTGAAATCGTTGCTCTGAATT
GCTGAGAAATGGACAAACACATCTTTGCTGCCATCTTTCGGCGTGATGAAACCAAAACCTTTTTCAGGGTTAAACCATTT
CACTAAACCAGTCATTTTGTTAGACATAATTATTACCTTTTGAAGAAATTAGCCCTTGGGCAGAATGGTCCGAAAAAAAA
TATCAGAGAGAAAAACCAACAAGGAAATCTCAAGAGGTACAAATAATAAAATTATAACAATGACTGCTTCAGATAAATTT
GTAACAAACCAGAACACCATTAACGCATGATTAACCACCCATAGCAAGGATTACTTTTGTAAAGAAAAACACAGCAATGA
AAGAATAGCTTTATTTATTAATAAAACGTGTCATTCTGATTAAGACCTTTTATCTTACCCTTAAGATTTCAGGAATTTTG
GCTCATGGAAGAGTCCTTTTTATTTAAATTTTACATTCCGCGATGTAAATGTTCCGATTTAATATTACCCTACATTTGAT
GCTTTTTATCTCTTAAAGATTCATAGATCTGTTGACAAGTCACTCCTGCGATGTAGCGTTCGTCAGCAATTTCAGCATAA
AGCTGAGCTTCTGCTGCAATATCTCCGAGCATGTTGGTGAGCATTCCTTCGGCGGTTTTGGTTGTTTTGCCTCTGACGGC
AGCGGCAAGATCTGCGGTATGCTTCGCTGCGTCAAGGCGTATGGCATATTTTTTTGCTTCGGCACGCAACTGGTTAACAC
TATCAGACAGATAAGCAGCCCTGGCAGAAATTTCAGCAGATTTCTGTTGCGCATCTTTAACAGCCTCATCACGGGCTATA
GTTCGCCCCTGTTCAATTATTCGAGCAGCAAATTGAGCATTTACCTCTTGTGATAATGCGGCAGCATCACGTTCCGCCCA
TTTTTTTTGCCATCCTCGGTCGCTCCAGACATTTCCGACGATAAATCCTGACAACACGAGAAAAATCACCATGAATATCT
GATTCACTGTTCTATCCCCCAGCAGGTTAATGCGCTCTCCTGGTCACGACGAATAACCTGACCGTAACAGTTATTTGAAC

GAATGCGGCAATCGCGTCCGCCATCCTTAATCCACCAGCGAATCGCTTCGCATGCACCTTTACGATCACCAGCATTCAGC
CGCTTATAAAACGTCGACGGGAAACACTTACCGGGGCCAATGTTATAGGGACAAAATGACGCGATACCCGCTTTTTGTGG
TTCGGTCAGTGGTACTTTAATATTGCGCTCCACCCATGCCAGCGCCTTATCACGCTCAATGGCGTTGACCTGGTCGCATT
TTTCCTTCGACAGTTTCATATTGGGAAAAACGGTTTTTCCATCCACCACTGTGGCACCCCGACAGATGGTCCATATGCCA
GAACCATCGCGGTATGCCATTGTGTGGTTACCTTCTTTTTCGTCCAGAAACTGGTCAAGTATCTGAGGAGCAGATGCGCC
AGCACCAATCAGCGCCAGAACGGCAGCCGACAGGCCGTATCTGATTTTTGTGTTCATAGATATTTATGATGAGGACGCTC
GTGCTTATTGGCAGGATTTTCAATCTTAAAGGAGTACTGATGCTGCAGATAAGACTCAACTTTTTCTGACAATTTTTCTG
CTACTTCCAGGAAGACTTGCCGGACGCTCCTTCTGGCTGCTGCCTCATAAAACTCCAGCGCAGCTCCTTCAACACGGTCC
ATGGCGACATCCAGGTCAAAAATTTCACCGTCAAAGCGTTCTTTGTCCTGTAAGGCTACAGTTACCGTAACTTTATTCTC
AAAATTACGGACTCCTTTCACAACCAGTTCATAGTCTTGAGTCATTGGATTACTCTCCTCTCGCAGCCTTACGCCTGTCT
TCTTTAATCTTGAAATAAAGATTTGTCAGATACGTCAGCAGGCCAAAAACCAGGCTACCCAGCACACCGATTGCAGCCCA
CTGTGACGGAGTTACTTTATCGAGTAACTGCAATGCCCAGAAACCAGCATTACCCGCCGATGTGCCATAGGCAACACCTG
TTGTTAACTTATCCATTGATTTCATATCCTCACCCCGATGTACACGGATGGTGCAATATGTTTGAAAAGATCGGAGTCTA
CGGGGTAGTTTTGACAGCACACGTTGTTCTCAACGGCGCTAAAAAAACATACACATTAAAAATGTGGGTAATTATTTTGA
AAGAAAGTCATATATAAAATAATAATACGAGAAATGTTTTCATATTTAGTGTACTGTATACGGCCATTTATACAGGAAAA
GCCTATGTCAGAACGTAAAAACTCAAAATCACGCCGTAATTATCTCGTTAAATGTTCCTGCCCAAACTGCACCCAAGAGT
CAGAACACAGTTTTTCAAGAGTACAAAAAGGTGCCCTTTTGATCTGCCCTCATTGCAACAAAGTATTCCAGACAAATCTT
AAAGCTGTAGCCTGATTGATTTTATTAGTAACAAGTATTTTTTATATTTTAATAATATATTTAAAGCAGATAATAAAAAA
CCCGCCTGAGCGGGTTTGAGATTGTGGTGCTTTTTGTGGGAGTCATCCACTTACGCACTTTGTTTTGCCATGCCAGCAGT
TAGCTTCTGCTGTAAAACTATTCATGCAGCAAACCTGCACTTCACCACAATGGTTAGCATACTTTTCCTGATTAAGATTT
TGCCAAATATGCTAGCCATTGTTTCATGTATTGGACCTCCTTACTTTTTATTAAAGAGATCCAATATTCACTACTCTGTC
CGTATCTCTACTCAGGCATCAGCCTTCTTCGTTATCGTATACAGACGAGCGATGAATTTTAATCAGTAATGATGACATTT
GCTGCTGCAGGACCTTTAGCACCACTCTCTATAGAGAAGGTAACCTTTTGACCTTCAAATAAGGTTCGATAATTATCATT
CTGAATCGCAGAAAAATGCACAAACACATCTTTACTACCATCAACAGGAGAAATAAAGCCGAAACCTTTATCAGCGTTAA
ACCATTTTACTAAACCAGTCATTTTATTTGACATTCTACATTCCTTAACTTGAGCCTTTCGGCATAAATGGTTTGCATAA
CAGAAACGACTTCGTACTTAATTGGAGAGACTCAAAGAAGGAATAAGTGAATAACACCTGAAATGAGAACTGCTTTAGTA
AACTACTTCGTATATCGTCTGTTCTTCAAACCGACGCAATCATTAACGCATAGTTGAACATATGAAGCAATGTTTATTTT
AGACATCCAGCCATCTTCAACCCCATCAAAAAACTATAGCTTTCTTCAGGAACGTGTGTATAGTGCGCCAAGTTATCAGT
ATTAAGGAATTTTTTTGTCCCGTAAAATGACAGGAATTGTCAAAACCTTTGACGGCAAAAGCGGCAAGGGTCTTATCACC
CCATCCGATGGTCGTATCGATGTCCAGCTTCATGTTTCAGCGCTCAATCTCCGCGATGCAGAAGAAATTACCACCGGATT
ACGCGTGGAATTTTGCCGGATAAATGGTCTGCGTGGCCCTTCAGCTGCCAATGTTTACCTTTCATGAGCTATATTAAAGC
TTTAATTTCAGGCCCCATCGGATCACACATGGAGAGTTTTTATGAATAACCCCGTCTGTCTTGATGACTGGTTGATTGGC
TTTAAAAGCTTATGCTGTACTTTGGCCGTAATAGCTCTGCTAATAATATAATAAGCAGACTCATTGTGTTTAGGGACATT
GTACTGGAAGAAAACATTTTAAACATCAGGCAAATAACCAAGTCACCAGCTAAATAATAAGTTAACAGACATGAGTCCCG
GGATGAGATTCAACATTACCATTGCCCCATTTAAAGCACAAAACCCGCTCATCAGCGGGTTTTCTACTTTTTCTTAACGT
CGGGTATACAAAGCCCATCGTTGAAAAAATTTTATCCATATTTTTTGAAAAATGCAAACATCATGTCGCCATCTTCAGCA
AAAATCATTTATCTCGTCACCTTCCTCAATTGCGCTTCCGCGTATGCTTCTTCCTGCCAGCACTTTGTTACCAGTTTACC
AATGACGTCCGCATACCCCTTATACCACTGATAATCGGTCAGGTCTGGTACCAGCTTCTGGACATGACGTCGTGCCAGCG
TGGTCGGTAAACGACTAAACCGGTTTCCATTACAACGCCCACAAATCTTATATACCGGTACGCCATGAAACCGGGTTCTT
TTTTCATCCAGAACAATCCCTTTACCCTTACACCCTCTGCACGCTGTGCTGGCTTCGCCCTTACCATGGCAATGCTGACA
TAGTTCCTTCACCCATTCTTCCTTGATTACAGATTCCCCGCGTCTGTAGTGTTTCACCACTTCGCGCAATACATTATAAA
ATCCCGTACCTGAACAATGCTCACAGCGAGCCTTACTTGCCGCAGACCTGGAGTAATCAGCAAAGGCAAAACTCACGAGG
TAAGGAATAATCTGTAACCGGATTTCTTCACTCAATTTGTTCAATGTCGGGTTATCCAGTGCCATCGCGTAATTTAGCAG
GCCTTCAATCGCAAACTGAGGGTCCTGAACACCAACTTTTGCCAGAAATAAGGCCAACCCAAGTGGTGCTTTCGACTGCA
CCATCCCCTGCGCTGCCATTACATCCGTAATTGTTAAACAACCGGTGCCTGTCGCTGGAGCGTCATCGCTCAATTTTGGA
GATTTTGGGGAGTAATATTTTGGTAAGGCTTCAAGGTTCATGCTCGTTCTCCACTTACGCCAGTACGCCAATTGCCAGCG
CGCGATCGATAAAACGAAATATCAGCTCCAGTTGGGAGCCATACTTATCTTCAAATGCCACTGTATCCGTATGCAGCTCG
TTGTGATGCTTTCTGCACAAAGGCAACACAAAGAGATCATGTGCTTTTGTTCCCATTCCGCCCTGCCCGTGACCAATCAG
ATGATGCGGATCGTCGGCTGGCATACCGCAGCAAGCACACGGCTGTGTCTTAACCCAACGTGTGTATTTCTCCTTAACCC
AGCGGCGACGTTTAGGCAGCTTCATGAAAGATTCCGGAGACTCTGGATCAACGGTGATGCTTACCACCGTCTTTTCCTGT
GGTGATTTTTGTTGCTGGTGGGCGTAAGGCAACGGTGCAAGATTTTTTGTGCGTTGTTTCAATATGCTGGTGGCGGTCAG
CTCTCCCGGTACGATGTCGCTTTCGCGGTACACCGAGCAGATTTTTTCCGCTGGTAATCCCAGCGAACGACGCGATACAG
CCTCAGGTAGTGCATCCACCACCTGATTGCAGACCGCCCACCAGGATAATTCAGCCAAAGATAATTCCCGCTCCTGCGTA
CCGCTTATTGCGTGACGGATGACGTCAATCACCCATGCTGTCAGATTTTGTTGAGCAAGCAGCTCCAGTGATTCCGATGT
CTGGTCACGCAGTTGGTTGTCGCAGTGCCAGCACAACACCATTGCGCCGGTACCATAACGGTGAATGACTGTTTCAGTGT
GATGGTAATCGCCATTAGGCCACTGGCAGGATGTAACATGACGTAATAGCCAGTCGGACAATGCGCCAACGCCGCCAGCA

GCACGAATCACCCGTTCGTTACTAAAAAACGGCAGCAATGTTTTGTCTTCCGCCAGCGGCTGGCGAACGGCAGGAACGAC
TCCGGATGGCAGATTACGCATGCTTTTTGGTTCCGGTTCCACCAGCACTCGAGGATTATGAAATATCTGTATGGATTCAC
GGCCCGGCTTAAGGACCACCAGCCCAAGCTCAGGCACCAGAACAGGTCTAAGTAATACCCGCACGTTACCTCCAGATCCG
TTGCTGGAAAGTGCGGGACGCACGTGGTGGGCGTTCGGAATAAGGCAGCCTGACAGAGATTATCCAGTGCCGATAGTCGA
GACTGAGAGCTTTCTTAACCTCGAACCCGCGCCTGCGGTAAGAATGAATCAGCCATTCGGCCTGTTCTGCAGTGCATGGA
GGGTGCTGGAACCATTCAGACTTGAATGCGTGAGAATACCGCCCGTGCGTGCAGGCAAGAACGGGCGAATTATCAGAATT
GTAATATTTTGCGTTGCGTGCCATCGGTTTTCTCCGGTGGCACGGTGTTACTCAGCGGGAGTTCAGCCCCGCGCAAGATT
GTAGATGAGTTTATTCTCCTGAAAAAGCAGAAAAGCCAGCTTTTATTCCGATCTCTTTCAATGCCTGTAATGAAGTGACA
AACTCACCTTCGCGCAAGATAAATCCGTCCGTGACCCGAGCATCCACAAAATTAATTAACGCAGCCCCATTCTTTCGCAA
ACACATAATGCGGTAATGACTAACAAGATTTCCATTTTCAACGCACACAGCATAGAGGCCATCTTCACAAAAAATTTTAC
GCAGTTCTTCGATGTTCATCATCAGAATCCTTCCGGATAATTAGCTCTCCCCTTTAAGGGACCATCCCTCTTATCCCTGC
GCGCTACTTAAGTATTTTTGATTCTATTCCGGCACCGTCCAGAACTTCAAACGCGTTGAAAATAAAAACAAAAACCCGCC
GAAGCGGGTTAAGTGCGGGTGCGTTGAGGATGCCTGCCACATCAGAGGTGGCGAGGGATTTCTCCCCCGCCGGGTCTCTT
ACTCCTCAGGTTCGTAAGCTGTGAAGACAGCGACCTCCGTCTGGCCGGTTCGGATTCGTACCTCGCAGAGGTCTTTCCTC
GTTACCAGTGCCGTCACTATGACGGTTAAACAGATGACGATCAGGGCGATTAACATCGCCTTTTGCTGCTTCATAGCCTG
CTTCTCCTGTCAACGCAAAGCAGAAGTGTCACCTTCGGTGCGAAACAGAGATGTCATGCTTTGGTTCAGAGAATGCGTTT
GACCGCCTCGCTATATACTTCCGAGCGTTCTCTTTTCCCAACAGAAATCACGAAAACGACAACTTTCTCGTCTATAACCT
GGTATACAAGGCGATAGCCTGAAGACCGGAGCTTAATCTTGTAACAATCAGGCATACCACGGAGCTTGTTTGCTTCAATC
CGGGGTGACTCAAGTACTTCAACCAGCTTCTTTTTCAACTGTTCACGTACCGTCGAGCCCAGCTTTCGCCATTCCTTTAG
TGCCCGCTCGTCAAAATCCAGAAAATACGCCATCAGAGTTCATCCAGCGTCACACGTACTGGCTTAGGATTACGAAGCCG
TTCTTTCACTATCTCCACAAGTTCAGCATCTTCATCACTCAGGAGTGTCTGTTTGAACGGCAAGCGTTCATTGTCAGCGA
TATACTCGAGCATGAGACGAAGCGCTTCAGAAGGAGTTACACCCATTTTTTCAAGCGCGGCGTAAGAACGCGCTTTAAGT
TCATCGTCAATACGCAGGTTAATGCTACCCATGTCTTACACCTCTTGTAATTACAAATGTCATTACAAGTATCGCACTAC
AACATGCTTAGGGCAAGTCACGAAGGAAGTCAGAAAGTAGTCGTAAGAACGGTGATCACTGTCCGCTTTGTGCCAGGAGC
AGCCATTGCTAAGTCCATCCTGTATTGTGCAGGTCAGCTCGTTTTTAAAGAGTCCGGCCATCATCTTACTGGTACAGACA
CCATATACTTTGTGACGGTCAGGCTACATATGCACAACTCAACTTATTCATCTATTTTTTGCTTTAGCATGTCAGTGTTG
CTTTCTCGTCGGCGGGTGAGCGGTGACCTGACCTGTCGATAAAGGAACGTAACACGTTTTATGCAACACCCGCATGCGGC
AGAAAATTATTGCCGAACGTTTACCCCTGTCAACAAGCTTTACTTTCTGAGGCGCGCCAGCCCGCGAGGAAAACAATCTG
AACATCAAACAATTAATGACACAAGAAATACGATTAAAGATTTTTTTGTGCATGCCGATAGTGCTTTTTTAAAAGGAGAA
ATCTATGTCTGTCACAATTCAGGGAAATACCTCAACCGTTATTTCAAACAACTCCGCCCCGGAAGGAACATCAGAAATAG
CCAAAATCACAAGACAAATTCAGGTGCTGACTGAAAAGCTTGGGAAAATCTCATCGGAAGAGGGGATGACGACACAGCAG
AAAAAAGAAATGGCTGCATTGGTACAGAAGCAAATTGAAAGCCTCTGGGCTCAACTGGAGCAGTTGTTAAGGCAGCAGGC
AGAGAAAAAGAATGAAGACGCGACAGTTCAGCCTGATAAAAAAGAAGAGAAAAAAGACGATACAAATACCGCTGGCACCA
TTGATATTTACGTCTAAGTGACAGCCGTATTGTGGCCCTCATCGGGCCACTTTTCGCCATCAGCCTTTTCTTTAAAGACA
TATTATCTTTGTATCATTTCTGATAGTTAACATTACAAGATATAAGTAATGGACGCACTCCCAATTAGTCTATTTAAATC
GCCACGAGTTTAACTGACAACCCATGATCAATTATGAATTGCAACTATTTCTGTAGTCACTTTTGTGGGGACAGTCCACA
AAACTGCCAACTTCCGCTTCTTGCTCTTAGCGGACATTAGCATAGGCTATTTACCATAACGCCTCATTACGCGCACCGCC
CAGACTGACTCAGCGCGTTTCTGGCATATCCCCGGTAAAACAAGTAACAAACCACCCGAAAATGAACACCAGAAACGCGA
CTTAAGAATCTACCCTATGAATGGATATGCACTCAACCGAATCGATCTTGGTTTCAATCTTTTTTATCGGGATCAGGCTT
CTTTTTAGGTAACTTCGGGGGCTTAACTTGCTGATGACTTTGCGTTCGGCGCGTAAGCCAGGGATGGTCAGCTTTAGGTT
TAACATAGTATTTTGAGCGTAAATCAATACGGGCATTATCCACTCGTTCATGGACACTCTTTTCATCATCCAGTGGTAGC
CTCCATAATTGCAGGCACTAGCGCCGTGAACTTTTCACGCTTATCCCTGGTGTCGATAGCCTTCCAGCGTTCAAATATCT
TCACTCGATTAACGCCAAGCGCTCGCTGATCAATCGCGCCACCTTCATATGTGACACGCTGAACATCGATGTTCGGGCGC
TCTTTCAAAGCCCAGAATGCTTCAGTGATTAATATCGTCGCCTGCTCCTGTGTCATTCCTGGTCGACATATCCAGGCATC
CAGAGCCTCACGAGCCTGTTCAGGAGTGATTTTCATTGTTCAACCGCCCCGCCCGCTTCGTCTTACGATATTCATCATAA
ACTTTGGGATCATACTGAAGCTCCCCGCCAGATGCCTCCTGTAGACGCATCGCGCGACCTTCGGGAACTAAATCCCCTTT
CCAGCTATAAAGCGAAGCCAAACGAATACCTGCTGCTTGTGCAAGTTTTGTTTTTGAACCGAAATACAAAAGAGCGTCAG
TTTTAAGCATTTAAAACACCTTTATTGTTAGTCATAACTAACAAGATAGATGTTAACAAAAACATAGTCAATACGATTTA
GCATTAGCTAACTATGGAAACAAAAAATTTAACTATCGGCGAACGCATCAGGTATCGTCGGAAAAACCTCAAACACACCC
AAAGGTCTCTTGCTAAAGCCCTGAAAATCTCCCATGTGTCTGTATCACAATGGGAACGGGGTGATAGTGAACCTACAGGG
AAGAACCTTTTTGCCCTCAGTAAAGTATTGCAATGCTCACCAACATGGATTCTATTTGGCGATGAAGACAAGCAACCAAC
ACCACCTGTTGAGAAGCCAGTTGCCTTATCCCCCAAAGAACTAGAGCTCCTTGAGCTGTTTAATGCACTGCCAGAATCAG
AACAGGATACCCAGCTCGCCGAAATGCGAGCTCGAGTAAAAAACTTCAATAAACTCTTTGAAGAATTACTAAAAGCCCGT
CAGCGGACAAATAAAAGATAACATCATCAATGAGTTATCTTTTACCACATCAATTATGTTAGCTATAGCATACAAAATCA
CTTGACCGATATGTTAGTCATGGCTAATCTTGTTTGCATCAACACACCGCACGGTGTTCTCAGCAAACAGTTCCGCTACC
CCAGCGTTAAGGGGAATGAGGTCAGCATGGATACTATCGATCTTGGCAACAACGAATCTCTGGTGTACGGCGTGTTTCC

AAACCAGGACGGCACGTTCACCGCAATGACGTATACCAAAAGCAAAACGTTTAAAACCGAAAATGGTGCCCGTCGCTGGC
TGGAAAGAAACTCAGGTGAGTGATATGGATTTCGACACAATCATGGAAAAGGCTTACGAAGAATACTTCGAAGGCCTTGC
CGAAGGCGAAGAAGCTCTCAGCTTCAGTGAGTTTAAACAGGCGCTTTCCAGTTCGGCAAAATCTAACGGCTGATAAGCGA
AACAGCACCGCGAGGAATCAGTATGCAGAAACGAGAACCCGTCATCATCGCGCCAGACTATACCGATGATGAACTTTATG
AGTGGATGCGCCAGAAAATTAATGCAGCGCAGGATCTGAAATGGGCTAATGAAGCCAGGGCTAAGCAGGCTGAAAATCTG
TCCGCTCTGGAGCAGGATATCACCAATCTGGAAAAAGCAGCGGCATTAAGCATTGCCAGAATGATTACATACCCGCGTTA
GTAGCTAATCAACAAAGCTAAGGTTAGTAATTAAGGAGTTCTCCACGGGTGAGGTGGAGTGCGTGCGCCGGACACGGGTG
CGCATCCGGAACTGACAGTTTACTGAAAGGATATTTCCCTGAAAAGTCAGACCATAACGCGAAAGCGCATGGCGAGGTAG
CTGGTTCATAGATAGCCTGTCGTTAAATTTTCGTCGACCGTGCGCTTCCGGTTGTGGCAACCCGCGAAATGGCGCGGCGG
TAAGTATGGCGGGGTTATTCCTTCCCCGTTGAGGACACCGGGTTGTCAGGTTGACCATACGCTTAAGTGACAACCCCGCT
GCAACGCCCTCTGTTATCAATTTTCTGGTGACGTTTGGCGGTATCAGTTTTACTCCGTGACTGCTCTGCCGCCCTTTTTA
AAGTGAATTTTGTGATGTGGTGAATGCGGCTGAGCGCACGCGGAACAGTTAAAACCAAAAACAGTGTTATGGGTGGATTC
TCTGTATCCGGCGTTAATTGTTAACTGGTTAACGTCACCTGGAGGCACCAGGCACTGCATCACAAAATTCATTGTTGAGG
ACGCGATAATGAAAACGTTATTACCAAACGTTAATACGTCTGAAGGTTGTTTTGAAATTGGTGTCACTATCAGTAACCCA
GTATTTACTGAAGATGCCATTAACAAGAGAAAACAAGAACGGGAGCTATTAAATAAAATATGCATTGTTTCAATGCTGGC
TCGTTTACGTCTGATGCCAAAAGGATGTGCACAATGAATTCAGCATTTGTGCTTGTTCTGACAGTTTTTCTTGTTTCCGG
AGAGCCAGTTGATATTGCAGTCAGTGTTCACAGGACAATGCAGGAGTGTATGACTGCAGCAACCGAACAGAAAATTCCCG
GTAACTGTTACCCGGTCGATAAAGTTATTCACCAGGATAATATCGAAATCCCGGCAGGTCTTTAAAACAGTTCCGTAATA
AATATCCGGTTTCATTCTTATATGCCAGCAATGGCAGGGATTTGTTCATCCTTAAATCTGTCATGAGGTTAAAACAAATG
AGTAAAGTCTTTATTTGCGCCGCTATTCCTGACGAACTGGCAACAAGGGAAGAAGGCGCTGTGGCTGTAGCCACAGCCAT
TGAAGCTGGCGACGAACGCCGTGCTCGAGCAAAATTTCACTGGCAATTCCTGGAACATTATCCGGCTGCTCAGGACTGCG
CTTATAAATTTATTGTCTGCGAGGATAAACCTGGCATACCCCGCCCTGCCCTCGATTCATGGGATGCTGAATATATGCAG
GAAAACCGCTGGGATGAGGAGTCTGCTTCTTTTGTCCCGGTTGAGACTGAATCCGATCCGATGAACGTCACTTTTGACAA
GCTGGCCCCTGAAGTACAGAACGCTGTCATGGTTAAGTTCGACACATGTGAAAACATCACCGTTGATATGGTTATTAGCG
CACAGGAATTGTTGCAGGAAGACATGGCAACATTCGACGGACATATCGTTGAAGCGTTGATGAAAATGCCAGAAGTTAAC
GCCATGTATCCGGAGCTTAAGTTGCACGCCATTGGGTGGGTTAAGCATAAATGTATTCCTGGTGCTAAATGGCCCGAAAT
TCAGGCAGAGATGCGCATCTGGAAAAAACGTCGCGAAGGTGAACGCAAGGAAACCGGAAAATACACGTCTGTTGTTGATC
TCGCCCGCGCCAGAGCCAATCAACAGTACACTGAAAATTCAACAGGAAAAATCAGCCCGGTCATTGCTGCCATTCATCGC
GAATACAAGCAGACATGGAAAACACTGGATGACGAACTGGCCTATGGGCGCTGCTTCGCAGACAGGCAGAACTTGATGGT
ATGCCTGCGATCAATGCCAAACGTGTTTACCGGATCATGCGCCAGAATGCGCTGTTGCTTGAGCGAAAACCTGCTGTACC
GCCATCGAAACGGGCACATACAGGCAGAGTGGCCGTGAAAGAAAGCAATCAGCGATGGTGCTCTGACGGGTTCGAGTTCT
GCTGTGATAACGGAGAGAGACTGCGTGTCACGTTCGCGCTGGACTGCTGTGATCGTGAGGCACTGCACTGGGCGGTCACT
ACCGGCGGCTTCAACAGTGAAACAGTACAGGACGTCATGCTGGGAGCGGTGGAACGCCGCTTCGGCAACGATCTTCCGTC
GTCTCCAGTGGAGTGGCTGACGGATAATGGTTCATGCTACCGGGCTAATGAAACACGCCAGTTCGCCCGGATGTTGGGAC
TTGAACCGAAGAACACGGCGGTGCGGAGTCCGGAGAGTAACGGAATAGCAGAGAGCTTCGTGAAAACGATAAAGCGTGAC
TACATCAGTATCATGCCCAAACCAGACGGGTTAACGGCAGCAAAGAACCTTGCAGAGGCGTTCGAGCATTATAACGAATG
GCATCCGCATAGTGCGCTGGGTTATCGCTCGCCACGGGAATATCTGCGGCAGCGGGCTTGTAATGGGTTAAGTGATAACA
GATGTCTGGAAATATAGGGGCAAATCCACAGGTGAACTACGCTCCTCTGTTTGTTACGCAATAAAAACTGGTGTTTTCGA
CTATGCAAAACAGTTTCCCTCCTCACGCAATCTGGAAAAATTTGGTGAGGCCCGACAAGATTTAACCATAAAAGAACTGG
CTGAAAAATTTCTGGCACTGAAAGAAACTGAAGTCGCCAAAACATCACTCAACACATACCGTGCCGTCATCAAAAATATC
CTGAGCATAATCGGTGAAAAAAATCTTGCCTCATCGATTAATAAAGAAAAATTACTGGAGGTTCGTAAAGAGTTACTGAC
TGGATACCAGATCCCCAAAAGTAACTATATTGTTACACAACCAGGGAGATCGGCTGTAACTGTAAATAATTACATGACAA
ATCTTAACGCCGTGTTCCAGTTTGGTGTTGATAACGGTTACCTGGCAGATAATCCGTTTAAGGGGATCTCGCCATTAAAG
GAATCAAGAACCATTCCGGATCCTCTTTCGCGGGAAGAATTTATCCGTCTTATCGATGCGTGCAGAAATCAGCAAGCAAA
AAATTTATGGTGTGTTTCTGTTTATACTGGAGTTCGCCCTGGTGAGCTGTGTGCACTTGGATGGGAGGACATAGATCTGA
AAAATGGAACAATGATGATCAGGAGAAATTTAGCAAAAGACCGTTTCACGGTACCAAAAACACAGGCGGGAACCAATCGG
GTCATTCATCTTATTAAGCCAGCAATCGACGCTCTCCGGAGTCAGATGACATTAACGAGACTGAGCAAAGAGCATATCAT
TGATGTTCACTTCAGAGAGTATGGCAGAACAGAAAAACAAAAATGCACCTTTGTTTTTCAACCTGAAGTGTCAGCGAGAG
TAAAAAATTATGGTGACCATTTTACCGTTGACTCAATAAGGCAGATGTGGGACGCAGCGATAAAACGTGCCGGACTCCGC
CATCGAAAATCATATCAGTCGAGACATACTTATGCCTGCTGGTCGCTGACAGCTGGTGCTAACCCGGCATTTATAGCAAA
CCAGATGGGCCATGCAGATGCGCAAATGGTATTTCAGGTATACGGAAAATGGATGTCTGAAAACAATAATGCACAGGTAG
CTTTGTTAAATACACAGTTAAGCGAGTTTGCCCCAACCATGCCCCATAACGAAGCAATGAAAAATTAATTTAATATTTAT
CAAATAGTTAACACGCATGACTCTTGAAATCCATAAATTCAAGCGCAGTGCCCAGCCATCCCGATACTGCTGCTTTCACC
AAATCCTTAGTGCTTCTTTCGTGTTTTTCTATTGTCATAATGGTTATCTCTAAAAAAGAGGTAAGATGCGTACTACTTAC
TCGCCGTTATTGGTATTATTCAGAAAAAGTGAGTAAGACTTTGCAGCAATGTTTTTGATCCTGTTCAAATAAACTAATGG
CATCAGCAACATGCTGGAAATCAAACGTATGGGTAATTAATTTTTCTGGTTTAATTAACCCTTTACTTAACCAGTCGATA

ACGATCGGGAATTTATTTGCATTTAAGCGTGAAGAGAAAATAGAGAGTTCTTTTCCGGTAATTCCTTGCTGAATCACTTC
AGACGGTTCACTGGAGAACCCCATCAATACAATACGTGCCGCTGGAGAAGCCAGCGTTACGGCCTCTTTCAGGATAGAAG
GATGACAAGCCGCATCGATAATTAATGTCGGCTTGATGCCTTTTTCAGTGAAAATCTCGCCAAGCGGTGTCTGGCTGTTA
TTAATCGCCCAGTCAGCCCCGCTCTCTTTCGCTTTTTCCAGTCGTTCATCAATGCGATCGGCAACAATCACATTTTTAAC
GTTATAGACGCCTTTTAATACCTGAACGATCGTCAGGCCGATTGGACCGGCACCATAAACCAGAACGGTATCATTTTCAG
TCGGTTGACCATGTCCGGTTACGTTAGCCGCAATGGTAAAAGGTTCGATCATTACCGCATATTGATCGGCCACTGCTTCA
GGAATTTTCCACGCATTTTTTGCCGGAACCACGGCATATTCACTGAAACCACCGTCAGCGTGCACACCTAATACAGCCAG
TGTCGTACAAACGTTCGGTTTACCTATAGAGCACGGATAGCAATGCCCACAGCTGACCACCGGATCGACAGCAACACGTT
CACCGACTCTGGCGCTTTCCACGCCTTCACCCACTGCATCAATGACGCCAAAGAATTCATGACCAATGACGCGCGGATAT
TTCGCAAAAGGATTATGCCCACGATAAATATGGCTATCTGAACCACAAATTCCGGCAAGTTTCACTTTTACTCGTACTTC
ACCCGCTGACGGGGTGGGTATTTCACGTTCGACAATCGCCAGTTGATTCGGTTTTTCAATTAATATGCTTTTCATTATCT
TACTCCTTACCAGTTCCACAGCGTGCCATCTTCCAGACGTGCGACTGGTAGATAAGCAGGTTCATAGGGATATTTCGCCG
CCAGCTTTTCATCGAATTCGATACCAAGACCCGGTTTGTCTCCCGGATGCATATAGCCGTTATCGAAAGTCCAGTTGTGC
GGGAAGACTTCGAGCATTTGTTCGGAATAACCCATGTATTCCTGGACACCGAAATTGGGGACCCACAGATCAAAGTGCAG
CGCCGCAGCCATGCAGACTGGTGACAAATCGGAAGGACCGTGTGAGCCAGTACGTACCTGATACAGCGAAGCAAAATCGG
CAATCCGGCGCATACCGGTAATTCCGCCTGCATGGGTCAGCGTGGTGCGGATATAATCGATGAGTTGCTCTTCAATCAGT
TGTTTGCAGTCCCAGATGCTGTTGAAGACTTCACCCACTGCGATGGGTGTGACGGTATGTTGGCGAATGAGACGGAAGCA
TTCCTGGTTTTCCGCAGGCGTCGGGTCTTCCATCCAGAACATGCGATAATCTTCAATGCTTTTACCAAAGCGCGCCGCTT
CAATAGGCGTTAAGCGATGGTGCATGTCATGCAGCAAATGTTCATTAAAACCAAACTTGTTACGTACCGCGTCAAACAAT
TTCGGCATGAAATCGAGGTATTTCTCCGTCGACCACAGCTGCTCTTCCGGCCACTGTCCTTTGGTTGCGGGTTCATAAGC
CAGACCTTTACCTTTCGACATGCCGTAGGTGGTTTTCATACCAGGGATTCCGCACTGCACGCGGATGGCTTTGAATCCAA
GCTCTTGATGACGGGCATAATCATCCAGAGCTTCATCAATACTGTGACCGGTGGTATGGCAATAAACCATCACCCCTTCA
CGAGACGCGCCGCCGAGTAACTGGTAAAGCGGCATGTTGGCAGCTTTGGCTTTAATATCCCACAGCGCCATATCAACCGC
TGAAATGGCCGACATCGTAACCGGACCGCGACGCCAGTAAGCACCTTTATAGAAAAACTGCCAGATATCTTCGATACGGT
GCGCATCGCGACCAATAAGCTGCGGACAAAGGTGATCCTGCAAATAAGAGGCCACGGAAAGCTCACGTCCATTGAGGGTG
GCATCCCCAAGGCCCGTAATACCGTCCTCAGTGGTGATTTTTAATGTGACGAAATTACGCCCCGGACAGGTAACAAAAAC
TTCAGCCTTTACGATCTTCATGTTCGATTCCTTGCATCGCTTGTCGTGATGCATGAAATCTACGCAACTGAGCTACTACC
ATACAAGTATAAAGATCGAAAAAAGCCGGAGTGATCACAAAAAAAGGCGTATATTTGCGCTGTGAATGGTTGACAAAAGA
TGAAATAGAATACCTTTTGTCAGCTGACACTTCCTCTTATCTTATTGATAAAATGGATTTATGTTCCTACGTGCGCCCCC
AGCCCGCAACAATGATCAACATGCCGCAAAGCGCAATCAACGCACCCGTCCAGTCATAAAGAGTCAGTTTCACGCCATCC
ACAACGCGCAGCCACATCAACGCCGTGCAGACATAAACGCCACCATAAGCCGCGTAAACACGCCCACTCGCCGCTGGATG
CAACGTTAACAACCAGACAAACAGCGCCAGTGAAATCCCCGCCGGAAGCAACAGCCAGATACTGGCGTTTCGTTTTAACC
ACAACCAGGGCAGAAAGCATCCAATAATTTCACACAGCGCAGTAGCAAAAAATAGTAACGTTGTTTTAATCATCTTTGTC
TCTTATTGACATCATGTATAGTTATAGGGCGACATAATATCATCAATATAAACACCCTCCTGGTACGTTTTGCGTCCGCA
GTGGATGGTGTAGAATCACCTTTAATCATTCATACAGGGAATGAATTATGAAAATCACTCTCAGCAAACGAATCGGCCTG
CTCGCTATTCTGCTGCCTTGCGCACTGGCATTGAGCACAACTGTTCATGCCGAAACTAACAAACTGGTGATTGAGTCTGG
CGACAGTGCACAAAGCCGCCAGCACGCCGCTATGGAAAAAGAGCAATGGAATGACACGCGCAATCTGCGCCAGAAAGTGA
ATAAACGCACTGAAAAAGAGTGGGATAAAGCCGACGCCGCTTTTGATAACCGCGATAAATGTGAGCAAAGCGCCAACATC
AATGCCTACTGGGAGCCCAATACTTTGCGCTGCCTGGACCGTCGAACTGGCCGCGTTATTACCCCCTAACCTGTTATTGA
TTTAAGGAATGTAAGGACACGTTATGCCAAGCGCCCACAGTGTTAAGCTACGCCCGCTGGAGCGTGAAGATTTACGCTAT
GTACATCAACTCGACAATAACGCCAGTGTGATGCGTTACTGGTTTGAGGAACCCTACGAAGCCTTTGTTGAACTCTCTGA
TCTGTATGATAAGCATATTCACGATCAGAGCGAACGGCGCTTTGTGGTGGAATGTGACGGCGAAAAAGCCGGTCTGGTGG
AGCTGGTGGAAATTAACCATGTTCATCGCCGCGCAGAATTTCAGATAATTATCTCCCCGGAGTATCAGGGGAAAGGTCTG
GCAACCCGTGCCGCCAAATTAGCAATGGACTATGGCTTTACCGTTCTCAATCTCTATAAGCTGTATCTGATCGTTGATAA
AGAGAATGAAAAAGCGATTCACATTTACCGCAAGCTTGGCTTTTCGGTTGAAGGTGAATTGATGCACGAGTTCTTTATTA
ATGGTCAATATCGTAATGCCATTCGCATGTGTATATTCCAGCATCAGTATCTGGCAGAGCACAAAACACCGGGTCAGACT
CTCCTGAAGCCGACCGCACAATAGCATTAATAATAATCGATCGTATTTTTGATGGTGTAAACCCGTTCGACGGCGGGTTT
TACTCCTTCATCAACAATGATTAGCTGACAGTCCACCGGATTAGCGTGACTGTCATATTCACAGCTCTGTTTTACATTAC
CAACCCGTTGATTATTCAGTAAAGTAACCGCTGTGTAATCTAATTTTTTGATCGGATCCGTTGATGGCGTGGCGCTGACA
GATAATGTTTTGTCGTTACTTTTCGTGGTTTTACCCAGCGGATAACCCTGATCATCATAGCGATATTCCATCTGCATTTG
TTTGCTGCTGGCTTTAATCACGAAGCCATTATCATCGGTTTCCCAACTCACCCCGGCAGAAGGTAATTCTGCTAGCTGGC
ATTTTCCCTGTAAACGTACTCTCTTCTCCAGCGTCTCGGCATCACGGTAATAATTGGCGTCCAGTACCAGAGCGACCACG
GTATTATTTTCCAGATCCAGTAATTCGAGTGAATCAAAACAGCCTTCTTCCGACAAAGTCCCAGAAACACGTTTCGTCAC
TTCACCTTGCTCATCCATTAATGTCTGAGTGAAATCTTTTACCGGACCACGCAGCGGATCAAAATCGAATTCATTAGAGA
AACTGGCCATCTCAGGGGTAAATGAAAGTGTTACCTCTGTGCGGTCACATCCTGTGAGGAATATCGCGAGTAAGCATGGT
AGTAATTTGTATTTCACAACAGTCACCAGAGAGTAGAGATGATTCTCAATCATAGTAGCAAATACAGTACTTTACACGTT

AAATGCTATGCTTAAAGAAGTTATCTTCGCGTAAGGAGCTTATGATGAAACTCTCAACCTGCTGTGCCGCGCTTCTGCTC
GCCCTCGCCTCACCCGCGGTACTCGCCGCGCCGGGATCCTGTGAGCGCATACAGAGCGATATATCACAGCGCATTATCAA
TAATGGCGTACCCGAAAGCAGCTTCACGTTAAGTATTGTACCCAATGACCAGGTTGATCAGCCTGATTCCCAGGTCGTCG
GCCATTGCGCTAATGATACGCATAAAATTCTCTATACCCGCACCACCAGCGGTAACGTCTCTGCTCCCGCGCAGTCTAGC
CAGGATGGCGCGCCTGCCGAACCGCAGTAACACATTATCGACTGAACGCCGGATATGACAAATCCGGCGATTTGAACATA
CAACATAATCCCACCTTATTACTCATACCCTTCTATTGATATGGATTAATAATTCTTAACCCAAAATGGGTAGACTCCCT
CTATTGTTAGCGCGCTAAATATTCAATATATAAACTTTTATATAACGATAAAGAACAGGGAGTGAGTTATGTCCAAAAAT
GAACGAATGGTGGGCATCAGCCGCAGAACACTCGTTAAATCTACCGCGATAGGTTCTCTGGCGCTGGCTGCAGGCGGTTT
TTCTTTGCCGTTTACCCTGCGCAATGCAGCAGCAGCGGTACAACAGGCCCGCGAAAAAGTGGTCTGGGGTGCCTGTTCCG
TCAACTGTGGTAGCCGCTGTGCACTTCGTCTACATGTTAAAGATAATGAAGTGACCTGGGTGGAAACTGACAATACCGGC
AGCGATGAGTACGGCAACCATCAGGTACGCGCCTGTTTGCGCGGTCGCTCCATCCGCCGGCGTATTAATCATCCCGATCG
CTTGAATTACCCAATGAAACGCGTGGGCAAACGCGGCGAAGGCAAATTCGAACGGATTAGCTGGGATGAAGCCCTGGATA
CTATCGCCAGTAGCCTGAAGAAAACCGTCGAACAATATGGCAATGAGGCTGTATATATTCAGTACTCTTCGGGGATCGTT
GGCGGCAATATGACCCGCTCTTCGCCATCAGCCTCGGCGGTCAAACGCCTGATGAACTGCTACGGCGGTTCACTCAACCA
GTATGGCTCCTACAGCACTGCGCAAATTTCCTGTGCCATGCCCTACACCTACGGCAGTAATGATGGCAACAGCACCACGG
ATATTGAAAACAGCAAGCTGGTCGTGATGTTTGGTAACAACCCGGCAGAAACCCGCATGAGCGGTGGTGGCATCACTTAT
CTTCTTGAAAAAGCGCGCGAGAAATCGAACGCCAAAATGATTGTTATCGATCCGCGATATACCGATACGGCTGCCGGTCG
TGAAGACGAATGGCTCCCTATTCGCCCGGGCACCGATGCCGCGCTGGTTGCGGGTATTGCCTGGGTATTGATTAACGAAA
ATCTCGTTGATCAACCTTTTCTCGATAAATACTGCGTCGGCTATGACGAAAAAACCTTACCCGCAGATGCACCCAAAAAT
GGTCACTATAAAGCCTATATTCTTGGTGAAGGTGACGATAAAACAGCGAAAACGCCGCAGTGGGCTTCGCAAATTACCGG
TATCCCGGAGGACCGTATCATCAAACTGGCGCGTGAAATTGGCACAGCAAAACCCGCTTATATCTGCCAGGGCTGGGGGC
CACAACGCCAGGCAAACGGCGAACTGACTGCACGCGCTATTGCTATGTTACCTATTTTGACGGGCAATGTCGGCATCAGC
GGCGGAAATAGTGGCGCGCGTGAATCGACCTATACCATTACCATAGAACGCCTGCCGGTGCTGGATAATCCGGTCAAAAC
GTCAATCTCCTGCTTCAGCTGGACAGATGCTATCGATCATGGTCCGCAAATGACGGCAATCCGCGACGGCGTCCGCGGCA
AAGATAAACTGGATGTGCCCATTAAGTTCATCTGGAACTACGCGGGAAATACCCTCGTTAATCAGCATTCTGACATCAAC
AAAACGCATGAAATTCTGCAGGACGAATCGAAATGCGAAATGATTGTGGTCATCGAAAACTTTATGACCTCATCGGCAAA
ATATGCCGACATTCTGCTGCCAGACCTGATGACCGTTGAGCAGGAAGATATTATTCCTAACGACTACGCCGGTAACATGG
GATATCTCATTTTCCTCCAGCCTGTCACCAGCGAAAAATTCGAACGCAAACCGATTTACTGGATCCTGAGTGAAGTCGCG
AAACGTCTTGGACCTGACGTCTATCAAAAGTTCACAGAAGGTCGCACGCAGGAACAATGGTTACAACATCTGTACGCCAA
AATGCTTGCCAAAGATCCGGCGTTACCGTCTTACGACGAACTGAAAAAAATGGGTATCTATAAGCGTAAAGATCCCAATG
GCCATTTTGTCGCCTACAAAGCATTTCGTGACGACCCCGAGGCAAATCCACTTAAAACGCCTTCCGGTAAGATTGAAATT
TATTCCAGCAGGCTGGCGGAAATTGCCCGTACCTGGGAACTGGAAAAAGATGAAGTGATAAGCCCATTGCCAGTTTATGC
CTCAACCTTTGAAGGCTGGAACTCCCCTGAACGTAGAACCTTCCCACTGCAACTGTTTGGTTTCCATTACAAATCCCGTA
CTCACTCGACCTACGGCAATATTGATCTCCTGAAGGCTGCCTGCCGTCAGGAGGTGTGGATCAACCCTATAGATGCGCAG
AAACGTGGGATTGCCAACGGCGATATGGTGCGGGTGTTTAACCATCGTGGCGAAGTTCGGCTACCAGCCAAAGTAACACC
ACGTATTCTCCCTGGAGTTAGCGCTATGGGCCAGGGAGCCTGGCACGAGGCCAATATGTCTGGCGATAAAATCGACCATG
GCGGCTGTGTGAATACGCTAACCACTCTGCGCCCTTCACCACTGGCGAAGGGAAACCCGCAGCACACTAATCTGGTCGAG
ATCGAAAAAATATAACCCACGACAACCATAAATTCTGGCATGACATTTTGTTTGAAAAGCAATAAGTGAGTAATGATGAA
AATCCATACCACAGAGGCGCTCATGAAGGCTGAAATCAGCCGTAGAAGTCTGATGAAAACCTCCGCACTTGGCAGTCTTG
CGCTGGCAAGCAGTGCTTTCACTCTGCCATTTTCCCAAATGGTCCGGGCGGCAGAGGCTCCGGTAGAAGAGAAAGCGGTC
TGGAGTTCCTGCACCGTTAACTGCGGGAGCCGCTGTCTGTTACGTTTGCATGTGAAAGATGACACCGTGTACTGGGTGGA
GTCTGATACGACAGGTGACGACGTCTACGGTAATCATCAGGTTCGAGCGTGTTTACGCGGGCGCTCTATCCGCCGACGGA
TGAATCATCCTGATAGGTTGAAATATCCCATGAAGCGCGTCGGCAAGCGCGGTGAAGGTAAATTTGAACGGATAAGTTGG
GACGAAGCCCTGGATACCATCAGTGATAATCTTCGGCGGATCCTGAAAGATTACGGCAATGAGGCTGTACATGTCCTGTA
CGGAACAGGCGTAGATGGCGGAAACATCACCAACTCAAACGTCCCGTACCGTCTGATGAACTCTTGCGGTGGTTTTCTCA
GTCGCTATGGCAGCTACAGTACCGCACAGATCAGTGCCGCAATGAGTTATATGTTCGGTGCCAATGATGGCAACAGCCCG
GATGATATCGCCAATACGAAACTGGTCGTTATGTTCGGAAATAACCCGGCAGAAACGCGGATGAGCGGCGGTGGTGTCAC
TTACTACGTCGAGCAAGCCCGCGAACGTTCAAACGCACGCATGATCGTCATCGATCCACGTTATAACGACACTGCTGCCG
GGCGTGAAGATGAATGGCTGCCCATTCGCCCTGGCACCGATGGCGCACTGGCCTGTGCGATTGCCTGGGTACTGATTACT
GAAAACATGGTCGATCAGCCATTTCTCGACAAATATTGTGTTGGTTACGATGAAAAAACGCTGCCCGCCAACGCACCACG
TAACGCGCATTATAAAGCCTATATTCTGGGCGAAGGGCCTGACGGCATAGCTAAAACGCCGGAATGGGCAGCAAAAATCA
CCAGCATCCCGGCAGAAAAAATTATCCAGTTGGCACGAGAGATCGGTTCAGCAAAACCTGCTTATATTTGTCAGGGTTGG
GGGCCACAACGACATTCCAACGGCGAGCAAACATCCCGCGCTATTGCCATGCTTTCCGTTCTCACCGGCAACGTCGGCAT
AAACGGCGGCAACTCAGGCGTACGCGAAGGTAGCTGGGATCTGGGGGTAGAATGGTTCCCGATGCTCGAGAATCCTGTTA
AAACGCAGATTTCCGTCTTTACATGGACAGATGCCATCGACCATGGTACGGAAATGACCGCGACCCGTGATGGTGTTCGT
GGAAAAGAAAAACTGGATGTCCCCATCAAGTTTTTATGGTGCTACGCCAGTAACACATTGATCAATCAACATGGCGACAT

CAATCACACCCATGAGGTGCTTCAGGATGACAGCAAGTGCGAGATGATTGTTGGCATTGACCACTTCATGACGGCCTCGG
CTAAGTATTGCGATATCCTGTTGCCCGACCTGATGCCAACAGAGCAAGAAGACCTTATCTCTCATGAATCTGCAGGGAAT
ATGGGCTATGTGATCCTCGCCCAACCCGCAACCTCAGCAAAATTTGAACGCAAACCCATCTACTGGATGCTGAGTGAAGT
CGCCAAACGCTTAGGACCAGACGTTTATCAAACCTTTACTGAAGGTCGCAGTCAGCATGAATGGATCAAATATCTCCATG
CGAAAACGAAGGAACGTAACCCTGAGATGCCCGACTACGAGGAGATGAAAACGACCGGGATCTTTAAGAAAAAATGCCCG
GAAGAACACTACGTCGCTTTCCGCGCATTCCGTGAAGATCCACAGGCAAACCCGTTGAAAACACCTTCGGGGAAAATCGA
AATTTATTCTGAACGACTGGCGAAGATTGCAGATACCTGGGAATTGAAAAAAGATGAAATTATTCATCCCCTTCCTGCGT
ATACCCCAGGTTTTGATGGCTGGGACGATCCCCTGCGGAAAACCTATCCACTGCAGTTAACGGGCTTCCATTACAAAGCG
CGTACCCACTCCAGCTACGGCAATATTGATGTGTTACAGCAGGCCTGCCCACAAGAGGTGTGGATCAACCCCATTGATGC
TCAGGCACGCGGTATCCGTCATGGCGATACCGTGCGGGTATTTAACAATAATGGAGAAATGCTGATTGCCGCAAAAGTGA
CTCCGCGTATTCTGCCTGGCGTTACCGCCATCGGGCAAGGTGCGTGGCTTAAAGCGGATATGTTTGGTGACCGGGTCGAT
CACGGCGGCAGTATCAATATTCTGACCTCTCACCGCCCTTCACCGCTGGCAAAGGGAAACCCGTCGCACAGCAATCTTGT
CCAGATCGAAAAGGTTTAAGGAGTAGCCCATGACCACACAATATGGATTTTTTATCGATTCCAGCCGCTGTACTGGCTGT
AAAACCTGCGAACTGGCGTGCAAAGATTTTAAAGATCTTGGCCCGGAAGTCAGTTTCCGCCGTATTTATGAATACGCTGG
CGGCGACTGGCAGGAAGATAATGGCGTCTGGCACCAGAACGTGTTCGCTTATTATCTCTCCATCTCCTGTAACCATTGCG
ATGACCCCGCATGTACAAAAGTCTGCCCCAGCGGCGCAATGCATAAGCGTGAAGATGGCTTTGTGGTGGTTGATGAAGAT
GTTTGTATCGGCTGCCGCTACTGCCACATGGCTTGTCCGTACGGCGCGCCACAGTACAATGCTGAAAAAGGGCACATGAC
GAAGTGCGATGGTTGTTATTCGCGCGTCGCCGAGGGGAAACAACCCATATGTGTCGAATCCTGCCCGCTGCGTGCACTTG
AGTTCGGTCCAATTGAAGAGCTGCGCCAGAAGCACGGCACTCTTGCTGCCGTCGCGCCACTGCCGCGCGCGCATTTCACA
AAACCCAATATCGTTATCAAACCTAACGCCAACAGCCGCCCGACCGGCGATACCACTGGTTATCTGGCTAATCCGGAGGA
AGTGTAAAATGGGAAATGGATGGCATGAGTGGCCACTGGTTATCTTTACCGTATTGGGTCAATGCGTAGTAGGCGCGCTG
ATTGTTAGCGGAATTGGCTGGTTCGCCGCCAAAAATGACGCAGACCGTCAGCGTATTGTCCGTGGCATGTTTTTCCTCTG
GCTGTTGATGGGCGTCGGGTTTATTGCCTCTGTCATGCACCTGGGATCGCCACTGCGGGCGTTTAACTCCCTGAATCGAA
TCGGAGCTTCTGGTCTGAGTAACGAGATTGCTGCCGGTTCCATATTTTTTGCCGTTGGCGGATTATGGTGGCTGGTGGCA
GTCATCGGTAAAATGCCGCAAGCGTTGGGGAAACTCTGGTTACTTTTCAGCATGGCGCTCGGCGTCATTTTCGTCTGGAT
GATGACCTGCGTGTATCAAATCGACACCGTGCCAACCTGGCATAACGGTTACACTACGCTGGCTTTCTTCCTGACGGTAT
TGTTGAGCGGTCCAATACTTGCTGCCGCAATTCTGCGGGCAGCTCGCGTTACATTTAATACCACACCATTTGCCATCATT
AGCGTTCTGGCGCTGATCGCTTGTGCCGGGGTGATTGTGCTGCAAGGTTTGTCTCTGGCGTCTATTCACTCATCCGTGCA
ACAAGCCAGCGCACTGGTACCAGATTACGCCTCCTTACAGGTCTGGCGTGTGGTATTGTTATGCGCAGGTCTTGGATGCT
GGCTTTGCCCGCTCATTCGTCGCCGTGAACCTCACGTTGCCGGACTTATTCTGGGGCTAATCCTCATACTTGGCGGTGAG
ATGATCGGGCGTGTGCTCTTTTATGGCCTGCACATGACAGTAGGAATGGCTATTGCAGGTTAACAAATTGGTGCGGGGCA
ACCCGCACATTTCAGGATGTTAGGAATGACCCATTTTTCACAGCAAGATAATTTTTCTGTCGCGGCGCGCGTGTTGGGCG
CGCTGTTTTATTACGCTCCAGAGAGTGCGGAAGCCGCACCTCTGGTTGCGGTACTCACCAGTGACGGCTGGGAAACTCAG
TGGCCTCTACCAGAAGCGTCATTAGCGCCGCTGGTGACGGCTTTTCAGACACAGTGTGAAGAGACTCACGCCCAGGCCTG
GCAGCGTTTGTTTGTCGGCCCGTGGGCACTGCCGTCTCCGCCGTGGGGGTCGGTCTGGCTGGATCGCGAATCTGTGCTGT
TTGGCGATTCAACATTGGCACTTCGTCAGTGGATGCGCGAGAAAGGCATTCAGTTTGAAATGAAGCAAAACGAACCGGAA
GATCATTTTGGTTCACTGCTGTTGATGGCTGCGTGGTTGGCGGAGAATGGTCGCCAGACAGAATGCGAAGAATTGCTGGC
ATGGCACCTTTTTCCGTGGTCAACACGTTTTCTTGATGTTTTTATCGAAAAAGCAGAACACCCTTTCTACCGTGCACTGG
GTGAACTGGCTCGCTTAACGCTGGCGCAATGGCAATCACAACTGTTAATTCCTGTCGCGGTTAAACCGCTGTTTCGATAG
GATCACCGTAATATTGCCGGATGGTGACGATTACGCGCAATCCGGCAATAATAGGTTACAGTGTCACGTTTTTTTATCTC
TTAAAGCACGCACTGCTTTTGCGGCTGGCCTCTTTTGCCGCAAAATAGTCGCCCGTGTTTCATTGCCCATTTCTGCTCAT
GCATCATCTACACATCTATCCGGATCTGCGCACTATGTTCCACCGTCTGCTTATCGCAACAGTCGTCGGTATTCTCGCGG
CCTTTGCCGTTGCCGGGTTTCGTCATGCGATGCTGCTACTGGAGTGGTTGTTCCTCAATAATGACTCCGGCAGTCTGGTC
AATGCAGCGACAAACCTTTCCCCCTGGCGACGGTTGCTAACTCCGGCGCTCGGCGGACTGGCGGCGGGTTTGTTGCTGAT
GGGCTGGCAGAAATTTACCCAACAACGCCCTCATGCGCCGACCGATTATATGGAAGCGTTGCAAACCGATGGACAGTTCG
ATTACGCAGCAAGCCTGGTTAAATCGCTTGCCTCTCTGCTGGTAGTAACCAGCGGCAGTGCAATTGGTCGCGAAGGTGCG
ATGATTCTTTTAGCTGCCCTTGCCGCCTCCTGTTTTGCCCAACGTTTTACGCCACGCCAGGAGTGGAAATTATGGATCGC
CTGTGGGGCCGCGGCGGGAATGGCTGCGGCCTATCGTGCCCCGCTTGCTGGCAGTTTATTTATAGCCGAAGTGCTGTTTG
GCACTATGATGTTGGCCTCTCTCGGCCCGGTGATTATTTCCGCCGTCGTGGCATTGCTGGTTAGCAATCTGATTAATCAT
AGCGACGCGTTACTCTACAACGTACAACTCTCAGTGACGGTTCAGGCTCGTGACTATGCGCTGATTATCAGTACAGGTGT
GCTGGCAGGTCTGTGCGGACCACTGTTGTTAACGTTAATGAACGCCTGTCATCGTGGATTTGTGAGTCTCAAACTTGCGC
CGCCCTGGCAACTGGCACTAGGCGGGTTGATCGTGGGTCTGCTTTCCCTGTTCACACCTGCAGTGTGGGGCAACGGCTAT
AGCACCGTACAATCCTTTTTAACCGCCCCACCACTGTTAATGATCATTGCCGGGATCTTCCTTTGTAAACTGTGTGCCGT
GCTGGCGAGTAGCGGTTCCGGCGCACCCGGTGGGGTCTTTACACCGACGCTATTTATCGGTCTTGCCATTGGCATGTTGT
ATGGTCGTAGCCTGGGATTATGGTTCCCTGATGGCGAAGAAATTACACTTTTACTCGGATTGACCGGGATGGCGACACTG
TTGGCGGCGACCACGCACGCGCCGATTATGTCGACGTTGATGATATGTGAAATGACCGGGGAGTATCAGCTACTCCCCGG

TTTATTGATTGCCTGCGTAATTGCGTCGGTAATTTCGCGGACGTTACACCGTGACTCTATCTACCGCCAGCACACTGCGC
AGCATAGCTAAGCGGATGTATTGCCCCAGTTCACGCTGTTCGGCGCGCGGCAGATAAGGCAGTTCACCAATGAGCGGTGC
CGGAAGTTTTTTACCCAGCACATCAATGATTTCCGCATAATGCGCCAGTCCTGGGTTGATTCGGTTAGCCACCCAGCCAA
TGAGCGGCAGCCCGTCGTTGGCGATCGCCTGAGCTGTTAGCAGTGCATGGTTAATGCAACCTTCCTGAATACCGACAACC
ATCAACACCGGCAGTTGTTCCTGCACTACCCATTCAGAGAGTGGACGCAAATCATTCATCAGACTGCGCCAGCCGCCAGT
CCCTTCTACCACGACATGATCGACTTTTTCGGTCAGGTTTGCCAGGCCGTTTGAAATGAGGGTGTAATTGATTGGGCAAC
TGTGCGCCACGCTACTTTCTTCTTCGCTTAACGCGATAGGATTAACTGCTTCATAAGGCAGTTCGATGGTTGAAACACTC
TGCAACACCAGGGCATCTTTATTACGCAGCCCTTCGGGTGTCTCTTTGCTCCCCTTCGCTACGGGTTTATATCCCGCAAC
CGTTTTTCCCTGGGAGGCTAACGCTTGTAGCAATGCGCGGGAAACCACCGTTTTCCCTACAGAAGTGTCTGTACCGGTAA
TAAAGAAACGCTTCAGCATCACTAACTCCACCGTTATGCTTCACAAATATAAACCAGGAAAATAATTAACCTTGAAAGTC
TAAGTTATGCTTTCCTGGCCCAAATTGAGATAGCGCAAATTTTGGTAGAACAGTTAAAAAATGTTAACCCTGCAACAGAC
GAATCAACAAAGAACCGTTATACATCGCGTCTTTTACCAGTGCAGCGCCTGCCATCGTGCCCTGGTTAGAAAACTGAGTA
CTCTCAACGCTGATGTGCTGACTATACGCAGGAAGGGCCTGCTGACGGATGCTGTCTGAGATGACCGGGAAGAGGATATC
TGCCGCTTTACTTAACGGTGAGCCAATCAGTATTTTTTGTGGGTTAAATAAATTCACCATGATGGCAAGAATGCGCCCGA
CATGCGCGCCCACCCCGGTAATGATGTCTTTTGCCAGTAGATCGCCGCGCAATGCCGCCTGACACAATGAGTCCACGGTT
AACGGTTGTCCATGTAACATCGAGCTCATGGATTGATTAAGACGCAGCTGTGCCAGCTCAAGAATACTGTCCACGCTGGC
GATGGTTTCGAGGCAGCCGTGATTCCCGCAATAACAGCGTTTCCCATACGGGTCGACCTGTGTGTGGCCTATTTCCACGA
GACTACTGCTGCCTGCGTGTAGCAGATGACCATCGGTAATGACGCCCGCCCCCACGTTGTGATCGATAACCACCTGAATC
ACATCGCGCGCCCCGCGTGAGGCACCAAACAAGGCCTCTGCCATCGTCCATGCGCTGATATCATGCTGAATATAAACCGG
AACGCCGGTATGCTGCTCCAGCGCCTCGCCGAGCGGCATCTCTTTTACATCCTCGTAGAACGGCATGCGATGTACAATAC
CATTTTCCGTATCAATAATTCCCGGCAAGGTTATGGCAATCGAAGTTAGACGCTCAAGTTTTTTCTGGTGGCGGATAAAA
AACTGATCGATATGGGAAATAATACGATCCAGCAATGGCAAGTCATCTTTTAACGCCAGTTCCTGCGACTCTTCCACCAC
CAGTTTGCTGCTCAGATCGCGCAGAGCAAGGAAAATCTCCCCGCGACTAATGCGCAGAGAAAGATAGTGCCAGGCTTCAG
TTTCAACCACCAGCCCCACCGCCGGACGGCCACGGTTCCCCGCTTCTTTGATTTCCAGCTCTTGCACCAGGTGTGCTTCG
AGCATCTCACGGACAATTTTAGTGATACTGGCAGGAGCCAGTTGCGCCAGACGGGAAAGATCGATACGCGAGACTGGACC
AAGCTGATCAATCAGGCGATAAACCGCGCCCGCGTTGGTCTGCTTTATTTGATCAATGTGCCCAGGCTGGTTTTCAGCAA
CCACCGCATACTCCCTATATTTTCGCGCTCCGAAATAATCTGTAGGCTATGGTGAAGCACTTCAATACGTGTCGTCAAAT
TTTTACTTAGGCATGTGATTAACAGCACATTTTTCGGGCTTTTTCGCTGAAATTTCCCTCACCTGAGAGCATTCAGCAGA
TGAATACGAAAGTTACGCGCAGCCGGACTTTGTTCATGATGTTTCGGCCAGACCAACCACATTTCAGAAACCGCATCCTC
TTCAGCAATCGGCACCCAGCGCATTTCGTTGAGCTGAACACGTTTAAATGACGCAGGCAAAATTGAAACACCCAGACCGG
CGGAAACCAGACCGATGATGGTCATTGCCTCGCCCACCTCCTGAGTGATGACGGGCGTCAAATGGTAACGTCGCATCAGC
CCGAGAATATCGTCATACAGCCCTGTCCCGACGTGCGGATCAAAAAAGACAAAGGGTTCTTTCGCCAGTTCAGCCAGCGT
TACATTCGGGTTATTTGCCAGGGGATGATCGTGCGGGATCATCGCCATAAGCGGTTCATGGACGATGACTGCGTGTTCAA
GCGACTCCGGTAACGCTGTGTTACGCAGCAATCCCATATCCAGCGTTCCTTCAATGAGCGGAGCGATTTGCTCGCGAGTG
TTCATTTCGCGGGTTTGTAAATGGACATCAGGATAATCACGGCGAAACAGCGATAACGTATCGGACACCGCCCGAATAAA
AGGAGCCGACGAAGTAAAACCAATGCGCAACTCCCCCGCTTCACCCTGATGCAGCCTTTCAGCGCGAGCGGCAGCGTCAT
CCACCATAGACAGGATTTGCCGACTATCTGCAAGAAACTGTTTTCCTGCTGCCGTCAGCAATACACTGCGATTGGTTCGT
GCCAGCAGTCGGGCACCAATTTGTTGCTCCAGCGCCTGAATCTGCTGACTTAGCGGCGGTTGCGAAATATTCAGGCGGGC
AGCGGCGCGCCCGAAATGCAGCTCTTCCGCAACAGCAACAAAGTAACGCAGATGACGAAGTTCAATATTCATATTTAAAA
CATCTTATTTGAGATTATTAATATATTAGACAGAACAATTCGATTTTCCTACCCTATGTATAAGCCTGATCTACAGGCAT
ATTTAGCAAGGATTTCAAGTGAGCCGTACTACAACTGTTGATGGCGCTCCGGCAAGCGACACTGACAAGCAAAGCATTTC
TCAGCCAAATCAATTTATTAAACGCGGTACGCCGCAATTTATGCGCGTCACCCTGGCGCTGTTCTCTGCCGGACTGGCAA
CATTTGCACTTCTCTATTGTGTGCAGCCTATCCTTCCGGTGCTTTCGCAGGAGTTTGGCTTAACCCCCGCGAACAGTAGT
ATTTCACTGTCCATTTCCACGGCGATGTTGGCTATTGGTTTGCTGTTTACTGGCCCGCTATCCGATGCCATTGGTCGCAA
ACCAGTGATGGTCACGGCGCTACTGTTGGCCTCCATTTGTACGTTACTTTCGACAATGATGACCAGCTGGCACGGCATTT
TGATTATGCGCGCCTTGATTGGGCTTTCGTTAAGTGGCGTGGCAGCTGTTGGCATGACTTATCTTAGCGAGGAAATCCAT
CCCAGTTTCGTGGCCTTTTCAATGGGGTTGTATATCAGCGGCAACTCAATTGGCGGCATGAGCGGACGCTTAATTAGCGG
TGTCTTCACGGACTTTTTCAACTGGCGAATTGCTCTGGCGGCAATCGGTTGTTTCGCGCTGGCCTCGGCGTTGATGTTCT
GGAAAATCCTCCCTGAATCACGCCATTTTCGCCCGACTTCGCTGCGCCCTAAGACGTTGTTTATCAACTTTCGTCTGCAC
TGGCGTGACCGGGGATTACCGTTATTGTTCGCAGAAGGCTTTTTGCTGATGGGGTCGTTCGTCACGCTGTTTAATTACAT
CGGCTATCGGTTGATGCTCTCCCCCTGGCATGTCAGTCAGGCCGTGGTTGGCTTATTATCGCTGGCTTATTTGACCGGTA
CATGGAGCTCACCCAAAGCCGGAACCATGACCACCCGCTATGGGCGTGGTCCAGTGATGTTGTTTTCGACGGGGGTTATG
CTGTTTGGTTTACTGATGACCTTATTCAGCTCGCTGTGGCTGATCTTTGCCGGAATGTTACTCTTCTCAGCAGGATTCTT
CGCAGCCCACTCAGTAGCCAGCAGCTGGATCGGCCCCCGCGCAAAACGCGCTAAAGGCCAGGCCTCCTCGCTGTATCTGT
TCAGTTACTATCTGGGGTCGAGTATTGCCGGGACGCTGGGTGGTGTTTTCTGGCATAACTATGGCTGGAACGGCGTCGGC
GCATTTATTGCTCTGATGCTGGTCATTGCTCTGCTGGTCGGGACGCGTTTGCATCGTCGTCTGCACGCCTGAAAAATAAG

TCCGGACTGCGGTAAATACCCGTCCGGACTTATTGCCAGCTCAAACCAACGTTAATAGCCATCCTAAAATAGACGAAGCG
CCAGCCAATTCCCGCAGCGCGTCTAGCGTCATCAGGATTATAAGTACCCAAATAAACGGATTCATTTTGCTGTGTGTCAT
TTATTACTGATGCGCAGTTATTCTACTGCTTTGTAAGTAGTAAAATAGTTAACCCGATCAAGACTACTATTATTGGTAGC
TAAATTTCCCTTAAGTCACAATACGTTATTATCAACGCTGTAATTTATTCAGCGTTTGTACATATCGTTACACGCTGAAA
CCAACCACTCACGGAAGTCTGCCATTCCCAGGGATATAGTTATTTCAACGGCCCCGCAGTGGGGTTAAATGAAAAAACAA
ATTGAGGGTATGACAATGAAAAAAGTATTAGCTCTGGTTGTTGCCGCTGCTATGGGTCTGTCTTCTGCCGCCTTTGCTGC
AGAGACTACGACCACACCTGCTCCGACTGCGACGACCACCAAAGCAGCGCCGGCGAAAACTACACATCATAAAAAACAGC
ATAAAGCAGCACCTGCCCAGAAAGCGCAGGCGGCTAAAAAGCATCATAAAAATACGAAAGCTGAACAGAAAGCCCCTGAA
CAAAAAGCGCAGGCAGCGAAGAAACACGCCAAGAAACACAGCCATCAGCAACCGGCAAAACCTGCTGCACAACCCGCAGC
GTAAGTTTTCAACAGTAATGCTGGCGCGCCCCCTCGCGCCTGAAAATTACGGTGCTAAGCGGGTAACGTTTAGCACCGCC
TTTAGCGGAGGGTAGTATGGTGGGCCGTTATCGCTTTGAGTTCATTCTGATCATCCTTATTTTATGCGCACTGATTACCG
CCCGTTTTTATCTTTCCTGATTGTAGTTATCTGATTTTACTCCCACTTTCATCCCGTCCCGTCTATAGTATTTATGAGGG
TTTGCTTTTAATAATCATAATTACCCACCAGAGTGTGATATGCGTACAACCATTGCTGTAGTGTTGGGTGCAATTAGTTT
GACGTCTGCTTTTGTGTTTGCAGATAAACCAGACGTTGCCAGATCGGCAAACGATGAGGTCAGCACCCTGTTTTTTGGTC
ATGATGATCGTGTGCCAGTGAATGACACGACCCAATCACCGTGGGATGCGGTTGGGCAACTGGAAACGGCCAGCGGCAAT
TTATGTACGGCGACGCTGATTGCACCCAATCTGGCATTAACGGCAGGACACTGTTTATTGACACCTCCAAAGGGTAAAGC
CGATAAAGCAGTGGCGCTGCGTTTTGTGTCAAATAAAGGTCTTTGGCGCTATGAGATCCACGACATAGAAGGCCGCGTTG
ATCCGACACTGGGAAAGCGGTTAAAAGCAGATGGGGATGGTTGGATTGTACCTCCCGCAGCCGCGCCGTGGGACTTCGGA
TTGATTGTGCTACGTAATCCCCCTTCTGGCATTACGCCGTTGCCGTTATTTGAGGGAGATAAAGCCGCGCTTACCGCCGC
ATTAAAAGCGGCAGGTCGTAAAGTGACTCAGGCAGGCTACCCTGAAGATCATCTCGATACGTTGTACAGTCATCAAAACT
GTGAAGTGACTGGCTGGGCGCAAACGTCGGTGATGTCACATCAGTGCGATACCTTGCCGGGTGACAGCGGTTCGCCTCTG
ATGTTGCATACCGATGACGGCTGGCAATTAATTGGGGTGCAAAGTTCGGCTCCTGCCGCGAAAGATCGCTGGCGCGCCGA
TAACCGGGCCATTTCTGTTACCGGTTTTCGCGACAAGCTGGATCAACTGTCGCAAAAATAATGTTCAAACGCTGCCCGAC
AGCGCGGGCAGCGTCTTCATCAGGCAAGTTTCACCATGATCATTCCAGCCAACAGCAAGACCAGGCCAATCCAGCCTTTA
CGATTTAACCGTTGACCAAACAAGATCCAACCTGCGGCTAACGTGGCGGCAATACCAAACCCGCCCCACAATGCATAAGC
GACAGACAAGTCGATCCCTTTAACGGCTTGAGAAAGCGCACTAAAGGCAGCCAGCACCGCCGCCAGGGAGAGCAAGCCAA
ATATTTTGCGACGAAAGCCGTCAGAAAATTTCAAAAAGACGTTAGCAACGATTTCCAGCACGATTGCCAATGCCAGCCAG
GCGGCGTGAACCCATTCAAACTGCGCCATGGTTCACCTCCAGTTCAGGTTTACGCGCTTTACGGGTACCTGATTTTATCA
ACACAATCCCGGCGACCAGGGTGGTTAACCCGGCAATTTTCATCAGCGATAAACTTTCGTCGAATAACAAAACGCTAAAC
AAGGTAATAAATAAAATACCGATACCTTCCCACAGCGCATAAGCTACGCCTAAGGCGATTTTTTTAACGGCGAAAGAGAG
AAATATATACGACAGAGAAATCATCACCAGCATTAAAATAAAGCCGCCATTTCCCTCACTGACGCTCGCCCATTTCATTG
ACAGCGTACCGGTAATTTCTGTAGCAATAGCCAGACCTAATAAAATCCAATAAATATACATTGTCCTTCTCCTGCAAGAG
AATTATTTTAATTTTCGCTTAATTCAGCGAAACCAAAGTAAAGTGGTCAAGCCCCGCGCATAGCGCAAGCTTTGGCAGAA
GAAAGGACTAAAGCGCGTTGCGCCAGTGCTGCTCACCTACGAGCAAGATAGTGGATGAGGTACGAAGATGGGTAAATGTA
GAAAATAACGTCCTGAACAAATTGTCCATAATATTACAATTATCCGCAGTGTTGCTTCTCGTCATCGCGGATGATAATTG
TCCTCGGTAGTTGAACACGCCTGATTTGTATCATAGCTTAAGAATTAACTCAAAATATTTTCACTTCTTTACCTGAGCGG
TTTGATTTTCGTTATGATGACGGAGCGAAAAAGACATTATTATTAGCAAAGGAAGAAAAAACGGGGACAAGCATGGCAAA
GCCGATCATCACGCTCAATGGCCTAAAAATCGTCATTATGTTGGGAATGCTGGTCATTATTCTCTGCGGTATCCGTTTTG
CCGCCGAGATCATCGTGCCGTTTATTCTCGCATTATTTATTGCTGTTATTCTTAACCCGCTGGTGCAACACATGGTCCGC
TGGCGTGTGCCGCGTGTACTGGCGGTGTCGATTTTGATGACCATCATCGTGATGGCGATGGTGTTGCTATTAGCTTATCT
GGGTTCCGCGCTCAACGAGTTGACGCGGACGTTACCGCAATATCGCAACTCTATTATGACGCCGCTGCAAGCGCTTGAAC
CGTTGTTGCAACGCGTAGGGATTGACGTCTCAGTTGACCAGCTGGCGCATTATATTGATCCGAACGCGGCGATGACGTTG
CTCACCAACTTATTGACGCAGTTATCTAATGCCATGTCATCAATATTTTTATTGCTGCTGACGGTGCTGTTTATGCTGCT
CGAAGTGCCACAATTGCCCGGAAAAATTTCAGCAAATGATGGCGCGTCCGGTTGAAGGGATGGCGGCGATTCAACGTGCGA
TTGACAGTGTTTCTCATTATCTGGTGCTGAAAACAGCCATCAGCATCATCACCGGCCTGGTCGCCTGGGCGATGCTCGCC
GCACTCGATGTTCGCTTCGCTTTTGTCTGGGGATTGCTGGCCTTTGCGCTTAATTACATCCCGAATATTGGTTCAGTCCT
CGCGGCAATCCCCCCTATCGCTCAGGTACTGGTGTTTAATGGCTTCTACGAAGCGTTGCTGGTGCTGGCGGGATATCTGC
TGATTAATCTGGTCTTCGGCAATATTCTGGAGCCGCGTATCATGGGGCGTGGGCTGGGGCTTTCCACATTGGTGGTATTT
TTGTCGTTGATTTTTTGGGGATGGTTGTTAGGACCGGTGGGTATGCTGCTTTCCGTGCCGTTGACAATTATTGTCAAAAT
TGCGCTTGAACAAACAGCGGGAGGTCAAAGCATCGCCGTTCTGTTAAGCGATCTCAATAAAGAGTGACGGCCTCAGCAGA
GGCCGTCAGGGTTACAGAGCTTTCAGGATTGCATCCACGCTGGCTTTGGCGTCACCAAACAGCATGTGGGTGTTTTCCTT
GAAGAACAGCGGGTTTTGCACACCAGCATAGCCAGTGTTCATCGAACGTTTAAAGACAATCACGTTCTGCGCTTTCCACA
CTTCCAGCACAGGCATACCAGCAATCGGACTCTTCGGATCATCCTGCGCCGCCGGGTTAACCGTATCGTTAGCACCAATC
ACCAGTACGGTATCGGTATCAGCAAAGTCATCATTGATCTCGTCCATTTCCAGCACGATGTCATACGGTACTTTTGCTTC
AGCCAGCAATACGTTCATATGTCCAGGCAAACGCCCCGCGACCGGGTGGATACCGAAACGCACATTAATACCACGAGCGC
GCAATTTCTCAGTAATTTCAGCGACAGGATATTGCGCCTGCGCGACTGCCATGCCGTACCCCGGAGTAATGATCACTGAA

TGGGAGTTTTTCAGCAGTTCCGCTGTCTCTTCTGCGGTGATTTCGCGGTGCTCACCCACTTCCTGATCATCGCCAGTAGA
AGAGCCGTCGGTGCCGAAACCACCCGCAATAACGCTGATAAAGGAACGGTTCATCGCCTTACACATAATGTAAGAAAGGA
TAGCCCCCGAAGAACCGACCAGCGCACCGGTCACAATCAGCAGGTCGTTGCTGAGCATAAAGCCCGCAGCCGCAGCCGCC
CAGCCGGAGTACGAGTTCAGCATCGACACCACCACTGGCATATCTGCACCACCGATGGAGGCGACTAAATGCCAGCCGAA
TACCAGCGCAATTGCGGTCATTATCAGCAATGCCAGCACTTGCAGGCCGACGCTGTCCGTGCGAACAAATACAATCAGCA
GCAGGAAGGAAACGACCAGAGCCGCCAGGTTCATTTTGTGACGGTTTGGCAGCATCAATGGTTTAGACGAAATCTTGCCA
CACAGTTTGCCGAACGCCACCACCGAACCCGTGAACGTTACCGCCCCGATGAAGATACCGAGGAACACTTCCGTCAGGTG
AATATTGACCAGAATCGGTGCCATTCCCGCGTCATGATGCAGATAGCTGTTAAAGCCAACCAGCACTGCCGCCAGACCCA
CGAAGCTATGCAGGATCGCCACCAGTTCTGGCATTTCGGTCATTTCAACTTTCTTCGCCAGACGGATACCAATTGCCCCA
CCAATGACCATCGCCAGCAAGATCCAGCCAACATTACCCGTATCCGGTCCAAAAATGGTTGCGATTAACGCAATCGCCAT
CCCGGCGATACCGAAGTTGTTACCCTGGCGAGACGTTTCATGTTTCGAAAGACCGGCCAGACTGAAGATAAACAGGATCG
CGGCAACAATGTATGCAGCTGTAACTAATCCTCCAGACATATGTTACCCCTTAATTTTTGCGGAACATTTTCAGCATGCG
CTGAGTCACGGTGAAGCCACCGAAAATATTAATGCTGGCTATAAGCACCGCGATAAAACTAAGGAAGCTAACCCAGCCGC
CCTGGCCAATCTGCAACAGTGCTCCGACAACAATAATCCCTGAAATCGCGTTGGTGACCGACATCAACGGTGTATGCAGC
GCGTGCGATACATTCCACACCACGTAATAACCGACAACGCAGGCCAGCGCGAAAACGGTGAAGTGCCCAAGGAATTCTTT
CGGCGCAACGCTTGCCATCCAGCCAAAAAGAATGATTGCCAGCGCCATCAACGCGTATTTACGCCACGGTGAGCAGGTAC
ATTTTTCCTCAGTTTTCACTTCCGGTGCCGCTTTTTGTGCCGCCTGCGGCTGAGCTGATACCTGAATCGGCGGTGCCGGC
CAGGTAATTTCGCCCGCACGGATCACGGTCACGCCGCGAATCACCACATCATCAAAATCAACAGTGATATTGCCGTCTTT
CTCTTTGCACAACAGTTTCAGCAGATTAACGAGGTTTGTGCCGTAAAGCTGTGAGGATTGCGTCGGCAGACGGCCCGGAA
GATCGGTATAACCAATCACTTTGACACCATTTTCCGTAGTGAAGATTTCACCCGGCACGGTGTATTCACAGTTGCCGCCG
TTTTGGGCTGCCAGGTCGACAATCACACTGCCCGCCTTCATGGAGTCAACCATTTCACGGGTAATTAGCTTCGGCGCTGG
TTTGCCTGGAATAAGCGCGGTGGTGACAATGATATCGACCTCTTTTGCCTGGGCGGCAAAGAGTTCCATTTCCGCTTTGA
TGAACGCGTCCGACATCACTTTGGCATAGCCATCGCCGCTGCCAGCTTCCTCTTTAAAATCCAGCTCGAGGAATTCCGCG
CCCATACTTTGAACTTGTTCTTTCACTTCCGGGCGGGTGTCGAATGCACGCACAATCGCGCCGAGACTGTTTGCTGCGCC
AATGGCGGCCAGACCTGCAACACCCGCACCAATCACCATCACTTTTGCCGGTGGCACTTTCCCGGCCGCAGTAATTTGCC
CGGTAAAGAAGCGCCCAAATTCATGTGCCGCTTCAACAATGGCGCGATAACCGGCGATGTTCGCCATCGAGCTTAGTGCG
TCCAGCGATTGTGCGCGTGAGATACGCGGCACAGAGTCCATCGCCATCACGGTCACGTTACGTTCCGCAAGTTTTTGCAT
TAATTCCGGATTCTGCGCAGGCCAGATAAAACTCACCAGCGTTGTCCCAGGATTCAGTAACGCAATTTCATCATCTAACG
GCGCATTGACCTTCAGAATGATCTCTGACTGCCAGACGCTATTCCCTTCTACAATTTCAGCGCCCGCTTGCACAAACGCT
TTATCGTCAAAACTTGCCAGTTGACCCGCGCCGCTCTCTACCGCGACGGTAAAACCCAGTTTCAGCAGCTGTTCCACTGT
TTTTGGCGTTGCTGCAACACGGGTTTCATTGGTTAACCGTTCTCTTGGTATGCCAATTCGCATGATATTCCCTTCCATCG
GTTTTATTGATGATGGTTTGCCTGTGTCAGGAGCCACACAAGCTGCTCATGTACGAGCTAAATGTTACTCCGTTAAAATA
AATTAGTAACAAACGCCCTATAACGTACTGAAAATTATGCCTGTGATCTAGCGCCAAAAATCAGTATTTCGGCGTGAACT
CGCAAAATATTAACGATTCAGCCGTGATAGTGGGATAAACACCTTAGAACGCCGGATAAAGACTGATAATTGTCTTCGAC
GGTCGGGTAAAACGAGACATCGCCCCGGCACGAATCACTACTTAACATTAAATTAACTTATACAATTCAGTTGCTTCAGT
AGTAATGATGCTGATACGGCTGTTTTTTAAGCATAGACGGTCATTTGAGCAGGATTAAAATTGGCTTAAGGAATGTGATA
TGAAAAATGACGCAGACAGTTACACCGTTTAAATGCAATAATCAGCCACGTTTCTCGTTAATAACAATACCAGTACCTGG
TTTGCGCAAGGCGAAGGATTATTTTTATGAAGCTTAAGAACACCCTCCTGGCGTCGGCACTGCTTTCTGCTATGGCATTC
TCCGTTAACGCAGCAACAGAACTGACACCGGAGCAAGCGGCAGCGGTTAAACCTTTTGACCGTGTAGTGGTTACCGGTCG
TTTTAATGCTATTGGCGAAGCGGTGAAAGCCGTTTCTCGTCGCGCAGATAAAGAAGGTGCCGCCTCTTTTTATGTTGTCG
ACACTTCTGATTTTGGTAACAGCGGTAACTGGCGTGTGGTCGCTGACCTCTATAAAGCCGATGCTGAAAAAGCAGAAGAA
ACAAGTAATCGCGTAATTAACGGTGTTGTCGAACTGCCGAAAGATCAGGCTGTTCTGATTGAACCGTTTGACACGGTCAC
CGTCCAGGGCTTCTATCGTAGCCAGCCAGAAGTCAATGATGCCATCACCAAAGCGGCAAAAGCGAAAGGTGCCTACTCTT
TCTACATCGTTCGTCAAATCGATGCCAACCAGGGCGGCAACCAGCGTATTACTGCATTCATCTATAAAAAAGATGCTAAG
AAACGTATCGTCCAGAGCCCGGATGTGATCCCGGCAGATTCCGAAGCAGGACGTGCAGCTCTGGCTGCCGGTGGCGAAGC
CGCGAAGAAAGTTGAGATCCCGGGTGTTGCGACTACCGCATCACCAAGTTCTGAAGTCGGTCGCTTCTTTGAAACCCAGT
CATCAAAAGGCGGGCGTTACACCGTCACGCTCCCGGATGGCACTAAAGTCGAAGAACTGAACAAAGCGACCGCAGCGATG
ATGGTCCCGTTCGACAGCATCAAATTCTCTGGCAACTACGGCAACATGACCGAAGTCTCCTATCAGGTTGCGAAACGTGC
CGCGAAGAAAGGTGCTAAGTATTACCACATCACCCGCCAGTGGCAGGAACGTGGTAATAACCTGACCGTCAGCGCAGATC
TGTATAAATAACTATTCAGTTACTCCTATAAGGCGGCTTGATGCCGCCTTTCGCATTTTTAGTCACAAATTTCCCCGCGA
CCATTGCATGCAGACACATCTCTTCGTAAAATCCCGCGCTTCAGTGGCAAACTCCATTTTTATGCGTTTTGCCCTAATAT
TTATTCTTCTTATCACGTTTTAATCACTGGATATCGATGGAAAAGAAACTGGGACTGAGCGCACTCACCGCGCTGGTATT
AAGCTCAATGCTGGGCGCGGGTGTTTTCAGTCTGCCGCAAAATATGGCGGCAGTTGCCAGCCCGGCAGCACTGCTCATCG
GCTGGGGTATTACTGGCGCTGGCATTTTATTGCTGGCCTTTGCCATGCTGATCCTCACGCGCATTCGTCCCGAACTTGAC
GGCGGTATCTTTACCTATGCCCGCGAAGGGTTTGGCGAGCTAATCGGCTTTTGTTCCGCATGGGGATACTGGCTGTGCGC
AGTCATCGCCAACGTCTCCTATCTGGTGATCGTTTTTTCCGCGTTAAGCTTTTTTACGGACACGCCGGAATTGCGCCTGT

TTGGCGATGGTAATACCTGGCAATCGATAGTCGGTGCATCGGCTTTATTGTGGATTGTTCACTTTTTGATCCTGCGCGGG
GTGCAAACCGCTGCCAGCATTAACCTGGTGGCGACACTGGCAAAATTGTTGCCGCTGGGTCTGTTTGTTGTGCTGGCGAT
GATGATGTTCAAACTGGATACCTTCAAGCTCGACTTCACCGGACTTGCACTTGGCGTACCCGTTTGGGAACAGGTGAAAA
ACACCATGCTGATCACCCTGTGGGTTTTCATTGGTGTGGAAGGTGCCGTGGTTGTTTCTGCGCGTGCGCGTAATAAACGT
GATGTTGGCAAAGCGACACTGCTGGCGGTTCTCTCCGCTCTGGGCGTTTACTTGCTGGTAACGCTGCTTTCGCTGGGTGT
GGTGGCACGTCCTGAACTGGCTGAAATTCGTAACCCGTCAATGGCCGGTCTGATGGTGGAAATGATGGGACCATGGGGCG
AAATCATCATCGCTGCCGGTTTGATCGTTTCCGTTTGCGGCGCGTACTTAAGCTGGACCATCATGGCAGCAGAAGTACCG
TTCCTGGCCGCCACTCATAAAGCATTCCCGCGCATTTTTGCGCGCCAGAACGCACAAGCTGCGCCATCCGCCTCACTGTG
GTTGACCAACATCTGCGTGCAAATTTGTCTGGTACTCATCTGGCTGACCGGTTCCGATTACAACACGTTGCTGACCATCG
CCTCAGAAATGATTCTGGTGCCCTATTTCCTGGTCGGCGCGTTCTTGCTGAAAATCGCAACCCGTCCACTGCATAAAGCG
GTAGGTGTCGGTGCCTGCATTTATGGCTTATGGTTATTGTACGCCTCTGGTCCAATGCACTTGCTGTTATCCGTTGTTCT
GTATGCACCGGGGCTGCTGGTTTTCCTGTACGCGCGCAAAACGCATACCCATGATAATGTACTGAACCGTCAGGAAATGG
TGCTGATTGGCATGCTGTTAATTGCCTCAGTTCCGGCGACCTGGATGCTGGTGGGATAACGTTCATCCCATCGTTGGATA
GCAAAGGAGATAACGATGGGTAAAACCCAGCCCTTGCCAATATTAATTACTGGCGGAGGTCGTCGCATCGGCCTCGCCCT
CGCATGGCATTTCATTAATCAAAAGCAACCGGTGATTGTCAGCTATCGGACACACTATCCAGCCATTGATGGACTGATTA
ATGCAGGTGCGCAGTGTATTCAGGCTGATTTTTCGACCAACGACGGTGTGATGGCGTTTGCCGATGAAGTACTAAAAAGC
ACCCATGGTCTGCGTGCTATTTTGCATAACGCCAGTGCGTGGATGGCGGAAAAACCGGGTGCGCCACTGGCCGACGTACT
GGCTTGCATGATGCAGATCCACGTTAATACCCCATACCTGCTCAACCATGCGCTGGAAAGATTACTGCGTGGGCACGGAC
ACGCCGCCAGCGATATCATTCACTTTACCGATTATGTGGTGGAGCGCGGTAGCGACAAACATATTGCGTATGCTGCAAGC
AAAGCGGCACTGGATAATATGACCCGCTCGTTTGCCCGCAAGCTGGCACCGGAAGTGAAAGTGAATTCTATTGCGCCATC
GCTGATCCTGTTTAATGAACATGATGATGCCGAATATCGACAACAGGCGCTGAATAAATCACTGATGAAAACCGCGCCTG
GCGAGAAAGAAGTGATCGACCTGGTCGATTACTTACTTACCAGTTGCTTTGTCACCGGACGCAGTTTCCCACTTGATGGC
GGTCGTCATCTGCGTTAATGCAGCTTTATCCAGCATATAATCAACACCCATGCGCTTATCCCCCAACACGCAACAGAACC
AACAAAAGCGGCGGGCAAGCGCATCATTCCGGTGAAATACCACAGCGACACCAGGTAAACAAAATAGGGAATAATCGACC
ACATACTAAAAATGATGGTTGCGCGTAAGGCTTCAATGCCGCGTTCGCTGGCAACAATATAATGCGCGATAAGCGCAAAG
GTCGGGAAAAGTGGAATCAGCCCGGCGATATAATAATTTTTCGTTTTTGCTAAAACACCAATCAACAGCACTACCAGCGC
ACCGAGCGCGGCTTTGATTACCAGCCCCATCTTTTTACCTTAACACTTCCATAACAAGTCATCAGTAGAATACCTGATGA
AAACTTGTTTAGAAACGATTGATAGTAAGTAAAAACAGCGCGGTGTATTGTGACGTTTTTATATCTACCGTGAATGTTAT
GAACACTATCGTATTTGTGGAAGATGATGCGGAAGTCGGTTCACTGATTGCCGCGTACCTGGCAAAACATGATATGCAGG
TTACCGTAGAGCCGCGCGGCGACCAGGCCGAAGAAACCATTTTGCGAGAAAATCCGGATTTGGTGTTACTCGACATCATG
CTACCAGGCAAGGACGGCATGACCATTTGTCGTGATTTACGCGCAAAGTGGTCTGGACCGATTGTTCTTCTAACCTCTCT
CGATAGCGATATGAACCACATCCTGGCACTGGAAATGGGTGCCTGCGACTATATTCTCAAAACGACGCCCCCTGCTGTTT
TGCTAGCGCGTTTACGTTTGCATTTGCGTCAGAATGAGCAAGCCACACTGACCAAAGGTCTTCAGGAAACGTCTCTGACT
CCCTACAAAGCCCTGCATTTCGGCACGTTGACCATCGATCCCATCAACCGCGTAGTCACCCTGGCTAACACTGAAATCTC
GCTCTCGACAGCTGATTTCGAATTATTGTGGGAATTAGCTACCCATGCCGGGCAAATCATGGACCGCGATGCATTGCTGA
AAAATTTACGCGGCGTCAGTTATGACGGACTGGATCGTAGCGTGGACGTGGCTATTTCGCGGTTAAGAAAAAAACTGCTC
GATAACGCCGCAGAACCTTATCGCATTAAAACTGTGCGTAACAAAGGCTATCTTTTTGCGCCTCATGCATGGGAATAAGC
GATGAAAAAACTGTTTATCCAGTTTTACCTGTTATTGTTTGTCTGCTTCCTTGTGATGTCTCTGCTGGTTGGGCTGGTGT
ACAAATTTACCGCCGAACGCGCGGGCAAACAGTCGCTGGATGATTTGATGAACAGTTCGCTGTATCTGATGCGCAGCGAA
TTGCGTGAGATCCCCCCACACGACTGGGGTAAAACTCTGAAAGAGATGGATTTAAATCTCTCTTTCGATCTGCGTGTCGA
GCCACTGAGTAAATACCATCTTGATGATATTTCCATGCACCGACTGCGTGGCGGCGAAATTGTCGCCCTGGACGATCAGT
ACACGTTTTTGCAGCGTATCCCGCGCAGCCACTACGTGCTGGCAGTTGGTCCTGTTCCTTATCTTTATTATCTCCATCAG
ATGCGATTGCTGGATATCGCCCTGATCGCTTTTATTGCTATTTCCCTCGCCTTTCCGGTGTTTATCTGGATGCGTCCGCA
CTGGCAGGATATGTTAAAACTGGAAGCAGCGGCGCAACGATTTGGCGATGGGCATCTCAATGAACGTATCCACTTTGATG
AGGGTTCGAGCTTTGAACGACTTGGCGTCGCATTTAACCAGATGGCGGACAATATCAACGCCTTAATTGCCAGCAAAAAA
CAGCTTATTGACGGTATCGCTCACGAACTGCGAACACCGTTAGTGCGCCTGCGTTATCGACTGGAGATGAGCGATAACCT
GAGCGCCGCCGAATCCCAGGCGTTGAATCGTGATATCAGTCAACTTGAAGCTTTAATTGAAGAGCTGCTGACTTATGCCC
GACTCGATCGCCCACAAAACGAGCTTCATCTTAGCGAACCAGACCTGCCGTTGTGGCTGTCAACGCATCTGGCAGATATT
CAGGCAGTAACGCCCGATAAAACGGTACGGATAAAAACGCTCGTGCAAGGCCATTATGCGGCGTTGGATATGCGCTTAAT
GGAGCGCGTGCTGGATAATTTGCTCAATAACGCCCTGCGGTACTGCCATTCAACGGTTGAAACCAGCCTGCTACTGTCGG
GGAATAGAGCGACATTAATTGTTGAGGATGATGGCCCAGGGATTGCCCCAGAAAACCGCGAACATATCTTTGAACCTTTT
GTTCGCCTCGATCCCAGCCGGGATCGCTCAACCGGCGGCTGCGGGCTGGGGCTGGCAATTGTCCACTCTATAGCACTGGC
AATGGGCGGTACGGTTAATTGTGACACCAGCGAACTGGGTGGTGCCCGCTTCTCGTTTAGCTGGCCGTTATGGCATAACA
TCCCGCAATTTACCTCTGCCTGACACTACGCGCACGATGGTCAAGTCACCACGACTGTGCTATAAAATAAGTATGTTGTA
ACTAAAGTGGTTAATATTATGGCGCGTTACGATCTCGTAGACCGACTCAACACTACCTTTCGCCAGATGGAACAAGAGCT
GGCTATATTTGCCGCTCATCTTGAGCAACACAAGCTATTGGTTGCCCGCGTGTTCTCTTTGCCGGAGGTAAAAAAAGAGG

ATGAGCATAATCCGCTTAATCGTATTGAGGTAAAACAACATCTCGGCAACGACGCGCAGTCGCTGGCGTTGCGTCATTTC
CGCCATTTATTTATTCAACAACAGTCCGAAAATCGCAGCAGCAAGGCCGCTGTCCGTCTGCCTGGCGTGTTGTGTTACCA
GGTCGATAACCTTTCGCAAGCAGCGTTGGTCAGTCATATTCAGCACATCAATAAACTCAAGACCACGTTCGAGCATATCG
TCACGGTTGAATCAGAACTCCCCACCGCGGCACGTTTTGAATGGGTGCATCGTCATTTGCCGGGGCTGATCACCCTTAAT
GCTTACCGCACGCTCACCGTTCTGCACGACCCCGCCACTTTACGCTTTGGTTGGGCTAATAAACATATCATTAAGAATTT
ACATCGTGATGAAGTCCTGGCACAGCTGGAAAAAAGCCTGAAATCACCACGCAGTGTCGCACCGTGGACGCGCGAGGAGT
GGCAAAGAAAACTGGAGCGAGAGTATCAGGATATCGCTGCCCTGCCACAGAACGCGAAGTTAAAAATCAAACGTCCGGTG
AAGGTGCAGCCGATTGCCCGCGTCTGGTACAAAGGAGATCAAAAACAAGTCCAACACGCCTGCCCTACACCACTGATTGC
ACTGATTAATCGGGATAATGGCGCGGGCGTGCCGGACGTTGGTGAGTTGTTAAATTACGATGCCGACAATGTGCAGCACC
GTTATAAACCTCAGGCGCAGCCGCTTCGTTTGATCATTCCACGGCTGCACCTGTATGTTGCAGATTAACGCCCGGCTTTC
ATACTGCCGACCATCTGTTCTGGCCGTACCCAGCTGTCAAACTCGGCTTCGCTAAGATACCCCAGCGCAAGGGCCGCAGC
TTTTAAGGTCAGCCCTTCTTTATGCGCTTTTTTGGCGATCTCGGCGGCTTTGTCATAACCAATGTGGGTGTTAAGCGCAG
TCACCAGCATCAGCGATTCATTGAGTAATTGATTGATTCGCTCACGATTCGGTTCAATACCCACTGCGCAGTGTTTGTTA
AAACTTTCCATGCCATCTGCCAGCAAGCGCACCGATTGCAGGAAATTGTGGATCACCATTGGACGGAAGACGTTCAGTTC
AAAGTTACCGGAAGCGCCCCCCATGTTGATCGCCACGTCGTTCCCCATCACCTGACAGCAGAGCATGGTTAATGCCTCAC
ACTGTGTTGGGTTCACTTTCCCCGGCATGATTGAGCTGCCCGGCTCATTTTCCGGGATTGAGATTTCACCAATTCCGCAG
CGCGGGCCAGAGGCCAGCCAGCGGACATCATTGGCGATTTTCATCAGTGACGCAGCCAACCCTTTCAACGCGCCGTGCGC
CTGAACCAGGGCATCACAGGTCGCCAGCGCTTCAAATTTGTTCGGCGCGGTAACAAACGGTGCACAGGTAATGACTGCCA
GTTCATCTGCTACGCGACGCGCATACTCCGGATGGGTATTTAGTCCAGTACCCACCGCTGTACCGCCAAGAGCCAGTTCC
GCTACGTGAGGCAGGCTGTATTCGATATGTTTGAGATTATGCTCGAGCATCGCTACCCAGCCGGAAATCTCCTGCCCCAG
CGTTAACGGCGTGGCATCCTGCAAGTGAGTACGACCAATTTTGACGATATCGGCAAAAGCACGGGATTTCTCATTCAGTG
TCTGTGTCAGGGTTTTAAGCTGAGGAATGAGTTGCTTGCGCAGCGCCAGCAGCGCCGCAACGTGCATCGCCGTCGGAAAG
ACATCGTTGGAACTTTGGCTTTTGTTCACGTCGTCGTTAGGGTGAACTTTACGTTCCATCCCGCGCACACCGCCGAGTAA
TTCACTGGCCCGGTTAGCCAGCACTTCGTTCATGTTCATGTTACTTTGCGTGCCGGAGCCGGTCTGCCAGATAGCCAGCG
GGAATTCGTCGTCATGCTGTCCTGCCAGTACTTCATCCGCCGCCTGACGAATGGCGCTCGCTTTCTCTTCAGACAACAAG
CCTAAATCTTCATTAACTTTTGCCGCTGCACGCTTGGTTAGCGCCAGCGCATGAATCAGTGAGGTGGGCATTTTCTCCGT
CGAAATGCGGAAATGCTCCAGCGAGCGTTGAGTTTGTGCGCCCCACAGCTTATCTGCCGGGACATCAATCGCCCCCATCG
AATCTTTTTCGCTGCGTACTGTATTCATGACCTGCTCCTCACCTGATTAATTTTTTCTTTCTGTTTTGCTTTCGTTAAGC
AACTTTTAGCTCACTTATTATTTACCATTTGATAACAAATGTTTGGTCTTTCGTGCCATGTAAAAAAACCGCCCCGAAGG
GCGGCTCTGTTTATTTCACACAGCGGGTGCATTGTGTGAGTTGTATCTGCTGGAAGAAGTCATTTCCTTTATCATCCACA
AGGATAAACGCCGGGAAATCTTCCACTTCAATTTTCCAGATGGCTTCCATTCCCAGTTCCGGATATTCAACACATTCCAG
GCTCTTAATACTTCCCTGCGCCAATACAGCGGCCGGACCACCGATACTGCCAAGGTAGAAGCCGCCGTGTTTTTTACAGG
CATCCGTCACCTGCTGGCTGCGGTTGCCTTTCGCCAGCATGATCATACTTCCGCCCTGCGCTTGCAGTTGATCGACATAA
GAATCCATCCGTCCGGCGGTCGTTGGGCCAAGAGAACCGGAGGCATAACCTTCCGGCGTTTTGGCCGGACCCGCGTAGTA
AATCGGATGATCTTTGATGTACTGCGGCAGCCCTTCACCGTTATCCATCCGCTCTTTCAGTTTGGCGTGAGCAATATCAC
GACCGACGATAATCGTGCCGTTAAGCGATAAGCGTGTAGAAACGGGATACTGCGACAACTGTGCGAGGATCTCTTTCATC
GGACGGTTAAGGTCAACGCGCACCGCTTCGCCTTCTCCCGCTTTGCGCAGCTCTTCCGGGATATATTTGCCTGGATTATG
TTCCAGTTTTTCGATCCAGATCCCCTGACGGTTGATCTTCGCTTTGATATTACGGTCAGCAGAGCAGGAGACGCCCATAC
CGACCGGGCAGGATGCGCCGTGACGTGGCAGGCGAATCACGCGGATGTCGTGAGCGAAGTATTTACCACCAAACTGCGCA
CCCAGACCAAGATTTTGCGCTTCGATCAGCAATTCTTTTTCCAGTTCCACATCGCGGAACGCCTGACCGTGCTCATTCCC
TTCCGTTGGCAGTTCATCATAGTATTTCGCGGAAGCCAGTTTCACCGTTTTAAGGTTCGTTTCTGCAGAAGTTCCACCAA
TAACGAACGCAATATGATACGGAGGACAGGCCGCCGTACCCAGCGTGCGCATCTTCTCAACCAGGTAATTTTTCAGTTTC
CCCGGCGTCAGTAACGCTTTGGTTTCCTGATAGAGATACGTCTTGTTTGCCGAACCACCACCTTTGGCGATACAGAGGAA
TTTGTATTCGTCGCCATCAACGGCATAAAGATCGATCTGCGCTGGCAGATTGGTGCCGGTATTCACTTCTTTATACATAT
CCAGCGGCGCGTTTTGCGAGTAGCGCAGATTATCTTCGATATAAGTGTTATAGACACCGCGCGCCAGCGCCGCTTCATCA
CCACCACCGGTCCATACACGCTGCCCTTTTTTACCAACAATAATCGCGGTGCCGGTATCCTGACAGGTTGGCAGAACGCC
TTTCGCCGCGATGTCGGAGTTACGCAGGAATTGCAGCGCCACATATTTATCATTTTCGCTGGCCTCCGGGTCACGCAGAA
TGTCGGCCACCTGTTGTTGGTGCGCCGGACGCAGCATGAACGACGCATCATGAAACGCCTGGCGCGCCAACAGAGTTAAC
GCTTCGGGTGCGACTTTCAAAATCTCCTGCCCTTCAAATTCAGATACGCTAACGTGTTCGCTGGTTAGCAGGTAATACTC
AGTATCATCTTTTTTGAGTGGAAAAGGAGCCTGATAATGAAAGGGTTTGTTTGACATTGTTCTCTCACTTACTGCCTGGT
TTGGTTATGCTCTGGGCGGGTGTTCCGTTGCCCTGTTAAAAGCGAGTAACAATATCCTACACACTTTTTTAACAAAAACT
GAGACTAGTACGACTTTTTGCGGCTCCAGGTTACTTCCCGTAGGATTCTTGCTTTAATAGTGGGATTAATTTCCACATTA
AAACAGGGATTGATCATGCAAAAACTCATTAACTCAGTGCAAAACTATGCCTGGGGCAGCAAAACGGCGTTGACTGAACT
TTATGGTATGGAAAATCCGTCCAGCCAGCCGATGGCCGAGCTGTGGATGGGCGCACATCCGAAAAGCAGTTCACGAGTGC
AGAATGCCGCCGGAGATATCGTTTCACTGCGTGATGTGATTGAGAGTGATAAATCGACTCTGCTCGGAGAGGCCGTTGCC
AAACGCTTTGGCGAACTGCCTTTCCTGTTCAAAGTATTATGCGCAGCACAGCCACTCTCCATTCAGGTTCATCCAAACAA

ACACAATTCTGAAATCGGTTTTGCCAAAGAAAATGCCGCAGGTATCCCGATGGATGCCGCCGAGCGTAACTATAAAGATC
CTAACCACAAGCCGGAGCTGGTTTTTGCGCTGACGCCTTTCCTTGCGATGAACGCGTTTCGTGAATTTTCCGAGATTGTC
TCCCTACTCCAGCCGGTCGCAGGTGCACATCCGGCGATTGCTCACTTTTTACAACAGCCTGATGCCGAACGTTTAAGCGA
ACTGTTCGCCAGCCTGTTGAATATGCAGGGTGAAGAAAAATCCCGCGCGCTGGCGATTTTAAAATCGGCCCTCGATAGCC
AGCAGGGTGAACCGTGGCAAACGATTCGTTTAATTTCTGAATTTTACCCGGAAGACAGCGGTCTGTTCTCCCCGCTATTG
CTGAATGTGGTGAAATTGAACCCTGGCGAAGCGATGTTCCTGTTCGCTGAAACACCGCACGCTTACCTGCAAGGCGTGGC
GCTGGAAGTGATGGCAAACTCCGATAACGTGCTGCGTGCGGGTCTGACGCCTAAATACATTGATATTCCGGAACTGGTTG
CCAATGTGAAATTCGAAGCCAAACCGGCTAACCAGTTGTTGACCCAGCCGGTGAAACAAGGTGCAGAACTGGACTTCCCG
ATTCCAGTGGATGATTTTGCCTTCTCGCTGCATGACCTTAGTGATAAAGAAACCACCATTAGCCAGCAGAGTGCCGCCAT
TTTGTTCTGCGTCGAAGGCGATGCAACGTTGTGGAAAGGTTCTCAGCAGTTACAGCTTAAACCGGGTGAATCAGCGTTTA
TTGCCGCCAACGAATCACCGGTGACTGTCAAAGGCCACGGCCGTTTAGCGCGTGTTTACAACAAGCTGTAAGAGCTTACT
GAAAAAATTAACATCTCTTGCTAAGCTTATTAAAGGCTTATAACACCTTCAGGCGGCCAGTCCGCCTGATTTCATTTTAT
GGATAATCATTATGAATAAATCGCTGGTAGCGGTAGGCGTCATTGTTGCGCTAGGCGTAGTCTGGACAGGCGGCGCATGG
TATACAGGCAAGAAGATTGAAACCCATCTCGAAGACATGGTCGCGCAGGCGAACGCGCAACTCAAACTGACAGCTCCTGA
ATCCAACCTGGAAGTGAGTTATCAAAACTATCATCGCGGCGTATTCAGCAGCCAGTTGCAACTGTTGGTGAAACCCATTG
CCGGGAAAGAAAATCCGTGGATTAAAAGCGGTCAGAGCGTCATCTTCAACGAATCGGTTGATCATGGTCCCTTCCCGCTT
GCCCAGCTTAAAAAACTGAACCTGATCCCGTCGATGGCATCAATTCAAACCACGCTGGTTAATAACGAAGTAAGCAAACC
ACTGTTTGATATGGCAAAAGGTGAAACGCCTTTTGAGATTAACTCGCGCATTGGTTACAGCGGTGATTCCAGTTCCGATA
TTTCGCTCAAGCCACTGAATTACGAGCAAAAGGATGAAAAAGTCGCCTTTAGCGGCGGCGAGTTCCAGTTAAATGCTGAC
AGAGACGGCAAAGCCATCTCCCTTTCCGGGGAGGCGCAAAGTGGTCGGATAGACGCAGTTAACGAATACAACCAGAAAGT
GCAGTTGACCTTTAATAATCTGAAAACCGACGGTTCCAGCACGCTGGCAAGTTTTGGTGAGCGTGTAGGAAATCAAAAAC
TGTCACTGGAAAAAATGACCATTTCAGTGGAAGGCAAAGAACTGGCACTGCTGGAAGGCATGGAGATCAGCGGTAAATCG
GATCTGGTCAATGACGGTAAAACGATCAATAGCCAACTGGATTACTCGCTAAACAGCCTGAAGGTACAGAATCAGGATCT
GGGCAGCGGCAAGCTGACTTTAAAAGTCGGCCAGATTGATGGTGAAGCCTGGCATCAGTTTAGCCAGCAATATAACGCGC
AAACTCAGGCGCTGCTGGCACAGCCAGAAATTGCCAACAACCCCGAACTTTATCAGGAGAAAGTGACGGAAGCCTTCTTT
AGCGCCCTGCCGCTGATGCTGAAAGGCGATCCGGTGATTACTATCGCGCCGCTAAGCTGGAAAAACAGTCAGGGTGAAAG
TGCCCTGAATCTGTCGCTGTTCCTGAAAGATCCGGCAACGACTAAAGAAGCGCCGCAAACGCTGGCGCAGGAAGTAGATC
GTTCGGTTAAATCTCTGGATGCGAAACTGACCATTCCGGTGGATATGGCAACTGAGTTTATGACTCAGGTAGCGAAGCTG
GAAGGTTATCAGGAAGATCAAGCGAAAAAACTGGCGAAACAGCAAGTTGAAGGTGCATCAGCAATGGGGCAGATGTTCCG
TCTGACCACCTTGCAGGACAATACCATCACCACCAGCCTGCAATATGCTAACGGTCAGATAACGTTAAATGGGCAGAAAA
TGTCGTTAGAAGATTTTGTTGGCATGTTTGCGATGCCAGCTCTTAACGTTCCGGCTGTACCGGCAATTCCGCAGCAGTAA
TTCACGGCCAAAACGCCGGAGAATTTTCACTCCGGCGTTTTCATCTGAAAACAAACCAGAGATTGCCTGATGCGCTACGC
TTATCAGGCCTACCTTTTCCTGCAACACTTTGAATTTATGAGTTTTTGTAGGCTGGATAAGGCGTTTACGCCGCATCCGG
CATAAAAAACGCGCACTTTGTCAACAATCTGAAACGCCGGAGATTTTTCTCTCCGGCGTTATTTTTTACTTCAGCATAAA
GTCATACTGAATACTCATTTTCACATCCCAGTAATTCAGGCGATCGCCCAGCGTACTGCCATCGCGCTTTTCCCGCCCTT
TATCGGCAGCAAGAAAGAAATTCATCCCTTGTAACGGCCCTTCGCTGACCCGATAGTTCATACCACCTAAGATACCCGTG
CCTTCAATCCGCACATCTCGTTTACTGGATTCATAGCCGCGAGTCACAATCGGTGCAAATGTCAGCGTAAATTGCGGCGT
TAAACGATAGTTGACGCCCAATTGCCAGGACTGCATTCCTTCACGGTTACGATCAAGACTTAATGAAAAGGGATAGCCAA
TGTCGGTATCCACCACGGCACCATTCATTGCATGGCGCAAATAACCACCACTGCCAAATACACTTCCCCACGGCGCATTC
CAGGTCAATTGTCCACTAACCAACGCCGTTTCATTGGGCTGGCTGGTATTACGACTTAAACCTTCGAGCTGCGCGTAAAA
GCCGAGCAACTCCCCTTTCAACGTATAATTCGGGTCGAGCGGTATGCTTTTCTCGAGAATACTTTGTATCTGATTGGAAA
CATCTTTCTGTACCGATGCCCCCAGAGAATAGCGACTGGTATCATCATGCCAACTGGCGGCTAAAAAAGAACGTGCGCGA
TGTTTATCGTATTTTCCCGTCTGGCTGTTATACACCCCTTCATCACGAATACCGGTTGTTGAATCAGTCCATAAAAATAC
TCGTGAACGCCACATTCCCCACAGATCAATATTTGGTGTTAAAGCCCAATAACCTAATGCTCCTTCAGAAACTGTCGGTA
ATGTACGTTGCGAACTGCCAGCCATTAACGGAATACGATAAGGTAAGCTACCATAACTATAATCCGTCCCGAAGCGACCT
AATTTAAGCAGGCTATTTTCACTGGGTTTTATTTTTACTGCCCCCAGGGCAAAGCCAAAACTTTTATCACCATCAAGATA
CCACCGGGTACTCATATCAGCACGAGCACCTAATTTATAAACATAATACGCCCCGCCTTCAACGCCAATAATATTGGAAT
AATAACCACTATTGAAATCGAGCAATCCTCCCTGTACCCATGCGTAAATATCTCTTCCCGCGCCAGCACTGGGCTTATCC
GCCCTGCGCAATTCGTTTTTCAGTCTGATGCGTAGTCCCGCTTCATCGTCAGCCAGTTGCGCCGCATAAGCAGAGGTAAG
GCCAGAGGCAGCCGTCAGGCAAATAACGGCCATGGCCACTATTTTTCTCATAGTTGCACCTTTGATGTTCTGATTATTTT
TATTGAATATTAATTAGTGATATCGCTGATTAATTGCTGCTGCACTTTTTTACGATTATCAATTTCAACCACGATTTCTT
TGAATTTTTTATCCGTGAGCGGATAAAACCAGATAATAACGAATGCCAGTAGCATAAATCCGCAAGGTACTAAGGCAATT
GATGTGCGGATGCCCATAATAACTTCCGGCGTTTGCACCTGATTGGCGATATATCCGCTTAACCCCAAAATAAAGGCAGG
AATTGAACCTCCGATTGCCTGACCGCATTTACGGGTAAATGAGAATAGTGAATAGGTGAGCCCTTCAATTCGCACGCCGG
TCAGGTATTCACCGTATTCTACGGTATCAGCTTCCAGCGCCCACATCACGGTCATGGTAACGCCCTGACCAATTGAAGCG
ATGGCCAACGCAACCAACGCCACCGGCAGTGACCAGACGGAAACCCAGAAGAACAGCAGATAACCGCAGGTTCCCAGCAA

AGCGCCAATCAGGAAGGTATTCTTTTTACCGATCCTCGCGACCATCCCCGGCACCAGCGGTGCCGATGCCACAGTACCAA
CCAGGTTTTGCACCAGTACCAGCACAGTGAACAGCCCGGTATCATTTAACACATAGCGCACGTAGAACAACGACGAGGCG
CTGACCGCAAAGGTCGAAATCAGCACACACAGCGCACCGATGCACAACATAAACAGCGGGCGATTCCGTTTCAGGGTTTG
CAGACTGATATTCAATGACGGCTGCGCAACGATACGTACCACATTCTCACGCGTCGATTTGAAGCAGATGAAGTAAAGCA
CCATTCCGGCAATCGCCAGCACAATTGTCCAGAAATGGTATACCGACACCATCTCTTCCGGGCTGGAGTTCTTAATGCTC
GGTCCTATCAGAAATGCCAGGCAGACAAAGGTCAATGAAGCGGCAATCCCACGAGCCGCGCCCAGACGGGCGCGGGATTG
TGGTTGTTGGGTCATCGCGGTAGCAAGTGAACCATAAGGAATATTCACCAGGCTGTAGCAAAGCCCGAGGCCCATGTAGG
TCAAATATGCATACACCACTTTGCTACCATGGCTCCAGTCGGTCAGCACCCAGAATACCAGCACGCTGAAGATCATTAAC
GGCGCAGTACCGAAGAGTAAAAACGGGCGGAATTTTCCCCAGCGGGTATTCACACTGTCCACCACTCGTCCGGCAAAGAC
GTCGGCGAAGGCATCGAATACCCGCACCAGTAACAGCATGGTGCCCGCCGCAGCGGCACCGACGCCAGCGACGTCGGTGT
AGTAACTCAACAGGAAGAGCGCCCCCATTGCGAAGGCGAAGTTATTGGCGACGTCACCGAGGCTGTAGCCGACGATGGTG
CGCCAGGAGAGTTGTTGATTCATTGTTTGCCTCCCTGCTGCGGTTTTTCACCGAAGTTCATGCCAGTCCAGCGTTTTTGC
AGCAGAAAAGCCGCCGACTTCGGTTTGCGGTCGCGAGTGAAGATCCCTTTCTTGTTACCGCCAACGCGCAATATGCCTTG
CGAGGTCGCAAAATCGGCGAAATTCCATACCTGTTCACCGACGACGGCGCTGACGCGATCAAAGACGCGGTGATACATAT
CCAGCCATGCACACTGATACTCTTCACTCCACATGTCGGTGTACATTGAGTGCAGCCCGGCTAACGTATCCACGCCGTAT
TCGGTGATGATAATCGGCTGATGCAGTTTCTCCTGCCAGGCCAGAAGTTCTTTTTCCAGTACCTTCTCTGCCGTTTCCAA
ATCGCCGCTTTGGACATACCATCCGTAATAACGGTTCAGGCACAGCACATCAAAGAGATCGCTGATGGTATCGGTGTGAG
CGTCGCAGAACATTACATTGACGCAGGTGATCGGACGCGTCGGGTCGAGTTTACGCGTTGCTTCCGCCAGTGGCGCGAAA
TATTCCCGTGCACCTTGCGGACGGGTATCCGGTTCGTTGGCAATACTCCACATCACCACGCTTGGGTGGTTTTTGTCACG
CGCTATCAGCTCTTTAATCGCCTGTAAGTGCGCTTGCTGAGTTTCCCCGTTGACTGCCTCTTCGCTGTACAGTTCTTTCG
GCTTGTTGCCCGCTTCGAAACCAATGCCTAAAGAGAGGTTAAAGCCGACAGCAGCAGTTTCATCAATCACCACGATGCCA
TGTTCATCTGCCCAGTCGAGCATCTCTTCAGCGTAAGGGTAATGCGAGGTACGGTAGGAGTTGGCCCCAATCCAGTCCAT
TAATGCGTGGTCGTGCACCATCAGCACGTTATCGAATCCTTTGCCACGCAAGTCCGCATCTTCATGACGACCAAAGCCAG
TAAAGTAGAACGGTTTGTGGTTAATCAGGAACTGTTCGCCCTTCACTGCCACTGACCGGATGCCGACGCGAAGCGGGTAG
ATATCACACTCTGTCTGGCTTTTGGCTGTGACGCACAGTTCATAGAGATAACCTTCACCCGGTTGCCAGAGGTGCGGATT
CACCACTTGCAAAGTCCCGCTAGTGCCTTGTCCAGTTGCAACCACCTGTTGATCCGCATCACGCAGTTCAACGCTGACAT
CACCATTGGCCACCACCTGCCAGTCAACAGACGCGTGGTTACAGTCTTGCGCGACATGCGTCACCACGGTGATATCGTCC
ACCCAGGTGTTCGGCGTGGTGTAGAGCATTACGCTGCGATGGATCCCGGCATAGTTAAAGAAATCATGGAAGTAAGACTG
CTTTTTCTTGCCGTTTTCGTCGGTAATCACCATTCCCGGCGGGATAGTCTGCCAGTTCAGTTCGTTGTTCACACAAACGG
TGATACGTACACTTTTCCCGGCAATAACATACGGCGTGACATCGGCTTCAAATGGCGTATAGCCGCCCTGATGCTCCATC
ACTTCCTGATTATTGACCCACACTTTGCCGTAATGAGTGACCGCATCGAAACGCAGCACGATACGCTGGCCTGCCCAACC
TTTCGGTATAAAGACTTCGCGCTGATACCAGACGTTGCCCGCATAATTACGAATATCTGCATCGGCGAACTGATCGTTAA
AACTGCCTGGCACAGCAATTGCCCGGCTTTCTTGTAACGCGCTTTCCCACCAACGCTGATCAATTCCACAGTTTTCGCGA
TCCAGACTGAATGCCCACAGGCCGTCGAGTTTTTTGATTTCACGGGTTGGGGTTTCTACAGGACGTAACATAAGGGACTC
CTCATTAAGATAATAATACTGGTCAACCTTTAATCTGATTAGTTAACCAATTCTGTGTTGCACACAGTTATCTGTGAGAA
ACCCATGGAAGAGAAATGGAAATAAAAAGAACTGACACCGCGATCAAAAAACCAAAAACTGCAATACCCACAGCGTGATA
TAGATCGCATTAATCTTTAAAACAGCGTTGACAGAAGCCATAAAATAAGCAACCGAACGTTCGTTCGAGCGTATTGGGAA
CGATAAACTCTCATGACATATGGCTACAGTGAATATTTTGGGGGGAATTCTCCGAAGCCGGGAAGGATAATTTTTGCACA
GAGAAATTACGATATGAAACGCGGGCACAGTTATCACCAGCCCGCCGAAAAAGAGAGAGAATCAGGATGCGGTTAAGATA
CCGCCAATCATCGCGCGTAATCCCTGAGCAAACGACGCTTCACGCGCCAGGTCTTCCGCTTCGATATCGGCCAGTACACC
GTAGGTCAGATCCAGCAGTAAACGTGAAGTCATTGCCGTGTTGATGTCTGGGCTTATCTCGCCTTTTTGCTGGGCATCAC
GCATCCGCTGGGCAACAAATTCCGTGATCGTCATATGCTTATTTTTAAGCATGGCGGCGACCTGTGGGTTACGCATCCCT
TCCGCCATAATTTCAACCACCAGCGCCCGTTGTCCAAAGGCTTCATGGGTGAGAGAGACAATGGACTCGACCATATAGTC
AACGAAATGAATCCCTTCAATCGGTTCCCGGAAACGGGCCAGCGCCCTCTCCTGGTCCTGTAAGATAATCGCCTGAATCA
AGGCTTCTTTGGAGATGAAATGGTGATAGAGCGTCCCGGGACTAATGGCGCAAGATTTACAGATGGCTTTCATCGAGGCA
CTGTGAAATCCATTTTCTGAAAAAATCTCTCTGGCAGCATTGAGGATCCGGGTCCGTGTCGGTTGTGCTTCAGTCTGCAT
GTTATCCATCATTAAACCATTATAGTTAGCGTGAATAACTAGTTTACCGCGTTGTCAGGTGAGCGCCTATCATTGCTGTT
GAATAGTGCGAAGGCACACTCTATTCGTATACTTTTCACTCTGAAATTGGTTAACCAATAGCCGTGATTTGGCTGCCAGT
TATTTAGCAGAGACGTTGATGAGTTTATCCCGGTAAACAACACGCACGGTTGTTTACCGGTCCGTTAGTGTATTAATTGA
GCTCCTGTACCCCACCACCGGAGACGGTGAGAATTTGTCCGCTTACCCAGCTCGCAGCAGGCGAGCAAAGGAACAGCGCT
GCGTTAGCAATATCTTGCGGTTGGCCCAGACGTCTGATCGGCGTGTGCTGTAACATTTTTTGTTCAATTTCTGGTGTAAT
AACGGATTTCAGGGCATCGGTTAATATTGCCCCCGGCGCAATGCCATTTACCCGAATATTTTTTTCACCCAGGTCAAACG
CCATATTTCTGACCAGATGACTGGCCGCAGCTTTAGATGATGCATAGGAAGTCATGTTTATATTTTTATTTTCTGCCGCC
ATAGAAGTGATGGTCAGAATAACGCCACCGCCATTTTTTTCCATTTCTGGCGCAACAAGTTGTGACAGATGGAAAAAAGA
AAACACATTCAGTTCATAAGCACGGCGAAAATCCGCCATTGGCATATCAAACGGTTTAGGTCCACCGCCACCGGCGTTGT
TAACCAGAATATCAACTTTACCCAGCTTACTGATAGCAAAGTCTGCCAGTGCAGAGAGTTCCTGTTCGGAAGTAATATCA

CAACGGCAGGCAAATGCCTGACCACCCAGTTGTTGAATTTCGTCTACAACATGGTTAGCTGCGTCGGCGTTAATATCACT
GACCACCACAGATGCGCCAGCTGTCGCGAATGTAATGGCGATTTCTTTACCAATACCTGCACCCGCACCTGTGATGATGG
CGCATTTTCCGTCGAGTCTCAGGTTGTCAGAATTAAACACATAAACCTCCTGTGAACTTCAGTAAAAATAAAAGGCACCG
CCTGTGTGCCACATTTTTTATCATTAACCTTGAGGAAAGGACACTACCAGGTATAGCAGATAAAGAGAGGATTATTTCGC
TGCAATGAGCCGGGCGGGAATAATAAGATTGCGTGAATGCGTCTCTTCATGGGTGATTTTTTGCATCATGCGATCCGCAA
GTGTGATACCAAGTTCCCGCGCTGGCGTGCTGGCCCAGGTAACAGGTATATCATCAAGTGTGGTTGGTGTCGCATCGGTA
AATGCCGCCAGCGAAACCTGTTGCTCAAAGTAACGATCGACTCCGCTTTCCCCGCTTTGCCGCCCTGCTTTGAGCAAACC
AAACCATGCCCCCATCGCAATAGTTTCGTTATAGCAAACCACGGCACTGATGGTCGGGTTATGACGTAAAAGCGCCGTGA
TAGCTTCCGCGGCTTGCTTCTGGCTGGAAGTGCACTCCAACACCCAATCGCTATGAAACGGCAGGCCAAATTTTAGTAGA
GTTGCACAATAGCCCCCAACCCGTTCTGCACGGGTTAATGAGGAACTTTGCCCTCCCAGCCAGGCGATCCGCTGATGCCC
ATTGCGAATGAGATGCTCCGTCAACAACTGTGCAGCCTGCATGTTGTCCGGGCGAACCGTATCAACATCATCAAGATAAC
TGGCACGGGAAGCGAAAATCACCGGGATAGCTTTTTCTTCTGCCATCCGTCGCAGGTCATCGCTACTTCCTGCAGCTCCG
GCAATTACCACACCATCGACACCCTGATTCAGTAACAGTGAAAACCGCTGTGCCAGCTGCTCACCGTCTTTACCGCCGTG
AAGCAAAAAAACCATCCGTCCCTGCGCTTCCAGAGCTTCCGTCAATCCGGCCGTCAATTCGGCGTAAAACGGCGCAGATA
AATCACGGACGATCAAACCAATGACGCCGCTTTGCCCGCCGCGCAGCGCCGACGCCTGGCGATTGCGCACAAATCCCAGC
TCTTCAATGGCGGCGTTAACGCGTTCTCCTGTGGCGGTAGAGATTCGCCCTTTGCCACTAAGCACCAGCGAAACGGTACT
TACCGACACGCCCGCAGCCAGCGCAACATCATGAATGGTTATTTTTTTGGCGGTAGCCATGCAGATGACCTACTCCCTGA
TTATGTGACAGATAAAACGTTTTACCTTTTATTTATCTTATACCCGCTATTATCGTTGCGTAATGTGATTTATGCCTCAC
TAAAATTTGATAAAACGTTTTATCTTCTCGCGCAATTTACTGAATCCAGATTGTTCTCTACGAGGAGTCGTTTTATGACG
GCGAAAACAGCACCGAAAGTCACGCTGTGGGAGTTCTTCCAGCAGTTAGGCAAAACCTTCATGTTACCCGTGGCATTATT
GTCGTTCTGCGGCATTATGCTCGGCATTGGTAGTTCTCTTAGCAGCCATGATGTCATAACCCTGATCCCGGTCCTGGGCA
ACCCCGTGTTGCAGGCTATCTTTACCTGGATGAGTAAGATTGGCTCGTTTGCTTTTAGTTTCCTGCCTGTCATGTTCTGT
ATCGCCATCCCGCTGGGCCTGGCACGCGAAAATAAAGGCGTAGCGGCATTCGCTGGCTTCATCGGTTATGCGGTAATGAA
CCTCGCGGTAAACTTCTGGTTGACCAATAAAGGCATTCTGCCAACCACGGATGCCGCGGTTCTGAAAGCCAATAACATCC
AGAGCATTCTTGGGATCCAGTCGATCGATACCGGGATCCTCGGTGCGGTGATCGCCGGTATTATCGTCTGGATGCTGCAT
GAGCGTTTCCATAATATCCGCCTGCCGGATGCGCTGGCATTCTTCGGCGGTACGCGCTTCGTACCAATTATCTCCTCGCT
GGTGATGGGCCTTGTCGGCCTGGTGATTCCATTAGTCTGGCCGATTTTCGCCATGGGTATTAGCGGCTTGGGCCATATGA
TAAACAGCGCGGGTGATTTCGGACCGATGCTGTTTGGTACCGGTGAACGTCTGCTGTTGCCGTTTGGTCTGCATCACATT
CTGGTGGCATTAATTCGCTTTACCGACGCAGGCGGCACGCAGGAAGTCTGCGGTCAAACCGTCAGCGGCGCACTGACCAT
CTTCCAGGCGCAATTGAGTTGCCCGACCACTCACGGTTTTTCTGAAAGCGCCACGCGTTTCCTTTCGCAAGGTAAAATGC
CTGCGTTTCTCGGCGGTCTGCCAGGTGCAGCGTTAGCTATGTATCACTGCGCGCGCCCGGAAAATCGCCATAAAATTAAA
GGTCTGCTGATTTCTGGCCTGATCGCCTGCGTCGTTGGCGGCACTACCGAACCGCTGGAATTCCTGTTCCTGTTCGTAGC
GCCAGTTCTGTATGTCATCCACGCGCTGTTAACCGGCCTCGGCTTCACCGTCATGTCTGTGCTCGGCGTCACCATCGGTA
ATACCGACGGCAATATCATCGACTTCGTGGTGTTCGGTATTTTGCATGGTCTGTCAACCAAGTGGTACATGGTGCCAGTG
GTGGCGGCAATCTGGTTTGTCGTTTACTACGTCATCTTCCGTTTCGCTATCACCCGCTTCAATCTGAAAACCCCGGGGCG
CGATAGCGAAGTTGCCAGCTCAATCGAAAAAGCCGTTGCCGGTGCGCCGGGTAAATCAGGTTACAACGTTCCTGCAATCC
TCGAAGCATTAGGCGGTGCCGACAATATTGTCAGCCTCGATAACTGCATTACCCGTCTGCGTTTGTCTGTGAAAGATATG
TCGCTTGTTAATGTGCAGGCACTGAAGGACAATCGGGCAATTGGCGTAGTACAACTTAATCAACATAACCTGCAGGTTGT
TATCGGGCCACAAGTTCAGTCAGTAAAAGATGAAATGGCCGGTCTGATGCATACTGTCCAGGCATAAGGATAAGATATGT
TCGATTTTTCAAAGGTCGTGGATCGTCATGGCACATGGTGTACACAGTGGGATTATGTCGCTGACCGTTTCGGCACTGCT
GACCTGTTACCGTTCACGATTTCAGACATGGATTTTGCCACTGCCCCCTGCATTATCGAGGCGCTGAATCAGCGCCTGAT
GCACGGCGTATTTGGCTACAGCCGCTGGAAAAACGATGAGTTTCTCGCGGCTATTGCCCACTGGTTTTCCACCCAGCATT
ACACCGCCATCGATTCTCAGACGGTGGTGTATGGCCCTTCTGTCATCTATATGGTTTCAGAACTGATTCGTCAGTGGTCT
GAAACAGGTGAAGGCGTGGTGATCCACACACCCGCCTATGACGCATTTTACAAGGCCATTGAAGGTAACCAGCGCACAGT
AATGCCCGTTGCTTTAGAGAAGCAGGCTGATGGTTGGTTTTGCGATATGGGCAAGTTGGAAGCCGTGTTGGCGAAACCAG
AATGTAAAATTATGCTCCTGTGTAGCCCACAGAATCCTACCGGGAAAGTGTGGACGTGCGATGAGCTGGAGATCATGGCT
GACCTGTGCGAGCGTCATGGTGTGCGGGTTATTTCCGATGAAATCCATATGGATATGGTTTGGGGCGAGCAGCCGCATAT
TCCCTGGAGTAATGTGGCTCGCGGAGACTGGGCGTTGCTAACGTCGGGCTCGAAAAGTTTCAATATTCCCGCCCTGACCG
GTGCTTACGGGATTATAGAAAATAGCAGTAGCCGCGATGCCTATTTATCGGCACTGAAAGGCCGTGATGGGCTTTCTTCC
CCTTCGGTACTGGCGTTAACTGCCCATATCGCCGCCTATCAGCAAGGCGCGCCGTGGCTGGATGCCTTACGCATCTATCT
GAAAGATAACCTGACGTATATCGCAGATAAAATGAACGCCGCGTTTCCTGAACTCAACTGGCAGATCCCACAATCCACTT
ATCTGGCATGGCTTGATTTACGTCCGTTGAATATTGACGACAACGCGTTGCAAAAAGCACTTATCGAACAAGAAAAAGTC
GCGATCATGCCGGGGTATACCTACGGTGAAGAAGGTCGTGGTTTTGTCCGTCTCAATGCCGGCTGCCCACGTTCGAAACT
GGAAAAAGGTGTGGCTGGATTAATTAACGCCATCCGCGCTGTTCGTTAACCCCAATTGCGCAACGTAAAAAATCGTTGCG
CAATCGTGGATTTTTACCCTGCTTTGTTTTTATAATGGTGCGCACTTTTATATCCAGAAAAAGAGTGCGACCATGATTGA
TACCACCCTGCCATTAACTGATATCCATCGCCACCTTGATGGCAACATTCGTCCCCAGACCATTCTTGAACTTGGCCGCC

AGTATAATATCTCGCTTCCTGCACAATCCCTGGAAACACTGATTCCCCACGTTCAGGTCATTGCCAACGAACCCGATCTG
GTGAGCTTTCTGACCAAACTTGACTGGGGCGTTAAAGTTCTCGCCTCTCTTGATGCCTGTCGCCGCGTGGCATTTGAAAA
CATTGAAGATGCAGCCCGTCACGGCCTGCACTATGTCGAGCTGCGTTTTTCACCAGGCTACATGGCAATGGCACATCAGC
TGCCTGTAGCGGGTGTTGTCGAAGCGGTGATCGATGGCGTACGTGAAGGTTGCCGCACCTTTGGTGTGCAGGCGAAGCTT
ATCGGCATTATGAGCCGGACCTTCGGCGAAGCCGCCTGTCAGCAAGAGCTGGAGGCCTTTTTAGCCCACCGTGACCAGAT
TACCGCACTTGATTTAGCCGGTGATGAACTTGGTTTCCCGGGAAGTCTGTTCCTTTCTCACTTCAACCGCGCGCGTGATG
CGGGCTGGCATATTACCGTCCATGCAGGCGAAGCTGCCGGGCCGGAAAGCATCTGGCAGGCGATTCGTGAACTGGGTGCG
GAGCGTATTGGACATGGCGTAAAAGCCATTGAAGATCGGGCGCTGATGGATTTTCTCGCCGAGCAACAAATTGGTATTGA
ATCCTGTCTGACCTCCAATATTCAGACCAGCACCGTAGCAGAGCTGGCTGCACATCCGCTGAAAACGTTCCTTGAGCATG
GCATTCGTGCCAGCATTAACACTGACGATCCCGGCGTACAGGGAGTGGATATCATTCACGAATATACCGTTGCCGCGCCA
GCTGCTGGGTTATCCCGCGAGCAAATCCGCCAGGCACAGATTAATGGTCTGGAAATGGCTTTCCTCAGCGCTGAGGAAAA
ACGCGCACTGCGAGAAAAAGTCGCCGCGAAGTAACAAAATGGATGGTGCAAATGCACCATCCATTTTTCATGCAAGGCAC
AAAGTCGCGCGATGTTTGGCGGATTCGATGCCCAGCTCAATCAACTCCATTACCTGGATTGCCTGGCTTGCCGGAACCGG
ATTTTCACCATCGCCATTTAACGCATCACGAATAGCCGCATAGTAAGCCGGATAATTCCCAGGCACCGTCAACAGCGTTT
CTTCGACACGTTCCTCACCTTCCACGCGGGTAAGTACGCCATCACGCATATCGTAGCCCCAGTCTTCCTGCGGTAGACGC
TCGCCATTTTTCAGACGTTCTTCCTGTGGATCGAGGCCATATTTCACATAACTGCCTCGGGATCCATGCACGATATACCG
TGCTGACTCAGCGGCTGCCAGCATGGTACCGTGTAAAATGACTCGCCGCTGTGGATAGGACAAGATGGCGTGGAAATAAT
CGGTCGACTGCGCTCCGGGCCGTAACTGTGCCAAATCTACCGTCATGCTGACCGGTAAACCAAAAAGCGTAATGGCCTGA
TCAAGAAGATGTGGTGCTAAATCGTACCAGATACCGCTGCCTGGGCCGCCCTGTTCACGCCAACGATCGCGCACCTGCGG
ACGGAAGCGGTCAAAATGAGACTCAAAGTAAGCAACTTCACCCAGCACGCCTTCCGCGAGTAAACCTTTTAGCGTCAAGA
AATCGCTATCCCAGCGACGGTTATGGAATACAGACAGCACACGCCCCAGGCTTTTTGCCAGCGCATCCAGCTCTCGCGCT
TGTGACAGTGTCACGGTAAAGGGTTTATCAACGACCACATGTTTACCCGCCTCAAGCGCCGCTTTGGCTAACGGGAAATG
GGTATCGTTGGGTGTAGGAATGACAATCAGGTCTATGTTGGGATCGTTAAACAGATGCTTCGGCTCAGAGACAACCGTAA
CCGTTGGCCAGTCGGCTTTTACTTTTGTTTCATCACTGCTGGAGATTACCGCCAGTTCCTGCCCGGGCGTGCCCGCAATC
AGGGGCGCATGGAAGGTTTTGCTCGCATAACCATACCCAATCAACCCAACACGGATGTTGTCGCTCATGTCATTGCCTCT
CATTGAAGTATGATGGCTATTTGACACTATCCTTTACCCACGCTCAACAGTTTAATAACCTGCCAGCAATAAGGGATGTT
GTTTAACTTAAGTCAAAAAAATAGCGAATTTTCCAACGACAAAAGCTAAATATCGCAAAAACCTCAGTAAAAATCTTGCT
GGAGCTATTATTGCTAAGTAACATTTACCCCCTGAAGTTAATGGATCAATCAAGAGAGATGTGGGCTGTAATGAATCGTC
TTATTGAATTAACAGGTTGGATCGTTCTTGTCGTTTCAGTCATTCTTCTTGGCGTGGCGAGTCACATTGACAACTATCAG
CCACCTGAACAGAGTGCTTCGGTACAACACAAGTAAGCTCTGCACTTGTGGAGCGACATGCTGCCCGTCCGGGTGCATGT
TTTCACTTGTCGGATATTAAACCAGGAATTTATTATCTTGTTCGATGTTGTTGGTGATTGTCAGGGATAGTAAAGCGCGT
TACTCTTTTCAGAGCAGTTTTTCATCCCTGAATATTTGCCATCAGCGCATTCTTATTATTGTAATAACACAAGCGTTCAC
TTTTGGTGAAATTTTATCATCACTAATAATACCTACCCCTTTGGTATTAATTATTTTATGGACCTTTTATGACTGTTCAG
GACTACTTATTAAAATTTCGCAAAATCAGTTCACTCGAAAGTCTGGAAAAACTCTACGACCATCTTAATTACACCCTGAC
GGACGATCAGGAACTGATCAATATGTATCGTGCTGCCGATCACCGTCGCGCAGAGCTGGTTTCTGGCGGGCGTTTGTTTG
ACCTCGGCCAGGTACCGAAGTCCGTCTGGCACTATGTCCAATAAAGAAAGTAGCGATCGGTTATAAAAGCCTTATAATAA
CGCCCCTAAAATTAGATTTGCCTGTTTGCGTCGCTATTCTGGAGAACCTATGACCACCACAACGCCACAACGTATTGGAG
GCTGGTTGCTTGGCCCGTTGGCCTGGCTTTTAGTCGCTCTGTTGAGTACGACGCTGGCGTTGTTGCTGTATACCGCTGCG
TTATCTTCTCCGCAAACATTTCAAACGCTCGGCGGACAAGCACTGACGACACAAATCTTATGGGGCGTTTCTTTTATTAC
CGCTATCGCCTTGTGGTATTACACACTGTGGTTGACCATCGCTTTCTTTAAACGTCGGCGTTGTGTTCCTAAACACTATA
TTATCTGGCTGCTTATTTCCGTATTGCTGGCAGTAAAAGCATTCGCTTTTTCACCCGTGGAAGATGGCATAGCCGTTCGC
CAGTTGCTGTTTACTTTGTTGGCAACCGCATTGATCGTCCCCTATTTCAAACGCTCGTCGCGGGTAAAAGCGACGTTTGT
GAATCCGTAATAACCTTACAGTTAACCTGTTGTCGCCTGCTCTGGATTAACGGATAATAGGCGGCTTTTTTATTTCAGGC
CGAAAAATGACTGACTACCTGTTACTGTTTGTCGGAACTGTACTGGTCAATAACTTTGTACTGGTCAAGTTTCTCGGTCT
CTGTCCGTTTATGGGGGTTTCCAAAAAACTGGAAACCGCGATGGGCATGGGGCTGGCAACAACGTTTGTGATGACGCTGG
CGTCTATTTGCGCCTGGCTTATCGATACGTGGATTTTGATCCCACTTAATCTGATTTACCTGCGCACCCTGGCATTTATT
CTGGTGATTGCTGTGGTCGTGCAGTTCACCGAGATGGTGGTGCGCAAAACCAGCCCGGTGCTTTACCGTCTGCTGGGGAT
TTTTTTGCCGCTTATCACCACCAACTGTGCAGTGCTCGGCGTGGCGTTGCTGAATATCAATCTCGGGCACAATTTCTTGC
AGTCGGCGCTGTACGGTTTTTCCGCCGCTGTCGGTTTCTCGCTGGTGATGGTGCTCTTCGCCGCCATCCGCGAACGCCTT
GCTGTGGCTGATGTCCCGGCACCTTTTCGCGGTAATGCCATTGCGTTAATTACCGCAGGTCTTATGTCTCTGGCCTTTAT
GGGCTTTAGTGGTTTGGTGAAGTTGTAATGAATGCTATCTGGATTGCCGTTGCCGCCGTGAGCCTGCTGGGCCTGGCGTT
TGGCGCCATTCTGGGTTATGCCTCCCGCCGTTTTGCGGTGGAAGACGATCCGGTCGTTGAGAAAATTGACGAAATCTTAC
CGCAGAGCCAGTGTGGTCAGTGCGGTTATCCCGGCTGTCGCCCCTACGCGGAAGCCATCAGCTGTAACGGTGAAAAAATC
AACCGTTGCGCCCCAGGTGGCGAAGCTGTGATGCTAAAAATTGCCGAGTTGCTTAATGTCGAGCCGCAGCCGCTGGATGG
CGAAGCGCAAGAGATAACGCCTGCGCGGATGGTGGCGGTTATTGATGAAAATAACTGTATTGGCTGCACTAAATGTATTC
AGGCGTGTCCGGTAGACGCCATCGTTGGCGCTACCCGTGCCATGCATACGGTAATGAGTGATCTCTGTACGGGCTGCAAT

TTATGTGTTGATCCGTGCCCGACGCACTGCATCTCGTTGCAACCGGTCGCAGAAACACCTGACTCCTGGAAATGGGATCT
GAACACCATTCCCGTGCGTATCATTCCCGTGGAACACCATGCTTAAGTTATTCTCTGCATTCAGAAAAAATAAAATCTGG
GATTTCAACGGCGGCATCCATCCACCGGAGATGAAAACCCAGTCCAACGGTACACCCCTGCGCCAGGTACCCCTGGCGCA
GCGTTTTGTTATTCCACTGAAACAGCATATTGGCGCTGAAGGTGAGTTGTGCGTTAGCGTCGGCGATAAAGTATTGCGCG
GCCAGCCGCTTACCCGTGGTCGCGGCAAAATGCTGCCTGTTCACGCGCCCACCTCGGGTACCGTTACGGCTATTGCGCCC
CACTCTACGGCTCATCCTTCAGCTTTAGCTGAATTAAGCGTGATTATTGATGCCGATGGTGAAGACTGCTGGATCCCGCG
CGACGGCTGGGCCGATTATCGCACTCGCAGTCGCGAAGAGTTAATCGAGCGCATACATCAGTTTGGTGTTGCCGGGCTGG
GCGGTGCAGGATTCCCGACAGGCGTTAAATTGCAGGGTGGCGGAGATAAGATTGAAACGTTGATTATCAACGCGGCTGAG
TGCGAGCCGTACATTACCGCCGATGACCGTTTGATGCAGGATTGCGCGGCTCAGGTCGTAGAGGGTATTCGCATTCTTGC
GCATATTCTGCAGCCACGCGAAATTCTTATCGGCATTGAAGATAACAAACCGCAGGCGATTTCCATGCTGCGCGCGGTGC
TGGCGGACTCTAACGATATTTCTCTGCGGGTGATTCCAACCAAATATCCTTCTGGCGGTGCTAAACAATTAACCTACATT
CTGACCGGGAAGCAGGTTCCACATGGCGGGCGTTCATCCGATATCGGCGTATTAATGCAAAACGTCGGCACTGCTTATGC
AGTGAAACGTGCCGTTATTGATGGCGAGCCGATTACCGAGCGTGTTGTAACCCTGACTGGCGAAGCAATCGCTCGCCCGG
GCAACGTCTGGGCACGGCTGGGGACGCCAGTGCGTCATTTATTGAATGATGCCGGATTCTGCCCCTCTGCCGATCAAATG
GTGATTATGGGTGGCCCGCTAATGGGCTTTACCTTGCCATGGCTGGATGTCCCGGTCGTAAAGATTACCAACTGTCTGTT
GGCTCCCTCTGCCAATGAACTTGGCGAACCACAGGAAGAACAAAGCTGCATCCGGTGTAGCGCCTGTGCTGACGCCTGCC
CTGCGGATCTTTTGCCGCAACAGTTGTACTGGTTCAGCAAAGGTCAGCAACACGATAAAGCTACCACGCATAACATTGCT
GATTGCATTGAATGTGGGGCTTGCGCGTGGGTTTGCCCGAGCAATATTCCCCTGGTGCAATATTTCCGTCAGGAAAAAGC
TGAAATTGCGGCTATTCGTCAGGAAGAAAAGCGCGCCGCAGAAGCCAAAGCGCGTTTCGAAGCGCGCCAGGCTCGTCTGG
AGCGCGAAAAAGCGGCTCGCCTTGAACGACATAAGAGCGCAGCCGTTCAACCTGCAGCCAAAGATAAAGATGCGATTGCT
GCCGCTCTGGCGCGGGTGAAAGAGAAACAGGCCCAGGCTACACAGCCTATTGTGATTAAAGCGGGCGAACGCCCGGATAA
CAGTGCAATTATTGCAGCACGGGAAGCCCGTAAAGCGCAAGCCAGAGCGAAACAGGCAGAACTGCAGCAAACTAACGACG
CAGCAACCGTTGCTGATCCACGTAAAACTGCCGTTGAAGCAGCTATCGCCCGCGCCAAAGCGCGCAAGCTGGAACAGCAA
CAGGCTAATGCGGAACCAGAACAACAGGTCGATCCGCGCAAAGCCGCCGTCGAAGCCGCTATTGCCCGTGCCAAAGCGCG
CAAGCTGGAACAGCAACAGGCTAATGCGGAACCAGAAGAACAGGTCGATCCGCGCAAAGCCGCCGTCGAAGCCGCTATTG
CCCGTGCCAAAGCACGCAAGCTGGAACAGCAACAGGCTAATGCCGAGCCAGAACAACAGGTCGATCCGCGCAAAGCCGCC
GTCGAAGCCGCTATTGCCCGAGCCAAAGCGCGCAAACGGGAACAGCAACCGGCTAATGCGGAGCCAGAAGAACAGGTTGA
TCCGCGCAAAGCTGCCGTCGAAGCGGCTATTGCACGCGCCAAAGCACGCAAGCTGGAACAGCAACAGGCTAATGCGGTAC
CAGAAGAACAGGTTGATCCGCGCAAAGCGGCAGTTGCCGCGGCTATTGCCCGCGCTCAGGCCAAAAAAGCCGCCCAGCAG
AAGGTTGTAAACGAGGACTAAATGGTATTCAGAATAGCTAGCTCCCCTTATACCCATAACCAGCGCCAGACATCGCGCAT
TATGCTGTTGGTGTTGCTCGCAGCCGTGCCAGGAATCGCAGCGCAACTGTGGTTTTTTGGTTGGGGTACTCTCGTTCAGA
TCCTGTTGGCATCGGTTAGTGCTCTGTTAGCCGAAGCTCTCGTACTCAAACTACGCAAGCAGTCGGTAGCCGCAACGTTG
AAAGATAACTCAGCATTGCTGACAGGCTTATTGCTGGCGGTAAGTATTCCCCCCCTCGCGCCATGGTGGATGGTCGTGCT
GGGTACGGTGTTTGCGGTGATCATCGCTAAACAGTTGTATGGCGGTCTGGGACAAAACCCGTTTAATCCGGCAATGATTG
GTTATGTGGTCTTACTGATCTCCTTCCCCGTGCAGATGACCAGCTGGTTACCGCCACATGAAATTGCGGTCAACATCCCT
GGTTTTATCGACGCCATCCAGGTTATTTTTAGCGGTCATACCGCCAGTGGTGGTGATATGAACACACTACGCTTAGGTAT
TGATGGCATTAGTCAGGCGACACCGCTGGATACATTTAAAACCTCTGTCCGTGCCGGTCATTCGGTTGAACAGATTATGC
AATATCCGATCTACAGCGGTATTCTGGCGGGCGCTGGTTGGCAATGGGTAAATCTCGCCTGGCTGGCTGGCGGCGTATGG
TTGCTATGGCAGAAAGCGATTCGCTGGCATATTCCCCTCAGCTTCTTAGTAACGCTGGCGTTATGCGCAATGTTGGGCTG
GTTGTTCTCACCAGAAACACTGGCAGCACCGCAAATTCATCTGCTGTCTGGAGCGACCATGCTCGGCGCATTCTTTATTT
TGACTGACCCGGTTACCGCTTCTACGACCAATCGTGGTCGTCTTATTTTCGGCGCGCTTGCGGGCTTATTAGTCTGGTTG
ATCCGCAGTTTCGGCGGCTATCCTGACGGCGTGGCTTTTGCCGTCCTGCTGGCGAACATCACGGTTCCTCTGATCGATTA
CTACACGCGTCCGCGCGTCTACGGCCATCGCAAAGGGTAAACCATGCTGAAAACTATCCGAAAACACGGCATTACGTTGG
CGCTATTTGCAGCGGGTTCAACAGGGTTAACTGCGGCCATCAACCAGATGACCAAAACGACGATTGCTGAACAGGCCAGT
CTGCAACAAAAGGCGTTATTTGATCAGGTGCTGCCAGCCGAACGCTATAACAATGCGCTGGCACAGAGTTGCTATCTGGT
AACTGCGCCAGAGTTAGGTAAAGGTGAGCATCGGGTTTACATCGCCAAACAGGATGACAAACCGGTAGCCGCCGTTCTGG
AAGCAACCGCGCCAGATGGCTATTCCGGTGCGATTCAGCTGCTGGTGGGAGCCGATTTTAACGGCACGGTACTTGGCACG
CGCGTGACAGAGCACCACGAAACGCCAGGGCTTGGCGATAAAATCGAACTGCGCCTTTCTGACTGGATCACCCATTTTGC
GGGTAAAAAAATCAGTGGTGCAGATGATGCGCACTGGGCGGTGAAGAAAGATGGTGGTGATTTCGACCAGTTCACCGGCG
CGACGATTACTCCCCGCGCGGTGGTTAATGCGGTAAAACGCGCCGGATTGTACGCTCAGACGTTACCGGCACAACTTTCT
CAACTTCCTGCCTGTGGAGAATAAAGCGTGAGCGAAATTAAAGACGTTATTGTTCAGGGGTTGTGGAAAAACAACTCTGC
GCTGGTCCAGTTGCTCGGCCTTTGTCCTCTGTTGGCGGTCACGTCCACTGCCACTAACGCTCTGGGTTTAGGACTTGCGA
CTACGCTGGTACTGACGCTGACCAACCTGACCATTTCGACGCTGCGTCACTGGACGCCAGCCGAGATCCGCATTCCCATT
TACGTGATGATCATCGCCTCGGTGGTCAGCGCTGTACAGATGCTGATCAACGCCTACGCCTTTGGCCTGTATCAATCATT
AGGGATTTTTATTCCGCTGATTGTCACTAACTGTATCGTTGTGGGCCGCGCTGAAGCCTTCGCCGCCAAAAAAGGTCCGG
CGCTTTCGGCACTGGACGGCTTTTCAATTGGTATGGGCGCAACCTGCGCCATGTTCGTGCTGGGTTCACTACGCGAAATT

ATCGGCAATGGCACATTGTTTGACGGTGCAGATGCGCTGTTAGGTAGCTGGGCAAAAGTATTACGCGTGGAGATTTTCCA
CACCGACTCCCCTTTCCTGCTGGCGATGCTGCCACCAGGTGCATTTATTGGCCTGGGACTGATGCTGGCAGGAAAATACC
TGATTGATGAAAGAATGAAAAAGCGCCGTGCTGAAGCAGCTGCAGAACGTGCATTGCCAAACGGTGAAACAGGGAATGTC
TGATGAATAAAGCAAAACGCCTGGAGATCCTCACTCGCCTGCGTGAGAACAATCCTCATCCCACCACCGAGCTTAATTTC
AGTTCGCCTTTTGAATTGCTGATTGCCGTACTGCTTTCCGCTCAGGCGACCGATGTCAGTGTTAATAAGGCGACGGCGAA
ACTCTACCCGGTGGCGAATACGCCTGCAGCGATGCTTGAACTGGGCGTTGAAGGGGTGAAAACCTATATCAAAACGATTG
GGCTTTATAACAGCAAAGCAGAAAATATCATCAAAACCTGCCGTATCTTGCTGGAGCAGCATAATGGCGAGGTTCCGGAA
GATCGTGCTGCGCTTGAAGCCCTGCCCGGCGTAGGTCGTAAAACAGCCAACGTCGTATTAAACACTGCATTCGGCTGGCC
GACTATTGCTGTCGACACGCACATTTTCCGCGTTTGTAATCGTACTCAATTTGCGCCGGGGAAAAACGTCGAACAGGTAG
AAGAAAAGCTACTGAAAGTGGTTCCAGCAGAGTTTAAAGTCGACTGCCACCATTGGTTGATCCTGCACGGGCGTTATACC
TGCATTGCCCGCAAGCCCCGCTGTGGCTCTTGTATTATTGAAGATCTTTGTGAATACAAAGAGAAAGTTGACATCTGAAG
AAAAGGGGTAACACCGATTACCCCATTGATAACCTTTCTTTATCCTCTTTTAAAACATCTTTTTAACGTCAATGATGCCA
TTGCTTAGCGTTATCATCAGGTAATCCGTTTGCGGATAACCAAAAATGCAGGTTAATTGTTTTTTTAATAGCGAAATTTT
CTATTCATTCGTGATCAAGATCACGATGCTGTCGCAATGTAAAAACATTGCAAGAATCAGGTTAAAATCATTTAAATTTA
CACACGCAACAAATATTGACCTACAAAACATTACACTGGCTATTTTTCAGAAACTGGACTATCTCACTAGTAAAAACGCT
AAATAGCAGAACATATCGCCGTTAAGCCATCACCCCCCTCTCAGTGCAGTGAAAAAATCTGCCGTTACGTTTTTTGAAAA
ATTTAACGCTGGATAACATTTCCCGGAATGGTTGAATTCCCCGCCTCAGTTATATGTAACAGATTATTACAAAGGACTTG
TCTGAAAGTGCAAGATAGTGAACATTACCTGCCGTTTCCCCTCCCACTATAACAATTGCGCGTATGTTTTTTATACATAA
CGCGAGAAAGCACCCCCGTTAATATGGGATGTAAAAAAAGAGGTAAAAGTGTCCACTGCAAACCAAAAACCAACTGAAAG
CGTCAGTTTGAACGCTTTCAAACAACCGAAGGCGTTCTATCTCATCTTCTCGATTGAGTTATGGGAACGTTTTGGTTATT
ACGGCCTACAAGGAATTATGGCTGTTTACCTGGTTAAACAACTGGGTATGTCTGAAGCGGATTCAATCACCCTTTTCTCT
TCCTTTAGTGCCCTGGTTTATGGTCTGGTCGCTATCGGCGGCTGGTTAGGTGACAAGGTACTGGGTACTAAACGCGTAAT
TATGCTCGGCGCTATTGTGCTGGCGATTGGTTATGCTCTGGTTGCCTGGTCTGGTCACGACGCCGGTATCGTTTATATGG
GTATGGCGGCTATTGCGGTCGGTAACGGCCTGTTTAAAGCTAACCCGTCTTCTCTGCTTTCTACATGCTATGAGAAAAAC
GACCCGCGTCTGGACGGTGCATTCACCATGTACTACATGTCCGTCAACATCGGCTCTTTCTTCTCTATGATTGCTACGCC
GTGGCTGGCCGCGAAATACGGCTGGAGTGTTGCGTTTGCGTTGAGCGTTGTAGGCCTGCTGATCACTATCGTTAACTTCG
CCTTCTGCCAACGCTGGGTTAAACAGTACGGTTCAAAACCAGACTTCGAGCCTATCAACTACCGTAACCTGCTGCTGACC
ATTATTGGTGTTGTGGCACTGATCGCTATCGCCACCTGGCTGCTGCACAATCAGGAAGTTGCGCGTATGGCGCTGGGCGT
TGTTGCCTTCGGTATCGTGGTTATCTTCGGTAAAGAAGCCTTCGCGATGAAAGGTGCTGCGCGTCGTAAAATGATCGTTG
CCTTCATCCTGATGCTCGAAGCCATTATCTTCTTCGTGCTGTACAGCCAGATGCCAACGTCACTGAACTTCTTTGCGATT
CGTAACGTTGAGCACTCCATTCTGGGTCTGGCCGTAGAACCTGAGCAGTATCAGGCACTGAACCCGTTCTGGATCATCAT
CGGTAGTCCGATTCTGGCCGCTATCTATAACAAGATGGGCGATACCCTGCCGATGCCAACCAAGTTTGCAATCGGCATGG
TGATGTGTTCTGGTGCGTTCCTGATTCTGCCGCTGGGTGCGAAATTCGCGTCTGACGCTGGTATCGTGTCTGTAAGCTGG
CTGGTCGCAAGCTATGGCCTGCAGAGCATCGGGGAACTGATGATCTCTGGTCTGGGTCTGGCAATGGTTGCTCAACTCGT
TCCGCAGCGTCTGATGGGCTTCATTATGGGTAGCTGGTTCCTGACCACTGCCGGTGCAAACCTGATTGGTGGTTATGTTG
CGGGTATGATGGCTGTGCCGGATAACGTTACCGATCCGCTGATGTCACTGGAAGTCTATGGTCGCGTATTCTTGCAGATT
GGTGTCGCTACTGCCGTTATTGCAGTACTGATGCTGCTGACCGCGCCGAAACTGCACCGCATGACGCAGGATGACGCTGC
AGACAAAGCGGCGAAAGCAGCCGTAGCGTAAAATTCAGGGAAACTCTTTTACAAGCCGCTAACTTTTCGTTAGCGGCTTT
TTTTTTGTTCAGCAACGCACTACCATACTTTAAACCACAGACAAAAGGAGTTACCGATGAAATTGTTCTACAAACCGGGT
GCCTGCTCTCTCGCTTCCCATATCACCCTGCGTGAGAGCGGAAAGGATTTTACCCTCGTCAGTGTGGATTTAATGAAAAA
ACGTCTCGAAAACGGTGACGATTACTTTGCCGTTAACCCTAAGGGGCAGGTGCCTGCATTGCTGCTGGATGACGGTACTT
TGCTGACGGAAGGCGTAGCGATTATGCAGTATCTTGCCGACAGCGTCCCCGACCGCCAGTTGCTGGCACCGGTAAACAGT
ATTTCCCGCTATAAAACCATCGAATGGCTGAATTACATCGCCACCGAGCTGCATAAAGGTTTCACACCTCTGTTTCGCCC
TGATACACCGGAAGAGTACAAACCGACAGTTCGCGCGCAGCTGGAGAAGAAGCTGCAATATGTGAACGAGGCACTGAAGG
ATGAGCACTGGATCTGCGGGCAAAGATTTACAATTGCTGATGCCTATCTGTTTACGGTTCTGCGCTGGGCATACGCGGTG
AAACTGAATCTGGAAGGGTTAGAGCACATTGCAGCATTTATGCAACGTATGGCTGAACGTCCGGAAGTACAAGACGCGCT
GTCAGCGGAAGGCTTAAAGTAATAGCCATCGGCCCACAGGTCGTTGTGGGCCGAAATGAGATATTTCAGAGCTTTGTTGC
GCTGAAGTAATGTTCTGGTTTGGCAATACGATCCTGAGCAGCCACCACTTGCAGCTCATATTCCTGCATTGCTTTGGTGG
TCACCATGATTTCGTAGACTGCAGCGGTCACATGTTCCAGCGCCTCCTGCAGCGTTGCCCCCTGAAGCAGTTTCACCAGC
AGTAAACCGCTCGTCACATCACCAACACCTACCGGCTGGCGCATACCAAAATCCACCAGCGGACGGCTGATATGCCAGGC
TTCATCGGCGGTGACCAGCAGCATTTCAAAACGGTCACGGCTGTAGCCAGCTCGCGCCAGGTGTTTAACCAACACAATTT
GTGGCCCTTGCGCAATGAGTTCGCGCGCTGCCAGAACCGCTTCTTCGACGTTATTTACCGCATGCTCACAGAGTATTTCC
AGCTCAACCAGATTTGGCGCAATGATATCGCTGGCAGGCAAACCGTGCCGCACATGAAACTCTGCGACACCCGGTGCAAC
GATACAGCCTTTTTCCGGATGACCCATTACCGGATCGCAAAAATATTTCGCCTGCGGATTCGCGGCTTTCACCTGACGGA
CGATACCGAGGATATGTTCACCCTGCTCCGCCGATCCCAGATAGCCACTTAATACGGCATCACAGGTGTGTAATTTATCA
ATGGCGGCAATGCCTTGCACAATTTCGGTTAAATGGCTGGGCGGCATCACGCAGCCAGTCCATTTGCCGTATTGGGTGTG

ATTAGAAAATTGAACGGTGTTCAGCGGCCAGACGTTCGCGCCCAGGCGGCGCATCGGAAACTCTGCCGCACTGTTACCCG
CATGACCATAAACAACGTGAGACTGGATAGCGAGAATATTTTTCATCATGTTCCCTGTATAAAAACCAGGGGGAGTGATT
TCTCACTCCCCCTTTCCACTTAATGCATTATTTCCAGCAAATCAGACAGTAATTCTTTTTACCGCGACGCAGTAAGGTAA
AACGACCAAACAGACGATCTTCTTCTTTAAAGAAGTATTCAGGATCGGACTGTTTTTCACCGTTAATGGTGATGGCATTG
GAGGCGATAGTTTTACGTGCCTGACCACGGGAAGGTTGCAGTTCAGAATCGACCAGTGCCTGCATCAGGTCTGCGCCCTT
TTCCATCTCAACCATCGGTACGCCGTCCTGCGCCAGCTGTTCGAAGTCCGCTTCACTCAGCGCACTCAAAGAACCGCTGA
ACAGGCATTCGGTAATACGTTTTGCCGCCTGTAAACCTTCTTCACCGTGAACCAGACGAGTCACCTGCTCCGCCAGTACA
TACTGGGCGCGCGGTGCTTTACCGCTGTTTTTATCTTCTTCTTCCAGGGCGTTGATCTCTTCAATGCTCATAAAGGTGAA
GAACTTCAGGAAGCGGTAAACGTCGGCATCCGCAGTGTTGATCCAGAACTGGTAGAATTTGTACGGGCTGGTTTTCTTCG
GATCCAACCAGACTGCGCCGCCTTCAGTTTTACCAAATTTGGTGCCATCTGCTTTAGTGATCAGCGGAACGGTCAGGCCA
AACACCTGATTCTGATGCAGACGACGGGTCAGGTCGATACCAGAAGTGATGTTACCCCACTGGTCAGAACCACCAATTTG
CAGCACCACACCGTACTGTTTGTTCAGACAGGCGAAGTCATAACCCTGCAACAGGTTGTAGGAAAACTCAGTGAACGAAA
TCCCCTGATCTTCACGGTTGAGACGCTGCTTAACCGCTTCTTTGTTGATCATCTGGTTAACGGAGAAGTGTTTGCCAATA
TCGCGCAGGAAGGTCAGCACATTCATATTGCCGAACCAGTCATAGTTGTTCGCCGCGATAGCAGAGTTTTCTCCACAGTC
GAAATCGAGGAACGGGGCAACCTGCTTACGGATTTTGTCCACCCACTCCTGAACAGTTTCTTCGGTGTTCAGCTTACGCT
CGGCAGCTTTGAAGCTCGGGTCGCCAATCAGACCCGTCGCGCCGCCTACCAGCGCAACCGGCTTGTGGCCCGCCTGCTGG
AAGCGTTTCAGGCATAACAATGGAACAAGATGCCCCAAATGCAAGCTGTCAGCGGTAGGATCGAAGCCGCAATAGAGCGC
GATCGGGCCTTGCGCCAGTCGCTCTGCTAACGCTTCCTCGTCCGTCACCTGGGCTACCAGCCCCCGCTCTTGCAATTGTT
TAATCAAGTTACTGCTTGCCATCAAAATCTCCATGTATATAACGACTGCACCTTTGCCGGTACACGACTTTTCGCCAGAT
GCGAAAGAGACATAGAATAAAGTGCCAGAATCAGGAGTACCAGCGATTAAAGCAAGATTTTTGCATCTTTTCAGGGTGCA
AGACGATCAATCTTCCACGCATCATTTTCACGCTGGTACAAAAAGCGGTCATGCAGGCGATGCTCACCACCCTGCCAGAA
CTCAATCTGTTCAAGGCTGACGCGAAAACCGCCCCAAAAGCTCGGCAATGGCACTTCGCCCTGTTGAAACTTCTGCTTCA
GCTCCAGGAATTTACTTTCAAGGATACCGCGGGCAGAAATGCGACTGGACTGCTTCGAAACCCATGCACCAATCTGGCTA
TCACGCGGGCGGCTATGAAAATATTTCATCACTTCGAGAGTCGAAAGTCGTTCTGCTTTACCGATCACCATCACCTGGCG
CTCAAGGGTATGCCACGGGAACAGCAGGCTAACGCGCGGATTATTTTCGATTTGATGTGCTTTACGGCTGCCGAGGTTGG
TGTAAAACACCATGCCTTTTTCGTCGTAATGTTTGAGTAAAACGATGCGCTGATAAGGCTGACCATGTTCATCCACGGTA
GCGACCACCATCGCGGTAGGGTCCGCCAGTTTGGCTTCACAAGCCTGAGAGAGCCAACGTTCAAAAAGGGTTAATGGATC
GGCGGGAAGATCGCGGCGGCGTAACCCGCCTTTGGTGTATTCACGGCGCAGATGCGCGATTTGCTGCAATTCGTCGTTAT
CAGACATGGTTTTCTTTACGGATTGTCAGTGGGTGACGCTATTGTGCGCCGCCCCTGGAAAAATCTCAACGCTGTGGATT
TTGTAACTGACAGTTATTCAAGACGATGCGGTCGCGTTTATAGACAGTCGCTTCATCGCCTTTCGACCAGAAAACATAGA
TTCCGTCAGTGTAACGCGCGCCAGAGGCTGAAATGCCCTGTTTGAGATGCAGTAGTTGATTATCGTAAACAAAACTGACC
TCCTGGCGCGGATTATTCAGTTTGACCGTCAACGGTTTTTCATCACACTGGTATTCCAGCGTATCGGTTTGCATGCGCTC
AACCAGCTGATTAAAGGCGCTACAGCCGGAGAGAAGGACCGGCAAAATGATAATTAACAGTTTTTTCATAGTCATATCCC
GAAGACTTTCCTGGTCTGGAGGGCAATACGCCCTCCCTAACGTTCCAAGTGTAACGGCAGACGCGGTAAGAAAAAATTCAG
TTAACTCTGATATCACGGGTTAGCGGGGAAAATAGCCCCCAGTACCGTCTCCTGGCTTGCGCCAGTGACGGAAGGCAGAT
TTCCTGGTAATCCCGCCAGCGTCCGCCAGGCAAGCCAGGCGAAAGCCAATGCTTCCATGTCATCGCCACTAATGCCAACG
GCATCGGTGGTGGTGACTTCTGTGCCTGGCAGTAATGCCGCCAGACGCGCCATGAGTAGCGGATTACGACTACCTCCACC
ACATACCATCAATCGTTCGCAGCCACCGCTCAACAAAACTTGTTCAGAAATGGTCACGGCGGTGAGTTCTGCCAGTGTCG
CCTGCACATCTCGGGGATCAACACCCGGAAAATGGCGCAAATGGCGCTCCAGCCAACCATAGTTAAAGTATTCGCGTCCG
GTGCTTTTCGGTGCAGGTTGCGAGAAATACGGGTCGCTGAGCATATTTTGCAGCAGTGGGAGAATAACTTTACCCGCCCG
TGCCCACTCGGCATCTTTATCGTAAGGTTTACCGGCCTGACGCCAGATCCAGGCATCCATCAGCATGTTACCAGGACCGG
TATCGTAGCCCCCAACCGGCTGCCCAGGAATGAGCAGTGACAGATTGGCGATGCCGCCAATATTGAGCACCATTCGTCGC
TCGGTTGGGTGAGCCAGCAGCGCATGATGGAACGCAGGTACCAGCGGTGCGCCTTGTCCTCCCAAGGCAATATCGCGACG
GCGAAAATCACCGACAACCGTAATTCCGGTGCGTGCCACAATTTGATTGTTATCGCCAATCTGTAAAGTGTGTGGTGCCA
CGCCCGTCGGTTCATGCCAGACGGTTTGACCGTGACAACCGATCGCAACTATATCTCTTGCCTGCAGATTTTGTTCCTTA
AGCAAGGCATTGACCGCATCAGCAAAAAGTTGCCCGAGTTGAGTATCAAGCTGTCCAAACTGCGAAAGTGTAAGCTGCTG
GCCCTGGCAAATATCCAGTACAGCCTGTTTCAGAGATACCGGGATCGGCCAACTCAAACTTGCCAGCTGTGCGACCCGGT
GTTCATCAATTGTCGCCAACACAACATCAACACCATCAAGGCTGGTGCCTGACATAACGCCAATAAAGCGGCCCGATTTC
ATAGTTCATCCTTTTTCAATCTGACGTTTGCGCACCACTCAAACATAAACTTTTCGTGAATACCATGCGGAATGACCGAT
TTTTACCGTTGGTAGTAAAACATTATCTTCAAATCAATAATCATCATGAATGTTTTGTTTATAATTGGTTGATCCTACTT
TCATTATGATTTGCTCATATTTGGTAGAACATGTAACCATGGATTCACATATGCCATATACTTTGACCATGAGGGATGCT
TGCGTGGCGTTTCATGGTGAACAGGAGATTTTTCAATGATTAAACGCGTATTGGTTGTTTCAATGGTAGGTCTGTCTCTT
GTCGGTTGTGTTAATAACGACACCCTGTCAGGGGATGTTTATACCGCTTCTGAAGCGAAACAAGTACAGAATGTCAGCTA
TGGCACCATCGTTAACGTACGTCCGGTACAGATTCAGGGCGGTGATGATTCCAACGTTATCGGTGCAATTGGCGGTGCTG
TTCTTGGTGGTTTCCTGGGGAATACTGTTGGTGGCGGAACCGGGCGTTCTCTGGCTACTGCAGCAGGCGCTGTTGCAGGT
GGCGTAGCTGGTCAGGGCGTACAGAGTGCAATGAACAAAACGCAGGGTGTCGAGCTGGAAATTCGTAAAGACGATGGTAA

TACCATCATGGTGGTACAGAAACAAGGCAACACTCGTTTCTCTCCGGGCCAACGTGTCGTACTGGCCAGCAATGGCAGTC
AGGTGACCGTTTCTCCGCGCTAAATAAGTTTGCGTGTGGTCAGGTTACTGACCACACGCCCCCTTCATTTCACCCTTTGG
CCTGTAACTCAATGATATTATGCTCAAGTTTTGCGATGAGCGTAATCAGTTGCTCCAGTTCCTCTGCGGAGATGCCATGT
AATATTTCCGCGCGGGTTTTGTTAATAACAGCTTCCATTTCGCTGATCAGCGGCTCTGCCTTTTCCGTCAGTTTAATACG
TTTAGCCCGACGATCGCTGGCACAAGTTTGACGCGAAATTAACCCTTTTTCTTCCAGTTGGTCCAGAGTACGGACCAGTG
ATGGCTGCTCGATGCCAATCGCTTTTGCCAGTTGAATTTGCGACTGGTCTGGAGGTAACTGATGGATATTGTGTAACGTA
ACCCAATGGGTTTGTGTTAACTCCAGCGGTTTCAGGCGATGGTCTATCAGAGCACGCCATATGCGCACCAACCGTGCCAG
ATCAGAACCTAGTGGCGATTCCAATTTCATCTCCTTATAATTAGCTTGCTAAGATATTATGCGGCTTTTAGAATAGTGTG
CAGCAATTGTATTGCTAAAACAAATGTATTGCTGCATTTGGTTACCGTCAGACATATTTTTCAGAAATTGCGCGTAAATT
TTTCGCACTTAAAGAATATTTATTAATCTAACGCAATATATTCGGTCGTAAAGGAATCTACTTTGTGAAGTTTATGCTCA
ATGCAACAGGATTGCCCTTACAAGATCTGGTGTTCGGTGCGTCCGTCTACTTTCCTCCGTTTTTCAAAGCATTCGCGTTT
GGATTCGTCATCTGGCTTGTCGTACACCGCCTGCTTCGTGGCTGGATCTACGCCGGTGACATCTGGCATCCCTTGTTAAT
GGATTTATCGCTGTTTGCGATTTGCGTTTGCCTTGCTCTGGCAATACTGATTGCGTGGTAACTATGTCAATTAAAACAAT
TAAGTATTTCTCAACAATCATTGTAGCGGTAGTTGCGGTTCTTGCCGGATGGTGGCTGTGGAATTATTACATGCAATCTC
CGTGGACACGCGACGGGAAAATACGCGCAGAACAGGTTTCTATCACCCCACAGGTGTCGGGACGTATTGTTGAGCTGAAT
ATAAAAGACAACCAGCTGGTTAACGCGGGGGATCTTCTGCTCACGATCGATAAAACGCCCTTTCAGATCGCCGAGCTGAA
CGCACAGGCTCAGCTGGCGAAAGCGCAATCTGACCTCGCAAAAGCCAATAACGAAGCGAATCGTCGTCGTCATCTCTCAC
AAAATTTTATTTCTGCCGAAGAACTCGATACCGCTAACCTCAATGTTAAAGCGATGCAGGCCAGTGTTGATGCCGCACAG
GCGACGCTGAAACAGGCACAATGGCAACTGGCGCAAACGGAAATTCGCGCCCCGGTGAGTGGATGGGTGACTAACCTTAC
TACCCGCATCGGCGATTACGCTGATACCGGGAAACCGCTGTTTGCCCTTGTCGACAGCCACTCGTTTTACGTCATTGGTT
ATTTTGAAGAAACCAAGTTGCGCCATATCCGCGAAGGTGCACCAGCACAAATTACCCTCTATAGTGATAACAAAACGTTA
CAGGGTCACGTTTCCAGTATCGGTCGGGCGATTTATGATCAGAGCGTTGAAAGTGACTCAAGCCTTATTCCGGATGTTAA
ACCTAACGTCCCCTGGGTTCGACTCGCCCAACGCGTTCCCGTCCGCTTTGCGCTGGATAAAGTCCCAGGCGATGTCACGT
TGGTGTCGGGCACCACCTGTAGCATTGCCGTAGGTCAATAATGAACGCATCGTCATGGTCCTTGCGCAATTTGCCCTGGT
TCAGGGCCACGCTGGCGCAATGGCGTTATGCGTTACGCAATACCATTGCCATGTGTCTGGCGCTGACGGTTGCCTATTAT
TTAAATCTGGATGAACCCTATTGGGCGATGACCTCGGCTGCAGTGGTTAGCTTTCCCACCGTTGGCGGTGTTATCAGCAA
AAGCCTCGGACGCATCGCTGGCAGTTTGCTCGGAGCCATTGCGGCACTGCTTCTTGCCGGGCATACGCTCAATGAGCCGT
GGTTTTTTCTATTGAGCATGTCGGCGTGGCTTGGCTTTTGTACCTGGGCCTGTGCGCACTTCACGAATAACGTCGCGTAT
GCATTTCAACTGGCGGGCTACACGGCTGCCATCATCGCCTTTCCGATGGTTAATATTACTGAGGCCAGCCAGCTGTGGGA
TATCGCTCAGGCGCGCGTTTGCGAGGTAATAGTCGGTATTTTGTGCGGCGGCATGATGATGATGATCCTGCCGAGCAGTT
CCGATGCTACTGCCCTTTTAACCGCATTGAAAAACATGCACGCCCGATTACTGGAACATGCCAGTTTACTCTGGCAGCCT
GAAACAACCGATGCCATTCGTGCAGCACATGAAGGGGTGATTGGGCAGATACTGACCATGAATTTGCTGCGTATCCAGGC
TTTCTGGAGCCACTATCGTTTTCGCCAGCAAAACGCGCGCCTTAATGCGCTGCTCCACCAGCAATTACGTATGACCAGTG
TCATCTCCAGCCTGCGACGTATGTTGCTCAACTGGCCCTCACCGCCAGGTGCCACACGAGAAATTCTCGAACAGTTGCTG
ACGGCGCTCGCCAGTTCGCAAACAGATGTTTACACCGTCGCACGTATTATCGCCCCGCTACGCCCGACCAACGTCGCCGA
CTATCGGCACGTCGCCTTCTGGCAGCGACTACGTTATTTTTGCCGCCTTTATCTGCAAAGTAGTCAGGAATTACATCGTC
TGCAAAGCGGTGTAGATGATCATACCAGACTCCCACGGACATCCGGCCTGGCTCGTCATACCGATAACGCCGAAGCTATG
TGGAGCGGGCTGCGTACATTTTGTACGTTGATGATGATTGGCGCATGGAGTATTGCTTCGCAATGGGATGCCGGTGCCAA
TGCATTAACGCTGGCAGCAATTAGCTGCGTACTCTACTCCGCCGTCGCAGCACCGTTTAAGTCGTTGTCACTTCTGATGC
GCACGCTGGTGTTACTTTCGCTATTCAGCTTTGTGGTCAAATTTGGTCTGATGGTCCAGATTAGCGATCTGTGGCAATTT
TTACTGTTTCTCTTTCCACTGCTGGCGACAATGCAGCTTCTTAAATTGCAGATGCCAAAATTTGCCGCATTGTGGGGGCA
ACTGATTGTTTTTATGGGTTCTTTTATCGCTGTCACTAATCCCCCGGTGTATGATTTTGCTGATTTTCTTAACGATAATC
TGGCAAAAATCGTTGGCGTCGCGTTGGCGTGGTTAGCGTTCGCCATTCTGCGTCCAGGATCGGATGCTCGTAAAAGCCGC
CGCCATATTCGCGCGCTGCGCCGGGATTTTGTCGATCAGCTAAGCCGCCATCCAACACTGAGTGAAAGCGAATTTGAATC
GCTCACTTATCATCACGTCAGTCAGTTGAGTAACAGCCAGGATGCGCTGGCTCGCCGTTGGTTATTACGCTGGGGTGTAG
TGCTGCTGAACTGTTCTCATGTTGTCTGGCAATTGCGCGACTGGGAATCGCGTTCCGATCCGTTATCGCGAGTACGGGAT
AACTGTATTTCACTGTTGCGGGGAGTGATGAGTGAGCGTGGCGTTCAGCAAAAATCACTGGCGGCCACACTTGAAGAATT
ACAGCGGATTTGCGACAGCCTTGCCCGTCATCATCAACCTGCCGCCCGTGAGCTGGCGGCAATTGTCTGGCGGCTGTACT
GCTCGCTTTCGCAACTTGAGCAAGCACCACCGCAAGGTACGCTGGCCTCTTAATTACTTAATTACACCACAGGCATAGCG
TTCACCGCCACCGCCCAGCGGTTTAGGTTGATCGGACATATTATCGCCGCCAACGTGGACCATCAGCGCTTTGTCTTTGA
TTTCATCCAGTGATTTCAGACGAGGCGCGATGACGGCATCGGTAGCTTTGCCGTCATTATTGACGACCAGTGCAGGCAGA
TCGCCTAAATGCCCGGCACCTTCTGGCCCTTCATGTTTACCGGTATTTTGTGGATCAAGATGCCCGCCTGCGGATTCCGC
GGCGCTGGCTTTGCCATCTTTGGTGGCTGGCTGGCAGCTTCCTTTGGCATGAATATGGAAGCCATGTTCACCGGGGGGTA
ATGCTTTCAGATCGGGCGAAAACTCCAGACCTTTATCGGTTTCAGTAATGGTGACGCTACCAATTGACTGCCCTACCCCT
TGCGACGTGACGAGGTTCATCTCGACTTTTTCACTGGCAGCTTGTGCGCCGGTTGCAACAACCAGCGCCAGAATAGCCAG
ACTAAAACGTTTCATAGGACCTCCGTTCATTGTGCATTCCTGAATAAGTGTAAACCAGTGACACATTTTTGAACGGCGGC

TATTCCTAAAAGTGCTTACGGTACGTCGTACCCCAGTGCCGCTTTACGGATACGAAACCATTGTTGACGGGTCATTTTCA
GTGTTTCTGCTTCGACAGCTGCCCGTACGCGCTCAATTTTACCTGAACCGATAATTGGCAGCGGCTGCGATGGTAAACGT
AATACCCAGGCGTAAACCACCTGTTCAATCGAGCCCGCGTTTAACTCCTCTGCCACCACAGCCAGTTCATCACGCAGCGG
CTGGAAATAATCATCATTAAACAGACGACCACCACCAAGGCAGGACCACGCCATCGGACGAACACGCAGTTGTTGTAGTT
GGTCGAGCGTGCCATCCAGCAGTAACGGCTGATGCACCGGGGATATTTCCACCTGATTAGTGGCAAGGGTAAACGGCAGA
CGTGATTGCAACAGGGCAAATTGCGCAGGCGTAAAGTTCGATACGCCAAAATGACGCACTTTGCCGCTCTGATGCAGATG
TTTGAACGCGTCCGCCACTTCATCGGCATCCATTAACGGGTCTGGTCGGTGGATTAACAGCAAATCCAGATGATCGGTCG
CGAGATTAATTAGCGACTGTTCGGCGCTCTTAATGATGTGATCGCGGTCAGTGATGTAATGACCAATGACGTTTTCTTCA
CGCGCGGTCGTCGCGATACCGCATTTACTGACGATTTCCATCCGTTCACGCAGGTGAGGTGCCAGTTTCAGTGCCTCGCC
AAACGCCGCTTCGCACTGATAGCCACCATAAATATCAGCATGGTCCACGGTGGTCACGCCGAGATCCAGATGCTCTTCAA
TAAAACTGACCAGCTGGCGGGCGGACATATTCCAGTCCATCAATCGCCAGTAGCCCATCACAAAACGGGAAAACTCCGGG
CCTTGCGGCGCAATAGTAATACGCTGAACCATAATCGCTTCCTCTTATCAGATATGAGAGGAGTATACGCAAGATTAGGT
TCAAAAGAGTGATGGTTGCTCCGGTTCGTCTGATGACGCTGGCTTATTTGCGCGTAATTTGCGCATTAATCGCTGCCGAC
AAAGGCGCAGCACCTCTTGTTTTTCGCCATCGCTCATTTTATTCCAGTTAAAACGCTCATCACGACTACGAAAACAGCCG
CGACAAAACCCGCGTTCGTCAGACTGGCAAATTCCCCGGCACGGGCTCTGGACGGGAAAGAACTCTAATTGCTCCGCCAC
TTCGCCCTCCTCAGATAAGATTATTACCATTATTGAAGCTGTTAATGTCCAAAGTAGCAACTTTGCTTGCACTAGACCGA
CTGGTCTACTACACTCCAACGCATGAACAAACACACCGAACATGATACTCGCGAACATCTCCTGGCGACGGGCGAGCAAC
TTTGCCTGCAACGTGGATTCACCGGGATGGGGCTAAGCGAATTACTAAAAACCGCTGAAGTGCCGAAAGGGTCCTTCTAT
CACTACTTTCGCTCTAAAGAAGCGTTTGGCGTTGCCATGCTTGAGCGTCATTACGCCGCATATCACCAGCGACTGACTGA
GTTGCTGCAATCCGGCGAAGGTAACTACCGCGACCGCATACTGGCTTATTACCAGCAAACACTGAACCAGTTTTGCCAAC
ATGGAACCATCAGTGGTTGCCTGACAGTAAAACTCTCTGCCGAAGTGTGCGATCTGTCAGAAGATATGCGCAGCGCGATG
GATAAAGGTGCTCGCGGCGTGATCGCCCTGCTCTCTCAGGCGCTGGAAAATGGCCGTGAGAACCATTGTTTAACCTTTTG
TGGCGAACCGCTGCAACAGGCACAAGTGCTTTACGCACTGTGGCTTGGCGCGAATCTGCAGGCCAAAATTTCGCGCAGTT
TCGAGCCACTGGAAAACGCGCTGGCCCATGTAAAAAACATTATTGCGACGCCTGCCGTTTAGCAGGCATTTTTTATCACC
AGACGACCGGGAGCCTTTATGTCATCTGAAAAACTGTATTCCCCACTGAAAGTGGGCGCGATCACGGCGGCAAACCGTAT
TTTTATGGCACCGCTGACGCGTCTGCGCAGTATTGAACCGGGTGACATTCCTACCCCGTTGATGGCGGAATACTATCGCC
AACGTGCCAGTGCCGGTTTGATTATTAGTGAAGCCACGCAAATTTCTGCCCAGGCAAAGGATATGCAGGTGCGCCTGGC
ATCCATAGTCCGGAGCAAATTGCCGCATGGAAAAAAATCACCGCTGGCGTTCATGCTGAAAATGGTCATATGGCCGTGCA
GCTGTGGCACACCGGACGCATTTCTCACGCCAGCCTGCAACCTGGCGGTCAGGCACCGGTAGCGCCTTCAGCACTTAGCG
CGGGAACACGTACTTCTCTGCGCGATGAAAATGGTCAGGCGATCCGTGTTGAAACATCCATGCCGCGTGCGCTTGAACTG
GAAGAGATTCCAGGTATCGTCAATGATTTCCGTCAGGCCATTGCTAACGCGCGTGAAGCCGGTTTTGATCTGGTAGAGCT
CCACTCTGCTCACGGTTATTTGCTGCATCAGTTCCTTTCTCCTTCTTCAAACCATCGTACCGATCAGTACGGCGGCAGCG
TGGAAAATCGCGCACGTTTGGTACTGGAAGTGGTCGATGCCGGGATTGAAGAATGGGGTGCCGATCGCATTGGCATTCGC
GTTTCACCAATCGGTACTTTCCAGAACACAGATAACGGCCCGAATGAAGAAGCCGATGCACTGTATCTGATTGAACAACT
GGGTAAACGCGGCATTGCTTATCTGCATATGTCAGAACCAGATTGGGCGGGGGGTGAACCGTATACTGATGCGTTCCGCG
AAAAAGTACGCGCCCGTTTCCACGGTCCGATTATCGGCGCAGGTGCATACACAGTAGAAAAAGCTGAAACGCTGATCGGC
AAAGGGTTAATTGATGCGGTGGCATTTGGTCGTGACTGGATTGCGAACCCGGATCTGGTCGCCCGCTTGCAGCGCAAAGC
TGAGCTTAACCCACAGCGTGCCGAAAGTTTCTACGGTGGCGGCGCGGAAGGCTATACCGATTACCCGACGTTGTAATCCA
ACATTGCGAGCGGCGTAAAGCCGCCGCTATACTAAAACAACATTTTGAATCTGTTAGCCATTTTGAGGATAAAAAGATGC
GTCTTCTTCATACCATGCTGCGCGTTGGCGATTTGCAACGCTCCATCGATTTTTATACCAAAGTGCTGGGCATGAAACTG
CTGCGTACCAGCGAAAACCCGGAATACAAATACTCACTGGCGTTTGTTGGCTACGGCCCGGAAACCGAAGAAGCGGTGAT
TGAACTGACCTACAACTGGGGCGTGGATAAATACGAACTCGGCACTGCTTATGGTCACATCGCGCTTAGCGTAGATAACG
CCGCTGAAGCGTGCGAAAAAATCCGTCAAAACGGGGGTAACGTGACCCGTGAAGCGGGTCCGGTAAAAGGCGGTACTACG
GTTATCGCGTTTGTGGAAGATCCGGACGGTTACAAAATTGAGTTAATCGAAGAGAAAGACGCCGGTCGCGGTCTGGGCAA
CTAATCTCCTGCCGGGCGTGAACTCATCGCGCCCGCATCTTTACTGCATCGACAAGTAATATTTGTCATAATGCGCGCTG
CAATTTATCCGTATTAAGAGAATCAGATGTCCGATAACGCTCAACTTACCGGTCTGTGCGACCGTTTTCGTGGTTTTTAT
CCTGTTGTGATCGATGTTGAAACAGCCGGATTTAACGCCAAAACCGATGCGCTGCTTGAGATTGCCGCCATCACCCTGAA
AATGGATGAACAAGGCTGGCTGATGCCGGACACCACATTACATTTCCACGTCGAACCATTTGTCGGCGCAAATTTGCAAC
CAGAAGCCCTCGCCTTCAACGGCATTGACCCGAACGATCCCGATCGCGGCGCGGTCAGCGAATACGAGGCGCTGCACGAA
ATTTTTAAAGTTGTACGTAAAGGTATTAAAGCGAGCGGCTGTAACCGCGCCATTATGGTGGCGCACAATGCCAATTTTGA
TCACAGCTTTATGATGGCCGCCGCAGAACGCGCCTCACTGAAACGTAACCCGTTCCACCCTTTCGCCACTTTTGACACTG
CTGCACTGGCCGGGCTGGCACTCGGACAAACCGTATTGTCAAAGGCTTGCCAGACCGCTGGCATGGACTTCGACAGCACC
CAGGCGCACTCCGCGCTGTACGACACCGAACGCACTGCTGTGCTGTTTTGTGAAATCGTCAACCGCTGGAAACGTCTGGG
AGGCTGGCCGCTATCTGCCGCCGAAGAGGTGTAATCGAGTCGATGCCTGATGACATGCAATGATTCAGGCATCTATAGTG
AGGCTATTCCACGCATCCTGCATGATATTCACGGGGAATAGCGTTAATGGCAGATAATCCAGACCCTTCATCGCTCCTGC
CGGACGTGTTTTCACCGGCGACCCGCGACTGGTTTCTTCGCGCCTTTAAACAGCCGACCGCTGTCCAGCCGCAAACCTGG

CATGTGGCGGCGCGAAGCGAACATGCGCTGGTGATTGCACCGACCGGCTCCGGGAAAACGCTGGCAGCATTTCTCTACGC
CCTCGATCGGCTCTTCCGCGAAGGCGGCGAAGATACCCGCGAGGCGCATAAGCGTAAAACCTCACGCATCCTCTATATTT
CACCGATAAAAGCCCTGGGCACCGACGTTCAGCGCAACTTGCAGATCCCGTTGAAGGGTATTGCCGATGAACGGCGGCGG
CGCGGCGAAACGGAAGTCAATCTTCGCGTAGGGATCCGTACTGGCGATACGCCTGCACAGGAACGCAGCAAACTCACCCG
TAATCCGCCGGATATTCTGATCACCACACCCGAATCACTCTATCTGATGCTGACCTCCCGCGCGCGCGAAACGCTACGCG
GCGTCGAAACGGTAATTATTGATGAAGTCCACGCGGTAGCGGGCAGTAAACGTGGTGCGCATCTGGCGTTAAGTCTGGAG
CGGCTCGATGCGCTGCTCCACACCTCAGCACAGCGAATTGGCCTTTCTGCCACTGTGCGCTCAGCCAGCGATGTGGCAGC
ATTTCTTGGTGGCGATCGCCCGGTTACGGTAGTCAACCCGCCCGCAATGCGCCATCCGCAGATACGAATTGTCGTACCGG
TCGCCAATATGGATGATGTCTCATCGGTCGCCAGCGGCACCGGCGAAGACAGCCATGCCGGCCGGGAAGGCTCCATCTGG
CCATATATTGAAACGGGTATCCTTGATGAAGTGTTGCGCCATCGCTCGACCATTGTCTTTACTAATTCGCGGGGGCTGGC
GGAAAAACTGACGGCACGATTAAATGAGCTTTACGCCGCACGCTTACAGCGTTCCCCGTCTATCGCCGTTGATGCGGCCC
ATTTCGAGTCGACCTCCGGCGCAACCTCTAACCGTGTACAAAGTAGCGACGTTTTTATTGCCCGCTCACACCACGGCTCC
GTCTCTAAAGAACAACGAGCAATCACCGAACAGGCGCTGAAATCGGGTGAATTACGCTGCGTGGTCGCAACCTCCAGTCT
TGAACTGGGGATTGATATGGGCGCGGTGGATCTGGTGATTCAGGTGGCAACGCCGCTTTCTGTTGCCAGTGGGTTACAAC
GCATTGGTCGCGCCGGACATCAGGTTGGCGGTGTATCTAAAGGGCTGTTTTTCCCCCGTACCCGGCGTGATTTAGTCGAT
TCCGCAGTCATTGTAGAGTGTATGTTCGCAGGCAGGCTGGAAAACCTGACACCACCGCATAATCCTCTCGACGTCCTTGC
GCAGCAAACCGTTGCCGCCGCGGCGATGGATGCATTACAGGTAGACGAATGGTACTCCCGCGTACGCCGTGCCGCACCGT
GGAAAGATCTGCCAAGACGTGTTTTTGACGCCACGCTGGATATGCTTTCCGGGCGCTATCCCTCTGGCGATTTTTCTGCT
TTTCGCCCCAAACTGGTCTGGAACAGGGAGACCGGGATATTGACCGCCCGACCTGGCGCTCAATTGTTGGCGGTTACCAG
CGGCGGCACCATTCCGGATCGTGGCATGTATAGCGTGTTATTACCCGAAGGTGAAGAAAAGGCCGGTTCGCGGCGGGTGG
GTGAACTGGATGAGGAGATGGTATATGAGTCGCGGGTGAACGACATTATCACTCTCGGCGCTACCTCATGGCGGATCCAG
CAAATCACCCGCGATCAGGTGATTGTGACTCCTGCTCCGGGTCGTTCTGCCCGGCTCCCCTTCTGGCGTGGTGAAGGTAA
CGGACGTCCGGCTGAATTAGGCGAGATGATCGGCGATTTTCTTCATTTGCTGGCGGATGGCGCGTTCTTTTCCGGGACTA
TTCCCCCGTGGCTGGCAGAAGAAAATACGATCGCCAATATTCAGGGGTTGATTGAGGAGCAGCGCAACGCGACGGGCATC
GTTCCGGGGAGTCGCCATCTGGTCCTCGAACGGTGCCGTGATGAAATTGGTGACTGGCGTATTATTTTGCACTCTCCCTA
TGGAAGACGGGTGCATGAACCCTGGGCGGTGGCGATTGCCGGGCGAATACATGCGCTATGGGGCGCTGACGCGTCGGTGG
TCGCCAGTGATGACGGCATTGTTGCACGTATTCCTGACACCGATGGTAAATTGCCCGATGCCGCGATTTTTTTGTTTGAA
CCAGAAAAGTTGCTGCAAATTGTCCGCGAGGCGGTAGGCAGCTCGGCACTTTTCGCCGCCCGTTTTCGCGAATGCGCCGC
GCGGGCATTATTAATGCCGGGGCGCACTCCGGGCCATCGCACCCCGCTTTGGCAACAACGGCTGCGCGCCAGTCAGTTGC
TGGAAATCGCTCAGGGATATCCGGATTTTCCGGTCATTCTCGAAACCCTACGCGAATGTCTGCAAGATGTTTATGATCTT
CCCGCACTGGAACGTTTGATGCGTCGCCTGAACGGTGGCGAAATTCAAATATCCGATGTAACGACCACTACGCCCTCGCC
TTTCGCCACAAGTTTATTGTTCGGCTATGTCGCGGAATTTATGTACCAGAGCGACGCCCCGCTGGCAGAGCGCCGGGCAT
CCGTACTGTCGCTGGACAGCGAGTTACTGCGCAATCTACTCGGACAGGTCGATCCGGGGGAATTACTCGACCCGCAGGTC
ATTCGCCAGGTGGAAGAAGAGTTGCAACGACTGGCTCCTGGCAGAAGAGCGAAAGGTGAAGAAGGATTGTTCGACCTGCT
GCGCGAACTGGGGCCAATGACCGTTGAAGACCTGGCGCAACGGCATACAGGCAGCAGTGAAGAGGTTGCGTCGTATCTGG
AAAATCTTCTTGCAGTAAAACGAATCTTCCCAGCGATGATTAGCGGACAGGAGCGTCTTGCCTGTATGGATGATGCCGCC
AGGCTGCGTGATGCCCTCGGCGTACGACTACCAGAGTCATTGCCAGAGATTTATTTACATAGAGTCAGTTACCCGCTTCG
CGACCTCTTTCTGCGCTATCTCCGGGCTCATGCTCTGGTCACGGCTGAACAACTGGCTCATGAGTTTAGTCTCGGTATTG
CCATTGTCGAAGAGCAGCTTCAGCAACTGCGTGAACAGGGTCTGGTGATGAATCTGCAACAAGACATCTGGGTGAGCGAT
GAAGTATTTCGTCGTCTGCGTTTGCGCTCGCTGCAAGCCGCCAGAGAAGCGACGCGTCCCGTTGCAGCCACGACCTATGC
GCGATTGCTGCTGGAACGTCAGGGCGTATTACCCGCCACCGATGGTAGCCCGGCGCTCTTTGCCTCAACATCGCCAGGCG
TTTATGAGGGCGTAGATGGCGTGATGCGGGTGATCGAACAGCTTGCCGGAGTCGGTTTACCCGCCTCACTCTGGGAAAGC
CAGATCCTGCCTGCCCGCGTACGCGACTATTCGTCAGAAATGCTCGATGAATTACTGGCAACCGGTGCGGTTATCTGGTC
GGGGCAAAAAAAACTGGGTGAAGATGACGGCCTGGTGGCACTGCATCTACAGGAATATGCTGCAGAATCGTTCACTCCCG
CCGAAGCGGATCAGGCGAATCGTTCGGCGCTGCAACAAGCGATAGTCGCTGTTCTGGCTGACGGAGGAGCCTGGTTTGCA
CAACAAATCAGCCAACGGATACGCGACAAAATCGGCGAATCGGTTGATCTCTCTGCCCTGCAAGAGGCGTTATGGGCGCT
GGTCTGGCAAGGCGTCATCACCAGCGACATTTGGGCACCGTTACGCGCCCTCACCCGCAGCAGTTCCAACGCACGCACCT
CAACTCGCCGCAGTCACCGGGCTCGTCGTGGACGTCCTGTCTATGCGCAACCCGTCTCGCCGCGGGTATCTTACAACACA
CCAAATCTGGCTGGACGCTGGTCGTTATTGCAGGTGGAGCCACTAAACGATACCGAAAGGATGCTGGCGCTGGCGGAAAA
TATGCTCGACCGCTACGGCATCATCAGTCGTCAGGCGGTGATAGCCGAAAATATCCCTGGCGGGTTTCCATCGATGCAAA
CGCTTTGTCGAAGTATGGAAGACTCCGGGCGAATTATGCGAGGTCGTTTTGTAGAAGGTCTGGGTGGCGCGCAATTCGCT
GAACGTCTGACTATTGACCGATTGCGCGATCTGGCGACACAAGCCACGCAAACGCGCCACTATACACCAGTGGCGCTCTC
TGCCAACGATCCGGCTAATGTGTGGGGAAATCTTCTGCCCTGGCCTGCACATCCGGCAACGCTGGTTCCAACGCGTCGGG
CGGGTGCGCTGGTGGTCGTTTCTGGCGGCAAATTGTTACTCTATCTGGCGCAAGGTGGCAAAAAAATGCTGGTCTGGCAG
GAAAAAGAGGAATTACTCGCCCCAGAGGTTTTCCACGCGCTGACTACCGCACTGCGTCGCGAACCACGGCTGCGCTTTAC
GCTAACAGAAGTGAATGATCTACCGGTCCGGCAAACGCCGATGTTTACGCTGCTGCGTGAGGCGGGATTTTCAAGTTCGC

CACAAGGGCTGGATTGGGGATAGAGAAAGGACTGACGGATGCCCGTTCGCATCCGTCAGTATTGCAGGACGGATTATTCC
GCGTCCGGCTCTTCAGACTTGTATTTAGCGGCAGTTTCTTTGATCAGCTGCTGCAGTTCGCCACGCTGATACATTTCGAT
CACGATATCACAACCGCCGACCAGCTCGCCGTCAACCCACAGTTGCGGGAAGGTCGGCCAGTTAGCATATTTCGGCAGTT
CCGCACGAATGTCCGGATTCTGCAGAATATCAACATAGGCAAAACGTTCGCCACATGCGGCAAGCGCCTGGACTGCCTGG
GCAGAGAAACCGCAGCTCGGCAGTTTCGGTGAACCTTTCATGTACAGCAGGATCGGGTTTTCAGCAATCTGGCGTTGGAT
TTTTTCGATAGTGGTGCTCATTGTCTTGCTTCCTCAAACTTCTTTACGGCAGTAATCTGACATTGTAGCGGGTCAGTGCG
GCATCGGAAAATAACATTTTCATCACGCTTTTGCTATTTTATCCCTTTGCTCTATTTGTTGCATTTCAAATATTCGTTTC
TTCTCTGATGCCGGCAAACTGGCGTATTTATAACCATTTTTAGCTTTCACTGCTGCATTTTTTTGCGCTCGCCAACGAAA
CGTATTTTTTAACAATAAAAGCTATTAACTTTCTCTTCTTCTATGCATTAGAATCATCAAGTTTTGTAAATCAGACGCAG
GCATGATAGACCTGCCTTTACAGAGGGACGCTCAGTGGCGCGGATAAACCGTATTTCGATCACGCTCTGTGCTTTACTTT
TTACCACCCTGCCTTTAACGCCTATGGCCCATGCTTCAAAGCAAGCCAGGGAGAGTTCTGCTACCACTCATATCACCAAA
AAAGCAGATAAAAAGAAAAGCACGGCAACCACCAAAAAGACCCAGAAAACAGCGAAAAAAGCCGCCAGTAAAAGTACGAC
CAAAAGCAAAACCGCTTCTTCCGTTAAAAAATCTTCCATTACCGCTTCTAAAAACGCCAAAACTCGCAGCAAACACGCCG
TCAATAAAACGGCCTCAGCCAGCTTTACCGAAAAGTGTACCAAACGTAAGGGCTATAAATCGCATTGTGTGAAAGTCAAA
AATGCCGCGTCAGGAACTCTTGCCGACGCGCACAAAGCGAAGGTGCAAAAAGCTACGAAAGTGGCAATGAATAAACTGAT
GCAGCAAATTGGTAAGCCATATCGTTGGGGTGGCAGCTCACCGCGTACCGGTTTTGATTGCAGCGGCCTGGTTTATTACG
CTTATAAAGATTTGGTGAAAATTCGTATTCCGCGTACGGCGAATGAAATGTATCACCTGCGTGATGCAGCGCCAATCGAA
CGTAGTGAACTGAAAAACGGCGACCTGGTCTTTTTCCGTACTCAGGGACGCGGCACAGCCGATCATGTCGGCGTGTATGT
CGGCAACGGCAAATTTATTCAGTCACCGCGCACAGGTCAGGAAATTCAAATCACTTCTCTCAGTGAAGACTACTGGCAGC
GCCACTATGTTGGCGCTCGTCGGGTAATGACCCCAAAAACACTTCGCTAAAACTTTACCCTGTTGTTACGGCAACAGGGT
AAGTTCATCTTTTGTCTCACCTTTTAATTTGCTACCCTATCCATACGCACAATAAGGCTATTGTACGTATGCAAATTAAT
AATAAAGGAGAGTAGCAATGTCATTCGAATTACCTGCACTACCATATGCTAAAGATGCTCTGGCACCGCACATTTCTGCG
GAAACCATCGAGTATCACTACGGCAAGCACCATCAGACTTATGTCACTAACCTGAACAACCTGATTAAAGGTACCGCGTT
TGAAGGTAAATCACTGGAAGAGATTATTCGCAGCTCTGAAGGTGGCGTATTCAACAACGCAGCTCAGGTCTGGAACCATA
CTTTCTACTGGAACTGCCTGGCACCGAACGCCGGTGGCGAACCGACTGGAAAAGTCGCTGAAGCTATCGCCGCATCTTTT
GGCAGCTTTGCCGATTTCAAAGCGCAGTTTACTGATGCAGCGATCAAAAACTTTGGTTCTGGCTGGACCTGGCTGGTGAA
AAACAGCGATGGCAAACTGGCTATCGTTTCAACCTCTAACGCGGGTACTCCGCTGACCACCGATGCGACTCCGCTGCTGA
CCGTTGATGTCTGGGAACACGCTTATTACATCGACTATCGCAATGCACGTCCTGGCTATCTGGAGCACTTCTGGGCGCTG
GTGAACTGGGAATTCGTAGCGAAAAATCTCGCTGCATAATAACTGATGGCAAATGCAGCATTGCCTGAAGCGCTACGCTT
ATCAGGCCTACGCGGATCATCGATGTAGGTCGGATAAGGCACTCGCCGCATCCGGCAAGATAAATCGCACGTTGTCAGCA
ACTGTAACGCAGAAGGTTATCCTTCTGCGTTTTTGTTTAATTAGCTGTTAGCAACGCAAACTGTTTCAGGTTGTTTTCTG
GCTGACATAAACACCAGCAATAATGCCAGTCCCGCGACAATCGCTCCCATCACCGGCACAAAGCTGTATCCCAGCCCAGC
GGAAATTACCGCACCACCAGCAGCTGCTCCCAGCGCATTTCCAAGATTAAAGGCACCAATATTGACTGATGAAGACAGAC
CTGGCGCTTCACTGGCGACACGCATCACGCGCATCTGTAACGGCGGTACGACCGCAAAGGTTGCTGCGCCCCACACCACC
ATGCTAATAGCTGCGCCGAACTCATTGCGGGCCAGGAACGGGATTGCCAGCATAATCACCATCAACAACAACAAAAAGCC
TTTCAACGTGCCGTTAACTGAACGATCTGCCAGTTTGCCGCCGAGATAGTTACCGATAGAGAATCCGACACCAATCAGCA
CCAGCATTGCCGTGACGAACACCGGTGTTGCGTGGGTAATACTTTGCAGTACCGGAGAGATATAGGTGTAGAGAGTAAAC
ATTGCACCAGCTCCCAGTACCGTCGTCAGCAATGCAGACAGCACCTGCGGACGCATTAATACCGCCAGCTCTTTTTTCAC
TTCAGGTCGTGCCCCTGCACCACCTTTAGGTAATGAGAAGAACAGACTTACCATTGAAATCACTCCCAGCCCCGCCGTTG
CCAGAAATGACATCCGCCAGCCGATGGTTTCACCCAACCAGGTCGCCGCCGGCACGCCACCGATATTTGCCAGGGTTAAC
CCCATAAACATAGTGGCAACTGCGCTGGCCTGTTTATGTTTTGGCACCACGCTTGCGGCCACGACTGAACCCAAACCAAA
AAATGCTCCGTGATTCAGGCTGGTCAAAATGCGTGAAAGCATCAGGGTCATATAATCCGGCGCGATGGCGGAAAGTACGT
TGCCGAGCGTGAAAATTGCCATCAGGAAAATCAACGCACTGCGGCGGGCACGATGAGAAAGTAGAAGCGTCATCAGCGGC
GCGCCAACCATTACGCCAACTGCATAGGCACTGATTAACATTCCGGCAGCGGGAATCGAGACATCCACACCGCGCGCAAT
GACGGGCAACAAGCCCATTGGCGAGAACTCCGTTGTCCCGATACCAAACGCGCCAATCGCCAGCGCCAGCAACGGATAGT
TAATTTTCATGCCTTATCTCCACCTCTTCGCGTCATTACGCGATATTCATTAAAGTGGCGAAAGCATGACAGCAATCACA
AAAAAATGAAAATAACAAAAAGAGAAAACACTTTTGCCATTTTGCTAACAAACAGGAAGGAGATGCGAGGGAGAACGCGC
TCCCTCGAGAGGAAATCAGTGCAGCGCGGCAGTCAAACCCACGGCTACGATCAAACCGAGGACGATAATCGTTGTTACCA
GTGAAAATTTAAGGTCGGTGCTCATCAAGTTTTCTCCTTTTTTATTACCACACAAAAAGTGATATTACGCATTTTTACAC
ACTGTGATGAAAAAATCTCCCGTCATTTATAATGATAAGTGTTTTTACCACTTCCCCTTTTCGTCAAGATCGGCCAAAAT
TCCACGCTTACACTATTTGCGTACTGGCCATTGACCCCTTCCTGACGCTCCGTGTCGTTTTTCCGGCGTACCGCAACACT
TTTGTTGTGCGTAAGGTGTGTAAAGGCAAACGTTTACCTTGCGATTTTGCAGGAGCTGAAGTTAGGGTCTGGAGTGAAAT
GGAATGGCAACAATAAAAGATGTAGCGAAACGAGCAAACGTTTCCACTACAACTGTGTCACACGTGATCAACAAAACACG
TTTCGTCGCTGAAGAAACGCGCAACGCCGTGTGGGCAGCGATTAAAGAATTACACTACTCCCCTAGCGCGGTGGCGCGTA
GCCTGAAGGTTAACCACACCAAGTCTATCGGTTTGCTGGCGACCAGCAGCGAAGCGGCCTATTTTGCCGAGATCATTGAA
GCAGTTGAAAAAAATTGCTTCCAGAAAGGTTACACCCTGATTCTGGGCAATGCGTGGAACAATCTTGAGAAACAGCGGGC

TTATCTGTCGATGATGGCGCAAAAACGCGTCGATGGTCTGCTGGTGATGTGTTCTGAGTACCCAGAGCCGTTGCTGGCGA
TGCTGGAAGAGTATCGCCATATCCCAATGGTGGTCATGGACTGGGGTGAAGCAAAAGCTGACTTCACCGATGCGGTCATT
GATAACGCGTTCGAAGGCGGCTACATGGCCGGGCGTTATCTGATTGAACGCGGTCACCGCGAAATCGGCGTCATCCCCGG
CCCGCTGGAACGTAACACCGGCGCAGGCCGCCTTGCCGGTTTTATGAAGGCGATGGAAGAAGCGATGATCAAGGTGCCGG
AAAGCTGGATTGTGCAGGGTGACTTTGAACCTGAATCCGGTTATCGCGCCATGCAGCAAATCCTGTCGCAGCCGCATCGC
CCTACTGCCGTCTTCTGTGGTGGCGATATCATGGCAATGGGCGCACTTTGTGCTGCTGATGAAATGGGCCTGCGCGTCCC
GCAGGATGTTTCGCTGATCGGTTATGATAACGTGCGCAACGCGCGCTATTTTACGCCGGCGCTGACCACGATCCATCAGC
CAAAAGATTCGCTGGGTGAAACAGCGTTCAACATGCTGTTGGATCGTATCGTCAACAAACGTGAAGAACCGCAGTCTATT
GAAGTGCATCCGCGCTTGATTGAACGCCGCTCCGTGGCTGACGGCCCGTTCCGCGACTATCGTCGTTAATCACCCGTTGC
GGGAGTCTCTTCCGGCTCCCGCAGCCACTCCTTATTCAGCGTCTCACTATCGCCGAGATACTCAAGCAACCAGGTTAACG
CAGGCGACATATCATTTTGCTGCCATGTCAGACAACATGCCGAATCCGGAAAGGGGTTTTCCAGTTCTAATGCTACCCAC
TTCCCCTCATTAAGCCACGGTTTGGCGAAATGTGTTGGCACCATCCCTATGCATAATCCTGCCGAGATACAGGTTGCCGA
TGATTCCCAGTCAGGCACGACAACTCTTTTTTGGTTATCCAGCAACCAGGTAATACGTTTAGGTAGCGTTCGCGAGGTGT
CTTCGCGCACCAACGACGGCCAGTTGCGCAACGTATCATCGCTGAACGGGCCATCCATCAACGCCAGCGGGTGGTGACTG
GCAACAACGCAACTCCAGCTTAGCATCCCCATATCCCGGAAGGCATAACGACCGCCTACCGGAATCGCGCGTGTTGCGCC
AATCGCCAGTTCCACGCGCCCGTCGGAAAGCGCATCCCAGACACCGTTGAACACTTCCTGAAAGACAAGAAGTTCGACAT
CATCAAAATGGCGATAAAAATCAACGATCATCTGCCGTGTACGTTCTGGCCTGACAATATTATCCACTGCGATAGCTAAC
TGACCGCGCCAGCCGTTCGCTATCTGCTGACATTGCTGGCGGGTGATCTGCATTTTTTTGACAACAGAGCGCCCTTCTTT
GAGAAACCACGCTCCAGCAGCGGTCAGCTCCACATCACGGTGCCGTCGTTCAAAGAGCGGCACCGCCAGCCACTCTTCCA
GCTGACGCACGGTATAGCTGACCGCAGAAGGAACGCGATGCAGCTCCTGTGCCGCAGCGCTAAAACTACCATTACGCGCT
ACCGCATCAACAACTTCGAGTGAATATTCTGACCACATAGTCTGCCTGCAAAATTTTTGAAACCAGTCATCAAATATTAC
CGTTTCACAACACTAATTTCACTCCCTACACTTTGCGGCGGTGTTTAATTGAGAGATTTAGAGAATATACATGCAACCTG
GGAAAAGATTTTTAGTCTGGCTGGCGGGTTTGAGCGTACTCGGTTTTCTGGCAACCGATATGTATCTGCCTGCTTTCGCC
GCCATACAGGCCGACCTGCAAACGCCTGCGTCTGCTGTCAGTGCCAGCCTTAGTCTGTTCCTTGCCGGTTTTGCCGCAGC
CCAGCTTCTGTGGGGGCCGCTCTCCGACCGTTATGGTCGTAAACCGGTATTATTAATCGGCCTGACAATTTTTGCGTTAG
GTAGTCTGGGGATGCTGTGGGTAGAAAACGCCGCTACGCTGCTGGTATTGCGTTTTGTACAGGCTGTGGGTGTCTGCGCC
GCGGCGGTTATCTGGCAAGCATTAGTGACAGATTATTATCCTTCACAGAAAGTTAACCGTATTTTTGCGGCCATCATGCC
GCTGGTGGGTCTATCTCCGGCACTGGCTCCTCTGTTAGGAAGCTGGCTGCTGGTCCATTTTTCCTGGCAGGCGATTTTCG
CCACCCTGTTTGCCATTACCGTGGTGCTGATTCTGCCTATTTTCTGGCTCAAACCCACGACGAAGGCCCGTAACAATAGT
CAGGATGGTCTGACCTTTACCGACCTGCTACGTTCTAAAACCTATCGCGGCAACGTGCTGATATACGCAGCCTGTTCAGC
CAGTTTTTTTGCATGGCTGACCGGTTCACCGTTCATCCTTAGTGAAATGGGCTACAGCCCGGCAGTTATTGGTTTAAGTT
ATGTCCCGCAAACTATCGCGTTTCTGATTGGTGGTTATGGCTGTCGCGCCGCGCTGCAGAAATGGCAAGGCAAGCAGTTA
TTACCGTGGTTGCTGGTGCTGTTTGCTGTCAGCGTCATTGCGACCTGGGCTGCGGGCTTCATTAGCCATGTGTCGCTGGT
CGAAATCCTGATCCCATTCTGTGTGATGGCGATTGCCAATGGCGCGATCTACCCTATTGTTGTCGCCCAGGCGCTGCGTC
CCTTCCCACACGCAACTGGTCGCGCCGCAGCGTTGCAGAACACTCTTCAACTGGGTCTGTGCTTCCTCGCAAGTCTGGTA
GTTTCCTGGCTGATCAGTATCAGCACGCCATTGCTCACCACCACCAGCGTGATGTTATCAACAGTAGTGCTGGTCGCGCT
GGGTTACATGATGCAACGTTGTGAAGAAGTTGGCTGCCAGAATCATGGCAATGCCGAAGTCGCTCATAGCGAATCACACT
GATCTATATCGATATACTTATACTTAGGCTGCTAACAAAATTTTGTTGTATGATTGAAATTAGCGGCCTATACTAATTTC
GAGTTGTTAAAGCTACGATAAATATTATGTTTTTACGGGGACAGGATCGTTCCCGACTCACTATGGATAGTCATTTCGGC
AAGGGTTCCTCCTTTCCCTCTGTTCTACGTCGGATTATAGACTCGCGGTTTTTTCTGCGAGATTTCTCACAAAGCCCAAA
AAGCGTCTACGCTGTTTTAAGGTTCTGATCACCGACCAGTGATGGAGAAACTATGAGTTCATCGTGTATAGAAGAAGTCA
GTGTACCGGATGACAACTGGTACCGTATCGCCAACGAATTACTTAGCCGTGCCGGTATAGCCATTAACGGTTCTGCCCCG
GCGGATATTCGTGTGAAAAACCCCGATTTTTTTAAACGCGTTCTGCAAGAAGGCTCTTTGGGGTTAGGCGAAAGTTATAT
GGATGGCTGGTGGGAATGTGACCGACTGGATATGTTTTTTAGCAAAGTCTTACGCGCAGGTCTCGAGAACCAACTCCCCC
ATCATTTCAAAGACACGCTGCGTATTGCCGGCGCTCGTCTCTTCAATCTGCAGAGTAAAAAACGTGCCTGGATAGTCGGC
AAAGAGCATTACGATTTGGGTAATGACTTGTTCAGCCGCATGCTTGATCCCTTCATGCAATATTCCTGCGCTTACTGGAA
AGATGCCGATAATCTGGAATCTGCCCAGCAGGCGAAGCTCAAAATGATTTGTGAAAAATTGCAGTTAAAACCAGGGATGC
GCGTACTGGATATTGGCTGCGGCTGGGGCGGACTGGCACACTACATGGCATCTAATTATGACGTAAGCGTGGTGGGCGTC
ACCATTTCTGCCGAACAGCAAAAAATGGCTCAGGAACGCTGTGAAGGCCTGGATGTCACCATTTTGCTGCAAGATTATCG
TGACCTGAACGACCAGTTTGATCGTATTGTTTCTGTGGGGATGTTCGAGCACGTCGGACCGAAAAATTACGATACCTATT
TTGCGGTGGTGGATCGTAATTTGAAACCGGAAGGCATATTCCTGCTCCATACTATCGGTTCGAAAAAAACCGATCTGAAT
GTTGATCCCTGGATTAATAAATATATTTTTCCGAACGGTTGCCTGCCCTCTGTACGCCAGATTGCTCAGTCCAGCGAACC
CCACTTTGTGATGGAAGACTGGCATAACTTCGGTGCTGATTACGATACTACGTTGATGGCGTGGTATGAACGATTCCTCG
CCGCATGGCCAGAAATTGCGGATAACTATAGTGAACGCTTTAAACGAATGTTTACCTATTATCTGAATGCCTGTGCAGGT
GCTTTCCGCGCCCGTGATATTCAGCTCTGGCAGGTCGTGTTCTCACGCGGTGTTGAAAACGGCCTTCGAGTGGCTCGCTA
AAGGCTATTCTATCGCCCCCTCTCCGGGGGCGATTTCAGATCAGGCTTCTGTGCCTGGTTGATTCATGGCATTTTCTCGT

GCCGCCAGCACACGTTCTACCGTATCTACCACTGCCTGAGTTTGTGGATCGATTTCAATGTTGACGCGTGCGCCAAGTTT
TTTCTTCCCAAGAGTCGTGCGTTCCAGTGTTTCCGGAATTAAATGGACGCAAAAACGCGTTGGCGTGACTTCGCCGACGG
TCAGGCTAATACCGTCGATGCCAATAAATCCTTTGTACAGAATATATTTCATCAACTGACTATCCTGGACTTTAAACCAG
ATCTGGCGATTATTTTCTGAGGTTAATATTTTCGCCACTTCAGCAGTGGTCATAATATGACCTGACATTAAGTGTCCGCC
AATTTCATCACTGAATTTCGCCGCACGCTCAACGTTTACCCAATCCCCCACTTTTAAATCGCCAAGATTGGTAATGCGTA
ACGTTTCTTTCATCAGGTCAAAACTGACATGGTTGCCGTTAATTTCCGTCACGGTCAGGCAGCAACCGTTATGCGCCACG
GAAGCACCGGTTTCCAGGCCGTCCAGCATGTGGTCGGGTAACTCCACCACATGCGTACGAAAATTTGGTTTCTCGTCAAT
CGACACCAGTTTTGCGGTGCCCTGTACAATCCCCGTAAACATACTTACAACTCCTGAAATCAGTTAAGACATTCTGTTCA
GCACAATAGCAGGTGGAAAACGCCCTTACCAGTGAAGGGGTAAGAATGGCTATTTTTTCACTGGAGAATTAATAAATCCT
CGCTACAATAGACTGAATTTCCCCTGCTTCTTCTTTTTGCTGCCCATTCAGGCGGCTTTTTAGTCTCTCATATAACTACA
AATAAAAGGTGTTCACGTGCAGAAGTATATCAGTGAAGCGCGTCTGTTATTAGCATTAGCAATCCCGGTGATTCTCGCGC
AAATCGCCCAAACTGCGATGGGTTTTGTCGATACCGTGATGGCGGGCGGCTATAGTGCCACCGACATGGCGGCGGTCGCT
ATCGGTACTTCTATCTGGCTTCCGGCGATCCTCTTTGGTCACGGACTGCTGCTGGCATTAACGCCGGTTATCGCGCAATT
AAATGGTTCCGGTCGACGTGAGCGCATTGCGCATCAGGTGCGACAAGGTTTCTGGCTGGCAGGTTTTGTTTCCGTTCTCA
TTATGCTGGTGCTGTGGAATGCAGGTTACATTATCCGCTCCATGGAAAACATCGATCCGGCTCTGGCGGACAAAGCCGTG
GGTTATCTGCGTGCGTTGTTGTGGGGCGCGCCGGGATATCTGTTCTTCCAGGTTGCCCGTAACCAGTGTGAAGGTCTGGC
AAAAACCAAGCCGGGTATGGTAATGGGCTTTATCGGCCTGCTGGTGAACATCCCGGTGAACTATATCTTTATTTATGGTC
ATTTCGGTATGCCTGAGCTCGGTGGCGTTGGTTGTGGCGTGGCTACTGCGGCGGTGTATTGGGTCATGTTCCTTGCCATG
GTTTCTTACATTAAACGCGCCCGCTCCATGCGCGATATTCGTAACGAAAAAGGCACCGCAAAACCCGATCCTGCGGTTAT
GAAACGACTGATTCAACTCGGTTTGCCGATTGCGCTGGCACTGTTCTTTGAAGTGACACTGTTTGCCGTCGTGGCTCTGT
TAGTGTCTCCGCTCGGTATTGTTGATGTCGCAGGACACCAGATTGCCCTGAACTTTAGTTCACTAATGTTCGTGCTTCCA
ATGTCGCTGGCGGCAGCGGTAACTATCCGCGTAGGTTATCGTCTGGGTCAGGGCTCAACGCTGGATGCGCAAACCGCTGC
GCGGACCGGGCTTATGGTGGGTGTCTGTATGGCAACCCTGACGGCCATTTTCACGGTTTCACTGCGGGAGCAAATCGCCC
TGTTGTACAACGACAATCCCGAGGTTGTAACGCTGGCTGCGCATTTGATGTTGCTGGCGGCGGTATATCAGATTTCTGAC
TCAATCCAGGTGATTGGCAGTGGGATTTTGCGTGGTTATAAAGATACGCGTTCCATTTTCTATATTACCTTTACGGCTTA
CTGGGTGCTGGGCTTGCCAAGCGGCTATATTCTGGCACTGACCGATCTGGTCGTTGAACCTATGGGGCCAGCAGGCTTCT
GGATAGGCTTTATTATTGGCCTGACGTCGGCAGCCATTATGATGATGTTGCGTATGCGGTTCCTGCAACGTCTGCCGTCA
GCCATCATTCTGCAACGAGCATCCCGCTAATAAAGACAAGGCGCAACCTTCACGGGTTGCGCCTGTATTTTTACGCAGGC
TGGAGCGTTGCGCCAATCCCGTCTTCGTCTGGCTGTAATTTCAGAGCGTTACACAGAGTTTGCCGACTGGCATCTGTTAT
CTCAACGTGTATATCCCCGGTAACTTCCCCTTTCACATTGATGAAGTCAGGTGAAATTTCTTTTGCACTGGTTTGCAGGC
AAATCGCTCCCTCTCCGCGCAGGCGTTTCACTTTCAGTTGCACATGCGTTGCCGCATCAGTGATCAGCCACAGTTTTCCA
CCATTCAGATTGATGTTACCAACAATGAAAGTGACACTGTCACCTTTTACCGTACTGCCGTCTAGAATCAACACACCGCC
CTGCTGTACAGTAATCGTTGATTGTTGCAGCTCTGCGCCATAACCAATAATCATTGAAGCGCCGTCCAGTAATGCAACAC
TCCCGGTCAGTTCAACCGGTGCGCGATCTTCCTCTACGACAAAATTTTCGTCCGCGCTGGTGGGTGACAACAGGATCACT
GTCCCACCTTGTATTACCGCGTTTTCCAGCGTACCGGCATGCACTTCAGCCCGACCACCCACATTCACGGACAGGTTTTG
CGTCTTACCGGAACTGTAGAGCTGAAGGCCATCCGTCCCCAGACGATAAATGGATCCATTCTCAACAATAGTGTCAGAGT
CTTCACCACCGAGATTATTCATGGTGCCACCAGCAAGCAACGTATCACTGGCTTTCCCGGCCAGCATAAAAACGCCGCCC
TGATTAATCTCACAATTGCTGGCTTCCCCACCTGGATACACCAACAATTCACCACCTTCATCTACCACGACCGCTGAGGT
TGTCCCATTAACCAACAGGCCGCCCTCTTGATCGAGAATGATTTTTTCCGCGCGATGTCCTTCCAGTACACGCAGGTTAC
CGCCATTTTCCAGCAACAAACCGCAGGCATAACCTTTATCAACGCTGAATTCACCTTCGGGATGGCGGCCATTAACCGTA
GCGAGCGTAGAGAGACTAATTGCGCCTCCCTCGGACTGAACAATATCTGTGGCAATGGCACCATCGCTGACCGATAGCGT
GCCATTGTTACCGTACTGTGTGCATTTGGCGATCCCACCGGTTGCCACTTTCAACCAGCCAGCCCCCGTGACCACGGTAT
TTTCGGCTCTTCCGCCAGCTTCAACAATCAACTCGCCTTCAGTAAGTTGCGTCGCGCCAATGACCTCGCCGGTCTTAACA
ATTTGCACGTTCCCGTCGCTCATCAAGTTTTTCGCATCAGATCCCATGATTTATTCCTTTGCTGCATCTGTGTGCCTTTA
TTGCTACCTAAGTGTAAAGGCTACGGAGGATTTATCCACGACAGATTTGAGATGGTGGCAAACAACTCTGTTTAAACTCT
GATACACGAATTATTGGGTTGTATCAGATGTAAATGCGATCCTGAATAAAAATCACCCTTGCAAATCAACAAAATATAGC
CAATTTGCTTAAAAGCTCTGCAAACGGTCGAAATAGCGCAGAAAATTACGTTTTGCCTCTTGCCACCTTCCCACTCTGCC
GCTAATATTCGTCCCCGTTGTCACCTACAACGTTGCGTTCATAGCTCAGTTGGTTAGAGCACCACCTTGACATGGTGGGG
GTCGTTGGTTCGAGTCCAATTGAACGCACCATCCTGCGTCCGTAGCTCAGTTGGTTAGAGCACCACCTTGACATGGTGGG
GGTCGGTGGTTCGAGTCCACTCGGACGCACCAGATTTTCTTAATCTGGTCTTCTCCTTTTTCCCTCTGTTTCTTCTCTGT
ATCCAATACGTTAAAAGATTTACACTGTCTTCGTATGCGTTATCAGAAGGAGAATCGCTATGGCAACTTTGTTACAACTT
CATTTTGCTTTTAATGGCCCATTTGGCGACGCAATGGCTGAGCAGCTTAAACCACTTGCTGAGTCGATTAATCAGGAACC
TGGTTTTCTGTGGAAGGTATGGACAGAAAGTGAAAAGAACCACGAAGCCGGTGGGATCTACCTTTTCACTGATGAAAAAA
GCGCCCTTGCCTATCTGGAAAAACATACTGCCCGACTGAAAAATCTCGGCGTTGAGGAAGTTGTCGCCAAAGTTTTCGAT
GTCAATGAGCCACTTAGTCAAATCAATCAGGCAAAACTCGCCTGACAGAATTTAATCAAGGGCGGTTAGCGCCCTTTTCA
TCCCTGTCTGAAATTTCTCAAATTCTAAAAATCTCAACCAAACTTATCTGATAACACTAAATTCGAAAGAATGCGTACAG

GTAAGTAACAATGAAAAAAATTGCTATTGTGGGTGCCGGGCCTACGGGGATCTACACCTTATTCTCGCTTCTACAGCAAC
AAACTCCACTTTCTATTTCTATCTTCGAGCAGGCTGACGAGGCCGGTGTCGGGATGCCATACAGTGATGAGGAAAACTCA
AAAATGATGCTGGCAAATATTGCCAGTATTGAAATACCGCCGATTTATTGTACGTATCTCGAATGGCTACAAAAGCAAGA
AGACAGCCATCTCCAGCGTTATGGCGTTAAAAAAGAAACCTTGCACGATCGTCAGTTTTTACCGCGAATTCTGCTGGGCG
AATATTTCCGCGATCAATTTTTACGACTAGTAGACCAGGCACGACAGCAAAAATTTGCAGTGGCTGTTTATGAATCATGC
CAGGTTACCGATCTGCAAATTACAAATGCTGGCGTCATGCTCGCTACAAATCAGGATTTACCCAGCGAGACGTTTGATTT
AGCGGTGATCGCCACGGGTCACGTCTGGCCTGATGAAGAAGAAGCAACCCGAACGTATTTTCCCAGCCCGTGGTCAGGCC
TGATGGAAGCAAAGGTCGATGCGTGTAACGTGGGTATTATGGGAACATCCTTGAGCGGACTGGATGCGGCAATGGCAGTG
GCTATTCAGCATGGTTCGTTCATTGAAGATGATAAACAACACGTCGTTTTTCACCGCGATAACGCAAGTGAAAAGCTAAA
TATCACGTTGTTGTCGCGCACGGGTATTTTACCCGAAGCCGATTTCTATTGCCCTATTCCCTACGAGCCCTTACACATCG
TCACCGATCAGGCATTAAATGCTGAGATTCAAAAAGGCGAAGAGGGCCTTTTGGATCGGGTATTTAGATTGATAGTAGAG
GAAATCAAGTTTGCTGATCCAGACTGGAGTCAACGCATAGCCTTAGAGAGCCTGAATGTCGATTCCTTTGCTCAAGCCTG
GTTTGCCGAGCGCAAACAACGCGACCCATTTGACTGGGCAGAAAAAAATCTCCAGGAAGTCGAACGCAATAAACGAGAAA
AACATACTGTTCCCTGGCGTTATGTCATTCTGCGCCTGCATGAAGCCGTACAGGAAATTGTTCCACATCTGAATGAACAC
GACCATAAACGGTTCAGTAAAGGCCTTGCCCGGGTTTTCATCGATAATTATGCGGCAATCCCTTCAGAGTCTATTCGTCG
CCTACTTGCCTTACGTGAAGCGGGAATCATTCATATTCTCGCCCTCGGTGAAGACTACAAAATGGAAATTAATGAGTCGC
GCACCGTCCTGAAAACGGAAGACAACAGCTACTCGTTTGACGTTTTTATTGATGCCCGCGGACAACGTCCGCTTAAAGTG
AAAGATATCCCTTTCCCTGGGCTACGCGAGCAATTACAGAAAACAGGGGATGAAATCCCTGATGTTGGCGAAGATTATAC
GTTACAGCAACCCGAAGATATTCGTGGGCGCGTAGCGTTCGGCGCGTTGCCCTGGTTGATGCACGACCAGCCTTTCGTTC
AGGGACTTACGGCATGTGCAGAAATTGGTGAGGCGATGGCTCGGGCGGTCGTAAAGCCTGCATCCCGTGCTCGTCGGCGT
CTTTCGTTTGATTAAAGGTAAAGCTATCAGCACTCAACAAACTGATGCTGTTCATTAAGACGATAGCGGACTCCGGCTCG
AATATTGTTTTCACCTTCAATGGCGACGGCGAAGCGAACTCTTTCGCCGTCATGATATGCCAGTGCGGCGCTTCCTCCTG
GCCCGAGAATAATTGAGTCGACCACGCCCGTGCTCACTACCGTTGAATTTTCACCGCTGGCAATGATGTGTACATTGTCG
CCGCTGTTGGCAATTCTGGCGTTTGCGCCAAAGCTGGCGATTTGCACCAGGTCACCATTACTGGCGACATGACACCGTTC
ACCCAATGTACAAACGCGTACTCGCATTCCGGTATTCGCAATCCGACTGCTATCTCCAGCGCTGCTTATCCGCGAAGAAT
TACCGGTTACAGCAACCCGTGCGCGTTCACCGGAGCTGCCAATGTGACTGTTAAAGCCGACACTTCCAATCCGCACGCTA
TAACCCATACTGGCAATTTGCGCGGCATACCCAGCGGTTGCGATTCTGGCGTTATCCTCACAACAGCCGATGCGAGCATG
ATCGTCGCTGCGGGCAAACTGACTGCAAAATGGTCTCTCTCCGGGGTCTGTGGCCAGTTTTTGCATAGAGGAAACTTCCT
GATGCGCGAAGTTTTCATCCGCCAGCCATTTTTTCCACGCATACTCAACCAGACTCTCCGCCCAGTCCGTATATCCTTCC
TCAATCAGTGCATGATGAATATCGGCATAACTACCGCCGCGTGGGAAATGGCGCAGGAACCAGCGATACATCACCGCTCC
GATACGCCATTCTCGCAGGTCTGCTTGTGTAATGATCATTCGTTAGTGCCGGTGATAGCCATCGACCATGCTTTTAAAGG
TTTCGTGTGGCGTACGCCCCGTGGTGCAAAGATAAAGATGACCGAAGATAAAAAAGAGGCTTATAAATGCCAGAGCAAAA
TGTGTCTGCAATAACCAGTATCTTACGCCAGGAAACACATCTCCCACGGCTTGCGGATAGAGACACAGCAGCCCCGTCAA
TAGTAACAACGGCAGCAATCCATACATGACACCAACATAGGCGACCTGCTGTAAGGGATTAAATTTAGACTGGGTTGTTG
CCGGGAAAGGATGTTCTTCCCCCTGCATAATGCCAAACAAATAAAATCGCGTTTGTTTTGCCGCTCGTTCCAGCCACCCC
TGACGACGAATGCGATAGTGGTGACCATTATCCCCAACGGCATTGATCAGCACAAAGCCGAGCCAGCATGCCAGTAACAA
AAATCCGCAAACTTCATGCACCGCAACCAGACTTTTAACCGCAGTTGCGCCCACCATCGCAAAGTGATTTATCAGCCCAC
TGGCCAGCAACAATACAAAGAGTAACGCATTCGACCAGTGCCATAATCTGACCGCTTTGCTGTAAAGATAAATCTTCTCA
CCATGATCGGTGGCTGATTTCTTCACGCCACGAGCACGAAGCAAGGCATGCAGCCCCAGCACCAACCACATCCCAACCAG
CAATACTCCGGCAATGATCAACCACACCGGCCAAAATTCGGGAGTGAACTGTGGCACATAGTTCGCTAACTGGCTCTGAA
ACTGTTCAGCATGTTGCGACGGGTTCATACATTTTCCTTTTTAATCAAATGTTGACCGAACCGGCGATACAGATGCGGTT
TCCCGGCACCAGGTAGCTGATATTGATAGTATTTATTTTGCTGCAACCACGCCTGAATTTCTGGGCTATCTTCGCGACCA
AAGATCAATGCGTGTTCCGGGCAGGCGCTGACGCAAATGGGCGGAAAGCCTTTTGCCAGTCGGGACTCAGCGCAGAAATC
ACATTTATCTGCCACTTTAGTGACAGGGTTAAGGTAGCGAACCTGGTACGGACAAGCGCCAATGCAATAACTGCAGCCGA
TGCACTGCGATTTTTCTACCCGCACGATCCCCTGCTCATCACGCCACGACGCCCCCGTCGGGCAAACGTCGATGCACGGA
GCATCTTCGCAGTGCTGACATGACTGGCGAAAAAAGTGATACTGCGTTTCGTTGTCGTTATCGGTGACTGGAATGTGTGC
TATCGATAAACGGCTTCCCTGAGCGGGGACGTGGTTAGTTTTACGGCATGCGCGGGCGCAAATATTACAGCCATTGCACC
GTGACTCGTCATGAATCATGGCATACCGAACTTCCTTTTCTTGCCTCGTGTTCGCTAACAGAGAAGACGCAGAACCAGTA
AAAAAGATTACTGTTCCCATCCCCAGAACAAATTTGCGTCGAGTGAAGGACATCTGATTATCCCCTAATGCAGTGGCTGC
AACTGTCCACCCTATCCATGACATCTCCCTGAAATTTCAGGGGTTCTGAAACTGCTCTACCTCGAACCATTCGCATAAGC
CATAAGCCTTGCCATAGGTCATAAATCGCTCGCTAAACAAGTACAACACCGCCGTCGATGACTCCACCTGCCGTATATCG
GCATATTCAGGTGCAACATCCATCGCGGCGATCGCGGCTTCAATCTGCGCTTCCTGGAAGTAATATGGCGCTTGCATCAG
CATGGCGACTTTGTAAGGACGAGGATACGTCTGGCACTCAAAACGAACGGCGTGTGCAATGGCGCGACAAATGTCCTGCT
CCACAACTTGCAAAGACATCGCCGCGTAGTTTTCACTCATGGCCTGCGTTGAGTAATAGTAATCATCCTTGCTTCCCGTC
AAACAAGCGATATCCGCGTAGTCTCCGTTCTGCTTAATGCTTTCCAGCAAAACTGACAGTTCCTCTTCTGCAACCAAATA
GGGTGGCGAGAGAAACACGGCGCGCGCCACCAGCTGCCCAGCCGCAGAATGCTGGCGAATGAACTCAGCCAGGATTTCAG

CAGGTTCTGGTTCGGTTACCGTATCAACGGGGTGACGCATCCCCTGCAACCATTGCCGCTTCGCCTCGTCAACTTCGCTG
ACTTTCGCTAACGGCAATTCATCCTGATGGTTCATCTTACCCATTCCTTACGCAGGCAGTAGATTGTGTGCTGCCAGATC
GGCGGCAATATCTTCCAGCCCCAGACGCTGCAATGTTTCGCGGGTTGGACAACCAAGCTGTGGGTCCCAGCCCATTTCTT
TGTAGAACATCGTGAGCGAAGCATGCATATCGTCACGATCCATTTTGTCAGTACCTTCAGTAAAGACCGGGATCTGCGGA
TCCTTGTCGAATACCCAGGAACAGATAAGATCGTGTTCATTACGCATATCTTTGGTTTGCATCAGTTTTACCGTGTAGGC
ACGATGCAACGTAAAAATACGCTCTGCAGCTAAATCTAATTTTTCCTGAGTCATCTCTTCGCCGGTGATCGCTTTGAAGA
ATTTGGCTTCAAGCGCCAGATCGCCCCGGTAATTACGGCTTTTCAGCGGCGAAACGGTCATTGGCCAGACCCAGTTGCAC
AGGGTGACGGCGTTATGCAAACAGACCCGCAACAGCGACCACTTTGCATATTTAATTTTTGCGTCATTGATTGGCGTGTA
GTTTTTGGTTTCATCGTAAGCATCTTCAGAACCAAAAAGTTCTTTCGCCACTTCACGTTGCAGTTTCAATGGCAAGCCGG
AACCAATAAAGTTGATATGGGTATGCGTCATGCAGTCACGGTTGAACATACAGTTAACAATGGAACCGACCTGCGCTGAC
GCTTCATTGGCATGGTGAACCGGATAGCCAAACGGCGACCAGAGTTTATTTTTCGCGTAGCCCCAGTACTCTTCACCCAA
ATTCCAGCGTTCTGCGATGGCATATGAACCATCAGCCAGGTGACTCAGCTCACCCACACGATGCGCCAGACGGTAGTAAA
AATCTTTAATGAAGTTAACGTCACCCGCTTCCAGTTGATCCCAGCGAATTTCTGCATACTCTTCAGCTGGCAGAACACGC
TTGAACACACCTTTGCTGTAACAATAAGTAAAGTCGCGATGCAACTGCCCGTAGTTACACCATAGGCCGTAGTCATCGAA
CAAATTCAGACCCACCAGGTTACCAATCACACGACCATCGTCTTTATCTTCAAAATCTTTCGGCCCGTTCGGGAAGATGG
TGGTATGGACAAAGTTTGCTACACAAGTGTTACCACCTGTGCTGGGCACGCCAAACTCTTTCACCCGAGGAATATTCATT
TGGGTCATACAACGGATCGGGCAAGAGTGGCAGCCGCTCATTTTTACTGTGTATTTCTCTGCCGCCGGTCCTAAGTCAAA
AACGGATTTATAGGTACGAAAGCCGACCGTATTCTGATTGCCTGGCGGAATTTCACCCGTTTCAATCGGACCACCTTCAG
CCGCGCCCCAAAACAGCCCTTTACGTGCTGTCCAGCGTGACTTGGGATCTGAATACTCTGCCCACGATTGTGGCGTACTT
GGCACGACATGGTTGTTATTCGCACCAATAAGTTCAGTCATCATGTAATCATTGAGACGCTTCATCTCCTGACGATCGGC
AATGTTGACCCCTTTCGTCCCTTCAACCGCAATCGCTTTCAGGTTTTTCGAACCCATTATTGCGCCAGTTCCCGCACCGC
CGCTGTGGTTACGGCTATTCAACATGCCAGAGAGAGGAACAAGGTTTTCCCCAGCCTGACCAATAGCCGCCACACAGGTT
TCCGGACTGGTCAATCGACAAATTTCTTCCGTCGTTGCGCGCGTCCCTTTTCCCCATAAGAAATCGGCTTTTTCCAGGCT
AACTTTGTCATCTTTAATCTTCAGCCATACCGGTGATTTCGCCTTCCCTTCGATAATAATGACGTCGTATCCAGCGAATT
TCATTTGCGCTGCAAAAAAGCCACCCATATGGGCATCGACGACTAAATTTCCTTTGGTAAAAGTAGAAAGTGAGGTGATA
TTTACGCGAGAACTACAGGGGGCACCAGATCCAGTTAATGGGCCGGTAGCAAAGACTAATTTATTCGCTTCATCGAAAGG
TTTCGTGCCTGGCGGTACTTCGTCATACATAATTTTGTAGCCGAAGCCCATGCCACCGACAAAACTTTTAAACTTACTGG
AATCTTCGAGGGTAATATTTCCTGTCGTGAGATTGACTCTTAATATATTACCTGTCCAACCGTTAGCCATGATTTTTTCC
TTTGCAAGATTCACACAGTAATATCTTTCCACTCGATAATTTTTAACGCCCCTGTTGGGCAGGCGTTTGCGCATTCACCG
CATAACACACATTTCGAGGATTTTTTACTTTCGGTATTTACGGTGGCCATCATCCACGGGCATGCCGTAGTACAGGCGCT
ACAGCCAATACAGCGTTTATGATCGACGGTAATACAGCCTTCTTTCTGCTGCCAGGTAATCGCACCAATCGGGCAGACGT
TCATGCATTGCGGCTCTTTGCATTGACGGCAGGTGTCCGCGGTATAGTTGAGATCGCCATACAGGCCGCCGCCAGAGCCA
ACCCCGTTGTCGCCAAAGAAATAATTGCGATGGATTTTGATACGGGAGAAGAATGTCCCTACTGAGCCATCGTTGAAGTT
GGTACAAGAGATTTCACAGCGGTGGCAACCCGTGCATCGCGCTCGCTGGGTCACCAGCACCCCTTTGGGGGTATTTATCA
ACCCTACTGTGCCGCTATCAATATCTTCCTGTTTGCAACCCAAAAGTGACAGTAACGCAGGGGCAATTGTTAGACCAGCA
AGGCCTTTTCCTGATATACGCAAGAATTCTAATCGCGTTAAGCCAATATCTAATAGTGGACGATCAACCGGGTTCATTTA
TTTCGCATCCTCTTCGCAAACACAGTGCTGACAATTCAGCAGCCTGATATTAAGCATTCAACTAATACGCTGCTGACTCT
GTCCCCAACACCAGATATTTTCCCTAACCCGATAGTGTATGAGAAAAGATTAACGCCGATAAACTAATCCTTTATGGTTA
GGAGAAAAAATAATGTGATTATTCTGAGCCCTTAACATTGATCGTTATCAATTAAAATTACAAAGCAAGAAATTATAAGT
GAACTGATATTTATTATCATTTGAAATAAATTTAACTTAAGCGACAATTATTTGTGACTTTCATTGCAGAAAATAAAAAT
AACATTATCGCTATAAATTAAAATACATACCGAAATTCGTATAATTTCAGCAGTTGTTATTTAGAATGATTAACTGGACC
GCAACTGAAGCAAGAATATCAGGATGAAGCTTACCGCCTCATCCTGAATGGTCATTTTACCAGTGCGTTAATGACGGTTT
CCATCGCCTGCTTTTCCAGCTCACTGCGCTGAATGCGTTTATTGGCTAATGCTGTACGTAATACGCCTGCGATAACAATA
TGCTTTGGTTCCTGCCCCAGATCGCGCATTTCCAGCACAACTTTACCGACAACTCTGCACATTTCTCGGTACAGTTCATC
CTCTTTTGTCCGATTTCCCATTCTTTATACCTATTTATCATGCCAACTATCAGCATATATCAATCTAACCAATTAAACAA
AAATCAAACAAAATCAGACAAATAACGCGATAAATTATTTTAACTGTAGCAATTGAGCGATGATATATTTATACACCGGA
TGAACTTTCACTTATCCTCACACTGACAACTTCGGCACCAGACGTTGCGCAAACAGTGAAGTTTTTGCGTAACCTTTTCC
CTGGAACGTTAAATCTTTGATAACAATTTATTGTCTAACAAGTTGTATATTTTTTGAAACGCTGTTTTTGTTTTCCTTTT
GGATTAATTTCAGCGTATAATGCGCGCCAATTGACTCTTGAATGGTTTCAGCACTTTGGACTGTAGAACTCAACGACTCA
AAAACAGGCACTCACGTTGGGCTGAGACACAAGCACACATTCCTCTGCACGCTTTTTCGATGTCACCTATCCTTAGAGCG
AGGCACCACCACTTTCGTAATACCGGATTCGCTTTCCGGCAGTGCGCCCAGAAAGCAAGTTTCTCCCATCCTTCTCAACT
TAAAGACTAAGACTGTCATGAAAAAGACCAAAATTGTTTGCACCATCGGACCGAAAACCGAATCTGAAGAGATGTTAGCT
AAAATGCTGGACGCTGGCATGAACGTTATGCGTCTGAACTTCTCTCATGGTGACTATGCAGAACACGGTCAGCGCATTCA
GAATCTGCGCAACGTGATGAGCAAAACTGGTAAAACCGCCGCTATCCTGCTTGATACCAAAGGTCCGGAAATCCGCACCA
TGAAACTGGAAGGCGGTAACGACGTTTCTCTGAAAGCTGGTCAGACCTTTACTTTCACCACTGATAAATCTGTTATCGGC
AACAGCGAAATGGTTGCGGTAACGTATGAAGGTTTCACTACTGACCTGTCTGTTGGCAACACCGTACTGGTTGACGATGG

TCTGATCGGTATGGAAGTTACCGCCATTGAAGGTAACAAAGTTATCTGTAAAGTGCTGAACAACGGTGACCTGGGCGAAA
ACAAAGGTGTGAACCTGCCTGGCGTTTCCATTGCTCTGCCAGCACTGGCTGAAAAAGACAAACAGGACCTGATCTTTGGT
TGCGAACAAGGCGTAGACTTTGTTGCTGCTTCCTTTATTCGTAAGCGTTCTGACGTTATCGAAATCCGTGAGCACCTGAA
AGCGCACGGCGGCGAAAACATCCACATCATCTCCAAAATCGAAAACCAGGAAGGCCTCAACAACTTCGACGAAATCCTCG
AAGCCTCTGACGGCATCATGGTTGCGCGTGGCGACCTGGGTGTAGAAATCCCGGTAGAAGAAGTTATCTTCGCCCAGAAG
ATGATGATCGAAAAATGTATCCGTGCACGTAAAGTCGTTATCACTGCGACCCAGATGCTGGATTCCATGATCAAAAACCC
ACGCCCGACTCGCGCAGAAGCCGGTGACGTTGCAAACGCCATCCTCGACGGTACTGACGCAGTGATGCTGTCTGGTGAAT
CCGCAAAAGGTAAATACCCGCTGGAAGCGGTTTCTATCATGGCGACCATCTGCGAACGTACCGACCGCGTGATGAACAGC
CGTCTCGAGTTCAACAATGACAACCGTAAACTGCGCATTACCGAAGCGGTATGCCGTGGTGCCGTTGAAACTGCTGAAAA
ACTGGATGCTCCGCTGATCGTGGTTGCTACTCAGGGCGGTAAATCTGCTCGCGCAGTACGTAAATACTTCCCGGATGCCA
CCATCCTGGCACTGACCACCAACGAAAAAACGGCTCATCAGTTGGTACTGAGCAAAGGCGTTGTGCCGCAGCTTGTTAAA
GAGATCACTTCTACTGATGATTTCTACCGTCTGGGTAAAGAACTGGCTCTGCAGAGCGGTCTGGCACACAAAGGTGACGT
TGTAGTTATGGTTTCTGGTGCACTGGTACCGAGCGGCACTACTAACACCGCATCTGTTCACGTCCTGTAATATTGCTTTT
GTGAATTAATTTGTATATCGAAGCGCCCTGATGGGCGCTTTTTTTATTTAATCGATAACCAGAAGCAATAAAAAATCAAA
TCGGATTTCACTATATAATCTCACTTTATCTAAGATGAATCCGATGGAAGCATCCTGTTTTCTCTCAATTTTTTTATCTA
AAACCCAGCGTTCGATGCTTCTTTGAGCGAACGATCAAAAATAAGTGCCTTCCCATCAAAAAAATATTCTCAACATAAAA
AACTTTGTGTAATACTTGTAACGCTACATGGAGATTAACTCAATCTAGAGGGTATTAATAATGAAAGCTACTAAACTGGT
ACTGGGCGCGGTAATCCTGGGTTCTACTCTGCTGGCAGGTTGCTCCAGCAACGCTAAAATCGATCAGCTGTCTTCTGACG
TTCAGACTCTGAACGCTAAAGTTGACCAGCTGAGCAACGACGTGAACGCAATGCGTTCCGACGTTCAGGCTGCTAAAGAT
GACGCAGCTCGTGCTAACCAGCGTCTGGACAACATGGCTACTAAATACCGCAAGTAATAGTACCTGTGAAGTGAAAAATG
GCGCACATTGTGCGCCATTTTTTTTGTCTGCCGTTTACCGCTACTGCGTCACGCGTAACATATTCCCTTGCTCTGGTTCA
CCATTCTGCGCTGACTCTACTGAAGGCGCATTGCTGGCTGCGGGAGTTGCTCCACTGCTCACCGAAACCGGATACCCTGC
CCGACGATACAACGCTTTATCGACTAACTTCTGATCTACAGCCTTATTGTCTTTAAATTGCGTAAAGCCTGCTGGCAGTG
TGTATGGCATTGTCTGAACGTTCTGCTGTTCTTCTGCCGATAGTGGTCGATGTACTTCAACATAACGCATCCCGTTAGGC
TCCACGGAATATTTCACCGGTTCGTTGATCACTTTCACCGGCGTTCCCGTCCGCACGCTGGAGAACAAGGCTTTAATATC
CGGTGCATTCATGCGAATACACCCTGAACTGACGCGCAAACCGACGCTGTCCGGCGCACTGGTACCATGAATGAGGTATT
CGCCATTACCATGCGCGAGGCGCAGTGCGTAACGTCCTAGCGGGTTATTTGGTCCGGCAGGAACGACTGGCGGTAATTTA
ATGCCACGCTCCAGCGAACGCTGACGAATGCCTGCCGTAGGCGTCCAGGTTGGGTTAGGGATTTTCTGCCCAACACGCGT
TTCCATCACCGGCGTTTCCAGCCCCTGCAATCCAATACCTATTGGATAAACCTGCACAATATTTTCTCCCGGCGGATAAT
AATAAAGGCGCAGCTCTGCAAGGTTAACGATAATCCCCTGACGCGGTGCATCAGGTAATAACAGTTGTGAAGGAATAGTT
ATCGTCGTACCAGGTTTTGGCACCGGGGCGATAGTGTTATTGGCTTCAAGGATCAACATTGCCGCAGTATCAAAACGTCG
GGCAATAGCCTGAAGGTTTTTATCCCCTTCTTGCACCGTATACGTTTGATTTTGCCCAACCAGTCGGCTTCCGGTTGGTG
GTAGCGGATAATCAACCGCCCAGGCAGCCTGGATGGCGCTAAAAGCGCCGATAAGCGTGAGTGTAAGCAAAGACGCGCGT
TTCATTGTAAACCTCCTGTATTTGCCGGAGACTCACGCTGAAACGTCGGATGGCGCTTATGTTCACCTGAAACCAAAACA
CTCCTGTGCAGGTCAGTGTAAACATTGACCATCCGGCAATGTGAGCCAACCGGATGAAAGCTGTCCTTTTAGTTTAGCTA
AGTGCAGCGGCTTTGGCGCGAATTGCGCGAATCATCGCTTCCAGACCTTGTGAACGAGATGGGGTGAGATGTTGGGTGAG
CGCCATTTTTTCAAACCACGGACGCACATCGAAATTGACAATATCCTGCGGCGTCATCTGATCGTAGAGAATAAAGACGA
CCGCAATAAGCCCTTTCACAATCGCCGCATCGCTGTCGCCCTGTAATTCAATAATTCCCTGGGCATTCTGGCGCATGACA
ATCCACACCTGACTCTGACAGCCCTGAATGCTATTTTGTGGACTTCTGTCTTCGTCGCGTAATTCTGGCAGACGCTGGCC
CAGCTCAATAATGTAGAGATATTTCTCTTCCCAGTTGGCGCAGCGTAAAAAATTACGCAGCAACTTTTCTTTATCCGGCA
ATAAAGCCATAGTGCCTCCCTGTTATCCCAGCAAACGGTGAATACGTTGCAGGCCGGTCACCAGACGATCCACTTCTTCA
TGGGTGTTATACATGGCCAGCGACGCCCGACACATCGCAGGGACGTTGTAATAGGCCATCAATGGCATTGCGCAGTGATG
TCCGGTACGCACAGCAATGCCGTAATTATCGAGAAAACTGCCAACATCATAGGCGTGGTGTTTACCGAGATTAAAAGCAA
TAACGCCAAGCCTGTTTTGTGGGCCATAGAGAGTGAGATCCGGTACAGATTCCAGCTGTGATAGCGCATAATGCATCAGA
TTCTGTTCATACTCGGCTATGTTATTAAGCCCCAGCGCCGAAACATACTCCAGCGCCGCGCCAAGACCAATGATGCCCCC
GGTATTGGGTGTACCGGCTTCAAACCGCCATGGTGCTTTGGTCCAGGTAGTGCCTTCACTCAGGCTGACGGTGGCGATCA
TAGAACCGCCCCCTTCCCACGGCGGCATCTCCTGCAACAAGGCTTCTTTCACATAAAGAATGCCAATTCCGGTGGGGCCA
TACAGTTTATGCCCGGAGAACACGTAAAAGTCGCAATCCAGCGCCTGAACATCCACCGGATGATGCATCACCGCCTGAGC
GCCATCCACCAGCACTTTTGCGCCATGCTGGTGCGCAAGCGTGATCATTTCCGCCAGTGGATTTTCTGTGCCAAGCACGT
TGGAGACATGAGTAATTGCCAGCAGGCGAGTTTTCTCATCAAACAGCGTAGGCAGCGTCTCCAGTTGCAACGTACCATCG
GGATTGAGCGGGATCACACGCAGCTCTGCGCCAACGCGTGCGCAAAGCATCTGCCAGGGAACAATGTTAGCGTGGTGCTC
CATCTGACTGATGATGATGTTATCGCCCGCCCGCACGTTGCTGTTGCCCCAGCTATTGGCGACCAGATTGATCCCTTCCG
TCGTGCCGCGGACGAACACCAGCTCTTCCGCCGAACGGGCATTAATAAACAGCGATGCCCGCTTGCGCACGTTCTCCATT
TTCTCGGTCGCCTGGGCGCTTAAGGTATGAATACCACGATGCACCGCCGCGTAGCCATGACGATAAAACTCGGCCTCGGC
GTCAATCACCTGGCTCGGTTTCTGCGCACTGGCGGCGCTGTCGAGATAAGCCAGCGGCAAACCGTTTACCTCACGCGAAA
GCACCGGAAAGTCGGCCCGCACTTTGTCGACGGAAAAAATCATCTTGCACCTCCTGGCAGCCGTTGACCGATTCGGGCCA

GCACCTGCTGTTTAAGCCCCTCATCACGCAGTGCTTCCGTCAGTTCGGCAGCGAAGGCGTAAATGATCATCTGCTGGGCA
TCCTGCTGATTGATCCCGCGCGAGCGCAGATAGAATATCTGTTCATCATCAATACGCCCCACCGTCGCGCCGTGGCTGCA
TTTCACATCATCTGCATAGATTTCCAGCTGCGGTTTCGTATCCACTTCCGCCAGTTTGCCCATCAGCAGATTGTTGTTGG
TCATCTGACCATCCGTTTTGATGGCGTGCTGCGCGACGTTGATCAAACCGTTAAATACCGCGCGGCCTTTGTCGCTGACG
ATAGTTTTGTGCAACTGTCGGCTGTTACAAAAACCTTTATTGTGTTCCAGCCAGGTACGGGTATCACACACCTCGTTTTT
CACCGGCATCGCCAGGCTATTGATCCGCAGCGTGCTGTTTTCGCCATTGAGTTGCGTACTGGTGTTGTGTCGTAACACTG
CGCCACCCAGCAGGAAACTGTGGCTAAATGCGGTGGCATCCTCAGCCAGCAACAAATCGTTATGAGCAAAGTGGTGACTG
AGCGGGTTTTCAAACGCCAGCTTGATATGCTGCAAGTGGGCATTCGCTGCGACGTTGATAGTGAACCGTGCCCCGGTAAA
ATGACGAGCATCATTCAGGCTGACAAAATGTTCGATCACCGTTGCTTCGGCACCTTCCGCCAGATCCAGATGATGTCGGT
AATGGGCAGTGTTCACCTCTTCACCTGCCACGCCCTGGGTGATATGCATTAACAGCAATGGCTTTGCCGGCCGTTGACCG
CGCTTCACGGCGATATGCGTCACGCTTTGTGCCAGGCTTTCCGTCAAATGCAGAAACACTTCCGCCTGAATAGCGTCGGG
TAAACCCTGACGGTCGTCGTTAATGCTCACTTCATATCCGCTGCCTTCAGTTGCATCGCTCAGTGCGGGCACGTAACGCC
CATCGACAAACACCAGCCGCACGGAGTCTAACGTTAACGCTAAGGCATCACGCTGCTGTGGGGATATCTCTCCCGCAATG
CTGACAAACTGGCTATTGATCAGCCCTTCCAGCGGCGTATATTTCCAGTTTTCATGTTTACGTGTCGGCAGTCCGGTACG
CAGCAATTGTTGTAAATGCTGCTGTGCTTGCGGGGAGCGTTTTGTCCCTTCAGCTTCAAACAAGTGATGCCACTGTTGCA
GCGCGTTACTGCTGTTCGGTAAGCCAGCCATAACCCTGCTCCTCCAGTTGTTTGACCAACGTGAAATCGCCGGATTTCAC
AATTCGTCCCTGATATAGCACATGAACGTAATCAGGCTTGATGTAGTCGAGAATGCGTTGGTAGTGCGTAACAATGATGA
ATGAGCGCTTGCCATCACGCAGCGAGTTCACGCCATCGGCGACCACTTTTAATGCGTCAATATCCAGCCCGGAGTCCGAC
TCATCAAGAATGCATAACTCCGGTTCCAGCACCGCCATTTGCAAAATATCGTTGCGCTTTTTCTCGCCGCCGGAAAAACC
AACGTTTACCGAACGGGTTAATAAATCTTCCGGCATCTTCAGGAGAGCGATTTTCTCTTCCATCAAATCCTGAAAATCAA
AGCGGTCGAGCGTTTCCTGGCCGCGATAGCTGCGCACCGCATTAAGTGCCGTTTGCAGGAAAAACTGGTTACTGACACCT
GGAATCTCCACCGGATACTGGAAGGCCATAAAGATGCCTTCGCCCGCGCGATCTTCCGGCGACAGCGCAAGCAAATCTTT
GCCTTTGAACTCAACCGTGCCGCCCGTCACTTCATAATCTTCTCGCCCGGCAAGCGTTGCCGATAAGGTACTTTTGCCCG
AACCGTTTGGCCCCATAATGGCGTGAACTTCGCCGGGATGAACGTCGAGGCTTAATCCGCGCAGGATAGCTTTATCTTCC
ACGCTGACGTGTAAATCTTTAATACTTAACATGTTTATTCCTTATCCGACGCTGTGTTCAAGACTGATGGCGAGGAGTTT
TTGTGCTTCAACGGCAAATTCCAACGGCAGCTCCGAGAACACGTCTTTGCAGAAACCGTTAACAATCATCGAGATGGCGT
CTTCTTCGCTGATCCCGCGTTGCAGGCAGTAAAACAGTTGATCTTCACCAATACGTGATGTCGTTGCCTCGTGTTCCAGT
TGCGCACTATTGTTACGACACTCAACATACGGGAAGGTATGCGCCCCACAATTAGCGCCAATCAGCATTGAGTCGCACTG
AGTGAAATTGCGCGCATTGGTTGCCGTCGGCATGATTTTCACTAAGCCGCGATAACTGTTCTGACTATGTCCGGCAGAGA
TCCCTTTCGAGATAATGGTCGATTTGGTGTTTTTACCGATGTGGATCATCTTGGTGCCGGTATCCGCTTGCTGATGACCG
CTGGTCAGCGCCACTGAGTAAAACTCACCAATGGAGTTATCGCCGCGCAAAATGCAGCTGGGATATTTCCACGTAATCGC
TGACCCGGTTTCTGATTGCGTCCATGACATTTTGCTGTTTTCGCCTTCGCACAAAGCACGCTTGGTGACGAAGTTGAGAA
TACCGCCGGTGTTGTTATCGCCAGGAAACCAGTTTTGTACCGTGGAATATTTCACCTCGGCGTTTTTATGGATGATGACT
TCCACCACTGCCGCGTGTAACTGATAGCTGTCACGCACCGGAGCGGAACAGCCTTCAATGTAGCTGACGTAGCTGTCTTC
GTCGGCCACCAGAATGGTGCGCTCAAACTGCCCGGTTTTTTCTGCGTTAATGCGAAAATAGGTGGAAAGTTCCATCGGGC
AGCGCACGCCTTTAGGCACATAAATAAACGTACCATCAGAGGCTACCGCCGCATTAAGCGCGGCAAAGAAGTTGTCATTC
CCCGGCACCACGGTGCCGAGATATTTACGCACCAGTTCCGGGTGATCGTGGATCGCCTCACCAAAGGAACAGAAAATAAT
TCCCTGCTCCGCCAGTTTTTCGCGATAAGTAGTGGCAACCGAAACTGAGTCGAAAATGGCATCCACCGCCACCTCTTTGC
CTTCCCGCACGGGAACGCCCAACTGCTCAAACGCCGCCTCCACCTCTTTACTTAAAAAGGCGTTCGCGCCAGTTTGCTGC
ACCGCGCCAGGTTCAGACGCGCAAGTGTCGTCACAATTACCGCACGATGGTGCTGAGTAGTAGCTGTAATCCTGATAATT
CAGCTTGTCGTAGTGCGCTTTCAACCAGTGCGGTTCTTCCATCTCCAGCCATGCGCGATAGGCGTTTAGACGAAACTCCA
GCATCCACTCCGGCTCATTACGCTTCGCCGAAATTGCGCGCACCACCTCTTCGTTTATCCCCTTTGCCAGCTCATCGGTG
GCTAACTGGGTGAAGAATCCTTCTTTATAATTCAGCGGGCCGCCGGTCCAGGTTTTGACATCGTCAGTTGCTTCAGTATT
ACGAGACATAGTACCGCCTATACCCCAAAGCTTTCGCCACAGCCACATTCATTCTGGGCTTTAGGGTTGTGAAATTTGAA
TATCTGATTAAGTCCTTCACGAACGAAATCGACTTCCGTGCCATCAATAAACGGCATCGCTTGCAGCGGGACAAACAGCT
TCGCGCCGTCGTGTTCAAACAGCAGATCGTCTTTGTCCGGCTCGCTAACACTGTCGAGCACATAGCCAAAGCCCGCGCAG
CCCGTTTGCTTCACGCCTAAGCGCACGCCGACCATACCCGGCTGCTTTGCCACCAGCTCACGGATGTGTATCGCCGCTGC
GGGTGTCAGCGTTAAGCCTTGCCAGGCGAAATCTTGTGGGTTAAAGGTTCCTGAATGCATGTCCATCGATTTACCTCACT
TCATCGCTTTCAGCGTATAACAGCATGTTAGTGATAATGATTATCAGTTCAACCCAGCAAACGCAGGGGCTTTACCGTAA
AACATGCTTTTTGCCTGCTTTTAATAAGCATAGACCCTGATGTGTGGGTTAACAGGCACGCTAAGAATTAGGTATCTCAT
TGTTAGATAATGGTTATTATCTAAGGAGCATTAAGGCTGTGATGAAAAAGAAAAAGTTGTATTGAAAATGACTATTTAAG
AGATAGGTAAAAAAGTGCAGCGTTCAGAAATAAGAAAACCCTTAAGTCTGTGCGACACAGGCTTAAGGGTTTCTACCCCA
TCCGGCGCTTATCTCCGGCACTCTCAGTGGCTTAGCTCTTGAAGGGGCGATAAGAATAATCTCATAAAGCTAACCCGCCG
TTTTAACACAAACTGCGATTAGTATTATTTTTGAACAATATCAGGCGGTAGATAAGCAGTATTAAGAAGGTCATCGAACC
TGGACGGAGGTTAATCCAGGTCGATTTGGCGAACTTGCGGCATTAAGTCAGGATCAATGCACGCCCAGACGCCAGGCAAA
GTAGATTTCTTCTTTTAATTCAGCAGAAGAGAGAGTAAGCAGGTCAGCAAATTCAAGTTCTAGTTGTTTCAGACGTTTGA

GATATTGGGCAGGTGAAAGATTGCTCTGGTCACGGCGTAAAAATTCAATGGCCAGCTGGGTGGGATCAAGTTGAGTAGAC
ATAGCATCCTCGCTTTTAGACAAGACCTGCACAGTATACCACCGTTTACTGTGCAGATAATGACCAAAAGCAATATGCGT
CACACTTTTCTGGTGACAACGTCACAAAATGGCGGTCGTCAATCGTGACGAACAGCACAAACGCCCTTTCTCATCGAAGA
TTTCAATCTGCCAGACCTGGTGACGCGAACCGAGATGCAACGGTTTGCATACGCCGCGCACCCGCCCTTCTCGTGCCGAG
CGGACGTGGTTAGCATTGATTTCCAGACCAACCACTTTTTGCTCACCTTCGGTACATAAATAACCGGCAACGGAACCGAT
ACTTTCGGCCAGTACCACGGATGCTCCTCCATGCAGCAACCCGAAAGGCTGCTTTGTCCGCGAGTCTACTGGCATTGTCG
CTTCAAGGGTGTCATCACCAATATGTTCAAAGCGAATATCCAGGAACCCCACCATGTTTCCTTCACCCATAGCATTCAGT
GCTTCCAGGGTGATTTTCCGTTTCCATATCATTTAATAATCTCCAGTAAAGCCTGCACAGGATGGCGTACCCCCGTGCCT
TCAACCCGTTTTACCTGGCTACGGCAGGAATATCCGGTCGCCAGACAGCGGTTACGCGGCAGTCGCTGCATAGCCTGATG
CCAGGATAACTCATAGATCCCGAGCGAATTTTCATGGTTTTTCGCTTCATGTCCGTAAGTCCCTGCCATGCCGCAGCAAC
CCACGCTGACATTTTCCAGTTTCGCGCCAAAACGGGCAAATATCGCGGCCCATTGTGCTGGCGCACCCGGCAAGGCGGTA
ACTTCGGTACAGTGACCAAAGAAATACCATGATTCACCGCTGACTGTAGCCACTGGCTGTGACTCAAGTGCGCTTGCCAG
CCATTCATTCGCCAGTAAGACGTTAAACTCGCCACGCTCCTCGCCCAGCGCCAGTTTATATTCATCGCGATAACAAAGTA
CCAGCGCCGGATCGACGCCCACCATTGGCATACCCAGCTTCGCCATACGGTTGAGGAAATCCGCCGTCTTTTTCGCCGTC
TTCGCAAAACGATTAAGAAAACCTTTAATATGCTGGGCTTTGCCATTTGGCGAAAATGGCAGTAACACAGGCTGGAAACC
TAATTTTTCGACCAGACGGACAAAATCCGCCACCACTTGCGCATCGTAATAGCTGGTAAAGGGGTCCTGCACCACCAACA
CTGTGCGCGCTTTCTGCTCTGCATTGAGAGATTCAAGCTGTTCCAGCGTCATGTTTGCCGAGCGATGCCCCACCATTTGT
TGTTGTAGCGAGGGGACCGACAGCAGCGGCAAATCAACCATGCCGATATGTTTTTCCGAGAGTTTGCGCACCAGCGGCTG
GTTAATGAAGAAGTTAAAGGTTTTCGGCGCGCGTGCCATCAGCGGCGCGTAGCTCTCGACCGTAGCGACGAGGTGGTCGC
GCAGCGGGCGTAAATAACGGGTGTGATAGAGCTGCAGAAAACGAGAGCGAAACTCCGGCACATCAATTTTGATGGGGCAC
TGGGTCGAACACGCTTTACAGGCCAGACAGCCCGACATCGCCTCTTTGACTTCGTGTGAGAAGTCATATTCGCCTTTATT
CGCATGCCAGCTATTGCGCGTGCGGGCAATTAACGTCCGCAAACTGACGCCGGATTCAGGCAGTTCTTGTTCCAGTTTGA
GTGGATCAACGCCGCGATCCGCCAACAAACGCAGCCATTCACGCACCAGCGTTGCGCGCCCTTTCGGTGAATGAATCCGG
TTCTGGGTGATCTTCATCGACGGACACATCGGACTACGGGCATCAAAGTTGAAGCATAAACCGTTGCCGTTACACTCCAT
CGCACCGCGCCACTGCTGGCGTACCGCAATGGGGATCTGCCGATCGAATGTACCGCGCTTCACCGCGTCCACTTTCATCA
TCGGCGCATCGAGACCTTCTGGCGGGCAAATCTTCCCTGGGTTGAGTCGGTTATGCGGGTCAAATGCCGCTTTCACTTTG
CGCAGTTCTGCAAAAAGTTCCTCACCGAAAAACGCCGGGCTGTATTCAGCGCGAAAACCTTTGCCGTGCTCGCCCCACAA
CAAACCACCGTATTTCGCAGTCAGCGCCACCACGTCATCAGAGATTTGCTTCATCAAAATCTCTTGTTGAGGATCGCACA
TATCCAGCGCTGGACGGACGTGCAAGACACCTGCGTCGACGTGACCGAACATACCGTAGCTTAAGCCGTGGCTGTCGAGC
AGCGCGCGAAATTCAGCAATATAATCCGCCAGGTGTTCCGGCGGTACGCAGGTATCCTCAGCAAACGGAATTGGCTTAGC
GGCACCTTTGGCATTGCCAAGCAGACCAACGGCTTTTTTGCGCATTGCATAGATACGTTCAACCCCCGCCAGCTCACGGC
ACACCTGCCAGCCGATGACACCTGCTTGATGACTGGCGATCAGCTCATCCAACCGCGCACAGAGTGCATTTACCCGCTCA
TCAATCAGCGCCTCATCATCACCAGCAAATTCCACAATGTTCAGCCCGAGCATCTCTTGGTCAGGCACATCGGTAATCAA
CTCGCTGACGGAATGCCAGACAATATCTTCCCGCGCCAGATTCAGCACTTTTGAGTCCACCGTCTCTACCGAAAGCGCCC
GCGCCTCAACCATAAACGGCGCGTTACGCAGCGCAGAGTCAAAAGAGTCATATTTGACGTTCACCAGACGGCGCACTTTA
GGCAAGCGTGTAATATCCAGCCGCGCTTCGGTAATAAAGGCCAGCGTCCCTTCTGAACCCGTCAGAATGCGCGTCAGGTC
GAACTCGGTCATCTCATCGTTAAAGACATGACGCAGATCGTAACCGGTAAGAAAGCGGTTAAGTTTGGGGAAGTTGTCGA
TAATTAACTGGCGTTGCTGACGGCAACGTTGATAAACCGTGTTATAAATTCGCCCGATTGTGGTATTGGATTTACCCAGC
GTTTCCGCCAATTCGACGGGTAAAGGTTGCGTATCGAGAATATCGCCCCCCAACAACACCGCGCGTACGCCAAGTACGTG
ATCTGACGTTTTGCCATAGACCAGCGATCCCTGACCGGATGCATCGGTATTGATCATCCCACCGAGCGTTGCCCGGTTGC
TGGTCGAAAGTTCCGGCGCAAAAAAGTAGCCGAACGGTTTCAGGTACTGATTGAGTTGATCTTTTATCACCCCGGCCTCA
ACGCGCACCCAGCCCTCTTCAGGGTTAATTTCGATGATGCGGTTCATATGGCGGGACATATCAACAATAATCCCCTGGTT
GAGCGCCTGACCGTTAGTGCCGGTGCCGCCGCCGCGGGGGGTAAAGATCAGCGATGAATAGCGTTCCTGCGCGGCAAGAC
GGGCGATCAGCGCCACATCTGCGGTTGAACGCGGAAATACCACCGCATCGGGGAGAAGTTGGTAAATACTGTTGTCGGTC
GACATTGTCAGACGATCGGCATAACTTGTCGCCGTATCGCCGGTAAAACCTTGTTGCTCCAGCTCTTGCAAAAAATTAAG
CACCAGTTGAACGACGCCGGGTGCCTGGGAAATCTGTGGAATCATTATATTGACCCTTTCCTGCGGTCTGTGATGTAGGT
CGATACACTATTCTTTCAGGCTGCTGCAATAGCGCACTGAAAGGTGATGTTTGTTTACTCTATGGATTTCGTGTTGCAGG
AAGGCGGCAAGCGAGTGAACTCCAGGAGCTTACAATAGTAAGTGACTGGGGTGAACGAACGTAGCCGCAGCACATGCAAC
TTGAAATACGACGAGTAAATCGTTTGCGTGTTGCCTGAGTTGTTGTACCACATTTTTTTCTAACACGCCCATCAGAATTA
AGGGCAGAATCGGCCTGTTAAAAACCGCTGAAATTGCTCATCATTATGCAGGTGAGTTTCGCGTGTTCACGTCGCGTCGA
CGATTTGACGCACAAAAAAGGTGAAAAGTAGTTATGGTAAATGTTCGTCAGCCCAGGGATGTCGCACAAATTCTGCTTTC
GGTGCTGTTTTTAGCCATCATGATTGTGGCATGTCTGTGGATTGTTCAACCCTTTATTCTCGGCTTTGCATGGGCCGGTA
CGGTGGTTATCGCCACCTGGCCGGTATTGTTACGTTTGCAAAAGATCATGTTTGGCCGCCGCTCTCTCGCCGTTCTGGTG
ATGACGCTGTTATTAGTGATGGTGTTTATCATCCCTATTGCTTTGCTGGTTAACAGTATCGTCGACGGCAGCGGCCCGCT
AATTAAAGCCATTTCCAGCGGTGACATGACGTTACCCGATCTGGCGTGGCTTAATACCATTCCGGTGATTGGCGCGAAGC
TGTATGCAGGCTGGCACAACTTGCTGGATATGGGGGGGACGGCGATCATGGCGAAAGTCCGCCCTTATATTGGCACCACC

ACCACCTGGTTCGTTGGGCAGGCGGCGCATATCGGGCGCTTTATGGTGCATTGTGCGCTGATGCTTCTCTTCAGTGCCCT
GCTGTACTGGCGCGGTGAACAGGTGGCACAAGGCATTCGCCATTTTGCAACCCGTCTGGCAGGCGTTCGCGGAGATGCCG
CCGTGCTGCTGGCGGCACAGGCTATCCGCGCGGTGGCGCTGGGTGTGGTGGTGACGGCGTTAGTACAGGCAGTGCTTGGC
GGTATCGGCCTCGCCGTATCCGGCGTACCTTATGCAACGTTGCTAACGGTGTTAATGATCCTCTCCTGCCTTGTCCAGCT
TGGCCCGTTGCCGGTACTGATTCCGGCGATTATCTGGCTCTACTGGACTGGCGATACCACCTGGGGAACGGTATTGTTAG
TGTGGAGCGGTGTGGTTGGCACGCTGGATAACGTCATCCGCCCAATGTTAATTCGCATGGGTGCCGATTTACCGCTGATC
CTGATTCTCTCTGGCGTTATTGGTGGTTTGATTGCTTTCGGGATGATCGGTCTGTTTATTGGTCCGGTTCTGTTAGCCGT
TTCCTGGCGTCTGTTTGCCGCGTGGGTGGAAGAAGTCCCGCCGCCGACGGACCAACCGGAAGAAATTCTCGAAGAACTTG
GCGAAATCGAGAAACCGAATAAGTAATTTCTCATCAGGCGGCTCTGCCGCCTGATTGTTAACCACCGCTAATTGATATTT
GCTTGCTCTTCCCCATATTTTCCTGCTTACCGCCATTCAGCTGGTAGTACCTGTCGCAAATTCTTTACAGTTTTTAAACT
AATGAGACGAATCTGATCGACGCAAAAAGTCCGTATGCCTACTATTAGCTCACGGTTATAAATCAACATATTGATTTATA
AGCATGGAAATCCCCTGAGTGAAACAACGAATTGCTGTGTGTAGTCTTTGCCCATCTCCCACGATGGGCTTTTTTTTAAC
ATTTTTCCGCTTCGCTACCTCGCCCCTCACTCTTTCCAATAATGTTGCACAAGATACTGTGACTCTCTTCAAGCCGTCAG
CAGGCTGATAATGTTATTTATATTTTATTGTTTATTTCATGCAGCCATTACACGATGAACGCTTATGAACTCCAGGCACT
CCGCCATATTTTTGCCATGACTATTGATGAATGCGCGACCTGGATTGCCCAGACCGGTGACAGTGAAAGCTGGCGTCAGT
GGGAAAATGGCAAGTGTGCCATTCCTGATCGTGTAGTCGAGCAACTGTTGGCTATGCGTCAGCAAAGAAAAAAACATCTT
CATGCCATCATAGAGAAAATAAATAACCGCATCGGCAACAACACCATGCGTTTTTTCCCCGACTTAACCGCATTTCAACG
AGTCTACCCTGATGGCAATTTTATTGACTGGAAGATCTATCAATCGGTAGCTGCTGAATTATATGCACACGATCTGGAGC
GGCTTTGTTAATTTTTCCACAGAAAGGAATTGTCGTTGTTACAACAATAATGAACGGATGCTGACACAACATCGCTTCAC
TTTTTAAAGCACCTTTGCTAAGTAGAACCTATGAAAAATCCCTATTTCCCTACCGCACTTGGGTTGTATTTTAATTACCT
GGTGCATGGTATGGGCGTCCTTTTGATGAGCCTGAATATGGCCTCGCTGGAGACACTTTGGCAGACTAATGCCGCGGGTG
TCTCGATAGTTATCTCATCGCTGGGCATTGGTCGATTAAGTGTCTTGCTTTTTGCAGGATTATTATCCGATCGCTTTGGT
CGCCGCCCTTTTATCATGCTCGGGATGTGCTGCTATATGGCCTTCTTTTTTGGCATCCTGCAGACCAATAACATCATTAT
CGCTTATGTTTTTGGCTTTCTGGCGGGAATGGCAAACAGTTTTCTCGATGCAGGCACTTATCCCAGTTTGATGGAAGCTT
TTCCACGCTCACCTGGGACAGCCAATATTTTAATTAAAGCATTTGTTTCCAGCGGACAATTTTTATTACCGCTAATCATT
AGCCTGTTAGTGTGGGCTGAACTGTGGTTCGGTTGGTCCTTTATGATTGCTGCAGGCATTATGTTTATTAACGCTCTGTT
TTTATACCGTTGTACGTTCCCACCCCATCCGGGTCGTCGCTTACCTGTCATAAAGAAAACCACCAGCTCTACGGAACATC
GCTGTTCAATTATCGATTTAGCCAGTTATACCTTATATGGCTATATCTCAATGGCAACGTTTTATCTGGTTAGCCAGTGG
CTGGCACAGTACGGACAATTTGTTGCAGGCATGTCATACACTATGTCGATCAAACTACTCAGTATCTACACCGTGGGTTC
GCTGCTTTGTGTATTTATTACCGCTCCACTCATTCGTAATACCGTTCGCCCAACAACATTACTGATGCTGTACACCTTTA
TCTCATTTATTGCTCTGTTTACCGTCTGCCTGCATCCCACATTTTATGTGGTGATAATATTTGCTTTTGTCATTGGTTTT
ACCTCTGCTGGAGGTGTTGTGCAAATTGGCCTGACGTTAATGGCTGAACGTTTCCCTTACGCTAAAGGTAAAGCTACAGG
GATCTATTACAGTGCGGGCAGTATTGCGACCTTTACTATTCCGTTGATTACGGCTCATCTGTCCCAAAGAAGTATTGCCG
ATATTATGTGGTTCGATACCGCCATCGCTGCCATCGGTTTTTTACTGGCACTGTTTATCGGCTTACGCAGCCGCAAAAAA
ACGCGGCATCACTCGCTAAAGGAAAATGTCGCTCCGGGTGGGTAATGCAATATTCTTTTCAGGTCATGCAAGATCTTACG
GATAAATAACTCTTTCTGCGCTAACTAAGGAAAATCGCGATCAAAAACAAACTATGACATGCAATATTCCTGGAAACATA
AACTTTATGCCATGTACCCAGGGAAAATCATCTTCAGTATAGTAATTATGTAAACCGTCGGAGAACAATACGTACGGTAA
CGAAATTATCTTTCAGCAAGGAGCTGTGAAAATGTCTCAAAATAAGGCTTTCAGCACGCCATTTATCCTGGCTGTTCTTT
GTATTTACTTCAGCTACTTCCTGCACGGCATTAGTGTTATTACGCTTGCCCAAAATATGTCATCTCTGGCGGAAAAGTTT
TCCACTGACAACGCGGGCATTGCCTACTTAATTTCCGGTATCGGTTTGGGGCGATTGATCAGTATTTTATTCTTCGGTGT
GATCTCCGATAAGTTTGGTCGTCGGGCGGTGATATTAATGGCAGTAATAATGTATCTGCTATTCTTCTTTGGTATTCCCG
CTTGCCCGAATTTAACTCTCGCCTACGGTCTGGCAGTGTGCGTAGGTATCGCTAACTCAGCGCTGGATACGGGTGGCTAC
CCCGCGCTCATGGAATGCTTTCCGAAAGCCTCTGGTTCGGCGGTCATACTGGTTAAAGCGATGGTGTCATTTGGGCAAAT
GTTCTACCCAATGCTGGTGAGCTATATGTTGCTCAATAATATCTGGTACGGCTATGGGCTGATTATTCCGGGTATTCTAT
TTGTACTGATCACGCTGATGCTGTTGAAAAGCAAATTCCCCAGCCAGTTGGTGGACGCCAGCGTAACTAATGAATTACCG
CAAATGAACAGCAAACCGTTAGTCTGGCTGGAAGGTGTTTCATCGGTACTGTTCGGTGTAGCCGCATTCTCGACCTTTTA
TGTGATTGTGGTGTGGATGCCCAAATATGCGATGGCTTTTGCTGGTATGTCAGAAGCTGAGGCATTAAAAACCATCTCTT
ATTACAGTATGGGCTCGTTGGTCTGTGTCTTTATTTTTGCCGCACTACTGAAAAAAATGGTCCGGCCCATCTGGGCTAAT
GTATTTAACTCTGCACTGGCAACAATAACAGCAGCCATTATCTACCTGTACCCTTCTCCACTGGTGTGCAATGCCGGAGC
CTTTGTTATCGGTTTCTCAGCAGCTGGCGGCATTTTACAGCTCGGCGTTTCGGTCATGTCAGAGTTTTTTCCCAAAAGCA
AAGCCAAAGTCACCAGTATTTATATGATGATGGGTGGACTGGCTAACTTTGTTATTCCACTGATTACCGGTTATCTGTCG
AACATCGGCCTGCAATATATCATTGTTCTCGATTTTACTTTCGCGCTGCTGGCCCTGATTACCGCAATTATTGTTTTTAT
CCGCTATTACCGCGTTTTCATTATTCCTGAAAATGATGTGCGGTTTGGCGAGCGTAAATTTTGCACCCGGTTAAACACAA
TTAAGCATAGAGGTTAAAGGAGTTAATTATGGATGTTACCGCAAAATACGAATTGATTGGGTTGATGGCCTATCCTATCC
GCCACAGTTTATCGCCCGAAATGCAGAATAAAGCCTTAGAAAAAGCGGGATTGCCATTTACCTATATGGCCTTCGAAGTG
GATAACGATAGCTTTCCTGGAGCAATTGAAGGATTAAAAGCCCTCAAAATGCGCGGAACTGGTGTATCGATGCCGAACAA

ACAACTGGCGTGTGAATATGTTGATGAATTAACACCAGCTGCCAAACTGGTGGGGGCCATCAACACCATCGTTAATGATG
ATGGCTATCTGCGTGGCTATAACACCGACGGCACGGGCCATATTCGCGCCATTAAAGAGAGCGGTTTTGATATCAAAGGC
AAAACGATGGTGCTGTTAGGGGCCGGTGGTGCCTCAACGGCAATTGGCGCGCAGGGGGCAATTGAAGGTTTAAAAGAAAT
TAAACTCTTTAACCGTCGGGATGAGTTCTTCGATAAAGCCCTCGCCTTCGCGCAGCGGGTTAATGAAAACACCGATTGTG
TCGTCACGGTCACCGATCTCGCCGATCAGCAAGCCTTTGCTGAAGCCCTGGCTTCCGCCGACATTTTAACCAATGGCACA
AAAGTGGGTATGAAACCCCTTGAGAATGAATCATTGGTTAATGATATCAGTCTGTTACATCCGGGACTTCTGGTCACTGA
ATGCGTGTATAACCCGCATATGACGAAGTTATTGCAGCAGGCGCAACAAGCTGGTTGCAAAACGATTGATGGATACGGCA
TGTTGTTGTGGCAAGGGGCTGAACAGTTCACATTATGGACTGGCAAAGATTTCCCTCTGGAATATGTTAAACAGGTCATG
GGGTTCGGTGCCTGACAGGCTGACCGCGTGCAGAAAGGGTAAAAAATGAAAACCGTAACTGTAAAAGATCTCGTCATTGG
TACGGGCGCACCTAAAATCATCGTCTCGCTGATGGCGAAAGATATCGCCAGCGTGAAATCCGAAGCTCTCGCCTATCGTG
AAGCGGACTTTGATATTCTGGAATGGCGTGTGGACCACTATGCCGACCTCTCCAATGTGGAGTCTGTCATGGCGGCAGCA
AAAATTCTCCGTGAGACCATGCCAGAAAAACCGCTGCTGTTTACCTTCCGCAGTGCCAAAGAAGGCGGCGAGCAGGCGAT
TTCCACCGAGGCTTATATTGCACTCAATCGTGCAGCCATCGACAGCGGCCTGGTTGATATGATCGATCTGGAGTTATTTA
CCGGTGATGATCAGGTTAAAGAAACCGTCGCCTACGCCCACGCGCATGATGTGAAAGTAGTCATGTCCAACCATGACTTC
CATAAAACGCCGGAAGCCGAAGAAATCATTGCCCGTCTGCGCAAAATGCAATCCTTCGACGCCGATATTCCTAAGATTGC
GCTGATGCCGCAAAGTACCAGCGATGTGCTGACGTTGCTTGCCGCGACCCTGGAGATGCAGGAGCAGTATGCCGATCGTC
CAATTATCACGATGTCGATGGCAAAAACTGGCGTAATTTCTCGTCTGGCTGGTGAAGTATTTGGCTCGGCGGCAACTTTT
GGTGCGGTAAAAAAAGCGTCTGCGCCAGGGCAAATCTCGGTAAATGATTTGCGCACGGTATTAACTATTTTACACCAGGC
ATAAGCAATAATATTTCGGCGGGAACACCCTCCCCGCCGAACTAAAAAATATATTCAATCGTATTTAATAAAAATATTTC
GTGAGTCTCTGTGCGCTAATTCTCCATTTGGCGTAGGGAAAATCACATCTGAATCAGGAATTAACAATGAAACCTGTAAA
ACCACCTCGTATTAATGGACGAGTGCCGGTCCTGTCGGCACAGGAAGCGGTGAATTATATTCCCGACGAAGCAACACTTT
GTGTGTTAGGCGCTGGCGGCGGTATTCTGGAAGCCACCACGTTAATTACTGCTCTTGCTGATAAATATAAACAGACTCAA
ACACCACGTAATTTATCGATTATTAGTCCAACAGGGCTTGGCGATCGCGCCGACCGTGGTATTAGTCCTCTGGCGCAAGA
AGGTCTGGTGAAATGGGCATTATGTGGTCACTGGGGACAATCGCCGCGTATTTCTGAACTCGCAGAACAAAATAAAATTA
TTGCTTATAACTACCCACAAGGTGTACTTACACAAACCTTACGCGCCGCCGCAGCCCACCAGCCTGGTATTATTAGTGAT
ATTGGCATCGGGACATTTGTCGATCCACGCCAGCAAGGCGGCAAACTGAATGAAGTCACTAAAGAAGACCTGATTAAACT
GGTCGAGTTTGATAACAAAGAATATCTCTATTACAAAGCGATTGCGCCAGATATTGCCTTCATTCGCGCTACCACCTGCG
ACAGTGAAGGCTACGCCACTTTTGAAGATGAGGTGATGTATCTCGACGCATTGGTTATTGCCCAGGCGGTGCACAATAAC
GGCGGTATTGTGATGATGCAGGTGCAGAAAATGGTTAAGAAAGCCACGCTGCATCCTAAATCTGTCCGTATTCCGGGTTA
TCTGGTGGATATTGTGGTGGTCGATCCGGATCAAACCCAACTGTATGGCGGTGCACCGGTTAACCGCTTTATTTCTGGTG
ACTTCACCCTTGATGACAGTACCAAACTTAGCCTGCCCCTAAACCAACGTAAATTAGTTGCGCGGCGCGCATTATTCGAA
ATGCGTAAAGGCGCGGTGGGGAATGTCGGCGTCGGTATTGCTGACGGCATTGGCCTGGTCGCCCGAGAAGAAGGTTGTGC
TGATGACTTTATTCTGACGGTAGAAACAGGTCCGATTGGCGGAATTACTTCACAGGGGATCGCCTTTGGCGCGAACGTGA
ATACCCGTGCCATTCTGGATATGACGTCCCAGTTTGATTTTTATCACGGTGGCGGTCTGGATGTTTGTTATTTGAGTTTT
GCTGAAGTCGACCAGCACGGTAACGTCGGCGTGCATAAATTCAATGGTAAAATCATGGGCACCGGTGGATTTATTGATAT
CAGTGCCACTTCGAAGAAAATCATTTTCTGCGGCACATTAACTGCGGGCAGTTTAAAAACAGAAATTACCGACGGCAAAT
TAAATATCGTCCAGGAAGGACGGGTGAAGAAATTTATTCGGGAACTACCGGAAATTACTTTCAGCGGAAAAATCGCTCTC
GAGCGAGGGCTGGATGTTCGTTATATCACTGAGCGCGCAGTATTCACGCTGAAAGAAGACGGCCTGCATTTAATCGAAAT
CGCCCCTGGCGTCGATTTACAAAAAGATATTCTCGACAAAATGGATTTCACCCCAGTGATTTCGCCAGAACTCAAACTGA
TGGACGAAAGATTATTTATCGATGCGGCGATGGGTTTTGTCCTGCCTGAAGCGGCTCATTAACAGGAGTATAATGATGGA
TTTTTCTTTAACTGAAGAACAAGAACTGCTGCTGGCCAGTATTCGCGAACTGATTACGACTAACTTTCCGGAAGAGTATT
TCCGCACCTGCGATCAAAACGGGACATATCCGCGTGAGTTTATGCGGGCGCTGGCGGATAACGGTATTTCCATGCTTGGC
GTGCCGGAAGAATTTGGTGGTATCCCTGCGGATTACGTCACCCAAATGCTGGCGCTGATGGAAGTGTCAAAATGCGGTGC
TCCGGCATTTTTAATTACCAACGGTCAATGTATTCACAGTATGCGCCGTTTCGGTTCTGCAGAGCAGCTACGTAAAACGG
CAGAGAGCACCCTGGAAACGGGTGATCCCGCCTATGCCCTGGCGTTGACGGAACCAGGTGCTGGCTCAGATAACAACAGT
GCCACTACCACTTACACGCGTAAAAACGGCAAGGTCTACATCAACGGACAAAAAACCTTTATTACCGGTGCGAAAGAGTA
CCCGTATATGCTGGTGCTGGCGCGCGATCCGCAACCGAAAGATCCCAAAAAAGCCTTCACCCTGTGGTGGGTCGACTCCA
GTAAGCCCGGCATTAAGATTAACCCGCTGCATAAAATCGGCTGGCATATGCTCAGCACCTGCGAAGTCTATCTCGACAAC
GTGGAGGTTGAAGAGAGCGACATGGTGGGCGAAGAAGGAATGGGTTTCCTCAATGTGATGTACAACTTTGAGATGGAGCG
CCTGATCAACGCCGCGCGCAGCACCGGCTTTGCCGAATGCGCCTTTGAAGATGCCGCCCGCTATGCCAACCAACGTATCG
CTTTTGGTAAGCCCATTGGTCATAACCAGATGATCCAGGAAAAACTGGCGCTGATGGCGATTAAGATCGACAACATGCGC
AACATGGTGCTGAAAGTGGCATGGCAAGCCGATCAGCATCAGTCACTGCGCACCAGCGCGGCGCTGGCAAAACTGTACTG
TGCACGTACCGCAATGGAAGTCATTGATGATGCGATTCAAATCATGGGCGGTCTGGGCTATACCGATGAGGCGCGCGTCT
CCCGCTTCTGGCGTGATGTCCGTTGTGAACGTATCGGCGGCGGTACAGACGAAATTATGATTTACGTAGCAGGTCGGCAG
ATCCTGAAAGACTATCAGAACAAATAATCTGCAGGCGGCGCAGCTTCTTAACAAACTGCGCCGCCAGATTTATCCAACAA
GACTTACCGGTTGAGGAAATTCAGATAGGATTTCTTGTTCCGCAAAACAGTTTTTAAATTGTCTACGGTAATCGCTGGGC

GAACAACCGACATGGCGCAAAAACAGTTTGGCAAAGTGATCGACATTTTCATAACCCACACGCCAGGAGATCTCTGCCTG
TGATAATTCAGTATTAGTGAGTGACCATTTTGCTTCCGTCATACGCCGTTGAATAACATAGTTAATAGGGGAAATACGAT
ACTCTTTGGTAAATTCATGGCAAATATAACTGACGCTGGCACGAAATTTTTTCGATAACTGTTCTAAAGTGATTTTTTCG
CGATAATTATTATTCAGATAGAAAAGAATATCTTTTATCAGAACATCTTTTTTAATATACCCCTGCTCCGAACGATAAGC
ATTTTTAAAATTTTCGTAGTAAAGAATGGCTAACGTATAGGCAAATGCATCCCATAACGAAGATGTTTGGCTATTTTTAC
TTTGCGGCAAAATCACACTTAGCTCATTAAATAAGGTTTTTATGACTTCTTTTCCCTGCCCCGCGGCAATTACCGGACAA
GAATGCGGTTGCAGTAGCTGATTTTCCTCAGCCCCCTGAAACTGAAAGCCGTACAGCGCACAGGTACACGTCGTTGCCGG
ATCGTTAACGTCAGAGGCCACCGCATGCAGCCTGCCGCGTTCTATCACCACAATGTCATCCGCATGTGCGACATATAACG
AAGAATCGATGGTAAACCTTGCAACCCCTTTCTTGACGTAAATCAACTCGGTTTCATTGTCATGAACGTGATGTCCGGAC
TCCCATTTTGGATCATCGCTAAATGCAAAACGTGAAAGCCGTGGCGTTTTACCGGCGACAAACAGCGTTTCACTGGCGTT
ATCAAAACAGCGTTGATACATGACAACCTCCCTATTCCATGAGCAAGCAAAAACAATATATGCCGGATAAAATACGGCGT
GTATTGCGGTTATACAACCGCGTTTAGCGAGAGCTAAACAAGATTATTTACACTGAATGCAAGATTGTACGGTCATGGAA
TAACTATATTGCCGCAAATTGCACTTTTGTTTTAGCGATCGCATTTTTTTTGCAAGATTGTTGGCAAGGAAAACAGCTTG
CTCCGTCGAAAACCCCGCACCGCTATCGCACACTATTTTCAGGCCATTTTTACCTTCCATCGGAGATGGTTCCGTATGCG
ACTCACAGGAGAAATCATGAAAATAATAACCTGCTTTAAGCTGGTGCCTGAAGAACAGGACATTGTTGTCACTCCAGAAT
ACACCCTGAATTTCGACAATGCCGACGCCAAAATCAGCCAGTTCGATCTCAATGCCATTGAAGCTGCAAGCCAGCTCGCA
ACAGATGACGATGAGATAGCCGCGCTGACCGTTGGCGGCTCATTGTTGCAGAACTCGAAAGTGCGCAAAGACGTGCTATC
CCGCGGGCCGCACAGCCTGTATTTGGTGCAGGATGCGCAACTTGAACATGCACTGCCTCTCGATACCGCAAAAGCGCTGG
CGGCAGCAATTGAAAAGATCGGCTTCGATTTACTGATCTTTGGTGAAGGTTCCGGCGACCTTTATGCCCAGCAGGTTGGC
TTGCTGGTCGGAGAAATTCTGCAACTTCCGGTGATTAATGCAGTGAGTGCTATTCAGCGTCAGGGCAATACACTGGTGAT
TGAACGCACGCTTGAAGATGATGTTGAAGTTATTGAACTCTCTGTTCCAGCCGTGCTCTGCGTCACCTCCGATATTAACG
TGCCACGCATTCCTTCGATGAAAGCCATTCTCGGCGCGGGTAAAAAACCGGTAAATCAGTGGCAGGCAAGTGATATTGAC
TGGAGCCAGAGCGCGCCACTTGCTGAACTGGTCGGCATTCGCGTACCGCCGCAAACAGAACGTAAGCACATCATTATCGA
TAACGATTCGCCGGAGGCCATTGCCGAGCTGGCGGAACATCTGAAGAAAGCCCTGAACTGAAGCCTACGGAGAAGAAACG
ATGAGTCAATTAAACAGCGTCTGGGTCTTTAGCGATAATCCTGAACGTTATGCTGAACTGTTTGGCGGCGCTCAGCAATG
GGGCCAACAGGTGTATGCCATTGTACAAAATACCGACCAGGCGCAGGCAGTTATGCCTTATGGTCCAAAATGTCTTTATG
TTCTTGCGCAAAACGACGCGCTGCAACGCACTGAAAATTACGCCGAAAGCATTGCTGCCCTGCTGAAAGATAAACACCCC
GCTATGTTGCTGTTGGCCGCGACGAAACGTGGTAAAGCGCTGGCAGCACGGTTAAGTGTGCAACTGAATGCGGCGCTGGT
GAACGATGCCACGGCGGTGGATATTGTCGATGGTCACATTTGCGCCGAACACCGGATGTATGGCGGGTTAGCGTTCGCTC
AGGAAAAGATCAACAGCCCGCTGGCGATCATTACCCTTGCACCCGGTGTTCAGGAACCGTGCACCAGTGATACCTCTCAT
CAGTGCCCGACAGAAACGGTACCTTATGTTGCTCCGCGTCATGAAATTCTCTGTCGCGAACGCCGTGCGAAAGCCGCAAG
CAGCGTGGACCTGAGCAAAGCAAAACGTGTGGTTGGCGTCGGTCGTGGACTGGCGGCGCAGGATGACCTAAAAATGGTCC
ACGAACTGGCGGCGGTGCTGAATGCTGAAGTCGGCTGTTCACGTCCAATTGCCGAAGGCGAGAACTGGATGGAGCGTGAA
CGTTATATCGGTGTCTCCGGCGTGTTGCTGAAATCCGATCTCTACCTGACGCTGGGGATCTCCGGGCAGATCCAGCATAT
GGTTGGCGGCAACGGCGCAAAAGTGATTGTCGCCATCAATAAAGATAAAAATGCGCCAATCTTCAACTATGCCGACTACG
GTCTGGTGGGCGATATCTACAAAGTCGTCCCTGCCCTGATTAGCCAGTTGAGCCGCTAATTCCCCTTTCAACCACATCTC
CCGCTGTAAACGCAGCGGGAAGGGAGCATAACGCATGTCGGATGACAAATTTGATGCCATTGTGGTCGGTGCGGGCGTTG
CTGGTAGCGTTGCCGCACTGGTCATGGCACGAGCCGGGCTGGATGTCCTGGTGATAGAACGCGGCGACAGTGCCGGATGT
AAAAACATGACCGGCGGGCGTCTTTATGCCCACACACTTGAAGCAATCATTCCAGGCTTTGCAGTATCAGCGCCGGTAGA
ACGCAAGGTCACACGCGAGAAAATCTCCTTCTTAACCGAAGAAAGCGCCGTTACCCTCGATTTTCACCGCGAGCAGCCAG
ATGTTCCGCAACACGCATCTTATACCGTATTGCGTAATCGTCTGGACCCGTGGTTGATGGAACAAGCCGAGCAGGCGGGC
GCGCAGTTTATCCCAGGCGTTCGCGTCGACGCGCTGGTTCGTGAAGGAAACAAGGTCACTGGCGTGCAGGCTGGGGATGA
TATTCTCGAAGCGAATGTGGTGATTCTGGCTGATGGCGTTAACTCGATGCTTGGCCGCTCGCTGGGAATGGTTCCCGCTT
CCGATCCGCATCATTACGCTGTTGGTGTTAAAGAGGTTATTGGCCTCACACCAGAACAGATCAACGATCGCTTTAATATT
ACGGGCGAGGAAGGTGCCGCCTGGCTGTTTGCCGGTTCCCCTTCTGACGGCCTGATGGGCGGGGGATTTCTCTATACCAA
CAAGGATTCCATATCCTTGGGGCTGGTTTGTGGATTGGGTGATATCGCCCATGCGCAAAAAAGCGTGCCGCAAATGCTGG
AAGATTTTAAACAACACCCCGCCATTCGCCCGCTGATTAGCGGCGGCAAACTGCTTGAATATTCCGCGCATATGGTGCCG
GAAGGCGGTCTGGCAATGGTGCCGCAACTGGTTAACGAGGGCGTGATGATCGTTGGTGACGCCGCAGGCTTCTGCCTGAA
TTTGGGTTTTACGGTCCGCGGCATGGATTTAGCCATTGCATCGGCTCAGGCTGCCGCCACAACGGTGATCGCCGCCAAAG
AACGCGCGGATTTCTCCGCCAGCAGTCTGGCGCAATACAAACGTGAGCTGGAACAAAGCTGCGTCATGCGTGATATGCAG
CATTTTCGCAAGATCCCGGCGCTGATGGAAAACCCGCGCCTGTTTAGCCAATACCCACGAATGGTAGCCGACATCATGAA
CGAGATGTTCACCATCGACGGCAAACCAAACCAGCCGGTACGAAAAATGATCATGGGACATGCGAAGAAAATTGGGCTGA
TCAACTTGCTGAAAGATGGCATTAAGGGAGCAACCGCGCTATGAGCCAGAACGCTACGGTTAACGTCGACATCAAATTAG
GCGTCAATAAATTCCATGTTGATGAGGGCCACCCGCATATCATTCTGGCGGAAAATCCCGATATCAATGAATTCCATAAA
TTAATGAAAGCCTGCCCTGCCGGACTTTATAAGCAGGATGACGCAGGAAACATTCATTTTGATTCCGCCGGTTGTCTGGA
GTGCGGCACCTGTCGGGTGCTGTGCGGTAACACTATTCTCGAACAGTGGCAATATCCCGCAGGCACCTTCGGTATTGATT

TTCGCTACGGCTAATCATGCATCCCACAGGCCCGCATCTCGGGCCTGATGTTCTGTTTCGAGAGTCCAACATGAAAGTGA
CATTAACGTTTAACGAACAACGTCGTGCGGCGTATCGTCAGCAAGGGTTATGGGGCGATGCTTCGCTGGCCGATTACTGG
CAGCAGACCGCTCGTGCGATGCCAGACAAAATTGCCGTGGTCGATAATCATGGTGCATCGTACACCTATAGCGCGCTCGA
TCACGCCGCGAGCTGTCTGGCAAACTGGATGTTAGCGAAGGGTATTGAATCAGGCGATCGCATCGCATTTCAACTGCCTG
GCTGGTGTGAATTTACCGTTATCTATCTTGCCTGCCTGAAAATCGGTGCAGTTTCCGTGCCGCTGTTGCCTTCCTGGCGG
GAAGCAGAACTGGTGTGGGTGCTCAATAAGTGTCAGGCAAAAATGTTCTTTGCACCGACGTTGTTTAAACAAACGCGTCC
GGTAGATTTAATCCTGCCGCTGCAAAATCAGCTTCCACAACTACAACAAATTGTCGGCGTGGACAAACTGGCTCCCGCCA
CCTCTTCCCTCTCATTAAGTCAGATTATCGCCGACAATACCTCACTGACCACGGCGATAACGACCCACGGCGATGAATTA
GCTGCGGTGCTGTTTACCTCCGGAACCGAGGGTCTGCCAAAGGGCGTGATGCTAACGCATAACAATATTCTCGCCAGTGA
GCGGGCTTATTGCGCGCGACTGAATCTGACCTGGCAGGATGTCTTTATGATGCCTGCGCCACTTGGTCACGCAACGGGCT
TTCTGCATGGCGTAACGGCACCATTCTTAATTGGCGCTCGCAGCGTGTTGTTAGATATTTTCACTCCTGATGCGTGTCTC
GCGCTGCTTGAGCAGCAGCGTTGCACCTGTATGCTCGGCGCAACGCCGTTTGTCTATGATCTTTTGAATGTACTAGAGAA
ACAACCCGCGGACCTTTCAGCGCTGCGTTTCTTTCTTTGCGGCGGAACCACAATCCCCAAAAAAGTGGCGCGTGAATGCC
AGCAGCGCGGCATTAAATTATTAAGTGTTTATGGTTCCACAGAAAGTTCGCCGCATGCGGTGGTGAATCTCGATGATCCT
TTGTCGCGCTTTATGCACACCGATGGTTACGCTGCCGCAGGTGTAGAGATTAAAGTGGTCGATGACGCACGCAAGACCTT
ACCGCCAGGTTGCGAAGGTGAAGAAGCCTCGCGTGGCCCCAATGTGTTTATGGGGTATTTTGATGAACCTGAATTAACCG
CCCGTGCCCTGGATGAAGAAGGCTGGTATTACAGCGGCGATCTCTGCCGTATGGATGAGGCTGGCTATATAAAAATTACC
GGACGCAAAAAAGATATTATTGTCCGCGGCGGCGAAAATATTAGCAGCCGTGAAGTGGAAGATATTTTATTGCAGCATCC
TAAAATTCACGATGCCTGTGTGGTTGCAATGTCCGATGAACGTTTAGGTGAACGATCATGCGCTTATGTCGTGCTGAAAG
CGCCGCATCATTCATTATCGCTGGAAGAGGTAGTGGCTTTTTTTAGCCGTAAACGGGTCGCAAAATATAAATATCCTGAA
CATATCGTGGTAATCGAAAAACTACCGCGAACTACCTCAGGTAAAATACAAAAGTTTTTGTTAAGAAAAGATATTATGCG
GCGTTTAACGCAGGATGTCTGTGAAGAGATTGAATAAGTTTCATCTTCGGGGATCACATAACCCCGGCGACTAAACGCCG
CCGGGGATTTATTTTATTTCTTCAGTTCAGCCAGGCTTAACCAGGTTTGCACCACGGTGTCCGGGTTCAGAGACAGGCTA
TCGATCCCCTCTTCCATCAACCATGCGGCAAAGTCTTCGTGGTCGGACGGACCCTGACCGCAAATCCCGACATATTTGCC
CTGTTTCTTCGCGGCACGGATAGCCATCGACAGCAGTGCTTTCACCGCATCGTTGCGCTCATCGAACAATTCAGACACCA
CGCCGGAGTCACGGTCCAGACCGAGCGCCAGCTGCGTCATATCGTTTGAGCCAATTGAGAAGCCGTCGAAATATTCGAGG
AACTGCTCGGCCAGCAAGGCGTTGGACGGGATTTCACACATCATGATGATTTTCAGCCCGTTCTCGCCACGTTTCAGCCC
CTGACGCGCCAGTTCTTCAACCACCGCTTTCGCCTGATCTACGGTACGCACGAACGGGATCATGATCTCAACGTTGGTCA
GTCCCATGTCGTTGCGCACACGTTTCACTGCTTCACACTCCAGCGCGAAACAGTCGCGGAAGCTGTCGGAAACATAGCGG
CCCGCGCCACGGAAGCCGAGCATCGGGTTCTCTTCATCTGGCTCGTAACGCTCACCACCGACCAGGTTGGCATATTCGTT
CGATTTAAAATCAGAGAGACGGACAATGACGCGCTTCGGATAAAACGCGGCACCCAGCGTCGCGATCCCTTCAGTCAGAC
GACCAACGTAAAATTCACGCGGAGAATCAAAACCTTTCATCATCTCGCGGATTTCGTTTTGCAACTGCGGTTCCTGATCG
TCAAACTCAAGCAGTGCGCGTGGGTGGACGCCAATCATACGGTTGATGATAAATTCCAGACGCGCAAGGCCCACGCCTTC
GTTCGGTAGGCAGGCGAAGTCGAAAGCACGGTCCGGGTTACCGACGTTCATCATCACTTTCAACGGCAGATCCGGCATCG
TTTCTACGCTGGAGCTTTTCACGCTAAATTCCAGCAACTCCGCATAGACGTAACCGGTATCACCTTCGGCACAAGAAACA
GTGACGTTCTCACCGTCTTTCATCCGTTCTGTTGCATCTCCACAGCCCACTACCGCCGGAATGCCCAGTTCACGAGCGAT
GATCGCCGCGTGACAGGTACGACCGCCACGGTTGGTGACGATGGCAGATGCTTTCTTCATGATCGGTTCCCAGTCCGGGT
CGGTCATGTCAGTAACCAGCACGTCGCCAGGTTCGATGCGGTTCATTTCGCTGATGTCATGGATGACTTTCACCGGACCC
GCACCGATGCGATGACCGATAGCACGGCCTTCGGCGATAATCTTACCCTGTGAATGCAGCGTATAACGCTCCATGACCTG
ACCGCGTGAGCGCACGGTTTCCGGACGCGCCTGCACAATGAACAGTTTACCGGTGTGGCCATCTTTCGCCCACTCAATAT
CCATCGGGCGACCGTAGTGTTTCTCAATTTGTACGGCCTGTTTTGCCAGTTCCTGCACTTCTTCGTTGGTCAGCGAGAAG
ATGTCACGCTGTTCCTGCGGTACGTCTTCGATTTTAACCTGCTTGCCGTGCTCCTGGGTCGGCGCGTAAACCATGCGGAT
TTTTTTCGACCCCATGGTGCGGCGCACGATAGCCGGGCGATTCGCCGCCAGTGTCGGTTTATGCACGTAAAACTCATCCG
GGTTAACCGCACCCTGCACGACCATCTCACCAAGGCCCCATGCGGAAGTGATAAACACCACCTGGTCAAAGCCGGATTCG
GTATCAATGGAGAACATCACGCCAGATGATGCGAGGTCAGAGCGCACCATCCGTTGAACACCGGCGGAGAGCGCCACACC
ACGGTGATCGTAACCCTGGTGCACACGATAAGAGATGGCGCGATCGTTAAACAGAGAAGCAAATACATGTTTCACTGCCA
CGAGAACGGCGTCAAAACCCTGAACGTTGAGGAAGGTTTCCTGCTGACCGGCAAAAGAAGCGTCCGGCATATCTTCTGCG
GTGGCGGAGGAGCGCACCGCAAAAGAGGCGTTTTCGTCATCGGCGGAAAGCTGTGCATAGGCTTCGCGGATGGCGTTTTC
CAGCTCAGGCTGGAAGGGAGTGTCGATAATCCACTGGCGGATTTGCGCGCCCGCTTTCGCAAGCTGAGTAACATCGTCAA
TATCCGTTTTATCCAGCAGTTCATAAATGCGCTGGTTTACGCCGCTTTGGTCCAGAAACTGGTTAAACGCGTCGGCGGTT
GTGGCGAAACCATTCGGAACGGAAACACCCATTCCGGAAAGATTAGTAATCATTTCACCCAGGGAGGCATTTTTGCCCCC
AACCCTGTCTACATCATTCATGCCGAGTTGGTTATACCAAAGCACCAGCGGTGACGAGCCATTGTTGGACATCGAACAAT
CCTTTTGTGATAAATGAACGGTTTGAGAAACACATTTCTGCGCATTTATCTTTGCATATTTAACCGGATGAAAAAAACGG
TGAATCGTTCAAGCAAATATATTTTTTTACTTTTTAAGACTGATCCCAGCGTTGCGCAAATCTGCTCTCTTCGACAATTT
CCACATAAACCATCGGTATAAACGAAACCATAAAAATGAAATGCTGTTTTCATAAAAAATAAAATTGAAGGTTCATTTTA
TAAACCAGACATAACGTTCTACGCTTCTGTGCGTTTTTAATTTATGCTTTCATAGAATTATGTCTGCATCACGGGAAGAA

CAAAATGGATAATGCTGTTGATCGCCACGTTTTTTATATTTCTGATGGTACGGCAATAACTGCGGAGGTATTAGGACACG
CAGTAATGTCACAATTTCCCGTCACTATCAGCAGCATCACGCTGCCGTTTGTCGAAAATGAGAGCCGTGCACGGGCAGTG
AAGGATCAGATTGACGCAATTTATCACCAGACAGGCGTGCGCCCGCTGGTCTTCTACTCCATCGTGTTGCCGGAGATTCG
CGCCATCATCTTGCAAAGTGAAGGCTTTTGCCAGGATATCGTTCAGGCGCTGGTTGCCCCGCTACAACAAGAGATGAAAC
TGGATCCAACGCCGATTGCTCATCGTACCCATGGCCTTAACCCTAATAATCTCAATAAATATGATGCGCGCATTGCGGCG
ATTGATTACACCCTCGCCCACGATGACGGCATTTCGTTGCGCAATCTGGACCAGGCTCAGGTGATCCTGCTCGGTGTTTC
TCGCTGTGGTAAAACCCCCACCAGTCTGTATCTGGCAATGCAGTTTGGTATCCGCGCGGCAAACTACCCCTTTATTGCCG
ACGATATGGATAATCTGGTGCTACCCGCGTCGCTCAAACCGCTTCAGCATAAATTGTTCGGCCTGACTATCGACCCGGAA
CGTCTGGCGGCGATTCGCGAGGAACGTCGGGAGAACAGTCGCTATGCCTCGCTTCGTCAGTGCAGGATGGAAGTCGCGGA
AGTGGAAGCCTTGTACCGTAAAAATCAGATCCCGTGGATTAACAGTACCAATTATTCGGTAGAAGAGATTGCCACCAAGA
TCCTCGATATCATGGGCCTTAGTCGCCGAATGTACTAGAGAACTAGTGCATTAGCTTATTTTTTTGTTATCATGCTAACC
ACCCGGCGAGGTGTGACACACCTCGCACTTGAAATCAGCAGCGATTGGTTTATCGTGATGCGCATCACTTCCCGGCAGTC
CTGCCGTAGAAGCAACAAATTTCTGAGACTTGTAATGAACAGAACTGACGAACTCCGTACTGCGCGTATTGAGAGCCTGG
TAACGCCCGCCGAACTCGCGCTACGGTATCCCGTAACGCCTGGCGTCGCCACCCATGTCACCGACTCCCGCCGCAGAATT
GAAAAAATACTGAATGGTGAAGATAAGCGACTGTTGGTCATTATTGGCCCCTGCTCGATCCACGATCTCACCGCTGCAAT
GGAGTACGCCACCCGTCTGCAGTCGCTGCGCAACCAGTACCAGTCACGGCTGGAAATCGTAATGCGCACCTATTTTGAAA
AACCACGAACTGTTGTCGGCTGGAAAGGACTAATCTCCGATCCAGATTTAAACGGCAGCTATCGGGTAAATCACGGTCTG
GAGCTGGCGCGCAAATTACTTTTACAGGTAAATGAGCTGGGCGTCCCAACCGCGACCGAGTTCCTCGATATGGTGACCGG
TCAGTTTATTGCTGATTTAATCAGTTGGGGCGCGATTGGCGCACGTACTACCGAAAGTCAGATCCACCGCGAAATGGCTT
CGGCACTCTCCTGTCCGGTAGGTTTTAAAAATGGTACCGATGGCAATACGCGGATTGCTGTGGATGCTATCCGCGCAGCC
CGCGCCAGCCATATGTTCCTCTCGCCAGACAAAAATGGTCAGATGACCATCTATCAGACCAGCGGCAACCCGTATGGCCA
CATTATTATGCGTGGCGGCAAAAAACCGAATTATCATGCCGATGATATCGCCGCAGCCTGCGATACGCTGCACGAGTTTG
ATTTACCTGAACATCTGGTGGTGGATTTCAGCCACGGTAACTGCCAGAAGCAGCACCGTCGCCAGTTAGAAGTTTGTGAG
GATATTTGTCAGCAAATCCGCAATGGCTCTACGGCGATTGCTGGAATTATGGCGGAAAGTTTCCTGCGCGAAGGAACGCA
AAAAATCGTCGGCAGTCAGCCGCTCACTTACGGTCAATCCATTACCGACCCGTGTCTGGGCTGGGAGGATACCGAACGCC
TGGTCGAAAAACTCGCCTCTGCGGTAGATACCCGCTTCTGAATGCGTGCCCATTCCTGACGGAATGGGCATTTCTGCGCA
ACTTGTTGTCTTCTCAACAAATTACTGCTTGCTCTGGTCAGCCATAATATTGATAATAAGAATCATTGTTATATCAATTA
TTATTAATTTTTATGCGTTATACGGATAGCAGAAAACTCACGCCTGAAACGGATGCCAATCACAAGACCGCTTCCCCGCA
GCCTATTCGGCGAATTTCCAGCCAGACACTGTTAGGTCCGGATGGCAAACTGATTATCGATCATGACGGGCAAGAATATC
TGCTCCGTAAAACTCAGGCTGGCAAGCTGCTGTTGACCAAGTAGCCTTTAACTCGAGCAGCTGACTTCCAGCCGTTTACC
CCAGTCAGGTGGACGACTGACATAGTCATCATCTCTGTCGCTGAAAGGATTTCGCAACGCCTCATGCAGGCGGTGCAATT
CCGTCATATCACCCTTTTCTGCCGCTTCAATCGCCCGTTGCGCCAACCAGTTGCGCAATACCAGAGCGGGGTTAACGCTT
TGCATCAGTTGCTGACGCTCACTATCGCTAACCTCGTCTTGTTGCAAACGCCCCCGATAACGGGCAAACCAGTCATCAAA
TGCCGCACGATCAATAAACTCATCACGTAGCGGTGACGCCGCGCTGTGCTGCTCGGTCAGACTCAGCATGCGGAATGTGC
GGGTATAATCGCTGCGCTCTCGCGCCATCAGACTGAATAATTCATTCAGTAGCGCGTTATCCTCTTTTTGCTCCGTCATG
AAGCCCAGTTTCTGCCGCATCCGTTCTCCATAATGCGTCAACAAAACCTGCTGATAGCTGTCCAGGGCCTCATTCAGGGC
ATCTACGGCAACAAATGGCGACAATGTCTGCGCCAGACGCTGTAAATTCCACAACGCGACGGCAGGTTGATTATCAAAGC
TGTAACGCCCTTGATGATCCGAGTGATTACAAATAAAACCGGGTTCGTAATCATCAAGAAAACCAAACGGCCCGTAATCA
AGCGTCAGCCCCAGCAGCGACATGTTGTCGGTATTCATCACCCCATGAGCAAAGCCGACCGTCTGCCATTGGGCAATTAA
CGACGCGGTACGTGCGACAACATCGCTAAACCAGAGACGGTATTTGTCCTCATCATCTGCAAGATGTGACCAGTAATGAC
GAATAGCAAAGTCAGCTAACTGACGAACCTTTTCCGACTCGCGGCGGTAGTAAAAATGTTCGAAATGACCAAAGCGCAGA
TGACTTGGTGCCACACGCATCAGCATCGCGCCTGGCTCCGCCGTTTCCCGATACACTGGCGAATCGCTGGTGACGATACT
TAACGCGCGGGTCGTCGGAATGCCCAGATAATGCATCGCCTCACTGGCGAGACTTTCTCGTATCGTCGAACGTAAAACCG
CCCGTCCATCACCCATTCGCGAATAAGGCGTCAGGCCAGCACCTTTCAGATGCCAGTCCATTGTAGTGCCATCAGCAAGC
AGTTGTTCGCCGAGTAAAATGCCGCGCCCATCACCCAGTTGGCCCGCCCAGACGCCGAACTGATGACCACTGTAAACCTG
CGCCAGTGGTGACATACCAGGCAGTAGGGCTTCGCCGCCCCAGACACCTGCGCCATTTTTAAACAGCGACGATGGAATAC
TCAGCGTGTTAGCCAGTTCGGTATTATGCCAAATCAGCCGGGCATTATTTAAAGGCGTAGGGGAAAGTGCTGTATAGGTT
TCTGGCAATTCATCGCGCCAGCGGGTAACAAAAGACAGGGTCATAGATCCTCCTGTTTGATAGTGTAGACGGTTACTCTC
GTCTTAAACACCAGCAAACAGAAGGGTTATCGCTGAACCAACGTCGTTATCTGATTTATGGGAACGGCAGGCCACAGGCA
TCCCTGGAGAGCATGGAAATCGAAGGGGGTAATCTGTGCCAATATTTCTGCCGTATCAATGCCTCCCGCAATGATGCAAT
TGCAACAAGGGGAAATTTGCGCCTGGATCGCACGAATAAACGGTTCGAAGGAGCGGTGCGTGATTTGTTGCTGAATAAAA
CTTTTATCCAGCATGACGCGGGTGAAAAGACCATCAAAAACCGCTTTCATTGTGCTATTGCCTGCTCCCAAATTACCTAA
CACAAGGGGATACACTTGCGATAAGGATAAAAGACCGCGATTATCTTTTCCTTCATTGAGATGCGGGTAATTCTCATTAA
TCAACAGTTCAATAAACGGATATTTTAATAGTTCGCCTGCGTAATTATCCCTCTCTAATAATAATGTAGCCACTTGTGGC
GTTAAATTAAGCCAGGCAAAGAGTTTATGTTGAATAAAAAAATGTTGGCATGATTTTAGTAATTCCAACTGCTCTGAAAA
TAATTGCCAATGTTGCTCCTCGGTAAGTTGCGCTATAACCCGACTCGTCGGGATACGAACCGTACCATCTTCACTGGAGA

AATGGGTAATTAACTCAACGCCGACAAGGACTTGCTGGTTGTCTCTTATCGGCAGGAAATAGCAATCAGAATGATAAAGA
TTCTCCAAAAAAATCTTCATGGTAACCGTCCCTCTCGAAGGATGTTTTTCAGTATCCGGCTAAAAGGGATGAGGCCATAA
GACATAACAATTACAGAAGGAGTAACTTTCATTTGTTCCATGTTAACCACTTTTTCAGGGGTCCTTTTTTTAGATTATCC
TGATTATAAACGAATAATCCATTTACGGAATTTTTGTCTGCAAAATACTACTGTATTCAGGAGTAAATGGACGGCGATGT
ATCATCGCCGTCCATTTTTCGACATACTAGATTCGTCTCGCCTGCCAGAAATTTTTCTGCCAATAGACATTATCAAGTGA
GGAACGCATCACTCCCTTGCTGGTAGAGGCGTGGATAAATTGGTTGTTGGTATCGTAAATACCTACATGCAAACCATTTT
GTCCGGACCCCGTTTTGAAAAAGACCAGGTCACCTGGCAGCAACTCGTCTTTATCAATTTGCGTGCCAATAGAGGCTTGT
TGTTTGGTTTCACGGGGCAGCTGCAAATCGAAACGATCGCGCATCGTCACAACCACAAATCCCGAACAGTCCACACCGCG
CCGCGTCATGCCACCATAACGATACGGCGTGCCATGCCAGCTTTGTAGCTGGTCGTTCAAACCGGCAATAACGGTAATCG
AATCAGAAAGTCTGGCATTTGGCGGCGGTGCTTTATGGTGGCTACACCCGGCCAGAAGCAGTGCTGTGATCAAAATAAGG
CAGAAACGCATTCCGTACGGTTCCTCTGTTTTTTATTCTTGCATTAATTTAGCGTCGTAATTACCCGATTTTCAAGATAC
TAATGAAATCAGATGGTCGAAATCAGCATTCTGTGACCTTCGATATCCAGACGGCGAAAATTCATCCCATAGGCCTGCGC
CAGATTTGGCGGCGTGAGCACCTCTTCCCTGCGTCCACTGGCCAGCATTTTTCCACCTTTTAGCAACCACGCCCGATGCG
CATGACGCAATGTGTGGTTGAGATCGTGACTGCTCATCACAATCGCCAGTCCTTGCTGACACAGCGCGCTCAGAATTTTG
TCTAACGCACTTTGTTGCGCAACATCAAGACTGTTCATCGGCTCATCAAGAAGCAGCAATTGGCCTGCGGGATTGGCTTG
TGGTGTGATTTGCAACACCACCGCAGCAAGACGTACGCGTTGCCATTCACCGCCGGAAAGTTGATTGGTGCTACGTCCGA
GTTTGTCATCAAGAGCCAGCGCCCCTGCGACATCATTCAGTAGTTCGGTACGCGTTTTATCGTGCTGATGCAGTGTCAGG
TAGTGCCAGACCGGCGTTGCAAACGGCGGCGTCTGCTGTTGTGAAAGATAGGCGCGATGCAGCGCGAGTTTTGTTGCGGA
CCATGCTTCCAGTGGTTGCCCCGCGAACTGAATGCTTCCCTTACCGCTGGTCATTCCGGCCATTCGCGCCAGTAAGGTAC
TCTTACCCGCGCCATTCGGCCCCACCAGGTGCAGGATCTCCCCAGCCCGAACCTCGCCAGAAAGCGGCCCCAGGCGGGTA
GATTCCGCAACATCTTGTAACTGCATCACAATAGACATTATTTTGCCAACGCCAGTTTAATGCTTTCCATCACAATGGGA
TCTTCCGGCGTCATATCCGGGGAAAAACGCTGGATGACTTTTCCGTCCCTGCCAACCAGGAATTTTTCAAAATTCCATAA
AATATCATCCGGGTACAGCGGTGCACGGCCTTTGCTGACCATACGGGCATAGAATCCGCTCTCTTCCGGCGCGACTGCGG
TCGGCGCTGCGGCAATCAATTTTTGATACAGCGGATGGCGTCCTTCGCCATTAACTTCAATCTTACTGAACATCGGGAAC
GTCACCCCCCATGTGGTGGTACAGTAAGTTTTAATCTCTTCATCGCTGCCCGGTTCTTGTTCCAGAAACTGGTTGCACGG
GAATCCCAGCACCATAAAACCTCGATCGACCCAGGCTTTCTGAATATTCTCCAACTGCTCATATTGCGGCGTTAAGCCAC
ACTTTGAGGCGACATTGACAATCAACAGCACATTACCGGCGAACTTCTCCAGCGTGGTCACTTCACCGTCGATATCTTTC
ACTACGGTCGTCAGAATGGAATCTTGCATCGTTTCTCCTGGGTGTGGTCAGTAAAAATCTTAGCTTTTAATCATAGACCG
TCTTTTTGCGGCTAACGTCCTGCTTTTAACAATAACCAGATAAACACCGGCGCACCTAACGTTGCGGTGACCACGCCAAT
AGGCAGCTCTGCGGCAGCTAATGCCAGGCGCGCTACAATATCGGCCAGCAGCAATGCGCTCGCCCCTGCCAGCGCGCAGC
CGGGAAGTAATACGCGATGATCGGTTAAACCACACAACCGGAGAATATGGGGGATCACCAGACCAATAAAGCCGATAGCA
CCCGCCAGCGCCACACTGACGCCAACCATCCAGCCGGTCGCTGCCACCAGCACATTGCGCCAGAACCACAGGGGTAAACC
CAGTTGCCGCGCCGAGATCTCGCCAAGTGCTAACATATTCATCGGCCTGGACTGACAACAGATCCACAACAACACGGGGA
TCAATGCCAGCATCAGCCAGCTTTGCCGCCAGTCTACGCCGCCAAAACCGCCCATCATCCAGTACATCAGCTGACGCAAA
TCAACTGAGGTGGAAAAGTAGATAGCCCACGTCATTAGTGCGCTACAGATAATCCCTAATGCAACGCCAGCCAGCAATAA
CCGACTGGTCGAAAGATGACGACGGGCGAAACGTAAGAGTATTAAAGTGATGATAAGCGCGCCAGCAATCGCACACAGCC
CTAGCGCCCAGTTGGGGAGTTGCCCTTGCCCAAGCAATACCGCGGCGATAAGCCCCACGCCTGCGCCGTTAGAGACGCCA
AGTAGTCCAGGTTCTGCCAGAGGATTTTCAAACAACGCCTGCATTACAGCGCCGGATATAGCCAGCGCCGCACCAACCAG
CAATACAGCCAGCGTACGTGGCAGGCGAATTTGCCAGACGAACAGTTCGCCACGAGGAGTAAACCAGTCACCTGGCGAGA
TCCATTGTTCACCGGCGCAAAGGCTTAAGAGAAGCGCCAGCAGCATCAAAACTGACAGGCATAATAACCAGCGAATATTT
TGTCGCTGTTGTTGGCGGGCAAGTGTCAGCATGGTATCCGTTCTGCTGAAGTGTCATGGCGTTGATTTTACGGTGACTCT
TCGACAGTGAAAAGAAAAAAGGCCGCAGAGCGGCCTTTTTAGTTAGATCAGATTACTCGTCTTTGGGCGAAGCGTTTTCG
ACCCGGCTTTTTAACTTCTGCCCGGGTCTGAAGGTCACCACGCGCCGTGCTGTAATGGGAATATCCTCGCCCGTTTTCGG
GTTACGTCCCGGGCGTTGATTCTTATCACGCAGATCGAAGTTACCAAAACCAGAGAGTTTCACCTGTTCGCCGTTTTCCA
GAGCGCGACGGATCTCTTCGAAAAACAGTTCAACCAGTTCTTTGGCATCCCGCTTGCTAAGCCCAAGCTTATCAAACAGA
TATTCTGACATTTCAGCTTTTGTAAGCGCCATAGGTTCAATCCCTCAATGATGCCTGGAATCGCTCTTTTAATGCCTCTA
CACATTTGGCGACGGTAGCGGCAATCTCCTCTTCTTCGAGTGTACGGCTGGTATCTTGCAGGATCAGGCTTATGGCGAGG
CTCTTATACCCCTCCGCAACACCCTTACCGCGGTACACGTCAAATAAGTTTACGCCAACTACCTGATTTACGCCAACTTT
CTTACATTCGGATAAAATATCCGCTGCGGGAACGTTTTCTGCGACCACCACCGCGATGTCACGACGGTTCGCCGGGAAGC
GAGAAATCTCGCGCGCCTGAGGCACCACGCGGTCTGCGAGCTTGTTCCACTCCAGTTCGAACACCAGAGTGCGACCGTTA
AGATCCAGTTTACGTTCCAGTTCAGGATGAACAACCCCAACAAAACCAATACGTTCACCTTTCAGATAAATCGCTGCGGA
TTGCCCCGGATGCAGTGCCGGATTCGCTTCTGCACGGAACTCAACCTCATTCAGTTTACCGGTCAGGTCGAGAACGGATT
CAAGATCGCCTTTCAAATCATAGAAATCAACGGTCTCTTTTGCCAGGTTCCAGTGCTCTTCGTAACGGTTACCGCAAATC
ACACCGGCTAACATCAGATCCTGACGAATGCCCAACGGTGCCTGAGTATCTGGTACGAAACGCAGACCGCTTTCGAAAAT
GCGCACACGGTTCTGCTGACGGTTCTGGTTGTACACCACGGTTGCCAGCAGGCCAGTCCACAGAGAAAGACGCATTGCTG
ACATTTCAACAGAGATCGGGCTTGGCAGCAGTAAGGCTTCAACGCCTGGATGGATCATCTGCTGCACTTTCGGATCAACG

AAGCTGTAGGTGATCACTTCCTGATAGCCTTTGTCGTTGAGCAGCGTTTTCACGCGCTTGAGCGACAGGTCAGCTTCACG
GTGAGTACCCATAATCAGGCTTGCCTGTACCGGCTCATCCGGGATGTTGTTGTAGCCGTAAACACGCGCGACTTCTTCAA
CCAGATCTTCTTCAATCTCCATATCGAAACGCCAGCTCGGCGCAACTGCCTGCCATTCGTCTTTGCCTTCGGTCACTTCG
CAGCCGAGACGACGCAGAATGTCAGTTACCTGCTCATCCGCAATATGATGGCCGATCAGGCGATCCAGTTTGCTACGACG
TAGAGTGATGGTTGCACGCTTCGGCAGCGTTGCTTCGTTGGTGATATCAATTACCGGGCCAGCCTCACCACCGCAGATGT
CGATCAGCAGACGGGTCGCACGTTCCATCGCTTTGTGCTGCAGTGCCGGATCAACGCCACGCTCATAACGGTGAGACGCA
TCGGTATGCAGGCCATGACGACGAGCACGACCGGTGATAGACAGCGGGCTAAAGAACGCGCATTCCAGCAGCACGTTTTG
TGTTTCGTCATTCACGCCAGAGTGTTCGCCACCGAAGATGCCGCCCATCGCCAGCGCCTTGTTGTGGTCGGCGATGACCA
GAGTGTCAGCATTCAGCTTCGCTTCAGTACCGTCGAGCAGCACCAGCGTTTCGCCCTCTTTCGCCATCCGCACCACAATG
CCGCCTTCAATGCGATCTTTATCGAAAGCGTGCATCGGCTGGCCCAGTTCGAGCAGCACATAGTTGGTGACGTCAACAAC
TGCATCGATAGAACGGATCCCGCAACGACGCAGTTTTTCTTTCATCCACAGCGGAGTTGGCGCTTTAACGTTAATGCCTT
TTACCACACGGCCAAGATAACGCGGGCAGGCTTCCGGCGCTTCGACTGTAATCGGCAGCGTGTCGTCGATGGTCGCACCA
ACCGGAACGATTTCCGGTTGAACCAGCGGCAGCTGGTTCAGCACGGCAACGTCACGCGCAACACCAATGATGCCTAAGCA
GTCGGCACGGTTTGGCGTCACGCTGATTTCGATGGTGTTGTCATCAAGTTTCAGGTATTCACGGATATCGGTGCCAATCG
GCGCATCCGCAGGCAGTTCGATAATGCCGCTGTGATCGTCAGAAATGCCCAGTTCAGAGAAGGAGCACAGCATCCCTTCA
GACGGTTCGCCACGCAGTTTCGCCGCTTTAATTTTGAAATCACCCGGCAGAACAGCACCAATGGTCGCTACCGCTACACG
CAGGCCCTGACGGCAGTTTGGCGCACCGCAGACGATGTCCAGCAGGCGATCGCCGCCGACATTCACTTTTGTCACACGCA
GTTTGTCAGCGTTCGGATGCTGCGCACACTCAACCACTTCACCAACGACCACGCCGTGGAAGCTGCCGGCAACCGGTTCT
ACACCGTCAACTTCCAGGCCCGCCATAGTGATTTGATTTGCCAGCGCATCGCTATCAATCGCCGGGTTCACCCATTCGCG
TAACCACAGTTCACTGAATTTCATAATCTATTCCTGCCTTATTTAAACTGTTTGAGGAAACGCAGATCGTTTTCGAAGAA
TGAACGCAGGTCGGTGACGCCGTAACGCAACATAGTCAGACGCTCCATCCCCATCCCGAAGGCGAAACCAGAGTAAACTT
CCGGGTCGATGCCAACGTTACGCAACACGTTCGGATGCACCATCCCGCAGCCCAGCACTTCCAGCCATTTACCGTTTTTA
CCCATGACGTCCACTTCTGCAGAAGGTTCGGTAAACGGGAAGTAGGAAGGACGGAAGCGAATCTGCAAATCTTCCTCAAA
GAAGTTACGCAGGAAGTCGTGCAGCGTGCCTTTCAGGTTGGTAAAGCTGATGTTGGTATCAACAATCAGACCTTCCATCT
GATGGAACATCGGCGTGTGAGTCTGGTCGTAGTCGTTACGATAAACACGGCCAGGCGCGATGATACGAATCGGTGGCTGC
TGGGCTTTCATGGTGCGGATCTGTACGCCAGAGGTCTGGGTACGCAGCAGGCGGGTAGTGTCAAACCAGAAAGTGTCGTG
GTCAGCGCGCGCCGGGTGGTGACCAGGAATGTTCAGAGCATCGAAGTTATGATAATCGTCTTCGATTTCCGGCCCGGTTG
CCACGGTAAAGCCAAGCTCACCGAAGAAACTTTCGATACGGTCGATGGTACGGGTAACCGGATGCAGACCGCCGTTTTCA
ATGCGACGACCTGGCAGAGAGACATCAATCGTTTCCGCCGCCAGACGCGCATTCAGTGCAGCGCTTTCCAGTTCCGCTTT
ACGCGCATTCAGCGCCTGCTGAACCTGCTCTTTCGCTTCGTTGATAACCGCACCAGCTGCCGGACGCTCTTCTGGCGGCA
GCTCACGCAGGGTCGTCATCTGAAGGGTTAAGTGCCCTTTTTTACCCAAATATTCGACGCGCACATTATCTAACGCGGCA
ACATCTGACGCCTGGCTAATGGCCGCCTTCGCACTGGCAACCAGTTCTGCGAGATGTGACATGGTTTTCCTCATTGTGTC
AGTGGTGACACTGGTTCGTTGGACTTAGAGCCTATCCCATCAGGCTATTTTACTTGCCATTTTGGTCCCGGGCTGTGCTC
GAAATTCTCACGTACTTAAATACGCTCCGGTTTCTCCGCGCTGGCCGTGTCCAGTCTGGCTGCGACAATTACACCTGATG
AGACAGGCTTTTTATTTTTCAAAACGCGCATACAAAAAAAGCCTCCACTGGGAGGCTTTCAGGCGCTGTTTTCCGTTTCT
CTTCTCACGCGCTAGCCTCCTGGATTCAGGTGCTAAAGTAAAAAAAGAAGCGGAAAATAGCAGCATTCATTGCTTGCGTT
ACCTTTTGGTACTCTTCAAAAGACCTTTATTGAAAAGGCTACGGCGATAAAAGTCAATGTTTTGATGGCGTTGAAACGAA
AAGAGGGAGACTAGCTCCCTCTTTCAACTGGCTTATGCCAGAGCTGCTTTCGCTTTTTCAACCAGAGCGGTGAACGCTAC
TTTGTCGAATACTGCGATATCAGCCAGGATCTTACGGTCGATTTCAACAGAGGCTTTTTTCAGGCCATTGATGAATTTGC
TGTAAGAAATACCGTTCTGACGTGCTGCTGCGTTGATACGCGCAATCCACAGTTGACGGAACTGACGCTTACGTTGACGA
CGGTCACGGTAAGCATACTGACCAGCTTTGATAACAGCCTGGAAGGCAACGCGGTATACGCGAGAACGCGCACCGTAGTA
GCCTTTAGCTTGTTTCAAAATTTTCTTGTGACGTGCACGTGCAATAACACCACGTTTTACGCGAGCCATATGTGCTCTCC
TGTATCTATATTCTAATTAAAAAGTTAAAAACGTTAACGGCTTATGCGTACGGCAGGCACGCGATTACCAGGCCCAGATC
GCCTTTGGAAACCATGGCTTTCGGACGCAGGTGACGTTTACGTTTGGTCGCTTTTTTGGTCAGAATGTGACGCAGGTTAG
CGTGCTTGTGCTTAAAACCACCTTTACCGGTTTTTTTGAAGCGCTTAGCAGCACCGCGTACGGTCTTAATTTTTGGCATT
TTAATAACTTCCACTTCGCATTGTTAATAAACGAAACAAAGGCGAACAAAGCCTGTGAAGCCCGAAGGCTCCACAGACAG
TGCTACTTGAAGGCCTTACTGTTTCTTCTTAGGAGCGAGCACCATGATCATCTGGCGGCCTTCGATCTTCGTTGGGAAGG
ATTCGACCACTGCCAGTTCTTGCAAATCGTCTTTCACGCGATTAAGCACTTCCATACCGATTTGCTGGTGCGCCATCTCA
CGACCGCGGAAACGCAGCGTGATTTTGGCTTTATCACCCTCTTCGAGAAAGCGAATCAGGCTGCGGAGTTTTACCTGATA
GTCGCCTTCATCTGTACCAGGACGGAATTTAATTTCCTTAACCTGGATAACTTTTTGCTTTTTCTTCTGTTCCTTAGAAG
ACTTGCTCTTTTCATAGAGGAATTTGCCGTAATCCATTATACGACAAACCGGCGGCTCGGCGTTAGGGCTGATCTCGACT
AAGTCTACTCCGGCTTCTTCTGCTTTCTCCAGAGCTTCTCTCAGACTCACAATACCAAGCTGCTCGCCTTCCAGACCTGT
TAAGCGAACTTCCTGGGCGCGAATTTCGCCATTGATACGGTTAGGGCGCGCCGTTTGAACTCGTTTTCCGCCTTTAATAC
CTTATTCCTCCAATTGTTTAAGACTGCGGCTGCGAATCTCTTGTTGCAGCTTCTCGATCACTTCATTTACGTCCATGCTT
CCCAGGTCTTTACCACGGCGGGTGCGAACGGCAACTTTGCCTGATTCCACCTCTTTATCACCACAGACCAGCATATATGG
GACGCGACGCAAAGTGTGCTCGCGGATTTTAAAGCCAATCTTCTCATTTCTCAAGTCTGCTTTAACACGAATGCCCGCAT

TTGATAGTTTTTGCGTCAATTCGTTAACGTATTCAGACTGTGAATCGGTAATATTCATGATAACAACCTGAACCGGCGCA
AGCCAGGTCGGGAAGAAACCAGCGAACTCTTCGGTCAGGATACCGATGAAACGTTCCATCGACCCCAGAATTGCGCGGTG
AATCATTACCGGTACTTTACGTTCATTGTCTTCGCCTACATAAGAAGCGCTCAGACGAGACGGCAAAGAGAAGTCCAGCT
GTACTGTACCGCACTGCCATGCACGATCGAGGCAGTCATACAGGGTAAATTCAATTTTCGGACCGTAGAAAGCGCCTTCA
CCCAGTTGATATTCAAACGGGATGTTGTTTTCTTCCAGCGCAACCGCCAGGTCCGCCTCAGCACGATCCCACATTTCGTC
GCTGCCAATACGTTTTTCAGGACGAGTGGAGAGTTTGACGACGATCTTCTCGAAGCCAAAAGTGCTGTACATATCATAGA
CTAAACGGATACATCCGTTAACTTCATCGCGAATTTGTTCTTCAGTACAGAAGATATGCGCGTCATCCTGGGTAAATCCA
CGCACGCGCATCAGGCCATGCAGCGAACCTGACGGCTCGTTACGGTGGCAGCTACCAAACTCGGCCATACGCAGCGGCAG
ATCGCGATAAGACTTCAGCCCCTGGTTGAAAATTTGTACGTGACCCGGGCAGTTCATCGGCTTAATGCAGTATTCACGGT
TCTCAGAAGATGTGGTGAACATTGCATCTTTGTAGTTGTCCCAGTGACCGGTTTTTTCCCACAGGACACGGTCCATCATG
AACGGACCTTTAACTTCCTGATACTGGTACTCTTTCAGTTTAGAACGAACAAACACTTCCAGTTCACGGAAGATGGTCCA
GCCGTCGTTGTGCCAGAATACCATACCCGGCGCTTCTTCCTGCATATGGTACAGGTCGAGCTGTTTACCGATTTTACGGT
GGTCGCGTTTCGCGGCTTCTTCCAGGCGCTGCAGGTAAGCGTTAAGTGCTTTTTTGTCTGCCCACGCCGTACCGTAAATA
CGTTGCAACATTTTGTTGTTGCTGTCGCCACGCCAGTAAGCCCCTGCCGTTTTCATTAGTTTGAAATGATGGCAGAAACG
CATGTTCGGTACGTGCGGACCGCGGCACATATCGACATATTCTTCATGGAAGTACAGACCTGGCTTGTCATCATGGGCGA
TGTTTTCGTCAAGAATGGAGACTTTGTAGCTCTCCCCACGGTTGGCGAAAGTTTCACGCGCTTCGTGCCAGCTGACTTTC
TTCTTAATGACGTCGTAGTTTTTCTCAGCAAGCTCATGCATCCGCTTCTCGAGTGCTTCGACATCTTCCTGGGTTAACGT
GCGGTCAAGATCAACGTCGTAATAAAAACCGTTGTCAATAACCGGGCCGATTGCCATTTTGGTATGCGGCCAAAGTTGTT
TAATCGCGTGCCCTAACAGGTGCGCACAGGAGTGACGAATGATCTCCAGACCTTCTTCGTCTTTGGCGGTAATGATCGAC
AGTTGTGCGTCGTTTTCAATCAGATCGCAAGCATCAACCAGTTCGCCATTAACGCGCCCTGCGATACAGGCTTTCGCCAG
ACCTGGACCAATGTCCAGCGCAACATCCATGGGGCTTACAGCGTGATCGTAATGGCGTTGGCTGCCATCAGGAAGAGTTA
TAACAGGCATTTTATATCCTTATTTGCAGTGGTGACCCACACGAAAGATCACATACAAAGAAAAATTTGTTTATTAACAG
TTCATTGCGAAACCATCTAGCCAACAAATGCTAAATTGGTTCGCAATCAGGTACACAAATGAAAGACATTAACCTCTCAC
CTTCCACCCGGTTGAGCGATGTTAACACTAAAAAAAGGGAGATTGTACCTTTCCGTTTCACATTGATTCGATTCGCAATT
CGTTTGTAGTGAGTACACCAGCAAAACGCTTAAAGAACAACTGATAACATCACGGTGCGTCTGAAAGGTTTACTGCGAGC
GGATTTACAGCAGCATAAACTGAATCCAGCAAAGTTCGTTGCGTGATTTGGCGGTAGTATTTTAAACGCTCATGAGATTA
ATGACGAAGTGGTCATATCACAATGATAAAAGTGACACAATTCTTATAACAATTTTTCGTGCACATTTCGTTCTGGCGAT
AATAATTAATCATCATCCTCAAAACCTCCAGATATATATAAGGCGAACAGATTATGAGTCAAAACGATATCATTATCAGA
ACTCATTATAAGTCTCCTCATAGATTGCACATCGATAGCGACATACCAACACCTTCATCAGAGCCTATTAATCAATTTGC
GCGCCAGCTCATCACCCTACTTGATACCTCTGACTTAAGTTCGATGCTGTCATACTGTGTTACTCAGGAATTTACCGCAA
ACTGTCGAAAAATATCACAAAATTGTTATTCCACTGCCCTTTTTACCATTAACTTTGCCACTTCACCCATCCATACAGAA
AATATACTCATTACATTACACTATAAAAAAGAAATCATTTCCTTATTACTGGAAACCACGCCTATTAAAGCTAACCATTT
GCGAAGCATACTGGATTATATTGAACAGGAACAGTTAACTGCTGAAGATCGTAACCATTGTATGAAACTGTCTAAAAAAA
TCCATAGAGAAAAAAACTATACACCCAACAGTAAATCTCAATGGTAGTGCATGTTTTTTGCAATCTCCTTCTGACGCTAT
TTTTTGTCGCCATCTGTCATTGCAATACGCCCTTGATTCATTGAGAAATGGAAAAGGCAAAGTCAACCTGATTAAACATT
ACTCCTCCGTTGAATCCATACAGCAGCATGTCCCCTTAGTCCGGGACGCGGAGTTCAGAGCATTACTTCGCCATCCTCCT
GCAGGGAGTCGCGTTATCGCGAGTAAGGATTTTGGCTTCGCTTTAGATATTTTCTTTTGTCGAATGATGGCAAACAATGT
CAGTCATATGTCCGCGATTTTATATATAGACAATCATACTTTGTCAGTAAGGCTACGAATAAAGCAGTCAGTGTATGGAC
AATTAAATTATGTTGTGTCCGTTTACGACCCGAACGATACCAACGTTGCCGTCAGAGACACCCACAGGACAGCACGGGGC
TTTCTTTCGCTTGATAAGTTCATCAGTTCAGGTCCCGATGCTCAGACCTGGGCTGATAGATATGTTCGCAACTGTGCAAT
TGCTATTCTGCCCCTATTACCTGTGGGAGTTCCAGGGGCTATTTTCGCGGGTATTGCATCACGAATGCCATTTGCCCCTA
TACATCCATCGGCAATGTTGTTAATAATGGCTACAGGCCAGTCTCAACAGCTTATTACATTATTCAAACAGTTGCCCATA
CTCCCTGAAAAAGAAATCATTGAAATAATAACTGCGCAGAATAGCGTTGGTACACCTGCTTTATTTTTGGCTATGATGAA
CGGACATACTGACAACGTGAAAATATTTATGCAAGAAATTCAGTCACTGGTAGATAATCACATCATTCATGAAGATAATC
TGGTTAAATTACTGCAAACTAAAAGTGCTAACGAAACACCTGGACTTTATATCTCCATGTTGTATGGATTCGATGAAATA
ATCGATATCTTTCTGAATGCATTAACCACTCCAATAGCACAAGAGCTTTTAAACAAAAAACTGGTGATGAGTATTTTAGC
CATGAAAATACATGATGGTGAGCCAGGATTATACGCCGCAATGGAAAATAATCACCCTTTGTGTGTCACACGGTTCCTCT
CTAAAATTAATGGCATCGCCTTTAAATACAAGTTGAGCAAAGCTAACATCATGGATTTATTAAAAGGAGCTACAGCACAG
GGAACCCCTGCATTATACATCGCCATGAGCAAGGGTAATGAAGACGTCGTGTTATCTTATATATCGACGCTGGGTGCTTT
TGCAAAAAAACATTCTTTTAGTCAACATCAGTTATTTACACTATTGGCCGCTAAAAATCATGACAACATGTCAGCTGTTC
ATATAGCCATTCATCATAAGCATTATAAAACTGTAGAAACATATTATGCTGCTATTAATGCAATCAGCCAAAGCCTGAGT
TTTAGTGCTGATGAAATAAAGACGTATTTATAACCAGCTATTTACGATATATATCGATACATAATTTTATTTTTTTCATA
AAACGCAGAAACAGTGACCATGTGCACAACGCCACCCGACGGCTGTAATAACAAAAAATCCCTTGATGCCTGTCCCTTTT
GTTACACTCCGTTATCACGCACAAGAGATATGCAGGACACTGGTATGCCGACTAAACGCTTTGATAAAAAACACTGGAAG
ATGGTGGTGGTGCTACTGGCAATCTGTGGCGCTATGTTGTTGCTACGTTGGGCAGCAATGATTTGGGGCTGAGCAGTGAC
AAACGAAGACAGCCAGACCGCATAACACGGTCCGGCAGATAATTTTACATGCTGTAGCCCAGCGATAAGGTTGTCCGACG

GTCGGTATGTTCCGGTGCAGACTCTGGAGGTTCAGAGTTCCATGTGACGTTGTAAGCCACCTTCAGTCCAAAATGTTCAT
TAATAGCAACATTTAATGCGCTTTCGGAGTTCAGCGTTGTGTCTTCCGCGCCAAAGACCGAAACACCCTGCGTAAATTTA
GCGTTGTCAGTCAACTGCCATGCATAAGCCCCGGAGGCATAGCCCAGCGGCTGGGTTTCACTGGCATTGTCGGTATATTT
GTCGTAACGCACACCTGGACCGAATTCAAAGCGGAAACTGTGTACCGGGCCATTCAAAAACTGACGACCATAACCCGCGG
TCAACACATCGCGCTCTCGATAGCCGTTATAACGGTCTGTCAGCCAGCTTGCCTGACCAAATAAATAGTCATAATCAGTT
AAATTAAAACGGCTACGCCCGCCCGCCGCATATTTTTCTGAAGAACGCTCATCATTAGAAGAAGTATTACTGGCGTTCCC
CCACAGCGACCAGGCAGTGGTGTGTCCATACCAGGTCATGGTGGTGTCAGCCGTAAGTGAGGAGCTTTTCGTATTGCCTG
ATTGTGCAAGATATCCTGCGTTCAGATTACCTTCGAAAGGTTTTTTAGCGCTGGCAGGATCATCCATGACAGTAAAAACG
GAATCATCGGCAGCTGCATTCAGTGACGCAAACATGCCCCCCGCCAACATAACGATGGCAGGAACTGTCTTCAAAAGCTT
CATTTATCAAGAGTCCGTACAACAAAAAAAGAGACCATCGCGGTCCCGGAAACTTTCTTAAGGATCAAAGATTAGCGTCC
CTGGAAAGGTAACGAATTATAAAAAGGCGCGAATAACTTAGCAATGTATTCTTATTTCATTTTTTGAATAAGCATGTGGC
GAAAACAGATTTTTATTTATATATATTTATCTGCAAAATTTTAAATAAAGCTCCAATAAATCATATTGTTAATTTCTTCA
CTTTCCGCTGATTCGGTGCCAGACTGAAATCAGCCTATAGGAGGAAATGATGGTACGTATCTATACGTTGACACTTGCGC
CCTCTCTCGATAGCGCAACAATTACCCCGCAAATTTATCCCGAAGGAAAACTGCGCTGTACCGCACCGGTGTTCGAACCC
GGGGGCGGCGGCATCAACGTCGCCCGCGCCATTGCCCATCTTGGAGGCAGTGCCACAGCGATCTTCCCGGCGGGTGGCGC
GACCGGCGAACACCTGGTTTCACTGTTGGCGGATGAAAATGTCCCCGTCGCTACTGTAGAAGCCAAAGACTGGACCCGGC
AGAATTTACACGTACATGTGGAAGCAAGCGGTGAGCAGTATCGTTTTGTTATGCCAGGCGCGGCATTAAATGAAGATGAG
TTTCGCCAGCTTGAAGAGCAAGTTCTGGAAATTGAATCCGGGGCCATCCTGGTCATAAGCGGAAGCCTGCCGCCAGGTGT
GAAGCTGGAAAAAATTAACCCAACTGATTTCCGCTGCGCAAAAACAAGGGATCCGCTGCATCGTCGACAGTTCTGGCGAAG
CGTTAAGTGCAGCACTGGCAATTGGTAACATCGAGTTGGTTAAGCCTAACCAAAAAGAACTCAGTGCGCTGGTGAATCGC
GAACTCACCCAGCCGGACGATGTCCGCAAAGCCGCGCAGGAAATCGTTAATAGCGGCAAGGCCAAACGGGTTGTCGTTTC
CCTGGGTCCACAAGGAGCGCTGGGTGTTGATAGTGAAAACTGTATTCAGGTGGTGCCACCACCGGTGAAAAGCCAGAGTA
CCGTTGGCGCTGGTGACAGCATGGTCGGCGCGATGACACTGAAACTGGCAGAAAATGCCTCTCTTGAAGAGATGGTTCGT
TTTGGCGTAGCTGCGGGGAGTGCAGCCACACTCAATCAGGGAACACGTCTGTGCTCCCATGACGATACGCAAAAAATTTA
CGCTTACCTTTCCCGCTAACAAAAACATTCCCCCAGCATTGGGGGAATCATCACCAACCTGTCGGCAACGCGTTTCTCCG
ACTATGCTCAAAAGTCATGTGATAACAAAGGGGTGAACTATGGCCAGTGGCGATCTTGTCCGTTATGTCATAACCGTCAT
GTTGCATGAGGATACATTGACTGAAATTAACGAGTTGAATAATTACCTGACTCGCGACGGTTTTTTGCTCACTATGACGG
ATGATGAGGGAAATATCCATGAGCTGGGAACTAACACTTTTGGACTTATCAGTACCCAAAGTGAAGAAGAAATTAGAGAA
CTGGTTTCGGGGCTTACCCAAAGTGCAACCGGCAAAGATCCTGAAATCACCATCACGACCTGGGAGGAATGGAATAGCAA
CAGAAAATAAATGGTTTTTGGGCAATAATCAGTCTGTGGTGTGCGTTAGCTCGTGTTTTTACACCGCATTCTTGCGCTAA
CCTTATGATCTGGCAGACAACATGGGAGAGACATCATGTGGCAGGCAATCAGTCGTCTTTTGAGCGAGCAGTTAGGTGAA
GGCGAAATCGAACTGCGTAATGAACTGCCTGGCGGAGAAGTCCATGCCGCATGGCATTTGCGCTATGCAGGACATGATTT
TTTCGTCAAATGTGATGAAAGGGAACTGCTTCCCGGTTTTACCGCCGAAGCCGACCAACTGGAGTTACTGTCGCGTAGTA
AAACCGTCACCGTGCCTAAGGTTTGGGCAGTTGGCGCTGACCGTGACTACAGTTTTCTGGTGATGGATTATCTCCCACCT
CGTCCGCTGGATGCGCATAGCGCATTTATTCTTGGTCAGCAAATTGCGCGTTTACATCAATGGAGCGACCAACCACAATT
TGGCCTTGATTTCGATAACGCGCTCTCCACAACTCCACAGCCCAACACCTGGCAACGTCGCTGGTCAACGTTTTTTGCTG
AACAACGGATTGGCTGGCAGTTGGAACTGGCTGCAGAAAAAGGGATCGCTTTCGGCAATATCGACGCCATCGTCGAGCAT
ATTCAGCAGCGTCTGGCCTCACATCAACCGCAGCCTTCTCTGTTGCACGGCGATTTATGGTCCGGCAACTGTGCACTGGG
TCCGGATGGCCCGTACATTTTCGACCCGGCCTGCTACTGGGGTGACCGAGAGTGCGACCTGGCGATGTTACCGCTGCATA
CTGAACAGCCGCCACAAATCTATGACGGCTATCAGTCAGTATCCCCGCTACCTGCCGATTTCCTTGAACGTCAACCGGTT
TACCAACTCTACACGCTGTTAAATCGTGCAAGGTTATTTGGCGGTCAGCATTTGGTTATTGCTCAGCAGTCATTGGATAG
ATTATTAGCAGCATGATATGGGTTGAGGATAATGGCCGCTCCGTGCGGCCTTTTGATTAAATAAACCCAAGCAGAGAAAA
GAACACATAACCTGCAGCAATACAAATAACCGGCAGGATATACAGTGAGAAAAACTGCAGGAAAATAGTATGGTGCGGAA
CAACAATACGGGACTCAATTTGTTCACGCGTTAGCCCCTCTTCCCCTTTGGCTTTTTCCAGAATGAGTTGATCTTCAACA
CCTTCCCGCAAGAAGCGCGCCTGGCGACTCATCCGGGCACCGGAATCCTGCAACGCCAGTCCGACAAAAATCAGGATGAA
AATCACCCAGAACATAACGTTCACGCCACCATTAAAATTGGGCGTCGGGGAGTTATACCAGAATAGATTCAAAAAAGGCG
TATTCGCCTGCATCATATCGATCATGACATGAGTAAAGTCGAGCATGACCGCATTAATGCCTTCCTTGTTTTCCTGCCGG
GTATTCATAAATTTCAGCAAAGAAATCAATGTAGAGATCAATGCAGGTATAAAAATCACCCAACCCAAAATCCTTTTCAA
AACAGCAATGCGTCCAGCTTGTTGATACGTCATGAGTTCTCCTTGATTAAGACGCGTCGTTTCACTTAGTTTACCTGTAG
ATATCTGTTTTCGCCCATTCTTTAAAGGCGATATGATAGGCGCTTAATCATAAGCACGGCTTAATACCTTACACATAATG
CTCTAAAGGAGAGGTTGTAATGTCAACCCCGCGTCAGATTCTTGCTGCAATTTTTGATATGGATGGATTACTTATCGACT
CAGAACCTTTATGGGATCGAGCCGAACTGGATGTGATGGCAAGCCTGGGGGTGGATATCTCCCGTCGTAACGAGCTGCCG
GACACCTTAGGTTTACGCATCGATATGGTGGTCGATCTTTGGTACGCCCGGCAACCGTGGAATGGGCCAAGCCGTCAGGA
AGTAGTAGAACGGGTTATTGCCCGTGCCATTTCACTGGTTGAAGAGACACGTCCATTATTACCAGGCGTGCGCGAAGCCG
TTGCGTTATGCAAAGAACAAGGTTTATTGGTGGGACTGGCCTCCGCGTCACCACTACATATGCTGGAAAAAGTGTTGACC
ATGTTTGACTTACGCGACAGTTTCGATGCCCTCGCCTCGGCCGAAAAACTGCCTTACAGCAAGCCGCATCCGCAAGTATA

TCTCGACTGCGCAGCAAAACTGGGCGTTGACCCTCTGACCTGCGTAGCGCTGGAAGATTCGGTAAATGGCATGATCGCCT
CTAAAGCAGCCCGCATGCGTTCCATCGTCGTTCCTGCGCCAGAAGCGCAAAATGATCCACGTTTTGTATTAGCAGACGTC
AAACTTTCATCGCTGACAGAACTCACCGCAAAAGACCTTCTCGGTTAATGACCAGGGGCAGTGATCTCGCTGCCCCTGGT
TCTTTATCTGAAATTGCATTCAACTGACGGATTAATCGTCAATTTAAGAGAAAGAGTTACACCGTCACCACTTCCGTGCA
CTGTATAAAAATCCTATACTGTACGTATCGACAGTTTAGTGAGTTTTATCATGACGGCGGAAGGTCACCTTCTCTTTTCT
ATTGCTTGTGCGGTATTTGCCAAAAATGCCGAGCTGACGCCCGTGCTGGCACAGGGTGACTGGTGGCATATTGTCCCTTC
CGCAATCCTGACGTGTTTGTTACCGGACATCGATCACCCAAAGTCGTTTCTTGGGCAGCGATTAAAATGGATATCAAAAC
CGATCGCCCGCGCTTTTGGGCATCGTGGTTTTACCCACAGTCTGCTGGCGGTATTTGCGCTGCTGGCAACCTTTTACCTT
AAGGTTCCGGAAGGCTGGTTCATTCCGGCTGATGCGCTACAAGGAATGGTGCTGGGTTATTTGAGCCACATACTTGCCGA
TATGCTGACACCCGCCGGTGTTCCCCTGCTCTGGCCATGCCGCTGGCGTTTCCGCTTGCCTATCCTGGTTCCCCAAAAGG
GCAACCAACTGGAACGTTTTATCTGCATGGCATTATTTGTCTGGTCGGTATGGATGCCCCATTCATTACCCGAGAACAGC
GCTGTTCGTTGGTCATCGCAAATGATCAATACCTTGCAGATCCAGTTTCATCGGCTTATTAAGCATCAGGTTGAATACTA
AAAAGGCAAAAATCACCTTTCTGGAATAAGCAATTCCATTTGAATATAAGAGCCAGCTCACAGTTCTGTTAATCTTGCGC
CAACACTATGACTGCTACGCAGTGATAGAAATAATAAGATCAGGAGAACGGGGATGAACTTTCCATTAATTGCGAACATC
GTGGTGTTCGTTGTACTGCTGTTTGCGCTGGCTCAGACCCGCCATAAACAGTGGAGTCTGGCGAAAAAAGTGCTGGTGGG
TCTGGTGATGGGTGTGGTTTTTGGCCTTGCCCTGCATACCATTTATGGTTCTGACAGCCAGGTACTTAAAGATTCTGTAC
AGTGGTTTAACATCGTTGGTAACGGCTATGTTCAACTGCTGCAAATGATCGTTATGCCGTTAGTCTTCGCCTCTATTCTG
AGCGCGGTTGCCCGTCTGCATAACGCATCTCAGTTAGGCAAAATCAGTTTTCTGACCATCGGTACGCTTTTGTTTACCAC
GCTGATTGCGGCGCTGGTCGGTGTGCTGGTCACCAACCTGTTTGGTTTGACGGCTGAAGGTCTGGTTCAGGGTGGTGCAG
AAACTGCACGTCTGAACGCCATTGAAAGTAACTATGTTGGTAAAGTCTCTGATCTGAGCGTTCCGCAGCTGGTCTTGTCC
TTTATCCCGAAAAACCCGTTTGCCGATCTTACCGGAGCCAATCCGACGTCAATTATCAGCGTGGTAATTTTTGCCGCATT
CCTCGGCGTAGCTGCATTGAAACTGCTGAAGGATGATGCGCCGAAAGGTGAACGCGTCTTAGCCGCTATCGATACCCTAC
AAAGCTGGGTGATGAAACTGGTTCGCCTGGTCATGCAGTTGACCCCTTACGGCGTTCTGGCTCTAATGACCAAAGTGGTT
GCAGGTTCTAACTTGCAAGACATCATCAAACTGGGAAGTTTCGTTGTCGCGTCCTACCTCGGTCTGCTGATTATGTTTGC
AGTGCATGGCATTCTGCTGGGCATTAATGGCGTGAGTCCGCTGAAGTACTTCCGTAAGGTATGGCCTGTGCTGACGTTTG
CCTTTACCAGCCGTTCCAGTGCTGCGTCTATCCCACTGAATGTGGAAGCACAAACGCGTCGTCTGGGCGTTCCTGAATCC
ATCGCCAGTTTCGCCGCCTCTTTCGGTGCAACCATTGGTCAGAACGGCTGCGCCGGTTTGTATCCGGCAATGCTGGCGGT
GATGGTTGCGCCTACGGTTGGCATTAACCCGCTGGACCCGATGTGGATTGCGACGCTGGTCGGTATTGTTACCGTTAGTT
CCGCAGGCGTTGCCGGTGTCGGTGGTGGTGCAACTTTCGCCGCACTGATTGTACTGCCTGCGATGGGCCTGCCAGTAACC
CTGGTGGCGCTGTTAATCTCCGTTGAACCGCTTATCGACATGGGCCGTACGGCGTTAAACGTTAGTGGCTCGATGACAGC
TGGCACGCTGACCAGCCAGTGGCTGAAGCAAACCGATAAAGCCATTCTGGATAGCGAAGACGACGCCGAACTGGCACACC
ATTAATTCTTATGCTGGCAACGGTCCGTTTTGTATAGGGGCCGTTGCCTTACTTTTTAAATGTTCCATCGCGGCGATGCA
TACTGACTCATATTTAAACAGATCGTCAGCGAAAAAAATGTCCAACTTATCAATACATTCCTGGCTCAATGAATGTGCCT
GTAGCGAGAAGCTTAAAACCTCATACCCGAGACGTTTTCTAAGTGAACTATCTGGAATACTCATAATATCTTCAAGCACA
CCACTTCCCATTCCCGGAAGATTTAGTGGATTAAAAAGAAGATTGATATTAAGCATTGATATTATCGAAGGAAACTTTAG
GAGACTCTCATTTTTTTGAATCGCAGCAAAAATGCTCCCGACAGGATGTATATAATTTTGTTCATAGTATATAGTCCTTA
AATAGTGAATAACCAACCTGTTTTTTATACTGATCAAATTGGTCATTTTTTCTGGGCGAAATACAGAAGTGACATCCATT
CCAATAAAATCTTTATTTATACCTCTTATCCGAATTTCACTACCATAAAGATTATTAATTATTACTCTATATTTAACATT
GTCCTGCAGATATGTATCAAAACTAAGTGCTGGCGCTCCTGGCTTTGAGGCATCATAGTTAATGTTAGACATATAGGCAA
TCATATAATTAAAGTTTGATTCTGCTATTTCATCGCAGTCACATTGACCCGTTGATATAAAGAAAGTGAGTGCTGTCATA
GTAGACTGCAGCCTTTCTTTAAAAGAGTCGAAAGCCAGGCTTTTAATATTTAAATCACCATAATTACTCTGTATTAAGTT
TGTAGAAAACATCTCCCGCCTCATATTGTTAACAAAATTATTATCTCATTTAAATCTAAGTCATTTACAATATAAGTTTA
AGAGCGACGCCACAGGATGAACTATCAAAAATAGCTCATCATGATTAGCAAAACTTAACCATTTTAAAATAAATAAACAA
TTAAAGAAAAAAGATCACTTATTTATAGCAATAGATCGTCAAAGGCAGCTTTTTGTTACAGGTGGTTTGAATGAATGTAG
CAACGAAATACAGAATTTCAGGTCATGTAACTCCCGGCAAAACCGGGAGGTATGTAATCCTTACTCAGTCACTTCCCCTT
CCTGGCGGATCTGATTTGCCCAACGTTGGGCAGATTCAGGCACAGTAAACGCCGGTGAGCGCAGAAATGACTCTCCCATC
AGTACAAACGCAACATATTTGCCACGCAGCATCCAGACATCACGAAACGAATCCATCTTTATCGCATGTTCTGGCGGCGC
GGGTTCCGTGCGTGGGACATAGCTAATAATCTGGCGGTTTTGCTGGCGGAGCGGTTTCTTCATTACTGGCTTCACTAAAC
GCATATTAAAAATCAGAAAAACTGTAGTTTAGCCGATTTAGCCCCTGTACGTCCCGCTTTGCGTGTATTTCATAACACCG
TTTCCAGAATAGTCTCCGAAGCGGGATCTGGCTGGTGGTCTATAGTTAGAGAGTTTTTTGACCAAAACAGCGGCCCTTTC
AGTAATAAATTAAGGAGACGAGTTCAATGTCGCAACATAACGAAAAGAACCCACATCAGCACCAGTCACCACTACACGAT
TCCAGCGAAGCGAAACCGGGGATGGACTCACTGGCACCTGAGGACGGCTCTCATCGTCCAGCGGCTGAACCAACACCGCC
AGGTGCACAACCTACCGCCCCAGGGAGCCTGAAAGCCCCTGATACGCGTAACGAAAAACTTAATTCTCTGGAAGACGTAC
GCAAAGGCAGTGAAAATTATGCGCTGACCACTAATCAGGGCGTGCGCATCGCCGACGATCAAAACTCACTGCGTGCCGGT
AGCCGTGGTCCAACGCTGCTGGAAGATTTTATTCTGCGCGAGAAAATCACCCACTTTGACCATGAGCGCATTCCGGAACG
TATTGTTCATGCACGCGGATCAGCCGCTCACGGTTATTTCCAGCCATATAAAAGCTTAAGCGATATTACCAAAGCGGATT

TCCTCTCAGATCCGAACAAAATCACCCCAGTATTTGTACGTTTCTCTACCGTTCAGGGTGGTGCTGGCTCTGCTGATACC
GTGCGTGATATCCGTGGCTTTGCCACCAAGTTCTATACCGAAGAGGGTATTTTTGACCTCGTTGGCAATAACACGCCAAT
CTTCTTTATCCAGGATGCGCATAAATTCCCCGATTTTGTTCATGCGGTAAAACCAGAACCGCACTGGGCAATTCCACAAG
GGCAAAGTGCCCACGATACTTTCTGGGATTATGTTTCTCTGCAACCTGAAACTCTGCACAACGTGATGTGGGCGATGTCG
GATCGCGGCATCCCCCGCAGTTACCGCACCATGGAAGGCTTCGGTATTCACACCTTCCGCCTGATTAATGCCGAAGGGAA
GGCAACGTTTGTACGTTTCCACTGGAAACCACTGGCAGGTAAAGCCTCACTCGTTTGGGATGAAGCACAAAAACTCACCG
GACGTGACCCGGACTTCCACCGCCGCGAGTTGTGGGAAGCCATTGAAGCAGGCGATTTTCCGGAATACGAACTGGGCTTC
CAGTTGATTCCTGAAGAAGATGAATTCAAGTTCGACTTCGATCTTCTCGATCCAACCAAACTTATCCCGGAAGAACTGGT
GCCCGTTCAGCGTGTCGGCAAAATGGTGCTCAATCGCAACCCGGATAACTTCTTTGCTGAAAACGAACAGGCGGCTTTCC
ATCCTGGGCATATCGTGCCGGGACTGGACTTCACCAACGATCCGCTGTTGCAGGGACGTTTGTTCTCCTATACCGATACA
CAAATCAGTCGTCTTGGTGGGCCGAATTTCCATGAGATTCCGATTAACCGTCCGACCTGCCCTTACCATAATTTCCAGCG
TGACGGCATGCATCGCATGGGGATCGACACTAACCCGGCGAATTACGAACCGAACTCGATTAACGATAACTGGCCGCGCG
AAACACCGCCGGGGCCGAAACGCGGCGGTTTTGAATCATACCAGGAGCGCGTGGAAGGCAATAAAGTTCGCGAGCGCAGC
CCATCGTTTGGCGAATATTATTCCCATCCGCGTCTGTTCTGGCTAAGTCAGACGCCATTTGAGCAGCGCCATATTGTCGA
TGGTTTCAGTTTTGAGTTAAGCAAAGTCGTTCGTCCGTATATTCGTGAGCGCGTTGTTGACCAGCTGGCGCATATTGATC
TCACTCTGGCCCAGGCGGTGGCGAAAAATCTCGGTATCGAACTGACTGACGACCAGCTGAATATCACCCCACCTCCGGAC
GTCAACGGTCTGAAAAAGGATCCATCCTTAAGTTTGTACGCCATTCCTGACGGTGATGTGAAAGGTCGCGTGGTAGCGAT
TTTACTTAATGATGAAGTGAGATCGGCAGACCTTCTGGCCATTCTCAAGGCGCTGAAGGCCAAAGGCGTTCATGCCAAAC
TGCTCTACTCCCGAATGGGTGAAGTGACTGCGGATGACGGTACGGTGTTGCCTATAGCCGCTACCTTTGCCGGTGCACCT
TCGCTGACGGTCGATGCGGTCATTGTCCCTTGCGGCAATATCGCGGATATCGCTGACAACGGCGATGCCAACTACTACCT
GATGGAAGCCTACAAACACCTTAAACCGATTGCGCTGGCGGGTGACGCGCGCAAGTTTAAAGCAACAATCAAGATCGCTG
ACCAGGGTGAAGAAGGGATTGTGGAAGCTGACAGCGCTGACGGTAGTTTTATGGATGAACTGCTAACGCTGATGGCAGCA
CACCGCGTGTGGTCACGCATTCCTAAGATTGACAAAATTCCTGCCTGATGGGAGGCGGCGCAATTGCGCCGCCTCAAATG
ATTTACATAGTGCGCTTTGTTTATGCCGGATGCGGCGTGAACGCCTTATCCGGCCTACAAAACTGTGCAAATTCAATATA
TTGCAGGAAACACGTAGGCCTGATAAGCGAAGCGCATCAGGCAGTTTTGCGTTTGTCAGCAGTCTCAAGCGGCGCAGTTA
CGCCGCCTTTGTAGGAATTAATCGCCGGATGCAAGGTTCACGCCGCATCTGGCAAACATCCTCACTTACACATCCCGATA
ACTCCCCAACCGATAACCACGCTGAGCGATAGCACCTTTCAACGACGCTGATGTCAACACATCCAGCTCCGTTAAGCGTG
GGAAACAGTAAGCACTCTGACGGATAGTATTATCGATAAACGCCGGATGACACATCACCTCCAGCGAACGATCACCCCGA
TGGCCTGCGTCATCCAGCACTTGCAGGAACAGCGACTCACTGATCTCTTCACCGTAGAATGCACTGCTGAATCCCTGGGT
AGTGCGCAGGTTGACCGGCAAATCAAACGCCATCTGACGATCCGCCCGCAACGCAATGCCCTGCTCTGCCGCAAACCTGG
CAACAATCGGGAAAATCTGCGGGAACATATGCACATGATGATGGCTATCAAGATGCGTAGGTTTGCGTCCAAATAGCTCA
ATGAAACGCAAATACTGACTGACAAGCTCCTGAGTAATTTCTTCCAGCGGTAAAGCATCTTCTTCTGCCAACTGCCAGAT
CCATTTTCCCAGCACACCATCGCGGGTTAACCCCGGCATAGCTGTCAGTGGCTTACCCATAGTAAGGACAAAGTGCATCC
CTATGGCCAGACTCGGTTCATCACGACTCAACTGCACCGCATGGTCAATAGCCTGCCCATTCACCAGCGCCGTCGTCGAC
GTGACAATCCCATTGCGACAGGCCTCGATAATGCCGTAGTTCTGACCTTTGCTTAAGCCAAAATCATCGGCATTAACAAT
CAGTAAGCGTTCCATAATCAGCCTCGGTTAATGTGCTTTTTTAAGCTCTGCGATGCAGTCGGCAAAGTTTGGCAGCCATT
TCTCGTGCGCCAGAATCATCTCGCGTGCCAGCAGCTCAGCATCGCGATCGGAATGCACCAACGGACTAAGGTTTAGCGCC
AGTAACACATCGTTAAATTCTCCGCTAAGTGCGGCGTTACTGGCAGCAATCTCGAAGCCTTTAATGGTGTGAATCAGCCC
CATTACTTTATCATCGAAATGCGTAATGCGCGGATGTGGCGTCGCGCCATCGCGCCCCAGCTTACAGGTCATTTCTACTG
CCCAGTCTGCCGGAATATTATCAATCTGCCCATGATGCGGGATATTAACGTAATGTTCTGCTTGCTTGTCGTTGTAGATA
GCGTTGATCACTTCGCATGCTGCATCAGAGTAATAAGCCCCACCGCGCTGTTCCAGTTCTTTCGGCTTAACTTTCAGCTC
AGGATTTTTATACAGCTCAAAAAGTTGTTTCTCGACTTTCTGTACTACCTGCGCTCGTGCGCCGCCTTTGTAGTATTCGC
CCATTTCAATAGCCAGCATCTCTTTCTGCTTGAAGTAATACAGCAGATAAGAACATGGCAGCAGATTCAACGAACGAATT
AAGCCCTCACTAAATGGCAGATCGAAAATATTTTTTACAGAGGACGCTTTTAACTGCCCTGACGCCACACCATCAAGCAA
TTCGGCAAAGCGCGACTTGCCATTTATCAGCACATCCTTAATGAACACCATATGGTTGAGGCCGAACAAATCGATAGATA
AATCATCGCTGTCTTTCAGCATCAGAACATCGCGAATAAACATCTTCATGCCGATCGGAATATTACACACGCCGATAAAG
CGTTTAAATCCGGTATGACGATAAACGGCTTCAGTGACCATTCCCGCCGGGTTAGTGAAGTTAATCACCCATGCATTCGG
ACAAAGTTCTTCGACATCTTTTACGATGTCAAAAATCACCGGAATGGTACGCAGACCTTTAAACAAACCGCCCGCGCCGT
TGGTTTCCTGACCAAGATAACCATGACTTAATGGAATACGTTCATCCAGTTCACGCGCCGGTAATTGGCCAACGCGCAGT
TGGGTAGTAACGAAATCAGCATCTTTCAATGCTTCGCGGCGATCCAGCGTTTTATAAAGCTTCATCGGGACGCCAGCGTT
ATCAATCATCCGTTGGCAGAGATCGAAAATAATATCCAGTTTCGGTTTACCACCTTCGACATCCACCAGCCATAATTCGC
TGACCGGCAATTCGTGATAACGCTTAATAAATCCTTCCAGTAACTCCGGGGTATAGCTGCTCCCGCCACCAATAGTGACG
ACTTTTAATTTCTGGCTCATAATTTCTCCCTTCAGTACAGAATACTGATATCTGGCATATCTGCCCCCCCGGACATAAAT
AATCCAGCAACAGGACAGATATGTGAATTGTCAGGTATAACGACTTACTGATTAAATTCAGTCAATTTCTTACGATAGCT
CTTAGGCGTAAAGGACGTTAATTTCTTAAACGTTTTAATAAACAAACTCGGGCTACTATAACCGGCCTCAAACGCAATAT
CCGTCACTGAATAGTTGGTCATTTCCAGTTGTTTTTTGGCAAAATTAATACGGATTTCATTAATAATCTGCATTGGCGTT

TTGCCATAATATCGTTGAGTCGCTCGCGTCAAATATTCCTGTGATTTGGCTGACAACGCCACCATATTCTCCAGCGCCGA
TTCACTAAACTGCTCTTTATCATGCATCTTTTCTACCGTACTTTTCAGCCACTGCGGTACATCATCAATCACCTGTTCTT
CGCGGTAATGACGTAAACGGTTAATGACATAAAAAGTAACCATCTCAACAAACTCTTCCAGCCCTGTTTCGCGGAAATTC
AATGAAGAAATCACTGTTTCCACATAGGTGAGAAAAGCGTTATTGGTCCGGTATACCTGCGAAGCGACAAAGCAATAAGG
CAACAATGGCAGGTAATGCTGCTCAAAAAAGCGTTTACTGATCCCAACGTTCAATATGCGCGTGGCACCAAACTCATAAA
AACTTTGATGGTGCGAACCTAACGGAATAAAAACAAAATCGCCCCGTTCCAGTAACACGCGCTTACCGTTAATCTCCTGG
AAATAACGCCCGGTTAATACCAGAGTAAATTCATAATAGTCGTGCTGATGCAGTCCGCTGATACTCTCAGTTTTGTTATA
GATAAACACATGGAAGTTTTTGCCATTAAACAACTGCTGTTCTCGGGCAGTGGCAATTTCCGGCGCGTTAATCACTGGCT
GCATCATCGACTCCTTATGCCTTCAGTTTTTCATGAAGCTCAATTAATTCAGTAATCAGTTCACGCGCAAGCATGGACGT
CATTAAATGATCCTGAGCGTGGACCAGCACCAGACTCACTTTCATCTTACCTTCGCCCGCATCGCCTTCAATCAGTTTCG
TCTGTACCAGATGCGCTTCATTCAATGCCATCCGTGACTGATCCATCATGGCTTTTGCTGCGGCAAAATCGCCCTGCTTC
GCCTGTTTCAGTGCCGCATACGCCAGGCTGCGCGCTTGTCCGGAGTTGATGATCAGCCCCATCACCACTTCTTCCAGCTC
TTCAGCTTCCGTTTGCGTATCGGGAATGTTATCGAGATCCATCATACATCGTTCCTCTCTTTTCTTACCGGCACGATTAC
CCGTACCGGCATCGATTAAAATTTCAGGGCGTTAGCGATATCTTCTTCGCTCTCTTCTTTATCAATCGCATTTTGTGCTT
TGTTAGCCACCACAACAAAGGGCAGATAAATTAACGTTGCGATGCCAAGGTTGAAGAGTGCGACCAGCAATGCGGCGACG
CTACCGTTGGTGTTAAAGAAGGCTCCCAGACCGGTTGGCATGGTCCACGGTGCAATATTGGTCACCGGAGGAATAATGCC
CATGTAGTACGCTGCGAGGGTGATTGCCGCCAGAATCGGTTGTACCAGTACAAACGGGATAAACATCACCGGGTTCATGA
TAATTGGCAGACCAAACAGAATCGGTTCGTTAATCTGGAAGATGCCGGACGGCAGCGCCAGTTTTGCCACCTGACGATAA
TCAGCACGGCGAGAGGCGATAAAGATAGCCAGGATCAGGCCTAAAGTCGCACCACTGCCCCCAAGGAAAATAAAGGAGTC
CAGCATCGGCTTGGCCCAGATATGGAAGGTCTTACCGGCTGCCAGCGCCGCTTCGACGGAACCATATTGCTGATAGGTCG
CGATATTTTCCAGTGCCCACGGCGTCATAATGCCGTTGTCCAGTGCGGTCAGCGCCAGCGCGCCATGAATACCGAAGAAC
CAGAGCAGTGGAACAAAGATCACATAGGCCCAGCCCACCACGCTACCCAACGATGCCAGTGGGGTTGAGATGGTATCCAT
AATGATCTGATGGAAGTTGGTGCCCCAGGTATTCAACGCCCAGGCAATAATCCCCATCACGGAAAGAATAATAAAGCCGG
GAATTAATGCCGAGAAGGAACGCGATACTGAAGCAGGTACGCTGTCGGGCAGTTTAATGACCCAATTGCGGCGGACAATA
AAGGTAAACATTTCTGCCACCACCAGGCCAATAATAATCCCGGAGATGATATTCGCCCCACCTAACCAGTTTGCACCAAC
CGCATAGGCCTCACCGACACTATATGGGGTGACGGTCATAAATGCTGCAACGGATAACAACCCAGCCGCCAGCGCATCGA
CTTTACGCTCTTCTGCCAGCGCCATGCCAATAAAGAACGGTGCCATTAAAGACATTATTCCTAATGTTCCGTTATATACG
TTGCCGCCAATACCTTTCAGACCATTAAGTGTTTCAATGGTTGAGGCGTCGAGGCGAATACCTAAGGAATAAAAAAACGA
CCCCTCCCCAAAGCTTAGAAAAACGTTGTTAATTAATACAAACATGGCCCCCGCAAGGGTTAACGGCATTAAGCGAATAA
AGCCATTTTTGATTGCATTAACGTGTGGCTGCTTTCCTATTTTAACTGCAAAAGGGAGGAGTACCTTTTCAAGCGATGCA
ATAACATTACTCATAGAAAAATACCCTTAAAAACCGCAATTTAAATATTGCGGTATTGATTTATGAAATAACTCTTTGAC
GGGAAAATTTAAAATAAATTAATTTGCTGCGGCTTTTTTAATCGCTGCAACCGCAGCCTTAAGCACGCCTAAACCATCGA
CTTTGCCATAAAGCAGCGAGTCAATTACTTCAACCGGTTTGTTGGGTAACAAACGCTGGATTTCGGGCAACATATAAGCA
ATCTGCGGCCCTAATAACACGACATCGGCATTCTGACCTTTTTCACCAGCCAGTGTTTCCGGAAATGCTTCAATAATGAC
CGGAACTTCATATTTTTCTGCCTGTGCGCGCATTTTTGATACCAGTAAAGAGGTAGACATGCCCGCAGAACAAAACAGAT
AAATGTGTTTCTTTTCCATAAAACTGCCCTCATCGACGATTATCTGTCAGCCAGACACTCCGCAAGCCTTAACCTGCTTC
CATGCTCTGGGTAACTTGCGAAACCAACATGACTTTTTTTGTGGCTGAAAGGAGTATACGGTATAGACCTGAGCAATAGT
ATTTCTTTGACCTTATAAATTGTTTCTCCTTGACCTGTGGTTATGACCCTCTTCACATTTCGGGCAAATAATTCGCGAAG
ATAATTAAGAACTCAGGCCAAAAAAAACCGGCGCAATGGCCGGTTTCCGTTGTTACTCAAGCTTTCAGACGAATTGATTA
CTTCGCAGCCTGTGGATCAGTGTCGTATTCAGCACAGGTCTGATAACCGGAGTTGATGACATGTCCGGTATCATCTAACG
CGACAAAGTAGGTTTCTGCTTTACCATCACGTTGACCCAGGATGTAGGTCTGGCAAGTACCGCGAGCATGGATCATGCTC
ACTTCAGACGAAGGTTTACCCGCAATTTGTGCAACCTGCGCCCGGCTCATGCCTTTTTTGACGTCTTTCACCACAGGCTG
TACAAACTGGTCTTTGGTACGATCATAAGCCGTACAACCCGCCAGCATGGTTAATACCGCCGCTGCACTCAGAATTCCTG
CCATATTCTTGTTCATATTCCGTCCTCTTGTTTATCAGCGTGTTAGATAAGCCTGGAATACATTGGGCGCTTTTTCAAGC
CCGTGAACGAAACGGCTCCGCTTTCAGAGGATTCCTGTATGACGTTTTAACCACCATTCAGCCCGCTGTCGCTTGTCGTT
TCAGTAGCAACGGGTTAGCTTTAAGGAAGTTTTGTCTTTTCTGTCTGGAGGGGTTCAATGACATTGCAACAACAAATAAT
AAAGGCGCTGGGCGCAAAACCGCAGATTAATGCTGAAGAGGAAATTCGTCGTAGTGTCGATTTTCTGAAAAGCTACCTGC
AAACTTATCCGTTCATTAAATCACTGGTGCTCGGGATCAGCGGCGGTCAGGACTCCACGCTTGCCGGAAAGCTGTGCCAG
ATGGCGATTAATGAGCTGCGCCTGGAAACCGGCAACGAATCACTGCAATTTATTGCCGTACGCCTGCCCTATGGTGTTCA
GGCCGACGAACAAGATTGCCAGGATGCCATTGCCTTTATTCAACCGGATCGCGTATTAACCGTTAATATCAAGGGCGCGG
TATTGGCCAGCGAACAGGCATTGCGGGAAGCAGGCATTGAACTGAGCGATTTTGTCCGTGGCAATGAAAAAGCGCGTGAG
CGGATGAAAGCACAATATAGCATTGCGGGTATGACCAGCGGTGTCGTGGTGGGCACCGATCATGCAGCAGAAGCCATTAC
CGGATTCTTCACTAAATATGGTGACGGCGGTACGGACATTAATCCGCTGTATCGTCTCAACAAACGTCAGGGTAAACAGT
TACTGGCGGCATTAGCTTGCCCGGAACACCTTTATAAGAAAGCGCCAACGGCCGATCTGGAAGATGATCGCCCTTCTCTG
CCAGATGAAGTGGCACTCGGCGTGACCTATGACAATATCGACGACTATCTGGAAGGGAAAAACGTACCTCAACAGGTCGC
CAGAACAATAGAGAACTGGTATCTGAAAACCGAACATAAACGCCGTCCGCCAATTACCGTTTTCGATGATTTCTGGAAAA

AGTAATAATTTGCACATATTGGATTGTGCGAAAAAGAGTAATTTGTTCACGCCGGATGCGGCGTGAACGCCTTATTCGAC
CTATAAAACTATGCAAATTCAATACATTGCAGGAGTCGAATAGGCCTGACAGGCGTAGCACGTCAGACGGTGTAACCTTT
GTCATCGACCCGCCTCTTTTTTAATCGCTTCCCGCCTGTTACACTGGATAGATAACCAGCATTCGGAGTCAACAGTGGTA
CGGCGTTTAACTTCTCCGCGGCTCGAATTTGAAGCTGCGGCAATTTATGAATATCCCGAACATTTACGTTCATTCCTTAA
TGACTTACCCACCCGACCAGGGGTGTATCTGTTTCATGGTGAAAGTGACACCATGCCGCTCTATATCGGCAAAAGCGTTA
ACATCCGCAGCCGCGTCCTTTCTCATTTACGTACCCCGGATGAAGCCGCCATGCTACGGCAATCCCGACGGATCAGCTGG
ATATGTACCGCCGGTGAAATCGGCGCTCTGCTCCTTGAAGCGCGATTAATCAAAGAACAACAGCCGCTGTTTAATAAACG
GTTGCGCCGCAATCGCCAGCTCTGTGCCCTGCAATTAAATGAAAAGCGCGTCGATGTGGTGTATGCCAAAGAGGTGGATT
TTTCACGAGCCCCCAACCTGTTTGGCCTGTTTGCCAATAGGCGCGCAGCTTTGCAAGCATTGCAGACCATCGCTGATGAA
CAAAAACTTTGTTATGGCCTGCTGGGACTGGAACCGTTAAGTCGCGGTCGTGCATGTTTTCGTTCAGCGCTAAAACGTTG
CGCCGGAGCATGCTGCGGTAAAGAGAGCCATGAGGAACATGCGCTACGCTTGCGCCAGTCTCTGGAGCGTTTGCGGGTGG
TGTGTTGGCCTTGGCAAGGGGCGGTGGCGCTGAAAGAACAGCACCCGGAAATGACTCAATATCATATTATTCAAAACTGG
CTGTGGCTGGGGGCGGTTAATTCGCTGGAAGAAGCGACAACGTTAATTCGGACACCCGCCGGGTTTGATCACGACGGTTA
TAAAATTCTTTGTAAGCCGCTGCTTTCCGGTAACTATGAAATTACTGAACTTGATCCGGCGAATGACCAGCGAGCCAGTT
GATTTCACTGAACAACAGTTTACCTTGCGGTTGTAAAAGCCGCAGGGTGTGCCTGCCATCGAACCAGCACGCGCCCTGGT
CGGTAGTGAGCAATTTATCGCCCAGTTGCCACGCCCCGTTAATCACAAACACCACCCCGCCGCGTGAGCCAAAGGTAGTA
AACGTACGTTCGGCAATTCTGACTTTCGCCTTACAGACATCCAGGCGTGTCATGATGTTGAAATCCATCGACATCTGCCC
TGCCGTCAGTTTCGCTTTTACCACCTGGTCCGCTGCAAAGGCAAAAGGCTGGAACGGTTTTAAGGTATGGTTAAAGCGGT
CTGCGCTTTCAAGGAGCATCTCGCCGCCTTCCAGCAACGTCACTATCCTTTCCATGCCGGGAAACAAAGAAAACTCCCCA
TTCGCCGCGATGGATGCAATGCTGGCACGCCAGTAAAAATCACGTTTTGCCGGTGGGAACGTGCAAATTTCGCGCGTTTC
ACCGGCAGCATTTCGCCACAGATTCACCGACATTTTACGCATATCAAAGTATTCCATACTCGCTCCCGGCCCGTTCTTTT
CATTGTTATCGTTCAGGTAACCGTGGATGGTTATCGGCAACTCGGGTGATTTACGTTAGTGGTGATCAGGCTTTTTTTAC
AACTGTTTGAGAAGAGAAAAGAAAACCGCCGATCCTGTCCACCGCATTACTGCAAGGTAGTGGACAAGACCGGCGGTCTT
AAGTTTTTTGGCTGAAAGATTATTCAGCAGTTGCAGGCATTTTACCTTTTGCCGCTGGACGTTCTGTCAGACGCTTCTCA
AAATTAGCATTAAATTGCTTTTTCTGTTCCGGCGTCAGGATGTTGTAAATTTTGTTCTGGGTTTCCATGTGCGCCAGCAT
GTTAGCTTTGCGCTGTTCTTCCATTTTTGCGATCTGCGCTTCAGCTTTTACTTTATCGAAGGTATCGCTGGCAATGATGT
CATGCATTGCGCGGCGTTCTTCCAGCGGCGGACGTTTCATCTGGTCACGCTGGCCTTTCATGATTTCGCGGATCTGCTGT
TTCTGCGCGTCGGTCAGGTTCAGGTCTTTGAACATCATGTCCTGATGCGGACCGAACTTGCCTTTGTGGTGCATCATCGG
CTTCGCGTCAGCCGGTGCTGCGGTAGTGGTGTCTGCGGCATGGGCCAGGTTAGCCGCGCCAAGAGCCAGGGTAGAGGCAA
CAAACAGTGCAGTTAATTTACGCATATTCTATATCCTTCCTTTCAGTTATTTATTACGGCTTTCTTAAGTAGCGTGCCGT
GTTGACGAGATTAACTTTACTGACTTTAGCGTCAATTAATCAGAGCAACGGTAAAACAATGAAAGTGTAAAAAACACTTT
TGCGCCAATTATGGAGAAAAAAAGAAAATTTGATGGAGAGTGATGAGAGAATATTACAACACGATGATTTTGCAGAGATT
ATGAAGAACTATACCGGATGACTGGTGATAAATAAAGCAAATAACCAGGATTAATCTGTATTAATTTATAAGAAAGCAAC
TTAATACCCGCAGAATGATTTCTGCGGGTAAGTATTAGCTTATTTTTTCGAGCATTAATCCCGCGCGTAATCCCAACGCT
ACCAACGGATTAGGGAATAACACATACTCTACATCATGGGTTACGGTAAAACGTTCCTCTCCGTCCTGCGCCAGCAATGT
TCCTTTCTCAAACGGCATAAAATTCAGCGTGTCACTTGCCATATGCATTTCGAAGGACGGCGAGTGGCGAGTAATTTGCG
AAACCACCCGATAACGGAGCGGCGGTGTTCTCACGATACCGACACTCTCACCAGATAGCAGCGCAGCAATTGCGCTGGCA
GTTACTGCAAACTGGCGAAGATCGTTTTGCCCAAAGGGCAACGCTTTGCCAAGTTCCAGCGTACAGGCCAGCGCGCCAAA
ATGTCTGGCGCTGAAATGGGTAAACGTACCACCAGGTTCCTGATGGAACACCAGCGCCTCCAGCCCCGCCGCACCCAGCC
ACGTCAGAAATTTCTCGTCCCAGGGAATGTCGCGTTGCGGTAATACACCGAACTGCGGATGCAAGGAGCCACGAATTGCG
GTATGTAGATCAAGGTGCCAGCGCACAGATTCTTTGCCCTGGTCATAAAAATCTTCCAGGCACTGTTCCAGTTCGCGCGC
CCGACAGGTTTCTCCGCTTTCAGCAAATAGCTGCCAACGACCGCCAAACATTCGATTCATATCGCTATGGCAATAACGTT
TCCCTTGCTTCAGCGCAGGAGGATTCCCGAGGATCACCAGCAACCGCCAACGTAACGGGATCTCGCCGTGAGATATCGCG
CCAAGCAACGCGTCCAGCATCTCCACAGGTGCCGTCTCATTACCGTGTATTCCCGCTGAAATCACCAGTGCGCCTTGCGG
TGGCGTTAATGGCGTCAGTTCCAGCACACCATCGCCCAGCCAGCGCCAGCGAACGCCGTTGATTTCTCGCTCGGTGATAA
CCGGTTTTTTACCCGTTAAGGTCAGAGCAAGAAAATTATCCATTGCCCCCTCCCTCGCGCTGGAACGGATAAACCGAACC
GAGATTCAGTAATTGGCTCAATACATCCAGTGCTTCCCGCCCTTCGCGCAGCAATTGCGGGTCGGCCAGATCGGCAGCAG
TAAGGCGATCGCGGTAGTAACGATCCACCCAGTCATTGAGCGCATTAAACAGCGTATCGTTCATCATCACCGCCGGATTC
ACCGCCCGGCGTTCTTCTTCTGTCAATACCACCCGCAACCGCAGGCACGCCGGGCCGCCGCCATTCGCCATGCTTTCACG
TAAATCAAAGACTTTTAGTTCGCTAATCGGGTTGTCAGCGGCAAGGAGTTCATTGAGATAACCCCATACTCCGGCGTGTT
CCCGACACTCCTGAGGCAGCACCAACATCATGGAACCATCATCGCGGCTCAGCAGTTGGCTGTTAAACAGATAGGTAGAC
ACCGTATCAGACACGGAAACCTGAGTTGCCGGAACTTCTATCGCCATAAAACCATTGACCCGCGCACGCAGGTTTGCCAG
TAACTGTGACTGGCGAGCGAACGCCTGTTGGTGGCAAAACAGCACCTGGCGGTTACTCACGGCAATCACGTCATTATGAA
AAACGCCCTGGTCGATAACGTCCGGGTTTTGCTGGGCGAAAATCACCTGTTGGGGATTCACCTGATTCAGCCTTGCCACC
GCCTCGCTGGCTTCGCGAGTCTGTCGCGCCGGATAACGGGAAGGCCGGGTATCATTGCCTTCTTCTCGCCCGTAGACAAA
AAGTTGCATACCCGGTTCACCGTAATGACCGCCGAGACGATTGTGGTTTGCCGCCCCCTCATCACCGAGCAACGCTACCT

GTGGCAACGCCGAATGGACGCTAAATTTCTCTTCGTCGTTAAAAATCGCTTTTAACAGCGATTCAGTGACGGGCGCTTCC
AGCGAACGGTGAAATTTATTGTTCAGGTTGGCAACGGTGAGATGCACTTTGCCATCCAGCGTATCGGCAGATGGCGCGAT
CGTTGCCGCATTGGCTACCCACATTGGCGAAGCGGAACTGACGCTGGAAAGCCAGTGCGGTGCCTGGCGTGCAACTTTTT
CCAGTACCTGCTCATCGCTACCACTGAATCCCAACTGACGCAGCACCGGAATAAACGGACGCTCGTGCGGCGGGATCACG
GCCTGGGGGAATCCCGCATCGGCAAGGGCTTTCATTTTCAGTAAGCCCTGCTTCGCCGCCAGTCGCGGGTTAGACACCTG
AAAACGGTGACGGGTAGAGGCTTCATTACCAAACGACAGGCCCGCGTAATGATGCGTCAGCCCTACCAGCCCGTCGAAAT
TGACTTCCCAGGCGTTCATCGCACCACCTCATCGGAAAAATCCAGCCCGGGGTTAAGCGTGGCGGGCAATGTTAACGAGT
CCGACTCCAGGCTCGCCATCGGCCATGCGCAGTAATCTGCGGCATACCAGGCGCTGGGGCGATGGTTACCGGAAGCACCA
ATGCCGCCGAATGGCGCGGTACTGGCAGCACCGGTAAGCGGTTTGTTCCAGTTAACAATCCCCGCCCGCGCCTCCAGCAA
CAGTTGATCGAACTTTTCCCGCTCGGGGGAAACCAGACCGCAAGAGAGTCCGAAGCGAGTGTTATTCGCCATTCGAATCG
CTTCATCGAAAGTATCATAACGCCAGACGCGCAATAACGGTCCGAACACCTCTTCATCTGGTACGCCAGCAACGCCTGTC
ATTTCAATGATCCCCGGCGTCAGCAACGATGTCCCTGCTTGTAATAAGCGCGGCGCAAGCAGGGGTCGTCCGCCCATCGC
TTCCAGTTGCTGCCATGCAGTAACCACCTGCTGTGCGGCCTGTTCAGAAATCAGCCCGCCAATAAACGGCTGCGGTTCGT
CATCCCAGTTGCCCGGCGTTAATCGCTGGCTGACGGCAACCAGACGAGCAAGAAACGCATCGCCCTGCGCCCCGCTTTTC
AGCAATAAACGGCGGGCGCAGGTGCAGCGTTGACCGGCTGTGACAAACGCCGACTGAATGGTCAGATGGACAGCCGCGTC
GATATCCGCCACCTCATCGATAATTAGCGGATTATTACCGCCCATCTCAAGGGCGAGAATTTTCTCCGGCTGACCGGAGA
GCTGGCGATGCAACTGGTAGCCTGTATTGGCGCTACCGGTAAACAGCAAACCGTCGAGATCCTCCAGCGCACTCAGCGCC
TGACCCGTTTCACGCCCGCCCTGCACCAGGTTCAGCACGCCTGGCGGCAAGCCAGCCTGCTGCCATAAACGCATTACCGC
TTCGCCACTCCACGGTGTCAGTTCGCTGGGTTTAAAGATAATGGTGTTACCTGCCAGCAATGCCGGAACGATATGTCCGT
TCGGCAAATGACCAGGGAAATTATACGGCCCAAACACCGCCAGCACGCCGTGCGGGCGATGTCGCAGGCTCGCCGCGCCG
TCCGGCATTTCACTACGCTGCTCGCCGGTACGAACGTGATACGCCTTAATTGATATCGCGATTTTATTGATCATCGCCGT
CACTTCGGTTGCCGCTTCCCAGCGCGGCTTACCCGTTTCTCTGGCAATAATCGCGGTTAATTCGGCTTTATTGCTTTCCA
GCAGTGCGGCAAAGCGTTCGACAACGGCATGACGTTCAGCAAATGAAAGCCGCGCCCAGCGCGGAAACGCCGCACGGGCT
GCCCGACAAGCCTGCTCGACCTGAGCGGCATCGGCATCATTGCCTTGCCATAACACCTCGCCCGATACCGGATTACGCTT
CACACGCGATGCGCCCTGGCCCGTTATCCAGTCACCGTTAATCCATAAAGTCATGCTGTTTTCTCCTCTGCGCACAGGCG
CACCAGACGAACGCGATCCCCGGCGTGGCATTTGAGGGCATCCAGTTGTGCGGCGGTTAAAATCAAACGCTCGGTTGCCG
GATCGGTACGCACCAGCACCACGCGGAAATGGTGATAATTTTCATTGGCGACCAGGCAGGCTGGGAAATCGCCCTGCGCA
GGCTGCCCTTCTGCCACTTCCACCAGCCGACTTTTACGGATGGCGCGCACGCGGTCGATGTCACACTCAAGCGTCGGCCC
ACCGTCAAAGATGTCGATATAGTTACGGTAGCGAAAACCTTCTTTCTCCAGCACCGCGCGGGCAGGCGCGGTTTGCGGAT
GTACCTGACCGATGACGTCCTGGGCTTCCTGGGATAAAAAGTGGGTATAGATCGGATGTTTCGGCATCAGTTCTGCAATA
AATGCCTTTTGCCCGGTGCCGCAGAGAAAATCGGCGCGGCTAAAATCCATCGAAAAGAAGCGTTTACCGAGGCTTTGCCA
GAACGGTGAATAGCCGTGTTCGTCAATCACCCCGCGCATTTCAGCAACCACTTTGTCATTAAACTTGTCGCGAAAAGCCG
CCATAAACATAAAGCGCGATTTCGACAGCAAATAGCCGTTGCCCTCTTTGCGCCAGTCCGGGTCGAGAAACAGCGTGCAC
AGCTCGCTGCTGCCGGTGTGATCGTTACTGAGAAACAGCGTCGGCAATGCGTTATAGACATTCAGCTCTTTTGAGGCGTG
AACCAACGTGCCGACGCGATAGTTGTACCAGGGATCGTTCAGCCCAACCGCCACCTCAATGGCACAAATCCCCGCCACGG
TGCCTGTCTCGCTATCTTCCAGCACGAACACATAGCCCTGCTCACTTTTGGGCAGTTCGCCTTGCCAGGTTTTGATTGCC
CTTTCGATACGCGCCGAAAGCGTGGCTTCATTGGCGGGAAGCGACGTCAGGCCGCCGCCCGTTTTGCTGGCAAGCTGCAT
CAGCGCCGAGACATCTGATCGCTCAACGGGACGGATGACCATCATGATGAACCTCGGCTAACAAAGTGTTCGCAAGCAGC
TGCAAAGCGATCCAGTCCGGTCGTCACCTCTTCTTCGCTGACATTGAGCGCAGGCGCAAAACGCACCACGTTGCCACCCG
CAATCAGTACCATCACGCCTGCTTTCGCCGCTTCCTGAGAGATCTGTTTCGCTTGCCCGGCGTAATCGGCATTCAGTACA
CAGCCAATCAGCAAACCTAAGCCGCGAACTTCACTGAACAAACCATAGCGGTGATTAATAGTATTAAGACGCTCAACAAA
CCAGTCGTGACGCTGTTTAACGCCATTAAGCATCTCTGGTGTGTTGATGAGCTCCAGCACTTTGCCTGCCACCGCCGAGG
CCAGCGGATTACCGCCATAGGTGGTGCCATGAGTGCCAACGGTCATCACGCGGGCGCACTCTTCGGTTGCCAACAACGCA
CCGACCGGGAAACCGCCGCCCAGCGCTTTGGCGGTAGTTAACAGATCAGGCGTCACGCCGTAGTGCATATAGGCATACAG
TTCCCCGGTGCGCCCGACGCCGGTTTGTACTTCATCAAAAATCAACAGCGCATTGTGGCGGTTACACAATTCACGCAGAC
CTTGTAAAAACGCGTTGCTGGCTGGCACCACACCGCCTTCCCCCTGGATGGGTTCGACAATCACCGCACAGGTAGAGTCG
TCAATCAGCGCGCTGGCAGAGTTAATATCGTTATATGCAGCATGACGAATATCCGCCGGCAGTGGCGCAAAATCCTGTGA
ATAGGCTGGCTGCCCACCCGCACTGACAGTAAACAGCGTGCGACCATGAAACGCATTTTTGAACGCCACGATGCCGCTCT
TATGGCTGCCGTAGCGGTCGTGAGCGAATTTACGCGCCAGTTTTAGCGCCGCTTCGTTGGCTTCCGCACCGGAGTTACAA
AAGAAGACGCGATCGGCAAACGTGGCGTCGATCAATTTTTTCGCCAGTCGCAGTACCGGCTCGTTGGTGTAACCGTTGCC
GGTATGCCAGAACTTACTCGCCTGTTCGTTCAGCGCTTCACGCAGTTCCGGATGCGCATGGCCCAGCGCGTTCACCGCAA
TGCCACCCGCGAAGTCGATATACTCTTTCCCCTGCTGATCCCACAAGCGCGAACCTTCGCCACGTACCGGTATAAAGGGT
GCCGGAGCGTAAACAGGTATCATCCATTCATCAAAGTTTTCACGCGTAATTGGCTGAGACATAGCGACCTCTACAGTAAA
TAATTCGTTATTTATATGTTAATAATAAGTAATGTTTGCGCTGTAAATGTAGATTGCAGGGTTCGTGCCAGCCAGTGATA
AAAGTGCATAAACGGCGGAGGCTAACTGGAAATCAAGGAGTTATAACCAAACCATATGCATTTAAAGTGCATATAAAGTG
AATACGTTTGCGATGTGGGTGAATAAAAAGAATAAAAAACGCAATGTTATGCAGAAGTAAAATATAATTCTGGAATTGTG

ATCATTGACGAAATTTACTGGAAATTACTGCGCCATTCTGACGCAGCGCGCACCAAAAGCGGGCATTTTTTGCGCCATCG
TTGACATCATTAACAACCATCGATCAAATCACTTAACAACAGGCGGTAAGCAACGCGAAATTCTGCTACCATCCACGCAC
TCTTTATCTGAATAAATGGCAGCGACTATGAAATTTGTCTCTTTTAATATCAACGGCCTGCGCGCCAGACCTCACCAGCT
TGAAGCCATCGTCGAAAAGCACCAACCGGATGTGATTGGCCTGCAGGAGACAAAAGTTCATGACGATATGTTTCCGCTCG
AAGAGGTGGCGAAGCTCGGCTACAACGTGTTTTATCACGGGCAGAAAGGCCATTATGGCGTGGCGCTGCTGACCAAAGAG
ACGCCGATTGCCGTGCGTCGCGGCTTTCCCGGTGACGACGAAGAGGCGCAGCGGCGGATTATTATGGCGGAAATCCCCTC
ACTGCTGGGTAATGTCACCGTGATCAACGGTTACTTCCCGCAGGGTGAAAGCCGCGACCATCCGATAAAATTCCCGGCAA
AAGCGCAGTTTTATCAGAATCTGCAAAACTACCTGGAAACCGAACTCAAACGTGATAATCCGGTACTGATTATGGGCGAT
ATGAATATCAGCCCTACAGATCTGGATATCGGCATTGGCGAAGAAAACCGTAAGCGCTGGCTGCGTACCGGTAAATGCTC
TTTCCTGCCGGAAGAGCGCGAATGGATGGACAGGCTGATGAGCTGGGGGTTGGTCGATACCTTCCGCCATGCGAATCCGC
AAACAGCAGATCGTTTCTCATGGTTTGATTACCGCTCAAAAGGTTTTGACGATAACCGTGGTCTGCGCATCGACCTGCTG
CTCGCCAGCCAACCGCTGGCAGAATGTTGCGTAGAAACCGGCATCGACTATGAAATCCGCAGCATGGAAAAACCGTCCGA
TCACGCCCCCGTCTGGGCGACCTTCCGCCGCTAATTTAGCAGCTCTCCTGGCTCAAACTGGGTCAGGAGAATTAACCTTG
AGAAAAATCAACAAACTGTCAGTAATGATTTGTTGCCTGCCGTCCTTTGTTATACCGTCTCTGCGTTTTTAGTTGTCTGA
CCACTTCTCTATTATCAAGTTTGATATAGGAAACTCCACGATGAACGCTGAGCGTAAATTTCTTTTTGCCTGTCTTATTT
TTGCGCTGGTCATTTACGCTATCCACGCTTTCGGTTTATTCGATCTGCTCACCGATTTACCCCACTTACAGACACTCATC
CGCCAGAGCGGATTTTTCGGCTATAGCCTCTATATTCTGTTATTCATCATTGCCACCCTCTTGCTGTTACCAGGAAGCAT
ACTGGTGATCGCGGGAGGAATAGTTTTTGGCCCGCTCTTAGGGACACTACTCTCATTAATTGCCGCCACGCTGGCCTCCT
CGTGCTCATTCCTGCTGGCGCGCTGGCTGGGACGTGATTTACTGCTGAAATACGTTGGCCATAGCAATACCTTCCAGGCC
ATTGAAAAAGGCATTGCGCGTAACGGTATTGATTTTCTTATTCTGACCCGCTTAATCCCGTTGTTTCCTTACAATATTCA
AAATTACGCTTACGGATTAACCACAATCGCCTTCTGGCCTTATACCCTTATTTCGGCACTCACGACCCTACCCGGTATTG
TTATTTATACCGTGATGGCAAGCGATCTCGCCAATGAAGGCATTACGCTGCGCTTTATTTTACAACTCTGTCTGGCGGGC
CTGGCGCTGTTTATTCTCGTCCAGCTCGCAAAACTCTACGCCCGACACAAACATGTGGATCTGTCTGCTTCGCGCCGCAG
CCCACTTACTCACCCAAAAAATGAAGGATAGAACGATGTTGCAACATTATTCAGTGTCATGGAAAAAAGGACTGGCTGCA
CTCTGTTTACTGGCTGTTGCTGGGCTTAGCGGCTGCGATCAACAAGAGAATGCGGCGGCAAAAGTGGAATACGACGGACT
TTCGAACAGCCAACCGCTGCGTGTCGATGCCAATAACCATACGGTAACCATGCTGGTGCAAATTAATGGTCGTTTCCTCA
CCGACGACACTCGTCACGGTATTGTGTTTAAAGATGGCTCCAACGGACATAAATCGCTGTTTATGGGTTATGCGACCCCG
AAAGCATTTTATGAAGCCCTGAAAGAGGCAGGTGGTACGCCGGGCGAAAACATGACGATGGATAATAAAGAAACGACTCA
TGTCACAGGCAGCAAACTGGATATTTCGGTCAACTGGCAAGGGGCGGCAAAAGCGTATTCCTTCGATGAAGTGATTGTTG
ACAGTAATGGCAAGAAACTGGACATGCGCTTTGGCGGTAATTTAACGGCAGCAGAAGAGAAGAAAACGGGTTGCCTGGTG
TGTCTGGATAGCTGCCCGGTCGGCATCGTCAGCAATGCAACATACACTTATGGTGCGGTTGAAAAACGTGGTGAAGTTAA
ATTCAAAGGCAATGCCTCAGTTCTCCCGGCGGATAACACGCTGGCAACGGTTACCTTTAAAATCGCCGAATAAAGCCAGG
ATAAAGGATGATGATGATGCAATCGCGAAAAATCTGGTACTACCGTATAACCCTCATCATCCTGTTGTTCGCTATGCTGC
TGGCATGGGCGCTGCTTCCCGGCGTCCATGAGTTTATCAATCGCAGCGTTGCGGCGTTTGCCGCCGTGGACCAACAGGGT
ATAGAACGCTTTATTCAGTCTTACGGCGCACTGGCGGCAGTTGTCTCGTTCTTGTTGATGATTTTGCAGGCCATTGCTGC
ACCGCTACCTGCGTTTTTGATCACCTTTGCCAATGCGTCGCTGTTTGGCGCGTTCTGGGGCGGCTTGCTGTCGTGGACCA
GTTCGATGGCCGGCGCGGCGCTGTGCTTTTTTATCGCCAGAGTGATGGGCCGCGAAGTGGTGGAAAAATTAACCGGCAAA
ACCGTGCTTGACAGTATGGACGGCTTTTTCACTCGCTACGGCAAACACACCATTCTGGTCTGTCGGTTATTGCCTTTTGT
CCCTTTCGATCCAATCAGCTATGCTGCCGGTTTGACTTCAATACGTTTTCGCTCGTTTTTTATCGCCACCGGGCTTGGTC
AGTTACCGGCAACTATTGTTTATTCCTGGGCGGGCAGCATGTTAACAGGCGGTACTTTCTGGTTTGTCACCGGACTGTTT
ATTCTGTTTGCCCTGACCGTGGTGATTTTTATGGCGAAGAAAATATGGCTTGAACGCCAGAAGAGGAATGCCTGATGGGT
TTACCGCCGCTTAGCAAAATTCCTTTAATTTTACGTCCACAGGCGTGGCTGCATCGTCGCCATTACGGCGAGGTGCTAAG
CCCCATTCGCTGGTGGGGGCGGATCCCGTTTATCTTTTATCTGGTGTCGATGTTTGTTGGCTGGCTGGAGCGCAAACGCT
CACCGCTCGATCCGGTAGTACGATCGCTTGTCAGCGCGCGCATTGCGCAAATGTGCCTGTGTGAGTTTTGTGTGGATATC
ACCAGTATGAAAGTCGCCGAGCGCACCGGCAGCAGCGATAAACTGCTGGCAGTGGCTGACTGGCGGCAAAGCCCGCTCTT
TAGCGATGAAGAACGGCTGGCGCTGGAGTACGCCGAAGCCGCAAGCGTAACGCCGCCAACGGTCGATGATGCCCTGCGTA
CCCGACTGGCTGCGCATTTTGACGCTCAGGCGCTCACCGAACTGACGGCATTGATCGGCCTGCAAAATCTGTCAGCCCGT
TTTAATTCTGCCATGGACATTCCCGCTCAGGGGCTGTGCCGTATTCCTGAAAAACGTTCTTAAGGAGAGATGATGCGCCA
TTGTGGGTGGTTGCTGGGATTGTTATCGCTGTTTTCTCTGGCAACACATGCCAGTGACTGGCAAGAAATTAAAAATGAGG
CCAAAGGGCAAACCGTCTGGTTTAACGCCTGGGGCGGCGATACCGCAATTAACCGCTATCTCGACTGGGTGAGCGGCGAG
ATGAAAACCCATTACGCTATAAACCTGAAGATTGTCCGTCTGGCGGATGCCGCAGACGCGGTGAAGCGCATTCAGACCGA
AGCCGCAGCCGGACGTAAAACGGGCGGCTCGGTGGATCTGCTCTGGGTGAACGGCGAAAACTTCCGCACCTTAAAAGAGG
CCAATTTATTACAAACGGGCTGGGCGGAGACTCTGCCCAACTGGCGCTATGTCGACACACAGCTGCCGGTGCGGGAAGAT
TTTTCTGTGCCGACACAAGGTGCGGAATCGCCCTGGGGCGGCGCACAACTGACGTTTATCGCCCGCCGCGATGTTACGCC
ACAGCCACCACAAACGCCGCAAGCCTTACTGGAGTTTGCTAAAGCCAATCCCGGCACGGTTACCTATCCGCGCCCACCGG
ACTTTACCGGCACGGCGTTTCTTGAACAGTTGCTGATTATGCTGACGCCCGATCCCGCCGCATTAAAAGAAGCGCCGGAC

GATGCGACTTTCGCCCGTGTCACTGCTCCCTTGTGGCAATATCTTGATGTGCTGCATCCGTATTTGTGGCGCGAAGGAAA
GGATTTCCCGCCTTCACCCGCGCGGATGGATGCTCTGCTGAAAGCCGGCACATTGCGCCTGTCGCTGACCTTTAACCCCG
CGCATGCGCAGCAAAAAATCGCCAGCGGCGATTTGCCTGCAAGCAGTTACAGTTTTGGCTTTCGCGAGGGGATGATTGGC
AACGTGCATTTCGTCACCATTCCTGCCAACGCGAATGCCAGTGCTGCGGCGAAGGTAGTTGCCAATTTCCTGCTCTCACC
CGATGCGCAACTGCGTAAAGCAGATCCCGCTGTCTGGGGCGATCCTTCTGTTCTCGATCCGCAAAAACTGCCTGACGGGC
AGCGCGAATCATTGCAATCAAGAATGCCGCAGGATCTGCCGCCGGTACTGGCTGAACCGCACGCAGGTTGGGTAAATGCG
CTGGAACAAGAATGGCTACACCGTTACGGTACGCATTAATCTTTTTGCTGTGGGCGATGGTGGCGGTGATTTATGCACCG
CTGATCCCGGCAGCTCTCACGCTGATATCGCCTGCTTTGTCGTTGACACACTGGCAGGCGTTATTTGCCGATCCACAGTT
ACCGCAGGCATTACTGGCAACGCTGGTGTCGACAACCATCGCGGCGGTCGGGGCATTGTTGATTGCCCTGCTGGTGATTG
TGGCGCTGTGGCCTGGGCCGAAATGGCAGCGTATGTGCGCCCGTCTGCCGTGGCTGCTCGCCATTCCCCATGTGGCTTTT
GCCACCAGCGCCCTTCTGCTCTTTGCTGACGGAGGGCTGCTTTATGACTATTTCCCGTATTTCACTCCGCCAATGGACCG
ATTTGGCATCGGTCTGGGCCTCACCCTTGCAGTGAAAGAAAGCGCATTTCTGCTGTGGATCTTAGCAGCAGTATTGAGCG
AAAAATGGCTGTTGCAGCAGGTCATTGTGCTGGATTCGCTGGGCTACAGCCGCTGGCAATGCCTGAACTGGCTGCTGTTG
CCCTCCGTCGCGCCTGCGCTGGCAATGGCGATGCTGGCGATTGTTGCCTGGTCGCTGTCGGTCGTGGATGTGGCAATTAT
TCTCGGGCCAGGTAATCCCCCGACGCTGGCGGTAATTAGCTGGCAGTGGTTAACCCAGGGCGACATTGACCAACAAACAA
AAGGCGCGCTTGCCAGCCTGCTGTTGATGCTGTTACTCGCCGCCTACGTTTTGCTGAGCTATCTGCTATGGCGCAGCTGG
CGGCGCACTATTCCCCGCGTAGATGGCGTTCGCAAGCCTGCCACGCCTTTATTGCCGGGCAATACGCTGGCGATTTTTTT
ACCCTTAACCGGTGTGCTGTGTGTGGTTCTGCTGGCGATCCTCGCGGATCAGTCGACGATCAATAGTGAAGCGCTCATCA
ACAGCCTGACAATGGGGCTGGTGGCGACATTCATCGCTTTGCTCCTGTTACTGCTGTGGCTGGAATGGGGGCCACAGCGT
CGCCAGTTGTGGCTATGGTTGCCCATTTTATTACCTGCTCTGCCACTGGTGGCGGGCCAGTACACGCTGGCGCTATGGCT
GAAACTGGATGGAAGCTGGACGGCGGTGGTCTGGGGGCATCTGCTGTGGGTGATGCCGTGGATGCTGTTTATCCTGCAAC
CCGCCTGGCAGCGCATTGATTCACGGTTAATTTTGATTGCGCAAACACTGGGCTGGTCGCGGGCCAAAATCTTCTTTTAC
GTGAAATGCCCACTCATGTTGCGCCCTGTGCTGATTGCCTTCGCGGTGGGATTTGCAGTCGGTATTGCGCAGTATATGCC
AACGCTGTGGCTGGGCGCGGGGCGTTTTCCGACGCTCACCACTGAGGCGGTGGCATTAAGCAGCGGCGGCAGCAACGGTA
TTCTCGCCGCCCAGGCTTTATGGCAACTGCTATTACCGCTTATTATTTTTGCCCTGACCGCGTTAGTCGCAAAATGGGTA
GGTTATGTCAGACAAGGACTCCGCTAATGCTCTGCGTGAAAAATGTTTCGCTACGTTTACCAGAAAGCCGCTTGCTGACA
AACGTTAACTTTACGGTGGATAAAGGTGACATTGTCACGTTAATGGGGCCGTCTGGCTGTGGAAAATCCACTCTGTTTTC
ATGGATGATTGGTGCACTGGCCGAACAGTTTTCTTGTACAGGTGAGCTATGGCTCAATGAGCAACGGATTGACATCCTAC
CCACCGCACAGCGTCAGATTGGCATTCTTTTTCAGGATGCACTGTTATTTGACCAGTTCAGTGTCGGGCAAAATTTACTG
CTGGCGCTACCGGCGACACTTAAAGGGAATGCCCGACGTAATGCCGTGAATGATGCACTTGAGCGTTCAGGCCTTGAGGG
AGCTTTCCATCAGGATCCTGCCACTTTGTCTGGCGGTCAGCGAGCGCGCGTTGCTCTACTACGCGCCCTTCTCGCCCAAC
CAAAAGCGTTACTCCTGGATGAGCCATTCAGCCGTCTTGATGTGGCTCTGCGCGATAATTTTCGCCAGTGGGTGTTCAGC
GAAGTTCGCGCCCTGGCGATCCCCGTCGTTCAGGTAACGCACGATCTCCAGGATGTTCCTGCTGATAGTTCTGTTCTGGA
TATGGCGCAGTGGTCAGAAAATTACAACAAACTGCGATAACGCAAAGTTTTTCTCAATGCGTCAGTTCAGAATGGCGCAC
TCAAAACTACAATGTCGGGATTTTCGATGAAACGTGTTTCTCAAATGACCGCGCTGGCAATGGCTTTAGGGCTGGCTTGC
GCTTCTTCGTGGGCCGCTGAACTGGCGAAGCCTCTTACACTTGACCAGCTTCAACAACAAAATGGCAAAGCGATAGATAC
TCGCCCCAGCGCGTTTTATAACGGCTGGCCACAAACCTTAAATGGCCCTTCTGGTCATGAACTTGCCGCCTTAAACCTCT
CTGCCAGCTGGCTTGACAAAATGAGCACCGAACAGCTCAACGCGTGGATCAAGCAACATAACCTGAAAACCGATGCTCCG
GTGGCGCTGTACGGTAATGACAAAGATGTCGACGCCGTCAAAACGCGACTGCAAAAAGCAGGTTTAACGCATATCTCCAT
CCTGAGTGACGCGCTAAGCGAACCTTCCCGTCTGCAAAAACTGCCGCATTTTGAGCAGCTGGTTTATCCGCAATGGCTGC
ACGACCTGCAACAAGGTAAAGAGGTTACGGCGAAACCTGCCGGTGACTGGAAAGTCATTGAAGCGGCCTGGGGCGCTCCT
AAGCTTTACCTTATCAGCCATATTCCCGGCGCTGACTACATCGATACCAACGAAGTGGAAAGTGAACCGCTGTGGAACAA
AGTTTCTGATGAACAACTAAAAGCGATGCTGGCAAAACACGGCATTCGCCATGACACCACGGTCATTCTGTATGGGCGTG
ACGTATACGCTGCAGCGCGTGTGGCGCAGATTATGCTTTATGCTGGCGTGAAAGATGTGCGCCTGCTGGATGGCGGCTGG
CAAACCTGGTCCGACGCGGGACTGCCTGTTGAGCGCGGAACGCCACCGAAAGTGAAAGCGGAACCGGATTTCGGCGTGAA
GATCCCGGCACAACCGCAGTTGATGCTCGATATGGAACAGGCCCGTGGACTGCTGCATCGCCAGGATGCATCGCTGGTGA
GCATTCGTTCGTGGCCAGAATTTATCGGTACGACCAGCGGTTACAGCTATATTAAACCAAAAGGTGAAATAGCCGGCGCA
CGTTGGGGACACGCTGGTAGCGACTCGACGCATATGGAAGATTTTCATAACCCGGATGGCACCATGCGCAGCGCCGATGA
TATTACCGCTATGTGGAAAGCGTGGAATATCAAACCAGAGCAGCAAGTTTCATTCTACTGCGGCACCGGCTGGCGCGCGT
CCGAAACCTTTATGTACGCACGCGCCATGGGTTGGAAGAATGTTTCCGTGTATGACGGCGGCTGGTACGAATGGAGCAGC
GATCCAAAAAATCCGGTAGCAACCGGTGAACGCGGCCCGGACAGTAGCAAATAACATTGCAATTACTGACGCTGGAGCGA
CTTCAGCGTCAGATACCCACTCCACACCCGCGTAAAGGTCGTCATCCAGCACAACGCACCGAAGATCCACGCAAACCACG
CAAACCATGCAGGGAATAAACATCCCAGCACAAACAGTAAGATCGTTTCTGTGCCTTCGGTTAATCCCCCAGATAGTAA
AACGACTTATGTGCATAACCGGGGTTATCAATCTGATGTTTTGCTGCCAGTGCAGCAAAGGCGAGAAAACTGCTGCCCGT
GCCGATAAACGCAAACAACAACCAGCCGCCTGCCAGCGCATTTTGCTCCGGTGCAGCAAGAATAAAGCCAAACGGCACCA
GCGCGTAAAAGAGAAAATCGAGAGAAATATCAAGAAAGCCGCCCGCGTCGGTAAGCTCTCTGCGCCGCGCCAGCGCACCG

TCCAGACCATCAAGCAGCCTGTTCAACAAAATGACGACTAGCGCCGCCAGATACCAGCCCAGCGCCAGAAACGGCAGAGC
CAGCACGCCGATGGCAAATCCCACTAATGTTAAACCATCTGGCGTAATGCCCGGTTTATCAAGAACCCGCACGCACTGAT
GCAACAACGGTTTAATCCGGGGATGAAGATGGCGGTCTAGCACAGGCACTCCTTAAATATAAAGCCTTTCTGATTGAGCA
ACAGTGCGGATATTATGGCATTTTTCGCTTATCTGCCCGTGTGTAATTTATGAAAATGATTGAAGTTGTTGCCGCCATCA
TTGAACGTGATGGCAAAATTTTACTCGCGCAACGCCCCGCCCAGAGCGATCAGGCGGGATTATGGGAGTTTGCCGGTGGT
AAAGTCGAGCCGGATGAAAGTCAGCGGCAGGCGCTGGTGCGTGAGTTACGCGAAGAACTGGGCATCGAAGCAACTGTGGG
TGAATATGTTGCCAGCCATCAGCGAGAAGTTTCGGGGCGGATTATCCATCTTCATGCCTGGCACGTACCCGACTTCCACG
GGACGTTACAGGCACATGAACATCAGGCGCTGGTCTGGTGCTCACCTGAAGAGGCGCTGCAATATCCGCTGGCCCCTGCT
GACATTCCATTATTAGAGGCGTTTATGGCTTTACGCGCCGCCAGACCAGCGGATTAGTGCTAAGGGTTTTGTCATCACGC
TGGCATTGCAGCAGTATTCCTTCGGCTTTAATTACCGCCCCTTCAGAATAATTTTGATCCTGATAAACGCAGCACTGAGT
ACAGGGCTGCGCTGACTGCCCGCCTGAACTGAACACTTCTGGCGGTACGTTTACCTCCACGTCCGGACGATAATGCGGGT
TAGCCAGTGCAATTAATGGAAATGCTAATACTACGGCGAACAATGCTCGACTCACAGGGAACTCCTTAACGTTATTGTCT
CTGCTACTGATAACGGTAGCCGGGTGGCAAAACTTTAGCGTCTGAGTTATCGCATTTGGTTATGAGATTACTCTCGTTAT
TAATTTGCTTTCCTGGGTCATTTTTTTCTTGCTTACCGTCACATTCTTGATGGTATAGTCGAAAACTGCAAAAGCACATG
ACATAAACAACATAAGCACAATCGTATTAATATATAAGGGTTTTATATCTATGGATCAGACATATTCTCTGGAGTCATTC
CTCAACCATGTCCAAAAGCGCGACCCGAATCAAACCGAGTTCGCGCAAGCCGTTCGTGAAGTAATGACCACACTCTGGCC
TTTTCTTGAACAAAATCCAAAATATCGCCAGATGTCATTACTGGAGCGTCTGGTTGAACCGGAGCGCGTGATCCAGTTTC
GCGTGGTATGGGTTGATGATCGCAACCAGATACAGGTCAACCGTGCATGGCGTGTGCAGTTCAGCTCTGCCATCGGCCCG
TACAAAGGCGGTATGCGCTTCCATCCGTCAGTTAACCTTTCCATTCTCAAATTCCTCGGCTTTGAACAAACCTTCAAAAA
TGCCCTGACTACTCTGCCGATGGGCGGTGGTAAAGGCGGCAGCGATTTCGATCCGAAAGGAAAAAGCGAAGGTGAAGTGA
TGCGTTTTTGCCAGGCGCTGATGACTGAACTGTATCGCCACCTGGGCGCGGATACCGACGTTCCGGCAGGTGATATCGGG
GTTGGTGGTCGTGAAGTCGGCTTTATGGCGGGGATGATGAAAAAGCTCTCCAACAATACCGCCTGCGTCTTCACCGGTAA
GGGCCTTTCATTTGGCGGCAGTCTTATTCGCCCGGAAGCTACCGGCTACGGTCTGGTTTATTTCACAGAAGCAATGCTAA
AACGCCACGGTATGGGTTTTGAAGGGATGCGCGTTTCCGTTTCTGGCTCCGGCAACGTCGCCCAGTACGCTATCGAAAAA
GCGATGGAATTTGGTGCTCGTGTGATCACTGCGTCAGACTCCAGCGGCACTGTAGTTGATGAAAGCGGATTCACGAAAGA
GAAACTGGCACGTCTTATCGAAATCAAAGCCAGCCGCGATGGTCGAGTGGCAGATTACGCCAAAGAATTTGGTCTGGTCT
ATCTCGAAGGCCAACAGCCGTGGTCTCTACCGGTTGATATCGCCCTGCCTTGCGCCACCCAGAATGAACTGGATGTTGAC
GCCGCGCATCAGCTTATCGCTAATGGCGTTAAAGCCGTCGCCGAAGGGGCAAATATGCCGACCACCATCGAAGCGACTGA
ACTGTTCCAGCAGGCAGGCGTACTATTTGCACCGGGTAAAGCGGCTAATGCTGGTGGCGTCGCTACATCGGGCCTGGAAA
TGGCACAAAACGCTGCGCGCCTGGGCTGGAAAGCCGAGAAAGTTGACGCACGTTTGCATCACATCATGCTGGATATCCAC
CATGCCTGTGTTGAGCATGGTGGTGAAGGTGAGCAAACCAACTACGTGCAGGGCGCGAACATTGCCGGTTTTGTGAAGGT
TGCCGATGCGATGCTGGCGCAGGGTGTGATTTAAGTTGTAAATGCCTGATGGCGCTACGCTTATCAGGCCTACAAATGGG
CACAATTCATTGCAGTTACGCTCTAATGTAGGCCGGGCAAGCGCAGCGCCCCCGGCAAAATTTCAGGCGTTTATGAGTAT
TTAACGGATGATGCTCCCCACGGAACATTTCTTATGGGCCAACGGCATTTCTTACTGTAGTGCTCCCAAAACTGCTTGTC
GTAACGATAACACGCTTCAAGTTCAGCATCCGTTAACTTTCTGCGATAGCAGCAGATATGCCAGTAAAGAAATCCCATTT
GACTATTTTTTTGATAATCTTCTTCGCTTTCGAACAACTCGTGCGCCTTTCGAGAAGCAAGCATTATATAATGCCAGGCC
AGTTCTTCTTCAATTGTCCCGTTTTGAAAAGCTGTGCTTGATATCGAGATCATCCATGATAATTCCGCCGCCCATATTAG
CTTCGCCGAGGATTTACCGGAGCTATGATTAGCGCAATCAGAGATATAGTCTGAGGGAAAAACAGCAAATTTATTCAACA
AGGCGATAACCTGCTCTGGGGCTTCCTCCATGTTTGCTTTAAAGGTATTGGCTCCATGGTCGCCAGAAAGAAAATGCTCC
ATTAAGGCACAATAACTTTCGCTATCTTCGATACCCCATTGATCCTCTAAAGACTCGCGTCTTTTACTTATGATATCGAT
CGAGTCAAAAGGAAGCACATGATATTGGAAGGTATCTTTGCCAGGTTCAGGCTTTCGCGGCCAGAACTCCAGCGTTTCAG
ACCATTGCTTATGATAGAATCGATAAGGTGCGATCAATTGTAGCGCCTGTAACTTCTCGATACTGAGCGGCTCAATACCT
TTAGCCTGATAATAATGCAGTTGTTCTTTTTTTGCTTTAAAACCGGCCCGAACAATAAGCCCCATCATAATTAATAGATA
AAGAAAAGAGCATCCCGCGGTAATCAGGCCTCTTTCATTCAAACCGTTGGATGTTATCGCTGCGAACACAAACATTACAG
CGACAACACATGTTAAATAAAACCCCCACTTACAAAGCAGCATGGCCTTATTTTCTTTAATCATCCGTTCAAAATTACTA
TTAAATATTTCCCAGCCATTAAAAGAATACTTCTCGCTCCCAGGATGGTTTTGTAATAAAACTTTTTTCATCACATTCCC
TGTTATTACATACTTTAGAGGCGATAATTTTATCCATGCAAAAAAAATATCCAACGAATTTTCTTGATCCGCTGGATATT
TCAGAATATGACTCGAATAGCACGAAAGATTCACTCGCTTACGCTATCGCCCCGCTTCCGACTTCATCTGCTGGCGGACT
TTTTTTCGCACTACGTTTACGCGGTGCAGCCTTTTTCTTATCAGCACTGCCACCACTGCCCGGAGCCACAATGCCGCGAA
ACTGCCGCACCGGCGTACGTTTGGCTTGATCAATAAGCTGATATAGCGTCCCCACCAGCGGCTGCATAAAGTCCTGATAG
CGACACTGCTTTTCGCTGATTTGCGTCAGCACCGATTCCCAGTGCGCGGTCATGTCCGGTCGCGTCGCCATCTCCGGCAG
CGAATGGAATAGCGCTTTTCCGGCGTCGGTGGAGTGGATATAGCGCCCTTTTTTGGTCAGGAAACCACGCTTGAACAACA
GTTCAATAATCCCGGCACGCGTTGCCTCTGTCCCCAGACCATCGGTCGCACGGAGGATCTTTTTCAGATCTTTATCCTGC
ACAAAGCGCGCGATCCCGGTCATCGCCGAAAGCAGTGTTGCATCGGTAAAATGGCGCGGCGGCTGGGTTTGCCGCTCTAC
CACTTCACCTTTTTCACACAGCAACTCATCGCCTTTCGCCACCACAGGCAGTGGCGTGCCGTCGTTTTCTTCATCGCGCT
CTTTGCTGCCTAACAGCGTGCGCCAGCCTGCTTCAGCAAGAAAACGCGCTTTAGCGACAAATTTGCCTTTGGCAATGTCC

AGTTCGATAACACACTTGCGGAACACCGCATCCGGGCAGAATTGCATCAGATACTGACGGGCAATCAGGTTATAGACCTT
CGCTTCGTTCTCCGTCAGGTTGATCGCAGAACTCCGTGCGGTCGGAATGATGGCGTGGTGCGCATCGACCTTTTTGTCAT
CCCAACAGCGGTTGCGTATATCTGGATCTACCACTGGCTGCGGCAACAGATCCGGTGCATGAACACTGATGGCATTCATC
ACCGCGTGGCGTCCGGCAAAATGTTCTTCTGGCAAATAGCGACAATCAGAACGCGGATAAGTGATTAGCTTGTGCGTTTC
GTACAGTTTCTGGCAGATATCAAGCACGTTCTGCGCACTCAGACCAAAACGTTTTGCCGCTTCAATCTGCAACGCTGAAA
GCGAAAAAGGCAGCGGCGCGGATTCTGATTCCCGTTTATCGTTATAGCTGGTGACAATAGCCGGTTGACCACTAATGCGG
TTAACCACATGCTCCGCCAGTGGACGATGTAACAAGCGCCCTTCTTCATCCTGGTACGGTTCACACGCTTCGCTCGGTTG
CCAGATAGCGGTAAACCGCTCATCGGCAGGTGTCACGATATGTGCTTTGACTTCAAAGAAGTCTTTCGCCACGAAGTTTT
CAATCTCTTCATCGCGGCGCACCACCAGCCCAAGCACGGGCGTCTGCACGCGTCCCACGGAAAGTACGCCCTGATAACCG
GCATTGCGACCGAGAATGGTATACGCACGGGTCATATTGATGCCGTACAGCCAGTCGGCACGCGCTCGCGCCAGCGCAGA
AACGCACAGCGGTACAAACTCACTGTTGGAACGAAGACGGTCGATCGCCCGCTCAACCGCCTGCGGGTTCAGGTCGTTTA
TCAAGCAACGCTGTACCTGCTGGCGCTTTTCCGGTGCCAGTTGCAGATAGTCCAGCACTTCATCCACCAGCAATTGCCCT
TCACGATCCGGGTCCCCGGCGTGAACGATTTCGCTGGCTTCATGCAGGAACCGTTTGATGACGTTAAGTTGTTTGGTCAC
GGAGGGTCGGGGCTGTAATTGCCACTTTTCCGGGACAATCGGCAAATCCGCAAGATTCCAGCGCGCATAGCGGCTGTCGT
AGGCGTCTGGCTGCGCCTGCTCAAGCAGGTGACCGATACACCAGGTCACCACCTGACCATTACCGCACTCGATAAAGCCA
TCGCCTTTCCGGTGCGGTTTGGGCAGGACATCAGCAATGGCGCGCGCCAGACTCGGTTTTTCGGCAATAAACAACCGCAT
TGAATTAACGAATCTCAACCATGGCACGACCGCCGCGCGCTGGCACCAGTTCGCCAATTGCCGTCAGTTCAATGCCAAAC
TCGGCGGCTGTAGCTTTGACCTCATTTTCTGCTTCCGGCATGACCGCCAGCAGCAAACCGCCAGAAGTTTGCGGATCACA
CAGCAGATCGCGCACTTCACGCGGCATTTCACCCATCAGATGACCGTAGCTGGCAAAGTTACGTTCAGTGCCGCCAGGTA
CTGCGCCCAACTTAATGTACTCTTCAACACCGGGGAGTTTCGGGATCGCTTCATAGTCGACGCGTGCCTGCACACCAGCC
CCCTGACACATTTCGCTCAAGTGGCCCAGCAGACCAAAGCCCGTAACGTCGGTCATCGCTTTTACGCCTTCGATGTTGGC
AAAGGACGCGCCTGCGATGTTCATCCGGCACATCACTTCCGTCGCCAGTCCCTGATGTTCTGGTTTCAACAGTGATTTTT
TCTCAGCCGTGGTAAGAACGCCGATCCCCAGCGGTTTCGTCAGGAACAGTTTGCATCCGGCTTGTGCGGTACTGTTTTTC
TTCACCCGCTCGGTCGGTACGATCCCCGTTACCGCCAGACCAAAAATCGGCTCCGGCGCATCGATGGAGTGACCGCCAGC
CAGCGCAATACCCGCCTGACGACATGCATAGCGTCCACCTTCGGTCACTTCGCGGGCAATTTCTGGGGAAAGTTTGTTAA
TCGGCCAGCCGAGGATCGCAATCGCCATAATCGGTTTGCCCCCCATCGCGAAGATATCGCTGATGGCGTTAGTCGCCGCA
ATGCGGCCAAAATCGAAAGGATTATCAACGATCGGCATAAAGAAGTCGGTGGTACTGATAACGCTGGTGCCATTGCCCAG
ATCGTACACCGCCGCATCGTCGCGGGTTTCATTACCCACAAGCAAATTCGGATCAACAAACTTCGCCTGCTCACTATGCA
GGATGGTTTCCAACACTTTTGGGGAAATTTTACAGCCGCAACCAGCTCCGTGGCTGTATTGGGTCAAACGAATCGAGTTC
TCGCTCATGGACATCTCCTGTCAATGCAATCCGGGTATGGTAACCCTCATTCCGTGAAGTGATAAGTGAGAGTGTCTGAA
TTCCTGCGCCTTTGCTCACAATCCAGACAGTTTCGCGACAATTATCAGAAATAAGTTACAAACGGCGTCGGGTCCGGGAC
GTTAATCGACGTAGATGCTTTCAGCTGCGGCGTACCGAGGTAGAGAAAACCGACAATTTTATCCTGCTCACGGCAACCGA
ATGCTTCACGCACTACCGGACTTTCAGTTAATGCGCCACTGCGCCAGATGCCGCCAAACCCCTGGGCAACTGCTGCCATT
TGCATCGCCATGACCGCGCATCCGGCAGACATTTCCTGTTCCCAGCGCGGGACTTTATGATTCTCTTCGCATTTCGCCAC
CACCGTGATGATGAGCGGTGCGCGGAACGGCGCATTACGGGCTTTGTCGATAGCTTTGTCATCACTACCGGCAGCAATCG
CCCCCTGTTCCAGTACGGCGCTGAAACGCTCGCGCCCTTCCCCTTCAATCACAAAAAAATGCCACGGTTGCATGGACTTA
TGGTCCGGCGCACGCATACCCGCACGCAGGATGTTTTGCAGTTGTTCACCCGTTGGCGCGGGTTCAGCCAAGCGGGAGGC
GCTACGGCGATTGATCAATAGTTCGAGTGCATCCATTTGATTAACTCCTGTCGTGATATTTATTCACAAAATTAACACGA
GAGTGGATTTTGTTACAGCACAGTCCGCAATTCCTGCTGACAAGTACCGGTTGGGTCATTACGATAACCACATCTATTGC
GCCTGTGACAGGTGTGACCTTAAGTTGGGAGAATACATGCGAACCCTTTGGCGATTTATTGCCGGATTTTTTAAATGGAC
GTGGCGTCTGCTGAATTTCGTCCGTGAAATGGTACTTAACCTGTTCTTTATTTTCCTCGTACTGGTTGGTGTGGGGATTT
GGATGCAGGTCAGTGGTGGTGATTCGAAAGAAACGGCCAGTCGTGGCGCACTGCTGCTGGACATTTCTGGTGTGATCGTC
GATAAACCCGACAGTTCTCAGCGGTTTAGTAAATTAAGCCGCCAGCTGCTTGGTGCCAGTTCCGATCGTCTGCAGGAAAA
CTCACTGTTTGATATCGTCAACACTATTCGCCAGGCGAAGGACGACCGCAATATCACCGGTATTGTGATGGATCTGAAAA
ACTTCGCAGGCGGCGACCAACCGTCTATGCAGTACATCGGCAAAGCTCTGAAAGAGTTTCGTGACAGCGGGAAACCGGTT
TATGCCGTTGGCGAGAACTACAGCCAGGGGCAATATTATCTCGCCAGTTTCGCCAATAAAATTTGGCTGTCTCCGCAAGG
CGTGGTTGATCTGCACGGCTTTGCCACCAACGGTCTGTACTACAAATCGTTGCTGGATAAGCTGAAAGTTTCCACCCATG
TGTTCCGCGTGGGTACGTATAAATCTGCCGTTGAACCGTTTATTCGTGATGATATGTCACCGGCAGCCCGCGAAGCTGAC
AGCCGCTGGATTGGTGAGCTGTGGCAAAACTATCTGAATACTGTTGCCGCTAACCGGCAGATCCCTGCTGAGCAGGTATT
CCCTGGCGCGCAAGGGTTGCTTGAGGGTTTAACCAAAACCGGTGGCGATACCGCGAAATATGCACTGGAAAACAAGCTGG
TCGATGCACTGGCATCGAGTGCGGAAATCGAAAAAGCACTGACCAAAGAATTCGGCTGGAGTAAGACTGATAAAAATTAT
CGCGCCATCAGTTATTACGATTACGCATTGAAAACGCCGGCAGATACCGGTGACAGCATCGGTGTCGTCTTTGCTAATGG
CGCAATTATGGATGGCGAGGAAACTCAGGGGAATGTTGGCGGTGATACCACTGCGGCACAAATCCGCGACGCTCGCCTTG
ACCCGAAAGTGAAAGCGATTGTCCTGCGTGTTAATAGCCCAGGCGGCAGCGTTACCGCGTCTGAAGTGATTCGCGCTGAA
CTGGCAGCAGCCCGGGCAGCGGGTAAGCCTGTGGTTGTATCGATGGGCGGCATGGCGGCATCTGGTGGTTACTGGATTTC
CACGCCAGCTAATTACATTGTGGCTAACCCCAGCACCCTGACCGGTTCTATCGGTATCTTCGGCGTGATCACCACCGTAG

AAAATAGTCTGGATTCGATTGGTGTTCATACTGATGGTGTCTCAACTTCACCGCTGGCGGATGTTTCTATCACCAGGGCA
CTGCCGCCGGAAGCGCAGCTGATGATGCAGTTAAGCATTGAGAATGGCTATAAACGCTTTATCACGCTGGTTGCTGATGC
GCGTCATTCGACGCCGGAGCAGATTGATAAAATTGCCCAGGGCCACGTCTGGACCGGTCAGGATGCAAAAGCTAACGGGC
TGGTCGATAGTCTCGGGGATTTCGATGATGCGGTCGCCAAAGCAGCAGAGCTGGCAAAAGTGAAACAGTGGCATCTGGAA
TATTACGTTGATGAACCGACCTTCTTCGACAAAGTGATGGACAACATGTCTGGTTCTGTCCGGGCAATGTTGCCAGATGC
GTTCCAGGCCATGTTACCTGCACCGCTGGCCTCGGTAGCCTCTACCGTTAAAAGTGAAAGTGACAAGCTGGCCGCGTTTA
ATGACCCACAAAACCGTTATGCGTTTTGCCTGACCTGCGCCAACATGCGTTAAGTCTTGTACTGAGTGGCCGACAGATCG
TCGGCCACATTATTTTTTACGTCGACGAATCCTCTTCCCGCTGTTTCGCCCCATATTTCCTTATCCGCATAGTATCAGGT
GCGCTCCCCCTGCCTCACGTATATACTTTTGCTCTTTCGATATCATTCATATCAATATCATGCAAAAGAAATCAATTTAC
GTTGCCTACACGGGCGGGACCATCGGGATGCAGCGTTCCGAGCAGGGTTATATACCGGTGTCAGGTCATCTACAACGCCA
ACTGGCGCTGATGCCGGAATTCCATCGCCCGGAGATGCCAGATTTCACCATTCATGAATATACGCCGCTGATGGATTCTT
CAGATATGACGCCGGAAGACTGGCAGCATATTGCTGAAGATATTAAAGCGCACTATGACGACTATGATGGTTTTGTCATT
CTGCACGGCACCGACACGATGGCGTATACCGCCTCTGCGCTGTCGTTCATGCTCGAGAATCTCGGTAAACCGGTCATTGT
GACAGGGTCACAAATCCCGCTGGCTGAGTTACGCTCTGACGGACAAATTAATCTGCTGAATGCGTTGTACGTTGCGGCGA
ATTATCCGATCAACGAAGTAACGCTCTTTTTCAATAACCGATTGTATCGCGGCAACCGCACTACCAAAGCCCATGCCGAT
GGTTTTGATGCGTTTGCCTCTCCAAACCTTCCTCCGTTACTGGAAGCAGGTATCCATATTCGTCGTTTGAATACGCCACC
CGCCCCGCACGGTGAAGGGGAATTAATCGTTCATCCAATCACCCCACAACCAATTGGCGTAGTGACGATTTATCCAGGGA
TTTCTGCTGACGTCGTGCGCAATTTTCTGCGCCAACCGGTGAAAGCATTGATTCTGCGCTCCTATGGCGTGGGTAATGCG
CCACAAAACAAAGCCTTCCTGCAGGAATTACAAGAAGCCAGCGATCGCGGTATTGTGGTGGTCAACCTGACACAATGTAT
GTCCGGTAAAGTGAACATGGGTGGTTATGCCACCGGTAACGCCCTCGCCCATGCCGGCGTAATTGGCGGTGCAGATATGA
CTGTAGAAGCCACGCTAACCAAACTGCATTACCTGCTTAGCCAGGAACTGGATACTGAAACCATTCGCAAGGCCATGAGC
CAAAACCTGCGCGGCGAACTGACGCCGGATGATTAAGGAGACTGTAATGCCCCCTCGCGCCCTGTTACTGGTCGATTTAC
AAAATGATTTCTGTGCTGGTGGCGCGCTCGCCGTGCCGGAAGGTGACAGTACGGTGGATGTCGCTAACCGCCTGATTGAC
TGGTGCCAGTCGCGCGGTGAAGCGGTTATCGCCAGTCAGGACTGGCACCCGGCGAATCACGGCAGTTTTGCCAGTCAGCA
CGGTGTAGAGCCTTATACGCCAGGCCAACTCGACGGTTTGCCACAAACCTTCTGGCCAGATCACTGTGTGCAGAACAGTG
AAGGCGCACAATTACATCCGTTACTGCACCAAAAAGCGATCGCAGCGGTGTTCCATAAAGGCGAAAATCCTTTAGTTGAC
AGTTACAGTGCCTTTTTTGATAACGGCCGTCGGCAGAAAACCTCTCTCGATGACTGGTTACGCGATCATGAAATCGATGA
ATTGATCGTTATGGGCCTGGCTACTGACTATTGCGTGAAGTTTACCGTGCTGGACGCGTTACAGTTAGGTTATAAGGTAA
ACGTGATTACCGATGGTTGTCGTGGCGTGAATATCCAGCCCCAGGACAGTGCGCACGCGTTTATGGAGATGTCAGCAGCT
GGGGCAACGCTATATACGCTGGCAGACTGGGAAGAGACACAGGGGTAATTTTACGCTGGCCTACAATTCTGTACTGGCAT
TGTAGGCCAAATAAAACACGTCAGTGGCACATCTGGCAATTGATGCCATCAACGAAAGATTTAATTCACCTCAGAAATCT
CTTCCAACGACACCTTCCGCGTTTCGATGCCAAAAATGGAGAGAACCAGCGCACAGAGCAATAACATAACACCAAGTACC
ATAAACACCGTGATCGACCCATAATGTGTTAATAATGCCGCAACGCCATAGGGCGTGAAGACTGCGACGATCCGTCCGAC
GGCATTAACGAAACCCGAACCGCGCAGGCGTAAATGCGTTGGCCAAAGCTCCGGGATATAAACCGCCGACGCGAAGCAAA
CATACATGTATAAAAAGAAGATCATCACCAGTCCATAGATTAAAATCGCCCACTCTGTAGTCTGAATTGAATAGATATAG
CCTAACACGGCAATAATAATAAGTAAGGTGGAGCCAAATAACCGACGAGGAAAATGATCAATAATTAATGCCGCAATAAA
TATTCCTACCGGAGCGCCAATCATAATAACAGCGGTCATTAATATTGATTTATCGACATCAATGCCGGAGTTAACAAATA
TGGTCGGTATCCATACGGTGATGGTATAAAGCGAAATGTTCATTGCAATTAAAACAGTAATCGCGACTAATGTACGTCGT
AACATTTCACCTTTAAACAGGAGCCAGAAAGTACCCTTGATTACTTTAACCTTGCTGTTGCTCTGATACGAAGTAAGCGG
GGGTAAACGAATACTCTTCTCTCTTTCAATTTGCTGCTCTACTTCACGAAGTTGGCATTCTGCACCTGCGATTTGCCCTT
TCCCTGCCAGCCATCGTGGCGACTCGATAAAGTATTTACCTGAGAGAAACCAGGCTAACAGTATGCCAATACCACCCAGC
AGAAACATTATTCGCCAACTAAAAAAAGCGATAACCACCACGCCTATCGCCGCAGACAGCATGGGCGACCAGTTACCAAC
AAATGAGAGCCGCGCGGACCATTTTCCACGCACCGTCGCGGGGATAAACTCCGTAAATGAGGCATAGCCAACCATAATCA
GCGCCCCCATTCCTGTTCCCATCAGGAAGCGAAAGAAGATGAGCCAGTACATATCAGGGACAAAAGCGGCCCCTGTTGCA
GCAATACCGACGATGAGAAGATTTATGCGAAACGCCCTGCGCCGCCCAAAGTAGTCACCAATAAACCCACCAGTAAGTGA
GCCGATGAAATAACCAAACATTAATGCCGAGGTAAAGGCGGCATTGAGGAAATTATTTGACCATCCATTGCTTACCAGCT
TTGCTAAGACGACATTACCGGAGTAACTCAAAAACCCTGTTAATAACAGACTAAAGCTTATAATACCAAATATACGATAA
TGAAACCGGGCCAAAGGCAAGCGGTCCAGTCTTGCGCCAATTTGATCATATTGTTCCATTAGGTGCCTCGGATTTGCTAA
AAAGGTAGCAATTGCGGCAAATTCACTTGCCGCATCTTTATCATCATTTTATTTCAAGGCGCAGGAACAAATAATATTGA
CAACGTGTGCTTAAATTACCACACCAGCTGGATATTATTGTCTTTGCAAAATGCTATCCACTCTGCACCCGGTGATTTAT
CAGTTATTATGTAATTAATATGACTAAAATCAGCTAACTGGACAAAAGCTTTGCGATCAAACTTAGAGTGATCAACCAAT
AACGCAACTTCTGTCGCCTGACGGATCATTGTCTTTTTGATTTCAGCTTCCGCTTCATTAGAGTCCAGCGCGCCGCTGTT
AATATCAAGACCTTTACAACTCATTACCATGATATCGACATGATAGCGCCTGATGATCTCTTTAGTAATTCTTCCTTGCA
GTGAAAGTGTATTTTTGTTTAGTTCCCCACCCGTTGAAACGACTTTAATTTCTGACTGAGCCAGAACATGAATCGCTTCC
GCCGAGTTGGTTAGCAACGTCAGGCCACTACGGTCCTGTAACAATTTGAGCAATTCCATTACGGTACTACTGGAATCGGC
TGCCATGGTGGTTTTATTGTCGATAAAGGGTAGTGCCTTGCGTGCAATAAGCTGCTTCTCTTCATAAAACGATGAAGCGC

GCTTATAAAAATGGATATTCTCCGTCAACATCGCTGTATTTAAAACAGCACCACCATAGGTTCTGGTCAAAAAGCCTTCA
TCTTCCAGCTTCTCAAGATCGCGGCGAATGGTTTCTTCGGTTACCTGAAAAATCCCACTCAAATTTGAGACTGTCACCTT
TTTATCGTTGGCAACCATTTGCTTAATTGCCTGAATCCTGTCTTTTGCCGCCACGATTACACCCCTGTATCTTTTTACAT
CACATTAGCGCGATTATCGCATAACCGATGTTTACTTTCAAAATAACCTGTTTGAATCACAGATTTTCATCACAGTTTTC
ACAGAAACAGAGGTGAATCGTGTTGAGTATTTAACGCTCCAGGGCCTCTGCCATTTCCCTCATCAATGTTGCGTCTGCAT
CCGATAAGTTGATATTCAGTGCCGCGACATTTTCGCGTACCTGTTCCGGTGCAGTAGCCCCACTAAGAATGGAGATTAAA
TCACTCTGTTTTAATATCCACGCCAGTGCCAGAGTGGGAATTGTGCACTGATAACGAGCACAAAGTGGCTGCCACTGTTC
AAGCATATCAATCACTTTCAGCATGTTTTCACGCTGGAACCAGACTTTATTTGCCCGAGCGCCGCCCGGAACGTAATCAC
GAGTGATGGTGCCGGTCAACAATCCCTGCTCTAGCGGGGAATAAACCTGAACCACAATGCCATTATCACGACATAGTGGC
AGCAGTTCGTTTTCCATTGCCCGGTCGAGGATACTGTATTTCGCCTGAATAATATCCAGTTCACCATATTGCAGATACTC
GCGGATATGGTCAGCATCGACGTTAGCAGCGCCTATAGCGCGAATTTTCCCTTCAGACTTTAACTCATTAAGCACTGCGA
CAGTTTCAGCGATCGGCGTAAAAAATGGCGGCACCGACTGCCAGTGCGTCATGTAGATATCGATGTAATCAATACCCAGA
CGTTGCAAGCTCGCTGCTACCTCTTCGCGGATAGATTCCGGGGAAAGGTTTTTATACAACTGCCGATCGCCAACTTTGTT
GAATAAACTTCCTTTTCGTTCCCAGACAATGCCGCATTTGGTTTCTACTACAACCTGTTCACGGGGCAGTTTTTTTAACG
CCTGACCGACGATAACTTCACTATTGCCAAAGTTATATCCTGGCGCAGTATCAATCAGATTAATGCCACAACGATGGGCT
TCAAGAATCGTATCAATACATATTTGCCGATCGAGATCGCCATTCCATGCAGGACCGCCGCCAATGGCCCATGTCCCCAA
CCCCATTCGCGAAAGCGTAATATCCGTTGTGCCTAAAGGTATCTTTTTCATTTGCCTTCATTATCCTTCGTATTCTTCCA
GCAATTGTTCGACCAGCTTTCTGTTTTTTACGCCGGTGGTGGCACCGACGCTTAGAACCGAGATAGCCGCCGTTGCATTG
GCAAAGCGTGCGCATTCACGCAGATTTTTGCCTTCTAACAGTGCCGCAATAAAACCTGAAGCAAAGTTATCGCCCGCGCC
AATGGTGTCGATGGCGGTTATTCCTGCGACCGCCGGCACCTTCATCGTCATGTCACCACGCTTGATAAAGCAGCCGTCTT
TACCCGTTTTAATCACCACCGTTTTTACGCCGCACGCAAGAAAGCAGTCAGCAATTTCATCCAGTGTCTCTTTCCCGGTG
AGTAATTTTGCCTCGGCAAAATTAGGAAACAGATAATCGACATAACTCAATGCTTCGCAAATATCATCCAGCGTTTCATT
CAACCGCGGTTTGATCATATCGGCACAGATAATCATCTGCCGGGCTTTAGCTTGCGTAAAAATTTCTGTTAGCGCTTTAC
CATCCAATAGTGGACTGTTGAAAATACTGGCCAGTGATAATAATTTCGCCTGAGAAAACCGCGCAAAATCAACATCGTCA
ATATTCAATTTCCACAGACTGCCATTACGGTTTGTGACAAACGTCCGCTCGCCATCCTCCGTCACCAGTCCAACGTTAAT
AGAGGTATCTATGCTGACGTCCTGCTTCAGGCTTTGAATATCAATATTCTCTTTGCGGCAATGGTCGAGAATAAATTGCC
CTGCGGCATCTTTACCAATACGACTCATTAATGCTGTGCGATGGCCCAGACGAGAAATAATTGTTGCTTCATTTATTGCA
TCACCCCCGGTGGTCATTGCGATTCTTTCAAGAGGATAAGAATCCACATCAAAGATATTTTTACTGACCGGTTGCAATGG
AATATCAACAATAGCGGCACCTATACAAATAACGTCGAGATTATCCATATCATTATTCCGCTTTGCCATCAGAACCGAAC
AGTTTAATTTTTTCCAATGCCCGTTCTTTTACAGCTTTACGTACTTCACGTTCTAAATGCAGGAAAGGCTGATCCTGGTT
TTCTTTCACTGCAACCATTGCGGCCTGACAGAGCTCCGTATGAATGTTGATTTTCGCGATACCCAACGAAATTGCAGTTT
TAATGTCGGCATCACTGATCCCCGATGCACCGTGCAAAACCAGCGGAACAGAAACGGCATCGCGTACGCGTTTGACGACC
TCAAAGTTCAATTGTGGCTCTGACGTATAAACCCCATGCTGGTTGCCGATGGCGACAGCCAATGAATCACAGCCCGTACG
TTCAACAAATTCAGCCGCCTGGTCAGGATCGGTATAGTGATAGCCCGCCAGCGCTTCCTCATAAACGGTTTCATTACCGA
CATGCCCTAATTCCGCCTCTACCGGAATACCCAGCGGATGGAAGAAATCGACAGCCTCTTTGGTTAAACGAATATTTTCT
TCGAAATCAAACGCGGAAGCATCACGCATTAATGAATTCATACCATGAGTCCAGGCGTTATGAATAATCTCCATACTCCG
ACCATGATCCCAATGAGTTATTACCGGCACCGTTGCTTTTTGTGCCATTGATACCATCATGTGAGAGAAATCTTCAAATG
AGGTGTTACCGACAAAACCTGTACCAAAAGAAATAATAACCGGGGATTTCGCTTCTTCGGCTGCGTCGATAACGCCCATC
AACATTTCTGCATTCCATACGTTAAAATGGGCAATTGCATAATGTTTATTTGTGGCATCGTTTTCCCAATATCTGATATC
TGCGAGCATGTTCTGTTCTCCCGTATTAATTAATCGCTAATTTTAATAACGCCTTTAATAATGTCGCGTTTGTTGTTAAC
TGACTCTTCAAATGCCTGTTGTACATCCCGATAATCGTAAATATGCGTCACCATCGATTTCACATCGAATCGCCCTGAAG
AAATAGCTTCAATCGTGACCGGATAACGATTGGCATAGCGGAATACCGTCTGGATAGTGACTTCGCGATTGATTTTGAGG
AAATTGATTGCCGAATCGCCGGGTACAGTACCAACAATCATAATTTTACCGCCGCGCATTACCAGATAAGGTGCCTGTTT
AACGGTGACCGCAGAACCCGCTGTTTCGAAAACAATATCTGCGCCCATGTCTTCGGTAAATTGCTGACAGCGTGCAATAG
TGTCTTCTTTTGCGCCGTTAATAACCACTGTCGCACCAAGCTGTTCCGCCATTGCCAGACGTTTTTCCAGCACATCAACG
ACGGCAATTTCCGTTGCTCCCAGGCATTTGCACGCTTGCAACGTCATCAAACCAATACAACCTGCTCCCAGAATAATTAT
CTTCTTACCCGGTTTAACATCTGCCAGCATCGCGGCATGCATCCCGACTGCGGCAGGCTCCACCAGCGCCCCTTCCATCG
TGTCCATATTGTCGGGCAGTTTGTAAGTAAAGCTCTCCGGATGACACAGATAGTGCGTTAATGCGCCGCGGTAGTTGGGT
TGTGTCGCCATAAAATCAACGTCCGGGCAGATGTTATATTTGCCTTCCAGACAGTAACGACAGTGACCGCAAGGAACGCC
AGGTTCGATATTTACCCGATCCCCCGGTTTAAATTTGCGCACGCGGCTTCCCACAGCCACAACCGTCCCGGCGCATTCAT
GACCCAGGCCAATTTCTTGATTTGGGTCTTTAGGCGGAATAAACGGGCCTGATTCAAAACCATGTACATCTGAACCACAA
ATACCGACATATTCTACTTTAATCAAAACTTCATCTTCTTTAGGCACTGGTATTTCTGCTGAAATAATTTTCATTGTGCC
CGGCACCTGCAATATTGCTTTTGAATTTTTCATTTTGAATCCACCCGTATTATTTATTGGCTACTGCATCAATACTTAAA
CTTTCAACGGAGACACCTTTGGTTTCGATTCCAATGGTGGCGATAGCAATTGCGACAATAATTGAAACCGCCCCCAGAAG
AATAAAGACTCCCGTAACGCCATAACTACTGAGCAGCACTGCAACGGCATAAGGTGCGGCAATACCACTGATTCGCCCTA
CCGCATTCGCCAGACCGGAGCCACGGAGTTTGGCCTCTGTCGGCCAGATTTCAGGGACATACACTGCCGAGGCATAGCAA

ACGTACATATAGACGAAAGTAATCAGGAAGAAACCAATCAACGTTATGAGCAACATACTGGTTTGCAGTGAATAGATATA
TCCGAGCACCGCAATCAGGATTAATAGCCCCACACCCATTGTTTTACGCGGAATTTTATCCATCACCAGCATGGCAATAA
AAATACCAAATGGCGCGCCAAACATACTCATGGTATTTAAAACAATCGAGTCTTTTAAATTAATCCCCTGGGTCATGAAT
ATTGTTGGCAACCAGTTAATTAGTGTGTACTGCACAACGTTCATGGCAATCAGCACACAAGAGCCTAATATCACGCGTTT
CAGTAATACTCCTGTCAGTAAGGCTGAATACGGCACCGCTTGTGGCGCTTTACCGTCATCAGCAATAACCACAGGCGGCA
AAGGTTTACCCGTCTGGCGTATGACGCCTTCTTCTATACTCCGCATGACTTTCTCGGCTTCCTGATACCGTCCGCGCGAT
TCCAGCCAGCGCGGGGATTCAGGAAAGTAGCGCCAGGCCAGCGCCGTAGCGATAAGCGACAATATTGCAGGGATAAGCAG
TTGTACCCGCCAGTTCCACTCTGCACTAATCAGCGGCGTGAGTCCCATCGCTATCAATGAACAGAGCGGATATGACCAGT
TGCCAATAAAGGAAACCCGACTTGACCACGTTCCACGATTTCTACCGGGCATATATTCGGTGAAACCAGCAAACAGCGTA
ACCAGTAAAGCTCCCAGCCCAACTCCCATCACAAAACGGCAGGCAATAAGGAAATCCATATTCGGTGAGAAAGCACCGAC
AACCATCGAGGCAATATGAATGGCCTCATAGAGGATGAACGCATTTCTGCGACCGGTTTTGTCGCCAATGATGCCGCCCA
CCAGCGCACCGAGAAACATTCCGGCGGTCGTGATTGCTGAGAATGTGGCTGTGGTGGAATTATCTGTCCAGCCCAACGCT
TTCAGCTGCGCGAGGATCAAGCCACCAACGGCATTACTCCAGCAGACAAGCAAGCCAAACGCGACGATGGCAAACATTGA
TGAATGCCAGCGGCAATCCGGTAAGCGATCCAGCCGTGCACCACAATGCGGTTTTGTTATCTGTTCCATGCGATAACGTT
CCTTCAGGAGAGTGGTTATTCATCAAAGTCGTAAGTCATGATCACTTTGATTGCGGTTTTATCGACCATCGCATCAAACC
CTTCGCGCCATTGCGACAGGCCGATACGATGCGTGATCATCGGTTTGACTTTGATAGCGCCGCTGGCTAATAGCCTGATA
GCGTTACGCCATGAGGTGGAGTCATAGGCCATATGCCCAATGATGCTTTTGTTCCAGGCGGTAATGTCATTAATCGAGAA
ATCAAGAGGTTTGAAGCCCATTCCAACGCGTACCACTTCCCCGTTCGGGCGGAGCATTTCGATGGCTTGTTTCAGTGCGA
TATTGGCACCGGAGCATTCAATCACCAGTCCCAGATTGTCTTTGCCACAAATTTGCTGGCAGCGCGCCACCACATCTTCG
GTAGAACCATTTACTACTGCCGTCGCACCCAGTTCTTTTGCAACCGGGAAGCGGACCGCCACATCTTCTTGCAGACCAAC
GACGACGATATTTACCGCCCCCATAATTCGCGCCATTTGTACGGAGAACAGCCCGAGTGGGCCAGTGCCGATGACGACCA
CATCCTGACCAGGAAGGAATTTCGATTGCTGCGCGATGGATTTGTAGGCATTACAGATAGGGTCAAGTACGGCTGCGTCC
TCATAATCAACACCATCAGGGATTTCCCACAACGCATGACGATGAATTTTGAGAATTTCACCAGGAACCAGACAATATTT
GGAAAAACCACCGCCCCAGGTATTATTATCCAGACCAAGGTTTACTTTTTCTGTACAACACAGAAAATCACCTTGTTCAC
AGGCCGGACAAACACCGCAAACGTGACCGCTGTTATCCGATACGACGCGTTGCCCCACTTTCCAGTCTTTGACTTTTTCA
CCAACCTGCGCAATACAACCTGCGAACTCATGGCCGCGGATAGAGTTAAACTCATCAGAACCGCTATCGACATTGTAGTG
CTTCATGTCTGCGCCGCAGATTGCCGCGGCTTTAATTTCAATCACTACATCTTCCGGGCCACACATGGGTTGTGGGACAT
CAATCATCTTGTAGCCGCCAAAGGCCTTGCCAAACCGAGCCAGTGCTTTCATTGCGTCATCCTCTGTGTTTAGTTTTGCT
TAGATGGCCATCATACTTACCCACATGATTTTTGAGGTCAACATTAAATCTGTAAAACACAGATAATGATCTGCGTTTTA
CAACTCAGATCACAATTACGCAGAAAATGGCTGGGTCTGTTACAGGTTGATGGAAGGCGGGGCGCAAAAAGAGCAAATTC
GAGAGGGATTACCGGCCAAATAGGTCTGAGACTTAAGTTCAGGAGAAGTCATTCAGGTGTGAACGGGACGGCAAGAGATC
GGACTGGGACTTAGCCAGGTCATGGCGAGATAATTGAGATGAGTCGTGGGGTCATTGTTGGCTATCCCCTTCATAGCCGA
GGCAAAAAGGGGATGAACCACTTACTTAAACATGGCAATAGGCTTTGCTGAGATACCAAAATCTTCTTTTAACTGCTGTT
TGCTCTTCATGACCATCTGACCATTTGTGTCAATTGTCATATGCTGTGCTTCGATATTATGGCGGGCTTGCCACAGCATC
ACCAGTTGCAGGCAGTTCTCTTTTTGTTCCTCGGTCAACGCAACGCCATCAGGCCATTTCCCCAGTTCAACGGCGGTCGA
CAAACGCTGGTATACCTCAGGCATCATGCTGTTGATAATGTCATCAAGATTCATAATTTTTCCGCTCCTGTGGAATAAGT
TGCTGAATCGTTTTTTCAACCGTTGATTTCTTCGCCGTTTTCGCCATCGGTAAAGCGTAAAGAGGCAGAGTTAACACAAT
AACGTTCGCCCGTTGGCTGCGGCCCGTCGGGGAAGACATGCCCCAGATGGGCATCACAGTTACCGCAACGAATTTCTATG
CGCTGCATTCCATGTGACAAGTCTTTGATATAACGAATGGATTCTTCACTTACCGGTTCGTAGAAACTGGGCCAGCCACA
GCCGGAATCATACTTGGTTTGGGAATGAAACAGCGGGGCATCGCAGATCAAACAGTGATATACGCCGTCACGCTTGTTAT
GCAGTAAACGACCCGTAAATGGCGGCTCTGTCCCATGATTCTGCGTCACGTAAAACTGCATCTCGGACAAATTTTTTTTC
AGTTCTTCTGCCGAAGGTTTATTAGCCATTTGCTCACATCTCACTTTAATCGTGCTCACATTACGTGACTGATTCTAACA
AAACATTAACACCAACTGGCAAAATTTTGTCCTAAACTTGATCTCGACGAAATGGCTGCACCTAAATCGTGATGAAAATC
ACATTTTTATCGTAATTGCCCTTTAAAATTCGGGGCGCCGACCCCATGTGGTCTCAAGCCCAAAGGAAGAGTGAGGCGAG
TCAGTCGCGTAATGCTTAGGCACAGGATTGATTTGTCGCAATGATTGACACGATTCCGCTTGACGCTGCGTAAGGTTTTT
GTAATTTTACAGGCAACCTTTTATTCACTAACAAATAGCTGGTGGAATATATGACTATCAAAGTAGGTATCAACGGTTTT
GGCCGTATCGGTCGCATTGTTTTCCGTGCTGCTCAGAAACGTTCTGACATCGAGATCGTTGCAATCAACGACCTGTTAGA
CGCTGATTACATGGCATACATGCTGAAATATGACTCCACTCACGGCCGTTTCGACGGTACCGTTGAAGTGAAAGACGGTC
ATCTGATCGTTAACGGTAAAAAAATCCGTGTTACCGCTGAACGTGATCCGGCTAACCTGAAATGGGACGAAGTTGGTGTT
GACGTTGTCGCTGAAGCAACTGGTCTGTTCCTGACTGACGAAACTGCTCGTAAACACATCACCGCTGGTGCGAAGAAAGT
GGTTATGACTGGTCCGTCTAAAGACAACACTCCGATGTTCGTTAAAGGCGCTAACTTCGACAAATATGCTGGCCAGGACA
TCGTTTCCAACGCTTCCTGCACCACCAACTGCCTGGCTCCGCTGGCTAAAGTTATCAACGATAACTTCGGCATCATCGAA
GGTCTGATGACCACCGTTCACGCTACTACCGCTACTCAGAAAACCGTTGATGGCCCGTCTCACAAAGACTGGCGCGGCGG
CCGCGGCGCTTCCCAGAACATCATCCCGTCCTCTACCGGTGCTGCTAAAGCTGTAGGTAAAGTACTGCCAGAACTGAATG
GCAAACTGACTGGTATGGCGTTCCGCGTTCCGACCCCGAACGTATCTGTAGTTGACCTGACCGTTCGTCTGGAAAAAGCT
GCAACTTACGAGCAGATCAAAGCTGCCGTTAAAGCTGCTGCTGAAGGCGAAATGAAAGGCGTTCTGGGCTACACCGAAGA

TGACGTAGTATCTACCGATTTCAACGGCGAAGTTTGCACTTCCGTGTTCGATGCTAAAGCTGGTATCGCTCTGAACGACA
ACTTCGTGAAACTGGTATCCTGGTACGACAACGAAACCGGTTACTCCAACAAAGTTCTGGACCTGATCGCTCACATCTCC
AAATAAGTTGAGATGACACTGTGATCTAAAAAGAGCGACTTCGGTCGCTCTTTTTTTTACCTGATAAAATGAAGTTAAAG
GACTGCGTCATGATTAAGAAAATTTTTGCCCTTCCGGTCATCGAACAAATCTCCCCTGTCCTCTCCCGTCGTAAACTGGA
TGAACTGGACCTCATTGTGGTCGATCATCCCCAGGTAAAAGCCTCTTTTGCATTACAGGGCGCACACCTTCTCTCGTGGA
AACCTGCGGGTGAAGAAGAAGTTCTGTGGTTGAGCAACAACACACCGTTCAAAAATGGCGTCGCTATTCGCGGTGGCGTA
CCGGTTTGCTGGCCGTGGTTTGGTCCGGCGGCACAACAAGGTCTGCCTGCGCACGGTTTTGCCCGCAACCTGCCGTGGAC
GCTGAAATCACATCATGAAGATGCTGATGGCGTAGCGCTGACTTTTGAATTGACGCAAAGCGAAGAGACGAAAAAATTCT
GGCCGCACGACTTTACGCTGTTAGCGCATTTCCGCGTGGGTAAAACTTGTGAAATCGATCTTGAATCACATGGCGAATTT
GAAACCACCTCTGCCCTGCATACCTACTTTAACGTGGGTGATATCGCTAAGGTAAGCGTCAGTGGGCTGGGCGATCGCTT
CATTGATAAAGTGAATGACGCGAAAGAAAATGTACTGACCGATGGTATTCAGACCTTCCCTGACCGTACCGATCGCGTGT
ATCTGAATCCACAAGATTGCAGCGTGATTAATGATGAAGCGCTGAATCGTATTATCGCCGTAGGCCACCAGCATCATCTG
AACGTTGTCGGCTGGAACCCGGGACCGGCGCTTTCAATTAGCATGGGCGATATGCCGGATGATGGCTACAAAACATTTGT
TTGTGTAGAAACGGCTTACGCTTCAGAAACGCAAAAAGTGACCAAAGAGAAACCTGCACATCTGGCGCAATCCATTCGCG
TTGCGAAACGTTAATTTACGTTAATGTTGTGTGCCGGGTGCAATGCATCCGGCACACAACATCACACCATATCCAGCGCA
GTTTTTCCTTTTGGTGCCGGATATGCCTTATCCAGCATAGCTAATTCCGCTGAAGAAAGTTCGACCTCAAGCACAGCCGC
ATTTTGTTGGACATGGGCAATCGTGGCCGCTTTTGGAATCGCCATCACACCCTGATGACTGATCACCCACGCCAACAATA
CTTGTGCCGCGCTGATATTGTGAGCATGTGCAATTTCGTTGACTACCGCGTTTTTTAACAGTCCATTGCGCAACCGCCCG
GCCTGGGCTAACGGACTGTAAGCCATCACCGGCATCTGCTGTTGCTGGCACCAGGGGAGTAGATCGTACTCAATTCCTCG
TGAACCGAGATGGTAAAGCACCTGATTAGTGGCACACTGATTTCCCCCCGGCAGCTGCCAGAGTTCCTGCATATCAGCAT
AATCAAGGTTAGAAACGCCCCAGCGGCGGATTTTTCCCTGGGCGATCAATTTTTCCATCGCTGCGACAGTCTCTTCAAAA
GCGAAACTGCCAGACCAGTGTAATAAGTAAAGATCGAGATAATCAGTATTGAGACGGCGTAAACTGGCTTCGCATGCATT
TATCGCTTTTTGCCCGCCAGCATTCCACGGATAGACTTTAGAGACGAGAAAGACCTTCTCTCGCAGACCGGTTAATGCTT
CCCCAACCACCTTTTCGGCACCGCCATCGGCATACATTTCGGCGGTATCAATGAGGGTTAAACCGAGTTCAATGCCCGCG
CGTAGTGCAGCAACTTCTGTTTTGCGCTGACTGGCATCTTCGCCCATATACCATGTTCCCTGCCCTACGGCTGGCAGTGA
GACATCGCCACTAAATTGAATCATTTTTTGTTGCATTGTTTCCTCCCAGGTATTGCACCACCGTAATGCAAAACAGGGCG
TGACGCCCTGTTTTTATGCACAAAATGCCCTGGAAAGATGCATTATCAGAATTTGTAGGTGATCCCGGTAGAAATCAGGC
CAGTCCAGGATTTATCCACCATCGGGCTGTCAGTAACTTCATCAGACAGACGGGTGTAGCGTGCGGTACCGTAAACACTC
CAGTCGCCGAGGAAGTTGTAGCTGGCGCTCAGCTCCAGGTAAGGGCTCCAGCTGTCGTTCGGGTTATAGCCACGCAGACC
GCTGCGAGCGGACTCTTTGCGCGATACGCCATAATAGTATTCGTTCTGGTTTTCGCTGTTCCACTGCACACCAATACCCG
GAGTCACGGTCAGGCCACCGTTGGTGTAACGATACAACCAGGCCATATCCCAGACGATGCCGTTGCTGTTATCCAGGGTA
TCGCCAGCCAGGGTGGTACGCAGGTAACCGTACTGGGTAAAGTGAGCATAAGACAGACCAGCCATCATGGTGCTCTTACG
GTCATCCAGGTGACGCATTTGGTGATCGCCACTGTCTTTCGCTTTGAAGTAAAGCGGCGACCAGTAAGCGGTAATTGAAA
GTTTATCCGTTGCGTCATTCCACAGGTAGTAACCACCACCTAAGCCACGGAACCAGAAGTTATCGCCTTCATAGTTGATT
ACCGGTACTGGGTAAACATCGGTATCGTAATCTTTATATGGGTGTTCAACGACACCTACGCCTGCGCCCAGGGAAAATTT
ACCTTCAGCGTGCGCTACGCCTGCAGACGTTGCGATAAGCACTCCAAGTGCCAGAAGTTTGAGTTTGGTCACAATTAATC
ATTCCTTAAACAAATGTTTAGCGGGCGACAAAGTTTACCCGTCAATACATCGAAACCCAACCTTTTTACGTTTTCATTTT
TTAAAGTAACTGTTTAATTTTCCTGACGCGGATGACACCGCGCTTACAGCCAAATGAATTTAGCGTTACTGGCGAGCCTG
GTCTTTACATTAATTATGCAAAATTTATGGATGAGTTGTTGATATGCCATTGAAATTAAGAAAGCCGTGCAGGCAAGTTT
TCCATTTGCCATCTACGCTTAATTTTGAAGGTGTATCACCGGGCACGTTGTTCTCATCGTCGATAAAATGGCATGAGAGT
TGCTGTGTTTTAGCAAGAGACGTCGTTCAGTTTACCTCTTCCGGGAGCCTCTACTATTCATATGAACGGCTCTTAACCTG
TGCTAAAAAACGAAAGGACGGCATACCATGAATATATTCGATCACTATCGCCAGCGATATGAAGCTGCCAAGGACGAAGA
GTTCACACTGCAGGAGTTTCTTACCACTTGTCGGCAAGATCGCAGTGCTTATGCCAACGCGGCTGAGCGGCTATTGATGG
CTATCGGTGAGCCTGTCATGGTCGATACAGCCCAGGAACCCAGACTTTCTCGACTCTTTTCTAACCGGGTCATTGCACGT
TATCCGGCGTTTGAAGAGTTTTACGGCATGGAAGACGCGATTGAACAGATTGTCTCTTATCTGAAACACGCGGCTCAGGG
GCTGGAAGAGAAGAAACAAATCCTGTATCTGCTGGGGCCTGTGGGTGGGGGTAAATCATCGCTTGCTGAGCGACTGAAAT
CATTAATGCAGCTCGTACCGATTTATGTATTGAGCGCGAACGGTGAGCGTAGCCCGGTCAACGATCATCCGTTCTGTCTT
TTCAATCCGCAGGAAGATGCGCAGATTCTGGAAAAAGAGTATGGCATTCCTCGCCGTTATCTCGGCACCATCATGTCGCC
GTGGGCGGCAAAACGCCTGCATGAATTTGGTGGCGATATCACTAAGTTCCGGGTAGTGAAGGTCTGGCCGTCAATTCTGC
AACAAATTGCTATCGCCAAAACGGAACCCGGCGATGAGAACAACCAGGACATCTCCGCGCTGGTTGGGAAAGTCGATATT
CGTAAACTCGAACACTACGCGCAGAATGACCCGGACGCCTACGGCTATTCCGGTGCGCTGTGCCGCGCCAATCAGGGGAT
CATGGAATTCGTTGAGATGTTTAAAGCACCGATTAAAGTGCTGCATCCCTTGTTAACCGCCACTCAGGAAGGTAACTACA
ACGGGACGGAAGGTATCTCCGCCCTGCCGTTCAACGGGATTATTCTCGCACACTCGAACGAGTCCGAATGGGTCACTTTC
CGTAATAACAAAAACAACGAAGCCTTCCTCGATCGTGTTTACATCGTGAAGGTGCCGTATTGCTTGCGCATTTCCGAAGA
GATCAAAATCTACGAGAAATTGCTTAATCACAGTGAATTGACTCACGCCCCATGCGCCCCTGGCACGCTCGAAACACTGT
CACGTTTTTCCATTCTTTCGCGCCTGAAAGAGCCAGAAAACTCCAGCATTTATTCAAAGATGCGGGTTTATGATGGCGAA

AGTCTGAAAGACACCGATCCCAAAGCCAAGTCGTATCAGGAATATCGTGACTACGCCGGTGTCGATGAAGGGATGAACGG
TCTGTCGACGCGTTTTGCGTTTAAGATCCTCTCCCGCGTGTTCAACTTCGATCATGTAGAAGTGGCAGCAAACCCGGTCC
ATCTGTTCTACGTCCTGGAACAGCAGATTGAGCGCGAGCAGTTCCCACAAGAGCAGGCAGAACGCTATCTGGAGTTCCTG
AAAGGTTATCTGATCCCGAAATATGCCGAGTTTATCGGCAAAGAGATCCAGACGGCCTACCTTGAATCCTATTCCGAATA
TGGGCAAAACATTTTCGACCGTTATGTTACCTACGCGGATTTCTGGATTCAGGATCAGGAGTATCGCGATCCGGATACCG
GGCAGCTGTTTGACCGCGAGTCTCTTAACGCCGAGCTGGAGAAAATCGAGAAACCGGCGGGGATCAGTAATCCAAAAGAT
TTCCGCAACGAGATTGTTAACTTCGTACTGCGCGCCAGAGCGAATAACAGCGGACGCAATCCGAACTGGACCAGCTATGA
AAAACTGCGCACGGTCATCGAGAAGAAAATGTTCTCCAATACCGAGGAGCTGTTGCCGGTTATCTCGTTTAACGCCAAAA
CGTCAACCGACGAGCAGAAGAAACACGACGACTTTGTCGACCGTATGATGGAAAAAGGCTACACCCGTAAACAGGTGCGT
TTACTGTGCGAATGGTATTTGCGCGTACGTAAATCGTCTTAACAACCCTGGCCCGGTACACATGTTACCGGGCCTACAAC
GACAGCGAACCGTGGGCCTGAGAAGCGGCAACACAGGCGTAGCATACAGTTGGCAAATGTAGTACGGGGGGCATATGACC
TGGTTTATTGACCGGCGTCTGAACGGCAAAAACAAAAGCATGGTGAATCGCCAGCGTTTTTTACGCCGTTATAAAGCGCA
AATTAAACAGTCGATCTCCGAGGCCATTAATAAGCGTTCGGTGACTGACGTCGACAGCGGCGAATCCGTATCCATTCCCA
CGGAAGATATTAGCGAACCGATGTTTCATCAGGGGCGTGGCGGTCTGCGCCACCGCGTGCATCCGGGCAATGACCATTTC
GTCCAGAACGACCGAATTGAACGTCCCCAGGGTGGCGGCGGAGGTTCCGGCAGTGGTCAGGGCCAGGCCAGCCAGGATGG
TGAAGGTCAGGATGAATTTGTCTTTCAGATTTCGAAAGATGAGTATCTTGATCTGCTCTTTGAAGATTTGGCCTTACCGA
ATCTGAAACAAAACCAACAACGCCAGCTGACCGAATATAAAACGCATCGGGCGGGTTATACCGCTAACGGCGTTCCGGCC
AATATCAGCGTTGTGCGTTCATTGCAGAACTCACTGGCGCGACGCACAGCCATGACGGCAGGCAAGCGGCGGGAACTTCA
TGCACTGGAAGAGAATTTGGCCATCATCAGCAACAGTGAACCTGCGCAACTGCTGGAAGAGGAACGTCTGCGCAAAGAAA
TTGCAGAATTACGTGCCAAAATTGAACGCGTCCCTTTTATTGACACCTTCGATTTACGTTACAAGAACTACGAGAAGCGG
CCCGATCCCTCCAGCCAGGCAGTGATGTTTTGCCTGATGGACGTTTCCGGTTCAATGGATCAATCCACTAAAGATATGGC
TAAGCGTTTTTATATTCTGCTGTATCTGTTCCTCAGCAGAACGTATAAGAACGTGGAAGTCGTATACATCCGCCATCATA
CCCAGGCGAAAGAAGTCGATGAACATGAGTTTTTCTACTCGCAGGAAACAGGCGGCACCATTGTTTCCAGCGCCCTGAAA
CTGATGGATGAGGTAGTGAAAGAGCGTTATAACCCGGCACAGTGGAATATTTACGCTGCACAAGCATCGGACGGCGATAA
CTGGGCCGATGACTCTCCGCTTTGCCATGAAATCCTGGCGAAAAAATTATTACCTGTTGTTCGTTATTACAGCTATATCG
AAATTACCCGTCGTGCACATCAGACATTGTGGCGAGAATATGAGCATCTGCAATCTACTTTCGACAACTTTGCGATGCAG
CACATCCGCGACCAGGATGATATTTATCCGGTGTTCCGTGAACTGTTTCATAAACAAAATGCAACAGCTAAAGGCTAAAA
CTATCAGCCAGGTCATTATCGCCTGGCTGATTTTTAGCTTACTGTAAATTATCTCATTTATTACATACATTAGCTTACAA
TCGCTTTAAATATGACAGCATAACCTTTACATAATTTAGTTCCAGAAAACAATCATTCGGAAAAATGATTCAGTCAACAC
GTATTTCCATGGGGTTATTCTTTAAATATTTTTTATCGTTAACGAAAATTGATCCTGGTCAAAACTATATATCTCTGCCA
TCAATAAAATCCAGCACTCACATTGCTCTCCTTTTTATGGTTTCTATGGGTACACAAAAATTAAAAGCTCAAAGCTTTTT
TATTTTCAGTTTATTGCTGACGTTAATTTTATTTTGCATTACTACCTTATATAACGAAAACACAAATGTAAAACTCATCC
CACAGATGAATTACCTGATGGTTGTTGTGGCTTTGTTTTTCCTTAACGCCGTCATTTTTCTTTTCATGTTAATGAAATAT
TTCACTAACAAACAAATTTTACCAACACTCATTTTAAGCCTTGCATTTTTAAGTGGCCTTATCTATTTAGTTGAAACCAT
TGTAATTATCCATAAACCAATTAACGGCAGTACACTGATCCAGACAAAGTCGAATGATGTTTCTATTTTCTATATTTTCC
GCCAACTCAGTTTTATTTGTTTAACCTCGCTGGCGCTCTTTTGTTATGGAAAAGACAACATCCTTGACAACAATAAGAAA
AAAACGGGAATCCTGTTGCTGGCGCTGATCCCTTTTTTAGTTTTTCCCCTTCTGGCACACAATCTGAGCAGTTATAACGC
TGACTATTCTTTGTATGTCGTCGATTACTGTCCGGACAACCATACTGCGACCTGGGGAATCAACTATACAAAAATATTGG
TTTGTCTGTGGGCATTTTTACTGTTCTTTATTATCATGCGCACACGATTAGCCAGCGAACTATGGCCGTTAATAGCATTA
TTATGTCTGGCATCGCTATGCTGCAACTTACTTCTACTGACTCTGGATGAGTATAATTATACTATCTGGTATATCAGTCG
CGGGATTGAAGTTTCCAGTAAACTGTTTGTTGTGTCTTTTCTGATTTATAACATTTTTCAGGAGCTGCAACTCTCCAGCA
AACTGGCAGTTCATGATGTGCTGACCAATATTTATAATCGGCGCTACTTTTTCAACAGCGTAGAGTCATTATTGTCGCGA
CCTGTTGTTAAGGACTTCTGTGTCATGCTGGTTGATATTAATCAGTTCAAACGCATCAATGCCCAATGGGGACATCGTGT
GGGTGATAAAGTGCTGGTTTCAATTGTCGATATTATCCAGCAAAGCATCCGCCCCGATGATATTTTAGCGCGACTGGAGG
GTGAGGTGTTTGGCTTGCTATTTACCGAACTCAATAGTGCCCAGGCAAAAATCATTGCGGAACGTATGCGTAAAAATGTC
GAACTCCTGACCGGCTTTAGTAACAGATATGATGTTCCTGAACAAATGACCATCAGTATTGGCACGGTTTTTTCAACGGG
TGACACGCGTAATATCTCGCTTGTCATGACGGAAGCAGATAAAGCCTTACGCGAAGCGAAAAGCGAGGGGGGCAACAAAG
TGATTATTCATCATATTTAAGTGCAAAAATATTCAGAGCCATGCTTTTTGCGTGGCTTTTGCATACAATTTATTACTATT
ACCCCTAAATTTCCCCTCCCCAGCGGCGCAGAGATGAGTATAATTAGCGCCCCTGTGCCAGGCCGCAATCGAACTTTATC
TGGTTTTCTCGTTTCACTAACCGAAGGAGTGCCATTTATCATGAAATTGCACCATAGAATGCTCCGGCATTTTATCGCCG
CAAGTGTCATTGTGCTGACATCTTCCTTCCTTATTTTTGAACTTGTCGCCAGCGACAGAGCAATGAGTGCCTATCTGCGC
TATATCGTGCAGAAAGCAGACTCCTCCTTTCTTTATGATAAGTATCAGAATCAGAGTATTGCCGCGCATGTGATGCGCGC
TCTCGCTGCTGAGCAGTCGGAAGTGTCGCCAGAACAGCGGCGCGCCATCTGCGAGGCTTTTGAGTCTGCCAATAACACCC
ATGGCTTAAACCTGACTGCCCATAAATACCCGGGCTTACGCGGCACACTACAAACCGCATCCACTGACTGCGACACAATT
GTGGAAGCTGCAGCACTATTACCCGCTTTTGATCAGGCAGTGGAAGGCAACCGCCACCAGGATGATTACGGTTCAGGTCT
TGGGATGGCCGAAGAGAAATTTCACTATTATCTCGATCTCAATGACCGCTATGTCTATTTTTATGAGCCGGTTAATGTTG

AATACTTTGCGATGAATAACTGGTCCTTCCTGCAGTCAGGAAGTATTGGCATCGATCGCAAAGATATTGAAAAGGTATTT
ACCGGGCGTACCGTATTGTCGAGCATTTACCAGGATCAGCGTACTAAACAGAACGTGATGAGTTTGCTGACGCCGGTATA
TGTCGCAGGGCAGCTAAAAGGGATTGTGCTGCTGGATATTAACAAAAACAATCTGCGGAATATCTTTTATACTCATGACC
GCCCTCTCCTCTGGCGTTTTCTCAATGTCACGCTAACCGATACCGATTCGGGGCGCGACATTATCATCAACCAGAGCGAA
GATAATCTGTTCCAGTATGTCAGTTACGTCCATGACTTACCGGGCGGCATTCGTGTCTCGTTATCCATTGATATTCTTTA
CTTTATCACGTCTTCGTGGAAAAGCGTTCTGTTCTGGATTTTGACGGCGTTAATTTTGCTGAATATGGTGCGGATGCACT
TCCGTTTATACCAAAATGTGTCGCGAGAAAATATTAGTGATGCGATGACTGGACTGTATAATCGCAAAATTTTAACCCCT
GAACTGGAGCAGCGGTTGCAGAAACTGGTGCAATCCGGTTCTTCGGTGATGTTTATTGCTATTGACATGGACAAGTTAAA
GCAAATAAATGACACCCTCGGTCATCAGGAGGGGGATTTAGCGATTACGTTATTAGCTCAGGCGATTAAACAGTCGATTC
GTAAAAGTGATTATGCCATCCGACTCGGTGGCGATGAATTCTGCATCATTCTTGTCGATTCGACGCCGCAAATTGCAGCA
CAACTGCCTGAACGTATCGAAAAACGTCTGCAACATATCGCGCCGCAGAAAGAGATCGGCTTCTCTTCCGGTATTTACGC
GATGAAAGAAAACGATACGTTACATGATGCGTATAAAGCTTCCGATGAGCGTTTATATGTCAATAAGCAGAACAAAAACA
GCCGTTCATGATAACCTTCTGTGGTTGTTTGCTTGTAATCTCAGGAGCGTGAAATGACTGAAATGGCTAAAGGAAGCGTG
ACGCATCAGCGGTTAATCGCTTTATTATCACAAGAAGGTGCTGACTTCCGCGTCGTTACCCATGAAGCGGTAGGTAAATG
TGAAGCGGTGTCAGAAATTCGTGGCACCGCTCTAGGCCAGGGTGCTAAGGCACTGGTATGTAAAGTCAAAGGCAATGGCG
TAAATCAACATGTTCTGGCGATCCTCGCAGCCGATCAACAGGCCGATCTGAGCCAACTCGCCAGCCATATAGGCGGATTA
CGCGCTTCGTTGGCCAGCCCGGCGGAAGTCGATGAACTCACAGGCTGTGTCTTCGGCGCGATCCCCCCTTTCAGCTTCCA
TCCAAAACTCAAACTGGTTGCCGACCCTCTACTCTTCGAACGATTTGATGAAATCGCCTTCAATGCAGGCATGCTGGATA
AATCCGTTATTTTGAAAACCGCGGATTATTTGCGCATTGCGCAACCAGAACTGGTCAATTTCCGCCGCACTGCGTAACTA
GCCGGTCCGTTCGATAAGAAGAACGGACAAAACCAGTACAACAGCAATGGCAAAAAACGATGACGTGATAATCAGTGTCT
CGACAAACATTTGATCGTTCATAGCATGCCCCCGGGAGATAGCTGTTTTCACGTTATTGTTAGCGTGCACAAATGGCAGT
TTGATGACAGTTCGCCTGATTTTTATGCAAAAAACGTAAAGATTTTCTACTTCCTTCCTGCAGCAAGCGTAAAGTAAGC
AGGCTTATTATTTTTTGGCAAGGAAACCACGATGTTTGATGTCACTTTGCTGATCCTGCTCGGGTTAGCTGCGCTGGGCT
TTATCAGTCATAACACCACTGTCGCCGTTTCAATTCTGGTGTTAATCATTGTCCGCGTCACACCGTTAAGCACCTTTTTT
CCGTGGATTGAAAAACAGGGGCTAAGTATCGGGATAATCATTCTGACGATTGGCGTTATGGCCCCCATCGCCAGCGGGAC
GCTACCGCCCTCAACCTTGATTCACTCGTTTCTGAACTGGAAATCACTGGTGGCGATTGCAGTAGGCGTGATTGTCTCCT
GGCTGGGTGGGCGCGGCGTGACGTTAATGGGCAGCCAGCCGCAACTGGTCGCCGGGCTGCTGGTAGGCACTGTTTTAGGC
GTAGCGCTGTTTCGCGGCGTACCGGTTGGGCCGCTTATTGCCGCGGGTCTGGTTTCGCTGATTGTGGGGAAACAGTAGTT
AATCTCGCAATATATCGGCCTGGCGTTTGCCCCAGGCCTTTCTTAAACATAGTAATAAAGGCCGTTGTTGAATCATACCC
CAGCGTATGAGCAACTTTCTGTACAGTATCGCCTTTCACTAATCCCTGAAGCGCCATAATCAGCTGTAACTGCTGACGCC
ACTGGCGAAAACTCAACCCCGTCTCTTTTACTATCAGTCGGGCAAGATTGCGTTCACTCATCGCAAAAAAGCCAGCCCAT
TGCCCCAATGCCCCCCACTCGACAGGCCCTTTCGCCATCATCTCCACCATCGTGCGGATTTTAGGATGAGAAGAAACGGG
CAGGTGCAGCTGTTGTTGTGGCTGCTGAGGGAGTTCATCAAACAGGACCTGAATTAATCTGCGGGTCATTGGCTCAGCAC
GCTGAGTCGTCGTCCTGTTTGCCAGGGTTAAAATTAATTCCCGGCATAAGGGCGATATTTTCAATGTGCAACATGTTGTC
GGCATTGTCACTGCGCTGGGTTCGATAAACAGAAAACAGAGTTCAGCATTAGCTGTTACTTGATTGCTATGTTCCACCCC
GCCCGGTATCCAAACCGCATATTGTGGCGGCACCATCCACAAAGCATTTTCCACCGTACAGGTAATTGCACCATGTAGCG
CCAGTATCAGCTGTCCTTTGCGGTGCTGATGTACAGGACTGGTTAACTCATCCGTTCCGGCGTGAATACAAAATGCGACC
GCGGCTTCATGGTGTCGGTCGGGTTCATAGCCATTGAGATTCAACCTGTGCATCATTTTGTCCGAACTTAGCGATAATTT
GTCATTTTAGCTTGATTCAACATAACAATAAAAACGGTAAGGTACAGCCTCGTTTGTAACAATGAGAAGCATATGACCTG
TTCAACTTCATTAAGCGGCAAAAACAGGATTGTCCTTATCGCTGGCATTCTGATGATTGCCACAACATTACGCGTCACCT
TTACCGGCGCAGCACCGTTACTGGATACGATTCGTTCCGCTTACTCGCTGACGACAGCGCAAACCGGCTTATTGACCACC
CTGCCATTATTGGCCTTTGCGCTAATCTCACCTTTGGCTGCCCCGGTAGCGCGACGTTTTGGTATGGAACGTAGCCTGTT
TGCCGCGTTACTTTTGATCTGTGCTGGTATCGCAATTCGCTCTCTCCCTTCGCCTTACTTATTATTTGGCGGTACAGCGG
TCATTGGCGGTGGGATTGCATTAGGCAATGTCTTACTGCCAGGATTAATTAAACGCGATTTCCCTCATTCCGTCGCCAGA
CTTACCGGCGCATATTCCCTGACAATGGGAGCTGCAGCGGCACTGGGATCGGCTATGGTCGTGCCGCTGGCTTTGAACGG
TTTTGGCTGGCAAGGCGCGTTGCTCATGCTGATGTGTTTTCCTCTGCTGGCTCTTTTTTTATGGCTGCCACAGTGGCGAA
GTCAACAACATGCAAATTTGAGTACCTCGCGCGCCTTACATACTCGGGGTATCTGGCGTTCACCGCTTGCCTGGCAGGTC
ACATTGTTTCTTGGGATCAACTCACTGGTCTATTACGTGATTATTGGCTGGCTTCCGGCGATCCTCATCAGTCACGGCTA
TAGCGAAGCACAGGCGGGTTCACTGCATGGTTTGCTGCAACTAGCCACAGCAGCACCCGGTTTGCTGATCCCACTTTTCT
TACATCATGTGAAAGATCAGCGTGGTATTGCAGCGTTCGTTGCCTTGATGTGCGCAGTGGGCGCGGTTGGGCTCTGCTTT
ATGCCAGCGCACGCGATCACCTGGACTCTGCTTTTCGGTTTTGGTTCCGGCGCAACAATGATACTGGGGTTGACGTTCAT
TGGTCTGCGGGCTAGTTCTGCGCATCAGGCGGCGGCACTCTCGGGGATGGCACAATCCGTCGGGTATTTGTTGGCAGCCT
GTGGGCCGCCGCTGATGGGTAAAATACACGATGCTAACGGTAACTGGTCTGTACCACTTATGGGTGTTGCCATACTTTCA
CTACTGATGGCGATTTTCGGACTTTGCGCCGGGAGAGACAAAGAAATTCGCTAATATCCGGTGCTATAGTGACGTAACAA
ATCATGCGTGAAAGGGAGAACAAACACGATGAATATTCAGTGCAAACGCGTTTATGATCCGGCTGAACAGAGCGATGGTT
ATCGCATACTGGTCGACCGCCTCTGGCCGCGCGGTATCAAAAAAACCGATTTAGCCCTTGATGAGTGGGATAAAGAAATC

ACGCCGTCAACGGAACTGCGCAAAGCCTTTCACGGCGAAGTCGTCGATTATGCAACCTTTCGCGAGCAATATCTTGCAGA
ACTGGCGCAACACGAGCAAGAAGGAAAGCGGCTGGCGGACATCGCCAAAAAACAGCCGCTGACCCTGCTCTACTCAGCAA
AAAACACCACGCAGAACCATGCGCTGGTGCTGGCCGACTGGCTACGTAGCTTGTGATTTTAGTACAGCATCCGGCGGTTA
TTTTTCACCAGCCGGATGGTCACGCCGCCACAATGCCCATTCATCAATCGTTTCACCGCCCGGTAATTTGCAATTGTTGC
TGACCCCTTGCGCTGTCTGCACTGGAATGAGCGTCCCGCCCTTCTGCTGGCAATAGACCGACGCCGGATTTGCCATACCA
ATCTGCGGCGGTTTAGGTGCTTCTGGCTGAGAAGGGGTTGAACAACCAGCCAGGACCAGCAAGCAAGGCAGAACAAAACT
GATAATTTTCATTTATTGATCTCACATATTTATCCAAGATTAGAGTATCGCGGTATCGTTTTGTTTTGCAGCACTATTTT
TATTACATTCACTCAAAACATATTACGTCTTGTTTCATCTTTGTTGATGATGTTTTATCATGCCTGCAAAGATTAAATAA
TCAGCATTTACCCGCCGTATCCTGGAGTTGTTCCGTGTCAGATCAGATTATCGCCCGCGTCTCGCAATCCCTTGCCAAAG
AACAGTCACTGGAAAGTCTGGTCCGACAGCTTCTGGAGATGCTGGAAATGGTCACTGATATGGAATCAACCTACCTGACC
AAAGTGGATGTCGAAGCGCGCCTGCAGCATATTATGTTTGCCCGTAACAGCCAGAAAATGTACATCCCGGAGAATTTTAC
CGTCTCGTGGGATTACTCGTTATGCAAACGCGCCATTGATGAAAACTGCTTTTTCAGCGATGAAGTCCCCGACCGTTGGG
GTGACTGTATTGCGGCACGCAATCTTGGCATCACCACATTTCTGAGCACGCCAATTCACTTACCGGATGGATCATTCTAT
GGCACGCTTTGCGCCGCCAGCAGTGAGAAGCGCCAGTGGAGTGAACGCGCGGAACAGGTTTTACAGTTATTCGCCGGACT
GATTGCACAATATATTCAAAAAGAGGCACTGGTTGAACAGCTGCGCGAAGCCAATGCTGCGCTGATTGCGCAATCGTATA
CCGACTCGTTAACCGGGCTACCGAATCGGCGGGCGATTTTTGAAAATCTGACGACACTGTTTTCCCTCGCCCGGCATCTT
AACCATAAGATAATGATCGCGTTTATCGATCTGGATAACTTCAAATTAATCAATGATCGTTTTGGTCATAATAGTGGCGA
TCTGTTTCTCATTCAGGTTGGCGAGCGCCTTAATACGCTCCAGCAAAATGGCGAAGTTATTGGTCGTCTCGGCGGTGATG
AGTTTTTAGTTGTTTCACTAAACAACGAGAATGCGGATATTTCGTCGCTGCGAGAACGCATTCAGCAGCAAATACGTGGA
GAATATCACTTAGGTGATGTTGATTTGTATTATCCCGGTGCCAGTCTTGGCATAGTAGAAGTCGATCCTGAAACAACCGA
TGCAGACAGTGCCCTGCATGCTGCCGATATTGCGATGTATCAGGAGAAAAAACACAAACAGAAAACACCTTTTGTCGCGC
ATCCAGCGCTACATTCCTGAGGCGTATTCACATCCTTTTGATTGGTGATAACATGCGAATCGGTATTATTTTTCCGGTTG
TAATCTTCATTACAGCGGTCGTATTTTTAGCATGGTTTTTTATTGGCGGCTATGCTGCCCCGGGAGCATAAAGATGAAAA
AAACAACGATTATTATGATGGGTGTGGCGATTATTGTCGTACTCGGCACTGAGCTGGGATGGTGGTAACGTCACCTCTAA
AAAATAGCAAAGGCTGCCTGTGTGCAGCCTTTGTGCAATTTAAGCGTTAACTTTTAATCTTCCTGTAGATAAATAGCACG
ACAATCGCACCAATAACGGCAACCACGAAGCTGCCAAAATTGAAGCCATCGACTTTACCAAAGCCAAACAGCGTGCTGAT
CCATCCGCCGACTACGGCACCGACTATCCCCAGCAGGATAGTCATAAAGAATCCACCTCCATCTTTACCTGGCATGATCC
ACTTCGCCAGAATACCGGCAATAAGCCCAAAAATAATCCATGACAGAATGCCCATTGTTTCCTCACTTATCTGTTTTGCA
TTAGCGGGTTAGTCGCTGATAAAAAGCATAGCACAACATCGGGAGGGCAAGATTTGTGACGAGCATCACGGAGGTTTTTT
TTGCGATGGCGCAGAAATTGCGCCATCAACGATCAGTGATAATTACCAACCACAAACATCATGTTCGTTTTCCGTGTCAT
AAGAACGTACGGTATTCACCAGATCTTTTATCACTTCAGCCGCCACTTCTGGCACCAGCAAAGTCATCGGCGTCTCTGTT
TCATAATCGACAGAAACGCCATTGCTGTTATTGGTGACGGTCACGGTATACGTTGCTTTGCCCATGATTCATTTCCCGTT
ATGAATGACTTTCCGTTGTTGCGCACCTTCCATCAGGACTTCAGGAGCCACGAAGAAGTCAATGTTGAAATAAGTATCGT
CAGTCATGGCTTCAATGTTGTGCCACTTTTCTGGAGGGAACACCGCAAACTGCCCCGCTTCGATAAGGATCACCTGATCA
GGCTCTGCACTGTGTTCATCAGCGTAGCCGAGATATTTGACCGCCCCATGCATAACGGAAAGGCGTGGGTAAACCCCCGG
GCGCGTTCCTTTATCAAGATGACGTTCGAATATTCCGGCAGGTGCAGTTTGTTTATTCCAGAAAGGCGTTGAGCGCGTAT
GAATATAATTCTGTGGGATTTGAAGCATCCTTTTCCCTCCTTCGGTGAATGCGCTGAAAACGGTTTATTCCAGCCGTTTC
AGGGTACGCCTGATAATTTGCATTTTAAATACCATTTATTGGTTACTTTTTAGCACCATATCAGCGAAGAATCAGGGAGG
ATTATAGATGGGAAGCCCGCGACAACGCGGGCTGAAAGCATCAGGATTGCAGCGTCGCCAGTCGGGCAGCGAAACCCACG
AACATCAAACCAATCAGTGAGTTGCCAACTTTAGCCAGTTTCTTTTTGGTACGTATGTACTGCGTGACAAAAGCACCAGA
TATAATCAGGAAGCTCAAATAGCAGAAACTCACCAGTTCCAGCGTCGCCGCCAGAATAAAGAATGAAATTCCCGTATGTG
GGGCATTAACATCGATAAACTGTACGAAAAACGACACATAGAACAAAATGGCTTTCGGATTAGTCAGGCTCAAAATTAAC
GCGCGTTTAAAAATAGCACCGTATTGGGGCTCATCGGATTTGGCCTCGCTATTTTTACCCTTCAGGGTCGCGTAAAGAAT
TTTACTCCCCAGATAGAGCAAATAAAACGCACCAAGATAACGTACAATGTTGAATAATATCGGGGTGGTCTTAATTAATG
TCGCCACTCCAGCCCATGCCAGAAACATCAATACCGCATCGCCAATAAATACACCGCAGGCCGCAAGATAACCGCCTTTC
ATACCGCTACTGACGCTATTTTTGAGTACAAACAGGGTATTTGGCCCTGGCACCAACACAATAAAAATGGCCCCAACCAG
ATAGGTCCAGTAATTCAGAACCCCGTATTCAGCGAACACATTAACCTCTTTAATTATCTTTCGATCATGCGCGATTAAAG
GTGAATATGCTAACCAATCTGTAGCGGCTTAGAAAGGAGAAAATCAGGTTTTAACCTGATATCAACCCGATAATTGAATC
ATTAATCAGGCATGCTCCAGTGAAAAATTCGGGTAGTGCTCGGCAAAATACTGGCGTAAAAATTCTACCGTTATCCGCAC
TTTCGCTGACGTCGCCAGCCTTGAAACATAAACGGCCCAGACGTTCGCTGGCTGGTAATATTCCGGTAGCACTTGCACTA
AATGACCACTGGCAATGTTTTCGCTAACATCCCACCAGGAACGCAGAGCAATCCCCTGCCCATCGAGACACCACTGATGG
ACAATTTCTCCATGATTAGACGACAGTGGGCCGGTGACTTTAATCGCATGGGGGCCTTCTTTATTGCGTAATTGCCAGAC
ACCAAAAGGATGATCGCGCTCTTTAATGACCAGGCACGGCAATGCAGAAAGATCGGTTAAATGTTTCGGCGCGCCATGTT
GCGCAATAAATTCCGGCGACGCGCAGAGAATACGGTAATTGGTCGCCAGTTTGCGGGCGATTAAATTAGGGGCGATGTCA
TCGCCAATGCGAATATCGAGATCGACACCTTCATTGACCAAATCGACCAGTCGGTCTTCCACATCAAAACGTAATTCCAG
TTGCGGATACGCCTTCGCCAGCGCTAATAACGCCGGAGCCACCACCTGTCGTCCAAAACCAAAGCTGCTGATAATACGCA

GCATCCCCTGCGGCACCTGACGCACGTCAGAAAGTTCGTCCATCATCTGACCGACATCCTGCAAAATCCGCTGCGCCCAT
TCATAAATCCGCTCTCCTTCTTCGGTAATGGTGACGCGGCGGGTGGTGCGGTGTAACAACACCACGTTTAGCGTTTGCTC
CAGCAAGGCGATGCGCTTGCTGACGAACGCCGGTGAAACGCCCAGTTCTTCCGCCACGGCGGCAAAACCAGCCCGGCGAG
CCACCAGCATAAAGACGCGCAAATCATTCAGCAGCGGTAAATTATTCATGATTCGTGTTTTATGTTTCACCAGTTACGGG
GATTAATTCCTTTTCAGTCAATTATAGGATGGTGATGTTGTCAATTTTGATGGTCAGGAAGTGAGAACCCAATGATGAAA
ACGATGCGTATTGCTGCGATCCCGGGAGACGGGATTGGCAAAGAAGTCCTTCCTGAAGGGATTCGCGTGTTACAGGCTGC
CGCTGAGCGCTGGGGCTTCGCCTTGAGTTTTGAGCAAATGGAGTGGGCGAGCTGCGAGTATTACAGCCATCACGGTAAAA
TGATGCCGGACGACTGGCATGAGCAACTTAGCCGTTTCGACGCCATCTATTTTGGTGCCGTCGGCTGGCCGGATACCGTT
CCGGACCATATTTCGTTGTGGGGTTCGCTGCTGAAATTTCGTCGTGAATTCGACCAGTACGTCAACCTGCGCCCGGTTCG
TCTCTTTCCTGGCGTTCCCTGCCCGCTGGCGGGAAAACAGCCTGGCGACATCGATTTTTACGTGGTCAGGGAAAACACCG
AAGGCGAATATTCCTCGCTCGGCGGTAGAGTGAATGAAGGTACAGAGCATGAAGTCGTCATTCAGGAATCGGTATTTACC
CGCCGTGGTGTCGATCGCATTTTGCGTTATGCCTTCGAACTTGCGCAAAGCCGTCCACGTAAGACACTAACTTCTGCCAC
TAAATCGAACGGTTTAGCCATCAGCATGCCGTACTGGGATGAGCGAGTGGAAGCAATGGCCGAGAATTACCCGGAGATCC
GCTGGGACAAGCAGCATATTGATATTCTCTGCGCGCGTTTTGTGATGCAGCCGGAACGATTCGATGTGGTGGTGGCGTCC
AATTTGTTTGGCGATATCCTTTCCGATCTTGGCCCGGCCTGCACCGGCACCATTGGCATTGCCCCATCCGCCAACCTGAA
TCCGGAACGCACTTTCCCGTCGCTCTTCGAGCCTGTCCACGGTTCCGCGCCGGATATCTACGGGAAAAATATTGCTAACC
CTATCGCCACGATTTGGGCCGGGGCAATGATGCTCGATTTTCTCGGCAATGGCGATGAGCGTTTCCAGCAAGCGCATAAC
GGTATTCTGGCAGCAATTGAAGAAGTGATTGCTCACGGGCCGAAAACACCTGATATGAAAGGCAATGCCACCACGCCACA
GGTTGCCGACGCGATTTGCAAAATTATTTTGCGTTAAGGTCAAACCAGTTTATTTGAACCGCGTCACTGACGCGGTTTTT
TTATTCGTTCTTTGCAGTAAATAACCTGCGTCATTTCACCTTTTATTGTTTCCGTTTCGTGTTTTATGGCTTTCCGTATT
CTTAATTGTTTAATTTATGTAACATGCAAATTTTGTTACGCGTACGTTAGGTTCCGCCGTACAGGTATTGGTTTTGCTGG
CAATGGGACTGGTGATTTATTTAGCCACCAGTAAATACGGCAATATTCGTCTTGGCGAAGGAAAACCGGAATACAGCACG
CTCTCCTGGCTGTTTATGTTTATTTGTGCCGGTTTAGGTTCTTCTACGCTTTATTGGGGGGTTGCTGAATGGGCCTATTA
TTATCAAACACCTGGATTAAATATCGCACCGCGTTCACAACAGGCACTCGAATTTAGCGTTCCCTACTCTTTCTTCCACT
GGGGCATCAGCGCCTGGGCAACTTATACGCTGGCCTCATTAATCATGGCTTATCACTTTCATGTGCGGAAAAACAAAGGT
CTGAGCCTTTCCGGCATTATTGCTGCTATTACCGGCGTTCGCCCGCAAGGCCCATGGGGAAAACTGGTCGATTTGATGTT
CCTGATCGCCACTGTCGGCGCACTGACCATTTCCCTTGTTGTTACCGCAGCAACCTTTACCCGTGGGCTTTCCGCGCTGA
CCGGTTTACCCGATAACTTCACCGTGCAGGCATTTGTGATCCTGCTTTCCGGCGGCATTTTTTGCCTAAGCTCGTGGATT
GGTATCAACAACGGTTTGCAACGTCTGAGCAAAATGGTTGGCTGGGGCGCGTTCCTGCTGCCATTACTGGTGCTGATTGT
CGGCCCAACCGAATTTATTACCAACAGCATCATCAATGCCATCGGCCTGACCACGCAAAACTTCCTGCAAATGAGCTTAT
TCACCGATCCGCTTGGCGATGGTTCATTTACCCGCAACTGGACCGTTTTCTACTGGCTGTGGTGGATCTCATACACCCCT
GGCGTAGCAATGTTTGTCACCCGCGTTTCCCGCGGTCGTAAGATTAAAGAAGTTATCTGGGGACTGATCCTCGGCAGCAC
CGTCGGTTGCTGGTTCTTCTTTGGCGTAATGGAAAGCTATGCCATTCATCAGTTTATCAATGGCGTAATCAACGTCCCAC
AGGTGCTGGAAACACTGGGCGGCGAAACAGCTGTACAGCAAGTTCTGATGTCGTTGCCAGCCGGTAAATTGTTCCTCGCC
GCATACCTGGGCGTGATGATTATTTTCCTTGCCTCGCATATGGATGCAGTGGCCTACACCATGGCGGCGACCAGTACGCG
TAATCTCCAGGAAGGTGACGATCCTGACCGTGGGCTGCGTCTTTTCTGGTGCGTGGTGATCACTCTGATCCCGCTTTCCA
TCTTGTTTACCGGTGCTTCGCTGGAAACGATGAAAACCACCGTCGTGCTCACAGCCCTTCCCTTCCTCGTCATTTTACTG
GTGAAAGTCGGCGGGTTTATTCGCTGGCTGAAACAGGATTACGCCGACATTCCGGCTCATCAAGTTGAACATTATCTCCC
GCAGACACCGGTTGAAGCCCTGGAAAAAACGCCAGTGCTCCCTGCGGGAACCGTATTCAAAGGCGACAACTGAGCGCCAT
CATCCTAACGATAAAGGTATCCCTATGAGCAATCTGAGCCCTGACTTTGTACTACCCGAAAATTTTTGCGCTAACCCGCA
AGAGGCGTGGACCATTCCTGCCCGTTTTTATACCGATCAGAACGCGTTTGAACACGAAAAAGAGAACGTCTTCGCCAAAA
GCTGGATTTGCGTCGCTCACAGCAGCGAACTGGCGAATGCCAATGATTATGTGACGCGTGAGATCATTGGCGAAAGCATC
GTGCTGGTACGCGGTCGTGATAAGGTTTTGCGCGCGTTCTATAACGTGTGTCCGCACCGTGGTCATCAGTTGTTGAGCGG
TGAAGGAAAAGCAAAAAATGTGATTACCTGCCCGTATCACGCATGGGCATTCAAACTCGATGGCAACCTGGCCCATGCAC
GTAACTGCGAAAACGTCGCCAATTTCGATAGCGACAAAGCGCAACTGGTTCCGGTGCGTCTGGAAGAATATGCCGGATTC
GTCTTCATCAACATGGACCCCAACGCCACCAGCGTAGAAGATCAATTACCCGGCCTGGGCGCGAAAGTGCTGGAAGCCTG
CCCGGAAGTCCACGATCTGAAACTGGCGGCCCGCTTTACCACCCGCACGCCTGCCAACTGGAAGAACATTGTCGATAACT
ATCTCGAGTGCTATCACTGTGGTCCGGCGCATCCAGGTTTCTCCGACTCCGTACAGGTTGATCGTTACTGGCACACCATG
CACGGTAACTGGACGCTGCAATACGGTTTCGCCAAACCGTCCGAACAGTCGTTTAAATTTGAAGAGGGTACGGATGCGGC
ATTCCACGGTTTCTGGCTGTGGCCGTGCACGATGCTGAACGTCACCCCGATCAAAGGGATGATGACGGTCATTTATGAAT
TCCCGGTGGATTCTGAAACTACCCTGCAAAACTACGATATTTACTTCACCAATGAAGAGTTAACCGACGAGCAAAAATCG
CTGATTGAGTGGTATCGCGATGTGTTCCGTCCGGAAGATTTACGTCTGGTTGAAAGCGTACAGAAAGGGCTGAAATCGCG
TGGCTATCGTGGTCAGGGGCGCATCATGGCCGACAGTAGCGGTAGTGGCATTTCCGAACATGGTATCGCCCATTTCCATA
ATCTGCTGGCGCAGGTGTTTAAGGACTAATGACATCGGCGGCGGTATTTTCCGCCGCTGGGCTGATTTTTGATGGAGTAC
AGCAATGTCAGACTATCAAATGTTTGAAGTACAGGTGAGCCAGGTTGAACCCCTTACCGAACAGGTGAAACGCTTCACGC
TGGTGGCAACCGATGGCAAACCATTACCTGCGTTTACCGGAGGAAGTCACGTCATTGTGCAGATGAGCGATGGTGATAAC

CAGTACAGCAATGCGTATTCACTACTGAGTTCGCCGCATGACACCTCTTGTTATCAGATTGCCGTTCGGCTGGAGGAAAA
CTCGCGCGGCGGTTCCCGCTTTTTGCATCAGCAGGTAAAAGTGGGCGATCGGTTAACGATTTCAACGCCTAATAACCTGT
TTGCGCTAATTCCCTCAGCCAGAAAGCATCTGTTTATCGCGGGCGGTATTGGTATCACCCCTTTCCTGTCGCACATGGCA
GAGCTGCAACACAGCGACGTCGACTGGCAGCTACATTACTGCTCGCGAAATCCAGAAAGTTGCGCATTTCGTGATGAGCT
AGTCCAGCATCCGCAGGCTGAGAAAGTCCATTTGCATCATTCATCAACCGGAACACGACTGGAATTAGCGCGATTATTGG
CGGATATCGAACCTGGCACACACGTTTATACCTGTGGCCCCGAGGCGCTAATTGAAGCGGTAAGAAGTGAAGCTGCGCGT
CTGGACATCGCCGCCGATACGCTGCACTTTGAGCAATTTGCTATCGAAGACAAAACCGGCGATGCATTTACCCTGGTGCT
TGCCCGTTCCGGAAAAGAGTTTGTGGTGCCGGAAGAGATGACTATTTTGCAGGTTATTGAAAATAATAAAGCCGCGAAAG
TGGAATGTTTATGTCGTGAAGGGGTATGCGGAACCTGCGAAACAGCAATACTGGAAGGTGAAGCTGACCATCGGGATCAA
TATTTTAGCGATGAAGAGCGTGCCAGCCAGCAAAGTATGTTGATCTGTTGTTCGCGTGCGAAGGGTAAACGCCTGGTGTT
GGATTTGTAGTTTGCCGAAGCCGGATGTGGCGCTGAGCGCGCCCAGTCCGGCTTCGGAAGATTTTACTGCGGATATTCCT
GCAATAAATTGTGTAATGCTTCCGCCATCAGCTCACCACGCCAGCCGGAAATCAGCTCCGGCAAATTGTTCTGCGGTTTC
AGTTTCCAGTGCCAGTTCAGCAGTTGGTTGATTTGCCGACGCGATGCCAGCAATTCGGCGCTGATCTTATGCGTTTCGCT
CACGTCAGTAATCAGCGACTTAATCGCTTTAAACGCTTTACGATAACCCGGCATGTCCATCAGGTTAAGCATCGGCTGCG
GTAAGGCATCTTCCGGCAATGTCTGCGCTTTTTCCACCAGCGCTAGCAGCGTTTTACCGTGAAAGCGGATTTCGCTACCG
GATAAACCCAGGCTGTCCAGTTCGCCTAAACTTCCCGGCATATAACGCGCTACCGACCACAAATGCTCTTCACGCACGAC
AAAGTTCACCGCCAGATCGCGCTCTCGCGCCTTGCGCAGTCGCCAGTCGGCTAACAGTTGCAGACAGGCCAGTTGGCGTG
TGCGTAATTGCCAGGCATTGGTGATATCACGCCAGGCATCTTCCGGCGCAACGACTTCCTGACGACGCATTTGCATCAGG
CGGCATTCATCCAGCGCCGCAGGTAGCCAGCCGGAGGCCTCCGTTTCTACCATAAGCTTGGCGGTGATCGGTAACAGATA
CCAGACATCCGCCGCTGCGTATTCACACTGACGTTCGGTCAGCGGTCTGGCCAGCCAGTCGGTGCGCGATTCACTCTTGT
CCAGCGTAACGCCGGAATACTCTTCCACCATGGAAGCGAAACCCCATGACATCGGGCGTCCGCAGAAGGCAGCAAGGATT
TGCGTGTCAATCAAGGGTTGTGGTAATTCGCCAAAGACATTGAGGAACACTTCCAGATCTTCACTGCCTGCATGGAGAAA
TTTTGTGATGGACGGATCGCGCAGGATCGCTTTCAGCGGTGACCAGTCGGTGATCCCGAGTGGATCGATTAGCGCCAGAT
GCTCGCCATCGAAAAGTTGAATCAACCCCAGCTGCGGGTAATAAGTGCGCGTACGAACAAATTCAGTATCCAGGGCTATC
GCCGGAAAGGCACGGACGGCTTCACACAAAGAAGCCAGCGCATCGTCCGTGGTAATCATTTGGTAATTCAAATTGTTTTC
TCTTTAGTGGGCGTCAAAAAAAACGCCGGATTAACCGGCGTCTGACGACTGACTTAACGCTCAGGCTTTATTGTCCACTT
TGCCGCGCGCTTCGTCACGTAATTCTCGTCGCAAAATTTTTCCGACGTTAGATTTCGGTAACTCATCACGAAACTCCACC
AGCTTCGGTACTTTGTATCCCGTGAGCTGACGGCGGCAAAAAGTCACCAGTGACTCTTCGGTAAGCGATGGATCTTTTTT
CACTACGAAGATTTTCACCGCTTCACCACTGGAGCCGGAAGGTACGCCAACAGCCGCGACTTCCTGTACGCCAGGATGCT
GCATGACGACATCTTCAATCTCGTTGGGATAGACGTTAAAACCGGAAACCAGAATCATGTCTTTTTTACGATCGACAATG
CGCAGGAATCCTTCTTCATCCATTACCGCGATGTCGCCGGTGTGTAACCAGCCATTTTTGATGATTTCATCGGTAGCATC
GGGACGCTGCCAGTAACCCAGCATCACCTGCGGTCCTTTGACACAAAGCTCACCCGGTTGACCTGGTGGTACTTCATTAT
CATCATCATCCACCAGTTTGGCTTCCGTCGACGGCACCGGCAAACCGATGCTACCACTATGATAATCAATATCATATGGG
TTAACGCTGACCAGCGGCGCACACTCGGTAAGGCCATAGCCTTCCAGCAGATACTGTCCGGTCAGTTTCACCCAACGCTC
TGCCACCACTTGCTGCACTGGCATCCCACCGCCTGCGGAAAGATGCAGACTGGAGAAATCCAGCTGCTGGAACTCTTTAT
TGTTCAGCAACGCATTGAACAAGGTGTTAACGCCCGTGATAGCGGTAAACGGATATTTCGCTAACTCTTTTACCAACCCT
GGAATATCGCGCGGGTTAGTGATAAGCAGGTTCTGCCCACCCAGTTCGATAAACAGCAGGCAGTTAATGGTCAGGGCAAA
AATGTGATACAGCGGCAGCGCCGTCACCACCAGCTCTTTGCCCGGATGCAACAGCGGACCATAGGTCGCGTTAACCTGTT
CCAGGTTCGCCAGCATATTGCGGTGAGTCAGCATCGCGCCTTTCGCCACACCAGTGGTGCCGCCGGTGTATTGCAGAAAA
GCTAAATCTTCCGGCACCAGTTCGGGTTTGACGTACTGCATCCGGTAGCCGTTATGCAGTGCGCTACGAAATGAAATGGC
ATCTGGCAGATGGTATTTCGGCACCAAACGCTTGATGTATTTAACAACGAAATTGACTACCGTGCCTTTTGCCGTAGATA
GCTGATCGCCCATACGGGTCAGAATTACGTGCTGAACGGCGGTTTTATCAACCACTTTTTCCAGTGTGTGAGCAAAGTTA
GACACGATAACAATCGCCGATGCGCCGCTATCGTTAAGCTGATGCTCAAGCTCACGCGGGGTATACAACGGGTTAACGTT
TACGACGATCATCCCGGCACGCAAAATGCCAAACAGCGCCACCGGATATTGCAATAAATTAGGCATCATCAACGCAACGC
GATCGCCTTTCTTCAGCCCCAACCCTTGTTGCAAATAAGCGGCAAACGCGCGACTGCGTTCTTCCAGCTTGCGGAAGGTC
ATTACCTCCCCCATATTCACAAACGCAGGTTGATCGGCGTAGCGCGCGACCGACTGCTCAAACATATCTACCAGAGATTG
ATAACGGTCAGGGTTGATCTCCGTCGGAACGTCCGCGGGATAACGGTTAAGCCAAACCTTCTTCAATTCTTCACCTCTAA
AATGCGTGTTCGTCGTCATCGCAACCCCAAATGATATACATGCCGTTAACATAATATTAACTCATCATACCAGCTTGATA
ATTACCCAACGAAAAGGTTGCGAAGCGCGTCACTATTTATTTTTATCTTTACCGTAAGAATGCAGAAACAGCGGACCAGC
CGCTGTTTCTTTTTCTTTAAAAACAAGCAATTATTCAGTTACAACTGTTTGTACTCTCGCGGGGCCGGGATTATACCAGC
CCCATCCGCCGTAGCCATAGGGCCAGCCACGACCGCCATAAAACCATGGATCAATCGGCTGAGGCGGCATAATCACCTGC
TGGGTTAAATGCCAACGTTTGTAACCCGTTACTTGCATCACCATAAATTTATAGGGCGTATTGCCGATTTTGCCGTCAAC
CGCACCAGTGATTGGCCCGACTACCGTAACCAGTTGTCCACGGAAATCCACCGGGTCCAGAAAACCGTTCACATCGGCAT
AAATGCGACCGCGAGAAGGTTCTCCCAGCGTCGGTCTGGCTCCGCTGTCCAGCGGTACGGTAGCAATTTCCAGGCGAGTT
TTCCCTTGCTGGTTTTGTACCGCAACCACTTTGCCACCAAAGCGTGCCTCCTGACCAACGTACAGCTGCGGCGCACTCAT
CACCCGAACTAAATCTTGTTGCGGCGTGGGACTGCTGCCTTTAATGGCGTCCGGCACAGTGACACAACCGCTCAGCATTA

GCGCAAACGTACCTGCCAGTATGCCTTTGATAACATTCTTTTGAACCGCCATGGTGCGACTCCTTTTTTTCTCAGGGCAT
ACTCTTAAGATTCATTCTTTGCCCGGAAGTTTCTTCCATGCGACGTTGTTACGTAAATAAACCGGTTCGGCATGTTCCAC
CGCCACCGTTTTACCCTCAGCAAACATCTGACACGCAATCGGCAGCATATCTTCAGCAGCAGGCAGTAACACTTCGCCAT
CGCGCAAAACCAGCCCGCTCTCTTTACCGAGATCCGGCCAAGCTTGCCAGCCCGTCCCTACCGTCACCCATTCGCCGGAA
AGCTGTTGCATTCGTTCATGGACGATTTCGGGTTTGAGTACGGCTTCGGTTTCTTCACCGTGCCAGATACCGTTTTCATC
ACGCTGATATTCGGCCCAGTAAACTTCGCCCATTCGCGCGTCAATGGCTGCCAGCACGCGGGTTGCGCCGTTTTTGCGCC
ACGCCCCTTGCGCCATCGTCATTAGCGTGGAGACGCCAATCATCGGTAATTCCGCGCCAAGCGCCAGCCCTTGCGCGATG
CCAATACCAATGCGCACGCCAGTAAAGCTACCGGGGCCGCGCCCGTAAGCCAGAGCGTTAATATCAGTCAGGGAAGTTCC
GCTGGTGGTCAGGATATCCTGCACCATCGGTAAGATTCGTTGAGTATGTTCACGAGGGCAAAGCTCAAAATGAGCGTTGA
CAGTACCGTCGTTCCACAGGGCGACAGAGCAGGCCTCTGTCGCGGTATCGATAGCCAGAATTCGCATGGGTCTTCGTGCT
TAGATCAATAAAAAGGCGCGCATCATACCATACTCCGTAACAAATTACCTGGAGGATGGTATCGCAAGGAAACGAACCGC
ACGGGCAATGTCACGGGTGCGTGGCGCGGGCGGCAGACTGGCGAGAAACGTCGCGCCGTAAGGACGCATCACCAGCCGAT
TGTCACAAATCACCAGCACGCCACGATCGTCGGCGTCGCGAATCAGTCGCCCTACCCCCTGTTTGAGAGTAATGACGGCA
TCTGGTAGTTGCACTTCATCGAACGGGTCGCCACCGCGCAAACGACAATCTTCCATGCGCGCTTTTAACAGTGGATCATC
CGGCGAGGTAAACGGCAATTTGTCGATAATTACCAATGACAATGTATCGCCACGCACGTCCACCCCTTCCCAGAAACTGC
TGGTTGCCACAAGAAGCGCATTACCGGCGCTGACAAATTGCTGCAACAGTTGCCCTTTGCTGGTTTCCCCCTGCAACAAT
ACAGGAAGCGTCATGGTAGCGCGGAACTGCTCGGCCAGATCGCGCATCATGGCGTGCGAGGTACAAAGCATAAAACAACG
ACCGTTGTTAGCTTCGATGATCGGTCGCAGCATTGCCGCTAACTGGCGAGCAGAACCTGGCTGGTTGGTTTGCGGCAGAT
TGCGCAGCACACAGAGTAACGCCTGGCGGCTGTAATCAAATGGGCTGGGCAACAGCAACGACTCGGCCTGTTCGATGCCA
AGCCGCGAGGTGAAATGATGCAGATCGTCGTTCACCGACAGCGTTGCTGAGGTGAAGATCCAGCTACCGGGTTTTTGCGC
CATTAACTCTTTGAATTTATCCGCCACGCTGAGCGGCGTGAGAGCCAGAGTAAAATGGCGCGAAGTGCATTCGTACCAGT
AGCTGTAGCCCGGCTGATTGATCTCTTTTAGCCGCTTCAGCCGTGTGCGATACAACGTGGCGCGCTCAAATGCCGCATCC
AGCAAGGCGGAACGCCCCAGTGACAGTTTCGCCACGTCATAACAAAGTTCCAGGGTGTCATCGAGCAGTAAAAATGCCCG
CTGAATTTGCGGATTAGCTAACAGCTCACGCAGGTTACCGCGATAACCTGGCTCACCGAGTTGCAGACGAAAATCCTGCG
CACTCTGGGCAAGACGATCAGCGCACTTTTGTAACTGCTGGGTGTCTTTTAATTCGGTGCGGTAGGCGATGGTGATGTCT
TTTGCCAGGTCGAGCAGTTGTCGACTGGAGAGTGACTGACCAAAATACTGGCTGGCAATGTCCGGTAGCTGGTGGGCTTC
GTCGAAGATCATGACGTCCGCTTCCGGGATCAGTTCGCCAAATCCACTCTCTTTAACCACCATATCCGCCAGAAAGAGAT
GATGGTTTACCACCACCACATCGGCGTCCATCGCTTTTTTACGTGCTTTGACCACAAAGCAATCTTTATACATCGGGCAG
TCGCTGCCAAGACAGTTGTCGTTGGTGCTGGTGACCAGCGGCCACGCCTGTGAATCTTCCGCCACGCTGACGCAGGTGCT
GATATCACCATCGACTGTTTGATTAGACCAGGAGCGCAGCAGGATCACATCGCTTAAGATTTGTACCGGCAGATCGCCCC
CCGCCAGCGCCTGCTGTTCGAGACGTTCGAGGCAGAGGTAGTTTGAGCGCCCTTTCAGCAGCGCCACGTTGCCCGTATAT
TTCAATGCCTTTGAGACTGTTGGCAAATCGCGGCTGTAGAGCTGATCCTGCAACGCTTTTGAGCCGGTCGAGATAATGAC
TTTCTTTTTCGCCCGCAGCGCAGGAGCCAGGTAAGCGTAGGTTTTGCCCGTACCGGTTCCTGCTTCCACCACCAGCGGCT
GGCCTTTTTCTATCGCCTGGGTGACGGCTACCGCCATCTGTCGCTGTGGTTCTCGCGGCTTAAAGCCTGGTATCGCTTTC
GCCAGCTGACCGTCTGGTGCAAAATCGTCCGTCACACTACCCCCTGTTGATTTGAACAGGGATTATGTCAGGATGAGGGC
GCTTTCGCCAGTTGAAGTGGTGACGGCGACCTCACATTGTGGCAGTCTTTGCAGCACGAAAATGGAAGTATAATGAGGAC
AAAATGATGACTATCGTTCGTATCGATGCTGAAGCCCGCTGGTCTGATGTAGTAATCCACAACAACACGCTCTACTACAC
TGGTGTACCGGAAAACCTCGACGCCGATGCCTTTGAGCAAACCGCCAACACGCTGGCACAGATTGACGCCGTGCTGGAAA
AACAGGGCAGCAATAAATCGAGCATTCTGGATGCCACCATTTTCCTGGCCGATAAAAACGACTTCGCGGCGATGAATAAA
GCGTGGGATGCTTGGGTTGTCGCGGGTCATGCGCCGGTGCGCTGCACGGTACAAGCGGGTTTGATGAACCCGAAGTATAA
AGTTGAAATTAAGATTGTGGCTGCGGTGTAAGCTTTATCGAAGCAAAATAAGTCAGACGATAATTTATCGATAATACTGG
TCGGTTTTACATAAATCGACCAGAGAATAAGATATTACTGTTGATGCTTCTGCTAATTCATTATTTATATTTATAATTTC
AATTTTATCTATAAAACTAACTATATAAATAAACATAAACATGCCGTTGTGTTCGCCCTTTGATTAGCAACTCTGGTTTT
CTTTCTTAGCTGAACAATCTGGTTATAGCGTGCGGACTGACCGTTAAATTTCTCCATCTTACTGGATAACACACCATGAG
CTGCTACCTTTAAAACACCTGAATGTCAACAGGTTAACTCGTGCCATATCGTTATAATCCCTTTGATATTAGATGCAAAT
TAAGGTCATATATAGCCTTATCGATAACATGGTTAATTTTAAGGATAAGAATATGCCTGCTGTAATAGATAAAGCCCTGG
ATTTCATTGGTGCCATGGATGTATCAGCGCCAACACCAAGTTCGATGAATGAAAGCACGGCGAAGGGAATCTTTAAATAT
TTAAAAGAACTGGGAGTACCCGCGAGTGCCGCTGATATTACGGCGCGAGCTGACCAGGAAGGCTGGAACCCTGGGTTCAC
GGAAAAAAATGGTTGGATGGGCAAAAAAAATGGAGACAGGTGAACGTTCTGTGATTAAAAATCCTGAATACTTTTCAACAT
ATATGCAGGAAGAACTAAAAGCACTGGTCTGAGTTAAATTTATATCAGCATAAATGGGTCAGGACGCTTTTAATCACATA
TAAAAAGCGTCCTTTTTCTCACAATCAAATGATCATCGGCTTCTATAACGAAATCGATACGGATTAGTGTGTTACTCGTC
TTCATCCTCAAAACGCGCCACGATTCGCTCGCCGGAGTGGTTGGCACGCAGCTCTTCTGCCACCAGCGCGATTGCCTGGC
CGCTGCTCATCCCTTGTGCCATCAGTTCCTGGATCCGCTCGACAGCTTTTTGCTGCTGTTCATGGGTGAGTGAAGGTAAA
CCTGCAAACATTGTTAACTCCTGCTAAATTGTTGGCGCTAATTATTTCATGCTACCCGGCACATAGCCAGTAGAGTCAGG
ACTGATGAAGACGTTATCTCCCGCTGTGATTACTTTACTCTGGCGTCAGGACGCCGCTGAATTTTATTTCTCCCGCTTAA
GCCACCTGCCGTGGGCGATGCTTTTACACTCCGGCTATGCCGATCATCCGTATAGCCGCTTTGATATTGTGGTCGCCGAG

CCGATTTGCACTTTAACCACTTTCGGTAAAGAAACCGTTGTTAGTGAAAGCGAAAAACGCACAACGACCACTGATGACCC
GCTACAGGTGCTCCAGCAGGTGCTGGATCGCGCAGACATTCGCCCAACGCATAACGAAGATTTGCCATTTCAGGGCGGCG
CACTGGGGTTGTTTGGCTACGATCTGGGCCGCCGTTTTGAGTCACTGCCAGAAATTGCGGAACAAGATATCGTTCTGCCG
GATATGGCAGTGGGTATCTACGATTGGGCGCTCATTGTCGACCACCAGCGTCATACAGTTTCTTTGCTGAGTCATAATGA
TGTCAATGCCCGTCGGGCCTGGCTGGAAAGCCAGCAATTCTCGCCGCAGGAAGATTTCACGCTCACTTCCGACTGGCAAT
CCAATATGACCCGCGAGCAGTACGGCGAAAAATTTCGCCAGGTACAGGAATATCTGCACAGCGGTGATTGCTATCAGGTG
AATCTCGCCCAACGTTTTCATGCGACCTATTCTGGCGATGAATGGCAGGCATTCCTTCAGCTTAATCAGGCCAACCGCGC
GCCATTTAGCGCTTTTTTACGTCTTGAACAGGGTGCAATTTTAAGCCTTTCGCCAGAGCGGTTTATTCTTTGTGATAATA
GTGAAATCCAGACCCGCCCGATTAAAGGCACGCTACCACGCCTGCCCGATCCTCAGGAAGATAGCAAACAAGCAGTAAAA
CTGGCGAACTCAGCGAAAGATCGTGCCGAAAATCTGATGATTGTCGATTTAATGCGTAATGATATCGGTCGTGTTGCCGT
AGCAGGTTCGGTAAAAGTACCAGAGCTGTTCGTGGTGGAACCCTTCCCTGCCGTGCATCATCTGGTCAGCACCATAACGG
CGCAACTACCAGAACAGTTACACGCCAGCGATCTGCTGCGCGCAGCTTTTCCTGGTGGCTCAATAACCGGGGCTCCGAAA
GTACGGGCTATGGAAATTATCGACGAACTGGAACCGCAGCGACGCAATGCCTGGTGCGGCAGCATTGGCTATTTGAGCTT
TTGCGGCAACATGGATACCAGTATTACTATCCGCACGCTGACTGCCATTAACGGACAAATTTTCTGCTCTGCGGGCGGTG
GAATTGTCGCCGATAGCCAGGAAGAAGCGGAATATCAGGAAACTTTTGATAAAGTTAATCGTATCCTGAAGCAACTGGAG
AAGTAAGACGTGGAATACCGTAGCCTGACGCTTGATGATTTTTTATCGCGCTTTCAACTTTTGCGCCCGCAAATTAACCG
GGAAACCCTAAATCATCGTCAGGCTGCTGTGTTAATCCCCATCGTCCGTCGACCGCAACCGGGGTTGTTGCTGACTCAGC
GTTCGATTCATCTGCGTAAACACGCTGGACAAGTGGCATTCCCTGGAGGTGCAGTCGATGACACGGACGCATCAGCTATC
GCCGCCGCGCTGCGCGAAGCTGAAGAAGAGGTCGCTATACCGCCTTCCGCCGTTGAAGTTATCGGCGTGCTGCCGCCCGT
CGATAGCGTCACTGGCTACCAGGTAACCCCAGTGGTCGGCATTATCCCGCCCGATCTGCCGTATCGCGCCAGTGAAGATG
AAGTCTCGGCGGTGTTTGAAATGCCGCTCGCCCAGGCATTACATCTGGGTCGTTATCACCCTTTAGATATCTACCGCCGT
GGTGATTCACATCGGGTATGGCTGTCCTGGTACGAACAGTATTTTGTATGGGGAATGACCGCAGGCATAATTCGTGAGCT
GGCGCTGCAAATTGGTGTGAAACCCTGACTATACTTATCTTTACATCTACAAAACACTACTTGAGACAATCATCGCAATA
TTAGTTAAATCGCGGTTTTTGATTAGTTTAATTCATGTGAATAGTTAAGCCAGTCGCCGCGTTCCCTCTTACACTATGCG
CTGTTATTAGTTCGTTACTGGAAGTCCAGTCACCTTGTCAGGAGTATTATCGTGATTAGTCTATTCGACATGTTTAAGGT
GGGGATTGGTCCCTCATCTTCCCATACCGTAGGGCCTATGAAGGCAGGTAAACAGTTCGTCGATGATCTGGTCGAAAAAG
GCTTACTGGATAGCGTTACTCGCGTTGCCGTGGACGTTTATGGTTCACTGTCGCTGACGGGTAAAGGCCACCACACCGAT
ATCGCCATTATTATGGGTCTTGCAGGTAACGAACCTGCCACCGTGGATATCGACAGTATTCCCGGTTTTATTCGCGACGT
AGAAGAGCGCGAACGTCTGCTGCTGGCACAGGGACGGCATGAAGTGGATTTCCCGCGCGACAACGGGATGCGTTTTCATA
ACGGCAACCTGCCGCTGCATGAAAACGGTATGCAAATCCACGCCTATAACGGCGATGAAGTCGTCTACAGCAAAACTTAT
TATTCCATCGGCGGCGGTTTTATCGTCGATGAAGAACACTTTGGTCAGGATGCTGCCAACGAAGTAAGCGTGCCGTATCC
GTTCAAATCTGCCACCGAACTGCTCGCGTACTGTAATGAAACCGGCTATTCGCTGTCTGGTCTCGCTATGCAGAACGAAC
TGGCGCTGCACAGCAAGAAAGAGATCGACGAGTATTTCGCGCATGTCTGGCAAACCATGCAGGCATGTATCGATCGCGGG
ATGAACACCGAAGGTGTACTGCCAGGCCCGCTGCGCGTGCCACGTCGTGCGTCTGCCCTGCGCCGGATGCTGGTTTCCAG
CGATAAACTGTCTAACGATCCGATGAATGTCATTGACTGGGTAAACATGTTTGCGCTGGCAGTTAACGAAGAAAACGCCG
CCGGTGGTCGTGTGGTAACTGCGCCAACCAACGGTGCCTGCGGTATCGTTCCGGCAGTGCTGGCTTACTATGACCACTTT
ATTGAATCGGTCAGCCCGGACATCTATACCCGTTACTTTATGGCAGCGGGCGCGATTGGTGCATTGTATAAAATGAACGC
CTCTATTTCCGGTGCGGAAGTTGGTTGCCAGGGCGAAGTGGGTGTTGCCTGTTCAATGGCTGCTGCGGGTCTTGCAGAAC
TGCTGGGCGGTAGCCCGGAACAGGTTTGCGTGGCGGCGGAAATTGGCATGGAACACAACCTTGGTTTAACCTGCGACCCG
GTTGCAGGGCAGGTTCAGGTGCCGTGCATTGAGCGTAATGCCATTGCCTCTGTGAAGGCGATTAACGCCGCGCGGATGGC
TCTGCGCCGCACCAGTGCACCGCGCGTCTCGCTGGATAAGGTCATCGAAACGATGTACGAAACCGGTAAGGACATGAACG
CCAAATACCGCGAAACCTCACGCGGTGGTCTGGCAATCAAAGTCCAGTGTGACTAATACTTCTTACTCGCCCATCTGCAA
CGGATGGGCGAATTTATACCCGCTTTCTCGTCTGCTGTAATATTCCCCACTACACTTCCACTGTTGCGTCAGGCGTTTGT
CGCCATACGCTTACAGGGTGGCCCGCATGCAAAAAGCACAACGGATCATTAAAACCTATCGTCGTAATCGAATGATTGTT
TGTACGATTTGCGCACTCGTTACGCTCGCTTCGACCCTGAGCGTGCGATTTATTTCACAGCGTAACTTAAATCAACAACG
GGTAGTACAATTCGCCAATCACGCTGTAGAGGAATTAGATAAAGTACTGCTTCCCCTACAGGCAGGTAGCGAAGTCTTGC
TTCCGCTGATTGGTCTGCCCTGCTCTGTCGCCCATTTGCCATTACGTAAACAGGCGGCAAAACTCCAAACTGTGCGATCC
ATTGGCCTGGTGCAAGACGGCACACTTTATTGCTCCAGCATTTTTGGTTATCGCAATGTGCCCGTCGTGGACATTCTGGC
TGAACTTCCTGCACCGCAACCACTTTTACGCCTGACGATCGACCGTGCCCTGATTAAAGGCAGTCCGGTTTTGATTCAAT
GGACGCCTGCAGCGGGCAGTAGCAATGCTGGGGTCATGGAGATGATTAACATCGACTTACTGACGGCAATGCTGCTTGAG
CCACAACTGCCGCAAATCAGTAGCGCCAGCCTGACGGTGGACAAACGGCATTTGCTCTATGGTAATGGGCTGGTAGATTC
CCTTCCGCAACCTGAAGACAATGAAAACTATCAGGTTTCTTCGCAACGCTTTCCTTTTACCATTAACGTTAATGGTCCGG
GGGCTACGGCGCTGGCATGGCACTATCTTCCAACACAATTACCGCTGGCGGTGCTGCTAAGTTTACTGGTGGGCTACATC
GCCTGGCTGGCGACCGCTTACCGAATGAGCTTTTCCCGCGAAATCAATCTGGGCCTGGCGCAACATGAGTTCGAATTGTT
CTGTCAGCCTTTGCTTAATGCGCGCAGCCAGCAATGTATTGGTGTAGAGATTTTGCTGCGCTGGAACAATCCGCGTCAGG
GCTGGATTTCACCGGATGTGTTTATTCCTATCGCGGAAGAACATCATTTAATTGTGCCACTGACCCGCTATGTGATGGCA

GAAACCATTCGTCAGCGCCATGTTTTCCCGATGAGTAGTCAGTTTCATGTTGGCATTAACGTCGCACCCAGCCATTTTCG
CCGTGGTGTGCTGATAAAAGATCTCAATCAGTACTGGTTTAGCGCTCACCCGATTCAGCAACTGATCCTCGAAATCACCG
AACGCGATGCCTTACTGGATGTTGATTATCGGATTGCCCGCGAGCTGCATCGTAAAAACGTCAAACTGGCGATTGATGAC
TTCGGCACCGGCAACAGCTCGTTTTCCTGGCTTGAAACATTACGTCCTGACGTGCTGAAAATTGATAAGTCATTTACCGC
AGCTATAGGTTCTGACGCGGTTAACTCGACGGTGACCGATATCATCATCGCGCTGGGGCAAAGACTGAATATTGAACTGG
TGGCGGAGGGCGTGGAAACGCAAGAACAGGCGAAGTATTTGCGCCGTCATGGCGTGCATATTTTGCAAGGGTATTTGTAC
GCACAGCCGATGCCGCTACGTGATTTTCCCAAATGGCTGGCGGGCAGCCAACCGCCGCCCGCCCGGCATAATGGACATAT
CACGCCCATTATGCCGTTACGTTAACGCTTACTCATCTTCATCGTGCGCCGGTTGCTCTTTAACAATGCGAACCAGATCA
ACACGATAATCATTGGCTTCAATGATGGTGATATGCAGTGGCCCTACATCAATCACATCGCCCACACGGGGAATGTGACC
ATTTGCCGAGATCACGAGGCCCGCGACCGTCGCGATATCATCGTCATCGGCAAGGTGCTCAACATCAAGCGCCTGCTGCA
AGGCATGCAAATCTGTACCGCCTTTTACCAGCCAGCCGTCACCATCAGTAATGATTTCCGGCGTTTCGTCAGCGTCCGGG
AATTCACCCGCAATGGCTTCCAGCACATCCAGCGGCGTGACCAGACCTTGTACCACACCAAACTCGTTGGTCACGATAAC
AAAGCTCCCGCGAGCACGACGCAGCACGCCCAACAGGTTGATCGGATCGAGGGTTTCCGGGACGATAATCGCCGGAGACG
CCGAAGCAATCGCCGCCACATCAACGCCCTCTTCCAGCGCCACCAGCAGTTCTTTAGCACGTACAATACCGATGATTTCA
TCCAGTTCACCGCGACATACCGGGAACAGACTGTGCGGTGAAGAGAGCAGTTGCTCGCGGATTTCATCGACCCCGAGATT
AGCGTCAACCCAGCTTATTTCACCGCGCGGCGTCATGATCCCGCGCAGAGAACGCGACGCCAGCGTCAGTACGCCGTTAA
TCATGTAACGTTCTTCTTCGGCAAATGCACCTTCCGGGATCGGCATCGGCATCGGGTTATCGGCATCGTGCTGAACATTG
GCCTGACGTTTCCCGCCCATCAAACGCAGGATGGCATCGGCAGTACGCGCTCGCAGCGGCAAAGTCGACTGGTGGCGAAT
AAAGTTGCGACGCGCAATCTGGTTAAACACTTCGATGATGATCGAGAAGCCAATCGCGGCATACAGGTAACCTTTCGGAA
TGTGGAAACCGAAACCTTCTGCCACCAGACTCAGACCAATCATTAACAGGAAGCTCAGACAGAGCACCACCACCGTGGGG
TGCTGGTTAACGAATCGCGTCAGCGGTTTGGATGCCAGCAACATAACCGCCATCGCAATCACTACCGCCGCCATCATCAC
CGGCAGATGGTTAACCATCCCTACTGCAGTAATTACCGCATCCAACGAGAAGACGGCGTCAAGGATGACAATCTGTGTGA
CGACCACCCAGAAACTGGCGTAGCCTTTACCGTGGCCGGAATCATGATCGCGGTTTTCCAGCCGTTCATGCAGTTCGGTT
GTTGCTTTGAACAGCAAGAATATCCCCCCGAACAACATAATCAGGTCGCGTCCGGAGAAGGAGAAATCCATGACGGTAAA
TAGCGGTTTGGTCAGCGTGACCATCCATGAAATCAGCGACAGCAGCCCCAGACGCATAATCAGCGCCAGTGATAACCCCA
GCAAACGCGCTTTATCGCGTTGTTTTGGCGGCAGTTTGTCAGCAAGAATGGCGATGAAGACCAGGTTATCGATACCCAGC
ACAATTTCGAGAACAACAAGCGTGAGTAGCCCCGCCCAAATTGAGGGGTCCATTAAGAATTCCATGACAAGCTCCTGCTT
AAGGAATAGCTATTCGACGCCAGAAATAATGCAGGCGTAACGACAAAATGCAAACGAAAGGTGCGGCATAGAGTGCCAGA
AAGGCAGGCGTTAAAAGGCCTGATGCTGAAATGACGTCGGTGACGATCCATACTGCGGGCTACTGCCCTATACTCCATGG
TTGTTAAACGGGAGTTAAACATATCAGAGACGCCTCTGATTTGGCAAAGATTTACCTTCCTTTGCAAACGAATGTGACAA
GGATATTTTACCTTTCGAAATTTCTGCTAATCGAAAGTTAAATTACGGATCTTCATCACATAAAATAATTTTTTCGATAT
CTAAAATAAATCGCGAAACGCAGGGGTTTTTGGTTGTAGCCCTTATCTGAATCGATTCGATTGTGGACGACGATTCAAAA
ATACATCTGGCACGTTGAGGTGTTAACGATAATAAAGGAGGTAGCAAGTGACCATTGCTATTGTTATAGGCACACATGGT
TGGGCTGCAGAGCAGTTGCTTAAAACGGCAGAAATGCTGTTAGGCGAGCAGGAAAACGTCGGCTGGATCGATTTCGTTCC
AGGTGAAAATGCCGAAACGCTGATTGAAAAGTACAACGCTCAGTTGGCAAAACTCGACACCACTAAAGGCGTGCTGTTTC
TCGTTGATACATGGGGAGGCAGCCCGTTCAATGCTGCCAGCCGCATTGTCGTCGACAAAGAGCATTATGAAGTCATTGCA
GGCGTTAACATTCCAATGCTCGTGGAAACGTTAATGGCCCGTGATGATGACCCAAGCTTTGATGAACTGGTGGCACTGGC
AGTAGAAACAGGCCGTGAAGGCGTGAAAGCACTGAAAGCCAAACCGGTTGAAAAAGCCGCGCCAGCACCCGCTGCCGCAG
CACCAAAAGCGGCTCCAACTCCGGCAAAACCAATGGGGCCAAACGACTACATGGTTATTGGCCTTGCGCGTATCGACGAC
CGTCTGATTCACGGTCAGGTCGCCACCCGCTGGACCAAAGAAACCAATGTCTCCCGTATTATTGTTGTTAGTGATGAAGT
GGCTGCGGATACCGTTCGTAAGACACTGCTCACCCAGGTTGCACCTCCGGGCGTAACAGCACACGTAGTTGATGTTGCCA
AAATGATTCGCGTCTACAACAACCCGAAATATGCTGGCGAACGCGTAATGCTGTTATTTACCAACCCAACAGATGTAGAG
CGTCTCGTTGAAGGCGGCGTGAAAATCACCTCTGTTAACGTCGGTGGTATGGCATTCCGTCAGGGTAAAACCCAGGTGAA
TAACGCGGTTTCGGTTGATGAAAAAGATATCGAGGCGTTCAAGAAACTGAATGCGCGCGGTATTGAGCTGGAAGTCCGTA
AGGTTTCCACCGATCCGAAACTGAAAATGATGGATCTGATCAGCAAAATCGATAAGTAACGTATTGTGTTGATTATCACT
CAGTTTTCACACTTAAGTCTTACGTAAACAGGAGAAGTACAATGGAGATTACCACTCTTCAAATTGTGCTGGTATTTATC
GTAGCCTGTATCGCAGGTATGGGATCAATCCTCGATGAATTTCAGTTTCACCGTCCGCTAATCGCGTGTACCCTGGTGGG
TATCGTTCTTGGGGATATGAAAACCGGTATTATTATCGGTGGTACGCTGGAAATGATCGCGCTGGGCTGGATGAACATCG
GTGCTGCAGTTGCGCCTGACGCCGCTCTGGCTTCTATCATTTCTACCATTCTGGTTATCGCAGGTCATCAGAGCATTGGT
GCAGGTATCGCACTGGCAATCCCTCTGGCCGCTGCGGGCCAGGTACTGACCATCATCGTTCGTACTATTACCGTTGCTTT
CCAGCACGCTGCGGATAAGGCTGCTGATAACGGCAACCTGACAGCGATTTCCTGGATCCACGTTTCTTCTCTGTTCCTGC
AAGCAATGCGTGTGGCTATTCCGGCCGTCATCGTTGCGCTGTCTGTTGGTACCAGCGAAGTACAGAACATGCTGAATGCG
ATTCCGGAAGTGGTGACCAATGGTCTGAATATCGCCGGTGGCATGATCGTGGTGGTTGGTTATGCGATGGTTATCAACAT
GATGCGTGCTGGCTACCTGATGCCGTTCTTCTACCTCGGCTTCGTAACCGCAGCATTCACCAACTTTAACCTGGTTGCTC
TGGGTGTGATTGGTACTGTTATGGCAGTGCTCTACATCCAACTTAGCCCGAAATACAACCGCGTAGCCGGTGCGCCTGCT
CAGGCAGCTGGTAACAACGATCTCGATAACGAACTGGACTAACAGGTGAGCGAAATGGTTGATACAACTCAAACTACCAC

CGAGAAAAAACTCACTCAAAGTGATATTCGTGGCGTCTTCCTGCGTTCTAACCTCTTCCAGGGTTCATGGAACTTCGAAC
GTATGCAGGCACTGGGTTTCTGCTTCTCTATGGTACCGGCAATTCGTCGCCTCTACCCTGAGAACAACGAAGCTCGTAAA
CAAGCTATTCGCCGTCACCTGGAGTTCTTTAACACCCAGCCGTTCGTGGCTGCGCCGATTCTCGGCGTAACCCTGGCGCT
GGAAGAACAGCGTGCTAATGGCGCAGAGATCGACGACGGTGCTATCAACGGTATCAAAGTCGGTTTGATGGGGCCACTGG
CTGGTGTAGGCGACCCGATCTTCTGGGGAACCGTACGTCCGGTATTTGCAGCACTGGGTGCCGGTATCGCGATGAGCGGC
AGCCTGTTAGGTCCGCTGCTGTTCTTCATCCTGTTTAACCTGGTGCGTCTGGCAACCCGTTACTACGGCGTAGCGTATGG
TTACTCCAAAGGTATCGATATCGTTAAAGATATGGGTGGTGGCTTCCTGCAAAAACTGACGGAAGGGGCGTCTATCCTCG
GCCTGTTTGTCATGGGGGCATTGGTTAACAAGTGGACACATGTCAACATCCCGCTGGTTGTCTCTCGCATTACTGACCAG
ACGGGCAAAGAACACGTTACTACTGTCCAGACTATTCTGGACCAGTTAATGCCAGGCCTGGTACCACTGCTGCTGACCTT
TGCTTGTATGTGGCTACTGCGCAAAAAAGTTAACCCGCTGTGGATCATCGTTGGCTTCTTCGTCATCGGTATCGCTGGTT
ACGCTTGCGGCCTGCTGGGACTGTAAGACTGTTGTACACTACCGGGGCCTTTTGGCCCCGTTTTTTTATCTGGAGGATTA
ATGACAATCACGGACCTGGTACTGATTCTTTTCATCGCCGCACTCCTGGCCTTCGCGATCTACGATCAGTTCATCATGCC
CCGCCGTAACGGCCCCACCCTGCTGGCAATTCCTTTGCTCCGGCGTGGTCGCATCGATAGCGTTATCTTCGTCGGATTGA
TTGTCATTCTTATCTATAACAACGTCACGAATCATGGTGCGTTAATAACGACATGGTTATTAAGCGCACTGGCTCTGATG
GGTTTTTATATATTCTGGATCCGCGTTCCGAAGATCATCTTTAAACAAAAAGGTTTTTTCTTCGCCAATGTCTGGATTGA
ATATAGCCGAATCAAAGCGATGAACTTGTCGGAAGATGGCGTGTTGGTGATGCAATTAGAACAGCGTCGGCTGTTAATCC
GCGTTCGAAATATCGACGATCTGGAAAAAATTTATAAGCTTCTCGTTTCAACTCAATAAGTTATGAATTTAGCCAAAGCT
ATGTTTAGTGTATTTTTAATAATCAGACATAGCTTAGGCTATATTACCTCTTCCCTTATTTGTTATTTATTTTAACGTTT
CATTGATATATAAATCCAAATGAAAATCGTTATCAATAAAGCAATGAAATAATATATTCCAACAGTTGTTTTATATTCTC
AAAATATGTTAAGGTTGCGCCCTCATTTGGGGAGTAGCCGATTTCCAGATTCCGGAAATGTACGTGTCAACATACTCGTT
GCAAAACGTGGCACGTACGGACTGAATACTTTCAGTCAGGCGAGACCATATGCACATCAATCGCTATGCCTGCATGAACG
CGAAAACCATCACGGCTTGTTGTTCATGTATGCATGTTTGCTGGGGGCGATGATGTGTTTTATGGATACCCCGGTCAGGA
CATTGTCATGAATATCACTGCTACTGTTCTTCTTGCGTTTGGTATGTCGATGGATGCATTTGCTGCATCAATCGGTAAAG
GTGCCACCCTCCATAAACCGAAATTTTCTGAAGCATTGCGAACCGGCCTTATTTTTGGTGCCGTCGAAACCCTGACGCCG
CTGATCGGCTGGGGAATGGGCATGTTAGCCAGCCGGTTTGTCCTTGAATGGAACCACTGGATTGCGTTTGTGCTGCTGAT
ATTCCTCGGCGGGCGAATGATTATTGAGGGTTTTCGTGGCGCAGATGATGAAGATGAAGAGCCGCGCCGTCGACACGGTT
TCTGGCTACTGGTAACCACCGCGATTGCCACCAGCCTGGATGCCATGGCTGTGGGTGTTGGTCTTGCTTTCCTGCAGGTC
AACATTATCGCGACCGCATTGGCCATTGGTTGTGCAACCTTGATTATGTCAACATTAGGGATGATGGTTGGTCGCTTTAT
CGGCTCAATTATTGGGAAAAAAGCGGAAATTCTCGGCGGGCTGGTGCTGATCGGCATCGGCGTCCAGATCCTCTGGACGC
ACTTCCACGGTTAATAAGAACGCTGCCAGAGGTGAATATTAAAGTCCGTCTGGCAGTCGAACACTTCTTTTGCTGCCAGT
GTTTGCCAGACTTCTGGCTTCGCACGCCAGGCAAACGGCGTCATCTGCAATAATGCGACGGCTTCATCACCGCGAAGACG
CATCGGATAACACAACTCCGCACTCTGCTGTAATGTAAAACCTTCCAGTTGTTCTGCATGAGGTGCATGAAGATGTACTT
CATTGTAAATCAGCCCCTTCAGCTCCATCAAATGTCGCGGTCCCGGCGTGGCAGTAATGACCCAGCCGCCGGGCTTCACT
ACTCGTGCTAATTCTTCTGCTTTACACGGCGCGTAAATACGTATTATGGCGTCCATACTGGTATCGGAAAACGGCAAACG
GTGGCTGGAAGCGACACAAAAAGTGACCTGCGGATAGCGTTTCGCCGCCGCTTTTATCGCTACCTTCGAAACATCCAGAC
CAAACGTGGTGATTTCGGGCAACGCATCGGCAAATGCGTGTGTGTAATACCCTTCACCACAGCCAATATCCAGCACCGCC
GTGGCCTTATCATCAAGCCGTTCCCTCAGTTGGGCGACAATTGCATCACGCAGCGGCTGATAATGTCCGGCATCTAAGAA
TGCGCGGCGTGCTTGCATCATTTCCGCGCTGTCGCCCGGATCACGAGACCGTTTATGCTGAACGGGCAGCAGATTGACAT
ACCCTTCTTTCGCCATATCAAACTGATGTCGCTGGGGACAGATATAGCTGTTTTTTTCACGCGAAAGAGGCTGATGGCAA
AGTGGACAGGAAAAAGACATGACAACTCCGGCAGGATACTAAAGGCCGCAAGTGTAACGCGAATCACGCGGCGGGCAAAT
ATCCGAACGCCAGGAGCGAAGATAAAGCATAGTAAAAAGCCTCGCATTCGACGAGGCTTTATAGCGAGATTGAAGCGTAT
TCACACTTCAGATCAGTGGATTCGATCAGATAGCTGTTACGTTAACAGCTGCCGGACCTTTCTGGCCGTCCTGAATTTCG
AACTCAACGTTCTGACCTTCAGCCAGAGTTTTGAAGCCATTACCCTGGATAGCGGAGAAGTGTACGAACACATCTTTGCT
GCCATCAGCCGGAGTAATGAAGCCAAAACCTTTAGACTCGTTGAACCACTTAACCTGACCTTTAATCTTTGCCATTTGAA
AAATTCCTTAGATTGTTTTCTTCGCCCGCAGGCATAACATAGATAAAACTGACACATTACTGCATGAGGCACCAATATAA
GGCTCGGCAGAGAAGCGGTATTCAACGTCAACGTGTTTACTCAGGACTTCTTTACTGAAAATGCCACACATAAACAGAAC
TGTACCTCGTTTAACCCGAAATCTGTTATCACATACAACGTTAATTATGGCAAGCCATTTTTAAACATGTCTCGATCAGA
CGCACAAATCTCGATACGTCTTCCACTTTTTTTGCACACTTATGCAACGGAATACGCGCCGATATATCATTGTGCTTAAC
CTTGCCAGTTCAGGCAGATACTTAAACTGGCGTATTTTCTAACATAGTTCAATAAGTTCAGTTGTTCCAGGCCAGTGGGA
GAAGTTATTACATAGTGCGTGCAATATCACATTTTTTGCTATGCAATGAATAAAAAGTTATATCACTTTTTCTCATAAAA
CAGTCAGTTAACGGCTATTAATTACCCTAAAGAGAAGTCAATCCCCAAAGGGATTGTAAATTTAAAATAAGAAAAATTGA
TGAATGAGCAAAAAAATCAAGAGAGAAACGTTTCGCTAATAAATAATACCACGCTTAATCACAAATCCGATGTAAACATC
CTAATAAATTAATGGGGTAAATATCTTTTTCTGATATCCCGTTACGCAAATAAATAATATGATAATTATTTGAAGTCAGA
CCAGAGTTTTAATTTGAAGCAGTTGCACCATGACAGTGCGTATAATCAGGATGAATTGAGTAACCCTTCAACGACGGGGT
TACTCAAGATTGAGGAAGGATTATCGTTCGGCGACCAGACGAATAATATCTTCGTCTTCAACCGATTTTTGACTCTCAAC
GGTCTTTTTATCTGCTTTCTCTGGTTCGTTTGCTTCGCACAAACGACGTAACAGTGCATTTTGACGTTTTTGCTGATCCA

GCAACGCCTCAAGCAGTTCAATCTGTTCGTTAGTCCGTGAACTGGCACGATTGATAAAAAACCACAAGATGAGCCCGATA
AGAAGAACCACCACCGATACAACCAAAGACGCAATGTTCATCACGCCTGAATTTACAACTTCGTTCATTTCACCACCTCT
GAGTAGAGGCGCTATTCTACCACTGCTGGAGAGGAAGAAAATCTAGTGCTGAAAAAATGATATCACCACGGGATAAACTG
GTTAATGGCACAAATTCCGCTGAAAAATTGTACATCCTGATCGCACATCATGTTGAATACCTGCGCCCAAAGCAGCAAGC
AAGCCAACACCACGACAACCAGAACGACCCATCGAAACTTTTTCACTCCAATCTCCGTTTCACCTACCTTATGTCATTAA
ACTAGCATGCGTTAGTTAAACAGCGCCTAACTATGTCGATATTCGTGCTTTTTCGGAATGAGTCGCTTGCTTCAGCGCAT
ATTGCCGCTACGATTAAGCGAACATAAAAAGAGAAGAGGTTGTAATGCGATTCATCATTCGCACAGTTATGCTGATAGCA
CTGGTATGGATAGGATTATTACTCAGCGGATATGGCGTTTTGATTGGAAGCAAAGAGAATGCCGCAGGATTAGGTTTGCA
ATGTACCTATCTGACTGCCAGAGGCACCAGCACGGTACAATACTTGCACACTAAAAGTGGTTTTTTGGGGATAACAGATT
GCCCGCTGTTACGCAAAAGCAATATCGTGGTCGATAATGGTTAAGGGCAATAAATAACCAGATTCTCACTCCTGAGTAAA
ATACTCTTATAGCAAGCGCGTTTAATATAACGCCACCTTCAACTTTATTTTTCTCATGCTAAATAAAGAACCTGTCTGTT
TGATATTCCGACAGGTTCTTACTCTCATCAGAACGGATAGTCGTGATAACCCATTTGGGCAGAAATTTTGCGCGCTGCGG
TATGCAACATTGCGACATATTCCTGTAAACGCTCTTCAGAGAAACGCAACGTCGGGAAGGAGATGCTCAAACCGGCAATG
ACCACGCCAAAGCGATCAAATACCGGTACCGCAATGCATCGCAGCCCTTCTTCCTGCTCTTCATTATCTTCGCCATACCC
CTGCTCGCGCACCTGGTCCAGAACGGGTAATAACGCTTCTGTACTGGTGATGGTCCGCTCGGTACTGCGTTTATACTCCA
CGCCCTCAAGAATTTGCTTCACTTCATCGCGATCGCGCCATGCCAGCAGTACCTTACCAATCGCGGTGCTGTACAGCGGA
TTACGACGCCCAATCCGTGAATACATGCGCAAATTGTACATAGAGTCAATTTTGTGAATGTAAACAATACTGTCTTCGTC
CAGTGCGCCGAGGTGGATAGTTTCTTTGGTCAGGCGGGAGAGCTCACGCATCTGGATATCTGCGCTACGAATTAAATCGA
CGTTTTGTAACGCGCGAGCGCCCAGTTCAAACAATTTCAGGGTCAGGGAATATTTCTCCGACTCCCCTTCCTGCGCCACA
TAACCTAAGGTTTTCATGGTCTGTAAAAAGCGATAAACGGTGCTTTTTGACATCATGACGCGCTGCGACAGCTCGGTTAT
CCCTATTTCGCGCTCTTCACCCAGCGCCTGCAAAATGCCAAAAACTTTTAGCACGGAAGATACAGAATCAGGCTGTTTAT
CCAGATCTGCGTTAGCCATTTATCACCTCATTGCGAGTGTTTTATAAAAATCAGAACTGTTTTTTATTATAATTTCGCAC
CAGGGTGGTCGCAATCCATCTTTTGCCGGTTAGTTACAATTCTGCGACATCCACCGTGAATATCAGTGCTAGAATCATAC
CCCTGTTGATTATTCACCAAAGATATAAAATTCCTATGCCAAAAGTTCAGGCCGACGGCCTGCCATTGCCCCAGCGATAC
GGTGCGATATTAACCATTGTGATTGGTATTTCGATGGCCGTCCTTGACGGCGCAATCGCCAACGTCGCCCTGCCAACAAT
CGCCACGGACCTTCATGCCACGCCAGCCAGTTCCATCTGGGTAGTGAACGCCTATCAAATCGCCATTGTCATCTCCCTGC
TCTCGTTTTCGTTTCTGGGCGATATGTTTGGCTATCGACGTATTTATAAATGCGGTCTGGTCGTTTTTCTGTTGTCTTCA
CTGTTCTGCGCCCTTTCTGATTCGCTGCAAATGCTCACCCTTGCGCGTGTCATACAAGGTTTCGGCGGTGCAGCGTTGAT
GAGCGTTAATACCGCACTTATCCGCCTGATCTATCCACAACGTTTTCTGGGTAGAGGGATGGGCATAAACTCGTTTATTG
TTGCCGTCTCTTCTGCTGCCGGGCCGACAATTGCTGCAGCAATCCTCTCCATCGCATCCTGGAAATGGTTATTTTTAATC
AACGTACCGTTAGGTATTATCGCCCTGCTTCTGGCGATGCGTTTTCTGCCACCCAATGGTTCTCGCGCCAGTAAACCCCG
TTTCGACCTGCCCAGCGCCGTGATGAACGCGTTAACCTTCGGCCTGCTTATCACTGCGTTGAGTGGTTTCGCTCAGGGGC
AATCGCTGACGTTAATTGCTGCGGAACTGGTGGTAATGGTTGTTGTTGGTATTTTCTTTATTCGCCGCCAGCTTTCTCTT
CCCGTACCGCTGCTACCGGTGGATTTACTGCGTATCCCGCTGTTTTCACTTTCTATTTGCACATCTGTTTGCTCTTTCTG
CGCACAAATGCTGGCAATGGTTTCCCTGCCCTTTTACCTGCAAACCGTGCTCGGGCGTAGTGAAGTCGAAACAGGTTTAC
TTCTGACACCGTGGCCGTTAGCAACGATGGTGATGGCTCCGCTGGCAGGCTATTTGATTGAACGCGTACATGCAGGATTG
CTGGGGGCTTTAGGGTTGTTCATCATGGCTGCGGGGCTTTTTTCCCTGGTTCTGCTGCCCGCGTCACCTGCGGATATCAA
TATTATCTGGCCGATGATCTTATGTGGTGCTGGATTTGGCTTATTCCAGTCACCCAATAACCACACCATTATTACCTCCG
CGCCTCGCGAACGTAGCGGTGGAGCCAGTGGCATGTTAGGAACGGCTCGTCTACTGGGTCAGAGTAGCGGCGCGGCGCTG
GTGGCGCTGATGCTAAATCAGTTTGGAGATAATGGTACACACGTCTCGCTGATGGCTGCGGCTATTCTGGCAGTGATTGC
TGCCTGTGTCAGTGGTTTACGTATCACTCAGCCACGATCCAGGGCATAAAAAAAGCGCGTCGATCAGGACGCGCTTTTTA
GTATTTACTTCATATTACTTCAGGTATTCACCCGTACGCAGAGCTTCAATTCGTTTATCCAGCGGCGGGTGGGTCATGAA
CAACTCACTGAGCGATTTCGACTTACCGTTAATGCAGAGAGCCATCATGCTGGTTGCTTCTTGCGGTTCATAGCTGGTTT
TCAGGCGCTGCAGCGCGGCAATCATTTTCTCGCGACCAACCAGTTTTGCCGAACCGGCATCAGCATGGAATTCACGATGA
CGCGAGAACCACATGGTGATAATGCTCGCCAGAATACCAAACACCAGTTCCAGAACCGTTGCAACCGCAAAGTAGATCAG
CGGGTTGCCGTTGCTCTCTTCACCTTCATCACGATTTCCGCCCATAAAACCCGCGGCAAGCTGCGCCAGAATACGGGAAA
TAAAGATAACGAAGGTGTTCACCACGCCCTGAATCAGCGTCATGGTGACCATATCACCATTGGCGATGTGGCTGATTTCG
TGAGCAATTACCGCCTCGGCTTCATCCGGGCTCATGTTCTGCAGCAAACCGGTGCTGACAGCAACCAGAGAGGCATCACG
GCGCGCACCGGTTGCAAAAGCGTTGATGTCCGGCGCATGGTAGATAGCCACTTGCGGCATAGCGATCCCCGCCTGACGAG
CCTGGGTTGCTACAGTATTGACCAGCCAACGTTCCCTTTCGTTACGCGGTTGCTCGATCACTTCCCCGCCAACAGATCGT
AATGCCATCCATTTGGACATCAGAAGCGAAACGAAGGAACCACCAAAACCGAACAGCAAGGCCATGATCATCAGCCCCTG
AACGCTGCTCGACTGTATCCCTGTCAGGCTCAGTACCAGCCCGAAAACGACCATTACGGCCAGGTTCGTTAGCAGGAAGA
GCGCGATTCGCATCATAATTTTCTTTTTACCTCAGTTTAACAAAACGCAATATGCGATACCCACATCGTATGGGTTACGC
GACTATTTTCAAGTCTGGATAGTGCGTAAGTCACCAGAAAGACACAACTTTACATTTTGTAGCATCTGATTTACGGCATC
TTGTCGCTGTTAAAAAATCAGGCACAATTTCTTGTGCCTGATTGATATTACTTGACGGGAGCGGGTTGTTCCGCGGGTCT
GGCTTTTTCAAGCTTCGCCAGATCGAGTGCGATATTCACCGTCTCATCCAGATAAGGATCCGGCTCCTGGTAATCTTTCG

GTAGATCATCCAGTTTCTTCAACTCCGGTTTACCTTCGCGTTTAAAGCGTTCGTTCAAACGCGCCAGACGCGTCGCATCA
TCTTCATTATTCTCTTTCTCACGCACAGCGTAATTCAGAGAAACGATATTGCGCTTGTCCTTCATAGCGTTGAAGCGCGC
GATATCCTTCATGATGTTCTGGAACTCAGGATCTTTCGCGATACGCGCATTATGTTCCTTCAGCAGCTCCGGTTCAAAGG
CCGTTAAATCTCCTGATTTCACATAAGTCGCGGCATCAATGCTATCCCACGGCAGCGCGTTATCTTCGAATTTCTCACCC
GTTTCCGTTTCTTCATTACCCGTCGGCATGATGATGTCTGGCGTTACGCCTTTACGTTGCGTACTGCCGCCGTTAACGCG
ATAGAATTTCTGGATCGTGTACTGCACAGAACCCAGCGCTGGCCATTCAGGACGTAACATCTGATCGTAAATACGGTTCA
ATGAACGGTATTGCTGAACGGTGCCTTTACCAAACGTCGGTTCACCCACAACCAGCGCACGACCGTAATCCTGCATTGCC
GCGGCAAAGATTTCTGAAGCCGAAGCACTGAAGCGGTCAACCAGCACCACCAGCGGGCCTTTATAGAAAACCTGTCCGTC
GGTATCGCTATCTTCACGAACCTTGCCGTTGTTATCGCGGACCTGAACAATGGGACCCGCAGGAATAAACAGACCGGAGA
GCGATACGGCTTCAGTTAACGCCCCACCGCCATTGCTACGCAGGTCGATGATGACGCTGCTGACATTCTGTTTTTCCAGT
TTCTGCAGTTGCACTTTGACATCGTCTGTCAAACCCACATAGAAGCCCGGAATATCCAGCACGCCGACTTTCTCTTTACC
GACGGTCTTCACCGACATTTTAACCGCGCGGTCTTCGAGACGAATACGTTCACGGGTCAACGTTACAGTACGGGTCTTGG
TCCCTTTACCAGCAGGTAAAATTTCCAGACGAACTTTACTGCCCTTCGGCCCTTTAATTAAGGCAACCACATCATCAAGA
CGCCAGCCAATCACGTCAACCATCGGCTTGCCTGTTTGACCAACACCGACAATTTTGTCACCAACGCTGATAGCTTTACT
CTTCGCTGCCGGACCACCTGCCACCATCGAATTGATAACGGTGTAGTCATCATCCATTTGCAGCACTGCGCCAATACCTT
CCAGCGACAAACTCATTTCAGTGTTGAACTGTTCGGTATTACGCGGGGAAAGATAGTTGGTATGCGGGTCGATTTCACGC
GCAAACGCCGTCATTGCCAGCGAGAAAACATCTTCGCTGTTGGTTTGCGCCAGACGACGAATGGCAAATTTGTAGCGGCG
AGTCAGGGTTTCACGAATTTCTTTATCCGTTTTTCCTGTCAGCTTCAGGCTTAACTCGTCGAATTTGACTTTACTGTCCC
ACAGCGCGTTCAACTCAGCCTCGTTTTTCGGCCAGGGCGCTTTGCTGCGGTCAAGGTTATAAGTGTCGTTGCCGGTGAAA
TCCATCGGCTTTTCCAGTACCGACAAAGCGTACTGGTAACGCTCAAAACGGCGCTTTTGCGCCAGATTGTAGAGATCGTA
GAAAACGTCGAGTTTGCCTGAACGCAGTTCATCGCCTAACTCGGTTTTCTTTTTCGCGAACTGTTCAACATCGCTTGCCA
GCAGCACGTTGTGGCTGTAATCGAGCAGATTCAGGTAGCGGTCAAAGATTTTGGCCGAAAATGCCTGATCGAGGTCGAAC
TGGCGATAATGAGAACGGGTGAAGCGCGACGTTACGCGCTCACTTACCGTCGCATGCTGCGTCTCTTCCTTTAATACCGG
AATTTGATCAGCACGCGTGATATCTTCTACAGCGAAGGTCTGGCCTGCTATTGCAAGCAGGCCAGCTAACGCGGTAAGCC
TAAAAAACATGTTCATGCCTGGCCCGGCCTCCGTTTCAGAACACCAGGTGTTCTGCGCGCACAATCAAAGACATACCCGA
ATTCAGCTGGACGCGGACGCCGTCTTTGGTGATTTCTAATACGGTGGCATCCATCGCGTTTTGACCCGCTTTCACCTTCA
GGGCTTGTCCGACAGTCAGAGCTGAAATGTCAGAAACCGGGGTGTGCTGTTCTTCGCGAGGTGCTTTTACTGTTTTTGGC
GCTTTCTCTACCGGCTTTTGCGCACGAGGTTTACGTTCAGCGCCTTCTTTGCGGCGTGGCGTAGTCGGACGTGGCTTGCG
TTCGCGGCGCGGTGCGTCTTCTTTCTCACCAGCAGTTGCGGCAGCTTCGCGTTTTTTCGCTTGCTGTTCAGCACGCTGTG
CCTGAACACGCGCTTTCGCTTCTTCAAGCTGCTTGCGAGCATGCTCTACATGTTGCTCGTCCAGCTCACCGCATGGGTTG
CCGTCAAGATCGACACGCGTTGCGCCGGGTTTAACACCGTAAAGATAACGCCAGCTCGAAGTGTAGAGACGTAAAGCGGA
TCGCAATTGCGTTTTGCTCAGGTTCATTTCCCCAGCAACACGATCGACCAAATCCTGAAAAATACCGATTTTCAGCGGAC
GCGCTTCACCTTCCGCACTGAAACAGTGGGGAAAACGTTCGGCCAGAAACGCGATTACTTCTTTACTGCTATTCAACTTA
GGTTGATTTTCCATGAAATTTCCTGATTACAACGGACGTAGCCAACAAGCGCAGGCATGAACAGGCGTCATTATAATGAC
GCTATCAGTAAATGCTACGTTATCCGTTGATTATCCTGCGACGCTCGCAAAGAATTTTTTGTAATCCGTCGTTGCAAGCA
CTTTTTCAAGCTGTGCCACAAGCTGACGTAAGCCTTGCTCGTCCTCGTCTGTAAAGCGACCGAAGACGGTACTATCGATG
TCGAGAACACCAATAATCTGATTTTTCACCACCAGCGGCAGAACAATTTCAGAATTACTCGCCGCATCACAGGCAATATG
CCCGTCAAACACATGAACATCCTCGATACGCTGCACTTGATTGCGGGCAACCGCAGTGCCGCACACGCCGCGCCCGACGG
GTATCCGGACACAGGCAATTTTGCCCTGAAATGGTCCGAGTACCAGTGTATCGTCCTCAAGCAAATAAAAACCTGCCCAG
TTTATGTCAGTGAGACGCTCATATAACAACGCACTGGTGTTCGCAAGCGTTGCCAGAAAACTGGTTTCTCCCGCCATCAG
CGCGTTAAAGTCGCGATTTAAATCCGCGTAAAATTCTGTTTTGTTCATTATATAATCACTTGGTTGTCTTACCTGGATCT
GCCAGCCTATTAAAATAAGCATTAAATGCGTTAATGCTCAAGATCATTCCCATCATGGGTTAAGATTAATGTTAATTCTT
ATTACATTTGGCACGTCATGGCTCTTAACACACCACAAATTACGCCGACAAAAAAGATAACAGTGAGGGCAATCGGCGAG
GAACTGCCGCGTGGTGATTACCAACGTTGCCCGCAATGTGACATGCTGTTTAGCCTGCCCGAGATAAATTCTCATCAAAG
TGCCTATTGTCCGCGCTGTCAGGCAAAAATTCGTGACGGGCGCGACTGGTCGCTAACGCGCCTGGCGGCAATGGCCTTCA
CCATGCTGTTGCTTATGCCGTTTGCCTGGGGCGAACCGCTGTTGCATATCTGGCTGTTAGGCATTCGTATTGACGCCAAC
GTTATGCAAGGCATCTGGCAAATGACCAAACAGGGCGATGCGATAACGGGGTCGATGGTCTTTTTCTGCGTTATTGGTGC
CCCGCTCATTCTGGTGACCTCCATAGCTTATTTATGGTTTGGTAACCGACTGGGAATGAATTTACGTCCGGTACTGCTGA
TGCTTGAGCGACTTAAAGAGTGGGTAATGCTCGATATCTACCTGGTCGGCATTGGCGTTGCTTCTATAAAGGTACAGGAT
TATGCCCATATCCAGGCTGGTGTCGGCTTGTTCTCTTTTGTGGCGTTGGTGATTTTAACGACGGTGACGTTGTCACATCT
TAATGTCGAAGAACTGTGGGAGCGATTTTATCCGCAGCGCCCCGCTACGCGTAGGGACGAGAAACTTCGTGTCTGTCTTG
GGTGCCATTTTACCGGCTATCCAGATCAGCGTGGTCGCTGCCCGCGTTGCCATATCCCGCTACGCCTGCGTCGCCGTCAT
AGTCTGCAAAAATGCTGGGCGGCGCTGTTAGCGTCAATCGTTTTATTGTTACCTGCCAACCTGTTGCCTATTTCTATCAT
TTATCTGAACGGAGGACGGCAGGAAGATACAATTCTTTCCGGAATTATGTCGCTGGCAAGTAGCAACATTGCGGTTGCAG
GAATCGTGTTTATCGCCAGTATTCTGGTACCGTTTACTAAAGTGATCGTCATGTTCACTTTACTGTTGAGCATTCATTTT
AAATGCCAGCAAGGTTTACGCACACGCATTCTGTTACTGCGGATGGTGACCTGGATTGGTCGCTGGTCGATGCTCGACCT

GTTTGTCATATCTTTAACCATGTCGCTGATTAATCGCGATCAGATCCTCGCTTTTACTATGGGACCGGCTGCGTTTTATT
TCGGCGCAGCGGTAATTTTGACTATTCTTGCTGTGGAATGGCTGGACAGCCGCTTACTTTGGGATGCACATGAGTCAGGA
AACGCCCGCTTCGACGACTGAAGCGCAGATTAAAAATAAACGCCGTATCTCACCTTTCTGGCTGCTGCCGTTCATCGCGC
TAATGATTGCCAGTTGGCTGATTTGGGACAGTTATCAGGACCGGGGTAATACCGTCACCATCGACTTTATGTCGGCGGAT
GGTATTGTTCCAGGCCGTACGCCTGTTCGTTATCAGGGCGTTGAAGTCGGAACAGTGCAGGATATCAGCCTCAGCGACGA
TCTTCGTAAGATTGAAGTCAAGGTCAGCATCAAGTCCGATATGAAAGATGCGCTGCGCGAAGAGACTCAGTTCTGGCTGG
TGACGCCAAAAGCATCGTTGGCAGGTGTCTCCGGGCTGGACGCCCTCGTCGGTGGGAACTATATCGGCATGATGCCGGGT
AAAGGTAAAGAGCAGGATCACTTTGTCGCACTCGATACCCAACCGAAATATCGGCTGGACAATGGCGATCTGATGATCCA
CCTGCAAGCCCCCGATCTCGGTTCGCTGAACAGCGGTTCATTGGTCTATTTCCGCAAGATCCCGGTGGGAAAAGTCTACG
ACTATGCCATCAATCCCAACAAGCAAGGCGTGGTGATTGATGTCCTGATCGAGCGGCGTTTTACCGATCTGGTGAAAAAA
GGTAGCCGTTTCTGGAACGTTTCCGGCGTTGATGCCAACGTCAGTATCAGTGGCGCGAAGGTGAAACTGGAAAGCCTGGC
GGCACTGGTTAACGGTGCGATTGCCTTCGATTCACCAGAAGAGTCGAAACCTGCCGAGGCGGAAGATACCTTTGGTCTGT
ATGAAGATCTGGCCCACAGCCAGCGTGGCGTAATAATAAAACTGGAACTGCCGAGTGGGGCCGGATTAACCGCCGACTCG
ACGCCGTTAATGTATCAGGGGCTGGAAGTCGGACAGCTGACTAAACTGGATTTAAATCCTGGTGGTAAAGTCACAGGGGA
AATGACCGTTGATCCCAGCGTCGTTACCCTGCTTCGTGAAAATACCCGCATCGAATTACGCAACCCGAAATTATCCCTTA
GCGATGCTAATCTCAGCGCCCTGCTGACCGGCAAAACCTTCGAGCTGGTGCCCGGCGATGGCGAGCCACGCAAAGAGTTC
GTTGTTGTGCCAGGCGAAAAAGCACTGCTGCATGAACCTGATGTTCTGACGCTGACCCTGACCGCGCCGGAAAGTTACGG
TATTGATGCGGGTCAGCCGCTCATTCTTCACGGCGTGCAGGTAGGCCAGGTTATCGATCGTAAACTCACCAGCAAAGGCG
TCACCTTTACCGTCGCCATCGAGCCTCAGCATCGCGAACTGGTAAAAGGCGATAGCAAATTTGTCGTCAACAGCCGTGTC
GATGTGAAGGTGGGGCTGGATGGCGTTGAGTTTCTCGGTGCCAGCGCCTCAGAATGGATCAATGGCGGGATACGTATTCT
GCCGGGCGATAAAGGTGAGATGAAAGCCAGCTATCCACTGTATGCCAATTTGGAAAAAGCGCTGGAGAACAGCCTTAGCG
ATTTACCCACCACAACCGTGAGTTTGAGTGCAGAGACGCTGCCGGATGTGCAGGCAGGATCGGTAGTGCTGTACCGTAAA
TTTGAAGTTGGTGAAGTGATTACCGTGCGTCCGCGAGCTAACGCGTTTGATATCGATCTGCATATTAAGCCGGAGTATCG
CAACCTTCTGACCAGCAATAGCGTGTTCTGGGCAGAAGGCGGGGCGAAAGTTCAGCTGAATGGTAGTGGTCTGACCGTAC
AGGCATCCCCGCTCTCCAGAGCATTAAAGGGAGCCATTAGCTTCGACAACCTCAGCGGTGCCAGCGCCAGTCAGCGTAAA
GGCGATAAGCGTATTCTGTATGCTTCCGAAACAGCGGCCCGTGCGGTTGGCGGGCAGATTACGCTTCACGCTTTCGATGC
CGGAAAACTGGCGGTCGGGATGCCAATTCGCTATCTCGGTATTGATATCGGGCAAATCCAGACGCTGGATCTGATTACCG
CACGCAATGAAGTACAGGCAAAGGCGGTACTTTACCCGGAATATGTCCAGACCTTTGCTCGCGGTGGTACGCGCTTCTCA
GTGGTCACACCGCAAATTTCGGCAGCTGGCGTTGAGCATCTTGATACTATCCTCCAGCCGTATATCAACGTCGAACCAGG
CCGGGGCAATCCTCGCCGCGACTTTGAATTACAAGAGGCCACCATTACTGATTCGCGTTACCTGGATGGCTTAAGCATTA
TTGTTGAAGCGCCGGAAGCCGGTTCGTTAGGCATCGGTACGCCTGTGCTGTTCCGTGGTCTGGAAGTCGGTACGGTTACA
GGAATGACGCTGGGGACATTGTCAGATCGCGTGATGATTGCGATGCGCATCAGTAAACGCTATCAACACCTGGTGCGTAA
CAATTCCGTCTTCTGGTTGGCATCGGGTTACAGTCTGGACTTTGGTCTGACGGGCGGCGTAGTGAAAACCGGCACCTTTA
ACCAGTTTATCCGTGGCGGCATCGCCTTCGCCACGCCTCCGGGTACGCCACTGGCACCGAAAGCCCAGGAAGGCAAACAC
TTCCTGTTGCAGGAAAGTGAACCGAAAGAGTGGCGTGAATGGGGAACTGCGCTTCCCAAATAATGCCCACTGCTCCGGCG
TGCCTGCGCCGGAGCGTTTATGCTAAACTGCGCGCCTGTTTTTTTGCCAGTGGTACATGCTCGTGGCCCAACACACCGTT
TATTTCCCGGACGCCTTTCTGACACAAATGCGCGAAGCGATGCCTTCGACGCTCTCATTTGATGATTTTCTTGCCGCCTG
TCAGCGCCCGTTGCGCCGCAGCATTCGCGTTAATACGCTGAAAATCTCCGTTGCTGATTTCCTGCAATTAACCGCTCCTT
ATGGCTGGACGCTTACGCCAATTCCGTGGTGTGAAGAAGGTTTCTGGATTGAACGCGACAATGAAGATGCATTGCCATTG
GGTAGTACCGCCGAGCATTTAAGTGGCCTGTTTTATATTCAGGAAGCCAGTTCAATGTTGCCTGTTGCCGCCTTGTTTGC
TGACGGTAATGCACCACAGCGGGTGATGGATGTCGCTGCCGCGCCTGGCTCCAAAACGACGCAAATTTCCGCGCGGATGA
ATAACGAAGGGGCAATCCTTGCCAATGAGTTTTCCGCCAGTCGGGTAAAAGTGTTACATGCCAATATCAGCCGCTGTGGT
ATCAGTAATGTTGCGCTCACACATTTTGATGGCCGCGTGTTTGGTGCGGCAGTGCCAGAAATGTTCGATGCCATTTTGCT
GGACGCTCCCTGCTCTGGCGAAGGCGTGGTGCGTAAAGATCCCGATGCGCTAAAAAACTGGTCACCAGAAAGCAATCAGG
AAATCGCAGCTACACAACGGGAGCTTATCGACAGCGCCTTTCATGCATTACGTCCTGGTGGTACGCTGGTTTACTCGACC
TGTACCTTAAACCAGGAAGAAAACGAAGCCGTTTGCCTGTGGCTGAAAGAGACTTACCCCGACGCAGTAGAGTTTTTACC
ACTTGGCGATCTCTTCCCTGGTGCAAACAAAGCGCTGACCGAAGAAGGCTTTTTGCATGTTTTCCCACAAATTTACGACT
GCGAAGGCTTCTTCGTTGCTCGTCTGCGTAAAACTCAGGCGATTCCCGCCTTACCCGCCCCCAAATACAAAGTCGGTAAT
TTTCCGTTCAGCCCGGTGAAAGATCGCGAAGCTGGACAAATTCGTCAGGCGGCTACAGGTGTTGGCTTAAACTGGGATGA
AAACCTGCGCCTCTGGCAGCGTGACAAAGAACTGTGGTTGTTCCCGGTGGGCATTGAAGCCCTGATCGGTAAAGTCCGAT
TTTCTCGGTTGGGGATTAAACTTGCCGAGACGCACAACAAAGGTTATCGCTGGCAGCATGAAGCGGTTATTGCTCTTGCC
TCACCCGATAATATGAATGCATTCGAGCTGACACCGCAGGAAGCGGAGGAGTGGTATCGCGGGCGCGATGTTTACCCGCA
AGCCGCGCCAGTGGCGGATGATGTATTGGTTACTTTCCAGCATCAACCGATTGGTTTAGCCAAACGGATTGGTTCGCGAT
TGAAAAACAGCTATCCGCGTGAACTGGTGCGCGATGGGAAACTTTTTACCGGTAACGCCTGACAGCGCACAAAAAAAGCG
CACTTTTTGACTGGCACATTCGGCTGCCTCAACTAGGCTGAAAAATGGTGCGATCGGACTGGTCGTACCACAATCGGCAG
CTAAATGGAGAGCACGAAGATGAAAACCAGTGTGCGCATAGGCGCTTTTGAAATCGACGACGGCGAATTACACGGTGAAT

CGCCAGGTGATCGAACGTTAACCATTCCTTGTAAATCTGACCCCGATTTATGTATGCAACTGGATGCCTGGGATGCTGAA
ACCAGTATCCCTGCCCTGCTAAATGGCGAACACTCTGTCCTTTACCGTACCCGTTACGATCAACAATCTGATGCCTGGAT
TATGCGTCTTGCCTGATCCAAAAAGAACCCGTCGGCATGGCGGGTTATTTGTCCTGGTTATTCCCCCGTTGTAAAATCTC
TCCTAAACTTAACGGTACGGCACCACACTTCGGGGATGAAATGTTCGCGCTGGTACTTTTTGTTTGCTACCTGGATGGCG
GTTGTGAAGATATTGTTGTGGATGTCTACAACACGGAACAGCAGTGTCTTTATTCTATGAGCGATCAACGGATCCGCCAG
GGCGGTTGTTTTCCGATTGAGGATTTTATAGATGGTTTCTGGCGACCTGCACAGGAGTACGGTGATTTTTAATTATTGCA
ATTGCACAAGAGTCAGTTCGCCCCCAAAGACAGCACCGGTATCAATATAATGCAGGTTGCCAATATCCACGCGATGTCGC
AACGGTGTATGACCAAACCAGAAATGATCAGCACCTGTAATTCCCTGCCCTTTTTGGCGTTCACCTAATCGCGAGCGGCT
CCACAAGACCTGATGCAAATCAACGTCCTTTTGCCATTCATAAACATCATCTGGATAATCGGCATGAGCAATAACATGCT
TGCCGGTGCGACTGTGTACTTCAAGAATAAAGGGCAAATGCTGACATTTTTCCAGCGCCGTTTTCGCTTGTTTCTGTTGA
TTATCTGCCAGCGCAATAAACCAGTCGCCGCCATTCATCAACCACAAAGACATCTGCTGGGATGCCAGCGCATCCATCGC
CATCTGTTCATGATTGCCTCTTACCGCACAAACCCAATGTTGTTCCAGTAACTGCAGACAACGTAAACTTTGCGGCCCAC
GATCGATAACGTCTCCCACTGAGATAAGTAAATCTCGCCACGGATCAAAACGACAATGCCATAATTTGCGGCGCAACTGC
TCAAGACAACCGTGTATATCGCCAGAAAGCCAGATATGTCGCCATTGATGACCCGCAATTCTCTGATAAACGGGCGCAGG
CTGTTTCATCAATATTTTCCTCCCGCGCTAAAGATCACATAATCTTAACAAGAATGTTAAAAAACGCTGGACTCAGACAG
TAGAGTGTGTGTTATGGTTGACTATAAAGTCAGCGAAGGAAATGCTTCTGGCTTTTAACAGATAAAAAGAGACCGAACAC
GATTCCTGTATTCGGTCCAGGGAAATGGCTCTTGGGAGAGAGCCGTGCGCTAAAAGTTGGCATTAATGCAGGCTTAGTTG
CCTTGCCCTTTAAGAATAGATGACGACGCCAGGTTTTCCAGTTTGCGTGCAAAATGGTCAATAAAAAGCGTGGTGGTCAT
CAGCTGAAATGTTAAAAACCGCCCGTTCTGGTGAAAGAACTGAGGCGGTTTTTTTATTGGAAATCAAAAGGCTATTTTAG
GTAATTAACAGAGTTTTTCAGCTCGTTCTATAAACGGTGCCAGACTCATTTTTTCGCCGGGATTGTTAGGATCATCAATC
TGAATCACCGAAATGGGTTGGGCATTGGTCTTCCCACTGGCAACTTCCTTTTGTGCGATATCGTTTAAAGGATACTGCAC
GAGGGTACTCGGATTAATAACATACAAAGCATTACCCGGTCGGCAAGTCAGCATCACCTCTTCGCGATTAAACGCCCATT
TGTCTTTACCCACTTCAAAACGACTGACGGTAATCACCTGCGGTGCAGCCAGCGCCGCTGCAGAACTGGTGAGTAACAGA
AACGCCAGAATACTTTTTTTCATCATTTTCGTAATCCATCAAAAAGGTTCGAGAGTCGCAAGCAGGCTGATGATCACCAG
CACTACCGCACCTATCGCCCATTCGAGTTTAGTCATCCACACAAACCAGGGTGCCGCACGATCTTCATCCTGCCTCATGC
GCGGTACAAGAACATAACGATTAGCCAGCGCAATGACCACCATAATCATCACCAGGATGGCTTTGAGCAATAAGAGTTGG
CCCCAGTAAGTCGTAAGCGTGGGGGGAAATCCGGTAATCAACAATGCATTAAGCACGCCGCTTGCCAGTACGCCGATCAC
CGCAAAATGCCCGCACCAGGAAAAACGCATCAGCGCCTGAATAGCCTGGTGTCGCCAACGGCCTTTGATGAGTTGCATAC
ACCAGAGCACCGGCAGTAATCCCCCAAACCACGCCGCCGCACAAATCAGGTGAATCGCGTGATTAGTCTGATGGATTTTC
GCTGTTACCCCTTCATTCAGCGTCGCATGCCCCACTCCCGCCAGCAGAATAAATTGCGCGGTAGTGAGCATAAACAGCAA
ACGTGGCATATTCCGCGGTTGCATAAGGGCGACGATCAACGTTACGAGGGCGAGAACAATTTGCCATAACCAGATGCCAC
CAAACTGCGTTTGCAAAACAGCCTGCCAAATGTTTGGCGAAAATACATCTGTCCATCCCGTTCCCATCAACCCGCCCTGA
ACGGCGAGCATTGCAGTGGCACTGATCAAACTCCAGACGGCAGCGTGCTGTTGTAAACGCAGAAAACGCTTCGCCAGCAA
GCGACGAATCGTCAAGGGGGCCAGCCAGGCACCGTACATTGCGAAGCCAAAAACCAGCATCAGCGAGGTGAAATGGATAA
ATCGCAGCGCGATCCAGGTAAACGCCAGCATGATTTATTTCACGCTAAAGGTGTAGTGTCCTTTCGTTTTATGCCCGTCC
ACCGAAACAACATGCCAGTCAACGGTGTAGGTTCCGGGTTTCAGCGAATCGGCCAGTGGAACAATCAGTTGCTTTTGGTC
CTGCTCATTTCGCTTCGCCGGTAATGTTTTAATATTTTCGTTTTTTGGCCCCGTGATTTTTGCACCACTGAATCCTGTTT
CAACACCTTCCGAGAAGTTTAAAGTGATTGCCTGCGGTGCAGCTGTCACTTGCGCGTTTGCCGCAGGATACTGATGCGTT
AAATGCGCATGTGCCCAGACGGAAGGTGTCACCAACGAAGTGGTCAGGATTGCCAGCGCGTAGCGAAGGGAGCGTGCAGT
TGAAGCCATATTATCTATTCCTTTTTGTAATAACTTTTTTACAGAGCATAACCTTGTCTAATGTCTGAGTCGAGGATCAT
CAATTCCGGCTTGCCATCCTGGCTCACTCTTAGTAACTTTTGCCCGCGAATGATGAGGAGATTAAGAATGCTGAAGAATC
TGGCTAAACTGGATCAAACAGAAATGGATAAAGTGAATGTCGATTTGGCGGCGGCCGGGGTGGCATTTAAAGAACGCTAC
AATATGCCGGTGATCGCTGAAGCGGTTGAACGTGAACAGCCTGAACATTTGCGCAGCTGGTTTCGCGAGCGGCTTATTGC
CCACCGTTTGGCTTCGGTCAATCTGTCACGTTTACCTTACGAGCCCAAACTTAAATAAAACTTATACAGAGTTACACTTT
CTTACATAACGCCTGCTAAATTATGAGTATTTTCTAAACCGCACTCATAATTTGCAGTCATTTTGAAAAGGAAGTCATTA
TGTCGTTCTGGAAAGTTGCAGCAGCGCAATATGAGCCTCGTAAAACCTCACTCACCGAACAGGTAGCTCATCATCTGGAG
TTTGTGAGGGCCGCCGCCAGGCAACAATGTCAGCTGTTAGTTTTTCCATCGCTTTCTTTACTGGGGTGTGATTATTCGCG
ACGTGCCCTTCCTGCCCCACCCGATCTCTCACTGTTAGACCCGCTTTGTTATGCCGCAACGACCTGGCGAATGACCATCA
TTGCCGGCCTTCCCGTTGAATATAACGATCGCTTTATTCGTGGAATTGCGGTGTTCGCACCGTGGCGAAAAACGCCTGGG
ATCTACCATCAGAGTCATGGTGCATGTCTGGGCCGTCGTTCCAGAACCATCACGGTGGTCGATGAACAACCGCAAGGTAT
GGATATGGACCCGACCTGTTCACTGTTTACTACAGGGCAATGTCTGGGAGAACCCGACCTGCTGGCGTCGGCCCGTCGCT
TACAATTTTTTTCACATCAGTACTCGATTGCCGTGCTGATGGCAAATGCCCGTGGTAACAGTGCACTATGGGATGAATAT
GGTCGTCTCATCGTTCGCGCCGATCGTGGTTCATTATTGTTAGTCGGTCAGCGTTCATCACAGGGTTGGCAAGGCGATAT
CATTCCATTACGCTAGGCTTTTTCGGCCTGGAGCATGCCATGTTGCGCATTATCGATACAGAAACCTGCGGTTTGCAGGG
AGGGATCGTTGAGATTGCCTCTGTTGATGTCATTGACGGAAAAATCGTCAACCCCATGAGCCACCTGGTGCGCCCCGATC
GTCCTATTAGTCCACAAGCGATGGCGATTCATCGCATCACCGAAGCCATGGTCGCCGATAAACCGTGGATTGAAGATGTG

ATCCCACACTATTACGGTAGTGAATGGTATGTCGCGCATAACGCCAGCTTTGACCGCCGCGTACTGCCTGAGATGCCCGG
TGAGTGGATTTGCACTATGAAACTGGCCCGTCGTTTGTGGCCTGGGATCAAGTACAGCAATATGGCGTTATATAAAACAC
GCAAGCTCAATGTACAGACGCCGCCGGGCCTGCATCATCACCGCGCGTTGTATGACTGTTATATCACCGCCGCGTTGCTT
ATCGATATTATGAACACCTCCGGCTGGACGGCAGAACAGATGGCCGATATCACCGGACGTCCGTCGTTGATGACGACCTT
CACCTTTGGCAAATACCGTGGCAAAGCGGTTTCCGACGTTGCCGAACGCGATCCGGGCTATCTGCGCTGGTTATTTAATA
ACCTGGACAGCATGAGCCCGGAGCTGCGTTTAACACTGAAACATTATCTGGAAAATACTTAGTCCGCAGGCGTAGCGGGT
AATGTTCCCTGCGCCAGCGCGACCAGAAAAGCATATTCCATCGCTACGCCTTCGTACGATTTAAAGCGACCAGATTTACC
GCCATGGCCTGAGTCCATGTCGGTACAGAGCAATAAAAGATGGTCATCGGTTTTCAGCTCGCGCAATTTAGCGACCCATT
TTGCCGGTTCCCAATATTGCACCTGAGAATCGTGCAAACCGGTCGTTACCAGTAAATGCGGATAAGCCTGTGCGGTGACG
TTGTCATACGGGCTGTAGCTTTTCATGTACTCGTAATATTGCGGATCCTGCGGGTTACCCCACTCTTCAAACTCACCAGT
GGTAAGAGGAATTGATTCATCAAGCATCGTTGTTACAACATCAACAAACGGTACCTGGGCGATAACGCCGTGGAATAATT
CCGGGCGTTGATTAATTGCAACGCCCATCAACATGCCCCCCGCACTCCCGCCCATCGCATAACAAAGCGAAGGAGAGCCA
TAGCCCAGTTTTAACAATGCATCGCAGGCATCAAGATAATCATTAAACGTATTTTTCTTCTTCAGAAATTTTCCGTCTTC
GTACCATTGTTGCCCCAGCTCACCACCGCCGCGAACATGGACAATGGCGTAGACAAAGCCACGATCTAACAAACTCAAGC
GGCTAAAACTGAAATCGGCATCAATACTTGCGCCGTAAGAACCATAGCCATACACCAGCAACGGGTTGTGTCCTTTGCGA
AAATGTTTGCGATGGTAGACCAACGAAACCGGAACTTCGACGCCATCACGGGCGACTATCCACAGGTGTTCACTGCGGTA
ATTCGCCGCATAAAAACCAGGAACTTCCGTTTGTTTTAATACACGACGCTCACCGGTATCCATATCCAGTTCAAACAAAG
TGTCTGGTGTAGTCATGGAAGAATAACCATAACGCAATCGCGCGGTTTCAGGTTCTGGATTGTAGGCAATCCAGGTCACA
TAGGCCGGATCATCAAAGGCAATACCAATGACTTCCCGGGTCTTGCGGTTAATTTGGCGCAAACTGGTTAACCCGCGCTG
ACGCTCTTCAACCACCAGCCAGTCGGTAAACAGCGTAAACCCTTCCAGCATGATGTTTTCGCGTGGCGGAATTAACTCTT
CCCACTGTTGCTCATCACGCATACGGGTACGGTATAAGCCAAAGTTTTTGCCGTGGCGGTTGGAACGCAGATAAAAACGA
TGCTGGTAGTGATCAAGGCTGTATTCGTGATCTTTGCGGCGCGGCAGAAAAACAAACGGCTCGGCATCGGCCATTTCCGC
GTCCAGCAGGCGAACTTCACTGGTGGTGGCGCTGGCCAAATGAATGACTACATAGTGCTTCGAGGTCGTTTTATGCAGGC
TGACGTAATAGGTATCGTCTTTTTCTTCGTAGATCAGTTTATCTTGCGATGCTGGCGTACCGATGGCGTGACGCCAGACC
TGATAAGGCAGCAGCGTCACCGGATGCTTGCGAACATAGTAGAAAATCCAGGAGTCATTTGCCCAGACAAAGCTGGGTTC
AACGTTATCCAGCAGTTCCGGGTACCAGTTACCAGTTTCCAGATTACGAAAACGAATGCCGTACTGGCGTCGGGAAAGAA
AATCTTCTGCCAGCGCCATAATGGTGTTATCGGGCGTAATCGCCATTCCGCCCATCGAATAAAACTCACTATGAGCTGCG
CGCTTATTGGCATCGAGCAATGTTTCCCACTCATCCCACTCTTCACTGAATGCCGATTGACGCTGGTAGATAGCATATTC
ACAGCCTGGTTCATAAATATGCCGATAGCGGTAGCCATTTTTGATGTAGGGCGCAGAAACTTCTCGTTGCGGAATGCGGT
CGATGATTTCCTTTAAGATGCGATCCTGCAAGGCTTGTTGTGAGGCCATCACCCGATGACCGTAACTATTTTCTTGTTGC
AGGTAGTCCAGGACTTCTGGCTGAGAACGCGTATCGTCCCGCAGCCAGTAGTAATTATCGATGCGCGTATCGCCATGAAG
CGTCATGGCGTGGGGAATGCGGGCGGCTTTTGGTAGCATGTTATTGTTCTTTCTGGTTGAAACATCTTATAAGGGTGGCA
AAACTCACCGGGGATGCAAGCGAAACAGGGGAGTCATTGCTTAGATGATGACAGGTAATGGCGCGGATATCGAATGTTAT
GCAGACAGAGAAAATCAGCCTGTTCAAATGGCTGTGCGATTCTGGATAGCCCGAAATAGTCAACTTCAGGCTATCCAGAG
AGCGGAATTATTCCGCCAAAGTGCGTTTTTGCTGTTCGAGATCGCGTTCAATGCCGTCACGAACATCCTGGGGGATTTTC
AGCGCGTCACCCAGTGCATTCAGGTAACTGCGTTCCATAAAATGGTCAATATCAATAGCCGCGCAACTCAGGAAATAGAT
TTCCAGCGCCTCTTCTTCATTGCGGACCCCGGTAGCCAGGCGTTGTGGATCCAGCGGTTGTTCGATTGCCTGCTCAATGA
GTACACGCCCCTGCTCTTCCACGCCGGCTCCACGCAATTGCTGGTCGATTGCCGCACGTTCTTTGGCATCAATATGACCA
TCACTTTTAGCGGCAAAGACCAGCGCAAGGATCAAACGTGCTGTACGCTCATCCAGCGGCGTACTTTGCGCGCCAAACTG
CGGTTCGTCCTGATGCGCCGCGCGAATTTTATCTTTGTATTTATTCCACAGCACCGTACCCGCTACCGCTCCGCCGCCAA
CCAGTAACGCGTTGGTGCCATATTTCGTAAGAAGTTTACGTGCTGATTTATTTGCGACCAGCAGCCCAGCCAGTCCGCCT
AATGCGCCTGGCACTAACAGTTTGACCAATCCCTGATCCGCAGACGAGGAGGTAGAAGAACTGCTTTGCCCAAGCAGGGA
TTGCAGTTGATTTAACCAGTTAGCCATATTTGCTCCTCAATAACCATTTTTAGTGCCTGTAGCATAGCGGATGAGGATGT
CAGAAAATGTATATCGAGACAAAAGATGCGCAAATAAGCGGGGCGGTAGGCCGTTATTCAAAGAAATTCGCGCCAGGTAA
GGTATTTACTGTATATCAGGTGATAAGGGTATTTTAGGTGAAAGTAGTTTGTCGTTTTGAGTGGCATTGTTTGATGCTGA
TTGCCCGATGCGACGCTGTTGCGTCTTCTCAGGCCTACGATTGTGGTGCGTTCTGGGGCGGACAAGGCACTCGCGCCGCA
TCCGCCAACAGTCAATCGAATTATCAGAAGCCATTAACGCCGCTGATATTCCGCCATTCCCGCTTTGCAGTCCACGCTGA
CCTGGTAATGAATATCGGCACTTTTACCACGCACGGTTAGCGGTACTGACCATTTATCATCTTTACCCTGAATGTCCTGC
AAACTGACCCAAGCCACGGGATCGGCCTGACCGACAATTTTTTGATCATCTGCCCAACGCGCCACGCGATTTTGTTGATA
ATCACGTTTTACGCTCGCGGCAATTCCGGCAGCATCCAGATCTTCACACTTTGGGAAAGTGACCGACTTGCTGGTTTCAT
TATTGGCAGCGAAAACTGATGCGCAGGCAGAAACCAACAACAGCCCTAAAAACGCCCCTCTTTTTTTCATGTTTTTCTCC
ATAGCACAATGATTCAGGAGAAAGCATGGTACAAATTGTCAGGAGCGCAAGTTGCTTCAGGCCGCGTGTGAGGCATCTTC
CTCTTCGGATTCAGCACCGACGACCTGTGGGGACGGTAATTTGCCGGTTTTCAGAATGGTGCTCAGCACATCTTTTTGTT
CTGCCAGCCATAGCGAAAGGGCTTCACGTTGCTCGTCTTCCATTTGCACTGGAGAATTTGTCAGCCAGGTGTCAAGCAGA
TCCGCCGTATCAAGCATTTTGTCATAAGCATCGGCTTCCTTTTTGCTGGTAAACGACATTTTCTCTTCGCCCTCACGAAT
GACTACGTATTTAACTTCAACCGCCATTTGCAGCCTCTCATAATAACTGTGATTTTATACAGTATATTTCTTTTCGGTTG

AGAAATCAACATCAGCAATAAAGACACACGCAAACGTTTTCGTTTATACTGCGCGCGGAATTAATCAGGGGATATTCGTT
ATGACGTTATTAGGCACTGCGCTGCGTCCGGCAGCAACTCGCGTGATGTTATTAGGCTCCGGTGAACTGGGTAAAGAAGT
GGCAATCGAGTGTCAGCGTCTCGGCGTAGAGGTGATTGCCGTCGATCGCTATGCCGACGCACCAGCCATGCATGTCGCGC
ATCGCTCCCATGTCATTAATATGCTTGATGGTGATGCATTACGCCGTGTGGTTGAACTGGAAAAACCACATTATATCGTG
CCGGAGATCGAAGCTATTGCCACCGATATGCTGATCCAACTTGAAGAGGAAGGACTGAATGTTGTCCCCTGCGCTCGCGC
AACGAAATTAACGATGAATCGCGAGGGTATCCGTCGCCTGGCGGCAGAAGAGCTGCAGCTGCCCACTTCCACTTATCGTT
TTGCCGATAGCGAAAGCCTTTTCCGCGAGGCGGTTGCTGACATTGGCTATCCCTGCATTGTAAAACCGGTGATGAGCTCT
TCCGGCAAGGGGCAGACGTTTATTCGTTCTGCAGAGCAACTTGCTCAGGCATGGAAGTACGCTCAGCAAGGCGGTCGCGC
CGGAGCGGGCCGCGTAATTGTTGAAGGCGTCGTTAAGTTTGACTTCGAAATTACCCTGCTAACCGTCAGCGCGGTGGATG
GCGTCCATTTCTGTGCACCAGTAGGTCATCGCCAGGAAGATGGCGACTACCGTGAATCCTGGCAACCACAGCAAATGAGC
CCGCTTGCCCTTGAACGTGCGCAGGAGATTGCCCGTAAAGTGGTGCTGGCACTGGGCGGTTATGGGTTGTTTGGTGTCGA
GCTATTTGTCTGTGGTGATGAGGTGATTTTCAGTGAGGTCTCCCCTCGTCCACATGATACCGGGATGGTGACGTTAATTT
CTCAAGATCTCTCAGAGTTTGCCCTGCATGTACGTGCCTTCCTCGGACTTCCGGTTGGCGGGATCCGTCAGTATGGTCCT
GCAGCTTCTGCCGTTATTCTGCCACAACTGACCAGTCAGAATGTCACGTTTGATAATGTGCAGAATGCCGTAGGCGCAGA
TTTGCAGATTCGTTTATTTGGTAAGCCGGAAATTGATGGCAGCCGTCGTCTGGGGGTGGCACTGGCTACTGCAGAGAGTG
TTGTTGACGCCATTGAACGCGCGAAGCACGCCGCCGGACAGGTAAAAGTACAGGGTTAAACCCGGCAAAAAAACGCTACA
AAAATGCCCGATCCTCGATCGGGCATTTTGACTTTTACAGCTTAGCGCCTTCTACAGCTTCACGCGCCAGCTTAGTAATG
CGGTCGTAATCGCCCGCTTCCAGCGCATCTGCCGGAACCAGCCAGGAACCACCGATGCACAGCACGCTTTTCAGCGCCAG
GTAGTCACGGTAGTTAGCCGGAGAAATACCACCCGTCGGGCAGAAACGGACCTGGGAGAACGGACCCGCGATCGCCTGCA
GGGCTTTCACGCCGCCGTTAGCTTCAGCCGGGAAGAATTTGAACTCTTTCAAACCGTAGTCCATACCCAGCATCAGTTCG
GAAACAGTGCTGATCCCCGGAATCAGAGGAATAGTCCCTTCGGTAGCAGCTTTCAGCAGCGGCTCGGTCAGACCCGGGCT
AATTGCGAACTGTGCACCCGCTTCAGTGACTTCTGCCAGCTGCTGTGGATTCAGCACCGTACCGGCACCCACAATCGCTT
CAGGCACTTCTTTGGCGATAGCACGGATAGCGTCAACTGCACACTCGGTACGCAGAGTCACTTCCAGAACGCGCACCCCA
CCAGCAACCAACGCTTTTGCCATCGGCACCGCGTGTTCCAGTTTTTTTACCACGATAACCGGTACAACCGGGCCGGTGGT
CAGGATTGATTCTGCACTTGTTTTCCAGTTTTTCATCAGAGTTTTCTCTCGCCTGATTACAAATTTGTCGTCTTAAAAAG
TGATACAGGTTGCGCCCTGTTCGGCACCGGACAGTTTTTCACGCAAGGCGCTGAATAATTCACGTCCTGTTCCCACGCGT
GACGCGCTCAGGTCAGGAATGTGCGGTTCGCGAGCAGCCAGTTCCGCTTCGTCTACCAGCAGCGTCAGTTCGCCTGTCTG
TCCATTCACACGAATGATGTCCCCGTCGCGCACTTTTGCCAGCAGCCCGCCATCGTAGGCTTCTGGTGTTACGTGGATAG
CTGACGGCACTTTACCTGAAGCGCCGGAGAGTCGTCCATCGGTAACTAACGCAATTTTGAAACACCGGTCCAATAATACA
CCAAGTGGCGGCATGAGTTTATGTAATTCTGGCATTCCGTTCGCTTTTGGCCCCTGATGACGGACAACAACGACACAATC
GCGGTCCAGCAAACCCGCTTCAAAGGCCGGCATAACGTCATGCTGGCTTTCAAAAACAACCGCTGGCGCTTCAATCACCT
GGTTCTCAACCGGCACGGCAGAGGTTTTCATAACCGCACGGCCCAGGTTACCGCTTAACACTTTTGTCCCACCATGATGA
GAGAAAGGTTGTTCGAAGGAAGCGATCACATTGCTGTCGAGTGATTTTTCCGCCCCTTCCCGCCAGTCCAGTTCACCATT
ATTCAGCCATGGTTCAAGGGTATAACGAGACAGACCAAAACCTGCCACCGTATTGACATCTTCATGCAGCAGGCCTGCTT
TGAGCAGTTCACGCACCAGAACCGGTACGCCACCTGCCGCCTGGAAGTGGTTAATATCGGCCGGACCGTTCGGGTAGAGA
CGTGCCATCAGCGGTACAACATCAGAAAGGTCAGAGAAGTCATCCCAGTTAATCTGAATACCGGCCGCGCGCGCCATCGC
CACCAGGTGCATGGTGTGGTTAGTGGAACCACCGGTCGCCAGCAGTGCAACGATACCGTTCACCACCACTTTCTCATCGA
TCATCTTACCGATCGGCATCCATTCATTACCATTACCGGTCATGCGTGTAACCTGACGCGCAGCTGCGGCGGTCAAAGCA
TCGCGCAGCGGAGAATCCGGATGAACAAAAGAAGAGCCTGGCAACTGCATCCCCATAAACTCCACCACCATCTGGTTGGT
GTTGGCAGTACCGTAGAAAGTACATGTTCCCGGCGCATGGTAAGACGCGGCTTCTGACTCCAGTAAGGCCATGCGGTCCA
CTTTACCTTCGGCATAAAGCTGGCGAATACGCACTTTTTCTTTATTTGGCAAACCGCTTGCCATCGGTCCAGACGGCACA
AACACCGCAGGCAAATGACCAAACGACAGGGCTGCCATCGTCAGACCCGGGACAATCTTGTCGCACACACCGAGGAACAG
AGCACCATCAAACATGTTATGGGACAGCCCCACCGCCGCAGACATCGCTATCACTTCGCGGCTTAGCAGCGACAATTCCA
TTCCATCCTGCCCCTGGGTGACACCATCACACATCGCCGGAACACCGCCCGCAACCTGACCAACCGCATTCGCTTCATGC
AGGGCTTTACGAATGATTTCTGGATAGTGTTCATAAGGCTGGTGCGCGGAGAGCATGTCGTTATAGGAGGTGATGATGGC
GATATTGTTACGCAACATGCTTTTCAAAGAGGCTTTGTCTTCTGGCTGGCAGGCAGCGAAACCGTGTGCCAGGTTACCGC
ATGCCAACTGCGAACGATGAACGGTCGAAGTTTTCGCTTGTTCTATCCGGGCGAGATAAGCAGAGCGAGTCTCGCGCGAA
CGTTCAATGATTCGATTTGTTACGCGTAACAATTGTGGATTCATAAAGGCTCCTGAAATTGAGTTGTCAGAGCAGGATGA
TTCACAACGCGTTTCATTCAGAGGATTTATGACTGAAACGCCTGTAACCGGAGCTCATAGGGCAAAAACGTTTCAGTCAG
TGTAATAAAAAAAGCCTCGTGGGTGAATCCGCACGAGGCCTGAAAGTGTAAAAATTGTTCTACAATCTGCGCAAGATCAT
GTTACCGGTAAAATAACCATAAAGGATAAGCGCAGATATTACTCAAACTCATTCCAGGAACGACCATCACGGGTAATCAT
CGCCACCGAGGCAACGGGTCCCCAGGTTCCGGCCTGATACGGTTTCGGCGCATCATTGTCCATCGCCCACGCCTCAGTAA
TGGAGTCTACCCATTTCCAGGCTTCTTCCACTTCGTCGCGACGTACAAACAGTGCCTGAATACCACGCATGGTTTCCAGC
AGCAAACGTTCATAGGCATCCGCCAGATGCGTCTGATTAAAGGTTTCTGAATAGCTCAGATCCAGCTTGGTGATTTGCAG
GTTATGTTTGTGGTCAAGGCCAGGAACTTTATTCAGTACCTGGATATCCACGCCTTCATCAGGTTGCAGACGGATAGTCA
GTTTATTCTGCGGCAGATCCTGCCACGATTCTTTAAACAGATTCAGTTCAGGTGTTTTGAAATAGACCACGACTTCAGAA

CATTTGGTCGGCAGACGTTTACCAGTACGCAGGTAGAATGGCACACCGGCCCAGCGCCAGTTATCAATGTCGACGCGGAT
CGCCACGAAAGTTTCTGTATTGCTGCTCTTGTTCGCGCCCTCTTCTTCCAGATATCCCGGCACTTTTTTGCCCTGGGCGA
AGCCCGCAGTATATTGCCCGCGTACGGTTTTTTCGCGTACGTTGGAGCGGTCGATGCGGCGCAGAGACTTCAGTACTTTC
ACTTTTTCATCGCGGATGCTGTCTGCGCTCAGGTCAGACGGCGGAGACATCGCAATCATGCAAAGAATTTGCAGCAGGTG
GTTCTGGATCATGTCGCGCATCTGACCGGCTTTATCAAAATAGCCCCAGCGCCCTTCGATCCCCACTTCTTCTGCCACGG
TAATCTCAACATGATCAATGGTGCGATTGTCCCAGTTATTCACAAACAGGGAGTTAGCAAAACGCAGCGCCAACAGGTTC
AGCACCGTTTCTTTACCAAGATAGTGGTCGATACGGTAAACCTGGCACTCCTCGAAGTATTCGCCAACCTGATCATTGAT
TTCCTGCGAGGTCGCCAGCGACGTCCCCAGCGGTTTCTCCATGACTACGCGTGCCGGTTTAGCATTCAGTTTTGCCTCGC
CAAGCCCTTTGCAAATTGCGCCAAAAGTGCTGGGCGGCATGGCAAAGTAGTTAATGGTGATACGATTTTTTTGATCCAGC
ATCGCGCCGAGACGGCTGAATGCAGCAGTGTCATTGACATCGAGATTACAAAAATCCAGACGTGCACTCAGGGTGTCCCA
TAAACCTTCATCAATGGTTTCTTTCATGAAAGTTTCGAGCGCCTCGCGGACAACTTTGGTATATGCCGCTTTATCCCAGT
CAGCACGCCCTACGCCGATAATCCGGGTGTCCGGGTTGAGCTGACCGGCTTTTTCCAGTTGATACAGGGAAGGCAGCAAT
TTACGACGCGCAAGGTCGCCTTTCGCGCCGAAAATGACCAGGTCACAGGCCTGGGCTGTTTGCGTTACCGCCATGTCATT
CTCCTTAAGTTAACTAACCCGGTACTTAAGCCAGGGTATACTTGTAATTTTCTTACGGTGCACTGTACTGCTTTTACGAG
CTTGCGAAAACTGTAAACGCTTATCCACCCGTGCGATTACGGGAAAAGCGCGCAAAGTGCGGCAAAACTGATAAAAAAAT
CATCGTTTTCCATTGGGTAAAAATCTGACACTGATCATGTTATGAAAAAAAATAACAACTTTTTTATCTGCTTTTGTCAT
TAACACGGCACACAGGCGTAATATCTGACAAAACTGCATTTCGATTTCTTTCAGTGCGGAAATCGTCATTACCCGTGAGT
CTCTTTACATCATGAATATGCTGGAAAAAATCCAGTCTCAGCTGGAACATTTGAGCAAATCAGAGCGCAAAGTTGCCGAG
GTCATTCTGGCTTCGCCCGATAACGCGATCCATTCGAGTATTGCTGCTATGGCACTGGAAGCCAATGTTAGCGAACCGAC
GGTGAATCGTTTCTGTCGCAGCATGGACACGCGCGGTTTTCCTGATTTTAAACTTCATCTGGCACAGAGTCTGGCGAATG
GCACTCCCTATGTTAATCGCAATGTCAATGAAGATGACAGCGTTGAATCATACACAGGGAAAATATTTGAGTCCGCAATG
GCAACGCTTGATCATGTCCGTCATTCACTGGATAAATCTGCCATCAACCGCGCCGTCGACTTGCTCACTCAGGCAAAAAA
AATCGCCTTTTTCGGATTAGGCTCTTCAGCCGCCGTTGCCCACGATGCGATGAATAAGTTCTTTCGTTTTAATGTTCCGG
TGGTGTACTCCGATGATATCGTGCTGCAACGCATGAGTTGTATGAATTGTAGCGACGGAGACGTGGTGGTGCTGATTTCT
CACACTGGAAGAACAAAAAATCTGGTCGAGCTGGCGCAGCTGGCACGCGAAAACGACGCCATGGTGATTGCCCTCACCTC
TGCGGGTACCCCGCTCGCCCGGGAAGCAACGCTGGCAATTACCCTCGACGTACCGGAAGATACTGACATTTATATGCCCA
TGGTTTCTCGACTTGCACAGCTGACCGTGATAGATGTGCTGGCGACAGGATTTACTTTGCGACGCGGTGCAAAATTCAGA
GATAACTTGAAGCGGGTCAAAGAAGCGCTGAAGGAATCGCGTTTTGATAAGCAGTTACTTAATTTAAGTGACGATCGCTA
AAAACGACTGTCACTGTCCTAATCTTATACGACATCCGAATGAGATTAATTTATCGCCATCGCGGCGTTATTTCATTCGG
ATTTCATGTTCAAGCAACACCTGGTTGTTTCAGTCAACGGAGTATTACATGTCCAGAAGGCTTCGCAGAACAAAAATCGT
TACCACGTTAGGCCCAGCAACAGATCGCGATAATAATCTTGAAAAAGTTATCGCGGCGGGTGCCAACGTTGTACGTATGA
ACTTTTCTCACGGCTCGCCTGAAGATCACAAAATGCGCGCGGATAAAGTTCGTGAGATTGCCGCAAAACTGGGGCGTCAT
GTGGCTATTCTGGGTGACCTCCAGGGGCCCAAAATCCGTGTATCCACCTTTAAAGAAGGCAAAGTTTTCCTCAATATTGG
GGATAAATTCCTGCTCGACGCCAACCTGGGTAAAGGTGAAGGCGACAAAGAAAAAGTCGGTATCGACTACAAAGGCCTGC
CTGCTGACGTCGTGCCTGGTGACATCCTGCTGCTGGACGATGGTCGCGTCCAGTTAAAAGTACTGGAAGTTCAGGGCATG
AAAGTGTTCACCGAAGTCACCGTCGGTGGTCCCCTCTCCAACAATAAAGGTATCAACAAACTTGGCGGCGGTTTGTCGGC
TGAAGCGCTGACCGAAAAAGACAAAGCAGACATTAAGACTGCGGCGTTGATTGGCGTAGATTACCTGGCTGTCTCCTTCC
CACGCTGTGGCGAAGATCTGAACTATGCCCGTCGCCTGGCACGCGATGCAGGATGTGATGCGAAAATTGTTGCCAAGGTT
GAACGTGCGGAAGCCGTTTGCAGCCAGGATGCAATGGATGACATCATCCTCGCCTCTGACGTGGTAATGGTTGCACGTGG
CGACCTCGGTGTGGAAATTGGCGACCCGGAACTGGTCGGCATTCAGAAAGCGTTGATCCGTCGTGCGCGTCAGCTAAACC
GAGCGGTAATCACGGCGACCCAGATGATGGAGTCAATGATTACTAACCCGATGCCGACGCGTGCAGAAGTCATGGACGTA
GCAAACGCCGTTCTGGATGGTACTGACGCTGTGATGCTGTCTGCAGAAACTGCCGCTGGGCAGTATCCGTCAGAAACCGT
TGCAGCCATGGCGCGCGTTTGCCTGGGTGCGGAAAAAATCCCGAGCATCAACGTTTCTAAACACCGTCTGGACGTTCAGT
TCGACAATGTGGAAGAAGCTATTGCCATGTCAGCAATGTACGCAGCTAACCACCTGAAAGGCGTTACGGCGATCATCACC
ATGACCGAATCGGGTCGTACCGCGCTGATGACCTCCCGTATCAGCTCTGGTCTGCCAATTTTCGCCATGTCGCGCCATGA
ACGTACGCTGAACCTGACTGCTCTCTATCGTGGCGTTACGCCGGTGCACTTTGATAGCGCTAATGACGGCGTAGCAGCTG
CCAGCGAAGCGGTTAATCTGCTGCGCGATAAAGGTTACTTGATGTCTGGTGACCTGGTGATTGTCACCCAGGGCGACGTG
ATGAGTACCGTGGGTTCTACTAATACCACGCGTATTTTAACGGTAGAGTAAGTACGTTGCCGGATGCGGCGAAAACGCCA
CATCCGGCCTACAGTTCAATGATAGTTCAACAGATTTCGAATATTCTGAAGCAAACTTGAACTTATCATCAGGCGAAGGC
CTCTCCTCGCGAGAGGCTTTTTTATTTGATGGGATAAAGATCTTTGCGCTTATACGGCTGGATTTCGCCCGGTTTGCGAG
TTTTCAGCAATTTTAGTATCCAGGTGTATTGTTCTGGTCGCGGACCAACAAAAATCTCGACTTCTTCATTCATCCGCCGC
GCAATCGTATGATCATCCGCCTCTAACAGATCATCCATCGGTGGGCGCACCTGAATCGTCAGACGATGCGTCTTGCCATC
ATAAATCGGAAACAGCGGTACAACGCGCGCACGGCACACTTTCATCAAACGACCAATCGCGGGCAACGTCGCTTTATAGG
TGGCAAAGAAATCCACAAATTCGCTGTGCTCTGGGCCATGATCCTGATCGGGTAAATAATATCCCCAGTACCCCTGACGT
ACCGACTGGATGAATGGTTTAATACCGTCATTTCTCGCATGCAGACGACCGCCAAAGCGACGACGCACCGTGTTCCAGAC
ATAATCAAAAACCGGGTTGCCCTGATTATGGAACATCGCTGCCATTTTCTGCCCTTGCGAGGCCATCAGCATGGCAGGAA

TATCGACGGCCCAACCGTGCGGCACCAGAAAGATAACTTTCTCGTTATTACGCCGCATCTCTTCGATGATCTCCAGCCCT
TGCCAGTCAACGCGCGGCTGAATTTTCTCCGGCCCGCGTATTGCCAACTCAGCCATCATTGCCATCGCTTGCGGCGCGGT
GGCAAACATCTCATCAACAATCGCTTCGCGTTCAGCTTCACTACGTTCTGGAAAGCAGAGCGACAGATTGATTAACGCAC
GACGGCGTGAGCTTTTTCCCAGTCGTCCGGCAAAACGTCCCAGCCGTGCCAGAATGGGATCACGGAACTTTGGCGGCGTT
AAAGCGATACCCGCCATCGCTGCTACGCCCAGCCATGCTCCCCAGTAGCGCGGGTGGCGAAAGGATTTATCAAACTCAGG
AATGTATTCGCTATTATTTTTTTTCGTTTCCATGCTTTTCCAGTTTCGGATAAGGCAAAAATCAATCTGGTGATAGTGTA
GCGGCGCAACTTGCCCCGCACCAAATAAAAAAAGCCGGTACTGACTGCGTACCGGCTGCGAATGGATGTTAATTAATCAA
ACCGTAGCTGCGGCACAATCTCTTTGGCCTGCGCCAGGAATTCGCGACGATCGGAGCCGGTCAGCCCTTCGGTACGCGGC
AGTTTTGCCGTCAGCGGGTTTACGGCCTGCTGGTTTATCCATACTTCATAGTGCAGATGCGGCCCGGTTGAACGTCCGGT
ATTACCGGAAAGCGCGATACGGTCGCCACGTTTCACCTTCTGTCCCGGTTTCACCAGAATCTTGCGCAAGTGCATATAAC
GCGTGGTGTAGCTGCGACCATGACGAATAGCCACATAATAACCTGCTGCGCCACTGCGTTTGGCAACCACCACTTCACCG
TCACCCACTGAAAGCACTGGCGTACCCTGCGGCATGGCGAAATCAACACCTCTGTGTGGTGCAACGCGACCGGTCACCGG
ATTAGTACGACGCGGGTTAAAATTAGACGAGATACGGAACTGTTTCGCCGTCGGGAATCGCAAGAATCCTTTCGCCAGAC
CAGTACCGTTACGATCGTAGAATTTGCCATCTTCAGCGCGGATAGCGTAATAATCTTTACCTTCTGAACGCAAACGTACG
CCCAGCAGCTGGCTTTGCTCACGTTTACCATCAAGCATTTCTCGAGACATTAACACCGCAAATTCATCGCCTTTTTTCAG
TTTGCGGAAATCCATTTGCCACTGCATGGCTTTAATCACTGCGCTCACTTCGGCGCTGGTTAAACCGGCGTTTCTGGCGC
TGGCAACAAAGCTTCCCCCGACGGTACCTTTCAGCAGATTGTTAACCCACTCTCCTTGCTGCATTTCGCTGGTCATTTTA
AAACCGTTAGCGGCAGTACGGTCATAGGTTCGGGTTTCACGACGAGACACTTCCCAGGTGAGGCGCTGCAGTTCGCCGTC
CGCGGTTAATGTCCAGGAGAGTTGTTGACCGATTTTCAGGTTACGCAATTCTTTGTCGGCCGCAGCCAGTTGGGTGATAT
CACCCATATCAATACCATACTGATTGAGAATGCTGCTTAGCGTATCGCCAGTGGAAACAACATATTCATGCACGCCCGCT
TCACCGGCGATTTTGTCATCCAGTTCGTCCTGGGGAATGGCTTCATCTTCTTGTGCAGCTTGATCAATCGGCTCACTGGC
TTCAGGTAAGAGCGAACGAATTTCGTTCTGTTCCAGCTCAATGGTTTTGACAATTGGCGTGGCATCACGGTGATAAACAT
AAGGCCGCCAGACAGCGACGGCCAGAGTAAGAACGGTGAGCGACCCCAACATAACGCGGTGTGGTCGCGGTAAATTATTA
AACGCCAGGGCGACAGAGCGGGCTATCTGTTGCACGTAATCACTTCCTCATTAATCTCCTTTCAGGCAGCTCGCATACTG
GTTGGCTAATTGACTCAGGAATTCTGAATAGCTTGTTTTACCCAGTTTGATATTCGTCCCCAGGGGATCCAACGTTCCCA
TACGAACGGATGTCCCTCGTGCGACGCTCTCAACGACCGCTGGCCTGAACTGTGGCTCAGCAAAAACGCAGGTTGCTTTT
TGCTCAACCAACTGTGTTCTTATTTCATGTAAACGCTGCGCGCCAGGTTGAATCTCAGGGTTAACGGTAAAATGACCAAG
CGGTGTCAGTCCAAACTGTTTTTCGAAATAGCCGTAAGCATCGTGAAAAACGAAATAACCTTTCCCCTTGAGCGGCGCGA
GCTCGTTACCAACCTGCGTTTCGGTTGAGGCTAATTGTGCCTCAAAATCCTTCAGGTTGGCGTCAAGTTTGGCTCGACTT
TGCGGCATAAGTTCCACTAATTTTCCATGGATTGCAACCGCTGTAGCCCGCGCTATCTCTGGGGAAAGCCAAAGATGCAT
GTTGAAATCGCCGTGATGGTGATCTTCGTCACTTTTTTCCGCGTGGTCGTGATCATCATCATCGCCGTGAATACTTTTCA
TCAGTAGCGGTTTCACATCTTCAAGCTGCGCAATCGTTACCTGCTTCGCTCCTGGTAATTTGCTTACCGGTTTTTGCATA
AACGCTTCCATCTCCGGGCCAACCCAAACGACTAAGTCCGCGTTCTGTAAGCGTTTTACATCCGATGGGCGCAGTGAATA
ATCATGTTCTGAAGCGCCGTCAGGAAGTAAAACCTCTGTTTCTGTTACCCCATCAGCAATGGCAGAAGCGATGAACCCAA
CGGGTTTAAGCGAAGCGACAACGGCGGCATCTGCGGCCTGTGTTGCACCTCCCCAGAGAGCGGCGGATAATGCTGCGAAA
AGAAGCGTTTTTTTATGTAACATAATGCGACCAATAATCGTAATGAATATGAGAAGTGTGATATTATAACATTTCATGAC
TACTGCAAGACTAAAATTAACATGACAAGTCTGGTTTCCCTGGAAAATGTCTCGGTTTCTTTTGGCCAACGCCGCGTCCT
CTCTGATGTGTCGCTGGAACTTAAACCTGGAAAAATTTTGACTTTACTTGGGCCAAATGGCGCAGGTAAGTCGACACTGG
TACGGGTAGTGCTCGGGCTGGTAACACCCGATGAAGGGGTTATCAAGCGCAACGGAAAACTGCGCATCGGCTATGTACCG
CAGAAGCTGTATCTCGACACCACGTTGCCACTGACCGTAAACCGTTTTTTACGCTTACGCCCTGGTACACATAAAGAAGA
TATTTTGCCTGCACTGAAACGTGTCCAGGCCGGGCACCTGATTAACGCACCGATGCAAAAGCTCTCTGGTGGCGAAACGC
AGCGTGTACTATTAGCGCGAGCATTGTTAAATCGCCCGCAATTATTAGTGCTGGATGAACCCACTCAAGGCGTGGATGTA
AATGGCCAGGTGGCGTTATATGACCTTATTGACCAACTGCGTCGTGAACTGGATTGTGGCGTTTTAATGGTTTCTCACGA
TCTGCATCTGGTGATGGCAAAAACCGATGAAGTGCTGTGCCTGAATCACCACATTTGTTGTTCCGGCACACCGGAAGTTG
TTTCCCTGCATCCGGAGTTTATTTCAATGTTTGGTCCTCGTGGTGCTGAACAACTGGGTATCTATCGCCATCATCATAAC
CATCGTCACGATTTACAGGGACGAATTGTTTTGCGTCGGGGAAATGATCGCTCATGATTGAATTATTATTTCCCGGTTGG
TTAGCCGGGATCATGCTCGCCTGTGCCGCGGGTCCGCTGGGTTCGTTTGTAGTCTGGCGTCGTATGTCTTATTTCGGTGA
TACGCTGGCTCATGCCTCATTACTTGGCGTCGCGTTTGGTTTGTTGCTGGACGTGAATCCATTCTATGCGGTGATTGCCG
TTACGCTGCTGCTGGCGGGCGGTCTGGTATGGCTGGAGAAGCGTCCACAGCTGGCGATCGACACGTTATTAGGGATTATG
GCGCACAGTGCCCTGTCGCTGGGCCTGGTGGTCGTGAGTCTGATGTCTAATATTCGTGTTGATTTGATGGCTTACCTGTT
CGGTGATTTGCTGGCAGTGACGCCAGAAGATCTCATCTCTATTGCGATTGGCGTGGTCATCGTGGTGGCTATTTTGTTCT
GGCAATGGCGCAATTTGCTGTCAATGACGATAAGCCCGGATCTGGCGTTTGTTGATGGTGTGAAATTACAGCGAGTGAAA
TTGTTGTTGATGCTGGTGACGGCATTGACGATTGGTGTAGCGATGAAATTTGTCGGTGCGTTGATTATTACTTCGTTGCT
GATTATTCCTGCTGCTACTGCGCGTCGCTTTGCCCGCACGCCGGAACAGATGGCTGGTGTCGCTGTTTTGGTGGGGATGG
TGGCAGTGACTGGCGGTTTAACCTTTTCCGCGGTTTACGATACGCCGGCGGGTCCGTCGGTGGTGCTATGTGCGGCACTG
TTATTTATTCTCAGTATGATGAAAAAGCAGGCCAGCTAATCTATCGCTGAACACATTTGTCGGATGCGGCGCGAGCGCCT

TATCCGACCTACGGTTCGGTATCTCTGGTAGTCCTGGTAAGACGCGAACAGCGTCGCATCAGGCATATTGCCAGTGCCGG
ATGCGGCGCGAGCGACCAATCCGACTTACGGCATTTCTGGCGGCGTTATGCCAAAGTGATTCCACGCCCGCGTCGTCGCC
ATACGCCCACGCGGTGTACGCTGCAAAAAGCCTTGCTGAATCAAATAAGGTTCCAGCACATCCTCAATGGTTTCACGTTC
TTCGCCAATGGCTGCCGCCAGGTTATCCAGACCTACAGGTCCACCAAAGAACTTATCGATTACCGCCAGCAACAATTTGC
GGTCCATATAATCGAAACCTTCAGCATCGACATTCAACATATCCAGCGCCTGAGCAGCGATATCTGCCGAGATGGTGCCA
TCGTGCTTCACTTCGGCGAAATCACGCACTCGACGCAGCAGACGGTTGGCAATGCGCGGCGTACCGCGAGCGCGACGAGC
AACTTCCAGCGCGCCGTCATCACTCATCTCAAGCCCCATAAAGCGTGCGCTGCGACTGACGATATATTGCAGATCCGGCA
CCTGATAAAACTCCAGACGTTGCACAATACCAAAACGGTCGCGCAACGGTGATGTCAGCGAACCTGCGCGCGTGGTTGCA
CCAATCAGGGTAAACGGCGGCAAATCAATTTTAATGGAGCGTGCCGCCGGACCTTCACCAATCATGATATCCAGTTGGTA
GTCTTCCATTGCCGGGTACAGCACTTCTTCAACAACTGGCGATAGACGGTGGATCTCATCAATAAACAGCACGTCATGCG
GTTCAAGGTTAGTGAGCATCGCAGCCAAATCGCCCGCCTTTTCCAGCACCGGACCAGAAGTCGTGCGTAAATTAACGCCC
ATTTCATTGGCGACAATGTTGGCAAGCGTAGTTTTACCCAACCCCGGAGGACCAAAAATCAACAAATGATCGAGGGCATC
GCCGCGCAGTTTCGCTGCTTTGATGAAAATCTCCATCTGTGAACGAACCTGCGGCTGACCAACATACTCTTCCAGTAATT
TGGGGCGAATGGCGCGATCTGCTACATCTTCCGGCAAAGTGGTACCGGCAGAAATCAGACGGTCTGCTTCAATCATCCTT
TACCTCATAACGCGGCGCGTAGGGCTTCGCGAATTAAAGTTTCACTGCTGGCGTCAGGGCGAGCGATTTTGCTCACCATG
CGGCTTGCTTCTTGTGGTTTATAGCCCAGCGCCACCAGCGCGGCAACCGCTTCTTGTTCAGCATCGTCGGTCGCCGGGCT
GGCAGGAGACGTGAGTACCAGGTCGGCGGCTGGCGTAAAGAGATCGCCATGCAAACCTTTAAATCGGTCTTTCATTTCAA
CAATCAAGCGTTCGGCGGTTTTTTTGCCAATACCCGGCAGTTTCACCAGTGCCCCCACTTCTTCACGCTCAACGGCATTA
ACGAACTGCTGCGCTGACATTCCGGAGAGGATCGCCAGCGCCAACTTCGGGCCGACGCCGTTGGTTTTGATCAACTCTTT
GAACAATGTGCGCTCTTGTTTATTGTTAAAACCGTACAGCAGTTGCGCGTCTTCACGCACCACAAAGTGGGTGAAAACGA
TCGCTTCCTGACCCGCTTCAGGGAGTTCATAAAAACAGGTCATCGGCATATGCACTTCATAGCCTACGCCGCCCACTTCA
ATTAACACCAGCGGGGGTTGTTTTTCAATGATGATGCCTCTGAGTCTGCCTATCACATGACGCTCCTGCGTAATGAATCA
AAGATAATGCTGTATGATAAAAAAATGCTGGATAGATATCCAGCGAAGGATGAAGAAAACTTGCGAGGTGTCTCGATGAA
CTGAAAAATGGCACAGTATAATTTATTCTACAGGTTATATTGGAAGCAAATATTTTAATATTACATATTCAGTGAAGAAA
TGCGTAATAAAAATATACATTGCGCCTCCTGAAAAAATAAATTTTTTATGCTATTACGTATATTTGTATCTATTTCAATG
GAATGACAACGTGAATATTAATTACCCTGCTGAATATGAAATTGGAGATATCGTCTTTACATGTATAGGTGCTGCCTTAT
TTGGTCAAATATCAGCTGCATCAAATTGCTGGAGTAATCACGTCGGGATCATTATCGGTCATAACGGTGAAGACTTCCTG
GTTGCAGAAAGCCGGGTTCCCCTTTCAACCATCACCACGCTATCTCGTTTTATTAAACGTTCGTCTAATCAACGCTATGC
TATAAAGCGATTAGACGCTGGGCTGACAGAACGACAAAAACAACGAATTGTTGAACAGGTTCCCTCCCGGCTACGCAAAC
TTTACCACACCGGTTTTAAATACGAATCTTCGCGCCAGTTCTGTTCAAAATTTGTTTTTGATATTTATAAAGAAGCGCTA
TGTATTCCGGTGGGTGAAATAGAGACGTTTGGAGAATTGTTAAATAGCAATCCGAATGCAAAACTCACTTTCTGGAAATT
CTGGTTCCTGGGTTCTATTCCGTGGGAGCGTAAAACCGTCACGCCAGCCAGTTTGTGGCATCATCCGGGTTTGGTGTTGA
TTCATGCGGAGGGAGTGGAAACGCCTCAGCCGGAACTGACCGAGGCGGTATAACTTAACGCAGTCGCCCTCTCGCCAGGT
TCAGCCGCGATTCGCTCATCTGCATCGCATTCTGACTAACGTGGCAGTGGGTGATAGCAATCGCCAGCGCATCGGCGGCA
TCCGCCTGTGGATTAGCGGGCAGTTTCAGCAAGGTGCGGACCATATGCTGCACCTGGCTTTTTTCGGCACTGCCAATACC
TACCACCGTTTGCTTTACCTGACGTGCCGCGTATTCAAATACTGGCAACTCCTGATTCACCGCCGCCACAATCGCCACGC
CGCGCGCCTGGCCCAGTTTCAGGGCTGAGTCAGCGTTCTTTGCCATAAAGACTTGTTCAATGGCGAAATAATCAGGCTGG
AACTGGGTGATGATTTCCGTCACGCCCGCATAGATGAGTTTCAGACGAGACGGTAAATCATCCACTTTGGTGCGGATGCA
TCCGCTACCCAGGTAGGACAGTTGCCTACCTACCTGGCGGATGACGCCGTAGCCGGTCACGCGCGAACCCGGATCAATGC
CGAGAATAATAGCCATCACGCGTCTCCGTTTTGCTGTTTAGCAGGCCTCATCAGAGAGTCGCTGCGACCTCATCAGAGAT
TTCACCGTTATGGTAAACTTCCTGCACGTCGTCGCAATCTTCCAGCATATCGATCAGACGCATCAGTTTCGGTGCGGTTT
CTGCATCCATATCAGCTTTGGTAGACGGGATCATGGAAACTTCCGCGCTGTCTGCTTTCAGACCTGCCGCTTCCAGAGCG
TCGCGCACTTTACCCATTTCTTCCCATGCAGTGTAGACATCAATCGCGCCGTCATCATAGGTCACAACGTCTTCAGCACC
TGCTTCCAGTGCTGCTTCCATGATGGTGTCTTCATCGCCTTTCTCGAAGGAGATCACGCCTTTTTTGCTGAACAGATAGG
CAACGGAACCATCAGTACCGAGGTTACCGCCACATTTGCTAAATGCATGACGCACTTCAGCAACGGTACGGTTGCGGTTG
TCAGACAGACATTCAATCATGATTGCCGTGCCGCCAGGACCGTAACCTTCGTAGATGATGGTTTCCATGTTTGCATCATC
ATCACCGCCCACACCACGTGCAATTGCGCGGTTCAGTGTGTCACGGGTCATGTTGTTAGACAGTGCTTTATCCACCGCCG
CACGCAGACGCGGGTTAGCGTCCGGATCGCCACCGCCCAGCTTAGCCGCGGTTACCAGCTCACGAATGATTTTAGTGAAG
ATTTTACCGCGCTTAGCATCCTGCGCAGCTTTACGATGTCTGGTGTTGGCCCATTTACTATGACCTGCCATAAAAATATC
TCCAGATAGCCCTGCCTGTTCAGGCAGCGTTAATTACAAACTGTTCAATCGCCTGCCGGTTGCTCCAGGACTTAGTGAGC
GCCGCCGCAGCAGGCGCATCAAGCCACTTGTAAGCCAGATGTTCAGTGAAAACGATCTGCCGCTCGTGCGGAAGCGCAAG
ACAGAACCATGATTCCGTATTACGCGTCACGCCCGGCGCATAGCGATGACGTAAATGTGAAAAAATTTCAAACTCTACCG
TGCGCTGACAGTCAATTAAGGTCAGTTGTTCAGCGACAACATCAATGGTGACCTCTTCCTTTACTTCGCGCATGGCAGCT
TGCGGCGCGGTTTCACCCTCTTCCACGCTGCCGGTTACCGACTGCCAGAAATCGGGATCGTCACGCCGCTGCAACATCAG
CACCCGTTTCGTATCTTGTGCGTAGATGACCACTAAGATCGAAACGGGACGCTTATACACTTTATCCTTCACGCTGCCTC
TTCGTTGACTGCCTTCGCTCATCCCATTCACATAGTTATCTATGCTCATGGGAGTTCACTCAGTTGCCGCCTCGATGCAA

CGCGAATGATTTCGTGTATTTGAGTCATATCAGTTATTCTCAGCCTTCTTCACAACCTGAATGCTCAGCTCAGCCAGTGC
AGTCGGGTTAGCAAAGCTCGGTGCTTCAGTCATCAGACACGCTGCCGCCGTGGTTTTCGGGAAGGCGATAACGTCACGGA
TATTGTCGGTGCCGGTCAGCAGCATGGTCAGACGGTCAAGACCGAATGCCAGACCTGCGTGCGGCGGAGTACCGTATTTC
AGAGCGTCGAGCAGGAAGCCGAATTTCTCGCGCTGTTCCTCTTCGTTGATACCCAGAATACCAAACACCGTCTGCTGCAT
ATCACCATTATGGATACGTACTGAACCACCGCCCACTTCGTAACCATTGATGACCATATCGTAAGCGTTCGCCACCGCAT
TTTCCGGTGCAGCTTTCAGTTCTGCAGCCGTCATATCTTTCGGTGAGGTGAACGGATGGTGCATTGCCGTCAGGCCGCCT
TCACCGTCGTCTTCAAACATCGGGAAGTCGATAACCCACAGCGGTGCCCATTTGCTTTCGTCGGTCAGACCAAGGTCTTT
ACCCACTTTCAGGCGCAGTGCACCCATCGCGTCGGCAACAATTTTCTTGTTGTCGGCACCGAAGAAAATCATATCGCCAT
CTTGCGCGGCAGTACGATCCAGGATGTCTTCGATGATTTCTGCATTAAGGAACTTCGCTACCGGGCTGTTGATACCTTCC
AGACCTTTCGCGCGTTCGTTAACTTTGATGTAAGCCAGACCTTTCGCGCCGTAGATTTTAACGAAGTTACCGTATTCGTC
GATCTGCTTACGGGTCAGCGATGCGCCGCCCGGAACGCGCAGAGCCGCTACGCGACCTTTCGGATCGTTCGCCGGACCTG
CAAATACAGCAAACTCAACAGATTTCAGCAGATCAGCAACGTCAGTCAGTTCCATCGGGTTACGCAGATCCGGTTTATCA
GAACCATAACGGCGTTCTGCTTCCGCAAAGGTCATTACCGGGAAATCGCCCAGATCCACACCCTTCACTTCCAGCCACAG
ATGACGCACCAGCGCTTCCATCACTTCACGCACTTGCGGCGCGGTCATGAAAGAAGTTTCCACATCGATCTGAGTAAATT
CAGGCTGACGGTCAGCACGCAGGTCTTCGTCACGGAAGCATTTAACGATCTGATAGTAACGGTCAAAACCGGACATCATC
AGCAGCTGTTTGAACAACTGCGGGGATTGCGGCAGTGCGTAGAATTTACCTTTGTGCACACGAGAAGGCACCAGGTAGTC
ACGCGCGCCTTCCGGCGTGGCTTTGGTCAGCATCGGAGTTTCGATGTCGAGGAAGCCGTGGTCATCCATAAAACGGCGCA
CCAGGCTGGTGATTTTAGCGCGGGTTTTCAGGCGCTGAGCCATTTCCGGACGACGCAGGTCGAGGTAGCGGTATTTCAGA
CGCGCTTCTTCGGTGTTGACGTGGTTAGAGTCAAGCGGCAGAACATCTGCGCGGTTGATGATAGTCAGCGAGGACGCCAG
CACTTCGATTTCGCCGGTCGCCATATCGCGGTTAATATTTTTTTCGTCACGCGCACGTACGGTGCCCGTGACCTGAATGC
AGAACTCATTACGCAGTTCAGAGGCCAGCTTTAACGCGTCCGCACGATCCGGATCGAAAAATACCTGCACGATACCTTCG
CGGTCGCGCATATCGATGAAGATCAGGCTACCAAGATCACGACGACGGTTGACCCAACCACACAGAGTCACCTGCTGCCC
CACGTGGGACAAACGGAGCTGTCCACAATATTCTGTACGCATGAGATATCCCTTAACTTAGCTGCCGGCGGATGCCCCCT
GCTGCGCAGGTGACCAAGTCGCAGCGTTAGCTGTATGTCACAACTGAATGAAAAAAGGCGGCTATTATACTGGAAATTCT
GCCGCACCGTAAGAGCCTGGCCCGCGCTGGAACGCCTCGTTACCACTTTATATCGGGCCTGAAATCAGACTCTACGCCAG
TTTGCTATAAAGGTGTTGCCCGAACTCATAAAAATTAACAAAATTTGTCGTTCCGCCATCGGCTAATCGCATTAAGGTGT
GAGGCACGATTTTGTTTTTTCAGGAGTCATTATGCTTGAACTTAATGCTAAAACCACTGCGCTGGTGGTGATCGATTTAC
AAGAAGGCATCTTACCTTTTGCCGGTGGTCCACATACTGCCGATGAGGTAGTTAATCGCGCCGGGAAGCTGGCGGCGAAA
TTTCGCGCCAGCGGTCAGCCCGTGTTTCTGGTGCGCGTTGGCTGGTCTGCCGATTACGCTGAAGCATTAAAACAGCCGGT
CGATGCGCCCTCACCGGCTAAAGTGTTGCCCGAAAATTGGTGGCAACATCCTGCTGCATTAGGTGCAACCGACAGCGATA
TCGAAATCATCAAACGTCAATGGGGTGCGTTTTACGGTACGGATCTGGAGTTGCAATTACGCCGCCGAGGTATCGATACA
ATAGTGTTATGTGGGATCTCGACCAATATCGGTGTTGAATCCACCGCCCGCAATGCCTGGGAACTTGGCTTTAATCTGGT
GATTGCCGAAGACGCCTGTAGTGCCGCCAGCGCCGAGCAGCACAATAACAGTATCAACCATATCTACCCGCGCATCGCCC
GTGTGCGTAGCGTGGAAGAGATCCTCAACGCGTTATGATTTACATCGGTCTACCGCAATGGTCGCATCCTAAATGGGTGC
GGTTGGGGATCACCAGCCTTGAAGAGTATGCCCGCCACTTTAACTGCGTGGAGGGCAACACCACGCTTTACGCCCTGCCG
AAACCCGAGGTTGTCCTGCGCTGGCGTGAGCAGACCACAGATGACTTCCGCTTCTGTTTTAAGTTTCCGGCGACCATTTC
GCATCAGGCAGCATTACGGCATTGCGATGATTTAGTGACTGAATTTTTGACCCGCATGTCACCGTTGGCTCCGCGCATTG
GGCAATACTGGCTGCAACTGCCTGCCACATTCGGCCCACGGGAGCTGCCTGCGCTTTGGCATTTTCTCGATTCTCTTCCT
GGCGAATTTAATTATGGCGTGGAAGTCCGCCATCCACAGTTTTTCGCCAAAGGAGAAGAGGAACAAACGCTTAATCGCGG
TTTACATCAGCGCGGCGTTAATCGGGTGATTTTAGACAGCCGCCCGGTTCATGCAGCACGTCCACACAGTGAAGCTATTC
GCGACGCTCAACGAAAAAAACCTAAAGTTCCGGTACATGCTGTACTGACGGCGACAAATCCGCTGATCCGTTTTATCGGT
AGTGATGATATGACGCAAAACCGGGAATTATTTCAGGTCTGGTTACAAAAATTAGCGCAGTGGCATCAGACCACTACGCC
TTATCTTTTTTTACATACGCCAGACATTGCCCAGGCACCGGAACTGGTACATACCCTGTGGGAAGACTTACGTAAAACGC
TTCCAGAGATCGGAGCAGTTCCGGCTATTCCACAGCAATCTTCTCTTTTCTGAATTTGCCACCTATCATAGACAGGTGCC
ATCGGCCATTTTAAAGGGAGTTTGTATGGTAAGCGCGCTGTACGCCGTTTTAAGTGCGTTGTTATTAATGAAGTTCTCTT
TTGATGTCGTTCGCCTGCGAATGCAGTACCGCGTTGCCTATGGCGACGGCGGTTTTAGCGAACTGCAAAGCGCTATTCGC
ATTCATGGTAACGCGGTGGAATATATTCCCATCGCGATTGTGCTGATGCTGTTTATGGAAATGAATGGCGCAGAAACCTG
GATGGTGCATATTTGCGGCATCGTTTTGCTTGCTGGTCGTCTGATGCATTATTACGGTTTTCATCACCGTCTGTTCCGCT
GGCGACGTTCCGGCATGAGCGCCACCTGGTGTGCGCTGTTGCTGATGGTGCTGGCGAATCTTTGGTATATGCCCTGGGAG
TTGGTTTTCTCCCTGCGTTAGCGCACAATACGCCACTTTCTTTTTCCCGGATTTTTACGTTATGTCTCACCGCGACACGC
TATTTTCTGCCCCTATCGCCAGACTGGGCGACTGGACCTTTGATGAACGGGTAGCTGAAGTCTTCCCGGATATGATCCAG
CGTTCCGTACCCGGCTATTCCAATATTATTTCCATGATTGGTATGTTAGCCGAGCGCTTCGTTCAACCTGGTACGCAGGT
TTACGATCTGGGTTGTTCTCTGGGCGCGGCGACGCTCTCGGTGCGTCGCAACATTCATCATGATAATTGCAAAATTATTG
CCATCGACAACTCCCCGGCGATGATTGAACGCTGCCGTCGTCATATTGACGCCTATAAAGCCCCTACGCCAGTAGACGTC
ATTGAAGGTGATATTCGCGATATCGCCATTGAAAACGCATCGATGGTGGTGCTGAATTTTACCCTGCAATTCCTGGAACC
TTCCGAGCGCCAGGCGTTACTGGATAAAATTTATCAAGGGCTGAACCCCGGCGGCGCGCTGGTGCTTTCGGAAAAATTCA

GTTTCGAAGATGCCAAAGTTGGTGAACTGTTGTTCAACATGCACCACGACTTTAAACGTGCCAACGGTTACAGCGAACTG
GAGATCAGCCAGAAGCGCAGCATGCTGGAAAACGTGATGCTGACCGATTCCGTGGAAACCCATAAAGCACGCCTGCATAA
CGCCGGTTTTGAGCATAGTGAGCTGTGGTTCCAGTGCTTTAACTTTGGTTCACTGGTGGCATTAAAAGCAGAGGACGCTG
CATGATCGACTTTGGTAACTTTTATTCTCTGATTGCCAAAAATCATCTTTCACACTGGCTCGAAACGCTGCCCGCGCAGA
TTGCTAACTGGCAGCGCGAGCAGCAGCACGGGTTGTTTAAGCAGTGGTCAAATGCGGTGGAGTTTCTACCTGAAATTAAA
CCGTATCGTCTTGATTTATTGCATAGCGTTACGGCAGAAAGCGAAGAGCCACTGAGCGCAGGGCAAATTAAACGCATTGA
AACGCTGATGCGCAACCTGATGCCGTGGCGCAAAGGGCCGTTCTCACTGTATGGCGTCAACATCGATACCGAATGGCGTT
CCGACTGGAAATGGGATCGTGTTCTGCCCCATCTTTCTGATTTAACCGGACGCACCATTCTGGATGTCGGCTGCGGCAGC
GGTTATCACATGTGGCGCATGATTGGCGCAGGCGCGCACCTCGCGGTGGGGATCGACCCTACGCAACTGTTTCTGTGCCA
GTTTGAAGCGGTGCGTAAACTACTGGGTAATGATCAGCGCGCGCATTTGTTACCGTTAGGTATTGAACAACTTCCGGCAC
TGAAAGCCTTTGATACCGTCTTTTCGATGGGCGTGCTTTACCATCGCCGTTCACCGCTGGAGCATCTATGGCAGTTGAAA
GATCAACTGGTCAATGAAGGTGAACTGGTGCTGGAAACGCTGGTTATTGATGGTGATGAGAACACGGTGCTGGTACCAGG
CGATCGTTACGCGCAAATGCGTAATGTCTATTTCATCCCTTCCGCGCTGGCGCTGAAAAACTGGCTGAAGAAGTGTGGTT
TTGTAGATATCCGCATTGCGGATGTGAGCGTTACCACCACAGAAGAGCAGCGACGCACCGAATGGATGGTTACCGAATCG
CTGGCCGATTTCCTCGACCCACATGATCCGGGTAAAACGGTGGAAGGTTATCCGGCACCTAAACGCGCGGTGCTGATTGC
GCGCAAGCCGTAAAGGTCTGGTATTACTGCCGGATGCGGCGTGAACGCCTTATCCGGCCTACAAAACCTTGCTAATTCAA
TATATTGCAGGGACTATGTAGGCCTGATAAGCATAGCGCATCAGGCAGCTTTACGTTTGCATAACCTCAGCGCCCGTTTC
CGGGCGCTATTCACGTCTTACGCCTGAACAGCTGGCTGATCAAACGCCGTTAACTTCGGTGCATTGCCCGTATATTTTTC
GATATACACCAACGCTGAGTTACCGGCACAGGCATTTGCCAGCTGCGAGCTGGGGATATCCGCCGTTAACACGTTCGCAC
TGCCGTTTTTACACAAGCCATTTTCCAGATCTGGCCATGCACCTTCATGCACGCATACCACGCCTTTTTTGATCCCGTCA
GTCACCACCGCGCCTGTCAAAATCTGACCACGTTTGTTCCAGACACGCACCAGATCGCCATTCGCAATACCAAAGCGTGC
AGCATCTTCGGTGTGAATAGTGATTGGTTCACGATCGGCGATCGCATATTTTTTACGCAGTTCCGCATAGTTAAGCTGAC
TGTGTAAACGGTGTGCCGGATGTGCGGTCAGAAGCTGTAACTGCTTCTCGTCGGCGGTACCTTTCCATTCATCAGGAGCC
AGCCAGGTTGGGTGTGCCGGGCAATCCTTATAGCCAAATTTTTCCAACGTTTTTGAATAGATTTCAATTTTGCCGCTTGG
CGTACCCAGCGCATTTTTCACCGGATCGGCGCGGAAATCACCATAACGAACGTACTGTTCGTTCTTCTCGCTGTGGCGCA
TTTCGATCAGTTTATTTTGCTGCCAGAAGGCATTAAACATTGGCATAGTGACGCGTTGCGCACGGGCACCTTTCTGAGCG
GCATCATAGAAAAATTTCAGCCACGCCATTTCATCTTTACCTTCGGTATAGATCTCTTTTCCGCCAGGTTTGAGTAATTC
CGCAAGATCGGCAAACACGTCAAAATCGTTACGCGCTTCAAATTGCGGAGCAACAGCCTGCTTCATCGGCACAATATGCT
GGTTGCTGTAATCACCGGTCATCGTCAAGTCATTGCGCTCAAACGATGTGGTGATCGGTAATACGATATCTGCGTGTTTG
GCTGCTGCGGTCCAGTAGCATTCAGAAACGACGATCATCTCCGGTTTCTGCCATGCTTTAATCAGACGATTAGTATCCTG
GTGATGGGTAAAGTTACCGCCACCCGCCCACCAGATCATTTTGATATTCGGATACGTTTGTTCTTTACCGTTATGCTGAT
ATTTGCCGCCAGGATTTTCCAGCGCATCGACGATACGGGCAACAGGAATCGCCGTCATTCCGCCATCATCAGCGGCCTCG
CTGGCATGTCCGGCAATCGCCGCAGACATTTCCGGCAGCACGCCGCCAACACGCGTCGGGTTGCCACCGTTGGAGTAATG
ATAAGAGAAACCAAAACCGCCGCCCGGTGTACCAATCTGCCCTAACATCGCCGCCAGCGTCACCAGCATCCAGTGTTTCT
GTTCACCATATTGCTGGCGCTGAATTCCCCAGCCTGCCATCAGCATTGTGCGATTCGCCGCCATCAGTTCAGCGAGTTTG
ACTATCTGCGCTTCCGGTACGCCAGTAATTTCCGCCGCCCAGACCGCGCTCTTCGGCGTATTATCGCTCTTACCTGTCAG
ATACTCTTCAAACTGCGGATACCCGGTAGTGTATTTTTCAAGGAACACTTTATCGTGTTTGCCTTGTGTCATCAGGGTAT
GCGCAATCCCTAACATCAGTGCCACGTCGGTGCCCATATTCGGCGCGATCCAGGTGGCATTATCGTCAAAGAATTCGATG
GTTTCGGAGCGGATAGGATCAATGGCAATCACTGGTTTGCCAGATTTTTTCAGCTGATGGAAGTATTCCAGCCCTTGCTC
ATCGGTACTGCTCCAGGCAATTTTTAAGGTATTCAGCGGGTTCATTCCCCACAGCACCACAACCTGGCTGTTTTCCAGAA
TCAGCGGCCAGGAGGTCTGCTGTTCATACACCTCTACAGAACCGACCACATGCGGCATGATCACCTGTGCTGCCCCGGTT
GAATAATCGCCGCTATGCCCGGAATAACCGCCCGCCAGGTTCATATAACGTTGCAGTAAGGTTTGCGCTTTATGCAACAC
GCCAGAAGAGCGCCAGCCGTAAGATCCGGCAAAAATGGCCGATGGTCCGTTAGCTTTACGAATACGATCATGTTGCTCAT
GAATCAGTTTTAATGCCTGTTCCCAGCTCACCTGTACATAGGTATCTTCGCCACGACCTTTCGCCGGTTGCAGTGGATTA
TCGAGATAGCTTTTTCTCACCATCGGATGCTGAATACGCGCCGTGGTGTGTACCTGATCCGCCGCCGTAGACTGTAAGGA
ATTCGGTATGGTTTTCGCCAGCGCGCCTGTTGAAGAAACAATCTTGCCGTCCTTCACTTCTACGTTCATCGCTCCCCAAC
GTCCCGCGGTGAGGATTTTACCGCCCTTCTCTTCTGCCCATGCGGGGAGCGGTGCTGCCGATGTCACCACCAGCGCTCCA
GCGGCAATACCGCTGTGTTTAATAAATTCACGTCTTGTTAATGTCATAACTTCCTCCCTGATCAACGAGGATCACTGTTT
CTCGGTAATATCTTTGGCGTTGTACTGGAAATACCGCGTTAAAATGTCCAGTTCGTTTTCGCTCATGCTGGTTCGTGCCC
CCATTCCTTTGGCAATGGACGGCCACGCATTGACGGTGTAATGGTCGGCGGCAATAGGGGCATGACAACCAGCGCAATAG
GTATCGTCAAGTTTTTCAGCGTATTGCCATAGCGGTTTACGGTCCGCTAATGCGGGATCGGTAAGCGCACCCTGTAAAGA
CGCCTGACGCCATTGATTGCCGTATTCGTCAGCCTGCCATTCCCCGTTTACAGTGAGCGCCTTGATACCTTCTTCACTTA
ATGTGGCTAGCGCCAGCCGTTGACCTGCCGCCAGGTAGAGCGTGTTTTCACTGCCCTGCATTTGATAACCCTGCAACAGA
ACGATCGGCTGTTTGCCACTGGCATCAACGACGGTGAGATCGGTTCCAGGATTCACGGTAGCCAGCTCGCCTATGTGAGA
AGTTTTGAAAGGATAAATATGTGCGCCATTAGTAACTGAAGTAGCGGCCTGACTTTCCAGCTCATGCGCCGCGTTGTCAT
CCATTTTTATTTCTGGCGGAAAATGGGCAATGCCTTTATGACAATCGATACAGGTTTCGCTGTCCTTTTGTGCTTTGTTA

TGCATTTTCTGCGCAGATTCACTTTGCGAGGCAATATCCATGGCATCAAAAGAATGGCAACTACGGCACGTTGCAGAGTC
AGTGGCTTTTAATTCTTTCCATACTGTTTCGGCCATTTCCTGGCGATGAGCTTCGAACTTATCGTCACTGTCTATTTTGC
CGCTAACAAATTCATGATAAATATCTTTAGATGCCTTTAATTTAGCAAATAAATAATCCATCCCTGACTTTGGAATATGG
CAATCGGCACATTCCGCACGTATCCCTTTCTGGTTCGAAAAGTGGACAGTTCCCTGATATTCCTCAAAAGGTTTACTCAT
CGAGTGGCAGGAAAGGCAAAATGCTGTATCCGACGTTTTATGTAAGACTTTTTGCGCCAGCAATAACCCGCCGCCACCAA
CCACAACCGCTATCAGCAAAAACAATAACCCAATGCGTTTTTTCCCTCGCATAGATTCCTCTAATTAAATTACTGGACAC
CGAAATCATTGTATAATTAAATATACAACGAATTAACCGCTTGCATAATTAGGCACAACACTGCCTGAAACAATCGATAA
AGAATATGATTTATTACAATGTAATCATTAATTGCTAAGGAATAACCCAGTTGCTATTTAGAATATATATTTATCAGTTT
TAGTAATTTAAATCCCATAATTAATGTGAATATATACAATATTTTTAGCACAACGCTATGCGCACTGTCACCCGCTCAAC
AGATCACGGTTATCGTTCGTTTTTTATACTGTTCAGGGATAAAAAAAGGCCCCTGTTGAAATTGCAGGGGCCTGGTACGA
GCAAGCATCATATTGGGCGACATGATGCAACGGTAAAAATCATTTGGCCTGATGGCGTTCAATGATTCCTTTCATTTCAG
CAACCGCCGCCCCGTCTACGATATAACGCGAATACTCGTCCGCGTGTTCATCTGATGACATGGACAATCCTTGATTACGA
TAACGCATCGGTGAGGCTTGCCACGCTCCCGCGGAGCTATGGACTTCCAGCACTCCGGCATCGAGGAAGTGGTGCAGGTT
TTCTGCACGGACTCCTGCTCCGGCCATAATGATTGGAGCATCACGATGGGCAATAAGTTCCATAATTTTTGATAAACCTT
GCAGTGCGTCTGATTTTTGCCCTGATGTCAGTACCCGGGCAATGCCCAATTCCGCAAGATTATTGAGTGTATATAAAGGG
TTAGCGCACATATCGAAGGCGCGATGAAAAGTCACTGCCAGCGGACCGGCAGCAGCCATTATTTTTTCCATTCGTGGCAT
ATCGACATTCCCGTCAACATCGAGAACGCCCGTCACCAGTCCAGGAAAACCTAATTCGCGGACCGTGCGCACATCCTCAA
GAATGGCGGCAAACTCACCGTCGCTGTAGCAAAAATCACCACCGCGTGGGCGAATTATCGGATGCACAGGGATCGTCACC
CGCTGGCGCACGGATTTCAGTACACCCAACGACGGCGTTAAGCCCCCCTCTTTTGGGGCTGCGCATAATTCAACTCTGTC
TGCGCCGTTTTGCTGCGCCGTTAGTGCACATTCCATGCTGTAACAGCAAATTTCCAGTAATGCCATTTTTACTCCTTAAT
TACGCCGACTGCTCGCTGGCAACGATCTCTTCAATGGACCACGGATGAAACTTAATAGTTGTTTTGCCATCGGTGACCGC
CAGCGTCGGATTCGGTAATCGCTCATGCTCACCTTTTGGCGAACTGGTTTTCACTGAGATCCCCGGTAGGCTGAGTCCTT
CATCAGAAAGCAATGCCAGCGCACGGGCATTCAGCGTTTCTGGATCACCCGGCAGAACGATTTCAATATGTTCCCAACCT
TCGTGTGGGTAACGTTTTTCCCCGGGCCACGGTAGCTCCACAATAGAAAACTGCCAGTGCGCAACCTGTACCGGTTCATG
CAATTTAAACAGACAAATCGGTCTGCCATTGATCATATTTTCTGACAAAAGCTCGCCACACTGTTCAAACCCGCGACGCC
AGCGTTCAGCAGTGGCGTTTTGATGGCAACGCAAAGAAATGTGATCGGCAGTCAGCGGAGTGATATTCAACCCCAGACGG
CGGGAAAGTTCATCTAATGCGTGGATAAATCGCGGTAAATCCGATGCAATATCCTGCAGCTCGTCGATAGATTGCCAGTT
CGCCATAATCACTCTTCGTCTTTCAGTAAAAGCGTTAATTTACCCTGTTGCCCTGTGCCAACCAACCGCTGATTTCACGC
CGCTTCTGATGCAATAGTGAAAACGGCAATACGCCACGCGCACGTTGCTGACGAAAACAGCCATTTGCAGTATACTCCCG
CCCTAATTTCTTTAACTGGTGCGGGCAATTTTTGCTCGCTTCATCAATGTAAGGTATTCCGGTGAATATTCAGGCTCTTC
TCTCAGAAAAAGTCCGTCAGGCCATGATTGCGGCAGGCGCGCCTGCGGATTGCGAACCGCAGGTTCGTCAGTCAGCAAAA
GTTCAGTTCGGCGACTATCAGGCTAACGGCATGATGGCAGTTGCTAAAAAACTGGGTATGGCACCGCGACAATTAGCAGA
GCAGGTGCTGACTCATCTGGATCTTAACGGTATCGCCAGCAAAGTTGAGATCGCCGGTCCAGGCTTTATCAACATTTTCC
TTGATCCGGCATTCCTGGCTGAACATGTTCAGCAGGCGCTGGCGTCCGATCGTCTCGGTGTTGCTACGCCAGAAAAACAG
ACCATTGTGGTTGACTACTCTGCGCCAAACGTGGCGAAAGAGATGCATGTCGGTCACCTGCGCTCTACCATTATTGGTGA
CGCAGCAGTGCGTACTCTGGAGTTCCTCGGTCACAAAGTGATTCGCGCAAACCACGTCGGCGACTGGGGCACTCAGTTCG
GTATGCTGATTGCATGGCTGGAAAAGCAGCAGCAGGAAAACGCCGGTGAAATGGAGCTGGCTGACCTTGAAGGTTTCTAC
CGCGATGCGAAAAAGCATTACGATGAAGATGAAGAGTTCGCCGAGCGCGCACGTAACTACGTGGTAAAACTGCAAAGCGG
TGACGAATATTTCCGCGAGATGTGGCGCAAACTGGTCGACATCACCATGACGCAGAACCAGATCACCTACGATCGTCTCA
ACGTGACGCTGACCCGTGATGACGTGATGGGCGAAAGCCTCTACAACCCGATGCTGCCAGGAATTGTGGCGGATCTCAAA
GCCAAAGGTCTGGCAGTAGAAAGCGAAGGGGCGACCGTCGTATTCCTTGATGAGTTTAAAAACAAGGAAGGCGAACCGAT
GGGCGTGATCATTCAGAAGAAAGATGGCGGCTATCTCTACACCACCACTGATATCGCCTGTGCGAAATATCGTTATGAAA
CACTGCATGCCGATCGCGTGCTGTATTACATCGACTCCCGTCAGCATCAACACCTGATGCAGGCATGGGCGATCGTCCGT
AAAGCAGGCTATGTACCGGAATCCGTACCGCTGGAACACCACATGTTCGGCATGATGCTGGGTAAAGACGGCAAACCGTT
CAAAACCCGCGCGGGTGGTACAGTGAAACTGGCCGATCTGCTGGATGAAGCCCTGGAACGTGCACGCCGTCTGGTGGCAG
AAAAGAACCCGGATATGCCAGCCGACGAGCTGGAAAAACTGGCTAACGCGGTTGGTATTGGTGCGGTGAAATATGCGGAT
CTCTCCAAAAACCGCACCACGGACTACATCTTCGACTGGGACAACATGCTGGCGTTTGAGGGTAATACCGCGCCATACAT
GCAGTATGCATACACGCGTGTATTGTCCGTGTTCCGTAAAGCAGAAATTGACGAAGAGCAACTGGCTGCAGCTCCGGTTA
TCATCCGTGAAGATCGTGAAGCGCAACTGGCAGCTCGCCTGCTGCAGTTTGAAGAAACCCTCACCGTGGTTGCCCGTGAA
GGCACGCCGCATGTAATGTGTGCTTACCTGTACGATCTGGCCGGTCTGTTCTCTGGCTTCTACGAGCACTGCCCGATCCT
CAGCGCAGAAAACGAAGAAGTGCGTAACAGCCGTCTAAAACTGGCACAACTGACGGCGAAGACGCTGAAGCTGGGTCTGG
ATACGCTGGGTATTGAGACTGTAGAGCGTATGTAATCGATTTTTCGTGAGAGTGAAGCCTGATCAGGGTTAGCCGATCAG
GCTTTTTTATTGCCATCTAAATGTATTCTGAAAATGGACATGCCATTGTTTTCTCACTGTTGGATAAGAGGCCAGAAGCG
TAATATCCGGCCCCAGGGAAACGATAACGGTTGAATTTAAGGAATACCGCAGTGTTTAAATTTCTTGTATTAACATTAGG
CATTATCTCTTGCCAGGCTTACGCAGAAGATACGGTTATAGTAAACGACCATGACATTTCAGCCATCAAAGATTGTTGGC
AAAAAAATTCAGATGATGATACTGACGTTAACGTGATCAAATCATGCCTGCGACAAGAATACAATCTCGTCGATGCGCAA

TTAAATAAAGCCTATGGTGAAGCTTATCGTTATATAGAACAAGTGCCACGCACAGGTGTAAAAAAACCTGATACCGAACA
ACTTAACTTGCTTAAAAAATCACAGCGAGCCTGGCTGGATTTTAGGGACAAAGAATGTGAATTAATCCTTTCAAATGAGG
ACGTTCAGGATTTAAGTGACCCTTATTCTGAATCAGAATGGCTCTCATGTATGATCATACAGACCAATACGCGAACTCGC
CAGTTGCAGCTATACCGTAACTCTGAAGATTTTTATCCAAGCCCTTTGACAAGAGGATAATTCACATCTTTTTGGCATGT
TTTGTTGCAAGCTATTCCTGATAAATAATTGCAACAAGACATCGAGCCTTTTTCACTGAGTTATTAAACATACTCGCGAG
CGCGTAATTTTTTTGTCCTTCAGCGATAATTCACAATCACTTCATTACGTTGTACCTTTAGCGGTGGAATTAACCGCCCA
CCGCCTGGCACTTCCCAGATAAATCGCAACGGTTCTGCTGCCGCTATACCGGAAAAGGCCACGGTGGTTCCGCTCTGCCC
CTCTAATTCGACACAACGAGATTGCGAACACAAGCGAACCCGCAGTCCTGAAGGTGTCGGGCCGATAAGCTGATAACGCC
ACGCTACCAGCGTCATCAATCCTGAAGCAGGTTGTCGCGTAGAAAGAGGCGCAGACGACATCGACTCACCGCGATGATTT
AGCGTAATTCCTACACTACTTGCCTGCCACATCCCCTCCCCGGCGGCTTGCACCAGCAGCGGAAACAATAATATTGCTAA
TAAGGTTCTCATTATTTGCCGCCAATTGTCGCCGTCATGCGGATATGTCGGTTATCAGACAGTTCCAGATTCGACAGGAC
CACTAACTGCGGCAAGCTGCGGCGCAGGAAGCGAGACAATAATGGTCGCAGCGCGTGGTTCACCAACAATACTGGCGGCG
CACCCAGCATCTCCTGACGGGATAGCGCTTCCTGAGTTTGCGCCAGTAAACGATCCGCCAGCCCTGGCTCCAGTCCTCCC
CCGCCCTGCAGCGCCTGTAGTAACAAACGTTCCAGCGGTGTATCGAGGCCAATAACATGGACTTCATCTTTGCCAGGAAA
CCACTGCTGGGTAATCGCCCGTCCCAACGCCACGCGCACGACGGCGGTTAATTCATGTGGATCGCTTTGGATGGGCGCAT
GTTCCGCCAGCGTTTCGAGAATGGTGCGCATATCGCGAATCGGTACTTTTTCATCGAGGAGATTTTGCAGCACTTTATGC
AGTGTGGTGAGCGTGACGACGCCAGGAACGAGATCTTCCGTCAGCTTTGGCATCTCCTGGGCGACGCGATCCAACAGCTG
TTGCGCCTCCTGACGACCAAACAGCTCTGCGGCATGCTGGCTAATGAGGTGGTTAAGATGCGTTGCTACCACCGTGCTGG
CCTCAACCACTGTGTACCCCTGAATCTGCGCCTGTTCTTTTAGCGCACTTTCAATCCAGATAGCATTCAGGCCAAATGCC
GGATCGACGGTCGCCTCACCAGGTAACGTCCCGGCAGCGGTTCCAGGGTTAATCGCCAGCCAGCGCCCCGGATAAGCATC
ACCACTGCCAATCTCCACGCCTTTCATCAAAATGCGATAGCGGGCAGGTTGCAGATCCATATTGTCGCGAATGTGCACCA
CTGGCGGCAGAAATCCCATCTCCTGGGCAAATTTCTTGCGGATACTGCGTATACGGCCCAACAACTCACCATCCTGCTGG
AAATCGACCATCGGGATCAGTCGATAACCCACTTCCATTCCCAGAGAATCTTCCAGTTGTACATCGTTCCACGTCGCTTC
GACAACGGTATTATTCTCTGCCATTTTTACCGGTTTGGGTTCGGCAGGCGCTTTTTGTTCGCGTCCGCGTATCCACCAGG
CCAGCCCGAGCAATCCGGCAGTGAACAGCAAAAATACCAGGTTCGGCATTCCAGGCACCAGGCCGAGTAAACCGAGCACG
GCGGCGCTTAACAACATAACGCTTGGGTTACTGAAAAGCTGATTCACCATCTGCTCGCCAACATCCTGATCGGTGCTGAC
ACGCGTAACGATGACCCCCGCGGCGGTAGAAATCACCAGCGCCGGAATTTGTGCCACCAGACCGTCGCCAATGGTCAATA
GCGTATAACTTTCCGCCGCGTGTCCCATGCTCATGCCATGTTGCAGCACGCCGACCAGCAACCCGCCGACAATGTTAATG
ACCATGATGAGGATCCCGGCGATGGCATCGCCGCGAACAAACTTACTTGCCCCGTCCATTGAGCCGTAAAAATCGGCTTC
CTGAGTCACTTCGGAGCGGCGTTTTTTCGCCTCATCTTCACCAATCAATCCGGCGTTAAGGTCGGCGTCAATCGCCATCT
GCTTACCCGGCATACCATCGAGAACAAAGCGCGCACCCACTTCTGCGATACGCCCGGCACCTTTGGTAATGACCATAAAG
TTGATGATCACGAGAATGACAAACACCACGATACCGATAGCGAAATTGCCACCAACGAGGAAGTGACCGAACGCTTCGAC
CACCTTCCCTGCCGCCGCCGCGCCGGTATGCCCTTCCATTAAAATGATACGGGTTGAAGCCACGTTAAGTGCCAGACGCA
ACAGCGTGGTAAACAACAGAATGGTCGGAAACGCAGCAAACTCAAGCGTGCGCTGGGTAAACATCGCCACCAGCAACACC
ATGATCGACAAGGCAATATTGAAGGTAAACAACAGGTCGAGTATGAATGCGGGCAGTGGCAGCACCATCATCGACAAGAT
CAACAGGATCAAAATCGGTCCGGCAAGGATCTGCCATTGTGTCGATTTCAGGTTTGCGGGCAGGCGCAGCATCGCGGCCA
GATTACTCATGGGTCGGTTTCTCGTTAATAAAATCCAGGGCTTCCGGCACCGGAAGATGAGTAGGTTGTACAGGGCGCTG
TCCACCAGCCAGACGCCAGCGTTTCAGTTGCCAGACCCAGGCCAGCACTTCCGCCACCGCGGCGTACAGTTGACCCGGGA
TTTGTTGACCAATCTCCGCATGTCGATACAGCGCTCGCGCCAGCGGCGGCGCTTCAAGCGTCGGGACGTTATTTTCAGCG
CCAATTTCACGAATGCGCAGCGCGACCAGCCCTGCACCTTTAGCGACCACTTTCGGTGCGCTCATTTTGTTTTCGTCATA
CTGCAACGCTACCGAATAGTGGGTCGGGTTATTGACAATGACATCCGCTTTCGGCACATCGGCCATCATCCGACGCCGTG
CAGCAGCTCGCTGCATCTGACGGATCCGCCCTTTAACATGAGGGTCACCTTCGCTTTGTTTGAACTCATCACGAATATCC
TGCCGTGACATACGCAGCTTTTTCAGGTGGCTGAATATTTGGAAAAAGACGTCAAATCCCACCATTGGAATGACACCAAG
CACCACCAGCAGTGCGCATAGCCCTACCAAATCCATCGCATTACCCATGGCGGTAATCGGAGACTCGGCCATCAAGCGCA
TCATCTGCGGCCAGTGATGCCAGAGAAAAAACCCCGTCACGCTGCCAACCAGGATGGTTTTCAAAATTGCTTTAAGCAAC
TCCGCGCCAGTCTGAGCCGAGAACATCCGTTTAATGCCCGGTAGCGGGTTGAGTTTGGAAAACTTCGGCTGCAAGGATTT
GCCGCTAAATACCAGCCCTCCCAGCATGACCGGAGAAATGAGCGCCACCAGCACCACGCCGCTAATCAGCGGCAGCAGCG
CCAGCATGGCTTCTCTGATCAGCAGAATAATCTGCCCGAGGATCAGATTCGGGTCATTGATAATACTGTGATCAAAATGC
AGCCCAGCGGAGAGCATGCCCGACAATCGACGGGCCAGCGACACACCGCCAAACCAGATAACACTAACGCCCACCAGCAA
AATCAGCAGTGAGGTCAGTTCACGGGAACGCGGGATTTGCCCCTCTTCCCGCGCTTTTTCTAGTCGGTGAGGTGTGGGGG
CTTCTGTTTTGTCGTCGCTCTCGTCAGACACGTCGCCAATCCTGGAAAGAGTATTAAGCGTGAATGATGCCAGAGCGCAA
AGCGTTCAATGGTTTGAGTAAGGGGCAAAACAGGCGGGATTTAGGGCTTTTGCTGCCACACATCAAGCATAGTGTGCGTT
TGTCGGATGCGGCGTCATCGCCTTATCAGACCGCCTGATATGACGTGGTCACGCCACATCAGGCAATACAAATCAAAATC
CAAGACTATCCAACAAATCGTCCACCTGATCCTGACTGGCTACCACACCGGCTTTGCTGGTATCGACCTGAGGTCCATTA
AGCAAACTCTGGTTTTCACGTTTTGGACGCGACTCCTGTTCCGGGATGTTTTCCAACAGCACCATCAGCAACTGGCGTTC
GATCTCCTGAATGACATCCATCATCCGCTTAATGACCTGCCCGGTGAGATCCTGAAAATCCTGCGCCATCATGATTTCCA

GCAGTTGCGCGTTAGTAAAGCTGGTATGCGCGGGTACATCTGCCAGAAATTGTCGTGTATCTGTTACCAGTTCACGGGCG
TCGGCAAGGTCAATCGGATCGGCAAACCAGTCATCCCAACGTTGGGTTAACGCTTTTGCTGATTTCTCCATTTGATCCTG
ATGCGGTTGTGACGCCTCAACACTGTTCAGCGCCCGCTCCGCAGCCTGGGCGGTCATCTGCACAACATAGTACAAACGAT
CGCGCGCATCGGGGATGGCTTCCGCCGCTTCGGCAATGGCCTGATCCAGCCCCAGTTCCCGCAAACTGTCGCGCAGCATA
CGCGTCAGGCTGCCGATGCGCGCAATGATATCGCCAGCTGAATGCTCGTCAGCAGGTTTGATTGATGGTTGCATCATAGT
CGCATCCTCACATGCCCAGTTTCTCAAAGATTTTGTTGAGTTTTTCCTCCAGCGTCGCGGCGGTAAATGGCTTCACCACA
TAGCCACTGGCCCCCGCTTGCGCCGCAGCAATGATGTTCTCTTTCTTCGCTTCTGCAGTCACCATTAACACTGGCAATGC
CGACATCGCGCCATCCGCACGAATTGTTTTCAGCAATTCCAGGCCATCCATATTGGGCATGTTCCAGTCGGAGATAACAA
ATCCATAACCGCCTGCCTGCAACTTATTGAGAGCGTCGACGCCATCTTCCGCTTCCTCAACATTATTGAATCCCAGCTCT
TTCAGCAGGTTACGCACTATGCGTCGCATGGTGGAAAAGTCATCCACAACCAAAAATTTAAGTTCTTTATCCGCCATTTC
ACACTCCTGATTTAAATACGTATCGCCTGTCCGGCACTAATTTTTGCCAACATTTGCTGGCTTACCTGGCTAAGATCGAC
CACTTCGCAGACACCACCCATATTGATGGCCTCGCGCGGCATGCCGAACACCACGCAACTTGCTTCGTTTTGCGCAAGGG
TCCATGCCCCCGCCTGACGCATCGCCAACATTCCCGCCGCGCCGTCGTTGCCCATACCGGTCAGGATCACCCCAACCGCA
TTACGCCCCGCCTGTTTGGCGACAGAATGGAACAACACATCTACCGAAGGCCGATGACGGTTAACCGCCGGGCCATCGTG
AATTTTGATTTGGTAATTTGCGCCACTACGCGACAGCTCCATATGCCGATCGCCCGGCGCAATATAGGCATGCCCCGGCA
AGACACGTTCTCCGTCTTCGGCTTCTTTAACCCCGATCTGGCAAAGCTTATTAAGTCTGTCGGCAAAAGAGCGGGTGAAA
CCGGGCGGCATATGCTGGGTAATTAACAGTGCCGGGCTGGAAAGCGGCAACGGTTGCAGTACGTGACGAATTGCCTCAGT
TCCACCCGTTGAAGCACCAATCGCAATCAGTTTTTCAGAACTCAACAACGGCCCCGCCTTCAGCGTTGTCGGTGCCGACA
ATGGCTTATGTGCTGCAAGGCTCGCCTTTGCTGCCGTACGCACCTTTTCAGCAATCATTTCGTTATACGCCAGCATACCT
TCGCGAATACCCAGTTGCGGTTTGGTGACAAAATCTATCGCCCCCAGCTCCAGCGCGCGCAGCGTGACTTCTGACCCTTT
GCCGGTCAGGGAAGAAACCATCACAACGGGCATTGGACGCAAACGCATTAATTTTTCGAGGAAATCCAGTCCGTCCATCC
GCGGCATTTCAACATCCAGCGTCAGCACATCGGGATTGAATTTCTTAATCAAGTCACGCGCGACCAGCGGATCAGGCGCG
GTCGCCACCATTTCCATGTCGCTATGGCTGTTGATGATTTCTGTCATGATCTGGCGCATCAGTGCCGAATCATCGACAGA
TAACACCCTGATTTTGCTCATCGTTAATCCTTACTTAGCGCATACACCGTCTGACCACGCAGCGTGAAGCGGCGCTCAAG
GTGGCTAAAGTTTTCAGAGTGACCCGCAAACAGCAATCCGTCGGGTTTAAGGAGCGGAACAAAGCGGCGCAAAATCTCCT
GCTGGGTAGTTTGATCGAAGTAGATCATGACGTTACGACAGAAGATCGCATCAAACGGCCCCGGCACGGTGTACTGTTTC
GCCAGTAGATTCAGCGGGGCAAAATCAACATAGTTCGCCAGCTCCTGACGCACGCGTACCAGCCCTTCATGCGGCCCCGT
CCCTCGCATGAAATACCGTTGCAGTTGCTGCGGCGTCAGGTTTTTCAACTCTTCATGGCGATAGATACCGCTTCTGGCTT
TTTCCAGCACTTCGGTGTCGATATCACTGGCAAACACTTTCCAGCGTCCGGGCGCGGTGCCCAATGTGTCAGCCAGCGTC
ATCGCAATGCTGTACGGCTCTTCGCCGGTCGAAGCCGCCGCGCTCCATACGCGATACTCGCCAGAACGGCGACGTGCGTG
ATCCGCGAGCAGAGGGAAATGATGTGCCTCACGGAAAAATGCCGTCAGATTCGTGGTCAGCGAATTGATAAACGCCTGCC
ACTCACCGCTGTGCTGATTAGATTCCAGCAAGTTCAGATAATGACCGAAATCCGTCAGTCCCAGCGAACGCAAACGACGA
ACCAGTCGGTTGTAAACCATGTCGCGTTTATGGTCAGCCAGAACGATCCCGGCTCGTTGATAGATCAATTGACTTATCCG
CCGAAAATGCGCGTCGGAAAGCGCCAGGCGCTCGGTCATCTGTAACAATAAAGACGTTTGCCCACAGGGCAGAGATGAAG
TCATAGCGCCTTCTCAATCACTTCAGGATACCACTGGCGCAATTTGTAACTGCACCGACTCATGTCGTGCTACTTCATGT
TCTTCAAGGGTAAATACCGCCACACGCGACGAAAGATGGTCGGCCTGATTCGCCAGTTGTTCCGTTGCCACCGCCGCTTC
TTCTACCAGCGAGGCGTTCTGCTGAGTCACCTGATCCATCTGGCTGACAGCCTGTGCAACCTGCTCAATCCCCCGCTGTT
GTTCTTCCGACGCAGAGGCAATTTCTCCCATAATGTCGTTCACGCGGGTCACCGAACTGACAATATCGATCATGGTCGCG
GCGGCGTTATTCACCAGTTTCGAACCCTGCTGGACACGATTGACTGACTCTTCGATCAGCCCTTTGATCTCTTTTGCTGC
CTGCGCGCTACGGCTGGCAAGATTGCGCACTTCACCTGCCACTACCGCAAAACCACGCCCCTGCTCTCCGGCGCGAGCCG
CTTCCACTGCCGCATTCAGGGCCAGAATATTGGTCTGGAAAGCAATTCCGTCGATAACGCTGATAATGTCGCCAATTTTT
TGCGAGCTGGTGGCGATCTCCTGCATGGTGTGAGTCATGGTACTGACCTGAACACCGCCCGCCTGCGCCGTTGTCGCGGC
ATTTTTTGCCAGTTCCGACGCCTGTCGTGCGTTATCGGCGTTTTGCCCTACCGTGGCGGTTAATTGCTCCATACTGGCGG
CCGTTTGTGCCAGCGATGCCGCCTGCTGTTCGGTACGACTTGAGAGATCGTTATTGCCTGCGACAATCTCCGCGATACCA
ATGTGCATCTCCTGGCTTCCCTTACGCACATCACTTACCGTCCCACGCAAAGCCTGCTGCATGGTCTTCAGACTGGCAAA
AATGGCGGTGATCTCATTACGACCATATACCGCAATCGGACGCGCCAGATTACCCGCAGCAATGCTGTCAAAATGGCTAC
CGATAATGGCCAGTGGTTGAACAATCATCTTGCGCGTCCACCACAGCGCACTGCTGATGTAGATCGCCGCAACAATAATC
ATGCTGATAAACACCAGTGCCGAAATCTGATAGTTACGCTGGCTTTGCGCACTGGCGGCTTCCAGCACATGGTTGATCTC
CAGCTGCCAGGCCTCAAAGTTAACGTCAAACGCATTCTGTGATCCCTGCACCGGCGCAGTGAGGAAATCCGAAAGTTGAT
TACTTTCAAGCCAGGTCGCCTGATGTTCGAGATCGTTGTGCCAGCGGGCAAAACTTTTCTCCGTCTCTTTTTGCAATCCC
CTGACGTGCTCGTTGCCCGCAGTCATCGCCATAAAACTTTTAAACAGCGTGGTGGATTGCGTCAGACTGGCGCGCGCCGT
CGTCATCAACGTTTTAATGTCATCCGCCGGATAGCTAAGTGCCGTCAGAGTGCCCGCTTTGTTCAGCGCGGTACTGGCCT
GTAACATTACCGCCCGAGTTTGCGCTAATGCCGCACGTTGCTGATTGCTCTGCTCGACCTGATTCAGTCGTTGCAAATCG
TCGCGAAATGCCCAAAAAGACATGCCGTTACTGCCAATCTGCAAGATCCCGCAGAGAATCAAAATTAAAAACAGCGTGGT
CGAAATTCGAATACGATTAAACATCAACGCTCCCCATCAGGCGGCAATGACCGCGTTAGTAAATACTCGTCAAAATGTTT
CCCAGTTTGGATCTTGTTCAGCAATTCGCAGTCGTGGCTGAGCCGGTGGTTGCTCACTGGCAGGACGGGATGGTGTTTGC

GGTTTATTGGTGAGTGGGCTGGCTGCCAGACGGAACGCGGAAACTGCTTGCGTTAAACGACTCGCCTGTTCTTCCAGCGC
AGCGGCGGCGGCAGCTGATTCCTGCACCAGCGATGCGTTCTGTTGCGTGACGCGATCCATTTCCGAAACCGCCAATGCGA
CTTGATCGATGCCACGGCTCTGTTCATCCGATGCCGATGCAATCTCGCCCATAATGTCAGTCACGCGAGTGACAGCATTG
ACGATATTGTTCATTGTTTCCCCGGCGCTTTCGACCAGCACCGAACCGGTATCAACGCGTGAGACGGAGTCTTCAATGAG
GGCTTTGATCTCTTTTGCCGCCTGGGCGCTGCGACTGGCAAGATTACGCACTTCACCCGCCACCACGGCAAAACCACGGC
CCTGTTCACCCGCACGCGCGGCTTCAACCGCGGCATTCAGCGCGAGGATATTAGTCTGGAAGGCAATACCGTCGATAACG
CTGATAATGTCGGCAATTTTCTTCGAACTATCGGCGATCTCATGCATCGTTTTCACTACGCCATCCACCACTTTGCCGCC
GTGCTGGGCGGTGTCGGAGGCACTTTGCGCCAGTTGCGAGGCCTGGCGGGCGTTATCGGCGTTTTGCTTCACTGTCGCGG
TGAGCTGCTCCATGCTGGCGGCAGTTTCTTCCAGCGCGGATGCCTGCTGTTCAGTACGGGAGGAAAGATCGGTGTTGCCC
GCCGCAATTTCACGGGTACCGGCATAGATGGCATCTGAACCTTCGCGGACATGAGTGACGGTGTCAGTCAAAGAGCGTTG
CATATGTGAAACGCTCTGCGCCAGGTCGCCCATTTCACTGCGCCCGTCAATGGTCAGGGTATTCGCCAGGTTACCACCGG
CGATTTCGCGAATGTGAGCAATAATTTTTGCCAGCGGAGTAAGCAACATACGGCGAATGCCGTACCACGCCACCAGCAGA
ATCAATACCACCACCAGCGCGATAACCGCCAGTTGCCACTGGGCAAATCGGTAATCATCTGCGTTGTCAGTGACGATATC
GCGATACAGTTTTTCACTGCTGAGGGCGTACTGAGCAAACGCTTCGCCCATTGCATTTTGCATTCCCTGGGTTGGCTGAG
CGAAATAAGCTCCAGTATTGCCATAATCTAGATAATCAATCAGTTCAGTTAACGCTGTGTAATAGTTTTTATATTTTTCA
TCAATATTACGACTGGTAGCGACCATTTCAGGTAACGGTGCCATGCTTTTGAATTTTTTATAATGCGTCGCTGCCTGCGC
CAATGTTTTCCTGGCGCTATCGAGCAATTCAACTTTGGCGTTACTTTGTTGATTGGAGGAATCCATCATCATCCGTACCG
CTGAACGACTCAGGTTAATGCGCGTTTGCAGCATTAAATCCCAGGTTGACGTCAGCTCGCCCTGCTGTTCCCGTAATTGA
TTGGAAACCACAAAGCTCTTCTGGCTATGGTGAAGGGAAGAAAAAAACAGACTGCCGGAAATAAGCTGTAACAGTGCGAA
TACCCCCAGCACCATTACCAACAGCGTGACTACGCGGATACGGTTAATCATAAGGCACCTTCCTGAAAACAAGTTGATCT
CGTTATCGGCAAGGAGGGGGGAAACTTTATTGCTGATGCCACCCGCCGCGAAATTGAAATAAAAAACCCGATGCGCAGAT
CATCGGGTTCATTTCAATTGAGGAAATCGGGAGAATTACGCCACTTCTGACGCCGCGCTATCTAACAGCGCCATCTCTTC
GCTGTTCAGCAGTTTTTCGATGTTCACCAGAATCAACATCCGGTCGCCCAGTGCGCCCAGTCCAGTGAGATATTCTGTTG
AAAGCGTCACGGCAAATTCCGGTGCCGGACGAATTTGCTCCGCCGTCAATGAAAGCACGTCTGAGACGCCGTCAACCACG
ATGCCGACCACCCGCTGTCCGAGATTCAGGACGATAACTACCGTGTTGTCGTTATAGTCCACATCCACCTGGCTGAACTT
AATTCGTAAGTCAACAATCGGCACAATAACGCCGCGCAGATTCGTGACGCCTTTGATAAACGCTGGCGTGTTCGCAATCC
GTGTTACCTGATCGTAGCCACGGATCTCCTGCACTTTCAGGATATCAATACCGTACTCTTCATCACCAAGGGTAAATACC
AGAAATTCCTGGCCTGACGGCTCGCTGGCCAGCTTTGTTACATTCGTCATACCGGTCATATTGTTACCTTTTTACTCATT
CAGGCGGCGGTGTTCGCCATACGTTGTTCGCGGTTTATCGCCTGCAAGGCGGAGACATCAACAATCAGTGCCACGCTGCC
GTCGCCAAGAATGGTCGCAGCAGAAATGCCGGGGACTTTGCGATAGTTACTTTCAAGGTTTTTAACCACAACCTGGTGTT
GACCAATTAATTGATCCACCAGCAAGGCGTAGCGGCGACCGCCACTTTGTAAGATCACCACAATTCCCTGGGTGGCTTCG
GTTTTCGCGCCCGCGACGTTGAACACTTTCCACAGTTCGACGATGGGCAGATATTCACCCCGCACTTCCAGCACCCGCTC
GCCGCCGGCCAGTGGATGGAGATCGGCTTCACGGGGTTGCAGTGATTCCATAACAGCATTCAGCGGCAGAATGAAAACTT
CATCCGCAACGCGTACGGACATGCCGTCGAGGATGGCCAGCGTCAGCGGCAGTAAAATGCGGATCGTAGTGCCAGTACCC
TGCTTCGACTGGATTTCGACATGACCGCCCATCTTCTGGATATTACGTTTAACGACGTCCATGCCGACGCCGCGCCCGGA
GACGTCGGTGACCTGCTCTGCCGTGGAGAAGCCAGGTGCAAATATCAGCATCGCGACTTCGTCGTCGCTCATGTTTTCGC
TGACAGTCAAACCTTGCGAGGCCGCTTTTGCCAGAATTCGCTCACGGTTTAGCCCCGCCCCATCGTCGGTCACTTCAATG
CAAATGTTGCCGCCCTGATGTTCGGCAGACAGAATTAAATTTCCGACGCTGTTTTTACCTGCGGCGAGCCGTTTTTCTGG
CAGTTCAATACCGTGATCGAGGCTATTGCGTACCAGGTGGGTCAGCGGGTCGATAATGCGTTCTATCAGGCTTTTGTCGA
GTTCAGTAGAACTGCCCACCAGCGTCAGTTCTACCTGCTTGCCGAGTTTTCCCGCCAGATCACGCACCAGCCGGGGATAG
CGACTAAAAACATATTCCATCGGCATCATGCGAATCGACATCACTGATTCCTGCAAATCACGGGCGTTACGTTGTAACTG
CCCCATGCTGGTTATCAAATCACCATGATTAACCGGGTCCAGTTCGCTGGAACGCTGGGCAAGCATGGACTGGGTGATAA
CCAGCTCGCCGACGAGGTTAATTAATTGATCAACCTTTTCTACCGCTACACGGATGCTGGTGGATTCATTGCTGCGCGTC
GTTTTTTCCCGCTCCACGCGGCCGGTTGGCGCTTGTTCGGCTGCCAGTTTAAGCACTGGTGGGGTGGATATTTTTGGCGA
GACTTCTACTGTTTCAAAGGTAATCTGATCGGCTTCAATCACAAAACAGAGTACCGCTGTGATGTCATCTTCGGCGATGT
CGCCCGGTAATATTGCCGAGAGCGAATCCGCCCCTTTCACCACGTCAGTTAACGTTGTCAGATGTCCCAGTTCTTCTTCC
[truncated: 540,716 more chars]
